# Dexamethasone attenuates interferon-related cytokine hyperresponsiveness in COVID-19 patients

**DOI:** 10.3389/fimmu.2023.1233318

**Published:** 2023-08-08

**Authors:** Job J. Engel, Caspar I. van der Made, Nick Keur, Todia Setiabudiawan, Rutger J. Röring, Georgia Damoraki, Helga Dijkstra, Heidi Lemmers, Sofia Ioannou, Garyfallia Poulakou, Jos W. M. van der Meer, Evangelos J. Giamarellos-Bourboulis, Vinod Kumar, Frank L. van de Veerdonk, Mihai G. Netea, Athanasios Ziogas

**Affiliations:** ^1^ Department of Internal Medicine and Radboud Center for Infectious Diseases, Radboud University Medical Center, Nijmegen, Netherlands; ^2^ Department of Internal Medicine, National and Kapodistrian University of Athens, Medical School, Athens, Greece; ^3^ Department of Therapeutics, National and Kapodistrian University of Athens, Medical School, Athens, Greece; ^4^ Department of Genetics, University Medical Center Groningen, Groningen, Netherlands; ^5^ Department of Immunology and Metabolism, Life & Medical Sciences Institute, University of Bonn, Bonn, Germany

**Keywords:** COVID-19, SARS-CoV-2, dexamethasone, cytokine storm, interferon, ISG15

## Abstract

**Background:**

Dexamethasone improves the survival of COVID-19 patients in need of supplemental oxygen therapy. Although its broad immunosuppressive effects are well-described, the immunological mechanisms modulated by dexamethasone in patients hospitalized with COVID-19 remain to be elucidated.

**Objective:**

We combined functional immunological assays and an omics-based approach to investigate the *in vitro* and *in vivo* effects of dexamethasone in the plasma and peripheral blood mononuclear cells (PBMCs) of COVID-19 patients.

**Methods:**

Hospitalized COVID-19 patients eligible for dexamethasone therapy were recruited from the general care ward between February and July, 2021. Whole blood transcriptomic and targeted plasma proteomic analyses were performed before and after starting dexamethasone treatment. PBMCs were isolated from healthy individuals and COVID-19 patients and stimulated with inactivated SARS-CoV-2 *ex vivo* in the presence or absence of dexamethasone and transcriptome and cytokine responses were assessed.

**Results:**

Dexamethasone efficiently inhibited SARS-CoV-2-induced *in vitro* expression of chemokines and cytokines in PBMCs at the transcriptional and protein level. Dexamethasone treatment in COVID-19 patients resulted in down-regulation of genes related to type I and II interferon (IFN) signaling in whole blood immune cells. In addition, dexamethasone attenuated circulating concentrations of secreted interferon-stimulating gene 15 (ISG15) and pro-inflammatory cytokines and chemokines correlating with disease severity and lethal outcomes, such as tumor necrosis factor (TNF), interleukin-6 (IL-6), chemokine ligand 2 (CCL2), C-X-C motif ligand 8 (CXCL8), and C-X-C motif chemokine ligand 10 (CXCL10). In PBMCs from COVID-19 patients that were stimulated *ex vivo* with multiple pathogens or Toll-like receptor (TLR) ligands, dexamethasone efficiently inhibited cytokine responses.

**Conclusion:**

We describe the anti-inflammatory impact of dexamethasone on the pathways contributing to cytokine hyperresponsiveness observed in severe manifestations of COVID-19, including type I/II IFN signaling. Dexamethasone could have adverse effects in COVID-19 patients with mild symptoms by inhibiting IFN responses in early stages of the disease, whereas it exhibits beneficial effects in patients with severe clinical phenotypes by efficiently diminishing cytokine hyperresponsiveness.

## Introduction

The coronavirus disease 2019 (COVID-19) pandemic remains a significant threat to human health, resulting in more than 6.7 million deaths as of January 2023 (1). The majority of individuals infected with severe acute respiratory syndrome coronavirus 2 (SARS-CoV-2) develop mild COVID-19 with symptoms of cough, fever and dyspnea, or remain asymptomatic (2). However, an important minority of COVID-19 patients develop severe forms of COVID-19, characterized by life-threatening pneumonia, leading to acute respiratory distress syndrome (ARDS) and/or respiratory failure, often necessitating cardiopulmonary monitoring and mechanical ventilation in the Intensive Care Unit (ICU) (2, 3).

Immunological studies have indicated that severe COVID-19 is hallmarked by an initial failure to control viral replication, followed by a dysregulated hyperinflammatory response leading to cytopathic damage of the respiratory epithelium (4, 5). The hyperinflammatory response involves the excessive induction of several anti-viral and pro-inflammatory cytokines and chemokines (3–8), which trigger immune cell recruitment, cell damage (lung epithelium), microvascular thrombosis and hyperpermeability, and systemic inflammation with multiorgan damage (9).

The treatments that have so far proven most successful in severe or critically ill patients are mostly already known immunomodulatory drugs, with corticosteroids remaining the standard of care (10). The landmark RECOVERY trial revealed that treatment with dexamethasone, a synthetic corticosteroid that is considered as an immunosuppressive drug, reduced mortality of patients with severe COVID-19 (11). The greatest benefit was shown for patients receiving invasive mechanical ventilation compared to those on non-invasive oxygen support. A meta-analysis of the RECOVERY trial and subsequent studies support the beneficial effect on survival and ventilator-free days in patients with severe disease (12), but suggest that dexamethasone treatment in patients without oxygen support may be harmful (13).

An in-depth understanding of the immunological effects of dexamethasone in COVID-19 aids in the identification of immunological profiles in patients that will either likely benefit from this therapy or from additional immunomodulatory treatment, or on the contrary could be more prone to the adverse effects of the immunosuppressive effects, including secondary infections. To this end, we applied functional immunological assays and omics-based approaches to assess the *in vivo* immunological effects of dexamethasone in patients hospitalized with COVID-19, by comparing whole-blood and peripheral blood mononuclear cell (PBMC) transcriptome and plasma inflammatory proteins before and during treatment. In addition, we performed *in vitro* experiments to study the effects of dexamethasone on the immune response in PBMCs from COVID-19 patients challenged with various inflammatory stimuli and pathogens.

## Materials and methods

### Study design and ethical approval

This prospective, low-intervention study was conducted at Radboudumc, Nijmegen, the Netherlands and two medical centers in Greece (Thoracic Diseases General Hospital ‘Sotiria’, Athens, Greece and Alexandra General Hospital, Athens, Greece). There was no formal study intervention, but all patients included in the study received dexamethasone based on clinical indication according to the local treatment guidelines. The study protocol was approved by the local medical ethics committee of the Radboudumc and the National Ethics Committee of Greece (approval 38/20) and was conducted in accordance with Good Clinical Practice Guidelines and the Declaration of Helsinki. Verbal informed consent was obtained from all participants and documented in the medical records of the respective hospitals to which they were admitted.

### Patients, participants, and clinical data collection

After admission to the general care wards, patients were considered eligible for inclusion if 1) they had been admitted to the hospital with reverse transcriptase polymerase chain reaction (RT-PCR)-confirmed or high suspicion of COVID-19, and 2) there was an indication to start dexamethasone treatment according to WHO treatment guidelines, but they had not started this treatment yet. Patients who had already started dexamethasone treatment or had been treated with corticosteroids or other immunomodulatory drugs (e.g., TNF blockers, inhibitors of interleukin -1 or 6) within 30 days prior to screening were excluded from participation. RT-PCR was performed for all patients and negatively tested patients were to be excluded from the study. As controls, samples of healthy individuals who did not report or present with any clinical signs of SARS-CoV-2 infection were used; their characteristics are detailed in **Supplementary Table 1**. These samples were collected within the 200FG cohort of the Human Functional Genomics project (http://www.humanfunctionalgenomics.org). (14, 15).

### Interventions and sampling

The COVID-19 patients included in the study were treated with dexamethasone administered daily in a 6 mg dose, as recommended by COVID-19 treatment guidelines (12, 16). Dexamethasone was administered in a clinical treatment setting and was not considered a study intervention. Study samples were taken at two timepoints, just before and between three to four days after starting dexamethasone treatment (which were designated in analyses and figures as pre-Dex and post-Dex, respectively). Venous blood was collected into 3 x 10 mL sterile Ethylenediaminetetraacetic acid (EDTA) tubes, and an additional volume of 2.5 mL blood was transferred into a PAXgene Blood RNA tube (BD Biosciences) and remained at room temperature for a minimum of two hours before being stored at − 80°C.

### Plasma samples and PBMC isolation

EDTA anticoagulated blood was centrifuged at 2000 x g at room temperature for 10 min and plasma was stored at −80°C until analysis. PBMCs were obtained by individuals with gradient centrifugation over Ficoll-Paque Plus (GE Healthcare) in SepMate tubes (STEMCELL Technology) and immediately freshly used for *ex vivo* functional assays or lysed in RLT buffer (Qiagen) and stored at −80°C for subsequent RNA isolation.

### *Ex vivo* stimulation experiments

PBMCs were counted and resuspended in Dutch modified Roswell Park Memorial Institute (RPMI) medium (Life Technologies), supplemented with 50 µg/mL gentamicin (Thermo Fisher Scientific), 1 mM sodium pyruvate (Thermo Fisher Scientific), and 2 mM Glutamax (Thermo Fisher Scientific). 5 x 10^5^ PBMCs were cultured in a final volume of 200 μL/well in round bottom 96-well plates (Greiner Bio-one) and stimulated with of heat-inactivated SARS-CoV-2 Wuhan-Hu-1 virus variant (1.19 x 10^4^ TCID50/ml; NRW-42 isolate, kindly provided by Heiner Schaal, University Hospital Düsseldorf, Germany) (17), heat-inactivated *Staphylococcus aureus* (1 x 10^6^ CFU/mL; clinical isolate), heat-inactivated influenza virus H1N1 (3.2 x 10^5^ K/mL, kindly provided by Ortwin Adams, University Hospital Düsseldorf, Germany), Imiquimod/R837 (10 µg/ml; InvivoGen), Poly (I:C) (10 µg/mL; InvivoGen), R848 (10 µg/mL; InvivoGen), purified lipopolysaccharide (LPS) derived from *Escherichia coli* O55:B5 (10 ng/mL; Sigma-Aldrich), or recombinant human interleukin-1 alpha (IL-1α) (10 ng/mL; R&D Systems), in the presence of dexamethasone (10 nM or otherwise stated; Sigma-Aldrich) or vehicle control (dimethyl sulfoxide; DMSO). After 4h cells were lysed in RLT buffer (Qiagen) and stored at −80°C for subsequent RNA isolation. After 24 h or 7 days, supernatants were collected and stored at –80°C until analysis.

### RNA isolation, RNA-sequencing, and data processing

Total RNA was extracted from whole blood collected in PAXgene^®^ tubes (BD Biosciences) using PAXgene Blood RNA kit (QIAGEN) following the manufacturer’s instructions. Frozen PAXgene tube samples were thawed at room temperature overnight before RNA isolation steps to ensure complete lysis of red blood cells and precipitation of RNA. Total RNA was isolated from PBMCs, and on column DNase (QIAGEN) treated, using the RNeasy mini kit (QIAGEN) by following the manufacturer’s instructions. RNA concentrations and purity were assessed with NanoDrop spectrophotometer (Thermo Fisher Scientific). Samples were submitted to Beijing Genomics Institute (BGI), and RNA integrity was further evaluated with the Agilent 4200 system (Agilent Technologies).

The RNA-Sequencing library was prepared by using the MGIEasy RNA Library Prep kit (MGI Tech). Strand specific RNA sequencing was performed on a DNBSEQ platform (paired-end, read length 100 pb) and at least 30 million clean reads were generated per sample. After sequencing, raw reads were filtered using SOAPnuke (18). Filters include the removal of the adapter sequence, contamination check, and removal of low-quality reads (Q ≥ 33). Thereafter, processed data was assessed for quality control using FastQC (19) (version 0.11.9) and MultiQC (20) (version 1.14). Next, the remaining filtered data were aligned to the human genome GENCODE Release 39 (GRCh38.p13) using the STAR (21) (version 2.7.10a) aligner with STAR’s default parameters. The quantification of read counts per gene was performed using the –quantMode GeneCounts from STAR.

### ELISA measurements

Production of secreted cytokines in PBMC culture supernatants was measured using ELISA. IL-1β, IL-6, TNF, CXCL10, interleukin-10 (IL-10), interleukin-1 receptor antagonist (IL-1Ra), and interferon-gamma (IFN-γ) concentrations were determined with duoSet ELISA kits (R&D Systems). The VeriKine Human Interferon Alpha ELISA Kit (PBL Assay Science) was used to measure interferon-alfa (IFN-α). Supernatants from 7-day cultures were used for IFN-γ measurements, whereas the remaining cytokines were measured in 24-hour culture supernatants. Plasma concentrations of Interferon Stimulated Gene 15 (ISG15) were measured using the Human ISG15 ELISA kit from Elabscience (Elabscience). All tests were performed according to the instructions of the manufacturer.

### Multiplex cytokine assay

Plasma concentrations of IFN-γ, IL-6, TNF, CXCL8, IL-10, IL-1Ra, CXCL10, CCL2 were measured in Luminex MAGPIX system and performed using the Milliplex human cytokine/chemokine magnetic bead panel kit according to manufacturer’s instructions (Milliplex; Millipore). Data were analyzed with the Milliplex analyst software (Millipore).

### OLINK proteomic analysis

Inflammatory biomarkers were measured in EDTA plasma using the Olink Target 96 Inflammation panel (Olink Proteomics AB). This panel uses a proximity extension assay (PEA) developed by Olink Proteomics, containing 96 protein targets relating to inflammatory response (22). Olink’s PEA defines proteome expression in an arbitrary unit called normalized protein expressions (NPX), which are calculated from the inverse of cycle threshold (Ct) values required to detect specific proteins included in the assay. This value can be used for relative quantification of protein expression within a cohort measured at the same occasion. Because the samples were taken from COVID-19 patients, 45 µL of each sample was inactivated with 5 µL of a 10% TritonX-100 solution. Olink data were analyzed using RStudio (version 2021.09.1 Build 372, RStudio).

### Differential expression analysis, functional pathway analysis, and data visualization

The R package DESeq2 (23) package (version 1.38.1, RStudio) was utilized for differential expression analysis. We filtered lowly expressed genes by removal of genes with less than 5 read counts across all samples in each comparison. Thereafter, filtered counts were normalized using the DESeq2 method which directly accounts for differences in the library size. Samples were analyzed using paired comparisons between experimental groups and differentially expressed genes (DEGs) were determined by the Wald test using the Benjamini-Hochberg correction (FDR) < 0.05. Visualization of DEGs was done using Volcano plots which were generated using the R package Enhancedvolcano (version 1.16.0). Finally, the normalized counts were Log_2_ transformed and used for visualization and downstream analyses. Functional enrichment was performed using the R packages ClusterProfiler (24) (4.6.0) and enrichR (25) (version 3.1) based on information from GO terms or Reactome databases. Hierarchical clustering and generation of heatmaps were performed using the ComplexHeatmap (26) (version 2.14.0) and pheatmap packages in R (version 1.0.12). Other visualizations were created using ggplot2 (version 3.4.0) or Graph Prism 9 (GraphPad software).

### Statistical analysis

For descriptive statistics, demographic data were analyzed in SPSS Statistics (version 28.0.1.0., IBM software). Statistical analyses of multiplex cytokine assay and the ELISA data were performed in Graph Prism 9 (GraphPad software). Two-way ANOVA followed by Tukey’s multiple comparison test was used for multiple group comparisons. Wilcoxon signed-rank test was used for paired and Mann–Whitney *U* test was used for unpaired comparisons. A *P* value < 0.05 was considered to be statistically significant.

## Results

### Dexamethasone alters the transcriptome of SARS-CoV-2-stimulated PBMCs *in vitro*

**Figure 1:**
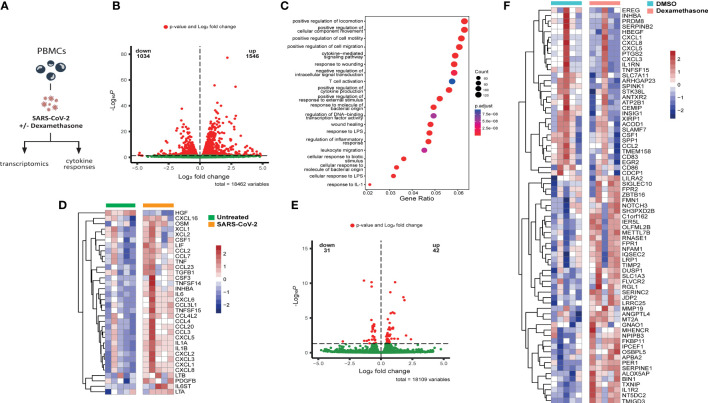
Dexamethasone alters the transcriptome of SARS-CoV-2 stimulated PBMCs *in vitro.* **(A)** Experimental *in vitro* setup for profiling the transcriptome and cytokine responses of PBMCs derived from healthy individuals stimulated with heat-inactivated SARS-CoV-2 in the absence (vehicle control DMSO) or presence of dexamethasone. **(B)** Volcano plot depicting the distribution of the adjusted *P* values (-Log_10_ *P*) and the fold changes (Log_2_ fold change) of differentially expressed genes in heat-inactivated SARS-CoV-2-treated PBMCs as compared to unstimulated cells 4h after exposure. Significantly altered genes are colored red (n=5 healthy individuals; FDR < 0.05). **(C)** Gene set enrichment analysis of top altered pathways in heat-inactivated SARS-CoV-2-treated PBMCs, as compared to unstimulated cells 4h after exposure. The counts indicate the total number of DEGs in the enriched pathway, whereas the color indicates the *P* value (n=5 healthy individuals; FDR < 0.05).**(D)** Heatmap showing statistically significant differentially expressed cytokine transcripts in PBMCs stimulated with heat-inactivated SARS-CoV-2, as compared to unstimulated cells 4h after exposure. Colors indicate the z-scores calculated per gene from the Log_2_ normalized counts (n=5 healthy individuals; red increased, blue decreased; FDR < 0.05). **(E)** Volcano plot depicting the distribution of the adjusted *P* values (-Log_10_ *P*) and the fold changes (Log_2_ fold change) of differentially expressed genes in heat-inactivated SARS-CoV-2-treated PBMCs as compared to cells treated with dexamethasone (10nM) 4h after exposure. Significantly altered genes are colored red (n=5 healthy individuals; FDR < 0.05). **(F)** Heatmap showing differentially expressed genes between untreated (DMSO) or dexamethasone (10nM)-treated PBMCs stimulated with heat-inactivated SARS-CoV-2 for 4h. Colors indicate the z-scores calculated per gene from the Log_2_ normalized counts (n=5 healthy individuals; red increased, blue decreased; FDR < 0.05).

First, we explored the direct *in vitro* effects of dexamethasone in SARS-CoV-2-stimulated leukocytes. To this end, PBMCs isolated from five healthy volunteers (**Supplementary Table 1**) were exposed to inactivated SARS-CoV-2 for four hours before analyzing their transcriptome profile (**Figure 1A**). Heat-inactivated SARS-CoV-2 was able to induce marked alterations in gene expression. Specifically, a total of 1546 genes underwent statistically significant upregulation, whereas 1034 genes were downregulated (**Figure 1B**). A list of all significant DEGs is shown in the supplementary data (**Supplementary File 1**). Pathway enrichment analysis revealed that SARS-CoV-2 stimulation resulted in upregulation of genes involved in inflammatory responses such as leukocyte locomotion and migration, as well as cytokine production and cytokine-mediated signaling (**Figure 1C**). Transcripts of several proinflammatory cytokines and chemokines were elevated (**Figure 1D**), among which mediators related to COVID-19 disease severity that can be medically targeted (27, 28).

We subsequently evaluated the *in vitro* anti-inflammatory activity of dexamethasone in PBMCs stimulated with SARS-CoV-2 and detected 42 upregulated DEGs after four hours exposure to the heat-inactivated virus, while 31 genes were downregulated (**Figure 1E**). Dexamethasone attenuated the expression of multiple genes previously observed to be upregulated in response to SARS-CoV-2, including several transcripts related to chemotaxis like *CCL2*, *CXCL1*, *CXCL3*, *CXCL5*, and *CXCL8*, as well as cytokine genes *CSF1*, *TNFSF15*, and *IL1RN*, and metabolic genes related to the immune response such as *PTGS2* and *ACOD1* (29, 30) (**Figure 1F**). Dexamethasone-treated cells additionally exhibited increased expression of genes with anti-inflammatory actions such as the decoy interleukin-1 receptor *IL1R2*, the product of which can reduce the activity of IL-1β (31), and *DUSP1*, which is implicated in the inhibition of proinflammatory signaling pathways (32–34) (**Figure 1F**).

These *in vitro* immune profiling data indicate that heat-inactivated SARS-CoV-2 can induce a potent transcriptional inflammatory response characterized by marked alterations in chemotaxis and cytokine responses, which is partially counteracted by dexamethasone treatment.

### Cytokine responses are inhibited by dexamethasone in PBMCs stimulated with SARS-CoV-2 *in vitro*

**Figure 2:**
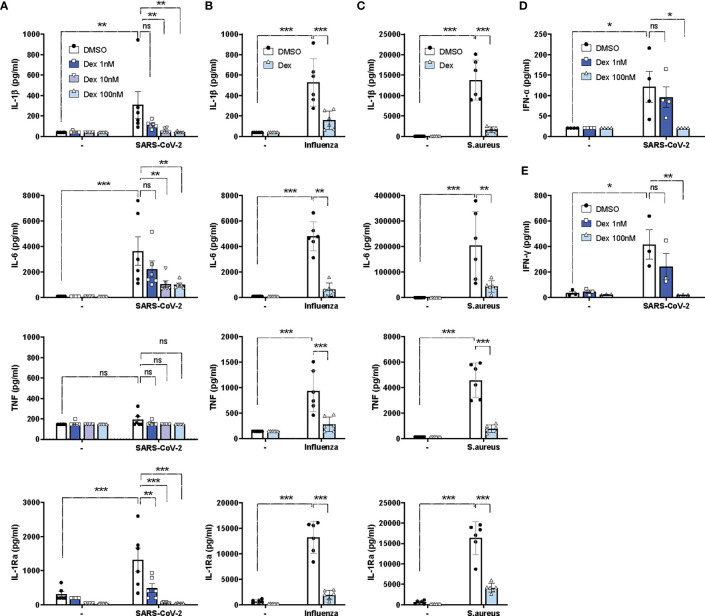
Dexamethasone efficiently inhibits cytokine responses in PBMCs upon stimulations with SARS-CoV-2, influenza H1N1 or S. aureus *in vitro.* **(A)** PBMCs were stimulated *in vitro* with heat-inactivated SARS-CoV-2 or left unstimulated for 24h in the absence (DMSO) or presence of different concentrations of dexamethasone, and the production of IL-1β, IL-6, TNF and IL-1Ra were assessed in supernatants (n=6 healthy individuals). **(B, C)** PBMCs were stimulated *in vitro* with heat-inactivated influenza H1N1 **(B)**, heat-inactivated S. aureus **(C)**, or left unstimulated for 24h in the absence (DMSO) or presence of dexamethasone (100nM) for 24h, and the production of IL-1β, IL-6, TNF and IL-1Ra were assessed in supernatants (n=6 healthy individuals). **(D)** PBMCs were stimulated *in vitro* with heat-inactivated SARS-CoV-2 or left unstimulated for 24h in the absence (DMSO) or presence of different concentrations of dexamethasone, and IFN-α was assessed in supernatants (n=4 healthy individuals). **(E)** PBMCs were stimulated *in vitro* with heat-inactivated SARS-CoV-2 or left unstimulated for 7d in the absence (DMSO) or presence of different concentrations of dexamethasone, and IFN-γ was assessed in supernatants (n=4 healthy individuals). Data are presented as mean ± SEM. Two-way ANOVA followed by Tukey’s multiple comparison test. *: *P* value < 0.05, **: *P* value < 0.01, ***: *P* value < 0.001, ns = not significant.

Given the excessive cytokine secretion in immune hyperactivation and COVID-19 severity (35), we investigated the *in vitro* effects of dexamethasone on SARS-CoV-2-induced cytokine production in PBMCs isolated from healthy individuals (**Supplementary Table 1**). SARS-CoV-2 stimulation alone led to a significant increase in the production of IL-1β, IL-6, and IL-1Ra, but not TNF, in healthy PBMCs (**Figure 2A**). As expected, dexamethasone diminished the production of IL-1β, IL-6, and IL-1Ra in a dose-dependent manner (1nM-100nM) and almost completely inhibited their production at the highest concentrations (100nM) (**Figure 2A**). Compared to heat-inactivated SARS-CoV-2, stimulation with other pathogens such as heat-inactivated influenza H1N1 or heat-inactivated *S. aureus* displayed stronger *in vitro* cytokine responses than with SARS-CoV-2, and these were also significantly decreased by dexamethasone (**Figures 2B, C**). Additionally, effective attenuation of the production of the anti-viral type-I and II IFNs, IFN-α and IFN-γ, respectively, was observed upon dexamethasone treatment in PBMCs stimulated with SARS-CoV-2 (**Figures 2D, E**).

### COVID-19 patient recruitment and characteristics

As dexamethasone exhibited direct *in vitro* anti-inflammatory effects in SARS-CoV-2-stimulated PBMCs, its efficiency in attenuating hyperinflammatory responses in COVID-19 patients was subsequently investigated. Thirteen patients hospitalized with COVID-19 were recruited in three participating medical centers in the Netherlands and Greece between the 4^th^ of February and the 1^st^ of July 2021. All patients were tested positive for SARS-CoV-2 by RT-PCR. None of the patients had been vaccinated against SARS-CoV-2. All patients required supplemental oxygen treatment and scored a 5 on the WHO clinical progression scale (WHO-CPS) at the time of enrolment, corresponding with moderate disease severity (36) (**Supplementary Table 2**). No secondary infections were reported and all patients included in the study survived. One patient required admission to the intensive care unit (ICU) and subsequently recovered.

Nine patients were enrolled and investigated before (pre-Dex) and three to four days after (post-Dex) starting dexamethasone treatment. Their demographic and clinical characteristics are summarized in **Table 1**. Mean age was 60.8 ( ± 6.9) and the majority of patients were men (77.8%). Patients were recruited a mean of 9.0 days ( ± 2.9) after the onset of symptoms. One patient was excluded from analyses because of inconsistent dosage of dexamethasone on one of the treatment days.

**Table 1:** Patient characteristics.

Patient characteristics	Expressed as			
**Demographic characteristics**				
Sex				
Female	Frequency	2 (22.2%)		
Male	Frequency	7 (77.8%)		
Age (years)	Mean ± SD	60.8 ± 6.9		
Length (cm)	Mean ± SD	175.8 ± 9.3		
Weight (kg)	Mean ± SD	85.2 ± 15.4		
BMI (kg/m2)	Mean ± SD	27.5 ± 4.9		
**Clinical characteristics**		**Timepoints**		
		Screening	Timepoint 1	Timepoint 2
Systolic blood pressure (mmHg)	Mean ± SD	137.1 ± 14.6	N.A.	N.A.
Diastolic blood pressure (mmHg)	Mean ± SD	80.4 ± 8.8	N.A.	N.A.
qSOFA score	Median (range)	0 (0–1)	N.A.	N.A.
Respiratory rate	Mean ± SD	20.8 ± 3.3	20.8 ± 3.3	18.9 ± 2.2
Duration of illness	Mean ± SD	9.0 ± 2.9	9.1 ± 2.9	11.8 ± 3.4
Supplemental oxygen volume (L/min)	Mean ± SD	4.1 ± 3.4	4.4 ± 3.5	4.6 ± 5.2
Type of supplemental oxygen				
Nasal cannula	Frequency	7 (77.8%)	6 (66.7%)	6 (66.7%)
Venturi mask (40%)	Frequency	0 (0%)	1 (11.1%)	2 (22.2%)
Venturi mask (60%)	Frequency	2 (22.2%)	2 (22.2%)	0 (0%)
Non-rebreathing mask	Frequency	0 (0%)	0 (0.0%)	0 (0%)
High-flow nasal cannula/Non-invasive ventilation mask	Frequency	0 (0%)	0 (0%)	1 (11.1%)
Mechanical ventilation	Frequency	0 (0%)	0 (0%)	0 (0%)
**Comedications during hospitalization**				
Remdesivir	Frequency	5 (55.6%)		
Anakinra	Frequency	1 (11.1%)		
Tocilizumab	Frequency	1 (11.1%)		
Baricitinib	Frequency	1 (11.1%)		
**Clinical outcome**				
Length of hospital stay (days)	Mean ± SD	8.8 ± 3.6		
Required ICU admission	Frequency	0 (0%)		
In-hospital mortality	Frequency	0 (0%)		

### Whole-blood transcriptome of COVID-19 patients treated with dexamethasone

**Figure 3:**
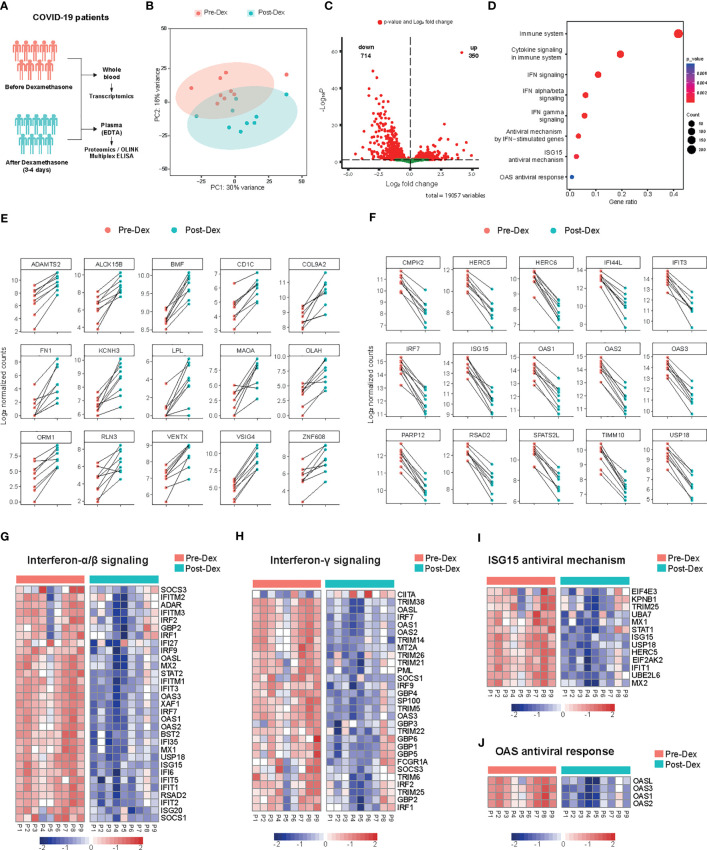
Whole blood transcriptome profile before and after dexamethasone treatment in hospitalized COVID-19 patients. **(A)** Schematic presentation of the study design. COVID-19 patients eligible for dexamethasone treatment were analyzed before and 3-4 days after starting dexamethasone treatment (n=9 COVID-19 patients). **(B)** Principal component analysis (PCA) plot of whole blood expressed genes in COVID-19 patients before (Pre-Dex) and after dexamethasone treatment (Post-Dex). The ellipses indicate the 95% confidence intervals (CI). **(C)** Volcano plot depicting the distribution of the adjusted *P* values (-Log_10_ *P*) and the fold changes (Log_2_ fold change) of whole blood DEGs in COVID-19 patients before and after dexamethasone treatment. Significantly altered genes are colored red (FDR < 0.05). **(D)** Gene set enrichment analysis of top altered pathways in whole blood transcriptome before versus after dexamethasone treatment. The counts indicate the total number of DEGs in the enriched pathway, whereas the color indicates the *P* value (FDR < 0.05). **(E)** Top 15 significantly upregulated protein-coding genes in whole blood transcriptome before versus after dexamethasone treatment. The y-axis indicates the Log_2_ normalized counts. **(F)** Top 15 significantly downregulated protein-coding genes in whole blood before versus after dexamethasone treatment. The y-axis indicates the Log_2_ normalized counts. **(G-J)** Heatmaps showing differentially expressed genes after dexamethasone treatment of IFN α/β signaling (*P* value = 1.41**·**10^-20^) **(G)**, IFN-γ signaling (*P* value = 6.42·10^−15^) **(H)**, ISG15 antiviral mechanism (*P* value = 7.42·10^−04^) **(I)**, or OAS antiviral response (*P* value = 7.95·10^−03^) **(J)**. Row scaling has been applied to each row (gene). The colors indicate the z-scores from the Log_2_ normalized counts (red increased, blue decreased; FDR < 0.05).

To delineate the molecular mechanisms underlying the immunomodulatory effects of dexamethasone in patients hospitalized with COVID-19, we performed whole-blood bulk RNA sequencing in nine patients, before and after dexamethasone treatment (**Figure 3A**). Unsupervised clustering using Principal Component Analysis (PCA) showed clear separation of the samples before and after treatment (**Figure 3B**). We detected 350 upregulated and 714 downregulated transcripts that were significantly differentially expressed after dexamethasone administration (**Figure 3C**; **Supplementary File 2**). Pathway enrichment analysis revealed that dexamethasone mostly affected pathways related to cytokine and IFN signaling as well as downstream IFN-stimulated pathways such as ISG15 and 2′-5′-Oligoadenylate Synthetase (OAS) antiviral responses (**Figure 3D**).

*VSIG4*, which promotes phagocytosis and acts as a negative regulator of T-cell and macrophage activation, was the most significantly induced gene upon dexamethasone treatment (37, 38) (**Figure 3E**). Among the most strongly upregulated DEGs were other negative regulators of inflammation like *MAOA* (39), genes related to lipid metabolism (*LPL*, *OLAH*, *ALOX15B*) (40), epithelial-to-mesenchymal transition (EMT) (*SHROOM2*, *ARGHAP29*, *FILIP1L*) (41, 42), dendritic cell differentiation (*VENTX*) (43) and vascular stability (*BMF*) (**Figure 3E**; **Supplementary File 2**). Genes related to phagocytosis and B-cell activation were also upregulated (**Supplementary File 2**).

Late in the clinical course of COVID-19, sustained IFN secretion correlates with worse disease outcomes (7, 44, 45). Pathway enrichment analysis indicated that interferon-stimulated genes (ISGs) (44) were strongly modulated by dexamethasone. The top downregulated DEGs mostly included genes involved in type-I IFN-signaling such as *ISG15*, *IRF7*, *RSAD2*, *USP18*, and *IFIT3* (**Figure 3F**). Both type-I and II IFN-related genes and genes related to upstream signaling such as *IRF7* and *STAT2* were consistently downregulated among patients treated with dexamethasone (**Figure 3G, H**). Additionally, gene clusters that constitute important pathways in the host defense against SARS-CoV-2, such as TLR7 and IFIH1/MDA5 signaling pathways were inhibited by dexamethasone (**Supplementary File 2**). ISG15 is implicated in antiviral responses by conjugating to viral proteins and additionally functioning as a cytokine (46), and several genes related to ISG15 antiviral response were decreased (**Figure 3I**). Of note, the top DEGs included genes related to ISG15-mediated antiviral signaling as well as the restriction of viral replication through OAS enzymes (**Figures 3F, I, J**) (47). Collectively, whole blood transcriptome analysis showed that dexamethasone predominantly affected IFN-related responses.

### Plasma cytokine and chemokine concentrations in COVID-19 patients treated with dexamethasone

**Figure 4:**
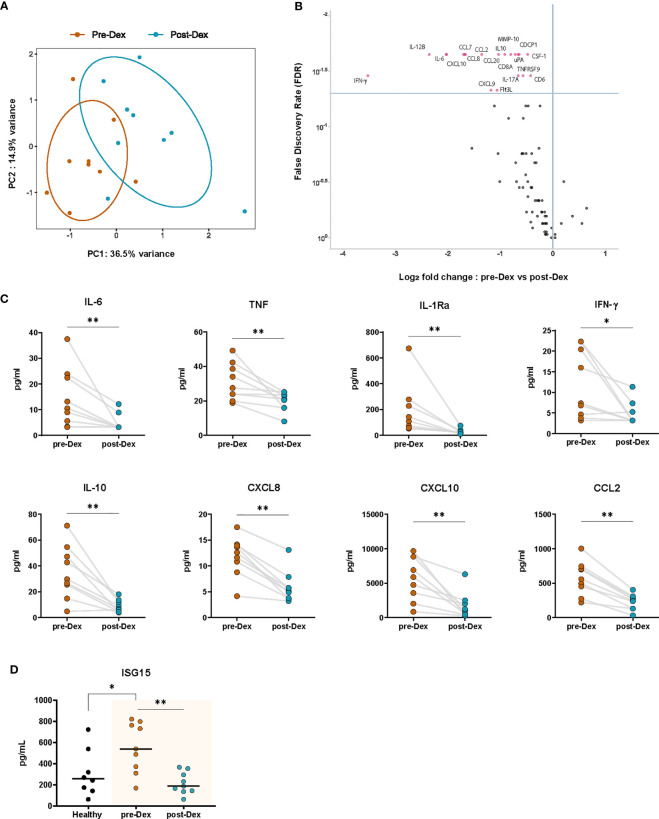
Dexamethasone treatment diminishes circulating inflammatory proteins in hospitalized COVID-19 patients. **(A)** PCA plot of normalized protein expression (NPX) values of circulating plasma proteins (inflammatory Olink panel) before (Pre-Dex) and 3-4 days after starting dexamethasone treatment (Post-Dex). The ellipses indicate the 95% CIs (n=9 COVID-19 patients). **(B)** Volcano plot depicting the distribution of the FDR and Log_2_ fold change in normalized protein expression values of inflammatory plasma proteins (inflammatory OLINK panel) in COVID-19 patients before versus after dexamethasone treatment. Significantly altered proteins (FDR < 0.05) are plotted in red (n=9, Wilcoxon signed-rank test). **(C)** Plasma concentrations of selected cytokines in COVID-19 patients before and after dexamethasone treatment as determined by Luminex multiplex assay (n=8-9). *: *P* value < 0.05, **: *P* value < 0.01, Wilcoxon signed-rank test. **(D)** Plasma concentrations of ISG15 in healthy individuals and in COVID-19 patients before and after dexamethasone treatment as determined by ELISA (n=8 healthy volunteers, n=9 patient samples before and after treatment). *: *P* value < 0.05, **: *P* value < 0.01, Mann-Whitney U test (healthy vs COVID-19 patients); Wilcoxon signed-rank test (pre-Dex vs post-Dex).

To assess the effects of dexamethasone on inflammatory biomarkers *in vivo*, we performed targeted proteomic analyses on EDTA plasma collected from nine COVID-19 patients before and after dexamethasone treatment by using the Olink Target 96 Inflammation panel (**Figure 3A**). A total of 80 of the 92 proteins included in the panel were detectable in >75% of all samples. The normalized protein expression (NPX) values of these 80 proteins were then compared pairwise between each timepoint for each patient. COVID-19 patients exhibited a distinct inflammatory biomarker profile upon dexamethasone treatment (**Figure 4A**). We observed 20 inflammatory proteins significantly decreased after dexamethasone treatment, including the cytokines IL-6, interleukin-17A (IL-17A), interleukin-12B (IL-12B), and IFN-γ, the chemokines CCL2, CXCL8, chemokine ligand 19 (CCL19), chemokine ligand 20 (CCL20), and CXCL10/IP-10, the proteases matrix metalloprotein-10 (MMP-10) and urokinase plasminogen activator (uPA), and the hematopoietic cytokines Fms-related tyrosine kinase 3 ligand (FLT3L) and colony stimulating factor 1 (CSF-1) (**Figure 4B**). Many of these mediators have previously been shown to correlate with COVID-19 disease severity (6, 48–53).

We further validated the relative expression results from Olink by using multiplex ELISA to measure patient plasma concentrations of key cytokines and chemokines strongly related to disease severity. Significant decreases in plasma concentrations of the pro-inflammatory cytokines IL-6, TNF, and IFN-γ, the leukocyte chemoattractants CXCL8, CXCL10, and CCL2, and the anti-inflammatory cytokines IL-10 and IL-1Ra were observed in COVID-19 patients during dexamethasone treatment (**Figure 4C**). Plasma concentrations of secreted ISG15, which were elevated in COVID-19 patients as compared to healthy volunteers, were significantly inhibited by dexamethasone (**Figure 4D**) (47).

### The effects of dexamethasone on *ex vivo* responses of PBMCs derived from COVID-19 patients

**Figure 5:**
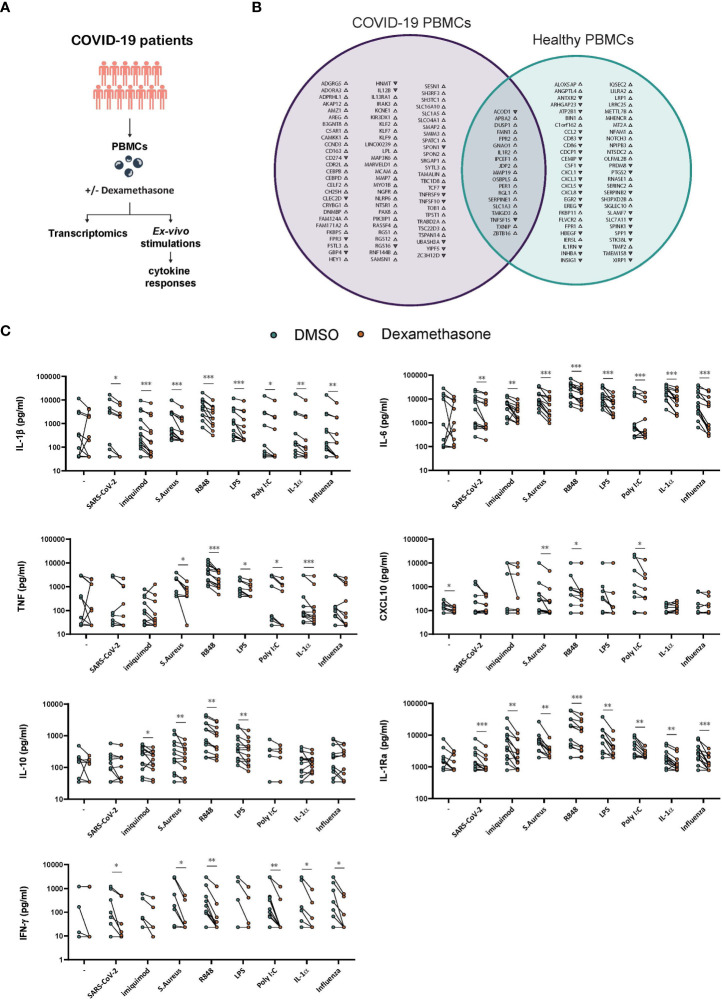
*Ex vivo* effects of dexamethasone in stimulated PBMCs derived from COVID-19 patients. **(A)** Experimental setup for profiling the *ex vivo* transcriptome and cytokine responses of stimulated PBMCs derived from COVID-19 patients in the absence (DMSO) or presence of dexamethasone. **(B)** PBMCs derived from COVID-19 patients or healthy individuals were stimulated *ex vivo* with heat-inactivated SARS-CoV-2 for 4h in the absence (DMSO) or presence of dexamethasone and analyzed for their transcriptome profile. In the Venn diagram the genes significantly altered by dexamethasone only in PBMCs from COVID-19 patients are depicted with purple whereas the genes significantly altered by dexamethasone only in PBMCs from healthy individuals (as referred in **Figure 1F**) are depicted with green. Gene upregulation or downregulation is indicated by the respective arrowheads. (n=5 COVID-19 patients, n=5 healthy individuals, FDR < 0.05). **(C)** PBMCs derived from COVID-19 patients were challenged *ex vivo* with heat-inactivated SARS-CoV-2, heat-inactivated influenza H1N1, heat-inactivated *S. Aureus*, the TLR ligands Imiquimod, R848, LPS or Poly I:C, the pro-inflammatory cytokine IL-1α, or left unstimulated for 24h in the absence (DMSO) or presence of dexamethasone (10nM), and the production of IL-1β, IL-6, TNF, CXCL10, IL-10 and IL-1Ra was assessed in supernatants after 24h (n=13 COVID-19 patients). The production of IFN-γ was assessed in the supernatants after 7d culture (n=12 COVID-19 patients). *: *P* value < 0.05, **: *P* value < 0.01, ***: *P* value < 0.001, Wilcoxon signed-rank test.

To further expand our understanding of the immunomodulatory effects of dexamethasone in COVID-19, we investigated the transcriptomic and cytokine profiles of *ex vivo* stimulated PBMCs derived from COVID-19 patients (**Figure 5A**; **Supplementary Table 3**). We first evaluated the effects of dexamethasone on the transcriptome of SARS-CoV-2-stimulated cells. The Venn diagram in **Figure 5B** displays the overlap and differences in the significantly altered transcripts upon treatment with dexamethasone in SARS-CoV-2-stimulated PBMCs derived from COVID-19 patients as compared to healthy individuals (previously described in **Figure 1F**). A total of 19 DEGs were significantly affected in PBMCs from both healthy individuals and COVID-19 patients, while 54 and 83 transcripts were only significantly altered in PBMCs from healthy volunteers and COVID-19 patients, respectively (**Figure 5B**).

Of the DEGs that overlapped between healthy and COVID-19 PBMCs, 17 were upregulated, among which *IL1R2*, whereas the genes encoding for the cytokine tumor necrosis factor superfamily member 15 (TNFSF15) and *ACOD1* were downregulated by dexamethasone (29, 54, 55). Of note, several chemokines were not significantly affected by dexamethasone in PBMCs from COVID-19 patients as compared to healthy individuals. Among others, cytokine-related genes *IL12B* and *TNFSF10* and members of the Krüppel-like factors (KLF) and CCAAT/enhancer-binding protein (CEBP) family of transcription factors as well as transcription factor 7 (TCF7) were affected only in COVID-19-derived PBMCs (**Figure 5B**).

We further evaluated the effects of dexamethasone in PBMCs from COVID-19 patients by examining their cytokine responses to several pathogens and inflammatory stimuli. To this end, PBMCs isolated from COVID-19 patients before receiving their first dose of dexamethasone, were exposed *ex vivo* to heat-inactivated SARS-CoV-2, heat-inactivated H1N1 Influenza, heat-inactivated *S. aureus*, the TLR4 ligand LPS, the TLR3 ligand poly I:C, the TLR7 ligand imiquimod, the TLR7/8 ligand R848, or the tissue damage-related cytokine IL-1α in the presence or absence of dexamethasone. The concentrations of IL-1β, IL-6, TNF, CXCL10, IL-10, IL-1Ra, and IFN-γ were evaluated in supernatants (**Figure 5C**). Dexamethasone was unable to significantly reduce the basal production of most tested cytokines except for CXCL10. Dexamethasone generally diminished IL-1β, IL-6, and IL-1Ra responses to all the tested stimuli, and was able to reduce the production of all cytokines when stimulated with *S. aureus* or R848 and IL-1β, IL-6, IL-1Ra, and IFN-γ in SARS-CoV-2- and influenza-stimulated cells. TNF, IL-10 and CXCL10 were not significantly modulated by dexamethasone in SARS-CoV-2-stimulated cells. Taken together, dexamethasone efficiently inhibited most cytokine responses in PBMCs isolated from SARS-Cov-2 infected individuals when exposed to various inflammatory stimuli.

## Discussion

The current study provides insights in the immunomodulatory mechanisms of dexamethasone in the context of COVID-19 and suggests that while dexamethasone can efficiently abrogate the immune hyperresponsiveness observed in hospitalized COVID-19 patients with increasing disease severity through the modulation of IFN-related signaling and cytokine secretion, the inhibition of IFN responses in early stages of the disease could have adverse effects in COVID-19 patients with mild symptoms.

Dysregulation of the IFN pathways is an important determinant of COVID-19 severity. A robust early IFN response is crucial to curb the progression of SARS-CoV-2 infection. SARS-CoV-2 actively interferes with its recognition by innate immune cells and subsequent IFN signaling and production (56) and elicits a lower type I IFN response compared to other viruses such as influenza (44, 57). The importance of intact type I and III IFN signaling is further illustrated by the observations that patients with inborn errors of TLR3 and TLR7 signaling or neutralizing antibodies against IFN-α and IFN-ω have an increased risk of developing severe disease (58–61). During later phases of the disease, when the viral activity has decreased, enhanced IFN signaling predominantly in the myeloid compartment contributes to the hyperinflammatory responses that characterize severe/critical COVID-19 and correlate with worse outcomes (7, 44, 45, 62). Interestingly, glucocorticoids have been shown to inhibit components of IFN signaling (63), although data from human studies is sparse. One of the underlying immunological mechanisms could be that plasmacytoid dendritic cells (pDCs) infiltrating the lungs during severe infection prime macrophages to become hyperresponsive by inducing pro-inflammatory transcriptional and epigenetic changes (64).

In the present study, we observed a strong modulation of type I and II IFN signaling in hospitalized COVID-19 patients treated with dexamethasone. In addition, dexamethasone was shown to inhibit circulating concentrations of IFN-inducible CXCL10, as wells as the production of IFN-α and IFN-γ by PBMCs upon *in vitro* stimulation with SARS-CoV-2. These findings align with a recent study showing suppression of IFN signaling by dexamethasone in neutrophils of patients with severe COVID-19, with *IFITM1, IFIT1* and *ISG15* as top downregulated genes similar to our data (65).

In hospitalized COVID-19 patients, ISG15 is among the strongest up-regulated genes in immune cells (52, 62, 66, 67) as well as in respiratory epithelial cells (68) and appears to be an important orchestrator of inflammation. Specifically in SARS-CoV-2 infection, secreted non-conjugated ISG15 acts as a cytokine that leads to sustained exacerbated inflammation (69). Through an increased concentration of non-conjugated ISG15 in macrophages, SARS-CoV-2 has been shown to induce a pro-inflammatory response that could contribute to the excessive proinflammatory cytokine response and related immunopathogenesis in severe COVID-19. Furthermore, it has been shown in patients, as well as a mouse model of ISG15 deficiency, that extracellular ISG15 is critically involved in the production of IFN-γ that confers resistance to tuberculosis (70, 71). Dexamethasone strongly inhibited plasma ISG15 concentration in patients with COVID-19, and thus demonstrated the capability of modulating cytokines and transcription factors that play a role in the antiviral host response but are also linked to cytokine hyperresponsiveness.

Subsequently, we observed that dexamethasone treatment attenuated plasma cytokine concentrations that were previously identified as strong independent predictors of disease severity and survival, including IL-6, CXCL8, and TNF (6, 52). Dexamethasone also inhibited the production of mediators associated with increased viral load and lung injury, such as CXCL10 and CCL7 (67, 72). Additionally, dexamethasone could effectively inhibit excessive leukocyte recruitment and influx as concentrations of key leukocyte chemokines such as CCL2, CXCL8 and CXCL10 were diminished. In contrast to the *in vivo* anti-inflammatory effects of dexamethasone in the systemic regulation of TNF, IL-10, and CXCL10, no direct effects were observed for these cytokines in *ex vivo* SARS-CoV-2 stimulated PBMCs, pointing out the regulatory role of dexamethasone in pathways induced by other inflammatory mediators or the involvement of other cell types. Interestingly, in a human model of endotoxin-induced pulmonary inflammation dexamethasone strongly inhibited systemic IL-6 concentrations, yet failed to inhibit local pulmonary IL-6 secretion (73). A previous bioinformatic analysis demonstrated that IL-6 is not a direct target of dexamethasone treatment in the context of COVID-19 (74). Similar to other studies we observed an anti-inflammatory systemic effect of IL-6 regulation by dexamethasone in COVID-19 (6, 12) but dexamethasone has also been shown to fail in the inhibition of IL-6, TNF and CXCL10 expression in the lung microenvironment (75), pointing out a possible inefficiency of regulating local hyperinflammatory response in the lungs.

Following the landmark RECOVERY trial that demonstrated a reduce in 28-day mortality in patients receiving supplemental oxygen in 2020 (11), dexamethasone has been a cornerstone in the treatment of severe COVID-19. Subsequent studies have confirmed its beneficial effects on ventilator-free days in critically ill patients, but not on mortality (12, 76, 77). Although dexamethasone is known to have broad immunosuppressive effects, treatments targeting only specific cytokines, such as IL-1 or IL-6 have also been used with clinical benefit (27, 28, 49, 50, 78). In our study, we show *in vivo* and *ex vivo* effects of dexamethasone on inflammatory pathways that are linked to COVID-19 disease severity, both on the transcriptomic and protein level. Dexamethasone decreased plasma concentrations of the IL-1 receptor antagonist IL-1Ra after *in vivo* treatment, while *ex vivo* dexamethasone treatment similarly led to downregulation of *IL1RN* transcripts while upregulating the *IL1R2* gene, which encodes for a decoy receptor for IL-1. In addition, *ex vivo* dexamethasone treatment downregulated the *ACOD1* gene in PBMCs derived from both healthy individuals and COVID-19 patients. Expression of *ACOD1* is specific to the monocyte-macrophage cell lineage and is modified through both the glucocorticoid receptor to which dexamethasone binds, and the JAK/STAT pathway (79).

It is currently unclear whether these observations would also extend to a decreased incidence of long COVID, which is characterized by persistently activated innate and adaptive immune cells, T cell exhaustion, as well as elevated levels of circulating cytokines such as type I and III IFNs (80, 81). A recent preprint study has observed that prior treatment with dexamethasone decreased persistent symptoms in hospitalized COVID-19 after 8 months of follow-up compared to a non-treated control group (82). It would therefore be of interest for further studies to interrogate whether dexamethasone treatment in patients with moderate-to-severe COVID-19 could limit the post-acute immunological sequelae observed in long COVID-19.

Despite the proven efficacy of dexamethasone in lowering mortality predominantly in severe or critical COVID-19 patients, a large observational cohort study has demonstrated that dexamethasone treatment had no mortality benefit on those receiving supplemental oxygen *via* low-flow nasal cannula in the first 48 hours, while it increased mortality in patients not receiving oxygen (13). Moreover, dexamethasone has treatment-related adverse events, including hyperglycemia, cardiovascular events, and bacterial superinfections (83, 84). These observations call for awareness of the risk-to-benefit ratio in initiating dexamethasone treatment in COVID-19. While dexamethasone is capable of reducing mortality risk when there is underlying inflammatory hyperresponsiveness, treatment could prove detrimental if started too early during the viral replication phase and when inflammation is not the predominant feature of the disease. The correct timing for corticosteroid treatment has also been a debated topic in other severe respiratory infectious diseases (85, 86). These findings underline the importance of patient selection and timing when initiating dexamethasone treatment in COVID-19 patients, while prompting the need for a deeper understanding of the molecular and cellular mechanisms underlying both its beneficial and detrimental effects.

This study has several limitations. First, the sample size of our patient cohort was relatively small. Still, we believe the results point to a set of consistent mechanisms. Second, our study lacked a control group for the *in vivo* experiments, since dexamethasone treatment was standard of care when this study was conducted and we could not ethically withhold an effective treatment from hospitalized COVID-19 patients. Therefore, we could not exactly distinguish whether changes observed before and after dexamethasone treatment of hospitalized patients were attributable solely to the immunomodulatory effects of dexamethasone itself and to what extent the natural clinical course of COVID-19 contributed. Additionally, even though healthy donors did not have COVID-19 symptoms and no proinflammatory phenotypes were observed at the molecular level, we cannot rule out the possibility of an asymptomatic infection since they were not tested for SARS-CoV-2 negativity with a RT-PCR test. Despite these limitations, we observed significant and biologically relevant results in the inflammatory proteome and transcriptome of patients before and after *in vivo* dexamethasone treatment and support the role of dexamethasone in these observed effects with *in vitro* experimental data.

Collectively, the current findings represent a molecular basis to support the notion of dexamethasone treatment to attenuate the inflammatory hyperresponsiveness underlying clinical disease in moderate to severe COVID-19 patients, while its detrimental effects mechanisms that are key to controlling viral replication call for caution when considering the treatment of mild COVID-19 patients or at an early stage of the disease.

## Data availability statement

The datasets presented in this study can be found in online repositories. The repository is publicly accessible and can be found through accession number GSE227585 or via this link: https://www.ncbi.nlm.nih.gov/geo/query/acc.cgi?acc=GSE227585.

## Ethics statement

The studies involving human participants were reviewed and approved by CMO Radboudumc en METC Oost-Nederland and the National Ethics Committee of Greece. Written informed consent for participation was not required for this study in accordance with the national legislation and the institutional requirements.

## Author contributions

Conceptualization: FV, MN, AZ. Data curation: JE, CM, NK, VK, AZ. Formal analysis: JE, CM, NK, VK, AZ. Funding acquisition: VK, FV, MN. Investigation: JE, CM, TS, RR, GD, HD, HL, AZ. Methodology: JE, CM, NK, VK, AZ. Project administration: AZ. Resources: JE, CM, SI, GP, EG-B, MN, AZ. Software: NK, VK. Supervision: FV, MN, AZ. Validation: FV, MN, AZ. Visualization: JE, CM, NK, AZ. Writing – Original draft preparation: JE, CM, AZ. Writing – Review & editing: All authors. All authors contributed to the article and approved the submitted version.
