# Supplementary material for: Dexamethasone attenuates interferon-related cytokine hyperresponsiveness in COVID-19 patients
Source: Front Immunol. 2023 Aug 8;14:1233318. doi: 10.3389/fimmu.2023.1233318 (PMC10442808; doi:10.3389/fimmu.2023.1233318)
Supplement: Supplementary file 1 [file DataSheet_1.zip › Supplementary File 1.pdf]

name	baseMean	log2FoldCl	lfcSE	stat	pvalue	padj	Symbol	entrez	name.y
ENSG0000	24899.36	2.183258	0.11364	19.21204	2.94E-82	5.44E-78	SERPINB2	5055	serpin family B member 2
ENSG0000	6453.791	1.1617	0.070814	16.4049	1.76E-60	1.64E-56	ADAM19	8728	ADAM metallopeptidase domain 19
ENSG0000	453.6922	2.836505	0.17475	16.23177	3.01E-59	1.86E-55	TNFAIP6	7130	TNF alpha induced protein 6
ENSG0000	5590.621	1.387787	0.096673	14.35542	9.85E-47	4.57E-43	MARCKS	4082	myristoylated alanine rich protein kinase C substrate
ENSG0000	2278.423	1.679397	0.120295	13.9607	2.71E-44	1.00E-40	EREG	2069	epiregulin
ENSG0000	8011.213	-1.44584	0.106552	-13.5694	6.09E-42	1.88E-38	CSF1R	1436	colony stimulating factor 1 receptor
ENSG0000	36455.72	1.218404	0.09136	13.33636	1.42E-40	3.77E-37	IER3	8870	immediate early response 3
ENSG0000	8053.662	1.322763	0.10087	13.11349	2.76E-39	6.39E-36	TRAF1	7185	TNF receptor associated factor 1
ENSG0000	3441.507	1.42402	0.110257	12.91542	3.68E-38	7.59E-35	BCL2A1	597	BCL2 related protein A1
ENSG0000	3146.455	2.860092	0.227688	12.56147	3.44E-36	6.38E-33	TNFSF15	9966	TNF superfamily member 15
ENSG0000	436.9784	2.12376	0.171197	12.40538	2.44E-35	4.12E-32	PMP22	5376	peripheral myelin protein 22
ENSG0000	1202.23	1.284498	0.104835	12.2526	1.63E-34	2.51E-31	ABCA1	19	ATP binding cassette subfamily A member 1
ENSG0000	5920.587	2.78129	0.229381	12.12519	7.77E-34	1.11E-30	CXCL5	6374	C-X-C motif chemokine ligand 5
ENSG0000	527.3579	1.528709	0.126598	12.07532	1.43E-33	1.89E-30	TBC1D7	51256	TBC1 domain family member 7
ENSG0000	2011.231	-1.49056	0.125713	-11.8569	1.98E-32	2.45E-29	PLXDC2	84898	plexin domain containing 2
ENSG0000	1167.51	1.381019	0.117812	11.7222	9.81E-32	1.14E-28	RNF144B	255488	ring finger protein 144B
ENSG0000	18526.64	0.868846	0.074459	11.6688	1.84E-31	2.01E-28	SERPINB9	5272	serpin family B member 9
ENSG0000	258.8978	2.341483	0.201431	11.62426	3.10E-31	3.20E-28	PRG4	10216	proteoglycan 4
ENSG0000	2608.796	1.504849	0.130558	11.52631	9.72E-31	9.49E-28	EMP1	2012	epithelial membrane protein 1
ENSG0000	375.9904	1.834771	0.160252	11.44931	2.37E-30	2.20E-27	PLPP3	8613	phospholipid phosphatase 3
ENSG0000	1476.199	1.157494	0.101687	11.3829	5.09E-30	4.49E-27	EIF1B	10289	eukaryotic translation initiation factor 1B
ENSG0000	5271.983	0.850804	0.074808	11.3731	5.69E-30	4.80E-27	WTAP	9589	WT1 associated protein
ENSG0000	67254.62	2.503155	0.220173	11.36903	5.96E-30	4.81E-27	CXCL8	3576	C-X-C motif chemokine ligand 8
ENSG0000	14370.43	1.038393	0.092336	11.24585	2.43E-29	1.88E-26	PNRC1	10957	proline rich nuclear receptor coactivator 1
ENSG0000	641.0355	-1.30146	0.116019	-11.2177	3.34E-29	2.48E-26	CD300LB	124599	CD300 molecule like family member b
ENSG0000	2065.439	1.035956	0.09246	11.20432	3.88E-29	2.77E-26	SESN2	83667	sestrin 2
ENSG0000	4275.382	1.349735	0.120727	11.18009	5.10E-29	3.51E-26	IRAK2	3656	interleukin 1 receptor associated kinase 2
ENSG0000	2641.049	1.349335	0.120905	11.16027	6.38E-29	4.23E-26	TXNRD1	7296	thioredoxin reductase 1
ENSG0000	1856.719	1.575001	0.143846	10.94922	6.70E-28	4.29E-25	CES1	1066	carboxylesterase 1
ENSG0000	8762.039	0.968685	0.088506	10.94491	7.03E-28	4.35E-25	ZC3H12A	80149	zinc finger CCCH-type containing 12A
ENSG0000	3790.782	1.014901	0.093127	10.89803	1.18E-27	7.05E-25	PIM2	11040	Pim-2 prot serine/threonine kinase
ENSG0000	5241.707	2.723216	0.252354	10.79125	3.79E-27	2.19E-24	PTGS2	5743	prostaglandin-endoperoxide synthase 2
ENSG0000	1346.409	-1.11832	0.103797	-10.7741	4.56E-27	2.56E-24	CAMK1D	57118	calcium/calmodulin dependent protein kinase ID
ENSG0000	948.6099	1.157932	0.107502	10.77126	4.71E-27	2.57E-24	RNF157	114804	ring finger protein 157
ENSG0000	781.2166	-1.38338	0.128874	-10.7344	7.02E-27	3.62E-24	FAM20C	56975	FAM20C golgi associated secretory pathway kinase
ENSG0000	499.7716	1.454326	0.135462	10.73604	6.89E-27	3.62E-24	TAF4B	6875	TATA-box binding protein associated factor 4b
ENSG0000	21209.57	1.012359	0.094869	10.67108	1.39E-26	6.97E-24	PPP1R15A	23645	protein phosphatase 1 regulatory subunit 15A
ENSG0000	12845.49	1.168355	0.109776	10.64311	1.88E-26	9.16E-24	NAMPT	10135	nicotinamide phosphoribosyltransferase
ENSG0000	5634.506	0.78747	0.0742	10.61283	2.60E-26	1.24E-23	SAMSN1	64092	SAM doma SH3 domain and nuclear localization signals 1
ENSG0000	87400.78	0.774318	0.073194	10.57903	3.73E-26	1.73E-23	FTL	2512	ferritin light chain
ENSG0000	7906.258	1.269727	0.120199	10.56352	4.40E-26	1.99E-23	BASP1	10409	brain abundant membrane attached signal protein 1
ENSG0000	16880.24	0.91458	0.086745	10.54331	5.45E-26	2.41E-23	NINJ1	4814	ninjurin 1
ENSG0000	361.9814	-1.81022	0.17219	-10.5129	7.53E-26	3.25E-23	PDK4	5166	pyruvate dehydrogenase kinase 4
ENSG0000	3455.977	5.269763	0.501779	10.50216	8.44E-26	3.55E-23	IL6	3569	interleukin 6
ENSG0000	5612.268	0.776511	0.073952	10.50015	8.62E-26	3.55E-23	PDE4A	5141	phosphodiesterase 4A
ENSG0000	3090.81	-0.91623	0.087583	-10.4613	1.30E-25	5.24E-23	SLC37A2	219855	solute carrier family 37 member 2
ENSG0000	1294.847	-0.99462	0.095567	-10.4075	2.29E-25	9.04E-23	CD84	8832	CD84 molecule
ENSG0000	4823.479	0.734799	0.070841	10.37253	3.31E-25	1.28E-22	ITK	3702	IL2 inducible T cell kinase
ENSG0000	15718.51	0.924937	0.089336	10.35347	4.04E-25	1.53E-22	PPIF	10105	peptidylprolyl isomerase F
ENSG0000	1584.891	2.738242	0.26506	10.33066	5.12E-25	1.90E-22	SLC7A11	23657	solute carrier family 7 member 11
ENSG0000	1361.797	1.198224	0.117245	10.21982	1.62E-24	5.88E-22	NRIP3	56675	nuclear receptor interacting protein 3
ENSG0000	333.9497	-1.87946	0.18601	-10.1041	5.30E-24	1.89E-21	TINAGL1	64129	tubulointerstitial nephritis antigen like 1
ENSG0000	6418.856	-1.2973	0.129592	-10.0106	1.37E-23	4.79E-21	PECAM1	5175	platelet and endothelial cell adhesion molecule 1
ENSG0000	696.2793	-1.29463	0.129653	-9.98535	1.77E-23	6.07E-21	NAV1	89796	neuron navigator 1
ENSG0000	10209.84	0.892977	0.090382	9.880066	5.08E-23	1.71E-20	NFKB1	4790	nuclear factor kappa B subunit 1
ENSG0000	4780.781	0.83153	0.084275	9.866894	5.79E-23	1.92E-20	ELL2	22936	elongation factor for RNA polymerase II 2
ENSG0000	4988.72	0.838175	0.085138	9.844891	7.21E-23	2.35E-20	VPS37B	79720	VPS37B subunit of ESCRT-I
ENSG0000	687.6694	1.44501	0.146938	9.834125	8.03E-23	2.57E-20	TXN	7295	thioredoxin
ENSG0000	14521.22	1.069712	0.109259	9.790597	1.24E-22	3.88E-20	PLAUR	5329	plasminog urokinase receptor
ENSG0000	3847.531	-0.98662	0.101112	-9.75763	1.71E-22	5.29E-20	STAB1	23166	stabilin 1
ENSG0000	559.0226	1.764089	0.181203	9.735433	2.13E-22	6.47E-20	MFSD2A	84879	major facilitator superfamily domain containing 2A
ENSG0000	3283.435	-0.85063	0.087396	-9.73302	2.18E-22	6.52E-20	VASH1	22846	vasohibin 1
ENSG0000	18564.28	1.926073	0.199943	9.633095	5.80E-22	1.71E-19	MMP19	4327	matrix metallopeptidase 19
ENSG0000	672.68	1.561604	0.162539	9.607577	7.43E-22	2.15E-19	SOWAHC	65124	sosondowah ankyrin repeat domain family member C
ENSG0000	7185.034	0.721825	0.075191	9.599903	8.00E-22	2.28E-19	SDCBP	6386	syndecan binding protein
ENSG0000	901.9249	1.17238	0.122252	9.589892	8.82E-22	2.48E-19	TMEM273	170371	transmembrane protein 273
ENSG0000	3879.039	0.81173	0.085168	9.530952	1.56E-21	4.31E-19	ATP2B1	490	ATPase plasma membrane Ca2+ transporting 1
ENSG0000	2228.723	0.832564	0.087422	9.523529	1.67E-21	4.57E-19	MTF1	4520	metal regulatory transcription factor 1
ENSG0000	2440.271	-0.93709	0.098485	-9.51503	1.82E-21	4.81E-19	ST14	6768	ST14 transmembrane serine protease matriptase
ENSG0000	374.2087	-1.52098	0.159828	-9.51634	1.79E-21	4.81E-19	A4GALT	53947	alpha 1 4-galactosyltransferase (P blood group)
ENSG0000	8208.161	1.01609	0.106956	9.5001	2.10E-21	5.48E-19	SLC2A3	6515	solute carrier family 2 member 3
ENSG0000	1491.141	1.245905	0.131457	9.477644	2.60E-21	6.70E-19	C15orf48	84419	chromosome 15 open reading frame 48
ENSG0000	1055.418	0.961802	0.101546	9.471545	2.76E-21	7.01E-19	NR1D1	9572	nuclear receptor subfamily 1 group D member 1
ENSG0000	18799.53	0.653828	0.069294	9.435631	3.89E-21	9.74E-19	PPP1R18	170954	protein phosphatase 1 regulatory subunit 18
ENSG0000	35142.15	0.883629	0.094279	9.372529	7.08E-21	1.75E-18	NFKBIA	4792	NFKB inhibitor alpha
ENSG0000	16517.82	0.962509	0.102924	9.35165	8.63E-21	2.11E-18	NFKB2	4791	nuclear factor kappa B subunit 2
ENSG0000	1083.877	1.038137	0.111174	9.33791	9.83E-21	2.37E-18	GCLM	2730	glutamate-cysteine ligase modifier subunit
ENSG0000	3460.886	0.855284	0.091951	9.301487	1.38E-20	3.29E-18	PGD	5226	phosphogluconate dehydrogenase
ENSG0000	2416.723	0.778853	0.083751	9.299658	1.41E-20	3.31E-18	ANTXR2	118429	ANTXR cell adhesion molecule 2
ENSG0000	3011.292	0.841056	0.091068	9.235433	2.57E-20	5.96E-18	CREM	1390	cAMP responsive element modulator
ENSG0000	17257.63	0.824216	0.089482	9.210998	3.23E-20	7.40E-18	SQSTM1	8878	sequestosome 1
ENSG0000	1657.043	0.979516	0.106737	9.176948	4.43E-20	1.00E-17	SMOX	54498	spermine oxidase
ENSG0000	7626.899	0.742799	0.081077	9.161682	5.11E-20	1.14E-17	ATP13A3	79572	ATPase 13A3
ENSG0000	1022.293	1.13704	0.124405	9.139819	6.26E-20	1.38E-17	FNIP2	57600	folliculin interacting protein 2
ENSG0000	1811.493	0.786972	0.086152	9.134699	6.56E-20	1.43E-17	CCRL2	9034	C-C motif chemokine receptor like 2
ENSG0000	644.2462	1.212763	0.133234	9.102488	8.83E-20	1.90E-17	PHACTR1	221692	phosphatase and actin regulator 1
ENSG0000	13126.46	3.252081	0.357353	9.10047	8.99E-20	1.92E-17	IL1A	3552	interleukin 1 alpha
ENSG0000	9867.556	0.711645	0.07829	9.089826	9.92E-20	2.09E-17	UBALD2	283991	UBA like domain containing 2
ENSG0000	11322.89	0.917043	0.101335	9.049647	1.43E-19	2.99E-17	AQP9	366	aquaporin 9
ENSG0000	15363.1	0.753326	0.083516	9.020115	1.88E-19	3.87E-17	GRINA	2907	glutamate ionotropic receptor NMDA type subunit associated protein 1
ENSG0000	871.6974	1.085883	0.120724	8.994735	2.37E-19	4.83E-17	IRGQ	126298	immunity related GTPase Q
ENSG0000	2862.199	-0.8045	0.089522	-8.98662	2.55E-19	5.14E-17	APBB1IP	54518	amyloid beta precursor protein binding family B member 1 interacting protein
ENSG0000	2091.056	0.779465	0.086762	8.983952	2.61E-19	5.21E-17	FOSL1	8061	FOS like 1 AP-1 transcription factor subunit
ENSG0000	327.2374	-1.34924	0.150717	-8.95216	3.49E-19	6.88E-17	SLC8A1	6546	solute carrier family 8 member A1
ENSG0000	24229.54	0.580951	0.065006	8.93695	4.00E-19	7.81E-17	FOSL2	2355	FOS like 2 AP-1 transcription factor subunit
ENSG0000	662.5026	1.027531	0.115097	8.927518	4.36E-19	8.42E-17	BATF	10538	basic leucine zipper ATF-like transcription factor
ENSG0000	2630.324	0.922344	0.103654	8.898311	5.67E-19	1.08E-16	IL1R1	3554	interleukin 1 receptor type 1
ENSG0000	1359.339	-0.89437	0.101062	-8.84965	8.78E-19	1.66E-16	CALHM2	51063	calcium homeostasis modulator family member 2
ENSG0000	8357.779	0.644187	0.072806	8.848041	8.91E-19	1.67E-16	NOTCH2	4853	notch receptor 2

ENSG0000	3061.805	-0.69695	0.078796	-8.84498	9.15E-19	1.70E-16	ITGB7	3695	integrin subunit beta 7
ENSG0000	3195.772	1.143894	0.130494	8.765873	1.85E-18	3.40E-16	NFKBIZ	64332	NFKB inhibitor zeta
ENSG0000	3955.857	0.70713	0.081511	8.675225	4.13E-18	7.50E-16	PLEKHB2	55041	pleckstrin homology domain containing B2
ENSG0000	1227.524	-0.9352	0.108133	-8.64858	5.21E-18	9.39E-16	NFAM1	150372	NFAT activating protein with ITAM motif 1
ENSG0000	1737.248	0.743518	0.086017	8.643822	5.44E-18	9.69E-16	CCND2	894	cyclin D2
ENSG0000	317.8505	-1.46335	0.16963	-8.62671	6.31E-18	1.12E-15	PLXNA4	91584	plexin A4
ENSG0000	5534.293	0.680791	0.079479	8.565704	1.07E-17	1.88E-15	XBP1	7494	X-box binding protein 1
ENSG0000	1867.952	1.121988	0.131184	8.55279	1.20E-17	2.07E-15	MSC	9242	musculin
ENSG0000	286.1006	1.572198	0.183833	8.552303	1.21E-17	2.07E-15	TRIB3	57761	tribbles pseudokinase 3
ENSG0000	4104.442	-1.08264	0.127419	-8.49667	1.95E-17	3.32E-15	HIVEP3	59269	HIVEP zinc finger 3
ENSG0000	321.2162	1.720123	0.203598	8.448635	2.95E-17	4.97E-15	LOC10041	1E+08	toll like receptor 2 pseudogene
ENSG0000	195.5124	2.423639	0.288138	8.411384	4.05E-17	6.77E-15	TNIP3	79931	TNFAIP3 interacting protein 3
ENSG0000	2973.575	0.753155	0.089582	8.407397	4.19E-17	6.94E-15	TP53BP2	7159	tumor protein p53 binding protein 2
ENSG0000	8331.889	0.781698	0.093183	8.388843	4.91E-17	8.06E-15	PFKFB3	5209	6-phosphc 6-biphosphatase 3
ENSG0000	3579.982	0.998707	0.119237	8.375795	5.49E-17	8.92E-15	OSM	5008	oncostatin M
ENSG0000	1198.737	1.001467	0.119799	8.359585	6.29E-17	1.02E-14	ZNF331	55422	zinc finger protein 331
ENSG0000	11240.3	0.650255	0.078317	8.302812	1.02E-16	1.63E-14	CD55	1604	CD55 molecule (Cromer blood group)
ENSG0000	10661.94	0.620389	0.074881	8.284945	1.18E-16	1.87E-14	HNRNPC	3183	heterogeneous nuclear ribonucleoprotein C
ENSG0000	139202.2	2.47654	0.300033	8.254214	1.53E-16	2.40E-14	IL1B	3553	interleukin 1 beta
ENSG0000	7279.614	-0.8007	0.097279	-8.23102	1.86E-16	2.89E-14	CYP1B1	1545	cytochrome P450 family 1 subfamily B member 1
ENSG0000	532.9295	1.172373	0.143124	8.191324	2.58E-16	3.99E-14	LOC10028	1E+08	uncharacterized LOC100288175
ENSG0000	2637.518	1.063217	0.13044	8.150993	3.61E-16	5.53E-14	AMPD3	272	adenosine monophosphate deaminase 3
ENSG0000	1483.594	-1.09822	0.135019	-8.13383	4.16E-16	6.32E-14	IQSEC2	23096	IQ motif and Sec7 domain ArfGEF 2
ENSG0000	3255.69	0.76414	0.093995	8.12957	4.31E-16	6.50E-14	GPR84	53831	G protein-coupled receptor 84
ENSG0000	6898.325	0.893218	0.110266	8.100596	5.47E-16	8.18E-14	PIM3	415116	Pim-3 prot serine/threonine kinase
ENSG0000	3041.908	2.451391	0.303523	8.076457	6.67E-16	9.89E-14	CCL20	6364	C-C motif chemokine ligand 20
ENSG0000	881.9672	1.005465	0.12454	8.073459	6.83E-16	1.01E-13	SMTN	6525	smoothelin
ENSG0000	724.6106	-1.10103	0.136422	-8.07076	6.99E-16	1.02E-13	FBP1	2203	fructose-bisphosphatase 1
ENSG0000	2808.755	0.636673	0.079025	8.056607	7.84E-16	1.14E-13	PCED1B	91523	PC-esterase domain containing 1B
ENSG0000	496.8061	1.51847	0.188853	8.040492	8.95E-16	1.29E-13	LIF	3976	LIF interleukin 6 family cytokine
ENSG0000	3635.403	0.782506	0.097548	8.021776	1.04E-15	1.49E-13	MAFG	4097	MAF bZIP transcription factor G
ENSG0000	20531.93	0.639755	0.080033	7.993612	1.31E-15	1.86E-13	SLC7A5	8140	solute carrier family 7 member 5
ENSG0000	3691.772	0.655169	0.082038	7.986149	1.39E-15	1.96E-13	ARL8B	55207	ADP ribosylation factor like GTPase 8B
ENSG0000	1299.183	0.955165	0.119757	7.975857	1.51E-15	2.11E-13	TNFRSF4	7293	TNF receptor superfamily member 4
ENSG0000	2937.912	-2.7127	0.340931	-7.95673	1.77E-15	2.45E-13	FN1	2335	fibronectin 1
ENSG0000	2057.53	-1.05844	0.133204	-7.94604	1.93E-15	2.65E-13	MYOF	26509	myoferlin
ENSG0000	746.2467	0.916778	0.115547	7.93421	2.12E-15	2.89E-13	CLIC4	25932	chloride intracellular channel 4
ENSG0000	5216.782	0.917228	0.115646	7.931328	2.17E-15	2.94E-13	MAFF	23764	MAF bZIP transcription factor F
ENSG0000	3803.592	0.65712	0.083	7.917136	2.43E-15	3.27E-13	ZHX2	22882	zinc fingers and homeoboxes 2
ENSG0000	1546.942	0.77291	0.098159	7.874066	3.43E-15	4.58E-13	MAP3K20	51776	mitogen-activated protein kinase kinase kinase 20
ENSG0000	1300.224	0.739165	0.093931	7.869204	3.57E-15	4.73E-13	AREG	374	amphiregulin
ENSG0000	3141.273	0.975404	0.125081	7.798187	6.28E-15	8.26E-13	CSTB	1476	cystatin B
ENSG0000	1832.26	0.702443	0.090172	7.790068	6.70E-15	8.75E-13	VAV1	7409	vav guanine nucleotide exchange factor 1
ENSG0000	461.4536	0.963973	0.124017	7.772923	7.67E-15	9.95E-13	ICOS	29851	inducible T cell costimulator
ENSG0000	2206.638	0.610307	0.07921	7.704943	1.31E-14	1.69E-12	STING1	340061	stimulator of interferon response cGAMP interactor 1
ENSG0000	1495.22	-0.8631	0.11229	-7.68642	1.51E-14	1.94E-12	SIPA1L3	23094	signal induced proliferation associated 1 like 3
ENSG0000	4474.94	0.911527	0.118607	7.685239	1.53E-14	1.94E-12	RELB	5971	RELB protc NF-kB subunit
ENSG0000	18150.8	0.526022	0.068485	7.680892	1.58E-14	1.99E-12	SMAP2	64744	small ArfGAP2
ENSG0000	907.2602	-1.18398	0.15424	-7.67626	1.64E-14	2.05E-12	CEBPA	1050	CCAAT enhancer binding protein alpha
ENSG0000	4556.841	0.63659	0.082949	7.674463	1.66E-14	2.07E-12	NFE2L2	4780	NFE2 like bZIP transcription factor 2
ENSG0000	1356.648	0.685264	0.089418	7.663637	1.81E-14	2.23E-12	TRIB2	28951	tribbles pseudokinase 2
ENSG0000	3812.971	-0.60505	0.07921	-7.63853	2.20E-14	2.70E-12	DOCK8	81704	dedicator of cytokinesis 8
ENSG0000	2922.067	-0.63177	0.082745	-7.6351	2.26E-14	2.75E-12	ARHGEF18	23370	Rho/Rac guanine nucleotide exchange factor 18
ENSG0000	231.7794	1.597938	0.209467	7.628599	2.37E-14	2.88E-12	NPIPB13	613037	nuclear pc member B13
ENSG0000	1101.656	-0.8022	0.105333	-7.61587	2.62E-14	3.15E-12	TBC1D22A	25771	TBC1 domain family member 22A
ENSG0000	580.5601	0.982766	0.129152	7.609353	2.75E-14	3.30E-12	DCUN1D3	123879	defective in cullin neddylation 1 domain containing 3
ENSG0000	679.9551	-2.609	0.343477	-7.59586	3.06E-14	3.64E-12	MERTK	10461	MER protc tyrosine kinase
ENSG0000	1418.75	0.662189	0.087197	7.594208	3.10E-14	3.66E-12	TANK	10010	TRAF family member associated NFKB activator
ENSG0000	423.4744	-1.29333	0.17175	-7.53028	5.06E-14	5.93E-12	EEPD1	80820	endonuclease/exonuclease/phosphatase family domain containing 1
ENSG0000	2100.947	0.740478	0.09834	7.529764	5.08E-14	5.93E-12	AZIN1	51582	antizyme inhibitor 1
ENSG0000	1569.112	-0.7544	0.100336	-7.51873	5.53E-14	6.41E-12	SULF2	55959	sulfatase 2
ENSG0000	6741.198	0.61011	0.081218	7.512017	5.82E-14	6.71E-12	TICAM1	148022	TIR domain containing adaptor molecule 1
ENSG0000	5121.16	0.75754	0.100964	7.503084	6.23E-14	7.14E-12	SNX9	51429	sorting nexin 9
ENSG0000	1967.75	0.749295	0.099887	7.501444	6.31E-14	7.18E-12	SH3BP5	9467	SH3 domain binding protein 5
ENSG0000	396.1048	1.299546	0.173749	7.479437	7.46E-14	8.44E-12	OLIG1	116448	oligodendrocyte transcription factor 1
ENSG0000	855.8329	0.815759	0.109321	7.462069	8.52E-14	9.57E-12	NA	NA	NA
ENSG0000	2109.31	0.619492	0.083029	7.46118	8.58E-14	9.58E-12	DNTTIP2	30836	deoxynucleotidyltransferase terminal interacting protein 2
ENSG0000	692.5411	1.038457	0.139604	7.438597	1.02E-13	1.13E-11	LAMB3	3914	laminin subunit beta 3
ENSG0000	8280.258	0.508109	0.068892	7.375474	1.64E-13	1.81E-11	PHF1	5252	PHD finger protein 1
ENSG0000	934.294	0.85133	0.115751	7.354859	1.91E-13	2.10E-11	GLA	2717	galactosidase alpha
ENSG0000	1475.577	0.627363	0.08534	7.35131	1.96E-13	2.14E-11	FKBP11	51303	FKBP prolyl isomerase 11
ENSG0000	5121.945	0.593053	0.080759	7.343491	2.08E-13	2.26E-11	CDK16	5127	cyclin dependent kinase 16
ENSG0000	2022.953	0.666701	0.090829	7.340195	2.13E-13	2.30E-11	HIVEP1	3096	HIVEP zinc finger 1
ENSG0000	439.0452	-0.96963	0.132194	-7.33485	2.22E-13	2.38E-11	ARHGAP18	93663	Rho GTPase activating protein 18
ENSG0000	2007.301	-0.67167	0.091788	-7.3176	2.52E-13	2.69E-11	CRTC1	23373	CREB regulated transcription coactivator 1
ENSG0000	654.9718	0.836573	0.114589	7.300621	2.86E-13	3.04E-11	SUPV3L1	6832	Suv3 like RNA helicase
ENSG0000	1981.206	0.821043	0.112678	7.28665	3.18E-13	3.35E-11	IFITM1	8519	interferon induced transmembrane protein 1
ENSG0000	2625.007	-0.68175	0.093713	-7.27484	3.47E-13	3.63E-11	GAS7	8522	growth arrest specific 7
ENSG0000	5728.489	0.607411	0.083688	7.258073	3.93E-13	4.09E-11	CSRNP1	64651	cysteine and serine rich nuclear protein 1
ENSG0000	5127.435	0.578782	0.079823	7.250854	4.14E-13	4.29E-11	PDE4B	5142	phosphodiesterase 4B
ENSG0000	4730.947	0.776281	0.107376	7.229548	4.85E-13	4.99E-11	SLC3A2	6520	solute carrier family 3 member 2
ENSG0000	7714.168	0.501801	0.069455	7.224848	5.02E-13	5.14E-11	B4GALT1	2683	beta-1 4-galactosyltransferase 1
ENSG0000	24333.54	0.589123	0.081559	7.223304	5.07E-13	5.17E-11	ARID5A	10865	AT-rich interaction domain 5A
ENSG0000	516.4827	1.356924	0.187963	7.219121	5.23E-13	5.30E-11	LYPD3	27076	LY6/PLAUR domain containing 3
ENSG0000	2246.654	0.837253	0.116101	7.211404	5.54E-13	5.58E-11	ACSL5	51703	acyl-CoA synthetase long chain family member 5
ENSG0000	1626.991	-0.60427	0.083836	-7.2078	5.69E-13	5.70E-11	DOCK4	9732	dedicator of cytokinesis 4
ENSG0000	1848.104	0.602741	0.083635	7.20679	5.73E-13	5.71E-11	IRAK3	11213	interleukin 1 receptor associated kinase 3
ENSG0000	4206.442	0.571452	0.079341	7.202472	5.91E-13	5.83E-11	DUSP4	1846	dual specificity phosphatase 4
ENSG0000	488.6517	0.9142	0.126926	7.202614	5.91E-13	5.83E-11	ZNRF1	84937	zinc and ring finger 1
ENSG0000	1587.659	-0.67596	0.093955	-7.19454	6.27E-13	6.15E-11	VIPR1	7433	vasoactive intestinal peptide receptor 1
ENSG0000	750.709	0.784842	0.109175	7.188833	6.53E-13	6.38E-11	GPAT3	84803	glycerol-3-phosphate acyltransferase 3
ENSG0000	2382.835	-0.6301	0.087706	-7.18422	6.76E-13	6.56E-11	ARHGEF40	55701	Rho guanine nucleotide exchange factor 40
ENSG0000	2034.914	-0.68372	0.095675	-7.14629	8.92E-13	8.61E-11	WDR81	124997	WD repeat domain 81
ENSG0000	17048.31	2.862592	0.400656	7.144767	9.01E-13	8.66E-11	CXCL1	2919	C-X-C motif chemokine ligand 1
ENSG0000	4180.043	0.648288	0.090821	7.138112	9.46E-13	9.05E-11	CD83	9308	CD83 molecule
ENSG0000	1395.778	0.81765	0.114591	7.135404	9.65E-13	9.18E-11	PALM2AK	445815	PALM2 and AKAP2 fusion
ENSG0000	432.7913	1.104464	0.155007	7.125265	1.04E-12	9.83E-11	TNFRSF9	3604	TNF receptor superfamily member 9
ENSG0000	2144.057	0.862669	0.12144	7.10367	1.21E-12	1.14E-10	ZSWIM4	65249	zinc finger SWIM-type containing 4
ENSG0000	2464.552	-0.65401	0.092233	-7.09084	1.33E-12	1.25E-10	STK38L	23012	serine/threonine kinase 38 like
ENSG0000	2650.531	-0.59492	0.084114	-7.07271	1.52E-12	1.42E-10	XYLT1	64131	xylosyltransferase 1

ENSG0000	14883.82	0.585619	0.082807	7.072103	1.53E-12	1.42E-10	EIF1	10209	eukaryotic translation initiation factor 1
ENSG0000	365.9474	-0.96821	0.136984	-7.06804	1.57E-12	1.45E-10	MEF2C	4208	myocyte enhancer factor 2C
ENSG0000	613.3947	0.982966	0.139644	7.039099	1.93E-12	1.78E-10	ADM	133	adrenomedullin
ENSG0000	287.9491	-1.16501	0.166233	-7.0083	2.41E-12	2.20E-10	CMKLR1	1240	chemerin chemokine-like receptor 1
ENSG0000	3072.296	-0.53148	0.075909	-7.0015	2.53E-12	2.30E-10	ALOX5	240	arachidonate 5-lipoxygenase
ENSG0000	3759.497	0.615645	0.088269	6.974688	3.07E-12	2.77E-10	ARF4	378	ADP ribosylation factor 4
ENSG0000	1264.445	0.770495	0.110595	6.966805	3.24E-12	2.92E-10	FAM177A	283635	family with sequence similarity 177 member A1
ENSG0000	573.5791	0.944638	0.135607	6.96602	3.26E-12	2.92E-10	SELENOK	58515	selenoprotein K
ENSG0000	2872.812	0.742699	0.106658	6.96337	3.32E-12	2.96E-10	KYNU	8942	kynureninase
ENSG0000	279.2301	-1.14753	0.164884	-6.95961	3.41E-12	3.01E-10	SCIMP	388325	SLP adaptor and CSK interacting membrane protein
ENSG0000	2511.978	0.642669	0.09234	6.959788	3.41E-12	3.01E-10	NPC1	4864	NPC intracellular cholesterol transporter 1
ENSG0000	9181.798	0.599628	0.086222	6.954472	3.54E-12	3.11E-10	TUBA4A	7277	tubulin alpha 4a
ENSG0000	811.567	-0.89077	0.128228	-6.94675	3.74E-12	3.27E-10	CD300LF	146722	CD300 molecule like family member f
ENSG0000	5203.838	1.790316	0.258231	6.933008	4.12E-12	3.59E-10	G0S2	50486	G0/G1 switch 2
ENSG0000	6957.754	1.386284	0.200139	6.926606	4.31E-12	3.74E-10	ETS2	2114	ETS proto- transcription factor
ENSG0000	6465.516	0.474321	0.068535	6.92084	4.49E-12	3.87E-10	JOSD1	9929	Josephin domain containing 1
ENSG0000	358.8191	-1.10522	0.160113	-6.90278	5.10E-12	4.36E-10	LIMS2	55679	LIM zinc finger domain containing 2
ENSG0000	7120.653	0.577788	0.083703	6.902871	5.10E-12	4.36E-10	PNP	4860	purine nucleoside phosphorylase
ENSG0000	10548.24	0.600621	0.087115	6.894602	5.40E-12	4.60E-10	ATF4	468	activating transcription factor 4
ENSG0000	5809.541	0.552964	0.080226	6.892613	5.48E-12	4.64E-10	SRSF3	6428	serine and arginine rich splicing factor 3
ENSG0000	313.1818	1.328454	0.192772	6.89131	5.53E-12	4.66E-10	NA	NA	NA
ENSG0000	304.2741	1.306201	0.189754	6.883663	5.83E-12	4.90E-10	ADTRP	84830	androgen dependent TFPI regulating protein
ENSG0000	3439.111	0.607178	0.088502	6.860644	6.86E-12	5.73E-10	EMD	2010	emerin
ENSG0000	32207.36	0.628898	0.091754	6.854205	7.17E-12	5.96E-10	FCER1G	2207	Fc epsilon receptor Ig
ENSG0000	411.2073	0.962634	0.14053	6.850044	7.38E-12	6.11E-10	NBPF14	25832	NBPF member 14
ENSG0000	347.3184	1.470406	0.214948	6.840751	7.88E-12	6.49E-10	ZMIZ1-AS1	283050	ZMIZ1 antisense RNA 1
ENSG0000	717.2265	-0.8515	0.124634	-6.83203	8.37E-12	6.86E-10	PRKCE	5581	protein kinase C epsilon
ENSG0000	257.6905	1.345904	0.197008	6.831714	8.39E-12	6.86E-10	C17orf107	1E+08	chromosome 17 open reading frame 107
ENSG0000	6686.355	0.573852	0.084242	6.81194	9.63E-12	7.81E-10	TUBB	203068	tubulin beta class I
ENSG0000	156.7543	-1.64802	0.241939	-6.8117	9.64E-12	7.81E-10	SMAD6	4091	SMAD family member 6
ENSG0000	2271.898	-0.55613	0.081887	-6.79146	1.11E-11	8.95E-10	TRIM14	9830	tripartite motif containing 14
ENSG0000	2005.827	0.842186	0.124056	6.788731	1.13E-11	9.08E-10	DUSP5	1847	dual specificity phosphatase 5
ENSG0000	26066.11	0.481061	0.070964	6.778898	1.21E-11	9.68E-10	CCR7	1236	C-C motif chemokine receptor 7
ENSG0000	152.9913	1.402543	0.207744	6.751316	1.47E-11	1.17E-09	MIR3142H	1.07E+08	MIR3142 host gene
ENSG0000	7288.588	-0.5415	0.080263	-6.7465	1.51E-11	1.20E-09	SORL1	6653	sortilin related receptor 1
ENSG0000	1791.487	0.591372	0.087778	6.737152	1.62E-11	1.27E-09	LPIN1	23175	lipin 1
ENSG0000	471.4088	0.980621	0.145733	6.728904	1.71E-11	1.34E-09	LOC10272	1.03E+08	salt inducible kinase 1B (putative)
ENSG0000	1365.263	0.753343	0.112016	6.725312	1.75E-11	1.37E-09	UBE2E1	7324	ubiquitin conjugating enzyme E2 E1
ENSG0000	2246.917	0.961671	0.142995	6.725198	1.75E-11	1.37E-09	SLC2A6	11182	solute carrier family 2 member 6
ENSG0000	36187.82	0.502852	0.074856	6.71762	1.85E-11	1.43E-09	TNFRSF1B	7133	TNF receptor superfamily member 1B
ENSG0000	45.53209	3.745079	0.557704	6.71517	1.88E-11	1.45E-09	NA	NA	NA
ENSG0000	106.368	1.952491	0.290821	6.713723	1.90E-11	1.46E-09	CEMIP	57214	cell migration inducing hyaluronidase 1
ENSG0000	2985.056	-0.58926	0.087848	-6.70776	1.98E-11	1.51E-09	SAMHD1	25939	SAM and HD domain containing deoxynucleoside triphosphate triphosphohydrolase 1
ENSG0000	1276.687	0.70038	0.104436	6.706282	2.00E-11	1.52E-09	FCAR	2204	Fc alpha receptor
ENSG0000	8388.949	0.552492	0.082412	6.70398	2.03E-11	1.54E-09	DENND5A	23258	DENN domain containing 5A
ENSG0000	882.7122	0.848283	0.12655	6.703151	2.04E-11	1.54E-09	SNHG16	1.01E+08	small nucleolar RNA host gene 16
ENSG0000	36200.5	0.505384	0.075476	6.69597	2.14E-11	1.62E-09	TNFAIP3	7128	TNF alpha induced protein 3
ENSG0000	726.9474	0.760775	0.114245	6.659148	2.75E-11	2.07E-09	GATA2	2624	GATA binding protein 2
ENSG0000	2341.196	-0.63182	0.094972	-6.65269	2.88E-11	2.15E-09	MAP4	4134	microtubule associated protein 4
ENSG0000	3523.078	-0.55132	0.082929	-6.64805	2.97E-11	2.21E-09	TBC1D9B	23061	TBC1 domain family member 9B
ENSG0000	3023.811	2.624754	0.394826	6.647869	2.97E-11	2.21E-09	CCL3L1	6349	C-C motif chemokine ligand 3 like 1
ENSG0000	1904.041	0.657612	0.09896	6.645259	3.03E-11	2.24E-09	SEC24A	10802	SEC24 hon COPII coat complex component
ENSG0000	4097.638	0.743981	0.112137	6.634575	3.25E-11	2.40E-09	NR4A2	4929	nuclear receptor subfamily 4 group A member 2
ENSG0000	414.4999	1.249951	0.189003	6.613384	3.76E-11	2.75E-09	LOC10012	1E+08	uncharacterized LOC100128059
ENSG0000	162.7661	1.435446	0.217149	6.610425	3.83E-11	2.80E-09	FAM184A	79632	family with sequence similarity 184 member A
ENSG0000	3432.486	0.7837	0.118637	6.605853	3.95E-11	2.87E-09	SLC39A8	64116	solute carrier family 39 member 8
ENSG0000	4046.813	0.555807	0.084258	6.596495	4.21E-11	3.05E-09	PDCD4	27250	programmed cell death 4
ENSG0000	7973.329	0.460828	0.069882	6.594371	4.27E-11	3.08E-09	TMBIM1	64114	transmembrane BAX inhibitor motif containing 1
ENSG0000	35075.57	1.454455	0.220673	6.590997	4.37E-11	3.14E-09	SOD2	6648	superoxide dismutase 2
ENSG0000	18767.6	1.815427	0.275577	6.587722	4.47E-11	3.20E-09	IL1RN	3557	interleukin 1 receptor antagonist
ENSG0000	998.8081	0.736047	0.111819	6.582494	4.63E-11	3.30E-09	MTHFD2	10797	methylene methenyltetrahydrofolate cyclohydrolase
ENSG0000	7150.78	0.444569	0.067579	6.57855	4.75E-11	3.36E-09	LPXN	9404	leupaxin
ENSG0000	49962.83	0.451597	0.068645	6.578751	4.74E-11	3.36E-09	BTG1	694	BTG anti-proliferation factor 1
ENSG0000	16360.78	1.309909	0.199265	6.573708	4.91E-11	3.46E-09	CTSL	1514	cathepsin L
ENSG0000	664.0612	0.819509	0.125066	6.552609	5.65E-11	3.97E-09	SLC11A2	4891	solute carrier family 11 member 2
ENSG0000	16257.89	2.536064	0.387147	6.550657	5.73E-11	4.01E-09	CXCL3	2921	C-X-C motif chemokine ligand 3
ENSG0000	1956.693	0.626542	0.095719	6.545666	5.92E-11	4.13E-09	CSGALNAC	55454	chondroitin sulfate N-acetylgalactosaminyltransferase 2
ENSG0000	543.9111	0.90853	0.139025	6.535023	6.36E-11	4.40E-09	FEZ1	9638	fasciculation and elongation protein zeta 1
ENSG0000	8418.004	0.561197	0.085869	6.535479	6.34E-11	4.40E-09	IL2RG	3561	interleukin 2 receptor subunit gamma
ENSG0000	4277.827	-0.53449	0.081802	-6.53388	6.41E-11	4.42E-09	PRR12	57479	proline rich 12
ENSG0000	6091.979	0.583985	0.089432	6.529916	6.58E-11	4.52E-09	MAP2K3	5606	mitogen-activated protein kinase kinase 3
ENSG0000	8341.224	0.541899	0.083054	6.524657	6.82E-11	4.66E-09	RIPOR1	79567	RHO family interacting cell polarization regulator 1
ENSG0000	198.8701	-1.18377	0.181449	-6.52397	6.85E-11	4.67E-09	PEAK3	374872	PEAK family member 3
ENSG0000	937.582	-0.71132	0.109074	-6.52143	6.96E-11	4.73E-09	FAM168A	23201	family with sequence similarity 168 member A
ENSG0000	1218.828	-0.86092	0.132189	-6.51277	7.38E-11	4.99E-09	IER5L	389792	immediate early response 5 like
ENSG0000	1731.479	0.581613	0.089453	6.501892	7.93E-11	5.35E-09	SEC22B	9554	SEC22 hon vesicle trafficking protein
ENSG0000	3668.159	0.543683	0.083719	6.494134	8.35E-11	5.61E-09	RAP1B	5908	RAP1B member of RAS oncogene family
ENSG0000	293.8453	-1.17557	0.181446	-6.47891	9.24E-11	6.19E-09	MFSD13A	79847	major facilitator superfamily domain containing 13A
ENSG0000	7139.441	0.603592	0.093189	6.4771	9.35E-11	6.24E-09	DOK3	79930	docking protein 3
ENSG0000	927.704	-0.77562	0.119789	-6.47487	9.49E-11	6.31E-09	NOG	9241	noggin
ENSG0000	4269.782	-0.488	0.075436	-6.46903	9.86E-11	6.53E-09	ADCY7	113	adenylate cyclase 7
ENSG0000	862.4601	0.732456	0.113631	6.44589	1.15E-10	7.59E-09	LAG3	3902	lymphocyte activating 3
ENSG0000	50763.27	0.534281	0.082901	6.444845	1.16E-10	7.61E-09	CD44	960	CD44 molecule (Indian blood group)
ENSG0000	7400.923	0.633713	0.098347	6.443613	1.17E-10	7.65E-09	ST3GAL1	6482	ST3 beta-g 3-sialyltransferase 1
ENSG0000	980.1749	1.762785	0.27417	6.429532	1.28E-10	8.36E-09	RIN2	54453	Ras and Rab interactor 2
ENSG0000	129.9624	1.490228	0.232314	6.414714	1.41E-10	9.18E-09	NIPAL4	348938	NIPA like domain containing 4
ENSG0000	225.0194	-1.10295	0.172186	-6.40554	1.50E-10	9.72E-09	MILR1	284021	mast cell immunoglobulin like receptor 1
ENSG0000	6307.892	0.54666	0.085364	6.40388	1.51E-10	9.76E-09	IVNS1ABP	10625	influenza virus NS1A binding protein
ENSG0000	550.9079	-0.90431	0.14121	-6.404	1.51E-10	9.76E-09	JAK2	3717	Janus kinase 2
ENSG0000	1123.33	-0.65542	0.102395	-6.40086	1.55E-10	9.92E-09	SCARB2	950	scavenger receptor class B member 2
ENSG0000	449.2317	1.007422	0.157449	6.39842	1.57E-10	1.00E-08	CMTM8	152189	CKLF like MARVEL transmembrane domain containing 8
ENSG0000	928.2567	0.846547	0.13233	6.397261	1.58E-10	1.01E-08	S1PR3	1903	sphingosine-1-phosphate receptor 3
ENSG0000	461.325	0.844998	0.132175	6.393024	1.63E-10	1.03E-08	SAV1	60485	salvador family WW domain containing protein 1
ENSG0000	974.6781	0.837678	0.131096	6.389785	1.66E-10	1.05E-08	LRG1	116844	leucine rich alpha-2-glycoprotein 1
ENSG0000	27907.04	0.536327	0.083961	6.387802	1.68E-10	1.06E-08	KDM6B	23135	lysine demethylase 6B
ENSG0000	18579.05	-0.54609	0.085928	-6.35527	2.08E-10	1.31E-08	MAFB	9935	MAF bZIP transcription factor B
ENSG0000	4733.108	0.539958	0.085077	6.346693	2.20E-10	1.38E-08	UBAP1	51271	ubiquitin associated protein 1
ENSG0000	1129.015	0.832447	0.131324	6.33887	2.31E-10	1.45E-08	HIP1	3092	huntingtin interacting protein 1
ENSG0000	1611.907	-0.67178	0.106066	-6.33361	2.39E-10	1.49E-08	ALCAM	214	activated leukocyte cell adhesion molecule
ENSG0000	169.1729	-1.43383	0.226643	-6.32638	2.51E-10	1.56E-08	SH3RF3	344558	SH3 domain containing ring finger 3

ENSG0000	1257.255	-0.78903	0.124912	-6.31667	2.67E-10	1.65E-08	ERC1	23085	ELKS/RAB6-interacting/CAST family member 1
ENSG0000	400.2848	1.320014	0.209103	6.312742	2.74E-10	1.69E-08	KMO	8564	kynurenine 3-monooxygenase
ENSG0000	1580.392	0.580988	0.092127	6.306391	2.86E-10	1.75E-08	TARS1	6897	threonyl-tRNA synthetase 1
ENSG0000	1330.064	0.644364	0.102229	6.303155	2.92E-10	1.78E-08	TENT5C	54855	terminal nucleotidyltransferase 5C
ENSG0000	782.5437	-0.77308	0.122653	-6.30296	2.92E-10	1.78E-08	NT5DC2	64943	5'-nucleotidase domain containing 2
ENSG0000	38.68883	3.613212	0.57326	6.302922	2.92E-10	1.78E-08	ADORA2A	135	adenosine A2a receptor
ENSG0000	10719.93	0.468609	0.074366	6.30142	2.95E-10	1.79E-08	H3-3B	3021	H3.3 histone B
ENSG0000	8419.632	0.425315	0.067529	6.298283	3.01E-10	1.82E-08	FNBP1	23048	formin binding protein 1
ENSG0000	3735.594	-0.51544	0.081906	-6.29302	3.11E-10	1.87E-08	DOCK10	55619	dedicator of cytokinesis 10
ENSG0000	46.47259	2.495196	0.397794	6.272586	3.55E-10	2.13E-08	GREM2	64388	gremlin 2 DAN family BMP antagonist
ENSG0000	135.1182	-1.64162	0.26172	-6.27241	3.56E-10	2.13E-08	ADAP2	55803	ArfGAP with dual PH domains 2
ENSG0000	1531.821	-0.54426	0.086814	-6.26919	3.63E-10	2.16E-08	CMSS1	84319	cms1 ribosomal small subunit homolog
ENSG0000	329.8863	-0.93435	0.14908	-6.26743	3.67E-10	2.18E-08	CRISPLD2	83716	cysteine rich secretory protein LCCL domain containing 2
ENSG0000	1507.453	0.614543	0.098185	6.259032	3.87E-10	2.30E-08	CCNH	902	cyclin H
ENSG0000	8673.633	0.593166	0.094777	6.258515	3.89E-10	2.30E-08	TLR2	7097	toll like receptor 2
ENSG0000	1343.74	0.582104	0.093026	6.257422	3.91E-10	2.30E-08	LDLR	3949	low density lipoprotein receptor
ENSG0000	690.7509	-0.72153	0.115493	-6.24741	4.17E-10	2.45E-08	DCHS1	8642	dachsous cadherin-related 1
ENSG0000	1465.18	-0.56826	0.091047	-6.2414	4.34E-10	2.54E-08	MEF2A	4205	myocyte enhancer factor 2A
ENSG0000	3080.128	0.529561	0.08486	6.240393	4.36E-10	2.55E-08	CRTC3	64784	CREB regulated transcription coactivator 3
ENSG0000	1160.353	0.639876	0.102645	6.233903	4.55E-10	2.65E-08	RHEB	6009	Ras homol mTORC1 binding
ENSG0000	1291.681	0.578985	0.092904	6.232083	4.60E-10	2.67E-08	SNHG1	23642	small nucleolar RNA host gene 1
ENSG0000	14703.11	2.425727	0.389305	6.230918	4.64E-10	2.68E-08	CXCL2	2920	C-X-C motif chemokine ligand 2
ENSG0000	10393.42	0.510427	0.081937	6.229511	4.68E-10	2.70E-08	CST7	8530	cystatin F
ENSG0000	3371.091	0.581813	0.093409	6.228627	4.71E-10	2.70E-08	PPP1R15B	84919	protein phosphatase 1 regulatory subunit 15B
ENSG0000	22152.64	0.533313	0.085641	6.227314	4.74E-10	2.72E-08	RAB7A	7879	RAB7A member RAS oncogene family
ENSG0000	495.8818	1.083642	0.17451	6.209615	5.31E-10	3.03E-08	TRIP10	9322	thyroid hormone receptor interactor 10
ENSG0000	9131.997	0.518388	0.083509	6.207593	5.38E-10	3.06E-08	NDRG1	10397	N-myc downstream regulated 1
ENSG0000	672.0518	-1.14162	0.183965	-6.20564	5.45E-10	3.09E-08	LRRK2	120892	leucine rich repeat kinase 2
ENSG0000	2300.226	0.702533	0.113377	6.196421	5.78E-10	3.27E-08	CDC42EP3	10602	CDC42 effector protein 3
ENSG0000	273.5622	-1.07622	0.173845	-6.19069	5.99E-10	3.38E-08	CDK14	5218	cyclin dependent kinase 14
ENSG0000	1781.404	0.595742	0.096328	6.184496	6.23E-10	3.50E-08	PEDS1	387521	plasmanylethanolamine desaturase 1
ENSG0000	802.7499	0.874727	0.141807	6.168416	6.90E-10	3.87E-08	SCARF1	8578	scavenger receptor class F member 1
ENSG0000	2371.652	0.553497	0.08978	6.165009	7.05E-10	3.94E-08	TNFAIP8	25816	TNF alpha induced protein 8
ENSG0000	7440.563	0.423658	0.068808	6.157147	7.41E-10	4.13E-08	R3HDM4	91300	R3H domain containing 4
ENSG0000	392.3378	0.941092	0.152906	6.154718	7.52E-10	4.18E-08	ENTPD7	57089	ectonucleoside triphosphate diphosphohydrolase 7
ENSG0000	8189.695	0.459008	0.074641	6.149581	7.77E-10	4.30E-08	BCL6	604	BCL6 transcription repressor
ENSG0000	1709.955	0.640626	0.104198	6.148136	7.84E-10	4.33E-08	SLAMF7	57823	SLAM family member 7
ENSG0000	3950.845	0.473737	0.077059	6.14769	7.86E-10	4.33E-08	USP11	8237	ubiquitin specific peptidase 11
ENSG0000	394.2165	-1.01229	0.164907	-6.13856	8.33E-10	4.57E-08	TRH	7200	thyrotropin releasing hormone
ENSG0000	1907.304	0.628859	0.102532	6.133284	8.61E-10	4.71E-08	SERPINB8	5271	serpin family B member 8
ENSG0000	4576.592	0.610376	0.099585	6.129186	8.83E-10	4.82E-08	HPSE	10855	heparanase
ENSG0000	4943.76	-0.68783	0.112349	-6.12222	9.23E-10	5.02E-08	CEBPD	1052	CCAAT enhancer binding protein delta
ENSG0000	2412.596	0.566574	0.092685	6.112925	9.78E-10	5.30E-08	YPEL5	51646	yippee like 5
ENSG0000	151.6122	1.309445	0.214223	6.112543	9.81E-10	5.30E-08	PDSS1	23590	decaprenyl diphosphate synthase subunit 1
ENSG0000	559.8898	-0.73914	0.120945	-6.11136	9.88E-10	5.33E-08	KATNIP	23247	katanin interacting protein
ENSG0000	818.6845	-0.67864	0.111105	-6.10808	1.01E-09	5.42E-08	USP5	8078	ubiquitin specific peptidase 5
ENSG0000	3847.348	0.507507	0.083248	6.096338	1.09E-09	5.82E-08	ITPRIP	85450	inositol 1 4 5-trisphosphate receptor interacting protein
ENSG0000	35.17093	4.002325	0.658363	6.079204	1.21E-09	6.46E-08	LOC10798	1.08E+08	uncharacterized LOC107984450
ENSG0000	2275.535	0.641115	0.105601	6.071096	1.27E-09	6.77E-08	PPP1CB	5500	protein phosphatase 1 catalytic subunit beta
ENSG0000	3882.236	0.480627	0.079241	6.06541	1.32E-09	6.99E-08	SRSF7	6432	serine and arginine rich splicing factor 7
ENSG0000	20277.85	0.501028	0.082638	6.062952	1.34E-09	7.08E-08	ANXA5	308	annexin A5
ENSG0000	1429.668	0.572549	0.094498	6.058851	1.37E-09	7.24E-08	PNKD	25953	PNKD metallo-beta-lactamase domain containing
ENSG0000	1140.215	-0.60961	0.100919	-6.04053	1.54E-09	8.09E-08	ARHGEF6	9459	Rac/Cdc42 guanine nucleotide exchange factor 6
ENSG0000	428.9127	0.876362	0.145222	6.034621	1.59E-09	8.37E-08	SH3PXD2B	285590	SH3 and PX domains 2B
ENSG0000	514.3774	0.865967	0.143881	6.018647	1.76E-09	9.21E-08	ZNF697	90874	zinc finger protein 697
ENSG0000	1855.971	-0.56525	0.093983	-6.01435	1.81E-09	9.42E-08	ZNF609	23060	zinc finger protein 609
ENSG0000	1527.268	0.534362	0.088851	6.014113	1.81E-09	9.42E-08	MIR22HG	84981	MIR22 host gene
ENSG0000	385.372	0.955174	0.158998	6.007444	1.88E-09	9.78E-08	IL2RA	3559	interleukin 2 receptor subunit alpha
ENSG0000	385.7619	-1.05834	0.176176	-6.00726	1.89E-09	9.78E-08	WDR7	23335	WD repeat domain 7
ENSG0000	5718.73	-0.60475	0.1008	-5.99945	1.98E-09	1.02E-07	C1orf162	128346	chromosome 1 open reading frame 162
ENSG0000	3802.649	0.516537	0.086153	5.995568	2.03E-09	1.04E-07	PRKCD	5580	protein kinase C delta
ENSG0000	856.4897	0.88439	0.147515	5.995234	2.03E-09	1.04E-07	FLT1	2321	fms related receptor tyrosine kinase 1
ENSG0000	4556.012	0.487133	0.08126	5.99476	2.04E-09	1.04E-07	GPR132	29933	G protein-coupled receptor 132
ENSG0000	329.9333	-0.97845	0.163366	-5.98933	2.11E-09	1.08E-07	NHSL2	340527	NHS like 2
ENSG0000	5009.294	0.599878	0.100275	5.982326	2.20E-09	1.12E-07	BIRC3	330	baculoviral IAP repeat containing 3
ENSG0000	371.6486	0.958433	0.16025	5.980858	2.22E-09	1.13E-07	CYP27A1	1593	cytochrome P450 family 27 subfamily A member 1
ENSG0000	5840.656	0.539431	0.09026	5.976383	2.28E-09	1.16E-07	DUSP2	1844	dual specificity phosphatase 2
ENSG0000	5572.501	0.481753	0.080673	5.971696	2.35E-09	1.19E-07	PIM1	5292	Pim-1 prot serine/threonine kinase
ENSG0000	3997.689	-0.46549	0.077969	-5.97018	2.37E-09	1.19E-07	LIMK1	3984	LIM domain kinase 1
ENSG0000	5013.265	0.464241	0.07779	5.967879	2.40E-09	1.21E-07	PTPN12	5782	protein tyrosine phosphatase non-receptor type 12
ENSG0000	6561.206	0.568755	0.095392	5.962286	2.49E-09	1.25E-07	SH2B3	10019	SH2B adaptor protein 3
ENSG0000	146.3793	1.370959	0.230218	5.955038	2.60E-09	1.30E-07	NA	NA	NA
ENSG0000	1233.258	-0.64229	0.10787	-5.95431	2.61E-09	1.30E-07	MTOR	2475	mechanistic target of rapamycin kinase
ENSG0000	5114.853	0.477931	0.080328	5.949765	2.69E-09	1.34E-07	ARL8A	127829	ADP ribosylation factor like GTPase 8A
ENSG0000	21023.81	-0.44428	0.074691	-5.94826	2.71E-09	1.34E-07	LSP1	4046	lymphocyte specific protein 1
ENSG0000	2505.946	-0.47752	0.080452	-5.93543	2.93E-09	1.45E-07	AOAH	313	acyloxyacyl hydrolase
ENSG0000	3412.184	0.450449	0.075892	5.935412	2.93E-09	1.45E-07	SUSD6	9766	sushi domain containing 6
ENSG0000	1470.79	-0.51455	0.08672	-5.93345	2.97E-09	1.46E-07	PPM1F	9647	protein ph Mg2+/Mn2+ dependent 1F
ENSG0000	2464.366	0.584719	0.098774	5.919762	3.22E-09	1.58E-07	SUB1	10923	SUB1 regulator of transcription
ENSG0000	1636.515	0.601877	0.101911	5.905881	3.51E-09	1.72E-07	KIR2DS4	3809	killer cell i two Ig domains and short cytoplasmic tail 4
ENSG0000	638.659	-0.81051	0.137389	-5.89941	3.65E-09	1.78E-07	GAL3ST4	79690	galactose-3-O-sulfotransferase 4
ENSG0000	1896.507	-0.53208	0.090357	-5.88864	3.89E-09	1.90E-07	MGAT5	4249	alpha-1 6-mannosylglycoprotein 6-beta-N-acetylglucosaminyltransferase
ENSG0000	879.954	0.847701	0.144275	5.875614	4.21E-09	2.05E-07	ITGA6	3655	integrin subunit alpha 6
ENSG0000	2063.605	-0.53566	0.091182	-5.87467	4.24E-09	2.05E-07	SND1	27044	staphylococcal nuclease and tudor domain containing 1
ENSG0000	152.5472	-1.25518	0.213766	-5.87173	4.31E-09	2.08E-07	MYO1D	4642	myosin ID
ENSG0000	1657.621	0.556418	0.094836	5.867171	4.43E-09	2.14E-07	RAB5A	5868	RAB5A member RAS oncogene family
ENSG0000	129.9363	-1.33745	0.228035	-5.86512	4.49E-09	2.15E-07	NLRC4	58484	NLR family CARD domain containing 4
ENSG0000	10468.18	0.456104	0.077767	5.865016	4.49E-09	2.15E-07	ATP6V1B2	526	ATPase H+ transporting V1 subunit B2
ENSG0000	1605.084	-0.79081	0.134842	-5.8647	4.50E-09	2.15E-07	ACP5	54	acid phosp tartrate resistant
ENSG0000	3706.374	0.543335	0.092657	5.863961	4.52E-09	2.15E-07	ADIPOR1	51094	adiponectin receptor 1
ENSG0000	2886.691	2.120134	0.361647	5.862436	4.56E-09	2.17E-07	MMP14	4323	matrix metallopeptidase 14
ENSG0000	13733.42	0.427756	0.073094	5.852163	4.85E-09	2.30E-07	RELA	5970	RELA prot NF-kB subunit
ENSG0000	370.0579	-0.82281	0.140722	-5.84705	5.00E-09	2.37E-07	MEGF8	1954	multiple EGF like domains 8
ENSG0000	10608.15	0.539216	0.092356	5.838462	5.27E-09	2.49E-07	UBE2D3	7323	ubiquitin conjugating enzyme E2 D3
ENSG0000	1033.009	-0.62657	0.107383	-5.8349	5.38E-09	2.53E-07	TCF12	6938	transcription factor 12
ENSG0000	1629.873	0.85208	0.146056	5.833921	5.41E-09	2.54E-07	CREG1	8804	cellular repressor of E1A stimulated genes 1
ENSG0000	4544.785	0.522648	0.089689	5.82734	5.63E-09	2.64E-07	RGS1	5996	regulator of G protein signaling 1
ENSG0000	5644.637	0.562778	0.096719	5.81872	5.93E-09	2.77E-07	HIVEP2	3097	HIVEP zinc finger 2
ENSG0000	415.0551	1.015229	0.174559	5.815974	6.03E-09	2.81E-07	ERRFI1	54206	ERBB receptor feedback inhibitor 1
ENSG0000	527.4775	0.718679	0.123604	5.814362	6.09E-09	2.83E-07	RAPGEF6	51735	Rap guanine nucleotide exchange factor 6

ENSG0000	279.5564	0.971809	0.167389	5.805701	6.41E-09	2.96E-07	HES1	3280	hes family bHLH transcription factor 1
ENSG0000	971.1663	-0.6043	0.104081	-5.80604	6.40E-09	2.96E-07	JAZF1	221895	JAZF zinc finger 1
ENSG0000	1084.21	-0.65148	0.112498	-5.79106	6.99E-09	3.23E-07	NACC2	138151	NACC family member 2
ENSG0000	1243.479	-0.5829	0.100723	-5.78711	7.16E-09	3.30E-07	CLEC16A	23274	C-type lectin domain containing 16A
ENSG0000	13981.27	0.393272	0.067988	5.784454	7.27E-09	3.34E-07	ACTN1	87	actinin alpha 1
ENSG0000	1668.351	-0.53042	0.091895	-5.77203	7.83E-09	3.59E-07	ANKRD44	91526	ankyrin repeat domain 44
ENSG0000	1052.514	0.62391	0.108174	5.767648	8.04E-09	3.67E-07	RIT1	6016	Ras like without CAAX 1
ENSG0000	1421.824	-0.61419	0.10653	-5.76543	8.14E-09	3.71E-07	TNIK	23043	TRAF2 and NCK interacting kinase
ENSG0000	1800.092	-0.60466	0.104912	-5.76345	8.24E-09	3.75E-07	SYK	6850	spleen associated tyrosine kinase
ENSG0000	1153.31	-0.58576	0.101757	-5.75647	8.59E-09	3.89E-07	MAST2	23139	microtubule associated serine/threonine kinase 2
ENSG0000	736.0937	-0.64863	0.112691	-5.75579	8.62E-09	3.90E-07	PFKFB4	5210	6-phosphc 6-biphosphatase 4
ENSG0000	12958.14	0.561519	0.097575	5.754725	8.68E-09	3.92E-07	BCL3	602	BCL3 transcription coactivator
ENSG0000	8217.167	2.237689	0.388894	5.753984	8.72E-09	3.92E-07	CCL3	6348	C-C motif chemokine ligand 3
ENSG0000	886.8924	0.592558	0.102992	5.753457	8.74E-09	3.93E-07	AP3M2	10947	adaptor related protein complex 3 subunit mu 2
ENSG0000	1127.89	-0.8093	0.141067	-5.73697	9.64E-09	4.32E-07	TANC2	26115	tetratricop ankyrin repeat and coiled-coil containing 2
ENSG0000	77727.39	2.1704	0.378504	5.734145	9.80E-09	4.38E-07	CCL2	6347	C-C motif chemokine ligand 2
ENSG0000	322.4455	-0.82935	0.144697	-5.73168	9.94E-09	4.42E-07	PPP1R12B	4660	protein phosphatase 1 regulatory subunit 12B
ENSG0000	2189.824	0.536962	0.093683	5.731693	9.94E-09	4.42E-07	NOP10	55505	NOP10 ribonucleoprotein
ENSG0000	602.4613	-0.82309	0.143627	-5.73075	1.00E-08	4.44E-07	GNB4	59345	G protein subunit beta 4
ENSG0000	4458.645	0.601008	0.104959	5.726103	1.03E-08	4.55E-07	TLNRD1	59274	talin rod domain containing 1
ENSG0000	12653.34	0.659518	0.115371	5.716481	1.09E-08	4.80E-07	SRC	6714	SRC proto- non-receptor tyrosine kinase
ENSG0000	1768.693	0.501932	0.087838	5.714298	1.10E-08	4.85E-07	CCDC93	54520	coiled-coil domain containing 93
ENSG0000	1215.035	0.666149	0.116764	5.705066	1.16E-08	5.11E-07	ASPH	444	aspartate beta-hydroxylase
ENSG0000	10270.88	0.476459	0.083568	5.701446	1.19E-08	5.21E-07	CCNI	10983	cyclin I
ENSG0000	1577.907	-0.65338	0.114779	-5.69249	1.25E-08	5.48E-07	BMP2K	55589	BMP2 inducible kinase
ENSG0000	4564.015	-0.44315	0.077875	-5.69056	1.27E-08	5.53E-07	FCGR3A	2214	Fc gamma receptor IIIa
ENSG0000	148.1694	1.500054	0.263659	5.689367	1.28E-08	5.55E-07	ELOVL7	79993	ELOVL fatty acid elongase 7
ENSG0000	19.3349	4.168474	0.733021	5.686705	1.30E-08	5.63E-07	IL21-AS1	1.01E+08	IL21 antisense RNA 1
ENSG0000	3414.98	-0.51954	0.09143	-5.68241	1.33E-08	5.76E-07	ABHD2	11057	abhydrola: acylglycerol lipase
ENSG0000	2998.375	0.631558	0.111268	5.675988	1.38E-08	5.96E-07	HBEGF	1839	heparin binding EGF like growth factor
ENSG0000	2783.976	0.445713	0.078673	5.665397	1.47E-08	6.33E-07	MAP1LC3E	81631	microtubule associated protein 1 light chain 3 beta
ENSG0000	1058.426	-0.59187	0.104604	-5.65821	1.53E-08	6.58E-07	NFIX	4784	nuclear factor I X
ENSG0000	356.1314	2.628015	0.465036	5.651207	1.59E-08	6.84E-07	INHBA	3624	inhibin subunit beta A
ENSG0000	786.7837	-0.6612	0.117085	-5.6472	1.63E-08	6.99E-07	IGSF9B	22997	immunoglobulin superfamily member 9B
ENSG0000	935.7502	0.704981	0.124955	5.641866	1.68E-08	7.17E-07	TRAF4	9618	TNF receptor associated factor 4
ENSG0000	5740.184	0.477163	0.084573	5.641998	1.68E-08	7.17E-07	JAK3	3718	Janus kinase 3
ENSG0000	1103.453	-0.68264	0.121069	-5.63841	1.72E-08	7.30E-07	RHOB	388	ras homolog family member B
ENSG0000	19660.74	0.361613	0.064178	5.634536	1.76E-08	7.45E-07	ATXN2L	11273	ataxin 2 like
ENSG0000	340452.1	0.610747	0.10843	5.632639	1.77E-08	7.52E-07	COX3	4514	cytochrome c oxidase subunit III
ENSG0000	1413.721	0.76009	0.135033	5.628903	1.81E-08	7.66E-07	SLCO4A1	28231	solute carrier organic anion transporter family member 4A1
ENSG0000	1132.956	-0.55756	0.099263	-5.61693	1.94E-08	8.19E-07	MDN1	23195	midasin AAA ATPase 1
ENSG0000	4261.277	-0.43766	0.077975	-5.61284	1.99E-08	8.35E-07	EP400	57634	E1A binding protein p400
ENSG0000	1310.394	-0.57836	0.103038	-5.61313	1.99E-08	8.35E-07	TOM1L2	146691	target of myb1 like 2 membrane trafficking protein
ENSG0000	4083.392	-0.43657	0.077787	-5.61243	2.00E-08	8.35E-07	CLSTN1	22883	calsyntenin 1
ENSG0000	2305.952	-0.53553	0.095536	-5.60553	2.08E-08	8.67E-07	ARID1B	57492	AT-rich interaction domain 1B
ENSG0000	1314.931	-0.61033	0.108974	-5.60071	2.13E-08	8.90E-07	ITPR1	3708	inositol 1 4 5-trisphosphate receptor type 1
ENSG0000	296.8294	0.909803	0.162592	5.595618	2.20E-08	9.14E-07	KBTBD8	84541	kelch repeat and BTB domain containing 8
ENSG0000	11819.69	0.468895	0.084083	5.576547	2.45E-08	1.02E-06	SELL	6402	selectin L
ENSG0000	1833.583	-0.5882	0.105496	-5.57559	2.47E-08	1.02E-06	ME2	4200	malic enzyme 2
ENSG0000	18260.69	0.481288	0.086476	5.565546	2.61E-08	1.08E-06	RPL27	6155	ribosomal protein L27
ENSG0000	3628.882	0.461685	0.083032	5.560334	2.69E-08	1.11E-06	SERPINB1	1992	serpin family B member 1
ENSG0000	774.1849	0.576263	0.103827	5.550205	2.85E-08	1.17E-06	ODC1	4953	ornithine decarboxylase 1
ENSG0000	611249.4	0.669348	0.120764	5.542604	2.98E-08	1.22E-06	ND4	4538	NADH dehydrogenase subunit 4
ENSG0000	1358.954	2.477192	0.447429	5.536498	3.09E-08	1.26E-06	F3	2152	coagulatio tissue factor
ENSG0000	178.4839	1.534075	0.277456	5.529078	3.22E-08	1.32E-06	AK4	205	adenylate kinase 4
ENSG0000	991.8569	-0.54058	0.097857	-5.52422	3.31E-08	1.35E-06	TBC1D8	11138	TBC1 domain family member 8
ENSG0000	14642.08	0.43629	0.079013	5.521728	3.36E-08	1.37E-06	SLC11A1	6556	solute carrier family 11 member 1
ENSG0000	955.5526	-0.56795	0.103074	-5.51008	3.59E-08	1.46E-06	WDFY4	57705	WDFY family member 4
ENSG0000	1757.511	0.679255	0.123357	5.506395	3.66E-08	1.48E-06	C1orf122	127687	chromosome 1 open reading frame 122
ENSG0000	5256.733	0.438786	0.079691	5.506096	3.67E-08	1.48E-06	ABCC1	4363	ATP binding cassette subfamily C member 1
ENSG0000	1304.101	-0.55989	0.101763	-5.50191	3.76E-08	1.51E-06	ADCY3	109	adenylate cyclase 3
ENSG0000	5807.418	0.406332	0.073953	5.494428	3.92E-08	1.58E-06	USP36	57602	ubiquitin specific peptidase 36
ENSG0000	2970.716	-0.49793	0.090672	-5.49152	3.98E-08	1.60E-06	NLRC3	197358	NLR family CARD domain containing 3
ENSG0000	2816.199	0.503043	0.091735	5.483661	4.17E-08	1.67E-06	PPP2CA	5515	protein phosphatase 2 catalytic subunit alpha
ENSG0000	1664.323	-0.56278	0.102646	-5.48273	4.19E-08	1.67E-06	ODF3B	440836	outer dense fiber of sperm tails 3B
ENSG0000	669.1565	0.663758	0.121181	5.477393	4.32E-08	1.72E-06	CD72	971	CD72 molecule
ENSG0000	1961.774	-0.49818	0.091099	-5.46857	4.54E-08	1.81E-06	HECTD4	283450	HECT domain E3 ubiquitin protein ligase 4
ENSG0000	423.4088	-0.83114	0.152046	-5.46638	4.59E-08	1.82E-06	SDC3	9672	syndecan 3
ENSG0000	1307.314	-0.52242	0.095653	-5.46163	4.72E-08	1.87E-06	TEP1	7011	telomerase associated protein 1
ENSG0000	724.7144	0.632543	0.11585	5.460027	4.76E-08	1.88E-06	TCAF2	285966	TRPM8 channel associated factor 2
ENSG0000	989.1031	0.63963	0.117188	5.458137	4.81E-08	1.90E-06	UBE2B	7320	ubiquitin conjugating enzyme E2 B
ENSG0000	11677.6	0.371747	0.068113	5.457795	4.82E-08	1.90E-06	TSPYL2	64061	TSPY like 2
ENSG0000	1373.621	-0.75228	0.137876	-5.45619	4.86E-08	1.91E-06	TMEM51	55092	transmembrane protein 51
ENSG0000	1213.025	0.612948	0.112405	5.45302	4.95E-08	1.94E-06	PELI1	57162	pellino E3 ubiquitin protein ligase 1
ENSG0000	10931.02	-0.3597	0.066037	-5.4469	5.13E-08	2.01E-06	IL10RA	3587	interleukin 10 receptor subunit alpha
ENSG0000	572.595	0.750893	0.13787	5.446374	5.14E-08	2.01E-06	PDCD1	5133	programmed cell death 1
ENSG0000	8118.905	0.516658	0.094911	5.443581	5.22E-08	2.03E-06	TREM1	54210	triggering receptor expressed on myeloid cells 1
ENSG0000	1613.164	0.467625	0.086059	5.433791	5.52E-08	2.15E-06	PHF20	51230	PHD finger protein 20
ENSG0000	423.2597	-0.72854	0.134179	-5.42961	5.65E-08	2.19E-06	WAKMAR:	1E+08	wound and keratinocyte migration associated lncRNA 2
ENSG0000	6078.577	-0.51247	0.09447	-5.42471	5.80E-08	2.25E-06	LRP1	4035	LDL receptor related protein 1
ENSG0000	11416.04	0.497946	0.091828	5.422575	5.87E-08	2.27E-06	KMT2E	55904	lysine methyltransferase 2E (inactive)
ENSG0000	2091.792	0.447848	0.082679	5.416688	6.07E-08	2.34E-06	HINT1	3094	histidine triad nucleotide binding protein 1
ENSG0000	1001.408	0.75544	0.139513	5.414822	6.13E-08	2.36E-06	DRAM1	55332	DNA damage regulated autophagy modulator 1
ENSG0000	3412.514	0.596639	0.110235	5.412445	6.22E-08	2.39E-06	SLC15A3	51296	solute carrier family 15 member 3
ENSG0000	4775.58	0.506726	0.09367	5.409697	6.31E-08	2.42E-06	CD63	967	CD63 molecule
ENSG0000	652.1001	0.750658	0.138814	5.407637	6.39E-08	2.44E-06	YRDC	79693	yrdC N6-threonylcarbamoyltransferase domain containing
ENSG0000	3994.355	-0.39203	0.072614	-5.3989	6.71E-08	2.56E-06	LBH	81606	LBH regulator of WNT signaling pathway
ENSG0000	846.1234	0.624461	0.115822	5.391571	6.98E-08	2.66E-06	NR1D2	9975	nuclear receptor subfamily 1 group D member 2
ENSG0000	1432.467	-0.49542	0.09195	-5.38794	7.13E-08	2.71E-06	NFIC	4782	nuclear factor I C
ENSG0000	1662.273	0.61901	0.114919	5.386488	7.18E-08	2.73E-06	PBX4	80714	PBX homeobox 4
ENSG0000	64.88905	1.687374	0.31387	5.376022	7.61E-08	2.88E-06	NA	NA	NA
ENSG0000	1577.079	0.596546	0.111113	5.368837	7.92E-08	2.99E-06	RASGEF1B	153020	RasGEF domain family member 1B
ENSG0000	562.9117	0.777956	0.144997	5.365315	8.08E-08	3.05E-06	PHLDA2	7262	pleckstrin homology like domain family A member 2
ENSG0000	3676.702	0.49646	0.092581	5.362469	8.21E-08	3.09E-06	S1PR5	53637	sphingosine-1-phosphate receptor 5
ENSG0000	1420.384	-0.6667	0.124384	-5.35999	8.32E-08	3.12E-06	HIC1	3090	HIC ZBTB transcriptional repressor 1
ENSG0000	4077.6	0.4614	0.08609	5.359512	8.34E-08	3.13E-06	CD48	962	CD48 molecule
ENSG0000	2205.366	-0.4562	0.085139	-5.35825	8.40E-08	3.14E-06	GALNT10	55568	polypeptide N-acetylgalactosaminyltransferase 10
ENSG0000	6453.405	0.421165	0.078683	5.35268	8.67E-08	3.23E-06	GTPBP1	9567	GTP binding protein 1
ENSG0000	1148.47	0.520479	0.097272	5.350748	8.76E-08	3.26E-06	KIR2DL1	3802	killer cell i two Ig domains and long cytoplasmic tail 1
ENSG0000	635.3299	0.925178	0.173766	5.324275	1.01E-07	3.77E-06	RUSC2	9853	RUN and SH3 domain containing 2

ENSG0000	506.1088	0.74121	0.139272	5.322024	1.03E-07	3.81E-06	PDK1	5163	pyruvate dehydrogenase kinase 1
ENSG0000	3222.853	0.549537	0.10345	5.312075	1.08E-07	4.01E-06	DDX39A	10212	DExD-box helicase 39A
ENSG0000	1506.49	-0.50144	0.094483	-5.3072	1.11E-07	4.11E-06	BLTP1	84162	bridge-like lipid transfer protein family member 1
ENSG0000	9132.505	1.270413	0.239442	5.305716	1.12E-07	4.14E-06	CCL4	6351	C-C motif chemokine ligand 4
ENSG0000	4056.009	0.38657	0.07287	5.304898	1.13E-07	4.15E-06	TRA2B	6434	transformer 2 beta homolog
ENSG0000	2145.17	-0.44449	0.083876	-5.29944	1.16E-07	4.27E-06	LDLRAD4	753	low density lipoprotein receptor class A domain containing 4
ENSG0000	5468.689	0.383807	0.07246	5.296827	1.18E-07	4.32E-06	GNG2	54331	G protein subunit gamma 2
ENSG0000	556.7876	1.979832	0.374109	5.292127	1.21E-07	4.42E-06	MIR3945H	731424	MIR3945 host gene
ENSG0000	1404.597	-0.59047	0.11163	-5.28949	1.23E-07	4.48E-06	RFLNB	359845	refilin B
ENSG0000	22206.12	0.480125	0.090812	5.287043	1.24E-07	4.53E-06	MCL1	4170	MCL1 apo| BCL2 family member
ENSG0000	131.2838	-1.33851	0.253181	-5.28676	1.25E-07	4.53E-06	MARVELD:	83742	MARVEL domain containing 1
ENSG0000	116.8522	-1.29724	0.245457	-5.28502	1.26E-07	4.56E-06	HTRA1	5654	HtrA serine peptidase 1
ENSG0000	41.01316	-3.04382	0.576182	-5.28274	1.27E-07	4.61E-06	HCRTR1	3061	hypocretin receptor 1
ENSG0000	1200.709	0.529988	0.100343	5.281776	1.28E-07	4.63E-06	OPTN	10133	optineurin
ENSG0000	304302.4	0.384477	0.072808	5.280693	1.29E-07	4.63E-06	FTH1	2495	ferritin heavy chain 1
ENSG0000	376.1768	-0.90633	0.171623	-5.28094	1.29E-07	4.63E-06	FLVCR2	55640	FLVCR heme transporter 2
ENSG0000	13129.3	0.552472	0.104621	5.280673	1.29E-07	4.63E-06	SBNO2	22904	strawberry notch homolog 2
ENSG0000	2560.433	-0.4635	0.087782	-5.28011	1.29E-07	4.63E-06	ABTB3	121551	ankyrin repeat and BTB domain containing 3
ENSG0000	2677.535	0.58418	0.110685	5.277881	1.31E-07	4.68E-06	TALDO1	6888	transaldolase 1
ENSG0000	16011.12	0.369068	0.069938	5.277093	1.31E-07	4.69E-06	JUND	3727	JunD prot(AP-1 transcription factor subunit
ENSG0000	1049.503	-0.53412	0.101242	-5.27568	1.32E-07	4.72E-06	RFX2	5990	regulatory factor X2
ENSG0000	3701.52	0.460693	0.087346	5.274362	1.33E-07	4.74E-06	LRRC8C	84230	leucine rich repeat containing 8 VRAC subunit C
ENSG0000	1602.672	0.509499	0.096721	5.267743	1.38E-07	4.91E-06	WDR74	54663	WD repeat domain 74
ENSG0000	241.664	1.029046	0.195466	5.26459	1.41E-07	4.98E-06	NA	NA	NA
ENSG0000	3297.975	0.409783	0.077925	5.258656	1.45E-07	5.14E-06	AKIRIN1	79647	akirin 1
ENSG0000	5499.316	0.403408	0.076851	5.249204	1.53E-07	5.39E-06	RPL37A	6168	ribosomal protein L37a
ENSG0000	575.5663	-0.6728	0.128175	-5.24906	1.53E-07	5.39E-06	FGD2	221472	FYVE RhoGEF and PH domain containing 2
ENSG0000	38545.91	-0.38522	0.073491	-5.2417	1.59E-07	5.60E-06	ENG	2022	endoglin
ENSG0000	3511.895	0.404292	0.077168	5.239091	1.61E-07	5.67E-06	RBM14	10432	RNA binding motif protein 14
ENSG0000	2074.933	-0.48077	0.091877	-5.23274	1.67E-07	5.86E-06	RBM6	10180	RNA binding motif protein 6
ENSG0000	1835.752	0.441692	0.08444	5.230845	1.69E-07	5.90E-06	RYBP	23429	RING1 and YY1 binding protein
ENSG0000	1590.126	-0.51189	0.097897	-5.22891	1.71E-07	5.96E-06	LGMN	5641	legumain
ENSG0000	136.961	1.204145	0.230306	5.228455	1.71E-07	5.96E-06	GCSAM	257144	germinal center associated signaling and motility
ENSG0000	423.4848	0.817152	0.156377	5.225517	1.74E-07	6.04E-06	SH3D21	79729	SH3 domain containing 21
ENSG0000	1185.083	0.573026	0.109675	5.224748	1.74E-07	6.06E-06	SMIM3	85027	small integral membrane protein 3
ENSG0000	822.906	-0.61012	0.116976	-5.21579	1.83E-07	6.35E-06	BTBD9	114781	BTB domain containing 9
ENSG0000	3719.94	0.444374	0.085204	5.21543	1.83E-07	6.35E-06	PLIN2	123	perilipin 2
ENSG0000	166.5612	-1.17042	0.224505	-5.21335	1.85E-07	6.41E-06	PHOSPHO:	162466	phosphoethanolamine/phosphocholine phosphatase 1
ENSG0000	2843.533	-0.49006	0.094024	-5.21203	1.87E-07	6.44E-06	HTT	3064	huntingtin
ENSG0000	1340.953	-0.67107	0.128864	-5.20757	1.91E-07	6.58E-06	KLF4	9314	KLF transcription factor 4
ENSG0000	3428.604	0.513434	0.098649	5.204672	1.94E-07	6.67E-06	IER5	51278	immediate early response 5
ENSG0000	2962.4	-0.45126	0.086707	-5.20446	1.95E-07	6.67E-06	MPRIP	23164	myosin phosphatase Rho interacting protein
ENSG0000	1184.377	-0.74313	0.142868	-5.20154	1.98E-07	6.76E-06	SAMD9L	219285	sterile alpha motif domain containing 9 like
ENSG0000	2384.602	0.408268	0.078533	5.1987	2.01E-07	6.85E-06	KPNA4	3840	karyopherin subunit alpha 4
ENSG0000	524.538	0.788929	0.151761	5.19848	2.01E-07	6.85E-06	BTG3	10950	BTG anti-proliferation factor 3
ENSG0000	457.4063	-0.75464	0.14525	-5.19549	2.04E-07	6.95E-06	HSPA6	3310	heat shock protein family A (Hsp70) member 6
ENSG0000	1066.456	-0.53735	0.103432	-5.19521	2.04E-07	6.95E-06	RTL5	340526	retrotransposon Gag like 5
ENSG0000	1201.397	-0.58657	0.113057	-5.18825	2.12E-07	7.20E-06	NKD1	85407	NKD inhibitor of WNT signaling pathway 1
ENSG0000	15632.18	0.380304	0.073311	5.18755	2.13E-07	7.21E-06	SRSF2	6427	serine and arginine rich splicing factor 2
ENSG0000	6719.813	0.423422	0.081792	5.176826	2.26E-07	7.62E-06	FLOT1	10211	flotillin 1
ENSG0000	2846.687	0.457599	0.088404	5.176257	2.26E-07	7.63E-06	BZW1	9689	basic leucine zipper and W2 domains 1
ENSG0000	6219.184	0.439969	0.085002	5.17596	2.27E-07	7.63E-06	CNOT6L	246175	CCR4-NOT transcription complex subunit 6 like
ENSG0000	27.93526	3.807686	0.735921	5.17404	2.29E-07	7.70E-06	NA	NA	NA
ENSG0000	460.6547	-2.38481	0.461419	-5.16841	2.36E-07	7.92E-06	CCR2	729230	C-C motif chemokine receptor 2
ENSG0000	8787.316	0.371114	0.071913	5.160569	2.46E-07	8.23E-06	SURF4	6836	surfeit 4
ENSG0000	1160.423	0.757901	0.146865	5.16054	2.46E-07	8.23E-06	PDGFB	5155	platelet derived growth factor subunit B
ENSG0000	3066.427	0.400283	0.07766	5.154308	2.55E-07	8.49E-06	CORO1C	23603	coronin 1C
ENSG0000	1238.689	-0.49676	0.096498	-5.14786	2.63E-07	8.76E-06	NDST1	3340	N-deacetylase and N-sulfotransferase 1
ENSG0000	9436.803	0.377853	0.073398	5.147997	2.63E-07	8.76E-06	PPP1R16B	26051	protein phosphatase 1 regulatory subunit 16B
ENSG0000	970.045	0.52161	0.101347	5.146755	2.65E-07	8.79E-06	NDUFB9	4715	NADH:ubiquinone oxidoreductase subunit B9
ENSG0000	765.6058	-0.63666	0.123724	-5.14583	2.66E-07	8.82E-06	ATP6V0E2	155066	ATPase H+ transporting V0 subunit e2
ENSG0000	618.5896	-0.62073	0.120724	-5.14173	2.72E-07	9.00E-06	TEX2	55852	testis expressed 2
ENSG0000	406.4857	-0.73801	0.143621	-5.13859	2.77E-07	9.14E-06	ASCC3	10973	activating signal cointegrator 1 complex subunit 3
ENSG0000	837.0085	0.562417	0.109463	5.137952	2.78E-07	9.15E-06	UBE2D1	7321	ubiquitin conjugating enzyme E2 D1
ENSG0000	12002.34	0.366834	0.071474	5.132439	2.86E-07	9.41E-06	STK40	83931	serine/threonine kinase 40
ENSG0000	1275.893	-0.60524	0.117952	-5.13123	2.88E-07	9.45E-06	CD27	939	CD27 molecule
ENSG0000	6220.511	0.503133	0.098258	5.120505	3.05E-07	9.98E-06	MAP4K4	9448	mitogen-activated protein kinase kinase kinase kinase 4
ENSG0000	1844.783	0.499334	0.09752	5.120329	3.05E-07	9.98E-06	RILPL2	196383	Rab interacting lysosomal protein like 2
ENSG0000	14302.16	0.388705	0.075929	5.119336	3.07E-07	1.00E-05	NKG7	4818	natural killer cell granule protein 7
ENSG0000	9086.747	0.403102	0.078772	5.117308	3.10E-07	1.01E-05	C15orf39	56905	chromosome 15 open reading frame 39
ENSG0000	860.4605	0.570371	0.11151	5.114966	3.14E-07	1.02E-05	NA	NA	NA
ENSG0000	853.8913	0.53333	0.104488	5.104213	3.32E-07	1.08E-05	IGFBP4	3487	insulin like growth factor binding protein 4
ENSG0000	1086.555	0.518139	0.101686	5.095463	3.48E-07	1.13E-05	DAB2	1601	DAB adaptor protein 2
ENSG0000	864.5848	0.545039	0.106987	5.094429	3.50E-07	1.13E-05	PPP1R3B	79660	protein phosphatase 1 regulatory subunit 3B
ENSG0000	520.9484	0.692159	0.135925	5.092208	3.54E-07	1.14E-05	ADCK2	90956	aarF domain containing kinase 2
ENSG0000	41.57192	1.902089	0.373597	5.091281	3.56E-07	1.15E-05	ITGB8	3696	integrin subunit beta 8
ENSG0000	189.2723	-1.33902	0.263025	-5.09086	3.56E-07	1.15E-05	SLC7A8	23428	solute carrier family 7 member 8
ENSG0000	7600.887	0.470617	0.092479	5.088878	3.60E-07	1.16E-05	MAPK1IP1	93487	mitogen-activated protein kinase 1 interacting protein 1 like
ENSG0000	25827.16	0.4354	0.085651	5.083408	3.71E-07	1.19E-05	YWHAZ	7534	tyrosine 3-monooxygenase/tryptophan 5-monooxygenase activation protein zeta
ENSG0000	521.5513	-0.67284	0.13245	-5.07992	3.78E-07	1.21E-05	CHD9	80205	chromodomain helicase DNA binding protein 9
ENSG0000	568.8832	-0.67925	0.133778	-5.07743	3.83E-07	1.22E-05	HERC2	8924	HECT and RLD domain containing E3 ubiquitin protein ligase 2
ENSG0000	340.4606	-1.18916	0.234309	-5.07517	3.87E-07	1.24E-05	CALHM6	441168	calcium homeostasis modulator family member 6
ENSG0000	1034.194	0.574147	0.113185	5.072661	3.92E-07	1.25E-05	SIGLEC14	1E+08	sialic acid binding Ig like lectin 14
ENSG0000	4596.794	-0.43452	0.085695	-5.07055	3.97E-07	1.26E-05	MRTFA	57591	myocardin related transcription factor A
ENSG0000	1650.155	0.434295	0.085825	5.060251	4.19E-07	1.33E-05	IRF4	3662	interferon regulatory factor 4
ENSG0000	3157.664	-0.41365	0.08177	-5.0587	4.22E-07	1.34E-05	ANKH	56172	ANKH inorganic pyrophosphate transport regulator
ENSG0000	924.6589	0.655866	0.129892	5.049327	4.43E-07	1.40E-05	CSF1	1435	colony stimulating factor 1
ENSG0000	43.99239	2.281226	0.452028	5.046644	4.50E-07	1.42E-05	GPRC5A	9052	G protein-coupled receptor class C group 5 member A
ENSG0000	2097.272	0.478911	0.095029	5.039614	4.66E-07	1.47E-05	PLAGL2	5326	PLAG1 like zinc finger 2
ENSG0000	2089.927	0.474016	0.094065	5.039246	4.67E-07	1.47E-05	ARL6IP1	23204	ADP ribosylation factor like GTPase 6 interacting protein 1
ENSG0000	198.2765	4.188508	0.831814	5.035392	4.77E-07	1.50E-05	CSF3	1440	colony stimulating factor 3
ENSG0000	1751.538	0.487138	0.096755	5.034773	4.78E-07	1.50E-05	BRAF	673	B-Raf prot serine/threonine kinase
ENSG0000	15572.93	0.47355	0.094095	5.032692	4.84E-07	1.52E-05	FERMT3	83706	FERM domain containing kindlin 3
ENSG0000	609.8491	0.63979	0.12716	5.031386	4.87E-07	1.52E-05	DHCR7	1717	7-dehydrocholesterol reductase
ENSG0000	484.2503	0.705908	0.140442	5.026345	5.00E-07	1.56E-05	EXOSC4	54512	exosome component 4
ENSG0000	4939.576	0.453047	0.090157	5.025091	5.03E-07	1.57E-05	SYTL3	94120	synaptotagmin like 3
ENSG0000	273768	0.577694	0.115012	5.022892	5.09E-07	1.58E-05	ATP6	4508	ATP synthase F0 subunit 6
ENSG0000	220.3645	-0.89907	0.179324	-5.01367	5.34E-07	1.66E-05	NLRX1	79671	NLR family member X1
ENSG0000	197.5719	1.193733	0.238344	5.008445	5.49E-07	1.70E-05	NA	NA	NA
ENSG0000	484.5641	-0.71027	0.141908	-5.00514	5.58E-07	1.73E-05	FABP5	2171	fatty acid binding protein 5

ENSG0000	7152.956	0.517174	0.103332	5.004978	5.59E-07	1.73E-05	IFITM2	10581	interferon induced transmembrane protein 2
ENSG0000	1479.059	0.531521	0.106368	4.997008	5.82E-07	1.80E-05	GPR171	29909	G protein-coupled receptor 171
ENSG0000	1969.884	-0.49208	0.098487	-4.99641	5.84E-07	1.80E-05	NSD1	64324	nuclear receptor binding SET domain protein 1
ENSG0000	183.9743	-1.06267	0.212788	-4.99405	5.91E-07	1.82E-05	KIAA1671	85379	KIAA1671
ENSG0000	594.5305	0.668206	0.133814	4.993533	5.93E-07	1.82E-05	MAD2L2	10459	mitotic arrest deficient 2 like 2
ENSG0000	121.2328	1.69498	0.339468	4.993052	5.94E-07	1.82E-05	OLIG2	10215	oligodendrocyte transcription factor 2
ENSG0000	1886.398	0.697893	0.139899	4.988553	6.08E-07	1.86E-05	GCH1	2643	GTP cyclohydrolase 1
ENSG0000	888.9335	-0.64315	0.129054	-4.98355	6.24E-07	1.91E-05	SRGAP2	23380	SLIT-ROBO Rho GTPase activating protein 2
ENSG0000	648.4623	0.602638	0.121132	4.975064	6.52E-07	1.99E-05	KDSR	2531	3-ketodihydrosphingosine reductase
ENSG0000	13698.44	-0.34969	0.07033	-4.97208	6.62E-07	2.01E-05	NBEAL2	23218	neurobeachin like 2
ENSG0000	146.6291	-1.18157	0.237638	-4.97213	6.62E-07	2.01E-05	PC	5091	pyruvate carboxylase
ENSG0000	3153.558	0.444108	0.089341	4.970941	6.66E-07	2.02E-05	CRYBG1	202	crystallin beta-gamma domain containing 1
ENSG0000	481.3072	-0.68995	0.138803	-4.97072	6.67E-07	2.02E-05	GRK3	157	G protein-coupled receptor kinase 3
ENSG0000	38.9218	3.128142	0.629451	4.969632	6.71E-07	2.03E-05	FAM124A	220108	family with sequence similarity 124 member A
ENSG0000	7960.17	0.381873	0.076921	4.96446	6.89E-07	2.08E-05	SSR2	6746	signal sequence receptor subunit 2
ENSG0000	56.93644	1.802102	0.363809	4.953428	7.29E-07	2.20E-05	GGT5	2687	gamma-glutamyltransferase 5
ENSG0000	1662.394	-0.48464	0.097939	-4.94837	7.48E-07	2.25E-05	FRYL	285527	FRY like transcription coactivator
ENSG0000	3322.468	0.508984	0.102883	4.94723	7.53E-07	2.26E-05	NCS1	23413	neuronal calcium sensor 1
ENSG0000	10800.28	-0.35421	0.071607	-4.94653	7.55E-07	2.27E-05	KLF13	51621	KLF transcription factor 13
ENSG0000	8139.55	0.373203	0.075558	4.939281	7.84E-07	2.35E-05	ATP6V1F	9296	ATPase H+ transporting V1 subunit F
ENSG0000	194.6888	-0.98572	0.199617	-4.93806	7.89E-07	2.36E-05	XXYLT1	152002	xyloside xylosyltransferase 1
ENSG0000	754.6349	-0.67271	0.136737	-4.91978	8.66E-07	2.59E-05	FXYD7	53822	FXYD domain containing ion transport regulator 7
ENSG0000	23844.52	0.931211	0.189342	4.918149	8.74E-07	2.61E-05	TNIP1	10318	TNFAIP3 interacting protein 1
ENSG0000	869.5782	0.512784	0.104304	4.916231	8.82E-07	2.63E-05	TBC1D15	64786	TBC1 domain family member 15
ENSG0000	990.0652	-0.5021	0.102142	-4.91573	8.85E-07	2.63E-05	WWC3	55841	WWC family member 3
ENSG0000	7470.738	0.326038	0.066511	4.902022	9.49E-07	2.81E-05	TRIR	79002	telomerase RNA component interacting RNase
ENSG0000	991.5753	-0.46713	0.09534	-4.89957	9.60E-07	2.85E-05	PEAK1	79834	pseudopodium enriched atypical kinase 1
ENSG0000	1368.263	0.521896	0.10662	4.894903	9.84E-07	2.91E-05	SLC20A1	6574	solute carrier family 20 member 1
ENSG0000	3709.773	-0.44662	0.091245	-4.89472	9.84E-07	2.91E-05	CABIN1	23523	calcineurin binding protein 1
ENSG0000	9178.553	0.402867	0.082349	4.892185	9.97E-07	2.93E-05	BHLHE40	8553	basic helix-loop-helix family member e40
ENSG0000	504.565	-0.65575	0.134035	-4.89236	9.96E-07	2.93E-05	TRERF1	55809	transcriptional regulating factor 1
ENSG0000	1072.469	0.452568	0.092495	4.892887	9.94E-07	2.93E-05	MAPK6	5597	mitogen-activated protein kinase 6
ENSG0000	144.42	-1.22491	0.250388	-4.89204	9.98E-07	2.93E-05	PCBP3	54039	poly(rC) binding protein 3
ENSG0000	3124.963	0.422075	0.086308	4.890317	1.01E-06	2.95E-05	CHMP1B	57132	charged multivesicular body protein 1B
ENSG0000	1623.072	-0.64773	0.132487	-4.889	1.01E-06	2.96E-05	EPSTI1	94240	epithelial stromal interaction 1
ENSG0000	25.27441	-3.10778	0.635715	-4.88864	1.02E-06	2.97E-05	CYP24A1	1591	cytochrome P450 family 24 subfamily A member 1
ENSG0000	562.7817	0.628976	0.128861	4.88106	1.06E-06	3.08E-05	CD22	933	CD22 molecule
ENSG0000	2478.957	0.562974	0.115494	4.874484	1.09E-06	3.18E-05	PCED1B-A	1E+08	PCED1B antisense RNA 1
ENSG0000	684.5991	-0.64995	0.133346	-4.87416	1.09E-06	3.18E-05	ST8SIA4	7903	ST8 alpha- 8-sialyltransferase 4
ENSG0000	15.08689	4.521481	0.927858	4.873032	1.10E-06	3.19E-05	GREM1	26585	gremlin 1 DAN family BMP antagonist
ENSG0000	1182.729	-0.70336	0.144394	-4.87115	1.11E-06	3.22E-05	FGL2	10875	fibrinogen like 2
ENSG0000	92.72298	1.373281	0.282007	4.869666	1.12E-06	3.23E-05	HEY1	23462	hes related family bHLH transcription factor with YRPW motif 1
ENSG0000	1883.505	0.423885	0.087072	4.868212	1.13E-06	3.25E-05	SLC35E1	79939	solute carrier family 35 member E1
ENSG0000	871.2787	-0.49831	0.102409	-4.86591	1.14E-06	3.29E-05	ADARB1	104	adenosine deaminase RNA specific B1
ENSG0000	7505.454	0.36919	0.07589	4.864784	1.15E-06	3.30E-05	ZNF207	7756	zinc finger protein 207
ENSG0000	5460.415	0.40359	0.082988	4.863237	1.15E-06	3.32E-05	NUP98	4928	nucleoporin 98 and 96 precursor
ENSG0000	5433.775	0.399313	0.082142	4.861232	1.17E-06	3.35E-05	EIF5	1983	eukaryotic translation initiation factor 5
ENSG0000	2627.319	-0.49177	0.101266	-4.85623	1.20E-06	3.43E-05	SLC38A10	124565	solute carrier family 38 member 10
ENSG0000	179.1752	-1.32069	0.272113	-4.85347	1.21E-06	3.47E-05	LDLRAD3	143458	low density lipoprotein receptor class A domain containing 3
ENSG0000	265.2605	-0.84497	0.174232	-4.84967	1.24E-06	3.53E-05	GPR160	26996	G protein-coupled receptor 160
ENSG0000	1662.592	0.434197	0.089542	4.849074	1.24E-06	3.54E-05	RAB1A	5861	RAB1A member RAS oncogene family
ENSG0000	136.2115	1.231556	0.254008	4.848492	1.24E-06	3.54E-05	MIR155HG	114614	MIR155 host gene
ENSG0000	930.4783	0.593787	0.122524	4.84627	1.26E-06	3.58E-05	DENND4A	10260	DENN domain containing 4A
ENSG0000	1929.997	0.46908	0.096896	4.841054	1.29E-06	3.67E-05	RAB8B	51762	RAB8B member RAS oncogene family
ENSG0000	271.425	-0.95527	0.197394	-4.83942	1.30E-06	3.69E-05	CTIF	9811	cap binding complex dependent translation initiation factor
ENSG0000	6621.728	-0.47558	0.098286	-4.83871	1.31E-06	3.70E-05	TNS1	7145	tensin 1
ENSG0000	6677.958	-0.51394	0.106255	-4.83689	1.32E-06	3.73E-05	MPEG1	219972	macrophage expressed 1
ENSG0000	159.0909	1.150725	0.238126	4.83243	1.35E-06	3.81E-05	SEMA3F	6405	semaphorin 3F
ENSG0000	3304.065	-0.3758	0.077779	-4.83163	1.35E-06	3.82E-05	LILRB3	11025	leukocyte immunoglobulin like receptor B3
ENSG0000	207.1405	-1.1686	0.241975	-4.82944	1.37E-06	3.85E-05	FTO	79068	FTO alpha-ketoglutarate dependent dioxygenase
ENSG0000	3851.237	0.378112	0.0783	4.829004	1.37E-06	3.86E-05	MTPN	136319	myotrophin
ENSG0000	42136.69	0.500674	0.103687	4.82872	1.37E-06	3.86E-05	JUNB	3726	JunB protc AP-1 transcription factor subunit
ENSG0000	487.6285	-0.61779	0.12797	-4.8276	1.38E-06	3.87E-05	ARRB1	408	arrestin beta 1
ENSG0000	2889.91	-0.55362	0.114733	-4.82523	1.40E-06	3.91E-05	TIMP2	7077	TIMP metallopeptidase inhibitor 2
ENSG0000	2129.835	-0.47541	0.098573	-4.82289	1.41E-06	3.95E-05	TMEM94	9772	transmembrane protein 94
ENSG0000	4827.708	0.450051	0.093408	4.818135	1.45E-06	4.04E-05	ISG20	3669	interferon stimulated exonuclease gene 20
ENSG0000	852.0137	-0.52904	0.109798	-4.81829	1.45E-06	4.04E-05	PRKCA	5578	protein kinase C alpha
ENSG0000	1259.217	-0.52461	0.108905	-4.81711	1.46E-06	4.05E-05	ARHGEF11	9826	Rho guanine nucleotide exchange factor 11
ENSG0000	4402.011	0.35741	0.074203	4.81664	1.46E-06	4.05E-05	GSPT1	2935	G1 to S phase transition 1
ENSG0000	2885.378	0.397379	0.082517	4.815724	1.47E-06	4.07E-05	ETF1	2107	eukaryotic translation termination factor 1
ENSG0000	941.0354	-0.57406	0.119325	-4.81086	1.50E-06	4.16E-05	H6PD	9563	hexose-6-phosphate dehydrogenase/glucose 1-dehydrogenase
ENSG0000	558.7741	-0.54453	0.113225	-4.80931	1.51E-06	4.19E-05	ALAD	210	aminolevulinate dehydratase
ENSG0000	6079.328	0.345272	0.07185	4.805449	1.54E-06	4.26E-05	SAMD4B	55095	sterile alpha motif domain containing 4B
ENSG0000	29216.84	0.320771	0.0668	4.801976	1.57E-06	4.33E-05	PFN1	5216	profilin 1
ENSG0000	344.6364	-0.68719	0.1432	-4.79881	1.60E-06	4.39E-05	HPCAL4	51440	hippocalcin like 4
ENSG0000	1532.914	0.54072	0.112693	4.79816	1.60E-06	4.40E-05	RHOH	399	ras homolog family member H
ENSG0000	2485.636	-0.42337	0.088397	-4.78937	1.67E-06	4.59E-05	CLASP1	23332	cytoplasmic linker associated protein 1
ENSG0000	610.2325	-0.54523	0.113912	-4.78647	1.70E-06	4.64E-05	PLAGL1	5325	PLAG1 like zinc finger 1
ENSG0000	3339.647	0.364824	0.076212	4.786951	1.69E-06	4.64E-05	NDEL1	81565	nudE neurodevelopment protein 1 like 1
ENSG0000	898.2769	0.48949	0.102267	4.786409	1.70E-06	4.64E-05	RAB22A	57403	RAB22A member RAS oncogene family
ENSG0000	276.4247	0.918368	0.19198	4.783668	1.72E-06	4.69E-05	ATP2B1-A	338758	ATP2B1 antisense RNA 1
ENSG0000	2096.138	-0.42198	0.08821	-4.78384	1.72E-06	4.69E-05	KIAA0930	23313	KIAA0930
ENSG0000	569.0005	-0.64815	0.135572	-4.78088	1.75E-06	4.75E-05	ITPR2	3709	inositol 1 4 5-trisphosphate receptor type 2
ENSG0000	10243.21	0.327536	0.068538	4.778921	1.76E-06	4.79E-05	MEF2D	4209	myocyte enhancer factor 2D
ENSG0000	160.3838	-1.17169	0.245395	-4.77471	1.80E-06	4.88E-05	DST	667	dystonin
ENSG0000	15951.77	0.331986	0.069574	4.771675	1.83E-06	4.95E-05	EFHD2	79180	EF-hand domain family member D2
ENSG0000	3392.939	0.614408	0.128914	4.766043	1.88E-06	5.08E-05	HBA1	3039	hemoglobin subunit alpha 1
ENSG0000	1487.324	0.47509	0.099707	4.764866	1.89E-06	5.10E-05	B3GNT7	93010	UDP-GlcN 3-N-acetylglucosaminyltransferase 7
ENSG0000	980.8162	0.552816	0.116028	4.764508	1.89E-06	5.10E-05	ZFYVE16	9765	zinc finger FYVE-type containing 16
ENSG0000	337.3197	0.685652	0.144059	4.759524	1.94E-06	5.22E-05	OSGIN2	734	oxidative stress induced growth inhibitor family member 2
ENSG0000	485.3627	-0.77241	0.162662	-4.74855	2.05E-06	5.51E-05	CD9	928	CD9 molecule
ENSG0000	750.1929	-0.54359	0.114501	-4.74748	2.06E-06	5.53E-05	DNASE1	1773	deoxyribonuclease 1
ENSG0000	579.5448	-0.58704	0.123703	-4.74553	2.08E-06	5.57E-05	PIGQ	9091	phosphatidylinositol glycan anchor biosynthesis class Q
ENSG0000	567.659	-0.57044	0.120214	-4.74524	2.08E-06	5.57E-05	PRAM1	84106	PML-RARA regulated adaptor molecule 1
ENSG0000	1647.839	0.462464	0.097477	4.744351	2.09E-06	5.59E-05	JMJD6	23210	jumonji dc arginine demethylase and lysine hydroxylase
ENSG0000	1467.777	0.506001	0.106671	4.743557	2.10E-06	5.60E-05	GAS5	60674	growth arrest specific 5
ENSG0000	268.8151	-0.73108	0.154192	-4.74135	2.12E-06	5.66E-05	FBXL16	146330	F-box and leucine rich repeat protein 16
ENSG0000	196.6649	-0.85293	0.179942	-4.74003	2.14E-06	5.69E-05	DAPK2	23604	death associated protein kinase 2
ENSG0000	5009.325	0.391882	0.082821	4.731657	2.23E-06	5.92E-05	CYTIP	9595	cytohesin 1 interacting protein
ENSG0000	26725.34	0.315429	0.066682	4.730347	2.24E-06	5.95E-05	IL2RB	3560	interleukin 2 receptor subunit beta

ENSG0000	1703.916	0.549249	0.116314	4.722126	2.33E-06	6.18E-05	KLHL21	9903	kelch like family member 21
ENSG0000	2792.68	-0.42557	0.090244	-4.71575	2.41E-06	6.37E-05	RCSD1	92241	RCSD domain containing 1
ENSG0000	3272.398	0.402978	0.085457	4.715574	2.41E-06	6.37E-05	LEPROTL1	23484	leptin receptor overlapping transcript like 1
ENSG0000	747.6921	0.744975	0.158001	4.715008	2.42E-06	6.38E-05	CD59	966	CD59 molecule (CD59 blood group)
ENSG0000	931.6448	0.588903	0.124936	4.713621	2.43E-06	6.41E-05	TGIF2	60436	TGFB induced factor homeobox 2
ENSG0000	31899.58	0.389593	0.082691	4.711454	2.46E-06	6.47E-05	CEBPB	1051	CCAAT enhancer binding protein beta
ENSG0000	24.48953	2.861792	0.607924	4.707484	2.51E-06	6.59E-05	CES1P1	51716	carboxylesterase 1 pseudogene 1
ENSG0000	996.5585	0.459657	0.097708	4.704416	2.55E-06	6.68E-05	ZFAND6	54469	zinc finger AN1-type containing 6
ENSG0000	205.3017	-0.95452	0.203255	-4.69617	2.65E-06	6.94E-05	EPHB2	2048	EPH receptor B2
ENSG0000	399.5693	0.634836	0.13523	4.694483	2.67E-06	6.99E-05	TMEM41B	440026	transmembrane protein 41B
ENSG0000	6407.611	0.333572	0.071092	4.6921	2.70E-06	7.06E-05	AGO2	27161	argonaute RISC catalytic component 2
ENSG0000	2349.268	0.575993	0.122784	4.691121	2.72E-06	7.09E-05	REL	5966	REL proto- NF-kB subunit
ENSG0000	1718.471	-0.53987	0.11509	-4.69082	2.72E-06	7.09E-05	STMN3	50861	stathmin 3
ENSG0000	4189.204	0.334269	0.071413	4.680786	2.86E-06	7.43E-05	BET1L	51272	Bet1 golgi vesicular membrane trafficking protein like
ENSG0000	142.9124	-0.93344	0.199477	-4.67942	2.88E-06	7.47E-05	MRTFB	57496	myocardin related transcription factor B
ENSG0000	7210.703	-0.33432	0.071553	-4.67233	2.98E-06	7.72E-05	NLRP1	22861	NLR family pyrin domain containing 1
ENSG0000	1221.629	0.450306	0.096415	4.670502	3.00E-06	7.78E-05	RAB21	23011	RAB21 member RAS oncogene family
ENSG0000	500.77	0.59985	0.128523	4.667242	3.05E-06	7.90E-05	EZH2	2146	enhancer of zeste 2 polycomb repressive complex 2 subunit
ENSG0000	8266.308	0.353769	0.075872	4.662734	3.12E-06	8.06E-05	CCNK	8812	cyclin K
ENSG0000	1964.615	0.385805	0.082764	4.661479	3.14E-06	8.10E-05	PPP1R2	5504	protein phosphatase 1 regulatory inhibitor subunit 2
ENSG0000	522.7604	0.638214	0.136936	4.660668	3.15E-06	8.12E-05	RHOU	58480	ras homolog family member U
ENSG0000	9.108617	5.525352	1.186387	4.657293	3.20E-06	8.24E-05	NMRAL2P	344887	NmrA like pseudogene
ENSG0000	219.3449	-0.82178	0.176482	-4.65647	3.22E-06	8.26E-05	KIAA0753	9851	KIAA0753
ENSG0000	987.4242	0.492967	0.105932	4.653615	3.26E-06	8.37E-05	TAF1D	79101	TATA-box RNA polymerase I subunit D
ENSG0000	1410.204	0.418898	0.090046	4.652066	3.29E-06	8.42E-05	DNAJC3	5611	DnaJ heat shock protein family (Hsp40) member C3
ENSG0000	36.88211	1.933638	0.416749	4.63981	3.49E-06	8.92E-05	FAM238A	645528	family with sequence similarity 238 member A
ENSG0000	369226.8	0.597355	0.128762	4.639226	3.50E-06	8.93E-05	CYTB	4519	cytochrome b
ENSG0000	684.1822	0.541874	0.116849	4.637397	3.53E-06	9.00E-05	PHLDB3	653583	pleckstrin homology like domain family B member 3
ENSG0000	14.13685	3.659642	0.789336	4.636352	3.55E-06	9.03E-05	GRK7	131890	G protein-coupled receptor kinase 7
ENSG0000	3387.527	0.468902	0.10116	4.635252	3.57E-06	9.07E-05	BID	637	BH3 interacting domain death agonist
ENSG0000	32968.83	0.3711	0.080099	4.632993	3.60E-06	9.16E-05	ITGAX	3687	integrin subunit alpha X
ENSG0000	1956.309	-0.52767	0.113909	-4.63235	3.62E-06	9.17E-05	ZDHHC8	29801	zinc finger DHHC-type palmitoyltransferase 8
ENSG0000	2371.035	0.399548	0.086282	4.630741	3.64E-06	9.23E-05	PNRC2	55629	proline rich nuclear receptor coactivator 2
ENSG0000	3883.618	0.337879	0.072972	4.630253	3.65E-06	9.24E-05	FASN	2194	fatty acid synthase
ENSG0000	5775.766	0.422465	0.091262	4.629146	3.67E-06	9.28E-05	IFNGR2	3460	interferon gamma receptor 2
ENSG0000	2578.872	-0.43982	0.095126	-4.62352	3.77E-06	9.52E-05	AQP3	360	aquaporin 3 (Gill blood group)
ENSG0000	272.9001	-0.97745	0.211436	-4.62292	3.78E-06	9.54E-05	NA	NA	NA
ENSG0000	244.7529	0.786724	0.170324	4.618972	3.86E-06	9.70E-05	TPPP	11076	tubulin polymerization promoting protein
ENSG0000	3828.568	0.409819	0.088743	4.618036	3.87E-06	9.74E-05	EIF4A2	1974	eukaryotic translation initiation factor 4A2
ENSG0000	1523.169	0.455671	0.098686	4.617377	3.89E-06	9.75E-05	MORF4L2	9643	mortality factor 4 like 2
ENSG0000	838.578	0.673227	0.145871	4.615219	3.93E-06	9.84E-05	ALDOA	226	aldolase fructose-bisphosphate A
ENSG0000	2011.754	-0.37448	0.081218	-4.61083	4.01E-06	1.00E-04	EHMT1	79813	euchromatic histone lysine methyltransferase 1
ENSG0000	154.784	1.062874	0.230901	4.603152	4.16E-06	1.04E-04	NA	NA	NA
ENSG0000	437.2092	-0.59734	0.129802	-4.60192	4.19E-06	1.04E-04	ALMS1	7840	ALMS1 centrosome and basal body associated protein
ENSG0000	770.2564	-0.54012	0.117438	-4.59916	4.24E-06	1.06E-04	VPS35L	57020	VPS35 endosomal protein sorting factor like
ENSG0000	4809.429	0.362263	0.078827	4.595664	4.31E-06	1.07E-04	G6PD	2539	glucose-6-phosphate dehydrogenase
ENSG0000	12372.84	0.32538	0.070821	4.594387	4.34E-06	1.08E-04	CRTC2	200186	CREB regulated transcription coactivator 2
ENSG0000	2091.217	0.508934	0.110822	4.59236	4.38E-06	1.09E-04	CEP170	9859	centrosomal protein 170
ENSG0000	23.32645	3.603086	0.784571	4.592427	4.38E-06	1.09E-04	ADORA2A-	646023	ADORA2A antisense RNA 1
ENSG0000	4932.247	0.406835	0.088624	4.590561	4.42E-06	1.09E-04	SPHK1	8877	sphingosine kinase 1
ENSG0000	2755.501	-0.40972	0.089439	-4.58101	4.63E-06	1.14E-04	TRANK1	9881	tetratricopeptide repeat and ankyrin repeat containing 1
ENSG0000	806.8559	0.51206	0.111776	4.58112	4.62E-06	1.14E-04	FAM222A	84915	family with sequence similarity 222 member A
ENSG0000	465.0832	-0.55319	0.121023	-4.57092	4.86E-06	1.20E-04	OGG1	4968	8-oxoguanine DNA glycosylase
ENSG0000	3208.829	1.245606	0.27257	4.569859	4.88E-06	1.20E-04	NLRP3	114548	NLR family pyrin domain containing 3
ENSG0000	1498.452	-0.41928	0.091782	-4.56826	4.92E-06	1.21E-04	ELMO1	9844	engulfment and cell motility 1
ENSG0000	2544.37	-0.36418	0.079774	-4.56522	4.99E-06	1.23E-04	ATG16L2	89849	autophagy related 16 like 2
ENSG0000	4717.287	0.332863	0.072944	4.563258	5.04E-06	1.24E-04	ARCN1	372	archain 1
ENSG0000	8287.937	0.439848	0.096477	4.559082	5.14E-06	1.26E-04	WAC	51322	WW domain containing adaptor with coiled-coil
ENSG0000	159.3555	-0.98622	0.216331	-4.55884	5.14E-06	1.26E-04	FUT8	2530	fucosyltransferase 8
ENSG0000	11673.79	0.34473	0.07561	4.55934	5.13E-06	1.26E-04	IL4R	3566	interleukin 4 receptor
ENSG0000	2237.84	0.423519	0.092969	4.555465	5.23E-06	1.28E-04	VHL	7428	von Hippel-Lindau tumor suppressor
ENSG0000	719.9162	-0.47236	0.103758	-4.55256	5.30E-06	1.29E-04	TTC9	23508	tetratricopeptide repeat domain 9
ENSG0000	7094.448	0.382958	0.084176	4.549484	5.38E-06	1.31E-04	PELATON	1.01E+08	plaque enriched lncRNA in atherosclerotic and inflammatory bowel macrophage regulation
ENSG0000	122.7722	1.160242	0.255131	4.547642	5.43E-06	1.32E-04	SGPP2	130367	sphingosine-1-phosphate phosphatase 2
ENSG0000	988.8071	-0.47299	0.104019	-4.54719	5.44E-06	1.32E-04	P2RX1	5023	purinergic receptor P2X 1
ENSG0000	7310.081	0.311053	0.068416	4.546468	5.46E-06	1.32E-04	CFLAR	8837	CASP8 and FADD like apoptosis regulator
ENSG0000	2555.745	-0.39162	0.08625	-4.54047	5.61E-06	1.36E-04	RIPOR2	9750	RHO family interacting cell polarization regulator 2
ENSG0000	318.692	0.714878	0.157474	4.539647	5.63E-06	1.36E-04	COL1A1	1277	collagen type I alpha 1 chain
ENSG0000	3084.765	0.401229	0.088416	4.53795	5.68E-06	1.37E-04	ACAP1	9744	ArfGAP wi ankyrin repeat and PH domains 1
ENSG0000	450.3821	-0.60591	0.133748	-4.53028	5.89E-06	1.42E-04	SPHK2	56848	sphingosine kinase 2
ENSG0000	1909.799	0.436369	0.096323	4.530283	5.89E-06	1.42E-04	RAP2C	57826	RAP2C member of RAS oncogene family
ENSG0000	585.0095	-0.6393	0.141137	-4.52963	5.91E-06	1.42E-04	NPTXR	23467	neuronal pentraxin receptor
ENSG0000	4178.566	-0.4223	0.093316	-4.52546	6.03E-06	1.45E-04	TBC1D10C	374403	TBC1 domain family member 10C
ENSG0000	1204.928	0.454381	0.100415	4.525044	6.04E-06	1.45E-04	CNNM4	26504	cyclin and CBS domain divalent metal cation transport mediator 4
ENSG0000	570.5806	-0.55474	0.122647	-4.52305	6.10E-06	1.46E-04	HVCN1	84329	hydrogen voltage gated channel 1
ENSG0000	244.1334	-0.94023	0.207955	-4.52132	6.15E-06	1.47E-04	CD101	9398	CD101 molecule
ENSG0000	117.5417	-1.12096	0.247946	-4.52098	6.16E-06	1.47E-04	UNC5B	219699	unc-5 netrin receptor B
ENSG0000	133.1022	1.215745	0.268929	4.520694	6.16E-06	1.47E-04	NA	NA	NA
ENSG0000	2109.302	0.458316	0.101401	4.519859	6.19E-06	1.48E-04	CLDND1	56650	claudin domain containing 1
ENSG0000	516.3864	0.552506	0.122614	4.506073	6.60E-06	1.57E-04	MDM2	4193	MDM2 proto-oncogene
ENSG0000	561.2332	-0.54882	0.121903	-4.50214	6.73E-06	1.60E-04	MAN1A2	10905	mannosidase alpha class 1A member 2
ENSG0000	2373.53	0.517738	0.115056	4.499871	6.80E-06	1.61E-04	CCDC71L	168455	coiled-coil domain containing 71 like
ENSG0000	1470.833	0.44714	0.099405	4.498174	6.85E-06	1.63E-04	CHRAC1	54108	chromatin accessibility complex subunit 1
ENSG0000	2769.948	0.389455	0.086674	4.493314	7.01E-06	1.66E-04	NCK2	8440	NCK adaptor protein 2
ENSG0000	105.7124	1.771526	0.395156	4.483107	7.36E-06	1.74E-04	EDN1	1906	endothelin 1
ENSG0000	1613.906	-0.47119	0.105129	-4.48206	7.39E-06	1.75E-04	ATP11A	23250	ATPase phospholipid transporting 11A
ENSG0000	1399.671	0.39324	0.087805	4.478581	7.51E-06	1.77E-04	CALU	813	calumenin
ENSG0000	452.0668	-0.5578	0.124678	-4.47395	7.68E-06	1.81E-04	KCNC3	3748	potassium voltage-gated channel subfamily C member 3
ENSG0000	8519.528	1.195957	0.267503	4.470821	7.79E-06	1.83E-04	SOCS3	9021	suppressor of cytokine signaling 3
ENSG0000	41.34197	1.726768	0.386331	4.469663	7.83E-06	1.84E-04	CXCL6	6372	C-X-C motif chemokine ligand 6
ENSG0000	565.4192	-0.82971	0.185768	-4.46639	7.96E-06	1.87E-04	FAM20A	54757	FAM20A golgi associated secretory pathway pseudokinase
ENSG0000	4753.413	-0.37682	0.084378	-4.46585	7.98E-06	1.87E-04	HDAC7	51564	histone deacetylase 7
ENSG0000	862.9601	-0.53128	0.119399	-4.44967	8.60E-06	2.01E-04	GTF2I	2969	general transcription factor IIi
ENSG0000	4709.057	0.346355	0.077856	4.448673	8.64E-06	2.02E-04	ERF	2077	ETS2 repressor factor
ENSG0000	12461.27	0.309821	0.06966	4.447629	8.68E-06	2.03E-04	IKZF1	10320	IKAROS family zinc finger 1
ENSG0000	6750.525	0.334259	0.075223	4.443592	8.85E-06	2.06E-04	YWHAE	7531	tyrosine 3-monooxygenase/tryptophan 5-monooxygenase activation protein epsilon
ENSG0000	1110.69	0.483127	0.108854	4.438317	9.07E-06	2.11E-04	SFMBT2	57713	Scm like with four mbt domains 2
ENSG0000	6677.175	0.383726	0.086475	4.437421	9.10E-06	2.12E-04	ARHGEF2	9181	Rho/Rac guanine nucleotide exchange factor 2
ENSG0000	15238.42	0.413241	0.093158	4.435912	9.17E-06	2.13E-04	LITAF	9516	lipopolysaccharide induced TNF factor
ENSG0000	1290.235	-0.42869	0.096644	-4.43579	9.17E-06	2.13E-04	ARHGAP3	2909	Rho GTPase activating protein 35

ENSG0000	3321.088	0.345815	0.077954	4.436128	9.16E-06	2.13E-04	MSL3	10943	MSL complex subunit 3
ENSG0000	260.3833	0.686902	0.154936	4.433449	9.27E-06	2.15E-04	MRPL1	65008	mitochondrial ribosomal protein L1
ENSG0000	3472.975	-0.42298	0.095465	-4.43076	9.39E-06	2.17E-04	PDE3B	5140	phosphodiesterase 3B
ENSG0000	3555.044	0.377219	0.085147	4.430211	9.41E-06	2.17E-04	IL21R	50615	interleukin 21 receptor
ENSG0000	4810.859	0.331984	0.075155	4.417336	9.99E-06	2.31E-04	PTPRE	5791	protein tyrosine phosphatase receptor type E
ENSG0000	7160.514	0.379796	0.086004	4.416006	1.01E-05	2.32E-04	PTPN1	5770	protein tyrosine phosphatase non-receptor type 1
ENSG0000	598.5335	-0.58175	0.131746	-4.41566	1.01E-05	2.32E-04	RASA1	5921	RAS p21 protein activator 1
ENSG0000	5431.071	0.393723	0.089245	4.411719	1.03E-05	2.36E-04	RPS13	6207	ribosomal protein S13
ENSG0000	708.6653	0.493089	0.111906	4.406296	1.05E-05	2.41E-04	MXI1	4601	MAX inter dimerization protein
ENSG0000	931.6895	-0.46818	0.106276	-4.40527	1.06E-05	2.42E-04	CXXC5	51523	CXXC finger protein 5
ENSG0000	1126.931	0.442789	0.100513	4.405288	1.06E-05	2.42E-04	NUP58	9818	nucleoporin 58
ENSG0000	2818.17	0.414921	0.094236	4.402982	1.07E-05	2.44E-04	DDX3Y	8653	DEAD-box helicase 3 Y-linked
ENSG0000	243.238	-0.79985	0.181754	-4.40075	1.08E-05	2.46E-04	ZBTB47	92999	zinc finger and BTB domain containing 47
ENSG0000	897.5541	0.463145	0.105289	4.398793	1.09E-05	2.48E-04	PNPLA8	50640	patatin like phospholipase domain containing 8
ENSG0000	1110.588	0.539696	0.12276	4.396347	1.10E-05	2.51E-04	PRDX1	5052	peroxiredoxin 1
ENSG0000	22009.33	0.306221	0.069671	4.395232	1.11E-05	2.52E-04	SELPLG	6404	selectin P ligand
ENSG0000	2596.467	-0.38489	0.087576	-4.39489	1.11E-05	2.52E-04	SZT2	23334	SZT2 subunit of KICSTOR complex
ENSG0000	10909.39	-0.42722	0.097265	-4.39237	1.12E-05	2.55E-04	QSOX1	5768	quiescin sulfhydryl oxidase 1
ENSG0000	1049.526	0.421636	0.096104	4.387291	1.15E-05	2.60E-04	SLC15A4	121260	solute carrier family 15 member 4
ENSG0000	619.1341	0.510076	0.116391	4.382419	1.17E-05	2.66E-04	ATP6V1A	523	ATPase H+ transporting V1 subunit A
ENSG0000	175.5921	1.324786	0.302359	4.381495	1.18E-05	2.67E-04	PRKAG2-A	644090	PRKAG2 antisense RNA 2
ENSG0000	250.6899	-0.74201	0.169505	-4.37752	1.20E-05	2.71E-04	C5AR2	27202	complement C5a receptor 2
ENSG0000	4581.437	0.411987	0.094192	4.373897	1.22E-05	2.75E-04	GNA15	2769	G protein subunit alpha 15
ENSG0000	264.3963	-0.71548	0.163578	-4.37393	1.22E-05	2.75E-04	DNAAF9	25943	dynein axonemal assembly factor 9
ENSG0000	969.8366	0.424607	0.097104	4.37273	1.23E-05	2.76E-04	KMT5A	387893	lysine methyltransferase 5A
ENSG0000	5910.814	0.366824	0.083896	4.37235	1.23E-05	0.000276	NPM1	4869	nucleophosmin 1
ENSG0000	548.0984	-0.55194	0.126264	-4.37132	1.23E-05	2.77E-04	EPHB6	2051	EPH receptor B6
ENSG0000	327.5649	-0.63485	0.14524	-4.37105	1.24E-05	2.77E-04	EVI5L	115704	ecotropic viral integration site 5 like
ENSG0000	991.0293	-0.43574	0.099724	-4.3695	1.25E-05	2.79E-04	ZFYVE26	23503	zinc finger FYVE-type containing 26
ENSG0000	7159.623	0.455103	0.1042	4.367582	1.26E-05	2.81E-04	METRNL	284207	meteorin l glial cell differentiation regulator
ENSG0000	453.5941	0.631137	0.144549	4.366246	1.26E-05	2.82E-04	PRR7	80758	proline ric synaptic
ENSG0000	626.7866	-0.56642	0.129759	-4.36515	1.27E-05	2.83E-04	ANKS1A	23294	ankyrin repeat and sterile alpha motif domain containing 1A
ENSG0000	1003.331	0.449957	0.103077	4.36524	1.27E-05	2.83E-04	TRGC2	6967	T cell receptor gamma constant 2
ENSG0000	1515.696	0.544392	0.124866	4.359809	1.30E-05	2.90E-04	PTGER2	5732	prostaglandin E receptor 2
ENSG0000	2083.542	0.412826	0.094693	4.35963	1.30E-05	2.90E-04	SREBF1	6720	sterol regulatory element binding transcription factor 1
ENSG0000	822.7659	0.468644	0.107566	4.356806	1.32E-05	2.93E-04	SLC30A7	148867	solute carrier family 30 member 7
ENSG0000	2601.395	-0.3369	0.077333	-4.35641	1.32E-05	2.93E-04	DOCK2	1794	dedicator of cytokinesis 2
ENSG0000	7834.47	0.355442	0.081676	4.35187	1.35E-05	2.99E-04	ATP1B3	483	ATPase Na+/K+ transporting subunit beta 3
ENSG0000	5389.962	0.343671	0.078987	4.351005	1.36E-05	3.00E-04	PLK3	1263	polo like kinase 3
ENSG0000	538.2031	0.75122	0.172652	4.351058	1.35E-05	3.00E-04	NRP2	8828	neuropilin 2
ENSG0000	544.2041	0.575411	0.132256	4.35075	1.36E-05	3.00E-04	SIGLEC5	8778	sialic acid binding Ig like lectin 5
ENSG0000	1755.425	-0.42677	0.098131	-4.34897	1.37E-05	3.01E-04	RGS14	10636	regulator of G protein signaling 14
ENSG0000	849.3463	-0.43561	0.100165	-4.34891	1.37E-05	3.01E-04	C2CD3	26005	C2 domain containing 3 centriole elongation regulator
ENSG0000	302.602	-0.77663	0.178591	-4.34866	1.37E-05	3.01E-04	CADM1	23705	cell adhesion molecule 1
ENSG0000	4525.672	-0.49849	0.114635	-4.34847	1.37E-05	3.01E-04	FOS	2353	Fos proto- AP-1 transcription factor subunit
ENSG0000	31.34494	2.943869	0.677131	4.347559	1.38E-05	3.02E-04	GJB2	2706	gap junction protein beta 2
ENSG0000	2458.841	0.352308	0.081031	4.347805	1.38E-05	3.02E-04	RPS6KA3	6197	ribosomal protein S6 kinase A3
ENSG0000	2003.603	-0.43932	0.101179	-4.34204	1.41E-05	3.08E-04	FOXN3	1112	forkhead box N3
ENSG0000	333.9965	-0.6626	0.152597	-4.34211	1.41E-05	3.08E-04	MYO9A	4649	myosin IXA
ENSG0000	586.0348	-0.57727	0.132937	-4.34244	1.41E-05	3.08E-04	SLC12A4	6560	solute carrier family 12 member 4
ENSG0000	6836.254	0.308368	0.071064	4.339272	1.43E-05	3.12E-04	LYN	4067	LYN proto- Src family tyrosine kinase
ENSG0000	166.2462	-0.88679	0.204392	-4.33867	1.43E-05	3.12E-04	NUDT16	131870	nudix hydrolase 16
ENSG0000	2098.089	-0.36965	0.085194	-4.33893	1.43E-05	3.12E-04	MAGED2	10916	MAGE family member D2
ENSG0000	12142.46	0.407258	0.093894	4.337441	1.44E-05	3.13E-04	STAT3	6774	signal transducer and activator of transcription 3
ENSG0000	1484.866	0.60842	0.140297	4.336642	1.45E-05	3.13E-04	PLAC8	51316	placenta associated 8
ENSG0000	3409.331	-0.33581	0.077432	-4.33686	1.45E-05	3.13E-04	NOL4L	140688	nucleolar protein 4 like
ENSG0000	8905.809	-0.36818	0.084894	-4.33698	1.44E-05	3.13E-04	TYMP	1890	thymidine phosphorylase
ENSG0000	1201.075	0.420123	0.096924	4.334563	1.46E-05	3.16E-04	KRAS	3845	KRAS prot GTPase
ENSG0000	1424.146	-0.40409	0.093255	-4.3332	1.47E-05	3.18E-04	GNAO1	2775	G protein subunit alpha o1
ENSG0000	1171.406	0.40372	0.093199	4.331806	1.48E-05	3.19E-04	STRAP	11171	serine/threonine kinase receptor associated protein
ENSG0000	96.99323	1.335181	0.30847	4.328405	1.50E-05	3.24E-04	DENND3-A	1.02E+08	DENND3 antisense RNA 1
ENSG0000	9.242464	-4.81176	1.111991	-4.32716	1.51E-05	3.25E-04	ENHO	375704	energy homeostasis associated
ENSG0000	163.0611	0.948634	0.219274	4.326258	1.52E-05	3.26E-04	CEACAM1	634	CEA cell adhesion molecule 1
ENSG0000	5501.287	0.330897	0.076563	4.321901	1.55E-05	3.32E-04	INSIG1	3638	insulin induced gene 1
ENSG0000	888.2288	-0.68604	0.158772	-4.32094	1.55E-05	3.34E-04	TLR1	7096	toll like receptor 1
ENSG0000	250.6764	-0.88118	0.204009	-4.3193	1.57E-05	3.36E-04	TMEM170	1E+08	transmembrane protein 170B
ENSG0000	14062.22	0.297758	0.068971	4.317156	1.58E-05	3.38E-04	TPM3	7170	tropomyosin 3
ENSG0000	1056.337	0.498414	0.115444	4.317361	1.58E-05	3.38E-04	SPAG4	6676	sperm associated antigen 4
ENSG0000	334.1768	-0.5995	0.138889	-4.31636	1.59E-05	3.39E-04	AMIGO1	57463	adhesion molecule with Ig like domain 1
ENSG0000	143527.9	0.418503	0.09699	4.314927	1.60E-05	3.41E-04	TIMP1	7076	TIMP metallopeptidase inhibitor 1
ENSG0000	2025.4	0.360372	0.08357	4.312211	1.62E-05	3.45E-04	SYAP1	94056	synapse associated protein 1
ENSG0000	913.4142	0.474562	0.110228	4.305286	1.67E-05	3.55E-04	RLIM	51132	ring finger LIM domain interacting
ENSG0000	1496.617	0.38863	0.090291	4.304213	1.68E-05	3.56E-04	PSMD11	5717	proteasom non-ATPase 11
ENSG0000	6436.427	0.391254	0.090924	4.303068	1.68E-05	3.58E-04	BAZ1A	11177	bromodomain adjacent to zinc finger domain 1A
ENSG0000	1982.976	0.470801	0.109447	4.301646	1.70E-05	3.60E-04	GARS1	2617	glycyl-tRNA synthetase 1
ENSG0000	272.2247	-0.68769	0.159884	-4.30122	1.70E-05	3.60E-04	GLT1D1	144423	glycosyltransferase 1 domain containing 1
ENSG0000	1297.945	-0.41577	0.096684	-4.30035	1.71E-05	3.61E-04	CC2D1A	54862	coiled-coil and C2 domain containing 1A
ENSG0000	1141.34	-0.47176	0.109834	-4.29522	1.75E-05	3.69E-04	SNAI3	333929	snail family transcriptional repressor 3
ENSG0000	2168.038	-0.37872	0.088298	-4.28913	1.79E-05	3.79E-04	SMARCA4	6597	SWI/SNF r matrix ass actin dep subfamily member 4
ENSG0000	363.3941	0.663715	0.154871	4.28561	1.82E-05	3.85E-04	KLF5	688	KLF transcription factor 5
ENSG0000	758.6376	-0.50741	0.118418	-4.28493	1.83E-05	3.85E-04	ZNF362	149076	zinc finger protein 362
ENSG0000	227.0462	0.807124	0.188362	4.284961	1.83E-05	3.85E-04	SOCS2	8835	suppressor of cytokine signaling 2
ENSG0000	14072.18	0.377126	0.088024	4.284343	1.83E-05	3.85E-04	PEA15	8682	proliferation and apoptosis adaptor protein 15
ENSG0000	9344.305	0.359918	0.084016	4.283923	1.84E-05	3.86E-04	DDIT4	54541	DNA damage inducible transcript 4
ENSG0000	1232.34	0.402884	0.094077	4.282505	1.85E-05	3.87E-04	PPP2R2A	5520	protein phosphatase 2 regulatory subunit Balpha
ENSG0000	2467.978	0.352264	0.08225	4.282857	1.85E-05	3.87E-04	SNHG29	125144	small nucleolar RNA host gene 29
ENSG0000	420.1596	0.696242	0.162578	4.282505	1.85E-05	3.87E-04	PTGIR	5739	prostaglandin I2 receptor
ENSG0000	2357.472	-0.3403	0.079504	-4.28026	1.87E-05	3.90E-04	ARAP3	64411	ArfGAP wi ankyrin repeat and PH domain 3
ENSG0000	3383.752	0.357653	0.083581	4.279122	1.88E-05	3.92E-04	PIK3AP1	118788	phosphoinositide-3-kinase adaptor protein 1
ENSG0000	143.3337	-1.04502	0.244257	-4.27837	1.88E-05	3.93E-04	CPEB3	22849	cytoplasmic polyadenylation element binding protein 3
ENSG0000	3631.47	-0.37003	0.086624	-4.27167	1.94E-05	4.04E-04	PFKL	5211	phosphofr liver type
ENSG0000	8710.571	0.297984	0.069756	4.271789	1.94E-05	4.04E-04	PGK1	5230	phosphoglycerate kinase 1
ENSG0000	744.3074	-0.458	0.107232	-4.27114	1.94E-05	4.04E-04	SUFU	51684	SUFU negative regulator of hedgehog signaling
ENSG0000	24.1003	2.84632	0.666442	4.270921	1.95E-05	4.04E-04	C1QTNF1	114897	C1q and TNF related 1
ENSG0000	2112.409	0.388543	0.09101	4.269252	1.96E-05	4.07E-04	GSTO1	9446	glutathione S-transferase omega 1
ENSG0000	2198.327	0.532322	0.124711	4.268457	1.97E-05	4.08E-04	TNFSF14	8740	TNF superfamily member 14
ENSG0000	1416.938	0.403984	0.094709	4.265524	1.99E-05	4.13E-04	HAPSTR1	29035	HUWE1 associated protein modifying stress responses
ENSG0000	2666.503	0.335168	0.078595	4.264518	2.00E-05	4.13E-04	RAB10	10890	RAB10 member RAS oncogene family
ENSG0000	2371.801	1.087746	0.255056	4.264741	2.00E-05	4.13E-04	RIPK2	8767	receptor interacting serine/threonine kinase 2
ENSG0000	266.9288	-0.69237	0.162357	-4.26448	2.00E-05	4.13E-04	ZNF518A	9849	zinc finger protein 518A

ENSG0000	12656.78	0.321215	0.07533	4.264112	2.01E-05	4.14E-04	RASSF5	83593	Ras association domain family member 5
ENSG0000	45.35391	-1.91791	0.450118	-4.2609	2.04E-05	4.19E-04	NA	NA	NA
ENSG0000	54079.8	0.311139	0.073028	4.260567	2.04E-05	4.19E-04	PKM	5315	pyruvate kinase M1/2
ENSG0000	2165.432	0.388181	0.091193	4.256712	2.07E-05	4.26E-04	ELOVL5	60481	ELOVL fatty acid elongase 5
ENSG0000	6203.607	0.316566	0.074405	4.25465	2.09E-05	4.30E-04	TAB2	23118	TGF-beta activated kinase 1 (MAP3K7) binding protein 2
ENSG0000	3873.062	-0.3734	0.087773	-4.25417	2.10E-05	4.30E-04	UCP2	7351	uncoupling protein 2
ENSG0000	1161.535	1.619401	0.380768	4.252982	2.11E-05	4.32E-04	MMP9	4318	matrix metallopeptidase 9
ENSG0000	2366.956	0.34586	0.081511	4.243118	2.20E-05	4.51E-04	GPBP1	65056	GC-rich promoter binding protein 1
ENSG0000	610.2919	-0.82355	0.194135	-4.24217	2.21E-05	4.52E-04	NA	NA	NA
ENSG0000	946.9253	-0.45598	0.107488	-4.24213	2.21E-05	4.52E-04	SH3BP5-A	1.01E+08	SH3BP5 antisense RNA 1
ENSG0000	1421.085	0.43053	0.101519	4.240903	2.23E-05	4.54E-04	ARID3A	1820	AT-rich interaction domain 3A
ENSG0000	1308.435	0.433259	0.102184	4.239984	2.24E-05	4.55E-04	KIR3DL1	3811	killer cell i three Ig domains and long cytoplasmic tail 1
ENSG0000	916.5405	0.444492	0.104916	4.236659	2.27E-05	4.61E-04	RAB18	22931	RAB18 member RAS oncogene family
ENSG0000	2432.006	0.394768	0.093197	4.235837	2.28E-05	4.63E-04	FGFR1	2260	fibroblast growth factor receptor 1
ENSG0000	15.7867	3.688458	0.871566	4.231988	2.32E-05	4.70E-04	NA	NA	NA
ENSG0000	6918.801	0.333039	0.078731	4.230094	2.34E-05	4.73E-04	SRRM1	10250	serine and arginine repetitive matrix 1
ENSG0000	541.2883	-0.55443	0.13108	-4.22971	2.34E-05	4.74E-04	EEFSEC	60678	eukaryotic selenocysteine-tRNA specific
ENSG0000	769.5236	-0.48305	0.114284	-4.22677	2.37E-05	4.79E-04	SEPHS2	22928	selenophosphate synthetase 2
ENSG0000	934.1144	0.483069	0.11431	4.225955	2.38E-05	4.81E-04	TBK1	29110	TANK binding kinase 1
ENSG0000	3873.678	0.355584	0.084175	4.224357	2.40E-05	4.83E-04	STAT4	6775	signal transducer and activator of transcription 4
ENSG0000	597.6532	0.565677	0.133911	4.224261	2.40E-05	4.83E-04	SMNDC1	10285	survival motor neuron domain containing 1
ENSG0000	6367.239	0.334207	0.079132	4.223402	2.41E-05	4.85E-04	TESK1	7016	testis associated actin remodelling kinase 1
ENSG0000	1989.605	0.426476	0.101088	4.218857	2.46E-05	4.94E-04	PDE4DIP	9659	phosphodiesterase 4D interacting protein
ENSG0000	13047.33	0.346762	0.082208	4.218122	2.46E-05	4.95E-04	PER1	5187	period circadian regulator 1
ENSG0000	647.205	-0.48008	0.11399	-4.2116	2.54E-05	5.09E-04	PLOD1	5351	procollage 2-oxoglutarate 5-dioxygenase 1
ENSG0000	296.2649	-0.65458	0.155472	-4.21029	2.55E-05	5.11E-04	ECHS1	1892	enoyl-CoA short chain 1
ENSG0000	160.6507	0.818303	0.19447	4.20787	2.58E-05	5.16E-04	INPP4B	8821	inositol polyphosphate-4-phosphatase type II B
ENSG0000	5311.344	0.330971	0.07866	4.207614	2.58E-05	5.16E-04	XPO6	23214	exportin 6
ENSG0000	2321.381	0.50037	0.118955	4.206395	2.59E-05	5.19E-04	NECTIN2	5819	nectin cell adhesion molecule 2
ENSG0000	3299.398	0.394468	0.093825	4.204318	2.62E-05	5.23E-04	NFKBID	84807	NFKB inhibitor delta
ENSG0000	6437.236	0.322741	0.076773	4.203825	2.62E-05	5.23E-04	SUPT5H	6829	SPT5 hom DSIF elongation factor subunit
ENSG0000	1236.075	-0.45679	0.108686	-4.20283	2.64E-05	5.25E-04	MTHFR	4524	methylenetetrahydrofolate reductase
ENSG0000	82.24645	-1.42605	0.339426	-4.20136	2.65E-05	5.28E-04	TMEM150	284417	transmembrane protein 150B
ENSG0000	1875.074	-0.43665	0.103982	-4.19925	2.68E-05	5.32E-04	NEK6	10783	NIMA related kinase 6
ENSG0000	3195.842	-0.485	0.115491	-4.19945	2.68E-05	5.32E-04	SNX20	124460	sorting nexin 20
ENSG0000	35495.16	0.366337	0.087244	4.19897	2.68E-05	5.32E-04	RPS27	6232	ribosomal protein S27
ENSG0000	142.0444	0.913117	0.217535	4.197566	2.70E-05	5.35E-04	MTCO3P1	1.07E+08	MT-CO3 pseudogene 12
ENSG0000	63.45373	1.496011	0.356652	4.1946	2.73E-05	5.41E-04	TRIM36	55521	tripartite motif containing 36
ENSG0000	205.8076	-0.9029	0.215281	-4.19408	2.74E-05	5.41E-04	DHX57	90957	DExH-box helicase 57
ENSG0000	1879.921	0.343716	0.081952	4.194135	2.74E-05	5.41E-04	GRK5	2869	G protein-coupled receptor kinase 5
ENSG0000	1445.376	-0.48598	0.115945	-4.19144	2.77E-05	5.47E-04	DOCK5	80005	dedicator of cytokinesis 5
ENSG0000	9264.264	0.294068	0.070178	4.190336	2.79E-05	5.49E-04	RPL23	9349	ribosomal protein L23
ENSG0000	148.7803	-1.18346	0.282457	-4.18987	2.79E-05	5.50E-04	CAVIN1	284119	caveolae associated protein 1
ENSG0000	1346.968	-0.45233	0.107975	-4.18916	2.80E-05	5.50E-04	EIF4G3	8672	eukaryotic translation initiation factor 4 gamma 3
ENSG0000	478.8396	0.667165	0.159257	4.189239	2.80E-05	5.50E-04	TRAF3IP2	10758	TRAF3 interacting protein 2
ENSG0000	9240.438	0.291883	0.069726	4.186153	2.84E-05	5.57E-04	RPL9	6133	ribosomal protein L9
ENSG0000	9991.778	0.336398	0.080375	4.185366	2.85E-05	5.58E-04	HSP90AB1	3326	heat shock protein 90 alpha family class B member 1
ENSG0000	11375.91	-0.29613	0.070764	-4.18478	2.85E-05	5.59E-04	EVL	51466	Enah/Vasp-like
ENSG0000	314.5571	-0.71758	0.171484	-4.18452	2.86E-05	5.59E-04	PRDM15	63977	PR/SET domain 15
ENSG0000	24543.23	0.324459	0.077556	4.183546	2.87E-05	5.61E-04	EIF4G2	1982	eukaryotic translation initiation factor 4 gamma 2
ENSG0000	1080.587	0.478107	0.114311	4.182533	2.88E-05	5.63E-04	DUSP8	1850	dual specificity phosphatase 8
ENSG0000	1176.883	0.418084	0.100044	4.179017	2.93E-05	5.71E-04	WDR48	57599	WD repeat domain 48
ENSG0000	551.1596	0.584299	0.139836	4.178445	2.94E-05	5.72E-04	CHIC2	26511	cysteine rich hydrophobic domain 2
ENSG0000	569.6535	0.470232	0.112547	4.178097	2.94E-05	5.72E-04	TATDN2	9797	TatD DNase domain containing 2
ENSG0000	2565.225	0.456922	0.109373	4.177665	2.95E-05	5.73E-04	B3GNT5	84002	UDP-GlcN 3-N-acetylglucosaminyltransferase 5
ENSG0000	104.1277	1.125986	0.269728	4.174524	2.99E-05	5.80E-04	NA	NA	NA
ENSG0000	1634.578	0.416918	0.099921	4.172472	3.01E-05	5.85E-04	POLR3E	55718	RNA polymerase III subunit E
ENSG0000	130.9875	-0.97724	0.234309	-4.17071	3.04E-05	5.87E-04	GPR162	27239	G protein-coupled receptor 162
ENSG0000	3589.153	0.308981	0.074077	4.171085	3.03E-05	5.87E-04	LGALS3	3958	galectin 3
ENSG0000	564.0745	0.520508	0.124803	4.170626	3.04E-05	5.87E-04	PSMA3	5684	proteasome 20S subunit alpha 3
ENSG0000	769.2369	-0.48578	0.116496	-4.16996	3.05E-05	5.89E-04	ATRN	8455	attractin
ENSG0000	1284.143	-0.3972	0.095321	-4.16701	3.09E-05	5.96E-04	LLGL1	3996	LLGL scribble cell polarity complex component 1
ENSG0000	8032.515	0.298982	0.07176	4.166426	3.09E-05	5.97E-04	MIDN	90007	midnolin
ENSG0000	5438.385	0.325656	0.078172	4.165873	3.10E-05	5.97E-04	RBM39	9584	RNA binding motif protein 39
ENSG0000	1992.644	0.407041	0.097785	4.162608	3.15E-05	6.05E-04	PDE4D	5144	phosphodiesterase 4D
ENSG0000	632.7098	-0.4926	0.118375	-4.16137	3.16E-05	6.08E-04	ANKMY1	51281	ankyrin repeat and MYND domain containing 1
ENSG0000	208.7641	-0.83425	0.20059	-4.15897	3.20E-05	6.14E-04	CD36	948	CD36 molecule
ENSG0000	11176.06	0.355914	0.085609	4.157433	3.22E-05	6.17E-04	HSPA5	3309	heat shock protein family A (Hsp70) member 5
ENSG0000	1440.883	-0.43743	0.105301	-4.15412	3.27E-05	6.26E-04	PRKDC	5591	protein kir DNA-activ catalytic subunit
ENSG0000	564.6153	-0.55551	0.133759	-4.15305	3.28E-05	6.28E-04	INCENP	3619	inner centromere protein
ENSG0000	1382.293	-0.39408	0.094937	-4.15093	3.31E-05	6.33E-04	VPS13D	55187	vacuolar protein sorting 13 homolog D
ENSG0000	414.4304	-0.55005	0.132522	-4.15063	3.32E-05	6.33E-04	ABCC5	10057	ATP binding cassette subfamily C member 5
ENSG0000	727.067	0.560747	0.135251	4.145962	3.38E-05	6.46E-04	IL12RB2	3595	interleukin 12 receptor subunit beta 2
ENSG0000	1255.244	-0.43554	0.105101	-4.14405	3.41E-05	6.50E-04	CAMK4	814	calcium/calmodulin dependent protein kinase IV
ENSG0000	991.507	0.492482	0.118864	4.143257	3.42E-05	6.52E-04	KLHL24	54800	kelch like family member 24
ENSG0000	1563.478	-0.43653	0.105369	-4.14287	3.43E-05	6.52E-04	RAB11FIP	9727	RAB11 family interacting protein 3
ENSG0000	6568.504	0.353014	0.085239	4.141475	3.45E-05	6.56E-04	UPP1	7378	uridine phosphorylase 1
ENSG0000	906.2803	-0.52106	0.125869	-4.13971	3.48E-05	6.60E-04	TECPR1	25851	tectonin beta-propeller repeat containing 1
ENSG0000	6036.882	0.329442	0.079593	4.139105	3.49E-05	6.61E-04	ACTR3	10096	actin related protein 3
ENSG0000	310.4002	0.74192	0.179334	4.13708	3.52E-05	6.66E-04	XCL1	6375	X-C motif chemokine ligand 1
ENSG0000	7691.481	0.358159	0.08658	4.136735	3.52E-05	6.67E-04	CXCL16	58191	C-X-C motif chemokine ligand 16
ENSG0000	3837.336	-0.36978	0.089395	-4.1365	3.53E-05	6.67E-04	LDLRAP1	26119	low density lipoprotein receptor adaptor protein 1
ENSG0000	12468.85	-0.32373	0.078284	-4.13526	3.55E-05	6.70E-04	PACS1	55690	phosphofurin acidic cluster sorting protein 1
ENSG0000	26.66858	-2.87741	0.695884	-4.1349	3.55E-05	6.70E-04	SRRM3	222183	serine/arginine repetitive matrix 3
ENSG0000	7746.582	1.766411	0.427632	4.130677	3.62E-05	6.82E-04	TNF	7124	tumor necrosis factor
ENSG0000	1070.087	0.446718	0.108167	4.129876	3.63E-05	6.83E-04	ANKRD9	122416	ankyrin repeat domain 9
ENSG0000	2998.061	0.425248	0.103072	4.125753	3.70E-05	6.95E-04	NFKBIB	4793	NFKB inhibitor beta
ENSG0000	538.6088	-0.58345	0.141434	-4.12522	3.70E-05	6.96E-04	SNPH	9751	syntaphilin
ENSG0000	305.6428	-0.61354	0.148756	-4.12446	3.72E-05	6.98E-04	DEPDC5	9681	DEP doma GATOR1 subcomplex subunit
ENSG0000	511470.5	0.440813	0.106973	4.120807	3.78E-05	7.08E-04	COX2	4513	cytochrome c oxidase subunit II
ENSG0000	149.5711	-0.9918	0.240737	-4.11984	3.79E-05	7.10E-04	MRC2	9902	mannose receptor C type 2
ENSG0000	1981.387	0.439166	0.106635	4.118406	3.82E-05	7.14E-04	MT2A	4502	metallothionein 2A
ENSG0000	2151.228	0.381136	0.092586	4.116573	3.85E-05	7.19E-04	ZBTB1	22890	zinc finger and BTB domain containing 1
ENSG0000	6961.526	-0.34527	0.083923	-4.11416	3.89E-05	7.26E-04	WAS	7454	WASP actin nucleation promoting factor
ENSG0000	1394.423	0.416197	0.101182	4.113374	3.90E-05	7.28E-04	GOLGA7	51125	golgin A7
ENSG0000	4229.453	0.324385	0.078913	4.110686	3.94E-05	7.35E-04	YY1AP1	55249	YY1 associated protein 1
ENSG0000	949.7179	0.45055	0.109763	4.104754	4.05E-05	7.54E-04	CD28	940	CD28 molecule
ENSG0000	1945.198	0.359482	0.087583	4.104472	4.05E-05	7.54E-04	H1-10	8971	H1.10 linker histone
ENSG0000	5615.334	0.331555	0.08082	4.102402	4.09E-05	7.60E-04	ORAI1	84876	ORAI calcium release-activated calcium modulator 1
ENSG0000	1817.784	-0.34932	0.08516	-4.1019	4.10E-05	7.60E-04	DCTN1	1639	dynactin subunit 1

ENSG0000	8735.541	-1.09985	0.26814	-4.10177	4.10E-05	7.60E-04	KCTD12	115207	potassium channel tetramerization domain containing 12
ENSG0000	5616.006	0.325035	0.079266	4.100549	4.12E-05	7.64E-04	IGKC	3514	immunoglobulin kappa constant
ENSG0000	4291.44	0.344518	0.084068	4.098061	4.17E-05	7.71E-04	MYC	4609	MYC protc bHLH transcription factor
ENSG0000	68.30503	-1.36167	0.332629	-4.09365	4.25E-05	7.85E-04	NA	NA	NA
ENSG0000	2216.771	-0.39583	0.096721	-4.09248	4.27E-05	7.88E-04	BMF	90427	Bcl2 modifying factor
ENSG0000	113.6016	0.931553	0.227821	4.088966	4.33E-05	8.00E-04	C11orf96	387763	chromosome 11 open reading frame 96
ENSG0000	2817.241	0.357465	0.087467	4.086855	4.37E-05	8.05E-04	LHFPL2	10184	LHFPL tetraspan subfamily member 2
ENSG0000	742.3596	0.482475	0.118057	4.086819	4.37E-05	8.05E-04	FCER2	2208	Fc epsilon receptor II
ENSG0000	4111.501	-1.26818	0.310367	-4.08608	4.39E-05	8.07E-04	CIITA	4261	class II major histocompatibility complex transactivator
ENSG0000	96838.46	0.420408	0.102975	4.082615	4.45E-05	8.19E-04	ZFP36	7538	ZFP36 ring finger protein
ENSG0000	338.2021	-0.73076	0.179065	-4.08097	4.48E-05	8.23E-04	RGS12	6002	regulator of G protein signaling 12
ENSG0000	11921.77	0.37046	0.090781	4.080815	4.49E-05	8.23E-04	PHLDA1	22822	pleckstrin homology like domain family A member 1
ENSG0000	850.4912	-0.43558	0.106746	-4.08053	4.49E-05	8.23E-04	PSMB8-AS	1.01E+08	PSMB8 antisense RNA 1 (head to head)
ENSG0000	1083.882	0.410622	0.10064	4.080126	4.50E-05	8.24E-04	STARD10	10809	StAR related lipid transfer domain containing 10
ENSG0000	1765.171	-0.33807	0.082871	-4.07949	4.51E-05	8.26E-04	MCM3AP	8888	minichromosome maintenance complex component 3 associated protein
ENSG0000	5099.035	0.379157	0.092973	4.078148	4.54E-05	8.30E-04	STAT5A	6776	signal transducer and activator of transcription 5A
ENSG0000	6354.896	0.292308	0.071685	4.077679	4.55E-05	8.30E-04	PITPNC1	26207	phosphatidylinositol transfer protein cytoplasmic 1
ENSG0000	1135.003	0.421608	0.103439	4.075905	4.58E-05	8.36E-04	ZC3H7A	29066	zinc finger CCCH-type containing 7A
ENSG0000	262.7569	-0.71375	0.175182	-4.07432	4.61E-05	8.41E-04	SLC16A5	9121	solute carrier family 16 member 5
ENSG0000	4809.973	-0.30802	0.075649	-4.07169	4.67E-05	8.49E-04	FHOD1	29109	formin homology 2 domain containing 1
ENSG0000	423.1514	0.577242	0.141809	4.070547	4.69E-05	8.53E-04	ELOC	6921	elongin C
ENSG0000	23.76294	1.965311	0.482898	4.069829	4.70E-05	8.55E-04	LINC0059!	414243	long intergenic non-protein coding RNA 595
ENSG0000	863.5345	-0.43998	0.108162	-4.06776	4.75E-05	8.61E-04	ATXN7L1	222255	ataxin 7 like 1
ENSG0000	6801.256	0.40515	0.099633	4.066428	4.77E-05	8.66E-04	HCK	3055	HCK proto Src family tyrosine kinase
ENSG0000	10149.71	0.321504	0.079131	4.062928	4.85E-05	8.76E-04	HDGF	3068	heparin binding growth factor
ENSG0000	218.4255	0.785958	0.193451	4.06282	4.85E-05	8.76E-04	ABHD17C	58489	abhydrola: depalmitoylase
ENSG0000	73.37013	-1.27891	0.314777	-4.0629	4.85E-05	8.76E-04	POLA1	5422	DNA polyn catalytic subunit
ENSG0000	2511.364	-0.52612	0.129522	-4.06205	4.86E-05	8.78E-04	NOD2	64127	nucleotide binding oligomerization domain containing 2
ENSG0000	105.5953	1.474689	0.3631	4.061389	4.88E-05	8.80E-04	NA	NA	NA
ENSG0000	1855.254	0.394302	0.097129	4.059554	4.92E-05	8.86E-04	ARPP19	10776	cAMP regulated phosphoprotein 19
ENSG0000	3636.515	0.321093	0.079104	4.059109	4.93E-05	8.87E-04	ESYT2	57488	extended synaptotagmin 2
ENSG0000	4509.658	0.510211	0.125714	4.058494	4.94E-05	8.88E-04	MXD1	4084	MAX dimerization protein 1
ENSG0000	990.3724	0.486491	0.119959	4.055478	5.00E-05	8.99E-04	ZNF503	84858	zinc finger protein 503
ENSG0000	1981.353	0.417104	0.102856	4.055243	5.01E-05	8.99E-04	LPAR2	9170	lysophosphatidic acid receptor 2
ENSG0000	5084.339	0.36486	0.090078	4.050471	5.11E-05	9.17E-04	VCP	7415	valosin containing protein
ENSG0000	3193.717	0.318966	0.078765	4.049587	5.13E-05	9.19E-04	BCL7B	9275	BAF chromatin remodeling complex subunit BCL7B
ENSG0000	6206.422	-0.34691	0.085668	-4.0495	5.13E-05	9.19E-04	TMC6	11322	transmembrane channel like 6
ENSG0000	145.8662	-0.87579	0.21629	-4.04917	5.14E-05	9.19E-04	OLFM2	93145	olfactomedin 2
ENSG0000	150.9801	-0.88415	0.21848	-4.04682	5.19E-05	9.28E-04	TMEM52B	120939	transmembrane protein 52B
ENSG0000	4313.557	0.356465	0.088205	4.041322	5.32E-05	9.49E-04	CLK1	1195	CDC like kinase 1
ENSG0000	3846.147	0.409758	0.101419	4.04026	5.34E-05	9.52E-04	AGAP3	116988	ArfGAP wi ankyrin repeat and PH domain 3
ENSG0000	15512.87	0.358266	0.088683	4.039842	5.35E-05	9.53E-04	ADGRE5	976	adhesion G protein-coupled receptor E5
ENSG0000	4632.489	-0.34344	0.085103	-4.03562	5.45E-05	9.69E-04	INTS1	26173	integrator complex subunit 1
ENSG0000	223.3528	-0.67445	0.167137	-4.03535	5.45E-05	9.70E-04	TTF2	8458	transcription termination factor 2
ENSG0000	1270.573	0.409089	0.101412	4.033911	5.49E-05	9.75E-04	KBTBD2	25948	kelch repeat and BTB domain containing 2
ENSG0000	4410.342	0.344401	0.085425	4.031616	5.54E-05	9.82E-04	PCBP2	5094	poly(rC) binding protein 2
ENSG0000	72.68326	-1.28551	0.318853	-4.03166	5.54E-05	9.82E-04	SIAH3	283514	siah E3 ubiquitin protein ligase family member 3
ENSG0000	528.8254	-0.48491	0.120421	-4.02679	5.65E-05	0.001002	ALDH2	217	aldehyde dehydrogenase 2 family member
ENSG0000	224.6926	-0.74862	0.185935	-4.02626	5.67E-05	0.001003	RPS6KA2	6196	ribosomal protein S6 kinase A2
ENSG0000	20.51542	-2.39084	0.593869	-4.02587	5.68E-05	0.001003	NA	NA	NA
ENSG0000	2339.984	0.388042	0.096382	4.026085	5.67E-05	0.001003	UBE2A	7319	ubiquitin conjugating enzyme E2 A
ENSG0000	123.6009	0.922419	0.229306	4.022652	5.75E-05	0.001016	IL1RL1	9173	interleukin 1 receptor like 1
ENSG0000	22444.07	-0.29496	0.073352	-4.02115	5.79E-05	0.001021	ARHGEF1	9138	Rho guanine nucleotide exchange factor 1
ENSG0000	512.0138	0.573496	0.142683	4.019366	5.84E-05	0.001028	SGTB	54557	small glutamine rich tetratricopeptide repeat co-chaperone beta
ENSG0000	4393.775	0.331576	0.082516	4.018332	5.86E-05	0.001031	RAB5C	5878	RAB5C member RAS oncogene family
ENSG0000	145.6652	-0.85368	0.21267	-4.01412	5.97E-05	0.001049	ENOX2	10495	ecto-NOX disulfide-thiol exchanger 2
ENSG0000	1135.371	-0.49153	0.122492	-4.01273	6.00E-05	0.001054	DAPK1	1612	death associated protein kinase 1
ENSG0000	155.0726	-1.03408	0.25777	-4.01166	6.03E-05	0.001057	NA	NA	NA
ENSG0000	970.8104	0.405406	0.101053	4.011799	6.03E-05	0.001057	OTULIN	90268	OTU deubiquitinase with linear linkage specificity
ENSG0000	453.0273	-0.50469	0.125832	-4.01086	6.05E-05	0.001059	CLIC5	53405	chloride intracellular channel 5
ENSG0000	197.0284	-0.7913	0.19728	-4.01106	6.04E-05	0.001059	ADK	132	adenosine kinase
ENSG0000	3024.208	0.306537	0.076458	4.009202	6.09E-05	0.001065	PSMB4	5692	proteasome 20S subunit beta 4
ENSG0000	1019.362	1.250322	0.311914	4.008548	6.11E-05	0.001065	PRDM8	56978	PR/SET domain 8
ENSG0000	85.24795	-1.09039	0.271996	-4.00885	6.10E-05	0.001065	MARCHF1	55016	membrane associated ring-CH-type finger 1
ENSG0000	5782.259	0.310082	0.077351	4.008743	6.10E-05	0.001065	MTCH1	23787	mitochondrial carrier 1
ENSG0000	672.369	0.558829	0.139468	4.006874	6.15E-05	0.001072	IL1RAP	3556	interleukin 1 receptor accessory protein
ENSG0000	396.4474	-0.58178	0.145271	-4.00482	6.21E-05	0.00108	C1QTNF6	114904	C1q and TNF related 6
ENSG0000	6903.746	0.297585	0.074317	4.004252	6.22E-05	0.001081	GABARAPI	23710	GABA type A receptor associated protein like 1
ENSG0000	2032.075	-0.39213	0.097953	-4.00322	6.25E-05	0.001085	INPP4A	3631	inositol polyphosphate-4-phosphatase type I A
ENSG0000	348.5543	-0.71947	0.179775	-4.00206	6.28E-05	0.001089	CDK19	23097	cyclin dependent kinase 19
ENSG0000	1177.035	-0.4101	0.102554	-3.99888	6.36E-05	0.001103	MCM5	4174	minichromosome maintenance complex component 5
ENSG0000	46.3487	-1.54357	0.386108	-3.99776	6.39E-05	0.001107	HGF	3082	hepatocyte growth factor
ENSG0000	6380.076	0.388867	0.097281	3.997365	6.41E-05	0.001108	RPS12	6206	ribosomal protein S12
ENSG0000	233.4813	-0.6571	0.164506	-3.9944	6.49E-05	0.001121	INKA2	55924	inka box actin regulator 2
ENSG0000	4485.889	-0.3731	0.093547	-3.98834	6.65E-05	0.001149	SLFN5	162394	schlafen family member 5
ENSG0000	17.05748	-2.92636	0.73385	-3.98768	6.67E-05	0.001151	NA	NA	NA
ENSG0000	236.1596	-0.80416	0.201791	-3.98514	6.74E-05	0.001162	NA	NA	NA
ENSG0000	4200.796	-0.32448	0.081443	-3.98416	6.77E-05	0.001166	PI4KA	5297	phosphatidylinositol 4-kinase alpha
ENSG0000	112.0596	0.921485	0.231351	3.983066	6.80E-05	0.00117	NBPF10	1E+08	NBPF member 10
ENSG0000	2398.84	-0.30803	0.077358	-3.98195	6.84E-05	0.001175	GIT2	9815	GIT ArfGAP 2
ENSG0000	3562.871	-0.33074	0.083091	-3.98047	6.88E-05	0.001181	WHRN	25861	whirlin
ENSG0000	520.5229	-0.49783	0.125075	-3.98027	6.88E-05	0.001181	SHTN1	57698	shootin 1
ENSG0000	6658.127	-0.40571	0.102003	-3.97739	6.97E-05	0.001194	GLUL	2752	glutamate-ammonia ligase
ENSG0000	784.3121	-0.4649	0.116967	-3.97465	7.05E-05	0.001205	THEM4	117145	thioesterase superfamily member 4
ENSG0000	916.8626	0.40017	0.100672	3.974983	7.04E-05	0.001205	RAP1GAP2	23108	RAP1 GTPase activating protein 2
ENSG0000	36.79046	2.046257	0.514819	3.974712	7.05E-05	0.001205	NA	NA	NA
ENSG0000	226.2694	-0.68206	0.171769	-3.97081	7.16E-05	0.001223	KLHL22	84861	kelch like family member 22
ENSG0000	87.6003	-1.23487	0.311037	-3.97017	7.18E-05	0.001225	KCNK13	56659	potassium two pore domain channel subfamily K member 13
ENSG0000	733.9442	0.47322	0.119242	3.968582	7.23E-05	0.001228	TCF7L2	6934	transcription factor 7 like 2
ENSG0000	550.4219	-0.48742	0.122796	-3.96938	7.21E-05	0.001228	PDK2	5164	pyruvate dehydrogenase kinase 2
ENSG0000	4218.146	0.316966	0.079861	3.968953	7.22E-05	0.001228	SEC14L1	6397	SEC14 like lipid binding 1
ENSG0000	735.3612	-0.49464	0.12464	-3.96856	7.23E-05	0.001228	DYM	54808	dymeclin
ENSG0000	389.8272	-0.52165	0.131446	-3.96859	7.23E-05	0.001228	ATXN10	25814	ataxin 10
ENSG0000	3110.188	-0.3483	0.087785	-3.9677	7.26E-05	0.001231	CLIC3	9022	chloride intracellular channel 3
ENSG0000	43599.2	0.294126	0.07418	3.965014	7.34E-05	0.001244	CXCR4	7852	C-X-C motif chemokine receptor 4
ENSG0000	1853.152	0.507041	0.1279	3.964346	7.36E-05	0.001247	SC5D	6309	sterol-C5-desaturase
ENSG0000	2642.419	-0.31714	0.080024	-3.96311	7.40E-05	0.001249	TMEM63A	9725	transmembrane protein 63A
ENSG0000	804.2005	-0.44461	0.112175	-3.96352	7.39E-05	0.001249	VGLL4	9686	vestigial like family member 4
ENSG0000	3092.773	0.328859	0.082982	3.963043	7.40E-05	0.001249	STK17A	9263	serine/threonine kinase 17a
ENSG0000	17512.13	0.315952	0.079717	3.963409	7.39E-05	0.001249	RPS16	6217	ribosomal protein S16

ENSG0000	458.1458	-0.599	0.151158	-3.96273	7.41E-05	0.001249	PPP1R16A	84988	protein phosphatase 1 regulatory subunit 16A
ENSG0000	1130.609	0.399414	0.100832	3.961202	7.46E-05	0.001256	SOD1	6647	superoxide dismutase 1
ENSG0000	1970.251	0.456233	0.11522	3.959662	7.51E-05	0.001263	CHST2	9435	carbohydrate sulfotransferase 2
ENSG0000	164.5483	1.076263	0.271848	3.959057	7.52E-05	0.001265	TRGV5P	6979	T cell receptor gamma variable 5P (pseudogene)
ENSG0000	3134.439	0.307907	0.077809	3.957203	7.58E-05	0.001273	RAF1	5894	Raf-1 prot serine/threonine kinase
ENSG0000	5754.159	0.297524	0.075185	3.95722	7.58E-05	0.001273	SREBF2	6721	sterol regulatory element binding transcription factor 2
ENSG0000	574.3095	0.454906	0.114985	3.956215	7.61E-05	0.001277	OAT	4942	ornithine aminotransferase
ENSG0000	703.2543	0.44658	0.112965	3.95326	7.71E-05	0.001292	MYLIP	29116	myosin regulatory light chain interacting protein
ENSG0000	4342.548	0.38527	0.097471	3.952677	7.73E-05	0.001294	CDC42SE2	56990	CDC42 small effector 2
ENSG0000	3000.165	0.327015	0.082741	3.952275	7.74E-05	0.001295	FUBP1	8880	far upstream element binding protein 1
ENSG0000	1177.638	0.366623	0.092776	3.951725	7.76E-05	0.001295	CYRIA	81553	CYFIP related Rac1 interactor A
ENSG0000	1985.839	-0.44554	0.112743	-3.95184	7.76E-05	0.001295	LILRA6	79168	leukocyte immunoglobulin like receptor A6
ENSG0000	2768.143	0.362835	0.091838	3.950816	7.79E-05	0.001299	TM9SF3	56889	transmembrane 9 superfamily member 3
ENSG0000	35.11215	1.768115	0.448147	3.945392	7.97E-05	0.001328	NA	NA	NA
ENSG0000	1249.011	-0.47756	0.121087	-3.94396	8.01E-05	0.001334	SLC35E4	339665	solute carrier family 35 member E4
ENSG0000	3405.701	-0.33686	0.085428	-3.94318	8.04E-05	0.001338	AP2A1	160	adaptor related protein complex 2 subunit alpha 1
ENSG0000	762.4575	-0.4802	0.121788	-3.94294	8.05E-05	0.001338	ZNF213	7760	zinc finger protein 213
ENSG0000	57.67509	-1.24988	0.317153	-3.94094	8.12E-05	0.001348	SLC2A13	114134	solute carrier family 2 member 13
ENSG0000	188.5389	0.788886	0.200253	3.939455	8.17E-05	0.001355	SPAG5	10615	sperm associated antigen 5
ENSG0000	7999.219	0.300187	0.076227	3.938077	8.21E-05	0.00136	YBX3	8531	Y-box binding protein 3
ENSG0000	224.729	0.698407	0.177342	3.938189	8.21E-05	0.00136	LYRM1	57149	LYR motif containing 1
ENSG0000	3979.831	0.322727	0.08199	3.936179	8.28E-05	0.00137	PABPC4	8761	poly(A) binding protein cytoplasmic 4
ENSG0000	1730.284	0.345342	0.087747	3.93564	8.30E-05	0.001372	WHAMM	123720	WASP hon golgi membranes and microtubules
ENSG0000	204.8092	0.77163	0.196207	3.932743	8.40E-05	0.001387	RIPK2-DT	1.02E+08	RIPK2 divergent transcript
ENSG0000	1004.445	-0.40112	0.102021	-3.93169	8.44E-05	0.001392	RTL6	84247	retrotransposon Gag like 6
ENSG0000	1051.237	0.435909	0.110889	3.93104	8.46E-05	0.001394	RHOC	389	ras homolog family member C
ENSG0000	339.9248	-0.77957	0.198358	-3.93013	8.49E-05	0.001395	FUCA1	2517	alpha-L-fucosidase 1
ENSG0000	11.28684	4.800178	1.221201	3.930702	8.47E-05	0.001395	NA	NA	NA
ENSG0000	509.5172	0.473116	0.120381	3.930161	8.49E-05	0.001395	TTYH2	94015	tweety family member 2
ENSG0000	112.8781	-0.92675	0.23579	-3.93039	8.48E-05	0.001395	DSN1	79980	DSN1 component of MIS12 kinetochore complex
ENSG0000	939.5835	0.481184	0.122528	3.927124	8.60E-05	0.001411	USP12	219333	ubiquitin specific peptidase 12
ENSG0000	196.6799	-0.78145	0.199087	-3.92519	8.67E-05	0.001421	RNF135	84282	ring finger protein 135
ENSG0000	309.1196	0.638206	0.162608	3.924825	8.68E-05	0.001422	SLC19A1	6573	solute carrier family 19 member 1
ENSG0000	1253.65	-0.41914	0.106857	-3.92246	8.76E-05	0.001434	INTS3	65123	integrator complex subunit 3
ENSG0000	5.177949	5.631578	1.43591	3.921957	8.78E-05	0.001434	NA	NA	NA
ENSG0000	16100.08	-0.28619	0.07297	-3.92195	8.78E-05	0.001434	SEPTIN9	10801	septin 9
ENSG0000	521.8548	0.566933	0.144547	3.92213	8.78E-05	0.001434	MAMLD1	10046	mastermind like domain containing 1
ENSG0000	234.8853	-0.70373	0.179467	-3.92124	8.81E-05	0.001436	TIMELESS	8914	timeless circadian regulator
ENSG0000	3822.186	-0.35505	0.090549	-3.92112	8.81E-05	0.001436	TSC2	7249	TSC complex subunit 2
ENSG0000	98.27495	-1.01832	0.259837	-3.91908	8.89E-05	0.001447	DISP1	84976	dispatched RND transporter family member 1
ENSG0000	45.57588	1.462809	0.37355	3.915962	9.00E-05	0.001465	GSDME	1687	gasdermin E
ENSG0000	808.6733	0.467941	0.119581	3.913182	9.11E-05	0.001481	JMY	133746	junction m p53 cofactor
ENSG0000	1851.246	-0.32662	0.083477	-3.91266	9.13E-05	0.001483	TBC1D1	23216	TBC1 domain family member 1
ENSG0000	106.0048	-0.93838	0.239856	-3.91228	9.14E-05	0.001484	CEP162	22832	centrosomal protein 162
ENSG0000	699.2269	-0.42257	0.10807	-3.9101	9.23E-05	0.001496	SLC27A3	11000	solute carrier family 27 member 3
ENSG0000	1068.904	-0.37848	0.096814	-3.90936	9.25E-05	0.001499	FAM234A	83986	family with sequence similarity 234 member A
ENSG0000	18380.81	0.287488	0.073567	3.907862	9.31E-05	0.001507	UBA1	7317	ubiquitin like modifier activating enzyme 1
ENSG0000	3019.926	-0.36559	0.093575	-3.90693	9.35E-05	0.001512	ESYT1	23344	extended synaptotagmin 1
ENSG0000	1144.863	-0.37377	0.095715	-3.90505	9.42E-05	0.001522	MAPKAP1	79109	MAPK associated protein 1
ENSG0000	18282.8	-0.29724	0.076165	-3.90259	9.52E-05	0.001536	SUN2	25777	Sad1 and UNC84 domain containing 2
ENSG0000	222.5802	0.791098	0.20272	3.90242	9.52E-05	0.001536	NA	NA	NA
ENSG0000	612.7039	0.496545	0.127292	3.900833	9.59E-05	0.001544	SBDS	51119	SBDS ribosome maturation factor
ENSG0000	1081.825	0.433214	0.111059	3.900739	9.59E-05	0.001544	TNFRSF10I	8795	TNF receptor superfamily member 10b
ENSG0000	13.70626	-3.31895	0.85093	-3.90038	9.60E-05	0.001545	MARVELD2	153562	MARVEL domain containing 2
ENSG0000	1570.112	0.345491	0.088586	3.900085	9.62E-05	0.001545	KCMF1	56888	potassium channel modulatory factor 1
ENSG0000	1004.407	0.402164	0.103211	3.896504	9.76E-05	0.001566	IDI1	3422	isopentenyl-diphosphate delta isomerase 1
ENSG0000	310.735	0.603422	0.154854	3.896706	9.75E-05	0.001566	PMAIP1	5366	phorbol-12-myristate-13-acetate-induced protein 1
ENSG0000	683.2462	-0.5176	0.132883	-3.89513	9.81E-05	0.001573	GIMAP8	155038	GTPase IMAP family member 8
ENSG0000	411.1391	0.612383	0.157251	3.894295	9.85E-05	0.001577	DDIT3	1649	DNA damage inducible transcript 3
ENSG0000	8453.661	0.267609	0.068745	3.892792	9.91E-05	0.001586	ZBTB4	57659	zinc finger and BTB domain containing 4
ENSG0000	173.2318	0.907028	0.233031	3.892303	9.93E-05	0.001588	SEPTIN10	151011	septin 10
ENSG0000	755.0048	-0.52594	0.135183	-3.89054	1.00E-04	0.001598	ZNF862	643641	zinc finger protein 862
ENSG0000	111.9865	1.068287	0.274627	3.889963	1.00E-04	0.001599	NA	NA	NA
ENSG0000	929.0548	-0.43461	0.111721	-3.89015	1.00E-04	0.001599	DNAJC1	64215	DnaJ heat shock protein family (Hsp40) member C1
ENSG0000	9861.423	0.304598	0.078314	3.889431	1.00E-04	0.001601	PPDPF	79144	pancreatic progenitor cell differentiation and proliferation factor
ENSG0000	52.12819	1.25348	0.322385	3.88814	1.01E-04	0.001608	LOC10013	1E+08	uncharacterized LOC100130357
ENSG0000	165.3909	-0.74803	0.192396	-3.88796	1.01E-04	0.001608	RHBDL3	162494	rhomboid like 3
ENSG0000	198.9185	0.957961	0.246508	3.886124	1.02E-04	0.001618	GADD45A	1647	growth arrest and DNA damage inducible alpha
ENSG0000	733.29	-0.47777	0.122944	-3.88605	1.02E-04	0.001618	CLMN	79789	calmin
ENSG0000	912.3119	-0.41893	0.107842	-3.88466	1.02E-04	0.001626	ITPRIPL2	162073	ITPRIP like 2
ENSG0000	3886.288	0.305849	0.078741	3.884213	1.03E-04	0.001627	AKIRIN2	55122	akirin 2
ENSG0000	20065.5	0.31331	0.080679	3.883424	1.03E-04	0.001631	CD53	963	CD53 molecule
ENSG0000	9142.656	0.304298	0.07836	3.883325	1.03E-04	0.001631	SF3B4	10262	splicing factor 3b subunit 4
ENSG0000	167.4421	0.981427	0.252976	3.879528	1.05E-04	0.001655	NA	NA	NA
ENSG0000	7.49332	4.301256	1.108922	3.878773	1.05E-04	0.001657	LINC01093	1.01E+08	long intergenic non-protein coding RNA 1093
ENSG0000	765.942	-0.49899	0.128648	-3.87873	1.05E-04	0.001657	DPEP2	64174	dipeptidase 2
ENSG0000	25.63606	1.935515	0.49932	3.876302	1.06E-04	0.001673	APRG1	339883	APRG1 tumor suppressor candidate
ENSG0000	56.66819	1.3159	0.339502	3.875969	1.06E-04	0.001674	LOC10012	1E+08	DnaJ heat shock protein family (Hsp40) member C7 pseudogene
ENSG0000	167.8825	0.987781	0.254905	3.875097	1.07E-04	0.001678	MTND4P1	1E+08	MT-ND4 pseudogene 12
ENSG0000	1162.941	0.373635	0.096446	3.874037	1.07E-04	0.001684	OFD1	8481	OFD1 centriole and centriolar satellite protein
ENSG0000	284.6086	-0.68404	0.176585	-3.87373	1.07E-04	0.001685	RNGTT	8732	RNA guanylyltransferase and 5'-phosphatase
ENSG0000	69.36798	-1.17195	0.302592	-3.87304	1.07E-04	0.001688	FZD2	2535	frizzled class receptor 2
ENSG0000	1336.045	-0.43013	0.11109	-3.87193	1.08E-04	0.001694	SORBS3	10174	sorbin and SH3 domain containing 3
ENSG0000	3710.842	0.308075	0.079628	3.86893	1.09E-04	0.001712	HNRNPA3	220988	heterogeneous nuclear ribonucleoprotein A3
ENSG0000	4041.739	0.31598	0.08167	3.869009	1.09E-04	0.001712	SERINC1	57515	serine incorporator 1
ENSG0000	170.4431	-0.81056	0.209639	-3.86643	1.10E-04	0.001729	TM6SF1	53346	transmembrane 6 superfamily member 1
ENSG0000	21296.79	0.267244	0.069142	3.865145	1.11E-04	0.001736	UBA52	7311	ubiquitin A-52 residue ribosomal protein fusion product 1
ENSG0000	305.7176	0.552726	0.143069	3.863344	1.12E-04	0.001748	MCTP1	79772	multiple C2 and transmembrane domain containing 1
ENSG0000	3494.379	-0.30714	0.079523	-3.86234	1.12E-04	0.001753	SH3KBP1	30011	SH3 domain containing kinase binding protein 1
ENSG0000	1192.992	0.464837	0.120371	3.8617	1.13E-04	0.001756	PILRA	29992	paired immunoglobin like type 2 receptor alpha
ENSG0000	2201.869	0.324168	0.083955	3.861213	1.13E-04	0.001758	ELK1	2002	ETS transcription factor ELK1
ENSG0000	52.63628	-1.51305	0.392184	-3.85802	1.14E-04	0.00178	NYNRIN	57523	NYN domain and retroviral integrase containing
ENSG0000	3731.406	-0.31658	0.082076	-3.85717	1.15E-04	0.001784	TAF1C	9013	TATA-box RNA polymerase I subunit C
ENSG0000	567.6612	0.487619	0.12642	3.85714	1.15E-04	0.001784	PSMD12	5718	proteasom non-ATPase 12
ENSG0000	26.41026	-2.09859	0.544338	-3.85531	1.16E-04	0.001795	A2M	2	alpha-2-macroglobulin
ENSG0000	2551.585	0.364267	0.094523	3.853759	1.16E-04	0.001805	SPAG9	9043	sperm associated antigen 9
ENSG0000	2803.935	0.435775	0.113184	3.850141	1.18E-04	0.001831	CHST15	51363	carbohydrate sulfotransferase 15
ENSG0000	415.8218	-0.55383	0.1439	-3.84874	1.19E-04	0.00184	GPX7	2882	glutathione peroxidase 7
ENSG0000	3901.455	0.303133	0.078779	3.847894	1.19E-04	0.001844	GSK3A	2931	glycogen synthase kinase 3 alpha
ENSG0000	3295.568	-0.29728	0.077376	-3.84204	1.22E-04	0.001887	ST6GAL1	6480	ST6 beta-g 6-sialyltransferase 1

ENSG0000	316.7391	0.652668	0.169904	3.841392	1.22E-04	0.001891	CLEC12A	160364	C-type lectin domain family 12 member A
ENSG0000	938.8825	-0.38392	0.099991	-3.83956	1.23E-04	0.001902	KIAA0319L	79932	KIAA0319 like
ENSG0000	1896.169	-0.35021	0.091208	-3.83973	1.23E-04	0.001902	SIN3B	23309	SIN3 transcription regulator family member B
ENSG0000	5140.68	-0.32478	0.08462	-3.83813	1.24E-04	0.001911	DBNL	28988	drebrin like
ENSG0000	139.2813	0.861474	0.224601	3.835572	1.25E-04	0.00193	YES1	7525	YES proto- Src family tyrosine kinase
ENSG0000	3916.209	-0.30894	0.080588	-3.83355	1.26E-04	0.001944	CUX1	1523	cut like homeobox 1
ENSG0000	20004.97	0.294531	0.076865	3.83182	1.27E-04	0.001955	RPS26	6231	ribosomal protein S26
ENSG0000	2729.295	0.361307	0.094294	3.831731	1.27E-04	0.001955	TP53INP2	58476	tumor protein p53 inducible nuclear protein 2
ENSG0000	2361.227	-0.99994	0.261068	-3.83019	1.28E-04	0.001965	MSR1	4481	macrophage scavenger receptor 1
ENSG0000	244.3959	-0.64009	0.167118	-3.83015	1.28E-04	0.001965	NECAB3	63941	N-terminal EF-hand calcium binding protein 3
ENSG0000	8815.281	0.341241	0.089101	3.829817	1.28E-04	0.001966	CDC37	11140	cell divisio HSP90 cochaperone
ENSG0000	4697.321	0.274889	0.071833	3.826755	1.30E-04	0.001989	UBE2Z	65264	ubiquitin conjugating enzyme E2 Z
ENSG0000	3562.175	0.304004	0.079446	3.826545	1.30E-04	0.001989	SH2D1B	117157	SH2 domain containing 1B
ENSG0000	641.9493	0.469954	0.122827	3.826142	1.30E-04	0.00199	TBC1D22B	55633	TBC1 domain family member 22B
ENSG0000	106091.5	0.280159	0.073227	3.825897	1.30E-04	0.001991	PABPC1	26986	poly(A) binding protein cytoplasmic 1
ENSG0000	971.6544	-0.40188	0.105087	-3.82421	1.31E-04	0.002003	NCAPD2	9918	non-SMC condensin I complex subunit D2
ENSG0000	1653.934	-0.3362	0.087936	-3.82328	1.32E-04	0.002009	ZBTB40	9923	zinc finger and BTB domain containing 40
ENSG0000	349.7036	-0.58152	0.152329	-3.81754	1.35E-04	0.002054	IQCE	23288	IQ motif containing E
ENSG0000	10415.83	0.281202	0.073683	3.816396	1.35E-04	0.002062	RHOG	391	ras homolog family member G
ENSG0000	2462.237	0.30663	0.080372	3.815125	1.36E-04	0.002071	CLU	1191	clusterin
ENSG0000	202.4143	-0.70282	0.184232	-3.81485	1.36E-04	0.002072	ACADSB	36	acyl-CoA dehydrogenase short/branched chain
ENSG0000	116.045	-0.85238	0.223518	-3.81347	1.37E-04	0.002081	ZNF704	619279	zinc finger protein 704
ENSG0000	10715.2	0.274905	0.072091	3.813276	1.37E-04	0.002081	SAT1	6303	spermidine/spermine N1-acetyltransferase 1
ENSG0000	4840.523	0.28465	0.07466	3.812595	1.38E-04	0.002085	RNF10	9921	ring finger protein 10
ENSG0000	52.54413	-1.26625	0.332211	-3.81158	1.38E-04	0.002092	CALD1	800	caldesmon 1
ENSG0000	778.9487	-0.45323	0.119019	-3.80802	1.40E-04	0.002114	CD38	952	CD38 molecule
ENSG0000	357.2143	-0.5348	0.140438	-3.80812	1.40E-04	0.002114	RAP1GDS1	5910	Rap1 GTPase-GDP dissociation stimulator 1
ENSG0000	641.198	-0.45309	0.118977	-3.80823	1.40E-04	0.002114	VPS13B	157680	vacuolar protein sorting 13 homolog B
ENSG0000	73.29414	-1.15994	0.304604	-3.80802	1.40E-04	0.002114	EPPK1	83481	epiplakin 1
ENSG0000	892.4805	-0.40365	0.105993	-3.80826	1.40E-04	0.002114	RSU1	6251	Ras suppressor protein 1
ENSG0000	2659.325	0.334663	0.087898	3.807409	1.40E-04	0.002118	YME1L1	10730	YME1 like 1 ATPase
ENSG0000	3456.4	-0.37939	0.099754	-3.8032	1.43E-04	0.002152	RERE	473	arginine-glutamic acid dipeptide repeats
ENSG0000	232.7493	-0.61039	0.160507	-3.80291	1.43E-04	0.002153	ACACA	31	acetyl-CoA carboxylase alpha
ENSG0000	401.6007	-0.55793	0.146815	-3.80021	1.45E-04	0.002173	CASP8AP2	9994	caspase 8 associated protein 2
ENSG0000	1978.38	-0.41466	0.109113	-3.80026	1.45E-04	0.002173	SAMD9	54809	sterile alpha motif domain containing 9
ENSG0000	2981.713	1.296215	0.341125	3.799826	1.45E-04	0.002175	CCL7	6354	C-C motif chemokine ligand 7
ENSG0000	2725.578	0.332765	0.087615	3.798037	1.46E-04	0.002189	TACC3	10460	transforming acidic coiled-coil containing protein 3
ENSG0000	2051.147	0.338372	0.089167	3.794809	1.48E-04	0.002214	TFG	10342	trafficking from ER to golgi regulator
ENSG0000	7185.66	0.411116	0.108337	3.794773	1.48E-04	0.002214	GRAMD1A	57655	GRAM domain containing 1A
ENSG0000	318.6628	-0.57396	0.151337	-3.79257	1.49E-04	0.00223	MAP3K12	7786	mitogen-activated protein kinase kinase kinase 12
ENSG0000	239.3208	-0.6839	0.180322	-3.79267	1.49E-04	0.00223	RCBTB2	1102	RCC1 and BTB domain containing protein 2
ENSG0000	2095.825	-0.31251	0.082417	-3.79177	1.50E-04	0.002235	RNF187	149603	ring finger protein 187
ENSG0000	93.54051	1.178556	0.310869	3.791166	1.50E-04	0.002239	NA	NA	NA
ENSG0000	516.9545	0.511385	0.135038	3.786978	1.52E-04	0.002274	CTPS1	1503	CTP synthase 1
ENSG0000	65068.96	0.262607	0.069343	3.78707	1.52E-04	0.002274	TPT1	7178	tumor pro translationally-controlled 1
ENSG0000	7563.367	0.282496	0.074617	3.785954	1.53E-04	0.002281	RAD23A	5886	RAD23 hoı nucleotide excision repair protein
ENSG0000	2459.227	-0.31223	0.082525	-3.7834	1.55E-04	0.002303	MAVS	57506	mitochondrial antiviral signaling protein
ENSG0000	422.1824	-0.59054	0.156101	-3.78304	1.55E-04	0.002304	SNX30	401548	sorting nexin family member 30
ENSG0000	19.29694	3.119825	0.824945	3.78186	1.56E-04	0.002313	FJX1	24147	four-jointed box kinase 1
ENSG0000	10791.61	0.284591	0.075256	3.781654	1.56E-04	0.002313	SLA2	84174	Src like adaptor 2
ENSG0000	17.36974	-2.75225	0.727879	-3.78119	1.56E-04	0.002316	SYT6	148281	synaptotagmin 6
ENSG0000	693.3462	-0.4408	0.116632	-3.77937	1.57E-04	0.00233	STAG1	10274	stromal antigen 1
ENSG0000	4095.336	0.315948	0.0836	3.779304	1.57E-04	0.00233	FAM117A	81558	family with sequence similarity 117 member A
ENSG0000	9304.208	0.290452	0.076888	3.777614	1.58E-04	0.002344	EZR	7430	ezrin
ENSG0000	202.9603	0.668474	0.177135	3.773812	1.61E-04	0.002378	NCR3	259197	natural cytotoxicity triggering receptor 3
ENSG0000	19145.23	-0.3297	0.087423	-3.77139	1.62E-04	0.002399	OLR1	4973	oxidized low density lipoprotein receptor 1
ENSG0000	1391.389	0.383905	0.10181	3.770812	1.63E-04	0.002403	NECAP1	25977	NECAP endocytosis associated 1
ENSG0000	189.1703	-0.716	0.189914	-3.77013	1.63E-04	0.002407	ARHGEF9	23229	Cdc42 guanine nucleotide exchange factor 9
ENSG0000	3896.19	0.326361	0.086574	3.769744	1.63E-04	0.002409	IKZF3	22806	IKAROS family zinc finger 3
ENSG0000	13861.49	0.300991	0.079898	3.767193	1.65E-04	0.002432	CLIC1	1192	chloride intracellular channel 1
ENSG0000	455.7018	-0.48673	0.129236	-3.76621	0.000166	0.00244	EXOC1	55763	exocyst complex component 1
ENSG0000	541.4902	-0.5031	0.133623	-3.76506	1.67E-04	0.002449	BAZ2B	29994	bromodomain adjacent to zinc finger domain 2B
ENSG0000	6763.118	-1.27678	0.339195	-3.76413	1.67E-04	0.002456	TGM2	7052	transglutaminase 2
ENSG0000	14742.43	0.279799	0.074342	3.763649	1.67E-04	0.002459	RHOA	387	ras homolog family member A
ENSG0000	801.1455	-0.43155	0.114757	-3.76054	1.70E-04	0.002488	ANKIB1	54467	ankyrin repeat and IBR domain containing 1
ENSG0000	12245.54	-0.267	0.07101	-3.7601	1.70E-04	0.00249	PTK2B	2185	protein tyrosine kinase 2 beta
ENSG0000	836.1857	-0.44407	0.118281	-3.75436	1.74E-04	0.002546	DAGLA	747	diacylglycerol lipase alpha
ENSG0000	16056.33	0.304323	0.081136	3.750789	1.76E-04	0.00258	CD93	22918	CD93 molecule
ENSG0000	832.4544	-0.44941	0.119828	-3.7505	1.76E-04	0.002581	MSI2	124540	musashi RNA binding protein 2
ENSG0000	2611.407	-0.35183	0.09387	-3.74803	1.78E-04	0.002605	CD151	977	CD151 molecule (Raph blood group)
ENSG0000	248.7298	0.619864	0.165406	3.747525	1.79E-04	0.002608	NA	NA	NA
ENSG0000	816.2255	-0.45765	0.122162	-3.74626	1.79E-04	0.002619	KLHL17	339451	kelch like family member 17
ENSG0000	286.779	-0.61484	0.164132	-3.74602	1.80E-04	0.00262	MTHFSD	64779	methenyltetrahydrofolate synthetase domain containing
ENSG0000	1018.109	0.414366	0.110689	3.743518	1.81E-04	0.002644	NA	NA	NA
ENSG0000	1547.861	0.352894	0.094294	3.742495	1.82E-04	0.002653	EIF2S3	1968	eukaryotic translation initiation factor 2 subunit gamma
ENSG0000	2131.286	0.302641	0.080873	3.742178	1.82E-04	0.002654	RSRP1	57035	arginine and serine rich protein 1
ENSG0000	922.8846	0.44647	0.119326	3.7416	1.83E-04	0.002658	B3GNT2	10678	UDP-GlcN/ 3-N-acetylglucosaminyltransferase 2
ENSG0000	766.4895	-0.39777	0.106318	-3.74133	1.83E-04	0.002658	LRRC14	9684	leucine rich repeat containing 14
ENSG0000	5790.353	0.263074	0.070322	3.740994	1.83E-04	0.002658	CALM1	801	calmodulin 1
ENSG0000	173.6008	-0.72512	0.193831	-3.74098	1.83E-04	0.002658	ACSF2	80221	acyl-CoA synthetase family member 2
ENSG0000	1055.544	-0.42563	0.113862	-3.73817	1.85E-04	0.002686	IL18BP	10068	interleukin 18 binding protein
ENSG0000	165.0921	0.789989	0.21143	3.736411	1.87E-04	0.002703	FAAH2	158584	fatty acid amide hydrolase 2
ENSG0000	24976.46	0.308915	0.082742	3.733479	1.89E-04	0.002732	TPM4	7171	tropomyosin 4
ENSG0000	90.9317	-1.10997	0.297357	-3.73277	1.89E-04	0.002738	RAB7B	338382	RAB7B member RAS oncogene family
ENSG0000	779.1166	0.426311	0.114223	3.732264	1.90E-04	0.002741	NA	NA	NA
ENSG0000	2807.117	-0.39565	0.106065	-3.73026	1.91E-04	0.002761	ARHGAP26	23092	Rho GTPase activating protein 26
ENSG0000	3148.571	0.365223	0.098009	3.726411	1.94E-04	0.002801	EIF3I	8668	eukaryotic translation initiation factor 3 subunit I
ENSG0000	1110.053	0.406547	0.109112	3.725963	1.95E-04	0.002804	CYSTM1	84418	cysteine rich transmembrane module containing 1
ENSG0000	142.9555	-0.92316	0.247775	-3.72579	1.95E-04	0.002804	SLC35B4	84912	solute carrier family 35 member B4
ENSG0000	1312.692	-0.40858	0.109693	-3.72477	1.95E-04	0.002813	TLR4	7099	toll like receptor 4
ENSG0000	440.1732	0.498759	0.133945	3.723621	1.96E-04	0.002824	SESTD1	91404	SEC14 and spectrin domain containing 1
ENSG0000	694.7722	-0.44441	0.119363	-3.72317	1.97E-04	0.002826	LINC01128	643837	long intergenic non-protein coding RNA 1128
ENSG0000	250.3877	0.654331	0.175849	3.720973	1.98E-04	0.002849	FRMD4B	23150	FERM domain containing 4B
ENSG0000	3318.443	0.322223	0.086648	3.718759	2.00E-04	0.002872	HLA-DQA1	3117	major hist class II DQ alpha 1
ENSG0000	454.4338	-0.50698	0.136341	-3.71848	2.00E-04	0.002873	GOLGA1	2800	golgin A1
ENSG0000	392.3811	-0.50165	0.134952	-3.71726	2.01E-04	0.002882	RMND5B	64777	required for meiotic nuclear division 5 homolog B
ENSG0000	8415.264	0.283496	0.076264	3.71732	2.01E-04	0.002882	SERF2	10169	small EDRK-rich factor 2
ENSG0000	1047.302	0.379963	0.102239	3.716426	2.02E-04	0.002888	UGP2	7360	UDP-glucose pyrophosphorylase 2
ENSG0000	321.6807	0.563331	0.151582	3.716333	2.02E-04	0.002888	RAB32	10981	RAB32 member RAS oncogene family
ENSG0000	5887.82	0.259662	0.069902	3.714666	2.03E-04	0.002905	SIK3	23387	SIK family kinase 3

ENSG0000	977.2152	0.418409	0.11269	3.712909	2.05E-04	0.002922	ALG2	85365	ALG2 alph 3-jan 6-mannosyltransferase
ENSG0000	585.6572	-0.46717	0.125826	-3.71284	2.05E-04	0.002922	NDRG3	57446	NDRG family member 3
ENSG0000	300.0892	-0.74935	0.201845	-3.71247	2.05E-04	0.002923	PALLD	23022	palladin cytoskeletal associated protein
ENSG0000	934.7679	0.445073	0.11989	3.712343	2.05E-04	0.002923	OSER1	51526	oxidative stress responsive serine rich 1
ENSG0000	162.3079	-2.08985	0.562997	-3.71201	2.06E-04	0.002925	FCGR1CP	1E+08	Fc gamma pseudogene
ENSG0000	41817.61	0.285272	0.076879	3.710655	2.07E-04	0.002936	RUNX3	864	RUNX family transcription factor 3
ENSG0000	2754.073	0.329169	0.088709	3.710673	2.07E-04	0.002936	ENSA	2029	endosulfine alpha
ENSG0000	1670.542	0.326044	0.087901	3.709209	2.08E-04	0.00295	RPL13AP5	728658	ribosomal protein L13a pseudogene 5
ENSG0000	2767.189	0.318545	0.085936	3.706786	2.10E-04	0.002976	ATP6V0B	533	ATPase H+ transporting V0 subunit b
ENSG0000	5484.975	-0.29455	0.079479	-3.70597	2.11E-04	0.002981	BIN1	274	bridging integrator 1
ENSG0000	17.63981	2.338112	0.630872	3.706158	2.10E-04	0.002981	TMEM255	55026	transmembrane protein 255A
ENSG0000	113.3856	-1.05802	0.285693	-3.70334	2.13E-04	0.00301	DCSTAMP	81501	dendrocyte expressed seven transmembrane protein
ENSG0000	218.5956	-0.71439	0.192932	-3.70281	2.13E-04	0.003014	IFT27	11020	intraflagellar transport 27
ENSG0000	2160.379	0.351052	0.09487	3.700357	2.15E-04	0.003041	FAM53C	51307	family with sequence similarity 53 member C
ENSG0000	12986.42	0.256203	0.069284	3.697876	0.000217	0.003069	SFPQ	6421	splicing factor proline and glutamine rich
ENSG0000	352.0182	-0.51686	0.139852	-3.69575	2.19E-04	0.003092	PAQR8	85315	progestin and adipoQ receptor family member 8
ENSG0000	88.78389	1.024666	0.277343	3.69458	2.20E-04	0.003104	ZC3H12C	85463	zinc finger CCCH-type containing 12C
ENSG0000	749.4982	-0.51569	0.139675	-3.69204	2.22E-04	0.003133	MAML3	55534	mastermind like transcriptional coactivator 3
ENSG0000	4407.2	0.321626	0.087174	3.689457	2.25E-04	0.003162	HNRNPA0	10949	heterogeneous nuclear ribonucleoprotein A0
ENSG0000	110.8088	1.08553	0.294335	3.688077	2.26E-04	0.003177	WNT9A	7483	Wnt family member 9A
ENSG0000	166.9234	0.760709	0.20631	3.687219	2.27E-04	0.003185	CITED4	163732	Cbp/p300 interacting transactivator with Glu/Asp rich carboxy-terminal domain 4
ENSG0000	787.1245	0.42572	0.115462	3.68709	2.27E-04	0.003185	ADNP2	22850	ADNP homeobox 2
ENSG0000	453.8331	-0.54296	0.147339	-3.68508	2.29E-04	0.003208	DPYD	1806	dihydropyrimidine dehydrogenase
ENSG0000	376.61	0.572623	0.155406	3.684697	2.29E-04	0.00321	GPR155	151556	G protein-coupled receptor 155
ENSG0000	1424.163	1.429889	0.388089	3.684436	2.29E-04	0.003211	CCL4L2	9560	C-C motif chemokine ligand 4 like 2
ENSG0000	14953.22	0.334528	0.090916	3.67953	2.34E-04	0.003271	RPL37	6167	ribosomal protein L37
ENSG0000	2663.511	0.341842	0.092934	3.678342	2.35E-04	0.003284	ST3GAL2	6483	ST3 beta-g 3-sialyltransferase 2
ENSG0000	5546.263	0.286836	0.077985	3.678081	2.35E-04	0.003284	STK4	6789	serine/threonine kinase 4
ENSG0000	25131.34	0.252191	0.068571	3.67778	2.35E-04	0.003286	RPS6	6194	ribosomal protein S6
ENSG0000	614.7725	-0.43886	0.119362	-3.67673	2.36E-04	0.003297	SASH1	23328	SAM and SH3 domain containing 1
ENSG0000	132.7091	-0.77882	0.21193	-3.6749	2.38E-04	0.003318	MINPP1	9562	multiple inositol-polyphosphate phosphatase 1
ENSG0000	624.0297	0.560023	0.152402	3.674636	2.38E-04	0.003319	SPSB1	80176	splA/ryanodine receptor domain and SOCS box containing 1
ENSG0000	3142.776	0.297614	0.081059	3.67159	2.41E-04	0.003356	MORF4L1	10933	mortality factor 4 like 1
ENSG0000	735.0664	0.617284	0.168171	3.670567	2.42E-04	0.003367	XCL2	6846	X-C motif chemokine ligand 2
ENSG0000	3628.246	-0.28517	0.077721	-3.66911	2.43E-04	0.003377	ZBTB7B	51043	zinc finger and BTB domain containing 7B
ENSG0000	1948.622	-0.32156	0.08764	-3.66914	2.43E-04	0.003377	PHF2	5253	PHD finger protein 2
ENSG0000	3766.311	-0.30378	0.082792	-3.66918	2.43E-04	0.003377	TBCD	6904	tubulin folding cofactor D
ENSG0000	8392.701	0.295619	0.080571	3.669054	2.43E-04	0.003377	PLEKHG2	64857	pleckstrin homology and RhoGEF domain containing G2
ENSG0000	531.8254	0.481515	0.13126	3.668419	2.44E-04	0.003383	SAR1B	51128	secretion associated Ras related GTPase 1B
ENSG0000	2478.893	0.335985	0.09165	3.664548	2.48E-04	0.003432	ZDHHC18	84243	zinc finger DHHC-type palmitoyltransferase 18
ENSG0000	668.5854	-0.48578	0.132574	-3.66419	2.48E-04	0.003434	PEPD	5184	peptidase D
ENSG0000	880.2313	-0.4751	0.129676	-3.66378	2.49E-04	0.003437	JAML	120425	junction adhesion molecule like
ENSG0000	2098.561	-0.31361	0.085611	-3.66326	2.49E-04	0.003442	PPIL2	23759	peptidylprolyl isomerase like 2
ENSG0000	949.0386	-0.41047	0.112089	-3.66204	2.50E-04	0.003455	BBX	56987	BBX high mobility group box domain containing
ENSG0000	2569.171	0.304318	0.083116	3.661365	2.51E-04	0.003462	LTB	4050	lymphotoxin beta
ENSG0000	1470.827	0.364096	0.099463	3.660613	2.52E-04	0.00347	P2RY10	27334	P2Y receptor family member 10
ENSG0000	1079.362	0.431679	0.117934	3.660346	2.52E-04	0.003471	CYCS	54205	cytochrom somatic
ENSG0000	39786.67	0.292739	0.07999	3.659681	2.53E-04	0.003477	RPS11	6205	ribosomal protein S11
ENSG0000	3119.629	-0.30728	0.084022	-3.65713	2.55E-04	0.003509	TRRAP	8295	transformation/transcription domain associated protein
ENSG0000	401.2688	0.467641	0.127878	3.656918	2.55E-04	0.00351	RIOK1	83732	RIO kinase 1
ENSG0000	3149.352	0.300525	0.082219	3.655186	2.57E-04	0.003531	STX5	6811	syntaxin 5
ENSG0000	3442.311	-0.27504	0.075276	-3.65372	2.58E-04	0.003548	TMEM131	23505	transmembrane protein 131
ENSG0000	159.7315	-0.78171	0.213989	-3.65303	2.59E-04	0.003554	WDR70	55100	WD repeat domain 70
ENSG0000	258.0887	-0.78589	0.215138	-3.65295	0.000259	0.003554	GCNT1	2650	glucosaminyl (N-acetyl) transferase 1
ENSG0000	5523.895	0.302517	0.082849	3.651407	2.61E-04	0.003573	TFE3	7030	transcription factor binding to IGHM enhancer 3
ENSG0000	11.56304	3.548875	0.972503	3.64922	2.63E-04	0.0036	MUCL1	118430	mucin like 1
ENSG0000	936.0855	-0.36816	0.100899	-3.64879	2.63E-04	0.003601	SUN1	23353	Sad1 and UNC84 domain containing 1
ENSG0000	813546.3	0.368356	0.100949	3.648952	2.63E-04	0.003601	COX1	4512	cytochrome c oxidase subunit I
ENSG0000	627.565	0.413879	0.113444	3.6483	2.64E-04	0.003605	NA	NA	NA
ENSG0000	11557.84	0.288876	0.079194	3.647433	2.65E-04	0.003615	MAPKAPK	9261	MAPK activated protein kinase 2
ENSG0000	406.1994	-0.48484	0.132962	-3.64647	2.66E-04	0.003626	DDHD2	23259	DDHD domain containing 2
ENSG0000	5122.123	-0.28711	0.07879	-3.64398	2.68E-04	0.003658	NISCH	11188	nischarin
ENSG0000	102.864	1.053465	0.289128	3.64359	2.69E-04	0.003661	LRRC32	2615	leucine rich repeat containing 32
ENSG0000	18362.71	0.281872	0.077368	3.643245	2.69E-04	0.003663	RPL15	6138	ribosomal protein L15
ENSG0000	22.45964	-2.17716	0.599085	-3.63414	2.79E-04	0.003793	BTBD8	284697	BTB domain containing 8
ENSG0000	508.4737	-0.49115	0.135209	-3.6325	2.81E-04	0.003814	TRAF5	7188	TNF receptor associated factor 5
ENSG0000	600.133	0.449321	0.123721	3.631728	2.82E-04	0.003822	ATP6V1D	51382	ATPase H+ transporting V1 subunit D
ENSG0000	166.1424	-0.73223	0.201696	-3.63035	2.83E-04	0.003837	NT5DC1	221294	5'-nucleotidase domain containing 1
ENSG0000	3590.937	0.304781	0.08395	3.630503	2.83E-04	0.003837	TOX4	9878	TOX high mobility group box family member 4
ENSG0000	138.1669	0.815759	0.22474	3.629797	2.84E-04	0.003843	ACAT2	39	acetyl-CoA acetyltransferase 2
ENSG0000	502.5245	-0.46302	0.127598	-3.62872	2.85E-04	0.003856	RASSF7	8045	Ras association domain family member 7
ENSG0000	13413.1	0.237474	0.065504	3.625332	2.89E-04	0.003904	PXN	5829	paxillin
ENSG0000	5031.503	0.311692	0.086012	3.623831	2.90E-04	0.003924	PRELID1	27166	PRELI domain containing 1
ENSG0000	325.961	0.652724	0.180161	3.622996	2.91E-04	0.003934	NA	NA	NA
ENSG0000	1090.849	0.40879	0.112864	3.621965	2.92E-04	0.003946	MTCO1P1:	1.07E+08	MT-CO1 pseudogene 12
ENSG0000	4214.906	0.327822	0.090513	3.621816	2.93E-04	0.003946	CYLD	1540	CYLD lysine 63 deubiquitinase
ENSG0000	729.5234	0.404387	0.111682	3.620888	2.94E-04	0.003957	USP14	9097	ubiquitin specific peptidase 14
ENSG0000	7672.03	0.245141	0.067709	3.620498	2.94E-04	0.003959	HNRNPK	3190	heterogeneous nuclear ribonucleoprotein K
ENSG0000	827.1217	0.44322	0.122422	3.620421	2.94E-04	0.003959	RPS15A	6210	ribosomal protein S15a
ENSG0000	59.0892	1.143575	0.315955	3.619418	2.95E-04	0.003971	NAMPTP1	646309	nicotinamide phosphoribosyltransferase pseudogene 1
ENSG0000	2809.164	0.289136	0.07989	3.619201	2.96E-04	0.003972	RBM22	55696	RNA binding motif protein 22
ENSG0000	1459.151	-0.43152	0.119251	-3.61857	2.96E-04	0.003978	SLC26A6	65010	solute carrier family 26 member 6
ENSG0000	696.8449	0.46822	0.129477	3.616239	2.99E-04	0.004012	MPIG6B	80739	megakaryocyte and platelet inhibitory receptor G6b
ENSG0000	133.2339	-0.87186	0.241168	-3.61517	3.00E-04	0.004025	RTN1	6252	reticulon 1
ENSG0000	159.0199	-0.75428	0.208685	-3.61443	3.01E-04	0.004034	PLXNB1	5364	plexin B1
ENSG0000	307.1648	0.874528	0.242014	3.613544	3.02E-04	0.004045	P2RY2	5029	purinergic receptor P2Y2
ENSG0000	11446.81	0.375099	0.103818	3.613047	3.03E-04	0.00405	RPL23A	6147	ribosomal protein L23a
ENSG0000	1067.534	0.414695	0.114813	3.611925	3.04E-04	0.004062	PAK1	5058	p21 (RAC1) activated kinase 1
ENSG0000	35.85481	1.821584	0.504333	3.611866	3.04E-04	0.004062	NA	NA	NA
ENSG0000	809.8056	-0.48423	0.134081	-3.6115	3.04E-04	0.004065	TAB1	10454	TGF-beta activated kinase 1 (MAP3K7) binding protein 1
ENSG0000	387.7733	0.483804	0.134059	3.608878	3.08E-04	0.004103	PVT1	5820	Pvt1 oncogene
ENSG0000	712.7461	-0.46941	0.130091	-3.60829	3.08E-04	0.004107	LIMD1	8994	LIM domain containing 1
ENSG0000	2560.976	0.37922	0.105096	3.608309	3.08E-04	0.004107	DDX21	9188	DExD-box helicase 21
ENSG0000	5550.941	-0.28416	0.078773	-3.60725	3.09E-04	0.00412	KCNAB2	8514	potassium voltage-gated channel subfamily A regulatory beta subunit 2
ENSG0000	20.52447	2.245147	0.622902	3.604335	3.13E-04	0.004164	BTG2-DT	730227	BTG2 divergent transcript
ENSG0000	964.1161	-0.38607	0.10726	-3.59942	3.19E-04	0.00424	SLX4	84464	SLX4 structure-specific endonuclease subunit
ENSG0000	12592.29	0.276811	0.076948	3.597361	3.21E-04	0.004271	RPL41	6171	ribosomal protein L41
ENSG0000	700.047	0.39159	0.108869	3.596906	3.22E-04	0.004272	CDK2	1017	cyclin dependent kinase 2
ENSG0000	16715.01	0.317473	0.08826	3.59702	3.22E-04	0.004272	VASP	7408	vasodilator stimulated phosphoprotein
ENSG0000	476.5157	-0.46996	0.130666	-3.59663	3.22E-04	0.004274	COG4	25839	component of oligomeric golgi complex 4

ENSG0000	52.03187	-1.22474	0.340802	-3.59369	3.26E-04	0.004319	ABLIM2	84448	actin binding LIM protein family member 2
ENSG0000	1380.844	-0.37197	0.103517	-3.59329	3.27E-04	0.004323	SELENON	57190	selenoprotein N
ENSG0000	111.8927	-0.84302	0.234707	-3.59177	3.28E-04	0.004344	CCDC149	91050	coiled-coil domain containing 149
ENSG0000	1179.652	0.383168	0.106682	3.591669	3.29E-04	0.004344	BUD31	8896	BUD31 homolog
ENSG0000	245.4579	-0.58564	0.163077	-3.59118	3.29E-04	0.004349	ENTPD1	953	ectonucleoside triphosphate diphosphohydrolase 1
ENSG0000	2269.812	0.289186	0.080599	3.587948	3.33E-04	0.0044	PPP6R3	55291	protein phosphatase 6 regulatory subunit 3
ENSG0000	3161.085	0.324593	0.090494	3.586893	3.35E-04	0.004414	MED13	9969	mediator complex subunit 13
ENSG0000	366.239	-0.48735	0.13592	-3.58558	3.36E-04	0.00443	ARSB	411	arylsulfatase B
ENSG0000	2276.446	0.374152	0.104345	3.585717	3.36E-04	0.00443	DCXR	51181	dicarbonyl and L-xylulose reductase
ENSG0000	6041.536	0.258505	0.072103	3.585235	3.37E-04	0.004433	MALAT1	378938	metastasis associated lung adenocarcinoma transcript 1
ENSG0000	4397.775	0.256245	0.071482	3.584758	3.37E-04	0.004438	IGF2R	3482	insulin like growth factor 2 receptor
ENSG0000	534.9057	-0.45165	0.126021	-3.58396	3.38E-04	0.004449	EXT2	2132	exostosin glycosyltransferase 2
ENSG0000	54.69082	-1.15471	0.322359	-3.58207	3.41E-04	0.004478	GPR25	2848	G protein-coupled receptor 25
ENSG0000	29.15743	-1.6962	0.473644	-3.58117	3.42E-04	0.00449	AMPH	273	amphiphysin
ENSG0000	418.4583	-0.65684	0.183443	-3.5806	3.43E-04	0.004497	SORT1	6272	sortilin 1
ENSG0000	2983.026	0.294409	0.082233	3.580186	3.43E-04	0.0045	RPS29	6235	ribosomal protein S29
ENSG0000	320.3813	-1.90623	0.532639	-3.57884	3.45E-04	0.00452	RNASE1	6035	ribonuclea pancreatic
ENSG0000	2418.511	-0.33899	0.094737	-3.57825	3.46E-04	0.004528	ITPK1	3705	inositol-tetrakisphosphate 1-kinase
ENSG0000	724.6078	-0.41877	0.117047	-3.57777	3.47E-04	0.004533	SNX29	92017	sorting nexin 29
ENSG0000	7897.027	0.261768	0.073177	3.577206	3.47E-04	0.004537	SEC61A1	29927	SEC61 translocon subunit alpha 1
ENSG0000	426.275	-0.5029	0.140589	-3.57712	3.47E-04	0.004537	MTHFD1L	25902	methylenetetrahydrofolate dehydrogenase (NADP+ dependent) 1 like
ENSG0000	6091.78	0.267377	0.074782	3.575406	3.50E-04	0.004564	EEF1B2	1933	eukaryotic translation elongation factor 1 beta 2
ENSG0000	696.8959	-0.42115	0.11781	-3.57485	3.50E-04	0.004571	GLB1	2720	galactosidase beta 1
ENSG0000	100.2697	-0.94243	0.263643	-3.57464	3.51E-04	0.004571	RSPH3	83861	radial spoke head 3
ENSG0000	439.2411	-0.46539	0.13023	-3.57363	3.52E-04	0.004585	MTIF3	219402	mitochondrial translational initiation factor 3
ENSG0000	3522.277	-0.28685	0.080274	-3.57338	3.52E-04	0.004587	CLSTN3	9746	calsyntenin 3
ENSG0000	3476.911	0.337588	0.094497	3.572487	3.54E-04	0.004599	NFKBIE	4794	NFKB inhibitor epsilon
ENSG0000	702.4324	-0.58275	0.163303	-3.56849	3.59E-04	0.004667	NCF1	653361	neutrophil cytosolic factor 1
ENSG0000	179.0091	0.716618	0.200858	3.567786	3.60E-04	0.004676	PSD	5662	pleckstrin and Sec7 domain containing
ENSG0000	780.9541	0.421558	0.11825	3.564969	3.64E-04	0.004723	TDP2	51567	tyrosyl-DNA phosphodiesterase 2
ENSG0000	4286.944	0.303754	0.085223	3.564217	3.65E-04	0.004733	RNF126	55658	ring finger protein 126
ENSG0000	429.732	-0.4732	0.132785	-3.56362	3.66E-04	0.004734	PHACTR2	9749	phosphatase and actin regulator 2
ENSG0000	483.4204	-0.52099	0.146195	-3.56365	3.66E-04	0.004734	LRRK1	79705	leucine rich repeat kinase 1
ENSG0000	225.8707	-0.69302	0.194454	-3.56394	3.65E-04	0.004734	ZHX3	23051	zinc fingers and homeoboxes 3
ENSG0000	231.0394	-0.71656	0.201125	-3.56278	3.67E-04	0.004746	SMCO4	56935	single-pass membrane protein with coiled-coil domains 4
ENSG0000	4278.095	0.343519	0.096554	3.557805	3.74E-04	0.004833	STK17B	9262	serine/threonine kinase 17b
ENSG0000	712.2268	-0.47625	0.133962	-3.55511	3.78E-04	0.00488	DNAJC13	23317	DnaJ heat shock protein family (Hsp40) member C13
ENSG0000	4802.467	0.273983	0.077091	3.554034	3.79E-04	0.004893	PTP4A2	8073	protein tyrosine phosphatase 4A2
ENSG0000	109.8296	-0.89611	0.252135	-3.5541	3.79E-04	0.004893	KANK2	25959	KN motif and ankyrin repeat domains 2
ENSG0000	938.783	-0.40263	0.11332	-3.55301	3.81E-04	0.004905	DNAJC11	55735	DnaJ heat shock protein family (Hsp40) member C11
ENSG0000	170.8895	-0.70928	0.199629	-3.553	3.81E-04	0.004905	CARMIL3	90668	capping protein regulator and myosin 1 linker 3
ENSG0000	1618.725	0.332934	0.093726	3.552205	3.82E-04	0.004917	AMZ2	51321	archaelysin family metallopeptidase 2
ENSG0000	1104.818	-0.33682	0.094827	-3.55192	3.82E-04	0.004918	LYL1	4066	LYL1 basic helix-loop-helix family member
ENSG0000	364.5572	-0.5199	0.146377	-3.55177	3.83E-04	0.004918	BORCS8	729991	BLOC-1 related complex subunit 8
ENSG0000	7842.77	0.290388	0.081768	3.551367	3.83E-04	0.004922	RPL34	6164	ribosomal protein L34
ENSG0000	6750.314	0.290922	0.081923	3.551161	3.84E-04	0.004923	PMEPA1	56937	prostate tı androgen induced 1
ENSG0000	6598.612	0.261383	0.073611	3.550863	3.84E-04	0.004925	MLF2	8079	myeloid leukemia factor 2
ENSG0000	5045.547	0.369456	0.104067	3.550192	3.85E-04	0.004934	RNF19B	127544	ring finger protein 19B
ENSG0000	4237.007	0.361839	0.102021	3.546706	3.90E-04	0.004996	IFITM3	10410	interferon induced transmembrane protein 3
ENSG0000	282.5005	0.578817	0.163207	3.546521	3.90E-04	0.004996	MIR4435-2	541471	MIR4435-2 host gene
ENSG0000	1816.698	-0.40081	0.113027	-3.54613	3.91E-04	0.005001	TRAF3IP3	80342	TRAF3 interacting protein 3
ENSG0000	2701.176	0.271478	0.07664	3.542252	3.97E-04	0.005071	FBL	2091	fibrillarin
ENSG0000	9055.834	0.314607	0.08882	3.542071	3.97E-04	0.005071	HNRNPA1	3178	heterogeneous nuclear ribonucleoprotein A1
ENSG0000	128.6612	-0.80867	0.228345	-3.54146	3.98E-04	0.005076	FBLN5	10516	fibulin 5
ENSG0000	2421.814	-0.30894	0.087232	-3.54152	3.98E-04	0.005076	ABCD1	215	ATP binding cassette subfamily D member 1
ENSG0000	249.4235	-0.61236	0.173042	-3.53882	4.02E-04	0.00512	PAPSS1	9061	3'-phosphoadenosine 5'-phosphosulfate synthase 1
ENSG0000	92.47521	-0.9417	0.266123	-3.53861	4.02E-04	0.00512	GATB	5188	glutamyl-tRNA amidotransferase subunit B
ENSG0000	248.3374	0.636749	0.179939	3.538694	4.02E-04	0.00512	ZFAS1	441951	ZNFX1 antisense RNA 1
ENSG0000	854.2009	-0.42524	0.120182	-3.53828	4.03E-04	0.005123	SNED1	25992	sushi nidogen and EGF like domains 1
ENSG0000	5634.733	0.262266	0.074133	3.537777	4.04E-04	0.005129	MECP2	4204	methyl-CpG binding protein 2
ENSG0000	135.4085	-0.73768	0.208531	-3.53751	4.04E-04	0.005131	WNT7A	7476	Wnt family member 7A
ENSG0000	2637.662	0.299244	0.084647	3.535211	4.07E-04	0.005169	YTHDC1	91746	YTH domain containing 1
ENSG0000	721.1846	0.397015	0.112302	3.535246	4.07E-04	0.005169	PRPS1	5631	phosphoribosyl pyrophosphate synthetase 1
ENSG0000	6130.908	0.345999	0.09789	3.534587	4.08E-04	0.005174	RPL35A	6165	ribosomal protein L35a
ENSG0000	1230.686	-0.52618	0.148859	-3.53476	4.08E-04	0.005174	NA	NA	NA
ENSG0000	5270.544	0.284805	0.080623	3.53256	4.12E-04	0.00521	SIRT2	22933	sirtuin 2
ENSG0000	3740.869	0.284784	0.080651	3.53107	4.14E-04	0.005236	ILF2	3608	interleukin enhancer binding factor 2
ENSG0000	504.2945	0.417382	0.118218	3.530599	4.15E-04	0.005242	COX7A2L	9167	cytochrome c oxidase subunit 7A2 like
ENSG0000	468.5085	-0.44414	0.125861	-3.5288	4.17E-04	0.005274	DPP3	10072	dipeptidyl peptidase 3
ENSG0000	911.26	-0.40962	0.116116	-3.52769	4.19E-04	0.005293	CEP164	22897	centrosomal protein 164
ENSG0000	669.0828	0.463381	0.131419	3.525991	4.22E-04	0.005323	MAP1A	4130	microtubule associated protein 1A
ENSG0000	1408.183	-0.33584	0.095251	-3.52582	4.22E-04	0.005323	RAB11FIP4	84440	RAB11 family interacting protein 4
ENSG0000	2706.84	0.302733	0.085867	3.525586	4.23E-04	0.005324	BACH2	60468	BTB domain and CNC homolog 2
ENSG0000	1374.662	0.376003	0.106698	3.52399	4.25E-04	0.005353	GLS	2744	glutaminase
ENSG0000	1837.63	0.299798	0.085106	3.522647	4.27E-04	0.005376	OXSR1	9943	oxidative stress responsive kinase 1
ENSG0000	695.6085	0.580995	0.164958	3.522075	4.28E-04	0.00538	TMEM88	92162	transmembrane protein 88
ENSG0000	150.8472	-0.81347	0.230958	-3.52214	4.28E-04	0.00538	PLCB1	23236	phospholipase C beta 1
ENSG0000	19670.15	0.661834	0.187951	3.521315	4.29E-04	0.005392	CDKN1A	1026	cyclin dependent kinase inhibitor 1A
ENSG0000	1385.581	-0.31847	0.090457	-3.52071	4.30E-04	0.005393	AGO1	26523	argonaute RISC component 1
ENSG0000	799.9665	0.406688	0.115511	3.520758	4.30E-04	0.005393	TRAT1	50852	T cell receptor associated transmembrane adaptor 1
ENSG0000	816.6299	-0.44145	0.125374	-3.52106	4.30E-04	0.005393	TRAPPC9	83696	trafficking protein particle complex subunit 9
ENSG0000	1059.21	0.346485	0.098423	3.520366	4.31E-04	0.005397	EPAS1	2034	endothelial PAS domain protein 1
ENSG0000	396.1141	0.477601	0.135697	3.519613	4.32E-04	0.005409	ADRB2	154	adrenoceptor beta 2
ENSG0000	2374.371	-0.32682	0.092885	-3.5185	4.34E-04	0.005428	AP2A2	161	adaptor related protein complex 2 subunit alpha 2
ENSG0000	2355.672	0.411855	0.117082	3.517661	4.35E-04	0.005437	EAF1	85403	ELL associated factor 1
ENSG0000	281.2902	-0.63238	0.179781	-3.5175	4.36E-04	0.005437	DIPK2A	205428	divergent protein kinase domain 2A
ENSG0000	393.1029	-0.62476	0.1776	-3.51781	4.35E-04	0.005437	CD163	9332	CD163 molecule
ENSG0000	13963.1	0.246575	0.070121	3.516423	4.37E-04	0.005456	RPL5	6125	ribosomal protein L5
ENSG0000	2957.955	0.410529	0.116813	3.514405	4.41E-04	0.005494	NUFIP2	57532	nuclear FMR1 interacting protein 2
ENSG0000	132.2395	1.975878	0.562259	3.514176	4.41E-04	0.005495	DNAAF1	123872	dynein axonemal assembly factor 1
ENSG0000	1789.684	-0.36102	0.102765	-3.5131	4.43E-04	0.005513	NA	NA	NA
ENSG0000	892.8975	0.428546	0.122041	3.511507	4.46E-04	0.005543	CORO7	79585	coronin 7
ENSG0000	9432.307	0.320471	0.09128	3.510858	4.47E-04	0.005552	SIRPA	140885	signal regulatory protein alpha
ENSG0000	827.343	0.387248	0.110369	3.508667	4.50E-04	0.005595	PPP2CB	5516	protein phosphatase 2 catalytic subunit beta
ENSG0000	1582.349	-0.3812	0.108655	-3.5083	4.51E-04	0.005599	APOL2	23780	apolipoprotein L2
ENSG0000	1749.075	-0.38264	0.109078	-3.50796	4.52E-04	0.005602	XAF1	54739	XIAP associated factor 1
ENSG0000	5.979056	4.528113	1.290947	3.50759	4.52E-04	0.005602	NA	NA	NA
ENSG0000	673.7398	-0.50467	0.143878	-3.50762	4.52E-04	0.005602	SCO2	9997	synthesis of cytochrome C oxidase 2
ENSG0000	812.7486	-0.40234	0.114755	-3.50609	4.55E-04	0.00563	PSKH1	5681	protein serine kinase H1
ENSG0000	1856.873	0.390207	0.111314	3.505475	4.56E-04	0.005639	LUCAT1	1.01E+08	lung cancer associated transcript 1

ENSG0000	1289.644	0.38232	0.109088	3.504678	4.57E-04	0.005653	USP32	84669	ubiquitin specific peptidase 32
ENSG0000	33442.15	0.26807	0.076539	3.502381	4.61E-04	0.005698	RACK1	10399	receptor for activated C kinase 1
ENSG0000	15.3716	2.28496	0.652641	3.501098	4.63E-04	0.005721	GPR19	2842	G protein-coupled receptor 19
ENSG0000	1213975	1.47162	0.420418	3.500374	4.65E-04	0.005733	NA	NA	NA
ENSG0000	182.1416	-0.6683	0.190945	-3.49994	4.65E-04	0.005739	SFXN5	94097	sideroflexin 5
ENSG0000	1915.476	0.356681	0.101961	3.498192	4.68E-04	0.005773	UXT	8409	ubiquitously expressed prefoldin like chaperone
ENSG0000	12869.83	0.281191	0.08039	3.497822	4.69E-04	0.005777	RBM38	55544	RNA binding motif protein 38
ENSG0000	49979.09	0.279364	0.079928	3.495185	4.74E-04	0.00583	SRGN	5552	serglycin
ENSG0000	1368.456	0.37417	0.107083	3.494218	4.75E-04	0.005848	ZFP91	80829	ZFP91 zinc atypical E3 ubiquitin ligase
ENSG0000	334.8864	0.578969	0.165739	3.493253	4.77E-04	0.005865	ST3GAL4	6484	ST3 beta-g 3-sialyltransferase 4
ENSG0000	253.7998	-0.55703	0.159468	-3.49303	4.78E-04	0.005866	ZNF37A	7587	zinc finger protein 37A
ENSG0000	25546.97	-1.14024	0.326528	-3.49202	4.79E-04	0.005873	TGFBI	7045	transforming growth factor beta induced
ENSG0000	1560.564	0.317148	0.090811	3.492415	4.79E-04	0.005873	SMURF1	57154	SMAD specific E3 ubiquitin protein ligase 1
ENSG0000	134.3444	0.850579	0.243572	3.492105	4.79E-04	0.005873	MYOM2	9172	myomesin 2
ENSG0000	1696.717	-0.32957	0.094371	-3.49229	4.79E-04	0.005873	SAMD1	90378	sterile alpha motif domain containing 1
ENSG0000	1612.595	0.304642	0.087244	3.491828	4.80E-04	0.005873	NOB1	28987	NIN1 (RPN12) binding protein 1 homolog
ENSG0000	2109.769	-0.35233	0.100914	-3.49143	4.80E-04	0.005878	EHMT2	10919	euchromatic histone lysine methyltransferase 2
ENSG0000	707.2849	0.521656	0.14943	3.49097	4.81E-04	0.005884	DGAT2	84649	diacylglycerol O-acyltransferase 2
ENSG0000	91.67164	0.944983	0.270842	3.489059	4.85E-04	0.005922	HEY2	23493	hes related family bHLH transcription factor with YRPW motif 2
ENSG0000	405.2504	-0.51175	0.146692	-3.48861	4.86E-04	0.005928	TCHP	84260	trichoplein keratin filament binding
ENSG0000	10169.27	0.271093	0.077717	3.488185	4.86E-04	0.005934	WBP2	23558	WW domain binding protein 2
ENSG0000	68.04232	1.46484	0.420106	3.486838	4.89E-04	0.00596	AKR1C1	1645	aldo-keto reductase family 1 member C1
ENSG0000	1141.126	-0.39899	0.11445	-3.4862	4.90E-04	0.00597	VPS13C	54832	vacuolar protein sorting 13 homolog C
ENSG0000	159.493	-0.72367	0.207621	-3.48552	4.91E-04	0.005978	PLPP6	403313	phospholipid phosphatase 6
ENSG0000	1058.068	0.388404	0.111433	3.485551	4.91E-04	0.005978	ZNF267	10308	zinc finger protein 267
ENSG0000	2428.061	0.280897	0.080606	3.484811	4.92E-04	0.00599	SLC25A44	9673	solute carrier family 25 member 44
ENSG0000	448.2632	0.488326	0.140168	3.48385	4.94E-04	0.006003	LINC00877	285286	long intergenic non-protein coding RNA 877
ENSG0000	55.60592	1.188512	0.341143	3.483912	4.94E-04	0.006003	ITGA2	3673	integrin subunit alpha 2
ENSG0000	131.4078	0.889124	0.255306	3.482579	4.97E-04	0.006024	NA	NA	NA
ENSG0000	775.8303	0.436236	0.125263	3.48256	4.97E-04	0.006024	KPNA2	3838	karyopherin subunit alpha 2
ENSG0000	1048.351	0.328992	0.094472	3.482419	4.97E-04	0.006024	KIR3DL2	3812	killer cell i three Ig domains and long cytoplasmic tail 2
ENSG0000	316.2896	-0.50211	0.144216	-3.48167	4.98E-04	0.006036	ZNF341	84905	zinc finger protein 341
ENSG0000	2758.774	-0.27806	0.079872	-3.48128	4.99E-04	0.006041	BIRC6	57448	baculoviral IAP repeat containing 6
ENSG0000	71.50244	1.105315	0.317555	3.480702	5.00E-04	0.00605	TNFRSF8	943	TNF receptor superfamily member 8
ENSG0000	196.2179	-0.79677	0.228921	-3.48056	5.00E-04	0.00605	SETBP1	26040	SET binding protein 1
ENSG0000	1778.767	0.337501	0.097116	3.475227	5.10E-04	0.006165	TC2N	123036	tandem C2 nuclear
ENSG0000	17788.52	0.25081	0.072174	3.475081	5.11E-04	0.006165	FBRS	64319	fibrosin
ENSG0000	122.9463	-0.9077	0.261211	-3.47497	5.11E-04	0.006165	SIRPB2	284759	signal regulatory protein beta 2
ENSG0000	28.76325	-1.79904	0.517871	-3.47391	5.13E-04	0.006186	ASGR1	432	asialoglycoprotein receptor 1
ENSG0000	1128.078	0.343987	0.099065	3.472326	5.16E-04	0.006218	SRA1	10011	steroid receptor RNA activator 1
ENSG0000	111.6179	1.052959	0.303346	3.471149	5.18E-04	0.006238	TRGV3	6976	T cell receptor gamma variable 3
ENSG0000	275.7646	-0.54275	0.156363	-3.47111	5.18E-04	0.006238	ALDH3B1	221	aldehyde dehydrogenase 3 family member B1
ENSG0000	1835.469	0.357122	0.102911	3.470214	5.20E-04	0.006255	HSH2D	84941	hematopoietic SH2 domain containing
ENSG0000	1803.995	0.360518	0.103902	3.469783	5.21E-04	0.00626	CSNK1A1	1452	casein kinase 1 alpha 1
ENSG0000	301.446	0.626914	0.180685	3.469648	5.21E-04	0.00626	KIR2DL3	3804	killer cell i two Ig domains and long cytoplasmic tail 3
ENSG0000	711.995	-0.40753	0.117583	-3.46595	5.28E-04	0.006343	PRR14L	253143	proline rich 14 like
ENSG0000	12188.09	-0.30506	0.088031	-3.46539	5.29E-04	0.006352	GBP2	2634	guanylate binding protein 2
ENSG0000	771.7861	0.431161	0.12447	3.463988	5.32E-04	0.006381	KLRB1	3820	killer cell lectin like receptor B1
ENSG0000	768.2139	0.367042	0.106006	3.462468	5.35E-04	0.006413	RPS4Y1	6192	ribosomal protein S4 Y-linked 1
ENSG0000	1087.063	-0.4837	0.13975	-3.46118	5.38E-04	0.00644	UNC93B1	81622	unc-93 hoi TLR signaling regulator
ENSG0000	94.1725	0.972651	0.281065	3.460594	5.39E-04	0.006449	FSTL3	10272	follistatin like 3
ENSG0000	97.23798	-1.27595	0.368814	-3.45959	5.41E-04	0.006469	MMP2	4313	matrix metallopeptidase 2
ENSG0000	6161.268	-0.26214	0.075781	-3.45923	5.42E-04	0.006474	LAIR1	3903	leukocyte associated immunoglobulin like receptor 1
ENSG0000	2020.603	-0.32846	0.094982	-3.45816	5.44E-04	0.006495	PARP4	143	poly(ADP-ribose) polymerase family member 4
ENSG0000	491.8007	-0.44048	0.127412	-3.45716	5.46E-04	0.006515	RALGAPA2	57186	Ral GTPase activating protein catalytic subunit alpha 2
ENSG0000	6489.315	-0.27243	0.078884	-3.45356	5.53E-04	0.006596	TNRC18	84629	trinucleotide repeat containing 18
ENSG0000	236.8216	-0.62908	0.182156	-3.4535	5.53E-04	0.006596	IDE	3416	insulin degrading enzyme
ENSG0000	3772.233	-0.32196	0.093261	-3.45228	5.56E-04	0.006617	PSD4	23550	pleckstrin and Sec7 domain containing 4
ENSG0000	774.2371	-0.35645	0.103252	-3.45228	5.56E-04	0.006617	PPP1R37	284352	protein phosphatase 1 regulatory subunit 37
ENSG0000	1825.541	-0.28345	0.082114	-3.45195	5.57E-04	0.006621	PYGB	5834	glycogen phosphorylase B
ENSG0000	1096.463	0.380544	0.110344	3.448708	5.63E-04	0.006697	ATP6V1C1	528	ATPase H+ transporting V1 subunit C1
ENSG0000	24.89252	-1.84469	0.535071	-3.44757	5.66E-04	0.006721	FAM238C	387644	family with sequence similarity 238 member C
ENSG0000	430.5296	-0.54325	0.1576	-3.44701	5.67E-04	0.00673	ZFHX3	463	zinc finger homeobox 3
ENSG0000	1182.274	0.351907	0.102101	3.446649	5.68E-04	0.006735	ETV3	2117	ETS variant transcription factor 3
ENSG0000	29.15467	2.059159	0.597621	3.445593	5.70E-04	0.006757	LOC12231	1.22E+08	uncharacterized LOC122319436
ENSG0000	12103.58	0.284092	0.082514	3.442975	5.75E-04	0.006819	TRIM28	10155	tripartite motif containing 28
ENSG0000	178.7912	0.640863	0.186174	3.442274	5.77E-04	0.006832	PLB1	151056	phospholipase B1
ENSG0000	2841.378	0.370502	0.107645	3.441884	5.78E-04	0.006837	TAMALIN	160622	trafficking regulator and scaffold protein tamalin
ENSG0000	221.0891	-0.63643	0.184918	-3.44169	5.78E-04	0.006838	CEP290	80184	centrosomal protein 290
ENSG0000	400.1455	-0.72223	0.209893	-3.44094	5.80E-04	0.006853	NLRP12	91662	NLR family pyrin domain containing 12
ENSG0000	209.6672	0.766776	0.222889	3.440161	5.81E-04	0.006868	SPRY2	10253	sprouty RTK signaling antagonist 2
ENSG0000	193.6775	-0.75838	0.220528	-3.43894	5.84E-04	0.006895	CAMK1	8536	calcium/calmodulin dependent protein kinase I
ENSG0000	1565.236	-0.3512	0.102188	-3.43674	5.89E-04	0.006946	MED24	9862	mediator complex subunit 24
ENSG0000	550.7108	-0.49909	0.145277	-3.43545	5.92E-04	0.006975	NCKIPSD	51517	NCK interacting protein with SH3 domain
ENSG0000	7587.153	0.262675	0.076472	3.434913	5.93E-04	0.006985	RPL24	6152	ribosomal protein L24
ENSG0000	444.0654	0.605201	0.176225	3.434255	5.94E-04	0.006995	ADGRB1	575	adhesion G protein-coupled receptor B1
ENSG0000	2907.857	0.304986	0.08881	3.434158	5.94E-04	0.006995	DDA1	79016	DET1 and DDB1 associated 1
ENSG0000	533.4517	0.414243	0.120659	3.433175	5.97E-04	0.007016	SNHG12	85028	small nucleolar RNA host gene 12
ENSG0000	763.484	0.454361	0.132371	3.432491	5.98E-04	0.007029	CTU2	348180	cytosolic thiouridylase subunit 2
ENSG0000	1437.458	-0.34008	0.099083	-3.43229	5.99E-04	0.00703	SMG6	23293	SMG6 nonsense mediated mRNA decay factor
ENSG0000	1399.094	0.371742	0.108378	3.43005	6.03E-04	0.007084	EIF2S2	8894	eukaryotic translation initiation factor 2 subunit beta
ENSG0000	18.0435	2.50082	0.729134	3.429852	6.04E-04	0.007085	NA	NA	NA
ENSG0000	394.4768	0.524723	0.15311	3.427087	6.10E-04	0.007153	COQ10B	80219	coenzyme Q10B
ENSG0000	460.7241	-0.42158	0.123079	-3.42528	6.14E-04	0.007196	ELMOD3	84173	ELMO domain containing 3
ENSG0000	885.4833	0.419992	0.122678	3.423532	6.18E-04	0.007229	NDUFS5	4725	NADH:ubiquinone oxidoreductase subunit S5
ENSG0000	416.0126	0.533272	0.155757	3.423745	6.18E-04	0.007229	SNHG15	285958	small nucleolar RNA host gene 15
ENSG0000	3302.08	0.272771	0.079674	3.423579	6.18E-04	0.007229	PRKX	5613	protein kinase cAMP-dependent X-linked catalytic subunit
ENSG0000	303.3482	0.525659	0.153568	3.422968	6.19E-04	0.007239	PGAP1	80055	post-GPI attachment to proteins inositol deacylase 1
ENSG0000	766.8341	0.42902	0.125352	3.422524	6.20E-04	0.007246	ZC3H12D	340152	zinc finger CCCH-type containing 12D
ENSG0000	4374.629	0.276458	0.080784	3.422194	6.21E-04	0.00725	CASC3	22794	CASC3 exon junction complex subunit
ENSG0000	4395.113	0.26005	0.076019	3.420872	6.24E-04	0.007281	TMEM184	25829	transmembrane protein 184B
ENSG0000	23434.28	-0.27898	0.08157	-3.42014	6.26E-04	0.007296	GRN	2896	granulin precursor
ENSG0000	16068.77	0.247428	0.072359	3.41946	6.27E-04	0.00731	DNM2	1785	dynamin 2
ENSG0000	1164.428	0.323513	0.094647	3.41811	6.31E-04	0.007342	PURB	5814	purine rich element binding protein B
ENSG0000	85.14456	0.897401	0.262603	3.417325	6.32E-04	0.00735	MET	4233	MET proto receptor tyrosine kinase
ENSG0000	49.32031	-1.23072	0.360144	-3.4173	6.32E-04	0.00735	PLLP	51090	plasmolipin
ENSG0000	3098.305	0.308959	0.09041	3.417313	6.32E-04	0.00735	DEDD2	162989	death effector domain containing 2
ENSG0000	2265.259	0.399299	0.116855	3.417063	6.33E-04	0.007352	SLC1A5	6510	solute carrier family 1 member 5
ENSG0000	2123.397	0.284794	0.083354	3.41668	6.34E-04	0.007357	TRA2A	29896	transformer 2 alpha homolog
ENSG0000	1288.47	-0.38306	0.112208	-3.41387	6.40E-04	0.007429	MDGA1	266727	MAM domain containing glycosylphosphatidylinositol anchor 1

ENSG0000	485.553	0.430725	0.126181	3.413555	6.41E-04	0.007433	THAP9-AS	1E+08	THAP9 antisense RNA 1
ENSG0000	476.0317	0.45129	0.132277	3.411694	6.46E-04	0.007476	MED10	84246	mediator complex subunit 10
ENSG0000	863.8805	-0.36898	0.108154	-3.41162	6.46E-04	0.007476	NEK9	91754	NIMA related kinase 9
ENSG0000	58.92806	-1.0751	0.315149	-3.41139	6.46E-04	0.007478	CROCCP3	114819	CROCC pseudogene 3
ENSG0000	98.48637	-1.02502	0.300641	-3.40946	6.51E-04	0.007526	ARHGAP2	58504	Rho GTPase activating protein 22
ENSG0000	13.81414	3.485197	1.022408	3.408812	6.52E-04	0.00754	RAPSN	5913	receptor associated protein of the synapse
ENSG0000	752.7782	-0.37193	0.109128	-3.40822	6.54E-04	0.007551	AP3B1	8546	adaptor related protein complex 3 subunit beta 1
ENSG0000	154.8619	0.718849	0.21095	3.407672	6.55E-04	0.007562	PRKAR1B-	1.02E+08	PRKAR1B antisense RNA 1
ENSG0000	5152.9	-0.2463	0.072285	-3.40739	6.56E-04	0.007565	IL16	3603	interleukin 16
ENSG0000	229.6478	0.590511	0.173316	3.407144	6.56E-04	0.007567	NGFR	4804	nerve growth factor receptor
ENSG0000	1145.724	0.449353	0.131914	3.406402	6.58E-04	0.007578	TMEM176	28959	transmembrane protein 176B
ENSG0000	2862.103	-0.29738	0.087301	-3.40641	6.58E-04	0.007578	ABCA7	10347	ATP binding cassette subfamily A member 7
ENSG0000	1157.213	0.356257	0.104609	3.405602	6.60E-04	0.007596	MANF	7873	mesencephalic astrocyte derived neurotrophic factor
ENSG0000	503.152	-0.41507	0.122099	-3.39947	6.75E-04	0.007759	WDR24	84219	WD repeat domain 24
ENSG0000	1712.052	0.346142	0.101821	3.399519	6.75E-04	0.007759	FAM110A	83541	family with sequence similarity 110 member A
ENSG0000	76.41044	-1.00159	0.294726	-3.39839	6.78E-04	0.007784	PSD3	23362	pleckstrin and Sec7 domain containing 3
ENSG0000	1384.185	0.305672	0.089985	3.396924	6.81E-04	0.007821	GNAI3	2773	G protein subunit alpha i3
ENSG0000	591.6439	0.43987	0.129501	3.396657	6.82E-04	0.007824	GCNT4	51301	glucosaminyl (N-acetyl) transferase 4
ENSG0000	17697.61	-0.87254	0.25692	-3.39614	6.83E-04	0.007834	VCAN	1462	versican
ENSG0000	1185.157	0.409769	0.120795	3.392266	6.93E-04	0.007937	UTP4	84916	UTP4 small subunit processome component
ENSG0000	296577.8	1.346439	0.396919	3.392222	6.93E-04	0.007937	NA	NA	NA
ENSG0000	636.0384	0.45065	0.132909	3.390677	6.97E-04	0.007977	UBASH3B	84959	ubiquitin associated and SH3 domain containing B
ENSG0000	17769.95	0.228668	0.067444	3.390485	6.98E-04	0.007978	ARPC2	10109	actin related protein 2/3 complex subunit 2
ENSG0000	293.8063	-0.54244	0.160009	-3.39003	6.99E-04	0.007986	KLHDC7B	113730	kelch domain containing 7B
ENSG0000	185.0944	-0.65925	0.194524	-3.38904	7.01E-04	0.00801	PELI3	246330	pellino E3 ubiquitin protein ligase family member 3
ENSG0000	946.5483	-0.43967	0.129742	-3.38883	7.02E-04	0.008011	MEGF9	1955	multiple EGF like domains 9
ENSG0000	1300.891	0.356176	0.105139	3.387662	7.05E-04	0.008041	RHOQ	23433	ras homolog family member Q
ENSG0000	312.5138	-0.5356	0.158145	-3.38676	7.07E-04	0.008057	SMARCAL	50485	SWI/SNF r matrix ass actin depe subfamily a like 1
ENSG0000	24.03967	-2.00495	0.591997	-3.38677	7.07E-04	0.008057	NA	NA	NA
ENSG0000	290.5766	0.718804	0.212261	3.386409	7.08E-04	0.008058	HES4	57801	hes family bHLH transcription factor 4
ENSG0000	1110.982	0.398302	0.117614	3.386507	7.08E-04	0.008058	PRKY	5616	protein kinase Y-linked (pseudogene)
ENSG0000	378.2641	-0.47827	0.141242	-3.38615	7.09E-04	0.00806	JRK	8629	Jrk helix-turn-helix protein
ENSG0000	806.1357	0.413753	0.122241	3.384748	7.12E-04	0.008097	RNF138	51444	ring finger protein 138
ENSG0000	101.7088	-0.87655	0.258998	-3.38438	7.13E-04	0.008102	BAZ2B-AS	643072	BAZ2B antisense RNA 1
ENSG0000	205.6431	-0.62515	0.184731	-3.3841	7.14E-04	0.008106	RECK	8434	reversion inducing cysteine rich protein with kazal motifs
ENSG0000	1167.597	-0.32455	0.095936	-3.38303	7.17E-04	0.008132	GFI1	2672	growth factor independent 1 transcriptional repressor
ENSG0000	1884.29	0.428029	0.126529	3.382852	7.17E-04	0.008133	GPRIN3	285513	GPRIN family member 3
ENSG0000	2387.027	0.324339	0.095947	3.380404	7.24E-04	0.0082	PAIP2	51247	poly(A) binding protein interacting protein 2
ENSG0000	741.2017	-0.41184	0.121856	-3.37968	7.26E-04	0.008217	ZMYM3	9203	zinc finger MYM-type containing 3
ENSG0000	148.6937	-0.70929	0.2099	-3.37919	7.27E-04	0.008227	SH3RF1	57630	SH3 domain containing ring finger 1
ENSG0000	269.8775	-0.56373	0.166851	-3.37866	7.28E-04	0.008233	BLTP3A	54887	bridge-like lipid transfer protein family member 3A
ENSG0000	1253.971	-0.30624	0.09064	-3.37866	7.28E-04	0.008233	NELL2	4753	neural EGFL like 2
ENSG0000	515.0149	0.406873	0.120441	3.378192	7.30E-04	0.008242	SAMD8	142891	sterile alpha motif domain containing 8
ENSG0000	1512.938	-0.49503	0.146545	-3.37801	7.30E-04	0.008242	OAS3	4940	2'-5'-oligoadenylate synthetase 3
ENSG0000	7796.035	-0.26826	0.079432	-3.37718	7.32E-04	0.008262	ABLIM1	3983	actin binding LIM protein 1
ENSG0000	2024.705	0.382172	0.113186	3.376495	7.34E-04	0.008277	HELZ	9931	helicase with zinc finger
ENSG0000	1474.347	-0.37145	0.11003	-3.37593	7.36E-04	0.008289	BRAT1	221927	BRCA1 associated ATM activator 1
ENSG0000	3480.854	0.260478	0.077171	3.375336	7.37E-04	0.008302	TGIF1	7050	TGFB induced factor homeobox 1
ENSG0000	3309.697	-0.26849	0.079588	-3.37355	7.42E-04	0.008351	MTSS1	9788	MTSS I-BAR domain containing 1
ENSG0000	1073.694	-0.32615	0.096788	-3.36977	7.52E-04	0.008446	DMAP1	55929	DNA methyltransferase 1 associated protein 1
ENSG0000	21.2778	-2.15194	0.638592	-3.36982	7.52E-04	0.008446	ST6GALNA	256435	ST6 N-acet 6-sialyltransferase 3
ENSG0000	3235.447	0.351772	0.104378	3.370162	7.51E-04	0.008446	CPD	1362	carboxypeptidase D
ENSG0000	560.8656	-0.45889	0.136167	-3.37006	7.52E-04	0.008446	APOL1	8542	apolipoprotein L1
ENSG0000	848.6538	0.343356	0.101911	3.369176	7.54E-04	0.008459	TAF9	6880	TATA-box binding protein associated factor 9
ENSG0000	444.3284	0.470701	0.139925	3.363945	7.68E-04	0.008616	DNAJB9	4189	DnaJ heat shock protein family (Hsp40) member B9
ENSG0000	14859.59	0.326337	0.097019	3.363631	7.69E-04	0.008621	RPS28	6234	ribosomal protein S28
ENSG0000	457.2501	0.524181	0.155852	3.363319	7.70E-04	0.008625	BMI1	648	BMI1 prot polycomb ring finger
ENSG0000	494.4494	-0.45096	0.134089	-3.36312	7.71E-04	0.008626	LRFN3	79414	leucine rich repeat and fibronectin type III domain containing 3
ENSG0000	305.9133	-0.55445	0.164913	-3.36205	7.74E-04	0.008655	AHCYL2	23382	adenosylhomocysteinase like 2
ENSG0000	776.7398	0.39227	0.116686	3.361755	7.74E-04	0.008658	SCML1	6322	Scm polycomb group protein like 1
ENSG0000	2969.905	0.255296	0.075991	3.359542	7.81E-04	0.008723	DENND3	22898	DENN domain containing 3
ENSG0000	2492.222	-0.32846	0.097813	-3.35808	7.85E-04	0.008764	BICRA	29998	BRD4 interacting chromatin remodeling complex associated protein
ENSG0000	535.1598	0.394369	0.117459	3.357501	7.87E-04	0.008772	STARD3NL	83930	STARD3 N-terminal like
ENSG0000	1035.943	-0.32166	0.095802	-3.35749	7.87E-04	0.008772	ZNF831	128611	zinc finger protein 831
ENSG0000	10584.49	0.299957	0.089378	3.356031	7.91E-04	0.008803	RPL14	9045	ribosomal protein L14
ENSG0000	253.5231	-0.77432	0.230722	-3.35605	7.91E-04	0.008803	EPHA1	2041	EPH receptor A1
ENSG0000	902.2127	0.395081	0.117716	3.356231	7.90E-04	0.008803	TMCC3	57458	transmembrane and coiled-coil domain family 3
ENSG0000	402.1151	0.467089	0.139199	3.355558	7.92E-04	0.008812	NXT1	29107	nuclear transport factor 2 like export factor 1
ENSG0000	271.2279	0.537752	0.160294	3.354774	7.94E-04	0.008832	SLC16A1	6566	solute carrier family 16 member 1
ENSG0000	25996.95	0.258106	0.076947	3.354348	7.96E-04	0.00884	RPS4X	6191	ribosomal protein S4 X-linked
ENSG0000	263.3647	-0.55526	0.165608	-3.35284	8.00E-04	0.008873	GALNT7	51809	polypeptide N-acetylgalactosaminyltransferase 7
ENSG0000	567.2145	0.447567	0.133481	3.353046	7.99E-04	0.008873	CLN8	2055	CLN8 transmembrane ER and ERGIC protein
ENSG0000	411.4692	-0.43568	0.12994	-3.3529	8.00E-04	0.008873	CRAMP1	57585	cramped chromatin regulator homolog 1
ENSG0000	238.2075	0.558353	0.166559	3.352276	8.02E-04	0.008885	ZDHHC9	51114	zinc finger DHHC-type palmitoyltransferase 9
ENSG0000	3508.219	0.273496	0.081591	3.352044	8.02E-04	0.008888	EHD4	30844	EH domain containing 4
ENSG0000	1307.272	0.327718	0.097815	3.350386	8.07E-04	0.008936	TMEM165	55858	transmembrane protein 165
ENSG0000	4177.622	-0.27392	0.081802	-3.34858	8.12E-04	0.008989	ORAI2	80228	ORAI calcium release-activated calcium modulator 2
ENSG0000	1984.626	-0.27825	0.083103	-3.34825	8.13E-04	0.008994	ANKFY1	51479	ankyrin repeat and FYVE domain containing 1
ENSG0000	1830.256	0.311305	0.093004	3.347217	8.16E-04	0.009022	GHITM	27069	growth hormone inducible transmembrane protein
ENSG0000	439.2915	0.443263	0.132463	3.346309	8.19E-04	0.009047	MED26	9441	mediator complex subunit 26
ENSG0000	28.77508	1.506956	0.450391	3.345884	8.20E-04	0.009055	NA	NA	NA
ENSG0000	2170.077	-0.27398	0.081952	-3.34321	8.28E-04	0.009132	EOMES	8320	eomesodermin
ENSG0000	62.15915	-1.06787	0.319428	-3.34305	8.29E-04	0.009132	CEP72	55722	centrosomal protein 72
ENSG0000	32.31375	1.715048	0.51302	3.343047	8.29E-04	0.009132	NA	NA	NA
ENSG0000	10524.65	0.24082	0.072044	3.342697	8.30E-04	0.009138	TAPBP	6892	TAP binding protein
ENSG0000	173.056	-0.63043	0.188753	-3.33996	8.38E-04	0.009223	NMT2	9397	N-myristoyltransferase 2
ENSG0000	44.73512	3.195886	0.956967	3.339598	8.39E-04	0.009229	NA	NA	NA
ENSG0000	505.0544	0.615314	0.184259	3.339389	8.40E-04	0.009231	NPIPB12	440353	nuclear pore complex interacting protein family member B12
ENSG0000	599.1128	-0.43684	0.130841	-3.33869	8.42E-04	0.009249	KCTD11	147040	potassium channel tetramerization domain containing 11
ENSG0000	98.75496	0.823607	0.246702	3.338465	8.42E-04	0.009251	PTX3	5806	pentraxin 3
ENSG0000	158.8391	0.724003	0.216889	3.338128	8.43E-04	0.009252	ARHGAP2	57636	Rho GTPase activating protein 23
ENSG0000	8807.038	0.364726	0.109262	3.338095	8.44E-04	0.009252	THBD	7056	thrombomodulin
ENSG0000	453.8704	0.430272	0.128929	3.337283	8.46E-04	0.009274	LINS1	55180	lines homolog 1
ENSG0000	1352.719	0.316518	0.094879	3.336019	8.50E-04	0.00931	VDAC2	7417	voltage dependent anion channel 2
ENSG0000	3143.853	0.29542	0.088576	3.33521	8.52E-04	0.009327	TSPYL1	7259	TSPY like 1
ENSG0000	10309.04	0.243707	0.073068	3.33534	8.52E-04	0.009327	SF3A1	10291	splicing factor 3a subunit 1
ENSG0000	291.8809	-0.63963	0.191794	-3.33498	8.53E-04	0.009329	FRY	10129	FRY microtubule binding protein
ENSG0000	547.9933	-0.43833	0.131441	-3.33481	8.54E-04	0.009329	TMEM104	54868	transmembrane protein 104
ENSG0000	333.4995	-0.47867	0.143552	-3.3345	8.55E-04	0.009332	TRAM2	9697	translocation associated membrane protein 2
ENSG0000	420.3359	-0.46515	0.139501	-3.33439	8.55E-04	0.009332	KRT72	140807	keratin 72

ENSG0000	1949.004	0.415943	0.124824	3.332242	8.61E-04	0.009399	HAPLN3	145864	hyaluronan and proteoglycan link protein 3
ENSG0000	1057.499	-0.33113	0.099401	-3.33126	8.65E-04	0.009427	PPHLN1	51535	periphilin 1
ENSG0000	16.13182	2.17793	0.653916	3.330597	8.67E-04	0.009443	PLEKHD1	400224	pleckstrin homology and coiled-coil domain containing D1
ENSG0000	1674.391	-0.31608	0.094906	-3.33044	8.67E-04	0.009443	C16orf54	283897	chromosome 16 open reading frame 54
ENSG0000	1739.833	0.321473	0.096532	3.330239	8.68E-04	0.009445	ARID3B	10620	AT-rich interaction domain 3B
ENSG0000	580.5582	0.427599	0.128501	3.327601	8.76E-04	0.009518	CCNJL	79616	cyclin J like
ENSG0000	2723.84	0.291987	0.087741	3.327816	8.75E-04	0.009518	ABI1	10006	abl interactor 1
ENSG0000	1104.176	0.366087	0.110015	3.327622	8.76E-04	0.009518	TBC1D25	4943	TBC1 domain family member 25
ENSG0000	528.0248	-0.48133	0.14471	-3.32619	8.80E-04	0.00956	RAB30	27314	RAB30 member RAS oncogene family
ENSG0000	5642.543	0.257986	0.07757	3.325855	8.81E-04	0.009566	HLA-DQB1	3119	major hist[illegible] class II DQ beta 1
ENSG0000	32.61788	-1.52262	0.458031	-3.32428	8.86E-04	0.009615	NA	NA	NA
ENSG0000	1128.137	0.382863	0.115222	3.322817	8.91E-04	0.00966	TENT4B	64282	terminal nucleotidyltransferase 4B
ENSG0000	19869.49	0.70851	0.213241	3.322575	8.92E-04	0.009663	EHD1	10938	EH domain containing 1
ENSG0000	1399.001	-0.30177	0.090843	-3.32189	8.94E-04	0.009681	SLFN13	146857	schlafen family member 13
ENSG0000	7.403389	4.238545	1.276424	3.320641	8.98E-04	0.009718	EHF	26298	ETS homologous factor
ENSG0000	6731.489	0.239928	0.072272	3.319793	9.01E-04	0.009742	ATP5F1B	506	ATP synthase F1 subunit beta
ENSG0000	884.5815	0.377462	0.113762	3.318007	9.07E-04	0.009799	SNHG7	84973	small nucleolar RNA host gene 7
ENSG0000	32.67781	-1.49474	0.450602	-3.3172	9.09E-04	0.009821	SLC6A12	6539	solute carrier family 6 member 12
ENSG0000	183.9778	0.616701	0.185949	3.316507	9.12E-04	0.00984	GCA	25801	grancalcin
ENSG0000	32.21722	2.006499	0.605042	3.316294	9.12E-04	0.009842	NA	NA	NA
ENSG0000	1493.844	0.35504	0.107111	3.314682	9.17E-04	0.009893	KLF16	83855	KLF transcription factor 16
ENSG0000	11.17489	-3.27572	0.988664	-3.31328	9.22E-04	0.009937	SLC46A2	57864	solute carrier family 46 member 2
ENSG0000	1392.235	0.378705	0.114341	3.312065	9.26E-04	0.009975	STX1A	6804	syntaxin 1A
ENSG0000	25.36687	-1.74554	0.527175	-3.31113	9.29E-04	0.010002	MAP2K6	5608	mitogen-activated protein kinase kinase 6
ENSG0000	8135.642	0.227629	0.068753	3.310831	9.30E-04	0.010007	PPIA	5478	peptidylprolyl isomerase A
ENSG0000	25522.43	0.27023	0.081632	3.310355	9.32E-04	0.010018	SARAF	51669	store-operated calcium entry associated regulatory factor
ENSG0000	444.4981	-0.46856	0.141565	-3.30986	9.33E-04	0.01003	NCEH1	57552	neutral cholesterol ester hydrolase 1
ENSG0000	57.12511	1.194548	0.361032	3.308703	9.37E-04	0.010066	MTND5P1	1.01E+08	MT-ND5 pseudogene 11
ENSG0000	2078.68	0.285053	0.086174	3.307875	9.40E-04	0.010084	MCMBP	79892	minichromosome maintenance complex binding protein
ENSG0000	664.9518	0.439817	0.132955	3.308009	9.40E-04	0.010084	SDC4	6385	syndecan 4
ENSG0000	459.7638	0.426523	0.129022	3.305806	9.47E-04	0.010153	PLA2G7	7941	phospholipase A2 group VII
ENSG0000	1405.588	0.319083	0.09654	3.305175	9.49E-04	0.01017	RNF185	91445	ring finger protein 185
ENSG0000	2084.879	-0.27031	0.081794	-3.30473	9.51E-04	0.01018	ACLY	47	ATP citrate lyase
ENSG0000	174.8461	-0.66591	0.201537	-3.30414	9.53E-04	0.010196	NBPF3	84224	NBPF member 3
ENSG0000	16661.02	0.262114	0.079364	3.302684	9.58E-04	0.010243	RPS8	6202	ribosomal protein S8
ENSG0000	343.1394	-0.48771	0.147738	-3.30117	9.63E-04	0.010293	LMO7	4008	LIM domain 7
ENSG0000	5172.134	0.274955	0.0833	3.300782	9.64E-04	0.010301	DDX24	57062	DEAD-box helicase 24
ENSG0000	457.9764	0.489485	0.14832	3.300187	9.66E-04	0.010317	S1PR2	9294	sphingosine-1-phosphate receptor 2
ENSG0000	1117.114	0.31812	0.096399	3.300016	9.67E-04	0.010317	ZRANB1	54764	zinc finger RANBP2-type containing 1
ENSG0000	28134.47	0.284869	0.086346	3.299148	9.70E-04	0.010343	ARHGDIA	396	Rho GDP dissociation inhibitor alpha
ENSG0000	8061.365	0.255328	0.077435	3.297305	9.76E-04	0.010405	LRP10	26020	LDL receptor related protein 10
ENSG0000	17.857	2.093981	0.635376	3.295655	9.82E-04	0.010461	LINC02561	1.11E+08	long intergenic non-protein coding RNA 2561
ENSG0000	10.38817	3.209789	0.974016	3.295417	9.83E-04	0.010463	CCL23	6368	C-C motif chemokine ligand 23
ENSG0000	436.6586	0.456144	0.138489	3.293723	9.89E-04	0.010521	RAB12	201475	RAB12 member RAS oncogene family
ENSG0000	303.1773	0.497839	0.151192	3.292764	9.92E-04	0.01055	CLEC4E	26253	C-type lectin domain family 4 member E
ENSG0000	328.7966	0.500255	0.151975	3.291699	9.96E-04	0.010584	NIBAN3	199786	niban apoptosis regulator 3
ENSG0000	442.0983	-0.43321	0.131614	-3.29151	9.97E-04	0.010585	ITGAE	3682	integrin subunit alpha E
ENSG0000	17.19244	-2.1258	0.646108	-3.29017	0.001001	0.01063	ATOH8	84913	atonal bHLH transcription factor 8
ENSG0000	14899.49	0.22124	0.067277	3.288516	0.001007	0.010687	CSNK1D	1453	casein kinase 1 delta
ENSG0000	1942.131	0.315952	0.09611	3.287382	0.001011	0.010717	EDEM1	9695	ER degradation enhancing alpha-mannosidase like protein 1
ENSG0000	472.3825	0.41241	0.125452	3.28739	0.001011	0.010717	SINHCAF	58516	SIN3-HDAC complex associated factor
ENSG0000	6000.193	-0.29825	0.090736	-3.28701	0.001013	0.010725	LMNA	4000	lamin A/C
ENSG0000	4883.4	0.269094	0.081881	3.286409	0.001015	0.010742	MOB1A	55233	MOB kinase activator 1A
ENSG0000	561.2833	0.394973	0.12031	3.282954	0.001027	0.010862	SFT2D1	113402	SFT2 domain containing 1
ENSG0000	599.7489	0.577558	0.175925	3.282982	0.001027	0.010862	CHST7	56548	carbohydrate sulfotransferase 7
ENSG0000	4861.369	0.259083	0.078957	3.281314	0.001033	0.010913	WDR26	80232	WD repeat domain 26
ENSG0000	2860.648	-0.34496	0.105128	-3.28135	0.001033	0.010913	CNDP2	55748	carnosine dipeptidase 2
ENSG0000	2289.783	-0.33347	0.101659	-3.28032	0.001037	0.010933	RABL6	55684	RAB member RAS oncogene family like 6
ENSG0000	159.1619	-0.66516	0.202765	-3.28047	0.001036	0.010933	NA	NA	NA
ENSG0000	113.946	-0.98238	0.299463	-3.28048	0.001036	0.010933	CDH5	1003	cadherin 5
ENSG0000	11.94686	-3.21384	0.980189	-3.27879	0.001043	0.010986	NA	NA	NA
ENSG0000	176.8557	-0.64861	0.198034	-3.27522	0.001056	0.01112	NUDT18	79873	nudix hydrolase 18
ENSG0000	780.2476	0.480022	0.146621	3.273888	0.001061	0.011166	SLC16A10	117247	solute carrier family 16 member 10
ENSG0000	31.22283	-1.89419	0.578676	-3.27332	0.001063	0.011182	CIBAR1	137392	CBY1 interacting BAR domain containing 1
ENSG0000	67.52593	-1.12718	0.344511	-3.27181	0.001069	0.011235	ABCC4	10257	ATP binding cassette subfamily C member 4
ENSG0000	1999.455	0.312119	0.095455	3.269797	0.001076	0.011309	ATP6V0E1	8992	ATPase H+ transporting V0 subunit e1
ENSG0000	2695.821	0.287031	0.08781	3.268764	0.00108	0.011344	ELMO2	63916	engulfment and cell motility 2
ENSG0000	1920.651	0.340597	0.104217	3.268138	0.001083	0.011363	CMTM7	112616	CKLF like MARVEL transmembrane domain containing 7
ENSG0000	726.0073	0.386495	0.118275	3.267772	0.001084	0.011371	FAS	355	Fas cell surface death receptor
ENSG0000	892.9557	-0.36654	0.112188	-3.26719	0.001086	0.011388	ZNF641	121274	zinc finger protein 641
ENSG0000	1566.448	0.297888	0.091192	3.266595	0.001088	0.011406	UBE2K	3093	ubiquitin conjugating enzyme E2 K
ENSG0000	1541.103	0.293396	0.089826	3.266269	0.00109	0.011412	FOXP3	50943	forkhead box P3
ENSG0000	10.98118	2.956704	0.905938	3.263695	0.0011	0.01151	NA	NA	NA
ENSG0000	229.1717	-0.63773	0.19555	-3.26123	0.001109	0.011604	HOMER2	9455	homer scaffold protein 2
ENSG0000	21.50588	-1.88617	0.578515	-3.26036	0.001113	0.011633	PITPNM3	83394	PITPNM family member 3
ENSG0000	554.2742	-0.39189	0.120227	-3.25956	0.001116	0.011646	UBR3	130507	ubiquitin protein ligase E3 component n-recognin 3
ENSG0000	17948.93	0.259059	0.079475	3.259617	0.001116	0.011646	RPS20	6224	ribosomal protein S20
ENSG0000	520.7855	-0.48997	0.15031	-3.25971	0.001115	0.011646	GPSM1	26086	G protein signaling modulator 1
ENSG0000	305.1914	1.308469	0.4015	3.258951	0.001118	0.011665	HCAR2	338442	hydroxycarboxylic acid receptor 2
ENSG0000	141.3464	-0.77145	0.236797	-3.25786	0.001123	0.011701	HS3ST1	9957	heparan sulfate-glucosamine 3-sulfotransferase 1
ENSG0000	376.1389	-0.47516	0.145857	-3.25775	0.001123	0.011701	CPPED1	55313	calcineurin like phosphoesterase domain containing 1
ENSG0000	740.4649	0.399372	0.122605	3.257395	0.001124	0.011709	MIF	4282	macrophage migration inhibitory factor
ENSG0000	8298.812	0.269253	0.082673	3.25683	0.001127	0.011726	RPL21	6144	ribosomal protein L21
ENSG0000	11.08211	-2.68985	0.826422	-3.25481	0.001135	0.011803	CXXC5-AS[illegible]	1.11E+08	CXXC5 antisense RNA 1
ENSG0000	2763.685	-0.28803	0.088505	-3.25438	0.001136	0.011814	GAA	2548	alpha glucosidase
ENSG0000	3427.307	0.290657	0.089319	3.254147	0.001137	0.011817	PTGER4	5734	prostaglandin E receptor 4
ENSG0000	789.8736	0.395724	0.121649	3.253007	0.001142	0.011852	NBN	4683	nibrin
ENSG0000	9.036336	-3.2943	1.012696	-3.253	0.001142	0.011852	MINAR1	23251	membrane integral NOTCH2 associated receptor 1
ENSG0000	5.419901	4.582716	1.408983	3.2525	0.001144	0.011866	ARHGEF37	389337	Rho guanine nucleotide exchange factor 37
ENSG0000	27.8426	-1.45121	0.446279	-3.2518	0.001147	0.011875	LINC0290[illegible]	401563	long intergenic non-protein coding RNA 2908
ENSG0000	1640.057	-0.29697	0.091324	-3.25188	0.001146	0.011875	SNX19	399979	sorting nexin 19
ENSG0000	310.7623	-0.57487	0.176773	-3.25201	0.001146	0.011875	DBP	1628	D-box binding PAR bZIP transcription factor
ENSG0000	55.50188	1.495298	0.460236	3.248985	0.001158	0.011987	NA	NA	NA
ENSG0000	10121.67	-0.26661	0.082092	-3.24773	0.001163	0.012033	CTSD	1509	cathepsin D
ENSG0000	320.1656	-0.49212	0.151567	-3.24692	0.001167	0.012061	GPA33	10223	glycoprotein A33
ENSG0000	3143.557	0.237708	0.073214	3.246739	0.001167	0.012062	TOB2	10766	transduce[illegible] 2
ENSG0000	136.7854	0.73304	0.225826	3.246043	0.00117	0.012085	NA	NA	NA
ENSG0000	3389.779	0.28548	0.087997	3.244203	0.001178	0.012156	STAU1	6780	staufen double-stranded RNA binding protein 1
ENSG0000	648.7779	0.385948	0.119021	3.242688	0.001184	0.01221	TSPAN32	10077	tetraspanin 32
ENSG0000	2804.538	0.254405	0.078456	3.242629	0.001184	0.01221	MAPRE1	22919	microtubule associated protein RP/EB family member 1

ENSG0000	1997.739	0.351077	0.108297	3.241802	0.001188	0.012239	PDE7A	5150	phosphodiesterase 7A
ENSG0000	1928.707	0.287403	0.088708	3.239893	0.001196	0.012314	VAPA	9218	VAMP associated protein A
ENSG0000	2209.827	0.325272	0.100411	3.239417	0.001198	0.012328	TOR4A	54863	torsin family 4 member A
ENSG0000	2424.448	0.284547	0.087869	3.238329	0.001202	0.012368	KCTD10	83892	potassium channel tetramerization domain containing 10
ENSG0000	1615.398	-0.32621	0.10075	-3.23776	0.001205	0.012386	IGF1R	3480	insulin like growth factor 1 receptor
ENSG0000	2651.527	0.293539	0.090675	3.237284	0.001207	0.0124	DDX6	1656	DEAD-box helicase 6
ENSG0000	5628.185	0.233402	0.072108	3.236844	0.001209	0.012412	SRSF5	6430	serine and arginine rich splicing factor 5
ENSG0000	679365.4	0.608897	0.188173	3.235829	0.001213	0.012449	ND1	4535	NADH dehydrogenase subunit 1
ENSG0000	615.5031	-0.41156	0.127262	-3.23398	0.001221	0.012523	SPTBN5	51332	spectrin b non-erythrocytic 5
ENSG0000	3374.936	0.299593	0.092654	3.233474	0.001223	0.012538	RPL36AL	6166	ribosomal protein L36a like
ENSG0000	84.84979	1.076492	0.332988	3.232823	0.001226	0.01256	CLIC2	1193	chloride intracellular channel 2
ENSG0000	3512.294	0.323003	0.099926	3.232437	0.001227	0.01257	SLC12A7	10723	solute carrier family 12 member 7
ENSG0000	495.9392	-0.62757	0.194167	-3.23214	0.001229	0.012576	TLR8	51311	toll like receptor 8
ENSG0000	261.6896	-0.5533	0.171287	-3.23023	0.001237	0.012652	SLC46A3	283537	solute carrier family 46 member 3
ENSG0000	486.6643	-0.44571	0.137985	-3.23011	0.001237	0.012652	XYLT2	64132	xylosyltransferase 2
ENSG0000	1153.849	0.36165	0.111978	3.229653	0.001239	0.012665	GNL3	26354	G protein nucleolar 3
ENSG0000	4806.487	0.290167	0.089888	3.228108	0.001246	0.012727	PPP4C	5531	protein phosphatase 4 catalytic subunit
ENSG0000	1119.025	-0.33604	0.10411	-3.2277	0.001248	0.012738	MYO5A	4644	myosin VA
ENSG0000	24.12985	-2.33969	0.725087	-3.22677	0.001252	0.012766	OLFML3	56944	olfactomedin like 3
ENSG0000	684.3464	0.360707	0.111784	3.226825	0.001252	0.012766	DDB2	1643	damage specific DNA binding protein 2
ENSG0000	4570.226	0.270212	0.083748	3.226495	0.001253	0.012771	TOM1	10043	target of myb1 membrane trafficking protein
ENSG0000	615.5102	0.487851	0.151215	3.226215	0.001254	0.012776	GSR	2936	glutathione-disulfide reductase
ENSG0000	101.9496	0.823661	0.255364	3.225441	0.001258	0.0128	LPAR1	1902	lysophosphatidic acid receptor 1
ENSG0000	86.93516	1.215006	0.376703	3.225367	0.001258	0.0128	IGLV1-44	28823	immunoglobulin lambda variable 1-44
ENSG0000	257.4734	0.65652	0.203595	3.224645	0.001261	0.012825	NA	NA	NA
ENSG0000	486.0533	-0.50619	0.157129	-3.2215	0.001275	0.01296	ADAM28	10863	ADAM metallopeptidase domain 28
ENSG0000	4570.106	-0.242	0.075127	-3.22118	0.001277	0.012967	SGSM2	9905	small G protein signaling modulator 2
ENSG0000	5321.898	0.240478	0.074661	3.220917	0.001278	0.012972	PPARD	5467	peroxisome proliferator activated receptor delta
ENSG0000	2865.455	0.264643	0.082177	3.22041	0.00128	0.012988	PLIN3	10226	perilipin 3
ENSG0000	60.63121	-1.1128	0.345753	-3.21847	0.001289	0.013069	CEP128	145508	centrosomal protein 128
ENSG0000	1334.982	-0.29956	0.093097	-3.21773	0.001292	0.013095	FYCO1	79443	FYVE and coiled-coil domain autophagy adaptor 1
ENSG0000	298.5459	-0.47641	0.148073	-3.21741	0.001294	0.013103	STN1	79991	STN1 subunit of CST complex
ENSG0000	180.3143	-0.6348	0.197342	-3.21677	0.001296	0.013125	PARG	8505	poly(ADP-ribose) glycohydrolase
ENSG0000	288.5898	-0.51168	0.159075	-3.21656	0.001297	0.013127	SLC30A9	10463	solute carrier family 30 member 9
ENSG0000	742.2169	-0.34672	0.107865	-3.21436	0.001307	0.013221	COMMD9	29099	COMM domain containing 9
ENSG0000	5308.114	0.226462	0.070459	3.214106	0.001309	0.013226	LPCAT1	79888	lysophosphatidylcholine acyltransferase 1
ENSG0000	33.38484	1.357688	0.422476	3.213643	0.001311	0.01324	PTGER3	5733	prostaglandin E receptor 3
ENSG0000	6811.316	-0.316	0.098388	-3.21182	0.001319	0.013317	NLRC5	84166	NLR family CARD domain containing 5
ENSG0000	612.253	0.383951	0.119589	3.210596	0.001325	0.013362	AEN	64782	apoptosis enhancing nuclease
ENSG0000	176.8611	-0.70221	0.21872	-3.21054	0.001325	0.013362	TANGO6	79613	transport and golgi organization 6 homolog
ENSG0000	3200.654	0.333235	0.103819	3.209767	0.001328	0.01339	ARIH1	25820	ariadne RBR E3 ubiquitin protein ligase 1
ENSG0000	1249.662	0.302871	0.09438	3.209069	0.001332	0.013416	FBXO34	55030	F-box protein 34
ENSG0000	2424.786	0.318818	0.09937	3.208385	0.001335	0.01344	DLST	1743	dihydrolipoamide S-succinyltransferase
ENSG0000	1708.923	-0.31579	0.098459	-3.20731	0.00134	0.013476	CACNA2D2	9254	calcium voltage-gated channel auxiliary subunit alpha2delta 2
ENSG0000	1320.892	0.318612	0.099335	3.207458	0.001339	0.013476	SNU13	4809	small nuclear ribonucleoprotein 13
ENSG0000	110.3232	-0.75704	0.236061	-3.20696	0.001341	0.013485	FAM131B	9715	family with sequence similarity 131 member B
ENSG0000	27.88311	1.72419	0.537811	3.205943	0.001346	0.013526	NA	NA	NA
ENSG0000	8375.641	0.249934	0.078007	3.20399	0.001355	0.01361	GZMB	3002	granzyme B
ENSG0000	444.7835	-0.48	0.149897	-3.20223	0.001364	0.013682	PPARG	5468	peroxisome proliferator activated receptor gamma
ENSG0000	29527.79	0.256054	0.079963	3.202167	0.001364	0.013682	ITGA5	3678	integrin subunit alpha 5
ENSG0000	1029.453	0.405568	0.126695	3.201141	0.001369	0.013723	STARD8	9754	StAR related lipid transfer domain containing 8
ENSG0000	8183.864	0.360799	0.112729	3.200588	0.001371	0.013742	OST4	1E+08	oligosacch non-catalytic
ENSG0000	865.1713	-0.3736	0.116739	-3.20032	0.001373	0.013747	TRPS1	7227	transcriptional repressor GATA binding 1
ENSG0000	2614.854	0.272681	0.085214	3.199967	0.001374	0.013757	DPF2	5977	double PHD fingers 2
ENSG0000	163.0501	-0.66201	0.206932	-3.19916	0.001378	0.013788	FAM149B1	317662	family with sequence similarity 149 member B1
ENSG0000	15437.86	-0.26444	0.08267	-3.19868	0.001381	0.013803	CORO1A	11151	coronin 1A
ENSG0000	352.1318	0.606378	0.189629	3.197702	0.001385	0.013828	NKX3-1	4824	NK3 homeobox 1
ENSG0000	23593.99	0.273623	0.085568	3.197736	0.001385	0.013828	SERPINA1	5265	serpin family A member 1
ENSG0000	1393.019	0.371847	0.116278	3.197917	0.001384	0.013828	NSMCE3	56160	NSE3 hom SMC5-SMC6 complex component
ENSG0000	1748.3	0.280353	0.087685	3.197272	0.001387	0.013841	ACVR1B	91	activin A receptor type 1B
ENSG0000	954.5988	-0.37885	0.118519	-3.19657	0.001391	0.013867	ZNF219	51222	zinc finger protein 219
ENSG0000	97.89831	-0.82596	0.258407	-3.19633	0.001392	0.013872	ASB13	79754	ankyrin repeat and SOCS box containing 13
ENSG0000	33.57477	-1.46416	0.458223	-3.19529	0.001397	0.013914	CSPG4	1464	chondroitin sulfate proteoglycan 4
ENSG0000	1746.302	0.295908	0.092619	3.194906	0.001399	0.013925	SLC1A4	6509	solute carrier family 1 member 4
ENSG0000	1492.561	-0.37335	0.116889	-3.19402	0.001403	0.013961	ICE1	23379	interactor of little elongation complex ELL subunit 1
ENSG0000	597.621	-0.36885	0.115505	-3.19334	0.001406	0.013986	TRAPPC11	60684	trafficking protein particle complex subunit 11
ENSG0000	12870.44	0.221222	0.069279	3.193188	0.001407	0.013986	PTBP1	5725	polypyrimidine tract binding protein 1
ENSG0000	1944.543	-0.29229	0.091578	-3.1917	0.001414	0.014051	ATP8B2	57198	ATPase phospholipid transporting 8B2
ENSG0000	509.2891	-0.39809	0.124736	-3.19149	0.001415	0.014053	MICU1	10367	mitochondrial calcium uptake 1
ENSG0000	171.2281	-0.66101	0.207152	-3.19095	0.001418	0.014072	LINC00996	285972	long intergenic non-protein coding RNA 996
ENSG0000	198.9467	0.587829	0.184232	3.190696	0.001419	0.014077	NA	NA	NA
ENSG0000	247.6611	-0.52975	0.166262	-3.18622	0.001441	0.014282	CHID1	66005	chitinase domain containing 1
ENSG0000	3742.784	0.27589	0.086589	3.186209	0.001442	0.014282	PATL1	219988	PAT1 hom processing body mRNA decay factor
ENSG0000	5229.975	-0.24863	0.078095	-3.18365	0.001454	0.014399	PLXND1	23129	plexin D1
ENSG0000	104.0809	-0.82938	0.260534	-3.18339	0.001456	0.014399	BAIAP2-DT	440465	BAIAP2 divergent transcript
ENSG0000	795.5319	-0.33982	0.106747	-3.18338	0.001456	0.014399	PROSER3	148137	proline and serine rich 3
ENSG0000	1254.16	-0.31724	0.099713	-3.18151	0.001465	0.014485	TRAK1	22906	trafficking kinesin protein 1
ENSG0000	234.8012	-0.51153	0.160806	-3.18102	0.001468	0.014501	EGLN1	54583	egl-9 family hypoxia inducible factor 1
ENSG0000	6.209773	4.044233	1.271672	3.180249	0.001471	0.014532	LOC10050	1.01E+08	uncharacterized LOC100505774
ENSG0000	2630.598	0.286155	0.089984	3.180054	0.001472	0.014533	APEX1	328	apurinic/apyrimidinic endodeoxyribonuclease 1
ENSG0000	1584.242	0.285135	0.089667	3.179922	0.001473	0.014533	TP53	7157	tumor protein p53
ENSG0000	4.332562	-4.87564	1.533488	-3.17944	0.001476	0.01455	ATP8B1	5205	ATPase phospholipid transporting 8B1
ENSG0000	232.2386	-0.59196	0.186401	-3.17573	0.001495	0.014729	TLR6	10333	toll like receptor 6
ENSG0000	33.5876	-1.5722	0.495157	-3.17516	0.001498	0.01475	ZNF236-DT	1E+08	ZNF236 divergent transcript
ENSG0000	107.9713	-0.77479	0.244066	-3.17453	0.001501	0.014775	RALGPS1	9649	Ral GEF with PH domain and SH3 binding motif 1
ENSG0000	117.7176	0.728587	0.229533	3.174212	0.001502	0.014783	NA	NA	NA
ENSG0000	7730.572	0.230411	0.072628	3.172474	0.001511	0.014864	SRRT	51593	serrate RNA effector molecule
ENSG0000	137.7202	0.667796	0.210522	3.1721	0.001513	0.014875	TMEM38B	55151	transmembrane protein 38B
ENSG0000	2085.581	0.326268	0.102907	3.170504	0.001522	0.014946	CDK17	5128	cyclin dependent kinase 17
ENSG0000	205.9737	-0.61571	0.194213	-3.17026	0.001523	0.014946	ZNF81	347344	zinc finger protein 81
ENSG0000	1831.796	-0.29087	0.091749	-3.17028	0.001523	0.014946	IL13RA1	3597	interleukin 13 receptor subunit alpha 1
ENSG0000	1445.743	0.316049	0.099727	3.169145	0.001529	0.014995	ZNF706	51123	zinc finger protein 706
ENSG0000	2257.015	0.269201	0.084975	3.167993	0.001535	0.015044	DDX27	55661	DEAD-box helicase 27
ENSG0000	295.9758	-0.52007	0.164171	-3.16789	0.001536	0.015044	ZNF75D	7626	zinc finger protein 75D
ENSG0000	11458.93	0.245873	0.077627	3.167363	0.001538	0.015064	HLA-DRB1	3123	major hist class II DR beta 1
ENSG0000	169.2099	0.613346	0.19367	3.166968	0.00154	0.015076	KLHL15	80311	kelch like family member 15
ENSG0000	35.48263	1.647419	0.5205	3.165068	0.00155	0.015167	COL4A2	1284	collagen type IV alpha 2 chain
ENSG0000	2234.958	0.272634	0.08615	3.164643	0.001553	0.015181	RNF149	284996	ring finger protein 149
ENSG0000	4914.547	-0.27799	0.087858	-3.1641	0.001556	0.015201	STAT2	6773	signal transducer and activator of transcription 2
ENSG0000	129.7142	-0.67484	0.213296	-3.16384	0.001557	0.015207	SHMT1	6470	serine hydroxymethyltransferase 1

ENSG0000	597.4128	-0.36194	0.114438	-3.16277	0.001563	0.015255	EXOC4	60412	exocyst complex component 4
ENSG0000	711.2063	0.33994	0.107548	3.160819	0.001573	0.015333	PMF1	11243	polyamine modulated factor 1
ENSG0000	4134.988	0.239623	0.075806	3.161011	0.001572	0.015333	MAT2A	4144	methionine adenosyltransferase 2A
ENSG0000	298.8072	0.621524	0.196632	3.160854	0.001573	0.015333	NA	NA	NA
ENSG0000	881.2422	0.339575	0.107439	3.160624	0.001574	0.015336	OAZ2	4947	ornithine decarboxylase antizyme 2
ENSG0000	320.4379	0.609253	0.192828	3.15956	0.00158	0.015384	HCAR3	8843	hydroxycarboxylic acid receptor 3
ENSG0000	77.11798	-1.0699	0.339301	-3.15325	0.001615	0.015712	LOC10334	1.03E+08	uncharacterized LOC103344931
ENSG0000	3025.023	0.280788	0.089067	3.152549	0.001619	0.015741	PSEN1	5663	presenilin 1
ENSG0000	118.8311	0.794811	0.252188	3.151654	0.001623	0.015781	SHD	56961	Src homology 2 domain containing transforming protein D
ENSG0000	745.5183	-0.35372	0.11225	-3.15118	0.001626	0.01579	SRPK2	6733	SRSF protein kinase 2
ENSG0000	1079.569	-0.31846	0.101056	-3.15128	0.001626	0.01579	PTPA	5524	protein phosphatase 2 phosphatase activator
ENSG0000	2062.591	0.26373	0.083724	3.149993	0.001633	0.015846	FBXO11	80204	F-box protein 11
ENSG0000	451.2892	-0.5251	0.166717	-3.14969	0.001634	0.015855	NUP93	9688	nucleoporin 93
ENSG0000	3585.026	0.233317	0.07408	3.14951	0.001635	0.015856	EIF4H	7458	eukaryotic translation initiation factor 4H
ENSG0000	4345.84	0.225605	0.071639	3.1492	0.001637	0.015865	ITPKB	3707	inositol-trisphosphate 3-kinase B
ENSG0000	1439.061	-0.31545	0.100285	-3.14547	0.001658	0.01606	ZNF638	27332	zinc finger protein 638
ENSG0000	182.6145	0.615992	0.195846	3.145294	0.001659	0.016061	CSNK2B	1460	casein kinase 2 beta
ENSG0000	2132.593	0.305792	0.097244	3.144602	0.001663	0.016082	GPAT4	137964	glycerol-3-phosphate acyltransferase 4
ENSG0000	157.0699	0.721347	0.229389	3.144638	0.001663	0.016082	NA	NA	NA
ENSG0000	62.67204	-1.00949	0.321063	-3.14421	0.001665	0.016095	ATAD5	79915	ATPase family AAA domain containing 5
ENSG0000	943.2585	0.401483	0.127703	3.143877	0.001667	0.016105	SEC61B	10952	SEC61 translocon subunit beta
ENSG0000	2896.061	0.320277	0.101902	3.143006	0.001672	0.016145	HERPUD1	9709	homocysteine inducible ER protein with ubiquitin like domain 1
ENSG0000	360.5473	-0.47675	0.151708	-3.14255	0.001675	0.016162	AKR7A2	8574	aldo-keto reductase family 7 member A2
ENSG0000	322.7823	0.614287	0.195484	3.142388	0.001676	0.016162	ABTB2	25841	ankyrin repeat and BTB domain containing 2
ENSG0000	2428.317	0.290817	0.092581	3.141219	0.001682	0.016219	MAPK7	5598	mitogen-activated protein kinase 7
ENSG0000	2232.746	0.327365	0.104229	3.140829	0.001685	0.016223	ZBTB17	7709	zinc finger and BTB domain containing 17
ENSG0000	4677.608	-0.25569	0.08141	-3.14084	0.001685	0.016223	CAPN1	823	calpain 1
ENSG0000	514.993	0.408914	0.13026	3.139213	0.001694	0.016288	PIK3CB	5291	phosphati 5-bisphosphate 3-kinase catalytic subunit beta
ENSG0000	623.5039	0.398536	0.126945	3.139445	0.001693	0.016288	TREML1	340205	triggering receptor expressed on myeloid cells like 1
ENSG0000	347.3273	0.447729	0.142622	3.139259	0.001694	0.016288	CHD4	1108	chromodomain helicase DNA binding protein 4
ENSG0000	1628.258	0.308701	0.098362	3.138417	0.001699	0.016324	PHF20L1	51105	PHD finger protein 20 like 1
ENSG0000	505.8064	0.419898	0.133863	3.136779	0.001708	0.016407	NEIL1	79661	nei like DNA glycosylase 1
ENSG0000	4477.905	0.335519	0.106981	3.136236	0.001711	0.01642	STX11	8676	syntaxin 11
ENSG0000	335.1137	-0.52166	0.16633	-3.13632	0.001711	0.01642	NEO1	4756	neogenin 1
ENSG0000	1037.669	-0.40164	0.128258	-3.13153	0.001739	0.016677	GVINP1	387751	GTPase very large interferon inducible pseudogene 1
ENSG0000	269.6468	0.668739	0.213597	3.13084	0.001743	0.016707	TRGV7	6981	T cell receptor gamma variable 7 (pseudogene)
ENSG0000	3667.312	-0.24103	0.077034	-3.12884	0.001755	0.016813	GLG1	2734	golgi glycoprotein 1
ENSG0000	30820.86	0.233012	0.07449	3.128084	0.001759	0.016847	RPLP0	6175	ribosomal protein lateral stalk subunit P0
ENSG0000	588.8326	0.400541	0.128061	3.12775	0.001761	0.016858	DERL2	51009	derlin 2
ENSG0000	313.5509	-0.4709	0.150587	-3.12709	0.001765	0.016887	TTLL5	23093	tubulin tyrosine ligase like 5
ENSG0000	2796.281	-0.23888	0.076406	-3.12641	0.00177	0.016908	SFXN3	81855	sideroflexin 3
ENSG0000	1198.815	0.306598	0.098068	3.126402	0.00177	0.016908	SPART	23111	spartin
ENSG0000	34.96208	1.495454	0.478397	3.125971	0.001772	0.016908	PRSS27	83886	serine protease 27
ENSG0000	6130.222	0.642224	0.205428	3.126269	0.00177	0.016908	TRIM25	7706	tripartite motif containing 25
ENSG0000	1126.53	0.350166	0.112018	3.125981	0.001772	0.016908	ZBTB21	49854	zinc finger and BTB domain containing 21
ENSG0000	15.86873	2.434606	0.778921	3.125615	0.001774	0.01692	LINC01537	1.02E+08	long intergenic non-protein coding RNA 1537
ENSG0000	1587.947	-0.32317	0.1034	-3.12543	0.001775	0.016922	SBK1	388228	SH3 domain binding kinase 1
ENSG0000	98.63616	-0.84789	0.271327	-3.12498	0.001778	0.016939	LYSMD4	145748	LysM domain containing 4
ENSG0000	9410.584	0.214427	0.068626	3.124552	0.001781	0.016955	SRPRA	6734	SRP receptor subunit alpha
ENSG0000	19.38633	2.062963	0.660421	3.123708	0.001786	0.016995	SLC22A1	6580	solute carrier family 22 member 1
ENSG0000	9369.279	-0.21102	0.067627	-3.12039	0.001806	0.017178	PRRC2B	84726	proline rich coiled-coil 2B
ENSG0000	481.6959	-0.44823	0.143668	-3.11988	0.001809	0.0172	TBL2	26608	transducin beta like 2
ENSG0000	9660.732	-0.26512	0.084994	-3.11934	0.001813	0.017214	RASGRP2	10235	RAS guanyl releasing protein 2
ENSG0000	24.50021	-1.82942	0.58646	-3.11942	0.001812	0.017214	ERBB3	2065	erb-b2 receptor tyrosine kinase 3
ENSG0000	197.4111	-0.5713	0.183171	-3.11895	0.001815	0.017228	PHF14	9678	PHD finger protein 14
ENSG0000	46.55721	-1.46978	0.471371	-3.11809	0.00182	0.017269	KIAA1549	57670	KIAA1549
ENSG0000	388.4214	-0.60612	0.194412	-3.11769	0.001823	0.017283	ASCL2	430	achaete-scute family bHLH transcription factor 2
ENSG0000	234.2709	0.665178	0.213397	3.117091	0.001826	0.017292	MTCO2P1	1.07E+08	MT-CO2 pseudogene 12
ENSG0000	7197.414	0.280137	0.089865	3.117312	0.001825	0.017292	RPS7	6201	ribosomal protein S7
ENSG0000	85.76212	-0.97717	0.313481	-3.11716	0.001826	0.017292	SIGLEC16	400709	sialic acid binding Ig like lectin 16
ENSG0000	2794.302	1.103914	0.354239	3.116294	0.001831	0.01733	ACOD1	730249	aconitate decarboxylase 1
ENSG0000	1372.908	-0.2808	0.090126	-3.11567	0.001835	0.017358	POLE	5426	DNA polyr catalytic subunit
ENSG0000	1634.424	-0.27609	0.08866	-3.11403	0.001845	0.017446	RANBP10	57610	RAN binding protein 10
ENSG0000	133.4024	-0.66755	0.214492	-3.11222	0.001857	0.017544	PPP1R26	9858	protein phosphatase 1 regulatory subunit 26
ENSG0000	994.6769	0.30667	0.098545	3.111987	0.001858	0.017549	NABP1	64859	nucleic acid binding protein 1
ENSG0000	9280.788	0.261333	0.08403	3.110003	0.001871	0.017658	MYO1G	64005	myosin IG
ENSG0000	2138.005	0.255845	0.082295	3.108887	0.001878	0.017716	HIGD2A	192286	HIG1 hypoxia inducible domain family member 2A
ENSG0000	3711.949	-0.33912	0.109086	-3.10874	0.001879	0.017716	KMT2C	58508	lysine methyltransferase 2C
ENSG0000	203.4569	-0.55242	0.177775	-3.10741	0.001887	0.017787	ADCK5	203054	aarF domain containing kinase 5
ENSG0000	340.1795	0.472374	0.152045	3.106798	0.001891	0.017815	LTA	4049	lymphotoxin alpha
ENSG0000	1274.801	-0.29905	0.096263	-3.10661	0.001892	0.017817	SLFN12L	1.01E+08	schlafen family member 12 like
ENSG0000	6.275207	3.549705	1.143064	3.105431	0.0019	0.017877	NA	NA	NA
ENSG0000	1426.864	0.333274	0.107324	3.105312	0.001901	0.017877	BNIP2	663	BCL2 interacting protein 2
ENSG0000	63561.61	0.594016	0.191338	3.104539	0.001906	0.017906	MTATP6P1	1.06E+08	MT-ATP6 pseudogene 1
ENSG0000	1288.678	0.326328	0.105115	3.104498	0.001906	0.017906	TMOD3	29766	tropomodulin 3
ENSG0000	239.7684	-0.64162	0.206681	-3.10438	0.001907	0.017906	CMTM1	113540	CKLF like MARVEL transmembrane domain containing 1
ENSG0000	15980.52	0.25938	0.083617	3.101988	0.001922	0.018037	ARF1	375	ADP ribosylation factor 1
ENSG0000	14545.19	0.247228	0.079701	3.101932	0.001923	0.018037	CD37	951	CD37 molecule
ENSG0000	2018.013	0.745811	0.2405	3.101089	0.001928	0.018079	ARFGAP3	26286	ADP ribosylation factor GTPase activating protein 3
ENSG0000	788.5798	0.365629	0.117988	3.098869	0.001943	0.018197	PITHD1	57095	PITH domain containing 1
ENSG0000	89.32926	0.819953	0.264598	3.098862	0.001943	0.018197	SFR1	119392	SWI5 dependent homologous recombination repair protein 1
ENSG0000	21.6789	1.812985	0.585111	3.098532	0.001945	0.018208	NA	NA	NA
ENSG0000	6188.656	0.231454	0.074728	3.097298	0.001953	0.018275	NRBP1	29959	nuclear receptor binding protein 1
ENSG0000	711.1816	-0.39208	0.126622	-3.09645	0.001959	0.018318	MORC2	22880	MORC family CW-type zinc finger 2
ENSG0000	895.2709	0.366849	0.118519	3.095282	0.001966	0.018381	ECD	11319	ecdysoneless cell cycle regulator
ENSG0000	1575.647	-0.30234	0.09769	-3.09491	0.001969	0.018395	TWF2	11344	twinfilin actin binding protein 2
ENSG0000	30311.1	0.210472	0.068009	3.094747	0.00197	0.018396	HNRNPUL	11100	heterogeneous nuclear ribonucleoprotein U like 1
ENSG0000	4805.587	0.680585	0.219957	3.094174	0.001974	0.018422	POU2F2	5452	POU class 2 homeobox 2
ENSG0000	94.89041	-0.93071	0.300856	-3.09355	0.001978	0.018452	NMUR1	10316	neuromedin U receptor 1
ENSG0000	7.961262	3.181893	1.028762	3.092934	0.001982	0.018481	OR2A20P	401428	olfactory receptor family 2 subfamily A member 20 pseudogene
ENSG0000	1027.689	0.370148	0.119682	3.092753	0.001983	0.018483	SEMA7A	8482	semaphorin 7A (John Milton Hagen blood group)
ENSG0000	16.92059	2.15865	0.698145	3.091979	0.001988	0.018484	IGKV1-16	28938	immunoglobulin kappa variable 1-16
ENSG0000	856.9329	-0.34123	0.110347	-3.09238	0.001986	0.018484	R3HDM1	23518	R3H domain containing 1
ENSG0000	1540.933	0.307629	0.099488	3.092125	0.001987	0.018484	SLBP	7884	stem-loop binding protein
ENSG0000	185.998	0.681457	0.220391	3.092032	0.001988	0.018484	THAP2	83591	THAP domain containing 2
ENSG0000	994.1599	0.329148	0.106441	3.092294	0.001986	0.018484	TXNL1	9352	thioredoxin like 1
ENSG0000	215.8124	0.553286	0.178965	3.091598	0.001991	0.01849	ARL4A	10124	ADP ribosylation factor like GTPase 4A
ENSG0000	15908.36	0.205355	0.066422	3.091677	0.00199	0.01849	NRGN	4900	neurogranin
ENSG0000	47.23518	-1.24245	0.401906	-3.09139	0.001992	0.018493	SLC4A11	83959	solute carrier family 4 member 11
ENSG0000	251.5853	-0.54336	0.175779	-3.09116	0.001994	0.018498	TNFRSF11	8792	TNF receptor superfamily member 11a

ENSG0000	354.7952	0.484729	0.156902	3.089366	0.002006	0.018601	HMGCS1	3157	3-hydroxy-3-methylglutaryl-CoA synthase 1
ENSG0000	653.9997	0.387577	0.125474	3.088889	0.002009	0.018622	PRR5	55615	proline rich 5
ENSG0000	400396.7	0.717856	0.232475	3.08788	0.002016	0.018676	ND5	4540	NADH dehydrogenase subunit 5
ENSG0000	741.8576	-0.50065	0.16221	-3.08644	0.002026	0.018757	RNASE6	6039	ribonuclease A family member k6
ENSG0000	305.5944	-0.46842	0.151821	-3.08532	0.002033	0.018819	TCEANC2	127428	transcription elongation factor A N-terminal and central domain containing 2
ENSG0000	1346.783	0.285194	0.092495	3.08335	0.002047	0.018934	NEU1	4758	neuraminidase 1
ENSG0000	3774.868	0.282513	0.091641	3.082836	0.00205	0.018953	DNAJC8	22826	DnaJ heat shock protein family (Hsp40) member C8
ENSG0000	66.75151	-0.90654	0.294068	-3.08275	0.002051	0.018953	TIGD2	166815	tigger transposable element derived 2
ENSG0000	1040.101	0.300203	0.097431	3.081194	0.002062	0.019043	DMTN	2039	dematin actin binding protein
ENSG0000	32.86742	1.30462	0.423617	3.079716	0.002072	0.019128	NA	NA	NA
ENSG0000	852.8503	0.332011	0.107838	3.078786	0.002078	0.019179	TMEM39A	55254	transmembrane protein 39A
ENSG0000	3596.896	-0.25221	0.081938	-3.07808	0.002083	0.019214	FAM78A	286336	family with sequence similarity 78 member A
ENSG0000	72.77872	0.978993	0.318103	3.077595	0.002087	0.019236	REG4	83998	regenerating family member 4
ENSG0000	403.45	-0.44869	0.145853	-3.0763	0.002096	0.019283	IL1R2	7850	interleukin 1 receptor type 2
ENSG0000	493.0469	-0.4209	0.136821	-3.07628	0.002096	0.019283	PLCD1	5333	phospholipase C delta 1
ENSG0000	30361.26	0.272602	0.088603	3.076682	0.002093	0.019283	RPL30	6156	ribosomal protein L30
ENSG0000	235.7932	-0.52285	0.169958	-3.07632	0.002096	0.019283	SMC2	10592	structural maintenance of chromosomes 2
ENSG0000	26144.14	0.218254	0.070976	3.075025	0.002105	0.019355	CFL1	1072	cofilin 1
ENSG0000	5.372802	3.849902	1.252512	3.073744	0.002114	0.019428	NA	NA	NA
ENSG0000	117.9939	-0.83017	0.270117	-3.07335	0.002117	0.019444	CDK5	1020	cyclin dependent kinase 5
ENSG0000	12876.84	-0.2523	0.082116	-3.07252	0.002123	0.019489	LILRB2	10288	leukocyte immunoglobulin like receptor B2
ENSG0000	227.8854	-0.52719	0.17162	-3.07185	0.002127	0.019523	CELSR2	1952	cadherin EGF LAG seven-pass G-type receptor 2
ENSG0000	724.0527	-0.43134	0.140444	-3.07126	0.002132	0.019552	ZMYND8	23613	zinc finger MYND-type containing 8
ENSG0000	599.9533	0.416579	0.135684	3.070211	0.002139	0.019606	BCL10	8915	BCL10 immune signaling adaptor
ENSG0000	9.945756	-2.55779	0.833158	-3.06999	0.002141	0.019606	MIR3936H	553103	MIR3936 host gene
ENSG0000	10552.8	0.229745	0.074834	3.070048	0.00214	0.019606	NONO	4841	non-POU domain containing octamer binding
ENSG0000	40.87817	1.159762	0.377859	3.069297	0.002146	0.019642	MIR9-1HG	10485	MIR9-1 host gene
ENSG0000	6326.872	0.306019	0.099712	3.069032	0.002148	0.01965	IRF2BPL	64207	interferon regulatory factor 2 binding protein like
ENSG0000	1001.681	0.367292	0.119685	3.068824	0.002149	0.019654	ACSL3	2181	acyl-CoA synthetase long chain family member 3
ENSG0000	303.3124	-0.48673	0.158622	-3.06848	0.002151	0.019667	UQCC1	55245	ubiquinol-cytochrome c reductase complex assembly factor 1
ENSG0000	1010.369	0.322252	0.105086	3.066558	0.002165	0.019784	IKZF5	64376	IKAROS family zinc finger 5
ENSG0000	1557.107	0.307411	0.100268	3.065893	0.00217	0.019811	CCR4	1233	C-C motif chemokine receptor 4
ENSG0000	289.8567	-0.4687	0.152877	-3.06585	0.002171	0.019811	MPP7	143098	MAGUK p55 scaffold protein 7
ENSG0000	1646.177	0.314134	0.102491	3.064984	0.002177	0.019859	SGK1	6446	serum/glucocorticoid regulated kinase 1
ENSG0000	1008.552	0.335084	0.109368	3.063806	0.002185	0.019928	STARD4	134429	StAR related lipid transfer domain containing 4
ENSG0000	187.8166	0.828873	0.270594	3.063156	0.00219	0.019961	HAS1	3036	hyaluronan synthase 1
ENSG0000	78.36076	1.026032	0.335018	3.062616	0.002194	0.019987	SENCR	1.01E+08	smooth muscle and endothelial cell enriched migration/differentiation-associated lncRNA
ENSG0000	151.2591	-0.64805	0.211625	-3.06228	0.002197	0.02	RHOBTB2	23221	Rho related BTB domain containing 2
ENSG0000	10.25012	-2.29968	0.751386	-3.06058	0.002209	0.020104	NA	NA	NA
ENSG0000	1768.009	-0.28325	0.092556	-3.0603	0.002211	0.020113	MCUB	55013	mitochondrial calcium uniporter dominant negative subunit beta
ENSG0000	375.3162	-0.43017	0.140584	-3.05988	0.002214	0.020131	LPAR5	57121	lysophosphatidic acid receptor 5
ENSG0000	861.7289	0.336454	0.110002	3.0586	0.002224	0.020198	TAPT1	202018	transmembrane anterior posterior transformation 1
ENSG0000	149.2501	-0.7253	0.237132	-3.05865	0.002223	0.020198	ADAMTS1'	170691	ADAM metallopeptidase with thrombospondin type 1 motif 17
ENSG0000	173.9397	-0.60648	0.198368	-3.05735	0.002233	0.020272	PYCR3	65263	pyrroline-5-carboxylate reductase 3
ENSG0000	3470.072	-0.30745	0.10058	-3.0568	0.002237	0.0203	ITGA4	3676	integrin subunit alpha 4
ENSG0000	8.517841	3.112677	1.018605	3.055824	0.002244	0.020356	NA	NA	NA
ENSG0000	5250.33	-0.23576	0.07716	-3.05541	0.002248	0.020374	INPPL1	3636	inositol polyphosphate phosphatase like 1
ENSG0000	5861.356	0.235552	0.077133	3.053832	0.002259	0.020471	SLC38A1	81539	solute carrier family 38 member 1
ENSG0000	2625.624	0.275852	0.090337	3.053596	0.002261	0.020477	MGAT4B	11282	alpha-1 3-mannosyl-glycoprotein 4-beta-N-acetylglucosaminyltransferase B
ENSG0000	1636.274	0.269899	0.088449	3.05147	0.002277	0.020613	UBE2H	7328	ubiquitin conjugating enzyme E2 H
ENSG0000	2250.19	0.24412	0.080039	3.049999	0.002288	0.020704	SH3GLB1	51100	SH3 doma endophilin B1
ENSG0000	8011.034	0.275551	0.090352	3.049749	0.00229	0.020711	BTF3	689	basic transcription factor 3
ENSG0000	712.8196	0.397118	0.130264	3.04856	0.002299	0.020783	LGR6	59352	leucine rich repeat containing G protein-coupled receptor 6
ENSG0000	1882.578	-0.27315	0.089607	-3.04836	0.002301	0.020787	GSE1	23199	Gse1 coiled-coil protein
ENSG0000	5934.334	0.252649	0.082891	3.047959	0.002304	0.020805	CAPZA1	829	capping actin protein of muscle Z-line subunit alpha 1
ENSG0000	6.072044	3.737353	1.226515	3.047131	0.00231	0.020852	NA	NA	NA
ENSG0000	32713.54	0.248396	0.081587	3.044538	0.00233	0.021002	RPS3	6188	ribosomal protein S3
ENSG0000	6.778343	-3.61542	1.187478	-3.04462	0.00233	0.021002	DHRS2	10202	dehydrogenase/reductase 2
ENSG0000	624.1676	0.340922	0.111977	3.044563	0.00233	0.021002	MYO1E	4643	myosin IE
ENSG0000	277.2205	-0.50853	0.167057	-3.04403	0.002334	0.021027	RUFY3	22902	RUN and FYVE domain containing 3
ENSG0000	3652.442	-0.25998	0.085429	-3.04318	0.002341	0.021076	CPSF1	29894	cleavage and polyadenylation specific factor 1
ENSG0000	105.1781	-0.70704	0.232393	-3.04242	0.002347	0.021106	ZNF268	10795	zinc finger protein 268
ENSG0000	479.753	-0.42393	0.139345	-3.04232	0.002348	0.021106	NTSR1	4923	neurotensin receptor 1
ENSG0000	370.5977	-0.44862	0.147446	-3.04259	0.002346	0.021106	DOP1B	9980	DOP1 leucine zipper like protein B
ENSG0000	28.6497	1.681782	0.552969	3.041366	0.002355	0.021163	F2RL2	2151	coagulation factor II thrombin receptor like 2
ENSG0000	534.8774	0.410018	0.134886	3.039732	0.002368	0.021267	CAMLG	819	calcium modulating ligand
ENSG0000	15.44452	2.045487	0.673051	3.039126	0.002373	0.0213	PDE6B-AS:	1.02E+08	PDE6B antisense RNA 1
ENSG0000	2865.243	0.377735	0.124318	3.038445	0.002378	0.021338	SOCS1	8651	suppressor of cytokine signaling 1
ENSG0000	2741.73	0.287082	0.094539	3.036651	0.002392	0.021455	YARS1	8565	tyrosyl-tRNA synthetase 1
ENSG0000	977.4289	0.328176	0.108119	3.035333	0.002403	0.021538	CISH	1154	cytokine inducible SH2 containing protein
ENSG0000	1366.985	0.338402	0.111498	3.035049	0.002405	0.021548	ALAS1	211	5'-aminolevulinate synthase 1
ENSG0000	2023.182	0.283816	0.093548	3.033906	0.002414	0.02162	SEC62	7095	SEC62 hon preprotein translocation factor
ENSG0000	12.10819	-2.16946	0.715182	-3.03343	0.002418	0.021633	RLN3	117579	relaxin 3
ENSG0000	731.4946	-0.37057	0.122163	-3.03346	0.002418	0.021633	TMEM164	84187	transmembrane protein 164
ENSG0000	26922.2	-0.21253	0.070073	-3.03304	0.002421	0.02165	BCL9L	283149	BCL9 like
ENSG0000	1342.221	-0.28542	0.094134	-3.03208	0.002429	0.021709	PITPNM2	57605	phosphatidylinositol transfer protein membrane associated 2
ENSG0000	343.1846	0.456184	0.150497	3.031175	0.002436	0.021764	LINC-PINT	378805	long interg p53 induced transcript
ENSG0000	16.6916	-1.97983	0.653239	-3.03079	0.002439	0.021781	NA	NA	NA
ENSG0000	13824.67	0.214028	0.070627	3.030382	0.002442	0.021794	FGR	2268	FGR proto- Src family tyrosine kinase
ENSG0000	1420.79	0.365627	0.120656	3.030313	0.002443	0.021794	CSNK1E	1454	casein kinase 1 epsilon
ENSG0000	2135.5	0.286644	0.094619	3.029437	0.00245	0.021847	RELT	84957	RELT TNF receptor
ENSG0000	9.624836	2.596137	0.857045	3.029172	0.002452	0.021856	MRPS24	64951	mitochondrial ribosomal protein S24
ENSG0000	625.6095	-0.33826	0.11169	-3.02857	0.002457	0.021889	KCNK6	9424	potassium two pore domain channel subfamily K member 6
ENSG0000	65.32259	0.955697	0.315688	3.027346	0.002467	0.021957	EML2-AS1	1E+08	EML2 antisense RNA 1
ENSG0000	98.65461	0.819611	0.270724	3.027476	0.002466	0.021957	MAPK11	5600	mitogen-activated protein kinase 11
ENSG0000	1182.151	-0.29592	0.097758	-3.02706	0.002469	0.021967	DAGLB	221955	diacylglycerol lipase beta
ENSG0000	496.3262	0.378416	0.125034	3.026503	0.002474	0.021997	SNHG17	388796	small nucleolar RNA host gene 17
ENSG0000	857.6246	-0.31309	0.103464	-3.02612	0.002477	0.022014	RNF26	79102	ring finger protein 26
ENSG0000	8561.48	0.251429	0.083097	3.025743	0.00248	0.022031	ZAP70	7535	zeta chain of T cell receptor associated protein kinase 70
ENSG0000	604.0575	0.3902	0.129004	3.02472	0.002489	0.022094	DCP2	167227	decapping mRNA 2
ENSG0000	6.613397	3.502528	1.158017	3.024592	0.00249	0.022094	COL15A1	1306	collagen type XV alpha 1 chain
ENSG0000	5084.657	0.22481	0.074335	3.024259	0.002492	0.022108	CHD2	1106	chromodomain helicase DNA binding protein 2
ENSG0000	915.2864	0.306089	0.101224	3.023878	0.002496	0.022125	PHF11	51131	PHD finger protein 11
ENSG0000	376.068	0.464869	0.153751	3.023506	0.002499	0.022142	POLR2K	5440	RNA polyn I and III subunit K
ENSG0000	683.0288	0.409385	0.135419	3.023104	0.002502	0.022151	RAB2B	84932	RAB2B member RAS oncogene family
ENSG0000	3896.685	-1.00321	0.331848	-3.02309	0.002502	0.022151	FPR3	2359	formyl peptide receptor 3
ENSG0000	453.8169	0.400245	0.132414	3.02268	0.002505	0.022165	SNHG8	1E+08	small nucleolar RNA host gene 8
ENSG0000	10.24714	2.898767	0.959072	3.022469	0.002507	0.022165	FOXD1	2297	forkhead box D1
ENSG0000	2922.066	0.24	0.079404	3.022512	0.002507	0.022165	UBE2D2	7322	ubiquitin conjugating enzyme E2 D2
ENSG0000	273.4627	-0.54629	0.18076	-3.02217	0.00251	0.022165	TMEM229	161145	transmembrane protein 229B

ENSG0000	3967.892	-0.22406	0.074137	-3.02221	0.002509	0.022165	NCOR1	9611	nuclear receptor corepressor 1
ENSG0000	3682.305	0.228505	0.075617	3.021888	0.002512	0.022176	IST1	9798	IST1 factor associated with ESCRT-III
ENSG0000	3699.895	-0.31867	0.105483	-3.02104	0.002519	0.022227	AP5B1	91056	adaptor related protein complex 5 subunit beta 1
ENSG0000	95.19663	-0.78948	0.261384	-3.02038	0.002525	0.022265	CSTPP1	79096	centriolar satellite-associated tubulin polyglutamylase complex regulator 1
ENSG0000	96.57602	0.822976	0.272519	3.019884	0.002529	0.022291	OPLAH	26873	5-oxoproli ATP-hydrolysing
ENSG0000	3326.652	0.251653	0.083367	3.018605	0.002539	0.022375	TMED5	50999	transmembrane p24 trafficking protein 5
ENSG0000	3117.633	-0.23958	0.079407	-3.01718	0.002551	0.022469	DNMT1	1786	DNA methyltransferase 1
ENSG0000	5354.316	0.252751	0.08378	3.016837	0.002554	0.022484	GYPC	2995	glycophorin C (Gerbich blood group)
ENSG0000	631.3	-0.3664	0.121511	-3.01537	0.002567	0.022583	GIPR	2696	gastric inhibitory polypeptide receptor
ENSG0000	235.5747	-0.54696	0.181427	-3.01477	0.002572	0.022616	ITFG1	81533	integrin alpha FG-GAP repeat containing 1
ENSG0000	267.014	0.588535	0.195269	3.013972	0.002579	0.022665	NA	NA	NA
ENSG0000	25.1485	2.161516	0.717494	3.012592	0.00259	0.022758	IGLV2-18	28814	immunoglobulin lambda variable 2-18
ENSG0000	66.17021	-0.91065	0.302484	-3.01056	0.002608	0.0229	NA	NA	NA
ENSG0000	1074.884	-0.35196	0.116939	-3.00976	0.002615	0.02295	TET3	200424	tet methylcytosine dioxygenase 3
ENSG0000	17.2391	2.292152	0.761899	3.008474	0.002626	0.023025	NA	NA	NA
ENSG0000	13.04903	2.786587	0.926202	3.008617	0.002624	0.023025	LOC10192	1.02E+08	uncharacterized LOC101927822
ENSG0000	357.7095	-0.45386	0.150892	-3.00785	0.002631	0.023062	CHCHD3	54927	coiled-coil-helix-coiled-coil-helix domain containing 3
ENSG0000	4825.806	0.218866	0.072773	3.007506	0.002634	0.023077	HNRNPDL	9987	heterogeneous nuclear ribonucleoprotein D like
ENSG0000	3764.32	-0.27763	0.092316	-3.00732	0.002636	0.02308	MAST3	23031	microtubule associated serine/threonine kinase 3
ENSG0000	386.2369	-0.52547	0.174771	-3.00661	0.002642	0.023123	RECQL5	9400	RecQ like helicase 5
ENSG0000	1378.542	-0.33106	0.110133	-3.00601	0.002647	0.023157	HDAC4	9759	histone deacetylase 4
ENSG0000	194.47	0.546681	0.181928	3.004941	0.002656	0.023221	RBKS	64080	ribokinase
ENSG0000	1450.018	-0.29576	0.098425	-3.00489	0.002657	0.023221	ODF2	4957	outer dense fiber of sperm tails 2
ENSG0000	134.9825	-0.7136	0.237517	-3.00442	0.002661	0.023246	NAALADL1	10004	N-acetylated alpha-linked acidic dipeptidase like 1
ENSG0000	5389.767	0.260999	0.086887	3.003885	0.002666	0.023276	PICALM	8301	phosphatidylinositol binding clathrin assembly protein
ENSG0000	17.93571	-1.71474	0.571027	-3.0029	0.002674	0.02334	FMO4	2329	flavin containing dimethylaniline monoxygenase 4
ENSG0000	1779.222	-0.26628	0.088694	-3.00219	0.00268	0.023373	COASY	80347	Coenzyme A synthase
ENSG0000	107.2418	0.706519	0.235331	3.00224	0.00268	0.023373	LINC0127(	284751	long intergenic non-protein coding RNA 1270
ENSG0000	1419.378	0.387858	0.129269	3.00039	0.002696	0.0235	BANP	54971	BTG3 associated nuclear protein
ENSG0000	2254.72	0.280797	0.093612	2.999575	0.002704	0.023552	YTHDF3	253943	YTH N6-methyladenosine RNA binding protein 3
ENSG0000	22029.44	0.197404	0.065853	2.997668	0.002721	0.023689	BRD2	6046	bromodomain containing 2
ENSG0000	118.6546	-0.75271	0.251276	-2.99554	0.00274	0.023834	RASAL2	9462	RAS protein activator like 2
ENSG0000	4008.313	0.234798	0.078394	2.995103	0.002744	0.023834	H3-3A	3020	H3.3 histone A
ENSG0000	3613.325	0.845238	0.282215	2.995017	0.002744	0.023834	TMEM158	25907	transmembrane protein 158
ENSG0000	16.21949	-2.02921	0.677545	-2.99495	0.002745	0.023834	RADIL	55698	Rap associating with DIL domain
ENSG0000	413.4654	0.457719	0.152812	2.99531	0.002742	0.023834	TMEM65	157378	transmembrane protein 65
ENSG0000	278.3596	-0.51236	0.171061	-2.9952	0.002743	0.023834	EIF2AK4	440275	eukaryotic translation initiation factor 2 alpha kinase 4
ENSG0000	52946.95	-0.24894	0.083141	-2.99416	0.002752	0.023885	PLEC	5339	plectin
ENSG0000	1309.444	0.326312	0.109055	2.99217	0.00277	0.02403	EIF4A3	9775	eukaryotic translation initiation factor 4A3
ENSG0000	454.6883	-0.4228	0.141387	-2.99036	0.002786	0.024161	DPY19L1	23333	dpy-19 like C-mannosyltransferase 1
ENSG0000	1804.774	0.311557	0.104205	2.989856	0.002791	0.02419	KHDC4	22889	KH domair pre-mRNA splicing factor
ENSG0000	3301.781	0.24635	0.082415	2.989135	0.002798	0.024236	PPRC1	23082	PPARG related coactivator 1
ENSG0000	4049.119	-0.23359	0.078161	-2.98863	0.002802	0.024265	RGS19	10287	regulator of G protein signaling 19
ENSG0000	2056.46	-0.26452	0.088533	-2.98779	0.00281	0.02432	CAPG	822	capping ac gelsolin like
ENSG0000	1423.858	0.272113	0.091084	2.987496	0.002813	0.024332	TOMM20	9804	translocase of outer mitochondrial membrane 20
ENSG0000	4696.509	0.250352	0.083828	2.986497	0.002822	0.0244	EIF3H	8667	eukaryotic translation initiation factor 3 subunit H
ENSG0000	717.5133	-0.35512	0.118922	-2.98617	0.002825	0.024415	NMI	9111	N-myc and STAT interactor
ENSG0000	2431.722	-0.2507	0.083959	-2.98598	0.002827	0.024418	IFFO1	25900	intermediate filament family orphan 1
ENSG0000	976.2822	0.902103	0.302168	2.985436	0.002832	0.024451	AKAP12	9590	A-kinase anchoring protein 12
ENSG0000	1035.935	0.288085	0.096518	2.984794	0.002838	0.024491	NECTIN1	5818	nectin cell adhesion molecule 1
ENSG0000	3841.659	0.223002	0.074726	2.984288	0.002842	0.02452	CLK3	1198	CDC like kinase 3
ENSG0000	106.493	0.7179	0.240571	2.984145	0.002844	0.02452	RPL14P1	144581	ribosomal protein L14 pseudogene 1
ENSG0000	1077.497	-0.3751	0.125721	-2.98362	0.002849	0.024551	XKR8	55113	XK related 8
ENSG0000	2511.869	-0.33217	0.111388	-2.98208	0.002863	0.024663	OAS2	4939	2'-5'-oligoadenylate synthetase 2
ENSG0000	374.3212	0.440877	0.147858	2.981751	0.002866	0.024667	WDR47	22911	WD repeat domain 47
ENSG0000	1005.94	-0.33286	0.111628	-2.98182	0.002865	0.024667	ABHD15	116236	abhydrolase domain containing 15
ENSG0000	1021.067	0.28272	0.094826	2.981472	0.002869	0.024678	MACO1	55219	macoilin 1
ENSG0000	485.625	-0.3656	0.122637	-2.98118	0.002871	0.02469	VSIG1	340547	V-set and immunoglobulin domain containing 1
ENSG0000	560.3656	-0.4033	0.13535	-2.97971	0.002885	0.024797	THOP1	7064	thimet oligopeptidase 1
ENSG0000	719.9048	-0.36352	0.122039	-2.97875	0.002894	0.024864	NPHP4	261734	nephrocystin 4
ENSG0000	774.929	-0.35155	0.118037	-2.9783	0.002899	0.024888	DNMT3A	1788	DNA methyltransferase 3 alpha
ENSG0000	722.1289	0.323154	0.10851	2.978103	0.0029	0.02489	SPRED2	200734	sprouty related EVH1 domain containing 2
ENSG0000	315.3223	0.464968	0.156135	2.977998	0.002901	0.02489	MGC1291(	84815	uncharacterized protein MGC12916
ENSG0000	1298.915	0.296123	0.099442	2.977849	0.002903	0.02489	TLE1	7088	TLE family transcriptional corepressor
ENSG0000	701.4493	0.345544	0.116066	2.977134	0.00291	0.024928	MCEMP1	199675	mast cell expressed membrane protein 1
ENSG0000	6886.979	0.260551	0.087518	2.977099	0.00291	0.024928	PRNP	5621	prion protein
ENSG0000	21.47543	1.651863	0.554953	2.97658	0.002915	0.024959	TMEM52	339456	transmembrane protein 52
ENSG0000	1945.841	-0.25491	0.085643	-2.97644	0.002916	0.024959	SLC35E2B	728661	solute carrier family 35 member E2B
ENSG0000	2131.874	-0.30856	0.103689	-2.97582	0.002922	0.024998	MYCBP2	23077	MYC binding protein 2
ENSG0000	713.2726	0.327484	0.110137	2.973417	0.002945	0.025183	INPP5A	3632	inositol polyphosphate-5-phosphatase A
ENSG0000	246.199	-0.50886	0.171192	-2.97246	0.002954	0.02525	PRKCZ	5590	protein kinase C zeta
ENSG0000	3789.826	0.23879	0.080341	2.972215	0.002957	0.025258	RAB35	11021	RAB35 member RAS oncogene family
ENSG0000	1898.317	0.27793	0.093542	2.971174	0.002967	0.025333	METTL9	51108	methyltransferase like 9
ENSG0000	14935.28	0.218934	0.073697	2.970728	0.002971	0.025358	ARHGAP9	64333	Rho GTPase activating protein 9
ENSG0000	1843.077	0.304802	0.102622	2.970155	0.002976	0.025393	S100A12	6283	S100 calcium binding protein A12
ENSG0000	6147.081	-0.24655	0.083016	-2.96996	0.002978	0.025398	CDC25B	994	cell division cycle 25B
ENSG0000	10645.74	-0.28153	0.094807	-2.96947	0.002983	0.025426	RNPEPL1	57140	arginyl aminopeptidase like 1
ENSG0000	1367.724	0.330809	0.111412	2.969242	0.002985	0.025434	BRK1	55845	BRICK1 subunit of SCAR/WAVE actin nucleating complex
ENSG0000	889.3596	-0.34438	0.116053	-2.96746	0.003003	0.02557	CEP250	11190	centrosomal protein 250
ENSG0000	4566.276	0.207945	0.070084	2.96707	0.003007	0.025591	ATP2A2	488	ATPase sarcoplasmic/endoplasmic reticulum Ca2+ transporting 2
ENSG0000	55.87788	1.003101	0.338101	2.966871	0.003008	0.025595	LINC0065(	200261	long intergenic non-protein coding RNA 656
ENSG0000	11.4651	-2.34969	0.79223	-2.96593	0.003018	0.025656	DZIP1L	199221	DAZ interacting zinc finger protein 1 like
ENSG0000	224.5882	-0.63685	0.214728	-2.96586	0.003018	0.025656	RIN1	9610	Ras and Rab interactor 1
ENSG0000	105.333	0.766707	0.258622	2.964587	0.003031	0.025751	PNPLA1	285848	patatin like phospholipase domain containing 1
ENSG0000	18208.19	0.290621	0.09805	2.964019	0.003036	0.025787	RPL27A	6157	ribosomal protein L27a
ENSG0000	4975.937	0.240516	0.081167	2.963218	0.003044	0.025842	POR	5447	cytochrome p450 oxidoreductase
ENSG0000	1060.918	-0.31645	0.106806	-2.96283	0.003048	0.025863	ELAC2	60528	elaC ribonuclease Z 2
ENSG0000	966.9959	-1.27432	0.430122	-2.96269	0.00305	0.025863	FCGR1A	2209	Fc gamma receptor Ia
ENSG0000	7077.315	0.210813	0.071199	2.960892	0.003067	0.02598	COPA	1314	COPI coat complex subunit alpha
ENSG0000	500.9977	-0.45595	0.153991	-2.96087	0.003068	0.02598	ZNF467	168544	zinc finger protein 467
ENSG0000	5.860219	3.973446	1.34188	2.961103	0.003065	0.02598	NA	NA	NA
ENSG0000	32.49671	-1.30004	0.439134	-2.96045	0.003072	0.026004	HKDC1	80201	hexokinase domain containing 1
ENSG0000	521.0657	0.45491	0.153689	2.959947	0.003077	0.026035	C18orf25	147339	chromosome 18 open reading frame 25
ENSG0000	1925.035	0.245667	0.083018	2.9592	0.003084	0.026074	LRRC41	10489	leucine rich repeat containing 41
ENSG0000	207.0123	-0.58818	0.198759	-2.95926	0.003084	0.026074	CWC27	10283	CWC27 spliceosome associated cyclophilin
ENSG0000	290.2528	-0.4424	0.149512	-2.95897	0.003087	0.026074	KLHL12	59349	kelch like family member 12
ENSG0000	2610.712	0.252359	0.085288	2.958917	0.003087	0.026074	MARCHF6	10299	membrane associated ring-CH-type finger 6
ENSG0000	122.8076	-0.70926	0.239757	-2.95824	0.003094	0.02612	TMIGD3	57413	transmembrane and immunoglobulin domain containing 3
ENSG0000	1413.645	0.280906	0.094994	2.957088	0.003106	0.026205	CASP8	841	caspase 8
ENSG0000	862.7885	-0.35321	0.119518	-2.95531	0.003124	0.026345	ERFL	390937	ETS repressor factor like

ENSG0000	1469.001	-0.2542	0.086028	-2.9548	0.003129	0.026364	VPS26B	112936	VPS26 retromer complex component B
ENSG0000	1740.571	-0.25407	0.085982	-2.95489	0.003128	0.026364	MYO18A	399687	myosin XVIIIA
ENSG0000	73126.04	0.21435	0.072549	2.954553	0.003131	0.026374	B2M	567	beta-2-microglobulin
ENSG0000	383.1175	-0.40264	0.136329	-2.95345	0.003142	0.026456	GLIS3	169792	GLIS family zinc finger 3
ENSG0000	9.428824	2.317673	0.78524	2.951549	0.003162	0.026607	LINC0196	1E+08	long intergenic non-protein coding RNA 1960
ENSG0000	270.567	0.532341	0.18046	2.949916	0.003179	0.026724	NA	NA	NA
ENSG0000	1735.655	0.292195	0.099052	2.94992	0.003179	0.026724	PSMA7	5688	proteasome 20S subunit alpha 7
ENSG0000	1795.011	-0.29519	0.100148	-2.9475	0.003204	0.026916	DGKQ	1609	diacylglycerol kinase theta
ENSG0000	590.002	0.403049	0.136746	2.947425	0.003204	0.026916	NA	NA	NA
ENSG0000	1066.77	0.28615	0.097091	2.947244	0.003206	0.02692	GPR65	8477	G protein-coupled receptor 65
ENSG0000	126.4943	-0.72023	0.244446	-2.94636	0.003215	0.026985	SPON1	10418	spondin 1
ENSG0000	63.32126	-1.16086	0.394018	-2.94622	0.003217	0.026985	KLHL29	114818	kelch like family member 29
ENSG0000	7.698859	-3.36897	1.143834	-2.94533	0.003226	0.02705	NA	NA	NA
ENSG0000	75.82823	0.908765	0.308617	2.944632	0.003233	0.027087	STRIP2	57464	striatin interacting protein 2
ENSG0000	91.55904	-0.72046	0.244668	-2.94464	0.003233	0.027087	CRYL1	51084	crystallin lambda 1
ENSG0000	1044.507	-0.33006	0.112139	-2.94329	0.003247	0.027192	RPS6KA4	8986	ribosomal protein S6 kinase A4
ENSG0000	310.9257	-0.49563	0.168423	-2.94274	0.003253	0.027203	PLXNA2	5362	plexin A2
ENSG0000	301.6742	-0.50324	0.171	-2.94292	0.003251	0.027203	GBE1	2632	1 4-alpha-glucan branching enzyme 1
ENSG0000	1213.346	0.295428	0.100391	2.942764	0.003253	0.027203	CCNT1	904	cyclin T1
ENSG0000	49.48608	-1.04787	0.356168	-2.94207	0.00326	0.027251	LOC12490	1.25E+08	uncharacterized LOC124904535
ENSG0000	949.737	-0.31538	0.107219	-2.94144	0.003267	0.027294	ARHGAP2	57584	Rho GTPase activating protein 21
ENSG0000	2332.026	0.264644	0.08999	2.940809	0.003274	0.027337	KCTD20	222658	potassium channel tetramerization domain containing 20
ENSG0000	179.8636	-0.63011	0.214413	-2.93876	0.003295	0.027506	LTBP2	4053	latent transforming growth factor beta binding protein 2
ENSG0000	649.0959	0.381806	0.12993	2.938551	0.003298	0.027512	FNIP1	96459	folliculin interacting protein 1
ENSG0000	190.6747	-0.5395	0.183634	-2.9379	0.003304	0.027546	DHTKD1	55526	dehydrogenase E1 and transketolase domain containing 1
ENSG0000	349.8722	0.414108	0.140954	2.937885	0.003305	0.027546	NA	NA	NA
ENSG0000	3905	-0.29072	0.098971	-2.93741	0.00331	0.027576	APOBR	55911	apolipoprotein B receptor
ENSG0000	1051.044	0.36436	0.124079	2.936512	0.003319	0.027644	SNRPD2	6633	small nuclear ribonucleoprotein D2 polypeptide
ENSG0000	1418.632	-0.36607	0.124695	-2.9357	0.003328	0.027704	LILRA2	11027	leukocyte immunoglobulin like receptor A2
ENSG0000	1482.487	0.420875	0.143383	2.935321	0.003332	0.027725	SLC44A1	23446	solute carrier family 44 member 1
ENSG0000	14470.74	0.23085	0.078671	2.934381	0.003342	0.027797	SH2D2A	9047	SH2 domain containing 2A
ENSG0000	2537.904	0.239264	0.081553	2.933848	0.003348	0.027832	LRRC59	55379	leucine rich repeat containing 59
ENSG0000	3757.289	0.218261	0.074419	2.932849	0.003359	0.027909	C1orf43	25912	chromosome 1 open reading frame 43
ENSG0000	693.0933	-0.346	0.118019	-2.93173	0.003371	0.027997	TULP4	56995	TUB like protein 4
ENSG0000	1296.021	-0.27895	0.095154	-2.93156	0.003373	0.028	TTC31	64427	tetratricopeptide repeat domain 31
ENSG0000	28.08981	1.456603	0.496931	2.9312	0.003377	0.02802	DGCR11	25786	DiGeorge syndrome critical region gene 11
ENSG0000	4255.975	0.244275	0.08337	2.930001	0.00339	0.028116	CD81	975	CD81 molecule
ENSG0000	607.8358	0.521671	0.178139	2.928454	0.003407	0.028244	ARL5B	221079	ADP ribosylation factor like GTPase 5B
ENSG0000	1026.277	0.287906	0.098343	2.927556	0.003416	0.028313	NIPA2	81614	NIPA magnesium transporter 2
ENSG0000	1201.279	0.305926	0.10457	2.925561	0.003438	0.028482	STK26	51765	serine/threonine kinase 26
ENSG0000	1090.773	0.294693	0.100752	2.924943	0.003445	0.028513	SWAP70	23075	switching B cell complex subunit SWAP70
ENSG0000	1547.439	0.268193	0.091688	2.925064	0.003444	0.028513	CSNK2A2	1459	casein kinase 2 alpha 2
ENSG0000	1317.877	0.275932	0.094372	2.923877	0.003457	0.028598	SLC35B2	347734	solute carrier family 35 member B2
ENSG0000	270.0207	-0.50501	0.172746	-2.92344	0.003462	0.028613	VPS8	23355	VPS8 subunit of CORVET complex
ENSG0000	383.0179	-0.46933	0.160541	-2.92345	0.003462	0.028613	TBC1D16	125058	TBC1 domain family member 16
ENSG0000	154.5988	0.671577	0.229783	2.92266	0.003471	0.02866	ITGB3	3690	integrin subunit beta 3
ENSG0000	1825.426	0.257445	0.088086	2.922654	0.003471	0.02866	CRKL	1399	CRK like pı adaptor protein
ENSG0000	1250.761	-0.33277	0.113893	-2.92174	0.003481	0.02872	RREB1	6239	ras responsive element binding protein 1
ENSG0000	1066.547	0.285323	0.097656	2.921725	0.003481	0.02872	CSRNP2	81566	cysteine and serine rich nuclear protein 2
ENSG0000	832.2762	-0.37046	0.126868	-2.92001	0.0035	0.028852	GOLM2	113201	golgi membrane protein 2
ENSG0000	13236.07	0.244901	0.083866	2.920135	0.003499	0.028852	CSNK1G2	1455	casein kinase 1 gamma 2
ENSG0000	202.5163	-0.61767	0.211626	-2.91867	0.003515	0.028964	GOLGA2P	55592	GOLGA2 pseudogene 5
ENSG0000	10054.16	0.245319	0.084079	2.917708	0.003526	0.029041	RPL7	6129	ribosomal protein L7
ENSG0000	677.2813	-0.32131	0.110151	-2.91701	0.003534	0.029086	PDE8A	5151	phosphodiesterase 8A
ENSG0000	23309.67	0.229171	0.078566	2.916939	0.003535	0.029086	FURIN	5045	furin paired basic amino acid cleaving enzyme
ENSG0000	619.011	0.37382	0.128166	2.91668	0.003538	0.029098	ZBTB43	23099	zinc finger and BTB domain containing 43
ENSG0000	104.1473	0.822172	0.281925	2.916281	0.003542	0.029122	MS4A3	932	membrane spanning 4-domains A3
ENSG0000	79.80661	-0.90723	0.311166	-2.91557	0.00355	0.029176	PCDH12	51294	protocadherin 12
ENSG0000	2080.263	-0.23383	0.080213	-2.91506	0.003556	0.02921	STK25	10494	serine/threonine kinase 25
ENSG0000	480.1803	0.450494	0.15455	2.914872	0.003558	0.029215	NEU4	129807	neuraminidase 4
ENSG0000	45.75563	-1.16037	0.398297	-2.91334	0.003576	0.029346	NINL	22981	ninein like
ENSG0000	426.3248	-0.41514	0.142519	-2.91288	0.003581	0.029376	NCF1B	654816	neutrophil cytosolic factor 1B pseudogene
ENSG0000	701.1885	-0.3052	0.10483	-2.91138	0.003598	0.029504	RABL2B	11158	RAB member of RAS oncogene family like 2B
ENSG0000	53.87784	1.011287	0.347455	2.91055	0.003608	0.02957	TEAD3	7005	TEA domain transcription factor 3
ENSG0000	775.871	0.374286	0.128662	2.909052	0.003625	0.029699	TRGC1	6966	T cell receptor gamma constant 1
ENSG0000	2540.165	-0.3039	0.104501	-2.90807	0.003637	0.029779	MIDEAS	91748	mitotic deacetylase associated SANT domain protein
ENSG0000	1374.689	-0.34345	0.118116	-2.90771	0.003641	0.0298	DICER1	23405	dicer 1 ribonuclease III
ENSG0000	37.09602	1.135008	0.390427	2.907094	0.003648	0.029846	CD80	941	CD80 molecule
ENSG0000	29739.95	0.208728	0.071826	2.906026	0.003661	0.029921	IL7R	3575	interleukin 7 receptor
ENSG0000	135.0786	0.724526	0.249316	2.906059	0.00366	0.029921	EPOP	1E+08	elongin BC and polycomb repressive complex 2 associated protein
ENSG0000	1649.333	0.329035	0.113273	2.904792	0.003675	0.030013	TNIP2	79155	TNFAIP3 interacting protein 2
ENSG0000	2285.406	-0.29286	0.100818	-2.90487	0.003674	0.030013	SPPL2B	56928	signal peptide peptidase like 2B
ENSG0000	3852.053	0.230875	0.079495	2.904293	0.003681	0.030039	RIC8A	60626	RIC8 guanine nucleotide exchange factor A
ENSG0000	202.238	-0.72185	0.248551	-2.90425	0.003681	0.030039	PKD1P3	339039	polycystin transient receptor potential channel interacting pseudogene 3
ENSG0000	1380.511	0.322775	0.111195	2.902788	0.003699	0.030159	CCNG1	900	cyclin G1
ENSG0000	837.5801	0.315913	0.108834	2.902719	0.003699	0.030159	SERTAD3	29946	SERTA domain containing 3
ENSG0000	980.8798	0.363895	0.125403	2.901804	0.00371	0.030234	C12orf57	113246	chromosome 12 open reading frame 57
ENSG0000	1361.572	0.290639	0.100179	2.901203	0.003717	0.030279	PHF10	55274	PHD finger protein 10
ENSG0000	1423.918	0.343716	0.11851	2.900311	0.003728	0.030352	TOMM7	54543	translocase of outer mitochondrial membrane 7
ENSG0000	20.12614	-1.64105	0.566099	-2.89888	0.003745	0.030464	CST6	1474	cystatin E/M
ENSG0000	386.8579	0.433966	0.149697	2.898961	0.003744	0.030464	SKAP1	8631	src kinase associated phosphoprotein 1
ENSG0000	7696.31	0.236725	0.081665	2.898725	0.003747	0.030466	MKNK2	2872	MAPK interacting serine/threonine kinase 2
ENSG0000	162.1759	-0.61134	0.210942	-2.89816	0.003754	0.030507	ZNF493	284443	zinc finger protein 493
ENSG0000	7153.275	-0.20047	0.069177	-2.89796	0.003756	0.030513	PBXIP1	57326	PBX homeobox interacting protein 1
ENSG0000	728.8877	0.348197	0.12017	2.89753	0.003761	0.030527	NRROS	375387	negative regulator of reactive oxygen species
ENSG0000	2226.565	-0.22866	0.078918	-2.89741	0.003763	0.030527	PRR5L	79899	proline rich 5 like
ENSG0000	141480.4	0.217967	0.075222	2.897637	0.00376	0.030527	TGFB1	7040	transforming growth factor beta 1
ENSG0000	5639.252	-0.20932	0.072252	-2.89714	0.003766	0.03054	MINK1	50488	misshapen like kinase 1
ENSG0000	11.5563	2.512058	0.867181	2.896807	0.00377	0.030546	PLAT	5327	plasminog tissue type
ENSG0000	8.115003	3.215734	1.110097	2.896805	0.00377	0.030546	GDF15	9518	growth differentiation factor 15
ENSG0000	11613.69	0.204881	0.070738	2.89633	0.003776	0.030579	BRD4	23476	bromodomain containing 4
ENSG0000	752.9971	-0.32251	0.111366	-2.89593	0.00378	0.030595	CYB561D1	284613	cytochrome b561 family member D1
ENSG0000	32.11216	1.602611	0.553435	2.895754	0.003782	0.030595	CHAC1	79094	ChaC glutathione specific gamma-glutamylcyclotransferase 1
ENSG0000	31.49055	-1.32531	0.457657	-2.89587	0.003781	0.030595	NA	NA	NA
ENSG0000	33.24411	-1.78086	0.615025	-2.89559	0.003784	0.030597	NA	NA	NA
ENSG0000	806.181	0.407045	0.140584	2.895384	0.003787	0.030604	TMEM176	55365	transmembrane protein 176A
ENSG0000	88.79225	-0.82419	0.284674	-2.89519	0.003789	0.030609	CRYBG3	131544	crystallin beta-gamma domain containing 3
ENSG0000	2218.497	0.261222	0.090232	2.89502	0.003791	0.030613	PID1	55022	phosphotyrosine interaction domain containing 1
ENSG0000	9.730491	2.692041	0.93013	2.894262	0.003801	0.030674	NA	NA	NA
ENSG0000	1615.922	0.249728	0.086323	2.892949	0.003816	0.030789	VAMP3	9341	vesicle associated membrane protein 3

ENSG0000	4130.488	0.229764	0.07945	2.891928	0.003829	0.030876	MAX	4149	MYC associated factor X
ENSG0000	543.153	0.356734	0.123409	2.890661	0.003844	0.030987	SELENOS	55829	selenoprotein S
ENSG0000	799.214	-0.40803	0.141163	-2.89051	0.003846	0.030988	IFI35	3430	interferon induced protein 35
ENSG0000	173.8651	0.574246	0.198684	2.890251	0.003849	0.031	NA	NA	NA
ENSG0000	532.7542	0.367298	0.12711	2.8896	0.003857	0.031051	EI24	9538	EI24 autophagy associated transmembrane protein
ENSG0000	158.1481	-0.59677	0.2066	-2.88851	0.003871	0.031146	ARSD	414	arylsulfatase D
ENSG0000	343.6439	-0.40705	0.140947	-2.88797	0.003877	0.031186	AGPS	8540	alkylglycerone phosphate synthase
ENSG0000	550.6458	0.444887	0.154057	2.887814	0.003879	0.031187	EVA1B	55194	eva-1 homolog B
ENSG0000	8808.687	0.217653	0.075385	2.887209	0.003887	0.031234	HYOU1	10525	hypoxia up-regulated 1
ENSG0000	6408.191	0.214646	0.074385	2.885603	0.003907	0.031376	KLF6	1316	KLF transcription factor 6
ENSG0000	266.3402	-0.51496	0.178463	-2.88551	0.003908	0.031376	MPV17L2	84769	MPV17 mitochondrial inner membrane protein like 2
ENSG0000	4475.611	-0.2286	0.079241	-2.88487	0.003916	0.031426	NEAT1	283131	nuclear paraspeckle assembly transcript 1
ENSG0000	92.11441	-0.87007	0.301625	-2.88462	0.003919	0.031437	GAREM2	150946	GRB2 associated regulator of MAPK1 subtype 2
ENSG0000	488.6128	-0.36707	0.127371	-2.8819	0.003953	0.031697	NXPH4	11247	neurexophilin 4
ENSG0000	10270.73	0.194758	0.067587	2.881602	0.003957	0.031702	PPP2R5C	5527	protein phosphatase 2 regulatory subunit B'gamma
ENSG0000	135.6172	-0.61686	0.214071	-2.88157	0.003957	0.031702	ERCC4	2072	ERCC excis endonuclease catalytic subunit
ENSG0000	3239.176	-0.22324	0.077482	-2.88119	0.003962	0.031727	DCAF5	8816	DDB1 and CUL4 associated factor 5
ENSG0000	615.2833	0.387824	0.134626	2.880748	0.003967	0.031757	NAF1	92345	nuclear assembly factor 1 ribonucleoprotein
ENSG0000	511.8378	-0.35874	0.124546	-2.88042	0.003971	0.031777	GABPB1-IT	55056	GABPB1 intronic transcript
ENSG0000	2023.069	0.241102	0.08371	2.88022	0.003974	0.031783	PSMC4	5704	proteasom ATPase 4
ENSG0000	40.89277	-1.46109	0.50732	-2.88001	0.003977	0.031791	ELFN2	114794	extracellular leucine rich repeat and fibronectin type III domain containing 2
ENSG0000	3134.856	0.26238	0.091159	2.878262	0.003999	0.031954	MARS1	4141	methionyl-tRNA synthetase 1
ENSG0000	788.7038	-0.3231	0.112306	-2.87698	0.004015	0.03207	PSMB10	5699	proteasome 20S subunit beta 10
ENSG0000	869.011	-0.3538	0.123004	-2.87631	0.004023	0.032124	CKAP5	9793	cytoskeleton associated protein 5
ENSG0000	983.6992	-0.29908	0.104006	-2.87562	0.004032	0.03218	CNTRL	11064	centriolin
ENSG0000	4.164796	4.001264	1.391874	2.874732	0.004044	0.032246	GSG1	83445	germ cell associated 1
ENSG0000	95750.42	0.261597	0.090999	2.874709	0.004044	0.032246	RPL10	6134	ribosomal protein L10
ENSG0000	965.6203	0.352739	0.122738	2.873919	0.004054	0.032313	BUD23	114049	BUD23 rRNA methyltransferase and ribosome maturation factor
ENSG0000	40.14212	-1.11116	0.386682	-2.87359	0.004058	0.032333	MMP28	79148	matrix metallopeptidase 28
ENSG0000	565.5803	-0.37079	0.12906	-2.87305	0.004065	0.032374	LOC72839	728392	uncharacterized LOC728392
ENSG0000	402.0713	0.382542	0.133207	2.871794	0.004081	0.032489	CD244	51744	CD244 molecule
ENSG0000	497.8266	-0.37616	0.130996	-2.87151	0.004085	0.032504	NAGPA	51172	N-acetylglucosamine-1-phosphodiester alpha-N-acetylglucosaminidase
ENSG0000	3482.178	0.295931	0.103073	2.871088	0.004091	0.032534	PTPN7	5778	protein tyrosine phosphatase non-receptor type 7
ENSG0000	18.59077	2.163136	0.753563	2.870545	0.004098	0.032576	IL5RA	3568	interleukin 5 receptor subunit alpha
ENSG0000	238.7274	-0.47544	0.165649	-2.8702	0.004102	0.032584	MMP17	4326	matrix metallopeptidase 17
ENSG0000	4805.473	0.223561	0.077888	2.870269	0.004101	0.032584	STAT5B	6777	signal transducer and activator of transcription 5B
ENSG0000	150.439	0.800184	0.278839	2.869702	0.004109	0.032621	EBI3	10148	Epstein-Barr virus induced 3
ENSG0000	23.54264	-1.66184	0.579222	-2.86909	0.004117	0.03267	NA	NA	NA
ENSG0000	1691.815	-0.26753	0.093251	-2.86895	0.004118	0.032671	TCF20	6942	transcription factor 20
ENSG0000	13028.95	0.188015	0.065538	2.86881	0.00412	0.032671	RPL6	6128	ribosomal protein L6
ENSG0000	1700.391	0.296089	0.103224	2.868409	0.004125	0.032698	MVD	4597	mevalonate diphosphate decarboxylase
ENSG0000	1436.046	-0.29554	0.103081	-2.86704	0.004143	0.032813	CSRP1	1465	cysteine and glycine rich protein 1
ENSG0000	523.7556	0.38324	0.133668	2.867107	0.004142	0.032813	SH2D3A	10045	SH2 domain containing 3A
ENSG0000	485.4441	0.344787	0.120272	2.866729	0.004147	0.03283	ITPRIPL1	150771	ITPRIP like 1
ENSG0000	94.38487	-1.9828	0.691826	-2.86604	0.004156	0.032888	ADORA3	140	adenosine A3 receptor
ENSG0000	244.5829	-0.52249	0.182404	-2.86447	0.004177	0.033037	ZNF446	55663	zinc finger protein 446
ENSG0000	257.465	0.542194	0.189349	2.863457	0.00419	0.033129	NA	NA	NA
ENSG0000	5018.091	0.229067	0.080002	2.863283	0.004193	0.033133	BRPF3	27154	bromodomain and PHD finger containing 3
ENSG0000	495.702	-0.39534	0.138116	-2.86241	0.004204	0.033211	SLC27A4	10999	solute carrier family 27 member 4
ENSG0000	1955.828	-0.23381	0.081695	-2.86199	0.00421	0.03324	CPT1A	1374	carnitine palmitoyltransferase 1A
ENSG0000	236.1288	-0.57284	0.200242	-2.86072	0.004227	0.033359	PRKAR1B	5575	protein kinase cAMP-dependent type I regulatory subunit beta
ENSG0000	2938.25	-0.23774	0.083128	-2.85989	0.004238	0.033433	OSBPL7	114881	oxysterol binding protein like 7
ENSG0000	1140.842	0.279682	0.097814	2.859316	0.004246	0.033479	ANKRD10	55608	ankyrin repeat domain 10
ENSG0000	1445.176	-0.27825	0.097326	-2.85898	0.00425	0.033501	SDC2	6383	syndecan 2
ENSG0000	46.63543	-1.31054	0.45844	-2.85868	0.004254	0.033517	NEBL	10529	nebulette
ENSG0000	115.0576	-0.66568	0.232941	-2.85771	0.004267	0.033606	RSRC1	51319	arginine and serine rich coiled-coil 1
ENSG0000	3064.054	0.300642	0.105217	2.857351	0.004272	0.03363	CARD19	84270	caspase recruitment domain family member 19
ENSG0000	1202.85	0.3142	0.110101	2.853728	0.004321	0.034001	RNF145	153830	ring finger protein 145
ENSG0000	5.326385	3.201796	1.122388	2.852665	0.004335	0.034101	THSD1	55901	thrombospondin type 1 domain containing 1
ENSG0000	6.047143	3.666666	1.285415	2.852514	0.004337	0.034102	NA	NA	NA
ENSG0000	168.2782	-0.52894	0.185479	-2.85177	0.004348	0.034154	LRFN4	78999	leucine rich repeat and fibronectin type III domain containing 4
ENSG0000	321.751	-0.45331	0.158953	-2.85188	0.004346	0.034154	ZSCAN29	146050	zinc finger and SCAN domain containing 29
ENSG0000	11.94465	2.472541	0.867669	2.849635	0.004377	0.034369	SNORD17	692086	small nucl C/D box 17
ENSG0000	5481.543	-0.27768	0.096749	-2.84946	0.004379	0.034373	MBOAT7	79143	membrane bound O-acyltransferase domain containing 7
ENSG0000	1325.432	-0.2829	0.099324	-2.84826	0.004396	0.034489	SH3BP1	23616	SH3 domain binding protein 1
ENSG0000	2436.739	0.23996	0.084264	2.847696	0.004404	0.03452	MTMR14	64419	myotubularin related protein 14
ENSG0000	299.5489	0.4339	0.152364	2.847788	0.004402	0.03452	KCTD9	54793	potassium channel tetramerization domain containing 9
ENSG0000	143.5211	-0.5857	0.205736	-2.84685	0.004415	0.034597	FBXL4	26235	F-box and leucine rich repeat protein 4
ENSG0000	777.156	-0.30696	0.107861	-2.8459	0.004429	0.034686	UBAC1	10422	UBA domain containing 1
ENSG0000	109.1191	-0.64917	0.228153	-2.84534	0.004436	0.034732	ELP4	26610	elongator acetyltransferase complex subunit 4
ENSG0000	286.9538	-0.46626	0.164046	-2.84222	0.00448	0.035034	ACBD6	84320	acyl-CoA binding domain containing 6
ENSG0000	456.026	0.352354	0.123973	2.842185	0.004481	0.035034	AGTPBP1	23287	ATP/GTP binding carboxypeptidase 1
ENSG0000	42377.02	0.215799	0.075921	2.842429	0.004477	0.035034	RPL13	6137	ribosomal protein L13
ENSG0000	2514.777	-0.26793	0.094377	-2.83895	0.004526	0.035333	SCRIB	23513	scribble planar cell polarity protein
ENSG0000	14089.92	0.293502	0.10338	2.839067	0.004525	0.035333	RPL35	11224	ribosomal protein L35
ENSG0000	7.950803	2.801564	0.986834	2.838941	0.004526	0.035333	C11orf91	1E+08	chromosome 11 open reading frame 91
ENSG0000	594.6073	-0.36011	0.126841	-2.83908	0.004524	0.035333	SARM1	23098	sterile alpha and TIR motif containing 1
ENSG0000	1635.769	0.259694	0.091491	2.838452	0.004533	0.035372	FAM118A	55007	family with sequence similarity 118 member A
ENSG0000	8786.865	-0.22253	0.078417	-2.83783	0.004542	0.035426	DGKZ	8525	diacylglycerol kinase zeta
ENSG0000	1675.028	0.245741	0.0866	2.837658	0.004545	0.03543	KLHL36	79786	kelch like family member 36
ENSG0000	2077.714	-0.25366	0.089425	-2.83654	0.004561	0.035539	BOD1L1	259282	biorientation of chromosomes in cell division 1 like 1
ENSG0000	46.84032	-1.13846	0.401418	-2.8361	0.004567	0.035574	ZSWIM5	57643	zinc finger SWIM-type containing 5
ENSG0000	5181.936	0.215425	0.075967	2.835773	0.004571	0.035595	STRN4	29888	striatin 4
ENSG0000	3857.988	0.619774	0.218602	2.835165	0.00458	0.035648	MTND2P2	1.01E+08	MT-ND2 pseudogene 28
ENSG0000	35.7989	1.222377	0.431189	2.834896	0.004584	0.035663	APOO	79135	apolipoprotein O
ENSG0000	235.5682	-0.54995	0.194029	-2.83439	0.004591	0.035704	CTSO	1519	cathepsin O
ENSG0000	823.2036	0.314974	0.11114	2.834019	0.004597	0.035731	CHRNE	1145	cholinergic receptor nicotinic epsilon subunit
ENSG0000	681.5169	-0.35044	0.1237	-2.83295	0.004612	0.035836	EDEM2	55741	ER degradation enhancing alpha-mannosidase like protein 2
ENSG0000	451.1083	0.36843	0.130066	2.832636	0.004617	0.035841	PAPSS2	9060	3'-phosphoadenosine 5'-phosphosulfate synthase 2
ENSG0000	92.83296	-0.78136	0.275835	-2.83269	0.004616	0.035841	IFT140	9742	intraflagellar transport 140
ENSG0000	16.34442	-1.83084	0.646638	-2.83132	0.004636	0.035959	NA	NA	NA
ENSG0000	9544.085	0.215672	0.076172	2.831377	0.004635	0.035959	RPL10A	4736	ribosomal protein L10a
ENSG0000	5442.008	-0.23392	0.082658	-2.82998	0.004655	0.036094	FAM120A	23196	family with sequence similarity 120A
ENSG0000	21043.21	0.210035	0.074222	2.829833	0.004657	0.036094	RPS14	6208	ribosomal protein S14
ENSG0000	1165.538	0.415619	0.146877	2.829715	0.004659	0.036094	CD69	969	CD69 molecule
ENSG0000	3.921835	-4.68761	1.656668	-2.82954	0.004661	0.036099	NA	NA	NA
ENSG0000	51.75352	1.026151	0.362684	2.829329	0.004665	0.036108	NA	NA	NA
ENSG0000	17684.03	0.187205	0.066247	2.825842	0.004716	0.036488	TMBIM6	7009	transmembrane BAX inhibitor motif containing 6
ENSG0000	1385.762	0.254067	0.089926	2.825281	0.004724	0.036536	SGMS1	259230	sphingomyelin synthase 1
ENSG0000	1038.961	0.278154	0.098457	2.825122	0.004726	0.036539	ZNF394	84124	zinc finger protein 394

ENSG0000	214.7397	-0.62479	0.221182	-2.82479	0.004731	0.036562	PTK2	5747	protein tyrosine kinase 2
ENSG0000	687.0852	-0.40503	0.143406	-2.82437	0.004737	0.036595	ABCB8	11194	ATP binding cassette subfamily B member 8
ENSG0000	164.2937	-0.52984	0.18771	-2.82268	0.004762	0.036773	HENMT1	113802	HEN methyltransferase 1
ENSG0000	4700.883	-0.21505	0.076203	-2.82208	0.004771	0.036826	CIZ1	25792	CDKN1A interacting zinc finger protein 1
ENSG0000	175.302	0.592338	0.21002	2.82039	0.004797	0.037005	DLL1	28514	delta like canonical Notch ligand 1
ENSG0000	5132.466	0.235911	0.083677	2.81931	0.004813	0.037091	CCNL1	57018	cyclin L1
ENSG0000	1549.221	-0.25165	0.08926	-2.81925	0.004814	0.037091	MAN2B2	23324	mannosidase alpha class 2B member 2
ENSG0000	15.28929	2.009222	0.712613	2.819514	0.00481	0.037091	NA	NA	NA
ENSG0000	599.5679	-0.38148	0.135323	-2.81902	0.004817	0.037102	RNF20	56254	ring finger protein 20
ENSG0000	2667.982	-0.25137	0.089186	-2.81852	0.004825	0.037144	ERAP1	51752	endoplasmic reticulum aminopeptidase 1
ENSG0000	3891.932	-0.63642	0.225947	-2.81667	0.004853	0.037344	CD86	942	CD86 molecule
ENSG0000	23.20618	-1.48503	0.527676	-2.81428	0.004889	0.037607	CA12	771	carbonic anhydrase 12
ENSG0000	552.0024	0.336815	0.119702	2.813791	0.004896	0.037648	WDR75	84128	WD repeat domain 75
ENSG0000	988.2102	-0.35681	0.12687	-2.81242	0.004917	0.037794	PIP4K2B	8396	phosphatidylinositol-5-phosphate 4-kinase type 2 beta
ENSG0000	1377.381	0.891223	0.316907	2.81226	0.004919	0.037797	SPP1	6696	secreted phosphoprotein 1
ENSG0000	888.7505	-0.3277	0.11654	-2.81186	0.004926	0.037812	TBC1D14	57533	TBC1 domain family member 14
ENSG0000	508.5141	0.343618	0.1222	2.811928	0.004925	0.037812	ZBTB25	7597	zinc finger and BTB domain containing 25
ENSG0000	577.7499	-0.34287	0.121957	-2.81136	0.004933	0.037856	EXOC2	55770	exocyst complex component 2
ENSG0000	25783.38	-0.18857	0.067106	-2.81008	0.004953	0.037991	NCOR2	9612	nuclear receptor corepressor 2
ENSG0000	316.4798	-0.51044	0.181662	-2.80982	0.004957	0.037997	FAF1	11124	Fas associated factor 1
ENSG0000	74.05567	-0.75447	0.268517	-2.80976	0.004958	0.037997	BORA	79866	BORA aurora kinase A activator
ENSG0000	51.65317	-0.9842	0.350533	-2.80771	0.004989	0.038224	NA	NA	NA
ENSG0000	477.1415	-0.46153	0.164429	-2.80684	0.005003	0.038311	IVD	3712	isovaleryl-CoA dehydrogenase
ENSG0000	7151.937	0.230367	0.08209	2.806281	0.005012	0.038362	NAP1L1	4673	nucleosome assembly protein 1 like 1
ENSG0000	117.8611	-0.61824	0.220399	-2.80512	0.00503	0.038485	SEMA3C	10512	semaphorin 3C
ENSG0000	244.2963	0.453619	0.161739	2.804629	0.005037	0.038528	LOC38990	389906	zinc finger protein 839 pseudogene
ENSG0000	2537.046	0.243263	0.086757	2.803938	0.005048	0.038581	ANKLE2	23141	ankyrin repeat and LEM domain containing 2
ENSG0000	675.9994	0.322565	0.115041	2.803913	0.005049	0.038581	QPCTL	54814	glutaminyl-peptide cyclotransferase like
ENSG0000	579.2628	0.31633	0.113011	2.799108	0.005124	0.039144	BLOC1S2	282991	biogenesis of lysosomal organelles complex 1 subunit 2
ENSG0000	102.9302	0.890436	0.318187	2.798471	0.005135	0.039205	LAIR2	3904	leukocyte associated immunoglobulin like receptor 2
ENSG0000	3.391174	4.855912	1.735475	2.79803	0.005142	0.039243	NA	NA	NA
ENSG0000	599.8615	-0.32365	0.115812	-2.79462	0.005196	0.039643	SKAP2	8935	src kinase associated phosphoprotein 2
ENSG0000	16954.96	0.254499	0.091114	2.793201	0.005219	0.039801	S100A9	6280	S100 calcium binding protein A9
ENSG0000	8632.785	0.198068	0.070935	2.792231	0.005235	0.039904	CDC42SE1	56882	CDC42 small effector 1
ENSG0000	3056.932	0.31057	0.111236	2.791993	0.005238	0.039917	IPO7	10527	importin 7
ENSG0000	250.6146	0.536545	0.192251	2.790862	0.005257	0.04004	RNASEK	440400	ribonuclease K
ENSG0000	21614.43	0.258843	0.092772	2.790094	0.005269	0.040119	RPL11	6135	ribosomal protein L11
ENSG0000	1101.753	-0.28629	0.102657	-2.78878	0.005291	0.040266	DOCK9	23348	dedicator of cytokinesis 9
ENSG0000	721.0585	0.386199	0.138499	2.788471	0.005296	0.040282	LY9	4063	lymphocyte antigen 9
ENSG0000	4602.009	0.2148	0.077034	2.788381	0.005297	0.040282	SSH2	85464	slingshot protein phosphatase 2
ENSG0000	29.36677	-1.27644	0.457966	-2.78721	0.005316	0.040403	RN7SL834I	1.06E+08	RNA 7SL cytoplasm pseudogene
ENSG0000	365.9367	-0.4336	0.155571	-2.78715	0.005317	0.040403	MICAL3	57553	microtubu calponin and LIM domain containing 3
ENSG0000	25.88799	1.378942	0.494823	2.786736	0.005324	0.040407	SLC4A5	57835	solute carrier family 4 member 5
ENSG0000	490.3844	0.379201	0.136072	2.786774	0.005324	0.040407	NT5C3A	51251	5'-nucleoti cytosolic IIIA
ENSG0000	46.71241	-1.15237	0.413563	-2.78645	0.005329	0.040407	NRCAM	4897	neuronal cell adhesion molecule
ENSG0000	660.8492	-0.3265	0.11717	-2.78653	0.005328	0.040407	PDP1	54704	pyruvate dehydrogenase phosphatase catalytic subunit 1
ENSG0000	619.0362	0.319251	0.114563	2.786682	0.005325	0.040407	CA5BP1	340591	carbonic anhydrase 5B pseudogene 1
ENSG0000	22.04602	1.803106	0.647235	2.785859	0.005339	0.040464	NA	NA	NA
ENSG0000	177.664	-0.58521	0.210084	-2.78561	0.005343	0.040478	ANKS3	124401	ankyrin repeat and sterile alpha motif domain containing 3
ENSG0000	7347.015	0.281521	0.101096	2.784695	0.005358	0.040576	RPL38	6169	ribosomal protein L38
ENSG0000	13075.8	0.211902	0.076131	2.783382	0.00538	0.040724	GNB1	2782	G protein subunit beta 1
ENSG0000	19.6097	-1.80509	0.6492	-2.78048	0.005428	0.041073	PTGFRN	5738	prostaglandin F2 receptor inhibitor
ENSG0000	1392.156	0.262522	0.09452	2.777428	0.005479	0.041444	RC3H1	149041	ring finger and CCCH-type domains 1
ENSG0000	523.6661	0.335479	0.120794	2.77728	0.005482	0.041446	BORCS5	118426	BLOC-1 related complex subunit 5
ENSG0000	1060.52	0.330384	0.119007	2.776176	0.0055	0.04157	NOP58	51602	NOP58 ribonucleoprotein
ENSG0000	734.436	-0.35928	0.129474	-2.77493	0.005521	0.041713	ALDH16A1	126133	aldehyde dehydrogenase 16 family member A1
ENSG0000	542.6819	0.387364	0.139616	2.774496	0.005529	0.041734	RUBCNL	80183	rubicon like autophagy enhancer
ENSG0000	152.9915	-0.8441	0.304228	-2.77455	0.005528	0.041734	CACNA1H	8912	calcium voltage-gated channel subunit alpha1 H
ENSG0000	429.6907	-0.36348	0.131017	-2.7743	0.005532	0.041742	TOX	9760	thymocyte selection associated high mobility group box
ENSG0000	62.86045	0.884426	0.318819	2.774072	0.005536	0.041755	PET100	1E+08	PET100 cytochrome c oxidase chaperone
ENSG0000	23.97565	1.766941	0.637058	2.773595	0.005544	0.041799	SPINK1	6690	serine peptidase inhibitor Kazal type 1
ENSG0000	760.0691	-0.32582	0.117481	-2.7734	0.005547	0.041807	NUBP1	4682	NUBP iron cytosolic
ENSG0000	12120.35	0.216804	0.078239	2.771032	0.005588	0.042095	RPS5	6193	ribosomal protein S5
ENSG0000	14.11318	-1.94136	0.700661	-2.77075	0.005593	0.042115	LDLRAD2	401944	low density lipoprotein receptor class A domain containing 2
ENSG0000	1305.19	0.247919	0.089484	2.770552	0.005596	0.042123	DESI1	27351	desumoylating isopeptidase 1
ENSG0000	1242.851	0.291458	0.105219	2.769997	0.005606	0.042178	SOAT1	6646	sterol O-acyltransferase 1
ENSG0000	241.004	-0.45234	0.163482	-2.76693	0.005659	0.042542	FBXL17	64839	F-box and leucine rich repeat protein 17
ENSG0000	849.8259	-0.41032	0.148295	-2.76694	0.005658	0.042542	SERPINE1	5054	serpin family E member 1
ENSG0000	27.84094	1.258555	0.454877	2.766804	0.005661	0.042542	AOC2	314	amine oxidase copper containing 2
ENSG0000	19.76004	-1.50823	0.545232	-2.76622	0.005671	0.0426	NA	NA	NA
ENSG0000	3143.028	0.224242	0.081075	2.765855	0.005677	0.042631	PSME4	23198	proteasome activator subunit 4
ENSG0000	5.390772	3.809314	1.377553	2.765276	0.005687	0.042689	LOC10537	1.05E+08	uncharacterized LOC105370525
ENSG0000	152.1985	-0.54429	0.196887	-2.76448	0.005701	0.042777	GTF2IRD2I	389524	GTF2I repeat domain containing 2B
ENSG0000	89.14856	-0.75132	0.271872	-2.76349	0.005719	0.042888	ZCCHC18	644353	zinc finger CCHC-type containing 18
ENSG0000	75.08631	-0.77553	0.280678	-2.76304	0.005727	0.04293	CBR4	84869	carbonyl reductase 4
ENSG0000	790.5396	0.326013	0.118	2.762815	0.005731	0.042943	GNG5	2787	G protein subunit gamma 5
ENSG0000	2909.863	0.233663	0.084602	2.761911	0.005746	0.043014	RNF11	26994	ring finger protein 11
ENSG0000	744.8616	0.365696	0.132408	2.761881	0.005747	0.043014	GSAP	54103	gamma-secretase activating protein
ENSG0000	1038.905	-0.72142	0.261193	-2.76201	0.005745	0.043014	OAS1	4938	2'-5'-oligoadenylate synthetase 1
ENSG0000	85.77966	-0.70214	0.254285	-2.76122	0.005759	0.043066	POLR3B	55703	RNA polymerase III subunit B
ENSG0000	1633.926	0.246003	0.089088	2.761347	0.005756	0.043066	PIP4P1	90809	phosphati 5-bisphosphate 4-phosphatase 1
ENSG0000	1138.129	-0.28828	0.104415	-2.76094	0.005763	0.043086	STK11IP	114790	serine/threonine kinase 11 interacting protein
ENSG0000	318.0195	0.395826	0.143404	2.760217	0.005776	0.043164	GZMK	3003	granzyme K
ENSG0000	3143.929	0.219175	0.079409	2.760084	0.005779	0.043164	PAFAH1B1	5048	platelet activating factor acetylhydrolase 1b regulatory subunit 1
ENSG0000	3821.553	-0.20002	0.072493	-2.75915	0.005795	0.04327	CALCOCO1	57658	calcium binding and coiled-coil domain 1
ENSG0000	3094.464	0.223689	0.081094	2.758393	0.005809	0.043353	MSL1	339287	MSL complex subunit 1
ENSG0000	350.8812	-0.42999	0.155908	-2.75796	0.005816	0.043376	QSER1	79832	glutamine and serine rich 1
ENSG0000	182.8998	-0.61736	0.223839	-2.75807	0.005814	0.043376	SOX8	30812	SRY-box transcription factor 8
ENSG0000	3413.182	0.232701	0.084393	2.757355	0.005827	0.043439	CHMP1A	5119	charged multivesicular body protein 1A
ENSG0000	21.63667	1.693105	0.614322	2.756054	0.00585	0.043577	C2CD4B	388125	C2 calcium dependent domain containing 4B
ENSG0000	156.8837	-0.64028	0.23231	-2.75615	0.005849	0.043577	GNB1L	54584	G protein subunit beta 1 like
ENSG0000	2141.438	0.252034	0.091493	2.754677	0.005875	0.043743	G3BP2	9908	G3BP stress granule assembly factor 2
ENSG0000	566.9203	-0.3396	0.123306	-2.75414	0.005885	0.043798	CCDC71	64925	coiled-coil domain containing 71
ENSG0000	70.68041	-0.9366	0.340111	-2.75382	0.00589	0.043822	GLCE	26035	glucuronic acid epimerase
ENSG0000	5.090484	3.845158	1.39691	2.752616	0.005912	0.043966	NA	NA	NA
ENSG0000	342.0749	0.447004	0.162487	2.751022	0.005941	0.044163	GPX3	2878	glutathione peroxidase 3
ENSG0000	477.0978	-0.4049	0.147203	-2.75059	0.005949	0.044201	PKP4	8502	plakophilin 4
ENSG0000	444.312	0.40196	0.146142	2.750481	0.005951	0.044201	UGCG	7357	UDP-glucose ceramide glucosyltransferase
ENSG0000	35.20317	-1.11786	0.406451	-2.75029	0.005954	0.044208	DNAAF10	116143	dynein axonemal assembly factor 10
ENSG0000	1224.661	-0.31597	0.114891	-2.75014	0.005957	0.044211	MAPKBP1	23005	mitogen-activated protein kinase binding protein 1

ENSG0000	1029.134	0.320817	0.116671	2.749762	0.005964	0.044245	SNRPB2	6629	small nuclear ribonucleoprotein polypeptide B2
ENSG0000	411.3443	-0.3834	0.13946	-2.7492	0.005974	0.044303	EP400P1	347918	EP400 pseudogene 1
ENSG0000	1767.056	-0.2477	0.090141	-2.74789	0.005998	0.044462	FAM43A	131583	family with sequence similarity 43 member A
ENSG0000	8.24686	3.152042	1.147281	2.747403	0.006007	0.044511	KCND3	3752	potassium voltage-gated channel subfamily D member 3
ENSG0000	23286.94	0.254713	0.092753	2.746149	0.00603	0.044663	RPLP1	6176	ribosomal protein lateral stalk subunit P1
ENSG0000	771.4828	0.324614	0.118223	2.745763	0.006037	0.044698	GFOD1	54438	glucose-fructose oxidoreductase domain containing 1
ENSG0000	4305.778	0.233981	0.085227	2.74538	0.006044	0.044732	SERP1	27230	stress associated endoplasmic reticulum protein 1
ENSG0000	1802.272	-0.24778	0.09032	-2.74332	0.006082	0.044996	PKD1	5310	polycystin transient receptor potential channel interacting
ENSG0000	2026.966	0.252536	0.092162	2.740138	0.006141	0.045398	SP3	6670	Sp3 transcription factor
ENSG0000	535.3616	-0.33917	0.123777	-2.74014	0.006141	0.045398	ERICH1	157697	glutamate rich 1
ENSG0000	3.201397	-4.59436	1.676931	-2.73974	0.006149	0.045435	CALHM3	119395	calcium homeostasis modulator 3
ENSG0000	187.6799	0.556091	0.203032	2.73893	0.006164	0.045529	H3C10	8357	H3 clustered histone 10
ENSG0000	1854.176	-0.30531	0.111484	-2.73862	0.00617	0.045549	TMEM250	90120	transmembrane protein 250
ENSG0000	1827.066	-0.23693	0.086518	-2.73852	0.006172	0.045549	CBX7	23492	chromobox 7
ENSG0000	59.98596	0.859194	0.313787	2.738145	0.006179	0.045583	CCR9	10803	C-C motif chemokine receptor 9
ENSG0000	72.31316	-0.92462	0.337813	-2.73706	0.006199	0.045707	JHY	79864	junctional cadherin complex regulator
ENSG0000	426.4227	-0.57757	0.211025	-2.73699	0.0062	0.045707	MIR223HG	1.16E+08	MIR223 host gene
ENSG0000	24.07976	1.473762	0.538541	2.736585	0.006208	0.045727	LINC0135	1.01E+08	long intergenic non-protein coding RNA 1353
ENSG0000	3321.181	-0.20195	0.073796	-2.7366	0.006208	0.045727	UBTF	7343	upstream binding transcription factor
ENSG0000	54.66663	-0.94389	0.344977	-2.73608	0.006217	0.045778	DPH5-DT	1.03E+08	DPH5 divergent transcript
ENSG0000	194.3279	0.695958	0.254506	2.734547	0.006247	0.045975	XIRP1	165904	xin actin binding repeat containing 1
ENSG0000	91099.34	0.230267	0.084234	2.733648	0.006264	0.046082	HLA-A	3105	major hist class I A
ENSG0000	19915.74	0.249155	0.09115	2.733452	0.006267	0.046091	LDHA	3939	lactate dehydrogenase A
ENSG0000	3825.562	0.213639	0.078169	2.733044	0.006275	0.04613	PAF1	54623	PAF1 hom Paf1/RNA polymerase II complex component
ENSG0000	5.811608	-3.22455	1.180033	-2.7326	0.006284	0.046156	PLCD4	84812	phospholipase C delta 4
ENSG0000	184.3602	-0.55784	0.204135	-2.73271	0.006282	0.046156	LOC40049	400499	putative uncharacterized protein LOC400499
ENSG0000	1998.763	0.266269	0.097458	2.732134	0.006293	0.046203	MAFK	7975	MAF bZIP transcription factor K
ENSG0000	568.7313	-0.34167	0.125103	-2.73112	0.006312	0.046326	MTMR11	10903	myotubularin related protein 11
ENSG0000	4292.119	0.205145	0.07514	2.730171	0.00633	0.046442	GIMAP4	55303	GTPase IMAP family member 4
ENSG0000	97.76696	1.031217	0.377785	2.729644	0.00634	0.046498	CDS1	1040	CDP-diacylglycerol synthase 1
ENSG0000	464.9462	-0.40226	0.147427	-2.72851	0.006362	0.046639	DLG1	1739	discs large MAGUK scaffold protein 1
ENSG0000	752.3083	0.349231	0.128046	2.727394	0.006384	0.046761	EMC6	83460	ER membrane protein complex subunit 6
ENSG0000	431.1513	-0.37987	0.139275	-2.72749	0.006382	0.046761	FN3KRP	79672	fructosamine 3 kinase related protein
ENSG0000	3646.922	0.235531	0.086377	2.726782	0.006396	0.046829	BIRC2	329	baculoviral IAP repeat containing 2
ENSG0000	1280.226	-0.27373	0.100433	-2.72552	0.00642	0.04699	LZTS3	9762	leucine zipper tumor suppressor family member 3
ENSG0000	913.3591	-0.35595	0.130661	-2.72425	0.006445	0.047153	ATP8A1	10396	ATPase phospholipid transporting 8A1
ENSG0000	112.6283	-0.66861	0.245477	-2.7237	0.006455	0.047198	SLC2A5	6518	solute carrier family 2 member 5
ENSG0000	1542.172	0.267482	0.098206	2.723668	0.006456	0.047198	TSG101	7251	tumor susceptibility 101
ENSG0000	22.03495	-1.50085	0.551172	-2.72302	0.006469	0.047272	CLDN12	9069	claudin 12
ENSG0000	2053.476	0.226055	0.083029	2.722614	0.006477	0.047312	HEIH	1.01E+08	hepatocellular carcinoma up-regulated EZH2-associated long non-coding RNA
ENSG0000	10.36307	2.539652	0.933133	2.721639	0.006496	0.047433	NA	NA	NA
ENSG0000	10.18658	-2.40158	0.882927	-2.72001	0.006528	0.047648	NA	NA	NA
ENSG0000	4.634461	3.642564	1.339321	2.71971	0.006534	0.047654	NA	NA	NA
ENSG0000	527.5063	0.347999	0.127949	2.719825	0.006532	0.047654	TTC19	54902	tetratricopeptide repeat domain 19
ENSG0000	9123.614	-0.22941	0.084361	-2.71939	0.00654	0.047663	KMT2D	8085	lysine methyltransferase 2D
ENSG0000	2380.843	0.288724	0.106169	2.719482	0.006538	0.047663	TRAF3	7187	TNF receptor associated factor 3
ENSG0000	2857.977	0.248958	0.091575	2.718613	0.006556	0.047756	RAD21	5885	RAD21 cohesin complex component
ENSG0000	444.5282	0.39371	0.144837	2.718309	0.006562	0.047781	ERO1B	56605	endoplasmic reticulum oxidoreductase 1 beta
ENSG0000	6187.554	-0.21226	0.078126	-2.71695	0.006589	0.04796	ADD1	118	adducin 1
ENSG0000	1179.16	-1.49736	0.551332	-2.7159	0.00661	0.048093	ANKRD22	118932	ankyrin repeat domain 22
ENSG0000	563.8236	-0.36829	0.135645	-2.71514	0.006625	0.048184	CACNA1I	8911	calcium voltage-gated channel subunit alpha1 I
ENSG0000	67.27584	-0.85981	0.316732	-2.71462	0.006635	0.048241	ZSCAN20	7579	zinc finger and SCAN domain containing 20
ENSG0000	275.0788	-0.49705	0.183155	-2.71382	0.006651	0.048339	SLC9A3R2	9351	SLC9A3 regulator 2
ENSG0000	287.6378	-0.5255	0.193704	-2.71289	0.00667	0.048439	THEM6	51337	thioesterase superfamily member 6
ENSG0000	3702.052	-0.25352	0.093449	-2.71287	0.00667	0.048439	LGALS9	3965	galectin 9
ENSG0000	2264.415	0.32243	0.118869	2.71249	0.006678	0.048476	IL6ST	3572	interleukin 6 cytokine family signal transducer
ENSG0000	2697.371	0.236554	0.087234	2.711716	0.006694	0.048571	PHF3	23469	PHD finger protein 3
ENSG0000	392.5903	-0.3696	0.136346	-2.71074	0.006713	0.048695	INTS9	55756	integrator complex subunit 9
ENSG0000	1208.21	-0.27748	0.102395	-2.70995	0.006729	0.048792	KAT6B	23522	lysine acetyltransferase 6B
ENSG0000	1431.364	0.27911	0.103013	2.709468	0.006739	0.048843	SHOC2	8036	SHOC2 leucine rich repeat scaffold protein
ENSG0000	1303.442	0.26146	0.096505	2.709277	0.006743	0.048853	CNOT9	9125	CCR4-NOT transcription complex subunit 9
ENSG0000	2839.887	0.228932	0.084534	2.708146	0.006766	0.048981	PASK	23178	PAS domain containing serine/threonine kinase
ENSG0000	12.69704	1.893515	0.699162	2.708266	0.006764	0.048981	IMMP2L	83943	inner mitochondrial membrane peptidase subunit 2
ENSG0000	322.69	-0.39343	0.145289	-2.70788	0.006771	0.048982	SLC25A20	788	solute carrier family 25 member 20
ENSG0000	1750.702	0.261265	0.09648	2.707981	0.006769	0.048982	RFFL	117584	ring finger and FYVE like domain containing E3 ubiquitin protein ligase
ENSG0000	581.0726	0.4045	0.149434	2.706881	0.006792	0.049111	CYTOR	112597	cytoskeleton regulator RNA
ENSG0000	6.998229	-3.09559	1.143681	-2.70669	0.006796	0.04912	NA	NA	NA
ENSG0000	113.2861	-0.62307	0.230277	-2.70574	0.006815	0.049241	SLC4A8	9498	solute carrier family 4 member 8
ENSG0000	6583.428	0.20462	0.075636	2.705335	0.006824	0.049282	SLC44A2	57153	solute carrier family 44 member 2
ENSG0000	904.8287	0.356813	0.131936	2.704441	0.006842	0.049396	DBI	1622	diazepam acyl-CoA binding protein
ENSG0000	1076.321	0.307839	0.113845	2.70401	0.006851	0.049441	SPTY2D1	144108	SPT2 chromatin protein domain containing 1
ENSG0000	9.342924	-2.535	0.937592	-2.70373	0.006857	0.049463	FRMD4A	55691	FERM domain containing 4A
ENSG0000	446.1986	-0.37718	0.139629	-2.70128	0.006907	0.04979	AFG3L1P	172	AFG3 like pseudogene
ENSG0000	323.4247	-0.50906	0.188447	-2.70134	0.006906	0.04979	PROCR	10544	protein C receptor
ENSG0000	120.2163	0.770517	0.285297	2.700748	0.006918	0.04985	NA	NA	NA
ENSG0000	87.93423	0.919249	0.340419	2.700341	0.006927	0.049855	MTND1P2	1.01E+08	MT-ND1 pseudogene 23
ENSG0000	3296.957	0.26426	0.097862	2.700332	0.006927	0.049855	ANKRD12	23253	ankyrin repeat domain 12
ENSG0000	1813.083	0.251701	0.09321	2.700366	0.006926	0.049855	NOP56	10528	NOP56 ribonucleoprotein
ENSG0000	427.5784	-0.3642	0.134895	-2.69991	0.006936	0.049898	POMGNT1	55624	protein O- 2-)
ENSG0000	2040.647	0.26355	0.097621	2.699718	0.00694	0.049908	GPI	2821	glucose-6-phosphate isomerase
ENSG0000	1460.761	0.265859	0.098493	2.699264	0.006949	0.049957	IFNAR1	3454	interferon alpha and beta receptor subunit 1
ENSG0000	520.0351	-0.37596	0.139319	-2.69856	0.006964	0.050043	NA	NA	NA
ENSG0000	62.68807	-0.89013	0.32995	-2.69776	0.006981	0.050144	FAHD2B	151313	fumarylacetoacetate hydrolase domain containing 2B
ENSG0000	914.4446	-0.30053	0.111437	-2.69684	0.007	0.050263	RNPEP	6051	arginyl aminopeptidase
ENSG0000	902.6148	-0.29019	0.107688	-2.69471	0.007045	0.050534	BICRAL	23506	BICRA like chromatin remodeling complex associated protein
ENSG0000	44.25291	1.008809	0.374364	2.694728	0.007045	0.050534	NA	NA	NA
ENSG0000	9.051489	-2.44391	0.906944	-2.69467	0.007046	0.050534	MCF2	4168	MCF.2 cell line derived transforming sequence
ENSG0000	732.4032	-0.32624	0.121143	-2.69299	0.007081	0.050764	COL7A1	1294	collagen type VII alpha 1 chain
ENSG0000	460.456	0.369009	0.137031	2.692893	0.007083	0.050764	HSPA13	6782	heat shock protein family A (Hsp70) member 13
ENSG0000	10.31052	-2.24862	0.83558	-2.69109	0.007122	0.051019	CHAD	1101	chondroadherin
ENSG0000	109.1009	-0.66836	0.248389	-2.69079	0.007128	0.051046	VANGL1	81839	VANGL planar cell polarity protein 1
ENSG0000	267.0025	-0.44639	0.165974	-2.68952	0.007156	0.051221	KCTD18	130535	potassium channel tetramerization domain containing 18
ENSG0000	37.49824	-1.19162	0.443135	-2.68907	0.007165	0.05127	ADAM22	53616	ADAM metallopeptidase domain 22
ENSG0000	319.968	-0.43516	0.161871	-2.68832	0.007181	0.051366	NA	NA	NA
ENSG0000	54.04138	-1.00565	0.374168	-2.6877	0.007195	0.051421	HPF1	54969	histone PARylation factor 1
ENSG0000	20076.48	-0.17158	0.063839	-2.68771	0.007194	0.051421	COTL1	23406	coactosin like F-actin binding protein 1
ENSG0000	2126.102	-0.25116	0.093521	-2.6856	0.00724	0.051725	WWP2	11060	WW domain containing E3 ubiquitin protein ligase 2
ENSG0000	3386.576	0.232608	0.086632	2.684995	0.007253	0.051799	HECA	51696	hdc homol cell cycle regulator
ENSG0000	122.1806	-0.61408	0.228779	-2.68419	0.007271	0.051905	DHX32	55760	DEAH-box helicase 32 (putative)
ENSG0000	243.794	0.510022	0.190063	2.683443	0.007287	0.052	SMPDL3A	10924	sphingomyelin phosphodiesterase acid like 3A

ENSG0000	1074.976	0.291679	0.108705	2.683219	0.007292	0.052015	SHMT2	6472	serine hydroxymethyltransferase 2
ENSG0000	113.0421	-0.61874	0.230624	-2.68288	0.007299	0.052048	NFS1	9054	NFS1 cysteine desulfurase
ENSG0000	5331.64	0.201934	0.075295	2.681902	0.00732	0.05218	CEMIP2	23670	cell migration inducing hyaluronidase 2
ENSG0000	1027.264	-0.29551	0.110204	-2.68149	0.007329	0.052224	FAAP100	80233	FA core complex associated protein 100
ENSG0000	837.0933	-0.34321	0.128043	-2.68047	0.007352	0.052364	BST2	684	bone marrow stromal cell antigen 2
ENSG0000	929.6258	-0.26384	0.09844	-2.68023	0.007357	0.052382	SPG11	80208	SPG11 ves spatacsin
ENSG0000	3138.586	0.204814	0.07643	2.679762	0.007367	0.052414	CS	1431	citrate synthase
ENSG0000	3872.703	0.199572	0.074473	2.679802	0.007367	0.052414	CLTC	1213	clathrin heavy chain
ENSG0000	897.5328	-0.34616	0.129189	-2.67945	0.007374	0.052442	L3MBTL2	83746	L3MBTL histone methyl-lysine binding protein 2
ENSG0000	10846.18	0.225816	0.084347	2.677241	0.007423	0.05277	HNRNPA2I	3181	heterogeneous nuclear ribonucleoprotein A2/B1
ENSG0000	12661.02	0.196013	0.073223	2.676949	0.00743	0.052796	ZBTB7A	51341	zinc finger and BTB domain containing 7A
ENSG0000	395.4354	-0.45151	0.168708	-2.67627	0.007445	0.052883	WDR90	197335	WD repeat domain 90
ENSG0000	259.0965	0.433022	0.161817	2.676008	0.00745	0.052904	POLB	5423	DNA polymerase beta
ENSG0000	1435.229	0.248998	0.093072	2.675324	0.007466	0.052991	RAB2A	5862	RAB2A member RAS oncogene family
ENSG0000	4.614098	-3.55836	1.330689	-2.67407	0.007494	0.05317	CCDC13	152206	coiled-coil domain containing 13
ENSG0000	260.8572	-0.48846	0.182685	-2.6738	0.0075	0.053191	NA	NA	NA
ENSG0000	1582.659	0.246012	0.09202	2.673471	0.007507	0.053224	MIAT	440823	myocardial infarction associated transcript
ENSG0000	398.5512	-0.38963	0.145765	-2.67302	0.007517	0.053269	MYO10	4651	myosin X
ENSG0000	3261.145	0.197797	0.074	2.672928	0.007519	0.053269	FBXO7	25793	F-box protein 7
ENSG0000	1582.647	-0.24315	0.090997	-2.67209	0.007538	0.053342	UGGT1	56886	UDP-glucose glycoprotein glucosyltransferase 1
ENSG0000	602.9353	0.321622	0.120362	2.67213	0.007537	0.053342	GRPEL1	80273	GrpE like 1 mitochondrial
ENSG0000	63.31221	0.818831	0.306439	2.672089	0.007538	0.053342	SEC14L2	23541	SEC14 like lipid binding 2
ENSG0000	71.86631	-0.76625	0.286785	-2.67188	0.007543	0.053355	PHETA2	150368	PH domain containing endocytic trafficking adaptor 2
ENSG0000	955.3755	-0.26101	0.097695	-2.67165	0.007548	0.053371	CARS2	79587	cysteinyl-t mitochondrial
ENSG0000	371.1538	0.379852	0.142195	2.671341	0.007555	0.0534	BLOC1S4	55330	biogenesis of lysosomal organelles complex 1 subunit 4
ENSG0000	1481.211	0.279849	0.104765	2.671211	0.007558	0.0534	EIF2A	83939	eukaryotic translation initiation factor 2A
ENSG0000	403.1897	0.435284	0.16299	2.670625	0.007571	0.053473	RBM27	54439	RNA binding motif protein 27
ENSG0000	4223.431	0.207053	0.077549	2.669957	0.007586	0.053559	MBP	4155	myelin basic protein
ENSG0000	265.338	-0.50363	0.188689	-2.66912	0.007605	0.053672	AXL	558	AXL receptor tyrosine kinase
ENSG0000	31.87698	-1.49924	0.561858	-2.66837	0.007622	0.053772	C17orf100	388327	chromosome 17 open reading frame 100
ENSG0000	200.0308	-0.52544	0.196936	-2.66806	0.007629	0.053784	LNPK	80856	lunapark ER junction formation factor
ENSG0000	4359.157	-0.19181	0.071894	-2.66804	0.00763	0.053784	RXRA	6256	retinoid X receptor alpha
ENSG0000	881.3573	0.287755	0.107903	2.666793	0.007658	0.053963	FOXP1	27086	forkhead box P1
ENSG0000	7.886458	2.669417	1.001596	2.665163	0.007695	0.054205	LINC0108	1.01E+08	long intergenic non-protein coding RNA 1088
ENSG0000	673.1853	-0.28678	0.107616	-2.66485	0.007702	0.054214	SLC35F6	54978	solute carrier family 35 member F6
ENSG0000	8.716735	-2.62306	0.984308	-2.66487	0.007702	0.054214	C9orf163	158055	chromosome 9 putative open reading frame 163
ENSG0000	49.1425	0.968536	0.363471	2.664684	0.007706	0.05422	SPATC1	375686	spermatogenesis and centriole associated 1
ENSG0000	169.4062	-0.57834	0.21706	-2.66443	0.007712	0.05424	SERPINH1	871	serpin family H member 1
ENSG0000	1477.724	0.345769	0.129786	2.664143	0.007718	0.054266	PPP3R1	5534	protein ph alpha
ENSG0000	4893.8	0.306268	0.114995	2.663315	0.007738	0.054379	NR4A3	8013	nuclear receptor subfamily 4 group A member 3
ENSG0000	11120.54	0.21229	0.07973	2.662602	0.007754	0.054474	SUPT6H	6830	SPT6 hom histone chaperone and transcription elongation factor
ENSG0000	22492.25	0.234637	0.088131	2.662366	0.007759	0.054492	DAZAP2	9802	DAZ associated protein 2
ENSG0000	1103.644	-0.26387	0.099178	-2.6606	0.0078	0.054757	TP53BP1	7158	tumor protein p53 binding protein 1
ENSG0000	662.1264	-0.31222	0.117378	-2.65996	0.007815	0.05484	TTL	150465	tubulin tyrosine ligase
ENSG0000	388.0061	-0.36803	0.13838	-2.65959	0.007823	0.05488	HERC2P2	400322	HERC2 pseudogene 2
ENSG0000	4676.507	0.191146	0.071874	2.659457	0.007827	0.054881	SETD5	55209	SET domain containing 5
ENSG0000	90.48477	-0.68407	0.257274	-2.65892	0.007839	0.054949	FZD5	7855	frizzled class receptor 5
ENSG0000	2384.747	0.255008	0.095925	2.65842	0.007851	0.055009	MRFAP1	93621	Morf4 family associated protein 1
ENSG0000	1054.043	-0.27001	0.101581	-2.65808	0.007859	0.055044	PRKACB	5567	protein kinase cAMP-activated catalytic subunit beta
ENSG0000	50.32776	0.998793	0.375789	2.657854	0.007864	0.05506	NA	NA	NA
ENSG0000	15.89008	1.935388	0.728674	2.65604	0.007906	0.055336	POLA2	23649	DNA polyr accessory subunit
ENSG0000	192.6871	0.465355	0.175395	2.653183	0.007974	0.055786	ACVR2A	92	activin A receptor type 2A
ENSG0000	7.761982	3.132104	1.1807	2.652751	0.007984	0.055836	SCUBE2	57758	signal pep CUB domain and EGF like domain containing 2
ENSG0000	1390.265	0.311579	0.117485	2.652088	0.008	0.055925	NDUFB4	4710	NADH:ubiquinone oxidoreductase subunit B4
ENSG0000	1863.635	0.261615	0.09866	2.651685	0.008009	0.05597	PITPNB	23760	phosphatidylinositol transfer protein beta
ENSG0000	233.4138	-0.50851	0.191802	-2.65125	0.008019	0.056021	ARL5A	26225	ADP ribosylation factor like GTPase 5A
ENSG0000	2958.465	0.211953	0.079991	2.649716	0.008056	0.056255	SRSF6	6431	serine and arginine rich splicing factor 6
ENSG0000	6286.374	0.196247	0.074073	2.649388	0.008064	0.056266	CD2	914	CD2 molecule
ENSG0000	21669.32	0.17321	0.06538	2.649272	0.008067	0.056266	LCP1	3936	lymphocyte cytosolic protein 1
ENSG0000	2300.284	0.291987	0.110213	2.649289	0.008066	0.056266	PDXK	8566	pyridoxal kinase
ENSG0000	129.2595	-0.60147	0.227049	-2.64907	0.008071	0.056278	LINC0086	439994	long intergenic non-protein coding RNA 863
ENSG0000	187.9503	-0.50805	0.191844	-2.64822	0.008092	0.056398	SUCLG2	8801	succinate-CoA ligase GDP-forming subunit beta
ENSG0000	1269.548	0.295134	0.111456	2.647985	0.008097	0.056417	STOM	2040	stomatin
ENSG0000	657.6829	0.329581	0.124497	2.647295	0.008114	0.056511	NA	NA	NA
ENSG0000	874.8844	-0.35588	0.134447	-2.64702	0.008121	0.056536	WDFY3	23001	WD repeat and FYVE domain containing 3
ENSG0000	135.0992	-0.55048	0.208017	-2.64632	0.008137	0.056631	FIG4	9896	FIG4 phosphoinositide 5-phosphatase
ENSG0000	3.20728	4.688478	1.773773	2.643223	0.008212	0.05713	TEKT5	146279	tektin 5
ENSG0000	1900.044	0.287043	0.108616	2.642731	0.008224	0.057192	RSRC2	65117	arginine and serine rich coiled-coil 2
ENSG0000	102.2803	-0.66051	0.249966	-2.6424	0.008232	0.057227	PPP3CB-A	1.02E+08	PPP3CB antisense RNA 1 (head to head)
ENSG0000	13.06197	-2.23147	0.845241	-2.64004	0.00829	0.057604	NUDT7	283927	nudix hydrolase 7
ENSG0000	18373.67	0.198108	0.075057	2.639427	0.008305	0.057688	CD6	923	CD6 molecule
ENSG0000	376.798	-0.35159	0.13322	-2.63916	0.008311	0.057712	RNF214	257160	ring finger protein 214
ENSG0000	553.252	-0.32266	0.122291	-2.63846	0.008328	0.057722	COG5	10466	component of oligomeric golgi complex 5
ENSG0000	1614.484	0.229575	0.087009	2.638529	0.008327	0.057722	ASB6	140459	ankyrin repeat and SOCS box containing 6
ENSG0000	230.5606	-0.51778	0.196214	-2.63887	0.008318	0.057722	AVPI1	60370	arginine vasopressin induced 1
ENSG0000	61.15645	-0.78516	0.297571	-2.63857	0.008326	0.057722	PGBD4	161779	piggyBac transposable element derived 4
ENSG0000	260.9147	0.435911	0.165209	2.638551	0.008326	0.057722	PIK3R6	146850	phosphoinositide-3-kinase regulatory subunit 6
ENSG0000	931.37	0.323946	0.122815	2.637686	0.008347	0.057833	POMP	51371	proteasome maturation protein
ENSG0000	17816.06	0.219989	0.083426	2.636933	0.008366	0.057897	RPL7A	6130	ribosomal protein L7a
ENSG0000	830.416	-0.34527	0.130925	-2.63715	0.008361	0.057897	TSPAN18	90139	tetraspanin 18
ENSG0000	2202.299	0.616604	0.233829	2.636986	0.008365	0.057897	GK	2710	glycerol kinase
ENSG0000	74.92827	-0.78922	0.299341	-2.63654	0.008376	0.057933	EHBP1	23301	EH domain binding protein 1
ENSG0000	16.40358	4.019211	1.524469	2.636467	0.008377	0.057933	TM4SF1	4071	transmembrane 4 L six family member 1
ENSG0000	1689.349	-0.26013	0.098674	-2.63625	0.008383	0.057949	MIB2	142678	MIB E3 ubiquitin protein ligase 2
ENSG0000	3464.496	0.255722	0.097009	2.636075	0.008387	0.057957	SERINC3	10955	serine incorporator 3
ENSG0000	12179.04	0.232537	0.08822	2.635869	0.008392	0.05797	ABHD17A	81926	abhydrola depalmitoylase
ENSG0000	7.803366	2.790067	1.058713	2.635339	0.008405	0.058018	SLC22A18	5003	SLC22A18 antisense RNA
ENSG0000	9.753489	-2.37696	0.901946	-2.63537	0.008405	0.058018	KLHL14	57565	kelch like family member 14
ENSG0000	576.4905	-0.31496	0.119533	-2.63491	0.008416	0.058069	MAPK9	5601	mitogen-activated protein kinase 9
ENSG0000	200.9751	-0.46717	0.177355	-2.63407	0.008437	0.058191	MDM1	56890	Mdm1 nuclear protein
ENSG0000	34.67384	1.144102	0.434479	2.633271	0.008457	0.058307	CD68	968	CD68 molecule
ENSG0000	935.7609	-0.27523	0.104545	-2.63269	0.008471	0.058366	RNF123	63891	ring finger protein 123
ENSG0000	6073.566	0.189649	0.072037	2.632674	0.008472	0.058366	RHBDD2	57414	rhomboid domain containing 2
ENSG0000	62.78419	-0.8025	0.304889	-2.63211	0.008486	0.058441	HAAO	23498	3-hydroxy 4-dioxygenase
ENSG0000	2347.326	0.201344	0.076508	2.631667	0.008497	0.05848	ISCU	23479	iron-sulfur cluster assembly enzyme
ENSG0000	20.70856	-1.49128	0.566675	-2.63164	0.008497	0.05848	NA	NA	NA
ENSG0000	16.69707	-1.62595	0.617989	-2.63104	0.008513	0.058561	NA	NA	NA
ENSG0000	22.72691	-1.4147	0.537761	-2.63072	0.00852	0.058593	TMEM54	113452	transmembrane protein 54
ENSG0000	307.8511	-0.41137	0.156428	-2.62976	0.008544	0.058738	TRIP4	9325	thyroid hormone receptor interactor 4
ENSG0000	70.65137	-0.75307	0.286504	-2.62848	0.008577	0.058937	SPRED1	161742	sprouty related EVH1 domain containing 1

ENSG0000	40.31209	-1.00602	0.382853	-2.6277	0.008596	0.059008	OBSL1	23363	obscurin like cytoskeletal adaptor 1
ENSG0000	711.7425	0.310421	0.118136	2.627663	0.008597	0.059008	LAMP3	27074	lysosomal associated membrane protein 3
ENSG0000	1126.501	0.249654	0.095013	2.62757	0.0086	0.059008	C6orf136	221545	chromosome 6 open reading frame 136
ENSG0000	58.90736	-0.91831	0.349479	-2.62765	0.008598	0.059008	TRBV12-4	28576	T cell receptor beta variable 12-4
ENSG0000	961.4108	-0.31992	0.121763	-2.62743	0.008603	0.059011	VPS53	55275	VPS53 subunit of GARP complex
ENSG0000	1022.482	-0.28032	0.106694	-2.6273	0.008607	0.059011	TOP3B	8940	DNA topoisomerase III beta
ENSG0000	134.518	0.53289	0.202877	2.626665	0.008623	0.05909	FTH1P10	2502	ferritin heavy chain 1 pseudogene 10
ENSG0000	745.9623	0.320439	0.121998	2.626594	0.008624	0.05909	PAFAH1B2	5049	platelet activating factor acetylhydrolase 1b catalytic subunit 2
ENSG0000	150.0824	-0.54885	0.209059	-2.62535	0.008656	0.059263	TMBIM4	51643	transmembrane BAX inhibitor motif containing 4
ENSG0000	148.7654	-0.52052	0.198265	-2.62536	0.008656	0.059263	AMER1	139285	APC membrane recruitment protein 1
ENSG0000	510476.6	0.599547	0.228597	2.622726	0.008723	0.059699	ND2	4536	NADH dehydrogenase subunit 2
ENSG0000	433.3799	0.41826	0.159532	2.621801	0.008747	0.059839	NBL1	4681	NBL1 DAN family BMP antagonist
ENSG0000	386.0653	-0.35742	0.136364	-2.62107	0.008766	0.05988	DENND1B	163486	DENN domain containing 1B
ENSG0000	4112.186	0.260922	0.099543	2.621208	0.008762	0.05988	GORASP2	26003	golgi reassembly stacking protein 2
ENSG0000	90.6597	-0.74909	0.285798	-2.62107	0.008766	0.05988	BCDIN3D	144233	BCDIN3 domain containing RNA methyltransferase
ENSG0000	307.686	-0.51263	0.195575	-2.62115	0.008763	0.05988	FCGBP	8857	Fc gamma binding protein
ENSG0000	15.03464	-1.7888	0.682566	-2.6207	0.008775	0.0599	CHST1	8534	carbohydrate sulfotransferase 1
ENSG0000	15.22292	1.742515	0.6649	2.620717	0.008775	0.0599	NA	NA	NA
ENSG0000	78.9064	-0.69298	0.264481	-2.62016	0.008789	0.059964	RNF170	81790	ring finger protein 170
ENSG0000	592.7021	0.303648	0.115893	2.620075	0.008791	0.059964	VPS26A	9559	VPS26 retromer complex component A
ENSG0000	1462.897	-0.2852	0.108855	-2.61996	0.008794	0.059964	NCLN	56926	nicalin
ENSG0000	222.0394	-0.46678	0.178188	-2.61957	0.008804	0.06001	SLC43A1	8501	solute carrier family 43 member 1
ENSG0000	49.54586	1.0348	0.395086	2.619177	0.008814	0.060018	NA	NA	NA
ENSG0000	26565.52	-0.20116	0.076806	-2.61904	0.008818	0.060018	EEIG1	399665	estrogen-induced osteoclastogenesis regulator 1
ENSG0000	885.9422	0.269015	0.102714	2.61908	0.008817	0.060018	CRY1	1407	cryptochrome circadian regulator 1
ENSG0000	745.3237	0.307148	0.117275	2.619028	0.008818	0.060018	ZNF101	94039	zinc finger protein 101
ENSG0000	3347.297	0.255116	0.097441	2.618146	0.008841	0.060107	VMP1	81671	vacuole membrane protein 1
ENSG0000	4.555793	-3.70926	1.416648	-2.61834	0.008836	0.060107	TMPRSS9	360200	transmembrane serine protease 9
ENSG0000	2906.24	-0.24689	0.094296	-2.6182	0.00884	0.060107	WIZ	58525	WIZ zinc finger
ENSG0000	52.23486	-0.90733	0.346576	-2.61798	0.008845	0.060114	STIL	6491	STIL centriolar assembly protein
ENSG0000	1614.103	-0.28418	0.108563	-2.61769	0.008853	0.060121	DHRS3	9249	dehydrogenase/reductase 3
ENSG0000	5216.955	-0.20644	0.078865	-2.61769	0.008853	0.060121	LTBP3	4054	latent transforming growth factor beta binding protein 3
ENSG0000	112.6462	0.670419	0.256277	2.615991	0.008897	0.060395	ADD2	119	adducin 2
ENSG0000	1128.85	0.265612	0.101538	2.61589	0.0089	0.060395	PER2	8864	period circadian regulator 2
ENSG0000	26.17722	1.177438	0.450136	2.615737	0.008904	0.0604	NA	NA	NA
ENSG0000	3751.581	-0.21591	0.082622	-2.61324	0.008969	0.060821	PTPRJ	5795	protein tyrosine phosphatase receptor type J
ENSG0000	5469.711	-0.2264	0.08664	-2.6131	0.008973	0.060824	CLCN7	1186	chloride voltage-gated channel 7
ENSG0000	397.7612	-0.40474	0.154977	-2.6116	0.009012	0.061069	HSD17B4	3295	hydroxysteroid 17-beta dehydrogenase 4
ENSG0000	233.1098	0.461442	0.17671	2.611288	0.00902	0.061102	TKTL1	8277	transketolase like 1
ENSG0000	9.274961	2.639281	1.010904	2.610812	0.009033	0.061165	PRRX2	51450	paired related homeobox 2
ENSG0000	4456.949	0.224388	0.085977	2.609872	0.009058	0.061311	CITED2	10370	Cbp/p300 interacting transactivator with Glu/Asp rich carboxy-terminal domain 2
ENSG0000	230.4345	-0.51016	0.195485	-2.60972	0.009062	0.061316	SLC45A3	85414	solute carrier family 45 member 3
ENSG0000	3911.652	-0.2259	0.086569	-2.6095	0.009067	0.061332	LCK	3932	LCK proto- Src family tyrosine kinase
ENSG0000	26.97818	1.296725	0.497011	2.60905	0.009079	0.061354	NA	NA	NA
ENSG0000	626.7719	0.315009	0.120739	2.609008	0.009081	0.061354	TIMM10B	26515	translocase of inner mitochondrial membrane 10B
ENSG0000	45.09685	0.919125	0.352262	2.609213	0.009075	0.061354	SPNS3	201305	sphingolipid transporter 3 (putative)
ENSG0000	4.418169	3.303252	1.266411	2.608358	0.009098	0.061412	SLC12A9-A	1.05E+08	SLC12A9 antisense RNA 1
ENSG0000	1831.815	0.233917	0.089682	2.608309	0.009099	0.061412	PRKAG2	51422	protein kinase AMP-activated non-catalytic subunit gamma 2
ENSG0000	735.517	-0.33508	0.12846	-2.60842	0.009096	0.061412	MLLT10	8028	MLLT10 histone lysine methyltransferase DOT1L cofactor
ENSG0000	2484.701	0.211067	0.08093	2.60803	0.009106	0.06144	LGALS8	3964	galectin 8
ENSG0000	2439.673	0.233683	0.089616	2.607592	0.009118	0.061496	ZCCHC14	23174	zinc finger CCHC-type containing 14
ENSG0000	2214.187	0.227143	0.087115	2.607379	0.009124	0.061512	COPB1	1315	COPI coat complex subunit beta 1
ENSG0000	793.9181	0.386669	0.148321	2.606967	0.009135	0.061541	TNFRSF18	8784	TNF receptor superfamily member 18
ENSG0000	239.848	0.448299	0.17196	2.607002	0.009134	0.061541	SPAG1	6674	sperm associated antigen 1
ENSG0000	5004.556	0.218987	0.084012	2.60663	0.009144	0.06158	RSL1D1	26156	ribosomal L1 domain containing 1
ENSG0000	1296.255	0.275932	0.105875	2.606211	0.009155	0.06161	NRAS	4893	NRAS prot GTPase
ENSG0000	6.918896	-2.609	1.001071	-2.60621	0.009155	0.06161	NA	NA	NA
ENSG0000	736.4864	0.345101	0.132421	2.60609	0.009158	0.06161	PWWP2B	170394	PWWP domain containing 2B
ENSG0000	3630.958	0.206152	0.079111	2.605867	0.009164	0.061628	BRD1	23774	bromodomain containing 1
ENSG0000	4329.327	-0.82148	0.315338	-2.60508	0.009185	0.061746	CCR1	1230	C-C motif chemokine receptor 1
ENSG0000	8.628002	2.35734	0.904996	2.604806	0.009193	0.061764	HMCN1	83872	hemicentin 1
ENSG0000	3336.888	0.231579	0.088907	2.604738	0.009194	0.061764	VAMP2	6844	vesicle associated membrane protein 2
ENSG0000	800.281	0.264272	0.101466	2.604544	0.0092	0.061776	PLPP5	84513	phospholipid phosphatase 5
ENSG0000	231.3698	0.507434	0.194839	2.604373	0.009204	0.061785	MASTL	84930	microtubule associated serine/threonine kinase like
ENSG0000	1400.998	-0.3036	0.116585	-2.60411	0.009211	0.061809	ABHD12	26090	abhydrola lysophospholipase
ENSG0000	541.7325	0.314819	0.120938	2.603156	0.009237	0.06196	SFXN1	94081	sideroflexin 1
ENSG0000	1204.182	-0.27964	0.107443	-2.60273	0.009249	0.062015	BRD3	8019	bromodomain containing 3
ENSG0000	106.7627	0.656111	0.252148	2.60209	0.009266	0.062108	PTTG1	9232	PTTG1 reg securin
ENSG0000	90.35189	0.672129	0.25842	2.600922	0.009297	0.062297	GMPR	2766	guanosine monophosphate reductase
ENSG0000	6.076951	3.015412	1.159433	2.600764	0.009302	0.062303	SNORD89	692205	small nucl C/D box 89
ENSG0000	44.67893	-1.10119	0.423507	-2.60017	0.009318	0.062349	CIP2A	57650	cellular inhibitor of PP2A
ENSG0000	6.078054	3.271725	1.25829	2.600137	0.009319	0.062349	TACR2	6865	tachykinin receptor 2
ENSG0000	782.1325	0.282364	0.108586	2.600363	0.009313	0.062349	SDF2	6388	stromal cell derived factor 2
ENSG0000	259.8909	-0.46071	0.177197	-2.60002	0.009322	0.062349	ZNF507	22847	zinc finger protein 507
ENSG0000	106.9753	-0.78067	0.300343	-2.59925	0.009343	0.062465	ZNF320	162967	zinc finger protein 320
ENSG0000	92.00296	0.654729	0.252052	2.597596	0.009388	0.062745	RPL21P28	1E+08	ribosomal protein L21 pseudogene 28
ENSG0000	10.11352	-2.34826	0.904268	-2.59687	0.009408	0.062839	TIMP4	7079	TIMP metallopeptidase inhibitor 4
ENSG0000	1058.519	-0.32584	0.125476	-2.59684	0.009409	0.062839	FES	2242	FES proto- tyrosine kinase
ENSG0000	1289.88	0.292881	0.112841	2.595513	0.009445	0.063058	PRKD3	23683	protein kinase D3
ENSG0000	115.7572	-0.60929	0.234762	-2.59534	0.00945	0.063067	SHROOM1	134549	shroom family member 1
ENSG0000	246.3954	-0.47367	0.18254	-2.59488	0.009462	0.063128	TRIM66	9866	tripartite motif containing 66
ENSG0000	258.1665	-0.4161	0.160433	-2.59364	0.009497	0.063335	JMJD4	65094	jumonji domain containing 4
ENSG0000	116.7735	-0.63802	0.246048	-2.59307	0.009512	0.063418	PMS2	5395	PMS1 hom mismatch repair system component
ENSG0000	321.8473	-0.37702	0.145422	-2.59259	0.009526	0.063483	GOLIM4	27333	golgi integral membrane protein 4
ENSG0000	6954.534	0.191443	0.073871	2.591597	0.009553	0.063643	GRB2	2885	growth factor receptor bound protein 2
ENSG0000	1110.146	0.291346	0.112443	2.591043	0.009569	0.063723	KLC1	3831	kinesin light chain 1
ENSG0000	3176.35	0.221236	0.085396	2.590706	0.009578	0.063762	APMAP	57136	adipocyte plasma membrane associated protein
ENSG0000	1460.16	-0.25458	0.0983	-2.58983	0.009602	0.063902	REEP4	80346	receptor accessory protein 4
ENSG0000	278.1548	-0.41061	0.158574	-2.58937	0.009615	0.063965	NA	NA	NA
ENSG0000	726.4405	0.303654	0.117314	2.588394	0.009642	0.064123	BCAT1	586	branched chain amino acid transaminase 1
ENSG0000	11.89029	2.496141	0.964726	2.58741	0.00967	0.064283	STON2	85439	stonin 2
ENSG0000	55.53324	0.905073	0.349844	2.587076	0.009679	0.064322	RABGEF1	27342	RAB guanine nucleotide exchange factor 1
ENSG0000	66858.91	0.203362	0.078635	2.58616	0.009705	0.064471	RPL28	6158	ribosomal protein L28
ENSG0000	2204.996	0.248766	0.096259	2.58434	0.009757	0.064766	USP15	9958	ubiquitin specific peptidase 15
ENSG0000	1161.712	0.279115	0.107998	2.584443	0.009754	0.064766	UBE2N	7334	ubiquitin conjugating enzyme E2 N
ENSG0000	27.44365	1.261739	0.488294	2.583972	0.009767	0.064811	NA	NA	NA
ENSG0000	5.782052	3.206606	1.241098	2.583684	0.009775	0.064842	MIR3685	1.01E+08	microRNA 3685
ENSG0000	252.0441	0.413861	0.160236	2.582819	0.0098	0.064982	RAB3IP	117177	RAB3A interacting protein
ENSG0000	104.0142	-0.62854	0.243409	-2.58223	0.009817	0.065071	SRBD1	55133	S1 RNA binding domain 1
ENSG0000	17430.97	-0.19916	0.077142	-2.58172	0.009831	0.065142	ZMIZ1	57178	zinc finger MIZ-type containing 1

ENSG0000	797.8551	-0.30788	0.119276	-2.58128	0.009843	0.065203	CDC42BPB	9578	CDC42 binding protein kinase beta
ENSG0000	4.006379	3.884682	1.505055	2.58109	0.009849	0.065215	NA	NA	NA
ENSG0000	4.972394	-3.42272	1.326201	-2.58085	0.009856	0.065225	NA	NA	NA
ENSG0000	866.8234	0.322409	0.124926	2.580793	0.009857	0.065225	LRRFIP2	9209	LRR binding FLII interacting protein 2
ENSG0000	2353.091	0.226804	0.087942	2.57901	0.009908	0.065539	RAN	5901	RAN member RAS oncogene family
ENSG0000	3170.841	-0.21011	0.081475	-2.57885	0.009913	0.065546	SPTBN1	6711	spectrin b non-erythrocytic 1
ENSG0000	306.4299	-0.42904	0.166387	-2.57857	0.009921	0.065576	PTCH1	5727	patched 1
ENSG0000	535.7658	0.354869	0.137631	2.578401	0.009926	0.065584	CASP3	836	caspase 3
ENSG0000	106.9285	0.758235	0.294119	2.577984	0.009938	0.06564	IFRD1	3475	interferon related developmental regulator 1
ENSG0000	81.59207	0.706632	0.274147	2.577562	0.00995	0.065674	LOC10099	1.01E+08	uncharacterized LOC100996437
ENSG0000	728.5562	-0.31323	0.12152	-2.57764	0.009948	0.065674	SLC25A19	60386	solute carrier family 25 member 19
ENSG0000	111.2427	-0.63863	0.247784	-2.57737	0.009956	0.065687	RFC5	5985	replication factor C subunit 5
ENSG0000	208.6944	-0.46189	0.179226	-2.57715	0.009962	0.065705	SH3BP4	23677	SH3 domain binding protein 4
ENSG0000	217.5175	-0.49778	0.193289	-2.5753	0.010015	0.065937	CPLANE1	65250	ciliogenesis and planar polarity effector complex subunit 1
ENSG0000	609.1773	0.334719	0.129958	2.575605	0.010006	0.065937	BTN2A2	10385	butyrophilin subfamily 2 member A2
ENSG0000	930.4051	0.249899	0.09704	2.575217	0.010018	0.065937	PSMB1	5689	proteasome 20S subunit beta 1
ENSG0000	2371.273	-0.21901	0.085037	-2.5755	0.01001	0.065937	TAOK3	51347	TAO kinase 3
ENSG0000	828.6013	-0.34272	0.133063	-2.57565	0.010005	0.065937	TMEM204	79652	transmembrane protein 204
ENSG0000	807.4432	-0.31474	0.12222	-2.5752	0.010018	0.065937	ZNF668	79759	zinc finger protein 668
ENSG0000	10.42524	-2.33059	0.905286	-2.57442	0.010041	0.066061	NA	NA	NA
ENSG0000	374.8007	0.367937	0.142946	2.573954	0.010054	0.066127	ADGRE1	2015	adhesion G protein-coupled receptor E1
ENSG0000	5.280269	-2.90468	1.129342	-2.57201	0.010111	0.066476	NA	NA	NA
ENSG0000	361.7733	-0.3958	0.153933	-2.57125	0.010133	0.066598	MSRA	4482	methionine sulfoxide reductase A
ENSG0000	182.3702	-0.51468	0.200211	-2.57071	0.010149	0.066678	EBF1	1879	EBF transcription factor 1
ENSG0000	17.19741	-1.59973	0.62253	-2.56972	0.010178	0.066798	ASPM	259266	assembly factor for spindle microtubules
ENSG0000	683.5275	-0.28272	0.110019	-2.56974	0.010177	0.066798	ABCB1	5243	ATP binding cassette subfamily B member 1
ENSG0000	89.53404	-0.66252	0.257799	-2.56992	0.010172	0.066798	TTLL11	158135	tubulin tyrosine ligase like 11
ENSG0000	4040.796	0.253705	0.09876	2.568903	0.010202	0.066873	HIPK1	204851	homeodomain interacting protein kinase 1
ENSG0000	1390.5	-0.23453	0.091297	-2.56891	0.010202	0.066873	FBH1	84893	F-box DNA helicase 1
ENSG0000	191.8693	0.581396	0.226326	2.568842	0.010204	0.066873	PARD6G	84552	par-6 family cell polarity regulator gamma
ENSG0000	110.5694	-0.6069	0.236233	-2.56909	0.010197	0.066873	PDE9A	5152	phosphodiesterase 9A
ENSG0000	1074.806	0.287466	0.111916	2.568596	0.010211	0.066897	CRK	1398	CRK proto adaptor protein
ENSG0000	9105.018	0.216465	0.08429	2.568104	0.010226	0.066969	OAZ1	4946	ornithine decarboxylase antizyme 1
ENSG0000	260.5868	0.43227	0.168339	2.567852	0.010233	0.066994	MOB1B	92597	MOB kinase activator 1B
ENSG0000	8652.438	0.194151	0.075625	2.567265	0.01025	0.067083	RTN4	57142	reticulon 4
ENSG0000	394.8365	-0.33572	0.130778	-2.56711	0.010255	0.06709	VKORC1L1	154807	vitamin K epoxide reductase complex subunit 1 like 1
ENSG0000	3303.963	-0.22367	0.08714	-2.56681	0.010264	0.067107	TEPSIN	146705	TEPSIN adaptor related protein complex 4 accessory protein
ENSG0000	70.60018	-0.71524	0.278652	-2.56677	0.010265	0.067107	LOC10272	1.03E+08	neuroblastoma breakpoint family member 1-like
ENSG0000	2305.667	0.293309	0.114322	2.565627	0.010299	0.067306	RPS21	6227	ribosomal protein S21
ENSG0000	1556.876	0.216441	0.084374	2.565257	0.01031	0.067354	PCGF3	10336	polycomb group ring finger 3
ENSG0000	212.6005	0.450119	0.17557	2.563756	0.010355	0.067622	DNPH1	10591	2'-deoxynucleoside 5'-phosphate N-hydrolase 1
ENSG0000	525.3831	-0.37115	0.144838	-2.56251	0.010392	0.067842	CSGALNAC	55790	chondroitin sulfate N-acetylgalactosaminyltransferase 1
ENSG0000	30992.04	0.199206	0.07776	2.561808	0.010413	0.067931	HLA-DRA	3122	major hist class II DR alpha
ENSG0000	65.14816	1.203605	0.469824	2.56182	0.010413	0.067931	NA	NA	NA
ENSG0000	498.3445	0.341024	0.13315	2.561201	0.010431	0.068026	IGF2BP2	10644	insulin like growth factor 2 mRNA binding protein 2
ENSG0000	7.882344	-2.58288	1.00853	-2.56104	0.010436	0.068034	MEOX1	4222	mesenchyme homeobox 1
ENSG0000	1486.077	-0.3285	0.128314	-2.56012	0.010464	0.068167	VRK2	7444	VRK serine/threonine kinase 2
ENSG0000	1829.246	0.25511	0.099648	2.560113	0.010464	0.068167	GNL1	2794	G protein nucleolar 1 (putative)
ENSG0000	5252.871	-0.18219	0.07117	-2.55991	0.01047	0.068183	CXCR3	2833	C-X-C motif chemokine receptor 3
ENSG0000	10540.72	0.203908	0.079659	2.559766	0.010474	0.068188	RPS3A	6189	ribosomal protein S3A
ENSG0000	81.82849	0.970451	0.379213	2.55912	0.010494	0.06829	JAKMIP2	9832	janus kinase and microtubule interacting protein 2
ENSG0000	8.082512	-2.68827	1.050962	-2.55792	0.01053	0.068479	B3GALNT1	8706	beta-1 3-N-acetylgalactosaminyltransferase 1 (globoside blood group)
ENSG0000	1347.815	-0.22592	0.08832	-2.55796	0.010529	0.068479	SMURF2	64750	SMAD specific E3 ubiquitin protein ligase 2
ENSG0000	158.6315	0.57762	0.225837	2.55768	0.010537	0.068502	ST6GALNA	55808	ST6 N-ace 6-sialyltransferase 1
ENSG0000	14834.39	0.226228	0.088457	2.557493	0.010543	0.068515	PRRC2C	23215	proline rich coiled-coil 2C
ENSG0000	1233.099	0.262878	0.102827	2.556511	0.010573	0.068667	MIER1	57708	MIER1 transcriptional regulator
ENSG0000	1339.712	0.29488	0.115357	2.556231	0.010581	0.068667	SBNO1	55206	strawberry notch homolog 1
ENSG0000	485.2562	-0.42368	0.165743	-2.55623	0.010581	0.068667	TCN2	6948	transcobalamin 2
ENSG0000	20804.89	0.207524	0.081182	2.556277	0.01058	0.068667	RAC2	5880	Rac family small GTPase 2
ENSG0000	841.1899	0.274069	0.107226	2.555987	0.010589	0.068691	SIRT1	23411	sirtuin 1
ENSG0000	1406.718	0.284565	0.111385	2.554798	0.010625	0.068902	CLTA	1211	clathrin light chain A
ENSG0000	5456.201	0.191652	0.075022	2.554611	0.010631	0.068915	GATAD2A	54815	GATA zinc finger domain containing 2A
ENSG0000	226.4336	-0.55246	0.216287	-2.55431	0.01064	0.068951	TNFSF13B	10673	TNF superfamily member 13b
ENSG0000	4402.485	0.19147	0.074977	2.553728	0.010658	0.069042	EIF3E	3646	eukaryotic translation initiation factor 3 subunit E
ENSG0000	1438.312	0.241741	0.094688	2.553009	0.01068	0.069161	CWC25	54883	CWC25 spliceosome associated protein homolog
ENSG0000	443.0718	-0.34059	0.133474	-2.55176	0.010718	0.069361	DNAAF5	54919	dynein axonemal assembly factor 5
ENSG0000	6505.863	0.187521	0.073487	2.551775	0.010718	0.069361	CERK	64781	ceramide kinase
ENSG0000	7.429139	2.496705	0.978582	2.551349	0.010731	0.069418	DEPP1	11067	DEPP autophagy regulator 1
ENSG0000	8017.364	0.179886	0.07054	2.550145	0.010768	0.069634	CYTH1	9267	cytohesin 1
ENSG0000	28670.43	0.224	0.08785	2.549788	0.010779	0.069681	RPL18	6141	ribosomal protein L18
ENSG0000	10.81463	-2.89761	1.136726	-2.54908	0.010801	0.069774	NA	NA	NA
ENSG0000	8.138061	2.633288	1.03301	2.549141	0.010799	0.069774	NA	NA	NA
ENSG0000	385.8162	0.425121	0.166784	2.548937	0.010805	0.069779	GNPDA1	10007	glucosamine-6-phosphate deaminase 1
ENSG0000	1884.274	-0.21547	0.084551	-2.54841	0.010822	0.06986	PTPRA	5786	protein tyrosine phosphatase receptor type A
ENSG0000	12.18436	-2.31607	0.908887	-2.54825	0.010826	0.069867	APOC1	341	apolipoprotein C1
ENSG0000	6.139416	3.118489	1.223973	2.547842	0.010839	0.069925	NA	NA	NA
ENSG0000	656.7049	-0.38121	0.149639	-2.54755	0.010848	0.069932	CAMSAP2	23271	calmodulin regulated spectrin associated protein family member 2
ENSG0000	153.0158	-0.533	0.20923	-2.54746	0.010851	0.069932	ZNF792	126375	zinc finger protein 792
ENSG0000	11.77989	1.784196	0.700388	2.54744	0.010852	0.069932	KCNJ14	3770	potassium inwardly rectifying channel subfamily J member 14
ENSG0000	6.427314	-2.85753	1.121897	-2.54705	0.010864	0.069986	NA	NA	NA
ENSG0000	11.69596	2.156251	0.846671	2.546739	0.010873	0.069988	NA	NA	NA
ENSG0000	174.6657	-0.4797	0.188345	-2.54692	0.010868	0.069988	ERLIN2	11160	ER lipid raft associated 2
ENSG0000	9074.167	0.214744	0.084323	2.546676	0.010875	0.069988	GDI1	2664	GDP dissociation inhibitor 1
ENSG0000	39.478	0.997054	0.391557	2.546383	0.010885	0.069999	OSBPL10	114884	oxysterol binding protein like 10
ENSG0000	976.0191	0.272806	0.107131	2.546467	0.010882	0.069999	MTMR6	9107	myotubularin related protein 6
ENSG0000	18.25555	-1.56088	0.613022	-2.54621	0.01089	0.070009	NME7	29922	NME/NM23 family member 7
ENSG0000	109.3524	-0.60539	0.237815	-2.54566	0.010907	0.070096	AVEN	57099	apoptosis and caspase activation inhibitor
ENSG0000	244.128	-0.42248	0.165996	-2.54509	0.010925	0.070176	CCDC142	84865	coiled-coil domain containing 142
ENSG0000	392.1661	0.338023	0.132818	2.545014	0.010927	0.070176	DTNBP1	84062	dystrobrevin binding protein 1
ENSG0000	285.9524	-0.41994	0.165013	-2.54489	0.010931	0.070178	TMED8	283578	transmembrane p24 trafficking protein family member 8
ENSG0000	2230.629	-0.24789	0.09742	-2.54453	0.010943	0.070225	ABTB1	80325	ankyrin repeat and BTB domain containing 1
ENSG0000	6.974384	-2.55146	1.002775	-2.5444	0.010947	0.070227	CCER2	643669	coiled-coil glutamate rich protein 2
ENSG0000	6.338352	-3.27168	1.286492	-2.5431	0.010987	0.070443	NA	NA	NA
ENSG0000	631.1362	0.315056	0.123887	2.543087	0.010988	0.070443	ACRBP	84519	acrosin binding protein
ENSG0000	370.2769	-0.37492	0.147493	-2.54196	0.011023	0.070645	SLC22A23	63027	solute carrier family 22 member 23
ENSG0000	766.1294	0.276842	0.108933	2.541397	0.011041	0.070696	LMO4	8543	LIM domain only 4
ENSG0000	28731.3	0.223342	0.087878	2.541512	0.011037	0.070696	RPLP2	6181	ribosomal protein lateral stalk subunit P2
ENSG0000	5495.498	0.217696	0.085662	2.541348	0.011043	0.070696	CHMP4B	128866	charged multivesicular body protein 4B
ENSG0000	285.4394	-0.39454	0.155257	-2.54121	0.011047	0.0707	SBF2	81846	SET binding factor 2
ENSG0000	456.2454	-0.39025	0.153692	-2.53918	0.011111	0.071088	MTFR1L	56181	mitochondrial fission regulator 1 like

ENSG0000	21.97323	-1.28628	0.506821	-2.53793	0.011151	0.071316	NA	NA	NA
ENSG0000	47.70376	1.056433	0.416635	2.53563	0.011225	0.071762	AK5	26289	adenylate kinase 5
ENSG0000	52.33311	-0.87758	0.346264	-2.53442	0.011263	0.071986	CARMIL1	55604	capping protein regulator and myosin 1 linker 1
ENSG0000	395.8661	0.412412	0.162758	2.533889	0.01128	0.07207	ZNF121	7675	zinc finger protein 121
ENSG0000	350.5934	-0.34548	0.136352	-2.53372	0.011286	0.07208	HOMEZ	57594	homeobox and leucine zipper encoding
ENSG0000	171.7156	-0.50466	0.199213	-2.53328	0.0113	0.072136	ITGB5	3693	integrin subunit beta 5
ENSG0000	2421.441	-0.22217	0.087704	-2.53321	0.011302	0.072136	CCDC92	80212	coiled-coil domain containing 92
ENSG0000	561.3283	-0.3391	0.133878	-2.53293	0.011311	0.072143	HDGFL2	84717	HDGF like 2
ENSG0000	4371.202	0.228651	0.090267	2.533042	0.011308	0.072143	RANGAP1	5905	Ran GTPase activating protein 1
ENSG0000	423.442	-0.3356	0.132511	-2.53265	0.01132	0.072176	RRAS	6237	RAS related
ENSG0000	101.3582	-0.61788	0.243978	-2.53252	0.011324	0.072177	CCDC126	90693	coiled-coil domain containing 126
ENSG0000	303.3868	-0.39249	0.154997	-2.53227	0.011333	0.072191	ATPAF1	64756	ATP synthase mitochondrial F1 complex assembly factor 1
ENSG0000	3754.675	0.186736	0.073753	2.53191	0.011344	0.072191	SERBP1	26135	SERPINE1 mRNA binding protein 1
ENSG0000	15974.49	0.162617	0.064225	2.532002	0.011341	0.072191	TCF7	6932	transcription factor 7
ENSG0000	285.3548	-0.42156	0.166488	-2.53209	0.011339	0.072191	VPS41	27072	VPS41 subunit of HOPS complex
ENSG0000	962.1056	-0.28278	0.111687	-2.53185	0.011346	0.072191	CDK5RAP2	55755	CDK5 regulatory subunit associated protein 2
ENSG0000	7703.545	0.212185	0.083827	2.531238	0.011366	0.072293	CD247	919	CD247 molecule
ENSG0000	9.759235	-3.00332	1.18675	-2.53071	0.011383	0.072376	NA	NA	NA
ENSG0000	275.4304	0.386623	0.152786	2.530482	0.011391	0.072399	MRTO4	51154	MRT4 hom ribosome maturation factor
ENSG0000	114.6322	-0.67604	0.267192	-2.53016	0.011401	0.072438	TK2	7084	thymidine kinase 2
ENSG0000	8031.051	0.177094	0.069996	2.530053	0.011405	0.072438	YWHAB	7529	tyrosine 3-monooxygenase/tryptophan 5-monooxygenase activation protein beta
ENSG0000	147922	0.570034	0.225322	2.529866	0.011411	0.072452	ND3	4537	NADH dehydrogenase subunit 3
ENSG0000	405.5886	0.404028	0.159745	2.52921	0.011432	0.072563	PANX2	56666	pannexin 2
ENSG0000	1401.804	-0.22786	0.090098	-2.529	0.011439	0.072582	PSIP1	11168	PC4 and SFRS1 interacting protein 1
ENSG0000	136.4427	-0.55306	0.218745	-2.52836	0.01146	0.072689	ENKD1	84080	enkurin domain containing 1
ENSG0000	12.17171	-2.21087	0.874808	-2.52726	0.011496	0.072892	TMEM200	114801	transmembrane protein 200A
ENSG0000	536.4016	0.411965	0.163035	2.526853	0.011509	0.072952	FSCN1	6624	fascin actin-bundling protein 1
ENSG0000	1411.627	-0.33253	0.131607	-2.52668	0.011515	0.072963	CORO1B	57175	coronin 1B
ENSG0000	135.5918	0.582282	0.230522	2.525931	0.011539	0.073093	ACHE	43	acetylcholinesterase (Cartwright blood group)
ENSG0000	65.50332	0.766054	0.303374	2.525118	0.011566	0.073238	GAS2L3	283431	growth arrest specific 2 like 3
ENSG0000	523.7948	-0.33407	0.132317	-2.52481	0.011576	0.073277	COQ8B	79934	coenzyme Q8B
ENSG0000	6348.321	0.228926	0.090686	2.524376	0.01159	0.073343	CTSW	1521	cathepsin W
ENSG0000	18.67366	-1.77365	0.703113	-2.52256	0.01165	0.073697	PANK1	53354	pantothenate kinase 1
ENSG0000	14.24076	-1.8823	0.746352	-2.52199	0.011669	0.073791	TSC22D1-/	641467	TSC22D1 antisense RNA 1
ENSG0000	2313.923	0.232784	0.092315	2.521642	0.011681	0.073839	IRS2	8660	insulin receptor substrate 2
ENSG0000	103.8645	-0.64001	0.253909	-2.52062	0.011715	0.074004	ZSWIM9	374920	zinc finger SWIM-type containing 9
ENSG0000	530.7217	0.361221	0.143301	2.520706	0.011712	0.074004	COX7B	1349	cytochrome c oxidase subunit 7B
ENSG0000	1814.841	0.236589	0.093904	2.519464	0.011753	0.074222	ACD	65057	ACD shelterin complex subunit and telomerase recruitment factor
ENSG0000	79.29215	-0.69251	0.274952	-2.51866	0.01178	0.074349	HCG11	493812	HLA complex group 11
ENSG0000	947.5335	-0.31063	0.123333	-2.51862	0.011782	0.074349	ATF7	11016	activating transcription factor 7
ENSG0000	18.33174	1.781438	0.707513	2.517887	0.011806	0.074479	MCOLN3	55283	mucolipin TRP cation channel 3
ENSG0000	4411.199	-0.19782	0.078588	-2.51721	0.011829	0.074598	ATF6B	1388	activating transcription factor 6 beta
ENSG0000	1824.555	0.212976	0.084628	2.516617	0.011849	0.074697	DEDD	9191	death effector domain containing
ENSG0000	1588.477	0.264377	0.105064	2.516329	0.011858	0.074733	SLC7A1	6541	solute carrier family 7 member 1
ENSG0000	522.4249	0.369476	0.146995	2.513521	0.011953	0.075305	CUTA	51596	cutA divalent cation tolerance homolog
ENSG0000	23951.2	0.213997	0.085155	2.513039	0.01197	0.075327	RPL29	6159	ribosomal protein L29
ENSG0000	241.7495	0.402781	0.160283	2.512938	0.011973	0.075327	POLR1F	221830	RNA polymerase I subunit F
ENSG0000	50.55863	0.834155	0.331899	2.513278	0.011962	0.075327	THAP7-AS:	439931	THAP7 antisense RNA 1
ENSG0000	541.8292	0.29242	0.116361	2.513042	0.01197	0.075327	SH2D1A	4068	SH2 domain containing 1A
ENSG0000	3.87714	3.808034	1.51554	2.512659	0.011983	0.075361	NA	NA	NA
ENSG0000	16.48229	-1.8659	0.742967	-2.51142	0.012025	0.075601	TMEM51-/	200197	TMEM51 antisense RNA 1
ENSG0000	413.1417	0.359236	0.143132	2.509821	0.012079	0.075918	C1orf52	148423	chromosome 1 open reading frame 52
ENSG0000	533.8775	-0.30633	0.122063	-2.50962	0.012086	0.075936	CASTOR3P	352954	CASTOR fa pseudogene
ENSG0000	76.72658	0.764396	0.304606	2.509459	0.012092	0.075944	SHB	6461	SH2 domain containing adaptor protein B
ENSG0000	1478.974	0.224361	0.089427	2.508875	0.012112	0.076044	ATF6	22926	activating transcription factor 6
ENSG0000	2151.031	-0.20696	0.082533	-2.50755	0.012157	0.076304	ZNF142	7701	zinc finger protein 142
ENSG0000	496.1245	-0.35218	0.140467	-2.50719	0.01217	0.076356	C2CD5	9847	C2 calcium dependent domain containing 5
ENSG0000	60.5376	0.759996	0.303195	2.506628	0.012189	0.076452	PSMA6	5687	proteasome 20S subunit alpha 6
ENSG0000	158.0969	0.552881	0.220596	2.506305	0.0122	0.076496	MTSS2	92154	MTSS I-BAR domain containing 2
ENSG0000	7244.662	0.232369	0.092728	2.505911	0.012214	0.076555	RPL31	6160	ribosomal protein L31
ENSG0000	21.94325	1.593889	0.636139	2.505566	0.012226	0.076604	BCYRN1	618	brain cytoplasmic RNA 1
ENSG0000	30460.3	0.181932	0.072637	2.50468	0.012256	0.07676	TAGLN2	8407	transgelin 2
ENSG0000	4370.309	0.237017	0.094632	2.504608	0.012259	0.07676	PAK2	5062	p21 (RAC1) activated kinase 2
ENSG0000	7441.783	0.17306	0.069108	2.504193	0.012273	0.076824	EIF5A	1984	eukaryotic translation initiation factor 5A
ENSG0000	350.5249	-0.51596	0.206116	-2.50323	0.012307	0.076982	VAMP5	10791	vesicle associated membrane protein 5
ENSG0000	1471.037	0.230935	0.092252	2.503308	0.012304	0.076982	HSPD1	3329	heat shock protein family D (Hsp60) member 1
ENSG0000	1126.959	0.248956	0.099461	2.503063	0.012312	0.076992	NCAPH2	29781	non-SMC condensin II complex subunit H2
ENSG0000	1007.178	-0.23655	0.094525	-2.50253	0.012331	0.077082	UBE3B	89910	ubiquitin protein ligase E3B
ENSG0000	10.24025	2.012088	0.804196	2.501987	0.01235	0.077148	RNU6-118	1.06E+08	RNA U6 small r pseudogene
ENSG0000	191.7825	-0.49169	0.196517	-2.50204	0.012348	0.077148	METTL7A	25840	methyltransferase like 7A
ENSG0000	1030.348	0.232814	0.093056	2.501867	0.012354	0.077148	TSPAN3	10099	tetraspanin 3
ENSG0000	502.3686	0.342326	0.136836	2.501724	0.012359	0.077153	ZBTB5	9925	zinc finger and BTB domain containing 5
ENSG0000	194.5203	-0.46448	0.185683	-2.50145	0.012369	0.077188	PGM2	55276	phosphoglucomutase 2
ENSG0000	2652.743	0.30997	0.123989	2.499985	0.01242	0.077481	ADA	100	adenosine deaminase
ENSG0000	16967.86	0.189427	0.075786	2.499493	0.012437	0.077545	ATP1A1	476	ATPase Na+/K+ transporting subunit alpha 1
ENSG0000	314.2372	-0.38265	0.153093	-2.49946	0.012438	0.077545	TSHZ2	128553	teashirt zinc finger homeobox 2
ENSG0000	749.2354	-0.30338	0.121421	-2.4986	0.012468	0.077705	NCAPD3	23310	non-SMC condensin II complex subunit D3
ENSG0000	4.950279	-3.2136	1.286693	-2.49756	0.012505	0.077896	AHRR	57491	aryl hydrocarbon receptor repressor
ENSG0000	1681.409	-0.27872	0.111601	-2.49749	0.012507	0.077896	YWHAH	7533	tyrosine 3-monooxygenase/tryptophan 5-monooxygenase activation protein eta
ENSG0000	4859.474	0.2039	0.081682	2.496254	0.012551	0.078094	WBP11	51729	WW domain binding protein 11
ENSG0000	9429.701	-0.18038	0.072259	-2.49632	0.012549	0.078094	MYO1F	4542	myosin IF
ENSG0000	331.4565	0.356914	0.142981	2.49624	0.012552	0.078094	EGLN2	112398	egl-9 family hypoxia inducible factor 2
ENSG0000	1059.868	0.271761	0.108887	2.495808	0.012567	0.078137	MKLN1	4289	muskelin 1
ENSG0000	1018.168	-0.25959	0.104005	-2.4959	0.012564	0.078137	RTL8C	8933	retrotransposon Gag like 8C
ENSG0000	2911.432	0.198342	0.0795	2.494859	0.012601	0.07832	FHIP2B	64760	FHF complex subunit HOOK interacting protein 2B
ENSG0000	35.88896	-1.12016	0.449045	-2.49454	0.012612	0.078363	UNC13B	10497	unc-13 homolog B
ENSG0000	346.6516	-0.40132	0.160946	-2.49352	0.012648	0.078556	RPS6KC1	26750	ribosomal protein S6 kinase C1
ENSG0000	186.5204	-0.49917	0.200193	-2.49343	0.012652	0.078556	NAGLU	4669	N-acetyl-alpha-glucosaminidase
ENSG0000	6.764364	3.032646	1.216317	2.493302	0.012656	0.078559	HNF1B	6928	HNF1 homeobox B
ENSG0000	88.2523	-0.79409	0.318508	-2.49315	0.012661	0.078565	NA	NA	NA
ENSG0000	2461.476	0.214205	0.085927	2.492871	0.012671	0.078575	NECAP2	55707	NECAP endocytosis associated 2
ENSG0000	840.7488	0.277221	0.111204	2.492905	0.01267	0.078575	RMC1	29919	regulator of MON1-CCZ1
ENSG0000	6.443143	2.839466	1.139657	2.49151	0.01272	0.07885	CFLAR-AS1	65072	CFLAR antisense RNA 1
ENSG0000	38.14162	-1.0249	0.411416	-2.49116	0.012733	0.078902	STAC3	246329	SH3 and cysteine rich domain 3
ENSG0000	1137.803	0.252154	0.101242	2.490599	0.012753	0.079	EIF6	3692	eukaryotic translation initiation factor 6
ENSG0000	483.7482	0.371389	0.149131	2.490353	0.012762	0.079002	PPIL4	85313	peptidylprolyl isomerase like 4
ENSG0000	100.8055	-0.86001	0.345325	-2.49043	0.012759	0.079002	CCL24	6369	C-C motif chemokine ligand 24
ENSG0000	3892.135	-0.31543	0.126763	-2.48833	0.012835	0.079427	PARP14	54625	poly(ADP-ribose) polymerase family member 14
ENSG0000	2561.922	0.228541	0.091852	2.488147	0.012841	0.079441	TSC22D2	9819	TSC22 domain family member 2
ENSG0000	267.65	-0.43485	0.174822	-2.48741	0.012868	0.079578	SIGLEC7	27036	sialic acid binding Ig like lectin 7

ENSG0000	266.6313	-0.4317	0.1736	-2.48675	0.012891	0.079699	PAM	5066	peptidylglycine alpha-amidating monooxygenase
ENSG0000	4.419163	3.335696	1.34201	2.485597	0.012933	0.079932	LOC10537	1.05E+08	uncharacterized LOC105370969
ENSG0000	4.024278	3.630171	1.460579	2.485432	0.012939	0.079943	ARRDC3-A	1E+08	ARRDC3 antisense RNA 1
ENSG0000	39.20777	-0.98091	0.394736	-2.48499	0.012956	0.080016	LOC10192	1.02E+08	uncharacterized LOC101929698
ENSG0000	8348.944	0.209457	0.084322	2.484003	0.012991	0.080211	DDX3X	1654	DEAD-box helicase 3 X-linked
ENSG0000	197.0405	0.52054	0.209718	2.482099	0.013061	0.080587	RELL1	768211	RELT like 1
ENSG0000	672.9414	-0.30903	0.124501	-2.48211	0.013061	0.080587	FAM111A	63901	FAM111 trypsin like peptidase A
ENSG0000	19326.37	0.868515	0.35001	2.481398	0.013087	0.080719	ICAM1	3383	intercellular adhesion molecule 1
ENSG0000	269.1548	-0.45867	0.184865	-2.48109	0.013098	0.080736	PADI2	11240	peptidyl arginine deiminase 2
ENSG0000	1040.203	0.259116	0.104437	2.481085	0.013098	0.080736	SLC41A1	254428	solute carrier family 41 member 1
ENSG0000	4.541569	4.18177	1.687439	2.478176	0.013206	0.081351	NA	NA	NA
ENSG0000	148937.4	-0.1963	0.079213	-2.47814	0.013207	0.081351	ACTB	60	actin beta
ENSG0000	5.212838	-3.26079	1.316288	-2.47726	0.013239	0.081524	NA	NA	NA
ENSG0000	3481.622	0.215354	0.086938	2.477094	0.013246	0.081536	CHST12	55501	carbohydrate sulfotransferase 12
ENSG0000	1638.142	0.250824	0.101266	2.476873	0.013254	0.081559	DCP1A	55802	decapping mRNA 1A
ENSG0000	1599.287	0.232367	0.093834	2.476364	0.013273	0.081649	PCNP	57092	PEST proteolytic signal containing nuclear protein
ENSG0000	3528.425	0.200222	0.08088	2.475533	0.013304	0.081812	FLI1	2313	Fli-1 proto ETS transcription factor
ENSG0000	138.332	-0.62746	0.253532	-2.47486	0.013329	0.081865	SRGAP2C	653464	SLIT-ROBO Rho GTPase activating protein 2C
ENSG0000	19.64423	-1.60776	0.649598	-2.47501	0.013323	0.081865	TRBJ1-2	28634	T cell receptor beta joining 1-2
ENSG0000	12374.25	0.183595	0.074185	2.474828	0.01333	0.081865	RASA3	22821	RAS p21 protein activator 3
ENSG0000	825.9222	-0.27441	0.110866	-2.47516	0.013318	0.081865	ZNF512B	57473	zinc finger protein 512B
ENSG0000	1645.947	-0.23239	0.093907	-2.47468	0.013336	0.081872	SNX17	9784	sorting nexin 17
ENSG0000	148.091	-0.61105	0.246952	-2.47438	0.013347	0.0819	PEX10	5192	peroxisomal biogenesis factor 10
ENSG0000	5258.214	0.186032	0.075185	2.474319	0.013349	0.0819	RAC1	5879	Rac family small GTPase 1
ENSG0000	149.9058	-0.66815	0.270102	-2.47369	0.013373	0.082017	FER	2241	FER tyrosine kinase
ENSG0000	4383.191	0.185287	0.074911	2.473416	0.013383	0.082053	ATP6AP1	537	ATPase H+ transporting accessory protein 1
ENSG0000	33126.4	0.177157	0.071632	2.473153	0.013393	0.082087	GNLY	10578	granulysin
ENSG0000	1638.335	-0.23285	0.094159	-2.47294	0.013401	0.082109	BAHD1	22893	bromo adjacent homology domain containing 1
ENSG0000	842.4029	0.266943	0.108027	2.471087	0.01347	0.082508	RCE1	9986	Ras converting CAAX endopeptidase 1
ENSG0000	928.4535	0.245834	0.099502	2.470639	0.013487	0.082575	LPCAT3	10162	lysophosphatidylcholine acyltransferase 3
ENSG0000	1362.064	0.261036	0.105664	2.470442	0.013495	0.082575	PPTC7	160760	protein phosphatase targeting COQ7
ENSG0000	613.2801	-0.32151	0.130144	-2.47046	0.013494	0.082575	BORCS6	54785	BLOC-1 related complex subunit 6
ENSG0000	2286.357	-0.20634	0.083578	-2.46885	0.013555	0.082917	PLCG1	5335	phospholipase C gamma 1
ENSG0000	232.8658	-0.45464	0.18419	-2.46835	0.013574	0.083005	COLGALT2	23127	collagen beta(1-O)galactosyltransferase 2
ENSG0000	2079.064	0.254487	0.103117	2.467955	0.013589	0.083069	HBP1	26959	HMG-box transcription factor 1
ENSG0000	85.20189	-0.72047	0.291951	-2.46777	0.013596	0.083084	SCAMP1-A	728769	SCAMP1 antisense RNA 1
ENSG0000	1143.344	0.274303	0.111173	2.46735	0.013612	0.083131	CREB3	10488	cAMP responsive element binding protein 3
ENSG0000	349.9901	0.377601	0.15304	2.467331	0.013612	0.083131	TEX10	54881	testis expressed 10
ENSG0000	20254.35	0.483964	0.196175	2.466999	0.013625	0.083152	BTG2	7832	BTG anti-proliferation factor 2
ENSG0000	166.3663	0.46321	0.187758	2.467065	0.013623	0.083152	UTP15	84135	UTP15 small subunit processome component
ENSG0000	178.8647	0.662486	0.268551	2.46689	0.013629	0.083152	GEM	2669	GTP binding protein overexpressed in skeletal muscle
ENSG0000	3567.447	-0.21373	0.086654	-2.46646	0.013646	0.083224	TTC3	7267	tetratricopeptide repeat domain 3
ENSG0000	423.1051	-0.37912	0.153741	-2.46595	0.013665	0.083315	SLC25A42	284439	solute carrier family 25 member 42
ENSG0000	1392.793	-0.23659	0.095955	-2.46558	0.013679	0.083347	SMPD1	6609	sphingomyelin phosphodiesterase 1
ENSG0000	751.7193	-0.29404	0.119252	-2.46568	0.013675	0.083347	PDS5B	23047	PDS5 cohesin associated factor B
ENSG0000	579.6615	0.333461	0.135259	2.465356	0.013688	0.083371	C9orf72	203228	C9orf72-SMCR8 complex subunit
ENSG0000	394.4317	-0.34221	0.13882	-2.4651	0.013697	0.083403	ABCC3	8714	ATP binding cassette subfamily C member 3
ENSG0000	848.8161	-0.26385	0.107069	-2.46428	0.013729	0.08354	MON2	23041	MON2 hor regulator of endosome-to-Golgi trafficking
ENSG0000	2132.772	0.210148	0.085276	2.464336	0.013727	0.08354	MARK3	4140	microtubule affinity regulating kinase 3
ENSG0000	2390.437	-0.20194	0.081966	-2.46375	0.013749	0.083635	GIMAP6	474344	GTPase IMAP family member 6
ENSG0000	80.65283	0.695313	0.282266	2.463325	0.013766	0.083708	CASP4LP	643733	caspase 4 pseudogene
ENSG0000	16697.32	0.222486	0.090333	2.462969	0.013779	0.083736	RPS18	6222	ribosomal protein S18
ENSG0000	963.1095	0.249604	0.10134	2.463032	0.013777	0.083736	RPRD1B	58490	regulation of nuclear pre-mRNA domain containing 1B
ENSG0000	112.692	0.648733	0.263426	2.462678	0.01379	0.083777	TPSAB1	7177	tryptase alpha/beta 1
ENSG0000	2492.415	-0.19083	0.077501	-2.46229	0.013805	0.083831	DENND2D	79961	DENN domain containing 2D
ENSG0000	36.06652	1.004987	0.408165	2.462209	0.013808	0.083831	NA	NA	NA
ENSG0000	221.9268	-0.47944	0.194735	-2.46203	0.013815	0.083845	ATXN7L3-A	1.02E+08	ATXN7L3 antisense RNA 1
ENSG0000	2414.75	0.214894	0.087294	2.461737	0.013827	0.083887	PRMT1	3276	protein arginine methyltransferase 1
ENSG0000	6654.574	0.178438	0.072488	2.461618	0.013831	0.083887	PDCD6IP	10015	programmed cell death 6 interacting protein
ENSG0000	18.33056	-1.68444	0.684568	-2.46058	0.013871	0.084029	NA	NA	NA
ENSG0000	5024.512	0.206681	0.083998	2.460544	0.013873	0.084029	LRRFIP1	9208	LRR binding FLII interacting protein 1
ENSG0000	40.27255	-1.09659	0.445628	-2.46077	0.013864	0.084029	ZNF425	155054	zinc finger protein 425
ENSG0000	127.4054	-0.5825	0.236728	-2.46064	0.013869	0.084029	ULK2	9706	unc-51 like autophagy activating kinase 2
ENSG0000	323.3902	0.376063	0.152868	2.460054	0.013892	0.084116	SLC26A2	1836	solute carrier family 26 member 2
ENSG0000	410.9916	-0.32667	0.132818	-2.45949	0.013913	0.08422	CUL7	9820	cullin 7
ENSG0000	284.8954	0.388793	0.158099	2.459181	0.013925	0.084266	CDC37L1	55664	cell divisio HSP90 cochaperone
ENSG0000	2443.149	0.225708	0.0918	2.458699	0.013944	0.084352	HSPA9	3313	heat shock protein family A (Hsp70) member 9
ENSG0000	51.35483	0.86463	0.351701	2.458424	0.013955	0.084389	AMN1	196394	antagonist of mitotic exit network 1 homolog
ENSG0000	528.202	-0.30416	0.123731	-2.45823	0.013962	0.084407	BMP1	649	bone morphogenetic protein 1
ENSG0000	750.4257	0.398583	0.162153	2.458058	0.013969	0.08441	NAB1	4664	NGFI-A binding protein 1
ENSG0000	370.0752	-0.39575	0.161007	-2.45798	0.013972	0.08441	GCHFR	2644	GTP cyclohydrolase I feedback regulator
ENSG0000	3139.222	0.223513	0.090949	2.457554	0.013989	0.084456	S100A8	6279	S100 calcium binding protein A8
ENSG0000	41.01545	-1.00867	0.410435	-2.45755	0.013989	0.084456	EML6	400954	EMAP like 6
ENSG0000	1508.248	-0.23262	0.094696	-2.45646	0.014031	0.084686	SYTL2	54843	synaptotagmin like 2
ENSG0000	109729	0.179918	0.073273	2.45543	0.014072	0.084901	HLA-B	3106	major hist class I B
ENSG0000	63.90493	-0.8633	0.351606	-2.45531	0.014076	0.084903	CEP126	57562	centrosomal protein 126
ENSG0000	804.1673	-0.25987	0.10585	-2.45505	0.014086	0.084936	CHMP6	79643	charged multivesicular body protein 6
ENSG0000	1386.235	-0.22322	0.09094	-2.45461	0.014104	0.084984	GIGYF2	26058	GRB10 interacting GYF protein 2
ENSG0000	7.370434	2.537476	1.033753	2.454624	0.014103	0.084984	ARHGAP8	23779	Rho GTPase activating protein 8
ENSG0000	265.9844	-0.39807	0.162216	-2.45393	0.01413	0.085035	NA	NA	NA
ENSG0000	1790.02	0.207738	0.084652	2.454018	0.014127	0.085035	PRPF3	9129	pre-mRNA processing factor 3
ENSG0000	326.44	0.387983	0.158104	2.453966	0.014129	0.085035	CD24	1E+08	CD24 molecule
ENSG0000	5841.227	0.276007	0.112474	2.453968	0.014129	0.085035	SLC38A2	54407	solute carrier family 38 member 2
ENSG0000	141.757	-0.52576	0.214292	-2.45349	0.014148	0.085111	CACFD1	11094	calcium channel flower domain containing 1
ENSG0000	352.2819	0.390238	0.159118	2.452506	0.014186	0.085317	RAB9A	9367	RAB9A member RAS oncogene family
ENSG0000	274.7034	-0.411	0.167619	-2.45199	0.014207	0.085398	DDX60	55601	DExD/H-box helicase 60
ENSG0000	36.88729	-1.02893	0.419661	-2.45181	0.014214	0.085398	MAP7	9053	microtubule associated protein 7
ENSG0000	1114.629	-0.23995	0.097862	-2.45193	0.014209	0.085398	CBFA2T2	9139	CBFA2/RUNX1 partner transcriptional co-repressor 2
ENSG0000	1315.36	-0.2885	0.117687	-2.4514	0.01423	0.085469	CPTP	80772	ceramide-1-phosphate transfer protein
ENSG0000	3801.114	-0.31171	0.127204	-2.45049	0.014266	0.085658	IGHA2	3494	immunoglobulin heavy constant alpha 2 (A2m marker)
ENSG0000	14.35538	-2.03784	0.831859	-2.44974	0.014296	0.085807	IGHV1-24	28467	immunoglobulin heavy variable 1-24
ENSG0000	78.26331	-0.71998	0.293959	-2.44926	0.014315	0.085894	TCTN2	79867	tectonic family member 2
ENSG0000	6.137926	2.707674	1.10579	2.448634	0.01434	0.085988	NA	NA	NA
ENSG0000	4753.689	0.191559	0.07823	2.448672	0.014338	0.085988	SYNGR2	9144	synaptogyrin 2
ENSG0000	167.4115	-0.46372	0.189421	-2.4481	0.014361	0.086087	TSPAN4	7106	tetraspanin 4
ENSG0000	888.4739	-0.26736	0.109222	-2.44785	0.014371	0.086119	GBA1	2629	glucosylceramidase beta 1
ENSG0000	125.1579	0.654936	0.267578	2.447645	0.014379	0.086124	CYB5A	1528	cytochrome b5 type A
ENSG0000	25.22967	1.335637	0.545692	2.447602	0.014381	0.086124	NA	NA	NA
ENSG0000	89.04384	-0.66693	0.272539	-2.44711	0.014401	0.086213	LIN52	91750	lin-52 DREAM MuvB core complex component
ENSG0000	5357.529	0.213513	0.087327	2.445	0.014485	0.086692	HNRNPM	4670	heterogeneous nuclear ribonucleoprotein M

ENSG0000	1728.842	0.206003	0.084261	2.444829	0.014492	0.086705	TCP1	6950	t-complex 1
ENSG0000	367.8446	0.334826	0.136973	2.444475	0.014506	0.086709	CIDECP1	152302	cell death inducing DFFA like effector c pseudogene 1
ENSG0000	109.0735	-0.6666	0.272698	-2.44446	0.014507	0.086709	BNC2	54796	basonuclin 2
ENSG0000	3.525304	-3.63242	1.485883	-2.44462	0.0145	0.086709	SNORD124	1.01E+08	small nucl C/D box 124
ENSG0000	385.1684	-0.34292	0.140346	-2.44337	0.014551	0.086944	PALD1	27143	phosphatase domain containing paladin 1
ENSG0000	14.89385	-1.73269	0.709302	-2.44281	0.014573	0.087051	SLC6A20	54716	solute carrier family 6 member 20
ENSG0000	1242.219	0.269122	0.11018	2.442558	0.014584	0.087084	GPR137	56834	G protein-coupled receptor 137
ENSG0000	333.9355	0.339544	0.139075	2.441444	0.014629	0.087325	TP53INP1	94241	tumor protein p53 inducible nuclear protein 1
ENSG0000	93.7313	0.625643	0.256301	2.441049	0.014645	0.087364	SYNGR3	9143	synaptogyrin 3
ENSG0000	532.5926	-0.31155	0.127628	-2.44106	0.014644	0.087364	ZNF652	22834	zinc finger protein 652
ENSG0000	175.9644	-0.58751	0.240722	-2.44062	0.014662	0.087384	INVS	27130	inversin
ENSG0000	29.59574	-1.14623	0.469643	-2.44064	0.014661	0.087384	GPR135	64582	G protein-coupled receptor 135
ENSG0000	434.0785	-0.3472	0.142253	-2.4407	0.014659	0.087384	DGCR6L	85359	DiGeorge syndrome critical region gene 6 like
ENSG0000	4269.143	-0.17903	0.073363	-2.44037	0.014672	0.087416	ARHGAP27	201176	Rho GTPase activating protein 27
ENSG0000	1316.703	0.227075	0.093096	2.439147	0.014722	0.087684	SMPD4BP	150776	sphingomy pseudogene
ENSG0000	4570.34	-0.22997	0.094303	-2.43864	0.014743	0.08778	AP1B1	162	adaptor related protein complex 1 subunit beta 1
ENSG0000	14.57061	-1.87786	0.770125	-2.43838	0.014753	0.087815	SCN8A	6334	sodium voltage-gated channel alpha subunit 8
ENSG0000	1906.342	-0.2167	0.088876	-2.43824	0.014759	0.08782	KIAA0513	9764	KIAA0513
ENSG0000	9624.302	-0.18683	0.076635	-2.43799	0.014769	0.087853	ANXA11	311	annexin A11
ENSG0000	260.0452	-0.38283	0.157104	-2.43677	0.014819	0.088094	ZNF253	56242	zinc finger protein 253
ENSG0000	57.47652	-0.83012	0.340653	-2.43685	0.014816	0.088094	RWDD2B	10069	RWD domain containing 2B
ENSG0000	367.1996	-0.35016	0.143714	-2.43651	0.01483	0.088129	WRAP53	55135	WD repeat containing antisense to TP53
ENSG0000	67.73935	-0.77297	0.317287	-2.4362	0.014842	0.088175	HERPUD2-	1.02E+08	HERPUD2 antisense RNA 1
ENSG0000	976.748	0.322266	0.132331	2.435299	0.014879	0.088339	CAMKK2	10645	calcium/calmodulin dependent protein kinase kinase 2
ENSG0000	307.8929	-0.39701	0.16302	-2.43532	0.014879	0.088339	TLE2	7089	TLE family transcriptional corepressor
ENSG0000	152.3572	-0.5317	0.218359	-2.43496	0.014894	0.088394	TMCO4	255104	transmembrane and coiled-coil domains 4
ENSG0000	1521.704	0.242476	0.099594	2.434637	0.014907	0.088444	PIP5K1A	8394	phosphatidylinositol-4-phosphate 5-kinase type 1 alpha
ENSG0000	441.4115	-0.31514	0.129468	-2.43408	0.01493	0.088552	WDR44	54521	WD repeat domain 44
ENSG0000	628.3871	0.287916	0.118324	2.433274	0.014963	0.088668	SENP5	205564	SUMO specific peptidase 5
ENSG0000	119.8544	0.778577	0.319983	2.433183	0.014967	0.088668	MSANTD3	91283	Myb/SANT DNA binding domain containing 3
ENSG0000	635.9645	-0.27029	0.111077	-2.43339	0.014958	0.088668	PBX3	5090	PBX homeobox 3
ENSG0000	4.092072	-3.35331	1.378182	-2.43314	0.014968	0.088668	LINC02724	1.01E+08	long intergenic non-protein coding RNA 2724
ENSG0000	9.323496	2.068167	0.850117	2.432803	0.014982	0.088694	NA	NA	NA
ENSG0000	1186.908	0.286059	0.117579	2.432901	0.014978	0.088694	POLR2C	5432	RNA polymerase II subunit C
ENSG0000	5.56341	3.282468	1.349862	2.431707	0.015028	0.088906	NA	NA	NA
ENSG0000	546.608	-0.37609	0.154657	-2.43175	0.015026	0.088906	TOGARAM	165186	TOG array regulator of axonemal microtubules 2
ENSG0000	215.4565	0.516074	0.212268	2.431234	0.015048	0.088994	COL9A2	1298	collagen type IX alpha 2 chain
ENSG0000	81.38376	0.650265	0.267527	2.430652	0.015072	0.089109	GAMT	2593	guanidinoacetate N-methyltransferase
ENSG0000	371.2273	0.368067	0.151482	2.42978	0.015108	0.089267	HAUS3	79441	HAUS augmin like complex subunit 3
ENSG0000	12.56916	-1.9838	0.816448	-2.42979	0.015108	0.089267	ATP6AP1-I	158960	ATP6AP1 divergent transcript
ENSG0000	459.2006	0.347679	0.143122	2.429246	0.01513	0.08937	LAMTOR5	10542	late endos MAPK and MTOR activator 5
ENSG0000	763.1327	0.26486	0.109054	2.428699	0.015153	0.089476	MFSD5	84975	major facilitator superfamily domain containing 5
ENSG0000	44.63014	-1.00618	0.414388	-2.4281	0.015178	0.089595	AMOT	154796	angiomotin
ENSG0000	1338.668	0.219858	0.090581	2.427195	0.015216	0.089791	VPS4A	27183	vacuolar protein sorting 4 homolog A
ENSG0000	7.774585	-2.60981	1.075471	-2.42667	0.015238	0.089892	NA	NA	NA
ENSG0000	1230.424	-0.25665	0.105772	-2.4264	0.015249	0.08993	TAPBPL	55080	TAP binding protein like
ENSG0000	210.5629	-0.4546	0.187373	-2.42617	0.015259	0.089958	NOD1	10392	nucleotide binding oligomerization domain containing 1
ENSG0000	2308.522	0.219106	0.090317	2.425971	0.015267	0.08998	TUBB1	81027	tubulin beta 1 class VI
ENSG0000	17.54338	-1.44421	0.595488	-2.42525	0.015298	0.090102	NA	NA	NA
ENSG0000	1498.389	0.216229	0.089157	2.42525	0.015298	0.090102	NARF	26502	nuclear prelamin A recognition factor
ENSG0000	125.3319	-0.53594	0.221014	-2.42493	0.015311	0.090152	ZNF180	7733	zinc finger protein 180
ENSG0000	2201.246	-0.2339	0.096483	-2.42423	0.015341	0.090288	NKTR	4820	natural killer cell triggering receptor
ENSG0000	65.40243	-0.80689	0.332854	-2.42415	0.015344	0.090288	SLC24A1	9187	solute carrier family 24 member 1
ENSG0000	328.5222	-0.35216	0.145291	-2.42384	0.015358	0.090338	ZBTB37	84614	zinc finger and BTB domain containing 37
ENSG0000	73.48144	-0.75785	0.312716	-2.42344	0.015374	0.090399	LOC72848	728488	POM121 membrane glycoprotein (rat) pseudogene
ENSG0000	13.72179	-1.73134	0.714437	-2.42336	0.015378	0.090399	ASIC1	41	acid sensing ion channel subunit 1
ENSG0000	265.5953	-0.37034	0.152887	-2.4223	0.015423	0.090617	DIS3L	115752	DIS3 like exosome 3'-5' exoribonuclease
ENSG0000	5071.711	0.217341	0.089727	2.422255	0.015425	0.090617	SF3A2	8175	splicing factor 3a subunit 2
ENSG0000	1436.542	-0.21589	0.089148	-2.42175	0.015446	0.090715	COPS7B	64708	COP9 signalosome subunit 7B
ENSG0000	976.2054	-0.39777	0.1643	-2.421	0.015478	0.090758	EEIG2	284611	EEIG family member 2
ENSG0000	504.1212	-0.31421	0.129782	-2.4211	0.015474	0.090758	MED27	9442	mediator complex subunit 27
ENSG0000	5159.843	0.17975	0.074234	2.421415	0.01546	0.090758	HNRNPF	3185	heterogeneous nuclear ribonucleoprotein F
ENSG0000	890.9154	-0.26451	0.109243	-2.4213	0.015465	0.090758	TSC22D1	8848	TSC22 domain family member 1
ENSG0000	736.482	0.27633	0.114131	2.421169	0.015471	0.090758	NA	NA	NA
ENSG0000	17.21694	-1.38099	0.570469	-2.4208	0.015486	0.090779	SEZ6L	23544	seizure related 6 homolog like
ENSG0000	4.012091	-3.68875	1.523939	-2.42054	0.015497	0.090787	NA	NA	NA
ENSG0000	5551.782	-0.1954	0.080724	-2.42061	0.015495	0.090787	TYK2	7297	tyrosine kinase 2
ENSG0000	8.054212	2.445784	1.010783	2.419691	0.015534	0.09097	PF4	5196	platelet factor 4
ENSG0000	1886.569	0.24623	0.101775	2.419367	0.015548	0.091023	SNRPB	6628	small nuclear ribonucleoprotein polypeptides B and B1
ENSG0000	1014.672	-0.27948	0.11555	-2.4187	0.015576	0.091105	IMPDH1	3614	inosine monophosphate dehydrogenase 1
ENSG0000	3864.028	0.19992	0.082654	2.41876	0.015574	0.091105	PCNX1	22990	pecanex 1
ENSG0000	7958.609	0.183573	0.07589	2.418925	0.015566	0.091105	NUMB	8650	NUMB endocytic adaptor protein
ENSG0000	3599.397	0.216892	0.089686	2.418345	0.015591	0.091164	ABCF1	23	ATP binding cassette subfamily F member 1
ENSG0000	1000.063	0.278792	0.115317	2.417625	0.015622	0.091311	KRT86	3892	keratin 86
ENSG0000	3258.76	0.201556	0.083373	2.417528	0.015626	0.091311	UBE2I	7329	ubiquitin conjugating enzyme E2 I
ENSG0000	150.6418	0.491464	0.203376	2.416532	0.015669	0.091532	KLRK1	22914	killer cell lectin like receptor K1
ENSG0000	46.33851	-0.87008	0.360106	-2.41617	0.015685	0.091595	NA	NA	NA
ENSG0000	1330.674	-0.29327	0.121412	-2.41547	0.015715	0.091742	MAP3K10	4294	mitogen-activated protein kinase kinase kinase 10
ENSG0000	428.2744	-0.35981	0.148975	-2.41524	0.015725	0.091771	ANAPC1	64682	anaphase promoting complex subunit 1
ENSG0000	37.68508	4.497768	1.862668	2.414691	0.015749	0.091881	MMP10	4319	matrix metallopeptidase 10
ENSG0000	210.7088	0.457464	0.189507	2.413968	0.01578	0.092034	PPCDC	60490	phosphopantothenoylcysteine decarboxylase
ENSG0000	98.72522	-0.59682	0.247298	-2.41336	0.015806	0.092158	NA	NA	NA
ENSG0000	20.30524	1.464695	0.607013	2.412956	0.015824	0.092203	NA	NA	NA
ENSG0000	105.8328	-0.57413	0.237937	-2.41296	0.015823	0.092203	ZNF416	55659	zinc finger protein 416
ENSG0000	13.57975	1.901044	0.787968	2.41259	0.01584	0.092267	NA	NA	NA
ENSG0000	48.13958	-0.91625	0.38003	-2.411	0.015909	0.092642	PDE4DIPP6	1E+08	PDE4DIP pseudogene 6
ENSG0000	15876.06	0.228552	0.094801	2.410855	0.015915	0.092649	HBB	3043	hemoglobin subunit beta
ENSG0000	620.3051	-0.27037	0.112163	-2.41047	0.015932	0.092718	MFGE8	4240	milk fat globule EGF and factor V/VIII domain containing
ENSG0000	8.55531	2.083617	0.864511	2.410168	0.015945	0.092765	TRBV10-2	28584	T cell receptor beta variable 10-2
ENSG0000	14.93386	1.788799	0.74253	2.40906	0.015994	0.093018	PLEKHA6	22874	pleckstrin homology domain containing A6
ENSG0000	1317.012	-0.23663	0.098239	-2.40873	0.016008	0.093074	NDOR1	27158	NADPH dependent diflavin oxidoreductase 1
ENSG0000	28.73041	-1.61896	0.672332	-2.40798	0.016041	0.093207	NA	NA	NA
ENSG0000	120.8163	-0.55044	0.228584	-2.40806	0.016037	0.093207	ZNF888	388559	zinc finger protein 888
ENSG0000	919.0525	0.243951	0.10135	2.407026	0.016083	0.093421	TERF1	7013	telomeric repeat binding factor 1
ENSG0000	18.92328	-1.38619	0.576194	-2.40577	0.016138	0.093713	TTC8	123016	tetratricopeptide repeat domain 8
ENSG0000	1138.223	-0.22523	0.093637	-2.40532	0.016158	0.093798	GMPPA	29926	GDP-mannose pyrophosphorylase A
ENSG0000	110.4687	-0.5907	0.245594	-2.40518	0.016165	0.093798	PPP1R32	220004	protein phosphatase 1 regulatory subunit 32
ENSG0000	24.62719	-1.13892	0.473545	-2.4051	0.016168	0.093798	MAPK8IP2	23542	mitogen-activated protein kinase 8 interacting protein 2
ENSG0000	18496.2	0.16808	0.069906	2.404373	0.0162	0.093954	FYN	2534	FYN proto- Src family tyrosine kinase
ENSG0000	322.9398	-0.40588	0.168829	-2.4041	0.016212	0.093994	VENTX	27287	VENT homeobox

ENSG0000	1360.464	0.218755	0.091	2.403896	0.016221	0.094018	IL18RAP	8807	interleukin 18 receptor accessory protein
ENSG0000	107.4355	0.65429	0.272195	2.403753	0.016228	0.094025	TNFSF9	8744	TNF superfamily member 9
ENSG0000	240.7285	-0.45901	0.190989	-2.40333	0.016246	0.094105	CD99L2	83692	CD99 molecule like 2
ENSG0000	294.6911	0.364646	0.151757	2.40282	0.016269	0.094178	B4GALT4	8702	beta-1 4-galactosyltransferase 4
ENSG0000	210.9212	-0.42844	0.178306	-2.40284	0.016268	0.094178	PCBD1	5092	pterin-4 alpha-carbinolamine dehydratase 1
ENSG0000	709.7229	-0.33711	0.140354	-2.40185	0.016312	0.094398	RPAP1	26015	RNA polymerase II associated protein 1
ENSG0000	537.9792	-0.3555	0.148097	-2.40044	0.016375	0.094733	NQO2	4835	N-ribosyldihydronicotinamide:quinone reductase 2
ENSG0000	63.61467	0.935472	0.38981	2.399816	0.016403	0.09484	LOC10798	1.08E+08	uncharacterized LOC107986639
ENSG0000	5906.932	-0.16697	0.069582	-2.39968	0.016409	0.09484	MLXIP	22877	MLX interacting protein
ENSG0000	1932.573	-0.20566	0.085701	-2.39977	0.016405	0.09484	LPIN2	9663	lipin 2
ENSG0000	21873.96	0.195019	0.081278	2.399393	0.016422	0.094886	RPL32	6161	ribosomal protein L32
ENSG0000	4464.774	0.215503	0.089838	2.398806	0.016449	0.095009	DSE	29940	dermatan sulfate epimerase
ENSG0000	3612.933	-0.22006	0.091748	-2.39849	0.016463	0.09506	MED13L	23389	mediator complex subunit 13L
ENSG0000	479.4904	-0.83278	0.347239	-2.3983	0.016471	0.095082	MNDA	4332	myeloid cell nuclear differentiation antigen
ENSG0000	2330.856	-0.22702	0.094669	-2.39801	0.016484	0.095125	SFI1	9814	SFI1 centrin binding protein
ENSG0000	2387.7	-0.19906	0.083016	-2.39779	0.016494	0.095153	TUBGCP2	10844	tubulin gamma complex associated protein 2
ENSG0000	25.048	-1.0967	0.457508	-2.39712	0.016525	0.095299	NHLRC4	283948	NHL repeat containing 4
ENSG0000	1987.313	-0.2202	0.091869	-2.39686	0.016536	0.095336	RPTOR	57521	regulatory associated protein of MTOR complex 1
ENSG0000	54.05484	-0.86521	0.361009	-2.39665	0.016546	0.095361	CPNE7	27132	copine 7
ENSG0000	291.1698	0.360447	0.150416	2.396329	0.01656	0.095386	RNF144A	9781	ring finger protein 144A
ENSG0000	4.507068	-3.11841	1.301295	-2.39639	0.016557	0.095386	RIMBP3B	440804	RIMS binding protein 3B
ENSG0000	378.8413	-0.37098	0.154824	-2.39617	0.016568	0.095399	HHEX	3087	hematopoietically expressed homeobox
ENSG0000	512.8672	0.308577	0.128793	2.39592	0.016579	0.095433	PIK3C2B	5287	phosphatidylinositol-4-phosphate 3-kinase catalytic subunit type 2 beta
ENSG0000	882.7114	0.261573	0.109209	2.395157	0.016613	0.095602	SUMO2	6613	small ubiquitin like modifier 2
ENSG0000	699.6442	0.28114	0.117394	2.394852	0.016627	0.095622	GTF2B	2959	general transcription factor IIB
ENSG0000	1612.32	0.269757	0.112635	2.394959	0.016622	0.095622	RANBP9	10048	RAN binding protein 9
ENSG0000	4.433256	3.120203	1.30294	2.394741	0.016632	0.095622	CABP7	164633	calcium binding protein 7
ENSG0000	1959.743	0.271282	0.113306	2.394242	0.016655	0.095722	GPR35	2859	G protein-coupled receptor 35
ENSG0000	14.93283	1.689599	0.705784	2.393932	0.016669	0.095774	GRIK5	2901	glutamate ionotropic receptor kainate type subunit 5
ENSG0000	679.7562	0.301406	0.125965	2.392774	0.016722	0.096017	EMC3	55831	ER membrane protein complex subunit 3
ENSG0000	692.5289	0.266858	0.111522	2.392871	0.016717	0.096017	DCAF16	54876	DDB1 and CUL4 associated factor 16
ENSG0000	7.035171	-2.39123	0.999422	-2.39262	0.016729	0.096029	NA	NA	NA
ENSG0000	451.1109	0.331296	0.138492	2.392175	0.016749	0.096051	BCAS2	10286	BCAS2 pre-mRNA processing factor
ENSG0000	590.2713	0.280604	0.117301	2.392173	0.016749	0.096051	TBC1D23	55773	TBC1 domain family member 23
ENSG0000	227.346	0.407438	0.170328	2.392077	0.016753	0.096051	SLC22A18	5002	solute carrier family 22 member 18
ENSG0000	498.0499	-0.33929	0.141826	-2.39232	0.016742	0.096051	RAB40C	57799	RAB40C member RAS oncogene family
ENSG0000	1368.93	0.234733	0.098166	2.391195	0.016794	0.096252	FAM13B	51306	family with sequence similarity 13 member B
ENSG0000	566.9022	0.346986	0.145168	2.390234	0.016838	0.096474	FHIT	2272	fragile histidine triad diadenosine triphosphatase
ENSG0000	2032.183	0.200413	0.083853	2.390068	0.016845	0.096488	CNOT2	4848	CCR4-NOT transcription complex subunit 2
ENSG0000	33968.66	0.168655	0.0706	2.388885	0.0169	0.09677	RPL4	6124	ribosomal protein L4
ENSG0000	86.8503	-0.68823	0.288156	-2.38838	0.016923	0.096872	LINC02481	339988	long intergenic non-protein coding RNA 2481
ENSG0000	45.66086	1.010775	0.42324	2.388185	0.016932	0.096878	SMAD1	4086	SMAD family member 1
ENSG0000	475.6198	0.339568	0.14219	2.388133	0.016934	0.096878	TMEM243	79161	transmembrane protein 243
ENSG0000	464.3328	-0.31411	0.131547	-2.38782	0.016949	0.096932	EFL1	79631	elongation factor like GTPase 1
ENSG0000	582.0979	-0.28093	0.117673	-2.38739	0.016968	0.097014	TUBGCP3	10426	tubulin gamma complex associated protein 3
ENSG0000	96.60573	0.68487	0.286907	2.387078	0.016983	0.097067	RNF31	55072	ring finger protein 31
ENSG0000	4148.668	-0.17814	0.074664	-2.38586	0.017039	0.097359	MAN2A2	4122	mannosidase alpha class 2A member 2
ENSG0000	215.2347	-0.43822	0.183693	-2.38562	0.01705	0.097393	ERMAP	114625	erythroblast membrane associated protein (Scianna blood group)
ENSG0000	44.91376	-0.92867	0.389391	-2.38494	0.017082	0.097543	UBL7-DT	440288	UBL7 divergent transcript
ENSG0000	13123.53	0.184191	0.077237	2.384756	0.01709	0.097561	UBB	7314	ubiquitin B
ENSG0000	163.8174	-0.47301	0.198369	-2.3845	0.017102	0.097599	LINC00324	284029	long intergenic non-protein coding RNA 324
ENSG0000	4.1343	-3.45698	1.450002	-2.38412	0.01712	0.097656	GRM4	2914	glutamate metabotropic receptor 4
ENSG0000	566.2976	-0.32654	0.136966	-2.38406	0.017123	0.097656	PLBD1	79887	phospholipase B domain containing 1
ENSG0000	1769.636	0.24503	0.102788	2.383842	0.017133	0.097684	SLC26A11	284129	solute carrier family 26 member 11
ENSG0000	861.5748	-0.2493	0.10461	-2.38317	0.017164	0.097832	SYNE3	161176	spectrin repeat containing nuclear envelope family member 3
ENSG0000	253.495	-0.39881	0.167358	-2.38295	0.017174	0.09786	RABL2A	11159	RAB member of RAS oncogene family like 2A
ENSG0000	386.6827	-0.36822	0.154532	-2.38283	0.01718	0.097862	ZFP90	146198	ZFP90 zinc finger protein
ENSG0000	70.13445	-0.79191	0.332366	-2.38264	0.017189	0.097883	TIGD1	200765	tigger transposable element derived 1
ENSG0000	447.7665	0.323793	0.135912	2.382374	0.017201	0.097923	GPRASP1	9737	G protein-coupled receptor associated sorting protein 1
ENSG0000	4556.201	-0.2255	0.094677	-2.3818	0.017228	0.098045	FGD3	89846	FYVE RhoGEF and PH domain containing 3
ENSG0000	7.838887	2.601541	1.092418	2.381451	0.017245	0.098109	NA	NA	NA
ENSG0000	2228.73	-0.23293	0.097865	-2.38015	0.017306	0.098426	HPCAL1	3241	hippocalcin like 1
ENSG0000	1934.182	-0.20762	0.08725	-2.37965	0.017329	0.098531	EXOC3	11336	exocyst complex component 3
ENSG0000	1779.364	0.195603	0.08221	2.379303	0.017345	0.098592	B4GALT3	8703	beta-1 4-galactosyltransferase 3
ENSG0000	910.0632	0.304038	0.127841	2.378252	0.017395	0.098817	SPCS2	9789	signal peptidase complex subunit 2
ENSG0000	791.2451	0.289851	0.121877	2.378235	0.017396	0.098817	FLCN	201163	folliculin
ENSG0000	245.1065	0.395102	0.166169	2.377707	0.017421	0.098929	SEC11C	90701	SEC11 hon signal peptidase complex subunit
ENSG0000	7.171878	2.626634	1.104944	2.377165	0.017446	0.099044	LOC11484	1.15E+08	Uncharacterized protein LOC114841035
ENSG0000	1688.139	0.212339	0.089349	2.376505	0.017478	0.099191	INTS6	26512	integrator complex subunit 6
ENSG0000	2.721114	4.472778	1.882255	2.376287	0.017488	0.099219	NA	NA	NA
ENSG0000	314.3778	0.39215	0.165072	2.375632	0.017519	0.099365	SIAH1	6477	siah E3 ubiquitin protein ligase 1
ENSG0000	8.432689	2.231972	0.939711	2.375169	0.017541	0.099459	MYMX	1.02E+08	myomixer myoblast fusion factor
ENSG0000	88.51547	0.750327	0.315938	2.37492	0.017553	0.099496	GLIS2	84662	GLIS family zinc finger 2
ENSG0000	2871.938	-0.18022	0.075899	-2.37445	0.017575	0.099591	ASAH1	427	N-acylsphingosine amidohydrolase 1
ENSG0000	6631.98	-0.24633	0.103817	-2.37277	0.017655	0.100016	FPR1	2357	formyl peptide receptor 1
ENSG0000	211.1631	0.453756	0.191257	2.372498	0.017668	0.100059	GAN	8139	gigaxonin
ENSG0000	22.19671	1.621091	0.683552	2.371569	0.017713	0.100177	LINC0200S	1.05E+08	long intergenic non-protein coding RNA 2009
ENSG0000	266.1318	0.396483	0.167181	2.371571	0.017713	0.100177	ZBTB10	65986	zinc finger and BTB domain containing 10
ENSG0000	510.8329	0.294922	0.124354	2.371631	0.01771	0.100177	CCDC59	29080	coiled-coil domain containing 59
ENSG0000	7152.83	0.224583	0.094699	2.37156	0.017713	0.100177	MOB3A	126308	MOB kinase activator 3A
ENSG0000	1341.245	0.217584	0.091749	2.371497	0.017716	0.100177	PQBP1	10084	polyglutamine binding protein 1
ENSG0000	3355.172	-0.18523	0.078117	-2.37115	0.017733	0.100241	CTNNA1	1495	catenin alpha 1
ENSG0000	13.7739	-1.74127	0.734429	-2.37092	0.017744	0.100242	ZNF436-AS	148898	ZNF436 antisense RNA 1
ENSG0000	5541.695	0.232149	0.097913	2.370982	0.017741	0.100242	ADRM1	11047	ADRM1 26S proteasome ubiquitin receptor
ENSG0000	36.00803	0.950802	0.401056	2.370748	0.017752	0.100259	NA	NA	NA
ENSG0000	982.1699	0.297454	0.125503	2.370105	0.017783	0.100402	ABCE1	6059	ATP binding cassette subfamily E member 1
ENSG0000	144.6209	-0.62187	0.262413	-2.36981	0.017797	0.100452	HSPG2	3339	heparan sulfate proteoglycan 2
ENSG0000	194.7085	0.496756	0.209701	2.368873	0.017842	0.100661	RGS16	6004	regulator of G protein signaling 16
ENSG0000	442.7878	-0.30719	0.12968	-2.36882	0.017845	0.100661	MYO19	80179	myosin XIX
ENSG0000	17.25172	1.733244	0.73185	2.368304	0.01787	0.10077	IL13	3596	interleukin 13
ENSG0000	4.153618	3.054708	1.289895	2.368182	0.017876	0.100772	NA	NA	NA
ENSG0000	3681.65	-0.19132	0.080791	-2.36805	0.017882	0.100778	HERC1	8925	HECT and RLD domain containing E3 ubiquitin protein ligase family member 1
ENSG0000	339.1573	0.520213	0.219805	2.366703	0.017947	0.101053	MPZL1	9019	myelin protein zero like 1
ENSG0000	2559.855	0.23159	0.097847	2.366863	0.01794	0.101053	MSL2	55167	MSL complex subunit 2
ENSG0000	1760.543	-0.19783	0.083586	-2.36677	0.017944	0.101053	ARHGAP1	392	Rho GTPase activating protein 1
ENSG0000	2892.945	0.26331	0.11127	2.366401	0.017962	0.101105	BACH1	571	BTB domain and CNC homolog 1
ENSG0000	2846.047	-0.18581	0.078608	-2.3638	0.018089	0.101788	ADAM10	102	ADAM metallopeptidase domain 10
ENSG0000	243.7146	-0.39708	0.168005	-2.36351	0.018103	0.101823	SP4	6671	Sp4 transcription factor
ENSG0000	100.5713	-0.655	0.277136	-2.36344	0.018106	0.101823	C17orf97	400566	chromosome 17 open reading frame 97
ENSG0000	39.47819	1.110202	0.469889	2.362691	0.018143	0.101999	EPHX3	79852	epoxide hydrolase 3

ENSG0000	235.8695	-0.42651	0.180614	-2.36145	0.018204	0.102308	JMJD7-PLA	8681	JMJD7-PLA2G4B readthrough
ENSG0000	1604.875	0.214082	0.090661	2.361343	0.018209	0.102308	TBC1D20	128637	TBC1 domain family member 20
ENSG0000	1211.337	-0.22207	0.094048	-2.3612	0.018216	0.102317	SNAP29	9342	synaptosome associated protein 29
ENSG0000	445.2512	-0.2958	0.125303	-2.36071	0.01824	0.10242	GTF2IP4	1E+08	general transcription factor IIi pseudogene 4
ENSG0000	78.80629	0.77657	0.329045	2.360074	0.018271	0.102566	VASH2	79805	vasohibin 2
ENSG0000	1489.12	0.254954	0.108065	2.359258	0.018311	0.102729	ELOA	6924	elongin A
ENSG0000	89.14123	2.194056	0.929969	2.359278	0.018311	0.102729	MMP1	4312	matrix metallopeptidase 1
ENSG0000	2292.56	-0.20643	0.087523	-2.35859	0.018345	0.102884	TRIM44	54765	tripartite motif containing 44
ENSG0000	758.951	-0.26995	0.114481	-2.35803	0.018372	0.102999	NUDCD3	23386	NudC domain containing 3
ENSG0000	11.15164	-2.30214	0.976331	-2.35795	0.018376	0.102999	NA	NA	NA
ENSG0000	316.2562	-0.37249	0.158023	-2.35721	0.018413	0.103172	GTDC1	79712	glycosyltransferase like domain containing 1
ENSG0000	563.8302	-0.28668	0.121639	-2.35684	0.018431	0.103245	POLD1	5424	DNA polyr catalytic subunit
ENSG0000	338.8314	-0.34294	0.145531	-2.35649	0.018449	0.103311	ARRDC1-A	85026	ARRDC1 antisense RNA 1
ENSG0000	6.163795	-2.7732	1.177167	-2.35583	0.018482	0.103465	LINC0175(	643355	long intergenic non-protein coding RNA 1750
ENSG0000	467.7083	0.305044	0.129502	2.355516	0.018497	0.10352	GFPT1	2673	glutamine--fructose-6-phosphate transaminase 1
ENSG0000	819.5163	0.259437	0.110146	2.355385	0.018503	0.103525	DYNLT1	6993	dynein light chain Tctex-type 1
ENSG0000	8467.809	0.19493	0.082777	2.354878	0.018529	0.103635	CD79A	973	CD79a molecule
ENSG0000	2535.746	0.211011	0.089617	2.354585	0.018543	0.103685	BCL2L11	10018	BCL2 like 11
ENSG0000	14.19715	-2.14472	0.911184	-2.35377	0.018584	0.103882	NA	NA	NA
ENSG0000	2460.44	0.21852	0.092848	2.353536	0.018596	0.103916	CBX4	8535	chromobox 4
ENSG0000	1818.723	0.297622	0.126472	2.35327	0.018609	0.103959	RORA	6095	RAR related orphan receptor A
ENSG0000	1036.839	0.246298	0.104699	2.352427	0.018651	0.104163	SAR1A	56681	secretion associated Ras related GTPase 1A
ENSG0000	498.5791	-0.31062	0.132061	-2.35213	0.018666	0.104215	BAIAP3	8938	BAI1 associated protein 3
ENSG0000	5.464945	-2.97333	1.264629	-2.35115	0.018715	0.104458	MIR657	724027	microRNA 657
ENSG0000	9.927975	1.746761	0.743126	2.350558	0.018745	0.104593	IMPG2	50939	interphotoreceptor matrix proteoglycan 2
ENSG0000	1008.445	-0.24591	0.104625	-2.35043	0.018752	0.104597	SMAP1	60682	small ArfGAP 1
ENSG0000	581.5931	0.274199	0.116716	2.34929	0.018809	0.104887	PRR3	80742	proline rich 3
ENSG0000	9.752879	-2.2656	0.964674	-2.34856	0.018846	0.10506	ZC3HAV1L	92092	zinc finger antiviral 1 like
ENSG0000	5.333943	2.535056	1.079503	2.348355	0.018857	0.105088	TCHH	7062	trichohyalin
ENSG0000	158.0817	0.476063	0.202739	2.348159	0.018866	0.105112	MAP3K13	9175	mitogen-activated protein kinase kinase kinase 13
ENSG0000	1151.1	0.246257	0.104892	2.347716	0.018889	0.105205	PLCB3	5331	phospholipase C beta 3
ENSG0000	16.89324	-1.80046	0.767029	-2.34732	0.018909	0.105286	KCNQ3	3786	potassium voltage-gated channel subfamily Q member 3
ENSG0000	64871.57	0.176783	0.075319	2.347119	0.018919	0.10531	ANPEP	290	alanyl ami membrane
ENSG0000	2118.225	0.257049	0.109549	2.346437	0.018954	0.105472	RGS2	5997	regulator of G protein signaling 2
ENSG0000	176.3358	0.456285	0.194512	2.34579	0.018987	0.105623	NA	NA	NA
ENSG0000	37.17531	-0.9965	0.424825	-2.34567	0.018993	0.105625	SYDE2	84144	synapse defective Rho GTPase homolog 2
ENSG0000	86.95854	-0.61897	0.263939	-2.34514	0.01902	0.105744	DTWD1	56986	DTW domain containing 1
ENSG0000	179.4974	-0.46482	0.198228	-2.3449	0.019032	0.105781	GDPD3	79153	glycerophosphodiester phosphodiesterase domain containing 3
ENSG0000	102086.6	0.192409	0.082105	2.343454	0.019106	0.10616	RPS2	6187	ribosomal protein S2
ENSG0000	3819.089	-0.18188	0.077621	-2.34321	0.019118	0.106196	SETD1A	9739	SET domai histone lysine methyltransferase
ENSG0000	213.5294	-0.43322	0.18496	-2.34224	0.019168	0.106442	VPS45	11311	vacuolar protein sorting 45 homolog
ENSG0000	726.1747	0.256786	0.109651	2.341837	0.019189	0.106525	RSL24D1	51187	ribosomal L24 domain containing 1
ENSG0000	8.03148	-2.17565	0.929226	-2.34136	0.019214	0.10663	RTN4IP1	84816	reticulon 4 interacting protein 1
ENSG0000	2637.888	0.198232	0.084683	2.34087	0.019239	0.106723	KPNA1	3836	karyopherin subunit alpha 1
ENSG0000	212.6774	0.390402	0.166781	2.340809	0.019242	0.106723	RRAGD	58528	Ras related GTP binding D
ENSG0000	189.2205	0.474069	0.202714	2.338603	0.019356	0.107323	SPIB	6689	Spi-B transcription factor
ENSG0000	54.93121	0.958219	0.409881	2.337797	0.019398	0.107523	SIAE	54414	sialic acid acetylesterase
ENSG0000	961.9612	-0.26334	0.112655	-2.33753	0.019412	0.107568	EID1	23741	EP300 interacting inhibitor of differentiation 1
ENSG0000	1022.584	-0.2262	0.096782	-2.33725	0.019426	0.107615	UBE2Q2	92912	ubiquitin conjugating enzyme E2 Q2
ENSG0000	5.497756	-3.20911	1.373217	-2.33693	0.019443	0.107677	RANBP17	64901	RAN binding protein 17
ENSG0000	4.344883	-3.3253	1.423262	-2.33639	0.019471	0.107798	OSCP1	127700	organic solute carrier partner 1
ENSG0000	316.1128	-0.38028	0.162779	-2.33618	0.019482	0.107827	SAMD3	154075	sterile alpha motif domain containing 3
ENSG0000	20019.24	0.186596	0.079928	2.334558	0.019567	0.108264	SF1	7536	splicing factor 1
ENSG0000	48.31294	0.833512	0.357074	2.334283	0.019581	0.10829	PI3	5266	peptidase inhibitor 3
ENSG0000	36.04644	0.957304	0.410113	2.334244	0.019583	0.10829	RPS2P7	86781	ribosomal protein S2 pseudogene 7
ENSG0000	369.3088	-0.31506	0.134983	-2.33409	0.019591	0.108303	SLC49A3	84179	solute carrier family 49 member 3
ENSG0000	59.09836	-0.80011	0.342883	-2.33348	0.019623	0.108446	RWDD2A	112611	RWD domain containing 2A
ENSG0000	31.53698	-1.27717	0.547368	-2.33329	0.019633	0.10847	CCDC9B	388115	coiled-coil domain containing 9B
ENSG0000	10425.85	-0.20075	0.08605	-2.33292	0.019652	0.108545	SASH3	54440	SAM and SH3 domain containing 3
ENSG0000	311.0624	-0.42119	0.18067	-2.33127	0.019739	0.108958	SFT2D3	84826	SFT2 domain containing 3
ENSG0000	929.2372	-0.24358	0.104478	-2.33138	0.019733	0.108958	APC	324	APC regulator of WNT signaling pathway
ENSG0000	591.3672	0.305543	0.131075	2.331057	0.01975	0.108988	ISY1	57461	ISY1 splicing factor homolog
ENSG0000	186.3486	-0.49383	0.211865	-2.33085	0.019761	0.109017	DCBLD1	285761	discoidin CUB and LCCL domain containing 1
ENSG0000	70.18353	-0.90464	0.388171	-2.33052	0.019778	0.109078	CLCN5	1184	chloride voltage-gated channel 5
ENSG0000	93.5892	-0.57616	0.247268	-2.33012	0.0198	0.109164	SWSAP1	126074	SWIM-type zinc finger 7 associated protein 1
ENSG0000	414.5638	0.401028	0.172151	2.32951	0.019832	0.109261	B3GAT1	27087	beta-1 3-glucuronyltransferase 1
ENSG0000	3478.867	0.210922	0.090546	2.329454	0.019835	0.109261	TPD52L2	7165	TPD52 like 2
ENSG0000	8872.663	0.802755	0.344598	2.329543	0.01983	0.109261	ATP8	4509	ATP synthase F0 subunit 8
ENSG0000	561.1721	-0.27238	0.116947	-2.32908	0.019855	0.109305	CEP68	23177	centrosomal protein 68
ENSG0000	5647.831	0.172781	0.074181	2.32918	0.01985	0.109305	MYL6	4637	myosin light chain 6
ENSG0000	985.115	0.672231	0.288669	2.328721	0.019874	0.109377	P2RY6	5031	pyrimidinergic receptor P2Y6
ENSG0000	595.0163	0.26772	0.115016	2.327666	0.01993	0.109625	SLC25A36	55186	solute carrier family 25 member 36
ENSG0000	3466.717	0.172419	0.074074	2.32765	0.019931	0.109625	GTPBP2	54676	GTP binding protein 2
ENSG0000	244.3066	0.59301	0.254801	2.327346	0.019947	0.109681	WNT5A	7474	Wnt family member 5A
ENSG0000	413.967	-0.34982	0.150346	-2.32679	0.019976	0.109811	SRCAP	10847	Snf2 related CREBBP activator protein
ENSG0000	3493.524	-0.2365	0.101675	-2.32608	0.020014	0.109987	VSIR	64115	V-set immunoregulatory receptor
ENSG0000	95.96451	-0.65025	0.279592	-2.32569	0.020035	0.110001	TRIT1	54802	tRNA isopentenyltransferase 1
ENSG0000	15790.45	0.165737	0.071263	2.325703	0.020034	0.110001	CCL5	6352	C-C motif chemokine ligand 5
ENSG0000	6345.397	0.165948	0.071358	2.325587	0.020041	0.110001	OTUD5	55593	OTU deubiquitinase 5
ENSG0000	24384.21	0.73691	0.316834	2.325853	0.020026	0.110001	ND4L	4539	NADH dehydrogenase subunit 4L
ENSG0000	568.1251	-0.2841	0.122174	-2.32532	0.020055	0.11004	TMEM87B	84910	transmembrane protein 87B
ENSG0000	119.0118	0.557884	0.239926	2.325232	0.02006	0.11004	HSF4	3299	heat shock transcription factor 4
ENSG0000	4.017987	3.234038	1.391195	2.324648	0.020091	0.110146	NA	NA	NA
ENSG0000	511.6276	0.318945	0.1372	2.324662	0.02009	0.110146	GNE	10020	glucosamine (UDP-N-acetyl)-2-epimerase/N-acetylmannosamine kinase
ENSG0000	2159.008	0.20685	0.088994	2.324299	0.020109	0.110216	MXRA7	439921	matrix remodeling associated 7
ENSG0000	433.6383	-0.32511	0.139883	-2.32415	0.020118	0.110228	COMT	1312	catechol-O-methyltransferase
ENSG0000	9086.976	0.184477	0.07939	2.323675	0.020143	0.110262	HNRNPH1	3187	heterogeneous nuclear ribonucleoprotein H1
ENSG0000	69.16331	-0.75266	0.323895	-2.32379	0.020137	0.110262	NA	NA	NA
ENSG0000	197.7941	-0.4215	0.181401	-2.32359	0.020148	0.110262	RAD52	5893	RAD52 ho DNA repair protein
ENSG0000	887.3294	-0.29211	0.12571	-2.32367	0.020143	0.110262	DGCR8	54487	DGCR8 microprocessor complex subunit
ENSG0000	7270.77	-0.20765	0.089389	-2.323	0.020179	0.110401	FLII	2314	FLII actin remodeling protein
ENSG0000	95.15107	-0.56717	0.244182	-2.32272	0.020194	0.110451	TSPAN2	10100	tetraspanin 2
ENSG0000	499.4232	-0.32485	0.1399	-2.32199	0.020234	0.110634	SCO1	6341	synthesis of cytochrome C oxidase 1
ENSG0000	61654.53	0.160033	0.068941	2.321291	0.020271	0.110807	PTMA	5757	prothymosin alpha
ENSG0000	4.314744	3.712743	1.599528	2.321149	0.020279	0.110817	LOC1053	1.05E+08	uncharacterized LOC105376478
ENSG0000	1807.516	0.197048	0.084921	2.320385	0.02032	0.110976	SLAIN2	57606	SLAIN motif family member 2
ENSG0000	1401.838	-0.21035	0.09065	-2.32042	0.020318	0.110976	MIER2	54531	MIER family member 2
ENSG0000	33.12946	1.033622	0.445515	2.320062	0.020338	0.111018	CRYGS	1427	crystallin gamma S
ENSG0000	13.66973	-1.69505	0.730618	-2.32002	0.02034	0.111018	PDZD2	23037	PDZ domain containing 2
ENSG0000	855.8826	0.307903	0.132793	2.318671	0.020413	0.111352	RCOR3	55758	REST corepressor 3

ENSG0000	38.24788	1.181713	0.509647	2.318691	0.020412	0.111352	NA	NA	NA
ENSG0000	2121.297	-0.19632	0.084691	-2.3181	0.020444	0.111457	DELE1	9812	DAP3 binding cell death enhancer 1
ENSG0000	5.225687	2.714164	1.17082	2.318174	0.02044	0.111457	NA	NA	NA
ENSG0000	6.007245	-2.4689	1.065226	-2.31773	0.020464	0.111533	FAM167B	84734	family with sequence similarity 167 member B
ENSG0000	9.996956	-2.61873	1.130357	-2.31672	0.020519	0.111798	NA	NA	NA
ENSG0000	7.296054	2.44586	1.05587	2.316441	0.020534	0.11185	NA	NA	NA
ENSG0000	1353.662	-0.26941	0.11635	-2.31552	0.020584	0.112086	ZKSCAN1	7586	zinc finger with KRAB and SCAN domains 1
ENSG0000	1585.198	0.216112	0.09334	2.315315	0.020596	0.112086	SRP68	6730	signal recognition particle 68
ENSG0000	2912.025	0.189087	0.081665	2.315396	0.020591	0.112086	LSM14A	26065	LSM14A mRNA processing body assembly factor
ENSG0000	48.99796	-0.82923	0.358477	-2.3132	0.020712	0.112684	SPEF2	79925	sperm flagellar 2
ENSG0000	427.3187	-0.3067	0.132627	-2.31246	0.020752	0.112873	SEC22C	9117	SEC22 hon vesicle trafficking protein
ENSG0000	1276.634	-0.22864	0.098899	-2.31183	0.020787	0.113028	TUBA1A	7846	tubulin alpha 1a
ENSG0000	500.0212	-0.28872	0.124898	-2.3116	0.0208	0.11303	KIF13A	63971	kinesin family member 13A
ENSG0000	74.9963	-0.66693	0.288512	-2.31161	0.020799	0.11303	ZNF439	90594	zinc finger protein 439
ENSG0000	5204.385	0.180469	0.078091	2.311014	0.020832	0.113173	MAF1	84232	MAF1 hon negative regulator of RNA polymerase III
ENSG0000	1762.208	0.205838	0.089073	2.310892	0.020839	0.113176	SPPL2A	84888	signal peptide peptidase like 2A
ENSG0000	392.7057	-0.32834	0.142097	-2.31071	0.020849	0.113197	PHKB	5257	phosphorylase kinase regulatory subunit beta
ENSG0000	3637.971	-0.24454	0.105853	-2.31017	0.020879	0.113326	IRF5	3663	interferon regulatory factor 5
ENSG0000	34.09498	-0.97687	0.423092	-2.30888	0.02095	0.113681	RASA4	10156	RAS p21 protein activator 4
ENSG0000	109.1052	-0.52095	0.22566	-2.30859	0.020967	0.113737	CDHR3	222256	cadherin related family member 3
ENSG0000	2331.714	0.212264	0.091976	2.30782	0.021009	0.113935	DDX41	51428	DEAD-box helicase 41
ENSG0000	14.05011	-1.53562	0.665643	-2.30697	0.021056	0.114157	CLDN11	5010	claudin 11
ENSG0000	14589.77	0.170933	0.074107	2.306561	0.021079	0.114248	PCBP1	5093	poly(rC) binding protein 1
ENSG0000	6789.536	-0.26426	0.114612	-2.30566	0.02113	0.114455	FYB1	2533	FYN binding protein 1
ENSG0000	280.2691	-0.38263	0.165947	-2.30571	0.021127	0.114455	SMIM15	643155	small integral membrane protein 15
ENSG0000	353.8994	0.33906	0.14708	2.305276	0.021151	0.114537	CCZ1	51622	CCZ1 hom vacuolar protein trafficking and biogenesis associated
ENSG0000	2100.81	-0.18731	0.081303	-2.30386	0.02123	0.114929	USP19	10869	ubiquitin specific peptidase 19
ENSG0000	1143.242	0.231677	0.100564	2.303765	0.021236	0.114929	ZPR1	8882	ZPR1 zinc finger
ENSG0000	1905.405	-0.21929	0.095207	-2.30331	0.021261	0.115033	DOCK11	139818	dedicator of cytokinesis 11
ENSG0000	28354.2	0.220504	0.095746	2.303004	0.021279	0.115093	CTSB	1508	cathepsin B
ENSG0000	36.00285	1.230241	0.53431	2.302487	0.021308	0.115217	ABCA13	154664	ATP binding cassette subfamily A member 13
ENSG0000	49.59864	0.767112	0.333214	2.302157	0.021326	0.115284	NA	NA	NA
ENSG0000	4676.82	0.187734	0.081564	2.301671	0.021354	0.115365	RBM5	10181	RNA binding motif protein 5
ENSG0000	30535.17	0.205431	0.089252	2.301687	0.021353	0.115365	THBS1	7057	thrombospondin 1
ENSG0000	1691.197	0.233102	0.10129	2.301325	0.021373	0.115437	POFUT2	23275	protein O-fucosyltransferase 2
ENSG0000	968.0244	0.282865	0.122936	2.300918	0.021396	0.115453	PARP15	165631	poly(ADP-ribose) polymerase family member 15
ENSG0000	1147.8	-0.26942	0.117088	-2.301	0.021392	0.115453	AKAP9	10142	A-kinase anchoring protein 9
ENSG0000	30.42586	-1.01251	0.440047	-2.30091	0.021397	0.115453	NEK3	4752	NIMA related kinase 3
ENSG0000	19.02205	1.239353	0.53868	2.300723	0.021407	0.115453	NA	NA	NA
ENSG0000	277.8925	-0.39295	0.17079	-2.30078	0.021404	0.115453	DBF4B	80174	DBF4 zinc finger B
ENSG0000	6.126106	2.732401	1.187733	2.300519	0.021419	0.115481	NA	NA	NA
ENSG0000	249.7831	0.422324	0.183596	2.300284	0.021432	0.115519	AMN	81693	amnion associated transmembrane protein
ENSG0000	3.376963	-3.40833	1.481886	-2.3	0.021448	0.115573	ELN-AS1	1.08E+08	ELN antisense RNA 1
ENSG0000	13.81788	-1.63471	0.710825	-2.29973	0.021463	0.115598	NA	NA	NA
ENSG0000	442.3762	-0.33831	0.147118	-2.29959	0.021472	0.115598	PLEKHG3	26030	pleckstrin homology and RhoGEF domain containing G3
ENSG0000	4647.35	0.262555	0.114171	2.299656	0.021468	0.115598	GNA13	10672	G protein subunit alpha 13
ENSG0000	570.0709	-0.28748	0.125028	-2.29931	0.021487	0.11565	SLC35C1	55343	solute carrier family 35 member C1
ENSG0000	991.7556	-0.30349	0.132018	-2.29888	0.021512	0.115746	NPIPB3	23117	nuclear pore complex interacting protein family member B3
ENSG0000	14.02256	-1.48754	0.647207	-2.29839	0.021539	0.115862	NA	NA	NA
ENSG0000	17003.9	0.723813	0.314942	2.298245	0.021548	0.115874	TNFAIP2	7127	TNF alpha induced protein 2
ENSG0000	991.2138	0.277162	0.120613	2.297952	0.021565	0.11593	HCST	10870	hematopoietic cell signal transducer
ENSG0000	95.3917	0.63558	0.276601	2.297826	0.021572	0.115935	NA	NA	NA
ENSG0000	4660.283	-0.17641	0.076778	-2.29767	0.02158	0.115948	UBE2R2	54926	ubiquitin conjugating enzyme E2 R2
ENSG0000	34.57631	-0.93855	0.408716	-2.29635	0.021656	0.11632	EXPH5	23086	exophilin 5
ENSG0000	121.4213	-0.53261	0.231954	-2.29618	0.021665	0.116337	NPR2	4882	natriuretic peptide receptor 2
ENSG0000	423.2278	0.326102	0.14204	2.295854	0.021684	0.116405	CDK4	1019	cyclin dependent kinase 4
ENSG0000	95.2715	0.573175	0.24967	2.295733	0.021691	0.116405	DNAJB4	11080	DnaJ heat shock protein family (Hsp40) member B4
ENSG0000	13.27419	1.874381	0.816498	2.295634	0.021697	0.116405	NA	NA	NA
ENSG0000	232.885	-0.54863	0.239008	-2.29546	0.021707	0.116424	ERMP1	79956	endoplasmic reticulum metallopeptidase 1
ENSG0000	1083.86	0.237655	0.103549	2.295103	0.021727	0.116467	FOXK1	221937	forkhead box K1
ENSG0000	16.33487	1.394876	0.607748	2.295155	0.021724	0.116467	EGLN3	112399	egl-9 family hypoxia inducible factor 3
ENSG0000	544.4352	-0.29339	0.127893	-2.29406	0.021787	0.116753	FAM167A	83648	family with sequence similarity 167 member A
ENSG0000	1381.603	-0.29601	0.129053	-2.29368	0.021809	0.116803	CCAR1	55749	cell division cycle and apoptosis regulator 1
ENSG0000	1256.012	0.213343	0.093011	2.293724	0.021806	0.116803	SRSF9	8683	serine and arginine rich splicing factor 9
ENSG0000	2109.777	-0.22095	0.096336	-2.2935	0.021819	0.116824	PPP6R2	9701	protein phosphatase 6 regulatory subunit 2
ENSG0000	377.1973	0.345037	0.150465	2.293142	0.02184	0.116901	PATL2	197135	PAT1 homolog 2
ENSG0000	22.85399	-1.27535	0.556232	-2.29284	0.021857	0.11696	NA	NA	NA
ENSG0000	1095.815	0.227324	0.099158	2.292542	0.021874	0.116985	USP39	10713	ubiquitin specific peptidase 39
ENSG0000	981.5445	0.226533	0.098809	2.292632	0.021869	0.116985	E2F3	1871	E2F transcription factor 3
ENSG0000	3941.368	-0.18078	0.078865	-2.29223	0.021892	0.117047	USP34	9736	ubiquitin specific peptidase 34
ENSG0000	19.52369	1.332387	0.581565	2.291038	0.021961	0.117382	CFAP298-T	1.1E+08	CFAP298-TCP10L readthrough
ENSG0000	80.81134	-0.63488	0.277177	-2.29054	0.02199	0.117502	SMIM30	401397	small integral membrane protein 30
ENSG0000	229.0482	-0.39367	0.171901	-2.29012	0.022014	0.117597	NSDHL	50814	NAD(P) dependent steroid dehydrogenase-like
ENSG0000	239.1782	0.388231	0.169536	2.289968	0.022023	0.117611	PGM3	5238	phosphoglucomutase 3
ENSG0000	2612.248	0.203795	0.089018	2.289361	0.022058	0.117765	TERF2IP	54386	TERF2 interacting protein
ENSG0000	235.403	-0.45173	0.197339	-2.28912	0.022073	0.117776	B3GLCT	145173	beta 3-glucosyltransferase
ENSG0000	649.1592	0.328034	0.143302	2.289108	0.022073	0.117776	TXLNG	55787	taxilin gamma
ENSG0000	8.51515	-1.91646	0.837259	-2.28897	0.022081	0.117786	LOC10798	1.08E+08	uncharacterized LOC107984850
ENSG0000	15.58607	1.4391	0.628812	2.288602	0.022102	0.117865	TMPRSS6	164656	transmembrane serine protease 6
ENSG0000	546.1381	-0.28466	0.124391	-2.28845	0.022111	0.117878	DPH7	92715	diphthamide biosynthesis 7
ENSG0000	223.5034	0.479841	0.209728	2.28792	0.022142	0.118009	PLAU	5328	plasminog urokinase
ENSG0000	1556.023	0.218787	0.095701	2.286151	0.022245	0.118525	MCOLN1	57192	mucolipin TRP cation channel 1
ENSG0000	288.486	-0.34172	0.149485	-2.28599	0.022255	0.118542	LOC93622	93622	Morf4 family associated protein 1 like 1
ENSG0000	2106.051	-0.2485	0.108721	-2.28564	0.022275	0.118616	PLPPR2	64748	phospholipid phosphatase related 2
ENSG0000	183.9205	0.484763	0.212128	2.285244	0.022299	0.118706	MTHFD2L	441024	methylenetetrahydrofolate dehydrogenase (NADP+ dependent) 2 like
ENSG0000	242.8135	-0.40999	0.179459	-2.2846	0.022336	0.118841	ATRIP	84126	ATR interacting protein
ENSG0000	2185.52	-0.18173	0.079548	-2.28459	0.022337	0.118841	MON1B	22879	MON1 hor secretory trafficking associated
ENSG0000	1390.719	-0.21603	0.094582	-2.28407	0.022367	0.11897	CAMSAP1	157922	calmodulin regulated spectrin associated protein 1
ENSG0000	2110.715	0.220642	0.096619	2.28362	0.022394	0.119048	CHD1	1105	chromodomain helicase DNA binding protein 1
ENSG0000	156.0658	0.488166	0.21377	2.283601	0.022395	0.119048	KCND1	3750	potassium voltage-gated channel subfamily D member 1
ENSG0000	14476.13	0.163344	0.071533	2.283482	0.022402	0.119051	DDX5	1655	DEAD-box helicase 5
ENSG0000	682.7095	-0.2557	0.112003	-2.28301	0.02243	0.119166	PIGG	54872	phosphatidylinositol glycan anchor biosynthesis class G
ENSG0000	5476.093	0.200316	0.087774	2.282188	0.022478	0.119388	PLEKHM2	23207	pleckstrin homology and RUN domain containing M2
ENSG0000	162.4399	-0.44961	0.197033	-2.2819	0.022495	0.119445	TMEM101	84336	transmembrane protein 101
ENSG0000	285.1867	-0.36669	0.160724	-2.28151	0.022518	0.119498	NA	NA	NA
ENSG0000	426.468	0.365841	0.160347	2.281557	0.022516	0.119498	NA	NA	NA
ENSG0000	50.57075	-0.81062	0.35532	-2.28137	0.022526	0.119507	CCDC136	64753	coiled-coil domain containing 136
ENSG0000	4.931224	3.295661	1.444743	2.28114	0.02254	0.119546	NA	NA	NA
ENSG0000	923.1698	-0.24089	0.105616	-2.28078	0.022561	0.11959	RIC1	57589	RIC1 homc RAB6A GEF complex partner 1
ENSG0000	226.9015	-0.39794	0.174473	-2.2808	0.022561	0.11959	MRPL54	116541	mitochondrial ribosomal protein L54

ENSG0000	922.996	-0.23466	0.102895	-2.28056	0.022574	0.119624	PNMA1	9240	PNMA family member 1
ENSG0000	1975.974	0.217931	0.095566	2.280433	0.022582	0.119631	POLR1D	51082	RNA polymerase I and III subunit D
ENSG0000	82.64066	-0.66023	0.289573	-2.28003	0.022606	0.119725	NA	NA	NA
ENSG0000	310.8119	0.387407	0.169986	2.279058	0.022664	0.119863	BMP6	654	bone morphogenetic protein 6
ENSG0000	226.9149	-0.38738	0.169979	-2.27898	0.022668	0.119863	FUCA2	2519	alpha-L-fucosidase 2
ENSG0000	3419.146	0.212834	0.09338	2.279234	0.022653	0.119863	AP5Z1	9907	adaptor related protein complex 5 subunit zeta 1
ENSG0000	1299.676	-0.21941	0.096255	-2.27945	0.02264	0.119863	GOLGA3	2802	golgin A3
ENSG0000	138.5439	-0.50399	0.22115	-2.27893	0.022671	0.119863	EARS2	124454	glutamyl-t mitochondrial
ENSG0000	877.4588	0.243666	0.106919	2.278982	0.022668	0.119863	ACOT9	23597	acyl-CoA thioesterase 9
ENSG0000	115.5923	-0.51848	0.227531	-2.27871	0.022684	0.119882	NUDT9	53343	nudix hydrolase 9
ENSG0000	591.6307	-0.27531	0.120831	-2.27845	0.0227	0.119882	KLC4	89953	kinesin light chain 4
ENSG0000	1429.028	-0.22949	0.100719	-2.27855	0.022694	0.119882	VAT1	10493	vesicle amine transport 1
ENSG0000	26.75603	-1.12449	0.493537	-2.27844	0.022701	0.119882	RIMBP3	85376	RIMS binding protein 3
ENSG0000	4.28166	3.581595	1.572623	2.277466	0.022758	0.120125	LOC10537	1.05E+08	uncharacterized LOC105376030
ENSG0000	115.3521	-0.55095	0.241917	-2.27745	0.02276	0.120125	PRRG4	79056	proline rich and Gla domain 4
ENSG0000	15.00523	-1.51596	0.66597	-2.27631	0.022827	0.120423	NA	NA	NA
ENSG0000	2682.827	0.210673	0.092551	2.276287	0.022829	0.120423	TMED2	10959	transmembrane p24 trafficking protein 2
ENSG0000	555.0458	0.295451	0.129801	2.276178	0.022835	0.120423	UAP1	6675	UDP-N-acetylglucosamine pyrophosphorylase 1
ENSG0000	751.4888	0.276278	0.12139	2.275957	0.022849	0.120458	SYS1	90196	SYS1 golgi trafficking protein
ENSG0000	1007.916	-0.327	0.143715	-2.27537	0.022883	0.120608	LPAR6	10161	lysophosphatidic acid receptor 6
ENSG0000	1444.595	-0.25981	0.114218	-2.27466	0.022926	0.120799	CCL22	6367	C-C motif chemokine ligand 22
ENSG0000	4.523342	3.382389	1.487179	2.274365	0.022944	0.120859	BEST2	54831	bestrophin 2
ENSG0000	816.73	-0.27196	0.119595	-2.27399	0.022967	0.120942	DCP1B	196513	decapping mRNA 1B
ENSG0000	6.871958	-2.25198	0.990368	-2.27388	0.022973	0.120942	IFT81	28981	intraflagellar transport 81
ENSG0000	142.8727	-0.48582	0.213782	-2.27252	0.023055	0.121307	SEMA6C	10500	semaphorin 6C
ENSG0000	73.75956	-0.62114	0.273324	-2.27255	0.023053	0.121307	WDR35	57539	WD repeat domain 35
ENSG0000	782.9011	-0.30449	0.133994	-2.27241	0.023062	0.121307	MPI	4351	mannose phosphate isomerase
ENSG0000	953.7341	0.275937	0.121473	2.271582	0.023112	0.121535	PPP4R2	151987	protein phosphatase 4 regulatory subunit 2
ENSG0000	132.0795	-0.50434	0.222066	-2.27114	0.023139	0.121642	ANKRD26	22852	ankyrin repeat domain containing 26
ENSG0000	167.2465	0.462179	0.203569	2.270382	0.023184	0.121848	GP1BA	2811	glycoprotein Ib platelet subunit alpha
ENSG0000	378.6855	-0.34249	0.15089	-2.26982	0.023219	0.121994	KRBA1	84626	KRAB-A domain containing 1
ENSG0000	28.28243	1.360892	0.599607	2.269639	0.023229	0.121998	NA	NA	NA
ENSG0000	71.88889	-0.70059	0.308687	-2.26959	0.023233	0.121998	CDC14B	8555	cell division cycle 14B
ENSG0000	66.63215	-0.66023	0.291002	-2.26882	0.023279	0.122207	SNHG5	387066	small nucleolar RNA host gene 5
ENSG0000	19.78725	-1.21138	0.534204	-2.26763	0.023352	0.122555	NA	NA	NA
ENSG0000	67.75957	-0.7206	0.317817	-2.26733	0.02337	0.122615	CEP41	95681	centrosomal protein 41
ENSG0000	270.8707	0.374311	0.16513	2.266767	0.023404	0.122726	DCTN6	10671	dynactin subunit 6
ENSG0000	23.16802	-1.08864	0.480256	-2.26679	0.023403	0.122726	CCND1	595	cyclin D1
ENSG0000	22207.69	0.48227	0.212774	2.26659	0.023415	0.122748	LCP2	3937	lymphocyte cytosolic protein 2
ENSG0000	2961.857	0.199607	0.08809	2.265956	0.023454	0.122917	FOXK2	3607	forkhead box K2
ENSG0000	1045.199	0.237941	0.105013	2.265831	0.023462	0.122922	ACVR1	90	activin A receptor type 1
ENSG0000	109.876	-0.61197	0.27015	-2.26531	0.023494	0.123037	C9orf64	84267	chromosome 9 open reading frame 64
ENSG0000	1288.396	-0.23709	0.104674	-2.26504	0.02351	0.123037	PHF19	26147	PHD finger protein 19
ENSG0000	175.3791	0.429264	0.189508	2.265148	0.023504	0.123037	AKR1C3	8644	aldo-keto reductase family 1 member C3
ENSG0000	1390.243	-0.21653	0.095586	-2.26526	0.023497	0.123037	TAF4	6874	TATA-box binding protein associated factor 4
ENSG0000	69.42439	0.695129	0.306941	2.264701	0.023531	0.123077	MAP3K6	9064	mitogen-activated protein kinase kinase kinase 6
ENSG0000	331.8167	-0.38151	0.168454	-2.26475	0.023528	0.123077	ILVBL	10994	ilvB acetolactate synthase like
ENSG0000	337.958	-0.34336	0.151646	-2.2642	0.023562	0.123192	NBAS	51594	NBAS subunit of NRZ tethering complex
ENSG0000	32.73695	1.107635	0.489211	2.264125	0.023566	0.123192	EEF1AKMT	1.11E+08	EEF1A lysine methyltransferase 4
ENSG0000	238.1877	-0.38018	0.16799	-2.26308	0.023631	0.123474	ABHD11	83451	abhydrolase domain containing 11
ENSG0000	37.11725	1.145302	0.506092	2.263033	0.023634	0.123474	BACE2	25825	beta-secretase 2
ENSG0000	3028.447	-0.18861	0.083378	-2.26216	0.023688	0.123605	AHDC1	27245	AT-hook DNA binding motif containing 1
ENSG0000	229.5033	-0.45104	0.199393	-2.26209	0.023692	0.123605	PIGM	93183	phosphatidylinositol glycan anchor biosynthesis class M
ENSG0000	1055.372	0.210732	0.093152	2.262246	0.023682	0.123605	ABL2	27	ABL proto- non-receptor tyrosine kinase
ENSG0000	55.11448	-0.75733	0.334761	-2.26231	0.023678	0.123605	FAM114A1	92689	family with sequence similarity 114 member A1
ENSG0000	4.915217	-3.35082	1.481084	-2.26241	0.023672	0.123605	NA	NA	NA
ENSG0000	8303.887	0.192825	0.085255	2.261739	0.023714	0.123682	RPS23	6228	ribosomal protein S23
ENSG0000	3158.43	0.173085	0.076536	2.261469	0.02373	0.123735	CDK9	1025	cyclin dependent kinase 9
ENSG0000	2.399569	4.439946	1.963773	2.260926	0.023764	0.12385	SNORA77	677843	small nucl H/ACA box 77
ENSG0000	1086.508	-0.21016	0.092959	-2.26081	0.023771	0.12385	TIA1	7072	TIA1 cytotoxic granule associated RNA binding protein
ENSG0000	605.1948	-0.27415	0.121264	-2.26079	0.023772	0.12385	NA	NA	NA
ENSG0000	4059.824	0.201549	0.089155	2.260663	0.02378	0.123856	QKI	9444	QKI KH domain containing RNA binding
ENSG0000	35.38857	-1.11195	0.49204	-2.25988	0.023829	0.124075	NA	NA	NA
ENSG0000	74.07406	0.754824	0.334046	2.259644	0.023843	0.124115	GSTM3	2947	glutathione S-transferase mu 3
ENSG0000	336.8812	0.374447	0.165798	2.258453	0.023917	0.124466	CCZ1B	221960	CCZ1 hom vacuolar protein trafficking and biogenesis associated
ENSG0000	16.91828	-1.44788	0.641218	-2.25802	0.023944	0.124571	NA	NA	NA
ENSG0000	218.6793	0.406965	0.180245	2.257847	0.023955	0.124593	NOCT	25819	nocturnin
ENSG0000	23.82666	-1.15529	0.511704	-2.25773	0.023963	0.124596	CCNA2	890	cyclin A2
ENSG0000	118.5013	0.608477	0.26954	2.257469	0.023979	0.124645	TMEM156	80008	transmembrane protein 156
ENSG0000	13.98846	-1.48929	0.659989	-2.25654	0.024037	0.124912	NA	NA	NA
ENSG0000	14.6154	1.583724	0.70203	2.25592	0.024076	0.125079	NA	NA	NA
ENSG0000	406.5704	0.358652	0.159074	2.254623	0.024157	0.125466	RPL23AP4	647099	ribosomal protein L23a pseudogene 42
ENSG0000	3.28396	4.278855	1.897973	2.254434	0.024169	0.125493	NA	NA	NA
ENSG0000	478.3713	0.291121	0.129147	2.25418	0.024185	0.125541	NDUFA4	4697	NDUFA4 mitochondrial complex associated
ENSG0000	1018.127	-0.21935	0.097318	-2.25398	0.024197	0.12557	TTC17	55761	tetratricopeptide repeat domain 17
ENSG0000	1339.333	0.208333	0.092437	2.253789	0.024209	0.125598	PTMS	5763	parathymosin
ENSG0000	212.0146	-0.40293	0.178791	-2.25361	0.024221	0.125621	ABHD8	79575	abhydrolase domain containing 8
ENSG0000	139.9701	0.538214	0.238865	2.253211	0.024246	0.125717	RASD1	51655	ras related dexamethasone induced 1
ENSG0000	398.1119	0.321602	0.142758	2.252779	0.024273	0.125823	TRBV29-1	28558	T cell receptor beta variable 29-1
ENSG0000	170.1526	-0.44941	0.199548	-2.25215	0.024313	0.125958	FANCG	2189	FA complementation group G
ENSG0000	1611.12	0.235392	0.104516	2.252212	0.024309	0.125958	HS3ST3B1	9953	heparan sulfate-glucosamine 3-sulfotransferase 3B1
ENSG0000	7825.064	0.200111	0.088866	2.251844	0.024332	0.126023	ECE1	1889	endothelin converting enzyme 1
ENSG0000	24.66426	-1.14252	0.507554	-2.25102	0.024384	0.126257	PCDHGC3	5098	protocadh 3
ENSG0000	1188.22	0.22583	0.100342	2.2506	0.024411	0.12633	SLU7	10569	SLU7 hom splicing factor
ENSG0000	3272.802	0.179276	0.079661	2.250476	0.024419	0.12633	ILRUN	64771	inflammation and lipid regulator with UBA-like and NBR1-like domains
ENSG0000	558.4531	-0.29427	0.130756	-2.25053	0.024415	0.12633	PPFIBP2	8495	PPFIA binding protein 2
ENSG0000	640.1643	0.254082	0.112926	2.25	0.024449	0.126451	SLC35F5	80255	solute carrier family 35 member F5
ENSG0000	4487.857	-0.19827	0.088145	-2.24941	0.024487	0.126611	PIEZO1	9780	piezo type mechanosensitive ion channel component 1
ENSG0000	10.70668	-1.65398	0.735341	-2.24927	0.024495	0.12662	AFG1L	246269	AFG1 like ATPase
ENSG0000	162.6785	0.464227	0.206404	2.249118	0.024505	0.126635	NA	NA	NA
ENSG0000	7.09903	-2.81687	1.252938	-2.24821	0.024563	0.126873	NA	NA	NA
ENSG0000	74.68515	-0.65525	0.291457	-2.24818	0.024565	0.126873	ALDH1B1	219	aldehyde dehydrogenase 1 family member B1
ENSG0000	1981.678	-0.2086	0.092808	-2.24769	0.024596	0.126928	PHYKPL	85007	5-phosphohydroxy-L-lysine phospho-lyase
ENSG0000	475.6906	0.274878	0.122297	2.247635	0.0246	0.126928	GCLC	2729	glutamate-cysteine ligase catalytic subunit
ENSG0000	2155.407	-0.19569	0.087068	-2.24758	0.024603	0.126928	GLUD1	2746	glutamate dehydrogenase 1
ENSG0000	2902.362	-0.17199	0.076516	-2.24783	0.024587	0.126928	SUGP2	10147	SURP and G-patch domain containing 2
ENSG0000	11.4432	2.24817	1.000486	2.247078	0.024635	0.127024	DYNLT2	6991	dynein light chain Tctex-type 2
ENSG0000	622.0337	0.292918	0.130354	2.247094	0.024634	0.127024	EIF1AX	1964	eukaryotic translation initiation factor 1A X-linked
ENSG0000	244.8305	-0.42015	0.187023	-2.2465	0.024672	0.127178	POLD4	57804	DNA polym accessory subunit
ENSG0000	163.1845	-0.49531	0.22053	-2.24601	0.024703	0.127306	ERCC2	2068	ERCC excis TFIIH core complex helicase subunit

ENSG0000	2.287674	-4.21238	1.875997	-2.24541	0.024742	0.127469	NA	NA	NA
ENSG0000	2523.087	0.21419	0.095439	2.244247	0.024817	0.127782	ZSWIM6	57688	zinc finger SWIM-type containing 6
ENSG0000	868.8001	-0.30345	0.135209	-2.24427	0.024815	0.127782	LMTK2	22853	lemur tyrosine kinase 2
ENSG0000	48.29678	0.858932	0.382745	2.244138	0.024824	0.127783	HR	55806	HR lysine demethylase and nuclear receptor corepressor
ENSG0000	83.77739	-0.66629	0.296941	-2.24386	0.024841	0.127808	SLX4IP	128710	SLX4 interacting protein
ENSG0000	1152.66	-0.25577	0.113989	-2.24385	0.024842	0.127808	NCF4	4689	neutrophil cytosolic factor 4
ENSG0000	3.608629	-3.3728	1.503301	-2.24359	0.024859	0.127857	CPED1	79974	cadherin like and PC-esterase domain containing 1
ENSG0000	23.66137	-1.23188	0.549499	-2.24183	0.024972	0.128406	ANG	283	angiogenin
ENSG0000	812.88	0.2569	0.114644	2.240854	0.025036	0.128696	WDFY1	57590	WD repeat and FYVE domain containing 1
ENSG0000	11.62554	1.923508	0.858793	2.239782	0.025105	0.129017	NA	NA	NA
ENSG0000	11352.81	0.209194	0.093441	2.238786	0.02517	0.129279	NIBAN2	64855	niban apoptosis regulator 2
ENSG0000	2693.792	0.227442	0.101589	2.23884	0.025166	0.129279	SAP30BP	29115	SAP30 binding protein
ENSG0000	113.101	0.531893	0.237677	2.237881	0.025229	0.129546	TMEM217	221468	transmembrane protein 217
ENSG0000	4502.429	0.228791	0.102248	2.237606	0.025247	0.129602	GPR183	1880	G protein-coupled receptor 183
ENSG0000	120.0029	-0.58404	0.261141	-2.23648	0.02532	0.129944	TAF1B	9014	TATA-box RNA polymerase I subunit B
ENSG0000	48.41683	0.811442	0.362846	2.236328	0.02533	0.129959	ENKUR	219670	enkurin TRPC channel interacting protein
ENSG0000	53.57701	2.226088	0.995624	2.235873	0.02536	0.130076	NQO1	1728	NAD(P)H quinone dehydrogenase 1
ENSG0000	2223.727	-0.20797	0.093048	-2.23505	0.025414	0.130317	TADA3	10474	transcriptional adaptor 3
ENSG0000	73.37921	-0.68158	0.305168	-2.23346	0.025519	0.130816	TMPO-AS1	1E+08	TMPO antisense RNA 1
ENSG0000	1265.677	0.233496	0.104577	2.232774	0.025564	0.131012	CASP1	834	caspase 1
ENSG0000	65.79053	0.761377	0.341022	2.232634	0.025573	0.131023	RASIP1	54922	Ras interacting protein 1
ENSG0000	407.1849	0.395696	0.177256	2.232345	0.025592	0.131085	RAMP1	10267	receptor activity modifying protein 1
ENSG0000	204.2105	0.412355	0.184759	2.231852	0.025625	0.131215	NA	NA	NA
ENSG0000	124.5261	-0.5658	0.253635	-2.23076	0.025697	0.131417	TDRKH	11022	tudor and KH domain containing
ENSG0000	83.77522	-0.64833	0.290601	-2.23099	0.025682	0.131417	MYO7A	4647	myosin VIIA
ENSG0000	12.04062	1.580932	0.70863	2.230968	0.025683	0.131417	A2M-AS1	144571	A2M antisense RNA 1
ENSG0000	5.794662	2.875298	1.288954	2.230722	0.0257	0.131417	SIGLEC15	284266	sialic acid binding Ig like lectin 15
ENSG0000	5239.861	0.195687	0.087723	2.230735	0.025699	0.131417	SH3GL1	6455	SH3 doma endophilin A2
ENSG0000	108.84	0.636074	0.285181	2.230424	0.025719	0.131482	PTGES	9536	prostaglandin E synthase
ENSG0000	116.6363	-0.52918	0.237355	-2.2295	0.025781	0.131759	ELMOD2	255520	ELMO domain containing 2
ENSG0000	4.387476	-3.05888	1.372304	-2.22901	0.025813	0.131806	FHAD1-AS	1.02E+08	FHAD1 antisense RNA 1
ENSG0000	358.1184	0.381926	0.171349	2.228935	0.025818	0.131806	NBPF9	400818	NBPF member 9
ENSG0000	59.60715	0.683477	0.306633	2.228973	0.025816	0.131806	CABP4	57010	calcium binding protein 4
ENSG0000	690.1086	-0.30159	0.135298	-2.22908	0.025809	0.131806	NAGA	4668	alpha-N-acetylgalactosaminidase
ENSG0000	3.723176	-3.166	1.421018	-2.22798	0.025882	0.132095	NA	NA	NA
ENSG0000	429.1045	-0.28812	0.129347	-2.22752	0.025912	0.132213	TRIM3	10612	tripartite motif containing 3
ENSG0000	1820.645	-0.18749	0.084189	-2.22703	0.025945	0.132346	TP53I11	9537	tumor protein p53 inducible protein 11
ENSG0000	2171.139	-0.18574	0.083426	-2.22644	0.025985	0.13251	STIM1	6786	stromal interaction molecule 1
ENSG0000	1636.765	0.190882	0.08575	2.226028	0.026012	0.132614	DLG5	9231	discs large MAGUK scaffold protein 5
ENSG0000	22522.02	-0.17186	0.07721	-2.22585	0.026024	0.132622	TXNIP	10628	thioredoxin interacting protein
ENSG0000	185.6085	0.403365	0.181223	2.225792	0.026028	0.132622	LIN7C	55327	lin-7 homc crumbs cell polarity complex component
ENSG0000	2035.757	0.225786	0.101466	2.225237	0.026065	0.132654	ATP6V1G1	9550	ATPase H+ transporting V1 subunit G1
ENSG0000	79.89854	-0.713	0.32042	-2.2252	0.026068	0.132654	B4GALNT3	283358	beta-1 4-N-acetyl-galactosaminyltransferase 3
ENSG0000	78.141	-0.6499	0.292066	-2.22517	0.02607	0.132654	KCNH3	23416	potassium voltage-gated channel subfamily H member 3
ENSG0000	231.8163	-0.4889	0.219704	-2.22527	0.026063	0.132654	RAPGEFL1	51195	Rap guanine nucleotide exchange factor like 1
ENSG0000	1026.621	0.211095	0.094867	2.225165	0.02607	0.132654	LSM7	51690	LSM7 hom U6 small nuclear RNA and mRNA degradation associated
ENSG0000	437.1857	0.363278	0.163295	2.224673	0.026103	0.132785	RBMXL1	494115	RBMX like 1
ENSG0000	7.153539	2.653434	1.193197	2.223803	0.026162	0.133047	NA	NA	NA
ENSG0000	60.70281	-0.76442	0.343896	-2.22283	0.026227	0.133259	UCN2	90226	urocortin 2
ENSG0000	2600.609	0.191017	0.085934	2.222827	0.026227	0.133259	NUP153	9972	nucleoporin 153
ENSG0000	4561.757	-0.23172	0.104253	-2.22265	0.026239	0.133259	PML	5371	PML nuclear body scaffold
ENSG0000	389.9072	-0.3253	0.146351	-2.22273	0.026234	0.133259	MVB12A	93343	multivesicular body subunit 12A
ENSG0000	2474.307	0.195863	0.088115	2.222813	0.026228	0.133259	SLC25A5	292	solute carrier family 25 member 5
ENSG0000	80.63502	-0.68203	0.306942	-2.222	0.026283	0.133444	PAM16	51025	presequence translocase associated motor 16
ENSG0000	14479.28	0.218826	0.098492	2.221764	0.026299	0.13349	RPS24	6229	ribosomal protein S24
ENSG0000	3239.699	0.173241	0.077978	2.221655	0.026307	0.133491	SLC25A3	5250	solute carrier family 25 member 3
ENSG0000	1236.215	0.217089	0.097723	2.221476	0.026319	0.133516	DEGS1	8560	delta 4-de sphingolipid 1
ENSG0000	1558.59	-0.24629	0.110901	-2.2208	0.026364	0.133565	MAN1C1	57134	mannosidase alpha class 1C member 1
ENSG0000	153.2122	-0.557	0.250804	-2.22084	0.026362	0.133565	TMEM25	84866	transmembrane protein 25
ENSG0000	1436.254	0.21959	0.098874	2.220902	0.026358	0.133565	EIF3J	8669	eukaryotic translation initiation factor 3 subunit J
ENSG0000	3502.538	-0.20962	0.094387	-2.22087	0.02636	0.133565	ACO2	50	aconitase 2
ENSG0000	156.8496	-0.42504	0.191382	-2.2209	0.026358	0.133565	SLC9A7	84679	solute carrier family 9 member A7
ENSG0000	33.31828	-0.9501	0.427858	-2.22061	0.026378	0.133593	ATP6V1E2	90423	ATPase H+ transporting V1 subunit E2
ENSG0000	1670.255	0.225731	0.101657	2.220506	0.026384	0.133593	RNF125	54941	ring finger protein 125
ENSG0000	297.3513	0.378973	0.170679	2.220391	0.026392	0.133596	SVIP	258010	small VCP interacting protein
ENSG0000	1000.869	0.236357	0.10646	2.220147	0.026409	0.133644	BFAR	51283	bifunctional apoptosis regulator
ENSG0000	626.485	-0.2546	0.114724	-2.21922	0.026472	0.133926	PDCD11	22984	programmed cell death 11
ENSG0000	1080.61	0.204579	0.09222	2.218377	0.026529	0.134056	SYF2	25949	SYF2 pre-mRNA splicing factor
ENSG0000	4162.843	0.757227	0.341299	2.218661	0.02651	0.134056	PTAFR	5724	platelet activating factor receptor
ENSG0000	1225.8	0.255242	0.115062	2.218311	0.026534	0.134056	RASA2	5922	RAS p21 protein activator 2
ENSG0000	2210.185	-0.20178	0.090947	-2.21863	0.026512	0.134056	FASTK	10922	Fas activated serine/threonine kinase
ENSG0000	584.5452	-0.29513	0.133038	-2.21835	0.026531	0.134056	PPP3CB	5532	protein phosphatase 3 catalytic subunit beta
ENSG0000	445.5432	0.275027	0.124002	2.217924	0.02656	0.134153	LAMTOR3	8649	late endos MAPK and MTOR activator 3
ENSG0000	1216.634	0.19919	0.089847	2.216999	0.026623	0.134435	DNAJA2	10294	DnaJ heat shock protein family (Hsp40) member A2
ENSG0000	100.2764	-0.54887	0.247586	-2.21688	0.026631	0.13444	GPC2	221914	glypican 2
ENSG0000	148.793	-0.4309	0.194401	-2.21655	0.026654	0.13449	CNPY4	245812	canopy FGF signaling regulator 4
ENSG0000	31245.82	0.166522	0.075128	2.216521	0.026656	0.13449	GNAS	2778	GNAS complex locus
ENSG0000	25.25399	1.346909	0.607721	2.216327	0.026669	0.134492	ANKRD44-	1.02E+08	ANKRD44 antisense RNA 1
ENSG0000	131.3037	0.519138	0.234236	2.216305	0.026671	0.134492	PXDC1	221749	PX domain containing 1
ENSG0000	511.4256	-0.28162	0.127073	-2.21619	0.026678	0.134493	PPME1	51400	protein phosphatase methylesterase 1
ENSG0000	69.21917	-0.65887	0.297369	-2.21565	0.026715	0.134644	MACROD1	28992	mono-ADP ribosylhydrolase 1
ENSG0000	5468.497	0.201436	0.090931	2.215253	0.026743	0.134745	RPL36	25873	ribosomal protein L36
ENSG0000	8.122389	2.360916	1.066098	2.214539	0.026792	0.134956	CTCF-DT	1.08E+08	CTCF divergent transcript
ENSG0000	352.1369	-0.31205	0.140917	-2.21443	0.026799	0.134958	C19orf54	284325	chromosome 19 open reading frame 54
ENSG0000	14.04415	-1.53756	0.69456	-2.21371	0.026849	0.135132	NA	NA	NA
ENSG0000	146.8575	-0.48436	0.218798	-2.21372	0.026848	0.135132	SIRPG	55423	signal regulatory protein gamma
ENSG0000	341.3319	-0.37782	0.170682	-2.21359	0.026857	0.135138	TENT5A	55603	terminal nucleotidyltransferase 5A
ENSG0000	144.8158	-0.46149	0.208548	-2.21289	0.026905	0.135345	SGMS2	166929	sphingomyelin synthase 2
ENSG0000	22.69562	1.271089	0.574592	2.21216	0.026956	0.135452	NA	NA	NA
ENSG0000	3.31263	4.033006	1.823017	2.21227	0.026948	0.135452	NPR3	4883	natriuretic peptide receptor 3
ENSG0000	4.664407	-2.91097	1.315897	-2.21216	0.026956	0.135452	ACTA2-AS:	1E+08	ACTA2 antisense RNA 1
ENSG0000	53.62781	-0.71067	0.321214	-2.21245	0.026936	0.135452	PRR11	55771	proline rich 11
ENSG0000	61.6936	-0.7302	0.330268	-2.21093	0.02704	0.135839	RCCD1	91433	RCC1 domain containing 1
ENSG0000	3.362007	3.648273	1.650829	2.209964	0.027108	0.136109	NA	NA	NA
ENSG0000	3542.899	0.186313	0.08431	2.209853	0.027115	0.136109	ILK	3611	integrin linked kinase
ENSG0000	115.0281	-0.49423	0.223649	-2.20984	0.027116	0.136109	BCAS4	55653	breast carcinoma amplified sequence 4
ENSG0000	209.8268	-0.60279	0.272802	-2.20964	0.02713	0.136143	LPL	4023	lipoprotein lipase
ENSG0000	137.4784	0.569269	0.257738	2.208706	0.027195	0.136432	SSX2IP	117178	SSX family member 2 interacting protein
ENSG0000	89.42773	-0.67681	0.30649	-2.20825	0.027227	0.136555	ZNF169	169841	zinc finger protein 169
ENSG0000	1382.1	-0.20626	0.093415	-2.20801	0.027244	0.136565	ZNF76	7629	zinc finger protein 76

ENSG0000	544.4954	0.281032	0.127278	2.208007	0.027244	0.136565	CDC42EP4	23580	CDC42 effector protein 4
ENSG0000	20.32555	1.225779	0.555232	2.207688	0.027266	0.13664	CA6	765	carbonic anhydrase 6
ENSG0000	334.5772	-0.36344	0.164705	-2.20664	0.027339	0.136969	NSL1	25936	NSL1 component of MIS12 kinetochore complex
ENSG0000	7.367571	-2.17226	0.984559	-2.20633	0.027361	0.137041	ENTPD3-A	285266	ENTPD3 antisense RNA 1
ENSG0000	128.0893	0.488757	0.221536	2.206217	0.027369	0.137044	SLC25A4	291	solute carrier family 25 member 4
ENSG0000	583.7824	0.34682	0.157237	2.205722	0.027403	0.13718	NA	NA	NA
ENSG0000	594.5518	0.265338	0.120302	2.205599	0.027412	0.137186	COMMD6	170622	COMM domain containing 6
ENSG0000	9.427048	2.009694	0.911292	2.205325	0.027431	0.137245	LOC10012	1E+08	small nuclear ribonucleoprotein polypeptide N pseudogene
ENSG0000	107.2073	0.582412	0.264148	2.20487	0.027463	0.137368	SELP	6403	selectin P
ENSG0000	498.1577	-0.27418	0.124363	-2.20471	0.027474	0.137385	YPEL1	29799	yippee like 1
ENSG0000	2145.723	0.204533	0.09278	2.204488	0.02749	0.137428	NSMF	26012	NMDA receptor synaptonuclear signaling and neuronal migration factor
ENSG0000	8.663608	2.488297	1.128991	2.204001	0.027524	0.137562	NA	NA	NA
ENSG0000	3170.018	-0.17737	0.080485	-2.20377	0.027541	0.137607	FHL3	2275	four and a half LIM domains 3
ENSG0000	1753.251	0.21499	0.097567	2.203507	0.027559	0.137662	PI4K2A	55361	phosphatidylinositol 4-kinase type 2 alpha
ENSG0000	183.688	-0.40908	0.185722	-2.20263	0.027621	0.137935	MKI67	4288	marker of proliferation Ki-67
ENSG0000	5.037551	-2.76723	1.256676	-2.20203	0.027664	0.138072	GUSBP2	387036	GUSB pseudogene 2
ENSG0000	120.3178	-0.52533	0.238567	-2.20202	0.027664	0.138072	BAHCC1	57597	BAH domain and coiled-coil containing 1
ENSG0000	450.3784	-0.29623	0.134586	-2.20104	0.027733	0.138324	TPRN	286262	taperin
ENSG0000	4.438201	-3.15952	1.435497	-2.201	0.027736	0.138324	NA	NA	NA
ENSG0000	520.2667	-0.30723	0.139584	-2.20106	0.027732	0.138324	BTK	695	Bruton tyrosine kinase
ENSG0000	5.774948	2.883262	1.310259	2.200528	0.027769	0.138437	EPDR1	54749	ependymin related 1
ENSG0000	102.0748	-0.61037	0.277382	-2.20047	0.027774	0.138437	TMEM254	80195	transmembrane protein 254
ENSG0000	2527.091	0.216787	0.098539	2.200024	0.027805	0.138555	FGFRL1	53834	fibroblast growth factor receptor like 1
ENSG0000	4571.861	-0.18783	0.085392	-2.1996	0.027835	0.138588	EPB41	2035	erythrocyte membrane protein band 4.1
ENSG0000	96.02743	0.697981	0.317329	2.19955	0.027839	0.138588	ST3GAL6	10402	ST3 beta-g 3-sialyltransferase 6
ENSG0000	765.4111	-0.25904	0.117773	-2.19951	0.027842	0.138588	RBM19	9904	RNA binding motif protein 19
ENSG0000	15.82812	-2.0247	0.920507	-2.19955	0.027839	0.138588	NA	NA	NA
ENSG0000	125.8478	-0.5122	0.232909	-2.19915	0.027867	0.138678	MLYCD	23417	malonyl-CoA decarboxylase
ENSG0000	117.2513	-0.61833	0.281208	-2.19882	0.02789	0.138756	SUOX	6821	sulfite oxidase
ENSG0000	2.412036	4.322802	1.966397	2.198336	0.027925	0.138892	NA	NA	NA
ENSG0000	2080.132	0.190643	0.08673	2.198126	0.02794	0.138929	STX4	6810	syntaxin 4
ENSG0000	131.8138	-0.55228	0.251311	-2.19762	0.027976	0.139072	CCNJ	54619	cyclin J
ENSG0000	903.7832	0.2358	0.107305	2.197468	0.027987	0.139088	CLK4	57396	CDC like kinase 4
ENSG0000	263.2487	0.447533	0.20368	2.197237	0.028004	0.139132	MOB4	25843	MOB fami phocein
ENSG0000	46.68006	-0.86968	0.39585	-2.19699	0.028021	0.139183	BMPR1A	657	bone morphogenetic protein receptor type 1A
ENSG0000	69.29226	-0.66192	0.301431	-2.19594	0.028096	0.139518	QRICH2	84074	glutamine rich 2
ENSG0000	3288.218	0.16154	0.073587	2.195216	0.028148	0.139738	ST13	6767	ST13 Hsp70 interacting protein
ENSG0000	1230.923	0.198314	0.090369	2.194495	0.0282	0.139808	SLC2A1	6513	solute carrier family 2 member 1
ENSG0000	1186.315	0.20955	0.095478	2.194762	0.028181	0.139808	BMAL1	406	basic helix-loop-helix ARNT like 1
ENSG0000	95.19856	0.529842	0.241414	2.19474	0.028182	0.139808	RPL6P27	645387	ribosomal protein L6 pseudogene 27
ENSG0000	1358.221	0.211602	0.096423	2.194511	0.028199	0.139808	RIOK3	8780	RIO kinase 3
ENSG0000	3655.733	0.196664	0.089616	2.194509	0.028199	0.139808	UBE2L3	7332	ubiquitin conjugating enzyme E2 L3
ENSG0000	171.7475	0.432667	0.19718	2.19427	0.028216	0.139837	NA	NA	NA
ENSG0000	39.44321	0.993169	0.452633	2.194205	0.028221	0.139837	CILP2	148113	cartilage intermediate layer protein 2
ENSG0000	2.714751	-4.13497	1.885124	-2.19347	0.028273	0.14006	ANGPT2	285	angiopoietin 2
ENSG0000	3.846386	-3.47938	1.586555	-2.19304	0.028304	0.140176	IGHJ4	28477	immunoglobulin heavy joining 4
ENSG0000	51.17297	-0.75682	0.345168	-2.19262	0.028334	0.140177	ATG4C	84938	autophagy related 4C cysteine peptidase
ENSG0000	2.598965	4.228885	1.928509	2.192826	0.02832	0.140177	RN7SKP70	1.07E+08	RN7SK pseudogene 70
ENSG0000	6.38752	2.91682	1.330212	2.192748	0.028326	0.140177	NA	NA	NA
ENSG0000	1132.126	0.239601	0.109276	2.192622	0.028335	0.140177	FUNDC2	65991	FUN14 domain containing 2
ENSG0000	1393.489	-0.1912	0.087209	-2.19246	0.028346	0.140197	PSME3IP1	80011	proteasome activator subunit 3 interacting protein 1
ENSG0000	2315.36	0.2612	0.119186	2.191532	0.028413	0.14049	PCGF5	84333	polycomb group ring finger 5
ENSG0000	41.22293	-0.90944	0.414999	-2.19143	0.028421	0.14049	GOLGA6L9	440295	golgin A6 family like 9
ENSG0000	6.498115	2.317284	1.057492	2.191302	0.02843	0.140498	LOC10537	1.05E+08	uncharacterized LOC105375924
ENSG0000	87.55886	-0.75306	0.343675	-2.19119	0.028438	0.1405	TMEM53	79639	transmembrane protein 53
ENSG0000	39.8418	0.902604	0.41202	2.190682	0.028475	0.140645	CRISP3	10321	cysteine rich secretory protein 3
ENSG0000	549.5406	-0.2753	0.125715	-2.18983	0.028536	0.140796	RBSN	64145	rabenosyn RAB effector
ENSG0000	3.711133	-3.14301	1.435316	-2.18977	0.028541	0.140796	NA	NA	NA
ENSG0000	1690.988	-0.20944	0.095631	-2.19009	0.028518	0.140796	HEXB	3074	hexosaminidase subunit beta
ENSG0000	37.79528	-1.08618	0.496055	-2.18963	0.028551	0.140796	RAPGEF3	10411	Rap guanine nucleotide exchange factor 3
ENSG0000	38.52085	-0.90697	0.414206	-2.18967	0.028548	0.140796	ADAMTS7	11173	ADAM metallopeptidase with thrombospondin type 1 motif 7
ENSG0000	11.72875	1.90886	0.871715	2.189775	0.028541	0.140796	SCML2	10389	Scm polycomb group protein like 2
ENSG0000	777.3808	-0.2399	0.109584	-2.18922	0.028581	0.140905	COQ4	51117	coenzyme Q4
ENSG0000	29.9241	-0.95957	0.438342	-2.1891	0.02859	0.140912	TRBJ1-3	28633	T cell receptor beta joining 1-3
ENSG0000	719.2188	-0.24525	0.112038	-2.18899	0.028598	0.140915	USP48	84196	ubiquitin specific peptidase 48
ENSG0000	130.2267	-0.45324	0.207107	-2.18845	0.028637	0.141071	NA	NA	NA
ENSG0000	2078.239	0.210914	0.096387	2.188194	0.028655	0.141124	SSR4	6748	signal sequence receptor subunit 4
ENSG0000	4.868223	-3.30725	1.511524	-2.18802	0.028668	0.141144	NA	NA	NA
ENSG0000	13.935	-1.63596	0.747718	-2.18793	0.028675	0.141144	ITGA9	3680	integrin subunit alpha 9
ENSG0000	919.3483	0.258497	0.118155	2.187778	0.028686	0.141161	MCTP2	55784	multiple C2 and transmembrane domain containing 2
ENSG0000	1682.211	-0.21267	0.097215	-2.18759	0.0287	0.141192	GSDMD	79792	gasdermin D
ENSG0000	479.1405	-0.28671	0.131078	-2.18736	0.028717	0.141223	PRPSAP1	5635	phosphoribosyl pyrophosphate synthetase associated protein 1
ENSG0000	226.5455	0.360103	0.164634	2.187291	0.028721	0.141223	RPL10P16	284393	ribosomal protein L10 pseudogene 16
ENSG0000	116.7445	-0.53825	0.246096	-2.18715	0.028732	0.141238	C20orf204	284739	chromosome 20 open reading frame 204
ENSG0000	124.0544	-0.53551	0.244948	-2.18621	0.0288	0.141536	PLEKHG4	25894	pleckstrin homology and RhoGEF domain containing G4
ENSG0000	1279.005	-0.22136	0.101266	-2.18595	0.028819	0.141591	MAPK3	5595	mitogen-activated protein kinase 3
ENSG0000	136.1038	-0.5339	0.244296	-2.18547	0.028854	0.141685	CRYZ	1429	crystallin zeta
ENSG0000	197.0826	-0.39685	0.181592	-2.18538	0.028861	0.141685	GRB10	2887	growth factor receptor bound protein 10
ENSG0000	6360.097	0.169985	0.077776	2.185581	0.028846	0.141685	HM13	81502	histocompatibility minor 13
ENSG0000	125.3972	-0.46088	0.211007	-2.18419	0.028949	0.142077	UMAD1	729852	UBAP1-MVB12-associated (UMA) domain containing 1
ENSG0000	3410.013	0.162105	0.074232	2.183774	0.028979	0.142188	RNF4	6047	ring finger protein 4
ENSG0000	2125.005	0.189545	0.086829	2.182969	0.029038	0.142441	UBE2M	9040	ubiquitin conjugating enzyme E2 M
ENSG0000	3361.264	0.172852	0.079188	2.182803	0.02905	0.142463	PLEKHM1	9842	pleckstrin homology and RUN domain containing M1
ENSG0000	9.38875	-1.90308	0.871964	-2.18252	0.029071	0.142528	DOCK1	1793	dedicator of cytokinesis 1
ENSG0000	970.1313	0.233689	0.107091	2.182154	0.029098	0.142591	CREB1	1385	cAMP responsive element binding protein 1
ENSG0000	161.9126	-0.45624	0.209081	-2.18214	0.0291	0.142591	APH1B	83464	aph-1 hom gamma-secretase subunit
ENSG0000	1527.275	0.189527	0.086858	2.182023	0.029108	0.142595	AIF1	199	allograft inflammatory factor 1
ENSG0000	532.3573	-0.30154	0.138206	-2.18184	0.029121	0.142622	PPP5C	5536	protein phosphatase 5 catalytic subunit
ENSG0000	507.259	0.310742	0.142433	2.181667	0.029134	0.142648	NUTM2B-A	1.01E+08	NUTM2B antisense RNA 1
ENSG0000	3813.569	0.186952	0.085702	2.18141	0.029153	0.142703	MMP24OS	1.01E+08	MMP24 opposite strand
ENSG0000	1096.617	-0.22305	0.102273	-2.18096	0.029186	0.142801	IPO13	9670	importin 13
ENSG0000	133.2372	0.576175	0.264188	2.180932	0.029188	0.142801	PSMD6-AS	1.01E+08	PSMD6 antisense RNA 2
ENSG0000	204.9298	-0.41108	0.188499	-2.18081	0.029198	0.142808	NA	NA	NA
ENSG0000	34.51027	-0.90037	0.412886	-2.18067	0.029208	0.142819	LAMC3	10319	laminin subunit gamma 3
ENSG0000	4.327649	2.66722	1.223413	2.180147	0.029247	0.142934	LOC1027	1.03E+08	WAS/WASL-interacting protein family member 2-like
ENSG0000	7.516886	-2.17241	0.996417	-2.18022	0.029241	0.142934	LOC12490	1.25E+08	uncharacterized LOC124904917
ENSG0000	13977.58	0.164591	0.07551	2.179729	0.029278	0.143025	ENO1	2023	enolase 1
ENSG0000	1411.575	0.211792	0.097166	2.179689	0.02928	0.143025	ATP6V1E1	529	ATPase H+ transporting V1 subunit E1
ENSG0000	1680.093	-0.22476	0.103133	-2.17938	0.029304	0.143039	PDIA4	9601	protein disulfide isomerase family A member 4
ENSG0000	3.275445	3.128972	1.435744	2.179338	0.029307	0.143039	NA	NA	NA

ENSG0000	2122.619	-0.21606	0.099133	-2.17946	0.029297	0.143039	DGCR2	9993	DiGeorge syndrome critical region gene 2	
ENSG0000	148.1505	-0.47964	0.220139	-2.17881	0.029346	0.143192	NPAS2	4862	neuronal PAS domain protein 2	
ENSG0000	6.954747	2.113081	0.970017	2.178397	0.029377	0.143267	TTK	7272	TTK protein kinase	
ENSG0000	10.88997	-1.77319	0.81396	-2.17848	0.029371	0.143267	CKLF	51192	chemokine like factor	
ENSG0000	21.52873	1.501475	0.689386	2.177988	0.029407	0.143377	CSF2	1437	colony stimulating factor 2	
ENSG0000	97.97617	0.542809	0.249237	2.177886	0.029415	0.143377	C16orf87	388272	chromosome 16 open reading frame 87	
ENSG0000	4.495021	2.920195	1.340922	2.177752	0.029424	0.143388	SPTA1	6708	spectrin al	erythrocytic 1
ENSG0000	11.15289	1.669231	0.766831	2.176792	0.029496	0.1437	CCDC162P	221262	coiled-coil	pseudogene
ENSG0000	45.05307	-0.77113	0.354297	-2.17652	0.029516	0.143761	SCUBE1	80274	signal pep	CUB domain and EGF like domain containing 1
ENSG0000	179.392	-0.40405	0.185677	-2.17609	0.029549	0.14388	ZNF696	79943	zinc finger protein 696	
ENSG0000	5.77808	-2.48214	1.141067	-2.17528	0.029609	0.144137	SYN2	6854	synapsin II	
ENSG0000	2339.609	0.229349	0.105463	2.174695	0.029653	0.144276	DAXX	1616	death domain associated protein	
ENSG0000	455.8525	-0.31286	0.143864	-2.17469	0.029653	0.144276	STX7	8417	syntaxin 7	
ENSG0000	4656.799	-0.15323	0.070468	-2.17446	0.02967	0.144322	TRIM8	81603	tripartite motif containing 8	
ENSG0000	3.071275	3.638122	1.673442	2.174036	0.029702	0.144439	RFPL2	10739	ret finger protein like 2	
ENSG0000	38.98825	-0.89206	0.410532	-2.17292	0.029786	0.144704	ZFYVE9	9372	zinc finger FYVE-type containing 9	
ENSG0000	425.783	-0.30064	0.138347	-2.17308	0.029774	0.144704	TUBG2	27175	tubulin gamma 2	
ENSG0000	1278.041	-0.20738	0.095441	-2.1729	0.029788	0.144704	MBD3	53615	methyl-CpG binding domain protein 3	
ENSG0000	577.2306	0.256405	0.117995	2.173011	0.029779	0.144704	TIAM1	7074	TIAM Rac1 associated GEF 1	
ENSG0000	403.7347	-0.32076	0.147653	-2.17242	0.029824	0.144841	DLAT	1737	dihydrolipoamide S-acetyltransferase	
ENSG0000	66.36525	-0.64672	0.297758	-2.17195	0.029859	0.144973	KLF8	11279	KLF transcription factor 8	
ENSG0000	2105.838	0.187154	0.086188	2.171479	0.029895	0.145107	UBE2O	63893	ubiquitin conjugating enzyme E2 O	
ENSG0000	655.9516	0.253725	0.11685	2.17138	0.029902	0.145107	ABCG1	9619	ATP binding cassette subfamily G member 1	
ENSG0000	17.09691	-1.37343	0.632555	-2.17124	0.029913	0.145109	CRYZL2P	730102	crystallin z	pseudogene
ENSG0000	803.6282	-0.24723	0.113874	-2.17108	0.029925	0.145109	NSD2	7468	nuclear receptor binding SET domain protein 2	
ENSG0000	184.8533	-0.43935	0.202365	-2.17106	0.029926	0.145109	MINDY2	54629	MINDY lysine 48 deubiquitinase 2	
ENSG0000	190.8997	-0.44626	0.205619	-2.1703	0.029984	0.145351	CCDC61	729440	coiled-coil domain containing 61	
ENSG0000	1796.162	-0.1959	0.090271	-2.17007	0.030001	0.145359	VPS39	23339	VPS39 subunit of HOPS complex	
ENSG0000	1719.986	-0.21525	0.099187	-2.17016	0.029995	0.145359	GPS1	2873	G protein pathway suppressor 1	
ENSG0000	8.925076	-1.9661	0.906071	-2.16992	0.030013	0.145377	MRPS31P5	1.01E+08	mitochondrial ribosomal protein S31 pseudogene 5	
ENSG0000	80.98904	-0.59513	0.27431	-2.16956	0.030041	0.145435	DOCK3	1795	dedicator of cytokinesis 3	
ENSG0000	7.19173	-2.47608	1.141269	-2.16958	0.030038	0.145435	NA	NA	NA	
ENSG0000	3.108585	-3.45971	1.595454	-2.16848	0.030122	0.145717	NA	NA	NA	
ENSG0000	14206.52	-0.17399	0.080231	-2.16864	0.03011	0.145717	PREX1	57580	phosphati	4 5-trisphosphate dependent Rac exchange factor 1
ENSG0000	306.8807	0.348753	0.160825	2.168523	0.030119	0.145717	RENBP	5973	renin binding protein	
ENSG0000	216.5334	-0.42321	0.195196	-2.16811	0.03015	0.145813	PPM1L	151742	protein ph	Mg2+/Mn2+ dependent 1L
ENSG0000	1037.061	0.221504	0.102178	2.167823	0.030172	0.145882	TMEM120	83862	transmembrane protein 120A	
ENSG0000	154.3214	-0.53914	0.24872	-2.16766	0.030185	0.145905	LINC0150	1.01E+08	long intergenic non-protein coding RNA 1503	
ENSG0000	325.2262	-0.34067	0.157185	-2.16732	0.03021	0.145952	PPOX	5498	protoporphyrinogen oxidase	
ENSG0000	122.5574	-0.46902	0.216399	-2.16741	0.030204	0.145952	TASL	80231	TLR adaptor interacting with endolysosomal SLC15A4	
ENSG0000	93.64912	-0.56343	0.259994	-2.16709	0.030228	0.145995	ZNF496-D	1.08E+08	ZNF496 divergent transcript	
ENSG0000	870.6715	0.259822	0.119899	2.167	0.030235	0.145995	BLTP3B	23074	bridge-like lipid transfer protein family member 3B	
ENSG0000	122.8129	-0.48217	0.222624	-2.16587	0.030321	0.146343	SCAT1	1.02E+08	S-phase cancer associated transcript 1	
ENSG0000	234.7481	0.383734	0.177175	2.165848	0.030323	0.146343	LOC10272	1.03E+08	periodic tryptophan protein 2 homolog	
ENSG0000	11.34656	1.53271	0.707714	2.165721	0.030332	0.146352	TXNDC2	84203	thioredoxin domain containing 2	
ENSG0000	4.474491	3.06004	1.413109	2.165466	0.030352	0.146408	VTRNA1-3	56662	vault RNA 1-3	
ENSG0000	102.4068	-0.55092	0.254422	-2.16536	0.03036	0.146408	LRRC45	201255	leucine rich repeat containing 45	
ENSG0000	4.204008	-3.44357	1.590389	-2.16524	0.03037	0.146417	SHISA2	387914	shisa family member 2	
ENSG0000	13.11507	-1.60537	0.741541	-2.16491	0.030395	0.146499	ZNF337-AS	1.03E+08	ZNF337 antisense RNA 1	
ENSG0000	1635.025	0.223123	0.103091	2.164321	0.03044	0.146678	CNOT4	4850	CCR4-NOT transcription complex subunit 4	
ENSG0000	548.29	-0.27103	0.125239	-2.16409	0.030457	0.146687	GORASP1	64689	golgi reassembly stacking protein 1	
ENSG0000	56.48001	-0.69411	0.32074	-2.1641	0.030456	0.146687	FUT7	2529	fucosyltransferase 7	
ENSG0000	436.9507	0.316979	0.146531	2.163225	0.030524	0.14697	BLVRB	645	biliverdin reductase B	
ENSG0000	348.513	0.328043	0.151687	2.162627	0.03057	0.147153	RBM15	64783	RNA binding motif protein 15	
ENSG0000	19.04679	-1.26184	0.583544	-2.16237	0.03059	0.14718	DOCK8-AS	157983	DOCK8 antisense RNA 1	
ENSG0000	5.768069	-2.31896	1.072428	-2.16235	0.030591	0.14718	NA	NA	NA	
ENSG0000	79.96568	0.578926	0.26775	2.162191	0.030603	0.1472	DNAJC25	548645	DnaJ heat shock protein family (Hsp40) member C25	
ENSG0000	5.577173	2.922754	1.352094	2.161649	0.030645	0.147363	SLC6A13	6540	solute carrier family 6 member 13	
ENSG0000	23.82283	-1.202	0.55611	-2.16144	0.030661	0.147402	WWC1	23286	WW and C2 domain containing 1	
ENSG0000	2927.49	0.200451	0.092747	2.161265	0.030675	0.147428	BAG3	9531	BAG cochaperone 3	
ENSG0000	144.7285	-0.43515	0.201355	-2.1611	0.030688	0.147434	DARS2	55157	aspartyl-tF	mitochondrial
ENSG0000	328.1915	-0.34276	0.158609	-2.16104	0.030692	0.147434	WDR54	84058	WD repeat domain 54	
ENSG0000	239.005	-0.41282	0.191075	-2.16052	0.030732	0.147551	SLC35A5	55032	solute carrier family 35 member A5	
ENSG0000	4.175977	-2.78068	1.287039	-2.16052	0.030732	0.147551	IL17D	53342	interleukin 17D	
ENSG0000	1399.722	0.247191	0.114433	2.160134	0.030762	0.147581	SRP9	6726	signal recognition particle 9	
ENSG0000	48.15408	0.772938	0.357803	2.160233	0.030755	0.147581	NA	NA	NA	
ENSG0000	7983.762	0.179644	0.083157	2.160305	0.030749	0.147581	KAT6A	7994	lysine acetyltransferase 6A	
ENSG0000	11507.83	0.149003	0.068984	2.159976	0.030775	0.147602	CAP1	10487	cyclase associated actin cytoskeleton regulatory protein 1	
ENSG0000	1566.787	0.20746	0.096057	2.159769	0.030791	0.14764	C11orf58	10944	chromosome 11 open reading frame 58	
ENSG0000	738.977	-0.23514	0.108886	-2.15954	0.030808	0.147686	THRA	7067	thyroid hormone receptor alpha	
ENSG0000	24.58577	1.104559	0.511578	2.159124	0.030841	0.147804	MILIP	92659	MYC inducible lncRNA inactivating p53	
ENSG0000	662.0182	0.306003	0.141753	2.158708	0.030873	0.147869	MARCO	8685	macrophage receptor with collagenous structure	
ENSG0000	2340.858	0.203629	0.094326	2.158778	0.030867	0.147869	DHX15	1665	DEAH-box helicase 15	
ENSG0000	2421.107	-0.18381	0.085151	-2.15864	0.030878	0.147869	IRF3	3661	interferon regulatory factor 3	
ENSG0000	795.0161	-0.28136	0.13036	-2.1583	0.030905	0.147919	ABCC10	89845	ATP binding cassette subfamily C member 10	
ENSG0000	2145.932	-0.19951	0.092439	-2.15833	0.030902	0.147919	DENND11	57189	DENN domain containing 11	
ENSG0000	2119.41	0.193748	0.089783	2.157959	0.030931	0.148008	CYTH2	9266	cytohesin 2	
ENSG0000	2862.275	-0.19735	0.091458	-2.15781	0.030943	0.148026	TRIM56	81844	tripartite motif containing 56	
ENSG0000	3.104597	-3.33345	1.545113	-2.15741	0.030973	0.148134	NA	NA	NA	
ENSG0000	132.6415	0.563166	0.261058	2.157242	0.030987	0.14816	GINS4	84296	GINS complex subunit 4	
ENSG0000	58.37103	-0.70104	0.325018	-2.15694	0.03101	0.148234	C19orf44	84167	chromosome 19 open reading frame 44	
ENSG0000	1365.875	-0.20412	0.094658	-2.15644	0.031049	0.148319	TOPBP1	11073	DNA topoisomerase II binding protein 1	
ENSG0000	661.6147	0.31175	0.14458	2.156238	0.031065	0.148319	SNX18	112574	sorting nexin 18	
ENSG0000	272.2017	-0.34661	0.16073	-2.1565	0.031045	0.148319	CORO2A	7464	coronin 2A	
ENSG0000	761.9604	0.237409	0.110095	2.156399	0.031053	0.148319	UBL5	59286	ubiquitin like 5	
ENSG0000	542.4479	0.317361	0.147185	2.156201	0.031068	0.148319	ITGB2-AS1	1.01E+08	ITGB2 antisense RNA 1	
ENSG0000	439.9702	-0.28113	0.130391	-2.15605	0.03108	0.148337	UBOX5	22888	U-box domain containing 5	
ENSG0000	345.7504	-0.32596	0.151202	-2.15582	0.031098	0.148384	MOSPD2	158747	motile sperm domain containing 2	
ENSG0000	2402.141	-0.17548	0.081417	-2.15536	0.031134	0.148517	MARF1	9665	meiosis regulator and mRNA stability factor 1	
ENSG0000	3714.594	0.181848	0.084389	2.15488	0.031171	0.148659	UBXN1	51035	UBX domain protein 1	
ENSG0000	2168.116	0.195015	0.090506	2.15471	0.031185	0.148684	AP1G1	164	adaptor related protein complex 1 subunit gamma 1	
ENSG0000	464.4714	0.265834	0.12344	2.153551	0.031275	0.149068	TFRC	7037	transferrin receptor	
ENSG0000	114.5101	0.679751	0.315653	2.153478	0.031281	0.149068	PLK2	10769	polo like kinase 2	
ENSG0000	1649.002	-0.21682	0.100748	-2.1521	0.03139	0.149469	XPC	7508	XPC compl	DNA damage recognition and repair factor
ENSG0000	561.8679	0.288163	0.133892	2.152196	0.031382	0.149469	NUDT4	11163	nudix hydrolase 4	
ENSG0000	26.05239	1.127747	0.523994	2.152212	0.031381	0.149469	ZC3H12B	340554	zinc finger CCCH-type containing 12B	
ENSG0000	63.89893	-0.76285	0.354528	-2.15173	0.031419	0.149571	NA	NA	NA	
ENSG0000	50954.01	0.183567	0.085358	2.150558	0.031511	0.149971	RPL13A	23521	ribosomal protein L13a	
ENSG0000	394.5484	0.354709	0.164986	2.149938	0.03156	0.150166	SNRPA1	6627	small nuclear ribonucleoprotein polypeptide A'	
ENSG0000	4830.482	0.191412	0.08907	2.149011	0.031634	0.150476	CD164	8763	CD164 molecule	

ENSG0000	351.478	-0.30373	0.141368	-2.14854	0.031671	0.150615	STK39	27347	serine/threonine kinase 39
ENSG0000	348.0587	-0.33454	0.15572	-2.14835	0.031686	0.15065	FMNL2	114793	formin like 2
ENSG0000	1732.734	0.188969	0.087967	2.148181	0.031699	0.150664	NSA2	10412	NSA2 ribosome biogenesis factor
ENSG0000	2263.465	0.188727	0.087857	2.148105	0.031705	0.150664	YKT6	10652	YKT6 v-SNARE homolog
ENSG0000	128.4193	-0.5189	0.241583	-2.14793	0.03172	0.150693	TMEM9	252839	transmembrane protein 9
ENSG0000	805.2734	-0.24032	0.111947	-2.1467	0.031817	0.151118	ATP10D	57205	ATPase phospholipid transporting 10D (putative)
ENSG0000	4.897745	-2.82872	1.317933	-2.14633	0.031847	0.151218	NA	NA	NA
ENSG0000	7.142381	-2.31555	1.078951	-2.14612	0.031864	0.151254	GNG12	55970	G protein subunit gamma 12
ENSG0000	298.1525	-0.36853	0.17173	-2.146	0.031873	0.151254	NLK	51701	nemo like kinase
ENSG0000	846.8363	0.224704	0.104712	2.14593	0.031879	0.151254	SUZ12	23512	SUZ12 polycomb repressive complex 2 subunit
ENSG0000	119.1885	-0.4817	0.224501	-2.14566	0.0319	0.151304	TRBV7-9	28589	T cell receptor beta variable 7-9
ENSG0000	614.2164	0.287099	0.133809	2.145594	0.031905	0.151304	UAP1L1	91373	UDP-N-acetylglucosamine pyrophosphorylase 1 like 1
ENSG0000	10.62625	2.009995	0.936932	2.145295	0.031929	0.151378	NA	NA	NA
ENSG0000	13658.74	0.18922	0.088215	2.14498	0.031954	0.15142	WDR1	9948	WD repeat domain 1
ENSG0000	192.3608	0.589219	0.274689	2.145038	0.03195	0.15142	IGLV2-14	28815	immunoglobulin lambda variable 2-14
ENSG0000	128.8756	0.52458	0.244584	2.144779	0.03197	0.151458	FAM89B	23625	family with sequence similarity 89 member B
ENSG0000	5.504418	-2.66335	1.242324	-2.14384	0.032046	0.151775	NA	NA	NA
ENSG0000	262.9634	0.524489	0.244706	2.143345	0.032085	0.151898	NBPF26	1.01E+08	NBPF member 26
ENSG0000	163.4624	-0.46082	0.215002	-2.14331	0.032088	0.151898	TMEM161	1.01E+08	TMEM161B divergent transcript
ENSG0000	779.5774	0.236248	0.110257	2.142693	0.032138	0.152095	SMAD4	4089	SMAD family member 4
ENSG0000	3147.18	0.169401	0.07908	2.142141	0.032182	0.152224	MIA3	375056	MIA SH3 domain ER export factor 3
ENSG0000	1391.988	-0.22617	0.105587	-2.14205	0.03219	0.152224	TRAFD1	10906	TRAF-type zinc finger domain containing 1
ENSG0000	18.307	-1.26871	0.592236	-2.14223	0.032175	0.152224	GATM	2628	glycine amidinotransferase
ENSG0000	120.5551	-0.51301	0.239533	-2.1417	0.032218	0.152278	ETAA1	54465	ETAA1 activator of ATR kinase
ENSG0000	209.0581	-0.40212	0.187755	-2.14174	0.032215	0.152278	USP30	84749	ubiquitin specific peptidase 30
ENSG0000	2427.37	0.216799	0.101246	2.141317	0.032248	0.152308	AFF4	27125	ALF transcription elongation factor 4
ENSG0000	711.3031	-0.23671	0.110537	-2.14141	0.032241	0.152308	AGFG2	3268	ArfGAP with FG repeats 2
ENSG0000	171.4367	-0.47536	0.221996	-2.14132	0.032249	0.152308	LIPE	3991	lipase E hormone sensitive type
ENSG0000	84.0446	-0.58707	0.27418	-2.14119	0.032259	0.152317	DIXDC1	85458	DIX domain containing 1
ENSG0000	6274.343	0.162546	0.075919	2.14105	0.03227	0.152332	PITPNA	5306	phosphatidylinositol transfer protein alpha
ENSG0000	8.961391	-1.80415	0.842873	-2.14047	0.032317	0.152495	MIR1244-	1E+08	microRNA 1244-3
ENSG0000	1280.627	0.2123	0.099186	2.140417	0.032321	0.152495	UBE2G1	7326	ubiquitin conjugating enzyme E2 G1
ENSG0000	6885.288	0.151543	0.07081	2.140152	0.032343	0.152558	ZC3H18	124245	zinc finger CCCH-type containing 18
ENSG0000	8.389872	2.07072	0.967774	2.139673	0.032381	0.152701	NA	NA	NA
ENSG0000	149.5573	-0.49969	0.233642	-2.1387	0.03246	0.153035	NA	NA	NA
ENSG0000	866.9307	0.238058	0.111317	2.138562	0.032471	0.153048	H2AZ1	3015	H2A.Z variant histone 1
ENSG0000	3.722023	3.05508	1.428722	2.13833	0.03249	0.153059	NA	NA	NA
ENSG0000	12.40536	-1.54362	0.721877	-2.13834	0.032489	0.153059	TSNAXIP1	55815	translin associated factor X interacting protein 1
ENSG0000	447.5396	-0.27584	0.129022	-2.1379	0.032525	0.153145	VIPAS39	63894	VPS33B in[illegible] apical-bas spe-39 homolog
ENSG0000	1478.487	0.204227	0.095525	2.13793	0.032522	0.153145	TNFRSF13	115650	TNF receptor superfamily member 13C
ENSG0000	415.9792	0.273643	0.128005	2.13776	0.032536	0.15316	ZNF134	7693	zinc finger protein 134
ENSG0000	6.007914	2.222314	1.039813	2.137226	0.03258	0.153298	CDH26	60437	cadherin 26
ENSG0000	1033.338	0.208994	0.097789	2.137196	0.032582	0.153298	TIMM17B	10245	translocase of inner mitochondrial membrane 17B
ENSG0000	26.16972	1.146889	0.536868	2.13626	0.032658	0.153578	CLEC1B	51266	C-type lectin domain family 1 member B
ENSG0000	318.3676	-0.334	0.156349	-2.13627	0.032658	0.153578	TPGS1	91978	tubulin polyglutamylase complex subunit 1
ENSG0000	74.4695	0.614858	0.287984	2.135041	0.032758	0.154007	RPL24P4	377381	RPL24 pseudogene 4
ENSG0000	134.3019	0.514908	0.24119	2.134867	0.032772	0.154035	BANK1	55024	B cell scaffold protein with ankyrin repeats 1
ENSG0000	40.90302	0.775273	0.363184	2.134655	0.032789	0.154077	NA	NA	NA
ENSG0000	2840.997	0.187445	0.087827	2.134268	0.032821	0.154175	HNRNPAB	3182	heterogeneous nuclear ribonucleoprotein A/B
ENSG0000	16.91391	-1.50853	0.706839	-2.1342	0.032827	0.154175	UBE2Q2P2	1E+08	UBE2Q2 pseudogene 2
ENSG0000	1692.797	-0.2105	0.098639	-2.13402	0.032841	0.154204	GYS1	2997	glycogen synthase 1
ENSG0000	336.5112	0.329258	0.154299	2.133897	0.032851	0.154212	CAMTA1	23261	calmodulin binding transcription activator 1
ENSG0000	23.97153	-1.19185	0.558657	-2.13342	0.032891	0.154358	FPGT	8790	fucose-1-phosphate guanylyltransferase
ENSG0000	5.801001	-2.45112	1.149079	-2.13311	0.032915	0.154435	PRELID3A	10650	PRELI domain containing 3A
ENSG0000	43.4011	0.90093	0.42252	2.132279	0.032984	0.154717	XPNPEP2	7512	X-prolyl aminopeptidase 2
ENSG0000	273.5982	0.321085	0.150598	2.13206	0.033002	0.154725	HELQ	113510	helicase POLQ like
ENSG0000	2034.49	0.173209	0.081242	2.132002	0.033007	0.154725	CMTM3	123920	CKLF like MARVEL transmembrane domain containing 3
ENSG0000	2020.068	0.19778	0.092769	2.131955	0.033011	0.154725	XRN2	22803	5'-3' exoribonuclease 2
ENSG0000	116.3989	-0.49776	0.233494	-2.13178	0.033025	0.154752	HCN3	57657	hyperpolarization activated cyclic nucleotide gated potassium channel 3
ENSG0000	149.7818	0.56681	0.265933	2.131398	0.033056	0.154822	PER3	8863	period circadian regulator 3
ENSG0000	5.150139	2.906463	1.363612	2.131444	0.033053	0.154822	NA	NA	NA
ENSG0000	11.28282	1.506838	0.707085	2.131056	0.033084	0.154915	ZNF410	57862	zinc finger protein 410
ENSG0000	1173.529	0.22123	0.10385	2.130282	0.033148	0.155175	SERTAD1	29950	SERTA domain containing 1
ENSG0000	50.52615	-0.80615	0.378727	-2.12859	0.033288	0.155684	FHAD1	114827	forkhead associated phosphopeptide binding domain 1
ENSG0000	14.01508	-1.59627	0.749928	-2.12856	0.033291	0.155684	EXO1	9156	exonuclease 1
ENSG0000	15.30605	1.274831	0.598914	2.128571	0.03329	0.155684	NA	NA	NA
ENSG0000	907.211	0.225759	0.106057	2.128657	0.033283	0.155684	KIR3DX1	90011	killer cell i three Ig domains X1 (pseudogene)
ENSG0000	102.2348	0.586773	0.275705	2.128264	0.033315	0.155759	CDKN2B	1030	cyclin dependent kinase inhibitor 2B
ENSG0000	4.270653	2.831359	1.331257	2.126831	0.033434	0.156276	MTCYBP2	1.07E+08	MT-CYB pseudogene 23
ENSG0000	126.3277	0.451974	0.212531	2.12662	0.033452	0.156318	NA	NA	NA
ENSG0000	502.385	-0.28419	0.133661	-2.1262	0.033487	0.156364	CLCN3	1182	chloride voltage-gated channel 3
ENSG0000	90.47301	-0.58369	0.274514	-2.12628	0.03348	0.156364	PRRT1	80863	proline rich transmembrane protein 1
ENSG0000	111.6461	-0.50488	0.23745	-2.12625	0.033482	0.156364	LRRC61	65999	leucine rich repeat containing 61
ENSG0000	163.4208	-0.45178	0.212516	-2.12588	0.033513	0.156413	STAU2	27067	staufen double-stranded RNA binding protein 2
ENSG0000	3.502995	3.101298	1.458842	2.125863	0.033515	0.156413	OR13A1	79290	olfactory receptor family 13 subfamily A member 1
ENSG0000	716.8328	-0.28583	0.134459	-2.12577	0.033522	0.156413	MSRB1	51734	methionine sulfoxide reductase B1
ENSG0000	689.8836	0.265252	0.12479	2.125599	0.033537	0.15644	ATP5MF	9551	ATP synthase membrane subunit f
ENSG0000	3.310373	-3.534	1.662776	-2.12536	0.033556	0.156492	NA	NA	NA
ENSG0000	5.36642	2.740612	1.28972	2.124967	0.033589	0.156607	NA	NA	NA
ENSG0000	1329.382	-0.20755	0.097721	-2.12388	0.03368	0.156991	PBRM1	55193	polybromo 1
ENSG0000	8.959687	-1.75782	0.828037	-2.12287	0.033764	0.157344	MAPK12	6300	mitogen-activated protein kinase 12
ENSG0000	343.3063	0.344744	0.162422	2.122515	0.033794	0.157444	SNHG3	8420	small nucleolar RNA host gene 3
ENSG0000	1155.074	0.199988	0.094229	2.12236	0.033807	0.157465	SKP1	6500	S-phase kinase associated protein 1
ENSG0000	200.8485	0.497428	0.234422	2.121935	0.033843	0.157592	C15orf61	145853	chromosome 15 open reading frame 61
ENSG0000	124.8423	-0.49159	0.231724	-2.12145	0.033884	0.157743	STAG3	10734	stromal antigen 3
ENSG0000	604.8075	0.245608	0.115816	2.120674	0.033949	0.158007	CDR2	1039	cerebellar degeneration related protein 2
ENSG0000	320.0293	-0.33726	0.159052	-2.12046	0.033967	0.15805	NEK4	6787	NIMA related kinase 4
ENSG0000	759.4011	0.352371	0.166197	2.120203	0.033989	0.158106	IGSF6	10261	immunoglobulin superfamily member 6
ENSG0000	100.097	0.653747	0.308369	2.120017	0.034005	0.158106	CD300H	1E+08	CD300H molecule (gene/pseudogene)
ENSG0000	17.52525	-1.18197	0.557516	-2.12007	0.034	0.158106	ZNF443	10224	zinc finger protein 443
ENSG0000	69.18483	-0.74552	0.351694	-2.1198	0.034023	0.158153	TRNP1	388610	TMF1 regulated nuclear protein 1
ENSG0000	1324.947	0.220934	0.104247	2.119319	0.034063	0.1583	SIAH2	6478	siah E3 ubiquitin protein ligase 2
ENSG0000	657.4152	-0.25929	0.122389	-2.11853	0.03413	0.15857	CPNE3	8895	copine 3
ENSG0000	145.2283	-0.41862	0.197743	-2.11701	0.034259	0.159127	SNX22	79856	sorting nexin 22
ENSG0000	1960.846	-0.18951	0.089531	-2.11673	0.034283	0.159201	SYNRG	11276	synergin gamma
ENSG0000	3.220528	3.637398	1.718618	2.116467	0.034305	0.159224	NPBWR1	2831	neuropeptides B and W receptor 1
ENSG0000	374.4323	-0.32159	0.151943	-2.11653	0.034299	0.159224	NA	NA	NA
ENSG0000	524.743	0.309576	0.146296	2.116094	0.034337	0.159329	MITD1	129531	microtubule interacting and trafficking domain containing 1
ENSG0000	7.188082	1.910705	0.902982	2.115996	0.034345	0.159329	P3H2	55214	prolyl 3-hydroxylase 2
ENSG0000	52.23383	-0.79217	0.374472	-2.11543	0.034393	0.159513	NA	NA	NA

ENSG0000	10673.73	-0.15984	0.075571	-2.11516	0.034416	0.159539	TRAC	28755	T cell receptor alpha constant
ENSG0000	3090.528	0.224506	0.10614	2.115191	0.034414	0.159539	RBM12	10137	RNA binding motif protein 12
ENSG0000	66.91391	-0.68199	0.322473	-2.11488	0.03444	0.15957	PODXL2	50512	podocalyxin like 2
ENSG0000	790.6003	-0.23282	0.110082	-2.11496	0.034434	0.15957	GLE1	2733	GLE1 RNA export mediator
ENSG0000	745.3337	0.254261	0.120261	2.114244	0.034494	0.159702	CRBN	51185	cereblon
ENSG0000	3615.247	0.161921	0.076582	2.114343	0.034486	0.159702	HNRNPL	3191	heterogeneous nuclear ribonucleoprotein L
ENSG0000	407.9973	0.374398	0.177078	2.114311	0.034489	0.159702	MAP1LC3A	84557	microtubule associated protein 1 light chain 3 alpha
ENSG0000	1631.347	0.202887	0.09597	2.114062	0.03451	0.159735	MAT2B	27430	methionine adenosyltransferase 2B
ENSG0000	14242.41	-0.17463	0.082626	-2.11351	0.034557	0.159873	ATP2A3	489	ATPase sarcoplasmic/endoplasmic reticulum Ca2+ transporting 3
ENSG0000	923.1668	-0.2321	0.109813	-2.11359	0.03455	0.159873	MARCHF2	51257	membrane associated ring-CH-type finger 2
ENSG0000	153.843	-0.43276	0.204782	-2.11327	0.034577	0.159887	HEATR5A	25938	HEAT repeat containing 5A
ENSG0000	491.9889	0.282333	0.133595	2.113346	0.034571	0.159887	LRRC37B	114659	leucine rich repeat containing 37B
ENSG0000	756.5244	-0.27886	0.131972	-2.11306	0.034596	0.159933	TRAPPC12	51112	trafficking protein particle complex subunit 12
ENSG0000	95.97277	-0.54309	0.257116	-2.11225	0.034665	0.160213	SCFD2	152579	sec1 family domain containing 2
ENSG0000	268.6996	0.347985	0.164793	2.111647	0.034717	0.160372	UBTD2	92181	ubiquitin domain containing 2
ENSG0000	1394.083	-0.21868	0.10356	-2.11165	0.034717	0.160372	MROH1	727957	maestro heat like repeat family member 1
ENSG0000	42.26079	0.805417	0.381435	2.111542	0.034726	0.160373	RND1	27289	Rho family GTPase 1
ENSG0000	124.6073	0.461553	0.218605	2.111359	0.034742	0.160406	RBPMS2	348093	RNA bindi mRNA processing factor 2
ENSG0000	1707.697	0.198242	0.093905	2.111085	0.034765	0.160475	UQCRB	7381	ubiquinol-cytochrome c reductase binding protein
ENSG0000	221.182	-0.40625	0.192451	-2.11095	0.034776	0.160487	ZFP64	55734	ZFP64 zinc finger protein
ENSG0000	1216.633	-0.23834	0.11298	-2.10961	0.034892	0.160979	BMPR2	659	bone morphogenetic protein receptor type 2
ENSG0000	450.2254	-0.3776	0.179029	-2.10913	0.034933	0.16113	KCNMB1	3779	potassium calcium-activated channel subfamily M regulatory beta subunit 1
ENSG0000	17.23124	-1.37995	0.654501	-2.1084	0.034996	0.161381	NA	NA	NA
ENSG0000	353.1325	-0.30038	0.142483	-2.1082	0.035014	0.161421	OCEL1	79629	occludin/ELL domain containing 1
ENSG0000	144.2833	-0.43191	0.204891	-2.10799	0.035031	0.161463	ACCS	84680	1-aminocyclopropane-1-carboxylate synthase homolog (inactive)
ENSG0000	263.1071	-0.36766	0.174468	-2.1073	0.035092	0.1617	TNFAIP8L2	79626	TNF alpha induced protein 8 like 2
ENSG0000	2.942377	-3.89126	1.84667	-2.10717	0.035102	0.16171	NA	NA	NA
ENSG0000	694.2203	0.26797	0.127205	2.1066	0.035152	0.161894	PPP3CC	5533	protein phosphatase 3 catalytic subunit gamma
ENSG0000	6.353461	-2.18003	1.0349	-2.10651	0.03516	0.161894	SFTPD	6441	surfactant protein D
ENSG0000	1615.604	-0.17839	0.084704	-2.10609	0.035197	0.162024	GARRE1	9710	granule associated Rac and RHOG effector 1
ENSG0000	20.74702	1.197441	0.568786	2.105258	0.035269	0.162316	HLF	3131	HLF transc PAR bZIP family member
ENSG0000	1877.296	0.181601	0.086287	2.10461	0.035325	0.162535	UBE2J1	51465	ubiquitin conjugating enzyme E2 J1
ENSG0000	266.2808	-0.32299	0.15352	-2.10391	0.035386	0.162776	MAP7D3	79649	MAP7 domain containing 3
ENSG0000	1539.813	0.226335	0.107605	2.103383	0.035432	0.162947	TMED9	54732	transmembrane p24 trafficking protein 9
ENSG0000	126.2952	-0.4583	0.217907	-2.10321	0.035448	0.162977	BICD1	636	BICD cargo adaptor 1
ENSG0000	97.18592	0.674855	0.320913	2.102924	0.035472	0.16301	MOB3B	79817	MOB kinase activator 3B
ENSG0000	346.974	0.294046	0.139823	2.102986	0.035467	0.16301	NA	NA	NA
ENSG0000	2274.706	-0.19172	0.0912	-2.10217	0.035539	0.163274	TRIOBP	11078	TRIO and F-actin binding protein
ENSG0000	414.7772	0.275553	0.131136	2.101274	0.035617	0.163593	MTURN	222166	maturin neural progenitor differentiation regulator homolog
ENSG0000	1024.873	0.237171	0.11292	2.100356	0.035698	0.163922	ENC1	8507	ectodermal-neural cortex 1
ENSG0000	8.28209	-2.1264	1.012543	-2.10006	0.035724	0.164003	MYLK-AS1	1.01E+08	MYLK antisense RNA 1
ENSG0000	3.931196	-3.04217	1.448821	-2.09975	0.03575	0.164084	KIAA1217	56243	KIAA1217
ENSG0000	790.1173	-0.22094	0.105236	-2.09943	0.035779	0.164173	PLPBP	11212	pyridoxal phosphate binding protein
ENSG0000	6.648089	-2.40523	1.145719	-2.09932	0.035789	0.16418	NA	NA	NA
ENSG0000	16.45199	1.646894	0.784559	2.099133	0.035805	0.164213	FFAR3	2865	free fatty acid receptor 3
ENSG0000	37314.91	0.158839	0.075682	2.098758	0.035838	0.164324	ARHGDIB	397	Rho GDP dissociation inhibitor beta
ENSG0000	593.0768	0.27747	0.132216	2.098605	0.035852	0.164346	GMFB	2764	glia maturation factor beta
ENSG0000	5.691313	-3.04706	1.452256	-2.09816	0.035891	0.164486	CECR2	27443	CECR2 histone acetyl-lysine reader
ENSG0000	2016.527	0.174468	0.083166	2.097829	0.03592	0.164578	FXR2	9513	FMR1 autosomal homolog 2
ENSG0000	8.388585	-1.95226	0.930845	-2.09729	0.035968	0.164714	NEK11	79858	NIMA related kinase 11
ENSG0000	16636.11	0.181338	0.08646	2.097356	0.035962	0.164714	RPL12	6136	ribosomal protein L12
ENSG0000	1400.572	0.188461	0.089881	2.096797	0.036012	0.164874	PSPC1	55269	paraspeckle component 1
ENSG0000	680.7253	-0.2426	0.11572	-2.09642	0.036045	0.164985	ADSS2	159	adenylosuccinate synthase 2
ENSG0000	179.3558	0.4231	0.201832	2.096296	0.036056	0.164996	MYBL2	4605	MYB proto-oncogene like 2
ENSG0000	5.442131	-2.82041	1.345514	-2.09616	0.036068	0.165012	LINC01359	1.02E+08	long intergenic non-protein coding RNA 1359
ENSG0000	100.1045	-0.547	0.260979	-2.09594	0.036088	0.165061	ROGDI	79641	rogdi atypical leucine zipper
ENSG0000	22.81685	-1.03051	0.491756	-2.09558	0.03612	0.165166	NA	NA	NA
ENSG0000	2851.517	-0.16577	0.079118	-2.09528	0.036146	0.165206	RNF216	54476	ring finger protein 216
ENSG0000	7.398684	-2.15202	1.027082	-2.09528	0.036146	0.165206	MIPEPP3	650794	mitochondrial intermediate peptidase pseudogene 3
ENSG0000	9548.673	-0.41914	0.200096	-2.09469	0.036199	0.165391	GBP4	115361	guanylate binding protein 4
ENSG0000	92.78852	-0.53066	0.253345	-2.09462	0.036205	0.165391	COMMD1	150684	copper metabolism domain containing 1
ENSG0000	40.07063	0.860708	0.410945	2.094459	0.036219	0.165416	SNAPC1	6617	small nuclear RNA activating complex polypeptide 1
ENSG0000	4.851442	-2.77433	1.324818	-2.09412	0.036249	0.165501	NA	NA	NA
ENSG0000	3833.843	-0.18189	0.086862	-2.09405	0.036256	0.165501	PHRF1	57661	PHD and ring finger domains 1
ENSG0000	186.2069	0.412569	0.197035	2.093892	0.03627	0.165525	IFT20	90410	intraflagellar transport 20
ENSG0000	4564.424	-0.19655	0.093876	-2.09375	0.036283	0.165543	HGSNAT	138050	heparan-alpha-glucosaminide N-acetyltransferase
ENSG0000	158.3558	-0.47172	0.225322	-2.09353	0.036302	0.165592	HS2ST1	9653	heparan sulfate 2-O-sulfotransferase 1
ENSG0000	842.5194	0.233681	0.111637	2.093224	0.036329	0.165651	MS4A1	931	membrane spanning 4-domains A1
ENSG0000	16173.79	0.186539	0.089118	2.093182	0.036333	0.165651	RPS25	6230	ribosomal protein S25
ENSG0000	15.33181	-1.26701	0.60534	-2.09306	0.036344	0.165659	NA	NA	NA
ENSG0000	3.222085	3.824222	1.827245	2.092889	0.036359	0.165688	MGP	4256	matrix Gla protein
ENSG0000	15.28761	1.435537	0.68598	2.092679	0.036378	0.165733	MIR17HG	407975	miR-17-92a-1 cluster host gene
ENSG0000	32.80307	-0.89167	0.426112	-2.09257	0.036388	0.165738	CDC20	991	cell division cycle 20
ENSG0000	18.4258	1.311046	0.626576	2.092399	0.036403	0.165766	TGFBR3L	1.01E+08	transforming growth factor beta receptor 3 like
ENSG0000	2832.599	-0.17135	0.081909	-2.09191	0.036446	0.165922	DLGAP4	22839	DLG associated protein 4
ENSG0000	4352.344	-0.19044	0.0911	-2.09041	0.036581	0.166496	MED25	81857	mediator complex subunit 25
ENSG0000	218.1854	0.383588	0.183525	2.090112	0.036608	0.166535	TAF13	6884	TATA-box binding protein associated factor 13
ENSG0000	6.945263	-2.16686	1.036685	-2.09018	0.036602	0.166535	NA	NA	NA
ENSG0000	7504.076	0.146693	0.07019	2.089938	0.036623	0.166565	KHSRP	8570	KH-type splicing regulatory protein
ENSG0000	192.4671	-0.49389	0.236387	-2.08933	0.036678	0.166772	C12orf4	57102	chromosome 12 open reading frame 4
ENSG0000	1061.04	-0.21271	0.101823	-2.08897	0.03671	0.166842	ZMYND11	10771	zinc finger MYND-type containing 11
ENSG0000	5146.55	0.165184	0.079075	2.088962	0.036711	0.166842	PRR14	78994	proline rich 14
ENSG0000	229.9726	-0.41303	0.197752	-2.08862	0.036742	0.166928	ABCB10	23456	ATP binding cassette subfamily B member 10
ENSG0000	892.2918	-0.22453	0.107505	-2.08855	0.036748	0.166928	DOK1	1796	docking protein 1
ENSG0000	27.09427	-0.99985	0.478367	-2.08803	0.036795	0.1671	TIFAB	497189	TIFA inhibitor
ENSG0000	1580.802	-0.24243	0.116152	-2.08718	0.036871	0.167406	TFEB	7942	transcription factor EB
ENSG0000	154.0327	-0.49387	0.236662	-2.08682	0.036904	0.167515	ANXA4	307	annexin A4
ENSG0000	167.1644	-0.55107	0.264089	-2.0867	0.036915	0.167524	KCNE3	10008	potassium voltage-gated channel subfamily E regulatory subunit 3
ENSG0000	24.09729	-1.17346	0.562404	-2.08651	0.036933	0.167561	MIR4420	1.01E+08	microRNA 4420
ENSG0000	8.674623	-2.15612	1.033422	-2.08639	0.036944	0.16757	HRK	8739	harakiri BCL2 interacting protein
ENSG0000	116.1265	0.496779	0.238128	2.086187	0.036962	0.167611	NA	NA	NA
ENSG0000	2843.437	-0.18274	0.087604	-2.08597	0.036982	0.16766	DIDO1	11083	death inducer-obliterator 1
ENSG0000	237.2905	0.330744	0.158598	2.085419	0.037031	0.167803	LINC01215	1.02E+08	long intergenic non-protein coding RNA 1215
ENSG0000	26.52038	1.18361	0.567561	2.085433	0.03703	0.167803	SOCS3-DT	1.02E+08	SOCS3 divergent transcript
ENSG0000	72.10708	0.662564	0.317788	2.084928	0.037076	0.167964	GHRLOS	1E+08	ghrelin opposite strand/antisense RNA
ENSG0000	26.66066	-1.13587	0.544925	-2.08446	0.037119	0.168076	NA	NA	NA
ENSG0000	182.2747	0.410697	0.197022	2.08452	0.037113	0.168076	RPL32P18	644907	ribosomal protein L32 pseudogene 18
ENSG0000	8.929966	-1.83878	0.882368	-2.08392	0.037168	0.168192	ANLN	54443	anillin actin binding protein
ENSG0000	230.2106	0.395382	0.189757	2.083627	0.037194	0.168192	UBE2W	55284	ubiquitin conjugating enzyme E2 W
ENSG0000	290.2574	-0.31229	0.149862	-2.08384	0.037175	0.168192	TONSL	4796	tonsoku lil DNA repair protein

ENSG0000	130.416	-0.47223	0.226613	-2.08385	0.037174	0.168192	SMUG1	23583	single-strand-selective monofunctional uracil-DNA glycosylase 1
ENSG0000	71.87738	-0.70281	0.33731	-2.08358	0.037199	0.168192	ZNF605	1E+08	zinc finger protein 605
ENSG0000	122.9567	-0.44831	0.21516	-2.08364	0.037193	0.168192	ZNF606	80095	zinc finger protein 606
ENSG0000	271.3761	0.339245	0.162834	2.083379	0.037217	0.168232	AXIN2	8313	axin 2
ENSG0000	8.600382	2.06904	0.993275	2.083049	0.037247	0.168263	MEF2C-AS	1.02E+08	MEF2C antisense RNA 1
ENSG0000	1153.893	0.235104	0.112879	2.082806	0.037269	0.168263	UBR2	23304	ubiquitin protein ligase E3 component n-recognin 2
ENSG0000	73.84295	0.834428	0.400598	2.082958	0.037255	0.168263	TPBG	7162	trophoblast glycoprotein
ENSG0000	287.9918	-0.32814	0.157538	-2.08291	0.037259	0.168263	BRD3OS	266655	BRD3 opposite strand
ENSG0000	160.955	-0.45209	0.21705	-2.08287	0.037263	0.168263	FANCI	55215	FA complementation group I
ENSG0000	16431.93	-0.67339	0.323379	-2.08237	0.037309	0.168322	GBP1	2633	guanylate binding protein 1
ENSG0000	678.3899	0.240274	0.115385	2.082365	0.037309	0.168322	SRPK1	6732	SRSF protein kinase 1
ENSG0000	23.57497	1.164289	0.559109	2.082401	0.037306	0.168322	NA	NA	NA
ENSG0000	886.9133	-0.20905	0.100415	-2.08186	0.037355	0.16849	TMX4	56255	thioredoxin related transmembrane protein 4
ENSG0000	59.75424	0.787265	0.378193	2.08165	0.037374	0.168535	MSC-AS1	1E+08	MSC antisense RNA 1
ENSG0000	823.9057	0.264128	0.12691	2.081223	0.037413	0.16867	PRELID3B	51012	PRELI domain containing 3B
ENSG0000	8126.799	0.143523	0.068972	2.080892	0.037444	0.16874	UBAP2L	9898	ubiquitin associated protein 2 like
ENSG0000	601.2286	-0.26616	0.127911	-2.08085	0.037447	0.16874	CPSF2	53981	cleavage and polyadenylation specific factor 2
ENSG0000	843.7261	-0.22539	0.108332	-2.0805	0.037479	0.168803	NEMF	9147	nuclear export mediator factor
ENSG0000	771.4382	0.238764	0.114759	2.08056	0.037474	0.168803	COPS2	9318	COP9 signalosome subunit 2
ENSG0000	220.8989	-0.39494	0.189864	-2.08012	0.037515	0.168894	NA	NA	NA
ENSG0000	3.155128	-3.50189	1.683535	-2.08008	0.037518	0.168894	NA	NA	NA
ENSG0000	104.7285	0.61718	0.296795	2.07948	0.037573	0.169102	NA	NA	NA
ENSG0000	339.8954	0.30595	0.147195	2.078534	0.03766	0.169453	SNRPG	6637	small nuclear ribonucleoprotein polypeptide G
ENSG0000	32.33476	0.930999	0.447941	2.078399	0.037673	0.169467	GALR2	8811	galanin receptor 2
ENSG0000	136.4294	-0.4735	0.227896	-2.07769	0.037738	0.169667	CYB561D2	11068	cytochrome b561 family member D2
ENSG0000	156.2206	0.523911	0.252169	2.07762	0.037744	0.169667	HPGD	3248	15-hydroxyprostaglandin dehydrogenase
ENSG0000	595.0199	-0.25853	0.124431	-2.0777	0.037737	0.169667	TRIP11	9321	thyroid hormone receptor interactor 11
ENSG0000	1814.946	-0.20567	0.099009	-2.07724	0.03778	0.169785	LTA4H	4048	leukotriene A4 hydrolase
ENSG0000	887.9468	-0.22305	0.107394	-2.0769	0.037811	0.169801	PRKCQ	5588	protein kinase C theta
ENSG0000	196.5209	0.379713	0.182819	2.076986	0.037803	0.169801	RPL26	6154	ribosomal protein L26
ENSG0000	882.5351	0.216058	0.104021	2.07706	0.037796	0.169801	TIMM50	92609	translocase of inner mitochondrial membrane 50
ENSG0000	8.908534	-1.65845	0.79861	-2.07668	0.037832	0.169853	TAS2R4	50832	taste 2 receptor member 4
ENSG0000	282.1443	0.418517	0.201548	2.076516	0.037846	0.169877	PFDN2	5202	prefoldin subunit 2
ENSG0000	6.874068	2.155032	1.037896	2.076346	0.037862	0.169907	CHST8	64377	carbohydrate sulfotransferase 8
ENSG0000	9392.244	-0.18189	0.087633	-2.0756	0.037931	0.170174	FBRSL1	57666	fibrosin like 1
ENSG0000	135.1344	-0.4551	0.219281	-2.07542	0.037948	0.17021	RUFY2	55680	RUN and FYVE domain containing 2
ENSG0000	297.6261	-0.33589	0.161862	-2.07518	0.03797	0.170269	AARS2	57505	alanyl-tRN mitochondrial
ENSG0000	347.834	-0.36404	0.17544	-2.07499	0.037987	0.170294	LMO2	4005	LIM domain only 2
ENSG0000	4046.98	0.17292	0.083338	2.074918	0.037994	0.170294	EIF3D	8664	eukaryotic translation initiation factor 3 subunit D
ENSG0000	16829.09	0.169968	0.081926	2.074642	0.03802	0.170368	RPS9	6203	ribosomal protein S9
ENSG0000	3612.055	0.184164	0.088789	2.074184	0.038062	0.170517	TES	26136	testin LIM domain protein
ENSG0000	741.234	0.257264	0.124041	2.074029	0.038077	0.17054	ZNF506	440515	zinc finger protein 506
ENSG0000	115.2816	0.593078	0.285987	2.073791	0.038099	0.170569	TUFT1	7286	tuftelin 1
ENSG0000	18.85134	1.407143	0.678546	2.073762	0.038101	0.170569	GOLGA8M	653720	golgin A8 family member M
ENSG0000	3.590154	3.131154	1.510091	2.073487	0.038127	0.170642	CDKL2	8999	cyclin dependent kinase like 2
ENSG0000	530.4978	0.244453	0.117937	2.072741	0.038196	0.170912	NSUN4	387338	NOP2/Sun RNA methyltransferase 4
ENSG0000	581.8662	-0.32706	0.157825	-2.07232	0.038236	0.171046	RECQL	5965	RecQ like helicase
ENSG0000	340.3841	-0.35718	0.172384	-2.07203	0.038262	0.171125	MTMR4	9110	myotubularin related protein 4
ENSG0000	4878.41	0.177593	0.085733	2.071482	0.038314	0.171271	TKT	7086	transketolase
ENSG0000	1159.165	0.309264	0.149293	2.071529	0.038309	0.171271	KCNN4	3783	potassium calcium-activated channel subfamily N member 4
ENSG0000	7258.777	0.185849	0.089737	2.071041	0.038355	0.171335	LEF1	51176	lymphoid enhancer binding factor 1
ENSG0000	577.6099	-0.31207	0.150685	-2.07103	0.038356	0.171335	POLL	27343	DNA polymerase lambda
ENSG0000	863.6106	-0.25459	0.122921	-2.0712	0.03834	0.171335	BCL7C	9274	BAF chromatin remodeling complex subunit BCL7C
ENSG0000	110.901	-0.51592	0.249131	-2.07089	0.038369	0.171353	LIMA1	51474	LIM domain and actin binding 1
ENSG0000	5.792646	-2.66132	1.285306	-2.07057	0.038399	0.171446	CC2D2A	57545	coiled-coil and C2 domain containing 2A
ENSG0000	1003.196	0.220293	0.106401	2.070416	0.038413	0.171469	ATG4D	84971	autophagy related 4D cysteine peptidase
ENSG0000	1828.474	0.17272	0.08343	2.070237	0.03843	0.171503	PGAM1	5223	phosphoglycerate mutase 1
ENSG0000	15.95996	-1.52211	0.735582	-2.06926	0.038522	0.171871	LINC02694	400359	long intergenic non-protein coding RNA 2694
ENSG0000	4059.542	-0.15011	0.072549	-2.06912	0.038534	0.171885	ZZEF1	23140	zinc finger ZZ-type and EF-hand domain containing 1
ENSG0000	4776.319	0.149507	0.072278	2.068493	0.038594	0.172067	ATP6V0D1	9114	ATPase H+ transporting V0 subunit d1
ENSG0000	1959.852	-0.17933	0.086696	-2.06855	0.038589	0.172067	ZC3H4	23211	zinc finger CCCH-type containing 4
ENSG0000	2.776541	3.675467	1.777028	2.068322	0.03861	0.172097	PROX1	5629	prospero homeobox 1
ENSG0000	187.948	0.415802	0.201051	2.068144	0.038626	0.17213	LINC0167S	1.02E+08	long intergenic non-protein coding RNA 1679
ENSG0000	698.0606	-0.25536	0.123483	-2.06796	0.038644	0.172165	TSEN34	79042	tRNA splicing endonuclease subunit 34
ENSG0000	316.678	-0.35439	0.171383	-2.06783	0.038656	0.172177	ACACB	32	acetyl-CoA carboxylase beta
ENSG0000	2862.616	-0.2019	0.097676	-2.06701	0.038734	0.172483	RBCK1	10616	RANBP2-type and C3HC4-type zinc finger containing 1
ENSG0000	4.443483	3.134682	1.516831	2.0666	0.038772	0.172577	NA	NA	NA
ENSG0000	3455.282	-0.18743	0.090696	-2.06658	0.038773	0.172577	RBL2	5934	RB transcriptional corepressor like 2
ENSG0000	358.2708	0.4287	0.207466	2.066364	0.038794	0.172628	HLA-DQA1	3117	major hist class II DQ alpha 1
ENSG0000	93.23632	0.630895	0.305336	2.066231	0.038807	0.172643	SMN1	6606	survival of telomeric
ENSG0000	196.3686	-0.3967	0.192006	-2.06609	0.03882	0.172658	ZNF557	79230	zinc finger protein 557
ENSG0000	41.3441	-0.97767	0.473235	-2.06593	0.038835	0.172684	PI16	221476	peptidase inhibitor 16
ENSG0000	2063.773	0.528088	0.255643	2.065728	0.038854	0.17273	CDCP1	64866	CUB domain containing protein 1
ENSG0000	34.00748	0.908884	0.440301	2.064234	0.038996	0.173192	CTH	1491	cystathionine gamma-lyase
ENSG0000	10160.03	0.164957	0.079908	2.064338	0.038986	0.173192	RPS27A	6233	ribosomal protein S27a
ENSG0000	360.688	-0.29434	0.142586	-2.06429	0.03899	0.173192	P2RY1	5028	purinergic receptor P2Y1
ENSG0000	53.52235	-0.73408	0.355576	-2.06447	0.038973	0.173192	IFT74	80173	intraflagellar transport 74
ENSG0000	2463.502	-0.17803	0.086264	-2.06378	0.039039	0.173342	CYTH4	27128	cytohesin 4
ENSG0000	215.3666	-0.43958	0.213095	-2.06281	0.03913	0.173707	NA	NA	NA
ENSG0000	54.02172	0.719499	0.348831	2.062603	0.03915	0.173755	UBE2T	29089	ubiquitin conjugating enzyme E2 T
ENSG0000	1041.701	-0.23433	0.113625	-2.06231	0.039178	0.173835	MICALL2	79778	MICAL like 2
ENSG0000	266.1891	0.343667	0.166684	2.061785	0.039228	0.174017	EXOSC5	56915	exosome component 5
ENSG0000	108.6446	-0.4911	0.238291	-2.06094	0.039309	0.174291	RTN4RL2	349667	reticulon 4 receptor like 2
ENSG0000	111.8681	-0.63704	0.309099	-2.06096	0.039307	0.174291	ZNF627	199692	zinc finger protein 627
ENSG0000	5.571568	-2.65568	1.288732	-2.0607	0.039332	0.174353	NA	NA	NA
ENSG0000	398.4455	-0.33025	0.160349	-2.05957	0.03944	0.174596	SMYD2	56950	SET and MYND domain containing 2
ENSG0000	5.824733	-2.9146	1.415009	-2.05978	0.03942	0.174596	TMEM30A	1.01E+08	TMEM30A divergent transcript
ENSG0000	176.6889	0.432337	0.20989	2.059831	0.039415	0.174596	COX6A1	1337	cytochrome c oxidase subunit 6A1
ENSG0000	121.837	-0.47303	0.229636	-2.05989	0.039409	0.174596	LINC00921	283876	long intergenic non-protein coding RNA 921
ENSG0000	556.1557	-0.25387	0.123264	-2.05953	0.039443	0.174596	ASPSCR1	79058	ASPSCR1 t UBX domain containing
ENSG0000	19.02587	-1.19405	0.579758	-2.05956	0.039441	0.174596	RBFOX2	23543	RNA binding fox-1 homolog 2
ENSG0000	740.4544	0.226093	0.109805	2.059045	0.03949	0.174698	BAG5	9529	BAG cochaperone 5
ENSG0000	1260.841	0.22221	0.107921	2.058995	0.039495	0.174698	MEX3C	51320	mex-3 RNA binding family member C
ENSG0000	692.079	-0.27743	0.134738	-2.05906	0.039488	0.174698	PCMTD2	55251	protein-L-isoaspartate (D-aspartate) O-methyltransferase domain containing 2
ENSG0000	8.228485	1.98634	0.964773	2.058867	0.039507	0.174711	NA	NA	NA
ENSG0000	812.6182	0.2102	0.102115	2.058463	0.039546	0.174832	WDR91	29062	WD repeat domain 91
ENSG0000	396.1877	-0.34547	0.16784	-2.05834	0.039557	0.174832	COQ9	57017	coenzyme Q9
ENSG0000	1183.075	0.21366	0.103805	2.058285	0.039563	0.174832	PISD	23761	phosphatidylserine decarboxylase
ENSG0000	1623.887	-0.18954	0.09212	-2.05749	0.039639	0.175129	PARP1	142	poly(ADP-ribose) polymerase 1
ENSG0000	279.3236	0.31803	0.154603	2.057078	0.039679	0.175261	CEPT1	10390	choline/ethanolamine phosphotransferase 1

ENSG0000	14.55157	1.485492	0.722301	2.056611	0.039724	0.175418	CCDC141	285025	coiled-coil domain containing 141
ENSG0000	1855.104	0.216459	0.105257	2.056481	0.039736	0.175428	APBA3	9546	amyloid beta precursor protein binding family A member 3
ENSG0000	20233.72	0.177745	0.086436	2.056392	0.039745	0.175428	CALR	811	calreticulin
ENSG0000	388.6721	0.274055	0.133281	2.05622	0.039761	0.175459	THOC1	9984	THO complex subunit 1
ENSG0000	6.533612	-2.2321	1.085662	-2.05598	0.039784	0.175518	NA	NA	NA
ENSG0000	1554.408	-0.1932	0.093996	-2.05545	0.039836	0.175704	CHD6	84181	chromodomain helicase DNA binding protein 6
ENSG0000	24.14652	-1.0858	0.528325	-2.05518	0.039862	0.175776	LINC00467	84791	long intergenic non-protein coding RNA 467
ENSG0000	4.221796	3.356388	1.633585	2.054615	0.039916	0.175975	NA	NA	NA
ENSG0000	3.769964	3.476143	1.692137	2.054291	0.039948	0.176071	CDH20	28316	cadherin 20
ENSG0000	5.926751	-2.58183	1.256979	-2.054	0.039976	0.176112	NA	NA	NA
ENSG0000	1176.251	-0.23248	0.11318	-2.05405	0.039971	0.176112	NF1	4763	neurofibromin 1
ENSG0000	7.837793	-1.85079	0.901175	-2.05375	0.039999	0.176174	TRAV26-2	28656	T cell receptor alpha variable 26-2
ENSG0000	2463.716	0.177762	0.08658	2.05316	0.040057	0.176326	GPBP1L1	60313	GC-rich promoter binding protein 1 like 1
ENSG0000	5841.418	0.151145	0.073618	2.053104	0.040062	0.176326	BICDL1	92558	BICD family like cargo adaptor 1
ENSG0000	291.26	-0.38548	0.187751	-2.05313	0.04006	0.176326	GIPC1	10755	GIPC PDZ domain containing family member 1
ENSG0000	7.195126	-2.4365	1.18694	-2.05276	0.040096	0.176432	CSRP1-AS1	1.08E+08	CSRP1 antisense RNA 1
ENSG0000	2167.091	0.186773	0.090991	2.052654	0.040106	0.176435	ATP5F1D	513	ATP synthase F1 subunit delta
ENSG0000	876.9695	-0.24329	0.11853	-2.05254	0.040117	0.17644	PDLIM5	10611	PDZ and LIM domain 5
ENSG0000	101.9095	0.623908	0.304013	2.052238	0.040147	0.176529	ESPL1	9700	extra spin(separase
ENSG0000	4.484662	2.862909	1.395658	2.051297	0.040238	0.176889	NA	NA	NA
ENSG0000	38.79932	-0.87181	0.425041	-2.05113	0.040254	0.176919	ZNF837	116412	zinc finger protein 837
ENSG0000	7.356868	-1.86225	0.908089	-2.05074	0.040293	0.177045	NA	NA	NA
ENSG0000	26.71595	0.973421	0.474787	2.050227	0.040342	0.177221	TCEAL9	51186	transcription elongation factor A like 9
ENSG0000	3.807248	-2.98147	1.454328	-2.05007	0.040358	0.177237	NA	NA	NA
ENSG0000	10.65438	1.493775	0.728672	2.049995	0.040365	0.177237	NA	NA	NA
ENSG0000	1253.482	0.226813	0.110669	2.049479	0.040415	0.177416	GALNT11	63917	polypeptide N-acetylgalactosaminyltransferase 11
ENSG0000	102.7327	-0.54796	0.267405	-2.04916	0.040446	0.177509	LRRC75A	388341	leucine rich repeat containing 75A
ENSG0000	2116.317	-0.1841	0.089881	-2.04822	0.040539	0.177832	CDK10	8558	cyclin dependent kinase 10
ENSG0000	322.9439	0.375552	0.18335	2.048284	0.040532	0.177832	DYNLT3	6990	dynein light chain Tctex-type 3
ENSG0000	35.78844	0.913744	0.446284	2.047447	0.040614	0.178121	ULBP2	80328	UL16 binding protein 2
ENSG0000	516.6568	0.284942	0.1392	2.047003	0.040658	0.17827	SMDT1	91689	single-pass membrane protein with aspartate rich tail 1
ENSG0000	23.7571	-1.34545	0.657315	-2.04689	0.040669	0.178277	FAT4	79633	FAT atypical cadherin 4
ENSG0000	7.460432	-1.99248	0.973468	-2.04679	0.040679	0.178279	NA	NA	NA
ENSG0000	525.2212	-0.24891	0.121647	-2.04616	0.040741	0.178431	DHDDS	79947	dehydrodolichyl diphosphate synthase subunit
ENSG0000	421.5711	0.266802	0.130389	2.046205	0.040736	0.178431	TRIM58	25893	tripartite motif containing 58
ENSG0000	142.123	0.423368	0.206911	2.046138	0.040743	0.178431	NPM3	10360	nucleophosmin/nucleoplasmin 3
ENSG0000	10.96799	-2.08287	1.018074	-2.04589	0.040767	0.178494	SPOCK1	6695	SPARC (os cwcv and kazal like domains proteoglycan 1
ENSG0000	118.6196	0.505595	0.247143	2.045761	0.04078	0.178509	NA	NA	NA
ENSG0000	755.6452	0.21742	0.106293	2.04548	0.040808	0.178588	MTMR1	8776	myotubularin related protein 1
ENSG0000	2277.964	-0.2019	0.098731	-2.04497	0.040858	0.178645	ZFYVE28	57732	zinc finger FYVE-type containing 28
ENSG0000	9031.118	0.161419	0.078928	2.045136	0.040841	0.178645	ARAP1	116985	ArfGAP wi ankyrin repeat and PH domain 1
ENSG0000	20.6893	-1.08663	0.531306	-2.04522	0.040834	0.178645	LOC28419	284191	uncharacterized LOC284191
ENSG0000	334.6972	0.282961	0.13837	2.044958	0.040859	0.178645	ZNF586	54807	zinc finger protein 586
ENSG0000	4010.657	-0.18939	0.092623	-2.04474	0.040881	0.178697	MACF1	23499	microtubule actin crosslinking factor 1
ENSG0000	62.5179	-0.70075	0.342739	-2.04456	0.040898	0.17873	C16orf74	404550	chromosome 16 open reading frame 74
ENSG0000	4.820806	2.734499	1.338116	2.043545	0.040999	0.179128	BASP1-AS1	285696	BASP1 antisense RNA 1
ENSG0000	4.534031	-2.82023	1.380501	-2.04291	0.041062	0.179362	PXT1	222659	peroxisomal testis enriched protein 1
ENSG0000	94.9569	-0.49229	0.24108	-2.04202	0.041149	0.179703	ANGPTL6	83854	angiopoietin like 6
ENSG0000	1074.974	-0.26924	0.13186	-2.0419	0.041162	0.179714	CCR5	1234	C-C motif chemokine receptor 5
ENSG0000	11483.17	-0.16812	0.082347	-2.0416	0.041191	0.179801	SBF1	6305	SET binding factor 1
ENSG0000	692.7169	-0.60717	0.297471	-2.04112	0.041239	0.179947	TNFSF10	8743	TNF superfamily member 10
ENSG0000	2178.875	0.179288	0.08784	2.041067	0.041244	0.179947	DERL1	79139	derlin 1
ENSG0000	11.55444	-1.67936	0.823001	-2.04054	0.041297	0.180106	LINC01962	1.03E+08	long intergenic non-protein coding RNA 1962
ENSG0000	34.66928	-0.87434	0.428492	-2.04051	0.0413	0.180106	ZNF546	339327	zinc finger protein 546
ENSG0000	27.03584	0.964616	0.472785	2.040287	0.041322	0.180106	UQCRHL	440567	ubiquinol-cytochrome c reductase hinge protein like
ENSG0000	26.76224	-1.05903	0.519037	-2.04037	0.041314	0.180106	NA	NA	NA
ENSG0000	365.7098	-0.31143	0.152649	-2.04018	0.041332	0.180106	ZNF469	84627	zinc finger protein 469
ENSG0000	129.0655	-0.47423	0.232453	-2.04012	0.041339	0.180106	RCN3	57333	reticulocalbin 3
ENSG0000	580.259	0.280597	0.13755	2.039969	0.041353	0.180127	ARPC5L	81873	actin related protein 2/3 complex subunit 5 like
ENSG0000	22.95442	1.125055	0.551614	2.039571	0.041393	0.180228	METTL7B	196410	methyltransferase like 7B
ENSG0000	12.35289	1.64474	0.806426	2.039543	0.041396	0.180228	LINC00494	284749	long intergenic non-protein coding RNA 494
ENSG0000	26.65284	-0.98538	0.483214	-2.03921	0.041429	0.180328	TTC34	1E+08	tetratricopeptide repeat domain 34
ENSG0000	2627.415	-0.21095	0.103485	-2.03845	0.041505	0.180453	ASH1L	55870	ASH1 like histone lysine methyltransferase
ENSG0000	21.20738	-1.36893	0.671459	-2.03874	0.041476	0.180453	OLFML2B	25903	olfactomedin like 2B
ENSG0000	7.004988	2.275492	1.11627	2.038479	0.041502	0.180453	NA	NA	NA
ENSG0000	342.3081	-0.28365	0.139149	-2.03844	0.041506	0.180453	ATE1	11101	arginyltransferase 1
ENSG0000	1433.066	-0.20723	0.101651	-2.03865	0.041485	0.180453	PHLDB1	23187	pleckstrin homology like domain family B member 1
ENSG0000	278.9339	0.354396	0.173881	2.038159	0.041534	0.180453	ZDBF2	57683	zinc finger DBF-type containing 2
ENSG0000	2841.566	-0.16823	0.082548	-2.03795	0.041555	0.180453	DGKD	8527	diacylglycerol kinase delta
ENSG0000	5.317281	2.320547	1.138617	2.038041	0.041546	0.180453	NA	NA	NA
ENSG0000	384.5799	0.314034	0.154092	2.037961	0.041554	0.180453	TRAPPC4	51399	trafficking protein particle complex subunit 4
ENSG0000	29.25967	1.000016	0.490605	2.038331	0.041517	0.180453	TMEM38A	79041	transmembrane protein 38A
ENSG0000	126.4591	-0.45445	0.223007	-2.03782	0.041568	0.180468	NA	NA	NA
ENSG0000	94.26134	-0.56366	0.276758	-2.03666	0.041684	0.18093	C14orf132	56967	chromosome 14 open reading frame 132
ENSG0000	78.89855	0.58996	0.289742	2.036153	0.041735	0.181066	FAM89A	375061	family with sequence similarity 89 member A
ENSG0000	7.178852	2.115592	1.039012	2.036158	0.041735	0.181066	CAP2P1	353163	cyclase associated actin cytoskeleton regulatory protein 2 pseudogene 1
ENSG0000	412.8667	-0.27349	0.134337	-2.03583	0.041767	0.181145	C8orf82	414919	chromosome 8 open reading frame 82
ENSG0000	2.64218	-3.74385	1.839025	-2.03578	0.041773	0.181145	TRARG1	286753	trafficking regulator of GLUT4 (SLC2A4) 1
ENSG0000	168.995	0.422003	0.207322	2.035494	0.041801	0.181226	ZNF595	152687	zinc finger protein 595
ENSG0000	4.740358	-2.85978	1.405044	-2.03537	0.041814	0.18124	NA	NA	NA
ENSG0000	154.4259	0.493932	0.242813	2.034205	0.041931	0.181704	IGKV3-20	28912	immunoglobulin kappa variable 3-20
ENSG0000	344.9584	-0.28528	0.140283	-2.03361	0.041991	0.18192	TIGIT	201633	T cell immunoreceptor with Ig and ITIM domains
ENSG0000	1405.236	-0.18257	0.089792	-2.03323	0.042029	0.182045	DUSP7	1849	dual specificity phosphatase 7
ENSG0000	3.686992	-2.9829	1.467222	-2.03303	0.04205	0.182091	NA	NA	NA
ENSG0000	3702.919	-0.20515	0.100931	-2.03263	0.04209	0.182222	ANKRD13D	338692	ankyrin repeat domain 13D
ENSG0000	3.305375	-3.05665	1.503866	-2.03253	0.0421	0.182225	LINC01512	1E+08	long intergenic non-protein coding RNA 1512
ENSG0000	1241.547	0.224283	0.110384	2.031849	0.042169	0.182238	NASP	4678	nuclear autoantigenic sperm protein
ENSG0000	1777.638	-0.17285	0.085067	-2.03197	0.042156	0.182238	GTF3C2	2976	general transcription factor IIIC subunit 2
ENSG0000	133.8012	-0.46913	0.230837	-2.03228	0.042125	0.182238	USP40	55230	ubiquitin specific peptidase 40
ENSG0000	33.56543	-0.86675	0.426521	-2.03214	0.042139	0.182238	ATP6V0E2	401431	ATP6V0E2 antisense RNA 1
ENSG0000	286.9259	-0.31698	0.156008	-2.03182	0.042172	0.182238	CEP170B	283638	centrosomal protein 170B
ENSG0000	3961.37	0.161519	0.079478	2.032257	0.042128	0.182238	TECR	9524	trans-2 3-enoyl-CoA reductase
ENSG0000	2.707614	-3.64684	1.794846	-2.03184	0.04217	0.182238	NA	NA	NA
ENSG0000	307.8454	0.326754	0.160828	2.031701	0.042184	0.182247	ZNF548	147694	zinc finger protein 548
ENSG0000	22.45171	-1.2313	0.606242	-2.03104	0.042251	0.182493	L3MBTL4	91133	L3MBTL histone methyl-lysine binding protein 4
ENSG0000	330.6611	-0.3301	0.162562	-2.03059	0.042297	0.182651	HERC4	26091	HECT and RLD domain containing E3 ubiquitin protein ligase 4
ENSG0000	37.07472	-0.80442	0.396219	-2.03023	0.042333	0.1827	ARHGAP39	80728	Rho GTPase activating protein 39
ENSG0000	138.0484	-0.44931	0.221296	-2.03035	0.042321	0.1827	NHLRC3	387921	NHL repeat containing 3
ENSG0000	10.51573	-2.14342	1.055776	-2.03018	0.042338	0.1827	IGLV5-45	28781	immunoglobulin lambda variable 5-45
ENSG0000	457.3878	-0.26333	0.129771	-2.02922	0.042436	0.183078	PWWP3A	84939	PWWP do DNA repair factor

ENSG0000	951.4911	-0.231	0.113857	-2.02886	0.042472	0.183151	AP3M1	26985	adaptor related protein complex 3 subunit mu 1
ENSG0000	2299.692	0.168649	0.083122	2.028932	0.042465	0.183151	CELF1	10658	CUGBP Elav-like family member 1
ENSG0000	163.4084	-0.43914	0.216487	-2.02849	0.04251	0.183272	KDM8	79831	lysine demethylase 8
ENSG0000	1650.748	0.200537	0.098866	2.028368	0.042523	0.183284	NA	NA	NA
ENSG0000	340.3201	-0.27903	0.137574	-2.02823	0.042537	0.183301	CEP85	64793	centrosomal protein 85
ENSG0000	3425.888	0.153748	0.075819	2.027826	0.042578	0.183436	WIPF2	147179	WAS/WASL interacting protein family member 2
ENSG0000	101.2181	-0.56131	0.276889	-2.02721	0.042641	0.183664	OSER1-DT	1.01E+08	OSER1 divergent transcript
ENSG0000	7.527746	-1.91646	0.945759	-2.02637	0.042727	0.183993	FSIP1	161835	fibrous sheath interacting protein 1
ENSG0000	978.1956	0.219924	0.108546	2.026091	0.042755	0.184073	NANS	54187	N-acetylneuraminate synthase
ENSG0000	28.46879	-1.14726	0.566525	-2.02508	0.042859	0.184477	BHLHE41	79365	basic helix-loop-helix family member e41
ENSG0000	1094.282	0.208931	0.103178	2.024954	0.042872	0.184489	CCT8	10694	chaperonin containing TCP1 subunit 8
ENSG0000	1507.875	-0.17195	0.084933	-2.02458	0.04291	0.184612	CCT6A	908	chaperonin containing TCP1 subunit 6A
ENSG0000	409.8998	0.300667	0.148522	2.024397	0.042929	0.18465	ODF2L	57489	outer dense fiber of sperm tails 2 like
ENSG0000	4.019854	-3.1368	1.550018	-2.02372	0.042999	0.184906	NA	NA	NA
ENSG0000	537.2941	-0.24358	0.120378	-2.02347	0.043025	0.184975	MAGED1	9500	MAGE family member D1
ENSG0000	1167.132	0.27455	0.1357	2.023216	0.043051	0.185044	CPEB4	80315	cytoplasmic polyadenylation element binding protein 4
ENSG0000	25.8373	1.030021	0.509198	2.022829	0.043091	0.185058	BRDT	676	bromodomain testis associated
ENSG0000	386.3964	0.308939	0.152711	2.023035	0.04307	0.185058	ATP6V1H	51606	ATPase H+ transporting V1 subunit H
ENSG0000	25.9729	0.923327	0.456497	2.022637	0.043111	0.185058	CD44-DT	1.01E+08	CD44 divergent transcript
ENSG0000	263.3305	0.359817	0.177888	2.022711	0.043103	0.185058	NA	NA	NA
ENSG0000	7.249864	-2.04799	1.012554	-2.0226	0.043114	0.185058	NA	NA	NA
ENSG0000	9194.41	0.157495	0.077862	2.022741	0.0431	0.185058	HSP90AA1	3320	heat shock protein 90 alpha family class A member 1
ENSG0000	60.63372	-0.69823	0.345294	-2.02213	0.043163	0.185225	ZNF347	84671	zinc finger protein 347
ENSG0000	395.2238	-0.27003	0.133556	-2.02185	0.043192	0.185306	FCSK	197258	fucose kinase
ENSG0000	1118.283	0.252369	0.12484	2.021544	0.043223	0.185399	CREBRF	153222	CREB3 regulatory factor
ENSG0000	65.22888	-0.64765	0.320453	-2.02105	0.043275	0.18557	TNFSF4	7292	TNF superfamily member 4
ENSG0000	593.2176	-0.26576	0.131499	-2.02097	0.043283	0.18557	ZNF445	353274	zinc finger protein 445
ENSG0000	9.130623	-1.75799	0.870351	-2.01987	0.043397	0.186015	SEMA3G	56920	semaphorin 3G
ENSG0000	248.1599	0.335079	0.165909	2.019654	0.043419	0.186067	CA2	760	carbonic anhydrase 2
ENSG0000	24894.18	0.128633	0.063718	2.018777	0.04351	0.186414	GNAI2	2771	G protein subunit alpha i2
ENSG0000	28.8592	0.911326	0.451561	2.018167	0.043574	0.186622	LINC00092	1E+08	long intergenic non-protein coding RNA 92
ENSG0000	721.0346	-0.24849	0.123128	-2.01812	0.043579	0.186622	DDHD1	80821	DDHD domain containing 1
ENSG0000	52.30952	-0.71614	0.354876	-2.01801	0.04359	0.186627	SGO2	151246	shugoshin 2
ENSG0000	25.99294	2.671676	1.324123	2.017695	0.043623	0.186724	IL24	11009	interleukin 24
ENSG0000	110.6508	-0.54639	0.27084	-2.01739	0.043655	0.186818	RASAL1	8437	RAS protein activator like 1
ENSG0000	20.83683	1.148778	0.569516	2.017115	0.043684	0.186897	NA	NA	NA
ENSG0000	2411.794	0.162253	0.080491	2.015803	0.043821	0.18744	CMTM6	54918	CKLF like MARVEL transmembrane domain containing 6
ENSG0000	12.89001	1.496984	0.742873	2.015127	0.043891	0.187687	PDLIM1P4	1E+08	PDZ and LIM domain 1 pseudogene 4
ENSG0000	629.3942	0.338375	0.167923	2.015059	0.043898	0.187687	INAFM1	255783	InaF motif containing 1
ENSG0000	353.5754	0.950327	0.471657	2.014869	0.043918	0.187728	IGLC2	3538	immunoglobulin lambda constant 2
ENSG0000	1127.874	-0.19127	0.094967	-2.01409	0.044	0.188033	APEX2	27301	apurinic/apyrimidinic endodeoxyribonuclease 2
ENSG0000	8.579614	-1.97084	0.97858	-2.01398	0.044012	0.188042	NA	NA	NA
ENSG0000	1760.778	0.171036	0.084953	2.013299	0.044083	0.188056	CASZ1	54897	castor zinc finger 1
ENSG0000	4798.502	-0.16435	0.081625	-2.01343	0.044069	0.188056	WDTC1	23038	WD and tetratricopeptide repeats 1
ENSG0000	11.88198	1.835088	0.911496	2.01327	0.044086	0.188056	NA	NA	NA
ENSG0000	660.1127	-0.24701	0.122675	-2.01352	0.04406	0.188056	AFDN	4301	afadin adherens junction formation factor
ENSG0000	716.2898	-0.2542	0.126236	-2.01367	0.044044	0.188056	FAM120B	84498	family with sequence similarity 120B
ENSG0000	1750.827	-0.19622	0.09746	-2.01336	0.044077	0.188056	GFUS	7264	GDP-L-fucose synthase
ENSG0000	1617.796	-0.17268	0.085747	-2.01383	0.044027	0.188056	PLEKHM1F	440456	pleckstrin homology and RUN domain containing M1 pseudogene 1
ENSG0000	1071.385	0.190521	0.094652	2.012853	0.04413	0.188071	SLC5A6	8884	solute carrier family 5 member 6
ENSG0000	6.375474	2.744385	1.363267	2.013094	0.044105	0.188071	IL36G	56300	interleukin 36 gamma
ENSG0000	130.1778	0.473546	0.235247	2.012972	0.044118	0.188071	RPS7P1	388363	ribosomal protein S7 pseudogene 1
ENSG0000	2041.583	0.199234	0.098981	2.012851	0.04413	0.188071	SUMO3	6612	small ubiquitin like modifier 3
ENSG0000	364.0297	-0.30687	0.152471	-2.01262	0.044154	0.18813	ZNF623	9831	zinc finger protein 623
ENSG0000	45.83836	-0.74994	0.372661	-2.0124	0.044178	0.188188	LIPT2-AS1	1E+08	LIPT2 antisense RNA 1
ENSG0000	12.87509	1.677679	0.833718	2.012287	0.04419	0.188194	NA	NA	NA
ENSG0000	36.45634	-0.85881	0.426818	-2.01211	0.044208	0.188231	TMEM141	85014	transmembrane protein 141
ENSG0000	1773.939	0.195387	0.097161	2.010969	0.044329	0.1887	RNASET2	8635	ribonuclease T2
ENSG0000	45.70331	0.730113	0.363098	2.010788	0.044348	0.188738	WLS	79971	Wnt ligand secretion mediator
ENSG0000	21.06585	1.017254	0.505935	2.010642	0.044363	0.18876	RPS13P2	729236	ribosomal protein S13 pseudogene 2
ENSG0000	1054.534	-0.21483	0.106865	-2.01026	0.044404	0.188862	SEMA4C	54910	semaphorin 4C
ENSG0000	2592.685	-0.17476	0.086938	-2.01018	0.044412	0.188862	CUL1	8454	cullin 1
ENSG0000	32.25881	-0.96239	0.478772	-2.01013	0.044418	0.188862	NA	NA	NA
ENSG0000	412.854	0.279408	0.13901	2.009979	0.044433	0.188885	N4BP2	55728	NEDD4 binding protein 2
ENSG0000	21.53944	1.12124	0.557954	2.009556	0.044478	0.189032	UPB1	51733	beta-ureidopropionase 1
ENSG0000	11.57273	-1.56432	0.778613	-2.00911	0.044526	0.189148	CNNM3-D	1.01E+08	CNNM3 divergent transcript
ENSG0000	29.66183	1.120982	0.557934	2.009166	0.04452	0.189148	TM4SF19	116211	transmembrane 4 L six family member 19
ENSG0000	754.5877	-0.21771	0.10837	-2.00894	0.044544	0.18918	NA	NA	NA
ENSG0000	269.8367	-0.36446	0.181455	-2.00854	0.044586	0.189272	ERI3	79033	ERI1 exoribonuclease family member 3
ENSG0000	27.83736	-1.03192	0.513764	-2.00855	0.044585	0.189272	NA	NA	NA
ENSG0000	1103.085	0.195949	0.097569	2.008309	0.04461	0.189334	ZCCHC8	55596	zinc finger CCHC-type containing 8
ENSG0000	131.6791	0.468983	0.233608	2.007563	0.04469	0.189627	NA	NA	NA
ENSG0000	19.44283	-1.28073	0.638041	-2.00728	0.044719	0.18971	BSN	8927	bassoon presynaptic cytomatrix protein
ENSG0000	212.2368	0.341074	0.170013	2.006168	0.044838	0.190171	OTUD3	23252	OTU deubiquitinase 3
ENSG0000	259.9384	-0.37428	0.18664	-2.00533	0.044928	0.190507	ZMYND15	84225	zinc finger MYND-type containing 15
ENSG0000	112.4659	0.489272	0.244148	2.003998	0.04507	0.191067	C17orf49	124944	chromosome 17 open reading frame 49
ENSG0000	4.968687	-2.89018	1.44249	-2.0036	0.045113	0.191203	NA	NA	NA
ENSG0000	1464.459	0.197995	0.098853	2.002933	0.045184	0.191351	IMPDH2	3615	inosine monophosphate dehydrogenase 2
ENSG0000	18.47458	1.094746	0.546571	2.002937	0.045184	0.191351	CDK1	983	cyclin dependent kinase 1
ENSG0000	92.43109	-0.49093	0.245106	-2.00294	0.045183	0.191351	JRKL	8690	JRK like
ENSG0000	1573.88	-0.19251	0.096117	-2.00289	0.045189	0.191351	CDIPT	10423	CDP-diacylglycerol--inositol 3-phosphatidyltransferase
ENSG0000	1997.267	0.17517	0.087505	2.001828	0.045303	0.191704	CDK11B	984	cyclin dependent kinase 11B
ENSG0000	34.40254	-0.91163	0.455381	-2.00191	0.045294	0.191704	EMILIN1	11117	elastin microfibril interfacer 1
ENSG0000	303.0131	-0.29063	0.14517	-2.00197	0.045287	0.191704	ERLIN1	10613	ER lipid raft associated 1
ENSG0000	18.0543	1.130779	0.564951	2.001554	0.045333	0.191785	TG	7038	thyroglobulin
ENSG0000	359.3704	0.29845	0.149161	2.000854	0.045408	0.19206	LY86	9450	lymphocyte antigen 86
ENSG0000	16.53194	-1.4596	0.729525	-2.00076	0.045419	0.192061	UGDH-AS1	1.01E+08	UGDH antisense RNA 1
ENSG0000	8489.196	-0.18465	0.092352	-1.99941	0.045564	0.19259	AHNAK	79026	AHNAK nucleoprotein
ENSG0000	736.1367	-0.26877	0.134424	-1.99945	0.04556	0.19259	DNASE2	1777	deoxyribo lysosomal
ENSG0000	5.06548	-2.63323	1.31709	-1.99928	0.045578	0.192603	DNAAF8	146562	dynein axonemal assembly factor 8
ENSG0000	353.1594	-0.31565	0.157903	-1.99899	0.045609	0.192677	ZNF37BP	1E+08	zinc finger pseudogene
ENSG0000	3.747539	-2.93927	1.470424	-1.99893	0.045616	0.192677	NA	NA	NA
ENSG0000	1090.559	0.198782	0.099455	1.998722	0.045638	0.192727	GMFG	9535	glia maturation factor gamma
ENSG0000	1528.66	-0.18026	0.090199	-1.99851	0.045662	0.192781	ATMIN	23300	ATM interactor
ENSG0000	17.99967	-1.16171	0.58136	-1.99826	0.045689	0.192807	SNX15	29907	sorting nexin 15
ENSG0000	4.474796	-2.71034	1.356301	-1.99833	0.045681	0.192807	ADAMTS7I	642935	ADAMTS7 pseudogene 4
ENSG0000	31.81756	1.016398	0.508736	1.99789	0.045729	0.192914	OR2W3	343171	olfactory receptor family 2 subfamily W member 3
ENSG0000	381.615	-0.28102	0.140664	-1.99783	0.045735	0.192914	CASP7	840	caspase 7
ENSG0000	121.4616	0.456861	0.228709	1.997564	0.045764	0.192993	MRPL38	64978	mitochondrial ribosomal protein L38
ENSG0000	148.1274	0.480634	0.240632	1.99738	0.045784	0.193034	C12orf29	91298	chromosome 12 open reading frame 29

ENSG0000	260.905	0.326027	0.163281	1.996721	0.045855	0.193291	NLE1	54475	notchless homolog 1
ENSG0000	2.551556	-3.80979	1.908734	-1.99598	0.045937	0.193589	RN7SL842I	1.06E+08	RNA 7SL cytoplasm pseudogene
ENSG0000	65.19689	0.732921	0.367221	1.995857	0.045949	0.193599	CFAP97D2	1.02E+08	CFAP97 domain containing 2
ENSG0000	204.1131	-0.38915	0.195006	-1.99559	0.045979	0.193637	EOGT	285203	EGF domain specific O-linked N-acetylglucosamine transferase
ENSG0000	54.91623	0.650676	0.326058	1.995583	0.045979	0.193637	DHRS12	79758	dehydrogenase/reductase 12
ENSG0000	133.8235	0.430284	0.215633	1.995444	0.045994	0.193657	NA	NA	NA
ENSG0000	960.1845	0.203709	0.102137	1.994475	0.0461	0.194058	MTRR	4552	5-methyltetrahydrofolate-homocysteine methyltransferase reductase
ENSG0000	1596.841	-0.19488	0.097714	-1.99434	0.046114	0.194074	APP	351	amyloid beta precursor protein
ENSG0000	4.50318	-2.60499	1.306363	-1.99408	0.046144	0.194153	HHIP-AS1	646576	HHIP antisense RNA 1
ENSG0000	4694.607	0.174151	0.08734	1.993941	0.046158	0.194171	EML4	27436	EMAP like 4
ENSG0000	8.688954	2.045414	1.026105	1.993378	0.04622	0.194384	NA	NA	NA
ENSG0000	1633.763	0.187951	0.094292	1.993287	0.04623	0.194384	BUB3	9184	BUB3 mitotic checkpoint protein
ENSG0000	275.6399	-0.35635	0.17881	-1.99288	0.046275	0.194528	OXLD1	339229	oxidoreductase like domain containing 1
ENSG0000	413.8257	0.258947	0.12995	1.992673	0.046297	0.194578	SBDSP1	155370	SBDS pseudogene 1
ENSG0000	229.7923	-0.38424	0.192848	-1.99246	0.04632	0.194631	LIPN	643418	lipase family member N
ENSG0000	332.4733	-0.30275	0.15198	-1.99208	0.046363	0.194722	ZNF251	90987	zinc finger protein 251
ENSG0000	43.9986	-0.72361	0.36324	-1.9921	0.046361	0.194722	B4GALT6	9331	beta-1 4-galactosyltransferase 6
ENSG0000	189.6854	-0.36452	0.183021	-1.99166	0.046408	0.194866	NMB	4828	neuromedin B
ENSG0000	224.7882	-0.33605	0.168734	-1.99157	0.046418	0.194866	FRMPD3	84443	FERM and PDZ domain containing 3
ENSG0000	1355.154	-0.18548	0.093157	-1.99108	0.046472	0.195004	UBE4B	10277	ubiquitination factor E4B
ENSG0000	2.741981	3.491103	1.753366	1.991087	0.046471	0.195004	ADAM20	8748	ADAM metallopeptidase domain 20
ENSG0000	130.2705	-0.53493	0.268777	-1.99022	0.046567	0.195356	LRRN3	54674	leucine rich repeat neuronal 3
ENSG0000	240.1484	-0.33848	0.170159	-1.9892	0.046679	0.195703	TTC22	55001	tetratricopeptide repeat domain 22
ENSG0000	4.526811	-2.24885	1.130541	-1.98918	0.046681	0.195703	NA	NA	NA
ENSG0000	962.6959	0.251678	0.126522	1.98921	0.046678	0.195703	NA	NA	NA
ENSG0000	616.2944	0.232353	0.116817	1.989037	0.046697	0.195727	NDUFA6	4700	NADH:ubiquinone oxidoreductase subunit A6
ENSG0000	1916.056	-0.16728	0.084123	-1.98853	0.046753	0.195916	RAI1	10743	retinoic acid induced 1
ENSG0000	40.2517	0.727649	0.365974	1.988253	0.046784	0.196001	RPL10P6	285176	ribosomal protein L10 pseudogene 6
ENSG0000	3.037741	-3.06997	1.544342	-1.98788	0.046825	0.196069	NA	NA	NA
ENSG0000	291.9602	-0.30681	0.154347	-1.98777	0.046837	0.196069	METTL14	57721	methyltra N6-adenosine-methyltransferase subunit
ENSG0000	1624.329	-0.19028	0.095727	-1.98772	0.046842	0.196069	ENO2	2026	enolase 2
ENSG0000	24.29138	-1.16201	0.584592	-1.98773	0.046841	0.196069	PGLYRP2	114770	peptidoglycan recognition protein 2
ENSG0000	3987.181	0.231637	0.116546	1.987509	0.046866	0.196125	FAU	2197	FAU ubiquitin like and ribosomal protein S30 fusion
ENSG0000	3.613222	2.8918	1.455144	1.987294	0.04689	0.19618	NA	NA	NA
ENSG0000	29.67867	0.920601	0.463367	1.986764	0.046949	0.196381	NA	NA	NA
ENSG0000	4204.676	0.170864	0.086025	1.986216	0.047009	0.196591	ZFAND5	7763	zinc finger AN1-type containing 5
ENSG0000	669.3746	0.242414	0.122064	1.985955	0.047038	0.196668	KCNA3	3738	potassium voltage-gated channel subfamily A member 3
ENSG0000	4.73669	-2.7175	1.369009	-1.98501	0.047143	0.197062	RPL13P8	1E+08	ribosomal protein L13 pseudogene 8
ENSG0000	5.820026	2.274634	1.146138	1.984607	0.047188	0.197206	SLFNL1	200172	schlafen like 1
ENSG0000	7.489806	1.821561	0.91799	1.984293	0.047223	0.197307	NA	NA	NA
ENSG0000	55.90445	-0.75906	0.382591	-1.98399	0.047257	0.197403	DDIAS	220042	DNA damage induced apoptosis suppressor
ENSG0000	82.93829	-0.54054	0.2725	-1.98364	0.047296	0.197485	RTP4	64108	receptor transporter protein 4
ENSG0000	646.0806	0.247195	0.124618	1.983626	0.047298	0.197485	TBC1D10A	83874	TBC1 domain family member 10A
ENSG0000	823.7087	0.231955	0.116944	1.983472	0.047315	0.197512	STX12	23673	syntaxin 12
ENSG0000	361.4911	0.284704	0.143565	1.983101	0.047356	0.197641	RBM7	10179	RNA binding motif protein 7
ENSG0000	104.1147	-0.51	0.257219	-1.98276	0.047394	0.19773	SCCPDH	51097	saccharopine dehydrogenase (putative)
ENSG0000	1032.792	0.293627	0.148093	1.982719	0.047399	0.19773	IL4I1	259307	interleukin 4 induced 1
ENSG0000	732.9392	-0.27524	0.138832	-1.98258	0.047415	0.197751	AGO4	192670	argonaute RISC component 4
ENSG0000	1849.219	0.168652	0.085074	1.982425	0.047432	0.197778	G3BP1	10146	G3BP stress granule assembly factor 1
ENSG0000	19.72277	1.021928	0.515561	1.982167	0.047461	0.197854	NA	NA	NA
ENSG0000	5.511112	-2.34759	1.184764	-1.98149	0.047537	0.198127	ESCO2	157570	establishment of sister chromatid cohesion N-acetyltransferase 2
ENSG0000	345.9034	0.281272	0.141991	1.980911	0.047601	0.198351	TM2D3	80213	TM2 domain containing 3
ENSG0000	20.68693	-1.05925	0.534846	-1.98048	0.04765	0.19851	RDH5	5959	retinol dehydrogenase 5
ENSG0000	14.6989	-1.35463	0.684044	-1.98033	0.047666	0.198534	LOC10050	1.01E+08	uncharacterized LOC100506606
ENSG0000	3399.191	0.187701	0.09479	1.980181	0.047683	0.198558	GATAD2B	57459	GATA zinc finger domain containing 2B
ENSG0000	2968.203	0.149442	0.075485	1.979765	0.04773	0.198664	KHDRBS1	10657	KH RNA bi signal transduction associated 1
ENSG0000	820.023	0.231437	0.116899	1.979801	0.047726	0.198664	COX7C	1350	cytochrome c oxidase subunit 7C
ENSG0000	1028.601	0.20123	0.101666	1.979324	0.04778	0.198737	RPL22	6146	ribosomal protein L22
ENSG0000	18.15062	-1.14714	0.579563	-1.97932	0.04778	0.198737	CCDC30	728621	coiled-coil domain containing 30
ENSG0000	1104.742	-0.18703	0.094489	-1.97943	0.047768	0.198737	ENGASE	64772	endo-beta-N-acetylglucosaminidase
ENSG0000	1230.188	0.192522	0.097291	1.978813	0.047837	0.198931	KPNA6	23633	karyopherin subunit alpha 6
ENSG0000	30.16143	-0.91594	0.462964	-1.97843	0.04788	0.198978	NA	NA	NA
ENSG0000	50.28786	-0.71712	0.362472	-1.97843	0.04788	0.198978	PTGDR2	11251	prostaglandin D2 receptor 2
ENSG0000	383.0269	0.314342	0.15887	1.978616	0.047859	0.198978	CPM	1368	carboxypeptidase M
ENSG0000	36.88066	-0.99145	0.501167	-1.97829	0.047897	0.199	NA	NA	NA
ENSG0000	180.0383	-0.38869	0.19651	-1.97797	0.047932	0.199101	LRP5	4041	LDL receptor related protein 5
ENSG0000	4780.177	-0.16031	0.081083	-1.97713	0.048027	0.199454	LSR	51599	lipolysis stimulated lipoprotein receptor
ENSG0000	653.1243	0.231707	0.117226	1.97658	0.048089	0.199666	GTF2H1	2965	general transcription factor IIH subunit 1
ENSG0000	298.3891	0.320333	0.162084	1.976335	0.048117	0.199737	NA	NA	NA
ENSG0000	23.13904	-1.06035	0.53669	-1.97572	0.048186	0.19998	LINC03033	400221	long intergenic non-protein coding RNA 3033
ENSG0000	2312.689	-0.16878	0.085435	-1.97557	0.048204	0.200008	CDK13	8621	cyclin dependent kinase 13
ENSG0000	440.5097	-0.30054	0.152148	-1.9753	0.048234	0.200087	FAM20B	9917	FAM20B glycosaminoglycan xylosylkinase
ENSG0000	220.2588	0.388516	0.196726	1.974915	0.048278	0.200225	RABAC1	10567	Rab acceptor 1
ENSG0000	8311.049	-0.14721	0.074553	-1.97456	0.048318	0.20026	THEMIS2	9473	thymocyte selection associated family member 2
ENSG0000	5.812992	-2.26968	1.149373	-1.97471	0.048301	0.20026	NA	NA	NA
ENSG0000	6.096799	2.298245	1.163915	1.974582	0.048316	0.20026	NA	NA	NA
ENSG0000	777.9645	-0.22889	0.115929	-1.97441	0.048335	0.200283	MFSD6	54842	major facilitator superfamily domain containing 6
ENSG0000	6347.815	0.166693	0.084436	1.974203	0.048359	0.200337	MYL12B	103910	myosin light chain 12B
ENSG0000	3.71087	-3.13231	1.586995	-1.97373	0.048412	0.200513	PHF24	23349	PHD finger protein 24
ENSG0000	356.647	-0.32015	0.162222	-1.97354	0.048434	0.200517	KCNC4	3749	potassium voltage-gated channel subfamily C member 4
ENSG0000	4059.723	0.167323	0.084781	1.973592	0.048428	0.200517	FKBP1A	2280	FKBP prolyl isomerase 1A
ENSG0000	186.3179	-0.41683	0.21122	-1.97343	0.048447	0.200524	PLEKHN1	84069	pleckstrin homology domain containing N1
ENSG0000	276.2642	0.320193	0.162308	1.972742	0.048525	0.200802	VMA21	203547	vacuolar ATPase assembly factor VMA21
ENSG0000	3.08212	-3.83792	1.945603	-1.97261	0.04854	0.200817	NPC1L1	29881	NPC1 like intracellular cholesterol transporter 1
ENSG0000	5.121285	2.635827	1.336375	1.972371	0.048567	0.200887	NA	NA	NA
ENSG0000	887.3054	-0.24633	0.124899	-1.97221	0.048586	0.200918	MRI1	84245	methylthioribose-1-phosphate isomerase 1
ENSG0000	24370.61	-0.70041	0.355213	-1.97181	0.048631	0.200955	GBP5	115362	guanylate binding protein 5
ENSG0000	450.044	0.307464	0.155922	1.971905	0.04862	0.200955	DIMT1	27292	DIM1 rRNA methyltransferase and ribosome maturation factor
ENSG0000	4842.336	-0.14451	0.073292	-1.97166	0.048649	0.200955	TESPA1	9840	thymocyte positive selection associated 1
ENSG0000	732.5347	-0.27118	0.137524	-1.97185	0.048626	0.200955	TELO2	9894	telomere maintenance 2
ENSG0000	14.74917	1.371166	0.695411	1.971734	0.04864	0.200955	NA	NA	NA
ENSG0000	67.06236	-0.63407	0.321647	-1.97134	0.048685	0.201062	GUSBP1	728411	GUSB pseudogene 1
ENSG0000	724.6998	-0.20652	0.104774	-1.97108	0.048714	0.201137	WDR11	55717	WD repeat domain 11
ENSG0000	3881.529	0.161952	0.082183	1.970628	0.048766	0.201307	ACTR1A	10121	actin related protein 1A
ENSG0000	1119.777	-0.19	0.096462	-1.96966	0.048878	0.201637	INPP5E	56623	inositol polyphosphate-5-phosphatase E
ENSG0000	14399.72	0.133106	0.067575	1.969746	0.048867	0.201637	CREBBP	1387	CREB binding protein
ENSG0000	447.1497	-0.26608	0.135091	-1.96965	0.048879	0.201637	PTPRS	5802	protein tyrosine phosphatase receptor type S
ENSG0000	22.22553	-0.96375	0.489447	-1.96906	0.048947	0.201871	TRAV12-3	28672	T cell receptor alpha variable 12-3
ENSG0000	99.31514	0.513922	0.261015	1.968933	0.048961	0.201885	GPR55	9290	G protein-coupled receptor 55
ENSG0000	8.3575	2.109986	1.071757	1.968717	0.048986	0.201942	CCM2L	140706	CCM2 like scaffold protein

ENSG0000	50.7865	0.656212	0.333472	1.967816	0.049089	0.202324	PSMC3IP	29893	PSMC3 interacting protein
ENSG0000	257.5804	-0.33635	0.170952	-1.96752	0.049123	0.20242	TRIM68	55128	tripartite motif containing 68
ENSG0000	34953.64	0.157266	0.079936	1.96739	0.049138	0.202436	RPL19	6143	ribosomal protein L19
ENSG0000	48.93081	0.730062	0.371131	1.96713	0.049168	0.20247	NA	NA	NA
ENSG0000	98.9584	0.541486	0.275257	1.967204	0.04916	0.20247	CKS2	1164	CDC28 protein kinase regulatory subunit 2
ENSG0000	111.8802	-0.58096	0.295377	-1.96683	0.049202	0.202552	CD248	57124	CD248 molecule
ENSG0000	10.86531	1.638682	0.833185	1.966768	0.04921	0.202552	WNT3	7473	Wnt family member 3
ENSG0000	1165.241	0.203768	0.103621	1.966473	0.049244	0.202647	RNF25	64320	ring finger protein 25
ENSG0000	3.159677	3.374163	1.716197	1.96607	0.049291	0.202793	DACT2	168002	dishevelled binding antagonist of beta catenin 2
ENSG0000	11.00176	-1.57707	0.802275	-1.96575	0.049328	0.202902	TJP2	9414	tight junction protein 2
ENSG0000	16247.17	-0.16339	0.083127	-1.96559	0.049346	0.202933	GPX1	2876	glutathione peroxidase 1
ENSG0000	680.1853	-0.21972	0.111801	-1.96531	0.049379	0.203022	COG3	83548	component of oligomeric golgi complex 3
ENSG0000	237.912	0.463544	0.235914	1.964886	0.049427	0.203176	GSTM1	2944	glutathione S-transferase mu 1
ENSG0000	4.270738	2.657979	1.353039	1.964452	0.049478	0.203338	EXOSC10-/	1.05E+08	EXOSC10 antisense RNA 1
ENSG0000	323.6622	-0.29657	0.151011	-1.96389	0.049543	0.203558	ATR	545	ATR serine/threonine kinase
ENSG0000	30.55324	-0.88751	0.451933	-1.9638	0.049553	0.203558	IGHV3-11	28450	immunoglobulin heavy variable 3-11
ENSG0000	703.4024	0.225857	0.115057	1.963005	0.049646	0.203892	STX3	6809	syntaxin 3
ENSG0000	10.50731	-1.65752	0.844649	-1.96238	0.049719	0.204056	RFX3-DT	1.02E+08	RFX3 divergent transcript
ENSG0000	2189.298	0.171652	0.087465	1.962517	0.049702	0.204056	CBL	867	Cbl proto-oncogene
ENSG0000	469.0853	-0.26472	0.134895	-1.9624	0.049716	0.204056	ZNF236	7776	zinc finger protein 236
ENSG0000	16.57705	-1.6903	0.861405	-1.96225	0.049733	0.20407	IGLV10-54	28772	immunoglobulin lambda variable 10-54
ENSG0000	1150.905	0.190181	0.096946	1.961725	0.049795	0.204205	TMEM222	84065	transmembrane protein 222
ENSG0000	225.7082	-0.34003	0.173334	-1.96169	0.049799	0.204205	ZNF407	55628	zinc finger protein 407
ENSG0000	333.1253	-0.27792	0.14166	-1.96187	0.049778	0.204205	TSHZ3	57616	teashirt zinc finger homeobox 3
ENSG0000	2.898706	-3.32412	1.694822	-1.96134	0.049839	0.204281	NA	NA	NA
ENSG0000	207.7356	-0.47177	0.240532	-1.96136	0.049837	0.204281	ARHGAP3?	9743	Rho GTPase activating protein 32
ENSG0000	19.52591	-1.16106	0.592107	-1.96089	0.049892	0.204429	GTF2H2	2966	general transcription factor IIH subunit 2
ENSG0000	1144.166	-0.25347	0.129265	-1.96084	0.049898	0.204429	TMEM106	113277	transmembrane protein 106A
ENSG0000	411.3099	0.376031	0.191792	1.960616	0.049924	0.204491	PPBP	5473	pro-platelet basic protein
ENSG0000	82.08723	0.652441	0.332796	1.960483	0.049939	0.204498	DNASE1L3	1776	deoxyribonuclease 1 like 3
ENSG0000	507.2973	0.24897	0.126999	1.960413	0.049948	0.204498	HTATIP2	10553	HIV-1 Tat interactive protein 2
ENSG0000	175.3916	-0.4038	0.206036	-1.95987	0.05001	0.20453	DUSP23	54935	dual specificity phosphatase 23
ENSG0000	157.2758	-0.38765	0.197791	-1.9599	0.050007	0.20453	FBLN2	2199	fibulin 2
ENSG0000	479.2834	0.260537	0.132912	1.960226	0.049969	0.20453	ZBTB24	9841	zinc finger and BTB domain containing 24
ENSG0000	193.2207	-0.34457	0.175805	-1.95996	0.050001	0.20453	PEMT	10400	phosphatidylethanolamine N-methyltransferase
ENSG0000	5.641475	1.887999	0.963196	1.960139	0.04998	0.20453	SNHG25	1.05E+08	small nucleolar RNA host gene 25
ENSG0000	211.1809	0.420598	0.214621	1.95973	0.050027	0.204554	MCOLN2	255231	mucolipin TRP cation channel 2
ENSG0000	7.149206	1.868683	0.953586	1.959636	0.050038	0.204554	NA	NA	NA
ENSG0000	1203.145	-0.23489	0.119903	-1.95899	0.050114	0.204776	PLOD3	8985	procollage 2-oxoglutarate 5-dioxygenase 3
ENSG0000	1306.1	-0.17101	0.087296	-1.95898	0.050115	0.204776	OIP5-AS1	729082	OIP5 antisense RNA 1
ENSG0000	145.4483	-0.39337	0.200846	-1.95855	0.050166	0.20494	MMUT	4594	methylmalonyl-CoA mutase
ENSG0000	6871.384	0.177024	0.090414	1.957934	0.050238	0.205188	GPX4	2879	glutathione peroxidase 4
ENSG0000	272.6938	0.321031	0.164028	1.957177	0.050327	0.205486	CSTA	1475	cystatin A
ENSG0000	94.56689	-0.65283	0.333568	-1.95712	0.050333	0.205486	CABLES1	91768	Cdk5 and Abl enzyme substrate 1
ENSG0000	285.3795	0.299754	0.153173	1.956962	0.050352	0.205508	ARL14EP	120534	ADP ribosylation factor like GTPase 14 effector protein
ENSG0000	576.6533	0.313484	0.160195	1.956889	0.05036	0.205508	ATG101	60673	autophagy related 101
ENSG0000	32.35302	-0.8009	0.409443	-1.95606	0.050458	0.205862	NA	NA	NA
ENSG0000	3410.002	0.200538	0.102537	1.955757	0.050494	0.205962	DNAJA1	3301	DnaJ heat shock protein family (Hsp40) member A1
ENSG0000	48.3344	-0.74052	0.378658	-1.95565	0.050507	0.205969	KATNAL1	84056	katanin catalytic subunit A1 like 1
ENSG0000	210.2759	-0.42842	0.219148	-1.95492	0.050592	0.206253	MRAS	22808	muscle RAS oncogene homolog
ENSG0000	695.377	-0.22042	0.112755	-1.95487	0.050599	0.206253	LRBA	987	LPS responsive beige-like anchor protein
ENSG0000	368.2594	-0.29721	0.15206	-1.95454	0.050637	0.206365	SMARCAD	56916	SWI/SNF-r matrix-as: subfamily containing DEAD/H box 1
ENSG0000	2051.957	0.207953	0.106407	1.954324	0.050663	0.206425	SRSF4	6429	serine and arginine rich splicing factor 4
ENSG0000	39.19082	0.753129	0.385458	1.953853	0.050719	0.206478	KTI12	112970	KTI12 chromatin associated homolog
ENSG0000	81.82426	0.541321	0.277055	1.953837	0.05072	0.206478	AK6	1.02E+08	adenylate kinase 6
ENSG0000	544.0199	-0.23909	0.12236	-1.95397	0.050705	0.206478	HMG20A	10363	high mobility group 20A
ENSG0000	391.8216	-0.30848	0.157867	-1.95406	0.050694	0.206478	MEX3D	399664	mex-3 RNA binding family member D
ENSG0000	504.9019	-1.16072	0.594194	-1.95343	0.050769	0.206583	APOL4	80832	apolipoprotein L4
ENSG0000	1151.299	-0.22855	0.117	-1.95345	0.050766	0.206583	ATRX	546	ATRX chromatin remodeler
ENSG0000	12.55504	-1.45919	0.747037	-1.9533	0.050784	0.206599	TRAV38-1	28644	T cell receptor alpha variable 38-1
ENSG0000	157.6841	0.440791	0.225687	1.953108	0.050807	0.20664	MRPL19	9801	mitochondrial ribosomal protein L19
ENSG0000	656.2339	0.270201	0.13835	1.95303	0.050816	0.20664	HOXB2	3212	homeobox B2
ENSG0000	8222.76	0.1488	0.0762	1.952757	0.050848	0.206726	MBD6	114785	methyl-CpG binding domain protein 6
ENSG0000	2702.272	0.165898	0.084961	1.952633	0.050863	0.206741	FAM168B	130074	family with sequence similarity 168 member B
ENSG0000	1709.484	0.659862	0.338108	1.951629	0.050982	0.207179	SNAI1	6615	snail family transcriptional repressor 1
ENSG0000	2277.586	0.162742	0.083428	1.950684	0.051095	0.207591	CLIP1	6249	CAP-Gly domain containing linker protein 1
ENSG0000	141.0046	-0.4779	0.245102	-1.9498	0.0512	0.207972	BBS4	585	Bardet-Biedl syndrome 4
ENSG0000	1275.597	0.255238	0.130913	1.949673	0.051215	0.207989	ELK4	2005	ETS transcription factor ELK4
ENSG0000	127.7203	-0.46822	0.240209	-1.94924	0.051267	0.208155	DYNC2I2	89891	dynein 2 intermediate chain 2
ENSG0000	351.4259	0.300727	0.154327	1.948643	0.051338	0.208397	COX8A	1351	cytochrome c oxidase subunit 8A
ENSG0000	1055.345	-0.18673	0.095834	-1.94851	0.051354	0.208416	COG1	9382	component of oligomeric golgi complex 1
ENSG0000	1599.237	0.186376	0.095656	1.948405	0.051366	0.208421	RAB27A	5873	RAB27A member RAS oncogene family
ENSG0000	280.0777	-0.32251	0.165557	-1.94803	0.051412	0.208519	ABHD6	57406	abhydrola: acylglycerol lipase
ENSG0000	3976.616	0.180403	0.092613	1.947921	0.051424	0.208519	HSF1	3297	heat shock transcription factor 1
ENSG0000	133.1988	0.575839	0.295615	1.947932	0.051423	0.208519	NA	NA	NA
ENSG0000	747.2587	0.217941	0.111903	1.947578	0.051465	0.20864	TXK	7294	TXK tyrosine kinase
ENSG0000	1607.537	0.179636	0.092248	1.94731	0.051498	0.208725	PRDX6	9588	peroxiredoxin 6
ENSG0000	1671.012	0.208407	0.107031	1.947174	0.051514	0.208746	ARID4B	51742	AT-rich interaction domain 4B
ENSG0000	299.1933	0.284281	0.146009	1.947006	0.051534	0.208781	PELI2	57161	pellino E3 ubiquitin protein ligase family member 2
ENSG0000	90.41939	0.586165	0.30109	1.946808	0.051558	0.208832	ICAM4	3386	intercellular adhesion molecule 4 (Landsteiner-Wiener blood group)
ENSG0000	3073.347	-0.17761	0.091267	-1.94605	0.051649	0.209157	AKT1	207	AKT serine/threonine kinase 1
ENSG0000	1409.51	0.16743	0.086052	1.945683	0.051693	0.209288	ANXA7	310	annexin A7
ENSG0000	4951.639	0.145294	0.074705	1.944898	0.051787	0.209624	UPF1	5976	UPF1 RNA helicase and ATPase
ENSG0000	1016.361	-0.18604	0.095666	-1.94465	0.051817	0.209651	C2orf68	388969	chromosome 2 open reading frame 68
ENSG0000	37.9946	-0.78218	0.40221	-1.94469	0.051812	0.209651	NA	NA	NA
ENSG0000	5.590317	-2.28682	1.176208	-1.94423	0.051868	0.209814	MIR5587	1.01E+08	microRNA 5587
ENSG0000	5.53399	-2.63637	1.356143	-1.94402	0.051893	0.20987	NA	NA	NA
ENSG0000	202.3159	0.421751	0.216981	1.943726	0.051929	0.209967	CRTAM	56253	cytotoxic and regulatory T cell molecule
ENSG0000	268.3226	-0.40836	0.21015	-1.94316	0.051996	0.210195	PVR	5817	PVR cell adhesion molecule
ENSG0000	42655.58	-0.14136	0.07276	-1.94285	0.052034	0.210281	PSAP	5660	prosaposin
ENSG0000	25.20633	-1.01425	0.522067	-1.94276	0.052045	0.210281	NA	NA	NA
ENSG0000	465.8261	0.279438	0.143839	1.942708	0.052051	0.210281	GNAL	2774	G protein subunit alpha L
ENSG0000	751.4614	-0.2348	0.120875	-1.9425	0.052077	0.210336	STX10	8677	syntaxin 10
ENSG0000	65.00359	-0.6031	0.310626	-1.94157	0.052189	0.210699	ZNF117	51351	zinc finger protein 117
ENSG0000	5.77913	-2.07176	1.067007	-1.94166	0.052179	0.210699	NA	NA	NA
ENSG0000	81.64185	-0.55095	0.28379	-1.94141	0.052208	0.210731	PTRH1	138428	peptidyl-tRNA hydrolase 1 homolog
ENSG0000	96.54989	-0.50483	0.260051	-1.94126	0.052226	0.210758	CYP2U1	113612	cytochrome P450 family 2 subfamily U member 1
ENSG0000	346.5974	-0.30362	0.156411	-1.94116	0.052239	0.210761	RBM12B	389677	RNA binding motif protein 12B
ENSG0000	1398.49	-0.20977	0.108086	-1.94074	0.05229	0.21083	NAPRT	93100	nicotinate phosphoribosyltransferase
ENSG0000	3.717298	-2.74874	1.416269	-1.94083	0.052278	0.21083	SCN4A	6329	sodium voltage-gated channel alpha subunit 4

ENSG0000	274.7619	0.340614	0.175502	1.940802	0.052282	0.21083	GPR174	84636	G protein-coupled receptor 174
ENSG0000	659.8037	-0.29264	0.150795	-1.94064	0.052302	0.210832	UBXN2B	137886	UBX domain protein 2B
ENSG0000	5.518784	-2.22423	1.14625	-1.94044	0.052327	0.210887	NA	NA	NA
ENSG0000	1459.688	0.2156	0.11114	1.939897	0.052392	0.211105	RBM17	84991	RNA binding motif protein 17
ENSG0000	9577.339	-0.1375	0.070884	-1.93977	0.052408	0.211123	SPTAN1	6709	spectrin al non-erythrocytic 1
ENSG0000	17.15194	1.212945	0.625478	1.939229	0.052473	0.211295	GRHL1	29841	grainyhead like transcription factor 1
ENSG0000	8.747547	-2.00061	1.031614	-1.9393	0.052464	0.211295	CRHBP	1393	corticotropin releasing hormone binding protein
ENSG0000	238.1259	0.324376	0.167297	1.938923	0.052511	0.211399	ZNF155	7711	zinc finger protein 155
ENSG0000	1137.867	0.210873	0.108789	1.938358	0.05258	0.21163	RPL13P12	388344	ribosomal protein L13 pseudogene 12
ENSG0000	48.38271	-0.68075	0.351259	-1.93802	0.05262	0.211748	GVQW3	1.01E+08	GVQW motif containing 3
ENSG0000	26.42225	1.109138	0.572432	1.93759	0.052673	0.211869	GHRL	51738	ghrelin and obestatin prepropeptide
ENSG0000	801.9608	-0.19818	0.102283	-1.93761	0.052671	0.211869	SDAD1	55153	SDA1 domain containing 1
ENSG0000	1245.168	-0.20674	0.106716	-1.93728	0.052711	0.211928	CROCCP2	84809	CROCC pseudogene 2
ENSG0000	22.23816	-0.9445	0.487519	-1.93736	0.052701	0.211928	DGCR6	8214	DiGeorge syndrome critical region gene 6
ENSG0000	2517.864	0.176231	0.09099	1.936817	0.052768	0.212111	RAB8A	4218	RAB8A member RAS oncogene family
ENSG0000	28.66756	-0.94892	0.490067	-1.93631	0.052829	0.212314	ANKRD42-	1.01E+08	ANKRD42 divergent transcript
ENSG0000	205.6375	0.347575	0.179517	1.936162	0.052848	0.212342	NA	NA	NA
ENSG0000	196.2333	-0.37381	0.193189	-1.93492	0.053	0.212868	ELAPOR1	57535	endosome-lysosome associated apoptosis and autophagy regulator 1
ENSG0000	43.9416	0.948877	0.490423	1.934813	0.053013	0.212868	RAB39A	54734	RAB39A member RAS oncogene family
ENSG0000	107.9256	-0.45344	0.234359	-1.93482	0.053013	0.212868	NA	NA	NA
ENSG0000	1811.719	-0.21691	0.112125	-1.93452	0.053049	0.212921	ADD3	120	adducin 3
ENSG0000	659.2354	0.218726	0.113062	1.934559	0.053044	0.212921	CEP95	90799	centrosomal protein 95
ENSG0000	7.552189	2.535894	1.311221	1.933995	0.053114	0.213133	GSTA1	2938	glutathione S-transferase alpha 1
ENSG0000	328.9727	0.290919	0.150493	1.9331	0.053224	0.213529	KBTBD4	55709	kelch repeat and BTB domain containing 4
ENSG0000	17855.29	-0.2242	0.115993	-1.9329	0.053248	0.21358	CD14	929	CD14 molecule
ENSG0000	41.73271	0.78676	0.407055	1.932809	0.05326	0.21358	NA	NA	NA
ENSG0000	8.772308	-1.75001	0.905534	-1.93257	0.053289	0.213651	NA	NA	NA
ENSG0000	11.07708	1.447174	0.749093	1.931902	0.053372	0.213936	NA	NA	NA
ENSG0000	45.01625	-0.79234	0.410161	-1.93176	0.053389	0.213958	CMTM4	146223	CKLF like MARVEL transmembrane domain containing 4
ENSG0000	147.4912	-0.43208	0.223731	-1.93126	0.053451	0.214162	EXOC6	54536	exocyst complex component 6
ENSG0000	617.7792	-0.25886	0.134079	-1.93066	0.053525	0.21441	ITFG2	55846	integrin alpha FG-GAP repeat containing 2
ENSG0000	420.2159	-0.30234	0.156616	-1.93047	0.053549	0.214414	PKN2	5586	protein kinase N2
ENSG0000	974.1619	0.185014	0.095837	1.930509	0.053544	0.214414	CIAO2B	51647	cytosolic iron-sulfur assembly component 2B
ENSG0000	3.502395	3.184643	1.649775	1.930351	0.053563	0.214427	LOC10537	1.05E+08	uncharacterized LOC105378353
ENSG0000	574.4498	-0.23064	0.119525	-1.92966	0.053649	0.214675	ZC3H10	84872	zinc finger CCCH-type containing 10
ENSG0000	2.928562	-3.37584	1.749426	-1.92969	0.053646	0.214675	NA	NA	NA
ENSG0000	181.3079	-0.44731	0.231821	-1.92955	0.053662	0.214684	ANK3	288	ankyrin 3
ENSG0000	19267.98	0.126902	0.06578	1.929195	0.053707	0.214722	YBX1	4904	Y-box binding protein 1
ENSG0000	6.253032	-2.15088	1.114804	-1.92938	0.053684	0.214722	NA	NA	NA
ENSG0000	2593.865	0.168061	0.087111	1.92928	0.053696	0.214722	PPP4R3A	55671	protein phosphatase 4 regulatory subunit 3A
ENSG0000	3933.49	0.170205	0.088233	1.929037	0.053726	0.214754	CNBP	7555	CCHC-type zinc finger nucleic acid binding protein
ENSG0000	4411.143	-0.14	0.072584	-1.92881	0.053754	0.214819	GGA3	23163	golgi assoc gamma ac ARF binding protein 3
ENSG0000	622.3056	0.234922	0.121805	1.928671	0.053772	0.214844	COQ10A	93058	coenzyme Q10A
ENSG0000	6332.538	0.198139	0.102743	1.928484	0.053795	0.21489	CD52	1043	CD52 molecule
ENSG0000	38.01064	0.755222	0.391693	1.9281	0.053843	0.215034	NA	NA	NA
ENSG0000	1670.682	0.202988	0.105287	1.927952	0.053861	0.215062	FNDC3A	22862	fibronectin type III domain containing 3A
ENSG0000	3679.494	0.159144	0.08255	1.927853	0.053873	0.215065	MTA2	9219	metastasis associated 1 family member 2
ENSG0000	2976.092	-0.16808	0.087191	-1.92766	0.053897	0.215114	UQCRC1	7384	ubiquinol-cytochrome c reductase core protein 1
ENSG0000	88.52464	0.543196	0.281823	1.927436	0.053925	0.215179	TSPAN13	27075	tetraspanin 13
ENSG0000	1436.968	-0.18139	0.094134	-1.92689	0.053993	0.215403	XPO7	23039	exportin 7
ENSG0000	605.211	0.247872	0.128655	1.926637	0.054025	0.215454	ISCA1	81689	iron-sulfur cluster assembly 1
ENSG0000	501.2464	-0.28821	0.149595	-1.92657	0.054033	0.215454	CLEC7A	64581	C-type lectin domain containing 7A
ENSG0000	124.6372	-0.53657	0.278518	-1.92651	0.054041	0.215454	CCNF	899	cyclin F
ENSG0000	414.6972	-0.26658	0.138438	-1.92563	0.05415	0.215845	ARHGEF12	23365	Rho guanine nucleotide exchange factor 12
ENSG0000	12.3867	1.348165	0.700284	1.925168	0.054208	0.215983	NA	NA	NA
ENSG0000	2.353005	-3.8508	2.000151	-1.92525	0.054197	0.215983	NA	NA	NA
ENSG0000	5.974159	-2.18727	1.136829	-1.92401	0.054353	0.216514	LYPD5	284348	LY6/PLAUR domain containing 5
ENSG0000	405.1666	0.257691	0.133956	1.923697	0.054393	0.216624	ZNF639	51193	zinc finger protein 639
ENSG0000	173.8785	-0.3532	0.183628	-1.92342	0.054427	0.216714	FRS3	10817	fibroblast growth factor receptor substrate 3
ENSG0000	107.436	-0.51083	0.265687	-1.92266	0.054523	0.217049	IL15	3600	interleukin 15
ENSG0000	152.117	-0.39324	0.204572	-1.92227	0.054572	0.217153	PGBD2	267002	piggyBac transposable element derived 2
ENSG0000	1023.895	0.202924	0.105562	1.922314	0.054566	0.217153	ZMYM2	7750	zinc finger MYM-type containing 2
ENSG0000	4.291629	-2.69883	1.404899	-1.92101	0.05473	0.217735	NA	NA	NA
ENSG0000	12.48219	-1.40305	0.730444	-1.92082	0.054755	0.217787	NA	NA	NA
ENSG0000	1275.763	0.213474	0.111157	1.92047	0.054799	0.217914	NUS1	116150	NUS1 dehydrodolichyl diphosphate synthase subunit
ENSG0000	47.01826	0.672376	0.350253	1.919689	0.054897	0.218221	NA	NA	NA
ENSG0000	106.6904	-0.4908	0.255689	-1.91951	0.05492	0.218221	TRAPPC13	80006	trafficking protein particle complex subunit 13
ENSG0000	566.7802	0.312432	0.162758	1.919615	0.054907	0.218221	NA	NA	NA
ENSG0000	102.2208	0.506313	0.263775	1.919486	0.054923	0.218221	TIAM2	26230	TIAM Rac1 associated GEF 2
ENSG0000	25.7148	0.977135	0.509161	1.919108	0.054971	0.218364	NA	NA	NA
ENSG0000	2196.569	-0.14975	0.078055	-1.9185	0.055048	0.218624	WBP1L	54838	WW domain binding protein 1 like
ENSG0000	10.18224	1.417771	0.739266	1.91781	0.055135	0.218763	RAD54L	8438	RAD54 like
ENSG0000	332.6284	-0.29289	0.152708	-1.91796	0.055116	0.218763	HEMK1	51409	HemK methyltransferase family member 1
ENSG0000	6.487873	2.351233	1.226076	1.917689	0.05515	0.218763	GCK	2645	glucokinase
ENSG0000	141.2748	0.473753	0.24704	1.917719	0.055147	0.218763	PGAP2	27315	post-GPI attachment to proteins 2
ENSG0000	722.1172	0.274248	0.143011	1.917664	0.055154	0.218763	SLC43A3	29015	solute carrier family 43 member 3
ENSG0000	4.001238	2.354339	1.227706	1.917673	0.055152	0.218763	NA	NA	NA
ENSG0000	1553.75	-0.20024	0.104432	-1.91744	0.055181	0.218826	NELFB	25920	negative elongation factor complex member B
ENSG0000	259.784	-0.33158	0.172952	-1.9172	0.055212	0.218901	SLC25A23	79085	solute carrier family 25 member 23
ENSG0000	21.12345	1.249352	0.65182	1.916714	0.055274	0.219101	EFCAB8	388795	EF-hand calcium binding domain 8
ENSG0000	376.4339	0.274905	0.143525	1.915387	0.055443	0.219714	POU2F1	5451	POU class 2 homeobox 1
ENSG0000	540.9868	-0.7178	0.374768	-1.91531	0.055453	0.219714	BATF2	116071	basic leucine zipper ATF-like transcription factor 2
ENSG0000	121.9212	-0.41952	0.219083	-1.9149	0.055505	0.219873	PPIP5K1	9677	diphosphoinositol pentakisphosphate kinase 1
ENSG0000	122.3192	-0.53194	0.277852	-1.91448	0.055559	0.220041	CEACAM6	4680	CEA cell adhesion molecule 6
ENSG0000	632.5614	0.251907	0.131593	1.914289	0.055583	0.22009	COX7A2	1347	cytochrome c oxidase subunit 7A2
ENSG0000	264.309	-0.33309	0.174017	-1.91411	0.055606	0.220131	AP5S1	55317	adaptor related protein complex 5 subunit sigma 1
ENSG0000	5.882768	-2.25207	1.176712	-1.91387	0.055637	0.22021	NA	NA	NA
ENSG0000	625.6865	-0.25353	0.13249	-1.91359	0.055672	0.220301	LZTS2	84445	leucine zipper tumor suppressor 2
ENSG0000	38.79217	-0.85326	0.446046	-1.91293	0.055757	0.220542	PECR	55825	peroxisomal trans-2-enoyl-CoA reductase
ENSG0000	26.23327	1.008726	0.527308	1.912972	0.055752	0.220542	PPP1R17	10842	protein phosphatase 1 regulatory subunit 17
ENSG0000	21.92905	-1.00588	0.525898	-1.91268	0.055789	0.22062	ASAH2	56624	N-acylsphingosine amidohydrolase 2
ENSG0000	4.807284	-2.39662	1.253097	-1.91256	0.055804	0.220636	NA	NA	NA
ENSG0000	2555.064	0.161527	0.084464	1.912369	0.055829	0.220686	NAA60	79903	N-alpha-ac NatF catalytic subunit
ENSG0000	998.6467	0.189203	0.098951	1.912093	0.055864	0.220779	ZNF131	7690	zinc finger protein 131
ENSG0000	17.12199	1.30692	0.683548	1.911965	0.055881	0.220796	RPS10	6204	ribosomal protein S10
ENSG0000	8.547363	1.831456	0.957999	1.911751	0.055908	0.220858	SLC16A4	9122	solute carrier family 16 member 4
ENSG0000	43.24505	-0.72843	0.38109	-1.91143	0.055949	0.220974	CLEC4F	165530	C-type lectin domain family 4 member F
ENSG0000	379.2129	0.280631	0.146826	1.911315	0.055964	0.220985	LILRA5	353514	leukocyte immunoglobulin like receptor A5
ENSG0000	1481.471	0.183235	0.095879	1.911101	0.055992	0.221046	CUL3	8452	cullin 3
ENSG0000	112.8939	-0.45142	0.236248	-1.91079	0.056032	0.22116	RNF208	727800	ring finger protein 208

ENSG0000	8.660754	1.824709	0.955404	1.909883	0.056148	0.221524	NA	NA	NA
ENSG0000	208.0958	-0.35878	0.18785	-1.90992	0.056144	0.221524	DKK3	27122	dickkopf WNT signaling pathway inhibitor 3
ENSG0000	241.8084	-0.38817	0.203326	-1.9091	0.056249	0.221873	LARS2	23395	leucyl-tRN mitochondrial
ENSG0000	60.79825	-0.59435	0.311339	-1.909	0.056262	0.221876	SLC15A2	6565	solute carrier family 15 member 2
ENSG0000	211.4056	0.377661	0.197841	1.908908	0.056274	0.221878	ARPC1B	10095	actin related protein 2/3 complex subunit 1B
ENSG0000	2070.978	-0.17623	0.092328	-1.90872	0.056298	0.221925	PRPF6	24148	pre-mRNA processing factor 6
ENSG0000	8142.704	0.146663	0.076852	1.908388	0.056341	0.222048	PIK3IP1	113791	phosphoinositide-3-kinase interacting protein 1
ENSG0000	7.323874	1.734768	0.909133	1.908156	0.056371	0.222119	NA	NA	NA
ENSG0000	784.3686	0.237412	0.124427	1.908047	0.056385	0.222127	LIX1L	128077	limb and CNS expressed 1 like
ENSG0000	7.8424	1.994695	1.045657	1.907599	0.056443	0.22225	PCOLCE-A!	1E+08	PCOLCE antisense RNA 1
ENSG0000	133.8862	0.433552	0.227285	1.907528	0.056452	0.22225	LOC65227(	652276	potassium channel tetramerization domain containing 5 pseudogene
ENSG0000	22.68252	-0.96961	0.508274	-1.90766	0.056435	0.22225	TMEM220	388335	transmembrane protein 220
ENSG0000	5.277498	-2.17582	1.140734	-1.90738	0.056471	0.222277	NA	NA	NA
ENSG0000	46.3066	0.822195	0.431109	1.907165	0.056499	0.222341	NA	NA	NA
ENSG0000	143.6308	-0.38685	0.202938	-1.90626	0.056617	0.222757	FAM114A2	10827	family with sequence similarity 114 member A2
ENSG0000	314.1689	-0.28385	0.148922	-1.906	0.05665	0.22284	GEMIN5	25929	gem nuclear organelle associated protein 5
ENSG0000	5.197051	-2.63527	1.383111	-1.90532	0.056738	0.22303	ODF3	113746	outer dense fiber of sperm tails 3
ENSG0000	13.85544	1.271146	0.667094	1.905498	0.056715	0.22303	SLC8A3	6547	solute carrier family 8 member A3
ENSG0000	224.6222	-0.43496	0.22828	-1.90536	0.056734	0.22303	ZFP3	124961	ZFP3 zinc finger protein
ENSG0000	3701.884	-0.16432	0.086247	-1.90526	0.056747	0.22303	MFNG	4242	MFNG O-fucosylpeptide 3-beta-N-acetylglucosaminyltransferase
ENSG0000	2232.388	-0.18582	0.097575	-1.90442	0.056855	0.223319	SCAP	22937	SREBF chaperone
ENSG0000	506.6931	-0.26193	0.137524	-1.90459	0.056834	0.223319	PIK3R4	30849	phosphoinositide-3-kinase regulatory subunit 4
ENSG0000	1240.033	-0.18803	0.098736	-1.90442	0.056856	0.223319	INTS15	79034	integrator complex subunit 15
ENSG0000	25.1144	-0.88507	0.464862	-1.90395	0.056917	0.223511	TRO	7216	trophinin
ENSG0000	90.99555	0.472733	0.248345	1.903535	0.056971	0.223674	SPACA6	147650	sperm acrosome associated 6
ENSG0000	5253.465	0.146524	0.076978	1.903442	0.056983	0.223675	PIK3R1	5295	phosphoinositide-3-kinase regulatory subunit 1
ENSG0000	604.9339	0.223768	0.117598	1.902825	0.057063	0.223943	AVL9	23080	AVL9 cell migration associated
ENSG0000	2860.36	0.415246	0.218247	1.902637	0.057088	0.223992	B4GALT5	9334	beta-1 4-galactosyltransferase 5
ENSG0000	53.5567	-0.65953	0.346698	-1.90232	0.05713	0.224108	LOC10012(	1E+08	uncharacterized LOC100129215
ENSG0000	1387.329	0.181137	0.095226	1.902181	0.057147	0.224131	POLR2J	5439	RNA polymerase II subunit J
ENSG0000	1582.304	-0.16783	0.088245	-1.90188	0.057187	0.224238	AATK	9625	apoptosis associated tyrosine kinase
ENSG0000	158.4937	0.374699	0.197068	1.90137	0.057254	0.224419	NA	NA	NA
ENSG0000	182.8749	0.397687	0.209161	1.901342	0.057257	0.224419	VSTM1	284415	V-set and transmembrane domain containing 1
ENSG0000	39.73907	-0.76389	0.401792	-1.90121	0.057275	0.22444	IPP	3652	intracisternal A particle-promoted polypeptide
ENSG0000	808.2559	0.20275	0.106669	1.900748	0.057335	0.224629	AKAP1	8165	A-kinase anchoring protein 1
ENSG0000	38.17761	0.870919	0.458248	1.900542	0.057362	0.22464	PBX1	5087	PBX homeobox 1
ENSG0000	20.84368	-1.13155	0.595372	-1.90058	0.057357	0.22464	NA	NA	NA
ENSG0000	243.565	0.312199	0.164315	1.900005	0.057432	0.224821	GPNMB	10457	glycoprotein nmb
ENSG0000	21.83726	-1.26271	0.664553	-1.90009	0.057422	0.224821	DACT3	147906	dishevelled binding antagonist of beta catenin 3
ENSG0000	2271.254	0.17765	0.093517	1.899651	0.057479	0.224955	TIPARP	25976	TCDD inducible poly(ADP-ribose) polymerase
ENSG0000	4942.248	0.135464	0.071315	1.899525	0.057496	0.224973	JADE2	23338	jade family PHD finger 2
ENSG0000	428.9903	-0.24492	0.128971	-1.899	0.057565	0.225042	SKIC3	9652	SKI3 subunit of superkiller complex
ENSG0000	14.0289	1.388702	0.731189	1.899239	0.057533	0.225042	KLRC3	3823	killer cell lectin like receptor C3
ENSG0000	114.4541	0.506177	0.26654	1.899061	0.057556	0.225042	HEBP1	50865	heme binding protein 1
ENSG0000	6.422285	-2.04285	1.075642	-1.89919	0.057539	0.225042	NA	NA	NA
ENSG0000	5.643515	-2.19016	1.153365	-1.89893	0.057574	0.225042	NA	NA	NA
ENSG0000	38.31192	-0.79007	0.416218	-1.89822	0.057667	0.225238	COL8A2	1296	collagen type VIII alpha 2 chain
ENSG0000	7.159647	1.77062	0.932774	1.898231	0.057666	0.225238	SLC30A3	7781	solute carrier family 30 member 3
ENSG0000	429.3546	-0.27803	0.146453	-1.89839	0.057644	0.225238	GLI4	2738	GLI family zinc finger 4
ENSG0000	957.5368	-0.21051	0.110907	-1.89809	0.057684	0.225238	TASOR2	54906	transcription activation suppressor family member 2
ENSG0000	4.145435	-2.33633	1.23095	-1.89799	0.057697	0.225238	NA	NA	NA
ENSG0000	2244.296	0.157079	0.082759	1.89803	0.057692	0.225238	KDELR1	10945	KDEL endoplasmic reticulum protein retention receptor 1
ENSG0000	734.9506	-0.21351	0.112526	-1.89747	0.057766	0.225414	PPP2R5E	5529	protein phosphatase 2 regulatory subunit B'epsilon
ENSG0000	2126.569	-0.17195	0.090618	-1.89752	0.057759	0.225414	KXD1	79036	KxDL motif containing 1
ENSG0000	5.380304	2.382337	1.255795	1.897075	0.057818	0.225568	PJVK	494513	pejvakin
ENSG0000	613.2663	0.232387	0.122521	1.896708	0.057867	0.22571	RRP36	88745	ribosomal RNA processing 36
ENSG0000	5.582318	2.100278	1.107736	1.896009	0.057959	0.226022	SLC22A14	9389	solute carrier family 22 member 14
ENSG0000	4.936252	-2.33112	1.22967	-1.89573	0.057996	0.226121	NA	NA	NA
ENSG0000	276.381	-0.31868	0.16814	-1.89535	0.058046	0.226266	SMG8	55181	SMG8 nonsense mediated mRNA decay factor
ENSG0000	116.6461	-0.51271	0.270548	-1.89507	0.058083	0.226317	ANXA2R-A	153684	ANXA2R antisense RNA 1
ENSG0000	9045.96	0.186044	0.098169	1.895144	0.058073	0.226317	GNB2	2783	G protein subunit beta 2
ENSG0000	27.33692	-1.03662	0.547107	-1.89473	0.058128	0.226444	ELOVL4	6785	ELOVL fatty acid elongase 4
ENSG0000	449.8387	0.260033	0.137272	1.894283	0.058187	0.226628	DLD	1738	dihydrolipoamide dehydrogenase
ENSG0000	3537.727	0.152428	0.080472	1.894167	0.058203	0.22664	PCIF1	63935	phosphorylated CTD interacting factor 1
ENSG0000	967.2606	-0.19492	0.102912	-1.89407	0.058216	0.226644	HPS6	79803	HPS6 biogenesis of lysosomal organelles complex 2 subunit 3
ENSG0000	8.709402	1.588397	0.838743	1.893782	0.058254	0.226653	HHLA2	11148	HERV-H LTR-associating 2
ENSG0000	487.2162	0.267302	0.141148	1.893775	0.058255	0.226653	CBFA2T3	863	CBFA2/RUNX1 partner transcriptional co-repressor 3
ENSG0000	2262.526	0.174597	0.092193	1.893818	0.058249	0.226653	STAG2	10735	stromal antigen 2
ENSG0000	75.37005	-0.51773	0.273436	-1.89341	0.058303	0.226794	APBB2	323	amyloid beta precursor protein binding family B member 2
ENSG0000	8.041562	1.592673	0.841264	1.893191	0.058332	0.226859	CALCA	796	calcitonin related polypeptide alpha
ENSG0000	349.7493	-0.27542	0.145507	-1.89284	0.058379	0.226994	ALDH18A1	5832	aldehyde dehydrogenase 18 family member A1
ENSG0000	6.016596	-2.33313	1.23282	-1.89252	0.058422	0.227112	GOLGA8Q	727909	golgin A8 family member Q
ENSG0000	39.14588	-0.78571	0.415193	-1.8924	0.058438	0.227127	MT1F	4494	metallothionein 1F
ENSG0000	7941.78	0.155409	0.08213	1.892239	0.058459	0.227161	HNRNPU	3192	heterogeneous nuclear ribonucleoprotein U
ENSG0000	45.39837	-0.68286	0.360949	-1.89185	0.058511	0.227314	ADPGK-AS	1E+08	ADPGK antisense RNA 1
ENSG0000	108.8634	0.434809	0.229879	1.891468	0.058562	0.227335	WBP1	23559	WW domain binding protein 1
ENSG0000	62.77486	-0.60833	0.321622	-1.89144	0.058565	0.227335	TRBV4-1	28617	T cell receptor beta variable 4-1
ENSG0000	939.7172	0.188262	0.099528	1.891541	0.058552	0.227335	FAM104A	84923	family with sequence similarity 104 member A
ENSG0000	4.79923	-2.6136	1.381799	-1.89145	0.058565	0.227335	NA	NA	NA
ENSG0000	188.8538	0.333739	0.17646	1.8913	0.058584	0.227362	RPL4P4	647276	ribosomal protein L4 pseudogene 4
ENSG0000	5.204609	-2.22487	1.176743	-1.8907	0.058665	0.227626	PHKA1	5255	phosphorylase kinase regulatory subunit alpha 1
ENSG0000	12.88431	-1.29291	0.683866	-1.89058	0.05868	0.227638	LINC0217!	283887	long intergenic non-protein coding RNA 2175
ENSG0000	2345.62	0.203668	0.10774	1.890362	0.058709	0.227705	ARID5B	84159	AT-rich interaction domain 5B
ENSG0000	21.56138	1.314521	0.695536	1.88994	0.058766	0.227877	NPHS1	4868	NPHS1 adl nephrin
ENSG0000	921.3399	-0.19039	0.100761	-1.88952	0.058823	0.228049	CAT	847	catalase
ENSG0000	131.9894	-0.41494	0.21974	-1.88831	0.058984	0.228578	PIGBOS1	1.02E+08	PIGB opposite strand 1
ENSG0000	2221.76	0.158933	0.084163	1.8884	0.058972	0.228578	ELL	8178	elongation factor for RNA polymerase II
ENSG0000	29.8187	-0.81779	0.433169	-1.88793	0.059035	0.228634	UBE3D	90025	ubiquitin protein ligase E3D
ENSG0000	779.2695	-0.24904	0.131908	-1.88801	0.059025	0.228634	SUSD3	203328	sushi domain containing 3
ENSG0000	2.64193	-3.38738	1.794122	-1.88805	0.05902	0.228634	NA	NA	NA
ENSG0000	1285.923	0.175085	0.092751	1.887692	0.059067	0.228704	FBXL5	26234	F-box and leucine rich repeat protein 5
ENSG0000	54.05494	-0.66372	0.351633	-1.88754	0.059087	0.228704	NINJ2	4815	ninjurin 2
ENSG0000	131.0017	-0.43872	0.232434	-1.88752	0.05909	0.228704	MTRF1	9617	mitochondrial translation release factor 1
ENSG0000	60.84493	-0.68914	0.365301	-1.88649	0.059229	0.229193	CNR2	1269	cannabinoid receptor 2
ENSG0000	740.3083	-0.21761	0.115366	-1.88623	0.059264	0.229232	DROSHA	29102	drosha ribonuclease III
ENSG0000	74.7557	-0.52639	0.279069	-1.88625	0.059262	0.229232	PIN4	5303	peptidylpr NIMA-interacting 4
ENSG0000	265.5718	0.377579	0.200231	1.885711	0.059334	0.229455	LSM1	27257	LSM1 hom mRNA degradation associated
ENSG0000	2452.984	-0.15539	0.082443	-1.88482	0.059454	0.229873	ASCC2	84164	activating signal cointegrator 1 complex subunit 2
ENSG0000	231.1313	0.361781	0.191976	1.88451	0.059496	0.229922	RPL27AP6	389435	ribosomal protein L27a pseudogene 6
ENSG0000	153.3584	0.432579	0.229543	1.884519	0.059495	0.229922	PTP4A3	11156	protein tyrosine phosphatase 4A3

ENSG0000	1634.009	0.172869	0.091735	1.884448	0.059504	0.229922	NSFL1C	55968	NSFL1 cofactor
ENSG0000	448.9945	0.233304	0.123871	1.88345	0.059639	0.230396	NTMT1	28989	N-terminal Xaa-Pro-Lys N-methyltransferase 1
ENSG0000	3.357398	3.043134	1.61592	1.883221	0.05967	0.23042	NA	NA	NA
ENSG0000	6.586417	-2.26392	1.202148	-1.88323	0.059669	0.23042	CYP2T1P	171523	cytochrom pseudogene
ENSG0000	28.40099	0.87244	0.463413	1.88264	0.059749	0.230509	NA	NA	NA
ENSG0000	471.72	-0.27297	0.145	-1.88253	0.059764	0.230509	PCNX2	80003	pecanex 2
ENSG0000	171.9283	-0.42325	0.224814	-1.88265	0.059747	0.230509	SEPSECS	51091	Sep (O-phosphoserine) tRNA:Sec (selenocysteine) tRNA synthase
ENSG0000	2.131313	-3.66642	1.947752	-1.88238	0.059784	0.230509	MANEA-D'	1.02E+08	MANEA divergent transcript
ENSG0000	235.8821	-0.32644	0.17342	-1.88237	0.059785	0.230509	DNAJC30	84277	DnaJ heat shock protein family (Hsp40) member C30
ENSG0000	54.76472	0.676984	0.359603	1.882586	0.059756	0.230509	TMEM63C	57156	transmembrane protein 63C
ENSG0000	1500.834	0.187014	0.099325	1.882842	0.059722	0.230509	DYNLL2	140735	dynein light chain LC8-type 2
ENSG0000	614.7959	0.265032	0.140801	1.882317	0.059793	0.230509	PSENEN	55851	presenilin gamma-secretase subunit
ENSG0000	711.8621	-0.21259	0.112987	-1.88156	0.059896	0.230571	GPR153	387509	G protein-coupled receptor 153
ENSG0000	265.4852	-0.30575	0.162496	-1.88157	0.059894	0.230571	TRMT1L	81627	tRNA methyltransferase 1 like
ENSG0000	654.6959	0.219626	0.116702	1.881944	0.059844	0.230571	PDCD10	11235	programmed cell death 10
ENSG0000	17.05522	-1.17773	0.62588	-1.88172	0.059874	0.230571	SOBP	55084	sine oculis binding protein homolog
ENSG0000	10.04265	1.708928	0.908101	1.88187	0.059854	0.230571	MIR93	407050	microRNA 93
ENSG0000	373.3734	-0.311	0.165286	-1.88159	0.059892	0.230571	FANCA	2175	FA complementation group A
ENSG0000	2.975114	-3.50666	1.863529	-1.88173	0.059872	0.230571	APCDD1L-I	149773	APCDD1L divergent transcript
ENSG0000	3.428878	-3.04521	1.618715	-1.88125	0.059938	0.230684	NA	NA	NA
ENSG0000	125.0955	0.409339	0.217704	1.880257	0.060073	0.231157	SLC19A2	10560	solute carrier family 19 member 2
ENSG0000	2110.983	-0.16459	0.08755	-1.87989	0.060123	0.231302	LRCH4	4034	leucine rich repeats and calponin homology domain containing 4
ENSG0000	10.29668	-1.71718	0.913531	-1.87972	0.060146	0.231341	NA	NA	NA
ENSG0000	35.95203	-0.78327	0.416761	-1.87942	0.060187	0.231356	NA	NA	NA
ENSG0000	606.1172	-0.24346	0.129531	-1.87954	0.060171	0.231356	EME2	197342	essential meiotic structure-specific endonuclease subunit 2
ENSG0000	1116.545	-0.18191	0.096788	-1.87948	0.060179	0.231356	LSM14B	149986	LSM family member 14B
ENSG0000	39.90256	-0.76222	0.40562	-1.87916	0.060223	0.231397	NA	NA	NA
ENSG0000	114.2148	-0.45162	0.240326	-1.87919	0.060219	0.231397	ZSCAN2	54993	zinc finger and SCAN domain containing 2
ENSG0000	292.5589	-0.28978	0.154234	-1.87885	0.060265	0.231422	SLC30A6	55676	solute carrier family 30 member 6
ENSG0000	3.123021	2.869013	1.527016	1.878836	0.060267	0.231422	NA	NA	NA
ENSG0000	505.1611	0.245408	0.130615	1.878876	0.060261	0.231422	RAE1	8480	ribonucleic acid export 1
ENSG0000	7386.374	-0.15067	0.08022	-1.87819	0.060355	0.231713	DENND4B	9909	DENN domain containing 4B
ENSG0000	410.821	-0.26124	0.139103	-1.87801	0.06038	0.231762	STX18	53407	syntaxin 18
ENSG0000	69.61052	0.537399	0.286218	1.877589	0.060437	0.231933	PDE6G	5148	phosphodiesterase 6G
ENSG0000	1284.155	-0.18492	0.098513	-1.87715	0.060498	0.231968	CD46	4179	CD46 molecule
ENSG0000	1214.204	0.216074	0.115113	1.877066	0.060509	0.231968	KIFC2	90990	kinesin family member C2
ENSG0000	143.0653	0.464307	0.24734	1.877199	0.060491	0.231968	NA	NA	NA
ENSG0000	240.1217	-0.34407	0.183297	-1.87709	0.060506	0.231968	ZNF84	7637	zinc finger protein 84
ENSG0000	58.03814	0.58395	0.311091	1.877106	0.060504	0.231968	FERMT2	10979	FERM domain containing kindlin 2
ENSG0000	41.27965	-0.76862	0.409536	-1.8768	0.060546	0.231974	ZBED6	1E+08	zinc finger BED-type containing 6
ENSG0000	48.72945	0.723676	0.385613	1.876691	0.060561	0.231974	LRRN1	57633	leucine rich repeat neuronal 1
ENSG0000	1558.818	-0.17868	0.095209	-1.87672	0.060556	0.231974	COPZ1	22818	COPI coat complex subunit zeta 1
ENSG0000	1907.211	-0.17126	0.091249	-1.87679	0.060547	0.231974	SLCO3A1	28232	solute carrier organic anion transporter family member 3A1
ENSG0000	67.64158	-0.60173	0.320693	-1.87635	0.060607	0.232103	ZNF429	353088	zinc finger protein 429
ENSG0000	13.30495	1.452847	0.774461	1.875947	0.060663	0.232269	NA	NA	NA
ENSG0000	225.9696	0.356936	0.190281	1.875835	0.060678	0.232279	ZNF250	58500	zinc finger protein 250
ENSG0000	8.543525	2.003767	1.0683	1.875661	0.060702	0.232285	SPRY4	81848	sprouty RTK signaling antagonist 4
ENSG0000	51.33788	0.662893	0.353422	1.875642	0.060705	0.232285	NIPAL2	79815	NIPA like domain containing 2
ENSG0000	1053.749	-0.18005	0.096008	-1.87535	0.060745	0.232392	SUSD1	64420	sushi domain containing 1
ENSG0000	592.8906	0.220683	0.117685	1.875204	0.060765	0.232419	RNF139	11236	ring finger protein 139
ENSG0000	60.52812	0.564258	0.301014	1.874525	0.060858	0.232729	NA	NA	NA
ENSG0000	230.4929	-0.34579	0.184507	-1.87411	0.060916	0.232853	ZNF691	51058	zinc finger protein 691
ENSG0000	284.0494	-0.30844	0.16458	-1.87413	0.060913	0.232853	HCN2	610	hyperpolarization activated cyclic nucleotide gated potassium and sodium channel 2
ENSG0000	84.44851	-0.53437	0.285182	-1.87378	0.06096	0.232975	SCIRT	1.02E+08	stem cell inhibitory RNA transcript
ENSG0000	2793.79	0.172996	0.092332	1.873629	0.060982	0.232988	ZNF598	90850	zinc finger E3 ubiquitin ligase
ENSG0000	1021.758	0.220064	0.117457	1.873578	0.060989	0.232988	DUSP3	1845	dual specificity phosphatase 3
ENSG0000	109.2018	-0.51233	0.27348	-1.87336	0.061018	0.233054	SWI5	375757	SWI5 homologous recombination repair protein
ENSG0000	683.4521	-0.23025	0.122954	-1.87263	0.061119	0.23339	ZDHHC12	84885	zinc finger DHHC-type palmitoyltransferase 12
ENSG0000	147.6484	-0.39115	0.208931	-1.87214	0.061187	0.233603	EIPR1	7260	EARP complex and GARP complex interacting protein 1
ENSG0000	7222.505	0.128182	0.068481	1.871798	0.061235	0.233735	EIF4B	1975	eukaryotic translation initiation factor 4B
ENSG0000	527.4718	0.272119	0.145431	1.871124	0.061328	0.233995	PLEKHF2	79666	pleckstrin homology and FYVE domain containing 2
ENSG0000	76.78588	-0.55368	0.295902	-1.87117	0.061322	0.233995	GSPT2	23708	G1 to S phase transition 2
ENSG0000	189.3693	0.368733	0.197109	1.870709	0.061385	0.234118	TARBP1	6894	TAR (HIV-1) RNA binding protein 1
ENSG0000	3.116207	-3.1452	1.681274	-1.87072	0.061383	0.234118	PPP1R36	145376	protein phosphatase 1 regulatory subunit 36
ENSG0000	657.8895	0.209642	0.112083	1.870419	0.061426	0.234223	EIF2AK3	9451	eukaryotic translation initiation factor 2 alpha kinase 3
ENSG0000	416.085	-0.30839	0.164892	-1.87023	0.061452	0.234276	PADI4	23569	peptidyl arginine deiminase 4
ENSG0000	103.6111	0.484798	0.259284	1.86976	0.061517	0.234476	ZNF567	163081	zinc finger protein 567
ENSG0000	124.0704	-0.48965	0.261958	-1.86919	0.061597	0.23473	ZBTB42	1E+08	zinc finger and BTB domain containing 42
ENSG0000	841.9212	-0.19516	0.104425	-1.86895	0.06163	0.234736	EPHA4	2043	EPH receptor A4
ENSG0000	2605.673	0.149069	0.079762	1.868923	0.061633	0.234736	SYNJ2	8871	synaptojanin 2
ENSG0000	58.67769	-0.75407	0.403482	-1.86891	0.061636	0.234736	UCP3	7352	uncoupling protein 3
ENSG0000	24.03755	0.87365	0.467524	1.868676	0.061668	0.234809	NA	NA	NA
ENSG0000	1031.001	-0.24248	0.12977	-1.86853	0.061688	0.234839	AGPAT2	10555	1-acylglycerol-3-phosphate O-acyltransferase 2
ENSG0000	404.2148	0.308079	0.164942	1.867798	0.06179	0.235178	RNF216P1	441191	ring finger protein 216 pseudogene 1
ENSG0000	317.1886	-0.3069	0.164319	-1.86769	0.061805	0.235187	DNAJC16	23341	DnaJ heat shock protein family (Hsp40) member C16
ENSG0000	1595.937	0.262745	0.140712	1.867246	0.061867	0.235375	TPRA1	131601	transmembrane protein adipocyte associated 1
ENSG0000	7.186247	1.942851	1.040691	1.866886	0.061918	0.235518	NA	NA	NA
ENSG0000	259.1872	0.372158	0.199415	1.866242	0.062007	0.235811	CLDND2	125875	claudin domain containing 2
ENSG0000	3748.071	0.156764	0.084008	1.866068	0.062032	0.235856	ARL6IP5	10550	ADP ribosylation factor like GTPase 6 interacting protein 5
ENSG0000	1053.606	0.253659	0.135988	1.865307	0.062138	0.236164	MAN2A1	4124	mannosidase alpha class 2A member 1
ENSG0000	53.42174	0.639936	0.343064	1.865354	0.062132	0.236164	FAM83D	81610	family with sequence similarity 83 member D
ENSG0000	1087.84	0.217874	0.11682	1.865048	0.062175	0.236254	EYA3	2140	EYA transcriptional coactivator and phosphatase 3
ENSG0000	484.7555	-0.25354	0.13596	-1.86478	0.062212	0.236347	ZDHHC11E	653082	zinc finger DHHC-type containing 11B
ENSG0000	181.1566	-0.34548	0.18529	-1.86456	0.062243	0.236416	PLA2G15	23659	phospholipase A2 group XV
ENSG0000	5.112908	2.014113	1.08037	1.864281	0.062282	0.236517	NA	NA	NA
ENSG0000	463.4708	-0.24584	0.131935	-1.86331	0.062418	0.236815	TAF12	6883	TATA-box binding protein associated factor 12
ENSG0000	321.7309	-0.32059	0.172031	-1.86353	0.062388	0.236815	ERCC6L2	375748	ERCC excision repair 6 like 2
ENSG0000	881.2095	-0.19317	0.103661	-1.8635	0.062391	0.236815	MAN1B1	11253	mannosidase alpha class 1B member 1
ENSG0000	619.6232	0.230757	0.123842	1.863313	0.062418	0.236815	SLC25A29	123096	solute carrier family 25 member 29
ENSG0000	4.629297	-2.34856	1.260452	-1.86327	0.062424	0.236815	IL11	3589	interleukin 11
ENSG0000	574.958	0.262129	0.140704	1.862978	0.062465	0.236921	MMGT1	93380	membrane magnesium transporter 1
ENSG0000	394.5427	-0.26857	0.144191	-1.86258	0.062521	0.237086	SOGA1	140710	suppresso autophagy associated 1
ENSG0000	190.1466	-0.40623	0.218118	-1.86245	0.06254	0.237107	NCALD	83988	neurocalcin delta
ENSG0000	142.5538	-0.47146	0.253165	-1.86228	0.062564	0.237148	AIM2	9447	absent in melanoma 2
ENSG0000	9.86375	-1.80109	0.967527	-1.86154	0.062669	0.237303	ASS1P1	442167	argininosuccinate synthetase 1 pseudogene 1
ENSG0000	5.298819	-2.16653	1.163618	-1.86189	0.062619	0.237303	NA	NA	NA
ENSG0000	4.536464	2.402439	1.290631	1.861446	0.062681	0.237303	LINC0272E	1.02E+08	long intergenic non-protein coding RNA 2728
ENSG0000	8.257759	1.937621	1.04082	1.86163	0.062655	0.237303	RHOT1P1	1E+08	ras homolog family member T1 pseudogene 1
ENSG0000	39.41569	-0.72604	0.38999	-1.8617	0.062646	0.237303	ZNF66	7617	zinc finger protein 66
ENSG0000	143.1334	-0.45765	0.245852	-1.86149	0.062675	0.237303	LAMA5	3911	laminin subunit alpha 5

ENSG0000	278.7299	0.291484	0.156655	1.860681	0.062789	0.237663	NGRN	51335	neugrin	neurite outgrowth associated	
ENSG0000	51.66416	-0.73113	0.392977	-1.86048	0.062817	0.237673	NA	NA	NA		
ENSG0000	114.9469	-0.43577	0.234216	-1.86055	0.062808	0.237673	TFR2	7036	transferrin receptor 2		
ENSG0000	14263.26	0.138676	0.074543	1.860335	0.062838	0.237703	RARA	5914	retinoic acid receptor alpha		
ENSG0000	944.8885	0.183468	0.098653	1.85974	0.062922	0.237807	TMEM183	92703	transmembrane protein 183A		
ENSG0000	438.9606	0.345244	0.185644	1.859708	0.062927	0.237807	SLC9B2	133308	solute carrier family 9 member B2		
ENSG0000	31.56979	-0.8675	0.466478	-1.85969	0.06293	0.237807	NA	NA	NA		
ENSG0000	74.83315	-0.58011	0.311921	-1.85981	0.062913	0.237807	CACNA1G	8913	calcium voltage-gated channel subunit alpha1 G		
ENSG0000	178.8822	-0.39269	0.211141	-1.85985	0.062906	0.237807	ITSN1	6453	intersectin 1		
ENSG0000	967.2211	0.189701	0.102075	1.858453	0.063105	0.23842	PSMB7	5695	proteasome 20S subunit beta 7		
ENSG0000	23.74962	0.973668	0.524031	1.858034	0.063164	0.238594	NA	NA	NA		
ENSG0000	343.4017	0.283552	0.152616	1.857948	0.063176	0.238594	PDCD5	9141	programmed cell death 5		
ENSG0000	336.0324	-0.27057	0.145651	-1.85768	0.063214	0.238686	KIZ	55857	kizuna centrosomal protein		
ENSG0000	2333.786	0.195222	0.105111	1.857292	0.06327	0.238703	RAP1A	5906	RAP1A	member of RAS oncogene family	
ENSG0000	15.30814	1.233697	0.664228	1.857338	0.063263	0.238703	NA	NA	NA		
ENSG0000	907.8551	0.534289	0.287644	1.857465	0.063245	0.238703	SLC7A7	9056	solute carrier family 7 member 7		
ENSG0000	33.84086	0.728752	0.392371	1.857304	0.063268	0.238703	ZNF490	57474	zinc finger protein 490		
ENSG0000	8254.677	0.127185	0.068505	1.856568	0.063373	0.239043	SEC16A	9919	SEC16 hon	endoplasmic reticulum export factor	
ENSG0000	21.08602	-0.93818	0.505366	-1.85643	0.063392	0.239069	WASL-DT	1.16E+08	WASL divergent transcript		
ENSG0000	2787.738	-0.18621	0.100312	-1.85629	0.063412	0.239094	PLD3	23646	phospholipase D family member 3		
ENSG0000	1519.564	0.174773	0.094165	1.856035	0.063449	0.239134	KAT7	11143	lysine acetyltransferase 7		
ENSG0000	7.788266	1.673346	0.901555	1.856067	0.063444	0.239134	NA	NA	NA		
ENSG0000	100.9836	0.526372	0.283626	1.855866	0.063473	0.239177	HLA-DQB2	3120	major hist	class II	DQ beta 2
ENSG0000	53.4896	0.615062	0.331722	1.85415	0.063718	0.239975	MYLK	4638	myosin light chain kinase		
ENSG0000	155.0321	0.425027	0.229225	1.854192	0.063712	0.239975	MZB1	51237	marginal zone B and B1 cell specific protein		
ENSG0000	24.86726	0.862368	0.465112	1.854111	0.063723	0.239975	HSF5	124535	heat shock transcription factor 5		
ENSG0000	16.92143	1.040676	0.561428	1.853622	0.063793	0.240141	NA	NA	NA		
ENSG0000	53.69873	-0.60872	0.32838	-1.85369	0.063783	0.240141	CLEC10A	10462	C-type lectin domain containing 10A		
ENSG0000	10.6056	1.597688	0.861974	1.853522	0.063808	0.240146	KCNJ2-AS1	400617	KCNJ2 antisense RNA 1		
ENSG0000	24140.22	-0.14053	0.075835	-1.85314	0.063862	0.240301	SEMA6B	10501	semaphorin 6B		
ENSG0000	5.986275	-1.92044	1.036504	-1.85281	0.06391	0.240433	PRMT5-DT	1.02E+08	PRMT5 divergent transcript		
ENSG0000	3056.269	0.212392	0.11464	1.852682	0.063928	0.240452	NPIPB5	1E+08	nuclear pore complex interacting protein family member B5		
ENSG0000	102.0421	-0.53119	0.286919	-1.85134	0.06412	0.240638	WDR5B	54554	WD repeat domain 5B		
ENSG0000	12.09028	1.311972	0.708594	1.851514	0.064096	0.240638	LINC0088(	339894	long intergenic non-protein coding RNA 880		
ENSG0000	3836.165	0.145104	0.078343	1.852168	0.064002	0.240638	HNRNPD	3184	heterogeneous nuclear ribonucleoprotein D		
ENSG0000	10.05498	-1.55192	0.837927	-1.85209	0.064013	0.240638	NA	NA	NA		
ENSG0000	2030.269	-0.16418	0.088671	-1.85159	0.064085	0.240638	KDM7A	80853	lysine demethylase 7A		
ENSG0000	3.083277	-3.05747	1.651454	-1.85138	0.064115	0.240638	ACTN3	89	actinin alpha 3		
ENSG0000	5.14048	-2.01813	1.09007	-1.85138	0.064116	0.240638	NA	NA	NA		
ENSG0000	5967.759	-0.12729	0.068744	-1.85168	0.064072	0.240638	GGA2	23062	golgi assoc	gamma ac	ARF binding protein 2
ENSG0000	990.7759	-0.1848	0.099789	-1.85192	0.064037	0.240638	RABEP2	79874	rabaptin	RAB GTPase binding effector protein 2	
ENSG0000	293.151	-0.29891	0.161449	-1.8514	0.064111	0.240638	DHX33	56919	DEAH-box helicase 33		
ENSG0000	42.22578	-0.68105	0.367851	-1.85143	0.064108	0.240638	DSC2	1824	desmocollin 2		
ENSG0000	758.2854	-0.22453	0.121404	-1.84945	0.064393	0.241614	NUDT3	11165	nudix hydrolase 3		
ENSG0000	193.3743	-0.36684	0.198363	-1.84932	0.064411	0.241633	MXD3	83463	MAX dimerization protein 3		
ENSG0000	960.0016	-0.21113	0.114187	-1.84894	0.064466	0.24179	GAB3	139716	GRB2 associated binding protein 3		
ENSG0000	12.35167	1.473391	0.796946	1.848798	0.064487	0.24182	CTRC	11330	chymotrypsin C		
ENSG0000	111.2367	-0.4708	0.25467	-1.84868	0.064504	0.241833	ZNF133	7692	zinc finger protein 133		
ENSG0000	506.1318	-0.22145	0.119831	-1.84805	0.064596	0.24213	TULP3	7289	TUB like protein 3		
ENSG0000	557.7214	0.236296	0.127873	1.847904	0.064616	0.242158	MOAP1	64112	modulator of apoptosis 1		
ENSG0000	434.8991	-0.2525	0.136664	-1.84762	0.064657	0.242263	TDRD7	23424	tudor domain containing 7		
ENSG0000	712.7082	-0.74714	0.404407	-1.84749	0.064676	0.242282	IFIT2	3433	interferon induced protein with tetratricopeptide repeats 2		
ENSG0000	814.9286	-0.20581	0.111472	-1.84628	0.064851	0.242617	VAV3	10451	vav guanine nucleotide exchange factor 3		
ENSG0000	77.58329	0.690568	0.374021	1.846335	0.064844	0.242617	HRH1	3269	histamine receptor H1		
ENSG0000	3.253082	-3.36334	1.821525	-1.84644	0.064828	0.242617	NA	NA	NA		
ENSG0000	30.97393	-0.94625	0.512485	-1.8464	0.064834	0.242617	DDX43	55510	DEAD-box helicase 43		
ENSG0000	236.665	-0.39664	0.214795	-1.84659	0.064807	0.242617	TTC39B	158219	tetratricopeptide repeat domain 39B		
ENSG0000	2194.904	-0.20632	0.111751	-1.84625	0.064856	0.242617	ZC3H13	23091	zinc finger CCCH-type containing 13		
ENSG0000	87.47826	-0.48897	0.264782	-1.8467	0.06479	0.242617	NA	NA	NA		
ENSG0000	14.4233	1.310628	0.710064	1.845789	0.064923	0.242717	NA	NA	NA		
ENSG0000	101.9086	-0.55573	0.301104	-1.84564	0.064944	0.242717	EMBP1	647121	embigin pseudogene 1		
ENSG0000	22.65648	-0.9833	0.532746	-1.84571	0.064934	0.242717	GMDS-DT	1.01E+08	GMDS divergent transcript		
ENSG0000	153.2786	-0.39185	0.212314	-1.84561	0.064949	0.242717	TDRD3	81550	tudor domain containing 3		
ENSG0000	574.6938	0.244179	0.132277	1.845967	0.064897	0.242717	MYL9	10398	myosin light chain 9		
ENSG0000	21.24322	1.170703	0.634435	1.845267	0.064999	0.242855	AKR1C7P	648947	aldo-keto	pseudogene	
ENSG0000	46.65765	-0.64532	0.34979	-1.84489	0.065054	0.243013	AKAP7	9465	A-kinase anchoring protein 7		
ENSG0000	5.357149	-2.46863	1.338165	-1.84479	0.065069	0.243018	LOC10537.	1.05E+08	uncharacterized LOC105375519		
ENSG0000	33.89714	-0.76994	0.417452	-1.84438	0.065128	0.243191	ZNF823	55552	zinc finger protein 823		
ENSG0000	846.0164	-0.19317	0.104788	-1.84341	0.065269	0.243668	WRAP73	49856	WD repea	antisense to TP73	
ENSG0000	71.66337	0.618184	0.335455	1.842824	0.065355	0.243896	ZC3H12A-I	728431	ZC3H12A divergent transcript		
ENSG0000	33.86197	-0.85229	0.462494	-1.84281	0.065356	0.243896	MAP10	54627	microtubule associated protein 10		
ENSG0000	1741.968	0.180428	0.097919	1.842627	0.065383	0.243948	MORC3	23515	MORC family CW-type zinc finger 3		
ENSG0000	12.02168	1.465679	0.795833	1.841691	0.06552	0.24441	LOC10537	1.05E+08	uncharacterized LOC105371899		
ENSG0000	1757.519	0.157996	0.085809	1.841253	0.065584	0.244551	GOLPH3	64083	golgi phosphoprotein 3		
ENSG0000	11477.91	0.174942	0.095012	1.841257	0.065584	0.244551	RPS19	6223	ribosomal protein S19		
ENSG0000	854.839	0.205062	0.111381	1.841078	0.06561	0.244597	GATC	283459	glutamyl-tRNA amidotransferase subunit C		
ENSG0000	5.999708	2.515525	1.366511	1.840838	0.065645	0.244679	CD81-AS1	1.02E+08	CD81 antisense RNA 1		
ENSG0000	78.42692	-0.58561	0.318176	-1.84051	0.065693	0.244807	NA	NA	NA		
ENSG0000	1508.053	0.186806	0.101512	1.840226	0.065735	0.244916	TMA7	51372	translation machinery associated 7 homolog		
ENSG0000	3722.657	-0.13425	0.072965	-1.83996	0.065775	0.244984	SSBP3	23648	single stranded DNA binding protein 3		
ENSG0000	2434.855	0.15108	0.082112	1.83992	0.06578	0.244984	ZNF655	79027	zinc finger protein 655		
ENSG0000	296.6775	-0.32059	0.174303	-1.83926	0.065877	0.245297	SMYD4	114826	SET and MYND domain containing 4		
ENSG0000	171.5888	-0.36165	0.196645	-1.83909	0.065902	0.24534	ACO1	48	aconitase 1		
ENSG0000	116.2738	-0.40656	0.221079	-1.839	0.065915	0.24534	RABEPK	10244	Rab9 effector protein with kelch motifs		
ENSG0000	201.729	-0.37712	0.205092	-1.8388	0.065945	0.245401	TXNRD2	10587	thioredoxin reductase 2		
ENSG0000	3832.404	0.146431	0.079674	1.837887	0.066079	0.245802	CERS2	29956	ceramide synthase 2		
ENSG0000	10.53289	-1.37163	0.746299	-1.83791	0.066075	0.245802	MAP6	4135	microtubule associated protein 6		
ENSG0000	325.0363	0.30853	0.167884	1.837757	0.066098	0.245824	SURF2	6835	surfeit 2		
ENSG0000	5.55598	2.301221	1.25239	1.837464	0.066141	0.245897	PRDM16-D	440556	PRDM16 divergent transcript		
ENSG0000	7.058119	1.827077	0.994357	1.837445	0.066144	0.245897	CATIP	375307	ciliogenesis associated TTC17 interacting protein		
ENSG0000	1351.358	-0.17249	0.093877	-1.83735	0.066158	0.245899	BRF1	2972	BRF1 RNA polymerase III transcription initiation factor subunit		
ENSG0000	1151.901	-0.19191	0.104456	-1.83725	0.066173	0.245904	ADAM15	8751	ADAM metallopeptidase domain 15		
ENSG0000	169.5738	0.384165	0.209181	1.836524	0.06628	0.246254	PTGDS	5730	prostaglandin D2 synthase		
ENSG0000	568.2642	-0.26818	0.146095	-1.83567	0.066406	0.246672	NDUFB7	4713	NADH:ubiquinone oxidoreductase subunit B7		
ENSG0000	36.1974	-0.79501	0.433126	-1.8355	0.066431	0.246715	NA	NA	NA		
ENSG0000	371.9715	-0.28543	0.15554	-1.83507	0.066496	0.246807	ACAD9	28976	acyl-CoA dehydrogenase family member 9		
ENSG0000	461.4284	-0.25553	0.139238	-1.83518	0.066479	0.246807	SARDH	1757	sarcosine dehydrogenase		
ENSG0000	980.5813	-0.19821	0.108007	-1.83515	0.066483	0.246807	AAAS	8086	aladin WD repeat nucleoporin		
ENSG0000	34.64646	-0.78036	0.425356	-1.83461	0.066564	0.247011	FLT3	2322	fms related receptor tyrosine kinase 3		
ENSG0000	902.9008	-0.20247	0.110365	-1.83452	0.066577	0.247011	OCIAD1	54940	OCIA domain containing 1		

ENSG0000	13.37593	-1.25897	0.686382	-1.83421	0.066622	0.247095	ZACN	353174	zinc activated ion channel
ENSG0000	101.8323	-0.4759	0.259463	-1.83418	0.066627	0.247095	ZNF738	148203	zinc finger protein 738
ENSG0000	114.3059	-0.4665	0.254376	-1.83392	0.066666	0.247193	EDAR	10913	ectodysplasin A receptor
ENSG0000	31.42273	-0.78236	0.426664	-1.83368	0.066702	0.247277	SLC4A4	8671	solute carrier family 4 member 4
ENSG0000	4.062888	-2.69877	1.471944	-1.83348	0.066732	0.247325	NA	NA	NA
ENSG0000	57.91901	-0.66485	0.36263	-1.83341	0.066742	0.247325	NA	NA	NA
ENSG0000	3641.282	0.132931	0.072519	1.833059	0.066794	0.247468	HCP5	10866	HLA complex P5
ENSG0000	494.3152	0.248965	0.135851	1.832634	0.066857	0.247653	SYPL1	6856	synaptophysin like 1
ENSG0000	352.1315	0.271901	0.148377	1.832497	0.066877	0.247679	RNF7	9616	ring finger protein 7
ENSG0000	513.0694	0.244395	0.133409	1.831919	0.066964	0.247948	FASTKD5	60493	FAST kinase domains 5
ENSG0000	340.9556	-0.31284	0.170817	-1.83147	0.067031	0.248149	RGL1	23179	ral guanine nucleotide dissociation stimulator like 1
ENSG0000	9.783506	1.779947	0.971936	1.831342	0.06705	0.248168	CENPW	387103	centromere protein W
ENSG0000	14811.27	-0.13292	0.072602	-1.83084	0.067125	0.248397	LENG8	114823	leukocyte receptor cluster member 8
ENSG0000	3827.264	0.161517	0.088228	1.830684	0.067148	0.248432	ATP5MC2	517	ATP synthase membrane subunit c locus 2
ENSG0000	671.5519	-0.1996	0.10905	-1.83034	0.067199	0.24857	TMEM50B	757	transmembrane protein 50B
ENSG0000	308.2693	0.282438	0.154317	1.830248	0.067213	0.248574	NFU1	27247	NFU1 iron-sulfur cluster scaffold
ENSG0000	224.6033	0.320803	0.175311	1.829914	0.067263	0.248709	LYRM4	57128	LYR motif containing 4
ENSG0000	1071.318	-0.19418	0.106123	-1.82977	0.067284	0.248738	D2HGDH	728294	D-2-hydroxyglutarate dehydrogenase
ENSG0000	4.502831	2.280263	1.246314	1.829606	0.067309	0.24878	KRT23	25984	keratin 23
ENSG0000	3094.231	0.18217	0.099581	1.829361	0.067346	0.248866	PSMD2	5708	proteasom non-ATPase 2
ENSG0000	2.708328	3.233136	1.768077	1.828618	0.067457	0.249195	FSCN2	25794	fascin acti retinal
ENSG0000	25.79689	-0.88962	0.486509	-1.82859	0.067461	0.249195	NA	NA	NA
ENSG0000	1099.08	0.195162	0.106737	1.828439	0.067484	0.249228	GID8	54994	GID complex subunit 8 homolog
ENSG0000	6036.852	-0.15201	0.083146	-1.82824	0.067514	0.249291	FNDC3B	64778	fibronectin type III domain containing 3B
ENSG0000	240.4405	-0.33985	0.185917	-1.82798	0.067552	0.249382	RIMKLB	57494	ribosomal modification protein rimK like family member B
ENSG0000	772.809	0.206823	0.113169	1.827555	0.067616	0.249519	EIF4E	1977	eukaryotic translation initiation factor 4E
ENSG0000	3.449745	-2.55482	1.397933	-1.82757	0.067614	0.249519	NA	NA	NA
ENSG0000	4.794023	-2.31966	1.269438	-1.82731	0.067653	0.249604	CCT8P1	644131	chaperonin containing TCP1 subunit 8 pseudogene 1
ENSG0000	2.868445	-2.93532	1.60698	-1.82661	0.067759	0.249845	NA	NA	NA
ENSG0000	250.1341	-0.31494	0.172416	-1.82665	0.067753	0.249845	MRPL42	28977	mitochondrial ribosomal protein L42
ENSG0000	509.8347	-0.25358	0.138821	-1.82666	0.067751	0.249845	VPS36	51028	vacuolar protein sorting 36 homolog
ENSG0000	38.83599	-0.68616	0.375729	-1.82622	0.067817	0.250011	IFT22	64792	intraflagellar transport 22
ENSG0000	1267.325	0.218675	0.11975	1.826092	0.067836	0.250032	F13A1	2162	coagulation factor XIII A chain
ENSG0000	2952.41	0.177664	0.09734	1.825201	0.067971	0.250478	LYPLA2	11313	lysophospholipase 2
ENSG0000	42.14261	0.657908	0.360524	1.824865	0.068021	0.250565	DDX47	51202	DEAD-box helicase 47
ENSG0000	8.318065	1.618169	0.886709	1.824916	0.068014	0.250565	LOC10042	1E+08	SHC binding and spindle associated 1 pseudogene
ENSG0000	369.9351	-0.28828	0.157983	-1.82475	0.068038	0.250577	FBXO41	150726	F-box protein 41
ENSG0000	3280.598	0.168838	0.092545	1.824395	0.068092	0.250727	UBALD1	124402	UBA like domain containing 1
ENSG0000	4419.901	0.15103	0.082793	1.824184	0.068124	0.25079	STK38	11329	serine/threonine kinase 38
ENSG0000	9.628034	-1.66868	0.914794	-1.8241	0.068137	0.25079	TBC1D8B	54885	TBC1 domain family member 8B
ENSG0000	80.64654	-0.51795	0.284009	-1.82372	0.068195	0.250955	FBN2	2201	fibrillin 2
ENSG0000	2111.251	-0.20395	0.111848	-1.82344	0.068236	0.251039	AFF1	4299	ALF transcription elongation factor 1
ENSG0000	2.412921	3.884242	2.130235	1.823386	0.068245	0.251039	NA	NA	NA
ENSG0000	304.9137	-0.26615	0.145995	-1.82303	0.068298	0.251185	DENND6B	414918	DENN domain containing 6B
ENSG0000	119.7901	-0.50795	0.278646	-1.82293	0.068315	0.251196	GIPC3	126326	GIPC PDZ domain containing family member 3
ENSG0000	1211.069	0.208502	0.114388	1.82275	0.068341	0.251244	CARS1	833	cysteinyl-tRNA synthetase 1
ENSG0000	2571.458	0.175718	0.096415	1.822525	0.068375	0.25132	LAPTM4A	9741	lysosomal protein transmembrane 4 alpha
ENSG0000	481.5058	-0.26596	0.145982	-1.8219	0.068471	0.251621	SNAPC2	6618	small nuclear RNA activating complex polypeptide 2
ENSG0000	146.637	0.362602	0.199052	1.821649	0.068508	0.251708	HSBP1L1	440498	heat shock factor binding protein 1 like 1
ENSG0000	1063.6	0.20177	0.110775	1.821435	0.068541	0.251778	TMEM175	84286	transmembrane protein 175
ENSG0000	213.3741	0.340137	0.186795	1.82091	0.06862	0.252021	ABCF2	10061	ATP binding cassette subfamily F member 2
ENSG0000	9.600319	-1.45366	0.798433	-1.82064	0.068661	0.25212	NA	NA	NA
ENSG0000	456.8277	-0.28634	0.157309	-1.82025	0.068721	0.25229	ZNF688	146542	zinc finger protein 688
ENSG0000	393.4547	0.270028	0.148361	1.820072	0.068748	0.252339	OXCT1	5019	3-oxoacid CoA-transferase 1
ENSG0000	26.08453	-1.08421	0.595749	-1.81991	0.068772	0.252379	TTC30B	150737	tetratricopeptide repeat domain 30B
ENSG0000	13.81708	1.192637	0.655406	1.819693	0.068806	0.252451	RIDA	10247	reactive intermediate imine deaminase A homolog
ENSG0000	20.84082	-1.07411	0.590489	-1.81902	0.068908	0.252778	TGM5	9333	transglutaminase 5
ENSG0000	12.40412	-1.65232	0.908408	-1.81892	0.068923	0.252783	NA	NA	NA
ENSG0000	38.81528	0.754604	0.414944	1.81857	0.068977	0.252849	C1orf115	79762	chromosome 1 open reading frame 115
ENSG0000	1007.171	-0.22631	0.124448	-1.81854	0.068982	0.252849	APEH	327	acylaminoacyl-peptide hydrolase
ENSG0000	11.49611	1.597123	0.878248	1.818534	0.068982	0.252849	NA	NA	NA
ENSG0000	2016.482	0.176484	0.097082	1.817883	0.069082	0.253164	MED29	55588	mediator complex subunit 29
ENSG0000	956.8485	-0.20118	0.110717	-1.81707	0.069206	0.25357	DCAF11	80344	DDB1 and CUL4 associated factor 11
ENSG0000	77.55519	0.577424	0.317796	1.816966	0.069222	0.253578	CDT1	81620	chromatin licensing and DNA replication factor 1
ENSG0000	207.4513	-0.34802	0.191564	-1.81673	0.069258	0.25366	TRAF3IP1	26146	TRAF3 interacting protein 1
ENSG0000	161.3093	0.36848	0.202842	1.816587	0.06928	0.253691	TMEM86B	255043	transmembrane protein 86B
ENSG0000	12.56314	-1.39689	0.769111	-1.81624	0.069334	0.253793	TNFSF18	8995	TNF superfamily member 18
ENSG0000	15.44255	-1.15916	0.638223	-1.81623	0.069336	0.253793	TSTD3	1E+08	thiosulfate sulfurtransferase like domain containing 3
ENSG0000	821.1735	0.256573	0.141275	1.816129	0.069351	0.253797	OGFRL1	79627	opioid growth factor receptor like 1
ENSG0000	4.134791	2.175552	1.197965	1.816039	0.069364	0.253798	RPS3AP44	646527	RPS3A pseudogene 44
ENSG0000	19.76716	-1.10467	0.60841	-1.81566	0.069422	0.253958	LOC1053	1.05E+08	uncharacterized LOC105376479
ENSG0000	1188.235	-0.20072	0.110593	-1.81494	0.069534	0.254071	PANK4	55229	pantothenate kinase 4 (inactive)
ENSG0000	5.598195	1.841379	1.014529	1.815008	0.069523	0.254071	NA	NA	NA
ENSG0000	652.5343	0.208393	0.114832	1.814765	0.06956	0.254071	PPM1K	152926	protein ph Mg2+/Mn2+ dependent 1K
ENSG0000	317.771	-0.282	0.155346	-1.81531	0.069476	0.254071	MRE11	4361	MRE11 ho double strand break repair nuclease
ENSG0000	4026.72	0.137152	0.075559	1.815168	0.069498	0.254071	VDR	7421	vitamin D receptor
ENSG0000	1145.786	0.19438	0.107097	1.814986	0.069526	0.254071	SNW1	22938	SNW domain containing 1
ENSG0000	110.5391	-0.4432	0.244228	-1.81471	0.069568	0.254071	DHRS7B	25979	dehydrogenase/reductase 7B
ENSG0000	1792.536	0.172618	0.09511	1.814923	0.069536	0.254071	SS18	6760	SS18 subunit of BAF chromatin remodeling complex
ENSG0000	11.79012	-1.33052	0.733207	-1.81466	0.069576	0.254071	OXT	5020	oxytocin/neurophysin I prepropeptide
ENSG0000	192.7007	-0.35847	0.19756	-1.81451	0.069599	0.254106	EXOG	9941	exo/endonuclease G
ENSG0000	5.012469	-2.44517	1.347703	-1.81433	0.069628	0.254159	STPG3-AS	1E+08	STPG3 antisense RNA 1
ENSG0000	69.07245	-0.56853	0.313489	-1.81354	0.069748	0.25455	SCRN3	79634	secernin 3
ENSG0000	242.2773	-0.29894	0.164887	-1.81298	0.069834	0.254813	MATCAP1	653319	microtubule associated tyrosine carboxypeptidase 1
ENSG0000	758.6066	-0.206	0.113649	-1.81258	0.069897	0.254929	DDI2	84301	DNA damage inducible 1 homolog 2
ENSG0000	329.6336	0.299953	0.165502	1.812379	0.069928	0.254929	OSTC	58505	oligosaccharyltransferase complex non-catalytic subunit
ENSG0000	7241.324	0.544241	0.300299	1.812333	0.069935	0.254929	ACSL1	2180	acyl-CoA synthetase long chain family member 1
ENSG0000	1904.428	0.17122	0.094472	1.812392	0.069926	0.254929	SPARC	6678	secreted protein acidic and cysteine rich
ENSG0000	766.7386	-0.20685	0.114119	-1.81258	0.069897	0.254929	MLLT3	4300	MLLT3 super elongation complex subunit
ENSG0000	904.4206	-0.20927	0.115482	-1.81216	0.069962	0.254978	FAM83G	644815	family with sequence similarity 83 member G
ENSG0000	4392.063	-0.14134	0.078003	-1.81201	0.069984	0.255009	FMNL3	91010	formin like 3
ENSG0000	3.923267	2.46602	1.361257	1.811575	0.070052	0.255206	VSTM2L	128434	V-set and transmembrane domain containing 2 like
ENSG0000	7.898322	1.868193	1.031622	1.810929	0.070152	0.255476	NA	NA	NA
ENSG0000	833.3288	-0.19002	0.104929	-1.81092	0.070154	0.255476	TFCP2	7024	transcription factor CP2
ENSG0000	395.8974	-0.2691	0.148647	-1.81035	0.070242	0.255747	PYCARD	29108	PYD and CARD domain containing
ENSG0000	41.1	0.71608	0.395613	1.810053	0.070288	0.255863	ARG2	384	arginase 2
ENSG0000	2944.257	0.161029	0.088983	1.809668	0.070347	0.25603	LST1	7940	leukocyte specific transcript 1
ENSG0000	334.6978	-0.2935	0.16221	-1.80937	0.070393	0.256146	RAB37	326624	RAB37 member RAS oncogene family
ENSG0000	132.653	0.398633	0.220378	1.808859	0.070473	0.256337	MED7	9443	mediator complex subunit 7
ENSG0000	125.3066	-0.39195	0.21667	-1.80895	0.07046	0.256337	ZNF517	340385	zinc finger protein 517

ENSG0000	912.2013	0.202664	0.112046	1.80876	0.070488	0.256342	UFM1	51569	ubiquitin fold modifier 1
ENSG0000	7475.56	-0.1487	0.082225	-1.80839	0.070546	0.256501	ADGRG1	9289	adhesion G protein-coupled receptor G1
ENSG0000	37.3882	-0.83961	0.464311	-1.80829	0.070562	0.25651	ADAMTS7I	390660	ADAMTS7 pseudogene 1
ENSG0000	5016.133	-0.14605	0.080811	-1.80736	0.070706	0.256984	CELF2	10659	CUGBP Elav-like family member 2
ENSG0000	3567.638	-0.1382	0.076468	-1.80726	0.070722	0.256991	AP2B1	163	adaptor related protein complex 2 subunit beta 1
ENSG0000	5.030131	-2.17044	1.201173	-1.80694	0.070772	0.257123	NA	NA	NA
ENSG0000	11.06624	-1.30741	0.723723	-1.8065	0.07084	0.257217	NA	NA	NA
ENSG0000	1063.883	0.212481	0.117616	1.806572	0.070829	0.257217	RSF1	51773	remodeling and spacing factor 1
ENSG0000	809.0352	0.217175	0.120212	1.806593	0.070826	0.257217	KIR2DL4	3805	killer cell i two Ig domains and long cytoplasmic tail 4
ENSG0000	462.4809	-0.27161	0.150365	-1.80634	0.070865	0.257247	DTHD1	401124	death domain containing 1
ENSG0000	25.46155	1.073167	0.594133	1.806273	0.070876	0.257247	NA	NA	NA
ENSG0000	15095.2	0.116649	0.064605	1.805579	0.070984	0.25754	CD8A	925	CD8a molecule
ENSG0000	979.5546	-0.18236	0.100996	-1.80563	0.070976	0.25754	UNKL	64718	unk like zinc finger
ENSG0000	436.2675	0.229562	0.127153	1.8054	0.071012	0.257591	ERH	2079	ERH mRNA splicing and mitosis factor
ENSG0000	315.7588	0.266172	0.147463	1.805002	0.071074	0.257766	TIMM17A	10440	translocase of inner mitochondrial membrane 17A
ENSG0000	6.119214	1.933268	1.07115	1.804852	0.071098	0.257801	NA	NA	NA
ENSG0000	119.9286	-0.43024	0.238422	-1.80451	0.071151	0.257915	PLK1	5347	polo like kinase 1
ENSG0000	21.66383	-0.8882	0.492226	-1.80445	0.071161	0.257915	DNAJC28	54943	DnaJ heat shock protein family (Hsp40) member C28
ENSG0000	181.4709	-0.34251	0.189819	-1.80438	0.071171	0.257915	MID2	11043	midline 2
ENSG0000	238.2255	0.332986	0.18459	1.803928	0.071243	0.258124	TNFRSF21	27242	TNF receptor superfamily member 21
ENSG0000	4.760186	-2.2689	1.257816	-1.80384	0.071257	0.258125	NA	NA	NA
ENSG0000	344.4498	-0.28688	0.159058	-1.8036	0.071294	0.258209	C1orf216	127703	chromosome 1 open reading frame 216
ENSG0000	162.7509	-0.38592	0.214004	-1.80334	0.071334	0.258278	MIGA1	374986	mitoguardin 1
ENSG0000	244.8567	-0.3035	0.1683	-1.8033	0.071341	0.258278	AGAP4	119016	ArfGAP wi ankyrin repeat and PH domain 4
ENSG0000	2737.258	0.199852	0.110843	1.80301	0.071387	0.258394	C19orf53	28974	chromosome 19 open reading frame 53
ENSG0000	5.532461	-2.08953	1.158982	-1.8029	0.071403	0.258404	NA	NA	NA
ENSG0000	299.3628	0.281461	0.156148	1.802526	0.071463	0.258568	COX6C	1345	cytochrome c oxidase subunit 6C
ENSG0000	2.367381	3.868122	2.146305	1.802224	0.07151	0.258689	SH3TC2-D1	255187	SH3TC2 divergent transcript
ENSG0000	177.0296	-0.35822	0.198791	-1.80197	0.07155	0.258733	STAG3L5P	1.02E+08	stromal antigen 3-like 5 pseudogene
ENSG0000	5.134772	-2.13016	1.182105	-1.80201	0.071544	0.258733	SCARA5	286133	scavenger receptor class A member 5
ENSG0000	5854.147	-0.3656	0.202925	-1.80164	0.071602	0.25882	STAT1	6772	signal transducer and activator of transcription 1
ENSG0000	52.99449	-0.65246	0.362144	-1.80167	0.071598	0.25882	NA	NA	NA
ENSG0000	56.79789	0.545605	0.302883	1.801371	0.071644	0.258922	GMNN	51053	geminin DNA replication inhibitor
ENSG0000	4.385713	2.320544	1.288559	1.800883	0.071721	0.259079	SPATA32	124783	spermatogenesis associated 32
ENSG0000	29456.08	0.156457	0.086877	1.800902	0.071718	0.259079	CD7	924	CD7 molecule
ENSG0000	559.5169	0.238781	0.132595	1.80083	0.07173	0.259079	mei-01	150365	meiotic double-stranded break formation protein 1
ENSG0000	4.476353	1.982239	1.100843	1.800656	0.071757	0.259128	NA	NA	NA
ENSG0000	548.3578	0.23345	0.129687	1.800097	0.071845	0.259366	ADI1	55256	acireductone dioxygenase 1
ENSG0000	2075.747	0.155208	0.086225	1.800033	0.071855	0.259366	LTF	4057	lactotransferrin
ENSG0000	838.7579	-0.18381	0.102117	-1.79997	0.071865	0.259366	MCM3	4172	minichromosome maintenance complex component 3
ENSG0000	7.374363	1.471202	0.81752	1.799591	0.071925	0.259482	EFCC1	79825	EF-hand and coiled-coil domain containing 1
ENSG0000	845.4997	0.223463	0.12417	1.799646	0.071916	0.259482	CXCR5	643	C-X-C motif chemokine receptor 5
ENSG0000	94.73206	-0.46361	0.257656	-1.79933	0.071967	0.259532	ANKRD36	375248	ankyrin repeat domain 36
ENSG0000	10.11252	-1.43112	0.79533	-1.7994	0.071955	0.259532	NA	NA	NA
ENSG0000	1288.432	-0.20916	0.116252	-1.79922	0.071984	0.259542	NPRL3	8131	NPR3 like GATOR1 complex subunit
ENSG0000	1005.447	0.232429	0.129192	1.799101	0.072003	0.25956	TRAP1	10131	TNF receptor associated protein 1
ENSG0000	10820.41	0.12724	0.070731	1.798936	0.072029	0.259603	CAPZB	832	capping actin protein of muscle Z-line subunit beta
ENSG0000	1193.825	0.175709	0.097679	1.798844	0.072043	0.259606	GPR107	57720	G protein-coupled receptor 107
ENSG0000	8.594155	1.511187	0.840148	1.798715	0.072064	0.259628	IGSF22	283284	immunoglobulin superfamily member 22
ENSG0000	16110.78	0.131131	0.072909	1.798572	0.072086	0.25966	RPSA	3921	ribosomal protein SA
ENSG0000	697.365	-0.20624	0.114708	-1.79798	0.07218	0.259933	MAP3K4	4216	mitogen-activated protein kinase kinase kinase 4
ENSG0000	5.127299	-2.14319	1.192042	-1.79791	0.07219	0.259933	PXDNL	137902	peroxidasin like
ENSG0000	1262.584	-0.20396	0.113451	-1.79776	0.072216	0.25995	MAN1A1	4121	mannosidase alpha class 1A member 1
ENSG0000	25.29201	-1.02671	0.571121	-1.79771	0.072223	0.25995	LINC03014	1.01E+08	long intergenic non-protein coding RNA 3014
ENSG0000	1552.936	-0.1744	0.097032	-1.7973	0.072289	0.260095	NDUFS8	4728	NADH:ubiquinone oxidoreductase core subunit S8
ENSG0000	11953.12	0.136407	0.075896	1.797278	0.072291	0.260095	ILF3	3609	interleukin enhancer binding factor 3
ENSG0000	117.9913	-0.42958	0.239056	-1.79697	0.072341	0.260165	MSH3	4437	mutS homolog 3
ENSG0000	22.94496	-0.9363	0.521068	-1.79689	0.072353	0.260165	GNG8	94235	G protein subunit gamma 8
ENSG0000	428.0796	0.267474	0.14884	1.797064	0.072325	0.260165	SLC6A8	6535	solute carrier family 6 member 8
ENSG0000	1572.586	0.174484	0.09712	1.796584	0.072402	0.26029	KIAA1191	57179	KIAA1191
ENSG0000	981.8447	0.1838	0.102315	1.796415	0.072429	0.260301	TRABD2A	129293	TraB domain containing 2A
ENSG0000	3.328011	3.019264	1.680743	1.796387	0.072433	0.260301	NA	NA	NA
ENSG0000	253.9474	0.284474	0.158378	1.796169	0.072468	0.260375	LOC10537	1.05E+08	uncharacterized LOC105372401
ENSG0000	458.4776	-0.2893	0.161077	-1.79607	0.072484	0.260384	DCAF6	55827	DDB1 and CUL4 associated factor 6
ENSG0000	44.22619	0.712725	0.396901	1.795727	0.072538	0.260527	HSPE1	3336	heat shock protein family E (Hsp10) member 1
ENSG0000	6818.689	0.352175	0.196246	1.794558	0.072724	0.261145	BRI3	25798	brain protein I3
ENSG0000	21.79026	1.14577	0.639255	1.792353	0.073076	0.262359	MIR5195	1.01E+08	microRNA 5195
ENSG0000	25.63495	-0.92008	0.513451	-1.79196	0.07314	0.262536	LOC10192	1.02E+08	uncharacterized LOC101929667
ENSG0000	1078.231	-0.18037	0.100667	-1.79171	0.073179	0.262576	AKT3	10000	AKT serine/threonine kinase 3
ENSG0000	46.12549	0.755322	0.421548	1.791783	0.073168	0.262576	ENPP2	5168	ectonucleotide pyrophosphatase/phosphodiesterase 2
ENSG0000	31.95813	0.803106	0.44827	1.791568	0.073202	0.262608	APOM	55937	apolipoprotein M
ENSG0000	1157.339	0.191407	0.106852	1.791324	0.073241	0.262697	AP1S2	8905	adaptor related protein complex 1 subunit sigma 2
ENSG0000	3.023728	3.486873	1.946979	1.790915	0.073307	0.262882	NA	NA	NA
ENSG0000	1352.559	0.164572	0.091909	1.79059	0.073359	0.263018	PSMD13	5719	proteasom non-ATPase 13
ENSG0000	107.3358	-0.45201	0.252467	-1.79038	0.073393	0.263089	ERBB2	2064	erb-b2 receptor tyrosine kinase 2
ENSG0000	981.6817	-0.18244	0.101923	-1.78997	0.073459	0.263276	TAF1	6872	TATA-box binding protein associated factor 1
ENSG0000	206.968	-0.37502	0.20959	-1.78931	0.073564	0.2636	BLNK	29760	B cell linker
ENSG0000	78.77269	-0.57066	0.319106	-1.7883	0.073727	0.263995	NAPEPLD	222236	N-acyl phosphatidylethanolamine phospholipase D
ENSG0000	2387.403	-0.17764	0.099322	-1.78848	0.073698	0.263995	ZNF710	374655	zinc finger protein 710
ENSG0000	6.873519	1.739908	0.973	1.788189	0.073746	0.263995	NA	NA	NA
ENSG0000	94.14659	-0.46563	0.260376	-1.7883	0.073727	0.263995	DUS2	54920	dihydrouridine synthase 2
ENSG0000	4.570694	-2.50303	1.399718	-1.78824	0.073737	0.263995	ASIP	434	agouti signaling protein
ENSG0000	6.1123	-2.2173	1.240289	-1.78773	0.073819	0.264208	SAMD15	161394	sterile alpha motif domain containing 15
ENSG0000	272.8641	-0.29301	0.163908	-1.78763	0.073836	0.264217	ZNF611	81856	zinc finger protein 611
ENSG0000	4.865106	-2.7882	1.559919	-1.7874	0.073873	0.264241	PPT2-EGFL	1.01E+08	PPT2-EGFL8 readthrough (NMD candidate)
ENSG0000	2210.191	0.167146	0.093517	1.787321	0.073886	0.264241	TXNDC11	51061	thioredoxin domain containing 11
ENSG0000	510.1896	-0.2371	0.132652	-1.78737	0.073877	0.264241	RINL	126432	Ras and Rab interactor like
ENSG0000	2956.301	0.153726	0.086015	1.787188	0.073907	0.264268	SOS2	6655	SOS Ras/Rho guanine nucleotide exchange factor 2
ENSG0000	7.972044	1.943229	1.087626	1.78667	0.073991	0.264516	PTGR1	22949	prostaglandin reductase 1
ENSG0000	2545.743	-0.17318	0.096942	-1.78644	0.074028	0.264597	ITPR3	3710	inositol 1 4 5-trisphosphate receptor type 3
ENSG0000	362.5927	-0.28791	0.161241	-1.7856	0.074165	0.264988	SLC27A1	376497	solute carrier family 27 member 1
ENSG0000	417.8743	-0.22652	0.126859	-1.78559	0.074166	0.264988	ATP7A	538	ATPase copper transporting alpha
ENSG0000	5.105781	-2.1385	1.197923	-1.78517	0.074233	0.265094	NA	NA	NA
ENSG0000	2173.525	0.181952	0.10193	1.785065	0.074251	0.265094	ATP11B	23200	ATPase phospholipid transporting 11B (putative)
ENSG0000	5287.488	0.131664	0.073756	1.785127	0.074241	0.265094	KPNB1	3837	karyopherin subunit beta 1
ENSG0000	51.21601	-0.67815	0.379905	-1.78505	0.074253	0.265094	MBTPS2	51360	membran site 2
ENSG0000	268.1809	-0.29667	0.166221	-1.7848	0.074294	0.265191	RHBDD1	84236	rhomboid domain containing 1
ENSG0000	4.332304	-2.54609	1.42678	-1.7845	0.074343	0.265263	LOC10537	1.05E+08	uncharacterized LOC105374736
ENSG0000	1746.388	0.1622	0.090891	1.78455	0.074334	0.265263	MLX	6945	MAX dimerization protein MLX
ENSG0000	24.15819	-0.95492	0.535368	-1.78367	0.074477	0.265475	EFNA5	1946	ephrin A5

ENSG0000	74.25569	-0.52698	0.295455	-1.78362	0.074486	0.265475	NA	NA	NA
ENSG0000	616.2023	-0.2063	0.115665	-1.78362	0.074485	0.265475	TRIM35	23087	tripartite motif containing 35
ENSG0000	215.4751	0.324282	0.181781	1.783917	0.074437	0.265475	MED6	10001	mediator complex subunit 6
ENSG0000	1663.334	0.171486	0.096146	1.783604	0.074488	0.265475	CSNK2A1	1457	casein kinase 2 alpha 1
ENSG0000	39.20342	-0.71494	0.400808	-1.78376	0.074463	0.265475	NA	NA	NA
ENSG0000	2141.365	-0.75899	0.425611	-1.78329	0.074539	0.265604	SERPING1	710	serpin family G member 1
ENSG0000	1630.146	-0.15517	0.087018	-1.78316	0.07456	0.265629	MHENCR	1.01E+08	melanoma highly expressed competing endogenous lncRNA for miR-425 and miR-489
ENSG0000	2413.535	0.170178	0.095459	1.782727	0.074631	0.265778	TMEM50A	23585	transmembrane protein 50A
ENSG0000	315.4132	-0.28657	0.160754	-1.78264	0.074645	0.265778	NPL	80896	N-acetylneuraminate pyruvate lyase
ENSG0000	109594.2	0.129657	0.072727	1.782795	0.07462	0.265778	GAPDH	2597	glyceraldehyde-3-phosphate dehydrogenase
ENSG0000	14.13479	1.445344	0.810958	1.782267	0.074706	0.265944	NA	NA	NA
ENSG0000	604.5835	0.236259	0.132588	1.781895	0.074766	0.266109	MICB	4277	MHC class I polypeptide-related sequence B
ENSG0000	33.99768	-0.83685	0.46967	-1.78177	0.074786	0.266119	LINC00511	400619	long intergenic non-protein coding RNA 511
ENSG0000	100.9156	-0.43599	0.244706	-1.7817	0.074798	0.266119	APOBEC3H	164668	apolipoprotein B mRNA editing enzyme catalytic subunit 3H
ENSG0000	69.37949	0.534131	0.299837	1.781403	0.074847	0.26619	BTLA	151888	B and T lymphocyte associated
ENSG0000	158.0156	-0.42625	0.239271	-1.78146	0.074838	0.26619	DHX35	60625	DEAH-box helicase 35
ENSG0000	30.86438	-0.81051	0.455067	-1.78108	0.0749	0.266327	NA	NA	NA
ENSG0000	8.097809	-1.75426	0.985194	-1.78063	0.074974	0.266534	ILDR1	286676	immunoglobulin like domain containing receptor 1
ENSG0000	45.13451	-0.66775	0.375026	-1.78055	0.074986	0.266534	NA	NA	NA
ENSG0000	141.1256	0.387994	0.217922	1.780427	0.075006	0.266553	CKS1B	1163	CDC28 protein kinase regulatory subunit 1B
ENSG0000	42.74172	-0.71362	0.400933	-1.77989	0.075094	0.266815	GPAM	57678	glycerol-3- mitochondrial
ENSG0000	254.4391	0.300146	0.168698	1.779198	0.075207	0.267114	MARCHF3	115123	membrane associated ring-CH-type finger 3
ENSG0000	15.85857	-1.4826	0.833288	-1.77921	0.075205	0.267114	ARHGAP11	89839	Rho GTPase activating protein 11B
ENSG0000	2419.453	0.161953	0.091032	1.779087	0.075225	0.267127	TARDBP	23435	TAR DNA binding protein
ENSG0000	4.244278	2.413227	1.356547	1.778948	0.075248	0.267157	LINC02274	1.01E+08	long intergenic non-protein coding RNA 2274
ENSG0000	1574.824	-0.17872	0.100487	-1.77857	0.07531	0.267326	ZNF106	64397	zinc finger protein 106
ENSG0000	248.8047	0.294161	0.165402	1.778459	0.075329	0.26734	INTS12	57117	integrator complex subunit 12
ENSG0000	625.5719	0.218192	0.122721	1.77795	0.075412	0.267387	C4orf3	401152	chromosome 4 open reading frame 3
ENSG0000	46.97193	-0.72658	0.408626	-1.77811	0.075386	0.267387	MAD2L1	4085	mitotic arrest deficient 2 like 1
ENSG0000	246.4733	-0.31445	0.176851	-1.77807	0.075392	0.267387	FOXRED1	55572	FAD dependent oxidoreductase domain containing 1
ENSG0000	3904.996	0.154087	0.08667	1.777857	0.075427	0.267387	FZR1	51343	fizzy and cell division cycle 20 related 1
ENSG0000	162.312	-0.39149	0.220206	-1.77785	0.075428	0.267387	EBF4	57593	EBF family member 4
ENSG0000	78.34619	0.527807	0.296823	1.778188	0.075373	0.267387	NA	NA	NA
ENSG0000	21.43835	0.95559	0.537635	1.777393	0.075504	0.267522	IL4	3565	interleukin 4
ENSG0000	259.7261	-0.36285	0.204149	-1.77736	0.075508	0.267522	SNTB1	6641	syntrophin beta 1
ENSG0000	3.432136	-2.69913	1.518624	-1.77736	0.07551	0.267522	SERHL2	253190	serine hydrolase like 2
ENSG0000	252.7583	0.296339	0.166806	1.776548	0.075643	0.267942	TXNDC9	10190	thioredoxin domain containing 9
ENSG0000	2.658708	3.547816	1.997129	1.776458	0.075657	0.267943	RAET1L	154064	retinoic acid early transcript 1L
ENSG0000	523.8546	0.210514	0.118532	1.776005	0.075732	0.268156	SFT2D2	375035	SFT2 domain containing 2
ENSG0000	258.815	-0.31856	0.179428	-1.7754	0.075831	0.268456	MCM9	254394	minichromosome maintenance 9 homologous recombination repair factor
ENSG0000	885.9105	-0.21874	0.12322	-1.77521	0.075864	0.26852	POLR1A	25885	RNA polymerase I subunit A
ENSG0000	486.3282	-0.25661	0.144618	-1.77439	0.075999	0.268947	MZF1	7593	myeloid zinc finger 1
ENSG0000	15.71095	-1.04944	0.591494	-1.77422	0.076027	0.268995	NA	NA	NA
ENSG0000	6.980928	-1.92453	1.084855	-1.774	0.076063	0.269071	NA	NA	NA
ENSG0000	21.96683	-1.08146	0.609798	-1.77348	0.076149	0.269254	LINC01134	1E+08	long intergenic non-protein coding RNA 1134
ENSG0000	905.2774	-0.17267	0.097371	-1.77337	0.076168	0.269254	ATXN7L2	127002	ataxin 7 like 2
ENSG0000	6.777725	1.902371	1.072868	1.773163	0.076202	0.269254	NA	NA	NA
ENSG0000	439.8416	-0.26147	0.147449	-1.77326	0.076185	0.269254	MEPCE	56257	methylphosphate capping enzyme
ENSG0000	5408.256	-0.1666	0.093942	-1.77339	0.076164	0.269254	BIN2	51411	bridging integrator 2
ENSG0000	77.36225	-0.48635	0.274281	-1.77317	0.076201	0.269254	MIR9-3HG	254559	MIR9-3 host gene
ENSG0000	27.90828	0.792791	0.447255	1.772568	0.0763	0.269551	NA	NA	NA
ENSG0000	8.523751	-1.82281	1.028407	-1.77246	0.076319	0.269564	NA	NA	NA
ENSG0000	9.186949	-1.52524	0.860693	-1.7721	0.076377	0.26972	CFAP61	26074	cilia and flagella associated protein 61
ENSG0000	43.98275	0.858952	0.484867	1.771522	0.076474	0.269958	GRIK4	2900	glutamate ionotropic receptor kainate type subunit 4
ENSG0000	225.2717	-0.30946	0.174681	-1.77155	0.076469	0.269958	AP4E1	23431	adaptor related protein complex 4 subunit epsilon 1
ENSG0000	1162.783	0.174825	0.098692	1.771426	0.07649	0.269963	GGNBP2	79893	gametogenetin binding protein 2
ENSG0000	14.39726	1.32929	0.750585	1.771006	0.07656	0.270056	NA	NA	NA
ENSG0000	2.848	-3.25488	1.837827	-1.77105	0.076553	0.270056	RPS10P1	394255	ribosomal protein S10 pseudogene 1
ENSG0000	921.2941	0.193675	0.109351	1.771135	0.076538	0.270056	CCDC9	26093	coiled-coil domain containing 9
ENSG0000	8007.206	0.126866	0.071639	1.770893	0.076579	0.270071	CSDE1	7812	cold shock domain containing E1
ENSG0000	8.040367	1.61554	0.912809	1.769857	0.076751	0.270628	RPL23AP5	1E+08	ribosomal protein L23a pseudogene 51
ENSG0000	342.1155	-0.24429	0.138038	-1.76972	0.076773	0.270654	PUSL1	126789	pseudouridine synthase like 1
ENSG0000	14.74751	-1.36138	0.769429	-1.76934	0.076838	0.270831	NA	NA	NA
ENSG0000	9.037907	-1.49077	0.842604	-1.76924	0.076854	0.270838	NA	NA	NA
ENSG0000	3976.907	0.169869	0.096025	1.76901	0.076892	0.270919	ARHGAP31	57514	Rho GTPase activating protein 31
ENSG0000	142.7608	-0.37007	0.209232	-1.76872	0.076941	0.270935	LTV1	84946	LTV1 ribosome biogenesis factor
ENSG0000	618.7358	-0.22507	0.127251	-1.76872	0.07694	0.270935	MAP4K5	11183	mitogen-activated protein kinase kinase kinase kinase 5
ENSG0000	238.0149	-0.29638	0.167556	-1.76884	0.07692	0.270935	KAT14	57325	lysine acetyltransferase 14
ENSG0000	324.7359	-0.25017	0.141498	-1.76802	0.077058	0.271296	PPIP5K2	23262	diphosphoinositol pentakisphosphate kinase 2
ENSG0000	121.0823	-0.39585	0.223965	-1.76745	0.077153	0.271529	ZNF354C	30832	zinc finger protein 354C
ENSG0000	4196.614	0.569977	0.32247	1.767537	0.077138	0.271529	EGR3	1960	early growth response 3
ENSG0000	241.5666	-0.32636	0.184683	-1.76712	0.077208	0.271647	TRMT44	152992	tRNA methyltransferase 44 homolog
ENSG0000	1448.496	-0.15882	0.08988	-1.76707	0.077216	0.271647	FHIP1B	84067	FHF complex subunit HOOK interacting protein 1B
ENSG0000	1859.542	-0.16425	0.092966	-1.76675	0.07727	0.271784	FKBP15	23307	FKBP prolyl isomerase family member 15
ENSG0000	5.608845	-1.92039	1.087241	-1.7663	0.077346	0.271978	LINC01547	84536	long intergenic non-protein coding RNA 1547
ENSG0000	41.88995	-0.7131	0.403735	-1.76625	0.077354	0.271978	NA	NA	NA
ENSG0000	1058.675	0.197622	0.111934	1.765529	0.077475	0.272352	LEMD3	23592	LEM domain containing 3
ENSG0000	14170.31	0.116243	0.065847	1.765356	0.077504	0.27237	JAK1	3716	Janus kinase 1
ENSG0000	53.01777	-0.57706	0.3269	-1.76524	0.077524	0.27237	NDUFB2-A	1E+08	NDUFB2 antisense RNA 1
ENSG0000	3.495696	-2.83491	1.605929	-1.76528	0.077517	0.27237	FGF13	2258	fibroblast growth factor 13
ENSG0000	12.69365	-1.35008	0.764865	-1.76512	0.077544	0.272387	ACTR3C	653857	actin related protein 3C
ENSG0000	10.08441	1.966154	1.113994	1.76496	0.077571	0.27243	LOC12490	1.25E+08	uncharacterized LOC124907871
ENSG0000	189.3627	-0.36587	0.207315	-1.76482	0.077594	0.272461	PUS7L	83448	pseudouridine synthase 7 like
ENSG0000	4032.088	0.136425	0.077327	1.764255	0.077689	0.272743	N4BP1	9683	NEDD4 binding protein 1
ENSG0000	444.2509	-0.2212	0.125396	-1.76402	0.077728	0.272828	COG2	22796	component of oligomeric golgi complex 2
ENSG0000	7022.721	0.132146	0.07492	1.763832	0.07776	0.272889	TSC22D4	81628	TSC22 domain family member 4
ENSG0000	25.84274	0.95906	0.543817	1.763572	0.077804	0.27294	SUGT1P4-S	1E+08	SUGT1P4-STRA6LP readthrough
ENSG0000	1214.235	0.189517	0.107461	1.763594	0.0778	0.27294	EIF1AD	84285	eukaryotic translation initiation factor 1A domain containing
ENSG0000	19.18113	1.00427	0.569527	1.763342	0.077843	0.273024	NA	NA	NA
ENSG0000	865.1032	-0.19801	0.11232	-1.76291	0.077916	0.273149	LARS1	51520	leucyl-tRNA synthetase 1
ENSG0000	2843.658	0.140754	0.079837	1.763028	0.077896	0.273149	DTX2	113878	deltex E3 ubiquitin ligase 2
ENSG0000	230.689	0.284044	0.161134	1.762782	0.077937	0.273149	MRPL17	63875	mitochondrial ribosomal protein L17
ENSG0000	2.758512	-2.78611	1.58046	-1.76285	0.077927	0.273149	TAS2R31	259290	taste 2 receptor member 31
ENSG0000	104.5897	-0.45621	0.25882	-1.76265	0.077959	0.273174	BAG2	9532	BAG cochaperone 2
ENSG0000	2065.905	0.164099	0.093104	1.762535	0.077979	0.273191	F2R	2149	coagulation factor II thrombin receptor
ENSG0000	8.223002	1.801674	1.022686	1.761708	0.078119	0.273629	CCL18	6362	C-C motif chemokine ligand 18
ENSG0000	52828.45	0.126362	0.071743	1.76131	0.078186	0.27371	SH3BGRL3	83442	SH3 domain binding glutamate rich protein like 3
ENSG0000	1049.746	-0.19237	0.10921	-1.76147	0.078159	0.27371	ZNF318	24149	zinc finger protein 318
ENSG0000	15.49669	1.128207	0.640524	1.761381	0.078174	0.27371	NA	NA	NA
ENSG0000	11.15177	1.43622	0.815579	1.760981	0.078242	0.27382	DRC7	84229	dynein regulatory complex subunit 7

ENSG0000	718.5721	-0.22536	0.127977	-1.76095	0.078247	0.27382	PIK3C3	5289	phosphatidylinositol 3-kinase catalytic subunit type 3
ENSG0000	18.64373	0.997866	0.566718	1.76078	0.078276	0.273869	RNF175	285533	ring finger protein 175
ENSG0000	7.457322	-1.71454	0.973936	-1.76043	0.078335	0.274026	NA	NA	NA
ENSG0000	818.1725	0.189028	0.107388	1.760231	0.078369	0.274091	NA	NA	NA
ENSG0000	16.91946	1.014695	0.576612	1.759754	0.07845	0.274322	DSTNP2	171220	DSTN pseudogene 2
ENSG0000	7.763646	-1.68822	0.95947	-1.75953	0.078487	0.2744	RN7SKP16	1.06E+08	RN7SK pseudogene 16
ENSG0000	954.1415	0.196717	0.111822	1.759203	0.078543	0.274546	CHCHD10	400916	coiled-coil-helix-coiled-coil-helix domain containing 10
ENSG0000	341.0375	-0.23811	0.135387	-1.75872	0.078625	0.274781	ECI1	1632	enoyl-CoA delta isomerase 1
ENSG0000	229.3695	0.295458	0.168007	1.758607	0.078644	0.274796	PNO1	56902	partner of NOB1 homolog
ENSG0000	857.8392	0.187352	0.106575	1.757927	0.07876	0.275096	DAD1	1603	defender against cell death 1
ENSG0000	15.51611	1.00565	0.572062	1.757941	0.078758	0.275096	MPP3	4356	MAGUK p55 scaffold protein 3
ENSG0000	24.67032	-0.87945	0.500428	-1.7574	0.078849	0.275357	PANO1	1.02E+08	proapoptotic nucleolar protein 1
ENSG0000	124.2975	-0.4787	0.272415	-1.75723	0.078878	0.275406	TNS2	23371	tensin 2
ENSG0000	3377.769	0.172367	0.098101	1.757035	0.078912	0.275471	PIAS1	8554	protein inhibitor of activated STAT 1
ENSG0000	275.8686	-0.30426	0.173197	-1.75676	0.07896	0.275586	PDGFRB	5159	platelet derived growth factor receptor beta
ENSG0000	1956.602	-0.2086	0.118756	-1.75653	0.078997	0.275666	GRAP	10750	GRB2 related adaptor protein
ENSG0000	215.849	-0.35075	0.199706	-1.75634	0.07903	0.275675	TYW1	55253	tRNA-yW synthesizing protein 1 homolog
ENSG0000	227.3969	-0.32162	0.183114	-1.75637	0.079025	0.275675	NA	NA	NA
ENSG0000	5.520026	2.150326	1.224893	1.755521	0.07917	0.276113	NA	NA	NA
ENSG0000	1853.186	0.150312	0.085635	1.755257	0.079215	0.276163	STRIP1	85369	striatin interacting protein 1
ENSG0000	649.9694	-0.2151	0.122546	-1.75523	0.07922	0.276163	ARMH3	79591	armadillo like helical domain containing 3
ENSG0000	45.55939	-0.75344	0.429265	-1.75518	0.079229	0.276163	ZNF846	162993	zinc finger protein 846
ENSG0000	6.345804	-1.8521	1.055311	-1.75503	0.079255	0.276201	NA	NA	NA
ENSG0000	126.1262	-0.42676	0.243223	-1.7546	0.079327	0.276401	DGKH	160851	diacylglycerol kinase eta
ENSG0000	3.307095	-2.8115	1.602611	-1.75432	0.079375	0.276515	NA	NA	NA
ENSG0000	24.78302	-1.16996	0.666944	-1.75421	0.079394	0.27653	PDCD1LG2	80380	programmed cell death 1 ligand 2
ENSG0000	10854.24	0.145989	0.083227	1.754091	0.079415	0.276551	ANKRD11	29123	ankyrin repeat domain containing 11
ENSG0000	7.16586	1.707183	0.97356	1.753547	0.079508	0.276699	CPNE9	151835	copine family member 9
ENSG0000	4032.203	0.16201	0.092392	1.753494	0.079517	0.276699	SKIL	6498	SKI like proto-oncogene
ENSG0000	23.71842	1.029846	0.587265	1.753629	0.079494	0.276699	ATP6V1G2	534	ATPase H+ transporting V1 subunit G2
ENSG0000	6.393795	-1.93861	1.105491	-1.75362	0.079496	0.276699	NA	NA	NA
ENSG0000	291.803	-0.29377	0.167595	-1.75288	0.079622	0.27687	POLK	51426	DNA polymerase kappa
ENSG0000	6966.001	0.13184	0.075214	1.75286	0.079626	0.27687	CANX	821	calnexin
ENSG0000	1009.574	0.187596	0.107019	1.752918	0.079616	0.27687	KDELR2	11014	KDEL endoplasmic reticulum protein retention receptor 2
ENSG0000	10.99007	1.344426	0.766903	1.75306	0.079592	0.27687	NA	NA	NA
ENSG0000	1692.149	-1.11004	0.633383	-1.75256	0.079677	0.276995	ATP1B2	482	ATPase Na+/K+ transporting subunit beta 2
ENSG0000	246.1383	0.372942	0.212879	1.751891	0.079793	0.277345	TMEM70	54968	transmembrane protein 70
ENSG0000	434.8714	-0.23509	0.134242	-1.75125	0.079903	0.277661	UROD	7389	uroporphyrinogen decarboxylase
ENSG0000	13.34063	1.223188	0.69849	1.751189	0.079913	0.277661	NDUFV2	4729	NADH:ubiquinone oxidoreductase core subunit V2
ENSG0000	2604.912	0.180846	0.103284	1.750966	0.079952	0.277742	RASGRP1	10125	RAS guanyl releasing protein 1
ENSG0000	4.684573	-2.59129	1.480048	-1.75081	0.079978	0.277781	SLC13A3	64849	solute carrier family 13 member 3
ENSG0000	1171.198	0.16585	0.094737	1.750637	0.080008	0.277835	ZDHHC6	64429	zinc finger DHHC-type palmitoyltransferase 6
ENSG0000	227.9094	0.297651	0.170056	1.750307	0.080065	0.277978	MARS2	92935	methionyl mitochondrial
ENSG0000	1278.601	-0.17096	0.097677	-1.75022	0.08008	0.277978	ARL2BP	23568	ADP ribosylation factor like GTPase 2 binding protein
ENSG0000	1330.96	0.20103	0.114871	1.750053	0.080109	0.278029	RB1CC1	9821	RB1 inducible coiled-coil 1
ENSG0000	3988.254	0.177977	0.101715	1.749755	0.080161	0.278155	NR1H2	7376	nuclear receptor subfamily 1 group H member 2
ENSG0000	4.549641	2.203899	1.259866	1.749313	0.080237	0.278368	NA	NA	NA
ENSG0000	102.9032	0.463475	0.264989	1.749036	0.080285	0.278482	DYRK3	8444	dual specificity tyrosine phosphorylation regulated kinase 3
ENSG0000	71.16044	-0.52423	0.299766	-1.7488	0.080326	0.278571	MAP9	79884	microtubule associated protein 9
ENSG0000	10.24213	-1.52314	0.871175	-1.74837	0.0804	0.278777	LINC01504	1.01E+08	long intergenic non-protein coding RNA 1504
ENSG0000	3.587205	2.436147	1.393723	1.747942	0.080474	0.278924	NA	NA	NA
ENSG0000	9.943545	-1.53234	0.876666	-1.74792	0.080478	0.278924	HINT2	84681	histidine triad nucleotide binding protein 2
ENSG0000	63.59234	-0.59518	0.340516	-1.74786	0.080487	0.278924	NDUFV2-A	1.02E+08	NDUFV2 antisense RNA 1
ENSG0000	20.23001	-1.08311	0.619889	-1.74726	0.080592	0.279236	NA	NA	NA
ENSG0000	2.972343	-3.02086	1.729636	-1.74653	0.080719	0.27962	NA	NA	NA
ENSG0000	223.5726	0.398891	0.228435	1.746194	0.080777	0.279771	ASB2	51676	ankyrin repeat and SOCS box containing 2
ENSG0000	83.40561	-0.48115	0.275595	-1.74585	0.080836	0.279881	PTK7	5754	protein tyrosine kinase 7 (inactive)
ENSG0000	7.851275	-1.55945	0.893237	-1.74584	0.080839	0.279881	RPL7AP43	644236	ribosomal protein L7a pseudogene 43
ENSG0000	1237.814	-0.17206	0.098566	-1.74561	0.080878	0.279963	DIP2B	57609	disco interacting protein 2 homolog B
ENSG0000	6.508407	-1.69981	0.973873	-1.74542	0.080912	0.28003	LOC84214	84214	Putative uncharacterized protein FLJ92257
ENSG0000	3528.857	0.148505	0.08509	1.745277	0.080937	0.280062	SIGLEC9	27180	sialic acid binding Ig like lectin 9
ENSG0000	21.4828	-0.9441	0.541023	-1.74503	0.08098	0.280159	ICAM5	7087	intercellular adhesion molecule 5
ENSG0000	93.17335	-0.49451	0.283442	-1.74467	0.081043	0.280271	TUBGCP5	114791	tubulin gamma complex associated protein 5
ENSG0000	2744.957	-0.14194	0.081356	-1.74469	0.08104	0.280271	CTC1	80169	CST telomere replication complex component 1
ENSG0000	28.60024	0.848655	0.486532	1.744294	0.081108	0.280445	DTL	51514	denticleless E3 ubiquitin protein ligase homolog
ENSG0000	188.3014	-0.33533	0.192342	-1.74339	0.081266	0.280939	ARHGAP12	94134	Rho GTPase activating protein 12
ENSG0000	18.16534	0.989793	0.567789	1.743241	0.081291	0.280975	RAB3IL1	5866	RAB3A interacting protein like 1
ENSG0000	9.590159	-1.39776	0.80194	-1.74297	0.081338	0.281032	PRDM16	63976	PR/SET domain 16
ENSG0000	49.18085	-0.62118	0.356379	-1.74303	0.081328	0.281032	SLC1A7	6512	solute carrier family 1 member 7
ENSG0000	330.554	0.274844	0.157744	1.74234	0.081449	0.281258	SMANTIS	1.08E+08	SMARCA4 interacting SWI/SNF chromatin remodeling complex scaffold lncRNA
ENSG0000	3.953945	-2.56096	1.469759	-1.74243	0.081433	0.281258	RNF43	54894	ring finger protein 43
ENSG0000	604.4543	-0.21278	0.122122	-1.74239	0.081441	0.281258	TRIM65	201292	tripartite motif containing 65
ENSG0000	3.739961	2.516229	1.444244	1.742247	0.081465	0.281261	NA	NA	NA
ENSG0000	122.4414	0.45219	0.25957	1.742077	0.081495	0.281312	LRP12	29967	LDL receptor related protein 12
ENSG0000	67.86892	0.604399	0.347007	1.741751	0.081552	0.281456	NA	NA	NA
ENSG0000	1064.701	0.185698	0.106637	1.741414	0.081611	0.281608	RNMT	8731	RNA guanine-7 methyltransferase
ENSG0000	17919.3	0.137547	0.079022	1.740609	0.081752	0.282042	VEGFA	7422	vascular endothelial growth factor A
ENSG0000	15.0367	1.134997	0.65217	1.740339	0.081799	0.282153	NBEA	26960	neurobeachin
ENSG0000	101.111	0.4099	0.235581	1.739956	0.081867	0.282201	RPS18P5	1E+08	ribosomal protein S18 pseudogene 5
ENSG0000	32.91543	-0.8692	0.499597	-1.73981	0.081893	0.282201	LOC12253	1.23E+08	RPSAP41-EIF3EP1
ENSG0000	71.44032	-0.50136	0.288137	-1.74001	0.081858	0.282201	NA	NA	NA
ENSG0000	158.023	-0.39464	0.226832	-1.73979	0.081896	0.282201	KDM7A-DT	1E+08	KDM7A divergent transcript
ENSG0000	3686.295	0.156808	0.09013	1.739798	0.081895	0.282201	PHB2	11331	prohibitin 2
ENSG0000	650.8271	0.197272	0.113392	1.739739	0.081905	0.282201	ARMCX3	51566	armadillo repeat containing X-linked 3
ENSG0000	122.4892	0.434838	0.249963	1.739606	0.081928	0.282229	CD200	4345	CD200 molecule
ENSG0000	165.269	-0.39631	0.227845	-1.73936	0.081972	0.282327	GEN1	348654	GEN1 Holliday junction 5' flap endonuclease
ENSG0000	192.2632	-0.31096	0.178853	-1.73866	0.082094	0.282696	TIMM10	26519	translocase of inner mitochondrial membrane 10
ENSG0000	559.9935	-0.20569	0.118316	-1.73849	0.082125	0.282751	USP28	57646	ubiquitin specific peptidase 28
ENSG0000	12.33711	1.627231	0.936106	1.738298	0.082158	0.282812	IFITM4P	340198	interferon induced transmembrane protein 4 pseudogene
ENSG0000	261.9984	-0.28366	0.163231	-1.73776	0.082253	0.283086	SHPRH	257218	SNF2 histone linker PHD RING helicase
ENSG0000	528.5967	0.256989	0.147922	1.737332	0.082329	0.283188	TFEC	22797	transcription factor EC
ENSG0000	20.42638	0.988963	0.569218	1.737407	0.082315	0.283188	TRBV14	28573	T cell receptor beta variable 14
ENSG0000	43.79882	0.792385	0.456059	1.737462	0.082306	0.283188	LINC01465	283416	long intergenic non-protein coding RNA 1465
ENSG0000	4.438308	-2.67766	1.541483	-1.73707	0.082375	0.283264	SLC25A27	9481	solute carrier family 25 member 27
ENSG0000	33.5734	0.835204	0.480958	1.736542	0.082468	0.283264	ENPP3	5169	ectonucleotide pyrophosphatase/phosphodiesterase 3
ENSG0000	6.25139	-1.73974	1.001673	-1.73684	0.082416	0.283264	FAM135B	51059	family with sequence similarity 135 member B
ENSG0000	561.1479	-0.21822	0.125652	-1.73673	0.082435	0.283264	PLEKHB1	58473	pleckstrin homology domain containing B1
ENSG0000	110.3671	-0.49314	0.283952	-1.7367	0.08244	0.283264	PDGFD	80310	platelet derived growth factor D
ENSG0000	4.663784	-2.25159	1.29647	-1.73671	0.082439	0.283264	NA	NA	NA
ENSG0000	2.886254	-3.08912	1.778921	-1.73651	0.082473	0.283264	USP3-AS1	1E+08	USP3 antisense RNA 1

ENSG0000	1752.111	-0.17708	0.101966	-1.73665	0.082449	0.283264	KSR1	8844	kinase suppressor of ras 1
ENSG0000	17.39201	-1.03769	0.597647	-1.73629	0.082513	0.283349	NA	NA	NA
ENSG0000	4.721642	-2.02329	1.165397	-1.73614	0.08254	0.283388	NA	NA	NA
ENSG0000	288.5861	-0.28697	0.165342	-1.7356	0.082634	0.283661	HSDL1	83693	hydroxysteroid dehydrogenase like 1
ENSG0000	1086.323	0.160904	0.092728	1.735234	0.082699	0.283831	PAN2	9924	poly(A) specific ribonuclease subunit PAN2
ENSG0000	324.5152	0.253401	0.146041	1.735134	0.082717	0.28384	AMZ2P1	201283	AMZ2 pseudogene 1
ENSG0000	4.360694	-2.76535	1.593957	-1.7349	0.082759	0.283931	PTPRH	5794	protein tyrosine phosphatase receptor type H
ENSG0000	460.9943	0.270509	0.155986	1.734187	0.082885	0.284294	PAICS	10606	phosphoribosylaminoimidazole carboxylase and phosphoribosylaminoimidazolesuccinocarboxamide synthase
ENSG0000	40.74202	-0.6511	0.375465	-1.73413	0.082895	0.284294	NA	NA	NA
ENSG0000	3.778668	2.684614	1.548336	1.73387	0.082941	0.284398	CFAP54	144535	cilia and flagella associated protein 54
ENSG0000	62.8175	-0.57557	0.331975	-1.73377	0.082959	0.284406	ST8SIA6	338596	ST8 alpha- 8-sialyltransferase 6
ENSG0000	3492.788	0.137373	0.07925	1.733421	0.083021	0.284566	WDR82	80335	WD repeat domain 82
ENSG0000	406.2244	0.259333	0.149677	1.732623	0.083163	0.285	POLR1E	64425	RNA polymerase I subunit E
ENSG0000	24.81852	-1.02828	0.593707	-1.73197	0.083278	0.285215	RBM33-DT	1.01E+08	RBM33 divergent transcript
ENSG0000	9.974622	1.414015	0.816429	1.73195	0.083282	0.285215	NA	NA	NA
ENSG0000	204.3024	0.328103	0.189444	1.731924	0.083287	0.285215	RDH10	157506	retinol dehydrogenase 10
ENSG0000	150.1837	-0.43413	0.250657	-1.73197	0.083279	0.285215	ZNF28	7576	zinc finger protein 28
ENSG0000	10.66313	-1.52722	0.881942	-1.73165	0.083335	0.285327	NA	NA	NA
ENSG0000	10.12658	-1.3631	0.78728	-1.73141	0.083379	0.285425	TTLL10	254173	tubulin tyrosine ligase like 10
ENSG0000	43.22777	-0.63484	0.366757	-1.73096	0.083459	0.285511	CLDN23	137075	claudin 23
ENSG0000	10.32956	-1.2677	0.732384	-1.73092	0.083466	0.285511	NA	NA	NA
ENSG0000	304.6804	-0.28484	0.164557	-1.73094	0.083463	0.285511	UBAP1L	390595	ubiquitin associated protein 1 like
ENSG0000	2119.603	0.182212	0.105274	1.730835	0.083481	0.285511	GPCPD1	56261	glycerophosphocholine phosphodiesterase 1
ENSG0000	184.0421	0.39821	0.230051	1.730964	0.083458	0.285511	NA	NA	NA
ENSG0000	71.12163	0.519979	0.300533	1.730194	0.083596	0.285587	LOC10099	1.01E+08	uncharacterized LOC100996756
ENSG0000	8.890978	-1.95471	1.129495	-1.73061	0.083522	0.285587	TREM2	54209	triggering receptor expressed on myeloid cells 2
ENSG0000	1218.32	-0.16463	0.095139	-1.73045	0.083551	0.285587	MARCHF8	220972	membrane associated ring-CH-type finger 8
ENSG0000	479.8897	-0.30111	0.174033	-1.7302	0.083595	0.285587	LACC1	144811	laccase domain containing 1
ENSG0000	7.838739	-1.62812	0.940946	-1.7303	0.083577	0.285587	NA	NA	NA
ENSG0000	226.7496	-0.32344	0.18692	-1.73034	0.08357	0.285587	GPATCH1	55094	G-patch domain containing 1
ENSG0000	570.7266	0.215755	0.124745	1.72956	0.083709	0.285868	RRP12	23223	ribosomal RNA processing 12 homolog
ENSG0000	5.299271	-2.09038	1.208599	-1.72959	0.083703	0.285868	LOC1053	1.05E+08	uncharacterized LOC105370500
ENSG0000	379.8413	-0.27795	0.160728	-1.72929	0.083758	0.285982	SPIDR	23514	scaffold protein involved in DNA repair
ENSG0000	433.1549	-0.29632	0.171395	-1.7289	0.083828	0.286063	HPS3	84343	HPS3 biogenesis of lysosomal organelles complex 2 subunit 1
ENSG0000	96.15464	0.520551	0.301072	1.728989	0.083811	0.286063	MED17	9440	mediator complex subunit 17
ENSG0000	66.81721	0.497394	0.287694	1.728898	0.083827	0.286063	NA	NA	NA
ENSG0000	372.5486	-0.25108	0.145241	-1.72869	0.083864	0.286134	SPAST	6683	spastin
ENSG0000	1535.362	0.152228	0.088083	1.728235	0.083946	0.286362	CNOT8	9337	CCR4-NOT transcription complex subunit 8
ENSG0000	1157.883	0.194863	0.112765	1.728049	0.083979	0.286423	BPNT2	54928	3'(2') 5'-bisphosphate nucleotidase 2
ENSG0000	9.955867	-3.60995	2.089318	-1.72781	0.084022	0.286514	PRUNE2	158471	prune homolog 2 with BCH domain
ENSG0000	67.94572	-0.64682	0.374386	-1.72769	0.084044	0.286539	MTUS1	57509	microtubule associated scaffold protein 1
ENSG0000	74.47407	-0.53307	0.308622	-1.72725	0.084122	0.286752	LCLAT1	253558	lysocardiolipin acyltransferase 1
ENSG0000	5449.552	0.123826	0.071708	1.726825	0.084199	0.286955	MARK2	2011	microtubule affinity regulating kinase 2
ENSG0000	81.23754	-0.54077	0.313172	-1.72675	0.084213	0.286955	RILPL1	353116	Rab interacting lysosomal protein like 1
ENSG0000	441.6177	-0.2194	0.127071	-1.72662	0.084237	0.286983	RCC1L	81554	RCC1 like
ENSG0000	400.7242	-0.25435	0.147323	-1.72649	0.084259	0.287005	EDRF1	26098	erythroid differentiation regulatory factor 1
ENSG0000	661.0245	-0.19824	0.114832	-1.72636	0.084283	0.287035	ARHGAP1	79658	Rho GTPase activating protein 10
ENSG0000	931.0203	-0.17732	0.102719	-1.72626	0.084301	0.287044	NOMO1	23420	NODAL modulator 1
ENSG0000	2252.133	0.140576	0.081438	1.726168	0.084317	0.287047	BSDC1	55108	BSD domain containing 1
ENSG0000	5384.411	-0.14833	0.08595	-1.72576	0.08439	0.287242	S1PR4	8698	sphingosine-1-phosphate receptor 4
ENSG0000	342.6173	0.250491	0.145162	1.725599	0.08442	0.28729	PFDN6	10471	prefoldin subunit 6
ENSG0000	1113.993	0.161434	0.093574	1.725193	0.084493	0.287486	STX16	8675	syntaxin 16
ENSG0000	2953.256	0.13993	0.081121	1.724954	0.084536	0.287525	PLEKHA2	59339	pleckstrin homology domain containing A2
ENSG0000	88.33854	-0.52283	0.30311	-1.72487	0.084551	0.287525	SAC3D1	29901	SAC3 domain containing 1
ENSG0000	56.09991	-0.56321	0.326504	-1.72498	0.08453	0.287525	IFT43	112752	intraflagellar transport 43
ENSG0000	20.59272	-1.02629	0.59521	-1.72426	0.084661	0.28784	L1TD1	54596	LINE1 type transposase domain containing 1
ENSG0000	42.10029	-0.69038	0.400412	-1.72419	0.084674	0.28784	IGHV4-39	28394	immunoglobulin heavy variable 4-39
ENSG0000	47.72434	-0.59804	0.346897	-1.72397	0.084712	0.287846	TEC	7006	tec protein tyrosine kinase
ENSG0000	40.01132	-0.66526	0.385903	-1.72392	0.084723	0.287846	PLK4	10733	polo like kinase 4
ENSG0000	7.340852	-1.78469	1.035249	-1.72393	0.084721	0.287846	FAM66B	1E+08	family with sequence similarity 66 member B
ENSG0000	12.32539	1.224385	0.7103	1.723757	0.084752	0.287892	NLRP7	199713	NLR family pyrin domain containing 7
ENSG0000	107.109	0.595378	0.345419	1.723641	0.084773	0.28791	KCNJ2	3759	potassium inwardly rectifying channel subfamily J member 2
ENSG0000	2469.922	-0.15228	0.088355	-1.72345	0.084808	0.287976	USP24	23358	ubiquitin specific peptidase 24
ENSG0000	145.0944	-0.45718	0.265378	-1.72276	0.084932	0.288347	NA	NA	NA
ENSG0000	274.0115	-0.28542	0.165685	-1.72267	0.084948	0.288349	ITPA	3704	inosine triphosphatase
ENSG0000	72.57533	-0.59935	0.348009	-1.72221	0.085031	0.288575	TCEA3	6920	transcription elongation factor A3
ENSG0000	153.6846	-0.39489	0.229309	-1.72209	0.085053	0.288597	CMAS	55907	cytidine monophosphate N-acetylneuraminic acid synthetase
ENSG0000	1824.5	-0.18284	0.106201	-1.72161	0.08514	0.288841	TAF15	8148	TATA-box binding protein associated factor 15
ENSG0000	220.1123	0.310161	0.180173	1.721456	0.085168	0.288883	GNG11	2791	G protein subunit gamma 11
ENSG0000	14.52364	1.078603	0.626615	1.721316	0.085194	0.288916	NME2	4831	NME/NM23 nucleoside diphosphate kinase 2
ENSG0000	4.101592	2.341712	1.360606	1.721081	0.085236	0.289008	NA	NA	NA
ENSG0000	14.07096	-1.40468	0.8163	-1.72079	0.085289	0.289083	PSEN2	5664	presenilin 2
ENSG0000	1099.347	0.188414	0.10949	1.720832	0.085281	0.289083	KDM6A	7403	lysine demethylase 6A
ENSG0000	3.77218	2.848822	1.655971	1.720334	0.085372	0.289264	NA	NA	NA
ENSG0000	384.2549	-0.24242	0.140915	-1.72032	0.085374	0.289264	LTO1	220064	LTO1 maturation factor of ABCE1
ENSG0000	3637.137	-0.13779	0.080106	-1.72004	0.085425	0.289331	ABL1	25	ABL proto- non-receptor tyrosine kinase
ENSG0000	549.4504	-0.20777	0.120793	-1.72009	0.085416	0.289331	ZNF768	79724	zinc finger protein 768
ENSG0000	1134.951	0.181289	0.105412	1.719821	0.085465	0.289414	NDUFA3	4696	NADH:ubiquinone oxidoreductase subunit A3
ENSG0000	64.77279	-0.74862	0.435378	-1.71946	0.085531	0.289584	IL27	246778	interleukin 27
ENSG0000	10.80341	1.203198	0.699902	1.719097	0.085597	0.289596	CA8	767	carbonic anhydrase 8
ENSG0000	1114.815	0.177174	0.103048	1.719329	0.085554	0.289596	TMEM71	137835	transmembrane protein 71
ENSG0000	15.33245	-1.07249	0.623827	-1.71921	0.085575	0.289596	UBE2Q2P1	388165	UBE2Q2 pseudogene 1
ENSG0000	5.674623	1.910832	1.111506	1.719138	0.085589	0.289596	NA	NA	NA
ENSG0000	109.0592	-0.42296	0.246118	-1.71852	0.085702	0.289708	NA	NA	NA
ENSG0000	3738.974	0.13608	0.079175	1.718727	0.085664	0.289708	ANKRD17	26057	ankyrin repeat domain 17
ENSG0000	9.521064	-1.49023	0.86717	-1.7185	0.085706	0.289708	KCNK17	89822	potassium two pore domain channel subfamily K member 17
ENSG0000	563.8299	-0.21387	0.124452	-1.71849	0.085708	0.289708	KLHL9	55958	kelch like family member 9
ENSG0000	1325.282	0.176729	0.102834	1.718582	0.08569	0.289708	SAP18	10284	Sin3A associated protein 18
ENSG0000	3.987258	2.316868	1.348747	1.717794	0.085834	0.290082	NA	NA	NA
ENSG0000	446.0314	-0.21865	0.127306	-1.71751	0.085886	0.290172	ERCC6	2074	ERCC excis chromatin remodeling factor
ENSG0000	40.29555	0.924064	0.538036	1.717476	0.085892	0.290172	LINC0232	1.02E+08	long intergenic non-protein coding RNA 2325
ENSG0000	1380.867	0.172155	0.100244	1.717353	0.085915	0.290195	CDIP1	29965	cell death inducing p53 target 1
ENSG0000	68003.89	0.120443	0.070143	1.717091	0.085963	0.290304	HLA-E	3133	major hist class I E
ENSG0000	14.99028	-1.03439	0.602454	-1.71696	0.085986	0.290329	SMIM1	388588	small integral membrane protein 1 (Vel blood group)
ENSG0000	18.1949	-1.04818	0.610747	-1.71622	0.086121	0.290734	NA	NA	NA
ENSG0000	280.145	-0.26341	0.153498	-1.71607	0.08615	0.290741	LAMTOR2	28956	late endos MAPK and MTOR activator 2
ENSG0000	7.521658	-1.77852	1.036412	-1.71604	0.086155	0.290741	ZNF578	147660	zinc finger protein 578
ENSG0000	8.933234	1.677073	0.978011	1.714779	0.086386	0.291467	ISL2	64843	ISL LIM homeobox 2
ENSG0000	4142.006	-0.18038	0.105206	-1.71457	0.086424	0.29147	KLF3	51274	KLF transcription factor 3
ENSG0000	12.8052	-1.33355	0.777721	-1.71469	0.086403	0.29147	B3GALT9	1E+08	beta-1 3-galactosyltransferase 9

ENSG0000	228.3648	-0.29402	0.171486	-1.71452	0.086434	0.29147	ZNF260	339324	zinc finger protein 260
ENSG0000	22.82257	0.984818	0.574487	1.714255	0.086482	0.29158	RGPD4	285190	RANBP2 like and GRIP domain containing 4
ENSG0000	30.61173	0.782223	0.456371	1.714007	0.086527	0.29168	NA	NA	NA
ENSG0000	32.3257	0.871027	0.508292	1.713636	0.086596	0.291857	NA	NA	NA
ENSG0000	75.78963	0.700779	0.409084	1.713045	0.086704	0.29217	NA	NA	NA
ENSG0000	162.5466	-0.41074	0.239844	-1.71252	0.0868	0.292388	ACKR3	57007	atypical chemokine receptor 3
ENSG0000	33.01475	-0.83938	0.49013	-1.71257	0.086792	0.292388	CENPE	1062	centromere protein E
ENSG0000	155.3937	-0.3832	0.223784	-1.71239	0.086826	0.29242	SIL1	64374	SIL1 nucleotide exchange factor
ENSG0000	476.7071	0.20448	0.119423	1.712229	0.086854	0.292464	ORMDL2	29095	ORMDL sphingolipid biosynthesis regulator 2
ENSG0000	121.1983	0.431456	0.252065	1.711687	0.086954	0.292747	NEURL1	9148	neuralized E3 ubiquitin protein ligase 1
ENSG0000	76.50855	0.515643	0.301271	1.711556	0.086979	0.292775	NA	NA	NA
ENSG0000	4.977854	2.479372	1.449237	1.710812	0.087116	0.293095	STIM1-AS1	1.05E+08	STIM1 antisense RNA 1
ENSG0000	938.1802	0.195423	0.11423	1.710784	0.087121	0.293095	ANKRD40	91369	ankyrin repeat domain 40
ENSG0000	4.418523	1.984412	1.159915	1.710825	0.087113	0.293095	NA	NA	NA
ENSG0000	38.28806	-0.72224	0.422207	-1.71062	0.087151	0.293144	PTPRN2	5799	protein tyrosine phosphatase receptor type N2
ENSG0000	642.2136	0.257211	0.150377	1.710445	0.087184	0.293199	INIP	58493	INTS3 and NABP interacting protein
ENSG0000	38.14112	-0.73277	0.428492	-1.71011	0.087245	0.293247	LRMDA	83938	leucine rich melanocyte differentiation associated
ENSG0000	83.4498	-0.57499	0.336227	-1.71013	0.087243	0.293247	ARHGAP1	84986	Rho GTPase activating protein 19
ENSG0000	6.884731	-1.92181	1.123772	-1.71014	0.08724	0.293247	COLEC12	81035	collectin subfamily member 12
ENSG0000	207.3819	0.28476	0.166525	1.710017	0.087263	0.293253	DMAC1	90871	distal membrane arm assembly component 1
ENSG0000	663.4544	-0.26391	0.154363	-1.70966	0.087329	0.293421	AKAP11	11215	A-kinase anchoring protein 11
ENSG0000	4.289209	-2.63624	1.542104	-1.70951	0.087356	0.293461	LOC10272	1.03E+08	uncharacterized LOC102723692
ENSG0000	8.831744	1.303707	0.762676	1.709384	0.08738	0.293487	NA	NA	NA
ENSG0000	1884.789	-0.14482	0.084734	-1.70914	0.087426	0.293587	TNRC6A	27327	trinucleotide repeat containing adaptor 6A
ENSG0000	837.6857	0.486657	0.284754	1.709041	0.087443	0.293594	RAB20	55647	RAB20 member RAS oncogene family
ENSG0000	10253.09	0.136863	0.080111	1.708411	0.08756	0.29372	FLNB	2317	filamin B
ENSG0000	4040.935	0.14905	0.087237	1.70857	0.087531	0.29372	HLA-DMA	3108	major hist class II DM alpha
ENSG0000	500.4011	-0.20902	0.122327	-1.70868	0.08751	0.29372	SMARCD3	6604	SWI/SNF r matrix ass actin depe subfamily member 3
ENSG0000	1420.773	0.152453	0.089231	1.708527	0.087539	0.29372	ABHD4	63874	abhydrola N-acyl phospholipase B
ENSG0000	1029.294	-0.18982	0.111107	-1.70848	0.087548	0.29372	ACSF3	197322	acyl-CoA synthetase family member 3
ENSG0000	310.0294	-0.25515	0.149449	-1.70726	0.087775	0.294333	RILP	83547	Rab interacting lysosomal protein
ENSG0000	765.9075	-0.24747	0.144947	-1.70728	0.08777	0.294333	LOC11226	1.12E+08	uncharacterized LOC112268269
ENSG0000	49.81974	-0.66127	0.387386	-1.707	0.087823	0.294396	PTCH2	8643	patched 2
ENSG0000	5720.76	0.148297	0.086876	1.706983	0.087825	0.294396	CIB1	10519	calcium and integrin binding 1
ENSG0000	819.3042	0.188487	0.110459	1.706399	0.087934	0.294698	JCHAIN	3512	joining chain of multimeric IgA and IgM
ENSG0000	774.509	-0.18535	0.108622	-1.70633	0.087947	0.294698	CAPRIN2	65981	caprin family member 2
ENSG0000	14024.88	0.171912	0.100761	1.706143	0.087981	0.29476	DUSP6	1848	dual specificity phosphatase 6
ENSG0000	53.41297	0.629212	0.36882	1.706011	0.088006	0.294789	NA	NA	NA
ENSG0000	3.918418	-2.47755	1.452405	-1.70583	0.08804	0.29485	GPR101	83550	G protein-coupled receptor 101
ENSG0000	3213.994	0.154749	0.090732	1.705562	0.08809	0.294963	TOB1	10140	transducer 1
ENSG0000	328.7778	0.24648	0.144532	1.70536	0.088127	0.295036	ZNF550	162972	zinc finger protein 550
ENSG0000	2446.92	0.162923	0.095598	1.704251	0.088334	0.295675	SLC39A13	91252	solute carrier family 39 member 13
ENSG0000	2219.071	0.155526	0.09127	1.704026	0.088376	0.295762	TRIM33	51592	tripartite motif containing 33
ENSG0000	474.4449	0.254152	0.14917	1.703775	0.088423	0.295866	ASPRV1	151516	aspartic peptidase retroviral like 1
ENSG0000	652.2921	-0.20385	0.119659	-1.70359	0.088457	0.295925	MLST8	64223	MTOR ass LST8 homolog
ENSG0000	159.0298	0.333686	0.195903	1.703323	0.088508	0.296042	ELAPOR2	222223	endosome-lysosome associated apoptosis and autophagy regulator family member 2
ENSG0000	37.72838	-0.6872	0.403606	-1.70265	0.088634	0.296412	TIMD4	91937	T cell immunoglobulin and mucin domain containing 4
ENSG0000	312.9305	0.269249	0.158175	1.702222	0.088714	0.296615	SDE2	163859	SDE2 telomere maintenance homolog
ENSG0000	50.74515	-0.65911	0.38722	-1.70215	0.088727	0.296615	RAB4B	53916	RAB4B member RAS oncogene family
ENSG0000	5.228202	2.112957	1.241524	1.701906	0.088773	0.296715	NA	NA	NA
ENSG0000	295.978	-0.27284	0.160329	-1.70176	0.0888	0.296752	NRDE2	55051	NRDE-2 necessary domain containing
ENSG0000	608.6375	0.197903	0.116335	1.701145	0.088916	0.297075	GP9	2815	glycoprotein IX platelet
ENSG0000	3016.724	0.13862	0.08149	1.701077	0.088929	0.297075	USP7	7874	ubiquitin specific peptidase 7
ENSG0000	1434.568	-0.17235	0.10133	-1.70091	0.088959	0.297124	RNF181	51255	ring finger protein 181
ENSG0000	42.7083	-0.71687	0.421492	-1.7008	0.08898	0.297139	RYR1	6261	ryanodine receptor 1
ENSG0000	82.73223	-0.52539	0.308941	-1.70061	0.089017	0.297208	AVIL	10677	advillin
ENSG0000	1036.381	0.181916	0.106978	1.700502	0.089036	0.297221	ARID4A	5926	AT-rich interaction domain 4A
ENSG0000	129.0221	-0.36222	0.213095	-1.6998	0.089168	0.297607	MTIF2	4528	mitochondrial translational initiation factor 2
ENSG0000	738.8325	-0.23164	0.136283	-1.6997	0.089187	0.297615	OSBPL11	114885	oxysterol binding protein like 11
ENSG0000	97.05766	-0.42042	0.247377	-1.69952	0.089221	0.297644	MXRA8	54587	matrix remodeling associated 8
ENSG0000	100.5925	-0.42318	0.249007	-1.69949	0.089227	0.297644	USP27X	389856	ubiquitin specific peptidase 27 X-linked
ENSG0000	503.6724	0.219418	0.129116	1.699387	0.089246	0.297653	PHF5A	84844	PHD finger protein 5A
ENSG0000	1522.595	0.153854	0.090543	1.699239	0.089274	0.297693	CHTOP	26097	chromatin target of PRMT1
ENSG0000	29.59268	0.770075	0.453229	1.699088	0.089303	0.297734	UBXN8	7993	UBX domain protein 8
ENSG0000	5.70987	-1.87208	1.101953	-1.69888	0.089343	0.297784	AS3MT	57412	arsenite methyltransferase
ENSG0000	6.158673	2.140594	1.260034	1.698838	0.08935	0.297784	LOC40055	400553	uncharacterized LOC400553
ENSG0000	1239.769	0.160083	0.094252	1.698467	0.08942	0.297964	KLC2	64837	kinesin light chain 2
ENSG0000	59.43649	0.647572	0.381412	1.697827	0.08954	0.298312	IGHV4-34	28395	immunoglobulin heavy variable 4-34
ENSG0000	2.881592	-2.77704	1.636093	-1.69736	0.089629	0.298553	C2orf66	401027	chromosome 2 open reading frame 66
ENSG0000	12.84752	-1.25716	0.740755	-1.69713	0.089672	0.298644	GNMT	27232	glycine N-methyltransferase
ENSG0000	2.362613	-3.62966	2.139038	-1.69686	0.089722	0.298651	TRPM8	79054	transient receptor potential cation channel subfamily M member 8
ENSG0000	5.749269	2.099135	1.23702	1.69693	0.08971	0.298651	CSPG4BP	727930	chondroiti pseudogene
ENSG0000	33.49816	-0.88791	0.523226	-1.69699	0.089699	0.298651	ELAC1	55520	elaC ribonuclease Z 1
ENSG0000	4.976789	2.047009	1.206567	1.696556	0.089781	0.298737	NA	NA	NA
ENSG0000	116.7492	-0.4236	0.249672	-1.69663	0.089766	0.298737	NA	NA	NA
ENSG0000	164.5401	-0.31289	0.184456	-1.6963	0.089829	0.298844	WDR4	10785	WD repeat domain 4
ENSG0000	31.40814	-0.85143	0.50198	-1.69614	0.089859	0.298891	PDXP	57026	pyridoxal phosphatase
ENSG0000	19.46077	1.162011	0.685268	1.695703	0.089942	0.29906	IL19	29949	interleukin 19
ENSG0000	5.750924	2.001613	1.180351	1.695778	0.089928	0.29906	NA	NA	NA
ENSG0000	5.283829	4.769887	2.813102	1.695597	0.089962	0.299073	NA	NA	NA
ENSG0000	21446.73	0.375037	0.221266	1.694959	0.090083	0.299422	POLR2A	5430	RNA polymerase II subunit A
ENSG0000	4.105912	2.271172	1.340213	1.694635	0.090145	0.299572	TLE1-DT	1.02E+08	TLE1 divergent transcript
ENSG0000	363.7835	0.243826	0.14391	1.694294	0.090209	0.299625	RABGGTB	5876	Rab geranylgeranyltransferase subunit beta
ENSG0000	137.5891	-0.46208	0.272711	-1.69438	0.090194	0.299625	LPAR3	23566	lysophosphatidic acid receptor 3
ENSG0000	313.0238	0.281172	0.165959	1.694229	0.090222	0.299625	CHKA	1119	choline kinase alpha
ENSG0000	8901.084	0.115499	0.068173	1.694211	0.090225	0.299625	DYNC1H1	1778	dynein cytoplasmic 1 heavy chain 1
ENSG0000	42.61729	-0.65559	0.386998	-1.69404	0.090258	0.299681	VSIG4	11326	V-set and immunoglobulin domain containing 4
ENSG0000	300.1437	-0.28332	0.167255	-1.69392	0.09028	0.2997	TARS3	123283	threonyl-tRNA synthetase 3
ENSG0000	165.6431	-0.32217	0.190209	-1.69378	0.090307	0.299737	DNAJC24	120526	DnaJ heat shock protein family (Hsp40) member C24
ENSG0000	12.39106	1.286837	0.759845	1.693551	0.090351	0.299827	RAB4A-AS	1.05E+08	RAB4A antisense RNA 1
ENSG0000	358.8801	-0.23544	0.13905	-1.69317	0.090424	0.300017	TAF6L	10629	TATA-box binding protein associated factor 6 like
ENSG0000	332.848	-0.25103	0.148279	-1.69296	0.090464	0.300058	SMC6	79677	structural maintenance of chromosomes 6
ENSG0000	39.462	-0.85078	0.502551	-1.69293	0.090469	0.300058	FOXJ1	2302	forkhead box J1
ENSG0000	170.3585	-0.32511	0.192073	-1.69264	0.090525	0.300184	AFF3	3899	ALF transcription elongation factor 3
ENSG0000	417.8517	-0.21242	0.125503	-1.69256	0.090539	0.300184	PIGO	84720	phosphatidylinositol glycan anchor biosynthesis class O
ENSG0000	953.8309	0.172242	0.101804	1.691905	0.090664	0.300491	DHX36	170506	DEAH-box helicase 36
ENSG0000	96.08229	-0.48376	0.285914	-1.69198	0.09065	0.300491	TTC12	54970	tetratricopeptide repeat domain 12
ENSG0000	153.786	-0.3393	0.200569	-1.69167	0.090708	0.300583	PKD1P4	353512	polycystin transient receptor potential channel interacting pseudogene 4
ENSG0000	19.19615	-0.96658	0.571407	-1.69158	0.090725	0.300587	TLN2	83660	talin 2
ENSG0000	100.7727	-0.45833	0.270986	-1.69135	0.090771	0.30063	ZBTB11-AS	1E+08	ZBTB11 antisense RNA 1

ENSG0000	3.457056	2.46465	1.457206	1.691353	0.090769	0.30063	NA	NA	NA
ENSG0000	18.75102	-1.11241	0.657787	-1.69114	0.090811	0.300709	NA	NA	NA
ENSG0000	37.49972	0.72377	0.428058	1.690825	0.09087	0.300852	ACP3	55	acid phosphatase 3
ENSG0000	544.6943	-0.22992	0.135992	-1.69071	0.090892	0.300869	PEX6	5190	peroxisomal biogenesis factor 6
ENSG0000	1046.192	-0.16274	0.096281	-1.69027	0.090977	0.300888	DUSP22	56940	dual specificity phosphatase 22
ENSG0000	1043.583	-0.18912	0.111899	-1.6901	0.091008	0.300888	CMTR1	23070	cap methyltransferase 1
ENSG0000	2.597246	3.252145	1.923861	1.690426	0.090946	0.300888	AKR1C2	1646	aldo-keto reductase family 1 member C2
ENSG0000	4.652935	-1.89689	1.122334	-1.69013	0.091003	0.300888	NA	NA	NA
ENSG0000	17.18712	1.105625	0.654233	1.689956	0.091036	0.300888	LOC1053€	1.05E+08	uncharacterized LOC105369344
ENSG0000	2665.186	-0.17336	0.102583	-1.69	0.091029	0.300888	YLPM1	56252	YLP motif containing 1
ENSG0000	604.0243	0.233103	0.137935	1.689942	0.091039	0.300888	TIMM44	10469	translocase of inner mitochondrial membrane 44
ENSG0000	118.3381	-0.4007	0.237113	-1.68992	0.091044	0.300888	ZNF780B	163131	zinc finger protein 780B
ENSG0000	196.5113	0.330997	0.195789	1.690585	0.090916	0.300888	ZNF576	79177	zinc finger protein 576
ENSG0000	320.3899	0.238254	0.141021	1.689488	0.091126	0.301095	MYNN	55892	myoneurin
ENSG0000	18.76146	0.937756	0.555103	1.689337	0.091155	0.301095	SLC16A11	162515	solute carrier family 16 member 11
ENSG0000	37.37308	1.952414	1.15571	1.689363	0.09115	0.301095	IGLV8-61	28774	immunoglobulin lambda variable 8-61
ENSG0000	37.12951	-0.76253	0.451404	-1.68925	0.091172	0.301098	NA	NA	NA
ENSG0000	4.911329	1.885604	1.116623	1.688667	0.091283	0.301412	NA	NA	NA
ENSG0000	51.88944	-0.55694	0.329846	-1.68849	0.091316	0.301468	C10orf143	387723	chromosome 10 open reading frame 143
ENSG0000	1700.53	0.180307	0.106823	1.687906	0.091429	0.301691	PEX14	5195	peroxisomal biogenesis factor 14
ENSG0000	7.087016	-1.69977	1.007041	-1.68789	0.091433	0.301691	CDC25A	993	cell division cycle 25A
ENSG0000	3.643513	2.214011	1.311627	1.687988	0.091414	0.301691	NA	NA	NA
ENSG0000	30.36974	-0.79178	0.469214	-1.68746	0.091515	0.301695	SLC45A1	50651	solute carrier family 45 member 1
ENSG0000	77.02283	-0.48662	0.288387	-1.68737	0.091532	0.301695	MYO7B	4648	myosin VIIB
ENSG0000	4.800826	-1.98643	1.176979	-1.68773	0.091462	0.301695	NA	NA	NA
ENSG0000	18.64017	-1.15447	0.68408	-1.68763	0.091483	0.301695	COL23A1	91522	collagen type XXIII alpha 1 chain
ENSG0000	6908.925	-0.15024	0.089025	-1.68757	0.091493	0.301695	TNFSF8	944	TNF superfamily member 8
ENSG0000	9.501031	1.473046	0.872957	1.68742	0.091523	0.301695	C11orf94	143678	chromosome 11 open reading frame 94
ENSG0000	4.691729	2.496394	1.479603	1.687205	0.091564	0.301724	NA	NA	NA
ENSG0000	6.899953	-2.00801	1.190172	-1.68716	0.091573	0.301724	SEPTIN3	55964	septin 3
ENSG0000	8.505405	-3.85159	2.283215	-1.68691	0.09162	0.301825	IGKV1D-16	28901	immunoglobulin kappa variable 1D-16
ENSG0000	6.872194	1.706693	1.011821	1.686754	0.091651	0.301873	HYKK	123688	hydroxylysine kinase
ENSG0000	3.547103	2.52298	1.495929	1.686564	0.091687	0.30194	CNGB1	1258	cyclic nucleotide gated channel subunit beta 1
ENSG0000	9611.072	0.143347	0.085011	1.686226	0.091752	0.3021	MAP3K11	4296	mitogen-activated protein kinase kinase kinase 11
ENSG0000	35.24952	-0.74389	0.44123	-1.68595	0.091805	0.30222	ZNF594	84622	zinc finger protein 594
ENSG0000	56.91046	-0.5433	0.322273	-1.68584	0.091827	0.302239	TFB1M	51106	transcripti mitochondrial
ENSG0000	4.47738	2.388924	1.41714	1.685736	0.091847	0.30225	NA	NA	NA
ENSG0000	115.6567	-0.42075	0.24964	-1.68541	0.091909	0.302293	GPR180	160897	G protein-coupled receptor 180
ENSG0000	6900.072	0.117856	0.069925	1.685465	0.091899	0.302293	ARF6	382	ADP ribosylation factor 6
ENSG0000	4945.88	-0.18395	0.109133	-1.68557	0.091879	0.302293	LILRB1	10859	leukocyte immunoglobulin like receptor B1
ENSG0000	1481.052	-0.14639	0.086881	-1.685	0.091989	0.302498	GABBR1	2550	gamma-aminobutyric acid type B receptor subunit 1
ENSG0000	1381.224	-0.17107	0.101531	-1.6849	0.092007	0.302498	SHARPIN	81858	SHANK associated RH domain interactor
ENSG0000	4.151734	2.385524	1.416012	1.684678	0.092051	0.302498	IGFALS	3483	insulin like growth factor binding protein acid labile subunit
ENSG0000	3.207324	-2.44834	1.453257	-1.68472	0.092042	0.302498	PSMA8	143471	proteasome 20S subunit alpha 8
ENSG0000	1352.576	0.153022	0.090832	1.684668	0.092053	0.302498	BLCAP	10904	BLCAP apoptosis inducing factor
ENSG0000	625.6881	0.190531	0.113115	1.684402	0.092104	0.302614	IPCEF1	26034	interaction protein for cytohesin exchange factors 1
ENSG0000	16.353	-1.16686	0.692783	-1.6843	0.092123	0.302623	NA	NA	NA
ENSG0000	582.5005	-0.20915	0.124202	-1.68396	0.09219	0.30279	CSNK1G1	53944	casein kinase 1 gamma 1
ENSG0000	6.511332	-1.99357	1.183931	-1.68386	0.092209	0.302799	NA	NA	NA
ENSG0000	3603.41	0.125654	0.074637	1.683532	0.092272	0.302901	NR3C1	2908	nuclear receptor subfamily 3 group C member 1
ENSG0000	770.6657	0.180992	0.107518	1.683358	0.092306	0.302901	VPS37A	137492	VPS37A subunit of ESCRT-I
ENSG0000	476.1076	0.219044	0.130112	1.683507	0.092277	0.302901	CWF19L1	55280	CWF19 like cell cycle control factor 1
ENSG0000	208.7609	-0.36119	0.214562	-1.68337	0.092303	0.302901	ISOC2	79763	isochorismatase domain containing 2
ENSG0000	298.8418	-0.2957	0.175667	-1.68327	0.092322	0.302902	PGPEP1	54858	pyroglutamyl-peptidase I
ENSG0000	361.6103	0.262888	0.156201	1.683019	0.092371	0.303009	TRMT10C	54931	tRNA meth mitochondrial RNase P subunit
ENSG0000	5930.327	0.1164	0.069166	1.682919	0.092391	0.303019	TMEM127	55654	transmembrane protein 127
ENSG0000	2.943225	-3.30862	1.966378	-1.6826	0.092453	0.30317	LOC10192	1.02E+08	uncharacterized LOC101929372
ENSG0000	720.665	-0.23265	0.138287	-1.68237	0.092497	0.303259	ANKRD27	84079	ankyrin repeat domain 27
ENSG0000	3291.328	-0.13443	0.079926	-1.68189	0.092591	0.303514	CENPB	1059	centromere protein B
ENSG0000	2653.891	0.157434	0.093612	1.681766	0.092614	0.303537	ACSL4	2182	acyl-CoA synthetase long chain family member 4
ENSG0000	359.6489	-0.24701	0.146898	-1.68148	0.092669	0.303609	ANAPC13	25847	anaphase promoting complex subunit 13
ENSG0000	452.4571	-0.22705	0.135023	-1.68155	0.092656	0.303609	FTX	1E+08	FTX transc XIST regulator
ENSG0000	3.175653	2.243546	1.334427	1.681281	0.092708	0.303685	RPS18P2	326320	ribosomal protein S18 pseudogene 2
ENSG0000	2491.615	0.165136	0.098244	1.68088	0.092786	0.303832	RAB6A	5870	RAB6A member RAS oncogene family
ENSG0000	74.39104	-0.47908	0.285006	-1.68094	0.092775	0.303832	KPTN	11133	kaptin actin binding protein
ENSG0000	16.9185	-1.08156	0.643586	-1.68052	0.092856	0.303899	IL17RE	132014	interleukin 17 receptor E
ENSG0000	256.8684	-0.29125	0.173295	-1.68068	0.092825	0.303899	UTP20	27340	UTP20 small subunit processome component
ENSG0000	299.0535	-0.25977	0.154568	-1.6806	0.09284	0.303899	ACOT8	10005	acyl-CoA thioesterase 8
ENSG0000	3496.566	0.133721	0.079583	1.680268	0.092905	0.304007	SCAF4	57466	SR-related CTD associated factor 4
ENSG0000	15.64461	-1.1889	0.707715	-1.67992	0.092973	0.304177	NA	NA	NA
ENSG0000	25.96737	-0.8474	0.504593	-1.67937	0.09308	0.3044	GSTCD	79807	glutathione S-transferase C-terminal domain containing
ENSG0000	693.2506	0.209995	0.125048	1.679313	0.093091	0.3044	CCDC32	90416	coiled-coil domain containing 32
ENSG0000	73052.78	0.145308	0.086522	1.679432	0.093068	0.3044	EEF2	1938	eukaryotic translation elongation factor 2
ENSG0000	605.2172	0.210221	0.125192	1.679185	0.093116	0.304429	UQCRH	7388	ubiquinol-cytochrome c reductase hinge protein
ENSG0000	141.1271	-0.34791	0.207214	-1.67897	0.093159	0.304515	SLC22A5	6584	solute carrier family 22 member 5
ENSG0000	2183.817	-0.14202	0.084602	-1.67871	0.093209	0.304626	SMC1A	8243	structural maintenance of chromosomes 1A
ENSG0000	172.0844	-0.32674	0.194664	-1.67846	0.093257	0.304727	FECH	2235	ferrochelatase
ENSG0000	9.274251	-1.41601	0.843842	-1.67805	0.093337	0.304938	TRAV25	28658	T cell receptor alpha variable 25
ENSG0000	2121.296	0.148163	0.088301	1.677928	0.093361	0.304961	PPP4R3B	57223	protein phosphatase 4 regulatory subunit 3B
ENSG0000	18.48301	-0.93927	0.560032	-1.67717	0.093509	0.305336	MTMR9LP	339483	myotubula pseudogene
ENSG0000	3995.573	0.128794	0.076792	1.677182	0.093507	0.305336	RBMX	27316	RNA binding motif protein X-linked
ENSG0000	31.14931	-0.75042	0.447487	-1.67697	0.093549	0.305412	ADAMTS6	11174	ADAM metallopeptidase with thrombospondin type 1 motif 6
ENSG0000	2133.151	0.162043	0.096651	1.676586	0.093623	0.305603	CD2BP2	10421	CD2 cytoplasmic tail binding protein 2
ENSG0000	1510.778	0.165549	0.098748	1.676474	0.093645	0.30562	CYC1	1537	cytochrome c1
ENSG0000	56.2842	0.527138	0.314615	1.6755	0.093836	0.306189	NA	NA	NA
ENSG0000	1566.088	-0.16214	0.096791	-1.6752	0.093894	0.306271	PPP1R13B	23368	protein phosphatase 1 regulatory subunit 13B
ENSG0000	3286.359	-0.13646	0.081456	-1.67524	0.093886	0.306271	DPP9	91039	dipeptidyl peptidase 9
ENSG0000	5263.551	-0.14494	0.086527	-1.67503	0.093929	0.306331	LFNG	3955	LFNG O-fucosylpeptide 3-beta-N-acetylglucosaminyltransferase
ENSG0000	157.8489	0.37823	0.225903	1.674303	0.094071	0.30674	MPZ	4359	myelin protein zero
ENSG0000	484.2395	-0.21396	0.12782	-1.67391	0.094147	0.306864	RNF169	254225	ring finger protein 169
ENSG0000	136.7223	0.358209	0.213985	1.67399	0.094132	0.306864	NA	NA	NA
ENSG0000	10283.33	-0.12601	0.075278	-1.67386	0.094159	0.306864	RNF166	115992	ring finger protein 166
ENSG0000	3.166104	2.812549	1.680562	1.673576	0.094214	0.30699	NA	NA	NA
ENSG0000	489.1727	0.249298	0.148979	1.67337	0.094255	0.307014	CERS4	79603	ceramide synthase 4
ENSG0000	2.927394	-2.73148	1.632284	-1.67341	0.094247	0.307014	NA	NA	NA
ENSG0000	3.296843	2.582196	1.543421	1.673034	0.094321	0.307176	NA	NA	NA
ENSG0000	9892.479	0.115748	0.069196	1.672757	0.094375	0.307299	KDM2A	22992	lysine demethylase 2A
ENSG0000	3.337399	-2.47254	1.478433	-1.67241	0.094444	0.307469	MAP2K4P1	139201	mitogen-activated protein kinase kinase 4 pseudogene 1
ENSG0000	839.9843	-0.22877	0.136826	-1.67196	0.094532	0.307684	SERINC2	347735	serine incorporator 2
ENSG0000	3.863899	-2.71409	1.62335	-1.6719	0.094543	0.307684	NA	NA	NA

ENSG0000	716.2257	-0.19278	0.115332	-1.6715	0.094623	0.307836	TFDP2	7029	transcription factor Dp-2
ENSG0000	11.08848	-1.73815	1.039866	-1.67151	0.094621	0.307836	SSTR2	6752	somatostatin receptor 2
ENSG0000	38.50468	-0.7112	0.425548	-1.67125	0.094672	0.307897	TRGV2	6974	T cell receptor gamma variable 2
ENSG0000	1687.135	-0.16802	0.100537	-1.67124	0.094675	0.307897	IGHG2	3501	immunoglobulin heavy constant gamma 2 (G2m marker)
ENSG0000	13.33988	-1.14094	0.683061	-1.67033	0.094854	0.308426	NA	NA	NA
ENSG0000	33.70767	-0.731	0.437661	-1.67024	0.094872	0.308429	NA	NA	NA
ENSG0000	95.09418	0.460909	0.275971	1.670131	0.094893	0.308445	MANEA	79694	mannosidase endo-alpha
ENSG0000	467.415	-0.26562	0.159068	-1.66988	0.094944	0.308554	ACSS2	55902	acyl-CoA synthetase short chain family member 2
ENSG0000	1274.154	0.167921	0.100571	1.669671	0.094985	0.308633	SSR3	6747	signal sequence receptor subunit 3
ENSG0000	281.8616	0.31735	0.190152	1.668922	0.095133	0.309008	MRPL52	122704	mitochondrial ribosomal protein L52
ENSG0000	50.61913	0.604948	0.362515	1.668751	0.095167	0.309008	NA	NA	NA
ENSG0000	1243.009	0.159766	0.095736	1.668817	0.095154	0.309008	SGF29	112869	SAGA complex associated factor 29
ENSG0000	38.19999	0.698595	0.418601	1.66888	0.095141	0.309008	PDCD2L	84306	programmed cell death 2 like
ENSG0000	5.182753	1.727868	1.035729	1.668263	0.095264	0.309269	NA	NA	NA
ENSG0000	2408.549	0.139099	0.083386	1.668136	0.095289	0.309296	GPR108	56927	G protein-coupled receptor 108
ENSG0000	14.23345	0.998295	0.598559	1.667831	0.095349	0.309415	PSPH	5723	phosphoserine phosphatase
ENSG0000	1753.405	-0.14922	0.089473	-1.66778	0.095359	0.309415	PDPR	55066	pyruvate dehydrogenase phosphatase regulatory subunit
ENSG0000	40.3786	0.61109	0.366525	1.667252	0.095464	0.309704	INE1	8552	inactivation escape 1
ENSG0000	28.43338	-0.86609	0.519512	-1.66713	0.095489	0.309729	NA	NA	NA
ENSG0000	612.5977	0.245178	0.147119	1.666526	0.095609	0.310064	TMED7	51014	transmembrane p24 trafficking protein 7
ENSG0000	150.218	-0.35441	0.212686	-1.66635	0.095643	0.310066	PAQR4	124222	progestin and adipoQ receptor family member 4
ENSG0000	8.043477	-1.58958	0.953885	-1.66642	0.095629	0.310066	ZNF763	284390	zinc finger protein 763
ENSG0000	313.0022	-0.3292	0.197577	-1.66618	0.095678	0.310125	HERC6	55008	HECT and RLD domain containing E3 ubiquitin protein ligase family member 6
ENSG0000	661.9432	-0.1812	0.108785	-1.66566	0.095781	0.310405	TANGO2	128989	transport and golgi organization 2 homolog
ENSG0000	270.8771	-0.34034	0.204345	-1.6655	0.095812	0.310453	ZCCHC7	84186	zinc finger CCHC-type containing 7
ENSG0000	673.2263	-0.19549	0.117412	-1.66498	0.095916	0.31066	HEATR5B	54497	HEAT repeat containing 5B
ENSG0000	29.28528	0.893663	0.53672	1.665045	0.095904	0.31066	NA	NA	NA
ENSG0000	18.75375	1.08796	0.653457	1.66493	0.095927	0.31066	SSC5D	284297	scavenger receptor cysteine rich family member with 5 domains
ENSG0000	40.65748	0.607342	0.364837	1.664697	0.095973	0.310757	SUCNR1	56670	succinate receptor 1
ENSG0000	3.834172	2.481637	1.490837	1.664593	0.095994	0.31077	FIBCD1	84929	fibrinogen C domain containing 1
ENSG0000	1385.742	-0.15661	0.094137	-1.66367	0.096178	0.311135	MCRS1	10445	microspherule protein 1
ENSG0000	14.9733	-0.98107	0.589688	-1.66372	0.096169	0.311135	PLEKHG7	440107	pleckstrin homology and RhoGEF domain containing G7
ENSG0000	5.679042	1.913937	1.15027	1.663903	0.096132	0.311135	BMP4	652	bone morphogenetic protein 4
ENSG0000	2043.227	-0.16664	0.100169	-1.66361	0.096191	0.311135	TP53I13	90313	tumor protein p53 inducible protein 13
ENSG0000	47.81305	-0.63042	0.378912	-1.66378	0.096157	0.311135	C2CD2	25966	C2 calcium dependent domain containing 2
ENSG0000	1154.07	0.153352	0.0922	1.663253	0.096262	0.311256	CD8B	926	CD8b molecule
ENSG0000	486.9481	0.25877	0.155577	1.66329	0.096254	0.311256	RRP1	8568	ribosomal RNA processing 1
ENSG0000	27.28998	-0.91224	0.548546	-1.66302	0.096308	0.311352	FUT10	84750	fucosyltransferase 10
ENSG0000	7.62637	-1.72802	1.039149	-1.66292	0.096328	0.311363	NA	NA	NA
ENSG0000	4.777678	-2.07417	1.24747	-1.6627	0.096373	0.311452	H2BC26	128312	H2B clustered histone 26
ENSG0000	9.73157	-1.4519	0.873367	-1.66242	0.096428	0.311577	MLXIPL	51085	MLX interacting protein like
ENSG0000	287.6545	-0.26553	0.159737	-1.6623	0.096453	0.311604	PGGT1B	5229	protein geranylgeranyltransferase type I subunit beta
ENSG0000	6.328927	-1.86107	1.119722	-1.66208	0.096497	0.311691	LOC14578	145783	uncharacterized LOC145783
ENSG0000	14.73055	1.130621	0.680473	1.661523	0.096608	0.311942	NA	NA	NA
ENSG0000	494.5001	0.215477	0.129684	1.661552	0.096603	0.311942	DUT	1854	deoxyuridine triphosphatase
ENSG0000	53.54316	-0.54616	0.328761	-1.66126	0.096662	0.31206	TMOD4	29765	tropomodulin 4
ENSG0000	281.2787	0.255276	0.153701	1.660855	0.096743	0.312266	ASF1A	25842	anti-silencing function 1A histone chaperone
ENSG0000	1682.822	-0.14683	0.088446	-1.66013	0.096889	0.312685	ZCCHC2	54877	zinc finger CCHC-type containing 2
ENSG0000	10.34328	1.422159	0.856758	1.65993	0.096928	0.312758	SPDYA	245711	speedy/RINGO cell cycle regulator family member A
ENSG0000	189.9284	-0.29301	0.176536	-1.65977	0.09696	0.31278	LCAT	3931	lecithin-cholesterol acyltransferase
ENSG0000	192.5392	0.30815	0.185663	1.659729	0.096969	0.31278	MPHOSPH	10200	M-phase phosphoprotein 6
ENSG0000	48.11346	-0.68092	0.410349	-1.65938	0.097039	0.312953	ZFHX2	85446	zinc finger homeobox 2
ENSG0000	1187.591	0.164882	0.099441	1.65809	0.097299	0.313736	MS4A7	58475	membrane spanning 4-domains A7
ENSG0000	255.8701	-0.26108	0.157482	-1.65784	0.09735	0.313844	NBPF11	200030	NBPF member 11
ENSG0000	2413.018	0.137742	0.083102	1.65751	0.097416	0.314004	DYRK1A	1859	dual specificity tyrosine phosphorylation regulated kinase 1A
ENSG0000	799.5895	-0.21545	0.130028	-1.65698	0.097523	0.314033	MEGF6	1953	multiple EGF like domains 6
ENSG0000	3.543234	-2.68146	1.618258	-1.657	0.097519	0.314033	GPR199P	653160	G protein- pseudogene
ENSG0000	80.51496	0.459712	0.277421	1.657088	0.097502	0.314033	NAP1L5	266812	nucleosome assembly protein 1 like 5
ENSG0000	137219.8	0.105605	0.063729	1.657085	0.097502	0.314033	EEF1A1	1915	eukaryotic translation elongation factor 1 alpha 1
ENSG0000	11.53524	1.249696	0.75423	1.656916	0.097536	0.314033	NA	NA	NA
ENSG0000	142.2381	-0.39714	0.239671	-1.65701	0.097517	0.314033	CDC42BPG	55561	CDC42 binding protein kinase gamma
ENSG0000	30.92609	0.850875	0.513557	1.656827	0.097554	0.314033	ZNF385C	201181	zinc finger protein 385C
ENSG0000	3652.127	0.153637	0.092731	1.656796	0.097561	0.314033	ICAM3	3385	intercellular adhesion molecule 3
ENSG0000	876.5879	0.19036	0.114903	1.656704	0.097579	0.314038	SMU1	55234	SMU1 DNA replication regulator and spliceosomal factor
ENSG0000	137.9325	0.36079	0.217791	1.656584	0.097604	0.314062	LINC0052	200298	long intergenic non-protein coding RNA 528
ENSG0000	1241.375	0.165972	0.100207	1.656282	0.097665	0.31415	CLEC2B	9976	C-type lectin domain family 2 member B
ENSG0000	31.78401	0.757041	0.45707	1.65629	0.097663	0.31415	MYO5C	55930	myosin VC
ENSG0000	653.6589	0.213485	0.128902	1.656184	0.097685	0.314159	USP16	10600	ubiquitin specific peptidase 16
ENSG0000	56.52865	-0.51163	0.30898	-1.65586	0.09775	0.314315	NA	NA	NA
ENSG0000	511.4068	0.230505	0.139247	1.655373	0.097849	0.314524	MTF2	22823	metal response element binding transcription factor 2
ENSG0000	26.22499	-0.94988	0.573797	-1.65542	0.097838	0.314524	NA	NA	NA
ENSG0000	227.043	0.360707	0.217931	1.655141	0.097896	0.31455	FTH1P2	2497	ferritin heavy chain 1 pseudogene 2
ENSG0000	21.56085	0.850889	0.514185	1.654831	0.097959	0.31455	GPR31	2853	G protein-coupled receptor 31
ENSG0000	19.41186	0.925069	0.558939	1.655044	0.097915	0.31455	AGBL3	340351	AGBL carboxypeptidase 3
ENSG0000	96.65253	0.448232	0.27086	1.654847	0.097955	0.31455	DIPK1B	138311	divergent protein kinase domain 1B
ENSG0000	1012.115	0.190269	0.11495	1.655231	0.097878	0.31455	STK35	140901	serine/threonine kinase 35
ENSG0000	10.52901	1.290734	0.779964	1.654863	0.097952	0.31455	NA	NA	NA
ENSG0000	379.4674	0.242039	0.146286	1.654563	0.098013	0.31467	PTRHD1	391356	peptidyl-tRNA hydrolase domain containing 1
ENSG0000	15.4129	1.179418	0.712867	1.654471	0.098032	0.314676	NA	NA	NA
ENSG0000	3617.106	-0.12543	0.07582	-1.65433	0.098061	0.314714	PTPN18	26469	protein tyrosine phosphatase non-receptor type 18
ENSG0000	4.026239	2.322845	1.404348	1.654038	0.09812	0.314849	FOXD4L1	200350	forkhead box D4 like 1
ENSG0000	761.4402	-0.22389	0.135381	-1.65375	0.098177	0.31498	IKBKE	9641	inhibitor of nuclear factor kappa B kinase subunit epsilon
ENSG0000	8.580378	-1.69972	1.027924	-1.65355	0.098219	0.31506	PARTICL	1.01E+08	promoter of MAT2A antisense radiation-induced circulating long non-coding RNA
ENSG0000	472.3303	0.233042	0.140982	1.65299	0.098333	0.315369	C19orf38	255809	chromosome 19 open reading frame 38
ENSG0000	3.439355	2.483005	1.502402	1.652691	0.098394	0.31551	NA	NA	NA
ENSG0000	497.8629	-0.23921	0.144751	-1.65257	0.098418	0.315532	ORC2	4999	origin recognition complex subunit 2
ENSG0000	257.7727	0.26229	0.158768	1.652032	0.098528	0.315831	CCDC102A	92922	coiled-coil domain containing 102A
ENSG0000	499.2406	0.223059	0.135051	1.651668	0.098602	0.315963	ZNF622	90441	zinc finger protein 622
ENSG0000	4.342972	-2.25112	1.362938	-1.65166	0.098603	0.315963	NA	NA	NA
ENSG0000	1505.011	0.17137	0.103783	1.651243	0.098689	0.316183	KDM5A	5927	lysine demethylase 5A
ENSG0000	139.3704	0.420343	0.254594	1.65103	0.098732	0.316268	MAMDC4	158056	MAM domain containing 4
ENSG0000	487.2785	-0.24855	0.150555	-1.65092	0.098756	0.316287	PGP	283871	phosphoglycolate phosphatase
ENSG0000	69.80994	-0.53153	0.322062	-1.65038	0.098865	0.316456	ZNF619	285267	zinc finger protein 619
ENSG0000	42.02983	-0.71352	0.43232	-1.65045	0.098851	0.316456	PDLIM4	8572	PDZ and LIM domain 4
ENSG0000	1879.077	-0.14019	0.084951	-1.65024	0.098894	0.316456	RNF130	55819	ring finger protein 130
ENSG0000	1183.822	0.16238	0.098391	1.650355	0.09887	0.316456	PPP2R2D	55844	protein phosphatase 2 regulatory subunit Bdelta
ENSG0000	72.75618	0.507905	0.307775	1.650249	0.098892	0.316456	RNASE3	6037	ribonuclease A family member 3
ENSG0000	9434.723	0.117261	0.071082	1.649653	0.099014	0.316787	HSP90B1	7184	heat shock protein 90 beta family member 1
ENSG0000	1076.599	0.159138	0.096486	1.649332	0.09908	0.316942	RAB11A	8766	RAB11A member RAS oncogene family
ENSG0000	232.695	-0.28779	0.174523	-1.64898	0.099152	0.317064	POLR2H	5437	RNA polyn I and III subunit H

ENSG0000	40.89445	-0.75299	0.456633	-1.649	0.099147	0.317064	TSSK6	83983	testis specific serine kinase 6
ENSG0000	51.36417	0.697179	0.422845	1.64878	0.099193	0.317115	MAFA	389692	MAF bZIP transcription factor A
ENSG0000	12.91761	1.100274	0.667345	1.648733	0.099202	0.317115	RPS15AP1	92682	ribosomal protein S15a pseudogene 1
ENSG0000	17.63245	-0.90862	0.55118	-1.64849	0.099252	0.31722	ZNF658	26149	zinc finger protein 658
ENSG0000	74.39678	-0.45566	0.276438	-1.64833	0.099286	0.317273	DNAJC6	9829	DnaJ heat shock protein family (Hsp40) member C6
ENSG0000	2682.917	0.150106	0.091096	1.647782	0.099397	0.317575	CANT1	124583	calcium activated nucleotidase 1
ENSG0000	5512.679	-0.12248	0.074341	-1.64759	0.099438	0.317649	CD96	10225	CD96 molecule
ENSG0000	12.54713	-1.24642	0.756823	-1.64691	0.099577	0.318039	FHL2	2274	four and a half LIM domains 2
ENSG0000	3468.918	0.180278	0.109519	1.646088	0.099746	0.318468	COX4I1	1327	cytochrome c oxidase subunit 4I1
ENSG0000	167.3833	-0.35257	0.214185	-1.64611	0.099741	0.318468	SETD4	54093	SET domain containing 4
ENSG0000	318.915	0.278114	0.168968	1.645956	0.099773	0.31847	MRPS10	55173	mitochondrial ribosomal protein S10
ENSG0000	4.69007	-2.08337	1.265777	-1.64592	0.099781	0.31847	NA	NA	NA
ENSG0000	101.8235	0.431273	0.262136	1.645225	0.099923	0.318871	PINLYP	390940	phospholipase A2 inhibitor and LY6/PLAUR domain containing
ENSG0000	220.1091	0.368187	0.223847	1.644819	0.100007	0.31903	NA	NA	NA
ENSG0000	563.3245	0.188746	0.114758	1.644733	0.100025	0.31903	C1orf174	339448	chromosome 1 open reading frame 174
ENSG0000	507.1714	-0.21093	0.128248	-1.64474	0.100023	0.31903	ASH2L	9070	ASH2 like histone lysine methyltransferase complex subunit
ENSG0000	727.5632	-0.19896	0.120998	-1.64434	0.100107	0.319236	TPCN1	53373	two pore segment channel 1
ENSG0000	732.987	-0.19505	0.118629	-1.6442	0.100136	0.319275	ZNF282	8427	zinc finger protein 282
ENSG0000	20.17744	-1.09163	0.663967	-1.64411	0.100154	0.319276	NA	NA	NA
ENSG0000	993.4373	-0.16869	0.102613	-1.64399	0.100178	0.319301	TASOR	23272	transcription activation suppressor
ENSG0000	116.8583	0.362194	0.220349	1.643734	0.100231	0.319359	MATN1-AS	1E+08	MATN1 antisense RNA 1
ENSG0000	10.62755	1.497232	0.910868	1.643742	0.100229	0.319359	NA	NA	NA
ENSG0000	9.397834	-1.72348	1.048702	-1.64344	0.100291	0.319495	NA	NA	NA
ENSG0000	10.17422	1.562715	0.951138	1.642995	0.100384	0.319571	NA	NA	NA
ENSG0000	8.1677	1.517906	0.923847	1.643027	0.100377	0.319571	SORCS3	22986	sortilin related VPS10 domain containing receptor 3
ENSG0000	1364.41	0.165423	0.100679	1.643082	0.100366	0.319571	VPS35	55737	VPS35 retromer complex component
ENSG0000	17.48658	-1.043	0.634785	-1.64308	0.100365	0.319571	RUNDC3A	10900	RUN domain containing 3A
ENSG0000	906.055	-0.16313	0.099321	-1.64246	0.100495	0.319871	NEURL4	84461	neuralized E3 ubiquitin protein ligase 4
ENSG0000	456.7648	0.212034	0.129104	1.642354	0.100517	0.319884	METTL22	79091	methyltra Kin17 lysine
ENSG0000	124.1971	-0.35017	0.213238	-1.64217	0.100556	0.319953	TRMT12	55039	tRNA methyltransferase 12 homolog
ENSG0000	401.262	0.273127	0.16633	1.642081	0.100573	0.319954	NA	NA	NA
ENSG0000	103.241	0.408872	0.249021	1.64192	0.100607	0.320005	LINC0029	339789	long intergenic non-protein coding RNA 299
ENSG0000	242.2789	-0.29249	0.17823	-1.64109	0.100779	0.320499	CCDC91	55297	coiled-coil domain containing 91
ENSG0000	11.92029	1.369587	0.834799	1.640619	0.100877	0.320657	NA	NA	NA
ENSG0000	11.53882	-1.22919	0.74932	-1.64041	0.100921	0.320657	LOC10050	1.01E+08	uncharacterized LOC100507403
ENSG0000	92.22599	-0.4367	0.266174	-1.64065	0.10087	0.320657	PEG13	359809	paternally expressed 13
ENSG0000	3377.116	-0.11973	0.072983	-1.64046	0.100909	0.320657	SFSWAP	6433	splicing factor SWAP
ENSG0000	48.18147	0.708005	0.431603	1.640409	0.10092	0.320657	IGHE	3497	immunoglobulin heavy constant epsilon
ENSG0000	783.6995	0.213572	0.130199	1.640349	0.100933	0.320657	MYDGF	56005	myeloid derived growth factor
ENSG0000	792.0857	-0.22019	0.134251	-1.64012	0.100981	0.320755	NAA80	24142	N-alpha-ac NatH catalytic subunit
ENSG0000	54.67552	-0.56278	0.343386	-1.63891	0.101232	0.321498	KRBA2	124751	KRAB-A domain containing 2
ENSG0000	4.214635	2.321625	1.416777	1.638666	0.101283	0.321504	NA	NA	NA
ENSG0000	238.2449	-0.29368	0.179246	-1.63842	0.101334	0.321504	ACTL6A	86	actin like 6A
ENSG0000	8.38994	-1.53647	0.937822	-1.63834	0.10135	0.321504	GCATP1	319140	glycine C-acetyltransferase pseudogene 1
ENSG0000	73.82885	-0.45588	0.278202	-1.63866	0.101285	0.321504	OTUD7A	161725	OTU deubiquitinase 7A
ENSG0000	3156.675	0.125881	0.07683	1.638434	0.101331	0.321504	RNF167	26001	ring finger protein 167
ENSG0000	11872.34	0.139159	0.084929	1.638542	0.101309	0.321504	EMP3	2014	epithelial membrane protein 3
ENSG0000	43.85768	-0.60704	0.370526	-1.63832	0.101355	0.321504	NEFH	4744	neurofilament heavy chain
ENSG0000	345.3686	-0.23781	0.1452	-1.63778	0.101467	0.32175	METTL25B	51093	methyltransferase like 25B
ENSG0000	21.54885	1.179403	0.720117	1.637793	0.101465	0.32175	NA	NA	NA
ENSG0000	91.74432	0.461541	0.281881	1.637362	0.101555	0.321972	EIF4BP3	1E+08	eukaryotic translation initiation factor 4B pseudogene 3
ENSG0000	343.0525	-1.11718	0.682384	-1.63718	0.101594	0.321985	FCGR1BP	2210	Fc gamma pseudogene
ENSG0000	57.23551	-0.5298	0.323588	-1.63725	0.101578	0.321985	PDGFA	5154	platelet derived growth factor subunit A
ENSG0000	5.213663	1.594656	0.974202	1.636884	0.101655	0.322124	RPL17P18	1E+08	ribosomal protein L17 pseudogene 18
ENSG0000	36.23217	0.734249	0.448636	1.636626	0.101709	0.322202	TAFA2	338811	TAFA chemokine like family member 2
ENSG0000	14.25168	-1.12165	0.685355	-1.6366	0.101714	0.322202	NA	NA	NA
ENSG0000	12.19561	-1.17481	0.718011	-1.6362	0.101798	0.322284	TTC30A	92104	tetratricopeptide repeat domain 30A
ENSG0000	328.4054	0.273683	0.167258	1.636286	0.10178	0.322284	RTP5	285093	receptor transporter protein 5 (putative)
ENSG0000	20.99649	-0.85543	0.522835	-1.63614	0.10181	0.322284	NA	NA	NA
ENSG0000	19.71948	-0.928	0.567118	-1.63635	0.101767	0.322284	ZNF711	7552	zinc finger protein 711
ENSG0000	445.9849	-0.22321	0.136449	-1.63588	0.101864	0.3224	SLC39A3	29985	solute carrier family 39 member 3
ENSG0000	13057.43	-0.17606	0.107632	-1.63577	0.101887	0.322419	RNF213	57674	ring finger protein 213
ENSG0000	7.606601	1.753826	1.072385	1.635445	0.101956	0.322472	NA	NA	NA
ENSG0000	194.4325	-0.29947	0.183097	-1.6356	0.101923	0.322472	COQ6	51004	coenzyme monooxygenase
ENSG0000	17.75461	-0.96781	0.59175	-1.6355	0.101943	0.322472	HERC2P7	1E+08	HERC2 pseudogene 7
ENSG0000	170.1346	-0.33104	0.202467	-1.63501	0.102047	0.322704	E2F1	1869	E2F transcription factor 1
ENSG0000	276.6326	0.274983	0.168212	1.634744	0.102103	0.322771	DCAF13	25879	DDB1 and CUL4 associated factor 13
ENSG0000	22.82059	1.118502	0.684206	1.634744	0.102103	0.322771	CYP2E1	1571	cytochrome P450 family 2 subfamily E member 1
ENSG0000	67.93068	0.563366	0.344657	1.634572	0.102139	0.32283	PDCD6IP-D	1.05E+08	PDCD6IP divergent transcript
ENSG0000	2.79483	-3.13148	1.916064	-1.63433	0.10219	0.322883	SATB2-AS1	150538	SATB2 antisense RNA 1
ENSG0000	50.40765	-0.56414	0.345171	-1.63439	0.102177	0.322883	NA	NA	NA
ENSG0000	19.71682	-1.26154	0.772027	-1.63406	0.102247	0.322951	NA	NA	NA
ENSG0000	32.39118	0.82544	0.505147	1.634058	0.102247	0.322951	CBARP	255057	CACN subunit beta associated regulatory protein
ENSG0000	4.187361	-2.50232	1.53148	-1.63392	0.102275	0.322985	NA	NA	NA
ENSG0000	4886.757	-0.12975	0.07942	-1.63375	0.102311	0.323045	ARPC5	10092	actin related protein 2/3 complex subunit 5
ENSG0000	15.45142	-0.98156	0.600855	-1.63361	0.102341	0.323084	CCNB2	9133	cyclin B2
ENSG0000	8.159739	1.446671	0.88562	1.633512	0.102361	0.323093	NA	NA	NA
ENSG0000	222.2629	-0.29141	0.178444	-1.63305	0.102458	0.323344	EHD3	30845	EH domain containing 3
ENSG0000	86.31232	0.44046	0.269758	1.632796	0.102512	0.323458	MYL5	4636	myosin light chain 5
ENSG0000	610.9947	-0.18724	0.114703	-1.63237	0.102602	0.323632	LMBR1	64327	limb development membrane protein 1
ENSG0000	2.41987	-3.04853	1.867524	-1.63239	0.102597	0.323632	APLP1	333	amyloid beta precursor like protein 1
ENSG0000	4524.613	0.122933	0.075314	1.632267	0.102623	0.323644	PSME3	10197	proteasome activator subunit 3
ENSG0000	1260.249	0.167895	0.102905	1.631564	0.102771	0.324001	SUMF2	25870	sulfatase modifying factor 2
ENSG0000	815.2122	-0.17501	0.107259	-1.63163	0.102758	0.324001	APPL2	55198	adaptor pr phosphotyrosine interacting with PH domain and leucine zipper 2
ENSG0000	3135.737	0.137712	0.084424	1.631206	0.102847	0.324184	ERN1	2081	endoplasmic reticulum to nucleus signaling 1
ENSG0000	163.3958	0.343524	0.210618	1.631029	0.102884	0.324247	ANKRD37	353322	ankyrin repeat domain 37
ENSG0000	10.63633	-1.45061	0.889545	-1.63074	0.102946	0.324387	NA	NA	NA
ENSG0000	336.6256	0.269832	0.165482	1.630581	0.102979	0.324435	MAP3K7CI	56911	MAP3K7 C-terminal like
ENSG0000	967.0679	-1.22555	0.751675	-1.63043	0.103011	0.324481	CXCL11	6373	C-X-C motif chemokine ligand 11
ENSG0000	14.18002	-1.3336	0.818126	-1.63006	0.103088	0.324668	TUBB2B	347733	tubulin beta 2B class IIb
ENSG0000	207.1318	-0.30781	0.188906	-1.62946	0.103217	0.325019	BLVRA	644	biliverdin reductase A
ENSG0000	20.39643	0.882701	0.541758	1.629328	0.103244	0.325049	GATA6	2627	GATA binding protein 6
ENSG0000	3.442922	-2.63598	1.618138	-1.62902	0.103308	0.325087	NA	NA	NA
ENSG0000	79.13344	0.449635	0.275992	1.62916	0.103279	0.325087	LMNTD2-A	692247	LMNTD2 antisense RNA 1
ENSG0000	97.31849	-0.44876	0.275469	-1.62908	0.103295	0.325087	MIEF2	125170	mitochondrial elongation factor 2
ENSG0000	185.3927	-0.29364	0.180332	-1.62833	0.103456	0.325495	TMEM143	55260	transmembrane protein 143
ENSG0000	3.661941	-2.63024	1.616024	-1.6276	0.10361	0.325869	NA	NA	NA
ENSG0000	404.5781	0.210498	0.129326	1.627655	0.103598	0.325869	MARCHF5	54708	membrane associated ring-CH-type finger 5
ENSG0000	8461.914	0.109891	0.067533	1.627217	0.103691	0.32607	ELF4	2000	E74 like ETS transcription factor 4
ENSG0000	15.28557	1.062512	0.653195	1.626637	0.103814	0.326402	NA	NA	NA

ENSG0000	246.7628	-0.28168	0.173227	-1.62606	0.103938	0.326679	ZNF512	84450	zinc finger protein 512
ENSG0000	6.264779	1.459389	0.897464	1.626125	0.103923	0.326679	NA	NA	NA
ENSG0000	1487.818	-0.14776	0.09089	-1.62569	0.104015	0.326866	TOR2A	27433	torsin family 2 member A
ENSG0000	2742.665	-0.15509	0.095417	-1.62541	0.104076	0.326948	MAP4K2	5871	mitogen-activated protein kinase kinase kinase kinase 2
ENSG0000	15.43576	-1.06618	0.655948	-1.62541	0.104076	0.326948	BCAS1	8537	brain enriched myelin associated protein 1
ENSG0000	447.2277	0.211447	0.130108	1.625156	0.104129	0.327059	GPN1	11321	GPN-loop GTPase 1
ENSG0000	8349.693	0.13281	0.081736	1.624864	0.104192	0.327144	HLA-F	3134	major hist class I F
ENSG0000	5.82228	1.737021	1.069005	1.624896	0.104185	0.327144	NA	NA	NA
ENSG0000	160.2177	-0.32255	0.198528	-1.62473	0.10422	0.327178	C1RL	51279	complement C1r subcomponent like
ENSG0000	5.767626	-2.19294	1.350197	-1.62416	0.104342	0.327505	CCDC81	60494	coiled-coil domain containing 81
ENSG0000	297.6303	-0.24919	0.153446	-1.62394	0.104388	0.327595	SIT1	27240	signaling threshold regulating transmembrane adaptor 1
ENSG0000	138.6105	0.435105	0.267963	1.623751	0.104429	0.327615	KLRG1	10219	killer cell lectin like receptor G1
ENSG0000	65.90086	-0.48341	0.297714	-1.62375	0.10443	0.327615	EID3	493861	EP300 interacting inhibitor of differentiation 3
ENSG0000	118.1853	-0.38911	0.2397	-1.62331	0.104524	0.327784	NDUFAF2	91942	NADH:ubiquinone oxidoreductase complex assembly factor 2
ENSG0000	402.9765	-0.23148	0.142612	-1.62316	0.104554	0.327784	C6orf120	387263	chromosome 6 open reading frame 120
ENSG0000	1799.616	-0.16448	0.101332	-1.62316	0.104554	0.327784	CD3G	917	CD3 gamma subunit of T-cell receptor complex
ENSG0000	2.451473	-2.98112	1.83651	-1.62325	0.104535	0.327784	PCOTH	542767	prostate and testis expressed opposite C1QTNF9B and MIPEP
ENSG0000	9812.192	0.121698	0.074997	1.622701	0.104653	0.327907	ZEB2	9839	zinc finger E-box binding homeobox 2
ENSG0000	306.7319	-0.23499	0.144804	-1.62281	0.10463	0.327907	DDX10	1662	DEAD-box helicase 10
ENSG0000	1264.167	0.177492	0.109375	1.622783	0.104636	0.327907	GDE1	51573	glycerophosphodiester phosphodiesterase 1
ENSG0000	160.1625	-0.3147	0.193945	-1.62265	0.104664	0.327907	CISD3	284106	CDGSH iron sulfur domain 3
ENSG0000	1466.69	0.148992	0.091845	1.622213	0.104758	0.328144	KCTD5	54442	potassium channel tetramerization domain containing 5
ENSG0000	126.2791	-0.36517	0.22515	-1.62189	0.104826	0.328304	GPBAR1	151306	G protein-coupled bile acid receptor 1
ENSG0000	159.3683	-0.33168	0.20452	-1.62176	0.104855	0.328337	COA3	28958	cytochrome c oxidase assembly factor 3
ENSG0000	188.662	-0.3339	0.205902	-1.62165	0.104877	0.328352	ZFTA	65998	zinc finger translocation associated
ENSG0000	14196.22	0.13643	0.084157	1.62115	0.104986	0.328622	ADAM8	101	ADAM metallopeptidase domain 8
ENSG0000	12996.42	0.148982	0.091902	1.621088	0.104999	0.328622	TPP1	1200	tripeptidyl peptidase 1
ENSG0000	4.64245	-1.97446	1.218054	-1.62099	0.105019	0.32863	FAM66C	440078	family with sequence similarity 66 member C
ENSG0000	4.03947	-2.25041	1.388555	-1.62069	0.105085	0.328726	NA	NA	NA
ENSG0000	1063.52	-0.155	0.095636	-1.62069	0.105085	0.328726	FBXO38	81545	F-box protein 38
ENSG0000	1016.571	-0.21861	0.134923	-1.62023	0.105183	0.32892	RFX7	64864	regulatory factor X7
ENSG0000	685.3908	-0.19516	0.120449	-1.62026	0.105175	0.32892	CIAO3	64428	cytosolic iron-sulfur assembly component 3
ENSG0000	414.6496	-0.22815	0.14085	-1.61982	0.105272	0.329142	PARN	5073	poly(A)-specific ribonuclease
ENSG0000	116.9938	-0.38188	0.235804	-1.61946	0.105348	0.329159	HYAL2	8692	hyaluronidase 2
ENSG0000	1362.052	0.146697	0.090574	1.619636	0.10531	0.329159	WDR55	54853	WD repeat domain 55
ENSG0000	5.850605	-1.71285	1.057618	-1.61954	0.105332	0.329159	NA	NA	NA
ENSG0000	81.46268	-0.43648	0.269514	-1.6195	0.10534	0.329159	LRRC27	80313	leucine rich repeat containing 27
ENSG0000	2663.096	-0.18294	0.112995	-1.61903	0.105441	0.32932	SP110	3431	SP110 nuclear body protein
ENSG0000	76.48137	-0.44095	0.272378	-1.61889	0.10547	0.32932	FAM174A	345757	family with sequence similarity 174 member A
ENSG0000	23.17005	-0.86979	0.537273	-1.61889	0.10547	0.32932	DNA2	1763	DNA replication helicase/nuclease 2
ENSG0000	209.2071	-0.29309	0.181031	-1.61899	0.10545	0.32932	LAGE3	8270	L antigen family member 3
ENSG0000	184.8387	-0.29164	0.180198	-1.61843	0.105569	0.329574	GABPB2	126626	GA binding protein transcription factor subunit beta 2
ENSG0000	313.4144	0.244623	0.151168	1.618218	0.105616	0.329607	MTRF1L	54516	mitochondrial translation release factor 1 like
ENSG0000	558.4456	-0.19583	0.121014	-1.61824	0.105611	0.329607	GTF3C4	9329	general transcription factor IIIC subunit 4
ENSG0000	13.33995	1.190328	0.73575	1.617843	0.105697	0.329804	IBSP	3381	integrin binding sialoprotein
ENSG0000	199.2068	-0.28966	0.179084	-1.61748	0.105775	0.329936	AGAP1	116987	ArfGAP wi ankyrin repeat and PH domain 1
ENSG0000	50.74027	0.530393	0.327898	1.617554	0.105759	0.329936	RPL26P19	441073	ribosomal protein L26 pseudogene 19
ENSG0000	291.6062	0.298177	0.184437	1.616694	0.105944	0.330147	C1D	10438	C1D nuclear receptor corepressor
ENSG0000	905.1433	0.162436	0.100474	1.616697	0.105944	0.330147	SEC31B	25956	SEC31 hon COPII coat complex component
ENSG0000	565.4484	-0.21319	0.131848	-1.6169	0.1059	0.330147	STYX	6815	serine/threonine/tyrosine interacting protein
ENSG0000	314.0265	-0.25463	0.157484	-1.61684	0.105913	0.330147	POMT2	29954	protein O-mannosyltransferase 2
ENSG0000	270.6725	-0.28351	0.17534	-1.61694	0.105891	0.330147	ZNF45	7596	zinc finger protein 45
ENSG0000	110.9182	-0.39847	0.246476	-1.61667	0.105949	0.330147	FAM120C	54954	family with sequence similarity 120C
ENSG0000	1527.999	-0.16271	0.100673	-1.61624	0.106042	0.330328	NFRKB	4798	nuclear factor related to kappaB binding protein
ENSG0000	7831.484	-0.12068	0.074665	-1.61624	0.106043	0.330328	ABR	29	ABR activator of RhoGEF and GTPase
ENSG0000	1568.569	-0.15371	0.095115	-1.61608	0.106076	0.330377	DNASE1L1	1774	deoxyribonuclease 1 like 1
ENSG0000	7.521497	-1.87403	1.159807	-1.61581	0.106135	0.330504	NA	NA	NA
ENSG0000	2526.862	0.150066	0.092883	1.615634	0.106173	0.330569	NOLC1	9221	nucleolar and coiled-body phosphoprotein 1
ENSG0000	670.8089	0.214169	0.132638	1.614687	0.106379	0.331152	DARS1	1615	aspartyl-tRNA synthetase 1
ENSG0000	251.1536	0.254898	0.15789	1.614403	0.10644	0.331232	RPL10P9	389342	ribosomal protein L10 pseudogene 9
ENSG0000	38.89009	0.599632	0.37142	1.614433	0.106434	0.331232	EEF1B2P3	644820	eukaryotic translation elongation factor 1 beta 2 pseudogene 3
ENSG0000	3665.872	0.141466	0.087644	1.614103	0.106505	0.331379	RBM3	5935	RNA binding motif protein 3
ENSG0000	2696.077	0.127627	0.079096	1.613558	0.106623	0.331691	DCAF15	90379	DDB1 and CUL4 associated factor 15
ENSG0000	43.68472	0.708081	0.438862	1.613449	0.106647	0.331709	ZBTB20	26137	zinc finger and BTB domain containing 20
ENSG0000	671.1561	0.214897	0.133219	1.613108	0.106721	0.331884	PHAF1	80262	phagosome assembly factor 1
ENSG0000	8699.476	-0.12287	0.076192	-1.61259	0.106833	0.33212	ARHGAP3	257106	Rho GTPase activating protein 30
ENSG0000	118.754	-0.39049	0.242147	-1.61264	0.106823	0.33212	RNF215	200312	ring finger protein 215
ENSG0000	36.31831	0.663938	0.411782	1.612352	0.106885	0.332227	AQP10	89872	aquaporin 10
ENSG0000	667.3558	0.239751	0.148739	1.611885	0.106987	0.332435	MELTF	4241	melanotransferrin
ENSG0000	4.416619	-1.9811	1.229063	-1.61188	0.106988	0.332435	NA	NA	NA
ENSG0000	22.55495	-1.00457	0.62357	-1.611	0.10718	0.33295	ZNF670	93474	zinc finger protein 670
ENSG0000	2.312281	-3.48702	2.164564	-1.61096	0.107189	0.33295	SULT1A2	6799	sulfotransferase family 1A member 2
ENSG0000	295.6143	0.307112	0.190654	1.610832	0.107216	0.332957	PACC1	55248	proton activated chloride channel 1
ENSG0000	4.026926	-2.24364	1.39296	-1.6107	0.107246	0.332957	PDE1C	5137	phosphodiesterase 1C
ENSG0000	2890.916	0.15554	0.096566	1.610716	0.107242	0.332957	SIN3A	25942	SIN3 transcription regulator family member A
ENSG0000	10.94805	-1.26207	0.783611	-1.61059	0.10727	0.332976	PYCR1	5831	pyrroline-5-carboxylate reductase 1
ENSG0000	367.3895	-0.24502	0.152185	-1.61003	0.107392	0.33326	YPEL4	219539	yippee like 4
ENSG0000	8.183991	-1.58159	0.98235	-1.61	0.107397	0.33326	NA	NA	NA
ENSG0000	99.57858	0.403052	0.250388	1.609708	0.107462	0.333405	MRPS33	51650	mitochondrial ribosomal protein S33
ENSG0000	3.182368	2.544818	1.581004	1.609622	0.10748	0.333407	ALDH1L2	160428	aldehyde dehydrogenase 1 family member L2
ENSG0000	2194.2	0.143671	0.089279	1.609242	0.107563	0.333608	MCM7	4176	minichromosome maintenance complex component 7
ENSG0000	5.368988	2.400392	1.491815	1.609041	0.107607	0.333689	NA	NA	NA
ENSG0000	2614.437	-0.13252	0.082369	-1.60889	0.10764	0.333736	SYMPK	8189	symplekin scaffold protein
ENSG0000	120.6531	0.412113	0.256176	1.608712	0.107679	0.3338	DDX11L16	727856	DEAD/H-box helicase 11 like 16 (pseudogene)
ENSG0000	1069.951	0.221164	0.13752	1.608225	0.107786	0.333959	BAK1	578	BCL2 antagonist/killer 1
ENSG0000	1497.469	0.152241	0.094668	1.608158	0.107801	0.333959	LUC7L2	51631	LUC7 like pre-mRNA splicing factor
ENSG0000	1361.066	0.145102	0.090229	1.608149	0.107802	0.333959	PSMD7	5713	proteasom non-ATPase 7
ENSG0000	36.38098	-0.72603	0.451418	-1.60834	0.107761	0.333959	GATD3	8209	glutamine amidotransferase class 1 domain containing 3
ENSG0000	51.36526	0.618896	0.384895	1.607959	0.107844	0.334033	BRMS1L	84312	BRMS1 like transcriptional repressor
ENSG0000	117.0339	-0.43378	0.269819	-1.60767	0.107907	0.33417	LINC0196	150967	long intergenic non-protein coding RNA 1963
ENSG0000	171.5328	-0.34805	0.216552	-1.60722	0.108006	0.334421	TMEM19	55266	transmembrane protein 19
ENSG0000	135.5169	-0.34872	0.216994	-1.60706	0.108042	0.334476	GALT	2592	galactose-1-phosphate uridylyltransferase
ENSG0000	4.731236	-1.88241	1.171547	-1.60677	0.108105	0.334617	NA	NA	NA
ENSG0000	2.379722	3.160426	1.967474	1.606336	0.1082	0.334855	NA	NA	NA
ENSG0000	65.58316	-0.59623	0.371212	-1.60617	0.108237	0.334913	ZFP30	22835	ZFP30 zinc finger protein
ENSG0000	15.24165	-1.19315	0.742947	-1.60596	0.108282	0.334996	SRD5A3	79644	steroid 5 alpha-reductase 3
ENSG0000	147.2351	0.329233	0.205173	1.604662	0.108568	0.335826	RPA3	6119	replication protein A3
ENSG0000	71.14375	-0.52441	0.326841	-1.60448	0.108609	0.335871	SNORD62	692093	small nucl C/D box 62B
ENSG0000	21.71139	-0.85046	0.530069	-1.60443	0.108619	0.335871	UHRF1	29128	ubiquitin like with PHD and ring finger domains 1
ENSG0000	48.02469	0.559857	0.349116	1.603643	0.108793	0.336352	ZNF280C	55609	zinc finger protein 280C

ENSG0000	446.8718	0.19974	0.124588	1.60321	0.108888	0.33656	ANKRD13C	81573	ankyrin repeat domain 13C
ENSG0000	1726.793	0.145894	0.091003	1.603174	0.108896	0.33656	IPO5	3843	importin 5
ENSG0000	7.339108	1.585752	0.989267	1.602957	0.108944	0.336653	ZBTB44-DT	646383	ZBTB44 divergent transcript
ENSG0000	193.7439	-0.31546	0.196845	-1.60259	0.109025	0.336678	OBI1	79596	ORC ubiquitin ligase 1
ENSG0000	2.748324	3.102967	1.935945	1.602818	0.108975	0.336678	NA	NA	NA
ENSG0000	549.7735	-0.23323	0.145522	-1.60268	0.109005	0.336678	CHTF18	63922	chromosome transmission fidelity factor 18
ENSG0000	36.87212	0.696612	0.434678	1.602593	0.109025	0.336678	TPTEP1	387590	TPTE pseudogene 1
ENSG0000	1809.773	0.165998	0.103617	1.602035	0.109148	0.337001	ZBTB18	10472	zinc finger and BTB domain containing 18
ENSG0000	1621.611	0.156358	0.097623	1.601645	0.109234	0.337155	RCAN3	11123	RCAN family member 3
ENSG0000	1233.714	0.711469	0.444211	1.601645	0.109234	0.337155	KANK1	23189	KN motif and ankyrin repeat domains 1
ENSG0000	35.30116	0.646514	0.4038	1.601074	0.109361	0.337489	NA	NA	NA
ENSG0000	240.4573	0.256976	0.160514	1.600954	0.109387	0.337515	ARL6IP6	151188	ADP ribosylation factor like GTPase 6 interacting protein 6
ENSG0000	651.9624	0.208855	0.130469	1.600807	0.10942	0.337559	LETMD1	25875	LETM1 domain containing 1
ENSG0000	86.05281	-0.51355	0.32084	-1.60063	0.10946	0.337626	LINC01550	388011	long intergenic non-protein coding RNA 1550
ENSG0000	539.3766	0.235527	0.147159	1.600493	0.109489	0.337661	MZT2A	653784	mitotic spindle organizing protein 2A
ENSG0000	5471.203	0.121612	0.075991	1.600351	0.109521	0.337703	ATG2A	23130	autophagy related 2A
ENSG0000	18.73652	0.944249	0.590205	1.599866	0.109628	0.337922	NA	NA	NA
ENSG0000	2.887505	-2.79868	1.7493	-1.59989	0.109624	0.337922	NA	NA	NA
ENSG0000	399.0849	-0.22645	0.141615	-1.59904	0.109811	0.338325	SCYL3	57147	SCY1 like pseudokinase 3
ENSG0000	37.78265	-0.65163	0.40751	-1.59906	0.109807	0.338325	NA	NA	NA
ENSG0000	3145.686	0.130694	0.081733	1.599031	0.109814	0.338325	NPLOC4	55666	NPL4 hom ubiquitin recognition factor
ENSG0000	979.2389	-0.17639	0.110318	-1.59894	0.109834	0.338332	KLF9	687	KLF transcription factor 9
ENSG0000	24850.53	0.124261	0.07773	1.598625	0.109904	0.33849	RPS15	6209	ribosomal protein S15
ENSG0000	2293.488	-0.14413	0.09017	-1.59841	0.109953	0.338584	CHMP7	91782	charged multivesicular body protein 7
ENSG0000	17.63243	0.918563	0.574864	1.59788	0.11007	0.338888	SLC12A5	57468	solute carrier family 12 member 5
ENSG0000	7.956078	1.852169	1.159409	1.597511	0.110152	0.33905	SSBP3-AS1	619518	SSBP3 antisense RNA 1
ENSG0000	13.91117	1.121835	0.702269	1.597443	0.110167	0.33905	MEX3A	92312	mex-3 RNA binding family member A
ENSG0000	953.4205	0.183921	0.115138	1.597397	0.110177	0.33905	APBB3	10307	amyloid beta precursor protein binding family B member 3
ENSG0000	1.995718	3.450001	2.160078	1.597165	0.110229	0.339153	NA	NA	NA
ENSG0000	407.7127	-0.26215	0.164231	-1.59624	0.110435	0.339452	SCMH1	22955	Scm polycomb group protein homolog 1
ENSG0000	31.99827	0.689522	0.431952	1.596295	0.110423	0.339452	NA	NA	NA
ENSG0000	2.446335	3.082351	1.931241	1.596046	0.110478	0.339452	SNORD19	692089	small nucl C/D box 19
ENSG0000	24.34479	0.770288	0.482531	1.596351	0.11041	0.339452	IL17RB	55540	interleukin 17 receptor B
ENSG0000	1428.057	0.168138	0.105349	1.596015	0.110485	0.339452	NOP14	8602	NOP14 nucleolar protein
ENSG0000	287.0581	-0.26149	0.163816	-1.59622	0.11044	0.339452	STK32C	282974	serine/threonine kinase 32C
ENSG0000	32.62616	-0.69548	0.435763	-1.59601	0.110486	0.339452	EEF1AKMT	25895	EEF1A lysine methyltransferase 3
ENSG0000	761.1281	0.164035	0.102779	1.595991	0.110491	0.339452	IDH3A	3419	isocitrate dehydrogenase (NAD(+)) 3 catalytic subunit alpha
ENSG0000	67.8413	-0.49516	0.310147	-1.59652	0.110373	0.339452	ZSCAN30	1E+08	zinc finger and SCAN domain containing 30
ENSG0000	599.2857	0.177008	0.110914	1.595906	0.11051	0.339454	ACP1	52	acid phosphatase 1
ENSG0000	1383.169	0.143321	0.089848	1.595155	0.110678	0.339632	PSMB2	5690	proteasome 20S subunit beta 2
ENSG0000	499.0159	0.207984	0.130371	1.595317	0.110641	0.339632	OXNAD1	92106	oxidoreductase NAD binding domain containing 1
ENSG0000	3683.151	0.169271	0.106105	1.595326	0.110639	0.339632	TRIM22	10346	tripartite motif containing 22
ENSG0000	3980.296	0.12283	0.076984	1.595533	0.110593	0.339632	TBC1D10B	26000	TBC1 domain family member 10B
ENSG0000	1419.956	0.196518	0.123192	1.595218	0.110664	0.339632	GZMM	3004	granzyme M
ENSG0000	47.62188	-0.61898	0.388011	-1.59527	0.110652	0.339632	HDAC8	55869	histone deacetylase 8
ENSG0000	733.8653	0.174764	0.10957	1.595004	0.110711	0.339679	TMEM185	79134	transmembrane protein 185B
ENSG0000	110.3508	0.409833	0.256966	1.594893	0.110736	0.339699	STAG3L4	64940	stromal antigen 3-like 4 (pseudogene)
ENSG0000	103.7557	-0.48273	0.302707	-1.5947	0.110779	0.339775	CA13	377677	carbonic anhydrase 13
ENSG0000	44.18255	0.589182	0.369557	1.594293	0.11087	0.339999	NA	NA	NA
ENSG0000	6.685186	1.842189	1.156506	1.592892	0.111185	0.340896	NA	NA	NA
ENSG0000	6.460812	1.553808	0.975505	1.592824	0.1112	0.340896	NA	NA	NA
ENSG0000	1355.778	0.15888	0.099757	1.592677	0.111233	0.340941	GMEB2	26205	glucocorticoid modulatory element binding protein 2
ENSG0000	9.391601	1.476373	0.927031	1.592582	0.111254	0.34095	ST18	9705	ST18 C2H2C-type zinc finger transcription factor
ENSG0000	9.873887	-1.28057	0.80414	-1.59247	0.111279	0.340971	NUDT16-D	339874	NUDT16 divergent transcript
ENSG0000	304.5064	-0.2675	0.167994	-1.59233	0.111311	0.341011	ZDHHC24	254359	zinc finger DHHC-type containing 24
ENSG0000	109.0903	0.413645	0.259821	1.59204	0.111376	0.341153	RGS9	8787	regulator of G protein signaling 9
ENSG0000	90.9343	0.441876	0.277579	1.59189	0.111409	0.3412	TUBD1	51174	tubulin delta 1
ENSG0000	867.3476	-0.16474	0.103503	-1.59162	0.111471	0.341231	PREP	5550	prolyl endopeptidase
ENSG0000	37.43239	-0.66775	0.419516	-1.59171	0.111449	0.341231	FANCC	2176	FA complementation group C
ENSG0000	60.52982	-0.49011	0.307938	-1.5916	0.111475	0.341231	NA	NA	NA
ENSG0000	27.46992	0.924913	0.581267	1.591202	0.111564	0.341392	FAM221A	340277	family with sequence similarity 221 member A
ENSG0000	1207.209	-0.15704	0.098692	-1.59121	0.111562	0.341392	CARM1	10498	coactivator associated arginine methyltransferase 1
ENSG0000	24.88152	-0.83435	0.524442	-1.59093	0.111625	0.341523	TRAV3	28690	T cell receptor alpha variable 3
ENSG0000	26.902	-0.72985	0.458823	-1.5907	0.111676	0.341623	NUBPL	80224	NUBP iron mitochondrial
ENSG0000	66.39117	-0.50991	0.320593	-1.59052	0.111717	0.341691	ZGRF1	55345	zinc finger GRF-type containing 1
ENSG0000	95.81636	0.394033	0.247766	1.590348	0.111756	0.341699	MGMT	4255	O-6-methylguanine-DNA methyltransferase
ENSG0000	1149.815	-0.16978	0.106759	-1.59035	0.111756	0.341699	PGLS	25796	6-phosphogluconolactonase
ENSG0000	3.33609	-2.40338	1.511718	-1.58984	0.111872	0.341996	NA	NA	NA
ENSG0000	16.79478	1.110046	0.698295	1.589652	0.111913	0.342066	NA	NA	NA
ENSG0000	15.22189	1.069611	0.672922	1.589501	0.111947	0.342113	MIR4458H	1.01E+08	MIR4458 host gene
ENSG0000	53.30281	0.577002	0.363077	1.589199	0.112015	0.342209	LOC15476	154761	family witl member C pseudogene
ENSG0000	885.6763	0.163371	0.102797	1.589268	0.112	0.342209	C14orf119	55017	chromosome 14 open reading frame 119
ENSG0000	305.509	0.286436	0.18028	1.588836	0.112097	0.342403	ZNF675	171392	zinc finger protein 675
ENSG0000	632.828	-0.24686	0.155407	-1.58847	0.11218	0.342575	LIPA	3988	lipase A lysosomal acid type
ENSG0000	12.95928	1.026682	0.646353	1.588423	0.112191	0.342575	NA	NA	NA
ENSG0000	1163.507	-0.15908	0.10016	-1.58829	0.112222	0.342614	LETM1	3954	leucine zipper and EF-hand containing transmembrane protein 1
ENSG0000	420.8311	0.238272	0.150062	1.587831	0.112325	0.342815	PHAX	51808	phosphorylated adaptor for RNA export
ENSG0000	7.054534	1.73401	1.092048	1.587852	0.11232	0.342815	P2RX2	22953	purinergic receptor P2X 2
ENSG0000	4.22755	-2.19978	1.385786	-1.58739	0.112424	0.343063	NA	NA	NA
ENSG0000	326.8875	0.238777	0.150455	1.587028	0.112506	0.343256	POLR1C	9533	RNA polymerase I and III subunit C
ENSG0000	224.8096	-0.27131	0.170964	-1.58694	0.112526	0.343261	DYNC2I1	55112	dynein 2 intermediate chain 1
ENSG0000	26.41956	-0.76058	0.479441	-1.58638	0.112653	0.343579	CD1D	912	CD1d molecule
ENSG0000	18022.4	0.122599	0.077285	1.586316	0.112668	0.343579	ANXA2	302	annexin A2
ENSG0000	2165.779	-0.13209	0.083289	-1.58588	0.112767	0.343824	GON4L	54856	gon-4 like
ENSG0000	5.077223	2.074133	1.308285	1.585384	0.112879	0.344016	TMEM269	1E+08	transmembrane protein 269
ENSG0000	300.1849	-0.30682	0.193544	-1.5853	0.112899	0.344016	FAM117B	150864	family with sequence similarity 117 member B
ENSG0000	173.8908	0.397115	0.250502	1.585275	0.112904	0.344016	NA	NA	NA
ENSG0000	1203.359	0.174235	0.109906	1.585313	0.112895	0.344016	RNF114	55905	ring finger protein 114
ENSG0000	420.4213	-0.27149	0.171286	-1.58503	0.11296	0.344132	ARMC6	93436	armadillo repeat containing 6
ENSG0000	3.764629	2.19984	1.388326	1.584528	0.113074	0.344421	NA	NA	NA
ENSG0000	73.77378	-0.48682	0.307291	-1.58424	0.113138	0.344561	ZNF816	125893	zinc finger protein 816
ENSG0000	2441.918	0.126857	0.080085	1.584035	0.113186	0.344649	YTHDF1	54915	YTH N6-methyladenosine RNA binding protein 1
ENSG0000	47.34343	-0.55292	0.34911	-1.58381	0.113238	0.34475	ZNF80	7634	zinc finger protein 80
ENSG0000	72.07948	0.48304	0.305038	1.583541	0.113298	0.344765	FOLR2	2350	folate receptor beta
ENSG0000	10.16632	1.222893	0.772187	1.583674	0.113268	0.344765	LOC12490	1.25E+08	uncharacterized LOC124902718
ENSG0000	2903.851	0.142064	0.08971	1.583595	0.113286	0.344765	EIF3K	27335	eukaryotic translation initiation factor 3 subunit K
ENSG0000	103.9001	-0.44174	0.278997	-1.58331	0.113351	0.344869	KANTR	1.03E+08	KANTR integral membrane protein
ENSG0000	292.3617	-0.27379	0.17298	-1.58278	0.113471	0.345178	VILL	50853	villin like
ENSG0000	469.9886	0.23286	0.147151	1.582458	0.113545	0.345346	PRPF39	55015	pre-mRNA processing factor 39
ENSG0000	2.188809	-3.03602	1.918906	-1.58216	0.113612	0.345402	LOC12490	1.25E+08	uncharacterized LOC124904048

ENSG0000	28.59226	-0.68446	0.432607	-1.58217	0.11361	0.345402	EID2B	126272	EP300 interacting inhibitor of differentiation 2B
ENSG0000	757.1692	-0.21948	0.138722	-1.58213	0.113619	0.345402	NA	NA	NA
ENSG0000	10.1327	-1.31512	0.831333	-1.58195	0.113662	0.345474	CELF5	60680	CUGBP Elav-like family member 5
ENSG0000	2499.976	-0.14456	0.091392	-1.58176	0.113703	0.345488	CNPY3	10695	canopy FGF signaling regulator 3
ENSG0000	1521.58	0.136831	0.086501	1.581843	0.113685	0.345488	UFD1	7353	ubiquitin recognition factor in ER associated degradation 1
ENSG0000	7.316848	-1.72328	1.089636	-1.58151	0.11376	0.345588	IFI27	3429	interferon alpha inducible protein 27
ENSG0000	554.5817	-0.22075	0.139584	-1.58146	0.113774	0.345588	CCDC137	339230	coiled-coil domain containing 137
ENSG0000	15.13415	-1.26387	0.799235	-1.58135	0.113798	0.345605	NECTIN4	81607	nectin cell adhesion molecule 4
ENSG0000	7.131781	1.764108	1.115687	1.581185	0.113836	0.345661	NA	NA	NA
ENSG0000	6151.732	0.139853	0.088453	1.581107	0.113854	0.345661	SMCHD1	23347	structural maintenance of chromosomes flexible hinge domain containing 1
ENSG0000	344.834	-0.25808	0.163322	-1.58016	0.11407	0.346261	CEACAM8	1088	CEA cell adhesion molecule 8
ENSG0000	110.2755	-0.37681	0.238509	-1.57985	0.114141	0.346419	C17orf67	339210	chromosome 17 open reading frame 67
ENSG0000	101.7604	-0.37717	0.23876	-1.5797	0.114175	0.346467	ALOX12-A!	1.01E+08	ALOX12 antisense RNA 1
ENSG0000	194.7146	-0.30375	0.192339	-1.57925	0.114278	0.346722	LOC10537	1.05E+08	uncharacterized LOC105377225
ENSG0000	40.07686	0.68467	0.433575	1.579128	0.114307	0.346753	NA	NA	NA
ENSG0000	1654.221	-0.17294	0.109526	-1.57903	0.11433	0.346767	RPUSD1	113000	RNA pseudouridine synthase domain containing 1
ENSG0000	643.2339	-0.21456	0.135891	-1.57893	0.114352	0.346777	ZNF414	84330	zinc finger protein 414
ENSG0000	73.67837	0.482157	0.305394	1.578802	0.114381	0.346809	RAI2	10742	retinoic acid induced 2
ENSG0000	192.1161	-0.34478	0.218466	-1.57817	0.114526	0.34719	PXYLP1	92370	2-phosphoxylose phosphatase 1
ENSG0000	2.983168	-2.2823	1.446464	-1.57785	0.114601	0.347286	NA	NA	NA
ENSG0000	1090.756	-0.15895	0.100736	-1.57789	0.114591	0.347286	DRG2	1819	developmentally regulated GTP binding protein 2
ENSG0000	155.9971	-0.31764	0.20132	-1.57779	0.114614	0.347286	TCFL5	10732	transcription factor like 5
ENSG0000	4265.048	0.12312	0.07805	1.577459	0.11469	0.347306	TNFRSF14	8764	TNF receptor superfamily member 14
ENSG0000	56.15947	-0.57543	0.36474	-1.57765	0.114647	0.347306	RBM45	129831	RNA binding motif protein 45
ENSG0000	310.0164	-0.2897	0.183653	-1.57744	0.114695	0.347306	GLT8D1	55830	glycosyltransferase 8 domain containing 1
ENSG0000	28.24403	-0.7946	0.503682	-1.57757	0.114663	0.347306	SIX5	147912	SIX homeobox 5
ENSG0000	1726.226	0.164071	0.104037	1.57704	0.114786	0.347525	TOLLIP	54472	toll interacting protein
ENSG0000	29.52139	-0.89617	0.568434	-1.57657	0.114895	0.347799	NAV2	89797	neuron navigator 2
ENSG0000	38.33427	0.641047	0.406632	1.576478	0.114916	0.347804	MFSD14A	64645	major facilitator superfamily domain containing 14A
ENSG0000	115.0299	-0.38953	0.247139	-1.57614	0.114992	0.347979	ZNF252P	286101	zinc finger pseudogene
ENSG0000	1181.658	0.184576	0.117134	1.575771	0.115078	0.348167	NELFA	7469	negative elongation factor complex member A
ENSG0000	859.3304	-0.1851	0.117473	-1.57571	0.115092	0.348167	DENND4C	55667	DENN domain containing 4C
ENSG0000	486.5402	0.212803	0.135087	1.575305	0.115186	0.348395	DHX40	79665	DEAH-box helicase 40
ENSG0000	3392.757	-0.13916	0.088369	-1.5748	0.115303	0.348439	ATP13A2	23400	ATPase cation transporting 13A2
ENSG0000	96.91745	-0.38535	0.244645	-1.57514	0.115224	0.348439	IFT172	26160	intraflagellar transport 172
ENSG0000	12.58961	-1.06352	0.675463	-1.57451	0.11537	0.348439	FST	10468	follistatin
ENSG0000	419.9715	-0.24977	0.158631	-1.57452	0.115368	0.348439	ZNF853	54753	zinc finger protein 853
ENSG0000	11.20355	1.348531	0.856408	1.574637	0.11534	0.348439	BCL2L14	79370	BCL2 like 14
ENSG0000	5.994265	1.682283	1.068097	1.575029	0.11525	0.348439	TNS2-AS1	283335	TNS2 antisense RNA 1
ENSG0000	3.554377	2.636211	1.673894	1.574897	0.11528	0.348439	LOC10537	1.05E+08	uncharacterized LOC105371734
ENSG0000	685.9716	-0.23645	0.150166	-1.57461	0.115347	0.348439	ZNF358	140467	zinc finger protein 358
ENSG0000	5.430109	-1.97753	1.255891	-1.57461	0.115347	0.348439	SMIM10L2	399668	small integral membrane protein 10 like 2A
ENSG0000	79.93469	-0.41977	0.26663	-1.57436	0.115404	0.348485	SLC25A35	399512	solute carrier family 25 member 35
ENSG0000	5422.073	0.164666	0.104621	1.573929	0.115504	0.348731	ALOX5AP	241	arachidonate 5-lipoxygenase activating protein
ENSG0000	1787.138	0.141968	0.090221	1.573554	0.115591	0.348936	LEMD2	221496	LEM domain nuclear envelope protein 2
ENSG0000	93.03519	-0.41093	0.261164	-1.57347	0.115611	0.34894	PTER	9317	phosphotriesterase related
ENSG0000	4.974717	-1.915	1.217323	-1.57312	0.11569	0.349089	NA	NA	NA
ENSG0000	321.691	-0.2376	0.15104	-1.57309	0.115698	0.349089	RHOT1	55288	ras homolog family member T1
ENSG0000	2639.127	0.128433	0.081655	1.57287	0.115749	0.349129	TAX1BP1	8887	Tax1 binding protein 1
ENSG0000	49.53573	-0.55253	0.351279	-1.5729	0.115743	0.349129	THAP8	199745	THAP domain containing 8
ENSG0000	516.9205	0.193955	0.123332	1.572623	0.115806	0.349245	PSMD5	5711	proteasom non-ATPase 5
ENSG0000	5091.691	0.136146	0.086584	1.572425	0.115852	0.349327	AXIN1	8312	axin 1
ENSG0000	473.7886	0.220241	0.140074	1.572316	0.115877	0.349346	C1QB	713	complement C1q B chain
ENSG0000	1670.821	0.144718	0.092064	1.571923	0.115968	0.349507	SELENOF	9403	selenoprotein F
ENSG0000	528.3171	-0.23658	0.150499	-1.57194	0.115965	0.349507	UFL1	23376	UFM1 specific ligase 1
ENSG0000	18.37675	0.865821	0.550909	1.571623	0.116038	0.34966	NA	NA	NA
ENSG0000	240.3289	-0.28417	0.180863	-1.5712	0.116137	0.349792	SMIM14	201895	small integral membrane protein 14
ENSG0000	631.7277	-0.18812	0.119733	-1.57119	0.116138	0.349792	PPP2R5B	5526	protein phosphatase 2 regulatory subunit B'beta
ENSG0000	76.43731	-0.57154	0.363751	-1.57125	0.116126	0.349792	CDADC1	81602	cytidine and dCMP deaminase domain containing 1
ENSG0000	32.37029	-0.65343	0.415949	-1.57095	0.116195	0.349907	SLC18A2	6571	solute carrier family 18 member A2
ENSG0000	249.1695	-0.24573	0.156452	-1.57063	0.11627	0.350073	TRIM74	378108	tripartite motif containing 74
ENSG0000	4133.188	0.133244	0.084854	1.570266	0.116353	0.350268	EMILIN2	84034	elastin microfibril interfacer 2
ENSG0000	44.91342	-0.57486	0.366162	-1.56997	0.116421	0.350417	ARHGAP6	395	Rho GTPase activating protein 6
ENSG0000	80.23145	-0.42461	0.270511	-1.56964	0.116499	0.350592	ZNF829	374899	zinc finger protein 829
ENSG0000	14385.95	0.11707	0.074592	1.569475	0.116537	0.350652	FCMR	9214	Fc mu receptor
ENSG0000	272.5057	-1.23919	0.789664	-1.56926	0.116587	0.350739	ETV7	51513	ETS variant transcription factor 7
ENSG0000	378.5941	-0.21805	0.138956	-1.56919	0.116604	0.350739	TUBGCP4	27229	tubulin gamma complex associated protein 4
ENSG0000	146.4529	0.350111	0.22314	1.56902	0.116643	0.3508	PF4V1	5197	platelet factor 4 variant 1
ENSG0000	1468.612	0.144296	0.09197	1.568936	0.116663	0.350802	RNF115	27246	ring finger protein 115
ENSG0000	2053.648	0.145297	0.092618	1.568774	0.116701	0.350859	MALT1	10892	MALT1 paracaspase
ENSG0000	222.2856	-0.27764	0.177015	-1.56847	0.116772	0.351017	BBIP1	92482	BBSome interacting protein 1
ENSG0000	447.9833	-0.23132	0.147491	-1.56836	0.116797	0.351034	CASS4	57091	Cas scaffold protein family member 4
ENSG0000	2211.183	-0.15481	0.098745	-1.56773	0.116945	0.351081	HECTD3	79654	HECT domain E3 ubiquitin protein ligase 3
ENSG0000	13.72644	-1.16801	0.745021	-1.56775	0.116939	0.351081	NA	NA	NA
ENSG0000	299.9765	-0.24455	0.155967	-1.56793	0.116897	0.351081	ANGEL2	90806	angel homolog 2
ENSG0000	235.3405	0.321758	0.205218	1.567882	0.116909	0.351081	BABAM2	9577	BRISC and BRCA1 A complex member 2
ENSG0000	10.25132	-1.36898	0.873046	-1.56804	0.116871	0.351081	NA	NA	NA
ENSG0000	612.3979	-0.2411	0.153757	-1.56807	0.116865	0.351081	LINC0304(	221416	long intergenic non-protein coding RNA 3040
ENSG0000	16.6507	0.992073	0.63276	1.567851	0.116916	0.351081	NA	NA	NA
ENSG0000	225.9843	-0.27204	0.17354	-1.56761	0.116972	0.351107	ZNF79	7633	zinc finger protein 79
ENSG0000	9.999857	-1.19526	0.762551	-1.56745	0.117009	0.35116	CEP295NL	1.01E+08	CEP295 N-terminal like
ENSG0000	3.578941	2.15058	1.372112	1.56735	0.117033	0.351174	RN7SL280I	1.06E+08	RNA 7SL cytoplasm pseudogene
ENSG0000	1184.638	0.187688	0.119763	1.56716	0.117077	0.351194	ADAM9	8754	ADAM metallopeptidase domain 9
ENSG0000	5.842632	1.595271	1.017934	1.567166	0.117076	0.351194	CERS3	204219	ceramide synthase 3
ENSG0000	5.353664	1.831803	1.16951	1.5663	0.117279	0.351741	NA	NA	NA
ENSG0000	423.9193	-0.21011	0.134186	-1.56584	0.117386	0.351948	CZIB	54987	CXXC motif containing zinc binding protein
ENSG0000	2514.668	-0.13032	0.083225	-1.56592	0.117368	0.351948	LARP4B	23185	La ribonucleoprotein 4B
ENSG0000	32.09754	-0.73533	0.469635	-1.56575	0.117406	0.351954	SHANK3	85358	SH3 and multiple ankyrin repeat domains 3
ENSG0000	41.64344	-0.80106	0.511691	-1.56551	0.117464	0.352022	NA	NA	NA
ENSG0000	13.2564	-1.31643	0.840945	-1.56541	0.117486	0.352022	LGR4	55366	leucine rich repeat containing G protein-coupled receptor 4
ENSG0000	22329.52	-0.10906	0.069668	-1.56544	0.117481	0.352022	HSPA8	3312	heat shock protein family A (Hsp70) member 8
ENSG0000	34.42289	0.730628	0.466774	1.56527	0.11752	0.352066	LINC0261(	151174	long intergenic non-protein coding RNA 2610
ENSG0000	1556.182	0.136962	0.087518	1.564946	0.117596	0.352236	STT3A	3703	STT3 oligosaccharyltransferase complex catalytic subunit A
ENSG0000	91.39079	-0.40488	0.258785	-1.56456	0.117686	0.352451	ZNF789	285989	zinc finger protein 789
ENSG0000	272.8671	-0.2796	0.178745	-1.56422	0.117767	0.352579	DSTYK	25778	dual serine/threonine and tyrosine protein kinase
ENSG0000	1858.898	0.150674	0.096321	1.564286	0.11775	0.352579	INPP5K	51763	inositol polyphosphate-5-phosphatase K
ENSG0000	2043.263	0.183294	0.117189	1.56409	0.117796	0.352594	NFAT5	10725	nuclear factor of activated T cells 5
ENSG0000	75.33819	-0.51578	0.329774	-1.56403	0.11781	0.352594	SDR42E1	93517	short chair member 1
ENSG0000	6.274197	1.415754	0.90548	1.563539	0.117926	0.352884	NA	NA	NA
ENSG0000	628.1738	-0.20293	0.129853	-1.56278	0.118105	0.353361	PLXNC1	10154	plexin C1

ENSG0000	36.06346	-0.64875	0.41525	-1.56232	0.118212	0.353625	CIT	11113	citron rho-interacting serine/threonine kinase
ENSG0000	20.80145	0.935482	0.598839	1.56216	0.11825	0.353683	RCVRN	5957	recoverin
ENSG0000	6924.317	0.109274	0.069955	1.562059	0.118274	0.353697	CPSF7	79869	cleavage and polyadenylation specific factor 7
ENSG0000	1143.789	-0.16938	0.108455	-1.56176	0.118346	0.353854	PSTPIP1	9051	proline-serine-threonine phosphatase interacting protein 1
ENSG0000	9.369	-1.38645	0.887912	-1.56148	0.118411	0.353993	CTF1	1489	cardiotrophin 1
ENSG0000	3222.586	0.122939	0.078759	1.56095	0.118536	0.354308	RPS2P5	1E+08	ribosomal protein S2 pseudogene 5
ENSG0000	5.823258	-1.46841	0.941007	-1.56047	0.11865	0.354535	NA	NA	NA
ENSG0000	2212.008	0.143973	0.092259	1.560519	0.118637	0.354535	ADNP	23394	activity dependent neuroprotector homeobox
ENSG0000	7.122519	1.675082	1.073514	1.560372	0.118672	0.354544	LINC01637	1.02E+08	long intergenic non-protein coding RNA 1637
ENSG0000	5.359999	1.919236	1.230472	1.559756	0.118818	0.354922	YBX1P4	1E+08	Y-box binding protein 1 pseudogene 4
ENSG0000	306.8046	0.236265	0.151498	1.559526	0.118872	0.355027	MMD	23531	monocyte to macrophage differentiation associated
ENSG0000	19.72832	-0.86914	0.557445	-1.55915	0.118962	0.355238	NA	NA	NA
ENSG0000	680.0488	0.218809	0.140378	1.558711	0.119065	0.355374	CHMP2B	25978	charged multivesicular body protein 2B
ENSG0000	102.4536	-0.39251	0.251813	-1.55874	0.119057	0.355374	AUH	549	AU RNA binding methylglutaconyl-CoA hydratase
ENSG0000	204.4657	0.755563	0.484725	1.558747	0.119056	0.355374	OSGIN1	29948	oxidative stress induced growth inhibitor 1
ENSG0000	2060.255	-0.15235	0.097804	-1.55772	0.119299	0.355784	DOK2	9046	docking protein 2
ENSG0000	80.52044	0.438517	0.281482	1.557889	0.11926	0.355784	SLC18A2-A	1.05E+08	SLC18A2 antisense RNA 1
ENSG0000	9.268234	1.522972	0.977634	1.557814	0.119277	0.355784	TRD-AS1	1.05E+08	TRD antisense RNA 1
ENSG0000	3.92034	-2.16431	1.389549	-1.55756	0.119336	0.355784	GOLGA8K	653125	golgin A8 family member K
ENSG0000	1293.214	0.155001	0.099493	1.557908	0.119255	0.355784	SNN	8303	stannin
ENSG0000	3.209026	-2.56064	1.643836	-1.55772	0.119299	0.355784	NA	NA	NA
ENSG0000	3573.085	0.131197	0.084228	1.557635	0.11932	0.355784	SGTA	6449	small glutamine rich tetratricopeptide repeat co-chaperone alpha
ENSG0000	17.3339	0.949589	0.609746	1.557352	0.119387	0.355877	NA	NA	NA
ENSG0000	189.8257	0.278809	0.179047	1.557184	0.119427	0.355939	PDE1B	5153	phosphodiesterase 1B
ENSG0000	5.235179	-1.80934	1.162345	-1.55663	0.119559	0.356162	NA	NA	NA
ENSG0000	12.12385	1.138194	0.731175	1.556664	0.11955	0.356162	NA	NA	NA
ENSG0000	227.8452	0.280962	0.180494	1.55663	0.119558	0.356162	ADAT3	113179	adenosine deaminase tRNA specific 3
ENSG0000	5.174032	2.173978	1.396693	1.556518	0.119585	0.356181	NA	NA	NA
ENSG0000	2022.589	-0.15741	0.101161	-1.55606	0.119694	0.356185	CEP350	9857	centrosomal protein 350
ENSG0000	6.968058	-1.64136	1.054698	-1.55623	0.119653	0.356185	NA	NA	NA
ENSG0000	115.6621	0.400255	0.257189	1.556268	0.119644	0.356185	METTL8	79828	methyltraı methylcytidine
ENSG0000	33.96308	0.731649	0.470098	1.556374	0.119619	0.356185	NA	NA	NA
ENSG0000	22.14091	-0.88711	0.570081	-1.55611	0.119682	0.356185	H4C16	121504	H4 histone 16
ENSG0000	33.14414	0.679789	0.436874	1.556029	0.119701	0.356185	SOCS2-AS1	144481	SOCS2 antisense RNA 1
ENSG0000	3308.74	0.119758	0.07697	1.555912	0.119729	0.35621	GSTK1	373156	glutathione S-transferase kappa 1
ENSG0000	1038.843	-0.18578	0.11941	-1.55578	0.11976	0.356245	TOMM40	10452	translocase of outer mitochondrial membrane 40
ENSG0000	7.604071	-1.46739	0.943241	-1.55569	0.119783	0.356255	NA	NA	NA
ENSG0000	7.686328	-1.3486	0.866984	-1.55551	0.119825	0.356266	NA	NA	NA
ENSG0000	143.4839	-0.36054	0.231773	-1.55558	0.119807	0.356266	ZNF18	7566	zinc finger protein 18
ENSG0000	1992.026	-0.1344	0.086425	-1.55508	0.119927	0.3564	PPM1M	132160	protein ph Mg2+/Mn2+ dependent 1M
ENSG0000	14.75098	0.90663	0.582966	1.555201	0.119898	0.3564	NA	NA	NA
ENSG0000	9.278484	1.385618	0.891012	1.555106	0.119921	0.3564	NA	NA	NA
ENSG0000	371.6354	-0.21737	0.1398	-1.55484	0.119984	0.356512	LINC00869	57234	long intergenic non-protein coding RNA 869
ENSG0000	1085.263	0.156097	0.100415	1.554526	0.120059	0.356677	TRMT112	51504	tRNA methyltransferase activator subunit 11-2
ENSG0000	8420.646	-0.12408	0.079831	-1.55433	0.120106	0.356707	USF2	7392	upstream c-fos interacting
ENSG0000	318.1314	0.238734	0.153594	1.554323	0.120107	0.356707	TOX2	84969	TOX high mobility group box family member 2
ENSG0000	7.427512	-1.85695	1.194851	-1.55412	0.120155	0.356791	NA	NA	NA
ENSG0000	6.33796	-1.53603	0.988487	-1.55392	0.120203	0.356876	NA	NA	NA
ENSG0000	4.413654	-2.43048	1.564464	-1.55355	0.120291	0.357064	C2orf74	339804	chromosome 2 open reading frame 74
ENSG0000	38.08006	-0.64849	0.417436	-1.5535	0.120305	0.357064	ZKSCAN7	55888	zinc finger with KRAB and SCAN domains 7
ENSG0000	50.90599	-0.55601	0.358005	-1.55308	0.120403	0.357299	ZNF57	126295	zinc finger protein 57
ENSG0000	177.3633	-0.32893	0.211842	-1.55269	0.120497	0.357519	CASK	8573	calcium/calmodulin dependent serine protein kinase
ENSG0000	182.8646	-0.32755	0.211017	-1.55226	0.120601	0.357739	PRMT6	55170	protein arginine methyltransferase 6
ENSG0000	268.8108	-0.28641	0.184517	-1.55222	0.120609	0.357739	VPS54	51542	VPS54 subunit of GARP complex
ENSG0000	3.183125	-2.50209	1.612488	-1.5517	0.120735	0.358054	NA	NA	NA
ENSG0000	40.56299	-0.74898	0.482844	-1.55118	0.120858	0.358354	NA	NA	NA
ENSG0000	3.338564	-2.40151	1.548485	-1.55088	0.120931	0.358354	NA	NA	NA
ENSG0000	475.8492	-0.2062	0.132951	-1.55092	0.120921	0.358354	IBTK	25998	inhibitor of Bruton tyrosine kinase
ENSG0000	321.1495	-0.22416	0.144536	-1.55087	0.120932	0.358354	PKD1P6	353511	polycystin transient receptor potential channel interacting pseudogene 6
ENSG0000	22.47932	-0.79496	0.512533	-1.55104	0.120892	0.358354	LRP3	4037	LDL receptor related protein 3
ENSG0000	1755.852	0.184308	0.118872	1.550476	0.121027	0.358577	PPP6C	5537	protein phosphatase 6 catalytic subunit
ENSG0000	445.2735	0.240898	0.155384	1.550342	0.12106	0.358616	LYSMD3	116068	LysM domain containing 3
ENSG0000	258.2893	-0.24246	0.156455	-1.5497	0.121213	0.358956	NME6	10201	NME/NM23 nucleoside diphosphate kinase 6
ENSG0000	40.75585	-0.60516	0.390493	-1.54972	0.121208	0.358956	IGHV3-7	28452	immunoglobulin heavy variable 3-7
ENSG0000	3776.429	-0.35076	0.226354	-1.54961	0.121236	0.358967	ATF5	22809	activating transcription factor 5
ENSG0000	29.03049	0.820272	0.529688	1.548595	0.121479	0.359629	NA	NA	NA
ENSG0000	61.23874	-0.4714	0.304443	-1.5484	0.121527	0.359712	MREG	55686	melanoregulin
ENSG0000	330.3538	-0.23098	0.149209	-1.54806	0.121609	0.359898	LINC01138	388685	long intergenic non-protein coding RNA 1138
ENSG0000	8.14258	-1.93493	1.250341	-1.54752	0.121737	0.360185	NA	NA	NA
ENSG0000	82.06966	-0.48637	0.314297	-1.54749	0.121745	0.360185	TCAF1	9747	TRPM8 channel associated factor 1
ENSG0000	70.73935	-0.46676	0.30168	-1.54722	0.121811	0.360323	ZNF710-AS	1.1E+08	ZNF710 antisense RNA 1
ENSG0000	33.35827	0.646113	0.41762	1.547132	0.121831	0.360326	PPP2R5CP	140921	protein phosphatase 2 regulatory subunit B'gamma pseudogene
ENSG0000	32.72084	-0.64262	0.415416	-1.54693	0.12188	0.360413	ADGRF3	165082	adhesion G protein-coupled receptor F3
ENSG0000	4.49551	-1.89289	1.223832	-1.54669	0.121938	0.360465	RNU6ATAC	1.06E+08	RNA U6atac sn pseudogene
ENSG0000	21.42833	-0.82843	0.535609	-1.54671	0.121933	0.360465	SEMA4G	57715	semaphorin 4G
ENSG0000	80.9901	-0.47296	0.305803	-1.54662	0.121956	0.360465	TMEM135	65084	transmembrane protein 135
ENSG0000	28.65606	-0.71463	0.462148	-1.54633	0.122024	0.360609	NA	NA	NA
ENSG0000	451.2661	-0.21976	0.142126	-1.5462	0.122057	0.360649	ZHX1	11244	zinc fingers and homeoboxes 1
ENSG0000	51.22864	0.601982	0.389364	1.546064	0.122089	0.360686	PRXL2A	84293	peroxiredoxin like 2A
ENSG0000	520.2952	0.202129	0.130748	1.545948	0.122117	0.360711	FBXO28	23219	F-box protein 28
ENSG0000	5.93814	1.860644	1.203646	1.54584	0.122143	0.360731	LINGO2	158038	leucine rich repeat and Ig domain containing 2
ENSG0000	820.8357	0.162849	0.105361	1.545621	0.122196	0.36083	ORMDL3	94103	ORMDL sphingolipid biosynthesis regulator 3
ENSG0000	6.136569	-1.89954	1.229126	-1.54544	0.122241	0.360904	EPHX4	253152	epoxide hydrolase 4
ENSG0000	1461.39	0.15816	0.102349	1.545298	0.122274	0.360931	FYTTD1	84248	forty-two-three domain containing 1
ENSG0000	87.33901	-0.42998	0.278261	-1.54524	0.122289	0.360931	PRTN3	5657	proteinase 3
ENSG0000	7.935165	-1.49844	0.969876	-1.54498	0.122352	0.361059	SLC25A10	1468	solute carrier family 25 member 10
ENSG0000	7238.687	-0.14284	0.092475	-1.54466	0.122428	0.361227	ABI3	51225	ABI family member 3
ENSG0000	6.510485	1.673445	1.083481	1.544508	0.122465	0.36128	ABCA4	24	ATP binding cassette subfamily A member 4
ENSG0000	6.598781	-1.52096	0.984817	-1.54441	0.12249	0.361294	TRAV24	28659	T cell receptor alpha variable 24
ENSG0000	15.38453	0.952997	0.617197	1.544073	0.122571	0.361367	LINC00298	339788	long intergenic non-protein coding RNA 298
ENSG0000	1023.05	0.166208	0.107641	1.54409	0.122566	0.361367	PANK3	79646	pantothenate kinase 3
ENSG0000	177.066	-0.29701	0.192357	-1.54406	0.122573	0.361367	CYP1A1	1543	cytochrome P450 family 1 subfamily A member 1
ENSG0000	9.354399	-1.61946	1.049005	-1.54381	0.122635	0.361489	NA	NA	NA
ENSG0000	227.1878	0.257508	0.166809	1.543732	0.122653	0.361489	PIGA	5277	phosphatidylinositol glycan anchor biosynthesis class A
ENSG0000	20.94093	0.819404	0.530825	1.543642	0.122675	0.361496	PTPRB	5787	protein tyrosine phosphatase receptor type B
ENSG0000	57.30785	-0.49572	0.321189	-1.54339	0.122737	0.361622	NINJ2-AS1	1E+08	NINJ2 antisense RNA 1
ENSG0000	44.67704	-0.56723	0.367769	-1.54235	0.122989	0.361879	SRGAP2B	647135	SLIT-ROBO Rho GTPase activating protein 2B
ENSG0000	15.97149	0.924037	0.598939	1.542791	0.122882	0.361879	THEM5	284486	thioesterase superfamily member 5
ENSG0000	4.319112	1.756525	1.138898	1.542302	0.123	0.361879	NA	NA	NA
ENSG0000	28.46771	-0.7327	0.475062	-1.54233	0.122993	0.361879	OCM	654231	oncomodulin

ENSG0000	171.6013	-0.33603	0.217835	-1.54259	0.122929	0.361879	NA	NA	NA
ENSG0000	176.8697	-0.28675	0.18587	-1.54274	0.122895	0.361879	NUDT14	256281	nudix hydrolase 14
ENSG0000	7315.012	0.109694	0.071113	1.542542	0.122942	0.361879	RNPS1	10921	RNA binding protein with serine rich domain 1
ENSG0000	279.7989	-0.27347	0.177312	-1.54232	0.122996	0.361879	CDYL2	124359	chromodomain Y like 2
ENSG0000	1306.302	0.159442	0.103365	1.542517	0.122948	0.361879	NDUFB11	54539	NADH:ubiquinone oxidoreductase subunit B11
ENSG0000	1406.074	-0.15693	0.101761	-1.54213	0.123043	0.361946	CACTIN	58509	cactin spliceosome C complex subunit
ENSG0000	466.391	-0.22309	0.144691	-1.54186	0.123106	0.362077	NF2	4771	NF2 moesin-ezrin-radixin like (MERLIN) tumor suppressor
ENSG0000	22.77357	-0.80655	0.523272	-1.54136	0.123229	0.362378	LOC15506	155060	AI894139 pseudogene
ENSG0000	30.23799	-0.6442	0.418041	-1.541	0.123318	0.36258	DDR2	4921	discoidin domain receptor tyrosine kinase 2
ENSG0000	1763.924	0.153753	0.09978	1.54092	0.123336	0.36258	UQCRC2	7385	ubiquinol-cytochrome c reductase core protein 2
ENSG0000	1224.336	-0.15946	0.103516	-1.54047	0.123445	0.362842	DCAF1	9730	DDB1 and CUL4 associated factor 1
ENSG0000	360.4267	0.24803	0.161068	1.539915	0.123581	0.363185	HAT1	8520	histone acetyltransferase 1
ENSG0000	1568.808	-0.1481	0.096185	-1.53978	0.123613	0.36322	TNRC6C	57690	trinucleotide repeat containing adaptor 6C
ENSG0000	4117.042	-0.13832	0.089841	-1.53963	0.12365	0.363272	NCOA6	23054	nuclear receptor coactivator 6
ENSG0000	866.7654	-0.25742	0.167218	-1.53942	0.123703	0.363369	IQCN	80726	IQ motif containing N
ENSG0000	1820.087	0.130176	0.084585	1.538995	0.123806	0.363614	NFYC	4802	nuclear transcription factor Y subunit gamma
ENSG0000	25.97378	0.804359	0.522826	1.538484	0.12393	0.36365	BATF3	55509	basic leucine zipper ATF-like transcription factor 3
ENSG0000	2553.285	0.149436	0.097132	1.538488	0.123929	0.36365	TMEM115	11070	transmembrane protein 115
ENSG0000	330.1987	-0.28121	0.182793	-1.53838	0.123955	0.36365	RAI14	26064	retinoic acid induced 14
ENSG0000	32.86722	0.653252	0.424633	1.538391	0.123953	0.36365	RPL21P75	728501	ribosomal protein L21 pseudogene 75
ENSG0000	252.6682	-0.25341	0.164687	-1.53873	0.123869	0.36365	RFK	55312	riboflavin kinase
ENSG0000	1231.024	0.158291	0.102894	1.538399	0.123951	0.36365	PTPN11	5781	protein tyrosine phosphatase non-receptor type 11
ENSG0000	119.2089	0.375508	0.244051	1.538649	0.12389	0.36365	NUFIP1	26747	nuclear FMR1 interacting protein 1
ENSG0000	16.94284	1.022765	0.665024	1.537937	0.124064	0.363912	DSCR9	257203	Down syndrome critical region 9
ENSG0000	561.4588	-0.18516	0.120414	-1.53768	0.124127	0.364038	PHC1	1911	polyhomeotic homolog 1
ENSG0000	260.9145	-0.24512	0.159436	-1.5374	0.124195	0.364182	CCDC28A	25901	coiled-coil domain containing 28A
ENSG0000	333.7376	-0.22075	0.143637	-1.53687	0.124325	0.364448	POMGNT2	84892	protein O- 4-)
ENSG0000	259.9483	0.286611	0.186489	1.53688	0.124323	0.364448	AGPAT5	55326	1-acylglycerol-3-phosphate O-acyltransferase 5
ENSG0000	578.3558	-0.20205	0.131495	-1.5366	0.124391	0.364584	TEX264	51368	testis expr ER-phagy receptor
ENSG0000	2273.734	-0.13262	0.086323	-1.53633	0.124458	0.364714	PRPF4B	8899	pre-mRNA processing factor 4B
ENSG0000	14.32102	1.046893	0.681456	1.536259	0.124475	0.364714	NUDT8	254552	nudix hydrolase 8
ENSG0000	29.59998	-0.69085	0.449809	-1.53587	0.12457	0.364934	PLS1	5357	plastin 1
ENSG0000	231.5217	-0.26215	0.170722	-1.53554	0.124651	0.365114	NVL	4931	nuclear VCP like
ENSG0000	103.7736	-0.39316	0.256072	-1.53534	0.1247	0.365201	SCARB1	949	scavenger receptor class B member 1
ENSG0000	218.8107	-0.26764	0.174343	-1.53511	0.124757	0.36531	CAPS	828	calcyphosine
ENSG0000	1390.721	-0.14597	0.095107	-1.5348	0.124834	0.365477	VOPP1	81552	VOPP1 WW domain binding protein
ENSG0000	9.051307	1.143736	0.745252	1.534698	0.124858	0.365489	NA	NA	NA
ENSG0000	7.238382	3.489155	2.274286	1.534176	0.124986	0.365691	MIR3671	1.01E+08	microRNA 3671
ENSG0000	508.1952	-0.20098	0.131007	-1.5341	0.125006	0.365691	AIDA	64853	axin intera dorsalization associated
ENSG0000	10.36366	-1.20285	0.784073	-1.53411	0.125004	0.365691	NA	NA	NA
ENSG0000	570.1539	-0.17901	0.116701	-1.53394	0.125045	0.365691	FBXO9	26268	F-box protein 9
ENSG0000	568.7977	0.19928	0.129896	1.53415	0.124993	0.365691	NTAN1	123803	N-terminal asparagine amidase
ENSG0000	43.43095	-0.6738	0.439259	-1.53394	0.125045	0.365691	RNF165	494470	ring finger protein 165
ENSG0000	2144.694	0.161923	0.105589	1.533527	0.125146	0.365928	USB1	79650	U6 snRNA biogenesis phosphodiesterase 1
ENSG0000	3059.836	0.137113	0.089417	1.533411	0.125175	0.365954	IL6R	3570	interleukin 6 receptor
ENSG0000	3638.517	-0.14245	0.092913	-1.53319	0.125228	0.366054	AGAP2	116986	ArfGAP wi ankyrin repeat and PH domain 2
ENSG0000	1337.069	0.157572	0.102797	1.532841	0.125315	0.366161	PYHIN1	149628	pyrin and HIN domain family member 1
ENSG0000	542.0557	0.179025	0.116796	1.532804	0.125324	0.366161	COPS3	8533	COP9 signalosome subunit 3
ENSG0000	690.7513	0.174157	0.113615	1.532872	0.125307	0.366161	RPS6KB1	6198	ribosomal protein S6 kinase B1
ENSG0000	16.62279	-0.91798	0.599035	-1.53243	0.125416	0.36637	DACH1	1602	dachshund family transcription factor 1
ENSG0000	516.9944	0.199095	0.129976	1.53178	0.125577	0.366725	NA	NA	NA
ENSG0000	594.712	-0.18583	0.121315	-1.53183	0.125565	0.366725	ZBP1	81030	Z-DNA binding protein 1
ENSG0000	4.041596	1.942531	1.268287	1.531618	0.125617	0.366784	NA	NA	NA
ENSG0000	2.436816	-2.83875	1.853592	-1.53148	0.12565	0.366823	VN1R1	57191	vomeronasal 1 receptor 1
ENSG0000	37.20764	-0.70563	0.460882	-1.53105	0.125757	0.366938	ST6GALNA	30815	ST6 N-acet 6-sialyltransferase 6
ENSG0000	12.61026	-1.29204	0.843919	-1.531	0.125768	0.366938	NUTM2E	283008	NUT family member 2E
ENSG0000	182.9573	-0.29408	0.192064	-1.53116	0.12573	0.366938	STARD9	57519	StAR related lipid transfer domain containing 9
ENSG0000	168.2774	-0.33598	0.219448	-1.53103	0.125763	0.366938	ILF3-DT	147727	ILF3 divergent transcript
ENSG0000	477.5752	-0.22605	0.147682	-1.53066	0.125853	0.367023	PCYOX1L	78991	prenylcysteine oxidase 1 like
ENSG0000	226814.3	0.108009	0.070571	1.530491	0.125895	0.367023	CD74	972	CD74 molecule
ENSG0000	4233.723	0.11132	0.072731	1.530577	0.125874	0.367023	CDKN1B	1027	cyclin dependent kinase inhibitor 1B
ENSG0000	2077.68	-0.13485	0.088107	-1.53049	0.125896	0.367023	KIF1C	10749	kinesin family member 1C
ENSG0000	838.3877	-0.15431	0.100812	-1.53063	0.125861	0.367023	HSD17B1-A	1.09E+08	HSD17B1 antisense RNA 1
ENSG0000	132.8507	-0.35024	0.228888	-1.53019	0.12597	0.367147	SLC35B3	51000	solute carrier family 35 member B3
ENSG0000	174.6422	-0.36205	0.236626	-1.53005	0.126005	0.367147	ZDHHC14	79683	zinc finger DHHC-type palmitoyltransferase 14
ENSG0000	220.0965	0.294061	0.192195	1.530014	0.126013	0.367147	INTS13	55726	integrator complex subunit 13
ENSG0000	25.67264	-0.74968	0.48999	-1.53	0.126018	0.367147	NA	NA	NA
ENSG0000	149.8028	0.312711	0.204401	1.52989	0.126044	0.367165	NA	NA	NA
ENSG0000	462.5115	0.192342	0.125752	1.529534	0.126132	0.36733	TXNL4B	54957	thioredoxin like 4B
ENSG0000	15.60365	-1.17167	0.766048	-1.5295	0.12614	0.36733	MMP24	10893	matrix metallopeptidase 24
ENSG0000	13.52415	-1.02023	0.667268	-1.52897	0.126271	0.367479	ZRANB3	84083	zinc finger RANBP2-type containing 3
ENSG0000	113.833	0.368652	0.241088	1.529116	0.126236	0.367479	COLQ	8292	collagen like tail subunit of asymmetric acetylcholinesterase
ENSG0000	734.7533	0.213158	0.139394	1.52918	0.12622	0.367479	SPATA2L	124044	spermatogenesis associated 2 like
ENSG0000	14.93153	1.114983	0.729208	1.529034	0.126256	0.367479	NA	NA	NA
ENSG0000	201.3925	-0.26073	0.17055	-1.52875	0.126327	0.367586	BCS1L	617	BCS1 hom ubiquinol-cytochrome c reductase complex chaperone
ENSG0000	5.043202	-2.02523	1.324911	-1.52858	0.12637	0.367651	WWTR1	25937	WW domain containing transcription regulator 1
ENSG0000	363.7145	0.251981	0.164886	1.528215	0.126459	0.367854	MGAM	8972	maltase-glucoamylase
ENSG0000	747.6841	-0.18968	0.124146	-1.5279	0.126538	0.368026	SLC38A7	55238	solute carrier family 38 member 7
ENSG0000	5.248492	2.039016	1.334887	1.527482	0.126641	0.368037	NA	NA	NA
ENSG0000	2108.391	0.169615	0.111027	1.527696	0.126588	0.368037	PROSER1	80209	proline and serine rich 1
ENSG0000	17.85391	0.853635	0.558815	1.527582	0.126616	0.368037	NA	NA	NA
ENSG0000	1124.331	-0.15707	0.102816	-1.52772	0.126581	0.368037	CNTROB	116840	centrobin centriole duplication and spindle assembly protein
ENSG0000	263.6326	-0.25556	0.1673	-1.52756	0.126621	0.368037	BCAS3	54828	BCAS3 microtubule associated cell migration factor
ENSG0000	13.15338	-1.01328	0.663462	-1.52726	0.126695	0.368107	NA	NA	NA
ENSG0000	2166.816	-0.13093	0.085729	-1.52723	0.126705	0.368107	SH3GLB2	56904	SH3 doma endophilin B2
ENSG0000	394.6594	-0.23167	0.151744	-1.52674	0.126825	0.368398	ZNF33B	7582	zinc finger protein 33B
ENSG0000	4.90525	-2.01176	1.318075	-1.52629	0.126938	0.368614	NA	NA	NA
ENSG0000	392.3793	-0.2239	0.146693	-1.52628	0.126939	0.368614	ADAT1	23536	adenosine deaminase tRNA specific 1
ENSG0000	2157.287	-0.14003	0.091768	-1.52597	0.127018	0.368649	RNF220	55182	ring finger protein 220
ENSG0000	1362.213	0.150832	0.098847	1.525921	0.12703	0.368649	RNF13	11342	ring finger protein 13
ENSG0000	638.7596	-0.17663	0.115736	-1.52614	0.126975	0.368649	THOC6	79228	THO complex subunit 6
ENSG0000	6.445944	-1.51701	0.994165	-1.52592	0.127031	0.368649	NA	NA	NA
ENSG0000	25.09045	-0.72389	0.474426	-1.52582	0.127055	0.368663	NA	NA	NA
ENSG0000	112.332	-0.37382	0.245031	-1.5256	0.127108	0.36876	IFT46	56912	intraflagellar transport 46
ENSG0000	7.111618	-1.54276	1.011421	-1.52534	0.127174	0.368845	NA	NA	NA
ENSG0000	605.1457	0.183844	0.120527	1.525326	0.127178	0.368845	KRR1	11103	KRR1 small subunit processome component homolog
ENSG0000	7.815919	1.398474	0.916978	1.52509	0.127237	0.368909	NA	NA	NA
ENSG0000	60.95657	-0.65158	0.42727	-1.52498	0.127263	0.368909	IGHV1-18	28468	immunoglobulin heavy variable 1-18
ENSG0000	102.6711	0.419979	0.275411	1.524919	0.127279	0.368909	STX8	9482	syntaxin 8
ENSG0000	6.391244	1.496054	0.981069	1.524923	0.127278	0.368909	TMSB15B	286527	thymosin beta 15B

ENSG0000	5.343858	1.95177	1.280083	1.524722	0.127329	0.368994	NUP62CL	54830	nucleoporin 62 C-terminal like
ENSG0000	1057.035	-0.1629	0.106845	-1.52463	0.127351	0.369001	KATNB1	10300	katanin regulatory subunit B1
ENSG0000	2283.551	-0.14936	0.097972	-1.52449	0.127386	0.369045	ZNF316	1E+08	zinc finger protein 316
ENSG0000	91.8518	-0.38618	0.25333	-1.52441	0.127406	0.369045	GAS8	2622	growth arrest specific 8
ENSG0000	3.294231	2.356999	1.546669	1.52392	0.127529	0.369286	NA	NA	NA
ENSG0000	1787.259	0.371057	0.243483	1.523958	0.127519	0.369286	OTUD1	220213	OTU deubiquitinase 1
ENSG0000	186.1435	-0.28807	0.189086	-1.5235	0.127634	0.369441	NSG1	27065	neuronal vesicle trafficking associated 1
ENSG0000	21.54573	0.74609	0.489732	1.523466	0.127642	0.369441	REELD1	345051	reeler domain containing 1
ENSG0000	29.88126	-0.66466	0.436272	-1.52349	0.127635	0.369441	ZNRF3	84133	zinc and ring finger 3
ENSG0000	32.13919	-0.68397	0.449007	-1.52329	0.127685	0.369508	DCST2	127579	DC-STAMP domain containing 2
ENSG0000	400.1001	0.203516	0.133624	1.523048	0.127747	0.369529	TMCO6	55374	transmembrane and coiled-coil domains 6
ENSG0000	267.5858	0.249391	0.163737	1.523118	0.127729	0.369529	TMEM126	55863	transmembrane protein 126B
ENSG0000	8.734293	1.527753	1.003104	1.523026	0.127752	0.369529	NA	NA	NA
ENSG0000	150.4836	-0.31772	0.208632	-1.52287	0.127791	0.369584	RPUSD2	27079	RNA pseudouridine synthase domain containing 2
ENSG0000	142.8255	-0.32913	0.216149	-1.52268	0.127838	0.369662	SLC40A1	30061	solute carrier family 40 member 1
ENSG0000	361.7829	-0.2582	0.169586	-1.52254	0.127874	0.369709	LRFN1	57622	leucine rich repeat and fibronectin type III domain containing 1
ENSG0000	5.730952	1.730132	1.136516	1.522313	0.127931	0.369723	IGHV3-13	28449	immunoglobulin heavy variable 3-13
ENSG0000	133.0458	-0.33587	0.220633	-1.52228	0.127939	0.369723	GALK1	2584	galactokinase 1
ENSG0000	918.8402	-0.18588	0.122102	-1.52234	0.127923	0.369723	VPS16	64601	VPS16 core subunit of CORVET and HOPS complexes
ENSG0000	509.822	0.185173	0.121679	1.521817	0.128055	0.370001	SNRPD3	6634	small nuclear ribonucleoprotein D3 polypeptide
ENSG0000	4.338391	-1.93999	1.275152	-1.52138	0.128164	0.370214	NA	NA	NA
ENSG0000	23.66174	0.842101	0.553575	1.521204	0.128209	0.370214	ZNF747-D'	1.08E+08	ZNF747 divergent transcript
ENSG0000	18344.46	0.129988	0.08545	1.521231	0.128202	0.370214	LIMD2	80774	LIM domain containing 2
ENSG0000	6.334374	-1.6514	1.085558	-1.52124	0.128198	0.370214	JAM2	58494	junctional adhesion molecule 2
ENSG0000	10.53782	-1.44475	0.949918	-1.52092	0.12828	0.370363	TMEM198	130612	transmembrane protein 198
ENSG0000	14.70215	-1.12115	0.73726	-1.5207	0.128336	0.370409	NA	NA	NA
ENSG0000	4137.188	0.129441	0.085117	1.520748	0.128323	0.370409	RNF40	9810	ring finger protein 40
ENSG0000	103.7385	0.361989	0.23812	1.520197	0.128461	0.37054	MLLT11	10962	MLLT11 transcription factor 7 cofactor
ENSG0000	8.034975	-1.32725	0.873051	-1.52025	0.128449	0.37054	XRCC2	7516	X-ray repair cross complementing 2
ENSG0000	16.88676	-1.041	0.684756	-1.52025	0.128449	0.37054	KIF18B	146909	kinesin family member 18B
ENSG0000	272.4312	-0.31681	0.208381	-1.52035	0.128423	0.37054	PCSK1N	27344	proprotein convertase subtilisin/kexin type 1 inhibitor
ENSG0000	2868.557	0.136729	0.089947	1.520113	0.128483	0.370544	BCLAF1	9774	BCL2 associated transcription factor 1
ENSG0000	107.4337	-0.39483	0.259753	-1.52002	0.128505	0.37055	ACSM3	6296	acyl-CoA synthetase medium chain family member 3
ENSG0000	1179.742	0.160435	0.105566	1.519754	0.128573	0.370689	ZFC3H1	196441	zinc finger C3H1-type containing
ENSG0000	127.4291	0.334537	0.220212	1.519159	0.128723	0.371063	WNT10B	7480	Wnt family member 10B
ENSG0000	859.6261	-0.18438	0.121392	-1.51892	0.128783	0.371123	MGA	23269	MAX dimerization protein MGA
ENSG0000	317.5046	0.238875	0.157264	1.518941	0.128777	0.371123	DMPK	1760	DM1 protein kinase
ENSG0000	41.99201	-0.61681	0.406158	-1.51864	0.128854	0.371269	APOBEC3D	140564	apolipoprotein B mRNA editing enzyme catalytic subunit 3D
ENSG0000	2.628355	2.699388	1.77764	1.518523	0.128883	0.371293	NA	NA	NA
ENSG0000	195.3364	0.273238	0.179964	1.51829	0.128941	0.371296	RAB33B	83452	RAB33B member RAS oncogene family
ENSG0000	576.3386	-0.18654	0.122863	-1.51829	0.12894	0.371296	REV3L	5980	REV3 like DNA directed polymerase zeta catalytic subunit
ENSG0000	79.25824	0.531492	0.350062	1.51828	0.128944	0.371296	CAPN12	147968	calpain 12
ENSG0000	19.61831	0.941843	0.620597	1.517641	0.129105	0.371601	ZP3	7784	zona pellucida glycoprotein 3
ENSG0000	237.6791	0.2918	0.192262	1.517721	0.129085	0.371601	CACNB3	784	calcium voltage-gated channel auxiliary subunit beta 3
ENSG0000	247.9903	0.238839	0.157377	1.517622	0.12911	0.371601	RAB5IF	55969	RAB5 interacting factor
ENSG0000	568.5085	-0.2005	0.132135	-1.51739	0.129169	0.371656	UPF3A	65110	UPF3A regulator of nonsense mediated mRNA decay
ENSG0000	1707.534	-0.14998	0.098842	-1.51739	0.129168	0.371656	INO80E	283899	INO80 complex subunit E
ENSG0000	23.53303	-0.74056	0.488083	-1.51729	0.129193	0.371669	BRCA1	672	BRCA1 DNA repair associated
ENSG0000	536.6169	-0.21623	0.142525	-1.51715	0.12923	0.371716	RFNG	5986	RFNG O-fucosylpeptide 3-beta-N-acetylglucosaminyltransferase
ENSG0000	794.4572	-0.17396	0.114675	-1.51697	0.129273	0.371725	TPGS2	25941	tubulin polyglutamylase complex subunit 2
ENSG0000	344.2406	0.255155	0.168199	1.516981	0.129271	0.371725	ALAS2	212	5'-aminolevulinate synthase 2
ENSG0000	257.2483	-0.25974	0.17124	-1.51684	0.129307	0.371764	RNF121	55298	ring finger protein 121
ENSG0000	997.9498	0.176359	0.116276	1.516727	0.129336	0.37179	STAM	8027	signal transducing adaptor molecule
ENSG0000	642.529	-0.18217	0.120152	-1.51615	0.12948	0.372055	ZMYM4	9202	zinc finger MYM-type containing 4
ENSG0000	31.19915	-0.69626	0.459237	-1.51612	0.129488	0.372055	CDCA7	83879	cell division cycle associated 7
ENSG0000	3976.043	-0.16	0.105528	-1.51622	0.129463	0.372055	RNH1	6050	ribonuclease/angiogenin inhibitor 1
ENSG0000	62.23527	0.468598	0.309141	1.515807	0.129568	0.372228	SLC35D2	11046	solute carrier family 35 member D2
ENSG0000	7.145958	-1.72361	1.137348	-1.51546	0.129655	0.372363	NA	NA	NA
ENSG0000	678.5122	0.161656	0.10667	1.515479	0.129651	0.372363	KIF3B	9371	kinesin family member 3B
ENSG0000	114.7275	-0.35749	0.235989	-1.51487	0.129804	0.372675	HACE1	57531	HECT domain and ankyrin repeat containing E3 ubiquitin protein ligase 1
ENSG0000	289.9008	0.255072	0.168377	1.514887	0.129801	0.372675	TMEM161	54929	transmembrane protein 161A
ENSG0000	127.4185	0.357996	0.23637	1.514558	0.129884	0.372847	GET4	51608	guided entry of tail-anchored proteins factor 4
ENSG0000	2411.46	0.120602	0.079653	1.514089	0.130003	0.373131	ATF7IP	55729	activating transcription factor 7 interacting protein
ENSG0000	234.8771	0.247917	0.163784	1.513689	0.130105	0.373364	EEF2K	29904	eukaryotic elongation factor 2 kinase
ENSG0000	17.87241	-0.83637	0.552753	-1.5131	0.130253	0.373624	RHEX	440712	regulator of hemoglobinization and erythroid cell expansion
ENSG0000	7.333606	1.473227	0.973652	1.513094	0.130256	0.373624	NA	NA	NA
ENSG0000	152.2269	0.335203	0.221519	1.513201	0.130229	0.373624	HPRT1	3251	hypoxanthine phosphoribosyltransferase 1
ENSG0000	3.871253	2.020911	1.336266	1.512357	0.130443	0.374104	SNORA73E	26768	small nucl H/ACA box 73B
ENSG0000	332.5986	0.25484	0.168594	1.511562	0.130645	0.374626	THAP1	55145	THAP domain containing 1
ENSG0000	5.535901	-1.62289	1.073989	-1.51109	0.130766	0.374855	FGD5	152273	FYVE RhoGEF and PH domain containing 5
ENSG0000	288.1286	-0.26669	0.176487	-1.51113	0.130756	0.374855	UROS	7390	uroporphyrinogen III synthase
ENSG0000	47.33057	-0.57475	0.380631	-1.50999	0.131047	0.375603	ARHGAP1:	9824	Rho GTPase activating protein 11A
ENSG0000	543.3707	-0.18038	0.119536	-1.50903	0.131292	0.376188	DENND1A	57706	DENN domain containing 1A
ENSG0000	1717.439	-0.16698	0.11065	-1.50907	0.131282	0.376188	FCHO1	23149	FCH and mu domain containing endocytic adaptor 1
ENSG0000	3219.177	0.14506	0.096134	1.508941	0.131314	0.376194	FPGS	2356	folylpolyglutamate synthase
ENSG0000	255.4294	0.303628	0.201238	1.508798	0.13135	0.37624	BCL7A	605	BAF chromatin remodeling complex subunit BCL7A
ENSG0000	30.27334	-0.6556	0.434597	-1.50852	0.131423	0.376346	LINC02608	1.02E+08	long intergenic non-protein coding RNA 2608
ENSG0000	18.79926	-0.8845	0.586349	-1.50849	0.131428	0.376346	FAM53A	152877	family with sequence similarity 53 member A
ENSG0000	409.9274	0.20659	0.136987	1.508103	0.131528	0.376459	CARD16	114769	caspase recruitment domain family member 16
ENSG0000	31.38758	-0.795	0.527121	-1.50819	0.131507	0.376459	APOLD1	81575	apolipoprotein L domain containing 1
ENSG0000	393.1777	0.268339	0.17793	1.508114	0.131525	0.376459	NA	NA	NA
ENSG0000	584.9288	0.197031	0.130656	1.508014	0.131551	0.376466	ZFX	7543	zinc finger protein X-linked
ENSG0000	785.0016	0.168278	0.111602	1.507841	0.131595	0.376534	PSMB6	5694	proteasome 20S subunit beta 6
ENSG0000	24.41847	-0.78751	0.522375	-1.50756	0.131667	0.376683	CCDC34	91057	coiled-coil domain containing 34
ENSG0000	5.346579	1.9124	1.268667	1.507408	0.131706	0.376735	UBXN10	127733	UBX domain protein 10
ENSG0000	12.0882	-1.2074	0.801081	-1.50722	0.131754	0.376816	NA	NA	NA
ENSG0000	8.583649	1.384081	0.918606	1.506719	0.131883	0.377066	NA	NA	NA
ENSG0000	40.88078	0.585631	0.388665	1.506775	0.131868	0.377066	SNRPN	6638	small nuclear ribonucleoprotein polypeptide N
ENSG0000	21.8693	0.775351	0.514826	1.506044	0.132056	0.377503	NA	NA	NA
ENSG0000	10.70248	1.177804	0.782267	1.505629	0.132162	0.377633	MMP23A	8511	matrix metallopeptidase 23A (pseudogene)
ENSG0000	249.7365	0.259077	0.172069	1.505665	0.132153	0.377633	LRRC8B	23507	leucine rich repeat containing 8 VRAC subunit B
ENSG0000	3.253094	-2.50828	1.6658	-1.50575	0.132131	0.377633	NA	NA	NA
ENSG0000	15.09395	1.041475	0.691778	1.505503	0.132195	0.377667	WASF3	10810	WASP family member 3
ENSG0000	4129.054	0.114657	0.076181	1.505056	0.13231	0.377879	TMEM214	54867	transmembrane protein 214
ENSG0000	194.7129	-0.29537	0.196248	-1.5051	0.132299	0.377879	NA	NA	NA
ENSG0000	6.255144	1.498649	0.996015	1.504645	0.132415	0.377921	PPM1J	333926	protein ph Mg2+/Mn2+ dependent 1J
ENSG0000	319.8219	-0.26707	0.17751	-1.50452	0.132447	0.377921	C4orf48	401115	chromosome 4 open reading frame 48
ENSG0000	805.7587	-0.18306	0.121664	-1.50466	0.13241	0.377921	TSPYL4	23270	TSPY like 4
ENSG0000	49.16268	-0.57688	0.383336	-1.5049	0.132349	0.377921	GOLGA8N	643699	golgin A8 family member N
ENSG0000	5668.181	-0.12481	0.082953	-1.50456	0.132437	0.377921	CSK	1445	C-terminal Src kinase

ENSG0000	1747.899	-0.1398	0.092911	-1.50471	0.1324	0.377921	CTCF	10664	CCCTC-binding factor
ENSG0000	913.7892	-0.18679	0.124196	-1.50402	0.132575	0.378217	RAPH1	65059	Ras association (RalGDS/AF-6) and pleckstrin homology domains 1
ENSG0000	55.04886	-0.49676	0.330337	-1.5038	0.132632	0.378217	EPM2A	7957	EPM2A glu laforin
ENSG0000	3.598556	-2.2764	1.513703	-1.50386	0.132616	0.378217	NA	NA	NA
ENSG0000	6637.057	-0.13111	0.087186	-1.50384	0.132622	0.378217	RAB11B	9230	RAB11B member RAS oncogene family
ENSG0000	1179.656	-0.14253	0.094788	-1.50371	0.132656	0.378228	TMEM129	92305	transmem E3 ubiquitin ligase
ENSG0000	172.9255	0.282942	0.188177	1.503595	0.132686	0.378254	CTLA4	1493	cytotoxic T-lymphocyte associated protein 4
ENSG0000	12.34588	1.357387	0.902838	1.503466	0.132719	0.37829	CAV1	857	caveolin 1
ENSG0000	356.7577	0.205958	0.137032	1.502991	0.132841	0.378582	WNT10A	80326	Wnt family member 10A
ENSG0000	71.40285	0.543223	0.361457	1.502872	0.132872	0.378611	NA	NA	NA
ENSG0000	345.8917	1.258996	0.837851	1.502648	0.13293	0.378717	CCL8	6355	C-C motif chemokine ligand 8
ENSG0000	81.67195	-0.41491	0.276141	-1.50252	0.132963	0.378752	SPEN-AS1	729614	SPEN antisense RNA 1
ENSG0000	250.0323	0.288916	0.1923	1.502419	0.132989	0.378769	MS4A14	84689	membrane spanning 4-domains A14
ENSG0000	130.3663	-0.32904	0.219039	-1.50222	0.133039	0.378855	VMAC	400673	vimentin type intermediate filament associated coiled-coil protein
ENSG0000	33.21919	-0.63665	0.423978	-1.50162	0.133196	0.379205	SLC46A1	113235	solute carrier family 46 member 1
ENSG0000	794.8893	-0.18569	0.123665	-1.50159	0.133203	0.379205	ZNF516	9658	zinc finger protein 516
ENSG0000	1622.626	-0.13061	0.087037	-1.50064	0.133449	0.379787	AATF	26574	apoptosis antagonizing transcription factor
ENSG0000	376.0988	-0.20215	0.134703	-1.50071	0.133431	0.379787	NT5C3B	115024	5'-nucleoti cytosolic IIIB
ENSG0000	2771.245	0.129959	0.086611	1.500491	0.133487	0.379838	SMARCA2	6595	SWI/SNF r matrix ass actin depe subfamily member 2
ENSG0000	6.405756	-1.68949	1.126767	-1.49942	0.133765	0.380572	NA	NA	NA
ENSG0000	5.901958	-1.78554	1.191232	-1.49891	0.133898	0.380785	CARD9	64170	caspase recruitment domain family member 9
ENSG0000	8.147239	1.313515	0.876325	1.498891	0.133902	0.380785	SNORD4A	26773	small nucl C/D box 4A
ENSG0000	4.118382	-2.08769	1.392714	-1.49901	0.133871	0.380785	NA	NA	NA
ENSG0000	1196.782	-0.15032	0.100323	-1.4984	0.134029	0.381088	TAFAZZIN	6901	tafazzin phospholipid-lysophospholipid transacylase
ENSG0000	94.74816	0.416936	0.278336	1.49796	0.134144	0.381292	PAX8	7849	paired box 8
ENSG0000	97.46516	-0.38248	0.255349	-1.49789	0.134163	0.381292	CDKAL1	54901	CDK5 regulatory subunit associated protein 1 like 1
ENSG0000	532.1395	-0.18973	0.126662	-1.49792	0.134154	0.381292	INTS4	92105	integrator complex subunit 4
ENSG0000	2.354499	3.156549	2.10761	1.497691	0.134213	0.381378	SMIM43	132332	small integral membrane protein 43
ENSG0000	6.644903	-1.4381	0.960321	-1.49752	0.134258	0.381447	NA	NA	NA
ENSG0000	235.0754	0.260184	0.17379	1.497113	0.134364	0.381684	KCNAB3	9196	potassium voltage-gated channel subfamily A regulatory beta subunit 3
ENSG0000	1335.767	0.143023	0.095537	1.497041	0.134383	0.381684	ZBED4	9889	zinc finger BED-type containing 4
ENSG0000	6.020373	1.536706	1.026608	1.496877	0.134425	0.381746	INPP5J	27124	inositol polyphosphate-5-phosphatase J
ENSG0000	453.279	0.23252	0.155428	1.495997	0.134654	0.382338	HPS5	11234	HPS5 biogenesis of lysosomal organelles complex 2 subunit 2
ENSG0000	1066.099	0.160247	0.107152	1.495513	0.134781	0.382373	ATF2	1386	activating transcription factor 2
ENSG0000	16.63177	-0.98087	0.655938	-1.49536	0.134819	0.382373	LOC10537	1.05E+08	uncharacterized LOC105375130
ENSG0000	891.8043	0.162833	0.108907	1.49516	0.134873	0.382373	PPP1R35	221908	protein phosphatase 1 regulatory subunit 35
ENSG0000	25.15567	-0.84102	0.562298	-1.49569	0.134735	0.382373	TRBV11-2	28581	T cell receptor beta variable 11-2
ENSG0000	12.64638	-1.09883	0.734857	-1.49529	0.134839	0.382373	DFFBP1	1E+08	DNA fragmentation factor subunit beta pseudogene 1
ENSG0000	238.2241	0.279936	0.187199	1.495393	0.134812	0.382373	TBC1D12	23232	TBC1 domain family member 12
ENSG0000	4.028067	1.997121	1.335445	1.495473	0.134791	0.382373	NA	NA	NA
ENSG0000	373.8195	0.209248	0.139939	1.495285	0.13484	0.382373	RPS2P46	125208	ribosomal protein S2 pseudogene 46
ENSG0000	5.239178	-2.206	1.475143	-1.49545	0.134798	0.382373	MIR635	693220	microRNA 635
ENSG0000	144.0481	0.345475	0.231054	1.495213	0.134859	0.382373	ZBTB32	27033	zinc finger and BTB domain containing 32
ENSG0000	1293.036	0.137107	0.091711	1.49499	0.134917	0.38244	PSMD1	5707	proteasom non-ATPase 1
ENSG0000	13.43531	-0.99685	0.666888	-1.49478	0.134972	0.382537	RNFT2	84900	ring finger transmembrane 2
ENSG0000	68.8918	-0.47011	0.31455	-1.49456	0.13503	0.382645	ZNNT1	1.16E+08	ZNF706 neighboring transcript 1
ENSG0000	6.163017	1.864449	1.247585	1.494447	0.135059	0.382651	NA	NA	NA
ENSG0000	1178.425	0.180615	0.120862	1.494389	0.135074	0.382651	RNF24	11237	ring finger protein 24
ENSG0000	15.37656	0.982474	0.65759	1.494053	0.135162	0.382783	NA	NA	NA
ENSG0000	84.57264	0.457605	0.306278	1.494084	0.135154	0.382783	S100P	6286	S100 calcium binding protein P
ENSG0000	427.271	0.195504	0.130896	1.493575	0.135287	0.383025	TAF11	6882	TATA-box binding protein associated factor 11
ENSG0000	3228.74	0.12433	0.083243	1.493568	0.135289	0.383025	TMUB1	83590	transmembrane and ubiquitin like domain containing 1
ENSG0000	41.21502	0.609593	0.408226	1.493272	0.135366	0.383186	RGMA	56963	repulsive guidance molecule BMP co-receptor a
ENSG0000	361.5648	0.252836	0.169344	1.493036	0.135428	0.383237	ATP5ME	521	ATP synthase membrane subunit e
ENSG0000	10.03049	1.50116	1.005385	1.49312	0.135406	0.383237	LDHD	197257	lactate dehydrogenase D
ENSG0000	58.78144	0.530514	0.355343	1.492966	0.135446	0.383237	YBEY	54059	ybeY metalloendoribonuclease
ENSG0000	1281.111	0.15835	0.106108	1.492342	0.135609	0.383641	UBE2S	27338	ubiquitin conjugating enzyme E2 S
ENSG0000	181.8775	-0.30829	0.206622	-1.49203	0.135691	0.383814	MTFR1	9650	mitochondrial fission regulator 1
ENSG0000	283.1053	-0.24825	0.166414	-1.49174	0.135768	0.383973	KLHL42	57542	kelch like family member 42
ENSG0000	208.6408	0.260899	0.174927	1.491474	0.135837	0.384109	PDCL	5082	phosducin like
ENSG0000	21.18459	0.993863	0.666435	1.491313	0.135879	0.384111	NA	NA	NA
ENSG0000	10.69502	-1.34324	0.900669	-1.49138	0.135862	0.384111	ACSBG1	23205	acyl-CoA synthetase bubblegum family member 1
ENSG0000	628.2612	-0.21285	0.14278	-1.49078	0.136019	0.384427	FAM229A	1E+08	family with sequence similarity 229 member A
ENSG0000	286.1323	0.265848	0.178339	1.490694	0.136042	0.384427	NA	NA	NA
ENSG0000	750.618	0.157631	0.105746	1.49065	0.136053	0.384427	PMPCB	9512	peptidase mitochondrial processing subunit beta
ENSG0000	45.61079	-0.57099	0.383119	-1.49037	0.136127	0.384577	ZWINT	11130	ZW10 interacting kinetochore protein
ENSG0000	5.322096	1.761504	1.182312	1.489881	0.136256	0.384882	CCDC171	203238	coiled-coil domain containing 171
ENSG0000	4.644548	-1.70639	1.14551	-1.48963	0.136321	0.38495	C3orf33	285315	chromosome 3 open reading frame 33
ENSG0000	12.33469	1.262035	0.847202	1.48965	0.136316	0.38495	NPTX2	4885	neuronal pentraxin 2
ENSG0000	36.6662	-0.64679	0.434281	-1.48934	0.136397	0.38504	NRAV	1.01E+08	negative regulator of antiviral response
ENSG0000	11.47699	1.155285	0.775671	1.489401	0.136382	0.38504	JPH4	84502	junctophilin 4
ENSG0000	1024.84	0.170642	0.114581	1.489273	0.136415	0.38504	SLC23A2	9962	solute carrier family 23 member 2
ENSG0000	12.89474	1.067476	0.716832	1.489159	0.136446	0.385066	NA	NA	NA
ENSG0000	112.4838	-0.34245	0.230044	-1.48864	0.136583	0.38539	POP1	10940	POP1 hom ribonuclease P/MRP subunit
ENSG0000	59.0698	-0.59419	0.399167	-1.48857	0.136602	0.38539	C9orf85	138241	chromosome 9 open reading frame 85
ENSG0000	14.11853	-0.95082	0.638794	-1.48846	0.136631	0.385413	LINC0203	1.05E+08	long intergenic non-protein coding RNA 2033
ENSG0000	495.5911	0.20066	0.134821	1.488353	0.136658	0.385431	SMPD2	6610	sphingomyelin phosphodiesterase 2
ENSG0000	3.142425	-2.47452	1.662802	-1.48816	0.136708	0.385513	KL	9365	klotho
ENSG0000	10.38125	1.319573	0.887035	1.487623	0.13685	0.385856	NA	NA	NA
ENSG0000	1359.284	-0.13363	0.089855	-1.48722	0.136958	0.386042	KIDINS220	57498	kinase D interacting substrate 220
ENSG0000	45.00964	0.520356	0.349871	1.48728	0.136941	0.386042	NA	NA	NA
ENSG0000	487.0065	-0.1999	0.134428	-1.48703	0.137007	0.386122	AP5M1	55745	adaptor related protein complex 5 subunit mu 1
ENSG0000	14.93858	1.099975	0.7398	1.486853	0.137054	0.386136	UBQLN1-A	1.05E+08	UBQLN1 antisense RNA 1
ENSG0000	36.20025	0.830226	0.558352	1.486922	0.137035	0.386136	LOC1053	1.05E+08	uncharacterized LOC105369363
ENSG0000	5.939833	1.702056	1.145035	1.486466	0.137156	0.386366	NA	NA	NA
ENSG0000	151.2861	-0.28687	0.193004	-1.48637	0.137182	0.386381	AP4S1	11154	adaptor related protein complex 4 subunit sigma 1
ENSG0000	78.94764	-0.45276	0.304683	-1.48602	0.137273	0.386579	DENND5B	160518	DENN domain containing 5B
ENSG0000	99.77391	-0.37642	0.253337	-1.48585	0.137318	0.386587	MSRB2	22921	methionine sulfoxide reductase B2
ENSG0000	6147.711	0.140195	0.094351	1.485882	0.13731	0.386587	ARHGAP4	393	Rho GTPase activating protein 4
ENSG0000	13.32307	-0.91792	0.617854	-1.48565	0.137371	0.38666	EIF2AK3-D	1.02E+08	EIF2AK3 divergent transcript
ENSG0000	26.31786	-0.75617	0.509	-1.4856	0.137385	0.38666	RAD50	10111	RAD50 double strand break repair protein
ENSG0000	100.4602	-0.38145	0.25679	-1.48545	0.137425	0.386703	WWOX	51741	WW domain containing oxidoreductase
ENSG0000	300.5203	-0.23884	0.160791	-1.48538	0.137443	0.386703	GTPBP3	84705	GTP bindir mitochondrial
ENSG0000	2744.923	0.13091	0.088145	1.485163	0.137501	0.386795	PUM2	23369	pumilio RNA binding family member 2
ENSG0000	4.657841	-1.92784	1.298121	-1.4851	0.137517	0.386795	NA	NA	NA
ENSG0000	177.9853	-0.30269	0.203944	-1.48418	0.137761	0.387387	IDH1	3417	isocitrate dehydrogenase (NADP(+)) 1
ENSG0000	391.6648	-0.25963	0.174935	-1.48415	0.137769	0.387387	MOSPD3	64598	motile sperm domain containing 3
ENSG0000	231.2282	0.262341	0.176822	1.483642	0.137904	0.38759	REXO2	25996	RNA exonuclease 2
ENSG0000	992.1239	-0.14784	0.09964	-1.48372	0.137882	0.38759	CDC27	996	cell division cycle 27
ENSG0000	9.693216	1.38577	0.934001	1.483692	0.137891	0.38759	PRR36	80164	proline rich 36

ENSG0000	1133.693	0.14439	0.097346	1.483264	0.138004	0.387813	ZNF740	283337	zinc finger protein 740
ENSG0000	300.6477	0.251308	0.169462	1.48298	0.13808	0.387966	GPR18	2841	G protein-coupled receptor 18
ENSG0000	4419.557	0.1085	0.07317	1.482843	0.138116	0.38801	PA2G4	5036	proliferation-associated 2G4
ENSG0000	418.8795	0.202123	0.136317	1.482745	0.138142	0.388024	CISD2	493856	CDGSH iron sulfur domain 2
ENSG0000	85.54992	-0.50575	0.341119	-1.48262	0.138176	0.388062	ITGB3BP	23421	integrin subunit beta 3 binding protein
ENSG0000	1998.303	0.125404	0.084593	1.482437	0.138224	0.38807	NAP1L4	4676	nucleosome assembly protein 1 like 4
ENSG0000	51.7552	0.610743	0.411987	1.482433	0.138225	0.38807	NA	NA	NA
ENSG0000	738.3296	0.182327	0.122997	1.482369	0.138242	0.38807	RP2	6102	RP2 activator of ARL3 GTPase
ENSG0000	78.31388	0.422032	0.28472	1.482273	0.138268	0.388074	EBPL	84650	EBP like
ENSG0000	5.621125	1.827162	1.232731	1.482206	0.138286	0.388074	NA	NA	NA
ENSG0000	55.77416	0.595686	0.40192	1.482102	0.138313	0.388093	FCRL5	83416	Fc receptor like 5
ENSG0000	382.9896	-0.20235	0.136551	-1.48185	0.138379	0.388219	CCDC66	285331	coiled-coil domain containing 66
ENSG0000	8.921781	1.225339	0.827109	1.481473	0.138481	0.388387	NEFM	4741	neurofilament medium chain
ENSG0000	198.1462	-0.28374	0.191523	-1.48151	0.138472	0.388387	KIAA1958	158405	KIAA1958
ENSG0000	1635.851	-0.1296	0.087489	-1.48139	0.138503	0.388392	TMUB2	79089	transmembrane and ubiquitin like domain containing 2
ENSG0000	270.605	-0.25228	0.170329	-1.48111	0.138579	0.388544	NMRAL1	57407	NmrA like redox sensor 1
ENSG0000	79.83807	1.111782	0.750709	1.480976	0.138613	0.388565	GCSAML	148823	germinal center associated signaling and motility like
ENSG0000	189.9811	0.267528	0.18065	1.480919	0.138628	0.388565	LOC72999	729998	eukaryotic translation elongation factor 1 gamma pseudogene
ENSG0000	43.79339	-0.56692	0.382851	-1.48077	0.138668	0.388617	NA	NA	NA
ENSG0000	2380.237	0.115695	0.078146	1.48049	0.138743	0.388651	TJAP1	93643	tight junction associated protein 1
ENSG0000	10.6752	1.399914	0.945553	1.480524	0.138733	0.388651	NA	NA	NA
ENSG0000	4.256295	-2.08029	1.405003	-1.48063	0.138705	0.388651	WSCD1	23302	WSC domain containing 1
ENSG0000	870.3049	-0.15759	0.106507	-1.47958	0.138985	0.389271	MAP3K7	6885	mitogen-activated protein kinase kinase kinase 7
ENSG0000	10.23046	-1.18277	0.799482	-1.47942	0.139028	0.389319	NA	NA	NA
ENSG0000	6.166492	-1.40443	0.949352	-1.47936	0.139044	0.389319	CHTF8	54921	chromosome transmission fidelity factor 8
ENSG0000	3.167093	2.485202	1.680037	1.479255	0.139072	0.389339	A1BG	1	alpha-1-B glycoprotein
ENSG0000	13.88071	1.222309	0.826593	1.478731	0.139212	0.389673	LINC0030	283860	long intergenic non-protein coding RNA 304
ENSG0000	2.172715	-2.66015	1.799393	-1.47836	0.139312	0.389893	GALNT18	374378	polypeptide N-acetylgalactosaminyltransferase 18
ENSG0000	727.4596	-0.16797	0.113635	-1.47814	0.13937	0.389939	OPA1	4976	OPA1 mitochondrial dynamin like GTPase
ENSG0000	760.5599	0.181022	0.122465	1.47815	0.139368	0.389939	CDCA4	55038	cell division cycle associated 4
ENSG0000	12.82534	1.019545	0.689946	1.477719	0.139483	0.390077	RPS2P20	645018	ribosomal protein S2 pseudogene 20
ENSG0000	9250.281	0.110359	0.074679	1.477773	0.139468	0.390077	PDLIM7	9260	PDZ and LIM domain 7
ENSG0000	53.48805	0.486643	0.329301	1.477807	0.139459	0.390077	ZNF487	642819	zinc finger protein 487
ENSG0000	5.461116	-1.73347	1.173275	-1.47747	0.139551	0.390208	PRKG2	5593	protein kinase cGMP-dependent 2
ENSG0000	3.498871	-2.28935	1.549762	-1.47722	0.139615	0.39033	NA	NA	NA
ENSG0000	75.25511	-0.50048	0.338843	-1.47702	0.139669	0.390421	ZNF287	57336	zinc finger protein 287
ENSG0000	250.9387	-0.26125	0.176889	-1.4769	0.139704	0.390458	SLX9	85395	SLX9 ribosome biogenesis factor
ENSG0000	171.5322	-0.30439	0.206126	-1.47671	0.139754	0.390542	CCDC85C	317762	coiled-coil domain containing 85C
ENSG0000	8.891864	-1.28506	0.870312	-1.47655	0.139797	0.390543	NA	NA	NA
ENSG0000	7221.73	-0.12265	0.083062	-1.47661	0.13978	0.390543	OS9	10956	OS9 endoplasmic reticulum lectin
ENSG0000	556.2764	-0.19757	0.133812	-1.47644	0.139825	0.390563	FAM199X	139231	family witl X-linked
ENSG0000	478.2135	0.187805	0.127239	1.476008	0.139942	0.390808	PHF13	148479	PHD finger protein 13
ENSG0000	8.076992	-1.39697	0.946636	-1.47572	0.140018	0.390808	CHST13	166012	carbohydrate sulfotransferase 13
ENSG0000	323.8581	-0.24	0.162631	-1.47574	0.140014	0.390808	KLHDC10	23008	kelch domain containing 10
ENSG0000	133.3415	-0.31781	0.215342	-1.47585	0.139983	0.390808	ANK1	286	ankyrin 1
ENSG0000	449.5996	0.183436	0.1243	1.475751	0.140011	0.390808	EMC4	51234	ER membrane protein complex subunit 4
ENSG0000	21.56942	-0.88739	0.601412	-1.47551	0.140076	0.39091	ZNF135	7694	zinc finger protein 135
ENSG0000	24.41914	-0.71599	0.485347	-1.47522	0.140154	0.390947	CHCHD6	84303	coiled-coil-helix-coiled-coil-helix domain containing 6
ENSG0000	3.904731	1.699379	1.152069	1.475067	0.140195	0.390947	ZNF876P	642280	zinc finger pseudogene
ENSG0000	243.6318	0.293696	0.199099	1.475124	0.140179	0.390947	NPDC1	56654	neural pro differentiation and control 1
ENSG0000	2044.349	0.132046	0.089508	1.475246	0.140146	0.390947	PIAS4	51588	protein inhibitor of activated STAT 4
ENSG0000	14.18261	-0.9745	0.66056	-1.47526	0.140143	0.390947	NEURL2	140825	neuralized E3 ubiquitin protein ligase 2
ENSG0000	5245.515	-0.11213	0.076038	-1.47465	0.140308	0.391204	NUP210	23225	nucleoporin 210
ENSG0000	26.91407	0.871519	0.591318	1.473858	0.14052	0.391736	RPP40	10799	ribonuclease P/MRP subunit p40
ENSG0000	863.4134	-0.18458	0.12526	-1.4736	0.14059	0.391873	EIF2AK2	5610	eukaryotic translation initiation factor 2 alpha kinase 2
ENSG0000	9.108074	-1.28402	0.871401	-1.47351	0.140614	0.39188	NA	NA	NA
ENSG0000	3134.591	-0.11984	0.081335	-1.47337	0.140652	0.391928	APBA2	321	amyloid beta precursor protein binding family A member 2
ENSG0000	292.7966	0.23249	0.157813	1.473205	0.140696	0.391991	ZMYM5	9205	zinc finger MYM-type containing 5
ENSG0000	12.31711	-1.12105	0.761139	-1.47286	0.140788	0.392029	NA	NA	NA
ENSG0000	57.67065	-0.55642	0.377755	-1.47296	0.140762	0.392029	C12orf42	374470	chromosome 12 open reading frame 42
ENSG0000	6.176955	-1.46106	0.991954	-1.47291	0.140774	0.392029	NA	NA	NA
ENSG0000	19.66439	0.776251	0.527043	1.472841	0.140794	0.392029	NA	NA	NA
ENSG0000	4.352623	2.250758	1.528262	1.472756	0.140817	0.392034	NA	NA	NA
ENSG0000	1333.22	0.148954	0.101158	1.472484	0.14089	0.392121	FCGR2A	2212	Fc gamma receptor IIa
ENSG0000	415.9163	0.199015	0.135153	1.472515	0.140882	0.392121	GNG7	2788	G protein subunit gamma 7
ENSG0000	9.989853	-1.42406	0.967256	-1.47227	0.140949	0.392224	TNFRSF17	608	TNF receptor superfamily member 17
ENSG0000	72.90535	-0.51077	0.34697	-1.47208	0.141	0.392309	KANK3	256949	KN motif and ankyrin repeat domains 3
ENSG0000	564.4656	-0.17815	0.121053	-1.47167	0.141111	0.392359	IARS2	55699	isoleucyl-t mitochondrial
ENSG0000	2.276302	-3.10854	2.11231	-1.47163	0.141121	0.392359	NA	NA	NA
ENSG0000	5.798824	1.496936	1.017312	1.471462	0.141166	0.392359	LINC0261	1.02E+08	long intergenic non-protein coding RNA 2614
ENSG0000	26.67988	-0.86777	0.589669	-1.47161	0.141125	0.392359	NA	NA	NA
ENSG0000	1430.365	0.149582	0.101637	1.471725	0.141095	0.392359	TBC1D13	54662	TBC1 domain family member 13
ENSG0000	202.1563	-0.358	0.243295	-1.47147	0.141164	0.392359	MEG3	55384	maternally expressed 3
ENSG0000	15.43669	0.940428	0.638974	1.471778	0.141081	0.392359	NA	NA	NA
ENSG0000	243.69	-0.24013	0.163198	-1.47138	0.141189	0.392362	NIPBL-DT	646719	NIPBL divergent transcript
ENSG0000	234.6509	-0.33574	0.228274	-1.47077	0.141353	0.392759	SLC16A7	9194	solute carrier family 16 member 7
ENSG0000	14532.65	0.116247	0.079049	1.470566	0.141408	0.392856	IGHA1	3493	immunoglobulin heavy constant alpha 1
ENSG0000	4.018239	-2.28383	1.553355	-1.47025	0.141493	0.392913	NA	NA	NA
ENSG0000	106.8079	-0.35168	0.239184	-1.47034	0.141471	0.392913	RECQL4	9401	RecQ like helicase 4
ENSG0000	2.500692	-2.69868	1.83547	-1.47029	0.141482	0.392913	IGLVI-70	28763	immunoglobulin lambda variable (I)-70 (pseudogene)
ENSG0000	52.85466	0.472387	0.321352	1.469996	0.141563	0.393049	INSRR	3645	insulin receptor related receptor
ENSG0000	749.0791	0.155329	0.105704	1.469475	0.141704	0.393352	PARK7	11315	Parkinsonism associated deglycase
ENSG0000	5.509888	1.987216	1.352366	1.469436	0.141714	0.393352	NA	NA	NA
ENSG0000	57.36773	0.493784	0.336056	1.469348	0.141738	0.39336	TREX2	11219	three prime repair exonuclease 2
ENSG0000	536.8273	-0.18162	0.123631	-1.46908	0.14181	0.393499	NAXD	55739	NAD(P)HX dehydratase
ENSG0000	437.3418	0.200543	0.136549	1.468653	0.141927	0.393765	COX20	116228	cytochrome c oxidase assembly factor COX20
ENSG0000	139.4716	-0.31037	0.211367	-1.46841	0.141994	0.393866	FZD1	8321	frizzled class receptor 1
ENSG0000	19.22126	-0.85942	0.585293	-1.46836	0.142006	0.393866	NA	NA	NA
ENSG0000	4793.64	-0.11887	0.080961	-1.46825	0.142035	0.393889	CCDC88C	440193	coiled-coil domain containing 88C
ENSG0000	228.2029	-0.24626	0.167755	-1.46796	0.142115	0.393934	FAM13A-A	285512	FAM13A antisense RNA 1
ENSG0000	4.482648	1.891509	1.288523	1.467966	0.142113	0.393934	NA	NA	NA
ENSG0000	3.766984	-1.91605	1.30518	-1.46804	0.142094	0.393934	NA	NA	NA
ENSG0000	3314.247	0.148764	0.101361	1.467667	0.142195	0.394064	UBE2Q1	55585	ubiquitin conjugating enzyme E2 Q1
ENSG0000	11.60237	1.193771	0.813454	1.467533	0.142231	0.394064	LINC0136	257358	long intergenic non-protein coding RNA 1366
ENSG0000	13.25651	-1.01432	0.691212	-1.46746	0.142251	0.394064	TRAV36DV	28646	T cell receptor alpha variable 36/delta variable 7
ENSG0000	4.358402	-2.10723	1.435913	-1.46752	0.142234	0.394064	NA	NA	NA
ENSG0000	216.8594	-0.26021	0.177329	-1.4674	0.142268	0.394064	EIF1AY	9086	eukaryotic translation initiation factor 1A Y-linked
ENSG0000	3.967914	2.003879	1.3657	1.467291	0.142297	0.394084	RSPH4A	345895	radial spoke head component 4A
ENSG0000	46.86493	-0.5366	0.365791	-1.46696	0.142386	0.394095	EFCAB2	84288	EF-hand calcium binding domain 2
ENSG0000	6256.613	-0.11971	0.0816	-1.467	0.142376	0.394095	SHISA5	51246	shisa family member 5

ENSG0000	778.2113	0.154412	0.105251	1.467083	0.142353	0.394095	ELP5	23587	elongator acetyltransferase complex subunit 5
ENSG0000	28.24111	-0.66999	0.456713	-1.46699	0.14238	0.394095	ANKRD24	170961	ankyrin repeat domain 24
ENSG0000	224.4317	0.268891	0.183342	1.466611	0.142482	0.394243	ZNF614	80110	zinc finger protein 614
ENSG0000	3.532019	2.201971	1.501326	1.466685	0.142462	0.394243	RAB9B	51209	RAB9B member RAS oncogene family
ENSG0000	250.7324	0.248361	0.16938	1.466294	0.142568	0.394423	CARNS1	57571	carnosine synthase 1
ENSG0000	1326.054	-0.15286	0.104295	-1.4657	0.14273	0.394813	BRMS1	25855	BRMS1 transcriptional repressor and anoikis regulator
ENSG0000	46.07444	-0.57576	0.392902	-1.46539	0.142814	0.394985	COPG2	26958	COPI coat complex subunit gamma 2
ENSG0000	26.64252	0.755871	0.515862	1.465257	0.142851	0.395028	TEX14	56155	testis expr intercellular bridge forming factor
ENSG0000	3.808387	2.154829	1.470759	1.465113	0.14289	0.395078	KRT18P31	646723	keratin 18 pseudogene 31
ENSG0000	107.2393	-0.40438	0.276043	-1.46492	0.142942	0.395164	HIBADH	11112	3-hydroxyisobutyrate dehydrogenase
ENSG0000	441.8898	-0.22883	0.156223	-1.4648	0.142977	0.3952	CEP192	55125	centrosomal protein 192
ENSG0000	4.454936	1.975243	1.348815	1.464429	0.143077	0.395417	NA	NA	NA
ENSG0000	8105.985	0.125826	0.085933	1.464234	0.14313	0.395505	PDIA3	2923	protein disulfide isomerase family A member 3
ENSG0000	712.0799	0.189991	0.129788	1.463855	0.143234	0.395733	USE1	55850	unconventional SNARE in the ER 1
ENSG0000	135.3528	-0.33433	0.228426	-1.46364	0.143292	0.395834	UBA6-DT	550112	UBA6 divergent transcript
ENSG0000	17.40451	-0.8478	0.579295	-1.4635	0.14333	0.39588	FARP1	10160	FERM ARH/RhoGEF and pleckstrin domain protein 1
ENSG0000	24.69329	0.684899	0.46808	1.463208	0.14341	0.396044	NA	NA	NA
ENSG0000	19.44786	1.018278	0.695991	1.463063	0.14345	0.396095	NA	NA	NA
ENSG0000	23.94679	0.791247	0.541214	1.461985	0.143745	0.396328	NA	NA	NA
ENSG0000	5.315981	1.699796	1.162795	1.461819	0.143791	0.396328	RN7SL145I	1.06E+08	RNA 7SL cytoplasm pseudogene
ENSG0000	372.1225	0.201502	0.137781	1.46248	0.14361	0.396328	TCTA	6988	T cell leukemia translocation altered
ENSG0000	12716.55	-0.61038	0.417341	-1.46254	0.143594	0.396328	CXCL10	3627	C-X-C motif chemokine ligand 10
ENSG0000	160.8737	0.311596	0.213102	1.462195	0.143688	0.396328	SDHA	6389	succinate dehydrogenase complex flavoprotein subunit A
ENSG0000	379.4307	-0.206	0.140847	-1.46257	0.143584	0.396328	CEP295	85459	centrosomal protein 295
ENSG0000	12320.09	0.098038	0.067051	1.462146	0.143701	0.396328	LASP1	3927	LIM and SH3 protein 1
ENSG0000	2386.623	-0.12126	0.082944	-1.46194	0.143757	0.396328	EFTUD2	9343	elongation factor Tu GTP binding domain containing 2
ENSG0000	2590.333	-0.13515	0.092431	-1.46212	0.143708	0.396328	STK11	6794	serine/threonine kinase 11
ENSG0000	317.9394	-0.22648	0.154928	-1.46187	0.143777	0.396328	GZF1	64412	GDNF inducible zinc finger protein 1
ENSG0000	10.48858	1.080607	0.739154	1.46195	0.143755	0.396328	NA	NA	NA
ENSG0000	1131.976	-0.15872	0.108534	-1.4624	0.143632	0.396328	THOC2	57187	THO complex subunit 2
ENSG0000	16.64033	-1.02794	0.703275	-1.46165	0.143836	0.396394	HAS3	3038	hyaluronan synthase 3
ENSG0000	1861.591	0.122773	0.084001	1.461563	0.143861	0.396403	AFTPH	54812	aftiphilin
ENSG0000	614.4447	0.19361	0.132476	1.461471	0.143886	0.396414	TMEM87A	25963	transmembrane protein 87A
ENSG0000	6.567465	-1.65147	1.130089	-1.46136	0.143916	0.396438	NA	NA	NA
ENSG0000	13.23629	1.324525	0.906526	1.4611	0.143988	0.396518	PKD1L1	168507	polycystin transient receptor potential channel interacting
ENSG0000	48.70416	-0.7511	0.514054	-1.46113	0.14398	0.396518	NA	NA	NA
ENSG0000	61.07906	0.452365	0.309676	1.460768	0.144079	0.396709	NA	NA	NA
ENSG0000	460.8977	0.203156	0.139092	1.460593	0.144127	0.396732	MAP3K5	4217	mitogen-activated protein kinase kinase kinase 5
ENSG0000	42.8419	0.607765	0.416111	1.460582	0.14413	0.396732	NA	NA	NA
ENSG0000	229.1589	-0.27257	0.186639	-1.4604	0.144181	0.396808	SNX14	57231	sorting nexin 14
ENSG0000	41.70863	-0.63839	0.437155	-1.46033	0.1442	0.396808	LIPC	3990	lipase C hepatic type
ENSG0000	568.8749	0.226172	0.154886	1.460244	0.144223	0.396812	HK2	3099	hexokinase 2
ENSG0000	326.1124	-0.22017	0.150799	-1.46002	0.144284	0.396919	SEPHS1	22929	selenophosphate synthetase 1
ENSG0000	3.103519	-2.83133	1.939455	-1.45986	0.144329	0.396986	RPL7AP64	728486	ribosomal protein L7a pseudogene 64
ENSG0000	3.385457	1.916893	1.313664	1.459196	0.144511	0.397427	ETFBKMT	254013	electron transfer flavoprotein subunit beta lysine methyltransferase
ENSG0000	66.41179	-0.42059	0.288378	-1.45847	0.144712	0.397861	COMMD8	54951	COMM domain containing 8
ENSG0000	7523.285	0.10628	0.07287	1.458486	0.144707	0.397861	SYVN1	84447	synoviolin 1
ENSG0000	326.5501	0.217741	0.149322	1.458196	0.144787	0.398008	P4HA1	5033	prolyl 4-hydroxylase subunit alpha 1
ENSG0000	18.73512	0.936477	0.642409	1.457758	0.144907	0.398281	IGKV1-9	28941	immunoglobulin kappa variable 1-9
ENSG0000	4.358425	2.273202	1.559718	1.457444	0.144994	0.398282	NA	NA	NA
ENSG0000	9.950854	-1.41851	0.973208	-1.45756	0.144961	0.398282	PDE7B	27115	phosphodiesterase 7B
ENSG0000	4.285927	-2.19021	1.502695	-1.45752	0.144973	0.398282	NA	NA	NA
ENSG0000	86.99052	-0.431	0.295686	-1.45761	0.144947	0.398282	ICOSLG	23308	inducible T cell costimulator ligand
ENSG0000	622.6535	0.187851	0.128912	1.457204	0.14506	0.398405	HTRA2	27429	HtrA serine peptidase 2
ENSG0000	301.02	-0.21975	0.15085	-1.45678	0.145176	0.398429	RPAP2	79871	RNA polymerase II associated protein 2
ENSG0000	50.27627	-0.57409	0.394067	-1.45682	0.145166	0.398429	SMYD3	64754	SET and MYND domain containing 3
ENSG0000	2.910521	-2.52316	1.731953	-1.45683	0.145164	0.398429	ACOXL	55289	acyl-CoA oxidase like
ENSG0000	103.5183	0.346079	0.23753	1.456991	0.145119	0.398429	SLC25A51	92014	solute carrier family 25 member 51
ENSG0000	5.264165	-1.53986	1.056871	-1.457	0.145117	0.398429	TECTA	7007	tectorin alpha
ENSG0000	5.586053	1.90834	1.310134	1.456599	0.145227	0.398451	MOXD1	26002	monooxygenase DBH like 1
ENSG0000	17.4957	-1.04888	0.720057	-1.45666	0.145209	0.398451	TINCR	257000	TINCR ubiquitin domain containing
ENSG0000	12.2436	-0.95201	0.653674	-1.4564	0.145283	0.398511	CDK13-DT	1.12E+08	CDK13 divergent transcript
ENSG0000	128.1196	0.370058	0.25411	1.456287	0.145313	0.398511	GON7	84520	GON7 subunit of KEOPS complex
ENSG0000	2.862056	2.401554	1.649065	1.456313	0.145306	0.398511	NA	NA	NA
ENSG0000	6.072987	-1.78929	1.229079	-1.4558	0.145448	0.398822	USP46-DT	643783	USP46 divergent transcript
ENSG0000	3775.656	-0.13181	0.090614	-1.45468	0.145757	0.399078	TTC7A	57217	tetratricopeptide repeat domain 7A
ENSG0000	555.9062	-0.19552	0.134381	-1.45494	0.145687	0.399078	P4HTM	54681	prolyl 4-hy transmembrane
ENSG0000	498.469	0.199549	0.137165	1.454817	0.14572	0.399078	GMPS	8833	guanine monophosphate synthase
ENSG0000	987.2188	0.149299	0.102619	1.454886	0.145701	0.399078	RWDD1	51389	RWD domain containing 1
ENSG0000	1693.994	-0.14023	0.096359	-1.45532	0.145582	0.399078	PCM1	5108	pericentriolar material 1
ENSG0000	23.30217	0.809372	0.556263	1.455015	0.145665	0.399078	TMEM132	54972	transmembrane protein 132A
ENSG0000	118.8541	-0.34928	0.240089	-1.45478	0.14573	0.399078	FAM118B	79607	family with sequence similarity 118 member B
ENSG0000	43.31704	0.512794	0.352483	1.454808	0.145722	0.399078	SPRYD7	57213	SPRY domain containing 7
ENSG0000	441.4383	-0.21139	0.145309	-1.45475	0.145739	0.399078	GHDC	84514	GH3 domain containing
ENSG0000	52.40572	0.569043	0.391033	1.455231	0.145605	0.399078	ADM5	199800	adrenomedullin 5 (putative)
ENSG0000	267.17	0.220994	0.15194	1.454484	0.145812	0.39917	CDC26	246184	cell division cycle 26
ENSG0000	413.5247	0.200636	0.137967	1.45423	0.145883	0.399304	GID4	79018	GID complex subunit 4 homolog
ENSG0000	1039.799	0.153279	0.105412	1.454092	0.145921	0.39935	UBA2	10054	ubiquitin like modifier activating enzyme 2
ENSG0000	5.822205	-1.76435	1.213971	-1.45337	0.146121	0.399513	LOC10192	1.02E+08	uncharacterized LOC101928728
ENSG0000	6147.936	0.102822	0.070749	1.453334	0.146131	0.399513	CTNNB1	1499	catenin beta 1
ENSG0000	480.9867	-0.18287	0.125803	-1.45363	0.146048	0.399513	OTULINL	54491	OTU deubiquitinase with linear linkage specificity like
ENSG0000	2681.661	0.12849	0.088392	1.453642	0.146046	0.399513	DNAJB6	10049	DnaJ heat shock protein family (Hsp40) member B6
ENSG0000	47.10113	-0.54426	0.374471	-1.45341	0.146109	0.399513	SCAI	286205	suppressor of cancer cell invasion
ENSG0000	5.108188	-1.53889	1.058847	-1.45337	0.146122	0.399513	MIR7152	1.02E+08	microRNA 7152
ENSG0000	1707.759	0.126189	0.086819	1.453472	0.146093	0.399513	AREL1	9870	apoptosis resistant E3 ubiquitin protein ligase 1
ENSG0000	396.7	-0.22446	0.154477	-1.45303	0.146215	0.399683	CA11	770	carbonic anhydrase 11
ENSG0000	6.091132	1.446225	0.995456	1.452827	0.146272	0.39978	NA	NA	NA
ENSG0000	504.3858	0.197015	0.13564	1.452482	0.146368	0.399983	SLC36A1	206358	solute carrier family 36 member 1
ENSG0000	172.9091	0.27743	0.191066	1.452012	0.146498	0.40028	NA	NA	NA
ENSG0000	307.879	-0.21481	0.147996	-1.45147	0.146649	0.400466	CPSF3	51692	cleavage and polyadenylation specific factor 3
ENSG0000	16.97364	-0.88832	0.612047	-1.45139	0.146672	0.400466	LINC0201ε	1.08E+08	long intergenic non-protein coding RNA 2018
ENSG0000	33.7994	0.613845	0.423013	1.451124	0.146745	0.400466	MIR570	693155	microRNA 570
ENSG0000	217.38	0.303878	0.209431	1.450971	0.146788	0.400466	RCHY1	25898	ring finger and CHY zinc finger domain containing 1
ENSG0000	4.876581	-1.901	1.310262	-1.45086	0.14682	0.400466	NA	NA	NA
ENSG0000	6.38869	-1.5583	1.07384	-1.45115	0.146738	0.400466	TRIM74	378108	tripartite motif containing 74
ENSG0000	1447.948	0.127011	0.087509	1.451397	0.146669	0.400466	NT5C2	22978	5'-nucleoti cytosolic II
ENSG0000	105.5821	-0.36699	0.25287	-1.45131	0.146694	0.400466	XRCC3	7517	X-ray repair cross complementing 3
ENSG0000	1230.081	0.137092	0.094436	1.451687	0.146589	0.400466	TRPM7	54822	transient receptor potential cation channel subfamily M member 7
ENSG0000	10.93838	-1.29774	0.89448	-1.45084	0.146825	0.400466	SKA1	220134	spindle and kinetochore associated complex subunit 1
ENSG0000	4.523738	1.897491	1.307728	1.450983	0.146785	0.400466	ZNF516-AS	1.02E+08	ZNF516 antisense RNA 1

ENSG0000	4299.677	0.116691	0.080423	1.450961	0.146791	0.400466	CTSA	5476	cathepsin A
ENSG0000	49.105	-0.50417	0.347519	-1.45076	0.146848	0.400468	NA	NA	NA
ENSG0000	2549.893	-0.11906	0.082085	-1.45044	0.146937	0.400653	ANAPC2	29882	anaphase promoting complex subunit 2
ENSG0000	10.08618	1.088758	0.750789	1.450152	0.147016	0.40081	NUSAP1	51203	nucleolar and spindle associated protein 1
ENSG0000	3.288624	2.133378	1.471499	1.449799	0.147114	0.400921	NA	NA	NA
ENSG0000	181.8244	-0.26149	0.180353	-1.44988	0.147092	0.400921	CTSF	8722	cathepsin F
ENSG0000	963.0883	0.150218	0.103615	1.449773	0.147122	0.400921	NME4	4833	NME/NM23 nucleoside diphosphate kinase 4
ENSG0000	121.9139	-0.32369	0.223299	-1.44957	0.147179	0.40094	SUMF1	285362	sulfatase modifying factor 1
ENSG0000	17.98767	-0.79638	0.54947	-1.44936	0.147237	0.40094	FAM86EP	348926	family witl member A pseudogene
ENSG0000	641.4483	0.168481	0.116229	1.449557	0.147182	0.40094	PLRG1	5356	pleiotropic regulator 1
ENSG0000	6.791773	-1.46282	1.00919	-1.4495	0.147198	0.40094	MTND5P1	1.01E+08	MT-ND5 pseudogene 14
ENSG0000	21.29267	0.805971	0.556066	1.449417	0.147221	0.40094	RPL9P7	6126	ribosomal protein L9 pseudogene 7
ENSG0000	3.678092	-2.13261	1.471681	-1.4491	0.147309	0.401079	AVPR2	554	arginine vasopressin receptor 2
ENSG0000	7973.84	0.132112	0.091202	1.44857	0.147458	0.401423	STK10	6793	serine/threonine kinase 10
ENSG0000	74.37686	-0.46534	0.321292	-1.44835	0.14752	0.401534	NRL	4901	neural retina leucine zipper
ENSG0000	3.626661	-2.25751	1.559696	-1.4474	0.147784	0.401545	MFAP2	4237	microfibril associated protein 2
ENSG0000	4.190891	-2.14239	1.480048	-1.44751	0.147753	0.401545	KIF17	57576	kinesin family member 17
ENSG0000	314.3645	0.255384	0.176377	1.447942	0.147633	0.401545	MAGOH	4116	mago hom exon junction complex subunit
ENSG0000	57.58541	0.546753	0.377595	1.447988	0.14762	0.401545	NA	NA	NA
ENSG0000	9.404828	1.180645	0.815365	1.447995	0.147619	0.401545	PHLDA3	23612	pleckstrin homology like domain family A member 3
ENSG0000	7.815911	-1.28805	0.889549	-1.44799	0.147621	0.401545	NA	NA	NA
ENSG0000	288.7198	-0.235	0.16236	-1.44741	0.147781	0.401545	TMCC1	23023	transmembrane and coiled-coil domain family 1
ENSG0000	143.5439	-0.30997	0.214124	-1.44761	0.147726	0.401545	PCCB	5096	propionyl-CoA carboxylase subunit beta
ENSG0000	35.15279	0.612229	0.42275	1.448207	0.147559	0.401545	SNHG26	1.1E+08	small nucleolar RNA host gene 26
ENSG0000	35.85174	0.617983	0.426933	1.447495	0.147758	0.401545	CCDC146	57639	coiled-coil domain containing 146
ENSG0000	137.1359	0.339312	0.234407	1.447535	0.147747	0.401545	HELB	92797	DNA helicase B
ENSG0000	709.506	-0.17482	0.120779	-1.44747	0.147766	0.401545	SLC22A17	51310	solute carrier family 22 member 17
ENSG0000	132.7884	-0.31451	0.217354	-1.447	0.147896	0.401792	MAGEE1	57692	MAGE family member E1
ENSG0000	3417.068	0.109263	0.075545	1.446342	0.148081	0.402236	CCNL2	81669	cyclin L2
ENSG0000	1313.609	0.153594	0.106221	1.445987	0.148181	0.402354	AAMP	14	angio associated migratory cell protein
ENSG0000	222.4853	-0.2479	0.171446	-1.44595	0.14819	0.402354	TTI2	80185	TELO2 interacting protein 2
ENSG0000	2373.668	0.130137	0.089996	1.446036	0.148167	0.402354	FOXO1	2308	forkhead box O1
ENSG0000	4006.361	0.110337	0.076312	1.445871	0.148213	0.402359	ZNF335	63925	zinc finger protein 335
ENSG0000	542.5378	0.192766	0.133356	1.445496	0.148319	0.402572	SLC30A5	64924	solute carrier family 30 member 5
ENSG0000	4.623689	1.908791	1.320609	1.445387	0.148349	0.402572	CCR6	1235	C-C motif chemokine receptor 6
ENSG0000	10120.89	-0.11481	0.07944	-1.44528	0.148379	0.402572	TTYH3	80727	tweety family member 3
ENSG0000	22.10255	-2.04093	1.412079	-1.44533	0.148364	0.402572	IGLV3-10	28803	immunoglobulin lambda variable 3-10
ENSG0000	2510.72	-0.13256	0.091732	-1.44507	0.148438	0.402616	PSME2	5721	proteasome activator subunit 2
ENSG0000	10.91154	-1.27267	0.880665	-1.44512	0.148424	0.402616	LINC0063	196872	long intergenic non-protein coding RNA 638
ENSG0000	3259.233	-0.14473	0.100157	-1.44499	0.148462	0.40262	PARP10	84875	poly(ADP-ribose) polymerase family member 10
ENSG0000	1093.177	0.16356	0.113213	1.444705	0.148541	0.402775	SPATA2	9825	spermatogenesis associated 2
ENSG0000	236.5873	-0.26998	0.186891	-1.44461	0.148567	0.402787	NA	NA	NA
ENSG0000	2453.591	-0.12707	0.087964	-1.44452	0.148593	0.4028	HNRNPR	10236	heterogeneous nuclear ribonucleoprotein R
ENSG0000	11382.26	0.100964	0.069919	1.444019	0.148734	0.403122	DIAPH1	1729	diaphanous related formin 1
ENSG0000	36.03703	-0.578	0.400362	-1.44369	0.148827	0.403283	CPLANE2	79363	ciliogenesis and planar polarity effector complex subunit 2
ENSG0000	136.9831	0.300264	0.20803	1.443365	0.148918	0.403283	FOXD2-AS	84793	FOXD2 adjacent opposite strand RNA 1
ENSG0000	7.529485	-1.80167	1.248093	-1.44353	0.14887	0.403283	RNU6-646	1.06E+08	RNA U6 small r pseudogene
ENSG0000	44.94263	-0.54246	0.375833	-1.44334	0.148924	0.403283	RASGEF1A	221002	RasGEF domain family member 1A
ENSG0000	1642.696	-0.1712	0.118607	-1.44343	0.148899	0.403283	ATM	472	ATM serine/threonine kinase
ENSG0000	5320.865	0.112516	0.077945	1.443537	0.148869	0.403283	KDM5C	8242	lysine demethylase 5C
ENSG0000	2.277391	-2.75148	1.906645	-1.4431	0.148992	0.40341	NA	NA	NA
ENSG0000	7297.278	-0.10564	0.073212	-1.44286	0.149059	0.403419	ERGIC1	57222	endoplasmic reticulum-golgi intermediate compartment 1
ENSG0000	82.67779	0.44494	0.308374	1.442857	0.149061	0.403419	ME3	10873	malic enzyme 3
ENSG0000	927.5081	0.16241	0.112558	1.442896	0.14905	0.403419	TSR2	90121	TSR2 ribosome maturation factor
ENSG0000	6.852708	-1.52536	1.057595	-1.44229	0.149221	0.403795	SLC35G5	83650	solute carrier family 35 member G5
ENSG0000	543.3159	-0.1899	0.131674	-1.44217	0.149255	0.403826	GUSBP11	91316	GUSB pseudogene 11
ENSG0000	56.85078	-0.47716	0.330898	-1.442	0.149302	0.403894	GALK2	2585	galactokinase 2
ENSG0000	1643.985	0.122132	0.084745	1.441178	0.149534	0.404343	ABCF3	55324	ATP binding cassette subfamily F member 3
ENSG0000	9.844839	-1.35997	0.943633	-1.44121	0.149526	0.404343	TCAF1P1	653199	TRPM8 channel associated factor 1 pseudogene 1
ENSG0000	913.4867	-0.1452	0.10077	-1.44093	0.149604	0.404343	MVB12B	89853	multivesicular body subunit 12B
ENSG0000	37.57628	-0.66266	0.459808	-1.44117	0.149537	0.404343	METTL15	196074	methyltransferase like 15
ENSG0000	17.15158	-0.81736	0.567299	-1.4408	0.149642	0.404343	NA	NA	NA
ENSG0000	5.427357	1.677523	1.164057	1.441101	0.149556	0.404343	EZHIP	340602	EZH inhibitory protein
ENSG0000	729.1437	0.172824	0.119947	1.440837	0.149631	0.404343	NBDY	550643	negative regulator of P-body association
ENSG0000	4.627357	-1.54928	1.075287	-1.4408	0.14964	0.404343	NA	NA	NA
ENSG0000	2.930173	-2.39115	1.659872	-1.44056	0.149708	0.404404	ZBTB7C	201501	zinc finger and BTB domain containing 7C
ENSG0000	18.47321	-0.89914	0.624151	-1.44059	0.149701	0.404404	ZNF667	63934	zinc finger protein 667
ENSG0000	1767.67	0.124747	0.086605	1.440415	0.14975	0.404458	NAGK	55577	N-acetylglucosamine kinase
ENSG0000	215.569	-0.27047	0.187869	-1.43968	0.149959	0.404964	KRT73	319101	keratin 73
ENSG0000	592.8524	-0.21684	0.150637	-1.4395	0.15001	0.405043	LPCAT2	54947	lysophosphatidylcholine acyltransferase 2
ENSG0000	131.4327	0.326863	0.227091	1.439345	0.150053	0.405099	NA	NA	NA
ENSG0000	123.0215	-0.35619	0.247512	-1.43909	0.150124	0.405172	PARP2	10038	poly(ADP-ribose) polymerase 2
ENSG0000	122.3158	-0.34579	0.240273	-1.43915	0.150107	0.405172	ZSWIM3	140831	zinc finger SWIM-type containing 3
ENSG0000	166.3179	0.28248	0.196313	1.438928	0.150171	0.40524	TMEM64	169200	transmembrane protein 64
ENSG0000	28.82516	0.636218	0.44221	1.438726	0.150228	0.405337	NA	NA	NA
ENSG0000	20.74465	-0.79701	0.554089	-1.43841	0.150316	0.405457	NA	NA	NA
ENSG0000	1287.929	0.138614	0.096362	1.438478	0.150298	0.405457	USP47	55031	ubiquitin specific peptidase 47
ENSG0000	3.227601	-2.23345	1.552951	-1.4382	0.150379	0.405472	MIR34AH	1.07E+08	MIR34A host gene
ENSG0000	1989.305	-0.13605	0.094601	-1.43816	0.150388	0.405472	CD300C	10871	CD300c molecule
ENSG0000	20.30356	-0.84051	0.584408	-1.43823	0.15037	0.405472	NA	NA	NA
ENSG0000	23.07228	0.768083	0.53423	1.437739	0.150508	0.405722	LINC0148	1.02E+08	long intergenic non-protein coding RNA 1480
ENSG0000	7.266111	-1.4888	1.035559	-1.43768	0.150524	0.405722	LINC01431	1.01E+08	long intergenic non-protein coding RNA 1431
ENSG0000	82.83228	-0.36911	0.256841	-1.43711	0.150686	0.406062	CHCHD4	131474	coiled-coil-helix-coiled-coil-helix domain containing 4
ENSG0000	957.679	0.14741	0.102576	1.437083	0.150694	0.406062	PSMD6	9861	proteasom non-ATPase 6
ENSG0000	458.9327	-0.20679	0.143906	-1.43697	0.150726	0.406087	IFNAR2	3455	interferon alpha and beta receptor subunit 2
ENSG0000	2.66722	-2.62322	1.825755	-1.43679	0.150778	0.406093	PPARGC1	10891	PPARG coactivator 1 alpha
ENSG0000	126.1449	-0.32581	0.226771	-1.43674	0.150793	0.406093	NA	NA	NA
ENSG0000	3876.418	0.144769	0.100757	1.436816	0.15077	0.406093	NACA	4666	nascent polypeptide associated complex subunit alpha
ENSG0000	32.90686	0.619283	0.431174	1.436273	0.150925	0.40627	CCNE2	9134	cyclin E2
ENSG0000	88.64497	0.397896	0.27702	1.436347	0.150904	0.40627	RARA-AS1	1.02E+08	RARA antisense RNA 1
ENSG0000	19650.01	0.119486	0.083183	1.436419	0.150883	0.40627	NOP53	29997	NOP53 ribosome biogenesis factor
ENSG0000	5.163184	-1.47789	1.029086	-1.43612	0.150969	0.406331	NA	NA	NA
ENSG0000	51.94406	0.546144	0.380347	1.43591	0.151028	0.40643	CDC42	998	cell division cycle 42
ENSG0000	37.22662	-0.59199	0.412352	-1.43565	0.151102	0.406569	BACE1	23621	beta-secretase 1
ENSG0000	7517.459	0.340772	0.237416	1.435337	0.151191	0.406751	PLEK	5341	pleckstrin
ENSG0000	727.5433	-0.16195	0.112852	-1.43506	0.151271	0.406803	ERCC3	2071	ERCC excis TFIIH core complex helicase subunit
ENSG0000	57.72323	-0.44493	0.31005	-1.43504	0.151276	0.406803	CENPP	401541	centromere protein P
ENSG0000	61.35976	0.465161	0.324116	1.435168	0.151239	0.406803	LYSET	26175	lysosomal enzyme trafficking factor
ENSG0000	325.9297	-0.24741	0.172458	-1.43463	0.151391	0.407053	SLC30A1	7779	solute carrier family 30 member 1
ENSG0000	36.90507	-0.59651	0.415913	-1.43422	0.151509	0.407094	GTF2H2C	728340	GTF2H2 family member C

ENSG0000	23925.06	0.108518	0.07566	1.434293	0.151489	0.407094	RPL8	6132	ribosomal protein L8
ENSG0000	10.01949	-1.29182	0.900722	-1.43421	0.151513	0.407094	NA	NA	NA
ENSG0000	83.26802	0.367731	0.256376	1.434342	0.151475	0.407094	NA	NA	NA
ENSG0000	5404.337	0.130482	0.090979	1.434196	0.151516	0.407094	OGT	8473	O-linked N-acetylglucosamine (GlcNAc) transferase
ENSG0000	515.7617	-0.17555	0.122415	-1.43404	0.151561	0.407155	PPCS	79717	phosphopantothenoylcysteine synthetase
ENSG0000	17.52801	0.942748	0.657509	1.433818	0.151624	0.407164	IGHEP2	3499	immunoglobulin heavy constant epsilon P2 (pseudogene)
ENSG0000	1342.572	0.131168	0.091474	1.433925	0.151594	0.407164	MRPL49	740	mitochondrial ribosomal protein L49
ENSG0000	5.133686	-1.86775	1.302797	-1.43364	0.151674	0.407164	ANKS1B	56899	ankyrin repeat and sterile alpha motif domain containing 1B
ENSG0000	5.157721	1.542498	1.075872	1.43372	0.151652	0.407164	NA	NA	NA
ENSG0000	254.4674	0.26394	0.184103	1.433652	0.151672	0.407164	VBP1	7411	VHL binding protein 1
ENSG0000	2425.8	0.135062	0.094241	1.433149	0.151815	0.407425	YWHAQ	10971	tyrosine 3-monooxygenase/tryptophan 5-monooxygenase activation protein theta
ENSG0000	328.0982	-0.20288	0.141561	-1.43316	0.151811	0.407425	GATAD1	57798	GATA zinc finger domain containing 1
ENSG0000	1.937989	-3.31306	2.312033	-1.43296	0.151868	0.407509	TRAJ31	28724	T cell receptor alpha joining 31
ENSG0000	1332.635	0.137528	0.09599	1.432731	0.151935	0.407628	NA	NA	NA
ENSG0000	1641.213	-0.13238	0.092408	-1.43253	0.151993	0.407665	TBRG1	84897	transforming growth factor beta regulator 1
ENSG0000	323.6063	-0.22178	0.154818	-1.43253	0.151993	0.407665	SOCS6	9306	suppressor of cytokine signaling 6
ENSG0000	3.464956	-2.04797	1.429882	-1.43227	0.152068	0.407808	NA	NA	NA
ENSG0000	442.8158	-0.19172	0.133868	-1.43215	0.1521	0.407836	NETO2	81831	neuropilin and tolloid like 2
ENSG0000	440.2553	0.198547	0.138645	1.432046	0.152131	0.407859	KLHDC2	23588	kelch domain containing 2
ENSG0000	298.5193	0.216321	0.151108	1.43157	0.152267	0.408106	IPPK	64768	inositol-pentakisphosphate 2-kinase
ENSG0000	343.7549	-0.21361	0.149216	-1.43158	0.152265	0.408106	EEA1	8411	early endosome antigen 1
ENSG0000	905.8152	0.148469	0.103727	1.431338	0.152333	0.408225	SDHB	6390	succinate dehydrogenase complex iron sulfur subunit B
ENSG0000	69.33862	0.522833	0.365338	1.431092	0.152404	0.408305	TTC32	130502	tetratricopeptide repeat domain 32
ENSG0000	2601.543	-0.11761	0.082183	-1.43108	0.152407	0.408305	ARHGEF3	50650	Rho guanine nucleotide exchange factor 3
ENSG0000	1214.579	-0.15003	0.104845	-1.43099	0.152432	0.408312	ZBTB38	253461	zinc finger and BTB domain containing 38
ENSG0000	2593.219	-0.14736	0.102994	-1.43081	0.152485	0.408395	ROCK1	6093	Rho associated coiled-coil containing protein kinase 1
ENSG0000	51.54584	-0.50602	0.3538	-1.43024	0.152648	0.408773	PRPF40B	25766	pre-mRNA processing factor 40 homolog B
ENSG0000	448.611	-0.20497	0.143384	-1.42954	0.152849	0.409092	SNAP47	116841	synaptosome associated protein 47
ENSG0000	312.2919	-0.24107	0.168622	-1.42963	0.152822	0.409092	GTF3C3	9330	general transcription factor IIIC subunit 3
ENSG0000	3.541492	2.39902	1.678045	1.429652	0.152817	0.409092	MTCO2P1:	1.07E+08	MT-CO2 pseudogene 11
ENSG0000	969.3225	-0.17553	0.122788	-1.42952	0.152855	0.409092	ZC3H14	79882	zinc finger CCCH-type containing 14
ENSG0000	2421.23	0.118782	0.083108	1.42924	0.152935	0.409131	SSU72	29101	SSU72 hor RNA polymerase II CTD phosphatase
ENSG0000	1510.746	-0.12913	0.090347	-1.42924	0.152936	0.409131	ANAPC5	51433	anaphase promoting complex subunit 5
ENSG0000	7.042658	-1.48472	1.038742	-1.42935	0.152905	0.409131	NOXRED1	122945	NADP dependent oxidoreductase domain containing 1
ENSG0000	262.798	-0.27435	0.191973	-1.42912	0.15297	0.409162	THADA	63892	THADA armadillo repeat containing
ENSG0000	90.68436	-0.38114	0.266728	-1.42894	0.153021	0.409239	NA	NA	NA
ENSG0000	315.3862	0.220876	0.15463	1.428417	0.153172	0.409584	LRP8	7804	LDL receptor related protein 8
ENSG0000	3179.502	-0.11683	0.081808	-1.42815	0.15325	0.409734	BTN3A1	11119	butyrophilin subfamily 3 member A1
ENSG0000	179.9723	-0.30297	0.212184	-1.42786	0.153333	0.409897	LDOC1	23641	LDOC1 regulator of NFKB signaling
ENSG0000	4.708357	1.594597	1.116932	1.427658	0.15339	0.409933	RPL23AP2	728481	ribosomal protein L23a pseudogene 21
ENSG0000	5.326234	-1.81898	1.274101	-1.42766	0.15339	0.409933	LINC0128	1.02E+08	long intergenic non-protein coding RNA 1284
ENSG0000	861.5065	0.152615	0.106926	1.4273	0.153494	0.410046	KDM5B	10765	lysine demethylase 5B
ENSG0000	41.26545	-0.55181	0.386595	-1.42736	0.153476	0.410046	NA	NA	NA
ENSG0000	82.21413	-0.42119	0.295102	-1.42728	0.153499	0.410046	SAAL1	113174	serum amyloid A like 1
ENSG0000	5347.188	0.129198	0.09054	1.426979	0.153586	0.410219	LGALS1	3956	galectin 1
ENSG0000	29.39092	-0.62846	0.440454	-1.42685	0.153622	0.410255	TNFRSF10	389641	TNFRSF10A divergent transcript
ENSG0000	8.307845	-1.32445	0.928294	-1.42676	0.153649	0.410269	MAMSTR	284358	MEF2 activating motif and SAP domain containing transcriptional regulator
ENSG0000	8.663345	-1.26439	0.886272	-1.42664	0.153685	0.410305	UTS2R	2837	urotensin 2 receptor
ENSG0000	34.71528	-0.61437	0.430723	-1.42637	0.153761	0.41045	TRAV8-6	28680	T cell receptor alpha variable 8-6
ENSG0000	31.05589	0.835949	0.586176	1.426104	0.153838	0.410597	NA	NA	NA
ENSG0000	333.4444	-0.27816	0.19506	-1.42601	0.153866	0.410611	SNTA1	6640	syntrophin alpha 1
ENSG0000	25.7513	-0.82154	0.576247	-1.42566	0.153965	0.410758	PRKCZ-AS:	1.01E+08	PRKCZ antisense RNA 1
ENSG0000	1014.972	0.163602	0.114755	1.425668	0.153964	0.410758	CTSC	1075	cathepsin C
ENSG0000	21.71976	0.779141	0.546599	1.425435	0.154031	0.410876	NAT8L	339983	N-acetyltransferase 8 like
ENSG0000	202.3843	-0.27336	0.191813	-1.42517	0.154109	0.410968	STRBP	55342	spermatid perinuclear RNA binding protein
ENSG0000	63.14222	0.451902	0.317088	1.425162	0.15411	0.410968	C19orf84	147646	chromosome 19 open reading frame 84
ENSG0000	225.2814	-0.24118	0.169283	-1.42469	0.154247	0.411058	MINDY1	55793	MINDY lysine 48 deubiquitinase 1
ENSG0000	1899.863	-0.73762	0.517821	-1.42446	0.154313	0.411058	SLAMF8	56833	SLAM family member 8
ENSG0000	280.7615	-0.22699	0.159339	-1.42458	0.154279	0.411058	IFT122	55764	intraflagellar transport 122
ENSG0000	3.432483	2.078129	1.458384	1.424953	0.154171	0.411058	FGF17	8822	fibroblast growth factor 17
ENSG0000	13.59234	1.005974	0.706172	1.424546	0.154289	0.411058	DPCD	25911	deleted in primary ciliary dyskinesia homolog (mouse)
ENSG0000	284.175	-0.26419	0.18547	-1.42443	0.154321	0.411058	JKAMP	51528	JNK1/MAPK8 associated membrane protein
ENSG0000	1198.026	0.160547	0.112677	1.424839	0.154204	0.411058	TSPOAP1-/	1.01E+08	TSPOAP1 SUPT4H1 and RNF43 antisense RNA 1
ENSG0000	5270.484	0.12109	0.084993	1.424706	0.154242	0.411058	DENND1C	79958	DENN domain containing 1C
ENSG0000	2.763586	2.741804	1.925014	1.424304	0.154359	0.411098	NA	NA	NA
ENSG0000	65.34622	0.44987	0.316118	1.42311	0.154704	0.411842	NA	NA	NA
ENSG0000	5.379638	-1.59997	1.124205	-1.4232	0.154678	0.411842	HOXA4	3201	homeobox A4
ENSG0000	3353.16	-0.10587	0.074387	-1.42318	0.154685	0.411842	NUP62	23636	nucleoporin 62
ENSG0000	188.5804	0.294338	0.206872	1.422804	0.154793	0.412019	GPR157	80045	G protein-coupled receptor 157
ENSG0000	18.39048	-0.8957	0.629682	-1.42246	0.154892	0.412222	PPFIA4	8497	PTPRF interacting protein alpha 4
ENSG0000	3193.727	-0.10854	0.076314	-1.42227	0.154947	0.41231	TAOK2	9344	TAO kinase 2
ENSG0000	603.1302	-0.18047	0.12692	-1.4219	0.155056	0.412353	GGCX	2677	gamma-glutamyl carboxylase
ENSG0000	68.10481	-0.43077	0.303013	-1.42163	0.155134	0.412353	TSEN2	80746	tRNA splicing endonuclease subunit 2
ENSG0000	14811.56	0.123271	0.086712	1.421605	0.155141	0.412353	EEF1D	1936	eukaryotic translation elongation factor 1 delta
ENSG0000	3556.287	-0.12082	0.084987	-1.42167	0.155121	0.412353	ANKRD52	283373	ankyrin repeat domain 52
ENSG0000	2.825869	2.443623	1.718609	1.421861	0.155067	0.412353	SCGB1C2	653486	secretoglobin family 1C member 2
ENSG0000	56.77908	-0.49454	0.347798	-1.42193	0.155047	0.412353	ZNF85	7639	zinc finger protein 85
ENSG0000	1123.99	0.155529	0.109383	1.421876	0.155062	0.412353	SNRPA	6626	small nuclear ribonucleoprotein polypeptide A
ENSG0000	17.09043	-0.89976	0.632871	-1.4217	0.155112	0.412353	MYT1	4661	myelin transcription factor 1
ENSG0000	37.79179	0.567125	0.398984	1.421422	0.155194	0.412435	KRT8P33	1E+08	keratin 8 pseudogene 33
ENSG0000	5865.5	-0.1141	0.080285	-1.42123	0.155249	0.412521	SCAMP2	10066	secretory carrier membrane protein 2
ENSG0000	28.84753	-0.62263	0.438167	-1.42099	0.155319	0.41265	NA	NA	NA
ENSG0000	3.073614	2.509024	1.765996	1.420742	0.155392	0.412783	NA	NA	NA
ENSG0000	41.06771	-0.56692	0.399087	-1.42055	0.155447	0.41287	CBLL1-AS1	1.02E+08	CBLL1 antisense RNA 1
ENSG0000	151.9952	-0.36029	0.253654	-1.4204	0.155491	0.412928	NA	NA	NA
ENSG0000	2.681402	-2.41943	1.70351	-1.42026	0.155531	0.412976	RB1-DT	1.01E+08	RB1 divergent transcript
ENSG0000	717.4637	-0.16707	0.117647	-1.4201	0.155578	0.413042	SLC25A11	8402	solute carrier family 25 member 11
ENSG0000	157.8017	-0.28676	0.201942	-1.42002	0.1556	0.413042	CDPF1	150383	cysteine rich DPF motif domain containing 1
ENSG0000	5.272647	-1.64472	1.158351	-1.41988	0.155641	0.413091	PLIN1	5346	perilipin 1
ENSG0000	10.4029	1.338385	0.942712	1.419717	0.15569	0.413102	LINC0181	1.02E+08	long intergenic non-protein coding RNA 1814
ENSG0000	3.206745	-2.61457	1.841527	-1.41978	0.155671	0.413102	MANSC4	1E+08	MANSC domain containing 4
ENSG0000	219.8347	0.263916	0.185931	1.41943	0.155774	0.413265	SRGAP1	57522	SLIT-ROBO Rho GTPase activating protein 1
ENSG0000	6.035578	1.380877	0.972936	1.419289	0.155815	0.413315	NA	NA	NA
ENSG0000	1166.819	0.136831	0.096448	1.418694	0.155988	0.413716	EPC2	26122	enhancer of polycomb homolog 2
ENSG0000	2120.322	0.136709	0.096385	1.418368	0.156083	0.413817	DR1	1810	down-regulator of transcription 1
ENSG0000	9.109805	-1.14081	0.804335	-1.41833	0.156093	0.413817	ROBO1	6091	roundabout guidance receptor 1
ENSG0000	10.05455	1.3064	0.921044	1.41839	0.156077	0.413817	PRKN	5071	parkin RBR E3 ubiquitin protein ligase
ENSG0000	257.7634	-0.24406	0.172104	-1.41812	0.156155	0.413915	NUP107	57122	nucleoporin 107
ENSG0000	489.5287	0.21711	0.153104	1.418055	0.156175	0.413915	ARRDC4	91947	arrestin domain containing 4
ENSG0000	3.974932	1.836527	1.295228	1.417918	0.156215	0.413961	DNAJC12	56521	DnaJ heat shock protein family (Hsp40) member C12

ENSG0000	54.12323	0.60204	0.424638	1.417774	0.156257	0.414014	TGFB1I1	7041	transforming growth factor beta 1 induced transcript 1
ENSG0000	7.904652	-3.2582	2.298389	-1.4176	0.156308	0.414089	MYT1L	23040	myelin transcription factor 1 like
ENSG0000	21.74504	0.738372	0.521121	1.41689	0.156515	0.414238	APLF	200558	aprataxin and PNKP like factor
ENSG0000	5.770535	-1.75804	1.240786	-1.41687	0.15652	0.414238	TMEM89	440955	transmembrane protein 89
ENSG0000	13.69634	1.052692	0.742853	1.417092	0.156456	0.414238	NA	NA	NA
ENSG0000	55.73859	-0.49166	0.346895	-1.41731	0.156392	0.414238	MYL6B	140465	myosin light chain 6B
ENSG0000	41.49735	-0.57227	0.40383	-1.41711	0.156451	0.414238	NA	NA	NA
ENSG0000	19038.11	-0.14302	0.100941	-1.41688	0.156517	0.414238	HMOX1	3162	heme oxygenase 1
ENSG0000	44.521	-0.54888	0.387297	-1.4172	0.156423	0.414238	TMLHE	55217	trimethyll epsilon
ENSG0000	22.89762	0.670413	0.473297	1.416474	0.156637	0.414467	UTF1	8433	undifferentiated embryonic cell transcription factor 1
ENSG0000	52.20382	-0.47457	0.335051	-1.41642	0.156651	0.414467	GSTZ1	2954	glutathione S-transferase zeta 1
ENSG0000	3.967643	-1.96358	1.387265	-1.41543	0.156942	0.415177	P2RY4	5030	pyrimidinergic receptor P2Y4
ENSG0000	501.9972	0.174321	0.123184	1.415134	0.157029	0.415289	SNX25	83891	sorting nexin 25
ENSG0000	2659.673	0.114505	0.080912	1.415177	0.157017	0.415289	USP9X	8239	ubiquitin specific peptidase 9 X-linked
ENSG0000	20.29828	0.875481	0.618746	1.414929	0.157089	0.415329	TRIP13	9319	thyroid hormone receptor interactor 13
ENSG0000	9.589032	-1.13199	0.799997	-1.41499	0.157071	0.415329	HMGN3-A	1E+08	HMGN3 antisense RNA 1
ENSG0000	600.0654	0.169267	0.119643	1.414766	0.157137	0.415341	PGRMC2	10424	progesterone receptor membrane component 2
ENSG0000	30.62883	0.722364	0.510591	1.414761	0.157138	0.415341	TDRD9	122402	tudor domain containing 9
ENSG0000	64.33678	0.433823	0.306681	1.414576	0.157193	0.415426	AIG1	51390	androgen induced 1
ENSG0000	19.99587	1.063687	0.7522	1.414102	0.157332	0.415675	NA	NA	NA
ENSG0000	66.03078	-0.51765	0.366052	-1.41413	0.157323	0.415675	NA	NA	NA
ENSG0000	233.6627	-0.24216	0.171264	-1.41398	0.157367	0.415707	SLAIN1	122060	SLAIN motif family member 1
ENSG0000	65.23581	0.420009	0.297101	1.413692	0.157452	0.415786	NA	NA	NA
ENSG0000	210.1009	-0.26892	0.190231	-1.41365	0.157464	0.415786	ARFIP1	27236	ADP ribosylation factor interacting protein 1
ENSG0000	1917.867	0.130461	0.092276	1.413801	0.15742	0.415786	TRIM38	10475	tripartite motif containing 38
ENSG0000	12.75877	-1.01779	0.720059	-1.41349	0.157512	0.415855	MTMR8	55613	myotubularin related protein 8
ENSG0000	38.23356	-0.64586	0.456991	-1.41329	0.157569	0.415887	TRAV8-3	28683	T cell receptor alpha variable 8-3
ENSG0000	70.48803	-0.41941	0.296761	-1.41329	0.157569	0.415887	HEATR9	256957	HEAT repeat containing 9
ENSG0000	81.74742	-0.45222	0.320036	-1.41303	0.157647	0.415998	BBS10	79738	Bardet-Biedl syndrome 10
ENSG0000	3.8487	-1.97622	1.398602	-1.413	0.157656	0.415998	METTL21E	121952	methyltra pseudogene
ENSG0000	1208.542	-0.16348	0.115719	-1.4127	0.157745	0.416172	MUS81	80198	MUS81 structure-specific endonuclease subunit
ENSG0000	10.98266	-1.17647	0.83292	-1.41247	0.157813	0.416234	NA	NA	NA
ENSG0000	61.46399	-0.431	0.305129	-1.41252	0.157796	0.416234	DNAL1	83544	dynein axonemal light chain 1
ENSG0000	14.10104	-1.00589	0.712323	-1.41213	0.157912	0.416436	NA	NA	NA
ENSG0000	12.53016	0.936566	0.66327	1.412042	0.157937	0.416444	NA	NA	NA
ENSG0000	2163.005	0.135145	0.095715	1.411959	0.157962	0.416449	MAP4K1	11184	mitogen-activated protein kinase kinase kinase kinase 1
ENSG0000	2.169419	-2.82804	2.003034	-1.41188	0.157985	0.416451	CALCB	797	calcitonin related polypeptide beta
ENSG0000	19.17167	-0.82401	0.583702	-1.4117	0.158038	0.41653	LOC10012	1E+08	uncharacterized LOC100128310
ENSG0000	1463.484	0.164271	0.116397	1.411295	0.158158	0.416788	PJA2	9867	praja ring finger ubiquitin ligase 2
ENSG0000	525.1915	-0.18172	0.128778	-1.41112	0.15821	0.416865	RARS1	5917	arginyl-tRNA synthetase 1
ENSG0000	2119.628	-0.36129	0.256165	-1.41038	0.158428	0.417109	LAP3	51056	leucine aminopeptidase 3
ENSG0000	173.6471	0.283488	0.200956	1.410695	0.158335	0.417109	DCUN1D4	23142	defective in cullin neddylation 1 domain containing 4
ENSG0000	2486.295	0.115421	0.081829	1.410518	0.158387	0.417109	IQGAP2	10788	IQ motif containing GTPase activating protein 2
ENSG0000	11683.22	0.10784	0.076457	1.410479	0.158398	0.417109	NOTCH1	4851	notch receptor 1
ENSG0000	171.3605	0.261576	0.185479	1.410271	0.15846	0.417109	NA	NA	NA
ENSG0000	2510.904	-0.16585	0.117592	-1.41035	0.158436	0.417109	OASL	8638	2'-5'-oligoadenylate synthetase like
ENSG0000	9.896975	1.219807	0.864931	1.410294	0.158453	0.417109	IGLC6	3542	immunoglobulin lambda constant 6
ENSG0000	15.28491	0.896239	0.635707	1.409831	0.15859	0.417392	RPL4P1	650808	ribosomal protein L4 pseudogene 1
ENSG0000	10.25538	1.223635	0.868143	1.409486	0.158692	0.417601	LINC02541	1.02E+08	long intergenic non-protein coding RNA 2541
ENSG0000	173.0102	-0.32846	0.233051	-1.40939	0.158719	0.417613	FLYWCH2	114984	FLYWCH family member 2
ENSG0000	1173.116	-0.14824	0.105188	-1.4093	0.158747	0.417628	POLM	27434	DNA polymerase mu
ENSG0000	38.893	0.569211	0.403951	1.409112	0.158802	0.417714	CYP4F3	4051	cytochrome P450 family 4 subfamily F member 3
ENSG0000	27.66936	-0.67893	0.482053	-1.40841	0.159008	0.418164	CAND2	23066	cullin associated and neddylation dissociated 2 (putative)
ENSG0000	66.63894	-0.4739	0.336487	-1.40838	0.159018	0.418164	TMEM45B	120224	transmembrane protein 45B
ENSG0000	3.719268	1.798512	1.277366	1.407985	0.159136	0.418326	NA	NA	NA
ENSG0000	762.1637	-0.15328	0.108869	-1.40797	0.159139	0.418326	FBXW4	6468	F-box and WD repeat domain containing 4
ENSG0000	15.77774	-0.83679	0.594363	-1.40787	0.15917	0.418326	GATD1-DT	171391	GATD1 divergent transcript
ENSG0000	21.12582	-0.82381	0.585139	-1.40788	0.159166	0.418326	GDPD1	284161	glycerophosphodiester phosphodiesterase domain containing 1
ENSG0000	58.51316	0.451093	0.320432	1.407765	0.159201	0.418347	PPFIBP1	8496	PPFIA binding protein 1
ENSG0000	1123.963	0.160482	0.114015	1.407551	0.159264	0.418455	LCOR	84458	ligand dependent nuclear receptor corepressor
ENSG0000	331.2155	0.225812	0.160459	1.407285	0.159343	0.418602	NA	NA	NA
ENSG0000	153.1639	-0.28692	0.203905	-1.40711	0.159396	0.418622	GTF2H3	2967	general transcription factor IIH subunit 3
ENSG0000	6.482434	-1.38401	0.983546	-1.40717	0.159378	0.418622	ZNF396	252884	zinc finger protein 396
ENSG0000	181.1595	-0.26941	0.19152	-1.40671	0.159514	0.418875	TOGARAM	23116	TOG array regulator of axonemal microtubules 1
ENSG0000	510.9104	-0.17933	0.127521	-1.40625	0.159651	0.419116	SLC25A24	29957	solute carrier family 25 member 24
ENSG0000	6.682124	-1.46282	1.040174	-1.40632	0.15963	0.419116	GEMIN7-A	1.05E+08	GEMIN7 antisense RNA 1
ENSG0000	8.434963	-1.33637	0.950482	-1.40599	0.159726	0.419252	NA	NA	NA
ENSG0000	47.71751	0.482986	0.343565	1.405809	0.159781	0.419278	NA	NA	NA
ENSG0000	8.377298	-1.43607	1.021489	-1.40585	0.159767	0.419278	RAB38	23682	RAB38 member RAS oncogene family
ENSG0000	474.5052	0.198442	0.141197	1.405426	0.159895	0.419404	FAM241A	132720	family with sequence similarity 241 member A
ENSG0000	221.3438	-0.25645	0.182468	-1.40547	0.159881	0.419404	TMOD2	29767	tropomodulin 2
ENSG0000	4004.27	0.158381	0.112693	1.405418	0.159897	0.419404	GADD45B	4616	growth arrest and DNA damage inducible beta
ENSG0000	333.0262	-0.23717	0.168816	-1.40493	0.160043	0.41965	CASD1	64921	CAS1 domain containing 1
ENSG0000	20.21017	0.816182	0.580996	1.404799	0.160081	0.41965	NA	NA	NA
ENSG0000	82.67323	-0.39083	0.278206	-1.40483	0.160072	0.41965	MMAB	326625	metabolism of cobalamin associated B
ENSG0000	133.3854	0.357305	0.254334	1.404864	0.160062	0.41965	C16orf91	283951	chromosome 16 open reading frame 91
ENSG0000	1960.822	-0.12177	0.086701	-1.40453	0.16016	0.419798	RALGAPB	57148	Ral GTPase activating protein non-catalytic subunit beta
ENSG0000	13.66912	-0.97973	0.697718	-1.40419	0.160263	0.419948	ITGA9-AS1	1.02E+08	ITGA9 antisense RNA 1
ENSG0000	555.8591	0.17258	0.122899	1.40425	0.160244	0.419948	NEMP1	23306	nuclear envelope integral membrane protein 1
ENSG0000	1.841407	-3.30921	2.357233	-1.40385	0.160362	0.42015	TULP1	7287	TUB like protein 1
ENSG0000	4.479426	-1.8048	1.285728	-1.40372	0.160402	0.420195	TAF1A-AS:	1.01E+08	TAF1A antisense RNA 1
ENSG0000	223.9734	-0.28529	0.203319	-1.40316	0.160569	0.420512	IFI44	10561	interferon induced protein 44
ENSG0000	243.2063	0.24845	0.177059	1.403209	0.160555	0.420512	FHL1	2273	four and a half LIM domains 1
ENSG0000	476.1047	-0.19574	0.139517	-1.40299	0.160619	0.420586	PRDX3	10935	peroxiredoxin 3
ENSG0000	3.341879	2.255106	1.607845	1.402564	0.160747	0.420682	LINC0257	1.11E+08	long intergenic non-protein coding RNA 2574
ENSG0000	4661.741	-0.11048	0.078767	-1.40262	0.160732	0.420682	CCDC69	26112	coiled-coil domain containing 69
ENSG0000	891.4485	0.168517	0.120145	1.402608	0.160734	0.420682	TBC1D4	9882	TBC1 domain family member 4
ENSG0000	1114.738	-0.14248	0.10158	-1.40268	0.160712	0.420682	PIN1	5300	peptidylpr NIMA-interacting 1
ENSG0000	5.995798	1.486654	1.060216	1.402218	0.16085	0.420892	ZG16B	124220	zymogen granule protein 16B
ENSG0000	203.7792	-0.26948	0.192214	-1.40197	0.160925	0.42103	ZCCHC9	84240	zinc finger CCHC-type containing 9
ENSG0000	331.6429	0.214378	0.152928	1.401829	0.160966	0.421078	EXOSC8	11340	exosome component 8
ENSG0000	5.496394	-1.83583	1.309675	-1.40174	0.160992	0.421085	PROM2	150696	prominin 2
ENSG0000	1301.301	0.132452	0.094512	1.401437	0.161084	0.421206	UCKL1	54963	uridine-cytidine kinase 1 like 1
ENSG0000	4.956132	-1.74626	1.246028	-1.40146	0.161077	0.421206	NA	NA	NA
ENSG0000	10.82548	-0.98996	0.706535	-1.40115	0.16117	0.421343	RABGAP1L	1.03E+08	RABGAP1L divergent transcript
ENSG0000	12.72124	1.242118	0.886525	1.401109	0.161181	0.421343	IGKV1-12	28940	immunoglobulin kappa variable 1-12
ENSG0000	508.4094	0.191685	0.136829	1.40091	0.161241	0.42144	KLRF1	51348	killer cell lectin like receptor F1
ENSG0000	688.5113	0.161424	0.115259	1.400528	0.161355	0.42162	NPIPB4	440345	nuclear pore complex interacting protein family member B4
ENSG0000	53.03762	-0.56938	0.406532	-1.40057	0.161342	0.42162	PCSK4	54760	proprotein convertase subtilisin/kexin type 4
ENSG0000	2062.904	0.13216	0.09438	1.400298	0.161424	0.42174	SOS1	6654	SOS Ras/Rac guanine nucleotide exchange factor 1

ENSG0000	6.493454	-1.94063	1.38629	-1.39987	0.161552	0.421955	MAK	4117	male germ cell associated kinase
ENSG0000	22.82481	0.937256	0.669523	1.399886	0.161547	0.421955	PVALB	5816	parvalbumin
ENSG0000	135.3772	0.333106	0.238074	1.39917	0.161762	0.422444	NA	NA	NA
ENSG0000	71.30494	0.381373	0.272603	1.399006	0.161811	0.422453	RFC4	5984	replication factor C subunit 4
ENSG0000	1745.307	0.126104	0.090134	1.399073	0.161791	0.422453	ITCH	83737	itchy E3 ubiquitin protein ligase
ENSG0000	2918.077	-0.70284	0.502417	-1.39891	0.161839	0.422467	CXCL9	4283	C-X-C motif chemokine ligand 9
ENSG0000	1288.982	-0.14006	0.100136	-1.39871	0.161901	0.422569	SETDB1	9869	SET domain bifurcated histone lysine methyltransferase 1
ENSG0000	14.37492	0.976062	0.698003	1.398364	0.162004	0.422778	LRRC7	57554	leucine rich repeat containing 7
ENSG0000	6.121316	1.820499	1.30199	1.398243	0.16204	0.422813	OPHN1	4983	oligophrenin 1
ENSG0000	7.689243	1.315172	0.940646	1.398158	0.162066	0.42282	ULBP1	80329	UL16 binding protein 1
ENSG0000	28.8756	-0.68198	0.487843	-1.39794	0.162131	0.422872	FAM86B3I	286042	family witl member A pseudogene
ENSG0000	55.561	0.48571	0.347433	1.397995	0.162115	0.422872	NA	NA	NA
ENSG0000	391.4254	-0.18529	0.132571	-1.39764	0.162222	0.423029	SUGT1	10910	SGT1 hom MIS12 kinetochore complex assembly cochaperone
ENSG0000	2278.082	-0.11349	0.081207	-1.39759	0.162237	0.423029	ADPGK	83440	ADP dependent glucokinase
ENSG0000	8.202024	1.207368	0.864135	1.397197	0.162354	0.423267	NA	NA	NA
ENSG0000	13.7164	0.999964	0.715726	1.397133	0.162373	0.423267	NA	NA	NA
ENSG0000	584.1838	-0.20301	0.145345	-1.39673	0.162493	0.42352	RHPN1	114822	rhophilin Rho GTPase binding protein 1
ENSG0000	317.5243	-0.23251	0.16649	-1.39653	0.162556	0.423587	LZIC	84328	leucine zipper and CTNNBIP1 domain containing
ENSG0000	686.7549	0.181441	0.129926	1.396497	0.162565	0.423587	STXBP3	6814	syntaxin binding protein 3
ENSG0000	6.527579	-1.32677	0.950132	-1.39641	0.162592	0.423597	NA	NA	NA
ENSG0000	3.300462	-2.12387	1.521071	-1.3963	0.162625	0.423624	NA	NA	NA
ENSG0000	53.23073	0.470823	0.337225	1.396169	0.162664	0.423666	NA	NA	NA
ENSG0000	39.32339	0.610477	0.437335	1.395903	0.162744	0.423815	NA	NA	NA
ENSG0000	9.012003	1.17995	0.845909	1.39489	0.163049	0.424431	NA	NA	NA
ENSG0000	8.562744	1.159392	0.831158	1.394911	0.163043	0.424431	NA	NA	NA
ENSG0000	17.7396	-0.89337	0.640408	-1.39501	0.163014	0.424431	DNAH10	196385	dynein axonemal heavy chain 10
ENSG0000	2530.228	0.126836	0.090947	1.394624	0.163129	0.42458	MARK4	57787	microtubule affinity regulating kinase 4
ENSG0000	286.662	-0.22737	0.163047	-1.39451	0.163163	0.424581	TMEM209	84928	transmembrane protein 209
ENSG0000	25240.77	-0.1054	0.075587	-1.39447	0.163175	0.424581	SLC43A2	124935	solute carrier family 43 member 2
ENSG0000	548.7236	-0.18717	0.134244	-1.39428	0.163233	0.424611	ZNF692	55657	zinc finger protein 692
ENSG0000	44.05806	0.511244	0.366669	1.394293	0.163229	0.424611	ILRUN-AS1	1.02E+08	ILRUN antisense RNA 1
ENSG0000	926.2102	0.165272	0.118574	1.393837	0.163367	0.424781	RTRAF	51637	RNA transı translation and transport factor
ENSG0000	5877.851	0.096857	0.069485	1.393941	0.163335	0.424781	PHF12	57649	PHD finger protein 12
ENSG0000	7.427599	1.417494	1.016929	1.393896	0.163349	0.424781	NA	NA	NA
ENSG0000	87.12816	0.353891	0.25397	1.393439	0.163487	0.424992	KLHL3	26249	kelch like family member 3
ENSG0000	10.37667	-1.11783	0.802251	-1.39336	0.16351	0.424992	LHFPL6	10186	LHFPL tetraspan subfamily member 6
ENSG0000	31.71404	-0.71001	0.509576	-1.39334	0.163517	0.424992	CD2BP2-D'	1.02E+08	CD2BP2 divergent transcript
ENSG0000	17.52485	0.878962	0.63088	1.393232	0.16355	0.425018	TMPRSS3	64699	transmembrane serine protease 3
ENSG0000	3.254535	-2.45403	1.761917	-1.39282	0.163675	0.42511	FAM27C	1E+08	family with sequence similarity 27 member C
ENSG0000	10.67587	-1.23207	0.884457	-1.39302	0.163614	0.42511	LINC0092S	503519	long intergenic non-protein coding RNA 929
ENSG0000	1261.631	0.134846	0.096807	1.392937	0.163639	0.42511	SLFN11	91607	schlafen family member 11
ENSG0000	366.6732	-0.21332	0.153155	-1.39281	0.163677	0.42511	ZNF224	7767	zinc finger protein 224
ENSG0000	2680.736	-0.123	0.088327	-1.39251	0.163767	0.425285	TAF6	6878	TATA-box binding protein associated factor 6
ENSG0000	6.518688	-1.37757	0.989424	-1.3923	0.163833	0.425336	KLRK1-AS1	1.02E+08	KLRK1 antisense RNA 1
ENSG0000	40.87575	0.632037	0.45394	1.392336	0.163821	0.425336	NA	NA	NA
ENSG0000	12.8168	-1.12808	0.810289	-1.39219	0.163865	0.425355	NA	NA	NA
ENSG0000	416.2657	0.192632	0.138373	1.392122	0.163885	0.425355	IL18R1	8809	interleukin 18 receptor 1
ENSG0000	5.894949	1.645528	1.182134	1.391998	0.163923	0.425393	NA	NA	NA
ENSG0000	393.3906	-0.22683	0.162988	-1.39172	0.164007	0.425495	PAFAH2	5051	platelet activating factor acetylhydrolase 2
ENSG0000	4.691433	1.435299	1.031316	1.391716	0.164008	0.425495	NA	NA	NA
ENSG0000	172.8621	-0.30163	0.216771	-1.39146	0.164085	0.425633	TTN	7273	titin
ENSG0000	1410.757	-0.12187	0.087602	-1.39117	0.164175	0.425766	CNOT11	55571	CCR4-NOT transcription complex subunit 11
ENSG0000	5.33479	-1.66752	1.198666	-1.39114	0.164182	0.425766	RAD9B	144715	RAD9 checkpoint clamp component B
ENSG0000	21.70929	-0.705	0.506843	-1.39096	0.164237	0.42585	DCBLD2	131566	discoidin CUB and LCCL domain containing 2
ENSG0000	89.29786	0.363771	0.261598	1.39057	0.164356	0.426057	NA	NA	NA
ENSG0000	3.805371	-2.18034	1.568083	-1.39045	0.164392	0.426057	NA	NA	NA
ENSG0000	313.476	-0.22447	0.161442	-1.39042	0.164402	0.426057	PEX1	5189	peroxisomal biogenesis factor 1
ENSG0000	303.9822	0.240473	0.172972	1.390245	0.164454	0.426057	NA	NA	NA
ENSG0000	12.09697	0.913048	0.656722	1.390312	0.164434	0.426057	RPS7P10	1E+08	ribosomal protein S7 pseudogene 10
ENSG0000	58.02224	-0.54259	0.390285	-1.39024	0.164455	0.426057	GINS3	64785	GINS complex subunit 3
ENSG0000	12.16054	-0.95229	0.685065	-1.39008	0.164506	0.426125	NA	NA	NA
ENSG0000	8.929339	1.18663	0.853733	1.38993	0.16455	0.426125	NA	NA	NA
ENSG0000	189.197	-0.26035	0.187304	-1.38998	0.164534	0.426125	ZNF577	84765	zinc finger protein 577
ENSG0000	216.5462	0.306004	0.220185	1.389758	0.164602	0.426201	FFAR2	2867	free fatty acid receptor 2
ENSG0000	379.6592	-0.21231	0.152809	-1.38941	0.164708	0.426416	ELMO3	79767	engulfment and cell motility 3
ENSG0000	19.40702	0.877111	0.631342	1.38928	0.164748	0.426458	LOXL4	84171	lysyl oxidase like 4
ENSG0000	212.5821	-0.29595	0.213106	-1.38874	0.164911	0.426647	FBXW8	26259	F-box and WD repeat domain containing 8
ENSG0000	5.229325	-4.14422	2.984092	-1.38877	0.164903	0.426647	NA	NA	NA
ENSG0000	3.038746	-1.99478	1.436328	-1.38881	0.164891	0.426647	NA	NA	NA
ENSG0000	31.26462	0.585695	0.421746	1.388738	0.164913	0.426647	EDA	1896	ectodysplasin A
ENSG0000	3064.872	-0.12002	0.086427	-1.38863	0.164945	0.426655	HP1BP3	50809	heterochromatin protein 1 binding protein 3
ENSG0000	416.8807	0.184511	0.132878	1.388577	0.164961	0.426655	CAPN1-AS	728975	CAPN1 antisense RNA 1
ENSG0000	6344.837	-0.10574	0.076169	-1.38826	0.165059	0.426846	CTDSP1	58190	CTD small phosphatase 1
ENSG0000	864.0711	-0.15603	0.112425	-1.38787	0.165176	0.42709	CUL9	23113	cullin 9
ENSG0000	211.8738	-0.77628	0.559483	-1.38749	0.165291	0.427117	GBP1P1	400759	guanylate binding protein 1 pseudogene 1
ENSG0000	26.3919	0.747398	0.538725	1.387347	0.165336	0.427117	NA	NA	NA
ENSG0000	4176.725	0.148233	0.106845	1.387362	0.165332	0.427117	TRIB1	10221	tribbles pseudokinase 1
ENSG0000	1072.2	-0.13726	0.098927	-1.38754	0.165276	0.427117	WDR37	22884	WD repeat domain 37
ENSG0000	1419.042	0.150942	0.108802	1.387309	0.165347	0.427117	DYRK2	8445	dual specificity tyrosine phosphorylation regulated kinase 2
ENSG0000	13.07819	-0.89258	0.643335	-1.38742	0.165314	0.427117	NA	NA	NA
ENSG0000	245.4958	0.219702	0.158345	1.387492	0.165292	0.427117	ZNF154	7710	zinc finger protein 154
ENSG0000	1286.883	-0.15313	0.110396	-1.3871	0.165412	0.427165	WASHC4	23325	WASH complex subunit 4
ENSG0000	3331.932	0.117649	0.084815	1.387122	0.165405	0.427165	MAP2K1	5604	mitogen-activated protein kinase kinase 1
ENSG0000	2.262981	2.676743	1.929952	1.386948	0.165458	0.42717	RNA5SP10	1.01E+08	RNA 5S ribosomal pseudogene 108
ENSG0000	2471.738	-0.11154	0.080425	-1.38694	0.16546	0.42717	COLGALT1	79709	collagen beta(1-O)galactosyltransferase 1
ENSG0000	9.054574	-1.21104	0.8734	-1.38658	0.165568	0.427331	NA	NA	NA
ENSG0000	6327.398	0.099132	0.07149	1.386651	0.165548	0.427331	ATN1	1822	atrophin 1
ENSG0000	102.7532	0.348188	0.251153	1.38636	0.165637	0.427448	NA	NA	NA
ENSG0000	212.3249	0.238853	0.172312	1.386164	0.165697	0.427458	PCGF3-AS:	1E+08	PCGF3 antisense RNA 1
ENSG0000	218.3961	-0.26126	0.188482	-1.38612	0.16571	0.427458	GTF2IP12	1.02E+08	general transcription factor IIi pseudogene 12
ENSG0000	96.07204	0.415337	0.299624	1.386194	0.165688	0.427458	NUP153-A	1.05E+08	NUP153 antisense RNA 1
ENSG0000	3.165483	-1.88502	1.360055	-1.38599	0.16575	0.427503	DIAPH3	81624	diaphanous related formin 3
ENSG0000	5.299879	1.4838	1.070762	1.385742	0.165826	0.427638	CNIH3	149111	cornichon family AMPA receptor auxiliary protein 3
ENSG0000	40.67788	-0.58714	0.423761	-1.38555	0.165885	0.42773	LTC4S	4056	leukotriene C4 synthase
ENSG0000	7.138224	-1.43816	1.038188	-1.38526	0.165972	0.427897	NA	NA	NA
ENSG0000	10.06379	1.290148	0.931611	1.384856	0.166096	0.428032	NA	NA	NA
ENSG0000	83.77396	0.398547	0.287774	1.384931	0.166074	0.428032	NUAK1	9891	NUAK family kinase 1
ENSG0000	13.96389	1.051742	0.75945	1.384874	0.166091	0.428032	ROCK1P1	727758	Rho associated coiled-coil containing protein kinase 1 pseudogene 1
ENSG0000	202.0244	0.263867	0.190547	1.384789	0.166117	0.428032	ZNF8	7554	zinc finger protein 8
ENSG0000	84.27603	-0.36533	0.263858	-1.38458	0.16618	0.428134	FANCM	57697	FA complementation group M

ENSG0000	3334.308	-0.11117	0.080304	-1.3844	0.166235	0.428156	TUT4	23318	terminal uridylyl transferase 4
ENSG0000	408.1348	-0.22461	0.162235	-1.38447	0.166214	0.428156	MCPH1	79648	microcephalin 1
ENSG0000	3.424028	2.207411	1.594763	1.384162	0.166309	0.428288	CCDC187	399693	coiled-coil domain containing 187
ENSG0000	172.6167	-0.26247	0.189693	-1.38366	0.166464	0.428627	RIC8B	55188	RIC8 guanine nucleotide exchange factor B
ENSG0000	252.9381	-0.24912	0.180076	-1.3834	0.166541	0.428766	CCDC14	64770	coiled-coil domain containing 14
ENSG0000	3663.043	-0.13984	0.101093	-1.38332	0.166566	0.42877	LILRB4	11006	leukocyte immunoglobulin like receptor B4
ENSG0000	23.32904	-0.72245	0.522415	-1.3829	0.166695	0.428855	LOC72986	729867	uncharacterized LOC729867
ENSG0000	507.1197	-0.17055	0.12333	-1.38284	0.166714	0.428855	XPR1	9213	xenotropic and polytropic retrovirus receptor 1
ENSG0000	116.8008	0.321027	0.232127	1.382979	0.166671	0.428855	COPS9	150678	COP9 signalosome subunit 9
ENSG0000	233.9588	-0.29032	0.209919	-1.38299	0.166668	0.428855	GTF2IP1	2970	general transcription factor IIi pseudogene 1
ENSG0000	668.7025	0.160567	0.116091	1.383111	0.166631	0.428855	APPBP2	10513	amyloid beta precursor protein binding protein 2
ENSG0000	21.11919	0.685345	0.495713	1.382543	0.166805	0.429029	RPS9P2	267009	ribosomal protein S9 pseudogene 2
ENSG0000	433.5591	0.195895	0.14173	1.382166	0.166921	0.429208	RNF2	6045	ring finger protein 2
ENSG0000	92.69512	0.483472	0.34978	1.382216	0.166905	0.429208	NA	NA	NA
ENSG0000	10.23694	-1.16732	0.844855	-1.38168	0.16707	0.429533	NA	NA	NA
ENSG0000	105.6564	-0.34065	0.24665	-1.3811	0.167248	0.429931	PARS2	25973	prolyl-tRN mitochondrial
ENSG0000	70.68799	-0.4062	0.294216	-1.38061	0.167399	0.430142	H1-2	3006	H1.2 linker cluster member
ENSG0000	924.9109	-0.15486	0.112168	-1.38061	0.1674	0.430142	EXTL3	2137	exostosin like glycosyltransferase 3
ENSG0000	25.05924	0.831719	0.602418	1.380634	0.167391	0.430142	BIK	638	BCL2 interacting killer
ENSG0000	60.84971	-0.44835	0.324779	-1.38047	0.167441	0.430188	CYB561D2	11068	cytochrome b561 family member D2
ENSG0000	31.59286	-0.7113	0.515433	-1.38001	0.167584	0.430258	IGFBP2	3485	insulin like growth factor binding protein 2
ENSG0000	378.6073	-0.18449	0.133683	-1.38007	0.167564	0.430258	SNX10	29887	sorting nexin 10
ENSG0000	355.4538	-0.22988	0.166563	-1.38016	0.167538	0.430258	RANBP6	26953	RAN binding protein 6
ENSG0000	25.62486	-0.6396	0.463421	-1.38016	0.167536	0.430258	NA	NA	NA
ENSG0000	11.80782	-0.96718	0.700739	-1.38023	0.167517	0.430258	ADRA2A	150	adrenoceptor alpha 2A
ENSG0000	39.77586	0.562279	0.407531	1.37972	0.167673	0.430426	NA	NA	NA
ENSG0000	7.545012	-1.65615	1.200464	-1.37959	0.167712	0.430467	NA	NA	NA
ENSG0000	3.003499	2.203638	1.597418	1.3795	0.167741	0.430481	NA	NA	NA
ENSG0000	2257.01	-0.12068	0.087492	-1.37936	0.167784	0.430481	GALNT2	2590	polypeptide N-acetylgalactosaminyltransferase 2
ENSG0000	20.79709	-0.79435	0.57589	-1.37935	0.167787	0.430481	TRAV29DV	28653	T cell receptor alpha variable 29/delta variable 5
ENSG0000	3.464852	-1.88739	1.36846	-1.3792	0.167832	0.430537	NA	NA	NA
ENSG0000	5.3106	1.464866	1.062315	1.378937	0.167914	0.430681	ESR1	2099	estrogen receptor 1
ENSG0000	4359.659	0.12185	0.08837	1.37887	0.167935	0.430681	TRAM1	23471	translocation associated membrane protein 1
ENSG0000	18.5739	-0.80179	0.581604	-1.37859	0.168021	0.430784	FAM161A	84140	FAM161 centrosomal protein A
ENSG0000	5.823139	-1.50964	1.095041	-1.37861	0.168014	0.430784	NA	NA	NA
ENSG0000	360.3837	0.209318	0.151849	1.378467	0.168059	0.430807	SF3B6	51639	splicing factor 3b subunit 6
ENSG0000	11.6463	1.048356	0.760704	1.378138	0.168161	0.430807	NA	NA	NA
ENSG0000	30.21535	0.615562	0.446635	1.378223	0.168134	0.430807	ZC2HC1A	51101	zinc finger C2HC-type containing 1A
ENSG0000	2.215436	-2.6004	1.886929	-1.37811	0.168169	0.430807	RHPN1-AS	78998	RHPN1 antisense RNA 1 (head to head)
ENSG0000	14.7332	-0.9533	0.691659	-1.37829	0.168115	0.430807	SRRM5	1E+08	serine/arginine repetitive matrix 5
ENSG0000	122.174	-0.34388	0.249493	-1.37831	0.168109	0.430807	NA	NA	NA
ENSG0000	78.32319	-0.3722	0.270246	-1.37728	0.168427	0.43117	NIFK-AS1	254128	NIFK antisense RNA 1
ENSG0000	523.7664	0.167834	0.121863	1.377239	0.168438	0.43117	CCNYL1	151195	cyclin Y like 1
ENSG0000	73.65275	-0.43163	0.313375	-1.37737	0.168399	0.43117	TXNDC5	81567	thioredoxin domain containing 5
ENSG0000	500.232	-0.17266	0.125341	-1.3775	0.168358	0.43117	DOP1A	23033	DOP1 leucine zipper like protein A
ENSG0000	370.6538	0.212019	0.153916	1.377499	0.168358	0.43117	CSAD	51380	cysteine sulfinic acid decarboxylase
ENSG0000	3.709361	-1.79746	1.305225	-1.37712	0.168474	0.43117	NA	NA	NA
ENSG0000	14.09779	0.972442	0.706121	1.37716	0.168463	0.43117	EMID1	129080	EMI domain containing 1
ENSG0000	2.932142	2.516773	1.828793	1.376194	0.168762	0.431787	LOC10192	1.02E+08	uncharacterized LOC101928059
ENSG0000	64.73824	-0.42206	0.306684	-1.3762	0.168759	0.431787	TBX6	6911	T-box transcription factor 6
ENSG0000	232.6196	0.234318	0.170284	1.376041	0.168809	0.431848	NR2C2AP	126382	nuclear receptor 2C2 associated protein
ENSG0000	459.6719	0.197909	0.143933	1.375004	0.16913	0.43261	ZZZ3	26009	zinc finger ZZ-type containing 3
ENSG0000	51.04161	0.469138	0.341221	1.374881	0.169168	0.432648	PLGRKT	55848	plasminogen receptor with a C-terminal lysine
ENSG0000	19.92316	0.889088	0.646933	1.374313	0.169345	0.433039	MTCYBP18	1.07E+08	MT-CYB pseudogene 18
ENSG0000	3.129524	2.356238	1.714626	1.374199	0.16938	0.43307	LRRC37A1	644397	leucine ric pseudogene
ENSG0000	307.5901	-0.2094	0.152399	-1.37405	0.169426	0.433129	BEST1	7439	bestrophin 1
ENSG0000	74.46391	0.368794	0.268476	1.373658	0.169548	0.433141	MRPL2	51069	mitochondrial ribosomal protein L2
ENSG0000	18.82427	0.883275	0.642914	1.373862	0.169485	0.433141	PITRM1-AS	1.01E+08	PITRM1 antisense RNA 1
ENSG0000	179.8285	-0.28477	0.207292	-1.37375	0.169519	0.433141	FUZ	80199	fuzzy planar cell polarity protein
ENSG0000	1739.475	-0.13068	0.095128	-1.37372	0.169528	0.433141	PES1	23481	pescadillo ribosomal biogenesis factor 1
ENSG0000	76.85943	-0.39862	0.290129	-1.37393	0.169464	0.433141	RRAGB	10325	Ras related GTP binding B
ENSG0000	77.81704	0.467027	0.340097	1.373217	0.169685	0.433221	GP5	2814	glycoprotein V platelet
ENSG0000	53.35442	-0.48354	0.352163	-1.37305	0.169737	0.433221	NA	NA	NA
ENSG0000	788.4967	0.147161	0.107157	1.373324	0.169652	0.433221	MRPS18B	28973	mitochondrial ribosomal protein S18B
ENSG0000	5.763578	-1.76172	1.282851	-1.37329	0.169663	0.433221	NA	NA	NA
ENSG0000	854.5524	0.15384	0.112022	1.373302	0.169658	0.433221	FGFR1OP2	26127	FGFR1 oncogene partner 2
ENSG0000	47.54056	0.545445	0.397271	1.372979	0.169759	0.433221	KANSL1-AS	644246	KANSL1 antisense RNA 1
ENSG0000	35.63442	-0.69671	0.507451	-1.37296	0.169766	0.433221	STXBP4	252983	syntaxin binding protein 4
ENSG0000	20.5514	-0.7293	0.531115	-1.37316	0.169703	0.433221	PTGER1	5731	prostaglandin E receptor 1
ENSG0000	116.2491	-0.3001	0.218632	-1.37263	0.169866	0.433347	NA	NA	NA
ENSG0000	3.816443	1.800679	1.311902	1.372571	0.169886	0.433347	NA	NA	NA
ENSG0000	259.1572	-0.21346	0.155508	-1.37269	0.169849	0.433347	NA	NA	NA
ENSG0000	8.871546	1.213945	0.884608	1.372298	0.169971	0.43347	NA	NA	NA
ENSG0000	15.71259	1.012152	0.737577	1.372266	0.169981	0.43347	PRKAR2A-	1.01E+08	PRKAR2A antisense RNA 1
ENSG0000	3.980743	-1.66553	1.213846	-1.37211	0.17003	0.433536	NA	NA	NA
ENSG0000	1651.163	-0.1194	0.087033	-1.37188	0.170101	0.433659	SMARCB1	6598	SWI/SNF r matrix ass actin depe subfamily member 1
ENSG0000	3.270674	-2.15282	1.56938	-1.37176	0.170137	0.43369	PRR15	222171	proline rich 15
ENSG0000	2.675779	-2.31961	1.691117	-1.37165	0.170174	0.433725	NA	NA	NA
ENSG0000	17.12688	-0.80305	0.585617	-1.37129	0.170284	0.433946	EFCAB11	90141	EF-hand calcium binding domain 11
ENSG0000	587.4756	-0.16237	0.118428	-1.37102	0.170369	0.434103	HEXA	3073	hexosaminidase subunit alpha
ENSG0000	24.24698	-0.64856	0.47319	-1.37061	0.170497	0.434309	MMRN1	22915	multimerin 1
ENSG0000	352.3229	0.20186	0.147276	1.370623	0.170492	0.434309	CLN8-AS1	1.02E+08	CLN8 antisense RNA 1
ENSG0000	21831.36	-0.11181	0.081589	-1.37045	0.170546	0.434375	KLF2	10365	KLF transcription factor 2
ENSG0000	500.3989	0.20541	0.149913	1.370192	0.170627	0.434521	CCDC85B	11007	coiled-coil domain containing 85B
ENSG0000	60.58357	-0.41815	0.305218	-1.37001	0.170684	0.434607	TMEM177	80775	transmembrane protein 177
ENSG0000	50.46366	-0.49902	0.364384	-1.36948	0.170848	0.434845	COA6-AS1	1.02E+08	COA6 antisense RNA 1
ENSG0000	341.3056	-0.23238	0.169664	-1.36963	0.170801	0.434845	TARBP2	6895	TARBP2 subunit of RISC loading complex
ENSG0000	19.38216	0.738161	0.538981	1.369551	0.170827	0.434845	SYNE4	163183	spectrin repeat containing nuclear envelope family member 4
ENSG0000	5.744707	1.887007	1.378365	1.369018	0.170994	0.435157	KIF26B	55083	kinesin family member 26B
ENSG0000	197.7001	0.244468	0.178588	1.368894	0.171032	0.435195	RRAS2	22800	RAS related 2
ENSG0000	181.5917	0.28306	0.206867	1.36832	0.171212	0.435484	NIT2	56954	nitrilase family member 2
ENSG0000	51.33359	-0.54514	0.398362	-1.36844	0.171173	0.435484	PSTK	118672	phosphoseryl-tRNA kinase
ENSG0000	76.18177	0.380201	0.277862	1.368306	0.171216	0.435484	LOC11974	1.2E+08	uncharacterized LOC119746555
ENSG0000	1003.079	0.148822	0.108782	1.368078	0.171288	0.435505	DIS3	22894	DIS3 homo exosome endoribonuclease and 3'-5' exoribonuclease
ENSG0000	99.11761	-0.34538	0.252468	-1.368	0.171313	0.435505	BRICD5	283870	BRICHOS domain containing 5
ENSG0000	47.43348	0.49895	0.364688	1.368155	0.171264	0.435505	NA	NA	NA
ENSG0000	569.133	-0.18492	0.135176	-1.36798	0.171319	0.435505	RTL10	79680	retrotransposon Gag like 10
ENSG0000	82.47229	0.371224	0.271465	1.367482	0.171474	0.435752	NR1H3	10062	nuclear receptor subfamily 1 group H member 3
ENSG0000	338.325	0.191211	0.139826	1.367499	0.171469	0.435752	PPP2R3C	55012	protein phosphatase 2 regulatory subunit B''gamma
ENSG0000	7.995704	-1.23441	0.902716	-1.36744	0.171486	0.435752	NA	NA	NA

ENSG0000	10.7734	-0.98426	0.720144	-1.36675	0.171703	0.436198	ATP1A1-A:	84852	ATP1A1 antisense RNA 1
ENSG0000	4539.784	0.096078	0.070297	1.366734	0.171709	0.436198	MADD	8567	MAP kinase activating death domain
ENSG0000	3.418358	-2.23823	1.638275	-1.36621	0.171872	0.436553	FLRT2	23768	fibronectin leucine rich transmembrane protein 2
ENSG0000	276.9576	-0.21256	0.155594	-1.3661	0.171906	0.43658	CD302	9936	CD302 molecule
ENSG0000	5.720074	1.380462	1.010585	1.366002	0.171938	0.436602	NA	NA	NA
ENSG0000	6.182908	1.768502	1.294844	1.365803	0.172001	0.436641	NA	NA	NA
ENSG0000	1466.31	-0.1705	0.124833	-1.36586	0.171983	0.436641	LINC00861	1E+08	long intergenic non-protein coding RNA 861
ENSG0000	142.2207	0.279281	0.204506	1.365637	0.172053	0.436714	E2F6	1876	E2F transcription factor 6
ENSG0000	11.28783	-0.97856	0.71703	-1.36474	0.172336	0.437339	LRRC20	55222	leucine rich repeat containing 20
ENSG0000	5.393167	1.58332	1.160194	1.364702	0.172347	0.437339	NA	NA	NA
ENSG0000	22.18973	0.827475	0.606403	1.364564	0.17239	0.43739	FZD6	8323	frizzled class receptor 6
ENSG0000	6608.95	0.104679	0.076722	1.364383	0.172447	0.437474	EIF3F	8665	eukaryotic translation initiation factor 3 subunit F
ENSG0000	4.968208	1.373285	1.007041	1.363683	0.172667	0.437952	RPL7AP50	87688	ribosomal protein L7a pseudogene 50
ENSG0000	117.4361	0.319718	0.23446	1.363635	0.172682	0.437952	DONSON	29980	DNA replication fork stabilization factor DONSON
ENSG0000	656.9181	-0.16878	0.123813	-1.36315	0.172835	0.438279	TMIGD2	126259	transmembrane and immunoglobulin domain containing 2
ENSG0000	167.443	0.26383	0.193573	1.362945	0.1729	0.438346	ATG9B	285973	autophagy related 9B
ENSG0000	44.39383	-0.48789	0.357974	-1.36292	0.172909	0.438346	NA	NA	NA
ENSG0000	118.4612	-0.32284	0.236993	-1.36222	0.173129	0.438445	GCFC2	6936	GC-rich sequence DNA-binding factor 2
ENSG0000	1216.611	-0.15638	0.114799	-1.36219	0.173137	0.438445	NDUFAF3	25915	NADH:ubiquinone oxidoreductase complex assembly factor 3
ENSG0000	237.8586	0.234081	0.171821	1.362356	0.173086	0.438445	HOPX	84525	HOP homeobox
ENSG0000	1271.358	0.143018	0.104987	1.362245	0.17312	0.438445	TNPO1	3842	transportin 1
ENSG0000	79.5708	-0.36922	0.271022	-1.36233	0.173093	0.438445	ACOT13	55856	acyl-CoA thioesterase 13
ENSG0000	170.6166	0.278283	0.204218	1.362675	0.172985	0.438445	PLEKHG1	57480	pleckstrin homology and RhoGEF domain containing G1
ENSG0000	280.4469	0.205555	0.150863	1.362529	0.173031	0.438445	DCUN1D2	55208	defective in cullin neddylation 1 domain containing 2
ENSG0000	388.1036	-0.19034	0.13969	-1.36255	0.173024	0.438445	ZNF41	7592	zinc finger protein 41
ENSG0000	3674.726	-0.10228	0.075092	-1.3621	0.173166	0.438455	ARHGEF7	8874	Rho guanine nucleotide exchange factor 7
ENSG0000	11.85412	-1.17307	0.861265	-1.36203	0.173188	0.438455	OR4D1	26689	olfactory receptor family 4 subfamily D member 1
ENSG0000	1691.68	-0.1256	0.092236	-1.36167	0.173302	0.438621	CD47	961	CD47 molecule
ENSG0000	4.235965	2.001765	1.470058	1.361691	0.173295	0.438621	LOC12490	1.25E+08	uncharacterized LOC124909451
ENSG0000	111.9763	0.336574	0.24719	1.361598	0.173325	0.438621	C4orf46	201725	chromosome 4 open reading frame 46
ENSG0000	1749.566	-0.11217	0.082407	-1.36117	0.173459	0.438761	PXK	54899	PX domain containing serine/threonine kinase like
ENSG0000	3.90145	2.067343	1.51885	1.361124	0.173475	0.438761	NA	NA	NA
ENSG0000	2305.033	0.123437	0.090675	1.361309	0.173416	0.438761	USP3	9960	ubiquitin specific peptidase 3
ENSG0000	3166.242	-0.1247	0.091612	-1.36114	0.173468	0.438761	MBD2	8932	methyl-CpG binding domain protein 2
ENSG0000	9.052403	1.168482	0.858662	1.360816	0.173572	0.438947	MIR212	406994	microRNA 212
ENSG0000	22.44525	0.720961	0.529985	1.360342	0.173722	0.439099	LOC12252	1.23E+08	uncharacterized LOC122526782
ENSG0000	75.97513	0.423852	0.311554	1.360447	0.173689	0.439099	CERCAM	51148	cerebral endothelial cell adhesion molecule
ENSG0000	129.842	-0.28539	0.209792	-1.36033	0.173726	0.439099	POGLUT3	143888	protein O-glucosyltransferase 3
ENSG0000	14.80888	-1.106	0.812929	-1.36051	0.173669	0.439099	CCDC40	55036	coiled-coil domain containing 40
ENSG0000	5.391972	1.814885	1.33426	1.360219	0.173761	0.439125	RPL23AP1	728134	ribosomal protein L23a pseudogene 18
ENSG0000	423.7466	0.198304	0.14583	1.359829	0.173884	0.439255	ATL2	64225	atlastin GTPase 2
ENSG0000	5848.632	0.120264	0.088445	1.359757	0.173907	0.439255	MXD4	10608	MAX dimerization protein 4
ENSG0000	26.86113	-0.67039	0.492981	-1.35987	0.173872	0.439255	PCDH9	5101	protocadherin 9
ENSG0000	961.5028	-0.16609	0.122127	-1.35998	0.173837	0.439255	MIA2	4253	MIA SH3 domain ER export factor 2
ENSG0000	88.97383	-0.35307	0.259694	-1.35956	0.17397	0.439354	COMMD1(	51397	COMM domain containing 10
ENSG0000	3.809459	-2.00754	1.476713	-1.35946	0.173999	0.439369	NA	NA	NA
ENSG0000	876.1192	-0.16345	0.120244	-1.3593	0.174053	0.439409	CAD	790	carbamoyl aspartate and dihydroorotase
ENSG0000	132.0823	-0.32035	0.23568	-1.35927	0.174063	0.439409	NA	NA	NA
ENSG0000	44.46647	-0.52503	0.386417	-1.35872	0.174234	0.439782	TRBV5-4	28611	T cell receptor beta variable 5-4
ENSG0000	5285.275	-0.09754	0.071809	-1.35838	0.174342	0.439996	CD4	920	CD4 molecule
ENSG0000	144.4612	-0.26763	0.197048	-1.3582	0.174399	0.440019	KIFAP3	22920	kinesin associated protein 3
ENSG0000	126.3468	-0.30599	0.225278	-1.35828	0.174376	0.440019	ZNF839	55778	zinc finger protein 839
ENSG0000	6.675624	1.328793	0.978415	1.358107	0.17443	0.440036	NA	NA	NA
ENSG0000	116.5102	0.383752	0.282641	1.357738	0.174547	0.440272	LINC0123	1.03E+08	long intergenic non-protein coding RNA 1238
ENSG0000	589.338	0.183276	0.135019	1.357407	0.174652	0.440418	SMYD5	10322	SMYD family member 5
ENSG0000	39.58463	-0.49177	0.36227	-1.35746	0.174636	0.440418	TASP1	55617	taspase 1
ENSG0000	6505.251	-0.12255	0.090294	-1.35721	0.174715	0.440517	CTSZ	1522	cathepsin Z
ENSG0000	8.290183	-1.28202	0.944859	-1.35683	0.174834	0.440757	NA	NA	NA
ENSG0000	11.23109	1.105598	0.814983	1.35659	0.174912	0.440892	NA	NA	NA
ENSG0000	36.49107	-0.55454	0.408836	-1.35639	0.174976	0.440995	MBOAT2	129642	membrane bound O-acyltransferase domain containing 2
ENSG0000	25.94314	0.710254	0.523708	1.356201	0.175035	0.441084	NA	NA	NA
ENSG0000	1180.723	-0.14301	0.105496	-1.3556	0.175226	0.441506	GOLGA2	2801	golgin A2
ENSG0000	510.5851	-0.18762	0.138437	-1.35526	0.175335	0.441719	STX17	55014	syntaxin 17
ENSG0000	185.1446	-0.28631	0.211293	-1.35505	0.175402	0.441829	PHF6	84295	PHD finger protein 6
ENSG0000	4.626936	1.696304	1.252177	1.354684	0.175518	0.442061	GCOM1	145781	GCOM1 MYZAP-POLR2M combined locus
ENSG0000	86.49879	-0.37734	0.278619	-1.35433	0.17563	0.442162	ZCCHC4	29063	zinc finger CCHC-type containing 4
ENSG0000	10.71848	1.212611	0.895279	1.35445	0.175593	0.442162	COCH	1690	cochlin
ENSG0000	2187.565	0.116234	0.085823	1.35434	0.175628	0.442162	CTSH	1512	cathepsin H
ENSG0000	166.0466	-0.26886	0.198556	-1.35405	0.17572	0.442328	ACP6	51205	acid phosp lysophosphatidic
ENSG0000	3250.805	0.104798	0.077414	1.353729	0.175823	0.442392	APH1A	51107	aph-1 hom gamma-secretase subunit
ENSG0000	515.1304	0.189765	0.140165	1.353866	0.175779	0.442392	NAA15	80155	N-alpha-ac NatA auxiliary subunit
ENSG0000	152.3154	-0.26849	0.198319	-1.35381	0.175798	0.442392	MTERF3	51001	mitochondrial transcription termination factor 3
ENSG0000	339.2477	-0.19761	0.145992	-1.3536	0.175863	0.442392	DNAJC15	29103	DnaJ heat shock protein family (Hsp40) member C15
ENSG0000	4.242657	-1.74715	1.290742	-1.3536	0.175864	0.442392	NA	NA	NA
ENSG0000	30.86809	-0.6475	0.478429	-1.35339	0.175932	0.442501	GAS1	2619	growth arrest specific 1
ENSG0000	1467.53	-0.12656	0.09353	-1.35319	0.175995	0.4426	NADK	65220	NAD kinase
ENSG0000	13.66284	-1.02154	0.755015	-1.353	0.176055	0.442691	NA	NA	NA
ENSG0000	511.3841	0.176162	0.130235	1.352643	0.17617	0.442877	PRKCQ-AS	439949	PRKCQ antisense RNA 1
ENSG0000	2156.579	0.112792	0.083388	1.352622	0.176177	0.442877	PCF11	51585	PCF11 cleavage and polyadenylation factor subunit
ENSG0000	72.76992	0.389321	0.287941	1.352085	0.176348	0.443249	LOXL1-AS1	1E+08	LOXL1 antisense RNA 1
ENSG0000	184.8371	-0.25329	0.187348	-1.35196	0.176388	0.443289	CDKL5	6792	cyclin dependent kinase like 5
ENSG0000	543.0637	0.178602	0.132125	1.351761	0.176452	0.443389	PCID2	55795	PCI domain containing 2
ENSG0000	284.8218	-0.21269	0.157368	-1.35152	0.176528	0.443521	HRAS	3265	HRas proto GTPase
ENSG0000	1492.555	-0.1228	0.090878	-1.35125	0.176616	0.443681	VPS52	6293	VPS52 subunit of GARP complex
ENSG0000	5.933077	1.536986	1.137586	1.351094	0.176665	0.443731	LOC10192	1.02E+08	uncharacterized LOC101927745
ENSG0000	39.03471	-0.50003	0.370108	-1.35104	0.176684	0.443731	HMGN5	79366	high mobility group nucleosome binding domain 5
ENSG0000	18.7654	-0.7424	0.549609	-1.35077	0.176769	0.443884	NA	NA	NA
ENSG0000	65.49416	-0.46193	0.342093	-1.35031	0.176915	0.444192	NA	NA	NA
ENSG0000	247.9134	-0.25333	0.187637	-1.35009	0.176987	0.444311	ZNF653	115950	zinc finger protein 653
ENSG0000	1180.438	0.149951	0.111078	1.349952	0.177031	0.444364	SYNCRIP	10492	synaptotagmin binding cytoplasmic RNA interacting protein
ENSG0000	14.51945	0.800335	0.593016	1.349603	0.177143	0.444524	CFL1P1	142913	cofilin 1 pseudogene 1
ENSG0000	30.5817	-0.66707	0.494244	-1.34967	0.177122	0.444524	NA	NA	NA
ENSG0000	4.48896	-1.67662	1.242475	-1.34942	0.177202	0.444611	NA	NA	NA
ENSG0000	7588.449	-0.10268	0.076099	-1.34934	0.177229	0.444618	SNRNP200	23020	small nuclear ribonucleoprotein U5 subunit 200
ENSG0000	213.5763	0.230021	0.170495	1.349136	0.177293	0.44472	CCNB1IP1	57820	cyclin B1 interacting protein 1
ENSG0000	819.4345	0.140226	0.103944	1.349055	0.177319	0.444725	DNAJB11	51726	DnaJ heat shock protein family (Hsp40) member B11
ENSG0000	63.66909	-0.42862	0.317759	-1.34888	0.177375	0.444804	ETFB	2109	electron transfer flavoprotein subunit beta
ENSG0000	9.564232	1.140864	0.845921	1.348665	0.177444	0.444919	ALDH8A1	64577	aldehyde dehydrogenase 8 family member A1
ENSG0000	1494.876	0.127649	0.094661	1.348484	0.177503	0.444944	GLTP	51228	glycolipid transfer protein
ENSG0000	7.705072	1.212888	0.899436	1.348497	0.177498	0.444944	OTOA	146183	otoancorin

ENSG0000	4.472857	-1.66771	1.23771	-1.34742	0.177846	0.445744	NA	NA	NA
ENSG0000	31.86355	-0.65888	0.489082	-1.34718	0.177922	0.445875	NA	NA	NA
ENSG0000	16.53792	-0.79558	0.590686	-1.34688	0.178019	0.446058	TNFAIP8L3	388121	TNF alpha induced protein 8 like 3
ENSG0000	6.780697	-1.71644	1.274648	-1.3466	0.178109	0.446224	NA	NA	NA
ENSG0000	120.4918	-0.31379	0.233043	-1.34651	0.178137	0.446233	PIGK	10026	phosphatidylinositol glycan anchor biosynthesis class K
ENSG0000	2270.569	0.153841	0.114289	1.346069	0.17828	0.446531	OSBPL8	114882	oxysterol binding protein like 8
ENSG0000	2913.084	0.111989	0.083217	1.345749	0.178384	0.44673	MAP3K3	4215	mitogen-activated protein kinase kinase kinase 3
ENSG0000	120.4581	0.318203	0.236489	1.345527	0.178455	0.446849	ROM1	6094	retinal outer segment membrane protein 1
ENSG0000	3.263074	1.779757	1.322795	1.345452	0.178479	0.446849	NA	NA	NA
ENSG0000	3.889095	2.104337	1.564429	1.345115	0.178588	0.447061	NA	NA	NA
ENSG0000	250.9603	0.220753	0.164137	1.344936	0.178646	0.447085	C3orf62	375341	chromosome 3 open reading frame 62
ENSG0000	838.7591	0.164578	0.122365	1.344976	0.178633	0.447085	NA	NA	NA
ENSG0000	3702.87	0.101476	0.075461	1.344744	0.178708	0.44718	CLASRP	11129	CLK4 associating serine/arginine rich protein
ENSG0000	5.250558	-1.53015	1.138011	-1.34458	0.17876	0.44725	NA	NA	NA
ENSG0000	3.926786	1.828871	1.360659	1.344107	0.178914	0.447574	NA	NA	NA
ENSG0000	214.2043	0.292879	0.217935	1.343883	0.178986	0.447695	GPR15	2838	G protein-coupled receptor 15
ENSG0000	447.4898	-0.20162	0.150051	-1.34367	0.179056	0.447748	ZNF621	285268	zinc finger protein 621
ENSG0000	8.204633	-1.2995	0.96712	-1.34368	0.179053	0.447748	GAS2	2620	growth arrest specific 2
ENSG0000	75.62682	0.372726	0.277477	1.343266	0.179186	0.447902	NA	NA	NA
ENSG0000	3012.782	0.106883	0.079571	1.343254	0.17919	0.447902	ANP32B	10541	acidic nuclear phosphoprotein 32 family member B
ENSG0000	2048.191	0.113724	0.084661	1.34329	0.179178	0.447902	SLTM	79811	SAFB like transcription modulator
ENSG0000	231.3275	-0.29368	0.21867	-1.34305	0.179257	0.448009	ATAD2	29028	ATPase family AAA domain containing 2
ENSG0000	24.99305	-0.86408	0.643443	-1.3429	0.179303	0.448064	KCNG2	26251	potassium voltage-gated channel modifier subfamily G member 2
ENSG0000	4.762331	1.547465	1.152858	1.342286	0.179503	0.4485	LOC12490	1.25E+08	uncharacterized LOC124902204
ENSG0000	8.86357	-1.17249	0.873545	-1.34222	0.179526	0.4485	C10orf105	414152	chromosome 10 open reading frame 105
ENSG0000	94.66076	-0.45794	0.341211	-1.34211	0.179561	0.448528	FNDC10	643988	fibronectin type III domain containing 10
ENSG0000	575.2183	0.166092	0.123772	1.341922	0.179621	0.448618	GART	2618	phosphori phosphor phosphoribosylaminoimidazole synthetase
ENSG0000	1189.841	0.123669	0.092171	1.341741	0.17968	0.448704	UQCRFS1	7386	ubiquinol- Rieske iron-sulfur polypeptide 1
ENSG0000	101.8867	0.356182	0.265519	1.341453	0.179773	0.448877	CTTN	2017	cortactin
ENSG0000	3.566733	1.716324	1.279604	1.341293	0.179825	0.448884	FNDC4	64838	fibronectin type III domain containing 4
ENSG0000	356.1273	-0.18973	0.141452	-1.34129	0.179827	0.448884	FARP2	9855	FERM ARH/RhoGEF and pleckstrin domain protein 2
ENSG0000	648.6165	-0.17039	0.127056	-1.34108	0.179894	0.448884	RAD54L2	23132	RAD54 like 2
ENSG0000	6.659935	1.492078	1.112602	1.341071	0.179897	0.448884	ALOX12B	242	arachidon 12R type
ENSG0000	87.34961	-0.34609	0.258042	-1.34122	0.179849	0.448884	SLC2A11	66035	solute carrier family 2 member 11
ENSG0000	83.91726	0.457346	0.34105	1.340991	0.179923	0.448888	NA	NA	NA
ENSG0000	115.337	0.311054	0.231977	1.340881	0.179959	0.448917	KLRC1	3821	killer cell lectin like receptor C1
ENSG0000	1090.606	-0.15415	0.114984	-1.34064	0.180037	0.44902	GOLGA4	2803	golgin A4
ENSG0000	766.4116	-0.14806	0.110442	-1.3406	0.180049	0.44902	GRHPR	9380	glyoxylate and hydroxypyruvate reductase
ENSG0000	50.76862	-0.46087	0.344049	-1.33954	0.180396	0.449463	LINC02611	1.02E+08	long intergenic non-protein coding RNA 2611
ENSG0000	123.5304	0.370801	0.276792	1.339637	0.180363	0.449463	AGA	175	aspartylglucosaminidase
ENSG0000	96.24065	-0.32674	0.243872	-1.33979	0.180314	0.449463	RMND1	55005	required for meiotic nuclear division 1 homolog
ENSG0000	219.1418	0.237261	0.177102	1.339683	0.180348	0.449463	VIM-AS1	1.01E+08	VIM antisense RNA 1
ENSG0000	16.55583	0.917054	0.684549	1.339648	0.18036	0.449463	E2F7	144455	E2F transcription factor 7
ENSG0000	395.4031	0.187365	0.139868	1.339584	0.180381	0.449463	ESCO1	114799	establishment of sister chromatid cohesion N-acetyltransferase 1
ENSG0000	412.8039	0.248905	0.18576	1.339926	0.180269	0.449463	RNF113A	7737	ring finger protein 113A
ENSG0000	23.56876	0.617181	0.460778	1.33943	0.180431	0.449489	ATAD3C	219293	ATPase family AAA domain containing 3C
ENSG0000	365.9212	-0.21864	0.163283	-1.33902	0.180565	0.449763	CFAP97	57587	cilia and flagella associated protein 97
ENSG0000	53.52336	-0.42486	0.317317	-1.33893	0.180594	0.449775	LIN9	286826	lin-9 DREAM MuvB core complex component
ENSG0000	4477.967	-0.14046	0.104916	-1.33882	0.180629	0.449798	S100A6	6277	S100 calcium binding protein A6
ENSG0000	194.8102	0.292169	0.21824	1.338751	0.180652	0.449798	CLEC17A	388512	C-type lectin domain containing 17A
ENSG0000	309.4759	-0.26752	0.199856	-1.33854	0.18072	0.449908	OLFM4	10562	olfactomedin 4
ENSG0000	90.66768	-0.37417	0.279555	-1.33846	0.180745	0.449911	PLVAP	83483	plasmalemma vesicle associated protein
ENSG0000	10.38879	-1.07386	0.802431	-1.33826	0.180811	0.450013	NA	NA	NA
ENSG0000	206.4126	0.261799	0.195667	1.337977	0.180904	0.450124	SLC35A3	23443	solute carrier family 35 member A3
ENSG0000	165.5859	-0.27561	0.20599	-1.33799	0.180899	0.450124	ZNF329	79673	zinc finger protein 329
ENSG0000	1128.057	-0.13754	0.102823	-1.33768	0.181001	0.450306	HGH1	51236	HGH1 homolog
ENSG0000	7.361209	-1.20897	0.903836	-1.3376	0.181026	0.450306	TIMP3	7078	TIMP metallopeptidase inhibitor 3
ENSG0000	450.7842	-0.17292	0.129341	-1.33693	0.181245	0.45073	NA	NA	NA
ENSG0000	2023.748	-0.14164	0.105939	-1.33696	0.181236	0.45073	HLA-DRB5	3127	major hist class II DR beta 5
ENSG0000	7.848287	-1.16819	0.87407	-1.33649	0.181388	0.45074	LYPLAL1-D	643723	LYPLAL1 divergent transcript
ENSG0000	3.556956	2.211662	1.654812	1.336503	0.181385	0.45074	IGKV1-39	28930	immunoglobulin kappa variable 1-39
ENSG0000	3.495588	-2.08323	1.558566	-1.33663	0.181343	0.45074	NA	NA	NA
ENSG0000	197.4255	0.240461	0.179922	1.336473	0.181395	0.45074	SNHG32	50854	small nucleolar RNA host gene 32
ENSG0000	12.42484	0.955985	0.715158	1.336746	0.181305	0.45074	NA	NA	NA
ENSG0000	379.2642	0.192332	0.14387	1.336843	0.181274	0.45074	DGKE	8526	diacylglycerol kinase epsilon
ENSG0000	2.668573	-2.23517	1.673075	-1.33597	0.18156	0.451075	SORD2P	653381	sorbitol de pseudogene
ENSG0000	4143.454	0.105768	0.079173	1.335912	0.181578	0.451075	MBD1	4152	methyl-CpG binding domain protein 1
ENSG0000	5.817711	-1.60514	1.201777	-1.33564	0.181668	0.451118	LRRC69	1E+08	leucine rich repeat containing 69
ENSG0000	1449.846	0.118423	0.088663	1.335651	0.181663	0.451118	PDXDC1	23042	pyridoxal dependent decarboxylase domain containing 1
ENSG0000	244.5079	0.238482	0.178542	1.335721	0.18164	0.451118	ZNF529	57711	zinc finger protein 529
ENSG0000	67.5175	0.392559	0.294028	1.33511	0.18184	0.451476	NA	NA	NA
ENSG0000	138.766	0.281764	0.211052	1.335046	0.181861	0.451476	SLC25A30	253512	solute carrier family 25 member 30
ENSG0000	53.47532	-0.4306	0.322602	-1.33477	0.181952	0.451642	TATDN3	128387	TatD DNase domain containing 3
ENSG0000	9.736432	1.087887	0.815317	1.334313	0.182101	0.451951	RPL26P32	400055	ribosomal protein L26 pseudogene 32
ENSG0000	1139.749	-0.13658	0.102369	-1.33423	0.182128	0.451956	BUD13	84811	BUD13 homolog
ENSG0000	525.8679	-0.20782	0.155768	-1.33416	0.182152	0.451957	PAXIP1	22976	PAX interacting protein 1
ENSG0000	20.93092	0.838334	0.628549	1.333762	0.182282	0.452218	VIL1	7429	villin 1
ENSG0000	4.225002	-1.59853	1.198809	-1.33343	0.182391	0.452378	NBPF25P	1.02E+08	NBPF men pseudogene
ENSG0000	211.0722	0.244179	0.183123	1.333416	0.182395	0.452378	NA	NA	NA
ENSG0000	8.583846	-1.24912	0.937135	-1.33292	0.182559	0.452725	FAM166B	730112	family with sequence similarity 166 member B
ENSG0000	76.7072	0.417534	0.313393	1.332299	0.182762	0.453167	LY96	23643	lymphocyte antigen 96
ENSG0000	976.6537	-0.16008	0.120183	-1.33193	0.182883	0.453405	INAFM2	1.01E+08	InaF motif containing 2
ENSG0000	6484.622	0.097254	0.07306	1.331154	0.183138	0.453978	SLC25A37	51312	solute carrier family 25 member 37
ENSG0000	79.57517	-0.43407	0.326173	-1.33078	0.18326	0.45422	TRBV30	28557	T cell receptor beta variable 30
ENSG0000	8.25938	1.227225	0.922293	1.330624	0.183313	0.454264	LOC12490	1.25E+08	uncharacterized LOC124904158
ENSG0000	1276.446	-0.13528	0.101671	-1.33057	0.18333	0.454264	PTPN9	5780	protein tyrosine phosphatase non-receptor type 9
ENSG0000	769.5328	0.1727	0.1298	1.330506	0.183351	0.454264	TUBB6	84617	tubulin beta 6 class V
ENSG0000	4.130381	-1.6666	1.252779	-1.33032	0.183412	0.454352	PRSS16	10279	serine protease 16
ENSG0000	10.28567	-1.07635	0.80934	-1.32991	0.183549	0.454632	NA	NA	NA
ENSG0000	45.72463	-0.47066	0.354033	-1.32943	0.183706	0.454728	NA	NA	NA
ENSG0000	418.0079	0.179332	0.134895	1.329422	0.183709	0.454728	TSTD2	158427	thiosulfate sulfurtransferase like domain containing 2
ENSG0000	1431.68	-0.12651	0.095152	-1.32956	0.183664	0.454728	NA	NA	NA
ENSG0000	241.813	-0.21241	0.159776	-1.32942	0.18371	0.454728	CEACAM2	90273	CEA cell adhesion molecule 21
ENSG0000	14592.33	-0.09295	0.069908	-1.32963	0.183639	0.454728	MED15	51586	mediator complex subunit 15
ENSG0000	6121.559	-0.10266	0.077238	-1.32909	0.183818	0.454933	IQSEC1	9922	IQ motif and Sec7 domain ArfGEF 1
ENSG0000	955.6081	-0.1547	0.116413	-1.32888	0.183888	0.455045	IL12RB1	3594	interleukin 12 receptor subunit beta 1
ENSG0000	16.69862	-1.13822	0.856583	-1.32879	0.183917	0.455058	MIR4537	1.01E+08	microRNA 4537
ENSG0000	5.196179	1.567248	1.179689	1.328526	0.184004	0.455152	NA	NA	NA
ENSG0000	9.502224	-1.12633	0.847771	-1.32858	0.183988	0.455152	GOLGA8H	728498	golgin A8 family member H
ENSG0000	27.9388	-0.63435	0.477848	-1.32752	0.184337	0.455859	POC1A	25886	POC1 centriolar protein A

ENSG0000	4.955862	-1.57556	1.186848	-1.32751	0.184339	0.455859	NA	NA	NA
ENSG0000	3.69877	-1.85153	1.394864	-1.32739	0.184379	0.455895	NA	NA	NA
ENSG0000	74.10531	-0.43188	0.325444	-1.32705	0.184493	0.456116	NA	NA	NA
ENSG0000	6.00283	-1.39698	1.053018	-1.32664	0.184627	0.456387	LOC64465	644656	uncharacterized LOC644656
ENSG0000	30.67305	0.669283	0.504549	1.326497	0.184675	0.456446	ZNF418	147686	zinc finger protein 418
ENSG0000	390.6399	0.193338	0.145808	1.325981	0.184846	0.456807	PKNOX1	5316	PBX/knotted 1 homeobox 1
ENSG0000	158.4103	-0.30266	0.228378	-1.32525	0.185087	0.457343	DNAL4	10126	dynein axonemal light chain 4
ENSG0000	572.0808	0.165401	0.124842	1.324883	0.18521	0.45758	EXOC8	149371	exocyst complex component 8
ENSG0000	356.112	0.202101	0.152559	1.32474	0.185257	0.45758	NA	NA	NA
ENSG0000	139.9031	0.308545	0.232898	1.324804	0.185236	0.45758	LOC10272	1.03E+08	trafficking protein particle complex subunit 10-like
ENSG0000	16.9224	0.992604	0.749351	1.324618	0.185298	0.457619	NA	NA	NA
ENSG0000	345.7606	-0.18456	0.139348	-1.32449	0.185341	0.457665	MFSD14CP	84278	major facil pseudogene
ENSG0000	1944.909	0.136729	0.103255	1.324195	0.185438	0.457844	THAP4	51078	THAP domain containing 4
ENSG0000	52.30518	-0.43825	0.331007	-1.32399	0.185505	0.457948	L3HYPDH	112849	trans-L-3-hydroxyproline dehydratase
ENSG0000	5.35146	-1.3653	1.031295	-1.32387	0.185546	0.457953	NA	NA	NA
ENSG0000	18.05148	-0.85187	0.643486	-1.32384	0.185556	0.457953	NA	NA	NA
ENSG0000	182.8688	-0.25821	0.19507	-1.32368	0.185609	0.458023	ZNF558	148156	zinc finger protein 558
ENSG0000	1934.32	0.11869	0.089672	1.323599	0.185636	0.458028	RAB14	51552	RAB14 member RAS oncogene family
ENSG0000	31.81346	-0.61645	0.465916	-1.32309	0.185807	0.458388	MRPL12	6182	mitochondrial ribosomal protein L12
ENSG0000	171.2838	-0.25755	0.194688	-1.32286	0.185881	0.45851	NA	NA	NA
ENSG0000	6.089714	-1.40146	1.059502	-1.32275	0.185918	0.45854	NA	NA	NA
ENSG0000	4.125427	-1.9809	1.497663	-1.32266	0.185948	0.458554	LINC03048	1E+08	long intergenic non-protein coding RNA 3048
ENSG0000	17.71889	0.714649	0.540502	1.322195	0.186103	0.458875	LDHB	3945	lactate dehydrogenase B
ENSG0000	59.79803	-0.48701	0.368365	-1.32209	0.186137	0.458897	SASS6	163786	SAS-6 centriolar assembly protein
ENSG0000	39.35915	-0.52054	0.393772	-1.32193	0.186191	0.45897	MCPH1-DT	1E+08	MCPH1 divergent transcript
ENSG0000	892.9438	-0.16579	0.125431	-1.32173	0.186259	0.459075	BDP1	55814	B double p subunit of RNA polymerase III transcription initiation factor IIIB
ENSG0000	22.61118	0.841076	0.636421	1.321573	0.18631	0.459081	CAMK1G	57172	calcium/calmodulin dependent protein kinase IG
ENSG0000	117.7855	0.34019	0.257403	1.321627	0.186292	0.459081	C11orf1	64776	chromosome 11 open reading frame 1
ENSG0000	12.35851	1.080492	0.817812	1.321199	0.186435	0.459327	MAP1LC3B	643246	microtubule associated protein 1 light chain 3 beta 2
ENSG0000	1342.445	-0.13187	0.099846	-1.3207	0.186602	0.459627	SNAPC4	6621	small nuclear RNA activating complex polypeptide 4
ENSG0000	956.1952	-0.13874	0.105052	-1.32068	0.186607	0.459627	WASHC2C	253725	WASH complex subunit 2C
ENSG0000	75.36714	0.39723	0.300816	1.320512	0.186664	0.459647	DRD4	1815	dopamine receptor D4
ENSG0000	233.6713	0.245247	0.185718	1.320531	0.186658	0.459647	PPP1R13L	10848	protein phosphatase 1 regulatory subunit 13 like
ENSG0000	3074.215	0.106039	0.080312	1.320344	0.18672	0.459724	ARHGAP17	55114	Rho GTPase activating protein 17
ENSG0000	44.01336	0.540896	0.409704	1.320213	0.186764	0.45977	NA	NA	NA
ENSG0000	763.979	0.133616	0.101236	1.31985	0.186885	0.459947	ACBD3	64746	acyl-CoA binding domain containing 3
ENSG0000	55.52546	0.454771	0.344543	1.319924	0.18686	0.459947	RPL21P16	729402	ribosomal protein L21 pseudogene 16
ENSG0000	2057.293	0.133473	0.101162	1.3194	0.187035	0.460255	SLC66A2	80148	solute carrier family 66 member 2
ENSG0000	13552.72	-0.09603	0.072792	-1.31923	0.187091	0.460331	ITGB2	3689	integrin subunit beta 2
ENSG0000	284.1212	0.216837	0.164388	1.319055	0.187151	0.460367	ALKBH1	8846	alkB homo histone H2A dioxygenase
ENSG0000	6.512545	1.79106	1.357849	1.319042	0.187155	0.460367	IGLV3-27	28791	immunoglobulin lambda variable 3-27
ENSG0000	3353.791	-0.1138	0.086289	-1.31887	0.187212	0.460446	CYRIB	51571	CYFIP related Rac1 interactor B
ENSG0000	386.2316	-0.18277	0.138643	-1.3183	0.187405	0.460859	CINP	51550	cyclin dependent kinase 2 interacting protein
ENSG0000	63.55052	-0.40544	0.307587	-1.31813	0.187461	0.460935	PCBD2	84105	pterin-4 alpha-carbinolamine dehydratase 2
ENSG0000	59.63293	0.48254	0.366107	1.31803	0.187494	0.460955	NA	NA	NA
ENSG0000	315.6834	0.235634	0.178819	1.317721	0.187597	0.461148	FAXDC2	10826	fatty acid hydroxylase domain containing 2
ENSG0000	680.7285	-0.15144	0.114971	-1.31717	0.187781	0.46154	TBCB	1155	tubulin folding cofactor B
ENSG0000	384.1865	0.197065	0.149626	1.317058	0.187819	0.461572	UPF3B	65109	UPF3B regulator of nonsense mediated mRNA decay
ENSG0000	85.35652	-0.33135	0.251832	-1.31576	0.188254	0.462579	SIRT5	23408	sirtuin 5
ENSG0000	2.989421	2.197033	1.66989	1.315675	0.188283	0.462589	LOC12490	1.25E+08	uncharacterized LOC124909384
ENSG0000	12.98786	1.147024	0.872105	1.315236	0.188431	0.462876	NA	NA	NA
ENSG0000	10.94707	-1.05237	0.800221	-1.31511	0.188475	0.462876	FAM187B2	1E+08	family witl member B pseudogene
ENSG0000	273.1855	-0.21157	0.160877	-1.31512	0.188469	0.462876	ZNF175	7728	zinc finger protein 175
ENSG0000	883.5584	0.157246	0.119611	1.314647	0.188628	0.463008	MAL	4118	mal T cell differentiation protein
ENSG0000	2.706834	2.10474	1.600984	1.314654	0.188626	0.463008	NA	NA	NA
ENSG0000	16.42051	-0.92	0.699687	-1.31487	0.188554	0.463008	NA	NA	NA
ENSG0000	28.72978	-0.56642	0.430832	-1.31472	0.188604	0.463008	RNF157-AS	1.01E+08	RNF157 antisense RNA 1
ENSG0000	1500.292	0.122519	0.09321	1.314441	0.188698	0.463018	UTRN	7402	utrophin
ENSG0000	499.3613	0.158746	0.120775	1.314392	0.188714	0.463018	CLNS1A	1207	chloride nucleotide-sensitive channel 1A
ENSG0000	26.00017	0.650501	0.494872	1.314483	0.188684	0.463018	EHD2	30846	EH domain containing 2
ENSG0000	3401.617	0.127491	0.097	1.314339	0.188732	0.463018	RBM10	8241	RNA binding motif protein 10
ENSG0000	2275.18	-0.12018	0.091457	-1.3141	0.188814	0.463095	ZNF687	57592	zinc finger protein 687
ENSG0000	583.8284	0.151879	0.115577	1.314097	0.188814	0.463095	PPA1	5464	inorganic pyrophosphatase 1
ENSG0000	4.71264	1.84105	1.401201	1.313909	0.188877	0.463189	MYO3B	140469	myosin IIIB
ENSG0000	42.46611	0.498323	0.379319	1.313731	0.188937	0.463214	ORC1	4998	origin recognition complex subunit 1
ENSG0000	5.619648	-1.24038	0.944123	-1.31379	0.188916	0.463214	NA	NA	NA
ENSG0000	3.283012	-2.19878	1.674579	-1.31303	0.189172	0.463728	NA	NA	NA
ENSG0000	4502.838	0.112903	0.08608	1.31161	0.189652	0.464129	ID2	3398	inhibitor of DNA binding 2
ENSG0000	39.86943	0.553296	0.421853	1.311584	0.189661	0.464129	ACVR1C	130399	activin A receptor type 1C
ENSG0000	11.18793	-1.01344	0.77261	-1.31171	0.189617	0.464129	CCDC152	1E+08	coiled-coil domain containing 152
ENSG0000	20632.15	-0.1094	0.083361	-1.3124	0.189386	0.464129	IRF1	3659	interferon regulatory factor 1
ENSG0000	6.919398	1.358169	1.035249	1.311925	0.189546	0.464129	NA	NA	NA
ENSG0000	3.038935	2.489198	1.896788	1.312323	0.189411	0.464129	NA	NA	NA
ENSG0000	464.1347	0.192336	0.14663	1.31171	0.189618	0.464129	STXBP5	134957	syntaxin binding protein 5
ENSG0000	493.529	0.183359	0.139789	1.311686	0.189626	0.464129	ZFAND2A	90637	zinc finger AN1-type containing 2A
ENSG0000	1179.408	0.180595	0.137653	1.311964	0.189532	0.464129	LCN2	3934	lipocalin 2
ENSG0000	780.9116	0.156952	0.119598	1.312325	0.18941	0.464129	TRAF6	7189	TNF receptor associated factor 6
ENSG0000	16.3671	-0.91421	0.69695	-1.31173	0.18961	0.464129	WFIKKN1	117166	WAP follistatin, immunog kunitz and netrin domain containing 1
ENSG0000	5.738394	-1.24235	0.946747	-1.31223	0.189441	0.464129	NA	NA	NA
ENSG0000	26.54453	-0.58237	0.443899	-1.31194	0.18954	0.464129	NA	NA	NA
ENSG0000	82.21856	-0.34177	0.260634	-1.3113	0.189756	0.464239	NA	NA	NA
ENSG0000	5.398819	-1.55918	1.188987	-1.31135	0.189738	0.464239	GRIP1	23426	glutamate receptor interacting protein 1
ENSG0000	2794.223	-0.12388	0.094481	-1.31112	0.189816	0.464326	NCOA3	8202	nuclear receptor coactivator 3
ENSG0000	33.73367	-0.55954	0.426862	-1.31082	0.189919	0.464517	C1QTNF12	388581	C1q and TNF related 12
ENSG0000	101.4522	0.391299	0.298571	1.310574	0.190002	0.464629	COBLL1	22837	cordon-bleu WH2 repeat protein like 1
ENSG0000	5927.435	0.113076	0.086283	1.310534	0.190015	0.464629	FUS	2521	FUS RNA binding protein
ENSG0000	2935.02	0.102858	0.07851	1.31012	0.190155	0.464911	YIPF3	25844	Yip1 domain family member 3
ENSG0000	66.96712	-0.46232	0.353028	-1.30957	0.19034	0.464941	NA	NA	NA
ENSG0000	71.38009	-0.4452	0.339864	-1.30993	0.19022	0.464941	LMLN	89782	leishmanolysin like peptidase
ENSG0000	5.280617	-1.80454	1.377968	-1.30956	0.190343	0.464941	TXLNB	167838	taxilin beta
ENSG0000	5.22005	-1.51863	1.159635	-1.30958	0.190339	0.464941	NA	NA	NA
ENSG0000	1947.534	-0.12912	0.098598	-1.30961	0.190329	0.464941	MTA1	9112	metastasis associated 1
ENSG0000	1036.444	-0.1449	0.110627	-1.30978	0.190269	0.464941	FTSJ3	117246	FtsJ RNA 2'-O-methyltransferase 3
ENSG0000	26.55309	-0.58992	0.45047	-1.30957	0.190341	0.464941	SPIN2B	474343	spindlin family member 2B
ENSG0000	1569.127	-0.12096	0.09241	-1.30893	0.190557	0.465341	GTF3A	2971	general transcription factor IIIA
ENSG0000	11.33652	-1.18347	0.90415	-1.30894	0.190556	0.465341	USP32P1	162632	ubiquitin specific peptidase 32 pseudogene 1
ENSG0000	1255.974	0.137388	0.104977	1.308746	0.19062	0.465373	HAX1	10456	HCLS1 associated protein X-1
ENSG0000	77.74924	0.380136	0.290453	1.308768	0.190613	0.465373	PFDN4	5203	prefoldin subunit 4
ENSG0000	316.3516	-0.20152	0.15404	-1.30824	0.190791	0.465727	GLRX5	51218	glutaredoxin 5
ENSG0000	35.27358	-0.64525	0.493419	-1.30772	0.190969	0.466013	FXN	2395	frataxin

ENSG0000	81.75072	-0.49421	0.377913	-1.30773	0.190965	0.466013	LENG8-AS:	1.04E+08	LENG8 antisense RNA 1
ENSG0000	849.0545	-0.17627	0.134796	-1.30768	0.190983	0.466013	SLC25A1	6576	solute carrier family 25 member 1
ENSG0000	1131.391	-0.17219	0.131693	-1.30752	0.191038	0.466085	IDH2	3418	isocitrate dehydrogenase (NADP(+)) 2
ENSG0000	7.669297	-1.19767	0.916154	-1.30728	0.191118	0.466101	NA	NA	NA
ENSG0000	10.07661	-1.25047	0.956478	-1.30737	0.191089	0.466101	PKD2L1	9033	polycystin transient receptor potential cation channel
ENSG0000	4.805278	1.563817	1.196243	1.307274	0.19112	0.466101	NA	NA	NA
ENSG0000	1235.285	0.139954	0.107074	1.307073	0.191188	0.466206	RNF5	6048	ring finger protein 5
ENSG0000	8258.367	-0.10396	0.079573	-1.30652	0.191377	0.466483	ANXA1	301	annexin A1
ENSG0000	242.2902	0.210479	0.161097	1.30654	0.191369	0.466483	BAZ1A-AS:	1.12E+08	BAZ1A antisense RNA 1
ENSG0000	167.2646	0.26146	0.200118	1.306533	0.191371	0.466483	NA	NA	NA
ENSG0000	17.74777	-0.79288	0.606946	-1.30634	0.191436	0.466537	NA	NA	NA
ENSG0000	24.66809	-0.72348	0.554015	-1.30589	0.19159	0.466537	ENPP5	59084	ectonucleotide pyrophosphatase/phosphodiesterase family member 5
ENSG0000	358.5357	0.181487	0.13897	1.305939	0.191573	0.466537	POMZP3	22932	POM121 and ZP3 fusion
ENSG0000	340.8954	0.209172	0.160164	1.305982	0.191559	0.466537	NMRK1	54981	nicotinamide riboside kinase 1
ENSG0000	141.1372	-0.27615	0.211426	-1.30614	0.191505	0.466537	ACAD10	80724	acyl-CoA dehydrogenase family member 10
ENSG0000	170.0642	-0.26897	0.205961	-1.30592	0.19158	0.466537	LINC0042€	1E+08	long intergenic non-protein coding RNA 426
ENSG0000	5901.84	0.095156	0.07286	1.305999	0.191553	0.466537	PAPOLA	10914	poly(A) polymerase alpha
ENSG0000	25.0184	0.692612	0.530388	1.305859	0.1916	0.466537	NA	NA	NA
ENSG0000	5.180058	1.668543	1.278093	1.305495	0.191724	0.466631	NA	NA	NA
ENSG0000	133.0443	-0.28006	0.214524	-1.30551	0.19172	0.466631	TCF19	6941	transcription factor 19
ENSG0000	343.9345	-0.20245	0.155074	-1.30549	0.191727	0.466631	RBAK	57786	RB associated KRAB zinc finger
ENSG0000	1680.205	0.116761	0.089445	1.305402	0.191756	0.466631	GZMH	2999	granzyme H
ENSG0000	28.80293	-0.66991	0.513193	-1.30538	0.191765	0.466631	NA	NA	NA
ENSG0000	204.0247	-0.25213	0.19318	-1.30514	0.191845	0.466765	NA	NA	NA
ENSG0000	21.74138	0.920693	0.705601	1.304835	0.191949	0.466893	NA	NA	NA
ENSG0000	24.61604	-0.59767	0.45803	-1.30487	0.191937	0.466893	RAD51B	5890	RAD51 paralog B
ENSG0000	10.64107	-1.1409	0.874452	-1.30471	0.191993	0.466893	NA	NA	NA
ENSG0000	107.8434	0.317787	0.243573	1.304691	0.191998	0.466893	ATP5PO	539	ATP synthase peripheral stalk subunit OSCP
ENSG0000	3.344856	1.992479	1.527531	1.304379	0.192105	0.46709	NA	NA	NA
ENSG0000	293.8404	-0.2204	0.169012	-1.30404	0.19222	0.467249	ZNF354A	6940	zinc finger protein 354A
ENSG0000	5.172376	-1.32047	1.012554	-1.3041	0.192201	0.467249	NA	NA	NA
ENSG0000	124.6982	-0.29861	0.229044	-1.30371	0.192332	0.46746	CSTF3	1479	cleavage stimulation factor subunit 3
ENSG0000	188.6427	-0.24665	0.189283	-1.30309	0.192542	0.467734	PNPT1	87178	polyribonucleotide nucleotidyltransferase 1
ENSG0000	372.7679	0.183375	0.140718	1.303135	0.192529	0.467734	FAM136A	84908	family with sequence similarity 136 member A
ENSG0000	10.38716	1.129109	0.866494	1.303078	0.192548	0.467734	SPICE1	152185	spindle and centriole associated protein 1
ENSG0000	805.6047	0.201208	0.1544	1.303167	0.192518	0.467734	ZNF107	51427	zinc finger protein 107
ENSG0000	1750.808	0.130395	0.100072	1.303011	0.192571	0.467734	POLR2L	5441	RNA polyn I and III subunit L
ENSG0000	3668.858	0.104252	0.080031	1.302633	0.1927	0.467986	CAPRIN1	4076	cell cycle associated protein 1
ENSG0000	491.1921	-0.16912	0.129854	-1.30235	0.192796	0.468156	ALDOC	230	aldolase fructose-bisphosphate C
ENSG0000	640.6411	0.167017	0.128255	1.302222	0.19284	0.468157	GABPB1	2553	GA binding protein transcription factor subunit beta 1
ENSG0000	7.319299	-1.35434	1.040033	-1.3022	0.192846	0.468157	RTL8B	441518	retrotransposon Gag like 8B
ENSG0000	755.3713	-0.13793	0.105956	-1.30178	0.192992	0.468449	DYNC1I2	1781	dynein cytoplasmic 1 intermediate chain 2
ENSG0000	1165.639	-0.62634	0.481261	-1.30146	0.193102	0.468654	IFIT3	3437	interferon induced protein with tetratricopeptide repeats 3
ENSG0000	443.6075	-0.17275	0.132801	-1.30085	0.193311	0.468899	NA	NA	NA
ENSG0000	5.759887	1.741254	1.338447	1.300951	0.193275	0.468899	NA	NA	NA
ENSG0000	572.089	-0.14974	0.115114	-1.3008	0.193327	0.468899	MFSD1	64747	major facilitator superfamily domain containing 1
ENSG0000	767.4803	-0.16728	0.128601	-1.30079	0.193329	0.468899	CEP120	153241	centrosomal protein 120
ENSG0000	137.5461	-0.2736	0.210284	-1.30108	0.193232	0.468899	HERC2P9	440248	HERC2 pseudogene 9
ENSG0000	4.33179	-1.66699	1.281854	-1.30046	0.193445	0.469119	NA	NA	NA
ENSG0000	2100.988	0.116883	0.089886	1.300342	0.193484	0.469152	HNRNPH3	3189	heterogeneous nuclear ribonucleoprotein H3
ENSG0000	106.6768	-0.31898	0.245372	-1.29997	0.193612	0.469401	ZNF765	91661	zinc finger protein 765
ENSG0000	1177.497	0.134226	0.103264	1.299825	0.193661	0.469459	RC3H2	54542	ring finger and CCCH-type domains 2
ENSG0000	179.314	-0.24887	0.191484	-1.29971	0.193701	0.469495	GTF2F2	2963	general transcription factor IIF subunit 2
ENSG0000	1322.664	-0.13825	0.106384	-1.29954	0.193758	0.469572	TSC1	7248	TSC complex subunit 1
ENSG0000	4.999206	1.289791	0.992631	1.299365	0.193819	0.469657	RPS4XP11	441550	ribosomal protein S4X pseudogene 11
ENSG0000	134.7932	0.28425	0.218835	1.298925	0.193969	0.469961	RAB33A	9363	RAB33A member RAS oncogene family
ENSG0000	2491.069	0.303756	0.23387	1.298823	0.194005	0.469985	RPL39	6170	ribosomal protein L39
ENSG0000	3491.616	0.113743	0.087582	1.298699	0.194047	0.470027	TRIM26	7726	tripartite motif containing 26
ENSG0000	37.35788	-0.56434	0.434577	-1.29859	0.194086	0.470059	NA	NA	NA
ENSG0000	220.7115	0.266512	0.205266	1.298374	0.194159	0.470174	ZNF277	11179	zinc finger protein 277
ENSG0000	1045.492	0.150586	0.115999	1.298168	0.19423	0.470228	KAT2B	8850	lysine acetyltransferase 2B
ENSG0000	4.435717	-1.74555	1.344632	-1.29816	0.194232	0.470228	METTL24	728464	methyltransferase like 24
ENSG0000	69.56593	0.412609	0.317951	1.297713	0.194386	0.47054	ST8SIA1	6489	ST8 alpha- 8-sialyltransferase 1
ENSG0000	63.52331	0.436095	0.336092	1.297546	0.194443	0.470617	LINC01451	401561	long intergenic non-protein coding RNA 1451
ENSG0000	10.69693	-0.95293	0.734847	-1.29678	0.194707	0.470954	ROR1	4919	receptor tyrosine kinase like orphan receptor 1
ENSG0000	134.7508	0.325331	0.250878	1.296772	0.19471	0.470954	EPHX1	2052	epoxide hydrolase 1
ENSG0000	150.1318	0.273564	0.210925	1.29697	0.194641	0.470954	SETMAR	6419	SET domain and mariner transposase fusion gene
ENSG0000	1622.49	-0.1237	0.095374	-1.29701	0.194626	0.470954	TSPOAP1	9256	TSPO associated protein 1
ENSG0000	155.0925	-0.25518	0.196761	-1.29691	0.194663	0.470954	CEP131	22994	centrosomal protein 131
ENSG0000	65.54929	0.390426	0.301105	1.296642	0.194754	0.471001	LINC01237	1.02E+08	long intergenic non-protein coding RNA 1237
ENSG0000	1373.41	0.1222	0.094263	1.296372	0.194847	0.471165	RNF41	10193	ring finger protein 41
ENSG0000	7.642537	1.163828	0.898036	1.29597	0.194986	0.471438	LOC10028!	1E+08	uncharacterized LOC100289495
ENSG0000	11165.03	0.107669	0.08309	1.295809	0.195041	0.471511	UBC	7316	ubiquitin C
ENSG0000	3814.224	0.130676	0.100856	1.295668	0.19509	0.471566	DAZAP1	26528	DAZ associated protein 1
ENSG0000	70.96451	-0.36084	0.278651	-1.29497	0.195331	0.472076	NUDT2	318	nudix hydrolase 2
ENSG0000	136.884	0.310463	0.239756	1.29491	0.195351	0.472076	CES4A	283848	carboxylesterase 4A
ENSG0000	10.63023	-1.37999	1.066409	-1.29405	0.195648	0.472424	NA	NA	NA
ENSG0000	75200.45	0.092375	0.071382	1.294092	0.195634	0.472424	HLA-C	3107	major hist class I C
ENSG0000	22.4841	0.785128	0.606649	1.294204	0.195595	0.472424	GARIN1A	346653	golgi associated RAB2 interactor 1A
ENSG0000	16.31052	-0.82385	0.636635	-1.29406	0.195644	0.472424	NA	NA	NA
ENSG0000	7.472572	-1.15052	0.888998	-1.29417	0.195605	0.472424	NA	NA	NA
ENSG0000	137.2122	0.353968	0.273513	1.294155	0.195612	0.472424	RRAD	6236	RRAD Ras related glycolysis inhibitor and calcium channel regulator
ENSG0000	298.007	0.197086	0.152318	1.293913	0.195695	0.472426	CRELD1	78987	cysteine rich with EGF like domains 1
ENSG0000	867.1066	0.129485	0.100079	1.293826	0.195725	0.472426	ATG12	9140	autophagy related 12
ENSG0000	131.2744	-0.31608	0.244292	-1.29384	0.195719	0.472426	TEDC1	283643	tubulin epsilon and delta complex 1
ENSG0000	55.41875	-0.4157	0.321402	-1.2934	0.195873	0.472722	NA	NA	NA
ENSG0000	3197.286	0.1147	0.088687	1.293312	0.195903	0.472732	GIMAP7	168537	GTPase IMAP family member 7
ENSG0000	1236.343	0.120908	0.0935	1.293141	0.195962	0.472813	GOSR1	9527	golgi SNAP receptor complex member 1
ENSG0000	3.457052	-2.15443	1.666858	-1.29251	0.196181	0.473161	NA	NA	NA
ENSG0000	17.94202	-0.72463	0.560599	-1.2926	0.196151	0.473161	NA	NA	NA
ENSG0000	2404.012	-0.11324	0.08761	-1.2925	0.196183	0.473161	TBC1D17	79735	TBC1 domain family member 17
ENSG0000	110.458	-0.30809	0.238408	-1.29229	0.196257	0.473278	SMG1P7	1.01E+08	SMG1 pseudogene 7
ENSG0000	109.4683	-0.35458	0.274407	-1.29215	0.196305	0.473333	RCN1	5954	reticulocalbin 1
ENSG0000	360.8227	0.196249	0.151896	1.291993	0.19636	0.473372	MIOS	54468	meiosis regulator for oocyte development
ENSG0000	8.510815	1.314844	1.017716	1.291955	0.196373	0.473372	MMP11	4320	matrix metallopeptidase 11
ENSG0000	308.7791	0.210535	0.16299	1.291704	0.19646	0.47352	LRRC37A2	474170	leucine rich repeat containing 37 member A2
ENSG0000	248.9169	-0.23694	0.183452	-1.29159	0.196501	0.473557	TMEM260	54916	transmembrane protein 260
ENSG0000	43.50035	0.459907	0.356162	1.291284	0.196605	0.473748	LRRC75B	388886	leucine rich repeat containing 75B
ENSG0000	4067.753	0.108918	0.084386	1.290717	0.196802	0.47416	KMT2A	4297	lysine methyltransferase 2A
ENSG0000	416.8542	0.17708	0.137208	1.2906	0.196842	0.47417	DANCR	57291	differentiation antagonizing non-protein coding RNA

ENSG0000	772.4769	0.143999	0.111579	1.290558	0.196857	0.47417	URI1	8725	URI1 prefoldin like chaperone
ENSG0000	2721.7	0.125058	0.096921	1.290305	0.196945	0.474258	SSR1	6745	signal sequence receptor subunit 1
ENSG0000	21.58212	-0.71952	0.557628	-1.29032	0.196941	0.474258	NA	NA	NA
ENSG0000	143.6475	-0.26402	0.204682	-1.28991	0.197081	0.474463	HMGCL	3155	3-hydroxy-3-methylglutaryl-CoA lyase
ENSG0000	8.039641	-1.29372	1.002928	-1.28994	0.19707	0.474463	NA	NA	NA
ENSG0000	22.37357	0.669279	0.519048	1.289434	0.197247	0.474801	IGKV2-30	28919	immunoglobulin kappa variable 2-30
ENSG0000	30.92091	-0.61994	0.48092	-1.28907	0.197373	0.47492	IQCC	55721	IQ motif containing C
ENSG0000	8.442062	1.341904	1.040943	1.289124	0.197355	0.47492	COQ3	51805	coenzyme methyltransferase
ENSG0000	337.7094	-0.19687	0.15271	-1.2892	0.197328	0.47492	SLC37A4	2542	solute carrier family 37 member 4
ENSG0000	4.648925	1.346276	1.044606	1.288789	0.197472	0.475095	NA	NA	NA
ENSG0000	22.9026	0.633982	0.492026	1.288512	0.197568	0.475203	NA	NA	NA
ENSG0000	4.5423	-1.90437	1.47792	-1.28854	0.197556	0.475203	CEP112	201134	centrosomal protein 112
ENSG0000	326.7506	0.217128	0.168557	1.28816	0.19769	0.47528	BOD1	91272	biorientation of chromosomes in cell division 1
ENSG0000	6.136414	1.509293	1.171574	1.288261	0.197655	0.47528	NA	NA	NA
ENSG0000	5533.328	-0.12308	0.095532	-1.28833	0.197632	0.47528	RESF1	55196	retroelement silencing factor 1
ENSG0000	330.2241	-0.22654	0.175871	-1.28813	0.197702	0.47528	CHST14	113189	carbohydrate sulfotransferase 14
ENSG0000	7071.604	-0.08592	0.066706	-1.28803	0.197735	0.475297	SMARCC2	6601	SWI/SNF r matrix ass actin dependent regulator of chromatin subfamily c member 2
ENSG0000	1156.489	-0.13402	0.104067	-1.28786	0.197796	0.475321	RGP1	9827	RGP1 hom RAB6A GEF complex partner 1
ENSG0000	220.6059	0.226166	0.175611	1.287876	0.197789	0.475321	PKIG	11142	cAMP-dependent protein kinase inhibitor gamma
ENSG0000	708.5338	0.176446	0.137018	1.287755	0.197831	0.475343	GMEB1	10691	glucocorticoid modulatory element binding protein 1
ENSG0000	200.0556	-0.26129	0.20293	-1.28758	0.197894	0.475432	DHRS1	115817	dehydrogenase/reductase 1
ENSG0000	415.1872	0.166355	0.129212	1.287457	0.197935	0.475469	HUS1	3364	HUS1 checkpoint clamp component
ENSG0000	561.3864	0.147303	0.114432	1.28726	0.198004	0.475573	TP53RK	112858	TP53 regulating kinase
ENSG0000	83.69354	-0.41531	0.322688	-1.28704	0.198082	0.475678	LDAH	60526	lipid droplet associated hydrolase
ENSG0000	771.5442	0.17167	0.133389	1.286987	0.198099	0.475678	COMMD5	28991	COMM domain containing 5
ENSG0000	254.1021	-0.22453	0.17451	-1.28661	0.19823	0.475933	MCM4	4173	minichromosome maintenance complex component 4
ENSG0000	179.3022	-0.24955	0.19399	-1.28639	0.198305	0.476051	OTUD7B	56957	OTU deubiquitinase 7B
ENSG0000	267.4189	-0.21954	0.170685	-1.28626	0.198353	0.476105	FGD4	121512	FYVE RhoGEF and PH domain containing 4
ENSG0000	40810.45	0.125236	0.097396	1.285845	0.198497	0.476203	S100A11	6282	S100 calcium binding protein A11
ENSG0000	67.76746	0.474064	0.368672	1.285869	0.198489	0.476203	GAL3ST2	64090	galactose-3-O-sulfotransferase 2
ENSG0000	6.747134	-1.31326	1.021307	-1.28586	0.198491	0.476203	APOA1	335	apolipoprotein A1
ENSG0000	281.3395	-0.21121	0.164242	-1.28597	0.198453	0.476203	SETDB2	83852	SET domain bifurcated histone lysine methyltransferase 2
ENSG0000	110.2717	-0.32393	0.251951	-1.28568	0.198556	0.476284	CEBPE	1053	CCAAT enhancer binding protein epsilon
ENSG0000	29335.06	0.089669	0.069804	1.284578	0.19894	0.47713	SPI1	6688	Spi-1 proto-oncogene
ENSG0000	105.5914	-0.32478	0.252845	-1.28452	0.198961	0.47713	CHRNB1	1140	cholinergic receptor nicotinic beta 1 subunit
ENSG0000	6.036172	1.525119	1.187539	1.284268	0.199048	0.477278	LINC0065§	1.01E+08	long intergenic non-protein coding RNA 659
ENSG0000	246.2732	-0.23725	0.184776	-1.28397	0.199151	0.477331	PEX13	5194	peroxisomal biogenesis factor 13
ENSG0000	9.902234	-1.21314	0.944749	-1.28409	0.19911	0.477331	NA	NA	NA
ENSG0000	5906.637	-0.12743	0.099247	-1.28395	0.19916	0.477331	TUBA1B	10376	tubulin alpha 1b
ENSG0000	4162.891	-0.13413	0.10447	-1.28391	0.199173	0.477331	SMG1	23049	SMG1 nonsense mediated mRNA decay associated PI3K related kinase
ENSG0000	627.4488	0.149908	0.116792	1.283541	0.199303	0.477418	GPATCH3	63906	G-patch domain containing 3
ENSG0000	2.746692	2.224197	1.732928	1.283491	0.19932	0.477418	RC3H1-DT	1.05E+08	RC3H1 divergent transcript
ENSG0000	96.16367	0.440884	0.343504	1.283489	0.199321	0.477418	IGKV3-11	28914	immunoglobulin kappa variable 3-11
ENSG0000	4.02681	-1.54534	1.204308	-1.28317	0.199431	0.477418	NA	NA	NA
ENSG0000	3889.389	0.107449	0.08374	1.283125	0.199448	0.477418	RFTN1	23180	raftlin lipid raft linker 1
ENSG0000	545.6707	-0.16656	0.129802	-1.28319	0.199424	0.477418	LYPLA1	10434	lysophospholipase 1
ENSG0000	3.223842	-2.08517	1.62473	-1.2834	0.199353	0.477418	MRPS28	28957	mitochondrial ribosomal protein S28
ENSG0000	3.736408	-1.70158	1.326021	-1.28322	0.199414	0.477418	PLAAT2	54979	phospholipase A and acyltransferase 2
ENSG0000	3.233162	-1.80409	1.406069	-1.28307	0.199467	0.477418	DDX19A-D	1.01E+08	DDX19A divergent transcript
ENSG0000	2135.951	-0.1434	0.111736	-1.28342	0.199345	0.477418	AP1M1	8907	adaptor related protein complex 1 subunit mu 1
ENSG0000	13.6522	0.829505	0.646632	1.282808	0.199559	0.477578	NA	NA	NA
ENSG0000	638.8457	-0.14183	0.110588	-1.28251	0.199662	0.477763	MPV17	4358	mitochondrial inner membrane protein MPV17
ENSG0000	552.3127	-0.1612	0.125749	-1.2819	0.199879	0.478017	MFF	56947	mitochondrial fission factor
ENSG0000	3.971178	-1.88937	1.473945	-1.28184	0.199898	0.478017	NA	NA	NA
ENSG0000	104.6991	-0.30576	0.238523	-1.28188	0.199885	0.478017	MIR600HG	81571	MIR600 host gene
ENSG0000	7.3804	1.031124	0.804364	1.281912	0.199873	0.478017	NA	NA	NA
ENSG0000	10.48256	-0.94192	0.734796	-1.28188	0.199885	0.478017	NA	NA	NA
ENSG0000	5.554807	-1.36756	1.067049	-1.28162	0.199974	0.478102	TRBJ2-1	28629	T cell receptor beta joining 2-1
ENSG0000	190.8078	0.251965	0.196614	1.281522	0.200011	0.478102	NA	NA	NA
ENSG0000	5740.988	-0.09942	0.077581	-1.28154	0.200003	0.478102	IL17RA	23765	interleukin 17 receptor A
ENSG0000	58.99909	-0.38663	0.301757	-1.28128	0.200096	0.478246	SNHG10	283596	small nucleolar RNA host gene 10
ENSG0000	521.6174	-0.18957	0.147961	-1.2812	0.200124	0.47825	DHX58	79132	DExH-box helicase 58
ENSG0000	4.71858	1.755599	1.370681	1.280823	0.200256	0.47846	MIR4635	1.01E+08	microRNA 4635
ENSG0000	1929.624	-0.11681	0.091197	-1.2808	0.200263	0.47846	FBXL19	54620	F-box and leucine rich repeat protein 19
ENSG0000	95.52201	-0.31317	0.24453	-1.2807	0.2003	0.478486	NADK2	133686	NAD kinas mitochondrial
ENSG0000	374.6145	-0.19149	0.149627	-1.27975	0.200633	0.479169	CCP110	9738	centriolar coiled-coil protein 110
ENSG0000	364.3273	-0.21974	0.171703	-1.27974	0.200637	0.479169	MIB1	57534	MIB E3 ubiquitin protein ligase 1
ENSG0000	251.587	-0.21382	0.16709	-1.27964	0.200671	0.479186	SCRN2	90507	secernin 2
ENSG0000	14.79239	0.877091	0.68549	1.279509	0.200718	0.479237	LAMA2	3908	laminin subunit alpha 2
ENSG0000	1.934203	2.905692	2.271101	1.27942	0.200749	0.47925	NA	NA	NA
ENSG0000	2.045474	-2.68937	2.102255	-1.27928	0.200798	0.479306	PODNL1	79883	podocan like 1
ENSG0000	24.61962	0.798814	0.624651	1.278817	0.200962	0.479588	NA	NA	NA
ENSG0000	2.799921	-2.15363	1.684108	-1.2788	0.200968	0.479588	IL17REL	400935	interleukin 17 receptor E like
ENSG0000	5.157522	-1.63994	1.282834	-1.27837	0.201118	0.479636	DAB1	1600	DAB adaptor protein 1
ENSG0000	292.335	0.202982	0.158771	1.278458	0.201088	0.479636	PDCL3	79031	phosducin like 3
ENSG0000	6925.537	-0.13271	0.103804	-1.27842	0.2011	0.479636	MYD88	4615	MYD88 innate immune signal transduction adaptor
ENSG0000	1944.476	0.114882	0.08985	1.278607	0.201036	0.479636	TIAL1	7073	TIA1 cytotoxic granule associated RNA binding protein like 1
ENSG0000	510.2625	0.182974	0.143126	1.278413	0.201104	0.479636	SDHD	6392	succinate dehydrogenase complex subunit D
ENSG0000	23.7831	-0.67567	0.528737	-1.2779	0.201285	0.479926	ITPR1-DT	1.01E+08	ITPR1 divergent transcript
ENSG0000	618.4144	-0.14622	0.114431	-1.27781	0.201317	0.479926	NA	NA	NA
ENSG0000	4205.996	0.100479	0.078632	1.277834	0.201308	0.479926	SMAD7	4092	SMAD family member 7
ENSG0000	562.7961	0.174774	0.136812	1.277478	0.201434	0.480029	PRKCI	5584	protein kinase C iota
ENSG0000	23.60766	-0.74057	0.579763	-1.27736	0.201475	0.480029	ULBP3	79465	UL16 binding protein 3
ENSG0000	85.78756	-0.39303	0.307679	-1.27741	0.201459	0.480029	ARL3	403	ADP ribosylation factor like GTPase 3
ENSG0000	348.3693	-0.18468	0.144583	-1.27732	0.20149	0.480029	CCS	9973	copper chaperone for superoxide dismutase
ENSG0000	5268.462	0.120694	0.094469	1.277598	0.201391	0.480029	RALY	22913	RALY heterogeneous nuclear ribonucleoprotein
ENSG0000	35.18849	0.520325	0.407512	1.276834	0.201661	0.480375	NA	NA	NA
ENSG0000	3.443924	1.797496	1.408246	1.276408	0.201811	0.480673	AFAP1-AS:	84740	AFAP1 antisense RNA 1
ENSG0000	223.1795	-0.23768	0.186238	-1.2762	0.201884	0.480683	MED18	54797	mediator complex subunit 18
ENSG0000	135.1214	-0.27148	0.212725	-1.27622	0.201876	0.480683	PAXIP1-DT	202781	PAXIP1 divergent transcript
ENSG0000	9.989919	1.075715	0.84294	1.276147	0.201903	0.480683	FOXB1	27023	forkhead box B1
ENSG0000	147.1029	-0.30158	0.236329	-1.2761	0.20192	0.480683	ZNF324B	388569	zinc finger protein 324B
ENSG0000	326.2583	-0.20611	0.161522	-1.27602	0.20195	0.480693	BCCIP	56647	BRCA2 and CDKN1A interacting protein
ENSG0000	27.64809	-0.58199	0.456169	-1.27582	0.20202	0.4808	TPSB2	64499	tryptase beta 2
ENSG0000	32.73815	0.605069	0.474372	1.275514	0.202127	0.480992	KCP	375616	kielin cysteine rich BMP regulator
ENSG0000	1729.219	0.105401	0.082663	1.275064	0.202287	0.48131	TENT4A	11044	terminal nucleotidyltransferase 4A
ENSG0000	57.18254	-0.44374	0.348067	-1.27487	0.202355	0.481348	MMS22L	253714	MMS22 lik DNA repair protein
ENSG0000	6.932016	1.275135	1.000199	1.274881	0.202351	0.481348	APC2	10297	APC regulator of WNT signaling pathway 2
ENSG0000	1662.778	0.120446	0.094482	1.274797	0.202381	0.481349	STARD7	56910	StAR related lipid transfer domain containing 7
ENSG0000	1919.752	0.107539	0.084365	1.274696	0.202417	0.481373	UBAP2	55833	ubiquitin associated protein 2

ENSG0000	22.07593	-0.63511	0.498378	-1.27434	0.202542	0.481608	CREB3L3	84699	cAMP responsive element binding protein 3 like 3
ENSG0000	42.29026	0.530522	0.416338	1.274258	0.202572	0.481618	H2BC5	3017	H2B clustered histone 5
ENSG0000	108.1575	-0.30315	0.237938	-1.27406	0.20264	0.481686	SLFN12	55106	schlafen family member 12
ENSG0000	148.447	0.286934	0.225231	1.273957	0.202679	0.481686	METTL4	64863	methyltrai N6-adenosine
ENSG0000	272.9474	-0.19449	0.152657	-1.27402	0.202658	0.481686	SMIM7	79086	small integral membrane protein 7
ENSG0000	7.705531	-1.29468	1.016403	-1.27378	0.20274	0.481757	MORN4	118812	MORN repeat containing 4
ENSG0000	271.7662	0.224788	0.17648	1.273727	0.20276	0.481757	EVA1C	59271	eva-1 homolog C
ENSG0000	14.18059	-0.87766	0.689161	-1.27352	0.202835	0.481873	NA	NA	NA
ENSG0000	907.9178	0.13279	0.104295	1.273221	0.20294	0.481936	CDC5L	988	cell division cycle 5 like
ENSG0000	132.3769	-0.31318	0.245956	-1.27333	0.202902	0.481936	NIPSNAP3	25934	nipsnap homolog 3A
ENSG0000	337.1722	-0.18946	0.1488	-1.27324	0.202933	0.481936	STX2	2054	syntaxin 2
ENSG0000	44.20717	-0.45537	0.35769	-1.27307	0.202993	0.481938	ZKSCAN3	80317	zinc finger with KRAB and SCAN domains 3
ENSG0000	17.0255	0.794027	0.623676	1.273141	0.202968	0.481938	NA	NA	NA
ENSG0000	16.09826	-0.91099	0.715876	-1.27255	0.203176	0.482313	LOC10798	1.08E+08	uncharacterized LOC107986412
ENSG0000	9.935226	0.995473	0.782357	1.272403	0.20323	0.482379	NA	NA	NA
ENSG0000	134.0485	0.296739	0.233239	1.272256	0.203282	0.482441	GALE	2582	UDP-galactose-4-epimerase
ENSG0000	1350.581	0.113328	0.089087	1.272098	0.203338	0.48245	TAF7	6879	TATA-box binding protein associated factor 7
ENSG0000	2514.56	-0.10238	0.080477	-1.27212	0.203329	0.48245	ZER1	10444	zyg-11 related cell cycle regulator
ENSG0000	3.442545	-2.17574	1.710645	-1.27188	0.203416	0.482572	NA	NA	NA
ENSG0000	4596.571	0.10342	0.081344	1.27139	0.20359	0.482842	CKAP4	10970	cytoskeleton associated protein 4
ENSG0000	49.47878	-0.49249	0.387371	-1.27137	0.203599	0.482842	WASH5P	375690	WASP fam pseudogene
ENSG0000	8.723489	-1.12602	0.885694	-1.27134	0.203608	0.482842	NA	NA	NA
ENSG0000	414.8111	0.188376	0.148319	1.270074	0.204058	0.483806	MRPS22	56945	mitochondrial ribosomal protein S22
ENSG0000	1661.111	-0.13016	0.102482	-1.27005	0.204066	0.483806	ECPAS	23392	Ecm29 proteasome adaptor and scaffold
ENSG0000	1912.591	0.120742	0.095076	1.269953	0.204101	0.483828	ADAM17	6868	ADAM metallopeptidase domain 17
ENSG0000	957.0731	0.154064	0.121326	1.26983	0.204145	0.48387	HSPB1	3315	heat shock protein family B (small) member 1
ENSG0000	14.1389	0.830232	0.653873	1.269715	0.204186	0.483878	RBM15-AS	440600	RBM15 antisense RNA 1
ENSG0000	14.93683	-0.91147	0.717878	-1.26967	0.204201	0.483878	NA	NA	NA
ENSG0000	50.42592	0.418071	0.32935	1.269382	0.204305	0.484063	PPM1N	147699	protein ph Mg2+/Mn2+ dependent 1N (putative)
ENSG0000	5.658572	-1.41337	1.113515	-1.26928	0.20434	0.484084	YY2	404281	YY2 transcription factor
ENSG0000	477.9047	-0.18194	0.143384	-1.26888	0.204483	0.484237	KRCC1	51315	lysine rich coiled-coil 1
ENSG0000	74.81358	-0.39727	0.313075	-1.26893	0.204466	0.484237	LINC0300	1E+08	long intergenic non-protein coding RNA 3009
ENSG0000	55.29909	-0.4417	0.348075	-1.26899	0.204444	0.484237	LOC10798	1.08E+08	SMAD specific E3 ubiquitin protein ligase 2 (SMURF2) pseudogene
ENSG0000	21.96296	0.718897	0.566784	1.268378	0.204663	0.484602	PPP1R14B	1.05E+08	PPP1R14B antisense RNA 1
ENSG0000	6.46079	1.436952	1.133336	1.267895	0.204835	0.484947	FZD4	8322	frizzled class receptor 4
ENSG0000	6.023808	-1.50532	1.187326	-1.26782	0.204861	0.484947	NA	NA	NA
ENSG0000	2150.616	0.112754	0.08896	1.267461	0.204991	0.485106	RBBP4	5928	RB binding chromatin remodeling factor
ENSG0000	196.1139	-0.25068	0.197781	-1.26747	0.204987	0.485106	TMEM41A	90407	transmembrane protein 41A
ENSG0000	77.14772	-0.3386	0.267156	-1.26742	0.205007	0.485106	MIRLET7B	400931	MIRLET7B host gene
ENSG0000	14.26594	-0.90998	0.718029	-1.26732	0.205039	0.485121	NA	NA	NA
ENSG0000	1998.079	0.105324	0.083135	1.266905	0.205189	0.48529	PPP2R5A	5525	protein phosphatase 2 regulatory subunit B'alpha
ENSG0000	2174.242	0.108665	0.085763	1.26703	0.205145	0.48529	DNAJB2	3300	DnaJ heat shock protein family (Hsp40) member B2
ENSG0000	66.80927	-0.38449	0.303395	-1.26695	0.205172	0.48529	IFT88	8100	intraflagellar transport 88
ENSG0000	1096.969	0.126573	0.099925	1.26668	0.20527	0.485419	MLKL	197259	mixed lineage kinase domain like pseudokinase
ENSG0000	116.0139	-0.27962	0.22079	-1.26644	0.205357	0.485563	APOOL	139322	apolipoprotein O like
ENSG0000	570.8586	-0.15016	0.118581	-1.2663	0.205406	0.485618	JPT1	51155	Jupiter microtubule associated homolog 1
ENSG0000	44.52923	-0.46092	0.364034	-1.26614	0.205465	0.485694	NA	NA	NA
ENSG0000	37.7117	-0.50685	0.400344	-1.26604	0.205499	0.485713	TTLL1	25809	TTL family tubulin polyglutamylase complex subunit L1
ENSG0000	1297.117	-0.13025	0.102911	-1.26566	0.205636	0.485852	MIGA2	84895	mitoguardin 2
ENSG0000	14.78609	-0.7884	0.622895	-1.26571	0.205617	0.485852	CCDC65	85478	coiled-coil domain containing 65
ENSG0000	72.76993	-0.42144	0.332952	-1.26577	0.205597	0.485852	ARL11	115761	ADP ribosylation factor like GTPase 11
ENSG0000	4.107874	1.679953	1.327506	1.265496	0.205694	0.485925	NA	NA	NA
ENSG0000	11.74699	0.95467	0.754533	1.265246	0.205783	0.486075	UTS2	10911	urotensin 2
ENSG0000	14.91366	-0.86628	0.684726	-1.26515	0.205816	0.486091	NA	NA	NA
ENSG0000	11.95427	-1.02036	0.80712	-1.26419	0.206161	0.486844	NA	NA	NA
ENSG0000	379.7718	0.19312	0.152792	1.26394	0.206252	0.486995	NFKBIL1	4795	NFKB inhibitor like 1
ENSG0000	4660.665	0.096222	0.076141	1.263743	0.206322	0.487101	ELF1	1997	E74 like ETS transcription factor 1
ENSG0000	219.5955	0.231295	0.183069	1.263427	0.206436	0.487252	NA	NA	NA
ENSG0000	53.37228	-0.46874	0.371012	-1.26339	0.206447	0.487252	SYT9-AS1	1.01E+08	SYT9 antisense RNA 1
ENSG0000	1556.819	0.116409	0.092143	1.263345	0.206465	0.487252	MRPL4	51073	mitochondrial ribosomal protein L4
ENSG0000	11.03462	1.226699	0.971068	1.263248	0.2065	0.487272	MYCT1	80177	MYC target 1
ENSG0000	698.3634	0.160635	0.127191	1.262942	0.20661	0.487407	URGCP	55665	upregulator of cell proliferation
ENSG0000	1040.995	0.135998	0.107682	1.262963	0.206602	0.487407	SIRT6	51548	sirtuin 6
ENSG0000	49.3765	-0.46361	0.367156	-1.2627	0.206696	0.487524	ADGRA3	166647	adhesion G protein-coupled receptor A3
ENSG0000	4.959668	1.409306	1.116142	1.262658	0.206712	0.487524	NA	NA	NA
ENSG0000	43.11991	-0.64444	0.510681	-1.26193	0.206974	0.488079	LINC0277	1.05E+08	long intergenic non-protein coding RNA 2777
ENSG0000	585.4597	-0.1537	0.121801	-1.26186	0.207	0.488079	ESRRA	2101	estrogen related receptor alpha
ENSG0000	11.82609	-1.02227	0.810217	-1.26172	0.207048	0.488107	PSRC1	84722	proline and serine rich coiled-coil 1
ENSG0000	858.1829	0.135275	0.107218	1.261679	0.207064	0.488107	PANK2	80025	pantothenate kinase 2
ENSG0000	847.2371	0.133707	0.106005	1.261332	0.207189	0.488277	NSMAF	8439	neutral sphingomyelinase activation associated factor
ENSG0000	10.48889	-1.26267	1.001035	-1.26136	0.207178	0.488277	NA	NA	NA
ENSG0000	11.42791	1.03021	0.816863	1.261178	0.207245	0.488346	NA	NA	NA
ENSG0000	99.83168	0.319937	0.253724	1.260968	0.20732	0.488462	LILRA4	23547	leukocyte immunoglobulin like receptor A4
ENSG0000	123.2176	-0.27024	0.214344	-1.26076	0.207394	0.488573	FAM30A	9834	family with sequence similarity 30 member A
ENSG0000	618.0466	-0.14837	0.117691	-1.26064	0.20744	0.48862	RUNDC1	146923	RUN domain containing 1
ENSG0000	232.1311	-0.20286	0.160937	-1.2605	0.207488	0.488627	MLH3	27030	mutL homolog 3
ENSG0000	174.2203	0.28085	0.222812	1.260481	0.207496	0.488627	NA	NA	NA
ENSG0000	162.9969	-0.26273	0.20852	-1.25999	0.207674	0.488924	TRBV4-2	28616	T cell receptor beta variable 4-2
ENSG0000	13.88362	0.87272	0.692643	1.259985	0.207675	0.488924	PLXNB3	5365	plexin B3
ENSG0000	14.65402	-0.84241	0.668754	-1.25966	0.207791	0.489135	MYH10	4628	myosin heavy chain 10
ENSG0000	8.3798	1.173294	0.931569	1.259481	0.207857	0.489228	ZSCAN10	84891	zinc finger and SCAN domain containing 10
ENSG0000	3.583554	1.711737	1.359512	1.259081	0.208001	0.48932	LINC02631	1.15E+08	long intergenic non-protein coding RNA 2631
ENSG0000	1941.975	0.125362	0.09955	1.259284	0.207928	0.48932	C9orf78	51759	chromosome 9 open reading frame 78
ENSG0000	5.80145	-1.46646	1.164711	-1.25908	0.208001	0.48932	NA	NA	NA
ENSG0000	1243.845	0.139499	0.110782	1.259226	0.207949	0.48932	UBL3	5412	ubiquitin like 3
ENSG0000	996.7021	0.193033	0.153332	1.258924	0.208058	0.489391	SNX8	29886	sorting nexin 8
ENSG0000	92.65342	-0.35781	0.28425	-1.25879	0.208107	0.489444	GPRIN1	114787	G protein regulated inducer of neurite outgrowth 1
ENSG0000	74.93268	0.440863	0.350288	1.258574	0.208184	0.489564	MUC1	4582	mucin 1 cell surface associated
ENSG0000	1099.197	0.125062	0.099377	1.25846	0.208225	0.489567	ABT1	29777	activator of basal transcription 1
ENSG0000	104.775	-0.30633	0.243441	-1.25835	0.208265	0.489567	ZNF93	81931	zinc finger protein 93
ENSG0000	14.31429	-0.91113	0.724193	-1.2584	0.208246	0.489567	TMEM191	728229	transmembrane protein 191B
ENSG0000	4920.783	-0.10244	0.081425	-1.25811	0.208353	0.48964	PCNX3	399909	pecanex 3
ENSG0000	376.411	-0.19898	0.158186	-1.25791	0.208425	0.48964	DCPS	28960	decapping scavenger
ENSG0000	58.00361	-0.4608	0.366324	-1.25791	0.208426	0.48964	IQSEC3	440073	IQ motif and Sec7 domain ArfGEF 3
ENSG0000	177.6211	-0.25551	0.203084	-1.25816	0.208335	0.48964	ZFYVE21	79038	zinc finger FYVE-type containing 21
ENSG0000	150.1568	0.257566	0.204759	1.2579	0.208428	0.48964	CMC2	56942	C-X9-C motif containing 2
ENSG0000	3.405361	-1.98343	1.577337	-1.25745	0.20859	0.489959	NA	NA	NA
ENSG0000	848.5254	-0.12713	0.101111	-1.25737	0.208621	0.48997	LONP2	83752	lon peptid peroxisomal
ENSG0000	48.79449	-0.44756	0.356085	-1.25688	0.208797	0.490322	NTHL1	4913	nth like DNA glycosylase 1
ENSG0000	15.79684	0.978749	0.778894	1.256589	0.208903	0.490507	LNCATV	1.02E+08	lncRNA negative regulator of antiviral signaling

ENSG0000	3.997422	1.884367	1.500112	1.256151	0.209061	0.490817	NA	NA	NA
ENSG0000	184.3608	-0.26039	0.207315	-1.25602	0.209109	0.490868	IL11RA	3590	interleukin 11 receptor subunit alpha
ENSG0000	11.16271	0.989079	0.787924	1.255298	0.209371	0.491245	MAP6D1	79929	MAP6 domain containing 1
ENSG0000	137.3483	-0.26356	0.209941	-1.25541	0.20933	0.491245	GASK1B	51313	golgi associated kinase 1B
ENSG0000	7.562033	1.152418	0.9179	1.255494	0.209299	0.491245	MIR7111	1.02E+08	microRNA 7111
ENSG0000	88.88797	0.354193	0.282178	1.255211	0.209402	0.491245	NA	NA	NA
ENSG0000	1040.011	0.138303	0.110181	1.255241	0.209391	0.491245	AARS1	16	alanyl-tRNA synthetase 1
ENSG0000	17.86492	0.769498	0.613205	1.25488	0.209522	0.491465	MIR181A2	1E+08	MIR181A2 host gene
ENSG0000	1972.832	0.113685	0.090614	1.254608	0.209621	0.491634	TAOK1	57551	TAO kinase 1
ENSG0000	1133.477	0.132637	0.10574	1.254369	0.209708	0.491776	FAM91A1	157769	family with sequence similarity 91 member A1
ENSG0000	1585.058	0.104726	0.083512	1.25402	0.209835	0.49201	MAGT1	84061	magnesium transporter 1
ENSG0000	3.059772	2.487366	1.983844	1.253811	0.209911	0.492127	NA	NA	NA
ENSG0000	749.2291	0.149397	0.119177	1.253567	0.21	0.492273	TBC1D2	55357	TBC1 domain family member 2
ENSG0000	22.20171	-0.69911	0.55783	-1.25326	0.210111	0.492471	BBS5	129880	Bardet-Biedl syndrome 5
ENSG0000	128.9432	0.268951	0.214634	1.253064	0.210182	0.492553	NCAPH	23397	non-SMC condensin I complex subunit H
ENSG0000	6.764449	1.452557	1.159246	1.253019	0.210199	0.492553	PCYT1B	9468	phosphate choline
ENSG0000	128.5479	-0.28858	0.230373	-1.25267	0.210324	0.492784	CETN2	1069	centrin 2
ENSG0000	72.10879	-0.37396	0.298582	-1.25246	0.210404	0.49291	PIGN	23556	phosphatidylinositol glycan anchor biosynthesis class N
ENSG0000	29.96083	0.536254	0.428243	1.252219	0.21049	0.493048	PFN2	5217	profilin 2
ENSG0000	3.858108	1.731615	1.383534	1.251588	0.21072	0.493525	NA	NA	NA
ENSG0000	150.7132	-0.25116	0.200727	-1.25123	0.210851	0.49377	DPYSL2	1808	dihydropyrimidinase like 2
ENSG0000	3.254303	-1.61925	1.294375	-1.25099	0.210938	0.493876	LINC0088(	730091	long intergenic non-protein coding RNA 886
ENSG0000	42.14025	0.523195	0.418235	1.250958	0.21095	0.493876	LINC02432	1.01E+08	long intergenic non-protein coding RNA 2432
ENSG0000	429.1029	0.196458	0.157145	1.250172	0.211237	0.494423	FRS2	10818	fibroblast growth factor receptor substrate 2
ENSG0000	735.0856	-0.1621	0.12966	-1.2502	0.211227	0.494423	RUVBL2	10856	RuvB like AAA ATPase 2
ENSG0000	46.82874	-0.4797	0.383764	-1.24998	0.211308	0.494527	IMPACT	55364	impact RWD domain protein
ENSG0000	20.00768	-0.85504	0.68416	-1.24977	0.211384	0.494643	IFITM10	402778	interferon induced transmembrane protein 10
ENSG0000	231.7067	-0.23033	0.184321	-1.24961	0.211441	0.494652	C15orf40	123207	chromosome 15 open reading frame 40
ENSG0000	989.686	-0.14507	0.116085	-1.24967	0.211419	0.494652	WDR13	64743	WD repeat domain 13
ENSG0000	146.8327	-0.24913	0.199438	-1.24914	0.211614	0.494833	RHOBTB3	22836	Rho related BTB domain containing 3
ENSG0000	9.576254	-1.21452	0.972304	-1.24911	0.211624	0.494833	NA	NA	NA
ENSG0000	273.0236	-0.20012	0.160212	-1.24911	0.211625	0.494833	RPP30	10556	ribonuclease P/MRP subunit p30
ENSG0000	692.1785	-0.14743	0.118027	-1.24912	0.211622	0.494833	GALNT1	2589	polypeptide N-acetylgalactosaminyltransferase 1
ENSG0000	29.21687	-0.62344	0.499191	-1.2489	0.211701	0.494948	APOE	348	apolipoprotein E
ENSG0000	563.8434	0.171303	0.137177	1.248771	0.211749	0.494997	CLUHP3	1E+08	clustered mitochondria homolog pseudogene 3
ENSG0000	143.8436	0.271946	0.217787	1.24868	0.211782	0.494997	ZSCAN12	9753	zinc finger and SCAN domain containing 12
ENSG0000	5.819659	1.381042	1.10605	1.248625	0.211802	0.494997	SIRPD	128646	signal regulatory protein delta
ENSG0000	31.45795	-0.5383	0.431256	-1.24821	0.211952	0.495161	NA	NA	NA
ENSG0000	4.744029	-1.85214	1.483791	-1.24825	0.211941	0.495161	LRRC17	10234	leucine rich repeat containing 17
ENSG0000	8527.925	-0.09419	0.075462	-1.24823	0.211948	0.495161	BAZ2A	11176	bromodomain adjacent to zinc finger domain 2A
ENSG0000	4.334751	-1.83297	1.469091	-1.24769	0.212145	0.495547	GSC	145258	goosecoid homeobox
ENSG0000	746.1194	-0.1451	0.116311	-1.24755	0.212197	0.495608	ID3	3399	inhibitor of DNA binding 3
ENSG0000	8118.922	0.107177	0.085933	1.24722	0.212317	0.495762	NCL	4691	nucleolin
ENSG0000	8.51746	-0.99353	0.796571	-1.24726	0.212301	0.495762	NA	NA	NA
ENSG0000	3794.907	0.106714	0.085581	1.246939	0.21242	0.495763	PRDM2	7799	PR/SET domain 2
ENSG0000	2886.537	-0.12027	0.096468	-1.24675	0.21249	0.495763	UBXN11	91544	UBX domain protein 11
ENSG0000	42.55011	0.514781	0.412855	1.246882	0.212441	0.495763	ABHD14A	25864	abhydrolase domain containing 14A
ENSG0000	1397.521	0.113638	0.091136	1.246903	0.212433	0.495763	PFKP	5214	phosphofr platelet
ENSG0000	1579.924	0.130091	0.104344	1.246752	0.212488	0.495763	SART3	9733	spliceoson U4/U6 recycling protein
ENSG0000	15.26875	0.820616	0.658226	1.246709	0.212504	0.495763	NA	NA	NA
ENSG0000	5.098865	1.542468	1.237193	1.246748	0.21249	0.495763	NA	NA	NA
ENSG0000	9.578817	-0.9429	0.75636	-1.24663	0.212532	0.495765	TRAV22	28661	T cell receptor alpha variable 22
ENSG0000	35.20176	0.499364	0.400705	1.246214	0.212686	0.496062	NDUFA6-D	1E+08	NDUFA6 divergent transcript
ENSG0000	106434.8	-0.10751	0.086288	-1.24593	0.212788	0.496238	FLNA	2316	filamin A
ENSG0000	20.81664	-0.65906	0.529018	-1.24582	0.212832	0.496278	LOC1004(	1E+08	nucleosome assembly protein 1 like 1 pseudogene
ENSG0000	1037.191	0.125868	0.101054	1.245551	0.212929	0.496442	FXR1	8087	FMR1 autosomal homolog 1
ENSG0000	575.1583	-0.15627	0.125483	-1.24535	0.213001	0.49646	TRIM52	84851	tripartite motif containing 52
ENSG0000	2455.436	0.10016	0.080422	1.245437	0.212971	0.49646	KTN1	3895	kinectin 1
ENSG0000	2.504139	-2.2061	1.771524	-1.24531	0.213017	0.49646	NA	NA	NA
ENSG0000	13.6749	-0.86644	0.695818	-1.24521	0.213053	0.496481	NA	NA	NA
ENSG0000	357.0116	0.193615	0.155505	1.245069	0.213107	0.496544	RPL9P8	254948	ribosomal protein L9 pseudogene 8
ENSG0000	336.8901	0.18744	0.150556	1.244982	0.213138	0.496556	ZNF324	25799	zinc finger protein 324
ENSG0000	11.6119	0.865226	0.69509	1.244769	0.213217	0.496675	RPL10P3	619445	ribosomal protein L10 pseudogene 3
ENSG0000	94.49783	-0.31748	0.25511	-1.24449	0.21332	0.496854	ZNF773	374928	zinc finger protein 773
ENSG0000	855.2941	-0.12834	0.103144	-1.24431	0.213386	0.496944	MTR	4548	5-methyltetrahydrofolate-homocysteine methyltransferase
ENSG0000	33.17598	-0.63175	0.507784	-1.24413	0.213452	0.497037	BBOF1	80127	basal body orientation factor 1
ENSG0000	9.692888	1.112167	0.894048	1.243969	0.213511	0.497112	LINC0288	1.05E+08	long intergenic non-protein coding RNA 2884
ENSG0000	4526.437	-0.11388	0.091567	-1.24365	0.213627	0.497284	CTBP1	1487	C-terminal binding protein 1
ENSG0000	23.00523	-0.61525	0.494726	-1.24362	0.213639	0.497284	WEE2-AS1	285962	WEE2 antisense RNA 1
ENSG0000	31.4609	0.513278	0.412761	1.243526	0.213674	0.497304	LOC12490	1.25E+08	uncharacterized LOC124901321
ENSG0000	3.095924	-1.97875	1.59209	-1.24287	0.213917	0.497784	SNX7	51375	sorting nexin 7
ENSG0000	33.04236	-0.53678	0.431909	-1.24282	0.213934	0.497784	MEAK7	57707	MTOR ass eak-7 homolog
ENSG0000	4874.769	0.094593	0.076118	1.242722	0.21397	0.497806	POM121	9883	POM121 transmembrane nucleoporin
ENSG0000	14.50618	-0.85991	0.692143	-1.24239	0.214091	0.498024	ARFGEF3	57221	ARFGEF family member 3
ENSG0000	8.654148	-1.40915	1.134448	-1.24215	0.214182	0.498173	TRPC2	7221	transient receptor potential cation channel subfamily C member 2 (pseudogene)
ENSG0000	2.282545	-2.49393	2.008099	-1.24194	0.214259	0.498291	NA	NA	NA
ENSG0000	4.118696	-1.56343	1.258957	-1.24184	0.214294	0.498309	DDX11L2	84771	DEAD/H-box helicase 11 like 2 (pseudogene)
ENSG0000	592.8589	-0.15623	0.125815	-1.24177	0.214321	0.49831	DYSF	8291	dysferlin
ENSG0000	54.80275	0.395751	0.318734	1.241635	0.214371	0.498364	NA	NA	NA
ENSG0000	4.941745	-1.45791	1.174291	-1.24153	0.214411	0.498393	SPTLC3	55304	serine palmitoyltransferase long chain base subunit 3
ENSG0000	276.6782	-0.2338	0.188346	-1.24135	0.214478	0.498486	DIAPH2	1730	diaphanous related formin 2
ENSG0000	180.4348	-0.23964	0.19311	-1.24095	0.214623	0.498516	THOC3	84321	THO complex subunit 3
ENSG0000	8.870445	0.979682	0.789463	1.240948	0.214625	0.498516	NA	NA	NA
ENSG0000	19.41739	0.773622	0.623355	1.241061	0.214583	0.498516	WWP1-AS	1.05E+08	WWP1 antisense RNA 1
ENSG0000	27.97419	0.664955	0.535824	1.240995	0.214608	0.498516	NA	NA	NA
ENSG0000	684.9184	0.133389	0.107489	1.240953	0.214623	0.498516	RIOX1	79697	ribosomal oxygenase 1
ENSG0000	4.514012	1.411514	1.137702	1.240671	0.214727	0.498691	NA	NA	NA
ENSG0000	90.74689	-0.3321	0.267751	-1.24034	0.214851	0.498916	DHCR24	1718	24-dehydrocholesterol reductase
ENSG0000	4.617872	1.685986	1.359413	1.24023	0.21489	0.498944	NA	NA	NA
ENSG0000	5.287039	1.638015	1.320941	1.240036	0.214962	0.499049	NA	NA	NA
ENSG0000	86.79601	0.345697	0.278817	1.239872	0.215023	0.499128	NA	NA	NA
ENSG0000	34.63924	-0.49473	0.399146	-1.23948	0.215168	0.499389	PON2	5445	paraoxonase 2
ENSG0000	101.8747	-0.32355	0.261077	-1.23928	0.215243	0.499389	CHMP4A	29082	charged multivesicular body protein 4A
ENSG0000	3.713177	-1.84653	1.489926	-1.23934	0.215218	0.499389	NA	NA	NA
ENSG0000	3.158787	-1.91401	1.544455	-1.23928	0.215242	0.499389	NA	NA	NA
ENSG0000	6.876975	1.237158	0.998365	1.239184	0.215277	0.499406	NA	NA	NA
ENSG0000	42.08182	-0.52703	0.425356	-1.23903	0.215336	0.499479	ZNF596	169270	zinc finger protein 596
ENSG0000	1588.636	0.156552	0.126374	1.238794	0.215422	0.499553	ATP5F1E	514	ATP synthase F1 subunit epsilon
ENSG0000	893.7446	0.126775	0.102336	1.238814	0.215414	0.499553	XIAP	331	X-linked inhibitor of apoptosis
ENSG0000	9480.44	0.093404	0.075404	1.238714	0.215451	0.49956	S100A10	6281	S100 calcium binding protein A10

ENSG0000	624.6504	-0.14468	0.116815	-1.2385	0.215529	0.499677	ICMT	23463	isoprenylcysteine carboxyl methyltransferase
ENSG0000	86.0931	-0.33585	0.271207	-1.23835	0.215587	0.49975	KIF16B	55614	kinesin family member 16B
ENSG0000	141.1981	0.279631	0.22585	1.238128	0.215669	0.499751	RPF2	84154	ribosome production factor 2 homolog
ENSG0000	2.44074	-2.11516	1.708319	-1.23815	0.215659	0.499751	LACTB2-AS	286190	LACTB2 antisense RNA 1
ENSG0000	1310.693	0.11863	0.095804	1.238253	0.215622	0.499751	CDC34	997	cell divisio ubiqiutin conjugating enzyme
ENSG0000	26.67138	-0.64783	0.523334	-1.23789	0.215756	0.499851	KIF24	347240	kinesin family member 24
ENSG0000	114.2463	-0.2821	0.227892	-1.23787	0.215766	0.499851	CEP152	22995	centrosomal protein 152
ENSG0000	1043.672	0.379569	0.306722	1.237502	0.215901	0.4999	ATF3	467	activating transcription factor 3
ENSG0000	70.76312	-0.39782	0.321484	-1.23745	0.215922	0.4999	ZNF124	7678	zinc finger protein 124
ENSG0000	2.311512	-2.29698	1.85586	-1.23769	0.215831	0.4999	TTC36	143941	tetratricopeptide repeat domain 36
ENSG0000	10.18915	0.958038	0.774189	1.237474	0.215911	0.4999	NA	NA	NA
ENSG0000	333.5843	0.194011	0.156759	1.237633	0.215852	0.4999	LSM12	124801	LSM12 homolog
ENSG0000	328.6826	-0.20963	0.169418	-1.23737	0.215949	0.499902	TARS2	80222	threonyl-t mitochondrial
ENSG0000	81.74685	-0.33996	0.274806	-1.23709	0.216054	0.50002	CNKSR1	10256	connector enhancer of kinase suppressor of Ras 1
ENSG0000	255.5551	0.219944	0.177785	1.237134	0.216037	0.50002	GOT1	2805	glutamic-oxaloacetic transaminase 1
ENSG0000	1651.685	-0.11489	0.092902	-1.23667	0.216209	0.500213	AHCYL1	10768	adenosylhomocysteinase like 1
ENSG0000	16.56911	-0.7627	0.616747	-1.23665	0.216219	0.500213	NA	NA	NA
ENSG0000	355.4791	0.184232	0.148976	1.236657	0.216214	0.500213	ASGR2	433	asialoglycoprotein receptor 2
ENSG0000	1259.544	0.123472	0.099852	1.236554	0.216253	0.500229	RETREG3	162427	reticulophagy regulator family member 3
ENSG0000	23.67995	-0.62868	0.508745	-1.23574	0.216556	0.500805	UFSP1	402682	UFM1 specific peptidase 1 (inactive)
ENSG0000	20.23277	-0.70785	0.572792	-1.23579	0.216538	0.500805	RTEL1	51750	regulator of telomere elongation helicase 1
ENSG0000	21.9685	-0.6958	0.563133	-1.23559	0.21661	0.500868	TRMT10A	93587	tRNA methyltransferase 10A
ENSG0000	1413.755	-0.13371	0.10823	-1.23543	0.216669	0.500943	RBM26	64062	RNA binding motif protein 26
ENSG0000	10.24653	0.851235	0.689158	1.235182	0.216763	0.501097	DDN-AS1	1.05E+08	DDN and PRKAG1 antisense RNA 1
ENSG0000	44.42276	-0.45043	0.364691	-1.2351	0.216792	0.501101	LYSMD1	388695	LysM domain containing 1
ENSG0000	18063.84	0.107899	0.087373	1.23493	0.216856	0.501188	GRK2	156	G protein-coupled receptor kinase 2
ENSG0000	57.06113	-0.41548	0.33658	-1.23442	0.217048	0.501505	NBPF20	1E+08	NBPF member 20
ENSG0000	766.9736	0.134553	0.108998	1.234453	0.217034	0.501505	HNRNPLL	92906	heterogeneous nuclear ribonucleoprotein L like
ENSG0000	1573.266	0.109777	0.088944	1.234232	0.217117	0.501579	PTDSS1	9791	phosphatidylserine synthase 1
ENSG0000	747.3899	0.130308	0.105582	1.234186	0.217133	0.501579	PLD2	5338	phospholipase D2
ENSG0000	27.348	-0.5649	0.457848	-1.23381	0.217273	0.501775	RGS13	6003	regulator of G protein signaling 13
ENSG0000	245.6924	0.221405	0.17944	1.233872	0.217251	0.501775	SNRPF	6636	small nuclear ribonucleoprotein polypeptide F
ENSG0000	9.500085	0.935506	0.758293	1.2337	0.217315	0.50181	NA	NA	NA
ENSG0000	57.26435	-0.43693	0.35419	-1.23361	0.217349	0.501827	CCDC112	153733	coiled-coil domain containing 112
ENSG0000	114.3175	-0.27767	0.225107	-1.2335	0.217388	0.501852	C14orf93	60686	chromosome 14 open reading frame 93
ENSG0000	719.4117	0.1591	0.12899	1.233433	0.217414	0.501852	ZNF274	10782	zinc finger protein 274
ENSG0000	626.8524	0.150053	0.12172	1.232772	0.217661	0.502334	ZNF589	51385	zinc finger protein 589
ENSG0000	5991.054	0.093026	0.075466	1.232693	0.21769	0.502334	NFATC2	4773	nuclear factor of activated T cells 2
ENSG0000	2097.391	0.132455	0.107455	1.232655	0.217704	0.502334	COL6A2	1292	collagen type VI alpha 2 chain
ENSG0000	51.62398	-0.44768	0.363231	-1.23248	0.217769	0.502422	CENPK	64105	centromere protein K
ENSG0000	102.6953	-0.33558	0.272329	-1.23226	0.217851	0.502485	NA	NA	NA
ENSG0000	1832.014	0.103247	0.083785	1.23228	0.217845	0.502485	SUPT16H	11198	SPT16 hon facilitates chromatin remodeling subunit
ENSG0000	16.70495	-0.7019	0.569686	-1.23209	0.217917	0.502575	NA	NA	NA
ENSG0000	1065.144	0.126398	0.102607	1.231861	0.218001	0.502706	HNRNPH2	3188	heterogeneous nuclear ribonucleoprotein H2
ENSG0000	1897.284	-0.10299	0.083607	-1.23178	0.21803	0.502711	KDM3B	51780	lysine demethylase 3B
ENSG0000	53.74039	0.387651	0.314945	1.230853	0.218378	0.50345	CNBD2	140894	cyclic nucleotide binding domain containing 2
ENSG0000	17.14838	0.668268	0.542977	1.230748	0.218417	0.503478	RPSAP15	220885	ribosomal protein SA pseudogene 15
ENSG0000	3.081694	2.010482	1.633968	1.23043	0.218536	0.503627	WNK3	65267	WNK lysine deficient protein kinase 3
ENSG0000	612.2987	-0.16157	0.131309	-1.23047	0.21852	0.503627	HTATSF1	27336	HIV-1 Tat specific factor 1
ENSG0000	538.8196	-0.15375	0.124982	-1.23021	0.218618	0.503753	FRAT1	10023	FRAT regulator of WNT signaling pathway 1
ENSG0000	319.2509	-0.1956	0.159055	-1.2298	0.218774	0.504049	CERS6	253782	ceramide synthase 6
ENSG0000	6.608982	-1.28319	1.043625	-1.22955	0.218866	0.504198	NA	NA	NA
ENSG0000	4.710638	-1.46709	1.193369	-1.22937	0.218935	0.504294	NA	NA	NA
ENSG0000	11.0008	0.97432	0.792859	1.228869	0.219121	0.504646	SCN3A	6328	sodium voltage-gated channel alpha subunit 3
ENSG0000	584.5636	0.153402	0.124837	1.228814	0.219142	0.504646	EIF2B2	8892	eukaryotic translation initiation factor 2B subunit beta
ENSG0000	16.82759	0.752891	0.612745	1.228718	0.219178	0.504666	LOC10099	1.01E+08	uncharacterized LOC100996583
ENSG0000	8508.163	0.088604	0.072134	1.22832	0.219327	0.504947	AP2M1	1173	adaptor related protein complex 2 subunit mu 1
ENSG0000	302.199	-0.21021	0.171146	-1.22824	0.219356	0.504951	PCBP4	57060	poly(rC) binding protein 4
ENSG0000	3.969484	1.446636	1.178288	1.227744	0.219543	0.505319	COPB2-DT	1.01E+08	COPB2 divergent transcript
ENSG0000	380.6136	0.176003	0.143363	1.227671	0.21957	0.50532	RBBP8	5932	RB binding endonuclease
ENSG0000	1537.486	-0.14133	0.115162	-1.22723	0.219736	0.505574	PAXX	286257	PAXX non-homologous end joining factor
ENSG0000	607.439	-0.14858	0.121066	-1.22725	0.219728	0.505574	METTL3	56339	methyltrai N6-adenosine-methyltransferase complex catalytic subunit
ENSG0000	73.86637	0.390109	0.317936	1.227005	0.219821	0.505708	SHF	90525	Src homology 2 domain containing F
ENSG0000	515.8968	-0.16913	0.137865	-1.22678	0.219904	0.505836	ANGEL1	23357	angel homolog 1
ENSG0000	58.65854	-0.43127	0.351609	-1.22657	0.219983	0.505956	TYMS	7298	thymidylate synthetase
ENSG0000	22.75006	0.660971	0.538929	1.226452	0.220029	0.505998	DBNDD1	79007	dysbindin domain containing 1
ENSG0000	353.737	-0.17271	0.140849	-1.22622	0.220114	0.506077	MPC1	51660	mitochondrial pyruvate carrier 1
ENSG0000	30.72092	0.544187	0.443794	1.226215	0.220118	0.506077	PLD6	201164	phospholipase D family member 6
ENSG0000	18.22402	0.655351	0.534599	1.225874	0.220246	0.50631	HPS1-AS1	1.02E+08	HPS1 antisense RNA 1
ENSG0000	30.43424	-0.61757	0.503986	-1.22536	0.220438	0.506689	COL6A3	1293	collagen type VI alpha 3 chain
ENSG0000	51.5836	-0.40331	0.329164	-1.22525	0.220483	0.506728	LARGE1	9215	LARGE xylosyl- and glucuronyltransferase 1
ENSG0000	298.6412	-0.56232	0.459033	-1.22502	0.220569	0.506752	CMPK2	129607	cytidine/uridine monophosphate kinase 2
ENSG0000	496.1828	-0.1499	0.12236	-1.2251	0.220539	0.506752	INPP1	3628	inositol polyphosphate-1-phosphatase
ENSG0000	7.756719	-1.16665	0.952367	-1.225	0.220575	0.506752	NA	NA	NA
ENSG0000	31.26399	0.630368	0.514761	1.224584	0.220732	0.506798	CPLX1	10815	complexin 1
ENSG0000	4015.594	0.097194	0.079365	1.22464	0.220711	0.506798	PPP1R11	6992	protein phosphatase 1 regulatory inhibitor subunit 11
ENSG0000	21.30363	0.686831	0.560863	1.224596	0.220727	0.506798	NA	NA	NA
ENSG0000	14.29694	0.853645	0.69699	1.224759	0.220666	0.506798	MROH8	140699	maestro heat like repeat family member 8
ENSG0000	13.58903	-0.85225	0.695851	-1.22476	0.220667	0.506798	KCNE5	23630	potassium voltage-gated channel subfamily E regulatory subunit 5
ENSG0000	22.31858	-0.61299	0.500659	-1.22436	0.220816	0.506929	USP43	124739	ubiquitin specific peptidase 43
ENSG0000	4540.658	0.094725	0.077384	1.224085	0.22092	0.507072	OTUB1	55611	OTU deubi ubiquitin aldehyde binding 1
ENSG0000	24.8024	-0.62698	0.512217	-1.22405	0.220933	0.507072	FBXL19-AS	283932	FBXL19 antisense RNA 1
ENSG0000	145.6783	-0.28726	0.234704	-1.22392	0.220983	0.507104	DYRK4	8798	dual specificity tyrosine phosphorylation regulated kinase 4
ENSG0000	1414.173	-0.12492	0.102071	-1.22387	0.221002	0.507104	ARSA	410	arylsulfatase A
ENSG0000	8.754462	-0.99396	0.812293	-1.22364	0.221086	0.507235	TUSC1	286319	tumor suppressor candidate 1
ENSG0000	54.60771	-0.40099	0.327757	-1.22343	0.221168	0.507314	MAN1B1-I	1E+08	MAN1B1 divergent transcript
ENSG0000	412.504	0.18118	0.148112	1.223266	0.221229	0.507314	STARD5	80765	StAR related lipid transfer domain containing 5
ENSG0000	38.88044	0.481454	0.393562	1.223322	0.221208	0.507314	NA	NA	NA
ENSG0000	32.48285	0.517751	0.423253	1.223264	0.22123	0.507314	ZNF682	91120	zinc finger protein 682
ENSG0000	43.87648	0.491425	0.401805	1.223043	0.221314	0.507443	CHIT1	1118	chitinase 1
ENSG0000	232.8705	-0.20726	0.169513	-1.2227	0.221444	0.507616	ASTE1	28990	asteroid homolog 1
ENSG0000	3.790077	1.467298	1.20004	1.222707	0.22144	0.507616	YAP1	10413	Yes1 associated transcriptional regulator
ENSG0000	37.95661	-0.49111	0.40202	-1.22161	0.221856	0.508184	NA	NA	NA
ENSG0000	688.9947	-0.17849	0.146091	-1.22174	0.221805	0.508184	ADAP1	11033	ArfGAP with dual PH domains 1
ENSG0000	623.5929	0.14079	0.115222	1.221895	0.221747	0.508184	STOML2	30968	stomatin like 2
ENSG0000	8.156808	-1.00524	0.822686	-1.2219	0.221746	0.508184	NA	NA	NA
ENSG0000	9720.443	0.1047	0.085693	1.221801	0.221783	0.508184	HUWE1	10075	HECT UBA and WWE domain containing E3 ubiquitin protein ligase 1
ENSG0000	150.1967	-0.27534	0.225386	-1.22165	0.221842	0.508184	ZFP92	139735	ZFP92 zinc finger protein
ENSG0000	512.6713	-0.1467	0.120103	-1.22143	0.221923	0.508274	SS18L1	26039	SS18L1 subunit of BAF chromatin remodeling complex
ENSG0000	150.5771	-0.29253	0.239554	-1.22115	0.22203	0.508457	TYW3	127253	tRNA-yW synthesizing protein 3 homolog

ENSG0000	21.63712	0.731928	0.599423	1.221054	0.222066	0.508476	IGHV1-46	28465	immunoglobulin heavy variable 1-46
ENSG0000	79.0312	-0.34399	0.281809	-1.22066	0.222213	0.508625	NA	NA	NA
ENSG0000	8.874638	1.110725	0.909896	1.220717	0.222193	0.508625	AGAP2-AS	1E+08	AGAP2 antisense RNA 1
ENSG0000	15.42501	-0.74806	0.612826	-1.22068	0.222207	0.508625	C17orf58	284018	chromosome 17 open reading frame 58
ENSG0000	794.16	0.146066	0.119695	1.220318	0.222344	0.508862	WDR18	57418	WD repeat domain 18
ENSG0000	333.8901	-0.1848	0.15145	-1.22019	0.222394	0.508913	ZBTB14	7541	zinc finger and BTB domain containing 14
ENSG0000	2.791135	-2.14707	1.760082	-1.21987	0.222514	0.509124	NA	NA	NA
ENSG0000	17201.07	0.092847	0.076143	1.219379	0.2227	0.509426	PIK3R5	23533	phosphoinositide-3-kinase regulatory subunit 5
ENSG0000	1176.411	0.126049	0.10337	1.2194	0.222692	0.509426	TPST2	8459	tyrosylprotein sulfotransferase 2
ENSG0000	108.459	0.307015	0.251874	1.218921	0.222874	0.509761	CDKN2AIP	91368	CDKN2A interacting protein N-terminal like
ENSG0000	5.596462	1.240091	1.017445	1.218829	0.222909	0.509778	NA	NA	NA
ENSG0000	4.023724	-1.52821	1.254092	-1.21858	0.223003	0.50993	PPM1K-DT	1.05E+08	PPM1K divergent transcript
ENSG0000	28.56146	0.533539	0.438006	1.218111	0.223182	0.510024	NA	NA	NA
ENSG0000	1289.572	-0.11725	0.096247	-1.21823	0.223136	0.510024	FAM120AO	158293	family with sequence similarity 120A opposite strand
ENSG0000	723.9461	-0.14188	0.116454	-1.21834	0.223093	0.510024	XPNPEP1	7511	X-prolyl aminopeptidase 1
ENSG0000	9.589077	1.11249	0.913171	1.218272	0.223121	0.510024	SOAT2	8435	sterol O-acyltransferase 2
ENSG0000	571.9386	-0.17312	0.142116	-1.21817	0.22316	0.510024	RELCH	57614	RAB11 bin coiled-coil and HEAT repeat containing
ENSG0000	3.293092	1.937142	1.590541	1.217914	0.223257	0.510132	NA	NA	NA
ENSG0000	279.7497	-0.19387	0.159207	-1.2177	0.223338	0.510255	EBNA1BP2	10969	EBNA1 binding protein 2
ENSG0000	171.0529	-0.25391	0.208528	-1.21761	0.223372	0.510261	CTNNBIP1	56998	catenin beta interacting protein 1
ENSG0000	29.43378	-0.77117	0.633379	-1.21755	0.223396	0.510261	S100A16	140576	S100 calcium binding protein A16
ENSG0000	74.03619	-0.3691	0.303236	-1.21721	0.223526	0.510433	FNTB	2342	farnesyltra CAAX box beta
ENSG0000	18.99822	-0.63683	0.523161	-1.21727	0.223501	0.510433	NA	NA	NA
ENSG0000	5.135834	-1.66703	1.370073	-1.21675	0.223701	0.510769	NA	NA	NA
ENSG0000	4.397638	-1.73758	1.429136	-1.21583	0.224051	0.511171	AQP1	358	aquaporin 1 (Colton blood group)
ENSG0000	1421.142	-0.13408	0.110289	-1.21572	0.224092	0.511171	NCOA2	10499	nuclear receptor coactivator 2
ENSG0000	1532.589	0.103692	0.085267	1.216076	0.223956	0.511171	PITRM1	10531	pitrilysin metallopeptidase 1
ENSG0000	134.2311	0.256216	0.210755	1.215706	0.224097	0.511171	RPS17	6218	ribosomal protein S17
ENSG0000	13.10503	-0.80341	0.660798	-1.21581	0.224057	0.511171	NA	NA	NA
ENSG0000	3187.37	0.097878	0.080498	1.215913	0.224018	0.511171	NFATC1	4772	nuclear factor of activated T cells 1
ENSG0000	5.692893	1.609254	1.323627	1.215791	0.224065	0.511171	IGLV4-60	28785	immunoglobulin lambda variable 4-60
ENSG0000	698.2244	0.142473	0.117172	1.215924	0.224014	0.511171	PGRMC1	10857	progesterone receptor membrane component 1
ENSG0000	4154.401	0.097405	0.080145	1.21536	0.224229	0.511346	MAML1	9794	mastermind like transcriptional coactivator 1
ENSG0000	725.39	-0.14307	0.11772	-1.21537	0.224226	0.511346	ARMC7	79637	armadillo repeat containing 7
ENSG0000	303.205	-0.25007	0.205808	-1.21505	0.224346	0.51155	ATG7	10533	autophagy related 7
ENSG0000	1490.323	-0.15775	0.129863	-1.21474	0.224464	0.511757	DMXL2	23312	Dmx like 2
ENSG0000	17.09523	-0.76914	0.633259	-1.21458	0.224526	0.51177	TLR3	7098	toll like receptor 3
ENSG0000	3.600353	1.664992	1.370813	1.214602	0.224518	0.51177	NA	NA	NA
ENSG0000	259.2085	-0.26139	0.215272	-1.21422	0.224662	0.512018	FCHO2	115548	FCH and mu domain containing endocytic adaptor 2
ENSG0000	36005.94	0.091642	0.075491	1.213945	0.224769	0.512199	RPL3	6122	ribosomal protein L3
ENSG0000	334.0603	0.185206	0.15259	1.213749	0.224844	0.512306	DPP4	1803	dipeptidyl peptidase 4
ENSG0000	15.17076	-0.8396	0.691876	-1.21351	0.224933	0.512447	LOC10798	1.08E+08	uncharacterized LOC107985216
ENSG0000	4.4288	-1.41403	1.165724	-1.21301	0.225128	0.512828	DLGAP5	9787	DLG associated protein 5
ENSG0000	120.2277	-0.31128	0.256646	-1.21287	0.225178	0.512836	MCCC1	56922	methylcrotonyl-CoA carboxylase subunit 1
ENSG0000	12.42856	-0.82668	0.681598	-1.21285	0.225187	0.512836	NPFF	8620	neuropeptide FF-amide peptide precursor
ENSG0000	20.60816	0.630375	0.519852	1.212606	0.225281	0.512987	NA	NA	NA
ENSG0000	250.2082	-0.19374	0.159804	-1.21234	0.225384	0.513096	GOPC	57120	golgi associated PDZ and coiled-coil motif containing
ENSG0000	7403.615	-0.08728	0.07199	-1.21236	0.225375	0.513096	LDB1	8861	LIM domain binding 1
ENSG0000	13.0225	-0.94446	0.779131	-1.2122	0.225435	0.51315	SMKR1	1E+08	small lysine rich protein 1
ENSG0000	27.17708	-0.53375	0.440395	-1.21198	0.225521	0.513281	ZNF772	400720	zinc finger protein 772
ENSG0000	21.01413	-0.6364	0.525201	-1.21173	0.225616	0.513435	NA	NA	NA
ENSG0000	336.0436	-0.18855	0.155661	-1.21129	0.225783	0.513753	WDR36	134430	WD repeat domain 36
ENSG0000	360.5893	0.184442	0.152283	1.211186	0.225824	0.513783	PRPS2	5634	phosphoribosyl pyrophosphate synthetase 2
ENSG0000	2.765883	-2.16517	1.787822	-1.21107	0.22587	0.513824	RN7SKP16	1.06E+08	RN7SK pseudogene 160
ENSG0000	25.86071	-0.59442	0.490937	-1.21078	0.22598	0.514011	TESMIN	9633	testis expressed metallothionein like protein
ENSG0000	2.002061	2.404814	1.986442	1.210614	0.226043	0.514093	NA	NA	NA
ENSG0000	10.55276	0.930419	0.769141	1.209686	0.226399	0.514342	NA	NA	NA
ENSG0000	5.733284	-1.52258	1.258647	-1.2097	0.226395	0.514342	NA	NA	NA
ENSG0000	5.41693	-1.23957	1.02471	-1.20968	0.226403	0.514342	SEC63P1	1E+08	SEC63 hon protein translocation regulator pseudogene 1
ENSG0000	20.83952	-0.74813	0.6183	-1.20997	0.226289	0.514342	ABCD2	225	ATP binding cassette subfamily D member 2
ENSG0000	22.43351	0.635174	0.524929	1.210018	0.226272	0.514342	DLEU1	10301	deleted in lymphocytic leukemia 1
ENSG0000	2664.136	0.132065	0.109157	1.209861	0.226332	0.514342	VEZF1	7716	vascular endothelial zinc finger 1
ENSG0000	137.2636	-0.27414	0.22653	-1.21016	0.226218	0.514342	ZNF426	79088	zinc finger protein 426
ENSG0000	4.658343	1.574761	1.301765	1.209712	0.226389	0.514342	HSD17B14	51171	hydroxysteroid 17-beta dehydrogenase 14
ENSG0000	844.3736	-0.13629	0.112648	-1.20984	0.226342	0.514342	INTS6L	203522	integrator complex subunit 6 like
ENSG0000	13.14344	-0.86268	0.713188	-1.2096	0.226431	0.514343	LOC10537	1.05E+08	uncharacterized LOC105371430
ENSG0000	486.9128	0.15902	0.131553	1.208789	0.226744	0.514752	NDUFB2	4708	NADH:ubiquinone oxidoreductase subunit B2
ENSG0000	142.2218	0.249148	0.206129	1.208702	0.226777	0.514752	TIMM8B	26521	translocase of inner mitochondrial membrane 8 homolog B
ENSG0000	7.230188	-1.32902	1.099519	-1.20873	0.226768	0.514752	CDH13-AS	1.03E+08	CDH13 antisense RNA 2
ENSG0000	5.798509	1.321962	1.093529	1.208895	0.226703	0.514752	PALM	5064	paralemmin
ENSG0000	36.47419	-0.53577	0.443173	-1.20894	0.226686	0.514752	TNNT1	7138	troponin T slow skeletal type
ENSG0000	5.482246	1.365644	1.129691	1.208866	0.226714	0.514752	TSPEAR-AS	54082	TSPEAR antisense RNA 1
ENSG0000	12.12571	0.806907	0.667707	1.208474	0.226865	0.514888	RPL3P2	116935	ribosomal protein L3 pseudogene 2
ENSG0000	21.25022	-0.66659	0.551672	-1.20831	0.226926	0.514964	TRAV12-1	28674	T cell receptor alpha variable 12-1
ENSG0000	232.9416	0.255876	0.211777	1.208234	0.226957	0.514971	GTPBP8	29083	GTP binding protein 8 (putative)
ENSG0000	16848.2	0.115953	0.09598	1.208093	0.227012	0.515032	CYBA	1535	cytochrome b-245 alpha chain
ENSG0000	5.471156	1.273107	1.053929	1.207962	0.227062	0.515083	RPS2P54	1E+08	ribosomal protein S2 pseudogene 54
ENSG0000	39.81025	-0.59639	0.493799	-1.20776	0.22714	0.515198	HASPIN	83903	histone H3 associated protein kinase
ENSG0000	3.210487	-2.02446	1.676422	-1.20761	0.227199	0.515204	NA	NA	NA
ENSG0000	51.68158	-0.39093	0.323714	-1.20764	0.227186	0.515204	FAM111A-	1.02E+08	FAM111A divergent transcript
ENSG0000	6.299741	1.475237	1.221839	1.20739	0.227282	0.515267	GUCY1B2	2974	guanylate cyclase 1 soluble subunit beta 2 (pseudogene)
ENSG0000	74.95521	0.328499	0.272071	1.2074	0.227278	0.515267	SLC30A4	7782	solute carrier family 30 member 4
ENSG0000	108.5503	0.28664	0.237424	1.207291	0.22732	0.51529	CCT6P1	643253	chaperonin containing TCP1 subunit 6 pseudogene 1
ENSG0000	101.7412	-0.31493	0.260911	-1.20702	0.227424	0.515463	CHROMR	1.02E+08	cholesterol induced regulator of metabolism RNA
ENSG0000	457.9777	-0.16293	0.135014	-1.20676	0.227524	0.515626	NSF	4905	N-ethylma vesicle fusing ATPase
ENSG0000	1015.368	0.17556	0.145505	1.206551	0.227605	0.51569	ARHGEF1C	55160	Rho guanine nucleotide exchange factor 10 like
ENSG0000	1685.893	-0.1166	0.096645	-1.20647	0.227635	0.51569	SUPT20H	55578	SPT20 hon SAGA complex component
ENSG0000	2.874577	-1.92959	1.599325	-1.20651	0.227623	0.51569	NA	NA	NA
ENSG0000	22.00522	-0.62707	0.519856	-1.20625	0.227723	0.515825	ZDHHC1	29800	zinc finger DHHC-type containing 1
ENSG0000	27.1346	-0.55494	0.460154	-1.20598	0.227825	0.515993	NA	NA	NA
ENSG0000	4064.239	0.102686	0.085172	1.205632	0.227959	0.516235	OXA1L	5018	OXA1L mitochondrial inner membrane protein
ENSG0000	22.26547	0.635529	0.527181	1.205523	0.228001	0.516267	HLA-G	3135	major hist class I G
ENSG0000	63.61835	-0.39579	0.328403	-1.2052	0.228124	0.516482	TMEM186	25880	transmembrane protein 186
ENSG0000	12.93051	0.842776	0.699522	1.204789	0.228285	0.516782	LOC12490	1.25E+08	uncharacterized LOC124902114
ENSG0000	10070.37	-0.08477	0.070401	-1.20406	0.228567	0.516853	SKI	6497	SKI proto-oncogene
ENSG0000	47.32145	-0.41516	0.34476	-1.2042	0.228511	0.516853	ARV1	64801	ARV1 hom fatty acid homeostasis modulator
ENSG0000	1158.594	-0.12707	0.105524	-1.20419	0.228516	0.516853	ZXDC	79364	ZXD family zinc finger C
ENSG0000	4.352808	1.586692	1.317589	1.204238	0.228497	0.516853	C9orf43	257169	chromosome 9 open reading frame 43
ENSG0000	30.73689	0.640588	0.53197	1.204181	0.22852	0.516853	NA	NA	NA
ENSG0000	2990.739	-0.1047	0.086938	-1.20428	0.228483	0.516853	RGCC	28984	regulator of cell cycle

ENSG0000	1427.467	0.133141	0.110543	1.204436	0.228421	0.516853	CHP1	11261	calcineurin like EF-hand protein 1
ENSG0000	224.7146	0.204725	0.170025	1.204094	0.228553	0.516853	LTK	4058	leukocyte receptor tyrosine kinase
ENSG0000	11.12301	0.999672	0.830227	1.204095	0.228553	0.516853	TEAD2	8463	TEA domain transcription factor 2
ENSG0000	510.6455	-0.18328	0.152306	-1.20337	0.228835	0.517396	SLC45A4	57210	solute carrier family 45 member 4
ENSG0000	960.37	0.12873	0.106983	1.203274	0.22887	0.517413	P2RX5	5026	purinergic receptor P2X 5
ENSG0000	161.6172	-0.24227	0.201425	-1.20277	0.229066	0.517792	WRN	7486	WRN RecQ like helicase
ENSG0000	936.584	0.131712	0.109546	1.202351	0.229228	0.518032	BAG1	573	BAG cochaperone 1
ENSG0000	71.4726	-0.41655	0.34644	-1.20237	0.22922	0.518032	CATSPER1	117144	cation channel sperm associated 1
ENSG0000	222.9732	0.210681	0.175265	1.20207	0.229336	0.518151	GPD1L	23171	glycerol-3-phosphate dehydrogenase 1 like
ENSG0000	120.0619	-0.2917	0.242648	-1.20213	0.229312	0.518151	CAMP	820	cathelicidin antimicrobial peptide
ENSG0000	1102.705	0.136145	0.11329	1.201736	0.229466	0.51838	NDE1	54820	nudE neurodevelopment protein 1
ENSG0000	63.70608	-0.38515	0.320524	-1.20162	0.229509	0.518415	TRBV15	28572	T cell receptor beta variable 15
ENSG0000	223.5426	-0.24678	0.205391	-1.20154	0.229543	0.518428	ABRAXAS1	84142	abraxas 1 BRCA1 A complex subunit
ENSG0000	274.7485	-0.19359	0.161136	-1.20142	0.229587	0.518464	WDR19	57728	WD repeat domain 19
ENSG0000	7250.344	-0.1138	0.094734	-1.20129	0.229637	0.51849	ARID1A	8289	AT-rich interaction domain 1A
ENSG0000	1082.571	-0.13615	0.11335	-1.20118	0.229682	0.51849	CHSY1	22856	chondroitin sulfate synthase 1
ENSG0000	1815.857	-0.1112	0.092575	-1.20124	0.229657	0.51849	PNKP	11284	polynucleotide kinase 3'-phosphatase
ENSG0000	20.70625	0.704969	0.586951	1.20107	0.229724	0.518522	CH25H	9023	cholesterol 25-hydroxylase
ENSG0000	2296.198	0.110756	0.092226	1.200915	0.229784	0.518531	AMPD2	271	adenosine monophosphate deaminase 2
ENSG0000	23.15067	-0.59284	0.493647	-1.20095	0.229772	0.518531	RUSC1-AS:	284618	RUSC1 antisense RNA 1
ENSG0000	442.6644	0.155996	0.129914	1.200764	0.229843	0.518537	FBXO32	114907	F-box protein 32
ENSG0000	2.354283	2.213634	1.843432	1.200822	0.22982	0.518537	NA	NA	NA
ENSG0000	4.833329	1.644808	1.370126	1.20048	0.229953	0.51866	ESYT3	83850	extended synaptotagmin 3
ENSG0000	258.5669	0.21406	0.178308	1.200505	0.229943	0.51866	CWF19L2	143884	CWF19 like cell cycle control factor 2
ENSG0000	5.791058	-1.23545	1.029319	-1.20026	0.230039	0.518728	C1orf220	400798	chromosome 1 putative open reading frame 220
ENSG0000	20.63921	-0.75263	0.627043	-1.20029	0.230028	0.518728	AKAP6	9472	A-kinase anchoring protein 6
ENSG0000	5.526404	-1.37296	1.143987	-1.20015	0.230081	0.518759	IBA57-DT	574432	IBA57 divergent transcript
ENSG0000	458.4191	-0.16723	0.139352	-1.20002	0.230131	0.518809	SOCS7	30837	suppressor of cytokine signaling 7
ENSG0000	753.5179	0.140334	0.116956	1.199891	0.230182	0.51886	HMGXB4	10042	HMG-box containing 4
ENSG0000	7.690047	-1.27431	1.062372	-1.1995	0.230334	0.519029	NA	NA	NA
ENSG0000	233.4161	-0.19916	0.166046	-1.19941	0.230367	0.519029	ST7	7982	suppression of tumorigenicity 7
ENSG0000	302.3054	0.187443	0.156288	1.199338	0.230396	0.519029	EXOSC1	51013	exosome component 1
ENSG0000	154.1551	-0.24699	0.205895	-1.19961	0.23029	0.519029	ALKBH3	221120	alkB homc alpha-ketoglutarate dependent dioxygenase
ENSG0000	522.0803	-0.146	0.121728	-1.19937	0.230386	0.519029	URB1	9875	URB1 ribosome biogenesis homolog
ENSG0000	16.77828	-0.79834	0.665725	-1.19921	0.230447	0.51908	NA	NA	NA
ENSG0000	254.6342	-0.20413	0.17024	-1.19906	0.230503	0.51908	ABITRAM	54942	actin binding transcription modulator
ENSG0000	114.6985	-0.30531	0.25462	-1.19907	0.230502	0.51908	NOMO3	408050	NODAL modulator 3
ENSG0000	32.44827	-0.54997	0.458722	-1.19891	0.230561	0.519148	SPATA5	166378	spermatogenesis associated 5
ENSG0000	592.8774	-0.14567	0.121525	-1.19871	0.230642	0.519266	ZFAT	57623	zinc finger and AT-hook domain containing
ENSG0000	218.9592	-0.20314	0.169515	-1.19834	0.230786	0.519464	POC5	134359	POC5 centriolar protein
ENSG0000	3.897481	1.645417	1.373002	1.198408	0.230758	0.519464	IGFBP6	3489	insulin like growth factor binding protein 6
ENSG0000	3.716302	-2.15287	1.797005	-1.19803	0.230905	0.519465	NTNG1	22854	netrin G1
ENSG0000	197.7042	-0.28655	0.239163	-1.19813	0.230867	0.519465	RNASEL	6041	ribonuclease L
ENSG0000	154.1676	-0.24373	0.203442	-1.19804	0.230901	0.519465	NA	NA	NA
ENSG0000	8.641133	-0.92617	0.773111	-1.19798	0.230926	0.519465	BAAT	570	bile acid-CoA:amino acid N-acyltransferase
ENSG0000	54.35597	-0.42951	0.358459	-1.19821	0.230835	0.519465	VSIG10L	147645	V-set and immunoglobulin domain containing 10 like
ENSG0000	1007.577	-0.12252	0.102287	-1.19779	0.231	0.519504	PINK1	65018	PTEN induced kinase 1
ENSG0000	124.1565	-0.34242	0.285874	-1.1978	0.230995	0.519504	ZSCAN21	7589	zinc finger and SCAN domain containing 21
ENSG0000	26.28461	0.702847	0.58685	1.197662	0.231049	0.519551	NA	NA	NA
ENSG0000	100.0356	0.339981	0.283964	1.197268	0.231202	0.519833	TMEM30B	161291	transmembrane protein 30B
ENSG0000	6191.216	0.088735	0.074152	1.196653	0.231442	0.520246	FNBP4	23360	formin binding protein 4
ENSG0000	505.4658	-0.17546	0.146617	-1.19669	0.231427	0.520246	GAS2L1	10634	growth arrest specific 2 like 1
ENSG0000	190.216	-0.2113	0.176619	-1.19633	0.231566	0.520282	BMP8A	353500	bone morphogenetic protein 8a
ENSG0000	38870.14	0.099555	0.083209	1.196441	0.231525	0.520282	HMGA1	3159	high mobility group AT-hook 1
ENSG0000	13.36096	-0.91462	0.764552	-1.19629	0.231585	0.520282	TRBV12-3	28577	T cell receptor beta variable 12-3
ENSG0000	5502.273	-0.09509	0.079477	-1.19643	0.231528	0.520282	SPN	6693	sialophorin
ENSG0000	2.338455	-2.37369	1.984269	-1.19625	0.231598	0.520282	RN7SL146I	1.06E+08	RNA 7SL cytoplasm pseudogene
ENSG0000	4.696138	1.405346	1.174992	1.196048	0.231678	0.520399	NA	NA	NA
ENSG0000	864.178	0.141271	0.118153	1.195662	0.231828	0.520673	RSBN1	54665	round spermatid basic protein 1
ENSG0000	135.9261	-0.26048	0.217872	-1.19557	0.231865	0.520685	CENPO	79172	centromere protein O
ENSG0000	38.47095	0.446745	0.37371	1.195433	0.231918	0.520685	PTPN13	5783	protein tyrosine phosphatase non-receptor type 13
ENSG0000	645.5837	0.152902	0.127904	1.195441	0.231915	0.520685	NOL7	51406	nucleolar protein 7
ENSG0000	17.15691	0.88817	0.743142	1.195155	0.232027	0.520861	NA	NA	NA
ENSG0000	126.738	-0.30303	0.253562	-1.19509	0.232052	0.520861	FSD1	79187	fibronectin type III and SPRY domain containing 1
ENSG0000	1162.603	0.136907	0.114584	1.19482	0.232158	0.520971	UXS1	80146	UDP-glucuronate decarboxylase 1
ENSG0000	2.068035	-2.39278	2.002596	-1.19484	0.232149	0.520971	GUCY2D	3000	guanylate retinal
ENSG0000	397.1105	-0.18997	0.159026	-1.1946	0.232245	0.521041	SSH3	54961	slingshot protein phosphatase 3
ENSG0000	33.72914	-0.55841	0.467433	-1.19462	0.232235	0.521041	OPRL1	4987	opioid related nociceptin receptor 1
ENSG0000	7.731069	1.092363	0.914586	1.19438	0.23233	0.521168	NA	NA	NA
ENSG0000	13.03898	0.907482	0.75986	1.194275	0.23237	0.521197	BSG-AS1	1.05E+08	BSG antisense RNA 1
ENSG0000	758.4519	0.39343	0.329529	1.193915	0.232511	0.521386	CD274	29126	CD274 molecule
ENSG0000	926.2933	0.143837	0.120473	1.193932	0.232505	0.521386	SIRPB1	10326	signal regulatory protein beta 1
ENSG0000	5853.557	0.418116	0.350229	1.193839	0.232541	0.52139	EGR2	1959	early growth response 2
ENSG0000	272.4841	-0.21938	0.183798	-1.19361	0.232631	0.52153	SLC66A1	54896	solute carrier family 66 member 1
ENSG0000	374.3439	-0.18642	0.156229	-1.19322	0.232782	0.521804	B3GALT4	8705	beta-1 3-galactosyltransferase 4
ENSG0000	1864.18	0.118469	0.099294	1.193112	0.232826	0.521839	SELENOT	51714	selenoprotein T
ENSG0000	8.895402	1.286702	1.078811	1.192704	0.232985	0.522134	MSLN	10232	mesothelin
ENSG0000	445.3576	0.15482	0.129853	1.192277	0.233153	0.522445	DESI2	51029	desumoylating isopeptidase 2
ENSG0000	807.3295	0.12873	0.107998	1.191972	0.233272	0.522651	HS6ST1	9394	heparan sulfate 6-O-sulfotransferase 1
ENSG0000	650.689	-0.15608	0.130975	-1.19166	0.233396	0.522864	RASGRP4	115727	RAS guanyl releasing protein 4
ENSG0000	13.67443	0.753494	0.632472	1.191347	0.233517	0.522945	RPS3P4	1E+08	ribosomal protein S3 pseudogene 4
ENSG0000	42.3679	-0.44407	0.372741	-1.19135	0.233516	0.522945	MOCS1	4337	molybdenum cofactor synthesis 1
ENSG0000	158.7161	-0.26658	0.223765	-1.19133	0.233523	0.522945	GPR146	115330	G protein-coupled receptor 146
ENSG0000	149.3728	-0.24926	0.209235	-1.19128	0.233545	0.522945	BBS2	583	Bardet-Biedl syndrome 2
ENSG0000	46.93022	-0.44764	0.375833	-1.19106	0.23363	0.522947	C2	717	complement C2
ENSG0000	12.1868	-0.88288	0.741229	-1.19111	0.233612	0.522947	NA	NA	NA
ENSG0000	1436.094	0.127858	0.107341	1.191143	0.233597	0.522947	ZNF24	7572	zinc finger protein 24
ENSG0000	921.9095	-0.13685	0.11491	-1.19093	0.23368	0.522995	CHAMP1	283489	chromosome alignment maintaining phosphoprotein 1
ENSG0000	158.3125	-0.24049	0.201966	-1.19075	0.233752	0.523094	EEF2KMT	196483	eukaryotic elongation factor 2 lysine methyltransferase
ENSG0000	18.04023	-0.71978	0.604544	-1.19062	0.233802	0.523142	NA	NA	NA
ENSG0000	223.7551	-0.21237	0.178395	-1.19043	0.233876	0.523182	ARL10	285598	ADP ribosylation factor like GTPase 10
ENSG0000	3004.239	-0.10687	0.089768	-1.19049	0.233853	0.523182	AGPAT3	56894	1-acylglycerol-3-phosphate O-acyltransferase 3
ENSG0000	3.38304	1.911904	1.606156	1.190361	0.233905	0.523183	CLIC6	54102	chloride intracellular channel 6
ENSG0000	538.2523	0.162449	0.136491	1.190186	0.233973	0.523273	NUDT16L1	84309	nudix hydrolase 16 like 1
ENSG0000	150.6965	0.25246	0.212133	1.190102	0.234006	0.523283	METTL21A	151194	methyltra HSPA lysine
ENSG0000	21.3123	-0.66172	0.556169	-1.18978	0.234135	0.523507	ADAM11	4185	ADAM metallopeptidase domain 11
ENSG0000	338.0522	-0.1711	0.14382	-1.18966	0.23418	0.523546	REEP3	221035	receptor accessory protein 3
ENSG0000	18.75062	0.664651	0.558733	1.189568	0.234216	0.523564	GRASLND	386597	glycosaminoglycan regulatory associated long non-coding RNA
ENSG0000	16.07628	0.810676	0.681608	1.189357	0.234299	0.523686	NA	NA	NA
ENSG0000	90.23124	-0.38064	0.320094	-1.18916	0.234379	0.523706	P2RY13	53829	purinergic receptor P2Y13

ENSG0000	1295.633	-0.12063	0.101431	-1.18925	0.23434	0.523706	PPP1R14B	26472	protein phosphatase 1 regulatory inhibitor subunit 14B
ENSG0000	4.573341	1.675935	1.409392	1.189119	0.234393	0.523706	NA	NA	NA
ENSG0000	279.2696	-0.18785	0.158039	-1.18861	0.234591	0.524087	ALG1	56052	ALG1 chitobiosyldiphosphodolichol beta-mannosyltransferase
ENSG0000	83.26921	0.350159	0.294647	1.188403	0.234675	0.52421	ZNF571	51276	zinc finger protein 571
ENSG0000	886.603	-0.12423	0.104549	-1.18825	0.234736	0.524284	UBIAD1	29914	UbiA prenyltransferase domain containing 1
ENSG0000	8.369841	1.436215	1.208868	1.188066	0.234808	0.524317	SLC22A13	9390	solute carrier family 22 member 13
ENSG0000	96.80546	-0.3149	0.265043	-1.18811	0.23479	0.524317	ANKRD55	79722	ankyrin repeat domain 55
ENSG0000	741.8127	0.128615	0.108285	1.187744	0.234934	0.524474	LTB4R	1241	leukotriene B4 receptor
ENSG0000	15.35333	-0.76875	0.647199	-1.18781	0.23491	0.524474	NA	NA	NA
ENSG0000	3.122642	1.850014	1.557933	1.18748	0.235038	0.524642	CDC42EP2	10435	CDC42 effector protein 2
ENSG0000	298.5593	-0.20404	0.171882	-1.18711	0.235185	0.524907	MRPS26	64949	mitochondrial ribosomal protein S26
ENSG0000	267.5421	-0.19263	0.162284	-1.18696	0.235242	0.524908	CCDC25	55246	coiled-coil domain containing 25
ENSG0000	6.129737	-1.29753	1.093114	-1.187	0.235227	0.524908	NA	NA	NA
ENSG0000	420.5106	-0.1758	0.148192	-1.18631	0.2355	0.525327	ABI2	10152	abl interactor 2
ENSG0000	103.4792	-0.32196	0.271377	-1.18639	0.23547	0.525327	RMDN1	51115	regulator of microtubule dynamics 1
ENSG0000	56.72979	0.364809	0.307526	1.186272	0.235515	0.525327	PRMT3	10196	protein arginine methyltransferase 3
ENSG0000	1436.577	-0.12664	0.106786	-1.18596	0.235637	0.52541	AGPAT4	56895	1-acylglycerol-3-phosphate O-acyltransferase 4
ENSG0000	18.70141	-0.72792	0.613735	-1.18605	0.235604	0.52541	CCDC180	1E+08	coiled-coil domain containing 180
ENSG0000	6.13486	-1.4408	1.214876	-1.18597	0.235636	0.52541	LAMA3	3909	laminin subunit alpha 3
ENSG0000	724.3425	0.129406	0.109139	1.1857	0.235741	0.525486	EIF2S1	1965	eukaryotic translation initiation factor 2 subunit alpha
ENSG0000	5.539811	1.54149	1.30004	1.185725	0.235731	0.525486	ACTG1P17	283693	actin gamma 1 pseudogene 17
ENSG0000	131.6151	0.241191	0.203423	1.185661	0.235756	0.525486	TMEM185	84548	transmembrane protein 185A
ENSG0000	30.88633	-0.50252	0.423905	-1.18545	0.235838	0.525605	NA	NA	NA
ENSG0000	34.49012	-0.49149	0.414636	-1.18536	0.235876	0.525626	NA	NA	NA
ENSG0000	9.028544	0.867383	0.731801	1.185273	0.23591	0.525639	XRCC6P2	389901	X-ray repair cross complementing 6 pseudogene 2
ENSG0000	362.7884	-0.1596	0.134684	-1.18499	0.236022	0.5257	RNF146	81847	ring finger protein 146
ENSG0000	9.633392	0.979162	0.826256	1.185058	0.235994	0.5257	PLAAT5	117245	phospholipase A and acyltransferase 5
ENSG0000	1962.032	0.122827	0.103647	1.185048	0.235999	0.5257	COL18A1	80781	collagen type XVIII alpha 1 chain
ENSG0000	23.52709	0.701429	0.592021	1.184804	0.236095	0.525798	NA	NA	NA
ENSG0000	32.35868	-0.53698	0.453287	-1.18464	0.236159	0.525879	TRAV8-4	28682	T cell receptor alpha variable 8-4
ENSG0000	3.637865	-1.81317	1.530767	-1.18448	0.236221	0.525954	NA	NA	NA
ENSG0000	23.5663	-0.60493	0.510804	-1.18426	0.236309	0.526023	NA	NA	NA
ENSG0000	8.821303	1.101617	0.930197	1.184284	0.236301	0.526023	NA	NA	NA
ENSG0000	744.0677	-0.12686	0.107141	-1.184	0.236413	0.526129	MR1	3140	major hist class I-related
ENSG0000	616.4937	0.156222	0.131937	1.18406	0.236389	0.526129	CCDC86	79080	coiled-coil domain containing 86
ENSG0000	11267.83	-0.08857	0.074816	-1.18385	0.236474	0.526201	AUTS2	26053	activator of transcription and developmental regulator AUTS2
ENSG0000	13.27473	-0.85972	0.726288	-1.18372	0.236526	0.526252	NA	NA	NA
ENSG0000	707.6066	-0.146	0.12336	-1.18351	0.236606	0.526313	GK5	256356	glycerol kinase 5
ENSG0000	3.194224	1.862931	1.574081	1.183504	0.23661	0.526313	H4C12	8362	H4 clustered histone 12
ENSG0000	8.128364	-1.1024	0.931762	-1.18313	0.236756	0.526577	CORO2B	10391	coronin 2B
ENSG0000	453.6535	-0.17404	0.147115	-1.183	0.23681	0.526593	DCK	1633	deoxycytidine kinase
ENSG0000	1499.958	-0.10968	0.09272	-1.18291	0.236844	0.526593	GOLGA8A	23015	golgin A8 family member A
ENSG0000	63.78409	-0.38366	0.32434	-1.1829	0.236849	0.526593	TACO1	51204	translational activator of cytochrome c oxidase I
ENSG0000	2.490292	-2.23421	1.889393	-1.1825	0.237008	0.526795	BMS1P10	728611	BMS1 pseudogene 10
ENSG0000	193.7115	0.217297	0.183767	1.182455	0.237025	0.526795	NA	NA	NA
ENSG0000	3625.199	0.103772	0.087753	1.182549	0.236988	0.526795	PRKAR1A	5573	protein kinase cAMP-dependent type I regulatory subunit alpha
ENSG0000	915.619	-0.12707	0.107498	-1.18211	0.237164	0.52704	OSTM1	28962	osteoclastogenesis associated transmembrane protein 1
ENSG0000	808.8986	0.120826	0.102228	1.181924	0.237236	0.527137	ARFRP1	10139	ADP ribosylation factor related protein 1
ENSG0000	850.7875	-0.12281	0.103924	-1.18177	0.237299	0.527214	MRNIP	51149	MRN complex interacting protein
ENSG0000	2535.189	0.11738	0.099349	1.181491	0.237408	0.527286	RALGAPA1	253959	Ral GTPase activating protein catalytic subunit alpha 1
ENSG0000	14.67752	-0.7367	0.623477	-1.1816	0.237364	0.527286	ERI2	112479	ERI1 exoribonuclease family member 2
ENSG0000	11.99933	1.005176	0.850785	1.181469	0.237417	0.527286	LINC0186	284365	long intergenic non-protein coding RNA 1869
ENSG0000	37.53865	0.497851	0.42144	1.181308	0.237481	0.527343	LTBP1	4052	latent transforming growth factor beta binding protein 1
ENSG0000	182.0971	-0.25437	0.215384	-1.18103	0.237591	0.527343	MITF	4286	melanocyte inducing transcription factor
ENSG0000	145.8051	-0.25426	0.215285	-1.18104	0.237588	0.527343	SHQ1	55164	SHQ1 H/ACA ribonucleoprotein assembly factor
ENSG0000	2.262815	2.102778	1.780545	1.180974	0.237613	0.527343	LRRC15	131578	leucine rich repeat containing 15
ENSG0000	3.913303	1.962973	1.662039	1.181063	0.237578	0.527343	NA	NA	NA
ENSG0000	7.215023	1.224395	1.036561	1.181209	0.23752	0.527343	CLDN9	9080	claudin 9
ENSG0000	149.5664	-0.27008	0.228731	-1.18078	0.237692	0.527433	DTYMK	1841	deoxythymidylate kinase
ENSG0000	110.5628	-0.36285	0.307324	-1.18068	0.237731	0.527433	TIGD6	81789	tigger transposable element derived 6
ENSG0000	2.985108	1.92213	1.628053	1.180631	0.237749	0.527433	GPRC5B	51704	G protein-coupled receptor class C group 5 member B
ENSG0000	975.7248	0.131672	0.111531	1.180586	0.237767	0.527433	ITGA2B	3674	integrin subunit alpha 2b
ENSG0000	16.45983	-0.7391	0.626101	-1.18048	0.23781	0.527464	LRRC43	254050	leucine rich repeat containing 43
ENSG0000	243.5444	-0.20327	0.172264	-1.18002	0.237993	0.527555	ECI2	10455	enoyl-CoA delta isomerase 2
ENSG0000	228.4654	-0.26471	0.224312	-1.18008	0.237968	0.527555	MYB	4602	MYB protc transcription factor
ENSG0000	637.7699	0.163166	0.13825	1.180224	0.237911	0.527555	ATP5PD	10476	ATP synthase peripheral stalk subunit d
ENSG0000	8076.169	-0.0818	0.069311	-1.18012	0.237953	0.527555	RHBDF2	79651	rhomboid 5 homolog 2
ENSG0000	210.8134	-0.21187	0.179535	-1.18009	0.237964	0.527555	CENPX	201254	centromere protein X
ENSG0000	303.9968	-0.17688	0.149922	-1.17984	0.238063	0.527647	CUEDC2	79004	CUE domain containing 2
ENSG0000	4.214682	-1.78878	1.516214	-1.17977	0.238093	0.52765	FAM86JP	1E+08	family witl member A pseudogene
ENSG0000	11.3072	0.818004	0.693666	1.179248	0.238299	0.528045	RPS7P3	440732	ribosomal protein S7 pseudogene 3
ENSG0000	2766.143	-0.10071	0.085411	-1.17909	0.238362	0.528057	KIAA2013	90231	KIAA2013
ENSG0000	7.471363	1.058404	0.897591	1.179161	0.238334	0.528057	RPS27AP5	1E+08	ribosomal protein S27a pseudogene 5
ENSG0000	7.260588	1.138367	0.9656	1.178922	0.238429	0.52809	STRCP1	554225	stereocilin pseudogene 1
ENSG0000	6.310296	1.238275	1.050355	1.178911	0.238434	0.52809	NA	NA	NA
ENSG0000	392.5946	-0.19078	0.161871	-1.17859	0.238562	0.528313	FADS2	9415	fatty acid desaturase 2
ENSG0000	18.93499	0.810416	0.687753	1.178353	0.238656	0.528331	NA	NA	NA
ENSG0000	301.6704	-0.19652	0.166763	-1.17844	0.238619	0.528331	NA	NA	NA
ENSG0000	3670.195	-0.0972	0.082491	-1.17837	0.238648	0.528331	EXOC7	23265	exocyst complex component 7
ENSG0000	6.506812	-1.17706	0.99927	-1.17792	0.238827	0.528645	NA	NA	NA
ENSG0000	637.7144	-0.15365	0.130469	-1.17769	0.238919	0.528787	CHMP3	51652	charged multivesicular body protein 3
ENSG0000	170.3306	-0.22972	0.195072	-1.17761	0.238953	0.528799	WDR41	55255	WD repeat domain 41
ENSG0000	12.93403	-0.88041	0.7479	-1.17717	0.239128	0.529013	FAM72B	653820	family with sequence similarity 72 member B
ENSG0000	32.94002	-0.51656	0.438847	-1.17708	0.239164	0.529013	OTOF	9381	otoferlin
ENSG0000	3.878314	-1.80083	1.52981	-1.17716	0.239131	0.529013	MIR6075	1.02E+08	microRNA 6075
ENSG0000	2182.934	-0.09681	0.082241	-1.17714	0.23914	0.529013	MAP3K14	9020	mitogen-activated protein kinase kinase kinase 14
ENSG0000	8.760664	0.937742	0.796748	1.176962	0.239211	0.529053	NA	NA	NA
ENSG0000	929.2659	-0.14158	0.120325	-1.17668	0.239325	0.529243	SMC3	9126	structural maintenance of chromosomes 3
ENSG0000	1254.585	-0.11608	0.098659	-1.17654	0.239379	0.52929	CCDC88A	55704	coiled-coil domain containing 88A
ENSG0000	424.9768	-0.16272	0.138319	-1.17641	0.239431	0.52929	NA	NA	NA
ENSG0000	849.7761	-0.13074	0.111137	-1.17641	0.239432	0.52929	WASHC2A	387680	WASH complex subunit 2A
ENSG0000	8.081858	1.204242	1.023915	1.176115	0.239549	0.529486	LOC10192	1.02E+08	uncharacterized LOC101927245
ENSG0000	88.33949	0.306179	0.260369	1.175943	0.239618	0.529524	NA	NA	NA
ENSG0000	77.32149	0.326027	0.27725	1.175929	0.239623	0.529524	SHANK1	50944	SH3 and multiple ankyrin repeat domains 1
ENSG0000	3054.016	0.094244	0.080156	1.175769	0.239687	0.529602	RBM23	55147	RNA binding motif protein 23
ENSG0000	144.7369	0.277902	0.236389	1.175609	0.239751	0.52968	SDHAF2	54949	succinate dehydrogenase complex assembly factor 2
ENSG0000	291.6843	-0.1707	0.145221	-1.17547	0.239807	0.52974	ATOX1	475	antioxidant 1 copper chaperone
ENSG0000	11.52026	0.900179	0.765901	1.17532	0.239867	0.529809	PLEKHH3	79990	pleckstrin MyTH4 and FERM domain containing H3
ENSG0000	87.60155	0.342454	0.291393	1.175233	0.239902	0.529823	INHA	3623	inhibin subunit alpha
ENSG0000	14.77736	0.712536	0.606397	1.175032	0.239982	0.529937	SPACDR	402573	sperm acrosome developmental regulator

ENSG0000	29.70027	-0.52311	0.445364	-1.17456	0.240171	0.530292	SCD5	79966	stearoyl-CoA desaturase 5
ENSG0000	64.12339	0.415134	0.353639	1.173892	0.240438	0.530733	EPHA2	1969	EPH receptor A2
ENSG0000	32.39749	0.585416	0.498716	1.173847	0.240456	0.530733	GPR161	23432	G protein-coupled receptor 161
ENSG0000	7.748271	1.073656	0.914642	1.173853	0.240454	0.530733	SGCE	8910	sarcoglycan epsilon
ENSG0000	412.0603	-0.18913	0.161176	-1.17346	0.24061	0.530946	TECPR2	9895	tectonin beta-propeller repeat containing 2
ENSG0000	1622.426	-0.11431	0.097407	-1.1735	0.240594	0.530946	CLPP	8192	caseinolytic mitochondrial matrix peptidase proteolytic subunit
ENSG0000	1970.738	0.167932	0.143138	1.173217	0.240709	0.5311	LIMK2	3985	LIM domain kinase 2
ENSG0000	7.502254	1.042777	0.889005	1.172971	0.240808	0.531255	PCDHGB5	56101	protocadh 5
ENSG0000	6.685666	1.078225	0.919632	1.172453	0.241015	0.531587	PAIP1P1	1E+08	PAIP1 pseudogene 1
ENSG0000	705.795	0.147585	0.125871	1.172512	0.240992	0.531587	ZNF581	51545	zinc finger protein 581
ENSG0000	75.03377	0.311912	0.266075	1.172271	0.241088	0.531684	RPS27AP1	643358	RPS27A pseudogene 16
ENSG0000	3.775687	1.598169	1.363463	1.172139	0.241141	0.531737	NA	NA	NA
ENSG0000	504.6879	-0.17951	0.15318	-1.17191	0.241235	0.53188	PTDSS2	81490	phosphatidylserine synthase 2
ENSG0000	3.373401	-2.06137	1.759243	-1.17174	0.241302	0.531967	NA	NA	NA
ENSG0000	17.78742	-0.71418	0.609546	-1.17166	0.241335	0.531975	NA	NA	NA
ENSG0000	4.147646	-1.39919	1.194391	-1.17146	0.241412	0.532005	NA	NA	NA
ENSG0000	7.930287	-1.12212	0.957921	-1.17141	0.241435	0.532005	DNM1P46	196968	dynamin 1 pseudogene 46
ENSG0000	25.32652	-0.56975	0.486334	-1.17151	0.241392	0.532005	MTCL1	23255	microtubule crosslinking factor 1
ENSG0000	1407.93	-0.11194	0.095599	-1.17098	0.241606	0.532013	MKNK1	8569	MAPK interacting serine/threonine kinase 1
ENSG0000	15.10505	-0.82055	0.700659	-1.17111	0.241555	0.532013	LINC0224!	400958	long intergenic non-protein coding RNA 2245
ENSG0000	100.2635	-0.29675	0.253418	-1.17097	0.24161	0.532013	TRBV19	28568	T cell receptor beta variable 19
ENSG0000	1504.23	-0.15913	0.135861	-1.1713	0.241479	0.532013	SLC31A2	1318	solute carrier family 31 member 2
ENSG0000	9.865352	1.050702	0.897234	1.171046	0.24158	0.532013	DUOX2	50506	dual oxidase 2
ENSG0000	341.7047	-0.19401	0.165656	-1.17115	0.241537	0.532013	B3GNTL1	146712	UDP-GlcN/ 3-N-acetylglucosaminyltransferase like 1
ENSG0000	7.621986	-1.12736	0.962965	-1.17072	0.241711	0.532172	TBX1	6899	T-box transcription factor 1
ENSG0000	21.66333	-0.6102	0.521322	-1.17049	0.241802	0.532183	NA	NA	NA
ENSG0000	2.436565	-2.17039	1.85421	-1.17052	0.241793	0.532183	NA	NA	NA
ENSG0000	105.5638	0.328725	0.28081	1.170633	0.241746	0.532183	METTL1	4234	methyltrai tRNA methylguanosine
ENSG0000	13.74335	0.854897	0.730508	1.170278	0.241889	0.532292	MANSC1	54682	MANSC domain containing 1
ENSG0000	694.5198	0.128933	0.110181	1.170195	0.241922	0.532292	LINC0092€	283663	long intergenic non-protein coding RNA 926
ENSG0000	10.43955	-0.87457	0.747393	-1.17016	0.241938	0.532292	NA	NA	NA
ENSG0000	4963.46	0.107051	0.091522	1.169672	0.242133	0.532469	TUBB4B	10383	tubulin beta 4B class IVb
ENSG0000	109.7573	0.316184	0.270307	1.169721	0.242113	0.532469	NRARP	441478	NOTCH regulated ankyrin repeat protein
ENSG0000	10.13679	-0.84489	0.722199	-1.16988	0.242047	0.532469	NA	NA	NA
ENSG0000	490.3515	0.16727	0.143002	1.1697	0.242122	0.532469	TNKS1BP1	85456	tankyrase 1 binding protein 1
ENSG0000	244.9615	-0.2223	0.190109	-1.16934	0.242268	0.532639	ZNF7	7553	zinc finger protein 7
ENSG0000	12.96494	-0.76365	0.653059	-1.16934	0.242266	0.532639	NA	NA	NA
ENSG0000	63.60175	-0.34816	0.297835	-1.16898	0.242413	0.532894	TMEM267	64417	transmembrane protein 267
ENSG0000	265.2985	0.206566	0.176731	1.168814	0.242479	0.532976	ABHD3	171586	abhydrola: phospholipase
ENSG0000	284.2401	-0.18051	0.154454	-1.16868	0.242531	0.533027	THUMPD3	25917	THUMP domain containing 3
ENSG0000	218.8042	-0.20341	0.174082	-1.16849	0.242609	0.533073	DHRS4-AS	55449	DHRS4 antisense RNA 1
ENSG0000	65.68101	0.36761	0.314596	1.168514	0.242599	0.533073	LOC11226	1.12E+08	uncharacterized LOC112268198
ENSG0000	18.16189	-0.66864	0.572327	-1.16828	0.242693	0.533161	FAM86C2I	645332	family witl member A pseudogene
ENSG0000	11.84956	-0.86476	0.740221	-1.16825	0.242707	0.533161	TRAV13-2	28670	T cell receptor alpha variable 13-2
ENSG0000	118.9186	-0.26671	0.228333	-1.1681	0.242768	0.53317	TSPAN5	10098	tetraspanin 5
ENSG0000	6.455707	1.075933	0.92109	1.168109	0.242763	0.53317	CFAP58-D	1.01E+08	CFAP58 divergent transcript
ENSG0000	799.9127	-0.18351	0.157125	-1.16792	0.24284	0.533265	LILRA1	11024	leukocyte immunoglobulin like receptor A1
ENSG0000	33.85781	-0.53092	0.454703	-1.16763	0.242957	0.533458	PRIM2	5558	DNA primase subunit 2
ENSG0000	4.072145	1.462909	1.253071	1.167459	0.243025	0.533545	PLGLB2	5342	plasminogen like B2
ENSG0000	24.37938	-0.65279	0.559264	-1.16724	0.243115	0.533679	PGBD1	84547	piggyBac transposable element derived 1
ENSG0000	70.05729	0.346754	0.297285	1.166403	0.243452	0.534355	L3MBTL1	26013	L3MBTL histone methyl-lysine binding protein 1
ENSG0000	2558.958	0.110839	0.09504	1.166235	0.243519	0.534377	MAPRE2	10982	microtubule associated protein RP/EB family member 2
ENSG0000	140.9741	-0.27546	0.236185	-1.16629	0.243495	0.534377	ZNF407-A!	400657	ZNF407 antisense RNA 1
ENSG0000	209.1939	-0.21626	0.185471	-1.16601	0.243611	0.534452	ABLIM3	22885	actin binding LIM protein family member 3
ENSG0000	3652.872	0.090205	0.077359	1.166057	0.243591	0.534452	CPNE1	8904	copine 1
ENSG0000	126.5943	-0.26727	0.229233	-1.16594	0.24364	0.534452	TMEM168	64418	transmembrane protein 168
ENSG0000	230.9076	-0.21731	0.1864	-1.16582	0.243687	0.534492	ZNF428	126299	zinc finger protein 428
ENSG0000	804.9729	-0.12094	0.103745	-1.16569	0.243739	0.534542	WDR59	79726	WD repeat domain 59
ENSG0000	1210.693	0.118778	0.101928	1.165307	0.243895	0.534758	RABEP1	9135	rabaptin RAB GTPase binding effector protein 1
ENSG0000	3771.544	-0.09558	0.082016	-1.16537	0.243869	0.534758	FLOT2	2319	flotillin 2
ENSG0000	21.99467	-0.64905	0.557052	-1.16515	0.243957	0.534831	ASAH2B	653308	N-acylsphingosine amidohydrolase 2B
ENSG0000	39.62763	-0.47617	0.408848	-1.16467	0.244152	0.535196	CDHR5	53841	cadherin related family member 5
ENSG0000	9.419503	0.976801	0.838789	1.164537	0.244206	0.535251	RPL27P1	326299	ribosomal protein L27 pseudogene 1
ENSG0000	68.85452	0.331807	0.284949	1.164442	0.244245	0.535272	SSTR3	6753	somatostatin receptor 3
ENSG0000	10.58333	0.80256	0.689394	1.164153	0.244362	0.535381	NA	NA	NA
ENSG0000	3.425478	2.062565	1.771802	1.164106	0.244381	0.535381	TAT-AS1	1E+08	TAT antisense RNA 1
ENSG0000	132.5728	-0.26332	0.22618	-1.16419	0.244346	0.535381	MKKS	8195	MKKS centrosomal shuttling protein
ENSG0000	1000.427	0.114376	0.098261	1.163999	0.244424	0.535412	OSBPL2	9885	oxysterol binding protein like 2
ENSG0000	80.90368	-0.31417	0.269926	-1.16392	0.244458	0.535424	ABCA3	21	ATP binding cassette subfamily A member 3
ENSG0000	29.5153	0.702537	0.60364	1.163836	0.244491	0.535431	NA	NA	NA
ENSG0000	6.694799	-1.11487	0.957999	-1.16375	0.244524	0.53544	ZNF501	115560	zinc finger protein 501
ENSG0000	342.6262	0.175131	0.150518	1.163523	0.244617	0.535582	SDHAP2	727956	SDHA pseudogene 2
ENSG0000	6.257802	1.172926	1.008404	1.163151	0.244768	0.535786	CRNDE	643911	colorectal neoplasia differentially expressed
ENSG0000	5.784238	1.357162	1.166752	1.163196	0.24475	0.535786	NA	NA	NA
ENSG0000	13.70703	1.08055	0.929208	1.162872	0.244881	0.535971	NA	NA	NA
ENSG0000	5287.034	0.084694	0.072873	1.162213	0.245149	0.536493	AKT2	208	AKT serine/threonine kinase 2
ENSG0000	395.2731	-0.17062	0.14682	-1.16211	0.245189	0.536518	CRIM1	51232	cysteine rich transmembrane BMP regulator 1
ENSG0000	158.5339	-0.25663	0.220883	-1.16185	0.245297	0.53669	INTS2	57508	integrator complex subunit 2
ENSG0000	721.9996	-0.13106	0.112832	-1.16153	0.245425	0.536907	RABGAP1	23637	RAB GTPase activating protein 1
ENSG0000	9.399769	-1.14024	0.981748	-1.16144	0.245462	0.536926	ACVRL1	94	activin A receptor like type 1
ENSG0000	14.8906	-0.78591	0.676844	-1.16114	0.245587	0.537135	NA	NA	NA
ENSG0000	262.9104	0.179671	0.154778	1.16083	0.245711	0.537342	BZW2	28969	basic leucine zipper and W2 domains 2
ENSG0000	808.8724	-0.12572	0.108316	-1.16065	0.245785	0.537442	DUS3L	56931	dihydrouridine synthase 3 like
ENSG0000	404.5128	0.164637	0.141868	1.160494	0.245848	0.537451	JAGN1	84522	jagunal homolog 1
ENSG0000	1311.148	0.113829	0.098086	1.160502	0.245844	0.537451	SLC39A7	7922	solute carrier family 39 member 7
ENSG0000	1680.139	-0.12503	0.107752	-1.16032	0.24592	0.537486	CREBZF	58487	CREB/ATF bZIP transcription factor
ENSG0000	5.340954	1.484574	1.279461	1.160312	0.245922	0.537486	CPB2-AS1	1.01E+08	CPB2 antisense RNA 1
ENSG0000	39.26593	-0.51667	0.445377	-1.16008	0.246017	0.537546	THAP9	79725	THAP domain containing 9
ENSG0000	17.1129	0.694176	0.598391	1.160071	0.24602	0.537546	NA	NA	NA
ENSG0000	6317.712	0.080259	0.069187	1.160032	0.246036	0.537546	PPP1R9B	84687	protein phosphatase 1 regulatory subunit 9B
ENSG0000	5.514547	1.226323	1.057548	1.159591	0.246215	0.537812	TP73	7161	tumor protein p73
ENSG0000	97.14706	-0.28224	0.2434	-1.15959	0.246216	0.537812	NA	NA	NA
ENSG0000	13.97897	-0.74176	0.639738	-1.15948	0.246261	0.537848	PLGLB1	5343	plasminogen like B1
ENSG0000	5.523008	1.738315	1.499542	1.159231	0.246362	0.53795	NA	NA	NA
ENSG0000	268.4663	-0.20181	0.174093	-1.15922	0.246366	0.53795	NIPSNAP1	8508	nipsnap homolog 1
ENSG0000	109.5601	-0.26807	0.23129	-1.15902	0.246447	0.53796	MMP23B	8510	matrix metallopeptidase 23B
ENSG0000	8692.751	-0.08384	0.072334	-1.15913	0.246402	0.53796	TRBC2	28638	T cell receptor beta constant 2
ENSG0000	289.0176	0.196013	0.169123	1.158996	0.246458	0.53796	TRIM37	4591	tripartite motif containing 37
ENSG0000	369.1745	-0.19287	0.166483	-1.15852	0.246651	0.53832	BTD	686	biotinidase
ENSG0000	7.473302	-1.13259	0.977882	-1.15821	0.246779	0.538509	SUCLG2-D	1.02E+08	SUCLG2 divergent transcript

ENSG0000	11.43504	-0.98331	0.849024	-1.15817	0.246796	0.538509	SHISAL1	85352	shisa like 1
ENSG0000	5519.455	-0.08734	0.075423	-1.15801	0.246862	0.538588	IRF2BP2	359948	interferon regulatory factor 2 binding protein 2
ENSG0000	5.754074	-1.40453	1.213036	-1.15786	0.24692	0.538588	NA	NA	NA
ENSG0000	33.57145	-0.52021	0.449275	-1.15789	0.246908	0.538588	IGHV2-5	28457	immunoglobulin heavy variable 2-5
ENSG0000	28.00807	-0.63813	0.551208	-1.15769	0.246992	0.538648	TICAM2	353376	TIR domain containing adaptor molecule 2
ENSG0000	26.04675	-0.7222	0.623844	-1.15765	0.247005	0.538648	EDN3	1908	endothelin 3
ENSG0000	510.0328	0.158842	0.137228	1.157506	0.247066	0.538717	VPS29	51699	VPS29 retromer complex component
ENSG0000	2588.198	-0.11116	0.096042	-1.15737	0.24712	0.538772	RPS6KA1	6195	ribosomal protein S6 kinase A1
ENSG0000	29.71827	0.503401	0.435061	1.157081	0.247239	0.538969	USP44	84101	ubiquitin specific peptidase 44
ENSG0000	26.9684	0.559647	0.483718	1.156969	0.247285	0.539005	NA	NA	NA
ENSG0000	53.13323	-0.41737	0.360865	-1.15658	0.247445	0.53929	TMEM218	219854	transmembrane protein 218
ENSG0000	8.771394	-1.26405	1.093443	-1.15603	0.247669	0.53955	NA	NA	NA
ENSG0000	65.8246	0.457189	0.395451	1.156121	0.247632	0.53955	LOC12490	1.25E+08	uncharacterized LOC124905349
ENSG0000	1061.16	0.113252	0.097961	1.156097	0.247642	0.53955	HMCES	56941	5-hydroxy ES cell specific
ENSG0000	807.8939	0.142943	0.123653	1.156001	0.247681	0.53955	DCTN4	51164	dynactin subunit 4
ENSG0000	36.20294	-0.51378	0.444493	-1.15589	0.247728	0.53959	STPG1	90529	sperm tail PG-rich repeat containing 1
ENSG0000	7.827347	-1.23405	1.067893	-1.15559	0.247848	0.539772	NA	NA	NA
ENSG0000	2348.215	0.095242	0.082423	1.155539	0.24787	0.539772	EIF3L	51386	eukaryotic translation initiation factor 3 subunit L
ENSG0000	4637.995	0.104017	0.090034	1.155307	0.247965	0.539915	MYADM	91663	myeloid associated differentiation marker
ENSG0000	23.90376	0.60046	0.519876	1.155005	0.248088	0.540121	RASSF8	11228	Ras association domain family member 8
ENSG0000	7.471192	1.068468	0.92518	1.154877	0.248141	0.540173	EPB42	2038	erythrocyte membrane protein band 4.2
ENSG0000	362.8599	-0.16223	0.140524	-1.15448	0.248305	0.540173	PEX11B	8799	peroxisomal biogenesis factor 11 beta
ENSG0000	6.154292	-1.23278	1.067694	-1.15462	0.248247	0.540173	NA	NA	NA
ENSG0000	3.66608	1.708255	1.479677	1.154479	0.248304	0.540173	DCDC1	341019	doublecortin domain containing 1
ENSG0000	920.7648	-0.12963	0.112266	-1.15471	0.248209	0.540173	SLC39A6	25800	solute carrier family 39 member 6
ENSG0000	5.334631	-1.54331	1.336838	-1.15445	0.248316	0.540173	UPK1A	11045	uroplakin 1A
ENSG0000	1446.515	0.111316	0.096413	1.154582	0.248262	0.540173	ZNF574	64763	zinc finger protein 574
ENSG0000	115.071	-0.302	0.261628	-1.15433	0.248366	0.540219	ZNF2	7549	zinc finger protein 2
ENSG0000	105.7792	-0.29526	0.255816	-1.15421	0.248415	0.540262	NA	NA	NA
ENSG0000	29.49896	0.523942	0.454128	1.153733	0.24861	0.540384	PMS2P2	5380	PMS1 hom mismatch repair system component pseudogene 2
ENSG0000	49.01318	-0.43321	0.375428	-1.15391	0.248536	0.540384	ZNF483	158399	zinc finger protein 483
ENSG0000	261.7533	-0.20015	0.173491	-1.15364	0.248646	0.540384	CLPB	81570	caseinolytic mitochondrial matrix peptidase chaperone subunit B
ENSG0000	86.34935	-0.30415	0.26358	-1.15393	0.248531	0.540384	ALKBH8	91801	alkB homc tRNA methyltransferase
ENSG0000	299.37	-0.21063	0.182575	-1.15367	0.248635	0.540384	ZNF785	146540	zinc finger protein 785
ENSG0000	5.163527	1.232755	1.068574	1.153645	0.248646	0.540384	NA	NA	NA
ENSG0000	74.56346	0.389897	0.338034	1.153428	0.248735	0.540514	TACSTD2	4070	tumor associated calcium signal transducer 2
ENSG0000	101.6764	-0.35907	0.311379	-1.15314	0.248851	0.540704	NA	NA	NA
ENSG0000	122.4187	0.252536	0.219096	1.152626	0.249064	0.540768	METTL5	29081	methyltra N6-adenosine
ENSG0000	3.915773	-1.73034	1.500784	-1.15296	0.248928	0.540768	SCN5A	6331	sodium voltage-gated channel alpha subunit 5
ENSG0000	80.09258	0.33897	0.294091	1.152602	0.249074	0.540768	LPAL2	80350	lipoprotei pseudogene
ENSG0000	762.8149	-0.15195	0.131807	-1.15281	0.248987	0.540768	ADO	84890	2-aminoethanethiol dioxygenase
ENSG0000	14075.75	0.096135	0.083409	1.152575	0.249085	0.540768	TMEM259	91304	transmembrane protein 259
ENSG0000	62.79952	-0.344	0.298396	-1.15282	0.248985	0.540768	ZNF793	390927	zinc finger protein 793
ENSG0000	48.50888	-0.47478	0.411924	-1.15258	0.249082	0.540768	NA	NA	NA
ENSG0000	2.926708	1.666307	1.44598	1.152372	0.249168	0.540857	UBE2R2-A	1.02E+08	UBE2R2 antisense RNA 1
ENSG0000	25.92276	-0.71326	0.618972	-1.15233	0.249184	0.540857	SLC9A5	6553	solute carrier family 9 member A5
ENSG0000	311.646	0.239323	0.207762	1.151909	0.249358	0.541171	TIFA	92610	TRAF interacting protein with forkhead associated domain
ENSG0000	4.637886	3.43505	2.982746	1.15164	0.249469	0.541285	DNAJC27-A	729723	DNAJC27 antisense RNA 1
ENSG0000	25.42593	0.616405	0.535215	1.151696	0.249446	0.541285	KLHDC7B-I	1.05E+08	KLHDC7B divergent transcript
ENSG0000	89.24187	0.438336	0.380666	1.151496	0.249528	0.54135	SIK1	150094	salt inducible kinase 1
ENSG0000	1464.789	-0.11754	0.102112	-1.15107	0.249705	0.54167	U2SURP	23350	U2 snRNP associated SURP domain containing
ENSG0000	17.29102	0.705388	0.612896	1.150911	0.249769	0.541746	NA	NA	NA
ENSG0000	150.6534	-0.22733	0.19755	-1.15074	0.249841	0.541839	SLC38A9	153129	solute carrier family 38 member 9
ENSG0000	79.86661	0.309699	0.269208	1.150409	0.249975	0.542003	RPL15P3	653232	ribosomal protein L15 pseudogene 3
ENSG0000	2.931114	-1.71099	1.487212	-1.15047	0.249952	0.542003	MIR589	693174	microRNA 589
ENSG0000	6523.874	0.089145	0.077505	1.150178	0.25007	0.542082	JARID2	3720	jumonji and AT-rich interaction domain containing 2
ENSG0000	416.1259	0.157844	0.137234	1.150184	0.250068	0.542082	RDX	5962	radixin
ENSG0000	933.561	0.119023	0.103533	1.149619	0.250301	0.542343	DPH2	1802	diphthamide biosynthesis 2
ENSG0000	1.870144	-2.63418	2.291	-1.1498	0.250228	0.542343	FBN3	84467	fibrillin 3
ENSG0000	1076.957	0.118739	0.103283	1.14965	0.250288	0.542343	VPS26C	10311	VPS26 endosomal protein sorting factor C
ENSG0000	1847.361	0.117386	0.10211	1.149602	0.250308	0.542343	SH3BGRL	6451	SH3 domain binding glutamate rich protein like
ENSG0000	2.615399	1.911906	1.663235	1.14951	0.250346	0.542362	ABCA6	23460	ATP binding cassette subfamily A member 6
ENSG0000	4535.214	-0.12197	0.106129	-1.14929	0.250438	0.542435	KLF10	7071	KLF transcription factor 10
ENSG0000	3442.82	-0.10074	0.087652	-1.14934	0.250417	0.542435	GLIPR1	11010	GLI pathogenesis related 1
ENSG0000	90.79981	0.28965	0.252074	1.149066	0.250529	0.542569	COA6	388753	cytochrome c oxidase assembly factor 6
ENSG0000	476.3101	-0.16721	0.145541	-1.14886	0.250613	0.542687	CHM	1121	CHM Rab escort protein
ENSG0000	2.943711	-1.76554	1.536895	-1.14877	0.25065	0.542705	LOC12490	1.25E+08	uncharacterized LOC124904076
ENSG0000	118.5256	-0.27413	0.238724	-1.14833	0.250833	0.542721	BOLA1	51027	bolA family member 1
ENSG0000	781.6271	0.120897	0.105253	1.148634	0.250707	0.542721	CWC22	57703	CWC22 spliceosome associated protein homolog
ENSG0000	60.07663	-0.39447	0.343492	-1.14842	0.250796	0.542721	PSMG3-AS	114796	PSMG3 antisense RNA 1 (head to head)
ENSG0000	623.5277	0.131706	0.114688	1.148382	0.250811	0.542721	ZNF395	55893	zinc finger protein 395
ENSG0000	345.9587	-0.1717	0.149504	-1.14844	0.250787	0.542721	SLC29A3	55315	solute carrier family 29 member 3
ENSG0000	133.7903	0.304838	0.265432	1.148457	0.25078	0.542721	CCPG1	9236	cell cycle progression 1
ENSG0000	893.6199	0.11988	0.104407	1.1482	0.250886	0.542772	GNL3L	54552	G protein nucleolar 3 like
ENSG0000	32.85702	0.525802	0.458107	1.147771	0.251063	0.543091	TMC4	147798	transmembrane channel like 4
ENSG0000	424.2735	0.174358	0.151973	1.147293	0.25126	0.543455	RRM1	6240	ribonucleotide reductase catalytic subunit M1
ENSG0000	6.118006	-1.34332	1.171122	-1.14704	0.251365	0.543619	NA	NA	NA
ENSG0000	806.9378	-0.11914	0.103879	-1.14687	0.251436	0.543709	TGFBRAP1	9392	transforming growth factor beta receptor associated protein 1
ENSG0000	1067.19	-0.13501	0.117734	-1.1467	0.251504	0.543792	SLMAP	7871	sarcolemma associated protein
ENSG0000	29.41736	0.490801	0.428111	1.146434	0.251616	0.543908	SPATA6	54558	spermatogenesis associated 6
ENSG0000	292.9024	0.177294	0.154649	1.146432	0.251617	0.543908	MSANTD7	1E+08	Myb/SANT DNA binding domain containing 7
ENSG0000	8.759123	0.927038	0.808859	1.146106	0.251751	0.544136	NA	NA	NA
ENSG0000	4.187244	-1.42312	1.242239	-1.14561	0.251957	0.544159	SNORD46	94161	small nucl C/D box 46
ENSG0000	10.80029	0.82494	0.719945	1.145838	0.251862	0.544159	LOC10065	1.01E+08	uncharacterized LOC100652758
ENSG0000	2.995299	-1.82029	1.588668	-1.1458	0.25188	0.544159	LRRC73	221424	leucine rich repeat containing 73
ENSG0000	403.2025	-0.16893	0.147406	-1.146	0.251793	0.544159	MICU2	221154	mitochondrial calcium uptake 2
ENSG0000	16.70049	-0.75135	0.655859	-1.1456	0.251961	0.544159	ATP8A2	51761	ATPase phospholipid transporting 8A2
ENSG0000	9.438698	0.978704	0.854328	1.145584	0.251967	0.544159	MKRN3	7681	makorin ring finger protein 3
ENSG0000	3016.822	0.090029	0.078586	1.145601	0.25196	0.544159	PHF23	79142	PHD finger protein 23
ENSG0000	51.21522	-0.38942	0.34003	-1.14527	0.252099	0.544317	NA	NA	NA
ENSG0000	1754.662	-0.11864	0.103588	-1.14527	0.252097	0.544317	PRPF19	27339	pre-mRNA processing factor 19
ENSG0000	844.0121	-0.11893	0.103853	-1.14517	0.25214	0.544341	FIZ1	84922	FLT3 interacting zinc finger 1
ENSG0000	243.4153	-0.23076	0.20158	-1.14476	0.252308	0.544641	LMF1	64788	lipase maturation factor 1
ENSG0000	55.48836	0.397522	0.347356	1.144424	0.252448	0.544879	NA	NA	NA
ENSG0000	21.90472	-0.55638	0.486342	-1.14401	0.252618	0.545184	MKLN1-AS	1.01E+08	MKLN1 antisense RNA
ENSG0000	7.133143	0.944717	0.826181	1.143474	0.252842	0.545423	RPS10P13	646785	ribosomal protein S10 pseudogene 13
ENSG0000	3.985345	-1.55894	1.363342	-1.14347	0.252842	0.545423	METTL27	155368	methyltransferase like 27
ENSG0000	12.45794	-0.76409	0.668227	-1.14346	0.252847	0.545423	TEPP	374739	testis prostate and placenta expressed
ENSG0000	54.95102	0.476505	0.41666	1.143628	0.252778	0.545423	GP6	51206	glycoprotein VI platelet
ENSG0000	26.73055	-0.53732	0.469969	-1.14331	0.252912	0.5455	RAB6B	51560	RAB6B member RAS oncogene family

ENSG0000	1529.695	-0.10945	0.095756	-1.14302	0.253028	0.545688	SPRYD3	84926	SPRY domain containing 3
ENSG0000	7.976776	-1.04106	0.91101	-1.14276	0.253139	0.545864	NA	NA	NA
ENSG0000	18.17496	0.648748	0.567928	1.142306	0.253327	0.546198	NA	NA	NA
ENSG0000	9.299531	-0.98077	0.858635	-1.14224	0.253353	0.546198	TRAV35	28647	T cell receptor alpha variable 35
ENSG0000	732.728	0.138687	0.121432	1.142097	0.253414	0.546265	MANBA	4126	mannosidase beta
ENSG0000	20.81592	-0.73608	0.64465	-1.14184	0.253522	0.546431	RMDN2	151393	regulator of microtubule dynamics 2
ENSG0000	120.9486	0.28953	0.25358	1.141769	0.25355	0.546431	TMEM8B	51754	transmembrane protein 8B
ENSG0000	11788.8	-0.10124	0.088698	-1.14137	0.253717	0.546727	ARHGAP4!	23526	Rho GTPase activating protein 45
ENSG0000	6.223386	-1.12108	0.982375	-1.1412	0.253789	0.546755	NA	NA	NA
ENSG0000	6.04112	1.177248	1.031537	1.141257	0.253763	0.546755	NA	NA	NA
ENSG0000	585.6753	-0.14545	0.127498	-1.14084	0.253938	0.547014	ENOSF1	55556	enolase superfamily member 1
ENSG0000	537.4814	-0.14431	0.126573	-1.14016	0.254218	0.547489	KIF1B	23095	kinesin family member 1B
ENSG0000	8.450646	-1.01719	0.892141	-1.14017	0.254214	0.547489	NA	NA	NA
ENSG0000	377.0578	-0.16614	0.145804	-1.13945	0.254515	0.547748	LINC0227:	1.01E+08	long intergenic non-protein coding RNA 2273
ENSG0000	470.9649	-0.16881	0.148126	-1.13964	0.254438	0.547748	DBN1	1627	drebrin 1
ENSG0000	32.3002	0.515081	0.452	1.13956	0.25447	0.547748	ZNF485	220992	zinc finger protein 485
ENSG0000	1751.459	0.09875	0.086656	1.139565	0.254467	0.547748	TFDP1	7027	transcription factor Dp-1
ENSG0000	95.83148	0.300285	0.263533	1.139458	0.254512	0.547748	CENPV	201161	centromere protein V
ENSG0000	110.9109	-0.28052	0.246135	-1.13968	0.254419	0.547748	TTC28-AS1	284900	TTC28 antisense RNA 1
ENSG0000	959.8371	-0.1144	0.100442	-1.13896	0.254722	0.547868	EDEM3	80267	ER degradation enhancing alpha-mannosidase like protein 3
ENSG0000	17.43091	-0.62834	0.551708	-1.13889	0.254747	0.547868	NA	NA	NA
ENSG0000	108.9929	0.253096	0.222244	1.138821	0.254778	0.547868	ALPK1	80216	alpha kinase 1
ENSG0000	162.3699	-0.2655	0.233115	-1.13893	0.254731	0.547868	CDK20	23552	cyclin dependent kinase 20
ENSG0000	6.406593	1.164648	1.022633	1.138872	0.254757	0.547868	MYCBPAP	84073	MYCBP associated protein
ENSG0000	3.181002	2.074894	1.821494	1.139116	0.254655	0.547868	NA	NA	NA
ENSG0000	2.896405	1.828846	1.605752	1.138934	0.254731	0.547868	NA	NA	NA
ENSG0000	1580.184	-0.11225	0.098585	-1.1386	0.254868	0.547999	BRD8	10902	bromodomain containing 8
ENSG0000	90.61954	0.338868	0.297753	1.138084	0.255085	0.548003	NA	NA	NA
ENSG0000	5188.727	0.082514	0.072491	1.138263	0.255011	0.548003	KIF21B	23046	kinesin family member 21B
ENSG0000	348.2868	0.201255	0.176778	1.138462	0.254928	0.548003	ASTL	431705	astacin like metalloendopeptidase
ENSG0000	1988.403	-0.09475	0.08323	-1.13843	0.254939	0.548003	TOP2B	7155	DNA topoisomerase II beta
ENSG0000	508.8551	-0.13983	0.122859	-1.13809	0.255081	0.548003	DAG1	1605	dystroglycan 1
ENSG0000	1238.097	-0.10249	0.090055	-1.1381	0.255078	0.548003	SLC12A9	56996	solute carrier family 12 member 9
ENSG0000	1484.035	-0.10743	0.094397	-1.13803	0.255107	0.548003	ULK3	25989	unc-51 like kinase 3
ENSG0000	450.4642	-0.14344	0.126015	-1.13825	0.255018	0.548003	G6PC3	92579	glucose-6-phosphatase catalytic subunit 3
ENSG0000	2.302032	-1.97598	1.736729	-1.13776	0.25522	0.548184	NA	NA	NA
ENSG0000	1540.945	-0.10998	0.096674	-1.13768	0.255255	0.548195	KLHDC4	54758	kelch domain containing 4
ENSG0000	181.8256	0.2155	0.189504	1.137175	0.255465	0.548583	CTNNAL1	8727	catenin alpha like 1
ENSG0000	44.46091	0.428074	0.376541	1.136859	0.255597	0.54862	NA	NA	NA
ENSG0000	10.02356	0.887368	0.78041	1.137053	0.255516	0.54862	NA	NA	NA
ENSG0000	5.048899	1.49284	1.313136	1.136851	0.255601	0.54862	NA	NA	NA
ENSG0000	10116.03	-0.0743	0.065352	-1.13696	0.255556	0.54862	CHD3	1107	chromodomain helicase DNA binding protein 3
ENSG0000	383.9067	0.151365	0.133209	1.136301	0.255831	0.54905	NUMBL	9253	NUMB like endocytic adaptor protein
ENSG0000	20.25719	0.749497	0.659675	1.136161	0.255889	0.549082	MPL	4352	MPL proto thrombopoietin receptor
ENSG0000	54.69572	-0.37956	0.334105	-1.13605	0.255934	0.549082	TRBJ1-5	28631	T cell receptor beta joining 1-5
ENSG0000	1019.669	-0.12273	0.108034	-1.13606	0.255932	0.549082	ZNF777	27153	zinc finger protein 777
ENSG0000	580.3151	0.133153	0.117247	1.135666	0.256096	0.549362	TDG	6996	thymine DNA glycosylase
ENSG0000	1356.759	-0.10051	0.088508	-1.1356	0.256124	0.549362	NIN	51199	ninein
ENSG0000	18.44678	-0.77893	0.686203	-1.13514	0.256319	0.549716	DBH	1621	dopamine beta-hydroxylase
ENSG0000	105.9897	-0.2684	0.23649	-1.13494	0.256402	0.549832	GLRX2	51022	glutaredoxin 2
ENSG0000	1204.068	-0.12515	0.110284	-1.13477	0.256471	0.549851	PACS2	23241	phosphofurin acidic cluster sorting protein 2
ENSG0000	52.56681	0.40251	0.354685	1.134836	0.256444	0.549851	CDR2L	30850	cerebellar degeneration related protein 2 like
ENSG0000	174.3082	-0.25395	0.223808	-1.13468	0.25651	0.549871	INSR	3643	insulin receptor
ENSG0000	31.74404	-0.5834	0.514281	-1.1344	0.256625	0.550055	DEFB1	1672	defensin beta 1
ENSG0000	1995.539	0.103893	0.091595	1.134257	0.256687	0.550124	SLC9A1	6548	solute carrier family 9 member A1
ENSG0000	4.379519	-1.36937	1.207559	-1.134	0.256794	0.550235	RPL17P50	729046	ribosomal protein L17 pseudogene 50
ENSG0000	445.134	0.158257	0.139558	1.133991	0.256798	0.550235	DPM1	8813	dolichyl-pl catalytic
ENSG0000	4039.188	-0.08757	0.077229	-1.13385	0.256857	0.550298	SYNE2	23224	spectrin repeat containing nuclear envelope protein 2
ENSG0000	164.4746	0.275444	0.242987	1.133573	0.256974	0.550484	SLC25A33	84275	solute carrier family 25 member 33
ENSG0000	211.8616	-0.21349	0.188368	-1.13334	0.25707	0.550564	GFM2	84340	GTP dependent ribosome recycling factor mitochondrial 2
ENSG0000	73.47334	-0.40367	0.356175	-1.13336	0.257065	0.550564	C21orf58	54058	chromosome 21 open reading frame 58
ENSG0000	508.3246	0.142771	0.125991	1.133185	0.257137	0.55062	ATP5MJ	9556	ATP synthase membrane subunit j
ENSG0000	366.0814	0.169397	0.149494	1.133139	0.257156	0.55062	NKAP	79576	NFKB activating protein
ENSG0000	5.855859	-1.19559	1.055202	-1.13304	0.257197	0.550645	CACTIN-A!	404665	CACTIN antisense RNA 1
ENSG0000	606.7462	-0.14228	0.125611	-1.13269	0.257343	0.550893	ATRAID	51374	all-trans retinoic acid induced differentiation factor
ENSG0000	692.2347	-0.13858	0.122369	-1.13246	0.257442	0.551033	CCDC12	151903	coiled-coil domain containing 12
ENSG0000	136.8741	-0.2879	0.254242	-1.1324	0.257468	0.551033	XPNPEP3	63929	X-prolyl aminopeptidase 3
ENSG0000	2083.534	-0.26549	0.234541	-1.13195	0.257655	0.55137	FPR2	2358	formyl peptide receptor 2
ENSG0000	6.438363	-1.18787	1.049849	-1.13146	0.25786	0.551745	DLGAP4-A	1.02E+08	DLGAP4 antisense RNA 1
ENSG0000	2880.237	0.085916	0.075943	1.131313	0.257923	0.551818	SMARCD1	6602	SWI/SNF r matrix ass actin depe subfamily member 1
ENSG0000	982.5426	-0.11166	0.098745	-1.13074	0.258163	0.552266	C1orf159	54991	chromosome 1 open reading frame 159
ENSG0000	37.12957	0.514513	0.455096	1.130559	0.258241	0.552369	SYCP2	10388	synaptonemal complex protein 2
ENSG0000	465.7092	-0.16308	0.144269	-1.13038	0.258317	0.552469	C2orf42	54980	chromosome 2 open reading frame 42
ENSG0000	107.2271	-0.36187	0.320267	-1.12991	0.258512	0.552759	LTB4R2	56413	leukotriene B4 receptor 2
ENSG0000	4627.385	0.097663	0.086433	1.129926	0.258507	0.552759	CHERP	10523	calcium homeostasis endoplasmic reticulum protein
ENSG0000	52.01502	-0.40192	0.355795	-1.12965	0.258625	0.552936	SGSM1	129049	small G protein signaling modulator 1
ENSG0000	2494.119	-0.09833	0.087073	-1.12932	0.258762	0.553165	ZC3H7B	23264	zinc finger CCCH-type containing 7B
ENSG0000	484.3245	0.144068	0.127582	1.129217	0.258806	0.553195	TFPT	29844	TCF3 fusion partner
ENSG0000	296.1997	-0.18068	0.16006	-1.12882	0.258975	0.553301	EXOSC9	5393	exosome component 9
ENSG0000	62.25915	-0.34725	0.30758	-1.12897	0.258909	0.553301	STOML1	9399	stomatin like 1
ENSG0000	3.493061	-1.88208	1.667284	-1.12883	0.25897	0.553301	NA	NA	NA
ENSG0000	10.3474	-0.95291	0.844084	-1.12892	0.25893	0.553301	NA	NA	NA
ENSG0000	82.90974	-0.30236	0.267903	-1.12861	0.259064	0.553427	SNHG11	128439	small nucleolar RNA host gene 11
ENSG0000	5.340917	1.232162	1.091907	1.128449	0.25913	0.553442	RN7SL473I	1.06E+08	RNA 7SL cytoplasm pseudogene
ENSG0000	8.301888	0.971898	0.861253	1.12847	0.259121	0.553442	NKAPP1	158801	NFKB activating protein pseudogene 1
ENSG0000	5.874886	-1.17514	1.041705	-1.1281	0.259279	0.553505	FAM124B	79843	family with sequence similarity 124 member B
ENSG0000	2099.773	-0.10679	0.094657	-1.12822	0.259228	0.553505	TUT7	79670	terminal uridylyl transferase 7
ENSG0000	14.19372	-1.03195	0.914714	-1.12817	0.259249	0.553505	ADRB1	153	adrenoceptor beta 1
ENSG0000	375.1071	-0.16518	0.146422	-1.12814	0.259262	0.553505	POLR2F	5435	RNA polyn I and III subunit F
ENSG0000	2603.701	-0.09457	0.083854	-1.1278	0.259405	0.553709	SMARCC1	6599	SWI/SNF r matrix ass actin dependent regulator of chromatin subfamily c member 1
ENSG0000	16.15094	0.853535	0.75703	1.127479	0.25954	0.553934	RHBDF1	64285	rhomboid 5 homolog 1
ENSG0000	246.4971	-0.18993	0.168497	-1.1272	0.25966	0.554126	FAM217B	63939	family with sequence similarity 217 member B
ENSG0000	584.9408	-0.15182	0.134698	-1.12709	0.259706	0.554161	APOBEC3F	200316	apolipoprotein B mRNA editing enzyme catalytic subunit 3F
ENSG0000	391.6018	-0.15886	0.140977	-1.12684	0.259811	0.554322	SPIRE1	56907	spire type actin nucleation factor 1
ENSG0000	3019.83	-0.08886	0.078863	-1.12674	0.259851	0.554344	APOBEC3C	27350	apolipoprotein B mRNA editing enzyme catalytic subunit 3C
ENSG0000	4067.878	-0.09685	0.085991	-1.1263	0.26004	0.554682	MAN2B1	4125	mannosidase alpha class 2B member 1
ENSG0000	50.12672	-0.4223	0.374982	-1.12618	0.260089	0.554722	C17orf75	64149	chromosome 17 open reading frame 75
ENSG0000	3.760758	1.646425	1.462345	1.12588	0.260216	0.554913	NA	NA	NA
ENSG0000	1557.664	0.098975	0.087913	1.125829	0.260238	0.554913	NPTN	27020	neuroplastin
ENSG0000	4444.239	-0.08819	0.078342	-1.1257	0.260295	0.55497	BTN3A2	11118	butyrophilin subfamily 3 member A2

ENSG0000	342.2403	0.165285	0.146881	1.1253	0.260462	0.555263	SLC35D1	23169	solute carrier family 35 member D1
ENSG0000	2.387143	2.027653	1.802168	1.125119	0.260539	0.555363	NA	NA	NA
ENSG0000	98.58242	0.315123	0.280202	1.12463	0.260746	0.55574	CD200R1	131450	CD200 receptor 1
ENSG0000	868.5186	0.133236	0.118482	1.124524	0.260791	0.555772	ZNF281	23528	zinc finger protein 281
ENSG0000	6.816788	-1.18565	1.054491	-1.12438	0.260853	0.555789	NA	NA	NA
ENSG0000	23.25537	-0.76601	0.681281	-1.12436	0.260859	0.555789	NA	NA	NA
ENSG0000	32.98248	-0.47039	0.418405	-1.12423	0.260914	0.555843	DYNC2H1	79659	dynein cytoplasmic 2 heavy chain 1
ENSG0000	103.4565	0.282322	0.251204	1.123872	0.261067	0.555979	FCGR2B	2213	Fc gamma receptor IIb
ENSG0000	1024.053	-0.12464	0.110892	-1.12396	0.261029	0.555979	GRSF1	2926	G-rich RNA sequence binding factor 1
ENSG0000	471.6553	-0.14108	0.125523	-1.12393	0.261043	0.555979	ACAD8	27034	acyl-CoA dehydrogenase family member 8
ENSG0000	4.734921	1.563246	1.39118	1.123684	0.261147	0.556025	NA	NA	NA
ENSG0000	4.155137	-1.49278	1.328818	-1.12339	0.261273	0.556025	NA	NA	NA
ENSG0000	47.9837	0.410019	0.364947	1.123504	0.261224	0.556025	PRSS57	400668	serine protease 57
ENSG0000	5763.281	0.099928	0.088959	1.123304	0.261308	0.556025	NUCB1	4924	nucleobindin 1
ENSG0000	3.370449	-1.83857	1.636727	-1.12332	0.261302	0.556025	NA	NA	NA
ENSG0000	3300.143	0.088634	0.078894	1.123446	0.261248	0.556025	ASXL1	171023	ASXL transcriptional regulator 1
ENSG0000	128.4372	-0.3238	0.288268	-1.12326	0.261329	0.556025	STS	412	steroid sulfatase
ENSG0000	1523.101	0.107747	0.095902	1.123505	0.261223	0.556025	LAMP2	3920	lysosomal associated membrane protein 2
ENSG0000	277.546	0.174436	0.155434	1.122247	0.261757	0.556213	PSMD14	10213	proteasom non-ATPase 14
ENSG0000	13.54573	-0.77909	0.693895	-1.12278	0.261531	0.556213	RBM44	375316	RNA binding motif protein 44
ENSG0000	3.511941	1.617509	1.440453	1.122917	0.261473	0.556213	LOC10192	1.02E+08	uncharacterized LOC101927237
ENSG0000	1496.327	-0.11269	0.100394	-1.12251	0.261647	0.556213	GLCCI1	113263	glucocorticoid induced 1
ENSG0000	21.79895	-0.6217	0.553817	-1.12258	0.261616	0.556213	NA	NA	NA
ENSG0000	8.349126	-1.02746	0.915023	-1.12288	0.261489	0.556213	TNFRSF10	8794	TNF receptor superfamily member 10c
ENSG0000	108.6975	-0.30135	0.268509	-1.12233	0.261723	0.556213	ANKRD46	157567	ankyrin repeat domain 46
ENSG0000	15.88268	-0.7291	0.649709	-1.1222	0.261777	0.556213	NA	NA	NA
ENSG0000	79.70338	0.33675	0.300055	1.122294	0.261738	0.556213	TNNT3	7140	troponin T fast skeletal type
ENSG0000	37.48439	0.485169	0.432208	1.122537	0.261634	0.556213	LINC00937	389634	long intergenic non-protein coding RNA 937
ENSG0000	196.8013	0.198944	0.177255	1.122364	0.261708	0.556213	IFI27L2	83982	interferon alpha inducible protein 27 like 2
ENSG0000	756.9121	-0.11482	0.102277	-1.12264	0.261589	0.556213	EDC3	80153	enhancer of mRNA decapping 3
ENSG0000	4811.164	0.086165	0.076814	1.121737	0.261974	0.556242	RPN1	6184	ribophorin I
ENSG0000	14.33533	-0.69768	0.621796	-1.12204	0.261846	0.556242	DENND2B	6764	DENN domain containing 2B
ENSG0000	532.7405	0.172701	0.153926	1.121976	0.261873	0.556242	PANX1	24145	pannexin 1
ENSG0000	440.9932	-0.14218	0.126738	-1.12184	0.26193	0.556242	CDC16	8881	cell division cycle 16
ENSG0000	558.2966	-0.13768	0.122738	-1.12178	0.261957	0.556242	TRAPPC1	58485	trafficking protein particle complex subunit 1
ENSG0000	95.07782	-0.34986	0.311866	-1.12182	0.261939	0.556242	ZNF714	148206	zinc finger protein 714
ENSG0000	4498.826	0.109078	0.097245	1.121675	0.262001	0.556242	BCR	613	BCR activator of RhoGEF and GTPase
ENSG0000	23.21817	-0.65428	0.58336	-1.12157	0.262047	0.556276	DPEP3	64180	dipeptidase 3
ENSG0000	17.74173	0.804308	0.717273	1.121342	0.262142	0.556352	KRT2	3849	keratin 2
ENSG0000	747.3873	0.134063	0.119553	1.121367	0.262132	0.556352	CRELD2	79174	cysteine rich with EGF like domains 2
ENSG0000	101.6093	-0.26688	0.238018	-1.12126	0.262175	0.556357	NA	NA	NA
ENSG0000	374.4555	0.154851	0.138122	1.121118	0.262238	0.556363	SRPRB	58477	SRP receptor subunit beta
ENSG0000	9.021577	-0.85628	0.763772	-1.12113	0.262234	0.556363	NA	NA	NA
ENSG0000	1751.948	0.126252	0.112645	1.120798	0.262374	0.556588	RNF38	152006	ring finger protein 38
ENSG0000	8.405257	-1.12014	0.999545	-1.12065	0.262436	0.556657	NA	NA	NA
ENSG0000	6.347331	-1.21853	1.087436	-1.12055	0.26248	0.556685	TMEM11-I	1.08E+08	TMEM11 divergent transcript
ENSG0000	17.41034	-0.73111	0.652626	-1.12026	0.262601	0.556783	LAMTOR5-	1.01E+08	LAMTOR5 antisense RNA 1
ENSG0000	3.413359	-1.53124	1.3669	-1.12023	0.262616	0.556783	C6orf163	206412	chromosome 6 open reading frame 163
ENSG0000	5.966754	-1.27816	1.140864	-1.12034	0.262567	0.556783	NA	NA	NA
ENSG0000	350.4203	-0.16297	0.145499	-1.1201	0.262672	0.556839	ZNF500	26048	zinc finger protein 500
ENSG0000	243.7776	0.197161	0.176043	1.11996	0.262731	0.556899	NCK1	4690	NCK adaptor protein 1
ENSG0000	332.5507	-0.16858	0.150541	-1.11981	0.262796	0.55691	SDK2	54549	sidekick cell adhesion molecule 2
ENSG0000	128.0995	0.25311	0.226022	1.119843	0.262781	0.55691	NA	NA	NA
ENSG0000	166.6861	0.266809	0.238294	1.119665	0.262857	0.556964	MRPS18C	51023	mitochondrial ribosomal protein S18C
ENSG0000	312.5431	-0.17676	0.157895	-1.1195	0.262926	0.556964	PDCD6	10016	programmed cell death 6
ENSG0000	14.02161	-0.83074	0.741999	-1.11959	0.262888	0.556964	NOS3	4846	nitric oxide synthase 3
ENSG0000	10.02545	1.117403	0.998221	1.119395	0.262972	0.556964	GATA3-AS	399717	GATA3 antisense RNA 1
ENSG0000	529.5162	0.16347	0.14603	1.119425	0.262959	0.556964	JUP	3728	junction plakoglobin
ENSG0000	4.269086	1.360397	1.215619	1.119099	0.263098	0.557168	NA	NA	NA
ENSG0000	193.2304	0.199812	0.178582	1.118881	0.263191	0.557174	RPLP0P6	220717	ribosomal protein lateral stalk subunit P0 pseudogene 6
ENSG0000	114.7897	-0.26175	0.233933	-1.11889	0.263186	0.557174	KRBOX5	124411	KRAB box domain containing 5
ENSG0000	7.317586	-1.0852	0.969779	-1.11901	0.263135	0.557174	NA	NA	NA
ENSG0000	4561.038	-0.10434	0.093265	-1.1187	0.263268	0.557273	OGFR	11054	opioid growth factor receptor
ENSG0000	1348.003	-0.09687	0.086604	-1.11857	0.263322	0.557325	ATXN7L3B	552889	ataxin 7 like 3B
ENSG0000	734.5179	0.132673	0.118636	1.118314	0.263433	0.557496	PEX16	9409	peroxisomal biogenesis factor 16
ENSG0000	14.9184	-0.67815	0.606464	-1.11821	0.263477	0.557526	SPAG16	79582	sperm associated antigen 16
ENSG0000	82.99217	-0.29659	0.265261	-1.11812	0.263517	0.557547	COQ5	84274	coenzyme methyltransferase
ENSG0000	188.3144	-0.63149	0.564856	-1.11797	0.263579	0.557614	IGLV1-40	28825	immunoglobulin lambda variable 1-40
ENSG0000	274.9419	0.172609	0.154436	1.117675	0.263706	0.557819	RPS15P4	401019	ribosomal protein S15 pseudogene 4
ENSG0000	373.5257	-0.16705	0.14952	-1.11726	0.263882	0.557852	NBPF12	149013	NBPF member 12
ENSG0000	3103.901	0.094243	0.084377	1.116925	0.264026	0.557852	PI4KB	5298	phosphatidylinositol 4-kinase beta
ENSG0000	1791.367	0.114667	0.102681	1.116731	0.26411	0.557852	PHC3	80012	polyhomeotic homolog 3
ENSG0000	65.80603	-0.35172	0.314809	-1.11724	0.26389	0.557852	SRFBP1	153443	serum response factor binding protein 1
ENSG0000	779.4821	0.119815	0.107227	1.117404	0.263822	0.557852	UCK1	83549	uridine-cytidine kinase 1
ENSG0000	71.67013	-0.37961	0.339756	-1.1173	0.263865	0.557852	KLRC4	8302	killer cell lectin like receptor C4
ENSG0000	164.7357	-0.2503	0.224053	-1.11715	0.26393	0.557852	ACADS	35	acyl-CoA dehydrogenase short chain
ENSG0000	24.48523	0.613592	0.549458	1.116723	0.264113	0.557852	NA	NA	NA
ENSG0000	474.2898	-0.13766	0.123211	-1.11726	0.263882	0.557852	CLPX	10845	caseinolytic mitochondrial matrix peptidase chaperone subunit X
ENSG0000	442.3446	0.143994	0.128934	1.116797	0.264081	0.557852	UBFD1	56061	ubiquitin family domain containing 1
ENSG0000	2139.948	0.099081	0.088693	1.117116	0.263945	0.557852	PELP1	27043	proline glutamate and leucine rich protein 1
ENSG0000	85.99994	0.318745	0.285401	1.116832	0.264066	0.557852	ZNF235	9310	zinc finger protein 235
ENSG0000	323.8369	0.155718	0.139412	1.116964	0.26401	0.557852	SAMM50	25813	SAMM50 sorting and assembly machinery component
ENSG0000	208.7588	0.202353	0.181349	1.115825	0.264497	0.558515	ALPL	249	alkaline pł biomineralization associated
ENSG0000	234.7602	-0.19893	0.17833	-1.11551	0.264632	0.558515	KIF3C	3797	kinesin family member 3C
ENSG0000	3.835386	1.692521	1.517064	1.115656	0.264569	0.558515	MUC4	4585	mucin 4 cell surface associated
ENSG0000	1430.122	-0.10382	0.093053	-1.11575	0.264528	0.558515	GUSB	2990	glucuronidase beta
ENSG0000	41.5378	0.520216	0.46632	1.115576	0.264604	0.558515	WDHD1	11169	WD repeat and HMG-box DNA binding protein 1
ENSG0000	635.7653	-0.17098	0.153273	-1.11552	0.264626	0.558515	CUL4B	8450	cullin 4B
ENSG0000	87.52729	0.345238	0.309492	1.115497	0.264638	0.558515	NA	NA	NA
ENSG0000	24.61342	-0.58861	0.527908	-1.11498	0.264858	0.558917	NA	NA	NA
ENSG0000	16.91271	0.65586	0.588335	1.114771	0.264948	0.559044	NA	NA	NA
ENSG0000	422.2581	0.170348	0.152834	1.114597	0.265023	0.559108	C2orf49	79074	chromosome 2 open reading frame 49
ENSG0000	5.548445	1.200618	1.077218	1.114555	0.265041	0.559108	MYO1A	4640	myosin IA
ENSG0000	42.48816	-0.41655	0.373762	-1.11449	0.265069	0.559108	KNL1	57082	kinetochore scaffold 1
ENSG0000	4.728726	1.102971	0.989841	1.114291	0.265154	0.559224	NA	NA	NA
ENSG0000	2676.989	-0.08595	0.077152	-1.11409	0.265242	0.559346	BTN3A3	10384	butyrophilin subfamily 3 member A3
ENSG0000	37.36516	0.506813	0.454991	1.113897	0.265323	0.559438	MIRLET7A	1.13E+08	miRlet-7a-1/let-7f-1/let-7d cluster host gene
ENSG0000	309.4741	0.209357	0.187971	1.113774	0.265376	0.559438	POLR2M	81488	RNA polymerase II subunit M
ENSG0000	1921.125	0.100724	0.090433	1.113797	0.265366	0.559438	BCAP31	10134	B cell receptor associated protein 31
ENSG0000	4.67072	-1.46074	1.311781	-1.11356	0.26547	0.559572	TEDC2	80178	tubulin epsilon and delta complex 2

ENSG0000	9.825462	-0.87969	0.790091	-1.1134	0.265537	0.55965	NA	NA	NA
ENSG0000	75.2505	-0.31412	0.282214	-1.11305	0.265687	0.559714	DPY19L4	286148	dpy-19 like 4
ENSG0000	1627.575	-0.11825	0.106232	-1.11316	0.265641	0.559714	APBB1	322	amyloid beta precursor protein binding family B member 1
ENSG0000	980.8879	0.108476	0.097443	1.113228	0.265611	0.559714	NAT10	55226	N-acetyltransferase 10
ENSG0000	5798.296	0.082346	0.073982	1.113047	0.265688	0.559714	CNOT3	4849	CCR4-NOT transcription complex subunit 3
ENSG0000	836.278	0.148181	0.133141	1.112967	0.265723	0.559723	IGHG3	3502	immunoglobulin heavy constant gamma 3 (G3m marker)
ENSG0000	2060.091	0.10683	0.096046	1.112275	0.26602	0.560222	PTBP3	9991	polypyrimidine tract binding protein 3
ENSG0000	5016.07	0.089975	0.08071	1.112309	0.266005	0.560222	SET	6418	SET nuclear proto-oncogene
ENSG0000	223.5622	-0.19931	0.179221	-1.1121	0.266093	0.560246	ZNF532	55205	zinc finger protein 532
ENSG0000	27.42565	0.543621	0.488852	1.112037	0.266122	0.560246	MIR646HG	284757	MIR646 host gene
ENSG0000	102.789	-0.28081	0.252502	-1.11209	0.266098	0.560246	ARMCX5	64860	armadillo repeat containing X-linked 5
ENSG0000	4.361823	1.252047	1.126425	1.111523	0.266343	0.560585	NA	NA	NA
ENSG0000	22.8041	-0.60022	0.539979	-1.11156	0.266327	0.560585	NA	NA	NA
ENSG0000	230.0972	-0.19291	0.173578	-1.11139	0.266403	0.560646	LRP5L	91355	LDL receptor related protein 5 like (pseudogene)
ENSG0000	3.557117	1.365899	1.229309	1.111111	0.26652	0.56083	HNRNPA3I	1E+08	heterogeneous nuclear ribonucleoprotein A3 pseudogene 12
ENSG0000	7.914374	-0.90309	0.812978	-1.11085	0.266635	0.561007	NA	NA	NA
ENSG0000	15.73473	-0.78108	0.703226	-1.11071	0.266693	0.561065	LRRC2	79442	leucine rich repeat containing 2
ENSG0000	426.22	-0.16636	0.149791	-1.11059	0.266745	0.561111	POLR3A	11128	RNA polymerase III subunit A
ENSG0000	4.240692	-1.28618	1.158414	-1.1103	0.266872	0.561124	OR1L8	138881	olfactory receptor family 1 subfamily L member 8
ENSG0000	36.76515	0.488865	0.440228	1.110482	0.266791	0.561124	NA	NA	NA
ENSG0000	23.28771	-0.59375	0.53471	-1.11041	0.266823	0.561124	NXN	64359	nucleoredoxin
ENSG0000	4356.664	0.084857	0.076425	1.110328	0.266858	0.561124	XRCC6	2547	X-ray repair cross complementing 6
ENSG0000	4.499229	1.506929	1.357531	1.110052	0.266977	0.561281	NA	NA	NA
ENSG0000	5.741834	1.117557	1.006896	1.109903	0.267041	0.561304	NA	NA	NA
ENSG0000	49.80844	0.441497	0.397826	1.109774	0.267096	0.561304	NDUFB8	4714	NADH:ubiquinone oxidoreductase subunit B8
ENSG0000	478.5386	-0.13985	0.126017	-1.10974	0.267109	0.561304	MAP2K5	5607	mitogen-activated protein kinase kinase 5
ENSG0000	102.6959	0.283116	0.255098	1.109831	0.267072	0.561304	NA	NA	NA
ENSG0000	422.8534	0.156808	0.141313	1.109646	0.267152	0.56133	MCU	90550	mitochondrial calcium uniporter
ENSG0000	61.44929	0.342589	0.3088	1.109422	0.267248	0.561469	SMIM4	440957	small integral membrane protein 4
ENSG0000	12.3673	0.824159	0.743025	1.109195	0.267346	0.561548	NA	NA	NA
ENSG0000	1049.303	-0.11435	0.103083	-1.10926	0.267319	0.561548	VAMP1	6843	vesicle associated membrane protein 1
ENSG0000	2052.464	-0.09621	0.086755	-1.10895	0.267452	0.561707	EXOSC10	5394	exosome component 10
ENSG0000	222.956	0.240636	0.217105	1.108388	0.267694	0.562152	PLEKHM3	389072	pleckstrin homology domain containing M3
ENSG0000	76.42303	-0.32372	0.292122	-1.10818	0.267785	0.562278	KCTD1	284252	potassium channel tetramerization domain containing 1
ENSG0000	53.34597	-0.43033	0.388412	-1.10792	0.267896	0.562447	GPAT2	150763	glycerol-3- mitochondrial
ENSG0000	44.40532	0.514855	0.464865	1.107535	0.268063	0.562671	LINC00243	401247	long intergenic non-protein coding RNA 243
ENSG0000	10.42237	-0.82244	0.742563	-1.10756	0.26805	0.562671	VSTM4	196740	V-set and transmembrane domain containing 4
ENSG0000	220.643	0.208304	0.188113	1.107332	0.26815	0.562788	SPRY1	10252	sprouty RTK signaling antagonist 1
ENSG0000	312.5221	-0.19538	0.176453	-1.10726	0.268179	0.562788	HLA-DRB6	3128	major hist class II DR beta 6 (pseudogene)
ENSG0000	7.96173	0.96531	0.871931	1.107095	0.268253	0.562879	NA	NA	NA
ENSG0000	48.96822	0.371415	0.335528	1.106957	0.268313	0.56294	CATSPERG	57828	cation channel sperm associated auxiliary subunit gamma
ENSG0000	693.2144	-0.13282	0.120014	-1.1067	0.268423	0.563109	MRPL43	84545	mitochondrial ribosomal protein L43
ENSG0000	14.01155	0.788967	0.71335	1.106004	0.268725	0.563487	CKAP2L	150468	cytoskeleton associated protein 2 like
ENSG0000	4.74852	3.086625	2.790325	1.106188	0.268645	0.563487	NA	NA	NA
ENSG0000	2230.239	-0.10376	0.093814	-1.10603	0.268713	0.563487	ATXN1	6310	ataxin 1
ENSG0000	7.089429	1.078431	0.975062	1.106013	0.268721	0.563487	UXT-AS1	1E+08	UXT antisense RNA 1
ENSG0000	27.40785	-0.59086	0.534271	-1.10591	0.268764	0.563505	MCF2L2	23101	MCF.2 cell line derived transforming sequence-like 2
ENSG0000	12.20794	0.811526	0.733902	1.105768	0.268827	0.563573	ASAP3	55616	ArfGAP wi ankyrin repeat and PH domain 3
ENSG0000	61.39949	-0.3543	0.320521	-1.10538	0.268993	0.563756	ARHGAP24	83478	Rho GTPase activating protein 24
ENSG0000	10.44692	0.804987	0.72817	1.105492	0.268946	0.563756	NA	NA	NA
ENSG0000	1403.364	-0.10892	0.098541	-1.10536	0.269005	0.563756	PGS1	9489	phosphatidylglycerophosphate synthase 1
ENSG0000	90.44315	-0.33025	0.29893	-1.10477	0.269259	0.56416	CLCN2	1181	chloride voltage-gated channel 2
ENSG0000	7314.046	-0.07564	0.068461	-1.10481	0.269243	0.56416	CTDSP2	10106	CTD small phosphatase 2
ENSG0000	50.32179	0.459164	0.415686	1.104594	0.269336	0.564193	SLC16A1-A	1.01E+08	SLC16A1 antisense RNA 1
ENSG0000	176.9464	0.240505	0.217721	1.104651	0.269311	0.564193	FAM153A	285596	family with sequence similarity 153 member A
ENSG0000	3.469582	-1.58702	1.436996	-1.1044	0.269419	0.56429	CFAP73	387885	cilia and flagella associated protein 73
ENSG0000	1857.723	-0.12216	0.110614	-1.10435	0.269443	0.56429	OSCAR	126014	osteoclast associated Ig-like receptor
ENSG0000	9.234903	-0.88871	0.804877	-1.10415	0.269527	0.564403	NA	NA	NA
ENSG0000	3.778061	1.527898	1.383977	1.103991	0.269597	0.564486	CMYA5	202333	cardiomyopathy associated 5
ENSG0000	57.47485	0.388765	0.352301	1.103502	0.269809	0.564866	ACSL6	23305	acyl-CoA synthetase long chain family member 6
ENSG0000	11.546	-0.97155	0.880583	-1.10331	0.269894	0.564967	DSC1	1823	desmocollin 1
ENSG0000	156.1415	0.223712	0.202775	1.103251	0.269918	0.564967	B3GNT8	374907	UDP-GlcN 3-N-acetylglucosaminyltransferase 8
ENSG0000	2949.387	0.125898	0.114123	1.103175	0.269951	0.564972	JMJD1C	221037	jumonji domain containing 1C
ENSG0000	663.7401	-0.14533	0.131759	-1.10303	0.270015	0.565043	CPSF4	10898	cleavage and polyadenylation specific factor 4
ENSG0000	30.60897	0.52422	0.475345	1.102821	0.270105	0.565062	RAB13	5872	RAB13 member RAS oncogene family
ENSG0000	6.195919	1.063152	0.964101	1.102739	0.270141	0.565062	SCGB1C1	147199	secretoglobin family 1C member 1
ENSG0000	7.787396	1.131773	1.02621	1.102866	0.270085	0.565062	NA	NA	NA
ENSG0000	21.51974	0.620993	0.563144	1.102726	0.270146	0.565062	NA	NA	NA
ENSG0000	9.64228	1.014993	0.920525	1.102624	0.27019	0.56509	NA	NA	NA
ENSG0000	5.672005	1.246394	1.130885	1.10214	0.270401	0.565467	NA	NA	NA
ENSG0000	14.37839	-0.70509	0.639795	-1.10205	0.270438	0.565477	RPSAP54	642641	ribosomal protein SA pseudogene 54
ENSG0000	19.49232	-0.61605	0.559034	-1.10199	0.270467	0.565477	GTSE1	51512	G2 and S-phase expressed 1
ENSG0000	955.9521	0.161768	0.146831	1.101732	0.270578	0.565638	UBXN7	26043	UBX domain protein 7
ENSG0000	589.6778	-0.13793	0.125203	-1.10167	0.270605	0.565638	ZBTB22	9278	zinc finger and BTB domain containing 22
ENSG0000	154.1013	-0.22924	0.208107	-1.10154	0.27066	0.56569	KRTCAP2	200185	keratinocyte associated protein 2
ENSG0000	11.69774	0.713454	0.647924	1.101138	0.270837	0.565995	NA	NA	NA
ENSG0000	863.1466	-0.12357	0.112229	-1.10104	0.270877	0.566016	TRIM24	8805	tripartite motif containing 24
ENSG0000	362.0093	-0.16652	0.151247	-1.10096	0.270916	0.566034	YIF1A	10897	Yip1 intera membrane trafficking protein
ENSG0000	20.03552	0.597478	0.542732	1.100871	0.270953	0.566047	OGFOD2	79676	2-oxoglutarate and iron dependent oxygenase domain containing 2
ENSG0000	8.47926	-1.05849	0.96174	-1.1006	0.271071	0.56623	NA	NA	NA
ENSG0000	86.9916	-0.32698	0.297161	-1.10035	0.271178	0.566389	NA	NA	NA
ENSG0000	582.5497	-0.13377	0.121588	-1.10023	0.271231	0.566437	NDUFS1	4719	NADH:ubiquinone oxidoreductase core subunit S1
ENSG0000	3.288489	1.720706	1.564347	1.099952	0.271353	0.566476	NA	NA	NA
ENSG0000	103.4434	0.273466	0.248611	1.099979	0.271341	0.566476	HSPA14	51182	heat shock protein family A (Hsp70) member 14
ENSG0000	586.1829	-0.12668	0.115178	-1.09986	0.271394	0.566476	ING4	51147	inhibitor of growth family member 4
ENSG0000	962.7238	0.116726	0.106126	1.099881	0.271384	0.566476	PRKAB1	5564	protein kinase AMP-activated non-catalytic subunit beta 1
ENSG0000	48.50031	-0.38319	0.348402	-1.09984	0.271403	0.566476	TRAV12-2	28673	T cell receptor alpha variable 12-2
ENSG0000	1005.104	-0.11224	0.10208	-1.09956	0.271524	0.566603	IGSF8	93185	immunoglobulin superfamily member 8
ENSG0000	1100.629	0.103721	0.09433	1.099558	0.271525	0.566603	SELENOW	6415	selenoprotein W
ENSG0000	252.9142	-0.20613	0.187502	-1.09935	0.271614	0.566662	ACOX3	8310	acyl-CoA c pristanoyl
ENSG0000	11.93483	-0.87419	0.795171	-1.09937	0.271606	0.566662	LOC10272	1.03E+08	immunoglobulin heavy variable 4-38-2-like
ENSG0000	5.293493	-1.13596	1.033839	-1.09877	0.271867	0.566682	NA	NA	NA
ENSG0000	228.5084	-0.18694	0.170144	-1.0987	0.271898	0.566682	WWC2	80014	WW and C2 domain containing 2
ENSG0000	8.179618	1.061024	0.965715	1.098693	0.271902	0.566682	MOCS2-DT	257396	MOCS2 divergent transcript
ENSG0000	3.516068	-1.79513	1.633405	-1.09901	0.271763	0.566682	PGAM2	5224	phosphoglycerate mutase 2
ENSG0000	7.017819	-1.09292	0.994525	-1.09894	0.271796	0.566682	MTND4P1	1.01E+08	MT-ND4 pseudogene 14
ENSG0000	2.27145	-2.15415	1.960243	-1.09892	0.271803	0.566682	NA	NA	NA
ENSG0000	82.38113	-0.29614	0.26954	-1.09867	0.27191	0.566682	N4BP2L2-I	116828	N4BPL2 intronic transcript 2
ENSG0000	26.82743	-0.51613	0.469548	-1.0992	0.271682	0.566682	NA	NA	NA
ENSG0000	7.635206	-1.02143	0.929598	-1.09879	0.27186	0.566682	ZNF460-AS	1.05E+08	ZNF460 antisense RNA 1

ENSG0000	7.476572	-1.03571	0.942728	-1.09863	0.271929	0.566682	SSR4P1	728039	signal sequence receptor subunit 4 pseudogene 1
ENSG0000	577.0803	0.135499	0.123399	1.098061	0.272178	0.567137	NOA1	84273	nitric oxide associated 1
ENSG0000	369.5824	-0.15955	0.145324	-1.0979	0.272247	0.567216	TOE1	114034	target of E exonuclease
ENSG0000	17.1184	0.619867	0.564687	1.097719	0.272327	0.567321	NA	NA	NA
ENSG0000	6.508493	-1.1093	1.011126	-1.0971	0.272599	0.567751	NA	NA	NA
ENSG0000	549.0719	0.16747	0.152657	1.097035	0.272626	0.567751	ACER3	55331	alkaline ceramidase 3
ENSG0000	536.1938	-0.14502	0.13218	-1.09716	0.27257	0.567751	ACTR10	55860	actin related protein 10
ENSG0000	5.611496	1.483931	1.352793	1.096938	0.272668	0.567776	NA	NA	NA
ENSG0000	13.19223	0.687967	0.627214	1.096861	0.272702	0.567782	FOXC1	2296	forkhead box C1
ENSG0000	4.936525	-1.56046	1.422759	-1.09678	0.272736	0.56779	NA	NA	NA
ENSG0000	65.46121	-0.33014	0.301194	-1.09609	0.273038	0.568354	ZNF138	7697	zinc finger protein 138
ENSG0000	804.5163	0.126176	0.115131	1.095936	0.273107	0.568434	TOR1B	27348	torsin family 1 member B
ENSG0000	248.735	-0.18369	0.16767	-1.09557	0.273267	0.568703	TKFC	26007	triokinase and FMN cyclase
ENSG0000	10.2521	0.816976	0.745874	1.095327	0.273373	0.56886	FAM171A2	284069	family with sequence similarity 171 member A2
ENSG0000	76.16314	-0.30225	0.275991	-1.09513	0.27346	0.568978	SHLD1	149840	shieldin complex subunit 1
ENSG0000	2215.037	-0.11512	0.105145	-1.09485	0.273584	0.569001	RBM47	54502	RNA binding motif protein 47
ENSG0000	414.0797	0.158765	0.145026	1.094738	0.273632	0.569001	SLC22A4	6583	solute carrier family 22 member 4
ENSG0000	198.8172	0.215477	0.196798	1.094918	0.273552	0.569001	KIF3A	11127	kinesin family member 3A
ENSG0000	1831.363	0.11435	0.104449	1.09479	0.273608	0.569001	NELFE	7936	negative elongation factor complex member E
ENSG0000	832.2081	-0.11235	0.102612	-1.09488	0.273568	0.569001	WDR46	9277	WD repeat domain 46
ENSG0000	115.6924	-0.24008	0.219311	-1.09468	0.273655	0.569001	ZNF34	80778	zinc finger protein 34
ENSG0000	100.5245	-0.35379	0.323214	-1.09459	0.273695	0.569008	TRAV8-2	28684	T cell receptor alpha variable 8-2
ENSG0000	538.3841	-0.1369	0.125079	-1.09453	0.27372	0.569008	UBASH3A	53347	ubiquitin associated and SH3 domain containing A
ENSG0000	488.1337	-0.16714	0.152719	-1.0944	0.273779	0.569066	THOC5	8563	THO complex subunit 5
ENSG0000	140.5742	0.227128	0.207607	1.094027	0.273943	0.569252	NA	NA	NA
ENSG0000	1954.199	0.094943	0.086785	1.093999	0.273955	0.569252	ARF3	377	ADP ribosylation factor 3
ENSG0000	83.78532	-0.32014	0.292635	-1.09399	0.27396	0.569252	LOC10272	1.03E+08	glutamine amidotransferase class 1 domain containing 3B
ENSG0000	8.252419	-1.02569	0.93785	-1.09366	0.274104	0.569477	CXCL14	9547	C-X-C motif chemokine ligand 14
ENSG0000	141.5646	-0.23877	0.218391	-1.09332	0.274253	0.569477	NA	NA	NA
ENSG0000	3803.889	-0.09437	0.0863	-1.09351	0.274171	0.569477	HIP1R	9026	huntingtin interacting protein 1 related
ENSG0000	139.1815	-0.26707	0.244244	-1.09345	0.274195	0.569477	KNOP1	400506	lysine rich nucleolar protein 1
ENSG0000	12.35149	0.737889	0.674898	1.093335	0.274247	0.569477	NA	NA	NA
ENSG0000	190.484	0.196549	0.179749	1.093464	0.27419	0.569477	ZNF551	90233	zinc finger protein 551
ENSG0000	942.4215	-0.14025	0.128316	-1.09304	0.274375	0.569618	NEK7	140609	NIMA related kinase 7
ENSG0000	4.771971	1.214478	1.111115	1.093026	0.274382	0.569618	RNY1P11	1.06E+08	RNY1 pseudogene 11
ENSG0000	36.73803	0.433978	0.397082	1.092919	0.27443	0.569652	PHGDH	26227	phosphoglycerate dehydrogenase
ENSG0000	16.99674	-0.62635	0.573192	-1.09274	0.274507	0.569686	NA	NA	NA
ENSG0000	10.94282	-0.83208	0.761426	-1.09279	0.274485	0.569686	NA	NA	NA
ENSG0000	20.00257	-0.59869	0.548039	-1.09243	0.274645	0.569909	NA	NA	NA
ENSG0000	182.6765	-0.21484	0.196721	-1.09212	0.274779	0.570059	ALS2	57679	alsin Rho guanine nucleotide exchange factor ALS2
ENSG0000	15.14571	0.693488	0.634977	1.092147	0.274769	0.570059	NA	NA	NA
ENSG0000	141.4895	0.222858	0.204111	1.091843	0.274902	0.570187	WNT1	7471	Wnt family member 1
ENSG0000	5340.818	-0.08411	0.077034	-1.09188	0.274886	0.570187	AP3D1	8943	adaptor related protein complex 3 subunit delta 1
ENSG0000	119.654	-0.27192	0.249079	-1.09169	0.274968	0.57026	CRLS1	54675	cardiolipin synthase 1
ENSG0000	917.5101	-0.11229	0.102868	-1.09159	0.275012	0.570286	PTGES2	80142	prostaglandin E synthase 2
ENSG0000	4.839153	1.247908	1.143319	1.091478	0.275063	0.570328	NA	NA	NA
ENSG0000	5.57535	-3.07048	2.813415	-1.09137	0.275109	0.57036	MIR124-1H	157627	MIR124-1 host gene
ENSG0000	134.346	-0.28193	0.258388	-1.09113	0.275217	0.57052	TLR7	51284	toll like receptor 7
ENSG0000	242.1831	0.225673	0.206883	1.090823	0.275351	0.570624	MED30	90390	mediator complex subunit 30
ENSG0000	280.6223	0.188996	0.173263	1.090804	0.275359	0.570624	ENDOG	2021	endonuclease G
ENSG0000	666.2586	0.123258	0.112987	1.090903	0.275315	0.570624	CLCF1	23529	cardiotrophin like cytokine factor 1
ENSG0000	8.083069	0.963154	0.88306	1.0907	0.275405	0.570649	STK32B	55351	serine/threonine kinase 32B
ENSG0000	5.471227	-1.33329	1.222488	-1.09064	0.275433	0.570649	MSMP	692094	microsemi prostate associated
ENSG0000	2895.727	-0.09237	0.084706	-1.09044	0.275519	0.570672	MAPKAPK	7867	MAPK activated protein kinase 3
ENSG0000	115.4163	-0.247	0.226522	-1.0904	0.275536	0.570672	TTC16	158248	tetratricopeptide repeat domain 16
ENSG0000	11.05496	0.963935	0.883921	1.090522	0.275483	0.570672	CYP11A1	1583	cytochrome P450 family 11 subfamily A member 1
ENSG0000	3.363532	1.402275	1.286393	1.090083	0.275676	0.570777	RPL17P36	729340	ribosomal protein L17 pseudogene 36
ENSG0000	298.1882	-0.17792	0.163215	-1.09008	0.275679	0.570777	LINC0064	1.01E+08	long intergenic non-protein coding RNA 649
ENSG0000	3324.964	0.101841	0.093425	1.090081	0.275677	0.570777	MED14	9282	mediator complex subunit 14
ENSG0000	100.1887	-0.29072	0.26673	-1.08995	0.275737	0.570783	TRBJ1-6	28630	T cell receptor beta joining 1-6
ENSG0000	25.69633	-0.57655	0.528981	-1.08993	0.275744	0.570783	AFF2	2334	ALF transcription elongation factor 2
ENSG0000	96.63378	-0.26368	0.241938	-1.08986	0.275775	0.570785	TEKTIP1	1E+08	tektin bundle interacting protein 1
ENSG0000	450.9793	-0.1474	0.135278	-1.08963	0.275874	0.570809	LPP	4026	LIM domain containing preferred translocation partner in lipoma
ENSG0000	8.940461	0.965102	0.885688	1.089664	0.275861	0.570809	NA	NA	NA
ENSG0000	8.907266	-0.99314	0.91145	-1.08962	0.275879	0.570809	TMEM272	283521	transmembrane protein 272
ENSG0000	14.56954	-0.6584	0.604356	-1.08942	0.275969	0.570931	PEX11A	8800	peroxisomal biogenesis factor 11 alpha
ENSG0000	338.4718	0.169335	0.155452	1.089302	0.276021	0.570974	COPS5	10987	COP9 signalosome subunit 5
ENSG0000	4.642952	-1.1799	1.083336	-1.08913	0.276095	0.571065	ZNF404	342908	zinc finger protein 404
ENSG0000	7.00277	1.277182	1.172902	1.088907	0.276195	0.571143	DNER	92737	delta/notch like EGF repeat containing
ENSG0000	448.0636	-0.13522	0.124179	-1.08893	0.276183	0.571143	EIF4E3	317649	eukaryotic translation initiation factor 4E family member 3
ENSG0000	86.89233	0.331925	0.304923	1.088553	0.276351	0.571402	CAMK2N2	94032	calcium/calmodulin dependent protein kinase II inhibitor 2
ENSG0000	424.1594	0.147176	0.135217	1.088441	0.276401	0.571441	RMDN3	55177	regulator of microtubule dynamics 3
ENSG0000	24.1187	-0.61126	0.561652	-1.08832	0.276454	0.571488	HOXA7	3204	homeobox A7
ENSG0000	1257.057	-0.12986	0.119401	-1.08763	0.276756	0.571795	MAP3K1	4214	mitogen-activated protein kinase kinase kinase 1
ENSG0000	56.90885	-0.35114	0.322816	-1.08773	0.276712	0.571795	LRRCC1	85444	leucine rich repeat and coiled-coil centrosomal protein 1
ENSG0000	95.07537	0.319466	0.29371	1.087691	0.276732	0.571795	NA	NA	NA
ENSG0000	608.7957	0.121548	0.111748	1.087692	0.276731	0.571795	ZNF791	163049	zinc finger protein 791
ENSG0000	31.67812	-0.47267	0.434472	-1.08791	0.276636	0.571795	ZNF850	342892	zinc finger protein 850
ENSG0000	133.88	-0.29601	0.272189	-1.08752	0.276809	0.571839	DPY19L3	147991	dpy-19 like C-mannosyltransferase 3
ENSG0000	789.5009	-0.16437	0.15118	-1.08726	0.27692	0.572006	ZNF644	84146	zinc finger protein 644
ENSG0000	8.151048	-1.13615	1.045312	-1.0869	0.277079	0.57227	NA	NA	NA
ENSG0000	225.9938	0.197421	0.18168	1.086646	0.277193	0.572339	UTP23	84294	UTP23 small subunit processome component
ENSG0000	513.6661	-0.15596	0.143557	-1.08643	0.277289	0.572339	HDHD3	81932	haloacid dehalogenase like hydrolase domain containing 3
ENSG0000	1230.53	-0.10191	0.093812	-1.08635	0.277323	0.572339	LRRC8A	56262	leucine rich repeat containing 8 VRAC subunit A
ENSG0000	348.1367	-0.16727	0.153962	-1.08644	0.277284	0.572339	TMEM80	283232	transmembrane protein 80
ENSG0000	12.63771	-0.87675	0.806982	-1.08646	0.277276	0.572339	AQP11	282679	aquaporin 11
ENSG0000	76.32657	-0.34508	0.317607	-1.08651	0.277252	0.572339	TGFB3	7043	transforming growth factor beta 3
ENSG0000	83.19752	0.316061	0.290941	1.08634	0.277329	0.572339	RDH13	112724	retinol dehydrogenase 13
ENSG0000	388.9641	0.18338	0.168875	1.085888	0.277528	0.572663	THAP3	90326	THAP domain containing 3
ENSG0000	3.525317	1.38186	1.27264	1.085822	0.277558	0.572663	NA	NA	NA
ENSG0000	1.683298	-2.46077	2.26637	-1.08578	0.277578	0.572663	NA	NA	NA
ENSG0000	13.20057	0.682122	0.628492	1.085331	0.277775	0.573006	WNK2	65268	WNK lysine deficient protein kinase 2
ENSG0000	10.02493	0.802808	0.739835	1.085118	0.27787	0.573009	NA	NA	NA
ENSG0000	114.8797	-0.26907	0.247934	-1.08524	0.277815	0.573009	ANKRD13B	124930	ankyrin repeat domain 13B
ENSG0000	1027.499	0.105577	0.097294	1.085139	0.27786	0.573009	TRMT1	55621	tRNA methyltransferase 1
ENSG0000	506.6109	0.132853	0.122444	1.085013	0.277916	0.573042	NOM1	64434	nucleolar protein with MIF4G domain 1
ENSG0000	277.5474	-0.17427	0.160643	-1.08484	0.277992	0.573072	PPIH	10465	peptidylprolyl isomerase H
ENSG0000	8.923026	-0.94401	0.870148	-1.08488	0.277974	0.573072	NA	NA	NA
ENSG0000	985.309	0.117195	0.108038	1.08475	0.278033	0.573091	SMIM12	113444	small integral membrane protein 12
ENSG0000	265.6617	0.168484	0.155433	1.083966	0.27838	0.573452	ZBED3	84327	zinc finger BED-type containing 3

ENSG0000	197.6907	0.200565	0.184989	1.084197	0.278277	0.573452	RP9	6100	RP9 pre-mRNA splicing factor
ENSG0000	55.40531	-0.39194	0.361591	-1.08394	0.278394	0.573452	GTSF1	121355	gametocyte specific factor 1
ENSG0000	895.6589	-0.11049	0.101929	-1.08394	0.278391	0.573452	XPOT	11260	exportin for tRNA
ENSG0000	241.4918	-0.223	0.205681	-1.08422	0.278266	0.573452	WIPI1	55062	WD repea phosphoinositide interacting 1
ENSG0000	55.3858	-0.34269	0.316126	-1.08402	0.278358	0.573452	NA	NA	NA
ENSG0000	20.42189	0.565211	0.521557	1.083701	0.278497	0.573603	LOC10798	1.08E+08	uncharacterized LOC107985688
ENSG0000	253.6585	-0.18329	0.169167	-1.08347	0.278598	0.57369	MGST3	4259	microsomal glutathione S-transferase 3
ENSG0000	9.100174	-0.83241	0.768327	-1.08341	0.278628	0.57369	ECI2-DT	1.01E+08	ECI2 divergent transcript
ENSG0000	11.31467	-0.93009	0.858497	-1.0834	0.278633	0.57369	ZNF208	7757	zinc finger protein 208
ENSG0000	70.45715	-0.30632	0.282771	-1.08327	0.278688	0.57374	ANKRD6	22881	ankyrin repeat domain 6
ENSG0000	4.335696	-1.71602	1.58431	-1.08313	0.278749	0.573792	NMNAT2	23057	nicotinamide nucleotide adenylyltransferase 2
ENSG0000	149.9545	-0.24263	0.224022	-1.08308	0.278775	0.573792	MRPL40	64976	mitochondrial ribosomal protein L40
ENSG0000	65.50106	-0.39461	0.364446	-1.08276	0.278916	0.574019	NA	NA	NA
ENSG0000	12.66568	-0.75021	0.693024	-1.08251	0.279025	0.574062	SGO1	151648	shugoshin 1
ENSG0000	25.73323	0.596737	0.55137	1.082281	0.279128	0.574062	TBC1D32	221322	TBC1 domain family member 32
ENSG0000	715.2726	-0.12751	0.117779	-1.0826	0.278985	0.574062	SLC39A9	55334	solute carrier family 39 member 9
ENSG0000	256.7224	-0.17387	0.160659	-1.08222	0.279154	0.574062	ZNF75A	7627	zinc finger protein 75a
ENSG0000	509.1953	-0.13413	0.123908	-1.08246	0.279049	0.574062	CARHSP1	23589	calcium regulated heat stable protein 1
ENSG0000	170.7629	-0.22729	0.210015	-1.08227	0.279135	0.574062	ZNF419	79744	zinc finger protein 419
ENSG0000	184.2081	0.216184	0.199747	1.082292	0.279123	0.574062	PDRG1	81572	p53 and DNA damage regulated 1
ENSG0000	67.91874	-0.47836	0.442098	-1.08203	0.279238	0.574163	ADARB2	105	adenosine deaminase RNA specific B2 (inactive)
ENSG0000	710.5078	-0.13084	0.120924	-1.08197	0.279265	0.574163	CDAN1	146059	codanin 1
ENSG0000	3.982706	-1.61435	1.492409	-1.08171	0.279383	0.574342	LOC10537	1.05E+08	uncharacterized LOC105375798
ENSG0000	2793.266	-0.08891	0.082239	-1.0811	0.279651	0.57483	MDH2	4191	malate dehydrogenase 2
ENSG0000	2879.361	-0.09985	0.092369	-1.08095	0.279718	0.574903	LAT2	7462	linker for activation of T cells family member 2
ENSG0000	106.9918	-0.29172	0.269942	-1.08068	0.279841	0.575092	FRA10AC1	118924	FRA10A associated CGG repeat 1
ENSG0000	35.55048	-0.46636	0.431679	-1.08034	0.279991	0.575226	NA	NA	NA
ENSG0000	1732.832	-0.1169	0.108193	-1.08044	0.279945	0.575226	PIK3CG	5294	phosphati 5-bisphosphate 3-kinase catalytic subunit gamma
ENSG0000	2.984154	-1.45921	1.350718	-1.08032	0.279999	0.575226	GSG1L	146395	GSG1 like
ENSG0000	286.4896	0.188676	0.174728	1.079828	0.280219	0.575576	RRAGC	64121	Ras related GTP binding C
ENSG0000	526.377	-0.14313	0.13255	-1.0798	0.280232	0.575576	DNAJC10	54431	DnaJ heat shock protein family (Hsp40) member C10
ENSG0000	370.3263	-0.15379	0.142458	-1.07956	0.280337	0.575728	MTCH2	23788	mitochondrial carrier 2
ENSG0000	3.689157	-1.43146	1.326153	-1.0794	0.280407	0.575746	ZNF230-D	1.02E+08	ZNF230 divergent transcript
ENSG0000	37.99496	-0.41006	0.379883	-1.07943	0.280398	0.575746	ACTL10	170487	actin like 10
ENSG0000	220.6024	0.212963	0.197319	1.079285	0.280461	0.575792	FH	2271	fumarate hydratase
ENSG0000	977.5782	0.117204	0.108634	1.078882	0.28064	0.576097	ZNF319	57567	zinc finger protein 319
ENSG0000	3.926453	1.331678	1.234702	1.078542	0.280792	0.576345	NA	NA	NA
ENSG0000	15.83578	-0.80371	0.745311	-1.07835	0.280877	0.576456	NA	NA	NA
ENSG0000	15.82349	-0.70484	0.654002	-1.07774	0.28115	0.576633	MSANTD1	345222	Myb/SANT DNA binding domain containing 1
ENSG0000	149.173	-0.23248	0.215682	-1.07789	0.281085	0.576633	NTNG2	84628	netrin G2
ENSG0000	6.259565	-1.0602	0.983695	-1.07777	0.281134	0.576633	RAG1	5896	recombination activating 1
ENSG0000	897.9885	-0.11191	0.103829	-1.07778	0.281132	0.576633	EIF2B1	1967	eukaryotic translation initiation factor 2B subunit alpha
ENSG0000	567.4885	0.128336	0.119058	1.07793	0.281065	0.576633	N4BP2L1	90634	NEDD4 binding protein 2 like 1
ENSG0000	8945.915	-0.08047	0.074664	-1.07778	0.281134	0.576633	ITGAL	3683	integrin subunit alpha L
ENSG0000	707.2042	-0.11455	0.106306	-1.07754	0.281241	0.576755	PABIR1	116224	PP2A Aalpha (PPP2R1A) and B55A (PPP2R2A) interacting phosphatase regulator 1
ENSG0000	9.296275	0.937508	0.870112	1.077457	0.281276	0.576764	PLEKHG4B	153478	pleckstrin homology and RhoGEF domain containing G4B
ENSG0000	3.746852	1.606084	1.49131	1.076962	0.281497	0.577153	NA	NA	NA
ENSG0000	121.7862	-0.24532	0.227803	-1.07689	0.281529	0.577155	COL5A3	50509	collagen type V alpha 3 chain
ENSG0000	81.78621	-0.29752	0.276329	-1.07669	0.281621	0.577279	NA	NA	NA
ENSG0000	2.023183	-2.41485	2.243167	-1.07654	0.281687	0.577287	LOC12490	1.25E+08	uncharacterized LOC124902005
ENSG0000	259.0146	0.189149	0.175696	1.076568	0.281674	0.577287	NAA38	84316	N-alpha-a NatC auxiliary subunit
ENSG0000	287.7012	-0.16706	0.155228	-1.07621	0.281831	0.577519	LYRM2	57226	LYR motif containing 2
ENSG0000	14.48241	-0.6607	0.614062	-1.07595	0.281948	0.577644	DISP3	57540	dispatched RND transporter family member 3
ENSG0000	2282.897	-0.09811	0.09119	-1.07591	0.281966	0.577644	POLRMT	5442	RNA polymerase mitochondrial
ENSG0000	1156.422	0.115733	0.107571	1.07587	0.281986	0.577644	KRI1	65095	KRI1 homolog
ENSG0000	4.71233	-1.14803	1.067385	-1.07556	0.282126	0.577867	BMS1P4	729096	BMS1 pseudogene 4
ENSG0000	1805.16	-0.10627	0.098825	-1.07531	0.282235	0.578004	PYGO2	90780	pygopus family PHD finger 2
ENSG0000	12.4013	-0.73932	0.687569	-1.07527	0.282255	0.578004	TRAV2	28691	T cell receptor alpha variable 2
ENSG0000	71.50728	-0.32465	0.301971	-1.07512	0.282322	0.578077	DAB2IP	153090	DAB2 interacting protein
ENSG0000	5.131663	-1.31681	1.224961	-1.07498	0.282383	0.578138	NA	NA	NA
ENSG0000	248.8401	0.185296	0.172387	1.074889	0.282424	0.578159	SOCS5	9655	suppressor of cytokine signaling 5
ENSG0000	3.936905	1.597665	1.486747	1.074604	0.282552	0.578293	NA	NA	NA
ENSG0000	12.56747	-0.84351	0.784931	-1.07462	0.282543	0.578293	RNF32-DT	1.01E+08	RNF32 divergent transcript
ENSG0000	3.443233	1.890619	1.759805	1.074334	0.282673	0.578417	NA	NA	NA
ENSG0000	228.7277	-0.17433	0.162275	-1.07431	0.282685	0.578417	INPP5F	22876	inositol polyphosphate-5-phosphatase F
ENSG0000	18.18469	-0.76731	0.714268	-1.07426	0.282706	0.578417	FNDC11	79025	fibronectin type III domain containing 11
ENSG0000	160.1787	0.221462	0.206184	1.074098	0.282779	0.578502	AGL	178	amylo-alpl 6-glucosic 4-alpha-glucanotransferase
ENSG0000	59.83569	0.340768	0.317403	1.073615	0.282995	0.578711	NA	NA	NA
ENSG0000	30.29615	0.470715	0.438443	1.073606	0.282999	0.578711	CLECL1P	160365	C-type lec pseudogene
ENSG0000	504.6873	0.146013	0.136001	1.073616	0.282995	0.578711	CCDC47	57003	coiled-coil domain containing 47
ENSG0000	5.320944	-1.35898	1.265829	-1.07359	0.283006	0.578711	HRH4	59340	histamine receptor H4
ENSG0000	135.8252	-0.24801	0.231036	-1.07347	0.28306	0.578757	MORN3	283385	MORN repeat containing 3
ENSG0000	54.51305	-0.39958	0.372261	-1.07339	0.283095	0.578766	CAB39L	81617	calcium binding protein 39 like
ENSG0000	8730.88	-0.07965	0.074217	-1.07321	0.283177	0.578805	SPEN	23013	spen family transcriptional repressor
ENSG0000	105.2243	-0.26402	0.246021	-1.07316	0.283201	0.578805	CDKN2C	1031	cyclin dependent kinase inhibitor 2C
ENSG0000	1051.34	0.129453	0.120631	1.073128	0.283214	0.578805	ZEB1	6935	zinc finger E-box binding homeobox 1
ENSG0000	21.02162	-0.59592	0.555345	-1.07307	0.283239	0.578805	GLS2	27165	glutaminase 2
ENSG0000	3.724387	1.462863	1.363495	1.072878	0.283326	0.578919	NA	NA	NA
ENSG0000	4404.085	-0.07791	0.07263	-1.07276	0.283378	0.578961	CAMTA2	23125	calmodulin binding transcription activator 2
ENSG0000	3.910974	1.559593	1.454168	1.072499	0.283496	0.579088	LINC02981	441204	long intergenic non-protein coding RNA 2981
ENSG0000	3.015964	-1.96167	1.829093	-1.07248	0.283502	0.579088	AOC1	26	amine oxidase copper containing 1
ENSG0000	481.4888	-0.14083	0.13136	-1.07209	0.283679	0.579386	ATP11C	286410	ATPase phospholipid transporting 11C
ENSG0000	5.565406	-1.02871	0.959711	-1.0719	0.283766	0.579434	PXMP2	5827	peroxisomal membrane protein 2
ENSG0000	4.65334	-1.21637	1.134742	-1.07194	0.283749	0.579434	C1QL1	10882	complement C1q like 1
ENSG0000	620.3514	-0.16595	0.154846	-1.07172	0.283844	0.579466	SDHC	6391	succinate dehydrogenase complex subunit C
ENSG0000	230.8774	-0.17489	0.163182	-1.07174	0.283838	0.579466	FAN1	22909	FANCD2 and FANCI associated nuclease 1
ENSG0000	47.40583	-0.3775	0.352268	-1.07164	0.283883	0.579475	CCDC125	202243	coiled-coil domain containing 125
ENSG0000	48.24486	0.395208	0.36881	1.071576	0.28391	0.579475	WNT11	7481	Wnt family member 11
ENSG0000	1274.971	0.097333	0.090854	1.071311	0.28403	0.579654	ELAVL1	1994	ELAV like RNA binding protein 1
ENSG0000	440.4484	-0.13813	0.128979	-1.07091	0.284209	0.579803	MAST4	375449	microtubule associated serine/threonine kinase family member 4
ENSG0000	79.59955	-0.29992	0.2801	-1.07077	0.284272	0.579803	ARHGAP2	1.09E+08	ARHGAP2 transcribed pseudogene
ENSG0000	15.26249	0.628832	0.587274	1.070765	0.284275	0.579803	EEF1DP1	126037	eukaryotic translation elongation factor 1 delta pseudogene 1
ENSG0000	454.0641	-0.14176	0.132394	-1.07073	0.28429	0.579803	MRPS12	6183	mitochondrial ribosomal protein S12
ENSG0000	635.1395	0.12747	0.119034	1.070876	0.284225	0.579803	UBL4A	8266	ubiquitin like 4A
ENSG0000	632.1191	0.178923	0.167101	1.070747	0.284283	0.579803	F8A1	8263	coagulation factor VIII associated 1
ENSG0000	11.04734	0.747844	0.698617	1.070464	0.284411	0.57997	ZNF687-AS	1.01E+08	ZNF687 antisense RNA 1
ENSG0000	29.65654	-0.46632	0.43565	-1.07041	0.284435	0.57997	TAPT1-AS1	202020	TAPT1 antisense RNA 1 (head to head)
ENSG0000	13.62002	0.664853	0.621221	1.070237	0.284513	0.580014	CFAP45	25790	cilia and flagella associated protein 45
ENSG0000	50.33167	0.393836	0.367994	1.070224	0.284519	0.580014	LOC10050	1.01E+08	uncharacterized LOC100506274
ENSG0000	3206.248	0.092386	0.086337	1.070064	0.284591	0.580097	PCSK7	9159	proprotein convertase subtilisin/kexin type 7

ENSG0000	326.6079	0.150151	0.14034	1.069904	0.284663	0.580098	SDHAP1	255812	SDHA pseudogene 1
ENSG0000	16.07128	-0.62822	0.587204	-1.06985	0.284685	0.580098	NA	NA	NA
ENSG0000	886.5023	-0.11042	0.103201	-1.06995	0.28464	0.580098	TAF3	83860	TATA-box binding protein associated factor 3
ENSG0000	901.2821	-0.11481	0.107367	-1.06936	0.284908	0.580234	CEP104	9731	centrosomal protein 104
ENSG0000	8.64855	-1.00682	0.941451	-1.06943	0.284876	0.580234	NA	NA	NA
ENSG0000	8624.329	-0.09242	0.086418	-1.06946	0.284864	0.580234	RIN3	79890	Ras and Rab interactor 3
ENSG0000	74.28999	-0.32997	0.308533	-1.06948	0.284855	0.580234	SMG1P1	641298	SMG1 pseudogene 1
ENSG0000	4.101801	-1.40231	1.311356	-1.06936	0.284907	0.580234	ZNF491	126069	zinc finger protein 491
ENSG0000	43.08053	-0.38062	0.355958	-1.06928	0.284945	0.580244	CBX8	57332	chromobox 8
ENSG0000	1638.215	-0.12813	0.119842	-1.06913	0.28501	0.580315	TBKBP1	9755	TBK1 binding protein 1
ENSG0000	10.49395	1.001339	0.937369	1.068245	0.28541	0.581065	CCN2	1490	cellular communication network factor 2
ENSG0000	705.8848	-0.12094	0.113228	-1.06813	0.285462	0.581107	AEBP1	165	AE binding protein 1
ENSG0000	72.40705	-0.30622	0.286751	-1.06791	0.285562	0.581246	NUDT1	4521	nudix hydrolase 1
ENSG0000	114.3837	0.25214	0.236161	1.06766	0.285674	0.581411	EPS8L2	64787	EPS8 like 2
ENSG0000	33.22103	0.61274	0.574011	1.067472	0.285759	0.58152	NA	NA	NA
ENSG0000	183.5228	-0.25322	0.237419	-1.06655	0.286175	0.582303	PLEKHG5	57449	pleckstrin homology and RhoGEF domain containing G5
ENSG0000	44.32426	0.398891	0.374065	1.066371	0.286256	0.582333	PODN	127435	podocan
ENSG0000	9.944227	-0.89804	0.842198	-1.06631	0.286284	0.582333	TMIE	259236	transmembrane inner ear
ENSG0000	86.44244	0.318641	0.298795	1.066418	0.286235	0.582333	TIMM23B	1.01E+08	translocase of inner mitochondrial membrane 23 homolog B
ENSG0000	21.44169	0.530276	0.497349	1.066204	0.286331	0.582365	RPL13P5	283345	ribosomal protein L13 pseudogene 5
ENSG0000	3.696092	-1.47024	1.379387	-1.06587	0.286483	0.582546	FAM72D	728833	family with sequence similarity 72 member D
ENSG0000	283.3479	-0.16024	0.150327	-1.06594	0.286452	0.582546	LCMT1	51451	leucine carboxyl methyltransferase 1
ENSG0000	2.945409	1.759345	1.650785	1.065763	0.286531	0.582579	HPCA	3208	hippocalcin
ENSG0000	737.3943	0.331658	0.311255	1.065552	0.286626	0.582645	HLX	3142	H2.0 like homeobox
ENSG0000	2.42798	-1.84435	1.730847	-1.06558	0.286615	0.582645	NA	NA	NA
ENSG0000	51.96311	0.450699	0.423208	1.064957	0.286895	0.583004	NA	NA	NA
ENSG0000	10.71194	0.960414	0.901837	1.064953	0.286897	0.583004	IGKV1-6	28943	immunoglobulin kappa variable 1-6
ENSG0000	3.37443	-1.51685	1.424166	-1.06508	0.28684	0.583004	NA	NA	NA
ENSG0000	199.8777	-0.20379	0.191371	-1.06488	0.28693	0.583007	SDHAP3	728609	SDHA pseudogene 3
ENSG0000	48.59112	0.364454	0.342295	1.064735	0.286996	0.583078	NA	NA	NA
ENSG0000	9.271216	-0.98098	0.921514	-1.06453	0.287088	0.5832	NA	NA	NA
ENSG0000	133.9357	-0.23819	0.223811	-1.06426	0.287209	0.583383	CENPJ	55835	centromere protein J
ENSG0000	7709.5	0.07339	0.068968	1.06412	0.287275	0.583452	NCOA4	8031	nuclear receptor coactivator 4
ENSG0000	10.47616	-0.87842	0.825586	-1.064	0.28733	0.5835	BORCS8-M	4207	BORCS8-MEF2B readthrough
ENSG0000	7.01811	-1.0417	0.97923	-1.06379	0.287422	0.583623	RN7SL181I	1.06E+08	RNA 7SL cytoplasm pseudogene
ENSG0000	5.737088	1.222967	1.149806	1.063629	0.287497	0.583712	NA	NA	NA
ENSG0000	8226.598	0.080255	0.075474	1.063336	0.28763	0.583853	CNOT1	23019	CCR4-NOT transcription complex subunit 1
ENSG0000	9.897031	0.759614	0.714361	1.063348	0.287624	0.583853	NA	NA	NA
ENSG0000	281.4367	-0.17062	0.160471	-1.06322	0.287683	0.58386	UVRAG	7405	UV radiation resistance associated
ENSG0000	3459.032	-0.08938	0.084071	-1.06319	0.287696	0.58386	ETV6	2120	ETS variant transcription factor 6
ENSG0000	21409.4	0.081967	0.077102	1.063095	0.287739	0.583883	TSC22D3	1831	TSC22 domain family member 3
ENSG0000	3.554803	-1.86445	1.754245	-1.06282	0.287863	0.583894	LOC10192!	1.02E+08	uncharacterized LOC101929691
ENSG0000	1686.384	-0.12193	0.114716	-1.06285	0.287848	0.583894	ANXA6	309	annexin A6
ENSG0000	947.6879	0.107443	0.101096	1.06278	0.287882	0.583894	CCT2	10576	chaperonin containing TCP1 subunit 2
ENSG0000	1892.752	0.113952	0.107225	1.062737	0.287901	0.583894	SCYL2	55681	SCY1 like pseudokinase 2
ENSG0000	11.97097	-0.7371	0.693527	-1.06282	0.287862	0.583894	DNAJC3-D	1E+08	DNAJC3 divergent transcript
ENSG0000	38.76198	0.43105	0.405699	1.062486	0.288015	0.583912	EDARADD	128178	EDAR associated death domain
ENSG0000	26.65508	-0.496	0.466766	-1.06263	0.287949	0.583912	NA	NA	NA
ENSG0000	1194.915	-0.10991	0.103446	-1.06244	0.288036	0.583912	LOC72874	728743	zinc finger domain-containing protein LOC728743
ENSG0000	5.96364	1.456439	1.370845	1.062439	0.288036	0.583912	NA	NA	NA
ENSG0000	2.405588	-1.96321	1.848111	-1.06228	0.288109	0.583995	LIMCH1	22998	LIM and calponin homology domains 1
ENSG0000	241.0274	0.178896	0.168541	1.061439	0.28849	0.584111	TMEM201	199953	transmembrane protein 201
ENSG0000	582.17	-0.12783	0.120469	-1.06112	0.288637	0.584111	SNRNP40	9410	small nuclear ribonucleoprotein U5 subunit 40
ENSG0000	91.64712	0.258765	0.24385	1.061162	0.288616	0.584111	LINC0098!	1.01E+08	long intergenic non-protein coding RNA 989
ENSG0000	14900.58	-0.06904	0.065044	-1.06149	0.288469	0.584111	RAPGEF1	2889	Rap guanine nucleotide exchange factor 1
ENSG0000	5.227162	1.228867	1.157222	1.061911	0.288276	0.584111	TPH1	7166	tryptophan hydroxylase 1
ENSG0000	8.296991	0.828745	0.780463	1.061863	0.288298	0.584111	RPL13AP2(	387841	ribosomal protein L13a pseudogene 20
ENSG0000	350.0322	0.19268	0.181535	1.061393	0.288511	0.584111	NFE2	4778	nuclear fa(erythroid 2
ENSG0000	32.42907	-0.47122	0.443998	-1.0613	0.288553	0.584111	L2HGDH	79944	L-2-hydroxyglutarate dehydrogenase
ENSG0000	3150.174	0.087885	0.082749	1.062061	0.288208	0.584111	CIRBP	1153	cold inducible RNA binding protein
ENSG0000	808.165	-0.1474	0.138907	-1.06111	0.288638	0.584111	PAK4	10298	p21 (RAC1) activated kinase 4
ENSG0000	3.720158	-1.69851	1.600338	-1.06135	0.288533	0.584111	NA	NA	NA
ENSG0000	623.3289	-0.14184	0.133586	-1.06179	0.288332	0.584111	AHCY	191	adenosylhomocysteinase
ENSG0000	1032.329	0.107147	0.100959	1.06129	0.288558	0.584111	CSE1L	1434	chromosome segregation 1 like
ENSG0000	332.9336	0.17891	0.168553	1.061446	0.288487	0.584111	ZNF74	7625	zinc finger protein 74
ENSG0000	474.7143	-0.1353	0.127413	-1.06192	0.288272	0.584111	PICK1	9463	protein interacting with PRKCA 1
ENSG0000	299.7153	-0.16563	0.156108	-1.06102	0.288682	0.584135	CPT2	1376	carnitine palmitoyltransferase 2
ENSG0000	17.33308	0.68896	0.649444	1.060846	0.28876	0.58423	PRR29-AS1	400612	PRR29 antisense RNA 1
ENSG0000	2978.153	0.095819	0.090338	1.06067	0.28884	0.584326	NAA50	80218	N-alpha-a(NatE catalytic subunit
ENSG0000	59.31202	0.334011	0.314926	1.060602	0.288871	0.584326	NA	NA	NA
ENSG0000	1157.303	0.124134	0.117054	1.060492	0.288921	0.584327	HSPA4	3308	heat shock protein family A (Hsp70) member 4
ENSG0000	144.0982	0.21479	0.202544	1.060462	0.288934	0.584327	MRPL15	29088	mitochondrial ribosomal protein L15
ENSG0000	4.634538	-1.38926	1.310551	-1.06006	0.289117	0.584569	NA	NA	NA
ENSG0000	631.3757	0.12693	0.119734	1.0601	0.289099	0.584569	TBCA	6902	tubulin folding cofactor A
ENSG0000	15.19835	0.658808	0.621552	1.059941	0.289171	0.584616	NA	NA	NA
ENSG0000	2618.386	-0.08906	0.08403	-1.05985	0.289212	0.584635	SH2B1	25970	SH2B adaptor protein 1
ENSG0000	179.7263	-0.19959	0.188344	-1.05971	0.289277	0.584702	FAAH	2166	fatty acid amide hydrolase
ENSG0000	15655.85	0.078413	0.074004	1.05958	0.289336	0.584755	ETS1	2113	ETS proto- transcription factor
ENSG0000	60.08862	-0.4366	0.412079	-1.05951	0.289367	0.584755	VWF	7450	von Willebrand factor
ENSG0000	599.4239	0.132018	0.124631	1.059278	0.289473	0.584907	PLXDC1	57125	plexin domain containing 1
ENSG0000	101.8739	0.267113	0.252219	1.05905	0.289577	0.585053	TRIAP1	51499	TP53 regulated inhibitor of apoptosis 1
ENSG0000	125.1147	-0.22741	0.214806	-1.05867	0.289751	0.585286	GTPBP10	85865	GTP binding protein 10
ENSG0000	13.84075	0.794218	0.750212	1.058659	0.289755	0.585286	IGHV4-61	28391	immunoglobulin heavy variable 4-61
ENSG0000	13.35516	0.737221	0.696592	1.058324	0.289908	0.585529	RET	5979	ret proto-oncogene
ENSG0000	330.6579	0.194041	0.183395	1.058044	0.290035	0.585724	RIMOC1	285636	RAB7A interacting MON1-CCZ1 complex subunit 1
ENSG0000	9.120758	-0.96584	0.912942	-1.05795	0.29008	0.58575	PCCA-DT	1.05E+08	PCCA divergent transcript
ENSG0000	23.29533	0.516069	0.488083	1.057337	0.290358	0.586247	ELOVL6	79071	ELOVL fatty acid elongase 6
ENSG0000	18.5243	0.579038	0.547786	1.057053	0.290487	0.586445	NA	NA	NA
ENSG0000	894.1467	-0.12815	0.12124	-1.05697	0.290525	0.586458	FAM3A	60343	FAM3 metabolism regulating signaling molecule A
ENSG0000	7.065297	-0.98363	0.9309	-1.05664	0.290676	0.586699	LINC02072	440446	long intergenic non-protein coding RNA 2072
ENSG0000	4.075655	-1.74021	1.647355	-1.05637	0.290801	0.586855	MIXL1	83881	Mix paired-like homeobox
ENSG0000	515.2621	-0.13978	0.132332	-1.05626	0.290849	0.586855	METAP1	23173	methionyl aminopeptidase 1
ENSG0000	8.571073	0.824261	0.780327	1.056302	0.29083	0.586855	CREB3L1	90993	cAMP responsive element binding protein 3 like 1
ENSG0000	13548.39	0.07914	0.074946	1.055966	0.290984	0.587059	GPSM3	63940	G protein signaling modulator 3
ENSG0000	3.360319	1.471366	1.393469	1.055902	0.291013	0.587059	POT1-AS1	401398	POT1 antisense RNA 1
ENSG0000	10.99015	-0.83145	0.78756	-1.05573	0.291092	0.587155	SLC22A16	85413	solute carrier family 22 member 16
ENSG0000	1773.372	-0.11199	0.106143	-1.05512	0.291372	0.587543	ACAP3	116983	ArfGAP wi ankyrin repeat and PH domains 3
ENSG0000	191.2043	0.209439	0.198524	1.054979	0.291435	0.587543	GORAB	92344	golgin RAB6 interacting
ENSG0000	2.20172	-1.91924	1.818906	-1.05516	0.291351	0.587543	NA	NA	NA
ENSG0000	10.51355	0.810861	0.768617	1.054962	0.291443	0.587543	NA	NA	NA

ENSG0000	4.00612	1.28536	1.218275	1.055066	0.291395	0.587543	NA	NA	NA
ENSG0000	268.5047	-0.17279	0.163825	-1.05469	0.291567	0.587601	GSTM4	2948	glutathione S-transferase mu 4
ENSG0000	8.728366	-0.87378	0.828419	-1.05475	0.291539	0.587601	NA	NA	NA
ENSG0000	78.83053	0.311467	0.295278	1.054827	0.291504	0.587601	TMEM238	388564	transmembrane protein 238
ENSG0000	909.6676	-0.11386	0.107975	-1.05448	0.291663	0.587731	MAML2	84441	mastermind like transcriptional coactivator 2
ENSG0000	12.5	-0.74069	0.702549	-1.05429	0.29175	0.587779	TBCE	6905	tubulin folding cofactor E
ENSG0000	13.49248	0.739216	0.701132	1.054317	0.291738	0.587779	BHLHE40-A	1.01E+08	BHLHE40 antisense RNA 1
ENSG0000	1777.976	0.095515	0.090628	1.053923	0.291918	0.588053	WSB2	55884	WD repeat and SOCS box containing 2
ENSG0000	8.190882	-0.94532	0.897462	-1.05333	0.29219	0.588537	NA	NA	NA
ENSG0000	3.397869	1.6006	1.520003	1.053024	0.29233	0.588755	SNCG	6623	synuclein gamma
ENSG0000	1697.341	-0.11166	0.106055	-1.0528	0.292432	0.588896	RAVER1	125950	ribonuclec PTB binding 1
ENSG0000	10.52134	-0.95717	0.90929	-1.05266	0.292497	0.588963	NPIPB15	440348	nuclear pore complex interacting protein family member B15
ENSG0000	22.51903	0.552184	0.525023	1.051734	0.292922	0.589053	CCDC74A	90557	coiled-coil domain containing 74A
ENSG0000	1313.335	0.111682	0.106155	1.052063	0.292771	0.589053	DDAH2	23564	dimethylarginine dimethylaminohydrolase 2
ENSG0000	239.0155	0.188258	0.17898	1.051836	0.292875	0.589053	PERP	64065	p53 apoptosis effector related to PMP22
ENSG0000	919.1265	-0.10778	0.102416	-1.05242	0.292608	0.589053	FBXW2	26190	F-box and WD repeat domain containing 2
ENSG0000	10.2202	0.807002	0.767308	1.051732	0.292923	0.589053	DLEU7	220107	deleted in lymphocytic leukemia 7
ENSG0000	151.8344	-0.23809	0.226356	-1.05186	0.292864	0.589053	SAMD4A	23034	sterile alpha motif domain containing 4A
ENSG0000	341.5926	-0.16215	0.154148	-1.05193	0.292834	0.589053	SETD6	79918	SET domai protein lysine methyltransferase
ENSG0000	5.742302	-1.17503	1.116676	-1.05226	0.29268	0.589053	SP6	80320	Sp6 transcription factor
ENSG0000	11396.91	-0.10909	0.103725	-1.05176	0.292911	0.589053	CD300E	342510	CD300e molecule
ENSG0000	9.677047	-0.91401	0.868437	-1.05248	0.29258	0.589053	GINS1	9837	GINS complex subunit 1
ENSG0000	41.62941	-0.48246	0.45863	-1.05196	0.292817	0.589053	IGLV6-57	28778	immunoglobulin lambda variable 6-57
ENSG0000	16.47104	0.605451	0.57541	1.052208	0.292704	0.589053	MTFP1	51537	mitochondrial fission process 1
ENSG0000	20.66001	-0.54344	0.517039	-1.05107	0.293226	0.589598	KIF2C	11004	kinesin family member 2C
ENSG0000	6.285739	-1.05981	1.008969	-1.05039	0.293539	0.589763	NA	NA	NA
ENSG0000	135.6572	0.322959	0.307478	1.050347	0.293558	0.589763	TMEM40	55287	transmembrane protein 40
ENSG0000	6.846705	1.006707	0.958193	1.050631	0.293428	0.589763	ARHGEF28	64283	Rho guanine nucleotide exchange factor 28
ENSG0000	538.1303	-0.12159	0.115765	-1.05035	0.293558	0.589763	CDC23	8697	cell division cycle 23
ENSG0000	19.48314	-0.60028	0.571338	-1.05066	0.293413	0.589763	HOXA10	3206	homeobox A10
ENSG0000	628.9013	0.141615	0.134821	1.050388	0.29354	0.589763	DNAJC2	27000	DnaJ heat shock protein family (Hsp40) member C2
ENSG0000	5.665221	-1.27189	1.210569	-1.05065	0.293417	0.589763	FAM227B	196951	family with sequence similarity 227 member B
ENSG0000	2215.26	-0.09218	0.087761	-1.05034	0.293562	0.589763	NCBP3	55421	nuclear cap binding subunit 3
ENSG0000	389.1176	-0.15652	0.149053	-1.05013	0.293657	0.589891	CHPF	79586	chondroitin polymerizing factor
ENSG0000	3.854957	1.516001	1.443983	1.049875	0.293776	0.590013	RBM24	221662	RNA binding motif protein 24
ENSG0000	8.19434	0.997143	0.949785	1.049862	0.293782	0.590013	B4GAT1-D	1.03E+08	B4GAT1 divergent transcript
ENSG0000	37.97843	-0.41576	0.396074	-1.04971	0.29385	0.590022	EVC2	132884	EvC ciliary complex subunit 2
ENSG0000	192.229	-0.20806	0.198201	-1.04972	0.293847	0.590022	INO80C	125476	INO80 complex subunit C
ENSG0000	4.025906	1.469647	1.40041	1.04944	0.293975	0.59021	NA	NA	NA
ENSG0000	66.03591	-0.31355	0.298797	-1.04936	0.294011	0.590217	NA	NA	NA
ENSG0000	8.324072	1.102134	1.050589	1.049062	0.294149	0.590432	NA	NA	NA
ENSG0000	159.3132	-0.2279	0.217266	-1.04892	0.294214	0.590435	FANCF	2188	FA complementation group F
ENSG0000	246.7766	-0.17341	0.165322	-1.04892	0.294215	0.590435	MRPL11	65003	mitochondrial ribosomal protein L11
ENSG0000	1961.614	0.085806	0.081819	1.04872	0.294307	0.590467	HMGN2	3151	high mobility group nucleosomal binding domain 2
ENSG0000	60.68603	-0.33758	0.321907	-1.04868	0.294326	0.590467	ANKRD34A	284615	ankyrin repeat domain 34A
ENSG0000	248.678	-0.17033	0.162421	-1.0487	0.294316	0.590467	HACD4	401494	3-hydroxyacyl-CoA dehydratase 4
ENSG0000	91.2763	0.275395	0.26265	1.048524	0.294397	0.590546	IMPA2	3613	inositol monophosphatase 2
ENSG0000	1670.916	0.090082	0.085922	1.048415	0.294448	0.590583	SRSF10	10772	serine and arginine rich splicing factor 10
ENSG0000	3.041962	1.604282	1.530337	1.048319	0.294492	0.590607	SNORA3B	677826	small nucl H/ACA box 3B
ENSG0000	624.7658	-0.13871	0.132325	-1.04824	0.29453	0.59062	FBXL6	26233	F-box and leucine rich repeat protein 6
ENSG0000	189.5248	0.198604	0.189499	1.048048	0.294616	0.59073	LINC00174	285908	long intergenic non-protein coding RNA 174
ENSG0000	7.231695	-1.34149	1.280399	-1.04771	0.294773	0.590852	NA	NA	NA
ENSG0000	4.482679	1.440385	1.374635	1.047831	0.294716	0.590852	ANGPT1	284	angiopoietin 1
ENSG0000	194.3607	-0.21607	0.206233	-1.04772	0.294769	0.590852	RTTN	25914	rotatin
ENSG0000	118.2025	-0.26262	0.250703	-1.04754	0.294851	0.590945	PMS1	5378	PMS1 hom mismatch repair system component
ENSG0000	73.94203	0.355201	0.339131	1.047386	0.294922	0.591022	ARSK	153642	arylsulfatase family member K
ENSG0000	76.75265	-0.34543	0.329861	-1.04719	0.295012	0.591139	PLAAT3	11145	phospholipase A and acyltransferase 3
ENSG0000	1114.474	0.105104	0.100382	1.047037	0.295082	0.591217	CAPZA2	830	capping actin protein of muscle Z-line subunit alpha 2
ENSG0000	438.0381	0.167964	0.160432	1.046949	0.295123	0.591223	KLF11	8462	KLF transcription factor 11
ENSG0000	811.1984	0.11782	0.11255	1.046823	0.295181	0.591223	IRAG2	4033	inositol 1 4 5-triphosphate receptor associated 2
ENSG0000	785.5495	0.130171	0.124346	1.046847	0.29517	0.591223	FMR1	2332	fragile X messenger ribonucleoprotein 1
ENSG0000	274.6638	0.16592	0.15851	1.046745	0.295217	0.591231	RINT1	60561	RAD50 interactor 1
ENSG0000	317.6378	0.17946	0.171526	1.046253	0.295444	0.591622	L3MBTL3	84456	L3MBTL histone methyl-lysine binding protein 3
ENSG0000	32.3203	-0.50015	0.478105	-1.04611	0.295512	0.591694	NA	NA	NA
ENSG0000	18673.06	0.070785	0.067683	1.04584	0.295635	0.591876	PRRC2A	7916	proline rich coiled-coil 2A
ENSG0000	163.3271	0.223106	0.213356	1.045699	0.2957	0.591943	BMT2	154743	base methyltransferase of 25S rRNA 2 homolog
ENSG0000	93.93422	0.287242	0.274709	1.045621	0.295736	0.59195	NA	NA	NA
ENSG0000	32.59524	0.436972	0.418051	1.04526	0.295903	0.592028	NA	NA	NA
ENSG0000	141.3409	-0.2116	0.202437	-1.04525	0.295907	0.592028	MTX1	4580	metaxin 1
ENSG0000	27.53196	-0.52522	0.502487	-1.04524	0.29591	0.592028	CD207	50489	CD207 molecule
ENSG0000	8.026826	-0.88157	0.843234	-1.04547	0.295808	0.592028	CXCL13	10563	C-X-C motif chemokine ligand 13
ENSG0000	2.236404	-1.74838	1.673013	-1.04505	0.296001	0.592028	NA	NA	NA
ENSG0000	109.9911	0.299832	0.286925	1.044985	0.29603	0.592028	CUBN	8029	cubilin
ENSG0000	7.215768	0.949093	0.908096	1.045146	0.295955	0.592028	NA	NA	NA
ENSG0000	11.15371	0.944156	0.903483	1.045017	0.296015	0.592028	CACNG6	59285	calcium voltage-gated channel auxiliary subunit gamma 6
ENSG0000	5.403181	1.066595	1.020793	1.044869	0.296084	0.592071	CBX2	84733	chromobox 2
ENSG0000	1102.243	0.096481	0.092362	1.044591	0.296212	0.592109	FBXW11	23291	F-box and WD repeat domain containing 11
ENSG0000	733.0213	0.133008	0.127336	1.044537	0.296237	0.592109	INTS10	55174	integrator complex subunit 10
ENSG0000	14.48879	0.638827	0.611621	1.044483	0.296262	0.592109	NA	NA	NA
ENSG0000	9.276016	0.778506	0.745298	1.044556	0.296228	0.592109	NA	NA	NA
ENSG0000	8.203867	1.012728	0.969524	1.044562	0.296226	0.592109	RPAP3-DT	1.05E+08	RPAP3 divergent transcript
ENSG0000	639.868	0.129086	0.123626	1.044166	0.296409	0.592147	IFRD2	7866	interferon related developmental regulator 2
ENSG0000	139.4839	-0.28477	0.272709	-1.04421	0.296388	0.592147	P2RY14	9934	purinergic receptor P2Y14
ENSG0000	2456.406	-0.09016	0.086338	-1.0443	0.296348	0.592147	HPS1	3257	HPS1 biogenesis of lysosomal organelles complex 3 subunit 1
ENSG0000	102.0786	0.258398	0.247429	1.044332	0.296332	0.592147	ADM2	79924	adrenomedullin 2
ENSG0000	25.81242	-0.5485	0.525435	-1.0439	0.296534	0.592205	FAM185A	222234	family with sequence similarity 185 member A
ENSG0000	34.75556	0.496718	0.475831	1.043896	0.296534	0.592205	NA	NA	NA
ENSG0000	951.7965	-0.10328	0.098927	-1.04402	0.296475	0.592205	CCDC57	284001	coiled-coil domain containing 57
ENSG0000	11.81035	0.695684	0.666665	1.04353	0.296703	0.592416	FKBP1C	642489	FKBP prolyl isomerase family member 1C
ENSG0000	17.51025	0.716269	0.686386	1.043538	0.296699	0.592416	HTRA4	203100	HtrA serine peptidase 4
ENSG0000	1138.701	0.100775	0.096593	1.043294	0.296812	0.59257	RBM8A	9939	RNA binding motif protein 8A
ENSG0000	10.02224	0.844021	0.809248	1.04297	0.296962	0.592806	CYP21A2	1589	cytochrome P450 family 21 subfamily A member 2
ENSG0000	6.447337	-1.245	1.194004	-1.04271	0.297084	0.592985	SNTG2	54221	syntrophin gamma 2
ENSG0000	425.6285	0.141244	0.13547	1.042618	0.297125	0.593004	NAA30	122830	N-alpha-ac NatC catalytic subunit
ENSG0000	196.8051	0.196093	0.188102	1.042481	0.297189	0.593067	SLC29A1	2030	solute carrier family 29 member 1 (Augustine blood group)
ENSG0000	696.5732	0.141075	0.135348	1.042314	0.297266	0.593157	TMX2	51075	thioredoxin related transmembrane protein 2
ENSG0000	257.1887	-0.49993	0.479731	-1.0421	0.297367	0.593166	IL31RA	133396	interleukin 31 receptor A
ENSG0000	257.9992	-0.18994	0.182269	-1.0421	0.297364	0.593166	UBE2V2	7336	ubiquitin conjugating enzyme E2 V2
ENSG0000	173.3896	-0.1939	0.186042	-1.04223	0.297305	0.593166	ZNG1A	55871	Zn regulated GTPase metalloprotein activator 1A
ENSG0000	524.6323	-0.14215	0.136424	-1.042	0.297413	0.593194	MON1A	84315	MON1 hor secretory trafficking associated

ENSG0000	377.4186	0.151115	0.145036	1.041916	0.297451	0.593206	ZCCHC10	54819	zinc finger CCHC-type containing 10
ENSG0000	2618.431	0.089841	0.086253	1.041608	0.297593	0.593329	GAB2	9846	GRB2 associated binding protein 2
ENSG0000	8.945305	-0.86582	0.831193	-1.04166	0.297569	0.593329	LOC10088	1.01E+08	acyl-CoA synthetase medium chain family member 4 pseudogene
ENSG0000	335.1365	-0.16061	0.154203	-1.04158	0.297608	0.593329	POFUT1	23509	protein O-fucosyltransferase 1
ENSG0000	59.79507	0.381505	0.366361	1.041334	0.297721	0.593489	STEAP3	55240	STEAP3 metalloreductase
ENSG0000	99.79932	0.256418	0.2463	1.041082	0.297838	0.593563	HLA-L	3139	major hist class I L (pseudogene)
ENSG0000	539.5775	-0.13538	0.13004	-1.04105	0.297854	0.593563	YIF1B	90522	Yip1 intera membrane trafficking protein
ENSG0000	158.1315	0.220098	0.211417	1.041063	0.297847	0.593563	LGALS2	3957	galectin 2
ENSG0000	107.1238	0.254424	0.244423	1.040916	0.297915	0.593621	ZNF10	7556	zinc finger protein 10
ENSG0000	202.1105	-0.17723	0.17027	-1.04084	0.297948	0.593623	ENDOV	284131	endonuclease V
ENSG0000	1245.246	-0.09996	0.096079	-1.04043	0.298142	0.593946	SUPT7L	9913	SPT7 like STAGA complex subunit gamma
ENSG0000	77.20414	0.37627	0.361699	1.040285	0.298207	0.593949	PLPP1	8611	phospholipid phosphatase 1
ENSG0000	290.0867	-0.154	0.148032	-1.0403	0.298202	0.593949	IKBKG	8517	inhibitor of nuclear factor kappa B kinase regulatory subunit gamma
ENSG0000	424.718	-0.15838	0.152313	-1.03985	0.29841	0.594228	SENP7	57337	SUMO specific peptidase 7
ENSG0000	54.07708	-0.40827	0.392653	-1.03978	0.298444	0.594228	KRTAP5-A	338651	KRTAP5-1/KRTAP5-2 antisense RNA 1
ENSG0000	15.72305	-0.65657	0.631416	-1.03984	0.298413	0.594228	MPZL2	10205	myelin protein zero like 2
ENSG0000	218.3246	0.198422	0.19085	1.039674	0.298491	0.594259	LINC0159	196913	long intergenic non-protein coding RNA 1599
ENSG0000	24055.19	0.093649	0.090084	1.039575	0.298538	0.594287	FCN1	2219	ficolin 1
ENSG0000	511.3145	-0.13281	0.127802	-1.03921	0.298706	0.594452	SOX13	9580	SRY-box transcription factor 13
ENSG0000	38.82092	-0.39225	0.377414	-1.03931	0.29866	0.594452	PDSS2	57107	decaprenyl diphosphate synthase subunit 2
ENSG0000	109.8396	-0.24607	0.236787	-1.03919	0.298716	0.594452	POLI	11201	DNA polymerase iota
ENSG0000	39.49275	-0.38967	0.375007	-1.0391	0.298758	0.594472	PAIP2B	400961	poly(A) binding protein interacting protein 2B
ENSG0000	166.2881	-0.21437	0.206345	-1.03887	0.298863	0.594617	KNTC1	9735	kinetochore associated 1
ENSG0000	409.2723	-0.15959	0.153674	-1.03847	0.29905	0.594902	SFMBT1	51460	Scm like with four mbt domains 1
ENSG0000	5.929591	1.204522	1.159947	1.038428	0.299071	0.594902	NA	NA	NA
ENSG0000	6.12034	1.156457	1.113981	1.03813	0.29921	0.595001	GSTM5	2949	glutathione S-transferase mu 5
ENSG0000	7.763195	1.013432	0.976224	1.038114	0.299217	0.595001	NA	NA	NA
ENSG0000	221.8599	-0.19668	0.189463	-1.03811	0.299217	0.595001	LNX2	222484	ligand of numb-protein X 2
ENSG0000	763.8858	0.113973	0.109819	1.037823	0.299353	0.595153	QSOX2	169714	quiescin sulfhydryl oxidase 2
ENSG0000	3935.197	-0.09978	0.096155	-1.03774	0.29939	0.595153	UNC13D	201294	unc-13 homolog D
ENSG0000	3093.365	-0.08979	0.086524	-1.03778	0.299372	0.595153	DIP2A	23181	disco interacting protein 2 homolog A
ENSG0000	4235.215	-0.08676	0.083625	-1.03748	0.299512	0.595333	MLLT1	4298	MLLT1 super elongation complex subunit
ENSG0000	760.795	0.116138	0.112029	1.036675	0.299888	0.595951	CEBPZ	10153	CCAAT enhancer binding protein zeta
ENSG0000	690.4524	0.116118	0.112008	1.036686	0.299882	0.595951	REST	5978	RE1 silencing transcription factor
ENSG0000	11.59907	1.056645	1.019612	1.03632	0.300053	0.596103	MLF1	4291	myeloid leukemia factor 1
ENSG0000	157.2316	0.246781	0.238133	1.036313	0.300056	0.596103	MB21D2	151963	Mab-21 domain containing 2
ENSG0000	407.3669	0.146072	0.140955	1.036305	0.30006	0.596103	CFAP20	29105	cilia and flagella associated protein 20
ENSG0000	661.1834	-0.13686	0.132088	-1.03615	0.300131	0.596179	ZBTB48	3104	zinc finger and BTB domain containing 48
ENSG0000	1537.908	0.113097	0.109163	1.036035	0.300186	0.596225	CALM2	805	calmodulin 2
ENSG0000	288.4401	-0.17924	0.173019	-1.03596	0.30022	0.596229	KIAA0586	9786	KIAA0586
ENSG0000	3.883621	-1.32831	1.282551	-1.03568	0.300353	0.59643	RHOD	29984	ras homolog family member D
ENSG0000	108.4866	-0.24638	0.237928	-1.03553	0.300422	0.596476	ADAT2	134637	adenosine deaminase tRNA specific 2
ENSG0000	7.766775	-1.02797	0.992743	-1.03549	0.300441	0.596476	ACER1	125981	alkaline ceramidase 1
ENSG0000	272.5286	-0.17235	0.166465	-1.03538	0.30049	0.59651	C10orf95-	1.01E+08	C10orf95 antisense RNA 1
ENSG0000	45.73645	0.348631	0.336752	1.035277	0.30054	0.596544	NA	NA	NA
ENSG0000	156.1249	0.211224	0.204129	1.034758	0.300782	0.596706	PPIL3	53938	peptidylprolyl isomerase like 3
ENSG0000	28.11225	-0.49895	0.482145	-1.03484	0.300742	0.596706	HTR7	3363	5-hydroxytryptamine receptor 7
ENSG0000	454.487	0.142607	0.137789	1.034961	0.300687	0.596706	TPCN2	219931	two pore segment channel 2
ENSG0000	3.476252	1.885297	1.821817	1.034844	0.300742	0.596706	NA	NA	NA
ENSG0000	5610.369	-0.08783	0.084878	-1.0348	0.300764	0.596706	MFSD12	126321	major facilitator superfamily domain containing 12
ENSG0000	5.2572	1.083979	1.047658	1.034669	0.300823	0.596724	SYTL4	94121	synaptotagmin like 4
ENSG0000	1012.218	-0.13475	0.130242	-1.03458	0.300864	0.596741	NA	NA	NA
ENSG0000	9.995836	0.785817	0.759792	1.034253	0.301018	0.596982	LINC0101	1.01E+08	long intergenic non-protein coding RNA 1012
ENSG0000	128.5612	-0.23689	0.229086	-1.03405	0.301111	0.596982	CDCA7L	55536	cell division cycle associated 7 like
ENSG0000	172.7825	0.244447	0.236397	1.034053	0.301111	0.596982	NA	NA	NA
ENSG0000	1214.35	0.104015	0.10059	1.034046	0.301114	0.596982	C19orf25	148223	chromosome 19 open reading frame 25
ENSG0000	21.03057	-0.54507	0.52716	-1.03397	0.301149	0.596988	HBQ1	3049	hemoglobin subunit theta 1
ENSG0000	3.787047	-1.69326	1.638046	-1.03371	0.301273	0.597031	RGS7	6000	regulator of G protein signaling 7
ENSG0000	290.6467	-0.15762	0.152482	-1.03367	0.301292	0.597031	ZNF212	7988	zinc finger protein 212
ENSG0000	5.457673	1.280073	1.238566	1.033513	0.301364	0.597031	NA	NA	NA
ENSG0000	28.71827	0.520119	0.503157	1.033711	0.301271	0.597031	MSRB3	253827	methionine sulfoxide reductase B3
ENSG0000	14.58015	-0.71121	0.688076	-1.03362	0.301314	0.597031	TRAV16	28667	T cell receptor alpha variable 16
ENSG0000	538.1693	-0.12455	0.120508	-1.03354	0.301351	0.597031	CEP89	84902	centrosomal protein 89
ENSG0000	8.081512	0.895569	0.866886	1.033087	0.301563	0.597361	IGSF3	3321	immunoglobulin superfamily member 3
ENSG0000	684.7028	0.123337	0.119411	1.032884	0.301658	0.597422	VAMP8	8673	vesicle associated membrane protein 8
ENSG0000	1955.735	-0.10002	0.096839	-1.03289	0.301656	0.597422	PTOV1	53635	PTOV1 extended AT-hook containing adaptor protein
ENSG0000	5.369768	1.183392	1.146514	1.032166	0.301995	0.598024	NA	NA	NA
ENSG0000	577.0892	0.137038	0.132802	1.031897	0.30212	0.598082	CCDC107	203260	coiled-coil domain containing 107
ENSG0000	42.27267	-0.41885	0.405899	-1.03192	0.30211	0.598082	NA	NA	NA
ENSG0000	34.71012	-1.30375	1.263338	-1.03199	0.302078	0.598082	IGLV4-69	28784	immunoglobulin lambda variable 4-69
ENSG0000	103.7674	0.280758	0.272207	1.031414	0.302347	0.598466	MED20	9477	mediator complex subunit 20
ENSG0000	248.845	-0.17128	0.166091	-1.03123	0.302433	0.598572	NOMO2	283820	NODAL modulator 2
ENSG0000	12.83477	-0.72413	0.702313	-1.03107	0.302509	0.598659	LINC0101	154092	long intergenic non-protein coding RNA 1010
ENSG0000	30.53921	0.466909	0.452885	1.030964	0.302558	0.598692	NA	NA	NA
ENSG0000	17.90768	0.607677	0.58948	1.03087	0.302602	0.598715	UCKL1-AS	1E+08	UCKL1 antisense RNA 1
ENSG0000	110.9917	-0.23447	0.227462	-1.0308	0.302634	0.598716	NXT2	55916	nuclear transport factor 2 like export factor 2
ENSG0000	17.85686	-0.56794	0.551021	-1.0307	0.302683	0.598747	MIPEP	4285	mitochondrial intermediate peptidase
ENSG0000	2377.819	-0.0917	0.089024	-1.03004	0.302992	0.599296	IPO9	55705	importin 9
ENSG0000	4833.489	0.077152	0.074931	1.029639	0.303179	0.599486	SRSF11	9295	serine and arginine rich splicing factor 11
ENSG0000	4843.748	0.076531	0.074326	1.029661	0.303169	0.599486	NIBAN1	116496	niban apoptosis regulator 1
ENSG0000	49.89087	0.353516	0.343344	1.029627	0.303185	0.599486	ACE	1636	angiotensin I converting enzyme
ENSG0000	35.76911	0.478407	0.464861	1.02914	0.303414	0.599746	TRAV8-1	28685	T cell receptor alpha variable 8-1
ENSG0000	180.2121	-0.22328	0.216954	-1.02918	0.303395	0.599746	TPM1	7168	tropomyosin 1
ENSG0000	4915.515	0.085343	0.082918	1.029241	0.303366	0.599746	PPP2R1A	5518	protein phosphatase 2 scaffold subunit Aalpha
ENSG0000	6.942461	-1.04923	1.020063	-1.02859	0.303671	0.600127	RASGRF2-	1.03E+08	RASGRF2 antisense RNA 1
ENSG0000	135.7295	0.286551	0.278576	1.028625	0.303656	0.600127	YEATS4	8089	YEATS domain containing 4
ENSG0000	162.2851	0.235652	0.229215	1.028086	0.303909	0.600531	C1orf109	54955	chromosome 1 open reading frame 109
ENSG0000	249.5246	-0.17291	0.168194	-1.02802	0.30394	0.600531	SS18L2	51188	SS18 like 2
ENSG0000	934.1742	0.136352	0.132698	1.027536	0.304168	0.600551	GNL2	29889	G protein nucleolar 2
ENSG0000	34.74577	-0.40847	0.397713	-1.02706	0.304393	0.600551	NA	NA	NA
ENSG0000	18.83874	0.73247	0.713048	1.027238	0.304308	0.600551	COL24A1	255631	collagen type XXIV alpha 1 chain
ENSG0000	120.5938	0.280093	0.272678	1.027195	0.304329	0.600551	RPL22L1	200916	ribosomal protein L22 like 1
ENSG0000	7.183931	-1.12761	1.097174	-1.02774	0.304071	0.600551	ANKRD18	348840	ankyrin re pseudogene
ENSG0000	8.293589	0.888105	0.864564	1.027229	0.304312	0.600551	PNLDC1	154197	PARN like ribonuclease domain containing exonuclease 1
ENSG0000	116.2542	-0.23934	0.232965	-1.02737	0.304245	0.600551	FIGNL1	63979	fidgetin like 1
ENSG0000	30.9049	-0.51994	0.505855	-1.02784	0.304023	0.600551	NA	NA	NA
ENSG0000	9.963802	0.869935	0.846756	1.027373	0.304245	0.600551	NA	NA	NA
ENSG0000	4122.941	-0.08435	0.082093	-1.02756	0.304159	0.600551	TLE4	7091	TLE family transcriptional corepressor
ENSG0000	3.467943	1.337136	1.301692	1.027229	0.304313	0.600551	NA	NA	NA
ENSG0000	1694.247	0.092797	0.090355	1.027027	0.304408	0.600551	TSEN54	283989	tRNA splicing endonuclease subunit 54

ENSG0000	713.014	0.110383	0.107467	1.027138	0.304356	0.600551	TRMT6	51605	tRNA methyltransferase 6 non-catalytic subunit
ENSG0000	7.532518	1.020626	0.99377	1.027024	0.304409	0.600551	AIRE	326	autoimmune regulator
ENSG0000	4263.127	-0.09295	0.090507	-1.02697	0.304436	0.600551	LMF2	91289	lipase maturation factor 2
ENSG0000	6876.499	-0.37785	0.367961	-1.02687	0.304483	0.600579	CSF2RB	1439	colony stimulating factor 2 receptor subunit beta
ENSG0000	29.11078	0.481423	0.468922	1.02666	0.304581	0.600619	MORN1	79906	MORN repeat containing 1
ENSG0000	8.649337	-0.85815	0.835974	-1.02653	0.304642	0.600619	EPHB3	2049	EPH receptor B3
ENSG0000	124.6893	-0.22655	0.220747	-1.02627	0.304762	0.600619	NA	NA	NA
ENSG0000	21.89269	-0.54958	0.535495	-1.02629	0.304753	0.600619	ORM1	5004	orosomucoid 1
ENSG0000	3227.443	0.095543	0.093091	1.026334	0.304734	0.600619	CCSER2	54462	coiled-coil serine rich protein 2
ENSG0000	48.16658	-0.33856	0.329883	-1.02632	0.304742	0.600619	ZNF382	84911	zinc finger protein 382
ENSG0000	284.7381	-0.16001	0.155888	-1.02646	0.304674	0.600619	ZNF350	59348	zinc finger protein 350
ENSG0000	16.70718	-0.63448	0.618162	-1.0264	0.304705	0.600619	AVP	551	arginine vasopressin
ENSG0000	10295.62	-0.07425	0.072361	-1.02608	0.304853	0.600733	ZMIZ2	83637	zinc finger MIZ-type containing 2
ENSG0000	149.5596	-0.22605	0.220552	-1.02495	0.305389	0.601663	DTNB	1838	dystrobrevin beta
ENSG0000	1666.622	-0.09091	0.088696	-1.02499	0.305367	0.601663	PLCG2	5336	phospholipase C gamma 2
ENSG0000	644.4265	0.124841	0.121842	1.024612	0.305546	0.601909	SLC25A38	54977	solute carrier family 25 member 38
ENSG0000	1956.154	0.092865	0.090743	1.023391	0.306123	0.602838	EIF5B	9669	eukaryotic translation initiation factor 5B
ENSG0000	8.287382	-1.06102	1.036689	-1.02347	0.306087	0.602838	NA	NA	NA
ENSG0000	106.3336	-0.24415	0.238573	-1.02339	0.306124	0.602838	TGDS	23483	TDP-gluco 6-dehydratase
ENSG0000	214.1037	0.178022	0.173962	1.023338	0.306148	0.602838	NDUFA1	4694	NADH:ubiquinone oxidoreductase subunit A1
ENSG0000	170.3886	-0.18918	0.18492	-1.02302	0.306299	0.603004	ZFP62	643836	ZFP62 zinc finger protein
ENSG0000	1069.856	-0.09701	0.094823	-1.02303	0.306293	0.603004	RRAGA	10670	Ras related GTP binding A
ENSG0000	7.073959	0.900832	0.88062	1.022953	0.30633	0.603004	NA	NA	NA
ENSG0000	1186.696	0.113561	0.111061	1.022511	0.306539	0.603292	PRKAR2A	5576	protein kinase cAMP-dependent type II regulatory subunit alpha
ENSG0000	75.09727	-0.2817	0.275519	-1.02244	0.306574	0.603292	NA	NA	NA
ENSG0000	84.43245	0.272796	0.266802	1.022466	0.30656	0.603292	NA	NA	NA
ENSG0000	4.376215	1.291797	1.263587	1.022326	0.306627	0.603332	SMIM10L2	644596	small integral membrane protein 10 like 2B
ENSG0000	200.4135	0.196313	0.192053	1.022182	0.306695	0.603337	AMIGO2	347902	adhesion molecule with Ig like domain 2
ENSG0000	63.08328	0.315061	0.308215	1.022212	0.306681	0.603337	CACNA1F	778	calcium voltage-gated channel subunit alpha1 F
ENSG0000	5259.408	-0.07665	0.075035	-1.02159	0.306977	0.603637	MACROH2	9555	macroH2A.1 histone
ENSG0000	1316.689	0.094592	0.092589	1.021635	0.306954	0.603637	SPINDOC	144097	spindlin interactor and repressor of chromatin binding
ENSG0000	946.2574	-0.10777	0.105487	-1.02161	0.306963	0.603637	PAN3	255967	poly(A) specific ribonuclease subunit PAN3
ENSG0000	1728.42	0.089092	0.087197	1.021733	0.306907	0.603637	EPS15L1	58513	epidermal growth factor receptor pathway substrate 15 like 1
ENSG0000	29.38135	0.571067	0.55905	1.021495	0.30702	0.603657	MPHOSPH	643802	MPHOSPH10 pseudogene 1
ENSG0000	281.9009	0.194644	0.190577	1.02134	0.307093	0.603723	SLC49A4	84925	solute carrier family 49 member 4
ENSG0000	2.896209	-1.67544	1.640518	-1.02129	0.307118	0.603723	NA	NA	NA
ENSG0000	3.045575	-1.6434	1.609787	-1.02088	0.30731	0.603986	TRGJP1	6971	T cell receptor gamma joining P1
ENSG0000	19.21763	0.785692	0.769633	1.020867	0.307318	0.603986	LINC02207	1.02E+08	long intergenic non-protein coding RNA 2207
ENSG0000	12.70149	-0.73699	0.722101	-1.02062	0.307433	0.604085	C4A	720	complement C4A (Rodgers blood group)
ENSG0000	685.2287	-0.14946	0.146433	-1.02068	0.307408	0.604085	PTAR1	375743	protein prenyltransferase alpha subunit repeat containing 1
ENSG0000	239.7078	-0.18249	0.178813	-1.02054	0.307472	0.604097	COMMD2	51122	COMM domain containing 2
ENSG0000	1445.251	0.094695	0.092803	1.020385	0.307546	0.604179	UBE3A	7337	ubiquitin protein ligase E3A
ENSG0000	104.2255	-0.26567	0.260441	-1.02008	0.307691	0.6044	PAAF1	80227	proteasomal ATPase associated factor 1
ENSG0000	91.16016	-0.27893	0.273587	-1.01952	0.307957	0.604441	CYBRD1	79901	cytochrome b reductase 1
ENSG0000	808.6394	0.136653	0.134037	1.019513	0.30796	0.604441	CRCP	27297	CGRP receptor component
ENSG0000	10.1571	0.825154	0.809413	1.019447	0.307991	0.604441	NA	NA	NA
ENSG0000	85.75762	-0.27288	0.26755	-1.01993	0.30776	0.604441	ZNF782	158431	zinc finger protein 782
ENSG0000	234.0265	0.169212	0.165989	1.019417	0.308005	0.604441	DCAF4	26094	DDB1 and CUL4 associated factor 4
ENSG0000	15.10835	-0.78284	0.767781	-1.01961	0.307914	0.604441	IGHV6-1	28385	immunoglobulin heavy variable 6-1
ENSG0000	471.9182	0.129978	0.127449	1.019845	0.307802	0.604441	TMEM170	124491	transmembrane protein 170A
ENSG0000	2221.648	-0.13631	0.133709	-1.01946	0.307983	0.604441	PSTPIP2	9050	proline-serine-threonine phosphatase interacting protein 2
ENSG0000	63.05404	-0.3151	0.309094	-1.01943	0.308001	0.604441	SPIN4	139886	spindlin family member 4
ENSG0000	5.132929	1.124595	1.103566	1.019055	0.308177	0.604714	NA	NA	NA
ENSG0000	13.72741	0.741018	0.727319	1.018835	0.308281	0.604855	CDC45	8318	cell division cycle 45
ENSG0000	1202.123	0.101382	0.099534	1.018567	0.308408	0.604912	RAB29	8934	RAB29 member RAS oncogene family
ENSG0000	7.769179	-1.13605	1.115268	-1.01863	0.308378	0.604912	NA	NA	NA
ENSG0000	2737.498	-0.09216	0.09047	-1.01866	0.308362	0.604912	ADA2	51816	adenosine deaminase 2
ENSG0000	3432.649	0.078379	0.07696	1.018439	0.30847	0.604941	TACC1	6867	transforming acidic coiled-coil containing protein 1
ENSG0000	8.042306	-0.91089	0.894489	-1.01834	0.308518	0.604941	C8orf88	1E+08	chromosome 8 open reading frame 88
ENSG0000	386.2166	0.147929	0.145266	1.01833	0.308521	0.604941	MED19	219541	mediator complex subunit 19
ENSG0000	832.2785	0.103811	0.101953	1.018233	0.308567	0.604968	NRBF2	29982	nuclear receptor binding factor 2
ENSG0000	7.781499	0.852816	0.838019	1.017657	0.308841	0.605261	MTARC2	54996	mitochondrial amidoxime reducing component 2
ENSG0000	8.780478	0.870786	0.855623	1.017721	0.30881	0.605261	NA	NA	NA
ENSG0000	335.1981	0.144691	0.142155	1.017837	0.308755	0.605261	NAPG	8774	NSF attachment protein gamma
ENSG0000	1680.915	0.125935	0.123752	1.017644	0.308847	0.605261	ERCC1	2067	ERCC excis endonuclease non-catalytic subunit
ENSG0000	3.495446	-1.57609	1.548914	-1.01754	0.308894	0.605289	CCDC194	1.11E+08	coiled-coil domain containing 194
ENSG0000	4.291619	1.563313	1.536736	1.017294	0.309013	0.605421	SLC44A3-A	1.02E+08	SLC44A3 antisense RNA 1
ENSG0000	5.94054	0.980952	0.964303	1.017266	0.309027	0.605421	NA	NA	NA
ENSG0000	18.0921	-0.6315	0.620924	-1.01703	0.309139	0.605512	STIMATE	375346	STIM activating enhancer
ENSG0000	10.09166	0.826976	0.813118	1.017042	0.309133	0.605512	NA	NA	NA
ENSG0000	62.94288	-0.36076	0.35478	-1.01685	0.309223	0.605613	HCG27	253018	HLA complex group 27
ENSG0000	222.2117	0.169996	0.16725	1.016423	0.309428	0.605951	MRPL21	219927	mitochondrial ribosomal protein L21
ENSG0000	976.1624	0.126953	0.124925	1.01623	0.30952	0.606067	ORMDL1	94101	ORMDL sphingolipid biosynthesis regulator 1
ENSG0000	586.8273	0.126357	0.12438	1.015891	0.309681	0.606319	ATP2C1	27032	ATPase secretory pathway Ca2+ transporting 1
ENSG0000	19.86584	0.546111	0.537678	1.015685	0.30978	0.606447	TRAV38-2I	28643	T cell receptor alpha variable 38-2/delta variable 8
ENSG0000	105.5478	0.265311	0.261276	1.015444	0.309894	0.606608	H2AC25	92815	H2A clustered histone 25
ENSG0000	20.0242	-0.67856	0.668345	-1.01528	0.30997	0.606693	NA	NA	NA
ENSG0000	34.00557	-0.42419	0.417908	-1.01503	0.310092	0.606867	PROC	5624	protein C inactivator of coagulation factors Va and VIIIa
ENSG0000	3.470694	-1.38927	1.368917	-1.01487	0.31017	0.606955	PCLAF	9768	PCNA clamp associated factor
ENSG0000	6.297609	1.268086	1.249816	1.014618	0.310288	0.607122	SUMO4	387082	small ubiquitin like modifier 4
ENSG0000	543.9836	0.136821	0.134867	1.014493	0.310347	0.60714	PGAM5	192111	PGAM far mitochondrial serine/threonine protein phosphatase
ENSG0000	19458.25	-0.07063	0.069622	-1.01446	0.310362	0.60714	ARRB2	409	arrestin beta 2
ENSG0000	398.8981	-0.16359	0.161272	-1.01435	0.310415	0.607178	FAM219A	203259	family with sequence similarity 219 member A
ENSG0000	524.8967	0.122429	0.120715	1.014198	0.310488	0.607258	NAT9	26151	N-acetyltransferase 9 (putative)
ENSG0000	380.3081	0.167891	0.1656	1.013833	0.310662	0.60747	MGST1	4257	microsomal glutathione S-transferase 1
ENSG0000	6.257349	1.263549	1.246247	1.013883	0.310638	0.60747	NA	NA	NA
ENSG0000	11.71128	-0.69856	0.689158	-1.01365	0.310751	0.60758	CEL	1056	carboxyl ester lipase
ENSG0000	7.807004	-1.04679	1.032853	-1.01349	0.310826	0.607661	NA	NA	NA
ENSG0000	4.87356	-1.10465	1.090252	-1.01321	0.31096	0.607713	KIF14	9928	kinesin family member 14
ENSG0000	76.40411	-0.28036	0.27668	-1.01332	0.310909	0.607713	ZNF624	57547	zinc finger protein 624
ENSG0000	3.501187	1.36311	1.345335	1.013212	0.310959	0.607713	NA	NA	NA
ENSG0000	42.94031	0.383109	0.378132	1.013162	0.310983	0.607713	ZNF280B	140883	zinc finger protein 280B
ENSG0000	4.167011	-1.38803	1.370318	-1.01293	0.311096	0.607869	ADAM20P	317760	ADAM metallopeptidase domain 20 pseudogene 1
ENSG0000	104.7992	-0.24112	0.238061	-1.01285	0.311132	0.607875	LSM5	23658	LSM5 hom U6 small nuclear RNA and mRNA degradation associated
ENSG0000	1186.329	0.097057	0.095862	1.012472	0.311312	0.608164	REPS1	85021	RALBP1 associated Eps domain containing 1
ENSG0000	810.6601	0.110263	0.108919	1.012342	0.311374	0.608221	PEX26	55670	peroxisomal biogenesis factor 26
ENSG0000	219.4289	-0.16788	0.165882	-1.01206	0.311508	0.608418	RBMS2	5939	RNA binding motif single stranded interacting protein 2
ENSG0000	510.5062	-0.13645	0.134883	-1.0116	0.31173	0.608764	ZNF767P	79970	zinc finger pseudogene
ENSG0000	84.24276	-0.26624	0.263196	-1.01156	0.311751	0.608764	DERA	51071	deoxyribose-phosphate aldolase
ENSG0000	21.63378	-0.52168	0.515912	-1.01117	0.311933	0.609055	NA	NA	NA

ENSG0000	5418.079	0.075053	0.074238	1.010981	0.312025	0.609172	MED12	9968	mediator complex subunit 12
ENSG0000	4.271043	-1.34869	1.334275	-1.01081	0.312109	0.609236	NA	NA	NA
ENSG0000	668.7629	-0.13144	0.130035	-1.01077	0.312126	0.609236	DHX37	57647	DEAH-box helicase 37
ENSG0000	54.64455	-0.34928	0.345581	-1.01071	0.312157	0.609236	MOSPD1	56180	motile sperm domain containing 1
ENSG0000	3.076299	1.380281	1.365863	1.010556	0.312229	0.609313	NA	NA	NA
ENSG0000	8.312936	-0.93285	0.923316	-1.01032	0.312342	0.609465	NA	NA	NA
ENSG0000	3619.478	0.081308	0.080493	1.010129	0.312433	0.609465	GATA3	2625	GATA binding protein 3
ENSG0000	16.36776	0.642878	0.636376	1.010217	0.312391	0.609465	CCDC184	387856	coiled-coil domain containing 184
ENSG0000	65.42483	0.347144	0.343667	1.010119	0.312438	0.609465	NME1	4830	NME/NM23 nucleoside diphosphate kinase 1
ENSG0000	4.011844	1.517846	1.502753	1.010044	0.312474	0.609471	NA	NA	NA
ENSG0000	486.6547	0.127668	0.126418	1.009886	0.31255	0.609554	MRPL34	64981	mitochondrial ribosomal protein L34
ENSG0000	1325.643	0.106208	0.105238	1.00922	0.312869	0.60995	CAPN10	11132	calpain 10
ENSG0000	6.3186	-1.00108	0.991966	-1.00919	0.312884	0.60995	MST1R	4486	macrophage stimulating 1 receptor
ENSG0000	14439.2	0.075936	0.075239	1.009267	0.312847	0.60995	HLA-DPA1	3113	major hist class II DP alpha 1
ENSG0000	130.015	-0.22474	0.222677	-1.00929	0.312838	0.60995	TMEM147	1.01E+08	TMEM147 antisense RNA 1
ENSG0000	61.95115	0.325032	0.32212	1.00904	0.312955	0.60999	MAIP1	79568	matrix AAA peptidase interacting protein 1
ENSG0000	3758.712	-0.09673	0.095864	-1.00901	0.31297	0.60999	CCDC88B	283234	coiled-coil domain containing 88B
ENSG0000	43.65075	-0.41286	0.409282	-1.00873	0.313104	0.610187	NA	NA	NA
ENSG0000	116.8612	0.228284	0.226343	1.008577	0.313177	0.610265	ASNSD1	54529	asparagine synthetase domain containing 1
ENSG0000	5.629076	-1.32272	1.311565	-1.00851	0.313211	0.610267	NA	NA	NA
ENSG0000	3.500735	-1.41077	1.399479	-1.00807	0.31342	0.61029	LOC34406	344065	zinc finger protein 570-like
ENSG0000	2123.768	-0.09168	0.090923	-1.00829	0.313315	0.61029	TLK1	9874	tousled like kinase 1
ENSG0000	249.3452	0.192123	0.19054	1.008308	0.313307	0.61029	SLC39A14	23516	solute carrier family 39 member 14
ENSG0000	606.3584	-0.12219	0.121197	-1.00821	0.313355	0.61029	YPEL2	388403	yippee like 2
ENSG0000	299.2524	0.151773	0.150552	1.008109	0.313402	0.61029	ZNF615	284370	zinc finger protein 615
ENSG0000	1198.696	0.09903	0.098233	1.008107	0.313403	0.61029	ESS2	8220	ess-2 splicing factor homolog
ENSG0000	26.597	-0.45518	0.451682	-1.00775	0.313577	0.610297	NA	NA	NA
ENSG0000	1951.284	-0.09309	0.09238	-1.00772	0.313589	0.610297	GOLGB1	2804	golgin B1
ENSG0000	4.62964	-1.19067	1.181487	-1.00778	0.313562	0.610297	NUDT12	83594	nudix hydrolase 12
ENSG0000	1114.736	-0.1077	0.106871	-1.00779	0.313553	0.610297	THAP12	5612	THAP domain containing 12
ENSG0000	481.6417	-0.12956	0.128539	-1.00792	0.313491	0.610297	HSD17B10	3028	hydroxysteroid 17-beta dehydrogenase 10
ENSG0000	17.20995	0.606008	0.601579	1.007362	0.313761	0.610568	RPL41P2	22970	ribosomal protein L41 pseudogene 2
ENSG0000	89.4907	0.335864	0.333452	1.007234	0.313822	0.610624	NA	NA	NA
ENSG0000	37.58504	0.402112	0.399269	1.00712	0.313877	0.610666	CENPH	64946	centromere protein H
ENSG0000	4.625651	-1.24803	1.239542	-1.00685	0.314007	0.610733	NA	NA	NA
ENSG0000	14.12711	-0.65562	0.651089	-1.00696	0.313955	0.610733	TXNRD3	114112	thioredoxin reductase 3
ENSG0000	183.187	0.200065	0.198705	1.006843	0.31401	0.610733	TM7SF2	7108	transmembrane 7 superfamily member 2
ENSG0000	1093.956	-0.11533	0.114562	-1.00674	0.314061	0.610767	OSBPL5	114879	oxysterol binding protein like 5
ENSG0000	29.2042	-0.46914	0.466046	-1.00664	0.314108	0.610795	OBSCN-AS	574407	OBSCN antisense RNA 1
ENSG0000	2184.14	-0.09015	0.089565	-1.00648	0.314183	0.610813	DAP	1611	death associated protein
ENSG0000	79.06858	0.279629	0.277827	1.006486	0.314182	0.610813	TNFSF13	8741	TNF superfamily member 13
ENSG0000	76.53288	-0.26839	0.26672	-1.00627	0.314286	0.610949	C14orf28	122525	chromosome 14 open reading frame 28
ENSG0000	37.59333	-0.47575	0.472859	-1.00612	0.314358	0.611024	MSS51	118490	MSS51 mitochondrial translational activator
ENSG0000	116.7444	0.224035	0.222704	1.005979	0.314426	0.611028	WDR53	348793	WD repeat domain 53
ENSG0000	5.459956	-1.24103	1.23365	-1.00598	0.314425	0.611028	NA	NA	NA
ENSG0000	3.700329	-1.33844	1.330606	-1.00588	0.314472	0.611053	LOC10537	1.05E+08	LOC105373289
ENSG0000	141.0015	-0.23451	0.233197	-1.00564	0.314588	0.611088	LMAN2L	81562	lectin mannose binding 2 like
ENSG0000	511.6359	0.127706	0.126984	1.005689	0.314565	0.611088	DDR1	780	discoidin domain receptor tyrosine kinase 1
ENSG0000	20.96538	-0.52752	0.524558	-1.00565	0.314583	0.611088	ACBD7	414149	acyl-CoA binding domain containing 7
ENSG0000	1761.33	0.082465	0.082046	1.005104	0.314847	0.611399	POGK	57645	pogo transposable element derived with KRAB domain
ENSG0000	77.52048	0.275391	0.273988	1.005122	0.314838	0.611399	KIF19	124602	kinesin family member 19
ENSG0000	183.5731	0.189819	0.188832	1.005225	0.314788	0.611399	HAUS8	93323	HAUS augmin like complex subunit 8
ENSG0000	2.534854	1.85286	1.843583	1.005032	0.314881	0.611401	NA	NA	NA
ENSG0000	465.8576	0.141293	0.140611	1.004851	0.314969	0.611452	EML2	24139	EMAP like 2
ENSG0000	77.89408	-0.31828	0.316751	-1.00484	0.314973	0.611452	HLCS	3141	holocarboxylase synthetase
ENSG0000	327.5288	-0.16932	0.168581	-1.00437	0.315201	0.611774	PGM1	5236	phosphoglucomutase 1
ENSG0000	22.30549	0.488508	0.486388	1.004359	0.315206	0.611774	RPL17P7	1E+08	ribosomal protein L17 pseudogene 7
ENSG0000	771.0558	0.113198	0.112731	1.004137	0.315313	0.611918	SMIM29	221491	small integral membrane protein 29
ENSG0000	25.89916	0.488989	0.487078	1.003924	0.315415	0.612053	ADRA2B	151	adrenoceptor alpha 2B
ENSG0000	6043.827	0.08492	0.084655	1.003129	0.315799	0.612216	PIK3CD	5293	phosphati 5-bisphosphate 3-kinase catalytic subunit delta
ENSG0000	871.797	-0.11352	0.113183	-1.003	0.315862	0.612216	NCDN	23154	neurochondrin
ENSG0000	169.6384	-0.24974	0.248973	-1.00309	0.315819	0.612216	STK36	27148	serine/threonine kinase 36
ENSG0000	263.7396	-0.16543	0.164859	-1.00349	0.315623	0.612216	LZTFL1	54585	leucine zipper transcription factor like 1
ENSG0000	396.8779	0.162574	0.162063	1.003155	0.315786	0.612216	CEP78	84131	centrosomal protein 78
ENSG0000	28.77525	-0.46886	0.467457	-1.003	0.315862	0.612216	FRMD3	257019	FERM domain containing 3
ENSG0000	4.644929	1.276306	1.271795	1.003547	0.315597	0.612216	NA	NA	NA
ENSG0000	3.005262	-1.45248	1.447939	-1.00314	0.315794	0.612216	NA	NA	NA
ENSG0000	170.042	0.200032	0.199384	1.003248	0.315741	0.612216	TMED1	11018	transmembrane p24 trafficking protein 1
ENSG0000	25.22387	0.482745	0.481289	1.003026	0.315848	0.612216	NA	NA	NA
ENSG0000	19.89857	-0.62924	0.627196	-1.00325	0.315739	0.612216	EPB41L1	2036	erythrocyte membrane protein band 4.1 like 1
ENSG0000	148.6393	-0.21236	0.211747	-1.0029	0.31591	0.612244	THAP6	152815	THAP domain containing 6
ENSG0000	391.6301	-0.15667	0.156284	-1.00245	0.316127	0.612593	STK16	8576	serine/threonine kinase 16
ENSG0000	818.9164	-0.11906	0.118778	-1.00239	0.316156	0.612593	SLC25A22	79751	solute carrier family 25 member 22
ENSG0000	525.6401	-0.14774	0.147404	-1.00231	0.316192	0.612598	TRIM5	85363	tripartite motif containing 5
ENSG0000	1109.908	0.118849	0.118624	1.001896	0.316394	0.612921	MZT2B	80097	mitotic spindle organizing protein 2B
ENSG0000	12.24241	-0.74405	0.742684	-1.00183	0.316424	0.612921	NA	NA	NA
ENSG0000	3696.728	0.095109	0.094994	1.001206	0.316727	0.613324	EDF1	8721	endothelial differentiation related factor 1
ENSG0000	126.6596	0.253309	0.25299	1.001261	0.316701	0.613324	ERP27	121506	endoplasmic reticulum protein 27
ENSG0000	571.6918	-0.1198	0.119654	-1.0012	0.316731	0.613324	CBX5	23468	chromobox 5
ENSG0000	313.4919	0.165672	0.16552	1.000914	0.316868	0.613474	GBGT1	26301	globoside 3-N-acetylgalactosaminyltransferase 1 (FORS blood group)
ENSG0000	439.744	0.125062	0.12495	1.000901	0.316875	0.613474	NAA16	79612	N-alpha-a NatA auxiliary subunit
ENSG0000	2084.178	0.094126	0.09406	1.000694	0.316975	0.613539	IL27RA	9466	interleukin 27 receptor subunit alpha
ENSG0000	3.245484	1.579649	1.578492	1.000733	0.316956	0.613539	PLCB4	5332	phospholipase C beta 4
ENSG0000	499.1978	-0.13235	0.132292	-1.00042	0.31711	0.613672	RAB3GAP2	25782	RAB3 GTPase activating non-catalytic protein subunit 2
ENSG0000	311.2552	0.163632	0.163555	1.000467	0.317084	0.613672	ABHD17B	51104	abhydrola depalmitoylase
ENSG0000	995.6434	-0.09641	0.096402	-1.00005	0.317288	0.613954	C11orf24	53838	chromosome 11 open reading frame 24
ENSG0000	43.53371	0.402113	0.402311	0.999506	0.317549	0.614213	TMEM81	388730	transmembrane protein 81
ENSG0000	179.9306	0.261854	0.262002	0.999433	0.317585	0.614213	PLCL1	5334	phospholipase C like 1 (inactive)
ENSG0000	14.57429	0.611396	0.611718	0.999474	0.317565	0.614213	NA	NA	NA
ENSG0000	75.85502	-0.3169	0.317032	-0.99958	0.317513	0.614213	CNR1	1268	cannabinoid receptor 1
ENSG0000	2041.658	-0.1044	0.104462	-0.99943	0.317588	0.614213	ADAMTS1	81794	ADAM metallopeptidase with thrombospondin type 1 motif 10
ENSG0000	6.015811	-1.08447	1.085274	-0.99926	0.317671	0.614309	NA	NA	NA
ENSG0000	3195.142	0.084115	0.084193	0.999078	0.317757	0.614327	FAM193A	8603	family with sequence similarity 193 member A
ENSG0000	303.5076	-0.16358	0.163739	-0.99903	0.317779	0.614327	TIRAP	114609	TIR domain containing adaptor protein
ENSG0000	102.8657	-0.28985	0.290122	-0.99906	0.317767	0.614327	MYEF2	50804	myelin expression factor 2
ENSG0000	25.62057	0.478216	0.478857	0.998662	0.317958	0.6145	MUTYH	4595	mutY DNA glycosylase
ENSG0000	2128.02	-0.08389	0.084008	-0.99857	0.318001	0.6145	AGPAT1	10554	1-acylglycerol-3-phosphate O-acyltransferase 1
ENSG0000	2945.883	0.089319	0.089444	0.998607	0.317985	0.6145	UBR5	51366	ubiquitin protein ligase E3 component n-recognin 5
ENSG0000	825.532	0.104687	0.104824	0.998694	0.317943	0.6145	CTNS	1497	cystinosin lysosomal cystine transporter
ENSG0000	666.4386	0.13087	0.131234	0.997219	0.318658	0.614871	GMPPB	29925	GDP-mannose pyrophosphorylase B

ENSG0000	2555.783	0.082229	0.082453	0.997289	0.318624	0.614871	CHCHD2	51142	coiled-coil-helix-coiled-coil-helix domain containing 2
ENSG0000	214.3547	0.19814	0.198672	0.99732	0.318609	0.614871	ASIC3	9311	acid sensing ion channel subunit 3
ENSG0000	67.82273	-0.29074	0.291547	-0.99724	0.318646	0.614871	ZNG1E	220869	Zn regulated GTPase metalloprotein activator 1E
ENSG0000	44.66874	-0.39301	0.39394	-0.99764	0.318454	0.614871	DNM1	1759	dynamin 1
ENSG0000	908.254	0.111787	0.112033	0.997805	0.318374	0.614871	MAPK8	5599	mitogen-activated protein kinase 8
ENSG0000	75.64173	0.27513	0.27572	0.997862	0.318346	0.614871	FGD6	55785	FYVE RhoGEF and PH domain containing 6
ENSG0000	60.51162	-0.35027	0.351101	-0.99765	0.318451	0.614871	NA	NA	NA
ENSG0000	96.15478	0.258251	0.258822	0.997794	0.318379	0.614871	NA	NA	NA
ENSG0000	19.30998	-1.40012	1.404009	-0.99723	0.318653	0.614871	EVPL	2125	envoplakin
ENSG0000	3850.34	-0.08861	0.088785	-0.998	0.318281	0.614871	SHFL	55337	shiftless antiviral inhibitor of ribosomal frameshifting
ENSG0000	77.01647	-0.29516	0.296004	-0.99715	0.318691	0.614871	ZNF677	342926	zinc finger protein 677
ENSG0000	54.23362	-0.3315	0.332438	-0.99719	0.318672	0.614871	ZNF813	126017	zinc finger protein 813
ENSG0000	790.9343	0.115824	0.116076	0.997826	0.318364	0.614871	LSS	4047	lanosterol synthase
ENSG0000	65.63152	0.327237	0.328034	0.997569	0.318488	0.614871	CBY1	25776	chibby fan beta catenin antagonist
ENSG0000	171.2713	0.222593	0.223385	0.996453	0.31903	0.615462	DNAAF2	55172	dynein axonemal assembly factor 2
ENSG0000	595.6125	0.11919	0.119624	0.996376	0.319067	0.61547	RRP8	23378	ribosomal RNA processing 8
ENSG0000	15.84392	-0.63591	0.638337	-0.99621	0.319151	0.615566	CA15P1	1.01E+08	CA15 pseudogene 1
ENSG0000	70.42303	0.330323	0.331731	0.995754	0.31937	0.615831	CPNE8	144402	copine 8
ENSG0000	433.652	0.142889	0.143503	0.995717	0.319388	0.615831	TWF1	5756	twinfilin actin binding protein 1
ENSG0000	1451.287	-0.0892	0.089581	-0.99579	0.319354	0.615831	MLEC	9761	malectin
ENSG0000	121.7168	-0.21788	0.218839	-0.99563	0.31943	0.615848	TCAIM	285343	T cell activ mitochondrial
ENSG0000	42.57772	0.388217	0.390051	0.995296	0.319592	0.616098	SNHG20	654434	small nucleolar RNA host gene 20
ENSG0000	69.82369	-0.31299	0.314535	-0.9951	0.319688	0.616113	TMCC2	9911	transmembrane and coiled-coil domain family 2
ENSG0000	1391.43	0.110499	0.111046	0.995075	0.3197	0.616113	IP6K2	51447	inositol hexakisphosphate kinase 2
ENSG0000	59.73078	0.328339	0.329941	0.995143	0.319667	0.616113	GABARAP	11337	GABA type A receptor-associated protein
ENSG0000	3392.769	0.077352	0.077754	0.994836	0.319816	0.616273	HERPUD2	64224	HERPUD family member 2
ENSG0000	7545.657	-0.08003	0.080454	-0.99476	0.319854	0.616283	HCLS1	3059	hematopoietic cell-specific Lyn substrate 1
ENSG0000	4.276102	1.131999	1.138062	0.994672	0.319896	0.616299	KIF4A	24137	kinesin family member 4A
ENSG0000	203.4025	-0.18701	0.188084	-0.99431	0.320074	0.616449	ABCD3	5825	ATP binding cassette subfamily D member 3
ENSG0000	289.9977	-0.16335	0.164278	-0.99435	0.320053	0.616449	TBC1D9	23158	TBC1 domain family member 9
ENSG0000	3457.218	0.074489	0.074911	0.99437	0.320043	0.616449	PRKCH	5583	protein kinase C eta
ENSG0000	8821.456	-0.0681	0.068491	-0.99424	0.320108	0.616451	TLE3	7090	TLE family transcriptional corepressor
ENSG0000	731.6006	-0.13632	0.137167	-0.99379	0.320323	0.616802	B3GALT6	126792	beta-1 3-galactosyltransferase 6
ENSG0000	309.5367	-0.15998	0.161013	-0.9936	0.320417	0.616892	BCL9	607	BCL9 transcription coactivator
ENSG0000	45.08001	0.389581	0.392106	0.993561	0.320437	0.616892	NA	NA	NA
ENSG0000	60.82382	0.33823	0.34056	0.993159	0.320632	0.617205	ZNF880	400713	zinc finger protein 880
ENSG0000	45.30733	-0.37105	0.373721	-0.99284	0.320787	0.617315	NA	NA	NA
ENSG0000	267.5762	-0.17045	0.171684	-0.99284	0.320789	0.617315	PKD2	5311	polycystin transient receptor potential cation channel
ENSG0000	672.3898	-0.16879	0.17	-0.9929	0.320757	0.617315	NOTCH3	4854	notch receptor 3
ENSG0000	10.06127	-0.73675	0.742161	-0.99271	0.32085	0.617367	NA	NA	NA
ENSG0000	15631.31	0.081947	0.082588	0.992237	0.321082	0.61775	PGGHG	80162	protein-glucosylgalactosylhydroxylysine glucosidase
ENSG0000	6.098326	0.898748	0.905917	0.992087	0.321155	0.617826	RPL23AP7	729617	ribosomal protein L23a pseudogene 74
ENSG0000	514.2845	0.129723	0.130773	0.99197	0.321212	0.617872	ELP2	55250	elongator acetyltransferase complex subunit 2
ENSG0000	3.678764	-1.30887	1.319784	-0.99173	0.321329	0.617996	GNB1-DT	1.05E+08	GNB1 divergent transcript
ENSG0000	10.25861	-0.85771	0.864886	-0.9917	0.321343	0.617996	CRIP3	401262	cysteine rich protein 3
ENSG0000	736.8912	-0.10825	0.109178	-0.99149	0.321449	0.618071	TRIM62	55223	tripartite motif containing 62
ENSG0000	185.3245	-0.19373	0.195387	-0.99154	0.32142	0.618071	TM7SF3	51768	transmembrane 7 superfamily member 3
ENSG0000	5.572686	-1.21005	1.220534	-0.99141	0.321485	0.618077	CHD5	26038	chromodomain helicase DNA binding protein 5
ENSG0000	2.286759	2.01659	2.034554	0.991171	0.321602	0.618238	LOC10537	1.05E+08	uncharacterized LOC105375523
ENSG0000	12.36785	-0.70867	0.71509	-0.99102	0.321676	0.618241	LOC12490	1.25E+08	uncharacterized LOC124900778
ENSG0000	48.88824	0.33921	0.342289	0.991003	0.321684	0.618241	NA	NA	NA
ENSG0000	168.3778	0.18583	0.187525	0.990962	0.321704	0.618241	WDR89	112840	WD repeat domain 89
ENSG0000	64.47939	-0.31323	0.316114	-0.99087	0.321747	0.61826	ANKRD16	54522	ankyrin repeat domain 16
ENSG0000	262.2137	0.152846	0.154275	0.990732	0.321816	0.618329	KIN	22944	Kin17 DNA and RNA binding protein
ENSG0000	20.63192	-0.52759	0.532735	-0.99034	0.322007	0.618392	NA	NA	NA
ENSG0000	352.4175	-0.15896	0.160494	-0.99043	0.321964	0.618392	MYBL1	4603	MYB proto-oncogene like 1
ENSG0000	20.39903	-0.67544	0.682039	-0.99032	0.322016	0.618392	CCDC144A	9720	coiled-coil domain containing 144A
ENSG0000	4.623482	1.371253	1.384402	0.990502	0.321929	0.618392	NA	NA	NA
ENSG0000	549.5633	-0.11895	0.120103	-0.99037	0.321993	0.618392	SOX12	6666	SRY-box transcription factor 12
ENSG0000	41.24544	-0.37513	0.37883	-0.99023	0.32206	0.618412	ZNF790	388536	zinc finger protein 790
ENSG0000	39.69962	0.399588	0.403611	0.99003	0.322159	0.618539	DGKK	139189	diacylglycerol kinase kappa
ENSG0000	476.183	-0.14251	0.143973	-0.98985	0.322247	0.61859	BTRC	8945	beta-transducin repeat containing E3 ubiquitin protein ligase
ENSG0000	108.6299	-0.29178	0.294775	-0.98984	0.322253	0.61859	ZNF449	203523	zinc finger protein 449
ENSG0000	3.775218	1.66127	1.678439	0.989771	0.322286	0.61859	LOC12490	1.25E+08	uncharacterized LOC124902371
ENSG0000	36.7783	0.38256	0.386672	0.989365	0.322485	0.618844	LOC72973	729732	uncharacterized LOC729732
ENSG0000	849.431	-0.12495	0.12629	-0.98942	0.322459	0.618844	SLC52A2	79581	solute carrier family 52 member 2
ENSG0000	16.70363	-0.6672	0.674642	-0.98897	0.322678	0.61907	NA	NA	NA
ENSG0000	4.084105	1.347138	1.362333	0.988846	0.322738	0.61907	NA	NA	NA
ENSG0000	954.5476	0.107057	0.10829	0.988615	0.322851	0.61907	NA	NA	NA
ENSG0000	416.1853	0.127695	0.129166	0.988612	0.322853	0.61907	PWP1	11137	PWP1 hon endonuclein
ENSG0000	228.6812	0.167573	0.169521	0.98851	0.322903	0.61907	SGPP1	81537	sphingosine-1-phosphate phosphatase 1
ENSG0000	43.63437	0.402709	0.407308	0.988708	0.322806	0.61907	TRPM4	54795	transient receptor potential cation channel subfamily M member 4
ENSG0000	89.19747	0.358511	0.362665	0.988545	0.322886	0.61907	OSBP2	23762	oxysterol binding protein 2
ENSG0000	2033.012	-0.08642	0.087406	-0.98866	0.32283	0.61907	CBX6	23466	chromobox 6
ENSG0000	8.945404	-0.77308	0.781891	-0.98873	0.322793	0.61907	FIRRE	286467	firre intergenic repeating RNA element
ENSG0000	57.48352	-0.34558	0.349627	-0.98843	0.32294	0.619077	SLC25A14	9016	solute carrier family 25 member 14
ENSG0000	3.0033	-1.67629	1.6963	-0.9882	0.323053	0.619166	LOC10042	1E+08	pleckstrin homology domain containing A1 pseudogene
ENSG0000	5.164522	-1.42742	1.444364	-0.98827	0.323021	0.619166	NA	NA	NA
ENSG0000	605.001	-0.1193	0.120779	-0.98778	0.323263	0.619504	PPIE	10450	peptidylprolyl isomerase E
ENSG0000	20.59596	-0.52274	0.52928	-0.98765	0.323324	0.619558	GLI1	2735	GLI family zinc finger 1
ENSG0000	313.9159	0.161492	0.16358	0.987232	0.323529	0.619835	PHLDB2	90102	pleckstrin homology like domain family B member 2
ENSG0000	26.95185	0.47797	0.484159	0.987217	0.323536	0.619835	NA	NA	NA
ENSG0000	17.63585	0.674645	0.683569	0.986945	0.323669	0.620027	ACOX2	8309	acyl-CoA oxidase 2
ENSG0000	10.98322	0.840938	0.852157	0.986835	0.323724	0.620066	NA	NA	NA
ENSG0000	10.24259	-0.93778	0.950544	-0.98658	0.32385	0.620245	LOC10798	1.08E+08	uncharacterized LOC107985211
ENSG0000	1539.049	0.102611	0.104022	0.986439	0.323918	0.62031	ZNF787	126208	zinc finger protein 787
ENSG0000	6.19792	-1.10617	1.122293	-0.98563	0.324313	0.620663	NA	NA	NA
ENSG0000	52.471	0.363195	0.368498	0.985608	0.324326	0.620663	GDAP1	54332	ganglioside induced differentiation associated protein 1
ENSG0000	3339.104	-0.08825	0.089525	-0.98573	0.324268	0.620663	SETX	23064	senataxin
ENSG0000	29.29289	0.430115	0.436309	0.985805	0.324229	0.620663	NDST2	8509	N-deacetylase and N-sulfotransferase 2
ENSG0000	43.80386	-0.33987	0.344841	-0.98559	0.324336	0.620663	HROB	78995	homologous recombination factor with OB-fold
ENSG0000	102.7489	0.2376	0.241001	0.985888	0.324188	0.620663	ZNF420	147923	zinc finger protein 420
ENSG0000	4.515514	1.034096	1.049074	0.985723	0.324269	0.620663	NA	NA	NA
ENSG0000	523.2591	0.117116	0.118849	0.985418	0.324419	0.620721	TOR1AIP2	163590	torsin 1A interacting protein 2
ENSG0000	261.3433	0.208355	0.211468	0.985278	0.324487	0.620721	MBD5	55777	methyl-CpG binding domain protein 5
ENSG0000	36.01713	1.046155	1.061938	0.985137	0.324557	0.620721	PROK2	60675	prokineticin 2
ENSG0000	520.8178	-0.11678	0.118539	-0.98518	0.324535	0.620721	MRPL41	64975	mitochondrial ribosomal protein L41
ENSG0000	206.8721	0.209871	0.213042	0.985115	0.324568	0.620721	ROBO3	64221	roundabout guidance receptor 3
ENSG0000	4.392492	-1.45369	1.475642	-0.98512	0.324564	0.620721	HOXB6	3216	homeobox B6
ENSG0000	2148.696	0.098409	0.099905	0.985032	0.324608	0.620735	ZMAT2	153527	zinc finger matrin-type 2

ENSG0000	822.7544	0.110608	0.11232	0.984756	0.324744	0.620821	PAPOLG	64895	poly(A) polymerase gamma
ENSG0000	42.96517	-0.34678	0.35215	-0.98475	0.324745	0.620821	BUB1	699	BUB1 mitotic checkpoint serine/threonine kinase
ENSG0000	6207.062	-0.07159	0.072708	-0.9846	0.32482	0.620821	HK1	3098	hexokinase 1
ENSG0000	14.43291	-0.59159	0.600762	-0.98473	0.324759	0.620821	CFAP251	144406	cilia and flagella associated protein 251
ENSG0000	666.1835	-0.11854	0.120396	-0.9846	0.324821	0.620821	PHKA2	5256	phosphorylase kinase regulatory subunit alpha 2
ENSG0000	301.3406	0.153623	0.156051	0.984443	0.324898	0.620905	OGFOD3	79701	2-oxoglutarate and iron dependent oxygenase domain containing 3
ENSG0000	26.65299	-0.51353	0.52172	-0.9843	0.32497	0.620915	KHK	3795	ketohexokinase
ENSG0000	21.24026	0.587132	0.596481	0.984327	0.324955	0.620915	LOC10537	1.05E+08	uncharacterized LOC105378268
ENSG0000	3399.437	-0.07689	0.078121	-0.98421	0.325013	0.620932	GDI2	2665	GDP dissociation inhibitor 2
ENSG0000	96.54911	-0.24403	0.248022	-0.98392	0.325155	0.62114	ZFP41	286128	ZFP41 zinc finger protein
ENSG0000	17.70767	-0.55804	0.567416	-0.98347	0.325376	0.62133	NA	NA	NA
ENSG0000	57.68505	0.407969	0.414885	0.983331	0.325445	0.62133	NA	NA	NA
ENSG0000	39.66705	-0.37345	0.379793	-0.98331	0.325455	0.62133	POLR2J3	548644	RNA polymerase II subunit J3
ENSG0000	268.7445	-0.16086	0.163582	-0.98336	0.325433	0.62133	RAB15	376267	RAB15 member RAS oncogene family
ENSG0000	7.363207	-1.00837	1.025169	-0.98361	0.325306	0.62133	SETBP1-DT	1.02E+08	SETBP1 divergent transcript
ENSG0000	20.5216	0.570702	0.580332	0.983407	0.325407	0.62133	TTTY14	83869	testis-spec Y-linked 14
ENSG0000	68.83073	-0.29314	0.298153	-0.98318	0.325519	0.621387	B4GAT1	11041	beta-1 4-glucuronyltransferase 1
ENSG0000	1515.459	0.095718	0.09739	0.982835	0.325689	0.621648	PSMB3	5691	proteasome 20S subunit beta 3
ENSG0000	5.522033	1.081383	1.10036	0.982754	0.325729	0.62166	LOC12490	1.25E+08	small nucleolar RNA ACA64
ENSG0000	12.5599	0.734378	0.747382	0.982601	0.325804	0.621739	NA	NA	NA
ENSG0000	5.562982	-1.11915	1.139109	-0.98248	0.325862	0.621787	NA	NA	NA
ENSG0000	24.17241	0.572202	0.582607	0.982141	0.326031	0.621916	LPP-AS2	339929	LPP antisense RNA 2
ENSG0000	296.9315	-0.16633	0.169342	-0.98224	0.32598	0.621916	TMED3	23423	transmembrane p24 trafficking protein 3
ENSG0000	18.96818	-0.66073	0.672738	-0.98215	0.326026	0.621916	NA	NA	NA
ENSG0000	13.75954	-0.72193	0.735162	-0.982	0.326101	0.621966	H2BC15	8341	H2B clustered histone 15
ENSG0000	35.63872	-0.41121	0.418765	-0.98195	0.326124	0.621966	NOL3	8996	nucleolar protein 3
ENSG0000	5.636499	1.071713	1.09189	0.981521	0.326336	0.621987	NA	NA	NA
ENSG0000	27.17355	0.478161	0.487114	0.981621	0.326287	0.621987	ZNF799	90576	zinc finger protein 799
ENSG0000	705.1073	-0.1203	0.122558	-0.98159	0.326304	0.621987	REX1BD	55049	required for excision 1-B domain containing
ENSG0000	152.5739	-0.21304	0.216995	-0.98176	0.326219	0.621987	ZNF552	79818	zinc finger protein 552
ENSG0000	34.73354	-0.46217	0.470749	-0.98177	0.326212	0.621987	DDTL	1E+08	D-dopachrome tautomerase like
ENSG0000	118.3323	-0.24113	0.245665	-0.98154	0.326329	0.621987	PNMA3	29944	PNMA family member 3
ENSG0000	2911.984	0.082987	0.084558	0.981428	0.326382	0.62201	UBP1	7342	upstream binding protein 1
ENSG0000	1683.388	0.097301	0.099157	0.981275	0.326457	0.62209	EIF3M	10480	eukaryotic translation initiation factor 3 subunit M
ENSG0000	3.635206	-1.46702	1.495363	-0.98105	0.326569	0.622239	NA	NA	NA
ENSG0000	110.8612	0.281334	0.286814	0.980896	0.326644	0.622318	HNRNPA1L	642659	heterogeneous nuclear ribonucleoprotein A1 like 3
ENSG0000	165.3674	-0.19344	0.197261	-0.98065	0.326765	0.622485	PLEKHA5	54477	pleckstrin homology domain containing A5
ENSG0000	471.8495	-0.12067	0.123079	-0.98043	0.326874	0.622501	TMCO3	55002	transmembrane and coiled-coil domains 3
ENSG0000	204.2595	0.190343	0.194122	0.980534	0.326823	0.622501	VCPKMT	79609	valosin containing protein lysine methyltransferase
ENSG0000	5.131148	-1.21003	1.234156	-0.98045	0.326862	0.622501	NA	NA	NA
ENSG0000	5.212717	1.152222	1.175438	0.980249	0.326963	0.622542	NA	NA	NA
ENSG0000	3078.457	0.074209	0.075699	0.980315	0.326931	0.622542	MTMR3	8897	myotubularin related protein 3
ENSG0000	10054.19	0.083015	0.084711	0.979977	0.327097	0.622734	PRF1	5551	perforin 1
ENSG0000	19.18343	0.634656	0.64788	0.979588	0.327289	0.623036	SLC9B1	150159	solute carrier family 9 member B1
ENSG0000	8.043822	-0.91554	0.934871	-0.97932	0.327423	0.623226	NA	NA	NA
ENSG0000	4.907063	-1.26832	1.295241	-0.97922	0.327473	0.623258	NA	NA	NA
ENSG0000	126.419	0.258119	0.263652	0.979012	0.327574	0.623378	ILDR2	387597	immunoglobulin like domain containing receptor 2
ENSG0000	59.411	0.313331	0.320067	0.978952	0.327603	0.623378	SMN1	6606	survival of telomeric
ENSG0000	25.47638	-0.48524	0.495783	-0.97874	0.327708	0.623512	TNS4	84951	tensin 4
ENSG0000	548.1036	-0.14296	0.146079	-0.97866	0.327746	0.623521	LIG1	3978	DNA ligase 1
ENSG0000	33.90199	-0.48943	0.500141	-0.97859	0.327784	0.623528	SPR	6697	sepiapterin reductase
ENSG0000	4171.666	-0.07878	0.080508	-0.97852	0.327817	0.623528	PARVG	64098	parvin gamma
ENSG0000	30.14563	0.524971	0.536644	0.978248	0.327952	0.623656	KRT18	3875	keratin 18
ENSG0000	3.900519	1.282771	1.311287	0.978254	0.327949	0.623656	SPATA41	388182	spermatogenesis associated 41
ENSG0000	8.494218	-1.04716	1.070605	-0.9781	0.328027	0.623735	NA	NA	NA
ENSG0000	4.51006	1.355145	1.385698	0.977951	0.328099	0.623744	NA	NA	NA
ENSG0000	458.0586	-0.13235	0.135328	-0.97798	0.328082	0.623744	IKZF4	64375	IKAROS family zinc finger 4
ENSG0000	324.3372	-0.16693	0.170712	-0.97784	0.328155	0.623788	AMDHD2	51005	amidohydrolase domain containing 2
ENSG0000	88.65207	0.249996	0.255708	0.977662	0.328241	0.623824	RPL17P6	645441	ribosomal protein L17 pseudogene 6
ENSG0000	670.7353	0.121551	0.124323	0.977709	0.328218	0.623824	SNRNP27	11017	small nuclear ribonucleoprotein U4/U6.U5 subunit 27
ENSG0000	2.736201	1.704394	1.743787	0.97741	0.328366	0.623997	MIR8075	1.02E+08	microRNA 8075
ENSG0000	14.32697	0.602132	0.616231	0.97712	0.32851	0.624141	FKBP7	51661	FKBP prolyl isomerase 7
ENSG0000	4.503131	1.129595	1.155985	0.977171	0.328485	0.624141	NA	NA	NA
ENSG0000	161.0066	0.2047	0.209512	0.977029	0.328555	0.624163	PABIR3	159091	PABIR family member 3
ENSG0000	3.893062	1.11218	1.138521	0.976864	0.328637	0.624191	NA	NA	NA
ENSG0000	3678.746	0.077171	0.078994	0.976924	0.328607	0.624191	SF3B3	23450	splicing factor 3b subunit 3
ENSG0000	39.60517	0.380521	0.389651	0.976569	0.328783	0.624404	KHDRBS3	10656	KH RNA bi signal transduction associated 3
ENSG0000	4.364592	1.124709	1.151929	0.97637	0.328881	0.624528	NA	NA	NA
ENSG0000	563.7286	-0.15203	0.155722	-0.97626	0.328934	0.624564	ADPRS	54936	ADP-ribosylserine hydrolase
ENSG0000	651.2393	0.133328	0.13658	0.976194	0.328968	0.624565	ATP5MC3	518	ATP synthase membrane subunit c locus 3
ENSG0000	762.8656	0.110096	0.112811	0.97593	0.329099	0.62475	CDC123	8872	cell division cycle 123
ENSG0000	2.590798	-1.69831	1.740634	-0.97568	0.329221	0.624916	NA	NA	NA
ENSG0000	889.8547	0.120654	0.123694	0.97542	0.329352	0.625101	ITM2A	9452	integral membrane protein 2A
ENSG0000	42.55126	-0.38031	0.389986	-0.97518	0.32947	0.625197	HIBCH	26275	3-hydroxyisobutyryl-CoA hydrolase
ENSG0000	18.29872	0.688604	0.706106	0.975214	0.329454	0.625197	IGHV4-4	28401	immunoglobulin heavy variable 4-4
ENSG0000	18.66265	-0.6026	0.618204	-0.97476	0.329678	0.625528	TRAV21	28662	T cell receptor alpha variable 21
ENSG0000	104.8735	-0.25309	0.259891	-0.97382	0.330148	0.626356	ZNF569	148266	zinc finger protein 569
ENSG0000	48.78105	-0.35246	0.362113	-0.97335	0.330381	0.626551	NMNAT1	64802	nicotinamide nucleotide adenylyltransferase 1
ENSG0000	329.3174	0.157033	0.161327	0.973384	0.330363	0.626551	DBF4	10926	DBF4 zinc finger
ENSG0000	41.61396	-0.36532	0.375432	-0.97306	0.330521	0.626551	MYORG	57462	myogenesis regulating glycosidase (putative)
ENSG0000	4.473105	0.984864	1.012077	0.973111	0.330498	0.626551	RPS3AP5	439992	RPS3A pseudogene 5
ENSG0000	417.0146	-0.16653	0.171124	-0.97315	0.330477	0.626551	ANKRD49	54851	ankyrin repeat domain 49
ENSG0000	833.9714	0.122889	0.126257	0.973326	0.330391	0.626551	ETNK1	55500	ethanolamine kinase 1
ENSG0000	289.6219	0.145714	0.149742	0.973101	0.330503	0.626551	EID2	163126	EP300 interacting inhibitor of differentiation 2
ENSG0000	47.7375	0.447044	0.459237	0.973449	0.33033	0.626551	PWP2	5822	PWP2 small subunit processome component
ENSG0000	320.9788	-0.17094	0.175711	-0.97285	0.330629	0.626564	AGRN	375790	agrin
ENSG0000	1457.151	-0.0825	0.084798	-0.97286	0.330623	0.626564	TPST1	8460	tyrosylprotein sulfotransferase 1
ENSG0000	15.4423	0.594189	0.610743	0.972895	0.330605	0.626564	CFAP161	161502	cilia and flagella associated protein 161
ENSG0000	1425.419	0.111805	0.114937	0.972753	0.330676	0.626589	MARCHF7	64844	membrane associated ring-CH-type finger 7
ENSG0000	7675.721	0.083056	0.085391	0.972651	0.330727	0.626621	SZRD1	26099	SUZ RNA binding domain containing 1
ENSG0000	8.066785	0.889293	0.914582	0.972349	0.330877	0.626841	SLFN14	342618	schlafen family member 14
ENSG0000	255.6555	0.162515	0.167169	0.972157	0.330972	0.626958	ZC3H6	376940	zinc finger CCCH-type containing 6
ENSG0000	54.2606	0.344626	0.35457	0.971955	0.331073	0.627051	DZIP3	9666	DAZ interacting zinc finger protein 3
ENSG0000	17.10602	0.548108	0.563942	0.971923	0.331089	0.627051	HLA-J	3137	major hist class I J (pseudogene)
ENSG0000	1345.37	0.101863	0.104837	0.971634	0.331232	0.627258	ADIPOR2	79602	adiponectin receptor 2
ENSG0000	1822.791	0.091399	0.094113	0.971157	0.33147	0.62758	SEC13	6396	SEC13 hon nuclear pore and COPII coat complex component
ENSG0000	848.3727	0.097582	0.100479	0.971168	0.331465	0.62758	FAF2	23197	Fas associated factor family member 2
ENSG0000	115.1712	-0.22253	0.229248	-0.97071	0.331695	0.627819	WFS1	7466	wolframin ER transmembrane glycoprotein
ENSG0000	4.817925	-1.25824	1.296223	-0.9707	0.331698	0.627819	NA	NA	NA
ENSG0000	835.6131	0.113242	0.116647	0.970808	0.331644	0.627819	OGFOD1	55239	2-oxoglutarate and iron dependent oxygenase domain containing 1

ENSG0000	12.96755	0.700219	0.721711	0.970221	0.331936	0.628206	NA	NA	NA
ENSG0000	52.56767	0.371202	0.382629	0.970136	0.331979	0.628223	NA	NA	NA
ENSG0000	55.6765	-0.33936	0.349892	-0.9699	0.332098	0.628384	CTNND1	1500	catenin delta 1
ENSG0000	4.349298	-1.26381	1.303371	-0.96965	0.332223	0.628556	PPP5D1P	1.01E+08	PPP5 tetra pseudogene
ENSG0000	525.0824	-0.15461	0.159483	-0.96945	0.332319	0.628598	FCRL3	115352	Fc receptor like 3
ENSG0000	365.2215	0.15618	0.16111	0.969398	0.332347	0.628598	USP37	57695	ubiquitin specific peptidase 37
ENSG0000	7720.018	-0.08291	0.085521	-0.96947	0.332311	0.628598	PITPNM1	9600	phosphatidylinositol transfer protein membrane associated 1
ENSG0000	3.293919	-1.41564	1.460685	-0.96916	0.332464	0.628691	NA	NA	NA
ENSG0000	8.267216	0.994369	1.025964	0.969204	0.332443	0.628691	NA	NA	NA
ENSG0000	409.3793	0.136659	0.141053	0.968849	0.332621	0.628791	VAMP4	8674	vesicle associated membrane protein 4
ENSG0000	34.72982	0.404539	0.417537	0.96887	0.33261	0.628791	LYPLAL1	127018	lysophospholipase like 1
ENSG0000	329.7959	0.153893	0.158831	0.96891	0.33259	0.628791	PTCRA	171558	pre T cell antigen receptor alpha
ENSG0000	467.0697	-0.13476	0.139125	-0.96865	0.33272	0.628791	TRPT1	83707	tRNA phosphotransferase 1
ENSG0000	13.72057	-0.75847	0.783009	-0.96866	0.332716	0.628791	CDON	50937	cell adhesi oncogene regulated
ENSG0000	1682.607	0.104999	0.108393	0.968685	0.332702	0.628791	SLC10A3	8273	solute carrier family 10 member 3
ENSG0000	7936.27	-0.07178	0.074134	-0.96824	0.332923	0.628899	SLA	6503	Src like adaptor
ENSG0000	32.64245	-0.44407	0.458654	-0.9682	0.332947	0.628899	PIP5K1B	8395	phosphatidylinositol-4-phosphate 5-kinase type 1 beta
ENSG0000	1152.872	0.090038	0.092994	0.968217	0.332936	0.628899	SRSF8	10929	serine and arginine rich splicing factor 8
ENSG0000	17.26593	0.601184	0.620867	0.968297	0.332896	0.628899	OVOS2	144203	alpha-2-macroglobulin like 1 pseudogene
ENSG0000	8.680632	-1.00307	1.035732	-0.96847	0.332812	0.628899	GOLGA6L1	642402	golgin A6 f pseudogene
ENSG0000	28.292	-0.43609	0.450574	-0.96786	0.333112	0.629042	VLDLR	7436	very low density lipoprotein receptor
ENSG0000	5.785229	-1.19955	1.239301	-0.96792	0.333084	0.629042	ARHGAP1:	1.14E+08	ARHGAP11A-SCG5 readthrough
ENSG0000	2.159377	2.037566	2.10527	0.967841	0.333124	0.629042	NA	NA	NA
ENSG0000	387.9469	-0.13527	0.139771	-0.96776	0.333163	0.629048	ANKRA2	57763	ankyrin repeat family A member 2
ENSG0000	6.812603	-1.0349	1.069444	-0.9677	0.333195	0.629048	FAM21FP	1E+08	family witl pseudogene
ENSG0000	10.52868	-0.69606	0.719389	-0.96757	0.333261	0.629071	NA	NA	NA
ENSG0000	2.468412	1.934351	1.999391	0.96747	0.333309	0.629071	SLC7A9	11136	solute carrier family 7 member 9
ENSG0000	157.2741	-0.19389	0.200403	-0.96752	0.333286	0.629071	LOC10272	1.03E+08	ICOS ligand
ENSG0000	130.4397	0.22918	0.236967	0.96714	0.333474	0.629319	WDR27	253769	WD repeat domain 27
ENSG0000	7.892686	-0.95557	0.988295	-0.96688	0.333603	0.62941	UNC79	57578	unc-79 hoı NALCN channel complex subunit
ENSG0000	799.9502	0.132122	0.136638	0.96695	0.333569	0.62941	CRIP2	1397	cysteine rich protein 2
ENSG0000	21.5222	0.491957	0.50883	0.96684	0.333624	0.62941	NDRG4	65009	NDRG family member 4
ENSG0000	56.00808	-0.35197	0.364093	-0.96671	0.33369	0.629469	LOC12253	1.23E+08	Zinc finger protein LOC122539214
ENSG0000	8.134216	0.922573	0.954565	0.966486	0.333801	0.629616	NA	NA	NA
ENSG0000	5.708974	1.139538	1.179581	0.966054	0.334017	0.629959	IGKV2-24	28923	immunoglobulin kappa variable 2-24
ENSG0000	7547.282	0.082104	0.084997	0.965968	0.33406	0.629976	ZSWIM8	23053	zinc finger SWIM-type containing 8
ENSG0000	85.96667	0.284466	0.294533	0.965819	0.334135	0.630052	ULK4	54986	unc-51 like kinase 4
ENSG0000	132.1241	-0.21192	0.219453	-0.96567	0.334208	0.630101	PSMG4	389362	proteasome assembly chaperone 4
ENSG0000	16.68261	-0.60865	0.630317	-0.96563	0.334229	0.630101	FKBP10	60681	FKBP prolyl isomerase 10
ENSG0000	1495.022	0.099419	0.102972	0.965491	0.334299	0.630105	TGFBR3	7049	transforming growth factor beta receptor 3
ENSG0000	13.54971	0.609926	0.631686	0.965553	0.334268	0.630105	NA	NA	NA
ENSG0000	172.3235	0.214229	0.222006	0.964968	0.334561	0.630472	SEM1	7979	SEM1 26S proteasome subunit
ENSG0000	5.495124	1.113574	1.153994	0.964974	0.334558	0.630472	UBQLNL	143630	ubiquilin like
ENSG0000	18.58514	-0.63455	0.657736	-0.96475	0.334672	0.630616	TRAV9-2	28677	T cell receptor alpha variable 9-2
ENSG0000	100.7251	0.229448	0.237854	0.964655	0.334718	0.630639	ZSCAN16-/	1E+08	ZSCAN16 antisense RNA 1
ENSG0000	358.2969	0.141526	0.146738	0.964483	0.334804	0.630737	ZNF200	7752	zinc finger protein 200
ENSG0000	14.19979	0.63466	0.658225	0.964199	0.334946	0.630749	THBS3-AS:	1.05E+08	THBS3 antisense RNA 1
ENSG0000	3.897109	-1.2953	1.343201	-0.96434	0.334877	0.630749	HPGDS	27306	hematopoietic prostaglandin D synthase
ENSG0000	3007.194	0.074447	0.077203	0.964301	0.334895	0.630749	SLC7A6	9057	solute carrier family 7 member 6
ENSG0000	2.869812	1.547569	1.604963	0.96424	0.334926	0.630749	DLL3	10683	delta like canonical Notch ligand 3
ENSG0000	44.40484	-0.35731	0.370623	-0.96407	0.335009	0.630804	NAGS	162417	N-acetylglutamate synthase
ENSG0000	284.1349	-0.17007	0.176439	-0.9639	0.335097	0.630905	LIG4	3981	DNA ligase 4
ENSG0000	26.0693	-0.54555	0.566033	-0.96381	0.335141	0.630923	NA	NA	NA
ENSG0000	30.09695	-0.46703	0.484631	-0.96368	0.335205	0.63098	NA	NA	NA
ENSG0000	5.623305	-1.17405	1.218527	-0.9635	0.335297	0.630989	NA	NA	NA
ENSG0000	8.96985	-0.91328	0.947908	-0.96347	0.335312	0.630989	WTIP	126374	WT1 interacting protein
ENSG0000	7.283728	0.9603	0.996645	0.963533	0.33528	0.630989	SLC8A2	6543	solute carrier family 8 member A2
ENSG0000	1093.515	0.106183	0.110251	0.963103	0.335496	0.631272	NOC4L	79050	nucleolar complex associated 4 homolog
ENSG0000	22.17988	0.509943	0.529521	0.963025	0.335535	0.631281	MFSD2B	388931	major facilitator superfamily domain containing 2B
ENSG0000	1331.081	0.097097	0.100843	0.962852	0.335622	0.631316	UFC1	51506	ubiquitin-fold modifier conjugating enzyme 1
ENSG0000	31.3411	-0.40963	0.425418	-0.96288	0.335608	0.631316	NA	NA	NA
ENSG0000	137.2305	0.579563	0.602081	0.9626	0.335748	0.63149	LINC0162	1.05E+08	long intergenic non-protein coding RNA 1629
ENSG0000	4.346947	1.197457	1.244321	0.962338	0.33588	0.63161	NA	NA	NA
ENSG0000	234.9031	-0.16283	0.169193	-0.9624	0.335848	0.63161	COMMD4	54939	COMM domain containing 4
ENSG0000	10.19949	0.903805	0.939348	0.962163	0.335968	0.631711	NA	NA	NA
ENSG0000	4.572692	-1.15191	1.197528	-0.96191	0.336096	0.631887	NA	NA	NA
ENSG0000	15.85688	0.593569	0.617166	0.961766	0.336167	0.631957	FAM111B	374393	FAM111 trypsin like peptidase B
ENSG0000	308.9557	-0.16253	0.169013	-0.96165	0.336225	0.632002	RAD17	5884	RAD17 checkpoint clamp loader component
ENSG0000	62.44005	0.304242	0.316437	0.961461	0.33632	0.632073	H2BC20P	337873	H2B cluste pseudogene
ENSG0000	14.10793	0.752221	0.782426	0.961396	0.336353	0.632073	EGF	1950	epidermal growth factor
ENSG0000	6.160095	0.912375	0.949033	0.961373	0.336365	0.632073	NA	NA	NA
ENSG0000	2530.946	0.093101	0.096857	0.961222	0.33644	0.632151	ZFR	51663	zinc finger RNA binding protein
ENSG0000	10.82117	-0.74043	0.770366	-0.96113	0.336484	0.63217	CDKN3	1033	cyclin dependent kinase inhibitor 3
ENSG0000	2443.523	-0.08543	0.088897	-0.961	0.33655	0.632201	CHPF2	54480	chondroitin polymerizing factor 2
ENSG0000	3.474632	-1.3756	1.431479	-0.96097	0.336569	0.632201	NA	NA	NA
ENSG0000	923.9529	0.113158	0.11779	0.960675	0.336716	0.632411	ATP5IF1	93974	ATP synthase inhibitory factor subunit 1
ENSG0000	497.9924	-0.11717	0.121981	-0.96058	0.336765	0.63244	ACOX1	51	acyl-CoA oxidase 1
ENSG0000	1209.336	-0.08807	0.091719	-0.96027	0.336918	0.632621	LEPROT	54741	leptin receptor overlapping transcript
ENSG0000	22.32994	0.48312	0.50312	0.96025	0.33693	0.632621	TRAM2-AS	401264	TRAM2 antisense RNA 1 (head to head)
ENSG0000	49.48933	-0.31437	0.327435	-0.96011	0.337001	0.632627	CCDC191	57577	coiled-coil domain containing 191
ENSG0000	2814.623	0.085417	0.088963	0.96014	0.336985	0.632627	XAB2	56949	XPA binding protein 2
ENSG0000	419.5661	-0.12089	0.125934	-0.95995	0.337082	0.632637	ACTR8	93973	actin related protein 8
ENSG0000	418.8596	-0.15228	0.158635	-0.95992	0.337096	0.632637	LAMTOR4	389541	late endos MAPK and MTOR activator 4
ENSG0000	7.600816	-0.92499	0.963632	-0.95989	0.337108	0.632637	ZNF471	57573	zinc finger protein 471
ENSG0000	23.8268	-0.49306	0.513753	-0.95972	0.337196	0.632738	RNA5SP38	1.01E+08	RNA 5S ribosomal pseudogene 383
ENSG0000	192.107	-0.2001	0.208565	-0.95943	0.337343	0.632806	ETFDH	2110	electron transfer flavoprotein dehydrogenase
ENSG0000	61.9031	-0.30113	0.313883	-0.95938	0.337369	0.632806	CCDC183-/	1E+08	CCDC183 antisense RNA 1
ENSG0000	5.910858	-1.07404	1.119389	-0.95949	0.337311	0.632806	CLEC4C	170482	C-type lectin domain family 4 member C
ENSG0000	81.10547	-0.25884	0.2698	-0.95939	0.33736	0.632806	ZFP14	57677	ZFP14 zinc finger protein
ENSG0000	50.68622	-0.32587	0.339778	-0.95908	0.33752	0.633026	SERGEF	26297	secretion regulating guanine nucleotide exchange factor
ENSG0000	407.6062	-0.13924	0.145212	-0.95885	0.337633	0.633141	RIPK3	11035	receptor interacting serine/threonine kinase 3
ENSG0000	83.69889	-0.25781	0.268878	-0.95882	0.33765	0.633141	ZNF440	126070	zinc finger protein 440
ENSG0000	444.9818	-0.12548	0.130903	-0.95859	0.337765	0.633293	MFN1	55669	mitofusin 1
ENSG0000	20.07359	-0.57782	0.603082	-0.95811	0.338009	0.633685	NA	NA	NA
ENSG0000	47.59595	-0.36869	0.384861	-0.95797	0.338077	0.633747	FTH1P22	1E+08	ferritin heavy chain 1 pseudogene 22
ENSG0000	10.64986	-0.7538	0.786926	-0.95791	0.33811	0.633747	KLRC2	3822	killer cell lectin like receptor C2
ENSG0000	126.59	-0.24375	0.254538	-0.9576	0.338264	0.633969	LOC1002	1E+08	uncharacterized LOC100294145
ENSG0000	197.6285	0.196989	0.205725	0.957536	0.338297	0.633969	ZNF805	390980	zinc finger protein 805
ENSG0000	3.180388	1.56815	1.638191	0.957245	0.338444	0.63418	TRIM45	80263	tripartite motif containing 45
ENSG0000	6.740429	-1.22051	1.27537	-0.95698	0.338575	0.634188	LMCD1	29995	LIM and cysteine rich domains 1

ENSG0000	1481.333	0.095206	0.099485	0.95699	0.338572	0.634188	ABHD14B	84836	abhydrolase domain containing 14B
ENSG0000	40.22981	-0.4075	0.425824	-0.95697	0.338584	0.634188	PRRT2	112476	proline rich transmembrane protein 2
ENSG0000	3.83656	1.356348	1.417285	0.957004	0.338565	0.634188	NA	NA	NA
ENSG0000	238.0103	-0.17502	0.182948	-0.95669	0.338725	0.634323	STYXL1	51657	serine/threonine/tyrosine interacting like 1
ENSG0000	2.650819	1.596572	1.668806	0.956715	0.338711	0.634323	IGLC5	3541	immunoglobulin lambda constant 5 (pseudogene)
ENSG0000	854.5974	-0.10566	0.110474	-0.95646	0.338837	0.634469	GNAQ	2776	G protein subunit alpha q
ENSG0000	101.3853	-0.24348	0.254682	-0.95602	0.339061	0.634823	TMEM68	137695	transmembrane protein 68
ENSG0000	61.33825	-0.31875	0.333482	-0.95583	0.339156	0.634938	ALDH6A1	4329	aldehyde dehydrogenase 6 family member A1
ENSG0000	7.688578	1.072989	1.123313	0.9552	0.339476	0.635241	CACNA1D	776	calcium voltage-gated channel subunit alpha1 D
ENSG0000	18.80494	-0.53491	0.560007	-0.95519	0.339484	0.635241	IL20RB	53833	interleukin 20 receptor subunit beta
ENSG0000	9.894099	-0.89091	0.932722	-0.95517	0.339492	0.635241	LINC0125‹	1.06E+08	long intergenic non-protein coding RNA 1259
ENSG0000	175.7394	-0.18871	0.197582	-0.95511	0.339524	0.635241	TMEM106	79022	transmembrane protein 106C
ENSG0000	47.7461	-0.34535	0.361525	-0.95525	0.339449	0.635241	ZNF540	163255	zinc finger protein 540
ENSG0000	93.75594	-0.28003	0.293182	-0.95514	0.339507	0.635241	CBR1	873	carbonyl reductase 1
ENSG0000	689.1382	-0.12625	0.132203	-0.95495	0.339601	0.635266	GOT2	2806	glutamic-oxaloacetic transaminase 2
ENSG0000	3690.513	0.074736	0.078262	0.954946	0.339605	0.635266	TRAPPC10	7109	trafficking protein particle complex subunit 10
ENSG0000	6.008085	-0.97641	1.022903	-0.95455	0.339807	0.635542	LNP1	348801	leukemia NUP98 fusion partner 1
ENSG0000	11.49202	-0.71407	0.748093	-0.95452	0.339821	0.635542	NA	NA	NA
ENSG0000	9.329379	0.883939	0.926305	0.954263	0.339951	0.635591	C10orf55	414236	chromosome 10 putative open reading frame 55
ENSG0000	4231.004	-0.0959	0.100482	-0.95437	0.339897	0.635591	UBE2L6	9246	ubiquitin conjugating enzyme E2 L6
ENSG0000	209.9435	0.174545	0.182899	0.954324	0.339919	0.635591	ZNF26	7574	zinc finger protein 26
ENSG0000	1618.527	0.084692	0.088782	0.95394	0.340114	0.635833	OGA	10724	O-GlcNAcase
ENSG0000	7.78191	0.861203	0.902927	0.953789	0.34019	0.6359	NA	NA	NA
ENSG0000	605.6389	0.121736	0.127641	0.953734	0.340218	0.6359	RRN3	54700	RRN3 hom RNA polymerase I transcription factor
ENSG0000	16.69775	0.597915	0.627012	0.953594	0.340289	0.635906	MTHFS	10588	methenyltetrahydrofolate synthetase
ENSG0000	1314.724	0.09686	0.101574	0.953592	0.34029	0.635906	MIEN1	84299	migration and invasion enhancer 1
ENSG0000	95.33376	0.243669	0.255565	0.953453	0.340361	0.635909	SH3BGRL2	83699	SH3 domain binding glutamate rich protein like 2
ENSG0000	9.500421	0.892058	0.93558	0.953481	0.340346	0.635909	NA	NA	NA
ENSG0000	205.2813	-0.16617	0.174305	-0.95335	0.340414	0.635945	KCTD7	154881	potassium channel tetramerization domain containing 7
ENSG0000	4.090204	-1.22051	1.280846	-0.9529	0.340642	0.636307	GIMAP3P	474345	GTPase IMAP family member 3 pseudogene
ENSG0000	11.32027	0.620636	0.651439	0.952716	0.340734	0.636309	NA	NA	NA
ENSG0000	8.951151	-0.99182	1.041126	-0.95264	0.340772	0.636309	INSYN2A	642938	inhibitory synaptic factor 2A
ENSG0000	84.99799	0.32342	0.339525	0.952567	0.34081	0.636309	TOP2A	7153	DNA topoisomerase II alpha
ENSG0000	242.0616	-0.16367	0.171802	-0.95264	0.340771	0.636309	ZNF44	51710	zinc finger protein 44
ENSG0000	114.8626	-0.21493	0.225633	-0.95256	0.340815	0.636309	REPS2	9185	RALBP1 associated Eps domain containing 2
ENSG0000	541.2413	-0.125	0.131274	-0.9522	0.340997	0.636584	ATAD3A	55210	ATPase family AAA domain containing 3A
ENSG0000	4.715126	1.334978	1.402142	0.952099	0.341047	0.636614	CCL17	6361	C-C motif chemokine ligand 17
ENSG0000	1726.41	-0.09011	0.094669	-0.95182	0.341189	0.636815	C6orf89	221477	chromosome 6 open reading frame 89
ENSG0000	25.22352	-0.48866	0.513495	-0.95164	0.341282	0.636831	HHAT	55733	hedgehog acyltransferase
ENSG0000	11.55752	0.79492	0.835346	0.951606	0.341297	0.636831	SLC12A8	84561	solute carrier family 12 member 8
ENSG0000	141.6239	0.224467	0.235884	0.951599	0.341301	0.636831	ALG8	79053	ALG8 alph 3-glucosyltransferase
ENSG0000	1798.124	0.10574	0.111139	0.951422	0.34139	0.636934	ELOB	6923	elongin B
ENSG0000	58.18489	0.28762	0.302419	0.951062	0.341573	0.637083	NA	NA	NA
ENSG0000	29.52993	0.473275	0.497603	0.95111	0.341549	0.637083	EFCAB12	90288	EF-hand calcium binding domain 12
ENSG0000	246.8406	-0.17128	0.18009	-0.95108	0.341562	0.637083	C17orf80	55028	chromosome 17 open reading frame 80
ENSG0000	3.722424	-1.18995	1.251335	-0.95094	0.341634	0.637132	NA	NA	NA
ENSG0000	3.76163	-1.493	1.570902	-0.95041	0.341904	0.637507	RPL23AP2!	653789	ribosomal protein L23a pseudogene 25
ENSG0000	1298.239	0.100409	0.105649	0.950401	0.341908	0.637507	UHRF2	115426	ubiquitin like with PHD and ring finger domains 2
ENSG0000	119.0277	0.222386	0.234007	0.950343	0.341938	0.637507	CLN5	1203	CLN5 intracellular trafficking protein
ENSG0000	791.2416	0.117903	0.124086	0.95017	0.342026	0.637607	BTBD7	55727	BTB domain containing 7
ENSG0000	316.5367	-0.14387	0.151454	-0.9499	0.342161	0.637777	MSTO1	55154	misato mitochondrial distribution and morphology regulator 1
ENSG0000	99.41696	-0.26761	0.281741	-0.94986	0.342186	0.637777	BCORP1	286554	BCL6 corepressor pseudogene 1
ENSG0000	6.831345	1.06867	1.125856	0.949207	0.342515	0.637836	TGFB2	7042	transforming growth factor beta 2
ENSG0000	2176.781	0.077254	0.081362	0.949516	0.342358	0.637836	ARIH2	10425	ariadne RBR E3 ubiquitin protein ligase 2
ENSG0000	137.1799	-0.20697	0.218034	-0.94927	0.342481	0.637836	TREML2	79865	triggering receptor expressed on myeloid cells like 2
ENSG0000	393.1502	0.142461	0.150025	0.949582	0.342325	0.637836	NSUN5P2	260294	NSUN5 pseudogene 2
ENSG0000	404.2299	0.145811	0.153556	0.94956	0.342336	0.637836	WASL	8976	WASP like actin nucleation promoting factor
ENSG0000	2.066149	-1.87173	1.972207	-0.94905	0.342593	0.637836	NA	NA	NA
ENSG0000	6.699281	-0.90926	0.957841	-0.94928	0.342479	0.637836	NA	NA	NA
ENSG0000	1856.199	-0.08994	0.094752	-0.94917	0.342535	0.637836	EMSY	56946	EMSY tran BRCA2 interacting
ENSG0000	171.8376	-0.17418	0.183454	-0.94945	0.342392	0.637836	PRSS23	11098	serine protease 23
ENSG0000	98.58881	0.239065	0.251942	0.94889	0.342677	0.637836	TRMT5	57570	tRNA methyltransferase 5
ENSG0000	21.22981	0.587676	0.6193	0.948936	0.342653	0.637836	TLCD2	727910	TLC domain containing 2
ENSG0000	1938.357	0.078535	0.082753	0.949026	0.342607	0.637836	ALKBH5	54890	alkB homc RNA demethylase
ENSG0000	495.245	0.112471	0.118534	0.948846	0.342699	0.637836	SRSF1	6426	serine and arginine rich splicing factor 1
ENSG0000	366.8359	-0.13017	0.137081	-0.94956	0.342338	0.637836	SPECC1L	23384	sperm antigen with calponin homology and coiled-coil domains 1 like
ENSG0000	92.38125	-0.2331	0.245705	-0.94868	0.342782	0.637928	ZNF514	84874	zinc finger protein 514
ENSG0000	14.8331	-0.64824	0.683419	-0.94853	0.342862	0.637961	CCNA1	8900	cyclin A1
ENSG0000	917.3752	0.117009	0.123361	0.948512	0.342869	0.637961	UQCR10	29796	ubiquinol- complex III subunit X
ENSG0000	482.2111	-0.13141	0.138576	-0.94831	0.342974	0.637963	NUAK2	81788	NUAK family kinase 2
ENSG0000	779.7896	0.109991	0.11598	0.948364	0.342944	0.637963	LRWD1	222229	leucine rich repeats and WD repeat domain containing 1
ENSG0000	17.3291	-0.54698	0.57674	-0.9484	0.342924	0.637963	NA	NA	NA
ENSG0000	1884.989	0.08395	0.088535	0.948209	0.343023	0.637991	NFATC2IP	84901	nuclear factor of activated T cells 2 interacting protein
ENSG0000	14.09923	-0.67933	0.716562	-0.94804	0.343107	0.638036	DISP2	85455	dispatched RND transporter family member 2
ENSG0000	156.7842	-0.18902	0.199379	-0.94803	0.343116	0.638036	ZNF737	1E+08	zinc finger protein 737
ENSG0000	93.96294	-0.22684	0.239372	-0.94764	0.343311	0.638335	TIMM21	29090	translocase of inner mitochondrial membrane 21
ENSG0000	20.58752	0.665219	0.702079	0.947498	0.343385	0.638408	TIE1	7075	tyrosine kinase with immunoglobulin like and EGF like domains 1
ENSG0000	143.8703	0.20996	0.221625	0.947366	0.343452	0.63847	PRMT9	90826	protein arginine methyltransferase 9
ENSG0000	1217.869	-0.10316	0.108912	-0.94721	0.343531	0.638551	TRAK2	66008	trafficking kinesin protein 2
ENSG0000	12.40841	-0.73944	0.781246	-0.94649	0.343897	0.639169	MZF1-AS1	1E+08	MZF1 antisense RNA 1
ENSG0000	70.48089	-0.30597	0.323331	-0.94631	0.343992	0.63928	CARF	79800	calcium responsive transcription factor
ENSG0000	695.5134	-0.10236	0.108188	-0.94616	0.344065	0.639353	CEP57	9702	centrosomal protein 57
ENSG0000	56.62742	0.303187	0.320467	0.946078	0.344109	0.639369	NA	NA	NA
ENSG0000	38.67357	-0.41454	0.438239	-0.94593	0.344186	0.639449	NA	NA	NA
ENSG0000	88.7634	-0.26341	0.278508	-0.9458	0.34425	0.639504	FAM83H	286077	family with sequence similarity 83 member H
ENSG0000	237.5505	-0.15397	0.162818	-0.94565	0.344327	0.639583	ACADM	34	acyl-CoA dehydrogenase medium chain
ENSG0000	30.73308	0.484082	0.512009	0.945456	0.344426	0.639703	ZFP37	7539	ZFP37 zinc finger protein
ENSG0000	4.158067	1.53453	1.623455	0.945225	0.344544	0.639858	SNURF	8926	SNRPN upstream open reading frame
ENSG0000	21.07732	-0.54735	0.579292	-0.94486	0.344729	0.639901	GJC2	57165	gap junction protein gamma 2
ENSG0000	89.47567	-0.24898	0.263537	-0.94475	0.344784	0.639901	SAP30	8819	Sin3A associated protein 30
ENSG0000	212.3645	-0.17434	0.184505	-0.94489	0.344713	0.639901	NA	NA	NA
ENSG0000	954.5767	-0.11042	0.116885	-0.94471	0.344809	0.639901	BLK	640	BLK proto- Src family tyrosine kinase
ENSG0000	11.37138	0.764452	0.808849	0.945111	0.344602	0.639901	NA	NA	NA
ENSG0000	127.8271	-0.27355	0.289555	-0.94471	0.344805	0.639901	LINC0244€	1.01E+08	long intergenic non-protein coding RNA 2446
ENSG0000	2027.041	-0.07481	0.079175	-0.94488	0.344718	0.639901	LUC7L	55692	LUC7 like
ENSG0000	10.58857	0.661372	0.700483	0.944165	0.345085	0.64035	NA	NA	NA
ENSG0000	8186.592	0.092164	0.097682	0.943508	0.345421	0.64087	PLEKHO1	51177	pleckstrin homology domain containing O1
ENSG0000	6004.573	-0.06535	0.069271	-0.94345	0.345451	0.64087	HIPK2	28996	homeodomain interacting protein kinase 2
ENSG0000	71.03173	0.305745	0.324083	0.943414	0.345469	0.64087	REM2	161253	RRAD and GEM like GTPase 2
ENSG0000	306.9806	0.144518	0.153204	0.943302	0.345526	0.640912	STRN3	29966	striatin 3

ENSG0000	16.61616	-0.53468	0.566924	-0.94312	0.345621	0.641024	LOC10537	1.05E+08	uncharacterized LOC105370259
ENSG0000	1078.851	0.095068	0.100818	0.942968	0.345697	0.641045	ATG9A	79065	autophagy related 9A
ENSG0000	7.768943	-0.92909	0.985296	-0.94296	0.345702	0.641045	HSD17B1	3292	hydroxysteroid 17-beta dehydrogenase 1
ENSG0000	4.340269	-1.36525	1.448531	-0.94251	0.345932	0.641361	NA	NA	NA
ENSG0000	5.529713	-1.03693	1.100206	-0.94249	0.345941	0.641361	GTSE1-DT	150384	GTSE1 divergent transcript
ENSG0000	920.8509	-0.09434	0.100126	-0.94219	0.346093	0.64138	HMGN4	10473	high mobility group nucleosomal binding domain 4
ENSG0000	75.5975	0.279404	0.296542	0.942206	0.346087	0.64138	MAP3K4-A	1.18E+08	MAP3K4 antisense RNA 1
ENSG0000	27.02532	-0.46528	0.493862	-0.94212	0.34613	0.64138	CROT	54677	carnitine O-octanoyltransferase
ENSG0000	619.3792	0.110363	0.117123	0.942285	0.346046	0.64138	CYB5B	80777	cytochrome b5 type B
ENSG0000	3025.457	-0.08421	0.089393	-0.94207	0.346159	0.64138	SLC25A39	51629	solute carrier family 25 member 39
ENSG0000	70.73099	-0.27003	0.286624	-0.9421	0.34614	0.64138	ZNF528	84436	zinc finger protein 528
ENSG0000	981.7621	0.114746	0.121822	0.941919	0.346234	0.641455	MYSM1	114803	Myb like SWIRM and MPN domains 1
ENSG0000	21.03384	-0.50181	0.53305	-0.94139	0.346507	0.641667	ATPSCKMT	134145	ATP synthase c subunit lysine N-methyltransferase
ENSG0000	294.4702	0.13916	0.14779	0.941608	0.346393	0.641667	NRM	11270	nurim
ENSG0000	204.0817	-0.18493	0.196437	-0.9414	0.346502	0.641667	SLC39A4	55630	solute carrier family 39 member 4
ENSG0000	111.9611	-0.2658	0.282338	-0.94143	0.346483	0.641667	COX10-DT	1.01E+08	COX10 divergent transcript
ENSG0000	30.24162	0.474872	0.504454	0.941358	0.346522	0.641667	EOLA1-DT	1E+08	EOLA1 divergent transcript
ENSG0000	440.0853	-0.14492	0.153955	-0.94129	0.346558	0.641671	CYP2S1	29785	cytochrome P450 family 2 subfamily S member 1
ENSG0000	260.4492	-0.16027	0.170282	-0.94119	0.346609	0.641693	UBA5	79876	ubiquitin like modifier activating enzyme 5
ENSG0000	22.48491	-0.48597	0.516373	-0.94113	0.346639	0.641693	ANKHD1-E	404734	ANKHD1-EIF4EBP3 readthrough
ENSG0000	173.1565	-0.17207	0.182878	-0.94092	0.346747	0.641828	CNNM2	54805	cyclin and CBS domain divalent metal cation transport mediator 2
ENSG0000	5.257579	-1.17515	1.249472	-0.94052	0.346951	0.642014	DNAI4	79819	dynein axonemal intermediate chain 4
ENSG0000	10.26477	-0.72261	0.768271	-0.94057	0.346925	0.642014	NA	NA	NA
ENSG0000	59.14216	0.340904	0.362433	0.940597	0.346912	0.642014	GATA1	2623	GATA binding protein 1
ENSG0000	3.118066	1.546294	1.64428	0.940408	0.347008	0.642043	NA	NA	NA
ENSG0000	138.3617	-0.24009	0.255322	-0.94035	0.347036	0.642043	ANKS6	203286	ankyrin repeat and sterile alpha motif domain containing 6
ENSG0000	5.427128	1.111755	1.182422	0.940236	0.347097	0.642091	CYP2J2	1573	cytochrome P450 family 2 subfamily J member 2
ENSG0000	17.63228	-0.53397	0.567978	-0.94012	0.347155	0.642135	CXXC4	80319	CXXC finger protein 4
ENSG0000	1580.71	0.086522	0.092052	0.939927	0.347255	0.642255	SPCS3	60559	signal peptidase complex subunit 3
ENSG0000	177.2753	0.173635	0.184812	0.939524	0.347462	0.642382	OPN3	23596	opsin 3
ENSG0000	2439.446	-0.0837	0.089082	-0.93955	0.347447	0.642382	IGHD	3495	immunoglobulin heavy constant delta
ENSG0000	18.09691	0.526927	0.560791	0.939615	0.347415	0.642382	NA	NA	NA
ENSG0000	19.89	-0.54371	0.578681	-0.93956	0.347441	0.642382	LINC0173	1.02E+08	long intergenic non-protein coding RNA 1730
ENSG0000	5.452807	-1.12642	1.199272	-0.93925	0.347601	0.642575	NA	NA	NA
ENSG0000	3894.022	0.08686	0.092485	0.939177	0.34764	0.642581	RANBP2	5903	RAN binding protein 2
ENSG0000	7.362454	1.107098	1.178878	0.939111	0.347674	0.642581	TMEM151	256472	transmembrane protein 151A
ENSG0000	77.12343	0.269827	0.287391	0.938884	0.347791	0.642605	TFB2M	64216	transcripti mitochondrial
ENSG0000	682.2633	-0.10363	0.110365	-0.93897	0.347745	0.642605	REV1	51455	REV1 DNA directed polymerase
ENSG0000	8.868559	0.82941	0.883378	0.938907	0.347779	0.642605	NA	NA	NA
ENSG0000	52.46151	-0.34009	0.362284	-0.93874	0.347863	0.642675	DPYSL4	10570	dihydropyrimidinase like 4
ENSG0000	8.996013	0.76168	0.811672	0.938409	0.348034	0.6428	TRBV21-1	28566	T cell receptor beta variable 21-1 (pseudogene)
ENSG0000	36.37587	0.412061	0.439087	0.938448	0.348014	0.6428	RPL13AP2	1E+08	ribosomal protein L13a pseudogene 25
ENSG0000	7.171628	-0.96294	1.026142	-0.93841	0.348033	0.6428	HAR1B	768097	highly accelerated region 1B
ENSG0000	13.71068	0.61689	0.657437	0.938326	0.348077	0.642814	NA	NA	NA
ENSG0000	3.471279	1.501509	1.600562	0.938113	0.348186	0.642952	LINC0057	1.01E+08	long intergenic non-protein coding RNA 570
ENSG0000	25.73865	0.454664	0.484874	0.937695	0.348401	0.643285	RPL35P5	441246	ribosomal protein L35 pseudogene 5
ENSG0000	1970.486	-0.07806	0.083271	-0.93742	0.348544	0.643356	IKBKB	3551	inhibitor of nuclear factor kappa B kinase subunit beta
ENSG0000	225.3169	-0.17196	0.18344	-0.93742	0.348541	0.643356	GANC	2595	glucosidas neutral C
ENSG0000	1400.676	-0.09385	0.100107	-0.93753	0.348487	0.643356	CES2	8824	carboxylesterase 2
ENSG0000	3.078617	-1.4649	1.56295	-0.93727	0.348621	0.643434	NA	NA	NA
ENSG0000	73.35587	-0.31495	0.336099	-0.93707	0.348721	0.643556	ZNF700	90592	zinc finger protein 700
ENSG0000	3670.869	0.068963	0.073617	0.936786	0.348869	0.643764	ZDHHC5	25921	zinc finger DHHC-type palmitoyltransferase 5
ENSG0000	3.83178	1.407014	1.502539	0.936424	0.349055	0.644043	GPRC5D	55507	G protein-coupled receptor class C group 5 member D
ENSG0000	11.91607	0.87803	0.937811	0.936254	0.349142	0.644059	NA	NA	NA
ENSG0000	10.36799	0.713116	0.761665	0.93626	0.349139	0.644059	EME1	146956	essential meiotic structure-specific endonuclease 1
ENSG0000	107.0808	0.220647	0.235682	0.936205	0.349168	0.644059	KIR2DP1	554300	killer cell i two Ig domains pseudogene 1
ENSG0000	25.96398	0.513338	0.548525	0.935851	0.34935	0.644212	NA	NA	NA
ENSG0000	94.43482	0.278652	0.297724	0.93594	0.349304	0.644212	NDUFS4	4724	NADH:ubiquinone oxidoreductase subunit S4
ENSG0000	3106.257	-0.0797	0.08516	-0.93584	0.349355	0.644212	NMT1	4836	N-myristoyltransferase 1
ENSG0000	2019.355	0.090717	0.09695	0.935704	0.349426	0.644279	SCAMP3	10067	secretory carrier membrane protein 3
ENSG0000	66.17046	-0.3039	0.324834	-0.93556	0.3495	0.644352	RAB27B	5874	RAB27B member RAS oncogene family
ENSG0000	3.534998	1.294058	1.383443	0.93539	0.349587	0.644449	NA	NA	NA
ENSG0000	22.19502	0.56584	0.605217	0.934937	0.349821	0.64447	PM20D1	148811	peptidase M20 domain containing 1
ENSG0000	3.674945	-1.55199	1.659885	-0.935	0.349789	0.64447	NA	NA	NA
ENSG0000	10.98724	0.706691	0.755643	0.935217	0.349676	0.64447	NA	NA	NA
ENSG0000	4.011539	-1.62548	1.738346	-0.93507	0.349751	0.64447	H1-4	3008	H1.4 linker cluster member
ENSG0000	5.019811	-1.09617	1.172305	-0.93505	0.349761	0.64447	JAKMIP3	282973	Janus kinase and microtubule interacting protein 3
ENSG0000	671.4532	-0.14601	0.156116	-0.93526	0.349655	0.64447	JDP2	122953	Jun dimerization protein 2
ENSG0000	477.7694	-0.11603	0.124106	-0.9349	0.349842	0.64447	DUSP18	150290	dual specificity phosphatase 18
ENSG0000	153.6228	-0.20021	0.214231	-0.93457	0.350008	0.644647	NPTX1	4884	neuronal pentraxin 1
ENSG0000	231.5215	-0.15825	0.169326	-0.9346	0.349993	0.644647	ZNF182	7569	zinc finger protein 182
ENSG0000	50.48499	-0.33427	0.357807	-0.93422	0.350192	0.644922	ZSCAN5A	79149	zinc finger and SCAN domain containing 5A
ENSG0000	5.224476	1.049043	1.123429	0.933787	0.350414	0.645173	NA	NA	NA
ENSG0000	2.836089	1.485291	1.590597	0.933794	0.35041	0.645173	MTND4LP	1.07E+08	MT-ND4L pseudogene 23
ENSG0000	55.25082	0.338313	0.362342	0.933684	0.350467	0.645173	SPTBN4	57731	spectrin b non-erythrocytic 4
ENSG0000	42.3858	-0.37524	0.401883	-0.93371	0.350455	0.645173	NA	NA	NA
ENSG0000	25.22	-0.4479	0.479818	-0.93348	0.35057	0.645298	NA	NA	NA
ENSG0000	53.73878	-0.34288	0.36738	-0.93332	0.350657	0.645317	ARL15	54622	ADP ribosylation factor like GTPase 15
ENSG0000	6.862595	-0.92262	0.988785	-0.93309	0.350774	0.645317	STEAP1B	256227	STEAP family member 1B
ENSG0000	18.07371	0.563654	0.60423	0.932848	0.350899	0.645317	NA	NA	NA
ENSG0000	25.99436	-0.42135	0.451572	-0.93307	0.350782	0.645317	PCMTD1-D	1.03E+08	PCMTD1 divergent transcript
ENSG0000	6.385848	0.948307	1.016357	0.933045	0.350797	0.645317	RPL23AP6	440027	ribosomal protein L23a pseudogene 65
ENSG0000	10.65468	-0.69666	0.746511	-0.93322	0.350706	0.645317	NA	NA	NA
ENSG0000	4.336261	1.419609	1.521039	0.933315	0.350657	0.645317	CDH15	1013	cadherin 15
ENSG0000	2.162561	1.989113	2.132179	0.932902	0.350871	0.645317	NCCRP1	342897	NCCRP1 F-box associated domain containing
ENSG0000	6.667401	0.920347	0.98666	0.932791	0.350928	0.645317	NA	NA	NA
ENSG0000	48.8758	-0.38171	0.409152	-0.93292	0.350862	0.645317	NAP1L3	4675	nucleosome assembly protein 1 like 3
ENSG0000	134.7262	-0.19311	0.207047	-0.93269	0.350978	0.645344	LY75	4065	lymphocyte antigen 75
ENSG0000	9.135853	0.889299	0.953653	0.932518	0.351069	0.645448	A3GALT2	127550	alpha 1 3-galactosyltransferase 2
ENSG0000	57.6348	-0.35519	0.380957	-0.93236	0.351148	0.645466	ITGA10	8515	integrin subunit alpha 10
ENSG0000	4.39175	-1.33014	1.426575	-0.9324	0.351128	0.645466	NA	NA	NA
ENSG0000	92.38663	0.239371	0.256804	0.932115	0.351277	0.645638	NDFIP2	54602	Nedd4 family interacting protein 2
ENSG0000	249.4805	-0.16423	0.176247	-0.93183	0.351425	0.645846	HOXB3	3213	homeobox B3
ENSG0000	8.097114	-0.94529	1.01482	-0.93148	0.351603	0.64611	FOXD4	2298	forkhead box D4
ENSG0000	302.3532	-0.14541	0.15615	-0.93125	0.351723	0.646203	RPL32P3	132241	ribosomal protein L32 pseudogene 3
ENSG0000	4156.483	0.07749	0.083209	0.931279	0.351709	0.646203	REXO1	57455	RNA exonuclease 1 homolog
ENSG0000	6.612676	0.939013	1.008726	0.930891	0.35191	0.646481	NA	NA	NA
ENSG0000	12.42005	-0.63647	0.683882	-0.93068	0.352019	0.646618	GGACT	87769	gamma-glutamylamine cyclotransferase
ENSG0000	298.6454	0.187467	0.201547	0.930139	0.352299	0.647068	NA	NA	NA
ENSG0000	2485.82	-0.07923	0.085254	-0.9294	0.352683	0.64717	PPT1	5538	palmitoyl-protein thioesterase 1

ENSG0000	179.2678	0.198172	0.213214	0.929448	0.352657	0.64717	CHML	1122	CHM like Rab escort protein
ENSG0000	305.5573	-0.16542	0.177927	-0.92968	0.352535	0.64717	SDHAP4	220729	SDHA pseudogene 4
ENSG0000	2.996036	1.939325	2.08631	0.929548	0.352605	0.64717	NA	NA	NA
ENSG0000	11.47611	0.681645	0.733247	0.929626	0.352565	0.64717	NA	NA	NA
ENSG0000	786.7852	-0.09928	0.106803	-0.92952	0.352619	0.64717	ZNF33A	7581	zinc finger protein 33A
ENSG0000	2963.897	0.079851	0.085865	0.929959	0.352392	0.64717	RPS6KB2	6199	ribosomal protein S6 kinase B2
ENSG0000	1281.066	-0.10392	0.111823	-0.92936	0.352705	0.64717	AIP	9049	aryl hydrocarbon receptor interacting protein
ENSG0000	275.3373	0.153704	0.165327	0.929696	0.352529	0.64717	MCAM	4162	melanoma cell adhesion molecule
ENSG0000	41.83742	-0.37261	0.400728	-0.92983	0.352458	0.64717	SRP14-DT	1E+08	SRP14 divergent transcript
ENSG0000	34.97609	0.397028	0.427238	0.929291	0.352738	0.64717	ALPK3	57538	alpha kinase 3
ENSG0000	363.513	0.14623	0.157371	0.929204	0.352784	0.647189	BBC3	27113	BCL2 binding component 3
ENSG0000	22.09462	-0.52927	0.569816	-0.92884	0.352974	0.647427	LYG1	129530	lysozyme g1
ENSG0000	1541.217	-0.08324	0.089616	-0.92882	0.352983	0.647427	VRK3	51231	VRK serine/threonine kinase 3
ENSG0000	2702.541	0.072135	0.07769	0.928502	0.353147	0.647599	HEXIM1	10614	HEXIM P-TEFb complex subunit 1
ENSG0000	121.7851	-0.20302	0.21864	-0.92856	0.353119	0.647599	ZNF230	7773	zinc finger protein 230
ENSG0000	2366.202	0.07761	0.083614	0.928192	0.353308	0.647694	MAEA	10296	macropha E3 ubiquitin ligase
ENSG0000	1765.85	0.101853	0.10974	0.928134	0.353338	0.647694	ATXN1L	342371	ataxin 1 like
ENSG0000	1042.184	0.109802	0.118294	0.928209	0.353299	0.647694	FBXO31	79791	F-box protein 31
ENSG0000	6.897921	-0.88915	0.957913	-0.92822	0.353293	0.647694	NA	NA	NA
ENSG0000	4.420888	1.16925	1.259945	0.928017	0.353399	0.647741	LINGO4	339398	leucine rich repeat and Ig domain containing 4
ENSG0000	8.103998	-0.87434	0.942282	-0.92789	0.353463	0.647795	NIPAL1	152519	NIPA like domain containing 1
ENSG0000	11.86445	0.672759	0.725428	0.927396	0.353721	0.648203	TMEM182	130827	transmembrane protein 182
ENSG0000	140.3635	-0.2322	0.250406	-0.92729	0.353774	0.648236	NUDCD1	84955	NudC domain containing 1
ENSG0000	22.43787	0.526898	0.568278	0.927184	0.353831	0.648277	LINC01801	400685	long intergenic non-protein coding RNA 1801
ENSG0000	5.987492	0.943795	1.018306	0.926828	0.354016	0.648551	DBIL5P	1E+08	diazepam pseudogene
ENSG0000	688.3679	0.107543	0.116063	0.926586	0.354142	0.648717	EXOSC6	118460	exosome component 6
ENSG0000	3.79271	1.387821	1.498082	0.926399	0.354239	0.648768	LINC0245	643770	long intergenic non-protein coding RNA 2453
ENSG0000	10.76033	0.708066	0.764298	0.926426	0.354225	0.648768	LOC12490	1.25E+08	uncharacterized LOC124903372
ENSG0000	12.91416	-0.69506	0.750454	-0.92619	0.354347	0.648902	AURKC	6795	aurora kinase C
ENSG0000	829.3404	0.110762	0.119654	0.925687	0.354609	0.649131	LSG1	55341	large 60S subunit nuclear export GTPase 1
ENSG0000	6.445713	1.158787	1.251758	0.925727	0.354588	0.649131	NA	NA	NA
ENSG0000	96.80318	-0.24512	0.264747	-0.92586	0.354519	0.649131	SAMD14	201191	sterile alpha motif domain containing 14
ENSG0000	7.998416	-0.83563	0.902718	-0.92568	0.354612	0.649131	NA	NA	NA
ENSG0000	827.6577	0.126713	0.136903	0.925566	0.354672	0.649139	YTHDC2	64848	YTH domain containing 2
ENSG0000	353.2035	0.133254	0.143975	0.925537	0.354687	0.649139	ZNF587	84914	zinc finger protein 587
ENSG0000	2994.173	0.085011	0.091861	0.925438	0.354738	0.649169	UBN1	29855	ubinuclein 1
ENSG0000	5.520015	0.857785	0.927677	0.924659	0.355143	0.649526	AK2P1	266920	adenylate kinase 2 pseudogene 1
ENSG0000	31.82067	-0.43372	0.469041	-0.9247	0.355121	0.649526	NA	NA	NA
ENSG0000	341.4047	0.132304	0.143049	0.924884	0.355026	0.649526	CACNA2D4	93589	calcium voltage-gated channel auxiliary subunit alpha2delta 4
ENSG0000	6571.163	-0.39565	0.427883	-0.92467	0.355137	0.649526	SECTM1	6398	secreted and transmembrane 1
ENSG0000	3.410953	1.287684	1.392369	0.924815	0.355062	0.649526	KATNAL2	83473	katanin catalytic subunit A1 like 2
ENSG0000	22.81309	0.45834	0.495638	0.924748	0.355097	0.649526	NANOS3	342977	nanos C2HC-type zinc finger 3
ENSG0000	4.663647	-0.99972	1.081403	-0.92447	0.355243	0.649645	F8A3	474384	coagulation factor VIII associated 3
ENSG0000	8.180702	-0.87673	0.948617	-0.92422	0.355371	0.649686	NA	NA	NA
ENSG0000	13.73326	0.747988	0.80931	0.924229	0.355367	0.649686	KRT17	3872	keratin 17
ENSG0000	21.54137	0.464698	0.502734	0.924341	0.355309	0.649686	CASTOR1	652968	cytosolic arginine sensor for mTORC1 subunit 1
ENSG0000	4604.149	0.079838	0.086391	0.92415	0.355408	0.64969	GUK1	2987	guanylate kinase 1
ENSG0000	603.6247	0.115254	0.124744	0.92393	0.355523	0.649772	ATF1	466	activating transcription factor 1
ENSG0000	4425.156	-0.06691	0.072424	-0.92393	0.355523	0.649772	APOBEC3G	60489	apolipoprotein B mRNA editing enzyme catalytic subunit 3G
ENSG0000	58.80434	0.29881	0.3235	0.923678	0.355654	0.649947	NA	NA	NA
ENSG0000	23.30742	0.527769	0.571473	0.923524	0.355734	0.649966	PDPN	10630	podoplanin
ENSG0000	29.2653	-0.39446	0.4271	-0.92358	0.355706	0.649966	MYOM1	8736	myomesin 1
ENSG0000	358.1327	-0.1293	0.140017	-0.92345	0.355771	0.649969	ANAPC7	51434	anaphase promoting complex subunit 7
ENSG0000	76.25938	0.252261	0.273204	0.923343	0.355828	0.65001	PFKM	5213	phosphofr muscle
ENSG0000	63.51011	-0.29716	0.321908	-0.92313	0.35594	0.650064	PLEKHA7	144100	pleckstrin homology domain containing A7
ENSG0000	451.0628	0.118929	0.128821	0.923214	0.355896	0.650064	CDKN2D	1032	cyclin dependent kinase inhibitor 2D
ENSG0000	1231.68	-0.09499	0.102908	-0.92308	0.355963	0.650064	CCDC124	115098	coiled-coil domain containing 124
ENSG0000	21.34366	0.504203	0.546257	0.923015	0.356	0.650066	LINC0216	1.05E+08	long intergenic non-protein coding RNA 2166
ENSG0000	6.815074	-0.85547	0.927004	-0.92283	0.356095	0.650113	RGS5	8490	regulator of G protein signaling 5
ENSG0000	4.542597	-1.13479	1.229682	-0.92283	0.356095	0.650113	LOC28509	285097	uncharacterized FLJ38379
ENSG0000	42.82536	-0.33026	0.358074	-0.92232	0.35636	0.650532	PTPRO	5800	protein tyrosine phosphatase receptor type O
ENSG0000	33.43216	0.431162	0.467721	0.921837	0.356614	0.650547	IL12A	3592	interleukin 12A
ENSG0000	6.13345	-1.07006	1.160727	-0.92189	0.356587	0.650547	NA	NA	NA
ENSG0000	500.2605	0.132425	0.143645	0.921891	0.356585	0.650547	CKAP2	26586	cytoskeleton associated protein 2
ENSG0000	96.57589	0.227933	0.247159	0.922213	0.356418	0.650547	WDR76	79968	WD repeat domain 76
ENSG0000	1190.84	0.09638	0.10453	0.92203	0.356513	0.650547	ANP32A	8125	acidic nuclear phosphoprotein 32 family member A
ENSG0000	2387.483	-0.11501	0.124755	-0.92192	0.356568	0.650547	VPS9D1	9605	VPS9 domain containing 1
ENSG0000	8.072333	0.832852	0.903447	0.921861	0.356601	0.650547	SYCE2	256126	synaptonemal complex central element protein 2
ENSG0000	10.53939	0.714314	0.774944	0.921761	0.356653	0.650555	TMEM119	338773	transmembrane protein 119
ENSG0000	13.12035	0.658818	0.714815	0.921662	0.356705	0.650586	NA	NA	NA
ENSG0000	74.11249	-0.26875	0.291789	-0.92103	0.357034	0.650865	CRACDL	343990	CRACD like
ENSG0000	5.513655	0.951304	1.032729	0.921155	0.356969	0.650865	C4orf50	389197	chromosome 4 open reading frame 50
ENSG0000	88.90463	-0.24851	0.269815	-0.92105	0.357026	0.650865	FAM135A	57579	family with sequence similarity 135 member A
ENSG0000	35.42416	0.371172	0.402883	0.92129	0.356899	0.650865	MICU3	286097	mitochondrial calcium uptake family member 3
ENSG0000	150.4418	0.180939	0.196446	0.92106	0.357019	0.650865	EIF4A1	1973	eukaryotic translation initiation factor 4A1
ENSG0000	36.35595	-0.37852	0.411224	-0.92046	0.357332	0.650953	FBXO2	26232	F-box protein 2
ENSG0000	1456.953	0.085787	0.093169	0.920764	0.357174	0.650953	YTHDF2	51441	YTH N6-methyladenosine RNA binding protein 2
ENSG0000	33.51557	-0.48768	0.529974	-0.9202	0.357471	0.650953	FLVCR1-DT	642946	FLVCR1 divergent transcript
ENSG0000	430.8443	-0.12079	0.131303	-0.91993	0.357609	0.650953	OLA1	29789	Obg like ATPase 1
ENSG0000	40.75104	0.42742	0.464592	0.91999	0.357578	0.650953	NA	NA	NA
ENSG0000	181.3738	0.16915	0.183766	0.920465	0.35733	0.650953	HACL1	26061	2-hydroxyacyl-CoA lyase 1
ENSG0000	1761.117	-0.12656	0.13752	-0.9203	0.357416	0.650953	XRN1	54464	5'-3' exoribonuclease 1
ENSG0000	30.28845	-0.48225	0.524164	-0.92003	0.357555	0.650953	NA	NA	NA
ENSG0000	102.9525	0.227453	0.247249	0.919933	0.357608	0.650953	SCART1	619207	scavenger receptor family member expressed on T cells 1
ENSG0000	2009.53	0.092684	0.100727	0.920152	0.357493	0.650953	H2AX	3014	H2A.X variant histone
ENSG0000	2456.856	-0.08757	0.095111	-0.92068	0.357217	0.650953	LTBR	4055	lymphotoxin beta receptor
ENSG0000	44.53002	-0.40205	0.436795	-0.92045	0.357337	0.650953	MOK	5891	MOK protein kinase
ENSG0000	4.881406	-1.12508	1.221932	-0.92074	0.357188	0.650953	NA	NA	NA
ENSG0000	302.6263	0.154952	0.168451	0.919865	0.357643	0.650953	NEDD4L	23327	NEDD4 like E3 ubiquitin protein ligase
ENSG0000	9202.206	0.076607	0.083255	0.920157	0.35749	0.650953	PRKCSH	5589	protein kinase C substrate 80K-H
ENSG0000	7.483951	-0.86893	0.943847	-0.92063	0.357245	0.650953	ZNF781	163115	zinc finger protein 781
ENSG0000	187.7346	0.191751	0.208533	0.919523	0.357822	0.651214	FAM169A	26049	family with sequence similarity 169 member A
ENSG0000	334.4033	0.17621	0.191673	0.919326	0.357925	0.651299	RASGRF2	5924	Ras protein specific guanine nucleotide releasing factor 2
ENSG0000	152.4435	-0.20148	0.219172	-0.9193	0.357939	0.651299	MRPS31	10240	mitochondrial ribosomal protein S31
ENSG0000	23.06689	-0.4466	0.485874	-0.91916	0.358011	0.651366	NA	NA	NA
ENSG0000	10.56024	-0.70348	0.765668	-0.91878	0.358212	0.651668	NA	NA	NA
ENSG0000	220.6307	-0.16716	0.181981	-0.91857	0.358319	0.651691	AGBL5	60509	AGBL carboxypeptidase 5
ENSG0000	128.6296	0.199251	0.216919	0.918552	0.35833	0.651691	NDUFB5	4711	NADH:ubiquinone oxidoreductase subunit B5
ENSG0000	224.1356	0.160128	0.174327	0.918552	0.35833	0.651691	CYP2R1	120227	cytochrome P450 family 2 subfamily R member 1
ENSG0000	1303.36	0.093146	0.101452	0.918132	0.35855	0.652004	METTL26	84326	methyltransferase like 26

ENSG0000	9.686311	0.730068	0.795204	0.918089	0.358572	0.652004	ZNF426-DT	1.02E+08	ZNF426 divergent transcript
ENSG0000	1924.654	0.082461	0.089826	0.918008	0.358615	0.652017	MKRN1	23608	makorin ring finger protein 1
ENSG0000	9524.669	0.063328	0.068992	0.91791	0.358666	0.652046	SEPTIN6	23157	septin 6
ENSG0000	59.48495	-0.28026	0.30538	-0.91775	0.358752	0.652139	MMP15	4324	matrix metallopeptidase 15
ENSG0000	137.8598	0.212866	0.231998	0.917532	0.358864	0.652151	NA	NA	NA
ENSG0000	37.98099	-0.37302	0.406529	-0.91758	0.358838	0.652151	POLRMTP1	284167	RNA polymerase mitochondrial pseudogene 1
ENSG0000	7.471078	-0.87736	0.956132	-0.91761	0.358822	0.652151	DRICH1	51233	aspartate rich 1
ENSG0000	3.343131	1.228285	1.338789	0.91746	0.358902	0.652156	NA	NA	NA
ENSG0000	4276.115	-0.07223	0.07874	-0.91737	0.35895	0.652179	GBF1	8729	golgi brefeldin A resistant guanine nucleotide exchange factor 1
ENSG0000	5.836892	-0.87503	0.954107	-0.91712	0.359081	0.652226	HEYL	26508	hes related family bHLH transcription factor with YRPW motif like
ENSG0000	342.8665	-0.1455	0.158639	-0.91718	0.359046	0.652226	TOMM40L	84134	translocase of outer mitochondrial membrane 40 like
ENSG0000	1933.068	0.095136	0.103729	0.917158	0.35906	0.652226	SMG9	56006	SMG9 nonsense mediated mRNA decay factor
ENSG0000	3.731623	1.290796	1.407869	0.916843	0.359225	0.652369	NA	NA	NA
ENSG0000	27.61949	0.452499	0.493546	0.916833	0.35923	0.652369	STX18-AS1	1.01E+08	STX18 antisense RNA 1 (head to head)
ENSG0000	5.700275	0.943962	1.029809	0.916638	0.359332	0.652491	NA	NA	NA
ENSG0000	459.1561	-0.1216	0.132691	-0.91643	0.359442	0.652625	TMX3	54495	thioredoxin related transmembrane protein 3
ENSG0000	16.1197	0.710303	0.775154	0.916338	0.35949	0.652648	SLC2A14	144195	solute carrier family 2 member 14
ENSG0000	12.01085	0.680995	0.743235	0.916258	0.359532	0.652661	TMPRSS5	80975	transmembrane serine protease 5
ENSG0000	7.23792	0.78013	0.851665	0.916005	0.359664	0.65271	NA	NA	NA
ENSG0000	1048.387	-0.10222	0.111591	-0.91606	0.359637	0.65271	LRPPRC	10128	leucine rich pentatricopeptide repeat containing
ENSG0000	1864.957	0.085734	0.09359	0.916066	0.359632	0.65271	PPFIA1	8500	PTPRF interacting protein alpha 1
ENSG0000	179.7237	-0.19559	0.213551	-0.91588	0.359727	0.65276	SRD5A1	6715	steroid 5 alpha-reductase 1
ENSG0000	159.1582	-0.17827	0.194674	-0.91573	0.359811	0.652848	MTG1	92170	mitochondrial ribosome associated GTPase 1
ENSG0000	755.8845	0.107503	0.117418	0.91556	0.359898	0.652941	FAM174C	55009	family with sequence similarity 174 member C
ENSG0000	1946.483	0.078663	0.085928	0.915445	0.359958	0.652987	HMGXB3	22993	HMG-box containing 3
ENSG0000	13.23797	0.556899	0.608459	0.91526	0.360055	0.653099	HNRNPKP4	644063	heterogeneous nuclear ribonucleoprotein K pseudogene 4
ENSG0000	83.51071	0.233278	0.254906	0.915151	0.360112	0.653139	PCGF6	84108	polycomb group ring finger 6
ENSG0000	2067.415	0.085602	0.093604	0.914514	0.360447	0.653682	CPSF6	11052	cleavage and polyadenylation specific factor 6
ENSG0000	5990.265	0.070371	0.076977	0.914184	0.36062	0.653762	AUP1	550	AUP1 lipid droplet regulating VLDL assembly factor
ENSG0000	2954.669	0.117832	0.128896	0.914162	0.360632	0.653762	PLSCR1	5359	phospholipid scramblase 1
ENSG0000	3.348982	1.618091	1.769798	0.91428	0.36057	0.653762	NA	NA	NA
ENSG0000	60.21831	0.339486	0.371285	0.914355	0.360531	0.653762	NA	NA	NA
ENSG0000	27.52491	0.491925	0.538177	0.914058	0.360686	0.653796	LINC01772	1.08E+08	long intergenic non-protein coding RNA 1772
ENSG0000	63.23546	0.299581	0.327787	0.913949	0.360744	0.653837	PIGW	284098	phosphatidylinositol glycan anchor biosynthesis class W
ENSG0000	2.796176	1.610241	1.762634	0.913542	0.360957	0.65416	MUC12-AS	1.03E+08	MUC12 antisense RNA 1
ENSG0000	4.667242	-0.99068	1.084536	-0.91346	0.361	0.654163	NA	NA	NA
ENSG0000	11.85004	-0.59928	0.656195	-0.91327	0.3611	0.654163	TRAV6	6956	T cell receptor alpha variable 6
ENSG0000	10.38962	-0.9151	1.001994	-0.91328	0.361095	0.654163	IGHV3-64D	1.03E+08	immunoglobulin heavy variable 3-64D
ENSG0000	274.905	0.144058	0.157718	0.91339	0.361038	0.654163	PHLPP2	23035	PH domain and leucine rich repeat protein phosphatase 2
ENSG0000	23.96294	0.494621	0.541914	0.91273	0.361385	0.654615	LRP6	4040	LDL receptor related protein 6
ENSG0000	55.51267	-0.28154	0.308493	-0.91261	0.361445	0.654661	ARMCX4	1E+08	armadillo repeat containing X-linked 4
ENSG0000	81.43732	0.25079	0.274872	0.912389	0.361564	0.65478	MAP3K2-D	1.01E+08	MAP3K2 divergent transcript
ENSG0000	247.5149	0.170799	0.187207	0.912355	0.361582	0.65478	HIGD1A	25994	HIG1 hypoxia inducible domain family member 1A
ENSG0000	305.2759	-0.15572	0.170698	-0.91228	0.361623	0.65479	HABP4	22927	hyaluronan binding protein 4
ENSG0000	5.749468	-0.90202	0.988826	-0.91221	0.361659	0.654792	TRBV5-5	28610	T cell receptor beta variable 5-5
ENSG0000	618.0302	0.123075	0.134947	0.912021	0.361758	0.654907	C8orf33	65265	chromosome 8 open reading frame 33
ENSG0000	4.091596	1.151962	1.263281	0.911881	0.361832	0.654913	STBD1	8987	starch binding domain 1
ENSG0000	39.41607	-0.44494	0.48792	-0.91192	0.361813	0.654913	GEMIN2	8487	gem nuclear organelle associated protein 2
ENSG0000	16.29784	-0.63972	0.701732	-0.91164	0.36196	0.655076	FGF18	8817	fibroblast growth factor 18
ENSG0000	373.5434	-0.13472	0.147786	-0.91158	0.361992	0.655076	ENDOD1	23052	endonuclease domain containing 1
ENSG0000	265.431	0.142804	0.156692	0.911373	0.362099	0.65514	LBX2-AS1	151534	LBX2 antisense RNA 1
ENSG0000	855.3442	-0.10104	0.110868	-0.91138	0.362095	0.65514	ZC3H15	55854	zinc finger CCCH-type containing 15
ENSG0000	8.746052	0.832823	0.91415	0.911035	0.362277	0.655335	UBE2QL1	134111	ubiquitin conjugating enzyme E2 Q family like 1
ENSG0000	362.0259	-0.13053	0.143271	-0.91108	0.362256	0.655335	PARP16	54956	poly(ADP-ribose) polymerase family member 16
ENSG0000	29.3591	0.450976	0.495433	0.910266	0.362682	0.65594	HECTD2	143279	HECT domain E3 ubiquitin protein ligase 2
ENSG0000	557.8317	0.113565	0.124752	0.910329	0.362649	0.65594	FBXO21	23014	F-box protein 21
ENSG0000	713.6413	0.102398	0.112535	0.909927	0.362861	0.6562	DUSP11	8446	dual specificity phosphatase 11
ENSG0000	199.3739	-0.16346	0.179658	-0.90985	0.362904	0.656213	LCMT2	9836	leucine carboxyl methyltransferase 2
ENSG0000	1382.154	0.086953	0.095589	0.909656	0.363004	0.656266	WAPL	23063	WAPL cohesin release factor
ENSG0000	355.4031	-0.14533	0.159757	-0.90968	0.36299	0.656266	APAF1	317	apoptotic peptidase activating factor 1
ENSG0000	32.27365	0.396008	0.435447	0.909428	0.363124	0.656368	NA	NA	NA
ENSG0000	467.7583	0.121536	0.133641	0.909415	0.363131	0.656368	SLC7A6OS	84138	solute carrier family 7 member 6 opposite strand
ENSG0000	5005.981	-0.06322	0.069538	-0.90907	0.363313	0.656633	QRICH1	54870	glutamine rich 1
ENSG0000	11.21145	0.793558	0.873388	0.908597	0.363563	0.656846	PXN-AS1	1.01E+08	PXN antisense RNA 1
ENSG0000	55.7679	0.296769	0.326581	0.908713	0.363502	0.656846	HDHD2	84064	haloacid dehalogenase like hydrolase domain containing 2
ENSG0000	32.57219	-0.41555	0.457366	-0.90858	0.363573	0.656846	RTEL1-TNF	1.01E+08	RTEL1-TNFRSF6B readthrough (NMD candidate)
ENSG0000	20.69245	-0.48409	0.532731	-0.9087	0.363509	0.656846	USP51	158880	ubiquitin specific peptidase 51
ENSG0000	25.41599	-0.43193	0.475733	-0.90793	0.363914	0.657202	NA	NA	NA
ENSG0000	55.51194	-0.28727	0.316492	-0.90767	0.364053	0.657202	RAB43	339122	RAB43 member RAS oncogene family
ENSG0000	1015.909	0.089823	0.098926	0.907988	0.363885	0.657202	C6orf47	57827	chromosome 6 open reading frame 47
ENSG0000	12.79455	-0.62814	0.691736	-0.90806	0.363846	0.657202	CYP51A1	1595	cytochrome P450 family 51 subfamily A member 1
ENSG0000	10.29305	-0.92247	1.016072	-0.90788	0.363944	0.657202	LCN8	138307	lipocalin 8
ENSG0000	1512.939	0.083539	0.092037	0.90767	0.364053	0.657202	NDUFV1	4723	NADH:ubiquinone oxidoreductase core subunit V1
ENSG0000	19.67975	-0.63478	0.699252	-0.90779	0.363989	0.657202	ZNF286B	729288	zinc finger protein 286B (pseudogene)
ENSG0000	27.23118	-0.46806	0.515607	-0.90779	0.363988	0.657202	NA	NA	NA
ENSG0000	45.70668	-0.3407	0.375439	-0.90747	0.364159	0.657329	KIF7	374654	kinesin family member 7
ENSG0000	2.00135	1.970393	2.172319	0.907046	0.364382	0.657668	NA	NA	NA
ENSG0000	110.5045	-0.23224	0.256128	-0.90671	0.364558	0.657921	NA	NA	NA
ENSG0000	6.735825	-0.86742	0.95718	-0.90622	0.364819	0.658072	NA	NA	NA
ENSG0000	42.2618	-0.34659	0.382363	-0.90645	0.364699	0.658072	ABCC2	1244	ATP binding cassette subfamily C member 2
ENSG0000	18.41226	-0.6182	0.682146	-0.90626	0.364796	0.658072	AFAP1L2	84632	actin filament associated protein 1 like 2
ENSG0000	7.396027	0.903415	0.996842	0.906277	0.364789	0.658072	NA	NA	NA
ENSG0000	3.613715	-1.27345	1.404979	-0.90638	0.364734	0.658072	NA	NA	NA
ENSG0000	25.74537	0.47466	0.524074	0.905712	0.365088	0.658266	LINC01857	1.03E+08	long intergenic non-protein coding RNA 1857
ENSG0000	4.307479	-1.19958	1.324506	-0.90568	0.365104	0.658266	FBXO38-DT	1.03E+08	FBXO38 divergent transcript
ENSG0000	454.0537	0.132356	0.146134	0.905717	0.365086	0.658266	DEFA3	1668	defensin alpha 3
ENSG0000	902.8284	0.106896	0.117994	0.905942	0.364967	0.658266	ATP5F1C	509	ATP synthase F1 subunit gamma
ENSG0000	8.850651	-0.81922	0.904498	-0.90571	0.365087	0.658266	LOC10042	1E+08	UTP18 small subı homolog (yeast) pseudogene
ENSG0000	20.1733	-0.66956	0.739348	-0.90561	0.365144	0.658275	NA	NA	NA
ENSG0000	2.586331	1.627447	1.797522	0.905383	0.365262	0.658331	MASP2	10747	MBL associated serine protease 2
ENSG0000	60.05038	-0.29324	0.323948	-0.90522	0.365349	0.658331	B3GALT2	8707	beta-1 3-galactosyltransferase 2
ENSG0000	3.493298	1.269649	1.402442	0.905313	0.365299	0.658331	WWC2-AS	152641	WWC2 antisense RNA 2
ENSG0000	14.73037	0.583195	0.644244	0.905239	0.365339	0.658331	DRGX	644168	dorsal root ganglia homeobox
ENSG0000	167.0428	-0.17509	0.19342	-0.90521	0.365353	0.658331	RCBTB1	55213	RCC1 and BTB domain containing protein 1
ENSG0000	10.8089	0.687255	0.759307	0.905108	0.365408	0.658367	CILP	8483	cartilage intermediate layer protein
ENSG0000	326.133	-0.15258	0.168607	-0.90495	0.365494	0.658393	SHLD2	54537	shieldin complex subunit 2
ENSG0000	9.016393	-0.74453	0.822725	-0.90495	0.365489	0.658393	LANCL3	347404	LanC like family member 3
ENSG0000	460.0485	0.110998	0.122713	0.904535	0.365712	0.658593	TNFRSF10I	8793	TNF receptor superfamily member 10d
ENSG0000	252.9869	-0.13973	0.154453	-0.90467	0.365641	0.658593	JOSD2	126119	Josephin domain containing 2
ENSG0000	22.21198	0.521218	0.576208	0.904565	0.365696	0.658593	NUDT11	55190	nudix hydrolase 11

ENSG0000	6.990623	-1.00923	1.116054	-0.90428	0.365845	0.658727	GRB14	2888	growth factor receptor bound protein 14
ENSG0000	3.984084	-1.18359	1.308904	-0.90426	0.365857	0.658727	NA	NA	NA
ENSG0000	783.4056	-0.10622	0.117519	-0.90387	0.366063	0.659034	USP33	23032	ubiquitin specific peptidase 33
ENSG0000	7.929427	0.891551	0.986473	0.903776	0.366114	0.659062	TMEM256	254863	transmembrane protein 256
ENSG0000	1574.36	0.07956	0.088045	0.903631	0.366191	0.659137	BTBD19	149478	BTB domain containing 19
ENSG0000	532.582	0.107852	0.119374	0.903481	0.366271	0.659152	WDR43	23160	WD repeat domain 43
ENSG0000	198.6074	-0.16224	0.179572	-0.9035	0.36626	0.659152	ZNF174	7727	zinc finger protein 174
ENSG0000	27.01363	-0.41185	0.455972	-0.90324	0.366398	0.65916	ADAMTS14	140766	ADAM metallopeptidase with thrombospondin type 1 motif 14
ENSG0000	5.055318	1.123897	1.24429	0.903243	0.366397	0.65916	FLJ13224	79857	uncharacterized LOC79857
ENSG0000	46.68345	0.315014	0.348757	0.903247	0.366395	0.65916	RNU7-40P	1E+08	RNA U7 small nuclear 40 pseudogene
ENSG0000	126.3912	-0.2231	0.247013	-0.90321	0.366417	0.65916	ADCK1	57143	aarF domain containing kinase 1
ENSG0000	10770.84	0.080726	0.089405	0.902926	0.366565	0.659258	MGAT1	4245	alpha-1 3-mannosyl-glycoprotein 2-beta-N-acetylglucosaminyltransferase
ENSG0000	98.13531	-0.26139	0.289502	-0.9029	0.366578	0.659258	LY6G5B	58496	lymphocyte antigen 6 family member G5B
ENSG0000	5414.387	-0.0801	0.088707	-0.90292	0.366568	0.659258	PTPN6	5777	protein tyrosine phosphatase non-receptor type 6
ENSG0000	739.7726	0.112617	0.124759	0.902678	0.366697	0.659394	RLF	6018	RLF zinc finger
ENSG0000	73.04936	-0.26661	0.295374	-0.90262	0.366725	0.659394	MACROH2	55506	macroH2A.2 histone
ENSG0000	69.61312	0.277179	0.30717	0.902365	0.366863	0.659578	ZCWPW1	55063	zinc finger CW-type and PWWP domain containing 1
ENSG0000	8.009364	-0.90848	1.007116	-0.90206	0.367024	0.659585	PIGZ	80235	phosphatidylinositol glycan anchor biosynthesis class Z
ENSG0000	3506.912	-0.06707	0.074356	-0.90206	0.367024	0.659585	SYNE1	23345	spectrin repeat containing nuclear envelope protein 1
ENSG0000	6.03548	0.974531	1.080357	0.902045	0.367033	0.659585	NA	NA	NA
ENSG0000	1573.603	-0.07815	0.086634	-0.90209	0.367007	0.659585	NBR1	4077	NBR1 autophagy cargo receptor
ENSG0000	1580.72	0.083826	0.092931	0.902023	0.367045	0.659585	SGSH	6448	N-sulfoglucosamine sulfohydrolase
ENSG0000	1643.205	-0.10193	0.11303	-0.90176	0.367182	0.659768	FLYWCH1	84256	FLYWCH-type zinc finger 1
ENSG0000	4142.134	-0.06941	0.076997	-0.90152	0.367311	0.659936	CHD8	57680	chromodomain helicase DNA binding protein 8
ENSG0000	875.0843	-0.09832	0.10909	-0.90126	0.367452	0.660017	MED23	9439	mediator complex subunit 23
ENSG0000	4.635755	-1.07442	1.192166	-0.90124	0.367463	0.660017	NA	NA	NA
ENSG0000	32.42785	0.358552	0.397834	0.901262	0.367449	0.660017	NA	NA	NA
ENSG0000	3.472137	-1.57277	1.745293	-0.90115	0.367508	0.660034	NA	NA	NA
ENSG0000	38.82936	-0.40533	0.449835	-0.90106	0.367557	0.660058	S100A13	6284	S100 calcium binding protein A13
ENSG0000	1043.738	0.113328	0.125802	0.900844	0.367671	0.660199	ALYREF	10189	Aly/REF export factor
ENSG0000	111.535	-0.2082	0.231167	-0.90066	0.367767	0.660308	ZNF254	9534	zinc finger protein 254
ENSG0000	57.14208	-0.28013	0.311126	-0.90036	0.367927	0.660379	NA	NA	NA
ENSG0000	710.3247	-0.11051	0.122744	-0.90034	0.36794	0.660379	NAAA	27163	N-acylethanolamine acid amidase
ENSG0000	537.4117	0.125456	0.139387	0.900054	0.368091	0.660379	AP3S1	1176	adaptor related protein complex 3 subunit sigma 1
ENSG0000	3.813998	-1.42291	1.580817	-0.90011	0.368062	0.660379	KRT79	338785	keratin 79
ENSG0000	10.52477	0.903165	1.003428	0.90008	0.368078	0.660379	LINC0039	1.01E+08	long intergenic non-protein coding RNA 398
ENSG0000	88.838	0.22306	0.247783	0.900223	0.368002	0.660379	DLEU2	8847	deleted in lymphocytic leukemia 2
ENSG0000	279.3168	-0.15294	0.169891	-0.90021	0.368006	0.660379	ITGA3	3675	integrin subunit alpha 3
ENSG0000	628.8811	0.119978	0.133169	0.900197	0.368015	0.660379	BPI	671	bactericidal permeability increasing protein
ENSG0000	226.6256	0.163551	0.18182	0.89952	0.368376	0.660761	TADA1	117143	transcriptional adaptor 1
ENSG0000	573.9754	0.105828	0.117649	0.899521	0.368375	0.660761	TSNARE1	203062	t-SNARE domain containing 1
ENSG0000	38.17079	-0.34698	0.385813	-0.89934	0.368472	0.660835	NA	NA	NA
ENSG0000	13.19188	-0.82391	0.916163	-0.89931	0.368489	0.660835	USP6	9098	ubiquitin specific peptidase 6
ENSG0000	112.9208	0.280149	0.311578	0.89913	0.368584	0.660836	SLC25A34	284723	solute carrier family 25 member 34
ENSG0000	1080.593	-0.09432	0.104904	-0.89908	0.368612	0.660836	ACTR1B	10120	actin related protein 1B
ENSG0000	4.374287	-1.2983	1.444095	-0.89904	0.368631	0.660836	NA	NA	NA
ENSG0000	4.228398	-1.24483	1.384372	-0.8992	0.368547	0.660836	IMPDH1P1	1.01E+08	inosine monophosphate dehydrogenase 1 pseudogene 10
ENSG0000	15.82701	0.530187	0.589876	0.89881	0.368754	0.660992	NA	NA	NA
ENSG0000	102.0585	-0.21674	0.241249	-0.89842	0.368961	0.6613	ACAT1	38	acetyl-CoA acetyltransferase 1
ENSG0000	3.790343	-1.19852	1.334294	-0.89824	0.369056	0.661405	LARP6	55323	La ribonuc translational regulator
ENSG0000	494.8064	0.11541	0.128522	0.89798	0.369196	0.661592	RBX1	9978	ring-box 1
ENSG0000	8.62495	0.907776	1.011312	0.897622	0.369387	0.661871	DMTF1-AS	1.02E+08	DMTF1 antisense RNA 1
ENSG0000	40.14167	0.467718	0.521127	0.897512	0.369446	0.661912	MACIR	90355	macrophage immunometabolism regulator
ENSG0000	26.22159	0.45322	0.50518	0.897146	0.369641	0.662197	SARNP	84324	SAP domain containing ribonucleoprotein
ENSG0000	5.608576	-1.22157	1.361777	-0.89704	0.369698	0.662236	NA	NA	NA
ENSG0000	50.92808	-0.31663	0.353107	-0.8967	0.369879	0.662433	ALG10B	144245	ALG10 alp 2-glucosyltransferase B
ENSG0000	8.806086	0.718326	0.801078	0.896699	0.36988	0.662433	NA	NA	NA
ENSG0000	5.720376	-1.00021	1.115556	-0.8966	0.369932	0.662464	DPRXP4	503645	divergent-paired related homeobox pseudogene 4
ENSG0000	24.56787	0.513494	0.572931	0.896259	0.370114	0.662534	LAD1	3898	ladinin 1
ENSG0000	6.224357	-1.02805	1.146841	-0.89642	0.370027	0.662534	NA	NA	NA
ENSG0000	5.089316	-1.14741	1.280116	-0.89633	0.370075	0.662534	TET1	80312	tet methylcytosine dioxygenase 1
ENSG0000	19.88013	0.593935	0.662672	0.896273	0.370107	0.662534	SPDYC	387778	speedy/RINGO cell cycle regulator family member C
ENSG0000	5.420484	-1.00646	1.123248	-0.89603	0.370238	0.662564	TSHR	7253	thyroid stimulating hormone receptor
ENSG0000	8.854898	0.698633	0.779697	0.896032	0.370236	0.662564	NA	NA	NA
ENSG0000	383.6919	0.131259	0.146486	0.896055	0.370224	0.662564	ZXDB	158586	zinc finger X-linked duplicated B
ENSG0000	180.4323	-0.16713	0.186564	-0.89585	0.370335	0.662608	IFNLR1	163702	interferon lambda receptor 1
ENSG0000	23.40451	0.516889	0.576975	0.895861	0.370327	0.662608	RPL13AP2	729212	ribosomal protein L13a pseudogene 26
ENSG0000	159.3564	-0.18107	0.202167	-0.89566	0.370437	0.662663	C1orf198	84886	chromosome 1 open reading frame 198
ENSG0000	221.7353	0.174463	0.194784	0.895678	0.370425	0.662663	MRPL36	64979	mitochondrial ribosomal protein L36
ENSG0000	187.0493	0.155838	0.174016	0.895538	0.3705	0.662712	H2AJ	55766	H2A.J histone
ENSG0000	15.82033	-0.6441	0.719561	-0.89513	0.370719	0.662913	CLHC1	130162	clathrin heavy chain linker domain containing 1
ENSG0000	45.10911	0.33585	0.375157	0.895227	0.370666	0.662913	IGHV3-33	28434	immunoglobulin heavy variable 3-33
ENSG0000	3.679701	-1.31358	1.467459	-0.89514	0.370711	0.662913	RSPO4	343637	R-spondin 4
ENSG0000	4.00616	1.41145	1.577201	0.894908	0.370836	0.663058	NA	NA	NA
ENSG0000	18.25692	-0.57067	0.637792	-0.89477	0.370913	0.66313	MADCAM1	8174	mucosal vascular addressin cell adhesion molecule 1
ENSG0000	2651.28	0.088759	0.099284	0.893992	0.371326	0.663678	ITSN2	50618	intersectin 2
ENSG0000	476.0104	-0.11901	0.133116	-0.89405	0.371296	0.663678	DMXL1	1657	Dmx like 1
ENSG0000	149.0664	-0.17499	0.195721	-0.89408	0.371279	0.663678	CA5B	11238	carbonic anhydrase 5B
ENSG0000	11.46376	0.685979	0.767609	0.893657	0.371506	0.663935	NA	NA	NA
ENSG0000	2131.384	0.081288	0.090977	0.893506	0.371586	0.664015	LRCH3	84859	leucine rich repeats and calponin homology domain containing 3
ENSG0000	7.320786	0.964531	1.079652	0.893372	0.371658	0.664019	S100A3	6274	S100 calcium binding protein A3
ENSG0000	135.0138	-0.24282	0.2718	-0.89336	0.371664	0.664019	ANO10	55129	anoctamin 10
ENSG0000	11.84933	0.709862	0.794658	0.893291	0.371701	0.664019	NRG2	9542	neuregulin 2
ENSG0000	9.282729	0.814131	0.911443	0.893234	0.371732	0.664019	KCNT1	57582	potassium sodium-activated channel subfamily T member 1
ENSG0000	22.58871	-0.50978	0.570939	-0.89288	0.371921	0.664293	LOC10537	1.05E+08	uncharacterized LOC105371795
ENSG0000	29.93309	0.38108	0.427093	0.892264	0.372252	0.664449	RPL10AP6	1E+08	ribosomal protein L10a pseudogene 6
ENSG0000	285.8772	0.158538	0.177684	0.892249	0.372259	0.664449	RUVBL1	8607	RuvB like AAA ATPase 1
ENSG0000	117.4115	-0.1989	0.222825	-0.89264	0.372053	0.664449	DOLPP1	57171	dolichyldiphosphatase 1
ENSG0000	505.6251	0.113033	0.126676	0.892294	0.372236	0.664449	ERG28	11161	ergosterol biosynthesis 28 homolog
ENSG0000	18.26047	0.614409	0.68855	0.892323	0.37222	0.664449	NA	NA	NA
ENSG0000	507.0941	-0.10436	0.116935	-0.89249	0.37213	0.664449	POP4	10775	POP4 hom ribonuclease P/MRP subunit
ENSG0000	21.94034	0.632021	0.708143	0.892505	0.372123	0.664449	NA	NA	NA
ENSG0000	40.05153	-0.3276	0.367398	-0.89168	0.372564	0.664865	HSPA1L	3305	heat shock protein family A (Hsp70) member 1 like
ENSG0000	199.184	0.162893	0.182673	0.89172	0.372543	0.664865	BNIP3	664	BCL2 interacting protein 3
ENSG0000	720.1342	-0.1175	0.131786	-0.89156	0.372628	0.664916	EIF4EBP1	1978	eukaryotic translation initiation factor 4E binding protein 1
ENSG0000	255.785	0.158241	0.177521	0.891393	0.372718	0.665006	UQCC2	84300	ubiquinol-cytochrome c reductase complex assembly factor 2
ENSG0000	208.4286	-0.16232	0.182112	-0.89133	0.372751	0.665006	MOCS3	27304	molybdenum cofactor synthesis 3
ENSG0000	347.7578	-0.13036	0.14627	-0.89124	0.372799	0.665028	DAAM1	23002	dishevelled associated activator of morphogenesis 1
ENSG0000	19290.42	0.060529	0.067923	0.891139	0.372854	0.665064	APLP2	334	amyloid beta precursor like protein 2
ENSG0000	33.48154	-0.35529	0.398866	-0.89074	0.373069	0.665237	KCTD3	51133	potassium channel tetramerization domain containing 3

ENSG0000	4429.719	0.090095	0.101135	0.890843	0.373013	0.665237	ZC3HAV1	56829	zinc finger antiviral 1
ENSG0000	1096.707	-0.08239	0.092505	-0.89069	0.373096	0.665237	CTR9	9646	CTR9 hom Paf1/RNA polymerase II complex component
ENSG0000	374.3251	0.134305	0.150781	0.890725	0.373077	0.665237	GOLT1B	51026	golgi transport 1B
ENSG0000	1402.745	-0.08042	0.090298	-0.89062	0.373131	0.665237	TMPO	7112	thymopoietin
ENSG0000	13.10062	0.550616	0.618401	0.890387	0.373258	0.6654	NA	NA	NA
ENSG0000	145.2901	0.184065	0.206766	0.890211	0.373352	0.665504	FAHD1	81889	fumarylacetoacetate hydrolase domain containing 1
ENSG0000	116.0282	0.221968	0.249362	0.890144	0.373389	0.665504	WEE1	7465	WEE1 G2 checkpoint kinase
ENSG0000	5.117715	-0.94927	1.066679	-0.88994	0.373501	0.66564	KLHL32	114792	kelch like family member 32
ENSG0000	222.7283	0.165176	0.185626	0.88983	0.373557	0.665676	TTC33	23548	tetratricopeptide repeat domain 33
ENSG0000	600.1357	0.110318	0.124019	0.889525	0.373721	0.665905	PSMC6	5706	proteasom ATPase 6
ENSG0000	6.544258	-0.98182	1.103867	-0.88943	0.37377	0.665927	C1orf116	79098	chromosome 1 open reading frame 116
ENSG0000	9.241974	0.809697	0.910417	0.889369	0.373805	0.665927	NA	NA	NA
ENSG0000	123.6852	-0.20812	0.234043	-0.88925	0.37387	0.665979	MRM1	79922	mitochondrial rRNA methyltransferase 1
ENSG0000	1272.973	0.100463	0.112993	0.889106	0.373946	0.66605	GPATCH2L	55668	G-patch domain containing 2 like
ENSG0000	11.04769	-0.76266	0.858023	-0.88885	0.374082	0.666229	ARHGEF35	445328	Rho guanine nucleotide exchange factor 35
ENSG0000	32.32717	-0.38628	0.434751	-0.8885	0.374271	0.666411	SANBR	84542	SANT and BTB domain regulator of CSR
ENSG0000	503.7967	0.109748	0.123528	0.888444	0.374302	0.666411	PIGX	54965	phosphatidylinositol glycan anchor biosynthesis class X
ENSG0000	6.91409	-0.78653	0.885342	-0.8884	0.374328	0.666411	PLPP7	84814	phospholipid phosphatase 7 (inactive)
ENSG0000	881.6183	0.091982	0.103523	0.888518	0.374262	0.666411	PDZD8	118987	PDZ domain containing 8
ENSG0000	275.7343	-0.15908	0.179132	-0.88809	0.374495	0.666643	CDK5RAP3	80279	CDK5 regulatory subunit associated protein 3
ENSG0000	7.081712	0.880736	0.991883	0.887943	0.374571	0.666651	SLC5A11	115584	solute carrier family 5 member 11
ENSG0000	91.70841	-0.2443	0.275128	-0.88796	0.374563	0.666651	SMG1P5	595101	SMG1 pseudogene 5
ENSG0000	6.759169	0.868374	0.978073	0.887841	0.374626	0.666683	NA	NA	NA
ENSG0000	169.1886	-0.17871	0.201305	-0.88778	0.374661	0.666683	VPS33A	65082	VPS33A core subunit of CORVET and HOPS complexes
ENSG0000	1270.685	0.091261	0.102835	0.887453	0.374835	0.666928	MOB2	81532	MOB kinase activator 2
ENSG0000	8.614044	-0.76049	0.857186	-0.88719	0.374974	0.667079	ARNT2	9915	aryl hydrocarbon receptor nuclear translocator 2
ENSG0000	131.4101	0.218976	0.246827	0.887162	0.374992	0.667079	NA	NA	NA
ENSG0000	1801.537	-0.09052	0.102044	-0.88704	0.375059	0.667135	PEF1	553115	penta-EF-hand domain containing 1
ENSG0000	15.58585	-0.67245	0.758486	-0.88657	0.375311	0.667396	ABHD1	84696	abhydrolase domain containing 1
ENSG0000	7073.35	-0.07292	0.082242	-0.88663	0.375279	0.667396	TGOLN2	10618	trans-golgi network protein 2
ENSG0000	3.961752	1.154895	1.302663	0.886564	0.375313	0.667396	NA	NA	NA
ENSG0000	38.10985	0.347085	0.391529	0.886484	0.375357	0.667408	AFTPH-DT	1.02E+08	AFTPH divergent transcript
ENSG0000	4849.602	0.063662	0.071821	0.886395	0.375405	0.66743	PTPN23	25930	protein tyrosine phosphatase non-receptor type 23
ENSG0000	5.320766	-1.07992	1.218477	-0.88629	0.375463	0.667469	NA	NA	NA
ENSG0000	32.06447	0.406546	0.458763	0.886179	0.375521	0.667487	LOC64236	642361	uncharacterized LOC642361
ENSG0000	4.073017	1.18628	1.338873	0.886028	0.375602	0.667487	RBP4	5950	retinol binding protein 4
ENSG0000	27.20216	0.52244	0.589595	0.886101	0.375563	0.667487	LOXL1	4016	lysyl oxidase like 1
ENSG0000	11.7261	-0.61383	0.692809	-0.886	0.375617	0.667487	F2RL3	9002	F2R like thrombin or trypsin receptor 3
ENSG0000	11.0169	-0.67303	0.759773	-0.88583	0.375709	0.667587	SYT7	9066	synaptotagmin 7
ENSG0000	2.996762	-1.34239	1.516579	-0.88515	0.376078	0.667746	NA	NA	NA
ENSG0000	136.2695	-0.20416	0.230608	-0.88532	0.375987	0.667746	LRRC42	115353	leucine rich repeat containing 42
ENSG0000	10.63993	-0.6194	0.699791	-0.88512	0.376093	0.667746	CSPG5	10675	chondroitin sulfate proteoglycan 5
ENSG0000	5.436586	1.079601	1.219801	0.885063	0.376122	0.667746	PRL	5617	prolactin
ENSG0000	37.5272	-0.3532	0.398917	-0.88539	0.375948	0.667746	H3C6	8353	H3 clustered histone 6
ENSG0000	81.1219	0.239795	0.270934	0.885067	0.376121	0.667746	PEX3	8504	peroxisomal biogenesis factor 3
ENSG0000	397.5238	0.129322	0.14604	0.885525	0.375874	0.667746	RHNO1	83695	RAD9-HUS1-RAD1 interacting nuclear orphan 1
ENSG0000	779.0434	-0.09025	0.101916	-0.88557	0.375848	0.667746	SPRING1	79794	SREBF pathway regulator in golgi 1
ENSG0000	43.54694	-0.34455	0.389143	-0.88541	0.375934	0.667746	NA	NA	NA
ENSG0000	393.3461	0.121234	0.137	0.884923	0.376198	0.667817	CEBPZOS	1.01E+08	CEBPZ opposite strand
ENSG0000	5.455956	-1.04513	1.181207	-0.8848	0.376267	0.667874	SLC35F3	148641	solute carrier family 35 member F3
ENSG0000	111.9998	-0.27827	0.314579	-0.88459	0.376377	0.668006	DLG3	1741	discs large MAGUK scaffold protein 3
ENSG0000	1172.53	0.085051	0.096233	0.883808	0.3768	0.668692	PSMA4	5685	proteasome 20S subunit alpha 4
ENSG0000	42.25986	-0.40243	0.455487	-0.88351	0.376963	0.668854	GPT2	84706	glutamic--pyruvic transaminase 2
ENSG0000	16.64858	0.713763	0.807865	0.883518	0.376957	0.668854	CCNP	79935	cyclin P
ENSG0000	52.7281	0.319504	0.361679	0.883391	0.377025	0.668901	RRM2	6241	ribonucleotide reductase regulatory subunit M2
ENSG0000	1498.178	0.085104	0.096361	0.88318	0.377139	0.669038	GET3	439	guided ent ATPase
ENSG0000	290.4915	0.138879	0.157285	0.882973	0.377251	0.669146	PPIEL	728448	peptidylprolyl isomerase E like pseudogene
ENSG0000	34.80504	-0.34948	0.395829	-0.88291	0.377286	0.669146	NA	NA	NA
ENSG0000	8.212834	-0.78046	0.884066	-0.88281	0.37734	0.669146	TMEM144	55314	transmembrane protein 144
ENSG0000	252.2444	-0.38847	0.440039	-0.8828	0.377344	0.669146	IDO1	3620	indoleami 3-dioxygenase 1
ENSG0000	11.40168	0.607814	0.688564	0.882726	0.377385	0.669154	HSP90AB3	3327	heat shock pseudogene
ENSG0000	366.0057	-0.14152	0.16033	-0.88265	0.377424	0.669161	UBTD1	80019	ubiquitin domain containing 1
ENSG0000	8.100287	0.675814	0.765935	0.882339	0.377594	0.669397	NA	NA	NA
ENSG0000	20.3133	0.4812	0.545488	0.882145	0.377698	0.669519	NA	NA	NA
ENSG0000	9.21601	-0.8135	0.922511	-0.88183	0.377869	0.669734	PIERCE1	138162	piercer of microtubule wall 1
ENSG0000	7.500064	-0.93106	1.055963	-0.88172	0.377928	0.669734	KLHL35	283212	kelch like family member 35
ENSG0000	15.96629	-0.54634	0.619609	-0.88175	0.377911	0.669734	TRAV41	28640	T cell receptor alpha variable 41
ENSG0000	2870.607	-0.07506	0.08517	-0.88131	0.378151	0.670001	FGFBP2	83888	fibroblast growth factor binding protein 2
ENSG0000	11.59474	-0.71213	0.80798	-0.88137	0.37812	0.670001	TMEM236	653567	transmembrane protein 236
ENSG0000	227.521	0.155414	0.17638	0.881129	0.378248	0.670108	FAM3C	10447	FAM3 metabolism regulating signaling molecule C
ENSG0000	6.168713	0.814847	0.924873	0.881037	0.378298	0.670133	NA	NA	NA
ENSG0000	1203.778	0.093754	0.106431	0.880892	0.378376	0.670207	TAB3	257397	TGF-beta activated kinase 1 (MAP3K7) binding protein 3
ENSG0000	13.48071	-0.74001	0.840193	-0.88076	0.37845	0.670275	NA	NA	NA
ENSG0000	17.02658	-0.57894	0.65742	-0.88062	0.378524	0.670296	NA	NA	NA
ENSG0000	1764.848	0.075466	0.085703	0.880545	0.378564	0.670296	IP6K1	9807	inositol hexakisphosphate kinase 1
ENSG0000	147.9752	-0.21424	0.243308	-0.88053	0.378574	0.670296	FAM172A	83989	family with sequence similarity 172 member A
ENSG0000	4.231658	-1.28814	1.463018	-0.88047	0.378607	0.670296	NA	NA	NA
ENSG0000	1808.513	0.0836	0.095024	0.879783	0.378977	0.670657	TPRG1L	127262	tumor protein p63 regulated 1 like
ENSG0000	50.16866	-0.30749	0.349515	-0.87977	0.378984	0.670657	KRT8P12	90133	keratin 8 pseudogene 12
ENSG0000	94.27283	-0.25545	0.290366	-0.87976	0.378992	0.670657	NA	NA	NA
ENSG0000	830.4933	-0.10284	0.116883	-0.87982	0.378954	0.670657	CHD7	55636	chromodomain helicase DNA binding protein 7
ENSG0000	292.4926	-0.13382	0.152075	-0.87999	0.378863	0.670657	PSMB5	5693	proteasome 20S subunit beta 5
ENSG0000	753.6163	0.100967	0.114784	0.879632	0.379059	0.670712	IL15RA	3601	interleukin 15 receptor subunit alpha
ENSG0000	6188.513	0.078679	0.089488	0.879214	0.379285	0.670793	TNFRSF25	8718	TNF receptor superfamily member 25
ENSG0000	1500.27	0.11743	0.133552	0.87928	0.37925	0.670793	TNS3	64759	tensin 3
ENSG0000	1017.536	0.084776	0.096398	0.879441	0.379162	0.670793	VCL	7414	vinculin
ENSG0000	4.122443	2.665821	3.031704	0.879314	0.379231	0.670793	LINC02381	400043	long intergenic non-protein coding RNA 2381
ENSG0000	10.31885	-0.69194	0.786897	-0.87932	0.379227	0.670793	RIBC1	158787	RIB43A domain with coiled-coils 1
ENSG0000	10.7712	-0.632	0.718959	-0.87905	0.379375	0.670888	NA	NA	NA
ENSG0000	283.8102	-0.16911	0.192405	-0.87894	0.379433	0.670926	FOLR3	2352	folate receptor gamma
ENSG0000	60.2147	0.261508	0.297691	0.878456	0.379696	0.671328	NA	NA	NA
ENSG0000	4194.505	0.066935	0.07621	0.878292	0.379785	0.671421	SEC31A	22872	SEC31 hon COPII coat complex component
ENSG0000	10260.98	0.066982	0.076295	0.877944	0.379974	0.671554	RAB1B	81876	RAB1B member RAS oncogene family
ENSG0000	4.163695	1.446081	1.64697	0.878026	0.37993	0.671554	RBFOX3	146713	RNA binding fox-1 homolog 3
ENSG0000	122.43	-0.19763	0.225103	-0.87796	0.379967	0.671554	ZNF181	339318	zinc finger protein 181
ENSG0000	1658.217	-0.09155	0.104287	-0.87789	0.380005	0.671554	XRCC1	7515	X-ray repair cross complementing 1
ENSG0000	2946.657	-0.07311	0.083295	-0.87769	0.380112	0.671589	INTS11	54973	integrator complex subunit 11
ENSG0000	8.690058	-0.72464	0.825715	-0.87759	0.380165	0.671589	NA	NA	NA
ENSG0000	2.536269	1.284057	1.462965	0.877708	0.380102	0.671589	NA	NA	NA
ENSG0000	1210.526	0.098343	0.112061	0.877583	0.38017	0.671589	PDZD4	57595	PDZ domain containing 4

ENSG0000	174.4656	-0.19255	0.219515	-0.87718	0.380387	0.671615	TRMT13	54482	tRNA methyltransferase 13 homolog
ENSG0000	6.695079	0.787042	0.897354	0.87707	0.380449	0.671615	SLC26A1	10861	solute carrier family 26 member 1
ENSG0000	4.366657	1.047786	1.194494	0.87718	0.380389	0.671615	LINC0261!	1.01E+08	long intergenic non-protein coding RNA 2615
ENSG0000	75.46917	0.279996	0.319268	0.876991	0.380491	0.671615	PUS7	54517	pseudouridine synthase 7
ENSG0000	20.60796	0.522745	0.59609	0.876956	0.38051	0.671615	NA	NA	NA
ENSG0000	16.57038	-0.63928	0.728919	-0.87702	0.380475	0.671615	LINC0054C	1.01E+08	long intergenic non-protein coding RNA 540
ENSG0000	21.61528	-0.52571	0.599198	-0.87736	0.380293	0.671615	RNF213-A!	1E+08	RNF213 antisense RNA 1
ENSG0000	2.945737	-1.25053	1.425255	-0.87741	0.380263	0.671615	NA	NA	NA
ENSG0000	28.30546	0.397624	0.453143	0.87748	0.380226	0.671615	NKPD1	284353	NTPase KAP family P-loop domain containing 1
ENSG0000	13551.73	-0.069	0.078692	-0.8768	0.380597	0.671703	NUMA1	4926	nuclear mitotic apparatus protein 1
ENSG0000	471.0771	-0.13467	0.153648	-0.87648	0.380768	0.671941	PDK3	5165	pyruvate dehydrogenase kinase 3
ENSG0000	2.35998	1.623488	1.852637	0.876312	0.38086	0.67204	NA	NA	NA
ENSG0000	17.38338	0.55001	0.627845	0.876028	0.381015	0.672249	H2AC20	8338	H2A clustered histone 20
ENSG0000	2621.451	-0.07214	0.08236	-0.87587	0.381098	0.672286	NCOA1	8648	nuclear receptor coactivator 1
ENSG0000	14.13058	0.541761	0.61855	0.875856	0.381108	0.672286	PSMC1P1	151645	proteasom ATPase 1 pseudogene 1
ENSG0000	88.1823	0.242354	0.276805	0.875539	0.381281	0.672498	NLN	57486	neurolysin
ENSG0000	36.54735	0.359271	0.410372	0.875475	0.381316	0.672498	LYPD2	137797	LY6/PLAUR domain containing 2
ENSG0000	250.4454	0.149826	0.171145	0.875435	0.381337	0.672498	NA	NA	NA
ENSG0000	263.3448	0.153155	0.174973	0.875307	0.381407	0.672557	RORC	6097	RAR related orphan receptor C
ENSG0000	7.361535	-0.92838	1.061049	-0.87496	0.381595	0.67271	RBPMS	11030	RNA bindi mRNA processing factor
ENSG0000	482.8184	-0.10648	0.121707	-0.87488	0.381639	0.67271	NAA40	79829	N-alpha-ac NatD catalytic subunit
ENSG0000	174.4235	-0.19538	0.223319	-0.87488	0.381638	0.67271	TESC	54997	tescalcin
ENSG0000	655.4528	0.113494	0.129706	0.875014	0.381566	0.67271	PPM1D	8493	protein ph Mg2+/Mn2+ dependent 1D
ENSG0000	1214.731	0.089424	0.10223	0.874735	0.381718	0.672786	DCAF12	25853	DDB1 and CUL4 associated factor 12
ENSG0000	899.99	0.08863	0.101384	0.8742	0.382009	0.672979	MRPL37	51253	mitochondrial ribosomal protein L37
ENSG0000	3759.096	-0.0637	0.07286	-0.87434	0.381933	0.672979	NCF2	4688	neutrophil cytosolic factor 2
ENSG0000	1869.533	-0.0897	0.102633	-0.874	0.382117	0.672979	CNNM3	26505	cyclin and CBS domain divalent metal cation transport mediator 3
ENSG0000	118.6319	-0.18799	0.21508	-0.87406	0.382086	0.672979	ZNF197	10168	zinc finger protein 197
ENSG0000	6.523958	1.105907	1.264769	0.874395	0.381903	0.672979	NA	NA	NA
ENSG0000	708.7034	0.111534	0.127623	0.873934	0.382154	0.672979	HDC	3067	histidine decarboxylase
ENSG0000	739.553	0.103325	0.118228	0.873943	0.382149	0.672979	GABARAPI	11345	GABA type A receptor associated protein like 2
ENSG0000	232.4411	-0.13856	0.158505	-0.8742	0.382011	0.672979	COX11	1353	cytochrome c oxidase copper chaperone COX11
ENSG0000	51.37554	0.325946	0.372879	0.874135	0.382045	0.672979	NA	NA	NA
ENSG0000	13.81425	0.578571	0.662172	0.873747	0.382256	0.67303	PCAT6	1.01E+08	prostate cancer associated transcript 6
ENSG0000	3217.422	-0.08142	0.093183	-0.8738	0.382229	0.67303	BCKDK	10295	branched chain keto acid dehydrogenase kinase
ENSG0000	34.48425	0.354078	0.405306	0.873608	0.382332	0.673036	NA	NA	NA
ENSG0000	23.52005	-0.43502	0.497943	-0.87364	0.382315	0.673036	C5orf63	401207	chromosome 5 open reading frame 63
ENSG0000	9.466601	-0.78703	0.901064	-0.87344	0.382423	0.673133	SH3TC2	79628	SH3 domain and tetratricopeptide repeats 2
ENSG0000	178.4601	-0.18349	0.21017	-0.87305	0.382636	0.673402	USP46	64854	ubiquitin specific peptidase 46
ENSG0000	296.1487	0.156033	0.178726	0.873026	0.382649	0.673402	PGLYRP1	8993	peptidoglycan recognition protein 1
ENSG0000	19.29496	-0.47065	0.539359	-0.87261	0.382877	0.67374	NA	NA	NA
ENSG0000	25.17057	0.473564	0.542791	0.872462	0.382956	0.673801	C1R	715	complement C1r
ENSG0000	19.33452	-0.52083	0.597003	-0.87241	0.382984	0.673801	EFHC2	80258	EF-hand domain containing 2
ENSG0000	44.21189	-0.33465	0.383635	-0.8723	0.383042	0.673839	CYP2D8P	1568	ccytochror pseudogene
ENSG0000	734.2314	-0.10867	0.124604	-0.87212	0.383144	0.673891	SSB	6741	small RNA binding exonuclease protection factor La
ENSG0000	8.39139	-0.78967	0.905468	-0.87212	0.383144	0.673891	NA	NA	NA
ENSG0000	51.28129	-0.39884	0.457446	-0.87189	0.383268	0.673944	DHFR	1719	dihydrofolate reductase
ENSG0000	2806.163	0.071173	0.081633	0.871863	0.383283	0.673944	CASP4	837	caspase 4
ENSG0000	180.5327	-0.17979	0.20621	-0.87187	0.383282	0.673944	LINC0066!	1.01E+08	long intergenic non-protein coding RNA 665
ENSG0000	32.0519	0.433493	0.497327	0.871646	0.383402	0.674088	IGLON5	402665	IgLON family member 5
ENSG0000	6.873825	-1.0324	1.184844	-0.87134	0.383571	0.674258	NA	NA	NA
ENSG0000	140.9542	0.215919	0.247793	0.871368	0.383553	0.674258	BEX2	84707	brain expressed X-linked 2
ENSG0000	591.4658	-0.11088	0.127282	-0.87113	0.383683	0.674391	ZNF827	152485	zinc finger protein 827
ENSG0000	12.18749	0.618628	0.710213	0.871046	0.383729	0.674407	NA	NA	NA
ENSG0000	36078.17	-0.06623	0.076047	-0.87084	0.383842	0.674542	FXYD5	53827	FXYD domain containing ion transport regulator 5
ENSG0000	15.17608	0.562331	0.64582	0.870723	0.383905	0.674589	NA	NA	NA
ENSG0000	856.0007	0.1037	0.119133	0.870458	0.38405	0.67478	DVL2	1856	dishevelled segment polarity protein 2
ENSG0000	555.2001	0.113187	0.130074	0.870171	0.384207	0.674992	CCDC157	550631	coiled-coil domain containing 157
ENSG0000	13.16183	-0.60813	0.699069	-0.86991	0.384349	0.675177	LINC01624	401289	long intergenic non-protein coding RNA 1624
ENSG0000	131.0549	0.200722	0.230813	0.869632	0.384501	0.675244	RDH14	57665	retinol dehydrogenase 14
ENSG0000	49.84176	0.329107	0.378449	0.86962	0.384508	0.675244	LINC00402	1.01E+08	long intergenic non-protein coding RNA 402
ENSG0000	6.19703	0.859019	0.987668	0.869745	0.38444	0.675244	MIR6774	1.02E+08	microRNA 6774
ENSG0000	129.8675	-0.1902	0.218725	-0.86958	0.384533	0.675244	BTBD3	22903	BTB domain containing 3
ENSG0000	1366.174	-0.08273	0.095163	-0.86936	0.384649	0.675385	COX5B	1329	cytochrome c oxidase subunit 5B
ENSG0000	32.82216	-0.35432	0.407657	-0.86916	0.38476	0.675388	ARMC12	221481	armadillo repeat containing 12
ENSG0000	870.2369	-0.09093	0.104615	-0.86921	0.384731	0.675388	KCTD21	283219	potassium channel tetramerization domain containing 21
ENSG0000	190.2468	0.163253	0.187803	0.869277	0.384696	0.675388	PDZD11	51248	PDZ domain containing 11
ENSG0000	11.4323	-0.65458	0.753326	-0.86893	0.384887	0.675547	LINC00987	1E+08	long intergenic non-protein coding RNA 987
ENSG0000	19.77099	0.626738	0.72155	0.868598	0.385067	0.675551	NA	NA	NA
ENSG0000	14.65238	-0.56451	0.649803	-0.86874	0.384987	0.675551	KIF15	56992	kinesin family member 15
ENSG0000	55.29696	0.350052	0.403043	0.868523	0.385108	0.675551	TRIQK	286144	triple QxxK/R motif containing
ENSG0000	185.0411	-0.1595	0.18358	-0.86881	0.38495	0.675551	NET1	10276	neuroepithelial cell transforming 1
ENSG0000	9717.959	-0.06364	0.073265	-0.86862	0.385057	0.675551	TSPAN14	81619	tetraspanin 14
ENSG0000	7922.071	-0.0806	0.092794	-0.86857	0.385083	0.675551	ARRDC2	27106	arrestin domain containing 2
ENSG0000	646.6924	0.135812	0.156402	0.868349	0.385203	0.675654	UBA6	55236	ubiquitin like modifier activating enzyme 6
ENSG0000	4.360102	-1.23088	1.417719	-0.86821	0.38528	0.675724	DEPDC1	55635	DEP domain containing 1
ENSG0000	464.4459	-0.13134	0.151335	-0.86787	0.385466	0.675944	NA	NA	NA
ENSG0000	374.9155	0.118731	0.136837	0.867683	0.385568	0.675944	RNF122	79845	ring finger protein 122
ENSG0000	10.58824	0.752002	0.866629	0.867732	0.385541	0.675944	NA	NA	NA
ENSG0000	1432.088	-0.0762	0.087828	-0.86765	0.385587	0.675944	CDK5R1	8851	cyclin dependent kinase 5 regulatory subunit 1
ENSG0000	82.98885	-0.26622	0.306794	-0.86775	0.385532	0.675944	CLIP3	25999	CAP-Gly domain containing linker protein 3
ENSG0000	295.0703	-0.12747	0.146957	-0.86741	0.385718	0.675987	MYCBP	26292	MYC binding protein
ENSG0000	195.966	0.160355	0.18486	0.867441	0.3857	0.675987	PPM1B	5495	protein ph Mg2+/Mn2+ dependent 1B
ENSG0000	13.41238	0.616711	0.710985	0.867403	0.385721	0.675987	ST20	400410	suppressor of tumorigenicity 20
ENSG0000	1175.271	-0.08265	0.095297	-0.86729	0.385783	0.676031	KCTD2	23510	potassium channel tetramerization domain containing 2
ENSG0000	9.888486	-0.8085	0.933	-0.86655	0.386186	0.676674	NA	NA	NA
ENSG0000	248.3422	-0.16886	0.194907	-0.86635	0.386299	0.676807	MED9	55090	mediator complex subunit 9
ENSG0000	89.92258	-0.22208	0.256442	-0.86602	0.38648	0.677061	CDKN1C	1028	cyclin dependent kinase inhibitor 1C
ENSG0000	96.25942	-0.21504	0.248413	-0.86566	0.386678	0.67728	AP1S1	1174	adaptor related protein complex 1 subunit sigma 1
ENSG0000	6.111291	0.811719	0.937654	0.865691	0.386659	0.67728	NA	NA	NA
ENSG0000	53.83611	-0.31045	0.358697	-0.8655	0.386766	0.677371	PLD1	5337	phospholipase D1
ENSG0000	33.26102	-0.40974	0.473603	-0.86515	0.386958	0.677642	TMEM67	91147	transmembrane protein 67
ENSG0000	96.29779	-0.2237	0.258596	-0.86507	0.387	0.677651	GMDS	2762	GDP-manr 6-dehydratase
ENSG0000	4.525435	-1.09417	1.265123	-0.86487	0.387111	0.677718	TAS2R20	259295	taste 2 receptor member 20
ENSG0000	134.3624	-0.20443	0.236368	-0.86489	0.387098	0.677718	HOXC4	3221	homeobox C4
ENSG0000	1116.61	0.085788	0.09933	0.863665	0.387772	0.677724	ATAD3B	83858	ATPase family AAA domain containing 3B
ENSG0000	20.58108	0.561599	0.650107	0.863856	0.387667	0.677724	NA	NA	NA
ENSG0000	1568.015	0.083707	0.096848	0.864311	0.387417	0.677724	DCAF8	50717	DDB1 and CUL4 associated factor 8
ENSG0000	181.7435	-0.17475	0.20228	-0.86391	0.387638	0.677724	NA	NA	NA
ENSG0000	5.229485	0.833135	0.963987	0.864259	0.387446	0.677724	NA	NA	NA

ENSG0000	3.196074	1.050829	1.216647	0.863709	0.387748	0.677724	LOC10028	1E+08	ribosomal protein S5 pseudogene
ENSG0000	456.421	-0.1134	0.131197	-0.86438	0.387377	0.677724	LSM8	51691	LSM8 hom U6 small nuclear RNA associated
ENSG0000	631.3924	-0.10964	0.126949	-0.86367	0.387769	0.677724	TNKS	8658	tankyrase
ENSG0000	6.646598	0.965404	1.117462	0.863925	0.387629	0.677724	LINC03022	1.02E+08	long intergenic non-protein coding RNA 3022
ENSG0000	396.6045	0.136478	0.157921	0.864221	0.387466	0.677724	NA	NA	NA
ENSG0000	7.001686	-0.7708	0.892237	-0.86389	0.387648	0.677724	NA	NA	NA
ENSG0000	1616.144	0.081269	0.09409	0.86374	0.387731	0.677724	VPS37C	55048	VPS37C subunit of ESCRT-I
ENSG0000	58.02307	-0.28806	0.33314	-0.86469	0.387207	0.677724	TMEM116	89894	transmembrane protein 116
ENSG0000	24.49697	0.437435	0.506312	0.863962	0.387609	0.677724	NA	NA	NA
ENSG0000	346.4199	-0.11932	0.138027	-0.8645	0.387313	0.677724	COIL	8161	coilin
ENSG0000	14.05692	-0.59117	0.68396	-0.86434	0.387403	0.677724	LCDR	1E+08	lysosome cell death regulator
ENSG0000	461.4695	0.111685	0.129227	0.864256	0.387448	0.677724	CD40	958	CD40 molecule
ENSG0000	13.59492	-0.6134	0.710284	-0.8636	0.387809	0.677724	ANKRD20A	391267	ankyrin re pseudogene
ENSG0000	140.3602	0.182123	0.210656	0.864552	0.387284	0.677724	AMMECR1	9949	AMMECR nuclear protein 1
ENSG0000	939.4265	0.103933	0.120383	0.863348	0.387946	0.677901	GPR137B	7107	G protein-coupled receptor 137B
ENSG0000	104.7648	-0.21619	0.250504	-0.86303	0.388121	0.678143	SLC12A2-D	644873	SLC12A2 divergent transcript
ENSG0000	904.6838	-0.09052	0.104893	-0.86293	0.388175	0.678172	EMC1	23065	ER membrane protein complex subunit 1
ENSG0000	2190.144	0.084511	0.097953	0.862771	0.388263	0.678263	ADGRG3	222487	adhesion G protein-coupled receptor G3
ENSG0000	132.1877	-0.18253	0.211579	-0.8627	0.388305	0.678272	ZNF780A	284323	zinc finger protein 780A
ENSG0000	303.2399	-0.12548	0.145544	-0.86215	0.388608	0.678737	MRPL45	84311	mitochondrial ribosomal protein L45
ENSG0000	4.68044	0.9855	1.143353	0.861938	0.388722	0.678872	NA	NA	NA
ENSG0000	494.7638	-0.13397	0.155472	-0.8617	0.388855	0.678983	CLIP4	79745	CAP-Gly domain containing linker protein family member 4
ENSG0000	682.9244	0.099078	0.114993	0.861601	0.388907	0.678983	RDH11	51109	retinol dehydrogenase 11
ENSG0000	78.01015	0.288583	0.334955	0.861556	0.388932	0.678983	SMIM5	643008	small integral membrane protein 5
ENSG0000	260.3261	-0.13606	0.157907	-0.86162	0.388899	0.678983	ZNF766	90321	zinc finger protein 766
ENSG0000	592.0477	-0.11521	0.133779	-0.86122	0.389118	0.679245	GSN	2934	gelsolin
ENSG0000	134.7801	0.196534	0.228243	0.861073	0.389198	0.67932	CD160	11126	CD160 molecule
ENSG0000	4450.905	0.064002	0.074341	0.860919	0.389283	0.679376	CDV3	55573	CDV3 homolog
ENSG0000	23.36584	0.409566	0.475752	0.860882	0.389303	0.679376	NRG1	3084	neuregulin 1
ENSG0000	2.435415	1.574376	1.829074	0.86075	0.389376	0.679438	MYBPH	4608	myosin binding protein H
ENSG0000	12.84439	0.534008	0.620591	0.860483	0.389523	0.679631	RPS7P11	644315	ribosomal protein S7 pseudogene 11
ENSG0000	427.9051	-0.12076	0.140392	-0.86016	0.389701	0.679878	CRAT	1384	carnitine O-acetyltransferase
ENSG0000	16.71964	0.518391	0.602751	0.860041	0.389766	0.679928	NA	NA	NA
ENSG0000	3.123935	1.591537	1.851508	0.85959	0.390015	0.680113	SPATA9	83890	spermatogenesis associated 9
ENSG0000	7.499606	-0.77269	0.898977	-0.85952	0.390056	0.680113	NA	NA	NA
ENSG0000	8.487959	0.765519	0.8906	0.859553	0.390035	0.680113	MYG1	60314	MYG1 exonuclease
ENSG0000	153.94	0.200903	0.23368	0.859735	0.389935	0.680113	FICD	11153	FIC domain protein adenylyltransferase
ENSG0000	12429.36	0.064136	0.074617	0.859534	0.390046	0.680113	CMIP	80790	c-Maf inducing protein
ENSG0000	43.83977	0.339429	0.394946	0.859433	0.390102	0.680129	KCNE1	3753	potassium voltage-gated channel subfamily E regulatory subunit 1
ENSG0000	39.19971	-0.40375	0.470024	-0.859	0.39034	0.680152	EFCAB7	84455	EF-hand calcium binding domain 7
ENSG0000	1618.015	0.079558	0.092664	0.858563	0.390582	0.680152	PTPN22	26191	protein tyrosine phosphatase non-receptor type 22
ENSG0000	45.11826	0.294169	0.342551	0.858759	0.390473	0.680152	NA	NA	NA
ENSG0000	231.0357	0.169933	0.19786	0.858851	0.390423	0.680152	CHI3L1	1116	chitinase 3 like 1
ENSG0000	164.9303	-0.16887	0.196648	-0.85872	0.390495	0.680152	B3GALNT2	148789	beta-1 3-N-acetylgalactosaminyltransferase 2
ENSG0000	15.11723	-0.78872	0.918485	-0.85871	0.390499	0.680152	LINC01891	1.05E+08	long intergenic non-protein coding RNA 1891
ENSG0000	2884.615	-0.06794	0.079099	-0.85895	0.39037	0.680152	RUBCN	9711	rubicon autophagy regulator
ENSG0000	6.572397	0.890946	1.036986	0.859169	0.390247	0.680152	NA	NA	NA
ENSG0000	28.39677	-0.49926	0.58154	-0.85852	0.390606	0.680152	DSP	1832	desmoplakin
ENSG0000	7.251146	-0.72493	0.843716	-0.85922	0.390221	0.680152	NA	NA	NA
ENSG0000	22.46353	0.522277	0.608344	0.858521	0.390605	0.680152	ADSS1	122622	adenylosuccinate synthase 1
ENSG0000	3.573483	-1.24643	1.451845	-0.85851	0.39061	0.680152	NA	NA	NA
ENSG0000	318.7931	0.130029	0.151465	0.858479	0.390628	0.680152	TMEM160	54958	transmembrane protein 160
ENSG0000	298.355	0.159128	0.185315	0.858692	0.390511	0.680152	RRP7BP	91695	ribosomal pseudogene
ENSG0000	51.36655	-0.29484	0.343571	-0.85816	0.390806	0.680269	ANKRD36C	400986	ankyrin repeat domain 36C
ENSG0000	71.11395	0.251005	0.292505	0.858124	0.390824	0.680269	ANKHD1	54882	ankyrin repeat and KH domain containing 1
ENSG0000	1927.043	0.083963	0.097841	0.858164	0.390802	0.680269	MARCHF9	92979	membrane associated ring-CH-type finger 9
ENSG0000	1285.757	-0.08966	0.104489	-0.85809	0.390842	0.680269	BCL2	596	BCL2 apoptosis regulator
ENSG0000	637.524	-0.10052	0.117152	-0.85801	0.390886	0.680282	MRPS15	64960	mitochondrial ribosomal protein S15
ENSG0000	15.64522	0.547506	0.638215	0.857871	0.390964	0.680289	GIHCG	1.01E+08	GIHCG inhibitor of miR-200b/200a/429 expression
ENSG0000	27.78176	-0.47605	0.554886	-0.85793	0.39093	0.680289	KLHDC1	122773	kelch domain containing 1
ENSG0000	19.00127	0.583145	0.679844	0.857763	0.391023	0.680329	NA	NA	NA
ENSG0000	4533.74	0.073861	0.086116	0.857691	0.391063	0.680335	PPIB	5479	peptidylprolyl isomerase B
ENSG0000	216.0567	-0.15839	0.184727	-0.85741	0.391218	0.680349	NDUFC1	4717	NADH:ubiquinone oxidoreductase subunit C1
ENSG0000	379.3795	0.127005	0.148119	0.857454	0.391194	0.680349	CHMP5	51510	charged multivesicular body protein 5
ENSG0000	3139.203	-0.10846	0.126495	-0.85744	0.391202	0.680349	IRF8	3394	interferon regulatory factor 8
ENSG0000	2.578481	-1.48436	1.730925	-0.85755	0.391139	0.680349	NA	NA	NA
ENSG0000	189.6863	0.152115	0.17762	0.856406	0.391773	0.681176	CAAP1	79886	caspase activity and apoptosis inhibitor 1
ENSG0000	646.4528	-0.09515	0.11112	-0.85627	0.39185	0.681176	SPG21	51324	SPG21 abh maspardin
ENSG0000	16.72176	-0.5165	0.603225	-0.85623	0.39187	0.681176	NA	NA	NA
ENSG0000	14.23292	-0.71266	0.832174	-0.85638	0.391786	0.681176	ITGB4	3691	integrin subunit beta 4
ENSG0000	34.32898	0.497138	0.580621	0.856218	0.391877	0.681176	CNFN	84518	cornifelin
ENSG0000	59.40708	0.255936	0.299029	0.855891	0.392058	0.681363	NDUFB6	4712	NADH:ubiquinone oxidoreductase subunit B6
ENSG0000	45.50354	-0.364	0.425278	-0.85591	0.392045	0.681363	ARL17A	51326	ADP ribosylation factor like GTPase 17A
ENSG0000	2.065664	-1.7585	2.055243	-0.85562	0.39221	0.681564	NA	NA	NA
ENSG0000	9427.617	0.071698	0.083825	0.855333	0.392367	0.68173	JUN	3725	Jun proto- AP-1 transcription factor subunit
ENSG0000	2.745296	-1.42868	1.670368	-0.85531	0.39238	0.68173	TMEM45A	55076	transmembrane protein 45A
ENSG0000	88.5757	0.244193	0.285621	0.854954	0.392577	0.681944	STAP1	26228	signal transducing adaptor family member 1
ENSG0000	3.176821	1.398197	1.635358	0.854979	0.392563	0.681944	NA	NA	NA
ENSG0000	523.638	0.12983	0.151896	0.854725	0.392703	0.6821	MLC1	23209	modulator of VRAC current 1
ENSG0000	43.95566	-0.30188	0.353254	-0.85458	0.392786	0.68218	TMEM202	1.05E+08	TMEM202 antisense RNA 1
ENSG0000	121.2867	-0.18869	0.220838	-0.85443	0.392865	0.682254	ORC5	5001	origin recognition complex subunit 5
ENSG0000	8.348328	0.753073	0.88148	0.854327	0.392924	0.682292	SLC22A20P	440044	solute carr pseudogene
ENSG0000	30.57892	-0.39572	0.463404	-0.85395	0.393135	0.682554	CENPQ	55166	centromere protein Q
ENSG0000	143.4762	0.174508	0.20436	0.853921	0.393149	0.682554	TVP23B	51030	trans-golgi network vesicle protein 23 homolog B
ENSG0000	213.2701	-0.15317	0.179396	-0.8538	0.393215	0.682606	RALGPS2	55103	Ral GEF with PH domain and SH3 binding motif 2
ENSG0000	3694.138	0.072039	0.084386	0.853674	0.393285	0.682664	ARGLU1	55082	arginine and glutamate rich 1
ENSG0000	2.864402	-1.60894	1.884887	-0.8536	0.393327	0.682673	RN7SL535P	1.06E+08	RNA 7SL cytoplasm pseudogene
ENSG0000	157.5392	-0.21068	0.24693	-0.85318	0.393559	0.683012	FMN1	342184	formin 1
ENSG0000	1252.237	0.077963	0.091415	0.852851	0.393742	0.683201	CCNY	219771	cyclin Y
ENSG0000	62.14432	0.264378	0.309982	0.85288	0.393726	0.683201	NA	NA	NA
ENSG0000	61.55262	0.275613	0.323367	0.852324	0.394034	0.683324	ERMN	57471	ermin
ENSG0000	25.83955	0.432438	0.507294	0.852442	0.393969	0.683324	CEP70	80321	centrosomal protein 70
ENSG0000	482.1142	-0.12198	0.143105	-0.85238	0.394003	0.683324	KLHL2	11275	kelch like family member 2
ENSG0000	62.19971	0.270542	0.317369	0.852453	0.393963	0.683324	ABCB9	23457	ATP binding cassette subfamily B member 9
ENSG0000	22.4483	0.405227	0.475299	0.852571	0.393897	0.683324	RPL26P4	283603	ribosomal protein L26 pseudogene 4
ENSG0000	523.2583	0.101408	0.118949	0.852534	0.393918	0.683324	TSR1	55720	TSR1 ribosome maturation factor
ENSG0000	13.38146	0.703898	0.826237	0.851933	0.394251	0.683394	RPS2P14	1E+08	ribosomal protein S2 pseudogene 14
ENSG0000	844.6028	-0.09536	0.111953	-0.85181	0.394317	0.683394	ZNF496	84838	zinc finger protein 496
ENSG0000	433.0113	-0.11429	0.134186	-0.85172	0.394369	0.683394	MROH6	642475	maestro heat like repeat family member 6
ENSG0000	9.448841	-0.73703	0.865156	-0.8519	0.39427	0.683394	IGHV3-53	28420	immunoglobulin heavy variable 3-53

ENSG0000	4.646137	-0.98835	1.159899	-0.8521	0.39416	0.683394	CATSPER2	440278	cation channel sperm associated 2 pseudogene 1
ENSG0000	452.5251	0.107445	0.126145	0.851757	0.394349	0.683394	GLOD4	51031	glyoxalase domain containing 4
ENSG0000	616.5064	-0.10456	0.122745	-0.85185	0.394296	0.683394	CD226	10666	CD226 molecule
ENSG0000	2.363476	-1.62203	1.903635	-0.85207	0.394176	0.683394	LINC0190!	1.02E+08	long intergenic non-protein coding RNA 1909
ENSG0000	159.4889	0.178139	0.209212	0.851477	0.394504	0.683469	SLC25A16	8034	solute carrier family 25 member 16
ENSG0000	1539.759	0.077534	0.091058	0.851483	0.394501	0.683469	OSBP	5007	oxysterol binding protein
ENSG0000	10.65397	-0.78575	0.922848	-0.85144	0.394523	0.683469	NA	NA	NA
ENSG0000	1466.384	-0.10351	0.121594	-0.85131	0.394596	0.683531	GAPT	202309	GRB2 bind transmembrane
ENSG0000	391.283	0.119425	0.140295	0.851243	0.394635	0.683534	LSM4	25804	LSM4 hom U6 small nuclear RNA and mRNA degradation associated
ENSG0000	9.843646	0.667616	0.784441	0.851072	0.394729	0.683635	NA	NA	NA
ENSG0000	20.16624	-0.50451	0.592895	-0.85092	0.394813	0.683672	NA	NA	NA
ENSG0000	374.3576	0.122736	0.144252	0.850846	0.394855	0.683672	CAMK2D	817	calcium/calmodulin dependent protein kinase II delta
ENSG0000	78.40331	0.292951	0.34431	0.850834	0.394861	0.683672	LIN7A	8825	lin-7 homc crumbs cell polarity complex component
ENSG0000	949.7538	-0.08621	0.101343	-0.85072	0.394924	0.683717	FOXO3	2309	forkhead box O3
ENSG0000	448.1042	0.17376	0.204289	0.850562	0.395013	0.683806	JAG1	182	jagged canonical Notch ligand 1
ENSG0000	2244.324	0.079711	0.093739	0.850347	0.395132	0.683949	CTDP1	9150	CTD phosphatase subunit 1
ENSG0000	9.658768	-0.75028	0.882614	-0.85007	0.395287	0.684154	NA	NA	NA
ENSG0000	4.018656	1.410506	1.659806	0.849802	0.395435	0.684346	FUT3	2525	fucosyltransferase 3 (Lewis blood group)
ENSG0000	46.57154	-0.32548	0.383063	-0.84967	0.395507	0.684407	NA	NA	NA
ENSG0000	13.10622	0.609595	0.717638	0.849445	0.395634	0.684558	NA	NA	NA
ENSG0000	30.13988	0.362521	0.426839	0.849317	0.395705	0.684558	TMSB4XP4	7118	TMSB4X pseudogene 4
ENSG0000	126.0591	-0.18351	0.216051	-0.84938	0.39567	0.684558	STRADA	92335	STE20 related adaptor alpha
ENSG0000	28.1337	0.386576	0.455296	0.849066	0.395844	0.684735	GNRH1	2796	gonadotropin releasing hormone 1
ENSG0000	110.8523	-0.20478	0.241231	-0.8489	0.395938	0.684773	ZKSCAN2	342357	zinc finger with KRAB and SCAN domains 2
ENSG0000	2.507618	-1.42861	1.682912	-0.84889	0.39594	0.684773	FGD1	2245	FYVE RhoGEF and PH domain containing 1
ENSG0000	1661.165	0.093953	0.1107	0.848719	0.396038	0.684877	PRKAA1	5562	protein kinase AMP-activated catalytic subunit alpha 1
ENSG0000	171.7801	0.171237	0.201819	0.848471	0.396176	0.684877	CDK7	1022	cyclin dependent kinase 7
ENSG0000	78.94158	-0.24308	0.286525	-0.84836	0.396236	0.684877	ZBTB3	79842	zinc finger and BTB domain containing 3
ENSG0000	9.599153	0.756959	0.892441	0.848189	0.396333	0.684877	ACTN1-DT	161159	ACTN1 divergent transcript
ENSG0000	16.44298	0.518448	0.611005	0.848517	0.39615	0.684877	ITGAD	3681	integrin subunit alpha D
ENSG0000	179.609	0.220476	0.259923	0.848234	0.396307	0.684877	ALOX12	239	arachidon: 12S type
ENSG0000	12.32563	0.573806	0.676221	0.848549	0.396133	0.684877	NA	NA	NA
ENSG0000	3.94877	1.18805	1.400373	0.848381	0.396226	0.684877	RANBP3-D	1E+08	RANBP3 divergent transcript
ENSG0000	8.066738	-0.79299	0.934894	-0.84821	0.396319	0.684877	NA	NA	NA
ENSG0000	21.05497	-0.43521	0.513286	-0.84789	0.3965	0.685102	ZNF594-DT	1E+08	ZNF594 divergent transcript
ENSG0000	852.6861	-0.11882	0.1402	-0.84751	0.396709	0.685273	ZNF292	23036	zinc finger protein 292
ENSG0000	27.03115	0.381113	0.449674	0.84753	0.3967	0.685273	NACAD	23148	NAC alpha domain containing
ENSG0000	7.257884	-0.75471	0.890502	-0.84751	0.39671	0.685273	NA	NA	NA
ENSG0000	68.99844	-0.25651	0.302843	-0.84702	0.396984	0.685537	MICOS10	440574	mitochondrial contact site and cristae organizing system subunit 10
ENSG0000	1354.425	0.088523	0.104513	0.847007	0.396991	0.685537	LUZP1	7798	leucine zipper protein 1
ENSG0000	239.4015	0.145395	0.171671	0.846938	0.397029	0.685537	ZNF703	80139	zinc finger protein 703
ENSG0000	6.198744	-0.97063	1.145728	-0.84717	0.3969	0.685537	NA	NA	NA
ENSG0000	54.41856	-0.27478	0.324446	-0.84691	0.397047	0.685537	SCAMP5	192683	secretory carrier membrane protein 5
ENSG0000	696.086	-0.09138	0.107947	-0.84651	0.397269	0.685747	MAPK14	1432	mitogen-activated protein kinase 14
ENSG0000	18.38436	0.455851	0.538531	0.846472	0.39729	0.685747	SUPT16HP	400011	SPT16 hon facilitates chromatin remodeling subunit pseudogene 1
ENSG0000	90.07091	-0.21907	0.258825	-0.84642	0.397321	0.685747	TMTC4	84899	transmembrane O-mannosyltransferase targeting cadherins 4
ENSG0000	64.46723	-0.27134	0.320514	-0.84657	0.397233	0.685747	NDRG2	57447	NDRG family member 2
ENSG0000	6.968778	-0.84448	0.997782	-0.84636	0.397354	0.685747	ZNF433	163059	zinc finger protein 433
ENSG0000	5.945259	0.95805	1.13213	0.846237	0.397421	0.685798	SMG1P6	1E+08	SMG1 pseudogene 6
ENSG0000	86.69184	-0.22269	0.263234	-0.84598	0.397562	0.685859	ITGA1	3672	integrin subunit alpha 1
ENSG0000	3031.647	0.066238	0.078298	0.845975	0.397567	0.685859	USP22	23326	ubiquitin specific peptidase 22
ENSG0000	3651.224	0.06717	0.079394	0.846035	0.397533	0.685859	KANSL1	284058	KAT8 regulatory NSL complex subunit 1
ENSG0000	187.1641	-0.15739	0.186128	-0.84558	0.397786	0.685897	TBPL1	9519	TATA-box binding protein like 1
ENSG0000	11.12315	-0.72653	0.859233	-0.84556	0.397799	0.685897	AAMDC	28971	adipogenesis associated Mth938 domain containing
ENSG0000	159.9446	0.18582	0.219755	0.845575	0.39779	0.685897	COX14	84987	cytochrome c oxidase assembly factor COX14
ENSG0000	286.9401	-0.1296	0.153265	-0.84562	0.397764	0.685897	PALB2	79728	partner and localizer of BRCA2
ENSG0000	20.57606	0.580261	0.686203	0.845611	0.39777	0.685897	TLCD1	116238	TLC domain containing 1
ENSG0000	820.3778	0.109983	0.130075	0.845538	0.397811	0.685897	RPS19BP1	91582	ribosomal protein S19 binding protein 1
ENSG0000	34.73401	0.329989	0.390383	0.845297	0.397945	0.686065	H2BC11	8970	H2B clustered histone 11
ENSG0000	113.7063	0.194184	0.229797	0.845024	0.398097	0.686264	NA	NA	NA
ENSG0000	1380.397	-0.08389	0.099337	-0.84454	0.398369	0.686546	ATG3	64422	autophagy related 3
ENSG0000	1382.053	-0.08691	0.102921	-0.84445	0.398415	0.686546	VEGFB	7423	vascular endothelial growth factor B
ENSG0000	1536.612	-0.09059	0.107269	-0.84452	0.398376	0.686546	GDPD5	81544	glycerophosphodiester phosphodiesterase domain containing 5
ENSG0000	3553.761	0.089445	0.105933	0.844358	0.39847	0.686546	KLRD1	3824	killer cell lectin like receptor D1
ENSG0000	853.494	-0.08423	0.099731	-0.84455	0.398361	0.686546	ATG2B	55102	autophagy related 2B
ENSG0000	6.384241	-0.85263	1.009825	-0.84433	0.398483	0.686546	ZNF229	7772	zinc finger protein 229
ENSG0000	127.5972	0.19925	0.236097	0.843931	0.398708	0.686615	MRPL33	9553	mitochondrial ribosomal protein L33
ENSG0000	5.679944	-0.99507	1.178913	-0.84405	0.398639	0.686615	NA	NA	NA
ENSG0000	2147.852	0.069948	0.082875	0.844016	0.398661	0.686615	SRP72	6731	signal recognition particle 72
ENSG0000	88.27007	-0.24311	0.288055	-0.84396	0.398694	0.686615	BEND3	57673	BEN domain containing 3
ENSG0000	3.753103	1.370833	1.623834	0.844195	0.39856	0.686615	NA	NA	NA
ENSG0000	78.86703	-0.26173	0.310192	-0.84375	0.398807	0.686658	PLAG1	5324	PLAG1 zinc finger
ENSG0000	4.093584	1.221955	1.448134	0.843813	0.398774	0.686658	HS3ST2	9956	heparan sulfate-glucosamine 3-sulfotransferase 2
ENSG0000	143.9199	0.184499	0.218713	0.843568	0.398911	0.686772	JTB	10899	jumping translocation breakpoint
ENSG0000	10592.3	0.060204	0.07139	0.843313	0.399053	0.686954	IQGAP1	8826	IQ motif containing GTPase activating protein 1
ENSG0000	376.163	-0.12671	0.150346	-0.8428	0.399341	0.687386	TTC13	79573	tetratricopeptide repeat domain 13
ENSG0000	270.8512	-0.15253	0.181009	-0.84268	0.399409	0.687439	SEPTIN11	55752	septin 11
ENSG0000	17.4366	-0.49215	0.584078	-0.8426	0.399449	0.687444	PLIN5	440503	perilipin 5
ENSG0000	44.48384	0.301189	0.3576	0.84225	0.399648	0.687467	LOC10537	1.05E+08	uncharacterized LOC105376805
ENSG0000	13.28874	0.637508	0.756877	0.842288	0.399627	0.687467	NA	NA	NA
ENSG0000	236.6618	-0.1371	0.162748	-0.84242	0.399551	0.687467	AGK	55750	acylglycerol kinase
ENSG0000	30.79063	-0.35595	0.422499	-0.84248	0.399522	0.687467	PRR22	163154	proline rich 22
ENSG0000	4.22173	1.219699	1.448073	0.842291	0.399625	0.687467	VN1R83P	1E+08	vomeronasal 1 receptor 83 pseudogene
ENSG0000	82.20495	0.300259	0.356634	0.841925	0.39983	0.687558	SPOCD1	90853	SPOC domain containing 1
ENSG0000	36.34955	0.385867	0.458333	0.841891	0.399849	0.687558	KIF9	64147	kinesin family member 9
ENSG0000	5.339983	-1.0749	1.276679	-0.84195	0.399817	0.687558	UACA	55075	uveal autoantigen with coiled-coil domains and ankyrin repeats
ENSG0000	5.876717	1.012872	1.202979	0.84197	0.399805	0.687558	LINC0197(	1.02E+08	long intergenic non-protein coding RNA 1970
ENSG0000	172.1366	-0.16306	0.19371	-0.84177	0.399918	0.687612	RPIA	22934	ribose 5-phosphate isomerase A
ENSG0000	5.486231	0.905547	1.076168	0.841456	0.400093	0.687773	TWF2-DT	1.02E+08	TWF2 divergent transcript
ENSG0000	397.4045	-0.11617	0.138049	-0.84152	0.400056	0.687773	GFM1	85476	G elongation factor mitochondrial 1
ENSG0000	557.4523	0.110248	0.131029	0.841403	0.400122	0.687773	CACUL1	143384	CDK2 associated cullin domain 1
ENSG0000	212.5225	-0.15206	0.18074	-0.8413	0.400179	0.687806	PARP3	10039	poly(ADP-ribose) polymerase family member 3
ENSG0000	3.537645	-1.44109	1.713687	-0.84093	0.400387	0.6881	KPNA4P1	1E+08	KPNA4 pseudogene 1
ENSG0000	11.11145	0.590248	0.701973	0.840841	0.400437	0.688123	NA	NA	NA
ENSG0000	530.2514	0.104615	0.124443	0.840661	0.400538	0.688232	PRKRA	8575	protein activator of interferon induced protein kinase EIF2AK2
ENSG0000	26.94738	-0.37397	0.444969	-0.84044	0.400662	0.688381	ZNF717	1E+08	zinc finger protein 717
ENSG0000	10342.45	0.074469	0.088617	0.840346	0.400714	0.688407	TMEM123	114908	transmembrane protein 123
ENSG0000	406.9639	-0.11582	0.137859	-0.84017	0.400815	0.688517	CYREN	78996	cell cycle regulator of NHEJ
ENSG0000	588.1017	-0.1083	0.128931	-0.84001	0.400902	0.688602	NA	NA	NA
ENSG0000	9.370291	0.783676	0.93352	0.839486	0.401197	0.688817	NA	NA	NA

ENSG0000	68.76927	-0.26784	0.318983	-0.83967	0.401094	0.688817	SLC25A15	10166	solute carrier family 25 member 15
ENSG0000	3822.666	0.078718	0.093771	0.839471	0.401205	0.688817	SRP14	6727	signal recognition particle 14
ENSG0000	205.1456	-0.14523	0.173001	-0.83946	0.401213	0.688817	WDR83	84292	WD repeat domain 83
ENSG0000	4.10636	-1.22971	1.464589	-0.83963	0.401116	0.688817	UMODL1-/	150147	UMODL1 antisense RNA 1
ENSG0000	2470.728	-0.079	0.094119	-0.83937	0.401261	0.688837	CARD8	22900	caspase recruitment domain family member 8
ENSG0000	49.22018	-0.3169	0.377837	-0.83871	0.401631	0.689398	DUS4L-BC/	1.15E+08	DUS4L-BCAP29 readthrough
ENSG0000	357.1266	0.115579	0.137814	0.838656	0.401663	0.689398	RPSAP58	388524	ribosomal protein SA pseudogene 58
ENSG0000	3664.438	0.083567	0.099727	0.837959	0.402054	0.690006	ARPC4	10093	actin related protein 2/3 complex subunit 4
ENSG0000	12.21932	0.682696	0.814901	0.837765	0.402163	0.690129	NA	NA	NA
ENSG0000	4.113728	-1.2414	1.482041	-0.83763	0.40224	0.690198	NA	NA	NA
ENSG0000	22.97237	0.418411	0.499661	0.83739	0.402373	0.690299	NA	NA	NA
ENSG0000	64.8965	-0.2445	0.291955	-0.83745	0.40234	0.690299	TCEAL1	9338	transcription elongation factor A like 1
ENSG0000	5.010476	-1.03927	1.241257	-0.83727	0.40244	0.690349	GTF2IP14	1.06E+08	general transcription factor IIi pseudogene 14
ENSG0000	48.551	-0.29067	0.347208	-0.83716	0.402505	0.690396	ECT2	1894	epithelial cell transforming 2
ENSG0000	3648.481	-0.10491	0.125361	-0.83688	0.402658	0.690596	APOL6	80830	apolipoprotein L6
ENSG0000	399.6645	-0.12107	0.144729	-0.83651	0.402868	0.690891	NBPF15	284565	NBPF member 15
ENSG0000	492.4449	-0.10439	0.124826	-0.8363	0.402988	0.69098	ATP9B	374868	ATPase phospholipid transporting 9B (putative)
ENSG0000	3.848067	-1.07966	1.291019	-0.83629	0.402994	0.69098	FAM83E	54854	family with sequence similarity 83 member E
ENSG0000	683.7239	0.100669	0.120537	0.835175	0.403619	0.691988	UTP25	27042	UTP25 small subunit processome component
ENSG0000	418.9388	-0.12457	0.14919	-0.83495	0.403748	0.692081	COG7	91949	component of oligomeric golgi complex 7
ENSG0000	51.48879	0.291927	0.349608	0.83501	0.403712	0.692081	NA	NA	NA
ENSG0000	58.86057	0.249061	0.298408	0.834632	0.403925	0.69232	C2orf74-D	339803	C2orf74 divergent transcript
ENSG0000	45.00574	0.412548	0.494347	0.83453	0.403983	0.692355	CAMSAP3	57662	calmodulin regulated spectrin associated protein family member 3
ENSG0000	2.105802	1.71195	2.051738	0.83439	0.404061	0.692426	NA	NA	NA
ENSG0000	167.1864	0.191159	0.229147	0.834223	0.404155	0.692523	YOD1	55432	YOD1 deubiquitinase
ENSG0000	9.448877	-0.64811	0.777087	-0.83403	0.404263	0.69258	NA	NA	NA
ENSG0000	6.715191	-0.96961	1.162507	-0.83406	0.404245	0.69258	EFCAB5	374786	EF-hand calcium binding domain 5
ENSG0000	3871.64	-0.06845	0.082096	-0.83374	0.404429	0.69261	ELOVL1	64834	ELOVL fatty acid elongase 1
ENSG0000	470.964	0.121705	0.145952	0.833871	0.404354	0.69261	NDUFA2	4695	NADH:ubiquinone oxidoreductase subunit A2
ENSG0000	144.3951	-0.18275	0.219196	-0.83374	0.40443	0.69261	JAG2	3714	jagged canonical Notch ligand 2
ENSG0000	2.38434	1.582441	1.897884	0.833792	0.404398	0.69261	RGS11	8786	regulator of G protein signaling 11
ENSG0000	10.70521	-0.68187	0.817942	-0.83364	0.404483	0.692636	NA	NA	NA
ENSG0000	3.285618	-1.48862	1.786157	-0.83342	0.404608	0.692722	SULT1C4	27233	sulfotransferase family 1C member 4
ENSG0000	117.653	0.197721	0.237233	0.833449	0.404591	0.692722	DDX19A	55308	DEAD-box helicase 19A
ENSG0000	1086.952	0.091875	0.11028	0.833104	0.404786	0.692948	MICAL2	9645	microtubu calponin and LIM domain containing 2
ENSG0000	8470.162	-0.07396	0.088777	-0.83305	0.404815	0.692948	TCIRG1	10312	T cell imm ATPase H+ transporting V0 subunit a3
ENSG0000	13.95612	0.504361	0.605648	0.832762	0.404979	0.693166	NA	NA	NA
ENSG0000	272.4196	-0.12602	0.151338	-0.83268	0.405028	0.693186	BAG4	9530	BAG cochaperone 4
ENSG0000	5.912193	-1.08004	1.297191	-0.8326	0.405073	0.693198	STAG3L5P-	1.02E+08	STAG3L5P-PVRIG2P-PILRB readthrough
ENSG0000	576.2098	-0.10047	0.120696	-0.83241	0.405175	0.693245	GLMP	112770	glycosylated lysosomal membrane protein
ENSG0000	51.55687	0.289197	0.347407	0.832447	0.405157	0.693245	KNDC1	85442	kinase non-catalytic C-lobe domain containing 1
ENSG0000	43.60407	0.315598	0.379171	0.832338	0.405218	0.693255	ZNF222	7673	zinc finger protein 222
ENSG0000	670.1677	0.104405	0.125467	0.832129	0.405336	0.693258	NPRL2	10641	NPR2 like GATOR1 complex subunit
ENSG0000	350.6943	-0.14189	0.170523	-0.83207	0.405369	0.693258	MMP25	64386	matrix metallopeptidase 25
ENSG0000	216.3311	-0.19487	0.234191	-0.83211	0.405346	0.693258	ALDH3A2	224	aldehyde dehydrogenase 3 family member A2
ENSG0000	1444.362	0.082552	0.099204	0.832147	0.405326	0.693258	ELOF1	84337	elongation factor 1
ENSG0000	9.752549	0.709958	0.853451	0.831867	0.405484	0.693326	SNORA5C	677796	small nucl H/ACA box 5C
ENSG0000	29.76184	0.442817	0.532317	0.831868	0.405484	0.693326	TRBV25-1	28562	T cell receptor beta variable 25-1
ENSG0000	3927.141	0.072833	0.087561	0.831799	0.405522	0.693328	PSMD8	5714	proteasom non-ATPase 8
ENSG0000	2.332119	1.588283	1.910454	0.831364	0.405768	0.693553	RAMP3	10268	receptor activity modifying protein 3
ENSG0000	373.1501	-0.12065	0.145114	-0.83138	0.40576	0.693553	PKIA	5569	cAMP-dependent protein kinase inhibitor alpha
ENSG0000	237.4696	-0.14532	0.174809	-0.8313	0.405803	0.693553	PNPLA7	375775	patatin like phospholipase domain containing 7
ENSG0000	75.84047	0.245134	0.294837	0.831422	0.405735	0.693553	LOC37444	374443	C-type lectin domain family 2 member D pseudogene
ENSG0000	40.97267	-0.31528	0.379652	-0.83046	0.406281	0.694113	LRRC1	55227	leucine rich repeat containing 1
ENSG0000	45.52367	-0.32998	0.397318	-0.83051	0.406252	0.694113	ZNF618	114991	zinc finger protein 618
ENSG0000	614.0351	0.102667	0.123622	0.830493	0.40626	0.694113	TMEM9B	56674	TMEM9 domain family member B
ENSG0000	7.311135	-0.84325	1.015183	-0.83064	0.406177	0.694113	CHRNA7	1139	cholinergic receptor nicotinic alpha 7 subunit
ENSG0000	75.36278	-0.24304	0.292824	-0.82998	0.40655	0.694509	LINC01011	401232	long intergenic non-protein coding RNA 1011
ENSG0000	1125.964	-0.08693	0.104745	-0.82988	0.406606	0.69454	NFATC3	4775	nuclear factor of activated T cells 3
ENSG0000	4986.811	0.0785	0.094609	0.829738	0.406687	0.694615	FAM53B	9679	family with sequence similarity 53 member B
ENSG0000	7.910263	-0.76131	0.917999	-0.82932	0.406924	0.694955	NA	NA	NA
ENSG0000	226.4881	-0.17525	0.211341	-0.82922	0.406981	0.694989	RPH3A	22895	rabphilin 3A
ENSG0000	926.5307	-0.09107	0.10987	-0.82885	0.40719	0.695042	PLCH2	9651	phospholipase C eta 2
ENSG0000	266.0883	0.141579	0.170795	0.828943	0.407137	0.695042	UBE2F	140739	ubiquitin conjugating enzyme E2 F (putative)
ENSG0000	97.657	0.209261	0.252453	0.828911	0.407155	0.695042	RPL7AP6	326310	ribosomal protein L7a pseudogene 6
ENSG0000	3.418081	1.119283	1.350163	0.828998	0.407105	0.695042	NA	NA	NA
ENSG0000	2036.825	0.084509	0.101962	0.828832	0.407199	0.695042	UBQLN2	29978	ubiquilin 2
ENSG0000	214.111	-0.14785	0.178418	-0.82868	0.407283	0.695121	WDR3	10885	WD repeat domain 3
ENSG0000	189.8799	-0.1549	0.186949	-0.82856	0.407352	0.695174	UMPS	7372	uridine monophosphate synthetase
ENSG0000	79.72879	-0.22065	0.266341	-0.82846	0.407412	0.695213	GTF2IRD1	9569	GTF2I repeat domain containing 1
ENSG0000	140.3187	-0.17698	0.213702	-0.82815	0.407586	0.695397	ZSCAN16	80345	zinc finger and SCAN domain containing 16
ENSG0000	15.69685	-0.58854	0.710764	-0.82804	0.407646	0.695397	NA	NA	NA
ENSG0000	345.0413	0.145636	0.175902	0.827935	0.407707	0.695397	RPRD1A	55197	regulation of nuclear pre-mRNA domain containing 1A
ENSG0000	74.85925	-0.24536	0.296349	-0.82794	0.407704	0.695397	C20orf96	140680	chromosome 20 open reading frame 96
ENSG0000	3.244427	-1.15383	1.393497	-0.82801	0.407665	0.695397	NA	NA	NA
ENSG0000	921.9842	-0.08653	0.104534	-0.82774	0.40782	0.695462	AHSA2P	130872	activator c pseudogene
ENSG0000	263.4425	-0.13568	0.163907	-0.82778	0.407795	0.695462	DUSP28	285193	dual specificity phosphatase 28
ENSG0000	20.16117	0.461836	0.55844	0.82701	0.408231	0.696099	SKOR1	390598	SKI family transcriptional corepressor 1
ENSG0000	47.6538	-0.29982	0.362702	-0.82664	0.408444	0.696269	NR6A1	2649	nuclear receptor subfamily 6 group A member 1
ENSG0000	99.51836	0.238364	0.28834	0.826677	0.40842	0.696269	CHRNA10	57053	cholinergic receptor nicotinic alpha 10 subunit
ENSG0000	619.9661	-0.10093	0.122098	-0.82664	0.40844	0.696269	POLR3H	171568	RNA polymerase III subunit H
ENSG0000	32.24628	0.3588	0.434234	0.826282	0.408644	0.696355	NA	NA	NA
ENSG0000	341.1693	-0.13402	0.162161	-0.82644	0.408557	0.696355	TRMO	51531	tRNA methyltransferase O
ENSG0000	6.14699	0.996878	1.206463	0.826281	0.408644	0.696355	NA	NA	NA
ENSG0000	3.838289	-0.99564	1.204832	-0.82637	0.408594	0.696355	ERCC6L	54821	ERCC excis spindle assembly checkpoint helicase
ENSG0000	999.2361	-0.08342	0.100964	-0.82621	0.408684	0.696359	CYB561A3	220002	cytochrome b561 family member A3
ENSG0000	5.020344	-1.09981	1.332285	-0.8255	0.409086	0.696979	LINC02021	1.05E+08	long intergenic non-protein coding RNA 2021
ENSG0000	132.0137	-0.19446	0.235699	-0.82503	0.409356	0.697375	RRP15	51018	ribosomal RNA processing 15 homolog
ENSG0000	18.62469	-0.47334	0.573989	-0.82465	0.40957	0.697612	SLC6A9	6536	solute carrier family 6 member 9
ENSG0000	14.38316	-0.53402	0.647536	-0.82469	0.409547	0.697612	NA	NA	NA
ENSG0000	1287.834	-0.07739	0.09388	-0.82436	0.409733	0.697826	HIF1AN	55662	hypoxia inducible factor 1 subunit alpha inhibitor
ENSG0000	268.1209	-0.12961	0.157269	-0.82416	0.409851	0.697962	ZNF397	84307	zinc finger protein 397
ENSG0000	1861.98	-0.07521	0.0913	-0.82376	0.410077	0.698283	CD3D	915	CD3 delta subunit of T-cell receptor complex
ENSG0000	71.77646	-0.27238	0.330692	-0.82368	0.410122	0.698295	PLIN4	729359	perilipin 4
ENSG0000	5.233641	0.963758	1.170312	0.823505	0.410221	0.698387	PPIL6	285755	peptidylprolyl isomerase like 6
ENSG0000	1685.048	0.089682	0.108909	0.823452	0.410251	0.698387	TNKS2	80351	tankyrase 2
ENSG0000	2344.104	0.088771	0.107818	0.82334	0.410315	0.698431	SNX3	8724	sorting nexin 3
ENSG0000	4999.352	-0.08884	0.107931	-0.82314	0.410426	0.698557	LY6E	4061	lymphocyte antigen 6 family member E
ENSG0000	1672.339	0.078743	0.095681	0.822973	0.410524	0.698658	DNMBP	23268	dynamin binding protein
ENSG0000	3.894311	1.460586	1.775161	0.822791	0.410627	0.698706	SLC23A1	9963	solute carrier family 23 member 1

ENSG0000	286.6081	-0.1352	0.164317	-0.8228	0.410624	0.698706	TNFRSF10/	8797	TNF receptor superfamily member 10a
ENSG0000	3.044995	1.285998	1.563349	0.822592	0.41074	0.698835	ZFX-AS1	1.01E+08	ZFX antisense RNA 1
ENSG0000	3.607041	1.000413	1.216425	0.822421	0.410837	0.698936	TUBAP2	399942	tubulin alpha pseudogene 2
ENSG0000	8.224782	0.923182	1.123257	0.82188	0.411145	0.699396	CBX3P2	645158	chromobox 3 pseudogene 2
ENSG0000	4.541125	1.172731	1.427529	0.821511	0.411355	0.699662	GNG3	2785	G protein subunit gamma 3
ENSG0000	273.7636	-0.12877	0.156752	-0.82147	0.411377	0.699662	LEO1	123169	LEO1 hom Paf1/RNA polymerase II complex component
ENSG0000	29.39323	-0.38195	0.465115	-0.8212	0.411532	0.699808	UCN	7349	urocortin
ENSG0000	11.49672	0.628478	0.765328	0.821189	0.411539	0.699808	IGF2BP3	10643	insulin like growth factor 2 mRNA binding protein 3
ENSG0000	976.5046	0.103147	0.12562	0.821101	0.411589	0.699829	CLIP2	7461	CAP-Gly domain containing linker protein 2
ENSG0000	125.4684	-0.2095	0.255232	-0.82082	0.411751	0.699931	NA	NA	NA
ENSG0000	954.3454	-0.09561	0.116496	-0.82073	0.411799	0.699931	SNRPC	6631	small nuclear ribonucleoprotein polypeptide C
ENSG0000	148.3635	-0.16462	0.200535	-0.8209	0.411701	0.699931	VRK1	7443	VRK serine/threonine kinase 1
ENSG0000	1514.166	0.080196	0.097711	0.820747	0.41179	0.699931	FAM50A	9130	family with sequence similarity 50 member A
ENSG0000	64.001	0.267545	0.326392	0.819703	0.412386	0.700056	HHLA3	11147	HERV-H LTR-associating 3
ENSG0000	6209.987	0.06684	0.081545	0.819669	0.412405	0.700056	SATB1	6304	SATB homeobox 1
ENSG0000	91.69611	-0.27792	0.33897	-0.81991	0.412268	0.700056	NREP	9315	neuronal regeneration related protein
ENSG0000	137.1955	0.178662	0.217982	0.819617	0.412435	0.700056	MRPL22	29093	mitochondrial ribosomal protein L22
ENSG0000	954.91	-0.09695	0.118174	-0.82042	0.411977	0.700056	RUNX2	860	RUNX family transcription factor 2
ENSG0000	209.6079	0.154973	0.189054	0.819729	0.412371	0.700056	MPLKIP	136647	M-phase specific PLK1 interacting protein
ENSG0000	22.34929	0.426304	0.520024	0.819777	0.412343	0.700056	NA	NA	NA
ENSG0000	1025.759	0.087573	0.106803	0.819955	0.412242	0.700056	ANAPC16	119504	anaphase promoting complex subunit 16
ENSG0000	249.9058	-0.14374	0.175239	-0.82024	0.412079	0.700056	NCR3LG1	374383	natural killer cell cytotoxicity receptor 3 ligand 1
ENSG0000	20.36642	0.419833	0.512026	0.819943	0.412248	0.700056	LOC10024	1E+08	uncharacterized LOC100240735
ENSG0000	2.078227	1.593959	1.943817	0.820015	0.412208	0.700056	LINC0106	1.04E+08	long intergenic non-protein coding RNA 1068
ENSG0000	287.9162	0.154223	0.188166	0.819609	0.412439	0.700056	RPS27L	51065	ribosomal protein S27 like
ENSG0000	10.68925	-0.75091	0.915504	-0.82021	0.412095	0.700056	NA	NA	NA
ENSG0000	4.559487	1.06689	1.300663	0.820266	0.412065	0.700056	TUBB8B	260334	tubulin beta 8B
ENSG0000	1385.587	0.079556	0.096962	0.820488	0.411938	0.700056	FTSJ1	24140	FtsJ RNA 2'-O-methyltransferase 1
ENSG0000	5.027482	0.942738	1.150731	0.819252	0.412643	0.700267	NA	NA	NA
ENSG0000	10.37189	0.780805	0.953018	0.819297	0.412617	0.700267	ITPKA	3706	inositol-trisphosphate 3-kinase A
ENSG0000	7.424653	-0.77574	0.946963	-0.81919	0.412677	0.700267	NA	NA	NA
ENSG0000	6.210503	-0.74743	0.912534	-0.81907	0.412748	0.700324	RRH	10692	retinal pigment epithelium-derived rhodopsin homolog
ENSG0000	555.9434	-0.10811	0.13201	-0.81895	0.412816	0.700367	AK3	50808	adenylate kinase 3
ENSG0000	146.5026	0.187241	0.228653	0.81889	0.412849	0.700367	GCNA	93953	germ cell nuclear acidic peptidase
ENSG0000	6090.244	0.07119	0.086956	0.818695	0.412961	0.700492	EPN1	29924	epsin 1
ENSG0000	893.8434	0.086603	0.105857	0.818113	0.413293	0.700868	HLA-DMB	3109	major hist class II DM beta
ENSG0000	875.2049	-0.10153	0.124099	-0.81811	0.413296	0.700868	OAF	220323	out at first homolog
ENSG0000	206.9754	0.148635	0.181671	0.818154	0.413269	0.700868	LLPH	84298	LLP homol long-term synaptic facilitation factor
ENSG0000	4.179645	-1.178	1.440697	-0.81766	0.413553	0.70124	ALOXE3	59344	arachidonate lipoxygenase 3
ENSG0000	1378.107	0.073418	0.089802	0.817552	0.413613	0.701278	TMED4	222068	transmembrane p24 trafficking protein 4
ENSG0000	381.0056	0.123166	0.150739	0.817083	0.413881	0.701667	ZNF330	27309	zinc finger protein 330
ENSG0000	79.56687	0.240593	0.294477	0.817018	0.413918	0.701667	MDK	4192	midkine
ENSG0000	24.89297	-0.407	0.4982	-0.81693	0.413967	0.701686	TFAP2E	339488	transcription factor AP-2 epsilon
ENSG0000	126.794	-0.2148	0.263064	-0.81654	0.41419	0.701935	ZNF35	7584	zinc finger protein 35
ENSG0000	2.575175	-1.24599	1.52588	-0.81657	0.414172	0.701935	TSPAN14-/	1.02E+08	TSPAN14 antisense RNA 1
ENSG0000	21.21979	-0.4459	0.546382	-0.81609	0.414449	0.702288	NA	NA	NA
ENSG0000	947.2954	0.092077	0.112833	0.816046	0.414474	0.702288	KEAP1	9817	kelch like ECH associated protein 1
ENSG0000	14.13494	0.504206	0.61799	0.81588	0.414569	0.702385	RPL4P5	158345	ribosomal protein L4 pseudogene 5
ENSG0000	4.967016	-1.01137	1.240638	-0.8152	0.414958	0.702602	C3orf20	84077	chromosome 3 open reading frame 20
ENSG0000	6.723874	-0.78887	0.967876	-0.81505	0.415044	0.702602	POLQ	10721	DNA polymerase theta
ENSG0000	531.477	-0.10262	0.125879	-0.81521	0.414952	0.702602	HMGCR	3156	3-hydroxy-3-methylglutaryl-CoA reductase
ENSG0000	8.878499	-0.62858	0.771266	-0.81499	0.415076	0.702602	GPER1	2852	G protein-coupled estrogen receptor 1
ENSG0000	735.21	0.090897	0.111462	0.8155	0.414786	0.702602	GLRX3	10539	glutaredoxin 3
ENSG0000	5125.127	-0.06455	0.079152	-0.81555	0.414759	0.702602	NCKAP1L	3071	NCK associated protein 1 like
ENSG0000	22.44722	-0.4412	0.541237	-0.81517	0.414974	0.702602	LINC0053	1.01E+08	long intergenic non-protein coding RNA 539
ENSG0000	722.0863	-0.0992	0.121673	-0.8153	0.414898	0.702602	ZNF689	115509	zinc finger protein 689
ENSG0000	2000.3	-0.07316	0.089731	-0.81536	0.414868	0.702602	ZDHHC7	55625	zinc finger DHHC-type palmitoyltransferase 7
ENSG0000	72.56416	0.268284	0.329143	0.8151	0.415015	0.702602	NA	NA	NA
ENSG0000	1186.949	-0.08714	0.106972	-0.8146	0.415303	0.702723	STRN	6801	striatin
ENSG0000	124.4248	-0.18327	0.224981	-0.8146	0.415299	0.702723	NA	NA	NA
ENSG0000	26.0819	-0.47004	0.577027	-0.81458	0.415311	0.702723	KRT8	3856	keratin 8
ENSG0000	1095.903	0.083896	0.103006	0.814472	0.415375	0.702723	ATP10A	57194	ATPase phospholipid transporting 10A (putative)
ENSG0000	12.53031	0.589732	0.724058	0.814482	0.415369	0.702723	LIPE-AS1	1.01E+08	LIPE antisense RNA 1
ENSG0000	41.79402	-0.41589	0.510612	-0.81448	0.415368	0.702723	LILRB5	10990	leukocyte immunoglobulin like receptor B5
ENSG0000	519.4778	-0.09981	0.122554	-0.8144	0.415417	0.702731	VPS13A	23230	vacuolar protein sorting 13 homolog A
ENSG0000	5.0873	1.08666	1.334438	0.81432	0.415462	0.702742	TCTE1	202500	t-complex-associated-testis-expressed 1
ENSG0000	1048.05	0.081266	0.099817	0.814152	0.415558	0.702841	TNFAIP1	7126	TNF alpha induced protein 1
ENSG0000	139.7772	0.187088	0.229916	0.813723	0.415804	0.703128	MSMO1	6307	methylsterol monooxygenase 1
ENSG0000	350.7087	0.115552	0.141993	0.813786	0.415767	0.703128	CRYBB2P1	1416	crystallin beta B2 pseudogene 1
ENSG0000	3.243331	-1.2927	1.588998	-0.81353	0.415912	0.703184	NA	NA	NA
ENSG0000	8.72344	0.656069	0.806407	0.813571	0.415891	0.703184	RPL13AP2	441632	ribosomal protein L13a pseudogene 22
ENSG0000	2985.819	0.079468	0.097697	0.813417	0.415979	0.703232	GGA1	26088	golgi assoc gamma ac ARF binding protein 1
ENSG0000	6.746814	0.902166	1.109632	0.813032	0.4162	0.703349	LOC1019	1.02E+08	uncharacterized LOC101927401
ENSG0000	8.045435	-0.72767	0.894896	-0.81313	0.416141	0.703349	NA	NA	NA
ENSG0000	11.54628	-0.65329	0.80351	-0.81305	0.416192	0.703349	FAM225A	286333	family with sequence similarity 225 member A
ENSG0000	24.84574	-0.38294	0.470915	-0.81319	0.41611	0.703349	AURKB	9212	aurora kinase B
ENSG0000	454.915	0.119999	0.147674	0.812599	0.416448	0.70364	DDX20	11218	DEAD-box helicase 20
ENSG0000	9.6516	-0.68306	0.840551	-0.81263	0.41643	0.70364	NA	NA	NA
ENSG0000	3064.317	0.066333	0.081639	0.812522	0.416492	0.703651	PTGES3	10728	prostaglandin E synthase 3
ENSG0000	2.212079	-1.53296	1.888156	-0.81188	0.416858	0.703732	MAB21L3	126868	mab-21 like 3
ENSG0000	26.69692	0.446689	0.550054	0.812081	0.416745	0.703732	NA	NA	NA
ENSG0000	157.5575	-0.1591	0.195917	-0.81208	0.416745	0.703732	MCM2	4171	minichromosome maintenance complex component 2
ENSG0000	5.743045	0.862513	1.062032	0.812135	0.416714	0.703732	TMEM183	653659	transmem pseudogene
ENSG0000	10.71824	0.724172	0.892307	0.811573	0.417037	0.703732	NA	NA	NA
ENSG0000	3145.728	-0.07352	0.090604	-0.81148	0.41709	0.703732	DEF6	50619	DEF6 guanine nucleotide exchange factor
ENSG0000	3551.373	-0.07254	0.089372	-0.81168	0.416975	0.703732	PNISR	25957	PNN interacting serine and arginine rich protein
ENSG0000	21.14001	0.497719	0.613293	0.811553	0.417048	0.703732	LINC0117	1.01E+08	long intergenic non-protein coding RNA 1176
ENSG0000	13.18365	-0.65538	0.806911	-0.81221	0.416669	0.703732	FAM88B	728673	family with sequence similarity 88 member B
ENSG0000	683.277	0.099502	0.122589	0.811669	0.416982	0.703732	CENATAC	338657	centrosomal AT-AC splicing factor
ENSG0000	25.25547	0.50273	0.61933	0.811733	0.416945	0.703732	NA	NA	NA
ENSG0000	4.109301	-1.26019	1.552986	-0.81146	0.417099	0.703732	NA	NA	NA
ENSG0000	80.53304	0.226287	0.278869	0.811447	0.417109	0.703732	FBXL8	55336	F-box and leucine rich repeat protein 8
ENSG0000	2425.863	0.069424	0.08554	0.811599	0.417022	0.703732	PSMD3	5709	proteasom non-ATPase 3
ENSG0000	505.643	0.119273	0.14693	0.811771	0.416923	0.703732	TXNL4A	10907	thioredoxin like 4A
ENSG0000	312.3327	0.114249	0.140815	0.811343	0.417169	0.703735	CNIH4	29097	cornichon family AMPA receptor auxiliary protein 4
ENSG0000	1048.343	0.089107	0.10983	0.811312	0.417187	0.703735	TMEM230	29058	transmembrane protein 230
ENSG0000	10.00481	-0.76568	0.944068	-0.81104	0.417342	0.703868	NA	NA	NA
ENSG0000	10.33912	-0.6555	0.808215	-0.81105	0.417339	0.703868	RPS2P32	256355	ribosomal protein S2 pseudogene 32
ENSG0000	9.876596	-0.71997	0.887969	-0.81081	0.417477	0.704032	TMEM169	92691	transmembrane protein 169
ENSG0000	5.484783	-0.76586	0.944872	-0.81054	0.417627	0.704174	NA	NA	NA

ENSG0000	556.603	0.124437	0.153539	0.81046	0.417676	0.704174	ZNF143	7702	zinc finger protein 143
ENSG0000	1344.467	0.093891	0.11584	0.810522	0.41764	0.704174	PPP1CC	5501	protein phosphatase 1 catalytic subunit gamma
ENSG0000	425.9304	0.112975	0.139408	0.810395	0.417713	0.704174	U2AF1L4	199746	U2 small nuclear RNA auxiliary factor 1 like 4
ENSG0000	575.7664	0.094976	0.117232	0.810149	0.417855	0.704207	MSH6	2956	mutS homolog 6
ENSG0000	892.3434	0.082374	0.101685	0.810097	0.417884	0.704207	PSMA3-AS	379025	PSMA3 antisense RNA 1
ENSG0000	11.06195	-0.65677	0.810565	-0.81027	0.417786	0.704207	TARM1	441864	T cell-inter activating receptor on myeloid cells 1
ENSG0000	41.93104	0.31081	0.38366	0.810119	0.417872	0.704207	CPXM1	56265	carboxype M14 family member 1
ENSG0000	53.09892	-0.25888	0.31982	-0.80946	0.41825	0.704407	NA	NA	NA
ENSG0000	23.95457	-0.40667	0.50225	-0.80969	0.418118	0.704407	NA	NA	NA
ENSG0000	4324.424	0.070427	0.087021	0.809309	0.418337	0.704407	SPON2	10417	spondin 2
ENSG0000	379.8483	0.131053	0.161974	0.809098	0.418459	0.704407	NDUFA5	4698	NADH:ubiquinone oxidoreductase subunit A5
ENSG0000	522.7293	0.098787	0.122035	0.8095	0.418227	0.704407	TGFBR1	7046	transforming growth factor beta receptor 1
ENSG0000	124.9444	0.202488	0.25025	0.809141	0.418434	0.704407	BSCL2	26580	BSCL2 lipic seipin
ENSG0000	126.6257	-0.19705	0.243489	-0.8093	0.418345	0.704407	LRR1	122769	leucine rich repeat protein 1
ENSG0000	8.286206	0.871441	1.076764	0.809315	0.418334	0.704407	NA	NA	NA
ENSG0000	4.181435	1.176029	1.453233	0.80925	0.418371	0.704407	TMEM132	124842	transmembrane protein 132E
ENSG0000	415.6804	0.136114	0.168217	0.80916	0.418423	0.704407	NA	NA	NA
ENSG0000	429.057	-0.12254	0.151404	-0.80936	0.418307	0.704407	PBDC1	51260	polysaccharide biosynthesis domain containing 1
ENSG0000	1551.232	-0.07699	0.09512	-0.80936	0.418311	0.704407	IDS	3423	iduronate 2-sulfatase
ENSG0000	1699.886	0.081914	0.101262	0.808932	0.418554	0.70444	MCRIP1	348262	MAPK regulated corepressor interacting protein 1
ENSG0000	278.9267	0.133729	0.165302	0.808997	0.418517	0.70444	PSMD10	5716	proteason non-ATPase 10
ENSG0000	2680.961	-0.07538	0.093204	-0.8088	0.418631	0.70445	GRK6	2870	G protein-coupled receptor kinase 6
ENSG0000	8797.353	0.057127	0.070633	0.808789	0.418636	0.70445	CIC	23152	capicua transcriptional repressor
ENSG0000	32.90032	-0.33186	0.410379	-0.80866	0.41871	0.704509	AGAP6	414189	ArfGAP wi ankyrin repeat and PH domain 6
ENSG0000	80.02936	0.225051	0.278364	0.808478	0.418815	0.70456	FAM161B	145483	FAM161 centrosomal protein B
ENSG0000	30.52368	0.373291	0.461685	0.808539	0.41878	0.70456	HAMP	57817	hepcidin antimicrobial peptide
ENSG0000	6.591751	-0.80512	0.996562	-0.8079	0.419149	0.704812	AMACR	23600	alpha-methylacyl-CoA racemase
ENSG0000	3.763718	-1.22126	1.511569	-0.80794	0.419126	0.704812	EPM2A-DT	1.01E+08	EPM2A divergent transcript
ENSG0000	26.27117	-0.35829	0.443382	-0.80807	0.419049	0.704812	GDPGP1	390637	GDP-D-glucose phosphorylase 1
ENSG0000	394.3176	0.127839	0.158239	0.807887	0.419155	0.704812	ZNF430	80264	zinc finger protein 430
ENSG0000	123.2505	-0.20357	0.251921	-0.80809	0.419041	0.704812	BCAT2	587	branched chain amino acid transaminase 2
ENSG0000	13.40746	0.581102	0.719413	0.807744	0.419238	0.704834	NA	NA	NA
ENSG0000	4.330833	0.94971	1.175773	0.807733	0.419244	0.704834	KRT18P4	391256	keratin 18 pseudogene 4
ENSG0000	838.693	-0.08834	0.109384	-0.80757	0.419337	0.704897	USO1	8615	USO1 vesicle transport factor
ENSG0000	545.1168	-0.11844	0.146678	-0.80749	0.419384	0.704897	ACAA2	10449	acetyl-CoA acyltransferase 2
ENSG0000	4416.539	-0.06302	0.07805	-0.80747	0.419396	0.704897	PIGT	51604	phosphatidylinositol glycan anchor biosynthesis class T
ENSG0000	352.8895	-0.11284	0.139923	-0.80641	0.420005	0.705026	TP73-AS1	57212	TP73 antisense RNA 1
ENSG0000	210.1181	-0.17518	0.217233	-0.80643	0.419997	0.705026	NBPF1	55672	NBPF member 1
ENSG0000	376.6439	-0.11432	0.141741	-0.80655	0.419923	0.705026	ZNF683	257101	zinc finger protein 683
ENSG0000	14.9784	0.548147	0.679192	0.807059	0.419633	0.705026	BCL2L15	440603	BCL2 like 15
ENSG0000	14.48161	0.551937	0.68414	0.806761	0.419804	0.705026	LINC0180	1.01E+08	long intergenic non-protein coding RNA 1806
ENSG0000	455.9891	0.104531	0.12958	0.806687	0.419847	0.705026	ANXA2R	389289	annexin A2 receptor
ENSG0000	23.44983	0.408427	0.506291	0.806705	0.419837	0.705026	MIR1244-2	1E+08	microRNA 1244-2
ENSG0000	2585.545	0.069192	0.085763	0.806778	0.419795	0.705026	NSD3	54904	nuclear receptor binding SET domain protein 3
ENSG0000	581.2977	-0.10309	0.127781	-0.80679	0.419788	0.705026	ELP1	8518	elongator acetyltransferase complex subunit 1
ENSG0000	199.5914	0.151794	0.188216	0.806484	0.419964	0.705026	BCL2L2	599	BCL2 like 2
ENSG0000	58.67561	-0.27216	0.337436	-0.80654	0.419931	0.705026	SLC27A2	11001	solute carrier family 27 member 2
ENSG0000	393.9556	-0.11505	0.142525	-0.8072	0.419552	0.705026	ZSCAN32	54925	zinc finger and SCAN domain containing 32
ENSG0000	174.2974	-0.16007	0.198368	-0.80694	0.419699	0.705026	ZNF226	7769	zinc finger protein 226
ENSG0000	252.2527	0.132737	0.164462	0.807098	0.41961	0.705026	NKRF	55922	NFKB repressing factor
ENSG0000	128.7897	0.191282	0.237236	0.806292	0.420074	0.705039	GPR75	10936	G protein-coupled receptor 75
ENSG0000	664.4243	0.10109	0.125411	0.806069	0.420203	0.705039	GCC1	79571	GRIP and coiled-coil domain containing 1
ENSG0000	3380.355	0.068087	0.084463	0.806107	0.420181	0.705039	ADGRE2	30817	adhesion G protein-coupled receptor E2
ENSG0000	153.1324	-0.16127	0.200042	-0.80621	0.420124	0.705039	ZNF585A	199704	zinc finger protein 585A
ENSG0000	16.66272	0.652591	0.809511	0.806155	0.420154	0.705039	NA	NA	NA
ENSG0000	6.721094	-0.79501	0.986471	-0.80592	0.420291	0.705123	CCDC122	160857	coiled-coil domain containing 122
ENSG0000	26.01879	-0.39512	0.490472	-0.80559	0.420478	0.705278	DYNC2LI1	51626	dynein cytoplasmic 2 light intermediate chain 1
ENSG0000	267.1495	-0.13379	0.166083	-0.80556	0.420498	0.705278	PDHX	8050	pyruvate dehydrogenase complex component X
ENSG0000	23.52451	-0.37327	0.463317	-0.80564	0.420451	0.705278	NA	NA	NA
ENSG0000	27.54463	0.386921	0.480398	0.805417	0.420579	0.705288	TRBV5-6	28609	T cell receptor beta variable 5-6
ENSG0000	4.347309	-0.89541	1.111656	-0.80547	0.420547	0.705288	NA	NA	NA
ENSG0000	10.35346	0.649914	0.807123	0.805224	0.420691	0.705295	NA	NA	NA
ENSG0000	289.9298	0.150288	0.186644	0.805211	0.420698	0.705295	SNAPC3	6619	small nuclear RNA activating complex polypeptide 3
ENSG0000	52.42949	0.319582	0.396849	0.805299	0.420647	0.705295	SYCE1L	1E+08	synaptonemal complex central element protein 1 like
ENSG0000	1486.14	0.080835	0.100475	0.804534	0.421089	0.705568	TMEM59	9528	transmembrane protein 59
ENSG0000	2.727438	-1.29021	1.603353	-0.80469	0.420996	0.705568	NA	NA	NA
ENSG0000	195.6979	0.160503	0.199485	0.804582	0.421061	0.705568	NA	NA	NA
ENSG0000	3.880958	-1.17377	1.458884	-0.80457	0.421071	0.705568	NA	NA	NA
ENSG0000	33.64252	-0.34454	0.428158	-0.8047	0.420991	0.705568	SDCBP2	27111	syndecan binding protein 2
ENSG0000	6.651217	0.810899	1.007581	0.804798	0.420936	0.705568	INGX	27160	inhibitor o X-linked (pseudogene)
ENSG0000	10.80792	0.632587	0.786639	0.804164	0.421302	0.705577	NA	NA	NA
ENSG0000	109.1583	-0.26427	0.328527	-0.80442	0.421152	0.705577	NA	NA	NA
ENSG0000	689.0807	-0.09575	0.119058	-0.80422	0.421271	0.705577	SCML4	256380	Scm polycomb group protein like 4
ENSG0000	13.81114	0.587656	0.730674	0.804266	0.421243	0.705577	NA	NA	NA
ENSG0000	788.3456	-0.10855	0.134996	-0.80413	0.421323	0.705577	TMEM134	80194	transmembrane protein 134
ENSG0000	2376.618	-0.06614	0.082248	-0.80416	0.421307	0.705577	EDC4	23644	enhancer of mRNA decapping 4
ENSG0000	160.1795	-0.15375	0.191234	-0.804	0.421398	0.70564	UQCR11	10975	ubiquinol- complex III subunit XI
ENSG0000	3.620244	1.088292	1.353766	0.803899	0.421455	0.705644	CAV2	858	caveolin 2
ENSG0000	101.4204	-0.19417	0.241547	-0.80386	0.421476	0.705644	NA	NA	NA
ENSG0000	24.5385	-0.44931	0.559138	-0.80357	0.421643	0.705731	WDR86	349136	WD repeat domain 86
ENSG0000	973.5079	0.080861	0.100623	0.803609	0.421623	0.705731	CSTF2T	23283	cleavage stimulation factor subunit 2 tau variant
ENSG0000	35.42025	-0.31872	0.396614	-0.80361	0.421624	0.705731	LINC0065	149837	long intergenic non-protein coding RNA 654
ENSG0000	2220.076	-0.07244	0.090164	-0.8034	0.421746	0.70584	TNRC6B	23112	trinucleotide repeat containing adaptor 6B
ENSG0000	480.5514	-0.10899	0.135686	-0.80322	0.421848	0.705947	NDUFV3	4731	NADH:ubiquinone oxidoreductase subunit V3
ENSG0000	313.4206	0.126924	0.158066	0.802981	0.421985	0.70605	KIAA1522	57648	KIAA1522
ENSG0000	852.8488	-0.08896	0.110776	-0.80304	0.421953	0.70605	SHISAL2A	348378	shisa like 2A
ENSG0000	1369.411	-0.11702	0.145879	-0.80214	0.422473	0.706457	LMNB1	4001	lamin B1
ENSG0000	6.174511	0.781119	0.973443	0.802429	0.422305	0.706457	C7orf57	136288	chromosome 7 open reading frame 57
ENSG0000	259.4539	-0.12796	0.159495	-0.80231	0.422372	0.706457	ACBD5	91452	acyl-CoA binding domain containing 5
ENSG0000	119.8447	-0.18688	0.232989	-0.8021	0.422495	0.706457	AGAP9	642517	ArfGAP wi ankyrin repeat and PH domain 9
ENSG0000	506.7568	-0.1175	0.146479	-0.80216	0.422461	0.706457	TMX1	81542	thioredoxin related transmembrane protein 1
ENSG0000	2.171763	-1.40491	1.750915	-0.80238	0.422331	0.706457	NA	NA	NA
ENSG0000	13007.11	-0.07161	0.089265	-0.80221	0.42243	0.706457	RASAL3	64926	RAS protein activator like 3
ENSG0000	8.31288	-0.75243	0.938183	-0.80201	0.422548	0.706481	LSMEM1	286006	leucine rich single-pass membrane protein 1
ENSG0000	967.0035	0.103112	0.128589	0.80187	0.422628	0.706488	SACM1L	22908	SAC1 like phosphatidylinositide phosphatase
ENSG0000	1835.877	-0.09186	0.114557	-0.80192	0.422601	0.706488	CHFR	55743	checkpoint with forkhead and ring finger domains
ENSG0000	1387.798	0.072672	0.090662	0.801565	0.422804	0.706528	VDAC1	7416	voltage dependent anion channel 1
ENSG0000	65.8207	-0.29853	0.37235	-0.80173	0.422708	0.706528	NHSL1	57224	NHS like 1
ENSG0000	2389.894	-0.0667	0.083209	-0.80162	0.422772	0.706528	NUB1	51667	negative regulator of ubiquitin like proteins 1
ENSG0000	6934.98	0.058701	0.073222	0.801696	0.422729	0.706528	EWSR1	2130	EWS RNA binding protein 1

ENSG0000	146.3501	0.172398	0.215115	0.801421	0.422888	0.706603	SNAPC5	10302	small nuclear RNA activating complex polypeptide 5
ENSG0000	10.5235	0.739416	0.922755	0.801313	0.422951	0.706645	REN	5972	renin
ENSG0000	7.497085	-0.8195	1.023021	-0.80106	0.423099	0.706812	DCAF4L1	285429	DDB1 and CUL4 associated factor 4 like 1
ENSG0000	260.5154	-0.12128	0.151412	-0.80101	0.423127	0.706812	USP31	57478	ubiquitin specific peptidase 31
ENSG0000	7.322016	0.825285	1.030547	0.800822	0.423234	0.706896	FBXO24	26261	F-box protein 24
ENSG0000	4.497621	1.072086	1.338897	0.800724	0.423292	0.706896	NA	NA	NA
ENSG0000	15.74616	-0.53355	0.666285	-0.80078	0.423259	0.706896	ZNF415	55786	zinc finger protein 415
ENSG0000	284.8148	-0.12528	0.156512	-0.80048	0.423433	0.707068	NA	NA	NA
ENSG0000	12.62413	-0.6449	0.805857	-0.80027	0.423554	0.707208	NA	NA	NA
ENSG0000	7.720957	-0.79466	0.993152	-0.80013	0.423633	0.707275	TMSB4Y	9087	thymosin beta 4 Y-linked
ENSG0000	209.6425	0.14894	0.186215	0.799831	0.423809	0.707314	CMTM5	116173	CKLF like MARVEL transmembrane domain containing 5
ENSG0000	41.76214	-0.35336	0.441831	-0.79976	0.423847	0.707314	FLJ40194	124871	uncharacterized FLJ40194
ENSG0000	15.07585	-0.5909	0.738827	-0.79979	0.423834	0.707314	SHC2	25759	SHC adaptor protein 2
ENSG0000	1264.393	0.0779	0.097399	0.799801	0.423826	0.707314	SEC23B	10483	SEC23 hon COPII coat complex component
ENSG0000	44.32706	0.341005	0.426296	0.799924	0.423755	0.707314	F8	2157	coagulation factor VIII
ENSG0000	4.133993	-1.14828	1.436057	-0.7996	0.42394	0.707349	TLCD4	148534	TLC domain containing 4
ENSG0000	221.9659	-0.13764	0.172135	-0.7996	0.423944	0.707349	NA	NA	NA
ENSG0000	7309.727	0.06236	0.078027	0.799211	0.424168	0.707659	SF3B1	23451	splicing factor 3b subunit 1
ENSG0000	195.9548	-0.14291	0.178851	-0.79907	0.42425	0.707709	DFFB	1677	DNA fragmentation factor subunit beta
ENSG0000	3.820264	-1.19768	1.499067	-0.79895	0.424317	0.707709	WDFY3-AS	404201	WDFY3 antisense RNA 2
ENSG0000	5062.508	-0.07243	0.090656	-0.79898	0.424299	0.707709	FBXW5	54461	F-box and WD repeat domain containing 5
ENSG0000	75.85299	0.241345	0.302099	0.798896	0.42435	0.707709	ZNF626	199777	zinc finger protein 626
ENSG0000	10.05365	0.574017	0.718649	0.798745	0.424438	0.707792	PPP1R2B	153743	PPP1R2 family member B
ENSG0000	4169.446	-0.06258	0.078387	-0.79833	0.424679	0.708049	SP100	6672	SP100 nuclear antigen
ENSG0000	440.1565	0.100459	0.125844	0.798281	0.424707	0.708049	TIMM23	1E+08	translocase of inner mitochondrial membrane 23
ENSG0000	6.2441	0.921633	1.154484	0.798307	0.424692	0.708049	NA	NA	NA
ENSG0000	3473.687	-0.05801	0.072681	-0.79821	0.424749	0.708055	SNX1	6642	sorting nexin 1
ENSG0000	866.3689	0.091966	0.115235	0.798074	0.424828	0.708059	BIN3	55909	bridging integrator 3
ENSG0000	6.15546	0.797246	0.998886	0.798135	0.424792	0.708059	GLDC	2731	glycine decarboxylase
ENSG0000	5.438482	0.838204	1.050673	0.797778	0.424999	0.708281	GARIN5A	112703	golgi associated RAB2 interactor 5A
ENSG0000	227.8715	-0.13785	0.172824	-0.79766	0.42507	0.708335	SUCLA2	8803	succinate-CoA ligase ADP-forming subunit beta
ENSG0000	6.781929	-0.70895	0.889055	-0.79742	0.425206	0.708403	FABP3	2170	fatty acid binding protein 3
ENSG0000	627.7062	-0.09356	0.117331	-0.79737	0.425239	0.708403	SYT11	23208	synaptotagmin 11
ENSG0000	3.371958	-1.19983	1.505093	-0.79718	0.425346	0.708403	NA	NA	NA
ENSG0000	193.527	0.146366	0.183543	0.79745	0.42519	0.708403	LOC10537	1.05E+08	uncharacterized LOC105375743
ENSG0000	374.1253	-0.10519	0.131939	-0.79723	0.425318	0.708403	MRPS16	51021	mitochondrial ribosomal protein S16
ENSG0000	13.95315	0.531136	0.666314	0.797126	0.425378	0.708403	KRT80	144501	keratin 80
ENSG0000	6731.955	-0.07018	0.08802	-0.79727	0.425291	0.708403	CAPN15	6650	calpain 15
ENSG0000	277.5155	-0.11979	0.150302	-0.79697	0.425468	0.708489	NAIF1	203245	nuclear apoptosis inducing factor 1
ENSG0000	4.606894	-1.01893	1.278618	-0.7969	0.42551	0.708495	AKR7A3	22977	aldo-keto reductase family 7 member A3
ENSG0000	261.4691	0.147524	0.185228	0.796448	0.425772	0.708741	BRIX1	55299	biogenesis of ribosomes BRX1
ENSG0000	77.83646	-0.22868	0.287087	-0.79657	0.425702	0.708741	ERCC8	1161	ERCC excis CSA ubiquitin ligase complex subunit
ENSG0000	7226.038	-0.05635	0.070754	-0.79645	0.425771	0.708741	HCFC1	3054	host cell factor C1
ENSG0000	1710.832	-0.09438	0.118523	-0.79633	0.425839	0.708788	TCERG1	10915	transcription elongation regulator 1
ENSG0000	537.2901	0.106069	0.133258	0.795968	0.426051	0.709078	ECSIT	51295	ECSIT signaling integrator
ENSG0000	5.077086	-1.01689	1.277847	-0.79579	0.426157	0.709127	NNAT	4826	neuronatin
ENSG0000	2.516352	-1.23443	1.551091	-0.79585	0.42612	0.709127	GPM6B	2824	glycoprotein M6B
ENSG0000	130.8781	-0.1863	0.23413	-0.7957	0.426205	0.709143	ZDHHC13	54503	zinc finger DHHC-type palmitoyltransferase 13
ENSG0000	6088.505	0.078161	0.098291	0.795203	0.426495	0.709144	IFI16	3428	interferon gamma inducible protein 16
ENSG0000	489.2423	0.106857	0.134335	0.795447	0.426353	0.709144	AIMP1	9255	aminoacyl tRNA synthetase complex interacting multifunctional protein 1
ENSG0000	17.14146	0.438908	0.552008	0.795112	0.426548	0.709144	NA	NA	NA
ENSG0000	27.88895	0.473887	0.596089	0.794993	0.426617	0.709144	NA	NA	NA
ENSG0000	423.6352	0.103394	0.130056	0.794994	0.426617	0.709144	CPQ	10404	carboxypeptidase Q
ENSG0000	126.5677	-0.19828	0.249414	-0.79498	0.426626	0.709144	LINC0091C	1E+08	long intergenic non-protein coding RNA 910
ENSG0000	279.2022	-0.16074	0.202114	-0.79532	0.42643	0.709144	MPO	4353	myeloperoxidase
ENSG0000	7.148134	-0.91778	1.153532	-0.79562	0.42625	0.709144	NA	NA	NA
ENSG0000	2.376666	1.797856	2.26028	0.795413	0.426373	0.709144	LOC10537	1.05E+08	uncharacterized LOC105372165
ENSG0000	75.22724	-0.22807	0.286793	-0.79524	0.426473	0.709144	GRIN3B	116444	glutamate ionotropic receptor NMDA type subunit 3B
ENSG0000	190.5132	-0.15367	0.193269	-0.79509	0.426562	0.709144	KCTD15	79047	potassium channel tetramerization domain containing 15
ENSG0000	3.382096	-1.32594	1.668543	-0.79467	0.426807	0.709379	C5orf52	1E+08	chromosome 5 open reading frame 52
ENSG0000	228.279	0.130499	0.164231	0.794605	0.426843	0.709379	NDUFAF8	284184	NADH:ubiquinone oxidoreductase complex assembly factor 8
ENSG0000	14.91273	0.531217	0.668655	0.794455	0.42693	0.709444	EFNA3	1944	ephrin A3
ENSG0000	10.17816	0.667672	0.840467	0.794406	0.426959	0.709444	NA	NA	NA
ENSG0000	441.7103	-0.11412	0.143671	-0.79432	0.427007	0.70946	ARHGAP3:	115703	Rho GTPase activating protein 33
ENSG0000	30.12569	0.414832	0.522397	0.794092	0.427142	0.70962	C8G	733	complement C8 gamma chain
ENSG0000	88.03246	-0.30518	0.384371	-0.79398	0.427207	0.709664	COL26A1	136227	collagen type XXVI alpha 1 chain
ENSG0000	59.98918	-0.27431	0.34556	-0.79382	0.427301	0.709715	FITM1	161247	fat storage inducing transmembrane protein 1
ENSG0000	5608.845	-0.16879	0.212631	-0.7938	0.427314	0.709715	CYBB	1536	cytochrome b-245 beta chain
ENSG0000	18.19315	0.439332	0.553688	0.793465	0.427507	0.709718	RPS3P3	440991	ribosomal protein S3 pseudogene 3
ENSG0000	42.05876	-0.33417	0.421116	-0.79354	0.427465	0.709718	UNC5CL	222643	unc-5 family C-terminal like
ENSG0000	109.2084	-0.18152	0.22876	-0.79351	0.427482	0.709718	KBTBD7	84078	kelch repeat and BTB domain containing 7
ENSG0000	2217.732	-0.06942	0.087494	-0.79347	0.427504	0.709718	KDM4B	23030	lysine demethylase 4B
ENSG0000	1706.198	-0.0861	0.108478	-0.79368	0.427384	0.709718	SLC2A4RG	56731	SLC2A4 regulator
ENSG0000	16.48796	0.497903	0.627603	0.793341	0.427579	0.709775	GNRHR2	114814	gonadotropin releasing hormone receptor 2 (pseudogene)
ENSG0000	15.08307	-0.56876	0.716987	-0.79326	0.427626	0.709789	NA	NA	NA
ENSG0000	42.38996	0.283272	0.357169	0.793104	0.427717	0.709866	ZFP69B	65243	ZFP69 zinc finger protein B
ENSG0000	49.65021	-0.28042	0.353598	-0.79305	0.427749	0.709866	VPS33B	26276	VPS33B late endosome and lysosome associated
ENSG0000	1132.311	0.096332	0.121497	0.792876	0.42785	0.709907	LATS1	9113	large tumor suppressor kinase 1
ENSG0000	4378.151	0.062273	0.078536	0.792915	0.427828	0.709907	YY1	7528	YY1 transcription factor
ENSG0000	566.0268	-0.0906	0.114277	-0.79279	0.427899	0.709924	PJA1	64219	praja ring finger ubiquitin ligase 1
ENSG0000	99.7173	-0.22182	0.279923	-0.79244	0.428106	0.710144	MAGEF1	64110	MAGE family member F1
ENSG0000	5.52864	0.763528	0.963524	0.792434	0.428108	0.710144	PLAC8L1	153770	PLAC8 like 1
ENSG0000	240.5671	0.137067	0.173048	0.792074	0.428318	0.710428	PELO	53918	pelota mRNA surveillance and ribosome rescue factor
ENSG0000	3.700189	-1.10903	1.400911	-0.79165	0.428565	0.710481	NA	NA	NA
ENSG0000	340.2756	0.137027	0.173096	0.791625	0.42858	0.710481	CSNK1G3	1456	casein kinase 1 gamma 3
ENSG0000	715.9994	0.084882	0.107184	0.79193	0.428402	0.710481	DNAJA3	9093	DnaJ heat shock protein family (Hsp40) member A3
ENSG0000	117.7894	-0.18222	0.230109	-0.79188	0.42843	0.710481	RAB11B-A	1.01E+08	RAB11B antisense RNA 1
ENSG0000	6.269774	0.997029	1.259447	0.79164	0.428571	0.710481	NA	NA	NA
ENSG0000	361.0884	-0.11783	0.14883	-0.79171	0.428528	0.710481	CABLES2	81928	Cdk5 and Abl enzyme substrate 2
ENSG0000	39.44351	-0.29057	0.367192	-0.79134	0.428745	0.710693	NA	NA	NA
ENSG0000	60.34941	0.257472	0.325416	0.79121	0.428822	0.710756	PAK6	56924	p21 (RAC1) activated kinase 6
ENSG0000	3.711933	1.298621	1.641711	0.791017	0.428934	0.710815	NA	NA	NA
ENSG0000	269.8281	-0.13139	0.166104	-0.79102	0.428931	0.710815	HEATR6	63897	HEAT repeat containing 6
ENSG0000	11.2238	0.604568	0.764568	0.790732	0.429101	0.710871	NA	NA	NA
ENSG0000	1078.749	-0.08093	0.102349	-0.79071	0.429115	0.710871	TRIM27	5987	tripartite motif containing 27
ENSG0000	4.637172	1.073044	1.35677	0.790881	0.429014	0.710871	GSTA4	2941	glutathione S-transferase alpha 4
ENSG0000	25.86354	0.376972	0.476789	0.790648	0.429149	0.710871	NA	NA	NA
ENSG0000	107.765	-0.18438	0.233208	-0.79063	0.429159	0.710871	ANKRD42	338699	ankyrin repeat domain 42
ENSG0000	137.5417	-0.16529	0.209135	-0.79037	0.429314	0.710985	ZBTB26	57684	zinc finger and BTB domain containing 26
ENSG0000	154.7851	-0.15983	0.202229	-0.79032	0.429343	0.710985	SYNJ2BP	55333	synaptojanin 2 binding protein

ENSG0000	435.0371	0.101548	0.128479	0.790382	0.429305	0.710985	BEX4	56271	brain expressed X-linked 4
ENSG0000	5.641667	-0.83699	1.059307	-0.79013	0.429453	0.710998	NA	NA	NA
ENSG0000	109.1522	0.211028	0.267088	0.790105	0.429466	0.710998	ALDH1A2	8854	aldehyde dehydrogenase 1 family member A2
ENSG0000	15.14024	-0.49469	0.626004	-0.79023	0.429391	0.710998	MORC2-AS	150291	MORC2 antisense RNA 1
ENSG0000	22.17913	-0.41113	0.520412	-0.79001	0.429523	0.711029	COPZ2	51226	COPI coat complex subunit zeta 2
ENSG0000	610.1484	0.090002	0.113936	0.789936	0.429565	0.711035	ALKBH7	84266	alkB homolog 7
ENSG0000	57.33397	-0.24607	0.311705	-0.78944	0.429857	0.711045	BMP8B	656	bone morphogenetic protein 8b
ENSG0000	146.7213	-0.16406	0.207726	-0.78979	0.429651	0.711045	FLVCR1	28982	FLVCR heme transporter 1
ENSG0000	493.6607	-0.12798	0.162054	-0.78975	0.429675	0.711045	CCDC167	154467	coiled-coil domain containing 167
ENSG0000	6451.706	-0.06824	0.086432	-0.78948	0.42983	0.711045	TRBC1	28639	T cell receptor beta constant 1
ENSG0000	4947.648	0.071483	0.090554	0.7894	0.429878	0.711045	EIF4EBP2	1979	eukaryotic translation initiation factor 4E binding protein 2
ENSG0000	4.362391	-2.37748	3.01108	-0.78958	0.429774	0.711045	NA	NA	NA
ENSG0000	1.820899	-1.64152	2.079365	-0.78943	0.429858	0.711045	NA	NA	NA
ENSG0000	2.318865	1.460507	1.850079	0.78943	0.429861	0.711045	TLCD3B	83723	TLC domain containing 3B
ENSG0000	92.96259	0.200593	0.254178	0.78918	0.430007	0.711194	NA	NA	NA
ENSG0000	9.478737	0.784699	0.994714	0.788869	0.430188	0.711305	SOX7	83595	SRY-box transcription factor 7
ENSG0000	933.2218	-0.07782	0.098643	-0.7889	0.43017	0.711305	ABCD4	5826	ATP binding cassette subfamily D member 4
ENSG0000	11.1774	-0.61658	0.781597	-0.78888	0.430184	0.711305	IGHV2-70	28454	immunoglobulin heavy variable 2-70
ENSG0000	5.875832	0.900411	1.14154	0.788768	0.430247	0.711327	NA	NA	NA
ENSG0000	4.073066	-1.06676	1.35255	-0.7887	0.430285	0.711327	LINC02415	1.07E+08	long intergenic non-protein coding RNA 2415
ENSG0000	292.339	0.119244	0.151201	0.788649	0.430317	0.711327	LINC00641	283624	long intergenic non-protein coding RNA 641
ENSG0000	9.193506	-0.71684	0.909042	-0.78857	0.430363	0.71134	LINC02910	284756	long intergenic non-protein coding RNA 2910
ENSG0000	150.1821	0.214516	0.272117	0.788323	0.430508	0.711515	NA	NA	NA
ENSG0000	53.50435	-0.25867	0.328204	-0.78813	0.430621	0.71164	ZNF541	84215	zinc finger protein 541
ENSG0000	22.284	-0.42027	0.533421	-0.78787	0.430772	0.711736	C3orf14	57415	chromosome 3 open reading frame 14
ENSG0000	211.1398	-0.14551	0.18468	-0.7879	0.430756	0.711736	NA	NA	NA
ENSG0000	25.79854	-0.39529	0.501746	-0.78783	0.430795	0.711736	NA	NA	NA
ENSG0000	2841.78	0.074285	0.094309	0.78767	0.43089	0.71183	UBXN4	23190	UBX domain protein 4
ENSG0000	238.5075	0.132099	0.167817	0.787163	0.431186	0.712066	IQCB1	9657	IQ motif containing B1
ENSG0000	549.6549	0.106855	0.135717	0.787336	0.431085	0.712066	VEZT	55591	vezatin adherens junctions transmembrane protein
ENSG0000	12.15903	0.683948	0.868827	0.787208	0.43116	0.712066	NA	NA	NA
ENSG0000	14.70727	0.506798	0.643753	0.787255	0.431132	0.712066	KLK14	43847	kallikrein related peptidase 14
ENSG0000	26.00182	0.36059	0.458135	0.787083	0.431233	0.71208	NA	NA	NA
ENSG0000	8.873479	0.659474	0.838024	0.786939	0.431317	0.712092	TMEM14A	28978	transmembrane protein 14A
ENSG0000	22.64726	-0.49498	0.628956	-0.78699	0.431287	0.712092	RBM26-AS	1.01E+08	RBM26 antisense RNA 1
ENSG0000	78.981	0.22868	0.290622	0.786862	0.431363	0.712103	TXNDC16	57544	thioredoxin domain containing 16
ENSG0000	23.80782	0.557746	0.709183	0.786464	0.431596	0.712425	NA	NA	NA
ENSG0000	548.2529	0.099315	0.126314	0.786256	0.431718	0.712563	RO60	6738	Ro60 Y RNA binding protein
ENSG0000	48.56738	0.326785	0.415678	0.78615	0.43178	0.712602	SELENBP1	8991	selenium binding protein 1
ENSG0000	1506.168	0.081914	0.104209	0.786054	0.431836	0.712631	TRIM11	81559	tripartite motif containing 11
ENSG0000	139.0617	0.184163	0.234394	0.785699	0.432044	0.712744	IGKV4-1	28908	immunoglobulin kappa variable 4-1
ENSG0000	2.421196	1.486378	1.891422	0.785852	0.431954	0.712744	NA	NA	NA
ENSG0000	977.893	0.079791	0.101557	0.785675	0.432058	0.712744	WRNIP1	56897	WRN helicase interacting protein 1
ENSG0000	277.6738	-0.11875	0.151117	-0.78579	0.431989	0.712744	EFNB1	1947	ephrin B1
ENSG0000	5.953308	0.956303	1.217545	0.785436	0.432198	0.712912	NA	NA	NA
ENSG0000	371.8267	0.119951	0.152766	0.785194	0.43234	0.713082	DNAJC9	23234	DnaJ heat shock protein family (Hsp40) member C9
ENSG0000	701.5071	0.084853	0.108102	0.784937	0.43249	0.713149	AGO3	192669	argonaute RISC catalytic component 3
ENSG0000	76.08468	0.21766	0.277294	0.784942	0.432488	0.713149	CNN3	1266	calponin 3
ENSG0000	7.452204	0.654644	0.834018	0.784928	0.432496	0.713149	EEF1A1P22	645693	eukaryotic translation elongation factor 1 alpha 1 pseudogene 22
ENSG0000	71.47425	-0.22304	0.284337	-0.78441	0.432801	0.713588	SEC22A	26984	SEC22 hon vesicle trafficking protein
ENSG0000	180.068	-0.16127	0.205701	-0.78398	0.433049	0.713836	FASTKD1	79675	FAST kinase domains 1
ENSG0000	461.5392	-0.09844	0.125583	-0.78389	0.433105	0.713836	UVSSA	57654	UV stimulated scaffold protein A
ENSG0000	7.61498	-0.72915	0.930078	-0.78397	0.433057	0.713836	NA	NA	NA
ENSG0000	400.1967	0.11671	0.148886	0.783893	0.433103	0.713836	RAB34	83871	RAB34 member RAS oncogene family
ENSG0000	64.49948	-0.23295	0.297272	-0.78364	0.433251	0.714013	TRAV13-1	28671	T cell receptor alpha variable 13-1
ENSG0000	425.9536	-0.11656	0.148766	-0.78351	0.433326	0.714073	SLC25A46	91137	solute carrier family 25 member 46
ENSG0000	101.3079	0.219825	0.280641	0.783295	0.433454	0.714221	NA	NA	NA
ENSG0000	292.5817	-0.12197	0.155769	-0.78303	0.43361	0.714415	CDK8	1024	cyclin dependent kinase 8
ENSG0000	4326.042	-0.06671	0.085243	-0.78261	0.433856	0.714693	PRKACA	5566	protein kinase cAMP-activated catalytic subunit alpha
ENSG0000	39.05836	-0.30235	0.386333	-0.78261	0.433854	0.714693	TPX2	22974	TPX2 microtubule nucleation factor
ENSG0000	5.696792	0.821557	1.049863	0.782538	0.433899	0.7147	LOC10013	1E+08	uncharacterized LOC100132249
ENSG0000	3.655592	-1.12201	1.434031	-0.78242	0.43397	0.714754	NA	NA	NA
ENSG0000	147.3325	-0.17085	0.218387	-0.78231	0.43403	0.714789	FBXO8	26269	F-box protein 8
ENSG0000	9.667404	-0.63508	0.81199	-0.78213	0.434138	0.714844	GTF2IRD2	84163	GTF2I repeat domain containing 2
ENSG0000	30.97679	-0.37943	0.48512	-0.78213	0.43414	0.714844	MPP2	4355	MAGUK p55 scaffold protein 2
ENSG0000	593.7676	-0.1073	0.137215	-0.78195	0.434241	0.714939	BROX	148362	BRO1 domain and CAAX motif containing
ENSG0000	659.6842	0.112887	0.144375	0.781897	0.434275	0.714939	COPS8	10920	COP9 signalosome subunit 8
ENSG0000	75.18603	-0.23774	0.304206	-0.7815	0.434509	0.715069	NCKAP1	10787	NCK associated protein 1
ENSG0000	115.8661	-0.23533	0.301118	-0.78153	0.434488	0.715069	CEP19	84984	centrosomal protein 19
ENSG0000	10.90737	-0.73929	0.945835	-0.78163	0.434435	0.715069	COBL	23242	cordon-bleu WH2 repeat protein
ENSG0000	397.8407	0.110432	0.141281	0.781647	0.434422	0.715069	MYRF	745	myelin regulatory factor
ENSG0000	192.543	0.1566	0.200448	0.781248	0.434657	0.715122	CMC1	152100	C-X9-C motif containing 1
ENSG0000	13.04427	-0.57397	0.734629	-0.78131	0.434619	0.715122	NCAPG	64151	non-SMC condensin I complex subunit G
ENSG0000	297.8108	0.117917	0.150923	0.781303	0.434625	0.715122	CTU1	90353	cytosolic thiouridylase subunit 1
ENSG0000	3.093492	1.271067	1.627574	0.780958	0.434827	0.71534	NA	NA	NA
ENSG0000	406.638	-0.10677	0.13676	-0.78075	0.434952	0.715418	PRDM11	56981	PR/SET domain 11
ENSG0000	94.42261	0.201756	0.258398	0.780795	0.434923	0.715418	CEP76	79959	centrosomal protein 76
ENSG0000	41.87515	0.326035	0.417839	0.780288	0.435221	0.715734	ADGRB2	576	adhesion G protein-coupled receptor B2
ENSG0000	45.54871	0.332693	0.426341	0.780344	0.435188	0.715734	COL11A2	1302	collagen type XI alpha 2 chain
ENSG0000	225.2643	0.145213	0.186121	0.780207	0.435269	0.715749	KLHL7	55975	kelch like family member 7
ENSG0000	4380.411	0.066312	0.08503	0.779865	0.43547	0.715931	ZNF384	171017	zinc finger protein 384
ENSG0000	114.8357	-0.18688	0.239651	-0.77982	0.435495	0.715931	ACOT2	10965	acyl-CoA thioesterase 2
ENSG0000	183.4733	0.162737	0.208653	0.779941	0.435425	0.715931	NA	NA	NA
ENSG0000	12.29187	0.520565	0.667763	0.779566	0.435647	0.716116	ATP7B	540	ATPase copper transporting beta
ENSG0000	5.180372	-0.91732	1.177292	-0.77918	0.435876	0.716285	NA	NA	NA
ENSG0000	5.315035	-0.77665	0.996754	-0.77918	0.435875	0.716285	NA	NA	NA
ENSG0000	10.7525	-0.61266	0.786165	-0.77931	0.435799	0.716285	PRC1-AS1	1.01E+08	PRC1 antisense RNA 1
ENSG0000	2.838297	-1.45621	1.869029	-0.77913	0.435904	0.716285	NA	NA	NA
ENSG0000	40.48702	-0.33584	0.431137	-0.77895	0.436007	0.716365	PRIMPOL	201973	primase and DNA directed polymerase
ENSG0000	839.4913	-0.07904	0.101479	-0.77892	0.43603	0.716365	DDX49	54555	DEAD-box helicase 49
ENSG0000	98.76494	-0.2053	0.263598	-0.77884	0.436074	0.716375	CYB5D2	124936	cytochrome b5 domain containing 2
ENSG0000	4.153578	-0.93639	1.203133	-0.77829	0.436397	0.716709	AARD	441376	alanine and arginine rich domain containing protein
ENSG0000	35.09427	-0.30342	0.38982	-0.77835	0.436361	0.716709	KBTBD3	143879	kelch repeat and BTB domain containing 3
ENSG0000	703.6511	0.082085	0.105465	0.778316	0.436383	0.716709	SLC35B1	10237	solute carrier family 35 member B1
ENSG0000	15.34597	0.470666	0.604789	0.778232	0.436432	0.716709	PLCG1-AS1	1.02E+08	PLCG1 antisense RNA 1
ENSG0000	677.3414	0.082791	0.106404	0.778078	0.436523	0.716794	MDM4	4194	MDM4 regulator of p53
ENSG0000	3397.461	-0.0667	0.085751	-0.77784	0.436663	0.716944	ZC3H3	23144	zinc finger CCCH-type containing 3
ENSG0000	21.01898	0.425123	0.546577	0.777793	0.436691	0.716944	RPL5P34	388907	ribosomal protein L5 pseudogene 34
ENSG0000	6.246862	-0.79532	1.0233	-0.77721	0.437035	0.717317	RSPH9	221421	radial spoke head component 9
ENSG0000	1027.897	-0.0867	0.111544	-0.77727	0.437002	0.717317	GATD1	347862	glutamine amidotransferase class 1 domain containing 1

ENSG0000	4729.043	-0.05701	0.073349	-0.77726	0.437005	0.717317	BPTF	2186	bromodomain PHD finger transcription factor	
ENSG0000	228.0156	0.133697	0.172043	0.777115	0.437091	0.717346	TM2D1	83941	TM2 domain containing 1	
ENSG0000	769.511	0.113711	0.146408	0.776672	0.437353	0.717422	TMEM33	55161	transmembrane protein 33	
ENSG0000	28.33306	0.351942	0.453099	0.776745	0.437309	0.717422	GLI3	2737	GLI family zinc finger 3	
ENSG0000	23.48648	-0.39368	0.506872	-0.77669	0.437343	0.717422	EEF1DP2	442429	eukaryotic translation elongation factor 1 delta pseudogene 2	
ENSG0000	12.00452	0.547231	0.704546	0.776715	0.437327	0.717422	NA	NA	NA	
ENSG0000	145.4702	0.166504	0.214337	0.776833	0.437257	0.717422	NA	NA	NA	
ENSG0000	6.825643	-0.81425	1.048428	-0.77664	0.437369	0.717422	NA	NA	NA	
ENSG0000	10.55809	-0.56966	0.733645	-0.77648	0.437465	0.717437	ALG14	199857	ALG14 UDP-N-acetylglucosaminyltransferase subunit	
ENSG0000	11.46391	-0.56317	0.725392	-0.77637	0.437533	0.717437	NA	NA	NA	
ENSG0000	4454.742	-0.06239	0.080336	-0.77656	0.437419	0.717437	KDM2B	84678	lysine demethylase 2B	
ENSG0000	14.27364	-0.51378	0.661748	-0.77639	0.437518	0.717437	MYO16	23026	myosin XVI	
ENSG0000	120.9264	-0.20661	0.266254	-0.77599	0.437758	0.717661	NA	NA	NA	
ENSG0000	7.349175	-0.72282	0.931543	-0.77594	0.437786	0.717661	NA	NA	NA	
ENSG0000	1225.131	0.073404	0.09459	0.776019	0.437738	0.717661	MYO1C	4641	myosin IC	
ENSG0000	8.604892	-0.7644	0.985298	-0.77581	0.437863	0.717724	SLC28A3	64078	solute carrier family 28 member 3	
ENSG0000	106.3177	0.202881	0.261531	0.775741	0.437902	0.717724	SIGLEC1	6614	sialic acid binding Ig like lectin 1	
ENSG0000	4.97458	-1.07774	1.389591	-0.77558	0.437995	0.717813	CAPNS2	84290	calpain small subunit 2	
ENSG0000	52.29101	0.256421	0.330752	0.775267	0.438182	0.717993	GPS2	2874	G protein pathway suppressor 2	
ENSG0000	1588.278	-0.07396	0.095393	-0.77527	0.438178	0.717993	ATP6AP2	10159	ATPase H+ transporting accessory protein 2	
ENSG0000	103.6509	0.189008	0.243842	0.775126	0.438265	0.718066	NEMP2	1E+08	nuclear envelope integral membrane protein 2	
ENSG0000	11.5736	0.498601	0.643448	0.77489	0.438405	0.718185	NA	NA	NA	
ENSG0000	725.7343	0.100384	0.129574	0.774725	0.438502	0.718185	SPCS1	28972	signal peptidase complex subunit 1	
ENSG0000	228.1718	-0.15033	0.19407	-0.77461	0.43857	0.718185	SOX4	6659	SRY-box transcription factor 4	
ENSG0000	123.9986	-0.22415	0.289344	-0.77467	0.438535	0.718185	TEKT4P2	1E+08	tektin 4 pseudogene 2	
ENSG0000	28.4696	-0.35686	0.46065	-0.7747	0.438518	0.718185	MGAT3	4248	beta-1	4-mannosyl-glycoprotein 4-beta-N-acetylglucosaminyltransferase
ENSG0000	196.8357	-0.15437	0.19923	-0.77483	0.43844	0.718185	MTM1	4534	myotubularin 1	
ENSG0000	357.4141	-0.11146	0.143945	-0.77433	0.438736	0.718393	AACS	65985	acetoacetyl-CoA synthetase	
ENSG0000	6.199459	0.787804	1.017546	0.774219	0.438801	0.718436	NDUFA7	4701	NADH:ubiquinone oxidoreductase subunit A7	
ENSG0000	821.6048	-0.08968	0.115856	-0.77409	0.438878	0.718498	P3H1	64175	prolyl 3-hydroxylase 1	
ENSG0000	134.2099	-0.17072	0.22057	-0.77398	0.438945	0.718544	CHCHD1	118487	coiled-coil-helix-coiled-coil-helix domain containing 1	
ENSG0000	16.93348	0.540227	0.698255	0.773681	0.43912	0.718573	RCAN3AS	1.01E+08	RCAN3 antisense RNA	
ENSG0000	11.98167	0.585369	0.756411	0.773877	0.439004	0.718573	NIM1K	167359	NIM1 serine/threonine protein kinase	
ENSG0000	6.875542	0.866905	1.120583	0.77362	0.439156	0.718573	PRSS35	167681	serine protease 35	
ENSG0000	1249.595	0.086188	0.111394	0.773715	0.439099	0.718573	FBXL3	26224	F-box and leucine rich repeat protein 3	
ENSG0000	76.57692	-0.30359	0.392403	-0.77367	0.439124	0.718573	HEXIM2	124790	HEXIM P-TEFb complex subunit 2	
ENSG0000	103.7742	-0.20266	0.262134	-0.77313	0.439448	0.718861	MTLN	205251	mitoregulin	
ENSG0000	41.25204	0.33024	0.427136	0.77315	0.439433	0.718861	ASNS	440	asparagine synthetase (glutamine-hydrolyzing)	
ENSG0000	346.3905	0.169658	0.219425	0.773192	0.439408	0.718861	NLRP6	171389	NLR family pyrin domain containing 6	
ENSG0000	4.756158	0.945969	1.224412	0.77259	0.439765	0.719252	IGHV1-58	28464	immunoglobulin heavy variable 1-58	
ENSG0000	2.980138	1.244005	1.610147	0.772603	0.439757	0.719252	NA	NA	NA	
ENSG0000	2593.774	0.061609	0.079772	0.772311	0.43993	0.719459	FADS3	3995	fatty acid desaturase 3	
ENSG0000	64.46391	-0.23911	0.309641	-0.77222	0.439984	0.719484	NAP1L2	4674	nucleosome assembly protein 1 like 2	
ENSG0000	2.243631	1.78141	2.307994	0.771843	0.440207	0.719665	NA	NA	NA	
ENSG0000	3633.964	0.061232	0.07933	0.771869	0.440192	0.719665	PRCC	5546	proline rich mitotic checkpoint control factor	
ENSG0000	11.09871	-0.58322	0.755821	-0.77164	0.440328	0.719665	NA	NA	NA	
ENSG0000	964.25	0.07778	0.100789	0.771704	0.44029	0.719665	MBD4	8930	methyl-Cp	DNA glycosylase
ENSG0000	108.2661	0.186168	0.24122	0.771779	0.440245	0.719665	TRMT11	60487	tRNA methyltransferase 11 homolog	
ENSG0000	13.40903	0.479808	0.621788	0.771659	0.440316	0.719665	TDRP	157695	testis development related protein	
ENSG0000	28.30249	-0.39782	0.516132	-0.77078	0.44084	0.720036	ATP6V1C2	245973	ATPase H+ transporting V1 subunit C2	
ENSG0000	26.35079	-0.36006	0.466893	-0.77119	0.440595	0.720036	PTPDC1	138639	protein tyrosine phosphatase domain containing 1	
ENSG0000	7.410109	-0.75776	0.983174	-0.77073	0.440866	0.720036	CDNF	441549	cerebral dopamine neurotrophic factor	
ENSG0000	702.2461	0.089491	0.116069	0.77102	0.440695	0.720036	ZNF408	79797	zinc finger protein 408	
ENSG0000	175.8947	0.167518	0.217324	0.770822	0.440812	0.720036	TIMM9	26520	translocase of inner mitochondrial membrane 9	
ENSG0000	266.9652	0.135881	0.176271	0.770867	0.440785	0.720036	LENG1	79165	leukocyte receptor cluster member 1	
ENSG0000	10.10029	0.572763	0.742773	0.771114	0.440639	0.720036	NA	NA	NA	
ENSG0000	5.909795	-0.7708	0.999751	-0.77099	0.440713	0.720036	NA	NA	NA	
ENSG0000	7.337788	-0.89742	1.164911	-0.77038	0.441076	0.720081	NEURL1B	54492	neuralized E3 ubiquitin protein ligase 1B	
ENSG0000	496.4136	0.091381	0.118595	0.770529	0.440986	0.720081	GDF11	10220	growth differentiation factor 11	
ENSG0000	272.849	-0.13013	0.168898	-0.77045	0.441031	0.720081	HOXB4	3214	homeobox B4	
ENSG0000	12.01795	-0.52038	0.675414	-0.77046	0.441029	0.720081	NA	NA	NA	
ENSG0000	11.28433	0.545955	0.708708	0.770352	0.441091	0.720081	CYYR1	116159	cysteine and tyrosine rich 1	
ENSG0000	2146.895	-0.1093	0.141899	-0.77029	0.441126	0.720081	MX1	4599	MX dynamin like GTPase 1	
ENSG0000	17.00977	-0.42025	0.545624	-0.77021	0.441174	0.720096	STON1	11037	stonin 1	
ENSG0000	1860.974	0.078978	0.102561	0.770064	0.441262	0.720176	LTBP4	8425	latent transforming growth factor beta binding protein 4	
ENSG0000	1181.567	0.086316	0.112154	0.769624	0.441523	0.720222	CASP2	835	caspase 2	
ENSG0000	252.4123	0.125769	0.163405	0.769672	0.441494	0.720222	POMK	84197	protein O-mannose kinase	
ENSG0000	6.598868	0.836861	1.087331	0.769647	0.441509	0.720222	OR7E14P	10819	olfactory receptor family 7 subfamily E member 14 pseudogene	
ENSG0000	2506.808	0.062648	0.081389	0.76973	0.44146	0.720222	RAB31	11031	RAB31	member RAS oncogene family
ENSG0000	28.00013	-0.40248	0.522849	-0.76978	0.44143	0.720222	BRSK1	84446	BR serine/threonine kinase 1	
ENSG0000	1742.245	0.070479	0.09154	0.769924	0.441345	0.720222	NUP50	10762	nucleoporin 50	
ENSG0000	942.2896	0.080073	0.104121	0.769044	0.441867	0.720277	PPP1R8	5511	protein phosphatase 1 regulatory subunit 8	
ENSG0000	7.122512	0.63649	0.827341	0.76932	0.441703	0.720277	NA	NA	NA	
ENSG0000	6.37317	0.940293	1.222194	0.769349	0.441686	0.720277	IQCH	64799	IQ motif containing H	
ENSG0000	27.11535	-0.38763	0.503999	-0.7691	0.441833	0.720277	AMH	268	anti-Mullerian hormone	
ENSG0000	113.3974	-0.19763	0.256974	-0.76906	0.44186	0.720277	ADGRE4P	326342	adhesion (	pseudogene
ENSG0000	4383.251	0.062993	0.081904	0.769101	0.441833	0.720277	TRPC4AP	26133	transient receptor potential cation channel subfamily C member 4 associated protein	
ENSG0000	25.06845	-0.42264	0.549551	-0.76907	0.44185	0.720277	URB1-AS1	84996	URB1 antisense RNA 1 (head to head)	
ENSG0000	75.75127	-0.21841	0.283878	-0.76938	0.441667	0.720277	TMEM187	8269	transmembrane protein 187	
ENSG0000	19.92886	-0.4097	0.532795	-0.76896	0.441914	0.72029	ANKAR	150709	ankyrin and armadillo repeat containing	
ENSG0000	956.5701	0.089596	0.116564	0.768642	0.442106	0.720539	SNX2	6643	sorting nexin 2	
ENSG0000	153.569	-0.17867	0.232499	-0.76846	0.442212	0.720649	PAK1IP1	55003	PAK1 interacting protein 1	
ENSG0000	28901.71	0.16502	0.214804	0.768236	0.442347	0.720806	HBA2	3040	hemoglobin subunit alpha 2	
ENSG0000	15.29178	0.551574	0.718287	0.767902	0.442546	0.720978	LOC10537	1.05E+08	uncharacterized LOC105378663	
ENSG0000	178.328	0.161226	0.209967	0.767862	0.442569	0.720978	MTHFD1	4522	methylene	cyclohydrolase and formyltetrahydrofolate synthetase 1
ENSG0000	24.53028	0.462993	0.602961	0.767865	0.442567	0.720978	IGHJ5	28476	immunoglobulin heavy joining 5	
ENSG0000	9.074037	0.594943	0.775144	0.767525	0.442769	0.721073	NA	NA	NA	
ENSG0000	13.58106	-0.62927	0.819757	-0.76763	0.442709	0.721073	BCAP29	55973	B cell receptor associated protein 29	
ENSG0000	299.1099	-0.12034	0.156805	-0.76744	0.442822	0.721073	DBH-AS1	138948	DBH antisense RNA 1	
ENSG0000	1759.957	-0.08323	0.108451	-0.76747	0.442803	0.721073	SLC25A28	81894	solute carrier family 25 member 28	
ENSG0000	1530.702	0.070224	0.091489	0.767567	0.442744	0.721073	DNAJC7	7266	DnaJ heat shock protein family (Hsp40) member C7	
ENSG0000	19.91154	-0.49204	0.641508	-0.76701	0.443078	0.721364	LRRC51	1.2E+08	leucine rich repeat containing 51	
ENSG0000	57.30233	-0.24704	0.322076	-0.76702	0.44307	0.721364	NDUFAF1	51103	NADH:ubiquinone oxidoreductase complex assembly factor 1	
ENSG0000	142.5517	0.155757	0.203105	0.76688	0.443153	0.721422	STK3	6788	serine/threonine kinase 3	
ENSG0000	1187.027	-0.07621	0.099392	-0.76676	0.443226	0.721478	POLDIP2	26073	DNA polymerase delta interacting protein 2	
ENSG0000	5.795962	-0.84127	1.097633	-0.76644	0.443416	0.721534	OXTR	5021	oxytocin receptor	
ENSG0000	163.9393	0.154713	0.201848	0.766485	0.443388	0.721534	ACTR3B	57180	actin related protein 3B	
ENSG0000	618.8252	0.093805	0.122366	0.766592	0.443324	0.721534	CCDC6	8030	coiled-coil domain containing 6	
ENSG0000	41.4111	0.323036	0.421445	0.766495	0.443382	0.721534	ZNF460	10794	zinc finger protein 460	
ENSG0000	92.66386	0.198038	0.258546	0.765971	0.443694	0.721922	PDZK1IP1	10158	PDZK1 interacting protein 1	

ENSG0000	93.92119	-0.21796	0.284631	-0.76576	0.443819	0.721998	HOOK1	51361	hook microtubule tethering protein 1
ENSG0000	4350.296	-0.08084	0.105557	-0.76579	0.443798	0.721998	MAF	4094	MAF bZIP transcription factor
ENSG0000	7.802526	-0.82057	1.071703	-0.76567	0.443872	0.722022	NA	NA	NA
ENSG0000	10.04291	-0.65052	0.849701	-0.76559	0.443922	0.72204	PTOV1-AS	1.01E+08	PTOV1 antisense RNA 1
ENSG0000	726.1837	0.085589	0.11183	0.765352	0.444062	0.722154	AGTRAP	57085	angiotensin II receptor associated protein
ENSG0000	71.02756	0.301119	0.393445	0.765338	0.44407	0.722154	IGLV1-47	28822	immunoglobulin lambda variable 1-47
ENSG0000	40.6122	-0.28741	0.375654	-0.76509	0.444217	0.722266	MIR4453H	54553	MIR4453 host gene
ENSG0000	19.00021	-0.43687	0.570992	-0.76511	0.444208	0.722266	EEF1AKMT	221143	EEF1A lysine methyltransferase 1
ENSG0000	2.420921	1.460688	1.910563	0.764533	0.44455	0.722532	NA	NA	NA
ENSG0000	29.95521	-0.34791	0.454938	-0.76475	0.444421	0.722532	MRC1	4360	mannose receptor C-type 1
ENSG0000	2.158124	1.780852	2.329463	0.76449	0.444575	0.722532	F7	2155	coagulation factor VII
ENSG0000	2.868611	-1.25675	1.643761	-0.76456	0.444537	0.722532	NA	NA	NA
ENSG0000	478.2137	0.093287	0.121998	0.764663	0.444472	0.722532	HIC2	23119	HIC ZBTB transcriptional repressor 2
ENSG0000	180.4243	0.138062	0.180625	0.76436	0.444653	0.722562	FAM220A	84792	family with sequence similarity 220 member A
ENSG0000	11.85424	0.562292	0.735668	0.764329	0.444671	0.722562	ZNF521	25925	zinc finger protein 521
ENSG0000	707.8361	0.086958	0.113813	0.764036	0.444846	0.722655	CAPN7	23473	calpain 7
ENSG0000	1907.225	-0.06239	0.081651	-0.76409	0.444817	0.722655	RAPGEF2	9693	Rap guanine nucleotide exchange factor 2
ENSG0000	56.19509	0.259987	0.340276	0.764046	0.44484	0.722655	LOC72855	728554	THO complex 3 pseudogene
ENSG0000	5.468569	0.894683	1.171113	0.76396	0.444891	0.722666	ARHGEF16	27237	Rho guanine nucleotide exchange factor 16
ENSG0000	1923.201	0.062738	0.082132	0.763868	0.444946	0.722692	ITPRID2	6744	ITPR interacting domain containing 2
ENSG0000	8.179938	-0.63957	0.837358	-0.76379	0.444992	0.722703	NA	NA	NA
ENSG0000	82.03743	0.200163	0.262094	0.763706	0.445042	0.722721	UBE2V1	7335	ubiquitin conjugating enzyme E2 V1
ENSG0000	234.864	0.133828	0.175327	0.763301	0.445284	0.723013	TGFA	7039	transforming growth factor alpha
ENSG0000	14.04532	-0.55347	0.725122	-0.76327	0.4453	0.723013	SLC4A10	57282	solute carrier family 4 member 10
ENSG0000	122.1601	-0.17328	0.227045	-0.7632	0.445345	0.723023	COX15	1355	cytochrome c oxidase assembly homolog COX15
ENSG0000	28.57417	-0.33075	0.433576	-0.76284	0.445557	0.72325	NA	NA	NA
ENSG0000	8.891358	0.707869	0.927947	0.762833	0.445563	0.72325	NEFL	4747	neurofilament light chain
ENSG0000	3.175993	-1.23218	1.616247	-0.76237	0.445839	0.723356	FAM81B	153643	family with sequence similarity 81 member B
ENSG0000	12.48917	-0.54176	0.710432	-0.76258	0.445712	0.723356	NA	NA	NA
ENSG0000	4.413888	1.217435	1.597126	0.762267	0.445901	0.723356	MUC12	10071	mucin 12 cell surface associated
ENSG0000	6.005468	0.823073	1.079709	0.76231	0.445875	0.723356	LINC02937	1.01E+08	long intergenic non-protein coding RNA 2937
ENSG0000	766.0517	-0.09354	0.122654	-0.76263	0.445687	0.723356	BMS1	9790	BMS1 ribosome biogenesis factor
ENSG0000	3.967189	1.243811	1.631309	0.762462	0.445784	0.723356	NA	NA	NA
ENSG0000	5.245938	0.814099	1.067835	0.762382	0.445832	0.723356	NA	NA	NA
ENSG0000	2832.69	-0.05766	0.075664	-0.76211	0.445997	0.723448	MAP2K7	5609	mitogen-activated protein kinase kinase 7
ENSG0000	131.2193	-0.17847	0.234316	-0.76167	0.446258	0.723623	TTC27	55622	tetratricopeptide repeat domain 27
ENSG0000	13.67692	-0.48173	0.632398	-0.76174	0.446212	0.723623	NA	NA	NA
ENSG0000	271.9949	-0.13474	0.17691	-0.76162	0.446287	0.723623	ISCA2	122961	iron-sulfur cluster assembly 2
ENSG0000	7.730975	0.772555	1.014379	0.761605	0.446296	0.723623	NA	NA	NA
ENSG0000	4.176715	-0.92445	1.213834	-0.7616	0.4463	0.723623	TUBA3FP	113691	tubulin alpha 3f pseudogene
ENSG0000	115.3516	0.189093	0.248343	0.761418	0.446408	0.723652	SEMA4F	10505	ssemaphorin 4F
ENSG0000	50.26216	-0.27944	0.367021	-0.76137	0.446438	0.723652	DTD2	112487	D-aminoacyl-tRNA deacylase 2
ENSG0000	88.6903	0.202212	0.265566	0.761438	0.446395	0.723652	MEX3B	84206	mex-3 RNA binding family member B
ENSG0000	43389.49	0.05707	0.074976	0.761176	0.446552	0.723652	FMNL1	752	formin like 1
ENSG0000	5.167515	1.012218	1.329726	0.761223	0.446524	0.723652	NA	NA	NA
ENSG0000	567.0221	0.106556	0.13997	0.761276	0.446492	0.723652	C21orf91	54149	chromosome 21 open reading frame 91
ENSG0000	10.77826	-0.62518	0.821425	-0.76109	0.446604	0.723674	NA	NA	NA
ENSG0000	9.724301	0.58241	0.765591	0.760732	0.446817	0.723776	RN7SL600	1.06E+08	RNA 7SL cytoplasm pseudogene
ENSG0000	37.56222	-0.30742	0.404112	-0.76072	0.446823	0.723776	NA	NA	NA
ENSG0000	38.1851	-0.28707	0.377309	-0.76084	0.446752	0.723776	CYB5D1	124637	cytochrome b5 domain containing 1
ENSG0000	6.642869	-0.6804	0.894325	-0.7608	0.446779	0.723776	CACNG8	59283	calcium voltage-gated channel auxiliary subunit gamma 8
ENSG0000	41.8979	-0.30527	0.401435	-0.76046	0.446982	0.723939	EFHD1	80303	EF-hand domain family member D1
ENSG0000	6.996638	-0.77427	1.018236	-0.76041	0.447012	0.723939	KLLN	1E+08	killin p53 regulated DNA replication inhibitor
ENSG0000	11.96788	-0.50153	0.659592	-0.76036	0.447041	0.723939	NA	NA	NA
ENSG0000	15.00449	-0.48886	0.643275	-0.75995	0.447285	0.723954	NA	NA	NA
ENSG0000	35.18705	-0.36008	0.473754	-0.76006	0.447216	0.723954	NA	NA	NA
ENSG0000	2.022141	-1.62832	2.142055	-0.76017	0.447156	0.723954	NA	NA	NA
ENSG0000	219.3764	-0.13037	0.171519	-0.76008	0.447207	0.723954	LINC0269	339263	long intergenic non-protein coding RNA 2693
ENSG0000	11.14966	-0.57797	0.760227	-0.76025	0.447103	0.723954	EFCAB13	124989	EF-hand calcium binding domain 13
ENSG0000	113.3252	0.190004	0.25	0.760014	0.447246	0.723954	ZC4H2	55906	zinc finger C4H2-type containing
ENSG0000	568.7122	0.09168	0.120654	0.759858	0.447339	0.723979	OCIAD2	132299	OCIA domain containing 2
ENSG0000	4.681378	0.945506	1.24457	0.759705	0.447431	0.724064	NA	NA	NA
ENSG0000	6281.921	0.053814	0.070844	0.759617	0.447484	0.724086	GIT1	28964	GIT ArfGAP 1
ENSG0000	42.78715	0.309203	0.407216	0.759308	0.447668	0.724322	LGALSL	29094	galectin like
ENSG0000	40.1213	0.303287	0.399519	0.75913	0.447775	0.724431	NEXN	91624	nexilin F-actin binding protein
ENSG0000	125.7063	0.17796	0.234479	0.758959	0.447877	0.724488	ST7L	54879	suppression of tumorigenicity 7 like
ENSG0000	4.96452	0.909316	1.198137	0.758941	0.447888	0.724488	NA	NA	NA
ENSG0000	2062.108	-0.08446	0.111295	-0.75886	0.447934	0.724499	JMJD8	339123	jumonji domain containing 8
ENSG0000	386.4646	0.104242	0.137399	0.758681	0.448043	0.724613	HCCS	3052	holocytochrome c synthase
ENSG0000	3.452641	-1.04833	1.38232	-0.75838	0.448222	0.724775	NA	NA	NA
ENSG0000	49.94791	0.276816	0.364994	0.758414	0.448203	0.724775	TMEM44	93109	transmembrane protein 44
ENSG0000	167.7301	0.155343	0.204911	0.7581	0.448391	0.724971	DNAJC27	51277	DnaJ heat shock protein family (Hsp40) member C27
ENSG0000	2011.809	0.069743	0.092009	0.758004	0.448448	0.724971	BTN2A1	11120	butyrophilin subfamily 2 member A1
ENSG0000	163.146	0.157118	0.207284	0.757985	0.44846	0.724971	POT1	25913	protection of telomeres 1
ENSG0000	3.5704	1.160098	1.530685	0.757895	0.448514	0.724995	COL20A1	57642	collagen type XX alpha 1 chain
ENSG0000	112.2884	-0.2017	0.266169	-0.7578	0.448574	0.725028	NA	NA	NA
ENSG0000	309.2182	0.120471	0.159006	0.757652	0.448659	0.72508	SEC61G	23480	SEC61 translocon subunit gamma
ENSG0000	3.124114	-1.26269	1.666673	-0.75761	0.448684	0.72508	NA	NA	NA
ENSG0000	221.6606	0.133397	0.176152	0.757288	0.448878	0.725203	NA	NA	NA
ENSG0000	121.1888	-0.17143	0.226356	-0.75735	0.448837	0.725203	MAPRE3	22924	microtubule associated protein RP/EB family member 3
ENSG0000	15.33228	0.51788	0.683814	0.75734	0.448846	0.725203	NA	NA	NA
ENSG0000	332.2362	0.117652	0.155378	0.757197	0.448932	0.725227	PPP1R3E	90673	protein phosphatase 1 regulatory subunit 3E
ENSG0000	3.873899	1.156465	1.527592	0.757051	0.44902	0.725306	NA	NA	NA
ENSG0000	6.887335	0.829423	1.095698	0.756981	0.449061	0.72531	COL17A1	1308	collagen type XVII alpha 1 chain
ENSG0000	200.8865	0.162863	0.215199	0.756799	0.44917	0.725423	ELANE	1991	elastase neutrophil expressed
ENSG0000	311.3204	0.117555	0.155391	0.756515	0.44934	0.725445	RRP9	9136	ribosomal U3 small nucleolar RNA binding protein
ENSG0000	188.4929	0.148529	0.196298	0.756652	0.449259	0.725445	NSMCE2	286053	NSE2 (MM SMC5-SMC6 complex SUMO ligase
ENSG0000	7.091514	-0.66893	0.884202	-0.75654	0.449328	0.725445	DEPDC7	91614	DEP domain containing 7
ENSG0000	13.9474	0.622344	0.822574	0.756581	0.449301	0.725445	TICRR	90381	TOPBP1 interacting checkpoint and replication regulator
ENSG0000	357.3493	-0.11234	0.148579	-0.75613	0.449572	0.725736	TMEM268	203197	transmembrane protein 268
ENSG0000	4.014439	1.095697	1.449173	0.756085	0.449599	0.725736	RNF222	643904	ring finger protein 222
ENSG0000	6.581058	0.668446	0.884348	0.755863	0.449731	0.725887	HDAC4-AS	1.02E+08	HDAC4 antisense RNA 1
ENSG0000	3.966295	-1.016	1.344341	-0.75576	0.449791	0.725891	GAL3ST1	9514	galactose-3-O-sulfotransferase 1
ENSG0000	13.06919	-0.51704	0.684157	-0.75573	0.449812	0.725891	NA	NA	NA
ENSG0000	112.3556	-0.19458	0.2575	-0.75565	0.449862	0.725908	POLR1HAS	80862	POLR1H ar pseudogene
ENSG0000	16.31299	0.481267	0.636994	0.755528	0.449932	0.725958	ARHGEF2-	1.08E+08	ARHGEF2 antisense RNA 2
ENSG0000	247.8967	-0.14807	0.19601	-0.7554	0.450009	0.72602	IFI44L	10964	interferon induced protein 44 like
ENSG0000	26.64785	0.327396	0.433481	0.755272	0.450086	0.72608	NA	NA	NA
ENSG0000	34.4805	-0.29721	0.393815	-0.7547	0.450427	0.726112	NA	NA	NA
ENSG0000	347.9825	0.12135	0.16083	0.754522	0.450536	0.726112	GUF1	60558	GTP binding elongation factor GUF1

ENSG0000	400.287	0.105369	0.139576	0.754922	0.450296	0.726112	FIP1L1	81608	factor interacting with PAPOLA and CPSF1
ENSG0000	68.55166	0.244066	0.3234	0.754688	0.450436	0.726112	ANXA3	306	annexin A3
ENSG0000	303.3438	0.111345	0.147565	0.754548	0.45052	0.726112	MOCS2	4338	molybdenum cofactor synthesis 2
ENSG0000	49.51733	-0.27202	0.360463	-0.75464	0.450465	0.726112	NA	NA	NA
ENSG0000	16.2793	0.439941	0.582629	0.755096	0.450191	0.726112	TIGD3	220359	tigger transposable element derived 3
ENSG0000	43.529	-0.2677	0.354495	-0.75517	0.450148	0.726112	CDIN1	84529	CDAN1 interacting nuclease 1
ENSG0000	584.2055	-0.10376	0.137496	-0.75464	0.450463	0.726112	COX5A	9377	cytochrome c oxidase subunit 5A
ENSG0000	5.700312	-1.04053	1.37811	-0.75504	0.450226	0.726112	NA	NA	NA
ENSG0000	222.5029	-0.12901	0.17095	-0.75464	0.450464	0.726112	BCL2L12	83596	BCL2 like 12
ENSG0000	97.53602	-0.1851	0.245398	-0.75429	0.450673	0.726268	CTSK	1513	cathepsin K
ENSG0000	2506.111	-0.07688	0.101939	-0.75419	0.450737	0.726309	ARFGAP1	55738	ADP ribosylation factor GTPase activating protein 1
ENSG0000	13.00015	-0.52448	0.695515	-0.75408	0.450798	0.726345	ZNF665	79788	zinc finger protein 665
ENSG0000	214.8119	-0.13935	0.184921	-0.75359	0.451097	0.726763	FANCE	2178	FA complementation group E
ENSG0000	889.4597	0.081847	0.108624	0.75349	0.451156	0.726794	MRFAP1L1	114932	Morf4 family associated protein 1 like 1
ENSG0000	2669.632	0.060359	0.080129	0.75327	0.451288	0.726944	CNPPD1	27013	cyclin Pas1/PHO80 domain containing 1
ENSG0000	76.68599	0.202202	0.268529	0.752996	0.451452	0.72702	NA	NA	NA
ENSG0000	30.92187	-0.35514	0.471619	-0.75303	0.451432	0.72702	TRIM16L	147166	tripartite motif containing 16 like
ENSG0000	7.744007	0.744234	0.98825	0.753083	0.4514	0.72702	BFSP1	631	beaded filament structural protein 1
ENSG0000	545.8488	0.099121	0.131692	0.752671	0.451647	0.727259	HSD17B11	51170	hydroxysteroid 17-beta dehydrogenase 11
ENSG0000	2856.73	-0.06328	0.084085	-0.75262	0.451679	0.727259	DDX54	79039	DEAD-box helicase 54
ENSG0000	3.658208	-0.93803	1.246726	-0.75239	0.451814	0.727412	SEMA6A	57556	semaphorin 6A
ENSG0000	2173.075	-0.07654	0.101751	-0.75226	0.451894	0.727479	ZNF646	9726	zinc finger protein 646
ENSG0000	255.9694	0.136122	0.180974	0.752163	0.451953	0.72751	KYAT3	56267	kynurenine aminotransferase 3
ENSG0000	291.6951	0.128503	0.170929	0.751791	0.452177	0.727747	PCYOX1	51449	prenylcysteine oxidase 1
ENSG0000	448.6199	-0.09901	0.1317	-0.75179	0.452179	0.727747	NXPE3	91775	neurexophilin and PC-esterase domain family member 3
ENSG0000	181.8381	0.16334	0.217299	0.751683	0.452242	0.727755	LRATD2	157638	LRAT domain containing 2
ENSG0000	4.110776	0.87471	1.163721	0.751649	0.452262	0.727755	TTC36-AS1	1.02E+08	TTC36 and KMT2A antisense RNA 1
ENSG0000	55.12096	0.269466	0.358568	0.751508	0.452347	0.727765	NA	NA	NA
ENSG0000	15.29335	0.503395	0.669808	0.751551	0.452321	0.727765	XKR7	343702	XK related 7
ENSG0000	183.0982	-0.14369	0.191258	-0.75131	0.452466	0.72788	TSNAX	7257	translin associated factor X
ENSG0000	5.944411	0.931702	1.240187	0.751259	0.452497	0.72788	NA	NA	NA
ENSG0000	4.66435	-0.9457	1.259384	-0.75093	0.452697	0.728137	NA	NA	NA
ENSG0000	4.461885	-0.94994	1.265128	-0.75086	0.452735	0.728137	IRX3	79191	iroquois homeobox 3
ENSG0000	615.4911	-0.09988	0.133046	-0.75075	0.452801	0.728143	FDPS	2224	farnesyl diphosphate synthase
ENSG0000	1250.207	-0.07591	0.101176	-0.75025	0.453106	0.728143	SLAMF6	114836	SLAM family member 6
ENSG0000	10.10958	0.65759	0.876775	0.75001	0.453249	0.728143	NA	NA	NA
ENSG0000	1470.34	-0.07578	0.100982	-0.75045	0.452985	0.728143	NDUFA10	4705	NADH:ubiquinone oxidoreductase subunit A10
ENSG0000	2741.508	-0.06804	0.090718	-0.75003	0.453237	0.728143	RAP2B	5912	RAP2B member of RAS oncogene family
ENSG0000	2.364486	-1.33159	1.774535	-0.75039	0.45302	0.728143	NA	NA	NA
ENSG0000	145.1425	-0.15756	0.209932	-0.75054	0.452932	0.728143	TMEM14C	51522	transmembrane protein 14C
ENSG0000	633.0338	0.087409	0.116525	0.75013	0.453176	0.728143	LMBRD1	55788	LMBR1 domain containing 1
ENSG0000	28.87101	-0.38005	0.506688	-0.75007	0.453212	0.728143	HDAC9	9734	histone deacetylase 9
ENSG0000	4.771686	0.890925	1.187447	0.750286	0.453083	0.728143	NA	NA	NA
ENSG0000	27.23614	-0.36478	0.485981	-0.7506	0.452896	0.728143	ZNF547	284306	zinc finger protein 547
ENSG0000	1085.985	-0.07639	0.10185	-0.75007	0.453214	0.728143	ZBTB45	84878	zinc finger and BTB domain containing 45
ENSG0000	11.36945	0.634646	0.845539	0.750582	0.452904	0.728143	NA	NA	NA
ENSG0000	4.352333	-1.02286	1.364049	-0.74987	0.453332	0.728213	NA	NA	NA
ENSG0000	352.8712	0.11881	0.158466	0.749749	0.453406	0.728269	MFAP3	4238	microfibril associated protein 3
ENSG0000	3.269209	-1.09929	1.467131	-0.74928	0.45369	0.728662	MFF-DT	654841	MFF divergent transcript
ENSG0000	562.4202	0.089707	0.11976	0.749057	0.453823	0.728714	RBBP5	5929	RB binding histone lysine methyltransferase complex subunit
ENSG0000	15.02222	-0.48071	0.641839	-0.74896	0.45388	0.728714	SEMA3B	7869	semaphorin 3B
ENSG0000	22.26255	0.436426	0.582683	0.748993	0.453861	0.728714	NA	NA	NA
ENSG0000	6.503641	-0.75931	1.013617	-0.74911	0.453789	0.728714	TMEM178	1.01E+08	transmembrane protein 178B
ENSG0000	36.27548	0.394334	0.526671	0.748729	0.454021	0.728873	GRM2	2912	glutamate metabotropic receptor 2
ENSG0000	4.898177	0.976711	1.304597	0.748669	0.454057	0.728873	IL6ST-DT	441072	IL6ST divergent transcript
ENSG0000	101.4018	-0.19594	0.262029	-0.74779	0.454588	0.729663	MKS1	54903	MKS transition zone complex subunit 1
ENSG0000	175.072	-0.14293	0.191208	-0.74753	0.454742	0.729744	ZSCAN26	7741	zinc finger and SCAN domain containing 26
ENSG0000	1626.242	0.072034	0.096365	0.747508	0.454757	0.729744	DMTF1	9988	cyclin D binding myb like transcription factor 1
ENSG0000	39.1532	-0.35196	0.47083	-0.74753	0.454745	0.729744	IDNK	414328	IDNK gluconokinase
ENSG0000	8472.844	0.060307	0.080763	0.746722	0.455231	0.730442	SLC6A6	6533	solute carrier family 6 member 6
ENSG0000	136.5026	-0.16248	0.217651	-0.7465	0.455367	0.730458	KANSL1L	151050	KAT8 regulatory NSL complex subunit 1 like
ENSG0000	6526.227	-0.06769	0.090674	-0.74649	0.455373	0.730458	CCND3	896	cyclin D3
ENSG0000	5.578682	-0.93804	1.256704	-0.74643	0.455407	0.730458	NA	NA	NA
ENSG0000	14.14732	-0.51711	0.692818	-0.74638	0.455436	0.730458	NA	NA	NA
ENSG0000	572.7851	-0.08662	0.116058	-0.74638	0.455438	0.730458	CBX1	10951	chromobox 1
ENSG0000	365.5337	0.1051	0.140828	0.746305	0.455483	0.730466	MRPL32	64983	mitochondrial ribosomal protein L32
ENSG0000	5.54138	-0.8397	1.125527	-0.74605	0.455636	0.730648	NA	NA	NA
ENSG0000	2.407089	-1.37164	1.838715	-0.74598	0.455682	0.730659	RNU4ATAC	1.06E+08	RNA U4atac sn pseudogene
ENSG0000	429.6121	-0.10333	0.138581	-0.74561	0.455903	0.730869	SLC31A1	1317	solute carrier family 31 member 1
ENSG0000	10.49816	-0.58125	0.779494	-0.74567	0.455866	0.730869	DEGS2	123099	delta 4-de sphingolipid 2
ENSG0000	2.56719	1.345499	1.804672	0.745564	0.455931	0.730869	NA	NA	NA
ENSG0000	322.2153	-0.11472	0.153897	-0.74546	0.455996	0.73091	MAP4K3	8491	mitogen-activated protein kinase kinase kinase kinase 3
ENSG0000	438.8164	0.107841	0.144762	0.744954	0.4563	0.731144	PHTF1	10745	putative homeodomain transcription factor 1
ENSG0000	3.907183	-0.96677	1.297698	-0.74499	0.45628	0.731144	NA	NA	NA
ENSG0000	2649.032	0.06197	0.083168	0.74512	0.456199	0.731144	BAZ1B	9031	bromodomain adjacent to zinc finger domain 1B
ENSG0000	3.163854	1.099244	1.475351	0.745072	0.456228	0.731144	MIR381HG	378881	MIR381 host gene
ENSG0000	17.7793	0.474736	0.63739	0.744812	0.456385	0.731218	LINC0130	1.02E+08	long intergenic non-protein coding RNA 1303
ENSG0000	6669.797	0.058933	0.079136	0.744705	0.45645	0.731259	TPI1	7167	triosephosphate isomerase 1
ENSG0000	301.1858	-0.11743	0.157797	-0.74416	0.45678	0.731455	NOL10	79954	nucleolar protein 10
ENSG0000	18.66323	-0.41768	0.561329	-0.74409	0.45682	0.731455	ZNF502	91392	zinc finger protein 502
ENSG0000	848.0709	-0.11249	0.151163	-0.74416	0.456779	0.731455	PRRC1	133619	proline rich coiled-coil 1
ENSG0000	36.48882	-0.29785	0.40034	-0.74398	0.456888	0.731455	TRBV6-1	28606	T cell receptor beta variable 6-1
ENSG0000	22.72648	-0.38365	0.515477	-0.74426	0.45672	0.731455	ADAMTSL2	9719	ADAMTS like 2
ENSG0000	216.0436	-0.15037	0.202026	-0.74432	0.456682	0.731455	RAD51-AS	1.01E+08	RAD51 antisense RNA 1
ENSG0000	15.18147	0.470489	0.632187	0.744225	0.45674	0.731455	PRSS22	64063	serine protease 22
ENSG0000	188.977	-0.13854	0.186195	-0.74404	0.45685	0.731455	C22orf46	79640	CTA-216E10.6
ENSG0000	62.4433	0.234542	0.315332	0.743793	0.457002	0.731499	NA	NA	NA
ENSG0000	333.0335	-0.11613	0.156128	-0.74383	0.456981	0.731499	RAP2A	5911	RAP2A member of RAS oncogene family
ENSG0000	5.074482	-0.90368	1.215051	-0.74374	0.457034	0.731499	NA	NA	NA
ENSG0000	76.6572	0.208326	0.280171	0.743569	0.457137	0.731601	ZNF286A	57335	zinc finger protein 286A
ENSG0000	288.743	0.12806	0.17226	0.743407	0.457235	0.731694	AASDHPPT	60496	aminoadipate-semialdehyde dehydrogenase-phosphopantetheinyl transferase
ENSG0000	987.1243	-0.07405	0.099635	-0.74322	0.457347	0.731811	METTL17	64745	methyltransferase like 17
ENSG0000	189.4564	-0.152	0.20457	-0.74304	0.457455	0.73192	TRMT10B	158234	tRNA methyltransferase 10B
ENSG0000	12.80915	0.477995	0.64357	0.742724	0.457649	0.732104	NA	NA	NA
ENSG0000	1085.065	-0.08329	0.112137	-0.74274	0.457637	0.732104	CRY2	1408	cryptochrome circadian regulator 2
ENSG0000	22888.44	-0.06289	0.084708	-0.74248	0.457794	0.732273	ZFP36L1	677	ZFP36 ring finger protein like 1
ENSG0000	161.3986	0.160673	0.216439	0.742349	0.457876	0.732341	EBP	10682	EBP cholestenol delta-isomerase
ENSG0000	4.163141	0.887411	1.195753	0.742136	0.458005	0.73242	NA	NA	NA
ENSG0000	7.29783	-0.65177	0.878442	-0.74196	0.458113	0.73242	SMG7-AS1	284649	SMG7 antisense RNA 1
ENSG0000	8.856622	-0.64974	0.875734	-0.74194	0.458123	0.73242	TMEM171	134285	transmembrane protein 171

ENSG0000	92.52983	-0.1912	0.257612	-0.74219	0.457971	0.73242	AKIP1	56672	A-kinase interacting protein 1
ENSG0000	42.68818	0.288932	0.389381	0.742028	0.45807	0.73242	TRPV3	162514	transient receptor potential cation channel subfamily V member 3
ENSG0000	18.95591	0.416876	0.561974	0.741806	0.458205	0.732488	NA	NA	NA
ENSG0000	3.933143	-0.99989	1.348191	-0.74165	0.458297	0.732509	MIR4645	1.01E+08	microRNA 4645
ENSG0000	124.514	-0.17396	0.234537	-0.74171	0.45826	0.732509	ERMARD	55780	ER membrane associated RNA degradation
ENSG0000	3.348517	-1.17606	1.586039	-0.74151	0.458385	0.732552	NA	NA	NA
ENSG0000	4827.479	0.056218	0.075818	0.74148	0.458403	0.732552	STXBP2	6813	syntaxin binding protein 2
ENSG0000	90.15474	0.229446	0.309572	0.741172	0.458589	0.732787	RTKN2	219790	rhotekin 2
ENSG0000	1087.727	0.078254	0.105626	0.74086	0.458778	0.733026	ATP5PB	515	ATP synthase peripheral stalk-membrane subunit b
ENSG0000	23.82537	-0.40385	0.545454	-0.74038	0.459068	0.733361	SH2D4A	63898	SH2 domain containing 4A
ENSG0000	83.1314	0.214509	0.289717	0.740411	0.459051	0.733361	ZNF544	27300	zinc finger protein 544
ENSG0000	2.342082	-1.49353	2.017549	-0.74027	0.459138	0.733411	NA	NA	NA
ENSG0000	116.9465	0.167938	0.226909	0.740113	0.459231	0.733497	PCOLCE	5118	procollagen C-endopeptidase enhancer
ENSG0000	43.06297	0.269408	0.364142	0.739842	0.459396	0.733697	C8orf44	56260	chromosome 8 putative open reading frame 44
ENSG0000	786.5278	0.079929	0.108074	0.739579	0.459555	0.733825	CREBL2	1389	cAMP responsive element binding protein like 2
ENSG0000	539.2428	0.089955	0.121629	0.739583	0.459553	0.733825	DENR	8562	density regulated re-initiation and release factor
ENSG0000	66.27006	0.247007	0.334053	0.739426	0.459648	0.733847	NA	NA	NA
ENSG0000	4.2765	-0.94583	1.279109	-0.73944	0.459638	0.733847	UMODL1	89766	uromodulin like 1
ENSG0000	7.018881	0.846086	1.144474	0.739279	0.459737	0.733926	IRF6	3664	interferon regulatory factor 6
ENSG0000	3.178581	1.208129	1.634885	0.738969	0.459926	0.734101	NA	NA	NA
ENSG0000	8.611078	0.68798	0.930953	0.739006	0.459903	0.734101	SCGB2B2	284402	secretoglobin family 2B member 2
ENSG0000	403.3375	-0.11608	0.15712	-0.73881	0.460021	0.734189	LARP4	113251	La ribonucleoprotein 4
ENSG0000	6.432987	0.746217	1.010565	0.738416	0.460262	0.73451	NA	NA	NA
ENSG0000	739.1411	0.084628	0.114665	0.738046	0.460487	0.734679	RAB11FIP1	80223	RAB11 family interacting protein 1
ENSG0000	1067.337	-0.26714	0.36195	-0.73806	0.460476	0.734679	P2RX7	5027	purinergic receptor P2X 7
ENSG0000	117.0719	-0.18618	0.252255	-0.73806	0.460476	0.734679	LOC10012	1E+08	uncharacterized LOC100128398
ENSG0000	480.1598	0.114982	0.155828	0.737876	0.46059	0.734771	RPL18AP3	390354	ribosomal protein L18a pseudogene 3
ENSG0000	16.84294	0.417398	0.565718	0.737821	0.460623	0.734771	SLC32A1	140679	solute carrier family 32 member 1
ENSG0000	2085.263	-0.06628	0.089875	-0.73745	0.460849	0.735004	SAP130	79595	Sin3A associated protein 130
ENSG0000	6.05435	-0.88119	1.194853	-0.73749	0.460824	0.735004	NA	NA	NA
ENSG0000	22.42157	0.408994	0.554799	0.737194	0.461004	0.735126	DGKG	1608	diacylglycerol kinase gamma
ENSG0000	49.00794	-0.26606	0.360896	-0.73721	0.460997	0.735126	BMERB1	89927	bMERB domain containing 1
ENSG0000	165.3527	-0.17483	0.237217	-0.73701	0.461115	0.735239	HSPA7	3311	heat shock protein family A (Hsp70) member 7 (pseudogene)
ENSG0000	133.8931	-0.17774	0.241214	-0.73687	0.4612	0.735311	TMEM120	144404	transmembrane protein 120B
ENSG0000	4.340123	-0.95975	1.302639	-0.73678	0.461258	0.73534	CLEC6A	93978	C-type lectin domain containing 6A
ENSG0000	105.7665	-0.17698	0.240285	-0.73655	0.461397	0.735499	B3GNT9	84752	UDP-GlcN/ 3-N-acetylglucosaminyltransferase 9
ENSG0000	4.644714	-0.99701	1.353814	-0.73644	0.461461	0.735537	ALG1L1P	200810	ALG1 like : pseudogene
ENSG0000	602.6477	0.099958	0.135767	0.736248	0.46158	0.7356	NA	NA	NA
ENSG0000	40.69448	0.277567	0.37697	0.736311	0.461541	0.7356	MRPS17	51373	mitochondrial ribosomal protein S17
ENSG0000	441.3523	-0.11242	0.152727	-0.73611	0.461666	0.735635	RNPC3	55599	RNA bindi RRM) containing 3
ENSG0000	11.12955	-0.55823	0.758451	-0.73602	0.46172	0.735635	NA	NA	NA
ENSG0000	74.75553	-0.21266	0.288927	-0.73602	0.46172	0.735635	ZNF525	170958	zinc finger protein 525
ENSG0000	6508.656	0.080485	0.109434	0.735468	0.462055	0.736104	DOT1L	84444	DOT1 like histone lysine methyltransferase
ENSG0000	12.04324	-0.52042	0.707681	-0.73539	0.462102	0.736117	LOC55420	554206	leucine carboxyl methyltransferase 1 pseudogene
ENSG0000	387.3691	0.100999	0.137365	0.735258	0.462182	0.736181	ATL3	25923	atlastin GTPase 3
ENSG0000	4450.009	0.064355	0.087554	0.735023	0.462325	0.736346	CARD11	84433	caspase recruitment domain family member 11
ENSG0000	707.577	0.09093	0.123813	0.734412	0.462697	0.736423	AMMECR1	83607	AMMECR1 like
ENSG0000	442.2748	0.095943	0.13061	0.734577	0.462597	0.736423	C5orf24	134553	chromosome 5 open reading frame 24
ENSG0000	3.026423	-1.35052	1.839054	-0.73436	0.462732	0.736423	NA	NA	NA
ENSG0000	11.69609	-0.51325	0.699061	-0.73419	0.462831	0.736423	LOC28319	283194	uncharacterized LOC283194
ENSG0000	14.43941	-0.45303	0.617068	-0.73416	0.46285	0.736423	CDCA3	83461	cell division cycle associated 3
ENSG0000	146.8086	-0.16492	0.224469	-0.73472	0.46251	0.736423	SALL2	6297	spalt like transcription factor 2
ENSG0000	1623.055	-0.06473	0.088167	-0.73418	0.462836	0.736423	PDPK1	5170	3-phosphoinositide dependent protein kinase 1
ENSG0000	29.76587	-0.36795	0.500902	-0.73457	0.4626	0.736423	DHODH	1723	dihydroorotate dehydrogenase (quinone)
ENSG0000	16.22977	-0.43024	0.58574	-0.73452	0.462634	0.736423	NA	NA	NA
ENSG0000	3.382679	1.159299	1.57823	0.734556	0.46261	0.736423	NA	NA	NA
ENSG0000	17103.34	0.07887	0.107414	0.734269	0.462785	0.736423	C5AR1	728	complement C5a receptor 1
ENSG0000	3165.813	-0.06259	0.085179	-0.73485	0.462429	0.736423	MICALL1	85377	MICAL like 1
ENSG0000	410.6072	-0.0944	0.128612	-0.73395	0.462979	0.736501	TRUB2	26995	TruB pseudouridine synthase family member 2
ENSG0000	75.15762	0.205123	0.279455	0.73401	0.462943	0.736501	PRX	57716	periaxin
ENSG0000	1811.359	-0.06438	0.087744	-0.73369	0.463135	0.736687	SUGP1	57794	SURP and G-patch domain containing 1
ENSG0000	155.3771	-0.16519	0.22524	-0.73338	0.463327	0.736823	LRRC8C-DT	400761	LRRC8C divergent transcript
ENSG0000	505.4191	0.11067	0.150923	0.733293	0.46338	0.736823	FAM131A	131408	family with sequence similarity 131 member A
ENSG0000	8534.775	0.049511	0.067515	0.733336	0.463354	0.736823	ACIN1	22985	apoptotic chromatin condensation inducer 1
ENSG0000	120.0446	-0.19005	0.25913	-0.73341	0.463307	0.736823	ENTPD5	957	ectonucleoside triphosphate diphosphohydrolase 5 (inactive)
ENSG0000	126.111	-0.17326	0.236407	-0.73287	0.463637	0.737042	HLTF	6596	helicase like transcription factor
ENSG0000	19.21033	-0.43349	0.591409	-0.73297	0.463576	0.737042	STXBP5-AS	729178	STXBP5 antisense RNA 1
ENSG0000	33.6876	-0.31802	0.433908	-0.73292	0.463606	0.737042	DPP3-DT	1.02E+08	DPP3 divergent transcript
ENSG0000	51.93107	-0.25872	0.353052	-0.7328	0.463682	0.73705	PRSS30P	124221	serine pro pseudogene
ENSG0000	104.3832	-0.19876	0.27129	-0.73266	0.463765	0.73706	NA	NA	NA
ENSG0000	1116.56	-0.07849	0.107129	-0.73266	0.463767	0.73706	DYNC1LI2	1783	dynein cytoplasmic 1 light intermediate chain 2
ENSG0000	33.71648	-0.31318	0.427652	-0.73233	0.463967	0.737179	RAB23	51715	RAB23 member RAS oncogene family
ENSG0000	91.65213	-0.22366	0.305471	-0.73218	0.464057	0.737179	ERV3-1	2086	endogeno envelope
ENSG0000	105.1237	0.176642	0.241237	0.732235	0.464025	0.737179	TOX-DT	1.01E+08	TOX divergent transcript
ENSG0000	3.903264	0.96912	1.323673	0.732144	0.46408	0.737179	OVOL1	5017	ovo like transcriptional repressor 1
ENSG0000	71.41243	-0.22748	0.31063	-0.73231	0.46398	0.737179	TYRO3	7301	TYRO3 protein tyrosine kinase
ENSG0000	2201.099	0.059914	0.081828	0.732197	0.464048	0.737179	GUCD1	83606	guanylyl cyclase domain containing 1
ENSG0000	15.2289	-0.44462	0.607383	-0.73202	0.464156	0.737236	ZNF285	26974	zinc finger protein 285
ENSG0000	378.1912	0.112027	0.153127	0.7316	0.464413	0.737517	ITGB1BP1	9270	integrin subunit beta 1 binding protein 1
ENSG0000	56.11264	0.24645	0.336841	0.731651	0.464382	0.737517	DYNLRB1	83658	dynein light chain roadblock-type 1
ENSG0000	59.56249	-0.22544	0.308276	-0.7313	0.464598	0.737749	XKR6	286046	XK related 6
ENSG0000	73.35432	0.207951	0.284509	0.730913	0.464832	0.738024	E2F5	1875	E2F transcription factor 5
ENSG0000	6.606302	0.756461	1.03509	0.730817	0.464891	0.738024	MALT1-AS	1.02E+08	MALT1 antisense RNA 1
ENSG0000	19.91435	-0.46249	0.632793	-0.73087	0.464858	0.738024	UBE2C	11065	ubiquitin conjugating enzyme E2 C
ENSG0000	7.741485	0.70341	0.962619	0.730726	0.464947	0.738049	IGLV3-9	28804	immunoglobulin lambda variable 3-9
ENSG0000	43.78921	-0.27471	0.376049	-0.7305	0.465082	0.738051	NEIL2	252969	nei like DNA glycosylase 2
ENSG0000	395.6207	0.096358	0.131911	0.730478	0.465098	0.738051	TTC9C	283237	tetratricopeptide repeat domain 9C
ENSG0000	5.144779	0.745528	1.020492	0.730558	0.465049	0.738051	NA	NA	NA
ENSG0000	2.720091	-1.32795	1.817961	-0.73046	0.465107	0.738051	LCA5L	150082	lebercilin LCA5 like
ENSG0000	3.386651	-1.12405	1.539032	-0.73036	0.465171	0.738089	C1orf127	148345	chromosome 1 open reading frame 127
ENSG0000	6.42399	0.864395	1.183722	0.730235	0.465247	0.738096	NA	NA	NA
ENSG0000	302.1808	0.120836	0.165483	0.7302	0.465268	0.738096	SLC12A2	6558	solute carrier family 12 member 2
ENSG0000	3.476788	-1.11256	1.523729	-0.73016	0.465295	0.738096	SYBU	55638	syntabulin
ENSG0000	702.5474	0.095094	0.130285	0.729895	0.465454	0.738223	CYB5R4	51167	cytochrome b5 reductase 4
ENSG0000	122.1257	-0.15552	0.21307	-0.72992	0.46544	0.738223	MTERF1	7978	mitochondrial transcription termination factor 1
ENSG0000	34.27297	-0.32526	0.445745	-0.7297	0.465574	0.738245	CHN1	1123	chimerin 1
ENSG0000	6.327575	-0.98018	1.34331	-0.72968	0.465587	0.738245	NUTM2B	729262	NUT family member 2B
ENSG0000	8313.427	-0.0591	0.080982	-0.72976	0.465539	0.738245	MAZ	4150	MYC associated zinc finger protein
ENSG0000	14.39838	0.480938	0.659174	0.729607	0.46563	0.73825	EEF1A1P4	1E+08	eukaryotic translation elongation factor 1 alpha 1 pseudogene 4
ENSG0000	169.2696	-0.15211	0.208619	-0.72911	0.465935	0.73837	TUBE1	51175	tubulin epsilon 1

ENSG0000	127.8296	-0.1661	0.227759	-0.72927	0.465839	0.73837	NA	NA	NA
ENSG0000	7.66656	0.657862	0.902256	0.72913	0.465922	0.73837	NA	NA	NA
ENSG0000	31.62742	-0.33733	0.462704	-0.72903	0.465981	0.73837	LOC73222!	732229	AN1-type zinc finger protein 5-like
ENSG0000	1151.564	0.069888	0.095864	0.729027	0.465985	0.73837	RBBP7	5931	RB binding chromatin remodeling factor
ENSG0000	370.6589	-0.10623	0.14571	-0.72905	0.465969	0.73837	MAGEH1	28986	MAGE family member H1
ENSG0000	12.96935	0.54692	0.750153	0.729077	0.465954	0.73837	GPRASP2	114928	G protein-coupled receptor associated sorting protein 2
ENSG0000	353.241	-0.10081	0.138301	-0.7289	0.466063	0.738379	NUP155	9631	nucleoporin 155
ENSG0000	2.991121	-1.02713	1.409176	-0.72889	0.46607	0.738379	NA	NA	NA
ENSG0000	4.133974	-1.01745	1.396733	-0.72845	0.466339	0.73858	NA	NA	NA
ENSG0000	2263.619	0.061123	0.083926	0.72829	0.466436	0.73858	KIF13B	23303	kinesin family member 13B
ENSG0000	5.09382	0.823638	1.130906	0.728299	0.466431	0.73858	MMP21	118856	matrix metallopeptidase 21
ENSG0000	20.39747	-0.49294	0.67675	-0.72839	0.466374	0.73858	GOLGA8R	1.01E+08	golgin A8 family member R
ENSG0000	13.77622	-0.46375	0.636554	-0.72853	0.466288	0.73858	MYL11	29895	myosin light chain 11
ENSG0000	2522.428	-0.06526	0.089601	-0.72831	0.466426	0.73858	MNT	4335	MAX network transcriptional repressor
ENSG0000	3.153529	1.157716	1.589953	0.728145	0.466525	0.738596	DPRXP3	503644	divergent-paired related homeobox pseudogene 3
ENSG0000	96.89376	0.180735	0.248213	0.728144	0.466526	0.738596	ZNF568	374900	zinc finger protein 568
ENSG0000	562.461	0.101662	0.139654	0.727952	0.466643	0.738718	TMEM181	57583	transmembrane protein 181
ENSG0000	744.9403	0.084893	0.116661	0.727694	0.466801	0.738856	MRPS25	64432	mitochondrial ribosomal protein S25
ENSG0000	4725.788	0.055679	0.076519	0.727641	0.466833	0.738856	PIP4K2A	5305	phosphatidylinositol-5-phosphate 4-kinase type 2 alpha
ENSG0000	466.0597	0.098703	0.135653	0.727615	0.466849	0.738856	HACD3	51495	3-hydroxyacyl-CoA dehydratase 3
ENSG0000	22.55542	-0.36125	0.496748	-0.72724	0.467079	0.738923	NA	NA	NA
ENSG0000	59.90926	0.269348	0.370282	0.727414	0.466972	0.738923	NA	NA	NA
ENSG0000	64.54549	-0.26378	0.362675	-0.72732	0.467033	0.738923	VIPR2	7434	vasoactive intestinal peptide receptor 2
ENSG0000	6351.831	-0.06298	0.086604	-0.72722	0.467091	0.738923	MAPK8IP3	23162	mitogen-activated protein kinase 8 interacting protein 3
ENSG0000	307.5647	-0.13419	0.184466	-0.72743	0.466963	0.738923	CAMKK1	84254	calcium/calmodulin dependent protein kinase kinase 1
ENSG0000	28.90675	-0.33666	0.463146	-0.72689	0.467292	0.739053	NA	NA	NA
ENSG0000	24.16213	-0.3537	0.486585	-0.72691	0.467281	0.739053	DPF3	8110	double PHD fingers 3
ENSG0000	14.59286	0.468962	0.645094	0.726967	0.467246	0.739053	PIPOX	51268	pipecolic acid and sarcosine oxidase
ENSG0000	408.4427	-0.1048	0.144216	-0.72669	0.467415	0.739184	PPARA	5465	peroxisome proliferator activated receptor alpha
ENSG0000	2012.939	0.061875	0.085159	0.726581	0.467483	0.739228	SNRK	54861	SNF related kinase
ENSG0000	6043.434	0.055269	0.076094	0.726324	0.46764	0.739413	CYB5R3	1727	cytochrome b5 reductase 3
ENSG0000	9.230135	0.573305	0.789762	0.725922	0.467887	0.739618	NA	NA	NA
ENSG0000	5.688886	0.902847	1.243731	0.725918	0.467889	0.739618	RPL39L	116832	ribosomal protein L39 like
ENSG0000	575.4424	-0.08493	0.116988	-0.72596	0.467863	0.739618	TTC1	7265	tetratricopeptide repeat domain 1
ENSG0000	149.3164	-0.14391	0.198265	-0.72584	0.467936	0.739629	NA	NA	NA
ENSG0000	336.9094	-0.11015	0.151796	-0.72566	0.468045	0.739739	C12orf43	64897	chromosome 12 open reading frame 43
ENSG0000	6095.66	0.055439	0.076431	0.725354	0.468235	0.739975	SCAF1	58506	SR-related CTD associated factor 1
ENSG0000	3198.549	-0.0871	0.120115	-0.72511	0.468385	0.740086	TAGAP	117289	T cell activation RhoGTPase activating protein
ENSG0000	47.91438	-0.25728	0.354789	-0.72515	0.468358	0.740086	NA	NA	NA
ENSG0000	1795.426	0.068642	0.094696	0.724867	0.468533	0.740132	TPP2	7174	tripeptidyl peptidase 2
ENSG0000	2216.533	0.058992	0.081379	0.724908	0.468508	0.740132	RTF1	23168	RTF1 hom Paf1/RNA polymerase II complex component
ENSG0000	40.0567	-0.27133	0.37428	-0.72493	0.468493	0.740132	ZNF284	342909	zinc finger protein 284
ENSG0000	171.3179	-0.14593	0.201341	-0.72478	0.468589	0.740157	CIPC	85457	CLOCK interacting pacemaker
ENSG0000	1550.654	0.066384	0.091633	0.724458	0.468785	0.740299	TADA2B	93624	transcriptional adaptor 2B
ENSG0000	171.2217	-0.1595	0.220174	-0.72444	0.468799	0.740299	MS4A4A	51338	membrane spanning 4-domains A4A
ENSG0000	5523.215	0.062198	0.085852	0.724482	0.46877	0.740299	SCAF11	9169	SR-related CTD associated factor 11
ENSG0000	11.96388	0.528023	0.729241	0.724071	0.469022	0.740336	RPL7AP16	1E+08	ribosomal protein L7a pseudogene 16
ENSG0000	111.458	-0.17604	0.2431	-0.72416	0.468967	0.740336	QDPR	5860	quinoid dihydropteridine reductase
ENSG0000	1008.763	-0.07449	0.10287	-0.72411	0.468997	0.740336	CNOT7	29883	CCR4-NOT transcription complex subunit 7
ENSG0000	283.2005	0.128151	0.176945	0.724243	0.468917	0.740336	NEDD8	4738	NEDD8 ubiquitin like modifier
ENSG0000	52.16304	-0.24228	0.334557	-0.72417	0.468959	0.740336	LRRC3	81543	leucine rich repeat containing 3
ENSG0000	668.6546	0.087104	0.120311	0.723993	0.46907	0.740349	PRPF4	9128	pre-mRNA processing factor 4
ENSG0000	65.13473	0.265562	0.366843	0.723912	0.46912	0.740364	SUSD4	55061	sushi domain containing 4
ENSG0000	16.45663	-0.52715	0.728495	-0.72362	0.469298	0.740582	NA	NA	NA
ENSG0000	17.58084	-0.44227	0.611329	-0.72346	0.469395	0.740673	NA	NA	NA
ENSG0000	14.74006	-0.45949	0.635197	-0.72337	0.46945	0.740696	HMMR	3161	hyaluronan mediated motility receptor
ENSG0000	11.50542	-0.52765	0.729652	-0.72316	0.469584	0.740845	TSFM	10102	Ts translat mitochondrial
ENSG0000	10.04238	-0.55212	0.764469	-0.72223	0.470156	0.741684	NA	NA	NA
ENSG0000	3.038419	-1.12969	1.564498	-0.72208	0.470247	0.741764	CPT1C	126129	carnitine palmitoyltransferase 1C
ENSG0000	2265.546	-0.07346	0.101743	-0.72201	0.47029	0.741769	SSBP4	170463	single stranded DNA binding protein 4
ENSG0000	226.0217	-0.13391	0.185503	-0.7219	0.470356	0.741811	MFSD8	256471	major facilitator superfamily domain containing 8
ENSG0000	18.38151	-0.38407	0.532119	-0.72178	0.470428	0.741861	MIF4GD-D	1E+08	MIF4GD divergent transcript
ENSG0000	15.66229	0.415835	0.576186	0.721703	0.470477	0.741875	NA	NA	NA
ENSG0000	64.20453	-0.24379	0.337839	-0.72163	0.470524	0.741887	ALKBH6	84964	alkB homolog 6
ENSG0000	489.6953	-0.09741	0.135065	-0.72119	0.47079	0.742179	OSBPL3	26031	oxysterol binding protein like 3
ENSG0000	26.92788	-0.37059	0.513827	-0.72124	0.470764	0.742179	TRDV2	28517	T cell receptor delta variable 2
ENSG0000	6.174934	0.771196	1.069558	0.721042	0.470884	0.742264	NA	NA	NA
ENSG0000	477.8323	0.099713	0.138394	0.720502	0.471216	0.742297	ALDH9A1	223	aldehyde dehydrogenase 9 family member A1
ENSG0000	63.39645	-0.20616	0.286148	-0.72045	0.471247	0.742297	NA	NA	NA
ENSG0000	82.23674	0.197703	0.274427	0.720423	0.471265	0.742297	CPA3	1359	carboxypeptidase A3
ENSG0000	607.8364	0.085547	0.118681	0.720819	0.471021	0.742297	MS4A6A	64231	membrane spanning 4-domains A6A
ENSG0000	522.0841	0.087424	0.121335	0.720514	0.471209	0.742297	MRPS7	51081	mitochondrial ribosomal protein S7
ENSG0000	731.0627	-0.07564	0.104995	-0.72045	0.471247	0.742297	TRAPPC8	22878	trafficking protein particle complex subunit 8
ENSG0000	4654.604	0.057257	0.07943	0.720849	0.471002	0.742297	PIP5K1C	23396	phosphatidylinositol-4-phosphate 5-kinase type 1 gamma
ENSG0000	4.656879	-0.90188	1.25158	-0.7206	0.471158	0.742297	NA	NA	NA
ENSG0000	863.1861	-0.07719	0.107135	-0.72049	0.471225	0.742297	BRWD1	54014	bromodomain and WD repeat domain containing 1
ENSG0000	22.18597	-0.38002	0.527792	-0.72003	0.471508	0.742554	NA	NA	NA
ENSG0000	3.545153	0.996625	1.384038	0.720085	0.471473	0.742554	NPAS1	4861	neuronal PAS domain protein 1
ENSG0000	2.630319	-1.39078	1.93182	-0.71993	0.471567	0.742584	NPIPB7	440350	nuclear pore complex interacting protein family member B7
ENSG0000	10.02407	0.496329	0.689474	0.719865	0.471608	0.742585	NA	NA	NA
ENSG0000	65.22357	0.22753	0.316218	0.719536	0.471811	0.742757	SMIM27	1E+08	small integral membrane protein 27
ENSG0000	38.49234	-0.27209	0.378199	-0.71943	0.471877	0.742757	ADAMTSL!	339366	ADAMTS like 5
ENSG0000	272.9923	-0.12218	0.169833	-0.71943	0.471873	0.742757	ZNF562	54811	zinc finger protein 562
ENSG0000	59.11157	-0.22257	0.309368	-0.71943	0.471873	0.742757	ZNF582	147948	zinc finger protein 582
ENSG0000	430.9858	0.099611	0.13849	0.719267	0.471976	0.74285	RNASE2	6036	ribonuclease A family member 2
ENSG0000	27.91761	-0.4043	0.562184	-0.71916	0.472045	0.742864	EPB41L4A	64097	erythrocyte membrane protein band 4.1 like 4A
ENSG0000	3.422454	1.049255	1.459076	0.719123	0.472065	0.742864	SLC34A1	6569	solute carrier family 34 member 1
ENSG0000	9.880806	0.811979	1.129332	0.718991	0.472147	0.742906	FTCDNL1	348751	formiminotransferase cyclodeaminase N-terminal like
ENSG0000	440.6447	0.103114	0.143424	0.718947	0.472174	0.742906	TTLL3	26140	tubulin tyrosine ligase like 3
ENSG0000	4.870844	0.960245	1.335744	0.718884	0.472212	0.742906	NA	NA	NA
ENSG0000	10.34644	-0.57788	0.804361	-0.71843	0.47249	0.743264	RBP5	83758	retinol binding protein 5
ENSG0000	112.3166	-0.1642	0.228571	-0.71839	0.47252	0.743264	C1GALT1C	29071	C1GALT1 specific chaperone 1
ENSG0000	187.2771	-0.13663	0.190252	-0.71815	0.472667	0.743432	UICLM	200772	up-regulated in colorectal cancer liver metastasis
ENSG0000	3.018546	1.082096	1.507078	0.718009	0.472752	0.743439	NA	NA	NA
ENSG0000	516.2015	-0.09516	0.132528	-0.71805	0.472724	0.743439	CASTOR2	729438	cytosolic arginine sensor for mTORC1 subunit 2
ENSG0000	7378.649	0.145607	0.202823	0.717901	0.472818	0.743482	NR4A1	3164	nuclear receptor subfamily 4 group A member 1
ENSG0000	2150.277	0.06189	0.086256	0.717524	0.473051	0.743721	BCL2L1	598	BCL2 like 1
ENSG0000	4.549925	0.935203	1.303364	0.71753	0.473047	0.743721	LOC10537.	1.05E+08	uncharacterized LOC105373175
ENSG0000	408.0123	0.105996	0.147789	0.717208	0.473246	0.743897	CXCR6	10663	C-X-C motif chemokine receptor 6
ENSG0000	20.577	-0.41927	0.584639	-0.71715	0.473283	0.743897	CLCN1	1180	chloride voltage-gated channel 1

ENSG0000	16.88116	-0.42653	0.594698	-0.71722	0.473235	0.743897	NA	NA	NA
ENSG0000	8.419953	-0.61888	0.863238	-0.71693	0.47342	0.744048	TRAV27	28655	T cell receptor alpha variable 27
ENSG0000	255.8345	-0.115	0.160477	-0.71662	0.47361	0.744095	NA	NA	NA
ENSG0000	4778.964	-0.05281	0.073688	-0.71668	0.47357	0.744095	ZNF276	92822	zinc finger protein 276
ENSG0000	152.6014	0.148666	0.207419	0.716744	0.473532	0.744095	UPRT	139596	uracil phosphoribosyltransferase homolog
ENSG0000	11.28506	0.484971	0.676692	0.71668	0.473572	0.744095	RAP2C-AS:	1.02E+08	RAP2C antisense RNA 1
ENSG0000	499.4057	-0.10453	0.145888	-0.71648	0.473693	0.744163	INTS8	55656	integrator complex subunit 8
ENSG0000	78.13767	0.233168	0.325557	0.716214	0.473859	0.744172	RPP14	11102	ribonuclease P/MRP subunit p14
ENSG0000	23.89046	-0.33466	0.467226	-0.71628	0.473819	0.744172	PPP2R3A	5523	protein phosphatase 2 regulatory subunit B''alpha
ENSG0000	7.427355	-0.73975	1.032786	-0.71627	0.473826	0.744172	TAS2R5	54429	taste 2 receptor member 5
ENSG0000	18490.12	0.075476	0.105359	0.716368	0.473764	0.744172	HIF1A	3091	hypoxia inducible factor 1 subunit alpha
ENSG0000	5.908766	-0.79544	1.110998	-0.71597	0.474009	0.744343	KLHL33	123103	kelch like family member 33
ENSG0000	87.7402	0.194401	0.271558	0.715872	0.47407	0.744377	NA	NA	NA
ENSG0000	323.1306	0.106738	0.14916	0.715596	0.474241	0.744456	GZMA	3001	granzyme A
ENSG0000	3.349096	-1.13303	1.583246	-0.71564	0.474215	0.744456	NA	NA	NA
ENSG0000	402.1026	0.098549	0.137708	0.715634	0.474217	0.744456	ZNF275	10838	zinc finger protein 275
ENSG0000	37.66941	0.277018	0.387204	0.715432	0.474342	0.744552	PIK3R3	8503	phosphoinositide-3-kinase regulatory subunit 3
ENSG0000	841.0134	0.071575	0.100064	0.715293	0.474428	0.744623	CCDC174	51244	coiled-coil domain containing 174
ENSG0000	9.244347	0.567238	0.793483	0.714871	0.474689	0.744926	LINC00964	157381	long intergenic non-protein coding RNA 964
ENSG0000	6.229111	-0.7164	1.002168	-0.71485	0.474701	0.744926	NA	NA	NA
ENSG0000	663.1257	-0.08497	0.118871	-0.71478	0.474746	0.744933	TSN	7247	translin
ENSG0000	15.5529	-0.52892	0.740228	-0.71453	0.474897	0.745087	NA	NA	NA
ENSG0000	4.687896	-0.95764	1.340318	-0.71449	0.474924	0.745087	NA	NA	NA
ENSG0000	53.37514	0.235236	0.32928	0.714395	0.474983	0.745116	LAPTM4B	55353	lysosomal protein transmembrane 4 beta
ENSG0000	198.4712	0.136819	0.19156	0.714233	0.475083	0.745188	NA	NA	NA
ENSG0000	100.7688	-0.18262	0.255731	-0.7141	0.475165	0.745188	BOK	666	BCL2 family apoptosis regulator BOK
ENSG0000	29.71591	0.326219	0.456798	0.714143	0.475139	0.745188	NA	NA	NA
ENSG0000	56.81166	-0.24234	0.339384	-0.71406	0.47519	0.745188	LOC12490	1.25E+08	EF-hand calcium-binding domain-containing protein 3-like
ENSG0000	464.7373	-0.10552	0.1478	-0.71393	0.475273	0.745193	NNT	23530	nicotinamide nucleotide transhydrogenase
ENSG0000	604.4242	0.092866	0.130067	0.713985	0.475236	0.745193	PLEKHJ1	55111	pleckstrin homology domain containing J1
ENSG0000	9.823206	-0.63454	0.888951	-0.71381	0.475347	0.745247	MYL4	4635	myosin light chain 4
ENSG0000	71.32983	-0.21126	0.296078	-0.71351	0.475529	0.745405	CCL28	56477	C-C motif chemokine ligand 28
ENSG0000	2.733206	-1.18515	1.660961	-0.71353	0.475516	0.745405	RDH12	145226	retinol dehydrogenase 12
ENSG0000	7.489998	0.710535	0.996226	0.713227	0.475705	0.745619	KCNN3	3782	potassium calcium-activated channel subfamily N member 3
ENSG0000	44.79187	0.28103	0.394433	0.71249	0.476162	0.746271	LINC02422	1.05E+08	long intergenic non-protein coding RNA 2422
ENSG0000	4.91744	0.956041	1.342057	0.71237	0.476236	0.746315	THBS4	7060	thrombospondin 4
ENSG0000	13.73057	-0.47066	0.660751	-0.71231	0.47627	0.746315	NA	NA	NA
ENSG0000	45.04794	-0.25978	0.36477	-0.71216	0.476364	0.746369	HEMGN	55363	hemogen
ENSG0000	69.40554	0.199234	0.279773	0.712129	0.476385	0.746369	RBFA	79863	ribosome binding factor A
ENSG0000	44.07074	-0.29728	0.417531	-0.71199	0.47647	0.746439	BBS9	27241	Bardet-Biedl syndrome 9
ENSG0000	191.9852	-0.15217	0.21378	-0.71182	0.476574	0.746539	MOSMO	730094	modulator of smoothened
ENSG0000	125.3326	-0.18729	0.263176	-0.71164	0.476685	0.746586	PDIK1L	149420	PDLIM1 interacting kinase 1 like
ENSG0000	10.76001	-0.52738	0.741057	-0.71165	0.476679	0.746586	NA	NA	NA
ENSG0000	89.9189	0.180792	0.254074	0.711574	0.476729	0.746592	LINC00852	84657	long intergenic non-protein coding RNA 852
ENSG0000	4.591702	-0.81846	1.150719	-0.71126	0.476921	0.74683	NA	NA	NA
ENSG0000	444.2049	0.100157	0.140929	0.71069	0.477276	0.747048	PAX8-AS1	654433	PAX8 antisense RNA 1
ENSG0000	1242.575	-0.06777	0.095357	-0.71069	0.477277	0.747048	SP140L	93349	SP140 nuclear body protein like
ENSG0000	8.645458	0.659362	0.927831	0.710648	0.477302	0.747048	GRAMD1C	54762	GRAM domain containing 1C
ENSG0000	844.9213	-0.07986	0.112352	-0.7108	0.47721	0.747048	SPTLC1	10558	serine palmitoyltransferase long chain base subunit 1
ENSG0000	616.2366	-0.08947	0.125852	-0.71087	0.477162	0.747048	GALNS	2588	galactosamine (N-acetyl)-6-sulfatase
ENSG0000	165.4108	-0.18188	0.255819	-0.71095	0.477114	0.747048	GNAZ	2781	G protein subunit alpha z
ENSG0000	705.2531	-0.09218	0.129756	-0.71042	0.477442	0.747143	BLMH	642	bleomycin hydrolase
ENSG0000	11.55474	0.522653	0.735762	0.710356	0.477483	0.747143	STAP2	55620	signal transducing adaptor family member 2
ENSG0000	7.550753	0.757738	1.066662	0.710382	0.477467	0.747143	NA	NA	NA
ENSG0000	7.396374	-0.67066	0.944478	-0.71008	0.477652	0.747344	LOC10537	1.05E+08	uncharacterized LOC105377623
ENSG0000	7.251519	-0.64106	0.903246	-0.70973	0.477872	0.747625	CAPS2	84698	calcyphosine 2
ENSG0000	137.0894	-0.15721	0.221535	-0.70965	0.477919	0.747636	NA	NA	NA
ENSG0000	472.357	-0.08699	0.122608	-0.70951	0.478011	0.747716	ZDHHC2	51201	zinc finger DHHC-type palmitoyltransferase 2
ENSG0000	15.44914	0.530487	0.747808	0.709389	0.478083	0.747765	SPTBN2	6712	spectrin b non-erythrocytic 2
ENSG0000	2.788765	1.112299	1.568141	0.709311	0.478132	0.747779	NA	NA	NA
ENSG0000	89.43312	-0.19326	0.272526	-0.70914	0.478239	0.747821	NA	NA	NA
ENSG0000	708.8012	0.085974	0.121232	0.70917	0.478219	0.747821	TMEM138	51524	transmembrane protein 138
ENSG0000	460.2008	0.090257	0.127297	0.709028	0.478307	0.747864	CDK6	1021	cyclin dependent kinase 6
ENSG0000	443.6634	-0.10113	0.142668	-0.70883	0.478432	0.747933	ZCCHC17	51538	zinc finger CCHC-type containing 17
ENSG0000	5.75004	0.752047	1.06092	0.708862	0.47841	0.747933	NA	NA	NA
ENSG0000	15.55463	-0.50984	0.719382	-0.70871	0.478502	0.747979	NA	NA	NA
ENSG0000	76.18963	-0.19052	0.268865	-0.7086	0.478572	0.748025	FAM104B	90736	family with sequence similarity 104 member B
ENSG0000	43.55824	-0.27014	0.381616	-0.70788	0.479021	0.748119	OSGEPL1	64172	O-sialoglycoprotein endopeptidase like 1
ENSG0000	23.35801	-0.39788	0.561724	-0.70832	0.478746	0.748119	CENPU	79682	centromere protein U
ENSG0000	90.91885	-0.18296	0.258543	-0.70766	0.479156	0.748119	IGIP	492311	IgA inducing protein
ENSG0000	845.4827	0.071549	0.101117	0.707581	0.479205	0.748119	DXO	1797	decapping exoribonuclease
ENSG0000	6.535724	-0.78	1.102161	-0.7077	0.47913	0.748119	TRBJ2-6	28623	T cell receptor beta joining 2-6
ENSG0000	22.2428	0.412781	0.582796	0.708276	0.478774	0.748119	RPSAP47	389672	ribosomal protein SA pseudogene 47
ENSG0000	4.49079	0.900058	1.272146	0.707512	0.479249	0.748119	NA	NA	NA
ENSG0000	27.39201	0.365326	0.516088	0.707876	0.479022	0.748119	KIF18A	81930	kinesin family member 18A
ENSG0000	19.96718	-0.38722	0.547337	-0.70747	0.479277	0.748119	AGBL2	79841	AGBL carboxypeptidase 2
ENSG0000	4325.08	-0.05497	0.077678	-0.70771	0.479124	0.748119	HMGB1	3146	high mobility group box 1
ENSG0000	11.03704	0.543305	0.767706	0.707699	0.479132	0.748119	AP3B2	8120	adaptor related protein complex 3 subunit beta 2
ENSG0000	5.09125	-0.94653	1.337353	-0.70777	0.47909	0.748119	NA	NA	NA
ENSG0000	4.32501	1.021128	1.442651	0.707814	0.479061	0.748119	UBBP4	23666	ubiquitin B pseudogene 4
ENSG0000	23.42127	-0.35311	0.498938	-0.70771	0.479123	0.748119	BRME1	79173	break repair meiotic recombinase recruitment factor 1
ENSG0000	4.655423	-0.83344	1.1769	-0.70817	0.478842	0.748119	NA	NA	NA
ENSG0000	2.408278	1.263908	1.786267	0.707569	0.479213	0.748119	HUNK	30811	hormonally up-regulated Neu-associated kinase
ENSG0000	3.584393	-1.13491	1.605276	-0.70699	0.479573	0.748392	RGPD1	400966	RANBP2 like and GRIP domain containing 1
ENSG0000	610.9598	-0.10948	0.15484	-0.70708	0.479517	0.748392	GLO1	2739	glyoxalase I
ENSG0000	2.844717	-0.98148	1.388139	-0.70705	0.479536	0.748392	LINC0228C	1.02E+08	long intergenic non-protein coding RNA 2280
ENSG0000	657.3633	-0.08744	0.123739	-0.70667	0.479775	0.748436	FLAD1	80308	flavin adenine dinucleotide synthetase 1
ENSG0000	1075.868	-0.09473	0.13404	-0.70676	0.479717	0.748436	PIKFYVE	200576	phosphoin FYVE-type zinc finger containing
ENSG0000	76.09893	0.29507	0.417669	0.706469	0.479896	0.748436	IER3-AS1	1.05E+08	IER3 antisense RNA 1
ENSG0000	92.58091	-0.17137	0.242541	-0.70654	0.47985	0.748436	C6orf226	441150	chromosome 6 open reading frame 226
ENSG0000	67.78675	-0.21429	0.303324	-0.70648	0.479888	0.748436	TYW1B	441250	tRNA-yW synthesizing protein 1 homolog B
ENSG0000	7.956971	0.713353	1.0099	0.70636	0.479964	0.748436	ASTN2	23245	astrotactin 2
ENSG0000	208.0569	0.127467	0.180326	0.70687	0.479647	0.748436	WBP4	11193	WW domain binding protein 4
ENSG0000	2574.614	-0.06981	0.098782	-0.70674	0.479729	0.748436	UBXN6	80700	UBX domain protein 6
ENSG0000	117.727	-0.18521	0.262173	-0.70642	0.479925	0.748436	SIGLEC17P	284367	sialic acid pseudogene
ENSG0000	10.04746	-0.62432	0.884231	-0.70606	0.480151	0.748664	CHADL	150356	chondroadherin like
ENSG0000	5.985808	0.810717	1.14852	0.70588	0.480263	0.748776	NA	NA	NA
ENSG0000	3.29333	0.999828	1.416783	0.705703	0.480373	0.748884	NA	NA	NA
ENSG0000	442.4992	0.10003	0.141782	0.705519	0.480487	0.749	BTF3L4	91408	basic transcription factor 3 like 4
ENSG0000	4.909064	0.863984	1.225019	0.705282	0.480634	0.749166	SMIM6	1E+08	small integral membrane protein 6

ENSG0000	159.9895	-0.14594	0.207008	-0.705	0.480809	0.749187	KIT	3815	KIT proto-(receptor tyrosine kinase
ENSG0000	710.4807	-0.07995	0.113399	-0.70504	0.480785	0.749187	TMEM198	440104	transmembrane protein 198B (pseudogene)
ENSG0000	1339.999	0.071287	0.101106	0.705071	0.480766	0.749187	ZCCHC3	85364	zinc finger CCHC-type containing 3
ENSG0000	4.736979	0.981307	1.391829	0.705048	0.48078	0.749187	NA	NA	NA
ENSG0000	31.61135	0.295091	0.41865	0.704862	0.480896	0.749259	PDE5A	8654	phosphodiesterase 5A
ENSG0000	1317.927	-0.06725	0.095439	-0.70461	0.481051	0.749438	RALBP1	10928	ralA binding protein 1
ENSG0000	74.02066	-0.18951	0.269121	-0.7042	0.481308	0.749649	EIF2B3	8891	eukaryotic translation initiation factor 2B subunit gamma
ENSG0000	703.165	-0.08543	0.121302	-0.70428	0.481258	0.749649	PRDM10	56980	PR/SET domain 10
ENSG0000	98.30354	-0.17856	0.253545	-0.70425	0.481276	0.749649	CPAMD8	27151	C3 and PZP like alpha-2-macroglobulin domain containing 8
ENSG0000	1075.634	-0.07051	0.100156	-0.70399	0.48144	0.749791	POU6F1	5463	POU class 6 homeobox 1
ENSG0000	3104.012	0.054459	0.077379	0.703799	0.481558	0.749854	POGZ	23126	pogo transposable element derived with ZNF domain
ENSG0000	395.1039	0.099896	0.14194	0.703794	0.481561	0.749854	POLR1B	84172	RNA polymerase I subunit B
ENSG0000	1974.271	0.06719	0.095509	0.703492	0.481749	0.750026	MGAT4A	11320	alpha-1 3-mannosyl-glycoprotein 4-beta-N-acetylglucosaminyltransferase A
ENSG0000	142.0471	-0.14834	0.210865	-0.70349	0.481752	0.750026	MRPL27	51264	mitochondrial ribosomal protein L27
ENSG0000	122.4108	-0.15024	0.213669	-0.70312	0.481979	0.750315	NDUFB3	4709	NADH:ubiquinone oxidoreductase subunit B3
ENSG0000	614.4094	0.099217	0.141157	0.702883	0.482129	0.750486	VTA1	51534	vesicle trafficking 1
ENSG0000	800.956	0.082633	0.117588	0.702735	0.482221	0.750566	CIDEB	27141	cell death inducing DFFA like effector b
ENSG0000	7.123236	-0.71266	1.014437	-0.70252	0.482357	0.750715	VWA3A	146177	von Willebrand factor A domain containing 3A
ENSG0000	37.91409	-0.26982	0.384214	-0.70228	0.482507	0.750767	NA	NA	NA
ENSG0000	272.0815	0.13797	0.196463	0.702269	0.482512	0.750767	SEC61A2	55176	SEC61 translocon subunit alpha 2
ENSG0000	21.64463	-0.37152	0.528972	-0.70234	0.482464	0.750767	ZKSCAN2-I	1.13E+08	ZKSCAN2 divergent transcript
ENSG0000	2135.226	-0.05731	0.081626	-0.70208	0.48263	0.750889	FCHSD1	89848	FCH and double SH3 domains 1
ENSG0000	2.728207	1.234278	1.758286	0.701978	0.482693	0.750923	ADD3-AS1	1.01E+08	ADD3 antisense RNA 1
ENSG0000	3083.838	0.068533	0.097679	0.701607	0.482925	0.751221	CLEC2D	29121	C-type lectin domain family 2 member D
ENSG0000	762.8272	-0.07327	0.104572	-0.70068	0.483501	0.751404	TAF5L	27097	TATA-box binding protein associated factor 5 like
ENSG0000	30.1789	0.3489	0.497838	0.700831	0.483408	0.751404	NA	NA	NA
ENSG0000	37.29918	0.35959	0.513068	0.700862	0.483389	0.751404	NA	NA	NA
ENSG0000	19.99544	-0.36073	0.514632	-0.70096	0.48333	0.751404	H2BC4	8347	H2B clustered histone 4
ENSG0000	12.7771	0.485314	0.692569	0.700744	0.483463	0.751404	ZNG1F	644019	Zn regulated GTPase metalloprotein activator 1F
ENSG0000	156.1126	-0.13747	0.19609	-0.70108	0.483255	0.751404	STXBP1	6812	syntaxin binding protein 1
ENSG0000	493.3169	-0.08973	0.12798	-0.70112	0.483226	0.751404	R3HDM2	22864	R3H domain containing 2
ENSG0000	83.65306	0.184735	0.263641	0.700708	0.483485	0.751404	NT5DC3	51559	5'-nucleotidase domain containing 3
ENSG0000	523.0834	0.094096	0.134189	0.701221	0.483165	0.751404	PABPN1	8106	poly(A) binding protein nuclear 1
ENSG0000	3.771679	-1.02158	1.456628	-0.70133	0.483098	0.751404	NA	NA	NA
ENSG0000	43.05801	-0.27508	0.392254	-0.70127	0.483134	0.751404	ARRDC5	645432	arrestin domain containing 5
ENSG0000	2.811244	1.106277	1.578955	0.700639	0.483529	0.751404	RPA4	29935	replication protein A4
ENSG0000	136.1607	-0.17649	0.251938	-0.70051	0.483608	0.751464	ZNF736	728927	zinc finger protein 736
ENSG0000	1575.909	-0.0641	0.091521	-0.70039	0.483681	0.751515	INO80	54617	INO80 complex ATPase subunit
ENSG0000	135.4962	-0.15707	0.224282	-0.70031	0.483736	0.751537	PTPRM	5797	protein tyrosine phosphatase receptor type M
ENSG0000	552.7951	-0.09601	0.137137	-0.70008	0.483877	0.751693	TIGD5	84948	tigger transposable element derived 5
ENSG0000	4.293182	-0.77612	1.109223	-0.6997	0.484115	0.751782	NA	NA	NA
ENSG0000	3.729693	0.98294	1.404583	0.699809	0.484046	0.751782	RUNDC3B	154661	RUN domain containing 3B
ENSG0000	108.1537	0.173213	0.247516	0.699806	0.484048	0.751782	SNX16	64089	sorting nexin 16
ENSG0000	20069.84	-0.16887	0.241351	-0.69966	0.484137	0.751782	WARS1	7453	tryptophanyl-tRNA synthetase 1
ENSG0000	3.699636	-1.38301	1.976588	-0.6997	0.484117	0.751782	ST8SIA5	29906	ST8 alpha- 8-sialyltransferase 5
ENSG0000	303.4943	0.107206	0.153262	0.699496	0.484242	0.75188	NA	NA	NA
ENSG0000	810.0516	-0.10323	0.147584	-0.69943	0.484281	0.75188	LMAN1	3998	lectin mannose binding 1
ENSG0000	29.85024	0.347374	0.496738	0.699311	0.484358	0.751936	RAC3	5881	Rac family small GTPase 3
ENSG0000	9.328036	-0.58636	0.838712	-0.69912	0.484477	0.752058	KCNMA1	3778	potassium calcium-activated channel subfamily M alpha 1
ENSG0000	22.20963	0.353385	0.505613	0.698924	0.4846	0.75206	DLK2	65989	delta like non-canonical Notch ligand 2
ENSG0000	8.737018	-0.58846	0.841807	-0.69904	0.484524	0.75206	NA	NA	NA
ENSG0000	2.314971	1.302469	1.863507	0.698934	0.484593	0.75206	SMIM10	644538	small integral membrane protein 10
ENSG0000	79.73723	0.197861	0.283199	0.698665	0.484761	0.752217	TAL1	6886	TAL bHLH erythroid differentiation factor
ENSG0000	344.5191	-0.09658	0.13841	-0.69778	0.485317	0.752217	LRIG2	9860	leucine rich repeats and immunoglobulin like domains 2
ENSG0000	24.72318	-0.47215	0.676701	-0.69772	0.485353	0.752217	IQGAP3	128239	IQ motif containing GTPase activating protein 3
ENSG0000	4.861406	-1.02398	1.466062	-0.69846	0.48489	0.752217	PKDCC	91461	protein kir cytoplasmic
ENSG0000	624.5778	0.078531	0.112548	0.697754	0.485331	0.752217	MKRN2	23609	makorin ring finger protein 2
ENSG0000	235.8944	0.112679	0.161333	0.698426	0.484911	0.752217	HERC5	51191	HECT and RLD domain containing E3 ubiquitin protein ligase 5
ENSG0000	6.848472	0.760526	1.089179	0.698256	0.485017	0.752217	LRIT3	345193	leucine ric Ig-like and transmembrane domains 3
ENSG0000	307.2673	-0.12119	0.173607	-0.69809	0.485122	0.752217	SMAD5	4090	SMAD family member 5
ENSG0000	21.32247	-0.40118	0.574628	-0.69815	0.485085	0.752217	EPHA1-AS	285965	EPHA1 antisense RNA 1
ENSG0000	34.67078	0.341161	0.489073	0.697568	0.485448	0.752217	NA	NA	NA
ENSG0000	220.1032	-0.12816	0.183735	-0.69753	0.485471	0.752217	DCUN1D5	84259	defective in cullin neddylation 1 domain containing 5
ENSG0000	309.8309	-0.11808	0.169273	-0.69758	0.485438	0.752217	MPHOSPH	10198	M-phase phosphoprotein 9
ENSG0000	449.322	0.109686	0.157249	0.697532	0.48547	0.752217	PLD4	122618	phospholipase D family member 4
ENSG0000	6.756024	0.790212	1.132021	0.698054	0.485143	0.752217	NA	NA	NA
ENSG0000	3.905623	-0.86039	1.233094	-0.69775	0.485334	0.752217	NA	NA	NA
ENSG0000	41.07318	0.28152	0.403109	0.698371	0.484945	0.752217	CD70	970	CD70 molecule
ENSG0000	315.7489	-0.1329	0.190238	-0.69862	0.484788	0.752217	NUDT19	390916	nudix hydrolase 19
ENSG0000	152.3602	0.135539	0.194097	0.698304	0.484987	0.752217	NA	NA	NA
ENSG0000	9168.049	0.059981	0.085979	0.697626	0.485411	0.752217	RUNX1	861	RUNX family transcription factor 1
ENSG0000	2796.812	-0.05852	0.08393	-0.69721	0.485671	0.752331	TCOF1	6949	treacle ribosome biogenesis factor 1
ENSG0000	600.2642	0.076436	0.109638	0.697167	0.485698	0.752331	SMIM10L1	1E+08	small integral membrane protein 10 like 1
ENSG0000	231.3228	-0.13152	0.18865	-0.69715	0.485707	0.752331	TADA2A	6871	transcriptional adaptor 2A
ENSG0000	32.5857	-0.29438	0.422259	-0.69717	0.485699	0.752331	LINC01281	286442	long intergenic non-protein coding RNA 1281
ENSG0000	1157.444	0.070822	0.101621	0.696921	0.485852	0.75243	CD58	965	CD58 molecule
ENSG0000	3337.739	0.070625	0.101333	0.696965	0.485824	0.75243	ARF5	381	ADP ribosylation factor 5
ENSG0000	864.8004	0.079201	0.113687	0.696659	0.486016	0.752621	PEX19	5824	peroxisomal biogenesis factor 19
ENSG0000	2069.975	0.071036	0.101986	0.696522	0.486102	0.752666	TMED10	10972	transmembrane p24 trafficking protein 10
ENSG0000	2.871915	1.147397	1.647415	0.696483	0.486126	0.752666	NA	NA	NA
ENSG0000	126.4356	-0.16914	0.242978	-0.6961	0.486364	0.752694	NA	NA	NA
ENSG0000	1072.975	0.066319	0.095332	0.695663	0.48664	0.752694	RFX5	5993	regulatory factor X5
ENSG0000	1150.424	0.073611	0.105907	0.69505	0.487024	0.752694	MCFD2	90411	multiple c(ER cargo receptor complex subunit
ENSG0000	130.7737	0.145166	0.208657	0.695717	0.486606	0.752694	METTL6	131965	methyltra methylcytidine
ENSG0000	25.86092	0.380566	0.546586	0.69626	0.486266	0.752694	LINC0088	1.01E+08	long intergenic non-protein coding RNA 888
ENSG0000	14.09626	-0.48366	0.69495	-0.69597	0.486447	0.752694	VWA5B2	90113	von Willebrand factor A domain containing 5B2
ENSG0000	35.9862	-0.29205	0.419473	-0.69623	0.486284	0.752694	KCNK5	8645	potassium two pore domain channel subfamily K member 5
ENSG0000	9.414092	0.542894	0.779805	0.696192	0.486308	0.752694	NA	NA	NA
ENSG0000	3949.338	0.064045	0.092112	0.695293	0.486872	0.752694	CD82	3732	CD82 molecule
ENSG0000	20.76368	-0.40438	0.581648	-0.69524	0.486908	0.752694	P3H3	10536	prolyl 3-hydroxylase 3
ENSG0000	3.229262	1.067638	1.533765	0.69609	0.486372	0.752694	LOC10050	1.01E+08	uncharacterized LOC100506071
ENSG0000	8.042993	0.650508	0.934757	0.695911	0.486485	0.752694	PROX2	283571	prospero homeobox 2
ENSG0000	9.311988	0.516762	0.742651	0.695834	0.486533	0.752694	RPL5P1	647436	ribosomal protein L5 pseudogene 1
ENSG0000	871.132	-0.07141	0.102763	-0.6949	0.487118	0.752694	TRIM69	140691	tripartite motif containing 69
ENSG0000	14.40956	-0.47475	0.683019	-0.69507	0.487012	0.752694	CETP	1071	cholesteryl ester transfer protein
ENSG0000	37.23705	-0.28245	0.406231	-0.6953	0.486869	0.752694	RPH3AL	9501	rabphilin 3A like (without C2 domains)
ENSG0000	78.6687	0.198766	0.285763	0.695565	0.486701	0.752694	TRIM47	91107	tripartite motif containing 47
ENSG0000	4.340152	-0.90075	1.295231	-0.69544	0.486781	0.752694	NA	NA	NA
ENSG0000	604.0495	0.096028	0.137986	0.695923	0.486477	0.752694	ZNF83	55769	zinc finger protein 83
ENSG0000	635.9173	-0.08161	0.117422	-0.695	0.487055	0.752694	RTCB	51493	RNA 2' 3'-cyclic phosphate and 5'-OH ligase

ENSG0000	3.588998	1.117998	1.606893	0.695751	0.486584	0.752694	NA	NA	NA
ENSG0000	3.472683	-0.85871	1.235721	-0.69491	0.487115	0.752694	NA	NA	NA
ENSG0000	5.274276	-0.84784	1.219763	-0.69508	0.487003	0.752694	XKRX	402415	XK related X-linked
ENSG0000	128.497	0.1658	0.238502	0.695171	0.486948	0.752694	OCRL	4952	OCRL inositol polyphosphate-5-phosphatase
ENSG0000	1324.708	0.077724	0.111895	0.694617	0.487295	0.752779	RABGAP1L	9910	RAB GTPase activating protein 1 like
ENSG0000	16.37977	-0.47168	0.679043	-0.69463	0.48729	0.752779	NA	NA	NA
ENSG0000	19.24011	-0.38829	0.558919	-0.69472	0.487232	0.752779	NA	NA	NA
ENSG0000	1502.613	-0.06559	0.094434	-0.69452	0.487357	0.752812	FBXL14	144699	F-box and leucine rich repeat protein 14
ENSG0000	15.21089	0.567476	0.817215	0.694403	0.48743	0.752861	RGS17	26575	regulator of G protein signaling 17
ENSG0000	93.36494	-0.19363	0.279047	-0.69391	0.48774	0.753227	DLGAP3	58512	DLG associated protein 3
ENSG0000	1286.655	0.069666	0.100408	0.693833	0.487787	0.753227	APRT	353	adenine phosphoribosyltransferase
ENSG0000	64.26787	0.21715	0.312973	0.693831	0.487788	0.753227	TMEM191	84222	transmembrane protein 191A (pseudogene)
ENSG0000	6.196328	-0.77119	1.111635	-0.69375	0.487842	0.753247	DET1	55070	DET1 partner of COP1 E3 ubiquitin ligase
ENSG0000	11.73847	-0.56122	0.809125	-0.69361	0.487929	0.753318	NA	NA	NA
ENSG0000	2193.083	0.055742	0.080398	0.693327	0.488104	0.753338	SHC1	6464	SHC adaptor protein 1
ENSG0000	16.33381	-0.40577	0.585239	-0.69334	0.488098	0.753338	NA	NA	NA
ENSG0000	6.065682	-0.75285	1.085741	-0.6934	0.488058	0.753338	ARSJ	79642	arylsulfatase family member J
ENSG0000	366.0803	-0.09655	0.139251	-0.69333	0.488102	0.753338	MED11	400569	mediator complex subunit 11
ENSG0000	2101.852	-0.07213	0.104068	-0.69309	0.488254	0.753496	RBPJ	3516	recombination signal binding protein for immunoglobulin kappa J region
ENSG0000	7.407602	-0.72645	1.04832	-0.69297	0.488328	0.753496	NA	NA	NA
ENSG0000	3.076823	-1.11335	1.606496	-0.69303	0.488291	0.753496	TMSB15B	286527	thymosin beta 15B
ENSG0000	28.44665	0.325196	0.469425	0.692753	0.488465	0.753547	CHAC2	494143	ChaC glutathione specific gamma-glutamylcyclotransferase 2
ENSG0000	61.9551	0.229532	0.331348	0.692723	0.488483	0.753547	MZT1	440145	mitotic spindle organizing protein 1
ENSG0000	26.87139	-0.39109	0.564483	-0.69283	0.488416	0.753547	IGHV1-69D	1.03E+08	immunoglobulin heavy variable 1-69D
ENSG0000	3.011914	-1.10594	1.597284	-0.69239	0.488695	0.753595	ZCWPW2	152098	zinc finger CW-type and PWWP domain containing 2
ENSG0000	24.55921	-0.34399	0.496764	-0.69246	0.488646	0.753595	SAP30-DT	1.05E+08	SAP30 divergent transcript
ENSG0000	94.61749	0.215464	0.311206	0.692351	0.488717	0.753595	F2RL1	2150	F2R like trypsin receptor 1
ENSG0000	1662.002	0.067547	0.097536	0.692538	0.4886	0.753595	ZNF746	155061	zinc finger protein 746
ENSG0000	3998.483	-0.08181	0.118124	-0.69261	0.488557	0.753595	HELZ2	85441	helicase with zinc finger 2
ENSG0000	54.44048	-0.22418	0.32383	-0.69228	0.488762	0.753601	FIGNL2	401720	fidgetin like 2
ENSG0000	7824.292	0.046206	0.066799	0.69172	0.489113	0.754003	DVL3	1857	dishevelled segment polarity protein 3
ENSG0000	5903.983	-0.05029	0.072704	-0.69164	0.489163	0.754003	AP1G2	8906	adaptor related protein complex 1 subunit gamma 2
ENSG0000	113.8371	-0.17205	0.248774	-0.69161	0.489185	0.754003	ZNF761	388561	zinc finger protein 761
ENSG0000	55.65418	0.227495	0.328913	0.691656	0.489153	0.754003	S100B	6285	S100 calcium binding protein B
ENSG0000	283.065	0.102774	0.148623	0.691507	0.489247	0.754036	FBXO30	84085	F-box protein 30
ENSG0000	3.524175	-0.93931	1.358622	-0.69137	0.489334	0.754055	IGKJ5	28946	immunoglobulin kappa joining 5
ENSG0000	240.4007	-0.11678	0.168908	-0.69136	0.489341	0.754055	HSBP1	3281	heat shock factor binding protein 1
ENSG0000	171.4821	-0.16063	0.232384	-0.69125	0.489411	0.754101	NA	NA	NA
ENSG0000	6.125013	0.794986	1.150961	0.690715	0.489745	0.754552	PLPPR3	79948	phospholipid phosphatase related 3
ENSG0000	903.7352	-0.06927	0.100314	-0.69056	0.489843	0.754578	XPO5	57510	exportin 5
ENSG0000	3.832063	0.971257	1.406375	0.69061	0.489811	0.754578	TRDJ1	28522	T cell receptor delta joining 1
ENSG0000	31.87383	-0.3034	0.439514	-0.69032	0.489996	0.75459	TFAP2E-AS	1.05E+08	TFAP2E antisense RNA 1
ENSG0000	22.40469	0.425749	0.616687	0.690381	0.489954	0.75459	NA	NA	NA
ENSG0000	762.0009	0.092017	0.133315	0.690223	0.490054	0.75459	SNF8	11267	SNF8 subunit of ESCRT-II
ENSG0000	1771.063	-0.05887	0.085278	-0.69032	0.489991	0.75459	ZNF266	10781	zinc finger protein 266
ENSG0000	176.5233	0.125762	0.182189	0.690284	0.490015	0.75459	ZNF71	58491	zinc finger protein 71
ENSG0000	2.670901	1.183031	1.714232	0.690123	0.490117	0.754624	NSMCE1-D	400512	NSMCE1 divergent transcript
ENSG0000	447.9014	0.137584	0.199453	0.689806	0.490316	0.754706	ISG15	9636	ISG15 ubiquitin like modifier
ENSG0000	10.69301	-0.5475	0.793689	-0.68981	0.490312	0.754706	NA	NA	NA
ENSG0000	4.22198	-0.86898	1.259795	-0.68978	0.490333	0.754706	NA	NA	NA
ENSG0000	140.1215	-0.1547	0.22424	-0.6899	0.490255	0.754706	ZMAT1	84460	zinc finger matrin-type 1
ENSG0000	3.45791	-0.98461	1.428027	-0.68949	0.490516	0.754924	RN7SL589P	1.06E+08	RNA 7SL cytoplasm pseudogene
ENSG0000	199.7594	-0.12684	0.184006	-0.68933	0.490617	0.754928	RNFT1	51136	ring finger transmembrane 1
ENSG0000	23.81945	-0.34113	0.4949	-0.68929	0.49064	0.754928	NA	NA	NA
ENSG0000	199.1954	0.134087	0.194521	0.689318	0.490623	0.754928	ZNF708	7562	zinc finger protein 708
ENSG0000	1074.003	-0.07202	0.104517	-0.68909	0.490768	0.755062	AKT1S1	84335	AKT1 substrate 1
ENSG0000	4.246305	1.023541	1.485534	0.689005	0.49082	0.755079	RPL34-DT	285456	RPL34 divergent transcript
ENSG0000	55.8759	0.219271	0.318274	0.688937	0.490863	0.755083	NA	NA	NA
ENSG0000	1742.146	-0.07081	0.102809	-0.68879	0.490959	0.755167	GNPTAB	79158	N-acetylglucosamine-1-phosphate transferase subunits alpha and beta
ENSG0000	441.6104	0.099308	0.144217	0.688601	0.491074	0.755274	DDX50	79009	DExD-box helicase 50
ENSG0000	65.74044	-0.22756	0.330494	-0.68855	0.491109	0.755274	HMGB3	3149	high mobility group box 3
ENSG0000	5.595376	0.73706	1.070593	0.68846	0.491163	0.755294	FSBP	1.01E+08	fibrinogen silencer binding protein
ENSG0000	30.50444	-0.29687	0.431307	-0.68831	0.491256	0.755375	NA	NA	NA
ENSG0000	10.73113	-0.50084	0.72774	-0.68821	0.491323	0.755414	SLC5A5	6528	solute carrier family 5 member 5
ENSG0000	77.30072	0.187561	0.272585	0.688082	0.491401	0.755472	PRPF18	8559	pre-mRNA processing factor 18
ENSG0000	3.739923	-1.04659	1.521274	-0.68797	0.491471	0.755517	NA	NA	NA
ENSG0000	2047.597	-0.07574	0.110176	-0.68746	0.491794	0.755625	UBA7	7318	ubiquitin like modifier activating enzyme 7
ENSG0000	277.5469	-0.1164	0.169288	-0.68758	0.491718	0.755625	SREK1IP1	285672	SREK1 interacting protein 1
ENSG0000	5.36245	0.744331	1.082784	0.687423	0.491816	0.755625	NA	NA	NA
ENSG0000	263.7101	-0.10739	0.156152	-0.68772	0.491631	0.755625	FAM204A	63877	family with sequence similarity 204 member A
ENSG0000	50.82545	-0.23166	0.336822	-0.68779	0.491584	0.755625	THTPA	79178	thiamine triphosphatase
ENSG0000	1560.184	0.079519	0.11568	0.687407	0.491827	0.755625	INF2	64423	inverted formin 2
ENSG0000	1152.753	-0.08002	0.11638	-0.68755	0.491735	0.755625	PRPF31	26121	pre-mRNA processing factor 31
ENSG0000	649.5691	-0.07881	0.114678	-0.68724	0.491933	0.755664	FIS1	51024	fission mitochondrial 1
ENSG0000	84.32141	-0.17321	0.252042	-0.68724	0.491932	0.755664	C10orf88	80007	chromosome 10 open reading frame 88
ENSG0000	629.3086	-0.08519	0.123975	-0.68716	0.491981	0.755675	ZNF398	57541	zinc finger protein 398
ENSG0000	18.86829	0.410256	0.597251	0.686906	0.492142	0.755859	SLC27A5	10998	solute carrier family 27 member 5
ENSG0000	447.2167	0.087323	0.127144	0.686804	0.492206	0.755875	MRPS21	54460	mitochondrial ribosomal protein S21
ENSG0000	6.821388	0.629512	0.916639	0.686761	0.492233	0.755875	NA	NA	NA
ENSG0000	1299.162	-0.06839	0.099605	-0.68661	0.492332	0.755963	GRAMD1B	57476	GRAM domain containing 1B
ENSG0000	3.47568	-1.07116	1.560447	-0.68645	0.492432	0.756055	SERPINA9	327657	serpin family A member 9
ENSG0000	607.5827	-0.08572	0.12492	-0.68622	0.492572	0.756206	KPNA3	3839	karyopherin subunit alpha 3
ENSG0000	682.5086	0.087234	0.127215	0.685721	0.492889	0.756317	MCM6	4175	minichromosome maintenance complex component 6
ENSG0000	4.779507	-0.8836	1.288423	-0.6858	0.492842	0.756317	NA	NA	NA
ENSG0000	172.6099	-0.14088	0.205358	-0.68601	0.492709	0.756317	GAB1	2549	GRB2 associated binding protein 1
ENSG0000	43.60792	-0.27918	0.407037	-0.68588	0.492786	0.756317	KCNMB4	27345	potassium calcium-activated channel subfamily M regulatory beta subunit 4
ENSG0000	89.34326	-0.16697	0.243478	-0.68576	0.492864	0.756317	CCDC78	124093	coiled-coil domain containing 78
ENSG0000	3.458858	-0.89401	1.30329	-0.68597	0.492735	0.756317	ATP2C2	9914	ATPase secretory pathway Ca2+ transporting 2
ENSG0000	56.76973	-0.24252	0.353743	-0.68559	0.492972	0.756338	KIF27	55582	kinesin family member 27
ENSG0000	552.3495	-0.09224	0.134538	-0.68557	0.492983	0.756338	GCDH	2639	glutaryl-CoA dehydrogenase
ENSG0000	208.4437	0.13106	0.191289	0.685142	0.493255	0.756628	METTL2B	55798	methyltra methylcytidine
ENSG0000	21.86293	0.409937	0.598318	0.68515	0.49325	0.756628	NA	NA	NA
ENSG0000	3.121974	1.02869	1.501784	0.684979	0.493357	0.756723	MICALL2-D	1.08E+08	MICALL2 divergent transcript
ENSG0000	1990.323	-0.06147	0.08975	-0.68485	0.493441	0.756789	NRDC	4898	nardilysin convertase
ENSG0000	23.65718	0.340021	0.496698	0.684563	0.49362	0.757	CASP5	838	caspase 5
ENSG0000	14.40288	0.47343	0.691786	0.684358	0.493749	0.757064	NA	NA	NA
ENSG0000	1188.764	-0.07164	0.104686	-0.6843	0.493784	0.757064	PATZ1	23598	POZ/BTB and AT hook containing zinc finger 1
ENSG0000	18.41891	-0.40798	0.596116	-0.6844	0.493722	0.757064	RPL4P6	1E+08	ribosomal protein L4 pseudogene 6
ENSG0000	74.87248	-0.18562	0.271322	-0.68415	0.493882	0.757089	BARD1	580	BRCA1 associated RING domain 1
ENSG0000	124.0493	-0.15146	0.221366	-0.6842	0.493847	0.757089	CFDP1	10428	craniofacial development protein 1

ENSG0000	421.3711	-0.09843	0.143932	-0.68387	0.494058	0.757297	SLC25A45	283130	solute carrier family 25 member 45
ENSG0000	2476.242	-0.0678	0.099152	-0.68379	0.49411	0.757313	MED16	10025	mediator complex subunit 16
ENSG0000	628.504	-0.09702	0.141934	-0.68358	0.49424	0.757451	PLCXD2	257068	phosphatidylinositol specific phospholipase C X domain containing 2
ENSG0000	3.912567	0.835646	1.222592	0.683504	0.494289	0.757463	NA	NA	NA
ENSG0000	10.14402	0.520432	0.761585	0.683354	0.494383	0.757482	NA	NA	NA
ENSG0000	2.219697	-1.37416	2.010774	-0.6834	0.494354	0.757482	TPI1P2	286016	triosephosphate isomerase 1 pseudogene 2
ENSG0000	7.644965	0.551202	0.806913	0.683101	0.494543	0.75754	EEF1A1P3!	389223	eukaryotic translation elongation factor 1 alpha 1 pseudogene 35
ENSG0000	111.2577	0.163645	0.23952	0.683221	0.494467	0.75754	ZDHHC11	79844	zinc finger DHHC-type containing 11
ENSG0000	7.197124	0.835603	1.223141	0.683162	0.494505	0.75754	SGCA	6442	sarcoglycan alpha
ENSG0000	217.8276	-0.11306	0.165567	-0.68285	0.494702	0.75772	ARL13B	200894	ADP ribosylation factor like GTPase 13B
ENSG0000	739.7273	0.074536	0.109189	0.682628	0.494842	0.757825	SEC11A	23478	SEC11 hon signal peptidase complex subunit
ENSG0000	23.21111	0.355938	0.521436	0.682612	0.494852	0.757825	PKD1P6-N	1.05E+08	PKD1P6-NPIPP1 readthrough
ENSG0000	34.37062	0.27118	0.39731	0.682541	0.494897	0.757831	FCRL2	79368	Fc receptor like 2
ENSG0000	765.0345	0.086537	0.126809	0.682419	0.494974	0.757887	ARRDC3	57561	arrestin domain containing 3
ENSG0000	5.639703	-0.84862	1.243975	-0.68218	0.495125	0.758055	NA	NA	NA
ENSG0000	2216.832	0.072294	0.106008	0.681968	0.495259	0.758198	KIF5B	3799	kinesin family member 5B
ENSG0000	154.5768	-0.13997	0.205272	-0.68186	0.495329	0.758243	AK1	203	adenylate kinase 1
ENSG0000	46.71595	-0.23879	0.35032	-0.68163	0.495473	0.758275	SLC2A9	56606	solute carrier family 2 member 9
ENSG0000	2003.756	-0.07128	0.104577	-0.68164	0.495466	0.758275	CARMIL2	146206	capping protein regulator and myosin 1 linker 2
ENSG0000	6479.951	-0.05648	0.082849	-0.68171	0.495425	0.758275	TMC8	147138	transmembrane channel like 8
ENSG0000	1508.639	0.075743	0.111188	0.681215	0.495736	0.758615	SEC24B	10427	SEC24 hon COPII coat complex component
ENSG0000	63.51903	-0.19986	0.293479	-0.681	0.495874	0.758701	CHI3L2	1117	chitinase 3 like 2
ENSG0000	37.76209	0.279138	0.40988	0.681025	0.495856	0.758701	RGS6	9628	regulator of G protein signaling 6
ENSG0000	2340.721	-0.05892	0.08653	-0.6809	0.495933	0.758728	RETREG2	79137	reticulophagy regulator family member 2
ENSG0000	7.737211	-0.58456	0.858677	-0.68077	0.496019	0.758798	NA	NA	NA
ENSG0000	135.7833	0.144888	0.212908	0.680516	0.496178	0.758865	NOXA1	10811	NADPH oxidase activator 1
ENSG0000	31.21135	0.313667	0.460902	0.680551	0.496156	0.758865	RPS6P25	729389	ribosomal protein S6 pseudogene 25
ENSG0000	17.16512	0.495586	0.728263	0.680504	0.496185	0.758865	SERPIND1	3053	serpin family D member 1
ENSG0000	381.6137	-0.10363	0.152375	-0.68012	0.496429	0.759174	NCF1C	654817	neutrophil cytosolic factor 1C pseudogene
ENSG0000	2.653259	-1.23216	1.812067	-0.67998	0.496519	0.759187	LINC0181¢	1.03E+08	long intergenic non-protein coding RNA 1819
ENSG0000	155.4028	-0.14259	0.209677	-0.68002	0.49649	0.759187	GEMIN7	79760	gem nuclear organelle associated protein 7
ENSG0000	231.4486	-0.1157	0.170206	-0.67978	0.496643	0.759308	DIS3L2	129563	DIS3 like 3'-5' exoribonuclease 2
ENSG0000	20.20844	0.453534	0.667233	0.679723	0.49668	0.759308	LIM2	3982	lens intrinsic membrane protein 2
ENSG0000	77.92797	0.180061	0.264965	0.679567	0.496779	0.759334	LINC0023¢	145200	long intergenic non-protein coding RNA 239
ENSG0000	4.834862	-1.00812	1.483387	-0.67961	0.496754	0.759334	NA	NA	NA
ENSG0000	9.757258	0.674264	0.99236	0.679455	0.496849	0.759337	TRAV10	28676	T cell receptor alpha variable 10
ENSG0000	4.317465	0.855209	1.258707	0.679434	0.496863	0.759337	NA	NA	NA
ENSG0000	14970.84	-0.05105	0.075149	-0.67928	0.496959	0.759421	OGDH	4967	oxoglutarate dehydrogenase
ENSG0000	182.9627	-0.14046	0.206792	-0.67922	0.497	0.759422	FBXO45	200933	F-box protein 45
ENSG0000	2184.236	-0.16067	0.236593	-0.67911	0.497068	0.759463	PARP9	83666	poly(ADP-ribose) polymerase family member 9
ENSG0000	2.168782	-1.4002	2.06221	-0.67898	0.497151	0.759527	DOK6	220164	docking protein 6
ENSG0000	51.25391	0.225622	0.3324	0.678767	0.497285	0.75967	TMEM163	81615	transmembrane protein 163
ENSG0000	12.63076	-0.45625	0.672376	-0.67856	0.497417	0.759808	RASGRF1	5923	Ras protein specific guanine nucleotide releasing factor 1
ENSG0000	10.57556	0.501829	0.739734	0.678391	0.497524	0.759846	TEN1-CDK:	1.01E+08	TEN1-CDK3 readthrough (NMD candidate)
ENSG0000	340.8941	-0.10007	0.147507	-0.67843	0.497497	0.759846	PCYT2	5833	phosphate ethanolamine
ENSG0000	9.555002	-0.52977	0.781388	-0.67799	0.49778	0.760175	CCDC183	84960	coiled-coil domain containing 183
ENSG0000	820.6612	-0.07709	0.113721	-0.67785	0.497867	0.760245	RASSF1	11186	Ras association domain family member 1
ENSG0000	11.99623	0.585559	0.864153	0.67761	0.498019	0.760414	NA	NA	NA
ENSG0000	215.4137	-0.13925	0.205568	-0.6774	0.498152	0.760555	ENO3	2027	enolase 3
ENSG0000	219.8701	-0.12032	0.177741	-0.67694	0.498441	0.760934	HSF2	3298	heat shock transcription factor 2
ENSG0000	191.7229	0.127513	0.188424	0.676736	0.498573	0.761073	TRAPPC6A	79090	trafficking protein particle complex subunit 6A
ENSG0000	3.669526	0.905327	1.338009	0.676622	0.498646	0.761121	POU5F1	5460	POU class 5 homeobox 1
ENSG0000	7.706082	0.63777	0.942942	0.676362	0.498811	0.761248	NFATC4	4776	nuclear factor of activated T cells 4
ENSG0000	367.2325	-0.10869	0.160698	-0.67639	0.498793	0.761248	MIS18BP1	55320	MIS18 binding protein 1
ENSG0000	111.7456	-0.16111	0.238242	-0.67625	0.498879	0.76129	TJP3	27134	tight junction protein 3
ENSG0000	69.86618	-0.18892	0.2794	-0.67615	0.498947	0.761331	TRBD1	28637	T cell receptor beta diversity 1
ENSG0000	9.428269	-0.5998	0.887309	-0.67597	0.499058	0.761374	KCTD21-A!	1E+08	KCTD21 antisense RNA 1
ENSG0000	15.36249	0.42357	0.626576	0.676007	0.499036	0.761374	DNMT3B	1789	DNA methyltransferase 3 beta
ENSG0000	7.482246	-0.75347	1.11532	-0.67557	0.499315	0.761516	CCDC150	284992	coiled-coil domain containing 150
ENSG0000	173.4491	0.137708	0.203827	0.675615	0.499285	0.761516	SLC35F2	54733	solute carrier family 35 member F2
ENSG0000	6.809567	0.724382	1.072187	0.675611	0.499287	0.761516	NA	NA	NA
ENSG0000	88.49917	-0.18154	0.268655	-0.67574	0.499207	0.761516	BAIAP2L2	80115	BAR/IMD domain containing adaptor protein 2 like 2
ENSG0000	4.148163	-0.81544	1.207159	-0.6755	0.499356	0.761516	GRTP1	79774	growth hormone regulated TBC protein 1
ENSG0000	5345.237	0.050216	0.07436	0.675308	0.49948	0.761642	MPP1	4354	MAGUK p55 scaffold protein 1
ENSG0000	966.3808	-0.07861	0.116433	-0.67514	0.499584	0.761739	YJU2	55702	YJU2 splicing factor homolog
ENSG0000	58.71322	0.211769	0.313711	0.675045	0.499647	0.761772	CNPY2	10330	canopy FGF signaling regulator 2
ENSG0000	12.71911	-0.45509	0.674358	-0.67485	0.499771	0.761898	NA	NA	NA
ENSG0000	249.528	0.113445	0.168137	0.674717	0.499855	0.761964	SNRPD1	6632	small nuclear ribonucleoprotein D1 polypeptide
ENSG0000	5.216852	0.834317	1.236741	0.67461	0.499924	0.762006	C20orf202	400831	chromosome 20 open reading frame 202
ENSG0000	151.4845	-0.17104	0.25363	-0.67438	0.500068	0.762038	SVBP	374969	small vasohibin binding protein
ENSG0000	79.05783	-0.20449	0.303208	-0.67442	0.500043	0.762038	ZFP82	284406	ZFP82 zinc finger protein
ENSG0000	3.176197	-0.96235	1.426804	-0.67448	0.500008	0.762038	NA	NA	NA
ENSG0000	88.60216	0.186516	0.276643	0.674213	0.500176	0.762093	ESPN	83715	espin
ENSG0000	3302.44	0.062259	0.092345	0.674196	0.500186	0.762093	PRPF38B	55119	pre-mRNA processing factor 38B
ENSG0000	682.5154	-0.09266	0.137485	-0.67398	0.500323	0.762238	ARMH1	339541	armadillo like helical domain containing 1
ENSG0000	273.3636	0.106424	0.157961	0.673738	0.500478	0.762412	PCSK5	5125	proprotein convertase subtilisin/kexin type 5
ENSG0000	82.51696	-0.22005	0.326747	-0.67345	0.500658	0.762561	RBM43	375287	RNA binding motif protein 43
ENSG0000	1171.473	0.076653	0.113819	0.673466	0.500651	0.762561	LACTB	114294	lactamase beta
ENSG0000	55.06661	0.215585	0.32021	0.67326	0.500782	0.762624	FAM50B	26240	family with sequence similarity 50 member B
ENSG0000	26.01817	-0.3012	0.447343	-0.67331	0.500753	0.762624	NA	NA	NA
ENSG0000	12.26051	-0.45828	0.680805	-0.67314	0.50086	0.76268	NA	NA	NA
ENSG0000	12.4541	-0.47253	0.702198	-0.67293	0.500992	0.762757	SAMD11	148398	sterile alpha motif domain containing 11
ENSG0000	4.128719	1.088678	1.617687	0.672984	0.500957	0.762757	CSMD1	64478	CUB and Sushi multiple domains 1
ENSG0000	1910.524	-0.05627	0.083663	-0.67253	0.501246	0.763027	BCL11B	64919	BAF chromatin remodeling complex subunit BCL11B
ENSG0000	182.3461	0.136914	0.203584	0.672521	0.501252	0.763027	SYNGR1	9145	synaptogyrin 1
ENSG0000	329.2615	0.128475	0.191094	0.67231	0.501386	0.763169	QTRT1	81890	queuine tRNA-ribosyltransferase catalytic subunit 1
ENSG0000	207.6122	0.116625	0.17352	0.672112	0.501512	0.763298	FEN1	2237	flap structure-specific endonuclease 1
ENSG0000	5.256483	0.703904	1.047548	0.671953	0.501613	0.763377	NA	NA	NA
ENSG0000	938.421	0.071336	0.10617	0.671901	0.501646	0.763377	TMEM11	8834	transmembrane protein 11
ENSG0000	81.18506	0.176953	0.263411	0.671776	0.501726	0.763436	C4orf33	132321	chromosome 4 open reading frame 33
ENSG0000	31.97904	-0.30689	0.456924	-0.67164	0.50181	0.7635	NA	NA	NA
ENSG0000	3.141287	0.871069	1.297077	0.671564	0.501862	0.763516	GNG5B	347687	G protein subunit gamma 5B
ENSG0000	15.03427	0.404035	0.601716	0.67147	0.501921	0.763544	SEMA6A-A	1.02E+08	SEMA6A antisense RNA 1
ENSG0000	192.2019	-0.13533	0.201591	-0.6713	0.502028	0.763581	ALS2CL	259173	ALS2 C-terminal like
ENSG0000	22.52615	-0.33458	0.498362	-0.67136	0.501993	0.763581	FBXL9P	26231	F-box and pseudogene
ENSG0000	15.67651	-0.37803	0.563213	-0.6712	0.502094	0.763619	UGGT2	55757	UDP-glucose glycoprotein glucosyltransferase 2
ENSG0000	99.9692	0.185676	0.276672	0.671105	0.502154	0.763648	FILIP1L	11259	filamin A interacting protein 1 like
ENSG0000	9.437253	0.48592	0.724196	0.670979	0.502234	0.763707	NA	NA	NA
ENSG0000	570.2016	0.077136	0.115001	0.670741	0.502386	0.763812	CNOT6	57472	CCR4-NOT transcription complex subunit 6
ENSG0000	153.9167	-0.14949	0.222861	-0.67076	0.502373	0.763812	METTL2A	339175	methyltraı methylcytidine

ENSG0000	1469.412	0.059903	0.089357	0.670378	0.502617	0.764102	BLOC1S3	388552	biogenesis of lysosomal organelles complex 1 subunit 3
ENSG0000	467.3614	-0.08984	0.134075	-0.67005	0.502828	0.764109	TMEM69	51249	transmembrane protein 69
ENSG0000	133.3266	-0.15512	0.231506	-0.67005	0.502824	0.764109	ZBTB41	360023	zinc finger and BTB domain containing 41
ENSG0000	227.2938	-0.12215	0.182253	-0.6702	0.502732	0.764109	ADPRH	141	ADP-ribosylarginine hydrolase
ENSG0000	169.9832	0.140223	0.209208	0.670257	0.502694	0.764109	NOC3L	64318	NOC3 like DNA replication regulator
ENSG0000	2.747708	1.137207	1.697159	0.670065	0.502816	0.764109	NA	NA	NA
ENSG0000	7.841809	-0.54996	0.820912	-0.66993	0.502901	0.764157	NA	NA	NA
ENSG0000	219.5032	-0.11871	0.177261	-0.66966	0.503071	0.764354	RCN2	5955	reticulocalbin 2
ENSG0000	11.15908	0.516012	0.770814	0.669439	0.503216	0.764448	NA	NA	NA
ENSG0000	10.15729	-0.64972	0.970488	-0.66948	0.503192	0.764448	NA	NA	NA
ENSG0000	3.723783	0.955473	1.428479	0.668874	0.503576	0.764831	MIR3605	1.01E+08	microRNA 3605
ENSG0000	787.509	0.07048	0.105375	0.668849	0.503592	0.764831	CNST	163882	consortin connexin sorting protein
ENSG0000	3759.337	-0.05199	0.077729	-0.66891	0.503554	0.764831	ATXN7L3	56970	ataxin 7 like 3
ENSG0000	813.7825	-0.09056	0.135446	-0.66861	0.503742	0.764923	TMEM140	55281	transmembrane protein 140
ENSG0000	7.108091	0.625901	0.936191	0.66856	0.503776	0.764923	ASAH1-AS	1.02E+08	ASAH1 antisense RNA 1
ENSG0000	62.12807	-0.21797	0.326016	-0.66858	0.503763	0.764923	EML5	161436	EMAP like 5
ENSG0000	63.44108	0.234627	0.350985	0.668482	0.503826	0.764936	SFXN4	119559	sideroflexin 4
ENSG0000	10.93982	0.471519	0.705687	0.66817	0.504025	0.765173	NA	NA	NA
ENSG0000	2477.54	-0.064	0.095799	-0.66811	0.504064	0.765173	ST6GALNA	27090	ST6 N-acet 6-sialyltransferase 4
ENSG0000	217.9812	-0.12798	0.191648	-0.66781	0.504257	0.765403	TMEM223	79064	transmembrane protein 223
ENSG0000	11.78104	0.550405	0.824867	0.667265	0.504603	0.765865	GPR63	81491	G protein-coupled receptor 63
ENSG0000	58.43024	0.212108	0.318053	0.666895	0.504839	0.766161	DISC1	27185	DISC1 scaffold protein
ENSG0000	62.98539	-0.19241	0.288673	-0.66653	0.505074	0.766455	SLC39A11	201266	solute carrier family 39 member 11
ENSG0000	117.6425	-0.16634	0.24964	-0.66631	0.505211	0.766599	LINC0221C	147081	long intergenic non-protein coding RNA 2210
ENSG0000	242.5084	-0.12394	0.186145	-0.66584	0.505511	0.766975	NEK1	4750	NIMA related kinase 1
ENSG0000	18.68572	0.510338	0.766532	0.665775	0.505555	0.766975	NA	NA	NA
ENSG0000	191.4587	-0.13458	0.202159	-0.66573	0.505583	0.766975	NA	NA	NA
ENSG0000	188.1406	-0.11831	0.177741	-0.66563	0.505648	0.767011	SLC2A8	29988	solute carrier family 2 member 8
ENSG0000	103.486	0.173135	0.260176	0.665451	0.505762	0.767122	MTX3	345778	metaxin 3
ENSG0000	1098.08	0.079833	0.120015	0.665186	0.505932	0.767191	SMC4	10051	structural maintenance of chromosomes 4
ENSG0000	4.121866	0.88931	1.336814	0.665246	0.505893	0.767191	NA	NA	NA
ENSG0000	95.24759	-0.17276	0.259661	-0.66531	0.505852	0.767191	SPATA33	124045	spermatogenesis associated 33
ENSG0000	956.2693	-0.0671	0.10094	-0.66478	0.506189	0.767506	EPS15	2060	epidermal growth factor receptor pathway substrate 15
ENSG0000	3.911745	-0.94864	1.427241	-0.66467	0.506262	0.767506	NA	NA	NA
ENSG0000	336.722	0.100349	0.151006	0.664538	0.506346	0.767506	SNAPIN	23557	SNAP associated protein
ENSG0000	10.42161	0.532654	0.801425	0.664633	0.506285	0.767506	NA	NA	NA
ENSG0000	191.0821	0.132045	0.198692	0.664571	0.506325	0.767506	PIGU	128869	phosphatidylinositol glycan anchor biosynthesis class U
ENSG0000	20.85231	-0.33722	0.507715	-0.6642	0.506565	0.767775	NA	NA	NA
ENSG0000	2.618591	1.126996	1.697191	0.664036	0.506667	0.767829	SNORD13E	1.1E+08	small nucl C/D box 13E
ENSG0000	117.3795	0.173064	0.260634	0.664011	0.506683	0.767829	ZNF461	92283	zinc finger protein 461
ENSG0000	4.1489	0.747012	1.125166	0.663913	0.506746	0.767861	HNRNPKP2	389053	heterogeneous nuclear ribonucleoprotein K pseudogene 2
ENSG0000	86.66736	-0.17614	0.265385	-0.66371	0.506877	0.767872	NA	NA	NA
ENSG0000	3.868388	-1.16701	1.758201	-0.66375	0.506851	0.767872	TTC23	64927	tetratricopeptide repeat domain 23
ENSG0000	20.58061	-0.43891	0.661295	-0.66371	0.506876	0.767872	MIR3176	1E+08	microRNA 3176
ENSG0000	73.94041	-0.1921	0.289537	-0.66349	0.507018	0.767897	EXOC6B	23233	exocyst complex component 6B
ENSG0000	3584.494	-0.05017	0.07561	-0.6635	0.507008	0.767897	LARP1	23367	La ribonuc translational regulator
ENSG0000	2831.187	-0.05038	0.075923	-0.66357	0.506969	0.767897	DCAF7	10238	DDB1 and CUL4 associated factor 7
ENSG0000	153.2946	0.165549	0.249564	0.663354	0.507104	0.767964	CCDC163	126661	CCDC163 homolog
ENSG0000	3.965164	0.902481	1.360844	0.663177	0.507217	0.768073	ETV3L	440695	ETS variant transcription factor 3 like
ENSG0000	30.57734	0.35505	0.535463	0.663072	0.507285	0.768112	PGLS-DT	1.01E+08	PGLS divergent transcript
ENSG0000	3.886798	-0.98457	1.485237	-0.66291	0.507391	0.768187	NA	NA	NA
ENSG0000	165.3605	0.139165	0.209945	0.662865	0.507417	0.768187	MIR3667H	348645	MIR3667 host gene
ENSG0000	6.225826	-0.6759	1.019909	-0.66271	0.507517	0.768226	NA	NA	NA
ENSG0000	77.88611	0.184031	0.277701	0.662696	0.507525	0.768226	CORO6	84940	coronin 6
ENSG0000	3.274202	-1.10793	1.672434	-0.66247	0.507672	0.768383	ATP6V0A4	50617	ATPase H+ transporting V0 subunit a4
ENSG0000	105.5427	0.180326	0.272246	0.662365	0.507737	0.768383	RPS3AP6	145767	RPS3A pseudogene 6
ENSG0000	31.06461	-0.28325	0.427656	-0.66234	0.507753	0.768383	SLC25A5-A	1E+08	SLC25A5 antisense RNA 1
ENSG0000	5.230206	0.854093	1.289681	0.662251	0.50781	0.768406	NA	NA	NA
ENSG0000	1319.761	-0.06357	0.096013	-0.66212	0.507893	0.768468	SETD3	84193	SET domai actin histidine methyltransferase
ENSG0000	3.069678	1.046204	1.580489	0.66195	0.508004	0.76856	GADL1	339896	glutamate decarboxylase like 1
ENSG0000	5.127814	1.861047	2.811682	0.661898	0.508036	0.76856	TFPI2	7980	tissue factor pathway inhibitor 2
ENSG0000	3.748642	-0.81417	1.230411	-0.66171	0.508159	0.768621	NA	NA	NA
ENSG0000	21.88756	-0.34503	0.521385	-0.66176	0.508128	0.768621	NA	NA	NA
ENSG0000	24.24119	-0.32382	0.489438	-0.66162	0.508217	0.768645	TLR10	81793	toll like receptor 10
ENSG0000	10.42223	-0.59598	0.901114	-0.66139	0.508365	0.768807	CDHR2	54825	cadherin related family member 2
ENSG0000	440.8644	0.092888	0.140468	0.661276	0.508435	0.76885	FBXO3	26273	F-box protein 3
ENSG0000	1091.143	-0.06258	0.094652	-0.66119	0.508493	0.768874	NFX1	4799	nuclear tra X-box binding 1
ENSG0000	412.0504	-0.09382	0.141927	-0.66104	0.508585	0.768889	SCPEP1	59342	serine carboxypeptidase 1
ENSG0000	11.84325	0.489127	0.739933	0.661042	0.508586	0.768889	NA	NA	NA
ENSG0000	5.702984	0.73632	1.114393	0.660736	0.508782	0.769024	NA	NA	NA
ENSG0000	257.6629	-0.11499	0.174033	-0.66071	0.508799	0.769024	ASCC1	51008	activating signal cointegrator 1 complex subunit 1
ENSG0000	1736.369	0.081708	0.123652	0.660795	0.508744	0.769024	HSPH1	10808	heat shock protein family H (Hsp110) member 1
ENSG0000	12.59168	-0.44924	0.680121	-0.66053	0.508913	0.769071	HNRNPD-I	1.02E+08	HNRNPD divergent transcript
ENSG0000	8.982685	-0.55668	0.84276	-0.66055	0.508904	0.769071	ACTG1P14	1E+08	actin gamma 1 pseudogene 14
ENSG0000	98.40531	-0.17567	0.266041	-0.66032	0.509048	0.76915	FASTKD3	79072	FAST kinase domains 3
ENSG0000	3.817561	0.964747	1.460952	0.660355	0.509026	0.76915	DIP2A-IT1	1.01E+08	DIP2A intronic transcript 1
ENSG0000	3.92307	0.843229	1.277198	0.660218	0.509114	0.769187	C12orf60	144608	chromosome 12 open reading frame 60
ENSG0000	40.49247	0.257267	0.389822	0.65996	0.50928	0.769284	ADHFE1	137872	alcohol dehydrogenase iron containing 1
ENSG0000	8.429484	-0.63473	0.961694	-0.66001	0.509247	0.769284	NA	NA	NA
ENSG0000	4.980191	0.903412	1.368964	0.659924	0.509303	0.769284	HIF1A-AS3	1.05E+08	HIF1A antisense RNA 3
ENSG0000	172.8285	0.13758	0.208508	0.659828	0.509364	0.769289	TMEM192	201931	transmembrane protein 192
ENSG0000	434.3616	-0.08714	0.132081	-0.65972	0.509431	0.769289	IRF9	10379	interferon regulatory factor 9
ENSG0000	23.3841	-0.38631	0.585517	-0.65977	0.509402	0.769289	PIF1	80119	PIF1 5'-to-3' DNA helicase
ENSG0000	711.7158	0.090918	0.137862	0.659489	0.509582	0.769455	PLEKHA1	59338	pleckstrin homology domain containing A1
ENSG0000	839.3976	-0.07051	0.107071	-0.65857	0.510172	0.769613	RPUSD3	285367	RNA pseudouridine synthase D3
ENSG0000	38.32928	0.254182	0.38579	0.65886	0.509986	0.769613	LOC10272	1.03E+08	uncharacterized LOC102723663
ENSG0000	453.6861	-0.09245	0.140402	-0.65845	0.510247	0.769613	DALRD3	55152	DALR anticodon binding domain containing 3
ENSG0000	440.3873	0.08504	0.129149	0.658463	0.510241	0.769613	TM2D2	83877	TM2 domain containing 2
ENSG0000	230.6153	-0.11382	0.172844	-0.6585	0.51022	0.769613	DECR1	1666	2 4-dienoyl-CoA reductase 1
ENSG0000	97.15481	-0.19029	0.288641	-0.65925	0.509733	0.769613	MTAP	4507	methylthioadenosine phosphorylase
ENSG0000	3.636931	0.757159	1.149677	0.658584	0.510163	0.769613	NA	NA	NA
ENSG0000	4503.251	-0.05212	0.079161	-0.65845	0.510247	0.769613	PPP1CA	5499	protein phosphatase 1 catalytic subunit alpha
ENSG0000	4.017832	-0.96057	1.458597	-0.65856	0.510179	0.769613	IPO4	79711	importin 4
ENSG0000	7.7383	-0.59201	0.898473	-0.6589	0.509957	0.769613	NA	NA	NA
ENSG0000	235.8302	0.117105	0.177684	0.659064	0.509855	0.769613	RRN3P3	1E+08	RRN3 pseudogene 3
ENSG0000	257.8875	0.123055	0.186893	0.658421	0.510268	0.769613	CNEP1R1	255919	CTD nuclear envelope phosphatase 1 regulatory subunit 1
ENSG0000	1119.644	0.062677	0.095176	0.658542	0.51019	0.769613	TXN2	25828	thioredoxin 2
ENSG0000	5.345714	-0.84764	1.286447	-0.6589	0.509958	0.769613	PRRG1	5638	proline rich and Gla domain 1
ENSG0000	4.900091	-0.93502	1.421116	-0.65795	0.510571	0.76982	LINC01762	1.02E+08	long intergenic non-protein coding RNA 1762
ENSG0000	5.41655	0.609381	0.926074	0.658026	0.510521	0.76982	NA	NA	NA

ENSG0000	177.1333	0.125097	0.190092	0.658088	0.510482	0.76982	GPALPP1	55425	GPALPP motifs containing 1
ENSG0000	210.1395	0.120757	0.183521	0.658001	0.510537	0.76982	LAT	27040	linker for activation of T cells
ENSG0000	712.5168	0.085136	0.129409	0.657879	0.510616	0.769825	RSAD1	55316	radical S-adenosyl methionine domain containing 1
ENSG0000	9.029279	0.530758	0.806993	0.657698	0.510732	0.769938	NA	NA	NA
ENSG0000	9.756059	0.600489	0.913242	0.657536	0.510837	0.770033	NA	NA	NA
ENSG0000	376.7785	-0.09082	0.138137	-0.65747	0.510879	0.770035	C22orf39	128977	chromosome 22 open reading frame 39
ENSG0000	6.972196	-0.74451	1.132835	-0.65721	0.511048	0.770227	SERPINI1	5274	serpin family I member 1
ENSG0000	565.364	0.093245	0.141909	0.657076	0.511132	0.770229	TRPV2	51393	transient receptor potential cation channel subfamily V member 2
ENSG0000	7.565254	0.574146	0.873791	0.657075	0.511132	0.770229	SP2-DT	1.03E+08	SP2 divergent transcript
ENSG0000	5.691424	-0.85877	1.307384	-0.65686	0.51127	0.770261	THNSL2	55258	threonine synthase like 2
ENSG0000	398.1029	0.093706	0.142661	0.656846	0.51128	0.770261	CCDC50	152137	coiled-coil domain containing 50
ENSG0000	2616.971	0.057285	0.087205	0.656903	0.511243	0.770261	PARP8	79668	poly(ADP-ribose) polymerase family member 8
ENSG0000	20.3003	-0.45816	0.697579	-0.65678	0.51132	0.770261	THBS4-AS	1.02E+08	THBS4 antisense RNA 1
ENSG0000	334.7805	-0.1158	0.176412	-0.65645	0.511537	0.770526	NA	NA	NA
ENSG0000	627.5771	-0.09056	0.138021	-0.65615	0.511726	0.770748	MIIP	60672	migration and invasion inhibitory protein
ENSG0000	83.22648	0.190626	0.29058	0.656018	0.511813	0.770815	NA	NA	NA
ENSG0000	56.69985	0.231867	0.3536	0.655735	0.511995	0.771027	EFHC1	114327	EF-hand domain containing 1
ENSG0000	10603.38	-0.05467	0.083378	-0.65564	0.512055	0.771055	ADAR	103	adenosine deaminase RNA specific
ENSG0000	3175.012	-0.05521	0.084213	-0.65557	0.512102	0.771063	ZNF865	1.01E+08	zinc finger protein 865
ENSG0000	44.28781	-0.2369	0.361507	-0.65532	0.51226	0.771238	NA	NA	NA
ENSG0000	7926.811	0.044905	0.068534	0.655217	0.512328	0.771278	SF3B2	10992	splicing factor 3b subunit 2
ENSG0000	22.29021	0.320573	0.489467	0.654942	0.512505	0.771362	BOLA3-DT	1.01E+08	BOLA3 divergent transcript
ENSG0000	826.7974	-0.07501	0.11452	-0.65499	0.512473	0.771362	SIGMAR1	10280	sigma non-opioid intracellular receptor 1
ENSG0000	30.42572	-0.32238	0.492225	-0.65494	0.512508	0.771362	NA	NA	NA
ENSG0000	103.1528	-0.17893	0.2733	-0.65471	0.512654	0.771519	GPHN	10243	gephyrin
ENSG0000	16.45453	0.410206	0.626654	0.654597	0.512727	0.771559	NA	NA	NA
ENSG0000	100.6495	-0.15624	0.238706	-0.65454	0.512764	0.771559	LAYN	143903	layilin
ENSG0000	190.296	-0.12269	0.187538	-0.65422	0.512968	0.771796	PPA2	27068	inorganic pyrophosphatase 2
ENSG0000	1565.944	-0.05963	0.09118	-0.65397	0.51313	0.771796	GPAA1	8733	glycosylphosphatidylinositol anchor attachment 1
ENSG0000	4.691328	-0.81316	1.243252	-0.65406	0.513073	0.771796	NA	NA	NA
ENSG0000	217.4031	0.131911	0.201696	0.65401	0.513105	0.771796	DTX1	1840	deltex E3 ubiquitin ligase 1
ENSG0000	1300.379	-0.05913	0.090392	-0.65414	0.513019	0.771796	AKAP8	10270	A-kinase anchoring protein 8
ENSG0000	33.67378	0.304254	0.465369	0.65379	0.513247	0.77191	MIR25	407014	microRNA 25
ENSG0000	20.55635	-0.34874	0.533509	-0.65367	0.513325	0.771965	AP1S3	130340	adaptor related protein complex 1 subunit sigma 3
ENSG0000	299.1186	0.095329	0.145885	0.653456	0.513463	0.771984	SCP2	6342	sterol carrier protein 2
ENSG0000	46.10503	-0.32096	0.491144	-0.65349	0.513442	0.771984	NA	NA	NA
ENSG0000	25.22011	-0.33166	0.507462	-0.65357	0.513391	0.771984	KCNQ1OT	10984	KCNQ1 opposite strand/antisense transcript 1
ENSG0000	3.135804	1.132794	1.733904	0.65332	0.51355	0.77199	NA	NA	NA
ENSG0000	3.861752	1.075045	1.645398	0.653365	0.513521	0.77199	INMT	11185	indolethylamine N-methyltransferase
ENSG0000	3.474843	0.91489	1.400649	0.65319	0.513634	0.772053	NA	NA	NA
ENSG0000	21.42082	0.330927	0.50682	0.652947	0.51379	0.772226	OSBPL1A	114876	oxysterol binding protein like 1A
ENSG0000	42.58359	0.226544	0.347178	0.652532	0.514058	0.772461	SULT1B1	27284	sulfotransferase family 1B member 1
ENSG0000	149.3745	0.167476	0.256639	0.652574	0.514031	0.772461	NDUFAF4	29078	NADH:ubiquinone oxidoreductase complex assembly factor 4
ENSG0000	7.192168	-0.69441	1.064213	-0.65251	0.514071	0.772461	GFRA2	2675	GDNF family receptor alpha 2
ENSG0000	166.535	-0.15201	0.232993	-0.65242	0.514128	0.772483	MBIP	51562	MAP3K12 binding inhibitory protein 1
ENSG0000	68.2889	0.196146	0.300707	0.652285	0.514218	0.772555	GUCY1B1	2983	guanylate cyclase 1 soluble subunit beta 1
ENSG0000	244.6968	0.161415	0.247514	0.652147	0.514306	0.772598	IL10	3586	interleukin 10
ENSG0000	261.2726	0.129965	0.199318	0.652047	0.514371	0.772598	FASTKD2	22868	FAST kinase domains 2
ENSG0000	4.994076	-0.73826	1.132227	-0.65205	0.514371	0.772598	LOC64666	646665	golgin A6 family member A pseudogene
ENSG0000	146.5855	-0.14879	0.228291	-0.65174	0.514567	0.772829	EBAG9	9166	estrogen receptor binding site associated antigen 9
ENSG0000	1779.68	-0.055	0.084401	-0.65165	0.514628	0.772859	HDAC1	3065	histone deacetylase 1
ENSG0000	18.02682	0.365519	0.561069	0.651468	0.514744	0.772908	NA	NA	NA
ENSG0000	3.529581	-0.84759	1.300951	-0.65152	0.514713	0.772908	RGS9BP	388531	regulator of G protein signaling 9 binding protein
ENSG0000	661.3497	-0.08608	0.132181	-0.65122	0.514904	0.773022	ZNF451	26036	zinc finger protein 451
ENSG0000	5.56588	0.632906	0.971791	0.651277	0.514868	0.773022	RPL26P6	1E+08	ribosomal protein L26 pseudogene 6
ENSG0000	920.8299	-0.07409	0.113784	-0.65113	0.514963	0.773049	MTG2	26164	mitochondrial ribosome associated GTPase 2
ENSG0000	25.59859	0.367368	0.564284	0.651034	0.515025	0.773079	NTAQ1	55093	N-terminal glutamine amidase 1
ENSG0000	102.7934	0.150487	0.231226	0.650823	0.515161	0.773221	EXD2	55218	exonuclease 3'-5' domain containing 2
ENSG0000	6761.517	-0.0524	0.08053	-0.65071	0.515236	0.773271	MICAL1	64780	microtubu calponin and LIM domain containing 1
ENSG0000	3.581585	0.998458	1.534851	0.650525	0.515353	0.773385	TRBJ2-2	28628	T cell receptor beta joining 2-2
ENSG0000	11.77281	0.510409	0.784965	0.650231	0.515543	0.773607	HJURP	55355	Holliday junction recognition protein
ENSG0000	28013.87	0.050077	0.077037	0.650044	0.515664	0.773725	TLN1	7094	talin 1
ENSG0000	52.63507	-0.20967	0.322631	-0.64987	0.515775	0.77383	SNX24	28966	sorting nexin 24
ENSG0000	85.26775	-0.16819	0.258858	-0.64973	0.515865	0.773847	ALDH4A1	8659	aldehyde dehydrogenase 4 family member A1
ENSG0000	217.6233	-0.115	0.176997	-0.64972	0.515871	0.773847	EXD3	54932	exonuclease 3'-5' domain containing 3
ENSG0000	869.9785	-0.07176	0.110472	-0.64957	0.515969	0.773928	TTI1	9675	TELO2 interacting protein 1
ENSG0000	14.79809	0.422383	0.650308	0.649511	0.516008	0.773928	NA	NA	NA
ENSG0000	690.1428	-0.07263	0.111872	-0.64925	0.51618	0.774124	WASH8P	1E+08	WAS prote pseudogene
ENSG0000	141.4235	0.138781	0.213977	0.648581	0.516609	0.774144	SLCO4C1	353189	solute carrier organic anion transporter family member 4C1
ENSG0000	11.10852	0.462652	0.713255	0.648648	0.516566	0.774144	TRGJP2	6972	T cell receptor gamma joining P2
ENSG0000	253.3387	0.117788	0.181449	0.649153	0.51624	0.774144	ARMC1	55156	armadillo repeat containing 1
ENSG0000	12.53439	-0.4237	0.653246	-0.6486	0.516595	0.774144	LINC0290	286122	long intergenic non-protein coding RNA 2904
ENSG0000	3.049913	-1.0407	1.603723	-0.64893	0.516387	0.774144	NA	NA	NA
ENSG0000	95.53238	-0.17805	0.274522	-0.64858	0.516611	0.774144	FAM216A	29902	family with sequence similarity 216 member A
ENSG0000	153.365	0.136599	0.21047	0.649021	0.516325	0.774144	PAFAH1B3	5050	platelet activating factor acetylhydrolase 1b catalytic subunit 3
ENSG0000	455.0806	0.103576	0.159625	0.648875	0.516419	0.774144	C19orf48	84798	multidrug resistance-related protein
ENSG0000	657.968	-0.08612	0.132764	-0.64868	0.516547	0.774144	SIGLEC12	89858	sialic acid binding Ig like lectin 12
ENSG0000	1186.783	-0.06351	0.097863	-0.64894	0.516379	0.774144	UBE2G2	7327	ubiquitin conjugating enzyme E2 G2
ENSG0000	180.2666	0.123617	0.190635	0.648447	0.516696	0.774209	FCRL6	343413	Fc receptor like 6
ENSG0000	141.0237	-0.14134	0.218024	-0.64826	0.516814	0.774323	HSPB11	51668	heat shock protein family B (small) member 11
ENSG0000	142.983	0.149927	0.231413	0.647874	0.517066	0.774639	LRRC57	255252	leucine rich repeat containing 57
ENSG0000	2.463568	-1.26634	1.955402	-0.64761	0.517236	0.774768	NA	NA	NA
ENSG0000	271.1718	-0.10545	0.16281	-0.64767	0.517197	0.774768	MAPKAPK	51275	MAPKAPK5 antisense RNA 1
ENSG0000	77.10412	0.183563	0.283512	0.647461	0.517334	0.774819	EPB41L5	57669	erythrocyte membrane protein band 4.1 like 5
ENSG0000	70.04912	-0.18511	0.285908	-0.64743	0.517354	0.774819	NPIPB2	729978	nuclear pore complex interacting protein family member B2
ENSG0000	6.365417	-0.59099	0.912912	-0.64737	0.517395	0.774819	SPATA25	128497	spermatogenesis associated 25
ENSG0000	28.28747	0.308316	0.476423	0.647146	0.517537	0.774865	LOC10192	1.02E+08	uncharacterized LOC101926907
ENSG0000	3.157568	0.924174	1.428125	0.647124	0.517552	0.774865	LCA5	167691	lebercilin LCA5
ENSG0000	4.749818	0.884861	1.367321	0.64715	0.517535	0.774865	ZNG1DP	653510	Zn regulat pseudogene
ENSG0000	125.9879	-0.16316	0.252337	-0.6466	0.517893	0.775314	ANKRD50	57182	ankyrin repeat domain containing 50
ENSG0000	320.1347	-0.10564	0.163405	-0.64647	0.517972	0.775369	TMEM234	56063	transmembrane protein 234
ENSG0000	20.31951	0.364773	0.564488	0.646202	0.518148	0.775571	PROS1	5627	protein S
ENSG0000	18.51401	-0.38485	0.595763	-0.64598	0.518292	0.775686	POLR3G	10622	RNA polymerase III subunit G
ENSG0000	3.447568	0.978194	1.514344	0.645952	0.51831	0.775686	NA	NA	NA
ENSG0000	66.39178	-0.21723	0.336325	-0.64589	0.518351	0.775686	PRSS21	10942	serine protease 21
ENSG0000	4.784592	-0.6891	1.067006	-0.64583	0.518393	0.775686	NA	NA	NA
ENSG0000	16.58476	0.473124	0.732754	0.645679	0.518487	0.775734	CDC42BPA	8476	CDC42 binding protein kinase alpha
ENSG0000	337.9396	-0.09883	0.153103	-0.64552	0.518593	0.775734	PHTF2	57157	putative homeodomain transcription factor 2
ENSG0000	723.6812	-0.07875	0.12199	-0.64555	0.518569	0.775734	AKR1B1	231	aldo-keto reductase family 1 member B
ENSG0000	21.6721	-0.32998	0.51109	-0.64564	0.518514	0.775734	TRAV19	28664	T cell receptor alpha variable 19

ENSG0000	304.2191	-0.11358	0.175972	-0.64543	0.518646	0.775734	TBC1D3	729873	TBC1 domain family member 3
ENSG0000	86.32669	0.188917	0.292719	0.645388	0.518676	0.775734	SCN1B	6324	sodium voltage-gated channel beta subunit 1
ENSG0000	2746.471	0.052859	0.081922	0.645241	0.518771	0.775814	TRIM21	6737	tripartite motif containing 21
ENSG0000	16.11315	0.476926	0.739308	0.645098	0.518864	0.77589	ELN	2006	elastin
ENSG0000	664.6861	0.069541	0.107863	0.644716	0.519111	0.776168	SRP54	6729	signal recognition particle 54
ENSG0000	26.39246	-0.38243	0.593213	-0.64468	0.519133	0.776168	SDCBP2-A!	1.01E+08	SDCBP2 antisense RNA 1
ENSG0000	7.161704	0.583144	0.904815	0.64449	0.519258	0.776292	NA	NA	NA
ENSG0000	118.1611	0.154715	0.240097	0.644384	0.519326	0.776332	MMAA	166785	metabolism of cobalamin associated A
ENSG0000	227.1649	-0.10767	0.167114	-0.64431	0.519374	0.77634	BRI3BP	140707	BRI3 binding protein
ENSG0000	11.32022	0.459995	0.714174	0.644094	0.519514	0.776488	ZCCHC14-I	1.02E+08	ZCCHC14 divergent transcript
ENSG0000	9.249618	0.522377	0.811373	0.643819	0.519693	0.776691	NA	NA	NA
ENSG0000	78.59882	0.176803	0.274785	0.643423	0.51995	0.776956	NA	NA	NA
ENSG0000	2.149692	-1.26256	1.962268	-0.64342	0.519954	0.776956	CDH4	1002	cadherin 4
ENSG0000	479.0426	0.094425	0.146773	0.643342	0.520002	0.776966	ZNF654	55279	zinc finger protein 654
ENSG0000	4908.646	-0.0604	0.093916	-0.64312	0.520148	0.777103	EFCAB14	9813	EF-hand calcium binding domain 14
ENSG0000	1495.649	0.055788	0.086752	0.643071	0.520178	0.777103	CCNDBP1	23582	cyclin D1 binding protein 1
ENSG0000	35.65284	-0.28345	0.441065	-0.64265	0.520451	0.77721	NA	NA	NA
ENSG0000	2739.935	0.080895	0.125889	0.64259	0.52049	0.77721	CBLL1	79872	Cbl proto-oncogene like 1
ENSG0000	238.4012	0.105351	0.163881	0.642847	0.520324	0.77721	PFDN5	5204	prefoldin subunit 5
ENSG0000	820.3228	0.07214	0.112267	0.642574	0.5205	0.77721	SPPL3	121665	signal peptide peptidase like 3
ENSG0000	16.57511	0.450142	0.700407	0.642687	0.520427	0.77721	NA	NA	NA
ENSG0000	6.669045	0.594024	0.92439	0.642612	0.520476	0.77721	NA	NA	NA
ENSG0000	4026.859	-0.05155	0.080273	-0.64215	0.520773	0.777503	ATG4B	23192	autophagy related 4B cysteine peptidase
ENSG0000	345.6995	-0.10384	0.161705	-0.64214	0.520781	0.777503	ZNF202	7753	zinc finger protein 202
ENSG0000	1677.924	0.058014	0.090411	0.641663	0.521092	0.777842	SIK2	23235	salt inducible kinase 2
ENSG0000	16.79533	-0.41312	0.64377	-0.64172	0.521056	0.777842	NA	NA	NA
ENSG0000	230.8649	0.125029	0.194938	0.641379	0.521277	0.777887	CCDC127	133957	coiled-coil domain containing 127
ENSG0000	463.9664	-0.0788	0.122868	-0.64136	0.52129	0.777887	NCOA7	135112	nuclear receptor coactivator 7
ENSG0000	7.475971	-0.56928	0.887518	-0.64143	0.521244	0.777887	FAM241B	219738	family with sequence similarity 241 member B
ENSG0000	9.24522	0.545515	0.850354	0.641515	0.521188	0.777887	IGHV3-49	28423	immunoglobulin heavy variable 3-49
ENSG0000	429.4048	-0.07925	0.123584	-0.64125	0.52136	0.777929	FAM8A1	51439	family with sequence similarity 8 member A1
ENSG0000	7.638332	0.597123	0.931428	0.641083	0.521469	0.778026	MELK	9833	maternal embryonic leucine zipper kinase
ENSG0000	13302.05	0.055203	0.086117	0.641022	0.521509	0.778026	ITM2B	9445	integral membrane protein 2B
ENSG0000	2796.549	0.062779	0.097973	0.640782	0.521665	0.778035	ATP2B4	493	ATPase plasma membrane Ca2+ transporting 4
ENSG0000	68.133	-0.19194	0.299511	-0.64083	0.521634	0.778035	NOTCH4	4855	notch receptor 4
ENSG0000	13.34102	-0.41373	0.645561	-0.64088	0.521598	0.778035	SNORD14/	26822	small nucl(C/D box 14A
ENSG0000	1626.001	0.063442	0.099011	0.640755	0.521682	0.778035	MYBBP1A	10514	MYB binding protein 1a
ENSG0000	13.45464	-0.45275	0.706795	-0.64057	0.521801	0.778149	INKA2-AS1	1.01E+08	INKA2 antisense RNA 1
ENSG0000	28.29812	-0.32963	0.514777	-0.64033	0.521959	0.778198	BCKDHB	594	branched chain keto acid dehydrogenase E1 subunit beta
ENSG0000	392.1564	0.085164	0.132975	0.640446	0.521883	0.778198	EXOSC2	23404	exosome component 2
ENSG0000	100.6467	-0.17314	0.270382	-0.64036	0.521936	0.778198	TTC5	91875	tetratricopeptide repeat domain 5
ENSG0000	314.6192	-0.10178	0.158973	-0.64025	0.522008	0.778208	NCAM1	4684	neural cell adhesion molecule 1
ENSG0000	64.75456	-0.22218	0.347136	-0.64003	0.522155	0.778295	OVGP1	5016	oviductal glycoprotein 1
ENSG0000	211.7775	-0.12082	0.188764	-0.64008	0.522124	0.778295	SSBP2	23635	single stranded DNA binding protein 2
ENSG0000	16.49526	0.389391	0.608544	0.639872	0.522256	0.778295	RAB19	401409	RAB19 member RAS oncogene family
ENSG0000	100.8368	0.167187	0.261321	0.639776	0.522318	0.778295	NA	NA	NA
ENSG0000	25.49851	0.332063	0.518991	0.639823	0.522287	0.778295	KRT10-AS1	147184	KRT10 antisense RNA 1
ENSG0000	675.3373	-0.08114	0.126823	-0.6398	0.5223	0.778295	ZNF628	89887	zinc finger protein 628
ENSG0000	4.690837	-0.79359	1.240954	-0.6395	0.522498	0.778501	NA	NA	NA
ENSG0000	327.8462	-0.10702	0.167374	-0.6394	0.522565	0.778538	FUT4	2526	fucosyltransferase 4
ENSG0000	5.131958	-0.89715	1.403916	-0.63904	0.5228	0.7787	NA	NA	NA
ENSG0000	324.9656	-0.0929	0.145376	-0.63904	0.522798	0.7787	PEX5	5830	peroxisomal biogenesis factor 5
ENSG0000	131.5598	-0.19584	0.306398	-0.63915	0.522723	0.7787	RAB3D	9545	RAB3D member RAS oncogene family
ENSG0000	815.3509	0.066938	0.104768	0.638917	0.522877	0.778752	SNX6	58533	sorting nexin 6
ENSG0000	62.84865	0.191863	0.300516	0.638447	0.523183	0.779145	NA	NA	NA
ENSG0000	7.048271	-0.5568	0.872716	-0.63801	0.523466	0.779217	NA	NA	NA
ENSG0000	20601.35	0.050075	0.078494	0.637945	0.52351	0.779217	TMSB10	9168	thymosin beta 10
ENSG0000	296.0576	-0.1021	0.16017	-0.63747	0.523818	0.779217	TIMMDC1	51300	translocase of inner mitochondrial membrane domain containing 1
ENSG0000	414.2847	-0.08876	0.139106	-0.63807	0.523429	0.779217	HARS2	23438	histidyl-tR mitochondrial
ENSG0000	70.83493	-0.18873	0.296051	-0.63749	0.523806	0.779217	USP45	85015	ubiquitin specific peptidase 45
ENSG0000	44.90123	0.237397	0.372334	0.637593	0.523738	0.779217	NA	NA	NA
ENSG0000	7642.824	0.072257	0.113225	0.638175	0.52336	0.779217	IRF7	3665	interferon regulatory factor 7
ENSG0000	2203.467	-0.05873	0.092043	-0.63812	0.523393	0.779217	TM9SF2	9375	transmembrane 9 superfamily member 2
ENSG0000	42.6038	0.226779	0.355547	0.637831	0.523584	0.779217	SLC38A6	145389	solute carrier family 38 member 6
ENSG0000	202.4139	0.118761	0.186182	0.637877	0.523554	0.779217	DUSP14	11072	dual specificity phosphatase 14
ENSG0000	32.40438	-0.25592	0.401457	-0.63747	0.52382	0.779217	ZNF566-A!	728752	ZNF566 antisense RNA 1
ENSG0000	66.51374	-0.18109	0.283939	-0.63777	0.523622	0.779217	ZNF470	388566	zinc finger protein 470
ENSG0000	890.1564	-0.07229	0.113384	-0.63761	0.523731	0.779217	TTLL12	23170	tubulin tyrosine ligase like 12
ENSG0000	52.28286	0.219152	0.343771	0.637494	0.523803	0.779217	CHKB	1120	choline kinase beta
ENSG0000	4.673755	0.867648	1.361611	0.637222	0.52398	0.779284	MYOCOS	1.11E+08	myocilin opposite strand
ENSG0000	442.6878	0.106058	0.166443	0.637206	0.52399	0.779284	POLR2D	5433	RNA polymerase II subunit D
ENSG0000	3.987382	0.977753	1.534201	0.637304	0.523927	0.779284	NA	NA	NA
ENSG0000	193.251	0.150504	0.236219	0.637137	0.524035	0.779288	NA	NA	NA
ENSG0000	16.68205	-0.43491	0.683043	-0.63673	0.524303	0.779373	PARP11-A!	1.05E+08	PARP11 antisense RNA 1
ENSG0000	633.6967	0.072289	0.113495	0.636938	0.524165	0.779373	EAPP	55837	E2F associated phosphoprotein
ENSG0000	1026.777	0.078984	0.124038	0.636774	0.524272	0.779373	TBL3	10607	transducin beta like 3
ENSG0000	367.0543	0.102589	0.161072	0.636916	0.524179	0.779373	NUDT21	11051	nudix hydrolase 21
ENSG0000	601.9951	0.076243	0.119728	0.636805	0.524252	0.779373	PTGR3	284273	prostaglandin reductase 3
ENSG0000	1581.963	-0.05292	0.083123	-0.63662	0.524373	0.779416	DHX16	8449	DEAH-box helicase 16
ENSG0000	449.1423	0.097211	0.152717	0.636543	0.524423	0.779427	NBPF8	728841	NBPF member 8
ENSG0000	29.17261	0.311947	0.490191	0.636378	0.52453	0.779524	NA	NA	NA
ENSG0000	569.1134	-0.08136	0.127987	-0.63572	0.524959	0.779537	PRXL2B	127281	peroxiredoxin like 2B
ENSG0000	16.816	0.391049	0.615123	0.635725	0.524956	0.779537	INO80B	83444	INO80 complex subunit B
ENSG0000	749.3959	0.069994	0.110077	0.635864	0.524865	0.779537	DYNC1LI1	51143	dynein cytoplasmic 1 light intermediate chain 1
ENSG0000	290.5714	-0.09791	0.154001	-0.63575	0.524939	0.779537	CEP135	9662	centrosomal protein 135
ENSG0000	26.14879	-0.28165	0.442811	-0.63604	0.524748	0.779537	PARM1	25849	prostate androgen-regulated mucin-like protein 1
ENSG0000	5.387546	0.708096	1.113397	0.635978	0.524791	0.779537	ZSCAN31	64288	zinc finger and SCAN domain containing 31
ENSG0000	191.9258	0.119856	0.188409	0.636147	0.52468	0.779537	UEVLD	55293	UEV and lactate/malate dehyrogenase domains
ENSG0000	4997.923	-0.05593	0.087922	-0.63608	0.524725	0.779537	TCF25	22980	transcription factor 25
ENSG0000	3116.17	0.057235	0.09001	0.635872	0.52486	0.779537	SAFB2	9667	scaffold attachment factor B2
ENSG0000	9.942146	0.579598	0.911399	0.635943	0.524813	0.779537	PRRG2	5639	proline rich and Gla domain 2
ENSG0000	1297.869	-0.07511	0.11817	-0.63562	0.525025	0.779572	ENTPD6	955	ectonucleoside triphosphate diphosphohydrolase 6
ENSG0000	87.60102	0.18246	0.287237	0.635223	0.525283	0.779632	NUDT17	200035	nudix hydrolase 17
ENSG0000	336.8184	0.097372	0.153249	0.635383	0.525178	0.779632	CHPT1	56994	choline phosphotransferase 1
ENSG0000	535.0037	-0.07605	0.1197	-0.63532	0.525219	0.779632	SACS	26278	sacsin molecular chaperone
ENSG0000	4.573302	0.718953	1.131907	0.63517	0.525318	0.779632	NA	NA	NA
ENSG0000	15.48821	0.47136	0.742071	0.635196	0.525301	0.779632	B9D1	27077	B9 domain containing 1
ENSG0000	1173.818	-0.17132	0.269636	-0.63538	0.525183	0.779632	APOBEC3A	200315	apolipoprotein B mRNA editing enzyme catalytic subunit 3A
ENSG0000	6.296404	0.652279	1.027277	0.634959	0.525455	0.779712	NA	NA	NA
ENSG0000	182.4836	-0.13463	0.212007	-0.63501	0.525424	0.779712	TCL1A	8115	TCL1 family AKT coactivator A

ENSG0000	17.77373	0.402421	0.633867	0.634866	0.525516	0.779723	PARD3	56288	par-3 family cell polarity regulator
ENSG0000	168.1689	-0.13261	0.208889	-0.63482	0.525547	0.779723	NA	NA	NA
ENSG0000	87.55021	-0.17173	0.270573	-0.63468	0.525635	0.779792	NA	NA	NA
ENSG0000	222.6307	-0.11956	0.188509	-0.63427	0.525908	0.780038	KDM1B	221656	lysine demethylase 1B
ENSG0000	5.066555	-0.76931	1.212925	-0.63426	0.525909	0.780038	NA	NA	NA
ENSG0000	1040.005	-0.06299	0.099322	-0.63423	0.525932	0.780038	DDX11	1663	DEAD/H-box helicase 11
ENSG0000	1706.886	0.069963	0.110323	0.634171	0.525969	0.780038	PNN	5411	pinin desmosome associated protein
ENSG0000	298.1276	-0.09974	0.157347	-0.63386	0.526171	0.780084	DUSP12	11266	dual specificity phosphatase 12
ENSG0000	117.2546	-0.15067	0.237632	-0.63405	0.52605	0.780084	MTA3	57504	metastasis associated 1 family member 3
ENSG0000	7.162621	0.511447	0.806748	0.633961	0.526106	0.780084	RPL17P39	645296	ribosomal protein L17 pseudogene 39
ENSG0000	748.4691	-0.09199	0.145139	-0.6338	0.526211	0.780084	DDRGK1	65992	DDRGK domain containing 1
ENSG0000	73.35232	0.753206	1.188366	0.633817	0.526201	0.780084	IGLV2-11	28816	immunoglobulin lambda variable 2-11
ENSG0000	12.42161	0.443599	0.700225	0.63351	0.526401	0.780303	TOLLIP-DT	255512	TOLLIP divergent transcript
ENSG0000	160.1817	0.145121	0.229128	0.633365	0.526495	0.780381	RHBDD3	25807	rhomboid domain containing 3
ENSG0000	4.287118	-0.79141	1.249953	-0.63315	0.526637	0.780428	NA	NA	NA
ENSG0000	9.167588	-0.66077	1.043985	-0.63293	0.52678	0.780428	CDH6	1004	cadherin 6
ENSG0000	1000.828	-0.07041	0.111239	-0.63293	0.526779	0.780428	TSSC4	10078	tumor suppressing subtransferable candidate 4
ENSG0000	351.8709	-0.10807	0.170738	-0.63296	0.52676	0.780428	ARL2	402	ADP ribosylation factor like GTPase 2
ENSG0000	5.428764	-0.70927	1.120139	-0.6332	0.526605	0.780428	FA2H	79152	fatty acid 2-hydroxylase
ENSG0000	38.35197	-0.32411	0.511952	-0.63309	0.526676	0.780428	LRRC46	90506	leucine rich repeat containing 46
ENSG0000	8.551421	0.664273	1.050128	0.632564	0.527019	0.78072	NA	NA	NA
ENSG0000	6.279492	0.651288	1.02984	0.632417	0.527115	0.7808	FAM209B	388799	family with sequence similarity 209 member B
ENSG0000	14.5039	0.415465	0.65721	0.632165	0.527279	0.780981	NA	NA	NA
ENSG0000	372.2949	-0.08787	0.139097	-0.63172	0.527572	0.781352	EPB41L2	2037	erythrocyte membrane protein band 4.1 like 2
ENSG0000	162.0401	-0.14449	0.228871	-0.63132	0.527831	0.781514	TMEM79	84283	transmembrane protein 79
ENSG0000	56.65166	-0.21288	0.337141	-0.63143	0.527759	0.781514	KRTCAP3	200634	keratinocyte associated protein 3
ENSG0000	91.27933	-0.17742	0.281037	-0.63129	0.527849	0.781514	PIGP	51227	phosphatidylinositol glycan anchor biosynthesis class P
ENSG0000	2412.065	-0.05211	0.082539	-0.63129	0.52785	0.781514	GRIPAP1	56850	GRIP1 associated protein 1
ENSG0000	776.5693	0.076034	0.120485	0.63107	0.527995	0.781666	USP38	84640	ubiquitin specific peptidase 38
ENSG0000	57.82155	0.190865	0.302555	0.630844	0.528142	0.781709	NA	NA	NA
ENSG0000	481.5609	-0.07714	0.122299	-0.63077	0.528192	0.781709	SCFD1	23256	sec1 family domain containing 1
ENSG0000	5.605157	-0.63087	1.000073	-0.63082	0.528157	0.781709	NA	NA	NA
ENSG0000	28.06427	0.307968	0.488177	0.630852	0.528137	0.781709	DTD1	92675	D-aminoacyl-tRNA deacylase 1
ENSG0000	1416.033	-0.07221	0.114503	-0.63064	0.528274	0.781768	C5orf15	56951	chromosome 5 open reading frame 15
ENSG0000	33.07192	0.308103	0.48865	0.630518	0.528356	0.781827	RASD2	23551	RASD family member 2
ENSG0000	30.87597	0.276059	0.437883	0.630439	0.528407	0.781841	DACT1	51339	dishevelled binding antagonist of beta catenin 1
ENSG0000	91.55298	-0.17482	0.277422	-0.63015	0.528597	0.782059	ZNF345	25850	zinc finger protein 345
ENSG0000	24871.64	0.057173	0.090767	0.629894	0.528764	0.782244	DUSP1	1843	dual specificity phosphatase 1
ENSG0000	5.012954	0.863358	1.370809	0.629817	0.528815	0.782256	CCR8	1237	C-C motif chemokine receptor 8
ENSG0000	15.32607	-0.36126	0.574049	-0.62932	0.529141	0.782676	XK	7504	X-linked K: Kell and VPS13A binding protein
ENSG0000	3.203914	1.115695	1.773393	0.62913	0.529264	0.782767	LOC10192	1.02E+08	uncharacterized LOC101927560
ENSG0000	18.98609	0.344661	0.547875	0.629086	0.529293	0.782767	WFDC21P	645638	WAP four- pseudogene
ENSG0000	2428.211	0.064078	0.101868	0.629031	0.529329	0.782767	SAFB	6294	scaffold attachment factor B
ENSG0000	31.59194	-0.28465	0.452944	-0.62844	0.529717	0.783153	NA	NA	NA
ENSG0000	670.7131	-0.08272	0.131615	-0.62846	0.529701	0.783153	IPO8	10526	importin 8
ENSG0000	77.82914	-0.19033	0.302818	-0.62853	0.529657	0.783153	EEF1A2	1917	eukaryotic translation elongation factor 1 alpha 2
ENSG0000	149.5146	-0.12769	0.20323	-0.62832	0.529796	0.783208	B9D2	80776	B9 domain containing 2
ENSG0000	21.80646	-0.37336	0.594377	-0.62815	0.529905	0.783307	NA	NA	NA
ENSG0000	4.513978	0.921569	1.468225	0.627675	0.530217	0.783625	NA	NA	NA
ENSG0000	18.09315	-0.37228	0.593145	-0.62763	0.530247	0.783625	AOPEP	84909	aminopeptidase O (putative)
ENSG0000	78.89004	-0.1746	0.278167	-0.62769	0.530209	0.783625	NA	NA	NA
ENSG0000	2061.962	-0.06285	0.100232	-0.62709	0.5306	0.783897	ARFGEF1	10565	ADP ribosylation factor guanine nucleotide exchange factor 1
ENSG0000	850.1941	0.074313	0.11847	0.627269	0.530483	0.783897	CDK5RAP1	51654	CDK5 regulatory subunit associated protein 1
ENSG0000	633.4141	-0.07008	0.111749	-0.62709	0.530599	0.783897	CSTF1	1477	cleavage stimulation factor subunit 1
ENSG0000	12.70365	-0.40399	0.644183	-0.62714	0.530567	0.783897	NA	NA	NA
ENSG0000	7590.318	-0.04247	0.067766	-0.62675	0.530821	0.783998	WASF2	10163	WASP family member 2
ENSG0000	80.60538	0.200705	0.32021	0.626793	0.530795	0.783998	GPR89B	51463	G protein-coupled receptor 89B
ENSG0000	163.7944	-0.13834	0.220737	-0.62673	0.530838	0.783998	SPATS2L	26010	spermatogenesis associated serine rich 2 like
ENSG0000	3.123387	-1.10808	1.767929	-0.62677	0.530813	0.783998	NA	NA	NA
ENSG0000	5.324015	0.711845	1.136169	0.626531	0.530967	0.784126	KBTBD11-/	1.02E+08	KBTBD11 antisense RNA 1
ENSG0000	4.718143	0.869684	1.388296	0.62644	0.531026	0.784152	SLC47A1	55244	solute carrier family 47 member 1
ENSG0000	132.5979	-0.13019	0.207855	-0.62635	0.531088	0.78418	ZNF248	57209	zinc finger protein 248
ENSG0000	5.547488	-0.67994	1.085945	-0.62612	0.531234	0.784326	TTC9-DT	1.02E+08	TTC9 divergent transcript
ENSG0000	26.3677	0.325113	0.519295	0.626067	0.531271	0.784326	DMD	1756	dystrophin
ENSG0000	5920.085	-0.05059	0.080816	-0.62595	0.531351	0.784381	SMAD3	4088	SMAD family member 3
ENSG0000	1088.233	0.071918	0.11492	0.625811	0.531439	0.784436	IWS1	55677	interacts v CTD assembly factor 1
ENSG0000	1743.49	-0.06548	0.104745	-0.62514	0.531882	0.784436	DHX30	22907	DExH-box helicase 30
ENSG0000	89.67214	-0.17821	0.284792	-0.62575	0.531481	0.784436	SLC35A1	10559	solute carrier family 35 member A1
ENSG0000	112.6638	-0.1498	0.239611	-0.6252	0.531843	0.784436	BRF2	55290	BRF2 RNA polymerase III transcription initiation factor subunit
ENSG0000	39.59195	-0.24792	0.396383	-0.62546	0.531667	0.784436	NA	NA	NA
ENSG0000	13.94334	0.397554	0.635808	0.625273	0.531792	0.784436	LOC12490	1.25E+08	uncharacterized LOC124902559
ENSG0000	16.5414	-0.39619	0.633792	-0.62511	0.531896	0.784436	NA	NA	NA
ENSG0000	114.9664	0.154419	0.246985	0.625218	0.531828	0.784436	INSL3	3640	insulin like 3
ENSG0000	402.5979	-0.09942	0.158956	-0.62548	0.531656	0.784436	C19orf12	83636	chromosome 19 open reading frame 12
ENSG0000	22.49498	-0.43377	0.69334	-0.62562	0.531565	0.784436	NA	NA	NA
ENSG0000	352.8565	0.104037	0.166397	0.625234	0.531817	0.784436	COL6A1	1291	collagen type VI alpha 1 chain
ENSG0000	614.4639	-0.08604	0.137585	-0.62538	0.531724	0.784436	CCDC22	28952	coiled-coil domain containing 22
ENSG0000	2.770306	-1.34659	2.154874	-0.6249	0.532035	0.784579	LOC12490	1.25E+08	uncharacterized LOC124909475
ENSG0000	509.0958	0.086602	0.138636	0.624676	0.532184	0.784714	JPT2	90861	Jupiter microtubule associated homolog 2
ENSG0000	307.3582	-0.10005	0.160183	-0.6246	0.532231	0.784714	ZNF160	90338	zinc finger protein 160
ENSG0000	4.764305	0.670102	1.073013	0.624505	0.532296	0.784714	LINC0169	1.05E+08	long intergenic non-protein coding RNA 1694
ENSG0000	8.47441	0.492682	0.788878	0.624535	0.532276	0.784714	FAM83F	113828	family with sequence similarity 83 member F
ENSG0000	26.70575	-0.28181	0.451526	-0.62412	0.532547	0.785006	PGBD5	79605	piggyBac transposable element derived 5
ENSG0000	2749.963	-0.056	0.089725	-0.62407	0.532578	0.785006	SMCR8	140775	SMCR8-C9orf72 complex subunit
ENSG0000	32.01326	-0.29399	0.47114	-0.624	0.532627	0.785016	SEPTIN4	5414	septin 4
ENSG0000	308.1303	0.101092	0.162234	0.623126	0.533202	0.785241	MRPL44	65080	mitochondrial ribosomal protein L44
ENSG0000	2.88166	-0.978	1.569509	-0.62312	0.533204	0.785241	NA	NA	NA
ENSG0000	209.0751	-0.1402	0.224923	-0.6233	0.533084	0.785241	SLC48A1	55652	solute carrier family 48 member 1
ENSG0000	4.236205	0.805244	1.291201	0.623639	0.532864	0.785241	NA	NA	NA
ENSG0000	75.63099	0.201545	0.323262	0.623472	0.532974	0.785241	TM9SF1	10548	transmembrane 9 superfamily member 1
ENSG0000	2.697405	-1.19516	1.91665	-0.62357	0.532911	0.785241	IGHVII-60-	28361	immunoglobulin heavy variable (II)-60-1 (pseudogene)
ENSG0000	11942.23	0.042059	0.067474	0.623334	0.533065	0.785241	PRPF8	10594	pre-mRNA processing factor 8
ENSG0000	132.9563	-0.13762	0.220834	-0.62316	0.533178	0.785241	NAPB	63908	NSF attachment protein beta
ENSG0000	5.876852	0.553063	0.887318	0.623298	0.533089	0.785241	NA	NA	NA
ENSG0000	16.50872	-0.36749	0.589459	-0.62344	0.532996	0.785241	CBR3-AS1	1.01E+08	CBR3 antisense RNA 1
ENSG0000	136.0553	-0.15432	0.247684	-0.62305	0.533253	0.785252	WARS2	10352	tryptopha mitochondrial
ENSG0000	6.909719	-0.70464	1.131119	-0.62296	0.53331	0.785274	EXOC3L2	90332	exocyst complex component 3 like 2
ENSG0000	588.9416	-0.09988	0.150744	-0.62281	0.53341	0.785359	CGAS	115004	cyclic GMP-AMP synthase
ENSG0000	33.80966	0.256583	0.412054	0.622693	0.533486	0.785396	ZNF684	127396	zinc finger protein 684
ENSG0000	5123.625	-0.04924	0.079077	-0.62264	0.533521	0.785396	SLC4A2	6522	solute carrier family 4 member 2

ENSG0000	444.7413	-0.09468	0.152388	-0.6213	0.5344	0.7854	IFI6	2537	interferon alpha inducible protein 6
ENSG0000	283.0406	-0.09905	0.159299	-0.62176	0.534101	0.7854	NDC1	55706	NDC1 transmembrane nucleoporin
ENSG0000	12.00813	-0.59817	0.962106	-0.62173	0.534121	0.7854	NA	NA	NA
ENSG0000	55.85313	-0.21573	0.347137	-0.62147	0.534293	0.7854	RTKN	6242	rhotekin
ENSG0000	9.697691	-0.5024	0.809035	-0.62099	0.534607	0.7854	NA	NA	NA
ENSG0000	126.3007	0.160155	0.257392	0.622222	0.533796	0.7854	NR3C2	4306	nuclear receptor subfamily 3 group C member 2
ENSG0000	9.485739	0.500511	0.8044	0.622217	0.533799	0.7854	LOC10192	1.02E+08	uncharacterized LOC101929200
ENSG0000	216.4396	-0.12539	0.201666	-0.62178	0.534089	0.7854	RGMB	285704	repulsive guidance molecule BMP co-receptor b
ENSG0000	9.622224	0.576251	0.926815	0.621754	0.534104	0.7854	SH3RF2	153769	SH3 domain containing ring finger 2
ENSG0000	619.1064	0.079773	0.12834	0.621575	0.534221	0.7854	TRIM39	56658	tripartite motif containing 39
ENSG0000	3.345896	-0.97316	1.565456	-0.62165	0.534174	0.7854	LINC0252	1.05E+08	long intergenic non-protein coding RNA 2528
ENSG0000	406.0398	0.082758	0.132987	0.622303	0.533743	0.7854	FBXL18	80028	F-box and leucine rich repeat protein 18
ENSG0000	46684.08	0.044385	0.071324	0.622301	0.533744	0.7854	AKNA	80709	AT-hook transcription factor
ENSG0000	3202.829	0.055704	0.089705	0.620969	0.53462	0.7854	DDB1	1642	damage specific DNA binding protein 1
ENSG0000	66.40926	0.188567	0.303202	0.621917	0.533997	0.7854	PTS	5805	6-pyruvoyltetrahydropterin synthase
ENSG0000	269.9167	0.130285	0.209474	0.621961	0.533968	0.7854	GXYLT1	283464	glucoside xylosyltransferase 1
ENSG0000	3326.51	-0.04788	0.077013	-0.62176	0.534098	0.7854	PLCB2	5330	phospholipase C beta 2
ENSG0000	1280.918	0.084124	0.135147	0.622459	0.53364	0.7854	MEFV	4210	MEFV inna pyrin
ENSG0000	4.129035	-1.89303	3.045749	-0.62153	0.534251	0.7854	ARHGAP4	9912	Rho GTPase activating protein 44
ENSG0000	4.500304	0.782377	1.259376	0.621242	0.534441	0.7854	NA	NA	NA
ENSG0000	16.04218	0.401405	0.645559	0.621794	0.534077	0.7854	NA	NA	NA
ENSG0000	2972.94	-0.05712	0.091946	-0.62118	0.53448	0.7854	EMC10	284361	ER membrane protein complex subunit 10
ENSG0000	166.5487	0.130342	0.209903	0.620963	0.534624	0.7854	ZNF841	284371	zinc finger protein 841
ENSG0000	27.28945	0.270554	0.434695	0.622401	0.533679	0.7854	ZNF8-DT	1.05E+08	ZNF8 divergent transcript
ENSG0000	616.5928	-0.07311	0.117705	-0.62111	0.534525	0.7854	TTC38	55020	tetratricopeptide repeat domain 38
ENSG0000	111.373	-0.15975	0.257192	-0.62112	0.53452	0.7854	CELSR1	9620	cadherin EGF LAG seven-pass G-type receptor 1
ENSG0000	3.397898	-0.81131	1.306693	-0.62089	0.534674	0.785411	NA	NA	NA
ENSG0000	821.402	-0.07219	0.116318	-0.62061	0.534854	0.785613	KIAA0232	9778	KIAA0232
ENSG0000	4.259112	0.684252	1.102746	0.620498	0.53493	0.785663	GLUD2	2747	glutamate dehydrogenase 2
ENSG0000	5.213056	0.775617	1.250343	0.620323	0.535045	0.785683	NA	NA	NA
ENSG0000	32.96303	-0.27899	0.449759	-0.62031	0.535052	0.785683	LOC1053	1.05E+08	uncharacterized LOC105374981
ENSG0000	29.44512	0.33689	0.543122	0.620284	0.535071	0.785683	LY6K	54742	lymphocyte antigen 6 family member K
ENSG0000	6.543432	0.601449	0.970261	0.619883	0.535335	0.785945	AP1M2	10053	adaptor related protein complex 1 subunit mu 2
ENSG0000	5255.511	-0.04446	0.071725	-0.61993	0.535302	0.785945	CALM3	808	calmodulin 3
ENSG0000	54.78968	-0.2099	0.338814	-0.61951	0.53558	0.786119	TRBV3-1	28619	T cell receptor beta variable 3-1
ENSG0000	4.370081	-0.6714	1.083736	-0.61953	0.53557	0.786119	DYNC1I2P	728532	dynein cytoplasmic 1 intermediate chain 2 pseudogene 1
ENSG0000	365.9219	-0.09076	0.146485	-0.61959	0.53553	0.786119	ADCY9	115	adenylate cyclase 9
ENSG0000	16.39577	-0.4405	0.711235	-0.61935	0.535687	0.786193	TTC39A	22996	tetratricopeptide repeat domain 39A
ENSG0000	50.94291	-0.21812	0.352203	-0.61931	0.535715	0.786193	TMEM107	84314	transmembrane protein 107
ENSG0000	17.61011	0.361605	0.584199	0.618976	0.535932	0.786221	NA	NA	NA
ENSG0000	5.641367	-0.64177	1.036995	-0.61887	0.535999	0.786221	OXER1	165140	oxoeicosanoid receptor 1
ENSG0000	97.89178	-0.15334	0.247641	-0.61919	0.535789	0.786221	TRIM23	373	tripartite motif containing 23
ENSG0000	755.7721	0.065949	0.106583	0.618762	0.536073	0.786221	CD109	135228	CD109 molecule
ENSG0000	164.3748	0.12257	0.198053	0.618875	0.535999	0.786221	ZNF597	146434	zinc finger protein 597
ENSG0000	12.3643	-0.51614	0.833896	-0.61895	0.535947	0.786221	PYCARD-A	1.01E+08	PYCARD antisense RNA 1
ENSG0000	4.796409	-0.68524	1.107297	-0.61884	0.536023	0.786221	RNU6-611	1.06E+08	RNA U6 small r pseudogene
ENSG0000	11.20696	0.455664	0.73635	0.618814	0.536039	0.786221	NA	NA	NA
ENSG0000	436.4304	0.093242	0.150715	0.618665	0.536137	0.786252	RBBP9	10741	RB binding serine hydrolase
ENSG0000	7.348191	-0.54838	0.886949	-0.61828	0.536389	0.786311	GOLGA4-A	152048	GOLGA4 antisense RNA 1
ENSG0000	129.7553	-0.15401	0.249082	-0.6183	0.536375	0.786311	LINC0100	1E+08	long intergenic non-protein coding RNA 1003
ENSG0000	404.0989	-0.09168	0.148265	-0.61833	0.536357	0.786311	THYN1	29087	thymocyte nuclear protein 1
ENSG0000	14.05354	-0.40294	0.651645	-0.61834	0.536352	0.786311	NA	NA	NA
ENSG0000	4.564604	0.747041	1.207865	0.61848	0.536259	0.786311	HSF2BP	11077	heat shock transcription factor 2 binding protein
ENSG0000	82.32413	-0.20905	0.338171	-0.61818	0.536458	0.786351	ZNF718	255403	zinc finger protein 718
ENSG0000	348.8353	0.100004	0.161807	0.618046	0.536545	0.786353	SNRNP48	154007	small nuclear ribonucleoprotein U11/U12 subunit 48
ENSG0000	151.9234	-0.13598	0.220012	-0.61808	0.536526	0.786353	ZXDA	7789	zinc finger X-linked duplicated A
ENSG0000	6.178195	0.647361	1.049049	0.617093	0.537173	0.786392	CALML6	163688	calmodulin like 6
ENSG0000	9.284648	-0.44675	0.724012	-0.61704	0.537207	0.786392	SEMA5A	9037	semaphorin 5A
ENSG0000	37.94753	0.237277	0.384322	0.617391	0.536977	0.786392	JARID2-AS	1.01E+08	JARID2 antisense RNA 1
ENSG0000	1027.047	0.073121	0.118416	0.617495	0.536908	0.786392	VARS1	7407	valyl-tRNA synthetase 1
ENSG0000	6417.655	-0.05262	0.085266	-0.61713	0.53715	0.786392	GNA12	2768	G protein subunit alpha 12
ENSG0000	297.3698	-0.09595	0.155483	-0.61714	0.537144	0.786392	POP7	10248	POP7 hom ribonuclease P/MRP subunit
ENSG0000	14.79894	0.374583	0.606301	0.617817	0.536696	0.786392	CLEC2L	154790	C-type lectin domain family 2 member L
ENSG0000	30.12509	-0.27075	0.438489	-0.61747	0.536928	0.786392	KCNIP2	30819	potassium voltage-gated channel interacting protein 2
ENSG0000	118.5304	-0.13938	0.225665	-0.61764	0.53681	0.786392	RIC3	79608	RIC3 acetylcholine receptor chaperone
ENSG0000	165.2651	0.122798	0.198995	0.617091	0.537174	0.786392	UNG	7374	uracil DNA glycosylase
ENSG0000	236.9588	0.119768	0.193925	0.617597	0.536841	0.786392	RN7SL1	6029	RNA component of signal recognition particle 7SL1
ENSG0000	131.9917	0.136542	0.22116	0.617389	0.536978	0.786392	PIGB	9488	phosphatidylinositol glycan anchor biosynthesis class B
ENSG0000	1907.479	-0.05523	0.089397	-0.61782	0.536695	0.786392	CLUH	23277	clustered mitochondria homolog
ENSG0000	14.57357	-0.4266	0.69111	-0.61727	0.537056	0.786392	SLC6A4	6532	solute carrier family 6 member 4
ENSG0000	15.27774	-0.42031	0.680826	-0.61735	0.537006	0.786392	NA	NA	NA
ENSG0000	5.167483	-0.72993	1.183238	-0.61689	0.537308	0.786477	NA	NA	NA
ENSG0000	5.907799	-0.67016	1.086508	-0.61681	0.537363	0.786495	CCDC103	388389	coiled-coil domain containing 103
ENSG0000	9.645402	0.556403	0.902423	0.616565	0.537521	0.786561	IFTAP	119710	intraflagellar transport associated protein
ENSG0000	917.7814	-0.07135	0.11573	-0.61655	0.537534	0.786561	DDX51	317781	DEAD-box helicase 51
ENSG0000	14.52133	-0.41618	0.675042	-0.61653	0.537545	0.786561	NA	NA	NA
ENSG0000	574.8749	-0.07739	0.125533	-0.61648	0.537577	0.786561	ZNF764	92595	zinc finger protein 764
ENSG0000	266.9396	0.103471	0.167875	0.616355	0.53766	0.786621	SDCCAG8	10806	SHH signaling and ciliogenesis regulator SDCCAG8
ENSG0000	17.75799	-0.38745	0.629095	-0.61589	0.537969	0.787009	NA	NA	NA
ENSG0000	2.218197	1.128166	1.832355	0.615692	0.538098	0.787098	NA	NA	NA
ENSG0000	2.627963	-1.19285	1.937512	-0.61566	0.538119	0.787098	MTUS2	23281	microtubule associated scaffold protein 2
ENSG0000	8.942462	-0.52185	0.847788	-0.61554	0.538198	0.787098	SLITRK5	26050	SLIT and NTRK like family member 5
ENSG0000	54.22975	0.215295	0.349768	0.615538	0.538199	0.787098	NPTN-IT1	1.01E+08	NPTN intronic transcript 1
ENSG0000	425.5425	0.089707	0.145813	0.615218	0.538411	0.787222	RNASEH1	246243	ribonuclease H1
ENSG0000	20.26697	-0.33626	0.546563	-0.61523	0.538405	0.787222	NA	NA	NA
ENSG0000	236.6009	0.102826	0.167115	0.615302	0.538355	0.787222	ZFP1	162239	ZFP1 zinc finger protein
ENSG0000	191.136	-0.11426	0.185751	-0.61511	0.538483	0.787265	METTL23	124512	methyltransferase like 23
ENSG0000	4.911457	-0.75225	1.223163	-0.61501	0.53855	0.787301	NA	NA	NA
ENSG0000	571.5136	-0.07452	0.121192	-0.61488	0.538633	0.787336	USP1	7398	ubiquitin specific peptidase 1
ENSG0000	143.2413	-0.13482	0.219275	-0.61484	0.538659	0.787336	TTC7B	145567	tetratricopeptide repeat domain 7B
ENSG0000	229.5657	-0.11272	0.183358	-0.61474	0.538728	0.787375	LINC0029	283267	long intergenic non-protein coding RNA 294
ENSG0000	1684.64	0.068385	0.111329	0.614258	0.539045	0.787776	FOXJ3	22887	forkhead box J3
ENSG0000	4.785189	0.689021	1.12199	0.614106	0.539145	0.787844	NA	NA	NA
ENSG0000	1264.273	0.059667	0.097167	0.614059	0.539176	0.787844	ZNF317	57693	zinc finger protein 317
ENSG0000	747.8129	-0.06852	0.111663	-0.6136	0.539479	0.787878	NIPAL3	57185	NIPA like domain containing 3
ENSG0000	342.5961	-0.10523	0.171626	-0.61314	0.539784	0.787878	CHRM3-AS	1.01E+08	CHRM3 antisense RNA 2
ENSG0000	43.46473	-0.25771	0.420408	-0.61299	0.539881	0.787878	ARL6	84100	ADP ribosylation factor like GTPase 6
ENSG0000	1555.314	-0.06849	0.111656	-0.6134	0.539612	0.787878	LNPEP	4012	leucyl and cystinyl aminopeptidase
ENSG0000	2.283784	1.293323	2.108818	0.613293	0.539683	0.787878	PRR18	285800	proline rich 18
ENSG0000	1549.193	-0.05729	0.093463	-0.61297	0.539898	0.787878	CPVL	54504	carboxypeptidase vitellogenic like

ENSG0000	8235.512	0.048428	0.078886	0.613904	0.539279	0.787878	CLEC5A	23601	C-type lectin domain containing 5A
ENSG0000	4.628715	0.884201	1.441521	0.613381	0.539625	0.787878	LINC0302(	1.02E+08	long intergenic non-protein coding RNA 3020
ENSG0000	105.9925	-0.14301	0.233022	-0.61371	0.539406	0.787878	ERCC6L2-A	1E+08	ERCC6L2 antisense RNA 1
ENSG0000	76.39782	-0.19685	0.320966	-0.6133	0.539675	0.787878	C1RL-AS1	283314	C1RL antisense RNA 1
ENSG0000	243.0626	-0.11755	0.191538	-0.61369	0.539419	0.787878	WASHC3	51019	WASH complex subunit 3
ENSG0000	266.6047	-0.12041	0.196146	-0.61389	0.539286	0.787878	SEH1L	81929	SEH1 like nucleoporin
ENSG0000	3.20109	-1.06879	1.743741	-0.61293	0.539922	0.787878	CYP4F12	66002	cytochrome P450 family 4 subfamily F member 12
ENSG0000	12.33498	0.418344	0.682021	0.613388	0.53962	0.787878	C19orf73	55150	chromosome 19 open reading frame 73
ENSG0000	15.25052	0.393046	0.641246	0.612941	0.539915	0.787878	ZNF582-D	386758	ZNF582 divergent transcript
ENSG0000	278.3831	-0.11415	0.186159	-0.61319	0.539751	0.787878	LZTR1	8216	leucine zipper like transcription regulator 1
ENSG0000	386.3018	-0.09477	0.154453	-0.61361	0.539475	0.787878	SLC25A53	401612	solute carrier family 25 member 53
ENSG0000	82.51526	-0.17865	0.291502	-0.61285	0.539978	0.787898	ZNF256	10172	zinc finger protein 256
ENSG0000	515.9267	0.073736	0.120343	0.612717	0.540063	0.787898	ANP32E	81611	acidic nuclear phosphoprotein 32 family member E
ENSG0000	89.64322	0.195041	0.31829	0.612779	0.540022	0.787898	PCSK6	5046	proprotein convertase subtilisin/kexin type 6
ENSG0000	1108.025	0.060488	0.098747	0.612552	0.540172	0.787996	PURA	5813	purine rich element binding protein A
ENSG0000	127.3825	0.185059	0.302183	0.612406	0.540269	0.788013	NA	NA	NA
ENSG0000	3.388776	0.905723	1.47892	0.612422	0.540259	0.788013	LINC0303	283710	long intergenic non-protein coding RNA 3034
ENSG0000	430.033	0.078873	0.12883	0.612224	0.54039	0.788127	TBC1D5	9779	TBC1 domain family member 5
ENSG0000	28.68956	-0.29936	0.489226	-0.6119	0.540602	0.788374	ZNF674	641339	zinc finger protein 674
ENSG0000	2.68213	-1.33555	2.184111	-0.61149	0.540878	0.788653	NA	NA	NA
ENSG0000	613.761	-0.0668	0.109229	-0.61151	0.54086	0.788653	NUDT22	84304	nudix hydrolase 22
ENSG0000	402.8783	0.100682	0.164672	0.611406	0.540931	0.788668	SPOPL	339745	speckle type BTB/POZ protein like
ENSG0000	402.7925	0.081573	0.133451	0.611259	0.541028	0.788747	IGFBP3	3486	insulin like growth factor binding protein 3
ENSG0000	3.84796	-0.78982	1.29231	-0.61117	0.54109	0.788775	C9orf24	84688	chromosome 9 open reading frame 24
ENSG0000	642.7428	0.073465	0.120243	0.610966	0.541222	0.788844	HEATR1	55127	HEAT repeat containing 1
ENSG0000	120.2805	-0.14612	0.239139	-0.61101	0.541194	0.788844	EXOSC7	23016	exosome component 7
ENSG0000	4.604669	-0.74822	1.225007	-0.61079	0.541341	0.788956	NA	NA	NA
ENSG0000	13250.92	-0.06257	0.102528	-0.61028	0.541675	0.788957	MARCKSL1	65108	MARCKS like 1
ENSG0000	5.86044	0.695682	1.139769	0.610371	0.541616	0.788957	NA	NA	NA
ENSG0000	427.3312	0.113055	0.185265	0.610236	0.541705	0.788957	IKZF2	22807	IKAROS family zinc finger 2
ENSG0000	6.123769	-0.65859	1.0789	-0.61042	0.541581	0.788957	NA	NA	NA
ENSG0000	2.63655	1.207487	1.978444	0.610321	0.541649	0.788957	NA	NA	NA
ENSG0000	40666.07	0.044404	0.072755	0.610331	0.541642	0.788957	SPOCK2	9806	SPARC (os cwcv and kazal like domains proteoglycan 2
ENSG0000	92.75889	0.158207	0.259293	0.610146	0.541765	0.788957	NA	NA	NA
ENSG0000	13.52375	0.48651	0.79737	0.610143	0.541767	0.788957	JMJD7	1E+08	jumonji domain containing 7
ENSG0000	42.01673	0.221786	0.363238	0.610581	0.541477	0.788957	NA	NA	NA
ENSG0000	578.9808	0.0727	0.119129	0.610257	0.541692	0.788957	SMS	6611	spermine synthase
ENSG0000	6.768196	0.775958	1.274934	0.608626	0.542772	0.788998	MMEL1	79258	membrane metalloendopeptidase like 1
ENSG0000	417.6549	-0.0848	0.139259	-0.60895	0.542558	0.788998	ZNF326	284695	zinc finger protein 326
ENSG0000	720.7631	0.073069	0.119806	0.609893	0.541933	0.788998	RNF103	7844	ring finger protein 103
ENSG0000	63.32836	-0.20182	0.330982	-0.60977	0.542013	0.788998	CCR3	1232	C-C motif chemokine receptor 3
ENSG0000	44.72275	-0.21946	0.360519	-0.60873	0.542704	0.788998	KALRN	8997	kalirin RhoGEF kinase
ENSG0000	3.253226	-0.93635	1.53747	-0.60902	0.54251	0.788998	CHSY3	337876	chondroitin sulfate synthase 3
ENSG0000	20.46238	0.331043	0.543486	0.609112	0.54245	0.788998	SAP30L-AS	386627	SAP30L antisense RNA 1 (head to head)
ENSG0000	91.3567	-0.16859	0.276852	-0.60894	0.542564	0.788998	NA	NA	NA
ENSG0000	5700.307	0.05371	0.088124	0.609486	0.542203	0.788998	SRF	6722	serum response factor
ENSG0000	199.4149	-0.11084	0.181957	-0.60913	0.542437	0.788998	SERAC1	84947	serine active site containing 1
ENSG0000	262.8704	-0.10107	0.166082	-0.60858	0.542804	0.788998	KIF20B	9585	kinesin family member 20B
ENSG0000	2044.883	-0.05313	0.087167	-0.60956	0.542155	0.788998	NADSYN1	55191	NAD synthetase 1
ENSG0000	352.6774	-0.10708	0.175604	-0.60978	0.54201	0.788998	XRRA1	143570	X-ray radiation resistance associated 1
ENSG0000	267.8419	0.106378	0.174803	0.60856	0.542816	0.788998	ZCRB1	85437	zinc finger CCHC-type and RNA binding motif containing 1
ENSG0000	176.9549	-0.13331	0.218611	-0.6098	0.541996	0.788998	SPINT1	6692	serine pep Kunitz type 1
ENSG0000	522.8668	-0.07534	0.123715	-0.60897	0.542547	0.788998	CEROX1	1.16E+08	cytoplasmic endogenous regulator of oxidative phosphorylation 1
ENSG0000	109.1641	0.154822	0.254127	0.609231	0.542372	0.788998	TIGD7	91151	tigger transposable element derived 7
ENSG0000	49.98646	-0.20806	0.34149	-0.60926	0.542354	0.788998	PRSS53	339105	serine protease 53
ENSG0000	3.073253	-0.94973	1.557721	-0.60969	0.542064	0.788998	NA	NA	NA
ENSG0000	7.078962	-0.6548	1.074088	-0.60963	0.542104	0.788998	PARD6G-A	1E+08	PARD6G antisense RNA 1
ENSG0000	8.528555	-0.50609	0.830287	-0.60953	0.542171	0.788998	NA	NA	NA
ENSG0000	901.2638	0.063477	0.104153	0.609457	0.542222	0.788998	RANBP1	5902	RAN binding protein 1
ENSG0000	185.0735	-0.11564	0.19	-0.60861	0.542783	0.788998	PPP1R3F	89801	protein phosphatase 1 regulatory subunit 3F
ENSG0000	299.8934	0.099032	0.162663	0.608814	0.542648	0.788998	ZFY	7544	zinc finger protein Y-linked
ENSG0000	8.743858	-0.50901	0.836833	-0.60825	0.54302	0.78917	OR7E7P	392752	olfactory receptor family 7 subfamily E member 7 pseudogene
ENSG0000	50.14164	-0.22562	0.370902	-0.6083	0.542989	0.78917	ZNF132	7691	zinc finger protein 132
ENSG0000	54.97698	-0.22285	0.366465	-0.60811	0.543113	0.789244	TATDN1	83940	TatD DNase domain containing 1
ENSG0000	657.5204	-0.07742	0.127361	-0.60786	0.543277	0.78942	GOLGA8B	440270	golgin A8 family member B
ENSG0000	9.947422	-0.45978	0.756486	-0.60779	0.543329	0.789433	NA	NA	NA
ENSG0000	230.423	-0.11702	0.192575	-0.60768	0.5434	0.789474	IFI30	10437	IFI30 lysosomal thiol reductase
ENSG0000	108.6183	0.155473	0.255923	0.607498	0.543521	0.789475	PLA2G12A	81579	phospholipase A2 group XIIA
ENSG0000	4.121396	0.694183	1.142636	0.607528	0.543501	0.789475	RPL23P6	646949	ribosomal protein L23 pseudogene 6
ENSG0000	963.0167	-0.06423	0.105735	-0.60749	0.543527	0.789475	SNAP23	8773	synaptosome associated protein 23
ENSG0000	3.680648	0.782517	1.288462	0.607327	0.543634	0.789568	RPS2P36	1E+08	ribosomal protein S2 pseudogene 36
ENSG0000	9.203626	-0.57347	0.944825	-0.60696	0.543878	0.78986	DTNA	1837	dystrobrevin alpha
ENSG0000	12.57728	0.426777	0.703541	0.606614	0.544107	0.790075	NA	NA	NA
ENSG0000	2424.015	0.057917	0.095514	0.606373	0.544267	0.790075	SH3TC1	54436	SH3 domain and tetratricopeptide repeats 1
ENSG0000	4240.942	0.055726	0.09188	0.606504	0.54418	0.790075	CCAR2	57805	cell cycle and apoptosis regulator 2
ENSG0000	12.50863	-0.43364	0.714892	-0.60658	0.544129	0.790075	NA	NA	NA
ENSG0000	269.1098	-0.1083	0.178597	-0.6064	0.544251	0.790075	CHAF1A	10036	chromatin assembly factor 1 subunit A
ENSG0000	9.462636	0.445193	0.734216	0.606351	0.544281	0.790075	RIPOR3	140876	RIPOR family member 3
ENSG0000	17.15761	-0.33312	0.549478	-0.60624	0.544355	0.790107	LINC0085	1.01E+08	long intergenic non-protein coding RNA 853
ENSG0000	348.4216	0.089584	0.147792	0.606153	0.544413	0.790107	H2BC21	8349	H2B clustered histone 21
ENSG0000	475.7255	0.07723	0.127453	0.605953	0.544546	0.790107	ARHGAP1!	55843	Rho GTPase activating protein 15
ENSG0000	8.179654	-0.54915	0.906166	-0.60602	0.544504	0.790107	NA	NA	NA
ENSG0000	2.514701	0.96455	1.59189	0.605915	0.544571	0.790107	NA	NA	NA
ENSG0000	58.21818	-0.204	0.336658	-0.60597	0.544536	0.790107	RPP25	54913	ribonuclease P and MRP subunit p25
ENSG0000	131.8141	-0.15866	0.261899	-0.60581	0.544644	0.790107	PEX12	5193	peroxisomal biogenesis factor 12
ENSG0000	24.9678	-0.38738	0.63943	-0.60582	0.544637	0.790107	NA	NA	NA
ENSG0000	11.44566	-0.43143	0.712382	-0.60562	0.544767	0.790174	NA	NA	NA
ENSG0000	573.4626	-0.07456	0.123114	-0.60561	0.544776	0.790174	GSS	2937	glutathione synthetase
ENSG0000	3.631608	0.85262	1.408571	0.605309	0.544974	0.790235	NA	NA	NA
ENSG0000	5.881268	-0.64637	1.067983	-0.60522	0.54503	0.790235	SCOC-AS1	1E+08	SCOC antisense RNA 1
ENSG0000	2.323967	-1.06368	1.757399	-0.60526	0.545007	0.790235	NA	NA	NA
ENSG0000	94.61418	0.176759	0.291971	0.605399	0.544914	0.790235	ING2	3622	inhibitor of growth family member 2
ENSG0000	4.810137	0.715739	1.182713	0.605167	0.545068	0.790235	NA	NA	NA
ENSG0000	8.652901	-0.48071	0.794345	-0.60516	0.545073	0.790235	NA	NA	NA
ENSG0000	25.81199	0.384862	0.636109	0.605025	0.545162	0.790302	KLK1	3816	kallikrein 1
ENSG0000	57.69534	0.231259	0.382328	0.604872	0.545264	0.790321	NA	NA	NA
ENSG0000	3.770913	-0.91758	1.517048	-0.60484	0.545282	0.790321	MRO	83876	maestro
ENSG0000	89.18015	-0.18666	0.308626	-0.60481	0.545303	0.790321	COL9A3	1299	collagen type IX alpha 3 chain
ENSG0000	63.67285	-0.17909	0.296195	-0.60464	0.545417	0.790424	PDGFC	56034	platelet derived growth factor C
ENSG0000	31.37188	0.260098	0.430225	0.604563	0.54547	0.790439	ZNF300	91975	zinc finger protein 300

ENSG0000	171.4569	-0.12002	0.198577	-0.60441	0.545569	0.790522	THBS3	7059	thrombospondin 3
ENSG0000	21.31335	-0.31477	0.520851	-0.60433	0.545625	0.790538	LOC10537	1.05E+08	uncharacterized LOC105377730
ENSG0000	273.9259	-0.1066	0.176419	-0.60427	0.545666	0.790538	BAD	572	BCL2 associated agonist of cell death
ENSG0000	57.10482	0.219681	0.363653	0.604093	0.545782	0.790644	PIANP	196500	PILR alpha associated neural protein
ENSG0000	52.99746	0.224409	0.37168	0.60377	0.545997	0.790846	PDIA5	10954	protein disulfide isomerase family A member 5
ENSG0000	16.66988	-0.33744	0.558898	-0.60376	0.546006	0.790846	NA	NA	NA
ENSG0000	7.369585	0.596582	0.988454	0.603551	0.546142	0.790981	DYNLT4	343521	dynein light chain Tctex-type 4
ENSG0000	1365.067	-0.05757	0.095401	-0.60342	0.546226	0.791041	SCAMP4	113178	secretory carrier membrane protein 4
ENSG0000	1445.416	0.05535	0.091751	0.603268	0.54633	0.79113	CCT4	10575	chaperonin containing TCP1 subunit 4
ENSG0000	20.48953	0.319945	0.530519	0.603079	0.546456	0.79125	SGO1-AS1	1.01E+08	SGO1 antisense RNA 1
ENSG0000	23.30397	0.306995	0.509158	0.602947	0.546544	0.791316	NA	NA	NA
ENSG0000	116.115	-0.14584	0.242081	-0.60246	0.546869	0.79143	MRPL48	51642	mitochondrial ribosomal protein L48
ENSG0000	5.542698	0.653799	1.085246	0.602444	0.546879	0.79143	NA	NA	NA
ENSG0000	36.75332	0.255782	0.424463	0.6026	0.546775	0.79143	IGHV3-21	28444	immunoglobulin heavy variable 3-21
ENSG0000	31.28049	0.256228	0.425228	0.602566	0.546797	0.79143	NA	NA	NA
ENSG0000	10.78069	-0.47827	0.793796	-0.60251	0.546837	0.79143	NA	NA	NA
ENSG0000	7.140806	-0.59859	0.993344	-0.6026	0.546772	0.79143	NA	NA	NA
ENSG0000	314.3695	-0.10299	0.170979	-0.60238	0.546923	0.791431	PCGF1	84759	polycomb group ring finger 1
ENSG0000	2.903075	1.005653	1.67006	0.602166	0.547064	0.791471	GYPB	2994	glycophorin B (MNS blood group)
ENSG0000	11.14999	0.448268	0.744372	0.602209	0.547035	0.791471	FAM183BP	340286	acyloxyacyl hydrolase (neutrophil)
ENSG0000	408.5662	0.079974	0.132815	0.602145	0.547078	0.791471	TGS1	96764	trimethylguanosine synthase 1
ENSG0000	703.8991	-0.07184	0.119369	-0.60184	0.547282	0.791471	TMCO1	54499	transmembrane and coiled-coil domains 1
ENSG0000	28755.98	0.060991	0.101348	0.601802	0.547306	0.791471	PTPRC	5788	protein tyrosine phosphatase receptor type C
ENSG0000	8.13389	0.51933	0.863157	0.601663	0.547398	0.791471	IPO9-AS1	1.01E+08	IPO9 antisense RNA 1
ENSG0000	1338.884	0.059488	0.098906	0.601457	0.547536	0.791471	SH3BP5L	80851	SH3 binding domain protein 5 like
ENSG0000	8.589235	-0.57291	0.951928	-0.60184	0.547279	0.791471	NA	NA	NA
ENSG0000	65.53634	0.190952	0.317327	0.601751	0.54734	0.791471	SDHAF3	57001	succinate dehydrogenase complex assembly factor 3
ENSG0000	839.4899	-0.06681	0.111074	-0.60149	0.547516	0.791471	CLDN15	24146	claudin 15
ENSG0000	42.07047	-0.22903	0.380756	-0.6015	0.547506	0.791471	MINCR	1.01E+08	MYC-induced long non-coding RNA
ENSG0000	8.145866	0.54604	0.907685	0.601574	0.547458	0.791471	SUGT1-DT	1.16E+08	SUGT1 divergent transcript
ENSG0000	11.02613	-0.57394	0.953551	-0.6019	0.547243	0.791471	NA	NA	NA
ENSG0000	3.020766	-0.78164	1.299619	-0.60144	0.547548	0.791471	NA	NA	NA
ENSG0000	11.44171	1.074074	1.786133	0.601341	0.547613	0.791504	GCAT	23464	glycine C-acetyltransferase
ENSG0000	90.44788	0.176744	0.293948	0.601276	0.547656	0.791505	MISP3	113230	MISP family member 3
ENSG0000	19.49189	-0.40373	0.672398	-0.60043	0.548222	0.791568	PXDN	7837	peroxidasin
ENSG0000	3.056714	1.0628	1.768889	0.600829	0.547954	0.791568	IGKV2D-28	28883	immunoglobulin kappa variable 2D-28
ENSG0000	38.77916	0.240674	0.400389	0.6011	0.547773	0.791568	CCDC138	165055	coiled-coil domain containing 138
ENSG0000	81.89327	-0.16683	0.27785	-0.60043	0.548219	0.791568	RNF217	154214	ring finger protein 217
ENSG0000	294.6429	-0.10182	0.16955	-0.60053	0.548154	0.791568	HDDC2	51020	HD domain containing 2
ENSG0000	2147.145	-0.05262	0.087634	-0.60049	0.54818	0.791568	TRAPPC14	55262	trafficking protein particle complex subunit 14
ENSG0000	287.8931	0.104586	0.174093	0.60075	0.548006	0.791568	CSPP1	79848	centrosome and spindle pole associated protein 1
ENSG0000	18.84232	0.401002	0.667352	0.600885	0.547917	0.791568	NA	NA	NA
ENSG0000	534.9315	0.078647	0.130922	0.600713	0.548031	0.791568	AEBP2	121536	AE binding protein 2
ENSG0000	56.52976	0.219348	0.36535	0.600378	0.548255	0.791568	SNUPN	10073	snurportin 1
ENSG0000	1194.248	-0.06297	0.104793	-0.60088	0.547923	0.791568	NUBP2	10101	NUBP iron cytosolic
ENSG0000	7.043065	0.56128	0.934603	0.600555	0.548136	0.791568	NA	NA	NA
ENSG0000	5.98732	-0.69493	1.156711	-0.60079	0.547983	0.791568	RPS6KA6	27330	ribosomal protein S6 kinase A6
ENSG0000	21.70274	-0.33578	0.559586	-0.60005	0.548473	0.791821	DOC2B	8447	double C2 domain beta
ENSG0000	10.73437	-0.42316	0.705478	-0.59982	0.548625	0.791831	ZNF660	285349	zinc finger protein 660
ENSG0000	543.5503	0.091042	0.151791	0.599784	0.54865	0.791831	GLRX	2745	glutaredoxin
ENSG0000	156.5999	0.132844	0.221455	0.599869	0.548594	0.791831	ADCY4	196883	adenylate cyclase 4
ENSG0000	5.613934	-0.59992	1.000207	-0.5998	0.54864	0.791831	PMFBP1	83449	polyamine modulated factor 1 binding protein 1
ENSG0000	11.90857	0.406035	0.677051	0.599711	0.548699	0.791839	TRIM17	51127	tripartite motif containing 17
ENSG0000	119.5653	-0.14979	0.249872	-0.59945	0.54887	0.791959	NOTCH2N	1.01E+08	notch 2 N-terminal like C
ENSG0000	138.8455	-0.12782	0.213217	-0.59948	0.548853	0.791959	MRPS14	63931	mitochondrial ribosomal protein S14
ENSG0000	987.2664	-0.06663	0.111166	-0.59939	0.54891	0.791959	USF3	205717	upstream transcription factor family member 3
ENSG0000	4.090389	0.709775	1.184297	0.599322	0.548958	0.791967	NA	NA	NA
ENSG0000	6.839461	0.607395	1.013795	0.59913	0.549086	0.79209	NA	NA	NA
ENSG0000	429.6168	-0.11303	0.188693	-0.59901	0.549167	0.792145	LSM3	27258	LSM3 hom U6 small nuclear RNA and mRNA degradation associated
ENSG0000	114.7855	0.156762	0.261765	0.598866	0.549262	0.792183	FOXO3B	2310	forkhead box O3B
ENSG0000	2214.167	-0.05303	0.088558	-0.59884	0.549279	0.792183	LUC7L3	51747	LUC7 like 3 pre-mRNA splicing factor
ENSG0000	224.6579	0.097656	0.163122	0.598667	0.549395	0.792228	ARMT1	79624	acidic residue methyltransferase 1
ENSG0000	5330.793	0.046011	0.076851	0.598707	0.549368	0.792228	RAB5B	5869	RAB5B member RAS oncogene family
ENSG0000	485.8271	0.079477	0.1328	0.598468	0.549528	0.792281	NIPSNAP2	2631	nipsnap homolog 2
ENSG0000	1158.089	0.062526	0.104485	0.598419	0.54956	0.792281	SVIL	6840	supervillin
ENSG0000	2618.666	-0.12957	0.216494	-0.59851	0.549496	0.792281	PLBD2	196463	phospholipase B domain containing 2
ENSG0000	26.00852	-0.3337	0.557975	-0.59806	0.549801	0.792566	TBC1D19	55296	TBC1 domain family member 19
ENSG0000	5.502073	0.772404	1.291962	0.597854	0.549938	0.792663	NA	NA	NA
ENSG0000	498.8746	0.075819	0.126823	0.59783	0.549953	0.792663	TOMM22	56993	translocase of outer mitochondrial membrane 22
ENSG0000	40.83243	0.239733	0.401205	0.597533	0.550151	0.792825	NA	NA	NA
ENSG0000	91.29453	0.170829	0.285888	0.597537	0.550149	0.792825	IGHG4	3503	immunoglobulin heavy constant gamma 4 (G4m marker)
ENSG0000	742.101	-0.06849	0.114634	-0.59746	0.550203	0.792838	YIPF2	78992	Yip1 domain family member 2
ENSG0000	659.5831	0.071066	0.119074	0.59682	0.550627	0.792841	RABIF	5877	RAB interacting factor
ENSG0000	23.5587	0.320661	0.537209	0.596902	0.550573	0.792841	NAT8B	51471	N-acetyltr gene/pseudogene)
ENSG0000	5.089955	-0.74711	1.251555	-0.59695	0.550542	0.792841	NA	NA	NA
ENSG0000	89.69277	0.150853	0.252596	0.597211	0.550367	0.792841	ANKMY2	57037	ankyrin repeat and MYND domain containing 2
ENSG0000	2.230068	1.268172	2.124338	0.596973	0.550526	0.792841	TRBV12-1	28579	T cell receptor beta variable 12-1 (pseudogene)
ENSG0000	508.7939	0.09147	0.153164	0.5972	0.550374	0.792841	ZNRD2	10534	zinc ribbon domain containing 2
ENSG0000	11.9456	0.452359	0.757671	0.597039	0.550481	0.792841	SUGT1P3	283507	SUGT1 pseudogene 3
ENSG0000	60.73381	0.227111	0.38036	0.597096	0.550444	0.792841	SHPK	23729	sedoheptulokinase
ENSG0000	181.5984	-0.11682	0.195706	-0.5969	0.550577	0.792841	PRPSAP2	5636	phosphoribosyl pyrophosphate synthetase associated protein 2
ENSG0000	6.780114	-0.72483	1.214549	-0.59679	0.550646	0.792841	NA	NA	NA
ENSG0000	1274.456	-0.06247	0.104679	-0.59675	0.550675	0.792841	TRMT2A	27037	tRNA methyltransferase 2 homolog A
ENSG0000	187.6853	0.113657	0.190495	0.59664	0.550748	0.792877	CELSR3	1951	cadherin EGF LAG seven-pass G-type receptor 3
ENSG0000	10.79942	-0.51041	0.855549	-0.59658	0.550786	0.792877	CDO1	1036	cysteine dioxygenase type 1
ENSG0000	4.561572	0.633184	1.061476	0.596513	0.550832	0.792882	EIF4A1P2	359792	eukaryotic translation initiation factor 4A1 pseudogene 2
ENSG0000	52.62494	0.212929	0.357087	0.596294	0.550979	0.793031	USP49	25862	ubiquitin specific peptidase 49
ENSG0000	925.124	0.061115	0.102511	0.596179	0.551056	0.793081	FBXL12	54850	F-box and leucine rich repeat protein 12
ENSG0000	96.53058	-0.14688	0.246501	-0.59588	0.551258	0.79331	PTCD2	79810	pentatricopeptide repeat domain 2
ENSG0000	270.1016	0.089809	0.150739	0.59579	0.551316	0.793331	TFAM	7019	transcripti mitochondrial
ENSG0000	24879.6	0.043142	0.072481	0.595215	0.5517	0.793584	WIPF1	7456	WAS/WASL interacting protein family member 1
ENSG0000	386.2868	0.084469	0.141915	0.595208	0.551705	0.793584	QTRT2	79691	queuine tRNA-ribosyltransferase accessory subunit 2
ENSG0000	257.6757	-0.10258	0.172316	-0.59532	0.551627	0.793584	PAX5	5079	paired box 5
ENSG0000	1470.189	0.062045	0.104238	0.595222	0.551695	0.793584	MMS19	64210	MMS19 hc cytosolic iron-sulfur assembly component
ENSG0000	12.52242	0.377258	0.633587	0.595433	0.551554	0.793584	NA	NA	NA
ENSG0000	311.549	-0.11278	0.189521	-0.59506	0.5518	0.79366	ZNF438	220929	zinc finger protein 438
ENSG0000	534.0256	0.076565	0.128718	0.594828	0.551958	0.793825	SERPINF1	5176	serpin family F member 1
ENSG0000	40.45909	-0.22933	0.385632	-0.59467	0.552061	0.793912	TRBV7-3	28595	T cell receptor beta variable 7-3
ENSG0000	195.4986	0.106701	0.17964	0.593973	0.55253	0.793921	COA5	493753	cytochrome c oxidase assembly factor 5
ENSG0000	2365.878	0.054666	0.092025	0.594028	0.552493	0.793921	PRPF40A	55660	pre-mRNA processing factor 40 homolog A

ENSG0000	5.658323	-0.75074	1.264033	-0.59392	0.552565	0.793921	KCNMB3	27094	potassium calcium-activated channel subfamily M regulatory beta subunit 3
ENSG0000	15860.3	-0.06701	0.112699	-0.59459	0.55212	0.793921	TAP1	6890	transporte ATP binding cassette subfamily B member
ENSG0000	6.850376	-0.51325	0.863707	-0.59424	0.552349	0.793921	C6orf132	647024	chromosome 6 open reading frame 132
ENSG0000	82.25816	-0.21685	0.36488	-0.59432	0.5523	0.793921	TRBV6-5	28602	T cell receptor beta variable 6-5
ENSG0000	178.2886	-0.12298	0.207053	-0.59396	0.552537	0.793921	DENND10	404636	DENN domain containing 10
ENSG0000	205.6648	0.118503	0.199379	0.59436	0.552271	0.793921	TMEM258	746	transmembrane protein 258
ENSG0000	317.7096	0.085079	0.143255	0.593897	0.552581	0.793921	RACGAP1	29127	Rac GTPase activating protein 1
ENSG0000	2.177022	-1.15635	1.944954	-0.59454	0.552153	0.793921	TSKS	60385	testis specific serine kinase substrate
ENSG0000	8.340994	0.534077	0.898504	0.594407	0.55224	0.793921	NA	NA	NA
ENSG0000	1435.304	-0.05778	0.097267	-0.594	0.552509	0.793921	ACSS1	84532	acyl-CoA synthetase short chain family member 1
ENSG0000	773.3223	0.061285	0.103225	0.593704	0.55271	0.794036	RPF1	80135	ribosome production factor 1 homolog
ENSG0000	147.9009	-0.12523	0.211002	-0.59349	0.552855	0.794036	LYRM7	90624	LYR motif containing 7
ENSG0000	45.20923	-0.27644	0.465816	-0.59346	0.552873	0.794036	NA	NA	NA
ENSG0000	27.26431	0.288912	0.48678	0.593517	0.552835	0.794036	NA	NA	NA
ENSG0000	947.704	0.06595	0.111129	0.593457	0.552876	0.794036	PTPN2	5771	protein tyrosine phosphatase non-receptor type 2
ENSG0000	17.6935	-0.33074	0.557373	-0.59339	0.552919	0.794037	EMP2	2013	epithelial membrane protein 2
ENSG0000	124.2836	-0.13126	0.221336	-0.59305	0.553145	0.794239	PRADC1	84279	protease associated domain containing 1
ENSG0000	318.1266	-0.09264	0.156204	-0.59307	0.553137	0.794239	DCAKD	79877	dephospho-CoA kinase domain containing
ENSG0000	919.2089	0.075162	0.126758	0.592952	0.553213	0.794275	PORCN	64840	porcupine O-acyltransferase
ENSG0000	17.10814	0.364066	0.614201	0.592747	0.55335	0.79441	NA	NA	NA
ENSG0000	56.42698	0.188676	0.318373	0.592625	0.553432	0.794467	NA	NA	NA
ENSG0000	7.768621	-0.48076	0.811423	-0.59248	0.553527	0.794499	TRBJ2-3	28626	T cell receptor beta joining 2-3
ENSG0000	14.91725	0.400704	0.676336	0.592463	0.553541	0.794499	TNNC2	7125	troponin C fast skeletal type
ENSG0000	5.355692	-0.73639	1.24368	-0.59211	0.553777	0.794601	LINC0111	339822	long intergenic non-protein coding RNA 1115
ENSG0000	11.01527	0.506513	0.855253	0.592237	0.553692	0.794601	MACC1	346389	MET transcriptional regulator MACC1
ENSG0000	869.4975	-0.06771	0.114375	-0.59199	0.55386	0.794601	NSUN5	55695	NOP2/Sun RNA methyltransferase 5
ENSG0000	35.41597	-0.24221	0.409034	-0.59215	0.553748	0.794601	PIWIL4	143689	piwi like RNA-mediated gene silencing 4
ENSG0000	9.399595	-0.47074	0.795371	-0.59186	0.553948	0.794601	TMEM92-	1.04E+08	TMEM92 antisense RNA 1
ENSG0000	28.78838	0.337945	0.570815	0.59204	0.553824	0.794601	NA	NA	NA
ENSG0000	84.43902	-0.19475	0.329049	-0.59186	0.553946	0.794601	PXMP4	11264	peroxisomal membrane protein 4
ENSG0000	725.7119	-0.07943	0.134215	-0.59185	0.553954	0.794601	BRWD3	254065	bromodomain and WD repeat domain containing 3
ENSG0000	10.14463	-0.41995	0.709982	-0.59149	0.554189	0.794604	BZW1-AS1	1.02E+08	BZW1 antisense RNA 1
ENSG0000	530.9815	-0.073	0.123388	-0.59163	0.554095	0.794604	PWWP2A	114825	PWWP domain containing 2A
ENSG0000	2760.22	0.047589	0.080445	0.591574	0.554136	0.794604	MDC1	9656	mediator of DNA damage checkpoint 1
ENSG0000	5.750624	-0.63211	1.06826	-0.59172	0.554036	0.794604	LOC11226	1.12E+08	uncharacterized LOC112268389
ENSG0000	127.7912	0.14011	0.236915	0.591394	0.554256	0.794604	IGHV3-23	28442	immunoglobulin heavy variable 3-23
ENSG0000	6.541044	0.52806	0.892831	0.591445	0.554222	0.794604	NA	NA	NA
ENSG0000	23.62606	-0.29495	0.498692	-0.59145	0.554218	0.794604	DSG2	1829	desmoglein 2
ENSG0000	3.410105	0.828176	1.401708	0.590834	0.554632	0.795019	NA	NA	NA
ENSG0000	6.41885	0.645057	1.091736	0.590855	0.554618	0.795019	NA	NA	NA
ENSG0000	4.550572	0.822527	1.392329	0.590756	0.554684	0.795033	ADAD2	161931	adenosine deaminase domain containing 2
ENSG0000	13.58882	0.396922	0.671982	0.590674	0.554739	0.79505	NA	NA	NA
ENSG0000	52.23184	0.235667	0.3994	0.590053	0.555155	0.795278	CXCR1	3577	C-X-C motif chemokine receptor 1
ENSG0000	243.2408	0.106833	0.180958	0.590372	0.554941	0.795278	FARSB	10056	phenylalanyl-tRNA synthetase subunit beta
ENSG0000	70.90837	-0.18639	0.315832	-0.59016	0.555083	0.795278	NIPSNAP3	55335	nipsnap homolog 3B
ENSG0000	2.132672	1.143069	1.936487	0.59028	0.555003	0.795278	NA	NA	NA
ENSG0000	203.8859	-0.1206	0.204366	-0.5901	0.555123	0.795278	TBC1D24	57465	TBC1 domain family member 24
ENSG0000	83.07318	-0.1641	0.278083	-0.59011	0.555119	0.795278	MCM8	84515	minichromosome maintenance 8 homologous recombination repair factor
ENSG0000	6.340992	0.552345	0.936202	0.589985	0.555201	0.795282	RPS2P29	646294	ribosomal protein S2 pseudogene 29
ENSG0000	38.54527	-0.23698	0.40179	-0.58982	0.555309	0.795376	XRCC4	7518	X-ray repair cross complementing 4
ENSG0000	303.6263	0.088688	0.150382	0.589753	0.555356	0.795382	ZBTB34	403341	zinc finger and BTB domain containing 34
ENSG0000	11.00748	-0.53679	0.910498	-0.58956	0.555485	0.795415	ADAMTS4	9507	ADAM metallopeptidase with thrombospondin type 1 motif 4
ENSG0000	49.18482	0.227458	0.385832	0.589526	0.555508	0.795415	CCNI2	645121	cyclin I family member 2
ENSG0000	13.9419	-0.40521	0.687229	-0.58963	0.555438	0.795415	TAGAP-AS	1.05E+08	TAGAP antisense RNA 1
ENSG0000	4.827613	0.768311	1.303623	0.589366	0.555616	0.795507	NA	NA	NA
ENSG0000	22.69508	-0.3943	0.669327	-0.5891	0.555795	0.795703	PARPBP	55010	PARP1 binding protein
ENSG0000	59.2503	-0.25884	0.439474	-0.58897	0.555879	0.795728	C18orf54	162681	chromosome 18 open reading frame 54
ENSG0000	179.3069	0.115612	0.196304	0.588944	0.555899	0.795728	ZNF584	201514	zinc finger protein 584
ENSG0000	53.0438	-0.55426	0.941445	-0.58873	0.556039	0.795868	RSPO3	84870	R-spondin 3
ENSG0000	13.4622	-0.46229	0.785382	-0.58861	0.556121	0.795923	NA	NA	NA
ENSG0000	299.3602	-0.0991	0.168474	-0.58822	0.556385	0.795993	S100PBP	64766	S100P binding protein
ENSG0000	89.38081	-0.17504	0.2976	-0.58816	0.556427	0.795993	ZNF322	79692	zinc finger protein 322
ENSG0000	230.4152	0.114507	0.194604	0.588408	0.556258	0.795993	RRS1	23212	ribosome biogenesis regulator 1 homolog
ENSG0000	1450.48	0.049873	0.084752	0.588465	0.55622	0.795993	LMBR1L	55716	limb development membrane protein 1 like
ENSG0000	903.2225	0.060202	0.102355	0.588162	0.556424	0.795993	ARMC5	79798	armadillo repeat containing 5
ENSG0000	2944.998	-0.04966	0.084425	-0.58827	0.556353	0.795993	TCF3	6929	transcription factor 3
ENSG0000	7.665787	-0.54365	0.924618	-0.58797	0.556551	0.79611	ZNF883	169834	zinc finger protein 883
ENSG0000	6.2077	-0.53713	0.9137	-0.58786	0.556623	0.796151	WBP2NL	164684	WBP2 N-terminal like
ENSG0000	249.4424	-0.10061	0.171173	-0.58776	0.556693	0.796189	RBM4	5936	RNA binding motif protein 4
ENSG0000	5.02041	-0.65733	1.118861	-0.5875	0.55687	0.7962	NA	NA	NA
ENSG0000	3.880889	0.905693	1.541637	0.587488	0.556876	0.7962	PGF	5228	placental growth factor
ENSG0000	283.1099	-0.10634	0.181033	-0.58743	0.556915	0.7962	NA	NA	NA
ENSG0000	768.0615	0.074499	0.126773	0.587657	0.556762	0.7962	DAPK3	1613	death associated protein kinase 3
ENSG0000	1394.569	0.0552	0.093949	0.587554	0.556832	0.7962	PI4KAP1	728233	phosphatidylinositol 4-kinase alpha pseudogene 1
ENSG0000	460.3981	0.082724	0.140848	0.587329	0.556983	0.796236	ATIC	471	5-aminoimidazole-4-carboxamide ribonucleotide formyltransferase/IMP cyclohydrolase
ENSG0000	5.590736	-0.67681	1.152983	-0.587	0.557201	0.796486	CMTM2	146225	CKLF like MARVEL transmembrane domain containing 2
ENSG0000	13.15264	-0.56269	0.960015	-0.58613	0.55779	0.79656	ANKRD23	200539	ankyrin repeat domain 23
ENSG0000	466.56	0.081842	0.139613	0.586208	0.557736	0.79656	C5orf22	55322	chromosome 5 open reading frame 22
ENSG0000	6.535887	0.56044	0.956089	0.58618	0.557754	0.79656	SMO	6608	smoothen frizzled class receptor
ENSG0000	90.59252	-0.16848	0.28734	-0.58636	0.557633	0.79656	TRBV5-1	28614	T cell receptor beta variable 5-1
ENSG0000	29.71091	-0.2539	0.432835	-0.5866	0.55747	0.79656	TRBV27	28560	T cell receptor beta variable 27
ENSG0000	460.3408	-0.07923	0.135264	-0.58578	0.558026	0.79656	ZNF783	1E+08	zinc finger protein 783
ENSG0000	306.3386	-0.08658	0.147772	-0.58589	0.55795	0.79656	ZNF510	22869	zinc finger protein 510
ENSG0000	2.723156	1.020195	1.738888	0.586694	0.557409	0.79656	OLFML2A	169611	olfactomedin like 2A
ENSG0000	957.9362	-0.05775	0.09855	-0.58602	0.557861	0.79656	UPF2	26019	UPF2 regulator of nonsense mediated mRNA decay
ENSG0000	17.77285	-0.38584	0.658434	-0.586	0.557877	0.79656	ZNF239	8187	zinc finger protein 239
ENSG0000	30.70662	-0.25779	0.439346	-0.58676	0.557364	0.79656	NUTM2D	728130	NUT family member 2D
ENSG0000	3178.748	-0.04928	0.084122	-0.58581	0.558004	0.79656	DDX23	9416	DEAD-box helicase 23
ENSG0000	5.86966	0.639915	1.091266	0.586397	0.557609	0.79656	CELA1	1990	chymotrypsin like elastase 1
ENSG0000	7.272192	-0.67792	1.156702	-0.58608	0.55782	0.79656	NA	NA	NA
ENSG0000	11.80015	-0.40992	0.699727	-0.58582	0.557995	0.79656	GINS2	51659	GINS complex subunit 2
ENSG0000	213.9919	-0.10718	0.182722	-0.58657	0.557491	0.79656	ZNF701	55762	zinc finger protein 701
ENSG0000	5436.621	0.046171	0.07872	0.586528	0.557521	0.79656	ZNFX1	57169	zinc finger NFX1-type containing 1
ENSG0000	756.1124	-0.06171	0.105269	-0.58618	0.557754	0.79656	CXorf38	159013	chromosome X open reading frame 38
ENSG0000	43.93573	-0.21002	0.358712	-0.58549	0.558217	0.796771	CIB2	10518	calcium and integrin binding family member 2
ENSG0000	241.3396	-0.09731	0.166239	-0.58539	0.558286	0.796809	CIAO2A	84191	cytosolic iron-sulfur assembly component 2A
ENSG0000	1070.437	-0.06412	0.109577	-0.58512	0.558468	0.797007	KAT8	84148	lysine acetyltransferase 8
ENSG0000	14.47812	0.35063	0.599341	0.585025	0.558531	0.797035	RPL7AP30	441034	ribosomal protein L7a pseudogene 30
ENSG0000	4.311651	0.726646	1.242906	0.584634	0.558794	0.797049	NA	NA	NA
ENSG0000	6.012069	0.681057	1.16488	0.584658	0.558777	0.797049	NA	NA	NA

ENSG0000	115.9896	-0.18902	0.323258	-0.58474	0.558726	0.797049	CDHR1	92211	cadherin related family member 1
ENSG0000	17.66993	-0.34241	0.585524	-0.58479	0.558687	0.797049	NA	NA	NA
ENSG0000	6.557051	0.504981	0.863584	0.584751	0.558715	0.797049	RPS10P26	1E+08	ribosomal protein S10 pseudogene 26
ENSG0000	29.80128	0.293678	0.502334	0.584628	0.558798	0.797049	SMIM24	284422	small integral membrane protein 24
ENSG0000	104.4901	0.148377	0.253955	0.584265	0.559042	0.797336	NA	NA	NA
ENSG0000	80.72983	0.163106	0.279362	0.583851	0.55932	0.797521	TMA16	55319	translation machinery associated 16 homolog
ENSG0000	16.64558	0.358161	0.613537	0.583764	0.559379	0.797521	TRAV23DV	28660	T cell receptor alpha variable 23/delta variable 6
ENSG0000	16.08955	-0.37338	0.639462	-0.5839	0.55929	0.797521	TMEM121	80757	transmembrane protein 121
ENSG0000	23.44049	-0.29638	0.507708	-0.58375	0.559387	0.797521	NA	NA	NA
ENSG0000	2.51768	1.173389	2.009959	0.583788	0.559363	0.797521	PNMA5	114824	PNMA family member 5
ENSG0000	5.06863	-0.6022	1.032411	-0.58329	0.559697	0.797794	NA	NA	NA
ENSG0000	77.37358	-0.17696	0.303389	-0.58328	0.559707	0.797794	TP53TG1	11257	TP53 target 1
ENSG0000	384.2676	0.084192	0.144341	0.583285	0.559702	0.797794	RITA1	84934	RBPJ interacting and tubulin associated 1
ENSG0000	5.542937	-0.65089	1.116275	-0.58309	0.559833	0.797911	TRAV5	28688	T cell receptor alpha variable 5
ENSG0000	22.77979	0.323551	0.555024	0.58295	0.559927	0.797984	NA	NA	NA
ENSG0000	12.98527	0.479397	0.822563	0.58281	0.560021	0.798057	NA	NA	NA
ENSG0000	1713.609	-0.04782	0.082091	-0.58252	0.560215	0.798149	NPEPPS	9520	aminopeptidase puromycin sensitive
ENSG0000	90.68997	-0.15804	0.27125	-0.58263	0.560145	0.798149	ASF1B	55723	anti-silencing function 1B histone chaperone
ENSG0000	1167.825	-0.05986	0.10275	-0.58254	0.560205	0.798149	ZNF146	7705	zinc finger protein 146
ENSG0000	18.5273	0.364957	0.626697	0.58235	0.560331	0.798254	NA	NA	NA
ENSG0000	35.69281	0.245258	0.421486	0.581889	0.560641	0.79841	C2orf92	728537	chromosome 2 open reading frame 92
ENSG0000	3749.971	-0.04291	0.073722	-0.58207	0.560522	0.79841	NUP214	8021	nucleoporin 214
ENSG0000	5.36382	-0.7855	1.349956	-0.58187	0.560656	0.79841	NA	NA	NA
ENSG0000	1631.189	-0.05096	0.08757	-0.58194	0.560608	0.79841	TSHZ1	10194	teashirt zinc finger homeobox 1
ENSG0000	83.43294	0.194146	0.333652	0.581882	0.560646	0.79841	SCARF2	91179	scavenger receptor class F member 2
ENSG0000	377.7287	-0.0787	0.135322	-0.58157	0.560855	0.798441	ACOT7	11332	acyl-CoA thioesterase 7
ENSG0000	707.3861	-0.07956	0.136809	-0.58152	0.560893	0.798441	INPP5B	3633	inositol polyphosphate-5-phosphatase B
ENSG0000	535.9222	0.092733	0.159418	0.581697	0.560771	0.798441	HBS1L	10767	HBS1 like translational GTPase
ENSG0000	55.04726	0.197426	0.339475	0.581563	0.560861	0.798441	CUX2	23316	cut like homeobox 2
ENSG0000	73.53848	0.163667	0.281449	0.581516	0.560893	0.798441	NA	NA	NA
ENSG0000	257.7005	0.101786	0.17509	0.581338	0.561013	0.79855	ENOPH1	58478	enolase-phosphatase 1
ENSG0000	163.5462	-0.13736	0.236407	-0.58103	0.561217	0.798779	VNN2	8875	vanin 2
ENSG0000	2.820705	-1.04809	1.805633	-0.58046	0.561608	0.799244	NA	NA	NA
ENSG0000	148.509	-0.12228	0.210673	-0.58042	0.561629	0.799244	POP5	51367	POP5 hom ribonuclease P/MRP subunit
ENSG0000	42.7696	-0.21993	0.379057	-0.58021	0.561776	0.799369	TNNI2	7136	troponin I: fast skeletal type
ENSG0000	6.372419	0.621391	1.07106	0.580164	0.561804	0.799369	NA	NA	NA
ENSG0000	117.4025	0.158353	0.273081	0.579875	0.561999	0.799463	SORCS2	57537	sortilin related VPS10 domain containing receptor 2
ENSG0000	3.754464	0.788387	1.359324	0.579985	0.561925	0.799463	NA	NA	NA
ENSG0000	169.7371	-0.12157	0.209638	-0.57991	0.561977	0.799463	STAMBPL1	57559	STAM binding protein like 1
ENSG0000	2592.111	-0.04606	0.07946	-0.57966	0.562146	0.79961	TMEM248	55069	transmembrane protein 248
ENSG0000	3750.242	-0.04471	0.077165	-0.57942	0.562307	0.799717	SMG5	23381	SMG5 nonsense mediated mRNA decay factor
ENSG0000	3612.653	0.049997	0.086281	0.579466	0.562275	0.799717	TOP1	7150	DNA topoisomerase I
ENSG0000	75.97217	-0.18892	0.32622	-0.57913	0.562502	0.799871	PPARGC1E	133522	PPARG coactivator 1 beta
ENSG0000	133.4417	0.134706	0.232582	0.579177	0.562469	0.799871	MED31	51003	mediator complex subunit 31
ENSG0000	1819.365	0.057478	0.099275	0.578971	0.562609	0.799962	UBE2J2	118424	ubiquitin conjugating enzyme E2 J2
ENSG0000	3.839211	-0.88696	1.532667	-0.57871	0.562787	0.800155	GPR17	2840	G protein-coupled receptor 17
ENSG0000	8.67213	0.577605	0.998834	0.578279	0.563076	0.800258	NA	NA	NA
ENSG0000	5.089455	-0.78956	1.364884	-0.57848	0.562938	0.800258	NA	NA	NA
ENSG0000	13.76946	0.428663	0.741205	0.578332	0.56304	0.800258	ST6GALNA	1.01E+08	ST6 N-acet 6-sialyltransferase 4 pseudogene 1
ENSG0000	9.381389	0.434265	0.750934	0.5783	0.563062	0.800258	NA	NA	NA
ENSG0000	4.96978	0.711616	1.230182	0.578464	0.562951	0.800258	PRKCG	5582	protein kinase C gamma
ENSG0000	639.6989	0.090161	0.155938	0.578184	0.56314	0.800287	ARMC8	25852	armadillo repeat containing 8
ENSG0000	18.4843	-0.39328	0.680865	-0.57762	0.563522	0.800522	FBXO36	130888	F-box protein 36
ENSG0000	5.96413	0.549716	0.951616	0.577666	0.563489	0.800522	NA	NA	NA
ENSG0000	55.69556	0.216704	0.375304	0.577411	0.563662	0.800522	SLC25A32	81034	solute carrier family 25 member 32
ENSG0000	359.388	0.089625	0.155148	0.577672	0.563485	0.800522	SLC41A2	84102	solute carrier family 41 member 2
ENSG0000	4.537168	0.715738	1.239652	0.57737	0.563689	0.800522	NA	NA	NA
ENSG0000	2806.267	0.043444	0.075237	0.577424	0.563653	0.800522	BRD7	29117	bromodomain containing 7
ENSG0000	8035.36	0.056616	0.098009	0.577666	0.56349	0.800522	EVI2B	2124	ecotropic viral integration site 2B
ENSG0000	551.0836	0.08531	0.147692	0.577618	0.563522	0.800522	CACNB1	782	calcium voltage-gated channel auxiliary subunit beta 1
ENSG0000	109.0532	-0.14088	0.244005	-0.57736	0.563693	0.800522	POLR1G	10849	RNA polymerase I subunit G
ENSG0000	123.9427	0.128584	0.222781	0.577175	0.563821	0.800643	NA	NA	NA
ENSG0000	9.532637	-0.49443	0.856853	-0.57703	0.563922	0.800666	GCNT2	2651	glucosaminyl (N-acetyl) transferase 2 (I blood group)
ENSG0000	8603.399	-0.03907	0.067702	-0.57702	0.563924	0.800666	GANAB	23193	glucosidase II alpha subunit
ENSG0000	32.75962	0.246595	0.427433	0.576922	0.563992	0.800702	EXTL2	2135	exostosin like glycosyltransferase 2
ENSG0000	3725.294	0.042943	0.074472	0.57663	0.564189	0.8009	HADHA	3030	hydroxyacyl-CoA dehydrogenase trifunctional multienzyme complex subunit alpha
ENSG0000	20.63659	-0.30852	0.535072	-0.57659	0.564219	0.8009	FDXR	2232	ferredoxin reductase
ENSG0000	37823.98	0.059205	0.102702	0.576477	0.564293	0.800944	RPL18A	6142	ribosomal protein L18a
ENSG0000	20.83451	0.308813	0.535828	0.576329	0.564393	0.800964	NA	NA	NA
ENSG0000	4.433315	0.835733	1.450039	0.576352	0.564377	0.800964	NA	NA	NA
ENSG0000	5.715065	-0.56203	0.975381	-0.57621	0.564472	0.801014	NA	NA	NA
ENSG0000	2.826675	0.96264	1.671875	0.575784	0.564761	0.801363	NA	NA	NA
ENSG0000	20.95363	-0.30331	0.526862	-0.57569	0.564821	0.801388	SYP	6855	synaptophysin
ENSG0000	558.1065	-0.08033	0.139571	-0.57553	0.564935	0.801487	BCAR3	8412	BCAR3 ada NSP family member
ENSG0000	23.87667	0.26749	0.464913	0.575354	0.565052	0.801592	ATP2A1	487	ATPase sarcoplasmic/endoplasmic reticulum Ca2+ transporting 1
ENSG0000	1264.041	0.063171	0.109814	0.575258	0.565117	0.801623	KIAA0040	9674	KIAA0040
ENSG0000	261.2482	0.088425	0.15376	0.575085	0.565234	0.801666	PIGV	55650	phosphatidylinositol glycan anchor biosynthesis class V
ENSG0000	2.395942	-0.95648	1.663028	-0.57515	0.565193	0.801666	FGF11	2256	fibroblast growth factor 11
ENSG0000	538.6831	0.067755	0.117847	0.574939	0.565332	0.801745	CIAPIN1	57019	cytokine induced apoptosis inhibitor 1
ENSG0000	430.9954	0.080151	0.139464	0.574709	0.565488	0.801905	NGDN	25983	neuroguidin
ENSG0000	409.481	0.092184	0.160502	0.574346	0.565734	0.802192	RPUSD4	84881	RNA pseudouridine synthase D4
ENSG0000	39.92737	0.221993	0.386612	0.574202	0.565831	0.802268	ZNF366	167465	zinc finger protein 366
ENSG0000	7973.741	-0.05135	0.089476	-0.57392	0.566024	0.80248	TRABD	80305	TraB domain containing
ENSG0000	10.91808	0.411728	0.717644	0.573721	0.566157	0.802607	RPS18P12	388339	ribosomal protein S18 pseudogene 12
ENSG0000	225.5948	-0.1026	0.178915	-0.57343	0.566354	0.80281	SNX4	8723	sorting nexin 4
ENSG0000	319.9451	-0.08818	0.153781	-0.57338	0.566386	0.80281	AAGAB	79719	alpha and gamma adaptin binding protein
ENSG0000	57.15363	0.192524	0.335883	0.573188	0.566518	0.802935	RPL36A	6173	ribosomal protein L36a
ENSG0000	5.356602	0.6653	1.161005	0.573038	0.566619	0.803017	GARNL3	84253	GTPase activating Rap/RanGAP domain like 3
ENSG0000	31.12542	0.271402	0.473756	0.572874	0.56673	0.803113	IGHV3-48	28424	immunoglobulin heavy variable 3-48
ENSG0000	3.45211	-0.78785	1.375861	-0.57263	0.566898	0.803222	KIAA1614	57710	KIAA1614
ENSG0000	99.31701	0.16458	0.287429	0.572593	0.56692	0.803222	EVC	2121	EvC ciliary complex subunit 1
ENSG0000	64.30857	-0.18016	0.31465	-0.57256	0.566941	0.803222	ZNF680	340252	zinc finger protein 680
ENSG0000	87.07292	-0.14861	0.259587	-0.5725	0.56698	0.803222	TEX30	93081	testis expressed 30
ENSG0000	86.62102	0.154037	0.269099	0.572419	0.567038	0.803243	SCLY	51540	selenocysteine lyase
ENSG0000	9404.037	-0.04295	0.075056	-0.57223	0.567168	0.803363	SETD1B	23067	SET domai histone lysine methyltransferase
ENSG0000	551.3502	-0.07963	0.139175	-0.57217	0.56721	0.803363	TXLNGY	246126	taxilin gam Y-linked
ENSG0000	387.1518	-0.08417	0.147281	-0.57151	0.567652	0.803928	ABRAXAS2	23172	abraxas 2 BRISC complex subunit
ENSG0000	3.648062	0.785085	1.3743	0.571262	0.567822	0.804108	NA	NA	NA
ENSG0000	1225.496	0.055064	0.096404	0.571185	0.567874	0.80412	NDUFB10	4716	NADH:ubiquinone oxidoreductase subunit B10
ENSG0000	9.11818	0.533291	0.933874	0.571052	0.567964	0.804125	MTARC1	64757	mitochondrial amidoxime reducing component 1

ENSG0000	46.54019	0.205644	0.360105	0.571067	0.567954	0.804125	NA	NA	NA
ENSG0000	243.627	-0.09015	0.15795	-0.57072	0.568188	0.804319	MRPL30	51263	mitochondrial ribosomal protein L30
ENSG0000	7.442646	-0.48501	0.849783	-0.57074	0.568175	0.804319	C22orf15	150248	chromosome 22 open reading frame 15
ENSG0000	8.179127	0.550629	0.964917	0.570649	0.568237	0.804327	SDK1	221935	sidekick cell adhesion molecule 1
ENSG0000	210.0646	0.118688	0.208027	0.57054	0.568311	0.804371	RCL1	10171	RNA terminal phosphate cyclase like 1
ENSG0000	44.71417	-0.19834	0.34777	-0.57033	0.568455	0.804442	LACTB2	51110	lactamase beta 2
ENSG0000	85.14	-0.19382	0.339875	-0.57027	0.568497	0.804442	SLC9A7P1	121456	solute carrier family 9 member 7 pseudogene 1
ENSG0000	3932.426	-0.04764	0.08355	-0.57025	0.568508	0.804442	CHST11	50515	carbohydrate sulfotransferase 11
ENSG0000	10.09523	0.421906	0.739914	0.57021	0.568536	0.804442	USP27X-DT	158572	USP27X divergent transcript
ENSG0000	13669.9	-0.04271	0.074916	-0.57014	0.568582	0.804446	AKAP13	11214	A-kinase anchoring protein 13
ENSG0000	725.0334	0.066634	0.11694	0.569812	0.568805	0.804653	SIVA1	10572	SIVA1 apoptosis inducing factor
ENSG0000	1078.603	0.059476	0.10438	0.569798	0.568815	0.804653	VPS18	57617	VPS18 core subunit of CORVET and HOPS complexes
ENSG0000	843.6978	-0.06097	0.107053	-0.56951	0.569007	0.804864	VIRMA	25962	vir like m6A methyltransferase associated
ENSG0000	338.3775	-0.0801	0.140675	-0.56936	0.56911	0.804947	SLC41A3	54946	solute carrier family 41 member 3
ENSG0000	7.798884	-0.57804	1.015519	-0.56921	0.569214	0.805033	MAG	4099	myelin associated glycoprotein
ENSG0000	35.67494	0.243197	0.427497	0.568885	0.569434	0.805258	P2RY12	64805	purinergic receptor P2Y12
ENSG0000	10.88102	0.384877	0.676591	0.568848	0.56946	0.805258	NA	NA	NA
ENSG0000	144.2832	0.133611	0.234984	0.568596	0.569631	0.805286	RPE	6120	ribulose-5-phosphate-3-epimerase
ENSG0000	1300.706	-0.05954	0.104707	-0.56862	0.569617	0.805286	ANO6	196527	anoctamin 6
ENSG0000	11.85574	-0.44442	0.781658	-0.56856	0.569653	0.805286	IGHV4-30-	28398	immunoglobulin heavy variable 4-30-2
ENSG0000	19.50928	0.346161	0.608766	0.568627	0.569609	0.805286	LOC10798	1.08E+08	putative uncharacterized protein LOC401522
ENSG0000	27.06555	0.308235	0.542319	0.568365	0.569787	0.8053	NFIA	4774	nuclear factor I A
ENSG0000	4.289709	-0.81813	1.439371	-0.5684	0.569766	0.8053	SCGB3A1	92304	secretoglobin family 3A member 1
ENSG0000	41.51512	0.217887	0.383363	0.568356	0.569793	0.8053	KIFC1	3833	kinesin family member C1
ENSG0000	7.509451	0.488772	0.860166	0.568229	0.569879	0.80536	NA	NA	NA
ENSG0000	259.4563	-0.10901	0.191864	-0.56814	0.56994	0.805384	CLOCK	9575	clock circadian regulator
ENSG0000	47.19405	-0.20114	0.354207	-0.56785	0.570136	0.805498	RNF207	388591	ring finger protein 207
ENSG0000	8.741113	-0.41965	0.739052	-0.56783	0.570152	0.805498	NA	NA	NA
ENSG0000	181.3917	0.105731	0.186175	0.567909	0.570096	0.805498	MALSU1	115416	mitochondrial assembly of ribosomal large subunit 1
ENSG0000	6.367681	0.59873	1.054537	0.567766	0.570194	0.805498	NA	NA	NA
ENSG0000	8.387296	-0.50618	0.892129	-0.56738	0.570457	0.805708	NA	NA	NA
ENSG0000	261.3614	-0.09876	0.174045	-0.56742	0.570428	0.805708	SLC39A10	57181	solute carrier family 39 member 10
ENSG0000	169.9951	-0.11286	0.19892	-0.56736	0.570473	0.805708	SDSL	113675	serine dehydratase like
ENSG0000	58.4487	-0.23565	0.415449	-0.56721	0.57057	0.805784	TRIM46	80128	tripartite motif containing 46
ENSG0000	11.72881	0.435767	0.768419	0.567095	0.57065	0.805784	KREMEN2	79412	kringle containing transmembrane protein 2
ENSG0000	288.5927	0.087357	0.154046	0.567085	0.570657	0.805784	ARMCX6	54470	armadillo repeat containing X-linked 6
ENSG0000	282.2848	0.095645	0.168774	0.566703	0.570916	0.805793	F11R	50848	F11 receptor
ENSG0000	3.893372	0.755747	1.332902	0.566993	0.570719	0.805793	NA	NA	NA
ENSG0000	435.4478	0.072319	0.127629	0.566637	0.570961	0.805793	ZNF830	91603	zinc finger protein 830
ENSG0000	514.8052	-0.07947	0.140218	-0.56675	0.570884	0.805793	ISYNA1	51477	inositol-3-phosphate synthase 1
ENSG0000	977.4013	-0.06209	0.109558	-0.56675	0.570881	0.805793	BCL2L13	23786	BCL2 like 13
ENSG0000	17.01533	-0.35915	0.633844	-0.56663	0.570967	0.805793	AIFM3	150209	apoptosis inducing factor mitochondria associated 3
ENSG0000	12865.53	0.041588	0.07338	0.566741	0.57089	0.805793	IRAK1	3654	interleukin 1 receptor associated kinase 1
ENSG0000	19.74749	0.382574	0.676116	0.565842	0.571501	0.806034	NA	NA	NA
ENSG0000	19.02461	0.299556	0.529386	0.565854	0.571493	0.806034	NA	NA	NA
ENSG0000	297.4027	0.096768	0.171067	0.565673	0.571616	0.806034	ZC3H8	84524	zinc finger CCCH-type containing 8
ENSG0000	28.3813	-0.25963	0.458911	-0.56575	0.571562	0.806034	LOC10028	1E+08	uncharacterized LOC100289230
ENSG0000	833.0478	-0.06049	0.10684	-0.56619	0.571265	0.806034	TBP	6908	TATA-box binding protein
ENSG0000	137.1067	-0.1343	0.23732	-0.56589	0.571471	0.806034	TRIM32	22954	tripartite motif containing 32
ENSG0000	12.9753	0.362993	0.641533	0.565822	0.571515	0.806034	EIF5AL1	143244	eukaryotic translation initiation factor 5A like 1
ENSG0000	12.94783	-0.35839	0.63304	-0.56614	0.571297	0.806034	RHEBL1	121268	RHEB like 1
ENSG0000	335.0669	0.086773	0.153338	0.565893	0.571466	0.806034	MAP3K9	4293	mitogen-activated protein kinase kinase kinase 9
ENSG0000	10.10261	-0.47936	0.847365	-0.5657	0.571595	0.806034	REXO5	81691	RNA exonuclease 5
ENSG0000	9.96796	0.437441	0.773298	0.565683	0.571609	0.806034	NA	NA	NA
ENSG0000	5.265709	0.763396	1.350157	0.565413	0.571793	0.806116	NA	NA	NA
ENSG0000	275.5886	-0.09931	0.175636	-0.56541	0.571792	0.806116	SERPINE2	5270	serpin family E member 2
ENSG0000	708.1412	-0.06428	0.113699	-0.56533	0.571848	0.806116	SLC20A2	6575	solute carrier family 20 member 2
ENSG0000	8.689343	-0.62583	1.107011	-0.56534	0.571844	0.806116	CRYM	1428	crystallin mu
ENSG0000	237.4215	-0.09436	0.167156	-0.56448	0.572429	0.806485	CLCC1	23155	chloride channel CLIC like 1
ENSG0000	284.9279	-0.09951	0.176248	-0.56458	0.572362	0.806485	ABHD5	51099	abhydrola: lysophosphatidic acid acyltransferase
ENSG0000	412.5508	0.078107	0.138279	0.56485	0.572176	0.806485	FCF1	51077	FCF1 rRNA-processing protein
ENSG0000	146.6465	-0.13265	0.235014	-0.56444	0.572457	0.806485	ANTKMT	65990	adenine nucleotide translocase lysine methyltransferase
ENSG0000	267.0913	0.084827	0.150215	0.564701	0.572277	0.806485	PIGL	9487	phosphatidylinositol glycan anchor biosynthesis class L
ENSG0000	23.78868	-0.33662	0.596042	-0.56477	0.572233	0.806485	NA	NA	NA
ENSG0000	106.1707	-0.14528	0.257393	-0.56444	0.572456	0.806485	NA	NA	NA
ENSG0000	30.58188	0.250184	0.443223	0.564466	0.572437	0.806485	LOC11226	1.12E+08	translation machinery-associated protein 7 -like
ENSG0000	1962.348	-0.0508	0.090016	-0.56434	0.572523	0.806516	PACSIN2	11252	protein kinase C and casein kinase substrate in neurons 2
ENSG0000	4257.85	-0.04155	0.073675	-0.56401	0.572744	0.80657	PPP1R10	5514	protein phosphatase 1 regulatory subunit 10
ENSG0000	292.738	0.10201	0.180852	0.564051	0.572719	0.80657	HSPA1B	3304	heat shock protein family A (Hsp70) member 1B
ENSG0000	497.0607	0.073613	0.130485	0.56415	0.572652	0.80657	UNC119B	84747	unc-119 lipid binding chaperone B
ENSG0000	2.94078	-0.97333	1.725865	-0.56396	0.572779	0.80657	NA	NA	NA
ENSG0000	92.819	-0.14087	0.24976	-0.56402	0.57274	0.80657	TMEM121	27439	transmembrane protein 121B
ENSG0000	2842.142	0.047899	0.084948	0.563859	0.57285	0.806608	BBLN	79095	bublin coiled coil protein
ENSG0000	7.440136	-0.68288	1.211624	-0.56361	0.573021	0.806727	TRPM6	140803	transient receptor potential cation channel subfamily M member 6
ENSG0000	4657.236	-0.05209	0.092408	-0.56367	0.572979	0.806727	ATP13A1	57130	ATPase 13A1
ENSG0000	946.8597	0.06337	0.112476	0.563404	0.57316	0.806861	TBCC	6903	tubulin folding cofactor C
ENSG0000	32.95127	0.261714	0.464932	0.562908	0.573497	0.807238	NA	NA	NA
ENSG0000	57.49722	0.180141	0.320033	0.562883	0.573514	0.807238	NA	NA	NA
ENSG0000	220.1189	0.10506	0.186671	0.562804	0.573568	0.807252	KATNBL1	79768	katanin regulatory subunit B1 like 1
ENSG0000	10.51432	0.523313	0.930161	0.562605	0.573704	0.807382	NTN1	9423	netrin 1
ENSG0000	24.13321	-0.29359	0.521932	-0.56252	0.573765	0.807407	SCNN1A	6337	sodium channel epithelial 1 subunit alpha
ENSG0000	426.744	-0.09451	0.168063	-0.56234	0.573883	0.807424	LRSAM1	90678	leucine rich repeat and sterile alpha motif containing 1
ENSG0000	2491.943	0.050566	0.089906	0.562433	0.573821	0.807424	ARPC3	10094	actin related protein 2/3 complex subunit 3
ENSG0000	100.2917	0.13629	0.242378	0.562306	0.573908	0.807424	CLC	1178	Charcot-Leyden crystal galectin
ENSG0000	88.15053	0.208814	0.371457	0.562148	0.574015	0.807514	NCR2	9436	natural cytotoxicity triggering receptor 2
ENSG0000	6.937621	-0.50617	0.900824	-0.5619	0.574183	0.807599	ITIH4	3700	inter-alpha-trypsin inhibitor heavy chain 4
ENSG0000	658.4378	0.068588	0.122052	0.561957	0.574145	0.807599	NOL11	25926	nucleolar protein 11
ENSG0000	309.2695	0.094495	0.168181	0.561868	0.574206	0.807599	C20orf27	54976	chromosome 20 open reading frame 27
ENSG0000	8.392269	0.606154	1.079177	0.561682	0.574333	0.807715	TBC1D29P	26083	TBC1 dom pseudogene
ENSG0000	11.29521	0.434764	0.774156	0.561597	0.574391	0.807736	ITGB1BP2	26548	integrin subunit beta 1 binding protein 2
ENSG0000	47.49115	-0.23177	0.412829	-0.56141	0.574516	0.80785	CEP44	80817	centrosomal protein 44
ENSG0000	38.55817	-0.22927	0.408455	-0.56132	0.574579	0.807878	COL5A1	1289	collagen type V alpha 1 chain
ENSG0000	674.9963	-0.08278	0.147531	-0.56109	0.574737	0.808038	CCDC186	55088	coiled-coil domain containing 186
ENSG0000	5.969084	-0.68525	1.221692	-0.5609	0.574863	0.808154	NA	NA	NA
ENSG0000	34.60121	-0.24621	0.439182	-0.5606	0.57507	0.808384	NA	NA	NA
ENSG0000	5.906167	-0.67355	1.202277	-0.56023	0.575325	0.808545	LINC0168	284648	long intergenic non-protein coding RNA 1686
ENSG0000	161.5239	0.112448	0.200759	0.560113	0.575402	0.808545	ATP5MG	10632	ATP synthase membrane subunit g
ENSG0000	3.434731	-0.96698	1.726244	-0.56016	0.575369	0.808545	NA	NA	NA
ENSG0000	12.77281	-0.4109	0.733279	-0.56037	0.57523	0.808545	NHIP	1.05E+08	neuronal h placenta associated
ENSG0000	621.3572	0.063247	0.112893	0.560241	0.575315	0.808545	SNX12	29934	sorting nexin 12

ENSG0000	361.6266	0.085994	0.153568	0.559977	0.575495	0.808615	RARS2	57038	arginyl-tRI	mitochondrial		
ENSG0000	3264.795	0.043496	0.077684	0.55991	0.575541	0.808617	TPR	7175	translocat	nuclear basket protein		
ENSG0000	3.128602	0.79654	1.423166	0.559696	0.575687	0.808761	RN7SL608I	1.06E+08	RNA	7SL	cytoplasm	pseudogene
ENSG0000	2314.991	0.045041	0.080501	0.559503	0.575818	0.808772	AGFG1	3267	ArfGAP with FG repeats 1			
ENSG0000	166.9752	-0.12886	0.230341	-0.55945	0.575854	0.808772	AP1AR	55435	adaptor related protein complex 1 associated regulatory protein			
ENSG0000	17.88031	-0.39705	0.709774	-0.5594	0.575886	0.808772	PDZD7	79955	PDZ domain containing 7			
ENSG0000	402.2278	-0.10598	0.189467	-0.55936	0.575913	0.808772	SNX33	257364	sorting nexin 33			
ENSG0000	42.41865	-0.20352	0.363805	-0.55942	0.575878	0.808772	PLSCR3	57048	phospholipid scramblase 3			
ENSG0000	19.54592	-0.32647	0.583848	-0.55918	0.576042	0.808831	NA	NA	NA			
ENSG0000	4049.765	-0.04892	0.087477	-0.55918	0.576037	0.808831	AKAP8L	26993	A-kinase anchoring protein 8 like			
ENSG0000	610.6579	0.06917	0.12373	0.559044	0.576132	0.808896	HYCC2	285172	hyccin PI4KA lipid kinase complex subunit 2			
ENSG0000	4.238426	0.79805	1.428042	0.558842	0.576269	0.809028	NA	NA	NA			
ENSG0000	14.20789	0.408059	0.730627	0.558504	0.5765	0.809229	SNORA33	594839	small nucl	H/ACA box 33		
ENSG0000	18.74547	0.344283	0.616392	0.558545	0.576472	0.809229	GLB1L3	112937	galactosidase beta 1 like 3			
ENSG0000	540.9051	0.064998	0.116393	0.558431	0.57655	0.809239	C1QBP	708	complement C1q binding protein			
ENSG0000	6998.809	0.045838	0.082153	0.557961	0.576871	0.809628	PPP1R12C	54776	protein phosphatase 1 regulatory subunit 12C			
ENSG0000	189.4631	-0.11797	0.211488	-0.5578	0.576983	0.809723	LINC0203E	1E+08	long intergenic non-protein coding RNA 2035			
ENSG0000	7.728736	-0.46548	0.834656	-0.55769	0.577053	0.809761	NA	NA	NA			
ENSG0000	328.0907	-0.08737	0.156827	-0.55713	0.577436	0.809793	NA	NA	NA			
ENSG0000	18.68459	-0.43569	0.781867	-0.55725	0.577359	0.809793	NA	NA	NA			
ENSG0000	3.095575	0.847749	1.520936	0.557386	0.577264	0.809793	CAPN11	11131	calpain 11			
ENSG0000	6.589543	-0.60981	1.09381	-0.55751	0.57718	0.809793	NA	NA	NA			
ENSG0000	3.622185	0.703777	1.263464	0.557022	0.577513	0.809793	NA	NA	NA			
ENSG0000	3.525604	0.707453	1.269634	0.55721	0.577384	0.809793	NA	NA	NA			
ENSG0000	496.5794	-0.06971	0.125114	-0.55718	0.577406	0.809793	UTP6	55813	UTP6 small subunit processome component			
ENSG0000	948.6712	0.054296	0.097466	0.557079	0.577474	0.809793	SPOP	8405	speckle type BTB/POZ protein			
ENSG0000	159.5819	-0.10817	0.194075	-0.55734	0.577297	0.809793	LMTK3	114783	lemur tyrosine kinase 3			
ENSG0000	5.970166	0.498548	0.894894	0.557102	0.577458	0.809793	MYBPC2	4606	myosin binding protein C2			
ENSG0000	105.5302	-0.15656	0.281119	-0.55692	0.577581	0.809827	DTX4	23220	deltex E3 ubiquitin ligase 4			
ENSG0000	4.27659	0.832921	1.495824	0.556831	0.577643	0.809853	ATP1A4	480	ATPase Na+/K+ transporting subunit alpha 4			
ENSG0000	10.58514	0.422291	0.75866	0.556627	0.577782	0.809868	TRBV11-3	28580	T cell receptor beta variable 11-3			
ENSG0000	619.4771	0.061571	0.110607	0.556664	0.577757	0.809868	CERS5	91012	ceramide synthase 5			
ENSG0000	82.22336	-0.15719	0.282403	-0.55662	0.577784	0.809868	HSD3B7	80270	hydroxy-d	3 beta- and steroid delta-isomerase 7		
ENSG0000	5.493381	0.518547	0.932648	0.555994	0.578215	0.81015	NA	NA	NA			
ENSG0000	670.9755	-0.07002	0.125944	-0.55596	0.578235	0.81015	TMEM167	153339	transmembrane protein 167A			
ENSG0000	20.03273	-0.30335	0.545592	-0.55601	0.578207	0.81015	SCIN	85477	scinderin			
ENSG0000	44.37947	-0.19698	0.354319	-0.55595	0.578248	0.81015	HSPA12A	259217	heat shock protein family A (Hsp70) member 12A			
ENSG0000	3.527906	0.798845	1.436839	0.555974	0.578229	0.81015	NA	NA	NA			
ENSG0000	448.7971	-0.07063	0.127047	-0.55595	0.578248	0.81015	ABHD13	84945	abhydrolase domain containing 13			
ENSG0000	960.2884	0.062769	0.112949	0.555734	0.578393	0.810292	PIK3CA	5290	phosphati	5-bisphosphate 3-kinase catalytic subunit alpha		
ENSG0000	15.63958	0.322374	0.5805	0.555339	0.578663	0.810609	H2AC8	3012	H2A clustered histone 8			
ENSG0000	1859.753	-0.0476	0.085737	-0.55521	0.578754	0.810613	SKIC2	6499	SKI2 subunit of superkiller complex			
ENSG0000	145.417	0.12271	0.220994	0.555265	0.578713	0.810613	NA	NA	NA			
ENSG0000	341.0856	0.08201	0.147807	0.554845	0.579001	0.810837	PTBP2	58155	polypyrimidine tract binding protein 2			
ENSG0000	279.8594	-0.09271	0.167084	-0.55485	0.579	0.810837	RNF14	9604	ring finger protein 14			
ENSG0000	43.68435	-0.21731	0.391785	-0.55468	0.579116	0.810938	NA	NA	NA			
ENSG0000	65.77885	-0.1619	0.292014	-0.55441	0.579299	0.811014	H2BC19P	337874	H2B cluste	pseudogene		
ENSG0000	133.274	0.128119	0.231089	0.554416	0.579294	0.811014	ETNK2	55224	ethanolamine kinase 2			
ENSG0000	19.85248	-0.28428	0.512764	-0.5544	0.579302	0.811014	ODAD1	93233	outer dynein arm docking complex subunit 1			
ENSG0000	3.197846	-0.81692	1.473714	-0.55433	0.579353	0.811025	RPL10P15	1E+08	ribosomal protein L10 pseudogene 15			
ENSG0000	356.6045	0.08263	0.149082	0.554257	0.579403	0.811033	UTP18	51096	UTP18 small subunit processome component			
ENSG0000	20.23204	-0.3084	0.556655	-0.55403	0.579559	0.811189	NA	NA	NA			
ENSG0000	59.16337	-0.18443	0.332972	-0.55389	0.579654	0.811262	C15orf62	643338	chromosome 15 open reading frame 62			
ENSG0000	27.11067	-0.2504	0.452197	-0.55373	0.579763	0.811353	PAXBP1-A!	1.01E+08	PAXBP1 antisense RNA 1			
ENSG0000	442.1863	0.08083	0.146019	0.553559	0.579881	0.811457	RALGDS	5900	ral guanine nucleotide dissociation stimulator			
ENSG0000	605.2334	-0.08193	0.148106	-0.55319	0.58013	0.811745	INO80D	54891	INO80 complex subunit D			
ENSG0000	3.60416	0.779544	1.409895	0.552909	0.580326	0.811745	FLNC	2318	filamin C			
ENSG0000	59.40361	0.189033	0.341881	0.552922	0.580317	0.811745	DCLRE1A	9937	DNA cross-link repair 1A			
ENSG0000	1099.814	-0.05843	0.10564	-0.55312	0.58018	0.811745	KAT2A	2648	lysine acetyltransferase 2A			
ENSG0000	9.167119	0.438092	0.792389	0.552875	0.580349	0.811745	LOC10537	1.05E+08	uncharacterized LOC105371967			
ENSG0000	425.6051	-0.07425	0.13427	-0.55298	0.58028	0.811745	ZNF431	170959	zinc finger protein 431			
ENSG0000	5.701673	-1.39728	2.527867	-0.55275	0.580435	0.811803	NA	NA	NA			
ENSG0000	62.43636	-0.1746	0.316083	-0.55239	0.580682	0.812084	ZNF678	339500	zinc finger protein 678			
ENSG0000	44.01009	-0.21879	0.396122	-0.55233	0.580723	0.812084	TRPV1	7442	transient receptor potential cation channel subfamily V member 1			
ENSG0000	580.0759	0.068127	0.123377	0.552182	0.580824	0.812163	ILKAP	80895	ILK associated serine/threonine phosphatase			
ENSG0000	233.4008	-0.10693	0.193693	-0.55204	0.580921	0.812177	SGK3	23678	serum/glucocorticoid regulated kinase family member 3			
ENSG0000	5824.128	0.048086	0.087101	0.552068	0.580902	0.812177	ELK3	2004	ETS transcription factor ELK3			
ENSG0000	5.761369	0.626132	1.134567	0.551869	0.581038	0.81228	LINC0049!	1.01E+08	long intergenic non-protein coding RNA 499			
ENSG0000	445.4219	-0.0764	0.138464	-0.55178	0.581097	0.8123	KDM4A	9682	lysine demethylase 4A			
ENSG0000	423.456	-0.08012	0.145215	-0.55172	0.581141	0.812301	R3HCC1	203069	R3H domain and coiled-coil containing 1			
ENSG0000	59.72605	-0.17337	0.314487	-0.55128	0.58144	0.812658	AFMID	125061	arylformamidase			
ENSG0000	4.981702	0.64966	1.17867	0.551181	0.58151	0.812694	TAX1BP3	30851	Tax1 binding protein 3			
ENSG0000	38.33462	0.219636	0.398849	0.550676	0.581856	0.812872	NA	NA	NA			
ENSG0000	10.65678	-0.44066	0.800025	-0.5508	0.58177	0.812872	TNNC1	7134	troponin C	slow skeletal and cardiac type		
ENSG0000	10.03343	0.418012	0.759033	0.550717	0.581828	0.812872	CCDC15	80071	coiled-coil domain containing 15			
ENSG0000	5.214117	-0.68403	1.242022	-0.55074	0.581815	0.812872	NFE2L1-DT	1.16E+08	NFE2L1 divergent transcript			
ENSG0000	14.27081	-0.33378	0.605968	-0.55082	0.581758	0.812872	NA	NA	NA			
ENSG0000	6.869532	-0.56417	1.024851	-0.55049	0.581984	0.812989	NA	NA	NA			
ENSG0000	10.16919	-0.40966	0.744564	-0.5502	0.58218	0.813018	COQ8A	56997	coenzyme Q8A			
ENSG0000	97.2203	-0.15055	0.273563	-0.55033	0.582095	0.813018	SLC9A9	285195	solute carrier family 9 member A9			
ENSG0000	256.957	-0.09622	0.174827	-0.55038	0.582056	0.813018	PFAS	5198	phosphoribosylformylglycinamidine synthase			
ENSG0000	532.4992	0.071087	0.129187	0.550266	0.582137	0.813018	PRICKLE3	4007	prickle planar cell polarity protein 3			
ENSG0000	7.233136	0.472871	0.859811	0.549971	0.582339	0.813179	NA	NA	NA			
ENSG0000	153.898	0.137186	0.249482	0.549885	0.582398	0.8132	NA	NA	NA			
ENSG0000	74.66417	0.15606	0.283846	0.549805	0.582453	0.813214	MMP7	4316	matrix metallopeptidase 7			
ENSG0000	18.54759	0.371502	0.675775	0.549743	0.582496	0.813214	LOC1053	1.05E+08	uncharacterized LOC105370941			
ENSG0000	161.9175	0.106154	0.193124	0.549666	0.582548	0.813226	TEF	7008	TEF transc	PAR bZIP family member		
ENSG0000	472.3445	-0.07337	0.133528	-0.54945	0.582694	0.813369	TSPAN17	26262	tetraspanin 17			
ENSG0000	6.027279	0.571475	1.040613	0.549171	0.582888	0.81354	MIR4292	1E+08	microRNA 4292			
ENSG0000	2.365724	-0.90223	1.64296	-0.54915	0.582904	0.81354	SHBG	6462	sex hormone binding globulin			
ENSG0000	100.4646	-0.13734	0.250151	-0.54901	0.582995	0.813606	SLC10A7	84068	solute carrier family 10 member 7			
ENSG0000	72.99152	-0.15193	0.276834	-0.5488	0.58314	0.813684	TMEM242	729515	transmembrane protein 242			
ENSG0000	20.19167	0.296926	0.541015	0.54883	0.583122	0.813684	NA	NA	NA			
ENSG0000	88.52179	-0.16507	0.300946	-0.54852	0.583335	0.813896	DPY19L2P2	349152	DPY19L2 pseudogene 2			
ENSG0000	554.1038	0.067805	0.123658	0.548324	0.583469	0.814022	TAF8	129685	TATA-box binding protein associated factor 8			
ENSG0000	393.6121	0.083079	0.151569	0.548128	0.583604	0.814149	RASSF4	83937	Ras association domain family member 4			
ENSG0000	7.31519	0.563984	1.029233	0.547965	0.583716	0.814243	NA	NA	NA			
ENSG0000	829.7519	0.066396	0.121203	0.547808	0.583823	0.814271	SEL1L3	23231	SEL1L family member 3			
ENSG0000	435.8303	-0.18451	0.336796	-0.54784	0.583804	0.814271	ERI1	90459	exoribonuclease 1			
ENSG0000	4.447711	-0.65001	1.186903	-0.54766	0.583929	0.814356	SORBS2	8470	sorbin and SH3 domain containing 2			

ENSG0000	681.6141	-0.06285	0.114821	-0.54734	0.584144	0.814595	MPG		4350	N-methylpurine DNA glycosylase
ENSG0000	9.528764	-0.39726	0.726026	-0.54717	0.58426	0.814696	RPS9P4		388556	ribosomal protein S9 pseudogene 4
ENSG0000	81.16289	-0.14755	0.269703	-0.5471	0.584308	0.814702	MAP3K21		84451	mitogen-activated protein kinase kinase kinase 21
ENSG0000	219.7545	0.110038	0.20117	0.546992	0.584384	0.814728	ABRACL		58527	ABRA C-terminal like
ENSG0000	15.73175	-0.35412	0.64745	-0.54695	0.584415	0.814728	NRTN		4902	neurturin
ENSG0000	8.597907	-0.60049	1.09808	-0.54685	0.584482	0.81476	NA	NA		NA
ENSG0000	3.958658	0.982426	1.797242	0.54663	0.584633	0.81491	CTAGE8		1E+08	CTAGE family member 8
ENSG0000	281.3864	-0.08672	0.158669	-0.54655	0.584687	0.814924	MRPS6		64968	mitochondrial ribosomal protein S6
ENSG0000	471.6482	0.074104	0.135707	0.546061	0.585024	0.815332	DENND6A		201627	DENN domain containing 6A
ENSG0000	132.2329	0.115841	0.212189	0.545931	0.585113	0.815334	SCLT1		132320	sodium channel and clathrin linker 1
ENSG0000	8.433397	0.432136	0.791499	0.545972	0.585085	0.815334	NA	NA		NA
ENSG0000	451.9138	-0.0883	0.161947	-0.54521	0.585608	0.815631	ROCK2		9475	Rho associated coiled-coil containing protein kinase 2
ENSG0000	152.0598	-0.10907	0.199997	-0.54538	0.585492	0.815631	GTF2E1		2960	general transcription factor IIE subunit 1
ENSG0000	13.67134	-0.42608	0.781966	-0.54488	0.585838	0.815631	NA	NA		NA
ENSG0000	30.15605	-0.22833	0.418549	-0.54552	0.585398	0.815631	NA	NA		NA
ENSG0000	76.46031	-0.15666	0.287421	-0.54506	0.58571	0.815631	KIAA1586		57691	KIAA1586
ENSG0000	1124.771	0.055782	0.102388	0.544812	0.585883	0.815631	TMEM30A		55754	transmembrane protein 30A
ENSG0000	23.25677	-0.30308	0.556381	-0.54473	0.585942	0.815631	ARC		23237	activity regulated cytoskeleton associated protein
ENSG0000	3.446521	-0.70999	1.303336	-0.54475	0.585927	0.815631	NA	NA		NA
ENSG0000	109.2864	-0.12623	0.231724	-0.54473	0.585938	0.815631	C11orf71		54494	chromosome 11 open reading frame 71
ENSG0000	32.68178	-0.21831	0.400729	-0.54478	0.585904	0.815631	RPS6KL1		83694	ribosomal protein S6 kinase like 1
ENSG0000	24.05015	0.282827	0.519064	0.544878	0.585837	0.815631	NA	NA		NA
ENSG0000	9.0008	0.437489	0.802919	0.544873	0.585841	0.815631	NA	NA		NA
ENSG0000	132.2595	-0.1245	0.228384	-0.54513	0.585664	0.815631	MCAT		27349	malonyl-CoA-acyl carrier protein transacylase
ENSG0000	5.580173	-0.60375	1.106971	-0.54541	0.585475	0.815631	CENPI		2491	centromere protein I
ENSG0000	211.1034	0.097576	0.179198	0.544517	0.586086	0.81577	SPTSSA		171546	serine palmitoyltransferase small subunit A
ENSG0000	842.1454	-0.06439	0.118305	-0.54424	0.586278	0.815942	GPN2		54707	GPN-loop GTPase 2
ENSG0000	3.175079	0.725284	1.333043	0.544081	0.586386	0.815942	CACNB4		785	calcium voltage-gated channel auxiliary subunit beta 4
ENSG0000	2491.257	0.044802	0.082341	0.5441	0.586373	0.815942	STT3B		201595	STT3 oligosaccharyltransferase complex catalytic subunit B
ENSG0000	169.6315	0.114235	0.209914	0.544202	0.586303	0.815942	SKIC8		80349	SKI8 subunit of superkiller complex
ENSG0000	595.7516	0.064093	0.117826	0.543961	0.586468	0.815992	SQLE		6713	squalene epoxidase
ENSG0000	16.68567	0.363604	0.668589	0.543838	0.586553	0.815992	NA	NA		NA
ENSG0000	7.007251	0.525764	0.966737	0.543854	0.586542	0.815992	NA	NA		NA
ENSG0000	513.8677	0.07173	0.131939	0.543658	0.586677	0.816095	PLAA		9373	phospholipase A2 activating protein
ENSG0000	115.4959	0.128065	0.235586	0.543602	0.586715	0.816095	TCAP		8557	titin-cap
ENSG0000	1312.743	0.050159	0.092306	0.543394	0.586858	0.816222	AURKAIP1		54998	aurora kinase A interacting protein 1
ENSG0000	177.0888	0.111954	0.206047	0.543342	0.586894	0.816222	NA	NA		NA
ENSG0000	30.70418	0.234794	0.432613	0.542735	0.587312	0.816496	HGD		3081	homogent 2-dioxygenase
ENSG0000	2.678903	1.01711	1.873475	0.5429	0.587198	0.816496	NA	NA		NA
ENSG0000	5.697302	-0.68534	1.262536	-0.54283	0.587248	0.816496	NA	NA		NA
ENSG0000	10.71258	0.469164	0.864306	0.542821	0.587253	0.816496	NA	NA		NA
ENSG0000	18.14805	-0.32376	0.596497	-0.54277	0.587288	0.816496	CLCN4		1183	chloride voltage-gated channel 4
ENSG0000	7.273153	-0.52599	0.969278	-0.54266	0.587365	0.816509	SDC1		6382	syndecan 1
ENSG0000	7166.391	0.039736	0.073242	0.542539	0.587447	0.816562	CLPTM1		1209	CLPTM1 regulator of GABA type A receptor forward trafficking
ENSG0000	44.83543	0.22883	0.421917	0.542358	0.587572	0.816613	NA	NA		NA
ENSG0000	1191.528	0.053973	0.099509	0.542394	0.587547	0.816613	PIH1D1		55011	PIH1 domain containing 1
ENSG0000	9.449736	-0.50497	0.931841	-0.54191	0.587883	0.816984	PKP2		5318	plakophilin 2
ENSG0000	24.88664	-0.2621	0.483743	-0.54181	0.587949	0.817014	LOC12490		1.25E+08	uncharacterized LOC124903386
ENSG0000	3520.304	-0.05108	0.094292	-0.54169	0.588033	0.81707	TUBGCP6		85378	tubulin gamma complex associated protein 6
ENSG0000	2292.169	0.049009	0.0905	0.541541	0.588135	0.817089	SARS1		6301	seryl-tRNA synthetase 1
ENSG0000	112.9568	0.128828	0.23787	0.541591	0.5881	0.817089	CALCRL		10203	calcitonin receptor like receptor
ENSG0000	3.777694	-0.77002	1.422121	-0.54146	0.588191	0.817106	NA	NA		NA
ENSG0000	482.0404	0.067685	0.125068	0.541187	0.588379	0.817209	FRG1		2483	FSHD region gene 1
ENSG0000	161.7962	0.111025	0.205161	0.541159	0.588398	0.817209	YJEFN3		374887	YjeF N-terminal domain containing 3
ENSG0000	1622.568	0.049934	0.092262	0.541221	0.588355	0.817209	DYRK1B		9149	dual specificity tyrosine phosphorylation regulated kinase 1B
ENSG0000	671.8457	-0.06462	0.119443	-0.54101	0.588502	0.817222	TMEM150		129303	transmembrane protein 150A
ENSG0000	899.2005	-0.05509	0.101862	-0.54083	0.588627	0.817222	PPP3CA		5530	protein phosphatase 3 catalytic subunit alpha
ENSG0000	381.7191	0.087763	0.162263	0.540867	0.588599	0.817222	LINC0084		729678	long intergenic non-protein coding RNA 847
ENSG0000	4.401648	0.744074	1.375749	0.54085	0.588611	0.817222	NDUFA4L2		56901	NDUFA4 mitochondrial complex associated like 2
ENSG0000	33.93896	-0.28197	0.521308	-0.54089	0.588584	0.817222	GPR34		2857	G protein-coupled receptor 34
ENSG0000	515.0796	0.071341	0.132001	0.540459	0.58888	0.817384	HS1BP3		64342	HCLS1 binding protein 3
ENSG0000	350.5635	-0.07815	0.144611	-0.54044	0.588897	0.817384	SLC25A26		115286	solute carrier family 25 member 26
ENSG0000	66.40856	0.163814	0.303095	0.540472	0.588871	0.817384	NA	NA		NA
ENSG0000	62.03418	-0.18516	0.342629	-0.5404	0.58892	0.817384	FOXM1		2305	forkhead box M1
ENSG0000	8.634121	0.499307	0.924186	0.540267	0.589013	0.817451	NA	NA		NA
ENSG0000	48.42134	-0.19683	0.364572	-0.53991	0.589262	0.817552	CD1C		911	CD1c molecule
ENSG0000	865.0833	-0.05644	0.104528	-0.53992	0.589252	0.817552	ZBTB11		27107	zinc finger and BTB domain containing 11
ENSG0000	24.67436	-0.24876	0.460687	-0.53998	0.589211	0.817552	FGFBP3		143282	fibroblast growth factor binding protein 3
ENSG0000	21.0216	-0.31186	0.577562	-0.53996	0.589226	0.817552	EEF1AKMT		399818	EEF1A lysine methyltransferase 2
ENSG0000	278.8707	-0.09083	0.168251	-0.53983	0.589317	0.817567	PDE6D		5147	phosphodiesterase 6D
ENSG0000	3013.842	0.045093	0.083569	0.539591	0.589479	0.8177	C6orf62		81688	chromosome 6 open reading frame 62
ENSG0000	15.0521	-0.36944	0.684703	-0.53956	0.5895	0.8177	HAUS7		55559	HAUS augmin like complex subunit 7
ENSG0000	551.5399	0.065453	0.121329	0.539464	0.589567	0.81773	SENP2		59343	SUMO specific peptidase 2
ENSG0000	6.914805	-0.53477	0.991416	-0.5394	0.589611	0.817731	HUS1B		135458	HUS1 checkpoint clamp component B
ENSG0000	20.5969	-0.29561	0.548248	-0.53918	0.589762	0.817817	NYAP1		222950	neuronal tyrosine phosphorylated phosphoinositide-3-kinase adaptor 1
ENSG0000	11.24195	0.372092	0.690058	0.539218	0.589736	0.817817	NA	NA		NA
ENSG0000	303.8211	0.086595	0.160639	0.539067	0.589841	0.817866	ZDHHC4		55146	zinc finger DHHC-type palmitoyltransferase 4
ENSG0000	13.75032	-0.39775	0.738443	-0.53863	0.590144	0.81799	LINC0140		1.05E+08	long intergenic non-protein coding RNA 1409
ENSG0000	108.7582	-0.15447	0.286822	-0.53856	0.590194	0.81799	NA	NA		NA
ENSG0000	341.7742	0.074479	0.138213	0.538871	0.589976	0.81799	TCP11L1		55346	t-complex 11 like 1
ENSG0000	3.415803	-0.8087	1.501123	-0.53873	0.590075	0.81799	NA	NA		NA
ENSG0000	244.8674	-0.10024	0.186056	-0.53876	0.590053	0.81799	COG8		84342	component of oligomeric golgi complex 8
ENSG0000	214.5684	0.097945	0.181866	0.538554	0.590195	0.81799	STX16-NPI		1.01E+08	STX16-NPEPL1 readthrough (NMD candidate)
ENSG0000	216.5748	0.096983	0.180133	0.538399	0.590302	0.818025	ZNF593		51042	zinc finger protein 593
ENSG0000	6.89338	0.68874	1.279259	0.53839	0.590308	0.818025	MFSD6L		162387	major facilitator superfamily domain containing 6 like
ENSG0000	25.02628	0.26395	0.490414	0.538218	0.590426	0.818066	NDUFA13		51079	NADH:ubiquinone oxidoreductase subunit A13
ENSG0000	532.0886	-0.06901	0.128197	-0.53828	0.590383	0.818066	KMT5C		84787	lysine methyltransferase 5C
ENSG0000	8.478428	0.52555	0.976929	0.537961	0.590604	0.818096	PTPRU		10076	protein tyrosine phosphatase receptor type U
ENSG0000	108.8039	0.140126	0.260477	0.537962	0.590603	0.818096	GGPS1		9453	geranylgeranyl diphosphate synthase 1
ENSG0000	6.093997	0.607469	1.129024	0.538048	0.590544	0.818096	LOC10537		1.05E+08	uncharacterized LOC105377663
ENSG0000	15.67273	0.369226	0.686676	0.537701	0.590783	0.818096	PIP5KL1		138429	phosphatidylinositol-4-phosphate 5-kinase like 1
ENSG0000	1351.315	-0.06898	0.128255	-0.53782	0.590704	0.818096	BANF1		8815	BAF nuclear assembly factor 1
ENSG0000	351.6234	-0.07829	0.145598	-0.53768	0.590796	0.818096	PUS1		80324	pseudouridine synthase 1
ENSG0000	210.4471	0.102193	0.190002	0.537852	0.59068	0.818096	RTN2		6253	reticulon 2
ENSG0000	3.924579	-0.89023	1.655703	-0.53768	0.590801	0.818096	NA	NA		NA
ENSG0000	626.7977	-0.07422	0.138077	-0.53756	0.590883	0.818149	PCMTD1		115294	protein-L-isoaspartate (D-aspartate) O-methyltransferase domain containing 1
ENSG0000	3.898039	-0.78211	1.455719	-0.53727	0.591081	0.818362	NA	NA		NA
ENSG0000	10.11049	-0.45498	0.847277	-0.53699	0.591275	0.81857	BCO2		83875	beta-carotene oxygenase 2
ENSG0000	18.57777	0.341851	0.636734	0.536882	0.591349	0.818611	NA	NA		NA
ENSG0000	16.17993	-0.36619	0.682858	-0.53626	0.59178	0.818793	SPATA24		202051	spermatogenesis associated 24

ENSG0000	3.462081	-0.63702	1.187813	-0.5363	0.591752	0.818793	CSNK2A3	283106	casein kinase 2 alpha 3
ENSG0000	248.3502	0.097898	0.182497	0.536436	0.591657	0.818793	NA	NA	NA
ENSG0000	539.3585	0.077453	0.1444	0.536379	0.591697	0.818793	SPTLC2	9517	serine palmitoyltransferase long chain base subunit 2
ENSG0000	4.610274	0.776364	1.447818	0.53623	0.5918	0.818793	NA	NA	NA
ENSG0000	3.407116	-0.92377	1.72262	-0.53626	0.591779	0.818793	NA	NA	NA
ENSG0000	90.72455	-0.15759	0.293702	-0.53657	0.591565	0.818793	TXNDC17	84817	thioredoxin domain containing 17
ENSG0000	11.2773	-0.41638	0.776562	-0.53618	0.591833	0.818793	RGL3	57139	ral guanine nucleotide dissociation stimulator like 3
ENSG0000	4.484186	-0.64489	1.204341	-0.53547	0.592326	0.819353	NA	NA	NA
ENSG0000	470.3897	0.070309	0.13129	0.535521	0.592289	0.819353	MMADHC	27249	metabolism of cobalamin associated D
ENSG0000	3.90501	-0.70312	1.313417	-0.53534	0.592418	0.819357	MIR762	1E+08	microRNA 762
ENSG0000	25.02119	0.267446	0.499548	0.535375	0.59239	0.819357	LGALS4	3960	galectin 4
ENSG0000	4.457217	0.633932	1.184901	0.535009	0.592644	0.819547	NA	NA	NA
ENSG0000	6.193262	0.565031	1.056069	0.535032	0.592627	0.819547	NA	NA	NA
ENSG0000	5.654517	-0.66999	1.252961	-0.53473	0.592838	0.819755	ZKSCAN8P	651302	ZKSCAN8 pseudogene 1
ENSG0000	4.228371	-0.6637	1.242315	-0.53425	0.593171	0.819909	LINC0279ε	1.08E+08	long intergenic non-protein coding RNA 2798
ENSG0000	20.15826	-0.30327	0.567635	-0.53427	0.593153	0.819909	NA	NA	NA
ENSG0000	52.61678	0.186044	0.34816	0.534363	0.59309	0.819909	CFAP44	55779	cilia and flagella associated protein 44
ENSG0000	241.8025	0.102789	0.19232	0.534468	0.593018	0.819909	MRPL47	57129	mitochondrial ribosomal protein L47
ENSG0000	396.2979	0.075403	0.141134	0.534262	0.59316	0.819909	DCLRE1C	64421	DNA cross-link repair 1C
ENSG0000	8.326112	0.571543	1.07011	0.534097	0.593274	0.819991	IGHV3-72	28410	immunoglobulin heavy variable 3-72
ENSG0000	8.466202	0.55751	1.044072	0.533976	0.593358	0.820046	PACSIN3	29763	protein kinase C and casein kinase substrate in neurons 3
ENSG0000	6813.239	-0.04097	0.076749	-0.53385	0.593447	0.820047	CAPN2	824	calpain 2
ENSG0000	3.813926	0.702773	1.31639	0.533864	0.593436	0.820047	NA	NA	NA
ENSG0000	17.31345	-0.30189	0.565753	-0.5336	0.593617	0.820052	LOC12490	1.25E+08	uncharacterized LOC124907726
ENSG0000	39.5256	-0.20383	0.382033	-0.53354	0.593659	0.820052	MEIS1	4211	Meis homeobox 1
ENSG0000	124.4919	-0.12136	0.227449	-0.53359	0.593624	0.820052	STRADB	55437	STE20 related adaptor beta
ENSG0000	6.600591	-0.49063	0.919667	-0.53349	0.593697	0.820052	NA	NA	NA
ENSG0000	4.992472	0.587402	1.100695	0.533665	0.593573	0.820052	H2BC17	8348	H2B clustered histone 17
ENSG0000	520.8761	0.075806	0.142102	0.533458	0.593717	0.820052	TCF4	6925	transcription factor 4
ENSG0000	11.27271	0.357002	0.669397	0.533318	0.593814	0.820125	NA	NA	NA
ENSG0000	203.2945	-0.0922	0.172955	-0.53308	0.593976	0.820288	MBOAT1	154141	membrane bound O-acyltransferase domain containing 1
ENSG0000	435.1716	0.073862	0.138637	0.532777	0.594188	0.820478	CDA	978	cytidine deaminase
ENSG0000	3436.438	-0.03919	0.073567	-0.53276	0.594202	0.820478	SSH1	54434	slingshot protein phosphatase 1
ENSG0000	193.9035	-0.11049	0.207494	-0.53252	0.594369	0.820648	NPIPB11	728888	nuclear pore complex interacting protein family member B11
ENSG0000	26.44189	-0.24763	0.465165	-0.53234	0.594491	0.820694	IGHV1-2	28474	immunoglobulin heavy variable 1-2
ENSG0000	8.307322	0.460225	0.864465	0.532381	0.594462	0.820694	TCEAL2	140597	transcription elongation factor A like 2
ENSG0000	402.6275	-0.07007	0.13172	-0.53197	0.594747	0.820968	DDX1	1653	DEAD-box helicase 1
ENSG0000	429.7935	0.070737	0.132982	0.531925	0.594778	0.820968	MTO1	25821	mitochondrial tRNA translation optimization 1
ENSG0000	679.6778	0.066391	0.124889	0.531598	0.595004	0.821219	TOMM34	10953	translocase of outer mitochondrial membrane 34
ENSG0000	6.323407	-0.49593	0.933167	-0.53145	0.595105	0.821298	LOC10272	1.03E+08	anaphase-promoting complex subunit 1-like
ENSG0000	4.069014	-0.60146	1.132432	-0.53113	0.595332	0.821368	PRELID2	153768	PRELI domain containing 2
ENSG0000	24.64446	-0.26861	0.505734	-0.53114	0.595324	0.821368	COL19A1	1310	collagen type XIX alpha 1 chain
ENSG0000	18.76379	-0.32242	0.607058	-0.53112	0.595333	0.821368	CYP4F22	126410	cytochrome P450 family 4 subfamily F member 22
ENSG0000	5.401596	0.495255	0.932172	0.531291	0.595217	0.821368	NA	NA	NA
ENSG0000	152.2152	-0.11292	0.212705	-0.53087	0.595512	0.821553	CYP4V2	285440	cytochrome P450 family 4 subfamily V member 2
ENSG0000	22.72117	0.294706	0.555272	0.530741	0.595598	0.821611	SERPINB1(	5273	serpin family B member 10
ENSG0000	26.98176	0.263374	0.496488	0.530475	0.595782	0.821743	NA	NA	NA
ENSG0000	188.503	0.098903	0.186427	0.53052	0.595752	0.821743	CHIC1	53344	cysteine rich hydrophobic domain 1
ENSG0000	340.6794	-0.07593	0.143209	-0.53023	0.59595	0.821912	PUM3	9933	pumilio RNA binding family member 3
ENSG0000	23.0725	0.329982	0.622416	0.530162	0.595999	0.82192	NA	NA	NA
ENSG0000	309.1361	-0.08821	0.166464	-0.5299	0.596182	0.822111	CEP85L	387119	centrosomal protein 85 like
ENSG0000	204.8528	0.102696	0.193831	0.529821	0.596236	0.822124	DPY30	84661	dpy-30 histone methyltransferase complex regulatory subunit
ENSG0000	5.787388	0.641314	1.210789	0.529666	0.596343	0.822158	CNTNAP2	26047	contactin associated protein 2
ENSG0000	3.724086	0.973731	1.838414	0.529658	0.596349	0.822158	NA	NA	NA
ENSG0000	21.38754	0.372164	0.703041	0.529363	0.596554	0.822197	MATN1	4146	matrilin 1
ENSG0000	2.186722	1.006635	1.901604	0.529361	0.596555	0.822197	NA	NA	NA
ENSG0000	90.31547	0.134489	0.254048	0.529383	0.59654	0.822197	HAUS1	115106	HAUS augmin like complex subunit 1
ENSG0000	134.0218	-0.1264	0.238755	-0.52943	0.59651	0.822197	WDR62	284403	WD repeat domain 62
ENSG0000	26.99593	0.234649	0.44351	0.529073	0.596755	0.822294	POPDC2	64091	popeye domain containing 2
ENSG0000	5.638172	-0.61681	1.165994	-0.529	0.596805	0.822294	KLKB1	3818	kallikrein B1
ENSG0000	1.948954	-1.28318	2.425644	-0.52901	0.596801	0.822294	TMEM232	642987	transmembrane protein 232
ENSG0000	1.808547	1.259374	2.379822	0.529188	0.596675	0.822294	GPR141BP	1E+08	G protein- pseudogene
ENSG0000	2212.889	-0.04525	0.085559	-0.52888	0.596891	0.822294	DGAT1	8694	diacylglycerol O-acyltransferase 1
ENSG0000	3.856648	0.663527	1.254577	0.528885	0.596885	0.822294	NA	NA	NA
ENSG0000	121.452	0.128775	0.243527	0.52879	0.596951	0.822315	SAYSD1	55776	SAYSVFN motif domain containing 1
ENSG0000	678.3982	0.070043	0.132509	0.528591	0.59709	0.822445	CTDSPL2	51496	CTD small phosphatase like 2
ENSG0000	631.2221	-0.06134	0.116066	-0.5285	0.59715	0.822467	RBM28	55131	RNA binding motif protein 28
ENSG0000	2675.025	-0.05125	0.097047	-0.52808	0.597447	0.822781	LYST	1130	lysosomal trafficking regulator
ENSG0000	14.05967	-0.39111	0.740678	-0.52805	0.597466	0.822781	LOC10041	1E+08	zinc finger protein 41 pseudogene
ENSG0000	227.016	-0.09444	0.179138	-0.5272	0.598052	0.822976	ZYG11B	79699	zyg-11 fam cell cycle regulator
ENSG0000	1591.15	0.051039	0.096721	0.527692	0.597713	0.822976	GCC2	9648	GRIP and coiled-coil domain containing 2
ENSG0000	2733.109	-0.05324	0.10096	-0.52731	0.597975	0.822976	SH2D3C	10044	SH2 domain containing 3C
ENSG0000	12.38174	-0.46535	0.882305	-0.52742	0.597901	0.822976	TRPM5	29850	transient receptor potential cation channel subfamily M member 5
ENSG0000	2.877178	-1.05061	1.992465	-0.52729	0.597991	0.822976	TENM4	26011	teneurin transmembrane protein 4
ENSG0000	119.0817	-0.13734	0.260498	-0.52723	0.598033	0.822976	FXYD6	53826	FXYD domain containing ion transport regulator 6
ENSG0000	770.3401	0.055656	0.105474	0.527674	0.597725	0.822976	RFWD3	55159	ring finger and WD repeat domain 3
ENSG0000	3.1297	0.91929	1.742635	0.527528	0.597827	0.822976	NA	NA	NA
ENSG0000	201.3729	0.10354	0.196361	0.527293	0.59799	0.822976	ROMO1	140823	reactive oxygen species modulator 1
ENSG0000	30.68186	-0.24918	0.472481	-0.52738	0.597928	0.822976	ERG	2078	ETS transcription factor ERG
ENSG0000	549.5151	0.06277	0.119096	0.527054	0.598156	0.823058	BAIAP2	10458	BAR/IMD domain containing adaptor protein 2
ENSG0000	16.29517	0.30538	0.579603	0.526879	0.598278	0.823103	TRBV13	28574	T cell receptor beta variable 13
ENSG0000	66.85985	-0.15139	0.287305	-0.52692	0.598247	0.823103	ZNF283	284349	zinc finger protein 283
ENSG0000	615.6595	-0.07608	0.144424	-0.52677	0.598356	0.823108	STMN1	3925	stathmin 1
ENSG0000	3.939293	0.847251	1.608622	0.526694	0.598406	0.823108	TENM2	57451	teneurin transmembrane protein 2
ENSG0000	225.5469	-0.09885	0.187679	-0.52668	0.598414	0.823108	MRS2	57380	magnesium transporter MRS2
ENSG0000	3.573939	0.72706	1.382548	0.525884	0.598969	0.823809	NA	NA	NA
ENSG0000	11.69849	0.480559	0.914319	0.525593	0.599171	0.824027	PRSS3	5646	serine protease 3
ENSG0000	5.129227	0.515776	0.982158	0.525145	0.599482	0.824213	NPM1P39	440577	nucleophosmin 1 pseudogene 39
ENSG0000	2246.747	-0.0675	0.128534	-0.52515	0.599476	0.824213	DTX3L	151636	deltex E3 ubiquitin ligase 3L
ENSG0000	2.155443	1.028112	1.957777	0.525143	0.599484	0.824213	NA	NA	NA
ENSG0000	497.3504	0.073785	0.1405	0.525162	0.599471	0.824213	ZNF526	116115	zinc finger protein 526
ENSG0000	1967.894	0.047111	0.089728	0.525045	0.599552	0.824245	IRF2BP1	26145	interferon regulatory factor 2 binding protein 1
ENSG0000	1812.785	-0.05687	0.108353	-0.5249	0.599653	0.824262	ASXL2	55252	ASXL transcriptional regulator 2
ENSG0000	378.9991	0.078103	0.148818	0.524826	0.599704	0.824262	NAA35	60560	N-alpha-ac NatC auxiliary subunit
ENSG0000	4.513287	-0.6122	1.166596	-0.52477	0.599742	0.824262	NA	NA	NA
ENSG0000	56.16658	-0.19456	0.370689	-0.52486	0.59968	0.824262	ZNF70	7621	zinc finger protein 70
ENSG0000	8.566932	0.476128	0.907768	0.524504	0.599928	0.824457	NME8	51314	NME/NM23 family member 8
ENSG0000	4.994819	-0.63798	1.216701	-0.52435	0.600032	0.824465	KAZN	23254	kazrin periplakin interacting protein
ENSG0000	1388.411	-0.04609	0.087896	-0.52434	0.600043	0.824465	DPM2	8818	dolichyl-pl regulatory
ENSG0000	8.32377	-0.56282	1.073469	-0.5243	0.600068	0.824465	MUC16	94025	mucin 16 cell surface associated

ENSG0000	32.5736	0.25198	0.480909	0.523966	0.600302	0.824685	FKBP14	55033	FKBP prolyl isomerase 14	
ENSG0000	3167.702	-0.0431	0.082266	-0.52394	0.600317	0.824685	MAPK1	5594	mitogen-activated protein kinase 1	
ENSG0000	14.00032	-0.35077	0.66976	-0.52372	0.600471	0.824836	MSANTD2	1.01E+08	MSANTD2 antisense RNA 1	
ENSG0000	680.0493	0.229257	0.437957	0.523469	0.600648	0.824956	IGLC3	3539	immunoglobulin lambda constant 3 (Kern-Oz+ marker)	
ENSG0000	4.863436	-0.65004	1.241775	-0.52347	0.600644	0.824956	ZCCHC12	170261	zinc finger CCHC-type containing 12	
ENSG0000	23.77654	0.267846	0.511739	0.523404	0.600693	0.824958	NA	NA	NA	
ENSG0000	2.304735	-0.98444	1.881497	-0.52322	0.600822	0.825073	NA	NA	NA	
ENSG0000	1047.654	-0.05839	0.111608	-0.52313	0.600881	0.825093	ZFAND2B	130617	zinc finger AN1-type containing 2B	
ENSG0000	11.3383	0.37457	0.716343	0.522892	0.601049	0.825264	NA	NA	NA	
ENSG0000	5.99901	0.609392	1.166187	0.52255	0.601287	0.825361	MCM10	55388	minichromosome maintenance 10 replication initiation factor	
ENSG0000	23.9062	-0.24369	0.466247	-0.52265	0.601215	0.825361	NA	NA	NA	
ENSG0000	44.33967	-0.21401	0.409559	-0.52254	0.601297	0.825361	ZBED5-AS1	729013	ZBED5 antisense RNA 1	
ENSG0000	31.72678	0.254694	0.48748	0.52247	0.601343	0.825361	CD276	80381	CD276 molecule	
ENSG0000	445.7512	-0.0736	0.140847	-0.52253	0.601305	0.825361	DEXI	28955	Dexi homolog	
ENSG0000	1579.453	0.045805	0.087684	0.522382	0.601405	0.825385	USP21	27005	ubiquitin specific peptidase 21	
ENSG0000	354.268	-0.08546	0.163724	-0.52195	0.601703	0.825672	USP6NL	9712	USP6 N-terminal like	
ENSG0000	3.12107	0.811958	1.555576	0.521966	0.601694	0.825672	LINC00896	150197	long intergenic non-protein coding RNA 896	
ENSG0000	3.474878	0.70169	1.344892	0.521744	0.601848	0.82581	HCG20	1.05E+08	HLA complex group 20	
ENSG0000	5.796189	-0.53968	1.034527	-0.52167	0.601903	0.825811	LEXM	163747	lymphocyte expansion molecule	
ENSG0000	102.3103	-0.14406	0.276853	-0.52034	0.602828	0.825811	SWT1	54823	SWT1 RNA endoribonuclease homolog	
ENSG0000	344.6093	0.088862	0.170599	0.520882	0.602449	0.825811	INTS7	25896	integrator complex subunit 7	
ENSG0000	749.1858	0.062661	0.120176	0.521414	0.602078	0.825811	EPRS1	2058	glutamyl-prolyl-tRNA synthetase 1	
ENSG0000	6.66079	0.467356	0.89829	0.520274	0.602873	0.825811	ANKRD44-	1.02E+08	ANKRD44 intronic transcript 1	
ENSG0000	955.5793	0.078767	0.151189	0.520984	0.602378	0.825811	RICTOR	253260	RPTOR independent companion of MTOR complex 2	
ENSG0000	443.7693	0.072548	0.139413	0.520384	0.602796	0.825811	RELL2	285613	RELT like 2	
ENSG0000	18.52684	-0.3279	0.629118	-0.52121	0.60222	0.825811	NA	NA	NA	
ENSG0000	370.4043	0.076725	0.147452	0.520341	0.602826	0.825811	PRKAR2B	5577	protein kinase cAMP-dependent type II regulatory subunit beta	
ENSG0000	6.674494	0.570958	1.09636	0.520776	0.602523	0.825811	NA	NA	NA	
ENSG0000	11.3473	0.356132	0.683856	0.520771	0.602526	0.825811	NA	NA	NA	
ENSG0000	3483.487	0.047319	0.090747	0.521444	0.602058	0.825811	ASAP1	50807	ArfGAP wi	ankyrin repeat and PH domain 1
ENSG0000	2.250611	-0.9975	1.916702	-0.52042	0.602768	0.825811	RNA5SP29	1.01E+08	RNA	5S ribosomal pseudogene 296
ENSG0000	930.4188	-0.05216	0.10011	-0.52098	0.60238	0.825811	SEC23IP	11196	SEC23 interacting protein	
ENSG0000	47.24324	0.172513	0.331378	0.520592	0.602651	0.825811	EFNB2	1948	ephrin B2	
ENSG0000	590.8655	0.065707	0.126067	0.521208	0.602222	0.825811	PSMC1	5700	proteasom	ATPase 1
ENSG0000	2933.084	-0.04531	0.087083	-0.5203	0.602856	0.825811	CENPT	80152	centromere protein T	
ENSG0000	10.69717	-0.38286	0.734817	-0.52102	0.60235	0.825811	CLDN7	1366	claudin 7	
ENSG0000	360.9788	-0.07172	0.137502	-0.52158	0.601963	0.825811	PIAS2	9063	protein inhibitor of activated STAT 2	
ENSG0000	136.6993	0.131314	0.252165	0.520747	0.602543	0.825811	IER3IP1	51124	immediate early response 3 interacting protein 1	
ENSG0000	228.0604	0.093281	0.17916	0.520658	0.602605	0.825811	NAA20	51126	N-alpha-ac	NatB catalytic subunit
ENSG0000	21.4527	-0.76846	1.476952	-0.5203	0.602854	0.825811	IGLV7-46	28775	immunoglobulin lambda variable 7-46	
ENSG0000	292.3593	-0.07559	0.144942	-0.52155	0.601983	0.825811	CCNQ	92002	cyclin Q	
ENSG0000	41.20888	-0.18778	0.361195	-0.51988	0.603144	0.826122	FHIP1A	729830	FHF complex subunit HOOK interacting protein 1A	
ENSG0000	8061.776	0.036307	0.069849	0.519793	0.603208	0.826148	U2AF2	11338	U2 small nuclear RNA auxiliary factor 2	
ENSG0000	49.1861	0.204355	0.393281	0.519615	0.603332	0.826202	TCTN1	79600	tectonic family member 1	
ENSG0000	17803.34	0.053786	0.103512	0.519609	0.603336	0.826202	PLP2	5355	proteolipid protein 2	
ENSG0000	27.13981	-0.24579	0.47323	-0.51939	0.603488	0.826349	PAPLN	89932	papilin	proteoglycan like sulfated glycoprotein
ENSG0000	2387.296	0.044326	0.085353	0.519322	0.603536	0.826354	ZFAND3	60685	zinc finger AN1-type containing 3	
ENSG0000	281.7372	-0.08176	0.157495	-0.51915	0.603656	0.826453	LARP1B	55132	La ribonucleoprotein 1B	
ENSG0000	9.576323	-0.3946	0.760276	-0.51903	0.603742	0.826453	TRDV1	28518	T cell receptor delta variable 1	
ENSG0000	128.4887	-0.12539	0.241566	-0.51906	0.603716	0.826453	TMEM62	80021	transmembrane protein 62	
ENSG0000	8.051167	0.410607	0.791328	0.518883	0.603842	0.826468	NA	NA	NA	
ENSG0000	201.4354	-0.09469	0.182478	-0.51891	0.603821	0.826468	STEEP1	63932	STING1 ER exit protein 1	
ENSG0000	3.505752	0.824404	1.589877	0.518533	0.604086	0.82674	NA	NA	NA	
ENSG0000	13.46545	-0.37614	0.725584	-0.51839	0.604185	0.826815	NA	NA	NA	
ENSG0000	28.66183	-0.26419	0.509701	-0.51832	0.604238	0.826826	NA	NA	NA	
ENSG0000	17.91128	-0.38582	0.744472	-0.51824	0.604288	0.826833	GOLGA2P7	388152	GOLGA2 pseudogene 7	
ENSG0000	10.54717	0.360355	0.695563	0.518077	0.604404	0.826871	NA	NA	NA	
ENSG0000	27.79782	-0.22379	0.431922	-0.51812	0.604378	0.826871	DEPDC1B	55789	DEP domain containing 1B	
ENSG0000	744.5803	-0.05895	0.113817	-0.51798	0.604476	0.826907	TTC14	151613	tetratricopeptide repeat domain 14	
ENSG0000	20.50783	-0.29391	0.567523	-0.51788	0.60454	0.826935	FSTL1	11167	follistatin like 1	
ENSG0000	8.498444	-0.52094	1.006123	-0.51776	0.604622	0.826986	NA	NA	NA	
ENSG0000	100.353	-0.12411	0.239771	-0.51761	0.604729	0.827071	ATP1B1	481	ATPase Na+/K+ transporting subunit beta 1	
ENSG0000	4.126337	-0.6179	1.195383	-0.51691	0.60522	0.827089	NA	NA	NA	
ENSG0000	8.126943	-0.51522	0.996138	-0.51721	0.605007	0.827089	BCAN	63827	brevican	
ENSG0000	166.132	-0.10909	0.210799	-0.51749	0.604813	0.827089	UCK2	7371	uridine-cytidine kinase 2	
ENSG0000	18.65113	-0.32023	0.619133	-0.51723	0.604998	0.827089	NA	NA	NA	
ENSG0000	7.435245	-0.48025	0.929857	-0.51648	0.60552	0.827089	PLAAT1	57110	phospholipase A and acyltransferase 1	
ENSG0000	18.7248	0.267323	0.517304	0.516762	0.605322	0.827089	RPL7P1	6174	ribosomal protein L7 pseudogene 1	
ENSG0000	137.2353	0.108592	0.210014	0.517068	0.605109	0.827089	SLC25A13	10165	solute carrier family 25 member 13	
ENSG0000	280.7057	0.082577	0.159632	0.517294	0.604951	0.827089	LRRC4	64101	leucine rich repeat containing 4	
ENSG0000	73.32742	-0.15647	0.302936	-0.51653	0.605486	0.827089	ENTPD1-A	728558	ENTPD1 antisense RNA 1	
ENSG0000	63.57177	0.168554	0.326375	0.516444	0.605545	0.827089	CRACR2B	283229	calcium release activated channel regulator 2B	
ENSG0000	209.0307	0.100682	0.19487	0.516662	0.605392	0.827089	SLC35E3	55508	solute carrier family 35 member E3	
ENSG0000	3.568788	-0.72805	1.409421	-0.51656	0.605461	0.827089	IQCD	115811	IQ motif containing D	
ENSG0000	1725.281	-0.0517	0.10009	-0.51656	0.605463	0.827089	CYFIP1	23191	cytoplasmic FMR1 interacting protein 1	
ENSG0000	946.4876	0.055402	0.107266	0.516496	0.605508	0.827089	PPP4R1	9989	protein phosphatase 4 regulatory subunit 1	
ENSG0000	3.948455	0.623718	1.2072	0.516665	0.60539	0.827089	RPL7AP66	388474	ribosomal protein L7a pseudogene 66	
ENSG0000	7.841081	0.405817	0.785774	0.516456	0.605536	0.827089	IGLL5	1E+08	immunoglobulin lambda like polypeptide 5	
ENSG0000	1192.305	0.050231	0.097263	0.51645	0.60554	0.827089	GRAMD4	23151	GRAM domain containing 4	
ENSG0000	45.77036	0.209029	0.404285	0.517034	0.605132	0.827089	L1CAM	3897	L1 cell adhesion molecule	
ENSG0000	55.57149	0.181837	0.352152	0.516359	0.605604	0.827108	PLTP	5360	phospholipid transfer protein	
ENSG0000	5.636826	-0.55627	1.079409	-0.51535	0.606308	0.827115	ZBTB8A	653121	zinc finger and BTB domain containing 8A	
ENSG0000	79.7104	-0.1535	0.297844	-0.51538	0.60629	0.827115	POLR3GL	84265	RNA polymerase III subunit GL	
ENSG0000	816.9382	0.057007	0.110656	0.515169	0.606435	0.827115	ADAMTSL4	54507	ADAMTS like 4	
ENSG0000	13.22277	-0.33153	0.643352	-0.51532	0.606327	0.827115	NA	NA	NA	
ENSG0000	1278.823	-0.05427	0.105175	-0.51596	0.605885	0.827115	BRD9	65980	bromodomain containing 9	
ENSG0000	423.5814	-0.06956	0.134907	-0.51563	0.606114	0.827115	ATG5	9474	autophagy related 5	
ENSG0000	28.84336	0.22383	0.434505	0.515139	0.606456	0.827115	DLC1	10395	DLC1 Rho GTPase activating protein	
ENSG0000	13.3185	-0.37163	0.721089	-0.51537	0.606291	0.827115	C5	727	complement C5	
ENSG0000	2912.863	-0.05028	0.097394	-0.5162	0.605712	0.827115	HIPK3	10114	homeodomain interacting protein kinase 3	
ENSG0000	2.175338	1.015815	1.969277	0.515832	0.605972	0.827115	NA	NA	NA	
ENSG0000	401.3281	-0.07488	0.145286	-0.5154	0.606273	0.827115	DDX55	57696	DEAD-box helicase 55	
ENSG0000	5889.284	0.039005	0.075691	0.515323	0.606327	0.827115	LAMP1	3916	lysosomal associated membrane protein 1	
ENSG0000	602.7772	0.066326	0.128752	0.515144	0.606452	0.827115	SYNM	23336	synemin	
ENSG0000	30.46776	-0.23695	0.459669	-0.51548	0.606217	0.827115	SRR	63826	serine racemase	
ENSG0000	5.642535	0.555207	1.077456	0.515294	0.606347	0.827115	C17orf113	1.11E+08	chromosome 17 open reading frame 113	
ENSG0000	23.13611	0.277967	0.539325	0.515399	0.606274	0.827115	ZNF565	147929	zinc finger protein 565	
ENSG0000	10.41216	0.478265	0.9264	0.516262	0.605671	0.827115	ZNF610	162963	zinc finger protein 610	
ENSG0000	181.7483	-0.10135	0.19668	-0.51529	0.606349	0.827115	ACTR5	79913	actin related protein 5	
ENSG0000	149.1829	-0.09987	0.193673	-0.51566	0.606091	0.827115	CRYZL1	9946	crystallin zeta like 1	

ENSG0000	3.535198	0.642928	1.24866	0.514894	0.606627	0.827288	NA	NA	NA
ENSG0000	4.792409	-0.6099	1.184998	-0.51468	0.606774	0.827322	ENTPD2	954	ectonucleoside triphosphate diphosphohydrolase 2
ENSG0000	826.1508	-0.06096	0.118447	-0.51467	0.606786	0.827322	INTS5	80789	integrator complex subunit 5
ENSG0000	2544.038	-0.04518	0.087787	-0.5147	0.60676	0.827322	PRKD2	25865	protein kinase D2
ENSG0000	623.5819	-0.07984	0.155163	-0.51456	0.606863	0.827366	FBXO6	26270	F-box protein 6
ENSG0000	98.37255	-0.14313	0.278299	-0.5143	0.607044	0.827553	PIBF1	10464	progesterone immunomodulatory binding factor 1
ENSG0000	5.222905	0.693979	1.349987	0.514063	0.607208	0.827593	NA	NA	NA
ENSG0000	13.1513	-0.37915	0.737498	-0.51411	0.607178	0.827593	BUB1B	701	BUB1 mitotic checkpoint serine/threonine kinase B
ENSG0000	2052.611	-0.04178	0.081259	-0.51415	0.607147	0.827593	PLA2G6	8398	phospholipase A2 group VI
ENSG0000	9.055315	-0.45125	0.877982	-0.51397	0.607275	0.827624	NA	NA	NA
ENSG0000	11.52847	0.424152	0.825475	0.513827	0.607373	0.827664	VEPH1	79674	ventricular zone expressed PH domain containing 1
ENSG0000	56.54712	-0.20913	0.407186	-0.5136	0.60753	0.827664	CLEC4D	338339	C-type lectin domain family 4 member D
ENSG0000	10.77699	-0.37775	0.735509	-0.51359	0.60754	0.827664	NA	NA	NA
ENSG0000	258.3186	0.092821	0.180681	0.513729	0.607442	0.827664	NDUFB1	4707	NADH:ubiquinone oxidoreductase subunit B1
ENSG0000	4.756086	0.636703	1.239713	0.513589	0.607539	0.827664	NA	NA	NA
ENSG0000	22.41529	-0.30752	0.598885	-0.51348	0.607614	0.827664	ZNF321P	399669	zinc finger pseudogene
ENSG0000	190.1383	0.096398	0.187735	0.513478	0.607617	0.827664	SLC38A5	92745	solute carrier family 38 member 5
ENSG0000	648.3459	-0.05967	0.116342	-0.51293	0.608003	0.828008	MCCC2	64087	methylcrotonyl-CoA carboxylase subunit 2
ENSG0000	83.80974	0.144004	0.280695	0.513024	0.607934	0.828008	MMP8	4317	matrix metallopeptidase 8
ENSG0000	2.113258	-1.21228	2.363178	-0.51299	0.607959	0.828008	NA	NA	NA
ENSG0000	17.58789	0.385567	0.752012	0.512714	0.608152	0.828149	NA	NA	NA
ENSG0000	14.79106	-0.30825	0.601338	-0.51261	0.608223	0.828176	NA	NA	NA
ENSG0000	125.4072	-0.14168	0.276423	-0.51256	0.608261	0.828176	VMO1	284013	vitelline membrane outer layer 1 homolog
ENSG0000	64.03312	0.165057	0.322338	0.512062	0.608608	0.828504	PAQR7	164091	progestin and adipoQ receptor family member 7
ENSG0000	4.903571	-0.69212	1.351742	-0.51202	0.608636	0.828504	LOC64596	645967	uncharacterized LOC645967
ENSG0000	49.62466	-0.18586	0.362968	-0.51206	0.608606	0.828504	NAT14	57106	N-acetyltransferase 14 (putative)
ENSG0000	6.844209	0.612214	1.196093	0.511845	0.60876	0.828612	B3GNT4	79369	UDP-GlcN 3-N-acetylglucosaminyltransferase 4
ENSG0000	4.232095	-0.72454	1.415747	-0.51177	0.608811	0.828619	NA	NA	NA
ENSG0000	725.6855	-0.06444	0.12593	-0.51171	0.608854	0.828619	MAP2K4	6416	mitogen-activated protein kinase kinase 4
ENSG0000	8.702278	0.435122	0.850456	0.511634	0.608907	0.82863	NA	NA	NA
ENSG0000	964.7917	0.057302	0.112058	0.51136	0.609099	0.82883	MEA1	4201	male-enhanced antigen 1
ENSG0000	6.252058	0.509069	0.996016	0.511105	0.609278	0.828986	IGHV2-26	28455	immunoglobulin heavy variable 2-26
ENSG0000	14.53546	-0.30827	0.603182	-0.51107	0.609303	0.828986	SARS2	54938	seryl-tRN mitochondrial
ENSG0000	5.274063	0.544678	1.066024	0.510943	0.609391	0.829045	NA	NA	NA
ENSG0000	4.738253	-0.58513	1.147469	-0.50993	0.610099	0.829056	NA	NA	NA
ENSG0000	82.14381	0.134268	0.263316	0.509911	0.610114	0.829056	CCDC18	343099	coiled-coil domain containing 18
ENSG0000	10.983	-0.40134	0.785653	-0.51083	0.609467	0.829056	NA	NA	NA
ENSG0000	54.4709	-0.16089	0.315196	-0.51044	0.60974	0.829056	USP13	8975	ubiquitin specific peptidase 13
ENSG0000	1931.074	0.048825	0.095591	0.510776	0.609508	0.829056	EPC1	80314	enhancer of polycomb homolog 1
ENSG0000	102.1811	-0.12768	0.250271	-0.51018	0.609927	0.829056	LARGE2	120071	LARGE xylosyl- and glucuronyltransferase 2
ENSG0000	7.785768	0.595874	1.16838	0.51	0.610051	0.829056	NA	NA	NA
ENSG0000	4123.255	0.03859	0.075641	0.510175	0.609929	0.829056	DUSP16	80824	dual specificity phosphatase 16
ENSG0000	533.2018	0.069452	0.136199	0.50993	0.610101	0.829056	ING1	3621	inhibitor of growth family member 1
ENSG0000	183.8365	-0.09953	0.195141	-0.51005	0.610013	0.829056	CGRRF1	10668	cell growth regulator with ring finger domain 1
ENSG0000	671.2162	-0.06046	0.118528	-0.51006	0.610013	0.829056	TDP1	55775	tyrosyl-DNA phosphodiesterase 1
ENSG0000	136.8767	0.103867	0.203634	0.510068	0.610004	0.829056	ZNF747	65988	zinc finger protein 747
ENSG0000	6.878433	0.497107	0.974765	0.509976	0.610068	0.829056	LINC0213	1.08E+08	long intergenic non-protein coding RNA 2132
ENSG0000	3.507533	0.875007	1.714195	0.510448	0.609738	0.829056	NA	NA	NA
ENSG0000	250.2562	0.080206	0.157124	0.510462	0.609728	0.829056	ZSCAN22	342945	zinc finger and SCAN domain containing 22
ENSG0000	15.9138	-0.35182	0.689832	-0.51	0.61005	0.829056	SGSM3	27352	small G protein signaling modulator 3
ENSG0000	10.87687	-0.37691	0.739295	-0.50983	0.610171	0.829072	NA	NA	NA
ENSG0000	572.8149	-0.05845	0.114745	-0.50939	0.610478	0.829227	STAMBP	10617	STAM binding protein
ENSG0000	914.3093	-0.05518	0.108344	-0.50935	0.610508	0.829227	LARP7	51574	La ribonuc transcriptional regulator
ENSG0000	72.86396	0.149615	0.293629	0.509538	0.610375	0.829227	ZNF165	7718	zinc finger protein 165
ENSG0000	14.2493	-0.32463	0.637124	-0.50952	0.610385	0.829227	BRIP1	83990	BRCA1 interacting helicase 1
ENSG0000	14.03741	0.3358	0.659263	0.509357	0.610502	0.829227	NA	NA	NA
ENSG0000	771.4906	-0.05387	0.105879	-0.50874	0.610931	0.829294	NAXE	128240	NAD(P)HX epimerase
ENSG0000	6.182714	-0.56485	1.110326	-0.50873	0.610943	0.829294	NUF2	83540	NUF2 component of NDC80 kinetochore complex
ENSG0000	17.14991	-0.30856	0.606042	-0.50915	0.61065	0.829294	NA	NA	NA
ENSG0000	1405.765	-0.04813	0.094601	-0.50873	0.610941	0.829294	TRIM41	90933	tripartite motif containing 41
ENSG0000	9.490444	-0.4304	0.845601	-0.50899	0.610758	0.829294	NA	NA	NA
ENSG0000	84.81642	-0.15098	0.296859	-0.50858	0.611049	0.829294	OTUD6B	51633	OTU deubiquitinase 6B
ENSG0000	4867.076	-0.04037	0.079339	-0.50879	0.610899	0.829294	FAM107B	83641	family with sequence similarity 107 member B
ENSG0000	309.945	-0.07635	0.150116	-0.50863	0.611009	0.829294	FDX1	2230	ferredoxin 1
ENSG0000	7.058341	0.435636	0.855591	0.509163	0.610638	0.829294	ALOX12P2	245	arachidonate 12-lipoxygenase pseudogene 2
ENSG0000	134.3274	-0.10416	0.204663	-0.50892	0.610809	0.829294	ZNF575	284346	zinc finger protein 575
ENSG0000	220.4133	0.095578	0.187926	0.508592	0.611039	0.829294	NA	NA	NA
ENSG0000	14.02285	0.34092	0.670998	0.508078	0.611398	0.829529	C2orf15	150590	chromosome 2 open reading frame 15
ENSG0000	36.62261	-0.23196	0.456613	-0.508	0.611452	0.829529	NA	NA	NA
ENSG0000	3.896781	0.793294	1.561574	0.508009	0.611447	0.829529	NA	NA	NA
ENSG0000	5.954898	0.600686	1.182273	0.508077	0.611399	0.829529	CNTF	1270	ciliary neurotrophic factor
ENSG0000	20.77744	-0.26597	0.523679	-0.5079	0.611526	0.829529	CACNA1C-	1.01E+08	CACNA1C antisense RNA 1
ENSG0000	655.8827	0.065863	0.129681	0.507883	0.611536	0.829529	PRMT5	10419	protein arginine methyltransferase 5
ENSG0000	18.70524	-0.36962	0.727394	-0.50814	0.611354	0.829529	CBR3	874	carbonyl reductase 3
ENSG0000	10.49576	0.378961	0.74638	0.507732	0.611641	0.829612	ALDH7A1	501	aldehyde dehydrogenase 7 family member A1
ENSG0000	8.810683	-1.3187	2.598504	-0.50748	0.611815	0.829764	NA	NA	NA
ENSG0000	2059.808	-0.05023	0.098991	-0.50742	0.611859	0.829764	NOC2L	26155	NOC2 like nucleolar associated transcriptional repressor
ENSG0000	143.5757	-0.11538	0.227441	-0.50728	0.611958	0.829764	ACVR2B	93	activin A receptor type 2B
ENSG0000	2463.73	0.043846	0.086433	0.507282	0.611957	0.829764	KHNYN	23351	KH and NYN domain containing
ENSG0000	23.65539	-0.25295	0.49867	-0.50725	0.611977	0.829764	NA	NA	NA
ENSG0000	19.23545	-0.27675	0.545768	-0.50708	0.612096	0.829785	SLC4A3	6508	solute carrier family 4 member 3
ENSG0000	478.7663	0.071163	0.140375	0.506949	0.61219	0.829785	TBL1XR1	79718	TBL1X/Y related 1
ENSG0000	3266.252	-0.04498	0.08869	-0.50712	0.612067	0.829785	NIPBL	25836	NIPBL cohesin loading factor
ENSG0000	879.8369	-0.05357	0.105663	-0.50698	0.612165	0.829785	MESD	23184	mesoderm development LRP chaperone
ENSG0000	3.638171	-0.77952	1.537778	-0.50691	0.612216	0.829785	NA	NA	NA
ENSG0000	5188.375	0.152931	0.301882	0.506592	0.612441	0.829868	EGR1	1958	early growth response 1
ENSG0000	11.78804	0.356623	0.703996	0.50657	0.612457	0.829868	NA	NA	NA
ENSG0000	633.2569	0.078215	0.154374	0.506661	0.612393	0.829868	CWC15	51503	CWC15 spliceosome associated protein homolog
ENSG0000	15.08528	0.344051	0.679144	0.506595	0.612439	0.829868	NA	NA	NA
ENSG0000	175.8425	-0.11192	0.221	-0.50643	0.612556	0.829942	USP54	159195	ubiquitin specific peptidase 54
ENSG0000	19.24641	0.280608	0.554241	0.506293	0.612651	0.830011	NA	NA	NA
ENSG0000	17.4089	0.333121	0.658861	0.505602	0.613136	0.830303	ARHGAP3	1.01E+08	ARHGAP31 antisense RNA 1
ENSG0000	113.1486	0.113172	0.223715	0.505875	0.612944	0.830303	HOMER1	9456	homer scaffold protein 1
ENSG0000	38.92624	-0.22366	0.442187	-0.50581	0.612994	0.830303	AASS	10157	aminoadipate-semialdehyde synthase
ENSG0000	10.51709	-0.46652	0.922379	-0.50578	0.61301	0.830303	NA	NA	NA
ENSG0000	286.0579	0.093294	0.184502	0.505655	0.613099	0.830303	MPPE1	65258	metallophosphoesterase 1
ENSG0000	29.74678	-0.23362	0.461955	-0.50573	0.613049	0.830303	CLTCL1	8218	clathrin heavy chain like 1
ENSG0000	3.98378	0.558497	1.104916	0.505466	0.613232	0.830312	RPL34P18	1E+08	ribosomal protein L34 pseudogene 18
ENSG0000	5.199137	-0.48586	0.961156	-0.5055	0.613211	0.830312	NA	NA	NA
ENSG0000	31.20639	0.221087	0.43762	0.505204	0.613416	0.830439	ANKRD39	51239	ankyrin repeat domain 39

ENSG0000	36.69292	0.200453	0.396745	0.505245	0.613387	0.830439	ZNF365	22891	zinc finger protein 365
ENSG0000	4.109827	0.646469	1.279835	0.505119	0.613475	0.830459	NA	NA	NA
ENSG0000	1480.073	-0.05562	0.11014	-0.50496	0.613584	0.830485	HK3	3101	hexokinase 3
ENSG0000	7.789803	-0.42075	0.833143	-0.50502	0.613544	0.830485	CCDC153	283152	coiled-coil domain containing 153
ENSG0000	55.85289	0.163669	0.324265	0.50474	0.613742	0.830578	MECR	51102	mitochondrial trans-2-enoyl-CoA reductase
ENSG0000	17.20393	0.338832	0.671247	0.504779	0.613714	0.830578	DPH6	89978	diphthamine biosynthesis 6
ENSG0000	3.345	-0.83923	1.662998	-0.50465	0.613804	0.830602	IDO2	169355	indoleami 3-dioxygenase 2
ENSG0000	16.63131	0.312767	0.619933	0.504518	0.613898	0.830668	LCN12	286256	lipocalin 12
ENSG0000	19.1924	0.258461	0.512536	0.504279	0.614065	0.830712	NA	NA	NA
ENSG0000	19.87651	-0.28768	0.570393	-0.50436	0.614007	0.830712	NA	NA	NA
ENSG0000	685.0186	-0.06363	0.126166	-0.50434	0.614022	0.830712	TNFRSF12/	51330	TNF receptor superfamily member 12A
ENSG0000	1749.909	-0.049	0.097181	-0.50419	0.614128	0.830737	GNPTG	84572	N-acetylglucosamine-1-phosphate transferase subunit gamma
ENSG0000	888.7107	-0.05341	0.105955	-0.50405	0.614229	0.830812	POMT1	10585	protein O-mannosyltransferase 1
ENSG0000	6.547624	0.48605	0.964521	0.503929	0.614311	0.830842	SEC24B-AS	1.01E+08	SEC24B antisense RNA 1
ENSG0000	2.613123	-0.87789	1.742239	-0.50389	0.61434	0.830842	NA	NA	NA
ENSG0000	3.530022	0.776162	1.540822	0.503733	0.614449	0.830863	LINC01191	440900	long intergenic non-protein coding RNA 1191
ENSG0000	3138.162	-0.04275	0.08488	-0.5037	0.61447	0.830863	POLG	5428	DNA polyr catalytic subunit
ENSG0000	7368.185	0.038734	0.076903	0.503674	0.61449	0.830863	HGS	9146	hepatocyte growth factor-regulated tyrosine kinase substrate
ENSG0000	72.52416	-0.15767	0.313125	-0.50355	0.614577	0.830919	FAM13A	10144	family with sequence similarity 13 member A
ENSG0000	66.55214	0.18491	0.367326	0.503394	0.614687	0.831008	NA	NA	NA
ENSG0000	39.90489	-0.19637	0.390152	-0.50331	0.614744	0.831024	RNF139-DT	1.02E+08	RNF139 divergent transcript
ENSG0000	8.781994	0.434298	0.863129	0.503167	0.614847	0.831103	CIART	148523	circadian associated repressor of transcription
ENSG0000	1002.206	0.06025	0.119774	0.503027	0.614945	0.831176	GALNT6	11226	polypeptide N-acetylgalactosaminyltransferase 6
ENSG0000	30.94653	0.221937	0.44132	0.502895	0.615038	0.831241	MRPL46	26589	mitochondrial ribosomal protein L46
ENSG0000	120.3469	-0.11102	0.220858	-0.50269	0.615182	0.83126	GPR141	353345	G protein-coupled receptor 141
ENSG0000	5.720936	0.551594	1.097588	0.502551	0.61528	0.83126	NA	NA	NA
ENSG0000	2.679116	0.892919	1.77698	0.502492	0.615321	0.83126	GPR176	11245	G protein-coupled receptor 176
ENSG0000	400.9282	-0.08567	0.170463	-0.50256	0.615275	0.83126	CD19	930	CD19 molecule
ENSG0000	1019.236	-0.05165	0.102714	-0.50281	0.615099	0.83126	MYPOP	339344	Myb relate partner of profilin
ENSG0000	198.3002	-0.09223	0.183526	-0.50256	0.615276	0.83126	RPGR	6103	retinitis pigmentosa GTPase regulator
ENSG0000	174.048	-0.09636	0.191836	-0.50231	0.615447	0.831369	MSANTD4	84437	Myb/SANT DNA binding domain containing 4 with coiled-coils
ENSG0000	15.81976	0.345585	0.688086	0.502241	0.615498	0.831377	NA	NA	NA
ENSG0000	42.66455	0.232739	0.463476	0.502158	0.615556	0.831395	DIP2C	22982	disco interacting protein 2 homolog C
ENSG0000	7.562579	0.553956	1.103355	0.502066	0.615621	0.831423	NA	NA	NA
ENSG0000	34.13315	0.232107	0.462454	0.501903	0.615736	0.831517	GDF7	151449	growth differentiation factor 7
ENSG0000	13.98944	0.319076	0.635927	0.501749	0.615844	0.831603	SPACA9	11092	sperm acrosome associated 9
ENSG0000	167.2375	-0.10386	0.207028	-0.50168	0.615894	0.831609	MTPAP	55149	mitochondrial poly(A) polymerase
ENSG0000	8.431296	0.402344	0.802174	0.501567	0.615972	0.831654	NA	NA	NA
ENSG0000	435.912	-0.06741	0.134421	-0.5015	0.616021	0.831659	B4GALT7	11285	beta-1 4-galactosyltransferase 7
ENSG0000	9.66146	0.359264	0.716568	0.501368	0.616112	0.831722	RPL7AP62	644029	ribosomal protein L7a pseudogene 62
ENSG0000	7.508086	0.439795	0.8774	0.501248	0.616197	0.831776	RYR3	6263	ryanodine receptor 3
ENSG0000	48.24033	-0.19433	0.387764	-0.50116	0.616262	0.831803	PIGF	5281	phosphatidylinositol glycan anchor biosynthesis class F
ENSG0000	9.678632	-0.43844	0.875033	-0.50106	0.616329	0.831834	MT1X	4501	metallothionein 1X
ENSG0000	26.33026	0.305674	0.610246	0.500902	0.61644	0.831862	ADCY6	112	adenylate cyclase 6
ENSG0000	2126.466	-0.04069	0.081237	-0.5009	0.61644	0.831862	MBTPS1	8720	membrane site 1
ENSG0000	1691.491	0.046884	0.093652	0.500619	0.616639	0.831896	ANKZF1	55139	ankyrin repeat and zinc finger peptidyl tRNA hydrolase 1
ENSG0000	384.1188	-0.07744	0.154773	-0.50036	0.616824	0.831896	GLYCTK	132158	glycerate kinase
ENSG0000	4.395664	0.674149	1.347301	0.50037	0.616815	0.831896	NA	NA	NA
ENSG0000	309.2463	0.073732	0.147255	0.500712	0.616574	0.831896	KIF21A	55605	kinesin family member 21A
ENSG0000	21.50535	-0.28489	0.569242	-0.50048	0.616739	0.831896	GOLGA8O	728047	golgin A8 family member O
ENSG0000	20.1564	-0.28505	0.569474	-0.50055	0.616686	0.831896	NA	NA	NA
ENSG0000	3173.856	-0.05116	0.102231	-0.50048	0.616737	0.831896	SEPTIN1	1731	septin 1
ENSG0000	15.28757	0.331052	0.66117	0.500706	0.616578	0.831896	NA	NA	NA
ENSG0000	19.80062	-0.30796	0.615565	-0.50028	0.616875	0.831904	TROAP	10024	trophinin associated protein
ENSG0000	24.80647	-0.26627	0.532351	-0.50017	0.616954	0.83195	NA	NA	NA
ENSG0000	13.53483	-0.40324	0.806385	-0.50005	0.617037	0.832002	ZNF726	730087	zinc finger protein 726
ENSG0000	198.0067	0.090827	0.18166	0.49998	0.617089	0.832011	KBTBD11	9920	kelch repeat and BTB domain containing 11
ENSG0000	13.27596	-0.33533	0.671343	-0.49949	0.617432	0.832173	ELF3	1999	E74 like ETS transcription factor 3
ENSG0000	7.640351	-0.46814	0.937749	-0.49922	0.617627	0.832173	THRB	7068	thyroid hormone receptor beta
ENSG0000	5.73436	-0.52636	1.053714	-0.49953	0.617407	0.832173	LRP2BP	55805	LRP2 binding protein
ENSG0000	577.193	-0.07036	0.140963	-0.49911	0.617702	0.832173	UBLCP1	134510	ubiquitin like domain containing CTD phosphatase 1
ENSG0000	208.9196	0.090728	0.181719	0.499277	0.617584	0.832173	TRBV20-1	28567	T cell receptor beta variable 20-1
ENSG0000	1052.032	0.052059	0.104286	0.499193	0.617643	0.832173	IARS1	3376	isoleucyl-tRNA synthetase 1
ENSG0000	5.432021	-0.54174	1.084908	-0.49934	0.617537	0.832173	NA	NA	NA
ENSG0000	222.3184	-0.09212	0.184493	-0.49932	0.617552	0.832173	NA	NA	NA
ENSG0000	292.6815	0.082141	0.164574	0.499115	0.617699	0.832173	ARL16	339231	ADP ribosylation factor like GTPase 16
ENSG0000	9.738168	0.466414	0.933533	0.499622	0.617341	0.832173	LOC10537	1.05E+08	uncharacterized LOC105372435
ENSG0000	95.41235	-0.1349	0.26996	-0.49972	0.617274	0.832173	PUDP	8226	pseudouridine 5'-phosphatase
ENSG0000	91.78296	0.144229	0.289095	0.4989	0.61785	0.832306	NA	NA	NA
ENSG0000	1419.949	0.04448	0.089171	0.498809	0.617914	0.832306	POLR2B	5431	RNA polymerase II subunit B
ENSG0000	208.9673	0.10041	0.201312	0.498778	0.617936	0.832306	MED21	9412	mediator complex subunit 21
ENSG0000	58.81633	-0.16988	0.340677	-0.49866	0.618016	0.832354	NA	NA	NA
ENSG0000	10.67035	0.367647	0.737423	0.498556	0.618092	0.832396	CCDC74B	91409	coiled-coil domain containing 74B
ENSG0000	98.49701	-0.13147	0.263813	-0.49834	0.618248	0.832545	CENPL	91687	centromere protein L
ENSG0000	312.9282	0.080041	0.160642	0.498259	0.618302	0.832557	R3HCC1L	27291	R3H domain and coiled-coil containing 1 like
ENSG0000	4.853415	-0.53838	1.080744	-0.49816	0.618374	0.832594	FAM186B	84070	family with sequence similarity 186 member B
ENSG0000	114.6179	0.155832	0.312889	0.498042	0.618454	0.832599	NA	NA	NA
ENSG0000	2.710453	0.825287	1.657124	0.498024	0.618467	0.832599	NA	NA	NA
ENSG0000	92.21898	0.148884	0.299004	0.497933	0.618531	0.832625	HTRA3	94031	HtrA serine peptidase 3
ENSG0000	130.1769	0.110031	0.221089	0.497677	0.618712	0.832807	NBEAL1	65065	neurobeachin like 1
ENSG0000	11.85892	0.409928	0.824295	0.497307	0.618972	0.832977	NA	NA	NA
ENSG0000	182.2437	-0.10512	0.21136	-0.49734	0.618953	0.832977	VPS50	55610	VPS50 subunit of EARP/GARPII complex
ENSG0000	95.02148	-0.15624	0.314105	-0.4974	0.618906	0.832977	EPS8	2059	epidermal growth factor receptor pathway substrate 8
ENSG0000	774.6346	0.052014	0.104626	0.49714	0.61909	0.833014	EIF2D	1939	eukaryotic translation initiation factor 2D
ENSG0000	9.035206	0.381647	0.767594	0.497198	0.619049	0.833014	SLC23A3	151295	solute carrier family 23 member 3
ENSG0000	68.27529	-0.18325	0.368775	-0.49691	0.619255	0.833176	DEFA4	1669	defensin alpha 4
ENSG0000	14.68841	0.313343	0.630767	0.496765	0.619355	0.833249	NA	NA	NA
ENSG0000	12.02566	-0.38493	0.775205	-0.49655	0.619507	0.833393	BIRC5	332	baculoviral IAP repeat containing 5
ENSG0000	40.03392	-0.19853	0.399888	-0.49647	0.619562	0.833408	SMIM20	389203	small integral membrane protein 20
ENSG0000	6.590646	0.592562	1.193931	0.496312	0.619674	0.833412	SLC35D3	340146	solute carrier family 35 member D3
ENSG0000	62.31561	0.152546	0.307382	0.496275	0.6197	0.833412	TSPAN9	10867	tetraspanin 9
ENSG0000	265.4306	0.087273	0.175821	0.496376	0.619629	0.833412	NA	NA	NA
ENSG0000	230.3321	-0.09832	0.198149	-0.49621	0.619749	0.833417	LANCL1	10314	LanC like glutathione S-transferase 1
ENSG0000	40.19694	0.189827	0.382809	0.49588	0.619979	0.833606	SHISA4	149345	shisa family member 4
ENSG0000	206.0349	0.114407	0.230715	0.49588	0.619979	0.833606	PDP2	57546	pyruvate dehydrogenase phosphatase catalytic subunit 2
ENSG0000	190.8431	0.093142	0.187872	0.495772	0.620055	0.833647	NDUFA8	4702	NADH:ubiquinone oxidoreductase subunit A8
ENSG0000	226.1112	-0.0825	0.16645	-0.49565	0.620144	0.833707	SPINT2	10653	serine pep Kunitz type 2
ENSG0000	3.544869	-0.66561	1.343319	-0.4955	0.620248	0.833786	KLHL13	90293	kelch like family member 13
ENSG0000	1603.18	0.04704	0.094989	0.495212	0.620451	0.833998	CCT3	7203	chaperonin containing TCP1 subunit 3
ENSG0000	21.14385	-0.25944	0.524128	-0.495	0.620603	0.834035	WDR5B-DT	1.03E+08	WDR5B divergent transcript

ENSG0000	2.935734	0.784922	1.585577	0.495038	0.620573	0.834035	NA	NA	NA
ENSG0000	13738.44	-0.0429	0.086662	-0.49498	0.620613	0.834035	DDX17	10521	DEAD-box helicase 17
ENSG0000	16.47882	-0.29373	0.593588	-0.49484	0.620716	0.834112	FAM149A	25854	family with sequence similarity 149 member A
ENSG0000	3874.702	0.040502	0.08193	0.494349	0.62106	0.834182	DHX9	1660	DExH-box helicase 9
ENSG0000	349.0763	-0.07547	0.152596	-0.49456	0.620913	0.834182	ZBTB49	166793	zinc finger and BTB domain containing 49
ENSG0000	13.18117	0.324118	0.655273	0.49463	0.620861	0.834182	NA	NA	NA
ENSG0000	43.26169	-0.18591	0.376174	-0.49421	0.621156	0.834182	TPK1	27010	thiamin pyrophosphokinase 1
ENSG0000	95.74957	-0.12975	0.262432	-0.49441	0.621017	0.834182	EPHX2	2053	epoxide hydrolase 2
ENSG0000	9.43279	0.434551	0.879322	0.494189	0.621173	0.834182	NA	NA	NA
ENSG0000	75.28609	-0.14371	0.29059	-0.49455	0.620915	0.834182	C11orf54	28970	chromosome 11 open reading frame 54
ENSG0000	79.99174	-0.12868	0.260359	-0.49425	0.621128	0.834182	DGKZP1	400126	diacylglycerol kinase zeta pseudogene 1
ENSG0000	68.91784	0.15479	0.313203	0.494217	0.621153	0.834182	NA	NA	NA
ENSG0000	226.7028	-0.09002	0.182318	-0.49376	0.621478	0.834531	GRPEL2	134266	GrpE like 2 mitochondrial
ENSG0000	417.3444	0.072047	0.145942	0.493671	0.621538	0.834553	MTRFR	91574	mitochondrial translation release factor in rescue
ENSG0000	9.204984	0.441942	0.895401	0.493569	0.621611	0.834589	NA	NA	NA
ENSG0000	40.7234	0.19127	0.387594	0.493481	0.621673	0.834612	SOX15	6665	SRY-box transcription factor 15
ENSG0000	4367.698	0.043096	0.087362	0.493301	0.6218	0.834723	RHOF	54509	ras homol filopodia associated
ENSG0000	4.60086	-0.57502	1.166144	-0.49309	0.621947	0.834859	NA	NA	NA
ENSG0000	34.99295	0.189566	0.384614	0.492875	0.622101	0.835006	NA	NA	NA
ENSG0000	1056.461	-0.04961	0.100763	-0.49232	0.62249	0.835094	LRRC8D	55144	leucine rich repeat containing 8 VRAC subunit D
ENSG0000	18.02557	0.313757	0.636975	0.492573	0.622314	0.835094	NA	NA	NA
ENSG0000	14.00846	-0.35619	0.72304	-0.49262	0.622278	0.835094	LOC12490	1.25E+08	uncharacterized LOC124901151
ENSG0000	205.1534	0.095377	0.193749	0.492273	0.622527	0.835094	TRIM52-A!	1.01E+08	TRIM52 antisense RNA 1 (head to head)
ENSG0000	2.707368	0.845415	1.717362	0.492275	0.622525	0.835094	TDRD1	56165	tudor domain containing 1
ENSG0000	49.99195	-0.17452	0.354543	-0.49224	0.622546	0.835094	NA	NA	NA
ENSG0000	18.45872	-0.28129	0.57093	-0.49269	0.622231	0.835094	NA	NA	NA
ENSG0000	83.44494	0.143989	0.292391	0.492452	0.6224	0.835094	NA	NA	NA
ENSG0000	6.160005	0.56633	1.150589	0.492209	0.622572	0.835094	MTCP1	4515	mature T cell proliferation 1
ENSG0000	4.753797	0.635823	1.292498	0.491934	0.622766	0.835294	NA	NA	NA
ENSG0000	9.946895	0.412152	0.838393	0.491598	0.623004	0.835431	NA	NA	NA
ENSG0000	27.63089	0.237779	0.483574	0.491712	0.622923	0.835431	HP	3240	haptoglobin
ENSG0000	38.75755	0.185075	0.376474	0.491601	0.623002	0.835431	TRAPPC5	126003	trafficking protein particle complex subunit 5
ENSG0000	540.7446	0.057538	0.117069	0.491487	0.623082	0.835475	ZNF264	9422	zinc finger protein 264
ENSG0000	160.1526	0.11226	0.228471	0.491354	0.623176	0.83553	KLHL8	57563	kelch like family member 8
ENSG0000	117.6971	0.107543	0.218894	0.491302	0.623213	0.83553	NA	NA	NA
ENSG0000	416.5443	0.074972	0.152647	0.49115	0.62332	0.835614	SETD7	80854	SET domai histone lysine methyltransferase
ENSG0000	559.3918	0.063379	0.129095	0.490949	0.623463	0.835745	RCC1	1104	regulator of chromosome condensation 1
ENSG0000	34.20796	0.206421	0.420511	0.490881	0.623511	0.835748	ATP8B1-A!	1.01E+08	ATP8B1 antisense RNA 1
ENSG0000	45.99962	0.197001	0.401639	0.490492	0.623786	0.836057	NA	NA	NA
ENSG0000	4309.248	0.042605	0.086916	0.490181	0.624006	0.836231	FOXP4	116113	forkhead box P4
ENSG0000	6.410557	-0.50304	1.026171	-0.49021	0.623983	0.836231	LINC0168	1.05E+08	long intergenic non-protein coding RNA 1684
ENSG0000	4.419199	-0.70797	1.445654	-0.48973	0.624328	0.8363	TRAV30	28652	T cell receptor alpha variable 30
ENSG0000	7.128504	0.463029	0.94503	0.489962	0.624161	0.8363	USP7-AS1	1.05E+08	USP7 antisense RNA 1
ENSG0000	219.2065	-0.10654	0.217543	-0.48974	0.624316	0.8363	SNTB2	6645	syntrophin beta 2
ENSG0000	104.1247	-0.13042	0.266242	-0.48984	0.62425	0.8363	PROCA1	147011	protein interacting with cyclin A1
ENSG0000	17.32189	-0.28919	0.590394	-0.48983	0.624252	0.8363	ZNF56P	7608	zinc finger pseudogene
ENSG0000	5.639716	0.653773	1.33473	0.489817	0.624264	0.8363	LINC0089	1E+08	long intergenic non-protein coding RNA 892
ENSG0000	57.5799	-0.16041	0.32764	-0.4896	0.624414	0.836355	C1orf112	55732	chromosome 1 open reading frame 112
ENSG0000	14.15129	0.314118	0.641871	0.489378	0.624574	0.836509	TOMM5	401505	translocase of outer mitochondrial membrane 5
ENSG0000	229.9385	-0.08484	0.173464	-0.48911	0.624764	0.836582	DGLUCY	80017	D-glutamate cyclase
ENSG0000	55.8435	-0.15659	0.320083	-0.48922	0.624687	0.836582	NA	NA	NA
ENSG0000	5.178017	0.529518	1.082532	0.489148	0.624737	0.836582	NA	NA	NA
ENSG0000	8.632401	0.471158	0.963561	0.488976	0.624859	0.836649	NA	NA	NA
ENSG0000	16.02263	0.336799	0.688885	0.488904	0.62491	0.836656	MGAM2	93432	maltase-glucoamylase 2 (putative)
ENSG0000	15023.09	0.033615	0.068776	0.488766	0.625007	0.836727	MAP7D1	55700	MAP7 domain containing 1
ENSG0000	20.22352	0.301811	0.617588	0.488694	0.625058	0.836734	COL13A1	1305	collagen type XIII alpha 1 chain
ENSG0000	63.24122	0.14359	0.293868	0.488621	0.62511	0.836743	LBHD1	79081	LBH domain containing 1
ENSG0000	365.1364	-0.06753	0.138261	-0.48844	0.62524	0.836856	NUTM2A-/	728190	NUTM2A antisense RNA 1
ENSG0000	488.2787	-0.06642	0.136065	-0.48817	0.625431	0.837029	POLR2G	5436	RNA polymerase II subunit G
ENSG0000	628.5631	-0.15509	0.317755	-0.48806	0.625504	0.837029	ANGPTL4	51129	angiopoietin like 4
ENSG0000	7.05096	0.433249	0.887624	0.4881	0.625479	0.837029	SMARCA1	6594	SWI/SNF r matrix ass actin depe subfamily member 1
ENSG0000	66.37611	-0.15072	0.308845	-0.488	0.625551	0.837032	TLCD3A	79850	TLC domain containing 3A
ENSG0000	3.688607	-0.7024	1.43973	-0.48787	0.625642	0.837065	MIR6730	1.02E+08	microRNA 6730
ENSG0000	7.113422	-0.4594	0.941716	-0.48784	0.625666	0.837065	RLN1	6013	relaxin 1
ENSG0000	11.81581	-0.3756	0.770037	-0.48777	0.625715	0.837069	PSME2P2	338099	proteasome activator subunit 2 pseudogene 2
ENSG0000	22.22653	0.26146	0.536421	0.487416	0.625964	0.837342	NA	NA	NA
ENSG0000	24.08051	-0.2924	0.600371	-0.48703	0.626236	0.837645	HELLS	3070	helicase lymphoid specific
ENSG0000	267.0898	0.088229	0.181208	0.486892	0.626335	0.837718	ZNF821	55565	zinc finger protein 821
ENSG0000	7.41321	-1.3466	2.766604	-0.48673	0.626448	0.837808	RPLP0P3	122589	ribosomal protein lateral stalk subunit P0 pseudogene 3
ENSG0000	73.84169	-0.13338	0.274075	-0.48666	0.6265	0.837818	BEGAIN	57596	brain enriched guanylate kinase associated
ENSG0000	2071.175	-0.04044	0.083253	-0.48574	0.62715	0.837844	SMPD4	55627	sphingomyelin phosphodiesterase 4
ENSG0000	1605.456	0.051992	0.1069	0.486359	0.626713	0.837844	PPIG	9360	peptidylprolyl isomerase G
ENSG0000	8.044586	-0.51516	1.060079	-0.48596	0.626995	0.837844	IRAK1BP1	134728	interleukin 1 receptor associated kinase 1 binding protein 1
ENSG0000	91.1084	0.124669	0.256261	0.486491	0.626619	0.837844	NDUFAF6	137682	NADH:ubiquinone oxidoreductase complex assembly factor 6
ENSG0000	5.143246	-0.66414	1.365334	-0.48643	0.626664	0.837844	ROR2	4920	receptor tyrosine kinase like orphan receptor 2
ENSG0000	760.4131	-0.0516	0.106181	-0.48599	0.626978	0.837844	ZDHHC16	84287	zinc finger DHHC-type palmitoyltransferase 16
ENSG0000	3907.399	-0.0393	0.080843	-0.48613	0.626873	0.837844	ULK1	8408	unc-51 like autophagy activating kinase 1
ENSG0000	10212.04	0.035219	0.072504	0.485755	0.627141	0.837844	STK24	8428	serine/threonine kinase 24
ENSG0000	5.128248	-0.57019	1.172833	-0.48616	0.626851	0.837844	IGHV3-43	28426	immunoglobulin heavy variable 3-43
ENSG0000	5.065346	0.613631	1.263293	0.48574	0.627152	0.837844	TVP23A	780776	trans-golgi network vesicle protein 23 homolog A
ENSG0000	230.6232	-0.09486	0.19497	-0.48655	0.626574	0.837844	NA	NA	NA
ENSG0000	2432.758	0.04493	0.092442	0.486034	0.626943	0.837844	SMARCD2	6603	SWI/SNF r matrix ass actin depe subfamily member 2
ENSG0000	1577.176	0.043656	0.089862	0.485811	0.627101	0.837844	WDR45B	56270	WD repeat domain 45B
ENSG0000	2.789059	0.886051	1.822135	0.486271	0.626775	0.837844	KLC3	147700	kinesin light chain 3
ENSG0000	66.98273	-0.14896	0.306788	-0.48555	0.627283	0.837898	ACTA2	59	actin alpha smooth muscle
ENSG0000	77.21789	-0.14179	0.291991	-0.48559	0.62726	0.837898	UQCC3	790955	ubiquinol-cytochrome c reductase complex assembly factor 3
ENSG0000	275.1455	-0.08706	0.179382	-0.48536	0.627423	0.837998	TTC21B	79809	tetratricopeptide repeat domain 21B
ENSG0000	5.396449	0.478676	0.986305	0.485322	0.627448	0.837998	NA	NA	NA
ENSG0000	17.59345	-0.74165	1.528464	-0.48522	0.627518	0.838031	XCR1	2829	X-C motif chemokine receptor 1
ENSG0000	32.91619	0.23145	0.477341	0.484874	0.627766	0.83804	KDF1	126695	keratinocyte differentiation factor 1
ENSG0000	4.204122	-0.59021	1.216814	-0.48505	0.627643	0.83804	NA	NA	NA
ENSG0000	115.119	-0.11122	0.229441	-0.48473	0.627866	0.83804	NFXL1	152518	nuclear tra X-box binding like 1
ENSG0000	2.742121	-0.88812	1.831598	-0.48489	0.627757	0.83804	NA	NA	NA
ENSG0000	143.7567	0.097361	0.200866	0.484704	0.627886	0.83804	NA	NA	NA
ENSG0000	76.72223	-0.16769	0.345759	-0.48498	0.627688	0.83804	IGHV5-51	28388	immunoglobulin heavy variable 5-51
ENSG0000	396.5613	-0.07482	0.154288	-0.48491	0.627739	0.83804	NA	NA	NA
ENSG0000	44.95949	0.221088	0.45602	0.48482	0.627804	0.83804	ZNF257	113835	zinc finger protein 257
ENSG0000	26.42283	0.237895	0.491326	0.484189	0.628251	0.838115	NA	NA	NA
ENSG0000	11.46042	0.361887	0.747108	0.484384	0.628113	0.838115	PRLR	5618	prolactin receptor
ENSG0000	4.135692	-0.54184	1.119235	-0.48412	0.628304	0.838115	SYNGAP1-	1.12E+08	SYNGAP1 antisense RNA 1

ENSG0000	8.305609	-0.43425	0.896735	-0.48426	0.628205	0.838115	KHDC1	80759	KH domain containing 1
ENSG0000	1515.927	-0.0458	0.094593	-0.48414	0.628288	0.838115	PLAAT4	5920	phospholipase A and acyltransferase 4
ENSG0000	13.2538	0.324805	0.670763	0.484233	0.628221	0.838115	NA	NA	NA
ENSG0000	18.16616	0.309185	0.638617	0.484147	0.628282	0.838115	MYO15A	51168	myosin XVA
ENSG0000	1339.936	-0.04834	0.09981	-0.48428	0.628184	0.838115	PIGS	94005	phosphatidylinositol glycan anchor biosynthesis class S
ENSG0000	11.76098	0.402508	0.831724	0.483945	0.628425	0.838217	TRAV39	28642	T cell receptor alpha variable 39
ENSG0000	29.4608	-0.21703	0.4488	-0.48359	0.62868	0.838497	TIMM8A	1678	translocase of inner mitochondrial membrane 8A
ENSG0000	154.7875	0.100376	0.207598	0.483514	0.628731	0.838503	TYW5	129450	tRNA-yW synthesizing protein 5
ENSG0000	174.0561	-0.09547	0.197488	-0.4834	0.62881	0.838548	ZGPAT	84619	zinc finger CCCH-type and G-patch domain containing
ENSG0000	1469.112	-0.05169	0.106953	-0.4833	0.628885	0.838588	EFR3A	23167	EFR3 homolog A
ENSG0000	145.5494	0.139975	0.289028	0.482913	0.629157	0.838831	TPM3P9	147804	tropomyosin 3 pseudogene 9
ENSG0000	4.288409	0.656025	1.358402	0.482938	0.629139	0.838831	CXADR	1525	CXADR Ig-like cell adhesion molecule
ENSG0000	297.9034	-0.07332	0.151906	-0.48268	0.629324	0.838993	NENF	29937	neudesin neurotrophic factor
ENSG0000	1038.839	-0.04883	0.101178	-0.48259	0.629385	0.839014	KDM1A	23028	lysine demethylase 1A
ENSG0000	35.55844	-0.20372	0.422241	-0.48248	0.629468	0.839065	METTL25	84190	methyltransferase like 25
ENSG0000	7.727899	-0.4486	0.930077	-0.48233	0.629574	0.839145	AATBC	284837	apoptosis associated transcript in bladder cancer
ENSG0000	307.84	0.07054	0.146363	0.48195	0.629841	0.839317	GOLPH3L	55204	golgi phosphoprotein 3 like
ENSG0000	12.58446	0.315919	0.655843	0.481699	0.63002	0.839317	KIF1A	547	kinesin family member 1A
ENSG0000	7.832179	-0.62127	1.289471	-0.4818	0.629946	0.839317	SLC35G2	80723	solute carrier family 35 member G2
ENSG0000	5.047067	-0.54312	1.127433	-0.48173	0.629996	0.839317	NA	NA	NA
ENSG0000	219.5861	0.084358	0.17512	0.481714	0.630009	0.839317	ZFAND1	79752	zinc finger AN1-type containing 1
ENSG0000	211.6143	0.091616	0.190165	0.48177	0.629969	0.839317	ADPRM	56985	ADP-ribos manganese dependent
ENSG0000	269.9877	-0.08059	0.167277	-0.48176	0.629979	0.839317	SLC9A6	10479	solute carrier family 9 member A6
ENSG0000	88.83789	0.177845	0.369268	0.481616	0.630079	0.839336	SLC4A1	6521	solute carrier family 4 member 1 (Diego blood group)
ENSG0000	1487.595	0.04383	0.09107	0.48128	0.630317	0.839338	DUSP10	11221	dual specificity phosphatase 10
ENSG0000	2.235582	-0.93111	1.934538	-0.48131	0.630299	0.839338	RBMS3	27303	RNA binding motif single stranded interacting protein 3
ENSG0000	94.70866	-0.13393	0.278241	-0.48135	0.630269	0.839338	DLEC1	9940	DLEC1 cilia and flagella associated protein
ENSG0000	9505.8	0.038555	0.080126	0.481185	0.630385	0.839338	HLA-DPB1	3115	major hist class II DP beta 1
ENSG0000	1.783946	1.03264	2.144445	0.481542	0.630132	0.839338	HOXA10-A	1.01E+08	HOXA10 antisense RNA
ENSG0000	7.125356	0.442555	0.919351	0.481378	0.630248	0.839338	NA	NA	NA
ENSG0000	42.84371	-0.22466	0.466896	-0.48117	0.630397	0.839338	FUNDC1	139341	FUN14 domain containing 1
ENSG0000	249.2761	-0.08553	0.177927	-0.48072	0.630715	0.839358	ODR4	54953	odr-4 GPCR localization factor homolog
ENSG0000	85.94367	-0.13915	0.289302	-0.481	0.63052	0.839358	CDK18	5129	cyclin dependent kinase 18
ENSG0000	219.5581	-0.08284	0.172305	-0.4808	0.630658	0.839358	DOLK	22845	dolichol kinase
ENSG0000	5.543339	-0.51705	1.07561	-0.4807	0.630729	0.839358	NA	NA	NA
ENSG0000	15.96681	-0.35716	0.74291	-0.48076	0.630684	0.839358	NA	NA	NA
ENSG0000	115.4368	0.111138	0.231165	0.480775	0.630676	0.839358	ZNF17	7565	zinc finger protein 17
ENSG0000	71.69693	-0.1493	0.310482	-0.48086	0.630615	0.839358	KCNG1	3755	potassium voltage-gated channel modifier subfamily G member 1
ENSG0000	5.33712	0.495674	1.03242	0.480109	0.63115	0.839617	NA	NA	NA
ENSG0000	45.79682	0.168896	0.351672	0.480265	0.631039	0.839617	NA	NA	NA
ENSG0000	59.83196	0.163288	0.34008	0.480146	0.631123	0.839617	NECTIN3	25945	nectin cell adhesion molecule 3
ENSG0000	1365.505	0.042291	0.08807	0.480199	0.631086	0.839617	CTBP2	1488	C-terminal binding protein 2
ENSG0000	14.17131	0.286109	0.59575	0.48025	0.63105	0.839617	NA	NA	NA
ENSG0000	107.838	0.129253	0.269312	0.479939	0.631271	0.839705	FAM234B	57613	family with sequence similarity 234 member B
ENSG0000	38.63711	0.192253	0.400619	0.479889	0.631306	0.839705	RFC3	5983	replication factor C subunit 3
ENSG0000	45.1239	0.17091	0.356528	0.479372	0.631674	0.840011	NKD2	85409	NKD inhibitor of WNT signaling pathway 2
ENSG0000	4.0274	-0.55775	1.163389	-0.47942	0.631643	0.840011	RPL32P1	6163	ribosomal protein L32 pseudogene 1
ENSG0000	127.69	-0.11215	0.23399	-0.47931	0.631718	0.840011	CENPBD1F	92806	CENPB DN pseudogene
ENSG0000	8.13775	-0.37878	0.790155	-0.47937	0.631676	0.840011	NA	NA	NA
ENSG0000	10.31882	-0.33239	0.693624	-0.47921	0.631788	0.840043	NA	NA	NA
ENSG0000	67.38285	0.148231	0.309417	0.479065	0.631893	0.840071	EFEMP2	30008	EGF containing fibulin extracellular matrix protein 2
ENSG0000	620.1617	-0.05373	0.112166	-0.47906	0.631899	0.840071	SUV39H1	6839	SUV39H1 histone lysine methyltransferase
ENSG0000	26.77114	-0.22325	0.466167	-0.4789	0.632012	0.840161	NA	NA	NA
ENSG0000	18.97909	0.363455	0.759188	0.478741	0.632123	0.840248	IGLV7-43	28776	immunoglobulin lambda variable 7-43
ENSG0000	3.880694	-0.57423	1.200212	-0.47844	0.632338	0.840414	OAZ3	51686	ornithine decarboxylase antizyme 3
ENSG0000	2.969731	-0.82242	1.718842	-0.47847	0.632313	0.840414	GHSR	2693	growth hormone secretagogue receptor
ENSG0000	214.6127	-0.0808	0.16896	-0.47824	0.632478	0.840419	CPOX	1371	coproporphyrinogen oxidase
ENSG0000	846.4079	0.058915	0.123189	0.478246	0.632475	0.840419	PFDN1	5201	prefoldin subunit 1
ENSG0000	504.0317	-0.06099	0.127515	-0.47833	0.632414	0.840419	ZNF211	10520	zinc finger protein 211
ENSG0000	26.09646	0.211906	0.44323	0.478095	0.632582	0.840497	CHEK1	1111	checkpoint kinase 1
ENSG0000	548.9145	-0.06235	0.130448	-0.47799	0.632659	0.84054	ZNF48	197407	zinc finger protein 48
ENSG0000	18.57223	-0.27177	0.569165	-0.4775	0.633008	0.840592	INAVA	55765	innate immunity activator
ENSG0000	358.5429	-0.07132	0.14928	-0.47773	0.63284	0.840592	GFPT2	9945	glutamine-fructose-6-phosphate transaminase 2
ENSG0000	70.68003	0.173397	0.36286	0.477862	0.632749	0.840592	MTRES1	51250	mitochondrial transcription rescue factor 1
ENSG0000	806.3413	0.053805	0.112725	0.47731	0.633141	0.840592	SESN1	27244	sestrin 1
ENSG0000	3.930631	0.542726	1.136807	0.477413	0.633068	0.840592	EEF1B2P6	647030	eukaryotic translation elongation factor 1 beta 2 pseudogene 6
ENSG0000	245.685	0.094138	0.197194	0.477388	0.633086	0.840592	TSPAN31	6302	tetraspanin 31
ENSG0000	742.2404	0.052496	0.109968	0.477372	0.633097	0.840592	ZNF263	10127	zinc finger protein 263
ENSG0000	799.4756	0.054089	0.113279	0.477485	0.633017	0.840592	TERF2	7014	telomeric repeat binding factor 2
ENSG0000	69.19712	0.13649	0.285812	0.47755	0.63297	0.840592	HSCB	150274	HscB mitochondrial iron-sulfur cluster cochaperone
ENSG0000	7.915416	-0.41768	0.875103	-0.47729	0.633152	0.840592	NA	NA	NA
ENSG0000	231.3172	-0.08828	0.185185	-0.47671	0.633571	0.841088	ATPAF2	91647	ATP synthase mitochondrial F1 complex assembly factor 2
ENSG0000	6.652162	-0.45221	0.949104	-0.47646	0.63375	0.84119	RIMKLA	284716	ribosomal modification protein rimK like family member A
ENSG0000	37.46171	-0.19777	0.415374	-0.47613	0.63398	0.84119	PDE6B	5158	phosphodiesterase 6B
ENSG0000	8.310968	-0.49004	1.02871	-0.47637	0.633814	0.84119	NA	NA	NA
ENSG0000	7.658527	-0.4322	0.90745	-0.47628	0.633871	0.84119	TMEM117	84216	transmembrane protein 117
ENSG0000	4.579258	0.575576	1.208588	0.476239	0.633904	0.84119	IGHV3-20	28445	immunoglobulin heavy variable 3-20
ENSG0000	106.2633	-0.13243	0.278168	-0.47609	0.63401	0.84119	SPNS1	83985	sphingolipid transporter 1 (putative)
ENSG0000	3.545474	-0.64237	1.349079	-0.47616	0.633963	0.84119	NA	NA	NA
ENSG0000	8.392826	-0.51002	1.070848	-0.47627	0.633879	0.84119	KLHL34	257240	kelch like family member 34
ENSG0000	83.18092	0.136051	0.285921	0.475834	0.634192	0.841267	AMY2B	280	amylase alpha 2B
ENSG0000	21.87452	-0.25703	0.540269	-0.47575	0.63425	0.841267	INKA1	389119	inka box actin regulator 1
ENSG0000	18.63376	-0.25844	0.543153	-0.47581	0.63421	0.841267	NA	NA	NA
ENSG0000	26.31637	0.246483	0.518659	0.475926	0.634127	0.841267	OTUB2	78990	OTU deubi ubiquitin aldehyde binding 2
ENSG0000	770.3899	0.051962	0.109258	0.475588	0.634368	0.841363	HOOK3	84376	hook microtubule tethering protein 3
ENSG0000	2740.486	-0.04801	0.101045	-0.47516	0.634672	0.841424	AAK1	22848	AP2 associated kinase 1
ENSG0000	7260.953	-0.0368	0.077447	-0.47515	0.634677	0.841424	SH3BP2	6452	SH3 domain binding protein 2
ENSG0000	42.15528	-0.21058	0.443214	-0.47512	0.634704	0.841424	SGCB	6443	sarcoglycan beta
ENSG0000	143.6148	-0.10766	0.226608	-0.4751	0.634718	0.841424	SNCA	6622	synuclein alpha
ENSG0000	297.1936	0.080434	0.169215	0.475338	0.634546	0.841424	PRXL2C	195827	peroxiredoxin like 2C
ENSG0000	3.609662	0.72303	1.521122	0.475327	0.634554	0.841424	MIR3677	1.01E+08	microRNA 3677
ENSG0000	730.6714	0.068757	0.144727	0.475078	0.634731	0.841424	ATP5PF	522	ATP synthase peripheral stalk subunit F6
ENSG0000	1.617999	-1.2316	2.592787	-0.47501	0.634781	0.841429	DDX10P1	1E+08	DEAD-box helicase 10 pseudogene 1
ENSG0000	377.5605	0.068192	0.143621	0.474803	0.634927	0.841503	NHP2	55651	NHP2 ribonucleoprotein
ENSG0000	182.2467	0.0884	0.186163	0.474852	0.634892	0.841503	MRM3	55178	mitochondrial rRNA methyltransferase 3
ENSG0000	30.87432	0.210292	0.443012	0.474688	0.635009	0.841521	NA	NA	NA
ENSG0000	11.6169	0.327891	0.690795	0.474657	0.635031	0.841521	APBA1	320	amyloid beta precursor protein binding family A member 1
ENSG0000	40.98593	-0.22462	0.473315	-0.47456	0.635098	0.841549	BIVM	54841	basic immunoglobulin-like variable motif containing
ENSG0000	12.35794	-0.32441	0.684164	-0.47417	0.635377	0.841752	CAP1P1	401076	CAP1 pseudogene 1
ENSG0000	606.8738	0.056819	0.119828	0.474168	0.63538	0.841752	SIMC1	375484	SUMO interacting motifs containing 1

ENSG0000	159.288	0.106309	0.224241	0.474086	0.635439	0.841752	ORC3	23595	origin recognition complex subunit 3
ENSG0000	65.92231	0.136891	0.288727	0.474117	0.635416	0.841752	PSMD9	5715	proteasom non-ATPase 9
ENSG0000	207.3822	0.088606	0.186921	0.47403	0.635478	0.841752	AKTIP	64400	AKT interacting protein
ENSG0000	11.19497	0.338328	0.714007	0.473844	0.635611	0.841868	NA	NA	NA
ENSG0000	4.534533	0.600583	1.268811	0.473343	0.635968	0.84204	AK3P3	1E+08	adenylate kinase 3 pseudogene 3
ENSG0000	2828.77	-0.03935	0.083124	-0.47338	0.635944	0.84204	MEN1	4221	menin 1
ENSG0000	9.618085	0.359677	0.759745	0.473418	0.635915	0.84204	NA	NA	NA
ENSG0000	7.664128	-0.42614	0.899932	-0.47353	0.635838	0.84204	NA	NA	NA
ENSG0000	53.99756	-0.16422	0.346882	-0.4734	0.635925	0.84204	PITPNA-AS	1E+08	PITPNA antisense RNA 1
ENSG0000	389.1437	-0.07449	0.157443	-0.47313	0.636119	0.84218	FAM98A	25940	family with sequence similarity 98 member A
ENSG0000	316.8626	0.081395	0.172067	0.473043	0.636182	0.842204	GDAP2	54834	ganglioside induced differentiation associated protein 2
ENSG0000	2529.53	0.040592	0.085843	0.472861	0.636312	0.842315	RAD23B	5887	RAD23 hor nucleotide excision repair protein
ENSG0000	503.1539	-0.05832	0.12338	-0.47271	0.636419	0.842397	WASHC1	1E+08	WASH complex subunit 1
ENSG0000	298.2845	0.081155	0.171788	0.472416	0.63663	0.842423	DPM3	54344	dolichyl-pl regulatory
ENSG0000	1782.749	-0.04197	0.088823	-0.47249	0.636579	0.842423	IMMT	10989	inner membrane mitochondrial protein
ENSG0000	4.603596	0.608917	1.288423	0.472606	0.636494	0.842423	LOC12490	1.25E+08	uncharacterized LOC124901990
ENSG0000	20.07577	-0.26682	0.564851	-0.47238	0.636655	0.842423	NA	NA	NA
ENSG0000	24.30184	-0.25709	0.544262	-0.47237	0.636666	0.842423	MAGIX	79917	MAGI fam X-linked
ENSG0000	2.612513	-0.87547	1.853894	-0.47223	0.636761	0.842488	MIR3153	1E+08	microRNA 3153
ENSG0000	38.20029	-0.19782	0.418979	-0.47215	0.636819	0.842506	NA	NA	NA
ENSG0000	3.967453	-0.6429	1.362293	-0.47192	0.636982	0.842512	CLEC20A	400797	C-type lectin domain containing 20A
ENSG0000	1431.709	0.04648	0.098483	0.471955	0.636959	0.842512	TBRG4	9238	transforming growth factor beta regulator 4
ENSG0000	6.638082	0.468553	0.992731	0.471984	0.636938	0.842512	DDX11L16	727856	DEAD/H-box helicase 11 like 16 (pseudogene)
ENSG0000	66.06034	0.142766	0.302541	0.471889	0.637006	0.842512	HOXB-AS1	1.01E+08	HOXB cluster antisense RNA 1
ENSG0000	48.74197	-0.18083	0.383413	-0.47164	0.637182	0.842561	SRGAP3	9901	SLIT-ROBO Rho GTPase activating protein 3
ENSG0000	123.8565	0.125943	0.267064	0.471582	0.637225	0.842561	CAPN5	726	calpain 5
ENSG0000	28.11222	0.219494	0.46527	0.471755	0.637102	0.842561	VKORC1	79001	vitamin K epoxide reductase complex subunit 1
ENSG0000	294.8363	-0.07103	0.150623	-0.47159	0.637221	0.842561	BABAM1	29086	BRISC and BRCA1 A complex member 1
ENSG0000	100.1731	0.118374	0.251162	0.471306	0.637422	0.842642	KCTD6	200845	potassium channel tetramerization domain containing 6
ENSG0000	13.04069	0.343243	0.728277	0.471309	0.63742	0.842642	UCHL3	7347	ubiquitin C-terminal hydrolase L3
ENSG0000	115.6341	-0.11201	0.237631	-0.47137	0.637377	0.842642	ZNF542P	147947	zinc finger pseudogene
ENSG0000	9290.521	-0.03403	0.072235	-0.47108	0.637581	0.842643	CSF3R	1441	colony stimulating factor 3 receptor
ENSG0000	61.8453	0.156432	0.332092	0.471051	0.637605	0.842643	NA	NA	NA
ENSG0000	6.046928	-0.46028	0.976894	-0.47117	0.63752	0.842643	NA	NA	NA
ENSG0000	2459.552	-0.04218	0.089529	-0.47111	0.637559	0.842643	CTDNEP1	23399	CTD nuclear envelope phosphatase 1
ENSG0000	22.15182	0.264148	0.561375	0.470538	0.637971	0.843067	NA	NA	NA
ENSG0000	73.50517	0.146843	0.312118	0.470472	0.638018	0.843069	LINC00662	148189	long intergenic non-protein coding RNA 662
ENSG0000	759.8615	0.061419	0.130636	0.470154	0.638245	0.843249	CARD6	84674	caspase recruitment domain family member 6
ENSG0000	28.39736	-0.2277	0.484253	-0.47021	0.638205	0.843249	SMIM8	57150	small integral membrane protein 8
ENSG0000	476.6427	-0.07077	0.150552	-0.47007	0.638304	0.843261	SLAMF1	6504	signaling lymphocytic activation molecule family member 1
ENSG0000	343.3595	-0.0658	0.140007	-0.46995	0.638391	0.843261	H2BC12	85236	H2B clustered histone 12
ENSG0000	397.4202	-0.06508	0.138479	-0.46999	0.638362	0.843261	ZNF778	197320	zinc finger protein 778
ENSG0000	3.958638	0.538513	1.146077	0.469875	0.638445	0.843272	TRAJ1	28754	T cell receptor alpha joining 1 (non-functional)
ENSG0000	73.59939	0.157137	0.334651	0.469555	0.638673	0.843334	PAQR6	79957	progestin and adipoQ receptor family member 6
ENSG0000	5.90247	-0.5159	1.098498	-0.46964	0.63861	0.843334	PTPRG	5793	protein tyrosine phosphatase receptor type G
ENSG0000	1310.82	0.050552	0.107646	0.469617	0.638629	0.843334	SF3B5	83443	splicing factor 3b subunit 5
ENSG0000	16.53566	0.297917	0.634342	0.469647	0.638607	0.843334	NA	NA	NA
ENSG0000	371.5539	0.07171	0.152768	0.469407	0.638779	0.843414	MCUR1	63933	mitochondrial calcium uniporter regulator 1
ENSG0000	8.321277	-0.38317	0.816749	-0.46913	0.638973	0.843608	KIF26A	26153	kinesin family member 26A
ENSG0000	34.22233	-0.20255	0.431802	-0.46907	0.639017	0.843608	MGAT5B	146664	alpha-1 6-mannosylglycoprotein 6-beta-N-acetylglucosaminyltransferase B
ENSG0000	45.80486	0.169417	0.361289	0.468922	0.639125	0.843691	SUSD2	56241	sushi domain containing 2
ENSG0000	511.817	0.061092	0.13039	0.468535	0.639402	0.843709	RAD1	5810	RAD1 checkpoint DNA exonuclease
ENSG0000	476.9486	0.067811	0.144647	0.468807	0.639208	0.843709	CUL2	8453	cullin 2
ENSG0000	1565.71	0.044785	0.095575	0.46858	0.63937	0.843709	C11orf68	83638	chromosome 11 open reading frame 68
ENSG0000	98.99069	-0.13199	0.281675	-0.4686	0.639352	0.843709	EIF3J-DT	645212	EIF3J divergent transcript
ENSG0000	15.58926	-0.32059	0.684245	-0.46853	0.639404	0.843709	RPH3AL-A	1.01E+08	RPH3AL antisense RNA 1
ENSG0000	509.8397	0.059376	0.126748	0.468458	0.639457	0.843709	DNTTIP1	116092	deoxynucleotidyltransferase terminal interacting protein 1
ENSG0000	6.264833	0.439758	0.938669	0.468491	0.639433	0.843709	PLS3	5358	plastin 3
ENSG0000	6.844678	0.463469	0.989989	0.468156	0.639673	0.843933	SMIM38	1.08E+08	small integral membrane protein 38
ENSG0000	69.94134	-0.1601	0.342248	-0.46779	0.639936	0.844002	LXN	56925	latexin
ENSG0000	5.645088	-0.49685	1.063074	-0.46738	0.640231	0.844002	LOC40112	401127	WD repeat domain 5 pseudogene
ENSG0000	40.41154	0.170341	0.364096	0.467846	0.639895	0.844002	NA	NA	NA
ENSG0000	3.954838	-0.66708	1.426839	-0.46752	0.640126	0.844002	VPS37D	155382	VPS37D subunit of ESCRT-I
ENSG0000	325.1467	-0.07177	0.153393	-0.46785	0.639891	0.844002	PGM2L1	283209	phosphoglucomutase 2 like 1
ENSG0000	6.096355	0.545081	1.164966	0.467894	0.63986	0.844002	GJB6	10804	gap junction protein beta 6
ENSG0000	5.035977	-0.53964	1.154331	-0.46749	0.640151	0.844002	NA	NA	NA
ENSG0000	84.92244	-0.13115	0.280441	-0.46764	0.640039	0.844002	NDUFAF5	79133	NADH:ubiquinone oxidoreductase complex assembly factor 5
ENSG0000	3.905256	0.661359	1.415178	0.467332	0.640262	0.844002	SGK2	10110	serum/glucocorticoid regulated kinase 2
ENSG0000	682.7854	0.054989	0.117611	0.467546	0.640109	0.844002	VAPB	9217	VAMP associated protein B and C
ENSG0000	1847.107	-0.0407	0.087024	-0.46763	0.640049	0.844002	KCNJ15	3772	potassium inwardly rectifying channel subfamily J member 15
ENSG0000	284.8605	-0.07823	0.167405	-0.46732	0.640271	0.844002	EOLA1	91966	endothelium and lymphocyte associated ASCH domain 1
ENSG0000	59.08688	-0.14278	0.305608	-0.46719	0.640361	0.844055	C2CD4D	1E+08	C2 calcium dependent domain containing 4D
ENSG0000	86.40501	0.136508	0.292232	0.467122	0.640412	0.844055	TLR5	7100	toll like receptor 5
ENSG0000	16.87889	-0.31928	0.683639	-0.46703	0.640477	0.844055	ALG1L13P	1.06E+08	ALG1 like pseudogene
ENSG0000	83.55864	-0.13659	0.292486	-0.46701	0.640493	0.844055	LRRC56	115399	leucine rich repeat containing 56
ENSG0000	4.466081	-0.65542	1.404574	-0.46663	0.640765	0.844307	NA	NA	NA
ENSG0000	1546.248	0.042994	0.092141	0.466614	0.640776	0.844307	ERP29	10961	endoplasmic reticulum protein 29
ENSG0000	6.10494	0.425992	0.913204	0.46648	0.640872	0.844314	KRT8P50	1.03E+08	keratin 8 pseudogene 50
ENSG0000	9.12987	0.420214	0.900809	0.466485	0.640868	0.844314	NAPSA	9476	napsin A aspartic peptidase
ENSG0000	20.1686	-0.28894	0.619603	-0.46633	0.640979	0.844336	NA	NA	NA
ENSG0000	16.06923	0.313937	0.673139	0.466378	0.640945	0.844336	NA	NA	NA
ENSG0000	20114.61	-0.03449	0.073974	-0.46625	0.641036	0.84435	MYO9B	4650	myosin IXB
ENSG0000	1745.952	0.043209	0.092727	0.465979	0.64123	0.844486	DDX56	54606	DEAD-box helicase 56
ENSG0000	4.713209	-0.67051	1.438844	-0.466	0.641213	0.844486	NA	NA	NA
ENSG0000	5.053002	-0.64879	1.392785	-0.46582	0.641344	0.844515	NA	NA	NA
ENSG0000	2741.215	-0.03793	0.081418	-0.46585	0.641319	0.844515	NPC2	10577	NPC intracellular cholesterol transporter 2
ENSG0000	21.07177	-0.26781	0.575503	-0.46535	0.64168	0.844898	LOC10012	1E+08	uncharacterized LOC100128494
ENSG0000	191.9991	-0.09489	0.204011	-0.46512	0.641843	0.844905	ORC4	5000	origin recognition complex subunit 4
ENSG0000	32.71661	-0.22494	0.483624	-0.46512	0.641843	0.844905	TRBJ1-4	28632	T cell receptor beta joining 1-4
ENSG0000	471.9393	0.061417	0.132054	0.465089	0.641868	0.844905	CAMK2G	818	calcium/calmodulin dependent protein kinase II gamma
ENSG0000	7.675792	0.471892	1.014346	0.465218	0.641776	0.844905	LPIN3	64900	lipin 3
ENSG0000	632.0878	-0.05391	0.115948	-0.46497	0.641956	0.844961	USPL1	10208	ubiquitin specific peptidase like 1
ENSG0000	1879.945	0.046859	0.100853	0.464628	0.642198	0.84516	CFD	1675	complement factor D
ENSG0000	58.03404	-0.14901	0.320697	-0.46464	0.642191	0.84516	N6AMT1	29104	N-6 adenine-specific DNA methyltransferase 1
ENSG0000	22.48403	-0.23696	0.510828	-0.46387	0.642741	0.845365	S100A2	6273	S100 calcium binding protein A2
ENSG0000	5.934032	0.469758	1.012374	0.464016	0.642636	0.845365	NA	NA	NA
ENSG0000	2.350736	0.960028	2.069749	0.463838	0.642764	0.845365	NA	NA	NA
ENSG0000	6.5455	-0.41626	0.897399	-0.46386	0.64275	0.845365	RPS15AP1	646819	ribosomal protein S15a pseudogene 19
ENSG0000	256.3585	0.07602	0.163801	0.464098	0.642578	0.845365	GFI1B	8328	growth factor independent 1B transcriptional repressor
ENSG0000	106.896	-0.14834	0.319564	-0.4642	0.642503	0.845365	TRAV26-1	28657	T cell receptor alpha variable 26-1

ENSG0000	3.584138	0.592068	1.27586	0.464054	0.642609	0.845365	NA	NA	NA
ENSG0000	50.78723	0.201587	0.434519	0.46393	0.642698	0.845365	PEX11G	92960	peroxisomal biogenesis factor 11 gamma
ENSG0000	116.7782	-0.12849	0.276782	-0.46422	0.642488	0.845365	ZNF343	79175	zinc finger protein 343
ENSG0000	47.96468	0.18848	0.406539	0.463621	0.642919	0.845509	HECW2-AS	1.02E+08	HECW2 antisense RNA 1
ENSG0000	34.32164	-0.18429	0.397716	-0.46337	0.643102	0.845691	GIN1	54826	gypsy retrotransposon integrase 1
ENSG0000	4.736099	-0.6455	1.393915	-0.46308	0.643305	0.845717	ENAH	55740	ENAH actin regulator
ENSG0000	4.987233	-0.52994	1.144105	-0.46319	0.643226	0.845717	OR2A7	401427	olfactory receptor family 2 subfamily A member 7
ENSG0000	623.4954	-0.05542	0.119677	-0.46309	0.643302	0.845717	COPS7A	50813	COP9 signalosome subunit 7A
ENSG0000	4.85974	-0.55702	1.202498	-0.46322	0.643209	0.845717	SLC5A10	125206	solute carrier family 5 member 10
ENSG0000	387.8391	0.061365	0.132573	0.46288	0.64345	0.845848	ZNF22	7570	zinc finger protein 22
ENSG0000	2623.418	0.041902	0.090555	0.462718	0.643566	0.845941	TUFM	7284	Tu translat mitochondrial
ENSG0000	11.98853	-0.42187	0.911883	-0.46264	0.643623	0.845955	CYP46A1	10858	cytochrome P450 family 46 subfamily A member 1
ENSG0000	1102.282	-0.04574	0.098896	-0.46256	0.643683	0.845974	YIPF5	81555	Yip1 domain family member 5
ENSG0000	221.1998	0.081828	0.176958	0.462416	0.643783	0.846045	SLC29A2	3177	solute carrier family 29 member 2
ENSG0000	10.77156	-0.34735	0.751664	-0.46211	0.644001	0.846213	TRPC1	7220	transient receptor potential cation channel subfamily C member 1
ENSG0000	20.58532	-0.23638	0.511683	-0.46196	0.644107	0.846213	NA	NA	NA
ENSG0000	8.195731	-0.39644	0.858023	-0.46204	0.644055	0.846213	ANKK1	255239	ankyrin repeat and kinase domain containing 1
ENSG0000	1637.249	0.061279	0.13266	0.461921	0.644138	0.846213	NAB2	4665	NGFI-A binding protein 2
ENSG0000	2919.914	-0.0392	0.084857	-0.46195	0.644116	0.846213	BLTP2	9703	bridge-like lipid transfer protein family member 2
ENSG0000	60.05085	0.15628	0.338497	0.461688	0.644305	0.846372	AK9	221264	adenylate kinase 9
ENSG0000	176.3158	-0.0906	0.196395	-0.46132	0.644568	0.846411	C1orf131	128061	chromosome 1 open reading frame 131
ENSG0000	355.2939	-0.06842	0.148309	-0.46137	0.644536	0.846411	MLH1	4292	mutL homolog 1
ENSG0000	9.746329	-0.34694	0.751969	-0.46138	0.644527	0.846411	NA	NA	NA
ENSG0000	366.5072	-0.06332	0.13725	-0.46136	0.644544	0.846411	TIMM22	29928	translocase of inner mitochondrial membrane 22
ENSG0000	52.72255	0.163692	0.354856	0.461292	0.644589	0.846411	CADM4	199731	cell adhesion molecule 4
ENSG0000	5.151708	-0.50612	1.097249	-0.46126	0.644609	0.846411	GPR82	27197	G protein-coupled receptor 82
ENSG0000	344.5926	0.062604	0.135788	0.461045	0.644766	0.846558	TMEM205	374882	transmembrane protein 205
ENSG0000	408.9421	0.069198	0.150232	0.460607	0.645081	0.846911	TMEM203	94107	transmembrane protein 203
ENSG0000	265.6277	-0.07509	0.163067	-0.46048	0.645173	0.846913	NAE1	8883	NEDD8 activating enzyme E1 subunit 1
ENSG0000	24.88872	0.258212	0.560698	0.460518	0.645144	0.846913	LINC0018	193629	long intergenic non-protein coding RNA 189
ENSG0000	17.58564	0.251717	0.546817	0.460332	0.645278	0.84699	RPL24P8	1E+08	RPL24 pseudogene 8
ENSG0000	73.03839	0.128712	0.279947	0.459774	0.645678	0.847106	NA	NA	NA
ENSG0000	1555.623	-0.04217	0.091669	-0.46006	0.645475	0.847106	CIAO1	9391	cytosolic iron-sulfur assembly component 1
ENSG0000	19.13394	-0.24651	0.536107	-0.45981	0.645654	0.847106	COL4A4	1286	collagen type IV alpha 4 chain
ENSG0000	17.67444	0.256531	0.557519	0.46013	0.645423	0.847106	NA	NA	NA
ENSG0000	6.139233	0.416442	0.905403	0.459952	0.645551	0.847106	NA	NA	NA
ENSG0000	8.541624	0.416406	0.905694	0.459764	0.645686	0.847106	CTSV	1515	cathepsin V
ENSG0000	318.9684	0.076639	0.166676	0.45981	0.645653	0.847106	ESAM	90952	endothelial cell adhesion molecule
ENSG0000	27.86731	-0.21709	0.472247	-0.45969	0.645736	0.847112	ZNF790-AS	284408	ZNF790 antisense RNA 1
ENSG0000	2613.174	-0.03517	0.076527	-0.4596	0.645806	0.847144	DNAJB12	54788	DnaJ heat shock protein family (Hsp40) member B12
ENSG0000	3.686958	0.733889	1.598511	0.459108	0.646157	0.847484	ZC2HC1C	79696	zinc finger C2HC-type containing 1C
ENSG0000	2937.395	-0.03615	0.078727	-0.45917	0.646113	0.847484	PHF8	23133	PHD finger protein 8
ENSG0000	27.30663	-0.21394	0.466134	-0.45896	0.646261	0.847561	GUCY1A1	2982	guanylate cyclase 1 soluble subunit alpha 1
ENSG0000	75.49741	-0.14	0.30518	-0.45875	0.646412	0.847642	SLC18B1	116843	solute carrier family 18 member B1
ENSG0000	7.320295	-0.45916	1.000886	-0.45875	0.646414	0.847642	LINC0086	643529	long intergenic non-protein coding RNA 865
ENSG0000	3.768512	0.648404	1.413935	0.458581	0.646535	0.84774	NA	NA	NA
ENSG0000	7.975284	0.36326	0.792374	0.458445	0.646633	0.847808	NA	NA	NA
ENSG0000	6.168259	0.579129	1.265785	0.457526	0.647293	0.848156	NA	NA	NA
ENSG0000	4.932241	0.627504	1.371485	0.457536	0.647286	0.848156	COL5A2	1290	collagen type V alpha 2 chain
ENSG0000	46.76978	-0.17359	0.379117	-0.45787	0.647043	0.848156	BBS12	166379	Bardet-Biedl syndrome 12
ENSG0000	10.29863	-0.31253	0.683181	-0.45747	0.647335	0.848156	NA	NA	NA
ENSG0000	20.63469	0.250037	0.546301	0.457691	0.647174	0.848156	VARS2	57176	valyl-tRNA mitochondrial
ENSG0000	1921.605	0.043303	0.094664	0.45744	0.647355	0.848156	SYNGAP1	8831	synaptic Ras GTPase activating protein 1
ENSG0000	8.041365	0.38798	0.847512	0.457787	0.647106	0.848156	NA	NA	NA
ENSG0000	2.297387	-0.89134	1.94768	-0.45764	0.647209	0.848156	NA	NA	NA
ENSG0000	1.801801	1.046173	2.284327	0.457979	0.646968	0.848156	NA	NA	NA
ENSG0000	35.43576	0.180328	0.393774	0.457947	0.64699	0.848156	IGHV3-74	28408	immunoglobulin heavy variable 3-74
ENSG0000	32.23154	0.206144	0.450933	0.457151	0.647563	0.848368	TENM1	10178	teneurin transmembrane protein 1
ENSG0000	12.21607	0.309676	0.678217	0.456603	0.647956	0.848824	NA	NA	NA
ENSG0000	1300.043	-0.04656	0.101989	-0.45652	0.648015	0.84884	RARG	5916	retinoic acid receptor gamma
ENSG0000	88.1563	0.130412	0.285873	0.456187	0.648255	0.849095	GRK4	2868	G protein-coupled receptor kinase 4
ENSG0000	9.591567	0.330812	0.72527	0.456123	0.648302	0.849096	LOC64445	644456	IK cytokine down-regulator of HLA II pseudogene
ENSG0000	5.511176	-0.48238	1.058031	-0.45592	0.648445	0.849154	RPL19P6	1E+08	ribosomal protein L19 pseudogene 6
ENSG0000	5.690303	-0.53146	1.165815	-0.45587	0.648483	0.849154	IFNG-AS1	1.01E+08	IFNG antisense RNA 1
ENSG0000	5.42878	-0.48894	1.072353	-0.45595	0.648425	0.849154	MESP1	55897	mesoderm posterior bHLH transcription factor 1
ENSG0000	1.867786	1.074104	2.357069	0.455695	0.64861	0.84926	MAS1	4142	MAS1 prot G protein-coupled receptor
ENSG0000	112.027	-0.10991	0.241252	-0.45556	0.648705	0.849324	CDH1	999	cadherin 1
ENSG0000	8.282888	0.456502	1.00285	0.455205	0.648962	0.849498	TCF7L1	83439	transcription factor 7 like 1
ENSG0000	12.41893	-0.30482	0.669667	-0.45519	0.648975	0.849498	NA	NA	NA
ENSG0000	3.176553	0.651245	1.430673	0.455202	0.648964	0.849498	NA	NA	NA
ENSG0000	2256.435	-0.03939	0.086555	-0.45511	0.649033	0.849514	MTDH	92140	metadherin
ENSG0000	11.89583	-0.32056	0.704565	-0.45497	0.649131	0.849583	NEXMIF	340533	neurite extension and migration factor
ENSG0000	4.798663	-0.51217	1.126031	-0.45485	0.649218	0.849636	TTTY10	246119	testis-spec Y-linked 10
ENSG0000	20.36156	-0.2347	0.516234	-0.45464	0.649368	0.849772	H2BC9	8345	H2B clustered histone 9
ENSG0000	391.7444	0.075388	0.165841	0.454577	0.649414	0.849772	HAUS6	54801	HAUS augmin like complex subunit 6
ENSG0000	440.2639	-0.06889	0.151633	-0.45432	0.649597	0.849952	ZSCAN18	65982	zinc finger and SCAN domain containing 18
ENSG0000	4.619749	0.559533	1.232088	0.454134	0.649732	0.850069	LOC10537	1.05E+08	uncharacterized LOC105373383
ENSG0000	1540.265	-0.04165	0.09181	-0.45365	0.650083	0.850469	ZBTB44	29068	zinc finger and BTB domain containing 44
ENSG0000	587.9141	-0.05074	0.111886	-0.4535	0.650192	0.85049	GYG1	2992	glycogenin 1
ENSG0000	163.018	0.096343	0.212433	0.453522	0.650173	0.85049	YARS2	51067	tyrosyl-tRNA synthetase 2
ENSG0000	977.0946	0.044334	0.097841	0.45312	0.650462	0.850784	ZFPL1	7542	zinc finger protein like 1
ENSG0000	2643.847	-0.03825	0.084442	-0.453	0.650546	0.850834	SEPTIN7	989	septin 7
ENSG0000	206.4936	0.087545	0.193287	0.452926	0.650602	0.850847	C12orf75	387882	chromosome 12 open reading frame 75
ENSG0000	5.422136	-0.48303	1.066785	-0.45279	0.650697	0.850912	GPT	2875	glutamic--pyruvic transaminase
ENSG0000	18.45774	-0.24927	0.550611	-0.45272	0.650752	0.850923	NA	NA	NA
ENSG0000	10.9466	0.366791	0.810475	0.452563	0.650864	0.85095	ABCB6	10058	ATP binding cassette subfamily B member 6 (Langereis blood group)
ENSG0000	6.409819	-0.38822	0.85775	-0.4526	0.650838	0.85095	ODF3L1	161753	outer dense fiber of sperm tails 3 like 1
ENSG0000	59.5083	-0.1872	0.41372	-0.45248	0.650925	0.85097	U2AF1	7307	U2 small nuclear RNA auxiliary factor 1
ENSG0000	246.3056	0.078296	0.173132	0.452234	0.6511	0.851139	EED	8726	embryonic ectoderm development
ENSG0000	20.86288	0.234215	0.518352	0.451845	0.651381	0.851445	NA	NA	NA
ENSG0000	667.9117	-0.05302	0.11736	-0.45174	0.651454	0.851481	DNHD1	144132	dynein heavy chain domain 1
ENSG0000	164.0454	-0.10857	0.240541	-0.45136	0.651727	0.851508	DOCK7	85440	dedicator of cytokinesis 7
ENSG0000	2.895817	0.843313	1.867802	0.4515	0.651629	0.851508	SLC38A11	151258	solute carrier family 38 member 11
ENSG0000	295.6813	-0.07386	0.163589	-0.45147	0.651648	0.851508	SCAMP1	9522	secretory carrier membrane protein 1
ENSG0000	1671.192	0.039324	0.08713	0.451332	0.65175	0.851508	CYTH3	9265	cytohesin 3
ENSG0000	7.718866	-0.44042	0.975745	-0.45136	0.651727	0.851508	NA	NA	NA
ENSG0000	5.911564	-0.43571	0.964766	-0.45163	0.651538	0.851508	NA	NA	NA
ENSG0000	18.72715	-0.2636	0.584132	-0.45126	0.651801	0.851515	GABRR2	2570	gamma-aminobutyric acid type A receptor subunit rho2
ENSG0000	2.556571	0.853082	1.891798	0.450937	0.652035	0.85176	NA	NA	NA
ENSG0000	21.00764	0.26213	0.58156	0.450736	0.652179	0.851769	UGT8	7368	UDP glycosyltransferase 8

ENSG0000	3.133613	-0.80044	1.775586	-0.4508	0.652133	0.851769	NA	NA	NA
ENSG0000	4.588101	0.512562	1.137114	0.450757	0.652164	0.851769	NA	NA	NA
ENSG0000	5.398089	-0.50801	1.127255	-0.45067	0.652231	0.851776	TRIM40	135644	tripartite motif containing 40
ENSG0000	11.56331	-0.31882	0.70758	-0.45058	0.65229	0.851793	TEX22	647310	testis expressed 22
ENSG0000	91.95477	0.116336	0.258347	0.450309	0.652487	0.851991	BDH2	56898	3-hydroxybutyrate dehydrogenase 2
ENSG0000	78.21791	-0.13331	0.296442	-0.4497	0.652928	0.852507	CASP6	839	caspase 6
ENSG0000	4.646245	-0.55533	1.235805	-0.44937	0.653165	0.852755	AKR1E2	83592	aldo-keto reductase family 1 member E2
ENSG0000	975.9753	-0.05594	0.124498	-0.44929	0.653226	0.852764	EBLN3P	1.01E+08	endogeno pseudogene
ENSG0000	153.7303	0.100765	0.224303	0.449234	0.653263	0.852764	RETN	56729	resistin
ENSG0000	11.52744	-0.32124	0.715439	-0.44902	0.65342	0.852872	MIA2-AS1	1E+08	MIA2 antisense RNA 1
ENSG0000	72424.03	-0.03108	0.069211	-0.44899	0.653437	0.852872	SRRM2	23524	serine/arginine repetitive matrix 2
ENSG0000	1378.963	0.041504	0.09249	0.448737	0.653622	0.852965	PRPF38A	84950	pre-mRNA processing factor 38A
ENSG0000	35.89582	-0.21526	0.479741	-0.4487	0.653647	0.852965	NA	NA	NA
ENSG0000	1223.165	-0.04499	0.100245	-0.44879	0.653583	0.852965	ADGRL1	22859	adhesion G protein-coupled receptor L1
ENSG0000	17.82343	-0.2479	0.552852	-0.44839	0.653868	0.853194	RALY-AS1	1.02E+08	RALY antisense RNA 1
ENSG0000	3.267419	0.534633	1.193387	0.447996	0.654156	0.853496	NA	NA	NA
ENSG0000	121.2966	-0.11336	0.253059	-0.44795	0.654192	0.853496	ARHGEF5	7984	Rho guanine nucleotide exchange factor 5
ENSG0000	7.227437	0.415113	0.927249	0.447683	0.654382	0.853637	TRBV11-1	28582	T cell receptor beta variable 11-1
ENSG0000	17.89296	0.26293	0.587381	0.447631	0.654419	0.853637	DSEL	92126	dermatan sulfate epimerase like
ENSG0000	53.27166	0.16228	0.362552	0.447605	0.654438	0.853637	NANP	140838	N-acetylneuraminic acid phosphatase
ENSG0000	40.83551	-0.17819	0.398214	-0.44746	0.654539	0.853649	TRBV24-1	28563	T cell receptor beta variable 24-1
ENSG0000	217.5624	-0.09107	0.203519	-0.44749	0.654521	0.853649	SPSB2	84727	splA/ryanodine receptor domain and SOCS box containing 2
ENSG0000	96.9478	-0.1359	0.303783	-0.44736	0.654615	0.853687	SCAPER	49855	S-phase cyclin A associated protein in the ER
ENSG0000	834.8711	-0.04987	0.111503	-0.44722	0.654715	0.853757	STX6	10228	syntaxin 6
ENSG0000	541.7315	-0.06871	0.153666	-0.44713	0.654781	0.853784	ZNF185	7739	zinc finger protein 185 with LIM domain
ENSG0000	5.014619	-0.4807	1.075308	-0.44703	0.654851	0.853811	TRBJ1-1	28635	T cell receptor beta joining 1-1
ENSG0000	382.4304	0.063665	0.142436	0.446974	0.654894	0.853811	NAA25	80018	N-alpha-ac NatB auxiliary subunit
ENSG0000	25.10178	0.205651	0.460578	0.446507	0.655231	0.854131	FZD8	8325	frizzled class receptor 8
ENSG0000	141.9442	-0.12129	0.271635	-0.44653	0.655211	0.854131	ACTR6	64431	actin related protein 6
ENSG0000	196.4894	-0.08811	0.197418	-0.4463	0.65538	0.854205	PATJ	10207	PATJ crumbs cell polarity complex component
ENSG0000	3.140129	-0.63203	1.416146	-0.44631	0.655376	0.854205	LINC00562	1.01E+08	long intergenic non-protein coding RNA 562
ENSG0000	840.82	-0.04797	0.107567	-0.44596	0.655625	0.854205	MRPL20	55052	mitochondrial ribosomal protein L20
ENSG0000	1052.071	-0.05121	0.114823	-0.44597	0.655618	0.854205	MFSD14B	84641	major facilitator superfamily domain containing 14B
ENSG0000	3.639597	-0.6777	1.519587	-0.44597	0.655616	0.854205	SLIT1	6585	slit guidance ligand 1
ENSG0000	6.796019	-0.46581	1.044522	-0.44596	0.65563	0.854205	NA	NA	NA
ENSG0000	504.1953	-0.0565	0.126698	-0.44592	0.655657	0.854205	DDX52	11056	DExD-box helicase 52
ENSG0000	3.129614	0.595179	1.334675	0.445935	0.655644	0.854205	NA	NA	NA
ENSG0000	719.4784	0.06037	0.13547	0.445636	0.65586	0.854411	TSPAN33	340348	tetraspanin 33
ENSG0000	1258.644	-0.04824	0.108283	-0.44548	0.655974	0.854413	PSMB9	5698	proteasome 20S subunit beta 9
ENSG0000	59.81055	0.134546	0.302064	0.445424	0.656014	0.854413	MAD1L1	8379	mitotic arrest deficient 1 like 1
ENSG0000	977.2695	-0.04462	0.10017	-0.4454	0.656033	0.854413	RNASEH2C	84153	ribonuclease H2 subunit C
ENSG0000	2.397597	-0.94733	2.127021	-0.44538	0.656047	0.854413	SLC14A2	8170	solute carrier family 14 member 2
ENSG0000	143.9806	-0.09133	0.205295	-0.44487	0.656413	0.85483	CRADD	8738	CASP2 and RIPK1 domain containing adaptor with death domain
ENSG0000	24.16963	0.240628	0.541073	0.444724	0.656519	0.854878	CRABP2	1382	cellular retinoic acid binding protein 2
ENSG0000	49.47786	0.155359	0.349413	0.444629	0.656588	0.854878	SUPT3H	8464	SPT3 hom SAGA and STAGA complex component
ENSG0000	37.60291	-0.18184	0.408934	-0.44466	0.656565	0.854878	GRIN2D	2906	glutamate ionotropic receptor NMDA type subunit 2D
ENSG0000	29.81683	0.240889	0.542076	0.444384	0.656765	0.854978	LYRM4-AS	1E+08	LYRM4 antisense RNA 1
ENSG0000	15.21418	-0.82153	1.848673	-0.44439	0.656762	0.854978	NA	NA	NA
ENSG0000	1670.157	0.044352	0.099818	0.444332	0.656803	0.854978	GRAP2	9402	GRB2 related adaptor protein 2
ENSG0000	10.46217	-0.3303	0.743876	-0.44402	0.657027	0.855088	POLN	353497	DNA polymerase nu
ENSG0000	89.18969	0.12087	0.272255	0.443959	0.657072	0.855088	NCAPG2	54892	non-SMC condensin II complex subunit G2
ENSG0000	2.989024	0.781758	1.760665	0.444013	0.657033	0.855088	LOC10050	1.01E+08	uncharacterized LOC100505915
ENSG0000	48.06885	0.170402	0.3837	0.444102	0.656969	0.855088	CHEK2	11200	checkpoint kinase 2
ENSG0000	4.792098	0.523113	1.178521	0.443872	0.657135	0.85511	NA	NA	NA
ENSG0000	3.345423	0.658691	1.484502	0.443712	0.657251	0.855153	NUDT6	11162	nudix hydrolase 6
ENSG0000	12.64171	-0.28101	0.633324	-0.4437	0.65726	0.855153	NA	NA	NA
ENSG0000	15.76164	-0.32034	0.722102	-0.44362	0.657319	0.85517	LRFN2	57497	leucine rich repeat and fibronectin type III domain containing 2
ENSG0000	19.01353	0.25276	0.570151	0.443321	0.657534	0.855329	NA	NA	NA
ENSG0000	89.75901	-0.11129	0.251022	-0.44336	0.657506	0.855329	IGFLR1	79713	IGF like family receptor 1
ENSG0000	19.27278	-0.24652	0.556381	-0.44307	0.657715	0.855505	NA	NA	NA
ENSG0000	106.7945	0.100342	0.226515	0.44298	0.65778	0.855525	FCRL1	115350	Fc receptor like 1
ENSG0000	3.823009	-0.66811	1.508419	-0.44292	0.657823	0.855525	GPR52	9293	G protein-coupled receptor 52
ENSG0000	57.25715	0.146825	0.331681	0.442669	0.658005	0.855702	ADAM12	8038	ADAM metallopeptidase domain 12
ENSG0000	8.152484	-0.37893	0.856165	-0.44259	0.658065	0.85572	NA	NA	NA
ENSG0000	25.18962	0.222433	0.502813	0.442376	0.658217	0.855858	ZBED2	79413	zinc finger BED-type containing 2
ENSG0000	39.7446	-0.21648	0.48987	-0.44192	0.658551	0.855924	AGMAT	79814	agmatinase
ENSG0000	503.5195	0.060639	0.137395	0.441349	0.65896	0.855924	SNIP1	79753	Smad nuclear interacting protein 1
ENSG0000	5.30438	-0.5053	1.143843	-0.44176	0.658666	0.855924	KCNK1	3775	potassium two pore domain channel subfamily K member 1
ENSG0000	3.361699	0.554309	1.255123	0.441637	0.658752	0.855924	NA	NA	NA
ENSG0000	1337.621	-0.04036	0.091431	-0.44143	0.658903	0.855924	WDR33	55339	WD repeat domain 33
ENSG0000	12.95471	0.307332	0.695339	0.441988	0.658498	0.855924	LRRC37A1	1.06E+08	leucine ric pseudogene
ENSG0000	11.75299	0.292331	0.661936	0.44163	0.658757	0.855924	RPL21P80	1E+08	ribosomal protein L21 pseudogene 80
ENSG0000	303.6314	0.069761	0.157813	0.442048	0.658454	0.855924	MSANTD2	79684	Myb/SANT DNA binding domain containing 2
ENSG0000	472.1329	0.055436	0.125484	0.441781	0.658648	0.855924	UBR7	55148	ubiquitin protein ligase E3 component n-recognin 7
ENSG0000	138.4396	0.092857	0.210298	0.441549	0.658816	0.855924	RPL3P4	326307	ribosomal protein L3 pseudogene 4
ENSG0000	439.3725	-0.06275	0.142049	-0.44177	0.658658	0.855924	TTBK2	146057	tau tubulin kinase 2
ENSG0000	9.937943	0.360848	0.816141	0.442139	0.658389	0.855924	LOC61303	613038	SAGA complex associated factor 29 pseudogene
ENSG0000	3.051905	-0.72643	1.645703	-0.44141	0.658916	0.855924	CLEC18B	497190	C-type lectin domain family 18 member B
ENSG0000	21.20456	-0.25943	0.58721	-0.4418	0.658633	0.855924	GTSCR1	220158	Gilles de la candidate 1
ENSG0000	3.215165	-0.65308	1.479321	-0.44148	0.658869	0.855924	TFIP11-DT	1.01E+08	TFIP11 divergent transcript
ENSG0000	123.1983	-0.11942	0.271082	-0.44054	0.659549	0.856268	TPRKB	51002	TP53RK binding protein
ENSG0000	46.93303	-0.20536	0.465866	-0.44081	0.659352	0.856268	ADAMDEC	27299	ADAM like decysin 1
ENSG0000	59.96858	-0.15019	0.340908	-0.44057	0.659524	0.856268	IL17C	27189	interleukin 17C
ENSG0000	3100.352	0.0381	0.086481	0.440562	0.65953	0.856268	CDK12	51755	cyclin dependent kinase 12
ENSG0000	5053.977	0.035438	0.080432	0.440597	0.659505	0.856268	DNAJB1	3337	DnaJ heat shock protein family (Hsp40) member B1
ENSG0000	214.7939	0.074699	0.16947	0.440781	0.659372	0.856268	ZNF468	90333	zinc finger protein 468
ENSG0000	9.747744	-0.42644	0.967891	-0.44059	0.659511	0.856268	GGT1	2678	gamma-glutamyltransferase 1
ENSG0000	36.81074	-0.18894	0.429218	-0.4402	0.65979	0.856402	ECEL1	9427	endothelin converting enzyme like 1
ENSG0000	3.85322	-0.559	1.269516	-0.44033	0.659701	0.856402	NA	NA	NA
ENSG0000	142.8477	-0.09468	0.215076	-0.44023	0.659771	0.856402	ZNF649	65251	zinc finger protein 649
ENSG0000	34.1155	0.177139	0.402683	0.439897	0.660012	0.85657	RPSAP19	730029	ribosomal protein SA pseudogene 19
ENSG0000	9.013735	-0.40156	0.912751	-0.43995	0.659975	0.85657	SEC1P	653677	secretory l pseudogene
ENSG0000	4.539121	-0.61594	1.400953	-0.43966	0.660187	0.856617	PCDHGA1(	56106	protocadh 10
ENSG0000	2.660047	0.773616	1.759357	0.439715	0.660143	0.856617	DUOXA1	90527	dual oxidase maturation factor 1
ENSG0000	8.158992	0.437843	0.995676	0.439744	0.660123	0.856617	HOXB9	3219	homeobox B9
ENSG0000	3.655776	0.539808	1.228161	0.439525	0.660281	0.856619	NA	NA	NA
ENSG0000	67.52966	-0.12448	0.283219	-0.43954	0.660273	0.856619	ZNF280D	54816	zinc finger protein 280D
ENSG0000	235.4953	0.084797	0.192998	0.439366	0.660396	0.856709	PDE2A	5138	phosphodiesterase 2A
ENSG0000	2.47341	0.81678	1.859327	0.439288	0.660453	0.856723	IGHV2-70I	1.03E+08	immunoglobulin heavy variable 2-70D
ENSG0000	28.96018	-0.21095	0.480439	-0.43908	0.660604	0.856859	NA	NA	NA

ENSG0000	168.2217	0.084362	0.192197	0.438937	0.660707	0.856932	JAM3	83700	junctional adhesion molecule 3
ENSG0000	146.608	0.095395	0.217381	0.438835	0.660781	0.856968	SKA2	348235	spindle and kinetochore associated complex subunit 2
ENSG0000	6.314282	-0.41387	0.944469	-0.4382	0.661239	0.857063	NA	NA	NA
ENSG0000	7.978266	0.470124	1.0732	0.438058	0.661344	0.857063	NA	NA	NA
ENSG0000	11.2303	0.315636	0.719606	0.438624	0.660934	0.857063	STIMATE-I	1.01E+08	STIMATE-MUSTN1 readthrough
ENSG0000	302.0775	-0.06326	0.144416	-0.43803	0.661363	0.857063	NMD3	51068	NMD3 ribosome export adaptor
ENSG0000	967.6256	-0.04588	0.10465	-0.4384	0.661097	0.857063	SECISBP2	79048	SECIS binding protein 2
ENSG0000	8.101622	0.370315	0.844891	0.438299	0.661169	0.857063	NA	NA	NA
ENSG0000	6.506839	0.393086	0.896475	0.43848	0.661038	0.857063	PIPSL	266971	PIP5K1A and PSMD4 like (pseudogene)
ENSG0000	3162.784	0.037489	0.085569	0.438112	0.661305	0.857063	SLC8B1	80024	solute carrier family 8 member B1
ENSG0000	2.363527	-0.93542	2.132872	-0.43857	0.660971	0.857063	MT1L	4500	metallothi pseudogene
ENSG0000	17.06721	0.313375	0.714737	0.438449	0.661061	0.857063	NA	NA	NA
ENSG0000	578.2647	0.053908	0.12302	0.438203	0.661239	0.857063	DSTN	11034	destrin actin depolymerizing factor
ENSG0000	5.298652	0.462228	1.055866	0.437772	0.661552	0.857189	NA	NA	NA
ENSG0000	5.295027	0.424209	0.968926	0.437813	0.661522	0.857189	NA	NA	NA
ENSG0000	1998.114	-0.03711	0.084819	-0.43753	0.661728	0.857357	SPG7	6687	SPG7 matr paraplegin
ENSG0000	20.68528	0.238409	0.544983	0.437461	0.661777	0.857361	NA	NA	NA
ENSG0000	6.23496	0.518011	1.186203	0.436697	0.662331	0.85763	LINC0279	1.05E+08	long intergenic non-protein coding RNA 2793
ENSG0000	35.02205	0.203696	0.466483	0.436664	0.662355	0.85763	RNF212	285498	ring finger protein 212
ENSG0000	3.918085	-0.68398	1.565169	-0.437	0.662111	0.85763	UBD	10537	ubiquitin D
ENSG0000	45.84063	0.161449	0.369705	0.436696	0.662332	0.85763	VNN1	8876	vanin 1
ENSG0000	18.48321	-0.25203	0.576996	-0.4368	0.662254	0.85763	AOAH-IT1	1.01E+08	AOAH intronic transcript 1
ENSG0000	89.00317	0.117973	0.269987	0.436958	0.662142	0.85763	ALDH1A1	216	aldehyde dehydrogenase 1 family member A1
ENSG0000	5.87237	-0.49845	1.141269	-0.43675	0.662292	0.85763	MTND5P3	1.07E+08	MT-ND5 pseudogene 32
ENSG0000	1475.888	0.039218	0.089732	0.437058	0.66207	0.85763	MRPL10	124995	mitochondrial ribosomal protein L10
ENSG0000	22.00079	0.228835	0.524563	0.436239	0.662663	0.857703	NA	NA	NA
ENSG0000	1614.862	-0.04161	0.095415	-0.43609	0.662775	0.857703	C1orf21	81563	chromosome 1 open reading frame 21
ENSG0000	5.45633	0.553415	1.269897	0.435795	0.662986	0.857703	NA	NA	NA
ENSG0000	22.93881	-0.23531	0.539877	-0.43586	0.662939	0.857703	CYP1B1-A	285154	CYP1B1 antisense RNA 1
ENSG0000	336.2639	-0.08065	0.185034	-0.43584	0.66295	0.857703	MIER3	166968	MIER family member 3
ENSG0000	4.918014	-0.49181	1.128721	-0.43572	0.663037	0.857703	OR7E38P	389537	olfactory receptor family 7 subfamily E member 38 pseudogene
ENSG0000	2.126402	-0.86553	1.985044	-0.43603	0.662819	0.857703	PTGES2-A	389791	PTGES2 antisense RNA 1 (head to head)
ENSG0000	82.36587	0.129809	0.297935	0.435695	0.663058	0.857703	KICS2	144577	KICSTOR subunit 2
ENSG0000	975.9597	0.042834	0.098218	0.436109	0.662757	0.857703	RNF34	80196	ring finger protein 34
ENSG0000	26.51722	-0.21458	0.491914	-0.43621	0.662685	0.857703	CDKL1	8814	cyclin dependent kinase like 1
ENSG0000	904.3772	0.045733	0.104835	0.436239	0.662664	0.857703	TRADD	8717	TNFRSF1A associated via death domain
ENSG0000	2751.313	0.03624	0.083141	0.435889	0.662917	0.857703	NAPA	8775	NSF attachment protein alpha
ENSG0000	226.9727	-0.07281	0.166937	-0.43612	0.662747	0.857703	H1-0	3005	H1.0 linker histone
ENSG0000	35.16021	0.188158	0.431258	0.436301	0.662618	0.857703	BEX5	340542	brain expressed X-linked 5
ENSG0000	491.4189	0.065879	0.151345	0.435293	0.66335	0.857999	FBXO44	93611	F-box protein 44
ENSG0000	8.091288	0.409721	0.941531	0.435164	0.663443	0.857999	TPSD1	23430	tryptase delta 1
ENSG0000	31.69253	0.199506	0.458473	0.435152	0.663452	0.857999	FLT3LG	2323	fms related receptor tyrosine kinase 3 ligand
ENSG0000	485.3567	0.056284	0.129351	0.435124	0.663472	0.857999	EOLA2	541578	endothelium and lymphocyte associated ASCH domain 2
ENSG0000	5.711243	0.523043	1.202595	0.434929	0.663614	0.858123	NA	NA	NA
ENSG0000	19.52073	0.224895	0.517309	0.43474	0.663751	0.858208	NA	NA	NA
ENSG0000	1196.519	0.055192	0.126982	0.434646	0.663819	0.858208	LONP1	9361	lon peptid mitochondrial
ENSG0000	46.12847	0.193268	0.444653	0.434649	0.663817	0.858208	NA	NA	NA
ENSG0000	928.3428	0.043241	0.099507	0.434548	0.66389	0.85824	CIR1	9541	corepress CIR1
ENSG0000	140.891	-0.09622	0.221537	-0.43433	0.66405	0.858322	HYI	81888	hydroxypyruvate isomerase (putative)
ENSG0000	60.79055	-0.17241	0.397016	-0.43427	0.664092	0.858322	NA	NA	NA
ENSG0000	1084.75	-0.04307	0.099174	-0.43433	0.664048	0.858322	TNPO3	23534	transportin 3
ENSG0000	356.7369	-0.06688	0.154042	-0.43416	0.664174	0.858368	CMTR2	55783	cap methyltransferase 2
ENSG0000	94.58146	-0.11515	0.265435	-0.43382	0.66442	0.858533	TTC24	164118	tetratricopeptide repeat domain 24
ENSG0000	21.18767	0.227496	0.524377	0.433841	0.664404	0.858533	NA	NA	NA
ENSG0000	128.2538	0.092689	0.213673	0.433791	0.66444	0.858533	NA	NA	NA
ENSG0000	13.19041	0.296909	0.684606	0.433694	0.664511	0.858563	NA	NA	NA
ENSG0000	4.657462	0.55679	1.284758	0.433381	0.664738	0.858677	SUGCT-AS	1.02E+08	SUGCT antisense RNA 1
ENSG0000	15.49549	0.26446	0.610185	0.43341	0.664717	0.858677	RHPN2	85415	rhophilin Rho GTPase binding protein 2
ENSG0000	20.97176	-0.23564	0.543684	-0.43341	0.664719	0.858677	CCDC116	164592	coiled-coil domain containing 116
ENSG0000	98.62119	0.112432	0.259699	0.432933	0.665064	0.859039	EFNA4	1945	ephrin A4
ENSG0000	15.80184	-0.32728	0.756662	-0.43253	0.665354	0.859275	NHLRC1	378884	NHL repeat containing E3 ubiquitin protein ligase 1
ENSG0000	353.6355	-0.07998	0.184898	-0.43257	0.665326	0.859275	TMEM106	54664	transmembrane protein 106B
ENSG0000	11.75076	0.386821	0.894407	0.432489	0.665386	0.859275	LINC01252	338817	long intergenic non-protein coding RNA 1252
ENSG0000	1224.027	-0.0399	0.092308	-0.43229	0.665534	0.85937	SENP6	26054	SUMO specific peptidase 6
ENSG0000	1186.998	0.045274	0.104737	0.43226	0.665552	0.85937	PSMC3	5702	proteasom ATPase 3
ENSG0000	529.9025	-0.05311	0.12293	-0.43205	0.665707	0.859409	MTREX	23517	Mtr4 exosome RNA helicase
ENSG0000	4.458884	-0.53414	1.236357	-0.43203	0.665721	0.859409	NA	NA	NA
ENSG0000	175.4293	-0.11021	0.255102	-0.43204	0.665712	0.859409	MPND	84954	MPN domain containing
ENSG0000	1990.81	-0.03937	0.091159	-0.43186	0.665844	0.859507	URM1	81605	ubiquitin related modifier 1
ENSG0000	5.249398	0.436728	1.011505	0.431761	0.665915	0.85954	NA	NA	NA
ENSG0000	17.74112	-0.23988	0.555865	-0.43154	0.666073	0.85964	ITGA6-AS1	1.02E+08	ITGA6 antisense RNA 1
ENSG0000	35.60935	-0.18319	0.424508	-0.43153	0.666085	0.85964	TREML3P	340206	triggering pseudogene
ENSG0000	19.74658	0.235103	0.545051	0.431341	0.66622	0.859754	SLFNL1-AS	1.01E+08	SLFNL1 antisense RNA 1
ENSG0000	47.28778	-0.16691	0.387047	-0.43125	0.666289	0.859783	GADD45G	10912	growth arrest and DNA damage inducible gamma
ENSG0000	89.91459	-0.12296	0.285288	-0.43099	0.666475	0.859963	MFSD4B	91749	major facilitator superfamily domain containing 4B
ENSG0000	9.210618	-0.33839	0.785659	-0.43071	0.666679	0.860166	NA	NA	NA
ENSG0000	9.82196	0.391059	0.908264	0.430557	0.666791	0.860247	NEURL3	93082	neuralized E3 ubiquitin protein ligase 3
ENSG0000	2155.449	0.03656	0.084956	0.430334	0.666953	0.860247	PCYT1A	5130	phosphate choline
ENSG0000	20.75497	0.261063	0.606695	0.430303	0.666975	0.860247	NSUN7	79730	NOP2/Sun RNA methyltransferase family member 7
ENSG0000	210.0215	0.081224	0.188712	0.430411	0.666897	0.860247	ZNF140	7699	zinc finger protein 140
ENSG0000	1134.564	0.043965	0.102186	0.430242	0.66702	0.860247	PSMC5	5705	proteasom ATPase 5
ENSG0000	21.17317	0.284088	0.659934	0.43048	0.666847	0.860247	PLA2G4C	8605	phospholipase A2 group IVC
ENSG0000	715.7154	0.05318	0.12369	0.429944	0.667236	0.860351	ASB1	51665	ankyrin repeat and SOCS box containing 1
ENSG0000	356.2338	0.073952	0.172056	0.429813	0.667332	0.860351	NUP54	53371	nucleoporin 54
ENSG0000	10.64149	-0.34707	0.80713	-0.43001	0.667188	0.860351	HCG25	414765	HLA complex group 25
ENSG0000	62.6932	-0.14827	0.344961	-0.42982	0.667323	0.860351	NA	NA	NA
ENSG0000	763.8565	-0.05803	0.134981	-0.42992	0.667253	0.860351	DPH1	1801	diphthamide biosynthesis 1
ENSG0000	18.12197	-0.26863	0.625262	-0.42963	0.667464	0.860402	TBCE	6905	tubulin folding cofactor E
ENSG0000	730.5926	0.052756	0.12278	0.429679	0.667429	0.860402	SH2B2	10603	SH2B adaptor protein 2
ENSG0000	330.6617	-0.06963	0.162112	-0.42954	0.667532	0.860429	RRM2B	50484	ribonucleotide reductase regulatory TP53 inducible subunit M2B
ENSG0000	133.0761	-0.09915	0.230887	-0.42945	0.667595	0.860451	ZKSCAN4	387032	zinc finger with KRAB and SCAN domains 4
ENSG0000	8.461001	-0.37735	0.87945	-0.42907	0.667872	0.860686	ZNF662	389114	zinc finger protein 662
ENSG0000	7.144741	-0.41651	0.970717	-0.42907	0.667869	0.860686	NA	NA	NA
ENSG0000	213.6935	-0.07779	0.18133	-0.42901	0.667917	0.860686	NPIPP1	1.01E+08	nuclear pore complex interacting protein pseudogene 1
ENSG0000	23.37342	-0.22122	0.515857	-0.42883	0.668045	0.860792	FMO5	2330	flavin containing dimethylaniline monoxygenase 5
ENSG0000	521.8656	0.054372	0.126866	0.428583	0.668227	0.860966	GALM	130589	galactose mutarotase
ENSG0000	1914.766	-0.03702	0.086429	-0.42837	0.668378	0.861101	VPS11	55823	VPS11 core subunit of CORVET and HOPS complexes
ENSG0000	22.22784	0.241673	0.564381	0.428209	0.668499	0.861197	TSGA10	80705	testis specific 10
ENSG0000	31.00716	-0.17835	0.416632	-0.42808	0.668596	0.861261	GGN	199720	gametogenetin
ENSG0000	4049.639	0.04933	0.115262	0.427984	0.668663	0.861288	SESN3	143686	sestrin 3

ENSG0000	20.04341	-0.2336	0.54611	-0.42775	0.668831	0.861445	NA	NA	NA
ENSG0000	291.0812	-0.07125	0.166672	-0.4275	0.669015	0.861622	CHCHD7	79145	coiled-coil-helix-coiled-coil-helix domain containing 7
ENSG0000	2198.071	-0.03872	0.090637	-0.42724	0.669201	0.861802	LAMTOR1	55004	late endos MAPK and MTOR activator 1
ENSG0000	14.87482	0.276876	0.648256	0.42711	0.669299	0.861869	NA	NA	NA
ENSG0000	62.85087	0.136114	0.31885	0.426889	0.66946	0.861976	EVI5	7813	ecotropic viral integration site 5
ENSG0000	10.80064	-0.29564	0.692573	-0.42687	0.669476	0.861976	IFNAR2	3455	interferon alpha and beta receptor subunit 2
ENSG0000	260.1041	0.07909	0.185548	0.426253	0.669924	0.862134	UNC50	25972	unc-50 inner nuclear membrane RNA binding protein
ENSG0000	207.3504	-0.07874	0.184704	-0.4263	0.669887	0.862134	WASH2P	375260	WASP fam pseudogene
ENSG0000	12.87325	0.280311	0.657127	0.42657	0.669692	0.862134	RPL17P28	1E+08	ribosomal protein L17 pseudogene 28
ENSG0000	1051.189	0.042205	0.098986	0.426372	0.669837	0.862134	GAPVD1	26130	GTPase activating protein and VPS9 domains 1
ENSG0000	7.91422	-0.40548	0.950994	-0.42637	0.669836	0.862134	NA	NA	NA
ENSG0000	1012.586	-0.04273	0.100214	-0.42635	0.669854	0.862134	TTC39C	125488	tetratricopeptide repeat domain 39C
ENSG0000	129.1218	-0.10019	0.234905	-0.42652	0.669729	0.862134	TBXA2R	6915	thromboxane A2 receptor
ENSG0000	6.606907	-0.45058	1.057435	-0.42611	0.670031	0.862173	NA	NA	NA
ENSG0000	7.737594	-0.50694	1.189949	-0.42602	0.670093	0.862173	NA	NA	NA
ENSG0000	336.6471	-0.06525	0.153129	-0.42608	0.670047	0.862173	ITPKC	80271	inositol-trisphosphate 3-kinase C
ENSG0000	12.32333	-0.3369	0.791011	-0.42591	0.670177	0.86222	CHDH	55349	choline dehydrogenase
ENSG0000	1041.179	-0.04227	0.099331	-0.42559	0.670403	0.862272	LRRC47	57470	leucine rich repeat containing 47
ENSG0000	299.0442	-0.06426	0.150976	-0.42564	0.670371	0.862272	PEX2	5828	peroxisomal biogenesis factor 2
ENSG0000	5.761475	-0.4108	0.964892	-0.42574	0.670295	0.862272	DPY19L2	283417	dpy-19 like 2
ENSG0000	114.2186	0.113731	0.267172	0.425685	0.670337	0.862272	ADGRL1-A	1.01E+08	ADGRL1 antisense RNA 1
ENSG0000	2.180768	-0.83122	1.95361	-0.42548	0.670487	0.862321	SHE	126669	Src homology 2 domain containing E
ENSG0000	9.313815	-0.31571	0.74215	-0.4254	0.670546	0.862337	NA	NA	NA
ENSG0000	21.80749	0.267636	0.629275	0.425308	0.670612	0.862361	RTN4RL1	146760	reticulon 4 receptor like 1
ENSG0000	3.933156	0.554629	1.304396	0.4252	0.670691	0.862403	COX6B2	125965	cytochrome c oxidase subunit 6B2
ENSG0000	3.798811	-0.62725	1.475855	-0.42501	0.67083	0.862463	LINC0103	1.04E+08	long intergenic non-protein coding RNA 1036
ENSG0000	748.2673	-0.04848	0.114041	-0.42507	0.670785	0.862463	ADGRG5	221188	adhesion G protein-coupled receptor G5
ENSG0000	31.91941	-0.18762	0.441644	-0.42483	0.670961	0.862571	LRGUK	136332	leucine rich repeats and guanylate kinase domain containing
ENSG0000	213.996	-0.08195	0.192986	-0.42466	0.671082	0.862668	NHLRC2	374354	NHL repeat containing 2
ENSG0000	62.50327	-0.13218	0.311343	-0.42455	0.671165	0.862714	PRORP	9692	protein only RNase P catalytic subunit
ENSG0000	8891.313	0.032595	0.076809	0.424363	0.671301	0.862829	CNN2	1265	calponin 2
ENSG0000	8.306275	0.363328	0.856414	0.424244	0.671388	0.862877	NA	NA	NA
ENSG0000	4.225036	-0.57561	1.356989	-0.42418	0.671431	0.862877	ESM1	11082	endothelial cell specific molecule 1
ENSG0000	646.0605	-0.05074	0.119689	-0.42391	0.671634	0.863078	RUSC1	23623	RUN and SH3 domain containing 1
ENSG0000	2.818734	-0.64508	1.522561	-0.42368	0.6718	0.863112	TM4SF19-	1.01E+08	TM4SF19 antisense RNA 1
ENSG0000	1560.954	-0.05159	0.121765	-0.4237	0.671785	0.863112	AMD1	262	adenosylmethionine decarboxylase 1
ENSG0000	5.697646	-0.49239	1.16192	-0.42377	0.671731	0.863112	ZNF295-A	150142	ZNF295 antisense RNA 1
ENSG0000	777.1262	0.049147	0.116042	0.423529	0.671909	0.863132	NDUFA12	55967	NADH:ubiquinone oxidoreductase subunit A12
ENSG0000	6.245884	0.500562	1.181838	0.423546	0.671897	0.863132	PPP4R4	57718	protein phosphatase 4 regulatory subunit 4
ENSG0000	828.3203	-0.0528	0.124697	-0.42343	0.671981	0.863165	TAF2	6873	TATA-box binding protein associated factor 2
ENSG0000	8.120003	-0.36149	0.854521	-0.42303	0.672271	0.863298	NA	NA	NA
ENSG0000	25.39541	-0.2433	0.575079	-0.42307	0.672246	0.863298	TRPV4	59341	transient receptor potential cation channel subfamily V member 4
ENSG0000	117.9595	0.108589	0.256671	0.423068	0.672246	0.863298	DHRS4L2	317749	dehydrogenase/reductase 4 like 2
ENSG0000	694.775	-0.06179	0.146028	-0.42315	0.672185	0.863298	ERAL1	26284	Era like 12S mitochondrial rRNA chaperone 1
ENSG0000	77.33366	0.11941	0.282375	0.422876	0.672386	0.863352	GAR1	54433	GAR1 ribonucleoprotein
ENSG0000	22.11957	-0.21708	0.513497	-0.42276	0.672472	0.863352	NA	NA	NA
ENSG0000	971.5868	-0.04107	0.097144	-0.42279	0.672451	0.863352	METAP2	10988	methionyl aminopeptidase 2
ENSG0000	6136.189	-0.03764	0.089044	-0.42272	0.672499	0.863352	E2F4	1874	E2F transcription factor 4
ENSG0000	5.45199	0.537951	1.273271	0.422495	0.672664	0.863503	NA	NA	NA
ENSG0000	495.3558	-0.0586	0.138971	-0.42165	0.673278	0.863527	GSTM2	2946	glutathione S-transferase mu 2
ENSG0000	44.20821	-0.14929	0.353686	-0.4221	0.672955	0.863527	LBX2	85474	ladybird homeobox 2
ENSG0000	17.32124	0.321116	0.761311	0.421794	0.673176	0.863527	NA	NA	NA
ENSG0000	3.12031	-0.61055	1.44738	-0.42183	0.673148	0.863527	NA	NA	NA
ENSG0000	9.867732	-0.36629	0.868475	-0.42176	0.673198	0.863527	PCGF2	7703	polycomb group ring finger 2
ENSG0000	953.0902	-0.04888	0.115838	-0.42201	0.673017	0.863527	HEXD	284004	hexosaminidase D
ENSG0000	228.5891	-0.0887	0.210119	-0.42214	0.672926	0.863527	DOHH	83475	deoxyhypusine hydroxylase
ENSG0000	21.70397	-0.22655	0.537134	-0.42178	0.673182	0.863527	ETV2	2116	ETS variant transcription factor 2
ENSG0000	1183.412	0.041248	0.097828	0.42164	0.673288	0.863527	PCED1A	64773	PC-esterase domain containing 1A
ENSG0000	1069.831	0.046274	0.109691	0.421858	0.673129	0.863527	CTNNBL1	56259	catenin beta like 1
ENSG0000	1568.05	-0.04015	0.095075	-0.42226	0.672833	0.863527	PCNT	5116	pericentrin
ENSG0000	3.634748	0.669373	1.587181	0.421737	0.673217	0.863527	VN1R110P	1E+08	vomeronasal 1 receptor 110 pseudogene
ENSG0000	30.93532	-0.20087	0.475701	-0.42226	0.672839	0.863527	SOWAHD	347454	sosondowah ankyrin repeat domain family member D
ENSG0000	542.2539	-0.05276	0.125159	-0.42153	0.673369	0.863571	EMC7	56851	ER membrane protein complex subunit 7
ENSG0000	24.42331	-0.19345	0.459197	-0.42127	0.673557	0.863699	SUV39H2	79723	SUV39H2 histone lysine methyltransferase
ENSG0000	24.07592	0.211071	0.501041	0.421265	0.673562	0.863699	EIF4A1P1	1E+08	eukaryotic translation initiation factor 4A1 pseudogene 10
ENSG0000	186.1573	0.077168	0.183264	0.421077	0.673699	0.863716	ZNG1B	150472	Zn regulated GTPase metalloprotein activator 1B
ENSG0000	7.725208	-0.36758	0.872984	-0.42106	0.673714	0.863716	NA	NA	NA
ENSG0000	10.62765	-0.38162	0.90614	-0.42115	0.673646	0.863716	LINC0276	283140	long intergenic non-protein coding RNA 2762
ENSG0000	7.005085	0.400216	0.950708	0.420966	0.67378	0.86374	LOC10537	1.05E+08	uncharacterized LOC105379362
ENSG0000	536.9458	-0.05394	0.128177	-0.42085	0.673865	0.863744	CDYL	9425	chromodomain Y like
ENSG0000	12.86671	-0.29073	0.690838	-0.42083	0.673876	0.863744	HLA-F-AS1	285830	HLA-F antisense RNA 1
ENSG0000	1190.255	-0.04231	0.100586	-0.42065	0.674009	0.863854	DDX18	8886	DEAD-box helicase 18
ENSG0000	1939.143	-0.03886	0.092397	-0.42059	0.674055	0.863854	UBE4A	9354	ubiquitination factor E4A
ENSG0000	811.3184	0.046298	0.110212	0.420083	0.674425	0.864268	TCEA1	6917	transcription elongation factor A1
ENSG0000	26.9122	0.227315	0.54126	0.419974	0.674505	0.864311	PKHD1L1	93035	PKHD1 like 1
ENSG0000	4.669825	0.645737	1.538112	0.419825	0.674614	0.864391	NA	NA	NA
ENSG0000	973.9992	0.045387	0.108163	0.419618	0.674765	0.864448	RMND5A	64795	required for meiotic nuclear division 5 homolog A
ENSG0000	341.3525	-0.06342	0.151144	-0.41957	0.674798	0.864448	COX10	1352	cytochrome c oxidase assembly factor heme A:farnesyltransferase COX10
ENSG0000	39.77498	0.156697	0.373413	0.419635	0.674752	0.864448	NA	NA	NA
ENSG0000	8.322863	0.399071	0.951826	0.419268	0.67502	0.864673	NA	NA	NA
ENSG0000	5.48426	0.3949	0.942142	0.419151	0.675105	0.864709	RPS3AP21	402287	RPS3A pseudogene 21
ENSG0000	475.9094	-0.05855	0.139697	-0.4191	0.675141	0.864709	PILRB	29990	paired immunoglobin like type 2 receptor beta
ENSG0000	414.6275	-0.06095	0.145458	-0.41901	0.675205	0.864731	MBNL2	10150	muscleblind like splicing regulator 2
ENSG0000	764.0498	-0.04684	0.111835	-0.41885	0.675326	0.864772	NIT1	4817	nitrilase 1
ENSG0000	3892.334	0.040424	0.096542	0.418716	0.675424	0.864772	NDFIP1	80762	Nedd4 family interacting protein 1
ENSG0000	1448.627	0.051268	0.122439	0.418728	0.675415	0.864772	ARID2	196528	AT-rich interaction domain 2
ENSG0000	27.65058	-0.19506	0.465757	-0.4188	0.675359	0.864772	ZNF563	147837	zinc finger protein 563
ENSG0000	25.28317	-0.20282	0.484624	-0.41851	0.675575	0.864906	NA	NA	NA
ENSG0000	99478.11	0.031403	0.075147	0.417894	0.676025	0.865347	VIM	7431	vimentin
ENSG0000	48.10692	-0.14891	0.356372	-0.41785	0.676059	0.865347	NA	NA	NA
ENSG0000	8.24056	-0.41626	0.995916	-0.41796	0.675974	0.865347	NA	NA	NA
ENSG0000	1030	0.042604	0.102029	0.417565	0.676265	0.865426	VPS72	6944	vacuolar protein sorting 72 homolog
ENSG0000	864.1465	0.058696	0.140573	0.417548	0.676278	0.865426	F5	2153	coagulation factor V
ENSG0000	7.100274	0.431703	1.03398	0.417516	0.676301	0.865426	CRACD	57482	capping protein inhibiting regulator of actin dynamics
ENSG0000	475.5849	0.055332	0.132532	0.417499	0.676313	0.865426	ATAT1	79969	alpha tubulin acetyltransferase 1
ENSG0000	137.5036	-0.08933	0.213997	-0.41744	0.676354	0.865426	HSDL2	84263	hydroxysteroid dehydrogenase like 2
ENSG0000	4.579524	-0.59995	1.437662	-0.41731	0.676454	0.865493	DES	1674	desmin
ENSG0000	22.42212	-0.21053	0.504705	-0.41714	0.676573	0.865586	NA	NA	NA
ENSG0000	773.207	-0.04378	0.105041	-0.41677	0.676849	0.86588	RFXANK	8625	regulatory factor X associated ankyrin containing protein
ENSG0000	12.30665	-0.32514	0.780339	-0.41667	0.676921	0.865912	HTR6	3362	5-hydroxytryptamine receptor 6

ENSG0000	3.850657	0.546522	1.312628	0.416357	0.677149	0.865919	C1orf21-D	1.19E+08	C1orf21 divergent transcript
ENSG0000	12.29011	-0.35287	0.847543	-0.41634	0.67716	0.865919	VWA7	80737	von Willebrand factor A domain containing 7
ENSG0000	377.7669	0.056875	0.136579	0.416426	0.677098	0.865919	MAD2L1BI	9587	MAD2L1 binding protein
ENSG0000	14.1263	-0.28947	0.695177	-0.41639	0.677124	0.865919	MIR4489	1.01E+08	microRNA 4489
ENSG0000	7.572367	-0.34074	0.818086	-0.41651	0.677034	0.865919	DLG2	1740	discs large MAGUK scaffold protein 2
ENSG0000	40.18178	0.202557	0.487142	0.415807	0.677551	0.866181	NA	NA	NA
ENSG0000	329.7755	0.07712	0.185396	0.415974	0.677429	0.866181	SCD	6319	stearoyl-CoA desaturase
ENSG0000	447.1578	0.051864	0.124697	0.415919	0.677469	0.866181	KANSL2	54934	KAT8 regulatory NSL complex subunit 2
ENSG0000	440.8937	-0.06145	0.147787	-0.41578	0.677573	0.866181	ESD	2098	esterase D
ENSG0000	4428.377	0.04858	0.116852	0.415742	0.677599	0.866181	FOSB	2354	FosB protc AP-1 transcription factor subunit
ENSG0000	1082.751	0.046561	0.112039	0.415577	0.67772	0.866257	FASLG	356	Fas ligand
ENSG0000	127.2501	0.09721	0.23394	0.415534	0.677751	0.866257	KRT5	3852	keratin 5
ENSG0000	7.41682	-0.37859	0.91136	-0.41541	0.677844	0.866316	KCNK12	56660	potassium two pore domain channel subfamily K member 12
ENSG0000	13.86637	0.274064	0.659875	0.415328	0.677902	0.86633	NA	NA	NA
ENSG0000	6677.324	0.035509	0.085524	0.415196	0.677998	0.866334	SDF4	51150	stromal cell derived factor 4
ENSG0000	603.7791	0.046016	0.110817	0.415238	0.677967	0.866334	RTL8A	26071	retrotransposon Gag like 8A
ENSG0000	22.99624	-0.26289	0.633369	-0.41507	0.678093	0.866395	RCAN2	10231	regulator of calcineurin 2
ENSG0000	41.88158	0.154387	0.372439	0.414528	0.678487	0.866839	NA	NA	NA
ENSG0000	2.644995	-0.77665	1.87395	-0.41444	0.678549	0.866858	PCDHGB4	8641	protocadh 4
ENSG0000	5.844314	-0.39295	0.94849	-0.41429	0.678661	0.866942	NA	NA	NA
ENSG0000	3.261412	-0.68445	1.653096	-0.41404	0.678844	0.867039	IGKV5-2	28907	immunoglobulin kappa variable 5-2
ENSG0000	76.84762	0.124808	0.301459	0.414012	0.678865	0.867039	NA	NA	NA
ENSG0000	150.9544	-0.08465	0.204483	-0.414	0.678877	0.867039	ZNF25	219749	zinc finger protein 25
ENSG0000	10.79801	-0.36213	0.8751	-0.41381	0.67901	0.867121	NA	NA	NA
ENSG0000	9539.896	0.03193	0.077166	0.41378	0.679035	0.867121	SON	6651	SON DNA and RNA binding protein
ENSG0000	9.596826	0.397724	0.961392	0.413696	0.679097	0.86714	NA	NA	NA
ENSG0000	3.63778	-0.50336	1.217748	-0.41335	0.679347	0.867365	NSRP1P1	1E+08	nuclear speckle splicing regulatory protein 1 pseudogene 1
ENSG0000	51.1286	-0.14043	0.339751	-0.41333	0.679367	0.867365	TRBV9	28586	T cell receptor beta variable 9
ENSG0000	5.889468	0.434837	1.052456	0.413164	0.679486	0.867458	NA	NA	NA
ENSG0000	8.041625	-0.324	0.784399	-0.41306	0.679563	0.867496	NA	NA	NA
ENSG0000	19.28618	0.219385	0.531204	0.412996	0.679609	0.867496	KCNH8	131096	potassium voltage-gated channel subfamily H member 8
ENSG0000	59.24123	-0.13044	0.315961	-0.41285	0.679716	0.867572	FANCL	55120	FA complementation group L
ENSG0000	73.56516	0.118718	0.287763	0.412555	0.679933	0.867789	ZNF554	115196	zinc finger protein 554
ENSG0000	11.18684	0.286339	0.695081	0.41195	0.680376	0.86791	NOS1AP	9722	nitric oxide synthase 1 adaptor protein
ENSG0000	34.21048	-0.18375	0.446079	-0.41192	0.680399	0.86791	GALNT7-D	1.02E+08	GALNT7 divergent transcript
ENSG0000	137.1584	0.101147	0.245344	0.412268	0.680143	0.86791	GKAP1	80318	G kinase anchoring protein 1
ENSG0000	620.6323	-0.05166	0.125361	-0.41209	0.680276	0.86791	FADD	8772	Fas associated via death domain
ENSG0000	7.582994	-0.34082	0.827211	-0.41201	0.680333	0.86791	NA	NA	NA
ENSG0000	135.8008	0.102012	0.247652	0.411915	0.680402	0.86791	NOPCHAP	121053	NOP protein chaperone 1
ENSG0000	4.665671	0.529432	1.285207	0.411943	0.680381	0.86791	MIR3613	1.01E+08	microRNA 3613
ENSG0000	59.57155	-0.1498	0.363476	-0.41213	0.680247	0.86791	LGALS9B	284194	galectin 9B
ENSG0000	545.2696	-0.05436	0.132028	-0.41171	0.680553	0.868044	PSMG2	56984	proteasome assembly chaperone 2
ENSG0000	2120.341	0.035515	0.086313	0.41147	0.680728	0.868207	ANKRD13/	88455	ankyrin repeat domain 13A
ENSG0000	125.3247	0.093635	0.227654	0.411304	0.680849	0.868235	EXOSC3	51010	exosome component 3
ENSG0000	15.83271	-0.30052	0.730855	-0.41119	0.680935	0.868235	LOC10192	1.02E+08	uncharacterized LOC101929107
ENSG0000	11.17107	0.386176	0.939179	0.411185	0.680937	0.868235	KRTAP16-:	1.01E+08	keratin associated protein 16-1
ENSG0000	4.20099	0.531013	1.29132	0.411217	0.680913	0.868235	NA	NA	NA
ENSG0000	3.385625	-0.53126	1.293938	-0.41058	0.681383	0.868397	RPL35P1	440737	ribosomal protein L35 pseudogene 1
ENSG0000	2.764658	0.754704	1.839648	0.410244	0.681627	0.868397	NA	NA	NA
ENSG0000	39.68099	0.192614	0.468935	0.410748	0.681257	0.868397	TMEM237	65062	transmembrane protein 237
ENSG0000	58.07593	-0.13301	0.324325	-0.41012	0.681721	0.868397	NKIRAS1	28512	NFKB inhibitor interacting Ras like 1
ENSG0000	209.991	-0.07009	0.170688	-0.41065	0.681328	0.868397	SCRN1	9805	secernin 1
ENSG0000	1389.621	-0.03754	0.091551	-0.41005	0.681767	0.868397	NOL6	65083	nucleolar protein 6
ENSG0000	380.1374	0.060306	0.146939	0.410413	0.681503	0.868397	ZMYND19	116225	zinc finger MYND-type containing 19
ENSG0000	815.8771	-0.04733	0.115416	-0.41008	0.681748	0.868397	FIBP	9158	FGF1 intracellular binding protein
ENSG0000	186.3098	-0.08439	0.205355	-0.41093	0.681124	0.868397	GNPNAT1	64841	glucosamine-phosphate N-acetyltransferase 1
ENSG0000	4.142029	0.608899	1.482235	0.410798	0.681221	0.868397	CYP4F35P	284233	cytochrom pseudogene
ENSG0000	15.24703	-0.26544	0.646288	-0.41072	0.681281	0.868397	NA	NA	NA
ENSG0000	11.99683	0.287605	0.701106	0.410216	0.681647	0.868397	RN7SL368I	1.06E+08	RNA 7SL cytoplasm pseudogene
ENSG0000	6.226961	0.440451	1.073613	0.410251	0.681622	0.868397	NA	NA	NA
ENSG0000	828.8642	0.041205	0.100385	0.410473	0.681459	0.868397	RTF2	51507	replication termination factor 2
ENSG0000	5.579181	0.502622	1.225701	0.410069	0.681755	0.868397	SNORA11	677799	small nucl H/ACA box 11
ENSG0000	635.884	-0.04596	0.112135	-0.40984	0.681924	0.868478	NRBP2	340371	nuclear receptor binding protein 2
ENSG0000	19.22	-0.23005	0.56133	-0.40984	0.681924	0.868478	NA	NA	NA
ENSG0000	7.431917	-0.45349	1.106967	-0.40967	0.682048	0.868482	NA	NA	NA
ENSG0000	945.2698	-0.04946	0.120718	-0.40969	0.682037	0.868482	PHKG2	5261	phosphorylase kinase catalytic subunit gamma 2
ENSG0000	35.88315	0.18038	0.440334	0.409643	0.682068	0.868482	HDX	139324	highly divergent homeobox
ENSG0000	15.13901	-0.24889	0.607714	-0.40956	0.682131	0.868503	FGF9	2254	fibroblast growth factor 9
ENSG0000	8.955693	-0.34878	0.85175	-0.40948	0.682184	0.868511	NA	NA	NA
ENSG0000	41.68765	0.174956	0.427402	0.409348	0.682285	0.868579	NA	NA	NA
ENSG0000	8.535454	0.338162	0.826314	0.409242	0.682362	0.868619	SKA3	221150	spindle and kinetochore associated complex subunit 3
ENSG0000	51.61837	0.145377	0.355292	0.409176	0.68241	0.86862	NA	NA	NA
ENSG0000	243.5259	0.073428	0.179537	0.408989	0.682548	0.868641	THUMPD3	440944	THUMPD3 antisense RNA 1
ENSG0000	396.548	0.062391	0.15256	0.408963	0.682567	0.868641	DPH3	285381	diphthamide biosynthesis 3
ENSG0000	2.442056	-0.76611	1.873042	-0.40902	0.682524	0.868641	NA	NA	NA
ENSG0000	231.7466	-0.07869	0.192521	-0.40871	0.682751	0.868816	ASB16-AS:	339201	ASB16 antisense RNA 1
ENSG0000	17.89938	0.275254	0.673702	0.40857	0.682855	0.868888	NA	NA	NA
ENSG0000	13.45175	0.255977	0.626712	0.408444	0.682948	0.868946	NA	NA	NA
ENSG0000	327.9983	0.059868	0.146625	0.408305	0.683049	0.869016	ATG14	22863	autophagy related 14
ENSG0000	108.5819	0.096455	0.236351	0.408102	0.683199	0.869147	ARSG	22901	arylsulfatase G
ENSG0000	308.9298	0.062897	0.154224	0.407832	0.683397	0.86928	CAMK2N1	55450	calcium/calmodulin dependent protein kinase II inhibitor 1
ENSG0000	915.1051	-0.0442	0.108371	-0.40784	0.683393	0.86928	KCTD17	79734	potassium channel tetramerization domain containing 17
ENSG0000	201.5365	0.069262	0.169872	0.407732	0.683471	0.869314	DHRS13	147015	dehydrogenase/reductase 13
ENSG0000	13.03209	-0.34373	0.843257	-0.40762	0.683551	0.869325	AJAP1	55966	adherens junctions associated protein 1
ENSG0000	47.1941	-0.14895	0.36545	-0.40759	0.683573	0.869325	PHF7	51533	PHD finger protein 7
ENSG0000	2.660144	0.741967	1.823704	0.406846	0.684121	0.869773	CYS1	192668	cystin 1
ENSG0000	837.9983	-0.04655	0.114406	-0.40692	0.684063	0.869773	SP140	11262	SP140 nuclear body protein
ENSG0000	1338.95	0.04374	0.1075	0.40688	0.684096	0.869773	SSNA1	8636	SS nuclear autoantigen 1
ENSG0000	3.220982	-0.58129	1.428453	-0.40693	0.684056	0.869773	PCBP2-OT:	1.02E+08	PCBP2 overlapping transcript 1
ENSG0000	117.2974	0.106426	0.261623	0.406792	0.684161	0.869773	SPATC1L	84221	spermatogenesis and centriole associated 1 like
ENSG0000	134.7494	-0.09487	0.233299	-0.40664	0.684271	0.869854	CR1	1378	complement C3b/C4b receptor 1 (Knops blood group)
ENSG0000	21.81651	-0.2175	0.535006	-0.40654	0.684343	0.869886	NA	NA	NA
ENSG0000	41.37592	-0.15481	0.381061	-0.40627	0.684543	0.870049	IL1RL2	8808	interleukin 1 receptor like 2
ENSG0000	7.771511	-0.32364	0.796677	-0.40624	0.684565	0.870049	EIF3CL	728689	eukaryotic translation initiation factor 3 subunit C like
ENSG0000	7.113281	0.356627	0.878066	0.40615	0.684632	0.870056	NA	NA	NA
ENSG0000	368.8673	-0.0613	0.150943	-0.40611	0.684664	0.870056	RBIS	401466	ribosomal biogenesis factor
ENSG0000	54.08466	-0.1498	0.369136	-0.40581	0.68488	0.87027	VPS9D1-A:	1E+08	VPS9D1 antisense RNA 1
ENSG0000	99.5638	0.13598	0.335427	0.405393	0.685189	0.870544	PRRT4	401399	proline rich transmembrane protein 4
ENSG0000	544.8853	0.059308	0.146288	0.405419	0.685169	0.870544	CDK2AP1	8099	cyclin dependent kinase 2 associated protein 1
ENSG0000	603.3603	-0.05156	0.127252	-0.40522	0.685319	0.87059	OSBPL9	114883	oxysterol binding protein like 9

ENSG0000	6.870377	-0.39697	0.979518	-0.40527	0.685277	0.87059	SPAG8	26206	sperm associated antigen 8
ENSG0000	4.567273	-0.41851	1.033038	-0.40512	0.685388	0.870618	NA	NA	NA
ENSG0000	14.30164	0.248311	0.613264	0.404901	0.68555	0.870764	SERF1A	8293	small EDRK-rich factor 1A
ENSG0000	11.40534	0.31783	0.785549	0.404597	0.685774	0.870989	DPPA4	55211	developmental pluripotency associated 4
ENSG0000	679.4326	-0.04964	0.12273	-0.40445	0.685883	0.871068	CAVIN2	8436	caveolae associated protein 2
ENSG0000	710.4968	-0.04334	0.107241	-0.40417	0.68609	0.871213	NATD1	256302	N-acetyltransferase domain containing 1
ENSG0000	2878.56	0.031038	0.076796	0.404165	0.686092	0.871213	PTTG1IP	754	PTTG1 interacting protein
ENSG0000	42.11646	-0.1493	0.369733	-0.40381	0.68635	0.871378	C1orf56	54964	chromosome 1 open reading frame 56
ENSG0000	27.26512	-0.21396	0.529703	-0.40392	0.686273	0.871378	RIGI	23586	RNA sensor RIG-I
ENSG0000	700.4143	-0.05609	0.138902	-0.4038	0.686362	0.871378	FBXL20	84961	F-box and leucine rich repeat protein 20
ENSG0000	942.7966	-0.04254	0.105452	-0.40337	0.686673	0.871522	RPA2	6118	replication protein A2
ENSG0000	1730.143	0.035979	0.089211	0.4033	0.686727	0.871522	PARP12	64761	poly(ADP-ribose) polymerase family member 12
ENSG0000	10.64235	-0.32662	0.809561	-0.40345	0.686619	0.871522	NA	NA	NA
ENSG0000	4.821352	0.453163	1.123371	0.403396	0.686657	0.871522	CLEC12B	387837	C-type lectin domain family 12 member B
ENSG0000	28.32668	0.190398	0.472148	0.403259	0.686758	0.871522	SSPN	8082	sarcospan
ENSG0000	4.851919	0.526933	1.306304	0.403377	0.686671	0.871522	RPL23P2	54028	ribosomal protein L23 pseudogene 2
ENSG0000	59.33495	0.126752	0.314472	0.403064	0.686901	0.871645	PHYH	5264	phytanoyl-CoA 2-hydroxylase
ENSG0000	1553.152	-0.03961	0.098377	-0.40266	0.687202	0.871689	NUDC	10726	nuclear di: dynein complex regulator
ENSG0000	5780.843	0.02898	0.071951	0.402767	0.68712	0.871689	ARL4C	10123	ADP ribosylation factor like GTPase 4C
ENSG0000	7.931947	0.347122	0.861896	0.402742	0.687138	0.871689	DOK7	285489	docking protein 7
ENSG0000	6.301646	-0.47125	1.170279	-0.40268	0.68718	0.871689	ZNF252P-/	286103	ZNF252P antisense RNA 1
ENSG0000	34.22859	-0.17941	0.445338	-0.40287	0.687045	0.871689	ADAMTS1:	11093	ADAM metallopeptidase with thrombospondin type 1 motif 13
ENSG0000	1621.869	-0.0381	0.094633	-0.40263	0.687218	0.871689	KAT5	10524	lysine acetyltransferase 5
ENSG0000	14.88191	-0.25799	0.640887	-0.40255	0.687278	0.871706	NA	NA	NA
ENSG0000	209.0366	0.076743	0.190793	0.402233	0.687513	0.871943	GPD2	2820	glycerol-3-phosphate dehydrogenase 2
ENSG0000	232.9241	-0.07405	0.184148	-0.40211	0.687606	0.872002	ABCB7	22	ATP binding cassette subfamily B member 7
ENSG0000	5.924404	0.452841	1.127959	0.40147	0.688074	0.872241	NA	NA	NA
ENSG0000	17.61452	0.235271	0.586478	0.401159	0.688303	0.872241	CR2	1380	complement C3d receptor 2
ENSG0000	76.36921	-0.13226	0.329264	-0.40167	0.687926	0.872241	HYAL3	8372	hyaluronidase 3
ENSG0000	19.70031	0.221964	0.553014	0.401371	0.688147	0.872241	EEF1A1P1!	1E+08	eukaryotic translation elongation factor 1 alpha 1 pseudogene 19
ENSG0000	1297.354	-0.0411	0.102445	-0.40118	0.68829	0.872241	DDX46	9879	DEAD-box helicase 46
ENSG0000	31.16396	0.209254	0.521538	0.401224	0.688255	0.872241	CA1	759	carbonic anhydrase 1
ENSG0000	2.566733	-0.81674	2.033829	-0.40158	0.687995	0.872241	NA	NA	NA
ENSG0000	97.20435	0.109414	0.272581	0.401399	0.688126	0.872241	ZNF16	7564	zinc finger protein 16
ENSG0000	4.948731	0.523005	1.302789	0.40145	0.688089	0.872241	NA	NA	NA
ENSG0000	6.945263	0.425389	1.060413	0.401154	0.688307	0.872241	NA	NA	NA
ENSG0000	21.53671	0.215444	0.53707	0.401147	0.688312	0.872241	AARSD1	80755	alanyl-tRNA synthetase domain containing 1
ENSG0000	6.952846	-0.38019	0.948414	-0.40087	0.688513	0.872298	LINC0086z	554279	long intergenic non-protein coding RNA 862
ENSG0000	4.217968	0.607494	1.515589	0.40083	0.688545	0.872298	LEKR1	389170	leucine glutamate and lysine rich 1
ENSG0000	12.85753	-0.31547	0.786846	-0.40093	0.688469	0.872298	BAIAP2L1	55971	BAR/IMD domain containing adaptor protein 2 like 1
ENSG0000	3551.42	-0.03268	0.081516	-0.4009	0.688496	0.872298	NACC1	112939	nucleus accumbens associated 1
ENSG0000	3.687811	0.596797	1.489506	0.400667	0.688665	0.872381	FOXI1	2299	forkhead box I1
ENSG0000	85.98271	-0.10891	0.271899	-0.40057	0.688738	0.872381	LINC0141C	1.03E+08	long intergenic non-protein coding RNA 1410
ENSG0000	371.0014	-0.08396	0.209621	-0.40055	0.688752	0.872381	CUL5	8065	cullin 5
ENSG0000	848.7325	-0.0447	0.111736	-0.40008	0.689095	0.872399	NFYA	4800	nuclear transcription factor Y subunit alpha
ENSG0000	245.2111	0.069951	0.174808	0.400162	0.689037	0.872399	KPNA5	3841	karyopherin subunit alpha 5
ENSG0000	31.50958	0.201083	0.502391	0.400252	0.688971	0.872399	TRGV4	6977	T cell receptor gamma variable 4
ENSG0000	5.482418	0.431363	1.078144	0.400097	0.689085	0.872399	TRBV7-4	28594	T cell receptor beta variable 7-4
ENSG0000	47.25207	0.14204	0.354937	0.400183	0.689021	0.872399	COMMD3	23412	COMM domain containing 3
ENSG0000	6.907611	0.371104	0.927556	0.400088	0.689092	0.872399	NA	NA	NA
ENSG0000	3.876195	-0.50477	1.261167	-0.40024	0.688981	0.872399	ZNF223	7766	zinc finger protein 223
ENSG0000	15.34422	0.247229	0.618181	0.39993	0.689208	0.872482	SPATA7	55812	spermatogenesis associated 7
ENSG0000	76.50694	0.114957	0.287633	0.399664	0.689404	0.872671	LOC10031(	1E+08	uncharacterized LOC100310756
ENSG0000	92.9195	0.117584	0.294468	0.399309	0.689666	0.872905	LOC10272	1.03E+08	uncharacterized LOC102723566
ENSG0000	51.17817	-0.12844	0.321683	-0.39928	0.689683	0.872905	ADAL	161823	adenosine deaminase like
ENSG0000	74.00542	-0.11536	0.289194	-0.39889	0.689974	0.87301	MST1	4485	macrophage stimulating 1
ENSG0000	6.152755	-0.40484	1.014967	-0.39887	0.689988	0.87301	EPHB1	2047	EPH receptor B1
ENSG0000	229.7475	-0.06485	0.16276	-0.39841	0.690329	0.87301	PSMG3	84262	proteasome assembly chaperone 3
ENSG0000	50.57124	-0.13599	0.341161	-0.39861	0.69018	0.87301	RELA-DT	1.05E+08	RELA divergent transcript
ENSG0000	24.20435	-0.19313	0.484166	-0.39889	0.689972	0.87301	DNAJB6P1	387820	DNAJB6 pseudogene 1
ENSG0000	113.5431	-0.13048	0.327136	-0.39886	0.689995	0.87301	PLEK2	26499	pleckstrin 2
ENSG0000	334.228	0.063978	0.160482	0.398665	0.69014	0.87301	NA	NA	NA
ENSG0000	34.70397	0.194274	0.487549	0.39847	0.690283	0.87301	NA	NA	NA
ENSG0000	19.57534	-0.21452	0.538457	-0.39841	0.690331	0.87301	NA	NA	NA
ENSG0000	1475.166	0.043375	0.108775	0.398755	0.690073	0.87301	ECH1	1891	enoyl-CoA hydratase 1
ENSG0000	177.0529	0.074675	0.187397	0.398483	0.690274	0.87301	IFT52	51098	intraflagellar transport 52
ENSG0000	178.3754	0.073675	0.184759	0.398764	0.690067	0.87301	LINC0020!	642852	long intergenic non-protein coding RNA 205
ENSG0000	621.3172	-0.04628	0.116209	-0.39826	0.690438	0.873086	HAUS5	23354	HAUS augmin like complex subunit 5
ENSG0000	12.58797	-0.33875	0.850717	-0.39819	0.690489	0.873091	MSH5	4439	mutS homolog 5
ENSG0000	6.814117	0.42519	1.067991	0.398122	0.69054	0.873097	NA	NA	NA
ENSG0000	11.47566	-0.31854	0.800247	-0.39805	0.690595	0.873106	RAD51	5888	RAD51 recombinase
ENSG0000	30.8748	0.167044	0.419889	0.397828	0.690757	0.873132	STAG3L1	54441	stromal antigen 3-like 1 (pseudogene)
ENSG0000	847.5366	-0.05534	0.139074	-0.39795	0.690668	0.873132	VCPIP1	80124	valosin containing protein interacting protein 1
ENSG0000	254.5713	0.068916	0.173219	0.397853	0.690739	0.873132	MCTS1	28985	MCTS1 re-initiation and release factor
ENSG0000	14.17107	-0.28394	0.713883	-0.39774	0.69082	0.873153	NA	NA	NA
ENSG0000	4.355663	1.212155	3.049103	0.397545	0.690966	0.873277	NA	NA	NA
ENSG0000	69.60567	-0.11611	0.292728	-0.39666	0.69162	0.873447	ALG6	29929	ALG6 alph 3-glucosyltransferase
ENSG0000	2302.142	0.033855	0.085317	0.396818	0.691502	0.873447	MOGS	7841	mannosyl-oligosaccharide glucosidase
ENSG0000	2.972992	-0.57973	1.460496	-0.39694	0.691412	0.873447	SNORD10C	594838	small nucl C/D box 100
ENSG0000	51.76709	0.150327	0.378816	0.396835	0.691489	0.873447	NA	NA	NA
ENSG0000	47.74272	-0.1529	0.3851	-0.39705	0.691331	0.873447	NA	NA	NA
ENSG0000	4409.888	0.033992	0.085609	0.397059	0.691324	0.873447	EIF3A	8661	eukaryotic translation initiation factor 3 subunit A
ENSG0000	4.290263	0.471953	1.189682	0.396705	0.691585	0.873447	NA	NA	NA
ENSG0000	4.891264	0.432501	1.090521	0.3966	0.691662	0.873447	SERP2	387923	stress associated endoplasmic reticulum protein family member 2
ENSG0000	1261.308	0.04035	0.101622	0.397057	0.691325	0.873447	MGAT2	4247	alpha-1 6-mannosyl-glycoprotein 2-beta-N-acetylglucosaminyltransferase
ENSG0000	977.5453	-0.04269	0.107648	-0.39654	0.691708	0.873447	TOP3A	7156	DNA topoisomerase III alpha
ENSG0000	113.9854	-0.09742	0.245307	-0.39715	0.69126	0.873447	LGALS9C	654346	galectin 9C
ENSG0000	5.534152	0.493121	1.242133	0.396995	0.691371	0.873447	HPN	3249	hepsin
ENSG0000	6.225184	0.479648	1.209605	0.396533	0.691712	0.873447	SHROOM4	57477	shroom family member 4
ENSG0000	15.03029	-0.24739	0.624336	-0.39624	0.691928	0.873635	CRYBG2	55057	crystallin beta-gamma domain containing 2
ENSG0000	4.948914	-0.50162	1.266116	-0.39619	0.691967	0.873635	NA	NA	NA
ENSG0000	2.48647	-0.6902	1.742306	-0.39614	0.692003	0.873635	NA	NA	NA
ENSG0000	720.9953	0.043627	0.110181	0.395955	0.692139	0.873676	ENY2	56943	ENY2 transcription and export complex 2 subunit
ENSG0000	3.735801	-0.62064	1.567918	-0.39584	0.692223	0.873676	FRMPD1	22844	FERM and PDZ domain containing 1
ENSG0000	931.3783	0.039673	0.100205	0.395917	0.692166	0.873676	ARFIP2	23647	ADP ribosylation factor interacting protein 2
ENSG0000	521.7122	-0.05103	0.128893	-0.3959	0.692176	0.873676	METTL16	79066	methyltrar N6-methyladenosine
ENSG0000	3.499924	0.606011	1.531424	0.395717	0.692314	0.873731	CHRNA5	1138	cholinergic receptor nicotinic alpha 5 subunit
ENSG0000	53.73677	0.12394	0.3134	0.395468	0.692498	0.873903	C1orf50	79078	chromosome 1 open reading frame 50
ENSG0000	228.6662	0.070885	0.179273	0.395404	0.692545	0.873903	NA	NA	NA
ENSG0000	137.9286	0.081592	0.206459	0.3952	0.692696	0.873915	BPNT1	10380	3'(2') 5'-bisphosphate nucleotidase 1

ENSG0000	45.78693	0.144926	0.366714	0.395202	0.692694	0.873915	PTPMT1	114971	protein tyrosine phosphatase mitochondrial 1
ENSG0000	69.66401	0.113297	0.286672	0.395213	0.692686	0.873915	KLHL11	55175	kelch like family member 11
ENSG0000	6.250674	-0.44218	1.119502	-0.39498	0.692858	0.874001	RNU6-322	1.07E+08	RNA U6 small r pseudogene
ENSG0000	14.69266	-0.2559	0.647845	-0.395	0.692843	0.874001	ZNF90	7643	zinc finger protein 90
ENSG0000	62.01442	0.122225	0.309529	0.394874	0.692936	0.87404	RASL11A	387496	RAS like family 11 member A
ENSG0000	9.752309	-0.31496	0.798388	-0.3945	0.693215	0.874332	MIR342	442909	microRNA 342
ENSG0000	72.43389	0.148147	0.375652	0.394373	0.693306	0.874347	ZNF804A	91752	zinc finger protein 804A
ENSG0000	231.5363	0.072432	0.183673	0.394352	0.693321	0.874347	COPS4	51138	COP9 signalosome subunit 4
ENSG0000	9.971784	-0.34764	0.881693	-0.39429	0.693369	0.874348	KLF15	28999	KLF transcription factor 15
ENSG0000	42.39085	-0.14908	0.378172	-0.39422	0.693418	0.874351	TSPYL5	85453	TSPY like 5
ENSG0000	8.201446	-0.34162	0.866814	-0.39411	0.6935	0.874394	RBM20	282996	RNA binding motif protein 20
ENSG0000	742.9326	-0.0494	0.125393	-0.39393	0.693634	0.87442	JADE1	79960	jade family PHD finger 1
ENSG0000	2.402255	0.786941	1.997646	0.393934	0.69363	0.87442	NA	NA	NA
ENSG0000	78.3371	0.109541	0.278099	0.393891	0.693661	0.87442	NUP50-DT	1.01E+08	NUP50 divergent transcript
ENSG0000	487.5737	-0.05518	0.140119	-0.39382	0.693715	0.874428	ATAD2B	54454	ATPase family AAA domain containing 2B
ENSG0000	9.853506	0.324971	0.825543	0.393645	0.693843	0.87453	LOC10192	1.02E+08	uncharacterized LOC101928415
ENSG0000	39.36243	-0.15727	0.399941	-0.39323	0.69415	0.874781	MTX2	10651	metaxin 2
ENSG0000	2810.936	0.030009	0.076324	0.393184	0.694183	0.874781	QARS1	5859	glutaminyl-tRNA synthetase 1
ENSG0000	417.8665	0.061398	0.156118	0.393279	0.694113	0.874781	SOCS4	122809	suppressor of cytokine signaling 4
ENSG0000	14.39475	-0.29926	0.761965	-0.39275	0.694503	0.874917	NCMAP	400746	non-compact myelin associated protein
ENSG0000	6.938158	-0.42095	1.071585	-0.39283	0.694447	0.874917	LOC10272	1.03E+08	uncharacterized LOC102723446
ENSG0000	13.56419	-0.30994	0.788997	-0.39283	0.694446	0.874917	NA	NA	NA
ENSG0000	46.67051	0.163423	0.416144	0.392731	0.694519	0.874917	TRAV14DV	28669	T cell receptor alpha variable 14/delta variable 4
ENSG0000	65.34588	0.127605	0.324929	0.392718	0.694528	0.874917	NA	NA	NA
ENSG0000	343.1345	-0.0695	0.177032	-0.39259	0.694619	0.874973	CD180	4064	CD180 molecule
ENSG0000	2419.918	0.040038	0.102016	0.392465	0.694715	0.874974	MFSD10	10227	major facilitator superfamily domain containing 10
ENSG0000	1182.986	0.040206	0.102439	0.392486	0.694699	0.874974	FKBP4	2288	FKBP prolyl isomerase 4
ENSG0000	170.3959	0.080338	0.205124	0.391656	0.695312	0.875084	SNRPE	6635	small nuclear ribonucleoprotein polypeptide E
ENSG0000	173.1971	-0.10036	0.256312	-0.39156	0.695384	0.875084	CHST10	9486	carbohydrate sulfotransferase 10
ENSG0000	39.6916	0.163187	0.41668	0.391636	0.695327	0.875084	ERICH6-AS	1.02E+08	ERICH6 antisense RNA 1
ENSG0000	139.7498	0.081118	0.2071	0.391687	0.695289	0.875084	SEPTIN8	23176	septin 8
ENSG0000	11.01035	0.312648	0.798236	0.391673	0.695299	0.875084	LOC1019	1.02E+08	uncharacterized LOC101929719
ENSG0000	1249.805	-0.12421	0.316934	-0.39192	0.695115	0.875084	HSPA1A	3303	heat shock protein family A (Hsp70) member 1A
ENSG0000	19.28708	0.235826	0.601479	0.392076	0.695002	0.875084	ZFAND2A-	1.02E+08	ZFAND2A divergent transcript
ENSG0000	456.2788	0.056803	0.145085	0.391514	0.695417	0.875084	AJM1	389813	apical junction component 1 homolog
ENSG0000	44.86409	0.140775	0.359808	0.391251	0.695612	0.875084	NA	NA	NA
ENSG0000	77.14081	-0.11032	0.281346	-0.3921	0.694981	0.875084	CYSLTR2	57105	cysteinyl leukotriene receptor 2
ENSG0000	278.0519	0.07117	0.181696	0.391698	0.695282	0.875084	KLHL28	54813	kelch like family member 28
ENSG0000	1152.607	0.042308	0.108127	0.391283	0.695588	0.875084	BTBD1	53339	BTB domain containing 1
ENSG0000	1524.886	-0.03551	0.090731	-0.39136	0.695531	0.875084	KIF22	3835	kinesin family member 22
ENSG0000	663.3347	0.044485	0.11362	0.391527	0.695408	0.875084	RSPRY1	89970	ring finger and SPRY domain containing 1
ENSG0000	4.966113	-0.51044	1.303294	-0.39165	0.695315	0.875084	LOC10192	1.02E+08	uncharacterized LOC101927556
ENSG0000	7.235422	-0.34917	0.89272	-0.39113	0.695698	0.875084	LOC12252	1.23E+08	uncharacterized LOC122526780
ENSG0000	90.50843	-0.10845	0.276512	-0.39222	0.694898	0.875084	P3H4	10609	prolyl 3-hydroxylase family member 4 (inactive)
ENSG0000	132.7656	-0.10636	0.271899	-0.39119	0.695655	0.875084	CUEDC1	404093	CUE domain containing 1
ENSG0000	141.086	0.093518	0.238965	0.391345	0.695542	0.875084	KRBOX4	55634	KRAB box domain containing 4
ENSG0000	138.3046	-0.08461	0.216409	-0.39097	0.695823	0.875106	RIOX2	84864	ribosomal oxygenase 2
ENSG0000	20.0327	-0.2055	0.525722	-0.39089	0.695881	0.875106	LEAP2	116842	liver enriched antimicrobial peptide 2
ENSG0000	6.954628	-0.34851	0.891334	-0.39099	0.695803	0.875106	KIF20A	10112	kinesin family member 20A
ENSG0000	193.9986	-0.07893	0.201953	-0.39085	0.695905	0.875106	ZNF549	256051	zinc finger protein 549
ENSG0000	13.06071	0.268646	0.687727	0.390629	0.696072	0.875231	NA	NA	NA
ENSG0000	321.8187	0.061941	0.158627	0.390481	0.696181	0.875231	MRPL50	54534	mitochondrial ribosomal protein L50
ENSG0000	36.11676	0.174431	0.446716	0.390474	0.696186	0.875231	NA	NA	NA
ENSG0000	75.6846	0.112728	0.288701	0.390465	0.696193	0.875231	IGLV2-23	28813	immunoglobulin lambda variable 2-23
ENSG0000	580.5699	-0.05388	0.138066	-0.39024	0.696356	0.875258	LSM10	84967	LSM10 U7 small nuclear RNA associated
ENSG0000	23.47153	-0.21196	0.543114	-0.39027	0.696339	0.875258	ME1	4199	malic enzyme 1
ENSG0000	55.76616	0.152478	0.390655	0.390313	0.696305	0.875258	YAE1	57002	YAE1 maturation factor of ABCE1
ENSG0000	2478.514	-0.03257	0.08349	-0.39016	0.696421	0.875281	KANSL3	55683	KAT8 regulatory NSL complex subunit 3
ENSG0000	3.539823	-0.53735	1.378097	-0.38992	0.696592	0.875345	HORMAD1	84072	HORMA domain containing 1
ENSG0000	780.7809	0.054006	0.138513	0.389896	0.696613	0.875345	HEBP2	23593	heme binding protein 2
ENSG0000	38.58407	-0.15668	0.40175	-0.39	0.69654	0.875345	TRBV10-3	28583	T cell receptor beta variable 10-3
ENSG0000	15.77368	-0.2708	0.694803	-0.38976	0.696716	0.875414	ETV5	2119	ETS variant transcription factor 5
ENSG0000	13.37303	0.279052	0.716324	0.389561	0.696862	0.875538	PRR29	92340	proline rich 29
ENSG0000	2402.909	0.03595	0.092369	0.389206	0.697124	0.875808	TMEM109	79073	transmembrane protein 109
ENSG0000	800.5012	0.043136	0.111116	0.388209	0.697861	0.876145	PTCD3	55037	pentatricopeptide repeat domain 3
ENSG0000	3.797506	-0.58051	1.49434	-0.38847	0.697665	0.876145	CHMP3-AS	1.05E+08	CHMP3 and RNF103 antisense RNA 1
ENSG0000	122.8305	-0.08513	0.219078	-0.38857	0.697595	0.876145	LINC0068	1.07E+08	long intergenic non-protein coding RNA 680
ENSG0000	2501.227	-0.03247	0.083579	-0.38853	0.697626	0.876145	YWHAG	7532	tyrosine 3-monooxygenase/tryptophan 5-monooxygenase activation protein gamma
ENSG0000	3.036918	-0.64535	1.660677	-0.38861	0.697567	0.876145	NA	NA	NA
ENSG0000	162.286	0.07985	0.20547	0.388622	0.697556	0.876145	RPL36AP3	729362	ribosomal protein L36a pseudogene 37
ENSG0000	1781.885	0.036718	0.094597	0.388153	0.697903	0.876145	FRMD8	83786	FERM domain containing 8
ENSG0000	5439.933	-0.03114	0.08022	-0.38819	0.697876	0.876145	RBM25	58517	RNA binding motif protein 25
ENSG0000	28.93094	0.174698	0.450099	0.388133	0.697918	0.876145	MGC1627	85001	uncharacterized protein MGC16275
ENSG0000	1608.955	0.036121	0.093076	0.388077	0.697959	0.876145	CD300A	11314	CD300a molecule
ENSG0000	1152.414	0.04183	0.107776	0.388115	0.697931	0.876145	PRDX2	7001	peroxiredoxin 2
ENSG0000	477.1393	0.051213	0.131885	0.388319	0.69778	0.876145	CENPBD2F	65996	CENPB DN pseudogene
ENSG0000	5.781355	-0.39197	1.01025	-0.388	0.698019	0.876162	TAB2	23118	TGF-beta activated kinase 1 (MAP3K7) binding protein 2
ENSG0000	8.012055	0.377596	0.973399	0.387915	0.698079	0.876177	LGALS17A	400696	galectin 14 pseudogene
ENSG0000	2.600005	-0.79878	2.061551	-0.38746	0.698413	0.876537	IGSF10	285313	immunoglobulin superfamily member 10
ENSG0000	4.864018	-0.47377	1.223198	-0.38732	0.69852	0.876553	PIK3CD-AS	1.02E+08	PIK3CD antisense RNA 2
ENSG0000	78.4643	0.120918	0.312174	0.38734	0.698505	0.876553	RBP7	116362	retinol binding protein 7
ENSG0000	30.35528	-0.17689	0.456807	-0.38723	0.698586	0.876576	MYLK4	340156	myosin light chain kinase family member 4
ENSG0000	2423.601	-0.03639	0.094019	-0.38708	0.698697	0.876656	CCM2	83605	CCM2 scaffold protein
ENSG0000	1195.749	-0.04603	0.119013	-0.38676	0.698936	0.876896	N4BP2L2	10443	NEDD4 binding protein 2 like 2
ENSG0000	395.7029	0.056177	0.145283	0.38667	0.699	0.87691	ECHDC2	55268	enoyl-CoA hydratase domain containing 2
ENSG0000	2.21792	-0.77997	2.01743	-0.38662	0.699041	0.87691	NA	NA	NA
ENSG0000	19.74471	-0.20873	0.540051	-0.38651	0.699123	0.876938	NA	NA	NA
ENSG0000	539.3842	0.044996	0.116433	0.386456	0.699159	0.876938	SURF6	6838	surfeit 6
ENSG0000	35.85247	0.192933	0.499317	0.386393	0.699205	0.876938	NA	NA	NA
ENSG0000	17.58898	0.248021	0.642759	0.385869	0.699593	0.877108	ARHGEF4	50649	Rho guanine nucleotide exchange factor 4
ENSG0000	685.881	0.046914	0.121514	0.386077	0.69944	0.877108	SUMO1	7341	small ubiquitin like modifier 1
ENSG0000	4.378777	-0.4219	1.09336	-0.38587	0.699589	0.877108	HPX	3263	hemopexin
ENSG0000	88.26165	-0.09652	0.249984	-0.38609	0.699432	0.877108	C12orf76	400073	chromosome 12 open reading frame 76
ENSG0000	83.28583	-0.0989	0.256329	-0.38581	0.699634	0.877108	EPN2	22905	epsin 2
ENSG0000	31.9923	-0.2017	0.522857	-0.38576	0.699672	0.877108	ZNF519	162655	zinc finger protein 519
ENSG0000	5.927119	-0.49168	1.273988	-0.38594	0.699541	0.877108	NA	NA	NA
ENSG0000	13.85033	0.246827	0.640081	0.385619	0.699779	0.877183	CIRBP-AS1	148046	CIRBP antisense RNA 1
ENSG0000	2.889755	-0.70586	1.831492	-0.3854	0.69994	0.877326	GTF2IP5	1E+08	general transcription factor IIi pseudogene 5
ENSG0000	41.49069	-0.15068	0.391192	-0.38518	0.700102	0.877328	TRBV18	28569	T cell receptor beta variable 18
ENSG0000	289.9643	-0.06935	0.180059	-0.38514	0.700131	0.877328	FAM210B	116151	family with sequence similarity 210 member B

ENSG0000	32.01598	0.18905	0.49066	0.385297	0.700017	0.877328	NKILA	1.05E+08	NF-kappaB interacting lncRNA
ENSG0000	4.063471	-0.46932	1.218568	-0.38514	0.700131	0.877328	CCNB3	85417	cyclin B3
ENSG0000	2371.924	-0.04654	0.120968	-0.38469	0.700467	0.877457	ITGAV	3685	integrin subunit alpha V
ENSG0000	345.9922	0.06603	0.171626	0.384734	0.700434	0.877457	TTF1	7270	transcription termination factor 1
ENSG0000	1666.115	0.03424	0.089008	0.384685	0.700471	0.877457	PTEN	5728	phosphatase and tensin homolog
ENSG0000	79.21238	-0.12015	0.31226	-0.38479	0.700394	0.877457	CHAF1B	8208	chromatin assembly factor 1 subunit B
ENSG0000	2495.932	0.03487	0.090607	0.384852	0.700347	0.877457	TUG1	55000	taurine up-regulated 1
ENSG0000	621.7389	-0.04707	0.122449	-0.3844	0.700683	0.877551	MED8	112950	mediator complex subunit 8
ENSG0000	68.52853	0.140381	0.365317	0.384271	0.700778	0.877551	CYB5RL	606495	cytochrome b5 reductase like
ENSG0000	742.3618	-0.04202	0.109346	-0.38426	0.700782	0.877551	SLC4A1AP	22950	solute carrier family 4 member 1 adaptor protein
ENSG0000	888.118	-0.04066	0.105815	-0.38427	0.700778	0.877551	MRPL28	10573	mitochondrial ribosomal protein L28
ENSG0000	2.991704	0.676676	1.760661	0.384331	0.700733	0.877551	MIR5190	1.01E+08	microRNA 5190
ENSG0000	9.587275	-0.32022	0.833678	-0.3841	0.700904	0.877592	LOC10192	1.02E+08	uncharacterized LOC101928626
ENSG0000	1577.092	0.039943	0.10401	0.384029	0.700957	0.877592	SEMA4A	64218	semaphorin 4A
ENSG0000	132.1896	-0.08779	0.228575	-0.38407	0.700926	0.877592	CRIP1	1396	cysteine rich protein 1
ENSG0000	568.4107	-0.04495	0.117079	-0.38393	0.701032	0.877626	NCBP1	4686	nuclear cap binding protein subunit 1
ENSG0000	306.6507	0.05717	0.149081	0.383484	0.701361	0.877979	OTUD6B-A	1.01E+08	OTUD6B antisense RNA 1 (head to head)
ENSG0000	154.113	0.079513	0.207733	0.382765	0.701894	0.87835	PCDH1	5097	protocadherin 1
ENSG0000	4.291136	-0.61703	1.611533	-0.38288	0.701808	0.87835	VIRMA-DT	1.02E+08	VIRMA divergent transcript
ENSG0000	15.48221	-0.26655	0.696286	-0.38282	0.701852	0.87835	TNFSF11	8600	TNF superfamily member 11
ENSG0000	69.72455	0.114119	0.297989	0.382963	0.701747	0.87835	CFL2	1073	cofilin 2
ENSG0000	2257.083	0.039556	0.103323	0.382836	0.701842	0.87835	MED1	5469	mediator complex subunit 1
ENSG0000	1469.794	-0.03601	0.094189	-0.3823	0.702241	0.878401	TXLNA	200081	taxilin alpha
ENSG0000	97.5776	-0.09266	0.242491	-0.38212	0.702373	0.878401	MANEAL	149175	mannosidase endo-alpha like
ENSG0000	6.498083	-0.34399	0.900655	-0.38194	0.702508	0.878401	NA	NA	NA
ENSG0000	184.0521	0.069522	0.181894	0.382213	0.702303	0.878401	IRS1	3667	insulin receptor substrate 1
ENSG0000	18.78837	0.267145	0.699407	0.38196	0.702491	0.878401	LOC10050	1.01E+08	uncharacterized LOC100507053
ENSG0000	21.71346	0.199167	0.521269	0.382081	0.702401	0.878401	LRRC24	441381	leucine rich repeat containing 24
ENSG0000	297.009	0.060856	0.159099	0.382502	0.702089	0.878401	DDX31	64794	DEAD-box helicase 31
ENSG0000	9.018227	-0.31948	0.836221	-0.38206	0.702419	0.878401	NOXO1	124056	NADPH oxidase organizer 1
ENSG0000	4.885289	-0.44126	1.155455	-0.38189	0.702544	0.878401	NA	NA	NA
ENSG0000	8.525484	-0.34373	0.899086	-0.38231	0.702229	0.878401	GPRC5C	55890	G protein-coupled receptor class C group 5 member C
ENSG0000	214.2954	-0.06349	0.166198	-0.38203	0.702442	0.878401	ZNF302	55900	zinc finger protein 302
ENSG0000	13.74068	-0.28456	0.74505	-0.38194	0.702508	0.878401	MIR642A	693227	microRNA 642a
ENSG0000	8.408104	-0.42402	1.11035	-0.38188	0.70255	0.878401	NA	NA	NA
ENSG0000	1947.707	0.036273	0.095049	0.38163	0.702736	0.878543	LINC0108	338799	long intergenic non-protein coding RNA 1089
ENSG0000	13.15571	0.251414	0.658843	0.381599	0.702759	0.878543	ZNF19	7567	zinc finger protein 19
ENSG0000	3.552107	0.547049	1.434359	0.381389	0.702914	0.878633	VAV3-AS1	1.01E+08	VAV3 antisense RNA 1
ENSG0000	8.803836	-0.34594	0.907099	-0.38137	0.702925	0.878633	NA	NA	NA
ENSG0000	141.9203	-0.07905	0.207377	-0.38121	0.703049	0.878669	NA	NA	NA
ENSG0000	5.494782	0.434017	1.138516	0.381213	0.703045	0.878669	LINC0143	728228	long intergenic non-protein coding RNA 1433
ENSG0000	271.557	-0.06214	0.163104	-0.38096	0.703234	0.878782	GPATCH11	253635	G-patch domain containing 11
ENSG0000	243.0195	-0.0631	0.165624	-0.38101	0.703198	0.878782	ZNF473	25888	zinc finger protein 473
ENSG0000	343.3464	-0.05425	0.142502	-0.38069	0.703431	0.878874	CDC40	51362	cell division cycle 40
ENSG0000	270.1176	-0.07321	0.192323	-0.38068	0.70344	0.878874	RBM4B	83759	RNA binding motif protein 4B
ENSG0000	782.0747	0.04207	0.110516	0.380668	0.70345	0.878874	GEMIN4	50628	gem nuclear organelle associated protein 4
ENSG0000	259.8698	-0.06348	0.166978	-0.38016	0.703826	0.879285	PYROXD1	79912	pyridine nucleotide-disulphide oxidoreductase domain 1
ENSG0000	144.4625	0.078954	0.207774	0.379997	0.703948	0.879378	GEMIN8	54960	gem nuclear organelle associated protein 8
ENSG0000	7.954609	0.391697	1.031684	0.379667	0.704192	0.879565	NA	NA	NA
ENSG0000	46.65966	-0.17485	0.460503	-0.3797	0.704168	0.879565	ALG10	84920	ALG10 alp 2-glucosyltransferase
ENSG0000	17.62751	-0.22079	0.581731	-0.37954	0.704286	0.879611	NA	NA	NA
ENSG0000	7.577934	0.350381	0.923414	0.379441	0.70436	0.879611	CYP21A1P	1590	cytochrom pseudogene
ENSG0000	64.80046	0.112846	0.297411	0.379426	0.704372	0.879611	ZNF567-D	1.02E+08	ZNF567 divergent transcript
ENSG0000	15.54213	-0.22772	0.600372	-0.3793	0.704464	0.879667	NA	NA	NA
ENSG0000	5.576825	0.399991	1.054864	0.379188	0.704549	0.879694	NA	NA	NA
ENSG0000	37.28252	0.460751	1.215237	0.379145	0.70458	0.879694	IGLC7	28834	immunoglobulin lambda constant 7
ENSG0000	102.7924	-0.09533	0.25153	-0.37899	0.704698	0.879781	KRIT1	889	KRIT1 ankyrin repeat containing
ENSG0000	3.312276	0.543685	1.434981	0.37888	0.704777	0.879821	RNU6-2	1.04E+08	RNA U6 small nuclear 2
ENSG0000	20.75029	-0.22774	0.601998	-0.3783	0.705208	0.879984	NA	NA	NA
ENSG0000	13.81145	0.251283	0.664209	0.378319	0.705194	0.879984	NA	NA	NA
ENSG0000	26.46911	-0.20362	0.538008	-0.37847	0.705082	0.879984	TRBV6-6	28601	T cell receptor beta variable 6-6
ENSG0000	8.329708	0.328541	0.86838	0.378338	0.705179	0.879984	NA	NA	NA
ENSG0000	15.16453	-0.27472	0.726271	-0.37826	0.705239	0.879984	ZNF205	7755	zinc finger protein 205
ENSG0000	51.23985	0.150603	0.39779	0.378599	0.704986	0.879984	HS3ST3A1	9955	heparan sulfate-glucosamine 3-sulfotransferase 3A1
ENSG0000	4594.488	0.033143	0.087583	0.378412	0.705125	0.879984	PNPLA6	10908	patatin like phospholipase domain containing 6
ENSG0000	3030.841	0.029436	0.077849	0.378114	0.705346	0.879998	NKIRAS2	28511	NFKB inhibitor interacting Ras like 2
ENSG0000	412.634	0.054212	0.143365	0.378142	0.705325	0.879998	RBMX2	51634	RNA binding motif protein X-linked 2
ENSG0000	16.72524	-0.21201	0.56087	-0.37801	0.705425	0.880038	NA	NA	NA
ENSG0000	6892.931	-0.03357	0.08887	-0.37771	0.705643	0.880251	TNFRSF1A	7132	TNF receptor superfamily member 1A
ENSG0000	145.7754	0.090499	0.239838	0.377333	0.705926	0.88032	TBX19	9095	T-box transcription factor 19
ENSG0000	106.2002	-0.10374	0.2749	-0.37738	0.705893	0.88032	PIP4P2	55529	phosphati 5-bisphosphate 4-phosphatase 2
ENSG0000	16.59257	-0.29109	0.771477	-0.37731	0.705943	0.88032	SAMD12	401474	sterile alpha motif domain containing 12
ENSG0000	199.2304	0.06827	0.180965	0.377255	0.705984	0.88032	BORCS7	119032	BLOC-1 related complex subunit 7
ENSG0000	122.8801	-0.08948	0.23704	-0.37749	0.705808	0.88032	ATP8B4	79895	ATPase phospholipid transporting 8B4 (putative)
ENSG0000	10.85199	-0.2843	0.75319	-0.37746	0.70583	0.88032	NA	NA	NA
ENSG0000	3.524486	0.537819	1.426353	0.377059	0.70613	0.880443	NA	NA	NA
ENSG0000	282.2775	0.064633	0.171487	0.3769	0.706248	0.880506	SPATA5L1	79029	spermatogenesis associated 5 like 1
ENSG0000	8.846594	0.315482	0.837125	0.376863	0.706275	0.880506	ZNF225-A	1E+08	ZNF225 and ZNF224 antisense RNA 1
ENSG0000	8.036449	-0.38135	1.012677	-0.37658	0.706488	0.880571	ZFP2	80108	ZFP2 zinc finger protein
ENSG0000	6.562722	0.443916	1.179037	0.376507	0.70654	0.880571	NA	NA	NA
ENSG0000	45.15144	-0.13088	0.347463	-0.37668	0.706413	0.880571	CDCA5	113130	cell division cycle associated 5
ENSG0000	683.0267	0.044455	0.118083	0.376473	0.706565	0.880571	CHORDC1	26973	cysteine and histidine rich domain containing 1
ENSG0000	2.63017	-0.56397	1.497998	-0.37648	0.706557	0.880571	NA	NA	NA
ENSG0000	7.639192	0.303411	0.8069	0.376021	0.706901	0.880931	NA	NA	NA
ENSG0000	63.15048	-0.11902	0.316618	-0.37592	0.706974	0.880963	ZNF367	195828	zinc finger protein 367
ENSG0000	24.48772	0.201804	0.536994	0.375803	0.707063	0.880986	LINC0134	731275	long intergenic non-protein coding RNA 1347
ENSG0000	10.66092	0.285895	0.760824	0.37577	0.707088	0.880986	NA	NA	NA
ENSG0000	3.54783	0.681255	1.816698	0.374997	0.707663	0.881525	NA	NA	NA
ENSG0000	397.5646	-0.05206	0.138838	-0.375	0.707663	0.881525	WDR73	84942	WD repeat domain 73
ENSG0000	6.998154	0.38177	1.018049	0.375002	0.707659	0.881525	MIR222	407007	microRNA 222
ENSG0000	3597.87	-0.0325	0.086827	-0.37432	0.708167	0.882093	BAP1	8314	BRCA1 associated protein 1
ENSG0000	4.711002	-0.44963	1.202865	-0.3738	0.708551	0.882165	NA	NA	NA
ENSG0000	66.29798	0.134682	0.360272	0.373835	0.708527	0.882165	C1orf54	79630	chromosome 1 open reading frame 54
ENSG0000	494.8067	0.050716	0.135633	0.373922	0.708463	0.882165	SUCO	51430	SUN domain containing ossification factor
ENSG0000	279.098	-0.0569	0.15208	-0.37416	0.708282	0.882165	ANAPC4	29945	anaphase promoting complex subunit 4
ENSG0000	34.36463	0.153954	0.412131	0.373556	0.708735	0.882165	HOXA1	3198	homeobox A1
ENSG0000	18.97834	0.261322	0.699198	0.373745	0.708594	0.882165	NA	NA	NA
ENSG0000	7.493128	0.337873	0.904118	0.373705	0.708624	0.882165	PTENP1	11191	phosphatase and tensin homolog pseudogene 1
ENSG0000	15.71452	0.23086	0.617386	0.373931	0.708455	0.882165	OR5B21	219968	olfactory receptor family 5 subfamily B member 21
ENSG0000	5.083548	0.427876	1.145198	0.373626	0.708683	0.882165	HPD	3242	4-hydroxyphenylpyruvate dioxygenase

ENSG0000	27.64269	-0.17965	0.480373	-0.37398	0.70842	0.882165	FAAP24	91442	FA core complex associated protein 24
ENSG0000	244.0435	-0.06436	0.172293	-0.37354	0.708747	0.882165	ZNF671	79891	zinc finger protein 671
ENSG0000	36.20603	0.163435	0.437649	0.373438	0.708823	0.882199	ASH1L-AS1	645676	ASH1L antisense RNA 1
ENSG0000	888.2787	-0.04302	0.115323	-0.37308	0.709092	0.882416	OBSCN	84033	obscurin cytoskeletal calmodulin and titin-interacting RhoGEF
ENSG0000	94.66404	-0.11631	0.311749	-0.3731	0.709072	0.882416	PPP2R2B	5521	protein phosphatase 2 regulatory subunit Bbeta
ENSG0000	12.99272	0.354519	0.950548	0.372963	0.709176	0.882462	NA	NA	NA
ENSG0000	176.8649	-0.07536	0.202096	-0.3729	0.709224	0.882462	AZU1	566	azurocidin 1
ENSG0000	32.63794	-0.17491	0.469274	-0.37272	0.709356	0.882507	SLAMF9	89886	SLAM family member 9
ENSG0000	2.37634	0.735314	1.972555	0.372772	0.709318	0.882507	KCTD19	146212	potassium channel tetramerization domain containing 19
ENSG0000	4.65897	-0.47171	1.266047	-0.37258	0.70946	0.882578	MAP1B	4131	microtubule associated protein 1B
ENSG0000	63.37468	0.137654	0.36971	0.372331	0.709647	0.882751	SLC1A3	6507	solute carrier family 1 member 3
ENSG0000	56.21712	0.140446	0.377491	0.372051	0.709855	0.882886	ATP6V1FN	1E+08	ATP6V1F neighbor
ENSG0000	267.7835	-0.05817	0.156365	-0.37199	0.709898	0.882886	BTBD10	84280	BTB domain containing 10
ENSG0000	47.80817	0.147446	0.396363	0.371998	0.709894	0.882886	SLC14A1	6563	solute carrier family 14 member 1 (Kidd blood group)
ENSG0000	55.47176	-0.13505	0.363334	-0.3717	0.710117	0.88303	GALNT12	79695	polypeptide N-acetylgalactosaminyltransferase 12
ENSG0000	1197.526	0.034813	0.093673	0.371646	0.710157	0.88303	CDS2	8760	CDP-diacylglycerol synthase 2
ENSG0000	145.5123	0.077438	0.20831	0.371741	0.710086	0.88303	PMM1	5372	phosphomannomutase 1
ENSG0000	1960.224	0.034205	0.092061	0.371547	0.71023	0.883062	TINF2	26277	TERF1 interacting nuclear factor 2
ENSG0000	1426.129	-0.04392	0.118266	-0.37136	0.710368	0.883174	PSMD4	5710	proteasom non-ATPase 4
ENSG0000	64.80611	0.109523	0.295005	0.371257	0.710446	0.883174	NBR2	10230	neighbor of BRCA1 lncRNA 2
ENSG0000	8.299238	-0.28956	0.779981	-0.37123	0.710463	0.883174	NA	NA	NA
ENSG0000	31.91424	0.216632	0.583789	0.37108	0.710578	0.883199	TMTC1	83857	transmembrane O-mannosyltransferase targeting cadherins 1
ENSG0000	13.87442	0.241682	0.651329	0.37106	0.710593	0.883199	NA	NA	NA
ENSG0000	68.6038	0.116504	0.314015	0.371016	0.710626	0.883199	GET1	7485	guided entry of tail-anchored proteins factor 1
ENSG0000	6.41735	0.406579	1.096792	0.370698	0.710862	0.883434	CCDC42	146849	coiled-coil domain containing 42
ENSG0000	96.60049	-0.13164	0.355287	-0.37051	0.711001	0.883547	NA	NA	NA
ENSG0000	14.8414	0.235188	0.635037	0.370354	0.711119	0.883583	C1QTNF3	114899	C1q and TNF related 3
ENSG0000	8.110236	-0.30871	0.833571	-0.37035	0.711125	0.883583	TLK2P1	646202	tousled like kinase 2 pseudogene 1
ENSG0000	8.577533	-0.34862	0.941571	-0.37025	0.711196	0.883611	NEK2	4751	NIMA related kinase 2
ENSG0000	13.91657	-0.27314	0.738047	-0.37008	0.711322	0.883678	DCAF8-DT	1E+08	DCAF8 divergent transcript
ENSG0000	15.52447	0.264626	0.715303	0.36995	0.71142	0.883678	NA	NA	NA
ENSG0000	9.937892	0.311669	0.842339	0.370004	0.71138	0.883678	NA	NA	NA
ENSG0000	5.408925	0.452057	1.22203	0.369923	0.71144	0.883678	NA	NA	NA
ENSG0000	29.78183	0.18138	0.491198	0.36926	0.711934	0.883758	MGST2	4258	microsomal glutathione S-transferase 2
ENSG0000	20.18741	0.191207	0.5178	0.369268	0.711928	0.883758	GGCT	79017	gamma-glutamylcyclotransferase
ENSG0000	3.870777	-0.47264	1.279019	-0.36953	0.711733	0.883758	ANKRD18A	253650	ankyrin repeat domain 18A
ENSG0000	21.07548	0.202943	0.549329	0.369437	0.711802	0.883758	FSD1L	83856	fibronectin type III and SPRY domain containing 1 like
ENSG0000	147.247	-0.10097	0.273348	-0.36939	0.711839	0.883758	RNF141	50862	ring finger protein 141
ENSG0000	2.972576	-0.55158	1.493567	-0.36931	0.711899	0.883758	TUNAR	1.01E+08	TCL1 upstream neural differentiation-associated RNA
ENSG0000	3.490593	0.463652	1.254713	0.369528	0.711734	0.883758	IGHV4-28	28400	immunoglobulin heavy variable 4-28
ENSG0000	2.353804	-0.65956	1.786146	-0.36926	0.711932	0.883758	C16orf95	1.01E+08	chromosome 16 open reading frame 95
ENSG0000	41.66157	0.138747	0.375554	0.369445	0.711796	0.883758	LOC12245	1.22E+08	testis-specific serine/proline-rich protein
ENSG0000	33.76727	-0.16501	0.447753	-0.36852	0.712484	0.884146	FCRLB	127943	Fc receptor like B
ENSG0000	4.386596	-0.45387	1.231112	-0.36867	0.712375	0.884146	PDC-AS1	1.03E+08	PDC antisense RNA 1
ENSG0000	260.3193	-0.05911	0.160376	-0.36856	0.712459	0.884146	CCDC90B	60492	coiled-coil domain containing 90B
ENSG0000	100.2306	-0.10418	0.282618	-0.36862	0.712408	0.884146	CEACAM4	1089	CEA cell adhesion molecule 4
ENSG0000	95.48033	-0.08865	0.240543	-0.36852	0.712484	0.884146	ZNF225	7768	zinc finger protein 225
ENSG0000	47.50188	-0.14011	0.38037	-0.36836	0.712603	0.884234	PRCD	768206	photoreceptor disc component
ENSG0000	31.51107	-0.22119	0.600974	-0.36805	0.712834	0.884402	CATIP-AS1	1.02E+08	CATIP antisense RNA 1
ENSG0000	13.53631	-0.31522	0.856399	-0.36808	0.712813	0.884402	SLC12A3	6559	solute carrier family 12 member 3
ENSG0000	8.16114	-0.35827	0.974076	-0.36781	0.713017	0.884455	DYNLT5	200132	dynein light chain Tctex-type family member 5
ENSG0000	5.345174	0.395124	1.074279	0.367804	0.713019	0.884455	TEX9	374618	testis expressed 9
ENSG0000	12047.84	-0.02936	0.079837	-0.36781	0.713019	0.884455	PPP6R1	22870	protein phosphatase 6 regulatory subunit 1
ENSG0000	7.430866	0.34342	0.934277	0.367579	0.713187	0.884545	NA	NA	NA
ENSG0000	2.254158	-0.72174	1.963375	-0.3676	0.713172	0.884545	INSM1	3642	INSM transcriptional repressor 1
ENSG0000	8.112085	-0.28651	0.779911	-0.36736	0.713351	0.884647	BRD7P2	647076	bromodomain containing 7 pseudogene 2
ENSG0000	994.6656	-0.03719	0.101236	-0.36734	0.713365	0.884647	SEC63	11231	SEC63 hon protein translocation regulator
ENSG0000	58.165	-0.12244	0.333483	-0.36715	0.713506	0.884752	MIX23	131076	mitochondrial matrix import factor 23
ENSG0000	257.5325	0.064099	0.174609	0.367099	0.713545	0.884752	POLR1H	30834	RNA polymerase I subunit H
ENSG0000	58.86444	-0.12514	0.341112	-0.36685	0.713729	0.884921	NOL12	79159	nucleolar protein 12
ENSG0000	66.27917	0.131237	0.357973	0.36661	0.71391	0.885054	RTN4R	65078	reticulon 4 receptor
ENSG0000	7310.226	-0.02682	0.073151	-0.36658	0.713932	0.885054	PLXNB2	23654	plexin B2
ENSG0000	4.868368	-0.40904	1.116825	-0.36626	0.714174	0.885173	NA	NA	NA
ENSG0000	1544.561	-0.03369	0.092016	-0.36617	0.714239	0.885173	KLF7	8609	KLF transcription factor 7
ENSG0000	3.191366	-0.54573	1.490057	-0.36625	0.71418	0.885173	NA	NA	NA
ENSG0000	7.639581	0.371857	1.015158	0.366304	0.714138	0.885173	LOC10099	1.01E+08	uncharacterized LOC100996660
ENSG0000	13.42525	0.282846	0.772526	0.366132	0.714267	0.885173	NA	NA	NA
ENSG0000	704.4647	0.039399	0.107645	0.366005	0.714361	0.885231	POLR3C	10623	RNA polymerase III subunit C
ENSG0000	10.16348	-0.28605	0.781782	-0.36589	0.714447	0.885278	NA	NA	NA
ENSG0000	3.214288	0.471174	1.288352	0.365719	0.714575	0.885319	ARF4-AS1	1.06E+08	ARF4 antisense RNA 1
ENSG0000	49.82789	0.155827	0.426059	0.365741	0.714558	0.885319	IGLV3-21	28796	immunoglobulin lambda variable 3-21
ENSG0000	12.74142	0.294946	0.8068	0.365575	0.714682	0.885392	CHKB-DT	1E+08	CHKB divergent transcript
ENSG0000	17.75643	-0.23551	0.64445	-0.36544	0.714781	0.885455	TEX45	374877	testis expressed 45
ENSG0000	7.662637	0.339438	0.929196	0.365302	0.714886	0.885467	NA	NA	NA
ENSG0000	1585.562	-0.0353	0.096619	-0.36534	0.714858	0.885467	DCTN2	10540	dynactin subunit 2
ENSG0000	6.696195	0.428927	1.174474	0.365207	0.714957	0.885496	PRICKLE2	166336	prickle planar cell polarity protein 2
ENSG0000	100.5227	-0.10916	0.299022	-0.36507	0.71506	0.885565	IL23A	51561	interleukin 23 subunit alpha
ENSG0000	80.16103	0.104335	0.286012	0.364794	0.715265	0.885701	ATP1A3	478	ATPase Na+/K+ transporting subunit alpha 3
ENSG0000	7.238871	0.359203	0.984587	0.364826	0.715241	0.885701	DBNDD2	55861	dysbindin domain containing 2
ENSG0000	3.119322	-0.4961	1.360885	-0.36454	0.715453	0.885814	NA	NA	NA
ENSG0000	27.94161	0.165044	0.45267	0.364602	0.715408	0.885814	NA	NA	NA
ENSG0000	10.21319	-0.25719	0.705747	-0.36442	0.715546	0.885871	NA	NA	NA
ENSG0000	5.763288	0.424293	1.164537	0.364344	0.715601	0.88588	PRR33	1.03E+08	proline rich 33
ENSG0000	140.979	-0.08246	0.226443	-0.36415	0.715742	0.885882	PODXL	5420	podocalyxin like
ENSG0000	74.47994	-0.108	0.29649	-0.36426	0.715663	0.885882	FKTN	2218	fukutin
ENSG0000	2965.234	-0.02814	0.077299	-0.36401	0.715848	0.885882	SIDT2	51092	SID1 transmembrane family member 2
ENSG0000	2722.112	-0.02967	0.081521	-0.36399	0.715865	0.885882	NCKAP5L	57701	NCK associated protein 5 like
ENSG0000	892.4157	0.040337	0.11083	0.363958	0.715889	0.885882	TLK2	11011	tousled like kinase 2
ENSG0000	184.4157	-0.06728	0.184807	-0.36405	0.715822	0.885882	SRRD	402055	SRR1 domain containing
ENSG0000	1920.392	0.033843	0.093107	0.363488	0.71624	0.886257	PRKCB	5579	protein kinase C beta
ENSG0000	152.8553	-0.07262	0.19992	-0.36325	0.716415	0.886355	CCDC17	149483	coiled-coil domain containing 17
ENSG0000	41.99743	-0.13873	0.381904	-0.36327	0.716406	0.886355	NA	NA	NA
ENSG0000	257.2649	-0.06346	0.174743	-0.36319	0.716466	0.88636	IPMK	253430	inositol polyphosphate multikinase
ENSG0000	6.558344	-0.3574	0.984586	-0.36299	0.716609	0.886477	YWHAH-A	25775	YWHAH antisense RNA 1
ENSG0000	5.57058	-0.4368	1.204482	-0.36264	0.716871	0.8867	PFN1P2	767846	profilin 1 pseudogene 2
ENSG0000	12.96185	0.275812	0.760601	0.362624	0.716885	0.8867	FGFR2	2263	fibroblast growth factor receptor 2
ENSG0000	229.326	-0.06096	0.168164	-0.36248	0.716994	0.886716	ZNF184	7738	zinc finger protein 184
ENSG0000	3.785899	0.479996	1.324005	0.362534	0.716953	0.886716	NA	NA	NA
ENSG0000	8.348805	-0.33471	0.923825	-0.36231	0.717123	0.886817	NA	NA	NA
ENSG0000	92.8754	-0.09549	0.263647	-0.3622	0.717206	0.88686	MYBPC3	4607	myosin binding protein C3

ENSG0000	32.94985	0.149846	0.413797	0.362123	0.71726	0.886868	NA	NA	NA
ENSG0000	88.73484	-0.09873	0.27271	-0.36204	0.717319	0.886882	NAT1	9	N-acetyltransferase 1
ENSG0000	641.049	0.04362	0.120583	0.361745	0.717542	0.886951	AZI2	64343	5-azacytidine induced 2
ENSG0000	2.928761	0.624327	1.725918	0.361736	0.717549	0.886951	NA	NA	NA
ENSG0000	7.630717	0.328716	0.908774	0.361713	0.717566	0.886951	NA	NA	NA
ENSG0000	230.682	-0.06858	0.189516	-0.36187	0.71745	0.886951	EPOR	2057	erythropoietin receptor
ENSG0000	21.61629	-0.20878	0.577689	-0.36141	0.717791	0.887138	NA	NA	NA
ENSG0000	332.6767	0.058826	0.162786	0.361372	0.717821	0.887138	MRPL51	51258	mitochondrial ribosomal protein L51
ENSG0000	15.0947	0.244975	0.678039	0.3613	0.717875	0.887138	PPFIA3	8541	PTPRF interacting protein alpha 3
ENSG0000	3.535518	0.683913	1.893159	0.361255	0.717909	0.887138	NA	NA	NA
ENSG0000	5.974819	0.333	0.925689	0.359732	0.719047	0.887252	MIR6859-1	1.02E+08	microRNA 6859-1
ENSG0000	2704.46	0.032586	0.090519	0.359994	0.718852	0.887252	SYTL1	84958	synaptotagmin like 1
ENSG0000	1712.47	-0.03942	0.109384	-0.3604	0.718551	0.887252	PHACTR4	65979	phosphatase and actin regulator 4
ENSG0000	41.55811	-0.12798	0.35554	-0.35996	0.718876	0.887252	OMA1	115209	OMA1 zinc metallopeptidase
ENSG0000	9.542094	-0.29634	0.822531	-0.36028	0.718636	0.887252	FCGR3B	2215	Fc gamma receptor IIIb
ENSG0000	103.8748	-0.09587	0.265938	-0.36051	0.718467	0.887252	IGKV1-5	28299	immunoglobulin kappa variable 1-5
ENSG0000	351.6432	0.05708	0.158188	0.360838	0.718221	0.887252	DCUN1D1	54165	defective in cullin neddylation 1 domain containing 1
ENSG0000	377.3435	0.052135	0.144857	0.359904	0.718919	0.887252	AFAP1	60312	actin filament associated protein 1
ENSG0000	20.49174	0.257416	0.715095	0.359974	0.718866	0.887252	LINC00689	154822	long intergenic non-protein coding RNA 689
ENSG0000	14.16319	0.259988	0.720717	0.360735	0.718297	0.887252	CHRNA2	1135	cholinergic receptor nicotinic alpha 2 subunit
ENSG0000	30.34879	-0.18538	0.515345	-0.35972	0.719053	0.887252	PNOC	5368	prepronociceptin
ENSG0000	323.7257	-0.05507	0.1528	-0.36042	0.71853	0.887252	GOLM1	51280	golgi membrane protein 1
ENSG0000	399.5504	0.06057	0.168215	0.360078	0.718789	0.887252	TRAF2	7186	TNF receptor associated factor 2
ENSG0000	490.0932	-0.04705	0.130454	-0.36069	0.718333	0.887252	NSMCE4A	54780	NSE4 hom SMC5-SMC6 complex component
ENSG0000	471.7142	-0.05054	0.140318	-0.36021	0.71869	0.887252	CRACR2A	84766	calcium release activated channel regulator 2A
ENSG0000	45.19793	0.129501	0.35972	0.360004	0.718844	0.887252	OVCH1-AS	1.01E+08	OVCH1 antisense RNA 1
ENSG0000	787.1089	0.040163	0.111243	0.361035	0.718073	0.887252	PRR13	54458	proline rich 13
ENSG0000	12.29763	0.305156	0.848193	0.359771	0.719018	0.887252	SRCIN1	80725	SRC kinase signaling inhibitor 1
ENSG0000	62.98422	-0.10458	0.290294	-0.36026	0.718654	0.887252	SLC6A16	28968	solute carrier family 6 member 16
ENSG0000	29.60042	-0.16133	0.447799	-0.36027	0.718644	0.887252	LAMP5	24141	lysosomal associated membrane protein family member 5
ENSG0000	8.515938	-0.29625	0.822721	-0.36009	0.71878	0.887252	PTK6	5753	protein tyrosine kinase 6
ENSG0000	162.4242	-0.07767	0.215531	-0.36038	0.718564	0.887252	BRCC3	79184	BRCA1/BRCA2-containing complex subunit 3
ENSG0000	5652.522	-0.0406	0.112927	-0.35954	0.719194	0.887307	MAP3K8	1326	mitogen-activated protein kinase kinase kinase 8
ENSG0000	24.46134	-0.18226	0.506876	-0.35957	0.719169	0.887307	LOXHD1	125336	lipoxygenase homology PLAT domains 1
ENSG0000	54.04978	0.14788	0.411537	0.359335	0.719344	0.887393	FLJ20021	90024	uncharacterized LOC90024
ENSG0000	52.07557	0.139997	0.389622	0.359316	0.719359	0.887393	CLDN5	7122	claudin 5
ENSG0000	144.2928	-0.07925	0.22064	-0.3592	0.719443	0.887438	RNF8	9025	ring finger protein 8
ENSG0000	113.7541	-0.09852	0.274331	-0.35912	0.719504	0.887453	MBLAC2	153364	metallo-beta-lactamase domain containing 2
ENSG0000	208.2588	-0.07813	0.217601	-0.35906	0.719553	0.887455	ALKBH4	54784	alkB homc lysine demethylase
ENSG0000	1405.188	0.038942	0.108477	0.358989	0.719603	0.887458	PLXNA1	5361	plexin A1
ENSG0000	9.380593	-0.34628	0.964768	-0.35893	0.719651	0.887458	NA	NA	NA
ENSG0000	23.58136	0.179972	0.501951	0.358544	0.719936	0.887463	NA	NA	NA
ENSG0000	9.556658	-0.27705	0.772656	-0.35857	0.719913	0.887463	CLEC1A	51267	C-type lectin domain family 1 member A
ENSG0000	9.11274	-0.30304	0.844523	-0.35883	0.719724	0.887463	RNF112	7732	ring finger protein 112
ENSG0000	5.836047	0.479625	1.337039	0.358722	0.719803	0.887463	NA	NA	NA
ENSG0000	547.8371	-0.05732	0.159871	-0.35854	0.719942	0.887463	SLC5A3	6526	solute carrier family 5 member 3
ENSG0000	131.2555	0.084903	0.236739	0.358637	0.719867	0.887463	FOXRED2	80020	FAD dependent oxidoreductase domain containing 2
ENSG0000	13.98746	0.236771	0.660518	0.358462	0.719998	0.887472	ADNP-AS1	1.02E+08	ADNP antisense RNA 1
ENSG0000	309.7516	0.068534	0.191272	0.358307	0.720114	0.887556	ZDHHC17	23390	zinc finger DHHC-type palmitoyltransferase 17
ENSG0000	59.21044	-0.11601	0.323852	-0.35822	0.720176	0.887574	SHISA8	440829	shisa family member 8
ENSG0000	824.8068	-0.03579	0.099999	-0.35786	0.720448	0.887733	IFFO2	126917	intermediate filament family orphan 2
ENSG0000	2643.037	0.032026	0.089492	0.35787	0.720441	0.887733	SMG7	9887	SMG7 nonsense mediated mRNA decay factor
ENSG0000	526.4938	0.051342	0.14342	0.357983	0.720356	0.887733	LTN1	26046	listerin E3 ubiquitin protein ligase 1
ENSG0000	11.226	0.258682	0.723142	0.35772	0.720553	0.887803	FSTL4	23105	follistatin like 4
ENSG0000	156.0082	0.07304	0.204394	0.35735	0.72083	0.887858	GRAMD2B	65983	GRAM domain containing 2B
ENSG0000	752.1937	-0.04896	0.136948	-0.35749	0.720725	0.887858	FRAT2	23401	FRAT regulator of WNT signaling pathway 2
ENSG0000	5.047373	-0.36942	1.03363	-0.3574	0.720795	0.887858	ZNF442	79973	zinc finger protein 442
ENSG0000	105.068	0.088067	0.246451	0.35734	0.720837	0.887858	ZNF587B	1E+08	zinc finger protein 587B
ENSG0000	5.949613	-0.39402	1.102093	-0.35752	0.720702	0.887858	SAMSN1-A	1.01E+08	SAMSN1 antisense RNA 1
ENSG0000	72.70854	0.10271	0.287556	0.357184	0.720954	0.887884	ADCY10P1	221442	ADCY10 pseudogene 1
ENSG0000	1233.582	0.034788	0.09739	0.357198	0.720943	0.887884	RRP7A	27341	ribosomal RNA processing 7 homolog A
ENSG0000	15.02237	0.221742	0.621017	0.357062	0.721045	0.887937	BTNL9	153579	butyrophilin like 9
ENSG0000	65.57248	-0.11359	0.318585	-0.35653	0.721441	0.888248	LOC12490	1.25E+08	uncharacterized LOC124900513
ENSG0000	549.9073	-0.04722	0.132422	-0.35658	0.721409	0.888248	ANKRD28	23243	ankyrin repeat domain 28
ENSG0000	119.2943	-0.08417	0.236069	-0.35654	0.721436	0.888248	CDC42EP1	11135	CDC42 effector protein 1
ENSG0000	100.1785	0.093482	0.262355	0.356321	0.7216	0.888267	RFX3	5991	regulatory factor X3
ENSG0000	5.753251	0.377955	1.060591	0.356363	0.721569	0.888267	NA	NA	NA
ENSG0000	6.956275	0.386045	1.08305	0.356442	0.721509	0.888267	NA	NA	NA
ENSG0000	9.722116	-0.27054	0.759708	-0.35611	0.721757	0.888342	CACNA2D3	55799	calcium voltage-gated channel auxiliary subunit alpha2delta 3
ENSG0000	7.215139	0.343357	0.964166	0.356118	0.721752	0.888342	CDRT4	284040	CMT1A duplicated region transcript 4
ENSG0000	9325.894	-0.02652	0.074517	-0.35591	0.72191	0.888471	UBR4	23352	ubiquitin protein ligase E3 component n-recognin 4
ENSG0000	1726.896	-0.03604	0.101276	-0.35582	0.721973	0.88849	FLJ32255	643977	uncharacterized LOC643977
ENSG0000	8.805911	0.304765	0.85667	0.355756	0.722024	0.888493	GNAI1	2770	G protein subunit alpha i1
ENSG0000	719.7817	0.038998	0.109809	0.355141	0.722484	0.888593	PRUNE1	58497	prune exopolyphosphatase 1
ENSG0000	40.68861	-0.13498	0.379934	-0.35528	0.722378	0.888593	PRDX6-AS1	1.02E+08	PRDX6 antisense RNA 1
ENSG0000	27.17596	0.160172	0.451018	0.355136	0.722488	0.888593	HMGB1P5	10354	high mobility group box 1 pseudogene 5
ENSG0000	3139.508	-0.02742	0.077169	-0.35528	0.722381	0.888593	GBA2	57704	glucosylceramidase beta 2
ENSG0000	4.045534	-0.46345	1.303482	-0.35555	0.722177	0.888593	ATP2C2-AS	1.05E+08	ATP2C2 antisense RNA 1
ENSG0000	545.9215	-0.05333	0.150097	-0.35531	0.722355	0.888593	EVI2A	2123	ecotropic viral integration site 2A
ENSG0000	1534.128	-0.0324	0.09115	-0.35547	0.722234	0.888593	FARSA	2193	phenylalanyl-tRNA synthetase subunit alpha
ENSG0000	508.4354	-0.05165	0.145425	-0.35516	0.72247	0.888593	LONRF3	79836	LON peptidase N-terminal domain and ring finger 3
ENSG0000	14.88978	-0.26435	0.74459	-0.35503	0.722568	0.888633	SIX4	51804	SIX homeobox 4
ENSG0000	2721.48	0.029084	0.081934	0.354964	0.722616	0.888633	ITGAM	3684	integrin subunit alpha M
ENSG0000	491.8564	0.057757	0.16284	0.354683	0.722827	0.888833	GPKOW	27238	G-patch domain and KOW motifs
ENSG0000	3.028707	-0.6084	1.715676	-0.35461	0.722879	0.888838	HOXB7	3217	homeobox B7
ENSG0000	19.88534	-0.18771	0.529625	-0.35441	0.723029	0.888846	RHOBTB1	9886	Rho related BTB domain containing 1
ENSG0000	63.39538	0.104221	0.294039	0.354447	0.723004	0.888846	RRN3P2	653390	RRN3 pseudogene 2
ENSG0000	963.0261	-0.03413	0.096302	-0.35445	0.723003	0.888846	NUP88	4927	nucleoporin 88
ENSG0000	42.94324	0.147103	0.415141	0.354345	0.72308	0.88885	NA	NA	NA
ENSG0000	24260.76	-0.02982	0.084184	-0.35421	0.72318	0.888913	TMSB4X	7114	thymosin beta 4 X-linked
ENSG0000	549.7562	0.041873	0.118271	0.354045	0.723305	0.889009	BRAP	8315	BRCA1 associated protein
ENSG0000	36.72885	0.165251	0.467035	0.353831	0.723466	0.889019	TFCP2L1	29842	transcription factor CP2 like 1
ENSG0000	157.4551	-0.07234	0.204518	-0.35372	0.723546	0.889019	ELP6	54859	elongator acetyltransferase complex subunit 6
ENSG0000	24.91354	0.183298	0.51821	0.353714	0.723553	0.889019	ANAPC10	10393	anaphase promoting complex subunit 10
ENSG0000	25.99151	0.170867	0.482962	0.353789	0.723497	0.889019	SBF2-AS1	283104	SBF2 antisense RNA 1
ENSG0000	10931.01	-0.02982	0.084285	-0.35377	0.72351	0.889019	SIPA1	6494	signal-induced proliferation-associated 1
ENSG0000	3184.061	-0.03536	0.099999	-0.35361	0.723631	0.889056	FCGRT	2217	Fc gamma receptor and transporter
ENSG0000	14.2562	0.256872	0.726763	0.353447	0.723753	0.889108	NA	NA	NA
ENSG0000	125.1034	-0.08011	0.226663	-0.35343	0.723769	0.889108	NA	NA	NA
ENSG0000	304.8945	0.055446	0.156964	0.353237	0.72391	0.889152	RFC2	5982	replication factor C subunit 2

ENSG0000	6.329069	-0.35241	0.997794	-0.35319	0.723949	0.889152	GJA3	2700	gap junction protein alpha 3
ENSG0000	3.197322	-0.51025	1.444272	-0.35329	0.723868	0.889152	PIK3R2	5296	phosphoinositide-3-kinase regulatory subunit 2
ENSG0000	7.021253	0.329606	0.933425	0.353114	0.724003	0.889159	CHD1L	9557	chromodomain helicase DNA binding protein 1 like
ENSG0000	13.71626	0.251594	0.713277	0.35273	0.724291	0.889395	NUTM2A	728118	NUT family member 2A
ENSG0000	214.047	0.065773	0.186468	0.352733	0.724289	0.889395	TUT1	64852	terminal u U6 snRNA-specific
ENSG0000	2007.81	0.029729	0.08433	0.352538	0.724435	0.889465	NR2C2	7182	nuclear receptor subfamily 2 group C member 2
ENSG0000	81.4677	-0.09944	0.282082	-0.35253	0.724444	0.889465	CFAP119	90835	cilia and flagella associated protein 119
ENSG0000	144.9067	-0.08551	0.242778	-0.35221	0.724683	0.889549	FBXO48	554251	F-box protein 48
ENSG0000	4.904143	0.422489	1.200283	0.351991	0.724845	0.889549	NA	NA	NA
ENSG0000	276.2151	-0.05925	0.168308	-0.35203	0.724813	0.889549	ZNF346	23567	zinc finger protein 346
ENSG0000	4.443414	0.499985	1.41861	0.352369	0.724561	0.889549	NA	NA	NA
ENSG0000	13.09724	0.289766	0.823229	0.351987	0.724848	0.889549	MIR3677H	1.07E+08	MIR3677 and MIR940 host gene
ENSG0000	98.10603	0.088363	0.250861	0.352241	0.724657	0.889549	NA	NA	NA
ENSG0000	2405.356	0.033131	0.094109	0.352047	0.724803	0.889549	ZNF217	7764	zinc finger protein 217
ENSG0000	373.2535	0.053202	0.151212	0.351838	0.72496	0.889612	VTI1A	143187	vesicle transport through interaction with t-SNAREs 1A
ENSG0000	7.639616	0.312861	0.889337	0.351791	0.724995	0.889612	NA	NA	NA
ENSG0000	256.6212	-0.06802	0.193472	-0.35156	0.725165	0.889762	FAM133B	257415	family with sequence similarity 133 member B
ENSG0000	22.78761	-0.22058	0.627915	-0.35128	0.725375	0.889842	CD34	947	CD34 molecule
ENSG0000	21.21373	-0.23384	0.665548	-0.35134	0.72533	0.889842	HBM	3042	hemoglobin subunit mu
ENSG0000	1639.907	0.032212	0.091696	0.351289	0.725372	0.889842	IDH3B	3420	isocitrate dehydrogenase (NAD(+)) 3 non-catalytic subunit beta
ENSG0000	1507.867	-0.03429	0.09764	-0.3512	0.725442	0.889866	GSK3B	2932	glycogen synthase kinase 3 beta
ENSG0000	24.47889	0.16818	0.479311	0.35088	0.725679	0.890097	NA	NA	NA
ENSG0000	209.7093	0.066189	0.188737	0.350697	0.725816	0.890207	UCHL5	51377	ubiquitin C-terminal hydrolase L5
ENSG0000	4.984908	0.416998	1.190661	0.350224	0.726171	0.890583	APCDD1L	164284	APC down-regulated 1 like
ENSG0000	529.5541	0.048026	0.13719	0.350068	0.726288	0.890668	GOSR2	9570	golgi SNAP receptor complex member 2
ENSG0000	9.206665	0.271536	0.776314	0.349776	0.726507	0.890878	NA	NA	NA
ENSG0000	495.6515	0.054965	0.157271	0.349493	0.726719	0.89102	PREPL	9581	prolyl endopeptidase like
ENSG0000	15.59646	-0.23407	0.669643	-0.34955	0.726678	0.89102	ADGRD1	283383	adhesion G protein-coupled receptor D1
ENSG0000	28.20113	-0.17372	0.497367	-0.34928	0.72688	0.891159	NA	NA	NA
ENSG0000	5.495178	-0.3919	1.12247	-0.34914	0.726986	0.891213	LINC0181€	1E+08	long intergenic non-protein coding RNA 1816
ENSG0000	35.12351	0.141179	0.404417	0.349092	0.72702	0.891213	TRAIP	10293	TRAF interacting protein
ENSG0000	5.29579	-0.46394	1.329258	-0.34902	0.727073	0.891219	LRRC37A6	387646	leucine ric pseudogene
ENSG0000	1.978892	-0.68363	1.959342	-0.34891	0.727157	0.891262	NA	NA	NA
ENSG0000	3.06727	0.474612	1.360704	0.348799	0.72724	0.891305	RPL7AP39	646476	ribosomal protein L7a pseudogene 39
ENSG0000	8.70256	0.275002	0.788613	0.348716	0.727303	0.891323	HNRNPA3I	387933	heterogeneous nuclear ribonucleoprotein A3 pseudogene 5
ENSG0000	348.7034	0.051888	0.149093	0.348023	0.727823	0.891347	BLZF1	8548	basic leucine zipper nuclear factor 1
ENSG0000	326.8726	-0.05176	0.148736	-0.34799	0.727851	0.891347	DHX29	54505	DExH-box helicase 29
ENSG0000	28.61721	0.155125	0.44517	0.348462	0.727493	0.891347	CCT6P3	643180	chaperonin containing TCP1 subunit 6 pseudogene 3
ENSG0000	2150.298	0.028136	0.08084	0.348051	0.727802	0.891347	ENTPD4	9583	ectonucleoside triphosphate diphosphohydrolase 4
ENSG0000	408.7303	0.052518	0.150822	0.348209	0.727683	0.891347	FBXO33	254170	F-box protein 33
ENSG0000	123.8833	-0.08617	0.247346	-0.34838	0.727557	0.891347	NA	NA	NA
ENSG0000	3.419642	0.512432	1.470949	0.348368	0.727564	0.891347	NA	NA	NA
ENSG0000	3.279828	-0.55445	1.592604	-0.34814	0.727734	0.891347	KIRREL2	84063	kirre like nephrin family adhesion molecule 2
ENSG0000	1282.516	0.032152	0.092266	0.348469	0.727488	0.891347	CHMP2A	27243	charged multivesicular body protein 2A
ENSG0000	6.491784	0.338266	0.970802	0.34844	0.72751	0.891347	SOX18	54345	SRY-box transcription factor 18
ENSG0000	36.86934	0.155605	0.447067	0.348058	0.727796	0.891347	NA	NA	NA
ENSG0000	6.744375	0.331595	0.95415	0.347529	0.728194	0.891519	GALNT14	79623	polypeptide N-acetylgalactosaminyltransferase 14
ENSG0000	807.3259	0.037969	0.109335	0.347269	0.728389	0.891519	HCG18	414777	HLA complex group 18
ENSG0000	73.25461	-0.09833	0.283136	-0.3473	0.728367	0.891519	KCNH2	3757	potassium voltage-gated channel subfamily H member 2
ENSG0000	1373.516	-0.03625	0.104239	-0.34773	0.728043	0.891519	KIAA2026	158358	KIAA2026
ENSG0000	711.9963	0.040392	0.116335	0.347206	0.728436	0.891519	MRPL57	78988	mitochondrial ribosomal protein L57
ENSG0000	45.7904	0.124798	0.35948	0.347164	0.728468	0.891519	ATP6V0D1	1.02E+08	ATP6V0D1 divergent transcript
ENSG0000	22.47156	-0.16834	0.484701	-0.34732	0.728355	0.891519	KIF1C-AS1	1.03E+08	KIF1C antisense RNA 1
ENSG0000	4.229153	-0.45015	1.295762	-0.3474	0.728292	0.891519	NA	NA	NA
ENSG0000	73.94181	-0.115	0.330997	-0.34744	0.728263	0.891519	TMEM97	27346	transmembrane protein 97
ENSG0000	654.4203	-0.04184	0.120509	-0.34716	0.728472	0.891519	LAS1L	81887	LAS1 like ribosome biogenesis factor
ENSG0000	7.304491	-0.36146	1.041588	-0.34702	0.728573	0.891584	NUDT10	170685	nudix hydrolase 10
ENSG0000	128.5835	0.074486	0.214745	0.346859	0.728698	0.891619	NTPCR	84284	nucleoside cancer-related
ENSG0000	1.947519	0.808548	2.331013	0.346865	0.728692	0.891619	PARK7P1	1E+08	PARK7 pseudogene 1
ENSG0000	1595.972	0.03492	0.100763	0.346551	0.728929	0.891725	ARAP2	116984	ArfGAP wi ankyrin repeat and PH domain 2
ENSG0000	29.32444	0.154774	0.446573	0.346582	0.728905	0.891725	ANKDD1A	348094	ankyrin repeat and death domain containing 1A
ENSG0000	8.41213	-0.3147	0.908026	-0.34657	0.728913	0.891725	NA	NA	NA
ENSG0000	60.68851	-0.10818	0.312448	-0.34622	0.729175	0.891794	DHFR2	200895	dihydrofolate reductase 2
ENSG0000	4591.892	-0.02497	0.072131	-0.34622	0.729178	0.891794	FAM193B	54540	family with sequence similarity 193 member B
ENSG0000	7.556493	-0.40043	1.156399	-0.34628	0.729134	0.891794	LOC12490	1.25E+08	uncharacterized LOC124902026
ENSG0000	65.80427	0.10348	0.298801	0.346316	0.729105	0.891794	HSD17B7P	158160	hydroxysteroid 17-beta dehydrogenase 7 pseudogene 2
ENSG0000	1048.416	-0.03592	0.10379	-0.34611	0.729259	0.891828	SCNM1	79005	sodium channel modifier 1
ENSG0000	3.562631	-0.43052	1.244085	-0.34605	0.729301	0.891828	RPL15P18	728088	ribosomal protein L15 pseudogene 18
ENSG0000	133.2411	-0.07576	0.218981	-0.34595	0.72938	0.891866	ZNF511	118472	zinc finger protein 511
ENSG0000	14.91344	-0.26256	0.759155	-0.34586	0.729447	0.891888	ATG10	83734	autophagy related 10
ENSG0000	515.6941	0.042418	0.122696	0.345712	0.729559	0.891966	NSMCE1	197370	NSE1 hom SMC5-SMC6 complex component
ENSG0000	258.999	0.064757	0.187392	0.34557	0.729666	0.891979	CCNG2	901	cyclin G2
ENSG0000	50.28685	-0.11913	0.344672	-0.34563	0.729624	0.891979	CEACAM1!	56971	CEA cell adhesion molecule 19
ENSG0000	160.8965	-0.07667	0.222045	-0.34529	0.729879	0.892078	TRNT1	51095	tRNA nucleotidyl transferase 1
ENSG0000	2040.745	0.029314	0.084947	0.34508	0.730034	0.892078	COPB2	9276	COPI coat complex subunit beta 2
ENSG0000	1601.14	0.030858	0.089421	0.345085	0.730031	0.892078	CLTB	1212	clathrin light chain B
ENSG0000	8.353235	0.295596	0.856763	0.345015	0.730083	0.892078	DNAJC9-A!	414245	DNAJC9 and MRPS16 antisense RNA 1
ENSG0000	7.921837	-0.29346	0.850519	-0.34504	0.730067	0.892078	NA	NA	NA
ENSG0000	10.84032	-0.27493	0.796301	-0.34526	0.729897	0.892078	GOLGA6L4	643707	golgin A6 family like 4
ENSG0000	932.9029	0.041549	0.120421	0.345031	0.730071	0.892078	GADD45GI	90480	GADD45G interacting protein 1
ENSG0000	35.53923	-0.13594	0.394332	-0.34474	0.730289	0.892095	FZD3	7976	frizzled class receptor 3
ENSG0000	530.1979	0.043697	0.126721	0.34483	0.730222	0.892095	SLC25A25	114789	solute carrier family 25 member 25
ENSG0000	12.93232	-0.25331	0.734672	-0.34479	0.730253	0.892095	NA	NA	NA
ENSG0000	5.331841	0.368257	1.068129	0.344768	0.730269	0.892095	NA	NA	NA
ENSG0000	141.8364	0.073078	0.21211	0.344527	0.73045	0.892232	CLN6	54982	CLN6 transmembrane ER protein
ENSG0000	284.9978	-0.05453	0.158381	-0.34431	0.730612	0.892371	ECHDC1	55862	ethylmalonyl-CoA decarboxylase 1
ENSG0000	1513.105	-0.03458	0.100462	-0.34422	0.73068	0.892395	STUB1	10273	STIP1 homology and U-box containing protein 1
ENSG0000	51.81521	0.112165	0.325962	0.344105	0.730767	0.892418	NA	NA	NA
ENSG0000	607.6101	-0.04352	0.126473	-0.34407	0.730794	0.892418	ELF2	1998	E74 like ETS transcription factor 2
ENSG0000	40.65178	-0.14778	0.429969	-0.34369	0.731076	0.892527	EFR3B	22979	EFR3 homolog B
ENSG0000	3.614449	-0.51863	1.508967	-0.3437	0.731074	0.892527	NA	NA	NA
ENSG0000	20.78273	-0.17155	0.499094	-0.34372	0.731055	0.892527	DNLZ	728489	DNL-type zinc finger
ENSG0000	22.92338	-0.16317	0.474561	-0.34383	0.730973	0.892527	NA	NA	NA
ENSG0000	936.0385	-0.04088	0.119059	-0.34335	0.731337	0.892554	FBXO42	54455	F-box protein 42
ENSG0000	37.5494	-0.13272	0.386359	-0.34351	0.731215	0.892554	KRT8P46	1E+08	keratin 8 pseudogene 46
ENSG0000	3.979699	0.479106	1.395359	0.343357	0.73133	0.892554	NA	NA	NA
ENSG0000	3.762251	-0.42808	1.246779	-0.34334	0.731339	0.892554	NA	NA	NA
ENSG0000	1366.7	-0.03716	0.108192	-0.34346	0.731256	0.892554	SYNJ1	8867	synaptojanin 1
ENSG0000	175.1777	-0.08393	0.244589	-0.34316	0.731474	0.892661	PEG10	23089	paternally expressed 10
ENSG0000	6.754592	0.339892	0.991447	0.342824	0.731731	0.892748	STARD4-A!	1.01E+08	STARD4 antisense RNA 1

ENSG0000	3.169686	0.590858	1.723873	0.34275	0.731786	0.892748	GRID2IP	392862	Grid2 interacting protein
ENSG0000	5.761137	0.397535	1.159111	0.342965	0.731625	0.892748	MAGI2	9863	membran WW and PDZ domain containing 2
ENSG0000	242.1625	-0.06079	0.17734	-0.34277	0.731768	0.892748	CEP20	123811	centrosomal protein 20
ENSG0000	1510.23	0.029361	0.085679	0.342685	0.731835	0.892748	DHX8	1659	DEAH-box helicase 8
ENSG0000	1229.935	0.038802	0.113225	0.342699	0.731825	0.892748	YDJC	150223	YdjC chitooligosaccharide deacetylase homolog
ENSG0000	189.9136	-0.06495	0.189659	-0.34247	0.732	0.892891	PI4K2B	55300	phosphatidylinositol 4-kinase type 2 beta
ENSG0000	1332.673	-0.03558	0.104025	-0.342	0.732352	0.89297	RIF1	55183	replication timing regulatory factor 1
ENSG0000	796.8142	0.039329	0.115102	0.341687	0.732586	0.89297	CLASP2	23122	cytoplasmic linker associated protein 2
ENSG0000	133.0697	-0.0724	0.211675	-0.34204	0.73232	0.89297	TRIM59	286827	tripartite motif containing 59
ENSG0000	2.21961	0.759383	2.221793	0.341788	0.73251	0.89297	HMGB1P1	1E+08	high mobility group box 1 pseudogene 17
ENSG0000	5.329194	-0.37915	1.108686	-0.34198	0.732368	0.89297	LHFPL3-AS	723809	LHFPL3 antisense RNA 2
ENSG0000	1457.057	0.038072	0.11136	0.341883	0.732439	0.89297	ZFYVE27	118813	zinc finger FYVE-type containing 27
ENSG0000	8439	-0.02596	0.075925	-0.34194	0.732396	0.89297	WNK1	65125	WNK lysine deficient protein kinase 1
ENSG0000	16.18318	-0.24703	0.72262	-0.34185	0.732466	0.89297	RNASE4	6038	ribonuclease A family member 4
ENSG0000	52.13584	-0.11916	0.348813	-0.34163	0.732633	0.89297	LOC10798	1.08E+08	uncharacterized LOC107985074
ENSG0000	8.019708	0.267755	0.782347	0.342246	0.732166	0.89297	NA	NA	NA
ENSG0000	21219.88	0.023622	0.069021	0.342234	0.732175	0.89297	ACTN4	81	actinin alpha 4
ENSG0000	91.83344	-0.09358	0.273923	-0.34161	0.732642	0.89297	NA	NA	NA
ENSG0000	4.341356	0.481458	1.409943	0.341474	0.732747	0.893038	RN7SL521I	1.06E+08	RNA 7SL cytoplasm pseudogene
ENSG0000	4.80486	-0.42908	1.256958	-0.34136	0.73283	0.89308	RPS26P21	1E+08	ribosomal protein S26 pseudogene 21
ENSG0000	4.55769	-0.36334	1.064611	-0.34129	0.732885	0.89309	TMEM240	339453	transmembrane protein 240
ENSG0000	475.1508	-0.04401	0.128991	-0.3412	0.732951	0.893111	RETREG1	54463	reticulophagy regulator 1
ENSG0000	9.429649	0.271646	0.79677	0.340934	0.733153	0.893263	EXTL3-AS1	1.02E+08	EXTL3 antisense RNA 1
ENSG0000	1248.162	0.033764	0.09904	0.340909	0.733172	0.893263	CBFB	865	core-binding factor subunit beta
ENSG0000	80.15186	0.093485	0.274287	0.340829	0.733232	0.893278	GGTA1	2681	glycoprotein alpha-galactosyltransferase 1 (inactive)
ENSG0000	29.11414	-0.17061	0.500894	-0.3406	0.733403	0.893416	LEF1-AS1	641518	LEF1 antisense RNA 1
ENSG0000	24.63013	0.168223	0.494067	0.340487	0.73349	0.893416	NA	NA	NA
ENSG0000	87.91397	0.0907	0.266351	0.340529	0.733458	0.893416	SPECC1	92521	sperm antigen with calponin homology and coiled-coil domains 1
ENSG0000	107.5757	0.092637	0.272309	0.340191	0.733713	0.893516	EGFL7	51162	EGF like domain multiple 7
ENSG0000	23.11241	0.187347	0.550524	0.340307	0.733625	0.893516	CCDC144B	284047	coiled-coil pseudogene
ENSG0000	178.3428	0.065117	0.191415	0.340186	0.733717	0.893516	NA	NA	NA
ENSG0000	143.4858	0.069073	0.203129	0.340045	0.733823	0.893586	HIKESHI	51501	heat shock protein nuclear import factor hikeshi
ENSG0000	26.93406	-0.19039	0.560469	-0.3397	0.734084	0.893589	NHLH1	4807	nescient helix-loop-helix 1
ENSG0000	282.5207	0.052429	0.154234	0.339933	0.733907	0.893589	CFAP36	112942	cilia and flagella associated protein 36
ENSG0000	118.2026	0.082478	0.242761	0.339752	0.734043	0.893589	OXSM	54995	3-oxoacyl- mitochondrial
ENSG0000	332.4405	-0.05009	0.147404	-0.3398	0.734005	0.893589	DTX3	196403	deltex E3 ubiquitin ligase 3
ENSG0000	271.8111	-0.0559	0.164668	-0.33949	0.734241	0.893589	ACBD4	79777	acyl-CoA binding domain containing 4
ENSG0000	9.217098	0.257124	0.757289	0.339533	0.734208	0.893589	TTC39C-AS	1.03E+08	TTC39C antisense RNA 1
ENSG0000	174.1566	-0.07178	0.211453	-0.33947	0.734259	0.893589	ZNF136	7695	zinc finger protein 136
ENSG0000	3.828598	0.477991	1.407966	0.339491	0.73424	0.893589	NA	NA	NA
ENSG0000	625.8706	-0.04199	0.123634	-0.33962	0.734144	0.893589	CFAP410	755	cilia and flagella associated protein 410
ENSG0000	9.724524	-0.24703	0.727996	-0.33933	0.734364	0.8936	H2AC11	8969	H2A clustered histone 11
ENSG0000	26.39314	-0.15254	0.44948	-0.33937	0.734327	0.8936	DUS4L	11062	dihydrouridine synthase 4 like
ENSG0000	100.7972	-0.09348	0.275607	-0.33919	0.734469	0.89361	PRRT3	285368	proline rich transmembrane protein 3
ENSG0000	13.46473	0.244629	0.721208	0.339194	0.734464	0.89361	MATCAP2	23366	microtubule associated tyrosine carboxypeptidase 2
ENSG0000	661.6398	0.041311	0.12184	0.339056	0.734567	0.893671	WDR45	11152	WD repeat domain 45
ENSG0000	40.69537	-0.12691	0.374425	-0.33894	0.734654	0.893718	KLHDC8B	200942	kelch domain containing 8B
ENSG0000	311.2503	0.051172	0.151052	0.33877	0.734783	0.893816	NA	NA	NA
ENSG0000	1133.614	-0.03277	0.0968	-0.33858	0.734925	0.893872	CREB3L2	64764	cAMP responsive element binding protein 3 like 2
ENSG0000	43.89368	-0.16511	0.487618	-0.3386	0.734913	0.893872	ADPRHL1	113622	ADP-ribosylhydrolase like 1
ENSG0000	1405.424	0.036455	0.107782	0.33823	0.73519	0.894077	PHB1	5245	prohibitin 1
ENSG0000	54.76701	0.105736	0.312567	0.338284	0.735149	0.894077	ZNF529-AS	1.02E+08	ZNF529 antisense RNA 1
ENSG0000	214.6553	-0.06147	0.181791	-0.33814	0.735256	0.894099	SMAGP	57228	small cell adhesion glycoprotein
ENSG0000	201.8496	0.065385	0.193442	0.338007	0.735358	0.894164	MRPL35	51318	mitochondrial ribosomal protein L35
ENSG0000	3454.839	0.031023	0.091807	0.337918	0.735425	0.894187	MYL12A	10627	myosin light chain 12A
ENSG0000	4.536853	0.478276	1.416186	0.337721	0.735573	0.894294	IGKV2D-40	28878	immunoglobulin kappa variable 2D-40
ENSG0000	441.7324	0.049609	0.146914	0.337674	0.735609	0.894294	TOP1MT	116447	DNA topoisomerase I mitochondrial
ENSG0000	14.02322	-0.25967	0.769481	-0.33746	0.735768	0.894428	NA	NA	NA
ENSG0000	148.6579	-0.06958	0.206457	-0.337	0.736114	0.894439	ARHGEF19	128272	Rho guanine nucleotide exchange factor 19
ENSG0000	5.567203	0.360885	1.070752	0.337039	0.736088	0.894439	NBPF19	1.01E+08	NBPF member 19
ENSG0000	14.17755	0.206391	0.611846	0.337324	0.735873	0.894439	C10orf88B	399815	C10orf88B (pseudogene)
ENSG0000	769.0828	-0.03598	0.10676	-0.33704	0.736085	0.894439	NUP160	23279	nucleoporin 160
ENSG0000	2312.518	-0.03049	0.090474	-0.33704	0.736088	0.894439	STIP1	10963	stress induced phosphoprotein 1
ENSG0000	11.89299	0.230063	0.682227	0.337224	0.735948	0.894439	NA	NA	NA
ENSG0000	1146.66	-0.03217	0.095452	-0.33704	0.736084	0.894439	RRP1B	23076	ribosomal RNA processing 1B
ENSG0000	1609.547	-0.02949	0.087592	-0.33664	0.736388	0.894514	RER1	11079	retention in endoplasmic reticulum sorting receptor 1
ENSG0000	41.06979	-0.13055	0.387846	-0.3366	0.736417	0.894514	NA	NA	NA
ENSG0000	8444.184	-0.02393	0.07109	-0.33664	0.736386	0.894514	INPP5D	3635	inositol polyphosphate-5-phosphatase D
ENSG0000	7.601027	-0.28313	0.841148	-0.3366	0.736417	0.894514	LOC12490	1.25E+08	uncharacterized LOC124904009
ENSG0000	28.53468	0.15439	0.458351	0.336838	0.736239	0.894514	RFPL3S	10737	RFPL3 antisense
ENSG0000	58.7971	0.105488	0.313524	0.336457	0.736526	0.894587	LINC0161	256021	long intergenic non-protein coding RNA 1619
ENSG0000	2.636882	-0.51141	1.52047	-0.33635	0.736607	0.894628	VWDE	221806	von Willebrand factor D and EGF domains
ENSG0000	7.394528	0.311136	0.925638	0.336131	0.736772	0.894717	NA	NA	NA
ENSG0000	13.96115	-0.28431	0.845842	-0.33612	0.736777	0.894717	ZNF22-AS1	220979	ZNF22 antisense RNA 1
ENSG0000	609.9799	0.037941	0.112934	0.335961	0.7369	0.894808	HAGH	3029	hydroxyacylglutathione hydrolase
ENSG0000	219.3674	0.071575	0.213207	0.335707	0.737092	0.894981	BDH1	622	3-hydroxybutyrate dehydrogenase 1
ENSG0000	6.080431	0.323748	0.964833	0.335548	0.737212	0.895069	NA	NA	NA
ENSG0000	121.9626	-0.07419	0.221185	-0.33542	0.737306	0.895124	MTFMT	123263	mitochondrial methionyl-tRNA formyltransferase
ENSG0000	2.939379	-0.6649	1.983789	-0.33516	0.737501	0.895185	CPS1	1373	carbamoyl-phosphate synthase 1
ENSG0000	11.0592	0.322937	0.963345	0.335225	0.737455	0.895185	PTPRK	5796	protein tyrosine phosphatase receptor type K
ENSG0000	58.3706	0.111667	0.333157	0.335177	0.737492	0.895185	AMOTL1	154810	angiomotin like 1
ENSG0000	23.65101	0.18446	0.551384	0.33454	0.737972	0.89523	ECE1-AS1	1.01E+08	ECE1 antisense RNA 1
ENSG0000	471.1463	-0.04415	0.131957	-0.33458	0.737945	0.89523	CYB5R1	51706	cytochrome b5 reductase 1
ENSG0000	125.9363	-0.07656	0.228547	-0.335	0.737624	0.89523	NA	NA	NA
ENSG0000	40.39319	0.124353	0.371522	0.334712	0.737843	0.89523	THNSL1	79896	threonine synthase like 1
ENSG0000	41.76805	0.119696	0.357495	0.33482	0.737761	0.89523	PAWR	5074	pro-apoptotic WT1 regulator
ENSG0000	417.9837	-0.19621	0.585897	-0.33489	0.737706	0.89523	SDS	10993	serine dehydratase
ENSG0000	199.0889	-0.08131	0.242887	-0.33475	0.737817	0.89523	COG6	57511	component of oligomeric golgi complex 6
ENSG0000	92.29011	0.103741	0.309811	0.334853	0.737736	0.89523	NA	NA	NA
ENSG0000	1355.278	-0.03096	0.092501	-0.33465	0.737887	0.89523	SEMA4B	10509	semaphorin 4B
ENSG0000	1696.852	0.032265	0.09659	0.334045	0.738346	0.895625	RGL2	5863	ral guanine nucleotide dissociation stimulator like 2
ENSG0000	1033.983	0.034376	0.103	0.333749	0.738569	0.895719	TTLL4	9654	tubulin tyrosine ligase like 4
ENSG0000	214.9161	0.059307	0.177665	0.333812	0.738522	0.895719	TFAP4	7023	transcription factor AP-4
ENSG0000	204.0774	-0.06157	0.184441	-0.33382	0.738514	0.895719	ZNF100	163227	zinc finger protein 100
ENSG0000	582.1398	-0.04072	0.122115	-0.33349	0.738766	0.895842	UTP3	57050	UTP3 small subunit processome component
ENSG0000	119.0145	0.083889	0.25154	0.3335	0.738757	0.895842	PM20D2	135293	peptidase M20 domain containing 2
ENSG0000	2.841107	0.469961	1.410195	0.33326	0.738938	0.895991	NA	NA	NA
ENSG0000	13.27138	0.244669	0.73461	0.333059	0.73909	0.895999	SGIP1	84251	SH3GL interacting endocytic adaptor 1
ENSG0000	12.03696	0.232753	0.698741	0.333103	0.739056	0.895999	PEX7	5191	peroxisomal biogenesis factor 7
ENSG0000	32.10057	-0.14378	0.43159	-0.33314	0.73903	0.895999	PARD6B	84612	par-6 family cell polarity regulator beta

ENSG0000	4.237456	0.480998	1.444896	0.332895	0.739214	0.896031	NA	NA	NA
ENSG0000	51.56471	-0.1385	0.416249	-0.33274	0.73933	0.896031	EFNA1	1942	ephrin A1
ENSG0000	1017.163	0.040896	0.122967	0.332577	0.739454	0.896031	NPAT	4863	nuclear pr coactivator of histone transcription
ENSG0000	81.33089	-0.09593	0.288329	-0.3327	0.739362	0.896031	ALG11	440138	ALG11 alp 2-mannosyltransferase
ENSG0000	27.81482	-0.14351	0.431502	-0.33258	0.739454	0.896031	RPGRIP1L	23322	RPGRIP1 like
ENSG0000	17.70913	-0.21117	0.634775	-0.33267	0.739386	0.896031	NA	NA	NA
ENSG0000	29.77317	0.144117	0.432852	0.332947	0.739174	0.896031	SRXN1	140809	sulfiredoxin 1
ENSG0000	32.71603	-0.14398	0.433224	-0.33234	0.739632	0.89613	LINC0033S	29092	long intergenic non-protein coding RNA 339
ENSG0000	66.00446	0.098801	0.297244	0.332389	0.739595	0.89613	NA	NA	NA
ENSG0000	2306.129	0.028971	0.087212	0.332195	0.739742	0.896205	RNF19A	25897	ring finger RBR E3 ubiquitin protein ligase
ENSG0000	142.9724	-0.08302	0.250008	-0.33208	0.739828	0.89625	MGME1	92667	mitochondrial genome maintenance exonuclease 1
ENSG0000	7.149066	-0.27494	0.828151	-0.33199	0.739895	0.896273	NA	NA	NA
ENSG0000	44.91106	-0.11969	0.360704	-0.33182	0.740027	0.896309	EAF2	55840	ELL associated factor 2
ENSG0000	1256.127	0.032932	0.099265	0.331761	0.74007	0.896309	CNP	1267	2' 3'-cyclic nucleotide 3' phosphodiesterase
ENSG0000	241.3158	-0.06268	0.188905	-0.33178	0.740054	0.896309	TRPM2	7226	transient receptor potential cation channel subfamily M member 2
ENSG0000	228.5148	-0.05585	0.168507	-0.33141	0.740334	0.896453	RAD18	56852	RAD18 E3 ubiquitin protein ligase
ENSG0000	264.1074	-0.07369	0.222318	-0.33146	0.740299	0.896453	DPYSL3	1809	dihydropyrimidinase like 3
ENSG0000	11.39204	-0.22781	0.687335	-0.33145	0.740308	0.896453	CACNA1C-	1.01E+08	CACNA1C antisense RNA 2
ENSG0000	102.8529	0.081323	0.245487	0.331272	0.740439	0.89652	ZNF484	83744	zinc finger protein 484
ENSG0000	6.288262	0.376102	1.13554	0.33121	0.740486	0.89652	HTR3A	3359	5-hydroxytryptamine receptor 3A
ENSG0000	4.532757	-0.33017	0.997464	-0.33101	0.740636	0.896523	DUTP6	1.01E+08	deoxyuridine triphosphatase pseudogene 6
ENSG0000	979.486	-0.03909	0.118102	-0.33097	0.740668	0.896523	MAPK13	5603	mitogen-activated protein kinase 13
ENSG0000	332.7951	-0.04929	0.148909	-0.33102	0.740631	0.896523	SLF2	55719	SMC5-SMC6 complex localization factor 2
ENSG0000	60.39756	0.118034	0.356651	0.330951	0.740682	0.896523	ZNF543	125919	zinc finger protein 543
ENSG0000	152.7977	-0.06533	0.197594	-0.33062	0.740934	0.896547	SLC66A3	130814	solute carrier family 66 member 3
ENSG0000	40.13256	-0.1402	0.424066	-0.3306	0.740943	0.896547	TMTC2	160335	transmembrane O-mannosyltransferase targeting cadherins 2
ENSG0000	327.9623	0.057863	0.174893	0.330845	0.740761	0.896547	TRIM13	10206	tripartite motif containing 13
ENSG0000	37.71375	-0.15176	0.458835	-0.33075	0.740835	0.896547	STX1B	112755	syntaxin 1B
ENSG0000	611.816	-0.04053	0.122563	-0.33067	0.740891	0.896547	AFG3L2	10939	AFG3 like matrix AAA peptidase subunit 2
ENSG0000	5.100293	0.362486	1.097931	0.330154	0.741284	0.896842	CCT6B	10693	chaperonin containing TCP1 subunit 6B
ENSG0000	300.705	-0.05728	0.173497	-0.33017	0.741274	0.896842	POLG2	11232	DNA polyr accessory subunit
ENSG0000	112.2883	0.077344	0.234467	0.32987	0.741498	0.896873	RPL7P9	653702	ribosomal protein L7 pseudogene 9
ENSG0000	10.22231	0.254465	0.771126	0.329992	0.741406	0.896873	NA	NA	NA
ENSG0000	1544.96	0.031307	0.094905	0.329879	0.741491	0.896873	THAP11	57215	THAP domain containing 11
ENSG0000	54.17651	-0.1238	0.375307	-0.32986	0.741503	0.896873	RPL17	6139	ribosomal protein L17
ENSG0000	6.648723	0.296189	0.898177	0.329767	0.741576	0.896903	LINC0054{	400123	long intergenic non-protein coding RNA 548
ENSG0000	1265.294	-0.04366	0.132424	-0.3297	0.741624	0.896903	RB1	5925	RB transcriptional corepressor 1
ENSG0000	7784.119	-0.02287	0.069382	-0.32957	0.741724	0.896907	SLC9A3R1	9368	SLC9A3 regulator 1
ENSG0000	866.8794	-0.03329	0.101002	-0.32963	0.741679	0.896907	AAR2	25980	AAR2 splicing factor
ENSG0000	3.040209	-0.6067	1.842434	-0.32929	0.741934	0.897092	NA	NA	NA
ENSG0000	47.93711	-0.12436	0.377708	-0.32924	0.741974	0.897092	NA	NA	NA
ENSG0000	5.099886	0.359692	1.09293	0.329108	0.742074	0.897108	LHX4	89884	LIM homeobox 4
ENSG0000	2.869627	-0.55644	1.690828	-0.32909	0.742084	0.897108	NA	NA	NA
ENSG0000	120.438	0.076242	0.231804	0.328904	0.742228	0.897224	ABCA5	23461	ATP binding cassette subfamily A member 5
ENSG0000	3.614561	-0.45927	1.396897	-0.32878	0.742323	0.89728	NA	NA	NA
ENSG0000	1013.744	-0.03478	0.105818	-0.32867	0.742406	0.897321	SAE1	10055	SUMO1 activating enzyme subunit 1
ENSG0000	101.8724	0.078272	0.23822	0.328572	0.742479	0.897352	RXYLT1	10329	ribitol xylosyltransferase 1
ENSG0000	13.33765	0.20914	0.63686	0.328393	0.742615	0.897457	NA	NA	NA
ENSG0000	217.9767	-0.05552	0.169156	-0.32824	0.742734	0.897543	NEK8	284086	NIMA related kinase 8
ENSG0000	64.46062	-0.12093	0.369033	-0.32769	0.743144	0.897605	ASAP2	8853	ArfGAP wi ankyrin repeat and PH domain 2
ENSG0000	8.044829	-0.30955	0.946025	-0.32721	0.743511	0.897605	EIF4EBP3	8637	eukaryotic translation initiation factor 4E binding protein 3
ENSG0000	790.2954	-0.03971	0.121151	-0.32781	0.743057	0.897605	SAP30L	79685	SAP30 like
ENSG0000	3510.049	-0.07041	0.215169	-0.32721	0.743509	0.897605	NEDD9	4739	neural pre developmentally down-regulated 9
ENSG0000	3076.853	-0.03207	0.097925	-0.32754	0.743259	0.897605	DEK	7913	DEK proto-oncogene
ENSG0000	6.760251	-0.33482	1.022919	-0.32732	0.743427	0.897605	CAHM	1.01E+08	colon adenocarcinoma hypermethylated
ENSG0000	82.15005	-0.09188	0.28074	-0.32728	0.743453	0.897605	LETM2	137994	leucine zipper and EF-hand containing transmembrane protein 2
ENSG0000	75.25796	-0.09032	0.275922	-0.32734	0.743412	0.897605	TMEM126	84233	transmembrane protein 126A
ENSG0000	15623.67	0.023413	0.071385	0.327976	0.74293	0.897605	STAT6	6778	signal transducer and activator of transcription 6
ENSG0000	13.74802	-0.23491	0.716596	-0.32782	0.743048	0.897605	VSIG10	54621	V-set and immunoglobulin domain containing 10
ENSG0000	11.47004	0.22605	0.689448	0.327871	0.743009	0.897605	NA	NA	NA
ENSG0000	375.568	0.053991	0.164948	0.327319	0.743427	0.897605	NDUFA11	126328	NADH:ubiquinone oxidoreductase subunit A11
ENSG0000	1741.484	-0.02911	0.088932	-0.32736	0.743394	0.897605	TNPO2	30000	transportin 2
ENSG0000	3.171171	-0.50144	1.531888	-0.32733	0.743416	0.897605	HSPB6	126393	heat shock protein family B (small) member 6
ENSG0000	127.1636	-0.07158	0.218636	-0.32741	0.743358	0.897605	POLR3F	10621	RNA polymerase III subunit F
ENSG0000	425.4152	-0.05515	0.168597	-0.32709	0.743601	0.897655	TAGLN	6876	transgelin
ENSG0000	179.6157	-0.0598	0.182963	-0.32686	0.743772	0.897696	PSMA1	5682	proteasome 20S subunit alpha 1
ENSG0000	1243.261	-0.03417	0.104507	-0.32695	0.743708	0.897696	AQR	9716	aquarius intron-binding spliceosomal factor
ENSG0000	272.1039	-0.0518	0.158493	-0.32685	0.743781	0.897696	HEATR3	55027	HEAT repeat containing 3
ENSG0000	4.424903	0.380057	1.164268	0.326434	0.744096	0.898018	NA	NA	NA
ENSG0000	108.9391	-0.07601	0.232941	-0.32631	0.744188	0.898071	MRPL39	54148	mitochondrial ribosomal protein L39
ENSG0000	11.90309	0.282711	0.866756	0.326171	0.744295	0.898142	ACY3	91703	aminoacylase 3
ENSG0000	130.2084	-0.07455	0.228748	-0.32593	0.74448	0.898223	LINC0095	400946	long intergenic non-protein coding RNA 954
ENSG0000	7.623536	0.350276	1.07504	0.325826	0.744556	0.898223	TTC41P	253724	tetratricop pseudogene
ENSG0000	9.661378	0.262146	0.804509	0.325846	0.744541	0.898223	SULT1A3	6818	sulfotransferase family 1A member 3
ENSG0000	4.568732	-0.40309	1.236805	-0.32591	0.744493	0.898223	VSTM2B	342865	V-set and transmembrane domain containing 2B
ENSG0000	593.6728	-0.03728	0.114463	-0.32572	0.744636	0.898261	TOMM70	9868	translocase of outer mitochondrial membrane 70
ENSG0000	426.5767	0.045252	0.13898	0.325603	0.744724	0.898309	SIKE1	80143	suppressor of IKBKE 1
ENSG0000	166.1746	-0.0752	0.231119	-0.32537	0.744901	0.898314	ISOC1	51015	isochorismatase domain containing 1
ENSG0000	1231.704	-0.03297	0.101345	-0.32535	0.744917	0.898314	TBXAS1	6916	thromboxane A synthase 1
ENSG0000	982.4327	0.036106	0.110999	0.325281	0.744968	0.898314	DNAJC4	3338	DnaJ heat shock protein family (Hsp40) member C4
ENSG0000	48.04879	0.109269	0.335927	0.325278	0.744971	0.898314	RFXAP	5994	regulatory factor X associated protein
ENSG0000	671.8403	-0.04028	0.123745	-0.32547	0.744823	0.898314	PARVB	29780	parvin beta
ENSG0000	598.689	-0.03992	0.122851	-0.32491	0.745249	0.898591	GOLGA5	9950	golgin A5
ENSG0000	4.445331	-0.42703	1.316862	-0.32428	0.745729	0.89865	H4C14	8370	H4 clustered histone 14
ENSG0000	62.44438	0.101622	0.313067	0.324601	0.745483	0.89865	ZMYND10	51364	zinc finger MYND-type containing 10
ENSG0000	917.0509	0.033414	0.102987	0.324448	0.745599	0.89865	GIMAP2	26157	GTPase IMAP family member 2
ENSG0000	470.6183	0.043138	0.132957	0.324449	0.745598	0.89865	NDUFS3	4722	NADH:ubiquinone oxidoreductase core subunit S3
ENSG0000	19.16746	-0.17726	0.546077	-0.32461	0.745474	0.89865	ATL1	51062	atlastin GTPase 1
ENSG0000	49.28889	-0.11912	0.367609	-0.32405	0.745902	0.89865	CAPN3	825	calpain 3
ENSG0000	1499.574	-0.03524	0.108638	-0.32435	0.74567	0.89865	UBL7	84993	ubiquitin like 7
ENSG0000	2.87124	-0.53912	1.662789	-0.32422	0.745768	0.89865	NA	NA	NA
ENSG0000	53.75226	-0.12194	0.376205	-0.32414	0.745833	0.89865	CEACAM3	1084	CEA cell adhesion molecule 3
ENSG0000	117.9312	0.075467	0.232868	0.324075	0.745881	0.89865	LENG9	94059	leukocyte receptor cluster member 9
ENSG0000	3553.927	-0.02746	0.084738	-0.32401	0.745928	0.89865	RRBP1	6238	ribosome binding protein 1
ENSG0000	137.1848	0.090735	0.279578	0.324544	0.745526	0.89865	SLPI	6590	secretory leukocyte peptidase inhibitor
ENSG0000	934.737	-0.10641	0.327778	-0.32464	0.745457	0.89865	KREMEN1	83999	kringle containing transmembrane protein 1
ENSG0000	41.0693	0.129514	0.399869	0.323891	0.746021	0.898704	WDPCP	51057	WD repeat containing planar cell polarity effector
ENSG0000	2123.361	-0.03025	0.093445	-0.32377	0.746116	0.89876	SSRP1	6749	structure specific recognition protein 1
ENSG0000	3.101269	0.547938	1.692745	0.323698	0.746167	0.898763	BRWD1-A!	1.03E+08	BRWD1 antisense RNA 2
ENSG0000	895.7613	0.036612	0.113243	0.3233	0.746468	0.898902	ATG16L1	55054	autophagy related 16 like 1

ENSG0000	4.803787	0.382246	1.182112	0.323358	0.746424	0.898902	FZD9	8326	frizzled class receptor 9
ENSG0000	3.873684	-0.4123	1.275332	-0.32329	0.746476	0.898902	LINC0228!	1.02E+08	long intergenic non-protein coding RNA 2285
ENSG0000	2823.886	-0.0275	0.085045	-0.32338	0.746409	0.898902	CYBC1	79415	cytochrome b-245 chaperone 1
ENSG0000	1516.247	0.033831	0.104741	0.323002	0.746694	0.899106	RCOR1	23186	REST corepressor 1
ENSG0000	4.102078	-0.41224	1.276556	-0.32293	0.746745	0.899109	NA	NA	NA
ENSG0000	9.182868	-0.28807	0.892337	-0.32282	0.746829	0.899152	NA	NA	NA
ENSG0000	314.196	-0.04927	0.152672	-0.32273	0.746898	0.899176	RASGRP3	25780	RAS guanyl releasing protein 3
ENSG0000	150.5394	-0.06887	0.21352	-0.32255	0.747039	0.899242	ALDH5A1	7915	aldehyde dehydrogenase 5 family member A1
ENSG0000	1367.061	-0.03127	0.096958	-0.32249	0.747083	0.899242	ARRDC1	92714	arrestin domain containing 1
ENSG0000	179.0096	-0.06123	0.189875	-0.32247	0.747097	0.899242	C18orf32	497661	chromosome 18 open reading frame 32
ENSG0000	5.490016	0.435804	1.352341	0.322259	0.747256	0.899258	DUSP19	142679	dual specificity phosphatase 19
ENSG0000	42.34254	-0.13151	0.408048	-0.32229	0.747233	0.899258	CCNB1	891	cyclin B1
ENSG0000	106.0274	0.078717	0.244192	0.322357	0.747183	0.899258	IL18	3606	interleukin 18
ENSG0000	2370.51	0.027897	0.086606	0.322116	0.747365	0.899295	RBBP6	5930	RB binding ubiquitin ligase
ENSG0000	1525.811	0.03895	0.120928	0.322091	0.747384	0.899295	SCAND1	51282	SCAN domain containing 1
ENSG0000	188.1717	0.067604	0.209996	0.321929	0.747506	0.899371	ABHD10	55347	abhydrola: depalmitoylase
ENSG0000	106.6962	-0.085	0.264078	-0.32188	0.747544	0.899371	PKD1P5	348156	polycystin transient receptor potential channel interacting pseudogene 5
ENSG0000	32.34341	-0.14695	0.457214	-0.32141	0.747899	0.899731	NA	NA	NA
ENSG0000	3.468328	-0.47071	1.464767	-0.32136	0.747941	0.899731	MIR210HC	1.01E+08	MIR210 host gene
ENSG0000	6.714848	0.30143	0.938299	0.321252	0.748019	0.899768	NA	NA	NA
ENSG0000	3.260892	-0.53927	1.679774	-0.32104	0.748181	0.899787	NA	NA	NA
ENSG0000	90.55611	0.091564	0.285188	0.321064	0.748162	0.899787	NA	NA	NA
ENSG0000	29.92417	0.193626	0.603018	0.321095	0.748138	0.899787	BEX1	55859	brain expressed X-linked 1
ENSG0000	257.842	-0.05358	0.167031	-0.32077	0.748381	0.899911	IGFBP7	3490	insulin like growth factor binding protein 7
ENSG0000	63.65415	-0.09925	0.309373	-0.3208	0.748365	0.899911	RAB3A	5864	RAB3A member RAS oncogene family
ENSG0000	21.38448	-0.17032	0.531326	-0.32055	0.74855	0.900056	LOC28563	285638	uncharacterized LOC285638
ENSG0000	727.2319	0.039579	0.12357	0.320296	0.748744	0.90023	REC8	9985	REC8 meiotic recombination protein
ENSG0000	6.267196	0.41834	1.306883	0.320105	0.748889	0.900346	SLC26A8	116369	solute carrier family 26 member 8
ENSG0000	6.767761	0.325823	1.01857	0.319883	0.749057	0.900437	NA	NA	NA
ENSG0000	129.5535	0.089313	0.279211	0.319877	0.749061	0.900437	NA	NA	NA
ENSG0000	12.16488	-0.21863	0.683683	-0.31978	0.749137	0.900469	NA	NA	NA
ENSG0000	6.530028	0.337282	1.055412	0.319573	0.749292	0.900537	NA	NA	NA
ENSG0000	3.245231	0.493581	1.544439	0.319586	0.749282	0.900537	NA	NA	NA
ENSG0000	3.347569	-0.44588	1.395774	-0.31945	0.749387	0.900537	NA	NA	NA
ENSG0000	8.231834	0.294486	0.92175	0.319485	0.749359	0.900537	LINC01841	1.05E+08	long intergenic non-protein coding RNA 1841
ENSG0000	2789.398	0.027884	0.08736	0.319178	0.749591	0.900724	ERGIC3	51614	ERGIC and golgi 3
ENSG0000	2.6486	-0.57514	1.803967	-0.31882	0.749861	0.90099	NPY	4852	neuropeptide Y
ENSG0000	4530.15	0.025726	0.08078	0.318465	0.750132	0.901257	RHOT2	89941	ras homolog family member T2
ENSG0000	141.3357	-0.07102	0.223128	-0.31831	0.750248	0.901337	METRN	79006	meteorin glial cell differentiation regulator
ENSG0000	61.1763	0.100209	0.315024	0.3181	0.750409	0.901433	CREB3L4	148327	cAMP responsive element binding protein 3 like 4
ENSG0000	245.4	-0.06199	0.194876	-0.31808	0.750425	0.901433	HES6	55502	hes family bHLH transcription factor 6
ENSG0000	14.42583	0.223692	0.703694	0.317882	0.750574	0.901504	SPATA6L	55064	spermatogenesis associated 6 like
ENSG0000	14.9782	0.212388	0.668152	0.317873	0.750581	0.901504	SEC14L5	9717	SEC14 like lipid binding 5
ENSG0000	6.748613	-0.30934	0.974033	-0.31759	0.750795	0.901691	MIR760	1E+08	microRNA 760
ENSG0000	2.348729	-0.66809	2.103953	-0.31754	0.750834	0.901691	NA	NA	NA
ENSG0000	150.9059	0.072035	0.227039	0.31728	0.751031	0.90187	GAS6	2621	growth arrest specific 6
ENSG0000	254.9179	0.052988	0.167052	0.317196	0.751095	0.901888	SIRT3	23410	sirtuin 3
ENSG0000	211.8289	-0.05366	0.169279	-0.31697	0.751263	0.902032	PIGH	5283	phosphatidylinositol glycan anchor biosynthesis class H
ENSG0000	2.188367	-0.70311	2.220136	-0.3167	0.751474	0.902105	NA	NA	NA
ENSG0000	48.46134	0.116418	0.367788	0.316537	0.751595	0.902105	POMC	5443	proopiomelanocortin
ENSG0000	1.976906	-0.62836	1.98426	-0.31667	0.751491	0.902105	TEX41	401014	testis expressed 41
ENSG0000	1726.763	0.040594	0.128307	0.316381	0.751713	0.902105	AHR	196	aryl hydrocarbon receptor
ENSG0000	8296.979	0.026241	0.082857	0.316696	0.751474	0.902105	ITGB1	3688	integrin subunit beta 1
ENSG0000	6.900213	0.344056	1.086984	0.316523	0.751605	0.902105	NA	NA	NA
ENSG0000	4886.986	-0.02526	0.079836	-0.31644	0.751669	0.902105	PGAP6	58986	post-GPI attachment to proteins 6
ENSG0000	8.177687	0.283877	0.897097	0.316439	0.751669	0.902105	CCDC102B	79839	coiled-coil domain containing 102B
ENSG0000	2.618182	-0.52799	1.669959	-0.31617	0.751875	0.902133	NA	NA	NA
ENSG0000	54.92009	0.103534	0.327474	0.316158	0.751883	0.902133	NA	NA	NA
ENSG0000	70.98948	0.106555	0.336975	0.316212	0.751841	0.902133	TCEANC	170082	transcription elongation factor A N-terminal and central domain containing
ENSG0000	4.030836	-0.43214	1.3675	-0.31601	0.751996	0.90221	NA	NA	NA
ENSG0000	3.154862	0.507587	1.6066	0.315939	0.752049	0.902215	TMEM59L	25789	transmembrane protein 59 like
ENSG0000	288.8893	0.058083	0.183879	0.315875	0.752098	0.902216	PCK2	5106	phosphoe mitochondrial
ENSG0000	14.19976	-0.20185	0.639253	-0.31575	0.75219	0.902268	TOB1-AS1	400604	TOB1 antisense RNA 1
ENSG0000	1851.534	-0.02923	0.092652	-0.31552	0.752364	0.902327	KLHL6	89857	kelch like family member 6
ENSG0000	12.95777	-0.32093	1.016822	-0.31562	0.75229	0.902327	NA	NA	NA
ENSG0000	3469.648	-0.02445	0.077517	-0.31543	0.752434	0.902327	CXXC1	30827	CXXC finger protein 1
ENSG0000	42.16757	-0.12698	0.402509	-0.31548	0.7524	0.902327	ADAMTS1	9510	ADAM metallopeptidase with thrombospondin type 1 motif 1
ENSG0000	2.831728	0.535282	1.697834	0.315274	0.752554	0.902354	NA	NA	NA
ENSG0000	833.3985	-0.03791	0.12022	-0.31533	0.752509	0.902354	FOXO4	4303	forkhead box O4
ENSG0000	1219.657	-0.03296	0.104677	-0.31492	0.752826	0.902616	NDUFS7	374291	NADH:ubiquinone oxidoreductase core subunit S7
ENSG0000	4.634633	0.388499	1.233886	0.314858	0.75287	0.902616	NA	NA	NA
ENSG0000	171.4222	-0.06592	0.209546	-0.31457	0.753086	0.902649	RABL3	285282	RAB member of RAS oncogene family like 3
ENSG0000	29.87362	-0.17243	0.548165	-0.31457	0.753092	0.902649	LOC10272	1.03E+08	uncharacterized LOC102723878
ENSG0000	8.893668	-0.23392	0.743456	-0.31464	0.753038	0.902649	NA	NA	NA
ENSG0000	298.6903	0.049267	0.15658	0.314642	0.753034	0.902649	CD40LG	959	CD40 ligand
ENSG0000	70.01883	-0.10256	0.326372	-0.31423	0.753346	0.902895	ITGA7	3679	integrin subunit alpha 7
ENSG0000	13.90915	0.201834	0.642839	0.313973	0.753541	0.902921	RAB25	57111	RAB25 member RAS oncogene family
ENSG0000	35.86748	-0.12442	0.39629	-0.31396	0.753551	0.902921	BPHL	670	biphenyl hydrolase like
ENSG0000	1594.376	0.032386	0.103159	0.313941	0.753566	0.902921	DNAJB5	25822	DnaJ heat shock protein family (Hsp40) member B5
ENSG0000	19.20429	-0.18717	0.596174	-0.31396	0.753553	0.902921	NA	NA	NA
ENSG0000	18.90804	-0.20224	0.644322	-0.31388	0.753611	0.902921	NA	NA	NA
ENSG0000	461.6436	-0.0385	0.122767	-0.31359	0.753832	0.903127	ATP6V0A2	23545	ATPase H+ transporting V0 subunit a2
ENSG0000	97.65468	-0.08352	0.266453	-0.31345	0.753942	0.903202	ZNF616	90317	zinc finger protein 616
ENSG0000	29.14068	-0.15826	0.505035	-0.31337	0.753998	0.90321	NA	NA	NA
ENSG0000	48.4206	0.115822	0.369751	0.313244	0.754096	0.90321	FBXO4	26272	F-box protein 4
ENSG0000	1018.336	0.031507	0.100576	0.313268	0.754077	0.90321	MN1	4330	MN1 protc transcriptional regulator
ENSG0000	32.67237	-0.13401	0.429021	-0.31236	0.754766	0.903233	DNM3	26052	dynamin 3
ENSG0000	6.008776	0.352923	1.130024	0.312314	0.754802	0.903233	NA	NA	NA
ENSG0000	38.01565	0.135317	0.433797	0.311937	0.755088	0.903233	NA	NA	NA
ENSG0000	42.34471	0.123324	0.393988	0.313016	0.754269	0.903233	NA	NA	NA
ENSG0000	234.3939	0.060568	0.193542	0.312946	0.754322	0.903233	CILK1	22858	ciliogenesis associated kinase 1
ENSG0000	161.7735	-0.07091	0.226964	-0.31244	0.754704	0.903233	ZUP1	221302	zinc finger containing ubiquitin peptidase 1
ENSG0000	17.76615	-0.18328	0.586143	-0.31269	0.754515	0.903233	SLC29A4	222962	solute carrier family 29 member 4
ENSG0000	3.645298	-0.37598	1.203974	-0.31229	0.754823	0.903233	NA	NA	NA
ENSG0000	48.20002	0.112264	0.359406	0.312361	0.754766	0.903233	NA	NA	NA
ENSG0000	158.8813	-0.07011	0.224643	-0.31212	0.754952	0.903233	EMC2	9694	ER membrane protein complex subunit 2
ENSG0000	4.134628	-0.35886	1.149696	-0.31213	0.75494	0.903233	NDST2	8509	N-deacetylase and N-sulfotransferase 2
ENSG0000	33.66572	0.136558	0.437115	0.312407	0.754731	0.903233	NA	NA	NA
ENSG0000	141.5641	0.097048	0.311026	0.312026	0.755021	0.903233	BMAL2	56938	basic helix-loop-helix ARNT like 2
ENSG0000	52.6004	-0.13439	0.429422	-0.31296	0.754311	0.903233	AHSP	51327	alpha hemoglobin stabilizing protein

ENSG0000	5.487421	-0.36622	1.170554	-0.31286	0.754386	0.903233	NA	NA	NA	
ENSG0000	34.11147	0.192315	0.616491	0.311952	0.755077	0.903233	NA	NA	NA	
ENSG0000	47.10379	-0.12027	0.385389	-0.31209	0.754974	0.903233	RAB40B	10966	RAB40B	member RAS oncogene family
ENSG0000	5.268361	-0.34096	1.092823	-0.312	0.755039	0.903233	NA	NA	NA	
ENSG0000	6.901587	0.342134	1.093816	0.312789	0.754441	0.903233	SNORD83A	116937	small nucl	C/D box 83A
ENSG0000	1020.646	-0.03579	0.114696	-0.31202	0.755023	0.903233	USP9Y	8287	ubiquitin specific peptidase 9 Y-linked	
ENSG0000	5.790808	0.424164	1.363698	0.31104	0.755771	0.903509	LRRIQ3	127255	leucine rich repeats and IQ motif containing 3	
ENSG0000	674.6554	-0.03622	0.116494	-0.31088	0.75589	0.903509	PTPN4	5775	protein tyrosine phosphatase non-receptor type 4	
ENSG0000	6.642032	0.298836	0.959759	0.311365	0.755523	0.903509	IL17RC	84818	interleukin 17 receptor C	
ENSG0000	22.09673	0.169022	0.542672	0.311464	0.755448	0.903509	NA	NA	NA	
ENSG0000	13.39693	0.205272	0.660315	0.31087	0.755899	0.903509	NA	NA	NA	
ENSG0000	18.72384	0.189221	0.608729	0.310845	0.755918	0.903509	NA	NA	NA	
ENSG0000	39.42324	-0.13213	0.424313	-0.3114	0.755494	0.903509	CD63-AS1	1.05E+08	CD63 antisense RNA 1	
ENSG0000	366.3865	0.049717	0.159959	0.310812	0.755944	0.903509	KLHL25	64410	kelch like family member 25	
ENSG0000	19.86401	0.161343	0.518788	0.311	0.7558	0.903509	LOC44031	440311	NOP53 ribosome biogenesis factor pseudogene	
ENSG0000	25.14548	-0.1478	0.475161	-0.31105	0.755761	0.903509	NA	NA	NA	
ENSG0000	58.4535	-0.10409	0.33471	-0.311	0.755803	0.903509	PPAN	56342	peter pan homolog	
ENSG0000	165.6778	-0.06532	0.210171	-0.3108	0.755952	0.903509	CD33	945	CD33 molecule	
ENSG0000	472.3385	-0.04269	0.137117	-0.31134	0.755539	0.903509	HDHD5	27440	haloacid dehalogenase like hydrolase domain containing 5	
ENSG0000	117.5554	-0.07862	0.253066	-0.31068	0.756041	0.903514	ADORA2B	136	adenosine A2b receptor	
ENSG0000	66.90301	0.102415	0.329662	0.310667	0.756054	0.903514	RAD51C	5889	RAD51 paralog C	
ENSG0000	33.5373	0.137898	0.444124	0.310494	0.756185	0.903573	RPS11P5	727826	ribosomal protein S11 pseudogene 5	
ENSG0000	97.17439	-0.07976	0.256903	-0.31047	0.756201	0.903573	ZNF559	84527	zinc finger protein 559	
ENSG0000	83.90781	-0.0889	0.286596	-0.31018	0.756423	0.90378	TPD52	7163	tumor protein D52	
ENSG0000	7.778986	0.294917	0.951664	0.309896	0.75664	0.903981	NA	NA	NA	
ENSG0000	3.530168	0.41282	1.332714	0.309759	0.756744	0.903989	BRCC3P1	1E+08	BRCA1/BRCA2-containing complex subunit 3 pseudogene 1	
ENSG0000	188.9594	0.061253	0.197732	0.30978	0.756729	0.903989	HID1	283987	HID1 domain containing	
ENSG0000	9059.38	-0.02467	0.079706	-0.30951	0.756937	0.904161	BSG	682	basigin (Ok blood group)	
ENSG0000	23.33496	-0.16996	0.549455	-0.30932	0.757076	0.904269	ICA1L	130026	islet cell autoantigen 1 like	
ENSG0000	54.99871	-0.10487	0.339321	-0.30906	0.757273	0.9043	HNMT	3176	histamine N-methyltransferase	
ENSG0000	3243.569	-0.02517	0.081456	-0.30897	0.757346	0.9043	LMAN2	10960	lectin	mannose binding 2
ENSG0000	3.538687	0.560417	1.813116	0.309091	0.757253	0.9043	NA	NA	NA	
ENSG0000	1174.631	-0.03322	0.107444	-0.3092	0.757172	0.9043	DEAF1	10522	DEAF1 transcription factor	
ENSG0000	9.239387	0.345245	1.117587	0.30892	0.757382	0.9043	TMEM92	162461	transmembrane protein 92	
ENSG0000	144.5247	-0.06101	0.197519	-0.3089	0.757395	0.9043	ZNF585B	92285	zinc finger protein 585B	
ENSG0000	2339.719	0.025916	0.083974	0.308623	0.757608	0.904497	ATP5F1A	498	ATP synthase F1 subunit alpha	
ENSG0000	3084.334	-0.0275	0.089173	-0.30837	0.757798	0.904665	SLC12A6	9990	solute carrier family 12 member 6	
ENSG0000	84.21876	-0.0882	0.286096	-0.3083	0.757855	0.904675	PPAT	5471	phosphoribosyl pyrophosphate amidotransferase	
ENSG0000	14.98159	0.194712	0.631769	0.308201	0.75793	0.904706	TIPIN	54962	TIMELESS interacting protein	
ENSG0000	99.63593	-0.09124	0.296113	-0.30812	0.757988	0.904717	PTCD1	26024	pentatricopeptide repeat domain 1	
ENSG0000	24.33635	0.151444	0.491622	0.30805	0.758044	0.904726	CLYBL	171425	citramalyl-CoA lyase	
ENSG0000	176.9761	0.062867	0.204126	0.307984	0.758095	0.904728	ZNHIT6	54680	zinc finger HIT-type containing 6	
ENSG0000	219.6222	-0.06508	0.211499	-0.30771	0.758305	0.904803	CHCHD5	84269	coiled-coil-helix-coiled-coil-helix domain containing 5	
ENSG0000	151.3334	0.069774	0.2268	0.307645	0.758353	0.904803	PINX1	54984	PIN2 (TERF1) interacting telomerase inhibitor 1	
ENSG0000	64.70516	0.101257	0.329131	0.307649	0.758349	0.904803	ST6GALNA	30815	ST6 N-acet	6-sialyltransferase 6
ENSG0000	299.2138	0.048744	0.158354	0.307817	0.758221	0.904803	TMEM199	147007	transmembrane protein 199	
ENSG0000	3.909833	-0.40261	1.309165	-0.30753	0.758438	0.904847	NA	NA	NA	
ENSG0000	5.400833	0.373983	1.217083	0.307278	0.758631	0.904961	RETREG1-A	1.02E+08	RETREG1 antisense RNA 1	
ENSG0000	40.58871	-0.10921	0.355383	-0.30729	0.758623	0.904961	AIFM2	84883	apoptosis inducing factor mitochondria associated 2	
ENSG0000	663.4981	-0.03317	0.108044	-0.30705	0.758806	0.905054	UBE2E3	10477	ubiquitin conjugating enzyme E2 E3	
ENSG0000	580.5963	0.039318	0.128047	0.307059	0.758798	0.905054	LHPP	64077	phospholysine phosphohistidine inorganic pyrophosphate phosphatase	
ENSG0000	1042.157	-0.02954	0.096413	-0.30639	0.75931	0.905297	MEAF6	64769	MYST/Esa1 associated factor 6	
ENSG0000	132.4979	0.072356	0.236091	0.306475	0.759243	0.905297	MYCL	4610	MYCL prot	bHLH transcription factor
ENSG0000	175.1432	0.065707	0.214984	0.305636	0.759882	0.905297	SPRTN	83932	SprT-like N-terminal domain	
ENSG0000	44.21757	-0.13043	0.426448	-0.30586	0.759714	0.905297	CD8B2	927	CD8b2 molecule	
ENSG0000	130.0843	0.063788	0.20813	0.306481	0.759239	0.905297	INSIG2	51141	insulin induced gene 2	
ENSG0000	35.9673	-0.15373	0.502302	-0.30605	0.759568	0.905297	IQCG	84223	IQ motif containing G	
ENSG0000	9.192973	-0.25803	0.844045	-0.30571	0.759825	0.905297	PKIB	5570	cAMP-dependent protein kinase inhibitor beta	
ENSG0000	273.904	0.046883	0.153193	0.306038	0.759576	0.905297	SLC37A3	84255	solute carrier family 37 member 3	
ENSG0000	23.41644	-0.14377	0.469475	-0.30624	0.759419	0.905297	C8orf76	84933	chromosome 8 open reading frame 76	
ENSG0000	54.56374	0.102036	0.333309	0.306129	0.759506	0.905297	ABO	28	ABO	alpha 1-3-N-acetylgalactosaminyltransferase and alpha 1-3-galactosyltransferase
ENSG0000	11759.54	-0.02545	0.083252	-0.30571	0.759827	0.905297	PNPLA2	57104	patatin like phospholipase domain containing 2	
ENSG0000	189.1285	-0.06378	0.208122	-0.30644	0.759269	0.905297	KRT81	3887	keratin 81	
ENSG0000	24.89359	0.138091	0.450256	0.306694	0.759077	0.905297	RPS8P1	192133	ribosomal protein S8 pseudogene 1	
ENSG0000	2681.983	0.032295	0.105611	0.305788	0.759766	0.905297	MAN2C1	4123	mannosidase alpha class 2C member 1	
ENSG0000	148.414	-0.07324	0.239164	-0.30623	0.759433	0.905297	PARD6A	50855	par-6 family cell polarity regulator alpha	
ENSG0000	5712.238	0.027852	0.090963	0.306195	0.759456	0.905297	MAP2K2	5605	mitogen-activated protein kinase kinase 2	
ENSG0000	45.43657	-0.11598	0.379368	-0.30573	0.759812	0.905297	FBXW9	84261	F-box and WD repeat domain containing 9	
ENSG0000	5.784104	-0.35468	1.1605	-0.30563	0.759889	0.905297	NA	NA	NA	
ENSG0000	11.87892	0.249104	0.815495	0.305463	0.760013	0.905387	NETO1	81832	neuropilin and tolloid like 1	
ENSG0000	186.9093	-0.06126	0.200713	-0.30523	0.760187	0.905473	MSH2	4436	mutS homolog 2	
ENSG0000	12.47555	-0.19612	0.64278	-0.30511	0.760281	0.905473	HOXA3	3200	homeobox A3	
ENSG0000	4.918223	0.368654	1.207548	0.305291	0.760144	0.905473	NA	NA	NA	
ENSG0000	15.44132	0.239319	0.784347	0.305119	0.760276	0.905473	NA	NA	NA	
ENSG0000	9.432312	0.236932	0.776887	0.304976	0.760385	0.905538	NA	NA	NA	
ENSG0000	9.402429	0.27655	0.907721	0.304665	0.760622	0.905762	S100A5	6276	S100 calcium binding protein A5	
ENSG0000	907.1961	0.038298	0.125812	0.304404	0.76082	0.905766	FAAP20	199990	FA core complex associated protein 20	
ENSG0000	12.91734	0.215502	0.707683	0.304518	0.760733	0.905766	NA	NA	NA	
ENSG0000	33.4511	0.159648	0.524226	0.30454	0.760717	0.905766	LOC10192	1.02E+08	uncharacterized LOC101928834	
ENSG0000	88.8347	-0.08949	0.293971	-0.30441	0.760817	0.905766	TCEAL4	79921	transcription elongation factor A like 4	
ENSG0000	2.693392	0.577338	1.897281	0.304298	0.760901	0.905804	UNC13A	23025	unc-13 homolog A	
ENSG0000	8.108923	0.321193	1.055894	0.304191	0.760983	0.905843	LY6G6C	80740	lymphocyte antigen 6 family member G6C	
ENSG0000	128.5697	-0.06429	0.211468	-0.30401	0.761119	0.90585	WDR12	55759	WD repeat domain 12	
ENSG0000	3.290172	-0.43944	1.44558	-0.30399	0.761135	0.90585	NA	NA	NA	
ENSG0000	279.4462	-0.04928	0.162091	-0.30401	0.761123	0.90585	DDX19B	11269	DEAD-box helicase 19B	
ENSG0000	5.96128	0.296088	0.97471	0.303771	0.761303	0.905934	NA	NA	NA	
ENSG0000	3.726313	0.456677	1.503088	0.303826	0.761261	0.905934	NID2	22795	nidogen 2	
ENSG0000	51.3877	-0.09962	0.328264	-0.30347	0.761534	0.905996	IGKV3-15	28913	immunoglobulin kappa variable 3-15	
ENSG0000	1762.717	-0.02661	0.087704	-0.30338	0.761599	0.905996	ZDHHC3	51304	zinc finger DHHC-type palmitoyltransferase 3	
ENSG0000	83.92378	0.104317	0.343563	0.303632	0.761408	0.905996	SLF1	84250	SMC5-SMC6 complex localization factor 1	
ENSG0000	532.8153	0.039851	0.131346	0.303407	0.761579	0.905996	TCP11L2	255394	t-complex 11 like 2	
ENSG0000	39.70605	0.111941	0.368799	0.303529	0.761487	0.905996	DOCK6	57572	dedicator of cytokinesis 6	
ENSG0000	5.111022	0.341586	1.127965	0.302833	0.762017	0.906309	RAPGEF5	9771	Rap guanine nucleotide exchange factor 5	
ENSG0000	182.1115	-0.09054	0.299017	-0.30278	0.762058	0.906309	STEAP4	79689	STEAP4 metalloreductase	
ENSG0000	19.04288	0.161992	0.534738	0.302938	0.761937	0.906309	NA	NA	NA	
ENSG0000	1467.037	0.026374	0.087092	0.302833	0.762017	0.906309	PARP6	56965	poly(ADP-ribose) polymerase family member 6	
ENSG0000	27.64213	0.135745	0.449163	0.302216	0.762487	0.906651	RPL18AP6	285053	ribosomal protein L18a pseudogene 6	
ENSG0000	2.324277	0.650214	2.152093	0.302131	0.762552	0.906651	LINC01485	1.02E+08	long intergenic non-protein coding RNA 1485	
ENSG0000	431.8654	0.043108	0.142729	0.302024	0.762634	0.906651	UIMC1	51720	ubiquitin interaction motif containing 1	
ENSG0000	12.04041	0.223155	0.73904	0.301953	0.762688	0.906651	NA	NA	NA	

ENSG0000	43.98885	-0.12629	0.417812	-0.30226	0.762457	0.906651	FAM110B	90362	family with sequence similarity 110 member B
ENSG0000	15.55128	-0.18437	0.610456	-0.30203	0.762631	0.906651	FKBP2	2286	FKBP prolyl isomerase 2
ENSG0000	4.449342	0.317632	1.051715	0.302013	0.762642	0.906651	HES7	84667	hes family bHLH transcription factor 7
ENSG0000	68.10176	-0.09264	0.307106	-0.30167	0.762903	0.906776	ERCC5	2073	ERCC excis endonuclease
ENSG0000	3.050111	0.512377	1.698733	0.301623	0.762939	0.906776	LOC33905	339059	uncharacterized LOC339059
ENSG0000	100.7887	0.0831	0.275461	0.301675	0.7629	0.906776	ZNF486	90649	zinc finger protein 486
ENSG0000	58.54159	-0.10125	0.335941	-0.30138	0.763122	0.906934	NA	NA	NA
ENSG0000	32.73324	-0.13437	0.446174	-0.30117	0.763287	0.906968	NA	NA	NA
ENSG0000	1.855619	0.663336	2.202644	0.301154	0.763297	0.906968	CCL26	10344	C-C motif chemokine ligand 26
ENSG0000	12.91895	-0.20594	0.683804	-0.30116	0.763291	0.906968	LOC10537	1.05E+08	uncharacterized LOC105370174
ENSG0000	347.4134	0.047874	0.159021	0.301053	0.763374	0.907002	ATAD1	84896	ATPase family AAA domain containing 1
ENSG0000	29.55492	-0.14068	0.467409	-0.30098	0.76343	0.90701	NA	NA	NA
ENSG0000	6.399487	-0.30114	1.000888	-0.30087	0.763515	0.907053	RN7SL832	1.06E+08	RNA 7SL cytoplasm pseudogene
ENSG0000	1775.567	-0.03278	0.109043	-0.3006	0.763722	0.907183	CLINT1	9685	clathrin interactor 1
ENSG0000	13.3596	0.195753	0.651095	0.300652	0.76368	0.907183	NA	NA	NA
ENSG0000	41.93547	-0.11631	0.387324	-0.3003	0.763948	0.907277	CAMKMT	79823	calmodulin-lysine N-methyltransferase
ENSG0000	2440.475	0.029179	0.097149	0.300352	0.763909	0.907277	USP4	7375	ubiquitin specific peptidase 4
ENSG0000	38.26996	0.126475	0.421041	0.300387	0.763882	0.907277	HTR7P1	93164	5-hydroxytryptamine receptor 7 pseudogene 1
ENSG0000	411.0376	-0.04256	0.141965	-0.29981	0.764324	0.907612	RPP38	10557	ribonuclease P/MRP subunit p38
ENSG0000	29.30943	-0.14301	0.477002	-0.2998	0.764328	0.907612	LOC10537	1.05E+08	uncharacterized LOC105371414
ENSG0000	25.52085	0.143331	0.478221	0.299717	0.764393	0.907631	NA	NA	NA
ENSG0000	462.5621	0.047249	0.157849	0.299328	0.76469	0.907639	PAIP1	10605	poly(A) binding protein interacting protein 1
ENSG0000	25.76088	-0.1687	0.563558	-0.29935	0.76467	0.907639	NA	NA	NA
ENSG0000	815.583	0.031502	0.10519	0.299482	0.764572	0.907639	PRCP	5547	prolylcarboxypeptidase
ENSG0000	53.63716	-0.09326	0.31159	-0.29931	0.764707	0.907639	LOC73018	730183	uncharacterized LOC730183
ENSG0000	4.344162	0.422561	1.412024	0.299259	0.764743	0.907639	NA	NA	NA
ENSG0000	117.0208	0.07283	0.243081	0.299611	0.764474	0.907639	CD79B	974	CD79b molecule
ENSG0000	485.9455	0.037215	0.124233	0.299562	0.764511	0.907639	UTP14A	10813	UTP14A small subunit processome component
ENSG0000	5.020337	0.354568	1.185439	0.299103	0.764862	0.907681	NA	NA	NA
ENSG0000	125.062	0.073907	0.247109	0.299084	0.764876	0.907681	CCDC106	29903	coiled-coil domain containing 106
ENSG0000	18.48633	0.168964	0.565322	0.29888	0.765031	0.907808	CABYR	26256	calcium binding tyrosine phosphorylation regulated
ENSG0000	23.76816	0.183704	0.615677	0.298377	0.765416	0.908089	FAM106A	80039	family with sequence similarity 106 member A
ENSG0000	343.7669	-0.04376	0.146661	-0.29838	0.765415	0.908089	ZNF333	84449	zinc finger protein 333
ENSG0000	26.1775	0.150405	0.503972	0.298438	0.765369	0.908089	BMP2	650	bone morphogenetic protein 2
ENSG0000	10.65554	-0.26198	0.878928	-0.29806	0.765654	0.908308	NA	NA	NA
ENSG0000	2026.814	0.029855	0.100183	0.298007	0.765698	0.908308	TUBA1C	84790	tubulin alpha 1c
ENSG0000	12.9931	0.207031	0.694986	0.297893	0.765785	0.908353	NA	NA	NA
ENSG0000	33.29855	-0.13038	0.437971	-0.29769	0.765943	0.908483	H4C8	8365	H4 clustered histone 8
ENSG0000	101.0309	0.072031	0.242328	0.297245	0.766279	0.908709	SPDL1	54908	spindle apparatus coiled-coil protein 1
ENSG0000	18.14906	0.187004	0.629182	0.297218	0.7663	0.908709	EEF1E1	9521	eukaryotic translation elongation factor 1 epsilon 1
ENSG0000	577.2962	0.036245	0.121911	0.297304	0.766234	0.908709	FHIP2A	57700	FHF complex subunit HOOK interacting protein 2A
ENSG0000	6.279274	0.34657	1.166198	0.297179	0.76633	0.908709	NA	NA	NA
ENSG0000	1183.034	-0.03394	0.114255	-0.29708	0.766403	0.908738	TENT2	167153	terminal nucleotidyltransferase 2
ENSG0000	17.55521	-0.18252	0.614696	-0.29693	0.766523	0.908764	ENPP1	5167	ectonucleotide pyrophosphatase/phosphodiesterase 1
ENSG0000	27.64158	-0.13768	0.463643	-0.29695	0.766502	0.908764	CNTLN	54875	centlein
ENSG0000	34.34472	-0.11802	0.398005	-0.29653	0.766826	0.908834	RGPD2	729857	RANBP2 like and GRIP domain containing 2
ENSG0000	388.3776	0.045897	0.15475	0.296587	0.766782	0.908834	LONRF1	91694	LON peptidase N-terminal domain and ring finger 1
ENSG0000	13.1974	0.907229	3.058163	0.296658	0.766727	0.908834	NA	NA	NA
ENSG0000	28.88942	0.152639	0.514563	0.296637	0.766743	0.908834	ALPK2	115701	alpha kinase 2
ENSG0000	730.2695	0.03763	0.126902	0.296527	0.766828	0.908834	CCDC159	126075	coiled-coil domain containing 159
ENSG0000	10.25026	0.228657	0.771723	0.296294	0.767005	0.90896	NA	NA	NA
ENSG0000	4.701813	0.416942	1.407351	0.29626	0.767032	0.90896	LOC10537	1.05E+08	uncharacterized LOC105372321
ENSG0000	662.5379	-0.03714	0.125431	-0.29612	0.767139	0.90903	FBXL15	79176	F-box and leucine rich repeat protein 15
ENSG0000	7.116978	0.263827	0.891353	0.295985	0.767242	0.909064	LINC0245	728084	long intergenic non-protein coding RNA 2458
ENSG0000	15.54643	0.207631	0.70157	0.295952	0.767266	0.909064	NA	NA	NA
ENSG0000	16.45286	-0.19116	0.646451	-0.2957	0.767457	0.909232	RP9P	441212	RP9 pseudogene
ENSG0000	2.487263	-0.62346	2.109061	-0.29561	0.767529	0.909259	NA	NA	NA
ENSG0000	9398.876	0.020745	0.070223	0.295413	0.767679	0.90932	DDX39B	7919	DExD-box helicase 39B
ENSG0000	23.91769	-0.19431	0.657711	-0.29543	0.767664	0.90932	TBC1D30	23329	TBC1 domain family member 30
ENSG0000	5.998132	0.313041	1.061213	0.294984	0.768006	0.909359	NA	NA	NA
ENSG0000	58.76126	0.10145	0.343704	0.295168	0.767866	0.909359	GEMIN6	79833	gem nuclear organelle associated protein 6
ENSG0000	8456.92	-0.02244	0.076036	-0.29511	0.767906	0.909359	RNF44	22838	ring finger protein 44
ENSG0000	22.59122	0.154581	0.523976	0.295016	0.767981	0.909359	CDC6	990	cell division cycle 6
ENSG0000	60.51571	-0.14061	0.476221	-0.29527	0.767788	0.909359	LRRC37A	9884	leucine rich repeat containing 37A
ENSG0000	1305.318	0.027034	0.091622	0.295062	0.767947	0.909359	UNK	85451	unk zinc finger
ENSG0000	10.6381	-0.21483	0.729216	-0.2946	0.768297	0.909646	NA	NA	NA
ENSG0000	7.265854	0.286069	0.972097	0.294281	0.768543	0.909864	RGPD6	729540	RANBP2 like and GRIP domain containing 6
ENSG0000	7.242477	-0.27425	0.93268	-0.29404	0.768726	0.909864	NA	NA	NA
ENSG0000	2368.511	0.025724	0.08743	0.294224	0.768587	0.909864	HDAC5	10014	histone deacetylase 5
ENSG0000	9.812684	0.282852	0.961841	0.294073	0.768702	0.909864	NA	NA	NA
ENSG0000	1416.271	0.026239	0.08922	0.294099	0.768683	0.909864	SELENOO	83642	selenoprotein O
ENSG0000	77.58167	0.104074	0.355055	0.293122	0.769429	0.910057	C1QA	712	complement C1q A chain
ENSG0000	1325.228	-0.03192	0.108812	-0.29337	0.769243	0.910057	ARNT	405	aryl hydrocarbon receptor nuclear translocator
ENSG0000	7.209661	0.26569	0.905597	0.293386	0.769227	0.910057	NUDT16L2	152195	nudix hydr pseudogene
ENSG0000	729.5442	0.04244	0.144771	0.293152	0.769406	0.910057	FEM1C	56929	fem-1 homolog C
ENSG0000	9.453171	-0.24748	0.843005	-0.29356	0.769091	0.910057	ZNG1C	445571	Zn regulated GTPase metalloprotein activator 1C
ENSG0000	6.730414	0.342225	1.165986	0.293507	0.769135	0.910057	PRTFDC1	56952	phosphoribosyl transferase domain containing 1
ENSG0000	3955.571	-0.02592	0.088286	-0.29365	0.769027	0.910057	SCYL1	57410	SCY1 like pseudokinase 1
ENSG0000	17.5709	0.176277	0.601125	0.293246	0.769334	0.910057	NA	NA	NA
ENSG0000	65.54395	-0.09637	0.328429	-0.29341	0.769207	0.910057	NA	NA	NA
ENSG0000	797.7203	0.033841	0.115439	0.293153	0.769405	0.910057	LRCH1	23143	leucine rich repeats and calponin homology domain containing 1
ENSG0000	738.1958	-0.03125	0.106522	-0.29336	0.769243	0.910057	LINC0127	92249	long intergenic non-protein coding RNA 1278
ENSG0000	746.3138	0.030349	0.103564	0.293046	0.769487	0.910067	SNX5	27131	sorting nexin 5
ENSG0000	69.24323	-0.08994	0.307057	-0.2929	0.769599	0.910084	GNG4	2786	G protein subunit gamma 4
ENSG0000	61.08915	-0.09503	0.324371	-0.29295	0.769559	0.910084	FRMD6	122786	FERM domain containing 6
ENSG0000	8.080512	0.237221	0.810128	0.292819	0.76966	0.910098	MIR4432H	1.07E+08	MIR4432 host gene
ENSG0000	503.7164	0.039931	0.136399	0.29275	0.769713	0.910103	SUCLG1	8802	succinate-CoA ligase GDP/ADP-forming subunit alpha
ENSG0000	365.7361	-0.04208	0.143869	-0.29251	0.769896	0.910203	NDUFS6	4726	NADH:ubiquinone oxidoreductase subunit S6
ENSG0000	3.295833	-0.41983	1.435254	-0.29251	0.769896	0.910203	ST7-AS1	93653	ST7 antisense RNA 1
ENSG0000	996.5324	0.032447	0.110987	0.292352	0.770018	0.910289	LPCAT4	254531	lysophosphatidylcholine acyltransferase 4
ENSG0000	2.403326	0.490711	1.680729	0.291963	0.770315	0.910292	NA	NA	NA
ENSG0000	44.54947	0.103644	0.354925	0.292015	0.770275	0.910292	HLA-DOB	3112	major hist class II DO beta
ENSG0000	2791.929	0.024415	0.083607	0.292022	0.77027	0.910292	GLIPR2	152007	GLI pathogenesis related 2
ENSG0000	231.9423	0.049556	0.169723	0.29198	0.770302	0.910292	HAUS2	55142	HAUS augmin like complex subunit 2
ENSG0000	151.3702	0.072227	0.247192	0.292189	0.770142	0.910292	NT5M	56953	5' 3'-nucleoti mitochondrial
ENSG0000	539.1053	0.036246	0.124105	0.292061	0.77024	0.910292	HSPBP1	23640	HSPA (Hsp70) binding protein 1
ENSG0000	2.592543	-0.53627	1.838182	-0.29174	0.770485	0.910435	NA	NA	NA
ENSG0000	117.6893	0.076313	0.261665	0.291646	0.770558	0.910463	HYPK	25764	huntingtin interacting protein K
ENSG0000	259.5194	-0.05087	0.174501	-0.29152	0.770656	0.910521	ZC3HC1	51530	zinc finger C3HC-type containing 1
ENSG0000	3872.883	-0.02505	0.08602	-0.29123	0.770878	0.910677	ABCA2	20	ATP binding cassette subfamily A member 2

ENSG0000	4.767244	-0.32662	1.121589	-0.29122	0.770887	0.910677	SNORD104	692227	small nucl C/D box 104
ENSG0000	203.31	0.05118	0.175994	0.290804	0.771201	0.910961	CRIPT	9419	CXXC repeat containing interactor of PDZ3 domain
ENSG0000	64.72106	0.113772	0.391273	0.290773	0.771225	0.910961	IL10RB	3588	interleukin 10 receptor subunit beta
ENSG0000	467.5168	-0.0369	0.127023	-0.29053	0.771414	0.911068	NKAPD1	55216	NKAP domain containing 1
ENSG0000	526.2988	-0.03924	0.135056	-0.29058	0.771375	0.911068	MRPS11	64963	mitochondrial ribosomal protein S11
ENSG0000	110.3272	0.067794	0.233418	0.290442	0.771478	0.911086	PMVK	10654	phosphomevalonate kinase
ENSG0000	4.070763	0.365866	1.260371	0.290284	0.771599	0.91111	NA	NA	NA
ENSG0000	93.25383	-0.08591	0.296461	-0.28978	0.771985	0.91111	CTDSPL	10217	CTD small phosphatase like
ENSG0000	4.328082	-0.38098	1.31562	-0.28958	0.772137	0.91111	LOC10537	1.05E+08	uncharacterized LOC105377294
ENSG0000	16.32644	-0.20461	0.705582	-0.28999	0.771826	0.91111	NA	NA	NA
ENSG0000	114.9384	0.068121	0.235129	0.289719	0.772031	0.91111	TRBV28	28559	T cell receptor beta variable 28
ENSG0000	91.30702	0.07249	0.249678	0.290334	0.77156	0.91111	TRUB1	142940	TruB pseudouridine synthase family member 1
ENSG0000	1745.496	0.027832	0.095958	0.290038	0.771787	0.91111	SEL1L	6400	SEL1L adaptor subunit of ERAD E3 ubiquitin ligase
ENSG0000	3.619355	0.414871	1.432607	0.289591	0.772129	0.91111	FBXO15	201456	F-box protein 15
ENSG0000	234.9615	0.048741	0.168014	0.290102	0.771739	0.91111	CD320	51293	CD320 molecule
ENSG0000	46.24776	0.127856	0.441072	0.289877	0.771911	0.91111	NTN5	126147	netrin 5
ENSG0000	65.34145	0.096221	0.331928	0.289885	0.771904	0.91111	CFAP298	56683	cilia and flagella associated protein 298
ENSG0000	7.430229	-0.25188	0.868884	-0.28989	0.771902	0.91111	RN7SKP80	1.06E+08	RN7SK pseudogene 80
ENSG0000	41.07453	0.158973	0.548821	0.289663	0.772074	0.91111	MRPL23	6150	mitochondrial ribosomal protein L23
ENSG0000	36.09043	-0.11531	0.398431	-0.28941	0.772269	0.911208	ODAD3	115948	outer dynein arm docking complex subunit 3
ENSG0000	41.79899	0.105653	0.365338	0.289191	0.772435	0.911229	PSMA2	5683	proteasome 20S subunit alpha 2
ENSG0000	9.684765	-0.2229	0.770745	-0.2892	0.772432	0.911229	SDAD1P1	157489	SDA1 domain containing 1 pseudogene 1
ENSG0000	2.895915	-0.48756	1.685535	-0.28926	0.772382	0.911229	EFNA2	1943	ephrin A2
ENSG0000	576.4291	-0.0383	0.132499	-0.28907	0.772531	0.911285	TNFSF12	8742	TNF superfamily member 12
ENSG0000	97.99179	-0.07481	0.259133	-0.2887	0.772811	0.911557	BEST4	266675	bestrophin 4
ENSG0000	386.6894	0.044871	0.155661	0.28826	0.773147	0.911673	PLEKHA3	65977	pleckstrin homology domain containing A3
ENSG0000	3.993707	0.412359	1.429826	0.288398	0.773042	0.911673	C3orf80	401097	chromosome 3 open reading frame 80
ENSG0000	9.965708	0.209485	0.726113	0.288502	0.772963	0.911673	PRECSIT	283487	p53 regulated carcinoma associated Stat3 activating long intergenic non-protein coding transcript
ENSG0000	440.5851	0.039506	0.137054	0.288251	0.773155	0.911673	MFAP1	4236	microfibril associated protein 1
ENSG0000	391.8493	0.046049	0.159726	0.2883	0.773117	0.911673	COMMD7	149951	COMM domain containing 7
ENSG0000	101.9615	0.068816	0.239053	0.28787	0.773446	0.911726	B4GALT2	8704	beta-1 4-galactosyltransferase 2
ENSG0000	22.91461	-0.15595	0.541493	-0.28799	0.773353	0.911726	RWDD3	25950	RWD domain containing 3
ENSG0000	18.2862	-0.16847	0.584904	-0.28802	0.77333	0.911726	SPDYE18	1.01E+08	speedy/RINGO cell cycle regulator family member E18
ENSG0000	193.9444	0.06215	0.215852	0.28793	0.7734	0.911726	RPS6KA5	9252	ribosomal protein S6 kinase A5
ENSG0000	954.0502	0.028737	0.099764	0.288049	0.773309	0.911726	THUMPD1	55623	THUMP domain containing 1
ENSG0000	13.38715	0.206349	0.717189	0.287719	0.773562	0.911805	NA	NA	NA
ENSG0000	123.4604	0.069441	0.241465	0.28758	0.773668	0.911872	SCX	642658	scleraxis bHLH transcription factor
ENSG0000	7.586005	-0.32003	1.114747	-0.28709	0.774043	0.912256	RAP1GAP	5909	RAP1 GTPase activating protein
ENSG0000	107.6565	0.06594	0.229931	0.286783	0.774278	0.912475	PALS2	51678	protein as MAGUK p55 family member
ENSG0000	24.38435	-0.13768	0.480211	-0.28671	0.774337	0.912486	PLEKHA8P	51054	pleckstrin homology domain containing A8 pseudogene 1
ENSG0000	208.1295	0.056569	0.197349	0.286643	0.774385	0.912486	SPRYD4	283377	SPRY domain containing 4
ENSG0000	2226.879	-0.03401	0.118794	-0.2863	0.774646	0.912527	TET2	54790	tet methylcytosine dioxygenase 2
ENSG0000	41739.93	0.022546	0.078737	0.286346	0.774613	0.912527	ZYX	7791	zyxin
ENSG0000	6.268077	-0.36118	1.260576	-0.28652	0.774479	0.912527	NA	NA	NA
ENSG0000	543.0849	-0.04196	0.146571	-0.28628	0.774667	0.912527	GNB5	10681	G protein subunit beta 5
ENSG0000	5.610062	0.33665	1.175872	0.286298	0.77465	0.912527	RN7SL454I	1.06E+08	RNA 7SL cytoplasm pseudogene
ENSG0000	114.4258	0.07043	0.246227	0.286036	0.77485	0.912686	MIS18A	54069	MIS18 kinetochore protein A
ENSG0000	4.281608	-0.39729	1.389663	-0.28589	0.774964	0.912749	RN7SL172I	1.06E+08	RNA 7SL cytoplasm pseudogene
ENSG0000	5.687497	-0.28805	1.007727	-0.28584	0.775003	0.912749	NA	NA	NA
ENSG0000	43.86205	-0.11134	0.389718	-0.28569	0.775115	0.912823	DND1	373863	DND microRNA-mediated repression inhibitor 1
ENSG0000	54.84998	-0.12069	0.422617	-0.28559	0.775194	0.912859	RPL23AP7	118433	ribosomal protein L23a pseudogene 7
ENSG0000	3.634297	-0.41493	1.453542	-0.28546	0.775292	0.912883	C6orf52	347744	chromosome 6 open reading frame 52
ENSG0000	36.30053	0.12048	0.422097	0.285432	0.775313	0.912883	TRBV23-1	28564	T cell receptor beta variable 23-1 (non-functional)
ENSG0000	16.31083	0.180126	0.631214	0.285364	0.775365	0.912886	LILRP2	79166	leukocyte immunoglobulin-like receptor pseudogene 2
ENSG0000	3.464243	-0.51806	1.817432	-0.28505	0.775606	0.913053	NA	NA	NA
ENSG0000	5.229086	-0.29882	1.048299	-0.28505	0.775605	0.913053	NA	NA	NA
ENSG0000	187.7352	-0.0511	0.179341	-0.28494	0.775689	0.913094	JPX	554203	JPX transc XIST activator
ENSG0000	1013.023	-0.03071	0.107844	-0.28473	0.775847	0.913106	MOB3C	148932	MOB kinase activator 3C
ENSG0000	2.945969	-0.41554	1.458757	-0.28486	0.775754	0.913106	NAIPP3	643784	NAIP pseudogene 3
ENSG0000	203.0979	-0.05282	0.185487	-0.28479	0.775808	0.913106	RAB39B	116442	RAB39B member RAS oncogene family
ENSG0000	272.7578	-0.0481	0.169023	-0.28456	0.775978	0.913202	RAMAC	83640	RNA guanine-7 methyltransferase activating subunit
ENSG0000	17.9926	-0.23	0.808911	-0.28434	0.776151	0.913232	RORB	6096	RAR related orphan receptor B
ENSG0000	163.3244	-0.05937	0.208791	-0.28434	0.776148	0.913232	BLOC1S1	2647	biogenesis of lysosomal organelles complex 1 subunit 1
ENSG0000	101.7232	-0.07533	0.26489	-0.28439	0.776109	0.913232	ZIK1	284307	zinc finger protein interacting with K protein 1
ENSG0000	741.3605	0.029908	0.105257	0.284142	0.776301	0.913351	RNF168	165918	ring finger protein 168
ENSG0000	3.28998	0.416805	1.477795	0.282045	0.777909	0.91341	NA	NA	NA
ENSG0000	582.709	-0.03422	0.121183	-0.28241	0.777629	0.91341	CDC14A	8556	cell division cycle 14A
ENSG0000	37.13265	-0.13417	0.475964	-0.2819	0.778023	0.91341	NA	NA	NA
ENSG0000	1818.964	0.026485	0.093709	0.282626	0.777464	0.91341	KDM3A	55818	lysine demethylase 3A
ENSG0000	11.8188	0.227118	0.803158	0.282781	0.777345	0.91341	KLHL23	151230	kelch like family member 23
ENSG0000	5.465602	0.402761	1.419829	0.283668	0.776664	0.91341	EPGN	255324	epithelial mitogen
ENSG0000	311.1762	0.043086	0.152399	0.282719	0.777393	0.91341	RWDD4	201965	RWD domain containing 4
ENSG0000	26.219	0.147844	0.524331	0.281966	0.777969	0.91341	CETN3	1070	centrin 3
ENSG0000	2.930628	0.415522	1.474366	0.281831	0.778073	0.91341	TRIM10	10107	tripartite motif containing 10
ENSG0000	2061.363	0.025159	0.088977	0.282759	0.777361	0.91341	PSMB8	5696	proteasome 20S subunit beta 8
ENSG0000	384.5079	0.036864	0.130356	0.282792	0.777336	0.91341	HLA-DOA	3111	major hist class II DO alpha
ENSG0000	508.2793	0.040901	0.144537	0.282978	0.777194	0.91341	CPNE5	57699	copine 5
ENSG0000	74.12396	-0.08252	0.291957	-0.28265	0.777444	0.91341	ARPC1A	10552	actin related protein 2/3 complex subunit 1A
ENSG0000	234.6283	-0.04726	0.166849	-0.28328	0.776965	0.91341	FBXO10	26267	F-box protein 10
ENSG0000	490.9656	-0.03412	0.120895	-0.28224	0.777757	0.91341	NUDT5	11164	nudix hydrolase 5
ENSG0000	50.04717	-0.0976	0.346206	-0.28191	0.778009	0.91341	CISD1	55847	CDGSH iron sulfur domain 1
ENSG0000	1999.074	0.024567	0.086592	0.283705	0.776636	0.91341	API5	8539	apoptosis inhibitor 5
ENSG0000	570.1235	-0.03542	0.125217	-0.28289	0.777263	0.91341	FCHSD2	9873	FCH and double SH3 domains 2
ENSG0000	9.792363	0.229767	0.810411	0.283519	0.776779	0.91341	NOP2	4839	NOP2 nucleolar protein
ENSG0000	5.320312	0.362642	1.286297	0.281927	0.777999	0.91341	NA	NA	NA
ENSG0000	249.1067	0.050094	0.176917	0.283148	0.777063	0.91341	NA	NA	NA
ENSG0000	18.35665	0.147096	0.519786	0.282992	0.777183	0.91341	NA	NA	NA
ENSG0000	1.88567	-0.66305	2.349482	-0.28221	0.777782	0.91341	MINDY2-D	1.02E+08	MINDY2 divergent transcript
ENSG0000	85.76714	-0.0859	0.304811	-0.28183	0.778075	0.91341	PDXDC2P-	283970	PDXDC2P-NPIPB14P readthrough
ENSG0000	120.3373	0.06547	0.230686	0.283808	0.776558	0.91341	NA	NA	NA
ENSG0000	27.67465	-0.17242	0.611266	-0.28207	0.777892	0.91341	NA	NA	NA
ENSG0000	4.201704	0.411625	1.454089	0.283081	0.777115	0.91341	NA	NA	NA
ENSG0000	16.15275	-0.17929	0.634838	-0.28241	0.777626	0.91341	CTXN1	404217	cortexin 1
ENSG0000	111.1911	-0.06565	0.232236	-0.28267	0.77743	0.91341	ZNF234	10780	zinc finger protein 234
ENSG0000	109.5478	0.072511	0.255631	0.283654	0.776675	0.91341	LIN7B	64130	lin-7 homc crumbs cell polarity complex component
ENSG0000	65.81363	-0.09363	0.331118	-0.28278	0.777348	0.91341	TSPY26P	128854	testis spec pseudogene
ENSG0000	77.96353	0.082051	0.289301	0.283618	0.776703	0.91341	SNX21	90203	sorting nexin family member 21
ENSG0000	484.4884	-0.03589	0.126677	-0.28334	0.776915	0.91341	GABPA	2551	GA binding protein transcription factor subunit alpha
ENSG0000	31.99707	0.166157	0.585104	0.283978	0.776427	0.91341	APOBEC3E	9582	apolipoprotein B mRNA editing enzyme catalytic subunit 3B
ENSG0000	459.1701	-0.05695	0.201756	-0.28227	0.777735	0.91341	CCDC134	79879	coiled-coil domain containing 134

ENSG0000	4.790772	0.407031	1.445177	0.281647	0.778214	0.913428	NA	NA	NA
ENSG0000	50.44663	0.116327	0.41307	0.281615	0.778239	0.913428	WDR11-D	283089	WDR11 divergent transcript
ENSG0000	5.851413	-0.28813	1.023021	-0.28165	0.778215	0.913428	UBAC2-AS	1E+08	UBAC2 antisense RNA 1
ENSG0000	2.33045	0.575083	2.044574	0.281273	0.778501	0.913447	TMEM139	135932	transmembrane protein 139
ENSG0000	5.454129	-0.28382	1.008665	-0.28138	0.77842	0.913447	NA	NA	NA
ENSG0000	235.8819	0.054464	0.193496	0.281473	0.778348	0.913447	MAK16	84549	MAK16 homolog
ENSG0000	8.105487	0.226463	0.804999	0.281321	0.778464	0.913447	CHRM4	1132	cholinergic receptor muscarinic 4
ENSG0000	74.99705	-0.08105	0.288047	-0.28139	0.778412	0.913447	ZNF891	1.01E+08	zinc finger protein 891
ENSG0000	399.3579	-0.04959	0.176362	-0.28121	0.778551	0.913448	NPEPL1	79716	aminopeptidase like 1
ENSG0000	26.8269	0.137721	0.489933	0.281102	0.778632	0.913486	NA	NA	NA
ENSG0000	4.695501	-0.35108	1.252627	-0.28028	0.779264	0.913661	NA	NA	NA
ENSG0000	83.61507	-0.07275	0.259317	-0.28053	0.779071	0.913661	ZMYM1	79830	zinc finger MYM-type containing 1
ENSG0000	524.1399	0.034467	0.122935	0.280368	0.779195	0.913661	MPHOSPH	10199	M-phase phosphoprotein 10
ENSG0000	461.8753	-0.04523	0.161129	-0.28069	0.778946	0.913661	DHRS9	10170	dehydrogenase/reductase 9
ENSG0000	3.832504	0.375139	1.337363	0.280507	0.779089	0.913661	HNRNPA1	344697	heterogeneous nuclear ribonucleoprotein A1 pseudogene 21
ENSG0000	37.8807	0.114901	0.409601	0.28052	0.779079	0.913661	CKMT2-AS	1E+08	CKMT2 antisense RNA 1
ENSG0000	12.94818	-0.23328	0.830753	-0.2808	0.778863	0.913661	NA	NA	NA
ENSG0000	5.34302	-0.33615	1.199336	-0.28028	0.779262	0.913661	NA	NA	NA
ENSG0000	19.4477	0.167371	0.597203	0.280259	0.779279	0.913661	NA	NA	NA
ENSG0000	3.685725	0.388908	1.385888	0.28062	0.779002	0.913661	KDM4D	55693	lysine demethylase 4D
ENSG0000	279.6799	0.042578	0.151957	0.2802	0.779324	0.913661	PYGL	5836	glycogen phosphorylase L
ENSG0000	26.55542	0.136398	0.487089	0.280026	0.779458	0.913704	NA	NA	NA
ENSG0000	62.2648	-0.08384	0.299492	-0.27994	0.779521	0.913704	C3orf86P	1.03E+08	long intergenic non-protein coding RNA 694
ENSG0000	36.80913	0.106613	0.38099	0.279832	0.779607	0.913704	RPS3AP26	644972	RPS3A pseudogene 26
ENSG0000	57.53585	0.087794	0.313603	0.279954	0.779513	0.913704	MTERF2	80298	mitochondrial transcription termination factor 2
ENSG0000	2009.827	-0.02913	0.10408	-0.27984	0.7796	0.913704	NOSIP	51070	nitric oxide synthase interacting protein
ENSG0000	7.664263	0.289698	1.03591	0.279656	0.779742	0.913804	NA	NA	NA
ENSG0000	7.264358	-0.33382	1.195922	-0.27913	0.780145	0.914104	NA	NA	NA
ENSG0000	194.395	-0.05084	0.182128	-0.27917	0.780116	0.914104	HADH	3033	hydroxyacyl-CoA dehydrogenase
ENSG0000	10.51793	0.226438	0.811016	0.279203	0.780089	0.914104	NA	NA	NA
ENSG0000	9.800741	-0.21388	0.766794	-0.27892	0.780305	0.914118	NA	NA	NA
ENSG0000	3.944541	-0.36968	1.325244	-0.27895	0.780281	0.914118	TUT1	64852	terminal u U6 snRNA-specific
ENSG0000	2.863673	-0.45054	1.61459	-0.27904	0.780212	0.914118	RNU6-638	1.06E+08	RNA U6 small r pseudogene
ENSG0000	292.2637	0.043305	0.155468	0.278548	0.780592	0.914281	PPP1R21	129285	protein phosphatase 1 regulatory subunit 21
ENSG0000	33.48043	0.136873	0.491303	0.278592	0.780558	0.914281	RMI2	116028	RecQ mediated genome instability 2
ENSG0000	3069.246	0.022664	0.081351	0.278594	0.780556	0.914281	WSB1	26118	WD repeat and SOCS box containing 1
ENSG0000	99.99203	-0.07505	0.269691	-0.27829	0.780787	0.914393	LIAS	11019	lipoic acid synthetase
ENSG0000	8.934416	-0.22618	0.812636	-0.27832	0.780763	0.914393	NA	NA	NA
ENSG0000	1542.942	-0.02647	0.095162	-0.27812	0.780922	0.914494	EIF4E2	9470	eukaryotic translation initiation factor 4E family member 2
ENSG0000	27.15819	-0.13547	0.487248	-0.27802	0.780994	0.914521	APTR	1.01E+08	Alu-mediated CDKN1A/p21 transcriptional regulator
ENSG0000	9.998841	-0.22216	0.799273	-0.27795	0.781048	0.914526	FBN1	2200	fibrillin 1
ENSG0000	9.946865	-0.21408	0.770698	-0.27778	0.781182	0.914625	HILPDA	29923	hypoxia inducible lipid droplet associated
ENSG0000	912.2606	0.030948	0.111536	0.277473	0.781417	0.914727	PIAS3	10401	protein inhibitor of activated STAT 3
ENSG0000	75.34229	0.078214	0.281872	0.277479	0.781412	0.914727	NA	NA	NA
ENSG0000	59.46805	-0.09675	0.348621	-0.27753	0.781376	0.914727	LIME1	54923	Lck interacting transmembrane adaptor 1
ENSG0000	7.718741	0.281816	1.016274	0.277304	0.781547	0.914822	FFAR1	2864	free fatty acid receptor 1
ENSG0000	11.23368	-0.21309	0.768808	-0.27717	0.781647	0.914881	NA	NA	NA
ENSG0000	8.26208	0.256457	0.92622	0.276886	0.781868	0.915082	NA	NA	NA
ENSG0000	580.1836	-0.03875	0.140027	-0.27671	0.782005	0.915178	NEPRO	25871	nucleolus and neural progenitor protein
ENSG0000	107.6692	-0.33463	1.209572	-0.27665	0.782048	0.915178	FOXQ1	94234	forkhead box Q1
ENSG0000	4.073534	-0.32854	1.190298	-0.27602	0.782537	0.91546	RPS16P1	441876	ribosomal protein S16 pseudogene 1
ENSG0000	55432.52	0.020324	0.073612	0.276092	0.782478	0.91546	ZFP36L2	678	ZFP36 ring finger protein like 2
ENSG0000	458.2474	-0.03777	0.13681	-0.27611	0.782463	0.91546	MTMR12	54545	myotubularin related protein 12
ENSG0000	45.88055	-0.10039	0.363628	-0.27608	0.782483	0.91546	NAIP	4671	NLR family apoptosis inhibitory protein
ENSG0000	987.7972	-0.02874	0.104116	-0.27604	0.782519	0.91546	AP2S1	1175	adaptor related protein complex 2 subunit sigma 1
ENSG0000	3265.69	-0.02555	0.092594	-0.27593	0.782604	0.915481	NFE2L1	4779	NFE2 like bZIP transcription factor 1
ENSG0000	151.5029	-0.05595	0.202878	-0.27577	0.782728	0.915569	NA	NA	NA
ENSG0000	41.51594	-0.12287	0.445731	-0.27565	0.782818	0.915576	LOXL3	84695	lysyl oxidase like 3
ENSG0000	15.62234	0.181984	0.66025	0.275629	0.782833	0.915576	ARL6IP4	51329	ADP ribosylation factor like GTPase 6 interacting protein 4
ENSG0000	272.1132	-0.04915	0.178418	-0.2755	0.782932	0.915577	GSKIP	51527	GSK3B interacting protein
ENSG0000	1492.565	0.02842	0.103137	0.275557	0.782889	0.915577	FAM222B	55731	family with sequence similarity 222 member B
ENSG0000	317.2276	-0.04491	0.163081	-0.27539	0.783016	0.915617	TRG-AS1	1.01E+08	T cell receptor gamma locus antisense RNA 1
ENSG0000	59.95811	0.087668	0.318429	0.275314	0.783075	0.915628	TST	7263	thiosulfate sulfurtransferase
ENSG0000	2573.18	0.025994	0.094476	0.275135	0.783213	0.915731	COX6B1	1340	cytochrome c oxidase subunit 6B1
ENSG0000	115.3482	0.065598	0.238691	0.274824	0.783452	0.915935	ETFRF1	144363	electron transfer flavoprotein regulatory factor 1
ENSG0000	461.6645	0.035543	0.12935	0.27478	0.783485	0.915935	PYM1	84305	PYM homc exon junction complex associated factor
ENSG0000	10.91244	0.199902	0.727747	0.274686	0.783557	0.915961	TPBGL	1.01E+08	trophoblast glycoprotein like
ENSG0000	237.4116	0.047582	0.173296	0.274572	0.783645	0.915995	TWNK	56652	twinkle mtDNA helicase
ENSG0000	9.83534	-0.23	0.83803	-0.27446	0.783734	0.915995	DMKN	93099	dermokine
ENSG0000	391.0963	0.037391	0.136239	0.274455	0.783735	0.915995	CARD8-AS	1.01E+08	CARD8 antisense RNA 1
ENSG0000	275.4555	-0.04649	0.169452	-0.27434	0.783823	0.91604	MICA	1.01E+08	MHC class I polypeptide-related sequence A
ENSG0000	23.48365	0.190269	0.694058	0.274141	0.783977	0.916162	NA	NA	NA
ENSG0000	2.624096	0.485909	1.77343	0.273994	0.784089	0.916236	ANKRD61	1E+08	ankyrin repeat domain 61
ENSG0000	46.20806	0.094986	0.34688	0.273829	0.784216	0.916327	CCDC24	149473	coiled-coil domain containing 24
ENSG0000	621.2145	0.037139	0.135848	0.273389	0.784554	0.916439	PSMA5	5686	proteasome 20S subunit alpha 5
ENSG0000	167.1683	0.054474	0.19926	0.273382	0.78456	0.916439	C2orf88	84281	chromosome 2 open reading frame 88
ENSG0000	90.89273	-0.06818	0.249256	-0.27354	0.784435	0.916439	AASDH	132949	aminoadipate-semialdehyde dehydrogenase
ENSG0000	20.59938	-0.16131	0.589858	-0.27348	0.784486	0.916439	HACD1	9200	3-hydroxyacyl-CoA dehydratase 1
ENSG0000	81.6965	-0.07906	0.289115	-0.27345	0.784504	0.916439	FAM86C1	55199	family witl pseudogene
ENSG0000	3390.068	0.020076	0.073466	0.273275	0.784642	0.916478	CCDC97	90324	coiled-coil domain containing 97
ENSG0000	23.65251	-0.13346	0.489124	-0.27286	0.784965	0.916682	NA	NA	NA
ENSG0000	71.93263	0.079246	0.290432	0.272857	0.784963	0.916682	NA	NA	NA
ENSG0000	8.96352	-0.25136	0.921238	-0.27286	0.784964	0.916682	FAM182A	284800	family with sequence similarity 182 member A
ENSG0000	8.951426	-0.22117	0.811646	-0.2725	0.785238	0.91677	IL7	3574	interleukin 7
ENSG0000	48.33101	-0.09741	0.357384	-0.27256	0.785193	0.91677	TMEM9B-	493900	TMEM9B antisense RNA 1
ENSG0000	812.1992	-0.03101	0.113788	-0.27253	0.785213	0.91677	KMT5B	51111	lysine methyltransferase 5B
ENSG0000	6.617176	0.287124	1.053655	0.272503	0.785235	0.91677	LOC10537	1.05E+08	uncharacterized LOC105371814
ENSG0000	113.6955	0.064269	0.236051	0.272268	0.785416	0.91692	MMACHC	25974	metabolism of cobalamin associated C
ENSG0000	6347.594	-0.0229	0.084171	-0.2721	0.785546	0.917014	GAK	2580	cyclin G associated kinase
ENSG0000	3.045593	0.41723	1.535681	0.27169	0.78586	0.917265	NAALADL	254827	N-acetylated alpha-linked acidic dipeptidase like 2
ENSG0000	78.45819	0.08348	0.30721	0.271738	0.785824	0.917265	BRCA2	675	BRCA2 DNA repair associated
ENSG0000	3284.809	0.022294	0.082183	0.271276	0.786178	0.917579	EIF2AK1	27102	eukaryotic translation initiation factor 2 alpha kinase 1
ENSG0000	9.743273	-0.25445	0.940612	-0.27051	0.786765	0.918148	NA	NA	NA
ENSG0000	79.28367	0.075179	0.277854	0.270572	0.78672	0.918148	NA	NA	NA
ENSG0000	124.2866	0.05859	0.216707	0.270365	0.78688	0.918167	NA	NA	NA
ENSG0000	3212.279	-0.02506	0.092684	-0.27038	0.786872	0.918167	NUP188	23511	nucleoporin 188
ENSG0000	4.82581	-0.30062	1.112686	-0.27018	0.787024	0.918243	NA	NA	NA
ENSG0000	5.070143	-0.36669	1.357682	-0.27009	0.787093	0.918243	NEIL3	55247	nei like DNA glycosylase 3
ENSG0000	47.7261	-0.10566	0.391175	-0.27011	0.787073	0.918243	FAR2	55711	fatty acyl-CoA reductase 2
ENSG0000	6.699445	0.656152	2.432611	0.269732	0.787367	0.918388	IGKV3D-2	28874	immunoglobulin kappa variable 3D-20

ENSG0000	109.1726	0.07485	0.277449	0.269779	0.787331	0.918388	MRPL13	28998	mitochondrial ribosomal protein L13
ENSG0000	7.502536	-0.25051	0.928646	-0.26976	0.787343	0.918388	BMS1P1	399761	BMS1 pseudogene 1
ENSG0000	10.2753	-0.22041	0.818227	-0.26937	0.787641	0.918632	LY86-AS1	285780	LY86 antisense RNA 1
ENSG0000	1862.087	-0.0228	0.084665	-0.26933	0.787675	0.918632	RUSF1	64755	RUS family member 1
ENSG0000	6.279522	-0.27557	1.023474	-0.26925	0.787734	0.918643	NA	NA	NA
ENSG0000	23.60251	0.145497	0.54064	0.26912	0.787837	0.918706	NA	NA	NA
ENSG0000	8.82101	0.231999	0.86287	0.268869	0.78803	0.918708	NA	NA	NA
ENSG0000	16.24919	0.17603	0.655348	0.268606	0.788233	0.918708	BAMBI	25805	BMP and activin membrane bound inhibitor
ENSG0000	7.532762	0.253361	0.942562	0.268801	0.788083	0.918708	BBS1	582	Bardet-Biedl syndrome 1
ENSG0000	9.446305	0.218286	0.812231	0.268749	0.788123	0.918708	RDH16	8608	retinol dehydrogenase 16
ENSG0000	7.131834	-0.24597	0.915744	-0.2686	0.788235	0.918708	LOC10537(	1.05E+08	uncharacterized LOC105370532
ENSG0000	498.9377	0.034379	0.12791	0.268777	0.788102	0.918708	SPATA20	64847	spermatogenesis associated 20
ENSG0000	863.2257	-0.02886	0.107439	-0.26865	0.788202	0.918708	PAXBP1	94104	PAX3 and PAX7 binding protein 1
ENSG0000	637.6346	-0.03287	0.122296	-0.2688	0.788087	0.918708	TRMU	55687	tRNA mitochondrial 2-thiouridylase
ENSG0000	1794.016	-0.02367	0.088197	-0.26834	0.788435	0.918826	LRIG1	26018	leucine rich repeats and immunoglobulin like domains 1
ENSG0000	683.7728	0.032915	0.122658	0.268346	0.788433	0.918826	LYSMD2	256586	LysM domain containing 2
ENSG0000	15.79265	0.155321	0.579376	0.268083	0.788635	0.919001	SNORC	389084	secondary ossification center associated regulator of chondrocyte maturation
ENSG0000	287.4111	-0.04667	0.17438	-0.26765	0.78897	0.919166	C1orf35	79169	chromosome 1 open reading frame 35
ENSG0000	9.102756	0.232678	0.869898	0.267477	0.789102	0.919166	ABCG2	9429	ATP binding cassette subfamily G member 2 (Junior blood group)
ENSG0000	1842.987	0.024797	0.092692	0.267523	0.789066	0.919166	ERBIN	55914	erbb2 interacting protein
ENSG0000	5.124429	-0.36943	1.381649	-0.26738	0.789173	0.919166	SEMA3A	10371	semaphorin 3A
ENSG0000	16.02989	0.179823	0.672458	0.267411	0.789153	0.919166	LINC0116З	1.02E+08	long intergenic non-protein coding RNA 1163
ENSG0000	1151.413	-0.02466	0.092067	-0.26781	0.788846	0.919166	IGHMBP2	3508	immunoglobulin mu DNA binding protein 2
ENSG0000	105.587	0.063893	0.238947	0.267392	0.789167	0.919166	TIGAR	57103	TP53 induced glycolysis regulatory phosphatase
ENSG0000	9.510897	-0.221	0.825605	-0.26768	0.788948	0.919166	RASL10B	91608	RAS like family 10 member B
ENSG0000	2.943916	0.459857	1.721459	0.267132	0.789368	0.919334	IL21R-AS1	283888	IL21R antisense RNA 1
ENSG0000	8.399162	-0.24618	0.922148	-0.26697	0.789496	0.919368	PHYHD1	254295	phytanoyl-CoA dioxygenase domain containing 1
ENSG0000	924.2783	0.040197	0.15055	0.267	0.789469	0.919368	KLF12	11278	KLF transcription factor 12
ENSG0000	326.9589	0.042304	0.158517	0.26687	0.789569	0.919396	ZNF91	7644	zinc finger protein 91
ENSG0000	3.779612	0.384761	1.443013	0.266637	0.789748	0.919547	HSD17B13	345275	hydroxysteroid 17-beta dehydrogenase 13
ENSG0000	436.9719	-0.0366	0.137382	-0.26638	0.78995	0.919723	ZSWIM1	90204	zinc finger SWIM-type containing 1
ENSG0000	13.21121	0.230753	0.866619	0.266268	0.790033	0.919763	EPS8L1	54869	EPS8 like 1
ENSG0000	3.379036	0.393419	1.478751	0.266048	0.790202	0.919902	DYTN	391475	dystrotelin
ENSG0000	109.7706	0.061831	0.232543	0.26589	0.790324	0.919986	ZMYM6	9204	zinc finger MYM-type containing 6
ENSG0000	351.0872	0.035724	0.134408	0.265788	0.790402	0.920019	ELP3	55140	elongator acetyltransferase complex subunit 3
ENSG0000	31.46267	-0.14291	0.5379	-0.26567	0.79049	0.920064	NA	NA	NA
ENSG0000	16.53422	0.165662	0.623768	0.265582	0.790561	0.920089	LINC0027٤	1.01E+08	long intergenic non-protein coding RNA 278
ENSG0000	358.3699	0.038588	0.145444	0.265308	0.790772	0.920277	YIPF6	286451	Yip1 domain family member 6
ENSG0000	2246.799	0.030073	0.113456	0.26506	0.790963	0.920415	ZDHHC20	253832	zinc finger DHHC-type palmitoyltransferase 20
ENSG0000	69.81205	-0.09632	0.36342	-0.26502	0.79099	0.920415	SERPINF2	5345	serpin family F member 2
ENSG0000	8.433831	0.246857	0.93252	0.26472	0.791225	0.92063	NA	NA	NA
ENSG0000	11.72782	-0.21583	0.815719	-0.26458	0.791329	0.920657	SLC1A2	6506	solute carrier family 1 member 2
ENSG0000	1007.523	0.030578	0.115579	0.264562	0.791347	0.920657	RAD9A	5883	RAD9 checkpoint clamp component A
ENSG0000	80.36041	0.077339	0.292451	0.264452	0.791432	0.920686	NA	NA	NA
ENSG0000	1937.436	0.022517	0.085161	0.264401	0.791471	0.920686	USP8	9101	ubiquitin specific peptidase 8
ENSG0000	6.832996	-0.25577	0.972238	-0.2641	0.791705	0.920805	NKAPL	222698	NFKB activating protein like
ENSG0000	70.10599	-0.07745	0.293337	-0.26401	0.791769	0.920805	PYROXD2	84795	pyridine nucleotide-disulphide oxidoreductase domain 2
ENSG0000	3418.499	0.025089	0.095	0.26409	0.791711	0.920805	VPS51	738	VPS51 subunit of GARP complex
ENSG0000	106.6112	-0.06373	0.241376	-0.26401	0.791772	0.920805	NPEPPSP1	440434	NPEPPS pseudogene 1
ENSG0000	4.582622	0.320087	1.21316	0.263845	0.791899	0.920837	NA	NA	NA
ENSG0000	542.3865	-0.03742	0.141811	-0.26387	0.791882	0.920837	CRNKL1	51340	crooked neck pre-mRNA splicing factor 1
ENSG0000	1203.592	0.029385	0.111514	0.263509	0.792158	0.921032	TMF1	7110	TATA element modulatory factor 1
ENSG0000	3.865209	-0.32639	1.238695	-0.2635	0.792166	0.921032	NA	NA	NA
ENSG0000	57.27379	0.090187	0.342478	0.263337	0.79229	0.921061	FKBPL	63943	FKBP prolyl isomerase like
ENSG0000	16.41867	0.166773	0.633458	0.263273	0.79234	0.921061	ARHGEF39	84904	Rho guanine nucleotide exchange factor 39
ENSG0000	30.66799	-0.11263	0.427706	-0.26334	0.792287	0.921061	SPTB	6710	spectrin b(erythrocytic
ENSG0000	5266.513	-0.02397	0.091158	-0.26293	0.792607	0.921101	PHC2	1912	polyhomeotic homolog 2
ENSG0000	186.207	0.051929	0.197435	0.263015	0.792539	0.921101	DPH5	51611	diphthamide biosynthesis 5
ENSG0000	3.234499	-0.37758	1.436894	-0.26278	0.792722	0.921101	H2BK1	1.14E+08	H2B.K variant histone 1
ENSG0000	15.75126	0.160867	0.61208	0.262821	0.792689	0.921101	BAALC	79870	BAALC binder of MAP3K1 and KLF4
ENSG0000	2.374573	-0.56572	2.151516	-0.26294	0.792597	0.921101	OR1F1	4992	olfactory receptor family 1 subfamily F member 1
ENSG0000	127.7167	-0.06022	0.229116	-0.26284	0.792671	0.921101	CLN3	1201	CLN3 lysos battenin
ENSG0000	372.7018	0.040446	0.15386	0.262874	0.792648	0.921101	TTPAL	79183	alpha tocopherol transfer protein like
ENSG0000	87.37282	0.067138	0.255637	0.26263	0.792836	0.921175	LSM6	11157	LSM6 hom U6 small nuclear RNA and mRNA degradation associated
ENSG0000	141.3708	0.06106	0.232559	0.262556	0.792893	0.921184	BCLAF3	256643	BCLAF1 and THRAP3 family member 3
ENSG0000	66.99565	0.07601	0.289582	0.262482	0.79295	0.921188	NA	NA	NA
ENSG0000	455.0308	-0.03602	0.137278	-0.26242	0.792996	0.921188	VPS25	84313	vacuolar protein sorting 25 homolog
ENSG0000	6.354973	-0.28574	1.089567	-0.26225	0.793131	0.9212	ANAPC1P1	1E+08	ANAPC1 pseudogene 1
ENSG0000	17.72732	0.161568	0.616163	0.262216	0.793155	0.9212	NA	NA	NA
ENSG0000	953.8715	-0.02692	0.102632	-0.26229	0.793099	0.9212	ZNF664	144348	zinc finger protein 664
ENSG0000	33.01208	-0.11277	0.430295	-0.26208	0.793263	0.921268	NA	NA	NA
ENSG0000	76.48372	-0.07379	0.281622	-0.262	0.79332	0.921276	ZNF607	84775	zinc finger protein 607
ENSG0000	2645.998	0.02304	0.08799	0.261844	0.793442	0.921303	IFNGR1	3459	interferon gamma receptor 1
ENSG0000	493.5008	-0.03521	0.13443	-0.26189	0.793405	0.921303	COA4	51287	cytochrome c oxidase assembly factor 4 homolog
ENSG0000	9.100524	-0.2105	0.804417	-0.26167	0.793572	0.921396	SGCD	6444	sarcoglycan delta
ENSG0000	140.8228	-0.06686	0.255711	-0.26148	0.793725	0.921476	CENPF	1063	centromere protein F
ENSG0000	544.328	0.034294	0.131184	0.261423	0.793767	0.921476	ZSCAN25	221785	zinc finger and SCAN domain containing 25
ENSG0000	10.33608	0.213004	0.815082	0.261328	0.79384	0.921476	C1QTNF4	114900	C1q and TNF related 4
ENSG0000	11.36723	-0.20691	0.791583	-0.26139	0.793792	0.921476	STAC2	342667	SH3 and cysteine rich domain 2
ENSG0000	3208.801	0.024473	0.093738	0.261082	0.794029	0.921639	RBM15B	29890	RNA binding motif protein 15B
ENSG0000	9.374372	-0.18847	0.722593	-0.26083	0.794226	0.921752	NA	NA	NA
ENSG0000	2.618513	0.439684	1.68572	0.260828	0.794225	0.921752	LINC0234٤	1.02E+08	long intergenic non-protein coding RNA 2348
ENSG0000	73.41199	-0.07913	0.303466	-0.26076	0.794278	0.921754	ZNF527	84503	zinc finger protein 527
ENSG0000	1190.675	-0.02444	0.093748	-0.26066	0.794354	0.921785	PPM1A	5494	protein ph Mg2+/Mn2+ dependent 1A
ENSG0000	214.1839	0.047304	0.18155	0.260555	0.794436	0.921822	GSDMB	55876	gasdermin B
ENSG0000	9.23837	-0.2257	0.867793	-0.26009	0.794797	0.921987	H2BC18	440689	H2B clustered histone 18
ENSG0000	7.830502	0.230186	0.8869	0.25954	0.795219	0.921987	NA	NA	NA
ENSG0000	552.8118	0.036256	0.139529	0.259849	0.79498	0.921987	TIPRL	261726	TOR signaling pathway regulator
ENSG0000	27.08307	0.117127	0.451161	0.259612	0.795163	0.921987	MAP4K3-D	728730	MAP4K3 divergent transcript
ENSG0000	38.06132	0.113526	0.436557	0.260049	0.794826	0.921987	NMNAT3	349565	nicotinamide nucleotide adenylyltransferase 3
ENSG0000	210.3784	-0.05054	0.194437	-0.25995	0.794906	0.921987	RIOK2	55781	RIO kinase 2
ENSG0000	5.858779	0.238114	0.917116	0.259633	0.795147	0.921987	OR52N4	390072	olfactory receptor family 52 subfamily N member 4
ENSG0000	63.49251	0.091234	0.350796	0.260076	0.794805	0.921987	HSPA2	3306	heat shock protein family A (Hsp70) member 2
ENSG0000	693.1124	-0.03088	0.118635	-0.26029	0.794643	0.921987	GPR68	8111	G protein-coupled receptor 68
ENSG0000	19.53043	-0.16282	0.627012	-0.25968	0.795112	0.921987	NA	NA	NA
ENSG0000	3.251079	-0.42503	1.636949	-0.25965	0.795135	0.921987	NA	NA	NA
ENSG0000	6.698841	0.254373	0.9791	0.259803	0.795016	0.921987	NA	NA	NA
ENSG0000	27.3499	0.113049	0.435587	0.259532	0.795224	0.921987	NA	NA	NA
ENSG0000	233.4323	0.045349	0.174941	0.259222	0.795464	0.922067	TESK2	10420	testis associated actin remodelling kinase 2
ENSG0000	407.3205	-0.03999	0.154241	-0.25925	0.795439	0.922067	TRMT61A	115708	tRNA methyltransferase 61A

ENSG0000	743.5534	-0.03442	0.132831	-0.25912	0.795542	0.922067	UBR1	197131	ubiquitin protein ligase E3 component n-recognin 1
ENSG0000	1733.528	-0.02414	0.093082	-0.25929	0.795411	0.922067	NUTF2	10204	nuclear transport factor 2
ENSG0000	230.1372	-0.04493	0.173375	-0.25918	0.7955	0.922067	ZNF776	284309	zinc finger protein 776
ENSG0000	9.888577	-0.18952	0.732135	-0.25886	0.795742	0.922184	NA	NA	NA
ENSG0000	17.57139	-0.19736	0.76238	-0.25887	0.795732	0.922184	PAK3	5063	p21 (RAC1) activated kinase 3
ENSG0000	3.894456	0.367554	1.420818	0.258692	0.795873	0.922278	PPP1R14C	81706	protein phosphatase 1 regulatory inhibitor subunit 14C
ENSG0000	3.226976	-0.38895	1.506309	-0.25821	0.796244	0.922558	WNK4	65266	WNK lysine deficient protein kinase 4
ENSG0000	1097.224	0.02469	0.095629	0.258186	0.796264	0.922558	TNFAIP8L1	126282	TNF alpha induced protein 8 like 1
ENSG0000	10.46381	0.200582	0.776582	0.258289	0.796184	0.922558	NA	NA	NA
ENSG0000	14.7059	-0.2451	0.950377	-0.25789	0.796489	0.92265	NA	NA	NA
ENSG0000	3.753059	0.309935	1.202417	0.25776	0.796592	0.92265	NA	NA	NA
ENSG0000	11.87274	0.230457	0.89395	0.257796	0.796564	0.92265	OMG	4974	oligodendrocyte myelin glycoprotein
ENSG0000	4.615273	-0.39283	1.522601	-0.258	0.796407	0.92265	MTDHP3	1E+08	metadherin pseudogene 3
ENSG0000	3.414147	0.439491	1.704695	0.257812	0.796552	0.92265	RIMBP3C	150221	RIMS binding protein 3C
ENSG0000	69.55814	0.078411	0.304448	0.257551	0.796754	0.92278	RPL6P10	642828	ribosomal protein L6 pseudogene 10
ENSG0000	824.8388	0.030005	0.117041	0.256364	0.79767	0.92283	GBP3	2635	guanylate binding protein 3
ENSG0000	3.779887	-0.36424	1.419326	-0.25663	0.797466	0.92283	NA	NA	NA
ENSG0000	533.5124	-0.03185	0.124009	-0.25686	0.797288	0.92283	RETSAT	54884	retinol saturase
ENSG0000	421.1635	-0.03963	0.154679	-0.25623	0.797771	0.92283	POGLUT1	56983	protein O-glucosyltransferase 1
ENSG0000	908.3543	0.034889	0.13598	0.256575	0.797507	0.92283	ZNF148	7707	zinc finger protein 148
ENSG0000	487.6341	-0.03173	0.123742	-0.25638	0.797655	0.92283	NA	NA	NA
ENSG0000	321.5515	0.036245	0.140997	0.257065	0.797129	0.92283	UQCRQ	27089	ubiquinol-cytochrome c reductase complex III subunit VII
ENSG0000	226.9134	0.056708	0.221336	0.256206	0.797792	0.92283	HRH2	3274	histamine receptor H2
ENSG0000	7.267502	0.212932	0.829329	0.256752	0.79737	0.92283	NA	NA	NA
ENSG0000	4973.625	-0.02058	0.080011	-0.2572	0.797023	0.92283	GIGYF1	64599	GRB10 interacting GYF protein 1
ENSG0000	2819.269	-0.02606	0.101405	-0.25697	0.797203	0.92283	SIGIRR	59307	single Ig and TIR domain containing
ENSG0000	11.56561	-0.20774	0.808729	-0.25687	0.797282	0.92283	NA	NA	NA
ENSG0000	280.6655	-0.03875	0.151198	-0.25625	0.797755	0.92283	ZW10	9183	zw10 kinetochore protein
ENSG0000	9.939204	0.189251	0.737293	0.256683	0.797424	0.92283	NA	NA	NA
ENSG0000	163.1147	0.048749	0.190023	0.256543	0.797531	0.92283	CBLN3	643866	cerebellin 3 precursor
ENSG0000	13.19708	0.179999	0.700347	0.257013	0.797168	0.92283	DICER1-AS	400242	DICER1 antisense RNA 1
ENSG0000	3.199752	-0.40757	1.584776	-0.25718	0.797043	0.92283	LOC12490	1.25E+08	uncharacterized LOC124903659
ENSG0000	180.6852	0.04954	0.193197	0.256422	0.797625	0.92283	SMG1P3	1E+08	SMG1 pseudogene 3
ENSG0000	78.33258	-0.07691	0.299276	-0.25698	0.797197	0.92283	PPP1R14A	94274	protein phosphatase 1 regulatory inhibitor subunit 14A
ENSG0000	8.614946	0.277378	1.079313	0.256995	0.797183	0.92283	ERVH48-1	90625	endogenous retrovirus group 48 member 1
ENSG0000	4861.911	-0.02506	0.097904	-0.25592	0.79801	0.922847	MBNL1	4154	muscleblind like splicing regulator 1
ENSG0000	610.8378	-0.0291	0.113729	-0.2559	0.798031	0.922847	YEATS2	55689	YEATS domain containing 2
ENSG0000	50.19483	-0.09274	0.362469	-0.25586	0.798056	0.922847	SHLD3	1.12E+08	shieldin complex subunit 3
ENSG0000	19.10216	-0.13543	0.528917	-0.25605	0.797913	0.922847	NA	NA	NA
ENSG0000	122.856	-0.05616	0.219256	-0.25612	0.797858	0.922847	ZNF232	7775	zinc finger protein 232
ENSG0000	15.31846	0.146236	0.572213	0.255562	0.798289	0.923003	NA	NA	NA
[truncated: 308,246 more chars]
