# Supplementary material for: Dexamethasone attenuates interferon-related cytokine hyperresponsiveness in COVID-19 patients
Source: Front Immunol. 2023 Aug 8;14:1233318. doi: 10.3389/fimmu.2023.1233318 (PMC10442808; doi:10.3389/fimmu.2023.1233318)
Supplement: Supplementary file 1 [file DataSheet_1.zip › Supplementary File 2.pdf]

name	baseMean	log2FoldCl	lfcSE	pvalue	padj	Symbol	entrez	name.y
ENSG0000	424.3723	4.150604	0.252328	1.97E-64	3.61E-60	VSIG4	11326	V-set and immunoglobulin domain containing 4
ENSG0000	474.5654	-3.00476	0.199457	4.29E-54	3.93E-50	USP18	11274	ubiquitin specific peptidase 18
ENSG0000	10600.09	-2.69098	0.185576	2.46E-50	1.50E-46	OAS3	4940	2'-5'-oligoadenylate synthetase 3
ENSG0000	7863.993	-3.32379	0.250392	3.05E-44	1.40E-40	ISG15	9636	ISG15 ubiquitin like modifier
ENSG0000	11148.1	-2.35143	0.182055	1.79E-40	6.56E-37	OAS2	4939	2'-5'-oligoadenylate synthetase 2
ENSG0000	3072.931	-3.33008	0.265695	2.90E-40	8.86E-37	RSAD2	91543	radical S-adenosyl methionine domain containing 2
ENSG0000	1004.348	-2.7318	0.219079	1.48E-38	3.87E-35	SPATS2L	26010	spermatogenesis associated serine rich 2 like
ENSG0000	675.2001	-2.15413	0.174368	6.05E-37	1.31E-33	HERC6	55008	HECT and RLD domain containing E3 ubiquitin protein ligase family member 6
ENSG0000	2651.105	-1.7825	0.143771	6.44E-37	1.31E-33	PARP12	64761	poly(ADP-ribose) polymerase family member 12
ENSG0000	1188.952	-2.5025	0.208707	1.18E-35	2.17E-32	HERC5	51191	HECT and RLD domain containing E3 ubiquitin protein ligase 5
ENSG0000	538.6839	-2.19292	0.187511	1.26E-33	2.10E-30	TIMM10	26519	translocase of inner mitochondrial membrane 10
ENSG0000	11198.39	-1.9982	0.177489	2.16E-31	3.29E-28	OAS1	4938	2'-5'-oligoadenylate synthetase 1
ENSG0000	5364.242	-2.96545	0.278474	2.95E-30	3.98E-27	IFI44L	10964	interferon induced protein 44 like
ENSG0000	15252.69	-2.38071	0.219457	3.04E-30	3.98E-27	IRF7	3665	interferon regulatory factor 7
ENSG0000	9783.322	-2.56926	0.238523	5.38E-30	6.57E-27	IFIT3	3437	interferon induced protein with tetratricopeptide repeats 3
ENSG0000	1117.758	-2.41977	0.224567	1.20E-29	1.35E-26	CMPK2	129607	cytidine/uridine monophosphate kinase 2
ENSG0000	5933.888	-2.07511	0.190969	1.25E-29	1.35E-26	OASL	8638	2'-5'-oligoadenylate synthetase like
ENSG0000	3216.503	-2.44741	0.231542	8.28E-29	8.43E-26	IFIT2	3433	interferon induced protein with tetratricopeptide repeats 2
ENSG0000	385.1383	-2.31657	0.222846	1.16E-27	1.11E-24	RTP4	64108	receptor transporter protein 4
ENSG0000	1579.664	-1.99144	0.190696	1.43E-27	1.31E-24	DHX58	79132	DExH-box helicase 58
ENSG0000	674.2421	-1.72216	0.164205	2.26E-27	1.97E-24	IFIH1	64135	interferon induced with helicase C domain 1
ENSG0000	2864.416	-2.74409	0.273503	4.06E-27	3.38E-24	IFIT1	3434	interferon induced protein with tetratricopeptide repeats 1
ENSG0000	160.5957	-2.86643	0.292909	1.17E-25	9.30E-23	EXOC3L1	283849	exocyst complex component 3 like 1
ENSG0000	488.4468	-1.46184	0.145508	3.70E-25	2.83E-22	TRIM5	85363	tripartite motif containing 5
ENSG0000	7862.333	-2.38204	0.247013	8.21E-25	6.02E-22	MT2A	4502	metallothionein 2A
ENSG0000	18237.25	-2.38768	0.247984	1.07E-24	7.50E-22	MX1	4599	MX dynamin like GTPase 1
ENSG0000	2849.003	-1.99359	0.205251	1.91E-24	1.30E-21	MOV10	4343	Mov10 RISC complex RNA helicase
ENSG0000	655.7331	2.750995	0.292132	3.53E-24	2.31E-21	ADAMTS2	9509	ADAM metallopeptidase with thrombospondin type 1 motif 2
ENSG0000	2340.253	-2.01388	0.20892	4.11E-24	2.59E-21	PLSCR1	5359	phospholipid scramblase 1
ENSG0000	18372.42	-2.63456	0.281962	4.24E-24	2.59E-21	SERPING1	710	serpin family G member 1
ENSG0000	6215.554	-2.17824	0.228225	4.87E-24	2.88E-21	XAF1	54739	XIAP associated factor 1
ENSG0000	165.2354	-3.07371	0.335243	1.81E-23	1.04E-20	LAMP3	27074	lysosomal associated membrane protein 3
ENSG0000	308.59	-2.16595	0.229637	2.90E-23	1.61E-20	MDK	4192	midkine
ENSG0000	292.1255	-2.51959	0.272668	6.62E-23	3.57E-20	NA	NA	NA
ENSG0000	4475.605	-1.50506	0.159652	1.08E-22	5.65E-20	PARP14	54625	poly(ADP-ribose) polymerase family member 14
ENSG0000	3906.315	-1.28839	0.137983	3.99E-22	2.03E-19	SHFL	55337	shiftless antiviral inhibitor of ribosomal frameshifting
ENSG0000	296.7005	-2.466	0.274551	4.93E-22	2.44E-19	NA	NA	NA
ENSG0000	491.2852	-1.45486	0.158476	1.38E-21	6.66E-19	BISPR	1.05E+08	BST2 interferon stimulated positive regulator
ENSG0000	11159.53	-1.50874	0.167135	4.17E-21	1.96E-18	MX2	4600	MX dynamin like GTPase 2
ENSG0000	77.96428	-2.05541	0.227674	5.10E-21	2.34E-18	RMI2	116028	RecQ mediated genome instability 2
ENSG0000	728.4127	-2.28916	0.262279	5.72E-21	2.56E-18	ETV7	51513	ETS variant transcription factor 7
ENSG0000	7599.629	-1.55069	0.174559	1.33E-20	5.81E-18	UBE2L6	9246	ubiquitin conjugating enzyme E2 L6
ENSG0000	348.3622	-1.58403	0.178498	1.86E-20	7.91E-18	AGRN	375790	agrin
ENSG0000	9919.712	-1.67207	0.190194	2.21E-20	9.21E-18	PARP10	84875	poly(ADP-ribose) polymerase family member 10
ENSG0000	1327.612	-1.33531	0.150381	2.43E-20	9.88E-18	TRIM69	140691	tripartite motif containing 69
ENSG0000	767.9463	-1.67214	0.189264	2.74E-20	1.09E-17	GALM	130589	galactose mutarotase
ENSG0000	3209.116	-2.03963	0.236585	3.15E-20	1.23E-17	IFI6	2537	interferon alpha inducible protein 6
ENSG0000	281.0449	-2.51565	0.295955	3.26E-20	1.24E-17	SEPTIN4	5414	septin 4
ENSG0000	3424.126	-1.80639	0.208487	4.58E-20	1.71E-17	SAMD9L	219285	sterile alpha motif domain containing 9 like
ENSG0000	37.14632	-3.72043	0.427534	5.09E-20	1.86E-17	CCL2	6347	C-C motif chemokine ligand 2
ENSG0000	6902.609	-1.56215	0.179031	5.31E-20	1.91E-17	HELZ2	85441	helicase with zinc finger 2
ENSG0000	53.43383	-2.74349	0.321057	1.23E-19	4.34E-17	LINC0257	1.11E+08	long intergenic non-protein coding RNA 2574
ENSG0000	2257.413	-1.44329	0.16656	1.26E-19	4.34E-17	DTX3L	151636	deltex E3 ubiquitin ligase 3L
ENSG0000	641.4002	-1.19377	0.14019	7.74E-19	2.63E-16	NA	NA	NA
ENSG0000	680.6808	-1.12262	0.133774	2.49E-18	8.29E-16	PHF11	51131	PHD finger protein 11
ENSG0000	805.803	-1.44319	0.173722	2.70E-18	8.83E-16	TOR1B	27348	torsin family 1 member B
ENSG0000	3221.08	-1.77868	0.220399	7.74E-18	2.49E-15	GBP1	2633	guanylate binding protein 1
ENSG0000	950.2072	-1.21506	0.147848	9.56E-18	3.02E-15	CMTR1	23070	cap methyltransferase 1
ENSG0000	3529.096	-1.35276	0.167363	1.88E-17	5.85E-15	STAT2	6773	signal transducer and activator of transcription 2
ENSG0000	111056	-1.96236	0.252008	3.14E-17	9.59E-15	LY6E	4061	lymphocyte antigen 6 family member E
ENSG0000	90.02762	-2.6187	0.340927	4.06E-17	1.22E-14	CCDC194	1.11E+08	coiled-coil domain containing 194
ENSG0000	97.98308	-1.92806	0.245199	6.51E-17	1.92E-14	UBQLNL	143630	ubiquilin like
ENSG0000	62.05633	-2.92225	0.387704	6.88E-17	2.00E-14	KCTD14	65987	potassium channel tetramerization domain containing 14
ENSG0000	10791.01	-1.27827	0.162152	1.06E-16	3.03E-14	RNF213	57674	ring finger protein 213
ENSG0000	4285.495	-1.7629	0.22842	1.08E-16	3.04E-14	CCR1	1230	C-C motif chemokine receptor 1
ENSG0000	2840.939	-1.77237	0.22792	1.14E-16	3.18E-14	EIF2AK2	5610	eukaryotic translation initiation factor 2 alpha kinase 2
ENSG0000	91.84528	-3.08175	0.427959	1.33E-16	3.63E-14	TNNT1	7138	troponin T slow skeletal type
ENSG0000	55.57229	-2.34487	0.301969	1.37E-16	3.69E-14	ATF3	467	activating transcription factor 3
ENSG0000	1690.528	-1.60788	0.210966	3.72E-16	9.87E-14	H2BC21	8349	H2B clustered histone 21
ENSG0000	2987.742	-1.73771	0.230608	4.49E-16	1.18E-13	IFI35	3430	interferon induced protein 35
ENSG0000	59.56019	2.470551	0.325242	4.78E-16	1.23E-13	NA	NA	NA
ENSG0000	2412.835	-1.50498	0.197741	5.21E-16	1.31E-13	PLAC8	51316	placenta associated 8
ENSG0000	9305.694	-1.76957	0.234605	5.14E-16	1.31E-13	IFITM1	8519	interferon induced transmembrane protein 1
ENSG0000	1623.383	-1.92515	0.260425	7.32E-16	1.81E-13	BATF2	116071	basic leucine zipper ATF-like transcription factor 2
ENSG0000	3637.724	-1.49396	0.199057	9.77E-16	2.39E-13	ZBP1	81030	Z-DNA binding protein 1
ENSG0000	1471.404	-1.09181	0.143011	1.10E-15	2.64E-13	NUB1	51667	negative regulator of ubiquitin like proteins 1
ENSG0000	133.3387	3.047084	0.437806	1.76E-15	4.19E-13	ORM1	5004	orosomucoid 1
ENSG0000	387.1125	-1.44801	0.195002	3.01E-15	7.06E-13	CCR3	1232	C-C motif chemokine receptor 3
ENSG0000	151.1852	-1.62145	0.218973	3.10E-15	7.19E-13	NA	NA	NA
ENSG0000	309.6574	2.363475	0.33605	3.57E-15	8.18E-13	ALOX15B	247	arachidonate 15-lipoxygenase type B
ENSG0000	31.03743	-2.67083	0.374821	1.39E-14	3.15E-12	FBXO39	162517	F-box protein 39
ENSG0000	1951.244	-1.52498	0.215913	2.59E-14	5.79E-12	GBP4	115361	guanylate binding protein 4
ENSG0000	614.4926	-1.09256	0.151275	2.62E-14	5.79E-12	NA	NA	NA
ENSG0000	100.4973	-1.69421	0.238121	3.30E-14	7.20E-12	G0S2	50486	G0/G1 switch 2
ENSG0000	9004.001	-1.59984	0.232404	6.85E-14	1.48E-11	PML	5371	PML nuclear body scaffold
ENSG0000	3742.735	-1.30732	0.187495	8.19E-14	1.74E-11	PARP9	83666	poly(ADP-ribose) polymerase family member 9
ENSG0000	6154.794	-1.15847	0.165185	8.97E-14	1.89E-11	IFI16	3428	interferon gamma inducible protein 16
ENSG0000	509.1061	-1.4174	0.204385	9.65E-14	2.01E-11	NA	NA	NA
ENSG0000	45.70544	4.526593	0.733353	1.14E-13	2.35E-11	FN1	2335	fibronectin 1
ENSG0000	47.44401	-3.20213	0.495277	1.28E-13	2.60E-11	AXL	558	AXL receptor tyrosine kinase
ENSG0000	238.4372	-2.20433	0.340672	1.94E-13	3.91E-11	PLVAP	83483	plasmalemma vesicle associated protein
ENSG0000	74.1514	-2.87658	0.456017	2.23E-13	4.43E-11	GBP1P1	400759	guanylate binding protein 1 pseudogene 1
ENSG0000	35168.77	-1.24528	0.182152	2.46E-13	4.85E-11	SHISA5	51246	shisa family member 5
ENSG0000	1163.228	-1.33977	0.197181	2.87E-13	5.53E-11	FBXO6	26270	F-box protein 6
ENSG0000	74.56401	-1.81982	0.269643	2.86E-13	5.53E-11	H2BC18	440689	H2B clustered histone 18

ENSG0000	867.029	-2.1956	0.347405	3.37E-13	6.43E-11	IFI44	10561	interferon induced protein 44
ENSG0000	153.0579	-1.36606	0.200979	4.00E-13	7.56E-11	AIM2	9447	absent in melanoma 2
ENSG0000	84.26329	-2.10619	0.32274	4.14E-13	7.73E-11	LOC12490	1.25E+08	uncharacterized LOC124904411
ENSG0000	3404.564	-0.91142	0.132871	4.47E-13	8.27E-11	TRIM38	10475	tripartite motif containing 38
ENSG0000	255.0827	-1.54804	0.233394	6.09E-13	1.12E-10	CD274	29126	CD274 molecule
ENSG0000	962.7185	-1.03709	0.154453	8.09E-13	1.47E-10	TAP2	6891	transporte ATP binding cassette subfamily B member
ENSG0000	772.68	-1.45815	0.222941	1.14E-12	2.06E-10	HBD	3045	hemoglobin subunit delta
ENSG0000	55.26866	-2.54236	0.407776	1.46E-12	2.60E-10	NA	NA	NA
ENSG0000	163.4435	3.758582	0.64158	1.57E-12	2.77E-10	OLAH	55301	oleoyl-ACP hydrolase
ENSG0000	186.9178	-1.55997	0.241081	1.88E-12	3.27E-10	CACNA1A	773	calcium voltage-gated channel subunit alpha1 A
ENSG0000	2707.017	-1.29544	0.198852	1.89E-12	3.27E-10	APOL6	80830	apolipoprotein L6
ENSG0000	2181.106	-1.3028	0.200072	3.01E-12	5.16E-10	BST2	684	bone marrow stromal cell antigen 2
ENSG0000	4743.754	-1.41927	0.222706	3.31E-12	5.61E-10	TMEM140	55281	transmembrane protein 140
ENSG0000	308.279	1.862319	0.299828	3.80E-12	6.39E-10	KCNH3	23416	potassium voltage-gated channel subfamily H member 3
ENSG0000	7616.318	-0.96155	0.148411	5.06E-12	8.42E-10	HSH2D	84941	hematopoietic SH2 domain containing
ENSG0000	512.6948	-1.69354	0.275574	7.10E-12	1.17E-09	DDX60	55601	DExD/H-box helicase 60
ENSG0000	2500.642	-1.01511	0.158501	7.51E-12	1.23E-09	TRIM14	9830	tripartite motif containing 14
ENSG0000	306.7516	-1.77199	0.292661	8.66E-12	1.40E-09	HES4	57801	hes family bHLH transcription factor 4
ENSG0000	2910.624	-1.33509	0.21601	9.91E-12	1.59E-09	EPSTI1	94240	epithelial stromal interaction 1
ENSG0000	2893.099	-0.93348	0.146428	1.07E-11	1.70E-09	GTPBP2	54676	GTP binding protein 2
ENSG0000	7947.169	-1.057	0.172524	1.57E-11	2.48E-09	IL1RN	3557	interleukin 1 receptor antagonist
ENSG0000	169.3013	1.991161	0.336183	1.75E-11	2.75E-09	ZNF608	57507	zinc finger protein 608
ENSG0000	3492.599	-1.69497	0.285766	2.06E-11	3.20E-09	LGALS3BP	3959	galectin 3 binding protein
ENSG0000	323.2932	-1.19322	0.192453	2.23E-11	3.43E-09	TDRD7	23424	tudor domain containing 7
ENSG0000	25.31621	-2.33298	0.3832	2.37E-11	3.62E-09	NA	NA	NA
ENSG0000	655.6927	0.849473	0.13659	3.17E-11	4.79E-09	BMF	90427	Bcl2 modifying factor
ENSG0000	397.2946	-1.1052	0.179458	3.31E-11	4.97E-09	GRAMD1B	57476	GRAM domain containing 1B
ENSG0000	260.4509	1.398069	0.231686	3.90E-11	5.81E-09	VENTX	27287	VENT homeobox
ENSG0000	11161.38	-1.04388	0.170815	4.27E-11	6.31E-09	TAP1	6890	transporte ATP binding cassette subfamily B member
ENSG0000	130.0987	-1.495	0.251129	5.90E-11	8.64E-09	RUFY4	285180	RUN and FYVE domain containing 4
ENSG0000	11.32286	-3.81678	0.636953	6.24E-11	9.08E-09	KRT5	3852	keratin 5
ENSG0000	1645.15	-1.00855	0.16676	7.02E-11	1.01E-08	TRAFD1	10906	TRAF-type zinc finger domain containing 1
ENSG0000	593.9678	-1.11573	0.185469	7.32E-11	1.05E-08	FANCA	2175	FA complementation group A
ENSG0000	4340.836	-1.479	0.254683	7.99E-11	1.14E-08	HBM	3042	hemoglobin subunit mu
ENSG0000	5961.246	-1.04485	0.174851	9.40E-11	1.33E-08	SP110	3431	SP110 nuclear body protein
ENSG0000	2489.883	-1.58717	0.278819	1.15E-10	1.60E-08	CEACAM1	634	CEA cell adhesion molecule 1
ENSG0000	458.506	-1.56749	0.277259	1.76E-10	2.44E-08	H2BC5	3017	H2B clustered histone 5
ENSG0000	1920.732	-1.12602	0.194507	2.28E-10	3.14E-08	ZCCHC2	54877	zinc finger CCHC-type containing 2
ENSG0000	7380.586	-1.3446	0.236442	2.52E-10	3.44E-08	FFAR2	2867	free fatty acid receptor 2
ENSG0000	60209.64	-1.07657	0.185799	2.66E-10	3.61E-08	SELL	6402	selectin L
ENSG0000	92.01451	-1.93354	0.354638	3.17E-10	4.27E-08	NA	NA	NA
ENSG0000	1616.915	-0.72958	0.123476	3.27E-10	4.37E-08	MIA3	375056	MIA SH3 domain ER export factor 3
ENSG0000	1363.101	-1.28521	0.229093	4.44E-10	5.89E-08	TNFSF13B	10673	TNF superfamily member 13b
ENSG0000	11906.22	-0.97539	0.171381	5.22E-10	6.88E-08	LGALS9	3965	galectin 9
ENSG0000	156.85	-1.25083	0.221044	5.46E-10	7.15E-08	RPAP3-DT	1.05E+08	RPAP3 divergent transcript
ENSG0000	117.74	5.127099	1.201061	5.66E-10	7.35E-08	MAOA	4128	monoamine oxidase A
ENSG0000	2650.481	-0.79284	0.137717	6.13E-10	7.90E-08	SP100	6672	SP100 nuclear antigen
ENSG0000	29.65941	-3.3152	0.587106	7.92E-10	1.01E-07	NKAIN1	79570	sodium/potassium transporting ATPase interacting 1
ENSG0000	48.02376	1.42898	0.254132	8.31E-10	1.06E-07	CD1C	911	CD1c molecule
ENSG0000	14.61385	-3.24946	0.616612	9.45E-10	1.19E-07	SHD	56961	Src homology 2 domain containing transforming protein D
ENSG0000	248.071	-1.39508	0.255778	1.15E-09	1.44E-07	CCRL2	9034	C-C motif chemokine receptor like 2
ENSG0000	43.70053	-1.75342	0.322343	1.20E-09	1.49E-07	NA	NA	NA
ENSG0000	184.2752	-1.08295	0.194676	1.24E-09	1.54E-07	SSTR3	6753	somatostatin receptor 3
ENSG0000	4187.048	-1.109	0.201444	1.26E-09	1.54E-07	STAT1	6772	signal transducer and activator of transcription 1
ENSG0000	1405.151	-0.72791	0.128678	1.28E-09	1.56E-07	BTN3A3	10384	butyrophilin subfamily 3 member A3
ENSG0000	603.9303	-1.27277	0.234883	1.59E-09	1.93E-07	H3C10	8357	H3 clustered histone 10
ENSG0000	2422.478	-1.43501	0.269185	1.65E-09	1.99E-07	SCO2	9997	synthesis of cytochrome C oxidase 2
ENSG0000	90.76287	-1.86269	0.358679	2.18E-09	2.61E-07	ACHE	43	acetylcholinesterase (Cartwright blood group)
ENSG0000	13.54205	4.304418	0.862834	2.52E-09	2.99E-07	LPL	4023	lipoprotein lipase
ENSG0000	4175.272	-1.36493	0.259775	2.55E-09	3.01E-07	GBP5	115362	guanylate binding protein 5
ENSG0000	1221.96	-1.45005	0.280362	2.87E-09	3.37E-07	APOL1	8542	apolipoprotein L1
ENSG0000	932.9796	1.400368	0.270445	3.10E-09	3.62E-07	COL9A2	1298	collagen type IX alpha 2 chain
ENSG0000	39.52352	-1.74142	0.335844	3.89E-09	4.51E-07	MPZL2	10205	myelin protein zero like 2
ENSG0000	4166.154	-0.74306	0.136501	4.26E-09	4.91E-07	BTN3A1	11119	butyrophilin subfamily 3 member A1
ENSG0000	97.00099	-1.30871	0.248836	4.31E-09	4.94E-07	FRMD3	257019	FERM domain containing 3
ENSG0000	108.3322	-1.66762	0.328266	4.45E-09	5.06E-07	KLHDC7B-	1.05E+08	KLHDC7B divergent transcript
ENSG0000	109.8583	-1.93552	0.395985	4.53E-09	5.12E-07	P2RY6	5031	pyrimidinergic receptor P2Y6
ENSG0000	151.632	-1.70055	0.344196	5.98E-09	6.72E-07	ANKRD22	118932	ankyrin repeat domain 22
ENSG0000	4994.468	-1.20915	0.23484	6.28E-09	7.02E-07	NTNG2	84628	netrin G2
ENSG0000	113.1661	-1.65646	0.332402	6.36E-09	7.06E-07	CLEC5A	23601	C-type lectin domain containing 5A
ENSG0000	685.0099	-1.33526	0.262732	6.63E-09	7.32E-07	KLHDC8B	200942	kelch domain containing 8B
ENSG0000	7.233718	-5.35157	1.105907	6.71E-09	7.36E-07	NA	NA	NA
ENSG0000	20.36142	-2.18027	0.42923	7.06E-09	7.70E-07	TNFSF15	9966	TNF superfamily member 15
ENSG0000	812.7311	-1.18013	0.229306	7.20E-09	7.80E-07	SP140	11262	SP140 nuclear body protein
ENSG0000	2683.459	-0.84689	0.160766	8.11E-09	8.74E-07	GADD45B	4616	growth arrest and DNA damage inducible beta
ENSG0000	927.0457	-0.67003	0.12513	8.89E-09	9.52E-07	ANKFY1	51479	ankyrin repeat and FYVE domain containing 1
ENSG0000	8552.877	-0.99189	0.194441	1.22E-08	1.30E-06	RBCK1	10616	RANBP2-type and C3HC4-type zinc finger containing 1
ENSG0000	275.1495	-1.37156	0.276284	1.24E-08	1.31E-06	H4C8	8365	H4 clustered histone 8
ENSG0000	81.54099	2.229404	0.493318	1.25E-08	1.32E-06	RLN3	117579	relaxin 3
ENSG0000	1981.802	-0.84278	0.162329	1.28E-08	1.34E-06	ZC3HAV1	56829	zinc finger antiviral 1
ENSG0000	18.40286	-3.09519	0.689222	1.37E-08	1.43E-06	LIF	3976	LIF interleukin 6 family cytokine
ENSG0000	481.3747	0.850928	0.165163	1.56E-08	1.62E-06	MFGE8	4240	milk fat globule EGF and factor V/VIII domain containing
ENSG0000	109.3506	-1.67174	0.351879	1.78E-08	1.84E-06	NA	NA	NA
ENSG0000	1566.413	-0.74591	0.14373	1.86E-08	1.90E-06	DRAP1	10589	DR1 associated protein 1
ENSG0000	1374.897	-0.86936	0.171626	2.04E-08	2.05E-06	RUBCN	9711	rubicon autophagy regulator
ENSG0000	11.38043	-2.89451	0.610471	2.04E-08	2.05E-06	SCT	6343	secretin
ENSG0000	36.49125	-1.45618	0.289622	2.02E-08	2.05E-06	LOC10537	1.05E+08	uncharacterized LOC105376995
ENSG0000	29812.46	-1.29669	0.26844	2.21E-08	2.21E-06	ALAS2	212	5'-aminolevulinate synthase 2
ENSG0000	11270.2	-0.90789	0.181525	2.85E-08	2.83E-06	ADAR	103	adenosine deaminase RNA specific
ENSG0000	48.16605	-1.55627	0.321228	2.86E-08	2.83E-06	IL27	246778	interleukin 27
ENSG0000	1134.009	-1.10161	0.225191	3.07E-08	3.03E-06	LAP3	51056	leucine aminopeptidase 3
ENSG0000	359.2996	-1.09863	0.225565	3.46E-08	3.37E-06	ZNF496	84838	zinc finger protein 496
ENSG0000	2039.811	-1.07976	0.221403	3.46E-08	3.37E-06	APOL2	23780	apolipoprotein L2
ENSG0000	194.2735	-0.88643	0.177249	3.53E-08	3.42E-06	MATCAP1	653319	microtubule associated tyrosine carboxypeptidase 1
ENSG0000	50.7538	1.972827	0.433811	3.66E-08	3.53E-06	NRG1	3084	neuregulin 1
ENSG0000	585.2326	-1.18697	0.247729	3.88E-08	3.72E-06	DHRS9	10170	dehydrogenase/reductase 9

ENSG0000	51.48282	1.569697	0.32976	4.78E-08	4.56E-06	FER1L4	80307	fer-1 like family member 4 (pseudogene)
ENSG0000	741.4182	-0.93715	0.191743	5.07E-08	4.81E-06	SNCA	6622	synuclein alpha
ENSG0000	256.6279	-2.67166	0.648898	5.15E-08	4.87E-06	METTL7B	196410	methyltransferase like 7B
ENSG0000	2939.12	-1.04023	0.216455	5.18E-08	4.87E-06	TRANK1	9881	tetratricopeptide repeat and ankyrin repeat containing 1
ENSG0000	1018.727	-0.75736	0.153665	6.06E-08	5.64E-06	MOB3C	148932	MOB kinase activator 3C
ENSG0000	39.31223	-1.79233	0.388385	6.07E-08	5.64E-06	NA	NA	NA
ENSG0000	139.5755	-1.58947	0.350893	6.12E-08	5.67E-06	FCGR1CP	1E+08	Fc gamma pseudogene
ENSG0000	524.6059	-1.54594	0.345312	6.29E-08	5.79E-06	IFIT5	24138	interferon induced protein with tetratricopeptide repeats 5
ENSG0000	17.67205	-2.16621	0.473922	7.10E-08	6.50E-06	KY	339855	kyphoscoliosis peptidase
ENSG0000	68.43484	2.358216	0.579661	7.86E-08	7.16E-06	AMPH	273	amphiphysin
ENSG0000	14.11827	-2.2428	0.483438	8.16E-08	7.40E-06	NA	NA	NA
ENSG0000	1266.446	-1.1122	0.238008	8.32E-08	7.50E-06	DBN1	1627	drebrin 1
ENSG0000	2372.349	1.452278	0.330822	8.59E-08	7.72E-06	NA	NA	NA
ENSG0000	64.70389	-1.57531	0.349949	9.44E-08	8.44E-06	SAMD4A	23034	sterile alpha motif domain containing 4A
ENSG0000	472.63	-0.72705	0.148999	9.58E-08	8.52E-06	SCARB2	950	scavenger receptor class B member 2
ENSG0000	39.18042	-1.47808	0.314494	9.80E-08	8.67E-06	LGALS9B	284194	galectin 9B
ENSG0000	211.0837	-1.06659	0.225891	1.02E-07	8.98E-06	SMIM5	643008	small integral membrane protein 5
ENSG0000	380.3731	0.992531	0.211071	1.04E-07	9.11E-06	MARVELD	83742	MARVEL domain containing 1
ENSG0000	413.4907	1.307344	0.289906	1.04E-07	9.11E-06	ALDH2	217	aldehyde dehydrogenase 2 family member
ENSG0000	257.2538	1.322226	0.291412	1.06E-07	9.24E-06	LINC0228	1.03E+08	long intergenic non-protein coding RNA 2289
ENSG0000	5115.04	-1.16929	0.256336	1.13E-07	9.80E-06	CEACAM3	1084	CEA cell adhesion molecule 3
ENSG0000	306.2106	-1.24397	0.270717	1.14E-07	9.80E-06	GBP3	2635	guanylate binding protein 3
ENSG0000	1509.703	-0.67142	0.138987	1.17E-07	1.00E-05	PATL1	219988	PAT1 hom processing body mRNA decay factor
ENSG0000	1519.402	0.717491	0.149105	1.21E-07	1.03E-05	RNASE6	6039	ribonuclease A family member k6
ENSG0000	3785.418	-1.06773	0.231384	1.26E-07	1.07E-05	HSPA6	3310	heat shock protein family A (Hsp70) member 6
ENSG0000	103.4185	-1.25488	0.27456	1.43E-07	1.21E-05	KPNB1-DT	1.12E+08	KPNB1 divergent transcript
ENSG0000	1473.203	-1.3905	0.319117	1.43E-07	1.21E-05	ESPN	83715	espin
ENSG0000	11.11	-3.06252	0.707192	1.48E-07	1.24E-05	PPM1K-D	1.05E+08	PPM1K divergent transcript
ENSG0000	224.5006	0.852706	0.18063	1.53E-07	1.27E-05	RAB44	401258	RAB44 member RAS oncogene family
ENSG0000	55.70804	1.7781	0.41167	1.52E-07	1.27E-05	CTSG	1511	cathepsin G
ENSG0000	56.76591	-1.34171	0.296629	1.68E-07	1.38E-05	VSIG10L	147645	V-set and immunoglobulin domain containing 10 like
ENSG0000	242.8191	1.380861	0.314878	1.78E-07	1.46E-05	SDC1	6382	syndecan 1
ENSG0000	3279.759	-0.776	0.165287	1.81E-07	1.48E-05	GTPBP1	9567	GTP binding protein 1
ENSG0000	7415.787	-1.65265	0.408252	1.93E-07	1.57E-05	IFI27	3429	interferon alpha inducible protein 27
ENSG0000	14.51087	2.333418	0.534612	1.95E-07	1.58E-05	GFRA2	2675	GDNF family receptor alpha 2
ENSG0000	3108.731	-0.92405	0.201604	2.04E-07	1.64E-05	NAPA	8775	NSF attachment protein alpha
ENSG0000	4490.096	-0.79521	0.171173	2.22E-07	1.79E-05	TRIM56	81844	tripartite motif containing 56
ENSG0000	3860.097	-2.60013	0.705807	2.40E-07	1.92E-05	OTOF	9381	otoferlin
ENSG0000	993.7688	0.772116	0.167155	2.47E-07	1.96E-05	METTL7A	25840	methyltransferase like 7A
ENSG0000	605.9378	2.868071	0.808406	2.76E-07	2.19E-05	DAAM2	23500	dishevelled associated activator of morphogenesis 2
ENSG0000	606.6992	-0.80899	0.176645	2.88E-07	2.27E-05	DYNLT1	6993	dynein light chain Tctex-type 1
ENSG0000	119.2253	-1.1312	0.253666	2.95E-07	2.32E-05	XK	7504	X-linked K: Kell and VPS13A binding protein
ENSG0000	4377.044	-0.82408	0.181875	3.36E-07	2.63E-05	ISG20	3669	interferon stimulated exonuclease gene 20
ENSG0000	112.1749	1.119581	0.253303	3.52E-07	2.74E-05	CPM	1368	carboxypeptidase M
ENSG0000	14.31554	2.854724	0.64019	3.61E-07	2.80E-05	ENTPD2	954	ectonucleoside triphosphate diphosphohydrolase 2
ENSG0000	18.63202	-1.97117	0.466013	3.97E-07	3.07E-05	TMEM255	55026	transmembrane protein 255A
ENSG0000	49.63071	-2.02438	0.526915	4.36E-07	3.36E-05	TMEM92	162461	transmembrane protein 92
ENSG0000	248.6494	1.432556	0.349133	4.57E-07	3.49E-05	GPER1	2852	G protein-coupled estrogen receptor 1
ENSG0000	430.0314	-0.68899	0.150646	4.57E-07	3.49E-05	ACOT9	23597	acyl-CoA thioesterase 9
ENSG0000	5.207146	-4.4108	1.231018	4.79E-07	3.64E-05	NA	NA	NA
ENSG0000	737.3999	-0.84537	0.190699	5.10E-07	3.86E-05	TMEM229	161145	transmembrane protein 229B
ENSG0000	36.67268	-1.49397	0.352349	5.19E-07	3.91E-05	PCYT1B	9468	phosphate choline
ENSG0000	948.8028	-0.72089	0.160248	5.26E-07	3.95E-05	PLAAT4	5920	phospholipase A and acyltransferase 4
ENSG0000	79.88154	1.044142	0.237343	5.28E-07	3.95E-05	ARPIN	348110	actin related protein 2/3 complex inhibitor
ENSG0000	19.18413	-1.90548	0.453371	5.43E-07	4.04E-05	NA	NA	NA
ENSG0000	43.93706	-1.16952	0.267764	5.58E-07	4.14E-05	RIGI	23586	RNA sensor RIG-I
ENSG0000	1300.848	-0.90543	0.208342	5.65E-07	4.17E-05	SH2D2A	9047	SH2 domain containing 2A
ENSG0000	8.966968	-2.61792	0.624327	5.77E-07	4.25E-05	TMEM123	1.02E+08	TMEM123 divergent transcript
ENSG0000	269.7782	-0.75307	0.168731	5.91E-07	4.33E-05	MT1F	4494	metallothionein 1F
ENSG0000	3732.062	-0.86512	0.197733	6.06E-07	4.42E-05	ZNFX1	57169	zinc finger NFX1-type containing 1
ENSG0000	1147.147	-0.87444	0.198761	6.26E-07	4.55E-05	CNP	1267	2' 3'-cyclic nucleotide 3' phosphodiesterase
ENSG0000	107.4649	-1.04286	0.241365	6.50E-07	4.70E-05	NA	NA	NA
ENSG0000	278.6091	1.100067	0.258952	6.51E-07	4.70E-05	OLIG1	116448	oligodendrocyte transcription factor 1
ENSG0000	68.22213	-1.3379	0.319931	6.90E-07	4.96E-05	NA	NA	NA
ENSG0000	324.7299	1.639348	0.421896	6.96E-07	4.96E-05	GRB10	2887	growth factor receptor bound protein 10
ENSG0000	3725.727	-0.60758	0.134606	6.96E-07	4.96E-05	SNX20	124460	sorting nexin 20
ENSG0000	18.24168	-3.0585	0.909814	7.09E-07	5.04E-05	HDC	3067	histidine decarboxylase
ENSG0000	326.1102	-1.03594	0.245476	7.13E-07	5.05E-05	ACTA2	59	actin alpha smooth muscle
ENSG0000	23.5177	1.56896	0.372528	7.80E-07	5.49E-05	EBI3	10148	Epstein-Barr virus induced 3
ENSG0000	508.4882	0.770748	0.175728	7.87E-07	5.50E-05	VIPR1	7433	vasoactive intestinal peptide receptor 1
ENSG0000	1205.198	-0.99666	0.234871	7.85E-07	5.50E-05	AHSP	51327	alpha hemoglobin stabilizing protein
ENSG0000	651.5806	1.205566	0.293797	8.07E-07	5.62E-05	CD163	9332	CD163 molecule
ENSG0000	374.9195	-0.71168	0.160755	8.37E-07	5.79E-05	ERAP2	64167	endoplasmic reticulum aminopeptidase 2
ENSG0000	237.5369	1.0053	0.236476	8.37E-07	5.79E-05	CASS4	57091	Cas scaffold protein family member 4
ENSG0000	4911.909	-0.85871	0.199918	8.70E-07	5.99E-05	TRIM21	6737	tripartite motif containing 21
ENSG0000	516.8166	1.22407	0.30338	9.46E-07	6.49E-05	BAIAP3	8938	BAI1 associated protein 3
ENSG0000	724.0412	-0.80777	0.187184	9.65E-07	6.60E-05	ANKRD9	122416	ankyrin repeat domain 9
ENSG0000	90.4093	-1.32554	0.327227	9.92E-07	6.76E-05	GATA2	2624	GATA binding protein 2
ENSG0000	5605.858	-2.09012	0.583208	1.01E-06	6.86E-05	SIGLEC1	6614	sialic acid binding Ig like lectin 1
ENSG0000	208.9957	-1.15856	0.281194	1.02E-06	6.86E-05	NA	NA	NA
ENSG0000	572.9013	-0.68504	0.156278	1.04E-06	6.99E-05	MICB	4277	MHC class I polypeptide-related sequence B
ENSG0000	933.0856	0.726747	0.167498	1.05E-06	7.07E-05	NUDT3	11165	nudix hydrolase 3
ENSG0000	45.0248	1.484509	0.366907	1.06E-06	7.09E-05	PID1	55022	phosphotyrosine interaction domain containing 1
ENSG0000	3241.506	-0.5877	0.131933	1.07E-06	7.15E-05	BTN3A2	11118	butyrophilin subfamily 3 member A2
ENSG0000	2035.632	-0.99347	0.239369	1.11E-06	7.39E-05	GZMB	3002	granzyme B
ENSG0000	1662.064	-1.18035	0.294825	1.16E-06	7.68E-05	DDX60L	91351	DExD/H-box 60 like
ENSG0000	1833.407	1.526405	0.4167	1.31E-06	8.62E-05	IGLV3-10	28803	immunoglobulin lambda variable 3-10
ENSG0000	3531.676	-0.762	0.17809	1.32E-06	8.70E-05	KIAA0319	79932	KIAA0319 like
ENSG0000	8.225478	3.79152	0.955073	1.39E-06	9.09E-05	MPV17L	255027	MPV17 mitochondrial inner membrane protein like
ENSG0000	157.6368	-1.32674	0.33879	1.40E-06	9.13E-05	MAP1A	4130	microtubule associated protein 1A
ENSG0000	28.78812	-2.08085	0.562931	1.44E-06	9.31E-05	NA	NA	NA
ENSG0000	84.2886	-1.28954	0.32341	1.44E-06	9.31E-05	H2BC6	8344	H2B clustered histone 6
ENSG0000	3278.896	-0.71793	0.168198	1.47E-06	9.46E-05	PSMB9	5698	proteasome 20S subunit beta 9
ENSG0000	262.1234	-1.14706	0.28794	1.49E-06	9.60E-05	EFCAB2	84288	EF-hand calcium binding domain 2
ENSG0000	97.16281	1.807383	0.490514	1.57E-06	1.00E-04	DEFA4	1669	defensin alpha 4
ENSG0000	267.9388	-1.44789	0.383097	1.60E-06	1.02E-04	NA	NA	NA

ENSG0000	136.0225	-1.06411	0.261659	1.61E-06	1.03E-04	NA	NA	NA
ENSG0000	88.53363	0.740169	0.172763	1.64E-06	1.04E-04	ZCCHC14	23174	zinc finger CCHC-type containing 14
ENSG0000	3920.921	-0.63823	0.148493	1.65E-06	1.04E-04	CX3CR1	1524	C-X3-C motif chemokine receptor 1
ENSG0000	193.0723	-0.93126	0.22568	1.69E-06	1.06E-04	HSPA7	3311	heat shock protein family A (Hsp70) member 7 (pseudogene)
ENSG0000	166.0503	2.249496	0.666089	1.71E-06	1.07E-04	ITGB4	3691	integrin subunit beta 4
ENSG0000	5.881677	5.17792	1.471191	1.73E-06	1.08E-04	CFAP77	389799	cilia and flagella associated protein 77
ENSG0000	332.5428	-0.73415	0.173316	1.72E-06	1.08E-04	DBF4B	80174	DBF4 zinc finger B
ENSG0000	1233.112	-0.74989	0.17858	1.83E-06	1.14E-04	IL1B	3553	interleukin 1 beta
ENSG0000	45.09789	-1.40761	0.357638	1.86E-06	1.15E-04	LINC00487	400941	long intergenic non-protein coding RNA 487
ENSG0000	15.55595	2.169473	0.56405	1.95E-06	1.20E-04	OLFML2A	169611	olfactomedin like 2A
ENSG0000	573.5844	0.680182	0.16114	2.13E-06	1.31E-04	EIF3L	51386	eukaryotic translation initiation factor 3 subunit L
ENSG0000	49.34972	-1.27443	0.324653	2.17E-06	1.33E-04	RPL17P28	1E+08	ribosomal protein L17 pseudogene 28
ENSG0000	52960.35	1.328004	0.361833	2.23E-06	1.36E-04	IGHG1	3500	immunoglobulin heavy constant gamma 1 (G1m marker)
ENSG0000	2890.262	-1.2158	0.320506	2.29E-06	1.39E-04	HSPB1	3315	heat shock protein family B (small) member 1
ENSG0000	657.5058	-0.5263	0.12224	2.35E-06	1.43E-04	MR1	3140	major hist class I-related
ENSG0000	481.6427	1.515697	0.433779	2.43E-06	1.47E-04	ELANE	1991	elastase neutrophil expressed
ENSG0000	39.52523	-1.12157	0.278343	2.48E-06	1.50E-04	RSPH9	221421	radial spoke head component 9
ENSG0000	68.09745	-1.42714	0.383808	2.94E-06	1.77E-04	ZNF496-D	1.08E+08	ZNF496 divergent transcript
ENSG0000	197.9945	-1.13796	0.296456	2.99E-06	1.79E-04	NA	NA	NA
ENSG0000	101.8547	0.806461	0.197682	3.15E-06	1.88E-04	ASB13	79754	ankyrin repeat and SOCS box containing 13
ENSG0000	111.7451	0.963688	0.242418	3.37E-06	2.00E-04	KBTBD11	9920	kelch repeat and BTB domain containing 11
ENSG0000	1216.492	0.611051	0.148193	3.53E-06	2.09E-04	CRTAP	10491	cartilage associated protein
ENSG0000	5.671221	5.110505	1.377631	3.60E-06	2.13E-04	SCN5A	6331	sodium voltage-gated channel alpha subunit 5
ENSG0000	2462.984	-1.01402	0.265545	3.71E-06	2.19E-04	TNFSF10	8743	TNF superfamily member 10
ENSG0000	1319.29	-0.88437	0.225768	3.86E-06	2.27E-04	CDKN1A	1026	cyclin dependent kinase inhibitor 1A
ENSG0000	616.117	-0.72322	0.178979	4.11E-06	2.41E-04	IRF9	10379	interferon regulatory factor 9
ENSG0000	448.334	-1.074	0.284112	4.17E-06	2.43E-04	NA	NA	NA
ENSG0000	65.58223	-1.52474	0.427463	4.22E-06	2.45E-04	LILRA4	23547	leukocyte immunoglobulin like receptor A4
ENSG0000	1656.152	-0.9864	0.258635	4.25E-06	2.47E-04	KLHDC7B	113730	kelch domain containing 7B
ENSG0000	8.194232	4.924196	1.338876	4.49E-06	2.59E-04	SHROOM2	357	shroom family member 2
ENSG0000	787.6254	-1.07377	0.288581	4.66E-06	2.68E-04	SOCS1	8651	suppressor of cytokine signaling 1
ENSG0000	349.6543	-0.71157	0.177544	4.68E-06	2.69E-04	TENT5A	55603	terminal nucleotidyltransferase 5A
ENSG0000	50.95613	-1.37795	0.380335	5.17E-06	2.96E-04	LOC10537	1.05E+08	uncharacterized LOC105377156
ENSG0000	3910.055	-1.04271	0.281971	5.32E-06	3.01E-04	TRIM58	25893	tripartite motif containing 58
ENSG0000	21.14007	-1.52884	0.407431	5.29E-06	3.01E-04	LINC0221	1.03E+08	long intergenic non-protein coding RNA 2218
ENSG0000	426.9684	0.963327	0.253685	5.31E-06	3.01E-04	TXNDC5	81567	thioredoxin domain containing 5
ENSG0000	428.9548	-1.08212	0.292105	5.48E-06	3.10E-04	LAG3	3902	lymphocyte activating 3
ENSG0000	5519.396	-0.92335	0.243912	5.51E-06	3.10E-04	ODF3B	440836	outer dense fiber of sperm tails 3B
ENSG0000	6027.103	-0.9345	0.246726	5.61E-06	3.15E-04	KCNJ15	3772	potassium inwardly rectifying channel subfamily J member 15
ENSG0000	246.7655	-1.15956	0.319707	5.84E-06	3.27E-04	PHACTR1	221692	phosphatase and actin regulator 1
ENSG0000	10.36238	2.728126	0.804975	5.88E-06	3.28E-04	NA	NA	NA
ENSG0000	100.8113	-1.20167	0.331005	5.99E-06	3.33E-04	NA	NA	NA
ENSG0000	21.97946	-1.8494	0.541894	6.04E-06	3.35E-04	NA	NA	NA
ENSG0000	75.07759	-1.09283	0.288218	6.14E-06	3.40E-04	MYBPH	4608	myosin binding protein H
ENSG0000	95.12675	-0.99051	0.261372	6.24E-06	3.44E-04	NA	NA	NA
ENSG0000	42.89485	-1.7236	0.50958	6.67E-06	3.67E-04	GBP6	163351	guanylate binding protein family member 6
ENSG0000	964.1931	0.845375	0.222845	7.07E-06	3.88E-04	RBM3	5935	RNA binding motif protein 3
ENSG0000	264.0036	-0.72125	0.185237	7.43E-06	4.06E-04	NOD1	10392	nucleotide binding oligomerization domain containing 1
ENSG0000	253.1158	-0.87934	0.233399	7.85E-06	4.28E-04	NA	NA	NA
ENSG0000	112.9965	0.690491	0.175645	7.88E-06	4.28E-04	SUCLA2	8803	succinate-CoA ligase ADP-forming subunit beta
ENSG0000	39.62312	-1.41843	0.40153	7.94E-06	4.29E-04	CDHR5	53841	cadherin related family member 5
ENSG0000	65.69443	-1.07811	0.290851	7.92E-06	4.29E-04	ABTB2	25841	ankyrin repeat and BTB domain containing 2
ENSG0000	1255.726	0.853569	0.227694	8.12E-06	4.37E-04	PER1	5187	period circadian regulator 1
ENSG0000	331.7572	-0.77102	0.201978	8.25E-06	4.43E-04	STX17	55014	syntaxin 17
ENSG0000	455.6482	-1.00278	0.276719	8.33E-06	4.46E-04	KCND1	3750	potassium voltage-gated channel subfamily D member 1
ENSG0000	167.1058	-1.27548	0.366793	8.37E-06	4.47E-04	MIR3945H	731424	MIR3945 host gene
ENSG0000	116.7557	1.011871	0.275099	8.53E-06	4.54E-04	INSR	3643	insulin receptor
ENSG0000	59.27167	1.619402	0.505674	8.69E-06	4.61E-04	SPATA6	54558	spermatogenesis associated 6
ENSG0000	66.34957	-0.99036	0.266065	8.96E-06	4.74E-04	NA	NA	NA
ENSG0000	235.1154	-1.29226	0.387291	9.02E-06	4.76E-04	RNU7-40P	1E+08	RNA U7 small nuclear 40 pseudogene
ENSG0000	239.3098	1.5019	0.484737	9.51E-06	5.01E-04	AZU1	566	azurocidin 1
ENSG0000	18.93288	-1.47646	0.411175	9.61E-06	5.03E-04	BAMBI	25805	BMP and activin membrane bound inhibitor
ENSG0000	989.7444	0.526164	0.131905	9.60E-06	5.03E-04	MHENCR	1.01E+08	melanoma highly expressed competing endogenous lncRNA for miR-425 and miR-489
ENSG0000	13.18084	-2.33071	0.728856	9.80E-06	5.11E-04	GRIN3A	116443	glutamate ionotropic receptor NMDA type subunit 3A
ENSG0000	274.4364	-0.87447	0.236863	1.02E-05	5.30E-04	RAB24	53917	RAB24 member RAS oncogene family
ENSG0000	2593.375	-0.68991	0.180449	1.06E-05	5.52E-04	FCGR3A	2214	Fc gamma receptor IIIa
ENSG0000	4.835519	5.00445	1.830275	1.09E-05	5.63E-04	ANGPT1	284	angiopoietin 1
ENSG0000	24026.83	-0.80062	0.216408	1.10E-05	5.67E-04	FCGR3B	2215	Fc gamma receptor IIIb
ENSG0000	1496.165	-0.89468	0.247121	1.11E-05	5.70E-04	H2AC6	8334	H2A clustered histone 6
ENSG0000	19971.99	-0.84381	0.230525	1.12E-05	5.73E-04	LITAF	9516	lipopolysaccharide induced TNF factor
ENSG0000	172.3086	1.140893	0.329819	1.14E-05	5.86E-04	ECHDC3	79746	enoyl-CoA hydratase domain containing 3
ENSG0000	490.2793	0.969006	0.271505	1.16E-05	5.90E-04	NT5DC2	64943	5'-nucleotidase domain containing 2
ENSG0000	58.18015	-0.9895	0.271697	1.17E-05	5.95E-04	RPS10P1	394255	ribosomal protein S10 pseudogene 1
ENSG0000	95.95776	0.960869	0.264296	1.18E-05	5.99E-04	SOX4	6659	SRY-box transcription factor 4
ENSG0000	621.5924	-0.5491	0.140761	1.20E-05	6.06E-04	CNOT4	4850	CCR4-NOT transcription complex subunit 4
ENSG0000	54.26519	-0.91827	0.247839	1.22E-05	6.15E-04	H2AC8	3012	H2A clustered histone 8
ENSG0000	16.6883	2.160887	0.659011	1.26E-05	6.35E-04	DES	1674	desmin
ENSG0000	220.6703	0.718043	0.191371	1.33E-05	6.66E-04	DAPK1	1612	death associated protein kinase 1
ENSG0000	168.183	-0.83759	0.229027	1.34E-05	6.69E-04	KIR2DL1	3802	killer cell i two Ig domains and long cytoplasmic tail 1
ENSG0000	9.260093	-2.91737	1.030167	1.38E-05	6.87E-04	KCTD19	146212	potassium channel tetramerization domain containing 19
ENSG0000	5.3203	2.886747	0.814172	1.39E-05	6.90E-04	NBEA	26960	neurobeachin
ENSG0000	13.41102	1.962655	0.588573	1.43E-05	7.09E-04	LINC0181	1.03E+08	long intergenic non-protein coding RNA 1819
ENSG0000	115.9605	-0.87802	0.242869	1.56E-05	7.73E-04	PTGDS	5730	prostaglandin D2 synthase
ENSG0000	135.109	1.313508	0.405878	1.63E-05	8.03E-04	TMEM119	338773	transmembrane protein 119
ENSG0000	154.8184	-0.8921	0.250642	1.66E-05	8.17E-04	KIAA1958	158405	KIAA1958
ENSG0000	87.93116	-1.12692	0.330295	1.69E-05	8.30E-04	NA	NA	NA
ENSG0000	34.7766	1.916494	0.708499	1.77E-05	8.65E-04	GPR34	2857	G protein-coupled receptor 34
ENSG0000	271.4185	-0.78036	0.216749	1.80E-05	8.77E-04	JPX	554203	JPX transc XIST activator
ENSG0000	14751.76	0.48846	0.126653	1.83E-05	8.94E-04	PABPC1	26986	poly(A) binding protein cytoplasmic 1
ENSG0000	2326.84	0.685564	0.1874	1.86E-05	9.02E-04	AHNAK	79026	AHNAK nucleoprotein
ENSG0000	19.6005	1.940204	0.657645	1.93E-05	9.35E-04	ADGRD1	283383	adhesion G protein-coupled receptor D1
ENSG0000	4434.1	-0.674	0.18486	1.96E-05	9.45E-04	UNC93B1	81622	unc-93 ho TLR signaling regulator
ENSG0000	7.013018	3.020901	0.97217	1.97E-05	9.47E-04	DPEP1	1800	dipeptidase 1
ENSG0000	165.977	-0.58312	0.154859	1.99E-05	9.57E-04	SLC9A9	285195	solute carrier family 9 member A9
ENSG0000	354.0778	-0.55098	0.145826	2.01E-05	9.65E-04	ADPRS	54936	ADP-ribosylserine hydrolase
ENSG0000	8.128045	-2.36567	0.778528	2.02E-05	9.65E-04	NA	NA	NA

ENSG0000	176.7196	-1.19641	0.374244	2.08E-05	9.91E-04	NA	NA	NA
ENSG0000	5244.465	1.056734	0.32125	2.08E-05	9.91E-04	IGLV3-19	28797	immunoglobulin lambda variable 3-19
ENSG0000	27.86488	1.262986	0.36308	2.10E-05	9.96E-04	UTS2R	2837	urotensin 2 receptor
ENSG0000	81.15282	1.252216	0.390066	2.19E-05	0.001037	SOCS5	9655	suppressor of cytokine signaling 5
ENSG0000	3696.421	-0.54387	0.145225	2.24E-05	0.001056	NLRC5	84166	NLR family CARD domain containing 5
ENSG0000	389.4418	-0.73131	0.20459	2.27E-05	0.00107	PPM1K	152926	protein ph Mg2+/Mn2+ dependent 1K
ENSG0000	11.47487	-1.77783	0.548788	2.28E-05	0.001071	NA	NA	NA
ENSG0000	105.6531	-0.93262	0.271025	2.29E-05	0.001075	PNPT1	87178	polyribonucleotide nucleotidyltransferase 1
ENSG0000	217.537	0.827514	0.23281	2.34E-05	0.001091	NA	NA	NA
ENSG0000	1481.433	-0.73985	0.208594	2.36E-05	0.001095	CSRNP1	64651	cysteine and serine rich nuclear protein 1
ENSG0000	861.7103	-0.7303	0.205573	2.36E-05	0.001095	RILP	83547	Rab interacting lysosomal protein
ENSG0000	227.2833	1.180005	0.363545	2.35E-05	0.001095	IGLV1-36	28826	immunoglobulin lambda variable 1-36
ENSG0000	77.40762	1.139377	0.34796	2.47E-05	0.001142	PDK4	5166	pyruvate dehydrogenase kinase 4
ENSG0000	273.3926	-0.58166	0.157595	2.48E-05	0.001143	MAD2L1BI	9587	MAD2L1 binding protein
ENSG0000	52.33301	1.17632	0.351335	2.53E-05	0.001163	UTF1	8433	undifferentiated embryonic cell transcription factor 1
ENSG0000	2797.668	-0.50999	0.136953	2.56E-05	0.001176	IRF2	3660	interferon regulatory factor 2
ENSG0000	5526.27	-0.99152	0.304536	2.68E-05	0.001226	TRIM22	10346	tripartite motif containing 22
ENSG0000	986.1307	-0.9411	0.283601	2.71E-05	0.00124	NA	NA	NA
ENSG0000	2045.394	0.502905	0.134138	2.78E-05	0.001265	EIF3F	8665	eukaryotic translation initiation factor 3 subunit F
ENSG0000	62.53755	-0.94166	0.275471	2.89E-05	0.001314	NA	NA	NA
ENSG0000	735.5833	-0.90159	0.269638	2.91E-05	0.00132	ESAM	90952	endothelial cell adhesion molecule
ENSG0000	11.2805	-2.12748	0.735816	3.01E-05	0.001363	NA	NA	NA
ENSG0000	7.36277	-2.48258	0.815189	3.02E-05	0.001363	LOC12490	1.25E+08	uncharacterized LOC124900731
ENSG0000	318.5862	-0.59209	0.162657	3.04E-05	0.00137	UBASH3B	84959	ubiquitin associated and SH3 domain containing B
ENSG0000	967.2244	-0.98714	0.304178	3.08E-05	0.001379	FCGR1A	2209	Fc gamma receptor Ia
ENSG0000	93.98575	-1.14771	0.359075	3.07E-05	0.001379	DUSP13	51207	dual specificity phosphatase 13
ENSG0000	69.69293	-0.78147	0.222822	3.09E-05	0.001382	TKTL1	8277	transketolase like 1
ENSG0000	313.8624	-0.66626	0.187664	3.11E-05	0.001384	LYRM1	57149	LYR motif containing 1
ENSG0000	5517.89	-0.68156	0.194157	3.22E-05	0.001431	CD300A	11314	CD300a molecule
ENSG0000	41.31924	-2.5618	1.072689	3.25E-05	0.001443	PDCD1LG2	80380	programmed cell death 1 ligand 2
ENSG0000	441.3951	0.969156	0.297744	3.28E-05	0.001447	IGKV2-30	28919	immunoglobulin kappa variable 2-30
ENSG0000	6.453079	2.79806	0.833839	3.28E-05	0.001447	TMEM108	66000	transmembrane protein 108
ENSG0000	8.476934	-2.96623	0.961135	3.32E-05	0.00146	LINC02068	1.05E+08	long intergenic non-protein coding RNA 2068
ENSG0000	1524.919	-0.51936	0.141674	3.35E-05	0.001473	DGLUCY	80017	D-glutamate cyclase
ENSG0000	16.78307	-1.52215	0.489894	3.44E-05	0.001508	NA	NA	NA
ENSG0000	73.88474	-1.11275	0.34956	3.49E-05	0.001528	NA	NA	NA
ENSG0000	38.97934	-1.30553	0.417387	3.51E-05	0.001529	NA	NA	NA
ENSG0000	735.8333	0.818796	0.2435	3.54E-05	0.001535	CAMK1D	57118	calcium/calmodulin dependent protein kinase ID
ENSG0000	118.0833	1.281414	0.423821	3.53E-05	0.001535	RTN1	6252	reticulon 1
ENSG0000	104.4928	-0.93081	0.282907	3.74E-05	0.001619	PARP11	57097	poly(ADP-ribose) polymerase family member 11
ENSG0000	236.801	0.9579	0.296073	3.75E-05	0.001621	ABCB9	23457	ATP binding cassette subfamily B member 9
ENSG0000	1694.489	-0.59234	0.166825	3.78E-05	0.001628	AKIRIN2	55122	akirin 2
ENSG0000	356.2893	-0.59574	0.167035	3.82E-05	0.001645	LYSMD2	256586	LysM domain containing 2
ENSG0000	70.02796	0.884189	0.26234	3.84E-05	0.001646	NFIA	4774	nuclear factor I A
ENSG0000	296.0825	-0.84844	0.255664	3.85E-05	0.001646	GAS6	2621	growth arrest specific 6
ENSG0000	133.3964	0.91312	0.277526	3.86E-05	0.001648	GLCCI1	113263	glucocorticoid induced 1
ENSG0000	44.14892	-1.15642	0.363444	3.87E-05	0.001648	CIB2	10518	calcium and integrin binding family member 2
ENSG0000	162.2736	0.841708	0.25153	3.88E-05	0.00165	MS4A14	84689	membrane spanning 4-domains A14
ENSG0000	125.4307	0.68611	0.196033	3.92E-05	0.001664	FAAH	2166	fatty acid amide hydrolase
ENSG0000	6036.933	0.583327	0.16409	3.99E-05	0.001688	RPL3	6122	ribosomal protein L3
ENSG0000	1331.503	-0.83216	0.253629	4.01E-05	0.001693	HCAR2	338442	hydroxycarboxylic acid receptor 2
ENSG0000	100.7132	-0.91369	0.278825	4.07E-05	0.001715	NA	NA	NA
ENSG0000	1275.122	0.576505	0.162992	4.31E-05	0.00181	QARS1	5859	glutaminyl-tRNA synthetase 1
ENSG0000	4059.636	-0.51026	0.14155	4.33E-05	0.001812	PLEK	5341	pleckstrin
ENSG0000	21.20595	1.748839	0.611776	4.33E-05	0.001812	HTRA1	5654	HtrA serine peptidase 1
ENSG0000	58.32347	0.83632	0.247789	4.46E-05	0.001858	LAMC1	3915	laminin subunit gamma 1
ENSG0000	41.4153	-1.07397	0.332045	4.46E-05	0.001858	NA	NA	NA
ENSG0000	114.7845	-0.86427	0.262942	4.48E-05	0.001859	RBM43	375287	RNA binding motif protein 43
ENSG0000	83.8965	-0.91064	0.276755	4.52E-05	0.001873	IL12RB2	3595	interleukin 12 receptor subunit beta 2
ENSG0000	88.94435	1.106898	0.359311	4.56E-05	0.001886	ALDH1A1	216	aldehyde dehydrogenase 1 family member A1
ENSG0000	139.3047	0.769716	0.228575	4.63E-05	0.001906	ARHGAP24	83478	Rho GTPase activating protein 24
ENSG0000	184.2477	-1.0307	0.333644	4.63E-05	0.001906	OSM	5008	oncostatin M
ENSG0000	105.6813	-0.99871	0.316266	4.66E-05	0.001913	CTSL	1514	cathepsin L
ENSG0000	3039.496	-0.60022	0.171668	4.70E-05	0.001927	UBA7	7318	ubiquitin like modifier activating enzyme 7
ENSG0000	13742.26	-0.61466	0.175241	4.87E-05	0.001992	UBALD2	283991	UBA like domain containing 2
ENSG0000	153.7996	1.0637	0.345951	4.92E-05	0.002006	PLXNA2	5362	plexin A2
ENSG0000	900.9588	-0.68644	0.203914	4.97E-05	0.002022	ASPRV1	151516	aspartic peptidase retroviral like 1
ENSG0000	722.9336	-0.49005	0.136243	4.98E-05	0.002024	FLJ32255	643977	uncharacterized LOC643977
ENSG0000	22387.53	0.511438	0.14364	5.14E-05	0.002084	RPL10	6134	ribosomal protein L10
ENSG0000	6.960078	-2.48917	0.773323	5.17E-05	0.002089	LINC02577	1.11E+08	long intergenic non-protein coding RNA 2577
ENSG0000	682.6674	-0.87528	0.273387	5.18E-05	0.002089	KRT1	3848	keratin 1
ENSG0000	177.3827	-0.89665	0.279519	5.26E-05	0.002117	TCN1	6947	transcobalamin 1
ENSG0000	1160.285	-0.83481	0.259638	5.33E-05	0.00214	HBQ1	3049	hemoglobin subunit theta 1
ENSG0000	628.7722	-0.60221	0.174478	5.41E-05	0.002167	RHOC	389	ras homolog family member C
ENSG0000	195.4266	-0.70013	0.207572	5.47E-05	0.002181	BMPR2	659	bone morphogenetic protein receptor type 2
ENSG0000	10446.39	-0.69777	0.206923	5.46E-05	0.002181	FYB1	2533	FYN binding protein 1
ENSG0000	8.570323	-2.29499	0.844415	5.50E-05	0.002189	MS4A2	2206	membrane spanning 4-domains A2
ENSG0000	8.250734	3.035648	1.177972	5.61E-05	0.002231	MYO1B	4430	myosin IB
ENSG0000	342.7375	-0.94351	0.301742	5.78E-05	0.002291	NA	NA	NA
ENSG0000	155.7798	1.011408	0.329733	5.83E-05	0.002307	MYO7A	4647	myosin VIIA
ENSG0000	3242.828	-0.98095	0.327255	5.90E-05	0.002328	CHI3L1	1116	chitinase 3 like 1
ENSG0000	16283.32	-0.78815	0.24508	5.91E-05	0.002328	TYMP	1890	thymidine phosphorylase
ENSG0000	61.46942	-0.94131	0.295206	5.98E-05	0.002346	H2BC9	8345	H2B clustered histone 9
ENSG0000	21.89464	-1.42538	0.479804	5.98E-05	0.002346	TRIM6	117854	tripartite motif containing 6
ENSG0000	118.7261	-1.4451	0.573497	6.09E-05	0.002383	SPDYC	387778	speedy/RINGO cell cycle regulator family member C
ENSG0000	49.7109	1.289377	0.448819	6.14E-05	0.002398	OSBPL1A	114876	oxysterol binding protein like 1A
ENSG0000	50.34456	-1.02431	0.32495	6.28E-05	0.002446	SLC1A7	6512	solute carrier family 1 member 7
ENSG0000	9220.245	-0.90133	0.293054	6.29E-05	0.002447	SIGLEC5	8778	sialic acid binding Ig like lectin 5
ENSG0000	76.43057	1.136495	0.385855	6.46E-05	0.002505	NA	NA	NA
ENSG0000	47.7457	-0.92844	0.290995	6.47E-05	0.002505	NA	NA	NA
ENSG0000	3158.31	-0.48813	0.138665	6.54E-05	0.002526	KPNB1	3837	karyopherin subunit beta 1
ENSG0000	479.5082	0.602315	0.177894	6.59E-05	0.002541	AGAP3	116988	ArfGAP wi ankyrin repeat and PH domain 3
ENSG0000	193.0564	0.778873	0.239668	6.71E-05	0.002583	NA	NA	NA
ENSG0000	1981.885	-0.63035	0.188742	6.83E-05	0.002625	GSDMD	79792	gasdermin D
ENSG0000	38.84471	1.331291	0.456152	6.93E-05	0.002649	CAV1	857	caveolin 1
ENSG0000	1051.431	-0.47192	0.134102	6.91E-05	0.002649	SLC25A28	81894	solute carrier family 25 member 28

ENSG0000	162.6715	-0.8285	0.261102	7.22E-05	0.002756	NA	NA	NA
ENSG0000	7101.032	-0.87605	0.287017	7.27E-05	0.002768	CLU	1191	clusterin
ENSG0000	403.6862	-0.86281	0.277609	7.32E-05	0.002774	FCGR1BP	2210	Fc gamma pseudogene
ENSG0000	900.3328	-0.64144	0.193457	7.33E-05	0.002774	BST1	683	bone marrow stromal cell antigen 1
ENSG0000	193.6473	-1.75868	0.752613	7.36E-05	0.002774	FAM20A	54757	FAM20A golgi associated secretory pathway pseudokinase
ENSG0000	54.29863	-1.13274	0.387279	7.36E-05	0.002774	CLC	1178	Charcot-Leyden crystal galectin
ENSG0000	183.8059	-0.72249	0.221863	7.35E-05	0.002774	KPTN	11133	kaptin actin binding protein
ENSG0000	67.47336	1.067651	0.359202	7.44E-05	0.002797	HDAC9	9734	histone deacetylase 9
ENSG0000	104.239	-0.93133	0.304112	7.67E-05	0.002878	IGFBP4	3487	insulin like growth factor binding protein 4
ENSG0000	1718.849	1.121454	0.405711	7.79E-05	0.002916	IGLV3-25	28793	immunoglobulin lambda variable 3-25
ENSG0000	77.94249	0.695386	0.210391	7.80E-05	0.002917	RPS6KA2	6196	ribosomal protein S6 kinase A2
ENSG0000	94.69873	-0.82547	0.26106	8.38E-05	0.003125	MYLK	4638	myosin light chain kinase
ENSG0000	303.2522	-0.52037	0.152033	8.42E-05	0.003136	GSDMB	55876	gasdermin B
ENSG0000	4743.21	-1.00584	0.354437	8.63E-05	0.003206	JUP	3728	junction plakoglobin
ENSG0000	53.19494	-1.02736	0.345266	9.04E-05	0.003353	H4C4	8360	H4 clustered histone 4
ENSG0000	6.662291	2.773173	0.911264	9.21E-05	0.00341	ARHGAP2(	9411	Rho GTPase activating protein 29
ENSG0000	243.5916	-0.78269	0.251432	9.25E-05	0.003416	GCH1	2643	GTP cyclohydrolase 1
ENSG0000	290.7214	0.493702	0.144516	9.30E-05	0.003427	GRAMD4	23151	GRAM domain containing 4
ENSG0000	91.01831	-0.80923	0.252969	9.37E-05	0.003446	ABO	28	ABO alpha 1-3-N-acetylgalactosaminyltransferase and alpha 1-3-galactosyltransferase
ENSG0000	148.6756	-0.54909	0.163747	9.97E-05	0.003658	GNGT2	2793	G protein subunit gamma transducin 2
ENSG0000	1531.639	-0.91027	0.318265	1.01E-04	0.003698	CCDC71L	168455	coiled-coil domain containing 71 like
ENSG0000	27.91344	-1.34455	0.492554	1.01E-04	0.0037	CDCP1	64866	CUB domain containing protein 1
ENSG0000	454.1062	0.690196	0.219292	1.02E-04	0.003734	KLF10	7071	KLF transcription factor 10
ENSG0000	102.2014	-0.70126	0.219101	1.03E-04	0.003743	BEX3	27018	brain expressed X-linked 3
ENSG0000	24138.73	-0.81759	0.272145	1.06E-04	0.003859	CXCR1	3577	C-X-C motif chemokine receptor 1
ENSG0000	610.3807	-0.71822	0.230752	1.08E-04	0.003923	VAMP5	10791	vesicle associated membrane protein 5
ENSG0000	25.26877	-1.246	0.439331	1.09E-04	0.003956	RHOBTB1	9886	Rho related BTB domain containing 1
ENSG0000	1774.306	-0.57591	0.175979	1.11E-04	0.003992	BAK1	578	BCL2 antagonist/killer 1
ENSG0000	1019.835	-0.65162	0.20587	1.11E-04	0.003992	ADM	133	adrenomedullin
ENSG0000	223.5702	0.561143	0.170261	1.12E-04	0.004032	SNRPD3	6634	small nuclear ribonucleoprotein D3 polypeptide
ENSG0000	594.4892	-0.55149	0.16685	1.17E-04	0.004206	LMNB1	4001	lamin B1
ENSG0000	87.61031	-0.61336	0.189573	1.21E-04	0.004318	NEXN	91624	nexilin F-actin binding protein
ENSG0000	6.19676	-0.13981	0.299308	1.21E-04	0.004318	NA	NA	NA
ENSG0000	746.2694	-0.68876	0.222108	1.22E-04	0.004365	NMI	9111	N-myc and STAT interactor
ENSG0000	3365.221	-0.70642	0.230101	1.26E-04	0.004494	TRIM25	7706	tripartite motif containing 25
ENSG0000	185.5693	-0.69068	0.222384	1.31E-04	0.004653	CARD16	114769	caspase recruitment domain family member 16
ENSG0000	89.37233	-0.64107	0.201495	1.32E-04	0.004677	ZNF155	7711	zinc finger protein 155
ENSG0000	359.3341	-0.83414	0.286035	1.32E-04	0.004689	DAB2	1601	DAB adaptor protein 2
ENSG0000	148312.2	-0.75649	0.253089	1.34E-04	0.004737	IFITM2	10581	interferon induced transmembrane protein 2
ENSG0000	356.6614	-0.45694	0.134739	1.37E-04	0.004837	DCAF10	79269	DDB1 and CUL4 associated factor 10
ENSG0000	3.884467	3.853725	1.484684	1.41E-04	0.004964	MIR124-1I	157627	MIR124-1 host gene
ENSG0000	84.58604	-0.83095	0.280316	1.41E-04	0.004964	IGSF9B	22997	immunoglobulin superfamily member 9B
ENSG0000	15.97073	1.663597	0.662847	1.43E-04	0.005002	CAPN13	92291	calpain 13
ENSG0000	3.408345	-5.10763	1.945966	1.43E-04	0.005017	COL26A1	136227	collagen type XXVI alpha 1 chain
ENSG0000	19.72545	-1.14515	0.394399	1.44E-04	0.005029	TRBV5-6	28609	T cell receptor beta variable 5-6
ENSG0000	117.7555	0.856537	0.292984	1.45E-04	0.005045	LAMA5	3911	laminin subunit alpha 5
ENSG0000	19.79193	-1.22584	0.437122	1.46E-04	0.005078	TRBD1	28637	T cell receptor beta diversity 1
ENSG0000	5.077116	2.554078	1.07452	1.46E-04	0.005078	FILIP1L	11259	filamin A interacting protein 1 like
ENSG0000	436.6478	0.44337	0.132022	1.47E-04	0.005089	NA	NA	NA
ENSG0000	27.70481	-1.22523	0.434566	1.47E-04	0.005099	ESRG	790952	embryonic stem cell related
ENSG0000	25.91893	1.02325	0.350842	1.48E-04	0.005099	NA	NA	NA
ENSG0000	9.067811	1.998847	0.81946	1.52E-04	0.005217	CALCRL	10203	calcitonin receptor like receptor
ENSG0000	581.4673	-0.7218	0.239094	1.51E-04	0.005217	S1PR5	53637	sphingosine-1-phosphate receptor 5
ENSG0000	10.68753	-1.42193	0.499452	1.53E-04	0.005274	NA	NA	NA
ENSG0000	116.9585	0.623393	0.199867	1.55E-04	0.005307	ATRN	8455	attractin
ENSG0000	26.94539	-1.85697	0.985171	1.59E-04	0.005455	PRSS33	260429	serine protease 33
ENSG0000	9.587039	-1.72052	0.648316	1.62E-04	0.005547	CUX2	23316	cut like homeobox 2
ENSG0000	1054.997	0.531591	0.167027	1.63E-04	0.005574	SLC25A5	292	solute carrier family 25 member 5
ENSG0000	11.12198	-2.01439	1.055974	1.65E-04	0.005612	TIFAB	497189	TIFA inhibitor
ENSG0000	19.63583	-1.35696	0.536084	1.69E-04	0.005729	NA	NA	NA
ENSG0000	10.20019	-1.8421	0.768042	1.70E-04	0.005783	H4C14	8370	H4 clustered histone 14
ENSG0000	5.466696	-2.35809	1.024785	1.76E-04	0.005965	NA	NA	NA
ENSG0000	33.42149	1.004159	0.35113	1.77E-04	0.005988	ATOH8	84913	atonal bHLH transcription factor 8
ENSG0000	53.71191	-1.02474	0.378244	1.78E-04	0.005988	NA	NA	NA
ENSG0000	1034.382	-0.52721	0.165916	1.78E-04	0.005988	TRIB1	10221	tribbles pseudokinase 1
ENSG0000	1971.72	-0.9473	0.359077	1.78E-04	0.005988	TMEM123	114908	transmembrane protein 123
ENSG0000	66.27808	1.088355	0.411008	1.78E-04	0.005988	NA	NA	NA
ENSG0000	23.81234	-1.13964	0.406656	1.79E-04	0.006008	EXOC3L2	90332	exocyst complex component 3 like 2
ENSG0000	634.0026	0.904857	0.338398	1.81E-04	0.006061	CD302	9936	CD302 molecule
ENSG0000	3608.516	-0.50817	0.159015	1.83E-04	0.006106	CCDC97	90324	coiled-coil domain containing 97
ENSG0000	497.3864	-0.73353	0.251923	1.85E-04	0.006162	SMANTIS	1.08E+08	SMARCA4 interacting SWI/SNF chromatin remodeling complex scaffold lncRNA
ENSG0000	130137.5	-1.57972	0.625206	1.86E-04	0.0062	IFITM3	10410	interferon induced transmembrane protein 3
ENSG0000	183.9836	-0.67306	0.224025	1.90E-04	0.006292	ASPHD2	57168	aspartate beta-hydroxylase domain containing 2
ENSG0000	20.47995	1.22482	0.446731	1.91E-04	0.006303	CCDC170	80129	coiled-coil domain containing 170
ENSG0000	1015.529	0.806051	0.286692	1.91E-04	0.006303	BHLHA15	168620	basic helix-loop-helix family member a15
ENSG0000	20.79922	1.310431	0.510747	1.94E-04	0.006387	SH3BP4	23677	SH3 domain binding protein 4
ENSG0000	23.08314	-1.0792	0.38844	1.94E-04	0.006403	C19orf84	147646	chromosome 19 open reading frame 84
ENSG0000	79.22849	0.879724	0.312868	1.96E-04	0.006453	OSBPL10	114884	oxysterol binding protein like 10
ENSG0000	459.8475	1.411053	0.615218	1.99E-04	0.006522	DEFA3	1668	defensin alpha 3
ENSG0000	11.01999	-1.70477	0.708033	1.99E-04	0.006522	NA	NA	NA
ENSG0000	148.0687	-0.58644	0.187843	1.99E-04	0.006522	NA	NA	NA
ENSG0000	415.5164	-0.45419	0.140131	2.01E-04	0.006549	NUMBL	9253	NUMB like endocytic adaptor protein
ENSG0000	40.62539	-0.86955	0.304393	2.03E-04	0.006624	MYOM1	8736	myomesin 1
ENSG0000	745.264	-0.90782	0.338549	2.04E-04	0.006647	SLC26A8	116369	solute carrier family 26 member 8
ENSG0000	2042.664	-0.66224	0.223977	2.12E-04	0.006897	MARCKS	4082	myristoylated alanine rich protein kinase C substrate
ENSG0000	304.5925	0.594798	0.195143	2.15E-04	0.006956	ACBD6	84320	acyl-CoA binding domain containing 6
ENSG0000	3819.751	-0.47801	0.150692	2.16E-04	0.006963	PPP1R11	6992	protein phosphatase 1 regulatory inhibitor subunit 11
ENSG0000	361.1699	-0.76879	0.265611	2.15E-04	0.006963	NA	NA	NA
ENSG0000	8.266703	-1.59518	0.60814	2.16E-04	0.006963	SLC12A3	6559	solute carrier family 12 member 3
ENSG0000	97.75503	0.839892	0.29796	2.17E-04	0.006981	IGKV1-8	28942	immunoglobulin kappa variable 1-8
ENSG0000	144.3941	0.950102	0.360134	2.18E-04	0.006994	CDK5R1	8851	cyclin dependent kinase 5 regulatory subunit 1
ENSG0000	427.1521	0.486682	0.152653	2.18E-04	0.006994	ALKBH7	84266	alkB homolog 7
ENSG0000	1525.578	-0.49114	0.153002	2.23E-04	0.007127	DENND2D	79961	DENN domain containing 2D
ENSG0000	25.99231	1.213073	0.477756	2.23E-04	0.007127	OVGP1	5016	oviductal glycoprotein 1
ENSG0000	8.325872	-1.51511	0.56448	2.23E-04	0.007127	SLC12A8	84561	solute carrier family 12 member 8
ENSG0000	540.1211	0.72527	0.253555	2.25E-04	0.007163	RHOQ	23433	ras homolog family member Q

ENSG0000	52.79462	1.105888	0.415237	2.25E-04	0.007163	NA	NA	NA	
ENSG0000	7.490826	-2.17784	0.984205	2.28E-04	0.007237	NA	NA	NA	
ENSG0000	1812.871	-0.62054	0.208559	2.34E-04	0.007404	CASP1	834	caspase 1	
ENSG0000	496.0103	-0.44721	0.140004	2.39E-04	0.007551	RNF114	55905	ring finger protein 114	
ENSG0000	18.13074	1.271729	0.487269	2.41E-04	0.007604	A3GALT2	127550	alpha 1 3-galactosyltransferase 2	
ENSG0000	316.8379	-0.5291	0.171035	2.42E-04	0.007604	LINC0113	388685	long intergenic non-protein coding RNA 1138	
ENSG0000	118.7723	-0.99112	0.381612	2.41E-04	0.007604	NA	NA	NA	
ENSG0000	212.838	-0.57665	0.190026	2.43E-04	0.007604	TSC22D1	8848	TSC22 domain family member 1	
ENSG0000	25.08988	-0.99239	0.362004	2.43E-04	0.007604	NA	NA	NA	
ENSG0000	23484.19	0.521915	0.168788	2.42E-04	0.007604	EEF2	1938	eukaryotic translation elongation factor 2	
ENSG0000	2728.043	-0.63748	0.215617	2.45E-04	0.007667	FPR2	2358	formyl peptide receptor 2	
ENSG0000	18127.42	-0.74087	0.266208	2.47E-04	0.007707	SECTM1	6398	secreted and transmembrane 1	
ENSG0000	6832.305	-0.64447	0.221134	2.47E-04	0.007707	APOBEC3A	200315	apolipoprotein B mRNA editing enzyme catalytic subunit 3A	
ENSG0000	1722.675	-0.51098	0.164813	2.50E-04	0.007782	TUBB1	81027	tubulin beta 1 class VI	
ENSG0000	298.7372	1.013822	0.418391	2.51E-04	0.007782	CEACAM8	1088	CEA cell adhesion molecule 8	
ENSG0000	249.607	-1.04578	0.432031	2.51E-04	0.007792	HP	3240	haptoglobin	
ENSG0000	1612.383	0.474984	0.150922	2.52E-04	0.007797	EIF4B	1975	eukaryotic translation initiation factor 4B	
ENSG0000	45.61917	-0.78636	0.275174	2.53E-04	0.007813	NA	NA	NA	
ENSG0000	555.8423	1.417033	0.722772	2.54E-04	0.007819	IGHV2-70	28454	immunoglobulin heavy variable 2-70	
ENSG0000	4646.247	-0.61084	0.209147	2.54E-04	0.007819	SIGLEC14	1E+08	sialic acid binding Ig like lectin 14	
ENSG0000	38.2229	1.17413	0.486471	2.56E-04	0.007876	CFAP45	25790	cilia and flagella associated protein 45	
ENSG0000	59.76702	0.816348	0.288772	2.57E-04	0.007898	ZP3	7784	zona pellucida glycoprotein 3	
ENSG0000	43.69437	0.805226	0.284523	2.67E-04	0.008154	NA	NA	NA	
ENSG0000	9356.686	-0.67044	0.235304	2.66E-04	0.008154	IL2RG	3561	interleukin 2 receptor subunit gamma	
ENSG0000	350.0321	0.534358	0.175242	2.70E-04	0.008253	CLINT1	9685	clathrin interactor 1	
ENSG0000	9.824274	-1.96903	0.997674	2.72E-04	0.008286	ALMS1P1	200420	ALMS1 pseudogene 1	
ENSG0000	1403.275	-0.61431	0.210278	2.74E-04	0.008352	PIK3AP1	118788	phosphoinositide-3-kinase adaptor protein 1	
ENSG0000	18.49117	1.23567	0.483574	2.78E-04	0.008431	LINC0123	1.03E+08	long intergenic non-protein coding RNA 1238	
ENSG0000	16.81361	-1.15782	0.44468	2.87E-04	0.008693	NA	NA	NA	
ENSG0000	10019.61	-0.63939	0.223815	2.98E-04	0.009021	NKG7	4818	natural killer cell granule protein 7	
ENSG0000	953.5825	0.469633	0.151016	2.99E-04	0.009029	HVCN1	84329	hydrogen voltage gated channel 1	
ENSG0000	26.78522	0.939842	0.344589	2.99E-04	0.009029	NA	NA	NA	
ENSG0000	814.4373	-0.56754	0.191344	3.00E-04	0.009031	SCARF1	8578	scavenger receptor class F member 1	
ENSG0000	31.16083	-0.80435	0.287236	3.08E-04	0.009259	NA	NA	NA	
ENSG0000	1050.239	-0.76276	0.285471	3.08E-04	0.009264	NA	NA	NA	
ENSG0000	3905.557	0.466692	0.150886	3.10E-04	0.009295	RPS5	6193	ribosomal protein S5	
ENSG0000	2536.395	-0.61572	0.214278	3.15E-04	0.00937	QPCT	25797	glutaminyl-peptide cyclotransferase	
ENSG0000	10304.21	-0.56434	0.191941	3.14E-04	0.00937	IRF1	3659	interferon regulatory factor 1	
ENSG0000	12.33674	-1.80912	0.834115	3.15E-04	0.00937	TRAV39	28642	T cell receptor alpha variable 39	
ENSG0000	82.37535	0.991436	0.400338	3.13E-04	0.00937	IGHV3-20	28445	immunoglobulin heavy variable 3-20	
ENSG0000	155.4975	-0.60566	0.208411	3.14E-04	0.00937	TMEM62	80021	transmembrane protein 62	
ENSG0000	19.93979	-1.28913	0.533724	3.19E-04	0.009456	RTEL1P1	1E+08	regulator of telomere elongation helicase 1 pseudogene 1	
ENSG0000	6872.58	0.577843	0.197682	3.19E-04	0.009456	RPL4	6124	ribosomal protein L4	
ENSG0000	31.15096	0.921361	0.3434	3.22E-04	0.00952	NA	NA	NA	
ENSG0000	673.5346	0.806946	0.310088	3.25E-04	0.009604	SLPI	6590	secretory leukocyte peptidase inhibitor	
ENSG0000	320.4238	-0.8323	0.323707	3.35E-04	0.009838	NT5C3A	51251	5'-nucleot cytosolic IIIA	
ENSG0000	337.0184	-0.66501	0.237552	3.34E-04	0.009838	STAC3	246329	SH3 and cysteine rich domain 3	
ENSG0000	10.33883	1.425248	0.580774	3.34E-04	0.009838	NA	NA	NA	
ENSG0000	166.1228	-0.87673	0.350365	3.36E-04	0.009864	CHMP5	51510	charged multivesicular body protein 5	
ENSG0000	285.8032	-0.80683	0.311714	3.37E-04	0.009864	EGFL7	51162	EGF like domain multiple 7	
ENSG0000	282.9642	-0.74631	0.273582	3.38E-04	0.009889	TRGC2	6967	T cell receptor gamma constant 2	
ENSG0000	16563.2	-0.52751	0.177805	3.40E-04	0.009947	GYPC	2995	glycophorin C (Gerbich blood group)	
ENSG0000	63.04353	1.040681	0.44071	3.46E-04	0.01009	PRRT4	401399	proline rich transmembrane protein 4	
ENSG0000	6.04059	-0.13016	0.288351	3.53E-04	0.010249	FAP	2191	fibroblast activation protein alpha	
ENSG0000	7.967769	2.032315	1.076041	3.53E-04	0.010249	PROS1	5627	protein S	
ENSG0000	469.8368	-0.56148	0.193008	3.53E-04	0.010249	ZFYVE26	23503	zinc finger FYVE-type containing 26	
ENSG0000	679.7571	0.64877	0.232546	3.60E-04	0.010372	RPS23	6228	ribosomal protein S23	
ENSG0000	490.2395	-0.44467	0.144077	3.60E-04	0.010372	IL15RA	3601	interleukin 15 receptor subunit alpha	
ENSG0000	1185.663	-0.70801	0.262398	3.59E-04	0.010372	SLC3A2	6520	solute carrier family 3 member 2	
ENSG0000	99.79318	-0.93571	0.381114	3.60E-04	0.010372	PLEK2	26499	pleckstrin 2	
ENSG0000	217.5393	-0.6941	0.251794	3.59E-04	0.010372	RARA-AS1	1.02E+08	RARA antisense RNA 1	
ENSG0000	14.06713	1.395512	0.580938	3.62E-04	0.010403	SASH1	23328	SAM and SH3 domain containing 1	
ENSG0000	4.737372	-0.13276	0.291264	3.63E-04	0.010425	APOBEC3E	1.01E+08	APOBEC3B antisense RNA 1	
ENSG0000	8.001014	2.041767	1.168642	3.64E-04	0.010436	IRX3	79191	iroquois homeobox 3	
ENSG0000	10.00235	1.949075	0.907648	3.67E-04	0.010491	WFDC1	58189	WAP four-disulfide core domain 1	
ENSG0000	411.8783	-0.56857	0.197694	3.81E-04	0.010899	LINC0087	285286	long intergenic non-protein coding RNA 877	
ENSG0000	23.55894	-0.87246	0.324754	3.89E-04	0.011086	MIR4477B	1.01E+08	microRNA 4477b	
ENSG0000	2.751838	3.368918	1.516119	3.94E-04	0.011234	RPL31P43	392382	ribosomal protein L31 pseudogene 43	
ENSG0000	11.95914	2.232685	1.198234	3.95E-04	0.011249	KLF14	136259	KLF transcription factor 14	
ENSG0000	725.8169	-0.47076	0.15647	3.97E-04	0.011279	EIF1B	10289	eukaryotic translation initiation factor 1B	
ENSG0000	3508.186	0.669771	0.248576	3.98E-04	0.011279	IGHD	3495	immunoglobulin heavy constant delta	
ENSG0000	75.32013	-0.79974	0.307351	4.02E-04	0.011386	KIR2DL4	3805	killer cell i two Ig domains and long cytoplasmic tail 4	
ENSG0000	42.30517	1.278185	0.612726	4.04E-04	0.011395	IGF1	3479	insulin like growth factor 1	
ENSG0000	307.6144	1.314122	0.670516	4.03E-04	0.011395	PRTN3	5657	proteinase 3	
ENSG0000	3.069085	2.866492	1.449886	4.20E-04	0.011827	NA	NA	NA	
ENSG0000	309.9838	0.681397	0.253296	4.22E-04	0.011861	QPCTL	54814	glutaminyl-peptide cyclotransferase like	
ENSG0000	32.28965	0.979727	0.393479	4.24E-04	0.011924	LINC0049	284749	long intergenic non-protein coding RNA 494	
ENSG0000	17.53532	1.194758	0.49794	4.28E-04	0.012005	NA	NA	NA	
ENSG0000	2.774042	-3.88355	1.63072	4.33E-04	0.012123	LOC10798	1.08E+08	uncharacterized LOC107985211	
ENSG0000	6.068028	1.812387	0.779918	4.44E-04	0.012403	ELF3	1999	E74 like ETS transcription factor 3	
ENSG0000	18.19698	1.425878	0.764205	4.50E-04	0.012556	BCAR3	8412	BCAR3 ada NSP family member	
ENSG0000	16.95938	1.856296	0.752445	4.59E-04	0.012799	PCSK9	255738	proprotein convertase subtilisin/kexin type 9	
ENSG0000	136.828	1.016201	0.464366	4.67E-04	0.012948	LOC10192	1.02E+08	uncharacterized LOC101929667	
ENSG0000	116.0785	0.511905	0.174979	4.65E-04	0.012948	FARP2	9855	FERM ARH/RhoGEF and pleckstrin domain protein 2	
ENSG0000	933.3567	-0.60395	0.221367	4.66E-04	0.012948	EIF4E3	317649	eukaryotic translation initiation factor 4E family member 3	
ENSG0000	365.5279	-0.72138	0.2776	4.72E-04	0.01307	GATA1	2623	GATA binding protein 1	
ENSG0000	215.7849	-0.81831	0.336719	4.77E-04	0.013189	NCOA7	135112	nuclear receptor coactivator 7	
ENSG0000	35.22712	-0.85266	0.335276	4.87E-04	0.013447	NA	NA	NA	
ENSG0000	30.65545	0.995503	0.416467	4.91E-04	0.013537	RAB11B-A	1.01E+08	RAB11B antisense RNA 1	
ENSG0000	212.9181	0.874507	0.369704	4.93E-04	0.013574	IGLV3-9	28804	immunoglobulin lambda variable 3-9	
ENSG0000	629.0155	0.786057	0.319319	4.94E-04	0.013597	IRS2	8660	insulin receptor substrate 2	
ENSG0000	9914.552	0.569897	0.205189	4.98E-04	0.013673	RPLP0	6175	ribosomal protein lateral stalk subunit P0	
ENSG0000	582.2273	0.427318	0.142385	5.09E-04	0.01395	CCNY	219771	cyclin Y	
ENSG0000	517.5897	-0.58589	0.2125	5.11E-04	0.013991	SQOR	58472	sulfide quinone oxidoreductase	
ENSG0000	2245.894	-0.65193	0.248484	5.12E-04	0.013991	SELENBP1	8991	selenium binding protein 1	
ENSG0000	635.8719	0.697261	0.26942	5.15E-04	0.014061	IGKV1-6	28943	immunoglobulin kappa variable 1-6	

ENSG0000	78.79995	0.705534	0.268384	5.23E-04	0.014257	AFF3	3899	ALF transcription elongation factor 3
ENSG0000	34.14449	-1.02409	0.44973	5.29E-04	0.014401	NA	NA	NA
ENSG0000	4560.278	0.423928	0.142162	5.31E-04	0.014438	NOP53	29997	NOP53 ribosome biogenesis factor
ENSG0000	12.79127	1.135314	0.463893	5.34E-04	0.014492	ICA1L	130026	islet cell autoantigen 1 like
ENSG0000	313.7239	-0.65774	0.250179	5.45E-04	0.014781	NA	NA	NA
ENSG0000	17220.62	-0.59478	0.22028	5.49E-04	0.014852	FCER1G	2207	Fc epsilon receptor Ig
ENSG0000	3431.733	-0.68372	0.268183	5.53E-04	0.014946	PRF1	5551	perforin 1
ENSG0000	64.04359	0.907133	0.38409	5.63E-04	0.015184	KLHL14	57565	kelch like family member 14
ENSG0000	64.33837	0.654787	0.244656	5.65E-04	0.015216	CHKA	1119	choline kinase alpha
ENSG0000	229.1612	-0.74879	0.30478	5.66E-04	0.015234	NA	NA	NA
ENSG0000	235.3967	0.516446	0.182683	5.75E-04	0.015429	RBP7	116362	retinol binding protein 7
ENSG0000	525.1926	0.562324	0.204744	5.75E-04	0.015429	SLC27A1	376497	solute carrier family 27 member 1
ENSG0000	6.119686	-1.66493	0.75875	5.78E-04	0.015468	H2AC13	8329	H2A clustered histone 13
ENSG0000	565.2646	-0.66442	0.257354	5.79E-04	0.015472	MYL4	4635	myosin light chain 4
ENSG0000	11.79102	-1.12441	0.467398	5.80E-04	0.015489	MEF2C-AS	1.02E+08	MEF2C antisense RNA 1
ENSG0000	1137.364	-0.37764	0.124543	5.87E-04	0.015647	CALCOCO	10241	calcium binding and coiled-coil domain 2
ENSG0000	42.0371	0.691693	0.260442	5.89E-04	0.015674	NA	NA	NA
ENSG0000	298.6179	-0.62762	0.238096	5.92E-04	0.01574	SELP	6403	selectin P
ENSG0000	990.981	-0.59716	0.223892	5.96E-04	0.01581	GMPR	2766	guanosine monophosphate reductase
ENSG0000	103.2143	1.05591	0.552562	5.97E-04	0.015824	KLF9	687	KLF transcription factor 9
ENSG0000	65.64756	0.804597	0.330644	6.00E-04	0.015856	ADORA3	140	adenosine A3 receptor
ENSG0000	10.1773	-1.49569	0.778082	5.99E-04	0.015856	IDO1	3620	indoleami 3-dioxygenase 1
ENSG0000	194.8883	-0.44905	0.154252	6.14E-04	0.016139	TNIK	23043	TRAF2 and NCK interacting kinase
ENSG0000	27.94796	-0.89427	0.364445	6.13E-04	0.016139	GADD45G	10912	growth arrest and DNA damage inducible gamma
ENSG0000	24.48596	-1.09335	0.508175	6.13E-04	0.016139	LINC0130	1.02E+08	long intergenic non-protein coding RNA 1303
ENSG0000	3.710942	-2.99323	1.387513	6.12E-04	0.016139	HSPB9	94086	heat shock protein family B (small) member 9
ENSG0000	182.5144	0.662431	0.255269	6.18E-04	0.016213	CSGALNAC	55790	chondroitin sulfate N-acetylgalactosaminyltransferase 1
ENSG0000	18.24597	1.234731	0.583066	6.20E-04	0.016236	FMO5	2330	flavin containing dimethylaniline monoxygenase 5
ENSG0000	474.1458	-0.52689	0.189663	6.22E-04	0.016278	APOBEC3F	200316	apolipoprotein B mRNA editing enzyme catalytic subunit 3F
ENSG0000	108.7636	0.935147	0.431384	6.23E-04	0.016285	OTX1	5013	orthodenticle homeobox 1
ENSG0000	6.213886	1.958757	1.087071	6.26E-04	0.016304	ORM2	5005	orosomucoid 2
ENSG0000	177.4412	1.275587	0.76105	6.25E-04	0.016304	OLFM4	10562	olfactomedin 4
ENSG0000	237.1125	-0.63398	0.242045	6.31E-04	0.016408	LINC01011	401232	long intergenic non-protein coding RNA 1011
ENSG0000	23.08031	1.177069	0.570567	6.33E-04	0.016458	GRAMD1C	54762	GRAM domain containing 1C
ENSG0000	254.6292	-0.4569	0.159573	6.46E-04	0.01676	TXNL4B	54957	thioredoxin like 4B
ENSG0000	147.2346	0.559757	0.205561	6.48E-04	0.016787	ZNF219	51222	zinc finger protein 219
ENSG0000	14.42883	-1.02898	0.435375	6.52E-04	0.016847	NA	NA	NA
ENSG0000	64.04919	-0.91615	0.406236	6.52E-04	0.016847	NA	NA	NA
ENSG0000	113.1572	-0.68592	0.270352	6.54E-04	0.016865	H2BC19P	337874	H2B cluste pseudogene
ENSG0000	2038.095	-0.78483	0.338258	6.58E-04	0.016941	SOCS3	9021	suppressor of cytokine signaling 3
ENSG0000	125.8797	0.619296	0.235323	6.59E-04	0.016946	TBC1D9	23158	TBC1 domain family member 9
ENSG0000	633.5306	-0.41989	0.143509	6.63E-04	0.017029	C2CD3	26005	C2 domain containing 3 centriole elongation regulator
ENSG0000	1066.725	-0.566	0.212138	6.79E-04	0.017427	RUNX1	861	RUNX family transcription factor 1
ENSG0000	190.6931	0.209637	0.384088	6.82E-04	0.017435	CNTNAP3	79937	contactin associated protein family member 3
ENSG0000	3066.323	-0.53088	0.195027	6.82E-04	0.017435	CASP4	837	caspase 4
ENSG0000	49.58885	-1.05596	0.534498	6.81E-04	0.017435	CCNA1	8900	cyclin A1
ENSG0000	161.7128	0.946762	0.451108	6.95E-04	0.017724	TNFRSF17	608	TNF receptor superfamily member 17
ENSG0000	268.7209	-0.56491	0.209783	7.09E-04	0.018064	KLC3	147700	kinesin light chain 3
ENSG0000	411.2995	-0.53021	0.195168	7.10E-04	0.018074	DEGS1	8560	delta 4-de sphingolipid 1
ENSG0000	6.294195	-1.67748	0.839887	7.21E-04	0.018317	CPA3	1359	carboxypeptidase A3
ENSG0000	1848.735	-0.66726	0.270154	7.22E-04	0.018317	SPON2	10417	spondin 2
ENSG0000	8.124214	1.851565	0.875678	7.24E-04	0.018319	CACNA2D	55799	calcium voltage-gated channel auxiliary subunit alpha2delta 3
ENSG0000	1301.462	-0.56413	0.210179	7.25E-04	0.018319	MXD3	83463	MAX dimerization protein 3
ENSG0000	740.975	-0.49924	0.180856	7.23E-04	0.018319	PPCDC	60490	phosphopantothenoylcysteine decarboxylase
ENSG0000	32.41246	-0.90451	0.391313	7.30E-04	0.018393	NA	NA	NA
ENSG0000	29.71586	1.09319	0.523944	7.31E-04	0.018393	RGMA	56963	repulsive guidance molecule BMP co-receptor a
ENSG0000	20.13539	-1.27716	0.682164	7.30E-04	0.018393	SPATA32	124783	spermatogenesis associated 32
ENSG0000	11.21217	1.532094	0.956866	7.35E-04	0.018467	CNR1	1268	cannabinoid receptor 1
ENSG0000	6990.634	-0.50281	0.186573	7.37E-04	0.018506	H3-3B	3021	H3.3 histone B
ENSG0000	53.08339	1.07147	0.497123	7.41E-04	0.018566	INHBB	3625	inhibin subunit beta B
ENSG0000	2.572689	-0.13709	0.295971	7.43E-04	0.018588	LINC0252	1.05E+08	long intergenic non-protein coding RNA 2528
ENSG0000	107.0521	-0.77654	0.331258	7.45E-04	0.018615	CARMIL3	90668	capping protein regulator and myosin 1 linker 3
ENSG0000	280.5879	-0.47228	0.168573	7.56E-04	0.018876	NA	NA	NA
ENSG0000	207.6596	-0.60207	0.231529	7.64E-04	0.019046	CD40LG	959	CD40 ligand
ENSG0000	2688.191	-0.38483	0.13255	7.69E-04	0.019137	TRAF3IP3	80342	TRAF3 interacting protein 3
ENSG0000	22.2664	-0.96307	0.427481	7.77E-04	0.019323	NA	NA	NA
ENSG0000	2007.857	0.465207	0.167173	7.82E-04	0.019361	RPS8	6202	ribosomal protein S8
ENSG0000	539.7131	-0.71426	0.293973	7.81E-04	0.019361	XRN1	54464	5'-3' exoribonuclease 1
ENSG0000	6.824024	1.608338	0.76986	7.81E-04	0.019361	PRR15	222171	proline rich 15
ENSG0000	238.8357	-0.5482	0.206398	7.92E-04	0.019543	NA	NA	NA
ENSG0000	179.7701	-0.6089	0.23843	7.91E-04	0.019543	GPD2	2820	glycerol-3-phosphate dehydrogenase 2
ENSG0000	42.04147	-0.8503	0.369583	7.97E-04	0.01964	NA	NA	NA
ENSG0000	15.20957	1.109921	0.500498	8.03E-04	0.019762	RTEL1-TNF	1.01E+08	RTEL1-TNFRSF6B readthrough (NMD candidate)
ENSG0000	61.67074	-0.7444	0.309704	8.13E-04	0.019984	NA	NA	NA
ENSG0000	70.64494	0.809593	0.351789	8.15E-04	0.020017	TIAM2	26230	TIAM Rac1 associated GEF 2
ENSG0000	79.80723	-0.82254	0.367128	8.21E-04	0.020051	NA	NA	NA
ENSG0000	227.5277	-0.48106	0.174299	8.18E-04	0.020051	TMEM79	84283	transmembrane protein 79
ENSG0000	1038.201	-0.48779	0.171715	8.21E-04	0.020051	CISH	1154	cytokine inducible SH2 containing protein
ENSG0000	97.86594	-0.82427	0.373763	8.20E-04	0.020051	KIR3DL2	3812	killer cell i three Ig domains and long cytoplasmic tail 2
ENSG0000	39.82955	0.826161	0.352468	8.38E-04	0.020419	LAMB2	3913	laminin subunit beta 2
ENSG0000	37.14448	0.884932	0.396239	8.38E-04	0.020419	GNMT	27232	glycine N-methyltransferase
ENSG0000	547.7913	-0.45077	0.159916	8.45E-04	0.020547	PIP4K2C	79837	phosphatidylinositol-5-phosphate 4-kinase type 2 gamma
ENSG0000	168.679	0.460748	0.165216	8.46E-04	0.020547	UPF3A	65110	UPF3A regulator of nonsense mediated mRNA decay
ENSG0000	99.77777	-0.61115	0.239812	8.50E-04	0.020635	H2BC11	8970	H2B clustered histone 11
ENSG0000	173.0367	-0.55036	0.209162	8.56E-04	0.020716	CYTOR	112597	cytoskeleton regulator RNA
ENSG0000	246.763	0.615994	0.245129	8.55E-04	0.020716	GPR162	27239	G protein-coupled receptor 162
ENSG0000	620.1546	-0.73625	0.327095	8.75E-04	0.021139	NECTIN2	5819	nectin cell adhesion molecule 2
ENSG0000	142.299	0.576619	0.222912	8.84E-04	0.021265	NOXA1	10811	NADPH oxidase activator 1
ENSG0000	63.75483	-0.73583	0.311271	8.82E-04	0.021265	NA	NA	NA
ENSG0000	772.3082	-0.36491	0.124452	8.85E-04	0.021265	IL12RB1	3594	interleukin 12 receptor subunit beta 1
ENSG0000	18.68806	1.162922	0.572038	8.83E-04	0.021265	FPR3	2359	formyl peptide receptor 3
ENSG0000	85.19993	-0.57956	0.222481	8.94E-04	0.021432	PBX1	5087	PBX homeobox 1
ENSG0000	2750.838	0.581791	0.22913	8.93E-04	0.021432	ZFP36L2	678	ZFP36 ring finger protein like 2
ENSG0000	8087.466	-0.44375	0.160281	9.06E-04	0.021694	LCP2	3937	lymphocyte cytosolic protein 2
ENSG0000	215.4263	-0.57419	0.222979	9.16E-04	0.021916	TMOD1	7111	tropomodulin 1
ENSG0000	709.1831	-0.55949	0.218246	9.20E-04	0.021972	FCGR2B	2213	Fc gamma receptor IIb

ENSG0000	3552.041	-0.43912	0.156826	9.21E-04	0.021972	HCP5	10866	HLA complex P5
ENSG0000	21.42252	-0.99657	0.467496	9.28E-04	0.022098	LOC28319	283194	uncharacterized LOC283194
ENSG0000	1352.112	0.709031	0.312252	9.32E-04	0.022181	DERL3	91319	derlin 3
ENSG0000	4717.908	-0.52918	0.203411	9.38E-04	0.022252	AGPAT1	10554	1-acylglycerol-3-phosphate O-acyltransferase 1
ENSG0000	186.3852	-0.61223	0.24515	9.38E-04	0.022252	NA	NA	NA
ENSG0000	5253.644	-0.52195	0.199672	9.41E-04	0.022307	IER2	9592	immediate early response 2
ENSG0000	334.6482	0.469344	0.172674	9.44E-04	0.022312	CD1D	912	CD1d molecule
ENSG0000	90.72411	-0.60024	0.23718	9.43E-04	0.022312	LRRC37A3	374819	leucine rich repeat containing 37 member A3
ENSG0000	90.84029	0.66277	0.267226	9.50E-04	0.022386	ZBED3	84327	zinc finger BED-type containing 3
ENSG0000	462.2374	0.442162	0.159824	9.49E-04	0.022386	CXXC5	51523	CXXC finger protein 5
ENSG0000	4.173045	-0.15427	0.310928	9.52E-04	0.022408	LINC0175(	643355	long intergenic non-protein coding RNA 1750
ENSG0000	155.5366	0.770215	0.345883	9.59E-04	0.022542	CYBRD1	79901	cytochrome b reductase 1
ENSG0000	8.129504	-1.44962	0.777156	9.64E-04	0.02264	NA	NA	NA
ENSG0000	209.6898	-0.73138	0.325917	9.74E-04	0.022833	TMEM158	25907	transmembrane protein 158
ENSG0000	7.992287	1.396222	0.66769	9.76E-04	0.022868	LOC10272	1.03E+08	uncharacterized LOC102723701
ENSG0000	6.773933	-2.01014	1.192459	0.000984	0.023018	CACNG6	59285	calcium voltage-gated channel auxiliary subunit gamma 6
ENSG0000	177.9198	0.525023	0.200582	9.87E-04	0.023071	ZNF395	55893	zinc finger protein 395
ENSG0000	5370.103	-0.44014	0.155102	9.89E-04	0.023077	MYL12A	10627	myosin light chain 12A
ENSG0000	12.62181	-1.66358	0.833459	9.92E-04	0.023103	SLC6A19	340024	solute carrier family 6 member 19
ENSG0000	15.00253	1.456921	0.80058	9.96E-04	0.023103	PRL	5617	prolactin
ENSG0000	63.85024	-0.81555	0.377407	9.93E-04	0.023103	H2BC17	8348	H2B clustered histone 17
ENSG0000	7016.534	0.424783	0.15277	9.96E-04	0.023103	RPS3	6188	ribosomal protein S3
ENSG0000	47.05448	1.07228	0.680297	9.96E-04	0.023103	IGLV3-16	28799	immunoglobulin lambda variable 3-16
ENSG0000	1008.363	-0.63156	0.26486	0.001008	0.023353	SMIM1	388588	small integral membrane protein 1 (Vel blood group)
ENSG0000	17.32677	0.91435	0.394246	0.00101	0.023358	NA	NA	NA
ENSG0000	3210.967	1.137056	0.710195	0.001021	0.023579	IL1R2	7850	interleukin 1 receptor type 2
ENSG0000	119.6894	0.862971	0.438153	0.001029	0.023749	IGLL5	1E+08	immunoglobulin lambda like polypeptide 5
ENSG0000	86.10178	-0.51998	0.199141	0.001039	0.02393	ST7L	54879	suppression of tumorigenicity 7 like
ENSG0000	17.13402	-0.91495	0.403268	0.001042	0.023974	FBXO38-D	1.03E+08	FBXO38 divergent transcript
ENSG0000	130.426	-0.60983	0.248687	0.001052	0.024168	NA	NA	NA
ENSG0000	33.89514	0.771918	0.333007	0.001053	0.02418	RPLP0P6	220717	ribosomal protein lateral stalk subunit P0 pseudogene 6
ENSG0000	246.8131	0.538416	0.210084	0.001064	0.024398	ARSG	22901	arylsulfatase G
ENSG0000	9.774809	-1.18221	0.5465	0.001068	0.024463	CYP2J2	1573	cytochrome P450 family 2 subfamily J member 2
ENSG0000	87.47789	0.712282	0.312024	0.001079	0.024673	NA	NA	NA
ENSG0000	6510.751	0.468809	0.176437	0.001083	0.024736	RPS4X	6191	ribosomal protein S4 X-linked
ENSG0000	28.56614	-0.79411	0.346793	0.001091	0.024868	CLEC1B	51266	C-type lectin domain family 1 member B
ENSG0000	151.9837	0.834328	0.414371	0.00109	0.024868	IGLV3-27	28791	immunoglobulin lambda variable 3-27
ENSG0000	147.4454	-0.64872	0.27321	0.001102	0.025078	DNAJA4	55466	DnaJ heat shock protein family (Hsp40) member A4
ENSG0000	1346.455	-0.40604	0.146425	0.001108	0.025115	LGALS8	3964	galectin 8
ENSG0000	202.9712	-0.63634	0.26132	0.001107	0.025115	MDGA1	266727	MAM domain containing glycosylphosphatidylinositol anchor 1
ENSG0000	20.53086	-1.31184	0.913908	0.001105	0.025115	NA	NA	NA
ENSG0000	1598.808	0.576932	0.234737	0.00111	0.02513	RPL5	6125	ribosomal protein L5
ENSG0000	12083.12	0.761117	0.366006	0.001113	0.025156	JCHAIN	3512	joining chain of multimeric IgA and IgM
ENSG0000	8.028591	-1.42773	0.941529	0.001114	0.025156	NA	NA	NA
ENSG0000	3.955288	2.807683	1.435524	0.001115	0.025156	CD300LD	1E+08	CD300 molecule like family member d
ENSG0000	3.383184	-2.10177	1.276365	0.001131	0.025493	RBPMS	11030	RNA bindi mRNA processing factor
ENSG0000	1182.473	-0.49828	0.191518	0.00114	0.025648	UBE2S	27338	ubiquitin conjugating enzyme E2 S
ENSG0000	29.11666	1.013387	0.53214	0.001153	0.025904	RNASE4	6038	ribonuclease A family member 4
ENSG0000	129.37	0.554008	0.219766	0.001154	0.025904	CLEC17A	388512	C-type lectin domain containing 17A
ENSG0000	51.06613	0.912159	0.459876	0.00116	0.026008	SGMS2	166929	sphingomyelin synthase 2
ENSG0000	735.6383	-0.47572	0.181024	0.001168	0.026156	TTC38	55020	tetratricopeptide repeat domain 38
ENSG0000	15.72743	-1.18637	0.705944	0.001176	0.02626	NA	NA	NA
ENSG0000	4004.459	0.437641	0.16241	0.001174	0.02626	NLRP1	22861	NLR family pyrin domain containing 1
ENSG0000	147.6662	0.739592	0.339937	0.001177	0.026267	RELL1	768211	RELT like 1
ENSG0000	116.4384	-0.57702	0.23315	0.001179	0.026267	NKX3-1	4824	NK3 homeobox 1
ENSG0000	62.66654	-0.69933	0.30312	0.001183	0.026334	MYRF	745	myelin regulatory factor
ENSG0000	4108.444	0.700668	0.32353	0.001189	0.026358	IGKV1-5	28299	immunoglobulin kappa variable 1-5
ENSG0000	46.01144	0.871819	0.440729	0.001189	0.026358	HES1	3280	hes family bHLH transcription factor 1
ENSG0000	46.18338	0.823727	0.389294	0.00119	0.026358	C16orf74	404550	chromosome 16 open reading frame 74
ENSG0000	353.0757	0.405796	0.147056	0.001186	0.026358	PICK1	9463	protein interacting with PRKCA 1
ENSG0000	3.676517	1.898606	1.025426	0.001201	0.026578	RPL4P5	158345	ribosomal protein L4 pseudogene 5
ENSG0000	144.1984	0.854504	0.457403	0.001207	0.02667	FLT3	2322	fms related receptor tyrosine kinase 3
ENSG0000	337.0057	0.665736	0.293304	0.001208	0.02667	IGHV3-15	28448	immunoglobulin heavy variable 3-15
ENSG0000	18.98185	0.850499	0.38263	0.001216	0.026747	CD1A	909	CD1a molecule
ENSG0000	1197.694	-0.66232	0.293305	0.001221	0.026747	CYSTM1	84418	cysteine rich transmembrane module containing 1
ENSG0000	38.48832	0.743726	0.326896	0.001216	0.026747	NFE2L3	9603	NFE2 like bZIP transcription factor 3
ENSG0000	1144.445	-0.44053	0.16405	0.001218	0.026747	DCAF11	80344	DDB1 and CUL4 associated factor 11
ENSG0000	153.1864	-0.55629	0.223701	0.00122	0.026747	NA	NA	NA
ENSG0000	283.3484	0.822192	0.430412	0.001217	0.026747	NECAB2	54550	N-terminal EF-hand calcium binding protein 2
ENSG0000	55.2212	-0.70376	0.309111	0.001225	0.026815	LOC10013	1E+08	uncharacterized LOC100130357
ENSG0000	301.5156	-0.58187	0.241441	0.001231	0.026905	SERTAD1	29950	SERTA domain containing 1
ENSG0000	23.55545	-0.83759	0.380049	0.001235	0.02691	SH3TC2	79628	SH3 domain and tetratricopeptide repeats 2
ENSG0000	718.3009	-0.64189	0.282244	0.001235	0.02691	PSTPIP2	9050	proline-serine-threonine phosphatase interacting protein 2
ENSG0000	19.44737	-1.03956	0.559194	0.001235	0.02691	HSPB6	126393	heat shock protein family B (small) member 6
ENSG0000	2306.232	-0.4775	0.184493	0.001253	0.02719	TRIM26	7726	tripartite motif containing 26
ENSG0000	104.2921	-0.83462	0.421164	0.001251	0.02719	RAB3IL1	5866	RAB3A interacting protein like 1
ENSG0000	142.5597	0.856083	0.459631	0.001252	0.02719	GPRC5D	55507	G protein-coupled receptor class C group 5 member D
ENSG0000	182.0368	-0.64627	0.282548	0.001274	0.02762	PDZK1IP1	10158	PDZK1 interacting protein 1
ENSG0000	132.2007	0.775864	0.377311	0.001276	0.027638	DSC2	1824	desmocollin 2
ENSG0000	5.272054	-0.14873	0.30438	0.001279	0.027662	ERICH3	127254	glutamate rich 3
ENSG0000	718.1304	-0.42526	0.161686	0.001286	0.027781	GNG5	2787	G protein subunit gamma 5
ENSG0000	81.78406	-0.61511	0.258389	0.001295	0.027934	DCUN1D3	123879	defective in cullin neddylation 1 domain containing 3
ENSG0000	86.43983	0.678997	0.303292	0.001303	0.028087	SLC2A5	6518	solute carrier family 2 member 5
ENSG0000	90.76574	0.79005	0.388128	0.001308	0.028118	IGKV1-39	28930	immunoglobulin kappa variable 1-39
ENSG0000	22191.12	0.581338	0.244985	0.001309	0.028118	EEF1A1	1915	eukaryotic translation elongation factor 1 alpha 1
ENSG0000	148.8885	0.79729	0.406602	0.001309	0.028118	PIAS2	9063	protein inhibitor of activated STAT 2
ENSG0000	1670.961	0.489	0.190166	0.001325	0.028421	RPL6	6128	ribosomal protein L6
ENSG0000	1832.263	-0.36739	0.132398	0.001352	0.028976	CARS2	79587	cysteinyl-t mitochondrial
ENSG0000	2.324601	0.144972	0.303681	0.001363	0.029162	NA	NA	NA
ENSG0000	440.2995	-0.51936	0.211564	0.00137	0.029282	WLS	79971	Wnt ligand secretion mediator
ENSG0000	1170.831	-0.32646	0.114535	0.001377	0.029389	DESI1	27351	desumoylating isopeptidase 1
ENSG0000	2106.539	0.995623	0.655678	0.001387	0.029588	PI3	5266	peptidase inhibitor 3
ENSG0000	819.1501	0.536629	0.220019	0.001393	0.029604	HLA-DMB	3109	major hist class II DM beta
ENSG0000	642.3693	-0.5923	0.255061	0.001392	0.029604	ZNF438	220929	zinc finger protein 438
ENSG0000	947.0993	-0.43394	0.164459	0.00139	0.029604	SLA2	84174	Src like adaptor 2
ENSG0000	1406.637	-0.48984	0.194209	0.001405	0.029744	ZNF107	51427	zinc finger protein 107

ENSG0000	137.3945	-0.56321	0.231533	0.001403	0.029744	DENND3-A	1.02E+08	DENND3 antisense RNA 1
ENSG0000	5228.791	-0.511	0.206215	0.001404	0.029744	TUBA1A	7846	tubulin alpha 1a
ENSG0000	216.9081	-0.57171	0.24045	0.001407	0.029749	ADPRH	141	ADP-ribosylarginine hydrolase
ENSG0000	529.1009	-0.48821	0.192518	0.001408	0.029749	AMBRA1	55626	autophagy and beclin 1 regulator 1
ENSG0000	93.11133	-0.80889	0.413579	0.001419	0.029954	NA	NA	NA
ENSG0000	465.6866	-0.38898	0.143502	0.001424	0.029973	KLHL6	89857	kelch like family member 6
ENSG0000	16.64574	0.922901	0.445959	0.001422	0.029973	PXN-AS1	1.01E+08	PXN antisense RNA 1
ENSG0000	51.01251	0.890864	0.485464	0.001434	0.030168	YPEL3-DT	1.02E+08	YPEL3 divergent transcript
ENSG0000	66.75792	0.913144	0.548825	0.001443	0.030315	BCAT1	586	branched chain amino acid transaminase 1
ENSG0000	3017.706	0.469412	0.183876	0.001448	0.030354	RPS6	6194	ribosomal protein S6
ENSG0000	12.89724	-1.2066	0.780798	0.001448	0.030354	KLHL35	283212	kelch like family member 35
ENSG0000	95.34201	0.550835	0.22599	0.001462	0.030611	ERP27	121506	endoplasmic reticulum protein 27
ENSG0000	230.9807	-0.43913	0.167917	0.001465	0.030626	TOMM40L	84134	translocase of outer mitochondrial membrane 40 like
ENSG0000	2186.153	0.404774	0.152632	0.001466	0.030626	PHB2	11331	prohibitin 2
ENSG0000	2.639051	2.324379	1.294917	0.001483	0.030911	CC2D2A	57545	coiled-coil and C2 domain containing 2A
ENSG0000	19.04953	0.99136	0.518112	0.001482	0.030911	VSTM2L	128434	V-set and transmembrane domain containing 2 like
ENSG0000	571.062	-0.45186	0.174917	0.001488	0.030975	DNASE1	1773	deoxyribonuclease 1
ENSG0000	1393.948	-0.50955	0.207468	0.001497	0.03113	SLC27A3	11000	solute carrier family 27 member 3
ENSG0000	87.77974	0.509774	0.203469	0.001501	0.031164	DCLRE1B	64858	DNA cross-link repair 1B
ENSG0000	2098.436	-0.49211	0.197561	0.001515	0.031431	RNF19B	127544	ring finger protein 19B
ENSG0000	314.3096	-0.4455	0.173064	0.00153	0.031701	BLVRA	644	biliverdin reductase A
ENSG0000	103.5437	0.757537	0.371894	0.001537	0.031818	RAB36	9609	RAB36 member RAS oncogene family
ENSG0000	730.7238	-0.39767	0.153388	0.001541	0.031862	TOR1A	1861	torsin family 1 member A
ENSG0000	147.5929	-0.57487	0.246094	0.001566	0.0323	NA	NA	NA
ENSG0000	6.852825	-0.18116	0.342117	0.001565	0.0323	SLC8A2	6543	solute carrier family 8 member A2
ENSG0000	2777.096	0.467122	0.184126	0.001572	0.032399	UBXN11	91544	UBX domain protein 11
ENSG0000	189.6345	-0.43866	0.169012	0.001582	0.032569	PRKCA	5578	protein kinase C alpha
ENSG0000	3.332009	0.113606	0.274105	0.001586	0.032613	NA	NA	NA
ENSG0000	940.5375	0.720285	0.365172	0.001593	0.032698	IGHV2-5	28457	immunoglobulin heavy variable 2-5
ENSG0000	131.6987	0.4763	0.18818	0.001594	0.032698	PRDM15	63977	PR/SET domain 15
ENSG0000	253.2113	0.688622	0.333364	0.001606	0.032906	DPEP3	64180	dipeptidase 3
ENSG0000	30.74514	-0.95051	0.57902	0.001627	0.033293	NA	NA	NA
ENSG0000	37.62166	-0.86113	0.463478	0.00163	0.033296	RAB19	401409	RAB19 member RAS oncogene family
ENSG0000	8.264929	0.252255	0.473412	0.001629	0.033296	ZC2HC1A	51101	zinc finger C2HC-type containing 1A
ENSG0000	11949.05	0.449745	0.176709	0.001633	0.033301	RPL13A	23521	ribosomal protein L13a
ENSG0000	370.9768	0.405122	0.153504	0.001654	0.033695	LSM4	25804	LSM4 hom U6 small nuclear RNA and mRNA degradation associated
ENSG0000	632.6913	0.486822	0.197349	0.001668	0.033943	LINC0092€	283663	long intergenic non-protein coding RNA 926
ENSG0000	182.9305	-0.40749	0.15452	0.001677	0.034066	MICA	1.01E+08	MHC class I polypeptide-related sequence A
ENSG0000	5911.282	-0.45279	0.179491	0.001677	0.034066	ELF4	2000	E74 like ETS transcription factor 4
ENSG0000	135.8566	0.678261	0.323075	0.001689	0.034221	AHR	196	aryl hydrocarbon receptor
ENSG0000	24.58167	0.786583	0.376243	0.001688	0.034221	TXNDC16	57544	thioredoxin domain containing 16
ENSG0000	202.9812	-0.67761	0.327367	0.001699	0.034395	CD300H	1E+08	CD300H molecule (gene/pseudogene)
ENSG0000	33.18551	0.690978	0.314082	0.001708	0.034539	LINC00852	84657	long intergenic non-protein coding RNA 852
ENSG0000	602.2754	-0.40385	0.154003	0.001712	0.03454	NIT1	4817	nitrilase 1
ENSG0000	9.851524	-1.1115	0.592685	0.001711	0.03454	NA	NA	NA
ENSG0000	5.010285	1.976071	1.093315	0.001721	0.034679	TRPV6	55503	transient receptor potential cation channel subfamily V member 6
ENSG0000	369.1909	-0.49517	0.206346	0.001733	0.034878	USP25	29761	ubiquitin specific peptidase 25
ENSG0000	79.77744	-0.79507	0.416291	0.001736	0.034906	GYPB	2994	glycophorin B (MNS blood group)
ENSG0000	183.7878	-0.58698	0.259998	0.001738	0.034906	TLR7	51284	toll like receptor 7
ENSG0000	2201.217	-0.45934	0.184115	0.001742	0.03495	STX11	8676	syntaxin 11
ENSG0000	9783.461	-0.49776	0.206542	0.001748	0.035036	NADK	65220	NAD kinase
ENSG0000	4081.977	-0.48218	0.195744	0.001762	0.035179	GBP2	2634	guanylate binding protein 2
ENSG0000	337.2939	0.610615	0.278931	0.001757	0.035179	MEF2C	4208	myocyte enhancer factor 2C
ENSG0000	186.9843	-0.57486	0.252226	0.001759	0.035179	H2BC4	8347	H2B clustered histone 4
ENSG0000	4174.168	-0.61174	0.284301	0.001763	0.035179	TBKBP1	9755	TBK1 binding protein 1
ENSG0000	9.497091	1.426728	1.053515	0.001781	0.035502	NA	NA	NA
ENSG0000	1318.668	-0.45303	0.18262	0.001783	0.035506	HEXD	284004	hexosaminidase D
ENSG0000	186.7564	-0.69232	0.344494	0.001796	0.035714	SPOCD1	90853	SPOC domain containing 1
ENSG0000	10.60589	0.963147	0.470578	0.001798	0.035714	LOC10065	1.01E+08	uncharacterized LOC100652758
ENSG0000	19.99611	-0.9176	0.504174	0.001802	0.035769	CEL	1056	carboxyl ester lipase
ENSG0000	24.10263	-1.04564	0.846394	0.001822	0.036121	SUCNR1	56670	succinate receptor 1
ENSG0000	304.3091	-0.46827	0.189485	0.001826	0.036145	IGF2BP2	10644	insulin like growth factor 2 mRNA binding protein 2
ENSG0000	30.06193	0.663143	0.297038	0.001829	0.036145	GAN	8139	gigaxonin
ENSG0000	13.65046	-1.07339	0.677393	0.001829	0.036145	SOCS3-DT	1.02E+08	SOCS3 divergent transcript
ENSG0000	27.77972	0.769014	0.367867	0.001838	0.036247	PPFIA4	8497	PTPRF interacting protein alpha 4
ENSG0000	87.71286	-0.66955	0.32083	0.001838	0.036247	MASTL	84930	microtubule associated serine/threonine kinase like
ENSG0000	2.492478	0.128752	0.287825	0.001842	0.036272	PTPRG	5793	protein tyrosine phosphatase receptor type G
ENSG0000	35.31378	0.922215	0.54493	0.001849	0.036353	EAF2	55840	ELL associated factor 2
ENSG0000	8207.124	-0.51674	0.219719	0.00185	0.036353	FRAT2	23401	FRAT regulator of WNT signaling pathway 2
ENSG0000	7.772255	-0.19985	0.367217	0.001856	0.036439	TTC39A	22996	tetratricopeptide repeat domain 39A
ENSG0000	1198.24	0.489924	0.202652	0.001869	0.036652	C20orf27	54976	chromosome 20 open reading frame 27
ENSG0000	891.2557	-0.48952	0.203676	0.001874	0.036673	PPP1R12B	4660	protein phosphatase 1 regulatory subunit 12B
ENSG0000	4346.39	-0.58498	0.264442	0.001876	0.036673	LILRA5	353514	leukocyte immunoglobulin like receptor A5
ENSG0000	2095.066	0.336102	0.122597	0.001874	0.036673	MFNG	4242	MFNG O-fucosylpeptide 3-beta-N-acetylglucosaminyltransferase
ENSG0000	354.7933	0.383911	0.145637	0.001883	0.036733	HDHD5	27440	haloacid dehalogenase like hydrolase domain containing 5
ENSG0000	51.35603	0.863153	0.527162	0.001883	0.036733	NA	NA	NA
ENSG0000	4658.119	-0.56231	0.252987	0.001893	0.036893	LIMK2	3985	LIM domain kinase 2
ENSG0000	195.6935	-0.47531	0.194114	0.001905	0.037091	NA	NA	NA
ENSG0000	11.29194	0.138043	0.293659	0.00192	0.037341	PHF24	23349	PHD finger protein 24
ENSG0000	18.52215	-0.82343	0.412088	0.001927	0.037426	MT1X	4501	metallothionein 1X
ENSG0000	433.5676	-0.36401	0.13683	0.001931	0.037442	SLC20A1	6574	solute carrier family 20 member 1
ENSG0000	1724.119	-0.55747	0.252061	0.001934	0.037442	TREML1	340205	triggering receptor expressed on myeloid cells like 1
ENSG0000	35.51521	-0.803	0.421563	0.001933	0.037442	WDR11-DT	283089	WDR11 divergent transcript
ENSG0000	28.17856	-0.86259	0.479398	0.001962	0.037957	PTPRS	5802	protein tyrosine phosphatase receptor type S
ENSG0000	528.8825	-0.34261	0.125752	0.001966	0.03798	HNRNPH3	3189	heterogeneous nuclear ribonucleoprotein H3
ENSG0000	493.8085	-0.5644	0.25336	0.002001	0.03863	ADCY4	196883	adenylate cyclase 4
ENSG0000	390.5698	-0.43596	0.174018	0.002004	0.038638	NA	NA	NA
ENSG0000	860.9701	-0.43277	0.172722	0.00202	0.038914	UBQLN2	29978	ubiquilin 2
ENSG0000	2.905031	1.947557	1.469946	0.002032	0.039008	NA	NA	NA
ENSG0000	545.0746	-0.56731	0.259152	0.00203	0.039008	H1-2	3006	H1.2 linker cluster member
ENSG0000	2654.269	-0.45566	0.190197	0.002029	0.039008	FAM210B	116151	family with sequence similarity 210 member B
ENSG0000	2084.04	-0.61072	0.294178	0.002046	0.039246	GBA1	2629	glucosylceramidase beta 1
ENSG0000	71.31682	0.931035	0.915901	0.002053	0.039341	IGHV2-70D	1.03E+08	immunoglobulin heavy variable 2-70D
ENSG0000	23.863	-0.80418	0.408959	0.00206	0.03939	OR2B6	26212	olfactory receptor family 2 subfamily B member 6
ENSG0000	5.722993	-0.13018	0.285433	0.002058	0.03939	LGSN	51557	lengsin lens protein with glutamine synthetase domain
ENSG0000	16.27438	-0.85145	0.442483	0.002091	0.039926	NA	NA	NA

ENSG0000	71.63826	-0.68462	0.341232	0.002092	0.039926	FOSL1	8061	FOS like 1 AP-1 transcription factor subunit
ENSG0000	11622.71	-0.49273	0.210524	0.002098	0.039988	NINJ1	4814	ninjurin 1
ENSG0000	355.2952	-0.4979	0.212128	0.002114	0.040253	CASZ1	54897	castor zinc finger 1
ENSG0000	4.180449	1.605384	1.034642	0.00212	0.040287	TDRKH-AS	1.1E+08	TDRKH antisense RNA 1
ENSG0000	5.63835	-1.58217	1.019653	0.00212	0.040287	LINC0208(	1.02E+08	long intergenic non-protein coding RNA 2080
ENSG0000	3.127613	2.470542	1.465469	0.002128	0.040403	TP63	8626	tumor protein p63
ENSG0000	45.17422	-0.69445	0.340267	0.002136	0.040456	NA	NA	NA
ENSG0000	126.156	-0.55694	0.247939	0.002134	0.040456	NA	NA	NA
ENSG0000	204.5447	-0.64685	0.316908	0.00214	0.040488	KIR2DS4	3809	killer cell i two Ig domains and short cytoplasmic tail 4
ENSG0000	368.3999	-0.72428	0.422716	0.002146	0.040567	RNF208	727800	ring finger protein 208
ENSG0000	596.254	-0.46943	0.196113	0.002151	0.040585	FNDC3B	64778	fibronectin type III domain containing 3B
ENSG0000	4.42448	-0.12917	0.284793	0.00215	0.040585	LRRC73	221424	leucine rich repeat containing 73
ENSG0000	8.04783	-1.14112	0.746817	0.002165	0.040802	LOC12490	1.25E+08	uncharacterized LOC124902559
ENSG0000	297.9732	-0.39177	0.152728	0.002177	0.040934	PAX8-AS1	654433	PAX8 antisense RNA 1
ENSG0000	4360.971	0.352148	0.132863	0.002176	0.040934	RNF44	22838	ring finger protein 44
ENSG0000	10.04359	-1.10056	0.792658	0.002188	0.041116	H3C12	8356	H3 clustered histone 12
ENSG0000	5.161545	1.487549	0.943972	0.002191	0.041127	NA	NA	NA
ENSG0000	5241.682	0.407817	0.163438	0.002206	0.041323	RPL7A	6130	ribosomal protein L7a
ENSG0000	25.78902	-0.87469	0.513153	0.002206	0.041323	SLFN14	342618	schlafen family member 14
ENSG0000	69.81843	-0.70462	0.371314	0.00223	0.04138	CHROMR	1.02E+08	cholesterol induced regulator of metabolism RNA
ENSG0000	103.8186	-0.46993	0.195551	0.002236	0.04138	TBCK	93627	TBC1 domain containing kinase
ENSG0000	2951.706	0.427683	0.172967	0.002216	0.04138	RPS14	6208	ribosomal protein S14
ENSG0000	2.498334	-0.17089	0.33201	0.002231	0.04138	DELEC1	50514	deleted in esophageal cancer 1
ENSG0000	242.2132	0.376062	0.145512	0.002213	0.04138	SLC35E3	55508	solute carrier family 35 member E3
ENSG0000	783.4121	-0.58693	0.279441	0.002235	0.04138	CMTM5	116173	CKLF like MARVEL transmembrane domain containing 5
ENSG0000	97.47381	0.493159	0.207756	0.002225	0.04138	GSTZ1	2954	glutathione S-transferase zeta 1
ENSG0000	157.864	0.814808	0.50065	0.002226	0.04138	IGHV1-69	28461	immunoglobulin heavy variable 1-69
ENSG0000	1617.954	0.451485	0.181942	0.002223	0.04138	CIITA	4261	class II major histocompatibility complex transactivator
ENSG0000	3558.057	-0.45233	0.187634	0.002223	0.04138	SLC66A2	80148	solute carrier family 66 member 2
ENSG0000	3275.018	-0.36383	0.139695	0.00222	0.04138	RAB8A	4218	RAB8A member RAS oncogene family
ENSG0000	380.6779	-0.61781	0.305185	0.002234	0.04138	CDC42EP1	11135	CDC42 effector protein 1
ENSG0000	221.1056	-0.52539	0.232235	0.00224	0.041401	E2F1	1869	E2F transcription factor 1
ENSG0000	166.5243	-0.69475	0.37489	0.002265	0.04183	CASP5	838	caspase 5
ENSG0000	2507.542	0.372077	0.143945	0.002295	0.042331	OXA1L	5018	OXA1L mitochondrial inner membrane protein
ENSG0000	38.41968	0.589574	0.26486	0.002298	0.042349	TTC34	1E+08	tetratricopeptide repeat domain 34
ENSG0000	105.2373	-0.43561	0.17612	0.002306	0.042446	TBX19	9095	T-box transcription factor 19
ENSG0000	147.1084	-0.62101	0.311102	0.002311	0.042498	KIR3DL1	3811	killer cell i three Ig domains and long cytoplasmic tail 1
ENSG0000	93.63388	0.512697	0.223421	0.002315	0.042527	FAM117B	150864	family with sequence similarity 117 member B
ENSG0000	193.9594	-0.62676	0.309513	0.002318	0.042548	IGHEP2	3499	immunoglobulin heavy constant epsilon P2 (pseudogene)
ENSG0000	5.606363	-1.30877	1.114353	0.002334	0.042798	NA	NA	NA
ENSG0000	46.83426	0.703388	1.212488	0.00234	0.042867	LINC0220;	1.02E+08	long intergenic non-protein coding RNA 2207
ENSG0000	5808.424	0.469448	0.199246	0.002346	0.042935	CABIN1	23523	calcineurin binding protein 1
ENSG0000	16.29414	0.942308	0.559163	0.002352	0.043005	LINC0173(	1.02E+08	long intergenic non-protein coding RNA 1736
ENSG0000	693.231	0.329416	0.123759	0.002365	0.043189	DELE1	9812	DAP3 binding cell death enhancer 1
ENSG0000	369.4213	-0.38419	0.151357	0.002368	0.043213	BBX	56987	BBX high mobility group box domain containing
ENSG0000	1140.363	0.367573	0.142945	0.002378	0.043346	SLC25A3	5250	solute carrier family 25 member 3
ENSG0000	2989.42	-0.55472	0.260016	0.002384	0.043413	SAT1	6303	spermidine/spermine N1-acetyltransferase 1
ENSG0000	1753.69	-0.42918	0.176234	0.002388	0.043442	PSME2	5721	proteasome activator subunit 2
ENSG0000	88036.76	0.488481	0.212196	0.002398	0.043587	COX3	4514	cytochrome c oxidase subunit III
ENSG0000	1443.506	-0.53792	0.248738	0.002422	0.043968	H2BC12	85236	H2B clustered histone 12
ENSG0000	11.23318	0.989847	1.297076	0.00243	0.044077	LOC10192	1.02E+08	uncharacterized LOC101929719
ENSG0000	86.42309	-0.20008	0.363528	0.002444	0.044284	LINC0109:	1.01E+08	long intergenic non-protein coding RNA 1093
ENSG0000	348.0389	0.363224	0.138949	0.002449	0.044316	HSD17B4	3295	hydroxysteroid 17-beta dehydrogenase 4
ENSG0000	66.8974	0.579924	0.268366	0.002451	0.044316	FUOM	282969	fucose mutarotase
ENSG0000	8.724771	-1.02908	0.608589	0.002454	0.044331	NA	NA	NA
ENSG0000	99.67134	-0.51593	0.227174	0.002464	0.044475	BRCA1	672	BRCA1 DNA repair associated
ENSG0000	43.82947	-0.62805	0.301331	0.002479	0.04466	NA	NA	NA
ENSG0000	1604.556	-0.5146	0.232013	0.002478	0.04466	ACRBP	84519	acrosin binding protein
ENSG0000	606.5465	0.439677	0.183571	0.002485	0.044707	GNG7	2788	G protein subunit gamma 7
ENSG0000	578.3241	-0.43055	0.179922	0.002498	0.04491	LINC0141(	1.03E+08	long intergenic non-protein coding RNA 1410
ENSG0000	73.00942	-0.67173	0.348623	0.002514	0.045145	KIR2DL3	3804	killer cell i two Ig domains and long cytoplasmic tail 3
ENSG0000	5.497779	-1.53184	1.030547	0.002522	0.045203	DNASE1L3	1776	deoxyribonuclease 1 like 3
ENSG0000	4353.2	-0.54247	0.251272	0.002521	0.045203	SLC2A3	6515	solute carrier family 2 member 3
ENSG0000	828.7925	0.340475	0.129992	0.002529	0.045292	SGSH	6448	N-sulfoglucosamine sulfohydrolase
ENSG0000	109.9116	-0.46803	0.197901	0.002543	0.0454	GPR137B	7107	G protein-coupled receptor 137B
ENSG0000	3.499286	1.754246	1.784325	0.002542	0.0454	LINC0111(	1E+08	long intergenic non-protein coding RNA 1119
ENSG0000	102.0133	0.522089	0.23263	0.002539	0.0454	LANCL2	55915	LanC like glutathione S-transferase 2
ENSG0000	497.9731	-0.55556	0.263497	0.002552	0.045513	STYXL1	51657	serine/threonine/tyrosine interacting like 1
ENSG0000	415.7536	0.345757	0.133015	0.002565	0.045708	FAM168B	130074	family with sequence similarity 168 member B
ENSG0000	13.80976	0.854606	0.468113	0.002568	0.045713	SATB2	23314	SATB homeobox 2
ENSG0000	3203.49	0.632367	0.335963	0.002573	0.045717	ITM2C	81618	integral membrane protein 2C
ENSG0000	68.32823	0.531582	0.237515	0.002572	0.045717	FAM204A	63877	family with sequence similarity 204 member A
ENSG0000	118.8555	-0.61232	0.29975	0.002576	0.045732	TRGC1	6966	T cell receptor gamma constant 1
ENSG0000	313.6751	-0.42464	0.175416	0.002598	0.046074	ATP10A	57194	ATPase phospholipid transporting 10A (putative)
ENSG0000	41.50757	0.851303	0.656083	0.002608	0.046158	NA	NA	NA
ENSG0000	708.643	-0.32599	0.123515	0.00261	0.046158	TBC1D20	128637	TBC1 domain family member 20
ENSG0000	1775.422	0.716026	0.470207	0.002611	0.046158	IGLV4-69	28784	immunoglobulin lambda variable 4-69
ENSG0000	137.2072	-0.63323	0.327768	0.002638	0.046606	TIFA	92610	TRAF interacting protein with forkhead associated domain
ENSG0000	575.9134	0.634388	0.337664	0.002641	0.046611	IGHV4-59	28392	immunoglobulin heavy variable 4-59
ENSG0000	39.5283	0.834805	0.536515	0.002648	0.046687	TMIGD3	57413	transmembrane and immunoglobulin domain containing 3
ENSG0000	2085.988	-0.54093	0.256576	0.002659	0.046785	B4GALT5	9334	beta-1 4-galactosyltransferase 5
ENSG0000	21440.3	-0.52643	0.236311	0.002656	0.046785	PPDPF	79144	pancreatic progenitor cell differentiation and proliferation factor
ENSG0000	2508.174	-0.47866	0.211114	0.002684	0.047136	C11orf68	83638	chromosome 11 open reading frame 68
ENSG0000	4831.528	-0.5717	0.281136	0.002683	0.047136	SLC4A1	6521	solute carrier family 4 member 1 (Diego blood group)
ENSG0000	1507.203	-0.37286	0.148381	0.002689	0.047177	APOBEC3(	60489	apolipoprotein B mRNA editing enzyme catalytic subunit 3G
ENSG0000	11376.61	-0.54319	0.258738	0.002703	0.047385	MXD1	4084	MAX dimerization protein 1
ENSG0000	1192.668	-0.32754	0.125448	0.002712	0.047502	STMP1	647087	short transmembrane mitochondrial protein 1
ENSG0000	12647.93	-0.40476	0.16646	0.00274	0.047942	MYD88	4615	MYD88 innate immune signal transduction adaptor
ENSG0000	10.45448	0.963282	0.567928	0.002755	0.048149	NEURL2	140825	neuralized E3 ubiquitin protein ligase 2
ENSG0000	1597.3	-0.41509	0.171564	0.002783	0.048493	CD247	919	CD247 molecule
ENSG0000	1774.707	-0.68911	0.413855	0.002781	0.048493	PROK2	60675	prokineticin 2
ENSG0000	60.5947	-0.61604	0.305936	0.002783	0.048493	TRBV6-5	28602	T cell receptor beta variable 6-5
ENSG0000	201.5916	0.49373	0.219301	0.002785	0.048493	FADS3	3995	fatty acid desaturase 3
ENSG0000	35.33331	0.560179	0.253274	0.002788	0.048498	TBC1D16	125058	TBC1 domain family member 16
ENSG0000	17622.53	-0.52319	0.244691	0.002799	0.048541	ALOX5AP	241	arachidonate 5-lipoxygenase activating protein
ENSG0000	276.2741	0.678687	0.396299	0.002797	0.048541	IGHG4	3503	immunoglobulin heavy constant gamma 4 (G4m marker)

ENSG0000	105.5617	0.587211	0.289819	0.002801	0.048541	CPAMD8	27151	C3 and PZP like alpha-2-macroglobulin domain containing 8
ENSG0000	2689.923	-0.46266	0.200208	0.002796	0.048541	TMEM184	25829	transmembrane protein 184B
ENSG0000	405.1389	-0.48963	0.220127	0.00281	0.048584	RNASEL	6041	ribonuclease L
ENSG0000	271.7967	0.534585	0.250533	0.002811	0.048584	TOMM20	9804	translocase of outer mitochondrial membrane 20
ENSG0000	625.9877	-0.34483	0.139775	0.002807	0.048584	ACSL5	51703	acyl-CoA synthetase long chain family member 5
ENSG0000	39.14123	0.738006	0.414971	0.002819	0.048673	FMN1	342184	formin 1
ENSG0000	1257.255	-0.4106	0.170613	0.002833	0.048857	SUPT20H	55578	SPT20 hon SAGA complex component
ENSG0000	184.4679	0.401559	0.164763	0.002841	0.048951	COQ9	57017	coenzyme Q9
ENSG0000	12.78388	0.976288	0.622792	0.002877	0.049532	DMRTC2	63946	DMRT like family C2
ENSG0000	295.5946	-0.64117	0.35158	0.002914	0.050028	TNFAIP6	7130	TNF alpha induced protein 6
ENSG0000	263.6069	-0.49314	0.222081	0.002909	0.050028	CYB561	1534	cytochrome b561
ENSG0000	3128.747	0.454849	0.198454	0.002911	0.050028	PPM1F	9647	protein ph Mg2+/Mn2+ dependent 1F
ENSG0000	32.97933	0.839686	0.563927	0.002923	0.050136	WNT5B	81029	Wnt family member 5B
ENSG0000	122.6403	0.758581	0.52495	0.002935	0.050289	LOC10192	1.02E+08	uncharacterized LOC101929707
ENSG0000	3.011307	0.11994	0.27726	0.00296	0.050673	NA	NA	NA
ENSG0000	7325.568	-0.43538	0.187402	0.002972	0.05083	NA	NA	NA
ENSG0000	66.9866	-0.24441	0.449229	0.002978	0.050895	SGIP1	84251	SH3GL interacting endocytic adaptor 1
ENSG0000	396.2186	-0.50756	0.235376	0.002986	0.050955	LOC12490	1.25E+08	uncharacterized LOC124903659
ENSG0000	14.08483	-0.12275	0.277351	0.002988	0.050955	ALOX15	246	arachidonate 15-lipoxygenase
ENSG0000	94.58632	-0.70044	0.422153	0.002991	0.050973	MS4A4A	51338	membrane spanning 4-domains A4A
ENSG0000	734.2467	-0.40022	0.163587	0.002995	0.050991	CLDN15	24146	claudin 15
ENSG0000	33.91355	-0.81256	0.681047	0.003012	0.05118	NA	NA	NA
ENSG0000	10.18504	-1.01984	1.035264	0.00301	0.05118	GNAL	2774	G protein subunit alpha L
ENSG0000	25.92569	0.805073	0.511517	0.003016	0.051195	ESF1	51575	ESF1 nucleolar pre-rRNA processing protein homolog
ENSG0000	6.14508	1.41418	1.13333	0.00307	0.052073	HRK	8739	harakiri BCL2 interacting protein
ENSG0000	3.612494	0.154654	0.309121	0.003074	0.052097	IGHD3-22	28497	immunoglobulin heavy diversity 3-22
ENSG0000	17.01338	-0.29606	0.572242	0.003095	0.052394	NMRAL2P	344887	NmrA like pseudogene
ENSG0000	300.9844	-0.47599	0.214292	0.00312	0.052722	TMEM268	203197	transmembrane protein 268
ENSG0000	216.6716	0.546963	0.266714	0.00312	0.052722	CYP2S1	29785	cytochrome P450 family 2 subfamily S member 1
ENSG0000	291.0439	-0.43578	0.189141	0.00314	0.052965	LINC00847	729678	long intergenic non-protein coding RNA 847
ENSG0000	20.74269	-0.78795	0.474762	0.003137	0.052965	TRD-AS1	1.05E+08	TRD antisense RNA 1
ENSG0000	3.919328	0.148985	0.303204	0.003149	0.053072	S100A7	6278	S100 calcium binding protein A7
ENSG0000	488.0759	-0.54736	0.271836	0.003155	0.053118	CMTM1	113540	CKLF like MARVEL transmembrane domain containing 1
ENSG0000	69.51868	1.267137	0.67256	0.00316	0.053146	IGHV1-69-	28458	immunoglobulin heavy variable 1-69-2
ENSG0000	1503.016	0.293155	0.111631	0.003211	0.053956	UBE2I	7329	ubiquitin conjugating enzyme E2 I
ENSG0000	50.91127	-0.57366	0.278861	0.003214	0.053965	CLEC1A	51267	C-type lectin domain family 1 member A
ENSG0000	33.79457	-0.57643	0.276417	0.003223	0.054073	CD63-AS1	1.05E+08	CD63 antisense RNA 1
ENSG0000	12249.15	0.356706	0.14384	0.003231	0.054155	RPL13	6137	ribosomal protein L13
ENSG0000	122.2091	0.505894	0.234843	0.003239	0.054231	YPEL2	388403	yippee like 2
ENSG0000	22.51382	-0.8277	0.60322	0.003244	0.05427	H2BC7	8343	H2B clustered histone 7
ENSG0000	43.53988	0.568967	0.273537	0.003259	0.054417	ZNF16	7564	zinc finger protein 16
ENSG0000	17.80585	0.859171	0.691351	0.003258	0.054417	LOC12490	1.25E+08	uncharacterized LOC124903386
ENSG0000	674.4573	0.362668	0.146957	0.003265	0.054465	SYNE3	161176	spectrin repeat containing nuclear envelope family member 3
ENSG0000	235.6365	0.407251	0.17309	0.003285	0.054759	MCRIP2	84331	MAPK regulated corepressor interacting protein 2
ENSG0000	14.99334	-0.80776	0.483326	0.003314	0.055132	PPM1J	333926	protein ph Mg2+/Mn2+ dependent 1J
ENSG0000	4070.569	-0.53048	0.261613	0.003314	0.055132	SLA	6503	Src like adaptor
ENSG0000	2.967854	-0.11972	0.278172	0.003323	0.055235	CCL8	6355	C-C motif chemokine ligand 8
ENSG0000	4.942598	-0.18714	0.348088	0.003331	0.055262	LOC10192	1.02E+08	uncharacterized LOC101927164
ENSG0000	56.47221	0.34677	0.686049	0.003329	0.055262	MACIR	90355	macrophage immunometabolism regulator
ENSG0000	45.5806	-0.54948	0.263169	0.003344	0.055439	LINC0029	339789	long intergenic non-protein coding RNA 299
ENSG0000	13315.96	0.369882	0.151833	0.003361	0.05567	RACK1	10399	receptor for activated C kinase 1
ENSG0000	457.9468	-0.40633	0.173863	0.003367	0.055719	CUL1	8454	cullin 1
ENSG0000	9.961455	-1.15587	0.974727	0.003373	0.055768	NA	NA	NA
ENSG0000	25765.1	-0.41758	0.180872	0.003411	0.056346	MNDA	4332	myeloid cell nuclear differentiation antigen
ENSG0000	121.0865	-0.60335	0.324215	0.003414	0.056346	P2RY2	5029	purinergic receptor P2Y2
ENSG0000	1620.251	-0.53252	0.26589	0.003432	0.056591	CYP4F3	4051	cytochrome P450 family 4 subfamily F member 3
ENSG0000	107.698	0.4318	0.186787	0.003443	0.056716	PKP4	8502	plakophilin 4
ENSG0000	18834.87	-0.57553	0.307036	0.003482	0.0573	NFE2	4778	nuclear fa erythroid 2
ENSG0000	37.89143	-0.63219	0.331153	0.003502	0.057583	PODN	127435	podocan
ENSG0000	7886.344	-0.57157	0.307751	0.003517	0.057759	NPRL3	8131	NPR3 like GATOR1 complex subunit
ENSG0000	427.9561	-0.45868	0.208614	0.003519	0.057759	FLCN	201163	folliculin
ENSG0000	108.3618	-0.47648	0.217869	0.003528	0.057855	NA	NA	NA
ENSG0000	20.96232	-0.75109	0.454783	0.003534	0.057868	CAMK2N1	55450	calcium/calmodulin dependent protein kinase II inhibitor 1
ENSG0000	1711.692	0.38063	0.159298	0.003535	0.057868	RPL37A	6168	ribosomal protein L37a
ENSG0000	350.5491	0.65222	0.411131	0.003556	0.058163	TPST1	8460	tyrosylprotein sulfotransferase 1
ENSG0000	726.4497	0.42668	0.189376	0.003567	0.058289	BLK	640	BLK proto- Src family tyrosine kinase
ENSG0000	220.3491	-0.44415	0.197759	0.003572	0.058324	ZNF200	7752	zinc finger protein 200
ENSG0000	194.1141	-0.48578	0.226706	0.003577	0.05834	STOML1	9399	stomatin like 1
ENSG0000	156.1379	-0.46387	0.210639	0.003622	0.059027	SPSB1	80176	splA/ryanodine receptor domain and SOCS box containing 1
ENSG0000	21.43263	-0.65475	0.342593	0.003627	0.059052	NA	NA	NA
ENSG0000	601.7507	-0.36431	0.150562	0.003633	0.059088	TSPOAP1-/	1.01E+08	TSPOAP1 SUPT4H1 and RNF43 antisense RNA 1
ENSG0000	157.1366	-0.44177	0.196518	0.003635	0.059088	CBR1	873	carbonyl reductase 1
ENSG0000	836.8511	-0.35734	0.14681	0.003639	0.059089	CIR1	9541	corepress CIR1
ENSG0000	1126.987	-0.51911	0.259012	0.003644	0.05909	HCAR3	8843	hydroxycarboxylic acid receptor 3
ENSG0000	17.4356	-0.73795	0.413169	0.003645	0.05909	NA	NA	NA
ENSG0000	3732.004	-0.34845	0.140349	0.003649	0.059102	CCNK	8812	cyclin K
ENSG0000	6135.907	-0.47697	0.225161	0.003707	0.059931	PRKD2	25865	protein kinase D2
ENSG0000	105.9548	-0.57712	0.302911	0.003707	0.059931	CYP4F29P	54055	cytochrom pseudogene
ENSG0000	964.4722	0.420167	0.183492	0.003758	0.060695	PABPC4	8761	poly(A) binding protein cytoplasmic 4
ENSG0000	29.66701	-0.67124	0.376646	0.003761	0.060695	NKD1	85407	NKD inhibitor of WNT signaling pathway 1
ENSG0000	272.351	-0.3891	0.164251	0.003772	0.06082	RAB3GAP1	22930	RAB3 GTPase activating protein catalytic subunit 1
ENSG0000	137.7699	-0.55987	0.291414	0.00378	0.060852	EPHB1	2047	EPH receptor B1
ENSG0000	2642.284	-0.48284	0.227578	0.003779	0.060852	TRBC1	28639	T cell receptor beta constant 1
ENSG0000	9661.126	-0.52357	0.264974	0.003794	0.060968	AP5B1	91056	adaptor related protein complex 5 subunit beta 1
ENSG0000	87.90643	-0.59041	0.31628	0.003793	0.060968	KIR3DX1	90011	killer cell i three Ig domains X1 (pseudogene)
ENSG0000	123.1632	-0.63566	0.381062	0.003833	0.061446	C2	717	complement C2
ENSG0000	2.507636	0.101533	0.264776	0.003834	0.061446	SLITRK5	26050	SLIT and NTRK like family member 5
ENSG0000	21910.08	0.405127	0.176416	0.003832	0.061446	RPS2	6187	ribosomal protein S2
ENSG0000	1328.261	0.463484	0.217591	0.003856	0.061746	RFLNB	359845	refilin B
ENSG0000	23.14636	0.744859	0.449052	0.003864	0.061813	PLCB1	23236	phospholipase C beta 1
ENSG0000	5.697583	1.076329	1.17745	0.003885	0.062107	NA	NA	NA
ENSG0000	55.13342	0.534246	0.262035	0.003893	0.062124	DLEC1	9940	DLEC1 cilia and flagella associated protein
ENSG0000	90.39963	-0.49273	0.231486	0.003892	0.062124	NT5DC3	51559	5'-nucleotidase domain containing 3
ENSG0000	269.9678	0.428955	0.190609	0.003907	0.062283	FAM30A	9834	family with sequence similarity 30 member A
ENSG0000	8.053446	0.231542	0.41653	0.003918	0.062407	NA	NA	NA
ENSG0000	386.1715	-0.52554	0.266574	0.003948	0.062835	MAP2K6	5608	mitogen-activated protein kinase kinase 6

ENSG0000	3746.015	-0.41924	0.186693	0.003954	0.062873	SAMHD1	25939	SAM and HD domain containing deoxynucleoside triphosphate triphosphohydrolase 1
ENSG0000	129.2482	0.552311	0.28836	0.003971	0.063089	DLGAP3	58512	DLG associated protein 3
ENSG0000	524.5838	-0.45519	0.212166	0.003978	0.063098	SYTL3	94120	synaptotagmin like 3
ENSG0000	13.98478	0.824195	1.453028	0.003976	0.063098	NA	NA	NA
ENSG0000	1772.039	-0.37754	0.159724	0.003986	0.063163	GNS	2799	glucosamine (N-acetyl)-6-sulfatase
ENSG0000	652.6095	-0.31821	0.127295	0.004	0.063274	SMIM29	221491	small integral membrane protein 29
ENSG0000	630.0874	-0.29248	0.116308	0.003997	0.063274	BRD7	29117	bromodomain containing 7
ENSG0000	6.871517	-0.96868	0.652758	0.004019	0.063523	NA	NA	NA
ENSG0000	609.2714	-0.35575	0.14882	0.004036	0.06374	CHST2	9435	carbohydrate sulfotransferase 2
ENSG0000	2287.658	-0.49175	0.236987	0.004045	0.063814	SPARC	6678	secreted protein acidic and cysteine rich
ENSG0000	100.3062	0.587615	0.324309	0.004053	0.063887	TOX2	84969	TOX high mobility group box family member 2
ENSG0000	3.196083	0.170936	0.330724	0.004081	0.064279	NA	NA	NA
ENSG0000	11.57969	0.891496	0.792526	0.004098	0.064484	NA	NA	NA
ENSG0000	191.8605	-0.62814	0.395672	0.004104	0.064531	NA	NA	NA
ENSG0000	9.230758	0.918726	0.637376	0.004134	0.064948	LOC10272	1.03E+08	ICOS ligand
ENSG0000	94.26721	0.451798	0.206936	0.004171	0.065419	NAB2	4665	NGFI-A binding protein 2
ENSG0000	6.768297	0.254008	0.464057	0.004171	0.065419	NA	NA	NA
ENSG0000	136.8013	-0.54883	0.288315	0.004203	0.065864	FRMD4A	55691	FERM domain containing 4A
ENSG0000	5.037046	-0.17599	0.33256	0.004222	0.066084	LINC02217	1.02E+08	long intergenic non-protein coding RNA 2217
ENSG0000	129.758	-0.46867	0.221981	0.004224	0.066084	NA	NA	NA
ENSG0000	2.630504	0.162072	0.319547	0.004228	0.066088	RPL7AP43	644236	ribosomal protein L7a pseudogene 43
ENSG0000	4.597559	0.292981	0.589568	0.004264	0.066593	PLPPR3	79948	phospholipid phosphatase related 3
ENSG0000	27.18393	-0.57169	0.290376	0.00428	0.066778	EPB41L4A	64097	erythrocyte membrane protein band 4.1 like 4A
ENSG0000	23022.31	-0.36099	0.153643	0.004322	0.067386	ZFP36	7538	ZFP36 ring finger protein
ENSG0000	401.2206	0.47236	0.228386	0.004341	0.067566	SNHG29	125144	small nucleolar RNA host gene 29
ENSG0000	38.45625	-0.68071	0.427902	0.004341	0.067566	ANKRD20/	391267	ankyrin re pseudogene
ENSG0000	618.808	-0.5186	0.265823	0.004352	0.067668	TMEM150	284417	transmembrane protein 150B
ENSG0000	36.63585	0.666162	0.395974	0.004356	0.067674	IGHJ3P	28478	immunoglobulin heavy joining 3P (pseudogene)
ENSG0000	48.67774	0.656117	0.412848	0.00437	0.067842	B9D1	27077	B9 domain containing 1
ENSG0000	52.91085	0.544236	0.279739	0.004446	0.068964	GPR157	80045	G protein-coupled receptor 157
ENSG0000	81.95627	-0.41699	0.186482	0.004455	0.069043	PABIR3	159091	PABIR family member 3
ENSG0000	162.5039	-0.51304	0.264153	0.004516	0.069921	TTC7B	145567	tetratricopeptide repeat domain 7B
ENSG0000	1678.54	-0.52242	0.275319	0.004538	0.070214	STOM	2040	stomatin
ENSG0000	17.18108	0.762226	0.542175	0.004559	0.070354	PPM1H	57460	protein ph Mg2+/Mn2+ dependent 1H
ENSG0000	64.48564	0.479044	0.229744	0.004554	0.070354	ZNF629	23361	zinc finger protein 629
ENSG0000	59.44084	-0.4543	0.211793	0.004558	0.070354	ZNF585A	199704	zinc finger protein 585A
ENSG0000	650.6314	-0.54842	0.304866	0.004605	0.071011	KLF1	10661	KLF transcription factor 1
ENSG0000	17.66398	0.776261	0.54662	0.004614	0.071086	TRAM2-AS	401264	TRAM2 antisense RNA 1 (head to head)
ENSG0000	3.431522	0.185462	0.346607	0.004645	0.071504	TMEM220	1.01E+08	TMEM220 antisense RNA 1
ENSG0000	78.69841	-0.5259	0.273937	0.004659	0.0716	PUS3	83480	pseudouridine synthase 3
ENSG0000	26.11211	0.603025	0.325549	0.004659	0.0716	NA	NA	NA
ENSG0000	11.42254	0.839981	0.646677	0.004668	0.071678	RAPH1	65059	Ras association (RalGDS/AF-6) and pleckstrin homology domains 1
ENSG0000	32.4844	-0.7409	0.490607	0.004679	0.071783	DNAAF1	123872	dynein axonemal assembly factor 1
ENSG0000	373.1229	0.553256	0.310843	0.004687	0.071852	IGHV1-3	28473	immunoglobulin heavy variable 1-3
ENSG0000	4.275341	0.117343	0.276737	0.004693	0.071854	NA	NA	NA
ENSG0000	4.001327	0.159901	0.313921	0.004695	0.071854	SH2D4A	63898	SH2 domain containing 4A
ENSG0000	401.187	0.390424	0.173563	0.004734	0.072384	UAP1L1	91373	UDP-N-acetylglucosamine pyrophosphorylase 1 like 1
ENSG0000	1963.218	0.504677	0.265602	0.00476	0.072658	RPS18	6222	ribosomal protein S18
ENSG0000	2.567073	0.150078	0.306076	0.00476	0.072658	NA	NA	NA
ENSG0000	5.94358	0.976603	1.269923	0.004764	0.07266	ZC3H12C	85463	zinc finger CCCH-type containing 12C
ENSG0000	46.6724	0.668679	0.470561	0.004784	0.072911	ARMC12	221481	armadillo repeat containing 12
ENSG0000	38.90809	0.561213	0.299696	0.004805	0.07292	NA	NA	NA
ENSG0000	47.334	0.776505	0.491138	0.004801	0.07292	HBG1	3047	hemoglobin subunit gamma 1
ENSG0000	653.7577	-0.39907	0.179624	0.004805	0.07292	RIPK3	11035	receptor interacting serine/threonine kinase 3
ENSG0000	9296.642	-0.48625	0.247822	0.004809	0.07292	MSRB1	51734	methionine sulfoxide reductase B1
ENSG0000	8.093987	-1.00996	0.736118	0.004809	0.07292	PPP4R1-AS	1.02E+08	PPP4R1 antisense RNA 1
ENSG0000	26239.97	-0.4879	0.250234	0.004797	0.07292	FPR1	2357	formyl peptide receptor 1
ENSG0000	41.35275	-0.61721	0.371619	0.004819	0.073008	NA	NA	NA
ENSG0000	164.7631	-0.44625	0.212515	0.004832	0.073149	RIN2	54453	Ras and Rab interactor 2
ENSG0000	12.40082	-1.00494	0.796079	0.004859	0.0735	SLC2A14	144195	solute carrier family 2 member 14
ENSG0000	8.332372	0.890113	0.829772	0.00489	0.073904	CABP4	57010	calcium binding protein 4
ENSG0000	92.1696	0.514787	0.267546	0.00491	0.07415	CAPN12	147968	calpain 12
ENSG0000	12.02024	-0.28872	0.536707	0.004916	0.074183	NA	NA	NA
ENSG0000	23.21239	-0.68705	0.431214	0.004924	0.074243	PTPRU	10076	protein tyrosine phosphatase receptor type U
ENSG0000	274.9625	-0.34225	0.144784	0.004932	0.074294	TRAF3IP2	10758	TRAF3 interacting protein 2
ENSG0000	11489.09	-0.45963	0.226644	0.00495	0.074488	FGD3	89846	FYVE RhoGEF and PH domain containing 3
ENSG0000	52.8282	0.639286	0.853005	0.004953	0.074488	RCVRN	5957	recoverin
ENSG0000	239.8164	0.453865	0.219468	0.004971	0.074706	BAIAP2-DT	440465	BAIAP2 divergent transcript
ENSG0000	4.624964	0.167207	0.321324	0.004981	0.07479	CXCL9	4283	C-X-C motif chemokine ligand 9
ENSG0000	9.12382	0.86911	0.884567	0.005017	0.075264	NA	NA	NA
ENSG0000	22.67306	0.456203	1.098037	0.005029	0.075382	SRGAP1	57522	SLIT-ROBO Rho GTPase activating protein 1
ENSG0000	6016.007	-0.39955	0.180534	0.005092	0.076207	KIAA0040	9674	KIAA0040
ENSG0000	54.21167	0.194675	0.360644	0.005091	0.076207	KCNMA1	3778	potassium calcium-activated channel subfamily M alpha 1
ENSG0000	2691.911	-0.40443	0.186846	0.005108	0.076296	PHF1	5252	PHD finger protein 1
ENSG0000	325.4959	0.327534	0.13717	0.005111	0.076296	HOOK3	84376	hook microtubule tethering protein 3
ENSG0000	281.3845	-0.30745	0.126091	0.005103	0.076296	NUTM2A-/	728190	NUTM2A antisense RNA 1
ENSG0000	331.6008	-0.48092	0.247188	0.005135	0.076601	CCL4	6351	C-C motif chemokine ligand 4
ENSG0000	130.3115	-0.54256	0.304199	0.005163	0.07695	SLC6A12	6539	solute carrier family 6 member 12
ENSG0000	605.9394	-0.42094	0.199046	0.005243	0.078085	OPTN	10133	optineurin
ENSG0000	642.1226	0.451753	0.223095	0.005248	0.078088	TPCN1	53373	two pore segment channel 1
ENSG0000	969.1765	0.379489	0.170405	0.005252	0.078094	RPS2P5	1E+08	ribosomal protein S2 pseudogene 5
ENSG0000	496.0562	-0.42859	0.205275	0.005263	0.078185	AFF1	4299	ALF transcription elongation factor 1
ENSG0000	119.9679	-0.60489	0.420946	0.005278	0.078226	TPM2	7169	tropomyosin 2
ENSG0000	13.5863	0.74186	0.49537	0.005276	0.078226	LIPT2	387787	lipoyl(octanoyl) transferase 2
ENSG0000	67.70255	0.559232	0.320545	0.005272	0.078226	PIGL	9487	phosphatidylinositol glycan anchor biosynthesis class L
ENSG0000	4.549798	0.250675	0.465682	0.005298	0.07845	NA	NA	NA
ENSG0000	13.94423	0.765416	0.555639	0.005316	0.078657	COCH	1690	cochlin
ENSG0000	894.2844	-0.38669	0.176146	0.005323	0.078696	GRIPAP1	56850	GRIP1 associated protein 1
ENSG0000	1112.727	-0.47461	0.243251	0.005338	0.078789	DDAH2	23564	dimethylarginine dimethylaminohydrolase 2
ENSG0000	742.5762	0.558106	0.339342	0.005337	0.078789	IGHV1-24	28467	immunoglobulin heavy variable 1-24
ENSG0000	25.41107	-0.6342	0.390465	0.00535	0.078906	NA	NA	NA
ENSG0000	7521.513	0.338105	0.145443	0.005362	0.079023	RPL19	6143	ribosomal protein L19
ENSG0000	25.79992	0.554034	0.294529	0.005393	0.079343	DNAAF2	55172	dynein axonemal assembly factor 2
ENSG0000	13310.82	0.577823	0.413411	0.005389	0.079343	IGLC3	3539	immunoglobulin lambda constant 3 (Kern-Oz+ marker)
ENSG0000	106.2734	0.447791	0.21684	0.005405	0.079397	NLN	57486	neurolysin
ENSG0000	3.00591	0.165029	0.322005	0.005403	0.079397	NA	NA	NA

ENSG0000	2.848336	-0.10749	0.267804	0.005462	0.080166	NA	NA	NA
ENSG0000	224.2007	-0.45263	0.223418	0.005489	0.080497	NA	NA	NA
ENSG0000	31.11019	0.57999	0.328404	0.005518	0.080868	PCMTD1-D	1.03E+08	PCMTD1 divergent transcript
ENSG0000	55.17966	0.540401	0.299603	0.005555	0.080881	PLEKHG5	57449	pleckstrin homology and RhoGEF domain containing G5
ENSG0000	106.416	0.567503	0.69341	0.005545	0.080881	NSUN7	79730	NOP2/Sun RNA methyltransferase family member 7
ENSG0000	93115.39	-0.38021	0.173468	0.005533	0.080881	HLA-A	3105	major hist class I A
ENSG0000	905.4613	-0.51522	0.286166	0.005547	0.080881	TMEM176	28959	transmembrane protein 176B
ENSG0000	243.2411	0.374943	0.168392	0.005551	0.080881	SNHG7	84973	small nucleolar RNA host gene 7
ENSG0000	11.28849	0.761584	0.537568	0.005547	0.080881	LOC61303	613038	SAGA complex associated factor 29 pseudogene
ENSG0000	3063.774	-0.41172	0.195538	0.005538	0.080881	N4BP1	9683	NEDD4 binding protein 1
ENSG0000	201.1278	0.503771	0.2715	0.005539	0.080881	STS	412	steroid sulfatase
ENSG0000	3806.437	0.423689	0.18692	0.005573	0.080974	RPL15	6138	ribosomal protein L15
ENSG0000	97.2138	0.55804	0.338804	0.005573	0.080974	DAGLA	747	diacylglycerol lipase alpha
ENSG0000	200.534	0.38526	0.174876	0.005574	0.080974	CCDC106	29903	coiled-coil domain containing 106
ENSG0000	54.55715	0.586653	0.358851	0.005579	0.080984	SPNS3	201305	sphingolipid transporter 3 (putative)
ENSG0000	7.466186	0.880643	0.637694	0.005585	0.081003	NA	NA	NA
ENSG0000	163.3402	-0.50999	0.280625	0.005604	0.081207	CDK18	5129	cyclin dependent kinase 18
ENSG0000	1170.747	0.350512	0.154454	0.00561	0.081243	KLF3	51274	KLF transcription factor 3
ENSG0000	5.890991	0.945854	0.855023	0.005627	0.081384	LOC10537	1.05E+08	LOC105373289
ENSG0000	12.30137	0.791586	0.645053	0.005629	0.081384	FNDC11	79025	fibronectin type III domain containing 11
ENSG0000	5.112399	0.218541	0.392735	0.005686	0.082137	COQ8A	56997	coenzyme Q8A
ENSG0000	44.74485	0.581152	0.354176	0.005707	0.082375	DNAJB5	25822	DnaJ heat shock protein family (Hsp40) member B5
ENSG0000	2688.904	-0.40471	0.190877	0.005716	0.08244	PLAUR	5329	plasminog urokinase receptor
ENSG0000	120.6939	0.302257	0.561575	0.005742	0.08272	GLIS2	84662	GLIS family zinc finger 2
ENSG0000	2772.425	-0.37315	0.169406	0.005744	0.08272	NR1H2	7376	nuclear receptor subfamily 1 group H member 2
ENSG0000	65.15562	0.527993	0.290691	0.005768	0.082999	ABCC6	368	ATP binding cassette subfamily C member 6
ENSG0000	13.69224	0.727283	0.523724	0.005812	0.083568	MITF	4286	melanocyte inducing transcription factor
ENSG0000	53.71005	0.524315	0.289639	0.005821	0.083625	TCFL5	10732	transcription factor like 5
ENSG0000	10.53036	-0.2916	0.53807	0.005837	0.083733	NA	NA	NA
ENSG0000	184.5299	-0.46564	0.239609	0.005835	0.083733	OSER1-DT	1.01E+08	OSER1 divergent transcript
ENSG0000	116.4171	0.524098	0.297783	0.005844	0.083771	ADAM28	10863	ADAM metallopeptidase domain 28
ENSG0000	8893.426	-0.29467	0.119037	0.005859	0.08392	TPM3	7170	tropomyosin 3
ENSG0000	19.36403	-0.83435	1.405131	0.005906	0.08439	NA	NA	NA
ENSG0000	14.50534	-0.73525	0.636064	0.005904	0.08439	NA	NA	NA
ENSG0000	439.2749	0.490277	0.266994	0.005897	0.08439	PAX5	5079	paired box 5
ENSG0000	10.517	0.806164	0.662368	0.005936	0.084747	UGGT2	55757	UDP-glucose glycoprotein glucosyltransferase 2
ENSG0000	187.264	0.420989	0.202442	0.00596	0.084977	SEC63	11231	SEC63 hon protein translocation regulator
ENSG0000	17.17622	0.687284	0.469246	0.005961	0.084977	UNC13B	10497	unc-13 homolog B
ENSG0000	4.161919	-0.11055	0.270724	0.005976	0.08509	GSDMC	56169	gasdermin C
ENSG0000	47.45212	-0.61161	0.425065	0.005982	0.08509	LOC12490	1.25E+08	uncharacterized LOC124902947
ENSG0000	7.785171	0.825868	0.93573	0.005983	0.08509	LINC0065S	1.01E+08	long intergenic non-protein coding RNA 659
ENSG0000	1022.127	-0.43521	0.218329	0.006007	0.085371	MICALL1	85377	MICAL like 1
ENSG0000	96.95292	-0.39898	0.186653	0.006014	0.085392	JTB	10899	jumping translocation breakpoint
ENSG0000	827.9767	0.315033	0.134956	0.006022	0.085426	SRSF11	9295	serine and arginine rich splicing factor 11
ENSG0000	200.7344	-0.37941	0.174338	0.006025	0.085426	NA	NA	NA
ENSG0000	4.650896	-1.08853	1.318573	0.006034	0.08548	IFITM9P	390218	interferon induced transmembrane protein 9 pseudogene
ENSG0000	7.252926	0.137376	0.292092	0.006045	0.085556	OR7E14P	10819	olfactory receptor family 7 subfamily E member 14 pseudogene
ENSG0000	87.79171	0.449276	0.224884	0.006048	0.085556	NA	NA	NA
ENSG0000	18.62697	0.68435	0.505899	0.006069	0.085668	C2orf92	728537	chromosome 2 open reading frame 92
ENSG0000	2813.499	0.314878	0.134734	0.006065	0.085668	KLF13	51621	KLF transcription factor 13
ENSG0000	1529.046	0.374378	0.172395	0.00607	0.085668	ERF	2077	ETS2 repressor factor
ENSG0000	13.08027	0.778238	0.684107	0.00609	0.085812	SEMA3G	56920	semaphorin 3G
ENSG0000	42.43009	0.643404	0.508399	0.006086	0.085812	FAM216A	29902	family with sequence similarity 216 member A
ENSG0000	4146.124	-0.47355	0.253707	0.006115	0.086095	WARS1	7453	tryptophanyl-tRNA synthetase 1
ENSG0000	190.7326	-0.33401	0.146035	0.006129	0.086224	SLC16A13	201232	solute carrier family 16 member 13
ENSG0000	62.95158	-0.53908	0.315413	0.006168	0.086714	SLC25A34	284723	solute carrier family 25 member 34
ENSG0000	157.1806	-0.53825	0.324861	0.006201	0.087011	NA	NA	NA
ENSG0000	24.85879	0.637044	1.185167	0.006206	0.087011	C4BPA	722	complement component 4 binding protein alpha
ENSG0000	1260.564	-0.52919	0.9455	0.006208	0.087011	SAMD9	54809	sterile alpha motif domain containing 9
ENSG0000	79.81433	0.52283	0.295718	0.006201	0.087011	RPL7AP64	728486	ribosomal protein L7a pseudogene 64
ENSG0000	7.918043	0.191426	0.356284	0.00624	0.087388	GLDN	342035	gliomedin
ENSG0000	72.81629	0.554525	0.334485	0.006253	0.087499	TPK1	27010	thiamin pyrophosphokinase 1
ENSG0000	10.24057	0.249406	0.443853	0.006294	0.087895	SPINK8	646424	serine peptidase inhibitor Kazal type 8 (putative)
ENSG0000	245.2892	-0.35941	0.162458	0.006295	0.087895	DTNBP1	84062	dystrobrevin binding protein 1
ENSG0000	8.281794	-0.96968	1.216369	0.006292	0.087895	NA	NA	NA
ENSG0000	36.22486	-0.49783	0.262115	0.006303	0.087935	MICB-DT	1.03E+08	MICB divergent transcript
ENSG0000	13.77271	0.712523	0.571557	0.006326	0.088192	NA	NA	NA
ENSG0000	36.44882	-0.602	0.399905	0.006366	0.088683	PTGDR2	11251	prostaglandin D2 receptor 2
ENSG0000	8756.941	-0.46853	0.254807	0.006373	0.088701	TPP1	1200	tripeptidyl peptidase 1
ENSG0000	8.803606	-0.78582	0.665565	0.006377	0.088701	NA	NA	NA
ENSG0000	1962.854	-0.31113	0.134449	0.006398	0.08892	PSMB8	5696	proteasome 20S subunit beta 8
ENSG0000	13.63461	0.281502	0.52298	0.006463	0.089626	NA	NA	NA
ENSG0000	5.830139	0.905168	0.908891	0.006455	0.089626	ACOT1	641371	acyl-CoA thioesterase 1
ENSG0000	286.5616	0.507746	0.294285	0.006459	0.089626	PLIN5	440503	perilipin 5
ENSG0000	74.23682	0.598959	0.584542	0.006485	0.089861	CEACAM6	4680	CEA cell adhesion molecule 6
ENSG0000	25020.28	-0.46221	0.249389	0.006506	0.090085	CXCR2	3579	C-X-C motif chemokine receptor 2
ENSG0000	174.8453	-0.38958	0.182899	0.00655	0.090626	TRIP6	7205	thyroid hormone receptor interactor 6
ENSG0000	1084.585	-0.31591	0.141006	0.006562	0.090723	EDEM2	55741	ER degradation enhancing alpha-mannosidase like protein 2
ENSG0000	4.862739	-0.2177	0.391398	0.006591	0.090981	KCND3	3752	potassium voltage-gated channel subfamily D member 3
ENSG0000	314.6756	0.387854	0.183807	0.00659	0.090981	SPIDR	23514	scaffold protein involved in DNA repair
ENSG0000	169.7425	-0.56198	0.393128	0.006634	0.091501	NA	NA	NA
ENSG0000	32.53417	0.624904	0.459624	0.006647	0.091623	ALG14	199857	ALG14 UDP-N-acetylglucosaminyltransferase subunit
ENSG0000	24.70983	0.652975	0.497172	0.006697	0.09217	PERP	64065	p53 apoptosis effector related to PMP22
ENSG0000	127.4014	0.502321	0.292569	0.006696	0.09217	STX16-NPE	1.01E+08	STX16-NPEPL1 readthrough (NMD candidate)
ENSG0000	26.64744	0.609306	0.785032	0.006708	0.092247	NA	NA	NA
ENSG0000	74.29222	0.496245	0.273867	0.006725	0.092418	WDR90	197335	WD repeat domain 90
ENSG0000	93.72491	-0.48436	0.265007	0.006755	0.092729	LOC12490	1.25E+08	small nucleolar RNA ACA64
ENSG0000	4964.745	0.377569	0.178545	0.006766	0.092729	HLA-DRA	3122	major hist class II DR alpha
ENSG0000	1405.859	-0.36011	0.16518	0.006763	0.092729	ILRUN	64771	inflammation and lipid regulator with UBA-like and NBR1-like domains
ENSG0000	2.967021	0.144254	0.297791	0.006768	0.092729	NA	NA	NA
ENSG0000	813.0472	0.343219	0.154294	0.006799	0.093075	IRF2BP2	359948	interferon regulatory factor 2 binding protein 2
ENSG0000	241.2753	-0.52283	0.328711	0.006804	0.093075	RAB20	55647	RAB20 member RAS oncogene family
ENSG0000	1587.385	0.366108	0.170633	0.006817	0.093195	RPL10A	4736	ribosomal protein L10a
ENSG0000	1394.514	0.445874	0.235763	0.006857	0.093661	RPL11	6135	ribosomal protein L11
ENSG0000	165.3842	0.414336	0.20531	0.006889	0.094036	UBE2E2	7325	ubiquitin conjugating enzyme E2 E2
ENSG0000	1780.408	0.414827	0.208479	0.006909	0.094117	RPS20	6224	ribosomal protein S20

ENSG0000	19.31584	0.621921	0.824301	0.00691	0.094117	KNDC1	85442	kinase non-catalytic C-lobe domain containing 1
ENSG0000	64.83424	0.522479	0.307673	0.006911	0.094117	BACE1	23621	beta-secretase 1
ENSG0000	5.218985	0.149016	0.300662	0.00692	0.094171	PLAAT1	57110	phospholipase A and acyltransferase 1
ENSG0000	10965.67	-0.33256	0.149424	0.006925	0.094171	CD53	963	CD53 molecule
ENSG0000	844.2333	-0.42128	0.2159	0.006933	0.09421	STRADB	55437	STE20 related adaptor beta
ENSG0000	205.6931	-0.45033	0.239526	0.006967	0.0946	PEAR1	375033	platelet endothelial aggregation receptor 1
ENSG0000	3.602504	0.137165	0.291589	0.006972	0.0946	NA	NA	NA
ENSG0000	79.195	-0.57505	0.567611	0.007001	0.094883	ATP1B2	482	ATPase Na+/K+ transporting subunit beta 2
ENSG0000	4.196396	-0.11838	0.275326	0.007003	0.094883	NA	NA	NA
ENSG0000	280.2032	-0.44882	0.242113	0.007012	0.094918	MPZL3	196264	myelin protein zero like 3
ENSG0000	24.90706	0.641455	0.511739	0.007016	0.094918	NA	NA	NA
ENSG0000	5.581154	-0.90879	1.554656	0.007028	0.094941	NA	NA	NA
ENSG0000	3370.567	0.399772	0.196955	0.007027	0.094941	GAA	2548	alpha glucosidase
ENSG0000	97.35142	-0.47739	0.263836	0.007038	0.094947	PDGFRB	5159	platelet derived growth factor receptor beta
ENSG0000	16.4019	0.688146	0.584284	0.007044	0.094947	NA	NA	NA
ENSG0000	5613.908	0.517595	0.336163	0.00704	0.094947	IGLV1-40	28825	immunoglobulin lambda variable 1-40
ENSG0000	13.64332	-0.65866	0.841003	0.007083	0.095378	LINC0099€	285972	long intergenic non-protein coding RNA 996
ENSG0000	123.2688	-0.37717	0.178439	0.007086	0.095378	NA	NA	NA
ENSG0000	289.095	0.343832	0.156607	0.007093	0.095399	CFAP410	755	cilia and flagella associated protein 410
ENSG0000	1161.678	-0.26778	0.112301	0.007101	0.095438	ERP44	23071	endoplasmic reticulum protein 44
ENSG0000	47.24095	-0.49827	0.281001	0.00711	0.095489	TAGAP-AS	1.05E+08	TAGAP antisense RNA 1
ENSG0000	9.23603	0.724144	0.858541	0.007137	0.095713	LINC0114	400752	long intergenic non-protein coding RNA 1144
ENSG0000	22.85555	-0.61033	0.413807	0.007137	0.095713	ASS1P1	442167	argininosuccinate synthetase 1 pseudogene 1
ENSG0000	94.7559	0.570579	0.427851	0.007147	0.095767	GLDC	2731	glycine decarboxylase
ENSG0000	184.6101	-0.41839	0.21142	0.007164	0.095934	NSUN3	63899	NOP2/Sun RNA methyltransferase 3
ENSG0000	1319.51	-0.32617	0.146222	0.007173	0.095974	CYTIP	9595	cytohesin 1 interacting protein
ENSG0000	13.41505	0.167304	0.317256	0.007224	0.096597	LOC12490	1.25E+08	uncharacterized LOC124901310
ENSG0000	140.2453	-0.42822	0.219649	0.007257	0.096964	PSMD6-AS	1.01E+08	PSMD6 antisense RNA 2
ENSG0000	103.6468	-0.4679	0.25529	0.007279	0.09719	CEP63	80254	centrosomal protein 63
ENSG0000	1579.831	0.451494	0.246562	0.007299	0.097243	GLIPR1	11010	GLI pathogenesis related 1
ENSG0000	199.858	0.41694	0.210692	0.007299	0.097243	BRI3BP	140707	BRI3 binding protein
ENSG0000	29.47362	-0.6141	0.407883	0.007299	0.097243	TRDV1	28518	T cell receptor delta variable 1
ENSG0000	65.74204	-0.4494	0.233742	0.007311	0.097312	PRSS23	11098	serine protease 23
ENSG0000	7.990161	0.138853	0.292774	0.007315	0.097312	LHX1	3975	LIM homeobox 1
ENSG0000	33.19252	-0.58605	0.411241	0.007333	0.097479	ITPKB-IT1	1.01E+08	ITPKB intronic transcript 1
ENSG0000	1687.142	0.441942	0.237126	0.007356	0.097655	AMPD2	271	adenosine monophosphate deaminase 2
ENSG0000	8.218749	0.342376	0.715363	0.007357	0.097655	NA	NA	NA
ENSG0000	9.939879	-0.31295	0.602113	0.007379	0.09774	TNFRSF21	27242	TNF receptor superfamily member 21
ENSG0000	6.97404	0.598241	1.652722	0.007371	0.09774	NA	NA	NA
ENSG0000	930.9666	0.387924	0.188491	0.007377	0.09774	NACC2	138151	NACC family member 2
ENSG0000	3469.824	-0.38001	0.184824	0.007426	0.098191	PSME1	5720	proteasome activator subunit 1
ENSG0000	1069.898	-0.42174	0.218207	0.007429	0.098191	FMNL1-DT	339192	FMNL1 divergent transcript
ENSG0000	68.78787	-0.47825	0.263843	0.007425	0.098191	NA	NA	NA
ENSG0000	2.76934	0.197455	0.363631	0.007441	0.098267	PMFBP1	83449	polyamine modulated factor 1 binding protein 1
ENSG0000	10118.34	-0.43666	0.233373	0.007453	0.098292	JAK3	3718	Janus kinase 3
ENSG0000	6.109341	0.119526	0.274843	0.007453	0.098292	NA	NA	NA
ENSG0000	293.4363	-0.39907	0.198536	0.007483	0.098609	LTA	4049	lymphotoxin alpha
ENSG0000	35.33001	-0.58693	0.436036	0.007501	0.098783	H2BC8	8339	H2B clustered histone 8
ENSG0000	409.8152	-0.41412	0.211832	0.007516	0.098898	DISC1	27185	DISC1 scaffold protein
ENSG0000	625.689	-0.34791	0.156866	0.007521	0.098898	NA	NA	NA
ENSG0000	30.26617	-0.59886	0.469299	0.007576	0.099506	VIL1	7429	villin 1
ENSG0000	394.6003	-0.3306	0.151966	0.007578	0.099506	SDF2	6388	stromal cell derived factor 2
ENSG0000	534.1329	-0.39116	0.193585	0.007614	0.09978	XPO1	7514	exportin 1
ENSG0000	3.497451	0.130934	0.285152	0.007612	0.09978	CD163L1	283316	CD163 molecule like 1
ENSG0000	15.34231	-0.63073	0.432694	0.007615	0.09978	LOC10192	1.02E+08	uncharacterized LOC101927702
ENSG0000	646.3615	-0.2893	0.125745	0.007646	0.100115	AP1G1	164	adaptor related protein complex 1 subunit gamma 1
ENSG0000	9.802177	-0.69847	0.60747	0.007712	0.100901	NA	NA	NA
ENSG0000	284.4928	-0.3869	0.190225	0.007717	0.100902	TAL1	6886	TAL bHLH erythroid differentiation factor
ENSG0000	5.690847	-0.57073	2.15725	0.007728	0.100972	NA	NA	NA
ENSG0000	447.8904	0.346817	0.161565	0.007734	0.100983	CD99L2	83692	CD99 molecule like 2
ENSG0000	11.0272	0.68577	0.863739	0.007747	0.101081	NA	NA	NA
ENSG0000	586.0943	0.479978	0.285329	0.007757	0.101136	IRF4	3662	interferon regulatory factor 4
ENSG0000	12.45056	0.68681	0.722731	0.007788	0.101468	SEMA3C	10512	semaphorin 3C
ENSG0000	1154.379	-0.38448	0.190155	0.007811	0.101698	PRR13	54458	proline rich 13
ENSG0000	5.216156	0.354939	0.819339	0.007841	0.102001	GPR150	285601	G protein-coupled receptor 150
ENSG0000	8.565646	0.13542	0.285784	0.007846	0.102001	GGT5	2687	gamma-glutamyltransferase 5
ENSG0000	116.154	0.39579	0.196146	0.007852	0.102003	ZBED4	9889	zinc finger BED-type containing 4
ENSG0000	7.922242	-0.28042	0.516971	0.00787	0.102175	NA	NA	NA
ENSG0000	83.06	0.557522	0.502609	0.007889	0.102345	ARRDC4	91947	arrestin domain containing 4
ENSG0000	48.92999	-0.48069	0.271027	0.007902	0.102437	PPT2	9374	palmitoyl-protein thioesterase 2
ENSG0000	13.37065	-0.70384	0.515343	0.007915	0.102535	RHD	6007	Rh blood group D antigen
ENSG0000	798.4689	-0.38723	0.190784	0.007931	0.102601	FAM222B	55731	family with sequence similarity 222 member B
ENSG0000	20.31334	-0.60376	0.777499	0.007928	0.102601	NA	NA	NA
ENSG0000	9.74237	-0.6965	0.713015	0.007982	0.10312	TSSK4	283629	testis specific serine kinase 4
ENSG0000	4558.544	-0.48587	0.296566	0.007983	0.10312	MIR223HG	1.16E+08	MIR223 host gene
ENSG0000	5.390437	0.138367	0.291132	0.008003	0.10324	FAM149A	25854	family with sequence similarity 149 member A
ENSG0000	118.7623	-0.44879	0.246097	0.008003	0.10324	ANKRD55	79722	ankyrin repeat domain 55
ENSG0000	518.1389	-0.31178	0.139989	0.008009	0.10324	DYNLL2	140735	dynein light chain LC8-type 2
ENSG0000	4538.308	-0.39735	0.201815	0.00802	0.103313	NAGK	55577	N-acetylglucosamine kinase
ENSG0000	19.06628	-0.61581	0.513963	0.008033	0.103412	ZNF684	127396	zinc finger protein 684
ENSG0000	218.2381	0.438117	0.238952	0.008042	0.103456	TMCO3	55002	transmembrane and coiled-coil domains 3
ENSG0000	145.416	0.357837	0.169539	0.00805	0.103472	ING5	84289	inhibitor of growth family member 5
ENSG0000	48.32568	0.493588	0.287555	0.008055	0.103472	MOB3B	79817	MOB kinase activator 3B
ENSG0000	1771.003	0.297438	0.134997	0.008078	0.103486	MXD4	10608	MAX dimerization protein 4
ENSG0000	909.2344	-0.48453	0.29558	0.008073	0.103486	TMEM176	55365	transmembrane protein 176A
ENSG0000	156.0768	0.348845	0.166491	0.008065	0.103486	CACNA2D	93589	calcium voltage-gated channel auxiliary subunit alpha2delta 4
ENSG0000	1762.003	-0.31555	0.143579	0.008079	0.103486	AP2B1	163	adaptor related protein complex 2 subunit beta 1
ENSG0000	184.814	-0.24378	0.421777	0.008101	0.103698	BICDL2	146439	BICD family like cargo adaptor 2
ENSG0000	9.858051	-0.69954	0.582527	0.008147	0.104149	NA	NA	NA
ENSG0000	971.4854	0.333718	0.156152	0.008148	0.104149	ERCC1	2067	ERCC excis endonuclease non-catalytic subunit
ENSG0000	36.02405	-0.49703	0.288031	0.008193	0.104583	CRYBG2	55057	crystallin beta-gamma domain containing 2
ENSG0000	211.4023	0.420563	0.222174	0.008189	0.104583	TIMM13	26517	translocase of inner mitochondrial membrane 13
ENSG0000	4013.387	-0.33339	0.153692	0.008215	0.104723	ARL8A	127829	ADP ribosylation factor like GTPase 8A
ENSG0000	464.0786	0.298086	0.132231	0.008212	0.104723	TMEM198	440104	transmembrane protein 198B (pseudogene)
ENSG0000	207.4586	0.503654	0.331462	0.008237	0.104931	MYO1D	4642	myosin ID
ENSG0000	46.46281	0.519025	0.318887	0.008286	0.105483	NOMO3	408050	NODAL modulator 3

ENSG0000	17.37107	-0.5936	0.702173	0.008349	0.106182	H3C6	8353	H3 clustered histone 6
ENSG0000	15.77407	-0.63638	0.555856	0.008353	0.106182	LINC03016	1.02E+08	long intergenic non-protein coding RNA 3016
ENSG0000	62.33644	-0.51647	0.333322	0.008362	0.106225	RGS6	9628	regulator of G protein signaling 6
ENSG0000	6.196139	0.94439	1.251733	0.008382	0.106366	ITLN1	55600	intelectin 1
ENSG0000	595.5757	0.328913	0.151902	0.008385	0.106366	NAAA	27163	N-acylethanolamine acid amidase
ENSG0000	57.42425	0.547329	0.58519	0.008406	0.106567	SDK2	54549	sidekick cell adhesion molecule 2
ENSG0000	21.61001	0.63114	0.563061	0.008419	0.106649	TSHR	7253	thyroid stimulating hormone receptor
ENSG0000	440.147	-0.33781	0.157857	0.008457	0.106933	ELMOD3	84173	ELMO domain containing 3
ENSG0000	67.60587	0.535413	0.365823	0.008457	0.106933	CRISP3	10321	cysteine rich secretory protein 3
ENSG0000	460.2461	-0.3183	0.143551	0.008463	0.106933	CREB3L2	64764	cAMP responsive element binding protein 3 like 2
ENSG0000	540.6132	-0.47831	0.3	0.008465	0.106933	GK	2710	glycerol kinase
ENSG0000	82.5885	-0.46814	0.264033	0.008522	0.107585	NA	NA	NA
ENSG0000	3.457188	0.171623	0.325711	0.008565	0.108055	NA	NA	NA
ENSG0000	3.577829	-0.25571	0.466368	0.008587	0.108257	NA	NA	NA
ENSG0000	102.6435	-0.41247	0.215656	0.008628	0.108698	NA	NA	NA
ENSG0000	134.7801	0.390073	0.19626	0.008653	0.108868	GSTM2	2946	glutathione S-transferase mu 2
ENSG0000	53.84681	-0.48485	0.287918	0.008652	0.108868	NA	NA	NA
ENSG0000	7.944296	-0.20482	0.365686	0.00866	0.108879	BCL2L14	79370	BCL2 like 14
ENSG0000	1285.159	0.421827	0.231719	0.008682	0.109076	SEL1L3	23231	SEL1L family member 3
ENSG0000	2025.721	0.380952	0.197991	0.008703	0.109265	ADAM15	8751	ADAM metallopeptidase domain 15
ENSG0000	13.35913	0.293228	0.514772	0.008738	0.109633	GOLGA8N	643699	golgin A8 family member N
ENSG0000	4371.43	-0.32687	0.152879	0.008808	0.110433	MLLT6	4302	MLLT6 PHD finger containing
ENSG0000	264.4928	0.338626	0.159587	0.008814	0.110437	SMDT1	91689	single-pass membrane protein with aspartate rich tail 1
ENSG0000	149.5135	-0.42296	0.22887	0.008835	0.110616	NA	NA	NA
ENSG0000	24.36353	-0.58173	0.582789	0.008843	0.110642	KRT8	3856	keratin 8
ENSG0000	185.2019	-0.33256	0.155838	0.008856	0.110734	MBD5	55777	methyl-CpG binding domain protein 5
ENSG0000	153.7657	-0.39205	0.207107	0.008875	0.110842	DTNB	1838	dystrobrevin beta
ENSG0000	5.704735	0.214629	0.381821	0.008877	0.110842	NA	NA	NA
ENSG0000	52.0503	-0.53928	0.6007	0.008895	0.110988	P2RY14	9934	purinergic receptor P2Y14
ENSG0000	94.45058	-0.36197	0.177179	0.008907	0.111069	LMAN2L	81562	lectin mannose binding 2 like
ENSG0000	2302.54	-0.44832	0.261255	0.008916	0.11111	SLC38A5	92745	solute carrier family 38 member 5
ENSG0000	34.10253	-0.52362	0.632565	0.008943	0.111367	EMP1	2012	epithelial membrane protein 1
ENSG0000	49.76059	0.528128	0.374235	0.00899	0.111868	TMEM168	64418	transmembrane protein 168
ENSG0000	1073.173	-0.30385	0.136645	0.008996	0.111868	TLNRD1	59274	talin rod domain containing 1
ENSG0000	14089.17	0.342288	0.158813	0.009019	0.112085	RPL18A	6142	ribosomal protein L18a
ENSG0000	51.84749	-0.53617	0.403213	0.009088	0.112863	LOC10798	1.08E+08	uncharacterized LOC107984948
ENSG0000	1554.406	-0.35405	0.17358	0.009119	0.113025	GLMP	112770	glycosylated lysosomal membrane protein
ENSG0000	4156.087	0.477165	0.314372	0.009126	0.113025	MZB1	51237	marginal zone B and B1 cell specific protein
ENSG0000	2.923843	0.124359	0.282829	0.009113	0.113025	ANKS1B	56899	ankyrin repeat and sterile alpha motif domain containing 1B
ENSG0000	829.6638	-0.38733	0.202012	0.009132	0.113025	GZMM	3004	granzyme M
ENSG0000	14.6897	-0.36075	0.687342	0.009122	0.113025	RN7SL491	1.06E+08	RNA 7SL cytoplasm pseudogene
ENSG0000	753.7217	0.380827	0.194019	0.009161	0.113163	CPVL	54504	carboxypeptidase vitellogenic like
ENSG0000	161.7751	-0.39414	0.204503	0.009161	0.113163	DDIT3	1649	DNA damage inducible transcript 3
ENSG0000	5.621181	0.160613	0.315064	0.00916	0.113163	KCNS1	3787	potassium voltage-gated channel modifier subfamily S member 1
ENSG0000	281.6822	-0.30446	0.139066	0.009243	0.11402	C2orf42	54980	chromosome 2 open reading frame 42
ENSG0000	828.0391	0.35864	0.176638	0.009249	0.11402	FBL	2091	fibrillarin
ENSG0000	307.3232	0.34771	0.169948	0.00925	0.11402	FUZ	80199	fuzzy planar cell polarity protein
ENSG0000	70.88968	0.511627	0.350768	0.009302	0.114355	STAG3	10734	stromal antigen 3
ENSG0000	36.97777	0.550945	0.431955	0.009297	0.114355	ARHGAP22	58504	Rho GTPase activating protein 22
ENSG0000	11.31074	0.641135	0.530455	0.009295	0.114355	SKOR1	390598	SKI family transcriptional corepressor 1
ENSG0000	56.66292	0.443008	0.243681	0.009288	0.114355	TEF	7008	TEF transc PAR bZIP family member
ENSG0000	14.71839	-0.59906	0.597413	0.009407	0.115571	PCAT1	1.01E+08	prostate cancer associated transcript 1
ENSG0000	18.78469	-0.59214	0.510059	0.009424	0.115697	FAM21FP	1E+08	family witl pseudogene
ENSG0000	808.1293	-0.30914	0.143174	0.009458	0.11604	LINC01278	92249	long intergenic non-protein coding RNA 1278
ENSG0000	423.9423	0.360536	0.179071	0.009465	0.116049	RPL13P12	388344	ribosomal protein L13 pseudogene 12
ENSG0000	460.8329	-0.47745	0.321501	0.009497	0.116283	GPR146	115330	G protein-coupled receptor 146
ENSG0000	3289.668	0.329364	0.156838	0.009496	0.116283	RPL27A	6157	ribosomal protein L27a
ENSG0000	69100.61	-0.43901	0.2596	0.009532	0.116478	S100A11	6282	S100 calcium binding protein A11
ENSG0000	2.747646	-0.11226	0.272364	0.009525	0.116478	OVOL1-AS	1.02E+08	OVOL1 antisense RNA 1
ENSG0000	21.2747	-0.55497	0.390762	0.009531	0.116478	LINC01841	1.05E+08	long intergenic non-protein coding RNA 1841
ENSG0000	380.3539	-0.46408	0.295398	0.00956	0.116667	ADCY3	109	adenylate cyclase 3
ENSG0000	3182.507	-0.35682	0.177917	0.009559	0.116667	LINC00861	1E+08	long intergenic non-protein coding RNA 861
ENSG0000	66.04512	0.42759	0.234005	0.009607	0.117159	NOP16	51491	NOP16 nucleolar protein
ENSG0000	3.579528	0.097386	0.261038	0.009623	0.117278	LGI4	163175	leucine rich repeat LGI family member 4
ENSG0000	73.51502	0.512879	0.354733	0.009634	0.117334	AFDN	4301	afadin adherens junction formation factor
ENSG0000	102.258	0.417688	0.227774	0.009641	0.11734	CDCA7L	55536	cell division cycle associated 7 like
ENSG0000	7.851498	0.677073	0.715836	0.009658	0.117475	NA	NA	NA
ENSG0000	57.41719	0.420773	0.227769	0.009738	0.117862	CREB3L4	148327	cAMP responsive element binding protein 3 like 4
ENSG0000	75.34455	-0.4614	0.275888	0.0097	0.117862	RGL1	23179	ral guanine nucleotide dissociation stimulator like 1
ENSG0000	63.54922	-0.51254	0.73724	0.009735	0.117862	ERFE	151176	erythroferrone
ENSG0000	15193.79	-0.39106	0.207914	0.009754	0.117862	TNFRSF1A	7132	TNF receptor superfamily member 1A
ENSG0000	31.16398	-0.54739	0.515823	0.009747	0.117862	SDSL	113675	serine dehydratase like
ENSG0000	1783.394	0.229094	0.098076	0.009739	0.117862	RHOT2	89941	ras homolog family member T2
ENSG0000	1065.773	0.284638	0.129171	0.009754	0.117862	COX4I1	1327	cytochrome c oxidase subunit 4I1
ENSG0000	182.1385	0.475065	0.307031	0.009729	0.117862	TNFRSF13I	23495	TNF receptor superfamily member 13B
ENSG0000	6661.256	-0.41571	0.235289	0.009752	0.117862	LILRA6	79168	leukocyte immunoglobulin like receptor A6
ENSG0000	2403.82	-0.31502	0.146115	0.009743	0.117862	UBE2L3	7332	ubiquitin conjugating enzyme E2 L3
ENSG0000	383.8783	-0.31843	0.150583	0.009847	0.118906	SERTAD3	29946	SERTA domain containing 3
ENSG0000	135.0186	0.492827	0.346687	0.009857	0.118942	PDIA5	10954	protein disulfide isomerase family A member 5
ENSG0000	148.857	-0.41578	0.229575	0.009895	0.119098	FOXD2-AS	84793	FOXD2 adjacent opposite strand RNA 1
ENSG0000	6.294231	0.234667	0.411343	0.009882	0.119098	PARTICL	1.01E+08	promoter of MAT2A antisense radiation-induced circulating long non-coding RNA
ENSG0000	9.32747	0.263524	0.484677	0.009884	0.119098	IGHV3-64	28414	immunoglobulin heavy variable 3-64
ENSG0000	20.50995	-0.55972	0.412522	0.009896	0.119098	CDH13-AS	1.03E+08	CDH13 antisense RNA 2
ENSG0000	44.827	-0.46047	0.270956	0.009932	0.11933	NA	NA	NA
ENSG0000	11868.92	-0.45282	0.285526	0.009922	0.11933	NCF1C	654817	neutrophil cytosolic factor 1C pseudogene
ENSG0000	643.1763	0.488377	0.452506	0.009941	0.11933	IGHV1-46	28465	immunoglobulin heavy variable 1-46
ENSG0000	25.13422	-0.50605	0.678903	0.009939	0.11933	KLK1	3816	kallikrein 1
ENSG0000	251.623	0.433603	0.247703	0.009958	0.119455	KCNC3	3748	potassium voltage-gated channel subfamily C member 3
ENSG0000	327.1888	-0.38194	0.200298	0.009975	0.119577	GCC1	79571	GRIP and coiled-coil domain containing 1
ENSG0000	372.4333	0.294559	0.136516	0.009997	0.11974	NUBP1	4682	NUBP iron cytosolic
ENSG0000	64.93632	-0.47272	0.293273	0.010001	0.11974	NA	NA	NA
ENSG0000	17.52266	0.465824	0.812726	0.010039	0.1198	NA	NA	NA
ENSG0000	256.5526	0.416614	0.236858	0.010033	0.1198	SCARB1	949	scavenger receptor class B member 1
ENSG0000	4.070086	-0.14029	0.290777	0.010028	0.1198	ACOD1	730249	aconitate decarboxylase 1
ENSG0000	263.4672	-0.44313	0.267285	0.010024	0.1198	CHSY1	22856	chondroitin sulfate synthase 1
ENSG0000	4.114911	-0.20292	0.364823	0.010032	0.1198	CLN3	1201	CLN3 lysos battenin

ENSG0000	8794.94	-0.36981	0.189893	0.010052	0.119876	CYBC1	79415	cytochrome b-245 chaperone 1
ENSG0000	16.5863	-0.54167	0.360935	0.01009	0.120255	NA	NA	NA
ENSG0000	176.4405	-0.43041	0.253576	0.010129	0.1206	H2BC20P	337873	H2B cluste pseudogene
ENSG0000	10.18281	-0.21834	0.378127	0.010138	0.1206	SLC4A3	6508	solute carrier family 4 member 3
ENSG0000	268.0945	0.431	0.256442	0.010146	0.1206	CD24	1E+08	CD24 molecule
ENSG0000	721.5332	-0.30172	0.139989	0.010165	0.1206	SCML4	256380	Scm polycomb group protein like 4
ENSG0000	4709.764	0.326776	0.157374	0.010153	0.1206	RPL12	6136	ribosomal protein L12
ENSG0000	46.67092	0.441183	0.251114	0.010164	0.1206	FGD6	55785	FYVE RhoGEF and PH domain containing 6
ENSG0000	592.8598	-0.45718	0.287953	0.010161	0.1206	NA	NA	NA
ENSG0000	255.649	-0.49497	0.398497	0.010179	0.12061	C1GALT1	56913	core 1 syn glycoprotein-N-acetylgalactosamine 3-beta-galactosyltransferase 1
ENSG0000	112.4658	0.42894	0.24411	0.010177	0.12061	NA	NA	NA
ENSG0000	16.72386	-0.58311	0.579085	0.010193	0.120699	IRAG1-AS1	1E+08	IRAG1 antisense RNA 1
ENSG0000	23.0922	-0.51837	0.341685	0.010222	0.120729	SGO1-AS1	1.01E+08	SGO1 antisense RNA 1
ENSG0000	11.21907	-0.35345	0.694401	0.010211	0.120729	CREB3L1	90993	cAMP responsive element binding protein 3 like 1
ENSG0000	5.770334	0.230271	0.403618	0.010222	0.120729	ACSBG1	23205	acyl-CoA synthetase bubblegum family member 1
ENSG0000	253.0523	0.347696	0.172255	0.010211	0.120729	NAGLU	4669	N-acetyl-alpha-glucosaminidase
ENSG0000	2641.613	-0.40741	0.223408	0.010232	0.120764	UBAP1	51271	ubiquitin associated protein 1
ENSG0000	70.94414	0.394219	0.207774	0.010258	0.120834	UQCC3	790955	ubiquinol-cytochrome c reductase complex assembly factor 3
ENSG0000	39.3021	-0.46075	0.271873	0.010254	0.120834	ST6GALNA	1.01E+08	ST6 N-acet 6-sialyltransferase 4 pseudogene 1
ENSG0000	258.4825	0.317904	0.149693	0.010255	0.120834	TOMM22	56993	translocase of outer mitochondrial membrane 22
ENSG0000	38.76715	0.322223	0.53507	0.010282	0.120974	IGHV1-58	28464	immunoglobulin heavy variable 1-58
ENSG0000	3.770583	-0.15583	0.30797	0.010283	0.120974	NA	NA	NA
ENSG0000	45.36063	0.437405	0.246978	0.010297	0.121062	GTF2IRD2I	389524	GTF2I repeat domain containing 2B
ENSG0000	276.3413	0.464773	0.304995	0.010321	0.121273	RPS21	6227	ribosomal protein S21
ENSG0000	2.665702	0.136001	0.289489	0.010351	0.121422	NA	NA	NA
ENSG0000	197.9668	0.427452	0.24277	0.010349	0.121422	SAPCD2	89958	suppressor APC domain containing 2
ENSG0000	6.088362	-0.20289	0.361302	0.010354	0.121422	NA	NA	NA
ENSG0000	8.83303	0.317688	0.600088	0.010376	0.121601	TEX45	374877	testis expressed 45
ENSG0000	45.41863	-0.46577	0.281257	0.010386	0.121644	GAREM2	150946	GRB2 associated regulator of MAPK1 subtype 2
ENSG0000	4606.639	-0.38411	0.202275	0.010409	0.121839	KLF6	1316	KLF transcription factor 6
ENSG0000	206.1414	-0.4693	0.30462	0.010429	0.12199	IFIT1B	439996	interferon induced protein with tetratricopeptide repeats 1B
ENSG0000	14383.28	-0.28833	0.132843	0.010446	0.122081	WIPF1	7456	WAS/WASL interacting protein family member 1
ENSG0000	36.05131	-0.48928	0.310573	0.01045	0.122081	H2BC15	8341	H2B clustered histone 15
ENSG0000	84.98513	-0.42299	0.240378	0.010475	0.122299	SLC6A16	28968	solute carrier family 6 member 16
ENSG0000	3.374126	-0.10285	0.265806	0.01049	0.122369	LINC01267	1.02E+08	long intergenic non-protein coding RNA 1267
ENSG0000	83.57534	-0.37998	0.198434	0.010501	0.122369	C12orf76	400073	chromosome 12 open reading frame 76
ENSG0000	511.3405	0.327418	0.157287	0.0105	0.122369	SLC25A29	123096	solute carrier family 25 member 29
ENSG0000	112.8623	-0.4706	0.30441	0.010542	0.12277	PHACTR2	9749	phosphatase and actin regulator 2
ENSG0000	3876.17	-0.38523	0.208501	0.010609	0.123469	LTB	4050	lymphotoxin beta
ENSG0000	3.167823	-0.15884	0.310045	0.010617	0.123477	NA	NA	NA
ENSG0000	3.095778	0.140665	0.293626	0.01064	0.123666	NA	NA	NA
ENSG0000	57.05847	-0.48359	0.310371	0.010657	0.123688	NA	NA	NA
ENSG0000	18386.09	-0.47663	0.393002	0.010676	0.123688	CD14	929	CD14 molecule
ENSG0000	347.3724	0.313931	0.149176	0.010658	0.123688	RREB1	6239	ras responsive element binding protein 1
ENSG0000	19.1691	-0.55174	0.644412	0.010682	0.123688	TMEM252	169693	transmembrane protein 252
ENSG0000	53.82552	0.400367	0.213778	0.010678	0.123688	ZNF26	7574	zinc finger protein 26
ENSG0000	8.397026	-0.49434	1.08781	0.010662	0.123688	CREB3L3	84699	cAMP responsive element binding protein 3 like 3
ENSG0000	18.1683	-0.57244	0.634969	0.010697	0.123779	TRBV6-3	28604	T cell receptor beta variable 6-3
ENSG0000	37.28543	0.488098	0.314016	0.010707	0.12382	ZNF837	116412	zinc finger protein 837
ENSG0000	3.081082	-0.14854	0.299866	0.01073	0.123931	LINC02798	1.08E+08	long intergenic non-protein coding RNA 2798
ENSG0000	4.981115	-0.25999	0.463745	0.01073	0.123931	NA	NA	NA
ENSG0000	8.752548	0.543046	1.079949	0.010766	0.124269	COL4A3	1285	collagen type IV alpha 3 chain
ENSG0000	18737.14	0.284091	0.47136	0.010922	0.125989	IGHG3	3502	immunoglobulin heavy constant gamma 3 (G3m marker)
ENSG0000	21.50301	-0.54067	0.540635	0.010933	0.126032	GUCY1A1	2982	guanylate cyclase 1 soluble subunit alpha 1
ENSG0000	115.1955	0.447373	0.277971	0.010973	0.126266	CTSK	1513	cathepsin K
ENSG0000	16.11909	-0.34782	0.619532	0.010972	0.126266	LINC0057(	1.01E+08	long intergenic non-protein coding RNA 570
ENSG0000	18.25501	-0.48532	0.836441	0.010974	0.126266	PVALB	5816	parvalbumin
ENSG0000	2009.247	0.388944	0.213398	0.011001	0.126506	RPL37	6167	ribosomal protein L37
ENSG0000	226.429	0.313862	0.151728	0.011032	0.126778	LUC7L2	51631	LUC7 like 2 pre-mRNA splicing factor
ENSG0000	3.694455	0.126108	0.280836	0.011088	0.12734	NA	NA	NA
ENSG0000	1535.539	0.304867	0.14403	0.011117	0.127449	HIGD2A	192286	HIG1 hypoxia inducible domain family member 2A
ENSG0000	29.21839	-0.5262	0.418671	0.011118	0.127449	NA	NA	NA
ENSG0000	50.66492	0.422452	0.238433	0.011118	0.127449	CCDC183-/	1E+08	CCDC183 antisense RNA 1
ENSG0000	10686.4	0.289634	0.135113	0.011144	0.127666	RPS11	6205	ribosomal protein S11
ENSG0000	125.0272	0.371521	0.194435	0.011178	0.12797	GINM1	116254	glycosylated integral membrane protein 1
ENSG0000	71.912	-0.42596	0.247521	0.011193	0.127999	RAB15	376267	RAB15 member RAS oncogene family
ENSG0000	5.311559	0.161668	0.312292	0.011194	0.127999	NA	NA	NA
ENSG0000	108.6222	-0.47946	0.350187	0.011267	0.128708	CD2AP	23607	CD2 associated protein
ENSG0000	296.1872	-0.47063	0.460704	0.01127	0.128708	GPR84	53831	G protein-coupled receptor 84
ENSG0000	17.33528	0.346484	0.618442	0.011303	0.129004	RSPH14	27156	radial spoke head 14 homolog
ENSG0000	2298.973	-0.2809	0.130236	0.011321	0.129132	KMT2E	55904	lysine methyltransferase 2E (inactive)
ENSG0000	3258.562	0.464129	0.33167	0.011329	0.129139	ARHGEF4C	55701	Rho guanine nucleotide exchange factor 40
ENSG0000	231.2288	0.434226	0.272184	0.011384	0.129686	DNAJC10	54431	DnaJ heat shock protein family (Hsp40) member C10
ENSG0000	154.9258	-0.44085	0.275716	0.01143	0.130133	CACNA2D2	9254	calcium voltage-gated channel auxiliary subunit alpha2delta 2
ENSG0000	1433.071	-0.33832	0.170891	0.011464	0.130432	MLKL	197259	mixed lineage kinase domain like pseudokinase
ENSG0000	6.533726	0.092122	0.257725	0.01153	0.131105	NA	NA	NA
ENSG0000	5.131184	0.179064	0.330069	0.011575	0.131527	KATNAL2	83473	katanin catalytic subunit A1 like 2
ENSG0000	481.4305	-0.42615	0.264353	0.011613	0.131886	SLC22A4	6583	solute carrier family 22 member 4
ENSG0000	84.23609	0.47283	0.326338	0.011669	0.132434	DENND5B	160518	DENN domain containing 5B
ENSG0000	58.24259	-0.48864	0.402247	0.011696	0.132666	CFAP58-D'	1.01E+08	CFAP58 divergent transcript
ENSG0000	8.181296	0.380905	0.753337	0.01171	0.132743	NA	NA	NA
ENSG0000	152.2225	0.352732	0.181707	0.011721	0.132783	PTRHD1	391356	peptidyl-tRNA hydrolase domain containing 1
ENSG0000	3.950179	0.090332	0.255476	0.011736	0.132863	NA	NA	NA
ENSG0000	79.4401	-0.44035	0.271922	0.011769	0.133154	PSPN	5623	persephin
ENSG0000	3.809321	0.083741	0.252866	0.011843	0.133659	LINC0111E	388948	long intergenic non-protein coding RNA 1118
ENSG0000	691.9912	-0.33711	0.171331	0.01183	0.133659	C6orf47	57827	chromosome 6 open reading frame 47
ENSG0000	131.3154	-0.32411	0.160385	0.01185	0.133659	C8orf58	541565	chromosome 8 open reading frame 58
ENSG0000	4557.465	-0.3495	0.177461	0.011829	0.133659	RHBDF2	79651	rhomboid 5 homolog 2
ENSG0000	535.0635	-0.45265	0.310693	0.011836	0.133659	INSL3	3640	insulin like 3
ENSG0000	6.457827	0.180408	0.330096	0.011903	0.134177	NA	NA	NA
ENSG0000	4.074104	0.170135	0.320296	0.011931	0.134415	NA	NA	NA
ENSG0000	267.5055	-0.40224	0.235143	0.011951	0.134555	SCN1B	6324	sodium voltage-gated channel beta subunit 1
ENSG0000	36.32788	0.513043	0.434678	0.01197	0.134682	CEP128	145508	centrosomal protein 128
ENSG0000	13264.24	-0.36945	0.207133	0.011994	0.13487	TAPBP	6892	TAP binding protein
ENSG0000	408.3994	0.447684	0.308118	0.012004	0.134897	DDIT4	54541	DNA damage inducible transcript 4
ENSG0000	300.5425	-0.26675	0.123208	0.012033	0.135139	ZNF101	94039	zinc finger protein 101

ENSG0000	122.384	-0.42859	0.262224	0.012078	0.135563	PIK3CD-AS	644997	PIK3CD antisense RNA 1
ENSG0000	24.3893	0.517356	0.406814	0.012122	0.135983	CCDC78	124093	coiled-coil domain containing 78
ENSG0000	1395.887	0.35285	0.185302	0.012136	0.136048	HLA-DQB1	3119	major hist class II DQ beta 1
ENSG0000	577.5421	0.376487	0.206005	0.012145	0.136071	NUDT16	131870	nudix hydrolase 16
ENSG0000	6.048401	0.31379	0.58931	0.012195	0.136544	SNORD17	692086	small nucl C/D box 17
ENSG0000	5.309044	-0.21027	0.370317	0.012217	0.136674	C9orf43	257169	chromosome 9 open reading frame 43
ENSG0000	39.27543	-0.39703	0.606355	0.012221	0.136674	DEPP1	11067	DEPP autophagy regulator 1
ENSG0000	22.50942	0.530296	0.417515	0.012261	0.137036	FAM131B	9715	family with sequence similarity 131 member B
ENSG0000	255.7117	-0.43015	0.273796	0.012271	0.137066	LDLR	3949	low density lipoprotein receptor
ENSG0000	22.05601	-0.52108	0.581834	0.012286	0.137066	MAFA	389692	MAF bZIP transcription factor A
ENSG0000	7302.144	-0.35405	0.188071	0.012281	0.137066	ABCA7	10347	ATP binding cassette subfamily A member 7
ENSG0000	26.7348	-0.50458	0.53976	0.012296	0.137085	EMILIN1	11117	elastin microfibril interfacer 1
ENSG0000	53.17826	0.495506	0.407258	0.012327	0.137351	BHLHE41	79365	basic helix-loop-helix family member e41
ENSG0000	9082.098	-0.34474	0.180325	0.012355	0.137582	RBM38	55544	RNA binding motif protein 38
ENSG0000	921.3791	-0.44539	0.306855	0.012371	0.137677	DHRS13	147015	dehydrogenase/reductase 13
ENSG0000	8.093347	-0.57706	0.704375	0.012443	0.138229	NA	NA	NA
ENSG0000	44.7969	0.486106	0.517468	0.012439	0.138229	NA	NA	NA
ENSG0000	9.832716	0.571577	0.691033	0.012443	0.138229	NA	NA	NA
ENSG0000	1137.151	0.264782	0.122266	0.012455	0.138269	IFFO1	25900	intermediate filament family orphan 1
ENSG0000	14.58286	0.213818	0.369316	0.012491	0.138535	ANKRD34B	340120	ankyrin repeat domain 34B
ENSG0000	101.289	0.36737	0.196279	0.012494	0.138535	STARD5	80765	StAR related lipid transfer domain containing 5
ENSG0000	9.976701	0.555554	0.674195	0.012508	0.138608	TBC1D12	23232	TBC1 domain family member 12
ENSG0000	4.604037	0.147881	0.296806	0.012528	0.138749	IGKV1D-43	28891	immunoglobulin kappa variable 1D-43
ENSG0000	315.9045	-0.3623	0.197967	0.012545	0.13885	MFSD13A	79847	major facilitator superfamily domain containing 13A
ENSG0000	20123.61	-0.42753	0.28057	0.012635	0.139761	XPO6	23214	exportin 6
ENSG0000	563.1345	-0.27398	0.127378	0.012702	0.140339	IP6K2	51447	inositol hexakisphosphate kinase 2
ENSG0000	12.8469	-0.50416	0.722415	0.012695	0.140339	LOC11226	1.12E+08	uncharacterized LOC112267968
ENSG0000	49.90943	0.421402	0.249249	0.012715	0.140389	DNAAF9	25943	dynein axonemal assembly factor 9
ENSG0000	159.7327	-0.41736	0.257206	0.012751	0.140488	HHLA3	11147	HERV-H LTR-associating 3
ENSG0000	3898.66	-0.39475	0.232908	0.012754	0.140488	RNF149	284996	ring finger protein 149
ENSG0000	21.92646	0.491312	0.333483	0.012748	0.140488	MBNL1-AS	401093	MBNL1 antisense RNA 1
ENSG0000	43.61286	0.468668	0.315865	0.01274	0.140488	NA	NA	NA
ENSG0000	88.64452	0.430352	0.27171	0.012765	0.140524	NDRG2	57447	NDRG family member 2
ENSG0000	36.5816	-0.49018	0.385281	0.012834	0.141194	NA	NA	NA
ENSG0000	52.77951	0.442624	0.280922	0.012868	0.141491	CEACAM19	56971	CEA cell adhesion molecule 19
ENSG0000	5.977757	-0.15905	0.308032	0.012899	0.141741	TAF11L2	391742	TATA-box binding protein associated factor 11 like 2
ENSG0000	6.650571	-0.28512	0.502015	0.012919	0.141866	COL23A1	91522	collagen type XXIII alpha 1 chain
ENSG0000	78.74949	0.449634	0.303408	0.012928	0.141866	HAGHL	84264	hydroxyacylglutathione hydrolase like
ENSG0000	131.2248	0.364154	0.196607	0.012933	0.141866	AKAP1	8165	A-kinase anchoring protein 1
ENSG0000	263.1649	-0.36146	0.196087	0.012943	0.141883	NA	NA	NA
ENSG0000	1077.308	-0.40741	0.249293	0.012986	0.142107	GYG1	2992	glycogenin 1
ENSG0000	3052.819	-0.40129	0.243343	0.012984	0.142107	FBXL5	26234	F-box and leucine rich repeat protein 5
ENSG0000	154.0086	0.323252	0.162558	0.012987	0.142107	TAF4	6874	TATA-box binding protein associated factor 4
ENSG0000	46.72891	-0.47224	0.347496	0.013049	0.1427	LRRC75B	388886	leucine rich repeat containing 75B
ENSG0000	40.58774	0.493064	0.435515	0.013078	0.14294	IGKV3D-11	28876	immunoglobulin kappa variable 3D-11
ENSG0000	143.384	-0.41429	0.256907	0.013136	0.143312	CXCL1	2919	C-X-C motif chemokine ligand 1
ENSG0000	278.2434	-0.3491	0.184241	0.013131	0.143312	DLEU2	8847	deleted in lymphocytic leukemia 2
ENSG0000	85.90865	0.462651	0.342511	0.013131	0.143312	CORO2B	10391	coronin 2B
ENSG0000	9456.419	0.324274	0.16936	0.013204	0.143973	RPL18	6141	ribosomal protein L18
ENSG0000	13121.28	-0.31738	0.160837	0.013222	0.144079	SASH3	54440	SAM and SH3 domain containing 3
ENSG0000	798.0221	0.380049	0.217359	0.013247	0.144093	TCL1A	8115	TCL1 family AKT coactivator A
ENSG0000	6.767455	0.10411	0.262156	0.013246	0.144093	SYCE2	256126	synaptonemal complex central element protein 2
ENSG0000	29.23009	-0.49828	0.484201	0.013237	0.144093	NA	NA	NA
ENSG0000	198.1486	-0.44723	0.321643	0.013256	0.14411	GALNT3	2591	polypeptide N-acetylgalactosaminyltransferase 3
ENSG0000	1207.819	-0.40321	0.248653	0.013309	0.144512	PTGS1	5742	prostaglandin-endoperoxide synthase 1
ENSG0000	97.40523	-0.37785	0.211431	0.013306	0.144512	BSCL2	26580	BSCL2 lipi seipin
ENSG0000	192.4047	0.358033	0.193002	0.01333	0.144624	ANAPC15	25906	anaphase promoting complex subunit 15
ENSG0000	3.181388	-0.1244	0.279392	0.013335	0.144624	SLC52A1	55065	solute carrier family 52 member 1
ENSG0000	266.2903	-0.31847	0.161124	0.013361	0.144822	ZNF512	84450	zinc finger protein 512
ENSG0000	69.85718	-0.47701	0.48619	0.013373	0.14486	HPD	3242	4-hydroxyphenylpyruvate dioxygenase
ENSG0000	153.6806	0.431713	0.29075	0.013386	0.144917	SNX22	79856	sorting nexin 22
ENSG0000	2230.981	-0.3573	0.198833	0.013431	0.145235	MIIP	60672	migration and invasion inhibitory protein
ENSG0000	40.28904	-0.24906	0.405885	0.013424	0.145235	NA	NA	NA
ENSG0000	20.64446	-0.51002	0.450478	0.013504	0.145939	EGF	1950	epidermal growth factor
ENSG0000	191.405	0.400736	0.243353	0.013553	0.146217	VAV2	7410	vav guanine nucleotide exchange factor 2
ENSG0000	2968.222	-0.36358	0.204616	0.013559	0.146217	ITPRIP	85450	inositol 1 4 5-trisphosphate receptor interacting protein
ENSG0000	1771.759	0.331114	0.172252	0.013553	0.146217	VPS51	738	VPS51 subunit of GARP complex
ENSG0000	105.5953	-0.37896	0.21287	0.013562	0.146217	DYNLRB1	83658	dynein light chain roadblock-type 1
ENSG0000	18.99838	-0.52986	0.478289	0.013625	0.146815	NA	NA	NA
ENSG0000	47.87865	0.418311	0.251469	0.013639	0.146873	FBXW9	84261	F-box and WD repeat domain containing 9
ENSG0000	350.0903	-0.41634	0.268105	0.013697	0.14736	SLC49A3	84179	solute carrier family 49 member 3
ENSG0000	30.31378	0.474103	0.340959	0.0137	0.14736	UBTD2	92181	ubiquitin domain containing 2
ENSG0000	6.378322	0.192924	0.345132	0.013751	0.147734	KCNH8	131096	potassium voltage-gated channel subfamily H member 8
ENSG0000	170.9684	0.379332	0.217573	0.013743	0.147734	MS4A7	58475	membrane spanning 4-domains A7
ENSG0000	821.6317	0.413974	0.271183	0.013798	0.14816	IGLV2-8	28817	immunoglobulin lambda variable 2-8
ENSG0000	869.3514	-0.31493	0.160376	0.013818	0.148277	VAMP3	9341	vesicle associated membrane protein 3
ENSG0000	7.337893	-0.3005	0.543265	0.013846	0.148492	NA	NA	NA
ENSG0000	4.994378	0.093281	0.256654	0.013862	0.148585	RBM44	375316	RNA binding motif protein 44
ENSG0000	11.59286	0.534099	0.56832	0.01391	0.148886	AZIN2	113451	antizyme inhibitor 2
ENSG0000	61.41697	-0.45348	0.323589	0.013916	0.148886	KIAA1614-	1.03E+08	KIAA1614 antisense RNA 1
ENSG0000	8617.465	-0.36898	0.210616	0.013923	0.148886	YBX3	8531	Y-box binding protein 3
ENSG0000	118.0453	0.358622	0.195884	0.013904	0.148886	GABPB1-IT	55056	GABPB1 intronic transcript
ENSG0000	34.79347	0.479328	0.367692	0.013937	0.148892	PLEKHH3	79990	pleckstrin MyTH4 and FERM domain containing H3
ENSG0000	600.0216	0.378383	0.218996	0.01394	0.148892	ERN1	2081	endoplasmic reticulum to nucleus signaling 1
ENSG0000	241.4539	0.298505	0.147415	0.013982	0.149251	WDR91	29062	WD repeat domain 91
ENSG0000	893.7421	-0.25338	0.11778	0.014032	0.149592	CDK12	51755	cyclin dependent kinase 12
ENSG0000	125.7459	-0.41498	0.265612	0.014036	0.149592	MMD	23531	monocyte to macrophage differentiation associated
ENSG0000	548.3182	0.383414	0.227495	0.014038	0.149592	MYDGF	56005	myeloid derived growth factor
ENSG0000	170.0398	-0.38387	0.224731	0.014047	0.149604	POLB	5423	DNA polymerase beta
ENSG0000	25.46242	-0.45987	0.308061	0.01409	0.149968	NA	NA	NA
ENSG0000	63134.29	0.43153	0.322276	0.014121	0.150211	IGKC	3514	immunoglobulin kappa constant
ENSG0000	70.11642	-0.45799	0.435669	0.014133	0.150256	SPACDR	402573	sperm acrosome developmental regulator
ENSG0000	74.56722	0.362351	0.194102	0.014153	0.150384	NDUFAF8	284184	NADH:ubiquinone oxidoreductase complex assembly factor 8
ENSG0000	8.767951	0.504757	0.733049	0.014173	0.150504	LACC1	144811	laccase domain containing 1
ENSG0000	148.4617	0.420028	0.270403	0.01421	0.150815	SPATS2	65244	spermatogenesis associated serine rich 2
ENSG0000	229.3771	0.152415	0.296579	0.014245	0.151022	ARG1	383	arginase 1

ENSG0000	12.72823	0.450332	0.671745	0.014246	0.151022	KCNMB4	27345	potassium calcium-activated channel subfamily M regulatory beta subunit 4
ENSG0000	481.1221	0.307578	0.155912	0.01426	0.151077	CPSF6	11052	cleavage and polyadenylation specific factor 6
ENSG0000	120.2949	-0.45307	0.407439	0.014268	0.151081	TMC4	147798	transmembrane channel like 4
ENSG0000	50.15648	-0.48175	0.334992	0.014284	0.151139	CPA5	93979	carboxypeptidase A5
ENSG0000	14.50083	-0.45577	0.628604	0.01429	0.151139	FIRRE	286467	firre intergenic repeating RNA element
ENSG0000	8.046259	-0.2256	0.39127	0.014306	0.151211	HTR3B	9177	5-hydroxytryptamine receptor 3B
ENSG0000	9.491359	-0.18854	0.33613	0.014337	0.15137	WNK4	65266	WNK lysine deficient protein kinase 4
ENSG0000	1676.525	-0.29281	0.145228	0.014336	0.15137	ELMO2	63916	engulfment and cell motility 2
ENSG0000	71.15081	0.317681	0.519177	0.014367	0.15144	IGKV2-29	28920	immunoglobulin kappa variable 2-29
ENSG0000	376.9916	0.301846	0.151161	0.014369	0.15144	CHRAC1	54108	chromatin accessibility complex subunit 1
ENSG0000	6.350591	0.213142	0.37711	0.014367	0.15144	PRR33	1.03E+08	proline rich 33
ENSG0000	25.26748	0.491373	0.429875	0.0144	0.151672	DCST2	127579	DC-STAMP domain containing 2
ENSG0000	3.363535	0.123464	0.277766	0.014415	0.151672	NA	NA	NA
ENSG0000	67.9933	-0.43116	0.285753	0.014412	0.151672	RPL13AP2(	729212	ribosomal protein L13a pseudogene 26
ENSG0000	99.35045	0.362767	0.201094	0.014429	0.151728	COPG2	26958	COPI coat complex subunit gamma 2
ENSG0000	1500.573	0.299248	0.150437	0.014448	0.151839	SLC7A7	9056	solute carrier family 7 member 7
ENSG0000	65.12118	0.456139	0.354352	0.014462	0.151902	MRPL15	29088	mitochondrial ribosomal protein L15
ENSG0000	138.0075	0.33638	0.179444	0.014474	0.151941	SLC11A2	4891	solute carrier family 11 member 2
ENSG0000	2180.7	0.34074	0.176912	0.014489	0.152011	LTA4H	4048	leukotriene A4 hydrolase
ENSG0000	582.8313	-0.28977	0.142653	0.014498	0.152013	RIPK1	8737	receptor interacting serine/threonine kinase 1
ENSG0000	1103.503	-0.3789	0.225249	0.014534	0.152308	TBX21	30009	T-box transcription factor 21
ENSG0000	2149.075	-0.39457	0.247441	0.014574	0.152644	LILRB4	11006	leukocyte immunoglobulin like receptor B4
ENSG0000	91.00498	0.412829	0.262759	0.014631	0.153144	MYO1E	4643	myosin IE
ENSG0000	10.86833	-0.49768	0.654117	0.014673	0.153496	TRAV6	6956	T cell receptor alpha variable 6
ENSG0000	14.61774	-0.41441	0.657955	0.01482	0.154955	NA	NA	NA
ENSG0000	19.51971	-0.47765	0.348206	0.01486	0.155279	CEBPB-AS:	1.02E+08	CEBPB antisense RNA 1
ENSG0000	24.32527	0.478933	0.490546	0.014876	0.155358	DPY19L4	286148	dpy-19 like 4
ENSG0000	5.208147	-0.29781	0.5296	0.014945	0.155991	LOC11530	1.15E+08	uncharacterized LOC115308161
ENSG0000	11.14906	0.284338	0.47852	0.014988	0.156355	SPAG8	26206	sperm associated antigen 8
ENSG0000	9.751816	-0.16507	0.309395	0.015	0.156384	NA	NA	NA
ENSG0000	4.175521	0.07287	0.247767	0.015022	0.156525	NA	NA	NA
ENSG0000	17.50074	-0.39859	0.589005	0.015038	0.156608	PRMT5-DT	1.02E+08	PRMT5 divergent transcript
ENSG0000	10602.31	-0.39295	0.249724	0.015058	0.156721	SBNO2	22904	strawberry notch homolog 2
ENSG0000	179.7978	-0.3819	0.231711	0.015077	0.156832	NA	NA	NA
ENSG0000	152.3644	-0.42335	0.454869	0.015097	0.156884	GPR171	29909	G protein-coupled receptor 171
ENSG0000	9.24859	0.234746	0.397044	0.015099	0.156884	NA	NA	NA
ENSG0000	13.98729	-0.48644	0.579826	0.015135	0.15708	NA	NA	NA
ENSG0000	73.96101	-0.45196	0.360858	0.015131	0.15708	NA	NA	NA
ENSG0000	2498.928	0.282399	0.447141	0.015171	0.157362	IGHV4-34	28395	immunoglobulin heavy variable 4-34
ENSG0000	619.3802	0.41094	0.273847	0.015185	0.157417	IGKV1-9	28941	immunoglobulin kappa variable 1-9
ENSG0000	465.8257	0.299357	0.155103	0.015217	0.157569	AKR1B1	231	aldo-keto reductase family 1 member B
ENSG0000	3.63298	-0.13225	0.283238	0.015208	0.157569	MIR3176	1E+08	microRNA 3176
ENSG0000	75.33995	0.424887	0.285762	0.015288	0.158082	COBLL1	22837	cordon-bleu WH2 repeat protein like 1
ENSG0000	45.72528	0.443766	0.322679	0.015277	0.158082	MAN1B1-I	1E+08	MAN1B1 divergent transcript
ENSG0000	14.43467	0.50973	0.500331	0.015292	0.158082	NA	NA	NA
ENSG0000	45.37159	0.462396	0.375131	0.015312	0.158112	LINC01948	1.02E+08	long intergenic non-protein coding RNA 1948
ENSG0000	59.56693	0.391118	0.233536	0.015308	0.158112	C16orf86	388284	chromosome 16 open reading frame 86
ENSG0000	34.2963	-0.4512	0.325	0.015342	0.15833	NA	NA	NA
ENSG0000	2006.764	-0.28311	0.14065	0.015407	0.158817	USF1	7391	upstream transcription factor 1
ENSG0000	5.250563	0.117684	0.271263	0.015405	0.158817	TCTE1	202500	t-complex-associated-testis-expressed 1
ENSG0000	550.6193	-0.39137	0.251235	0.015421	0.158872	ANK1	286	ankyrin 1
ENSG0000	3.555016	0.098396	0.260873	0.015508	0.159685	SPAG16	79582	sperm associated antigen 16
ENSG0000	103.3169	-0.40223	0.255359	0.015554	0.160065	TIGIT	201633	T cell immunoreceptor with Ig and ITIM domains
ENSG0000	287.2325	0.345316	0.192712	0.015588	0.160203	SNX8	29886	sorting nexin 8
ENSG0000	48.98391	0.423058	0.278707	0.015594	0.160203	SIAH1	6477	siah E3 ubiquitin protein ligase 1
ENSG0000	4.434188	-0.16787	0.317666	0.01558	0.160203	NA	NA	NA
ENSG0000	19.08179	0.425411	0.610074	0.01562	0.160384	IGHD3-3	28501	immunoglobulin heavy diversity 3-3
ENSG0000	10.24035	-0.50267	0.600811	0.015679	0.160897	NA	NA	NA
ENSG0000	1593.482	-0.31366	0.164337	0.015707	0.161095	TMEM131	23240	transmembrane 131 like
ENSG0000	653.6983	0.354961	0.203653	0.015737	0.161314	SSR3	6747	signal sequence receptor subunit 3
ENSG0000	4.347	0.247857	0.432961	0.015766	0.161432	NA	NA	NA
ENSG0000	101.7381	0.460281	0.396135	0.015765	0.161432	LOC10272	1.03E+08	immunoglobulin heavy variable 4-38-2-like
ENSG0000	1068.002	0.363651	0.210119	0.015788	0.161565	RPS13	6207	ribosomal protein S13
ENSG0000	20.91017	0.481229	0.507702	0.015868	0.162258	MOXD1	26002	monooxygenase DBH like 1
ENSG0000	254.0647	0.394251	0.253179	0.015873	0.162258	PNOC	5368	prepronociceptin
ENSG0000	654.5913	0.326706	0.175982	0.015886	0.162301	EIF4EBP1	1978	eukaryotic translation initiation factor 4E binding protein 1
ENSG0000	12.44492	-0.50937	0.515838	0.015901	0.162365	HGD	3081	homogent 2-dioxygenase
ENSG0000	4.997424	0.161101	0.308157	0.015917	0.16243	NA	NA	NA
ENSG0000	7454.887	0.277382	0.139298	0.015974	0.162869	RPL29	6159	ribosomal protein L29
ENSG0000	16.06791	0.501069	0.473921	0.015978	0.162869	RASA4	10156	RAS p21 protein activator 4
ENSG0000	123.4548	-0.41659	0.294563	0.016018	0.163193	GPR18	2841	G protein-coupled receptor 18
ENSG0000	4.518718	-0.20467	0.367142	0.016114	0.164076	NA	NA	NA
ENSG0000	796.5302	-0.37748	0.244831	0.016163	0.164308	BAZ2B-AS:	643072	BAZ2B antisense RNA 1
ENSG0000	3217.528	-0.37362	0.230338	0.016163	0.164308	TREML2	79865	triggering receptor expressed on myeloid cells like 2
ENSG0000	4.443979	-0.23117	0.400089	0.016164	0.164308	NA	NA	NA
ENSG0000	11.88383	0.503172	0.562813	0.016239	0.164983	PITPNM3	83394	PITPNM family member 3
ENSG0000	1222.05	0.398236	0.267372	0.01628	0.165156	PTP4A3	11156	protein tyrosine phosphatase 4A3
ENSG0000	48.34972	-0.42228	0.285027	0.016278	0.165156	NA	NA	NA
ENSG0000	5545.627	-0.36553	0.220185	0.016283	0.165156	LYL1	4066	LYL1 basic helix-loop-helix family member
ENSG0000	17.21321	0.471271	0.526651	0.016316	0.165219	NA	NA	NA
ENSG0000	214.3436	0.322463	0.170862	0.016313	0.165219	FUCA2	2519	alpha-L-fucosidase 2
ENSG0000	79.98047	0.418568	0.303061	0.016303	0.165219	TOMM7	54543	translocase of outer mitochondrial membrane 7
ENSG0000	719.9633	0.317378	0.169042	0.016328	0.165247	BBC3	27113	BCL2 binding component 3
ENSG0000	93.25198	0.409056	0.491583	0.016376	0.165554	SLC5A9	200010	solute carrier family 5 member 9
ENSG0000	4585.811	0.357249	0.201906	0.016371	0.165554	RPSA	3921	ribosomal protein SA
ENSG0000	56.61605	-0.42662	0.302049	0.016408	0.165784	SMPDL3B	27293	sphingomyelin phosphodiesterase acid like 3B
ENSG0000	1184.85	-0.41089	0.338863	0.016424	0.16585	FAM8A1	51439	family with sequence similarity 8 member A1
ENSG0000	136.7824	0.350352	0.198166	0.016445	0.165972	PCCB	5096	propionyl-CoA carboxylase subunit beta
ENSG0000	6168.221	0.289632	0.147509	0.016465	0.166082	RPLP1	6176	ribosomal protein lateral stalk subunit P1
ENSG0000	8.416149	-0.46242	0.703974	0.016475	0.166092	HBZP1	3051	hemoglobin subunit zeta pseudogene 1
ENSG0000	278.2019	-0.31855	0.168188	0.016587	0.167133	CCDC93	54520	coiled-coil domain containing 93
ENSG0000	120.4713	0.430085	0.378456	0.01662	0.167373	PXYLP1	92370	2-phosphoxylose phosphatase 1
ENSG0000	3.003644	0.140267	0.290867	0.016672	0.167803	KAZALD1	81621	Kazal type serine peptidase inhibitor domain 1
ENSG0000	175.6505	-0.39491	0.257702	0.016692	0.167915	CERCAM	51148	cerebral endothelial cell adhesion molecule
ENSG0000	6992.392	-0.31791	0.17234	0.016762	0.168525	GUK1	2987	guanylate kinase 1
ENSG0000	11.55634	-0.49528	0.549725	0.016797	0.168782	ZNF385C	201181	zinc finger protein 385C

ENSG0000	7.202849	0.296767	0.510161	0.016821	0.168928	GSTCD	79807	glutathione S-transferase C-terminal domain containing
ENSG0000	6.546862	0.15076	0.296855	0.016896	0.169455	NA	NA	NA
ENSG0000	6.710737	-0.34319	0.59768	0.016901	0.169455	GDF15	9518	growth differentiation factor 15
ENSG0000	18.12457	-0.47821	0.39628	0.016889	0.169455	CPT1B	1375	carnitine palmitoyltransferase 1B
ENSG0000	43.45385	0.396287	0.50709	0.016925	0.169607	PCBP3	54039	poly(rC) binding protein 3
ENSG0000	55.05438	-0.44473	0.390979	0.016952	0.169778	HBZ	3050	hemoglobin subunit zeta
ENSG0000	3450.599	0.301887	0.15587	0.016964	0.169811	RPS16	6217	ribosomal protein S16
ENSG0000	7.585855	0.2086	0.359902	0.016992	0.169999	CALY	50632	calcyon neuron specific vesicular protein
ENSG0000	31.48796	0.457134	0.432535	0.017066	0.170585	IGKV3D-15	28875	immunoglobulin kappa variable 3D-15
ENSG0000	1033.275	-0.40465	0.286074	0.01707	0.170585	ST3GAL4	6484	ST3 beta-g 3-sialyltransferase 4
ENSG0000	190.3151	0.39791	0.267849	0.017129	0.171088	PIGX	54965	phosphatidylinositol glycan anchor biosynthesis class X
ENSG0000	3.376683	0.114917	0.270538	0.01714	0.171099	SLC4A9	83697	solute carrier family 4 member 9
ENSG0000	133.1679	-0.41256	0.303744	0.017155	0.171161	KIAA1522	57648	KIAA1522
ENSG0000	80.40741	-0.37351	0.226489	0.017195	0.171462	CD27-AS1	678655	CD27 antisense RNA 1
ENSG0000	317.9075	-0.34693	0.200515	0.017273	0.171954	FCRL3	115352	Fc receptor like 3
ENSG0000	184.4103	-0.40004	0.270193	0.01731	0.171954	NA	NA	NA
ENSG0000	3241.531	-0.35819	0.217826	0.017305	0.171954	IGF2R	3482	insulin like growth factor 2 receptor
ENSG0000	1886.175	-0.38488	0.256604	0.017294	0.171954	PTMS	5763	parathymosin
ENSG0000	38.02539	0.409478	0.272512	0.017296	0.171954	UBL7-DT	440288	UBL7 divergent transcript
ENSG0000	12.22391	-0.32631	0.524893	0.017284	0.171954	NA	NA	NA
ENSG0000	4.014984	0.192745	0.346888	0.017298	0.171954	AOC4P	90586	amine oxic pseudogene
ENSG0000	5.006003	0.184693	0.335271	0.017338	0.172141	ADD3-AS1	1.01E+08	ADD3 antisense RNA 1
ENSG0000	8.334134	0.149956	0.299666	0.017352	0.172188	NA	NA	NA
ENSG0000	1111.991	0.411176	0.370431	0.017403	0.172569	IRAK3	11213	interleukin 1 receptor associated kinase 3
ENSG0000	80.91696	-0.37341	0.227294	0.017417	0.172569	RNF31	55072	ring finger protein 31
ENSG0000	3.668963	0.168368	0.317932	0.017419	0.172569	GOLGA8H	728498	golgin A8 family member H
ENSG0000	265.0141	0.294194	0.157398	0.017436	0.172643	ZNF318	24149	zinc finger protein 318
ENSG0000	3215.469	0.388916	0.263599	0.017471	0.172898	IGKV3-20	28912	immunoglobulin kappa variable 3-20
ENSG0000	2310.079	-0.27569	0.139127	0.017518	0.173245	PCGF5	84333	polycomb group ring finger 5
ENSG0000	10.04852	-0.31271	0.520463	0.017525	0.173245	LOC10537	1.05E+08	uncharacterized LOC105371967
ENSG0000	3.622587	0.150233	0.298594	0.017575	0.173316	NA	NA	NA
ENSG0000	162.2038	-0.36674	0.224381	0.017561	0.173316	NA	NA	NA
ENSG0000	39.91979	0.422531	0.300237	0.01756	0.173316	NA	NA	NA
ENSG0000	105.6641	0.318279	0.171548	0.017572	0.173316	ATXN7L3-A	1.02E+08	ATXN7L3 antisense RNA 1
ENSG0000	199.5158	-0.31358	0.167709	0.017579	0.173316	ZNF701	55762	zinc finger protein 701
ENSG0000	21.43872	0.463752	0.408069	0.017629	0.173714	GGACT	87769	gamma-glutamylamine cyclotransferase
ENSG0000	1978.973	-0.40056	0.301292	0.017713	0.174448	SMCHD1	23347	structural maintenance of chromosomes flexible hinge domain containing 1
ENSG0000	40.60719	-0.38875	0.243817	0.017895	0.176131	NA	NA	NA
ENSG0000	78.08794	0.343968	0.195554	0.017913	0.176131	ADK	132	adenosine kinase
ENSG0000	511.7507	-0.3096	0.165994	0.017912	0.176131	KCTD21	283219	potassium channel tetramerization domain containing 21
ENSG0000	24.70391	-0.45707	0.414294	0.017943	0.176329	TRBV4-1	28617	T cell receptor beta variable 4-1
ENSG0000	196.4343	-0.40084	0.287168	0.017969	0.176495	SNPH	9751	syntaphilin
ENSG0000	141.1552	0.40759	0.416511	0.018006	0.17676	IGHV3-13	28449	immunoglobulin heavy variable 3-13
ENSG0000	12.57384	0.477289	0.505546	0.018029	0.176895	NA	NA	NA
ENSG0000	3.031168	0.128666	0.281274	0.018091	0.177398	NA	NA	NA
ENSG0000	49.31618	-0.39844	0.260927	0.0181	0.177398	TRAV12-2	28673	T cell receptor alpha variable 12-2
ENSG0000	83.66936	-0.41555	0.367162	0.018153	0.177826	YJEFN3	374887	YjeF N-terminal domain containing 3
ENSG0000	907.114	-0.35724	0.218413	0.018193	0.17812	SBK1	388228	SH3 domain binding kinase 1
ENSG0000	37.99864	0.418523	0.296348	0.018242	0.178285	B4GAT1	11041	beta-1 4-glucuronyltransferase 1
ENSG0000	774.942	-0.24126	0.116571	0.018221	0.178285	MAPRE2	10982	microtubule associated protein RP/EB family member 2
ENSG0000	3.086827	0.123945	0.279194	0.018248	0.178285	NA	NA	NA
ENSG0000	52863.06	-0.34479	0.205048	0.018249	0.178285	RAC2	5880	Rac family small GTPase 2
ENSG0000	830.5638	0.387179	0.277285	0.018261	0.178311	PLIN4	729359	perilipin 4
ENSG0000	4.431675	0.133396	0.285292	0.018303	0.178625	NA	NA	NA
ENSG0000	626.7072	0.287815	0.150908	0.018339	0.178784	EIF3M	10480	eukaryotic translation initiation factor 3 subunit M
ENSG0000	9802.346	-0.3806	0.25782	0.018331	0.178784	BCL3	602	BCL3 transcription coactivator
ENSG0000	8.228753	0.235956	0.399434	0.018376	0.178962	WWC2	80014	WW and C2 domain containing 2
ENSG0000	656.4322	0.349497	0.209373	0.018377	0.178962	PARVB	29780	parvin beta
ENSG0000	341.6504	-0.37363	0.23909	0.018418	0.179263	PPFIBP2	8495	PPFIA binding protein 2
ENSG0000	3.906584	-0.20108	0.3561	0.018457	0.179555	NA	NA	NA
ENSG0000	4548.11	-0.32398	0.183181	0.018519	0.180056	RERE	473	arginine-glutamic acid dipeptide repeats
ENSG0000	8729.928	-0.37464	0.272977	0.018548	0.180148	BRI3	25798	brain protein I3
ENSG0000	6.266142	0.219772	0.37696	0.018541	0.180148	SGCA	6442	sarcoglycan alpha
ENSG0000	146.5989	0.414353	0.33024	0.018587	0.18043	TIGD3	220359	tigger transposable element derived 3
ENSG0000	71.47765	0.422256	0.359782	0.018642	0.180808	STEAP3	55240	STEAP3 metalloreductase
ENSG0000	1103.348	-0.29681	0.156483	0.018645	0.180808	MIEN1	84299	migration and invasion enhancer 1
ENSG0000	103.0082	0.395401	0.275937	0.01867	0.18095	CHD9	80205	chromodomain helicase DNA binding protein 9
ENSG0000	642.2225	-0.26785	0.140299	0.018703	0.181082	RAD9A	5883	RAD9 checkpoint clamp component A
ENSG0000	35.48707	0.424625	0.438933	0.018703	0.181082	CELSR1	9620	cadherin EGF LAG seven-pass G-type receptor 1
ENSG0000	113.6995	-0.39574	0.280714	0.018726	0.181204	USP49	25862	ubiquitin specific peptidase 49
ENSG0000	368.9394	0.387896	0.425961	0.01874	0.181249	IL1R1	3554	interleukin 1 receptor type 1
ENSG0000	123.9506	-0.3697	0.234606	0.018801	0.181744	CYSLTR1	10800	cysteinyl leukotriene receptor 1
ENSG0000	46.65392	-0.37544	0.232641	0.018863	0.182241	TDRKH	11022	tudor and KH domain containing
ENSG0000	299.3263	0.277364	0.14302	0.018894	0.182253	ZBTB40	9923	zinc finger and BTB domain containing 40
ENSG0000	140.3421	0.349632	0.211359	0.018889	0.182253	RPL13AP5	728658	ribosomal protein L13a pseudogene 5
ENSG0000	271.3732	0.283975	0.146746	0.018884	0.182253	WDR45B	56270	WD repeat domain 45B
ENSG0000	38.17757	0.358121	0.498433	0.018927	0.182284	ST6GALNA	256435	ST6 N-acet 6-sialyltransferase 3
ENSG0000	7881.163	0.276075	0.14432	0.018937	0.182284	RPL8	6132	ribosomal protein L8
ENSG0000	55.99274	-0.39984	0.277578	0.018936	0.182284	CDKL1	8814	cyclin dependent kinase like 1
ENSG0000	33.68896	-0.40338	0.464867	0.018922	0.182284	NA	NA	NA
ENSG0000	1886.242	-0.38127	0.26214	0.018961	0.182421	PPP1R3B	79660	protein phosphatase 1 regulatory subunit 3B
ENSG0000	6.008946	0.129001	0.279725	0.018985	0.182554	SLC41A2	84102	solute carrier family 41 member 2
ENSG0000	304.183	-0.34628	0.20756	0.019003	0.182634	FCRL6	343413	Fc receptor like 6
ENSG0000	27.45479	-0.42015	0.299577	0.019053	0.18278	NA	NA	NA
ENSG0000	388.5364	-0.32371	0.183981	0.019074	0.18278	RAB2B	84932	RAB2B member RAS oncogene family
ENSG0000	103.9602	0.414899	0.307766	0.019078	0.18278	IGHV3-73	28409	immunoglobulin heavy variable 3-73
ENSG0000	38.86758	-0.38859	0.253034	0.01906	0.18278	PLD6	201164	phospholipase D family member 6
ENSG0000	36.56589	-0.26062	0.407615	0.019044	0.18278	FFAR3	2865	free fatty acid receptor 3
ENSG0000	10.85654	0.436234	0.585722	0.019054	0.18278	RPS2P7	86781	ribosomal protein S2 pseudogene 7
ENSG0000	137.2965	0.324457	0.182568	0.019118	0.183062	FUBP3	8939	far upstream element binding protein 3
ENSG0000	32.98494	-0.43079	0.344786	0.019276	0.184389	NA	NA	NA
ENSG0000	7.453358	0.153659	0.298275	0.019276	0.184389	NA	NA	NA
ENSG0000	1143.57	-0.29999	0.161087	0.019295	0.184444	SLAMF6	114836	SLAM family member 6
ENSG0000	871.6903	0.377935	0.255354	0.019302	0.184444	MYO7B	4648	myosin VIIB
ENSG0000	7.361812	0.173924	0.318686	0.019406	0.184498	NEXN-AS1	374987	NEXN antisense RNA 1
ENSG0000	173.7844	0.338351	0.198041	0.019325	0.184498	PDE4DIP	9659	phosphodiesterase 4D interacting protein

ENSG0000	1858.547	0.238511	0.118637	0.019427	0.184498	RCSD1	92241	RCSD domain containing 1
ENSG0000	1460.611	-0.28036	0.14635	0.019348	0.184498	PRDX6	9588	peroxiredoxin 6
ENSG0000	53.01984	0.402925	0.294491	0.019424	0.184498	GEN1	348654	GEN1 Holliday junction 5' flap endonuclease
ENSG0000	859.7716	0.284858	0.149393	0.019391	0.184498	CDV3	55573	CDV3 homolog
ENSG0000	16.16727	-0.45153	0.436778	0.019449	0.184498	NA	NA	NA
ENSG0000	2.987284	0.102536	0.262755	0.019362	0.184498	NA	NA	NA
ENSG0000	440.8274	-0.34284	0.205035	0.019424	0.184498	MSRB2	22921	methionine sulfoxide reductase B2
ENSG0000	9.926901	0.327919	0.534415	0.019374	0.184498	NA	NA	NA
ENSG0000	3841.622	-0.33694	0.201519	0.019445	0.184498	CTSW	1521	cathepsin W
ENSG0000	1967.421	-0.3588	0.228608	0.019411	0.184498	ZNF213	7760	zinc finger protein 213
ENSG0000	69.07439	0.388949	0.260645	0.019407	0.184498	NA	NA	NA
ENSG0000	201.945	0.347771	0.209142	0.019435	0.184498	CENPX	201254	centromere protein X
ENSG0000	1018.238	0.389879	0.377657	0.01946	0.184509	IGHV1-18	28468	immunoglobulin heavy variable 1-18
ENSG0000	13.01282	-0.40516	0.552717	0.019536	0.185034	IFITM4P	340198	interferon induced transmembrane protein 4 pseudogene
ENSG0000	28824.33	-0.38873	0.298428	0.019533	0.185034	NCF1	653361	neutrophil cytosolic factor 1
ENSG0000	3614.996	-0.27941	0.146449	0.019556	0.185073	GIMAP4	55303	GTPase IMAP family member 4
ENSG0000	229.658	0.396374	0.302149	0.01956	0.185073	HID1	283987	HID1 domain containing
ENSG0000	855.6349	-0.35382	0.212411	0.019606	0.185338	SMPD1	6609	sphingomyelin phosphodiesterase 1
ENSG0000	9.508634	0.315075	0.516507	0.019618	0.185338	CEP83	51134	centrosomal protein 83
ENSG0000	1264.787	0.281849	0.148637	0.019613	0.185338	RSL1D1	26156	ribosomal L1 domain containing 1
ENSG0000	9.111667	0.412715	0.617003	0.019631	0.185358	UGDH-AS1	1.01E+08	UGDH antisense RNA 1
ENSG0000	12.55642	-0.18831	0.331565	0.019675	0.185579	FCER1A	2205	Fc epsilon receptor Ia
ENSG0000	6232.461	-0.27411	0.142021	0.019669	0.185579	TNK2	10188	tyrosine kinase non receptor 2
ENSG0000	1045.252	-0.3045	0.166246	0.019684	0.185579	GRAP2	9402	GRB2 related adaptor protein 2
ENSG0000	59.26264	-0.39369	0.267009	0.019696	0.185592	RIPOR3	140876	RIPOR family member 3
ENSG0000	3.09973	-0.08768	0.255084	0.019725	0.185767	KCNK17	89822	potassium two pore domain channel subfamily K member 17
ENSG0000	157.7257	-0.31606	0.177185	0.019798	0.186365	SNTB1	6641	syntrophin beta 1
ENSG0000	843.1135	0.281996	0.148662	0.019889	0.187125	MTDH	92140	metadherin
ENSG0000	2.721569	0.117217	0.275263	0.019944	0.187543	RBP5	83758	retinol binding protein 5
ENSG0000	172.7466	0.383681	0.265544	0.019961	0.187611	TSPAN4	7106	tetraspanin 4
ENSG0000	2003.182	0.26856	0.13853	0.019985	0.187736	AKT1	207	AKT serine/threonine kinase 1
ENSG0000	50.4324	-0.36501	0.225433	0.020024	0.187907	NRL	4901	neural retina leucine zipper
ENSG0000	6528.682	-0.35436	0.234122	0.020017	0.187907	SLC16A3	9123	solute carrier family 16 member 3
ENSG0000	462.8756	-0.34573	0.212324	0.020066	0.188207	MPZ	4359	myelin protein zero
ENSG0000	23008.87	-0.38924	0.339724	0.020122	0.188638	CTSD	1509	cathepsin D
ENSG0000	63.47763	0.373733	0.460506	0.020152	0.188817	CHAD	1101	chondroadherin
ENSG0000	302.9475	-0.39301	0.329217	0.020214	0.189304	CNIH4	29097	cornichon family AMPA receptor auxiliary protein 4
ENSG0000	125.0134	-0.33013	0.191539	0.020243	0.189447	CAPN5	726	calpain 5
ENSG0000	90.32848	0.39466	0.274639	0.02025	0.189447	MRPL40	64976	mitochondrial ribosomal protein L40
ENSG0000	6.850685	0.334719	0.558334	0.020295	0.18977	TTC23	64927	tetratricopeptide repeat domain 23
ENSG0000	165.0599	-0.36586	0.239227	0.02035	0.190186	IGFLR1	79713	IGF like family receptor 1
ENSG0000	152.8934	0.280488	0.147709	0.020363	0.190216	CHCHD3	54927	coiled-coil-helix-coiled-coil-helix domain containing 3
ENSG0000	62715.64	-0.36472	0.243552	0.020405	0.190506	S100A9	6280	S100 calcium binding protein A9
ENSG0000	21.54604	0.436232	0.377584	0.020477	0.190696	NA	NA	NA
ENSG0000	31.71478	-0.40891	0.30104	0.020447	0.190696	NA	NA	NA
ENSG0000	350.1584	0.339443	0.205162	0.020477	0.190696	IER5L	389792	immediate early response 5 like
ENSG0000	33.18485	0.235224	0.377996	0.020452	0.190696	PLAAT2	54979	phospholipase A and acyltransferase 2
ENSG0000	16822.17	-0.33686	0.204925	0.020465	0.190696	WBP2	23558	WW domain binding protein 2
ENSG0000	699.2522	0.35621	0.228709	0.020516	0.190958	NIBAN3	199786	niban apoptosis regulator 3
ENSG0000	250.3992	-0.32479	0.187331	0.020532	0.191014	PARP3	10039	poly(ADP-ribose) polymerase family member 3
ENSG0000	25511.38	-0.3527	0.226572	0.020578	0.191056	DMTN	2039	dematin actin binding protein
ENSG0000	230.5772	-0.27318	0.142472	0.020565	0.191056	TCHP	84260	trichoplein keratin filament binding
ENSG0000	8.618166	-0.28917	0.472858	0.020551	0.191056	NA	NA	NA
ENSG0000	1095.415	0.267253	0.138675	0.020575	0.191056	CERK	64781	ceramide kinase
ENSG0000	29.09524	0.419305	0.363634	0.02061	0.191255	EVI5	7813	ecotropic viral integration site 5
ENSG0000	18.72767	-0.38149	0.506518	0.02065	0.191529	RPS29P14	1E+08	ribosomal protein S29 pseudogene 14
ENSG0000	26.72381	0.422363	0.418623	0.020791	0.192441	CRYZL2P	730102	crystallin z pseudogene
ENSG0000	2.558338	0.157263	0.307089	0.02079	0.192441	NA	NA	NA
ENSG0000	10.13054	0.38693	0.584161	0.020782	0.192441	NA	NA	NA
ENSG0000	55.26344	0.414198	0.342668	0.020779	0.192441	ZNF532	55205	zinc finger protein 532
ENSG0000	1567.509	-0.27428	0.144662	0.02081	0.192524	TMEM63A	9725	transmembrane protein 63A
ENSG0000	5990	-0.33566	0.202381	0.020893	0.193197	TFE3	7030	transcription factor binding to IGHM enhancer 3
ENSG0000	10.94552	-0.36797	0.553249	0.020918	0.193233	H4C12	8362	H4 clustered histone 12
ENSG0000	13.70731	-0.26853	0.435638	0.020908	0.193233	CLEC4C	170482	C-type lectin domain family 4 member C
ENSG0000	14.43188	0.416919	0.521664	0.020975	0.193554	FUT10	84750	fucosyltransferase 10
ENSG0000	16.19475	-0.44379	0.417428	0.020985	0.193554	WNT11	7481	Wnt family member 11
ENSG0000	2.81014	-0.14118	0.292675	0.020973	0.193554	ADAM20P	317760	ADAM metallopeptidase domain 20 pseudogene 1
ENSG0000	414.3913	0.316963	0.180944	0.021005	0.193646	DPYD	1806	dihydropyrimidine dehydrogenase
ENSG0000	253.0507	0.327786	0.193205	0.021103	0.19445	ARHGAP31	57514	Rho GTPase activating protein 31
ENSG0000	13.23942	0.446393	0.449256	0.021124	0.194544	NA	NA	NA
ENSG0000	128.0185	-0.39528	0.336384	0.021198	0.195127	FASLG	356	Fas ligand
ENSG0000	3.959256	0.169923	0.317274	0.02121	0.195139	DDN	23109	dendrin
ENSG0000	6.112963	-0.15383	0.298805	0.021238	0.195299	FAM124A	220108	family with sequence similarity 124 member A
ENSG0000	415.2958	0.27653	0.146067	0.021295	0.195501	EIF2D	1939	eukaryotic translation initiation factor 2D
ENSG0000	7.68754	0.196594	0.346902	0.021278	0.195501	NA	NA	NA
ENSG0000	16.02337	0.374289	0.520828	0.021313	0.195501	DLEU1	10301	deleted in lymphocytic leukemia 1
ENSG0000	11.49468	0.413348	0.601045	0.021284	0.195501	PRAMENP	649179	PRAME N- pseudogene
ENSG0000	36.81067	0.250116	0.396411	0.021304	0.195501	IGLV1-41	28824	immunoglobulin lambda variable 1-41 (pseudogene)
ENSG0000	3.929473	0.199	0.350021	0.021445	0.19661	RPSAP15	220885	ribosomal protein SA pseudogene 15
ENSG0000	501.0695	0.29536	0.162765	0.021515	0.197051	MEGF6	1953	multiple EGF like domains 6
ENSG0000	10773.92	-0.36162	0.249214	0.021511	0.197051	NCF1B	654816	neutrophil cytosolic factor 1B pseudogene
ENSG0000	5.299988	0.167147	0.313154	0.021566	0.19742	NA	NA	NA
ENSG0000	729.0684	-0.33657	0.204838	0.02159	0.197542	DUSP3	1845	dual specificity phosphatase 3
ENSG0000	5.200369	0.204952	0.356502	0.021638	0.197665	PLEKHG7	440107	pleckstrin homology and RhoGEF domain containing G7
ENSG0000	571.3761	-0.29237	0.161216	0.021636	0.197665	PRKAB1	5564	protein kinase AMP-activated non-catalytic subunit beta 1
ENSG0000	386.5581	0.26662	0.139539	0.021657	0.197665	PGP	283871	phosphoglycolate phosphatase
ENSG0000	52.09334	0.354684	0.222139	0.021656	0.197665	IZUMO4	113177	IZUMO family member 4
ENSG0000	41.40685	0.417932	0.348391	0.021631	0.197665	NA	NA	NA
ENSG0000	3.861081	-0.16856	0.318757	0.021699	0.197951	NA	NA	NA
ENSG0000	219.1996	0.331498	0.199591	0.021732	0.198155	DANCR	57291	differentiation antagonizing non-protein coding RNA
ENSG0000	53.04841	0.345891	0.210344	0.021744	0.198166	HLCS	3141	holocarboxylase synthetase
ENSG0000	294.7259	-0.38919	0.309994	0.021801	0.198335	OR2W3	343171	olfactory receptor family 2 subfamily W member 3
ENSG0000	429.4428	-0.34624	0.221576	0.021782	0.198335	CEP63	80254	centrosomal protein 63
ENSG0000	115.9543	0.315597	0.182671	0.021786	0.198335	MGMT	4255	O-6-methylguanine-DNA methyltransferase
ENSG0000	1041.94	-0.3686	0.263946	0.021806	0.198335	INAFM1	255783	InaF motif containing 1
ENSG0000	3.713972	0.107641	0.265035	0.021836	0.198507	LINC02021	1.05E+08	long intergenic non-protein coding RNA 2021

ENSG0000	20.97198	0.425474	0.36915	0.021875	0.198762	NA	NA	NA
ENSG0000	6.905834	0.214957	0.366933	0.021892	0.198818	NA	NA	NA
ENSG0000	263.187	-0.35416	0.233752	0.021938	0.199142	ITGB3	3690	integrin subunit beta 3
ENSG0000	4.434393	-0.17543	0.323135	0.022011	0.199415	NA	NA	NA
ENSG0000	70.62218	0.353177	0.22305	0.022012	0.199415	COQ2	27235	coenzyme polyprenyltransferase
ENSG0000	3.344425	0.145582	0.294891	0.022	0.199415	NA	NA	NA
ENSG0000	22.15073	0.421507	0.329127	0.021985	0.199415	NHS	4810	NHS actin remodeling regulator
ENSG0000	3.634813	-0.1046	0.263535	0.02203	0.199478	NA	NA	NA
ENSG0000	2841.691	0.294066	0.164074	0.022041	0.199483	RPL23A	6147	ribosomal protein L23a
ENSG0000	6505.813	-0.33871	0.209052	0.022103	0.199645	GNLY	10578	granulysin
ENSG0000	3652.794	0.301477	0.170649	0.022085	0.199645	RPL32	6161	ribosomal protein L32
ENSG0000	4027.501	-0.33925	0.226497	0.022085	0.199645	ABCA2	20	ATP binding cassette subfamily A member 2
ENSG0000	47.78224	-0.3807	0.26533	0.022099	0.199645	LDAF1	57146	lipid droplet assembly factor 1
ENSG0000	600.7284	0.300321	0.169557	0.022129	0.199779	BICD2	23299	BICD cargo adaptor 2
ENSG0000	239.6523	0.353693	0.218565	0.022219	0.200401	AP3S2	10239	adaptor related protein complex 3 subunit sigma 2
ENSG0000	495.218	-0.32163	0.200818	0.022214	0.200401	SPATA2L	124044	spermatogenesis associated 2 like
ENSG0000	458.256	0.36673	0.26676	0.022275	0.200802	LMAN1	3998	lectin mannose binding 1
ENSG0000	704.7022	0.308567	0.179063	0.0223	0.200842	EDEM1	9695	ER degradation enhancing alpha-mannosidase like protein 1
ENSG0000	274.9107	-0.29702	0.166518	0.022315	0.200842	MTURN	222166	maturin neural progenitor differentiation regulator homolog
ENSG0000	8166.326	-0.30124	0.171755	0.022323	0.200842	YWHAZ	7534	tyrosine 3-monooxygenase/tryptophan 5-monooxygenase activation protein zeta
ENSG0000	1558.122	-0.3501	0.231624	0.022317	0.200842	JPT1	51155	Jupiter microtubule associated homolog 1
ENSG0000	494.6254	0.321823	0.191346	0.022349	0.200979	SRPK2	6733	SRSF protein kinase 2
ENSG0000	23.3322	0.376965	0.471706	0.022367	0.201041	GCM1	8521	glial cells missing transcription factor 1
ENSG0000	251.2617	0.375269	0.367892	0.02241	0.201329	CHIT1	1118	chitinase 1
ENSG0000	177.8286	0.282068	0.153203	0.022425	0.201362	SCRN2	90507	secernin 2
ENSG0000	51.23197	0.386245	0.278096	0.022442	0.201415	TP73	7161	tumor protein p73
ENSG0000	170.4668	0.28592	0.155859	0.022464	0.201517	CYREN	78996	cell cycle regulator of NHEJ
ENSG0000	172.4473	0.325768	0.19727	0.022478	0.201543	GNL2	29889	G protein nucleolar 2
ENSG0000	201.196	-0.30253	0.172531	0.022505	0.201587	NMRK1	54981	nicotinamide riboside kinase 1
ENSG0000	52.61929	-0.38019	0.423801	0.0225	0.201587	JPH4	84502	junctophilin 4
ENSG0000	309.3393	0.300167	0.16961	0.022574	0.202108	SGK1	6446	serum/glucocorticoid regulated kinase 1
ENSG0000	41.96504	0.404004	0.339471	0.022654	0.202724	FITM2	128486	fat storage inducing transmembrane protein 2
ENSG0000	9.280902	0.322065	0.504759	0.022678	0.202836	NIPAL4	348938	NIPA like domain containing 4
ENSG0000	54.51123	0.372088	0.259485	0.022699	0.202925	SGK3	23678	serum/glucocorticoid regulated kinase family member 3
ENSG0000	69.095	0.33758	0.207205	0.022742	0.203217	ZNF544	27300	zinc finger protein 544
ENSG0000	8.649111	0.26423	0.427253	0.022758	0.203257	SOCS6	9306	suppressor of cytokine signaling 6
ENSG0000	1257.087	-0.27477	0.148975	0.0228	0.203536	PLAGL2	5326	PLAG1 like zinc finger 2
ENSG0000	5.937759	-0.2582	0.440097	0.022889	0.204039	NA	NA	NA
ENSG0000	8.849252	0.26882	0.435837	0.022887	0.204039	LOC10192	1.02E+08	uncharacterized LOC101927245
ENSG0000	41.07316	-0.38193	0.274745	0.02289	0.204039	NA	NA	NA
ENSG0000	30.19421	0.397372	0.438398	0.022905	0.20407	OXSM	54995	3-oxoacyl- mitochondrial
ENSG0000	34.58906	-0.40392	0.34913	0.022932	0.204212	ELOVL7	79993	ELOVL fatty acid elongase 7
ENSG0000	5.716004	-0.21768	0.371668	0.023004	0.204755	NA	NA	NA
ENSG0000	2249.146	-0.24698	0.124721	0.023051	0.205077	MAX	4149	MYC associated factor X
ENSG0000	851.3678	-0.23418	0.118658	0.023091	0.205334	SNX19	399979	sorting nexin 19
ENSG0000	645.3771	-0.32303	0.19864	0.023137	0.205541	ATXN7	6314	ataxin 7
ENSG0000	2375.01	0.37175	0.291671	0.023137	0.205541	IGLV2-11	28816	immunoglobulin lambda variable 2-11
ENSG0000	33.18145	-0.24263	0.379912	0.023236	0.205872	DSP	1832	desmoplakin
ENSG0000	53.26615	-0.36477	0.24378	0.023234	0.205872	LOC10272	1.03E+08	uncharacterized LOC102723878
ENSG0000	56.16599	-0.38301	0.28664	0.023239	0.205872	MIR9-3HG	254559	MIR9-3 host gene
ENSG0000	4.524788	0.140553	0.288902	0.023242	0.205872	ZNF286B	729288	zinc finger protein 286B (pseudogene)
ENSG0000	75.09595	-0.34676	0.430042	0.023195	0.205872	METTL4	64863	methyltra N6-adenosine
ENSG0000	1136.055	-0.28117	0.154885	0.023225	0.205872	C19orf25	148223	chromosome 19 open reading frame 25
ENSG0000	8918.818	-0.33281	0.211723	0.023273	0.206053	CSF2RB	1439	colony stimulating factor 2 receptor subunit beta
ENSG0000	34.17637	0.348085	0.448054	0.023309	0.206166	REELD1	345051	reeler domain containing 1
ENSG0000	11891.69	-0.32403	0.199611	0.023308	0.206166	CD3E	916	CD3 epsilon subunit of T-cell receptor complex
ENSG0000	188.1663	0.361827	0.254254	0.023375	0.206653	BANK1	55024	B cell scaffold protein with ankyrin repeats 1
ENSG0000	79.65614	-0.32452	0.19434	0.023393	0.206711	GGPS1	9453	geranylgeranyl diphosphate synthase 1
ENSG0000	26.9902	0.398445	0.426898	0.023413	0.206713	AFTPH-DT	1.02E+08	AFTPH divergent transcript
ENSG0000	2081.675	-0.25774	0.136446	0.023416	0.206713	NOSIP	51070	nitric oxide synthase interacting protein
ENSG0000	127.4114	0.341979	0.218326	0.023446	0.206715	SETD7	80854	SET domai histone lysine methyltransferase
ENSG0000	14.70882	0.279667	0.435955	0.023445	0.206715	SPOCK1	6695	SPARC (os cwcv and kazal like domains proteoglycan 1
ENSG0000	68.82558	-0.38209	0.290858	0.02345	0.206715	MTSS2	92154	MTSS I-BAR domain containing 2
ENSG0000	20.2359	0.346126	0.486054	0.023491	0.206878	SLC16A14	151473	solute carrier family 16 member 14
ENSG0000	11.40942	0.242757	0.393026	0.023495	0.206878	LINC0190	151475	long intergenic non-protein coding RNA 1907
ENSG0000	9.671353	0.099595	0.25889	0.023502	0.206878	UACA	55075	uveal autoantigen with coiled-coil domains and ankyrin repeats
ENSG0000	8.505371	-0.10906	0.264163	0.023535	0.207063	RBP4	5950	retinol binding protein 4
ENSG0000	2.616919	-0.11005	0.267519	0.023548	0.207086	SLC44A3-	1.02E+08	SLC44A3 antisense RNA 1
ENSG0000	356.4141	0.283232	0.156365	0.023594	0.207386	KIF13B	23303	kinesin family member 13B
ENSG0000	699.7908	-0.35695	0.252994	0.023616	0.20748	H1-0	3005	H1.0 linker histone
ENSG0000	2315.599	-0.3341	0.213951	0.023655	0.20772	TSEN34	79042	tRNA splicing endonuclease subunit 34
ENSG0000	36.76838	-0.37732	0.265737	0.023703	0.207956	EPS8L1	54869	EPS8 like 1
ENSG0000	3.434654	-0.06499	0.244439	0.023704	0.207956	NA	NA	NA
ENSG0000	119.9817	0.356234	0.246225	0.0238	0.207996	SLC30A1	7779	solute carrier family 30 member 1
ENSG0000	14.27053	0.331004	0.484174	0.023797	0.207996	COL6A3	1293	collagen type VI alpha 3 chain
ENSG0000	6.566469	0.247744	0.41033	0.023785	0.207996	LINC0088	1.01E+08	long intergenic non-protein coding RNA 888
ENSG0000	355.8289	0.312533	0.184835	0.023789	0.207996	UVSSA	57654	UV stimulated scaffold protein A
ENSG0000	140.1539	0.36904	0.269754	0.023733	0.207996	SLC25A40	55972	solute carrier family 25 member 40
ENSG0000	6.578248	0.200658	0.347985	0.023757	0.207996	SPTBN2	6712	spectrin b non-erythrocytic 2
ENSG0000	110.7862	0.366324	0.406455	0.023753	0.207996	KATNBL1	79768	katanin regulatory subunit B1 like 1
ENSG0000	813.5791	-0.35207	0.240001	0.023757	0.207996	OPRL1	4987	opioid related nociceptin receptor 1
ENSG0000	49.86267	0.371712	0.263021	0.023825	0.208116	MAGEF1	64110	MAGE family member F1
ENSG0000	2.752406	0.116787	0.271773	0.023864	0.208364	RPS3P6	645000	ribosomal protein S3 pseudogene 6
ENSG0000	110.6725	0.383686	0.362218	0.023933	0.20886	IGKV6-21	28906	immunoglobulin kappa variable 6-21 (non-functional)
ENSG0000	1554.104	-0.35721	0.255552	0.023944	0.20886	LUCAT1	1.01E+08	lung cancer associated transcript 1
ENSG0000	902.7049	-0.31373	0.188632	0.023965	0.20894	STX16	8675	syntaxin 16
ENSG0000	3.033126	-0.05886	0.242609	0.023989	0.209055	NA	NA	NA
ENSG0000	7407.227	-0.3556	0.254565	0.024032	0.209094	TNFRSF10	8794	TNF receptor superfamily member 10c
ENSG0000	1184.719	0.353002	0.387539	0.024022	0.209094	IGHV3-33	28434	immunoglobulin heavy variable 3-33
ENSG0000	4.948684	0.198454	0.345621	0.024039	0.209094	NA	NA	NA
ENSG0000	67.05225	0.356396	0.238931	0.024022	0.209094	ENOX2	10495	ecto-NOX disulfide-thiol exchanger 2
ENSG0000	5303.333	-0.30533	0.180335	0.024061	0.209185	EVL	51466	Enah/Vasp-like
ENSG0000	30.23158	-0.38582	0.423191	0.024081	0.209259	NAMPTP1	646309	nicotinamide phosphoribosyltransferase pseudogene 1
ENSG0000	32.98582	0.393691	0.311785	0.024103	0.209348	ZDHHC1	29800	zinc finger DHHC-type containing 1
ENSG0000	4.817093	-0.18146	0.326615	0.024188	0.209985	MORN4	118812	MORN repeat containing 4
ENSG0000	8.500766	0.33227	0.513914	0.024245	0.210382	NA	NA	NA

ENSG0000	106868.1	-0.28703	0.162829	0.024299	0.210754	FTL	2512	ferritin light chain
ENSG0000	214.2055	0.271646	0.145984	0.024345	0.210805	TGFBRAP1	9392	transforming growth factor beta receptor associated protein 1
ENSG0000	11.47451	-0.27837	0.43586	0.024318	0.210805	LINC00885	401109	long intergenic non-protein coding RNA 885
ENSG0000	398.9363	0.352382	0.233377	0.024351	0.210805	HLA-DOA	3111	major hist class II DO alpha
ENSG0000	75.55327	-0.33677	0.21166	0.024351	0.210805	ACTR3B	57180	actin related protein 3B
ENSG0000	3.675372	0.107437	0.265268	0.024526	0.211023	FHAD1	114827	forkhead associated phosphopeptide binding domain 1
ENSG0000	54.70524	-0.36009	0.418044	0.024484	0.211023	RN7SL473	1.06E+08	RNA 7SL cytoplasm pseudogene
ENSG0000	78.227	0.366894	0.264443	0.024432	0.211023	SFT2D3	84826	SFT2 domain containing 3
ENSG0000	1791.247	0.165966	0.30778	0.024423	0.211023	ZDHHC19	131540	zinc finger DHHC-type palmitoyltransferase 19
ENSG0000	39.94163	-0.38284	0.292364	0.024452	0.211023	CEP162	22832	centrosomal protein 162
ENSG0000	173.1617	-0.31181	0.185448	0.024497	0.211023	TFR2	7036	transferrin receptor 2
ENSG0000	538.2955	-0.36624	0.314749	0.024455	0.211023	ABCA1	19	ATP binding cassette subfamily A member 1
ENSG0000	217.8396	-0.29644	0.168277	0.024446	0.211023	NUTM2B-A	1.01E+08	NUTM2B antisense RNA 1
ENSG0000	56.11805	0.344542	0.425741	0.024492	0.211023	RRAS2	22800	RAS related 2
ENSG0000	14253.04	-0.3558	0.261316	0.024487	0.211023	AQP9	366	aquaporin 9
ENSG0000	1226.899	-0.36013	0.266699	0.024392	0.211023	CD300E	342510	CD300e molecule
ENSG0000	42.51855	0.374229	0.268589	0.024506	0.211023	ZNF253	56242	zinc finger protein 253
ENSG0000	62.90569	0.366041	0.255878	0.024522	0.211023	MGAT3	4248	beta-1 4-mannosyl-glycoprotein 4-beta-N-acetylglucosaminyltransferase
ENSG0000	71.37188	0.381768	0.325859	0.02458	0.211329	NA	NA	NA
ENSG0000	117.4949	0.351903	0.237956	0.024585	0.211329	CRYL1	51084	crystallin lambda 1
ENSG0000	2121.823	-0.27804	0.152852	0.024658	0.211765	ZDHHC12	84885	zinc finger DHHC-type palmitoyltransferase 12
ENSG0000	222.0552	0.297564	0.17055	0.024649	0.211765	RAI1	10743	retinoic acid induced 1
ENSG0000	130.6611	0.369323	0.281397	0.024696	0.211986	SHTN1	57698	shootin 1
ENSG0000	81.29137	0.384583	0.320665	0.024731	0.212187	ISL2	64843	ISL LIM homeobox 2
ENSG0000	761.7473	-0.30586	0.181521	0.024776	0.212473	MTF1	4520	metal regulatory transcription factor 1
ENSG0000	972.3144	-0.29519	0.168511	0.024808	0.212648	NFE2L2	4780	NFE2 like bZIP transcription factor 2
ENSG0000	122.3412	0.341274	0.223637	0.024846	0.212875	UQCRH	7388	ubiquinol-cytochrome c reductase hinge protein
ENSG0000	30.85194	0.306551	0.433293	0.024907	0.213146	CCDC13-A	729083	CCDC13 antisense RNA 2
ENSG0000	4.249732	-0.07876	0.250328	0.02489	0.213146	TSKU	25987	tsukushi small leucine rich proteoglycan
ENSG0000	70.50147	0.37836	0.286803	0.024912	0.213146	TAMALIN	160622	trafficking regulator and scaffold protein tamalin
ENSG0000	43.16073	-0.38627	0.349116	0.024928	0.213178	LINC01816	1E+08	long intergenic non-protein coding RNA 1816
ENSG0000	120.7253	0.306466	0.180129	0.024948	0.213252	TCP11L1	55346	t-complex 11 like 1
ENSG0000	930.2393	0.247167	0.131216	0.024983	0.213406	MTA1	9112	metastasis associated 1
ENSG0000	11.61221	0.153959	0.295711	0.024989	0.213406	AFF2	2334	ALF transcription elongation factor 2
ENSG0000	430.9272	-0.25325	0.139225	0.02504	0.213613	SP140L	93349	SP140 nuclear body protein like
ENSG0000	8.600746	0.277105	0.442945	0.025049	0.213613	SLC35G1	159371	solute carrier family 35 member G1
ENSG0000	55.11444	0.386799	0.323898	0.025047	0.213613	VRK1	7443	VRK serine/threonine kinase 1
ENSG0000	232.8263	0.368586	0.29809	0.025073	0.213649	LINC03034	283710	long intergenic non-protein coding RNA 3034
ENSG0000	934.1195	-0.2417	0.126744	0.025076	0.213649	RNF185	91445	ring finger protein 185
ENSG0000	79.18383	-0.32751	0.203896	0.025115	0.21388	SLC45A3	85414	solute carrier family 45 member 3
ENSG0000	3.842949	-0.12647	0.278474	0.025236	0.214809	NTRK1	4914	neurotrophic receptor tyrosine kinase 1
ENSG0000	434.7238	-0.27664	0.154553	0.025252	0.214851	TMCO6	55374	transmembrane and coiled-coil domains 6
ENSG0000	440.0699	0.349498	0.247164	0.025362	0.21568	EEF1B2	1933	eukaryotic translation elongation factor 1 beta 2
ENSG0000	1049.937	0.35394	0.257191	0.025375	0.215692	MPZL1	9019	myelin protein zero like 1
ENSG0000	32.82839	-0.29784	0.428156	0.025393	0.215749	NA	NA	NA
ENSG0000	16.29787	0.356674	0.482433	0.025428	0.215867	SPR	6697	sepiapterin reductase
ENSG0000	611.8565	-0.2769	0.154907	0.025431	0.215867	MVB12A	93343	multivesicular body subunit 12A
ENSG0000	673.0738	0.269831	0.14698	0.025451	0.215937	EIF3A	8661	eukaryotic translation initiation factor 3 subunit A
ENSG0000	94.12871	0.324345	0.201959	0.025483	0.216106	NDUFS5	4725	NADH:ubiquinone oxidoreductase subunit S5
ENSG0000	475.6114	0.289707	0.166482	0.0255	0.216157	PRKDC	5591	protein kir DNA-activ catalytic subunit
ENSG0000	4979.089	-0.26757	0.147683	0.025544	0.21643	H3-3A	3020	H3.3 histone A
ENSG0000	36.06006	0.386713	0.322272	0.025571	0.21656	COMMD10	51397	COMM domain containing 10
ENSG0000	9.32005	-0.14762	0.290332	0.025672	0.217168	DEPDC1	55635	DEP domain containing 1
ENSG0000	18.91109	0.233336	0.371805	0.025689	0.217168	NOCT	25819	nocturnin
ENSG0000	44.55002	-0.36158	0.256587	0.025691	0.217168	GMDS-DT	1.01E+08	GMDS divergent transcript
ENSG0000	649.9578	0.366045	0.334481	0.02569	0.217168	RFX2	5990	regulatory factor X2
ENSG0000	1352.225	0.272563	0.151668	0.025736	0.21745	APEX1	328	apurinic/apyrimidinic endodeoxyribonuclease 1
ENSG0000	221.0088	-0.34316	0.245723	0.025791	0.217544	TNFRSF4	7293	TNF receptor superfamily member 4
ENSG0000	30.5833	0.384346	0.408193	0.025797	0.217544	NXPH4	11247	neurexophilin 4
ENSG0000	584.7293	-0.3011	0.180524	0.025804	0.217544	GLRX5	51218	glutaredoxin 5
ENSG0000	230.8733	0.273249	0.15146	0.025807	0.217544	TRAPPC2L	51693	trafficking protein particle complex subunit 2L
ENSG0000	140.6867	-0.34076	0.233856	0.025804	0.217544	SPACA6	147650	sperm acrosome associated 6
ENSG0000	41.16297	0.377823	0.295201	0.025844	0.217739	TMA16	55319	translation machinery associated 16 homolog
ENSG0000	4.337361	0.207787	0.359944	0.025867	0.217739	PEG10	23089	paternally expressed 10
ENSG0000	78.68555	0.379237	0.341134	0.025877	0.217739	TPD52	7163	tumor protein D52
ENSG0000	33.38292	0.384703	0.374343	0.025857	0.217739	MRTFB	57496	myocardin related transcription factor B
ENSG0000	9.040173	0.195084	0.33614	0.025921	0.21801	TRIM16L	147166	tripartite motif containing 16 like
ENSG0000	5.410508	-0.21206	0.360292	0.026044	0.218944	NA	NA	NA
ENSG0000	244.3906	-0.31843	0.200647	0.026089	0.219121	NA	NA	NA
ENSG0000	2871.249	-0.30989	0.194285	0.026086	0.219121	NDEL1	81565	nudE neurodevelopment protein 1 like 1
ENSG0000	26.66604	0.341843	0.438783	0.026157	0.219491	ARMCX5	64860	armadillo repeat containing X-linked 5
ENSG0000	805.8933	0.24846	0.131508	0.026156	0.219491	RBMX	27316	RNA binding motif protein X-linked
ENSG0000	12519.63	-0.30358	0.184383	0.026204	0.219785	JUNB	3726	JunB protc AP-1 transcription factor subunit
ENSG0000	843.7485	-0.25567	0.139187	0.026223	0.219842	HERPUD2	64224	HERPUD family member 2
ENSG0000	15.33531	0.261707	0.406343	0.026264	0.219981	NA	NA	NA
ENSG0000	43.21364	0.352365	0.239254	0.026253	0.219981	PIGBOS1	1.02E+08	PIGB opposite strand 1
ENSG0000	503.8391	0.334683	0.225047	0.026316	0.22032	ELL2	22936	elongation factor for RNA polymerase II 2
ENSG0000	7.327324	0.1988	0.341599	0.026342	0.220433	IQCK	124152	IQ motif containing K
ENSG0000	453.7344	-0.35003	0.257697	0.026401	0.220825	DNAJA1	3301	DnaJ heat shock protein family (Hsp40) member A1
ENSG0000	104.5207	0.337811	0.39957	0.026435	0.220913	IGKV1D-39	28893	immunoglobulin kappa variable 1D-39
ENSG0000	9.971237	0.298834	0.459803	0.026429	0.220913	NA	NA	NA
ENSG0000	51.49482	-0.36173	0.262978	0.02648	0.221187	CCDC30	728621	coiled-coil domain containing 30
ENSG0000	665.6334	0.250984	0.134932	0.026492	0.221187	MPRIP	23164	myosin phosphatase Rho interacting protein
ENSG0000	13.41335	0.234836	0.374727	0.026519	0.221311	NA	NA	NA
ENSG0000	9.624742	0.182897	0.323117	0.026602	0.221899	NA	NA	NA
ENSG0000	40.0987	0.381007	0.367864	0.026642	0.22203	ERMN	57471	ermin
ENSG0000	136.9236	-0.35055	0.254798	0.026666	0.22203	UBE2F	140739	ubiquitin conjugating enzyme E2 F (putative)
ENSG0000	166.3733	0.327767	0.212986	0.026655	0.22203	XXYLT1	152002	xyloside xylosyltransferase 1
ENSG0000	11.56086	0.234184	0.376851	0.026641	0.22203	NA	NA	NA
ENSG0000	2.682434	0.076471	0.249258	0.026715	0.222277	NA	NA	NA
ENSG0000	159.1992	-0.28891	0.168455	0.02672	0.222277	RAB5IF	55969	RAB5 interacting factor
ENSG0000	202.9092	0.343136	0.248492	0.026734	0.222292	TMEM39A	55254	transmembrane protein 39A
ENSG0000	3.441959	-0.12784	0.279868	0.02679	0.222581	IRF6	3664	interferon regulatory factor 6
ENSG0000	26.52886	0.363995	0.425819	0.026804	0.222581	LINC01237	1.02E+08	long intergenic non-protein coding RNA 1237
ENSG0000	122.1029	0.297207	0.175335	0.026805	0.222581	NEU3	10825	neuraminidase 3
ENSG0000	26.10074	0.390805	0.351443	0.02685	0.222855	NA	NA	NA

ENSG0000	23.10627	-0.37302	0.409688	0.026862	0.222855	HSD17B13	345275	hydroxysteroid 17-beta dehydrogenase 13
ENSG0000	4717.701	-0.33142	0.2256	0.026886	0.22295	STAT3	6774	signal transducer and activator of transcription 3
ENSG0000	120.0846	0.316995	0.20132	0.026976	0.223393	NT5C3B	115024	5'-nucleot cytosolic IIIB
ENSG0000	5.270949	-0.10954	0.264914	0.026964	0.223393	LYPD5	284348	LY6/PLAUR domain containing 5
ENSG0000	317.4348	-0.31981	0.20233	0.026971	0.223393	GPKOW	27238	G-patch domain and KOW motifs
ENSG0000	134.5055	-0.36191	0.32071	0.026994	0.223444	TRBV7-2	28596	T cell receptor beta variable 7-2
ENSG0000	42.23528	-0.36584	0.270604	0.027104	0.224157	NA	NA	NA
ENSG0000	20.78859	0.394706	0.408819	0.027105	0.224157	NA	NA	NA
ENSG0000	1705.304	0.254931	0.136083	0.02713	0.224163	HNRNPA1	3178	heterogeneous nuclear ribonucleoprotein A1
ENSG0000	2.619082	0.130713	0.281275	0.027121	0.224163	NA	NA	NA
ENSG0000	16.44772	0.374166	0.443927	0.027156	0.22419	NA	NA	NA
ENSG0000	1072.404	-0.29451	0.17576	0.027158	0.22419	MTG2	26164	mitochondrial ribosome associated GTPase 2
ENSG0000	128.3322	-0.32703	0.213116	0.027174	0.224222	TTN-AS1	1.01E+08	TTN antisense RNA 1
ENSG0000	405.77	-0.32358	0.211592	0.027206	0.224384	TGM2	7052	transglutaminase 2
ENSG0000	5.545091	0.237898	0.394156	0.02723	0.224479	NA	NA	NA
ENSG0000	351.0334	0.267695	0.149758	0.027295	0.224919	EPRS1	2058	glutamyl-prolyl-tRNA synthetase 1
ENSG0000	76.06552	0.31862	0.197627	0.027322	0.225039	MMAB	326625	metabolism of cobalamin associated B
ENSG0000	54.58105	0.361665	0.269784	0.027339	0.225077	SLC35A3	23443	solute carrier family 35 member A3
ENSG0000	7.056995	0.152869	0.295764	0.02737	0.225227	NA	NA	NA
ENSG0000	175.2587	-0.3576	0.346343	0.027395	0.225233	NFE4	58160	nuclear fa(erythroid 4
ENSG0000	1105.698	0.331966	0.221219	0.027395	0.225233	KCNQ1	3784	potassium voltage-gated channel subfamily Q member 1
ENSG0000	16.82743	0.397189	0.405824	0.027413	0.225284	RPL23AP6	440027	ribosomal protein L23a pseudogene 65
ENSG0000	3.599206	-0.12041	0.273507	0.027427	0.225286	GNAI1	2770	G protein subunit alpha i1
ENSG0000	46.89384	0.364286	0.273056	0.027438	0.225286	ZNF324B	388569	zinc finger protein 324B
ENSG0000	87.73253	0.292036	0.170479	0.02752	0.225754	SOCS7	30837	suppressor of cytokine signaling 7
ENSG0000	5854.182	-0.35284	0.284368	0.027516	0.225754	LRG1	116844	leucine rich alpha-2-glycoprotein 1
ENSG0000	80.01968	0.351989	0.444052	0.027559	0.22597	TXLNGY	246126	taxilin gan Y-linked
ENSG0000	696.7711	-0.27893	0.161165	0.027576	0.226011	GORASP1	64689	golgi reassembly stacking protein 1
ENSG0000	18.81894	0.361881	0.433099	0.027696	0.226874	SMIM13	221710	small integral membrane protein 13
ENSG0000	2.568828	-0.06183	0.243844	0.027706	0.226874	NA	NA	NA
ENSG0000	70.23045	-0.36092	0.286756	0.027804	0.227475	HEG1	57493	heart development protein with EGF like domains 1
ENSG0000	3312.862	-0.35019	0.276336	0.027792	0.227475	RNASE2	6036	ribonuclease A family member 2
ENSG0000	2823.728	-0.20424	0.103037	0.027822	0.22752	CALM1	801	calmodulin 1
ENSG0000	58.06905	0.367999	0.328784	0.027868	0.227688	CEP85L	387119	centrosomal protein 85 like
ENSG0000	82.61566	-0.34939	0.253701	0.027863	0.227688	SMPD3	55512	sphingomyelin phosphodiesterase 3
ENSG0000	135.8532	0.329152	0.218602	0.027885	0.227729	LINC0042(	1E+08	long intergenic non-protein coding RNA 426
ENSG0000	4.095193	0.166362	0.312595	0.027953	0.228002	IGKV2D-2(	28884	immunoglobulin kappa variable 2D-26
ENSG0000	910.605	-0.34703	0.266379	0.027956	0.228002	FUT7	2529	fucosyltransferase 7
ENSG0000	76.39376	0.362571	0.292619	0.027952	0.228002	CATSPER1	117144	cation channel sperm associated 1
ENSG0000	15.59974	-0.35438	0.445702	0.027992	0.228012	LINC0277	1.05E+08	long intergenic non-protein coding RNA 2777
ENSG0000	309.1879	-0.29691	0.176612	0.027984	0.228012	ANXA4	307	annexin A4
ENSG0000	286.4076	-0.33818	0.23952	0.028011	0.228012	BPGM	669	bisphosphoglycerate mutase
ENSG0000	17.20319	-0.2629	0.404653	0.028019	0.228012	PHLDA2	7262	pleckstrin homology like domain family A member 2
ENSG0000	5.487873	-0.15769	0.303352	0.028014	0.228012	MEIS3	56917	Meis homeobox 3
ENSG0000	252.8034	0.291189	0.175128	0.028057	0.228221	ACCS	84680	1-aminocyclopropane-1-carboxylate synthase homolog (inactive)
ENSG0000	6.3254	-0.12867	0.277121	0.028081	0.228312	H4C5	8367	H4 clustered histone 5
ENSG0000	12.07834	0.390811	0.455078	0.028104	0.228402	GDAP1	54332	ganglioside induced differentiation associated protein 1
ENSG0000	3.355328	-0.12144	0.273732	0.02813	0.228511	NA	NA	NA
ENSG0000	1084.102	-0.29423	0.17935	0.028256	0.22933	DNAJB2	3300	DnaJ heat shock protein family (Hsp40) member B2
ENSG0000	7.125573	0.208988	0.352051	0.02825	0.22933	SEPTIN3	55964	septin 3
ENSG0000	4600.139	-0.29455	0.179644	0.028388	0.2303	CD7	924	CD7 molecule
ENSG0000	2073.136	-0.24306	0.131619	0.028428	0.230356	SIAH2	6478	siah E3 ubiquitin protein ligase 2
ENSG0000	97.17001	0.346728	0.252601	0.028433	0.230356	TLE1	7088	TLE family transcriptional corepressor
ENSG0000	407.9404	-0.26953	0.15434	0.02843	0.230356	TMX2	51075	thioredoxin related transmembrane protein 2
ENSG0000	384.6388	0.337149	0.249243	0.028446	0.230356	SLC17A9	63910	solute carrier family 17 member 9
ENSG0000	5.001361	0.168995	0.311864	0.028487	0.230587	NA	NA	NA
ENSG0000	718.6581	-0.29624	0.179584	0.028502	0.230606	ZMAT5	55954	zinc finger matrin-type 5
ENSG0000	410.3662	-0.29792	0.182758	0.028527	0.230706	HTATIP2	10553	HIV-1 Tat interactive protein 2
ENSG0000	52.17574	-0.34817	0.384709	0.028576	0.230823	NA	NA	NA
ENSG0000	20.91489	-0.37373	0.401184	0.028583	0.230823	NA	NA	NA
ENSG0000	82.8331	0.341485	0.239562	0.028616	0.230823	ENC1	8507	ectodermal-neural cortex 1
ENSG0000	62.78063	-0.35919	0.280496	0.028595	0.230823	AK1	203	adenylate kinase 1
ENSG0000	14.79407	0.381611	0.436842	0.028617	0.230823	NA	NA	NA
ENSG0000	32.50624	0.37071	0.383708	0.028598	0.230823	DOCK6	57572	dedicator of cytokinesis 6
ENSG0000	10.25146	-0.26474	0.408469	0.028774	0.231787	LINC0303	400221	long intergenic non-protein coding RNA 3033
ENSG0000	6.217666	0.258011	0.413651	0.028766	0.231787	NA	NA	NA
ENSG0000	91.66709	-0.33107	0.223355	0.028773	0.231787	PPP1R3F	89801	protein phosphatase 1 regulatory subunit 3F
ENSG0000	4.473181	-0.10665	0.265671	0.028808	0.231957	CLEC4F	165530	C-type lectin domain family 4 member F
ENSG0000	563.5198	-0.23609	0.126801	0.028858	0.23206	FAM120A(	158293	family with sequence similarity 120A opposite strand
ENSG0000	52.12482	-0.36	0.28106	0.028844	0.23206	KLRG1	10219	killer cell lectin like receptor G1
ENSG0000	41.25191	0.365119	0.289378	0.028859	0.23206	EIF3C	8663	eukaryotic translation initiation factor 3 subunit C
ENSG0000	8.337697	-0.20443	0.346189	0.028903	0.232315	CRACD	57482	capping protein inhibiting regulator of actin dynamics
ENSG0000	34858.23	-0.32185	0.219022	0.028927	0.232409	SLC25A39	51629	solute carrier family 25 member 39
ENSG0000	96.86814	0.35784	0.323459	0.02895	0.232485	MIS18BP1	55320	MIS18 binding protein 1
ENSG0000	256.2764	-0.24121	0.130673	0.028972	0.232562	CSTF2T	23283	cleavage stimulation factor subunit 2 tau variant
ENSG0000	33.70578	-0.37112	0.324505	0.028988	0.23259	NA	NA	NA
ENSG0000	1959.306	-0.23144	0.124005	0.029002	0.232597	SRF	6722	serum response factor
ENSG0000	67.05631	-0.36329	0.332203	0.029019	0.232633	EPAS1	2034	endothelial PAS domain protein 1
ENSG0000	411.5313	-0.2897	0.173171	0.029053	0.232807	RAB29	8934	RAB29 member RAS oncogene family
ENSG0000	23.99263	0.327971	0.430008	0.029067	0.232817	KCNQ5	56479	potassium voltage-gated channel subfamily Q member 5
ENSG0000	284.8591	-0.32749	0.226073	0.029095	0.23294	CORO2A	7464	coronin 2A
ENSG0000	7.193914	-0.09259	0.255615	0.029203	0.233697	NA	NA	NA
ENSG0000	17.50811	0.30271	0.426818	0.029216	0.233703	DLEU7	220107	deleted in lymphocytic leukemia 7
ENSG0000	3.583827	0.092674	0.25585	0.029231	0.233717	LINC0220	1.02E+08	long intergenic non-protein coding RNA 2202
ENSG0000	29.553	-0.37204	0.361171	0.029271	0.233837	PHLDA3	23612	pleckstrin homology like domain family A member 3
ENSG0000	42.28288	0.368684	0.322047	0.02926	0.233837	RPL10P9	389342	ribosomal protein L10 pseudogene 9
ENSG0000	101.2565	0.245766	0.366823	0.029316	0.234092	DGKH	160851	diacylglycerol kinase eta
ENSG0000	12.90946	0.350002	0.462716	0.029386	0.234553	FAM72B	653820	family with sequence similarity 72 member B
ENSG0000	34.9362	0.352493	0.254957	0.029448	0.234838	C1orf115	79762	chromosome 1 open reading frame 115
ENSG0000	1513.923	-0.33369	0.245057	0.029443	0.234838	ADGRG1	9289	adhesion G protein-coupled receptor G1
ENSG0000	1268.549	-0.27538	0.160445	0.029466	0.234879	TMEM50A	23585	transmembrane protein 50A
ENSG0000	11.64416	0.198717	0.334838	0.029489	0.234964	SLC25A5-/	1E+08	SLC25A5 antisense RNA 1
ENSG0000	37.62198	0.36315	0.301302	0.029516	0.234971	NA	NA	NA
ENSG0000	86.86592	-0.33852	0.229502	0.02951	0.234971	TMEM9B-/	493900	TMEM9B antisense RNA 1
ENSG0000	9.459794	0.295778	0.455897	0.029615	0.235154	LOC10050	1.01E+08	uncharacterized LOC100505774
ENSG0000	85.32379	0.393114	0.288	0.029617	0.235154	BTNL3	10917	butyrophilin like 3

ENSG0000	152.656	0.309485	0.197852	0.029615	0.235154	SUN1	23353	Sad1 and UNC84 domain containing 1
ENSG0000	31.10417	-0.36765	0.383664	0.029591	0.235154	IGF2BP3	10643	insulin like growth factor 2 mRNA binding protein 3
ENSG0000	2.688193	-0.08962	0.254598	0.029594	0.235154	NA	NA	NA
ENSG0000	2.437478	0.066491	0.245223	0.029573	0.235154	NA	NA	NA
ENSG0000	908.4313	-0.20628	0.337361	0.029629	0.235154	NA	NA	NA
ENSG0000	16.76576	-0.1783	0.312488	0.029709	0.235692	CXCL10	3627	C-X-C motif chemokine ligand 10
ENSG0000	126.286	0.343898	0.365023	0.029781	0.235958	MAK	4117	male germ cell associated kinase
ENSG0000	208.7366	-0.31022	0.200554	0.029762	0.235958	ARID5B	84159	AT-rich interaction domain 5B
ENSG0000	3642.859	0.196433	0.100328	0.029771	0.235958	MAPK8IP3	23162	mitogen-activated protein kinase 8 interacting protein 3
ENSG0000	347.0111	-0.27271	0.151681	0.02981	0.235978	RELL2	285613	RELT like 2
ENSG0000	47.20274	-0.34372	0.243801	0.029799	0.235978	XPNPEP2	7512	X-prolyl aminopeptidase 2
ENSG0000	91.05112	-0.30526	0.191423	0.029871	0.23626	RBMXL1	494115	RBMX like 1
ENSG0000	3.932439	-0.17325	0.317834	0.029861	0.23626	NA	NA	NA
ENSG0000	20.99923	0.243739	0.377494	0.030013	0.236593	CTRC	11330	chymotrypsin C
ENSG0000	37.56864	-0.35135	0.257082	0.030013	0.236593	ABHD16A	7920	abhydrola: phospholipase
ENSG0000	85.02233	-0.31613	0.205372	0.029938	0.236593	AIG1	51390	androgen induced 1
ENSG0000	2.463102	0.128463	0.279882	0.030029	0.236593	SNORA5C	677796	small nucl(H/ACA box 5C
ENSG0000	82.02726	0.341978	0.253886	0.029952	0.236593	MIR600HG	81571	MIR600 host gene
ENSG0000	528.3093	-0.31123	0.2011	0.03001	0.236593	FBXW4	6468	F-box and WD repeat domain containing 4
ENSG0000	127.2662	0.313985	0.203979	0.029964	0.236593	HEATR3	55027	HEAT repeat containing 3
ENSG0000	3092.74	-0.3156	0.212085	0.030007	0.236593	USB1	79650	U6 snRNA biogenesis phosphodiesterase 1
ENSG0000	122.5773	0.295461	0.180881	0.030029	0.236593	KAT14	57325	lysine acetyltransferase 14
ENSG0000	508.8066	-0.27771	0.164397	0.030092	0.236785	KDM1B	221656	lysine demethylase 1B
ENSG0000	64.69313	0.324397	0.215999	0.030093	0.236785	RP9	6100	RP9 pre-mRNA splicing factor
ENSG0000	499.26	0.275517	0.161994	0.030131	0.236785	SMARCA2	6595	SWI/SNF r matrix ass actin depe subfamily member 2
ENSG0000	75.75109	-0.34309	0.253179	0.030077	0.236785	C1R	715	complement C1r
ENSG0000	22.15598	-0.367	0.291873	0.030127	0.236785	PRKAG1	5571	protein kinase AMP-activated non-catalytic subunit gamma 1
ENSG0000	9.909962	-0.30045	0.443317	0.030117	0.236785	NA	NA	NA
ENSG0000	25.91404	0.375923	0.334253	0.03015	0.236826	C2orf69	205327	chromosome 2 open reading frame 69
ENSG0000	264.3305	0.345347	0.28484	0.030188	0.237029	EID1	23741	EP300 interacting inhibitor of differentiation 1
ENSG0000	1788.96	-0.33358	0.252301	0.030274	0.2375	SH3GLB1	51100	SH3 doma endophilin B1
ENSG0000	181.5166	0.29381	0.188485	0.030272	0.2375	TEX2	55852	testis expressed 2
ENSG0000	2106.403	-0.30059	0.191219	0.030313	0.2377	ETV6	2120	ETS variant transcription factor 6
ENSG0000	37.25564	0.358756	0.284786	0.030346	0.237857	FXN	2395	frataxin
ENSG0000	9.023	0.05829	0.241675	0.030455	0.238609	IL1RL1	9173	interleukin 1 receptor like 1
ENSG0000	5.255684	0.202913	0.348217	0.030469	0.238619	DOC2A	8448	double C2 domain alpha
ENSG0000	324.4246	-0.34259	0.27526	0.030503	0.238782	EPB42	2038	erythrocyte membrane protein band 4.2
ENSG0000	379.8465	-0.30988	0.20424	0.030555	0.239087	MILR1	284021	mast cell immunoglobulin like receptor 1
ENSG0000	855.8671	-0.33452	0.254816	0.030625	0.239329	GLRX	2745	glutaredoxin
ENSG0000	45.68339	-0.35038	0.265615	0.030623	0.239329	LINC02904	286122	long intergenic non-protein coding RNA 2904
ENSG0000	37.63331	0.319574	0.420752	0.030608	0.239329	CLEC12B	387837	C-type lectin domain family 12 member B
ENSG0000	1391.125	-0.29635	0.186226	0.030665	0.239543	CDKN2D	1032	cyclin dependent kinase inhibitor 2D
ENSG0000	46.44197	-0.35161	0.269588	0.030724	0.239873	ECM1	1893	extracellular matrix protein 1
ENSG0000	1388.12	0.324093	0.23173	0.030747	0.239873	MS4A6A	64231	membrane spanning 4-domains A6A
ENSG0000	4.333649	0.161251	0.3062	0.030739	0.239873	NA	NA	NA
ENSG0000	103.8596	0.308749	0.199747	0.030796	0.240155	HTATSF1	27336	HIV-1 Tat specific factor 1
ENSG0000	19.93858	0.325553	0.428869	0.030814	0.240193	NA	NA	NA
ENSG0000	17.93399	0.219905	0.353785	0.030842	0.240312	XRCC4	7518	X-ray repair cross complementing 4
ENSG0000	625.5245	-0.33252	0.257891	0.030901	0.240669	SPATA13	221178	spermatogenesis associated 13
ENSG0000	44.94772	-0.34928	0.272669	0.031009	0.241407	NAPB	63908	NSF attachment protein beta
ENSG0000	220.5186	-0.28775	0.370765	0.031069	0.24177	MGAM2	93432	maltase-glucoamylase 2 (putative)
ENSG0000	126.4818	0.340746	0.280041	0.031125	0.241921	DENND6A	201627	DENN domain containing 6A
ENSG0000	2891.764	-0.28092	0.171367	0.031128	0.241921	CSTB	1476	cystatin B
ENSG0000	3.013011	0.10891	0.265157	0.031104	0.241921	NA	NA	NA
ENSG0000	283.665	0.315156	0.209398	0.031161	0.24197	UBR5	51366	ubiquitin protein ligase E3 component n-recognin 5
ENSG0000	106.6702	0.296734	0.18476	0.031156	0.24197	BTBD7	55727	BTB domain containing 7
ENSG0000	2803.959	-0.27586	0.165441	0.031176	0.241987	PIM1	5292	Pim-1 prot serine/threonine kinase
ENSG0000	36.30803	-0.3247	0.403986	0.031223	0.242198	LY6G6C	80740	lymphocyte antigen 6 family member G6C
ENSG0000	1219.774	-0.26626	0.155631	0.03123	0.242198	CCDC92	80212	coiled-coil domain containing 92
ENSG0000	3.545439	0.072621	0.246554	0.031271	0.242353	TTC30A	92104	tetratricopeptide repeat domain 30A
ENSG0000	14.65828	-0.26569	0.398794	0.031276	0.242353	LOC10537	1.05E+08	uncharacterized LOC105375924
ENSG0000	318.7766	-0.33862	0.26775	0.031293	0.242378	NBN	4683	nibrin
ENSG0000	9.288994	0.191089	0.330618	0.031338	0.242624	XRCC2	7516	X-ray repair cross complementing 2
ENSG0000	93.88364	0.349342	0.310782	0.031354	0.242649	PCMTD2	55251	protein-L-isoaspartate (D-aspartate) O-methyltransferase domain containing 2
ENSG0000	7.337042	0.187523	0.328174	0.031384	0.242779	CYGB	114757	cytoglobin
ENSG0000	1098.777	-0.24443	0.137222	0.031497	0.243172	PEF1	553115	penta-EF-hand domain containing 1
ENSG0000	64.72606	0.323203	0.219541	0.031502	0.243172	POLR1E	64425	RNA polymerase I subunit E
ENSG0000	153.0672	-0.33946	0.325705	0.031491	0.243172	CTTN	2017	cortactin
ENSG0000	128.6983	0.304312	0.45086	0.031471	0.243172	IGHV7-4-1	57289	immunoglobulin heavy variable 7-4-1
ENSG0000	7065.784	-0.30612	0.210863	0.031484	0.243172	PLEKHO2	80301	pleckstrin homology domain containing O2
ENSG0000	256.3098	-0.33189	0.25154	0.031544	0.243305	GVINP1	387751	GTPase very large interferon inducible pseudogene 1
ENSG0000	165.3622	0.33405	0.254894	0.031545	0.243305	THBS1	7057	thrombospondin 1
ENSG0000	8.061004	-0.20636	0.346567	0.031625	0.243814	ENPP2	5168	ectonucleotide pyrophosphatase/phosphodiesterase 2
ENSG0000	11.03212	0.364584	0.441758	0.031651	0.243914	NA	NA	NA
ENSG0000	35.73544	0.34755	0.261641	0.031721	0.244252	EFHC1	114327	EF-hand domain containing 1
ENSG0000	72.38571	-0.31575	0.209273	0.031712	0.244252	NA	NA	NA
ENSG0000	237.9302	0.263919	0.154155	0.031753	0.244368	ALDH9A1	223	aldehyde dehydrogenase 9 family member A1
ENSG0000	610.4844	-0.29993	0.194314	0.031764	0.244368	MXI1	4601	MAX inter dimerization protein
ENSG0000	103.8478	0.291302	0.1798	0.031777	0.244368	NXT1	29107	nuclear transport factor 2 like export factor 1
ENSG0000	1063.265	-0.29893	0.196271	0.031831	0.24468	GM2A	2760	ganglioside GM2 activator
ENSG0000	42.91913	0.339033	0.244952	0.031901	0.244915	CRABP2	1382	cellular retinoic acid binding protein 2
ENSG0000	40.19262	0.356735	0.3392	0.031918	0.244915	SNX25	83891	sorting nexin 25
ENSG0000	19.26069	0.170546	0.305438	0.031924	0.244915	ZNF138	7697	zinc finger protein 138
ENSG0000	76.91329	0.307677	0.199612	0.031911	0.244915	CDC42BPG	55561	CDC42 binding protein kinase gamma
ENSG0000	150.5399	-0.24569	0.13785	0.031928	0.244915	AAGAB	79719	alpha and gamma adaptin binding protein
ENSG0000	6.206608	-0.22383	0.366267	0.031965	0.244936	NA	NA	NA
ENSG0000	1415.693	-0.28279	0.173924	0.031953	0.244936	NOD2	64127	nucleotide binding oligomerization domain containing 2
ENSG0000	43.7496	0.339913	0.246163	0.031971	0.244936	LYPD3	27076	LY6/PLAUR domain containing 3
ENSG0000	31.06156	0.360499	0.311414	0.031985	0.244938	NSMCE2	286053	NSE2 (MM SMC5-SMC6 complex SUMO ligase
ENSG0000	592.6372	0.326151	0.238834	0.032005	0.244989	RPS27A	6233	ribosomal protein S27a
ENSG0000	16.86756	0.273246	0.400301	0.032026	0.245052	NA	NA	NA
ENSG0000	8.368767	0.189105	0.32802	0.032058	0.245114	SLC38A11	151258	solute carrier family 38 member 11
ENSG0000	22.97575	0.370673	0.332037	0.032065	0.245114	BCKDHB	594	branched chain keto acid dehydrogenase E1 subunit beta
ENSG0000	111.1068	-0.34891	0.304534	0.032074	0.245114	NA	NA	NA
ENSG0000	4.42166	0.172134	0.31794	0.032111	0.245292	IGKV2-26	28922	immunoglobulin kappa variable 2-26 (pseudogene)
ENSG0000	5.877128	-0.26009	0.416397	0.032159	0.245439	NA	NA	NA

ENSG0000	345.0075	0.290015	0.181664	0.03217	0.245439	AHCY	191	adenosylhomocysteinase
ENSG0000	2.644286	-0.14611	0.292902	0.032153	0.245439	AR	367	androgen receptor
ENSG0000	5.732202	0.175644	0.317091	0.032233	0.245813	CEP70	80321	centrosomal protein 70
ENSG0000	5861.333	-0.32183	0.236199	0.032253	0.245867	MVP	9961	major vault protein
ENSG0000	1123.99	0.325145	0.239548	0.032294	0.246076	IMPA2	3613	inositol monophosphatase 2
ENSG0000	655.6843	-0.32832	0.335345	0.03235	0.24619	ARRDC3	57561	arrestin domain containing 3
ENSG0000	18.08442	0.311212	0.418471	0.032345	0.24619	ADAMTS1	11093	ADAM metallopeptidase with thrombospondin type 1 motif 13
ENSG0000	2.543871	0.142276	0.291897	0.032332	0.24619	TFIP11-DT	1.01E+08	TFIP11 divergent transcript
ENSG0000	1304.071	0.235322	0.130723	0.032365	0.246207	DGKD	8527	diacylglycerol kinase delta
ENSG0000	302.3314	0.335946	0.263479	0.032449	0.246744	LRP3	4037	LDL receptor related protein 3
ENSG0000	9.237396	0.294285	0.434984	0.032471	0.246804	ALDH8A1	64577	aldehyde dehydrogenase 8 family member A1
ENSG0000	2.788683	0.11013	0.267231	0.032497	0.246905	MIR1244-3	1E+08	microRNA 1244-3
ENSG0000	415.0359	-0.26778	0.158894	0.032551	0.247002	PATL2	197135	PAT1 homolog 2
ENSG0000	4.67402	0.206626	0.353034	0.032537	0.247002	ALDH1A3-	1.02E+08	ALDH1A3 antisense RNA 1
ENSG0000	902.4821	-0.32623	0.246257	0.032541	0.247002	NA	NA	NA
ENSG0000	142.4135	0.330652	0.242849	0.032634	0.247187	MRPL30	51263	mitochondrial ribosomal protein L30
ENSG0000	126.3373	0.328011	0.240325	0.032593	0.247187	OPA1	4976	OPA1 mitochondrial dynamin like GTPase
ENSG0000	19.71396	-0.33669	0.427367	0.032656	0.247187	TRBV24-1	28563	T cell receptor beta variable 24-1
ENSG0000	2.396903	0.072413	0.248737	0.032639	0.247187	NA	NA	NA
ENSG0000	141.2855	-0.29614	0.187628	0.032648	0.247187	SMAGP	57228	small cell adhesion glycoprotein
ENSG0000	72.86418	0.300902	0.19034	0.032605	0.247187	CCDC157	550631	coiled-coil domain containing 157
ENSG0000	9.935097	-0.24989	0.388414	0.032684	0.247298	PIP5KL1	138429	phosphatidylinositol-4-phosphate 5-kinase like 1
ENSG0000	185.9311	-0.2773	0.167993	0.032698	0.247303	ERBB2	2064	erb-b2 receptor tyrosine kinase 2
ENSG0000	84.12269	-0.33681	0.260235	0.032759	0.247658	TGFBR3	7049	transforming growth factor beta receptor 3
ENSG0000	47.70408	-0.34913	0.291531	0.032813	0.247962	C1orf112	55732	chromosome 1 open reading frame 112
ENSG0000	328.2911	0.274355	0.36675	0.032898	0.248503	IGHV4-4	28401	immunoglobulin heavy variable 4-4
ENSG0000	4.053136	0.125777	0.27526	0.033015	0.249186	TIMM23B-	1.13E+08	TIMM23B-AGAP6 readthrough (NMD candidate)
ENSG0000	148.8932	0.299937	0.193608	0.033003	0.249186	TRAPPC6A	79090	trafficking protein particle complex subunit 6A
ENSG0000	124.7675	0.27275	0.164156	0.033079	0.249465	FRS3	10817	fibroblast growth factor receptor substrate 3
ENSG0000	255.839	-0.32457	0.240291	0.033073	0.249465	DHRS12	79758	dehydrogenase/reductase 12
ENSG0000	8.305296	0.157208	0.296824	0.033137	0.249702	NA	NA	NA
ENSG0000	78.6953	-0.32081	0.223724	0.033145	0.249702	NA	NA	NA
ENSG0000	68.93102	-0.25062	0.369902	0.033152	0.249702	TMPRSS9	360200	transmembrane serine protease 9
ENSG0000	16.6906	-0.35854	0.392343	0.033223	0.250136	ENKUR	219670	enkurin TRPC channel interacting protein
ENSG0000	8131.271	0.305822	0.217527	0.033246	0.250203	FCGRT	2217	Fc gamma receptor and transporter
ENSG0000	7.12276	0.169571	0.310379	0.033315	0.25055	IGLV3-22	28795	immunoglobulin lambda variable 3-22
ENSG0000	60.40016	-0.33089	0.243359	0.033319	0.25055	BIK	638	BCL2 interacting killer
ENSG0000	309.016	0.249438	0.1427	0.033391	0.250989	ZNF706	51123	zinc finger protein 706
ENSG0000	712.9019	0.312992	0.220451	0.033416	0.251069	OGFRL1	79627	opioid growth factor receptor like 1
ENSG0000	2.909204	0.06928	0.2456	0.03347	0.251357	NA	NA	NA
ENSG0000	5.990852	0.09634	0.256844	0.033483	0.251357	NA	NA	NA
ENSG0000	7.703502	0.130781	0.276963	0.033506	0.251357	SDC2	6383	syndecan 2
ENSG0000	182.9185	0.264805	0.160632	0.033509	0.251357	TIGD5	84948	tigger transposable element derived 5
ENSG0000	44.0699	0.345867	0.337369	0.03356	0.25164	DNAJC24	120526	DnaJ heat shock protein family (Hsp40) member C24
ENSG0000	21.55795	0.28744	0.400468	0.033594	0.251793	SEPTIN10	151011	septin 10
ENSG0000	101.0609	0.340739	0.339658	0.033631	0.251968	FXYD7	53822	FXYD domain containing ion transport regulator 7
ENSG0000	135.766	0.278073	0.173221	0.03368	0.252125	SLC25A25	114789	solute carrier family 25 member 25
ENSG0000	409.0012	0.281515	0.366709	0.033671	0.252125	RPL23	9349	ribosomal protein L23
ENSG0000	22.28444	-0.36771	0.352481	0.033751	0.25221	GTF2H2	2966	general transcription factor IIH subunit 2
ENSG0000	2026.22	-0.32541	0.319393	0.033727	0.25221	MPIG6B	80739	megakaryocyte and platelet inhibitory receptor G6b
ENSG0000	5094.8	-0.30355	0.198973	0.03376	0.25221	SEMA4D	10507	semaphorin 4D
ENSG0000	118.1395	0.26819	0.159371	0.03372	0.25221	DNMBP	23268	dynamin binding protein
ENSG0000	285.7627	0.30298	0.202933	0.033742	0.25221	PAQR4	124222	progestin and adipoQ receptor family member 4
ENSG0000	13.90281	0.179141	0.31234	0.033817	0.25253	C1orf127	148345	chromosome 1 open reading frame 127
ENSG0000	20.65598	-0.34263	0.388336	0.033881	0.252807	OR52K1	390036	olfactory receptor family 52 subfamily K member 1
ENSG0000	15.86131	-0.25404	0.382261	0.033868	0.252807	NA	NA	NA
ENSG0000	59.53026	-0.33579	0.260216	0.033928	0.252866	SCCPDH	51097	saccharopine dehydrogenase (putative)
ENSG0000	639.8925	-0.31267	0.224726	0.033928	0.252866	CARD6	84674	caspase recruitment domain family member 6
ENSG0000	49.32266	-0.34184	0.385359	0.033931	0.252866	NA	NA	NA
ENSG0000	4.304482	-0.09593	0.256597	0.033965	0.252884	TMEM52B	120939	transmembrane protein 52B
ENSG0000	93.63369	0.317888	0.223392	0.033975	0.252884	KDM8	79831	lysine demethylase 8
ENSG0000	36.9197	-0.33644	0.375475	0.03395	0.252884	MORC2-AS	150291	MORC2 antisense RNA 1
ENSG0000	63.75844	0.320479	0.225807	0.034057	0.253306	SLC25A16	8034	solute carrier family 25 member 16
ENSG0000	16.67722	-0.33011	0.414797	0.034059	0.253306	NA	NA	NA
ENSG0000	2.408257	0.095812	0.258762	0.034098	0.253492	NA	NA	NA
ENSG0000	121.4544	-0.31943	0.232343	0.034143	0.253723	ENDOD1	23052	endonuclease domain containing 1
ENSG0000	2895.38	-0.24002	0.140745	0.034239	0.254333	DCAF12	25853	DDB1 and CUL4 associated factor 12
ENSG0000	136.4457	-0.31064	0.21522	0.034309	0.254754	ABL2	27	ABL proto- non-receptor tyrosine kinase
ENSG0000	44.32052	0.346134	0.28912	0.034344	0.254909	MMP19	4327	matrix metallopeptidase 19
ENSG0000	102.1174	-0.32184	0.233548	0.034364	0.254952	NA	NA	NA
ENSG0000	19.05949	-0.35484	0.366432	0.034504	0.255531	NA	NA	NA
ENSG0000	206.0284	0.330561	0.266752	0.034512	0.255531	ATP2B1	490	ATPase plasma membrane Ca2+ transporting 1
ENSG0000	20.81479	-0.34618	0.383787	0.034485	0.255531	ZFHX2	85446	zinc finger homeobox 2
ENSG0000	1259.496	-0.32985	0.27089	0.034495	0.255531	NFIX	4784	nuclear factor I X
ENSG0000	7.287348	0.176409	0.314049	0.034458	0.255531	GEMIN7-A	1.05E+08	GEMIN7 antisense RNA 1
ENSG0000	554.0688	-0.26821	0.168276	0.034636	0.256085	ARHGEF3	50650	Rho guanine nucleotide exchange factor 3
ENSG0000	119.2902	0.300202	0.19867	0.034656	0.256085	GOLGA2P5	55592	GOLGA2 pseudogene 5
ENSG0000	37.93893	0.344006	0.275023	0.034647	0.256085	TRPV1	7442	transient receptor potential cation channel subfamily V member 1
ENSG0000	3223.581	-0.28693	0.185828	0.034635	0.256085	CANT1	124583	calcium activated nucleotidase 1
ENSG0000	3.842207	-0.14855	0.292221	0.034652	0.256085	NXF3	56000	nuclear RNA export factor 3
ENSG0000	6.807625	0.167081	0.305812	0.034676	0.256126	KLHL11	55175	kelch like family member 11
ENSG0000	44.56317	0.346254	0.289506	0.034695	0.256168	ZNF503	84858	zinc finger protein 503
ENSG0000	13.19778	0.322015	0.424057	0.034756	0.256247	RAVER2	55225	ribonuclec PTB binding 2
ENSG0000	232.4614	-0.3219	0.239131	0.034736	0.256247	MTMR11	10903	myotubularin related protein 11
ENSG0000	54.95131	-0.34201	0.304106	0.034766	0.256247	TRBV5-1	28614	T cell receptor beta variable 5-1
ENSG0000	674.5614	0.261049	0.155584	0.03475	0.256247	RAB40C	57799	RAB40C member RAS oncogene family
ENSG0000	290.1021	-0.32826	0.310558	0.034776	0.256247	MAP1LC3A	84557	microtubule associated protein 1 light chain 3 alpha
ENSG0000	29.48652	-0.35087	0.294277	0.034805	0.256358	NA	NA	NA
ENSG0000	8.371407	-0.26677	0.419103	0.034861	0.256559	FAM225A	286333	family with sequence similarity 225 member A
ENSG0000	40.30244	-0.34483	0.301429	0.034849	0.256559	HELB	92797	DNA helicase B
ENSG0000	3.160094	0.115676	0.269715	0.034874	0.256559	NA	NA	NA
ENSG0000	3.60777	0.127141	0.277051	0.034889	0.256562	RUFY1-AS1	1.02E+08	RUFY1 antisense RNA 1
ENSG0000	63.55402	0.327405	0.246523	0.034963	0.257002	SETBP1	26040	SET binding protein 1
ENSG0000	444.8456	-0.32094	0.243948	0.03504	0.257465	MYBPC3	4607	myosin binding protein C3
ENSG0000	8086.974	-0.27844	0.175682	0.035073	0.257603	BCL2L1	598	BCL2 like 1
ENSG0000	3.430349	0.098768	0.259286	0.035097	0.257682	IGSF9	57549	immunoglobulin superfamily member 9

ENSG0000	85.85396	-0.33321	0.271825	0.035146	0.257937	DIPK1B	138311	divergent protein kinase domain 1B	
ENSG0000	44.15116	0.330703	0.357483	0.035174	0.258034	GPATCH11	253635	G-patch domain containing 11	
ENSG0000	337.3429	-0.27555	0.1718	0.03523	0.258342	ORAI3	93129	ORAI calcium release-activated calcium modulator 3	
ENSG0000	31.57109	0.33894	0.35213	0.035273	0.258449	ZNG1A	55871	Zn regulated GTPase metalloprotein activator 1A	
ENSG0000	3.976395	0.167427	0.312469	0.035261	0.258449	FAM106A	80039	family with sequence similarity 106 member A	
ENSG0000	34.50567	-0.34531	0.350439	0.035291	0.258482	FZD5	7855	frizzled class receptor 5	
ENSG0000	3.291445	0.131905	0.282092	0.035327	0.25864	STKLD1	169436	serine/threonine kinase like domain containing 1	
ENSG0000	8331.444	-0.32828	0.293316	0.035381	0.258935	ADGRG3	222487	adhesion G protein-coupled receptor G3	
ENSG0000	40.70103	0.311558	0.381748	0.035406	0.259013	CATIP-AS1	1.02E+08	CATIP antisense RNA 1	
ENSG0000	4.177207	-0.10289	0.263072	0.035497	0.259461	ITLN2	142683	intelectin 2	
ENSG0000	4.37664	0.149708	0.29415	0.035485	0.259461	ZNF541	84215	zinc finger protein 541	
ENSG0000	435.8363	-0.23426	0.132812	0.03551	0.259461	MANBAL	63905	mannosidase beta like	
ENSG0000	554.9264	-0.28995	0.193501	0.03566	0.260317	PYHIN1	149628	pyrin and HIN domain family member 1	
ENSG0000	5150.936	-0.28751	0.187008	0.03567	0.260317	TRBC2	28638	T cell receptor beta constant 2	
ENSG0000	560.2469	-0.28144	0.177293	0.035648	0.260317	IL18BP	10068	interleukin 18 binding protein	
ENSG0000	717.6255	-0.31013	0.222746	0.035692	0.260374	SIRPG	55423	signal regulatory protein gamma	
ENSG0000	233.0384	0.318946	0.248381	0.035745	0.260658	MDM2	4193	MDM2 proto-oncogene	
ENSG0000	12.68375	0.253187	0.380436	0.035815	0.261065	PCGF6	84108	polycomb group ring finger 6	
ENSG0000	43.35119	0.337293	0.273204	0.035845	0.261181	DHX32	55760	DEAH-box helicase 32 (putative)	
ENSG0000	61.87751	-0.30466	0.204524	0.035884	0.26122	POMC	5443	proopiomelanocortin	
ENSG0000	192.0744	0.266807	0.161503	0.03587	0.26122	TMEM150	129303	transmembrane protein 150A	
ENSG0000	620.8284	0.300546	0.197477	0.035902	0.26122	RPL35A	6165	ribosomal protein L35a	
ENSG0000	2184.826	-0.29606	0.204906	0.035907	0.26122	LILRB1	10859	leukocyte immunoglobulin like receptor B1	
ENSG0000	4.570512	0.121137	0.272333	0.036026	0.261666	LINC01305	285084	long intergenic non-protein coding RNA 1305	
ENSG0000	4.861538	-0.15018	0.292708	0.036025	0.261666	HCP5B	352990	HLA complex P5B	
ENSG0000	29.05234	0.244088	0.363391	0.035987	0.261666	OLR1	4973	oxidized low density lipoprotein receptor 1	
ENSG0000	897.7495	-0.27003	0.167769	0.036015	0.261666	TMEM219	124446	transmembrane protein 219	
ENSG0000	320.9093	-0.28145	0.180619	0.036079	0.261845	STAT4	6775	signal transducer and activator of transcription 4	
ENSG0000	1470.038	-0.28451	0.187508	0.036078	0.261845	CD300LF	146722	CD300 molecule like family member f	
ENSG0000	112.3811	-0.32107	0.245389	0.036116	0.261998	TTC21A	199223	tetratricopeptide repeat domain 21A	
ENSG0000	32185.19	-0.28065	0.18154	0.036129	0.261998	ITM2B	9445	integral membrane protein 2B	
ENSG0000	8.402334	0.189595	0.329734	0.036177	0.262245	ASIC1	41	acid sensing ion channel subunit 1	
ENSG0000	2432.459	0.219112	0.121855	0.036223	0.26247	IRAK1	3654	interleukin 1 receptor associated kinase 1	
ENSG0000	23536.75	-0.31214	0.237108	0.036287	0.262833	LRP10	26020	LDL receptor related protein 10	
ENSG0000	318.0369	0.313271	0.235249	0.036309	0.26289	ARHGEF10	55160	Rho guanine nucleotide exchange factor 10 like	
ENSG0000	31.33824	-0.34066	0.328121	0.036333	0.262956	SERPINB2	5055	serpin family B member 2	
ENSG0000	233.2926	-0.32664	0.296247	0.036388	0.263255	KIF3C	3797	kinesin family member 3C	
ENSG0000	3.10152	0.062903	0.244165	0.03646	0.26367	APC2	10297	APC regulator of WNT signaling pathway 2	
ENSG0000	10975.68	-0.32079	0.276786	0.036481	0.263718	CDA	978	cytidine deaminase	
ENSG0000	2.996458	0.090084	0.254907	0.036548	0.264038	NA	NA	NA	
ENSG0000	8.632029	0.125086	0.272352	0.036554	0.264038	DZANK1	55184	double zinc ribbon and ankyrin repeat domains 1	
ENSG0000	21.32985	-0.34282	0.365122	0.036603	0.264286	FAM151B	167555	family with sequence similarity 151 member B	
ENSG0000	235.4802	-0.32139	0.256885	0.036639	0.264337	TMOD2	29767	tropomodulin 2	
ENSG0000	20.62363	-0.34103	0.361491	0.036635	0.264337	ZNF763	284390	zinc finger protein 763	
ENSG0000	42.65656	-0.30017	0.382906	0.036747	0.264937	TMEM51	55092	transmembrane protein 51	
ENSG0000	3.217878	-0.13649	0.283422	0.036754	0.264937	H3C8	8355	H3 clustered histone 8	
ENSG0000	6.147772	0.176241	0.314681	0.036766	0.264937	NA	NA	NA	
ENSG0000	186.5312	0.281833	0.178921	0.036785	0.264975	RPL22	6146	ribosomal protein L22	
ENSG0000	292.7088	-0.26575	0.163458	0.036861	0.265309	RBBP5	5929	RB binding	histone lysine methyltransferase complex subunit
ENSG0000	507.856	-0.29123	0.189828	0.036857	0.265309	CAPN1-AS	728975	CAPN1 antisense RNA 1	
ENSG0000	5.163127	0.119304	0.271017	0.036917	0.265611	IGHD6-25	28485	immunoglobulin heavy diversity 6-25	
ENSG0000	445.3241	-0.32266	0.273008	0.036981	0.265866	PNPLA1	285848	patatin like phospholipase domain containing 1	
ENSG0000	944.7184	-0.25611	0.153083	0.036977	0.265866	SPTLC2	9517	serine palmitoyltransferase long chain base subunit 2	
ENSG0000	3.850145	0.131149	0.280462	0.037144	0.266931	FKBP7	51661	FKBP prolyl isomerase 7	
ENSG0000	9353.868	-0.31877	0.264413	0.037212	0.267314	ACSL1	2180	acyl-CoA synthetase long chain family member 1	
ENSG0000	5359.263	-0.27521	0.176727	0.037255	0.267514	PIM2	11040	Pim-2 prot	serine/threonine kinase
ENSG0000	8428.949	-0.31529	0.301465	0.037305	0.267667	NAMPT	10135	nicotinamide phosphoribosyltransferase	
ENSG0000	454.9409	-0.31789	0.258002	0.03732	0.267667	NA	NA	NA	
ENSG0000	17.25936	-0.12925	0.272392	0.037303	0.267667	NA	NA	NA	
ENSG0000	31.51502	0.305902	0.381893	0.03734	0.267706	NA	NA	NA	
ENSG0000	130.4472	-0.30451	0.217715	0.037412	0.268118	SRBD1	55133	S1 RNA binding domain 1	
ENSG0000	56.02849	0.329344	0.269132	0.037431	0.268147	MSH3	4437	mutS homolog 3	
ENSG0000	123314.3	-0.26823	0.16917	0.037453	0.268205	HLA-B	3106	major hist class I	B
ENSG0000	16.96983	0.3016	0.40141	0.037534	0.268681	ZNF425	155054	zinc finger protein 425	
ENSG0000	115.62	0.27429	0.17297	0.03757	0.268723	NCBP2	22916	nuclear cap binding protein subunit 2	
ENSG0000	36.26112	0.338372	0.318136	0.03756	0.268723	HMGB3	3149	high mobility group box 3	
ENSG0000	251.9597	0.26185	0.160387	0.037644	0.269065	MRPL55	128308	mitochondrial ribosomal protein L55	
ENSG0000	1162.284	0.318584	0.259625	0.037647	0.269065	PLXND1	23129	plexin D1	
ENSG0000	1739.882	-0.22208	0.125572	0.037755	0.26952	SMG7	9887	SMG7 nonsense mediated mRNA decay factor	
ENSG0000	45.05432	-0.30786	0.36671	0.037745	0.26952	CDKN1C	1028	cyclin dependent kinase inhibitor 1C	
ENSG0000	2.360831	0.104565	0.26294	0.037749	0.26952	CCDC42	146849	coiled-coil domain containing 42	
ENSG0000	49.71342	-0.3225	0.246653	0.037779	0.269586	MACROH2	55506	macroH2A.2 histone	
ENSG0000	107.5783	0.269446	0.167669	0.037846	0.269925	PAXIP1	22976	PAX interacting protein 1	
ENSG0000	2.747278	0.064242	0.245383	0.037856	0.269925	HIF3A	64344	hypoxia inducible factor 3 subunit alpha	
ENSG0000	7.47605	0.216971	0.35279	0.037962	0.270545	NUDT6	11162	nudix hydrolase 6	
ENSG0000	194.4151	0.324054	0.272326	0.037972	0.270545	LARGE2	120071	LARGE xylosyl- and glucuronyltransferase 2	
ENSG0000	74.47268	-0.32937	0.279527	0.038007	0.270687	PF4V1	5197	platelet factor 4 variant 1	
ENSG0000	21.93113	-0.24411	0.363431	0.038064	0.270993	NA	NA	NA	
ENSG0000	717.059	-0.24915	0.149162	0.03808	0.270996	WSB2	55884	WD repeat and SOCS box containing 2	
ENSG0000	13476.56	-0.30296	0.223842	0.038126	0.271223	CCL5	6352	C-C motif chemokine ligand 5	
ENSG0000	15.26999	0.177116	0.309901	0.038144	0.271239	ESCO2	157570	establishment of sister chromatid cohesion N-acetyltransferase 2	
ENSG0000	4.969729	0.120524	0.272862	0.038179	0.271383	LINC01252	338817	long intergenic non-protein coding RNA 1252	
ENSG0000	689.247	-0.3097	0.238233	0.038199	0.271421	CD36	948	CD36 molecule	
ENSG0000	128.9662	0.324426	0.296069	0.038245	0.271507	WFS1	7466	wolframin ER transmembrane glycoprotein	
ENSG0000	102.6473	-0.31887	0.321876	0.038255	0.271507	DOCK4	9732	dedicator of cytokinesis 4	
ENSG0000	237.6151	-0.31472	0.250039	0.038226	0.271507	NA	NA	NA	
ENSG0000	68.15991	-0.2879	0.385595	0.038379	0.272035	THEM5	284486	thioesterase superfamily member 5	
ENSG0000	218.0878	-0.28302	0.184719	0.038376	0.272035	LETM2	137994	leucine zipper and EF-hand containing transmembrane protein 2	
ENSG0000	4.013089	0.106718	0.262387	0.03835	0.272035	SPINT1-AS	1.03E+08	SPINT1 antisense RNA 1	
ENSG0000	136.7526	0.282363	0.185207	0.038389	0.272035	SHMT1	6470	serine hydroxymethyltransferase 1	
ENSG0000	162735.2	-0.28868	0.198788	0.038405	0.272044	HLA-C	3107	major hist class I	C
ENSG0000	86445.9	-0.31244	0.256627	0.038459	0.272217	SPI1	6688	Spi-1 proto-oncogene	
ENSG0000	911.8638	-0.31336	0.254598	0.038455	0.272217	WSB1	26118	WD repeat and SOCS box containing 1	
ENSG0000	67.03198	-0.32512	0.275814	0.038521	0.272443	SCAMP1-A	728769	SCAMP1 antisense RNA 1	
ENSG0000	421.1752	0.318436	0.275339	0.038508	0.272443	IGHV6-1	28385	immunoglobulin heavy variable 6-1	
ENSG0000	7.415122	-0.22895	0.368447	0.038575	0.27272	NA	NA	NA	

ENSG0000	76.46634	0.312279	0.231391	0.038608	0.272784	HACL1	26061	2-hydroxyacyl-CoA lyase 1
ENSG0000	15.59448	0.274128	0.38698	0.038614	0.272784	ALKBH8	91801	alkB homc tRNA methyltransferase
ENSG0000	36.0743	-0.32327	0.350684	0.038639	0.272854	MMRN1	22915	multimerin 1
ENSG0000	33.34724	-0.30747	0.368664	0.038659	0.272894	XCL2	6846	X-C motif chemokine ligand 2
ENSG0000	207.9526	-0.29379	0.340026	0.038797	0.273575	UGCG	7357	UDP-glucose ceramide glucosyltransferase
ENSG0000	233.1225	-0.31359	0.269812	0.0388	0.273575	B3GAT1	27087	beta-1 3-glucuronyltransferase 1
ENSG0000	1657.522	-0.24952	0.147556	0.038792	0.273575	ITGB2-AS1	1.01E+08	ITGB2 antisense RNA 1
ENSG0000	11.18673	0.264446	0.397463	0.038838	0.273735	NA	NA	NA
ENSG0000	67.34775	-0.32267	0.276869	0.038862	0.273801	S1PR2	9294	sphingosine-1-phosphate receptor 2
ENSG0000	22.24532	0.30836	0.38584	0.038949	0.274302	LDLRAD2	401944	low density lipoprotein receptor class A domain containing 2
ENSG0000	54.71078	-0.29169	0.195297	0.039066	0.274919	C9orf40	55071	chromosome 9 open reading frame 40
ENSG0000	566.5858	0.274227	0.177809	0.039066	0.274919	TOMM40	10452	translocase of outer mitochondrial membrane 40
ENSG0000	1248.372	-0.30308	0.216086	0.039115	0.275159	TLR1	7096	toll like receptor 1
ENSG0000	1686.423	0.237806	0.140545	0.03917	0.27544	LFNG	3955	LFNG O-fucosylpeptide 3-beta-N-acetylglucosaminyltransferase
ENSG0000	1375.542	0.310531	0.312272	0.039226	0.275725	IGKV3-15	28913	immunoglobulin kappa variable 3-15
ENSG0000	110.5048	0.312541	0.334181	0.039241	0.275728	NA	NA	NA
ENSG0000	35.19994	-0.33038	0.287284	0.039348	0.276371	GTF2H2C	728340	GTF2H2 family member C
ENSG0000	118.001	-0.29664	0.209378	0.03944	0.276804	RBM48	84060	RNA binding motif protein 48
ENSG0000	6747.692	0.312914	0.284479	0.039429	0.276804	IGLV2-14	28815	immunoglobulin lambda variable 2-14
ENSG0000	5676.139	-0.29354	0.210391	0.039488	0.276968	DEDD2	162989	death effector domain containing 2
ENSG0000	4.098886	0.122923	0.272618	0.039493	0.276968	NA	NA	NA
ENSG0000	3920.84	-0.26249	0.165852	0.03951	0.276975	TADA3	10474	transcriptional adaptor 3
ENSG0000	33.57818	-0.33258	0.318517	0.039575	0.277328	LINC0203	1.05E+08	long intergenic non-protein coding RNA 2033
ENSG0000	4.727053	0.146441	0.291416	0.039674	0.27783	NA	NA	NA
ENSG0000	269.8094	-0.31743	0.274886	0.039677	0.27783	MYOF	26509	myoferlin
ENSG0000	27.57757	-0.33157	0.344626	0.03984	0.278122	FHL2	2274	four and a half LIM domains 2
ENSG0000	35.29923	-0.25505	0.362422	0.039796	0.278122	NA	NA	NA
ENSG0000	642.525	-0.30834	0.253164	0.039792	0.278122	HACD4	401494	3-hydroxyacyl-CoA dehydratase 4
ENSG0000	74.81684	0.299673	0.349737	0.039785	0.278122	VCPKMT	79609	valosin containing protein lysine methyltransferase
ENSG0000	278.2079	-0.27374	0.178795	0.039792	0.278122	SLFN13	146857	schlafen family member 13
ENSG0000	10.99236	0.199303	0.331302	0.03981	0.278122	NA	NA	NA
ENSG0000	514.5576	-0.2957	0.341195	0.039834	0.278122	MCEMP1	199675	mast cell expressed membrane protein 1
ENSG0000	68.80634	-0.32188	0.27528	0.039776	0.278122	CYP2T1P	171523	cytochrom pseudogene
ENSG0000	14.37786	0.297423	0.392463	0.040025	0.279307	NA	NA	NA
ENSG0000	28.9803	0.320276	0.353324	0.040064	0.279401	AGL	178	amylo-alpl 6-glucosic 4-alpha-glucanotransferase
ENSG0000	9.430467	-0.20973	0.341532	0.040069	0.279401	TBX1	6899	T-box transcription factor 1
ENSG0000	435.1478	-0.26004	0.16267	0.040088	0.279426	PRUNE1	58497	prune exopolyphosphatase 1
ENSG0000	121.1542	-0.25141	0.152784	0.040129	0.279605	VPS33A	65082	VPS33A core subunit of CORVET and HOPS complexes
ENSG0000	30.95774	0.332399	0.31706	0.040148	0.279633	FAM114A1	92689	family with sequence similarity 114 member A1
ENSG0000	60.53789	0.324191	0.309803	0.040238	0.279774	MTCO3P1	1.07E+08	MT-CO3 pseudogene 12
ENSG0000	507.3274	-0.2764	0.18149	0.040221	0.279774	LPP	4026	LIM domain containing preferred translocation partner in lipoma
ENSG0000	32.43827	0.320738	0.344329	0.040229	0.279774	APOM	55937	apolipoprotein M
ENSG0000	36.43426	-0.3158	0.347792	0.040227	0.279774	HARBI1	283254	harbinger transposase derived 1
ENSG0000	616.1902	-0.25815	0.161618	0.040245	0.279774	PPP2R5B	5526	protein phosphatase 2 regulatory subunit B'beta
ENSG0000	148.0817	0.289355	0.201029	0.040267	0.279798	DHX57	90957	DExH-box helicase 57
ENSG0000	140.0411	0.29182	0.203501	0.040287	0.279798	ADGRL1-A	1.01E+08	ADGRL1 antisense RNA 1
ENSG0000	281.2245	0.288576	0.198873	0.040294	0.279798	SLC25A42	284439	solute carrier family 25 member 42
ENSG0000	10.77152	0.21073	0.340198	0.040317	0.279849	CDC20-DT	1.05E+08	CDC20 divergent transcript
ENSG0000	2664.868	-0.25512	0.159023	0.040357	0.279916	ATP6V0E1	8992	ATPase H+ transporting V0 subunit e1
ENSG0000	52220.78	-0.30808	0.255587	0.040352	0.279916	SERPINA1	5265	serpin family A member 1
ENSG0000	38.31229	0.327465	0.302024	0.040481	0.280562	LTB4R2	56413	leukotriene B4 receptor 2
ENSG0000	113.546	0.260388	0.350219	0.040468	0.280562	IGHV3-53	28420	immunoglobulin heavy variable 3-53
ENSG0000	787.4685	0.240372	0.143963	0.040536	0.280732	CORO1C	23603	coronin 1C
ENSG0000	119.6165	0.255491	0.15619	0.040525	0.280732	EXOSC6	118460	exosome component 6
ENSG0000	179.823	-0.2722	0.177126	0.040558	0.28075	NA	NA	NA
ENSG0000	7.339094	-0.17949	0.315358	0.040585	0.28075	NA	NA	NA
ENSG0000	740.8936	-0.30298	0.245593	0.040578	0.28075	SIGLEC7	27036	sialic acid binding Ig like lectin 7
ENSG0000	38.83917	-0.29668	0.35522	0.040606	0.28075	PDE5A	8654	phosphodiesterase 5A
ENSG0000	1334.76	-0.20224	0.112527	0.040615	0.28075	VAMP1	6843	vesicle associated membrane protein 1
ENSG0000	3.014357	0.138312	0.286823	0.040752	0.281274	SEMA3F	6405	semaphorin 3F
ENSG0000	158.0989	0.282837	0.192567	0.040751	0.281274	SSBP2	23635	single stranded DNA binding protein 2
ENSG0000	13.70301	0.31558	0.388756	0.04071	0.281274	GGTA1	2681	glycoprotein alpha-galactosyltransferase 1 (inactive)
ENSG0000	429.8315	-0.28624	0.198476	0.040746	0.281274	LPAR5	57121	lysophosphatidic acid receptor 5
ENSG0000	8.671524	0.253695	0.384895	0.040786	0.281402	ZMIZ1-AS1	283050	ZMIZ1 antisense RNA 1
ENSG0000	10.98525	-0.26902	0.384767	0.040886	0.281981	NA	NA	NA
ENSG0000	3.776088	-0.07263	0.245939	0.040928	0.282167	NA	NA	NA
ENSG0000	2373.856	-0.27679	0.18659	0.040982	0.282434	RGS3	5998	regulator of G protein signaling 3
ENSG0000	17.19736	-0.26354	0.372445	0.041051	0.282617	RNF207	388591	ring finger protein 207
ENSG0000	808.0878	0.215251	0.123005	0.041099	0.282617	ZNF362	149076	zinc finger protein 362
ENSG0000	290.4619	0.273285	0.181519	0.041057	0.282617	NXPE3	91775	neurexophilin and PC-esterase domain family member 3
ENSG0000	13.00819	-0.20952	0.33703	0.041069	0.282617	CAV2	858	caveolin 2
ENSG0000	72.65014	0.318076	0.313619	0.041117	0.282617	NOMO2	283820	NODAL modulator 2
ENSG0000	1981.739	-0.27384	0.182928	0.041103	0.282617	ZNF646	9726	zinc finger protein 646
ENSG0000	20.77044	0.219789	0.344367	0.041097	0.282617	ICOSLG	23308	inducible T cell costimulator ligand
ENSG0000	45.0229	0.323094	0.291883	0.041141	0.282679	BPHL	670	biphenyl hydrolase like
ENSG0000	819.4014	-0.28462	0.204797	0.041209	0.283039	MAGED2	10916	MAGE family member D2
ENSG0000	26.52648	0.274249	0.372483	0.041247	0.283193	LDHD	197257	lactate dehydrogenase D
ENSG0000	311.0002	-0.23557	0.140146	0.04128	0.283318	MTMR4	9110	myotubularin related protein 4
ENSG0000	37.14511	0.285244	0.369215	0.041331	0.283562	ARL4A	10124	ADP ribosylation factor like GTPase 4A
ENSG0000	114.1784	0.308187	0.234428	0.041378	0.283777	LOC10537	1.05E+08	proline-rich protein 18-like
ENSG0000	306.4206	0.215591	0.121912	0.041423	0.283974	AKAP8	10270	A-kinase anchoring protein 8
ENSG0000	2.656679	-0.11544	0.269788	0.04148	0.284155	NA	NA	NA
ENSG0000	179.9471	-0.29915	0.218621	0.04147	0.284155	CLN3	1201	CLN3 lysos battenin
ENSG0000	47.0472	0.321908	0.285251	0.041553	0.284444	CENPF	1063	centromere protein F
ENSG0000	23.5201	0.310643	0.370615	0.041544	0.284444	NA	NA	NA
ENSG0000	221.1119	0.25629	0.159909	0.041578	0.284457	ZNF398	57541	zinc finger protein 398
ENSG0000	466.7015	-0.24606	0.149577	0.041586	0.284457	ASCL2	430	achaete-scute family bHLH transcription factor 2
ENSG0000	296.6842	0.280468	0.19115	0.041621	0.284573	POLR2B	5431	RNA polymerase II subunit B
ENSG0000	2389.741	-0.28294	0.197256	0.041649	0.284573	CD6	923	CD6 molecule
ENSG0000	2330.023	-0.26116	0.167234	0.04165	0.284573	TPST2	8459	tyrosylprotein sulfotransferase 2
ENSG0000	16.22609	0.311315	0.386717	0.041693	0.284762	TYW1B	441250	tRNA-yW synthesizing protein 1 homolog B
ENSG0000	2.542103	0.056077	0.241621	0.041775	0.285217	HSD17B14	51171	hydroxysteroid 17-beta dehydrogenase 14
ENSG0000	3.155308	0.106999	0.263113	0.041799	0.285275	ARMC9	80210	armadillo repeat containing 9
ENSG0000	3.981304	-0.09942	0.258456	0.041842	0.285462	ZNF80	7634	zinc finger protein 80
ENSG0000	81.86178	0.318671	0.29397	0.041894	0.285609	DGKG	1608	diacylglycerol kinase gamma
ENSG0000	1591.502	-0.25929	0.164134	0.041895	0.285609	ZDHHC5	25921	zinc finger DHHC-type palmitoyltransferase 5

ENSG0000	24230.48	-0.28985	0.211869	0.041911	0.28561	PPP1R18	170954	protein phosphatase 1 regulatory subunit 18
ENSG0000	3.377631	0.153957	0.297488	0.04193	0.285637	INO80B	83444	INO80 complex subunit B
ENSG0000	15.7351	-0.29445	0.382941	0.041983	0.285796	NA	NA	NA
ENSG0000	118.4481	-0.30286	0.231743	0.041985	0.285796	SLF2	55719	SMC5-SMC6 complex localization factor 2
ENSG0000	157.1582	0.298806	0.223425	0.042051	0.286037	RASGEF1A	221002	RasGEF domain family member 1A
ENSG0000	85.78672	-0.30128	0.227599	0.042041	0.286037	NGRN	51335	neugrin neurite outgrowth associated
ENSG0000	732.9368	-0.29759	0.235858	0.042258	0.286927	WDFY3	23001	WD repeat and FYVE domain containing 3
ENSG0000	11.09068	-0.20735	0.342279	0.042255	0.286927	ABCG2	9429	ATP binding cassette subfamily G member 2 (Junior blood group)
ENSG0000	165.6355	0.303424	0.23936	0.042237	0.286927	NA	NA	NA
ENSG0000	511.7749	-0.30464	0.250591	0.042261	0.286927	NA	NA	NA
ENSG0000	720.4819	-0.30149	0.239123	0.042232	0.286927	SRC	6714	SRC proto· non-receptor tyrosine kinase
ENSG0000	5.746758	-0.13394	0.279311	0.04235	0.287425	LOC12490	1.25E+08	uncharacterized LOC124902388
ENSG0000	1339.243	-0.29544	0.215826	0.042437	0.28771	HS1BP3	64342	HCLS1 binding protein 3
ENSG0000	2220.697	-0.24771	0.154113	0.042427	0.28771	NPC2	10577	NPC intracellular cholesterol transporter 2
ENSG0000	69.52707	0.30667	0.238945	0.042439	0.28771	ABCB7	22	ATP binding cassette subfamily B member 7
ENSG0000	21.72185	0.256402	0.363811	0.042481	0.287891	NA	NA	NA
ENSG0000	3.617913	0.08318	0.250805	0.042606	0.288618	MGP	4256	matrix Gla protein
ENSG0000	13076.81	-0.22987	0.137104	0.042635	0.288618	UBC	7316	ubiquitin C
ENSG0000	2.899597	0.129833	0.278112	0.042636	0.288618	C21orf91-(	246312	C21orf91 overlapping transcript 1
ENSG0000	4.952005	0.09482	0.255194	0.042652	0.288624	NA	NA	NA
ENSG0000	10.23685	0.227662	0.355654	0.042768	0.28927	MIX23	131076	mitochondrial matrix import factor 23
ENSG0000	9301.962	-0.29895	0.228778	0.042794	0.28927	TRAC	28755	T cell receptor alpha constant
ENSG0000	24.76153	-0.32882	0.325065	0.042795	0.28927	CYP4F22	126410	cytochrome P450 family 4 subfamily F member 22
ENSG0000	6.367536	0.14972	0.28999	0.042829	0.289395	POGLUT2	79070	protein O-glucosyltransferase 2
ENSG0000	23.02458	0.324873	0.345998	0.042862	0.2894	PLEKHG1	57480	pleckstrin homology and RhoGEF domain containing G1
ENSG0000	53.67558	0.311996	0.249726	0.042849	0.2894	NRIP1	8204	nuclear receptor interacting protein 1
ENSG0000	18.33794	0.24692	0.361262	0.042909	0.289614	MIR5195	1.01E+08	microRNA 5195
ENSG0000	150.3736	0.274844	0.184926	0.042979	0.28998	SDHAF1	644096	succinate dehydrogenase complex assembly factor 1
ENSG0000	16.0735	0.167118	0.299311	0.043231	0.291374	KDF1	126695	keratinocyte differentiation factor 1
ENSG0000	13.08305	0.252473	0.367848	0.043234	0.291374	CKMT2-AS	1E+08	CKMT2 antisense RNA 1
ENSG0000	38.24713	0.312035	0.244881	0.043209	0.291374	TUBGCP5	114791	tubulin gamma complex associated protein 5
ENSG0000	6.818831	0.233874	0.3675	0.043341	0.291882	NA	NA	NA
ENSG0000	1666.425	0.255431	0.164449	0.043327	0.291882	BTF3	689	basic transcription factor 3
ENSG0000	655.6607	0.262995	0.175801	0.043389	0.292072	PMF1	11243	polyamine modulated factor 1
ENSG0000	419.6848	-0.22831	0.132577	0.043401	0.292072	NA	NA	NA
ENSG0000	497.7175	0.278855	0.331434	0.043454	0.29232	IGHV3-48	28424	immunoglobulin heavy variable 3-48
ENSG0000	5.46124	-0.1938	0.33365	0.043556	0.2929	RPL21P35	1E+08	ribosomal protein L21 pseudogene 35
ENSG0000	81.11064	0.309296	0.254546	0.043592	0.293033	UBA5	79876	ubiquitin like modifier activating enzyme 5
ENSG0000	13.22692	0.188596	0.316792	0.043611	0.293056	BEST4	266675	bestrophin 4
ENSG0000	249.7707	0.269513	0.180217	0.043664	0.293089	NA	NA	NA
ENSG0000	4.224758	-0.11835	0.270634	0.043653	0.293089	MYZAP	1.01E+08	myocardial zonula adherens protein
ENSG0000	3354.134	-0.27025	0.182413	0.043642	0.293089	OGFR	11054	opioid growth factor receptor
ENSG0000	112.2014	0.305719	0.299131	0.043723	0.29312	SYNPO	11346	synaptopodin
ENSG0000	33.14581	-0.31863	0.274961	0.043742	0.29312	SAP30L-AS	386627	SAP30L antisense RNA 1 (head to head)
ENSG0000	276.2903	0.226205	0.133879	0.043749	0.29312	ENTR1	10807	endosome associated trafficking regulator 1
ENSG0000	651.7238	-0.27782	0.197213	0.043705	0.29312	GDPD5	81544	glycerophosphodiester phosphodiesterase domain containing 5
ENSG0000	3.010928	0.084085	0.251387	0.043743	0.29312	TYRO3	7301	TYRO3 protein tyrosine kinase
ENSG0000	6.289457	-0.15763	0.295938	0.043768	0.293141	SPATA1	1.01E+08	spermatogenesis associated 1
ENSG0000	69.16024	-0.3125	0.26812	0.043802	0.29326	TUFT1	7286	tuftelin 1
ENSG0000	5.462719	0.106626	0.261629	0.043832	0.293352	NA	NA	NA
ENSG0000	6.726702	0.138869	0.280938	0.043896	0.293675	DFFBP1	1E+08	DNA fragmentation factor subunit beta pseudogene 1
ENSG0000	17.82824	0.203819	0.327063	0.043932	0.293807	TMEM253	643382	transmembrane protein 253
ENSG0000	236.277	0.267136	0.176593	0.043952	0.293834	MICOS13	125988	mitochondrial contact site and cristae organizing system subunit 13
ENSG0000	8.341935	-0.20439	0.336436	0.044006	0.294013	REG4	83998	regenerating family member 4
ENSG0000	80.17299	0.311094	0.283918	0.04401	0.294013	CKAP2	26586	cytoskeleton associated protein 2
ENSG0000	5.389167	0.158816	0.299324	0.044104	0.294531	MIR155HG	114614	MIR155 host gene
ENSG0000	11.73063	-0.24951	0.370886	0.044201	0.29507	NA	NA	NA
ENSG0000	49.30656	0.313349	0.310508	0.044255	0.295246	LZTFL1	54585	leucine zipper transcription factor like 1
ENSG0000	88.6274	0.303389	0.317611	0.04426	0.295246	PMEPA1	56937	prostate tı androgen induced 1
ENSG0000	6.446761	0.182596	0.316761	0.044315	0.295507	NA	NA	NA
ENSG0000	900.2609	0.289802	0.217351	0.044404	0.295755	TENT5C	54855	terminal nucleotidyltransferase 5C
ENSG0000	2324.771	0.285134	0.207184	0.044416	0.295755	HLA-DMA	3108	major hist· class II DM alpha
ENSG0000	121.5525	0.274096	0.189059	0.044403	0.295755	KLHDC2	23588	kelch domain containing 2
ENSG0000	3.209348	0.099259	0.258437	0.044378	0.295755	NA	NA	NA
ENSG0000	581.3938	-0.2106	0.121377	0.044465	0.295971	PLPBP	11212	pyridoxal phosphate binding protein
ENSG0000	1287.768	0.29795	0.241145	0.0445	0.295989	RPS25	6230	ribosomal protein S25
ENSG0000	86.33934	0.270815	0.181014	0.044489	0.295989	DCUN1D2	55208	defective in cullin neddylation 1 domain containing 2
ENSG0000	82.90536	-0.30797	0.252738	0.044604	0.296416	CD160	11126	CD160 molecule
ENSG0000	31.8762	0.314267	0.313382	0.044616	0.296416	STAG3L4	64940	stromal antigen 3-like 4 (pseudogene)
ENSG0000	4.834244	-0.09709	0.258499	0.044629	0.296416	CYP19A1	1588	cytochrome P450 family 19 subfamily A member 1
ENSG0000	5.6144	0.130413	0.276061	0.044622	0.296416	NA	NA	NA
ENSG0000	126.0975	0.305397	0.291343	0.044673	0.296471	FCRL1	115350	Fc receptor like 1
ENSG0000	199.4361	0.286268	0.207333	0.044667	0.296471	NUP93	9688	nucleoporin 93
ENSG0000	988.6034	0.223706	0.13199	0.044702	0.296471	SPG7	6687	SPG7 matr paraplegin
ENSG0000	43.58781	0.315294	0.297245	0.044691	0.296471	NA	NA	NA
ENSG0000	22.68662	-0.2418	0.350227	0.044835	0.29703	NA	NA	NA
ENSG0000	52.86898	-0.30698	0.250126	0.044815	0.29703	RASGRF2	5924	Ras protein specific guanine nucleotide releasing factor 2
ENSG0000	627.2179	0.266499	0.179066	0.044829	0.29703	LONP2	83752	lon peptid peroxisomal
ENSG0000	9.532997	-0.08272	0.250701	0.044883	0.29724	NA	NA	NA
ENSG0000	175.0249	0.304388	0.283497	0.044935	0.29748	PARM1	25849	prostate androgen-regulated mucin-like protein 1
ENSG0000	443.8746	-0.26849	0.18184	0.045002	0.297569	DIP2B	57609	disco interacting protein 2 homolog B
ENSG0000	157.3048	-0.24899	0.158085	0.044989	0.297569	MFAP1	4236	microfibril associated protein 1
ENSG0000	333.6203	0.231894	0.14211	0.045014	0.297569	MAP2K1	5604	mitogen-activated protein kinase kinase 1
ENSG0000	149.6829	0.240673	0.147786	0.044982	0.297569	ZNF75A	7627	zinc finger protein 75a
ENSG0000	46.41807	0.27832	0.353941	0.045064	0.297689	CLEC2L	154790	C-type lectin domain family 2 member L
ENSG0000	56.54664	-0.29273	0.332784	0.045058	0.297689	NA	NA	NA
ENSG0000	13.171	0.245887	0.360708	0.045166	0.297715	NDUFAF2	91942	NADH:ubiquinone oxidoreductase complex assembly factor 2
ENSG0000	166.4633	-0.28728	0.328511	0.045146	0.297715	HSPA1B	3304	heat shock protein family A (Hsp70) member 1B
ENSG0000	61.49648	-0.30844	0.274287	0.045151	0.297715	SMPDL3A	10924	sphingomyelin phosphodiesterase acid like 3A
ENSG0000	16.48225	0.205596	0.328835	0.045151	0.297715	NA	NA	NA
ENSG0000	10.04359	0.193166	0.321928	0.045138	0.297715	NA	NA	NA
ENSG0000	13320.31	0.288805	0.32286	0.045125	0.297715	IGLC2	3538	immunoglobulin lambda constant 2
ENSG0000	2811.949	0.266061	0.185914	0.045191	0.29777	SYK	6850	spleen associated tyrosine kinase
ENSG0000	3.694403	0.141809	0.287493	0.045342	0.298089	NA	NA	NA
ENSG0000	845.8031	-0.25007	0.161652	0.045272	0.298089	NEU1	4758	neuraminidase 1
ENSG0000	962.9478	-0.21463	0.126008	0.045335	0.298089	RNF5	6048	ring finger protein 5

ENSG0000	11.80594	0.257714	0.375329	0.045313	0.298089	NA	NA	NA
ENSG0000	35.6122	0.314487	0.266134	0.045304	0.298089	WNT10B	7480	Wnt family member 10B
ENSG0000	38.29911	-0.31234	0.255075	0.045353	0.298089	NA	NA	NA
ENSG0000	1726.325	0.246848	0.161134	0.0453	0.298089	EIF3K	27335	eukaryotic translation initiation factor 3 subunit K
ENSG0000	3.69939	0.085706	0.251739	0.045429	0.298201	NA	NA	NA
ENSG0000	949.9935	0.256953	0.168126	0.045468	0.298201	AIF1	199	allograft inflammatory factor 1
ENSG0000	20.11426	0.278354	0.364884	0.045412	0.298201	MYEOV	26579	myeloma overexpressed
ENSG0000	36.35758	0.310903	0.258789	0.045452	0.298201	NEK3	4752	NIMA related kinase 3
ENSG0000	11.83922	0.207287	0.333198	0.045448	0.298201	NA	NA	NA
ENSG0000	19.22941	0.221415	0.340781	0.045438	0.298201	LOC12490	1.25E+08	uncharacterized LOC124904009
ENSG0000	2465.394	0.288768	0.213477	0.045554	0.298664	MAN2B1	4125	mannosidase alpha class 2B member 1
ENSG0000	5.786465	0.124323	0.272391	0.045619	0.298898	NA	NA	NA
ENSG0000	6.234406	0.138173	0.2816	0.045623	0.298898	NA	NA	NA
ENSG0000	2248.572	-0.24257	0.153004	0.045669	0.299091	STARD3	10948	StAR related lipid transfer domain containing 3
ENSG0000	629.5766	-0.27524	0.187429	0.045739	0.299228	PFKP	5214	phosphofr platelet
ENSG0000	4281.165	0.262972	0.178193	0.045733	0.299228	NFKBIA	4792	NFKB inhibitor alpha
ENSG0000	77.28241	0.297815	0.239869	0.045737	0.299228	SNRNP25	79622	small nuclear ribonucleoprotein U11/U12 subunit 25
ENSG0000	32.02221	-0.31689	0.293622	0.045767	0.299306	NA	NA	NA
ENSG0000	6405.633	-0.30078	0.267571	0.045808	0.29947	MALAT1	378938	metastasis associated lung adenocarcinoma transcript 1
ENSG0000	77.90635	-0.23636	0.332959	0.045884	0.299759	RN7SL600	1.06E+08	RNA 7SL cytoplasm pseudogene
ENSG0000	182.3469	0.302714	0.263271	0.045885	0.299759	NA	NA	NA
ENSG0000	3.729232	0.058565	0.241505	0.045987	0.300211	NA	NA	NA
ENSG0000	1845.49	0.285723	0.215325	0.045979	0.300211	RPL35	11224	ribosomal protein L35
ENSG0000	925.6954	-0.26131	0.175634	0.046041	0.300348	ELF1	1997	E74 like ETS transcription factor 1
ENSG0000	1922.894	0.294112	0.290468	0.046039	0.300348	IGLV1-47	28822	immunoglobulin lambda variable 1-47
ENSG0000	128.8777	0.265365	0.175543	0.046085	0.300517	TSR1	55720	TSR1 ribosome maturation factor
ENSG0000	297.3227	0.293141	0.231175	0.0461	0.300517	ASF1B	55723	anti-silencing function 1B histone chaperone
ENSG0000	149.7418	0.257777	0.169231	0.04627	0.301327	NA	NA	NA
ENSG0000	704.6562	0.227603	0.138487	0.046262	0.301327	GLI4	2738	GLI family zinc finger 4
ENSG0000	5.348664	0.197924	0.331291	0.046273	0.301327	RPS11P5	727826	ribosomal protein S11 pseudogene 5
ENSG0000	23.83817	0.316425	0.326362	0.046369	0.301841	ZNF697	90874	zinc finger protein 697
ENSG0000	751.2284	-0.2501	0.161789	0.046459	0.302298	APBB3	10307	amyloid beta precursor protein binding family B member 3
ENSG0000	42.34795	0.306593	0.315683	0.046472	0.302298	MACROD1	28992	mono-ADP ribosylhydrolase 1
ENSG0000	137.6017	0.301606	0.261679	0.04653	0.302532	ANP32E	81611	acidic nuclear phosphoprotein 32 family member E
ENSG0000	2372.303	-0.25473	0.162386	0.046557	0.302532	PIM3	415116	Pim-3 prot serine/threonine kinase
ENSG0000	620.7992	0.306486	0.262917	0.046551	0.302532	ATP8	4509	ATP synthase F0 subunit 8
ENSG0000	25.53324	0.317694	0.294899	0.046588	0.302627	GLUD1P3	2749	glutamate dehydrogenase 1 pseudogene 3
ENSG0000	39.4748	0.306108	0.250432	0.04665	0.302917	COPRS	55352	coordinator of PRMT5 and differentiation stimulator
ENSG0000	912.6109	-0.27718	0.200765	0.046667	0.302919	PRR5	55615	proline rich 5
ENSG0000	355.5031	-0.29343	0.24336	0.046761	0.303423	BLTP1	84162	bridge-like lipid transfer protein family member 1
ENSG0000	166.5011	-0.26806	0.184701	0.046878	0.304074	ARHGEF12	23365	Rho guanine nucleotide exchange factor 12
ENSG0000	1571.825	-0.25637	0.171377	0.046946	0.304305	NDRG1	10397	N-myc downstream regulated 1
ENSG0000	1015.035	-0.29796	0.276447	0.046932	0.304305	SLFN5	162394	schlafen family member 5
ENSG0000	30.19807	-0.31016	0.310311	0.046986	0.304452	LYSMD1	388695	LysM domain containing 1
ENSG0000	4.52841	0.102723	0.259522	0.047035	0.304662	NA	NA	NA
ENSG0000	44.25659	0.283469	0.342244	0.047056	0.304688	COX6C	1345	cytochrome c oxidase subunit 6C
ENSG0000	987.8365	-0.28564	0.232799	0.047145	0.30473	RSRP1	57035	arginine and serine rich protein 1
ENSG0000	111.5401	-0.27279	0.191198	0.047139	0.30473	NA	NA	NA
ENSG0000	98.49397	0.303925	0.28956	0.047104	0.30473	MADCAM1	8174	mucosal vascular addressin cell adhesion molecule 1
ENSG0000	72.6032	0.295082	0.229606	0.047117	0.30473	PVR	5817	PVR cell adhesion molecule
ENSG0000	214.8124	-0.25708	0.170273	0.047084	0.30473	TRIB3	57761	tribbles pseudokinase 3
ENSG0000	66.94683	0.267524	0.180836	0.047225	0.305141	CENPBD1P	92806	CENPB DN pseudogene
ENSG0000	61.22992	0.280216	0.198914	0.047276	0.305326	MST1	4485	macrophage stimulating 1
ENSG0000	161.2586	0.139111	0.278039	0.047287	0.305326	IGHV1-69D	1.03E+08	immunoglobulin heavy variable 1-69D
ENSG0000	19.49823	0.310289	0.339979	0.047339	0.305551	NA	NA	NA
ENSG0000	377.4059	0.22956	0.144971	0.047377	0.305687	R3HDM1	23518	R3H domain containing 1
ENSG0000	2765.944	0.265669	0.184013	0.047419	0.305743	HLA-DPB1	3115	major hist class II DP beta 1
ENSG0000	288.9405	-0.26454	0.183683	0.047413	0.305743	SFT2D1	113402	SFT2 domain containing 1
ENSG0000	850.1707	0.238739	0.156929	0.047474	0.305989	SPCS1	28972	signal peptidase complex subunit 1
ENSG0000	14.12784	0.303247	0.365823	0.047537	0.306231	ARHGEF4	50649	Rho guanine nucleotide exchange factor 4
ENSG0000	6.518329	-0.14549	0.287369	0.047545	0.306231	NDST1-AS	1.03E+08	NDST1 antisense RNA 1
ENSG0000	477.2411	-0.25973	0.179126	0.04758	0.306351	EML2	24139	EMAP like 2
ENSG0000	61.85513	0.254143	0.343946	0.047602	0.306388	HAT1	8520	histone acetyltransferase 1
ENSG0000	14.95104	-0.22935	0.346257	0.04762	0.306392	ADAMTS14	140766	ADAM metallopeptidase with thrombospondin type 1 motif 14
ENSG0000	50.38591	-0.2886	0.332288	0.047686	0.306675	LOC12252	1.23E+08	uncharacterized LOC122526782
ENSG0000	1291.081	0.29086	0.242736	0.047747	0.306675	SND1	27044	staphylococcal nuclease and tudor domain containing 1
ENSG0000	10.75062	-0.2125	0.336436	0.0477	0.306675	NA	NA	NA
ENSG0000	139.7244	-0.29851	0.262753	0.047731	0.306675	MAMDC4	158056	MAM domain containing 4
ENSG0000	75.26048	0.284058	0.208513	0.047743	0.306675	ZHX3	23051	zinc fingers and homeoboxes 3
ENSG0000	146.7623	0.286973	0.218451	0.047788	0.30683	SNTB2	6645	syntrophin beta 2
ENSG0000	9.884652	0.193044	0.322218	0.047877	0.30686	NA	NA	NA
ENSG0000	6.607517	-0.13438	0.278111	0.047848	0.30686	NA	NA	NA
ENSG0000	3917.963	-0.25919	0.185332	0.04786	0.30686	AP1G2	8906	adaptor related protein complex 1 subunit gamma 2
ENSG0000	156.8943	-0.26415	0.180776	0.047844	0.30686	NA	NA	NA
ENSG0000	8789.708	0.249541	0.1632	0.04781	0.30686	RPS28	6234	ribosomal protein S28
ENSG0000	5.00555	-0.16988	0.306155	0.047927	0.307074	OR7E100P	81430	olfactory receptor family 7 subfamily E member 100 pseudogene
ENSG0000	1239.157	-0.28367	0.219668	0.048003	0.307453	GZMH	2999	granzyme H
ENSG0000	18.25013	0.274755	0.36422	0.048081	0.307502	EXO1	9156	exonuclease 1
ENSG0000	8.319283	-0.16286	0.29909	0.048076	0.307502	ACRP1	645529	ACR pseudogene 1
ENSG0000	655.6623	-0.28777	0.230484	0.048083	0.307502	GPR27	2850	G protein-coupled receptor 27
ENSG0000	57.01118	0.285648	0.210075	0.048094	0.307502	LIN54	132660	lin-54 DREAM MuvB core complex component
ENSG0000	39.48914	0.306345	0.260846	0.048063	0.307502	SLC25A13	10165	solute carrier family 25 member 13
ENSG0000	42.47031	0.308703	0.291498	0.048284	0.308192	TNFRSF9	3604	TNF receptor superfamily member 9
ENSG0000	1015.857	-0.24576	0.159147	0.048257	0.308192	SRGAP2	23380	SLIT-ROBO Rho GTPase activating protein 2
ENSG0000	2.433076	0.058164	0.242564	0.04826	0.308192	LINC02453	643770	long intergenic non-protein coding RNA 2453
ENSG0000	45.32142	-0.25031	0.336198	0.048265	0.308192	TMEM272	283521	transmembrane protein 272
ENSG0000	5.053077	-0.09487	0.255762	0.048286	0.308192	EDA2R	60401	ectodysplasin A2 receptor
ENSG0000	577.3098	0.231816	0.139683	0.048322	0.308311	ASXL1	171023	ASXL transcriptional regulator 1
ENSG0000	191.5824	-0.29392	0.25171	0.048372	0.308498	SDC3	9672	syndecan 3
ENSG0000	277.8509	0.255412	0.169795	0.048392	0.308498	POMT1	10585	protein O-mannosyltransferase 1
ENSG0000	1414.648	-0.27523	0.203542	0.048419	0.308498	TESPA1	9840	thymocyte positive selection associated 1
ENSG0000	1522.901	0.248779	0.162751	0.048404	0.308498	ERGIC3	51614	ERGIC and golgi 3
ENSG0000	7.991995	0.157469	0.293464	0.048649	0.30969	SPDYE18	1.01E+08	speedy/RINGO cell cycle regulator family member E18
ENSG0000	27.93293	-0.28648	0.344046	0.048656	0.30969	FAM110B	90362	family with sequence similarity 110 member B
ENSG0000	13.23099	0.174966	0.307687	0.048636	0.30969	IQSEC3	440073	IQ motif and Sec7 domain ArfGEF 3
ENSG0000	28.26228	0.3027	0.325416	0.048779	0.310242	PRMT3	10196	protein arginine methyltransferase 3

ENSG0000	444.9383	-0.27088	0.187375	0.048792	0.310242	PRKCH	5583	protein kinase C eta
ENSG0000	33.71127	0.274382	0.33858	0.048794	0.310242	LARGE1	9215	LARGE xylosyl- and glucuronyltransferase 1
ENSG0000	2.75428	0.121534	0.272038	0.04887	0.31062	RNA5SP30	1.01E+08	RNA 5S ribosomal pseudogene 307
ENSG0000	5.061844	0.111998	0.263389	0.048896	0.310678	TECTA	7007	tectorin alpha
ENSG0000	7.715048	0.211142	0.337816	0.048926	0.310759	HAUS7	55559	HAUS augmin like complex subunit 7
ENSG0000	196.863	-0.28141	0.214734	0.048976	0.310969	PRKCQ	5588	protein kinase C theta
ENSG0000	244.1479	-0.2726	0.327354	0.049011	0.311082	NA	NA	NA
ENSG0000	8.774188	0.127885	0.272501	0.049124	0.311334	TMEM237	65062	transmembrane protein 237
ENSG0000	23.86816	0.196458	0.316005	0.049137	0.311334	ELFN1-AS1	1.02E+08	ELFN1 antisense RNA 1
ENSG0000	559.0649	-0.28934	0.259291	0.049073	0.311334	FADS2	9415	fatty acid desaturase 2
ENSG0000	6738.097	0.237156	0.151144	0.049153	0.311334	RPS26	6231	ribosomal protein S26
ENSG0000	279.9634	-0.24439	0.158593	0.049131	0.311334	ATP6V0D1	1.02E+08	ATP6V0D1 divergent transcript
ENSG0000	741.4104	0.244229	0.158458	0.049143	0.311334	LOC72839	728392	uncharacterized LOC728392
ENSG0000	25.65484	-0.30405	0.317827	0.049328	0.311941	ITGA10	8515	integrin subunit alpha 10
ENSG0000	10.2903	0.12102	0.268441	0.049361	0.311941	CHML	1122	CHM like Rab escort protein
ENSG0000	8.356267	0.224123	0.348457	0.049291	0.311941	STIMATE-I	1.01E+08	STIMATE-MUSTN1 readthrough
ENSG0000	2144.46	-0.20803	0.124066	0.049381	0.311941	ARCN1	372	archain 1
ENSG0000	1855.821	-0.26425	0.18654	0.049332	0.311941	ICAM2	3384	intercellular adhesion molecule 2
ENSG0000	1441.019	-0.1877	0.10787	0.049276	0.311941	DDA1	79016	DET1 and DDB1 associated 1
ENSG0000	63.86747	-0.28294	0.327855	0.049385	0.311941	CADM4	199731	cell adhesion molecule 4
ENSG0000	29.66539	0.302893	0.335089	0.049336	0.311941	RAI2	10742	retinoic acid induced 2
ENSG0000	8416.525	-0.26832	0.188261	0.049495	0.312528	CCND3	896	cyclin D3
ENSG0000	52.092	-0.30219	0.258806	0.04956	0.312726	TMEM45B	120224	transmembrane protein 45B
ENSG0000	23.83283	0.174795	0.301458	0.049556	0.312726	NFYB	4801	nuclear transcription factor Y subunit beta
ENSG0000	370.8165	-0.28844	0.24224	0.049613	0.31295	TMEM86B	255043	transmembrane protein 86B
ENSG0000	458.8903	0.290572	0.285455	0.049703	0.313411	IGF1R	3480	insulin like growth factor 1 receptor
ENSG0000	1434.82	-0.28981	0.26692	0.049721	0.313415	CR1	1378	complement C3b/C4b receptor 1 (Knops blood group)
ENSG0000	2.792263	-0.06968	0.246988	0.049796	0.313785	EPHA2	1969	EPH receptor A2
ENSG0000	9.125667	0.169434	0.301697	0.049817	0.313807	NA	NA	NA
ENSG0000	22.32387	0.303447	0.327347	0.050087	0.315176	PLXNB1	5364	plexin B1
ENSG0000	7.28399	0.199332	0.329247	0.050098	0.315176	SETD9	133383	SET domain containing 9
ENSG0000	555.0697	0.249147	0.166292	0.050115	0.315176	LIPA	3988	lipase A lysosomal acid type
ENSG0000	5.092089	-0.11673	0.266534	0.05012	0.315176	NA	NA	NA
ENSG0000	5.310736	0.161338	0.298789	0.050109	0.315176	WFDC21P	645638	WAP four- pseudogene
ENSG0000	9373.694	0.27673	0.215601	0.05015	0.315255	ERGIC1	57222	endoplasmic reticulum-golgi intermediate compartment 1
ENSG0000	1883.043	-0.23609	0.152188	0.050167	0.315255	AP5Z1	9907	adaptor related protein complex 5 subunit zeta 1
ENSG0000	231.1698	-0.23404	0.14784	0.050284	0.315771	ARSB	411	arylsulfatase B
ENSG0000	9.901301	0.138711	0.278767	0.050269	0.315771	NA	NA	NA
ENSG0000	42.96015	0.244417	0.334396	0.050325	0.315916	LINC02631	1.15E+08	long intergenic non-protein coding RNA 2631
ENSG0000	92.70765	0.29293	0.258576	0.050388	0.31621	BAG4	9530	BAG cochaperone 4
ENSG0000	2829.551	-0.26114	0.182239	0.050478	0.316555	SH3BP5L	80851	SH3 binding domain protein 5 like
ENSG0000	3.754034	-0.07422	0.247332	0.050467	0.316555	NA	NA	NA
ENSG0000	20.93807	-0.28135	0.346713	0.050519	0.316598	IGFBP3	3486	insulin like growth factor binding protein 3
ENSG0000	3.140634	-0.09633	0.257112	0.050517	0.316598	SOCS5P4	1E+08	suppressor of cytokine signaling 5 pseudogene 4
ENSG0000	3.80398	0.071749	0.245608	0.050604	0.317022	LEFTY2	7044	left-right determination factor 2
ENSG0000	72.53511	0.294989	0.283747	0.050689	0.317441	SYNJ2BP	55333	synaptojanin 2 binding protein
ENSG0000	93.58782	0.277907	0.207136	0.050775	0.317507	FANCD2	2177	FA complementation group D2
ENSG0000	133.2453	-0.27744	0.213279	0.050724	0.317507	NA	NA	NA
ENSG0000	88166.6	-0.25679	0.178741	0.050749	0.317507	HLA-E	3133	major hist class I E
ENSG0000	92.39918	-0.29095	0.302069	0.050754	0.317507	MRC2	9902	mannose receptor C type 2
ENSG0000	42.69916	0.297254	0.252098	0.050786	0.317507	TIMM21	29090	translocase of inner mitochondrial membrane 21
ENSG0000	5.193893	-0.1766	0.311139	0.050806	0.317523	PDZD2	23037	PDZ domain containing 2
ENSG0000	3.600783	0.092447	0.254559	0.050991	0.318574	NA	NA	NA
ENSG0000	4.653786	-0.14313	0.284779	0.051061	0.318731	DDX59-AS	1.02E+08	DDX59 antisense RNA 1
ENSG0000	48.87353	0.293925	0.289058	0.051069	0.318731	ARHGAP1(	79658	Rho GTPase activating protein 10
ENSG0000	816.6254	-0.22384	0.136471	0.051039	0.318731	SPG11	80208	SPG11 ves spatacsin
ENSG0000	131.2818	-0.28567	0.238409	0.051114	0.318905	NA	NA	NA
ENSG0000	31.1898	0.302986	0.264958	0.0512	0.319332	NA	NA	NA
ENSG0000	4.126776	-0.09299	0.254902	0.051235	0.31944	KIF26B	55083	kinesin family member 26B
ENSG0000	1572.063	-0.26617	0.194189	0.05127	0.319555	CARM1	10498	coactivator associated arginine methyltransferase 1
ENSG0000	7.065773	-0.16054	0.297486	0.051322	0.319765	NA	NA	NA
ENSG0000	5.16282	0.068127	0.245418	0.051354	0.319859	NA	NA	NA
ENSG0000	5.581634	0.09003	0.25285	0.051385	0.319942	LHX4	89884	LIM homeobox 4
ENSG0000	6665.668	-0.28546	0.241954	0.051418	0.320041	RNF24	11237	ring finger protein 24
ENSG0000	3.580016	-0.10158	0.260262	0.051475	0.320287	ZNF114	163071	zinc finger protein 114
ENSG0000	26.27564	0.251777	0.347956	0.051561	0.320599	LRRN3	54674	leucine rich repeat neuronal 3
ENSG0000	4.752088	0.095322	0.256431	0.051544	0.320599	TLE6	79816	TLE family subcortical maternal complex member
ENSG0000	35.86405	0.296812	0.313062	0.051716	0.321458	FBN2	2201	fibrillin 2
ENSG0000	18.22821	0.214364	0.331705	0.051825	0.321958	HTRA3	94031	HtrA serine peptidase 3
ENSG0000	2554.024	-0.27408	0.211264	0.051832	0.321958	VPS9D1	9605	VPS9 domain containing 1
ENSG0000	8.715465	-0.18267	0.316478	0.051854	0.321963	LOC10012	1E+08	uncharacterized LOC100129066
ENSG0000	218.4249	0.249058	0.170172	0.051868	0.321963	TRUB2	26995	TruB pseudouridine synthase family member 2
ENSG0000	70.15421	-0.29132	0.248432	0.05193	0.322242	BNIPL	149428	BCL2 interacting protein like
ENSG0000	494.4464	0.269752	0.19313	0.051981	0.322341	FOXO3	2309	forkhead box O3
ENSG0000	2.521898	0.074668	0.248517	0.051966	0.322341	NA	NA	NA
ENSG0000	28.14293	0.153017	0.284457	0.052025	0.322503	TAF9B	51616	TATA-box binding protein associated factor 9b
ENSG0000	927.9058	0.25858	0.180879	0.052069	0.322669	SHMT2	6472	serine hydroxymethyltransferase 2
ENSG0000	2430.73	0.274763	0.218486	0.052155	0.322872	TBXAS1	6916	thromboxane A synthase 1
ENSG0000	5.09242	0.136287	0.278918	0.052138	0.322872	UBAC2-AS	1E+08	UBAC2 antisense RNA 1
ENSG0000	25121.88	-0.28253	0.26482	0.052131	0.322872	MBOAT7	79143	membrane bound O-acyltransferase domain containing 7
ENSG0000	18.86975	0.304945	0.320106	0.052232	0.323129	NA	NA	NA
ENSG0000	2.333442	0.116234	0.269735	0.052217	0.323129	NA	NA	NA
ENSG0000	1160.853	0.246372	0.315404	0.052276	0.32329	IGHV5-51	28388	immunoglobulin heavy variable 5-51
ENSG0000	7.013093	0.116055	0.270048	0.052338	0.323566	OLFM1	10439	olfactomedin 1
ENSG0000	3.009463	0.130495	0.277365	0.052386	0.323752	CCDC144B	284047	coiled-coil pseudogene
ENSG0000	62.81363	0.294407	0.267323	0.052415	0.323756	ACO1	48	aconitase 1
ENSG0000	323.2035	-0.28557	0.259173	0.052422	0.323756	SPECC1	92521	sperm antigen with calponin homology and coiled-coil domains 1
ENSG0000	33.92511	-0.28943	0.312374	0.052442	0.323773	LINC0134	731275	long intergenic non-protein coding RNA 1347
ENSG0000	1615.799	-0.24707	0.167624	0.052464	0.323798	TMEM131	23505	transmembrane protein 131
ENSG0000	9.05402	0.164828	0.297064	0.05258	0.324406	NA	NA	NA
ENSG0000	17.80076	-0.27087	0.349889	0.052632	0.324616	NA	NA	NA
ENSG0000	12.01724	0.252845	0.354633	0.052835	0.325753	MUTYH	4595	mutY DNA glycosylase
ENSG0000	255.7902	-0.26095	0.313892	0.052852	0.325753	SPTB	6710	spectrin b erythrocytic
ENSG0000	26.23466	-0.29735	0.314621	0.052911	0.32601	PDLIM1P4	1E+08	PDZ and LIM domain 1 pseudogene 4
ENSG0000	74.12804	-0.2923	0.282725	0.052958	0.326171	PTPRN	5798	protein tyrosine phosphatase receptor type N
ENSG0000	18.04006	0.286574	0.343145	0.052991	0.326171	RNF217	154214	ring finger protein 217

ENSG0000	2.963048	0.105754	0.262782	0.052988	0.326171	NR5A1	2516	nuclear receptor subfamily 5 group A member 1	
ENSG0000	45.42657	-0.28581	0.309961	0.053012	0.326195	NA	NA	NA	
ENSG0000	24.75701	0.270253	0.339038	0.053051	0.32632	TVP23C	201158	trans-golgi network vesicle protein 23 homolog C	
ENSG0000	3.032506	0.110563	0.263941	0.05317	0.326917	NA	NA	NA	
ENSG0000	379.6212	-0.21426	0.132466	0.053183	0.326917	CEACAM2	90273	CEA cell adhesion molecule 21	
ENSG0000	83.11717	0.285287	0.233283	0.053208	0.326961	PAQR7	164091	progestin and adipoQ receptor family member 7	
ENSG0000	26.56155	0.282538	0.331363	0.05333	0.327487	ACVR2A	92	activin A receptor type 2A	
ENSG0000	23.69779	0.287416	0.327506	0.053315	0.327487	NA	NA	NA	
ENSG0000	88.10973	-0.2784	0.216535	0.053412	0.32777	SHISA4	149345	shisa family member 4	
ENSG0000	432.625	0.234149	0.152568	0.053399	0.32777	KCTD20	222658	potassium channel tetramerization domain containing 20	
ENSG0000	15.02438	-0.28122	0.351885	0.053439	0.32783	DIABLO	56616	diablo IAP-binding mitochondrial protein	
ENSG0000	7199.243	0.242616	0.163259	0.053489	0.328026	PTMA	5757	prothymosin alpha	
ENSG0000	20.05581	0.276381	0.343727	0.053542	0.328243	C6orf163	206412	chromosome 6 open reading frame 163	
ENSG0000	15.83988	-0.27153	0.363509	0.053563	0.328258	NA	NA	NA	
ENSG0000	87.93607	0.28263	0.225914	0.053591	0.328318	TMEM63C	57156	transmembrane protein 63C	
ENSG0000	2.56518	0.068738	0.244833	0.053654	0.328515	TUBB2B	347733	tubulin beta 2B class IIb	
ENSG0000	6.511227	0.135588	0.277156	0.053659	0.328515	NA	NA	NA	
ENSG0000	240.1862	0.279513	0.230423	0.053691	0.328525	ERLEC1	27248	endoplasmic reticulum lectin 1	
ENSG0000	351.8654	-0.24364	0.164145	0.053696	0.328525	IL10RB	3588	interleukin 10 receptor subunit beta	
ENSG0000	26.6072	0.302836	0.300659	0.053716	0.328535	NA	NA	NA	
ENSG0000	153.6561	0.241485	0.160472	0.05378	0.328822	ATP9B	374868	ATPase phospholipid transporting 9B (putative)	
ENSG0000	689.299	-0.21902	0.138002	0.053932	0.329529	LSM10	84967	LSM10	U7 small nuclear RNA associated
ENSG0000	11.56469	0.273236	0.35843	0.053921	0.329529	PRCD	768206	photoreceptor disc component	
ENSG0000	3329.401	-0.28009	0.253911	0.053973	0.329668	TOM1	10043	target of myb1 membrane trafficking protein	
ENSG0000	528.701	0.281117	0.245007	0.054052	0.330042	MAGED1	9500	MAGE family member D1	
ENSG0000	24.77541	-0.11177	0.260793	0.054078	0.330091	SLED1	643036	proteoglyc	pro eosinophil major basic protein 2 pseudogene
ENSG0000	50.89539	0.29258	0.265504	0.054102	0.330128	BRCC3	79184	BRCA1/BRCA2-containing complex subunit 3	
ENSG0000	7.818826	0.179076	0.308656	0.054198	0.330604	EGR3	1960	early growth response 3	
ENSG0000	45.80461	0.277278	0.21048	0.054344	0.33105	MATN1-AS	1E+08	MATN1 antisense RNA 1	
ENSG0000	4.127435	0.096482	0.256401	0.054314	0.33105	NA	NA	NA	
ENSG0000	316.0371	-0.27663	0.225023	0.054295	0.33105	HAPLN3	145864	hyaluronan and proteoglycan link protein 3	
ENSG0000	10.11238	-0.24239	0.351507	0.054329	0.33105	PRR29	92340	proline rich 29	
ENSG0000	3065.471	-0.24918	0.172385	0.054425	0.331433	NCSTN	23385	nicastrin	
ENSG0000	7160.357	-0.27732	0.237112	0.054456	0.331514	OSCAR	126014	osteoclast associated Ig-like receptor	
ENSG0000	942.0352	0.257075	0.183777	0.054518	0.33178	SKI	6497	SKI proto-oncogene	
ENSG0000	4.493741	-0.14083	0.283634	0.054546	0.33184	NA	NA	NA	
ENSG0000	5512.335	-0.24769	0.172893	0.054643	0.332323	S100A6	6277	S100 calcium binding protein A6	
ENSG0000	2.815646	0.153638	0.295394	0.054688	0.332483	NA	NA	NA	
ENSG0000	2.31585	0.092418	0.255571	0.054724	0.332593	NA	NA	NA	
ENSG0000	114.5133	0.251037	0.319218	0.054807	0.33266	KCNN3	3782	potassium calcium-activated channel subfamily N member 3	
ENSG0000	552.5408	-0.21317	0.133268	0.054808	0.33266	TNIP2	79155	TNFAIP3 interacting protein 2	
ENSG0000	75.34081	-0.26652	0.196637	0.054794	0.33266	TMEM60	85025	transmembrane protein 60	
ENSG0000	90.68014	0.279296	0.2224	0.054795	0.33266	REEP6	92840	receptor accessory protein 6	
ENSG0000	2.803524	-0.08385	0.250675	0.054829	0.332679	NA	NA	NA	
ENSG0000	1731.052	-0.24948	0.179081	0.054847	0.33268	DLGAP4	22839	DLG associated protein 4	
ENSG0000	2.807037	0.071358	0.247139	0.054867	0.332686	NA	NA	NA	
ENSG0000	25694.98	-0.24096	0.163963	0.055031	0.332911	EFHD2	79180	EF-hand domain family member D2	
ENSG0000	1443.521	-0.2624	0.313484	0.055015	0.332911	C3orf86P	1.03E+08	long intergenic non-protein coding RNA 694	
ENSG0000	60.40603	-0.27357	0.209492	0.055024	0.332911	TNFRSF10I	8793	TNF receptor superfamily member 10d	
ENSG0000	21.80688	0.192471	0.311517	0.054991	0.332911	MRPL13	28998	mitochondrial ribosomal protein L13	
ENSG0000	1152.856	0.233961	0.154577	0.055019	0.332911	MYC	4609	MYC protc	bHLH transcription factor
ENSG0000	1992.811	0.235041	0.156835	0.055008	0.332911	POLR2E	5434	RNA polyn	I and III subunit E
ENSG0000	302.0795	0.244935	0.167686	0.05499	0.332911	mei-01	150365	meiotic double-stranded break formation protein 1	
ENSG0000	49.39608	0.290341	0.282124	0.055095	0.333101	CCDC14	64770	coiled-coil domain containing 14	
ENSG0000	1290.23	0.247906	0.172706	0.055117	0.333101	RASSF4	83937	Ras association domain family member 4	
ENSG0000	17550.69	-0.27797	0.240119	0.055114	0.333101	CST7	8530	cystatin F	
ENSG0000	4.139438	0.085887	0.251424	0.055181	0.333313	OXTR	5021	oxytocin receptor	
ENSG0000	11.759	0.202154	0.332315	0.055188	0.333313	ZFY	7544	zinc finger protein Y-linked	
ENSG0000	12.689	0.208189	0.32561	0.055247	0.33334	RAD50	10111	RAD50 double strand break repair protein	
ENSG0000	182.0675	0.282533	0.290764	0.055228	0.33334	FAM20C	56975	FAM20C golgi associated secretory pathway kinase	
ENSG0000	612.02	-0.2388	0.160712	0.055246	0.33334	UNK	85451	unk zinc finger	
ENSG0000	4.060772	-0.13675	0.279706	0.055362	0.333566	FAM72D	728833	family with sequence similarity 72 member D	
ENSG0000	5097.229	-0.22581	0.150068	0.05531	0.333566	RASSF5	83593	Ras association domain family member 5	
ENSG0000	94.85257	-0.2579	0.180072	0.055394	0.333566	FAM241A	132720	family with sequence similarity 241 member A	
ENSG0000	11.44705	-0.25806	0.353987	0.055379	0.333566	NA	NA	NA	
ENSG0000	526.4785	0.234228	0.154942	0.055375	0.333566	MRPS34	65993	mitochondrial ribosomal protein S34	
ENSG0000	51.34775	0.285497	0.235424	0.055347	0.333566	TRIP10	9322	thyroid hormone receptor interactor 10	
ENSG0000	1506.14	0.276034	0.245963	0.055427	0.333654	IGKV3-11	28914	immunoglobulin kappa variable 3-11	
ENSG0000	936.9946	-0.25104	0.177666	0.055526	0.333752	YIPF1	54432	Yip1 domain family member 1	
ENSG0000	158.6645	-0.23831	0.159076	0.055534	0.333752	ACP6	51205	acid phosp	lysophosphatidic
ENSG0000	27.16588	0.272453	0.328955	0.05547	0.333752	NA	NA	NA	
ENSG0000	96.95009	0.258448	0.184772	0.055498	0.333752	ULK2	9706	unc-51 like autophagy activating kinase 2	
ENSG0000	613.8848	0.214715	0.134726	0.05551	0.333752	CCDC57	284001	coiled-coil domain containing 57	
ENSG0000	70.67056	0.280134	0.229386	0.055662	0.334191	DUSP28	285193	dual specificity phosphatase 28	
ENSG0000	312.0498	0.223536	0.141011	0.055648	0.334191	NOA1	84273	nitric oxide associated 1	
ENSG0000	336.2189	0.211908	0.132613	0.055658	0.334191	TUBGCP3	10426	tubulin gamma complex associated protein 3	
ENSG0000	399.6669	-0.26828	0.206909	0.05574	0.334441	RNF169	254225	ring finger protein 169	
ENSG0000	28.06605	0.285406	0.319408	0.055736	0.334441	NA	NA	NA	
ENSG0000	9.696499	0.139872	0.27835	0.055821	0.334735	APRG1	339883	APRG1 tumor suppressor candidate	
ENSG0000	93.68156	0.280722	0.234576	0.055826	0.334735	BRICD5	283870	BRICHOS domain containing 5	
ENSG0000	2.917979	0.073612	0.246493	0.056088	0.336195	LINC0204	1.03E+08	long intergenic non-protein coding RNA 2043	
ENSG0000	56.09331	0.285961	0.244892	0.056121	0.336283	N4BP3	23138	NEDD4 binding protein 3	
ENSG0000	484.2906	0.150933	0.285324	0.05616	0.33641	NA	NA	NA	
ENSG0000	96.60606	0.27751	0.295765	0.056195	0.33651	NUGGC	389643	nuclear G1	germinal center associated
ENSG0000	251.2634	0.238734	0.16086	0.056273	0.336867	GPATCH2L	55668	G-patch domain containing 2 like	
ENSG0000	4.354266	0.12031	0.269179	0.056364	0.337079	IMPG2	50939	interphotoreceptor matrix proteoglycan 2	
ENSG0000	2.763115	0.08757	0.252169	0.05633	0.337079	NA	NA	NA	
ENSG0000	7.643517	-0.17487	0.306491	0.05636	0.337079	NA	NA	NA	
ENSG0000	9145.244	-0.26765	0.233388	0.056409	0.337238	GRAMD1A	57655	GRAM domain containing 1A	
ENSG0000	474.2278	-0.26761	0.248827	0.056434	0.337276	FAM111A	63901	FAM111 trypsin like peptidase A	
ENSG0000	4550.632	-0.25646	0.192239	0.056456	0.337302	PHF21A	51317	PHD finger protein 21A	
ENSG0000	8.046108	-0.13779	0.277576	0.056495	0.337421	NA	NA	NA	
ENSG0000	55.45172	0.286588	0.264945	0.056591	0.337888	COQ7	10229	coenzyme	hydroxylase
ENSG0000	56.53656	0.275417	0.214983	0.056657	0.338109	LRCH1	23143	leucine rich repeats and calponin homology domain containing 1	
ENSG0000	3.465038	0.113992	0.266011	0.056665	0.338109	DLL4	54567	delta like canonical Notch ligand 4	
ENSG0000	6.809776	-0.19104	0.319021	0.056724	0.338269	AFAP1-AS	84740	AFAP1 antisense RNA 1	

ENSG0000	20.84745	0.297667	0.302015	0.056729	0.338269	ZNF135	7694	zinc finger protein 135
ENSG0000	232.5862	-0.25302	0.181053	0.056774	0.338339	SENP2	59343	SUMO specific peptidase 2
ENSG0000	220.9408	0.214856	0.135693	0.056778	0.338339	INTS10	55174	integrator complex subunit 10
ENSG0000	1875.879	-0.26788	0.215255	0.056932	0.339146	FGL2	10875	fibrinogen like 2
ENSG0000	202.4173	-0.22231	0.142864	0.056997	0.339319	ZNF622	90441	zinc finger protein 622
ENSG0000	208.9863	-0.26954	0.203795	0.056998	0.339319	PRKY	5616	protein kinase Y-linked (pseudogene)
ENSG0000	3.100143	0.114469	0.26713	0.057091	0.339499	NA	NA	NA
ENSG0000	651.1439	0.199382	0.12197	0.057071	0.339499	ANAPC16	119504	anaphase promoting complex subunit 16
ENSG0000	60.22368	0.281762	0.286547	0.057071	0.339499	KIF11	3832	kinesin family member 11
ENSG0000	235.0615	0.2187	0.139467	0.057102	0.339499	OSGEP	55644	O-sialoglycoprotein endopeptidase
ENSG0000	90.33576	0.274047	0.223665	0.057129	0.339548	RBM4	5936	RNA binding motif protein 4
ENSG0000	57.89496	0.087386	0.250217	0.057225	0.339901	HPGD	3248	15-hydroxyprostaglandin dehydrogenase
ENSG0000	33.07001	0.29036	0.260709	0.057222	0.339901	THAP7-AS	439931	THAP7 antisense RNA 1
ENSG0000	2458.825	-0.18267	0.108675	0.05727	0.340057	NXF1	10482	nuclear RNA export factor 1
ENSG0000	82.59329	-0.28223	0.280228	0.057354	0.340441	SLC6A9	6536	solute carrier family 6 member 9
ENSG0000	17.39764	-0.25842	0.338727	0.057583	0.341561	PDGFA	5154	platelet derived growth factor subunit A
ENSG0000	16.2751	0.266712	0.34255	0.057598	0.341561	DUSP4	1846	dual specificity phosphatase 4
ENSG0000	1891.558	-0.23937	0.165221	0.057594	0.341561	ABHD4	63874	abhydrola N-acyl phospholipase B
ENSG0000	23.51567	0.259173	0.332627	0.057629	0.341587	GORAB	92344	golgin RAB6 interacting
ENSG0000	26.42647	0.233718	0.327122	0.05764	0.341587	POLR2J4	84820	RNA polyn pseudogene
ENSG0000	10.05785	-0.15806	0.291277	0.057751	0.342133	CHKB-CPT	386593	CHKB-CPT1B readthrough (NMD candidate)
ENSG0000	66.70153	0.285332	0.278588	0.057776	0.342171	SIGLEC11	114132	sialic acid binding Ig like lectin 11
ENSG0000	5053.143	-0.25811	0.195465	0.057926	0.34295	RUNX3	864	RUNX family transcription factor 3
ENSG0000	15.07299	0.210954	0.322764	0.057987	0.34295	USP13	8975	ubiquitin specific peptidase 13
ENSG0000	143.9723	-0.22497	0.14683	0.057954	0.34295	RMDN3	55177	regulator of microtubule dynamics 3
ENSG0000	6.541826	-0.11621	0.26506	0.057982	0.34295	RNF43	54894	ring finger protein 43
ENSG0000	53.7346	-0.27485	0.219055	0.058001	0.34295	APOBEC3H	164668	apolipoprotein B mRNA editing enzyme catalytic subunit 3H
ENSG0000	582.6836	0.274273	0.208721	0.058031	0.343019	CERS4	79603	ceramide synthase 4
ENSG0000	401.6845	-0.27115	0.225665	0.058093	0.343273	NA	NA	NA
ENSG0000	14.89491	-0.24461	0.338872	0.058127	0.343362	LINC02175	283887	long intergenic non-protein coding RNA 2175
ENSG0000	5.539953	0.123916	0.270849	0.058203	0.343702	SCGB2B2	284402	secretoglobin family 2B member 2
ENSG0000	2.807352	-0.08448	0.250744	0.058264	0.343848	NA	NA	NA
ENSG0000	17.85505	0.132031	0.273343	0.058265	0.343848	NA	NA	NA
ENSG0000	285.5856	0.274283	0.289759	0.058297	0.343927	CCDC88A	55704	coiled-coil domain containing 88A
ENSG0000	38.82424	0.276242	0.306812	0.058356	0.344162	MARS2	92935	methionyl mitochondrial
ENSG0000	761.9832	0.25051	0.181506	0.058382	0.344162	NPM1	4869	nucleophosmin 1
ENSG0000	172.2345	0.235731	0.159437	0.058394	0.344162	ZNF689	115509	zinc finger protein 689
ENSG0000	248.2952	0.229566	0.156831	0.058471	0.344335	RNF115	27246	ring finger protein 115
ENSG0000	368.2564	0.260623	0.203162	0.058451	0.344335	TKFC	26007	triokinase and FMN cyclase
ENSG0000	6.979978	0.072869	0.245237	0.058479	0.344335	ST8SIA5	29906	ST8 alpha- 8-sialyltransferase 5
ENSG0000	208.8462	0.209111	0.13381	0.058507	0.344386	TMEM203	94107	transmembrane protein 203
ENSG0000	459.0254	0.220972	0.14488	0.058607	0.344672	PANK4	55229	pantothenate kinase 4 (inactive)
ENSG0000	7.656756	0.175094	0.304099	0.058601	0.344672	HOXA10	3206	homeobox A10
ENSG0000	2.477989	0.065826	0.243785	0.058612	0.344672	NA	NA	NA
ENSG0000	23.24954	0.293611	0.276387	0.058663	0.34486	NRAV	1.01E+08	negative regulator of antiviral response
ENSG0000	51.34395	-0.28094	0.258318	0.058813	0.345635	CHRNB1	1140	cholinergic receptor nicotinic beta 1 subunit
ENSG0000	67.90187	0.267814	0.207281	0.058874	0.34588	EPN2	22905	epsin 2
ENSG0000	176.0881	0.246384	0.173882	0.058976	0.346369	MRTO4	51154	MRT4 hom ribosome maturation factor
ENSG0000	29.67572	0.288765	0.278445	0.059052	0.346505	FAHD2B	151313	fumarylacetoacetate hydrolase domain containing 2B
ENSG0000	12.37551	0.144057	0.27971	0.059073	0.346505	EPS8	2059	epidermal growth factor receptor pathway substrate 8
ENSG0000	22.89281	-0.27307	0.327137	0.059075	0.346505	TRAV12-3	28672	T cell receptor alpha variable 12-3
ENSG0000	359.2418	0.201967	0.124299	0.059055	0.346505	DYM	54808	dymeclin
ENSG0000	8.237675	0.1689	0.299755	0.059153	0.346646	NA	NA	NA
ENSG0000	1351.444	-0.22599	0.150397	0.059181	0.346646	PTPN7	5778	protein tyrosine phosphatase non-receptor type 7
ENSG0000	2.787258	-0.0649	0.245404	0.059144	0.346646	PAX3	5077	paired box 3
ENSG0000	387.6	0.265769	0.21403	0.059187	0.346646	AP1G2-AS	1.03E+08	AP1G2 antisense RNA 1
ENSG0000	107.4266	-0.26339	0.301338	0.059194	0.346646	TRAPPC5	126003	trafficking protein particle complex subunit 5
ENSG0000	25.85211	0.287626	0.251017	0.059217	0.346672	MIPEP	4285	mitochondrial intermediate peptidase
ENSG0000	9098.655	-0.23529	0.185247	0.05932	0.347162	FCGR2A	2212	Fc gamma receptor IIa
ENSG0000	676.5904	-0.21466	0.138544	0.059418	0.347404	LMO2	4005	LIM domain only 2
ENSG0000	603.5748	-0.19783	0.123225	0.059401	0.347404	TEP1	7011	telomerase associated protein 1
ENSG0000	37.3683	-0.28547	0.266794	0.059382	0.347404	STON2	85439	stonin 2
ENSG0000	3242.927	-0.2176	0.140332	0.059582	0.348144	NRBP1	29959	nuclear receptor binding protein 1
ENSG0000	31.23715	-0.28606	0.285113	0.059581	0.348144	NA	NA	NA
ENSG0000	3087.932	-0.22324	0.15249	0.059608	0.348184	TPD52L2	7165	TPD52 like 2
ENSG0000	5.632406	0.116658	0.26727	0.059647	0.348188	EML6	400954	EMAP like 6
ENSG0000	18.13338	0.240907	0.333774	0.059641	0.348188	HS3ST1	9957	heparan sulfate-glucosamine 3-sulfotransferase 1
ENSG0000	146.577	0.272253	0.268964	0.059667	0.348192	NIPSNAP2	2631	nipsnap homolog 2
ENSG0000	3890.279	-0.23421	0.163622	0.059693	0.348234	UBE2D3	7323	ubiquitin conjugating enzyme E2 D3
ENSG0000	223.0706	0.274764	0.247152	0.059743	0.348302	MAML3	55534	mastermind like transcriptional coactivator 3
ENSG0000	552.0687	0.237874	0.16411	0.059735	0.348302	HNRNPA0	10949	heterogeneous nuclear ribonucleoprotein A0
ENSG0000	405.9045	0.248744	0.175221	0.059834	0.34865	NA	NA	NA
ENSG0000	12.86774	0.254103	0.341006	0.05984	0.34865	NA	NA	NA
ENSG0000	3958.504	-0.27043	0.253102	0.059927	0.348868	S100A12	6283	S100 calcium binding protein A12
ENSG0000	157.4384	-0.26836	0.218299	0.059964	0.348868	TSPAN5	10098	tetraspanin 5
ENSG0000	851.0934	-0.26511	0.221257	0.059943	0.348868	KLRD1	3824	killer cell lectin like receptor D1
ENSG0000	466.9272	0.230938	0.15631	0.059973	0.348868	EIF2B1	1967	eukaryotic translation initiation factor 2B subunit alpha
ENSG0000	4458.834	-0.12636	0.268776	0.059961	0.348868	CD177	57126	CD177 molecule
ENSG0000	397.637	0.271598	0.261139	0.060116	0.349589	RNF144B	255488	ring finger protein 144B
ENSG0000	3.333487	0.095659	0.256933	0.060142	0.349628	NA	NA	NA
ENSG0000	108.8	0.264286	0.207259	0.060168	0.349667	IKZF4	64375	IKAROS family zinc finger 4
ENSG0000	2687.554	-0.25143	0.18669	0.060239	0.349925	MOB1A	55233	MOB kinase activator 1A
ENSG0000	28.42439	0.183703	0.302394	0.06025	0.349925	IGKV1-33	28933	immunoglobulin kappa variable 1-33
ENSG0000	1661.352	0.269014	0.231041	0.060278	0.349978	CD93	22918	CD93 molecule
ENSG0000	282.6404	0.254361	0.190548	0.060354	0.35024	SEC24A	10802	SEC24 hon COPII coat complex component
ENSG0000	9.307593	-0.15512	0.289036	0.060362	0.35024	NA	NA	NA
ENSG0000	6.998224	0.127939	0.271413	0.060465	0.35073	SPRED1	161742	sprouty related EVH1 domain containing 1
ENSG0000	637.3267	0.238795	0.167138	0.060591	0.351166	KPNA1	3836	karyopherin subunit alpha 1
ENSG0000	10.3766	0.141855	0.27824	0.060598	0.351166	NA	NA	NA
ENSG0000	389.8739	0.231073	0.157876	0.060595	0.351166	NDUFV3	4731	NADH:ubiquinone oxidoreductase subunit V3
ENSG0000	202.4763	0.227563	0.151865	0.060659	0.35141	TRIM3	10612	tripartite motif containing 3
ENSG0000	5.147935	0.09319	0.253093	0.060747	0.351805	LINC01664	1.01E+08	long intergenic non-protein coding RNA 1664
ENSG0000	10.32048	-0.18461	0.307394	0.060786	0.351812	TSGA10	80705	testis specific 10
ENSG0000	30.91237	0.279952	0.307044	0.060783	0.351812	HERC2P3	283755	HERC2 pseudogene 3
ENSG0000	24.40807	-0.2505	0.325291	0.060927	0.352404	NA	NA	NA
ENSG0000	7.965998	-0.08564	0.250486	0.060926	0.352404	SCUBE1	80274	signal pep CUB domain and EGF like domain containing 1

ENSG0000	128.2549	0.279864	0.259294	0.060984	0.352523	EIF2AK3	9451	eukaryotic translation initiation factor 2 alpha kinase 3
ENSG0000	54.56714	0.266979	0.209667	0.060986	0.352523	COPS9	150678	COP9 signalosome subunit 9
ENSG0000	44.44581	0.232058	0.329252	0.061165	0.353442	LCN8	138307	lipocalin 8
ENSG0000	422.3337	0.217009	0.142412	0.061265	0.353912	MYO5A	4644	myosin VA
ENSG0000	2.576487	-0.07149	0.246237	0.061366	0.354382	NA	NA	NA
ENSG0000	3.832673	0.11339	0.267177	0.061491	0.354991	FOXO6	1E+08	forkhead box O6
ENSG0000	8.370701	0.147936	0.284817	0.061682	0.355869	HOOK1	51361	hook microtubule tethering protein 1
ENSG0000	49.0564	0.278493	0.268862	0.06168	0.355869	LIMA1	51474	LIM domain and actin binding 1
ENSG0000	5.647719	0.144692	0.283553	0.061825	0.356024	NA	NA	NA
ENSG0000	11.95089	0.216776	0.326901	0.061734	0.356024	SOWAHC	65124	sosondowah ankyrin repeat domain family member C
ENSG0000	2884.441	-0.23542	0.165709	0.061809	0.356024	SLC35A4	113829	solute carrier family 35 member A4
ENSG0000	280.3372	0.214321	0.138638	0.061797	0.356024	R3HCC1	203069	R3H domain and coiled-coil containing 1
ENSG0000	3212.914	0.256828	0.201677	0.061798	0.356024	HSP90B1	7184	heat shock protein 90 beta family member 1
ENSG0000	42.89087	0.277044	0.240732	0.061804	0.356024	RPL10P16	284393	ribosomal protein L10 pseudogene 16
ENSG0000	151.871	-0.24656	0.310822	0.061974	0.356767	SPATC1	375686	spermatogenesis and centriole associated 1
ENSG0000	4.122902	0.131227	0.276894	0.06211	0.357438	LIPT2-AS1	1E+08	LIPT2 antisense RNA 1
ENSG0000	58.15939	0.263189	0.300271	0.062143	0.357518	RPGR	6103	retinitis pigmentosa GTPase regulator
ENSG0000	8.176876	-0.1416	0.278522	0.062189	0.357668	NA	NA	NA
ENSG0000	80.1259	0.259288	0.200147	0.062212	0.357688	NCBP2AS2	152217	NCBP2 antisense 2 (head to head)
ENSG0000	121.0545	-0.26595	0.220543	0.06226	0.357852	NA	NA	NA
ENSG0000	958.1607	-0.26498	0.230684	0.062324	0.357998	NFIL3	4783	nuclear fac interleukin 3 regulated
ENSG0000	9.38302	0.104187	0.257527	0.062317	0.357998	NETO1	81832	neuropilin and tolloid like 1
ENSG0000	18.91572	0.282599	0.309271	0.062382	0.358215	GPR89A	653519	G protein-coupled receptor 89A
ENSG0000	56.8901	-0.27349	0.275607	0.062464	0.358368	CMC1	152100	C-X9-C motif containing 1
ENSG0000	5.408242	0.160994	0.294778	0.062445	0.358368	EHHADH	1962	enoyl-CoA hydratase and 3-hydroxyacyl CoA dehydrogenase
ENSG0000	12188.95	-0.23069	0.173349	0.062467	0.358368	ZFP36L1	677	ZFP36 ring finger protein like 1
ENSG0000	2.416513	-0.09656	0.256779	0.062491	0.358392	NA	NA	NA
ENSG0000	20.34105	0.252195	0.34519	0.062532	0.358516	LOC10537	1.05E+08	uncharacterized LOC105377225
ENSG0000	3008.184	0.26699	0.245134	0.062564	0.358545	INKA2	55924	inka box actin regulator 2
ENSG0000	34.93736	0.279912	0.258915	0.062599	0.358545	BBS9	27241	Bardet-Biedl syndrome 9
ENSG0000	261.5981	-0.21293	0.139437	0.062577	0.358545	TTC9C	283237	tetratricopeptide repeat domain 9C
ENSG0000	49.73544	0.267006	0.215686	0.062615	0.358545	NKRF	55922	NFKB repressing factor
ENSG0000	58.89841	-0.27268	0.287027	0.062669	0.35874	NA	NA	NA
ENSG0000	587.6096	-0.26553	0.264974	0.062758	0.359136	ZNF683	257101	zinc finger protein 683
ENSG0000	62.69586	0.274249	0.252883	0.062797	0.359136	AFMID	125061	arylformamidase
ENSG0000	2410.059	-0.24769	0.18655	0.062794	0.359136	CDC34	997	cell divisio ubiqiutin conjugating enzyme
ENSG0000	98.90562	0.26724	0.218236	0.062941	0.359846	BEGAIN	57596	brain enriched guanylate kinase associated
ENSG0000	69.99611	-0.26955	0.228675	0.062998	0.359948	AP1M2	10053	adaptor related protein complex 1 subunit mu 2
ENSG0000	365.6671	0.251307	0.19874	0.062998	0.359948	TXNRD2	10587	thioredoxin reductase 2
ENSG0000	12.03371	-0.24255	0.332861	0.063058	0.360179	ZNF620	253639	zinc finger protein 620
ENSG0000	143.2835	-0.26009	0.208787	0.063117	0.360403	NUDT16L2	152195	nudix hydr pseudogene
ENSG0000	1128.604	-0.26451	0.277176	0.063204	0.360788	CDK5RAP2	55755	CDK5 regulatory subunit associated protein 2
ENSG0000	6.883016	0.173114	0.301465	0.06325	0.360859	ARC	23237	activity regulated cytoskeleton associated protein
ENSG0000	216.0277	0.263474	0.222115	0.063256	0.360859	SPATC1L	84221	spermatogenesis and centriole associated 1 like
ENSG0000	70.80463	0.26093	0.206627	0.063311	0.361062	LOC72999	729998	eukaryotic translation elongation factor 1 gamma pseudogene
ENSG0000	16.12025	0.174246	0.296689	0.063332	0.361071	RPL13P5	283345	ribosomal protein L13 pseudogene 5
ENSG0000	17.72224	0.237118	0.327528	0.063393	0.361302	LOC72813	728138	KIAA2013 pseudogene
ENSG0000	582.0519	0.211524	0.138372	0.063457	0.361331	FAM229A	1E+08	family with sequence similarity 229 member A
ENSG0000	460.034	-0.24774	0.186275	0.063443	0.361331	EVA1B	55194	eva-1 homolog B
ENSG0000	6.807596	0.152823	0.286642	0.063438	0.361331	MTFP1	51537	mitochondrial fission process 1
ENSG0000	92.08626	-0.2702	0.244757	0.0635	0.361464	SH3D21	79729	SH3 domain containing 21
ENSG0000	44.48998	0.243549	0.313288	0.063578	0.361798	PRPF39	55015	pre-mRNA processing factor 39
ENSG0000	22.88467	0.250298	0.3211	0.063611	0.361869	LINC02245	400958	long intergenic non-protein coding RNA 2245
ENSG0000	11.24894	-0.19619	0.313373	0.063657	0.362018	ADAM22	53616	ADAM metallopeptidase domain 22
ENSG0000	1031.016	0.194364	0.122009	0.063679	0.362033	YY1	7528	YY1 transcription factor
ENSG0000	51.55817	-0.27096	0.240786	0.063734	0.36209	SDE2	163859	SDE2 telomere maintenance homolog
ENSG0000	803.5207	0.194701	0.124035	0.063742	0.36209	GTF3C2	2976	general transcription factor IIIC subunit 2
ENSG0000	690.9101	-0.26421	0.244465	0.063748	0.36209	PTCRA	171558	pre T cell antigen receptor alpha
ENSG0000	588.548	-0.25018	0.198748	0.063783	0.362108	E2F2	1870	E2F transcription factor 2
ENSG0000	3.243968	0.072831	0.246026	0.063791	0.362108	STK33	65975	serine/threonine kinase 33
ENSG0000	41.11455	0.271646	0.223245	0.063894	0.362134	NA	NA	NA
ENSG0000	2.773923	0.099919	0.257803	0.063844	0.362134	SNORA33	594839	small nucl H/ACA box 33
ENSG0000	4.893922	-0.09257	0.254258	0.063871	0.362134	SPAG6	9576	sperm associated antigen 6
ENSG0000	5223.467	-0.23283	0.166325	0.063834	0.362134	RNF167	26001	ring finger protein 167
ENSG0000	182.8879	0.215065	0.141036	0.06389	0.362134	CHD6	84181	chromodomain helicase DNA binding protein 6
ENSG0000	19.39153	0.263276	0.321564	0.063917	0.362148	LSM5	23658	LSM5 hom U6 small nuclear RNA and mRNA degradation associated
ENSG0000	137.3769	-0.25305	0.293658	0.064042	0.362745	ARAP2	116984	ArfGAP wi ankyrin repeat and PH domain 2
ENSG0000	121.2531	-0.25373	0.19522	0.064063	0.362756	C2orf88	84281	chromosome 2 open reading frame 88
ENSG0000	56.9088	0.270782	0.286263	0.06412	0.362962	RPGRIP1	57096	RPGR interacting protein 1
ENSG0000	4548.928	-0.25771	0.216078	0.064188	0.363013	CD55	1604	CD55 molecule (Cromer blood group)
ENSG0000	309.4083	0.254339	0.196772	0.064168	0.363013	EME2	197342	essential meiotic structure-specific endonuclease subunit 2
ENSG0000	836.7832	-0.24574	0.184893	0.064174	0.363013	NPIPB5	1E+08	nuclear pore complex interacting protein family member B5
ENSG0000	126.7918	0.245612	0.180448	0.064231	0.36303	EEF2K	29904	eukaryotic elongation factor 2 kinase
ENSG0000	236.0041	-0.24942	0.200874	0.064227	0.36303	ZNF429	353088	zinc finger protein 429
ENSG0000	56.69362	0.270526	0.240934	0.064305	0.363335	FAM98B	283742	family with sequence similarity 98 member B
ENSG0000	1524.958	-0.25811	0.22128	0.064338	0.363414	CD5	921	CD5 molecule
ENSG0000	879.6192	-0.23944	0.176747	0.064456	0.363852	C7orf50	84310	chromosome 7 open reading frame 50
ENSG0000	104.7687	0.247077	0.183596	0.064454	0.363852	KCTD15	79047	potassium channel tetramerization domain containing 15
ENSG0000	869.2648	0.21661	0.144638	0.064539	0.363986	IER3	8870	immediate early response 3
ENSG0000	317.7104	0.207816	0.135014	0.0645	0.363986	UROS	7390	uroporphyrinogen III synthase
ENSG0000	71.62411	0.259056	0.204404	0.064537	0.363986	TMEM218	219854	transmembrane protein 218
ENSG0000	213.4341	0.235152	0.163645	0.064631	0.364391	ATXN10	25814	ataxin 10
ENSG0000	2153.267	-0.25943	0.269318	0.064725	0.364809	HLX	3142	H2.0 like homeobox
ENSG0000	188.7629	0.252676	0.196576	0.064814	0.365198	XYLT1	64131	xylosyltransferase 1
ENSG0000	33.82548	0.277124	0.268552	0.064916	0.365663	ACY3	91703	aminoacylase 3
ENSG0000	4.663199	0.117612	0.267184	0.064942	0.365696	NA	NA	NA
ENSG0000	51.0899	0.271417	0.249081	0.065013	0.365985	SMIM15	643155	small integral membrane protein 15
ENSG0000	349.9949	0.241937	0.176236	0.065075	0.36622	CD79B	974	CD79b molecule
ENSG0000	236.1499	-0.23791	0.175467	0.065156	0.366561	PTPRN2	5799	protein tyrosine phosphatase receptor type N2
ENSG0000	1356.792	-0.22233	0.152022	0.065177	0.366569	VRK3	51231	VRK serine/threonine kinase 3
ENSG0000	255.8119	0.239586	0.172784	0.065201	0.366593	SREK1	140890	splicing regulatory glutamic acid and lysine rich protein 1
ENSG0000	2.41801	0.081859	0.249742	0.065307	0.366995	NA	NA	NA
ENSG0000	22.61781	0.253078	0.321301	0.065527	0.366995	FBXO4	26272	F-box protein 4
ENSG0000	183.4296	-0.25602	0.283203	0.065485	0.366995	SLC37A3	84255	solute carrier family 37 member 3
ENSG0000	424.8094	0.241818	0.179319	0.06534	0.366995	MTSS1	9788	MTSS I-BAR domain containing 1
ENSG0000	827.961	-0.23182	0.164616	0.065341	0.366995	FADD	8772	Fas associated via death domain

ENSG0000	233.1309	-0.22781	0.159602	0.065511	0.366995	CRACR2A	84766	calcium release activated channel regulator 2A
ENSG0000	4.507205	0.112552	0.263469	0.065497	0.366995	NA	NA	NA
ENSG0000	53.46649	0.261739	0.207916	0.065461	0.366995	PAGR1	79447	PAXIP1 associated glutamate rich protein 1
ENSG0000	229.8569	0.228191	0.158793	0.065449	0.366995	RAB34	83871	RAB34 member RAS oncogene family
ENSG0000	2.39437	0.069585	0.245077	0.065447	0.366995	DTNA	1837	dystrobrevin alpha
ENSG0000	88.43217	-0.25374	0.197383	0.065533	0.366995	LINC0298	1.02E+08	long intergenic non-protein coding RNA 2987
ENSG0000	24.03831	0.268518	0.304982	0.065421	0.366995	ZNF235	9310	zinc finger protein 235
ENSG0000	72.98076	-0.26753	0.269379	0.065367	0.366995	UCKL1-AS	1E+08	UCKL1 antisense RNA 1
ENSG0000	36.10337	-0.26133	0.300219	0.065555	0.367006	NA	NA	NA
ENSG0000	61.42552	0.254059	0.300903	0.065655	0.367267	ZZZ3	26009	zinc finger ZZ-type containing 3
ENSG0000	46.58963	0.241335	0.310721	0.065662	0.367267	RIOK2	55781	RIO kinase 2
ENSG0000	17.08037	0.163662	0.289579	0.065636	0.367267	NA	NA	NA
ENSG0000	875.6939	-0.22552	0.157092	0.0657	0.367367	F13A1	2162	coagulation factor XIII A chain
ENSG0000	852.5672	-0.26204	0.246751	0.06576	0.36748	VNN3P	55350	vanin 3 pseudogene
ENSG0000	618.6046	0.24585	0.176477	0.065754	0.36748	MIDEAS	91748	mitotic deacetylase associated SANT domain protein
ENSG0000	36.72189	0.27058	0.284629	0.065828	0.367653	RABGGTB	5876	Rab geranylgeranyltransferase subunit beta
ENSG0000	415.8494	-0.255	0.201766	0.065858	0.367653	GPR155	151556	G protein-coupled receptor 155
ENSG0000	92.85119	-0.26736	0.250479	0.065836	0.367653	TRBV7-9	28589	T cell receptor beta variable 7-9
ENSG0000	62.38261	0.273537	0.287594	0.065872	0.367653	LINC0067	1.01E+08	long intergenic non-protein coding RNA 672
ENSG0000	7.967817	-0.14296	0.278626	0.065962	0.368044	RPS2P47	644388	ribosomal protein S2 pseudogene 47
ENSG0000	3.200627	0.086976	0.252747	0.066068	0.368525	CDH1	999	cadherin 1
ENSG0000	605.0661	-0.23256	0.167414	0.066091	0.368538	NFKBIE	4794	NFKB inhibitor epsilon
ENSG0000	276.8629	-0.24635	0.187762	0.066126	0.36862	MMADHC	27249	metabolism of cobalamin associated D
ENSG0000	465.1286	0.209485	0.138612	0.06616	0.368701	TAGLN	6876	transgelin
ENSG0000	533.713	-0.20582	0.135237	0.066285	0.369287	PPP1R3E	90673	protein phosphatase 1 regulatory subunit 3E
ENSG0000	160.1417	0.253624	0.201701	0.066333	0.36944	OLA1	29789	Obg like ATPase 1
ENSG0000	273.475	0.211102	0.140184	0.066397	0.369685	SIVA1	10572	SIVA1 apoptosis inducing factor
ENSG0000	193.492	-0.25149	0.199596	0.066486	0.370069	SPART	23111	spartin
ENSG0000	20.79445	-0.24068	0.319239	0.066542	0.370269	CA12	771	carbonic anhydrase 12
ENSG0000	127.265	0.246178	0.183763	0.066617	0.370574	TDP1	55775	tyrosyl-DNA phosphodiesterase 1
ENSG0000	9.044722	-0.18455	0.309146	0.066663	0.370712	HESX1	8820	HESX homeobox 1
ENSG0000	387.0828	-0.23453	0.169125	0.066702	0.370716	EIF4G3	8672	eukaryotic translation initiation factor 4 gamma 3
ENSG0000	3.55388	0.109765	0.262415	0.066724	0.370716	RPSAP54	642641	ribosomal protein SA pseudogene 54
ENSG0000	925.817	0.215906	0.146004	0.066711	0.370716	THRA	7067	thyroid hormone receptor alpha
ENSG0000	236.5087	0.225478	0.158973	0.066857	0.370751	DARS1	1615	aspartyl-tRNA synthetase 1
ENSG0000	11.5112	0.209473	0.320056	0.066805	0.370751	ZNF391	346157	zinc finger protein 391
ENSG0000	17.75702	0.275229	0.301124	0.066816	0.370751	RDH10	157506	retinol dehydrogenase 10
ENSG0000	92.83169	0.262907	0.255966	0.066839	0.370751	ALG5	29880	ALG5 dolichyl-phosphate beta-glucosyltransferase
ENSG0000	13.63361	0.250871	0.328893	0.066816	0.370751	NA	NA	NA
ENSG0000	351.3744	0.224348	0.156918	0.066872	0.370751	GGNBP2	79893	gametogenetin binding protein 2
ENSG0000	4.162134	0.095367	0.255218	0.066839	0.370751	NA	NA	NA
ENSG0000	2723.201	-0.22452	0.175908	0.06693	0.370958	CD2	914	CD2 molecule
ENSG0000	16.44619	-0.17449	0.296085	0.067014	0.370979	RPS2P14	1E+08	ribosomal protein S2 pseudogene 14
ENSG0000	117.2169	0.259472	0.21931	0.066963	0.370979	KCTD7	154881	potassium channel tetramerization domain containing 7
ENSG0000	780.6555	0.236025	0.294195	0.067014	0.370979	IGHV1-2	28474	immunoglobulin heavy variable 1-2
ENSG0000	3.853738	0.094605	0.253934	0.067008	0.370979	NA	NA	NA
ENSG0000	23.65483	0.276198	0.272924	0.06721	0.371002	KTI12	112970	KTI12 chromatin associated homolog
ENSG0000	3.898216	0.122224	0.268816	0.067232	0.371002	MEX3A	92312	mex-3 RNA binding family member A
ENSG0000	78.26322	0.248791	0.189226	0.067262	0.371002	SLC2A9	56606	solute carrier family 2 member 9
ENSG0000	3.392199	0.089097	0.252293	0.067044	0.371002	NA	NA	NA
ENSG0000	38.66229	0.272032	0.263951	0.067237	0.371002	WDR27	253769	WD repeat domain 27
ENSG0000	147.0751	-0.26047	0.227883	0.067164	0.371002	ADGRA2	25960	adhesion G protein-coupled receptor A2
ENSG0000	127.09	0.234721	0.170185	0.067248	0.371002	PWP1	11137	PWP1 hon endonuclein
ENSG0000	1775.59	0.159681	0.284377	0.06709	0.371002	IGHV3-30	28439	immunoglobulin heavy variable 3-30
ENSG0000	2068.313	-0.2303	0.167334	0.067113	0.371002	USP10	9100	ubiquitin specific peptidase 10
ENSG0000	59.93686	0.254778	0.200228	0.067251	0.371002	FOSB	2354	FosB protc AP-1 transcription factor subunit
ENSG0000	1216.146	-0.23921	0.179355	0.067197	0.371002	TOP1	7150	DNA topoisomerase I
ENSG0000	128.5628	0.225387	0.156641	0.067162	0.371002	DKC1	1736	dyskerin pseudouridine synthase 1
ENSG0000	55.69647	-0.27329	0.250047	0.067452	0.371079	NHLH1	4807	nescient helix-loop-helix 1
ENSG0000	1450.618	-0.23663	0.18159	0.067339	0.371079	DOK1	1796	docking protein 1
ENSG0000	11.56559	0.228931	0.328144	0.067509	0.371079	NA	NA	NA
ENSG0000	1690.265	0.224744	0.15753	0.067333	0.371079	SCAP	22937	SREBF chaperone
ENSG0000	7.921441	0.173527	0.299166	0.067384	0.371079	GGCT	79017	gamma-glutamylcyclotransferase
ENSG0000	166.7521	0.262673	0.251769	0.067483	0.371079	BLTP3B	23074	bridge-like lipid transfer protein family member 3B
ENSG0000	337.0738	-0.26062	0.241382	0.067519	0.371079	BICDL1	92558	BICD family like cargo adaptor 1
ENSG0000	56.71575	0.259836	0.212423	0.067448	0.371079	GALK2	2585	galactokinase 2
ENSG0000	1761.899	-0.25137	0.222043	0.067481	0.371079	RUNDC3A	10900	RUN domain containing 3A
ENSG0000	233.6518	0.253201	0.200736	0.067334	0.371079	SEC11C	90701	SEC11 hon signal peptidase complex subunit
ENSG0000	5.707334	-0.12494	0.269505	0.067499	0.371079	NA	NA	NA
ENSG0000	8.239665	-0.19574	0.316489	0.067452	0.371079	CENPI	2491	centromere protein I
ENSG0000	792.1705	-0.25502	0.218351	0.067578	0.371294	FAXDC2	10826	fatty acid hydroxylase domain containing 2
ENSG0000	130.2206	-0.23032	0.164162	0.067601	0.37131	TM2D2	83877	TM2 domain containing 2
ENSG0000	14561.37	-0.24707	0.281869	0.067726	0.371663	DYSF	8291	dysferlin
ENSG0000	45.17151	0.258408	0.206887	0.067703	0.371663	SFMBT1	51460	Scm like with four mbt domains 1
ENSG0000	385.7628	-0.22121	0.153549	0.067721	0.371663	H2AJ	55766	H2A.J histone
ENSG0000	216.7244	0.220013	0.161505	0.067831	0.372125	GLYCTK	132158	glycerate kinase
ENSG0000	35.52947	-0.20232	0.303221	0.067874	0.372252	NA	NA	NA
ENSG0000	116.3566	0.254567	0.207403	0.068057	0.373033	DNPH1	10591	2'-deoxynucleoside 5'-phosphate N-hydrolase 1
ENSG0000	330.1691	0.257414	0.230472	0.068049	0.373033	NORAD	647979	non-coding RNA activated by DNA damage
ENSG0000	11.10676	-0.21749	0.32068	0.06808	0.373044	LOC10012	1E+08	uncharacterized LOC100128059
ENSG0000	1054.291	0.258502	0.23219	0.068158	0.37336	TGFBI	7045	transforming growth factor beta induced
ENSG0000	39.89577	-0.26451	0.288043	0.06824	0.3737	LOC10537	1.05E+08	uncharacterized LOC105378268
ENSG0000	51.90889	-0.23995	0.306242	0.068268	0.373741	BOK	666	BCL2 family apoptosis regulator BOK
ENSG0000	361.223	-0.21605	0.147976	0.068472	0.374524	CISD3	284106	CDGSH iron sulfur domain 3
ENSG0000	4.042769	0.065388	0.243188	0.068473	0.374524	NA	NA	NA
ENSG0000	40.82686	-0.24014	0.309974	0.068455	0.374524	NA	NA	NA
ENSG0000	512.7402	-0.25885	0.260363	0.068498	0.374553	FKBP4	2288	FKBP prolyl isomerase 4
ENSG0000	219.9907	-0.25945	0.240606	0.068566	0.374715	NA	NA	NA
ENSG0000	37.41089	0.266906	0.253502	0.06861	0.374715	COA7	65260	cytochrome c oxidase assembly factor 7
ENSG0000	4501.06	-0.22947	0.16663	0.06857	0.374715	HPS1	3257	HPS1 biogenesis of lysosomal organelles complex 3 subunit 1
ENSG0000	4.801656	-0.10766	0.260322	0.068602	0.374715	DPY19L3-D	400684	DPY19L3 divergent transcript
ENSG0000	5.927106	-0.08726	0.250501	0.068665	0.374796	TRBV5-5	28610	T cell receptor beta variable 5-5
ENSG0000	57.8668	-0.26106	0.222654	0.068661	0.374796	C2CD2	25966	C2 calcium dependent domain containing 2
ENSG0000	1690.717	-0.20655	0.138247	0.068687	0.374804	TOX4	9878	TOX high mobility group box family member 4
ENSG0000	72.26887	0.254005	0.20318	0.068726	0.374903	NA	NA	NA
ENSG0000	6.73322	-0.1678	0.295927	0.068792	0.375059	NA	NA	NA

ENSG0000	66.87821	-0.25854	0.214957	0.068816	0.375059	YPEL4	219539	yippee like 4
ENSG0000	383.9906	-0.21449	0.146985	0.068814	0.375059	IFNAR2	3455	interferon alpha and beta receptor subunit 2
ENSG0000	21.64184	0.269968	0.290388	0.068864	0.37521	NA	NA	NA
ENSG0000	80.17768	0.250156	0.293453	0.06897	0.375676	EMID1	129080	EMI domain containing 1
ENSG0000	70.86437	0.212464	0.301253	0.069049	0.37599	NA	NA	NA
ENSG0000	98.49085	0.241453	0.181044	0.06912	0.376043	NPHP4	261734	nephrocystin 4
ENSG0000	429.1239	0.19184	0.123143	0.069104	0.376043	DDX42	11325	DEAD-box helicase 42
ENSG0000	248.0632	-0.24068	0.185979	0.069114	0.376043	ADARB1	104	adenosine deaminase RNA specific B1
ENSG0000	123.5895	-0.25459	0.279082	0.069283	0.376707	TSPAN9	10867	tetraspanin 9
ENSG0000	10.38266	-0.15671	0.285423	0.069281	0.376707	NA	NA	NA
ENSG0000	2.926163	-0.07792	0.248612	0.069327	0.376803	RN7SL441	1.06E+08	RNA 7SL cytoplasm pseudogene
ENSG0000	42.97243	0.263533	0.226712	0.069342	0.376803	ZSCAN2	54993	zinc finger and SCAN domain containing 2
ENSG0000	127.4199	0.255536	0.213707	0.069365	0.376817	STK32C	282974	serine/threonine kinase 32C
ENSG0000	335.4	0.248945	0.196135	0.069427	0.377044	SIGLEC16	400709	sialic acid binding Ig like lectin 16
ENSG0000	9.890757	-0.12775	0.269859	0.069485	0.377134	RNU6-37P	1.06E+08	RNA U6 small r pseudogene
ENSG0000	13.24489	0.248198	0.324142	0.069497	0.377134	COA6-AS1	1.02E+08	COA6 antisense RNA 1
ENSG0000	3183.417	-0.25021	0.211452	0.069506	0.377134	SDCBP	6386	syndecan binding protein
ENSG0000	178.0821	0.206747	0.134796	0.069542	0.377146	CPSF3	51692	cleavage and polyadenylation specific factor 3
ENSG0000	200.5537	-0.22694	0.162541	0.069549	0.377146	IKBKG	8517	inhibitor of nuclear factor kappa B kinase regulatory subunit gamma
ENSG0000	3.543277	0.085681	0.251753	0.069585	0.377231	NA	NA	NA
ENSG0000	134.8868	0.243859	0.289775	0.069645	0.377441	NR6A1	2649	nuclear receptor subfamily 6 group A member 1
ENSG0000	4.414385	-0.13566	0.276342	0.069678	0.377508	LZTS1	11178	leucine zipper tumor suppressor 1
ENSG0000	897.4976	0.191316	0.124078	0.069736	0.37771	NACA	4666	nascent polypeptide associated complex subunit alpha
ENSG0000	735.7583	-0.21849	0.153861	0.06982	0.377996	CC2D1B	200014	coiled-coil and C2 domain containing 1B
ENSG0000	2.513668	0.063176	0.242794	0.069871	0.377996	NA	NA	NA
ENSG0000	10.51772	0.1788	0.300522	0.069845	0.377996	XYLB	9942	xylulokinase
ENSG0000	3.533172	-0.08262	0.24908	0.069862	0.377996	NA	NA	NA
ENSG0000	7.086642	0.198062	0.317097	0.069904	0.378065	LOC10537	1.05E+08	uncharacterized LOC105373383
ENSG0000	2626.912	-0.24893	0.201453	0.070028	0.37843	ARHGEF11	9826	Rho guanine nucleotide exchange factor 11
ENSG0000	19.19009	0.221968	0.3156	0.070034	0.37843	FKBP14	55033	FKBP prolyl isomerase 14
ENSG0000	5.916549	0.109026	0.262152	0.07	0.37843	ZNF215	7762	zinc finger protein 215
ENSG0000	24465.13	-0.24927	0.210353	0.070115	0.378759	VASP	7408	vasodilator stimulated phosphoprotein
ENSG0000	1064.788	-0.25015	0.202511	0.070144	0.378801	DCXR	51181	dicarbonyl and L-xylulose reductase
ENSG0000	662.1464	-0.20512	0.137027	0.070247	0.379133	UBE3B	89910	ubiquitin protein ligase E3B
ENSG0000	5.038433	-0.12416	0.269476	0.070235	0.379133	NA	NA	NA
ENSG0000	7682.642	-0.24528	0.205399	0.070305	0.37922	SH2D3C	10044	SH2 domain containing 3C
ENSG0000	3.734208	0.06744	0.243122	0.070325	0.37922	ZC2HC1C	79696	zinc finger C2HC-type containing 1C
ENSG0000	2365.488	0.256673	0.2408	0.070287	0.37922	IRF2BPL	64207	interferon regulatory factor 2 binding protein like
ENSG0000	174.8998	0.230429	0.16802	0.070404	0.379305	BCL9	607	BCL9 transcription coactivator
ENSG0000	24.97081	-0.26971	0.268609	0.07041	0.379305	DND1	373863	DND microRNA-mediated repression inhibitor 1
ENSG0000	16496.73	-0.25412	0.234553	0.070368	0.379305	GRINA	2907	glutamate ionotropic receptor NMDA type subunit associated protein 1
ENSG0000	8.526391	-0.14611	0.279961	0.070424	0.379305	LINC0023	145200	long intergenic non-protein coding RNA 239
ENSG0000	8.016149	0.148697	0.282036	0.070478	0.379443	NA	NA	NA
ENSG0000	542.7637	0.236402	0.178656	0.07049	0.379443	P2RX5	5026	purinergic receptor P2X 5
ENSG0000	449.0266	0.223361	0.158828	0.070573	0.379662	PRXL2B	127281	peroxiredoxin like 2B
ENSG0000	127.169	-0.24977	0.208412	0.070569	0.379662	TBXA2R	6915	thromboxane A2 receptor
ENSG0000	5582.056	-0.24065	0.189227	0.070608	0.379739	MIDN	90007	midnolin
ENSG0000	205.2533	-0.25642	0.260275	0.070689	0.37997	LNCATV	1.02E+08	lncRNA negative regulator of antiviral signaling
ENSG0000	4935.559	0.243644	0.184975	0.070692	0.37997	PECAM1	5175	platelet and endothelial cell adhesion molecule 1
ENSG0000	102.0456	0.245192	0.302749	0.07073	0.380063	DDX3Y	8653	DEAD-box helicase 3 Y-linked
ENSG0000	3.582022	0.074921	0.247136	0.070767	0.380152	LINC01331	1.04E+08	long intergenic non-protein coding RNA 1331
ENSG0000	501.2754	0.185776	0.119419	0.070802	0.380225	H2AZ2	94239	H2A.Z variant histone 2
ENSG0000	3.359898	-0.04445	0.238245	0.070963	0.38031	NA	NA	NA
ENSG0000	138.4235	-0.24626	0.196356	0.07088	0.38031	CGAS	115004	cyclic GMP-AMP synthase
ENSG0000	4.363028	0.127632	0.272616	0.070902	0.38031	TMEM200	114801	transmembrane protein 200A
ENSG0000	8.3917	0.175849	0.300087	0.070889	0.38031	ZNF485	220992	zinc finger protein 485
ENSG0000	37195.84	-0.25237	0.248459	0.070937	0.38031	NRGN	4900	neurogranin
ENSG0000	76.2769	0.250051	0.197946	0.070892	0.38031	GEMIN8	54960	gem nuclear organelle associated protein 8
ENSG0000	3.905443	0.101339	0.257209	0.070945	0.38031	XKRX	402415	XK related X-linked
ENSG0000	644.6334	0.208846	0.141898	0.070989	0.380341	MPG	4350	N-methylpurine DNA glycosylase
ENSG0000	114.6104	0.236866	0.176665	0.071043	0.38047	TMEM208	29100	transmembrane protein 208
ENSG0000	32.62342	0.267739	0.261451	0.071055	0.38047	SUSD2	56241	sushi domain containing 2
ENSG0000	315.9694	0.201201	0.134272	0.071106	0.380632	CRCP	27297	CGRP receptor component
ENSG0000	711.3373	0.191065	0.122525	0.071141	0.380707	TBC1D2B	23102	TBC1 domain family member 2B
ENSG0000	3.040716	0.086912	0.251539	0.071188	0.380849	NA	NA	NA
ENSG0000	2.517081	0.088159	0.254108	0.071289	0.381278	LOC12490	1.25E+08	uncharacterized LOC124909384
ENSG0000	4.032379	0.060661	0.241863	0.071323	0.381348	TMEM117	84216	transmembrane protein 117
ENSG0000	5.759697	-0.14915	0.283707	0.071453	0.381932	NA	NA	NA
ENSG0000	349.2471	0.19128	0.124192	0.071614	0.382011	DPM3	54344	dolichyl-pł regulatory
ENSG0000	113.6244	-0.25139	0.225328	0.071534	0.382011	LGR6	59352	leucine rich repeat containing G protein-coupled receptor 6
ENSG0000	2.40731	0.041734	0.236998	0.071603	0.382011	ECRG4	84417	ECRG4 augurin precursor
ENSG0000	52.63177	-0.25842	0.272954	0.071601	0.382011	ITGA1	3672	integrin subunit alpha 1
ENSG0000	25.75684	-0.22654	0.309105	0.071567	0.382011	NA	NA	NA
ENSG0000	19.28848	0.157349	0.28423	0.071529	0.382011	PCDH9	5101	protocadherin 9
ENSG0000	4.977388	0.123895	0.270192	0.071569	0.382011	FBXL22	283807	F-box and leucine rich repeat protein 22
ENSG0000	3.067968	-0.08914	0.254078	0.071644	0.382061	H1-6	3010	H1.6 linker cluster member
ENSG0000	12.75426	-0.20117	0.308804	0.071692	0.382185	NA	NA	NA
ENSG0000	354.5985	-0.24414	0.196774	0.071709	0.382185	IL21R	50615	interleukin 21 receptor
ENSG0000	96.30736	0.218478	0.153776	0.071734	0.382205	PMS2	5395	PMS1 hom mismatch repair system component
ENSG0000	91.31159	0.262088	0.262177	0.071823	0.382346	NOL3	8996	nucleolar protein 3
ENSG0000	1051.765	-0.22722	0.164261	0.071812	0.382346	PSMB10	5699	proteasome 20S subunit beta 10
ENSG0000	1819.854	-0.24156	0.19328	0.071808	0.382346	RBM10	8241	RNA binding motif protein 10
ENSG0000	15.79579	-0.14244	0.277976	0.071862	0.3824	LOC15727	157273	uncharacterized LOC157273
ENSG0000	6.464162	0.150233	0.283519	0.071875	0.3824	NA	NA	NA
ENSG0000	27.49196	0.257526	0.294715	0.071925	0.382444	LINC0300	1E+08	long intergenic non-protein coding RNA 3009
ENSG0000	1152.875	0.244858	0.193744	0.071912	0.382444	NDUFV1	4723	NADH:ubiquinone oxidoreductase core subunit V1
ENSG0000	26.38231	0.222967	0.307249	0.072111	0.382798	LINC01342	254099	long intergenic non-protein coding RNA 1342
ENSG0000	6.888323	-0.13491	0.27438	0.072055	0.382798	SYNPO2	171024	synaptopodin 2
ENSG0000	12.47042	0.190741	0.305751	0.072074	0.382798	COL11A2	1302	collagen type XI alpha 2 chain
ENSG0000	6.749821	-0.17043	0.299016	0.072117	0.382798	NA	NA	NA
ENSG0000	610.9353	-0.23056	0.186095	0.072029	0.382798	PDE4A	5141	phosphodiesterase 4A
ENSG0000	6.356656	0.176601	0.301746	0.072085	0.382798	NA	NA	NA
ENSG0000	9.064638	0.111655	0.260615	0.072196	0.382998	APLF	200558	aprataxin and PNKP like factor
ENSG0000	410.453	-0.21758	0.153598	0.072191	0.382998	EPG5	57724	ectopic P-granules 5 autophagy tethering factor
ENSG0000	23.10008	0.199852	0.302836	0.072231	0.383071	NA	NA	NA
ENSG0000	430.9728	-0.22414	0.16118	0.072302	0.383074	RAB1A	5861	RAB1A member RAS oncogene family

ENSG0000	7.30291	-0.12435	0.26833	0.072267	0.383074	NA	NA	NA
ENSG0000	9.54905	0.222916	0.32403	0.072305	0.383074	NA	NA	NA
ENSG0000	190.1255	0.234585	0.176855	0.072315	0.383074	MRPL54	116541	mitochondrial ribosomal protein L54
ENSG0000	209.4827	-0.22429	0.162118	0.072424	0.383318	ATOX1	475	antioxidant 1 copper chaperone
ENSG0000	117.8567	0.250851	0.210976	0.072391	0.383318	TMEM243	79161	transmembrane protein 243
ENSG0000	77.34743	0.23861	0.29407	0.07241	0.383318	MBNL2	10150	muscleblind like splicing regulator 2
ENSG0000	193.2954	0.250579	0.210525	0.07247	0.383451	CLNS1A	1207	chloride nucleotide-sensitive channel 1A
ENSG0000	191.2679	-0.25516	0.245556	0.072516	0.383586	ITGB5	3693	integrin subunit beta 5
ENSG0000	1813.447	0.219935	0.160001	0.072645	0.384156	ATP5F1A	498	ATP synthase F1 subunit alpha
ENSG0000	107.2495	0.25725	0.260076	0.072692	0.384199	NT5DC1	221294	5'-nucleotidase domain containing 1
ENSG0000	179.7614	-0.23679	0.279009	0.072695	0.384199	LY96	23643	lymphocyte antigen 96
ENSG0000	19.55449	-0.22025	0.309683	0.072729	0.384267	NA	NA	NA
ENSG0000	460.1177	0.242783	0.276186	0.072881	0.384512	IGKV1-17	28937	immunoglobulin kappa variable 1-17
ENSG0000	24.34429	0.237561	0.311796	0.072879	0.384512	ACOXL	55289	acyl-CoA oxidase like
ENSG0000	2.851278	-0.05797	0.241106	0.072872	0.384512	NA	NA	NA
ENSG0000	7.976033	0.161465	0.289613	0.072863	0.384512	NA	NA	NA
ENSG0000	1291.587	0.252935	0.24744	0.07288	0.384512	PANX2	56666	pannexin 2
ENSG0000	136.1772	0.243019	0.192701	0.072921	0.384615	SELENOS	55829	selenoprotein S
ENSG0000	194.1515	-0.25148	0.223102	0.072973	0.384671	TRMT1L	81627	tRNA methyltransferase 1 like
ENSG0000	8.173773	0.14624	0.280502	0.072974	0.384671	LOC10042	1E+08	SHC binding and spindle associated 1 pseudogene
ENSG0000	13.60065	-0.10869	0.258223	0.073026	0.384779	RNU7-45P	1E+08	RNA U7 small nuclear 45 pseudogene
ENSG0000	158.9406	0.240068	0.186952	0.073036	0.384779	FBXW8	26259	F-box and WD repeat domain containing 8
ENSG0000	287.0741	0.209384	0.144196	0.07306	0.384793	B3GALT6	126792	beta-1 3-galactosyltransferase 6
ENSG0000	140.2454	-0.24872	0.233457	0.073193	0.385383	NPIPA1	9284	nuclear pore complex interacting protein family member A1
ENSG0000	286.1388	0.222759	0.160891	0.073223	0.385432	NDUFS3	4722	NADH:ubiquinone oxidoreductase core subunit S3
ENSG0000	10.39933	0.172372	0.295927	0.073248	0.385449	C17orf107	1E+08	chromosome 17 open reading frame 107
ENSG0000	33.43474	0.244841	0.296951	0.073352	0.385888	NA	NA	NA
ENSG0000	850.2899	-0.19172	0.125854	0.073473	0.386415	CRYBG1	202	crystallin beta-gamma domain containing 1
ENSG0000	8173.19	-0.24057	0.196337	0.073615	0.386846	TUBA4A	7277	tubulin alpha 4a
ENSG0000	3.980592	0.101449	0.257016	0.073628	0.386846	TNNC1	7134	troponin C slow skeletal and cardiac type
ENSG0000	49.95894	0.260086	0.240309	0.073672	0.386846	CTC-338M	1.02E+08	uncharacterized LOC101928649
ENSG0000	14.91255	0.257619	0.306536	0.073649	0.386846	NA	NA	NA
ENSG0000	4.573797	0.109461	0.262476	0.073645	0.386846	MYO16-AS	1.01E+08	MYO16 antisense RNA 1
ENSG0000	40.19525	-0.25931	0.266322	0.073682	0.386846	UPK3A	7380	uroplakin 3A
ENSG0000	2.965501	-0.04411	0.238274	0.073703	0.386848	MIR328	442901	microRNA 328
ENSG0000	6.856251	0.139134	0.276178	0.073873	0.387491	HHLA2	11148	HERV-H LTR-associating 2
ENSG0000	4.111819	0.123002	0.268927	0.07386	0.387491	LINC0196	1.03E+08	long intergenic non-protein coding RNA 1962
ENSG0000	362.7476	-0.23753	0.185237	0.073889	0.387491	ACER3	55331	alkaline ceramidase 3
ENSG0000	415.981	0.222611	0.161897	0.073935	0.387619	TELO2	9894	telomere maintenance 2
ENSG0000	203.2688	0.239729	0.285715	0.07396	0.387641	IGHV2-26	28455	immunoglobulin heavy variable 2-26
ENSG0000	46.60915	0.234622	0.305901	0.07399	0.387683	VWF	7450	von Willebrand factor
ENSG0000	79.50799	-0.12158	0.266165	0.074074	0.388017	CAVIN3	112464	caveolae associated protein 3
ENSG0000	24.36196	-0.22339	0.307661	0.074121	0.388152	LINC0063	196872	long intergenic non-protein coding RNA 638
ENSG0000	9.838205	0.153416	0.282822	0.074159	0.388184	NA	NA	NA
ENSG0000	16.59092	0.233824	0.312926	0.07417	0.388184	ELAC1	55520	elaC ribonuclease Z 1
ENSG0000	7756.242	-0.23412	0.183301	0.074195	0.388204	LYN	4067	LYN proto- Src family tyrosine kinase
ENSG0000	107.735	-0.24514	0.275257	0.074235	0.388301	C1orf21	81563	chromosome 1 open reading frame 21
ENSG0000	1578.527	-0.20594	0.142869	0.074288	0.388469	ZNF672	79894	zinc finger protein 672
ENSG0000	304.9255	-0.23525	0.18332	0.074389	0.388888	RCBTB2	1102	RCC1 and BTB domain containing protein 2
ENSG0000	626.4869	-0.21909	0.157367	0.074498	0.389236	GMPR2	51292	guanosine monophosphate reductase 2
ENSG0000	3.032242	-0.07139	0.245386	0.074495	0.389236	MTND4LP	1.07E+08	MT-ND4L pseudogene 23
ENSG0000	5.001339	0.10808	0.260281	0.074521	0.389243	IGFALS	3483	insulin like growth factor binding protein acid labile subunit
ENSG0000	2632.023	-0.21568	0.15588	0.074582	0.389342	CHMP1A	5119	charged multivesicular body protein 1A
ENSG0000	7.004657	0.138971	0.276497	0.074578	0.389342	ADAMTS1	9510	ADAM metallopeptidase with thrombospondin type 1 motif 1
ENSG0000	127.031	0.225578	0.293646	0.074656	0.389615	ZFHX3	463	zinc finger homeobox 3
ENSG0000	533.4188	-0.20503	0.141294	0.074704	0.389753	PHACTR4	65979	phosphatase and actin regulator 4
ENSG0000	2.804414	-0.0922	0.254516	0.074817	0.390234	NA	NA	NA
ENSG0000	6.701349	0.089233	0.251174	0.074866	0.390268	HOXA9	3205	homeobox A9
ENSG0000	316.3691	0.217119	0.295383	0.074851	0.390268	IGHV3-11	28450	immunoglobulin heavy variable 3-11
ENSG0000	99.07158	-0.24933	0.213286	0.074928	0.390447	GOLGA7B	401647	golgin A7 family member B
ENSG0000	993.3639	0.199243	0.140787	0.074943	0.390447	COMMD9	29099	COMM domain containing 9
ENSG0000	2.715529	0.046837	0.238155	0.07499	0.390577	RPLP0P10	390578	ribosomal protein lateral stalk subunit P0 pseudogene 10
ENSG0000	8.92359	0.161082	0.288465	0.075096	0.391018	LDHB	3945	lactate dehydrogenase B
ENSG0000	9.989999	0.17374	0.294943	0.075271	0.391164	BOLA3-DT	1.01E+08	BOLA3 divergent transcript
ENSG0000	342.1976	0.241663	0.199628	0.075253	0.391164	TMEM185	79134	transmembrane protein 185B
ENSG0000	50.47529	0.253879	0.269152	0.075201	0.391164	TYW5	129450	tRNA-yW synthesizing protein 5
ENSG0000	224.7404	0.219015	0.15929	0.075298	0.391164	AIMP2	7965	aminoacyl tRNA synthetase complex interacting multifunctional protein 2
ENSG0000	9.171375	0.140379	0.276109	0.075162	0.391164	SHB	6461	SH2 domain containing adaptor protein B
ENSG0000	96.46427	-0.25062	0.213265	0.075337	0.391164	NA	NA	NA
ENSG0000	513.035	0.205445	0.141735	0.07529	0.391164	ZNF609	23060	zinc finger protein 609
ENSG0000	157.8809	0.226505	0.168393	0.075334	0.391164	LDLRAD4	753	low density lipoprotein receptor class A domain containing 4
ENSG0000	93.59372	-0.25546	0.249871	0.075173	0.391164	TUBB4A	10382	tubulin beta 4A class IVa
ENSG0000	15018.09	-0.24303	0.206866	0.075322	0.391164	NCF4	4689	neutrophil cytosolic factor 4
ENSG0000	869.5071	-0.1922	0.126762	0.0754	0.391379	TCTA	6988	T cell leukemia translocation altered
ENSG0000	3.652796	-0.0927	0.253721	0.075466	0.391612	GTSCR1	220158	Gilles de la candidate 1
ENSG0000	129.5187	0.081815	0.249093	0.07555	0.391826	IGHV1-45	28466	immunoglobulin heavy variable 1-45
ENSG0000	4.710387	-0.07037	0.244293	0.075539	0.391826	NTN1	9423	netrin 1
ENSG0000	325.581	-0.20243	0.138256	0.075605	0.392	OGFOD1	55239	2-oxoglutarate and iron dependent oxygenase domain containing 1
ENSG0000	7071.042	-0.24707	0.236247	0.075672	0.392234	CTSA	5476	cathepsin A
ENSG0000	4581.353	-0.24343	0.21045	0.075775	0.392658	DHX34	9704	DExH-box helicase 34
ENSG0000	21.53098	-0.24813	0.30231	0.075812	0.392736	LINC0085	1.01E+08	long intergenic non-protein coding RNA 853
ENSG0000	53.13405	0.238442	0.289553	0.075842	0.392781	NA	NA	NA
ENSG0000	17.00685	0.2562	0.295745	0.075895	0.392917	RN7SL832	1.06E+08	RNA 7SL cytoplasm pseudogene
ENSG0000	333.4448	-0.21272	0.151624	0.075911	0.392917	TOMM34	10953	translocase of outer mitochondrial membrane 34
ENSG0000	158.4091	0.249823	0.244666	0.075955	0.392924	KLHL5	51088	kelch like family member 5
ENSG0000	24.71214	-0.2043	0.303647	0.075953	0.392924	NA	NA	NA
ENSG0000	2431.171	0.23241	0.181364	0.075984	0.392963	MLXIP	22877	MLX interacting protein
ENSG0000	3690.037	-0.23238	0.182856	0.076102	0.393463	IL2RB	3560	interleukin 2 receptor subunit beta
ENSG0000	233.1632	0.242124	0.274036	0.076164	0.39356	NA	NA	NA
ENSG0000	3.866167	0.12485	0.270122	0.076161	0.39356	TEX29	121793	testis expressed 29
ENSG0000	329.7668	0.243032	0.202024	0.076244	0.393749	SNX29	92017	sorting nexin 29
ENSG0000	4.296903	-0.09341	0.253817	0.076239	0.393749	HES7	84667	hes family bHLH transcription factor 7
ENSG0000	12.60336	-0.19238	0.303409	0.076642	0.394215	NA	NA	NA
ENSG0000	17.57329	-0.18626	0.29787	0.07644	0.394215	NCKAP1	10787	NCK associated protein 1
ENSG0000	42.36937	0.217391	0.299104	0.076657	0.394215	IFT57	55081	intraflagellar transport 57
ENSG0000	4.327247	0.123321	0.271224	0.076666	0.394215	CAPN11	11131	calpain 11

ENSG0000	174.3265	0.24092	0.197301	0.076498	0.394215	BACH2	60468	BTB domain and CNC homolog 2
ENSG0000	7.03091	0.184906	0.304231	0.076697	0.394215	NA	NA	NA
ENSG0000	40.37552	-0.25551	0.261025	0.076654	0.394215	NA	NA	NA
ENSG0000	1192.268	-0.22139	0.164256	0.076466	0.394215	OSTF1	26578	osteoclast stimulating factor 1
ENSG0000	3.316166	0.085076	0.249973	0.076403	0.394215	NA	NA	NA
ENSG0000	21.9948	0.250755	0.298188	0.076715	0.394215	PALD1	27143	phosphatase domain containing paladin 1
ENSG0000	182.8596	0.215475	0.153934	0.076721	0.394215	ZC3H14	79882	zinc finger CCCH-type containing 14
ENSG0000	1301.295	-0.24572	0.24609	0.076702	0.394215	MCTP2	55784	multiple C2 and transmembrane domain containing 2
ENSG0000	10.04386	-0.19005	0.304555	0.076509	0.394215	SLC6A4	6532	solute carrier family 6 member 4
ENSG0000	3.712389	0.082643	0.248541	0.076689	0.394215	NA	NA	NA
ENSG0000	1627.886	-0.24367	0.197265	0.076642	0.394215	CARD8	22900	caspase recruitment domain family member 8
ENSG0000	2.712449	0.11667	0.26948	0.076444	0.394215	NA	NA	NA
ENSG0000	4.685012	0.076154	0.245702	0.076642	0.394215	NA	NA	NA
ENSG0000	4.277775	-0.10615	0.259199	0.076662	0.394215	PIR	8544	pirin
ENSG0000	1712.692	-0.22987	0.172959	0.076769	0.394352	LAMP2	3920	lysosomal associated membrane protein 2
ENSG0000	15.24927	-0.24934	0.308519	0.076822	0.394404	NPAS2	4862	neuronal PAS domain protein 2
ENSG0000	693.8139	-0.23053	0.17994	0.076812	0.394404	MYLIP	29116	myosin regulatory light chain interacting protein
ENSG0000	157.8915	0.225925	0.168896	0.076913	0.394648	WDR11	55717	WD repeat domain 11
ENSG0000	15.40955	0.188113	0.301803	0.076892	0.394648	NA	NA	NA
ENSG0000	169.2565	-0.19033	0.130735	0.076975	0.394743	SYNJ2	8871	synaptojanin 2
ENSG0000	6848.274	-0.23427	0.188481	0.076967	0.394743	GLIPR2	152007	GLI pathogenesis related 2
ENSG0000	91.6186	0.246169	0.279514	0.077049	0.394921	IGHJ5	28476	immunoglobulin heavy joining 5
ENSG0000	21.41218	0.23303	0.303562	0.077053	0.394921	IMPACT	55364	impact RWD domain protein
ENSG0000	324.5094	-0.2461	0.248363	0.077086	0.394985	CAMP	820	cathelicidin antimicrobial peptide
ENSG0000	5.523011	0.102585	0.256957	0.077124	0.395067	MST1L	11223	macrophage stimulating 1 like (pseudogene)
ENSG0000	1649.24	-0.18877	0.12459	0.077204	0.395256	PIP4K2A	5305	phosphatidylinositol-5-phosphate 4-kinase type 2 alpha
ENSG0000	323.9432	0.219209	0.163731	0.07719	0.395256	GRK3	157	G protein-coupled receptor kinase 3
ENSG0000	2000.955	-0.21903	0.154269	0.077235	0.395306	WWC3	55841	WWC family member 3
ENSG0000	1205.545	-0.23423	0.276759	0.077367	0.39587	PFKFB3	5209	6-phosphc 6-biphosphatase 3
ENSG0000	95.94593	0.248802	0.218881	0.077531	0.396489	KLF11	8462	KLF transcription factor 11
ENSG0000	633.7812	-0.22101	0.164918	0.07753	0.396489	SPATA2	9825	spermatogenesis associated 2
ENSG0000	1262.961	-0.1727	0.111492	0.077607	0.396617	NECAP2	55707	NECAP endocytosis associated 2
ENSG0000	11.44514	-0.17856	0.29646	0.07773	0.396617	DNAJC27-A	729723	DNAJC27 antisense RNA 1
ENSG0000	7.176414	-0.16192	0.288932	0.077868	0.396617	ANKRD53	79998	ankyrin repeat domain 53
ENSG0000	621.1462	-0.19849	0.136632	0.077918	0.396617	DCP1A	55802	decapping mRNA 1A
ENSG0000	8.179236	0.153304	0.283343	0.077812	0.396617	NA	NA	NA
ENSG0000	2027.199	-0.21484	0.157071	0.077739	0.396617	FCHSD1	89848	FCH and double SH3 domains 1
ENSG0000	2.806529	0.070476	0.244928	0.077673	0.396617	LINC02696	1.02E+08	long intergenic non-protein coding RNA 2696
ENSG0000	3.781282	-0.1096	0.260781	0.077904	0.396617	MAJIN	283129	membrane anchored junction protein
ENSG0000	149.366	-0.2475	0.229443	0.077859	0.396617	NA	NA	NA
ENSG0000	568.2572	0.238275	0.196626	0.077797	0.396617	PEBP1	5037	phosphatidylethanolamine binding protein 1
ENSG0000	12.99034	0.147454	0.278075	0.077706	0.396617	NA	NA	NA
ENSG0000	825.4346	-0.1936	0.133111	0.077916	0.396617	TERF2IP	54386	TERF2 interacting protein
ENSG0000	241.7508	-0.25012	0.238957	0.077925	0.396617	NA	NA	NA
ENSG0000	363.6209	0.209247	0.148345	0.07774	0.396617	RPA1	6117	replication protein A1
ENSG0000	8385.664	-0.24729	0.232118	0.077767	0.396617	NUCB1	4924	nucleobindin 1
ENSG0000	8.626381	-0.20069	0.31074	0.077756	0.396617	FBXW4P1	26226	F-box and WD repeat domain containing 4 pseudogene 1
ENSG0000	136.6355	0.248133	0.230771	0.077663	0.396617	NDUFA6	4700	NADH:ubiquinone oxidoreductase subunit A6
ENSG0000	92.35506	-0.24778	0.217928	0.078	0.396818	RRAGC	64121	Ras related GTP binding C
ENSG0000	3.460104	-0.11525	0.264219	0.078007	0.396818	CFAP141	388701	cilia and flagella associated protein 141
ENSG0000	3.07341	0.108355	0.263766	0.078275	0.398068	NA	NA	NA
ENSG0000	321.4453	-0.19763	0.135762	0.078323	0.398201	CBFA2T2	9139	CBFA2/RUNX1 partner transcriptional co-repressor 2
ENSG0000	963.387	-0.22373	0.170903	0.078416	0.398564	HAGH	3029	hydroxyacylglutathione hydrolase
ENSG0000	29.37495	-0.23765	0.295563	0.078445	0.398601	LCN12	286256	lipocalin 12
ENSG0000	3.348378	0.069399	0.244479	0.078493	0.398733	NA	NA	NA
ENSG0000	7.919897	-0.14385	0.276896	0.078531	0.398814	JMJD1C-AS	84989	JMJD1C antisense RNA 1
ENSG0000	5.358097	0.060116	0.240785	0.078626	0.399104	PTTG2	10744	pituitary tumor-transforming 2
ENSG0000	562.3332	-0.24458	0.218189	0.078631	0.399104	ZNF408	79797	zinc finger protein 408
ENSG0000	997.9663	0.239764	0.216822	0.078796	0.399649	KLF7	8609	KLF transcription factor 7
ENSG0000	43.84923	-0.25079	0.246382	0.07885	0.399649	NUDT9	53343	nudix hydrolase 9
ENSG0000	7.259236	0.151656	0.282812	0.078828	0.399649	NA	NA	NA
ENSG0000	3.568797	-0.12	0.26723	0.078787	0.399649	NA	NA	NA
ENSG0000	1108.617	-0.21623	0.160236	0.078861	0.399649	SETX	23064	senataxin
ENSG0000	3762.073	0.238141	0.200038	0.07887	0.399649	ULK1	8408	unc-51 like autophagy activating kinase 1
ENSG0000	9084.308	-0.243	0.23315	0.078937	0.399883	APBB1IP	54518	amyloid beta precursor protein binding family B member 1 interacting protein
ENSG0000	7.18649	-0.1398	0.275286	0.079036	0.400271	NA	NA	NA
ENSG0000	8673.183	-0.24504	0.220099	0.079105	0.400397	MCL1	4170	MCL1 apo BCL2 family member
ENSG0000	5.373097	0.130386	0.271521	0.07909	0.400397	SATB2-AS1	150538	SATB2 antisense RNA 1
ENSG0000	491.4858	0.220965	0.169214	0.079184	0.400686	NA	NA	NA
ENSG0000	9776.912	0.238306	0.265246	0.079231	0.400818	TSC22D3	1831	TSC22 domain family member 3
ENSG0000	335.524	0.244712	0.225616	0.079278	0.400942	PPP3CA	5530	protein phosphatase 3 catalytic subunit alpha
ENSG0000	454.5782	0.210625	0.28773	0.079334	0.401117	RPL21	6144	ribosomal protein L21
ENSG0000	30.6025	0.239234	0.293607	0.079369	0.401148	PHF7	51533	PHD finger protein 7
ENSG0000	136.8188	0.209556	0.150697	0.079384	0.401148	EXD3	54932	exonuclease 3'-5' domain containing 3
ENSG0000	35.39061	0.247891	0.271015	0.079441	0.401215	SHPRH	257218	SNF2 histone linker PHD RING helicase
ENSG0000	39.30155	0.253201	0.226779	0.079435	0.401215	RDX	5962	radixin
ENSG0000	208.6837	0.230852	0.182926	0.079565	0.401364	PBRM1	55193	polybromo 1
ENSG0000	3.558581	0.091918	0.253271	0.07958	0.401364	TAFA1	407738	TAFA chemokine like family member 1
ENSG0000	16.43492	0.257525	0.287266	0.079511	0.401364	NA	NA	NA
ENSG0000	13.67301	0.217439	0.308711	0.079566	0.401364	DAB2IP	153090	DAB2 interacting protein
ENSG0000	6.079333	0.154197	0.285589	0.079566	0.401364	NA	NA	NA
ENSG0000	4.745972	0.159493	0.290802	0.079616	0.401433	NA	NA	NA
ENSG0000	1938.289	0.225233	0.170548	0.07967	0.401597	TCF25	22980	transcription factor 25
ENSG0000	45.16351	0.230843	0.291044	0.07974	0.401632	LSM3	27258	LSM3 hom U6 small nuclear RNA and mRNA degradation associated
ENSG0000	24.95166	0.186257	0.29516	0.079743	0.401632	CABP5	56344	calcium binding protein 5
ENSG0000	22.36615	0.217469	0.302216	0.079707	0.401632	BEND2	139105	BEN domain containing 2
ENSG0000	4.365543	0.114285	0.263046	0.079775	0.401681	ZNF563	147837	zinc finger protein 563
ENSG0000	1460.268	-0.24231	0.229863	0.079858	0.401881	UBXN2B	137886	UBX domain protein 2B
ENSG0000	12.80397	0.226091	0.316559	0.07985	0.401881	NYNRIN	57523	NYN domain and retroviral integrase containing
ENSG0000	9.20065	0.149718	0.280614	0.079968	0.402322	NA	NA	NA
ENSG0000	51.06415	0.240319	0.276045	0.08	0.402362	NA	NA	NA
ENSG0000	4256.084	0.180384	0.118744	0.08002	0.402362	RPS9	6203	ribosomal protein S9
ENSG0000	18.01737	-0.25327	0.286028	0.080047	0.402389	FOXN3-AS	400236	FOXN3 antisense RNA 1
ENSG0000	50.43208	-0.24812	0.222995	0.080304	0.402687	SEMA4F	10505	ssemaphorin 4F
ENSG0000	2.786321	0.064107	0.242653	0.080415	0.402687	NA	NA	NA
ENSG0000	14.63492	-0.21772	0.308149	0.080342	0.402687	NA	NA	NA

ENSG0000	11.86265	0.123905	0.265605	0.080399	0.402687	FOXC1	2296	forkhead box C1
ENSG0000	182.5932	-0.23675	0.194313	0.080326	0.402687	RRAGD	58528	Ras related GTP binding D
ENSG0000	560.0502	-0.1945	0.134047	0.080364	0.402687	ZHX2	22882	zinc fingers and homeoboxes 2
ENSG0000	1552.085	-0.21861	0.160256	0.080227	0.402687	TESK1	7016	testis associated actin remodelling kinase 1
ENSG0000	169.6752	0.232136	0.186844	0.080448	0.402687	TAF6L	10629	TATA-box binding protein associated factor 6 like
ENSG0000	419.1888	-0.22965	0.186239	0.080317	0.402687	ARL11	115761	ADP ribosylation factor like GTPase 11
ENSG0000	34.79201	0.228418	0.293144	0.080237	0.402687	DLGAP5	9787	DLG associated protein 5
ENSG0000	5.359409	0.110708	0.2603	0.080245	0.402687	MIR193BH	1E+08	MIR193b-365a host gene
ENSG0000	45374.57	-0.23429	0.264747	0.080332	0.402687	GRN	2896	granulin precursor
ENSG0000	31.06426	-0.24574	0.279152	0.080458	0.402687	LIN37	55957	lin-37 DREAM MuvB core complex component
ENSG0000	44.08722	0.24952	0.265428	0.080455	0.402687	LINC0148(	1.02E+08	long intergenic non-protein coding RNA 1480
ENSG0000	10273.35	-0.24183	0.237255	0.080375	0.402687	LILRB3	11025	leukocyte immunoglobulin like receptor B3
ENSG0000	24.61226	-0.21107	0.299683	0.080232	0.402687	KCNE5	23630	potassium voltage-gated channel subfamily E regulatory subunit 5
ENSG0000	7.77772	0.189645	0.305277	0.0805	0.402787	CNBD2	140894	cyclic nucleotide binding domain containing 2
ENSG0000	188.0134	-0.20332	0.143446	0.080524	0.402796	KBTBD4	55709	kelch repeat and BTB domain containing 4
ENSG0000	307.3026	-0.19733	0.137757	0.080571	0.402921	RPP25L	138716	ribonuclease P/MRP subunit p25 like
ENSG0000	2318.403	-0.23388	0.195546	0.080597	0.402939	WIPF2	147179	WAS/WASL interacting protein family member 2
ENSG0000	118.964	-0.23626	0.258741	0.080679	0.403243	GPX3	2878	glutathione peroxidase 3
ENSG0000	28.31904	0.25522	0.233998	0.080718	0.403324	EBF4	57593	EBF family member 4
ENSG0000	1182.533	0.23887	0.208408	0.080759	0.40342	RPL24	6152	ribosomal protein L24
ENSG0000	45.92947	0.251701	0.228467	0.080836	0.403696	DMAC2L	27109	distal membrane arm assembly component 2 like
ENSG0000	147.8237	-0.22421	0.172289	0.080861	0.403708	COLQ	8292	collagen like tail subunit of asymmetric acetylcholinesterase
ENSG0000	32.07883	-0.25334	0.239012	0.080891	0.403748	STX5-DT	1.05E+08	STX5 divergent transcript
ENSG0000	262.6594	0.205316	0.145884	0.080948	0.403889	WDR5	11091	WD repeat domain 5
ENSG0000	5965.228	-0.174	0.116959	0.080963	0.403889	SERF2	10169	small EDRK-rich factor 2
ENSG0000	18.12806	-0.22526	0.302326	0.081006	0.403981	LINC01762	1.02E+08	long intergenic non-protein coding RNA 1762
ENSG0000	10.77817	0.151697	0.280034	0.081026	0.403981	NA	NA	NA
ENSG0000	6.421898	0.127304	0.268449	0.081116	0.404242	KCTD21-A:	1E+08	KCTD21 antisense RNA 1
ENSG0000	5.557974	-0.13276	0.272462	0.081134	0.404242	NA	NA	NA
ENSG0000	6.758317	-0.11742	0.264129	0.081144	0.404242	NA	NA	NA
ENSG0000	6.675129	0.142159	0.277928	0.081205	0.404433	LOC10192	1.02E+08	uncharacterized LOC101928718
ENSG0000	6.488522	-0.09764	0.253945	0.081234	0.404471	ASS1	445	argininosuccinate synthase 1
ENSG0000	172.2614	0.224671	0.172628	0.081358	0.404744	PUSL1	126789	pseudouridine synthase like 1
ENSG0000	33.3892	0.241874	0.289514	0.081378	0.404744	NA	NA	NA
ENSG0000	402.4836	0.238599	0.203921	0.081332	0.404744	DNAJC3	5611	DnaJ heat shock protein family (Hsp40) member C3
ENSG0000	323.6647	-0.20437	0.143442	0.081344	0.404744	SYS1	90196	SYS1 golgi trafficking protein
ENSG0000	9.947811	-0.15984	0.28648	0.081417	0.404833	NA	NA	NA
ENSG0000	625.1278	0.234895	0.198586	0.081469	0.404977	HIPK2	28996	homeodomain interacting protein kinase 2
ENSG0000	8.260041	0.116589	0.261845	0.081499	0.40502	NA	NA	NA
ENSG0000	2.664047	0.069825	0.247424	0.081609	0.405363	EPCAM	4072	epithelial cell adhesion molecule
ENSG0000	437.1706	0.181561	0.123077	0.081648	0.405363	ACTR1B	10120	actin related protein 1B
ENSG0000	1215.029	-0.22403	0.173885	0.081657	0.405363	NOP10	55505	NOP10 ribonucleoprotein
ENSG0000	2.602698	0.064241	0.243801	0.08162	0.405363	NA	NA	NA
ENSG0000	868.1102	-0.21036	0.152885	0.081722	0.405577	BIN3	55909	bridging integrator 3
ENSG0000	313.1677	0.218825	0.165468	0.081772	0.405606	DUS3L	56931	dihydrouridine synthase 3 like
ENSG0000	427.3903	0.239111	0.226551	0.081762	0.405606	DBP	1628	D-box binding PAR bZIP transcription factor
ENSG0000	62.31276	0.240032	0.201905	0.081895	0.405994	PECR	55825	peroxisomal trans-2-enoyl-CoA reductase
ENSG0000	546.3135	-0.1827	0.122613	0.081892	0.405994	TFDP1	7027	transcription factor Dp-1
ENSG0000	103.4732	-0.23587	0.19591	0.081963	0.406223	PHC1	1911	polyhomeotic homolog 1
ENSG0000	11.49932	-0.22018	0.30913	0.082029	0.406285	ITGA9-AS1	1.02E+08	ITGA9 antisense RNA 1
ENSG0000	79.43056	0.228466	0.285085	0.082042	0.406285	ALCAM	214	activated leukocyte cell adhesion molecule
ENSG0000	342.7277	-0.18337	0.128101	0.082005	0.406285	HPS3	84343	HPS3 biogenesis of lysosomal organelles complex 2 subunit 1
ENSG0000	70.66092	0.243464	0.211026	0.08211	0.406436	SEMA6C	10500	semaphorin 6C
ENSG0000	198.9827	0.20645	0.148269	0.082184	0.406436	GMPS	8833	guanine monophosphate synthase
ENSG0000	58.81477	0.247784	0.251735	0.082122	0.406436	KNSTRN	90417	kinetochore localized astrin (SPAG5) binding protein
ENSG0000	5.124025	0.093726	0.252713	0.082165	0.406436	MIR635	693220	microRNA 635
ENSG0000	925.684	0.211742	0.154835	0.082154	0.406436	SPPL2B	56928	signal peptide peptidase like 2B
ENSG0000	8.956618	-0.09753	0.254948	0.082231	0.406561	NA	NA	NA
ENSG0000	4.119732	0.11309	0.262703	0.082483	0.407698	ZNF682	91120	zinc finger protein 682
ENSG0000	24.85541	-0.24956	0.267087	0.082549	0.407747	FMO4	2329	flavin containing dimethylaniline monoxygenase 4
ENSG0000	23.11003	-0.19376	0.293925	0.082565	0.407747	SERPINE1	5054	serpin family E member 1
ENSG0000	712.4159	-0.20032	0.141409	0.082649	0.407747	SLC39A13	91252	solute carrier family 39 member 13
ENSG0000	1255.293	-0.22866	0.187502	0.082637	0.407747	GNG2	54331	G protein subunit gamma 2
ENSG0000	604.9084	0.174803	0.115635	0.082644	0.407747	SLTM	79811	SAFB like transcription modulator
ENSG0000	3039.373	-0.20766	0.157006	0.082643	0.407747	VAMP2	6844	vesicle associated membrane protein 2
ENSG0000	2.863563	0.089211	0.25153	0.082559	0.407747	LOC12490	1.25E+08	uncharacterized LOC124904611
ENSG0000	120.8236	-0.24291	0.230824	0.082762	0.40775	CHI3L2	1117	chitinase 3 like 2
ENSG0000	7.547425	-0.18893	0.303447	0.08276	0.40775	XXYLT1-AS	1.01E+08	XXYLT1 antisense RNA 2
ENSG0000	847.9071	0.198711	0.140388	0.082805	0.40775	VOPP1	81552	VOPP1 WW domain binding protein
ENSG0000	5443.609	-0.21318	0.160097	0.082773	0.40775	TSPAN14	81619	tetraspanin 14
ENSG0000	13.24112	0.19076	0.298625	0.082697	0.40775	NA	NA	NA
ENSG0000	8.471315	0.201229	0.308773	0.082699	0.40775	SLC9A5	6553	solute carrier family 9 member A5
ENSG0000	33.34419	-0.23065	0.290515	0.082793	0.40775	NA	NA	NA
ENSG0000	12.48178	-0.18806	0.29917	0.082852	0.407755	NA	NA	NA
ENSG0000	193.5431	0.2197	0.167443	0.082833	0.407755	TCERG1	10915	transcription elongation regulator 1
ENSG0000	1369.422	0.212835	0.162966	0.082873	0.407755	LGALS3	3958	galectin 3
ENSG0000	4.217517	-0.10608	0.259926	0.082989	0.408109	AGBL3	340351	AGBL carboxypeptidase 3
ENSG0000	4.703389	-0.07834	0.246775	0.08299	0.408109	FAM83E	54854	family with sequence similarity 83 member E
ENSG0000	16.47579	0.11151	0.258726	0.083025	0.408171	NA	NA	NA
ENSG0000	11.10949	0.169521	0.288981	0.083178	0.408283	EPHB3	2049	EPH receptor B3
ENSG0000	417.7006	-0.18425	0.124001	0.083146	0.408283	TSPAN17	26262	tetraspanin 17
ENSG0000	85.96847	-0.24517	0.238787	0.083181	0.408283	HINT3	135114	histidine triad nucleotide binding protein 3
ENSG0000	5.556329	-0.08622	0.249147	0.083106	0.408283	NA	NA	NA
ENSG0000	28.57951	0.250623	0.231439	0.083131	0.408283	ZSCAN5A	79149	zinc finger and SCAN domain containing 5A
ENSG0000	235.5133	0.230131	0.276331	0.083139	0.408283	NTSR1	4923	neurotensin receptor 1
ENSG0000	316.3987	0.217322	0.16486	0.083325	0.408768	KDM1A	23028	lysine demethylase 1A
ENSG0000	1306.663	0.210739	0.283531	0.083319	0.408768	IGHV4-39	28394	immunoglobulin heavy variable 4-39
ENSG0000	366.6123	0.207815	0.282395	0.083367	0.408869	ZBTB16	7704	zinc finger and BTB domain containing 16
ENSG0000	487.6458	0.212218	0.158744	0.083438	0.408996	AAMP	14	angio associated migratory cell protein
ENSG0000	88.69666	0.239424	0.204273	0.083434	0.408996	CYTH3	9265	cytohesin 3
ENSG0000	4.804756	0.086565	0.249614	0.083506	0.409218	NA	NA	NA
ENSG0000	52.52476	0.247742	0.234469	0.083556	0.409353	THG1L	54974	tRNA-histidine guanylyltransferase 1 like
ENSG0000	281.0919	0.229597	0.17666	0.083701	0.409518	FBXO41	150726	F-box protein 41
ENSG0000	16.25272	0.191644	0.296584	0.083651	0.409518	ANKRD39	51239	ankyrin repeat domain 39
ENSG0000	154.8948	0.22092	0.170449	0.083687	0.409518	ZNF821	55565	zinc finger protein 821
ENSG0000	1216.678	-0.23384	0.202887	0.0837	0.409518	CNDP2	55748	carnosine dipeptidase 2

ENSG0000	686.4941	-0.23857	0.231989	0.083635	0.409518	ATF5	22809	activating transcription factor 5
ENSG0000	528.4187	0.236195	0.233933	0.083874	0.409708	SRP72	6731	signal recognition particle 72
ENSG0000	10.56715	0.191499	0.301635	0.083809	0.409708	SPRY1	10252	sprouty RTK signaling antagonist 1
ENSG0000	83.9111	0.243595	0.24484	0.083806	0.409708	STRBP	55342	spermatid perinuclear RNA binding protein
ENSG0000	14.94387	-0.18576	0.296059	0.083771	0.409708	TRAV1-2	28692	T cell receptor alpha variable 1-2
ENSG0000	19.84445	0.232162	0.300851	0.083864	0.409708	IGHJ4	28477	immunoglobulin heavy joining 4
ENSG0000	70.87959	-0.24442	0.235166	0.083843	0.409708	LOC10537	1.05E+08	uncharacterized LOC105372401
ENSG0000	2.653497	0.092281	0.253685	0.083926	0.40978	FMC1	154791	formation of mitochondrial complex V assembly factor 1 homolog
ENSG0000	525.0276	-0.23608	0.24943	0.083934	0.40978	ALOX12	239	arachidon: 12S type
ENSG0000	5.298115	0.087567	0.250136	0.084011	0.409978	KIT	3815	KIT proto-(receptor tyrosine kinase
ENSG0000	170.4	0.2249	0.177969	0.084041	0.409978	SEPHS1	22929	selenophosphate synthetase 1
ENSG0000	8.471565	-0.17067	0.29217	0.084041	0.409978	MIR657	724027	microRNA 657
ENSG0000	5.854819	0.124646	0.266847	0.084106	0.410062	NA	NA	NA
ENSG0000	397.7531	-0.22001	0.274425	0.084082	0.410062	GP1BA	2811	glycoprotein Ib platelet subunit alpha
ENSG0000	16.34678	0.218283	0.314084	0.084126	0.410062	NA	NA	NA
ENSG0000	14.75783	-0.20729	0.303561	0.084151	0.410076	NA	NA	NA
ENSG0000	224.9249	-0.23967	0.232047	0.084371	0.410318	NA	NA	NA
ENSG0000	315.5865	0.195006	0.135673	0.084307	0.410318	PIAS3	10401	protein inhibitor of activated STAT 3
ENSG0000	1473.408	0.177857	0.119617	0.084236	0.410318	EIF3H	8667	eukaryotic translation initiation factor 3 subunit H
ENSG0000	29.89446	0.223934	0.291387	0.08438	0.410318	C12orf42	374470	chromosome 12 open reading frame 42
ENSG0000	22.44717	0.228474	0.290591	0.084293	0.410318	RPS6KL1	83694	ribosomal protein S6 kinase like 1
ENSG0000	2.595046	0.067723	0.244185	0.084374	0.410318	NA	NA	NA
ENSG0000	6.304196	-0.16514	0.290434	0.084379	0.410318	NA	NA	NA
ENSG0000	2.882589	0.070536	0.244583	0.084259	0.410318	DMC1	11144	DNA meiotic recombinase 1
ENSG0000	186.3685	-0.23497	0.202985	0.084454	0.410358	LINC0227:	1.01E+08	long intergenic non-protein coding RNA 2273
ENSG0000	1149.099	0.218399	0.168658	0.084438	0.410358	MAN2C1	4123	mannosidase alpha class 2C member 1
ENSG0000	131.4828	0.213287	0.159345	0.084455	0.410358	STX8	9482	syntaxin 8
ENSG0000	237.7508	0.238883	0.225711	0.084612	0.411011	ZFAND6	54469	zinc finger AN1-type containing 6
ENSG0000	12.03997	-0.21071	0.30635	0.084702	0.411342	C1orf226	400793	chromosome 1 open reading frame 226
ENSG0000	145.4684	0.221847	0.173161	0.084763	0.411416	TSPAN3	10099	tetraspanin 3
ENSG0000	10.64159	-0.12346	0.266841	0.084745	0.411416	LINC0173(	1.02E+08	long intergenic non-protein coding RNA 1730
ENSG0000	169.2947	0.199294	0.141727	0.084828	0.411621	SLC31A1	1317	solute carrier family 31 member 1
ENSG0000	21.63398	0.23899	0.290705	0.084898	0.411855	CR2	1380	complement C3d receptor 2
ENSG0000	8.752231	0.156893	0.28383	0.084944	0.411967	RCAN3AS	1.01E+08	RCAN3 antisense RNA
ENSG0000	187.7094	0.181437	0.28217	0.085009	0.412175	RPL34	6164	ribosomal protein L34
ENSG0000	26.50261	-0.24931	0.262377	0.085142	0.412712	EGLN3	112399	egl-9 family hypoxia inducible factor 3
ENSG0000	842.4123	-0.22189	0.263961	0.085172	0.412744	GFI1B	8328	growth factor independent 1B transcriptional repressor
ENSG0000	9834.945	-0.2279	0.191686	0.085214	0.412842	ITPK1	3705	inositol-tetrakisphosphate 1-kinase
ENSG0000	15.18514	0.166474	0.285243	0.085314	0.413203	SASS6	163786	SAS-6 centriolar assembly protein
ENSG0000	267.4048	-0.19929	0.14722	0.085334	0.413203	ZNF397	84307	zinc finger protein 397
ENSG0000	309.4462	0.229494	0.264518	0.085368	0.413255	IGKV2-24	28923	immunoglobulin kappa variable 2-24
ENSG0000	61.8343	-0.24163	0.218011	0.08539	0.413255	SIAE	54414	sialic acid acetylesterase
ENSG0000	96.24692	-0.22359	0.280938	0.085458	0.413461	NA	NA	NA
ENSG0000	244.874	0.194912	0.137918	0.085478	0.413461	SLC12A4	6560	solute carrier family 12 member 4
ENSG0000	15.08426	0.208314	0.301743	0.085585	0.41387	RASSF1-AS	1.02E+08	RASSF1 antisense RNA 1
ENSG0000	16836.89	0.237579	0.244803	0.085685	0.414246	SLC11A1	6556	solute carrier family 11 member 1
ENSG0000	3.366574	-0.0483	0.239773	0.085737	0.414389	COBL	23242	cordon-bleu WH2 repeat protein
ENSG0000	1925.701	-0.23138	0.202597	0.08578	0.414463	SIPA1L1	26037	signal induced proliferation associated 1 like 1
ENSG0000	1300.98	0.217157	0.168312	0.085798	0.414463	SRSF2	6427	serine and arginine rich splicing factor 2
ENSG0000	111.088	0.234502	0.194922	0.085919	0.41494	TMEM147	1.01E+08	TMEM147 antisense RNA 1
ENSG0000	137.5791	0.226054	0.183846	0.086096	0.415676	SLC12A2-D	644873	SLC12A2 divergent transcript
ENSG0000	851.2485	0.228264	0.257256	0.086139	0.415676	IGHV3-23	28442	immunoglobulin heavy variable 3-23
ENSG0000	6.931258	-0.10052	0.25594	0.08614	0.415676	LOC12490	1.25E+08	uncharacterized LOC124905179
ENSG0000	170.7885	-0.19113	0.136698	0.086198	0.415847	PIGC	5279	phosphatidylinositol glycan anchor biosynthesis class C
ENSG0000	4.669367	0.098436	0.255045	0.08627	0.416087	NA	NA	NA
ENSG0000	83.58131	-0.22898	0.187414	0.08636	0.416088	ARHGEF19	128272	Rho guanine nucleotide exchange factor 19
ENSG0000	4902.463	-0.21972	0.172482	0.08631	0.416088	TMEM127	55654	transmembrane protein 127
ENSG0000	3.415736	0.126524	0.270419	0.086361	0.416088	CHDH	55349	choline dehydrogenase
ENSG0000	1323.792	0.221873	0.177917	0.086336	0.416088	BRAT1	221927	BRCA1 associated ATM activator 1
ENSG0000	49.59879	0.243498	0.230328	0.08667	0.417138	ANKRD16	54522	ankyrin repeat domain 16
ENSG0000	376.386	0.210103	0.157797	0.086642	0.417138	NARS1	4677	asparaginyl-tRNA synthetase 1
ENSG0000	3.750934	0.100173	0.257368	0.086633	0.417138	DM1-AS	1.1E+08	DM1 locus antisense RNA
ENSG0000	5.278396	0.103644	0.256789	0.086665	0.417138	NA	NA	NA
ENSG0000	299.6903	-0.22456	0.186097	0.086707	0.417206	SIDT1	54847	SID1 transmembrane family member 1
ENSG0000	3.653464	-0.11807	0.266771	0.08677	0.417401	SERHL	94009	serine hydrolase like (pseudogene)
ENSG0000	9.126394	0.165461	0.287924	0.08696	0.418203	SLC2A1-DT	440584	SLC2A1 divergent transcript
ENSG0000	482.056	0.215823	0.176801	0.086984	0.418207	ATP5MC3	518	ATP synthase membrane subunit c locus 3
ENSG0000	230.5895	-0.21615	0.168027	0.087159	0.418391	CASP9	842	caspase 9
ENSG0000	14.01448	0.158923	0.282561	0.087228	0.418391	PLCL1	5334	phospholipase C like 1 (inactive)
ENSG0000	63.40928	-0.24053	0.243837	0.087181	0.418391	B3GNT7	93010	UDP-GlcN/ 3-N-acetylglucosaminyltransferase 7
ENSG0000	18.08798	0.193681	0.295683	0.087074	0.418391	NA	NA	NA
ENSG0000	5.191038	0.100972	0.255246	0.087071	0.418391	NA	NA	NA
ENSG0000	37.67193	0.241306	0.263453	0.087103	0.418391	SLC46A1	113235	solute carrier family 46 member 1
ENSG0000	3169.783	0.228545	0.259493	0.087139	0.418391	SIGLEC10	89790	sialic acid binding Ig like lectin 10
ENSG0000	4.638457	0.063655	0.243173	0.087184	0.418391	TLDC2	140711	TBC/LysM-associated domain containing 2
ENSG0000	4804.317	-0.20055	0.144467	0.087212	0.418391	SEPTIN6	23157	septin 6
ENSG0000	203.6592	0.201411	0.150801	0.087297	0.418612	HDAC2	3066	histone deacetylase 2
ENSG0000	22.68907	0.239726	0.283239	0.087356	0.418677	MAPK8IP1	9479	mitogen-activated protein kinase 8 interacting protein 1
ENSG0000	2.726473	-0.06787	0.244351	0.087337	0.418677	NA	NA	NA
ENSG0000	81.26425	0.240471	0.245052	0.087395	0.418756	LTC4S	4056	leukotriene C4 synthase
ENSG0000	524.9991	-0.19181	0.135718	0.087503	0.419162	ACOT8	10005	acyl-CoA thioesterase 8
ENSG0000	25.61747	0.199818	0.293326	0.087528	0.419168	NA	NA	NA
ENSG0000	121.4477	0.236532	0.235014	0.08755	0.419168	ARHGAP19	84986	Rho GTPase activating protein 19
ENSG0000	3.869905	-0.13621	0.274979	0.087586	0.41923	NA	NA	NA
ENSG0000	97.72453	0.237001	0.220161	0.087664	0.419493	MRS2	57380	magnesium transporter MRS2
ENSG0000	102.9958	-0.23761	0.228298	0.087693	0.419525	IRAK2	3656	interleukin 1 receptor associated kinase 2
ENSG0000	10.60845	-0.17953	0.29289	0.087737	0.4196	H2AC15	8330	H2A clustered histone 15
ENSG0000	298.0975	-0.23185	0.2268	0.087755	0.4196	AJM1	389813	apical junction component 1 homolog
ENSG0000	156.2708	0.233894	0.261116	0.087799	0.419639	KLHL8	57563	kelch like family member 8
ENSG0000	2.556514	0.068683	0.24464	0.087827	0.419639	DNAJC9-A	414245	DNAJC9 and MRPS16 antisense RNA 1
ENSG0000	301.7086	0.217437	0.171994	0.087831	0.419639	IPO5	3843	importin 5
ENSG0000	15.69251	0.17516	0.287951	0.087925	0.419867	CCDC65	85478	coiled-coil domain containing 65
ENSG0000	132.2799	0.228163	0.190492	0.087919	0.419867	CLPX	10845	caseinolytic mitochondrial matrix peptidase chaperone subunit X
ENSG0000	17.17638	0.220359	0.295233	0.08799	0.420066	GUSBP1	728411	GUSB pseudogene 1
ENSG0000	84.80613	-0.22861	0.269524	0.088075	0.420256	NA	NA	NA
ENSG0000	349.496	0.237977	0.258933	0.088059	0.420256	THBD	7056	thrombomodulin

ENSG0000	472.1321	-0.20945	0.156916	0.088106	0.420295	CCDC71	64925	coiled-coil domain containing 71
ENSG0000	46.97039	0.234131	0.269764	0.088141	0.420341	NCEH1	57552	neutral cholesterol ester hydrolase 1
ENSG0000	421.4331	-0.22718	0.195639	0.088162	0.420341	PLEKHF1	79156	pleckstrin homology and FYVE domain containing 1
ENSG0000	100.1294	0.231848	0.264282	0.088309	0.420932	SCARF2	91179	scavenger receptor class F member 2
ENSG0000	47.5438	0.166899	0.281274	0.088356	0.421045	MS4A3	932	membrane spanning 4-domains A3
ENSG0000	6.10057	0.147692	0.279519	0.088487	0.421561	NA	NA	NA
ENSG0000	4.627808	0.14902	0.282488	0.088545	0.42173	NA	NA	NA
ENSG0000	38.42023	0.243546	0.236725	0.088577	0.421772	MTFR1	9650	mitochondrial fission regulator 1
ENSG0000	4.570953	0.099634	0.255732	0.088668	0.422092	NA	NA	NA
ENSG0000	1046.812	-0.2265	0.20249	0.088796	0.422596	ZNF319	57567	zinc finger protein 319
ENSG0000	387.8508	0.185823	0.131116	0.088871	0.42284	FCHSD2	9873	FCH and double SH3 domains 2
ENSG0000	1414.327	-0.22595	0.193647	0.088949	0.422885	PNKD	25953	PNKD metallo-beta-lactamase domain containing
ENSG0000	2327.881	-0.19439	0.133719	0.088925	0.422885	FYN	2534	FYN proto- Src family tyrosine kinase
ENSG0000	50.07972	-0.2391	0.222437	0.08893	0.422885	ARPC1A	10552	actin related protein 2/3 complex subunit 1A
ENSG0000	38.64016	0.177007	0.282813	0.089046	0.423233	KIF20B	9585	kinesin family member 20B
ENSG0000	2.751471	0.100315	0.257301	0.089092	0.423345	NA	NA	NA
ENSG0000	104.4222	0.216286	0.168944	0.089254	0.423474	UTP11	51118	UTP11 small subunit processome component
ENSG0000	82.57891	0.237154	0.228322	0.089258	0.423474	DNAJC21	134218	DnaJ heat shock protein family (Hsp40) member C21
ENSG0000	263.076	-0.1917	0.13704	0.089236	0.423474	SMU1	55234	SMU1 DNA replication regulator and spliceosomal factor
ENSG0000	477.295	-0.22339	0.187325	0.089168	0.423474	AHSA1	10598	activator of HSP90 ATPase activity 1
ENSG0000	3.872079	0.097665	0.255412	0.089222	0.423474	NA	NA	NA
ENSG0000	100.5154	-0.23604	0.225205	0.089236	0.423474	TRIM47	91107	tripartite motif containing 47
ENSG0000	8.630785	0.165702	0.288228	0.089291	0.42352	VEPH1	79674	ventricular zone expressed PH domain containing 1
ENSG0000	615.8938	0.225695	0.191114	0.089328	0.423587	CCR2	729230	C-C motif chemokine receptor 2
ENSG0000	12.18679	0.216068	0.301873	0.089354	0.423599	GLMN	11146	glomulin FKBP associated protein
ENSG0000	3848.298	-0.20977	0.162495	0.089425	0.423825	CASC3	22794	CASC3 exon junction complex subunit
ENSG0000	2582.944	-0.22927	0.207652	0.089615	0.424288	ARHGAP26	23092	Rho GTPase activating protein 26
ENSG0000	1888.516	-0.22811	0.205059	0.089594	0.424288	PCED1B	91523	PC-esterase domain containing 1B
ENSG0000	9.314567	-0.20609	0.302834	0.089552	0.424288	TSC22D1-/	641467	TSC22D1 antisense RNA 1
ENSG0000	2.994494	-0.09226	0.252682	0.089613	0.424288	NA	NA	NA
ENSG0000	544.9898	-0.19821	0.145742	0.089691	0.4243	LIMS1	3987	LIM zinc finger domain containing 1
ENSG0000	39.0571	0.233182	0.269744	0.089646	0.4243	PMS1	5378	PMS1 hom mismatch repair system component
ENSG0000	6.720336	0.139407	0.273788	0.089745	0.4243	HILPDA	29923	hypoxia inducible lipid droplet associated
ENSG0000	34.44412	-0.23924	0.263095	0.089689	0.4243	CEP170B	283638	centrosomal protein 170B
ENSG0000	834.9428	-0.20612	0.157428	0.089757	0.4243	RTF2	51507	replication termination factor 2
ENSG0000	29.36924	0.1927	0.297334	0.089719	0.4243	EIF1AY	9086	eukaryotic translation initiation factor 1A Y-linked
ENSG0000	3.335952	0.059367	0.241689	0.089861	0.424684	TMEM37	140738	transmembrane protein 37
ENSG0000	416.4431	-0.18661	0.132015	0.089912	0.424813	PPP2R2A	5520	protein phosphatase 2 regulatory subunit Balpha
ENSG0000	13.6342	0.140976	0.271555	0.089994	0.424983	LINC01191	440900	long intergenic non-protein coding RNA 1191
ENSG0000	90.98369	0.224335	0.183765	0.089989	0.424983	IFT52	51098	intraflagellar transport 52
ENSG0000	39.32127	-0.18414	0.282994	0.090031	0.425047	STK3	6788	serine/threonine kinase 3
ENSG0000	113.1353	-0.22592	0.264855	0.090091	0.425224	GNAO1	2775	G protein subunit alpha o1
ENSG0000	472.7298	-0.22979	0.248067	0.090201	0.42563	ADGRE1	2015	adhesion G protein-coupled receptor E1
ENSG0000	7.765729	0.131845	0.269322	0.090226	0.425641	LOC15506	155060	AI894139 pseudogene
ENSG0000	54.87658	-0.2388	0.238378	0.090297	0.425864	POC1A	25886	POC1 centriolar protein A
ENSG0000	1044.254	0.197092	0.142367	0.090361	0.426059	HNRNPH1	3187	heterogeneous nuclear ribonucleoprotein H1
ENSG0000	6.594983	-0.16078	0.285401	0.090396	0.426113	NA	NA	NA
ENSG0000	6.936632	0.091379	0.250992	0.090484	0.426418	IGHV3-69-	28402	immunoglobulin heavy variable 3-69-1 (pseudogene)
ENSG0000	9.72041	0.147026	0.276753	0.090564	0.426685	PLCG1-AS:	1.02E+08	PLCG1 antisense RNA 1
ENSG0000	9.55732	0.179573	0.292988	0.09059	0.4267	MIR25	407014	microRNA 25
ENSG0000	55.96849	0.229153	0.253231	0.090632	0.426784	KLHL7	55975	kelch like family member 7
ENSG0000	4.44749	-0.06238	0.241457	0.090683	0.426918	LOC10537	1.05E+08	uncharacterized LOC105370525
ENSG0000	49.40239	0.230353	0.194482	0.090825	0.427385	TFAP2E-AS	1.05E+08	TFAP2E antisense RNA 1
ENSG0000	521.4584	-0.16084	0.107249	0.090832	0.427385	ZNF394	84124	zinc finger protein 394
ENSG0000	24372.82	-0.22723	0.205548	0.090853	0.427385	SLC25A37	51312	solute carrier family 25 member 37
ENSG0000	810.8558	0.165717	0.113051	0.090933	0.427653	SUGP2	10147	SURP and G-patch domain containing 2
ENSG0000	464.4799	-0.22402	0.197193	0.09123	0.428828	C4orf3	401152	chromosome 4 open reading frame 3
ENSG0000	3522.269	0.198391	0.146982	0.091208	0.428828	RPS19	6223	ribosomal protein S19
ENSG0000	4.305885	0.089312	0.250958	0.091353	0.429298	LOC12490	1.25E+08	uncharacterized LOC124902204
ENSG0000	9.639931	0.165975	0.285856	0.091514	0.429916	CCDC121	79635	coiled-coil domain containing 121
ENSG0000	8.79754	0.129287	0.267162	0.091532	0.429916	ERCC5	2073	ERCC excis endonuclease
ENSG0000	8.693356	-0.13006	0.267577	0.091555	0.429918	ODF3	113746	outer dense fiber of sperm tails 3
ENSG0000	58.96672	-0.23535	0.256239	0.091689	0.430126	PLEKHN1	84069	pleckstrin homology domain containing N1
ENSG0000	10.34034	-0.17222	0.288103	0.091858	0.430126	AK4	205	adenylate kinase 4
ENSG0000	4.143755	-0.09318	0.253499	0.091746	0.430126	NA	NA	NA
ENSG0000	31.7164	0.234761	0.268008	0.09185	0.430126	SMIM4	440957	small integral membrane protein 4
ENSG0000	22.44171	0.233753	0.278399	0.091753	0.430126	DST	667	dystonin
ENSG0000	502.8537	-0.22983	0.230267	0.091654	0.430126	EXOSC4	54512	exosome component 4
ENSG0000	25.19295	0.230199	0.280579	0.09184	0.430126	CARD9	64170	caspase recruitment domain family member 9
ENSG0000	3.026316	-0.05993	0.24232	0.091834	0.430126	NA	NA	NA
ENSG0000	455.9238	-0.22167	0.187563	0.091762	0.430126	ZFYVE1	53349	zinc finger FYVE-type containing 1
ENSG0000	541.4528	-0.19904	0.148786	0.091851	0.430126	RAB11A	8766	RAB11A member RAS oncogene family
ENSG0000	125.9697	0.226494	0.193143	0.0917	0.430126	MICAL3	57553	microtubu calponin and LIM domain containing 3
ENSG0000	16.76593	0.174647	0.285995	0.091911	0.430265	BBS7	55212	Bardet-Biedl syndrome 7
ENSG0000	57.16519	0.197323	0.28198	0.091945	0.430313	LDLRAD3	143458	low density lipoprotein receptor class A domain containing 3
ENSG0000	496.5979	-0.23109	0.222773	0.091979	0.430362	CLIC3	9022	chloride intracellular channel 3
ENSG0000	257.3586	-0.22997	0.21024	0.092081	0.430732	CEP19	84984	centrosomal protein 19
ENSG0000	16.0971	0.208709	0.29446	0.092204	0.431195	SCX	642658	scleraxis bHLH transcription factor
ENSG0000	2224.088	-0.22467	0.247357	0.09227	0.431393	SMOX	54498	spermine oxidase
ENSG0000	199.7596	-0.22898	0.208346	0.092348	0.43165	RIMOC1	285636	RAB7A interacting MON1-CCZ1 complex subunit 1
ENSG0000	60.84795	0.234875	0.249082	0.092381	0.431692	ARL5A	26225	ADP ribosylation factor like GTPase 5A
ENSG0000	2451.742	-0.18965	0.137612	0.092428	0.431804	RAB35	11021	RAB35 member RAS oncogene family
ENSG0000	80.49025	-0.22465	0.189646	0.092463	0.431855	GCNT1	2650	glucosaminyl (N-acetyl) transferase 1
ENSG0000	14.22652	0.159375	0.280233	0.092499	0.431912	NA	NA	NA
ENSG0000	4.13177	0.121541	0.266771	0.092549	0.432039	NA	NA	NA
ENSG0000	3.665501	0.085391	0.249044	0.092657	0.432432	NA	NA	NA
ENSG0000	13.8458	0.168603	0.285228	0.092832	0.4326	SPTA1	6708	spectrin al erythrocytic 1
ENSG0000	1261.16	-0.22837	0.21494	0.092763	0.4326	CREG1	8804	cellular repressor of E1A stimulated genes 1
ENSG0000	18.92702	0.11186	0.257019	0.092835	0.4326	TMEM45A	55076	transmembrane protein 45A
ENSG0000	136.8397	0.22821	0.218507	0.092835	0.4326	NA	NA	NA
ENSG0000	208.0435	0.229621	0.22563	0.092796	0.4326	SENP6	26054	SUMO specific peptidase 6
ENSG0000	353.779	-0.18922	0.136642	0.092757	0.4326	DCLRE1C	64421	DNA cross-link repair 1C
ENSG0000	848.8526	-0.19364	0.142553	0.09289	0.432746	MYPOP	339344	Myb relate partner of profilin
ENSG0000	355.09	-0.21126	0.166455	0.092989	0.433096	MFSD5	84975	major facilitator superfamily domain containing 5
ENSG0000	100.547	-0.19731	0.146598	0.093105	0.433529	TSTD1	1E+08	thiosulfate sulfurtransferase like domain containing 1
ENSG0000	4027.731	0.207148	0.271325	0.093135	0.433559	IGLV6-57	28778	immunoglobulin lambda variable 6-57

ENSG0000	55.85295	-0.22536	0.267902	0.093272	0.433689	NA	NA	NA	
ENSG0000	3.704685	0.075977	0.246107	0.093242	0.433689	KLHDC9	126823	kelch domain containing 9	
ENSG0000	1689.513	0.223906	0.260121	0.093282	0.433689	CCNJL	79616	cyclin J like	
ENSG0000	3508.225	-0.22667	0.211552	0.093267	0.433689	ZER1	10444	zyg-11 related cell cycle regulator	
ENSG0000	618.3576	-0.22925	0.235997	0.093278	0.433689	OSBP2	23762	oxysterol binding protein 2	
ENSG0000	6.442831	0.140292	0.27442	0.093341	0.433855	RNF157-A!	1.01E+08	RNF157 antisense RNA 1	
ENSG0000	3966.34	0.227499	0.237505	0.093445	0.434227	IGKV4-1	28908	immunoglobulin kappa variable 4-1	
ENSG0000	11.77849	-0.14084	0.270767	0.093628	0.434967	EYS	346007	eyes shut homolog	
ENSG0000	29.00343	0.176734	0.284322	0.09366	0.435004	SSPN	8082	sarcospan	
ENSG0000	3.960082	-0.1056	0.258154	0.093699	0.435078	CD276	80381	CD276 molecule	
ENSG0000	22.0727	-0.18196	0.284314	0.093732	0.435122	LINC01624	401289	long intergenic non-protein coding RNA 1624	
ENSG0000	2876.425	-0.21256	0.17219	0.093821	0.4352	ZAP70	7535	zeta chain of T cell receptor associated protein kinase 70	
ENSG0000	52.55656	-0.23326	0.218954	0.093805	0.4352	ZSCAN16	80345	zinc finger and SCAN domain containing 16	
ENSG0000	2104.999	-0.22686	0.212658	0.093869	0.4352	HCG27	253018	HLA complex group 27	
ENSG0000	533.2223	0.202528	0.27347	0.093789	0.4352	EIF3E	3646	eukaryotic translation initiation factor 3 subunit E	
ENSG0000	247.0837	-0.19114	0.140194	0.093868	0.4352	ORMDL2	29095	ORMDL sphingolipid biosynthesis regulator 2	
ENSG0000	36.67553	0.236838	0.220614	0.093892	0.4352	MRPL12	6182	mitochondrial ribosomal protein L12	
ENSG0000	8379.709	-0.2221	0.195974	0.093971	0.435237	WDTC1	23038	WD and tetratricopeptide repeats 1	
ENSG0000	2.85702	0.079929	0.24788	0.093969	0.435237	POU5F1P6	1E+08	POU class 5 homeobox 1 pseudogene 6	
ENSG0000	212.9718	0.189421	0.136893	0.093959	0.435237	SPRING1	79794	SREBF pathway regulator in golgi 1	
ENSG0000	10.08961	0.194474	0.296112	0.094118	0.435805	HAS3	3038	hyaluronan synthase 3	
ENSG0000	6.466213	0.173149	0.292057	0.094162	0.435902	NA	NA	NA	
ENSG0000	310.5969	-0.18891	0.137275	0.094289	0.436272	SLC4A1AP	22950	solute carrier family 4 member 1 adaptor protein	
ENSG0000	3.61658	0.110147	0.260145	0.094305	0.436272	ARHGAP1:	1.14E+08	ARHGAP11A-SCG5 readthrough	
ENSG0000	4.508679	0.107189	0.257931	0.094314	0.436272	PKD1P6-N	1.05E+08	PKD1P6-NPIPP1 readthrough	
ENSG0000	34.12092	0.237315	0.22903	0.094471	0.436778	GXYLT1	283464	glucoside xylosyltransferase 1	
ENSG0000	241.5562	-0.22698	0.244453	0.094464	0.436778	FSTL3	10272	follistatin like 3	
ENSG0000	111.9971	0.23115	0.215998	0.094516	0.436879	TRIM7	81786	tripartite motif containing 7	
ENSG0000	9.578145	0.209371	0.300723	0.094644	0.436971	NA	NA	NA	
ENSG0000	4.075278	-0.07825	0.2465	0.094628	0.436971	NA	NA	NA	
ENSG0000	176.0543	0.221017	0.186748	0.094608	0.436971	ASL	435	argininosuccinate lyase	
ENSG0000	4247.178	-0.21574	0.180188	0.094631	0.436971	RELA	5970	RELA proto	NF-kB subunit
ENSG0000	12.73036	0.210121	0.296483	0.094655	0.436971	MAST1	22983	microtubule associated serine/threonine kinase 1	
ENSG0000	346.7572	-0.18916	0.138232	0.094692	0.437027	TRAF6	7189	TNF receptor associated factor 6	
ENSG0000	484.2163	0.220136	0.181984	0.094766	0.437261	NAA80	24142	N-alpha-ac	NatH catalytic subunit
ENSG0000	2537.698	-0.18865	0.13482	0.094875	0.437328	CAPN2	824	calpain 2	
ENSG0000	17.49218	0.160774	0.279441	0.094882	0.437328	USP46	64854	ubiquitin specific peptidase 46	
ENSG0000	25.5834	0.225309	0.278218	0.094842	0.437328	KATNAL1	84056	katanin catalytic subunit A1 like 1	
ENSG0000	32.64441	0.213312	0.281579	0.0949	0.437328	SLC25A15	10166	solute carrier family 25 member 15	
ENSG0000	34.259	-0.16103	0.276394	0.094894	0.437328	TARM1	441864	T cell-inter	activating receptor on myeloid cells 1
ENSG0000	243.8472	0.20209	0.153834	0.094958	0.437404	MRPS27	23107	mitochondrial ribosomal protein S27	
ENSG0000	522.7879	-0.21066	0.168139	0.094964	0.437404	RNF157	114804	ring finger protein 157	
ENSG0000	66.48418	0.21824	0.177881	0.095047	0.437548	SLC35B4	84912	solute carrier family 35 member B4	
ENSG0000	682.5758	0.169457	0.118909	0.095061	0.437548	ANXA7	310	annexin A7	
ENSG0000	21.47542	0.237916	0.26734	0.095067	0.437548	ZNF577	84765	zinc finger protein 577	
ENSG0000	1205.014	0.188687	0.139793	0.095213	0.437781	EIF3I	8668	eukaryotic translation initiation factor 3 subunit I	
ENSG0000	207.263	0.201344	0.153539	0.095201	0.437781	UBE2E3	10477	ubiquitin conjugating enzyme E2 E3	
ENSG0000	202.5173	0.194298	0.143906	0.095164	0.437781	PSPC1	55269	paraspeckle component 1	
ENSG0000	35.02786	-0.21975	0.274569	0.095179	0.437781	NA	NA	NA	
ENSG0000	11.97422	0.088152	0.248259	0.095307	0.43788	PGAM2	5224	phosphoglycerate mutase 2	
ENSG0000	9.884893	-0.14837	0.275473	0.095296	0.43788	NA	NA	NA	
ENSG0000	2.78981	-0.06612	0.243623	0.095289	0.43788	NA	NA	NA	
ENSG0000	3.663676	-0.08665	0.249746	0.095397	0.438185	NA	NA	NA	
ENSG0000	2.540176	0.082903	0.250721	0.095434	0.438245	HHATL	57467	hedgehog acyltransferase like	
ENSG0000	897.8163	-0.17302	0.121086	0.095467	0.438285	PNP	4860	purine nucleoside phosphorylase	
ENSG0000	1184.446	-0.2222	0.201648	0.095493	0.438285	NT5C2	22978	5'-nucleoti	cytosolic II
ENSG0000	867.4336	-0.22114	0.189353	0.095514	0.438285	APOL3	80833	apolipoprotein L3	
ENSG0000	63.53168	0.218607	0.260425	0.095558	0.438375	ATP2A1	487	ATPase sarcoplasmic/endoplasmic reticulum Ca2+ transporting 1	
ENSG0000	51.40907	0.23567	0.233619	0.095643	0.438547	AMN	81693	amnion associated transmembrane protein	
ENSG0000	6.595443	0.102351	0.257083	0.095632	0.438547	NA	NA	NA	
ENSG0000	44.96457	0.217262	0.275593	0.095693	0.438667	ND6	4541	NADH dehydrogenase subunit 6	
ENSG0000	739.5278	0.219651	0.191764	0.095776	0.438936	ZEB2	9839	zinc finger E-box binding homeobox 2	
ENSG0000	46290.38	-0.21641	0.184353	0.095873	0.43927	GPSM3	63940	G protein signaling modulator 3	
ENSG0000	15.73188	-0.15479	0.276043	0.095917	0.43936	NA	NA	NA	
ENSG0000	96.84416	0.214161	0.173287	0.095959	0.439446	MGME1	92667	mitochondrial genome maintenance exonuclease 1	
ENSG0000	8.169778	0.117689	0.261951	0.096335	0.439782	C1orf167	284498	chromosome 1 open reading frame 167	
ENSG0000	27893.64	-0.2198	0.194932	0.096368	0.439782	TNFRSF1B	7133	TNF receptor superfamily member 1B	
ENSG0000	107.6745	-0.22778	0.195361	0.096413	0.439782	GALE	2582	UDP-galactose-4-epimerase	
ENSG0000	4975.938	-0.22369	0.235837	0.096325	0.439782	RGS2	5997	regulator of G protein signaling 2	
ENSG0000	148.6512	0.201282	0.153315	0.096167	0.439782	IARS2	55699	isoleucyl-t	mitochondrial
ENSG0000	18.6352	0.133936	0.267688	0.096351	0.439782	KBTBD8	84541	kelch repeat and BTB domain containing 8	
ENSG0000	770.2908	-0.18194	0.12764	0.096416	0.439782	TFG	10342	trafficking from ER to golgi regulator	
ENSG0000	3.559633	0.110979	0.260489	0.096079	0.439782	EGFL8	80864	EGF like domain multiple 8	
ENSG0000	48.82298	0.230553	0.255155	0.096211	0.439782	ANKRD6	22881	ankyrin repeat domain 6	
ENSG0000	50.68779	0.226809	0.258679	0.096236	0.439782	CDKN2B	1030	cyclin dependent kinase inhibitor 2B	
ENSG0000	80.98741	-0.21015	0.275322	0.096252	0.439782	LINC02649	399715	long intergenic non-protein coding RNA 2649	
ENSG0000	558.9875	0.213661	0.184854	0.096417	0.439782	AMPD3	272	adenosine monophosphate deaminase 3	
ENSG0000	2434.306	0.221495	0.189175	0.096377	0.439782	GSTP1	2950	glutathione S-transferase pi 1	
ENSG0000	10.7407	0.178309	0.290458	0.0964	0.439782	GALR2	8811	galanin receptor 2	
ENSG0000	28.34038	0.181986	0.282883	0.096308	0.439782	APCDD1	147495	APC down-regulated 1	
ENSG0000	4324.831	-0.21901	0.191793	0.096286	0.439782	RASAL3	64926	RAS protein activator like 3	
ENSG0000	7508.2	-0.2221	0.244869	0.09648	0.439851	TP53I11	9537	tumor protein p53 inducible protein 11	
ENSG0000	413.2569	-0.21541	0.180128	0.09647	0.439851	DUSP18	150290	dual specificity phosphatase 18	
ENSG0000	10.32929	-0.20927	0.296649	0.096522	0.439931	NA	NA	NA	
ENSG0000	216.718	0.192771	0.143142	0.096577	0.440075	ANKMY1	51281	ankyrin repeat and MYND domain containing 1	
ENSG0000	13.35267	0.136271	0.268997	0.096644	0.44027	TRIM9	114088	tripartite motif containing 9	
ENSG0000	598.4625	0.16182	0.111534	0.096719	0.440503	NDUFA10	4705	NADH:ubiquinone oxidoreductase subunit A10	
ENSG0000	65.61043	0.228278	0.206607	0.096814	0.440824	PLAAT3	11145	phospholipase A and acyltransferase 3	
ENSG0000	4.654553	0.077993	0.245917	0.096866	0.44095	MRC1	4360	mannose receptor C-type 1	
ENSG0000	438.8544	0.213565	0.176602	0.09692	0.441088	ZNF768	79724	zinc finger protein 768	
ENSG0000	29.57961	0.233587	0.274529	0.096986	0.441277	FAM167A	83648	family with sequence similarity 167 member A	
ENSG0000	17.77016	0.168616	0.282164	0.097088	0.441292	NA	NA	NA	
ENSG0000	104.4788	0.228637	0.216535	0.097089	0.441292	WDR54	84058	WD repeat domain 54	
ENSG0000	2091.749	-0.19719	0.153857	0.097019	0.441292	TMUB1	83590	transmembrane and ubiquitin like domain containing 1	
ENSG0000	536.8427	0.165111	0.114397	0.097097	0.441292	UBAC1	10422	UBA domain containing 1	
ENSG0000	288.8142	-0.21384	0.268298	0.097128	0.441292	PLD4	122618	phospholipase D family member 4	

ENSG0000	17.30276	0.180636	0.285562	0.097133	0.441292	ARHGAP1:	9824	Rho GTPase activating protein 11A
ENSG0000	177.6019	-0.2258	0.217462	0.097195	0.441381	MTRR	4552	5-methyltetrahydrofolate-homocysteine methyltransferase reductase
ENSG0000	245.5911	-0.20786	0.16823	0.097225	0.441381	POMP	51371	proteasome maturation protein
ENSG0000	102.0184	0.225675	0.204014	0.097204	0.441381	GOLGA8A	23015	golgin A8 family member A
ENSG0000	37.70822	-0.23107	0.250708	0.09725	0.441382	CCL3	6348	C-C motif chemokine ligand 3
ENSG0000	2592.158	-0.22403	0.227032	0.097414	0.442018	LRFN1	57622	leucine rich repeat and fibronectin type III domain containing 1
ENSG0000	9994.663	-0.20409	0.163845	0.097505	0.442121	PTK2B	2185	protein tyrosine kinase 2 beta
ENSG0000	6.361829	-0.09271	0.251319	0.097581	0.442121	LOC12490	1.25E+08	uncharacterized LOC124902371
ENSG0000	782.6866	-0.21773	0.185311	0.09756	0.442121	OSBPL5	114879	oxysterol binding protein like 5
ENSG0000	1695894	-0.22301	0.215429	0.097521	0.442121	HBA2	3040	hemoglobin subunit alpha 2
ENSG0000	56.97352	0.221397	0.259292	0.097549	0.442121	MIS12	79003	MIS12 kinetochore complex component
ENSG0000	719.9406	-0.21455	0.186646	0.09752	0.442121	TRPV2	51393	transient receptor potential cation channel subfamily V member 2
ENSG0000	2574.101	-0.22364	0.220053	0.097661	0.442263	ZC3H3	23144	zinc finger CCCH-type containing 3
ENSG0000	2.960893	0.082032	0.248112	0.097654	0.442263	P2RX6	9127	purinergic receptor P2X 6
ENSG0000	7.899005	-0.17927	0.292814	0.097872	0.443107	KLHL30	377007	kelch like family member 30
ENSG0000	14.24279	0.17739	0.285031	0.097908	0.443161	AOAH-IT1	1.01E+08	AOAH intronic transcript 1
ENSG0000	975.4972	-0.18425	0.137589	0.097977	0.443366	CDH23	64072	cadherin related 23
ENSG0000	6.598344	0.167996	0.287994	0.09812	0.443903	NA	NA	NA
ENSG0000	23.97877	-0.225	0.272946	0.098198	0.444144	RNU6-125	1.06E+08	RNA U6 small r pseudogene
ENSG0000	288.7877	0.222156	0.193478	0.098243	0.444242	IDS	3423	iduronate 2-sulfatase
ENSG0000	2657.054	-0.19316	0.144629	0.09842	0.44493	EXOC7	23265	exocyst complex component 7
ENSG0000	73.31191	-0.22305	0.257218	0.098572	0.445507	CHORDC1	26973	cysteine and histidine rich domain containing 1
ENSG0000	214.9246	-0.2077	0.165881	0.098734	0.446085	TNF	7124	tumor necrosis factor
ENSG0000	83.39378	-0.22598	0.247288	0.098748	0.446085	NPIPB11	728888	nuclear pore complex interacting protein family member B11
ENSG0000	197.9659	0.220837	0.203461	0.098781	0.446122	LAX1	54900	lymphocyte transmembrane adaptor 1
ENSG0000	12319.13	-0.21943	0.203457	0.098871	0.446417	ZDHHC18	84243	zinc finger DHHC-type palmitoyltransferase 18
ENSG0000	47.69155	0.230579	0.245802	0.098911	0.446488	NA	NA	NA
ENSG0000	4.590415	-0.12009	0.264031	0.098936	0.446491	NA	NA	NA
ENSG0000	728.9226	0.219712	0.246031	0.099139	0.446645	CHST13	166012	carbohydrate sulfotransferase 13
ENSG0000	2.385917	-0.04483	0.237928	0.099025	0.446645	LOC10028	1E+08	prefoldin subunit 1 pseudogene
ENSG0000	9929.109	-0.21875	0.197576	0.099141	0.446645	FLOT1	10211	flotillin 1
ENSG0000	127.1225	0.221716	0.199198	0.099127	0.446645	VIM-AS1	1.01E+08	VIM antisense RNA 1
ENSG0000	2.862033	0.095186	0.2534	0.099086	0.446645	NA	NA	NA
ENSG0000	4.99651	-0.09114	0.250742	0.099072	0.446645	NA	NA	NA
ENSG0000	294.8333	0.230801	0.217331	0.099038	0.446645	VPREB3	29802	V-set pre-B cell surrogate light chain 3
ENSG0000	68.21876	-0.20593	0.165659	0.099185	0.446735	EXOSC3	51010	exosome component 3
ENSG0000	56.40268	-0.22817	0.241558	0.099253	0.446821	MAPK11	5600	mitogen-activated protein kinase 11
ENSG0000	905.6029	0.193961	0.14652	0.099248	0.446821	TAFAZZIN	6901	tafazzin phospholipid-lysophospholipid transacylase
ENSG0000	79.06977	0.222188	0.194649	0.099437	0.447211	ABHD6	57406	abhydrola: acylglycerol lipase
ENSG0000	139.919	0.209635	0.170607	0.099411	0.447211	FBXW7	55294	F-box and WD repeat domain containing 7
ENSG0000	13.38702	-0.18277	0.290888	0.09943	0.447211	NA	NA	NA
ENSG0000	410.5124	-0.18451	0.135345	0.099416	0.447211	PITPNB	23760	phosphatidylinositol transfer protein beta
ENSG0000	3.658102	-0.08214	0.247996	0.099643	0.448021	RWDD3-D	1.02E+08	RWDD3 divergent transcript
ENSG0000	4285.081	-0.21518	0.188048	0.099725	0.448021	H1-10	8971	H1.10 linker histone
ENSG0000	506.609	0.110904	0.256404	0.09973	0.448021	RPS3A	6189	ribosomal protein S3A
ENSG0000	8.543811	-0.17593	0.288342	0.099764	0.448021	AKAP3	10566	A-kinase anchoring protein 3
ENSG0000	4.15013	0.104189	0.258665	0.099756	0.448021	KL	9365	klotho
ENSG0000	33.09454	0.23264	0.236439	0.099708	0.448021	COA8	84334	cytochrome c oxidase assembly factor 8
ENSG0000	2.389652	0.072379	0.244934	0.099819	0.448048	NA	NA	NA
ENSG0000	249.3375	0.2256	0.211068	0.099812	0.448048	ROGDI	79641	rogdi atypical leucine zipper
ENSG0000	466.5229	0.118115	0.259118	0.099867	0.448155	RPL7	6129	ribosomal protein L7
ENSG0000	2.481472	0.076699	0.248005	0.099976	0.448534	SOX7	83595	SRY-box transcription factor 7
ENSG0000	1039.518	-0.19835	0.155162	0.100032	0.448676	SIRT7	51547	sirtuin 7
ENSG0000	31.22058	0.186866	0.281477	0.100059	0.448687	TAF4B	6875	TATA-box binding protein associated factor 4b
ENSG0000	2197.558	0.203581	0.266718	0.100122	0.44875	RPS27	6232	ribosomal protein S27
ENSG0000	357.1463	0.201041	0.161516	0.100115	0.44875	SNX5	27131	sorting nexin 5
ENSG0000	245.3924	-0.21943	0.235205	0.100225	0.449101	ALG1L13P	1.06E+08	ALG1 like : pseudogene
ENSG0000	235.4985	0.206559	0.166953	0.100317	0.449239	GTF2I	2969	general transcription factor IIi
ENSG0000	1048.811	0.186823	0.134454	0.100323	0.449239	MCRS1	10445	microspherule protein 1
ENSG0000	4694.889	-0.20551	0.167809	0.100345	0.449239	UBXN6	80700	UBX domain protein 6
ENSG0000	18.01679	0.174478	0.28223	0.100354	0.449239	CCL25	6370	C-C motif chemokine ligand 25
ENSG0000	3321.196	-0.20735	0.171693	0.10065	0.450455	PLEKHM1	9842	pleckstrin homology and RUN domain containing M1
ENSG0000	5.934487	0.127468	0.266451	0.100811	0.450821	CPNE9	151835	copine family member 9
ENSG0000	230.7819	-0.22085	0.205152	0.100829	0.450821	FGFRL1	53834	fibroblast growth factor receptor like 1
ENSG0000	344.2835	-0.22264	0.220299	0.100845	0.450821	PALM2AK/	445815	PALM2 and AKAP2 fusion
ENSG0000	61.26936	0.224523	0.251618	0.10085	0.450821	TCTN1	79600	tectonic family member 1
ENSG0000	157.0594	0.222631	0.197068	0.100855	0.450821	RPS4Y1	6192	ribosomal protein S4 Y-linked 1
ENSG0000	231.1748	0.193309	0.147565	0.100894	0.450885	TPGS1	91978	tubulin polyglutamylase complex subunit 1
ENSG0000	140.7553	0.21176	0.175663	0.10092	0.450891	NA	NA	NA
ENSG0000	16.00466	0.19974	0.289612	0.100952	0.450926	DNAJC25	548645	DnaJ heat shock protein family (Hsp40) member C25
ENSG0000	320.2824	-0.22016	0.20655	0.101322	0.452355	PRKAR1B	5575	protein kinase cAMP-dependent type I regulatory subunit beta
ENSG0000	300.1891	0.221736	0.209124	0.101319	0.452355	GTF2IP4	1E+08	general transcription factor IIi pseudogene 4
ENSG0000	9.208692	0.091679	0.249559	0.101419	0.452681	ESR1	2099	estrogen receptor 1
ENSG0000	1044.682	-0.18456	0.138461	0.101451	0.45271	HEIH	1.01E+08	hepatocellular carcinoma up-regulated EZH2-associated long non-coding RNA
ENSG0000	28.92007	-0.22934	0.255922	0.10152	0.452907	NA	NA	NA
ENSG0000	5.987583	0.09391	0.250516	0.10155	0.452933	NA	NA	NA
ENSG0000	7.065991	0.134926	0.269625	0.101602	0.453055	NA	NA	NA
ENSG0000	43.334	-0.22786	0.221975	0.101636	0.453095	PDE9A	5152	phosphodiesterase 9A
ENSG0000	28.76891	-0.19472	0.284698	0.101727	0.45317	NA	NA	NA
ENSG0000	976.8376	-0.18059	0.135504	0.101679	0.45317	TMEM179	374395	transmembrane protein 179B
ENSG0000	4.108639	-0.09063	0.250991	0.10171	0.45317	PARP4P1	347613	poly(ADP-ribose) polymerase family member 4 pseudogene 1
ENSG0000	4.780969	-0.07601	0.246355	0.10179	0.453338	PRDM16	63976	PR/SET domain 16
ENSG0000	32.48707	-0.17444	0.277113	0.10184	0.453455	LINC0135	1.02E+08	long intergenic non-protein coding RNA 1359
ENSG0000	61.72953	0.224753	0.231158	0.101889	0.453525	ITGAE	3682	integrin subunit alpha E
ENSG0000	47.61363	-0.20264	0.273326	0.101931	0.453525	CA15P1	1.01E+08	CA15 pseudogene 1
ENSG0000	1856.337	0.221533	0.221152	0.101928	0.453525	IL13RA1	3597	interleukin 13 receptor subunit alpha 1
ENSG0000	5.351305	0.139241	0.273328	0.101985	0.453658	NA	NA	NA
ENSG0000	18.6042	-0.20951	0.282385	0.102031	0.453753	GNG8	94235	G protein subunit gamma 8
ENSG0000	30.06365	0.151267	0.271208	0.10209	0.453793	COL4A4	1286	collagen type IV alpha 4 chain
ENSG0000	2602.794	-0.21878	0.208959	0.102088	0.453793	RNF40	9810	ring finger protein 40
ENSG0000	1917.411	-0.21849	0.227459	0.102266	0.454071	ADAMTSL	54507	ADAMTS like 4
ENSG0000	58.02471	0.204205	0.273226	0.102335	0.454071	SH3PXD2E	285590	SH3 and PX domains 2B
ENSG0000	421.9851	0.194006	0.148389	0.102351	0.454071	LY86	9450	lymphocyte antigen 86
ENSG0000	14.6121	0.171798	0.282363	0.102334	0.454071	C17orf100	388327	chromosome 17 open reading frame 100
ENSG0000	12.02388	0.182475	0.286138	0.102336	0.454071	SOX15	6665	SRY-box transcription factor 15
ENSG0000	5.705491	-0.1112	0.259735	0.102203	0.454071	BTBD17	388419	BTB domain containing 17

ENSG0000	2.653021	0.074208	0.244976	0.102221	0.454071	NA	NA	NA
ENSG0000	2.533064	0.06738	0.244405	0.102242	0.454071	ZNF404	342908	zinc finger protein 404
ENSG0000	53.64909	-0.22098	0.253643	0.102381	0.454095	NA	NA	NA
ENSG0000	7.771749	0.141164	0.272053	0.102486	0.454451	ETV5	2119	ETS variant transcription factor 5
ENSG0000	6.392869	-0.16975	0.287417	0.102531	0.45454	TUSC1	286319	tumor suppressor candidate 1
ENSG0000	275.2064	0.214523	0.184371	0.10263	0.454741	MCFD2	90411	multiple co ER cargo receptor complex subunit
ENSG0000	4.954241	0.086759	0.248897	0.102651	0.454741	NA	NA	NA
ENSG0000	5.234282	0.134332	0.271032	0.102641	0.454741	RPS23P8	653658	ribosomal protein S23 pseudogene 8
ENSG0000	14.80352	-0.20565	0.289375	0.1028	0.455073	FMNL2	114793	formin like 2
ENSG0000	11.87397	-0.14301	0.270308	0.10277	0.455073	NDUFA4L2	56901	NDUFA4 mitochondrial complex associated like 2
ENSG0000	42.89212	0.222146	0.258673	0.102799	0.455073	LOC10272	1.03E+08	glutamine amidotransferase class 1 domain containing 3B
ENSG0000	79.85609	0.19592	0.269684	0.102831	0.455102	DESI2	51029	desumoylating isopeptidase 2
ENSG0000	6.895561	-0.11206	0.259285	0.10291	0.455181	LGALS8-AS	1E+08	LGALS8 antisense RNA 1
ENSG0000	77.74399	-0.2246	0.225574	0.10289	0.455181	NA	NA	NA
ENSG0000	2449.041	-0.18948	0.144093	0.102924	0.455181	CLK3	1198	CDC like kinase 3
ENSG0000	8.504747	0.187124	0.291417	0.103006	0.455434	GRM2	2912	glutamate metabotropic receptor 2
ENSG0000	117.7291	-0.20695	0.170588	0.103102	0.455472	ARHGEF2-	1.08E+08	ARHGEF2 antisense RNA 2
ENSG0000	4.211313	-0.11734	0.262173	0.103124	0.455472	LOC10537	1.05E+08	uncharacterized LOC105377623
ENSG0000	220.8136	-0.21917	0.230306	0.103164	0.455472	LHFPL2	10184	LHFPL tetraspan subfamily member 2
ENSG0000	9.251896	-0.14279	0.271982	0.103135	0.455472	PTGES	9536	prostaglandin E synthase
ENSG0000	4304.148	-0.20983	0.181099	0.103161	0.455472	CHST15	51363	carbohydrate sulfotransferase 15
ENSG0000	151.9702	0.201563	0.160981	0.103154	0.455472	EIF2AK4	440275	eukaryotic translation initiation factor 2 alpha kinase 4
ENSG0000	2921.846	-0.20535	0.168191	0.103218	0.455602	TAGAP	117289	T cell activation RhoGTPase activating protein
ENSG0000	54.18933	0.206362	0.269839	0.103275	0.455746	MKKS	8195	MKKS centrosomal shuttling protein
ENSG0000	14.92911	0.205993	0.287208	0.103322	0.455803	ATPSCKMT	134145	ATP synthase c subunit lysine N-methyltransferase
ENSG0000	4009.011	-0.21791	0.213294	0.103338	0.455803	STEAP4	79689	STEAP4 metalloreductase
ENSG0000	73.54086	0.223957	0.23007	0.103398	0.455956	MYL5	4636	myosin light chain 5
ENSG0000	133.7704	-0.19519	0.150339	0.103434	0.456006	USP35	57558	ubiquitin specific peptidase 35
ENSG0000	20.74026	0.188611	0.282712	0.10351	0.456122	UBE2T	29089	ubiquitin conjugating enzyme E2 T
ENSG0000	2.370667	0.062926	0.243123	0.103486	0.456122	STARD13	90627	StAR related lipid transfer domain containing 13
ENSG0000	39.18154	0.179598	0.278333	0.103909	0.456965	RNU6-176	1.06E+08	RNA U6 small r pseudogene
ENSG0000	11.24173	0.088768	0.248627	0.103823	0.456965	NA	NA	NA
ENSG0000	10.64709	0.201902	0.289834	0.103795	0.456965	NA	NA	NA
ENSG0000	144.3175	0.198033	0.155856	0.103951	0.456965	C12orf43	64897	chromosome 12 open reading frame 43
ENSG0000	21.47295	0.211119	0.278363	0.103858	0.456965	NA	NA	NA
ENSG0000	434.1977	-0.18583	0.136966	0.103784	0.456965	VIPAS39	63894	VPS33B int apical-bas spe-39 homolog
ENSG0000	49.84209	0.223066	0.250287	0.103813	0.456965	PKD1P5	348156	polycystin transient receptor potential channel interacting pseudogene 5
ENSG0000	242.4189	0.213796	0.187775	0.103944	0.456965	COPS3	8533	COP9 signalosome subunit 3
ENSG0000	156.26	0.191565	0.148884	0.103901	0.456965	PRPSAP1	5635	phosphoribosyl pyrophosphate synthetase associated protein 1
ENSG0000	1991.028	-0.21826	0.22127	0.103842	0.456965	PLIN3	10226	perilipin 3
ENSG0000	33308.16	-0.19445	0.153524	0.104026	0.457186	LCP1	3936	lymphocyte cytosolic protein 1
ENSG0000	182.8675	-0.19735	0.159971	0.104095	0.457332	PPP1R21	129285	protein phosphatase 1 regulatory subunit 21
ENSG0000	15.12095	0.21624	0.280711	0.104109	0.457332	NA	NA	NA
ENSG0000	5.307659	-0.14898	0.277927	0.104225	0.457343	NAP1L5	266812	nucleosome assembly protein 1 like 5
ENSG0000	646.4267	0.21392	0.183537	0.104192	0.457343	JARID2	3720	jumonji and AT-rich interaction domain containing 2
ENSG0000	12111.79	-0.20737	0.177292	0.104237	0.457343	TSC22D4	81628	TSC22 domain family member 4
ENSG0000	6.855199	-0.15575	0.279545	0.104167	0.457343	FAM241B	219738	family with sequence similarity 241 member B
ENSG0000	8.575267	0.133262	0.267341	0.104231	0.457343	NA	NA	NA
ENSG0000	111.3358	-0.19322	0.150417	0.104315	0.457576	PPP2R1B	5519	protein phosphatase 2 scaffold subunit Abeta
ENSG0000	272.1011	0.200428	0.157053	0.104423	0.457943	ECI1	1632	enoyl-CoA delta isomerase 1
ENSG0000	3.508992	0.105073	0.257236	0.104503	0.457964	SELENOP	6414	selenoprotein P
ENSG0000	11.28908	-0.19194	0.288323	0.104461	0.457964	NA	NA	NA
ENSG0000	1596.49	-0.20008	0.162574	0.104481	0.457964	AGPAT3	56894	1-acylglycerol-3-phosphate O-acyltransferase 3
ENSG0000	19.8411	0.16685	0.277581	0.104568	0.458137	CPED1	79974	cadherin like and PC-esterase domain containing 1
ENSG0000	30.1901	-0.17441	0.275905	0.104668	0.458466	LRATD2	157638	LRAT domain containing 2
ENSG0000	8.862293	-0.17533	0.287554	0.104702	0.458508	NA	NA	NA
ENSG0000	2795.071	-0.21094	0.186641	0.104765	0.458564	SEMA4B	10509	semaphorin 4B
ENSG0000	17.70848	0.187404	0.28502	0.10476	0.458564	NA	NA	NA
ENSG0000	3.47068	0.110348	0.260393	0.10487	0.458915	AMZ1	155185	archaelysin family metallopeptidase 1
ENSG0000	501.0849	0.215516	0.19766	0.104914	0.458997	TNFRSF13(	115650	TNF receptor superfamily member 13C
ENSG0000	11.03429	0.10805	0.256105	0.104966	0.459002	NEB	4703	nebulin
ENSG0000	24.34885	-0.22683	0.255281	0.104956	0.459002	KCNK13	56659	potassium two pore domain channel subfamily K member 13
ENSG0000	7.458664	0.111414	0.257683	0.105049	0.459258	ARHGAP44	9912	Rho GTPase activating protein 44
ENSG0000	7.134959	0.161035	0.281165	0.10514	0.459327	NA	NA	NA
ENSG0000	39.56127	-0.18407	0.276418	0.105134	0.459327	BRCA2	675	BRCA2 DNA repair associated
ENSG0000	1327.711	-0.20437	0.171363	0.105139	0.459327	NFKBIB	4793	NFKB inhibitor beta
ENSG0000	113.8295	-0.19686	0.155184	0.105368	0.459666	ANXA2R-C	648987	ANXA2R overlapping transcript
ENSG0000	4.843226	0.131627	0.269417	0.105321	0.459666	NA	NA	NA
ENSG0000	188.4198	0.202848	0.166251	0.105299	0.459666	SEC23IP	11196	SEC23 interacting protein
ENSG0000	58.8425	0.218963	0.195643	0.10531	0.459666	NA	NA	NA
ENSG0000	1645.778	-0.18674	0.143386	0.105363	0.459666	CMTM3	123920	CKLF like MARVEL transmembrane domain containing 3
ENSG0000	1592.164	-0.17495	0.128944	0.105342	0.459666	PTPN1	5770	protein tyrosine phosphatase non-receptor type 1
ENSG0000	3.106699	-0.04355	0.237964	0.105606	0.459722	SLC9A4	389015	solute carrier family 9 member A4
ENSG0000	1658.541	-0.2126	0.234164	0.105564	0.459722	GP9	2815	glycoprotein IX platelet
ENSG0000	23.20215	0.170306	0.277379	0.105412	0.459722	ANKRD50	57182	ankyrin repeat domain containing 50
ENSG0000	10.29184	-0.20126	0.289136	0.10544	0.459722	NA	NA	NA
ENSG0000	14.64691	0.177666	0.281926	0.105518	0.459722	NA	NA	NA
ENSG0000	4.645905	0.10685	0.257026	0.105614	0.459722	NA	NA	NA
ENSG0000	323.2917	0.197875	0.158517	0.105607	0.459722	C1QBP	708	complement C1q binding protein
ENSG0000	5.699327	0.090569	0.250233	0.105632	0.459722	NA	NA	NA
ENSG0000	650.2753	0.218125	0.231541	0.105518	0.459722	TMX4	56255	thioredoxin related transmembrane protein 4
ENSG0000	615.9457	0.173721	0.126541	0.105484	0.459722	UXT	8409	ubiquitously expressed prefoldin like chaperone
ENSG0000	832.3785	-0.19961	0.168693	0.105728	0.460031	ARMC7	79637	armadillo repeat containing 7
ENSG0000	614.038	0.184988	0.14056	0.105812	0.460287	BHLHE40	8553	basic helix-loop-helix family member e40
ENSG0000	170.551	-0.21675	0.203471	0.105846	0.460327	LY6G5C	80741	lymphocyte antigen 6 family member G5C
ENSG0000	12571.69	-0.21663	0.229876	0.105932	0.460587	BASP1	10409	brain abundant membrane attached signal protein 1
ENSG0000	61.80148	-0.18956	0.272402	0.106034	0.460923	NA	NA	NA
ENSG0000	8.968889	0.119244	0.260519	0.106084	0.46103	SBF2-AS1	283104	SBF2 antisense RNA 1
ENSG0000	21.05958	-0.2105	0.27587	0.106157	0.461239	LINC02608	1.02E+08	long intergenic non-protein coding RNA 2608
ENSG0000	826.5181	-0.18867	0.147769	0.106286	0.461486	TRIM11	81559	tripartite motif containing 11
ENSG0000	2.355711	0.063302	0.242589	0.10627	0.461486	LOC64596	645967	uncharacterized LOC645967
ENSG0000	3083.366	-0.20974	0.183033	0.106301	0.461486	PAK1	5058	p21 (RAC1) activated kinase 1
ENSG0000	3.638759	0.078707	0.245725	0.106315	0.461486	MEX3B	84206	mex-3 RNA binding family member B
ENSG0000	10.55387	0.103825	0.253787	0.106543	0.462367	PDZD3	79849	PDZ domain containing 3
ENSG0000	18.06709	0.185686	0.281348	0.106654	0.462741	NDUFS4	4724	NADH:ubiquinone oxidoreductase subunit S4
ENSG0000	230.9736	-0.21642	0.228429	0.106698	0.46282	CLEC2B	9976	C-type lectin domain family 2 member B

ENSG0000	7.315292	0.179611	0.288558	0.10675	0.462937	NUDT13	25961	nudix hydrolase 13
ENSG0000	19.2325	-0.20288	0.279766	0.106788	0.462994	LINC0276	283140	long intergenic non-protein coding RNA 2762
ENSG0000	6.090506	-0.0763	0.244063	0.10683	0.463064	AJUBA	84962	ajuba LIM protein
ENSG0000	15.48026	0.210644	0.279745	0.106913	0.463186	ZNF852	285346	zinc finger protein 852
ENSG0000	49.4691	-0.21854	0.251497	0.106938	0.463186	SEMA3B	7869	semaphorin 3B
ENSG0000	38.8963	0.224838	0.228592	0.1069	0.463186	PAXIP1-D	202781	PAXIP1 divergent transcript
ENSG0000	47.59927	0.223125	0.234352	0.106959	0.463186	ADGRB1	575	adhesion G protein-coupled receptor B1
ENSG0000	23.03832	0.182115	0.279567	0.107057	0.46339	NA	NA	NA
ENSG0000	7.755067	0.10443	0.254382	0.107041	0.46339	ADSS1	122622	adenylosuccinate synthase 1
ENSG0000	3330.351	0.183503	0.139912	0.107088	0.463416	RPL14	9045	ribosomal protein L14
ENSG0000	6245.993	-0.21049	0.190907	0.107236	0.463945	MARK2	2011	microtubule affinity regulating kinase 2
ENSG0000	13.92295	-0.18193	0.281109	0.10729	0.464072	NA	NA	NA
ENSG0000	3.786884	0.0698	0.245369	0.107344	0.464195	ITGA8	8516	integrin subunit alpha 8
ENSG0000	2806.212	0.185727	0.143046	0.107422	0.464423	CTSH	1512	cathepsin H
ENSG0000	148.7306	-0.21792	0.208786	0.107586	0.464823	CTDSPL	10217	CTD small phosphatase like
ENSG0000	133.221	0.221161	0.20867	0.107549	0.464823	SLC49A4	84925	solute carrier family 49 member 4
ENSG0000	407.0403	-0.21436	0.203131	0.107591	0.464823	DAPP1	27071	dual adaptor of phosphotyrosine and 3-phosphoinositides 1
ENSG0000	20492.58	-0.21186	0.192674	0.10765	0.464967	PXN	5829	paxillin
ENSG0000	6.588332	-0.09574	0.253232	0.107765	0.465357	NA	NA	NA
ENSG0000	31.63867	-0.17904	0.274337	0.107843	0.465583	NA	NA	NA
ENSG0000	21.43773	0.209644	0.276804	0.107951	0.46594	ZNF490	57474	zinc finger protein 490
ENSG0000	18.00653	-0.15217	0.2706	0.107994	0.466017	TRAV12-1	28674	T cell receptor alpha variable 12-1
ENSG0000	10.73134	0.157404	0.278723	0.10806	0.466192	NUF2	83540	NUF2 component of NDC80 kinetochore complex
ENSG0000	153.2175	0.209109	0.182565	0.108204	0.466481	PPIH	10465	peptidylprolyl isomerase H
ENSG0000	78.33258	-0.21557	0.248459	0.108195	0.466481	NA	NA	NA
ENSG0000	6.718813	0.130128	0.26595	0.108191	0.466481	NA	NA	NA
ENSG0000	5.080458	-0.10433	0.256039	0.108268	0.466499	NA	NA	NA
ENSG0000	4359.721	0.210088	0.187308	0.108265	0.466499	PPIB	5479	peptidylprolyl isomerase B
ENSG0000	4.083785	-0.06865	0.243625	0.108285	0.466499	DOK6	220164	docking protein 6
ENSG0000	4.509865	-0.08569	0.248238	0.108344	0.466574	NA	NA	NA
ENSG0000	100.1153	0.215861	0.202	0.108353	0.466574	SKIC8	80349	SKI8 subunit of superkiller complex
ENSG0000	60.80179	0.220861	0.235028	0.108447	0.466766	TADA1	117143	transcriptional adaptor 1
ENSG0000	7.603163	0.123023	0.263251	0.108448	0.466766	NA	NA	NA
ENSG0000	91.09995	0.20568	0.174176	0.108555	0.467114	LRP5L	91355	LDL receptor related protein 5 like (pseudogene)
ENSG0000	11.2336	0.150508	0.272618	0.108621	0.46729	LY86-AS1	285780	LY86 antisense RNA 1
ENSG0000	25.69557	-0.19954	0.274065	0.108755	0.467361	NA	NA	NA
ENSG0000	89.6149	0.211464	0.254767	0.108765	0.467361	DNM1	1759	dynamin 1
ENSG0000	9.962112	-0.19631	0.284757	0.10871	0.467361	ADAM12	8038	ADAM metallopeptidase domain 12
ENSG0000	2.675724	-0.08109	0.249243	0.108735	0.467361	SCUBE2	57758	signal pep CUB domain and EGF like domain containing 2
ENSG0000	610.9206	0.164508	0.12112	0.108689	0.467361	GPATCH8	23131	G-patch domain containing 8
ENSG0000	38.77773	-0.20662	0.264961	0.108876	0.467388	PLCXD2	257068	phosphatidylinositol specific phospholipase C X domain containing 2
ENSG0000	410.9202	-0.21455	0.22582	0.108847	0.467388	SENCR	1.01E+08	smooth muscle and endothelial cell enriched migration/differentiation-associated lncRNA
ENSG0000	153.9105	0.20886	0.168849	0.108855	0.467388	DHRS4L2	317749	dehydrogenase/reductase 4 like 2
ENSG0000	54.14344	-0.21915	0.23971	0.108912	0.467388	DNASE1L2	1775	deoxyribonuclease 1 like 2
ENSG0000	2.434575	0.057297	0.240639	0.108943	0.467388	NA	NA	NA
ENSG0000	30654.45	-0.21434	0.20269	0.10895	0.467388	CD37	951	CD37 molecule
ENSG0000	1840.552	-0.18689	0.146854	0.108889	0.467388	TANGO2	128989	transport and golgi organization 2 homolog
ENSG0000	24901.13	0.201599	0.169593	0.10902	0.467578	NA	NA	NA
ENSG0000	95.23718	0.218159	0.21851	0.109051	0.467603	MTFMT	123263	mitochondrial methionyl-tRNA formyltransferase
ENSG0000	4.54692	0.101635	0.254692	0.10909	0.467661	NA	NA	NA
ENSG0000	6.168721	-0.08119	0.245876	0.10917	0.467895	ATE1-AS1	1E+08	ATE1 antisense RNA 1
ENSG0000	4.055809	0.080571	0.246679	0.109205	0.467936	LOC33926	339260	uncharacterized LOC339260
ENSG0000	332.6732	-0.19738	0.164088	0.109232	0.467941	MLC1	23209	modulator of VRAC current 1
ENSG0000	291.3102	-0.16803	0.122504	0.109407	0.468257	NUDT18	79873	nudix hydrolase 18
ENSG0000	727.5778	-0.20888	0.181169	0.109382	0.468257	PRRG4	79056	proline rich and Gla domain 4
ENSG0000	18.7598	-0.13707	0.267116	0.109408	0.468257	MT1L	4500	metallothi pseudogene
ENSG0000	23.4577	-0.12557	0.259846	0.109349	0.468257	PMAIP1	5366	phorbol-12-myristate-13-acetate-induced protein 1
ENSG0000	103.4958	0.215156	0.238757	0.10966	0.46865	UBR3	130507	ubiquitin protein ligase E3 component n-recognin 3
ENSG0000	766.057	-0.17408	0.130002	0.109663	0.46865	LRCH3	84859	leucine rich repeats and calponin homology domain containing 3
ENSG0000	1924.938	-0.21082	0.203936	0.109659	0.46865	MAPK14	1432	mitogen-activated protein kinase 14
ENSG0000	12.28594	-0.19652	0.28364	0.109679	0.46865	NA	NA	NA
ENSG0000	10794.33	-0.21227	0.227524	0.109669	0.46865	ACTN1	87	actinin alpha 1
ENSG0000	97.74862	0.218109	0.222428	0.109586	0.46865	BIRC5	332	baculoviral IAP repeat containing 5
ENSG0000	365.2074	0.190491	0.148924	0.10957	0.46865	PHF20	51230	PHD finger protein 20
ENSG0000	468.88	0.173086	0.128358	0.109713	0.468656	D2HGDH	728294	D-2-hydroxyglutarate dehydrogenase
ENSG0000	348.6649	-0.21333	0.231951	0.110009	0.468656	GNB4	59345	G protein subunit beta 4
ENSG0000	37.24014	-0.10947	0.252798	0.109912	0.468656	CXCL8	3576	C-X-C motif chemokine ligand 8
ENSG0000	28.10858	-0.18779	0.27404	0.110002	0.468656	NA	NA	NA
ENSG0000	106.693	-0.21661	0.210769	0.109802	0.468656	LOC28563	285638	uncharacterized LOC285638
ENSG0000	4.850616	0.111152	0.259158	0.109843	0.468656	ARMH2	1.02E+08	armadillo like helical domain containing 2
ENSG0000	39.84469	-0.22117	0.215997	0.109894	0.468656	MCPH1-D	1E+08	MCPH1 divergent transcript
ENSG0000	71.62328	-0.21637	0.212367	0.11	0.468656	PTK2	5747	protein tyrosine kinase 2
ENSG0000	106.2436	0.206938	0.177158	0.109992	0.468656	LINC0029	283267	long intergenic non-protein coding RNA 294
ENSG0000	42.57874	-0.21723	0.249793	0.109758	0.468656	CACNA1H	8912	calcium voltage-gated channel subunit alpha1 H
ENSG0000	4446.184	-0.1684	0.124379	0.109735	0.468656	EIF1	10209	eukaryotic translation initiation factor 1
ENSG0000	148.4641	-0.20004	0.258548	0.110013	0.468656	LOC10537	1.05E+08	uncharacterized LOC105371795
ENSG0000	366.0963	0.194241	0.156064	0.109838	0.468656	ARHGAP3	2909	Rho GTPase activating protein 35
ENSG0000	816.749	0.189575	0.149458	0.110106	0.468835	RBBP4	5928	RB binding chromatin remodeling factor
ENSG0000	42.68062	-0.20604	0.261881	0.110097	0.468835	NA	NA	NA
ENSG0000	9467.771	-0.21012	0.189452	0.110214	0.468964	STK40	83931	serine/threonine kinase 40
ENSG0000	6.734157	0.080345	0.24556	0.110176	0.468964	NA	NA	NA
ENSG0000	262.1773	-0.19704	0.162109	0.110205	0.468964	IFI27L2	83982	interferon alpha inducible protein 27 like 2
ENSG0000	15.75715	0.174481	0.278678	0.110281	0.469034	MAP10	54627	microtubule associated protein 10
ENSG0000	6.673441	0.135193	0.268538	0.110261	0.469034	NA	NA	NA
ENSG0000	106.6457	-0.2112	0.246935	0.110316	0.469073	FAM238A	645528	family with sequence similarity 238 member A
ENSG0000	1077.289	-0.19594	0.163127	0.110451	0.46954	MAP1LC3	81631	microtubule associated protein 1 light chain 3 beta
ENSG0000	145.2117	0.215577	0.213178	0.110696	0.470037	MYCL	4610	MYCL prot bHLH transcription factor
ENSG0000	343.1982	0.203155	0.174239	0.110636	0.470037	NUCKS1	64710	nuclear casein kinase and cyclin dependent kinase substrate 1
ENSG0000	26.59602	0.196814	0.27374	0.110689	0.470037	TRMT61B	55006	tRNA methyltransferase 61B
ENSG0000	186.7809	-0.19364	0.156364	0.110601	0.470037	PSMB5	5693	proteasome 20S subunit beta 5
ENSG0000	2421.266	-0.20784	0.186059	0.110668	0.470037	NACC1	112939	nucleus accumbens associated 1
ENSG0000	15.90554	-0.18889	0.279506	0.110819	0.470338	SPATA5	166378	spermatogenesis associated 5
ENSG0000	1902.41	-0.19846	0.158109	0.110804	0.470338	TMUB2	79089	transmembrane and ubiquitin like domain containing 2
ENSG0000	267.5089	-0.18365	0.146397	0.110886	0.470515	CLN8-AS1	1.02E+08	CLN8 antisense RNA 1
ENSG0000	193.4683	0.223908	0.24742	0.111149	0.470867	GSTM1	2944	glutathione S-transferase mu 1
ENSG0000	200.7383	0.214205	0.206979	0.111147	0.470867	ZSWIM6	57688	zinc finger SWIM-type containing 6

ENSG0000	5.037472	0.132121	0.269107	0.111086	0.470867	NAV2	89797	neuron navigator 2
ENSG0000	510.2522	-0.2025	0.174044	0.111013	0.470867	NA	NA	NA
ENSG0000	2459.692	-0.20708	0.244056	0.111027	0.470867	PEAK3	374872	PEAK family member 3
ENSG0000	19012.57	-0.20677	0.188217	0.111116	0.470867	C5AR1	728	complement C5a receptor 1
ENSG0000	3416.683	-0.20451	0.172144	0.111137	0.470867	PPP1R15A	23645	protein phosphatase 1 regulatory subunit 15A
ENSG0000	6.932913	0.117056	0.259766	0.111189	0.470928	CFAP418	157657	cilia and flagella associated protein 418
ENSG0000	2225.004	-0.18581	0.144723	0.111323	0.471388	PIAS1	8554	protein inhibitor of activated STAT 1
ENSG0000	6.435078	0.140391	0.271118	0.111382	0.471525	C1QTNF3	114899	C1q and TNF related 3
ENSG0000	318.7094	-0.19885	0.160833	0.111468	0.471782	DNAJC13	23317	DnaJ heat shock protein family (Hsp40) member C13
ENSG0000	28.92408	-0.21544	0.257766	0.111528	0.471928	CTF1	1489	cardiotrophin 1
ENSG0000	6.863774	0.14494	0.272873	0.111706	0.472354	FSCN3	29999	fascin actin-bundling protein 3
ENSG0000	21716.45	-0.21135	0.222971	0.111696	0.472354	FLOT2	2319	flotillin 2
ENSG0000	4228.662	0.204972	0.18384	0.111684	0.472354	XBP1	7494	X-box binding protein 1
ENSG0000	69.70929	0.215582	0.205708	0.111855	0.472874	TIMM9	26520	translocase of inner mitochondrial membrane 9
ENSG0000	4.596945	0.109107	0.257829	0.111891	0.472915	LOC10050	1.01E+08	uncharacterized LOC100507250
ENSG0000	54.43721	0.215651	0.24219	0.11204	0.473439	SFXN2	118980	sideroflexin 2
ENSG0000	3.31686	-0.04743	0.237747	0.112069	0.47345	OR52B4	143496	olfactory receptor family 52 subfamily B member 4
ENSG0000	79.68731	0.190441	0.268381	0.112099	0.473467	SMARCAD	56916	SWI/SNF-r matrix-as: subfamily containing DEAD/H box 1
ENSG0000	21.86653	0.205791	0.272861	0.112127	0.473476	NA	NA	NA
ENSG0000	18.71805	0.183221	0.277495	0.112236	0.473794	KIF9	64147	kinesin family member 9
ENSG0000	77.39073	-0.21576	0.220441	0.112254	0.473794	RETREG1	54463	reticulophagy regulator 1
ENSG0000	115.6296	-0.20325	0.17766	0.11236	0.474132	SCAPER	49855	S-phase cyclin A associated protein in the ER
ENSG0000	54.6229	0.18433	0.269866	0.112588	0.474984	SLC36A4	120103	solute carrier family 36 member 4
ENSG0000	5.086294	-0.10689	0.256707	0.113017	0.475665	NA	NA	NA
ENSG0000	240.4988	0.210852	0.199103	0.112778	0.475665	GLS	2744	glutaminase
ENSG0000	609.3027	-0.17532	0.133245	0.113035	0.475665	ERAP1	51752	endoplasmic reticulum aminopeptidase 1
ENSG0000	133.1469	0.195569	0.160063	0.112852	0.475665	MAP3K4	4216	mitogen-activated protein kinase kinase kinase 4
ENSG0000	8.626535	-0.12714	0.265775	0.113027	0.475665	C9orf152	401546	chromosome 9 open reading frame 152
ENSG0000	4.05842	-0.10679	0.260761	0.112932	0.475665	ANXA8	653145	annexin A8
ENSG0000	2.710808	-0.10002	0.255384	0.112938	0.475665	OOSP3	1.13E+08	oocyte secreted protein family member 3
ENSG0000	126.9212	-0.20458	0.251252	0.112942	0.475665	CDK17	5128	cyclin dependent kinase 17
ENSG0000	28.42156	0.213669	0.257856	0.112896	0.475665	PCCA	5095	propionyl-CoA carboxylase subunit alpha
ENSG0000	56.47764	-0.21138	0.191766	0.112985	0.475665	TRAV13-1	28671	T cell receptor alpha variable 13-1
ENSG0000	79.69146	0.218915	0.260706	0.113016	0.475665	MRPL23	6150	mitochondrial ribosomal protein L23
ENSG0000	16.70902	0.126338	0.260832	0.113069	0.475684	LOC10537	1.05E+08	uncharacterized LOC105370152
ENSG0000	2185.931	0.21089	0.20567	0.113091	0.475684	PLEKHG3	26030	pleckstrin homology and RhoGEF domain containing G3
ENSG0000	22.95256	-0.20989	0.265402	0.113152	0.47583	NA	NA	NA
ENSG0000	3.89376	0.079693	0.246409	0.113365	0.47629	C4orf50	389197	chromosome 4 open reading frame 50
ENSG0000	296.72	-0.20485	0.245412	0.11334	0.47629	NAIP	4671	NLR family apoptosis inhibitory protein
ENSG0000	24.97481	-0.20626	0.267664	0.113363	0.47629	LOC10012	1E+08	uncharacterized LOC100129215
ENSG0000	7.771275	-0.1003	0.252514	0.113338	0.47629	FAM215A	23591	family with sequence similarity 215 member A
ENSG0000	7.404101	-0.1472	0.272946	0.113597	0.477155	LOC10272	1.03E+08	uncharacterized LOC102724593
ENSG0000	85.85983	0.210666	0.194975	0.113775	0.477794	QRSL1	55278	glutaminyl-tRNA amidotransferase subunit QRSL1
ENSG0000	4.849596	-0.0963	0.252632	0.113894	0.478182	NFATC4	4776	nuclear factor of activated T cells 4
ENSG0000	23.94906	0.206417	0.268364	0.114007	0.478547	PALLD	23022	palladin cytoskeletal associated protein
ENSG0000	488.5822	0.215282	0.199488	0.114046	0.478599	MAN1A1	4121	mannosidase alpha class 1A member 1
ENSG0000	686.3985	0.187137	0.150775	0.114073	0.478603	REEP5	7905	receptor accessory protein 5
ENSG0000	10.02989	0.1329	0.266415	0.114351	0.479515	LIN9	286826	lin-9 DREAM MuvB core complex component
ENSG0000	186.5138	-0.20703	0.239394	0.114395	0.479515	TMEM260	54916	transmembrane protein 260
ENSG0000	379.7639	0.164561	0.121689	0.114388	0.479515	PSMD7	5713	proteasom non-ATPase 7
ENSG0000	98.91825	-0.19816	0.259249	0.114359	0.479515	NA	NA	NA
ENSG0000	31.9937	0.171325	0.271961	0.114535	0.479707	SNRPG	6637	small nuclear ribonucleoprotein polypeptide G
ENSG0000	24.95031	0.170932	0.273696	0.114624	0.479707	RPL26L1	51121	ribosomal protein L26 like 1
ENSG0000	33191.67	-0.20625	0.238684	0.114563	0.479707	DOK3	79930	docking protein 3
ENSG0000	16.54581	-0.15504	0.270262	0.114502	0.479707	NA	NA	NA
ENSG0000	4.362758	-0.05452	0.240084	0.114585	0.479707	STOX1	219736	storkhead box 1
ENSG0000	1677.725	-0.19053	0.15626	0.114602	0.479707	NAA60	79903	N-alpha-a NatF catalytic subunit
ENSG0000	137.1948	0.191213	0.154901	0.114596	0.479707	MED9	55090	mediator complex subunit 9
ENSG0000	42.76121	0.208393	0.259491	0.114728	0.479922	EEA1	8411	early endosome antigen 1
ENSG0000	2235.648	0.207764	0.172822	0.114717	0.479922	ATP5F1D	513	ATP synthase F1 subunit delta
ENSG0000	67.30521	0.198925	0.166293	0.114771	0.479993	UBXN2A	165324	UBX domain protein 2A
ENSG0000	6601.199	-0.20493	0.191197	0.115002	0.480741	RNF10	9921	ring finger protein 10
ENSG0000	66.6666	0.21215	0.198789	0.114979	0.480741	CRAMP1	57585	cramped chromatin regulator homolog 1
ENSG0000	15.34892	0.152566	0.270638	0.115103	0.480864	NA	NA	NA
ENSG0000	66.94347	0.211409	0.242599	0.11511	0.480864	ARL1	400	ADP ribosylation factor like GTPase 1
ENSG0000	106.0515	0.205365	0.18235	0.115076	0.480864	NA	NA	NA
ENSG0000	83.96872	0.192515	0.156327	0.115174	0.480865	NOM1	64434	nucleolar protein with MIF4G domain 1
ENSG0000	1666.646	0.217603	0.259797	0.115173	0.480865	NLRP6	171389	NLR family pyrin domain containing 6
ENSG0000	66.30043	0.203057	0.257592	0.115189	0.480865	HSPA13	6782	heat shock protein family A (Hsp70) member 13
ENSG0000	308.6494	-0.18002	0.140606	0.115221	0.480886	RTCB	51493	RNA 2' 3'-cyclic phosphate and 5'-OH ligase
ENSG0000	3.722879	0.075715	0.244356	0.115261	0.480946	NA	NA	NA
ENSG0000	187.7846	0.200856	0.167452	0.115291	0.48096	MEGF8	1954	multiple EGF like domains 8
ENSG0000	16.48292	0.171072	0.273768	0.115377	0.481103	FGFBP3	143282	fibroblast growth factor binding protein 3
ENSG0000	3.934292	0.063877	0.241685	0.115351	0.481103	NA	NA	NA
ENSG0000	9.746116	-0.13879	0.267384	0.115451	0.481302	NA	NA	NA
ENSG0000	25.82158	0.196785	0.271732	0.115485	0.481332	TRAIP	10293	TRAF interacting protein
ENSG0000	4.921599	-0.12466	0.264577	0.115546	0.48138	NA	NA	NA
ENSG0000	183.5584	0.181487	0.142132	0.115549	0.48138	LTO1	220064	LTO1 maturation factor of ABCE1
ENSG0000	58.61881	-0.19998	0.259532	0.115576	0.481383	NA	NA	NA
ENSG0000	48.12979	-0.21123	0.19952	0.115691	0.481753	CEP135	9662	centrosomal protein 135
ENSG0000	3604.852	-0.20593	0.194925	0.11575	0.481888	ST3GAL2	6483	ST3 beta-g 3-sialyltransferase 2
ENSG0000	111.0561	0.209628	0.206712	0.115779	0.481901	ELP6	54859	elongator acetyltransferase complex subunit 6
ENSG0000	892.1862	-0.18789	0.147586	0.11595	0.482099	RAB10	10890	RAB10 member RAS oncogene family
ENSG0000	770.568	-0.15817	0.115603	0.11609	0.482099	AFTPH	54812	aftiphilin
ENSG0000	26.39679	0.203667	0.265009	0.116027	0.482099	CD200	4345	CD200 molecule
ENSG0000	71.01844	-0.21377	0.224044	0.115868	0.482099	TRBV19	28568	T cell receptor beta variable 19
ENSG0000	64.86286	0.209688	0.196239	0.115939	0.482099	ZBTB5	9925	zinc finger and BTB domain containing 5
ENSG0000	30.85226	0.175583	0.273551	0.116049	0.482099	FAM90A1	55138	family with sequence similarity 90 member A1
ENSG0000	10.8636	0.141654	0.269064	0.115973	0.482099	A2M	2	alpha-2-macroglobulin
ENSG0000	347.4494	-0.16851	0.125445	0.116066	0.482099	ZC3H10	84872	zinc finger CCCH-type containing 10
ENSG0000	190.2456	-0.20834	0.202193	0.115885	0.482099	CDC42EP4	23580	CDC42 effector protein 4
ENSG0000	258.0371	-0.18092	0.141647	0.115989	0.482099	TTC39C	125488	tetratricopeptide repeat domain 39C
ENSG0000	79.23736	0.196844	0.164363	0.11629	0.482821	CDCA4	55038	cell division cycle associated 4
ENSG0000	510.4697	0.119298	0.257579	0.116358	0.482883	RPS7	6201	ribosomal protein S7
ENSG0000	4.715051	0.117156	0.261983	0.116339	0.482883	NAP1L2	4674	nucleosome assembly protein 1 like 2
ENSG0000	325.4437	-0.18517	0.261287	0.116603	0.482954	FAM157A	728262	family with sequence similarity 157 member A

ENSG0000	15.8432	0.198917	0.274617	0.116567	0.482954	NA	NA	NA
ENSG0000	382.9447	0.176926	0.137052	0.116551	0.482954	YWHAG	7532	tyrosine 3-monooxygenase/tryptophan 5-monooxygenase activation protein gamma
ENSG0000	33.03708	0.211751	0.250242	0.116477	0.482954	ZNF487	642819	zinc finger protein 487
ENSG0000	19.10127	-0.1659	0.272673	0.11658	0.482954	RNA5SP38	1.01E+08	RNA 5S ribosomal pseudogene 383
ENSG0000	11.4613	0.177281	0.279685	0.11661	0.482954	NA	NA	NA
ENSG0000	1448.764	-0.1823	0.14474	0.116493	0.482954	ARHGAP1	55114	Rho GTPase activating protein 17
ENSG0000	749.3172	-0.18621	0.151494	0.116612	0.482954	VPS53	55275	VPS53 subunit of GARP complex
ENSG0000	191.2742	0.19717	0.16828	0.11648	0.482954	NAA38	84316	N-alpha-a NatC auxiliary subunit
ENSG0000	1912.52	-0.20866	0.204047	0.116648	0.48299	DENND1A	57706	DENN domain containing 1A
ENSG0000	64.93781	0.212534	0.207299	0.116691	0.48306	WDR97	340390	WD repeat domain 97
ENSG0000	824.3782	-0.15655	0.115015	0.116817	0.483476	ZFYVE27	118813	zinc finger FYVE-type containing 27
ENSG0000	15.67534	0.1786	0.278137	0.116924	0.483697	TCTN2	79867	tectonic family member 2
ENSG0000	112.6718	-0.19235	0.260026	0.116919	0.483697	HSD3B7	80270	hydroxy-d 3 beta- and steroid delta-isomerase 7
ENSG0000	11.31315	0.183405	0.279642	0.117093	0.484289	ZNF280B	140883	zinc finger protein 280B
ENSG0000	4.589346	0.056583	0.239031	0.117283	0.484856	NA	NA	NA
ENSG0000	7.318921	0.071534	0.242755	0.117258	0.484856	NA	NA	NA
ENSG0000	312.5759	-0.19945	0.176072	0.117511	0.48493	IDH1	3417	isocitrate dehydrogenase (NADP(+)) 1
ENSG0000	9.871959	0.140522	0.268079	0.117552	0.48493	RPL39L	116832	ribosomal protein L39 like
ENSG0000	208.5245	0.205309	0.242514	0.117366	0.48493	STXBP5	134957	syntaxin binding protein 5
ENSG0000	123.0524	-0.20594	0.193137	0.117383	0.48493	STX1A	6804	syntaxin 1A
ENSG0000	349.4314	0.204375	0.185979	0.1175	0.48493	PRAG1	157285	PEAK1 rela kinase-activating pseudokinase 1
ENSG0000	142.3445	0.194075	0.162561	0.117454	0.48493	MAPKAPK	8550	MAPK activated protein kinase 5
ENSG0000	2603.171	0.213985	0.18915	0.117343	0.48493	RIPOR1	79567	RHO family interacting cell polarization regulator 1
ENSG0000	94.61614	0.204941	0.184693	0.117566	0.48493	ENKD1	84080	enkurin domain containing 1
ENSG0000	1816.099	-0.17051	0.130853	0.117411	0.48493	RAB5C	5878	RAB5C member RAS oncogene family
ENSG0000	418.3895	0.194323	0.167464	0.117515	0.48493	SPATA20	64847	spermatogenesis associated 20
ENSG0000	301.2026	-0.18648	0.150916	0.117806	0.485024	TOR1AIP2	163590	torsin 1A interacting protein 2
ENSG0000	34.92974	0.180784	0.271046	0.117752	0.485024	ODR4	54953	odr-4 GPCR localization factor homolog
ENSG0000	185.5936	-0.20904	0.216134	0.117788	0.485024	DNAAF11	23639	dynein axonemal assembly factor 11
ENSG0000	740.6913	-0.18037	0.145153	0.117713	0.485024	CCDC85B	11007	coiled-coil domain containing 85B
ENSG0000	354.0563	-0.1737	0.133531	0.117684	0.485024	SNW1	22938	SNW domain containing 1
ENSG0000	34.9339	0.187035	0.266008	0.117827	0.485024	HROB	78995	homologous recombination factor with OB-fold
ENSG0000	4025.31	-0.18532	0.151267	0.117721	0.485024	IFNGR2	3460	interferon gamma receptor 2
ENSG0000	18.42986	0.186681	0.271889	0.117823	0.485024	CYP2D8P	1568	ccytochror pseudogene
ENSG0000	159.5406	0.189354	0.156083	0.117665	0.485024	CCNQ	92002	cyclin Q
ENSG0000	4.189998	0.107721	0.260138	0.117932	0.485171	HAVCR1	26762	hepatitis A virus cellular receptor 1
ENSG0000	66.44818	-0.2061	0.188496	0.117923	0.485171	POLR1HAS	80862	POLR1H ar pseudogene
ENSG0000	29.90555	-0.18586	0.268523	0.117942	0.485171	ZFP82	284406	ZFP82 zinc finger protein
ENSG0000	427.219	0.20588	0.191884	0.118026	0.485408	SLC37A2	219855	solute carrier family 37 member 2
ENSG0000	5.66269	0.131204	0.266219	0.11806	0.485438	MAP6D1	79929	MAP6 domain containing 1
ENSG0000	58.25042	0.20633	0.187097	0.118097	0.485482	LIAS	11019	lipoic acid synthetase
ENSG0000	624.2441	-0.18485	0.151276	0.118176	0.485589	CCND2	894	cyclin D2
ENSG0000	4.741627	-0.09411	0.252323	0.118152	0.485589	NA	NA	NA
ENSG0000	149.0338	-0.18979	0.157662	0.118204	0.485595	TMEM87A	25963	transmembrane protein 87A
ENSG0000	58.34187	-0.20713	0.244532	0.118267	0.485744	NA	NA	NA
ENSG0000	299.3073	0.197937	0.173727	0.118333	0.485872	MRPS25	64432	mitochondrial ribosomal protein S25
ENSG0000	3118.335	-0.20543	0.202438	0.118351	0.485872	LAMP1	3916	lysosomal associated membrane protein 1
ENSG0000	229.2587	0.194024	0.164746	0.118485	0.48631	ATXN1L	342371	ataxin 1 like
ENSG0000	112.9896	-0.20936	0.203563	0.118537	0.486415	ZNF271P	10778	zinc finger pseudogene
ENSG0000	11.66702	-0.18049	0.277418	0.118594	0.48654	NA	NA	NA
ENSG0000	4.869473	-0.05778	0.240558	0.118629	0.486576	RGS1	5996	regulator of G protein signaling 1
ENSG0000	214.1458	-0.2034	0.243016	0.118683	0.48669	EPHB2	2048	EPH receptor B2
ENSG0000	7.136684	-0.08152	0.246348	0.118752	0.486757	FLJ42393	401105	uncharacterized LOC401105
ENSG0000	5.649786	0.126144	0.264017	0.118753	0.486757	RPL13AP2	441632	ribosomal protein L13a pseudogene 22
ENSG0000	185.2833	-0.16992	0.269415	0.11881	0.486773	AOC1	26	amine oxidase copper containing 1
ENSG0000	4.184843	-0.08148	0.246572	0.118791	0.486773	NA	NA	NA
ENSG0000	8.104813	0.129544	0.264355	0.118965	0.486862	IGKV2-28	28921	immunoglobulin kappa variable 2-28
ENSG0000	158.4256	-0.176	0.138044	0.118952	0.486862	RFT1	91869	RFT1 homolog
ENSG0000	28.96166	0.211546	0.247976	0.118904	0.486862	MYB	4602	MYB protc transcription factor
ENSG0000	154.0648	0.208094	0.21538	0.118901	0.486862	BAIAP2	10458	BAR/IMD domain containing adaptor protein 2
ENSG0000	12.59744	0.134845	0.266861	0.118931	0.486862	TMEM191	728229	transmembrane protein 191B
ENSG0000	5519.889	-0.19588	0.172313	0.119043	0.486965	GLUL	2752	glutamate-ammonia ligase
ENSG0000	7.702231	-0.1273	0.262692	0.119039	0.486965	TLR3	7098	toll like receptor 3
ENSG0000	2.393598	0.063374	0.242112	0.119129	0.486989	NA	NA	NA
ENSG0000	287.4217	0.207257	0.214274	0.119099	0.486989	RUNX2	860	RUNX family transcription factor 2
ENSG0000	76.52324	-0.20895	0.229011	0.119112	0.486989	ZNF493	284443	zinc finger protein 493
ENSG0000	83.44958	0.210351	0.22018	0.119252	0.487383	TMEM14C	51522	transmembrane protein 14C
ENSG0000	46.28601	0.190105	0.264207	0.119338	0.487517	CYP2R1	120227	cytochrome P450 family 2 subfamily R member 1
ENSG0000	5.240664	-0.09038	0.249055	0.119325	0.487517	CAPS2	84698	calcyphosine 2
ENSG0000	612.346	-0.19122	0.162203	0.1194	0.487551	B3GALT4	8705	beta-1 3-galactosyltransferase 4
ENSG0000	28.97604	0.21504	0.230494	0.119452	0.487551	HERPUD2-	1.02E+08	HERPUD2 antisense RNA 1
ENSG0000	4349.816	0.204454	0.20762	0.119443	0.487551	RPL30	6156	ribosomal protein L30
ENSG0000	76998.14	0.200121	0.194426	0.119383	0.487551	CYTB	4519	cytochrome b
ENSG0000	25.37884	-0.18033	0.270461	0.119571	0.487926	NA	NA	NA
ENSG0000	41.81129	0.21393	0.216095	0.119602	0.487942	NA	NA	NA
ENSG0000	193.5069	0.181695	0.144846	0.119727	0.488103	NA	NA	NA
ENSG0000	2733.93	-0.19751	0.176522	0.119801	0.488103	SHARPIN	81858	SHANK associated RH domain interactor
ENSG0000	5.081574	0.118352	0.26108	0.119777	0.488103	NA	NA	NA
ENSG0000	292.5827	-0.1997	0.169777	0.119762	0.488103	AEN	64782	apoptosis enhancing nuclease
ENSG0000	23.99332	-0.20131	0.26788	0.119777	0.488103	NA	NA	NA
ENSG0000	18.26454	-0.20082	0.267482	0.11968	0.488103	CCDC116	164592	coiled-coil domain containing 116
ENSG0000	116.2046	0.197845	0.171387	0.119846	0.48818	TMEM120	144404	transmembrane protein 120B
ENSG0000	7.889024	0.131379	0.26471	0.119934	0.488429	RPL41P2	22970	ribosomal protein L41 pseudogene 2
ENSG0000	15.98045	0.165367	0.272373	0.119985	0.488528	NUBPL	80224	NUBP iron mitochondrial
ENSG0000	414.4169	0.18707	0.154688	0.120073	0.488602	NKTR	4820	natural killer cell triggering receptor
ENSG0000	840.5157	-0.19728	0.177633	0.120137	0.488602	GPR137	56834	G protein-coupled receptor 137
ENSG0000	587.1076	-0.17384	0.136659	0.120123	0.488602	CEP164	22897	centrosomal protein 164
ENSG0000	471.5489	0.201141	0.240895	0.120112	0.488602	LINC0127	284751	long intergenic non-protein coding RNA 1270
ENSG0000	14.98755	0.173939	0.273878	0.120131	0.488602	DGCR11	25786	DiGeorge syndrome critical region gene 11
ENSG0000	5144.152	0.207269	0.210073	0.120205	0.488773	PLD3	23646	phospholipase D family member 3
ENSG0000	60.49845	-0.20935	0.229464	0.12031	0.488874	NA	NA	NA
ENSG0000	3.20145	0.07765	0.245853	0.120286	0.488874	RNASE13	440163	ribonuclease A family member 13 (inactive)
ENSG0000	9.233624	-0.14031	0.267951	0.120276	0.488874	PROCR	10544	protein C receptor
ENSG0000	26.8199	-0.13745	0.261834	0.120442	0.488893	NA	NA	NA
ENSG0000	51.9795	-0.19365	0.260123	0.120474	0.488893	FBXL13	222235	F-box and leucine rich repeat protein 13
ENSG0000	30.15022	-0.19876	0.263989	0.120412	0.488893	TRBV18	28569	T cell receptor beta variable 18

ENSG0000	2.425167	0.080553	0.246966	0.120386	0.488893	NA	NA	NA
ENSG0000	130.4246	0.20701	0.214186	0.120475	0.488893	NA	NA	NA
ENSG0000	51.59302	0.21099	0.208578	0.120438	0.488893	LOC73018	730183	uncharacterized LOC730183
ENSG0000	1739.734	-0.19044	0.155532	0.12057	0.48915	TRAPPC14	55262	trafficking protein particle complex subunit 14
ENSG0000	4.033018	0.09168	0.251237	0.120619	0.48915	SLCO5A1	81796	solute carrier organic anion transporter family member 5A1
ENSG0000	9.302598	-0.11386	0.25748	0.120605	0.48915	SYTL4	94121	synaptotagmin like 4
ENSG0000	12.14886	0.131387	0.262665	0.120703	0.489274	SRRM2-AS	1E+08	SRRM2 antisense RNA 1
ENSG0000	59.09133	0.210129	0.217471	0.120677	0.489274	SLC9A7	84679	solute carrier family 9 member A7
ENSG0000	388.8402	-0.1665	0.126087	0.120768	0.489431	KMT5A	387893	lysine methyltransferase 5A
ENSG0000	3.756339	0.091559	0.251716	0.120844	0.489596	CD1E	913	CD1e molecule
ENSG0000	1439.304	0.191524	0.174706	0.120862	0.489596	PTOV1	53635	PTOV1 extended AT-hook containing adaptor protein
ENSG0000	19.4746	0.160931	0.270221	0.120972	0.489825	NA	NA	NA
ENSG0000	24.6586	0.210005	0.259905	0.120966	0.489825	HMMR	3161	hyaluronan mediated motility receptor
ENSG0000	393.315	-0.19681	0.175368	0.121108	0.490265	ADGRL1	22859	adhesion G protein-coupled receptor L1
ENSG0000	5.548948	0.076721	0.244538	0.121328	0.490938	LOC10798	1.08E+08	uncharacterized LOC107985323
ENSG0000	113.403	0.204535	0.196174	0.121314	0.490938	ELP4	26610	elongator acetyltransferase complex subunit 4
ENSG0000	298.4208	0.176065	0.260936	0.121355	0.490941	SLC8A1	6546	solute carrier family 8 member A1
ENSG0000	9.290748	-0.11844	0.258242	0.121385	0.490954	SLC14A2	8170	solute carrier family 14 member 2
ENSG0000	9.470452	-0.18281	0.280722	0.121465	0.491002	NA	NA	NA
ENSG0000	16.70292	-0.17826	0.272189	0.121477	0.491002	HISLA	283587	HIF1A stabilizing long noncoding RNA
ENSG0000	17.75689	0.210635	0.257236	0.121465	0.491002	IQCH-AS1	1.01E+08	IQCH antisense RNA 1
ENSG0000	17.97455	0.196839	0.269305	0.121524	0.491083	KBTBD6	89890	kelch repeat and BTB domain containing 6
ENSG0000	87.69501	-0.19545	0.169738	0.121673	0.491403	ACOT13	55856	acyl-CoA thioesterase 13
ENSG0000	144.1798	-0.19973	0.244744	0.121684	0.491403	NA	NA	NA
ENSG0000	967.7533	0.194384	0.172567	0.121645	0.491403	WDR81	124997	WD repeat domain 81
ENSG0000	1328.114	0.178838	0.143742	0.121716	0.491423	HERPUD1	9709	homocysteine inducible ER protein with ubiquitin like domain 1
ENSG0000	4.402766	0.064256	0.240665	0.121761	0.491496	PLA2G2C	391013	phospholipase A2 group IIC
ENSG0000	244.3046	0.19218	0.250425	0.121792	0.491514	ORMDL1	94101	ORMDL sphingolipid biosynthesis regulator 1
ENSG0000	45.48206	-0.20282	0.252291	0.121904	0.491859	PCDH1	5097	protocadherin 1
ENSG0000	320.3759	-0.20013	0.185044	0.122096	0.492526	UBASH3A	53347	ubiquitin associated and SH3 domain containing A
ENSG0000	48.22362	0.204091	0.246033	0.122132	0.492563	LOC65227	652276	potassium channel tetramerization domain containing 5 pseudogene
ENSG0000	34.14961	0.211528	0.214049	0.122202	0.492736	TMTC2	160335	transmembrane O-mannosyltransferase targeting cadherins 2
ENSG0000	187.6539	-0.20095	0.239431	0.122253	0.492832	PCSK1N	27344	proprotein convertase subtilisin/kexin type 1 inhibitor
ENSG0000	15.56197	0.125674	0.259654	0.122324	0.492908	SDC4	6385	syndecan 4
ENSG0000	11.52819	0.131538	0.263274	0.122326	0.492908	NA	NA	NA
ENSG0000	6.738236	0.058795	0.23944	0.12253	0.493404	NA	NA	NA
ENSG0000	4.741238	0.112848	0.258596	0.122523	0.493404	SLC18A2	6571	solute carrier family 18 member A2
ENSG0000	801.7024	0.19562	0.177295	0.122525	0.493404	PIK3IP1	113791	phosphoinositide-3-kinase interacting protein 1
ENSG0000	17.62633	0.168477	0.27209	0.122567	0.493448	AHI1	54806	Abelson helper integration site 1
ENSG0000	3.478704	0.092156	0.251354	0.12264	0.493632	URAD	646625	ureidoimidazoline (2-oxo-4-hydroxy-4-carboxy-5-) decarboxylase
ENSG0000	2.537396	0.072427	0.245137	0.122741	0.493737	SUCLG2-D	1.02E+08	SUCLG2 divergent transcript
ENSG0000	14.40012	-0.1169	0.256249	0.122716	0.493737	NA	NA	NA
ENSG0000	1862.499	-0.18038	0.147581	0.122747	0.493737	SNN	8303	stannin
ENSG0000	3.707333	0.077841	0.245338	0.122777	0.493751	NA	NA	NA
ENSG0000	506.6384	-0.19988	0.176899	0.122806	0.493757	LSS	4047	lanosterol synthase
ENSG0000	488.7476	-0.1671	0.129058	0.122942	0.493872	MAFK	7975	MAF bZIP transcription factor K
ENSG0000	41.29153	0.204925	0.247725	0.122888	0.493872	FOLR2	2350	folate receptor beta
ENSG0000	56.04528	-0.19637	0.254577	0.122917	0.493872	LOC10537	1.05E+08	uncharacterized LOC105370969
ENSG0000	26.15183	0.166575	0.269058	0.122928	0.493872	PRSS21	10942	serine protease 21
ENSG0000	25.61807	0.175828	0.269316	0.123007	0.494022	COMMD3	23412	COMM domain containing 3
ENSG0000	12.96612	0.153974	0.268686	0.123056	0.494114	NA	NA	NA
ENSG0000	4.011776	0.092447	0.250351	0.123231	0.49449	NA	NA	NA
ENSG0000	205.6701	-0.19559	0.181663	0.123228	0.49449	NUP205	23165	nucleoporin 205
ENSG0000	8.327843	-0.13444	0.264598	0.123227	0.49449	TDRP	157695	testis development related protein
ENSG0000	24.1797	-0.16409	0.267882	0.123316	0.494724	CPLANE1	65250	ciliogenesis and planar polarity effector complex subunit 1
ENSG0000	8.81596	0.113855	0.256839	0.123425	0.494943	TATDN1	83940	TatD DNase domain containing 1
ENSG0000	298.1732	0.198518	0.182021	0.123401	0.494943	TIMM44	10469	translocase of inner mitochondrial membrane 44
ENSG0000	3.835472	0.072936	0.244861	0.123502	0.495106	NA	NA	NA
ENSG0000	3184.341	-0.20249	0.211872	0.12352	0.495106	B3GNT8	374907	UDP-GlcN/ 3-N-acetylglucosaminyltransferase 8
ENSG0000	98.14526	0.175714	0.262023	0.12366	0.495562	SLC25A36	55186	solute carrier family 25 member 36
ENSG0000	619.7582	-0.17993	0.149956	0.123779	0.495823	FBXO44	93611	F-box protein 44
ENSG0000	4.122557	-0.07363	0.244865	0.12378	0.495823	NA	NA	NA
ENSG0000	117.7954	0.188453	0.256269	0.123849	0.495989	SAP30	8819	Sin3A associated protein 30
ENSG0000	36.6112	0.207535	0.242105	0.123876	0.495989	C17orf75	64149	chromosome 17 open reading frame 75
ENSG0000	3345.542	-0.17915	0.146749	0.123973	0.496134	KIF21B	23046	kinesin family member 21B
ENSG0000	7.749947	-0.08204	0.244881	0.124065	0.496134	CACNA1D	776	calcium voltage-gated channel subunit alpha1 D
ENSG0000	550.0493	-0.20408	0.202836	0.124058	0.496134	LNPEP	4012	leucyl and cystinyl aminopeptidase
ENSG0000	256.9455	-0.20432	0.208841	0.12409	0.496134	ABLIM3	22885	actin binding LIM protein family member 3
ENSG0000	1380.212	-0.20217	0.206649	0.124101	0.496134	NDST1	3340	N-deacetylase and N-sulfotransferase 1
ENSG0000	2.66088	0.068753	0.242829	0.124027	0.496134	NA	NA	NA
ENSG0000	16.31887	0.18517	0.27108	0.124076	0.496134	NA	NA	NA
ENSG0000	25.40986	0.207539	0.249417	0.124164	0.496251	SPACA9	11092	sperm acrosome associated 9
ENSG0000	3.986538	0.056674	0.239319	0.124185	0.496251	MYBPC2	4606	myosin binding protein C2
ENSG0000	502.5191	-0.18359	0.15296	0.124244	0.49638	ASB16-AS1	339201	ASB16 antisense RNA 1
ENSG0000	110.7566	0.206057	0.213373	0.124323	0.496587	GAS5	60674	growth arrest specific 5
ENSG0000	6.984588	0.087598	0.247772	0.124425	0.496714	UGT8	7368	UDP glycosyltransferase 8
ENSG0000	54.12058	-0.195	0.253171	0.124436	0.496714	CCDC146	57639	coiled-coil domain containing 146
ENSG0000	30.13804	0.210054	0.239758	0.124401	0.496714	SLIRP	81892	SRA stem-loop interacting RNA binding protein
ENSG0000	7.11446	0.14983	0.271746	0.124464	0.496715	SLC5A2	6524	solute carrier family 5 member 2
ENSG0000	1167.479	-0.18873	0.168012	0.124495	0.496732	PCED1B-A	1E+08	PCED1B antisense RNA 1
ENSG0000	119.2498	-0.20496	0.211457	0.124605	0.496924	RAB11FIP5	26056	RAB11 family interacting protein 5
ENSG0000	7.353073	0.10164	0.25226	0.124602	0.496924	P4HA2	8974	prolyl 4-hydroxylase subunit alpha 2
ENSG0000	12.14962	0.137786	0.264691	0.124706	0.496924	ME1	4199	malic enzyme 1
ENSG0000	106.0824	-0.19535	0.174378	0.124698	0.496924	RAMAC	83640	RNA guanine-7 methyltransferase activating subunit
ENSG0000	4.995604	-0.0765	0.244543	0.124695	0.496924	NA	NA	NA
ENSG0000	4180.369	-0.1924	0.171603	0.12463	0.496924	OTUD5	55593	OTU deubiquitinase 5
ENSG0000	304.67	-0.19326	0.171584	0.124757	0.497022	RCE1	9986	Ras converting CAAX endopeptidase 1
ENSG0000	3063.182	0.194273	0.174753	0.124912	0.49749	PPIA	5478	peptidylprolyl isomerase A
ENSG0000	1149.562	-0.17412	0.14403	0.124929	0.49749	MON1B	22879	MON1 hor secretory trafficking associated
ENSG0000	782.7749	-0.20236	0.227785	0.125047	0.497741	PAG1	55824	phosphoprotein membrane anchor with glycosphingolipid microdomains 1
ENSG0000	22.99441	0.144914	0.265361	0.125025	0.497741	IGHJ6	28475	immunoglobulin heavy joining 6
ENSG0000	4728.916	-0.19694	0.200613	0.125075	0.497745	TCF7	6932	transcription factor 7
ENSG0000	2.666477	0.094144	0.251948	0.12513	0.497855	RPS10P3	158104	ribosomal protein S10 pseudogene 3
ENSG0000	91.43391	0.206226	0.212657	0.125253	0.498236	ARFGEF2	10564	ADP ribosylation factor guanine nucleotide exchange factor 2
ENSG0000	17.28014	0.20471	0.260585	0.125286	0.498261	NA	NA	NA
ENSG0000	12.0702	-0.1906	0.274585	0.125318	0.498279	NA	NA	NA

ENSG0000	18.56378	-0.16073	0.267609	0.125362	0.498345	TRGV4	6977	T cell receptor gamma variable 4
ENSG0000	2.872537	0.071475	0.243899	0.125391	0.498353	RPL17P9	729301	ribosomal protein L17 pseudogene 9
ENSG0000	3.871906	0.112595	0.259522	0.125421	0.498364	NA	NA	NA
ENSG0000	58.5618	0.174382	0.261099	0.125471	0.49839	PTBP2	58155	polypyrimidine tract binding protein 2
ENSG0000	1221.757	-0.16856	0.131606	0.125509	0.49839	ATF7IP	55729	activating transcription factor 7 interacting protein
ENSG0000	33.10041	-0.19968	0.250974	0.125503	0.49839	COX16	51241	cytochrome c oxidase assembly factor COX16
ENSG0000	4140.747	-0.1678	0.133027	0.125756	0.499264	PRKCB	5579	protein kinase C beta
ENSG0000	15.31481	0.127954	0.260873	0.125919	0.499783	FZD3	7976	frizzled class receptor 3
ENSG0000	133.4393	0.203324	0.201475	0.125969	0.499783	MLLT10	8028	MLLT10 histone lysine methyltransferase DOT1L cofactor
ENSG0000	2.655194	0.085873	0.251264	0.125953	0.499783	OR13A1	79290	olfactory receptor family 13 subfamily A member 1
ENSG0000	46.19638	-0.18531	0.258866	0.126046	0.499979	TMEM126	55863	transmembrane protein 126B
ENSG0000	125.2159	0.184441	0.15366	0.126101	0.500089	RNASEH1	246243	ribonuclease H1
ENSG0000	2.399767	0.059927	0.24236	0.126152	0.500186	RPL17P6	645441	ribosomal protein L17 pseudogene 6
ENSG0000	61.23017	0.173258	0.263039	0.126311	0.500382	TXNDC9	10190	thioredoxin domain containing 9
ENSG0000	20.63488	-0.17048	0.271655	0.126281	0.500382	NA	NA	NA
ENSG0000	39.79757	0.208056	0.221195	0.126306	0.500382	LBHD1	79081	LBH domain containing 1
ENSG0000	30.07062	-0.20906	0.234762	0.126246	0.500382	ZNF542P	147947	zinc finger pseudogene
ENSG0000	2.501818	0.078031	0.245667	0.126355	0.500449	EEF1A1P2	645693	eukaryotic translation elongation factor 1 alpha 1 pseudogene 22
ENSG0000	986.6692	0.190113	0.165845	0.126412	0.500565	MAP3K14	9020	mitogen-activated protein kinase kinase kinase 14
ENSG0000	10.45202	0.132424	0.263215	0.126525	0.500799	DACT1	51339	dishevelled binding antagonist of beta catenin 1
ENSG0000	6.656269	-0.14498	0.270062	0.126526	0.500799	NA	NA	NA
ENSG0000	2.517106	0.04836	0.238027	0.126572	0.500875	GARIN5A	112703	golgi associated RAB2 interactor 5A
ENSG0000	3.711133	-0.06085	0.240273	0.126611	0.500921	LGALS17A	400696	galectin 14 pseudogene
ENSG0000	46.91373	-0.20702	0.227156	0.126695	0.500927	ARHGEF5	7984	Rho guanine nucleotide exchange factor 5
ENSG0000	9.592476	0.116673	0.257127	0.126674	0.500927	CYP2E1	1571	cytochrome P450 family 2 subfamily E member 1
ENSG0000	60.8724	0.20385	0.197569	0.126661	0.500927	SELENOH	280636	selenoprotein H
ENSG0000	312.8068	0.180064	0.151849	0.126929	0.501205	CNTRL	11064	centriolin
ENSG0000	20397.02	-0.19982	0.215712	0.126813	0.501205	RHOG	391	ras homolog family member G
ENSG0000	677.748	0.17822	0.146357	0.126911	0.501205	VPS37C	55048	VPS37C subunit of ESCRT-I
ENSG0000	6.506035	-0.09238	0.249346	0.126827	0.501205	LOC10798	1.08E+08	uncharacterized LOC107984551
ENSG0000	321.1415	0.191618	0.168384	0.126849	0.501205	RTRAF	51637	RNA trans translation and transport factor
ENSG0000	432.9381	0.195376	0.179409	0.126889	0.501205	THOC6	79228	THO complex subunit 6
ENSG0000	4.16712	0.073509	0.244572	0.126962	0.501225	RPS3AP6	145767	RPS3A pseudogene 6
ENSG0000	52.59115	0.206528	0.214746	0.127188	0.502011	CBX8	57332	chromobox 8
ENSG0000	6.958237	0.11262	0.25607	0.127348	0.502316	TLCD2	727910	TLC domain containing 2
ENSG0000	87.33587	-0.19508	0.173522	0.127332	0.502316	NA	NA	NA
ENSG0000	29.73118	0.205796	0.245498	0.127345	0.502316	NA	NA	NA
ENSG0000	19.7467	0.199631	0.26248	0.127394	0.502391	NA	NA	NA
ENSG0000	3.326015	0.100291	0.253446	0.127435	0.502426	RPL3P2	116935	ribosomal protein L3 pseudogene 2
ENSG0000	2.609598	0.061873	0.241834	0.127458	0.502426	ALOXE3	59344	arachidonate lipoxygenase 3
ENSG0000	237.6852	0.178242	0.151018	0.127521	0.502506	PI4K2A	55361	phosphatidylinositol 4-kinase type 2 alpha
ENSG0000	8.691313	0.147471	0.268869	0.127533	0.502506	ZNF77	58492	zinc finger protein 77
ENSG0000	3.877778	0.068514	0.2447	0.12772	0.50286	NA	NA	NA
ENSG0000	200.861	-0.0895	0.245741	0.12776	0.50286	BCL2A1	597	BCL2 related protein A1
ENSG0000	27.26829	0.197909	0.254586	0.127693	0.50286	SHPK	23729	sedoheptulokinase
ENSG0000	627.5176	-0.19204	0.167702	0.12774	0.50286	APEX2	27301	apurinic/apyrimidinic endodeoxyribonuclease 2
ENSG0000	3.655586	0.072819	0.244122	0.127704	0.50286	CCT4P2	1E+08	chaperonin containing TCP1 subunit 4 pseudogene 2
ENSG0000	147.5134	-0.19865	0.191653	0.127857	0.503131	ZNF44	51710	zinc finger protein 44
ENSG0000	71.01303	-0.20035	0.193275	0.128686	0.503583	TNFRSF18	8784	TNF receptor superfamily member 18
ENSG0000	122.5336	0.196139	0.249098	0.128561	0.503583	BTBD19	149478	BTB domain containing 19
ENSG0000	3.88595	-0.06577	0.241892	0.128156	0.503583	ADCY10	55811	adenylate cyclase 10
ENSG0000	6.637227	-0.12288	0.260629	0.128499	0.503583	LINC0189	1.05E+08	long intergenic non-protein coding RNA 1891
ENSG0000	14.12882	0.163923	0.270991	0.128187	0.503583	NA	NA	NA
ENSG0000	3.283012	0.053158	0.238951	0.128463	0.503583	NA	NA	NA
ENSG0000	4934.976	-0.19917	0.221191	0.128087	0.503583	HK3	3101	hexokinase 3
ENSG0000	629.7748	-0.17947	0.146467	0.128637	0.503583	GPANK1	7918	G-patch domain and ankyrin repeats 1
ENSG0000	22.8891	0.181857	0.253029	0.128549	0.503583	CRIP3	401262	cysteine rich protein 3
ENSG0000	34.67484	0.206729	0.232545	0.128219	0.503583	GTF2H5	404672	general transcription factor IIH subunit 5
ENSG0000	4034.258	-0.18178	0.154711	0.128642	0.503583	EIF2AK1	27102	eukaryotic translation initiation factor 2 alpha kinase 1
ENSG0000	10.95167	-0.16531	0.273792	0.128071	0.503583	TRBJ1-6	28630	T cell receptor beta joining 1-6
ENSG0000	131.4261	0.19908	0.192165	0.128671	0.503583	TOP1MT	116447	DNA topoisomerase I mitochondrial
ENSG0000	63.25492	-0.20171	0.232865	0.128397	0.503583	PLGRKT	55848	plasminogen receptor with a C-terminal lysine
ENSG0000	717.9618	0.182675	0.154954	0.128504	0.503583	PTGES2	80142	prostaglandin E synthase 2
ENSG0000	4.955323	-0.06877	0.242381	0.128195	0.503583	MIR7152	1.02E+08	microRNA 7152
ENSG0000	33.29746	0.196383	0.255264	0.128362	0.503583	ZFTA	65998	zinc finger translocation associated
ENSG0000	146.8983	0.185381	0.158369	0.128681	0.503583	SIK2	23235	salt inducible kinase 2
ENSG0000	3.175558	0.089467	0.249889	0.128219	0.503583	NA	NA	NA
ENSG0000	1279.476	-0.16656	0.14045	0.128436	0.503583	PARP4	143	poly(ADP-ribose) polymerase family member 4
ENSG0000	30.73818	0.190776	0.259509	0.128002	0.503583	MCF2L	23263	MCF.2 cell line derived transforming sequence like
ENSG0000	53.1823	0.206034	0.221154	0.128089	0.503583	ANG	283	angiogenin
ENSG0000	2.436476	0.045758	0.238122	0.128264	0.503583	LINC0064	400238	long intergenic non-protein coding RNA 642
ENSG0000	15362.73	-0.19798	0.200173	0.128647	0.503583	KDM6B	23135	lysine demethylase 6B
ENSG0000	1750.011	-0.18872	0.163541	0.128422	0.503583	IRF3	3661	interferon regulatory factor 3
ENSG0000	830.4956	-0.1478	0.109746	0.128552	0.503583	STAU1	6780	staufen double-stranded RNA binding protein 1
ENSG0000	22.37177	-0.20425	0.248417	0.128739	0.503682	LOC10798	1.08E+08	uncharacterized LOC107985688
ENSG0000	5.11391	0.092111	0.250826	0.128822	0.503791	PSCA	8000	prostate stem cell antigen
ENSG0000	2.627449	-0.04492	0.23727	0.128801	0.503791	NA	NA	NA
ENSG0000	140.496	-0.18435	0.155084	0.12892	0.504067	UBAP1L	390595	ubiquitin associated protein 1 like
ENSG0000	124.868	0.201674	0.201318	0.12907	0.504337	PAM	5066	peptidylglycine alpha-amidating monooxygenase
ENSG0000	7.346779	0.112313	0.256416	0.129071	0.504337	NA	NA	NA
ENSG0000	8.268384	0.112943	0.256235	0.129067	0.504337	ZNF594	84622	zinc finger protein 594
ENSG0000	105.9879	0.188438	0.164449	0.129156	0.504559	ACTL6A	86	actin like 6A
ENSG0000	313.214	0.181109	0.154287	0.129209	0.504661	ZNF587	84914	zinc finger protein 587
ENSG0000	497.0566	0.173536	0.141949	0.129382	0.504689	BCL2L11	10018	BCL2 like 11
ENSG0000	154.2661	0.188688	0.245971	0.129342	0.504689	TLR10	81793	toll like receptor 10
ENSG0000	62.98765	0.202209	0.210172	0.129356	0.504689	THOC3	84321	THO complex subunit 3
ENSG0000	2449.204	-0.19007	0.24197	0.12932	0.504689	CA1	759	carbonic anhydrase 1
ENSG0000	5.899219	-0.08956	0.249786	0.129357	0.504689	GLB1L3	112937	galactosidase beta 1 like 3
ENSG0000	140.3042	0.181305	0.151614	0.129364	0.504689	HDDC3	374659	HD domain containing 3
ENSG0000	73.05763	0.197519	0.182289	0.129568	0.505013	SMIM19	114926	small integral membrane protein 19
ENSG0000	96.06964	-0.19996	0.22902	0.129543	0.505013	CNIH2	254263	cornichon family AMPA receptor auxiliary protein 2
ENSG0000	395.5428	0.169613	0.136991	0.129603	0.505013	TMEM134	80194	transmembrane protein 134
ENSG0000	96.6314	-0.20011	0.22736	0.129587	0.505013	NA	NA	NA
ENSG0000	25.73866	-0.17978	0.263227	0.12954	0.505013	NA	NA	NA
ENSG0000	20.63069	0.154372	0.266909	0.129633	0.505022	PDIK1L	149420	PDLIM1 interacting kinase 1 like
ENSG0000	513.5687	0.162771	0.127346	0.129687	0.505126	TNPO2	30000	transportin 2

ENSG0000	619.2135	-0.1954	0.226058	0.129731	0.50519	NT5M	56953	5' 3'-nucleot mitochondrial
ENSG0000	15.6276	0.19741	0.263661	0.129803	0.505362	NA	NA	NA
ENSG0000	16.37981	-0.16266	0.267085	0.12995	0.505829	NUSAP1	51203	nucleolar and spindle associated protein 1
ENSG0000	108.8273	-0.20066	0.205633	0.130063	0.506114	WDR41	55255	WD repeat domain 41
ENSG0000	938.5172	0.183174	0.158853	0.130079	0.506114	FAU	2197	FAU ubiquitin like and ribosomal protein S30 fusion
ENSG0000	127.6853	0.199985	0.200249	0.130216	0.506362	ZCCHC17	51538	zinc finger CCHC-type containing 17
ENSG0000	3.031128	0.08109	0.247646	0.130202	0.506362	DYNLT4	343521	dynein light chain Tctex-type 4
ENSG0000	41.26485	0.177317	0.261673	0.130225	0.506362	ACADSB	36	acyl-CoA dehydrogenase short/branched chain
ENSG0000	890.8423	-0.19287	0.180774	0.130425	0.506536	C1QB	713	complement C1q B chain
ENSG0000	2.711623	0.038585	0.236446	0.13048	0.506536	TMEM89	440955	transmembrane protein 89
ENSG0000	10.34453	0.160951	0.271838	0.13045	0.506536	CAHM	1.01E+08	colon adenocarcinoma hypermethylated
ENSG0000	63.28922	0.189616	0.255561	0.130368	0.506536	GARIN1A	346653	golgi associated RAB2 interactor 1A
ENSG0000	1404.329	-0.1912	0.243498	0.130506	0.506536	IRAG1	10335	inositol 1 4 5-triphosphate receptor associated 1
ENSG0000	1069.698	0.192458	0.179402	0.130519	0.506536	DDX54	79039	DEAD-box helicase 54
ENSG0000	10.41486	0.156997	0.271878	0.130376	0.506536	NA	NA	NA
ENSG0000	6.786285	0.118429	0.258484	0.130487	0.506536	NA	NA	NA
ENSG0000	801.8676	0.193699	0.181288	0.130299	0.506536	SMARCB1	6598	SWI/SNF r matrix ass actin depe subfamily member 1
ENSG0000	359.4133	0.202575	0.208059	0.130571	0.50663	H2AZ1	3015	H2A.Z variant histone 1
ENSG0000	5.352065	0.085582	0.247865	0.130687	0.506972	SDAD1P1	157489	SDA1 domain containing 1 pseudogene 1
ENSG0000	293.6195	-0.19901	0.204389	0.130849	0.507352	TMEM234	56063	transmembrane protein 234
ENSG0000	11.36426	0.108947	0.253332	0.130844	0.507352	EFNA1	1942	ephrin A1
ENSG0000	35.50462	0.204715	0.234362	0.130895	0.507352	GLB1L	79411	galactosidase beta 1 like
ENSG0000	6.885192	0.110247	0.254983	0.13089	0.507352	SIRT4	23409	sirtuin 4
ENSG0000	942.8178	0.156693	0.121277	0.13115	0.508231	ARRDC2	27106	arrestin domain containing 2
ENSG0000	10.35392	0.152532	0.269612	0.131181	0.508243	DPY19L2P.	349152	DPY19L2 pseudogene 2
ENSG0000	3.999457	0.054063	0.238506	0.131296	0.508258	LINGO2	158038	leucine rich repeat and Ig domain containing 2
ENSG0000	5.408785	0.126498	0.265236	0.131272	0.508258	NA	NA	NA
ENSG0000	3.366072	-0.08377	0.247215	0.131284	0.508258	TSGA10IP	254187	testis specific 10 interacting protein
ENSG0000	8014.077	0.19376	0.186621	0.131265	0.508258	CD79A	973	CD79a molecule
ENSG0000	156.9999	0.18569	0.161636	0.131378	0.50847	BDH1	622	3-hydroxybutyrate dehydrogenase 1
ENSG0000	25.08269	-0.13803	0.260264	0.131551	0.508709	DNAJC27	51277	DnaJ heat shock protein family (Hsp40) member C27
ENSG0000	286.1902	0.198627	0.20791	0.131525	0.508709	GMPPB	29925	GDP-mannose pyrophosphorylase B
ENSG0000	15.15232	0.159837	0.267933	0.13147	0.508709	ASTN2	23245	astrotactin 2
ENSG0000	209.6981	0.180755	0.153039	0.131501	0.508709	GOLGA5	9950	golgin A5
ENSG0000	2.516963	0.087749	0.249568	0.13167	0.508952	PDE7B	27115	phosphodiesterase 7B
ENSG0000	22.77904	0.199217	0.254645	0.131654	0.508952	FBXL19-AS	283932	FBXL19 antisense RNA 1
ENSG0000	130.5649	0.196779	0.189416	0.131781	0.509228	FAM136A	84908	family with sequence similarity 136 member A
ENSG0000	279.2176	-0.19711	0.196536	0.131797	0.509228	PRAF2	11230	PRA1 domain family member 2
ENSG0000	8.469382	0.153382	0.270505	0.131871	0.509243	NA	NA	NA
ENSG0000	13.49702	0.118796	0.255878	0.13183	0.509243	NA	NA	NA
ENSG0000	4161.924	-0.16768	0.13692	0.131912	0.509243	FAM117A	81558	family with sequence similarity 117 member A
ENSG0000	648.334	0.176137	0.152026	0.131894	0.509243	TTC3	7267	tetratricopeptide repeat domain 3
ENSG0000	248.0004	0.184634	0.150017	0.131958	0.509316	NUP85	79902	nucleoporin 85
ENSG0000	193.0993	0.197243	0.18892	0.132057	0.50959	MCM4	4173	minichromosome maintenance complex component 4
ENSG0000	502.4741	0.162325	0.128221	0.132194	0.509828	MRPL43	84545	mitochondrial ribosomal protein L43
ENSG0000	425.4081	0.197128	0.214758	0.132154	0.509828	NOMO1	23420	NODAL modulator 1
ENSG0000	177.8658	0.178439	0.150086	0.132202	0.509828	LRRC45	201255	leucine rich repeat containing 45
ENSG0000	3.704867	0.07332	0.2448	0.132313	0.510135	NA	NA	NA
ENSG0000	337.9044	0.188471	0.237259	0.132397	0.510135	LINC02035	1E+08	long intergenic non-protein coding RNA 2035
ENSG0000	48.74555	-0.19043	0.246304	0.132418	0.510135	NUDT16-D	339874	NUDT16 divergent transcript
ENSG0000	28.04118	-0.19922	0.247661	0.132384	0.510135	WASH5P	375690	WASP fam pseudogene
ENSG0000	3984.601	0.182605	0.154386	0.132449	0.510135	STRN4	29888	striatin 4
ENSG0000	2.739138	0.047455	0.237183	0.132449	0.510135	NA	NA	NA
ENSG0000	38.73398	0.18366	0.258303	0.132496	0.510209	TTC33	23548	tetratricopeptide repeat domain 33
ENSG0000	99.857	0.199568	0.201097	0.132552	0.510306	ZYG11B	79699	zyg-11 fan cell cycle regulator
ENSG0000	4.199885	0.10721	0.25539	0.132577	0.510306	NA	NA	NA
ENSG0000	599.91	-0.17965	0.154062	0.132721	0.510751	OSER1	51526	oxidative stress responsive serine rich 1
ENSG0000	134.3911	0.201118	0.211866	0.132764	0.51081	RPS6KA5	9252	ribosomal protein S6 kinase A5
ENSG0000	49.64604	-0.19994	0.214559	0.132851	0.510826	AMIGO2	347902	adhesion molecule with Ig like domain 2
ENSG0000	1006.568	-0.2046	0.192656	0.132849	0.510826	GALNS	2588	galactosamine (N-acetyl)-6-sulfatase
ENSG0000	101.9227	-0.19978	0.203373	0.132827	0.510826	ZNF766	90321	zinc finger protein 766
ENSG0000	14.14331	0.168087	0.268415	0.132942	0.510958	S100A5	6276	S100 calcium binding protein A5
ENSG0000	1061.09	-0.18834	0.172233	0.132961	0.510958	ZBTB45	84878	zinc finger and BTB domain containing 45
ENSG0000	3.906562	-0.08134	0.245683	0.13297	0.510958	MYH7B	57644	myosin heavy chain 7B
ENSG0000	418.7975	-0.16281	0.128952	0.133008	0.510998	ASH2L	9070	ASH2 like histone lysine methyltransferase complex subunit
ENSG0000	102.3249	0.199899	0.206743	0.133056	0.511076	ATG14	22863	autophagy related 14
ENSG0000	392.7805	0.175012	0.1462	0.133314	0.511755	SPCS2	9789	signal peptidase complex subunit 2
ENSG0000	3167.601	-0.18258	0.160884	0.133289	0.511755	AGAP2	116986	ArfGAP wi ankyrin repeat and PH domain 2
ENSG0000	5.542894	-0.10691	0.254334	0.133317	0.511755	RNU6-611	1.06E+08	RNA U6 small r pseudogene
ENSG0000	56.13065	0.150367	0.260679	0.13345	0.511947	SMC2	10592	structural maintenance of chromosomes 2
ENSG0000	190.6869	0.194939	0.209303	0.133436	0.511947	KTN1	3895	kinectin 1
ENSG0000	230.6267	0.194701	0.190862	0.133399	0.511947	ATP5PF	522	ATP synthase peripheral stalk subunit F6
ENSG0000	95.93907	-0.19303	0.182296	0.133561	0.512262	CA11	770	carbonic anhydrase 11
ENSG0000	4.032831	0.077502	0.244551	0.133798	0.512571	C3orf20	84077	chromosome 3 open reading frame 20
ENSG0000	60.06793	-0.19878	0.229264	0.133715	0.512571	NA	NA	NA
ENSG0000	6.679931	0.127007	0.261502	0.133751	0.512571	LOC10537	1.05E+08	uncharacterized LOC105376306
ENSG0000	265.5939	0.196577	0.194064	0.133809	0.512571	PTDSS2	81490	phosphatidylserine synthase 2
ENSG0000	36.61033	0.196941	0.249958	0.133769	0.512571	CRLS1	54675	cardiolipin synthase 1
ENSG0000	90.86971	-0.19881	0.20525	0.133708	0.512571	CDKL5	6792	cyclin dependent kinase like 5
ENSG0000	573.0293	-0.19938	0.218323	0.133849	0.512616	FCAR	2204	Fc alpha receptor
ENSG0000	893.2422	0.161728	0.127294	0.133884	0.512645	POM121	9883	POM121 transmembrane nucleoporin
ENSG0000	4755.63	0.183611	0.245394	0.134128	0.513472	TPT1	7178	tumor pro translationally-controlled 1
ENSG0000	1673.999	-0.18948	0.1754	0.134179	0.51356	NCOA1	8648	nuclear receptor coactivator 1
ENSG0000	152.6862	-0.1862	0.2503	0.134284	0.513852	AMN1	196394	antagonist of mitotic exit network 1 homolog
ENSG0000	434.5077	-0.18172	0.156943	0.134341	0.513963	ECHDC2	55268	enoyl-CoA hydratase domain containing 2
ENSG0000	6.932369	0.140367	0.266655	0.134375	0.513986	OPHN1	4983	oligophrenin 1
ENSG0000	176.263	-0.17481	0.143024	0.134435	0.514	MRPL44	65080	mitochondrial ribosomal protein L44
ENSG0000	951.8831	-0.19292	0.186292	0.134408	0.514	TMEM63B	55362	transmembrane protein 63B
ENSG0000	1019.637	0.173649	0.146368	0.134479	0.514064	PNKP	11284	polynucleotide kinase 3'-phosphatase
ENSG0000	22.63852	0.186566	0.263569	0.134593	0.514283	LAMC3	10319	laminin subunit gamma 3
ENSG0000	33912.06	0.157533	0.12384	0.134574	0.514283	ND3	4537	NADH dehydrogenase subunit 3
ENSG0000	2.716989	-0.0565	0.240321	0.134684	0.514522	NA	NA	NA
ENSG0000	132.1556	0.197757	0.215966	0.13473	0.514594	SUCLG2	8801	succinate-CoA ligase GDP-forming subunit beta
ENSG0000	70.41805	-0.19192	0.240605	0.134906	0.514737	NA	NA	NA
ENSG0000	233.2385	-0.16719	0.1362	0.134816	0.514737	RNF216P1	441191	ring finger protein 216 pseudogene 1
ENSG0000	10.2036	0.116335	0.255549	0.134855	0.514737	LOC12490	1.25E+08	uncharacterized LOC124902718

ENSG0000	1459.637	0.197922	0.202332	0.134909	0.514737	KCTD12	115207	potassium channel tetramerization domain containing 12
ENSG0000	59.72368	0.188584	0.24827	0.134879	0.514737	RBM41	55285	RNA binding motif protein 41
ENSG0000	14.57869	0.173649	0.26878	0.134975	0.514798	NA	NA	NA
ENSG0000	57.3713	0.198869	0.222111	0.13498	0.514798	TARS3	123283	threonyl-tRNA synthetase 3
ENSG0000	497.952	0.190185	0.177262	0.135093	0.515119	HIP1R	9026	huntingtin interacting protein 1 related
ENSG0000	8.814812	-0.17251	0.272809	0.135283	0.515737	NA	NA	NA
ENSG0000	68.45941	0.196744	0.23457	0.135363	0.515935	FAM172A	83989	family with sequence similarity 172 member A
ENSG0000	10.62637	-0.1208	0.258306	0.135443	0.516043	PRG4	10216	proteoglycan 4
ENSG0000	673.6812	0.165029	0.25512	0.135448	0.516043	IGHV3-21	28444	immunoglobulin heavy variable 3-21
ENSG0000	142.2134	-0.1957	0.213487	0.135491	0.516099	SLC35A5	55032	solute carrier family 35 member A5
ENSG0000	2414.959	-0.18988	0.182766	0.135568	0.516286	APMAP	57136	adipocyte plasma membrane associated protein
ENSG0000	1509.076	-0.1724	0.144465	0.135633	0.516427	ZBTB4	57659	zinc finger and BTB domain containing 4
ENSG0000	376.8122	0.175768	0.149393	0.135783	0.516782	MRPS15	64960	mitochondrial ribosomal protein S15
ENSG0000	9.924607	0.153341	0.267741	0.135759	0.516782	TNFRSF11/	8792	TNF receptor superfamily member 11a
ENSG0000	1316.769	-0.17077	0.145557	0.136125	0.517974	SCNM1	79005	sodium channel modifier 1
ENSG0000	26.67327	0.188771	0.254682	0.136194	0.517993	RPL4P4	647276	ribosomal protein L4 pseudogene 4
ENSG0000	224.5055	-0.18876	0.174699	0.136207	0.517993	RBM18	92400	RNA binding motif protein 18
ENSG0000	104.714	0.197627	0.204766	0.136214	0.517993	ZNF552	79818	zinc finger protein 552
ENSG0000	27.41802	0.14668	0.262389	0.136453	0.518687	NA	NA	NA
ENSG0000	241.3404	-0.19375	0.192986	0.13645	0.518687	TBC1D3	729873	TBC1 domain family member 3
ENSG0000	44.60632	0.199747	0.21783	0.136489	0.518715	SERPINF2	5345	serpin family F member 2
ENSG0000	10.71403	-0.1062	0.252039	0.136541	0.518806	TSPO2	222642	translocator protein 2
ENSG0000	1278.838	0.180103	0.159813	0.136654	0.519019	MBD2	8932	methyl-CpG binding domain protein 2
ENSG0000	143.882	0.197931	0.215137	0.136633	0.519019	COL9A3	1299	collagen type IX alpha 3 chain
ENSG0000	206.4914	0.190047	0.242384	0.136755	0.519295	CNKSR1	10256	connector enhancer of kinase suppressor of Ras 1
ENSG0000	177.1146	0.188457	0.239078	0.13685	0.519502	GNL3	26354	G protein nucleolar 3
ENSG0000	716.5255	0.180011	0.157332	0.136887	0.519502	GNA12	2768	G protein subunit alpha 12
ENSG0000	264.4028	0.174267	0.148119	0.136914	0.519502	MRPS7	51081	mitochondrial ribosomal protein S7
ENSG0000	108.0564	0.191175	0.239084	0.136923	0.519502	PHF5A	84844	PHD finger protein 5A
ENSG0000	3.620767	-0.06603	0.241999	0.137033	0.51959	NA	NA	NA
ENSG0000	101.1728	0.196444	0.200841	0.137033	0.51959	MPHOSPH	10199	M-phase phosphoprotein 10
ENSG0000	119.9429	0.192973	0.187578	0.137088	0.51959	ITPR2	3709	inositol 1 4 5-trisphosphate receptor type 2
ENSG0000	1040.293	-0.15883	0.126354	0.137077	0.51959	TINF2	26277	TERF1 interacting nuclear factor 2
ENSG0000	1125.146	-0.18734	0.219564	0.13705	0.51959	SLC6A8	6535	solute carrier family 6 member 8
ENSG0000	342.3951	-0.16258	0.12956	0.13731	0.519879	NASP	4678	nuclear autoantigenic sperm protein
ENSG0000	734.9238	0.185962	0.161053	0.137301	0.519879	CYRIA	81553	CYFIP related Rac1 interactor A
ENSG0000	7.144286	0.134828	0.264002	0.137333	0.519879	ADGRF3	165082	adhesion G protein-coupled receptor F3
ENSG0000	130.4104	-0.19069	0.23657	0.137436	0.519879	CCR5	1234	C-C motif chemokine receptor 5
ENSG0000	988.0804	0.18891	0.178702	0.137446	0.519879	GAPT	202309	GRB2 bind transmembrane
ENSG0000	255.7872	0.178808	0.155668	0.137416	0.519879	TXNDC15	79770	thioredoxin domain containing 15
ENSG0000	2.995659	0.063029	0.240741	0.137448	0.519879	APBA1	320	amyloid beta precursor protein binding family A member 1
ENSG0000	560.7987	-0.19308	0.19491	0.137303	0.519879	GATA3	2625	GATA binding protein 3
ENSG0000	55.99767	-0.19831	0.206308	0.137218	0.519879	KCTD1	284252	potassium channel tetramerization domain containing 1
ENSG0000	11.79379	0.098907	0.25005	0.137418	0.519879	MAP3K15	389840	mitogen-activated protein kinase kinase kinase 15
ENSG0000	54.60757	0.167794	0.257796	0.137571	0.520237	DCAF13	25879	DDB1 and CUL4 associated factor 13
ENSG0000	4.412872	0.073876	0.243518	0.137764	0.520752	CNNM3-D	1.01E+08	CNNM3 divergent transcript
ENSG0000	60.88763	0.157141	0.259155	0.137757	0.520752	MRPL32	64983	mitochondrial ribosomal protein L32
ENSG0000	326.4355	-0.17795	0.24811	0.137935	0.52129	NA	NA	NA
ENSG0000	23.60346	0.180435	0.259441	0.138044	0.521378	KCNQ4	9132	potassium voltage-gated channel subfamily Q member 4
ENSG0000	43.25516	0.182005	0.253321	0.13803	0.521378	PTER	9317	phosphotriesterase related
ENSG0000	102.027	-0.19702	0.210627	0.137995	0.521378	MGC1627!	85001	uncharacterized protein MGC16275
ENSG0000	61.99144	-0.19244	0.182336	0.13809	0.521442	CCDC85C	317762	coiled-coil domain containing 85C
ENSG0000	7.005929	-0.13123	0.262473	0.138117	0.521442	NA	NA	NA
ENSG0000	304.0465	0.190026	0.180734	0.138146	0.521443	DENND6B	414918	DENN domain containing 6B
ENSG0000	18189.36	0.193052	0.198315	0.138269	0.521646	TKT	7086	transketolase
ENSG0000	2.83644	0.081414	0.246445	0.138285	0.521646	IL17C	27189	interleukin 17C
ENSG0000	12.03439	0.155856	0.26726	0.138282	0.521646	CYP2D6	1565	cytochrome P450 family 2 subfamily D member 6
ENSG0000	21.21946	-0.1908	0.255088	0.138318	0.521663	FEZ1	9638	fasciculation and elongation protein zeta 1
ENSG0000	7.981069	-0.09379	0.248045	0.138348	0.521668	NPW	283869	neuropeptide W
ENSG0000	21.85266	0.161138	0.264166	0.138421	0.521708	IGKV6D-21	28870	immunoglobulin kappa variable 6D-21 (non-functional)
ENSG0000	9.998103	0.147188	0.266051	0.138412	0.521708	NA	NA	NA
ENSG0000	1418.359	0.164854	0.135016	0.138444	0.521708	POM121C	1E+08	POM121 transmembrane nucleoporin C
ENSG0000	4.236948	0.087317	0.24733	0.138511	0.521852	NA	NA	NA
ENSG0000	9334.392	-0.16916	0.142135	0.138587	0.52195	CLIC1	1192	chloride intracellular channel 1
ENSG0000	214.4816	0.17168	0.142681	0.138594	0.52195	ECD	11319	ecdysoneless cell cycle regulator
ENSG0000	307.8392	-0.1954	0.192459	0.138649	0.522051	RSBN1	54665	round spermatid basic protein 1
ENSG0000	904.7686	0.192002	0.18901	0.138737	0.522276	NA	NA	NA
ENSG0000	4.260951	0.072953	0.244035	0.138913	0.522404	ZNF660	285349	zinc finger protein 660
ENSG0000	4.385216	-0.10425	0.253672	0.138903	0.522404	NA	NA	NA
ENSG0000	183.455	-0.19213	0.19868	0.138924	0.522404	PPA1	5464	inorganic pyrophosphatase 1
ENSG0000	46.86253	0.198219	0.230522	0.138943	0.522404	NA	NA	NA
ENSG0000	4.501099	0.066794	0.241102	0.138807	0.522404	NA	NA	NA
ENSG0000	205.4175	-0.18805	0.178253	0.138895	0.522404	TASL	80231	TLR adaptor interacting with endolysosomal SLC15A4
ENSG0000	452.0169	-0.18085	0.156861	0.13922	0.523126	UGP2	7360	UDP-glucose pyrophosphorylase 2
ENSG0000	3.624014	0.075942	0.244129	0.139205	0.523126	FAM88C	1.03E+08	family with sequence similarity 88 member C
ENSG0000	6395.512	-0.19177	0.220207	0.139181	0.523126	POLR2A	5430	RNA polymerase II subunit A
ENSG0000	12.61817	-0.11441	0.253445	0.139323	0.523296	NA	NA	NA
ENSG0000	65.36338	0.200446	0.213399	0.139309	0.523296	SIGLEC17F	284367	sialic acid pseudogene
ENSG0000	47.32537	0.15045	0.259074	0.139366	0.523316	LINC02432	1.01E+08	long intergenic non-protein coding RNA 2432
ENSG0000	10.17739	-0.13444	0.261063	0.139418	0.523316	MAP9	79884	microtubule associated protein 9
ENSG0000	2.632011	0.053596	0.238634	0.139442	0.523316	NA	NA	NA
ENSG0000	4.606317	0.081321	0.2459	0.139418	0.523316	HLF	3131	HLF transc PAR bZIP family member
ENSG0000	63.49186	-0.19301	0.235095	0.139565	0.523668	NPIPP1	1.01E+08	nuclear pore complex interacting protein pseudogene 1
ENSG0000	301.1654	0.187583	0.175625	0.139663	0.523715	RMND5A	64795	required for meiotic nuclear division 5 homolog A
ENSG0000	216.267	0.179842	0.159183	0.139646	0.523715	TRIM24	8805	tripartite motif containing 24
ENSG0000	2.942289	0.071442	0.243821	0.139612	0.523715	CES3	23491	carboxylesterase 3
ENSG0000	513.351	0.196316	0.214608	0.139822	0.523881	IQGAP2	10788	IQ motif containing GTPase activating protein 2
ENSG0000	10.93473	-0.11062	0.254174	0.13974	0.523881	CT70	1.08E+08	cancer/testis associated transcript 70
ENSG0000	19.0768	0.168154	0.268813	0.139816	0.523881	UNC5B	219699	unc-5 netrin receptor B
ENSG0000	1554.525	0.188423	0.180197	0.139782	0.523881	TUBGCP2	10844	tubulin gamma complex associated protein 2
ENSG0000	101.6859	0.19222	0.188417	0.139879	0.52399	ECI2	10455	enoyl-CoA delta isomerase 2
ENSG0000	59.40401	0.195823	0.19822	0.140071	0.524602	WDR3	10885	WD repeat domain 3
ENSG0000	50.53814	0.195988	0.211666	0.140109	0.524603	ZNF326	284695	zinc finger protein 326
ENSG0000	72.3842	-0.1957	0.205843	0.14036	0.524603	C1orf198	84886	chromosome 1 open reading frame 198
ENSG0000	8.453443	0.128558	0.260002	0.140263	0.524603	NA	NA	NA
ENSG0000	60.87183	0.19712	0.240348	0.140444	0.524603	SLC25A4	291	solute carrier family 25 member 4

ENSG0000	5611.83	-0.15012	0.135258	0.140441	0.524603	HLA-F		3134	major hist class I F
ENSG0000	4.165827	0.069613	0.244018	0.140334	0.524603	NA	NA		NA
ENSG0000	5.938304	-0.11442	0.256216	0.140228	0.524603	NA	NA		NA
ENSG0000	5.471307	0.106864	0.258722	0.14036	0.524603	TEK		7010	TEK receptor tyrosine kinase
ENSG0000	1846.239	0.193828	0.200962	0.14042	0.524603	NA	NA		NA
ENSG0000	1121.584	0.183348	0.168239	0.140268	0.524603	DDB1		1642	damage specific DNA binding protein 1
ENSG0000	20.34829	-0.19192	0.251262	0.140403	0.524603	CDH24		64403	cadherin 24
ENSG0000	337.34	0.184423	0.168414	0.140302	0.524603	MGAT2		4247	alpha-1 6-mannosyl-glycoprotein 2-beta-N-acetylglucosaminyltransferase
ENSG0000	6.55607	0.109845	0.254103	0.140364	0.524603	ZNF774		342132	zinc finger protein 774
ENSG0000	4.102806	0.114061	0.259649	0.140558	0.52492	LOC10192		1.02E+08	uncharacterized LOC101928059
ENSG0000	103.3793	0.1936	0.217326	0.140601	0.524976	NA	NA		NA
ENSG0000	36.3575	0.195522	0.236747	0.140708	0.52502	IPP		3652	intracisternal A particle-promoted polypeptide
ENSG0000	165.7297	-0.191	0.188514	0.140717	0.52502	HERC4		26091	HECT and RLD domain containing E3 ubiquitin protein ligase 4
ENSG0000	20.00367	0.178186	0.260923	0.140719	0.52502	ZNF567		163081	zinc finger protein 567
ENSG0000	62.22372	0.194006	0.186488	0.140728	0.52502	SS18L1		26039	SS18L1 subunit of BAF chromatin remodeling complex
ENSG0000	1885.066	-0.18138	0.165365	0.140849	0.525366	DENND5A		23258	DENN domain containing 5A
ENSG0000	2479.751	-0.18402	0.174637	0.140883	0.525388	BLVRB		645	biliverdin reductase B
ENSG0000	8.687287	-0.10589	0.252535	0.140969	0.525581	NA	NA		NA
ENSG0000	2.489622	-0.04364	0.236569	0.140993	0.525581	NA	NA		NA
ENSG0000	1722.768	0.162833	0.252554	0.141073	0.525772	LTF		4057	lactotransferrin
ENSG0000	9.863302	0.12558	0.258429	0.141218	0.526059	NA	NA		NA
ENSG0000	7.924913	-0.13815	0.263671	0.141363	0.526059	NA	NA		NA
ENSG0000	2304.665	-0.1902	0.212773	0.141336	0.526059	MME		4311	membrane metalloendopeptidase
ENSG0000	7.219562	0.118665	0.256878	0.141329	0.526059	NA	NA		NA
ENSG0000	798.7265	0.14597	0.112208	0.141315	0.526059	TOM1L2		146691	target of myb1 like 2 membrane trafficking protein
ENSG0000	539.8595	-0.15325	0.1224	0.141351	0.526059	HELZ		9931	helicase with zinc finger
ENSG0000	34.38895	0.197896	0.221092	0.141326	0.526059	SNHG20		654434	small nucleolar RNA host gene 20
ENSG0000	68.48257	-0.18418	0.245557	0.141379	0.526059	SHISA7		729956	shisa family member 7
ENSG0000	10.28052	0.14499	0.264218	0.141419	0.526098	DET1		55070	DET1 partner of COP1 E3 ubiquitin ligase
ENSG0000	7.958832	-0.10092	0.250661	0.14152	0.526367	LINC0086		643529	long intergenic non-protein coding RNA 865
ENSG0000	919.2202	0.157978	0.12739	0.14157	0.526448	SERBP1		26135	SERPINE1 mRNA binding protein 1
ENSG0000	132.4005	-0.19116	0.182793	0.141649	0.526529	MITD1		129531	microtubule interacting and trafficking domain containing 1
ENSG0000	31.80402	0.125488	0.254594	0.14163	0.526529	LOC10028		1E+08	cytoskeleton associated protein 2-like pseudogene
ENSG0000	7093.494	-0.18944	0.194989	0.141706	0.526548	JAML		120425	junction adhesion molecule like
ENSG0000	311.9641	0.167614	0.140461	0.141712	0.526548	CSNK1E		1454	casein kinase 1 epsilon
ENSG0000	1580.09	-0.19076	0.202561	0.141742	0.526551	ABCD1		215	ATP binding cassette subfamily D member 1
ENSG0000	27.46936	-0.16213	0.260641	0.141933	0.527102	CTSE		1510	cathepsin E
ENSG0000	129.1079	0.187106	0.177545	0.141948	0.527102	POLR2D		5433	RNA polymerase II subunit D
ENSG0000	99.28562	0.192594	0.202432	0.142149	0.527653	TBC1D24		57465	TBC1 domain family member 24
ENSG0000	68.34653	0.194062	0.196807	0.142153	0.527653	ZNF175		7728	zinc finger protein 175
ENSG0000	2140.496	0.189462	0.190258	0.142223	0.527804	KDM4B		23030	lysine demethylase 4B
ENSG0000	52.62157	0.196261	0.202177	0.142257	0.527824	FLVCR1		28982	FLVCR heme transporter 1
ENSG0000	213.4605	0.188166	0.179385	0.142393	0.528095	DYNC1LI2		1783	dynein cytoplasmic 1 light intermediate chain 2
ENSG0000	21.33227	0.153533	0.262061	0.142417	0.528095	LOC73222		732229	AN1-type zinc finger protein 5-like
ENSG0000	2.70813	0.087941	0.248534	0.142412	0.528095	NA	NA		NA
ENSG0000	6.777583	0.116051	0.25644	0.142577	0.528581	STPG4		285051	sperm-tail PG-rich repeat containing 4
ENSG0000	956.5308	-0.14286	0.108174	0.142732	0.528692	TMEM248		55069	transmembrane protein 248
ENSG0000	93.71206	0.19331	0.208982	0.142723	0.528692	NA	NA		NA
ENSG0000	3.691648	-0.08302	0.248892	0.142751	0.528692	SPON1		10418	spondin 1
ENSG0000	2.539543	-0.05993	0.24177	0.142707	0.528692	IPO4		79711	importin 4
ENSG0000	25.98992	-0.15982	0.260555	0.142659	0.528692	ST6GALNA		55808	ST6 N-ace 6-sialyltransferase 1
ENSG0000	125.5851	0.185902	0.174286	0.142907	0.528843	UCK2		7371	uridine-cytidine kinase 2
ENSG0000	59.21153	-0.19722	0.213041	0.142859	0.528843	CRACDL		343990	CRACD like
ENSG0000	14.8992	0.145429	0.261378	0.142905	0.528843	ACYP1		97	acylphosphatase 1
ENSG0000	6.114534	0.123812	0.259969	0.142875	0.528843	MIF4GD-D		1E+08	MIF4GD divergent transcript
ENSG0000	35.95821	-0.13787	0.255876	0.142985	0.529026	NA	NA		NA
ENSG0000	138.8697	-0.19189	0.210978	0.143098	0.529335	ACKR1		2532	atypical chemokine receptor 1 (Duffy blood group)
ENSG0000	1108.945	0.175228	0.157089	0.14316	0.529351	DNAJB12		54788	DnaJ heat shock protein family (Hsp40) member B12
ENSG0000	102.4998	-0.17383	0.151487	0.143153	0.529351	RAB3IP		117177	RAB3A interacting protein
ENSG0000	18.35228	0.139312	0.259267	0.143268	0.529537	NA	NA		NA
ENSG0000	16.25659	0.170472	0.262987	0.143262	0.529537	MAGEE1		57692	MAGE family member E1
ENSG0000	337.1859	-0.18905	0.191758	0.14336	0.529769	NFATC2		4773	nuclear factor of activated T cells 2
ENSG0000	34.69314	-0.19492	0.228282	0.143424	0.529793	LRRC24		441381	leucine rich repeat containing 24
ENSG0000	2134.082	0.180918	0.166109	0.143415	0.529793	SYVN1		84447	synoviolin 1
ENSG0000	17.8475	0.188294	0.254753	0.143487	0.529919	NA	NA		NA
ENSG0000	15.60014	-0.18854	0.254505	0.143674	0.530013	ANKRD36		57730	ankyrin repeat domain 36B
ENSG0000	132.6659	0.192555	0.199227	0.143668	0.530013	MAPK9		5601	mitogen-activated protein kinase 9
ENSG0000	24.32101	0.136417	0.257529	0.143579	0.530013	ANKRD46		157567	ankyrin repeat domain 46
ENSG0000	708.5291	0.17103	0.157709	0.143675	0.530013	MLST8		64223	MTOR ass LST8 homolog
ENSG0000	16.42529	-0.17392	0.261527	0.143686	0.530013	NA	NA		NA
ENSG0000	1324.942	0.183416	0.172181	0.143546	0.530013	CHCHD10		400916	coiled-coil-helix-coiled-coil-helix domain containing 10
ENSG0000	28.28602	-0.18235	0.251537	0.143823	0.530409	SV2A		9900	synaptic vesicle glycoprotein 2A
ENSG0000	421.5415	-0.1698	0.147273	0.143952	0.530779	RMC1		29919	regulator of MON1-CCZ1
ENSG0000	219.3652	0.171566	0.148336	0.144037	0.530987	MRPS16		51021	mitochondrial ribosomal protein S16
ENSG0000	2894.473	0.157978	0.128057	0.144215	0.53143	RPN1		6184	ribophorin I
ENSG0000	2.456637	0.041338	0.237762	0.144206	0.53143	NA	NA		NA
ENSG0000	263.5856	-0.18789	0.22347	0.144263	0.531499	STAM2		10254	signal transducing adaptor molecule 2
ENSG0000	5.884929	0.091245	0.247985	0.144309	0.531561	NA	NA		NA
ENSG0000	6.412323	-0.07141	0.242573	0.144415	0.531843	CFH		3075	complement factor H
ENSG0000	882.243	0.177356	0.159908	0.144478	0.531969	ZNF641		121274	zinc finger protein 641
ENSG0000	12.67376	-0.18254	0.260842	0.144512	0.531988	SPATA41		388182	spermatogenesis associated 41
ENSG0000	1980.262	-0.17473	0.239689	0.144599	0.532096	CD164		8763	CD164 molecule
ENSG0000	5.51716	-0.0473	0.237194	0.144574	0.532096	HEPACAM		253012	HEPACAM family member 2
ENSG0000	155.3745	0.183151	0.173206	0.144694	0.532135	PRADC1		84279	protease associated domain containing 1
ENSG0000	38.5059	0.187178	0.245236	0.144697	0.532135	NA	NA		NA
ENSG0000	928.484	0.162558	0.136159	0.144664	0.532135	FOXN3		1112	forkhead box N3
ENSG0000	50.09932	-0.19272	0.19353	0.144878	0.532254	STPG1		90529	sperm tail PG-rich repeat containing 1
ENSG0000	970.3542	-0.18338	0.174152	0.14478	0.532254	LRSAM1		90678	leucine rich repeat and sterile alpha motif containing 1
ENSG0000	207.7976	-0.18793	0.226741	0.144904	0.532254	HSPH1		10808	heat shock protein family H (Hsp110) member 1
ENSG0000	198.7641	-0.17998	0.16504	0.144839	0.532254	ZNF839		55778	zinc finger protein 839
ENSG0000	31.34006	-0.19067	0.24403	0.144827	0.532254	CLUAP1		23059	clusterin associated protein 1
ENSG0000	147.1324	0.177051	0.157378	0.144856	0.532254	SMYD4		114826	SET and MYND domain containing 4
ENSG0000	23.92813	0.191416	0.244077	0.144983	0.532437	ZNF439		90594	zinc finger protein 439
ENSG0000	3.840488	0.089829	0.24876	0.14505	0.532579	NA	NA		NA
ENSG0000	35.83379	-0.18102	0.2477	0.145239	0.532833	LINC0098		1.01E+08	long intergenic non-protein coding RNA 989
ENSG0000	4.784386	0.093736	0.248879	0.145184	0.532833	PANK1		53354	pantothenate kinase 1

ENSG0000	4.372158	0.107853	0.257821	0.145217	0.532833	COLCA1	399948	colorectal cancer associated 1
ENSG0000	11.43505	-0.12506	0.256427	0.145264	0.532833	PRR19	284338	proline rich 19
ENSG0000	4.277547	0.067651	0.241876	0.145265	0.532833	SMIM10	644538	small integral membrane protein 10
ENSG0000	8271.33	-0.1706	0.155353	0.145312	0.532896	RGS19	10287	regulator of G protein signaling 19
ENSG0000	25.50071	0.155031	0.256085	0.145439	0.533045	LSM11	134353	LSM11 U7 small nuclear RNA associated
ENSG0000	17.24876	-0.12121	0.254022	0.145387	0.533045	BTBD3	22903	BTB domain containing 3
ENSG0000	17.16334	-0.18691	0.253851	0.145439	0.533045	MAMLD1	10046	mastermind like domain containing 1
ENSG0000	640.6008	0.122915	0.254057	0.145561	0.533355	RPL9	6133	ribosomal protein L9
ENSG0000	13.41651	-0.13174	0.257726	0.145582	0.533355	NA	NA	NA
ENSG0000	43.31698	0.193306	0.228641	0.145642	0.533468	NT5E	4907	5'-nucleotidase ecto
ENSG0000	1805.656	-0.15908	0.133641	0.145687	0.533526	PTTG1IP	754	PTTG1 interacting protein
ENSG0000	13.9721	0.175146	0.26228	0.14575	0.533651	RPS3P4	1E+08	ribosomal protein S3 pseudogene 4
ENSG0000	18.55497	0.133989	0.258232	0.145882	0.534028	NA	NA	NA
ENSG0000	168.562	0.194066	0.215657	0.14595	0.534093	S1PR3	1903	sphingosine-1-phosphate receptor 3
ENSG0000	62.39672	-0.18919	0.186195	0.145958	0.534093	LINC0221(	147081	long intergenic non-protein coding RNA 2210
ENSG0000	316.3336	-0.18339	0.184028	0.146069	0.534197	NMUR1	10316	neuromedin U receptor 1
ENSG0000	91.35316	-0.18755	0.186754	0.14603	0.534197	INVS	27130	inversin
ENSG0000	1508.703	-0.18311	0.176825	0.146075	0.534197	TICAM1	148022	TIR domain containing adaptor molecule 1
ENSG0000	152.6046	0.189615	0.190593	0.146281	0.534689	RPL23AP4	647099	ribosomal protein L23a pseudogene 42
ENSG0000	4.816834	0.092584	0.248584	0.146297	0.534689	NA	NA	NA
ENSG0000	16.40544	0.137098	0.258029	0.146242	0.534689	NA	NA	NA
ENSG0000	58.20876	0.193123	0.208215	0.146341	0.534746	NA	NA	NA
ENSG0000	117.5648	0.180871	0.233208	0.146427	0.534952	UQCRQ	27089	ubiquinol-cytochrome c reductase complex III subunit VII
ENSG0000	88.37569	-0.1918	0.20959	0.146483	0.535049	ZNF350	59348	zinc finger protein 350
ENSG0000	286.0346	0.176899	0.1577	0.146528	0.535106	C12orf57	113246	chromosome 12 open reading frame 57
ENSG0000	181.4308	0.179518	0.164394	0.146591	0.535231	PELI3	246330	pellino E3 ubiquitin protein ligase family member 3
ENSG0000	79.98905	-0.18876	0.193695	0.146655	0.535359	ZC3H12A-I	728431	ZC3H12A divergent transcript
ENSG0000	9.704662	0.132185	0.259595	0.14678	0.535601	SCART1	619207	scavenger receptor family member expressed on T cells 1
ENSG0000	18056.38	-0.18453	0.187936	0.146765	0.535601	YPEL3	83719	yippee like 3
ENSG0000	79.16758	0.190664	0.197436	0.14686	0.535786	OXNAD1	92106	oxidoreductase NAD binding domain containing 1
ENSG0000	19.86479	-0.16661	0.262047	0.146953	0.536017	TRAV8-3	28683	T cell receptor alpha variable 8-3
ENSG0000	639.6323	-0.15502	0.12779	0.147089	0.536087	KCMF1	56888	potassium channel modulatory factor 1
ENSG0000	7.376215	-0.12395	0.258524	0.147033	0.536087	NA	NA	NA
ENSG0000	29.30679	0.168415	0.259394	0.147083	0.536087	LINC0148	1.02E+08	long intergenic non-protein coding RNA 1484
ENSG0000	324.8374	-0.18532	0.18905	0.147059	0.536087	CLDND2	125875	claudin domain containing 2
ENSG0000	6827.457	-0.17607	0.238327	0.147217	0.536108	PADI4	23569	peptidyl arginine deiminase 4
ENSG0000	30.33464	0.193109	0.229305	0.147257	0.536108	SMYD3	64754	SET and MYND domain containing 3
ENSG0000	3862.089	-0.18503	0.186778	0.147216	0.536108	LTBR	4055	lymphotoxin beta receptor
ENSG0000	28.06605	0.195706	0.232977	0.147185	0.536108	USP31	57478	ubiquitin specific peptidase 31
ENSG0000	136.8875	-0.188	0.194656	0.147194	0.536108	GABARAP	11337	GABA type A receptor-associated protein
ENSG0000	2971.316	-0.18552	0.189218	0.14727	0.536108	CCR7	1236	C-C motif chemokine receptor 7
ENSG0000	121.4838	0.190515	0.203541	0.147569	0.536361	PINK1-AS	1.01E+08	PINK1 antisense RNA
ENSG0000	992.2686	-0.17465	0.15896	0.147574	0.536361	RPRD2	23248	regulation of nuclear pre-mRNA domain containing 2
ENSG0000	38.3662	0.195432	0.206838	0.147371	0.536361	ZC3H8	84524	zinc finger CCCH-type containing 8
ENSG0000	52.88604	-0.19274	0.206426	0.147524	0.536361	PRRT1	80863	proline rich transmembrane protein 1
ENSG0000	2.536987	0.046162	0.238493	0.147476	0.536361	CRISP2	7180	cysteine rich secretory protein 2
ENSG0000	2967.896	-0.16688	0.13995	0.147503	0.536361	BAZ2A	11176	bromodomain adjacent to zinc finger domain 2A
ENSG0000	219.5776	-0.16415	0.140622	0.147452	0.536361	CSNK1G1	53944	casein kinase 1 gamma 1
ENSG0000	10.5421	-0.14338	0.261505	0.147533	0.536361	NA	NA	NA
ENSG0000	557.4597	0.175534	0.163297	0.147757	0.536919	NOB1	28987	NIN1 (RPN12) binding protein 1 homolog
ENSG0000	6.691633	0.081537	0.244987	0.147918	0.537398	KRT8P46	1E+08	keratin 8 pseudogene 46
ENSG0000	30.60886	0.184729	0.241109	0.148051	0.53756	TMEM144	55314	transmembrane protein 144
ENSG0000	261.2825	-0.17787	0.16179	0.148034	0.53756	FIG4	9896	FIG4 phosphoinositide 5-phosphatase
ENSG0000	1471.988	-0.18372	0.185934	0.148041	0.53756	FOS	2353	Fos proto- AP-1 transcription factor subunit
ENSG0000	20.92651	-0.18535	0.247061	0.148137	0.537662	ACTG1P17	283693	actin gamma 1 pseudogene 17
ENSG0000	6.227816	0.060775	0.238866	0.148118	0.537662	NA	NA	NA
ENSG0000	1459.013	-0.16279	0.143745	0.148179	0.537707	PSEN1	5663	presenilin 1
ENSG0000	6.524878	-0.11019	0.25469	0.148291	0.538007	TRAV1-1	28693	T cell receptor alpha variable 1-1
ENSG0000	84.09542	-0.18477	0.176939	0.148364	0.538164	BRF2	55290	BRF2 RNA polymerase III transcription initiation factor subunit
ENSG0000	3.404137	-0.05325	0.228758	0.148426	0.538283	SFTPD	6441	surfactant protein D
ENSG0000	329.1602	-0.17399	0.157206	0.148533	0.538564	SNRNP35	11066	small nuclear ribonucleoprotein U11/U12 subunit 35
ENSG0000	141.0509	0.143643	0.254456	0.148595	0.538575	IGHV4-30-	28398	immunoglobulin heavy variable 4-30-2
ENSG0000	12.32793	0.167298	0.26303	0.148569	0.538575	CLDN7	1366	claudin 7
ENSG0000	68.53079	-0.1877	0.221791	0.148655	0.538688	NA	NA	NA
ENSG0000	7.941332	-0.11239	0.253947	0.148736	0.538872	LOC10537	1.05E+08	uncharacterized LOC105371730
ENSG0000	77.11925	0.141817	0.253535	0.14893	0.539365	KIF27	55582	kinesin family member 27
ENSG0000	13.00918	0.135892	0.258729	0.14893	0.539365	TTC8	123016	tetratricopeptide repeat domain 8
ENSG0000	946.0962	0.179965	0.233704	0.149064	0.539529	RPL41	6171	ribosomal protein L41
ENSG0000	3.233799	-0.07169	0.24261	0.149036	0.539529	RN7SL364	1.06E+08	RNA 7SL cytoplasm pseudogene
ENSG0000	144.2917	0.1721	0.244924	0.149053	0.539529	IGLC6	3542	immunoglobulin lambda constant 6
ENSG0000	175.56	-0.18262	0.175637	0.149247	0.540086	CMKLR1	1240	chemerin chemokine-like receptor 1
ENSG0000	117.6934	-0.17709	0.162053	0.149441	0.540678	MFSD9	84804	major facilitator superfamily domain containing 9
ENSG0000	867.6773	-0.18374	0.183854	0.149517	0.540847	NAPSB	256236	napsin B a pseudogene
ENSG0000	32.23449	-0.17644	0.2504	0.149706	0.540891	GUCY1B1	2983	guanylate cyclase 1 soluble subunit beta 1
ENSG0000	134.5344	0.172651	0.155314	0.149601	0.540891	POMK	84197	protein O-mannose kinase
ENSG0000	32.24529	-0.15633	0.254841	0.149696	0.540891	NA	NA	NA
ENSG0000	113.7991	0.184424	0.230639	0.149687	0.540891	VWA8	23078	von Willebrand factor A domain containing 8
ENSG0000	29.78876	0.157973	0.25635	0.14963	0.540891	PGBD4	161779	piggyBac transposable element derived 4
ENSG0000	126.6867	-0.17561	0.160766	0.14956	0.540891	ARMCX6	54470	armadillo repeat containing X-linked 6
ENSG0000	171.6501	0.169449	0.148331	0.149779	0.541049	MRPL11	65003	mitochondrial ribosomal protein L11
ENSG0000	303.1807	0.183919	0.185079	0.149882	0.541313	JADE1	79960	jade family PHD finger 1
ENSG0000	1839.527	0.17182	0.15966	0.149929	0.541375	HADHA	3030	hydroxyacyl-CoA dehydrogenase trifunctional multienzyme complex subunit alpha
ENSG0000	4.135849	0.073485	0.242996	0.14998	0.541453	GPRASP2	114928	G protein-coupled receptor associated sorting protein 2
ENSG0000	19.41195	0.15477	0.25811	0.150024	0.541505	URB1-AS1	84996	URB1 antisense RNA 1 (head to head)
ENSG0000	86.06267	0.182564	0.236902	0.150446	0.542014	HSPG2	3339	heparan sulfate proteoglycan 2
ENSG0000	3464.287	-0.18581	0.206236	0.150212	0.542014	FOSL2	2355	FOS like 2 AP-1 transcription factor subunit
ENSG0000	102.2822	-0.18655	0.219806	0.150342	0.542014	TOGARAM	165186	TOG array regulator of axonemal microtubules 2
ENSG0000	208.7778	-0.17911	0.170028	0.15026	0.542014	REL	5966	REL proto- NF-kB subunit
ENSG0000	184.3057	-0.181	0.228587	0.15044	0.542014	GIMAP5	55340	GTPase IMAP family member 5
ENSG0000	82.87759	0.189235	0.205651	0.150491	0.542014	IL18	3606	interleukin 18
ENSG0000	2.587289	0.079154	0.247136	0.150313	0.542014	NA	NA	NA
ENSG0000	203.2639	-0.18511	0.193257	0.15038	0.542014	NA	NA	NA
ENSG0000	1360.927	0.144237	0.115343	0.15041	0.542014	TEPSIN	146705	TEPSIN adaptor related protein complex 4 accessory protein
ENSG0000	77.80018	0.181714	0.172142	0.150477	0.542014	RDH13	112724	retinol dehydrogenase 13
ENSG0000	500.3265	-0.16034	0.137082	0.1503	0.542014	XIAP	331	X-linked inhibitor of apoptosis
ENSG0000	14.40064	0.156637	0.260697	0.15056	0.542157	NA	NA	NA

ENSG0000	118.6967	-0.1847	0.191088	0.150626	0.542288	TNNI2	7136	troponin I2, fast skeletal type
ENSG0000	1038.84	0.157443	0.130854	0.151221	0.542808	ENSA	2029	endosulfine alpha
ENSG0000	222.5258	-0.18329	0.229575	0.151088	0.542808	MARCO	8685	macrophage receptor with collagenous structure
ENSG0000	66.78393	0.185673	0.186053	0.151103	0.542808	METTL21A	151194	methyltrai HSPA lysine
ENSG0000	12.08244	0.161217	0.262634	0.150987	0.542808	PACRGL	133015	parkin coregulated like
ENSG0000	14.1154	-0.14103	0.260189	0.151117	0.542808	PRDM5	11107	PR/SET domain 5
ENSG0000	85.4745	-0.16155	0.250409	0.150947	0.542808	NA	NA	NA
ENSG0000	650.6125	-0.15724	0.130741	0.151128	0.542808	ZNF862	643641	zinc finger protein 862
ENSG0000	3.694755	0.082177	0.246223	0.151115	0.542808	NA	NA	NA
ENSG0000	78.39136	-0.17806	0.166314	0.150987	0.542808	LOC10031	1E+08	uncharacterized LOC100310756
ENSG0000	67.13635	0.183633	0.23602	0.151306	0.542808	USP6NL	9712	USP6 N-terminal like
ENSG0000	151.5924	0.179829	0.170273	0.15136	0.542808	MRE11	4361	MRE11 ho double strand break repair nuclease
ENSG0000	3.501223	0.084072	0.247236	0.151363	0.542808	DDX47	51202	DEAD-box helicase 47
ENSG0000	1003.025	0.149465	0.122035	0.151197	0.542808	ANAPC5	51433	anaphase promoting complex subunit 5
ENSG0000	9.782656	0.058804	0.238327	0.151219	0.542808	SCNN1B	6338	sodium channel epithelial 1 subunit beta
ENSG0000	2.872332	-0.08449	0.246674	0.151305	0.542808	NA	NA	NA
ENSG0000	8100.266	-0.16736	0.147793	0.150854	0.542808	CALM3	808	calmodulin 3
ENSG0000	10327.08	-0.18309	0.188248	0.151081	0.542808	LILRA2	11027	leukocyte immunoglobulin like receptor A2
ENSG0000	45.71655	-0.19162	0.210001	0.151151	0.542808	NA	NA	NA
ENSG0000	10.7331	-0.14508	0.261523	0.151361	0.542808	NA	NA	NA
ENSG0000	31.88794	0.184401	0.242083	0.15094	0.542808	TCEAL4	79921	transcription elongation factor A like 4
ENSG0000	119.4155	0.19035	0.19839	0.151405	0.542853	LNPK	80856	lunapark ER junction formation factor
ENSG0000	100.4791	0.190711	0.198137	0.151435	0.542853	PLCD3	113026	phospholipase C delta 3
ENSG0000	209.1499	-0.16346	0.142595	0.15158	0.54322	HEBP1	50865	heme binding protein 1
ENSG0000	23.37037	-0.17917	0.249755	0.151596	0.54322	LIPC	3990	lipase C hepatic type
ENSG0000	19.43398	0.121464	0.253169	0.151717	0.543545	CEND1	51286	cell cycle exit and neuronal differentiation 1
ENSG0000	6.544331	0.137806	0.263071	0.15182	0.543702	NA	NA	NA
ENSG0000	101.6047	-0.17981	0.170322	0.151799	0.543702	NA	NA	NA
ENSG0000	40.46583	0.16551	0.253494	0.151913	0.543928	FA2H	79152	fatty acid 2-hydroxylase
ENSG0000	295.223	-0.16916	0.151325	0.151992	0.544104	IPCEF1	26034	interaction protein for cytohesin exchange factors 1
ENSG0000	7.877506	0.131516	0.259733	0.152023	0.54411	NA	NA	NA
ENSG0000	5.028218	0.090118	0.247445	0.152053	0.54411	CCDC187	399693	coiled-coil domain containing 187
ENSG0000	3.62994	-0.05872	0.23997	0.152208	0.544313	NA	NA	NA
ENSG0000	10.33374	0.122404	0.25559	0.152215	0.544313	IRAK1BP1	134728	interleukin 1 receptor associated kinase 1 binding protein 1
ENSG0000	3.377045	-0.05428	0.239004	0.152198	0.544313	TRBJ1-1	28635	T cell receptor beta joining 1-1
ENSG0000	51.63697	0.186063	0.223528	0.15225	0.544313	DDX19A	55308	DEAD-box helicase 19A
ENSG0000	18.02476	0.152589	0.260302	0.152258	0.544313	ATP2C2	9914	ATPase secretory pathway Ca2+ transporting 2
ENSG0000	716.3528	-0.18039	0.179458	0.152303	0.544366	PEX6	5190	peroxisomal biogenesis factor 6
ENSG0000	79.78055	0.188892	0.198942	0.152422	0.544686	ARL3	403	ADP ribosylation factor like GTPase 3
ENSG0000	2.927233	0.066686	0.241859	0.152489	0.544822	NA	NA	NA
ENSG0000	57.49946	-0.18785	0.196817	0.152578	0.545034	PEX13	5194	peroxisomal biogenesis factor 13
ENSG0000	189.3621	-0.18371	0.221891	0.152661	0.545222	ZDHHC2	51201	zinc finger DHHC-type palmitoyltransferase 2
ENSG0000	132.3746	0.184356	0.198067	0.1527	0.545255	ENDOG	2021	endonuclease G
ENSG0000	9.532204	0.158763	0.264775	0.152736	0.545278	LINC0197(	1.02E+08	long intergenic non-protein coding RNA 1970
ENSG0000	59.34879	0.180775	0.238684	0.152875	0.545561	PRKCI	5584	protein kinase C iota
ENSG0000	3.781973	0.102167	0.255419	0.152867	0.545561	NA	NA	NA
ENSG0000	1574.568	0.164334	0.14469	0.153069	0.546147	CS	1431	citrate synthase
ENSG0000	492.3317	-0.18219	0.222271	0.15315	0.54626	TBC1D8	11138	TBC1 domain family member 8
ENSG0000	54.10439	-0.13117	0.253487	0.15316	0.54626	IKZF2	22807	IKAROS family zinc finger 2
ENSG0000	5.169106	0.097217	0.251625	0.153227	0.546284	NRP2	8828	neuropilin 2
ENSG0000	207.444	-0.17424	0.154768	0.153218	0.546284	VTI1A	143187	vesicle transport through interaction with t-SNAREs 1A
ENSG0000	2061.917	0.1727	0.236427	0.153355	0.546633	IGLV2-23	28813	immunoglobulin lambda variable 2-23
ENSG0000	174.3752	0.17797	0.167012	0.153456	0.546889	KAT6B	23522	lysine acetyltransferase 6B
ENSG0000	40.23086	0.187204	0.230069	0.153499	0.546935	COX14	84987	cytochrome c oxidase assembly factor COX14
ENSG0000	17.6823	-0.18089	0.249532	0.15362	0.547136	NA	NA	NA
ENSG0000	11.0349	-0.12344	0.255505	0.153645	0.547136	NA	NA	NA
ENSG0000	260.0832	-0.1829	0.216166	0.153639	0.547136	NA	NA	NA
ENSG0000	791.5189	-0.17655	0.170368	0.153729	0.547328	CARS1	833	cysteinyl-tRNA synthetase 1
ENSG0000	110.3445	-0.17707	0.236376	0.153792	0.547449	SERPINF1	5176	serpin family F member 1
ENSG0000	33.03947	-0.18979	0.218853	0.153967	0.547578	NA	NA	NA
ENSG0000	1295.381	-0.16563	0.143912	0.154008	0.547578	SNX27	81609	sorting nexin 27
ENSG0000	6.218987	0.101492	0.250411	0.153859	0.547578	NA	NA	NA
ENSG0000	3.046782	-0.04119	0.236328	0.154006	0.547578	LINC0150!	1.01E+08	long intergenic non-protein coding RNA 1505
ENSG0000	3.198236	-0.08155	0.245306	0.153905	0.547578	C9orf163	158055	chromosome 9 putative open reading frame 163
ENSG0000	492.0163	-0.17916	0.182126	0.153931	0.547578	DMWD	1762	DM1 locus WD repeat containing
ENSG0000	232.8357	0.182441	0.183298	0.154106	0.547821	TENT4A	11044	terminal nucleotidyltransferase 4A
ENSG0000	12.54823	-0.09805	0.248724	0.154156	0.547891	NA	NA	NA
ENSG0000	524.839	0.182881	0.175991	0.154225	0.548029	HIPK1	204851	homeodomain interacting protein kinase 1
ENSG0000	9.370855	0.093995	0.247234	0.15432	0.548243	FAM167B	84734	family with sequence similarity 167 member B
ENSG0000	35.10473	0.188065	0.228647	0.154345	0.548243	MAP3K2-D	1.01E+08	MAP3K2 divergent transcript
ENSG0000	6.450307	0.111906	0.25382	0.154461	0.548549	SNHG19	1.01E+08	small nucleolar RNA host gene 19
ENSG0000	3068.376	0.170463	0.239654	0.154497	0.548572	RPL27	6155	ribosomal protein L27
ENSG0000	352.9787	-0.17356	0.161325	0.154649	0.549004	VPS13B	157680	vacuolar protein sorting 13 homolog B
ENSG0000	13.57059	0.166514	0.260142	0.154785	0.549211	NA	NA	NA
ENSG0000	3.742489	0.055807	0.238631	0.154778	0.549211	FZD9	8326	frizzled class receptor 9
ENSG0000	67.37026	-0.18064	0.233934	0.154797	0.549211	FITM1	161247	fat storage inducing transmembrane protein 1
ENSG0000	289.6366	0.174395	0.194839	0.154855	0.54931	GSTM4	2948	glutathione S-transferase mu 4
ENSG0000	13.64878	0.163747	0.259754	0.155091	0.550042	C17orf58	284018	chromosome 17 open reading frame 58
ENSG0000	658.9455	-0.18771	0.213389	0.155167	0.550205	CCDC9	26093	coiled-coil domain containing 9
ENSG0000	73.55163	-0.18503	0.195343	0.15545	0.550286	GBAP1	2630	glucosylceramidase beta pseudogene 1
ENSG0000	9.473521	-0.09763	0.248338	0.155348	0.550286	PITX1	5307	paired like homeodomain 1
ENSG0000	4.116281	0.072275	0.242252	0.155376	0.550286	NA	NA	NA
ENSG0000	28.28853	-0.16531	0.252263	0.155385	0.550286	LINC0117(	1.01E+08	long intergenic non-protein coding RNA 1176
ENSG0000	2.649368	0.060981	0.240016	0.155461	0.550286	NA	NA	NA
ENSG0000	961.9538	-0.15413	0.131789	0.155295	0.550286	KCTD13	253980	potassium channel tetramerization domain containing 13
ENSG0000	383.5412	-0.17743	0.227695	0.155405	0.550286	VSTM1	284415	V-set and transmembrane domain containing 1
ENSG0000	4.314093	0.082663	0.247435	0.155325	0.550286	NA	NA	NA
ENSG0000	118.527	0.174199	0.161088	0.155264	0.550286	ARHGEF9	23229	Cdc42 guanine nucleotide exchange factor 9
ENSG0000	51.33676	0.175301	0.242801	0.155571	0.550571	PDP1	54704	pyruvate dehydrogenase phosphatase catalytic subunit 1
ENSG0000	217.4963	0.177587	0.169495	0.155855	0.550892	IFT122	55764	intraflagellar transport 122
ENSG0000	21.54835	0.128228	0.25401	0.155796	0.550892	NA	NA	NA
ENSG0000	277.7704	-0.1762	0.16826	0.155838	0.550892	DTX3	196403	deltex E3 ubiquitin ligase 3
ENSG0000	17.81184	-0.14394	0.257996	0.155773	0.550892	ARG2	384	arginase 2
ENSG0000	157.014	-0.18152	0.22412	0.155728	0.550892	ST20	400410	suppressor of tumorigenicity 20
ENSG0000	233.8406	0.165438	0.148452	0.155881	0.550892	ACAA2	10449	acetyl-CoA acyltransferase 2
ENSG0000	8.817494	0.146207	0.261656	0.155869	0.550892	TASP1	55617	taspase 1

ENSG0000	204.5368	0.111445	0.25107	0.155902	0.550892	RPL39	6170	ribosomal protein L39
ENSG0000	176.0773	-0.1607	0.140725	0.156002	0.550926	THAP3	90326	THAP domain containing 3
ENSG0000	1241.391	-0.1795	0.2236	0.155998	0.550926	F5	2153	coagulation factor V
ENSG0000	34.69479	0.168083	0.249916	0.155945	0.550926	RPP14	11102	ribonuclease P/MRP subunit p14
ENSG0000	4.42881	0.057699	0.239129	0.156117	0.551224	CDH4	1002	cadherin 4
ENSG0000	7.909724	-0.06641	0.240234	0.156412	0.552054	SHANK1	50944	SH3 and multiple ankyrin repeat domains 1
ENSG0000	128.6123	0.170103	0.239979	0.156405	0.552054	ERVK3-1	1.05E+08	endogenous retrovirus group K3 member 1
ENSG0000	13.04819	0.158077	0.261096	0.156455	0.552097	RNF32-DT	1.01E+08	RNF32 divergent transcript
ENSG0000	2.514818	-0.05531	0.239157	0.156497	0.552139	NA	NA	NA
ENSG0000	17.97528	0.149644	0.259749	0.156641	0.55254	IGKV2D-3(	28881	immunoglobulin kappa variable 2D-30
ENSG0000	102.9338	0.180696	0.179599	0.156822	0.553074	ZNRF1	84937	zinc and ring finger 1
ENSG0000	7708.687	-0.17092	0.158469	0.156865	0.55312	TUBA1B	10376	tubulin alpha 1b
ENSG0000	48.131	-0.18728	0.195826	0.156946	0.553297	LOC10041	1E+08	toll like receptor 2 pseudogene
ENSG0000	25.09698	0.160077	0.253572	0.157192	0.554059	GPATCH2	55105	G-patch domain containing 2
ENSG0000	5.815739	0.080716	0.244253	0.157496	0.554489	NA	NA	NA
ENSG0000	1087.977	0.153664	0.129805	0.157377	0.554489	HNRNPD	3184	heterogeneous nuclear ribonucleoprotein D
ENSG0000	171.7569	0.182849	0.193882	0.157436	0.554489	CCDC125	202243	coiled-coil domain containing 125
ENSG0000	33.66973	0.171774	0.247596	0.157403	0.554489	NA	NA	NA
ENSG0000	3.488702	0.061	0.23934	0.157491	0.554489	NA	NA	NA
ENSG0000	2.904433	-0.06559	0.24211	0.157462	0.554489	NA	NA	NA
ENSG0000	2.693176	0.059925	0.240283	0.157542	0.554545	NA	NA	NA
ENSG0000	1065.949	0.139834	0.115039	0.157845	0.554605	MACF1	23499	microtubule actin crosslinking factor 1
ENSG0000	22.4869	0.142298	0.255348	0.157662	0.554605	WNT6	7475	Wnt family member 6
ENSG0000	180.6268	0.18286	0.202514	0.157846	0.554605	OTULINL	54491	OTU deubiquitinase with linear linkage specificity like
ENSG0000	18.62958	-0.12488	0.254262	0.157836	0.554605	NA	NA	NA
ENSG0000	19.34079	0.157695	0.25776	0.157862	0.554605	NA	NA	NA
ENSG0000	8.614625	-0.14381	0.260922	0.157849	0.554605	TRBV25-1	28562	T cell receptor beta variable 25-1
ENSG0000	3.384311	-0.08013	0.245947	0.157609	0.554605	NA	NA	NA
ENSG0000	2.892278	0.062165	0.243208	0.157849	0.554605	SLC25A41	284427	solute carrier family 25 member 41
ENSG0000	37.47526	0.181263	0.236214	0.157678	0.554605	HSCB	150274	HscB mitochondrial iron-sulfur cluster cochaperone
ENSG0000	226.7084	0.17574	0.163955	0.15775	0.554605	BRD1	23774	bromodomain containing 1
ENSG0000	19.78924	-0.17842	0.246464	0.157946	0.554793	TCAP	8557	titin-cap
ENSG0000	9.856047	-0.12687	0.255066	0.158257	0.55511	NA	NA	NA
ENSG0000	12.59094	-0.14251	0.257444	0.158252	0.55511	NA	NA	NA
ENSG0000	517.1556	0.166744	0.15045	0.158139	0.55511	PIDD1	55367	p53-induced death domain protein 1
ENSG0000	4.27668	0.094667	0.250905	0.158202	0.55511	KRT7	3855	keratin 7
ENSG0000	1500.199	-0.16544	0.14552	0.158177	0.55511	PHF23	79142	PHD finger protein 23
ENSG0000	11.13871	0.160075	0.26104	0.158278	0.55511	MTCO1P4(	1.07E+08	MT-CO1 pseudogene 40
ENSG0000	347.1492	-0.14626	0.121676	0.158177	0.55511	BCL2L13	23786	BCL2 like 13
ENSG0000	170.8785	0.178481	0.232591	0.158086	0.55511	IGLV4-60	28785	immunoglobulin lambda variable 4-60
ENSG0000	64.11318	0.164069	0.234838	0.158369	0.555322	ZNF75D	7626	zinc finger protein 75D
ENSG0000	326.1265	-0.17629	0.171433	0.158484	0.555371	F11R	50848	F11 receptor
ENSG0000	180.4948	0.169325	0.155011	0.158469	0.555371	NENF	29937	neudesin neurotrophic factor
ENSG0000	5.854061	0.071309	0.241047	0.158453	0.555371	SLC22A13	9390	solute carrier family 22 member 13
ENSG0000	18.97162	-0.16954	0.254315	0.158504	0.555371	NA	NA	NA
ENSG0000	20.00304	0.171776	0.251949	0.158583	0.55554	PHLPP2	23035	PH domain and leucine rich repeat protein phosphatase 2
ENSG0000	114.4975	-0.18198	0.216191	0.158677	0.555764	ADCY10P1	221442	ADCY10 pseudogene 1
ENSG0000	12.02941	-0.15078	0.260301	0.15872	0.555807	NA	NA	NA
ENSG0000	43.35576	0.183823	0.193634	0.158773	0.555814	PDCL	5082	phosducin like
ENSG0000	223.8363	-0.17518	0.169756	0.158782	0.555814	CCSER2	54462	coiled-coil serine rich protein 2
ENSG0000	224.1484	0.15751	0.136098	0.158855	0.555964	SLC26A11	284129	solute carrier family 26 member 11
ENSG0000	1002.51	-0.16145	0.143718	0.158954	0.556202	DDX23	9416	DEAD-box helicase 23
ENSG0000	73.14543	0.184394	0.201007	0.159056	0.556346	PDCL3	79031	phosducin like 3
ENSG0000	136.494	0.18282	0.195268	0.159053	0.556346	TRIO	7204	trio Rho guanine nucleotide exchange factor
ENSG0000	46.11119	-0.17701	0.238735	0.159116	0.556452	MUC1	4582	mucin 1 cell surface associated
ENSG0000	133.1099	0.181119	0.224064	0.159239	0.556484	MTREX	23517	Mtr4 exosome RNA helicase
ENSG0000	8.128931	-0.07467	0.241915	0.159177	0.556484	SOBP	55084	sine oculis binding protein homolog
ENSG0000	851.3544	-0.1489	0.125124	0.15922	0.556484	FIS1	51024	fission mitochondrial 1
ENSG0000	4.667388	0.092268	0.24837	0.159278	0.556484	LOC10272	1.03E+08	uncharacterized LOC102723692
ENSG0000	854.2214	0.150572	0.1329	0.159273	0.556484	CES2	8824	carboxylesterase 2
ENSG0000	50.21031	-0.17128	0.243199	0.159408	0.556832	TRBV9	28586	T cell receptor beta variable 9
ENSG0000	379.8789	0.169	0.239643	0.15957	0.557025	MARCHF1	55016	membrane associated ring-CH-type finger 1
ENSG0000	33.79894	-0.15206	0.253116	0.159564	0.557025	CNGA4	1262	cyclic nucleotide gated channel subunit alpha 4
ENSG0000	199.7101	-0.16028	0.140459	0.159563	0.557025	NA	NA	NA
ENSG0000	180.1772	0.184478	0.19785	0.159584	0.557025	PRDX4	10549	peroxiredoxin 4
ENSG0000	52.58643	0.184883	0.207966	0.159689	0.557163	IFT172	26160	intraflagellar transport 172
ENSG0000	2.600934	-0.08142	0.246863	0.159715	0.557163	MIR762	1E+08	microRNA 762
ENSG0000	22.31713	0.149479	0.255377	0.159658	0.557163	ZNF880	400713	zinc finger protein 880
ENSG0000	985.2854	0.180146	0.212939	0.159769	0.557246	C10orf105	414152	chromosome 10 open reading frame 105
ENSG0000	298.1982	0.17404	0.166576	0.159837	0.557377	PHETA1	144717	PH domain containing endocytic trafficking adaptor 1
ENSG0000	1182.161	0.16528	0.151458	0.159887	0.557436	CANX	821	calnexin
ENSG0000	31.20843	0.167648	0.255777	0.159915	0.557436	RPSAP47	389672	ribosomal protein SA pseudogene 47
ENSG0000	2.484156	0.047654	0.237606	0.160033	0.55774	NA	NA	NA
ENSG0000	1018.885	0.174388	0.167933	0.160103	0.557864	CLPTM1L	81037	CLPTM1 like
ENSG0000	18.10247	-0.15005	0.255821	0.160129	0.557864	NA	NA	NA
ENSG0000	342.1092	-0.15825	0.137241	0.160203	0.558015	NSF	4905	N-ethylma vesicle fusing ATPase
ENSG0000	8.519938	0.097842	0.247662	0.160239	0.558035	L1CAM	3897	L1 cell adhesion molecule
ENSG0000	183.4519	-0.17793	0.190673	0.160341	0.558284	NA	NA	NA
ENSG0000	57.60387	-0.18089	0.180307	0.160433	0.558496	PAX8	7849	paired box 8
ENSG0000	163.847	0.178377	0.218447	0.160561	0.558836	CASTOR2	729438	cytosolic arginine sensor for mTORC1 subunit 2
ENSG0000	12.32857	0.13021	0.255279	0.160621	0.558939	CC2D2B	387707	coiled-coil and C2 domain containing 2B
ENSG0000	3.60017	-0.06761	0.241753	0.160763	0.55922	NA	NA	NA
ENSG0000	361.089	-0.18363	0.201078	0.160763	0.55922	CD59	966	CD59 molecule (CD59 blood group)
ENSG0000	6.005801	0.075002	0.242909	0.160803	0.559255	LINC00887	1E+08	long intergenic non-protein coding RNA 887
ENSG0000	13.16786	0.165938	0.256514	0.160862	0.559354	ZNF280C	55609	zinc finger protein 280C
ENSG0000	7353.307	-0.17797	0.189861	0.160971	0.559456	AGTRAP	57085	angiotensin II receptor associated protein
ENSG0000	155.2686	0.173604	0.159323	0.160983	0.559456	TMEM258	746	transmembrane protein 258
ENSG0000	14.06548	0.168236	0.255227	0.160935	0.559456	CEP76	79959	centrosomal protein 76
ENSG0000	39.29643	0.144456	0.251719	0.161021	0.559482	ZCCHC10	54819	zinc finger CCHC-type containing 10
ENSG0000	5.32211	0.084508	0.246212	0.161101	0.559652	THRB	7068	thyroid hormone receptor beta
ENSG0000	353.2515	0.154457	0.132898	0.161239	0.560026	DUSP23	54935	dual specificity phosphatase 23
ENSG0000	65.14157	0.183165	0.188047	0.161344	0.560284	SNHG11	128439	small nucleolar RNA host gene 11
ENSG0000	108.7832	0.177281	0.23272	0.161437	0.5605	DNAJB9	4189	DnaJ heat shock protein family (Hsp40) member B9
ENSG0000	694.4264	-0.16314	0.239595	0.161622	0.560826	OXER1	165140	oxoeicosanoid receptor 1
ENSG0000	3.341749	-0.09846	0.250431	0.16159	0.560826	NA	NA	NA
ENSG0000	1782.131	0.160697	0.14317	0.16161	0.560826	SLC2A4RG	56731	SLC2A4 regulator

ENSG0000	1375.492	-0.16333	0.146217	0.161922	0.561346	OST4	1E+08	oligosacch non-catalytic
ENSG0000	13.09854	0.112161	0.250989	0.162103	0.561346	ARL13B	200894	ADP ribosylation factor like GTPase 13B
ENSG0000	155.0162	0.172878	0.166052	0.162089	0.561346	MTO1	25821	mitochondrial tRNA translation optimization 1
ENSG0000	245.2525	-0.18037	0.211942	0.161979	0.561346	TXN	7295	thioredoxin
ENSG0000	14.50164	-0.15329	0.257171	0.162101	0.561346	GNG3	2785	G protein subunit gamma 3
ENSG0000	12.67928	0.163346	0.258932	0.161982	0.561346	AMOTL1	154810	angiomotin like 1
ENSG0000	8.088678	0.108011	0.251464	0.162041	0.561346	ZNF10	7556	zinc finger protein 10
ENSG0000	636.7321	0.167529	0.22993	0.162043	0.561346	HMGB1	3146	high mobility group box 1
ENSG0000	49.83926	0.155554	0.250231	0.162087	0.561346	LINC0228	283575	long intergenic non-protein coding RNA 2288
ENSG0000	1179.091	0.168047	0.166462	0.16189	0.561346	JMJD8	339123	jumonji domain containing 8
ENSG0000	69.67524	0.18012	0.222804	0.16211	0.561346	ZNF331	55422	zinc finger protein 331
ENSG0000	36.85643	-0.17454	0.239278	0.162141	0.561351	NA	NA	NA
ENSG0000	11731.91	-0.17492	0.177346	0.162253	0.561526	LASP1	3927	LIM and SH3 protein 1
ENSG0000	7.181476	-0.12801	0.258031	0.162235	0.561526	MIR12136	1.13E+08	microRNA 12136
ENSG0000	7.192468	0.097631	0.248295	0.162377	0.561742	NA	NA	NA
ENSG0000	2.514464	0.071477	0.243849	0.162348	0.561742	ANO7	50636	anoctamin 7
ENSG0000	5.206715	-0.10965	0.252923	0.162416	0.56177	NA	NA	NA
ENSG0000	163.5229	-0.17759	0.181567	0.162489	0.561855	ZNF691	51058	zinc finger protein 691
ENSG0000	12.71207	-0.13236	0.255344	0.162502	0.561855	ZNF835	90485	zinc finger protein 835
ENSG0000	89.09945	-0.18227	0.206649	0.162569	0.561981	CLN5	1203	CLN5 intracellular trafficking protein
ENSG0000	36.04966	-0.18135	0.228738	0.162715	0.562381	ROM1	6094	retinal outer segment membrane protein 1
ENSG0000	37.68387	-0.17427	0.238438	0.162747	0.562386	NA	NA	NA
ENSG0000	794.1703	-0.15659	0.241935	0.162822	0.562536	BPI	671	bactericidal permeability increasing protein
ENSG0000	7.431045	0.112206	0.252543	0.162884	0.562644	RPS13P2	729236	ribosomal protein S13 pseudogene 2
ENSG0000	129.919	-0.17462	0.171116	0.162924	0.562677	NR1H3	10062	nuclear receptor subfamily 1 group H member 3
ENSG0000	32.24449	0.176929	0.238552	0.163002	0.562735	NA	NA	NA
ENSG0000	98.19957	-0.18101	0.204115	0.162979	0.562735	GNG11	2791	G protein subunit gamma 11
ENSG0000	475.0573	0.150097	0.128801	0.163139	0.5631	SMC1A	8243	structural maintenance of chromosomes 1A
ENSG0000	483.6016	0.164046	0.149868	0.163215	0.563258	PPP5C	5536	protein phosphatase 5 catalytic subunit
ENSG0000	4.944284	0.074546	0.242082	0.163259	0.563302	ZNF713	349075	zinc finger protein 713
ENSG0000	70.76212	-0.17708	0.232168	0.163512	0.563645	EPHX2	2053	epoxide hydrolase 2
ENSG0000	1070.236	0.171581	0.162478	0.163417	0.563645	ANAPC2	29882	anaphase promoting complex subunit 2
ENSG0000	367.8566	0.168903	0.157826	0.163431	0.563645	TPCN2	219931	two pore segment channel 2
ENSG0000	32.32691	0.183919	0.224837	0.163486	0.563645	RHBDL1	9028	rhomboid like 1
ENSG0000	186.5963	0.177786	0.215297	0.163495	0.563645	GALNT1	2589	polypeptide N-acetylgalactosaminyltransferase 1
ENSG0000	2.828885	0.060079	0.239617	0.163583	0.563783	ZNF883	169834	zinc finger protein 883
ENSG0000	8.859434	-0.07488	0.241372	0.163712	0.564121	TRAV5	28688	T cell receptor alpha variable 5
ENSG0000	12.06763	-0.16685	0.255183	0.163757	0.564173	POLN	353497	DNA polymerase nu
ENSG0000	161.2806	0.170182	0.161241	0.163833	0.564329	SEC22C	9117	SEC22 hon vesicle trafficking protein
ENSG0000	12717.59	-0.17439	0.177606	0.163912	0.564372	NCF2	4688	neutrophil cytosolic factor 2
ENSG0000	31.27482	-0.17372	0.240329	0.163888	0.564372	LOC10537	1.05E+08	uncharacterized LOC105370941
ENSG0000	19.70435	-0.16596	0.251466	0.163938	0.564372	NA	NA	NA
ENSG0000	28.27247	0.167375	0.248634	0.163984	0.564422	ECT2	1894	epithelial cell transforming 2
ENSG0000	1038.962	-0.17321	0.170612	0.164075	0.564629	RAB27A	5873	RAB27A member RAS oncogene family
ENSG0000	4.366485	0.083721	0.245692	0.164161	0.56482	NA	NA	NA
ENSG0000	47.72965	0.180291	0.226797	0.164288	0.56515	ZNF227	7770	zinc finger protein 227
ENSG0000	263.1494	0.169649	0.161364	0.164644	0.565294	FNDC10	643988	fibronectin type III domain containing 10
ENSG0000	10.38948	0.088803	0.244302	0.164609	0.565294	EXTL2	2135	exostosin like glycosyltransferase 2
ENSG0000	304.6459	0.172254	0.167559	0.16449	0.565294	FCRLA	84824	Fc receptor like A
ENSG0000	1404.538	-0.15174	0.132175	0.164546	0.565294	JADE2	23338	jade family PHD finger 2
ENSG0000	16.28949	0.164243	0.253298	0.164566	0.565294	RTN4IP1	84816	reticulon 4 interacting protein 1
ENSG0000	800.3738	-0.14502	0.123327	0.164725	0.565294	TSG101	7251	tumor susceptibility 101
ENSG0000	17.39173	0.092689	0.244638	0.164707	0.565294	ZNF280D	54816	zinc finger protein 280D
ENSG0000	1218.204	0.171261	0.178928	0.164731	0.565294	TXNDC11	51061	thioredoxin domain containing 11
ENSG0000	248.2345	-0.15985	0.142556	0.164663	0.565294	LRRC37A2	474170	leucine rich repeat containing 37 member A2
ENSG0000	676.1922	0.177866	0.204069	0.164565	0.565294	TMEM205	374882	transmembrane protein 205
ENSG0000	34.08737	-0.12506	0.249287	0.164403	0.565294	IL4I1	259307	interleukin 4 induced 1
ENSG0000	198.0219	-0.17421	0.17394	0.164487	0.565294	ZNF586	54807	zinc finger protein 586
ENSG0000	732.5302	0.151407	0.13081	0.164475	0.565294	UFD1	7353	ubiquitin recognition factor in ER associated degradation 1
ENSG0000	118.3136	0.173967	0.170083	0.164913	0.565813	PXMP4	11264	peroxisomal membrane protein 4
ENSG0000	13.98681	0.165695	0.255526	0.165018	0.565962	PANO1	1.02E+08	proapoptotic nucleolar protein 1
ENSG0000	35.86167	-0.18335	0.201076	0.165016	0.565962	CD2BP2-D	1.02E+08	CD2BP2 divergent transcript
ENSG0000	19.66157	-0.17296	0.246654	0.165082	0.566076	NA	NA	NA
ENSG0000	10.12548	0.147471	0.258777	0.165137	0.566158	NA	NA	NA
ENSG0000	3.941696	-0.09811	0.251468	0.165176	0.566158	TNIP3	79931	TNFAIP3 interacting protein 3
ENSG0000	130.7209	0.179798	0.217613	0.165199	0.566158	KNOP1	400506	lysine rich nucleolar protein 1
ENSG0000	147.836	0.171282	0.164856	0.165421	0.566593	ZMAT3	64393	zinc finger matrin-type 3
ENSG0000	9.532256	0.099828	0.24785	0.16538	0.566593	NA	NA	NA
ENSG0000	2750.504	-0.17222	0.221836	0.165449	0.566593	PTEN	5728	phosphatase and tensin homolog
ENSG0000	959.7981	-0.16439	0.150991	0.165405	0.566593	MPHOSPH	54737	M-phase phosphoprotein 8
ENSG0000	3.665104	0.063191	0.241262	0.165489	0.566622	GPNMB	10457	glycoprotein nmb
ENSG0000	196.5982	-0.17683	0.194961	0.165553	0.566629	TIPRL	261726	TOR signaling pathway regulator
ENSG0000	846.5156	0.13103	0.107029	0.165543	0.566629	RBM15B	29890	RNA binding motif protein 15B
ENSG0000	2.487917	-0.05017	0.238807	0.165668	0.566916	NA	NA	NA
ENSG0000	165.3958	0.178319	0.21671	0.165736	0.567044	ZFAS1	441951	ZNFX1 antisense RNA 1
ENSG0000	15.62897	0.09698	0.245695	0.16593	0.567603	NA	NA	NA
ENSG0000	689.7211	-0.14788	0.126001	0.165963	0.567607	NA	NA	NA
ENSG0000	362.8863	-0.16809	0.158678	0.166051	0.567754	MED8	112950	mediator complex subunit 8
ENSG0000	112.3932	-0.17505	0.175357	0.166068	0.567754	PNMA1	9240	PNMA family member 1
ENSG0000	140.6331	-0.17442	0.176721	0.166117	0.567817	TP53BP1	7158	tumor protein p53 binding protein 1
ENSG0000	5.013348	0.105324	0.251966	0.166179	0.567924	WNT2B	7482	Wnt family member 2B
ENSG0000	22.7363	0.088577	0.243433	0.16624	0.568025	LOC72850	728506	POM121 membrane glycoprotein (rat) pseudogene
ENSG0000	5.435146	0.105158	0.250928	0.166271	0.568025	FSCN2	25794	fascin acti retinal
ENSG0000	3.33021	0.091999	0.248394	0.166364	0.568133	NA	NA	NA
ENSG0000	86.31718	-0.1802	0.196932	0.166344	0.568133	CBLN3	643866	cerebellin 3 precursor
ENSG0000	258.0804	0.148051	0.126634	0.166453	0.56823	ZBTB22	9278	zinc finger and BTB domain containing 22
ENSG0000	999.1519	-0.16118	0.15125	0.166455	0.56823	B3GAT3	26229	beta-1 3-glucuronyltransferase 3
ENSG0000	168.1784	-0.17816	0.190034	0.166571	0.56852	TEKTIP1	1E+08	tektin bundle interacting protein 1
ENSG0000	23.26144	0.163588	0.249735	0.166657	0.568601	ZNF595	152687	zinc finger protein 595
ENSG0000	371.1958	0.170931	0.166964	0.166649	0.568601	TMED3	23423	transmembrane p24 trafficking protein 3
ENSG0000	424.112	0.174993	0.190499	0.166808	0.568669	MTCO1P1	1.07E+08	MT-CO1 pseudogene 12
ENSG0000	2145.072	-0.16295	0.153555	0.166726	0.568669	LYPLA2	11313	lysophospholipase 2
ENSG0000	1924.799	-0.14931	0.129586	0.166811	0.568669	RBM5	10181	RNA binding motif protein 5
ENSG0000	25.64492	0.158441	0.254742	0.166764	0.568669	GATD3	8209	glutamine amidotransferase class 1 domain containing 3
ENSG0000	76.81589	0.180446	0.200634	0.166832	0.568669	RNF215	200312	ring finger protein 215
ENSG0000	32.46789	-0.16716	0.24391	0.166883	0.568737	C9orf85	138241	chromosome 9 open reading frame 85

ENSG0000	62.86034	0.180353	0.199266	0.167105	0.569284	NA	NA	NA
ENSG0000	105.8932	-0.17637	0.178799	0.167086	0.569284	NDUFAF1	51103	NADH:ubiquinone oxidoreductase complex assembly factor 1
ENSG0000	82.80885	0.180187	0.195096	0.167199	0.569497	TAS1R3	83756	taste 1 receptor member 3
ENSG0000	19.36769	-0.14972	0.254307	0.167291	0.569703	TRAV8-1	28685	T cell receptor alpha variable 8-1
ENSG0000	14.73737	0.115173	0.249702	0.167423	0.569948	ILDR1	286676	immunoglobulin like domain containing receptor 1
ENSG0000	61.94409	0.180958	0.214747	0.167487	0.569948	FLJ20021	90024	uncharacterized LOC90024
ENSG0000	1751.219	-0.1715	0.176675	0.167427	0.569948	DPM2	8818	dolichyl-pl regulatory
ENSG0000	2.574615	0.059062	0.239653	0.167477	0.569948	MAP3K9-D	1.01E+08	MAP3K9 divergent transcript
ENSG0000	34.85879	0.175882	0.234716	0.167748	0.570729	EFCAB8	388795	EF-hand calcium binding domain 8
ENSG0000	32.40907	0.153381	0.250274	0.167829	0.570898	TOGARAM	23116	TOG array regulator of axonemal microtubules 1
ENSG0000	59.0175	0.153177	0.248406	0.167915	0.571086	CYP1B1-AS	285154	CYP1B1 antisense RNA 1
ENSG0000	2050.418	0.147438	0.127015	0.167959	0.571127	CMTM7	112616	CKLF like MARVEL transmembrane domain containing 7
ENSG0000	33.19495	0.169149	0.241428	0.168045	0.571299	MAIP1	79568	matrix AAA peptidase interacting protein 1
ENSG0000	105.5362	0.135819	0.247833	0.168102	0.571299	PHTF2	57157	putative homeodomain transcription factor 2
ENSG0000	610.3347	0.130115	0.110184	0.168084	0.571299	USP7	7874	ubiquitin specific peptidase 7
ENSG0000	15.31374	0.123159	0.25218	0.168286	0.571605	TTC32	130502	tetratricopeptide repeat domain 32
ENSG0000	970.7796	-0.16766	0.159814	0.168272	0.571605	ZFPL1	7542	zinc finger protein like 1
ENSG0000	342.1092	0.155231	0.137615	0.168229	0.571605	LSM14B	149986	LSM family member 14B
ENSG0000	70.4326	0.170774	0.233835	0.168324	0.571626	HEATR5A	25938	HEAT repeat containing 5A
ENSG0000	33.47467	-0.17701	0.231944	0.168379	0.571647	NA	NA	NA
ENSG0000	59.60817	-0.17786	0.180728	0.168732	0.571647	OTUD7B	56957	OTU deubiquitinase 7B
ENSG0000	1299.971	-0.14978	0.129324	0.168735	0.571647	TEX261	113419	testis expressed 261
ENSG0000	4.040566	0.078353	0.243602	0.168609	0.571647	GOLGA4-A	152048	GOLGA4 antisense RNA 1
ENSG0000	41.1038	0.180684	0.213471	0.168655	0.571647	BLOC1S5	63915	biogenesis of lysosomal organelles complex 1 subunit 5
ENSG0000	36.48795	0.177016	0.227677	0.168678	0.571647	JARID2-AS	1.01E+08	JARID2 antisense RNA 1
ENSG0000	3.076601	-0.06909	0.242386	0.168672	0.571647	RPL13AP1	1E+08	ribosomal protein L13a pseudogene 16
ENSG0000	3.479023	-0.04943	0.237356	0.168557	0.571647	SLCO5A1-/	1.21E+08	SLCO5A1 antisense RNA 1
ENSG0000	265.3451	0.176998	0.210959	0.168601	0.571647	CLPB	81570	caseinolytic mitochondrial matrix peptidase chaperone subunit B
ENSG0000	430.2557	-0.14327	0.121687	0.168397	0.571647	BUD13	84811	BUD13 homolog
ENSG0000	3.404806	-0.08332	0.245299	0.168646	0.571647	RN7SL606	1.06E+08	RNA 7SL cytoplasm pseudogene
ENSG0000	444.9462	0.154159	0.144452	0.168449	0.571647	UQCRFS1	7386	ubiquinol- Rieske iron-sulfur polypeptide 1
ENSG0000	717.8614	0.119658	0.249643	0.168627	0.571647	IGLC7	28834	immunoglobulin lambda constant 7
ENSG0000	256.1184	0.179123	0.200432	0.168771	0.571663	NSA2	10412	NSA2 ribosome biogenesis factor
ENSG0000	31.15916	0.181473	0.222303	0.168853	0.571834	URB2	9816	URB2 ribosome biogenesis homolog
ENSG0000	7.423337	0.072678	0.24133	0.168922	0.571963	NA	NA	NA
ENSG0000	15.25019	-0.16921	0.248509	0.169085	0.572407	SCAT1	1.02E+08	S-phase cancer associated transcript 1
ENSG0000	520.1207	-0.15118	0.136701	0.169205	0.572707	ZNF800	168850	zinc finger protein 800
ENSG0000	26.88838	0.164254	0.246214	0.169377	0.572925	LY6G5B	58496	lymphocyte antigen 6 family member G5B
ENSG0000	375.6104	-0.16083	0.149017	0.169394	0.572925	WASHC2A	387680	WASH complex subunit 2A
ENSG0000	5.894153	-0.11939	0.254655	0.169382	0.572925	PRSS8	5652	serine protease 8
ENSG0000	3.876711	0.07318	0.242399	0.16934	0.572925	RBFOX2	23543	RNA binding fox-1 homolog 2
ENSG0000	3.0599	0.091059	0.248691	0.169495	0.573071	VSIG8	391123	V-set and immunoglobulin domain containing 8
ENSG0000	51.05875	0.18144	0.20101	0.169531	0.573071	NA	NA	NA
ENSG0000	633.6015	-0.15238	0.137551	0.16953	0.573071	TBC1D25	4943	TBC1 domain family member 25
ENSG0000	292.0557	-0.16932	0.167087	0.169673	0.573339	SLC41A1	254428	solute carrier family 41 member 1
ENSG0000	26.84722	0.156791	0.249643	0.16965	0.573339	SPDL1	54908	spindle apparatus coiled-coil protein 1
ENSG0000	665.8093	0.17422	0.185121	0.169859	0.573756	DCPS	28960	decapping scavenger
ENSG0000	18.9512	-0.12792	0.251578	0.169851	0.573756	PLXNB3	5365	plexin B3
ENSG0000	285.9899	0.161341	0.148539	0.169918	0.573849	PPOX	5498	protoporphyrinogen oxidase
ENSG0000	6.602	0.112424	0.25243	0.169981	0.57385	NA	NA	NA
ENSG0000	1821.029	0.159052	0.146942	0.16996	0.57385	CAT	847	catalase
ENSG0000	817.65	-0.16013	0.149167	0.170106	0.57406	MED28	80306	mediator complex subunit 28
ENSG0000	29.17793	-0.17438	0.236031	0.17009	0.57406	NA	NA	NA
ENSG0000	4.463509	0.066369	0.24045	0.170234	0.574387	DSCC1	79075	DNA replication and sister chromatid cohesion 1
ENSG0000	1818.023	0.146666	0.128759	0.170274	0.574417	ILF3	3609	interleukin enhancer binding factor 3
ENSG0000	3.973287	0.069585	0.241683	0.170384	0.57447	NA	NA	NA
ENSG0000	171.8902	-0.1761	0.193978	0.170478	0.57447	HAUS4	54930	HAUS augmin like complex subunit 4
ENSG0000	499.611	-0.17492	0.19598	0.170379	0.57447	SPPL2A	84888	signal peptide peptidase like 2A
ENSG0000	10.97514	0.099757	0.24764	0.170468	0.57447	NPTN-IT1	1.01E+08	NPTN intronic transcript 1
ENSG0000	14.45306	-0.10938	0.248666	0.170454	0.57447	NA	NA	NA
ENSG0000	6.231876	0.094603	0.247413	0.170401	0.57447	ADAM11	4185	ADAM metallopeptidase domain 11
ENSG0000	508.0559	0.158841	0.146745	0.170552	0.57453	SEC61B	10952	SEC61 translocon subunit beta
ENSG0000	327.7329	-0.15175	0.134637	0.170559	0.57453	ERG28	11161	ergosterol biosynthesis 28 homolog
ENSG0000	6.917489	-0.10168	0.248612	0.170667	0.574784	VEGFC	7424	vascular endothelial growth factor C
ENSG0000	94.2901	0.174038	0.177453	0.170697	0.574784	CYB5D2	124936	cytochrome b5 domain containing 2
ENSG0000	225.3646	-0.15701	0.13937	0.170861	0.575219	GGCX	2677	gamma-glutamyl carboxylase
ENSG0000	2293.413	-0.17456	0.20395	0.170914	0.575219	SH2B2	10603	SH2B adaptor protein 2
ENSG0000	20.36661	-0.1519	0.251421	0.17092	0.575219	GSTO2	119391	glutathione S-transferase omega 2
ENSG0000	2714.624	-0.16721	0.165192	0.170997	0.575373	AKAP13	11214	A-kinase anchoring protein 13
ENSG0000	11.81102	0.148711	0.25328	0.171239	0.575552	ZRANB3	84083	zinc finger RANBP2-type containing 3
ENSG0000	6.104128	0.100737	0.24868	0.171207	0.575552	RNU4ATA(	1.06E+08	RNA U4atac sn pseudogene
ENSG0000	7.961739	-0.09776	0.247926	0.17122	0.575552	RADIL	55698	Rap associating with DIL domain
ENSG0000	1909.086	-0.13749	0.115066	0.171238	0.575552	PSME3IP1	80011	proteasome activator subunit 3 interacting protein 1
ENSG0000	379.0948	0.144623	0.121031	0.171104	0.575552	RBM12	10137	RNA binding motif protein 12
ENSG0000	79.86665	-0.17225	0.17332	0.171177	0.575552	TMEM187	8269	transmembrane protein 187
ENSG0000	1104.615	0.152664	0.139214	0.171402	0.575738	AK2	204	adenylate kinase 2
ENSG0000	238.9173	0.173933	0.184418	0.17139	0.575738	HSPBAP1	79663	HSPB1 associated protein 1
ENSG0000	78.62068	0.173027	0.172485	0.171337	0.575738	VASH1-AS	1.01E+08	VASH1 antisense RNA 1
ENSG0000	15.54602	0.148177	0.254274	0.17142	0.575738	STXBP4	252983	syntaxin binding protein 4
ENSG0000	31.9002	-0.17068	0.237967	0.171576	0.575895	LINC0177	1.08E+08	long intergenic non-protein coding RNA 1772
ENSG0000	4.97505	0.071402	0.241487	0.171889	0.575895	ARL6	84100	ADP ribosylation factor like GTPase 6
ENSG0000	7.668025	0.118866	0.253179	0.171721	0.575895	GPLD1	2822	glycosylphosphatidylinositol specific phospholipase D1
ENSG0000	925.0484	0.170261	0.221827	0.171507	0.575895	VARS1	7407	valyl-tRNA synthetase 1
ENSG0000	39.19796	0.170973	0.236215	0.171699	0.575895	GABRR2	2570	gamma-aminobutyric acid type A receptor subunit rho2
ENSG0000	350.1796	0.172838	0.184467	0.171984	0.575895	PSMB1	5689	proteasome 20S subunit beta 1
ENSG0000	160.3586	0.169109	0.165971	0.171991	0.575895	DNAAF5	54919	dynein axonemal assembly factor 5
ENSG0000	23.60909	-0.14205	0.252412	0.171986	0.575895	TRBV10-3	28583	T cell receptor beta variable 10-3
ENSG0000	85.00866	0.176523	0.191347	0.172002	0.575895	AGAP4	119016	ArfGAP wi ankyrin repeat and PH domain 4
ENSG0000	1116.442	-0.17314	0.192847	0.171859	0.575895	OGA	10724	O-GlcNAcase
ENSG0000	161.634	0.166098	0.16033	0.17186	0.575895	DHRS4-AS	55449	DHRS4 antisense RNA 1
ENSG0000	130.8105	-0.17137	0.222587	0.171947	0.575895	NA	NA	NA
ENSG0000	180.3172	0.172108	0.175285	0.171737	0.575895	TFAP4	7023	transcription factor AP-4
ENSG0000	426.0176	-0.1374	0.117054	0.171661	0.575895	GOSR1	9527	golgi SNAP receptor complex member 1
ENSG0000	2807.749	-0.16948	0.169257	0.171606	0.575895	SP2	6668	Sp2 transcription factor
ENSG0000	12.02898	0.145876	0.256496	0.171885	0.575895	MYCBPAP	84073	MYCBP associated protein
ENSG0000	2.604289	0.074358	0.243815	0.171681	0.575895	NA	NA	NA

ENSG0000	175.8448	0.142761	0.245684	0.172088	0.576081	TMEM170	1E+08	transmembrane protein 170B
ENSG0000	39.63557	0.177644	0.221576	0.172248	0.57651	EPHA1-AS	285965	EPHA1 antisense RNA 1
ENSG0000	23.82008	0.181542	0.219615	0.172338	0.576551	AOPEP	84909	aminopeptidase O (putative)
ENSG0000	433.5302	-0.16298	0.149063	0.172351	0.576551	PPME1	51400	protein phosphatase methylesterase 1
ENSG0000	1024.705	-0.17524	0.19453	0.172355	0.576551	HIF1A	3091	hypoxia inducible factor 1 subunit alpha
ENSG0000	96.25531	-0.17123	0.225316	0.172407	0.576622	RBM7	10179	RNA binding motif protein 7
ENSG0000	11.35146	-0.11369	0.249923	0.172522	0.576657	LOC10537	1.05E+08	uncharacterized LOC105378663
ENSG0000	32.50378	-0.16988	0.237437	0.172531	0.576657	MEIS1	4211	Meis homeobox 1
ENSG0000	324.4056	-0.16696	0.165532	0.17263	0.576657	PCBP4	57060	poly(rC) binding protein 4
ENSG0000	9.433137	-0.13532	0.256847	0.172638	0.576657	LINC0228(	1.02E+08	long intergenic non-protein coding RNA 2280
ENSG0000	63.76073	0.175943	0.219875	0.172546	0.576657	CEP20	123811	centrosomal protein 20
ENSG0000	25.32655	0.171923	0.238344	0.172601	0.576657	NA	NA	NA
ENSG0000	1400.516	-0.14987	0.133186	0.172462	0.576657	PLA2G6	8398	phospholipase A2 group VI
ENSG0000	400.6307	0.170571	0.173699	0.172698	0.576751	PSME4	23198	proteasome activator subunit 4
ENSG0000	74.47076	-0.17226	0.218672	0.172794	0.576757	BLZF1	8548	basic leucine zipper nuclear factor 1
ENSG0000	133.9904	-0.15347	0.24237	0.172775	0.576757	ITGA7	3679	integrin subunit alpha 7
ENSG0000	25.52203	0.181485	0.227282	0.172794	0.576757	PDXDC2P-	283970	PDXDC2P-NPIPB14P readthrough
ENSG0000	70.06791	0.17567	0.193528	0.173507	0.577734	LRRC42	115353	leucine rich repeat containing 42
ENSG0000	46.47415	0.178711	0.205652	0.173915	0.577734	VANGL1	81839	VANGL planar cell polarity protein 1
ENSG0000	1699.868	-0.16746	0.168198	0.173256	0.577734	INTS3	65123	integrator complex subunit 3
ENSG0000	9241.904	-0.17915	0.179387	0.17363	0.577734	ARHGAP3(	257106	Rho GTPase activating protein 30
ENSG0000	2.91801	0.035443	0.235991	0.173274	0.577734	B3GALT2	8707	beta-1 3-galactosyltransferase 2
ENSG0000	6.335663	0.097285	0.24766	0.173266	0.577734	LOC10013	1E+08	Putative uncharacterized protein FLJ44553
ENSG0000	8.724606	-0.11093	0.250849	0.173795	0.577734	PRRT3-AS:	1.01E+08	PRRT3 antisense RNA 1
ENSG0000	59.466	0.175181	0.18278	0.173703	0.577734	TAMM41	132001	TAM41 mitochondrial translocator assembly and maintenance homolog
ENSG0000	12.36546	-0.1388	0.254972	0.173963	0.577734	ERICH6-AS	1.02E+08	ERICH6 antisense RNA 1
ENSG0000	2.326729	0.055949	0.239452	0.173573	0.577734	STBD1	8987	starch binding domain 1
ENSG0000	1186.046	-0.13437	0.113265	0.173622	0.577734	RBM22	55696	RNA binding motif protein 22
ENSG0000	199.1728	0.172565	0.183929	0.173152	0.577734	CCDC167	154467	coiled-coil domain containing 167
ENSG0000	37.30255	0.17786	0.189944	0.173968	0.577734	ICA1	3382	islet cell autoantigen 1
ENSG0000	2.974091	0.057674	0.238838	0.173534	0.577734	ST7-AS1	93653	ST7 antisense RNA 1
ENSG0000	69.80111	0.177665	0.195009	0.173671	0.577734	MTAP	4507	methylthioadenosine phosphorylase
ENSG0000	616.8833	0.166462	0.164783	0.173721	0.577734	FNBP4	23360	formin binding protein 4
ENSG0000	13286.08	-0.16963	0.217322	0.17332	0.577734	C15orf39	56905	chromosome 15 open reading frame 39
ENSG0000	3.545921	-0.0654	0.240397	0.173898	0.577734	NA	NA	NA
ENSG0000	3.229641	0.072561	0.242193	0.173863	0.577734	RN7SKP12	1.06E+08	RN7SK pseudogene 127
ENSG0000	456.1899	0.125779	0.10512	0.173383	0.577734	PDPR	55066	pyruvate dehydrogenase phosphatase regulatory subunit
ENSG0000	2.787729	0.061388	0.240219	0.173724	0.577734	NA	NA	NA
ENSG0000	21.59649	-0.16332	0.246351	0.173596	0.577734	SYNE4	163183	spectrin repeat containing nuclear envelope family member 4
ENSG0000	16.08968	0.155349	0.252693	0.173159	0.577734	ZNF649	65251	zinc finger protein 649
ENSG0000	507.7115	-0.16687	0.166142	0.173733	0.577734	SLC37A1	54020	solute carrier family 37 member 1
ENSG0000	3.41117	0.066736	0.240375	0.173471	0.577734	NA	NA	NA
ENSG0000	2.899547	0.072496	0.243181	0.173968	0.577734	CXorf65	158830	chromosome X open reading frame 65
ENSG0000	71.72449	0.177214	0.206503	0.17397	0.577734	APOOL	139322	apolipoprotein O like
ENSG0000	10.72522	0.077772	0.241587	0.173393	0.577734	RADX	55086	RPA1 relat X-linked
ENSG0000	5.277106	0.078531	0.242708	0.174024	0.577809	NA	NA	NA
ENSG0000	180.5567	0.169681	0.170495	0.174064	0.577836	MRPL23	6150	mitochondrial ribosomal protein L23
ENSG0000	9.4475	0.127947	0.25456	0.174137	0.577842	NA	NA	NA
ENSG0000	911.3227	-0.16969	0.172938	0.17416	0.577842	SAFB	6294	scaffold attachment factor B
ENSG0000	188.6954	0.127562	0.245336	0.174132	0.577842	IGLV9-49	28773	immunoglobulin lambda variable 9-49
ENSG0000	25.8639	0.175373	0.235663	0.174323	0.577949	SORCS2	57537	sortilin related VPS10 domain containing receptor 2
ENSG0000	5.230748	-0.08148	0.243372	0.174262	0.577949	NA	NA	NA
ENSG0000	142.6394	-0.17328	0.188142	0.17435	0.577949	ATG9B	285973	autophagy related 9B
ENSG0000	1479.998	-0.16171	0.155215	0.174279	0.577949	TLE4	7091	TLE family transcriptional corepressor
ENSG0000	41.16471	-0.14191	0.247179	0.174304	0.577949	POGLUT3	143888	protein O-glucosyltransferase 3
ENSG0000	13.02199	-0.13761	0.252401	0.174404	0.578022	HLA-F-AS1	285830	HLA-F antisense RNA 1
ENSG0000	2806.715	-0.16589	0.174362	0.174563	0.578444	SLC9A8	23315	solute carrier family 9 member A8
ENSG0000	464.7513	-0.14076	0.123067	0.174607	0.578461	BTN2A2	10385	butyrophilin subfamily 2 member A2
ENSG0000	6.265814	-0.06949	0.240642	0.174631	0.578461	TRBV10-1	28585	T cell receptor beta variable 10-1
ENSG0000	229.5667	-0.17452	0.198749	0.174682	0.578526	BCL10	8915	BCL10 immune signaling adaptor
ENSG0000	153.8206	0.174177	0.197996	0.174867	0.578979	MRRF	92399	mitochondrial ribosome recycling factor
ENSG0000	42793	-0.17144	0.20455	0.174882	0.578979	TGFB1	7040	transforming growth factor beta 1
ENSG0000	6.411399	0.128654	0.256918	0.175081	0.579439	NA	NA	NA
ENSG0000	63.27448	-0.17408	0.193057	0.175084	0.579439	CDK8	1024	cyclin dependent kinase 8
ENSG0000	8.629102	-0.11659	0.255324	0.175229	0.579813	MTDHP3	1E+08	metadherin pseudogene 3
ENSG0000	224.1795	0.173435	0.19296	0.175269	0.579839	PDHB	5162	pyruvate dehydrogenase E1 subunit beta
ENSG0000	9564.577	-0.15625	0.142465	0.175441	0.579938	ADIPOR1	51094	adiponectin receptor 1
ENSG0000	300.3927	0.163396	0.158768	0.175439	0.579938	TNFRSF10I	8795	TNF receptor superfamily member 10b
ENSG0000	6.150644	0.081848	0.244259	0.175383	0.579938	NA	NA	NA
ENSG0000	147.1908	0.148187	0.242826	0.175414	0.579938	BTAF1	9044	B-TFIID TATA-box binding protein associated factor 1
ENSG0000	76.33164	0.175166	0.197465	0.175457	0.579938	NA	NA	NA
ENSG0000	38.66089	0.14889	0.24663	0.175661	0.580491	RPL15P3	653232	ribosomal protein L15 pseudogene 3
ENSG0000	10.1226	-0.13744	0.254359	0.175687	0.580491	CFL1P1	142913	cofilin 1 pseudogene 1
ENSG0000	47.23044	0.174875	0.222695	0.175782	0.580653	STARD9	57519	StAR related lipid transfer domain containing 9
ENSG0000	518.8938	0.149293	0.131454	0.1758	0.580653	ZNF581	51545	zinc finger protein 581
ENSG0000	3.290609	0.059291	0.239661	0.175913	0.580818	LOC12490	1.25E+08	uncharacterized LOC124904158
ENSG0000	11577.25	-0.16896	0.177746	0.175892	0.580818	CYTH4	27128	cytohesin 4
ENSG0000	4.362431	-0.07135	0.242665	0.176043	0.580865	NA	NA	NA
ENSG0000	77.87342	0.17335	0.181705	0.176054	0.580865	PSMG3-AS	114796	PSMG3 antisense RNA 1 (head to head)
ENSG0000	124.1097	-0.17	0.175532	0.176051	0.580865	TSPAN31	6302	tetraspanin 31
ENSG0000	19.76671	-0.15872	0.247164	0.175969	0.580865	LOC10099	1.01E+08	uncharacterized LOC100996660
ENSG0000	4.301588	-0.07067	0.240887	0.176095	0.580893	NA	NA	NA
ENSG0000	57.42425	-0.1715	0.17577	0.176141	0.580942	ZNF836	162962	zinc finger protein 836
ENSG0000	1873.067	-0.15313	0.140269	0.176178	0.580961	MECP2	4204	methyl-CpG binding protein 2
ENSG0000	5.846055	0.064229	0.239091	0.17625	0.581005	PDC-AS1	1.03E+08	PDC antisense RNA 1
ENSG0000	5.213964	0.096302	0.247673	0.176255	0.581005	NA	NA	NA
ENSG0000	14.83799	0.149407	0.252345	0.1763	0.581046	ZNF879	345462	zinc finger protein 879
ENSG0000	22.52561	-0.13822	0.249749	0.176523	0.581455	KIF5C	3800	kinesin family member 5C
ENSG0000	2.643302	0.0689	0.243063	0.176527	0.581455	NA	NA	NA
ENSG0000	858.4197	-0.16851	0.178355	0.176468	0.581455	CD151	977	CD151 molecule (Raph blood group)
ENSG0000	6.401143	0.078907	0.242757	0.176554	0.581455	THSD1	55901	thrombospondin type 1 domain containing 1
ENSG0000	68.55711	-0.17631	0.201969	0.176582	0.581455	QRICH2	84074	glutamine rich 2
ENSG0000	133.5937	0.162754	0.155583	0.17662	0.581475	HMGXB4	10042	HMG-box containing 4
ENSG0000	54.2406	0.175696	0.202337	0.176899	0.58208	SMAD5	4090	SMAD family member 5
ENSG0000	17.18416	0.142558	0.251093	0.176852	0.58208	ANGPTL6	83854	angiopoietin like 6
ENSG0000	15.14692	-0.11839	0.252281	0.176869	0.58208	DGKK	139189	diacylglycerol kinase kappa

ENSG0000	13.13566	0.075813	0.241649	0.176972	0.582214	COX6B2	125965	cytochrome c oxidase subunit 6B2
ENSG0000	122.6575	-0.17269	0.184771	0.17714	0.58235	FLVCR2	55640	FLVCR heme transporter 2
ENSG0000	745.8315	-0.15449	0.230107	0.177131	0.58235	TMEM88	92162	transmembrane protein 88
ENSG0000	3.517545	0.066902	0.241497	0.177088	0.58235	NA	NA	NA
ENSG0000	2886.536	0.161189	0.243447	0.177118	0.58235	IGLV3-21	28796	immunoglobulin lambda variable 3-21
ENSG0000	342.6658	0.165031	0.164191	0.177202	0.582429	DDX21	9188	DExD-box helicase 21
ENSG0000	30.84437	-0.14742	0.248148	0.177228	0.582429	CDADC1	81602	cytidine and dCMP deaminase domain containing 1
ENSG0000	3.867213	-0.08525	0.244946	0.177447	0.582942	CAMK2N2	94032	calcium/calmodulin dependent protein kinase II inhibitor 2
ENSG0000	2.480708	0.078419	0.244069	0.177445	0.582942	NA	NA	NA
ENSG0000	1461.852	-0.15074	0.13261	0.177527	0.583097	IST1	9798	IST1 factor associated with ESCRT-III
ENSG0000	60.08381	-0.15655	0.239743	0.177659	0.583112	FAM200B	285550	family with sequence similarity 200 member B
ENSG0000	13.05953	0.08093	0.241556	0.177641	0.583112	LRRCC1	85444	leucine rich repeat and coiled-coil centrosomal protein 1
ENSG0000	42.5033	-0.16695	0.231183	0.177642	0.583112	ZEB1-AS1	220930	ZEB1 antisense RNA 1
ENSG0000	89.61841	-0.17212	0.215769	0.177581	0.583112	LINC0155(	388011	long intergenic non-protein coding RNA 1550
ENSG0000	27.30715	-0.17446	0.226151	0.177701	0.583146	FGGY	55277	FGGY carbohydrate kinase domain containing
ENSG0000	26.98007	0.159356	0.243528	0.177839	0.583495	ZNF354B	117608	zinc finger protein 354B
ENSG0000	11.95929	0.09871	0.245671	0.177971	0.583614	NA	NA	NA
ENSG0000	37.88358	-0.16149	0.240685	0.177918	0.583614	TIGD7	91151	tigger transposable element derived 7
ENSG0000	484.3841	0.145326	0.129264	0.177961	0.583614	NELFCD	51497	negative elongation factor complex member C/D
ENSG0000	12.29864	-0.15484	0.251493	0.178169	0.584161	NA	NA	NA
ENSG0000	2.374539	0.056175	0.240709	0.178315	0.584331	NA	NA	NA
ENSG0000	10.49439	0.150194	0.253803	0.178324	0.584331	NA	NA	NA
ENSG0000	4.085661	0.085251	0.245682	0.178381	0.584331	IMMP1L	196294	inner mitochondrial membrane peptidase subunit 1
ENSG0000	610.9761	0.163797	0.160348	0.178375	0.584331	MED13L	23389	mediator complex subunit 13L
ENSG0000	107864.6	-0.16801	0.179374	0.17835	0.584331	FKBP8	23770	FKBP prolyl isomerase 8
ENSG0000	202.6546	-0.16635	0.166789	0.178562	0.584724	NA	NA	NA
ENSG0000	3.193914	0.036107	0.235138	0.178565	0.584724	USP3-AS1	1E+08	USP3 antisense RNA 1
ENSG0000	145.3417	0.168384	0.219895	0.178777	0.585314	RAB30	27314	RAB30 member RAS oncogene family
ENSG0000	78.90637	0.173217	0.206559	0.178876	0.585458	HDDC2	51020	HD domain containing 2
ENSG0000	14.45813	0.092678	0.244018	0.178884	0.585458	NA	NA	NA
ENSG0000	265.1623	0.16821	0.185015	0.17898	0.585511	TMEM165	55858	transmembrane protein 165
ENSG0000	214.9316	-0.164	0.225716	0.178997	0.585511	PRKAR2B	5577	protein kinase cAMP-dependent type II regulatory subunit beta
ENSG0000	542.6292	-0.12557	0.108897	0.178958	0.585511	RNF34	80196	ring finger protein 34
ENSG0000	3.87481	0.077609	0.243103	0.179098	0.585737	HOXA7	3204	homeobox A7
ENSG0000	201.6071	0.172037	0.20095	0.179184	0.58581	RANBP2	5903	RAN binding protein 2
ENSG0000	4.53828	0.109599	0.251549	0.17917	0.58581	RPS9P2	267009	ribosomal protein S9 pseudogene 2
ENSG0000	674.6015	0.164179	0.164678	0.179363	0.586288	ID2	3398	inhibitor of DNA binding 2
ENSG0000	3543.785	0.16846	0.218802	0.179394	0.586288	GAS7	8522	growth arrest specific 7
ENSG0000	28.99425	0.170377	0.234154	0.179473	0.58644	MMP11	4320	matrix metallopeptidase 11
ENSG0000	753.8691	0.153634	0.143175	0.179527	0.586513	CHP1	11261	calcineurin like EF-hand protein 1
ENSG0000	22.47818	-0.14033	0.25092	0.179602	0.586597	LOC72987	729870	uncharacterized LOC729870
ENSG0000	197.4833	0.148404	0.239273	0.179635	0.586597	GASK1B	51313	golgi associated kinase 1B
ENSG0000	330.4126	0.164113	0.162528	0.179649	0.586597	PLD2	5338	phospholipase D2
ENSG0000	16.47222	0.09682	0.245274	0.179754	0.586732	DEPDC1B	55789	DEP domain containing 1B
ENSG0000	12.43479	0.121202	0.250935	0.179749	0.586732	CCDC34	91057	coiled-coil domain containing 34
ENSG0000	154.0258	0.170007	0.181742	0.179798	0.586771	TSN	7247	translin
ENSG0000	1211.018	0.154084	0.143575	0.179896	0.586984	RPUSD1	113000	RNA pseudouridine synthase domain containing 1
ENSG0000	49.85765	0.17188	0.22038	0.179985	0.587019	NA	NA	NA
ENSG0000	4171.73	-0.15269	0.140333	0.180003	0.587019	CCM2	83605	CCM2 scaffold protein
ENSG0000	274.4467	0.163579	0.161783	0.179981	0.587019	KCNN4	3783	potassium calcium-activated channel subfamily N member 4
ENSG0000	25173.73	-0.16828	0.183489	0.180048	0.587063	TYROBP	7305	transmembrane immune signaling adaptor TYROBP
ENSG0000	7.932059	0.134084	0.254646	0.180109	0.587157	NA	NA	NA
ENSG0000	2.817425	0.067158	0.242169	0.180289	0.587396	RNA5SP82	1.01E+08	RNA 5S ribosomal pseudogene 82
ENSG0000	6.159606	-0.10307	0.249467	0.180279	0.587396	LINC0302(	1.02E+08	long intergenic non-protein coding RNA 3020
ENSG0000	159.5036	0.171347	0.190468	0.180297	0.587396	OTUD1	220213	OTU deubiquitinase 1
ENSG0000	375.6624	0.171169	0.199053	0.18031	0.587396	ERCC2	2068	ERCC excis TFIIH core complex helicase subunit
ENSG0000	1440.527	0.173808	0.180762	0.180409	0.587612	ZMIZ1	57178	zinc finger MIZ-type containing 1
ENSG0000	167.6291	0.173274	0.194328	0.180462	0.587679	UAP1	6675	UDP-N-acetylglucosamine pyrophosphorylase 1
ENSG0000	259.0489	0.153516	0.142124	0.180555	0.58788	INO80	54617	INO80 complex ATPase subunit
ENSG0000	828.8076	-0.15098	0.133998	0.180641	0.588056	PROSER1	80209	proline and serine rich 1
ENSG0000	886.955	0.132883	0.113019	0.180726	0.588122	RNF220	55182	ring finger protein 220
ENSG0000	250.9067	0.170349	0.205192	0.180712	0.588122	GLO1	2739	glyoxalase I
ENSG0000	42.23757	0.105285	0.243707	0.180883	0.588488	IGKV1D-16	28901	immunoglobulin kappa variable 1D-16
ENSG0000	40.93877	0.161376	0.238174	0.180903	0.588488	NR1D2	9975	nuclear receptor subfamily 1 group D member 2
ENSG0000	5476.657	0.168809	0.210565	0.181026	0.588575	PTAFR	5724	platelet activating factor receptor
ENSG0000	26.03465	-0.16838	0.237209	0.180996	0.588575	TRAV38-2I	28643	T cell receptor alpha variable 38-2/delta variable 8
ENSG0000	2.34601	0.042017	0.236155	0.180962	0.588575	NA	NA	NA
ENSG0000	3.407633	-0.03029	0.234157	0.181141	0.588739	UICLM	200772	up-regulated in colorectal cancer liver metastasis
ENSG0000	45.03531	-0.17277	0.219373	0.181199	0.588739	GHRLOS	1E+08	ghrelin opposite strand/antisense RNA
ENSG0000	565.8763	-0.15903	0.153937	0.181129	0.588739	ACP2	53	acid phosp lysosomal
ENSG0000	1191.168	-0.15878	0.15169	0.181205	0.588739	PPP2R5C	5527	protein phosphatase 2 regulatory subunit B'gamma
ENSG0000	5.418576	0.085435	0.244192	0.181253	0.588791	NA	NA	NA
ENSG0000	4.922038	-0.08788	0.245401	0.181356	0.588868	RHOD	29984	ras homolog family member D
ENSG0000	124.4294	0.16995	0.187881	0.181344	0.588868	RPUSD4	84881	RNA pseudouridine synthase D4
ENSG0000	117.9814	-0.16186	0.229477	0.181373	0.588868	TGFB1I1	7041	transforming growth factor beta 1 induced transcript 1
ENSG0000	24.10885	-0.1633	0.240252	0.181445	0.588891	TMEM121	80757	transmembrane protein 121
ENSG0000	6.520281	0.092913	0.245394	0.181426	0.588891	LOC10537	1.05E+08	uncharacterized LOC105371430
ENSG0000	14.01849	-0.13793	0.251823	0.181541	0.589101	H4C16	121504	H4 histone 16
ENSG0000	128.6603	0.164511	0.163318	0.18167	0.589414	LINC00667	339290	long intergenic non-protein coding RNA 667
ENSG0000	37.42629	-0.17463	0.201282	0.181803	0.589531	ATP6V1G2	534	ATPase H+ transporting V1 subunit G2
ENSG0000	64.50861	-0.17366	0.194886	0.181784	0.589531	TTC39B	158219	tetratricopeptide repeat domain 39B
ENSG0000	2.357031	0.063621	0.242474	0.181773	0.589531	TMC2	117532	transmembrane channel like 2
ENSG0000	2.430936	0.051581	0.237837	0.181922	0.589815	NA	NA	NA
ENSG0000	9.203371	0.094012	0.245254	0.182125	0.590328	NA	NA	NA
ENSG0000	43.37905	-0.17342	0.202265	0.182242	0.590328	NA	NA	NA
ENSG0000	2105.977	0.159525	0.156332	0.182237	0.590328	RABL6	55684	RAB member RAS oncogene family like 6
ENSG0000	24.15817	-0.13489	0.249141	0.182231	0.590328	NA	NA	NA
ENSG0000	263.1535	0.166448	0.174481	0.182164	0.590328	SAMM50	25813	SAMM50 sorting and assembly machinery component
ENSG0000	11.32752	0.11039	0.248689	0.1823	0.590413	MROH8	140699	maestro heat like repeat family member 8
ENSG0000	187.3719	-0.16102	0.231437	0.182428	0.590619	CACNA1E	777	calcium voltage-gated channel subunit alpha1 E
ENSG0000	9.980763	-0.07486	0.241143	0.182399	0.590619	PROC	5624	protein C inactivator of coagulation factors Va and VIIIa
ENSG0000	4.091132	0.104577	0.250323	0.182538	0.590803	CDNF	441549	cerebral dopamine neurotrophic factor
ENSG0000	20.03982	0.16039	0.242633	0.18255	0.590803	DPF3	8110	double PHD fingers 3
ENSG0000	140.6648	-0.16052	0.170157	0.182587	0.590821	INTS7	25896	integrator complex subunit 7
ENSG0000	25.69934	-0.16796	0.231791	0.182702	0.591038	ST3GAL3	6487	ST3 beta-g 3-sialyltransferase 3
ENSG0000	719.087	-0.16766	0.201925	0.182784	0.591038	CSF1	1435	colony stimulating factor 1

ENSG0000	12.36432	-0.12747	0.250563	0.182759	0.591038	FAM238C	387644	family with sequence similarity 238 member C
ENSG0000	18.17792	0.15648	0.246288	0.182736	0.591038	NA	NA	NA
ENSG0000	18.87553	-0.16951	0.234401	0.182906	0.591329	BMAL2	56938	basic helix-loop-helix ARNT like 2
ENSG0000	8.236612	0.081647	0.242143	0.18295	0.591366	LOC10042	1E+08	pleckstrin homology domain containing A1 pseudogene
ENSG0000	523.703	-0.15224	0.141791	0.183122	0.59182	LINC0052ε	200298	long intergenic non-protein coding RNA 528
ENSG0000	393.3115	0.159611	0.155618	0.183181	0.591904	BRD3	8019	bromodomain containing 3
ENSG0000	214.7038	0.166478	0.175354	0.183301	0.592098	SDHD	6392	succinate dehydrogenase complex subunit D
ENSG0000	3.444886	-0.05497	0.237681	0.183334	0.592098	NA	NA	NA
ENSG0000	4.189832	0.086344	0.245094	0.183338	0.592098	PTGR2	145482	prostaglandin reductase 2
ENSG0000	47.85839	0.171805	0.213674	0.183392	0.59217	CNPY2	10330	canopy FGF signaling regulator 2
ENSG0000	164.7467	0.165225	0.164327	0.183623	0.592648	CNR2	1269	cannabinoid receptor 2
ENSG0000	22792.46	0.169239	0.201658	0.183696	0.592648	SMAP2	64744	small ArfGAP2
ENSG0000	440.0228	0.138709	0.122614	0.183832	0.592648	DAP3	7818	death associated protein 3
ENSG0000	25.64298	0.158195	0.242186	0.183739	0.592648	IL17RC	84818	interleukin 17 receptor C
ENSG0000	39.8566	-0.1544	0.241115	0.1838	0.592648	NA	NA	NA
ENSG0000	77.68363	-0.14916	0.240234	0.183701	0.592648	TRBV20-1	28567	T cell receptor beta variable 20-1
ENSG0000	171.2528	0.162343	0.163441	0.183718	0.592648	SWAP70	23075	switching B cell complex subunit SWAP70
ENSG0000	7114.569	0.167356	0.210835	0.183639	0.592648	ZNF385A	25946	zinc finger protein 385A
ENSG0000	13.9086	-0.13592	0.251365	0.1838	0.592648	NA	NA	NA
ENSG0000	5.096488	-0.06073	0.238368	0.183986	0.592676	NA	NA	NA
ENSG0000	6.614141	0.107748	0.249087	0.183936	0.592676	NA	NA	NA
ENSG0000	31.28806	0.173756	0.206866	0.184002	0.592676	ZKSCAN2-I	1.13E+08	ZKSCAN2 divergent transcript
ENSG0000	1475.519	0.165164	0.173601	0.183878	0.592676	MAPK1	5594	mitogen-activated protein kinase 1
ENSG0000	29.49448	-0.13612	0.247248	0.183994	0.592676	FGF13	2258	fibroblast growth factor 13
ENSG0000	9.355871	0.081221	0.242956	0.184082	0.592726	LINC01781	1.02E+08	long intergenic non-protein coding RNA 1781
ENSG0000	136.8685	0.155406	0.148103	0.184075	0.592726	FN3KRP	79672	fructosamine 3 kinase related protein
ENSG0000	72.3018	-0.16293	0.229086	0.184312	0.593362	WASF1	8936	WASP family member 1
ENSG0000	81.73135	0.138858	0.245889	0.184407	0.593459	DENND1B	163486	DENN domain containing 1B
ENSG0000	36.55834	0.17455	0.196016	0.184396	0.593459	CBY1	25776	chibby fan beta catenin antagonist
ENSG0000	38.63853	-0.16704	0.228481	0.184513	0.593694	SYNGAP1-	1.12E+08	SYNGAP1 antisense RNA 1
ENSG0000	32.73463	0.136492	0.255694	0.184799	0.594511	NA	NA	NA
ENSG0000	20.64784	-0.1649	0.237198	0.184864	0.594617	NA	NA	NA
ENSG0000	82.46272	0.170164	0.187777	0.184966	0.594841	ZMYND19	116225	zinc finger MYND-type containing 19
ENSG0000	256.2774	-0.15624	0.1499	0.185004	0.594857	PDE6D	5147	phosphodiesterase 6D
ENSG0000	1005.836	0.161091	0.161526	0.185215	0.59509	H6PD	9563	hexose-6-phosphate dehydrogenase/glucose 1-dehydrogenase
ENSG0000	1300.101	-0.16419	0.176071	0.185178	0.59509	LAPTM4A	9741	lysosomal protein transmembrane 4 alpha
ENSG0000	10.645	0.069647	0.242699	0.185309	0.59509	IGKV1-13	28939	immunoglobulin kappa variable 1-13
ENSG0000	237.863	0.156786	0.151418	0.18546	0.59509	HS6ST1	9394	heparan sulfate 6-O-sulfotransferase 1
ENSG0000	1289.545	0.153627	0.148321	0.185407	0.59509	DUSP7	1849	dual specificity phosphatase 7
ENSG0000	26.99834	0.165887	0.23344	0.18518	0.59509	ZDHHC11	79844	zinc finger DHHC-type containing 11
ENSG0000	49.06793	-0.16275	0.214063	0.185207	0.59509	CD83	9308	CD83 molecule
ENSG0000	1342.812	-0.13683	0.125006	0.185375	0.59509	DCAF5	8816	DDB1 and CUL4 associated factor 5
ENSG0000	5.763685	-0.07901	0.242136	0.185419	0.59509	NA	NA	NA
ENSG0000	884.1314	-0.16618	0.182856	0.185466	0.59509	CD300LB	124599	CD300 molecule like family member b
ENSG0000	6.699168	-0.08357	0.243286	0.185431	0.59509	NA	NA	NA
ENSG0000	148.6326	-0.16156	0.162616	0.185426	0.59509	PDZD11	51248	PDZ domain containing 11
ENSG0000	284.7735	0.103394	0.245087	0.185633	0.595417	RPL31	6160	ribosomal protein L31
ENSG0000	184.498	0.163998	0.169811	0.185606	0.595417	SCFD1	23256	sec1 family domain containing 1
ENSG0000	14.95806	-0.13832	0.2507	0.185724	0.595605	NA	NA	NA
ENSG0000	4.107814	0.078875	0.242701	0.185833	0.595849	RPL23AP1	728134	ribosomal protein L23a pseudogene 18
ENSG0000	6.292447	0.07911	0.242096	0.185906	0.595978	RASL10A	10633	RAS like family 10 member A
ENSG0000	40.34988	0.168446	0.225287	0.185978	0.596104	NA	NA	NA
ENSG0000	8.39681	0.094871	0.245315	0.186043	0.596211	SLC39A1	27173	solute carrier family 39 member 1
ENSG0000	8.907436	0.121978	0.251007	0.186079	0.596221	TIPIN	54962	TIMELESS interacting protein
ENSG0000	35827.81	-0.16555	0.20796	0.186178	0.596417	FCN1	2219	ficolin 1
ENSG0000	369.0175	-0.16732	0.189864	0.186238	0.596417	BATF	10538	basic leucine zipper ATF-like transcription factor
ENSG0000	752.4702	-0.16357	0.219397	0.186231	0.596417	RGL4	266747	ral guanine nucleotide dissociation stimulator like 4
ENSG0000	55.05768	0.162339	0.231831	0.186336	0.596576	SNED1	25992	sushi nidogen and EGF like domains 1
ENSG0000	13.51233	0.121436	0.249607	0.186353	0.596576	ZNF674	641339	zinc finger protein 674
ENSG0000	24.61983	-0.1652	0.235378	0.186723	0.596824	NA	NA	NA
ENSG0000	463.2508	0.147648	0.142015	0.186591	0.596824	RPL7L1	285855	ribosomal protein L7 like 1
ENSG0000	7.615415	-0.11376	0.24991	0.186631	0.596824	NA	NA	NA
ENSG0000	10.39254	-0.14061	0.252546	0.186678	0.596824	NA	NA	NA
ENSG0000	15.47906	0.144742	0.248993	0.186616	0.596824	IL23A	51561	interleukin 23 subunit alpha
ENSG0000	7.620883	0.097071	0.246774	0.186672	0.596824	CFAP251	144406	cilia and flagella associated protein 251
ENSG0000	3.038603	-0.06481	0.239859	0.186584	0.596824	NA	NA	NA
ENSG0000	8.344511	0.101773	0.247346	0.186513	0.596824	TEX30	93081	testis expressed 30
ENSG0000	4837.816	-0.15726	0.156149	0.186693	0.596824	ATXN2L	11273	ataxin 2 like
ENSG0000	6.666453	0.109341	0.250031	0.186777	0.596892	PALM	5064	paralemmin
ENSG0000	9.13393	-0.11291	0.248878	0.186818	0.596918	NA	NA	NA
ENSG0000	307.4354	-0.1625	0.168521	0.186863	0.596957	HDHD3	81932	haloacid dehalogenase like hydrolase domain containing 3
ENSG0000	16.56481	0.159791	0.243695	0.186945	0.597032	RPL23AP7	729617	ribosomal protein L23a pseudogene 74
ENSG0000	87.91282	-0.17202	0.198689	0.186951	0.597032	NLRP2	55655	NLR family pyrin domain containing 2
ENSG0000	4.82761	0.082487	0.243313	0.187039	0.597209	CDK13-DT	1.12E+08	CDK13 divergent transcript
ENSG0000	639.7826	0.156707	0.161186	0.187098	0.597293	NPEPL1	79716	aminopeptidase like 1
ENSG0000	26.32366	0.165203	0.233275	0.187202	0.59752	NA	NA	NA
ENSG0000	24.13513	-0.17157	0.221635	0.187248	0.597564	SLC4A5	57835	solute carrier family 4 member 5
ENSG0000	52.30816	-0.16513	0.224182	0.187387	0.597902	CHMP4A	29082	charged multivesicular body protein 4A
ENSG0000	16.71712	-0.1298	0.24887	0.187551	0.598323	NA	NA	NA
ENSG0000	25.82969	0.161813	0.238282	0.18764	0.598502	RWDD2A	112611	RWD domain containing 2A
ENSG0000	60.78669	0.168284	0.209283	0.187757	0.598562	NA	NA	NA
ENSG0000	261.6521	0.155789	0.152077	0.187749	0.598562	NSD2	7468	nuclear receptor binding SET domain protein 2
ENSG0000	27.10061	0.163871	0.23358	0.187702	0.598562	FABP5	2171	fatty acid binding protein 5
ENSG0000	604.6608	-0.14668	0.13615	0.187837	0.598674	KPNA6	23633	karyopherin subunit alpha 6
ENSG0000	1135.947	0.147333	0.236465	0.187858	0.598674	IGLV7-43	28776	immunoglobulin lambda variable 7-43
ENSG0000	38.64333	-0.14457	0.246322	0.187973	0.598938	LOC10536	1.05E+08	uncharacterized LOC105369595
ENSG0000	487.1994	0.154226	0.151722	0.188046	0.598962	HSPA9	3313	heat shock protein family A (Hsp70) member 9
ENSG0000	29.83566	0.101843	0.244747	0.188027	0.598962	AP3B2	8120	adaptor related protein complex 3 subunit beta 2
ENSG0000	22.07144	0.163903	0.235496	0.188092	0.599005	ULK4	54986	unc-51 like kinase 4
ENSG0000	90.94841	-0.16562	0.171464	0.188154	0.599098	NPIPB4	440345	nuclear pore complex interacting protein family member B4
ENSG0000	315.0634	0.166488	0.196158	0.188189	0.599104	CACNA1I	8911	calcium voltage-gated channel subunit alpha1 I
ENSG0000	17.60696	0.157101	0.24417	0.1883	0.599354	IFT22	64792	intraflagellar transport 22
ENSG0000	13.45204	0.12198	0.248809	0.188408	0.59953	NA	NA	NA
ENSG0000	5905.098	-0.17096	0.188118	0.188421	0.59953	PLBD1	79887	phospholipase B domain containing 1
ENSG0000	141.5579	-0.16682	0.204147	0.188461	0.599553	SESTD1	91404	SEC14 and spectrin domain containing 1
ENSG0000	470.7154	0.146358	0.133727	0.188496	0.599561	NUDT5	11164	nudix hydrolase 5

ENSG0000	263.2026	-0.15728	0.156862	0.188553	0.599638	GNPDA1	10007	glucosamine-6-phosphate deaminase 1
ENSG0000	770.2988	0.161372	0.176574	0.188729	0.600095	CLUH	23277	clustered mitochondria homolog
ENSG0000	1224.974	-0.1686	0.192578	0.18884	0.60017	ALPK1	80216	alpha kinase 1
ENSG0000	49.40669	0.164195	0.229403	0.188839	0.60017	DCUN1D5	84259	defective in cullin neddylation 1 domain containing 5
ENSG0000	69.31204	-0.16889	0.197818	0.188851	0.60017	SIRPD	128646	signal regulatory protein delta
ENSG0000	118.5753	-0.16697	0.185	0.188925	0.600195	ANXA2R	389289	annexin A2 receptor
ENSG0000	695.8152	0.166703	0.18154	0.188894	0.600195	LINC0290€	401563	long intergenic non-protein coding RNA 2908
ENSG0000	717.1518	-0.16238	0.174573	0.188969	0.600232	N4BP2L2	10443	NEDD4 binding protein 2 like 2
ENSG0000	16.13107	-0.15699	0.244645	0.189027	0.600312	RTL8B	441518	retrotransposon Gag like 8B
ENSG0000	13.38796	-0.10712	0.246541	0.189079	0.600373	TFF3	7033	trefoil factor 3
ENSG0000	2372.586	0.162891	0.220109	0.189199	0.600649	DGAT2	84649	diacylglycerol O-acyltransferase 2
ENSG0000	11.08097	-0.095	0.244398	0.189355	0.600958	MYCT1	80177	MYC target 1
ENSG0000	29.64288	0.128998	0.245772	0.189396	0.600958	OTUD6B-A	1.01E+08	OTUD6B antisense RNA 1 (head to head)
ENSG0000	2548.039	-0.15995	0.164428	0.189427	0.600958	TUBB4B	10383	tubulin beta 4B class IVb
ENSG0000	9.893428	0.120896	0.250682	0.18941	0.600958	NA	NA	NA
ENSG0000	509.7714	0.152729	0.146885	0.189659	0.601029	UFC1	51506	ubiquitin-fold modifier conjugating enzyme 1
ENSG0000	3.010606	-0.08392	0.245731	0.189646	0.601029	DRC1	92749	dynein regulatory complex subunit 1
ENSG0000	390.0538	0.167281	0.205884	0.189642	0.601029	NAA50	80218	N-alpha-ac NatE catalytic subunit
ENSG0000	102.1454	0.150645	0.237707	0.189614	0.601029	HLA-DRB6	3128	major hist class II DR beta 6 (pseudogene)
ENSG0000	157.413	0.169121	0.204103	0.18968	0.601029	CDKN2A	1029	cyclin dependent kinase inhibitor 2A
ENSG0000	35.20106	-0.16172	0.233317	0.189579	0.601029	CETP	1071	cholesteryl ester transfer protein
ENSG0000	153.9794	0.166388	0.186035	0.189581	0.601029	ELP2	55250	elongator acetyltransferase complex subunit 2
ENSG0000	249.1383	0.148372	0.139727	0.189852	0.601473	SYNGR1	9145	synaptogyrin 1
ENSG0000	17.12842	-0.1444	0.246557	0.189956	0.601698	NA	NA	NA
ENSG0000	2595.641	0.155323	0.161312	0.190097	0.60204	MDH2	4191	malate dehydrogenase 2
ENSG0000	36.98554	0.171272	0.201721	0.19019	0.602229	RPL23AP3	130773	ribosomal protein L23a pseudogene 37
ENSG0000	6.649051	0.094751	0.245961	0.190263	0.602358	IL12A	3592	interleukin 12A
ENSG0000	10.56034	-0.13113	0.250044	0.190309	0.602399	PPFIBP1	8496	PPFIA binding protein 1
ENSG0000	430.0319	0.161847	0.178147	0.19036	0.60242	ATP5PB	515	ATP synthase peripheral stalk-membrane subunit b
ENSG0000	13.16506	-0.14226	0.248752	0.190381	0.60242	NA	NA	NA
ENSG0000	33.68768	-0.16368	0.230933	0.190503	0.602493	IER3-AS1	1.05E+08	IER3 antisense RNA 1
ENSG0000	830.179	-0.16424	0.194512	0.19047	0.602493	TMCC3	57458	transmembrane and coiled-coil domain family 3
ENSG0000	1587.186	-0.16328	0.179378	0.1905	0.602493	ZNF787	126208	zinc finger protein 787
ENSG0000	2.955041	-0.06853	0.241681	0.190597	0.602686	MOCOS	55034	molybdenum cofactor sulfurase
ENSG0000	679.7822	-0.15759	0.159474	0.19066	0.602747	ATG3	64422	autophagy related 3
ENSG0000	199.7316	-0.16296	0.174294	0.190682	0.602747	NA	NA	NA
ENSG0000	2220.9	-0.16397	0.196705	0.19074	0.602824	CHMP2A	27243	charged multivesicular body protein 2A
ENSG0000	112.7717	0.160205	0.224229	0.190839	0.603033	INCENP	3619	inner centromere protein
ENSG0000	58.55848	0.166083	0.217632	0.190936	0.603033	TIMM17A	10440	translocase of inner mitochondrial membrane 17A
ENSG0000	194.612	0.140649	0.237279	0.190951	0.603033	VWCE	220001	von Willebrand factor C and EGF domains
ENSG0000	1424.287	-0.15921	0.165177	0.19097	0.603033	CXCL16	58191	C-X-C motif chemokine ligand 16
ENSG0000	660.5498	-0.14948	0.142896	0.190914	0.603033	ME2	4200	malic enzyme 2
ENSG0000	4.710283	0.093906	0.246698	0.191027	0.603109	NPAS1	4861	neuronal PAS domain protein 1
ENSG0000	4.442925	-0.08764	0.244572	0.191093	0.603213	NA	NA	NA
ENSG0000	24.29695	0.169357	0.224529	0.191267	0.603296	PIGZ	80235	phosphatidylinositol glycan anchor biosynthesis class Z
ENSG0000	152.1507	0.160573	0.220552	0.191341	0.603296	SAR1B	51128	secretion associated Ras related GTPase 1B
ENSG0000	68.11146	-0.14713	0.237759	0.191397	0.603296	CALHM6	441168	calcium homeostasis modulator family member 6
ENSG0000	128.7956	0.130246	0.241056	0.19134	0.603296	ECHDC1	55862	ethylmalonyl-CoA decarboxylase 1
ENSG0000	15.41489	0.163752	0.238314	0.191207	0.603296	MRPS17	51373	mitochondrial ribosomal protein S17
ENSG0000	130.4918	0.154464	0.231504	0.191415	0.603296	SMC5	23137	structural maintenance of chromosomes 5
ENSG0000	3.20187	0.07365	0.242213	0.191153	0.603296	NA	NA	NA
ENSG0000	192.7172	0.155411	0.157107	0.19131	0.603296	CHTF18	63922	chromosome transmission fidelity factor 18
ENSG0000	17177.41	-0.16099	0.173107	0.191416	0.603296	EVI2B	2124	ecotropic viral integration site 2B
ENSG0000	2.967269	0.085324	0.247834	0.191561	0.603315	NA	NA	NA
ENSG0000	4369.183	-0.15913	0.180338	0.191652	0.603315	ARID5A	10865	AT-rich interaction domain 5A
ENSG0000	48.16267	0.1647	0.224969	0.191514	0.603315	AASDH	132949	aminoadipate-semialdehyde dehydrogenase
ENSG0000	106.007	-0.16531	0.197627	0.191644	0.603315	ZSCAN26	7741	zinc finger and SCAN domain containing 26
ENSG0000	383.6799	-0.15461	0.156799	0.191638	0.603315	LZTS2	84445	leucine zipper tumor suppressor 2
ENSG0000	12982.33	-0.15729	0.161127	0.191551	0.603315	RAB1B	81876	RAB1B member RAS oncogene family
ENSG0000	39.95658	0.154462	0.236339	0.191476	0.603315	ATP5PO	539	ATP synthase peripheral stalk subunit OSCP
ENSG0000	93.63862	0.166114	0.182436	0.191725	0.603439	PIP5K1B	8395	phosphatidylinositol-4-phosphate 5-kinase type 1 beta
ENSG0000	10.58372	-0.13083	0.250032	0.191892	0.60346	TRIM17	51127	tripartite motif containing 17
ENSG0000	2.926102	0.076847	0.245606	0.191962	0.60346	NA	NA	NA
ENSG0000	11.63733	0.128915	0.249859	0.191834	0.60346	NA	NA	NA
ENSG0000	2.529474	0.04787	0.236969	0.191951	0.60346	NA	NA	NA
ENSG0000	1048.202	-0.15488	0.164314	0.191801	0.60346	PPIF	10105	peptidylprolyl isomerase F
ENSG0000	591.7787	-0.15973	0.149486	0.191893	0.60346	ATF7	11016	activating transcription factor 7
ENSG0000	148.5791	-0.16228	0.214621	0.191939	0.60346	MPHOSPH	643802	MPHOSPH10 pseudogene 1
ENSG0000	216.2215	0.154359	0.150144	0.192105	0.603807	SLC41A3	54946	solute carrier family 41 member 3
ENSG0000	37.63963	0.160995	0.229185	0.192218	0.603954	NOPCHAP	121053	NOP protein chaperone 1
ENSG0000	177.381	0.157524	0.158464	0.19222	0.603954	ACADS	35	acyl-CoA dehydrogenase short chain
ENSG0000	34.02065	-0.12546	0.244483	0.192251	0.603954	NA	NA	NA
ENSG0000	156.1318	0.162854	0.170135	0.192369	0.603983	FH	2271	fumarate hydratase
ENSG0000	89.23439	0.167759	0.199237	0.192311	0.603983	WDR36	134430	WD repeat domain 36
ENSG0000	9.290885	-0.10064	0.24559	0.192392	0.603983	TLCD3A	79850	TLC domain containing 3A
ENSG0000	1204.879	-0.16389	0.187643	0.192354	0.603983	RRAS	6237	RAS related
ENSG0000	240.2309	-0.15581	0.156152	0.19246	0.603988	TTC22	55001	tetratricopeptide repeat domain 22
ENSG0000	996.7884	-0.14378	0.134294	0.19243	0.603988	CD2BP2	10421	CD2 cytoplasmic tail binding protein 2
ENSG0000	183.9024	0.15464	0.229353	0.19256	0.6042	KAZN	23254	kazrin periplakin interacting protein
ENSG0000	2.55695	0.04353	0.23571	0.192616	0.604273	NA	NA	NA
ENSG0000	78.63827	-0.16645	0.190787	0.192808	0.604628	SLC35D1	23169	solute carrier family 35 member D1
ENSG0000	2.727752	0.057887	0.239313	0.192841	0.604628	EREG	2069	epiregulin
ENSG0000	7.8997	-0.09516	0.244835	0.192988	0.604628	NA	NA	NA
ENSG0000	67.27	0.132346	0.244225	0.192862	0.604628	CCNC	892	cyclin C
ENSG0000	92.46208	0.166434	0.197427	0.192929	0.604628	DDHD2	23259	DDHD domain containing 2
ENSG0000	155.5238	-0.15744	0.159576	0.192923	0.604628	UBFD1	56061	ubiquitin family domain containing 1
ENSG0000	6.010629	-0.10649	0.248635	0.192846	0.604628	NA	NA	NA
ENSG0000	6.333112	0.068921	0.240107	0.192994	0.604628	HAR1B	768097	highly accelerated region 1B
ENSG0000	345.942	0.165855	0.184805	0.193084	0.604807	KIAA0232	9778	KIAA0232
ENSG0000	18.05544	0.161196	0.237459	0.193186	0.604902	RBM33-D1	1.01E+08	RBM33 divergent transcript
ENSG0000	492.042	-0.14626	0.13555	0.193213	0.604902	VPS4A	27183	vacuolar protein sorting 4 homolog A
ENSG0000	121.7089	-0.16453	0.199084	0.193211	0.604902	ZNF224	7767	zinc finger protein 224
ENSG0000	538.7684	-0.16388	0.188525	0.193321	0.605135	RIOK3	8780	RIO kinase 3
ENSG0000	14.93482	0.138159	0.248176	0.193616	0.605956	NA	NA	NA
ENSG0000	31.61005	0.147591	0.240774	0.193746	0.606061	TYW3	127253	tRNA-yW synthesizing protein 3 homolog
ENSG0000	4.116992	-0.07494	0.24169	0.193821	0.606061	CGN	57530	cingulin

ENSG0000	10.31268	0.13642	0.250919	0.193848	0.606061	FKBP1B	2281	FKBP prolyl isomerase 1B
ENSG0000	191.2505	0.160306	0.168373	0.193773	0.606061	STN1	79991	STN1 subunit of CST complex
ENSG0000	46.58233	-0.13162	0.241836	0.193803	0.606061	ACE	1636	angiotensin I converting enzyme
ENSG0000	27.5717	0.123036	0.245441	0.193691	0.606061	NEFH	4744	neurofilament heavy chain
ENSG0000	184.1445	0.160068	0.167392	0.193916	0.606073	DNAJC30	84277	DnaJ heat shock protein family (Hsp40) member C30
ENSG0000	84.90686	0.165933	0.202321	0.193951	0.606073	ZNF195	7748	zinc finger protein 195
ENSG0000	823.3184	0.153083	0.151474	0.193921	0.606073	GTF3A	2971	general transcription factor IIIA
ENSG0000	15185.13	-0.16251	0.203196	0.19399	0.606091	RARA	5914	retinoic acid receptor alpha
ENSG0000	52.32833	0.166842	0.207474	0.194121	0.60619	SGPP2	130367	sphingosine-1-phosphate phosphatase 2
ENSG0000	408.136	-0.15117	0.150309	0.194066	0.60619	GSTO1	9446	glutathione S-transferase omega 1
ENSG0000	321.0958	0.159871	0.169806	0.194121	0.60619	PEMT	10400	phosphatidylethanolamine N-methyltransferase
ENSG0000	95.29751	0.158406	0.164113	0.194164	0.606221	FBXO25	26260	F-box protein 25
ENSG0000	132.8992	0.165222	0.189553	0.194251	0.606286	TMEM201	199953	transmembrane protein 201
ENSG0000	313.3593	-0.16019	0.171661	0.194242	0.606286	ARSD	414	arylsulfatase D
ENSG0000	42.79568	-0.14759	0.238269	0.194323	0.606303	GRIK5	2901	glutamate ionotropic receptor kainate type subunit 5
ENSG0000	16.44347	-0.14746	0.24521	0.194294	0.606303	NA	NA	NA
ENSG0000	7.646481	-0.09294	0.244758	0.194404	0.60635	NA	NA	NA
ENSG0000	255.2514	0.162609	0.214777	0.19438	0.60635	FAR2	55711	fatty acyl-CoA reductase 2
ENSG0000	1316.034	0.163165	0.185445	0.194454	0.606403	PRMT1	3276	protein arginine methyltransferase 1
ENSG0000	6.219494	-0.1174	0.250689	0.194607	0.606587	NA	NA	NA
ENSG0000	7.739543	0.084288	0.242645	0.194607	0.606587	SMIM33	1.11E+08	small integral membrane protein 33
ENSG0000	2.705543	0.054632	0.238349	0.194613	0.606587	MTDHP1	1E+08	metadherin pseudogene 1
ENSG0000	17.33133	0.099535	0.243183	0.19469	0.606622	ST6GALNA	30815	ST6 N-acet 6-sialyltransferase 6
ENSG0000	1605.347	-0.14293	0.233106	0.194668	0.606622	ITGA2B	3674	integrin subunit alpha 2b
ENSG0000	2572.532	-0.13392	0.235124	0.194744	0.606688	HSPA1A	3303	heat shock protein family A (Hsp70) member 1A
ENSG0000	3.988419	-0.08383	0.243633	0.19484	0.606767	LOC10192	1.02E+08	uncharacterized LOC101929536
ENSG0000	1539.807	0.158357	0.165011	0.194869	0.606767	OAZ2	4947	ornithine decarboxylase antizyme 2
ENSG0000	1534.845	-0.15625	0.163229	0.194843	0.606767	TM9SF4	9777	transmembrane 9 superfamily member 4
ENSG0000	369.3536	-0.14659	0.138102	0.194994	0.606879	SYF2	25949	SYF2 pre-mRNA splicing factor
ENSG0000	1003.469	-0.16051	0.215924	0.19501	0.606879	TLR5	7100	toll like receptor 5
ENSG0000	56.33282	-0.16775	0.192248	0.195023	0.606879	NA	NA	NA
ENSG0000	3124.434	0.133731	0.120449	0.195037	0.606879	ATP5MC2	517	ATP synthase membrane subunit c locus 2
ENSG0000	5.992046	-0.07384	0.240456	0.195141	0.607099	SULT1C4	27233	sulfotransferase family 1C member 4
ENSG0000	4249.943	-0.12822	0.114678	0.195187	0.607138	SZRD1	26099	SUZ RNA binding domain containing 1
ENSG0000	5.836873	0.101858	0.252675	0.195274	0.607304	MAFIP	727764	MAFF interacting protein (pseudogene)
ENSG0000	2386.122	-0.15915	0.189739	0.195354	0.607333	DTX2	113878	deltex E3 ubiquitin ligase 2
ENSG0000	137.6768	0.164386	0.183832	0.19535	0.607333	NA	NA	NA
ENSG0000	44.79771	-0.15919	0.228715	0.195382	0.607333	ANKRD24	170961	ankyrin repeat domain 24
ENSG0000	8.683395	-0.11835	0.24991	0.195617	0.607752	CR1L	1379	complement C3b/C4b receptor 1 like
ENSG0000	50.33777	0.161504	0.22087	0.195589	0.607752	WDR12	55759	WD repeat domain 12
ENSG0000	119.931	0.15514	0.154906	0.195558	0.607752	TDG	6996	thymine DNA glycosylase
ENSG0000	14.99874	0.121457	0.249455	0.195764	0.607921	CENPE	1062	centromere protein E
ENSG0000	96.47473	-0.16377	0.202412	0.195771	0.607921	TBPL1	9519	TATA-box binding protein like 1
ENSG0000	7.038201	-0.10883	0.247648	0.195749	0.607921	H3-5	440093	H3.5 histone
ENSG0000	3.028451	0.04361	0.23571	0.196044	0.608056	NA	NA	NA
ENSG0000	45.68745	0.16095	0.227045	0.195992	0.608056	MRPL22	29093	mitochondrial ribosomal protein L22
ENSG0000	3.277222	0.081881	0.245291	0.196047	0.608056	SLC34A1	6569	solute carrier family 34 member 1
ENSG0000	924.9113	-0.15756	0.170348	0.19597	0.608056	LAMTOR4	389541	late endos MAPK and MTOR activator 4
ENSG0000	31.54492	0.159744	0.236789	0.196015	0.608056	PGM5	5239	phosphoglucomutase 5
ENSG0000	702.6637	-0.16161	0.198336	0.195977	0.608056	CYLD	1540	CYLD lysine 63 deubiquitinase
ENSG0000	20.66244	-0.07596	0.239171	0.195971	0.608056	IGLV5-48	28780	immunoglobulin lambda variable 5-48 (non-functional)
ENSG0000	345.2666	0.149742	0.143245	0.196299	0.608337	IER5	51278	immediate early response 5
ENSG0000	10.16738	0.087201	0.242326	0.196251	0.608337	MTBP	27085	MDM2 binding protein
ENSG0000	1570.339	0.153634	0.157102	0.196221	0.608337	PRDX5	25824	peroxiredoxin 5
ENSG0000	113.2591	0.161892	0.17514	0.19621	0.608337	MRGBP	55257	MRG domain binding protein
ENSG0000	249.5053	-0.1594	0.172146	0.196303	0.608337	LINC0064(	1.01E+08	long intergenic non-protein coding RNA 649
ENSG0000	138.0685	-0.16173	0.177112	0.196353	0.608388	NA	NA	NA
ENSG0000	2.410138	0.057366	0.238199	0.19647	0.608465	GCC2-AS1	644903	GCC2 antisense RNA 1
ENSG0000	9.25108	-0.11782	0.249293	0.196581	0.608465	TSPAN14-/	1.02E+08	TSPAN14 antisense RNA 1
ENSG0000	5.397256	0.073724	0.24043	0.196532	0.608465	LINC01465	283416	long intergenic non-protein coding RNA 1465
ENSG0000	34.32586	0.167457	0.213006	0.196438	0.608465	CRY1	1407	cryptochrome circadian regulator 1
ENSG0000	4794.572	0.163038	0.195977	0.196525	0.608465	DPEP2	64174	dipeptidase 2
ENSG0000	16.70247	-0.07796	0.239566	0.19661	0.608465	EHD2	30846	EH domain containing 2
ENSG0000	102.0884	0.160663	0.217167	0.196587	0.608465	EIF1AX	1964	eukaryotic translation initiation factor 1A X-linked
ENSG0000	1253.577	-0.13816	0.128592	0.196718	0.608594	MARK3	4140	microtubule affinity regulating kinase 3
ENSG0000	35.14421	0.154781	0.235918	0.19669	0.608594	ZNF205	7755	zinc finger protein 205
ENSG0000	31.64773	-0.13232	0.244208	0.19684	0.608857	NA	NA	NA
ENSG0000	623.4957	-0.14939	0.146674	0.19687	0.608857	ANKH	56172	ANKH inorganic pyrophosphate transport regulator
ENSG0000	2302.642	0.161711	0.208793	0.197034	0.609263	SULF2	55959	sulfatase 2
ENSG0000	965.7393	-0.16111	0.179756	0.197075	0.609286	TOR1AIP1	26092	torsin 1A interacting protein 1
ENSG0000	3.639504	-0.08703	0.245207	0.197152	0.609422	C2orf66	401027	chromosome 2 open reading frame 66
ENSG0000	66.99872	0.163803	0.209333	0.197211	0.609499	WDR43	23160	WD repeat domain 43
ENSG0000	20887.75	0.157924	0.166988	0.197245	0.609503	MTATP6P1	1.06E+08	MT-ATP6 pseudogene 1
ENSG0000	104.1097	0.161156	0.2208	0.197293	0.609547	IGKV3D-2(	28874	immunoglobulin kappa variable 3D-20
ENSG0000	10.38396	0.094379	0.243774	0.197663	0.609972	IGKV2D-2ł	28883	immunoglobulin kappa variable 2D-28
ENSG0000	51.85179	0.16602	0.19423	0.19752	0.609972	METTL5	29081	methyltrar N6-adenosine
ENSG0000	94.30862	0.162549	0.17823	0.19758	0.609972	RNF7	9616	ring finger protein 7
ENSG0000	11.57422	0.146999	0.247207	0.197533	0.609972	NA	NA	NA
ENSG0000	3.385284	0.062545	0.239902	0.197637	0.609972	IGHD1-26	28506	immunoglobulin heavy diversity 1-26
ENSG0000	39.09534	-0.16424	0.216368	0.197575	0.609972	KIF1C-AS1	1.03E+08	KIF1C antisense RNA 1
ENSG0000	198.6911	0.155896	0.15866	0.197626	0.609972	ALDH3A2	224	aldehyde dehydrogenase 3 family member A2
ENSG0000	69.01505	0.150957	0.23411	0.197709	0.610011	OXR1	55074	oxidation resistance 1
ENSG0000	76.14787	-0.16326	0.211514	0.19778	0.610128	NA	NA	NA
ENSG0000	347.6678	0.161552	0.18721	0.197814	0.610128	NEK6	10783	NIMA related kinase 6
ENSG0000	105.1482	0.162062	0.183263	0.19786	0.61017	COX11	1353	cytochrome c oxidase copper chaperone COX11
ENSG0000	4.086466	0.078323	0.242749	0.197962	0.610356	NA	NA	NA
ENSG0000	1711.484	-0.15379	0.157743	0.197987	0.610356	HCST	10870	hematopoietic cell signal transducer
ENSG0000	1089.326	-0.15676	0.164642	0.198091	0.610573	SIT1	27240	signaling threshold regulating transmembrane adaptor 1
ENSG0000	3.379273	0.092499	0.24692	0.198177	0.610633	NA	NA	NA
ENSG0000	5.450841	-0.09154	0.245701	0.198148	0.610633	NA	NA	NA
ENSG0000	10.0638	0.106053	0.246258	0.198244	0.610735	FAM24B	196792	family with sequence similarity 24 member B
ENSG0000	128.4902	0.16089	0.171971	0.198395	0.610892	SEC61G	23480	SEC61 translocon subunit gamma
ENSG0000	67.01659	-0.16381	0.186154	0.198374	0.610892	C8orf44	56260	chromosome 8 putative open reading frame 44
ENSG0000	21695.78	-0.15781	0.170978	0.19835	0.610892	AKNA	80709	AT-hook transcription factor
ENSG0000	11.81405	-0.12429	0.247353	0.198447	0.61095	HBEGF	1839	heparin binding EGF like growth factor
ENSG0000	53.89048	0.162753	0.220463	0.198629	0.611305	PROB1	389333	proline rich basic protein 1

ENSG0000	10.01166	0.146826	0.248239	0.198622	0.611305	STRIP2	57464	striatin interacting protein 2
ENSG0000	19.92872	0.144401	0.24377	0.19869	0.611359	LOC12490	1.25E+08	uncharacterized LOC124904535
ENSG0000	8381.956	-0.16486	0.208791	0.198713	0.611359	IL32	9235	interleukin 32
ENSG0000	49.5151	-0.13724	0.242305	0.198896	0.611819	DRC7	84229	dynein regulatory complex subunit 7
ENSG0000	11.36073	0.120803	0.247323	0.199064	0.61182	RMDN2	151393	regulator of microtubule dynamics 2
ENSG0000	323.5577	-0.16219	0.193809	0.199029	0.61182	MIR4435-2	541471	MIR4435-2 host gene
ENSG0000	1174.608	-0.16416	0.179296	0.198989	0.61182	CARD11	84433	caspase recruitment domain family member 11
ENSG0000	17.41501	0.153584	0.240517	0.199003	0.61182	NA	NA	NA
ENSG0000	21683.9	-0.14485	0.140252	0.199036	0.61182	LIMD2	80774	LIM domain containing 2
ENSG0000	544.027	0.145511	0.140042	0.199104	0.611842	WDFY2	115825	WD repeat and FYVE domain containing 2
ENSG0000	62.34269	-0.16078	0.172942	0.199268	0.61209	DDIAS	220042	DNA damage induced apoptosis suppressor
ENSG0000	5.44648	-0.09265	0.244645	0.199245	0.61209	NA	NA	NA
ENSG0000	4.064976	-0.04832	0.236423	0.199285	0.61209	TIAM1-AS	150051	TIAM1 antisense RNA 1
ENSG0000	25.36988	0.151932	0.237646	0.199365	0.612231	CTTNBP2N	55917	CTTNBP2 N-terminal like
ENSG0000	2.603917	0.039399	0.235403	0.199428	0.612322	DDX19A-D	1.01E+08	DDX19A divergent transcript
ENSG0000	7.916719	-0.07104	0.239768	0.199636	0.612784	IL6R-AS1	1.02E+08	IL6R antisense RNA 1
ENSG0000	11.9947	0.111315	0.246167	0.199645	0.612784	TPRG1	285386	tumor protein p63 regulated 1
ENSG0000	369.4823	0.152733	0.154881	0.199708	0.612875	C1orf159	54991	chromosome 1 open reading frame 159
ENSG0000	106.8534	0.152623	0.153083	0.19989	0.61333	ZCCHC24	219654	zinc finger CCHC-type containing 24
ENSG0000	6.687109	0.108409	0.247626	0.199958	0.613436	RPL6P10	642828	ribosomal protein L6 pseudogene 10
ENSG0000	66.59463	0.163575	0.18752	0.200032	0.613561	NA	NA	NA
ENSG0000	367.6506	-0.15566	0.160693	0.200134	0.613771	MAML2	84441	mastermind like transcriptional coactivator 2
ENSG0000	79.84607	0.163124	0.181524	0.200304	0.614032	TMEM53	79639	transmembrane protein 53
ENSG0000	130.4911	0.141131	0.236623	0.200454	0.614032	RALGPS2	55103	Ral GEF with PH domain and SH3 binding motif 2
ENSG0000	8347.819	-0.15544	0.165282	0.200368	0.614032	MGAT1	4245	alpha-1 3-mannosyl-glycoprotein 2-beta-N-acetylglucosaminyltransferase
ENSG0000	42.09936	-0.14132	0.238745	0.20042	0.614032	NA	NA	NA
ENSG0000	252.4086	-0.14568	0.140729	0.200425	0.614032	NAIF1	203245	nuclear apoptosis inducing factor 1
ENSG0000	3.07675	0.067746	0.24075	0.200406	0.614032	NA	NA	NA
ENSG0000	329.852	0.155947	0.158037	0.200294	0.614032	PGAP3	93210	post-GPI attachment to proteins phospholipase 3
ENSG0000	4.230044	-0.05155	0.236558	0.200579	0.614313	NA	NA	NA
ENSG0000	568.8516	-0.15964	0.183228	0.200622	0.614342	ZNF341	84905	zinc finger protein 341
ENSG0000	39.95148	-0.10465	0.242119	0.200784	0.614735	NA	NA	NA
ENSG0000	63.41064	-0.16286	0.209351	0.200824	0.614756	ZNF844	284391	zinc finger protein 844
ENSG0000	32.38052	-0.14299	0.243255	0.200898	0.614775	NA	NA	NA
ENSG0000	228.2682	0.155544	0.162427	0.200896	0.614775	THEM4	117145	thioesterase superfamily member 4
ENSG0000	33.37107	0.163491	0.214732	0.201036	0.614787	TMEM86A	144110	transmembrane protein 86A
ENSG0000	20.66672	0.102733	0.242932	0.201006	0.614787	NA	NA	NA
ENSG0000	3267.675	-0.15887	0.179752	0.20102	0.614787	PSTPIP1	9051	proline-serine-threonine phosphatase interacting protein 1
ENSG0000	21.82746	0.137461	0.244241	0.200967	0.614787	SIGLEC6	946	sialic acid binding Ig like lectin 6
ENSG0000	128.0104	0.153397	0.156005	0.201143	0.614838	PEX10	5192	peroxisomal biogenesis factor 10
ENSG0000	366.4242	0.162641	0.177261	0.201108	0.614838	PCGF3	10336	polycomb group ring finger 3
ENSG0000	40.2513	0.125857	0.243431	0.201153	0.614838	C3	718	complement C3
ENSG0000	1311.623	0.153332	0.169288	0.201309	0.615211	TUFM	7284	Tu translat mitochondrial
ENSG0000	280.0383	0.140102	0.135892	0.20136	0.615265	CPSF4	10898	cleavage and polyadenylation specific factor 4
ENSG0000	519.5397	-0.13864	0.232738	0.201418	0.615334	MIER1	57708	MIER1 transcriptional regulator
ENSG0000	3.095401	-0.05181	0.238835	0.201577	0.615334	LRRC2	79442	leucine rich repeat containing 2
ENSG0000	2.769521	-0.02557	0.234741	0.201527	0.615334	NA	NA	NA
ENSG0000	16.20358	-0.13299	0.245165	0.201584	0.615334	HOXA1	3198	homeobox A1
ENSG0000	472.4447	-0.15287	0.158366	0.201503	0.615334	JOSD2	126119	Josephin domain containing 2
ENSG0000	4.854622	-0.0726	0.241839	0.201471	0.615334	NA	NA	NA
ENSG0000	146.4896	-0.1541	0.221793	0.201628	0.615366	ADM2	79924	adrenomedullin 2
ENSG0000	12.75463	0.140302	0.246526	0.201931	0.616186	ZDHHC11E	653082	zinc finger DHHC-type containing 11B
ENSG0000	2.347546	-0.06277	0.239162	0.20217	0.616268	H2AC18	8337	H2A clustered histone 18
ENSG0000	336.8481	-0.13586	0.126186	0.202238	0.616268	CCDC174	51244	coiled-coil domain containing 174
ENSG0000	27.08398	0.153145	0.23604	0.202294	0.616268	CDC25A	993	cell division cycle 25A
ENSG0000	35.76838	0.141376	0.241665	0.202236	0.616268	FKBP9	11328	FKBP prolyl isomerase 9
ENSG0000	37.02	0.164736	0.198466	0.20214	0.616268	KRIT1	889	KRIT1 ankyrin repeat containing
ENSG0000	113.8971	0.160185	0.164008	0.202253	0.616268	FAM83H	286077	family with sequence similarity 83 member H
ENSG0000	21.44283	-0.11636	0.245134	0.202256	0.616268	UBQLN1-A	1.05E+08	UBQLN1 antisense RNA 1
ENSG0000	2.801484	-0.0416	0.236002	0.202276	0.616268	LINC00677	1.05E+08	long intergenic non-protein coding RNA 677
ENSG0000	151.4394	0.155629	0.22134	0.202264	0.616268	NA	NA	NA
ENSG0000	3.437736	0.067507	0.240098	0.202124	0.616268	ZNF726	730087	zinc finger protein 726
ENSG0000	8.513846	-0.13877	0.248843	0.202387	0.61645	LOC55420	554206	leucine carboxyl methyltransferase 1 pseudogene
ENSG0000	283.9578	-0.14941	0.151328	0.202561	0.61655	GPATCH3	63906	G-patch domain containing 3
ENSG0000	89.16851	0.160215	0.180111	0.202503	0.61655	CTPS1	1503	CTP synthase 1
ENSG0000	21.74776	0.137131	0.243475	0.202669	0.61655	CDHR2	54825	cadherin related family member 2
ENSG0000	117.9027	0.152351	0.160828	0.202689	0.61655	MPC1	51660	mitochondrial pyruvate carrier 1
ENSG0000	316.5568	0.155977	0.217703	0.20252	0.61655	AGTPBP1	23287	ATP/GTP binding carboxypeptidase 1
ENSG0000	2134.516	-0.15618	0.172349	0.202609	0.61655	FRAT1	10023	FRAT regulator of WNT signaling pathway 1
ENSG0000	62.89813	-0.1538	0.221573	0.202658	0.61655	PTGDR	5729	prostaglandin D2 receptor
ENSG0000	139.4808	0.163286	0.208985	0.202679	0.61655	ZNF83	55769	zinc finger protein 83
ENSG0000	23.88024	0.154497	0.23337	0.202735	0.616586	ZNF469	84627	zinc finger protein 469
ENSG0000	334.1519	0.155249	0.218458	0.202844	0.616805	CDC20	991	cell division cycle 20
ENSG0000	2.866988	-0.05884	0.239419	0.202987	0.616805	RN7SL589	1.06E+08	RNA 7SL cytoplasm pseudogene
ENSG0000	889.8452	0.134225	0.125006	0.202914	0.616805	MED22	6837	mediator complex subunit 22
ENSG0000	39.34583	0.166996	0.20691	0.202963	0.616805	RNFT2	84900	ring finger transmembrane 2
ENSG0000	308.8559	0.148967	0.149356	0.202916	0.616805	MAP2K5	5607	mitogen-activated protein kinase kinase 5
ENSG0000	4.991576	-0.08912	0.244309	0.203009	0.616805	NA	NA	NA
ENSG0000	117.6576	0.158833	0.180094	0.203056	0.616846	FEN1	2237	flap structure-specific endonuclease 1
ENSG0000	48.99133	-0.16337	0.194513	0.203179	0.616893	PRIM2	5558	DNA primase subunit 2
ENSG0000	16.21782	0.086351	0.240605	0.203206	0.616893	SSC4D	136853	scavenger receptor cysteine rich family member with 4 domains
ENSG0000	298.343	-0.1574	0.169372	0.203178	0.616893	TTC16	158248	tetratricopeptide repeat domain 16
ENSG0000	360.9782	0.15325	0.177845	0.203188	0.616893	ANKRD10	55608	ankyrin repeat domain 10
ENSG0000	3.707365	0.06217	0.238381	0.203265	0.616968	P2RY1	5028	purinergic receptor P2Y1
ENSG0000	351.8152	0.165266	0.207288	0.203443	0.617381	CAMK1	8536	calcium/calmodulin dependent protein kinase I
ENSG0000	16.53187	0.109007	0.244168	0.203468	0.617381	GRIN3B	116444	glutamate ionotropic receptor NMDA type subunit 3B
ENSG0000	214.5166	0.153779	0.155561	0.203513	0.617414	TMEM160	54958	transmembrane protein 160
ENSG0000	319.1764	0.146259	0.144398	0.203682	0.617723	CLCN6	1185	chloride voltage-gated channel 6
ENSG0000	116.6563	-0.1575	0.174074	0.20365	0.617723	BCLAF3	256643	BCLAF1 and THRAP3 family member 3
ENSG0000	1903.021	0.156987	0.168059	0.20386	0.618161	CPSF1	29894	cleavage and polyadenylation specific factor 1
ENSG0000	17.77159	0.116007	0.245084	0.203929	0.618268	ITGAV	3685	integrin subunit alpha V
ENSG0000	10658.15	-0.15726	0.19794	0.204064	0.618409	FERMT3	83706	FERM domain containing kindlin 3
ENSG0000	36.01001	-0.15001	0.235922	0.204099	0.618409	NA	NA	NA
ENSG0000	100.2377	0.160756	0.186386	0.204111	0.618409	TNFRSF12A	51330	TNF receptor superfamily member 12A
ENSG0000	7527.587	0.159423	0.192499	0.204014	0.618409	POU2F2	5452	POU class 2 homeobox 2
ENSG0000	202.7807	-0.15527	0.167089	0.204248	0.618547	THADA	63892	THADA armadillo repeat containing

ENSG0000	3.183324	0.0443	0.236262	0.204291	0.618547	LMCD1	29995	LIM and cysteine rich domains 1
ENSG0000	3.029595	0.046054	0.236053	0.204205	0.618547	KDM4D	55693	lysine demethylase 4D
ENSG0000	7.601405	0.133526	0.249486	0.204262	0.618547	NA	NA	NA
ENSG0000	5.346097	0.074952	0.241911	0.204376	0.618584	SCRG1	11341	stimulator of chondrogenesis 1
ENSG0000	42.81321	0.138427	0.239603	0.204473	0.618584	ARHGAP1:	94134	Rho GTPase activating protein 12
ENSG0000	238.4578	0.15898	0.182372	0.204438	0.618584	FOXRED1	55572	FAD dependent oxidoreductase domain containing 1
ENSG0000	210.7024	-0.14715	0.147548	0.204452	0.618584	KCTD11	147040	potassium channel tetramerization domain containing 11
ENSG0000	6.246263	-0.055	0.236967	0.204371	0.618584	NA	NA	NA
ENSG0000	6.726057	0.074817	0.24027	0.204566	0.618765	NA	NA	NA
ENSG0000	132.0823	-0.15414	0.162365	0.204653	0.618924	LENG1	79165	leukocyte receptor cluster member 1
ENSG0000	5.303546	0.068518	0.238976	0.204833	0.618953	NA	NA	NA
ENSG0000	4461.93	-0.15391	0.165876	0.20481	0.618953	YIPF3	25844	Yip1 domain family member 3
ENSG0000	4.430727	-0.09962	0.24718	0.204899	0.618953	CFAP418-/	1.01E+08	CFAP418 antisense RNA 1
ENSG0000	883.007	-0.1295	0.118366	0.204829	0.618953	TRADD	8717	TNFRSF1A associated via death domain
ENSG0000	8.725855	0.130701	0.249252	0.204897	0.618953	INCA1	388324	inhibitor o cyclin A1 interacting protein 1
ENSG0000	17.41744	0.144714	0.241726	0.204846	0.618953	ZIK1	284307	zinc finger protein interacting with K protein 1
ENSG0000	8.040966	0.074559	0.239698	0.204818	0.618953	NA	NA	NA
ENSG0000	29.47648	0.141954	0.239824	0.205024	0.619231	CAAP1	79886	caspase activity and apoptosis inhibitor 1
ENSG0000	158.1388	0.06945	0.237727	0.205073	0.619276	IGHV3-49	28423	immunoglobulin heavy variable 3-49
ENSG0000	143.2293	0.135338	0.237616	0.205346	0.619517	AQP10	89872	aquaporin 10
ENSG0000	37.85415	0.159887	0.21887	0.205192	0.619517	NA	NA	NA
ENSG0000	538.6848	0.144426	0.1415	0.205304	0.619517	GSR	2936	glutathione-disulfide reductase
ENSG0000	4.589861	-0.09143	0.244458	0.20526	0.619517	NA	NA	NA
ENSG0000	414.9225	0.157236	0.175701	0.205356	0.619517	PSIP1	11168	PC4 and SFRS1 interacting protein 1
ENSG0000	966.6587	0.16895	0.177375	0.205233	0.619517	TMED10	10972	transmembrane p24 trafficking protein 10
ENSG0000	293.0456	0.145951	0.148557	0.20542	0.619518	ACAD9	28976	acyl-CoA dehydrogenase family member 9
ENSG0000	180.2534	0.146246	0.146341	0.205433	0.619518	DDX28	55794	DEAD-box helicase 28
ENSG0000	124.9882	0.151942	0.154816	0.205458	0.619518	ZNF653	115950	zinc finger protein 653
ENSG0000	244.7204	-0.15711	0.176594	0.205548	0.619586	GPAT3	84803	glycerol-3-phosphate acyltransferase 3
ENSG0000	2.35054	0.081917	0.244202	0.205546	0.619586	RPL35P5	441246	ribosomal protein L35 pseudogene 5
ENSG0000	28.6892	0.127615	0.242863	0.205622	0.619607	C10orf88	80007	chromosome 10 open reading frame 88
ENSG0000	6.727287	0.086794	0.24342	0.20559	0.619607	ODAD4	83538	outer dynein arm docking complex subunit 4
ENSG0000	190.879	-0.15821	0.199066	0.205751	0.619689	DHRS3	9249	dehydrogenase/reductase 3
ENSG0000	333.2364	0.149485	0.152124	0.205725	0.619689	PREP	5550	prolyl endopeptidase
ENSG0000	4.42526	-0.05463	0.237788	0.205728	0.619689	LOC10192	1.02E+08	uncharacterized LOC101927018
ENSG0000	52.04841	0.145337	0.235132	0.20586	0.619813	ZNF84	7637	zinc finger protein 84
ENSG0000	11.17252	0.092374	0.242221	0.205859	0.619813	NA	NA	NA
ENSG0000	369.4518	-0.14885	0.151851	0.205957	0.620003	PSMA5	5686	proteasome 20S subunit alpha 5
ENSG0000	8730.084	-0.13672	0.12742	0.206016	0.620078	TMBIM6	7009	transmembrane BAX inhibitor motif containing 6
ENSG0000	355.6776	-0.15394	0.217286	0.206069	0.620136	PIK3R6	146850	phosphoinositide-3-kinase regulatory subunit 6
ENSG0000	28.4515	-0.15476	0.228668	0.206262	0.620616	ATP1A3	478	ATPase Na+/K+ transporting subunit alpha 3
ENSG0000	384.0118	0.158425	0.185859	0.206418	0.620779	SNX18	112574	sorting nexin 18
ENSG0000	853.2207	-0.16086	0.18265	0.206417	0.620779	REC8	9985	REC8 meiotic recombination protein
ENSG0000	253.3801	0.141045	0.137038	0.206366	0.620779	PTPN9	5780	protein tyrosine phosphatase non-receptor type 9
ENSG0000	4090.109	-0.15676	0.180285	0.206496	0.620911	MRTFA	57591	myocardin related transcription factor A
ENSG0000	2233.485	-0.15076	0.220902	0.206702	0.621423	NA	NA	NA
ENSG0000	13.73225	0.123115	0.245598	0.206734	0.621423	MMP28	79148	matrix metallopeptidase 28
ENSG0000	24.20155	0.154078	0.232495	0.206838	0.621533	YEATS4	8089	YEATS domain containing 4
ENSG0000	2.988678	0.056042	0.237886	0.206824	0.621533	NA	NA	NA
ENSG0000	14.6237	0.130894	0.245841	0.207001	0.621733	HPDL	84842	4-hydroxyphenylpyruvate dioxygenase like
ENSG0000	26.27272	0.128049	0.241839	0.207608	0.621733	ZBTB11-AS	1E+08	ZBTB11 antisense RNA 1
ENSG0000	43.92869	0.159524	0.21678	0.207549	0.621733	PODXL2	50512	podocalyxin like 2
ENSG0000	5.233687	0.058059	0.239343	0.207356	0.621733	NA	NA	NA
ENSG0000	3.359608	0.083151	0.243617	0.20733	0.621733	LOC10537	1.05E+08	uncharacterized LOC105377663
ENSG0000	185.4317	-0.13611	0.128736	0.207353	0.621733	HARS2	23438	histidyl-tR mitochondrial
ENSG0000	34.38817	0.163141	0.205105	0.207566	0.621733	PMS2P3	5387	PMS1 hom mismatch repair system component pseudogene 3
ENSG0000	40.91185	-0.16135	0.204783	0.207713	0.621733	TOX-DT	1.01E+08	TOX divergent transcript
ENSG0000	7.448534	0.095172	0.244236	0.207353	0.621733	SNX32	254122	sorting nexin 32
ENSG0000	12.7365	-0.126	0.245455	0.207565	0.621733	TRAV38-1	28644	T cell receptor alpha variable 38-1
ENSG0000	352.3659	-0.15638	0.207945	0.207623	0.621733	SNX6	58533	sorting nexin 6
ENSG0000	8.794947	0.124381	0.247819	0.207201	0.621733	NA	NA	NA
ENSG0000	204.5815	0.141597	0.137507	0.207099	0.621733	GANC	2595	glucosidas neutral C
ENSG0000	113.3181	0.146557	0.228898	0.207485	0.621733	FEM1B	10116	fem-1 homolog B
ENSG0000	156.1385	0.157274	0.180325	0.207245	0.621733	SNX33	257364	sorting nexin 33
ENSG0000	25.71217	0.156405	0.228954	0.20714	0.621733	RPL23AP5	729480	ribosomal protein L23a pseudogene 5
ENSG0000	46.38326	0.165201	0.196883	0.207166	0.621733	KRT10	3858	keratin 10
ENSG0000	1568.814	0.132514	0.121796	0.207681	0.621733	RPL38	6169	ribosomal protein L38
ENSG0000	1356.778	0.15502	0.189315	0.207371	0.621733	ZNF516	9658	zinc finger protein 516
ENSG0000	23804.99	-0.15616	0.201416	0.207419	0.621733	CNN2	1265	calponin 2
ENSG0000	14.01901	-0.12454	0.2456	0.207365	0.621733	NA	NA	NA
ENSG0000	299.9603	-0.15541	0.215674	0.207073	0.621733	SMIM24	284422	small integral membrane protein 24
ENSG0000	2629.981	-0.15114	0.16025	0.207719	0.621733	SAMD4B	55095	sterile alpha motif domain containing 4B
ENSG0000	6.440032	-0.08865	0.243102	0.207407	0.621733	LOC10192	1.02E+08	uncharacterized LOC101929372
ENSG0000	12.93063	0.142952	0.243612	0.207862	0.621855	NA	NA	NA
ENSG0000	3.391743	0.063257	0.238866	0.207861	0.621855	NA	NA	NA
ENSG0000	4.890225	-0.01832	0.234554	0.207816	0.621855	TNFAIP8L3	388121	TNF alpha induced protein 8 like 3
ENSG0000	26.54535	0.135194	0.240977	0.20792	0.621928	S100Z	170591	S100 calcium binding protein Z
ENSG0000	693.1224	0.152037	0.160086	0.208092	0.622239	TRAPPC12	51112	trafficking protein particle complex subunit 12
ENSG0000	110.8479	0.15142	0.156495	0.208067	0.622239	GATB	5188	glutamyl-tRNA amidotransferase subunit B
ENSG0000	121.3713	-0.15017	0.224958	0.208189	0.622303	AIDA	64853	axin intera dorsalization associated
ENSG0000	104.586	0.134088	0.236812	0.208216	0.622303	SESN1	27244	sestrin 1
ENSG0000	813.6232	0.133465	0.125118	0.208178	0.622303	XRN2	22803	5'-3' exoribonuclease 2
ENSG0000	4.496647	0.055414	0.236858	0.208402	0.622389	NA	NA	NA
ENSG0000	457.5817	-0.1546	0.157728	0.208482	0.622389	SOS1	6654	SOS Ras/Rac guanine nucleotide exchange factor 1
ENSG0000	1511.147	0.15764	0.190834	0.208431	0.622389	JDP2	122953	Jun dimerization protein 2
ENSG0000	576.1253	-0.15696	0.184526	0.208414	0.622389	GDPD3	79153	glycerophosphodiester phosphodiesterase domain containing 3
ENSG0000	3.314954	-0.04419	0.235896	0.208331	0.622389	NA	NA	NA
ENSG0000	3.53262	0.056788	0.237417	0.208448	0.622389	NA	NA	NA
ENSG0000	189.4246	0.15772	0.201851	0.208475	0.622389	BCOR	54880	BCL6 corepressor
ENSG0000	316.493	-0.15001	0.156019	0.208668	0.622659	CASTOR3P	352954	CASTOR fa pseudogene
ENSG0000	608.7783	-0.1302	0.121456	0.208675	0.622659	RRAGA	10670	Ras related GTP binding A
ENSG0000	1165.637	0.142686	0.138656	0.208655	0.622659	PLEKHG2	64857	pleckstrin homology and RhoGEF domain containing G2
ENSG0000	4.633124	0.064389	0.238746	0.208713	0.622673	SPATA25	128497	spermatogenesis associated 25
ENSG0000	133.1347	0.159839	0.207954	0.208891	0.623102	NA	NA	NA
ENSG0000	10.17721	-0.07594	0.239683	0.209095	0.623608	NA	NA	NA
ENSG0000	87.08379	0.157665	0.179051	0.209506	0.624113	ACTR8	93973	actin related protein 8

ENSG0000	20.0411	-0.10426	0.243853	0.209535	0.624113	CMBL	134147	carboxymethylenebutenolidase homolog
ENSG0000	76.49975	0.156805	0.17377	0.209327	0.624113	ZNF251	90987	zinc finger protein 251
ENSG0000	13.42061	-0.14224	0.243764	0.209449	0.624113	MTCO2P1	1.07E+08	MT-CO2 pseudogene 11
ENSG0000	977.6819	-0.15309	0.164683	0.209568	0.624113	ACD	65057	ACD shelterin complex subunit and telomerase recruitment factor
ENSG0000	197.9187	-0.14695	0.149108	0.209572	0.624113	TIMM22	29928	translocase of inner mitochondrial membrane 22
ENSG0000	49.50399	-0.15976	0.209281	0.209516	0.624113	LOC11226	1.12E+08	uncharacterized LOC112268198
ENSG0000	57.04051	0.162293	0.199142	0.209605	0.624113	PRSS57	400668	serine protease 57
ENSG0000	310.0274	0.152023	0.162014	0.209548	0.624113	ADNP	23394	activity dependent neuroprotector homeobox
ENSG0000	6.030361	-0.0554	0.23969	0.209546	0.624113	PRAME	23532	PRAME nuclear receptor transcriptional regulator
ENSG0000	13.04919	0.12131	0.246245	0.209755	0.624458	NA	NA	NA
ENSG0000	9.831066	0.084933	0.241105	0.209804	0.624501	NA	NA	NA
ENSG0000	2.335816	-0.04153	0.235579	0.209857	0.624547	NA	NA	NA
ENSG0000	4.009427	-0.07094	0.241139	0.209887	0.624547	LINC0191	1.05E+08	long intergenic non-protein coding RNA 1918
ENSG0000	235.233	0.148623	0.152599	0.209937	0.62458	ING1	3621	inhibitor of growth family member 1
ENSG0000	4.183122	0.066041	0.239333	0.209966	0.62458	NA	NA	NA
ENSG0000	4.911997	0.058021	0.237177	0.210058	0.624628	AMPD1	270	adenosine monophosphate deaminase 1
ENSG0000	2.925868	0.067917	0.241736	0.210145	0.624628	NA	NA	NA
ENSG0000	4.302685	0.080124	0.242032	0.210086	0.624628	NA	NA	NA
ENSG0000	18.49753	0.145773	0.238646	0.210211	0.624628	M1AP	130951	meiosis 1 associated protein
ENSG0000	68.65854	-0.15024	0.227281	0.210221	0.624628	HELQ	113510	helicase POLQ like
ENSG0000	2075.049	0.154517	0.207479	0.210158	0.624628	CSF1R	1436	colony stimulating factor 1 receptor
ENSG0000	11.25847	0.090566	0.241439	0.210212	0.624628	NHLRC1	378884	NHL repeat containing E3 ubiquitin protein ligase 1
ENSG0000	165.2191	0.141517	0.141208	0.210265	0.624658	BTD	686	biotinidase
ENSG0000	77.15448	0.156801	0.214032	0.210384	0.624807	IFNLR1	163702	interferon lambda receptor 1
ENSG0000	179.8274	0.156647	0.199421	0.210382	0.624807	DSE	29940	dermatan sulfate epimerase
ENSG0000	26.58233	-0.15081	0.232364	0.210634	0.625348	C17orf67	339210	chromosome 17 open reading frame 67
ENSG0000	725.1473	0.14267	0.142651	0.210605	0.625348	TPGS2	25941	tubulin polyglutamylase complex subunit 2
ENSG0000	19.07584	-0.156	0.227767	0.21072	0.625501	FBXO24	26261	F-box protein 24
ENSG0000	25.14899	0.148232	0.235583	0.210913	0.625771	FAM86B3	286042	family witl member A pseudogene
ENSG0000	2.685663	0.036446	0.235405	0.210907	0.625771	NA	NA	NA
ENSG0000	6.614819	0.111123	0.246928	0.210882	0.625771	NA	NA	NA
ENSG0000	527.4892	0.141098	0.139884	0.211109	0.62625	NSD3	54904	nuclear receptor binding SET domain protein 3
ENSG0000	31.0607	0.153283	0.226712	0.21126	0.626411	MIGA1	374986	mitoguardin 1
ENSG0000	112.6429	0.15673	0.181073	0.211283	0.626411	KCNC4	3749	potassium voltage-gated channel subfamily C member 4
ENSG0000	13.37362	-0.10555	0.243491	0.21139	0.626411	CCT6P3	643180	chaperonin containing TCP1 subunit 6 pseudogene 3
ENSG0000	29.50914	-0.12595	0.241087	0.211348	0.626411	TRBV15	28572	T cell receptor beta variable 15
ENSG0000	924.4915	-0.15458	0.177748	0.211342	0.626411	NRBF2	29982	nuclear receptor binding factor 2
ENSG0000	117.8185	0.156306	0.178477	0.211402	0.626411	BCCIP	56647	BRCA2 and CDKN1A interacting protein
ENSG0000	78.60784	0.152422	0.223266	0.211219	0.626411	GPALPP1	55425	GPALPP motifs containing 1
ENSG0000	57.86384	0.136691	0.236136	0.211665	0.626975	PPM1B	5495	protein ph Mg2+/Mn2+ dependent 1B
ENSG0000	88.00629	-0.15814	0.204024	0.21168	0.626975	SLC22A23	63027	solute carrier family 22 member 23
ENSG0000	2410.393	-0.14494	0.148296	0.211696	0.626975	SIRT2	22933	sirtuin 2
ENSG0000	405.9522	0.131247	0.125587	0.21174	0.627005	TRAF3	7187	TNF receptor associated factor 3
ENSG0000	553.805	0.136022	0.227371	0.211841	0.627204	RPS6KA3	6197	ribosomal protein S6 kinase A3
ENSG0000	2836.184	-0.15538	0.194936	0.211944	0.627257	MINDY1	55793	MINDY lysine 48 deubiquitinase 1
ENSG0000	83.23381	-0.14767	0.225809	0.212304	0.627257	LRRN1	57633	leucine rich repeat neuronal 1
ENSG0000	6.664124	0.090822	0.243147	0.212297	0.627257	LINC0201	1.08E+08	long intergenic non-protein coding RNA 2018
ENSG0000	38.84398	0.159316	0.209209	0.212175	0.627257	TBCCD1	55171	TBCC domain containing 1
ENSG0000	565.7325	-0.15448	0.178254	0.212302	0.627257	ZMAT2	153527	zinc finger matrin-type 2
ENSG0000	420.9889	0.156888	0.183524	0.21208	0.627257	KIF13A	63971	kinesin family member 13A
ENSG0000	1087.348	-0.13043	0.123753	0.21229	0.627257	LEMD2	221496	LEM domain nuclear envelope protein 2
ENSG0000	7.601028	0.099998	0.244795	0.212105	0.627257	NA	NA	NA
ENSG0000	235.2295	0.151866	0.164801	0.212244	0.627257	KMT5B	51111	lysine methyltransferase 5B
ENSG0000	738.1409	0.140829	0.140564	0.212164	0.627257	SPG21	51324	SPG21 ab maspardin
ENSG0000	4.862717	0.075934	0.240779	0.212035	0.627257	NA	NA	NA
ENSG0000	638.4488	0.142704	0.143396	0.211993	0.627257	OCEL1	79629	occludin/ELL domain containing 1
ENSG0000	5420.867	-0.1476	0.168084	0.212104	0.627257	PSMF1	9491	proteasome inhibitor subunit 1
ENSG0000	1252.387	-0.13522	0.132378	0.212364	0.627331	POLR2J	5439	RNA polymerase II subunit J
ENSG0000	1926.081	-0.14174	0.140822	0.212465	0.627529	NMT1	4836	N-myristoyltransferase 1
ENSG0000	8.230097	-0.08919	0.24236	0.212667	0.627628	H2BC26	128312	H2B clustered histone 26
ENSG0000	3.412017	0.046874	0.235987	0.212684	0.627628	NA	NA	NA
ENSG0000	5.889503	0.083095	0.241692	0.21265	0.627628	FAM124B	79843	family with sequence similarity 124 member B
ENSG0000	289.8478	-0.13928	0.136014	0.212738	0.627628	HAVCR2	84868	hepatitis A virus cellular receptor 2
ENSG0000	366.3878	0.142751	0.14518	0.212736	0.627628	CCT6A	908	chaperonin containing TCP1 subunit 6A
ENSG0000	2.736107	0.052768	0.237518	0.212696	0.627628	MIR106B	406900	microRNA 106b
ENSG0000	19.6494	-0.11314	0.242885	0.212593	0.627628	NA	NA	NA
ENSG0000	93.09172	0.145799	0.147542	0.212776	0.627637	SLC20A2	6575	solute carrier family 20 member 2
ENSG0000	6.160738	0.078254	0.240943	0.212975	0.627792	OLFML3	56944	olfactomedin like 3
ENSG0000	24.51427	-0.15737	0.221051	0.213016	0.627792	MELTF	4241	melanotransferrin
ENSG0000	19.30812	0.106188	0.242664	0.212963	0.627792	TTK	7272	TTK protein kinase
ENSG0000	66.94132	-0.15796	0.196769	0.213034	0.627792	SYTL2	54843	synaptotagmin like 2
ENSG0000	3.474159	0.063744	0.239871	0.212889	0.627792	NA	NA	NA
ENSG0000	18.2677	0.120017	0.242803	0.212973	0.627792	RCAN1	1827	regulator of calcineurin 1
ENSG0000	452.7905	-0.15659	0.194046	0.213305	0.62849	ITK	3702	IL2 inducible T cell kinase
ENSG0000	25.62454	0.124407	0.241929	0.213709	0.62883	INSIG2	51141	insulin induced gene 2
ENSG0000	7.099382	-0.07746	0.240078	0.213588	0.62883	TFPI	7035	tissue factor pathway inhibitor
ENSG0000	2.715864	0.033881	0.234838	0.213606	0.62883	NA	NA	NA
ENSG0000	15.89681	-0.14005	0.240589	0.213592	0.62883	TRBV6-6	28601	T cell receptor beta variable 6-6
ENSG0000	45.86964	0.131561	0.23887	0.21376	0.62883	RBIS	401466	ribosomal biogenesis factor
ENSG0000	3.347138	0.069004	0.239753	0.213583	0.62883	NA	NA	NA
ENSG0000	28.35107	0.148981	0.227645	0.213761	0.62883	LOC10798	1.08E+08	SMAD specific E3 ubiquitin protein ligase 2 (SMURF2) pseudogene
ENSG0000	10.12035	-0.12058	0.244455	0.213723	0.62883	NA	NA	NA
ENSG0000	63.0477	0.158796	0.176195	0.213764	0.62883	ZNF814	730051	zinc finger protein 814
ENSG0000	2101.002	0.155513	0.189353	0.213628	0.62883	PFKL	5211	phosphofr liver type
ENSG0000	839.9775	-0.14382	0.143109	0.214182	0.628948	ZBTB17	7709	zinc finger and BTB domain containing 17
ENSG0000	502.737	-0.1495	0.162622	0.214593	0.628948	SEC22B	9554	SEC22 hon vesicle trafficking protein
ENSG0000	36.79588	-0.15905	0.20591	0.214044	0.628948	TMEM81	388730	transmembrane protein 81
ENSG0000	156.2157	0.147087	0.150295	0.214233	0.628948	COG2	22796	component of oligomeric golgi complex 2
ENSG0000	405.1143	-0.12832	0.121124	0.213916	0.628948	SUPT7L	9913	SPT7 like STAGA complex subunit gamma
ENSG0000	106.5878	0.154445	0.211725	0.214069	0.628948	GMCL1	64395	germ cell-l spermatogenesis associated
ENSG0000	249.1114	0.092753	0.242072	0.214594	0.628948	IGKV1-12	28940	immunoglobulin kappa variable 1-12
ENSG0000	11140.3	-0.13511	0.1319	0.214473	0.628948	RAB7A	7879	RAB7A member RAS oncogene family
ENSG0000	4456.914	0.139228	0.135308	0.213982	0.628948	MACROH2	9555	macroH2A.1 histone
ENSG0000	4329.56	-0.15779	0.200253	0.214569	0.628948	PILRA	29992	paired immunoglobin like type 2 receptor alpha
ENSG0000	84.36088	-0.15716	0.20557	0.214561	0.628948	POLR2J3	548644	RNA polymerase II subunit J3
ENSG0000	40.51258	-0.15769	0.207617	0.214493	0.628948	CASP4LP	643733	caspase 4 pseudogene

ENSG0000	100.8519	0.158096	0.190431	0.214412	0.628948	YAF2	10138	YY1 associated factor 2
ENSG0000	325.2967	0.13611	0.136908	0.213859	0.628948	TTC9	23508	tetratricopeptide repeat domain 9
ENSG0000	91.85572	0.158592	0.201716	0.214569	0.628948	NPIPA5	1E+08	nuclear pore complex interacting protein family member A5
ENSG0000	28.07252	0.154999	0.223112	0.214284	0.628948	TEFM	79736	transcripti mitochondrial
ENSG0000	10.53294	-0.09124	0.241911	0.213944	0.628948	NA	NA	NA
ENSG0000	127.8319	0.155429	0.178348	0.214517	0.628948	SWSAP1	126074	SWIM-type zinc finger 7 associated protein 1
ENSG0000	69.17178	0.150826	0.221151	0.214263	0.628948	TRPM4	54795	transient receptor potential cation channel subfamily M member 4
ENSG0000	6.270266	0.059159	0.237177	0.214455	0.628948	LOC12253	1.23E+08	Zinc finger protein LOC122539214
ENSG0000	1790.045	0.152629	0.208586	0.214468	0.628948	MYBL2	4605	MYB proto-oncogene like 2
ENSG0000	143.8493	0.151834	0.166436	0.214082	0.628948	CA5B	11238	carbonic anhydrase 5B
ENSG0000	526.1224	0.145097	0.149369	0.214377	0.628948	ATP6AP2	10159	ATPase H+ transporting accessory protein 2
ENSG0000	379.3935	0.125424	0.116934	0.214644	0.628996	ELOA	6924	elongin A
ENSG0000	325.3437	0.142151	0.140166	0.214863	0.629536	LETMD1	25875	LETM1 domain containing 1
ENSG0000	14.94237	-0.1526	0.231849	0.214906	0.629562	ZNF284	342909	zinc finger protein 284
ENSG0000	7.367174	0.078573	0.240014	0.214964	0.62963	PCAT6	1.01E+08	prostate cancer associated transcript 6
ENSG0000	41.17685	-0.15592	0.215376	0.21512	0.629828	MANEAL	149175	mannosidase endo-alpha like
ENSG0000	2.845564	0.056785	0.237527	0.215135	0.629828	SLC9B1	150159	solute carrier family 9 member B1
ENSG0000	415.2498	0.136589	0.133588	0.215115	0.629828	HECTD4	283450	HECT domain E3 ubiquitin protein ligase 4
ENSG0000	54.9384	-0.1585	0.192788	0.215249	0.630064	NCALD	83988	neurocalcin delta
ENSG0000	2.939861	0.058356	0.238391	0.215565	0.630685	SPDYA	245711	speedy/RINGO cell cycle regulator family member A
ENSG0000	477.7939	0.149417	0.21741	0.215557	0.630685	SLC38A1	81539	solute carrier family 38 member 1
ENSG0000	11.32056	-0.11656	0.243936	0.215555	0.630685	NA	NA	NA
ENSG0000	13.29112	0.089644	0.240758	0.215771	0.631187	EFHC2	80258	EF-hand domain containing 2
ENSG0000	24.68665	0.117767	0.242057	0.215852	0.631263	MRPS33	51650	mitochondrial ribosomal protein S33
ENSG0000	104.0298	0.153993	0.213238	0.215866	0.631263	C11orf80	79703	chromosome 11 open reading frame 80
ENSG0000	65.91624	0.152852	0.218336	0.21591	0.63129	GOLGA8B	440270	golgin A8 family member B
ENSG0000	162.3962	0.154983	0.181677	0.215999	0.63145	IDE	3416	insulin degrading enzyme
ENSG0000	134.7704	-0.15492	0.183381	0.216123	0.631713	CCDC149	91050	coiled-coil domain containing 149
ENSG0000	5.709051	-0.08495	0.242647	0.216291	0.632101	NA	NA	NA
ENSG0000	437.5561	0.131964	0.127689	0.216384	0.632273	PHAF1	80262	phagosome assembly factor 1
ENSG0000	5.046898	0.080202	0.241274	0.216451	0.632368	GPD1	2819	glycerol-3-phosphate dehydrogenase 1
ENSG0000	102.0108	-0.15633	0.17454	0.216628	0.632783	SNAI3-AS1	197187	SNAI3 antisense RNA 1
ENSG0000	45.53876	-0.13252	0.235675	0.216806	0.632864	EMBP1	647121	embigin pseudogene 1
ENSG0000	309.1194	0.154225	0.182195	0.216766	0.632864	ATP6V1A	523	ATPase H+ transporting V1 subunit A
ENSG0000	16.35221	-0.13902	0.240076	0.216828	0.632864	NA	NA	NA
ENSG0000	6.894115	-0.08403	0.241752	0.216794	0.632864	NA	NA	NA
ENSG0000	14.81735	-0.14132	0.241027	0.216721	0.632864	NA	NA	NA
ENSG0000	387.8478	0.153448	0.196267	0.216879	0.632911	TNFAIP8	25816	TNF alpha induced protein 8
ENSG0000	987.4162	-0.15332	0.197071	0.216961	0.633023	STARD10	10809	StAR related lipid transfer domain containing 10
ENSG0000	205.0245	-0.15372	0.177647	0.216986	0.633023	CTIF	9811	cap binding complex dependent translation initiation factor
ENSG0000	110.9381	0.146849	0.223328	0.217128	0.633134	BMP8B	656	bone morphogenetic protein 8b
ENSG0000	148.7656	0.155731	0.199932	0.217097	0.633134	PYCR1	5831	pyrroline-5-carboxylate reductase 1
ENSG0000	3241.054	-0.14769	0.167127	0.2171	0.633134	GATAD2A	54815	GATA zinc finger domain containing 2A
ENSG0000	275.4757	0.149714	0.185086	0.217191	0.633143	NA	NA	NA
ENSG0000	353.3033	0.153219	0.174323	0.2172	0.633143	SLC35B1	10237	solute carrier family 35 member B1
ENSG0000	2.543917	-0.04737	0.236304	0.217265	0.63317	CIBAR1	137392	CBY1 interacting BAR domain containing 1
ENSG0000	31.41093	0.105711	0.240483	0.217278	0.63317	NA	NA	NA
ENSG0000	4.383099	0.086907	0.243158	0.217365	0.633321	NA	NA	NA
ENSG0000	2.63152	-0.06665	0.239475	0.217451	0.633472	NPC1L1	29881	NPC1 like intracellular cholesterol transporter 1
ENSG0000	89.16014	-0.15198	0.211058	0.217499	0.633509	ZNF230	7773	zinc finger protein 230
ENSG0000	1370.529	-0.15304	0.188248	0.217542	0.633535	PPP2R5A	5525	protein phosphatase 2 regulatory subunit B'alpha
ENSG0000	136.7258	0.145656	0.151751	0.217596	0.633593	KCNMB1	3779	potassium calcium-activated channel subfamily M regulatory beta subunit 1
ENSG0000	2.534861	0.041289	0.235186	0.217686	0.633752	NA	NA	NA
ENSG0000	1520.106	-0.15296	0.183924	0.217791	0.633959	FAM110A	83541	family with sequence similarity 110 member A
ENSG0000	51.04271	0.156089	0.20793	0.217883	0.634125	LOC93622	93622	Morf4 family associated protein 1 like 1
ENSG0000	4833.604	0.12821	0.122069	0.217974	0.634128	SSR2	6746	signal sequence receptor subunit 2
ENSG0000	404.111	0.131902	0.126863	0.217973	0.634128	CNTROB	116840	centrobin centriole duplication and spindle assembly protein
ENSG0000	2.811808	0.037837	0.234937	0.217988	0.634128	EPX	8288	eosinophil peroxidase
ENSG0000	64.31642	0.154368	0.176181	0.218158	0.634522	ATP6V1E2	90423	ATPase H+ transporting V1 subunit E2
ENSG0000	2.621795	0.052244	0.236882	0.218242	0.634602	NA	NA	NA
ENSG0000	11678.12	-0.13435	0.132572	0.218255	0.634602	DAZAP2	9802	DAZ associated protein 2
ENSG0000	1235.731	-0.1454	0.170279	0.218338	0.634742	LEF1	51176	lymphoid enhancer binding factor 1
ENSG0000	54.68219	0.156172	0.184437	0.218378	0.63476	POLA1	5422	DNA polyr catalytic subunit
ENSG0000	14.01042	-0.1297	0.242896	0.218466	0.634914	NA	NA	NA
ENSG0000	7.007716	0.08895	0.245706	0.218551	0.635061	FZD7	8324	frizzled class receptor 7
ENSG0000	94.30799	0.15474	0.192374	0.218624	0.63507	ATF1	466	activating transcription factor 1
ENSG0000	253.4363	-0.15285	0.182501	0.218606	0.63507	SYNJ1	8867	synaptojanin 1
ENSG0000	40.06963	-0.15385	0.214851	0.218728	0.635071	NA	NA	NA
ENSG0000	3.040067	0.048206	0.236575	0.218661	0.635071	FAM81A	145773	family with sequence similarity 81 member A
ENSG0000	7.083155	-0.09955	0.244155	0.218701	0.635071	NA	NA	NA
ENSG0000	441.8308	0.151308	0.210287	0.218767	0.63508	FBP1	2203	fructose-bisphosphatase 1
ENSG0000	21181.82	-0.08667	0.240395	0.218801	0.63508	MMP9	4318	matrix metallopeptidase 9
ENSG0000	2.480266	-0.04067	0.235735	0.218878	0.635204	CCL23	6368	C-C motif chemokine ligand 23
ENSG0000	17.24636	-0.1056	0.241442	0.21893	0.635253	H19	283120	H19 imprinted maternally expressed transcript
ENSG0000	31.07824	0.144249	0.23242	0.219	0.635257	NA	NA	NA
ENSG0000	37.08992	0.153602	0.215099	0.219415	0.635257	NA	NA	NA
ENSG0000	20.66495	0.108713	0.241987	0.219372	0.635257	NA	NA	NA
ENSG0000	2303.848	-0.14828	0.174284	0.219086	0.635257	MAP4K4	9448	mitogen-activated protein kinase kinase kinase kinase 4
ENSG0000	52.6553	-0.15491	0.179406	0.219451	0.635257	MED20	9477	mediator complex subunit 20
ENSG0000	26.20114	0.104712	0.240914	0.219012	0.635257	KPNA5	3841	karyopherin subunit alpha 5
ENSG0000	28.31546	0.142402	0.234984	0.2193	0.635257	RASA4B	1E+08	RAS p21 protein activator 4B
ENSG0000	1030.326	0.151712	0.17488	0.219439	0.635257	REEP4	80346	receptor accessory protein 4
ENSG0000	197.9521	0.141203	0.228215	0.219298	0.635257	LYPLA1	10434	lysophospholipase 1
ENSG0000	537.3199	-0.1521	0.178496	0.219282	0.635257	CHPT1	56994	choline phosphotransferase 1
ENSG0000	7.779299	0.076919	0.239953	0.219109	0.635257	GEMIN2	8487	gem nuclear organelle associated protein 2
ENSG0000	13.50172	0.147615	0.236529	0.219246	0.635257	SYCE1L	1E+08	synaptonemal complex central element protein 1 like
ENSG0000	2297.152	0.151414	0.182462	0.219192	0.635257	FCHO1	23149	FCH and mu domain containing endocytic adaptor 1
ENSG0000	57.58055	0.153556	0.212369	0.219445	0.635257	PRX	57716	periaxin
ENSG0000	28.85711	-0.15799	0.19658	0.219286	0.635257	NA	NA	NA
ENSG0000	5.593744	0.06625	0.238377	0.219564	0.635384	NA	NA	NA
ENSG0000	10.22657	0.101349	0.242923	0.219531	0.635384	NA	NA	NA
ENSG0000	3.061444	0.055543	0.237221	0.219624	0.635456	CALML4	91860	calmodulin like 4
ENSG0000	339.1835	-0.15335	0.16916	0.219805	0.635479	ARID4B	51742	AT-rich interaction domain 4B
ENSG0000	8.330051	-0.13042	0.244893	0.219804	0.635479	NA	NA	NA
ENSG0000	2.365258	0.044925	0.236371	0.219761	0.635479	EEF1A1P2	645715	eukaryotic translation elongation factor 1 alpha 1 pseudogene 24
ENSG0000	80.87955	0.15534	0.200184	0.21973	0.635479	GTF2IP1	2970	general transcription factor IIi pseudogene 1

ENSG0000	17.60845	0.120712	0.243028	0.219705	0.635479	ZNF254	9534	zinc finger protein 254
ENSG0000	6.628625	0.090441	0.243117	0.219858	0.63553	BFSP2	8419	beaded filament structural protein 2
ENSG0000	2.450316	0.04239	0.235647	0.219949	0.635692	NA	NA	NA
ENSG0000	14.57861	0.138532	0.241051	0.220016	0.635788	NA	NA	NA
ENSG0000	17.18029	-0.10631	0.241279	0.220066	0.63583	MAPK8IP2	23542	mitogen-activated protein kinase 8 interacting protein 2
ENSG0000	2.617574	0.065733	0.240384	0.22035	0.636551	NA	NA	NA
ENSG0000	4.936653	0.024431	0.23508	0.220451	0.636642	WNT4	54361	Wnt family member 4
ENSG0000	17.65811	0.144339	0.235125	0.220435	0.636642	ADAL	161823	adenosine deaminase like
ENSG0000	6.246813	-0.0912	0.243018	0.220641	0.636991	ZNF410	57862	zinc finger protein 410
ENSG0000	17.66163	0.120763	0.242871	0.220609	0.636991	NA	NA	NA
ENSG0000	564.4043	-0.12127	0.112482	0.220756	0.637221	GLE1	2733	GLE1 RNA export mediator
ENSG0000	9352.76	-0.14928	0.169868	0.220804	0.63726	ITGAL	3683	integrin subunit alpha L
ENSG0000	101.8607	-0.15393	0.19312	0.220869	0.637346	KIN	22944	Kin17 DNA and RNA binding protein
ENSG0000	9.130292	-0.08027	0.240319	0.220938	0.637399	OSBPL6	114880	oxysterol binding protein like 6
ENSG0000	4.784304	0.068693	0.23866	0.220992	0.637399	TREM2	54209	triggering receptor expressed on myeloid cells 2
ENSG0000	187.489	-0.0836	0.238846	0.220988	0.637399	CLEC4D	338339	C-type lectin domain family 4 member D
ENSG0000	117.4841	0.102003	0.238135	0.221137	0.637617	PFKFB2	5208	6-phosphc 6-biphosphatase 2
ENSG0000	2.561627	-0.03539	0.23561	0.221135	0.637617	NA	NA	NA
ENSG0000	4365.463	-0.14993	0.176783	0.221261	0.637633	ARPC4	10093	actin related protein 2/3 complex subunit 4
ENSG0000	506.4721	0.156931	0.177996	0.221281	0.637633	IMPDH2	3615	inosine monophosphate dehydrogenase 2
ENSG0000	308.563	-0.1519	0.189783	0.221217	0.637633	ZC3H12D	340152	zinc finger CCCH-type containing 12D
ENSG0000	3645.598	-0.14005	0.144443	0.221213	0.637633	SP1	6667	Sp1 transcription factor
ENSG0000	29.7525	0.146797	0.229493	0.221458	0.637666	NA	NA	NA
ENSG0000	8.477399	-0.11016	0.245215	0.221467	0.637666	SERPINI1	5274	serpin family I member 1
ENSG0000	987.7727	-0.14805	0.164662	0.221367	0.637666	LRWD1	222229	leucine rich repeats and WD repeat domain containing 1
ENSG0000	195.5342	0.146344	0.15179	0.221425	0.637666	NME3	4832	NME/NM23 nucleoside diphosphate kinase 3
ENSG0000	3.282697	0.063014	0.239678	0.221346	0.637666	ALOX12B	242	arachidon: 12R type
ENSG0000	180.7767	-0.14416	0.152227	0.221632	0.63774	PPID	5481	peptidylprolyl isomerase D
ENSG0000	4.609171	-0.08622	0.243619	0.221572	0.63774	TRBV12-5	28575	T cell receptor beta variable 12-5
ENSG0000	54.11004	0.155411	0.187976	0.221628	0.63774	KANTR	1.03E+08	KANTR integral membrane protein
ENSG0000	251474.9	0.150708	0.173615	0.221533	0.63774	COX1	4512	cytochrome c oxidase subunit I
ENSG0000	2552.749	-0.14955	0.205772	0.22178	0.637965	CAPG	822	capping ac gelsolin like
ENSG0000	5.46491	0.098093	0.24468	0.221757	0.637965	NA	NA	NA
ENSG0000	7.180863	0.08384	0.241582	0.221844	0.638049	RFESD	317671	Rieske Fe-S domain containing
ENSG0000	60.43159	0.155161	0.200718	0.221932	0.638202	NA	NA	NA
ENSG0000	4.513843	-0.06982	0.242615	0.222213	0.638868	PAQR9	344838	progestin and adipoQ receptor family member 9
ENSG0000	5.487685	0.073828	0.239672	0.222233	0.638868	SEMA4G	57715	semaphorin 4G
ENSG0000	2.530718	0.041404	0.236432	0.222401	0.639249	NA	NA	NA
ENSG0000	15.8459	0.124524	0.24187	0.22253	0.63952	NA	NA	NA
ENSG0000	3.603773	-0.05333	0.237679	0.222664	0.639712	NA	NA	NA
ENSG0000	2309.035	-0.14234	0.143594	0.222728	0.639712	RBM23	55147	RNA binding motif protein 23
ENSG0000	535.1421	-0.14515	0.157107	0.222736	0.639712	ARIH1	25820	ariadne RBR E3 ubiquitin protein ligase 1
ENSG0000	2.828498	-0.02531	0.233834	0.222686	0.639712	NA	NA	NA
ENSG0000	137.278	0.152614	0.181811	0.22287	0.639995	NDUFB2	4708	NADH:ubiquinone oxidoreductase subunit B2
ENSG0000	132.0678	-0.14554	0.219636	0.222947	0.640116	PACSIN1	29993	protein kinase C and casein kinase substrate in neurons 1
ENSG0000	7178.184	-0.14874	0.203804	0.22308	0.640309	P2RY13	53829	purinergic receptor P2Y13
ENSG0000	1171.661	-0.15145	0.186935	0.223084	0.640309	ST14	6768	ST14 transmembrane serine protease matriptase
ENSG0000	410.8018	0.151553	0.197838	0.223153	0.640322	BCLAF1	9774	BCL2 associated transcription factor 1
ENSG0000	333.8091	0.125731	0.120412	0.223221	0.640322	KMT5C	84787	lysine methyltransferase 5C
ENSG0000	44.00436	0.145854	0.226783	0.223161	0.640322	ZNF548	147694	zinc finger protein 548
ENSG0000	5.17719	0.071584	0.239431	0.223228	0.640322	HMGN5	79366	high mobility group nucleosome binding domain 5
ENSG0000	252.0324	-0.15188	0.201728	0.223354	0.640581	NA	NA	NA
ENSG0000	140.7541	0.147093	0.217675	0.223496	0.64089	TAF9	6880	TATA-box binding protein associated factor 9
ENSG0000	2529.821	-0.1473	0.212827	0.223634	0.641184	LOC72848	728488	POM121 membrane glycoprotein (rat) pseudogene
ENSG0000	22746.26	-0.14816	0.205852	0.223686	0.641234	WAS	7454	WASP actin nucleation promoting factor
ENSG0000	6709.473	-0.14576	0.16137	0.224033	0.641343	PSD4	23550	pleckstrin and Sec7 domain containing 4
ENSG0000	17.3341	0.108822	0.241916	0.224071	0.641343	NA	NA	NA
ENSG0000	781.6824	-0.14518	0.156858	0.223868	0.641343	RPUSD3	285367	RNA pseudouridine synthase D3
ENSG0000	785.1207	-0.1457	0.160597	0.223875	0.641343	SRA1	10011	steroid receptor RNA activator 1
ENSG0000	1511.366	-0.1391	0.144357	0.224015	0.641343	ZFAND3	60685	zinc finger AN1-type containing 3
ENSG0000	182.7754	0.151617	0.17976	0.223965	0.641343	GSAP	54103	gamma-secretase activating protein
ENSG0000	287.3574	-0.13754	0.141272	0.223881	0.641343	RNF20	56254	ring finger protein 20
ENSG0000	104.6675	0.141782	0.149386	0.224074	0.641343	ZNF511	118472	zinc finger protein 511
ENSG0000	5413.263	0.10838	0.098355	0.224074	0.641343	TMEM259	91304	transmembrane protein 259
ENSG0000	14.90439	-0.13754	0.238638	0.223925	0.641343	NA	NA	NA
ENSG0000	224.8551	0.141454	0.145935	0.224151	0.641461	TBL2	26608	transducin beta like 2
ENSG0000	7.846502	-0.10118	0.243148	0.224265	0.641653	MIR4432H	1.07E+08	MIR4432 host gene
ENSG0000	73.75839	-0.14469	0.224235	0.224363	0.641653	HLA-L	3139	major hist class I L (pseudogene)
ENSG0000	133.7561	0.156141	0.193972	0.224428	0.641653	PYCR3	65263	pyrroline-5-carboxylate reductase 3
ENSG0000	289.338	-0.14713	0.160533	0.2244	0.641653	POLD4	57804	DNA polyr accessory subunit
ENSG0000	92.75816	-0.15253	0.197944	0.224407	0.641653	NA	NA	NA
ENSG0000	18.82614	0.097059	0.241735	0.224291	0.641653	LOC10272	1.03E+08	potassium voltage-gated channel subfamily E regulatory subunit 1B
ENSG0000	188.6391	-0.14545	0.159892	0.224468	0.641666	RHPN1	114822	rhophilin Rho GTPase binding protein 1
ENSG0000	6.399713	0.104512	0.244867	0.224598	0.641841	NA	NA	NA
ENSG0000	19915.15	-0.14752	0.206549	0.224599	0.641841	MOB3A	126308	MOB kinase activator 3A
ENSG0000	65.80046	-0.15237	0.207435	0.224638	0.641852	LINC-PINT	378805	long interg p53 induced transcript
ENSG0000	5.070478	0.060757	0.237498	0.224715	0.641873	ITGA9	3680	integrin subunit alpha 9
ENSG0000	2.994761	0.041908	0.235327	0.224691	0.641873	NA	NA	NA
ENSG0000	68.59497	-0.1529	0.196923	0.224914	0.642093	METTL6	131965	methyltrai methylcytidine
ENSG0000	7.68586	0.116814	0.24547	0.224923	0.642093	PCOLCE-A:	1E+08	PCOLCE antisense RNA 1
ENSG0000	2004.087	-0.1451	0.159479	0.224958	0.642093	IKZF3	22806	IKAROS family zinc finger 3
ENSG0000	735.1956	0.123152	0.11737	0.224967	0.642093	FOXK2	3607	forkhead box K2
ENSG0000	1106.53	0.134699	0.135461	0.224915	0.642093	BID	637	BH3 interacting domain death agonist
ENSG0000	186.818	-0.14801	0.203962	0.225159	0.642204	USP33	23032	ubiquitin specific peptidase 33
ENSG0000	331.9565	0.132513	0.131397	0.225209	0.642204	FOXK1	221937	forkhead box K1
ENSG0000	127.5793	-0.15064	0.175676	0.225083	0.642204	WHRN	25861	whirlin
ENSG0000	37.92633	0.14398	0.226516	0.22514	0.642204	APIP	51074	APAF1 interacting protein
ENSG0000	145.368	0.143493	0.219333	0.225215	0.642204	RPS15A	6210	ribosomal protein S15a
ENSG0000	1686.571	0.142286	0.154486	0.225218	0.642204	PRDX2	7001	peroxiredoxin 2
ENSG0000	185.0511	-0.14762	0.161833	0.225287	0.642204	C19orf12	83636	chromosome 19 open reading frame 12
ENSG0000	189.3745	0.133496	0.229694	0.225275	0.642204	STK26	51765	serine/threonine kinase 26
ENSG0000	350.1392	0.134451	0.135303	0.225463	0.642325	ADI1	55256	acireductone dioxygenase 1
ENSG0000	379.6582	0.144332	0.155701	0.22559	0.642325	FBXL18	80028	F-box and leucine rich repeat protein 18
ENSG0000	114.5407	0.151496	0.200343	0.225566	0.642325	NUP42	11097	nucleoporin 42
ENSG0000	1338.641	-0.13322	0.133477	0.22551	0.642325	VPS39	23339	VPS39 subunit of HOPS complex
ENSG0000	21.12337	-0.12337	0.240354	0.225433	0.642325	USP7-AS1	1.05E+08	USP7 antisense RNA 1

ENSG0000	1515.064	-0.11924	0.120574	0.225673	0.642325	MLX	6945	MAX dimerization protein MLX
ENSG0000	6081.136	0.154297	0.176513	0.225668	0.642325	P4HB	5034	prolyl 4-hydroxylase subunit beta
ENSG0000	225.1855	0.148713	0.173068	0.22568	0.642325	EXOSC5	56915	exosome component 5
ENSG0000	73.88493	-0.13169	0.23294	0.225547	0.642325	KCNG1	3755	potassium voltage-gated channel modifier subfamily G member 1
ENSG0000	5.772518	0.057249	0.236496	0.225454	0.642325	CYYR1	116159	cysteine and tyrosine rich 1
ENSG0000	51.68893	0.146873	0.222494	0.225766	0.642469	TNK1	8711	tyrosine kinase non receptor 1
ENSG0000	24.50876	0.140196	0.233377	0.225968	0.642747	CENPP	401541	centromere protein P
ENSG0000	73.57886	0.155323	0.192116	0.225904	0.642747	ZNF432	9668	zinc finger protein 432
ENSG0000	1610.147	-0.1494	0.179488	0.22596	0.642747	MED12	9968	mediator complex subunit 12
ENSG0000	308.8675	0.148873	0.201731	0.226019	0.642791	HINT1	3094	histidine triad nucleotide binding protein 1
ENSG0000	327.2584	0.134336	0.133683	0.226067	0.642827	MTOR	2475	mechanistic target of rapamycin kinase
ENSG0000	15.00237	-0.1328	0.238834	0.22617	0.64302	ANKRD23	200539	ankyrin repeat domain 23
ENSG0000	587.623	-0.1376	0.142154	0.226355	0.643446	RINL	126432	Ras and Rab interactor like
ENSG0000	11.00667	-0.09731	0.241239	0.226414	0.643515	NA	NA	NA
ENSG0000	467.6157	0.137374	0.151681	0.226643	0.643667	LIMD1	8994	LIM domain containing 1
ENSG0000	12.72286	0.077328	0.238495	0.226603	0.643667	ZNF354C	30832	zinc finger protein 354C
ENSG0000	11.67203	0.128685	0.247143	0.226629	0.643667	LOC73010	730101	uncharacterized LOC730101
ENSG0000	222.7135	0.147442	0.16779	0.226578	0.643667	FOXO1	2308	forkhead box O1
ENSG0000	17.51667	-0.1466	0.229221	0.226566	0.643667	ABHD17C	58489	abhydrola: depalmitoylase
ENSG0000	24.36298	0.140101	0.23164	0.226759	0.643703	NA	NA	NA
ENSG0000	2.529153	-0.06848	0.241533	0.226797	0.643703	ITGA2	3673	integrin subunit alpha 2
ENSG0000	28.30789	-0.13736	0.233113	0.226694	0.643703	PRXL2A	84293	peroxiredoxin like 2A
ENSG0000	33.93679	-0.13534	0.23305	0.226766	0.643703	TRAV3	28690	T cell receptor alpha variable 3
ENSG0000	361.8555	0.150591	0.183731	0.226884	0.643851	LCOR	84458	ligand dependent nuclear receptor corepressor
ENSG0000	2174.819	0.143752	0.16547	0.227039	0.644006	DDOST	1650	dolichyl-diphosphooligosaccharide--protein glycosyltransferase non-catalytic subunit
ENSG0000	244.914	-0.15017	0.178924	0.227044	0.644006	NCR3	259197	natural cytotoxicity triggering receptor 3
ENSG0000	80.45089	0.124582	0.235799	0.226977	0.644006	NPIPB15	440348	nuclear pore complex interacting protein family member B15
ENSG0000	102.68	0.114542	0.235522	0.227346	0.644078	IGKV5-2	28907	immunoglobulin kappa variable 5-2
ENSG0000	142.05	0.147986	0.214505	0.227282	0.644078	CFAP92	57501	cilia and flagella associated protein 92 (putative)
ENSG0000	212.7394	0.150384	0.174111	0.227334	0.644078	RAP1GDS1	5910	Rap1 GTPase-GDP dissociation stimulator 1
ENSG0000	8.391053	-0.07187	0.237987	0.227349	0.644078	NA	NA	NA
ENSG0000	69.70081	-0.15248	0.192974	0.227351	0.644078	NDUFB6	4712	NADH:ubiquinone oxidoreductase subunit B6
ENSG0000	25.63333	0.154753	0.208918	0.227131	0.644078	SLC38A6	145389	solute carrier family 38 member 6
ENSG0000	107.9588	-0.15125	0.187732	0.227315	0.644078	FAN1	22909	FANCD2 and FANCI associated nuclease 1
ENSG0000	216.2499	0.142671	0.15236	0.227307	0.644078	ATPAF2	91647	ATP synthase mitochondrial F1 complex assembly factor 2
ENSG0000	13.47029	0.127129	0.241151	0.227468	0.644311	LYRM4-AS	1E+08	LYRM4 antisense RNA 1
ENSG0000	96.99373	0.149387	0.209403	0.227696	0.644801	IQCB1	9657	IQ motif containing B1
ENSG0000	4.70968	0.045216	0.23479	0.227774	0.644801	NA	NA	NA
ENSG0000	2104.509	-0.14698	0.208037	0.227817	0.644801	MANSC1	54682	MANSC domain containing 1
ENSG0000	257.1074	0.139978	0.146607	0.227778	0.644801	CBX1	10951	chromobox 1
ENSG0000	286.1035	0.150354	0.192144	0.227803	0.644801	BCORL1	63035	BCL6 corepressor like 1
ENSG0000	176.8585	0.150906	0.185391	0.227907	0.644957	RRP1	8568	ribosomal RNA processing 1
ENSG0000	981.571	0.114136	0.10677	0.227978	0.644958	CCNL2	81669	cyclin L2
ENSG0000	6.125249	-0.06007	0.236753	0.227954	0.644958	RAB25	57111	RAB25 member RAS oncogene family
ENSG0000	13.7248	0.138539	0.234548	0.228028	0.645001	ZNF66	7617	zinc finger protein 66
ENSG0000	6.754157	-0.10581	0.245905	0.228116	0.645149	CNNM1	26507	cyclin and CBS domain divalent metal cation transport mediator 1
ENSG0000	165.58	-0.14718	0.17833	0.228151	0.645149	TRAF4	9618	TNF receptor associated factor 4
ENSG0000	1716.633	-0.14548	0.162488	0.22826	0.645257	MFSD12	126321	major facilitator superfamily domain containing 12
ENSG0000	8384.081	-0.1489	0.186657	0.228241	0.645257	DENND1C	79958	DENN domain containing 1C
ENSG0000	63.08496	0.144049	0.227456	0.228398	0.645527	AGGF1	55109	angiogenic factor with G-patch and FHA domains 1
ENSG0000	13.89831	0.0847	0.238816	0.228426	0.645527	NA	NA	NA
ENSG0000	71.95968	0.152377	0.197274	0.228471	0.645556	MLH3	27030	mutL homolog 3
ENSG0000	125.8666	-0.14989	0.178762	0.228564	0.645717	SSB	6741	small RNA binding exonuclease protection factor La
ENSG0000	752.2178	-0.12834	0.126293	0.22877	0.645792	HAX1	10456	HCLS1 associated protein X-1
ENSG0000	2.431795	-0.03959	0.236244	0.228651	0.645792	NA	NA	NA
ENSG0000	3.265814	-0.04905	0.23666	0.228872	0.645792	SLC4A10	57282	solute carrier family 4 member 10
ENSG0000	90.56454	-0.143	0.216951	0.228858	0.645792	SAMD3	154075	sterile alpha motif domain containing 3
ENSG0000	26.56567	0.152801	0.2141	0.228763	0.645792	NA	NA	NA
ENSG0000	957.8695	0.150636	0.178543	0.228844	0.645792	NAP1L1	4673	nucleosome assembly protein 1 like 1
ENSG0000	5.807867	0.081692	0.240584	0.228846	0.645792	EID3	493861	EP300 interacting inhibitor of differentiation 3
ENSG0000	33.77176	0.152961	0.207964	0.228807	0.645792	SPIN3	169981	spindlin family member 3
ENSG0000	59.46852	0.149756	0.174004	0.228936	0.645873	PFDN5	5204	prefoldin subunit 5
ENSG0000	397.0063	0.147116	0.171241	0.229034	0.646051	DNHD1	144132	dynein heavy chain domain 1
ENSG0000	3.182312	0.047454	0.236434	0.229081	0.646083	FAM66B	1E+08	family with sequence similarity 66 member B
ENSG0000	156.643	0.154897	0.196002	0.229197	0.64631	APPL1	26060	adaptor pı phosphotyrosine interacting with PH domain and leucine zipper 1
ENSG0000	8.529851	0.108785	0.243928	0.229249	0.646359	PHLDB2	90102	pleckstrin homology like domain family B member 2
ENSG0000	831.0738	0.139411	0.14622	0.229286	0.646362	NSMF	26012	NMDA receptor synaptonuclear signaling and neuronal migration factor
ENSG0000	232.7928	0.135402	0.137799	0.229389	0.646554	MACO1	55219	macoilin 1
ENSG0000	144.5232	0.138875	0.144639	0.229647	0.647182	FAM20B	9917	FAM20B glycosaminoglycan xylosylkinase
ENSG0000	241.7951	0.149845	0.179401	0.229775	0.647443	LRPPRC	10128	leucine rich pentatricopeptide repeat containing
ENSG0000	18.07002	0.123891	0.239641	0.229877	0.647629	ADAM23	8745	ADAM metallopeptidase domain 23
ENSG0000	4.420237	-0.05125	0.235679	0.229956	0.647652	NA	NA	NA
ENSG0000	35.53571	0.131015	0.235301	0.229948	0.647652	HAUS1	115106	HAUS augmin like complex subunit 1
ENSG0000	550.7277	0.144348	0.160314	0.230205	0.64798	TTLL4	9654	tubulin tyrosine ligase like 4
ENSG0000	13.51045	0.123913	0.241245	0.230126	0.64798	CENPH	64946	centromere protein H
ENSG0000	1829.469	-0.13602	0.141918	0.230202	0.64798	ZFTRAF1	50626	zinc finger TRAF-type containing 1
ENSG0000	401.0374	-0.14395	0.159351	0.230214	0.64798	EPOR	2057	erythropoietin receptor
ENSG0000	12.52067	-0.10092	0.241643	0.230327	0.6481	NA	NA	NA
ENSG0000	26.27318	0.114837	0.238135	0.230326	0.6481	ABCA5	23461	ATP binding cassette subfamily A member 5
ENSG0000	506.4558	-0.14382	0.171059	0.230381	0.648153	RAB14	51552	RAB14 member RAS oncogene family
ENSG0000	2701.331	-0.13932	0.149417	0.230497	0.648181	PBX2	5089	PBX homeobox 2
ENSG0000	7.933611	0.105085	0.243367	0.230458	0.648181	PTPRK	5796	protein tyrosine phosphatase receptor type K
ENSG0000	187.7463	-0.1469	0.203011	0.230471	0.648181	NA	NA	NA
ENSG0000	6.337055	0.100654	0.243561	0.230555	0.648244	TRAV26-2	28656	T cell receptor alpha variable 26-2
ENSG0000	94.33611	0.144275	0.15959	0.230733	0.648444	C6orf226	441150	chromosome 6 open reading frame 226
ENSG0000	1487.909	-0.12997	0.133762	0.230673	0.648444	FDFT1	2222	farnesyl-diphosphate farnesyltransferase 1
ENSG0000	5.138199	0.079146	0.240174	0.230719	0.648444	LOC10192	1.02E+08	uncharacterized LOC101929698
ENSG0000	283.9873	0.126394	0.124259	0.230818	0.648584	NDUFS1	4719	NADH:ubiquinone oxidoreductase core subunit S1
ENSG0000	21.28102	0.141622	0.230463	0.230897	0.648606	NA	NA	NA
ENSG0000	20.4054	0.137891	0.233127	0.230891	0.648606	LINC02724	1.01E+08	long intergenic non-protein coding RNA 2724
ENSG0000	35.62082	-0.15155	0.208812	0.230961	0.648688	KIAA1671	85379	KIAA1671
ENSG0000	7.817982	-0.09373	0.242036	0.23119	0.648972	FHIP1A	729830	FHF complex subunit HOOK interacting protein 1A
ENSG0000	298.9349	0.130505	0.131076	0.231275	0.648972	BRD9	65980	bromodomain containing 9
ENSG0000	61.3263	0.150643	0.20286	0.231238	0.648972	EBAG9	9166	estrogen receptor binding site associated antigen 9
ENSG0000	81.55886	-0.14787	0.171694	0.231147	0.648972	SDHAF2	54949	succinate dehydrogenase complex assembly factor 2
ENSG0000	15.17047	0.102562	0.241867	0.231258	0.648972	IGHV4-55	28393	immunoglobulin heavy variable 4-55 (pseudogene)

ENSG0000	13579.95	-0.11816	0.112787	0.231116	0.648972	TMSB4X	7114	thymosin beta 4 X-linked
ENSG0000	33801.49	-0.14434	0.207844	0.231355	0.649099	FMNL1	752	formin like 1
ENSG0000	1784.786	-0.1349	0.140329	0.231402	0.64913	RIC8A	60626	RIC8 guanine nucleotide exchange factor A
ENSG0000	9.020213	0.084549	0.239749	0.231525	0.649291	CA13	377677	carbonic anhydrase 13
ENSG0000	140.5852	-0.1484	0.178981	0.231637	0.649291	NA	NA	NA
ENSG0000	46.90848	-0.14428	0.220076	0.231573	0.649291	IFI30	10437	IFI30 lysosomal thiol reductase
ENSG0000	11.26062	-0.12938	0.240013	0.231634	0.649291	ZNF460-AS	1.05E+08	ZNF460 antisense RNA 1
ENSG0000	5578.169	0.140769	0.146622	0.231619	0.649291	ND4L	4539	NADH dehydrogenase subunit 4L
ENSG0000	12.77655	-0.12535	0.240335	0.231681	0.649317	AAMDC	28971	adipogenesis associated Mth938 domain containing
ENSG0000	254.3918	0.146589	0.162454	0.231843	0.649473	OXSR1	9943	oxidative stress responsive kinase 1
ENSG0000	4792.509	-0.13805	0.173685	0.231811	0.649473	DEF6	50619	DEF6 guanine nucleotide exchange factor
ENSG0000	5.335796	-0.08171	0.240649	0.231803	0.649473	NA	NA	NA
ENSG0000	14.22681	-0.10468	0.242436	0.2319	0.649532	NA	NA	NA
ENSG0000	727.8891	0.141315	0.157088	0.232017	0.64976	GATD1	347862	glutamine amidotransferase class 1 domain containing 1
ENSG0000	14.18429	0.109103	0.240428	0.232145	0.65002	ZEB2-AS1	1E+08	ZEB2 antisense RNA 1
ENSG0000	65.71929	0.151653	0.192455	0.232192	0.650052	GCLC	2729	glutamate-cysteine ligase catalytic subunit
ENSG0000	46.64458	0.150811	0.201228	0.232234	0.650071	UTP20	27340	UTP20 small subunit processome component
ENSG0000	1071.027	-0.12064	0.118291	0.232273	0.650082	ISCU	23479	iron-sulfur cluster assembly enzyme
ENSG0000	5.926117	-0.06772	0.23795	0.232319	0.65011	ADAMTS6	11174	ADAM metallopeptidase with thrombospondin type 1 motif 6
ENSG0000	3.723516	0.084975	0.242139	0.232482	0.650466	NA	NA	NA
ENSG0000	1024.468	-0.14404	0.167216	0.232578	0.650637	MARCKSL1	65108	MARCKS like 1
ENSG0000	66.91431	0.148977	0.205734	0.232669	0.650792	SPEG	10290	striated muscle enriched protein kinase
ENSG0000	18.65129	-0.07693	0.23703	0.232957	0.651399	CYP21A2	1589	cytochrome P450 family 21 subfamily A member 2
ENSG0000	2580.215	-0.14325	0.16655	0.232925	0.651399	CORO1B	57175	coronin 1B
ENSG0000	564.6512	-0.12765	0.127439	0.233182	0.651558	RNF25	64320	ring finger protein 25
ENSG0000	109.8139	-0.15213	0.179605	0.233228	0.651558	CCR4	1233	C-C motif chemokine receptor 4
ENSG0000	60.89239	0.15078	0.196227	0.23308	0.651558	GBE1	2632	1 4-alpha-glucan branching enzyme 1
ENSG0000	145.6539	0.139683	0.221132	0.233186	0.651558	NUS1	116150	NUS1 dehydrodolichyl diphosphate synthase subunit
ENSG0000	14.09659	0.125045	0.239511	0.233195	0.651558	NA	NA	NA
ENSG0000	16.73294	-0.13189	0.23638	0.233217	0.651558	NA	NA	NA
ENSG0000	37.15612	0.149784	0.212899	0.233349	0.651797	HFE	3077	homeostatic iron regulator
ENSG0000	25674.61	-0.13828	0.217633	0.233416	0.651885	TLN1	7094	talin 1
ENSG0000	4.560293	0.080613	0.240865	0.233453	0.65189	CENPW	387103	centromere protein W
ENSG0000	1414.465	-0.11302	0.107239	0.233632	0.652291	MBD1	4152	methyl-CpG binding domain protein 1
ENSG0000	50.15231	0.132588	0.231354	0.233691	0.652311	MOSMO	730094	modulator of smoothened
ENSG0000	56.12582	0.129956	0.231856	0.233711	0.652311	ZNF486	90649	zinc finger protein 486
ENSG0000	234.4108	-0.14834	0.186524	0.233775	0.652391	EOMES	8320	eomesodermin
ENSG0000	1363.706	-0.12896	0.130783	0.233961	0.652809	ZNF276	92822	zinc finger protein 276
ENSG0000	442.3674	-0.13606	0.142945	0.234018	0.65287	ABCC10	89845	ATP binding cassette subfamily C member 10
ENSG0000	24.8105	0.117265	0.237701	0.23419	0.652951	TFB2M	64216	transcripti mitochondrial
ENSG0000	12.18078	0.080485	0.238297	0.234126	0.652951	NA	NA	NA
ENSG0000	8.620361	0.095342	0.241982	0.234093	0.652951	NA	NA	NA
ENSG0000	39.86596	-0.14169	0.225478	0.234173	0.652951	RNF165	494470	ring finger protein 165
ENSG0000	18.75951	-0.12301	0.238062	0.234299	0.653095	MELTF-AS	1.01E+08	MELTF antisense RNA 1
ENSG0000	104.2525	-0.14443	0.161811	0.234313	0.653095	SMG8	55181	SMG8 nonsense mediated mRNA decay factor
ENSG0000	58.87292	-0.12883	0.228722	0.23457	0.653132	CDC42	998	cell division cycle 42
ENSG0000	44.23137	0.103632	0.23776	0.234641	0.653132	SNX4	8723	sorting nexin 4
ENSG0000	104.3038	-0.14542	0.166349	0.234375	0.653132	CLP1	10978	cleavage factor polyribonucleotide kinase subunit 1
ENSG0000	34866.03	-0.14153	0.159002	0.234478	0.653132	CFL1	1072	cofilin 1
ENSG0000	2214.695	0.128221	0.129651	0.234602	0.653132	RPS6KB2	6199	ribosomal protein S6 kinase B2
ENSG0000	430.0082	-0.12096	0.117324	0.234416	0.653132	C14orf119	55017	chromosome 14 open reading frame 119
ENSG0000	48.89338	0.14959	0.183858	0.234642	0.653132	MEX3D	399664	mex-3 RNA binding family member D
ENSG0000	203.9755	-0.14906	0.181849	0.234488	0.653132	TMIGD2	126259	transmembrane and immunoglobulin domain containing 2
ENSG0000	23.07331	0.132048	0.23283	0.234647	0.653132	CDH26	60437	cadherin 26
ENSG0000	149.0039	-0.14727	0.183781	0.234762	0.653253	ROCK2	9475	Rho associated coiled-coil containing protein kinase 2
ENSG0000	3.843708	0.04971	0.235688	0.234738	0.653253	NA	NA	NA
ENSG0000	160.2738	0.150172	0.183326	0.234827	0.653337	GAL3ST4	79690	galactose-3-O-sulfotransferase 4
ENSG0000	162.1183	0.140439	0.220143	0.235092	0.653973	BTF3L4	91408	basic transcription factor 3 like 4
ENSG0000	7.42664	0.064852	0.237106	0.235132	0.653984	PTPDC1	138639	protein tyrosine phosphatase domain containing 1
ENSG0000	417.802	0.131408	0.13485	0.235211	0.654107	ITFG2	55846	integrin alpha FG-GAP repeat containing 2
ENSG0000	254.4615	0.136945	0.149643	0.235357	0.654412	MZT2A	653784	mitotic spindle organizing protein 2A
ENSG0000	8.713674	0.088004	0.240233	0.235523	0.654431	NA	NA	NA
ENSG0000	2.740752	0.043943	0.235364	0.235469	0.654431	NA	NA	NA
ENSG0000	223.8762	0.134841	0.139519	0.235542	0.654431	POLR3E	55718	RNA polymerase III subunit E
ENSG0000	368.5858	0.148691	0.168726	0.235413	0.654431	ARMC5	79798	armadillo repeat containing 5
ENSG0000	126.8738	0.147417	0.201961	0.235519	0.654431	NEDD4L	23327	NEDD4 like E3 ubiquitin protein ligase
ENSG0000	1621.397	-0.13452	0.149674	0.235605	0.654507	PDIA3	2923	protein disulfide isomerase family A member 3
ENSG0000	531.3768	-0.13245	0.141771	0.23567	0.654586	ZMYND8	23613	zinc finger MYND-type containing 8
ENSG0000	17.67534	-0.10593	0.238752	0.235814	0.654887	NA	NA	NA
ENSG0000	70.52559	0.150585	0.184899	0.235876	0.654918	UFSP2	55325	UFM1 specific peptidase 2
ENSG0000	55.41238	0.149572	0.194537	0.235896	0.654918	LYRM9	201229	LYR motif containing 9
ENSG0000	9.306123	-0.11398	0.24201	0.235959	0.654992	PTPRVP	148713	protein tyı pseudogene
ENSG0000	11.58932	-0.10866	0.240032	0.236189	0.655118	NA	NA	NA
ENSG0000	20.82386	0.126854	0.236537	0.236196	0.655118	BORCS7	119032	BLOC-1 related complex subunit 7
ENSG0000	62.1397	0.11801	0.234789	0.236189	0.655118	RAB11FIP2	22841	RAB11 family interacting protein 2
ENSG0000	15.15659	-0.13212	0.235407	0.236056	0.655118	TGFBR3L	1.01E+08	transforming growth factor beta receptor 3 like
ENSG0000	7.29667	0.091917	0.240976	0.236219	0.655118	PPP5D1P	1.01E+08	PPP5 tetra pseudogene
ENSG0000	163.1498	0.13482	0.141003	0.236151	0.655118	SRRD	402055	SRR1 domain containing
ENSG0000	777.6056	-0.13978	0.154388	0.236255	0.655121	SLC25A22	79751	solute carrier family 25 member 22
ENSG0000	4.171861	0.073608	0.239689	0.236547	0.655779	RPL7AP30	441034	ribosomal protein L7a pseudogene 30
ENSG0000	184.1803	0.139135	0.203866	0.236565	0.655779	TCP11L2	255394	t-complex 11 like 2
ENSG0000	6474.438	-0.13618	0.215706	0.236685	0.655993	PADI2	11240	peptidyl arginine deiminase 2
ENSG0000	12.7538	0.074321	0.237385	0.236733	0.655993	HACE1	57531	HECT domain and ankyrin repeat containing E3 ubiquitin protein ligase 1
ENSG0000	145.0175	0.148287	0.190443	0.236749	0.655993	SMURF2	64750	SMAD specific E3 ubiquitin protein ligase 2
ENSG0000	1454.434	-0.14602	0.186098	0.236785	0.655994	RALB	5899	RAS like proto-oncogene B
ENSG0000	410.0134	0.12974	0.132307	0.236961	0.656349	CNNM3	26505	cyclin and CBS domain divalent metal cation transport mediator 3
ENSG0000	12.3947	0.131468	0.237772	0.237021	0.656349	NA	NA	NA
ENSG0000	7.793296	-0.08718	0.240144	0.237001	0.656349	ZNF702P	79986	zinc finger pseudogene
ENSG0000	95.19817	0.145809	0.20965	0.237094	0.656353	TMEM18	129787	transmembrane protein 18
ENSG0000	54.4025	0.147606	0.207053	0.237062	0.656353	EIF4BP3	1E+08	eukaryotic translation initiation factor 4B pseudogene 3
ENSG0000	2400.178	-0.14475	0.171548	0.237221	0.656449	PRRC2C	23215	proline rich coiled-coil 2C
ENSG0000	5.057717	-0.07201	0.238654	0.237298	0.656449	NA	NA	NA
ENSG0000	7.095586	0.087809	0.241934	0.237354	0.656449	TRIM40	135644	tripartite motif containing 40
ENSG0000	6376.688	-0.14366	0.200251	0.237369	0.656449	VNN2	8875	vanin 2
ENSG0000	13.32427	-0.12329	0.241761	0.237329	0.656449	TRGV9	6983	T cell receptor gamma variable 9
ENSG0000	3.493057	0.050878	0.23565	0.23738	0.656449	LINC01801	400685	long intergenic non-protein coding RNA 1801

ENSG0000	311.0103	-0.14649	0.195574	0.23729	0.656449	KDM6A	7403	lysine demethylase 6A
ENSG0000	3410.074	-0.12916	0.132054	0.23774	0.657028	TNFRSF14	8764	TNF receptor superfamily member 14
ENSG0000	490.6261	-0.14353	0.194475	0.237768	0.657028	GNAI3	2773	G protein subunit alpha i3
ENSG0000	39.87554	0.147602	0.210107	0.237724	0.657028	WDSUB1	151525	WD repea sterile alpha motif and U-box domain containing 1
ENSG0000	3.843679	-0.04032	0.234411	0.237632	0.657028	NA	NA	NA
ENSG0000	6.156372	-0.07292	0.238691	0.237751	0.657028	NA	NA	NA
ENSG0000	1563.453	-0.13461	0.14242	0.23786	0.657084	CSRP1	1465	cysteine and glycine rich protein 1
ENSG0000	23.40222	0.147276	0.217152	0.23785	0.657084	PSPH	5723	phosphoserine phosphatase
ENSG0000	5.936701	0.016331	0.233883	0.237918	0.657146	NA	NA	NA
ENSG0000	316.626	-0.14589	0.170543	0.238011	0.657204	RAB11FIP3	9727	RAB11 family interacting protein 3
ENSG0000	2014.167	0.124441	0.12435	0.238002	0.657204	COX6B1	1340	cytochrome c oxidase subunit 6B1
ENSG0000	4.770495	0.049227	0.235854	0.23823	0.65771	LINC0225€	1.01E+08	long intergenic non-protein coding RNA 2256
ENSG0000	32.37705	-0.14403	0.217983	0.238368	0.657711	SLC25A33	84275	solute carrier family 25 member 33
ENSG0000	10.70319	0.122932	0.24175	0.238555	0.657711	ESPNL	339768	espin like
ENSG0000	40.25128	-0.14368	0.21616	0.239018	0.657711	RFC4	5984	replication factor C subunit 4
ENSG0000	14.63985	0.11228	0.240168	0.238888	0.657711	SH3RF1	57630	SH3 domain containing ring finger 1
ENSG0000	7.35978	0.084585	0.239988	0.238559	0.657711	EEF1A1P19	1E+08	eukaryotic translation elongation factor 1 alpha 1 pseudogene 19
ENSG0000	112.984	-0.14442	0.167449	0.238415	0.657711	PI16	221476	peptidase inhibitor 16
ENSG0000	16.39328	0.132799	0.234865	0.238829	0.657711	NA	NA	NA
ENSG0000	4.451669	0.05674	0.236295	0.238405	0.657711	ULBP3	79465	UL16 binding protein 3
ENSG0000	1154.48	0.11446	0.112382	0.238955	0.657711	POLM	27434	DNA polymerase mu
ENSG0000	222.8822	0.141918	0.159076	0.238932	0.657711	CDK5	1020	cyclin dependent kinase 5
ENSG0000	116.4692	0.146782	0.178637	0.23869	0.657711	ADCK5	203054	aarF domain containing kinase 5
ENSG0000	8.976455	0.101171	0.24228	0.238611	0.657711	LNCAROD	1.02E+08	lncRNA activating regulator of DKK1
ENSG0000	5.035785	0.064007	0.23812	0.238907	0.657711	HSPA12A	259217	heat shock protein family A (Hsp70) member 12A
ENSG0000	3.589908	0.057629	0.23714	0.238584	0.657711	NA	NA	NA
ENSG0000	45.60292	0.139396	0.223113	0.238427	0.657711	WFIKKN1	117166	WAP follistatin, immunog kunitz and netrin domain containing 1
ENSG0000	87.73429	0.148193	0.198508	0.238974	0.657711	RPS2P46	125208	ribosomal protein S2 pseudogene 46
ENSG0000	8.215867	0.07439	0.238279	0.238913	0.657711	RPS7P1	388363	ribosomal protein S7 pseudogene 1
ENSG0000	6.900345	-0.07094	0.237766	0.239021	0.657711	LOC10192	1.02E+08	uncharacterized LOC101927369
ENSG0000	598.6208	-0.13676	0.14866	0.23888	0.657711	SNX11	29916	sorting nexin 11
ENSG0000	394.6462	-0.14387	0.190114	0.238806	0.657711	RTN2	6253	reticulon 2
ENSG0000	145.9474	0.144298	0.205612	0.238645	0.657711	SNRPD2	6633	small nuclear ribonucleoprotein D2 polypeptide
ENSG0000	82.91814	-0.14757	0.197458	0.238855	0.657711	PNMA3	29944	PNMA family member 3
ENSG0000	45.92464	-0.1468	0.208822	0.239157	0.657888	FBXO8	26269	F-box protein 8
ENSG0000	201.5982	0.146078	0.177784	0.239146	0.657888	RAB21	23011	RAB21 member RAS oncogene family
ENSG0000	8.298276	0.080574	0.239035	0.239309	0.658209	LOC10272	1.03E+08	anaphase-promoting complex subunit 1-like
ENSG0000	6.075379	-0.09233	0.241487	0.239472	0.658556	CXCL3	2921	C-X-C motif chemokine ligand 3
ENSG0000	109.3063	-0.14276	0.210949	0.239521	0.658592	TLE2	7089	TLE family transcriptional corepressor
ENSG0000	686.3475	-0.13157	0.14228	0.239636	0.658614	SLC35F6	54978	solute carrier family 35 member F6
ENSG0000	66.4336	-0.14543	0.168202	0.239621	0.658614	NA	NA	NA
ENSG0000	37.8158	-0.14947	0.201606	0.239628	0.658614	RAB39B	116442	RAB39B member RAS oncogene family
ENSG0000	258.6694	-0.14445	0.199436	0.239919	0.658797	CCDC17	149483	coiled-coil domain containing 17
ENSG0000	3.666377	-0.06889	0.238992	0.23979	0.658797	B3GALNT1	8706	beta-1 3-N-acetylgalactosaminyltransferase 1 (globoside blood group)
ENSG0000	1199.842	-0.1372	0.13442	0.239911	0.658797	PHYKPL	85007	5-phosphohydroxy-L-lysine phospho-lyase
ENSG0000	38.09678	-0.13179	0.22938	0.239837	0.658797	VPS33B	26276	VPS33B late endosome and lysosome associated
ENSG0000	3777.3	-0.13786	0.151909	0.239867	0.658797	PHF12	57649	PHD finger protein 12
ENSG0000	28.75716	-0.12618	0.233221	0.23988	0.658797	LOC12490	1.25E+08	uncharacterized LOC124905134
ENSG0000	108.5749	-0.14106	0.157944	0.240102	0.6592	NBR2	10230	neighbor of BRCA1 lncRNA 2
ENSG0000	17.73269	0.135707	0.232382	0.240202	0.659254	GSTM3	2947	glutathione S-transferase mu 3
ENSG0000	4.48009	0.048072	0.23619	0.240199	0.659254	RPSAP22	1E+08	ribosomal protein SA pseudogene 22
ENSG0000	53.87996	-0.14163	0.21637	0.240229	0.659254	NA	NA	NA
ENSG0000	305.9797	-0.13067	0.139529	0.240416	0.659668	SYT11	23208	synaptotagmin 11
ENSG0000	15.25264	0.083446	0.238084	0.240476	0.659733	NA	NA	NA
ENSG0000	6.551705	-0.05726	0.235601	0.240565	0.65988	FAM171A2	284069	family with sequence similarity 171 member A2
ENSG0000	965.0969	-0.13513	0.146496	0.240691	0.660048	ARL4C	10123	ADP ribosylation factor like GTPase 4C
ENSG0000	136.3444	0.143234	0.164473	0.240699	0.660048	DLG1	1739	discs large MAGUK scaffold protein 1
ENSG0000	2.391926	-0.05704	0.237163	0.240744	0.660073	SEPTIN4-A	1.02E+08	SEPTIN4 antisense RNA 1
ENSG0000	12.6368	-0.09095	0.239883	0.240784	0.660083	NA	NA	NA
ENSG0000	303.0919	0.145043	0.200095	0.240859	0.660146	AHCTF1	25909	AT-hook containing transcription factor 1
ENSG0000	42.36031	-0.11125	0.235645	0.240879	0.660146	HPN	3249	hepsin
ENSG0000	156.1453	-0.13382	0.142661	0.241078	0.660593	ISCA2	122961	iron-sulfur cluster assembly 2
ENSG0000	148.7271	0.13599	0.145769	0.241122	0.660615	SIMC1	375484	SUMO interacting motifs containing 1
ENSG0000	2.39298	0.048564	0.236455	0.241194	0.660713	PARD6G	84552	par-6 family cell polarity regulator gamma
ENSG0000	4235.574	0.124413	0.133684	0.241321	0.660962	TRABD	80305	TraB domain containing
ENSG0000	2.558509	0.03627	0.23449	0.241555	0.661307	NA	NA	NA
ENSG0000	1120.748	0.125612	0.118197	0.241547	0.661307	SRSF5	6430	serine and arginine rich splicing factor 5
ENSG0000	122.0021	0.146007	0.179999	0.24152	0.661307	GTPBP3	84705	GTP bindir mitochondrial
ENSG0000	7.737193	-0.08985	0.241444	0.241618	0.661381	RASIP1	54922	Ras interacting protein 1
ENSG0000	13.6285	-0.10805	0.239287	0.241723	0.661472	NA	NA	NA
ENSG0000	3.077518	0.062396	0.239137	0.241703	0.661472	NA	NA	NA
ENSG0000	352.8868	0.129716	0.134648	0.242029	0.661473	CTNNBIP1	56998	catenin beta interacting protein 1
ENSG0000	554.6426	0.136011	0.147284	0.241927	0.661473	PRPF38B	55119	pre-mRNA processing factor 38B
ENSG0000	98.89653	0.146163	0.192591	0.241868	0.661473	ZNF621	285268	zinc finger protein 621
ENSG0000	15.01695	0.108641	0.239575	0.241978	0.661473	TRMT10A	93587	tRNA methyltransferase 10A
ENSG0000	59.33321	0.143391	0.213737	0.242085	0.661473	ERMARD	55780	ER membrane associated RNA degradation
ENSG0000	2630.356	-0.13849	0.156533	0.242064	0.661473	HSF1	3297	heat shock transcription factor 1
ENSG0000	69.65439	0.146983	0.197591	0.241868	0.661473	HABP4	22927	hyaluronan binding protein 4
ENSG0000	9807.596	-0.14372	0.183732	0.241932	0.661473	SIPA1	6494	signal-induced proliferation-associated 1
ENSG0000	252.849	0.142282	0.170952	0.241864	0.661473	CLN6	54982	CLN6 transmembrane ER protein
ENSG0000	96.93375	0.146425	0.176596	0.242021	0.661473	BACE2	25825	beta-secretase 2
ENSG0000	20.6684	0.137011	0.228492	0.242369	0.662098	ZNF687-A!	1.01E+08	ZNF687 antisense RNA 1
ENSG0000	926.9994	0.143064	0.159152	0.242386	0.662098	SEC13	6396	SEC13 hon nuclear pore and COPII coat complex component
ENSG0000	3.909135	-0.06289	0.237612	0.242434	0.66213	NA	NA	NA
ENSG0000	95.85668	0.143379	0.167152	0.242543	0.662146	LYRM4	57128	LYR motif containing 4
ENSG0000	69.34754	0.146742	0.181433	0.242484	0.662146	ZNF37BP	1E+08	zinc finger pseudogene
ENSG0000	121.8052	0.146241	0.188108	0.242548	0.662146	PABIR2	159090	PABIR family member 2
ENSG0000	2.663803	0.047883	0.239061	0.242602	0.662181	SFRP1	6422	secreted frizzled related protein 1
ENSG0000	41.49411	-0.1466	0.181878	0.242633	0.662181	LOXL1-AS1	1E+08	LOXL1 antisense RNA 1
ENSG0000	3.821869	0.064303	0.23922	0.242677	0.662201	ATP13A4	84239	ATPase 13A4
ENSG0000	109.2255	0.146589	0.210602	0.242772	0.662362	MRPL42	28977	mitochondrial ribosomal protein L42
ENSG0000	830.998	-0.14268	0.176501	0.24282	0.662394	MCOLN1	57192	mucolipin TRP cation channel 1
ENSG0000	2.42086	-0.03911	0.235854	0.24286	0.662403	LOC44123	441239	uncharacterized LOC441239
ENSG0000	54.18097	0.146014	0.200064	0.243037	0.662788	CCDC91	55297	coiled-coil domain containing 91
ENSG0000	311.1494	0.124572	0.127571	0.243216	0.662967	CNOT10	25904	CCR4-NOT transcription complex subunit 10
ENSG0000	114.4452	-0.14569	0.190375	0.243142	0.662967	ABCF2	10061	ATP binding cassette subfamily F member 2

ENSG0000	38.90834	0.143075	0.213729	0.243247	0.662967	GINS4	84296	GINS complex subunit 4
ENSG0000	692.3168	-0.12767	0.13285	0.243231	0.662967	PARP6	56965	poly(ADP-ribose) polymerase family member 6
ENSG0000	2.52635	0.04793	0.238193	0.243349	0.663146	AICDA	57379	activation induced cytidine deaminase
ENSG0000	672.4895	-0.131	0.13626	0.243473	0.663294	GPR107	57720	G protein-coupled receptor 107
ENSG0000	249.4625	0.143254	0.167814	0.243496	0.663294	LINC00921	283876	long intergenic non-protein coding RNA 921
ENSG0000	243.9335	-0.14011	0.207732	0.243512	0.663294	C18orf25	147339	chromosome 18 open reading frame 25
ENSG0000	46.16299	0.141324	0.218488	0.243555	0.663314	ARHGEF17	9828	Rho guanine nucleotide exchange factor 17
ENSG0000	118.2124	0.142578	0.206915	0.243646	0.663324	METAP1	23173	methionyl aminopeptidase 1
ENSG0000	3.518484	0.05847	0.239704	0.243704	0.663324	NA	NA	NA
ENSG0000	5.102762	0.07563	0.239067	0.243703	0.663324	NA	NA	NA
ENSG0000	12.54234	-0.12548	0.238372	0.243611	0.663324	NA	NA	NA
ENSG0000	562.828	-0.14087	0.182289	0.243749	0.663347	LOC72874	728743	zinc finger domain-containing protein LOC728743
ENSG0000	5.403427	0.063718	0.237509	0.243877	0.663599	EML5	161436	EMAP like 5
ENSG0000	13.77309	0.120114	0.238692	0.244072	0.663833	ABCB4	5244	ATP binding cassette subfamily B member 4
ENSG0000	21.49882	0.130016	0.232704	0.244037	0.663833	C1QTNF4	114900	C1q and TNF related 4
ENSG0000	9.961662	-0.11052	0.244121	0.244055	0.663833	LOC10798	1.08E+08	putative uncharacterized protein LOC401522
ENSG0000	226.9728	0.13552	0.148151	0.244146	0.663847	ERLIN2	11160	ER lipid raft associated 2
ENSG0000	172.0864	0.137146	0.15098	0.24415	0.663847	ATXN3	4287	ataxin 3
ENSG0000	568.3785	0.122591	0.229421	0.244186	0.663848	VNN1	8876	vanin 1
ENSG0000	245.2889	0.142085	0.167459	0.244262	0.66395	C1QA	712	complement C1q A chain
ENSG0000	154.9373	0.145044	0.195577	0.244297	0.66395	RHNO1	83695	RAD9-HUS1-RAD1 interacting nuclear orphan 1
ENSG0000	531.2556	-0.12177	0.123277	0.244391	0.664108	JOSD1	9929	Josephin domain containing 1
ENSG0000	3.369397	0.084724	0.242128	0.244461	0.664199	NA	NA	NA
ENSG0000	5.327642	0.086604	0.240926	0.244562	0.664278	LINC0088€	730091	long intergenic non-protein coding RNA 886
ENSG0000	142.9326	0.141616	0.200264	0.244548	0.664278	ENY2	56943	ENY2 transcription and export complex 2 subunit
ENSG0000	7.64511	0.083015	0.239342	0.244671	0.664422	MZT1	440145	mitotic spindle organizing protein 1
ENSG0000	64068.02	0.143426	0.179649	0.244688	0.664422	ATP6	4508	ATP synthase F0 subunit 6
ENSG0000	47.4885	-0.13303	0.224969	0.244835	0.664722	PGLS-DT	1.01E+08	PGLS divergent transcript
ENSG0000	835.0383	0.11677	0.230059	0.244981	0.664797	MBNL1	4154	muscleblind like splicing regulator 1
ENSG0000	135.0973	-0.14206	0.166275	0.244969	0.664797	FAM177A1	283635	family with sequence similarity 177 member A1
ENSG0000	114.4567	-0.1426	0.171879	0.244922	0.664797	ALOX12-A	1.01E+08	ALOX12 antisense RNA 1
ENSG0000	25.18408	0.138741	0.225554	0.245007	0.664797	HEATR9	256957	HEAT repeat containing 9
ENSG0000	769.2016	0.139431	0.160023	0.245166	0.665128	MAFB	9935	MAF bZIP transcription factor B
ENSG0000	273.3237	0.146268	0.197779	0.245266	0.665202	SLC1A4	6509	solute carrier family 1 member 4
ENSG0000	195.5336	0.1325	0.219797	0.24524	0.665202	PDE3B	5140	phosphodiesterase 3B
ENSG0000	522.3735	0.140091	0.162997	0.245373	0.665395	PSMC3	5702	proteasom ATPase 3
ENSG0000	1416.422	0.144715	0.160681	0.2458	0.665667	ACAP3	116983	ArfGAP wi ankyrin repeat and PH domains 3
ENSG0000	319.6992	0.132874	0.143415	0.245549	0.665667	TMEM69	51249	transmembrane protein 69
ENSG0000	27.66603	0.130077	0.232438	0.245785	0.665667	NA	NA	NA
ENSG0000	275.9445	-0.13073	0.139862	0.245775	0.665667	CIDECP1	152302	cell death inducing DFFA like effector c pseudogene 1
ENSG0000	44.28577	0.144073	0.209652	0.245775	0.665667	LINC03014	1.01E+08	long intergenic non-protein coding RNA 3014
ENSG0000	898.1002	0.137897	0.156456	0.245674	0.665667	FPGS	2356	folylpolyglutamate synthase
ENSG0000	48.37171	-0.13697	0.218767	0.245762	0.665667	ANKRD13E	124930	ankyrin repeat domain 13B
ENSG0000	267.4447	0.139754	0.161686	0.245674	0.665667	USE1	55850	unconventional SNARE in the ER 1
ENSG0000	330.2044	0.140313	0.162744	0.245574	0.665667	DOP1B	9980	DOP1 leucine zipper like protein B
ENSG0000	1266.738	0.133733	0.145261	0.245862	0.665736	HMG20B	10362	high mobility group 20B
ENSG0000	69.13492	0.144832	0.182109	0.245943	0.665856	METTL8	79828	methyltra methylcytidine
ENSG0000	5.045344	0.085891	0.240433	0.245995	0.665861	NA	NA	NA
ENSG0000	33.59034	0.126418	0.230754	0.246018	0.665861	NA	NA	NA
ENSG0000	12.8472	-0.10626	0.238718	0.246126	0.666057	NA	NA	NA
ENSG0000	51.9446	-0.14588	0.196556	0.246329	0.666292	RAB43	339122	RAB43 member RAS oncogene family
ENSG0000	101.1629	0.135201	0.219002	0.246358	0.666292	COMMD2	51122	COMM domain containing 2
ENSG0000	19.49743	0.105819	0.237865	0.246311	0.666292	PRR16	51334	proline rich 16
ENSG0000	5.545761	0.081601	0.239705	0.246255	0.666292	NA	NA	NA
ENSG0000	13.6867	0.126959	0.235849	0.246418	0.666355	UBE3D	90025	ubiquitin protein ligase E3D
ENSG0000	13.47179	-0.11862	0.236969	0.246528	0.666468	THNSL1	79896	threonine synthase like 1
ENSG0000	13.05848	0.113989	0.238247	0.246533	0.666468	C15orf62	643338	chromosome 15 open reading frame 62
ENSG0000	558.0067	-0.10949	0.107379	0.246572	0.666475	TRAPPC3	27095	trafficking protein particle complex subunit 3
ENSG0000	8.60413	-0.08289	0.238361	0.24682	0.666605	MSANTD1	345222	Myb/SANT DNA binding domain containing 1
ENSG0000	21.56722	-0.10296	0.236963	0.246838	0.666605	NRN1	51299	neuritin 1
ENSG0000	2.387283	0.041724	0.234859	0.246688	0.666605	MLXIPL	51085	MLX interacting protein like
ENSG0000	104.52	0.145002	0.194892	0.246784	0.666605	KNTC1	9735	kinetochore associated 1
ENSG0000	9.611291	0.093223	0.239793	0.246819	0.666605	NA	NA	NA
ENSG0000	8.22401	0.099905	0.240406	0.246758	0.666605	NA	NA	NA
ENSG0000	6.683335	0.060473	0.235713	0.246916	0.666716	MTCL1	23255	microtubule crosslinking factor 1
ENSG0000	18365.54	-0.14089	0.196022	0.246973	0.666773	BCL6	604	BCL6 transcription repressor
ENSG0000	142.9661	0.139565	0.160845	0.247218	0.667131	CLCC1	23155	chloride channel CLIC like 1
ENSG0000	127.2647	0.144271	0.190743	0.247251	0.667131	NUCB2	4925	nucleobindin 2
ENSG0000	11.1416	0.096329	0.239506	0.24722	0.667131	NA	NA	NA
ENSG0000	76.11507	0.141448	0.162985	0.247182	0.667131	MAP7D3	79649	MAP7 domain containing 3
ENSG0000	301.7232	-0.14049	0.206022	0.247307	0.667182	NA	NA	NA
ENSG0000	62.22678	0.139411	0.184046	0.247384	0.667293	RFC5	5985	replication factor C subunit 5
ENSG0000	59.41271	0.145625	0.189099	0.247441	0.667315	C18orf21	83608	chromosome 18 open reading frame 21
ENSG0000	130.5255	0.141491	0.197049	0.247502	0.667315	SLC39A6	25800	solute carrier family 39 member 6
ENSG0000	46.91022	0.138044	0.218811	0.247469	0.667315	PNPLA4	8228	patatin like phospholipase domain containing 4
ENSG0000	151.8362	0.137989	0.214422	0.24755	0.667347	THAP12	5612	THAP domain containing 12
ENSG0000	367.7887	0.126718	0.132371	0.247712	0.667438	MGAT5	4249	alpha-1 6-mannosylglycoprotein 6-beta-N-acetylglucosaminyltransferase
ENSG0000	25.64289	0.135297	0.22933	0.247729	0.667438	STAG3L1	54441	stromal antigen 3-like 1 (pseudogene)
ENSG0000	3.573028	0.08238	0.241038	0.247641	0.667438	LRP12	29967	LDL receptor related protein 12
ENSG0000	9.819503	0.084688	0.238342	0.247668	0.667438	P3H3	10536	prolyl 3-hydroxylase 3
ENSG0000	18.35813	-0.1334	0.229885	0.247783	0.667484	MMP15	4324	matrix metallopeptidase 15
ENSG0000	120.9781	0.139176	0.15865	0.247829	0.667509	GPATCH4	54865	G-patch domain containing 4 (gene/pseudogene)
ENSG0000	8.960852	-0.08504	0.239013	0.247967	0.667562	TMEM171	134285	transmembrane protein 171
ENSG0000	25.00907	0.127933	0.232138	0.247932	0.667562	TIGD6	81789	tigger transposable element derived 6
ENSG0000	23.57752	-0.11482	0.235177	0.247983	0.667562	UST	10090	uronyl 2-sulfotransferase
ENSG0000	21.03127	0.101206	0.237644	0.247994	0.667562	ZNF675	171392	zinc finger protein 675
ENSG0000	2.541244	0.038196	0.235414	0.248358	0.667674	SNORD99	692212	small nucl C/D box 99
ENSG0000	41.79911	-0.14639	0.19085	0.248621	0.667674	ATP1A1-A	84852	ATP1A1 antisense RNA 1
ENSG0000	507.2255	0.120805	0.123907	0.248211	0.667674	GON4L	54856	gon-4 like
ENSG0000	220.0481	-0.14053	0.169384	0.248268	0.667674	SCYL3	57147	SCY1 like pseudokinase 3
ENSG0000	24.24091	-0.13474	0.227087	0.248463	0.667674	GPC1	2817	glypican 1
ENSG0000	9.078147	0.103837	0.240811	0.248148	0.667674	ITIH1	3697	inter-alpha-trypsin inhibitor heavy chain 1
ENSG0000	40.50755	-0.13045	0.226133	0.248638	0.667674	EOGT	285203	EGF domain specific O-linked N-acetylglucosamine transferase
ENSG0000	36.35553	0.139976	0.217019	0.248655	0.667674	PAK1IP1	55003	PAK1 interacting protein 1
ENSG0000	1606.217	0.142199	0.204263	0.248256	0.667674	PDIA4	9601	protein disulfide isomerase family A member 4
ENSG0000	2.976098	-0.04003	0.234785	0.248163	0.667674	NA	NA	NA

ENSG0000	87.35479	-0.14079	0.173061	0.248464	0.667674	RELA-DT	1.05E+08	RELA divergent transcript
ENSG0000	3.271325	0.077703	0.240235	0.248502	0.667674	NA	NA	NA
ENSG0000	3.919805	-0.0814	0.240414	0.248403	0.667674	MIR3613	1.01E+08	microRNA 3613
ENSG0000	44.83694	-0.14441	0.177106	0.248415	0.667674	HYPK	25764	huntingtin interacting protein K
ENSG0000	7.631639	-0.08557	0.239133	0.248617	0.667674	PIERCE2	145788	piercer of microtubule wall 2
ENSG0000	6.34819	0.035111	0.23381	0.24859	0.667674	NA	NA	NA
ENSG0000	755.0612	-0.13302	0.146392	0.248588	0.667674	TP53I13	90313	tumor protein p53 inducible protein 13
ENSG0000	4.731607	-0.05908	0.237387	0.248845	0.668084	CECR2	27443	CECR2 histone acetyl-lysine reader
ENSG0000	122.8945	-0.14246	0.176102	0.249084	0.66863	ADAT3	113179	adenosine deaminase tRNA specific 3
ENSG0000	888.8612	0.141523	0.174901	0.249134	0.668664	PYGB	5834	glycogen phosphorylase B
ENSG0000	3603.651	-0.13629	0.163008	0.249243	0.668858	CAMTA2	23125	calmodulin binding transcription activator 2
ENSG0000	33.49111	0.136917	0.221379	0.249397	0.669071	GEMIN6	79833	gem nuclear organelle associated protein 6
ENSG0000	2.988815	-0.04662	0.235326	0.249565	0.669071	NA	NA	NA
ENSG0000	197.003	0.135652	0.156979	0.249568	0.669071	PRRC1	133619	proline rich coiled-coil 1
ENSG0000	4.144569	0.079025	0.23984	0.249397	0.669071	RPL10P3	619445	ribosomal protein L10 pseudogene 3
ENSG0000	1108.246	-0.12439	0.130426	0.249538	0.669071	KCTD2	23510	potassium channel tetramerization domain containing 2
ENSG0000	1150.221	0.138886	0.163191	0.249548	0.669071	SLC16A5	9121	solute carrier family 16 member 5
ENSG0000	25.13135	0.140361	0.217453	0.249578	0.669071	ETV2	2116	ETS variant transcription factor 2
ENSG0000	238.3388	-0.13417	0.149498	0.249616	0.669076	LPIN1	23175	lipin 1
ENSG0000	3.824879	-0.03755	0.235426	0.249668	0.669117	EDN3	1908	endothelin 3
ENSG0000	10.52285	-0.09641	0.239405	0.249731	0.669189	NEFL	4747	neurofilament light chain
ENSG0000	17.604	0.100591	0.23743	0.249902	0.669549	RIPK2-DT	1.02E+08	RIPK2 divergent transcript
ENSG0000	3.46098	0.048939	0.235557	0.249946	0.66957	C6orf132	647024	chromosome 6 open reading frame 132
ENSG0000	3148.391	-0.13072	0.132297	0.250105	0.669737	DENND4B	9909	DENN domain containing 4B
ENSG0000	155.4215	0.140932	0.20546	0.250104	0.669737	GPR25	2848	G protein-coupled receptor 25
ENSG0000	34.85989	0.099733	0.236744	0.250222	0.669737	ZBTB26	57684	zinc finger and BTB domain containing 26
ENSG0000	10272.16	0.131659	0.144283	0.250198	0.669737	RPLP2	6181	ribosomal protein lateral stalk subunit P2
ENSG0000	2552.26	-0.12885	0.17876	0.250195	0.669737	WWP2	11060	WW domain containing E3 ubiquitin protein ligase 2
ENSG0000	1807.159	0.13395	0.148912	0.25027	0.669737	MAP2K7	5609	mitogen-activated protein kinase kinase 7
ENSG0000	57.99424	-0.13665	0.21963	0.250301	0.669737	COL6A1	1291	collagen type VI alpha 1 chain
ENSG0000	437.2565	0.121513	0.124324	0.250257	0.669737	MED14	9282	mediator complex subunit 14
ENSG0000	14.06419	0.083649	0.237774	0.250377	0.669744	MAP4K3	8491	mitogen-activated protein kinase kinase kinase kinase 3
ENSG0000	22.29225	0.138183	0.220928	0.250375	0.669744	LINC0068(	1.07E+08	long intergenic non-protein coding RNA 680
ENSG0000	496.8471	-0.12363	0.129221	0.250414	0.669744	PLIN2	123	perilipin 2
ENSG0000	2.344385	0.033511	0.234428	0.250453	0.669753	NA	NA	NA
ENSG0000	5680.578	-0.1427	0.172472	0.250537	0.669879	EMP3	2014	epithelial membrane protein 3
ENSG0000	54.58842	0.134498	0.221025	0.250718	0.670214	NA	NA	NA
ENSG0000	74.57216	0.143779	0.188604	0.250742	0.670214	SENP3	26168	SUMO specific peptidase 3
ENSG0000	41.03935	0.144342	0.197966	0.250772	0.670214	NA	NA	NA
ENSG0000	38.65964	0.123324	0.232222	0.250995	0.67057	NA	NA	NA
ENSG0000	4207.745	-0.12807	0.137601	0.251035	0.67057	CHD3	1107	chromodomain helicase DNA binding protein 3
ENSG0000	7173.944	-0.13986	0.196805	0.251046	0.67057	CALR	811	calreticulin
ENSG0000	3.703484	0.057397	0.236522	0.251084	0.67057	NA	NA	NA
ENSG0000	7.6288	0.090693	0.240234	0.251088	0.67057	YWHAH-A	25775	YWHAH antisense RNA 1
ENSG0000	187.5492	0.135037	0.152591	0.251149	0.670633	UTP3	57050	UTP3 small subunit processome component
ENSG0000	1054.766	-0.13247	0.146033	0.251222	0.670651	PTGES3	10728	prostaglandin E synthase 3
ENSG0000	3.778298	0.061689	0.236873	0.251229	0.670651	NA	NA	NA
ENSG0000	212.1527	-0.13429	0.150876	0.251288	0.670711	BRAP	8315	BRCA1 associated protein
ENSG0000	3739.606	-0.14054	0.181911	0.251385	0.670873	NATD1	256302	N-acetyltransferase domain containing 1
ENSG0000	67.46778	0.142435	0.173999	0.251462	0.670882	RIOX2	84864	ribosomal oxygenase 2
ENSG0000	90.05891	0.143198	0.172697	0.251454	0.670882	SLC2A8	29988	solute carrier family 2 member 8
ENSG0000	6.900718	0.085508	0.238864	0.251558	0.671042	CAP1P1	401076	CAP1 pseudogene 1
ENSG0000	2844.461	-0.14146	0.18196	0.251601	0.671057	TNFSF14	8740	TNF superfamily member 14
ENSG0000	468.7759	-0.11629	0.116796	0.251726	0.671195	NEK9	91754	NIMA related kinase 9
ENSG0000	2.335017	-0.04823	0.23575	0.25172	0.671195	LOC12490	1.25E+08	uncharacterized LOC124905202
ENSG0000	188.7721	-0.13849	0.20624	0.252223	0.67126	ACOT7	11332	acyl-CoA thioesterase 7
ENSG0000	14181.62	-0.13747	0.145382	0.2523	0.67126	CAP1	10487	cyclase associated actin cytoskeleton regulatory protein 1
ENSG0000	19.93951	0.128178	0.231056	0.251866	0.67126	GPR89B	51463	G protein-coupled receptor 89B
ENSG0000	161.2187	-0.13361	0.146565	0.252089	0.67126	LINC0095⁄	400946	long intergenic non-protein coding RNA 954
ENSG0000	33.36698	0.115439	0.234358	0.252123	0.67126	LINC0200⁽	1.05E+08	long intergenic non-protein coding RNA 2009
ENSG0000	93.71796	0.117879	0.230972	0.252167	0.67126	MANEA	79694	mannosidase endo-alpha
ENSG0000	185.8434	0.133349	0.147439	0.252294	0.67126	SMPD2	6610	sphingomyelin phosphodiesterase 2
ENSG0000	2.75263	-0.06771	0.238862	0.252069	0.67126	NA	NA	NA
ENSG0000	56.32825	0.132265	0.222736	0.251812	0.67126	QSER1	79832	glutamine and serine rich 1
ENSG0000	19.05909	0.093079	0.23758	0.252283	0.67126	NA	NA	NA
ENSG0000	418.3158	-0.14005	0.177285	0.251951	0.67126	SLC46A3	283537	solute carrier family 46 member 3
ENSG0000	192.1089	0.138403	0.219813	0.25198	0.67126	ATOSA	56204	atos homolog A
ENSG0000	396.6499	-0.14199	0.1853	0.251927	0.67126	SLX4	84464	SLX4 structure-specific endonuclease subunit
ENSG0000	2996.852	0.125084	0.224251	0.252141	0.67126	PGLYRP1	8993	peptidoglycan recognition protein 1
ENSG0000	1169.068	0.137613	0.157865	0.251905	0.67126	TMEM164	84187	transmembrane protein 164
ENSG0000	4.102069	0.059008	0.236431	0.252343	0.671278	CKAP2LP1	1.01E+08	CKAP2L pseudogene 1
ENSG0000	1094.529	0.14391	0.183797	0.252462	0.6714	EIF3B	8662	eukaryotic translation initiation factor 3 subunit B
ENSG0000	1776.657	0.139895	0.172508	0.252445	0.6714	CD22	933	CD22 molecule
ENSG0000	26.53921	0.141495	0.215479	0.252692	0.671767	IPO11	51194	importin 11
ENSG0000	54.7944	-0.14415	0.185822	0.252645	0.671767	CREM	1390	cAMP responsive element modulator
ENSG0000	12.47352	-0.08742	0.238873	0.25271	0.671767	NA	NA	NA
ENSG0000	7.365154	0.075165	0.238411	0.252756	0.671791	GDF7	151449	growth differentiation factor 7
ENSG0000	2.713918	0.030182	0.233895	0.252933	0.671963	LNCTAM3⁄	1.03E+08	long non coding transcriptional activator of miR34a
ENSG0000	163.2121	0.141988	0.183625	0.252915	0.671963	SCP2	6342	sterol carrier protein 2
ENSG0000	264.8565	-0.12959	0.13392	0.252978	0.671963	OFD1	8481	OFD1 centriole and centriolar satellite protein
ENSG0000	7.507927	-0.07388	0.237441	0.253004	0.671963	LANCL3	347404	LanC like family member 3
ENSG0000	25.97373	0.14521	0.204954	0.252995	0.671963	UPRT	139596	uracil phosphoribosyltransferase homolog
ENSG0000	1491.681	0.14296	0.160949	0.253091	0.672095	ZNF316	1E+08	zinc finger protein 316
ENSG0000	1238.005	-0.11325	0.113947	0.253168	0.672203	ISG20L2	81875	interferon stimulated exonuclease gene 20 like 2
ENSG0000	4.258368	-0.06043	0.238718	0.253274	0.672386	SPTSSB	165679	serine palmitoyltransferase small subunit B
ENSG0000	74.90901	0.141237	0.209273	0.253336	0.672454	ZNF12	7559	zinc finger protein 12
ENSG0000	29.52047	-0.13443	0.225817	0.253442	0.672639	TRAV8-6	28680	T cell receptor alpha variable 8-6
ENSG0000	8.057755	-0.09829	0.240206	0.253479	0.67264	SLC7A11	23657	solute carrier family 7 member 11
ENSG0000	3.863177	-0.04969	0.235552	0.253646	0.672656	TJP2	9414	tight junction protein 2
ENSG0000	37.97108	-0.13839	0.217129	0.253568	0.672656	RDH5	5959	retinol dehydrogenase 5
ENSG0000	6.571489	-0.07251	0.237698	0.253603	0.672656	NA	NA	NA
ENSG0000	29.84952	-0.11005	0.234391	0.253669	0.672656	CHAC1	79094	ChaC glutathione specific gamma-glutamylcyclotransferase 1
ENSG0000	865.6392	-0.13296	0.203903	0.253659	0.672656	PPP4R1	9989	protein phosphatase 4 regulatory subunit 1
ENSG0000	58.02904	0.141898	0.207877	0.253711	0.672671	EED	8726	embryonic ectoderm development
ENSG0000	6.169302	0.077171	0.238441	0.253809	0.672832	LOC64465	644656	uncharacterized LOC644656
ENSG0000	66.09257	0.142666	0.182172	0.253861	0.672874	STEEP1	63932	STING1 ER exit protein 1

ENSG0000	8.541009	0.083823	0.237811	0.253955	0.673026	CTXN1	404217	cortexin 1
ENSG0000	205.3473	0.134854	0.15272	0.25405	0.673082	KRBA1	84626	KRAB-A domain containing 1
ENSG0000	10.3618	-0.10349	0.239579	0.254028	0.673082	GLS2	27165	glutaminase 2
ENSG0000	2.388312	0.018586	0.23381	0.254256	0.67353	BMP2	650	bone morphogenetic protein 2
ENSG0000	470.5245	0.138878	0.169976	0.254492	0.674058	RAB2A	5862	RAB2A member RAS oncogene family
ENSG0000	100.0673	0.143582	0.189814	0.254535	0.674075	FBXO32	114907	F-box protein 32
ENSG0000	1416.02	0.120809	0.124804	0.254603	0.674157	COPZ1	22818	COPI coat complex subunit zeta 1
ENSG0000	502.8186	0.140084	0.176202	0.254878	0.67444	CITED4	163732	Cbp/p300 interacting transactivator with Glu/Asp rich carboxy-terminal domain 4
ENSG0000	8.924842	-0.10619	0.239949	0.25501	0.67444	NA	NA	NA
ENSG0000	2.466147	-0.05645	0.238572	0.254929	0.67444	VIT	5212	vitrin
ENSG0000	393.111	-0.118	0.122014	0.255513	0.67444	DGUOK	1716	deoxyguanosine kinase
ENSG0000	135.189	0.138751	0.163672	0.255405	0.67444	BCS1L	617	BCS1 hom ubiquinol-cytochrome c reductase complex chaperone
ENSG0000	130.2915	0.140097	0.192532	0.255092	0.67444	LRRC58	116064	leucine rich repeat containing 58
ENSG0000	23.58641	0.089119	0.237288	0.25553	0.67444	FSTL4	23105	follistatin like 4
ENSG0000	8.636623	-0.07535	0.23766	0.255205	0.67444	NA	NA	NA
ENSG0000	21.48205	0.134783	0.22453	0.255106	0.67444	NA	NA	NA
ENSG0000	9.444784	0.10377	0.239109	0.255594	0.67444	FAM185A	222234	family with sequence similarity 185 member A
ENSG0000	207.5972	0.136454	0.157219	0.255252	0.67444	PMPCB	9512	peptidase mitochondrial processing subunit beta
ENSG0000	615.7539	-0.12338	0.131112	0.255149	0.67444	LEPROTL1	23484	leptin receptor overlapping transcript like 1
ENSG0000	11.94555	-0.1184	0.237128	0.25559	0.67444	NA	NA	NA
ENSG0000	2.634888	0.035681	0.234663	0.255507	0.67444	NA	NA	NA
ENSG0000	529.9579	-0.1244	0.134352	0.254772	0.67444	SUSD3	203328	sushi domain containing 3
ENSG0000	421.4524	-0.13233	0.172694	0.255184	0.67444	MFSD14B	84641	major facilitator superfamily domain containing 14B
ENSG0000	94.19011	0.14	0.169792	0.254798	0.67444	MTG1	92170	mitochondrial ribosome associated GTPase 1
ENSG0000	7.846367	0.105051	0.239953	0.254997	0.67444	ARL6IP4	51329	ADP ribosylation factor like GTPase 6 interacting protein 4
ENSG0000	513.6571	0.132236	0.155494	0.255278	0.67444	EP400	57634	E1A binding protein p400
ENSG0000	59.84163	-0.14204	0.206131	0.255476	0.67444	LOC10537	1.05E+08	uncharacterized LOC105372435
ENSG0000	18.15139	0.126274	0.231608	0.255436	0.67444	SPAG4	6676	sperm associated antigen 4
ENSG0000	1023.191	-0.1308	0.16152	0.255446	0.67444	OSBPL2	9885	oxysterol binding protein like 2
ENSG0000	164.0836	-0.13989	0.199474	0.255582	0.67444	GNAZ	2781	G protein subunit alpha z
ENSG0000	4188.8	0.140033	0.182126	0.255561	0.67444	TSPO	706	translocator protein
ENSG0000	241.4668	-0.12996	0.144057	0.255893	0.675037	CDC73	79577	cell division cycle 73
ENSG0000	65.30884	0.141491	0.196715	0.255893	0.675037	CASK	8573	calcium/calmodulin dependent serine protein kinase
ENSG0000	1832.104	-0.13654	0.156779	0.256133	0.675571	FKBP15	23307	FKBP prolyl isomerase family member 15
ENSG0000	19.47591	0.125244	0.232129	0.256315	0.675858	RPS18P5	1E+08	ribosomal protein S18 pseudogene 5
ENSG0000	705.5004	-0.1282	0.140833	0.256302	0.675858	KLC1	3831	kinesin light chain 1
ENSG0000	378.7064	0.126874	0.220827	0.256408	0.676005	AP1S2	8905	adaptor related protein complex 1 subunit sigma 2
ENSG0000	265.4731	-0.13009	0.143583	0.256679	0.676266	NA	NA	NA
ENSG0000	52.93236	0.142373	0.18211	0.256555	0.676266	CAPN10-D	1.02E+08	CAPN10 divergent transcript
ENSG0000	210.4564	-0.13885	0.173271	0.256684	0.676266	SCLT1	132320	sodium channel and clathrin linker 1
ENSG0000	2.506063	0.043133	0.235997	0.256625	0.676266	CLDN12	9069	claudin 12
ENSG0000	8.26436	0.084073	0.239103	0.256692	0.676266	KISS1R	84634	KISS1 receptor
ENSG0000	3.030347	-0.03525	0.235087	0.256787	0.676419	SGCD	6444	sarcoglycan delta
ENSG0000	171.7368	-0.13953	0.197866	0.256978	0.676651	CHMP2B	25978	charged multivesicular body protein 2B
ENSG0000	67.28953	-0.14126	0.179158	0.256952	0.676651	CDC14B	8555	cell division cycle 14B
ENSG0000	8.273195	0.067696	0.236586	0.256986	0.676651	NUTM2E	283008	NUT family member 2E
ENSG0000	1401.658	0.116846	0.117749	0.257096	0.676748	SERP1	27230	stress associated endoplasmic reticulum protein 1
ENSG0000	412.3948	-0.1286	0.142057	0.257065	0.676748	SELENOW	6415	selenoprotein W
ENSG0000	26.82057	0.140073	0.208216	0.257381	0.677303	PRMT9	90826	protein arginine methyltransferase 9
ENSG0000	1825.908	-0.13781	0.197019	0.257371	0.677303	UPP1	7378	uridine phosphorylase 1
ENSG0000	58.17038	0.139407	0.2013	0.257543	0.677326	SPEN-AS1	729614	SPEN antisense RNA 1
ENSG0000	90.64689	0.127753	0.221804	0.257612	0.677326	LINC0121	1.02E+08	long intergenic non-protein coding RNA 1215
ENSG0000	4.365149	0.059343	0.237066	0.257592	0.677326	NA	NA	NA
ENSG0000	2.859125	-0.02437	0.234405	0.257575	0.677326	RNU6-130	1.06E+08	RNA U6 small r pseudogene
ENSG0000	441.8048	0.122231	0.131768	0.25756	0.677326	SLC39A9	55334	solute carrier family 39 member 9
ENSG0000	491.7633	-0.13296	0.157628	0.257511	0.677326	LLGL2	3993	LLGL scribble cell polarity complex component 2
ENSG0000	47.0991	0.133099	0.215918	0.257657	0.677347	TMEM97	27346	transmembrane protein 97
ENSG0000	6.78569	0.082418	0.238531	0.257775	0.677388	NA	NA	NA
ENSG0000	123.7597	0.14193	0.199589	0.257783	0.677388	NA	NA	NA
ENSG0000	23.98379	0.143308	0.205662	0.257722	0.677388	NDUFV2-A	1.02E+08	NDUFV2 antisense RNA 1
ENSG0000	19.29552	0.134036	0.226015	0.257836	0.67743	HTR6	3362	5-hydroxytryptamine receptor 6
ENSG0000	1199.065	0.135285	0.162389	0.257971	0.677439	RPS12	6206	ribosomal protein S12
ENSG0000	567.8719	-0.13879	0.171276	0.258024	0.677439	STK17A	9263	serine/threonine kinase 17a
ENSG0000	104.7949	-0.13954	0.191416	0.258007	0.677439	CASP7	840	caspase 7
ENSG0000	244.6601	0.127177	0.139086	0.257994	0.677439	DCTPP1	79077	dCTP pyrophosphatase 1
ENSG0000	105.9596	0.137083	0.165619	0.257961	0.677439	WDR83	84292	WD repeat domain 83
ENSG0000	20.44721	-0.11938	0.231895	0.25808	0.677487	LINC0009	1E+08	long intergenic non-protein coding RNA 92
ENSG0000	7.556866	0.068063	0.237711	0.258315	0.677626	EFCAB7	84455	EF-hand calcium binding domain 7
ENSG0000	98.95027	0.14055	0.180082	0.258249	0.677626	PER2	8864	period circadian regulator 2
ENSG0000	359.4691	0.128142	0.217104	0.258338	0.677626	EIF2A	83939	eukaryotic translation initiation factor 2A
ENSG0000	25.30053	-0.12544	0.228681	0.258393	0.677626	EVC	2121	EvC ciliary complex subunit 1
ENSG0000	812.9528	0.124333	0.133104	0.25854	0.677626	VPS52	6293	VPS52 subunit of GARP complex
ENSG0000	1656.604	-0.12905	0.147378	0.258481	0.677626	ARID3B	10620	AT-rich interaction domain 3B
ENSG0000	553.2707	-0.12782	0.213055	0.258452	0.677626	LPCAT2	54947	lysophosphatidylcholine acyltransferase 2
ENSG0000	3.999029	-0.05297	0.236953	0.258409	0.677626	SEZ6	124925	seizure related 6 homolog
ENSG0000	8779.632	-0.12838	0.144513	0.258533	0.677626	ARHGAP2	201176	Rho GTPase activating protein 27
ENSG0000	5.607504	0.077254	0.238853	0.25835	0.677626	LOC61326	613266	uncharacterized LOC613266
ENSG0000	9.047648	0.09011	0.239062	0.258184	0.677626	NA	NA	NA
ENSG0000	3793.963	-0.12794	0.216078	0.25878	0.678095	S100A8	6279	S100 calcium binding protein A8
ENSG0000	101.2918	0.139631	0.197158	0.258792	0.678095	ZNF146	7705	zinc finger protein 146
ENSG0000	137.5207	0.138261	0.168562	0.25903	0.678231	USP40	55230	ubiquitin specific peptidase 40
ENSG0000	36.57386	0.090782	0.234738	0.25902	0.678231	USP53	54532	ubiquitin specific peptidase 53
ENSG0000	16.76543	-0.12901	0.230599	0.259022	0.678231	LOC10192	1.02E+08	uncharacterized LOC101928834
ENSG0000	85.68872	-0.13689	0.207062	0.259017	0.678231	REEP3	221035	receptor accessory protein 3
ENSG0000	102.1143	0.134431	0.154667	0.258897	0.678231	MYL6B	140465	myosin light chain 6B
ENSG0000	34.42743	0.141285	0.203911	0.259127	0.678292	GPSM2	29899	G protein signaling modulator 2
ENSG0000	591.2518	0.122113	0.130454	0.259095	0.678292	HDAC3	8841	histone deacetylase 3
ENSG0000	19.60775	0.124092	0.231055	0.259334	0.678542	ACVR2B	93	activin A receptor type 2B
ENSG0000	17.423	-0.10758	0.235735	0.259278	0.678542	STEAP1B	256227	STEAP family member 1B
ENSG0000	508.2053	0.124636	0.133893	0.259308	0.678542	GRHPR	9380	glyoxylate and hydroxypyruvate reductase
ENSG0000	7.161542	0.085257	0.238831	0.259378	0.678561	CCER2	643669	coiled-coil glutamate rich protein 2
ENSG0000	778.4852	0.131886	0.166058	0.259525	0.678631	SEPTIN2	4735	septin 2
ENSG0000	11.36531	-0.10237	0.238821	0.259549	0.678631	MIR7848	1.02E+08	microRNA 7848
ENSG0000	3.484582	0.053414	0.236455	0.25953	0.678631	NA	NA	NA
ENSG0000	479.6574	0.128564	0.143887	0.259553	0.678631	ENGASE	64772	endo-beta-N-acetylglucosaminidase
ENSG0000	160.4741	-0.1343	0.209697	0.259661	0.67876	ID3	3399	inhibitor of DNA binding 3

ENSG0000	170.1207	0.126821	0.151095	0.259676	0.67876	CDPF1	150383	cysteine rich DPF motif domain containing 1
ENSG0000	121.3546	0.137628	0.199642	0.259794	0.678954	CAMK2D	817	calcium/calmodulin dependent protein kinase II delta
ENSG0000	5.175701	-0.06354	0.236771	0.259825	0.678954	COL27A1	85301	collagen type XXVII alpha 1 chain
ENSG0000	31.74664	-0.13912	0.208631	0.259877	0.678993	NA	NA	NA
ENSG0000	8.227571	0.055917	0.236134	0.259991	0.679195	IGHJ3	28479	immunoglobulin heavy joining 3
ENSG0000	485.2286	0.124332	0.1378	0.260219	0.679499	PSMB7	5695	proteasome 20S subunit beta 7
ENSG0000	6.444773	0.077041	0.237847	0.260159	0.679499	LRRC43	254050	leucine rich repeat containing 43
ENSG0000	204.591	0.138506	0.175188	0.260212	0.679499	PRR11	55771	proline rich 11
ENSG0000	28.68295	-0.1368	0.215014	0.260358	0.679767	MTHFD2L	441024	methylenetetrahydrofolate dehydrogenase (NADP+ dependent) 2 like
ENSG0000	6037.425	-0.13865	0.169145	0.260582	0.680254	APLP2	334	amyloid beta precursor like protein 2
ENSG0000	13.16705	-0.09641	0.237385	0.260806	0.680741	POU5F2	134187	POU doma transcription factor 2
ENSG0000	21.46398	0.127359	0.228136	0.260951	0.681024	LOC72855	728554	THO complex 3 pseudogene
ENSG0000	3.387269	0.05654	0.236877	0.2611	0.681316	PNMA6A	84968	PNMA family member 6A
ENSG0000	6.779119	0.080722	0.238542	0.261246	0.681498	ZNF658	26149	zinc finger protein 658
ENSG0000	281.2589	0.129938	0.181804	0.261248	0.681498	NA	NA	NA
ENSG0000	19.56663	0.120512	0.230578	0.261282	0.681498	ZKSCAN2	342357	zinc finger with KRAB and SCAN domains 2
ENSG0000	437.8422	0.127291	0.140922	0.261363	0.681612	SLC35E1	79939	solute carrier family 35 member E1
ENSG0000	24.48714	-0.12257	0.228609	0.261586	0.682059	NA	NA	NA
ENSG0000	3.967329	0.06246	0.236485	0.261609	0.682059	NA	NA	NA
ENSG0000	134.204	0.134822	0.204754	0.261696	0.68219	DTX4	23220	deltex E3 ubiquitin ligase 4
ENSG0000	6.805444	-0.08917	0.238681	0.261788	0.682332	ZNRF3	84133	zinc and ring finger 3
ENSG0000	125.8889	0.138022	0.194973	0.261972	0.682715	GOT1	2805	glutamic-oxaloacetic transaminase 1
ENSG0000	32.95201	-0.13746	0.210836	0.262241	0.682809	MICOS10	440574	mitochondrial contact site and cristae organizing system subunit 10
ENSG0000	18.938	0.130015	0.226782	0.262254	0.682809	NA	NA	NA
ENSG0000	31.80213	-0.13998	0.203218	0.262269	0.682809	NA	NA	NA
ENSG0000	21.74331	-0.10568	0.234368	0.262238	0.682809	ZNF484	83744	zinc finger protein 484
ENSG0000	2.379114	-0.04317	0.235801	0.262248	0.682809	CCDC3	83643	coiled-coil domain containing 3
ENSG0000	290.8632	-0.13679	0.166414	0.262268	0.682809	SH2D3A	10045	SH2 domain containing 3A
ENSG0000	15.4037	-0.13085	0.228658	0.262199	0.682809	NA	NA	NA
ENSG0000	20.23762	0.092741	0.23546	0.262431	0.683134	NA	NA	NA
ENSG0000	896.6791	-0.13535	0.199115	0.262481	0.683167	CLDN9	9080	claudin 9
ENSG0000	9.295178	0.055534	0.235219	0.262549	0.683247	BMP8A	353500	bone morphogenetic protein 8a
ENSG0000	136.1335	0.140063	0.192397	0.262625	0.683349	MAN1A2	10905	mannosidase alpha class 1A member 2
ENSG0000	8.775432	-0.07618	0.236273	0.262828	0.683583	NA	NA	NA
ENSG0000	82.74557	0.138858	0.182516	0.262805	0.683583	MRM3	55178	mitochondrial rRNA methyltransferase 3
ENSG0000	3.031367	-0.05323	0.236422	0.262758	0.683583	C20orf144	128864	chromosome 20 open reading frame 144
ENSG0000	2926.054	-0.14148	0.181251	0.26296	0.683734	PNPLA6	10908	patatin like phospholipase domain containing 6
ENSG0000	5179.401	-0.13475	0.168728	0.262928	0.683734	MED15	51586	mediator complex subunit 15
ENSG0000	36.17468	-0.13987	0.200525	0.26304	0.683753	CKS1B	1163	CDC28 protein kinase regulatory subunit 1B
ENSG0000	13.88396	-0.12961	0.228285	0.26325	0.683753	NA	NA	NA
ENSG0000	104.7623	0.131465	0.163375	0.263671	0.683753	COMMD1	150684	copper metabolism domain containing 1
ENSG0000	213.9944	0.131555	0.150836	0.263053	0.683753	FAHD2A	51011	fumarylacetoacetate hydrolase domain containing 2A
ENSG0000	87.10921	0.137894	0.201039	0.263141	0.683753	NIT2	56954	nitrilase family member 2
ENSG0000	5.69623	0.086108	0.239308	0.263649	0.683753	DZIP1L	199221	DAZ interacting zinc finger protein 1 like
ENSG0000	27.95456	-0.12014	0.23076	0.263355	0.683753	NA	NA	NA
ENSG0000	1018.459	-0.12837	0.207809	0.263484	0.683753	C6orf62	81688	chromosome 6 open reading frame 62
ENSG0000	199.5302	-0.14265	0.186472	0.263433	0.683753	VTA1	51534	vesicle trafficking 1
ENSG0000	4.076721	0.066163	0.237028	0.263235	0.683753	CDKN2B-A	1E+08	CDKN2B antisense RNA 1
ENSG0000	8.067304	-0.07879	0.237529	0.263677	0.683753	NA	NA	NA
ENSG0000	3909.656	-0.12253	0.130773	0.26349	0.683753	RAB5B	5869	RAB5B member RAS oncogene family
ENSG0000	6.677286	-0.08514	0.238796	0.263647	0.683753	NA	NA	NA
ENSG0000	8.428656	0.093526	0.238639	0.263603	0.683753	FAM169BI	283777	protein FAM169B
ENSG0000	261.1548	0.132638	0.157843	0.263324	0.683753	METTL22	79091	methyltra Kin17 lysine
ENSG0000	1219.115	0.135983	0.169246	0.263227	0.683753	SREBF1	6720	sterol regulatory element binding transcription factor 1
ENSG0000	808.7857	0.110499	0.115316	0.263656	0.683753	BPTF	2186	bromodomain PHD finger transcription factor
ENSG0000	61.92957	-0.13332	0.214206	0.263149	0.683753	ADAMTSL!	339366	ADAMTS like 5
ENSG0000	469.7514	0.122768	0.139206	0.263372	0.683753	RANBP1	5902	RAN binding protein 1
ENSG0000	5.371407	0.066086	0.239006	0.264267	0.684033	LOC10798	1.08E+08	uncharacterized LOC107984850
ENSG0000	21.16755	0.082119	0.235677	0.264494	0.684033	PTPRF	5792	protein tyrosine phosphatase receptor type F
ENSG0000	115.7646	-0.13749	0.18763	0.263836	0.684033	NFU1	27247	NFU1 iron-sulfur cluster scaffold
ENSG0000	518.4866	0.131478	0.152592	0.264078	0.684033	STARD7	56910	StAR related lipid transfer domain containing 7
ENSG0000	97.9551	-0.13165	0.213375	0.26438	0.684033	SCRN3	79634	secernin 3
ENSG0000	186.7007	0.133023	0.156765	0.264301	0.684033	STX18	53407	syntaxin 18
ENSG0000	24.359	0.114167	0.232636	0.2639	0.684033	SNHG8	1E+08	small nucleolar RNA host gene 8
ENSG0000	49.86892	0.138419	0.185941	0.264312	0.684033	SNX14	57231	sorting nexin 14
ENSG0000	13.1667	-0.0983	0.236275	0.264493	0.684033	BSPRY	54836	B-box and SPRY domain containing
ENSG0000	626.4261	-0.13586	0.154768	0.264163	0.684033	TBC1D13	54662	TBC1 domain family member 13
ENSG0000	113.2506	-0.13799	0.182531	0.264178	0.684033	SMCO4	56935	single-pass membrane protein with coiled-coil domains 4
ENSG0000	5.023159	0.078521	0.238303	0.264448	0.684033	NA	NA	NA
ENSG0000	166.5237	0.138489	0.182515	0.264357	0.684033	METAP2	10988	methionyl aminopeptidase 2
ENSG0000	92.11193	-0.13621	0.166793	0.264483	0.684033	ENTPD5	957	ectonucleoside triphosphate diphosphohydrolase 5 (inactive)
ENSG0000	2.816794	0.047771	0.235763	0.264492	0.684033	PPL	5493	periplakin
ENSG0000	35.2725	0.124507	0.226942	0.263922	0.684033	LINC00908	284276	long intergenic non-protein coding RNA 908
ENSG0000	6128.437	-0.13801	0.180943	0.264298	0.684033	PKN1	5585	protein kinase N1
ENSG0000	468.2797	-0.13647	0.186672	0.264377	0.684033	PPP1R3D	5509	protein phosphatase 1 regulatory subunit 3D
ENSG0000	5157.717	-0.12633	0.148173	0.264176	0.684033	FBXO7	25793	F-box protein 7
ENSG0000	9.225949	-0.08505	0.237809	0.264548	0.684077	NA	NA	NA
ENSG0000	294.783	-0.13451	0.167407	0.26481	0.684561	MON1A	84315	MON1 hor secretory trafficking associated
ENSG0000	8122.274	-0.1308	0.19115	0.264796	0.684561	UNC13D	201294	unc-13 homolog D
ENSG0000	216.1433	-0.13339	0.159967	0.264868	0.684614	RIPK2	8767	receptor interacting serine/threonine kinase 2
ENSG0000	3.437454	-0.04943	0.235272	0.265155	0.685063	LOC10798	1.08E+08	lymphocyte specific protein 1 pseudogene
ENSG0000	46.50708	0.131945	0.214642	0.265192	0.685063	CFAP97	57587	cilia and flagella associated protein 97
ENSG0000	10.58234	-0.0867	0.237636	0.265173	0.685063	DLL1	28514	delta like canonical Notch ligand 1
ENSG0000	666.2374	0.129436	0.152853	0.26515	0.685063	GHDC	84514	GH3 domain containing
ENSG0000	29.2074	0.131397	0.221269	0.265295	0.685234	GLB1L2	89944	galactosidase beta 1 like 2
ENSG0000	173.2215	0.137263	0.193747	0.265553	0.685298	MCM2	4171	minichromosome maintenance complex component 2
ENSG0000	696.3544	-0.12661	0.15291	0.265479	0.685298	ZFAT	57623	zinc finger and AT-hook domain containing
ENSG0000	5.885598	-0.06533	0.23646	0.265421	0.685298	NA	NA	NA
ENSG0000	107.396	0.135275	0.207499	0.265502	0.685298	SPTSSA	171546	serine palmitoyltransferase small subunit A
ENSG0000	56.50611	-0.1364	0.203352	0.265438	0.685298	BBS4	585	Bardet-Biedl syndrome 4
ENSG0000	1532.616	0.135726	0.172376	0.265523	0.685298	IDH2	3418	isocitrate dehydrogenase (NADP(+)) 2
ENSG0000	261.9084	0.120188	0.218092	0.265582	0.685298	SAMD14	201191	sterile alpha motif domain containing 14
ENSG0000	2570.14	-0.13501	0.194462	0.265655	0.685341	CD48	962	CD48 molecule
ENSG0000	3.788945	-0.07829	0.23962	0.2657	0.685341	NA	NA	NA
ENSG0000	225.2428	0.136583	0.160467	0.265737	0.685341	ADAMTS1(	81794	ADAM metallopeptidase with thrombospondin type 1 motif 10
ENSG0000	4.57438	0.043329	0.234551	0.265748	0.685341	NA	NA	NA

ENSG0000	2325.234	-0.13587	0.205668	0.265877	0.685578	ITGB7	3695	integrin subunit beta 7
ENSG0000	161.4494	0.133851	0.159242	0.265955	0.685682	EBNA1BP2	10969	EBNA1 binding protein 2
ENSG0000	509.6651	0.136245	0.166225	0.266009	0.685724	APP	351	amyloid beta precursor protein
ENSG0000	105.8814	0.130501	0.149419	0.266175	0.686055	RNF168	165918	ring finger protein 168
ENSG0000	23.01124	-0.12219	0.228737	0.266263	0.686124	MKLN1-AS	1.01E+08	MKLN1 antisense RNA
ENSG0000	62.80894	0.134816	0.206773	0.266277	0.686124	IFT140	9742	intraflagellar transport 140
ENSG0000	48.2294	-0.13257	0.21505	0.266346	0.68613	EIF1B-AS1	440952	EIF1B antisense RNA 1
ENSG0000	2.400704	-0.0473	0.235224	0.266354	0.68613	PITRM1-A:	1.01E+08	PITRM1 antisense RNA 1
ENSG0000	82.42255	0.133166	0.166133	0.266436	0.686244	MYO9A	4649	myosin IXA
ENSG0000	106.8025	0.138091	0.183215	0.266499	0.686312	BAHCC1	57597	BAH domain and coiled-coil containing 1
ENSG0000	22.41672	-0.12415	0.227803	0.266585	0.686384	CDCA2	157313	cell division cycle associated 2
ENSG0000	25.16554	0.132694	0.218525	0.266602	0.686384	MC1R	4157	melanocortin 1 receptor
ENSG0000	1225.787	-0.13603	0.185691	0.266817	0.686785	LRRC4	64101	leucine rich repeat containing 4
ENSG0000	41.93801	0.111321	0.23108	0.266833	0.686785	ZFAND1	79752	zinc finger AN1-type containing 1
ENSG0000	12.97757	0.110341	0.236162	0.267006	0.68681	UBXN10	127733	UBX domain protein 10
ENSG0000	111.3653	0.134398	0.175325	0.267322	0.68681	MSTO1	55154	misato mitochondrial distribution and morphology regulator 1
ENSG0000	95.66273	0.135866	0.202875	0.267661	0.68681	NAV1	89796	neuron navigator 1
ENSG0000	8.601224	0.107608	0.237417	0.267353	0.68681	NA	NA	NA
ENSG0000	8.589645	-0.07582	0.236623	0.267735	0.68681	NA	NA	NA
ENSG0000	76.9055	0.136577	0.197899	0.267008	0.68681	RWDD4	201965	RWD domain containing 4
ENSG0000	21.64161	0.076448	0.233805	0.267688	0.68681	MBLAC2	153364	metallo-beta-lactamase domain containing 2
ENSG0000	7246.745	-0.13348	0.165429	0.267319	0.68681	GRK6	2870	G protein-coupled receptor kinase 6
ENSG0000	29.69398	-0.11278	0.231199	0.267579	0.68681	ADTRP	84830	androgen dependent TFPI regulating protein
ENSG0000	401.291	0.127339	0.21563	0.267078	0.68681	RPS24	6229	ribosomal protein S24
ENSG0000	22.80415	-0.12638	0.224723	0.267373	0.68681	AFAP1L2	84632	actin filament associated protein 1 like 2
ENSG0000	2403.852	-0.13891	0.174863	0.267583	0.68681	SIGIRR	59307	single Ig and TIR domain containing
ENSG0000	53.73541	0.129202	0.218866	0.267309	0.68681	SLC35F2	54733	solute carrier family 35 member F2
ENSG0000	133.1992	0.13791	0.181837	0.266991	0.68681	CD9	928	CD9 molecule
ENSG0000	492.6992	-0.13542	0.17937	0.267526	0.68681	APAF1	317	apoptotic peptidase activating factor 1
ENSG0000	397.7483	0.13171	0.158429	0.267743	0.68681	KDM2B	84678	lysine demethylase 2B
ENSG0000	3.472991	0.045993	0.236977	0.267624	0.68681	NA	NA	NA
ENSG0000	27.87343	0.090035	0.235405	0.267509	0.68681	WDR89	112840	WD repeat domain 89
ENSG0000	5.137023	0.05932	0.235713	0.26756	0.68681	TMEM220	388335	transmembrane protein 220
ENSG0000	870.2137	-0.13162	0.16939	0.267594	0.68681	RFNG	5986	RFNG O-fucosylpeptide 3-beta-N-acetylglucosaminyltransferase
ENSG0000	1693.825	0.133889	0.165234	0.267417	0.68681	CEBPA	1050	CCAAT enhancer binding protein alpha
ENSG0000	10.38638	0.068303	0.23623	0.267477	0.68681	SYCP2	10388	synaptonemal complex protein 2
ENSG0000	11.38299	-0.0664	0.235421	0.26717	0.68681	NA	NA	NA
ENSG0000	1338.056	0.123909	0.138463	0.266988	0.68681	TUBGCP6	85378	tubulin gamma complex associated protein 6
ENSG0000	11.54947	-0.11492	0.234852	0.267801	0.686863	ZNF516-AS	1.02E+08	ZNF516 antisense RNA 1
ENSG0000	4.036481	0.044786	0.236286	0.26789	0.6869	UGT2B17	7367	UDP glucuronosyltransferase family 2 member B17
ENSG0000	153.8951	-0.12317	0.136142	0.267853	0.6869	ZNF174	7727	zinc finger protein 174
ENSG0000	36.02269	-0.13798	0.208662	0.267945	0.686944	NA	NA	NA
ENSG0000	34.13639	0.083515	0.234596	0.268072	0.687175	SLC1A3	6507	solute carrier family 1 member 3
ENSG0000	172.533	0.128752	0.210811	0.268174	0.687244	NA	NA	NA
ENSG0000	16.43491	0.10395	0.235279	0.268166	0.687244	FAM111B	374393	FAM111 trypsin like peptidase B
ENSG0000	92.91502	0.114488	0.227157	0.268671	0.687357	HS2ST1	9653	heparan sulfate 2-O-sulfotransferase 1
ENSG0000	148.1183	0.13309	0.160153	0.268697	0.687357	DDX1	1653	DEAD-box helicase 1
ENSG0000	15.87974	-0.11048	0.234703	0.268484	0.687357	CPLX1	10815	complexin 1
ENSG0000	6.901471	-0.06589	0.236275	0.268641	0.687357	NA	NA	NA
ENSG0000	3.97998	0.070893	0.238374	0.268363	0.687357	NA	NA	NA
ENSG0000	52.46772	-0.13818	0.189755	0.268493	0.687357	RNF8	9025	ring finger protein 8
ENSG0000	2.622796	-0.03367	0.234049	0.268541	0.687357	GIMAP3P	474345	GTPase IMAP family member 3 pseudogene
ENSG0000	238.9494	0.125177	0.140325	0.268631	0.687357	REXO4	57109	REX4 hom 3'-5' exonuclease
ENSG0000	150.229	0.129192	0.149292	0.268523	0.687357	ACACB	32	acetyl-CoA carboxylase beta
ENSG0000	164.5378	0.136518	0.181081	0.268739	0.687357	AURKB	9212	aurora kinase B
ENSG0000	3.517924	0.043295	0.234737	0.268382	0.687357	NA	NA	NA
ENSG0000	3966.889	-0.1158	0.123689	0.268416	0.687357	AKT2	208	AKT serine/threonine kinase 2
ENSG0000	1888.19	-0.11508	0.124029	0.268744	0.687357	STK4	6789	serine/threonine kinase 4
ENSG0000	26.97435	0.128324	0.2236	0.268277	0.687357	MIS18A	54069	MIS18 kinetochore protein A
ENSG0000	1398.75	0.13357	0.161965	0.268793	0.687386	NRROS	375387	negative regulator of reactive oxygen species
ENSG0000	27.48132	-0.12466	0.225935	0.269107	0.687765	TMEM240	339453	transmembrane protein 240
ENSG0000	137.5719	-0.13087	0.154934	0.269129	0.687765	NA	NA	NA
ENSG0000	51.73942	-0.13783	0.191138	0.269023	0.687765	PRKAR1B-.	1.02E+08	PRKAR1B antisense RNA 1
ENSG0000	170.4338	0.135112	0.201234	0.269024	0.687765	VPS36	51028	vacuolar protein sorting 36 homolog
ENSG0000	2.625083	0.040756	0.234522	0.2691	0.687765	NA	NA	NA
ENSG0000	7.844724	0.066939	0.235937	0.269304	0.688118	NA	NA	NA
ENSG0000	3836.53	0.121008	0.132809	0.269361	0.688166	CSNK1G2	1455	casein kinase 1 gamma 2
ENSG0000	420.8225	-0.13443	0.177463	0.269402	0.688176	DCP1B	196513	decapping mRNA 1B
ENSG0000	2.813017	0.049068	0.23642	0.269527	0.688253	NA	NA	NA
ENSG0000	291.6866	0.132263	0.197477	0.269597	0.688253	MFSD1	64747	major facilitator superfamily domain containing 1
ENSG0000	2.962771	-0.03875	0.235156	0.269733	0.688253	CLDN11	5010	claudin 11
ENSG0000	12.2613	-0.10231	0.236464	0.269669	0.688253	NA	NA	NA
ENSG0000	1517.583	-0.13434	0.184689	0.269733	0.688253	STX3	6809	syntaxin 3
ENSG0000	3.165203	0.037391	0.234067	0.269551	0.688253	TEX14	56155	testis expr intercellular bridge forming factor
ENSG0000	7.450722	0.087893	0.237779	0.269732	0.688253	NA	NA	NA
ENSG0000	320.8727	0.132314	0.164577	0.269566	0.688253	SOX12	6666	SRY-box transcription factor 12
ENSG0000	11.38662	0.064624	0.235029	0.269861	0.688405	SCN3A	6328	sodium voltage-gated channel alpha subunit 3
ENSG0000	6.025406	-0.04119	0.23408	0.269868	0.688405	NA	NA	NA
ENSG0000	7068.26	-0.13165	0.161976	0.270005	0.688574	ECE1	1889	endothelin converting enzyme 1
ENSG0000	51.69031	0.137902	0.183438	0.27008	0.688574	NUDT17	200035	nudix hydrolase 17
ENSG0000	27.83754	0.107458	0.232077	0.270064	0.688574	KIAA0825	285600	KIAA0825
ENSG0000	38.74485	-0.13901	0.209878	0.270114	0.688574	TRBV28	28559	T cell receptor beta variable 28
ENSG0000	83.91837	0.135455	0.190965	0.270159	0.688574	TRPS1	7227	transcriptional repressor GATA binding 1
ENSG0000	9533.152	-0.13257	0.166564	0.270124	0.688574	PGGHG	80162	protein-glucosylgalactosylhydroxylysine glucosidase
ENSG0000	132.1129	-0.13586	0.190645	0.270328	0.688711	NA	NA	NA
ENSG0000	7.224853	0.08311	0.239389	0.270364	0.688711	NA	NA	NA
ENSG0000	3.653767	0.039243	0.234056	0.270315	0.688711	NA	NA	NA
ENSG0000	4.138688	0.040895	0.233987	0.2703	0.688711	NA	NA	NA
ENSG0000	1152.559	0.124052	0.146449	0.270573	0.689053	KLHDC3	116138	kelch domain containing 3
ENSG0000	295.9792	0.112793	0.226223	0.270567	0.689053	ASPH	444	aspartate beta-hydroxylase
ENSG0000	73.84626	0.132804	0.158427	0.270651	0.68906	COQ6	51004	coenzyme monooxygenase
ENSG0000	163.7117	0.133414	0.202285	0.270631	0.68906	ATP5MJ	9556	ATP synthase membrane subunit j
ENSG0000	150.4055	-0.12787	0.213504	0.270776	0.689074	MTHFD2	10797	methylene methenyltetrahydrofolate cyclohydrolase
ENSG0000	588.6031	-0.12442	0.140924	0.270921	0.689074	KPNA4	3840	karyopherin subunit alpha 4
ENSG0000	15.31586	0.090873	0.235807	0.270831	0.689074	NA	NA	NA
ENSG0000	27.24786	-0.10682	0.232929	0.270807	0.689074	SLC22A18/	5003	SLC22A18 antisense RNA

ENSG0000	89.01991	-0.13165	0.207987	0.270937	0.689074	CDCA5	113130	cell division cycle associated 5
ENSG0000	3610.709	-0.12369	0.130756	0.270933	0.689074	KDM2A	22992	lysine demethylase 2A
ENSG0000	3.982504	0.063136	0.237541	0.270882	0.689074	JAM2	58494	junctional adhesion molecule 2
ENSG0000	14.01564	0.114471	0.23367	0.270957	0.689074	JADE3	9767	jade family PHD finger 3
ENSG0000	1998.571	0.110662	0.117987	0.27113	0.689321	REPIN1	29803	replication initiator 1
ENSG0000	684.6467	0.127824	0.152124	0.271095	0.689321	PMM2	5373	phosphomannomutase 2
ENSG0000	1030.952	0.118818	0.13003	0.271226	0.689365	BLTP2	9703	bridge-like lipid transfer protein family member 2
ENSG0000	2.825225	0.036739	0.234925	0.27126	0.689365	RPS15AP1	92682	ribosomal protein S15a pseudogene 1
ENSG0000	29.56842	-0.11846	0.22876	0.271251	0.689365	ARMCX2	9823	armadillo repeat containing X-linked 2
ENSG0000	108.7686	0.134355	0.20353	0.2714	0.689625	RNF103	7844	ring finger protein 103
ENSG0000	10.31419	0.101072	0.237091	0.271619	0.690076	MOCS1	4337	molybdenum cofactor synthesis 1
ENSG0000	15.84767	0.112045	0.233706	0.271653	0.690076	BIVM	54841	basic immunoglobulin-like variable motif containing
ENSG0000	11.78008	0.102594	0.236038	0.271773	0.690085	SNHG26	1.1E+08	small nucleolar RNA host gene 26
ENSG0000	70934.86	-0.1332	0.189723	0.271781	0.690085	MMP25	64386	matrix metallopeptidase 25
ENSG0000	62.31925	0.135305	0.168248	0.271807	0.690085	ZSWIM7	125150	zinc finger SWIM-type containing 7
ENSG0000	3254.645	0.134387	0.175714	0.271779	0.690085	SIRPB2	284759	signal regulatory protein beta 2
ENSG0000	922.3395	0.127059	0.154213	0.271891	0.690202	HGSNAT	138050	heparan-alpha-glucosaminide N-acetyltransferase
ENSG0000	82.76778	0.116745	0.225657	0.271974	0.690317	SLC30A9	10463	solute carrier family 30 member 9
ENSG0000	2.725176	0.043812	0.234703	0.272038	0.690384	NA	NA	NA
ENSG0000	578.6617	0.12539	0.143243	0.272124	0.690405	NABP2	79035	nucleic acid binding protein 2
ENSG0000	8.962086	0.115315	0.237025	0.272159	0.690405	TRAV20	28663	T cell receptor alpha variable 20
ENSG0000	15.67782	-0.10844	0.234288	0.272098	0.690405	TRAV23DV	28660	T cell receptor alpha variable 23/delta variable 6
ENSG0000	474.789	0.122819	0.137869	0.272235	0.69044	PSMB6	5694	proteasome 20S subunit beta 6
ENSG0000	404.9728	0.133979	0.170682	0.272249	0.69044	SRSF6	6431	serine and arginine rich splicing factor 6
ENSG0000	191.7039	-0.12906	0.152898	0.272372	0.690465	LIMS2	55679	LIM zinc finger domain containing 2
ENSG0000	3.37628	0.033486	0.233513	0.272354	0.690465	NA	NA	NA
ENSG0000	4.007124	0.061845	0.237712	0.272483	0.690465	DNAH8	1769	dynein axonemal heavy chain 8
ENSG0000	57.9354	-0.13057	0.212506	0.272507	0.690465	GALT	2592	galactose-1-phosphate uridylyltransferase
ENSG0000	61.7035	0.137565	0.172709	0.272522	0.690465	EXD2	55218	exonuclease 3'-5' domain containing 2
ENSG0000	75.24836	-0.1136	0.226831	0.272329	0.690465	ALPK3	57538	alpha kinase 3
ENSG0000	296.0582	-0.13441	0.188047	0.272498	0.690465	DMPK	1760	DM1 protein kinase
ENSG0000	114.1976	-0.10522	0.229322	0.272672	0.69075	NA	NA	NA
ENSG0000	249.9082	0.120508	0.134433	0.272828	0.69105	PAN2	9924	poly(A) specific ribonuclease subunit PAN2
ENSG0000	4.158638	-0.06744	0.237358	0.272873	0.691067	LOC10050	1.01E+08	uncharacterized LOC100506207
ENSG0000	2.319121	0.032533	0.23482	0.272934	0.691126	NA	NA	NA
ENSG0000	43.87136	0.130606	0.206364	0.273016	0.691161	STX1B	112755	syntaxin 1B
ENSG0000	7.784934	0.083677	0.237093	0.273023	0.691161	BRIP1	83990	BRCA1 interacting helicase 1
ENSG0000	18.18117	0.102729	0.234973	0.273121	0.691314	ZNF322	79692	zinc finger protein 322
ENSG0000	56.057	0.135326	0.192519	0.273262	0.691417	PTCD2	79810	pentatricopeptide repeat domain 2
ENSG0000	18.54319	0.10867	0.233262	0.273268	0.691417	LOC37444	374443	C-type lectin domain family 2 member D pseudogene
ENSG0000	26.22168	0.129731	0.219671	0.273275	0.691417	NA	NA	NA
ENSG0000	261.6731	0.131318	0.195014	0.273563	0.691627	MTCO2P1	1.07E+08	MT-CO2 pseudogene 12
ENSG0000	121.8885	0.127571	0.148005	0.273495	0.691627	MSH6	2956	mutS homolog 6
ENSG0000	12.36082	0.098737	0.23698	0.273461	0.691627	CHAC2	494143	ChaC glutathione specific gamma-glutamylcyclotransferase 2
ENSG0000	171.4532	-0.11083	0.224165	0.273586	0.691627	WDFY1	57590	WD repeat and FYVE domain containing 1
ENSG0000	748.377	-0.12231	0.141268	0.273623	0.691627	MIGA2	84895	mitoguardin 2
ENSG0000	68.46705	0.131647	0.209359	0.273471	0.691627	RASL11A	387496	RAS like family 11 member A
ENSG0000	2.412596	-0.03237	0.234551	0.273616	0.691627	DSC1	1823	desmocollin 1
ENSG0000	489.5795	-0.11359	0.121476	0.273665	0.691638	UPF2	26019	UPF2 regulator of nonsense mediated mRNA decay
ENSG0000	293.6684	-0.12916	0.153742	0.273727	0.691699	COG7	91949	component of oligomeric golgi complex 7
ENSG0000	142.9409	0.118128	0.222523	0.273944	0.691962	KLHL2	11275	kelch like family member 2
ENSG0000	8.907491	0.071748	0.235834	0.273942	0.691962	TEX9	374618	testis expressed 9
ENSG0000	2735.067	-0.13592	0.178916	0.27388	0.691962	ATP13A1	57130	ATPase 13A1
ENSG0000	520.413	0.117352	0.12852	0.274091	0.692151	SIN3A	25942	SIN3 transcription regulator family member A
ENSG0000	545.7835	-0.10748	0.112507	0.274094	0.692151	POLR2C	5432	RNA polymerase II subunit C
ENSG0000	242.322	-0.1295	0.203201	0.27424	0.692161	SLC30A7	148867	solute carrier family 30 member 7
ENSG0000	36.08907	-0.13813	0.189491	0.274175	0.692161	SPRED2	200734	sprouty related EVH1 domain containing 2
ENSG0000	2259.56	0.132647	0.168916	0.274287	0.692161	NA	NA	NA
ENSG0000	84.42561	0.127114	0.162876	0.274177	0.692161	RACGAP1	29127	Rac GTPase activating protein 1
ENSG0000	2.37314	0.050319	0.236406	0.274259	0.692161	NA	NA	NA
ENSG0000	37.78274	0.129852	0.216438	0.274414	0.692386	USP54	159195	ubiquitin specific peptidase 54
ENSG0000	214.5265	-0.13256	0.199549	0.274501	0.692492	CD28	940	CD28 molecule
ENSG0000	59.26455	0.135829	0.179667	0.274532	0.692492	DSN1	79980	DSN1 component of MIS12 kinetochore complex
ENSG0000	18.60901	-0.12249	0.22695	0.274707	0.692839	NA	NA	NA
ENSG0000	92.70868	-0.13322	0.182854	0.274817	0.69302	C18orf32	497661	chromosome 18 open reading frame 32
ENSG0000	505.7375	-0.13162	0.200538	0.274976	0.693236	EIF4A2	1974	eukaryotic translation initiation factor 4A2
ENSG0000	9.580427	0.097072	0.237403	0.274978	0.693236	MIR570	693155	microRNA 570
ENSG0000	3.895073	0.064643	0.238158	0.27502	0.693245	NA	NA	NA
ENSG0000	4.299998	0.060961	0.235849	0.27511	0.693378	NA	NA	NA
ENSG0000	1330.137	0.130529	0.169874	0.275202	0.693513	CPNE2	221184	copine 2
ENSG0000	12917.98	-0.13393	0.16885	0.275324	0.693629	PLP2	5355	proteolipid protein 2
ENSG0000	45.19593	0.129264	0.215514	0.275316	0.693629	EOLA2-DT	1E+08	EOLA2 divergent transcript
ENSG0000	187373.9	-0.13239	0.183569	0.27544	0.693698	CSF3R	1441	colony stimulating factor 3 receptor
ENSG0000	57.19139	0.135376	0.177109	0.275462	0.693698	RIMS3	9783	regulating synaptic membrane exocytosis 3
ENSG0000	52.34246	0.136031	0.179092	0.275485	0.693698	WBP1	23559	WW domain binding protein 1
ENSG0000	8.27544	-0.09703	0.238289	0.275503	0.693698	LINC00896	150197	long intergenic non-protein coding RNA 896
ENSG0000	62.7065	0.130567	0.210374	0.275681	0.693992	ANKRD13C	81573	ankyrin repeat domain 13C
ENSG0000	497.67	0.112541	0.120222	0.275714	0.693992	PTDSS1	9791	phosphatidylserine synthase 1
ENSG0000	24.94484	0.118265	0.227142	0.275762	0.693992	POLRMTP1	284167	RNA polymerase mitochondrial pseudogene 1
ENSG0000	19.40716	-0.12037	0.226776	0.275771	0.693992	ZNF630	57232	zinc finger protein 630
ENSG0000	4.714519	0.073584	0.237533	0.275919	0.694175	NA	NA	NA
ENSG0000	578.7942	-0.12963	0.159148	0.275916	0.694175	PDK3	5165	pyruvate dehydrogenase kinase 3
ENSG0000	13.86709	0.124482	0.227783	0.276064	0.694254	SRGAP3	9901	SLIT-ROBO Rho GTPase activating protein 3
ENSG0000	2.72274	-0.06716	0.237756	0.276045	0.694254	AVPR1A	552	arginine vasopressin receptor 1A
ENSG0000	154.1273	0.132671	0.16827	0.275991	0.694254	SNHG17	388796	small nucleolar RNA host gene 17
ENSG0000	2741.898	0.134243	0.175902	0.276127	0.694318	SSH2	85464	slingshot protein phosphatase 2
ENSG0000	15.82335	0.115504	0.232099	0.276396	0.694455	C1orf116	79098	chromosome 1 open reading frame 116
ENSG0000	3439.42	0.133122	0.192655	0.276358	0.694455	HDLBP	3069	high density lipoprotein binding protein
ENSG0000	402.4152	0.133042	0.176963	0.276599	0.694455	MSL2	55167	MSL complex subunit 2
ENSG0000	34.02595	0.112496	0.228803	0.276486	0.694455	N4BP2	55728	NEDD4 binding protein 2
ENSG0000	491.4085	-0.12346	0.136753	0.276533	0.694455	TTC1	7265	tetratricopeptide repeat domain 1
ENSG0000	2.750015	0.066147	0.23762	0.276344	0.694455	NA	NA	NA
ENSG0000	1163.586	-0.12491	0.152533	0.276493	0.694455	FHIP1B	84067	FHF complex subunit HOOK interacting protein 1B
ENSG0000	198.9369	0.137578	0.188382	0.276563	0.694455	DENND4A	10260	DENN domain containing 4A
ENSG0000	183.8139	0.125167	0.210304	0.276504	0.694455	CLTCL1	8218	clathrin heavy chain like 1
ENSG0000	106.9471	-0.13232	0.213185	0.276557	0.694455	CLDN5	7122	claudin 5

ENSG0000	3334.2	-0.12518	0.147455	0.276421	0.694455	PISD	23761	phosphatidylserine decarboxylase
ENSG0000	320.7064	0.130471	0.161481	0.276673	0.694497	PMPCA	23203	peptidase mitochondrial processing subunit alpha
ENSG0000	14.48445	-0.1274	0.224898	0.276692	0.694497	NA	NA	NA
ENSG0000	21.84815	-0.11871	0.22752	0.276763	0.694581	SLC4A11	83959	solute carrier family 4 member 11
ENSG0000	8864.515	-0.12719	0.207071	0.277046	0.694847	PGD	5226	phosphogluconate dehydrogenase
ENSG0000	18.86448	0.094896	0.235103	0.276993	0.694847	PLA2G2D	26279	phospholipase A2 group IID
ENSG0000	61.34955	0.120486	0.223166	0.277132	0.694847	PEX1	5189	peroxisomal biogenesis factor 1
ENSG0000	565.6039	0.133563	0.17759	0.277135	0.694847	EPHB6	2051	EPH receptor B6
ENSG0000	3.865638	-0.06162	0.236305	0.277053	0.694847	NA	NA	NA
ENSG0000	1470.107	-0.11066	0.124415	0.276989	0.694847	STK24	8428	serine/threonine kinase 24
ENSG0000	374.7722	-0.13267	0.18681	0.277084	0.694847	PLXNA3	55558	plexin A3
ENSG0000	626.0354	-0.13279	0.175098	0.277435	0.695105	NCDN	23154	neurochondrin
ENSG0000	30.70941	0.129766	0.216653	0.277465	0.695105	PLA2G4A	5321	phospholipase A2 group IVA
ENSG0000	277.7545	-0.13081	0.201694	0.277366	0.695105	GPR141	353345	G protein-coupled receptor 141
ENSG0000	8.496997	0.061582	0.235143	0.277418	0.695105	SDR42E2	1E+08	short chai member 2
ENSG0000	9974.931	-0.12144	0.21466	0.277418	0.695105	APOBR	55911	apolipoprotein B receptor
ENSG0000	2523.984	0.120771	0.136462	0.277372	0.695105	ARHGEF18	23370	Rho/Rac guanine nucleotide exchange factor 18
ENSG0000	5.760281	-0.08825	0.23862	0.27762	0.695398	NA	NA	NA
ENSG0000	1335.055	-0.13019	0.16591	0.277751	0.695441	ATP2B4	493	ATPase plasma membrane Ca2+ transporting 4
ENSG0000	4.312114	-0.05454	0.235458	0.277701	0.695441	NA	NA	NA
ENSG0000	2.37958	-0.06112	0.236788	0.27775	0.695441	NA	NA	NA
ENSG0000	11.70151	0.100652	0.235358	0.278175	0.696311	PIK3CD-AS	1.02E+08	PIK3CD antisense RNA 2
ENSG0000	134.4668	-0.12336	0.140813	0.278156	0.696311	UQCR11	10975	ubiquinol- complex III subunit XI
ENSG0000	16.48609	-0.10669	0.233542	0.278214	0.696313	CCDC136	64753	coiled-coil domain containing 136
ENSG0000	235.6896	-0.13052	0.200479	0.278378	0.696368	RIT1	6016	Ras like without CAAX 1
ENSG0000	21.10917	0.109586	0.231861	0.278388	0.696368	FAM177B	400823	family with sequence similarity 177 member B
ENSG0000	2.583664	0.050272	0.237895	0.27832	0.696368	LOC10798	1.08E+08	uncharacterized LOC107985892
ENSG0000	68.56125	0.134412	0.18188	0.278286	0.696368	NGDN	25983	neuroguidin
ENSG0000	14.36795	0.079944	0.234612	0.278548	0.696589	MNAT1	4331	MNAT1 component of CDK activating kinase
ENSG0000	131.6262	0.1319	0.165707	0.278566	0.696589	NDUFAB1	4706	NADH:ubiquinone oxidoreductase subunit AB1
ENSG0000	37.68693	-0.12941	0.213345	0.27859	0.696589	DLG3	1741	discs large MAGUK scaffold protein 3
ENSG0000	28.73435	0.102325	0.232096	0.278716	0.696809	MTX3	345778	metaxin 3
ENSG0000	362.5331	0.103381	0.108314	0.278778	0.696869	UCK1	83549	uridine-cytidine kinase 1
ENSG0000	80.59507	-0.13328	0.187629	0.278849	0.696952	NA	NA	NA
ENSG0000	170.6901	-0.12565	0.148154	0.27894	0.697084	ANXA2R-A	153684	ANXA2R antisense RNA 1
ENSG0000	16052.76	0.132228	0.179071	0.279059	0.697286	CST3	1471	cystatin C
ENSG0000	4.268699	-0.07302	0.238763	0.279169	0.697464	NA	NA	NA
ENSG0000	76.98049	0.128838	0.209868	0.279229	0.69752	NIPAL2	79815	NIPA like domain containing 2
ENSG0000	238.5143	-0.1346	0.1956	0.279344	0.69757	PDCD1	5133	programmed cell death 1
ENSG0000	21.2206	0.123817	0.224081	0.279363	0.69757	CYP2U1	113612	cytochrome P450 family 2 subfamily U member 1
ENSG0000	3.043791	0.039028	0.236025	0.279301	0.69757	LOC11226	1.12E+08	uncharacterized LOC112268389
ENSG0000	2.659672	0.050002	0.237943	0.279414	0.697603	NA	NA	NA
ENSG0000	10.48189	0.062382	0.234017	0.279783	0.697764	NA	NA	NA
ENSG0000	394.1027	0.125215	0.15044	0.279599	0.697764	SLC39A4	55630	solute carrier family 39 member 4
ENSG0000	222.0556	0.131205	0.193864	0.279717	0.697764	MAP3K8	1326	mitogen-activated protein kinase kinase kinase 8
ENSG0000	195.6686	0.13116	0.191657	0.279596	0.697764	CARNS1	57571	carnosine synthase 1
ENSG0000	754.996	0.12028	0.132913	0.279737	0.697764	UQCRC2	7385	ubiquinol-cytochrome c reductase core protein 2
ENSG0000	1067.221	0.117298	0.130227	0.279759	0.697764	SGSM2	9905	small G protein signaling modulator 2
ENSG0000	63.3577	0.131042	0.201495	0.279649	0.697764	SIX5	147912	SIX homeobox 5
ENSG0000	6.691992	-0.09917	0.236981	0.279581	0.697764	NA	NA	NA
ENSG0000	40.08059	-0.11076	0.228377	0.279911	0.697987	FGFR4	2264	fibroblast growth factor receptor 4
ENSG0000	10.50824	0.099747	0.235429	0.280157	0.698314	ZNF232-AS	1.02E+08	ZNF232 antisense RNA 1
ENSG0000	70.6888	0.133294	0.196894	0.280087	0.698314	SEH1L	81929	SEH1 like nucleoporin
ENSG0000	11.88944	-0.07499	0.235595	0.280144	0.698314	MAOB	4129	monoamine oxidase B
ENSG0000	39.10241	0.136233	0.197617	0.280334	0.698662	BNIP1	662	BCL2 interacting protein 1
ENSG0000	260.3768	-0.12892	0.156713	0.280394	0.698716	EMSY	56946	EMSY tran BRCA2 interacting
ENSG0000	6.111892	0.084392	0.23752	0.280661	0.698752	AKR7A3	22977	aldo-keto reductase family 7 member A3
ENSG0000	9.962299	0.099214	0.236188	0.280603	0.698752	LEXM	163747	lymphocyte expansion molecule
ENSG0000	2.966169	-0.04601	0.235006	0.280714	0.698752	NA	NA	NA
ENSG0000	63.98845	-0.13226	0.202281	0.280569	0.698752	L3MBTL3	84456	L3MBTL histone methyl-lysine binding protein 3
ENSG0000	32.33059	-0.12206	0.223332	0.280528	0.698752	NA	NA	NA
ENSG0000	2.481299	0.038145	0.234972	0.280695	0.698752	GRAPL	400581	GRB2 related adaptor protein like
ENSG0000	1305.244	-0.1281	0.159491	0.280472	0.698752	RFXANK	8625	regulatory factor X associated ankyrin containing protein
ENSG0000	22.50207	-0.12204	0.224239	0.280694	0.698752	CHKB-DT	1E+08	CHKB divergent transcript
ENSG0000	337.6794	-0.12756	0.15583	0.280835	0.698959	DVL2	1856	dishevelled segment polarity protein 2
ENSG0000	300.2348	-0.12634	0.153098	0.281239	0.699588	SMIM3	85027	small integral membrane protein 3
ENSG0000	16.93851	0.103021	0.233643	0.281288	0.699588	NA	NA	NA
ENSG0000	76.23882	0.132434	0.175108	0.281335	0.699588	DYNC2I1	55112	dynein 2 intermediate chain 1
ENSG0000	6.50973	0.081973	0.242194	0.281372	0.699588	COL13A1	1305	collagen type XIII alpha 1 chain
ENSG0000	231.2936	0.13222	0.185205	0.281354	0.699588	CLMN	79789	calmin
ENSG0000	247.3586	-0.12148	0.141633	0.281394	0.699588	CYB5B	80777	cytochrome b5 type B
ENSG0000	567.1091	0.127249	0.155054	0.281187	0.699588	UBE2G1	7326	ubiquitin conjugating enzyme E2 G1
ENSG0000	4823.31	-0.11009	0.118722	0.281333	0.699588	ABHD17A	81926	abhydrola depalmitoylase
ENSG0000	34.66168	-0.13298	0.200251	0.281645	0.700023	LOC10050	1.01E+08	uncharacterized LOC100506606
ENSG0000	3.002196	-0.03738	0.23519	0.281613	0.700023	LOC10537	1.05E+08	uncharacterized LOC105371899
ENSG0000	19.66403	-0.11363	0.229371	0.281721	0.700117	PLAU	5328	plasminog urokinase
ENSG0000	32.60996	-0.12807	0.212504	0.281922	0.700167	PUS10	150962	pseudouridine synthase 10
ENSG0000	579.9074	-0.11269	0.220914	0.281913	0.700167	FAS	355	Fas cell surface death receptor
ENSG0000	3932.407	-0.12264	0.154655	0.281932	0.700167	HNRNPC	3183	heterogeneous nuclear ribonucleoprotein C
ENSG0000	118.8295	0.12208	0.216597	0.281924	0.700167	CIAO2A	84191	cytosolic iron-sulfur assembly component 2A
ENSG0000	32477.71	-0.1297	0.170213	0.281883	0.700167	ARRB2	409	arrestin beta 2
ENSG0000	6.208194	0.070176	0.235779	0.282072	0.700323	NA	NA	NA
ENSG0000	21.1645	0.091766	0.233932	0.282037	0.700323	IGLC5	3541	immunoglobulin lambda constant 5 (pseudogene)
ENSG0000	2.399369	0.035426	0.234567	0.282199	0.700545	EPO	2056	erythropoietin
ENSG0000	55.73335	0.130207	0.20642	0.282516	0.700953	ZNF639	51193	zinc finger protein 639
ENSG0000	7.704844	-0.08638	0.236661	0.282495	0.700953	NA	NA	NA
ENSG0000	1066.921	0.123573	0.149218	0.282514	0.700953	RASA3	22821	RAS p21 protein activator 3
ENSG0000	4.517936	-0.05323	0.235083	0.282463	0.700953	LOC10537	1.05E+08	uncharacterized LOC105371083
ENSG0000	62.07944	0.132993	0.190984	0.282583	0.701024	TWNK	56652	twinkle mtDNA helicase
ENSG0000	24.77493	-0.10779	0.23155	0.282752	0.701206	NA	NA	NA
ENSG0000	1076.081	0.129625	0.196909	0.282772	0.701206	FCER2	2208	Fc epsilon receptor II
ENSG0000	389.1831	0.122798	0.14384	0.282721	0.701206	PORCN	64840	porcupine O-acyltransferase
ENSG0000	3.092674	0.056597	0.235561	0.282819	0.701229	NA	NA	NA
ENSG0000	212.431	0.123226	0.212937	0.282921	0.701354	CERS6	253782	ceramide synthase 6
ENSG0000	57.89851	0.128884	0.209838	0.282946	0.701354	ATP5MG	10632	ATP synthase membrane subunit g
ENSG0000	10.52842	-0.08457	0.235709	0.283169	0.70181	IL5RA	3568	interleukin 5 receptor subunit alpha

ENSG0000	1865.898	0.120981	0.140336	0.283419	0.702097	CITED2	10370	Cbp/p300 interacting transactivator with Glu/Asp rich carboxy-terminal domain 2
ENSG0000	4.074212	0.041862	0.234503	0.283438	0.702097	OIT3	170392	oncoprotein induced transcript 3
ENSG0000	2.670219	-0.05136	0.235529	0.283394	0.702097	NA	NA	NA
ENSG0000	54.46151	0.093047	0.231251	0.283337	0.702097	ATP11C	286410	ATPase phospholipid transporting 11C
ENSG0000	2.378106	-0.04819	0.234932	0.283519	0.702204	MAGI2	9863	membrane WW and PDZ domain containing 2
ENSG0000	9932.604	-0.12307	0.147076	0.283582	0.702266	ZBTB7B	51043	zinc finger and BTB domain containing 7B
ENSG0000	8.646642	-0.08933	0.235899	0.283692	0.702347	PSEN2	5664	presenilin 2
ENSG0000	8.66667	-0.10348	0.235268	0.283676	0.702347	NA	NA	NA
ENSG0000	3.175845	-0.05656	0.237209	0.2841	0.703264	DNAH6	1768	dynein axonemal heavy chain 6
ENSG0000	90.00894	-0.13284	0.189559	0.284221	0.703468	UBR1	197131	ubiquitin protein ligase E3 component n-recognin 1
ENSG0000	35.70439	0.126269	0.216106	0.284308	0.703587	ZNF268	10795	zinc finger protein 268
ENSG0000	24.54683	-0.12438	0.218852	0.28437	0.703647	SMN1	6606	survival of telomeric
ENSG0000	27.14631	0.10715	0.229889	0.284516	0.703817	ZSCAN12	9753	zinc finger and SCAN domain containing 12
ENSG0000	199.6414	-0.1306	0.170293	0.284515	0.703817	ZNF575	284346	zinc finger protein 575
ENSG0000	10.43673	0.063935	0.235129	0.284711	0.70392	NA	NA	NA
ENSG0000	22.72034	0.115847	0.225978	0.284675	0.70392	PON2	5445	paraoxonase 2
ENSG0000	15.31555	-0.10786	0.231104	0.284621	0.70392	OGFOD2	79676	2-oxoglutarate and iron dependent oxygenase domain containing 2
ENSG0000	62.74157	-0.13314	0.17799	0.284638	0.70392	ZSWIM3	140831	zinc finger SWIM-type containing 3
ENSG0000	4.334893	0.035886	0.233707	0.284807	0.704061	RN7SL280	1.06E+08	RNA 7SL cytoplasm pseudogene
ENSG0000	771.9711	-0.13116	0.173253	0.28489	0.704171	LY9	4063	lymphocyte antigen 9
ENSG0000	145.0684	0.131317	0.17905	0.284978	0.704294	MAPRE3	22924	microtubule associated protein RP/EB family member 3
ENSG0000	480.9875	0.119011	0.139231	0.28511	0.704342	MFF	56947	mitochondrial fission factor
ENSG0000	4.803428	0.062489	0.235809	0.285042	0.704342	NA	NA	NA
ENSG0000	20.08917	-0.12712	0.217311	0.285113	0.704342	SSR4P1	728039	signal sequence receptor subunit 4 pseudogene 1
ENSG0000	14.94661	-0.12266	0.224403	0.285359	0.704664	NA	NA	NA
ENSG0000	1109.319	-0.1182	0.133034	0.285397	0.704664	AIP	9049	aryl hydrocarbon receptor interacting protein
ENSG0000	361.8548	0.124806	0.150503	0.285308	0.704664	PPFIA1	8500	PTPRF interacting protein alpha 1
ENSG0000	3.620059	-0.06327	0.236128	0.285347	0.704664	NA	NA	NA
ENSG0000	636.4072	-0.12964	0.192253	0.28544	0.704674	RIN1	9610	Ras and Rab interactor 1
ENSG0000	1332.904	-0.12932	0.168602	0.285651	0.704959	USP19	10869	ubiquitin specific peptidase 19
ENSG0000	11717.34	-0.1222	0.209445	0.285674	0.704959	PDLIM7	9260	PDZ and LIM domain 7
ENSG0000	99.23396	0.125732	0.149462	0.285709	0.704959	RIOK1	83732	RIO kinase 1
ENSG0000	33.51676	-0.13409	0.206087	0.285708	0.704959	MTRES1	51250	mitochondrial transcription rescue factor 1
ENSG0000	2.953171	0.044119	0.234262	0.285957	0.705475	LOC10041	1E+08	M-phase phosphoprotein 8 pseudogene
ENSG0000	19.45586	0.108312	0.230871	0.286033	0.705568	NA	NA	NA
ENSG0000	52.49466	0.121252	0.218451	0.286251	0.705574	JMY	133746	junction m p53 cofactor
ENSG0000	1195.855	-0.12858	0.156638	0.286344	0.705574	RUFY1	80230	RUN and FYVE domain containing 1
ENSG0000	2.584844	-0.0432	0.235514	0.286334	0.705574	GATA3-AS	399717	GATA3 antisense RNA 1
ENSG0000	182.6492	-0.12721	0.161686	0.286344	0.705574	C10orf95-,	1.01E+08	C10orf95 antisense RNA 1
ENSG0000	15.57353	0.116455	0.228685	0.286329	0.705574	NA	NA	NA
ENSG0000	28.79098	0.128959	0.2109	0.28618	0.705574	NA	NA	NA
ENSG0000	2.681405	0.068762	0.237601	0.286145	0.705574	TEN1-CDK	1.01E+08	TEN1-CDK3 readthrough (NMD candidate)
ENSG0000	6.829295	0.070755	0.235706	0.286382	0.705574	LOC10537	1.05E+08	uncharacterized LOC105372421
ENSG0000	802.4285	-0.1308	0.158682	0.286143	0.705574	PANK2	80025	pantothenate kinase 2
ENSG0000	7.536834	0.077455	0.236077	0.286546	0.705598	TFCP2L1	29842	transcription factor CP2 like 1
ENSG0000	9.796469	-0.07075	0.235477	0.286607	0.705598	LOC12490	1.25E+08	uncharacterized LOC124908011
ENSG0000	632.1056	0.121873	0.166345	0.286519	0.705598	TRAM2	9697	translocation associated membrane protein 2
ENSG0000	3.391519	0.039375	0.23529	0.286457	0.705598	NA	NA	NA
ENSG0000	2.378494	0.020797	0.233509	0.286573	0.705598	TUT1	64852	terminal u U6 snRNA-specific
ENSG0000	165.3485	-0.13027	0.17325	0.286623	0.705598	NA	NA	NA
ENSG0000	35.59366	0.11246	0.227752	0.286717	0.705735	NIPBL-DT	646719	NIPBL divergent transcript
ENSG0000	542.8789	0.105166	0.11288	0.286807	0.705862	TTC31	64427	tetratricopeptide repeat domain 31
ENSG0000	13.2833	-0.11827	0.227526	0.286883	0.705936	NA	NA	NA
ENSG0000	11.10939	0.11307	0.231647	0.286953	0.705936	CLIC5	53405	chloride intracellular channel 5
ENSG0000	432.1084	0.130082	0.18465	0.286943	0.705936	CYFIP1	23191	cytoplasmic FMR1 interacting protein 1
ENSG0000	2.321403	0.033882	0.236016	0.287191	0.706428	NA	NA	NA
ENSG0000	72.08875	0.122754	0.208673	0.287292	0.706582	CCDC25	55246	coiled-coil domain containing 25
ENSG0000	22.73319	0.129836	0.209862	0.287383	0.706683	C1orf50	79078	chromosome 1 open reading frame 50
ENSG0000	1460.904	-0.11021	0.125237	0.287411	0.706683	STK38	11329	serine/threonine kinase 38
ENSG0000	70.94886	0.131185	0.183143	0.287477	0.70675	NA	NA	NA
ENSG0000	558.6418	-0.12282	0.156861	0.287561	0.706772	GABARAPI	11345	GABA type A receptor associated protein like 2
ENSG0000	4.206288	0.073231	0.236811	0.287562	0.706772	NA	NA	NA
ENSG0000	19.43512	0.113823	0.227327	0.287694	0.707	S100A13	6284	S100 calcium binding protein A13
ENSG0000	255.0189	0.128904	0.166277	0.28799	0.707087	NOL9	79707	nucleolar protein 9
ENSG0000	13.85958	0.114671	0.230766	0.288024	0.707087	LINC01857	1.03E+08	long intergenic non-protein coding RNA 1857
ENSG0000	18.83731	-0.11712	0.225128	0.288052	0.707087	TRBV7-3	28595	T cell receptor beta variable 7-3
ENSG0000	272.9476	-0.12773	0.163485	0.287959	0.707087	MVB12B	89853	multivesicular body subunit 12B
ENSG0000	2409.553	-0.12431	0.150713	0.287812	0.707087	ACTR1A	10121	actin related protein 1A
ENSG0000	6316.863	-0.12786	0.191621	0.288077	0.707087	TLE3	7090	TLE family transcriptional corepressor
ENSG0000	3.975083	0.042319	0.234001	0.287891	0.707087	NA	NA	NA
ENSG0000	124.9585	-0.12798	0.159852	0.288022	0.707087	MAFF	23764	MAF bZIP transcription factor F
ENSG0000	6.37994	-0.05883	0.235373	0.288052	0.707087	FAM83F	113828	family with sequence similarity 83 member F
ENSG0000	2.61981	-0.02888	0.233972	0.288209	0.707316	NA	NA	NA
ENSG0000	3.555231	-0.05231	0.235767	0.2883	0.70735	PTGER4P2	442421	PTGER4P2 transcribed pseudogene
ENSG0000	94.88063	-0.12871	0.161847	0.288267	0.70735	SMAD7	4092	SMAD family member 7
ENSG0000	3.414442	0.031862	0.23353	0.288379	0.707402	MATN1	4146	matrilin 1
ENSG0000	429.9772	0.12576	0.203936	0.288456	0.707402	BPNT2	54928	3'(2') 5'-bisphosphate nucleotidase 2
ENSG0000	1799.925	-0.12021	0.13736	0.288463	0.707402	AXIN1	8312	axin 1
ENSG0000	3.381164	0.05954	0.235737	0.288476	0.707402	USP43	124739	ubiquitin specific peptidase 43
ENSG0000	17.76799	0.062551	0.233982	0.288588	0.707583	RPL22L1	200916	ribosomal protein L22 like 1
ENSG0000	227.4749	-0.12938	0.191018	0.288637	0.70761	TNFRSF8	943	TNF receptor superfamily member 8
ENSG0000	3874.566	0.125704	0.159579	0.288745	0.707685	CPPED1	55313	calcineurin like phosphoesterase domain containing 1
ENSG0000	1395.526	-0.11519	0.131624	0.288744	0.707685	NKIRAS2	28511	NFKB inhibitor interacting Ras like 2
ENSG0000	33.93739	0.133098	0.188382	0.288984	0.708175	TCEAL3	85012	transcription elongation factor A like 3
ENSG0000	43.66326	-0.13043	0.200325	0.289124	0.708423	DOCK8-AS	157983	DOCK8 antisense RNA 1
ENSG0000	16.56849	0.112047	0.228934	0.289394	0.708509	ANKAR	150709	ankyrin and armadillo repeat containing
ENSG0000	102.5379	0.129976	0.190352	0.289361	0.708509	DENND11	57189	DENN domain containing 11
ENSG0000	15.17103	-0.09628	0.233856	0.289499	0.708509	TRBV12-3	28577	T cell receptor beta variable 12-3
ENSG0000	92.87588	-0.13133	0.188974	0.289378	0.708509	NA	NA	NA
ENSG0000	24.85725	0.12709	0.213087	0.289507	0.708509	EEF1AKMT	25895	EEF1A lysine methyltransferase 3
ENSG0000	109.0641	0.127971	0.184548	0.289242	0.708509	MPHOSPH	10198	M-phase phosphoprotein 9
ENSG0000	8.205909	0.074381	0.235268	0.289354	0.708509	UCHL3	7347	ubiquitin C-terminal hydrolase L3
ENSG0000	29.47832	0.127092	0.198054	0.289366	0.708509	RPL7AP6	326310	ribosomal protein L7a pseudogene 6
ENSG0000	32.81032	0.12947	0.206429	0.289441	0.708509	CENPV	201161	centromere protein V
ENSG0000	196.7122	0.129292	0.171589	0.28957	0.708569	AGO3	192669	argonaute RISC catalytic component 3
ENSG0000	9813.935	-0.1275	0.189595	0.289665	0.708641	EHD1	10938	EH domain containing 1

ENSG0000	1367.325	0.124631	0.15457	0.289677	0.708641	CRTC1	23373	CREB regulated transcription coactivator 1
ENSG0000	54.64671	0.112343	0.223766	0.289732	0.708682	RYR1	6261	ryanodine receptor 1
ENSG0000	2307.519	-0.12497	0.157856	0.289868	0.708919	BBLN	79095	bublin coiled coil protein
ENSG0000	584.4193	-0.12544	0.173371	0.290007	0.709044	C1QC	714	complement C1q C chain
ENSG0000	29.12272	0.11899	0.221226	0.290032	0.709044	MMS22L	253714	MMS22 lik DNA repair protein
ENSG0000	4.978535	0.065632	0.235774	0.290035	0.709044	MSC-AS1	1E+08	MSC antisense RNA 1
ENSG0000	6.987147	0.065082	0.238618	0.290114	0.709141	NA	NA	NA
ENSG0000	4.391459	0.088733	0.237921	0.290246	0.709181	NA	NA	NA
ENSG0000	180.9106	-0.12792	0.163032	0.290241	0.709181	ATRIP	84126	ATR interacting protein
ENSG0000	191.7626	-0.12723	0.159785	0.29023	0.709181	KLHL26	55295	kelch like family member 26
ENSG0000	14.3402	0.089829	0.233679	0.290366	0.70919	KCNK5	8645	potassium two pore domain channel subfamily K member 5
ENSG0000	359.8722	0.118684	0.161044	0.290302	0.70919	CCDC86	79080	coiled-coil domain containing 86
ENSG0000	509.0372	-0.12147	0.147147	0.290339	0.70919	SPPL3	121665	signal peptide peptidase like 3
ENSG0000	6.258015	0.06165	0.235468	0.290414	0.709211	UPK2	7379	uroplakin 2
ENSG0000	815241.2	-0.06743	0.237287	0.290616	0.709574	HBB	3043	hemoglobin subunit beta
ENSG0000	152.236	0.129131	0.171176	0.290639	0.709574	NA	NA	NA
ENSG0000	76.0338	-0.11072	0.22364	0.290731	0.709652	STAP1	26228	signal transducing adaptor family member 1
ENSG0000	585.1624	-0.12518	0.158681	0.290749	0.709652	MBNL3	55796	muscleblind like splicing regulator 3
ENSG0000	454.7779	-0.1084	0.120116	0.290892	0.709907	DXO	1797	decapping exoribonuclease
ENSG0000	136.382	0.120196	0.14217	0.290991	0.710054	DNAJC17	55192	DnaJ heat shock protein family (Hsp40) member C17
ENSG0000	3.399958	0.043208	0.23484	0.291102	0.71023	NA	NA	NA
ENSG0000	324.6602	0.12784	0.19183	0.291403	0.710243	KDM3A	55818	lysine demethylase 3A
ENSG0000	12.12638	-0.08986	0.235639	0.29135	0.710243	NAIPP3	643784	NAIP pseudogene 3
ENSG0000	76.73549	0.092857	0.230203	0.29129	0.710243	FCHO2	115548	FCH and mu domain containing endocytic adaptor 2
ENSG0000	37.62537	-0.1154	0.22198	0.291456	0.710243	C5	727	complement C5
ENSG0000	17.09833	0.107388	0.230507	0.291449	0.710243	NA	NA	NA
ENSG0000	171.5366	-0.12004	0.142704	0.291415	0.710243	NA	NA	NA
ENSG0000	65.89049	-0.11427	0.223255	0.291228	0.710243	LOXHD1	125336	lipoxygenase homology PLAT domains 1
ENSG0000	767.2372	0.124917	0.155392	0.291256	0.710243	MIER2	54531	MIER family member 2
ENSG0000	598.9969	0.121555	0.170068	0.29131	0.710243	HSD17B10	3028	hydroxysteroid 17-beta dehydrogenase 10
ENSG0000	686.224	-0.12648	0.181617	0.291686	0.710425	IVNS1ABP	10625	influenza virus NS1A binding protein
ENSG0000	42.78662	0.126446	0.208177	0.291667	0.710425	CCDC112	153733	coiled-coil domain containing 112
ENSG0000	125.7914	0.130279	0.185685	0.291649	0.710425	PNPLA7	375775	patatin like phospholipase domain containing 7
ENSG0000	3.375874	-0.06987	0.23738	0.291634	0.710425	NA	NA	NA
ENSG0000	1059.889	-0.12147	0.147177	0.29182	0.710657	SLC25A11	8402	solute carrier family 25 member 11
ENSG0000	6.180643	0.059219	0.234774	0.292126	0.710815	RAB7B	338382	RAB7B member RAS oncogene family
ENSG0000	8.61245	0.084475	0.236026	0.291958	0.710815	EIF2AK3-D	1.02E+08	EIF2AK3 divergent transcript
ENSG0000	9.224448	-0.0913	0.235007	0.292049	0.710815	PGAP1	80055	post-GPI attachment to proteins inositol deacylase 1
ENSG0000	6.925447	0.071479	0.235357	0.291975	0.710815	LOC10042	1E+08	heat shock protein 90 alpha family class B member 1 pseudogene
ENSG0000	232.2421	-0.12554	0.162337	0.292234	0.710815	NA	NA	NA
ENSG0000	86.35247	0.119792	0.219229	0.292143	0.710815	KBTBD7	84078	kelch repeat and BTB domain containing 7
ENSG0000	15.0303	0.122443	0.221754	0.292017	0.710815	HNRNPA1L	642659	heterogeneous nuclear ribonucleoprotein A1 like 3
ENSG0000	5.36275	0.049503	0.234407	0.292201	0.710815	ZNF443	10224	zinc finger protein 443
ENSG0000	5.969621	0.081148	0.236908	0.29219	0.710815	NA	NA	NA
ENSG0000	3.242436	-0.02976	0.233102	0.292291	0.71086	NA	NA	NA
ENSG0000	8.203737	0.069724	0.236859	0.292357	0.710925	EYA2	2139	EYA transcriptional coactivator and phosphatase 2
ENSG0000	220.0987	0.127043	0.164665	0.292474	0.711099	RUSC2	9853	RUN and SH3 domain containing 2
ENSG0000	41.70704	0.119998	0.21787	0.292506	0.711099	HYLS1	219844	HYLS1 centriolar and ciliogenesis associated
ENSG0000	77.40632	0.12786	0.201521	0.292591	0.71121	WWP1	11059	WW domain containing E3 ubiquitin protein ligase 1
ENSG0000	6.986974	0.085276	0.236234	0.29269	0.711219	LOC10192	1.02E+08	uncharacterized LOC101928728
ENSG0000	5.618707	-0.06984	0.235708	0.292827	0.711219	LOC28468	284685	EWS RNA binding protein 1 pseudogene
ENSG0000	3.284676	-0.05438	0.235133	0.292662	0.711219	NA	NA	NA
ENSG0000	257.3716	-0.11901	0.141762	0.292826	0.711219	HOMEZ	57594	homeobox and leucine zipper encoding
ENSG0000	546.9074	0.110397	0.123882	0.292805	0.711219	CTCF	10664	CCCTC-binding factor
ENSG0000	6.141011	-0.04914	0.233659	0.292775	0.711219	NA	NA	NA
ENSG0000	4.379612	0.061673	0.235334	0.292919	0.711267	NA	NA	NA
ENSG0000	7.499585	-0.04711	0.232968	0.292925	0.711267	KBTBD3	143879	kelch repeat and BTB domain containing 3
ENSG0000	7.859093	0.067839	0.235177	0.292991	0.711332	GNRHR2	114814	gonadotropin releasing hormone receptor 2 (pseudogene)
ENSG0000	51.72531	0.129793	0.195589	0.293045	0.711369	ZNF383	163087	zinc finger protein 383
ENSG0000	253.9754	-0.12605	0.16038	0.293126	0.711379	ENO2	2026	enolase 2
ENSG0000	11.3474	0.105817	0.232295	0.293111	0.711379	NFILZ	1.05E+08	NFIL3 like basic leucine zipper
ENSG0000	7.824529	0.077509	0.235863	0.293299	0.711704	LOC12490	1.25E+08	uncharacterized LOC124902694
ENSG0000	360.8226	-0.11659	0.138047	0.293391	0.711834	CCDC107	203260	coiled-coil domain containing 107
ENSG0000	27.38206	-0.1298	0.201819	0.293438	0.711853	NUTM2D	728130	NUT family member 2D
ENSG0000	314.9229	-0.11971	0.143539	0.293562	0.711871	CPSF2	53981	cleavage and polyadenylation specific factor 2
ENSG0000	140.8087	0.128636	0.189928	0.293523	0.711871	NA	NA	NA
ENSG0000	1912.975	-0.10492	0.115627	0.29355	0.711871	ATXN7L3	56970	ataxin 7 like 3
ENSG0000	14.59997	0.087266	0.233574	0.293697	0.711948	RPL32P18	644907	ribosomal protein L32 pseudogene 18
ENSG0000	166.4206	0.126845	0.189282	0.293711	0.711948	ZCCHC7	84186	zinc finger CCHC-type containing 7
ENSG0000	21.19195	0.120402	0.223076	0.293681	0.711948	KANK3	256949	KN motif and ankyrin repeat domains 3
ENSG0000	117.2842	0.125651	0.159204	0.293999	0.712096	LINC0196	150967	long intergenic non-protein coding RNA 1963
ENSG0000	216.8313	-0.12503	0.160071	0.294044	0.712096	EAF1	85403	ELL associated factor 1
ENSG0000	102.7542	0.123161	0.206302	0.293981	0.712096	HYCC1	84668	hyccin PI4KA lipid kinase complex subunit 1
ENSG0000	5.142442	0.088262	0.23691	0.294007	0.712096	BAIAP2L1	55971	BAR/IMD domain containing adaptor protein 2 like 1
ENSG0000	1613.149	-0.11225	0.127328	0.293852	0.712096	MKRN1	23608	makorin ring finger protein 1
ENSG0000	2.577957	0.05378	0.23697	0.293912	0.712096	SYCE1	93426	synaptonemal complex central element protein 1
ENSG0000	2.889485	0.059669	0.236731	0.293833	0.712096	NA	NA	NA
ENSG0000	45.06893	-0.09488	0.229658	0.294086	0.712104	TRBV30	28557	T cell receptor beta variable 30
ENSG0000	20.41072	-0.12131	0.219651	0.294144	0.712152	NA	NA	NA
ENSG0000	998.4274	-0.12784	0.187099	0.294193	0.712175	CRAT	1384	carnitine O-acetyltransferase
ENSG0000	11.35227	0.091815	0.234546	0.29434	0.712342	NA	NA	NA
ENSG0000	9.037317	-0.08406	0.235606	0.294325	0.712342	NA	NA	NA
ENSG0000	26.07949	0.123211	0.216271	0.2944	0.712393	RPS17	6218	ribosomal protein S17
ENSG0000	9.875285	0.088099	0.234862	0.294511	0.712451	NA	NA	NA
ENSG0000	304.6407	0.124634	0.161146	0.294511	0.712451	DOCK10	55619	dedicator of cytokinesis 10
ENSG0000	58.2117	0.129967	0.184061	0.294541	0.712451	SRCAP	10847	Snf2 related CREBBP activator protein
ENSG0000	8.34658	-0.0527	0.233466	0.294641	0.712506	CHN1	1123	chimerin 1
ENSG0000	3.053918	-0.04593	0.234905	0.294613	0.712506	NA	NA	NA
ENSG0000	1129.147	-0.12496	0.157757	0.294821	0.712678	CPTP	80772	ceramide-1-phosphate transfer protein
ENSG0000	91.46521	-0.12864	0.184597	0.294829	0.712678	TTC21B	79809	tetratricopeptide repeat domain 21B
ENSG0000	3.401081	0.052923	0.235262	0.294762	0.712678	BTNL9	153579	butyrophilin like 9
ENSG0000	87.69001	-0.12536	0.201439	0.29498	0.712948	ANAPC4	29945	anaphase promoting complex subunit 4
ENSG0000	1047.301	0.119264	0.144075	0.295114	0.713084	SET	6418	SET nuclear proto-oncogene
ENSG0000	424.5981	0.12739	0.173944	0.295106	0.713084	TOB1	10140	transducer 1
ENSG0000	377.0108	-0.11547	0.214947	0.295254	0.713225	CD101	9398	CD101 molecule
ENSG0000	2612.776	0.117282	0.141134	0.296056	0.713225	S100A10	6281	S100 calcium binding protein A10

ENSG0000	14.1228	0.074945	0.233655	0.295729	0.713225	NA	NA	NA
ENSG0000	18.86461	0.119931	0.221335	0.296067	0.713225	MCEE	84693	methylmalonyl-CoA epimerase
ENSG0000	28.35357	0.100845	0.230035	0.295884	0.713225	COL7A1	1294	collagen type VII alpha 1 chain
ENSG0000	73.91413	0.128445	0.193028	0.295498	0.713225	ANKRA2	57763	ankyrin repeat family A member 2
ENSG0000	209.4651	-0.12738	0.176426	0.295428	0.713225	TCF19	6941	transcription factor 19
ENSG0000	1231.91	-0.1119	0.125571	0.295971	0.713225	BCL7B	9275	BAF chromatin remodeling complex subunit BCL7B
ENSG0000	247.1026	0.123901	0.156044	0.295556	0.713225	RCC1L	81554	RCC1 like
ENSG0000	112.5955	0.127921	0.172633	0.295657	0.713225	NA	NA	NA
ENSG0000	79.72558	-0.12586	0.201596	0.295621	0.713225	THAP1	55145	THAP domain containing 1
ENSG0000	42.5198	0.13008	0.195442	0.295996	0.713225	GGH	8836	gamma-glutamyl hydrolase
ENSG0000	451.8915	-0.11183	0.128697	0.295871	0.713225	DECR1	1666	2 4-dienoyl-CoA reductase 1
ENSG0000	174.1419	0.12766	0.181435	0.295214	0.713225	MFSD3	113655	major facilitator superfamily domain containing 3
ENSG0000	82.41947	-0.12849	0.178111	0.295648	0.713225	GOLM1	51280	golgi membrane protein 1
ENSG0000	5.872718	-0.08155	0.23678	0.295554	0.713225	ZNF503-AS	1E+08	ZNF503 antisense RNA 2
ENSG0000	5.069688	-0.05257	0.234511	0.295416	0.713225	NA	NA	NA
ENSG0000	43012.75	-0.12557	0.189476	0.295767	0.713225	SELPLG	6404	selectin P ligand
ENSG0000	646.8636	0.124425	0.200212	0.295331	0.713225	PPP1CC	5501	protein phosphatase 1 catalytic subunit gamma
ENSG0000	64.23263	-0.12791	0.19652	0.295966	0.713225	NA	NA	NA
ENSG0000	6895.438	0.12368	0.201171	0.295416	0.713225	IGHG2	3501	immunoglobulin heavy constant gamma 2 (G2m marker)
ENSG0000	6.075459	-0.05761	0.233977	0.296043	0.713225	NA	NA	NA
ENSG0000	169.4173	-0.12649	0.16705	0.295775	0.713225	LRRC37B	114659	leucine rich repeat containing 37B
ENSG0000	42.68983	0.086876	0.232987	0.296598	0.713345	ATP6V0CP	1.12E+08	ATPase H+ transporting V0 subunit c pseudogene 4
ENSG0000	822.3952	0.117765	0.137539	0.296368	0.713345	ATRAID	51374	all-trans retinoic acid induced differentiation factor
ENSG0000	1662.076	0.124853	0.159254	0.296535	0.713345	TTC7A	57217	tetratricopeptide repeat domain 7A
ENSG0000	42.14206	0.124183	0.208554	0.296326	0.713345	KCTD6	200845	potassium channel tetramerization domain containing 6
ENSG0000	4.244223	0.071194	0.236524	0.296486	0.713345	RPL24P4	377381	RPL24 pseudogene 4
ENSG0000	29.9317	-0.10376	0.228067	0.296352	0.713345	TRBV27	28560	T cell receptor beta variable 27
ENSG0000	7.964809	0.094669	0.235079	0.296591	0.713345	RNU6-892	1.06E+08	RNA U6 small r pseudogene
ENSG0000	1180.011	-0.12323	0.157897	0.296496	0.713345	KAT6A	7994	lysine acetyltransferase 6A
ENSG0000	9.807459	0.086505	0.234891	0.296662	0.713345	NA	NA	NA
ENSG0000	3.74908	0.055567	0.234848	0.296175	0.713345	VWA3A	146177	von Willebrand factor A domain containing 3A
ENSG0000	1828.657	-0.12461	0.161247	0.296636	0.713345	STAT5A	6776	signal transducer and activator of transcription 5A
ENSG0000	41.28789	0.120902	0.219447	0.296616	0.713345	LINGO3	645191	leucine rich repeat and Ig domain containing 3
ENSG0000	85.06581	0.129054	0.182994	0.296306	0.713345	RAB3A	5864	RAB3A member RAS oncogene family
ENSG0000	1265.556	0.125653	0.166057	0.296389	0.713345	CD33	945	CD33 molecule
ENSG0000	272.6755	-0.11757	0.212395	0.296741	0.713346	BIRC2	329	baculoviral IAP repeat containing 2
ENSG0000	65.7557	0.128546	0.194473	0.296721	0.713346	HSBP1L1	440498	heat shock factor binding protein 1 like 1
ENSG0000	13.4517	0.099086	0.233209	0.296799	0.713393	TEAD2	8463	TEA domain transcription factor 2
ENSG0000	185.2492	-0.12584	0.188321	0.296866	0.713459	HMGCR	3156	3-hydroxy-3-methylglutaryl-CoA reductase
ENSG0000	4.140086	0.05427	0.236026	0.296965	0.713605	NA	NA	NA
ENSG0000	25.61967	0.098695	0.228773	0.297399	0.713899	NBEAL1	65065	neurobeachin like 1
ENSG0000	463.1784	-0.12586	0.181041	0.297384	0.713899	FAM13A-A	285512	FAM13A antisense RNA 1
ENSG0000	91.56325	-0.13089	0.183091	0.297177	0.713899	CA2	760	carbonic anhydrase 2
ENSG0000	74.15917	-0.08247	0.230429	0.297307	0.713899	KLRF1	51348	killer cell lectin like receptor F1
ENSG0000	60.68649	-0.12846	0.188366	0.297257	0.713899	SOCS2	8835	suppressor of cytokine signaling 2
ENSG0000	10.13762	0.10719	0.231723	0.297288	0.713899	MIA2-AS1	1E+08	MIA2 antisense RNA 1
ENSG0000	4.322362	-0.05031	0.234528	0.297357	0.713899	NA	NA	NA
ENSG0000	3.155006	0.071978	0.237098	0.297384	0.713899	NA	NA	NA
ENSG0000	280.688	0.105178	0.226263	0.297685	0.714305	SRP9	6726	signal recognition particle 9
ENSG0000	3.6479	-0.05897	0.235238	0.297744	0.714305	NA	NA	NA
ENSG0000	18.60262	0.0973	0.231078	0.297764	0.714305	CARNMT1	138199	carnosine N-methyltransferase 1
ENSG0000	125.4361	-0.12643	0.180293	0.29772	0.714305	GPSM1	26086	G protein signaling modulator 1
ENSG0000	6.000238	0.077632	0.235842	0.297681	0.714305	METTL21E	121952	methyltra pseudogene
ENSG0000	89.61568	0.125273	0.198658	0.29786	0.714442	MEPCE	56257	methylphosphate capping enzyme
ENSG0000	4.358327	0.03611	0.233184	0.298087	0.714893	LOC10192	1.02E+08	uncharacterized LOC101929140
ENSG0000	291.1229	-0.1095	0.218913	0.298177	0.715017	GIMAP2	26157	GTPase IMAP family member 2
ENSG0000	217.4479	-0.1218	0.148561	0.298408	0.715289	SLC25A20	788	solute carrier family 25 member 20
ENSG0000	101.4265	-0.12476	0.16486	0.298386	0.715289	USP30	84749	ubiquitin specific peptidase 30
ENSG0000	4.778912	0.069779	0.236206	0.298341	0.715289	RBM26-AS	1.01E+08	RBM26 antisense RNA 1
ENSG0000	38.48047	0.129559	0.199936	0.29855	0.715442	NMB	4828	neuromedin B
ENSG0000	509.5744	-0.12306	0.158397	0.29853	0.715442	EIF6	3692	eukaryotic translation initiation factor 6
ENSG0000	17.03874	-0.11477	0.224276	0.298612	0.715466	NA	NA	NA
ENSG0000	201.7713	0.122563	0.152639	0.298638	0.715466	ANAPC11	51529	anaphase promoting complex subunit 11
ENSG0000	523.4393	0.113796	0.131255	0.298691	0.715499	XPC	7508	XPC compl DNA damage recognition and repair factor
ENSG0000	30.07046	-0.1111	0.22371	0.298835	0.715751	NA	NA	NA
ENSG0000	996.6565	-0.11546	0.137755	0.298977	0.715903	GPBP1L1	60313	GC-rich promoter binding protein 1 like 1
ENSG0000	729.2144	-0.12492	0.191063	0.29896	0.715903	RENBP	5973	renin binding protein
ENSG0000	15.70317	-0.11757	0.221704	0.299063	0.716017	NA	NA	NA
ENSG0000	533.2549	0.124888	0.168249	0.299119	0.716056	PDK2	5164	pyruvate dehydrogenase kinase 2
ENSG0000	281.5606	-0.1261	0.178293	0.299179	0.716105	DUSP5	1847	dual specificity phosphatase 5
ENSG0000	3.052087	-0.05959	0.235739	0.29927	0.716138	NA	NA	NA
ENSG0000	5.544165	-0.06139	0.234434	0.299249	0.716138	NA	NA	NA
ENSG0000	2.879926	0.038546	0.234217	0.299344	0.71622	HOMER1	9456	homer scaffold protein 1
ENSG0000	40.26878	0.126232	0.20405	0.299486	0.716253	CCDC191	57577	coiled-coil domain containing 191
ENSG0000	910.8472	0.124818	0.169035	0.29952	0.716253	MCM7	4176	minichromosome maintenance complex component 7
ENSG0000	10.11615	0.069482	0.234604	0.299545	0.716253	CCNE2	9134	cyclin E2
ENSG0000	23.28211	-0.10527	0.226987	0.299481	0.716253	NA	NA	NA
ENSG0000	7.371549	-0.01812	0.234201	0.299553	0.716253	SIGLEC8	27181	sialic acid binding Ig like lectin 8
ENSG0000	1544.095	0.121066	0.149184	0.299593	0.716256	ABHD14B	84836	abhydrolase domain containing 14B
ENSG0000	457.0393	-0.11678	0.14058	0.299695	0.716405	ALG12	79087	ALG12 alp 6-mannosyltransferase
ENSG0000	82.00606	0.127347	0.176691	0.299808	0.716582	TRMT44	152992	tRNA methyltransferase 44 homolog
ENSG0000	1216.457	-0.12064	0.151616	0.300022	0.717001	CHST12	55501	carbohydrate sulfotransferase 12
ENSG0000	8.21816	0.092359	0.235278	0.300091	0.717071	KCNJ14	3770	potassium inwardly rectifying channel subfamily J member 14
ENSG0000	125.9041	0.127225	0.184479	0.30015	0.717118	PUDP	8226	pseudouridine 5'-phosphatase
ENSG0000	438.7117	0.107662	0.132043	0.300881	0.717118	DVL1	1855	dishevelled segment polarity protein 1
ENSG0000	5.394513	0.069219	0.235247	0.301163	0.717118	MIR34AHC	1.07E+08	MIR34A host gene
ENSG0000	70.93781	0.125495	0.194809	0.301227	0.717118	MFSD2A	84879	major facilitator superfamily domain containing 2A
ENSG0000	18.58189	0.097005	0.230751	0.300988	0.717118	BEND5	79656	BEN domain containing 5
ENSG0000	357.7539	-0.10868	0.122681	0.302112	0.717118	PRPF38A	84950	pre-mRNA processing factor 38A
ENSG0000	19.49886	-0.09819	0.230383	0.300395	0.717118	NA	NA	NA
ENSG0000	47.87141	0.122788	0.207525	0.302342	0.717118	SPRTN	83932	SprT-like N-terminal domain
ENSG0000	8.774662	-0.08767	0.234036	0.300983	0.717118	NA	NA	NA
ENSG0000	135.0657	0.122766	0.200488	0.301495	0.717118	TAPT1	202018	transmembrane anterior posterior transformation 1
ENSG0000	4.175691	0.04798	0.234247	0.300486	0.717118	NA	NA	NA
ENSG0000	5.813342	0.046889	0.233431	0.302239	0.717118	NA	NA	NA
ENSG0000	359.9115	-0.12393	0.182539	0.301731	0.717118	CPEB4	80315	cytoplasmic polyadenylation element binding protein 4

ENSG0000	255.0847	0.122018	0.199986	0.30232	0.717118	SYNCRIP	10492	synaptotagmin binding cytoplasmic RNA interacting protein
ENSG0000	3.525063	0.055837	0.239116	0.301916	0.717118	LOC10537	1.05E+08	uncharacterized LOC105375130
ENSG0000	531.1034	0.122356	0.156966	0.301034	0.717118	KDELR2	11014	KDEL endoplasmic reticulum protein retention receptor 2
ENSG0000	3.571562	0.040758	0.235436	0.301213	0.717118	GCK	2645	glucokinase
ENSG0000	7.792804	0.091032	0.234363	0.301454	0.717118	TAS2R4	50832	taste 2 receptor member 4
ENSG0000	82.0271	0.126734	0.183687	0.30189	0.717118	ASIC3	9311	acid sensing ion channel subunit 3
ENSG0000	65.97584	0.127421	0.188375	0.301925	0.717118	MAK16	84549	MAK16 homolog
ENSG0000	5.688693	-0.077	0.236263	0.300661	0.717118	C8orf88	1E+08	chromosome 8 open reading frame 88
ENSG0000	7.499577	-0.09146	0.234655	0.302328	0.717118	LOC12490	1.25E+08	uncharacterized LOC124900254
ENSG0000	125.2891	-0.1218	0.15227	0.302109	0.717118	DENND4C	55667	DENN domain containing 4C
ENSG0000	17.76317	0.0777	0.233214	0.300494	0.717118	MELK	9833	maternal embryonic leucine zipper kinase
ENSG0000	211.9934	0.124379	0.185667	0.301918	0.717118	SLC44A1	23446	solute carrier family 44 member 1
ENSG0000	30.8276	-0.11374	0.221881	0.301087	0.717118	B3GALT9	1E+08	beta-1 3-galactosyltransferase 9
ENSG0000	4.38899	-0.04105	0.233975	0.302147	0.717118	ZNF365	22891	zinc finger protein 365
ENSG0000	102.5565	0.127841	0.179655	0.300877	0.717118	PPP3CB-A!	1.02E+08	PPP3CB antisense RNA 1 (head to head)
ENSG0000	77.8123	-0.12622	0.171141	0.301815	0.717118	SYT9-AS1	1.01E+08	SYT9 antisense RNA 1
ENSG0000	2.366994	0.040885	0.234022	0.302151	0.717118	SPTY2D1O	1.01E+08	SPTY2D1 opposite strand
ENSG0000	79447.1	-0.12428	0.17062	0.300457	0.717118	FTH1	2495	ferritin heavy chain 1
ENSG0000	944.191	-0.12553	0.189975	0.301229	0.717118	STIP1	10963	stress induced phosphoprotein 1
ENSG0000	10.89642	-0.0894	0.234303	0.30228	0.717118	NRXN2	9379	neurexin 2
ENSG0000	32.6257	0.124085	0.20814	0.300475	0.717118	NARS2	79731	asparaginy mitochondrial
ENSG0000	406.1765	0.105241	0.118891	0.300761	0.717118	CENATAC	338657	centrosomal AT-AC splicing factor
ENSG0000	3052.271	0.125999	0.180185	0.300932	0.717118	CAMKK2	10645	calcium/calmodulin dependent protein kinase kinase 2
ENSG0000	319.2412	0.120508	0.149917	0.300527	0.717118	METTL17	64745	methyltransferase like 17
ENSG0000	29.40001	0.126124	0.208462	0.300588	0.717118	HOMER2	9455	homer scaffold protein 2
ENSG0000	3.311194	0.050643	0.236703	0.300775	0.717118	NA	NA	NA
ENSG0000	1034.393	-0.11523	0.140413	0.30138	0.717118	MPDU1	9526	mannose-P-dolichol utilization defect 1
ENSG0000	20.47734	0.112768	0.224245	0.301747	0.717118	ZNF286A	57335	zinc finger protein 286A
ENSG0000	375.6943	-0.1119	0.130519	0.300591	0.717118	TNFAIP1	7126	TNF alpha induced protein 1
ENSG0000	100.7759	0.119984	0.159069	0.30173	0.717118	NA	NA	NA
ENSG0000	939.6736	0.115489	0.132485	0.30196	0.717118	PHB1	5245	prohibitin 1
ENSG0000	11.85417	-0.05755	0.233428	0.302032	0.717118	CDR2L	30850	cerebellar degeneration related protein 2 like
ENSG0000	3852.744	-0.12351	0.183071	0.300915	0.717118	SEC14L1	6397	SEC14 like lipid binding 1
ENSG0000	114.1885	0.12589	0.168607	0.300272	0.717118	ENDOV	284131	endonuclease V
ENSG0000	14786.24	-0.12436	0.177343	0.302159	0.717118	BSG	682	basigin (Ok blood group)
ENSG0000	812.7436	-0.12258	0.200828	0.301845	0.717118	GNA15	2769	G protein subunit alpha 15
ENSG0000	1288.786	-0.1235	0.192295	0.302248	0.717118	PAF1	54623	PAF1 hom Paf1/RNA polymerase II complex component
ENSG0000	1081.091	0.109044	0.12507	0.301751	0.717118	SUMO3	6612	small ubiquitin like modifier 3
ENSG0000	23.06702	0.128139	0.203561	0.300866	0.717118	NA	NA	NA
ENSG0000	27.18319	-0.11239	0.222928	0.301557	0.717118	ARHGAP6	395	Rho GTPase activating protein 6
ENSG0000	5.401415	-0.04552	0.233625	0.301925	0.717118	NA	NA	NA
ENSG0000	181.0773	-0.10586	0.220201	0.301559	0.717118	ZBTB33	10009	zinc finger and BTB domain containing 33
ENSG0000	318.5065	0.106477	0.121666	0.301076	0.717118	THOC2	57187	THO complex subunit 2
ENSG0000	1331.095	0.120525	0.151384	0.300464	0.717118	SSR4	6748	signal sequence receptor subunit 4
ENSG0000	7292.933	-0.1176	0.145483	0.302479	0.717351	CDC42SE1	56882	CDC42 small effector 1
ENSG0000	3.21064	-0.03118	0.233614	0.302574	0.717388	LINC02701	1.05E+08	long intergenic non-protein coding RNA 2701
ENSG0000	3.431632	-0.04188	0.234765	0.302561	0.717388	NA	NA	NA
ENSG0000	21.15853	0.125358	0.217239	0.302747	0.717707	C5orf63	401207	chromosome 5 open reading frame 63
ENSG0000	58.95258	0.119314	0.213268	0.302956	0.717831	ORC1	4998	origin recognition complex subunit 1
ENSG0000	365.1269	0.124385	0.167101	0.302946	0.717831	ELK3	2004	ETS transcription factor ELK3
ENSG0000	8128.752	0.124743	0.173464	0.302949	0.717831	PPP1R9B	84687	protein phosphatase 1 regulatory subunit 9B
ENSG0000	113.8887	-0.1254	0.171441	0.302912	0.717831	ZNF611	81856	zinc finger protein 611
ENSG0000	114.1285	-0.12023	0.155137	0.303051	0.717938	CCNYL1	151195	cyclin Y like 1
ENSG0000	185.676	-0.12502	0.174481	0.303119	0.717938	LRRC61	65999	leucine rich repeat containing 61
ENSG0000	165.8171	-0.12529	0.186143	0.303092	0.717938	ATP6V1D	51382	ATPase H+ transporting V1 subunit D
ENSG0000	16.00052	-0.09543	0.231736	0.303219	0.718056	NA	NA	NA
ENSG0000	15.55661	-0.08349	0.232553	0.303247	0.718056	CAVIN1	284119	caveolae associated protein 1
ENSG0000	34.91883	0.111187	0.221701	0.303537	0.718185	GRPEL2	134266	GrpE like 2 mitochondrial
ENSG0000	8.461399	0.093931	0.234137	0.30351	0.718185	CYSRT1	375791	cysteine rich tail 1
ENSG0000	193.1044	-0.11746	0.209555	0.303468	0.718185	FHIP2A	57700	FHF complex subunit HOOK interacting protein 2A
ENSG0000	154.0144	-0.12423	0.171978	0.303514	0.718185	TM2D3	80213	TM2 domain containing 3
ENSG0000	417.9508	-0.1166	0.140475	0.303502	0.718185	DCAKD	79877	dephospho-CoA kinase domain containing
ENSG0000	30.84523	-0.07181	0.232168	0.303491	0.718185	APOL4	80832	apolipoprotein L4
ENSG0000	12.36154	-0.11326	0.225915	0.303766	0.718633	NA	NA	NA
ENSG0000	4.101621	0.058646	0.235028	0.304151	0.718896	NA	NA	NA
ENSG0000	347.0733	-0.10445	0.117639	0.304051	0.718896	EIF1AD	84285	eukaryotic translation initiation factor 1A domain containing
ENSG0000	107.9093	-0.12545	0.175559	0.304103	0.718896	GPR68	8111	G protein-coupled receptor 68
ENSG0000	61.52927	0.126688	0.180439	0.304121	0.718896	JMJD7-PL/	8681	JMJD7-PLA2G4B readthrough
ENSG0000	827.607	0.120872	0.20453	0.303921	0.718896	DAPK2	23604	death associated protein kinase 2
ENSG0000	9.23405	0.077763	0.23432	0.304142	0.718896	TRPV3	162514	transient receptor potential cation channel subfamily V member 3
ENSG0000	34.50044	-0.11574	0.218407	0.30404	0.718896	PTK6	5753	protein tyrosine kinase 6
ENSG0000	131.3734	-0.12354	0.182473	0.3042	0.718918	C21orf58	54058	chromosome 21 open reading frame 58
ENSG0000	1689.101	-0.11841	0.145829	0.304568	0.719046	NFYC	4802	nuclear transcription factor Y subunit gamma
ENSG0000	2.974448	0.057868	0.237628	0.304536	0.719046	NA	NA	NA
ENSG0000	3.181148	0.0481	0.234555	0.304391	0.719046	NA	NA	NA
ENSG0000	141.0465	0.125503	0.173	0.304503	0.719046	TRIM74	378108	tripartite motif containing 74
ENSG0000	3.581691	-0.05065	0.235513	0.30456	0.719046	ZNG1DP	653510	Zn regulat pseudogene
ENSG0000	45179.32	-0.12	0.200317	0.304529	0.719046	LSP1	4046	lymphocyte specific protein 1
ENSG0000	553.3044	0.104621	0.116919	0.304547	0.719046	ANKLE2	23141	ankyrin repeat and LEM domain containing 2
ENSG0000	300.9364	-0.12259	0.162501	0.3045	0.719046	SPINT2	10653	serine pep Kunitz type 2
ENSG0000	1393.358	-0.12325	0.169441	0.304741	0.719096	HECTD3	79654	HECT domain E3 ubiquitin protein ligase 3
ENSG0000	1437.228	-0.12372	0.173675	0.304722	0.719096	CASP8	841	caspase 8
ENSG0000	22.04473	-0.1125	0.223601	0.304746	0.719096	LYNX1	66004	Ly6/neurotoxin 1
ENSG0000	4.568095	0.07102	0.235743	0.304689	0.719096	NA	NA	NA
ENSG0000	160.6058	0.122193	0.200536	0.304985	0.719566	SMC3	9126	structural maintenance of chromosomes 3
ENSG0000	11.78784	0.094002	0.232986	0.305031	0.719583	FAM153CI	653316	protein FAM153C
ENSG0000	169.5116	-0.12064	0.151252	0.305112	0.719682	PMM1	5372	phosphomannomutase 1
ENSG0000	460.4967	0.121403	0.197706	0.305219	0.719841	ALDH16A1	126133	aldehyde dehydrogenase 16 family member A1
ENSG0000	95.60785	0.124607	0.181144	0.305397	0.719895	CEBPZ	10153	CCAAT enhancer binding protein zeta
ENSG0000	2.662614	0.037758	0.235457	0.305399	0.719895	MET	4233	MET protc receptor tyrosine kinase
ENSG0000	3363.032	0.119268	0.15082	0.305366	0.719895	DPP7	29952	dipeptidyl peptidase 7
ENSG0000	554.484	0.112832	0.131182	0.305354	0.719895	ABCC1	4363	ATP binding cassette subfamily C member 1
ENSG0000	1782.94	-0.11917	0.153438	0.305673	0.719933	XKR8	55113	XK related 8
ENSG0000	832.7613	0.117931	0.206837	0.305773	0.719933	CHPF	79586	chondroitin polymerizing factor
ENSG0000	137.2459	0.114027	0.13605	0.305778	0.719933	PDE12	201626	phosphodiesterase 12
ENSG0000	187.1721	0.103849	0.224755	0.305601	0.719933	MRPL3	11222	mitochondrial ribosomal protein L3

ENSG0000	303.6554	0.123884	0.167775	0.305682	0.719933	SMARCA5	8467	SWI/SNF r matrix ass actin depe subfamily member 5
ENSG0000	89.97213	-0.12443	0.190446	0.305796	0.719933	HIKESHI	51501	heat shock protein nuclear import factor hikeshi
ENSG0000	3.486881	0.063957	0.238089	0.30556	0.719933	CCL3L1	6349	C-C motif chemokine ligand 3 like 1
ENSG0000	2.377477	0.036605	0.233987	0.305476	0.719933	NA	NA	NA
ENSG0000	6494.272	-0.11873	0.155352	0.30566	0.719933	MAP2K2	5605	mitogen-activated protein kinase kinase 2
ENSG0000	2.608487	0.031736	0.234091	0.305808	0.719933	NA	NA	NA
ENSG0000	19.30344	-0.09126	0.231075	0.305987	0.720262	NA	NA	NA
ENSG0000	67.31223	0.125835	0.173618	0.306042	0.720299	SLC35E4	339665	solute carrier family 35 member E4
ENSG0000	58.63739	-0.12041	0.208822	0.306107	0.720357	TRIM10	10107	tripartite motif containing 10
ENSG0000	14.36868	0.07026	0.233409	0.306204	0.720494	ALG10B	144245	ALG10 alp 2-glucosyltransferase B
ENSG0000	11.62351	0.071024	0.233706	0.306279	0.720578	CFAP54	144535	cilia and flagella associated protein 54
ENSG0000	16.4276	0.107819	0.22713	0.306319	0.720579	SNX24	28966	sorting nexin 24
ENSG0000	304.9208	0.118873	0.157553	0.306373	0.720613	PLPP5	84513	phospholipid phosphatase 5
ENSG0000	995.933	-0.11075	0.130185	0.306516	0.720765	COX5B	1329	cytochrome c oxidase subunit 5B
ENSG0000	215.9785	0.119153	0.146666	0.306478	0.720765	TRIM65	201292	tripartite motif containing 65
ENSG0000	157.6487	-0.11655	0.146813	0.306596	0.72086	GTF2A2	2958	general transcription factor IIA subunit 2
ENSG0000	234.4381	0.116889	0.144828	0.306639	0.720868	MSI2	124540	musashi RNA binding protein 2
ENSG0000	2.995617	0.055132	0.235544	0.3067	0.720921	NA	NA	NA
ENSG0000	7.788134	0.079992	0.23673	0.30675	0.720944	TIMP3	7078	TIMP metallopeptidase inhibitor 3
ENSG0000	17.02432	0.09104	0.231537	0.306791	0.720947	IFT80	57560	intraflagellar transport 80
ENSG0000	4.472572	-0.0536	0.23636	0.306829	0.720947	SCGB1C1	147199	secretoglobin family 1C member 1
ENSG0000	590.5459	0.122107	0.157214	0.306986	0.721083	TTLL3	26140	tubulin tyrosine ligase like 3
ENSG0000	2.981582	-0.04606	0.233986	0.307005	0.721083	NA	NA	NA
ENSG0000	3.071286	0.070341	0.236534	0.306929	0.721083	RAD9B	144715	RAD9 checkpoint clamp component B
ENSG0000	34.77812	-0.11668	0.216479	0.307111	0.721238	TRBV4-2	28616	T cell receptor beta variable 4-2
ENSG0000	916.1272	0.114737	0.136904	0.307165	0.721272	SCIMP	388325	SLP adaptor and CSK interacting membrane protein
ENSG0000	29.16712	-0.12271	0.210899	0.307303	0.721338	LOC10012	1E+08	small nuclear ribonucleoprotein polypeptide N pseudogene
ENSG0000	3332.694	0.086328	0.231233	0.307278	0.721338	FKBP5	2289	FKBP prolyl isomerase 5
ENSG0000	98.75743	0.118733	0.149638	0.307311	0.721338	TEX10	54881	testis expressed 10
ENSG0000	425.1508	-0.12337	0.161376	0.307365	0.721361	MKLN1	4289	muskelin 1
ENSG0000	1160.511	0.108041	0.126645	0.3074	0.721361	TAF1C	9013	TATA-box RNA polymerase I subunit C
ENSG0000	213.4647	0.129605	0.194363	0.307501	0.721415	PPIG	9360	peptidylprolyl isomerase G
ENSG0000	12502.59	-0.12094	0.16176	0.307463	0.721415	TCIRG1	10312	T cell imm ATPase H+ transporting V0 subunit a3
ENSG0000	562.9587	-0.11588	0.207899	0.307604	0.721563	NA	NA	NA
ENSG0000	3.809703	0.065964	0.236128	0.307663	0.72161	COL24A1	255631	collagen type XXIV alpha 1 chain
ENSG0000	746.4535	-0.11686	0.174432	0.307712	0.721633	PITPNM2	57605	phosphatidylinositol transfer protein membrane associated 2
ENSG0000	1065.289	-0.12316	0.171107	0.307776	0.721689	F8A1	8263	coagulation factor VIII associated 1
ENSG0000	1015.81	0.112678	0.139267	0.307922	0.721797	PEA15	8682	proliferation and apoptosis adaptor protein 15
ENSG0000	18.76508	0.107388	0.226121	0.307915	0.721797	NA	NA	NA
ENSG0000	19.97414	0.105328	0.227889	0.30794	0.721797	SLC26A2	1836	solute carrier family 26 member 2
ENSG0000	112.1173	-0.11733	0.144635	0.307983	0.721807	SPATA5L1	79029	spermatogenesis associated 5 like 1
ENSG0000	32.6124	0.120147	0.211255	0.308214	0.722234	PGBD2	267002	piggyBac transposable element derived 2
ENSG0000	3.754432	-0.04593	0.235072	0.308245	0.722234	NA	NA	NA
ENSG0000	3172.417	-0.12374	0.169754	0.308527	0.722804	LTB4R	1241	leukotriene B4 receptor
ENSG0000	78.26045	0.124621	0.176261	0.308637	0.722884	SNRPF	6636	small nuclear ribonucleoprotein polypeptide F
ENSG0000	166.8863	0.113588	0.212354	0.30864	0.722884	ZMYM2	7750	zinc finger MYM-type containing 2
ENSG0000	23.42555	0.106014	0.224749	0.308921	0.723448	TRIAP1	51499	TP53 regulated inhibitor of apoptosis 1
ENSG0000	9.577684	0.081912	0.233771	0.309056	0.723579	NA	NA	NA
ENSG0000	10.94262	0.088597	0.23269	0.309055	0.723579	ATP6AP1-I	158960	ATP6AP1 divergent transcript
ENSG0000	728.4605	0.123384	0.174356	0.309238	0.723727	AGO4	192670	argonaute RISC component 4
ENSG0000	4.255372	-0.05367	0.234407	0.309232	0.723727	FTCDNL1	348751	formiminotransferase cyclodeaminase N-terminal like
ENSG0000	63.20204	-0.12462	0.191204	0.309201	0.723727	KLF5	688	KLF transcription factor 5
ENSG0000	1089.239	-0.12057	0.158468	0.309322	0.723833	BTN2A1	11120	butyrophilin subfamily 2 member A1
ENSG0000	1830.253	0.122816	0.17183	0.309417	0.723869	NAPRT	93100	nicotinate phosphoribosyltransferase
ENSG0000	191.0359	-0.12272	0.170048	0.309405	0.723869	LATS2	26524	large tumor suppressor kinase 2
ENSG0000	151.6276	0.116657	0.144306	0.309464	0.723888	MRPL18	29074	mitochondrial ribosomal protein L18
ENSG0000	11.71384	-0.0772	0.233349	0.309541	0.723975	NA	NA	NA
ENSG0000	18.72878	0.037104	0.23502	0.309588	0.723992	LINC0130(	731779	long intergenic non-protein coding RNA 1300
ENSG0000	27.43098	-0.11087	0.220911	0.309628	0.723993	PSD	5662	pleckstrin and Sec7 domain containing
ENSG0000	116.7308	0.119311	0.151676	0.309697	0.724062	NLE1	54475	notchless homolog 1
ENSG0000	3.108592	0.031382	0.233042	0.309762	0.724089	NA	NA	NA
ENSG0000	776.3956	-0.12054	0.156711	0.309787	0.724089	FXR2	9513	FMR1 autosomal homolog 2
ENSG0000	12.74263	0.107252	0.229357	0.309862	0.724172	FBLN2	2199	fibulin 2
ENSG0000	970.1006	-0.11047	0.131729	0.310107	0.724467	SRSF4	6429	serine and arginine rich splicing factor 4
ENSG0000	31.09226	0.12057	0.209278	0.310107	0.724467	NA	NA	NA
ENSG0000	251.6184	0.125789	0.188922	0.310046	0.724467	COX7A2L	9167	cytochrome c oxidase subunit 7A2 like
ENSG0000	17.41451	-0.10461	0.227239	0.310292	0.724713	BEAN1-AS	1.02E+08	BEAN1 antisense RNA 1
ENSG0000	2710.979	-0.1222	0.165232	0.310275	0.724713	NHSL2	340527	NHS like 2
ENSG0000	8.794195	0.081193	0.23396	0.310371	0.724714	NA	NA	NA
ENSG0000	4.999083	-0.07569	0.235387	0.310342	0.724714	DHDH	27294	dihydrodiol dehydrogenase
ENSG0000	76.62374	0.122863	0.189993	0.310421	0.724739	MTMR9	66036	myotubularin related protein 9
ENSG0000	6.376596	0.079354	0.234884	0.310501	0.724746	NA	NA	NA
ENSG0000	2972.791	0.122842	0.188834	0.310504	0.724746	ADGRE2	30817	adhesion G protein-coupled receptor E2
ENSG0000	13.01039	-0.1046	0.228804	0.31068	0.725066	ACOT11	26027	acyl-CoA thioesterase 11
ENSG0000	2630.119	-0.1188	0.152494	0.310723	0.725073	SMG5	23381	SMG5 nonsense mediated mRNA decay factor
ENSG0000	144.3347	0.12366	0.177792	0.311	0.725355	SNX9	51429	sorting nexin 9
ENSG0000	249.7882	0.123913	0.188205	0.310988	0.725355	PSMC2	5701	proteason ATPase 2
ENSG0000	14.27252	0.100658	0.229571	0.311002	0.725355	CACNA1C-	1.01E+08	CACNA1C antisense RNA 1
ENSG0000	168265.7	0.121141	0.163275	0.310919	0.725355	COX2	4513	cytochrome c oxidase subunit II
ENSG0000	1513.978	0.10387	0.118661	0.311094	0.725478	FAM53B	9679	family with sequence similarity 53 member B
ENSG0000	35.05974	0.115974	0.215341	0.311177	0.725487	GCFC2	6936	GC-rich sequence DNA-binding factor 2
ENSG0000	212.1532	0.126688	0.188781	0.311145	0.725487	PDS5B	23047	PDS5 cohesin associated factor B
ENSG0000	3.742049	0.041488	0.233802	0.311317	0.725544	MYOCOS	1.11E+08	myocilin opposite strand
ENSG0000	2186.672	-0.12161	0.173059	0.311244	0.725544	ACTR3	10096	actin related protein 3
ENSG0000	10.14826	-0.08803	0.232447	0.311541	0.725544	IL20RB	53833	interleukin 20 receptor subunit beta
ENSG0000	11.01116	0.100594	0.23075	0.311715	0.725544	FAIM	55179	Fas apoptotic inhibitory molecule
ENSG0000	68.03286	-0.12102	0.192859	0.311757	0.725544	NA	NA	NA
ENSG0000	326.4059	0.122049	0.162777	0.311731	0.725544	NA	NA	NA
ENSG0000	691.7052	0.113121	0.129282	0.311743	0.725544	GHITM	27069	growth hormone inducible transmembrane protein
ENSG0000	199.435	0.121867	0.196154	0.311361	0.725544	PDZD8	118987	PDZ domain containing 8
ENSG0000	63.49342	0.124171	0.192674	0.311564	0.725544	ZBTB39	9880	zinc finger and BTB domain containing 39
ENSG0000	20.98049	0.122941	0.208276	0.311714	0.725544	SNHG10	283596	small nucleolar RNA host gene 10
ENSG0000	16.50942	-0.10661	0.226028	0.311612	0.725544	NA	NA	NA
ENSG0000	598.4002	-0.11958	0.159832	0.311517	0.725544	GALK1	2584	galactokinase 1
ENSG0000	62.88707	-0.12381	0.190342	0.311602	0.725544	FN3K	64122	fructosamine 3 kinase
ENSG0000	7.766308	-0.09908	0.232364	0.311716	0.725544	NA	NA	NA

ENSG0000	2073.123	-0.11508	0.164592	0.311853	0.725674	LCK	3932	LCK proto-	Src family tyrosine kinase
ENSG0000	49.59105	0.119379	0.204527	0.311892	0.725674	LSM6	11157	LSM6 hom	U6 small nuclear RNA and mRNA degradation associated
ENSG0000	2610.887	0.120386	0.196671	0.312001	0.725807	ARAP3	64411	ArfGAP wi	ankyrin repeat and PH domain 3
ENSG0000	4.585699	0.075786	0.235747	0.312028	0.725807	SETBP1-DT	1.02E+08	SETBP1 divergent transcript	
ENSG0000	12.4736	-0.0781	0.233089	0.312231	0.726026	SLC45A1	50651	solute carrier family 45 member 1	
ENSG0000	16.5129	0.105357	0.226939	0.312196	0.726026	NA	NA	NA	
ENSG0000	778.4308	0.122636	0.178242	0.312241	0.726026	OAF	220323	out at first homolog	
ENSG0000	1349.609	-0.12138	0.171793	0.312431	0.726135	PTPA	5524	protein phosphatase 2 phosphatase activator	
ENSG0000	7.962104	-0.0724	0.233962	0.312447	0.726135	NA	NA	NA	
ENSG0000	1720.534	0.09896	0.117742	0.312436	0.726135	LAIR1	3903	leukocyte associated immunoglobulin like receptor 1	
ENSG0000	83.66089	0.111772	0.215995	0.312344	0.726135	TENM1	10178	teneurin transmembrane protein 1	
ENSG0000	6.820717	0.056761	0.233573	0.312531	0.726166	NA	NA	NA	
ENSG0000	523.7215	-0.11996	0.162146	0.312539	0.726166	RARG	5916	retinoic acid receptor gamma	
ENSG0000	50.50079	-0.12282	0.198993	0.312591	0.726195	NA	NA	NA	
ENSG0000	445.4779	-0.11675	0.150647	0.312752	0.726284	MUL1	79594	mitochondrial E3 ubiquitin protein ligase 1	
ENSG0000	71.63362	0.121285	0.160405	0.312787	0.726284	MTA3	57504	metastasis associated 1 family member 3	
ENSG0000	414.3137	0.111818	0.138918	0.312788	0.726284	VDAC1	7416	voltage dependent anion channel 1	
ENSG0000	26.87241	0.117915	0.214774	0.312726	0.726284	ANK3	288	ankyrin 3	
ENSG0000	14.96202	0.095379	0.230357	0.313032	0.726757	ZNF678	339500	zinc finger protein 678	
ENSG0000	209.002	0.121527	0.169481	0.313074	0.726763	WRAP53	55135	WD repeat containing antisense to TP53	
ENSG0000	1034.797	0.114748	0.137662	0.313456	0.727307	SH3TC1	54436	SH3 domain and tetratricopeptide repeats 1	
ENSG0000	13.61948	0.08867	0.231587	0.313414	0.727307	SPATA24	202051	spermatogenesis associated 24	
ENSG0000	9.412832	0.090187	0.233175	0.313467	0.727307	NA	NA	NA	
ENSG0000	431.3119	-0.12061	0.165135	0.313438	0.727307	NPDC1	56654	neural pro	differentiation and control 1
ENSG0000	321.4455	-0.11057	0.134413	0.313662	0.727484	PEX19	5824	peroxisomal biogenesis factor 19	
ENSG0000	127.1745	-0.11936	0.155473	0.313661	0.727484	GNE	10020	glucosamine (UDP-N-acetyl)-2-epimerase/N-acetylmannosamine kinase	
ENSG0000	214.7734	0.110768	0.133283	0.31364	0.727484	ITPA	3704	inosine triphosphatase	
ENSG0000	2.524036	0.02308	0.233082	0.313776	0.727564	NA	NA	NA	
ENSG0000	7666.953	0.121621	0.1702	0.313766	0.727564	SIRPA	140885	signal regulatory protein alpha	
ENSG0000	10.00056	0.058399	0.233767	0.314072	0.727882	IGKV2D-24	28885	immunoglobulin kappa variable 2D-24 (non-functional)	
ENSG0000	891.7367	-0.12094	0.182738	0.314034	0.727882	FGFBP2	83888	fibroblast growth factor binding protein 2	
ENSG0000	6.588098	0.082612	0.234359	0.314059	0.727882	NA	NA	NA	
ENSG0000	34.34035	0.106875	0.22202	0.313989	0.727882	MRPL39	54148	mitochondrial ribosomal protein L39	
ENSG0000	3.383693	0.047397	0.234528	0.314136	0.727938	NA	NA	NA	
ENSG0000	74.93097	0.12179	0.170917	0.314178	0.727943	NUP107	57122	nucleoporin 107	
ENSG0000	350.9417	-0.10851	0.131742	0.314244	0.727961	RUSC1	23623	RUN and SH3 domain containing 1	
ENSG0000	9.821834	0.086343	0.232653	0.314385	0.727961	RAB4A-AS	1.05E+08	RAB4A antisense RNA 1	
ENSG0000	17.28794	0.091354	0.230603	0.314378	0.727961	ENHO	375704	energy homeostasis associated	
ENSG0000	122.5427	0.124763	0.177697	0.314377	0.727961	TRMO	51531	tRNA methyltransferase O	
ENSG0000	27.99895	0.083294	0.231181	0.314342	0.727961	OMG	4974	oligodendrocyte myelin glycoprotein	
ENSG0000	114.6143	0.121491	0.188126	0.31443	0.727975	CEP170	9859	centrosomal protein 170	
ENSG0000	4.933034	-0.0781	0.235381	0.314614	0.728021	NA	NA	NA	
ENSG0000	19.32979	-0.10379	0.225652	0.314649	0.728021	NA	NA	NA	
ENSG0000	1260.29	-0.12183	0.194029	0.314543	0.728021	ATN1	1822	atrophin 1	
ENSG0000	25.62516	0.101762	0.226192	0.314622	0.728021	ZNF773	374928	zinc finger protein 773	
ENSG0000	198.9941	0.102205	0.220733	0.314617	0.728021	IGLV5-45	28781	immunoglobulin lambda variable 5-45	
ENSG0000	737.1091	0.120811	0.170367	0.314747	0.728156	ANKRD12	23253	ankyrin repeat domain 12	
ENSG0000	283.7036	0.122169	0.156975	0.314799	0.728184	TNFAIP8L1	126282	TNF alpha induced protein 8 like 1	
ENSG0000	161.8302	0.112025	0.212828	0.314906	0.728327	CSTA	1475	cystatin A	
ENSG0000	97.85975	0.122306	0.173556	0.31494	0.728327	FBXL8	55336	F-box and leucine rich repeat protein 8	
ENSG0000	3.595233	-0.04137	0.233545	0.315063	0.728329	DCBLD2	131566	discoidin	CUB and LCCL domain containing 2
ENSG0000	3.394755	-0.06495	0.236065	0.3151	0.728329	NA	NA	NA	
ENSG0000	3.234474	-0.04092	0.234025	0.315056	0.728329	NA	NA	NA	
ENSG0000	17.08021	0.115666	0.217959	0.315009	0.728329	PDXP	57026	pyridoxal phosphatase	
ENSG0000	80.19433	0.123813	0.1814	0.315199	0.728464	NA	NA	NA	
ENSG0000	25.74573	0.074391	0.231561	0.315308	0.728476	NUDCD1	84955	NudC domain containing 1	
ENSG0000	590.4099	-0.11446	0.148997	0.315296	0.728476	SEPHS2	22928	selenophosphate synthetase 2	
ENSG0000	75.64923	0.113253	0.213192	0.315323	0.728476	PTRH2	51651	peptidyl-tRNA hydrolase 2	
ENSG0000	3.845372	0.055661	0.234989	0.315437	0.728585	B3GAT1-D	283177	B3GAT1 divergent transcript	
ENSG0000	14.68948	0.090154	0.231423	0.31545	0.728585	SULT1A2	6799	sulfotransferase family 1A member 2	
ENSG0000	155.1817	0.117711	0.150564	0.315518	0.728651	TRAPPC11	60684	trafficking protein particle complex subunit 11	
ENSG0000	6451.043	0.121044	0.187343	0.315559	0.728653	PRKCSH	5589	protein kinase C substrate 80K-H	
ENSG0000	36.59544	-0.1179	0.210073	0.315823	0.728729	FBXO2	26232	F-box protein 2	
ENSG0000	95.41118	0.12051	0.161945	0.315896	0.728729	ABTB3	121551	ankyrin repeat and BTB domain containing 3	
ENSG0000	614.5974	0.110901	0.130918	0.31595	0.728729	SDR39U1	56948	short chain dehydrogenase/reductase family 39U member 1	
ENSG0000	5.430973	0.045822	0.234286	0.315867	0.728729	IGHD3-9	28500	immunoglobulin heavy diversity 3-9	
ENSG0000	1142.818	-0.10601	0.125343	0.315708	0.728729	CIB1	10519	calcium and integrin binding 1	
ENSG0000	51.74064	0.122836	0.180485	0.31595	0.728729	DECR2	26063	2	4-dienoyl-CoA reductase 2
ENSG0000	3.629831	0.038595	0.233864	0.315782	0.728729	TSNAXIP1	55815	translin associated factor X interacting protein 1	
ENSG0000	2275.318	-0.11351	0.143179	0.315824	0.728729	RAB37	326624	RAB37	member RAS oncogene family
ENSG0000	186.4655	0.121981	0.161784	0.315801	0.728729	ZNF275	10838	zinc finger protein 275	
ENSG0000	105.7442	0.107023	0.217549	0.316202	0.729128	PEX2	5828	peroxisomal biogenesis factor 2	
ENSG0000	751.4188	-0.119	0.165608	0.316198	0.729128	TFIP11	24144	tuftelin interacting protein 11	
ENSG0000	47.40355	0.109769	0.215594	0.316282	0.729221	NCAPH	23397	non-SMC condensin I complex subunit H	
ENSG0000	12.69607	0.049537	0.233238	0.316586	0.729585	ZBTB41	360023	zinc finger and BTB domain containing 41	
ENSG0000	166.056	0.121476	0.17285	0.316631	0.729585	MTMR12	54545	myotubularin related protein 12	
ENSG0000	1696.062	-0.11364	0.150826	0.316689	0.729585	DNAJC4	3338	DnaJ heat shock protein family (Hsp40) member C4	
ENSG0000	1990.629	-0.11516	0.148235	0.316726	0.729585	DPF2	5977	double PHD fingers 2	
ENSG0000	28.55228	-0.1124	0.218075	0.31668	0.729585	THAP2	83591	THAP domain containing 2	
ENSG0000	1158.139	-0.12018	0.193141	0.316759	0.729585	VPS18	57617	VPS18 core subunit of CORVET and HOPS complexes	
ENSG0000	8.818561	0.092819	0.232632	0.316557	0.729585	TMEM256	254863	transmembrane protein 256	
ENSG0000	26.18347	0.097454	0.227677	0.316534	0.729585	JSRP1	126306	junctional sarcoplasmic reticulum protein 1	
ENSG0000	3.00745	0.029266	0.233532	0.316883	0.729736	NA	NA	NA	
ENSG0000	1922.174	0.120619	0.18521	0.316904	0.729736	MICAL2	9645	microtubu	calponin and LIM domain containing 2
ENSG0000	568.3961	0.101271	0.120965	0.316981	0.729821	BECN1	8678	beclin 1	
ENSG0000	18.82174	0.078939	0.231425	0.317086	0.729879	IFT88	8100	intraflagellar transport 88	
ENSG0000	752.9339	-0.11832	0.15588	0.317056	0.729879	PLCG1	5335	phospholipase C gamma 1	
ENSG0000	54.12254	-0.12027	0.200495	0.317379	0.730462	PRKCZ-AS1	1.01E+08	PRKCZ antisense RNA 1	
ENSG0000	415.5866	0.120596	0.146381	0.317496	0.730639	EIF2B5	8893	eukaryotic translation initiation factor 2B subunit epsilon	
ENSG0000	4.262837	0.058965	0.23403	0.317546	0.730662	ALG1L9P	285407	ALG1 like	pseudogene
ENSG0000	9.967535	-0.07115	0.233193	0.317726	0.730985	MIR4489	1.01E+08	microRNA 4489	
ENSG0000	30.65793	-0.11471	0.214832	0.31777	0.730995	DBH-AS1	138948	DBH antisense RNA 1	
ENSG0000	218.551	0.121539	0.173962	0.317865	0.731029	PPP3CB	5532	protein phosphatase 3 catalytic subunit beta	
ENSG0000	542.5235	-0.11955	0.166992	0.317858	0.731029	PPP1R16B	26051	protein phosphatase 1 regulatory subunit 16B	
ENSG0000	33.04894	-0.11456	0.208763	0.318159	0.731069	BTG2-DT	730227	BTG2 divergent transcript	
ENSG0000	5.875768	0.058778	0.233616	0.318078	0.731069	PLK2	10769	polo like kinase 2	

ENSG0000	65.7778	0.115757	0.209427	0.318006	0.731069	FZD1	8321	frizzled class receptor 1
ENSG0000	678.3607	0.124534	0.178665	0.318161	0.731069	IRAG2	4033	inositol 1 4 5-triphosphate receptor associated 2
ENSG0000	310.22	0.113774	0.142904	0.318124	0.731069	ELP5	23587	elongator acetyltransferase complex subunit 5
ENSG0000	5.188609	0.065646	0.234177	0.318115	0.731069	TBC1D29P	26083	TBC1 dom pseudogene
ENSG0000	571.8416	0.115974	0.149802	0.318104	0.731069	ACSS1	84532	acyl-CoA synthetase short chain family member 1
ENSG0000	3.111616	-0.05034	0.234327	0.318238	0.731154	RHBDF1	64285	rhomboid 5 homolog 1
ENSG0000	116.7185	-0.11876	0.192323	0.318466	0.731174	NBPF26	1.01E+08	NBPF member 26
ENSG0000	187.2113	0.119042	0.194917	0.318463	0.731174	PDK1	5163	pyruvate dehydrogenase kinase 1
ENSG0000	35.66691	-0.1127	0.214594	0.318307	0.731174	SCAT8	1.13E+08	S-phase cancer associated transcript 8
ENSG0000	55.1782	0.111353	0.215542	0.318493	0.731174	FAM3C	10447	FAM3 metabolism regulating signaling molecule C
ENSG0000	495.7486	0.102309	0.120517	0.318527	0.731174	LARP4B	23185	La ribonucleoprotein 4B
ENSG0000	3354.483	-0.11652	0.148568	0.3185	0.731174	YWHAB	7529	tyrosine 3-monooxygenase/tryptophan 5-monooxygenase activation protein beta
ENSG0000	348.5484	-0.12513	0.157804	0.31848	0.731174	MIAT	440823	myocardial infarction associated transcript
ENSG0000	255.6771	-0.11506	0.146318	0.318584	0.731214	PVT1	5820	Pvt1 oncogene
ENSG0000	73.92943	0.118791	0.201466	0.318826	0.731444	INTS13	55726	integrator complex subunit 13
ENSG0000	11.25547	-0.08384	0.231873	0.31875	0.731444	AMDHD1	144193	amidohydrolase domain containing 1
ENSG0000	60.57545	0.12195	0.187185	0.318844	0.731444	NA	NA	NA
ENSG0000	4962.638	-0.11367	0.181666	0.318806	0.731444	SF3A1	10291	splicing factor 3a subunit 1
ENSG0000	1209.777	0.106926	0.132828	0.318965	0.731593	ILF2	3608	interleukin enhancer binding factor 2
ENSG0000	47.32745	-0.07978	0.229679	0.318989	0.731593	NA	NA	NA
ENSG0000	3543.917	0.111022	0.137609	0.319387	0.731682	PPT1	5538	palmitoyl-protein thioesterase 1
ENSG0000	17.20067	-0.07359	0.232103	0.319306	0.731682	ZSWIM5	57643	zinc finger SWIM-type containing 5
ENSG0000	19.59162	-0.11652	0.21518	0.31934	0.731682	LINC00862	554279	long intergenic non-protein coding RNA 862
ENSG0000	119.7007	0.12151	0.173174	0.319177	0.731682	PLEKHM3	389072	pleckstrin homology domain containing M3
ENSG0000	5.047822	-0.05105	0.233353	0.319239	0.731682	NA	NA	NA
ENSG0000	156.4301	-0.12076	0.18416	0.319378	0.731682	MGST1	4257	microsomal glutathione S-transferase 1
ENSG0000	16.25332	-0.10894	0.218894	0.319303	0.731682	LINC02328	1.02E+08	long intergenic non-protein coding RNA 2328
ENSG0000	8.525245	0.071395	0.233613	0.319155	0.731682	ZNF519	162655	zinc finger protein 519
ENSG0000	618.0612	0.120144	0.184894	0.319147	0.731682	ZNF296	162979	zinc finger protein 296
ENSG0000	990.1676	-0.10773	0.132054	0.31956	0.731896	VPS72	6944	vacuolar protein sorting 72 homolog
ENSG0000	14.51879	-0.08693	0.231489	0.31954	0.731896	NA	NA	NA
ENSG0000	47.81714	0.089323	0.228319	0.3197	0.732124	G2E3	55632	G2/M-phase specific E3 ubiquitin protein ligase
ENSG0000	82.55366	-0.12129	0.19466	0.319814	0.732295	SCD	6319	stearoyl-CoA desaturase
ENSG0000	275.0096	0.115197	0.150431	0.319878	0.732305	PHPT1	29085	phosphohistidine phosphatase 1
ENSG0000	4.484086	-0.07074	0.236834	0.31992	0.732305	NA	NA	NA
ENSG0000	66.8979	0.125463	0.180498	0.319939	0.732305	ZNF587B	1E+08	zinc finger protein 587B
ENSG0000	414.4446	-0.11767	0.157499	0.320144	0.732656	SH3BP5	9467	SH3 domain binding protein 5
ENSG0000	40.48588	0.122953	0.180447	0.320172	0.732656	NA	NA	NA
ENSG0000	239.3791	-0.11669	0.154433	0.320243	0.732726	ANKRD54	129138	ankyrin repeat domain 54
ENSG0000	17.24539	0.111201	0.220484	0.320339	0.732855	GTF2IRD2	84163	GTF2I repeat domain containing 2
ENSG0000	2.602684	0.05095	0.236234	0.32065	0.733475	MSH2-OT1	644093	MSH2 overlapping transcript 1
ENSG0000	163.272	0.111037	0.133295	0.3208	0.733726	PRDM4	11108	PR/SET domain 4
ENSG0000	58.23726	0.117786	0.20384	0.320906	0.73377	MSC	9242	musculin
ENSG0000	23.69276	0.102583	0.224107	0.320932	0.73377	GLI1	2735	GLI family zinc finger 1
ENSG0000	4.982261	-0.05612	0.234264	0.320939	0.73377	NA	NA	NA
ENSG0000	106.0526	0.120865	0.175223	0.320993	0.733802	RNF14	9604	ring finger protein 14
ENSG0000	6.632632	-0.06292	0.235753	0.321208	0.734202	GCOM1	145781	GCOM1 MYZAP-POLR2M combined locus
ENSG0000	5.847617	-0.06236	0.233698	0.321576	0.734492	NA	NA	NA
ENSG0000	6.281582	0.078832	0.234351	0.321475	0.734492	LEF1-AS1	641518	LEF1 antisense RNA 1
ENSG0000	1592.128	-0.11837	0.167298	0.321537	0.734492	SNX3	8724	sorting nexin 3
ENSG0000	65.40927	-0.11996	0.181749	0.321532	0.734492	MINPP1	9562	multiple inositol-polyphosphate phosphatase 1
ENSG0000	16.7777	-0.08621	0.230784	0.321558	0.734492	NA	NA	NA
ENSG0000	3.635219	0.063712	0.234599	0.321536	0.734492	NA	NA	NA
ENSG0000	1229.758	-0.11491	0.151305	0.321871	0.734645	AURKAIP1	54998	aurora kinase A interacting protein 1
ENSG0000	4.34111	-0.05339	0.234308	0.321825	0.734645	NA	NA	NA
ENSG0000	2024.6	0.111969	0.141256	0.321873	0.734645	SETD1B	23067	SET domai histone lysine methyltransferase
ENSG0000	68.05184	0.11384	0.21109	0.321746	0.734645	NAA30	122830	N-alpha-ac NatC catalytic subunit
ENSG0000	3.063391	0.06066	0.236331	0.321883	0.734645	FOXB1	27023	forkhead box B1
ENSG0000	2.819909	0.028897	0.233705	0.321709	0.734645	NA	NA	NA
ENSG0000	796.1095	-0.11735	0.162659	0.322106	0.735008	ALAS1	211	5'-aminolevulinate synthase 1
ENSG0000	64.75561	0.120933	0.170191	0.322123	0.735008	ZNF696	79943	zinc finger protein 696
ENSG0000	179.2891	-0.09405	0.222447	0.322239	0.735024	TMSB4XP4	7118	TMSB4X pseudogene 4
ENSG0000	205.1492	0.120863	0.177155	0.32222	0.735024	APH1B	83464	aph-1 hom gamma-secretase subunit
ENSG0000	186.2201	-0.11372	0.15794	0.32225	0.735024	HOOK2	29911	hook microtubule tethering protein 2
ENSG0000	4.894622	0.07016	0.234718	0.322382	0.735131	NA	NA	NA
ENSG0000	6.781198	0.071826	0.23409	0.322418	0.735131	RNY1P16	1.06E+08	RNY1 pseudogene 16
ENSG0000	55.64936	0.120816	0.194341	0.322381	0.735131	ZNF70	7621	zinc finger protein 70
ENSG0000	183.3179	0.11465	0.208384	0.32248	0.735182	MAN2A1	4124	mannosidase alpha class 2A member 1
ENSG0000	2.794303	-0.04293	0.235173	0.322587	0.735334	HR	55806	HR lysine demethylase and nuclear receptor corepressor
ENSG0000	12.0565	-0.08954	0.230419	0.322655	0.735353	TRAV24	28659	T cell receptor alpha variable 24
ENSG0000	4.500802	0.061283	0.234551	0.322675	0.735353	NA	NA	NA
ENSG0000	1115.657	0.118609	0.188674	0.322789	0.735521	MCM5	4174	minichromosome maintenance complex component 5
ENSG0000	770.3681	-0.11937	0.184717	0.323002	0.735668	LBR	3930	lamin B receptor
ENSG0000	74.55659	0.106686	0.217605	0.323048	0.735668	CCSAP	126731	centriole cilia and spindle associated protein
ENSG0000	27.96278	0.105659	0.221921	0.323053	0.735668	NADK2	133686	NAD kinas mitochondrial
ENSG0000	3038.786	-0.11709	0.162355	0.322921	0.735668	MEFV	4210	MEFV inna pyrin
ENSG0000	18.54092	0.094446	0.228246	0.323054	0.735668	C18orf54	162681	chromosome 18 open reading frame 54
ENSG0000	2005.706	-0.11168	0.145099	0.323103	0.735687	WAC	51322	WW domain containing adaptor with coiled-coil
ENSG0000	4.431586	0.074248	0.234913	0.323239	0.735904	NA	NA	NA
ENSG0000	29.07702	0.10438	0.223309	0.323359	0.736088	PEX11G	92960	peroxisomal biogenesis factor 11 gamma
ENSG0000	2.546717	-0.04718	0.234168	0.323513	0.736164	PRELID1P1	728666	PRELID1 pseudogene 1
ENSG0000	1884.007	0.11923	0.172249	0.323506	0.736164	ABHD2	11057	abhydrola acylglycerol lipase
ENSG0000	2.316788	0.048729	0.234915	0.323508	0.736164	CACNG8	59283	calcium voltage-gated channel auxiliary subunit gamma 8
ENSG0000	999.3172	0.093216	0.106145	0.323578	0.736219	TRIM27	5987	tripartite motif containing 27
ENSG0000	160.746	0.118955	0.195244	0.323621	0.736225	NSMCE4A	54780	NSE4 hom SMC5-SMC6 complex component
ENSG0000	8070.31	-0.1111	0.141766	0.323797	0.736362	PCBP1	5093	poly(rC) binding protein 1
ENSG0000	816.1173	0.110066	0.138875	0.323895	0.736362	UGGT1	56886	UDP-glucose glycoprotein glucosyltransferase 1
ENSG0000	532.5639	0.107165	0.131207	0.323867	0.736362	TRAK1	22906	trafficking kinesin protein 1
ENSG0000	3.026648	0.045756	0.233978	0.323858	0.736362	NA	NA	NA
ENSG0000	2.72585	0.030769	0.23329	0.323922	0.736362	LOC10798	1.08E+08	uncharacterized LOC107984016
ENSG0000	5.193137	0.080145	0.234749	0.323842	0.736362	EEF1DP1	126037	eukaryotic translation elongation factor 1 delta pseudogene 1
ENSG0000	429.0697	0.118182	0.186261	0.323996	0.736439	ZNF217	7764	zinc finger protein 217
ENSG0000	301.387	0.110694	0.140024	0.32407	0.736515	MRPL57	78988	mitochondrial ribosomal protein L57
ENSG0000	3.707936	0.054073	0.233986	0.324194	0.736523	ACOXL-AS	400997	ACOXL antisense RNA 1
ENSG0000	6.932709	-0.06394	0.233431	0.324175	0.736523	SCARNA20	677681	small Cajal body-specific RNA 20
ENSG0000	66.00371	0.119565	0.183689	0.324162	0.736523	ZNF182	7569	zinc finger protein 182

ENSG0000	2.675468	-0.03785	0.233883	0.324341	0.736664	NA	NA	NA
ENSG0000	143.7204	0.116071	0.154696	0.324377	0.736664	CDAN1	146059	codanin 1
ENSG0000	180.7294	0.119489	0.172609	0.324336	0.736664	ILVBL	10994	ilvB acetolactate synthase like
ENSG0000	631.9652	-0.09969	0.117652	0.324474	0.736794	MRPL49	740	mitochondrial ribosomal protein L49
ENSG0000	5.630465	0.050591	0.233314	0.32455	0.736849	LOC11226	1.12E+08	uncharacterized LOC112268239
ENSG0000	73.87427	0.120602	0.181511	0.324653	0.736849	DARS2	55157	aspartyl-tF mitochondrial
ENSG0000	396.6997	0.105804	0.215625	0.324699	0.736849	TP53INP1	94241	tumor protein p53 inducible nuclear protein 1
ENSG0000	7.320254	0.067772	0.240028	0.324656	0.736849	TBC1D3F	84218	TBC1 domain family member 3F
ENSG0000	1206.005	-0.10819	0.134722	0.32466	0.736849	KANSL1	284058	KAT8 regulatory NSL complex subunit 1
ENSG0000	16.04871	0.103343	0.22498	0.324752	0.736878	NA	NA	NA
ENSG0000	20.58184	-0.10072	0.224591	0.325077	0.736978	NA	NA	NA
ENSG0000	97.85995	0.108248	0.166252	0.32503	0.736978	POLR1B	84172	RNA polymerase I subunit B
ENSG0000	2.777006	0.04278	0.235107	0.324962	0.736978	LINC0123٤	1.03E+08	long intergenic non-protein coding RNA 1238
ENSG0000	2108.43	-0.11635	0.193677	0.325219	0.736978	POR	5447	cytochrome p450 oxidoreductase
ENSG0000	40.10754	-0.12052	0.18762	0.32523	0.736978	ENTPD7	57089	ectonucleoside triphosphate diphosphohydrolase 7
ENSG0000	13.5776	0.089772	0.23054	0.325033	0.736978	RFXAP	5994	regulatory factor X associated protein
ENSG0000	31.02962	0.078385	0.229675	0.325066	0.736978	CGRRF1	10668	cell growth regulator with ring finger domain 1
ENSG0000	698.4813	-0.11232	0.146361	0.325216	0.736978	DNTTIP1	116092	deoxynucleotidyltransferase terminal interacting protein 1
ENSG0000	358.7346	-0.11477	0.126108	0.325224	0.736978	LOC11226	1.12E+08	uncharacterized LOC112268269
ENSG0000	649.7635	0.118164	0.147118	0.325239	0.736978	MCM3AP	8888	minichromosome maintenance complex component 3 associated protein
ENSG0000	3.434259	0.05902	0.234799	0.325218	0.736978	MIR659	724029	microRNA 659
ENSG0000	407.4451	-0.11487	0.181057	0.325358	0.736985	ZFC3H1	196441	zinc finger C3H1-type containing
ENSG0000	5.301236	0.045537	0.234268	0.325293	0.736985	SEZ6L	23544	seizure related 6 homolog like
ENSG0000	5.230305	0.035844	0.233634	0.325363	0.736985	NA	NA	NA
ENSG0000	10.6378	0.092966	0.230268	0.325546	0.737035	NA	NA	NA
ENSG0000	364.7421	-0.10418	0.128919	0.325541	0.737035	BUD31	8896	BUD31 homolog
ENSG0000	758.3072	-0.11748	0.173063	0.325443	0.737035	SLC7A5	8140	solute carrier family 7 member 5
ENSG0000	156.5572	-0.10843	0.211413	0.325495	0.737035	FMR1	2332	fragile X messenger ribonucleoprotein 1
ENSG0000	93.72192	0.116431	0.20138	0.325592	0.737048	ERH	2079	ERH mRNA splicing and mitosis factor
ENSG0000	8.013936	-0.07385	0.23271	0.325716	0.737238	GUCY2D	3000	guanylate retinal
ENSG0000	146.8665	0.11841	0.159422	0.325764	0.737256	AFG3L2	10939	AFG3 like matrix AAA peptidase subunit 2
ENSG0000	213.8191	-0.11328	0.146433	0.325808	0.737265	SUOX	6821	sulfite oxidase
ENSG0000	2.716747	0.040547	0.234196	0.325914	0.737413	NA	NA	NA
ENSG0000	79.87112	0.108976	0.213218	0.325969	0.737446	ACAT1	38	acetyl-CoA acetyltransferase 1
ENSG0000	1657.944	0.109886	0.138788	0.32608	0.737605	TBC1D17	79735	TBC1 domain family member 17
ENSG0000	4.523679	-0.0527	0.233489	0.326135	0.73764	CXCL5	6374	C-X-C motif chemokine ligand 5
ENSG0000	9.521933	-0.10594	0.22616	0.326319	0.737874	MIR9-1HG	10485	MIR9-1 host gene
ENSG0000	25.34846	0.106697	0.22058	0.326304	0.737874	KCP	375616	kielin cysteine rich BMP regulator
ENSG0000	7.48043	-0.05756	0.23262	0.326364	0.737885	NA	NA	NA
ENSG0000	3.575397	0.032792	0.233103	0.326412	0.737901	NA	NA	NA
ENSG0000	419.518	-0.10949	0.138167	0.326575	0.738003	C14orf93	60686	chromosome 14 open reading frame 93
ENSG0000	17.49541	-0.07469	0.237832	0.32654	0.738003	FAM3B	54097	FAM3 metabolism regulating signaling molecule B
ENSG0000	3.031678	0.040879	0.234154	0.326578	0.738003	MIR1249	1E+08	microRNA 1249
ENSG0000	14.19569	0.063433	0.232048	0.32662	0.738007	CRYBB1	1414	crystallin beta B1
ENSG0000	3.720157	0.065515	0.234539	0.32693	0.738616	NA	NA	NA
ENSG0000	465.4434	0.104897	0.128032	0.327062	0.738678	UROD	7389	uroporphyrinogen decarboxylase
ENSG0000	9.298258	-0.09271	0.23035	0.327035	0.738678	NA	NA	NA
ENSG0000	148.4248	0.112464	0.144019	0.327078	0.738678	EP400P1	347918	EP400 pseudogene 1
ENSG0000	16.11475	0.104346	0.224415	0.327185	0.7387	NA	NA	NA
ENSG0000	5.379513	0.048634	0.232987	0.327209	0.7387	NA	NA	NA
ENSG0000	115.7477	0.118128	0.180991	0.32716	0.7387	NTHL1	4913	nth like DNA glycosylase 1
ENSG0000	5.10475	0.071596	0.237939	0.327396	0.738976	NA	NA	NA
ENSG0000	32.89913	-0.10643	0.218551	0.327412	0.738976	F2RL3	9002	F2R like thrombin or trypsin receptor 3
ENSG0000	500.4274	0.116579	0.163042	0.327504	0.739092	FGD5-AS1	1.01E+08	FGD5 antisense RNA 1
ENSG0000	51.26041	-0.11859	0.17928	0.327632	0.739291	FLVCR1-D	642946	FLVCR1 divergent transcript
ENSG0000	13.00713	-0.05249	0.231595	0.327762	0.739493	TTC26	79989	tetratricopeptide repeat domain 26
ENSG0000	88.98563	-0.11712	0.197298	0.327818	0.739528	NA	NA	NA
ENSG0000	2.936059	0.022091	0.232745	0.327998	0.739844	SNORD12(	26765	small nucl C/D box 12C
ENSG0000	259.2611	-0.11528	0.146978	0.328095	0.739881	DNAJC1	64215	DnaJ heat shock protein family (Hsp40) member C1
ENSG0000	436.1987	-0.11529	0.155563	0.328082	0.739881	ABHD8	79575	abhydrolase domain containing 8
ENSG0000	13.99473	0.104931	0.224203	0.328244	0.739943	TRAV4	28689	T cell receptor alpha variable 4
ENSG0000	1068.638	-0.11496	0.15739	0.328221	0.739943	KIF1C	10749	kinesin family member 1C
ENSG0000	15.05973	0.106108	0.222893	0.32823	0.739943	NA	NA	NA
ENSG0000	110.7727	0.113043	0.205139	0.328783	0.740097	OSTC	58505	oligosaccharyltransferase complex non-catalytic subunit
ENSG0000	6.990344	-0.04558	0.233114	0.328533	0.740097	PNLDC1	154197	PARN like ribonuclease domain containing exonuclease 1
ENSG0000	2177.951	-0.10839	0.139712	0.328613	0.740097	STIM1	6786	stromal interaction molecule 1
ENSG0000	69.64036	0.119228	0.188438	0.328514	0.740097	RIC3	79608	RIC3 acetylcholine receptor chaperone
ENSG0000	217.4296	0.113426	0.147541	0.328781	0.740097	SNHG1	23642	small nucleolar RNA host gene 1
ENSG0000	65.62057	-0.1093	0.212482	0.328797	0.740097	N4BP2L2-I	116828	N4BPL2 intronic transcript 2
ENSG0000	3.3935	-0.0367	0.232492	0.328633	0.740097	UNC79	57578	unc-79 hoı NALCN channel complex subunit
ENSG0000	111.4934	0.112979	0.144717	0.328721	0.740097	PAM16	51025	presequence translocase associated motor 16
ENSG0000	341.271	-0.10754	0.13256	0.328761	0.740097	TERF2	7014	telomeric repeat binding factor 2
ENSG0000	111.5688	0.114971	0.154564	0.328702	0.740097	RPAIN	84268	RPA interacting protein
ENSG0000	700.0623	0.106869	0.211719	0.328598	0.740097	IGLV7-46	28775	immunoglobulin lambda variable 7-46
ENSG0000	1714.137	0.104031	0.125916	0.328426	0.740097	GGA1	26088	golgi assoc gamma ac ARF binding protein 1
ENSG0000	46.18154	0.12013	0.178565	0.328863	0.740153	TRIT1	54802	tRNA isopentenyltransferase 1
ENSG0000	2.611857	0.035281	0.233459	0.328974	0.740221	NA	NA	NA
ENSG0000	84.4896	0.112913	0.207159	0.32897	0.740221	NME1	4830	NME/NM23 nucleoside diphosphate kinase 1
ENSG0000	823.7515	0.110405	0.212467	0.329083	0.740375	CA4	762	carbonic anhydrase 4
ENSG0000	3.241015	0.041973	0.233332	0.32919	0.740527	RAB39A	54734	RAB39A member RAS oncogene family
ENSG0000	2.999984	-0.03344	0.232729	0.329356	0.740627	NA	NA	NA
ENSG0000	307.9633	0.109298	0.140388	0.329315	0.740627	SETD3	84193	SET domai actin histidine methyltransferase
ENSG0000	2.796223	0.047712	0.234015	0.329344	0.740627	EPB41L1	2036	erythrocyte membrane protein band 4.1 like 1
ENSG0000	1189.847	-0.11294	0.179129	0.32945	0.740682	BAZ1A	11177	bromodomain adjacent to zinc finger domain 1A
ENSG0000	113.0587	-0.11412	0.199962	0.329462	0.740682	TM6SF1	53346	transmembrane 6 superfamily member 1
ENSG0000	18.68865	-0.08969	0.227966	0.329612	0.740838	C14orf28	122525	chromosome 14 open reading frame 28
ENSG0000	34.84815	0.113836	0.209505	0.329588	0.740838	RRAGB	10325	Ras related GTP binding B
ENSG0000	53.41269	0.12021	0.185054	0.32972	0.74099	CDKN2AIP	91368	CDKN2A interacting protein N-terminal like
ENSG0000	2.90995	0.043482	0.236841	0.329866	0.741228	NA	NA	NA
ENSG0000	322.0309	0.113944	0.152139	0.330207	0.741812	ZNF668	79759	zinc finger protein 668
ENSG0000	1128.902	0.108786	0.143059	0.330181	0.741812	KAT8	84148	lysine acetyltransferase 8
ENSG0000	557.7493	-0.11553	0.161696	0.33025	0.741818	BAZ2B	29994	bromodomain adjacent to zinc finger domain 2B
ENSG0000	203.7785	0.106641	0.13126	0.330302	0.741843	DCTD	1635	dCMP deaminase
ENSG0000	544.3939	0.095517	0.22193	0.330471	0.742111	MAP3K2	10746	mitogen-activated protein kinase kinase kinase 2
ENSG0000	231.2337	-0.10424	0.142427	0.330509	0.742111	NGLY1	55768	N-glycanase 1
ENSG0000	96.42626	0.117929	0.167241	0.330543	0.742111	CSTF2	1478	cleavage stimulation factor subunit 2

ENSG0000	4.007069	-0.04056	0.2332	0.330847	0.742276	NA	NA	NA
ENSG0000	130.0998	0.109273	0.209136	0.330818	0.742276	ZC3H15	55854	zinc finger CCCH-type containing 15
ENSG0000	65.66555	0.115643	0.204211	0.330819	0.742276	GASK1B-A	285505	GASK1B antisense RNA 1
ENSG0000	82.70662	0.114866	0.195526	0.33086	0.742276	LYRM7	90624	LYR motif containing 7
ENSG0000	409.543	0.112589	0.197011	0.330676	0.742276	EEPD1	80820	endonuclease/exonuclease/phosphatase family domain containing 1
ENSG0000	9.173261	0.075921	0.232049	0.330838	0.742276	NA	NA	NA
ENSG0000	42.84675	-0.11977	0.186112	0.330928	0.74234	C17orf49	124944	chromosome 17 open reading frame 49
ENSG0000	720.5694	-0.11484	0.160679	0.331098	0.742426	CD96	10225	CD96 molecule
ENSG0000	5979.797	-0.11587	0.185346	0.331129	0.742426	ATP6V0D1	9114	ATPase H+ transporting V0 subunit d1
ENSG0000	22.99047	-0.07571	0.230545	0.331092	0.742426	RN7SL368	1.06E+08	RNA 7SL cytoplasm pseudogene
ENSG0000	37.55441	0.117463	0.199381	0.331031	0.742426	PAXBP1-A	1.01E+08	PAXBP1 antisense RNA 1
ENSG0000	447.3646	-0.10182	0.12947	0.331296	0.74271	KHDC4	22889	KH domai pre-mRNA splicing factor
ENSG0000	116.9461	-0.1289	0.191948	0.331501	0.742897	RASGRP3	25780	RAS guanyl releasing protein 3
ENSG0000	22.08478	0.095101	0.225427	0.331466	0.742897	GOLIM4	27333	golgi integral membrane protein 4
ENSG0000	66.33326	0.088458	0.225661	0.33143	0.742897	MYNN	55892	myoneurin
ENSG0000	525.2778	0.094967	0.112351	0.331603	0.743034	GPAT4	137964	glycerol-3-phosphate acyltransferase 4
ENSG0000	2.411887	0.035634	0.234397	0.331759	0.743112	NA	NA	NA
ENSG0000	66.48076	-0.11762	0.19339	0.331681	0.743112	CRELD1	78987	cysteine rich with EGF like domains 1
ENSG0000	593.8835	-0.11938	0.186383	0.331748	0.743112	TDP2	51567	tyrosyl-DNA phosphodiesterase 2
ENSG0000	122.0644	-0.10865	0.138166	0.331815	0.743145	GTF2E2	2961	general transcription factor IIE subunit 2
ENSG0000	27.09632	0.117023	0.204102	0.33228	0.743554	MDS2	259283	myelodysplastic syndrome 2 translocation associated
ENSG0000	7.723918	0.078775	0.232882	0.332775	0.743554	FAM178B	51252	family with sequence similarity 178 member B
ENSG0000	2363.411	0.109963	0.155095	0.332594	0.743554	LRRFIP1	9208	LRR binding FLII interacting protein 1
ENSG0000	145.9964	-0.11456	0.154652	0.332169	0.743554	PRRT3	285368	proline rich transmembrane protein 3
ENSG0000	162.965	0.117978	0.189015	0.332653	0.743554	ZBTB38	253461	zinc finger and BTB domain containing 38
ENSG0000	347.5358	-0.11097	0.147903	0.332458	0.743554	C4orf48	401115	chromosome 4 open reading frame 48
ENSG0000	353.5236	-0.10884	0.137936	0.332399	0.743554	BMP2K	55589	BMP2 inducible kinase
ENSG0000	67.88566	0.111729	0.208975	0.332471	0.743554	TMEM181	57583	transmembrane protein 181
ENSG0000	497.6403	-0.09585	0.119503	0.332863	0.743554	CDK13	8621	cyclin dependent kinase 13
ENSG0000	10509.03	-0.11533	0.167565	0.332433	0.743554	GNB2	2783	G protein subunit beta 2
ENSG0000	1115.758	0.102675	0.126058	0.332469	0.743554	ANP32B	10541	acidic nuclear phosphoprotein 32 family member B
ENSG0000	62.91915	0.117236	0.195311	0.33252	0.743554	NA	NA	NA
ENSG0000	3417.234	0.111613	0.153863	0.332434	0.743554	INPPL1	3636	inositol polyphosphate phosphatase like 1
ENSG0000	17.9132	-0.11141	0.215076	0.332738	0.743554	NUDT15	55270	nudix hydrolase 15
ENSG0000	419.4405	0.107541	0.143124	0.332931	0.743554	CCDC32	90416	coiled-coil domain containing 32
ENSG0000	48.07339	0.044863	0.232546	0.332699	0.743554	RSL24D1	51187	ribosomal L24 domain containing 1
ENSG0000	6.344671	0.089323	0.232494	0.332904	0.743554	RASGRF1	5923	Ras protein specific guanine nucleotide releasing factor 1
ENSG0000	2824.013	-0.11847	0.159106	0.332146	0.743554	CAPN15	6650	calpain 15
ENSG0000	3585.253	-0.11403	0.157788	0.332688	0.743554	PRR14	78994	proline rich 14
ENSG0000	90.92676	0.115788	0.153629	0.332226	0.743554	LINC00324	284029	long intergenic non-protein coding RNA 324
ENSG0000	54.74031	-0.1183	0.18388	0.332594	0.743554	CYB5A	1528	cytochrome b5 type A
ENSG0000	2.83233	-0.0364	0.234119	0.332887	0.743554	NA	NA	NA
ENSG0000	9.042644	-0.07934	0.231425	0.332083	0.743554	TUBA8	51807	tubulin alpha 8
ENSG0000	22.3722	-0.09494	0.224733	0.333113	0.743869	NA	NA	NA
ENSG0000	9.4504	-0.09342	0.229216	0.333442	0.743873	NA	NA	NA
ENSG0000	155.2377	0.113581	0.200652	0.333326	0.743873	DCHS1	8642	dachsous cadherin-related 1
ENSG0000	9.22365	0.065816	0.232654	0.333462	0.743873	E2F7	144455	E2F transcription factor 7
ENSG0000	78.2238	0.10951	0.209887	0.333254	0.743873	PSMC6	5706	proteasom ATPase 6
ENSG0000	59.51801	0.113013	0.205043	0.333204	0.743873	PALB2	79728	partner and localizer of BRCA2
ENSG0000	553.6034	-0.11065	0.145887	0.333311	0.743873	KAT2A	2648	lysine acetyltransferase 2A
ENSG0000	1408.182	0.112001	0.150729	0.333388	0.743873	AKAP8L	26993	A-kinase anchoring protein 8 like
ENSG0000	5782.258	-0.11571	0.176985	0.333439	0.743873	LPAR2	9170	lysophosphatidic acid receptor 2
ENSG0000	880.8841	0.104733	0.128104	0.33348	0.743873	ZDHHC8	29801	zinc finger DHHC-type palmitoyltransferase 8
ENSG0000	674.1267	-0.08984	0.103116	0.333676	0.74422	PIP4P1	90809	phosphati 5-bisphosphate 4-phosphatase 1
ENSG0000	1047.706	0.113151	0.149229	0.333828	0.744377	MLEC	9761	malectin
ENSG0000	1191.263	-0.11076	0.146591	0.333801	0.744377	SERINC3	10955	serine incorporator 3
ENSG0000	187.7379	0.117175	0.181994	0.333963	0.744487	KIAA1143	57456	KIAA1143
ENSG0000	67.25908	-0.11678	0.167137	0.333999	0.744487	ANKRD33E	651746	ankyrin repeat domain 33B
ENSG0000	13.86463	-0.08575	0.229867	0.333999	0.744487	TRBV7-6	28592	T cell receptor beta variable 7-6
ENSG0000	2.662174	-0.05002	0.233263	0.334154	0.744574	GPR199P	653160	G protein- pseudogene
ENSG0000	134.783	0.112966	0.155666	0.334323	0.744574	NUP133	55746	nucleoporin 133
ENSG0000	17.08867	0.106197	0.220175	0.33427	0.744574	NA	NA	NA
ENSG0000	389.2258	0.10953	0.142531	0.334156	0.744574	DDX46	9879	DEAD-box helicase 46
ENSG0000	304.0091	0.114286	0.16043	0.334271	0.744574	KLF4	9314	KLF transcription factor 4
ENSG0000	69.51434	-0.10936	0.209066	0.334212	0.744574	NA	NA	NA
ENSG0000	6.034867	-0.05618	0.232792	0.334319	0.744574	NA	NA	NA
ENSG0000	149.3718	0.118404	0.175058	0.33443	0.744724	ZCRB1	85437	zinc finger CCHC-type and RNA binding motif containing 1
ENSG0000	1795.113	0.103347	0.127268	0.334534	0.744772	CSDE1	7812	cold shock domain containing E1
ENSG0000	322.1581	0.107787	0.136524	0.334496	0.744772	CCAR1	55749	cell division cycle and apoptosis regulator 1
ENSG0000	31.72417	0.119	0.193663	0.334945	0.744833	NA	NA	NA
ENSG0000	13.65136	0.072572	0.231677	0.334908	0.744833	LINC02596	1.05E+08	long intergenic non-protein coding RNA 2596
ENSG0000	135.8	0.116808	0.167996	0.334643	0.744833	ADSS2	159	adenylosuccinate synthase 2
ENSG0000	142.3686	0.113729	0.154301	0.335032	0.744833	SYPL1	6856	synaptophysin like 1
ENSG0000	36.86332	0.119451	0.179105	0.334996	0.744833	XPA	7507	XPA DNA damage recognition and repair factor
ENSG0000	129.2396	0.117062	0.172512	0.33509	0.744833	UNC119B	84747	unc-119 lipid binding chaperone B
ENSG0000	45.34328	0.112637	0.207844	0.334991	0.744833	KLHL28	54813	kelch like family member 28
ENSG0000	230.2411	0.11179	0.145936	0.33482	0.744833	ETFA	2108	electron transfer flavoprotein subunit alpha
ENSG0000	10.06449	0.065618	0.232194	0.334848	0.744833	CD68	968	CD68 molecule
ENSG0000	280.0094	-0.11344	0.156768	0.334977	0.744833	CCDC137	339230	coiled-coil domain containing 137
ENSG0000	2195.15	0.094305	0.122768	0.335074	0.744833	RNF126	55658	ring finger protein 126
ENSG0000	1186.95	-0.10949	0.143227	0.334887	0.744833	ELOF1	84337	elongation factor 1
ENSG0000	950.9231	0.107006	0.140199	0.334902	0.744833	TAB1	10454	TGF-beta activated kinase 1 (MAP3K7) binding protein 1
ENSG0000	504.5096	0.099343	0.120498	0.335187	0.74496	VPS25	84313	vacuolar protein sorting 25 homolog
ENSG0000	89.22447	-0.11168	0.151013	0.335394	0.74533	TNFRSF10A	8797	TNF receptor superfamily member 10a
ENSG0000	1294.937	-0.11536	0.178854	0.33544	0.74534	SSH3	54961	slingshot protein phosphatase 3
ENSG0000	80.31013	0.113231	0.150633	0.335645	0.745546	CSPP1	79848	centrosome and spindle pole associated protein 1
ENSG0000	34.38488	0.107025	0.213346	0.335654	0.745546	DHRS11	79154	dehydrogenase/reductase 11
ENSG0000	8.559571	-0.10315	0.226609	0.335635	0.745546	MILIP	92659	MYC inducible lncRNA inactivating p53
ENSG0000	134842.4	0.115514	0.185157	0.335802	0.745566	CD74	972	CD74 molecule
ENSG0000	112.1127	0.117262	0.184559	0.335826	0.745566	EARS2	124454	glutamyl-t mitochondrial
ENSG0000	8.950561	0.073771	0.232463	0.335726	0.745566	NA	NA	NA
ENSG0000	828.9763	-0.10928	0.143438	0.335777	0.745566	MAFG	4097	MAF bZIP transcription factor G
ENSG0000	681.9039	0.109998	0.149273	0.335878	0.745589	IDH3B	3420	isocitrate dehydrogenase (NAD(+)) 3 non-catalytic subunit beta
ENSG0000	42.92186	-0.10829	0.212142	0.336035	0.745707	NA	NA	NA
ENSG0000	6.974853	0.077466	0.232483	0.336052	0.745707	AKR1E2	83592	aldo-keto reductase family 1 member E2
ENSG0000	48.49486	0.098327	0.222098	0.33601	0.745707	CIPC	85457	CLOCK interacting pacemaker

ENSG0000	15.05911	0.100696	0.224408	0.336158	0.74576	GYPE	2996	glycophorin E (MNS blood group)
ENSG0000	21.19529	-0.11689	0.202864	0.336148	0.74576	LIPE-AS1	1.01E+08	LIPE antisense RNA 1
ENSG0000	1455.451	-0.11396	0.162733	0.336217	0.7458	SCAMP4	113178	secretory carrier membrane protein 4
ENSG0000	520.636	0.108448	0.140942	0.33682	0.746044	PLK3	1263	polo like kinase 3
ENSG0000	41.69374	0.116579	0.195367	0.337067	0.746044	ZNHIT6	54680	zinc finger HIT-type containing 6
ENSG0000	52.33241	0.117933	0.183107	0.337278	0.746044	PRMT6	55170	protein arginine methyltransferase 6
ENSG0000	59.75899	0.105494	0.214205	0.336599	0.746044	DTL	51514	denticleless E3 ubiquitin protein ligase homolog
ENSG0000	169.7839	0.102592	0.129756	0.337304	0.746044	PCGF1	84759	polycomb group ring finger 1
ENSG0000	279.2565	-0.10084	0.126523	0.337232	0.746044	SMPD4BP	150776	sphingomy pseudogene
ENSG0000	595.8211	-0.0999	0.123036	0.337229	0.746044	DGKQ	1609	diacylglycerol kinase theta
ENSG0000	5155.825	-0.11464	0.180245	0.336839	0.746044	TREM1	54210	triggering receptor expressed on myeloid cells 1
ENSG0000	82.84129	-0.1169	0.187944	0.336507	0.746044	MRPL17	63875	mitochondrial ribosomal protein L17
ENSG0000	97.27119	0.117858	0.172761	0.33662	0.746044	RNF121	55298	ring finger protein 121
ENSG0000	1091.498	-0.11443	0.182707	0.336978	0.746044	CLEC7A	64581	C-type lectin domain containing 7A
ENSG0000	5.881351	0.047073	0.233174	0.337022	0.746044	NA	NA	NA
ENSG0000	15.42229	0.101943	0.224296	0.336763	0.746044	FANCM	57697	FA complementation group M
ENSG0000	11.7282	0.066369	0.231933	0.337276	0.746044	IGHV5-78	28387	immunoglobulin heavy variable 5-78 (pseudogene)
ENSG0000	332.3115	0.115935	0.176058	0.337043	0.746044	LMF1	64788	lipase maturation factor 1
ENSG0000	37105.28	-0.11439	0.171003	0.337268	0.746044	CORO1A	11151	coronin 1A
ENSG0000	860.4011	0.114499	0.168207	0.336946	0.746044	SETD1A	9739	SET domai histone lysine methyltransferase
ENSG0000	48.88312	0.114147	0.193791	0.336662	0.746044	CCDC43	124808	coiled-coil domain containing 43
ENSG0000	237.4893	0.115946	0.164078	0.337088	0.746044	RNF125	54941	ring finger protein 125
ENSG0000	79.27845	0.116976	0.19184	0.336455	0.746044	MEX3C	51320	mex-3 RNA binding family member C
ENSG0000	124.197	-0.11535	0.182632	0.336459	0.746044	ERFL	390937	ETS repressor factor like
ENSG0000	4.550156	0.074732	0.235046	0.336726	0.746044	KRT18P5	728300	keratin 18 pseudogene 5
ENSG0000	423.7139	0.115856	0.168618	0.337098	0.746044	CRELD2	79174	cysteine rich with EGF like domains 2
ENSG0000	211.6102	0.111939	0.148967	0.33716	0.746044	UTP14A	10813	UTP14A small subunit processome component
ENSG0000	8.618903	0.068303	0.232502	0.337378	0.746052	NA	NA	NA
ENSG0000	31.42128	0.106964	0.216534	0.337389	0.746052	ZNF761	388561	zinc finger protein 761
ENSG0000	920.6416	0.096575	0.11938	0.337552	0.746322	YJU2B	81576	YJU2 splicing factor homolog B
ENSG0000	381.8915	0.107536	0.153959	0.337938	0.746459	PRDX1	5052	peroxiredoxin 1
ENSG0000	6.493916	0.060269	0.234413	0.338341	0.746459	NA	NA	NA
ENSG0000	11.41223	0.097019	0.227038	0.338343	0.746459	CCDC138	165055	coiled-coil domain containing 138
ENSG0000	26.23612	0.106248	0.216454	0.33851	0.746459	ASNSD1	54529	asparagine synthetase domain containing 1
ENSG0000	1350.903	-0.11299	0.15825	0.338452	0.746459	TMEM115	11070	transmembrane protein 115
ENSG0000	5.31076	0.057129	0.233121	0.337964	0.746459	APBB2	323	amyloid beta precursor protein binding family B member 2
ENSG0000	57.17156	-0.11654	0.193075	0.337735	0.746459	PLA2G12A	81579	phospholipase A2 group XIIA
ENSG0000	15.3678	-0.10876	0.217775	0.338585	0.746459	NA	NA	NA
ENSG0000	140.5528	0.107725	0.210069	0.337868	0.746459	ZNF292	23036	zinc finger protein 292
ENSG0000	23.56096	0.111324	0.21183	0.338417	0.746459	MAP7	9053	microtubule associated protein 7
ENSG0000	158.6646	-0.11579	0.184319	0.338219	0.746459	SLC22A1	6580	solute carrier family 22 member 1
ENSG0000	82.05425	0.103095	0.215759	0.338486	0.746459	UQCRB	7381	ubiquinol-cytochrome c reductase binding protein
ENSG0000	25.53447	-0.11465	0.204527	0.33783	0.746459	NTAQ1	55093	N-terminal glutamine amidase 1
ENSG0000	436.3501	-0.10379	0.12517	0.338585	0.746459	RGP1	9827	RGP1 hom RAB6A GEF complex partner 1
ENSG0000	7.302886	0.062666	0.232976	0.338503	0.746459	NA	NA	NA
ENSG0000	2.987163	-0.02941	0.232933	0.338347	0.746459	NA	NA	NA
ENSG0000	20.59324	-0.08939	0.22685	0.338331	0.746459	CFAP58	159686	cilia and flagella associated protein 58
ENSG0000	17.67513	0.093764	0.22627	0.33829	0.746459	NA	NA	NA
ENSG0000	2.679715	0.038808	0.234592	0.337815	0.746459	PERCC1	1.05E+08	proline and glutamate rich with coiled coil 1
ENSG0000	808.5764	0.08891	0.107921	0.337982	0.746459	PIN1	5300	peptidylpr NIMA-interacting 1
ENSG0000	3.595763	-0.04042	0.234102	0.338287	0.746459	VSTM2B	342865	V-set and transmembrane domain containing 2B
ENSG0000	9.313384	-0.06919	0.233035	0.338369	0.746459	TSKS	60385	testis specific serine kinase substrate
ENSG0000	143.8419	-0.10897	0.206202	0.337685	0.746459	NCR1	9437	natural cytotoxicity triggering receptor 1
ENSG0000	1642.461	0.095923	0.135312	0.338592	0.746459	CENPB	1059	centromere protein B
ENSG0000	6.223445	0.078042	0.232899	0.338702	0.746553	DNLZ	728489	DNL-type zinc finger
ENSG0000	104.5146	0.08581	0.2219	0.338716	0.746553	CEP57	9702	centrosomal protein 57
ENSG0000	149.6815	0.112486	0.198591	0.338858	0.746665	NOP58	51602	NOP58 ribonucleoprotein
ENSG0000	502.5761	0.115178	0.181991	0.338863	0.746665	CALU	813	calumenin
ENSG0000	44.49069	0.116819	0.184687	0.338889	0.746665	FANCF	2188	FA complementation group F
ENSG0000	2326.465	-0.10969	0.157577	0.338998	0.746726	ZNF687	57592	zinc finger protein 687
ENSG0000	91.57659	0.11338	0.151885	0.338982	0.746726	PEX5	5830	peroxisomal biogenesis factor 5
ENSG0000	2568.43	0.08954	0.105004	0.339084	0.746773	CTBP1	1487	C-terminal binding protein 1
ENSG0000	9.009492	0.067352	0.233011	0.339101	0.746773	ORC6	23594	origin recognition complex subunit 6
ENSG0000	50.79447	-0.1026	0.216271	0.339207	0.746917	TRBV3-1	28619	T cell receptor beta variable 3-1
ENSG0000	140.3769	-0.1135	0.192077	0.339455	0.74716	SLAMF8	56833	SLAM family member 8
ENSG0000	32.06172	0.112282	0.207951	0.339522	0.74716	NA	NA	NA
ENSG0000	159.0964	0.100193	0.216509	0.339536	0.74716	PPP4R2	151987	protein phosphatase 4 regulatory subunit 2
ENSG0000	14.69004	-0.0541	0.231574	0.339562	0.74716	NA	NA	NA
ENSG0000	114.0012	-0.11423	0.167227	0.339376	0.74716	ZNF160	90338	zinc finger protein 160
ENSG0000	26496.6	-0.11394	0.176106	0.33949	0.74716	NFAM1	150372	NFAT activating protein with ITAM motif 1
ENSG0000	381.7299	-0.09664	0.116831	0.339671	0.747186	EYA3	2140	EYA transcriptional coactivator and phosphatase 3
ENSG0000	2184.541	-0.10435	0.155114	0.339673	0.747186	NUAK2	81788	NUAK family kinase 2
ENSG0000	328.5569	0.10459	0.131387	0.339697	0.747186	PARN	5073	poly(A)-specific ribonuclease
ENSG0000	73.57318	0.112723	0.153687	0.339788	0.747207	PARS2	25973	prolyl-tRN mitochondrial
ENSG0000	501.6023	0.109063	0.145956	0.339765	0.747207	MRPS18B	28973	mitochondrial ribosomal protein S18B
ENSG0000	8.149619	-0.06496	0.232581	0.339949	0.747237	NA	NA	NA
ENSG0000	26.54552	-0.10123	0.219181	0.339964	0.747237	LTBP1	4052	latent transforming growth factor beta binding protein 1
ENSG0000	978.8193	-0.11216	0.19407	0.339902	0.747237	TNS1	7145	tensin 1
ENSG0000	122.8747	0.116955	0.176491	0.340013	0.747237	CDC23	8697	cell division cycle 23
ENSG0000	32.77003	-0.10296	0.216654	0.340089	0.747237	CBLL1-AS1	1.02E+08	CBLL1 antisense RNA 1
ENSG0000	10.47948	-0.06101	0.231914	0.339966	0.747237	PLXNA4	91584	plexin A4
ENSG0000	233.7563	0.111365	0.151378	0.340128	0.747237	GATC	283459	glutamyl-tRNA amidotransferase subunit C
ENSG0000	8.206831	0.066013	0.232209	0.340102	0.747237	ALOX12P2	245	arachidonate 12-lipoxygenase pseudogene 2
ENSG0000	57.3937	-0.10569	0.210525	0.340225	0.747313	HOXB3	3213	homeobox B3
ENSG0000	335.4477	0.111233	0.143093	0.340244	0.747313	MIEF1	54471	mitochondrial elongation factor 1
ENSG0000	173.0397	0.113278	0.192523	0.340413	0.747415	TMF1	7110	TATA element modulatory factor 1
ENSG0000	15.60686	0.08389	0.229485	0.340405	0.747415	ANKRD42	338699	ankyrin repeat domain 42
ENSG0000	3.092925	-0.03489	0.234003	0.340403	0.747415	NA	NA	NA
ENSG0000	2726.851	0.113933	0.165942	0.340465	0.747439	TCF3	6929	transcription factor 3
ENSG0000	37.93425	0.114984	0.188402	0.340601	0.747523	PCBD2	84105	pterin-4 alpha-carbinolamine dehydratase 2
ENSG0000	1847.229	-0.1124	0.160146	0.340569	0.747523	PPP1R10	5514	protein phosphatase 1 regulatory subunit 10
ENSG0000	859.9988	-0.11274	0.189236	0.340645	0.747523	SKAP2	8935	src kinase associated phosphoprotein 2
ENSG0000	669.5659	0.112485	0.156659	0.340666	0.747523	SLC2A6	11182	solute carrier family 2 member 6
ENSG0000	2.546456	0.037673	0.233755	0.340807	0.747565	NA	NA	NA
ENSG0000	28.35303	0.089924	0.225038	0.340802	0.747565	ZNG1E	220869	Zn regulated GTPase metalloprotein activator 1E
ENSG0000	10.8653	-0.07097	0.23145	0.340775	0.747565	TMC5	79838	transmembrane channel like 5

ENSG0000	119.7542	-0.1099	0.200245	0.340975	0.747746	ARFIP1	27236	ADP ribosylation factor interacting protein 1
ENSG0000	13.02423	0.095207	0.235425	0.341005	0.747746	CNTNAP2	26047	contactin associated protein 2
ENSG0000	206.4686	0.106292	0.138063	0.341013	0.747746	ZNF446	55663	zinc finger protein 446
ENSG0000	63.6588	-0.10829	0.207994	0.341236	0.748146	SLC18B1	116843	solute carrier family 18 member B1
ENSG0000	6489.747	-0.10953	0.171416	0.341326	0.748234	PIK3CD	5293	phosphati 5-bisphosphate 3-kinase catalytic subunit delta
ENSG0000	33.04722	0.086767	0.225799	0.341399	0.748234	BARD1	580	BRCA1 associated RING domain 1
ENSG0000	17.53403	0.114004	0.208324	0.341397	0.748234	PHLDA1	22822	pleckstrin homology like domain family A member 1
ENSG0000	1084.106	-0.09751	0.119924	0.341516	0.748401	SAP30BP	29115	SAP30 binding protein
ENSG0000	34.70892	0.115083	0.195411	0.341716	0.748627	NA	NA	NA
ENSG0000	797.5647	-0.11329	0.183398	0.341823	0.748627	FOXP4	116113	forkhead box P4
ENSG0000	6.394958	0.075962	0.232393	0.341745	0.748627	C11orf94	143678	chromosome 11 open reading frame 94
ENSG0000	157.0217	0.109213	0.204356	0.341678	0.748627	TAF1D	79101	TATA-box RNA polymerase I subunit D
ENSG0000	15.86322	0.103648	0.221827	0.341811	0.748627	PDCD2L	84306	programmed cell death 2 like
ENSG0000	146.3874	0.106131	0.13689	0.341959	0.748714	SUMF1	285362	sulfatase modifying factor 1
ENSG0000	5.767164	-0.03966	0.233354	0.341986	0.748714	NA	NA	NA
ENSG0000	4.89879	0.048225	0.232878	0.34191	0.748714	NINL	22981	ninein like
ENSG0000	5.401264	-0.06846	0.233041	0.342095	0.748727	TBCE	6905	tubulin folding cofactor E
ENSG0000	122.4356	0.082857	0.226123	0.342482	0.748727	COX7C	1350	cytochrome c oxidase subunit 7C
ENSG0000	4.670397	-0.07522	0.233712	0.342452	0.748727	NA	NA	NA
ENSG0000	33.803	-0.11011	0.208134	0.342437	0.748727	TRBV5-4	28611	T cell receptor beta variable 5-4
ENSG0000	4076.726	0.110725	0.179648	0.342329	0.748727	NCOA4	8031	nuclear receptor coactivator 4
ENSG0000	138.6044	-0.10624	0.207877	0.342172	0.748727	BTBD10	84280	BTB domain containing 10
ENSG0000	18.50284	0.099724	0.221973	0.342252	0.748727	TGFB3	7043	transforming growth factor beta 3
ENSG0000	4.876647	-0.03901	0.233046	0.342301	0.748727	NA	NA	NA
ENSG0000	984.8034	-0.10754	0.141431	0.342174	0.748727	FAM174C	55009	family with sequence similarity 174 member C
ENSG0000	15.01169	-0.07859	0.229633	0.342219	0.748727	PGLYRP2	114770	peptidoglycan recognition protein 2
ENSG0000	18612.74	-0.10581	0.139058	0.342459	0.748727	UBA52	7311	ubiquitin A-52 residue ribosomal protein fusion product 1
ENSG0000	174.1241	0.11161	0.196043	0.342463	0.748727	URI1	8725	URI1 prefoldin like chaperone
ENSG0000	10.63416	0.086127	0.229742	0.342622	0.748765	EDN1	1906	endothelin 1
ENSG0000	540.7076	-0.11271	0.165305	0.342572	0.748765	ARL2	402	ADP ribosylation factor like GTPase 2
ENSG0000	4.117224	0.052238	0.237092	0.342584	0.748765	CLECL1P	160365	C-type lect pseudogene
ENSG0000	5.054962	0.028827	0.232625	0.342687	0.748819	CELF5	60680	CUGBP Elav-like family member 5
ENSG0000	38.36844	-0.10302	0.216077	0.342809	0.748995	OMA1	115209	OMA1 zinc metallopeptidase
ENSG0000	8.682321	0.092787	0.228452	0.343029	0.749387	NA	NA	NA
ENSG0000	134.2823	0.1139	0.171289	0.343118	0.749492	MRPS21	54460	mitochondrial ribosomal protein S21
ENSG0000	3.621449	0.039378	0.233122	0.343181	0.74954	CETN3	1070	centrin 3
ENSG0000	2528.598	-0.10939	0.150142	0.343415	0.749745	ARPC5	10092	actin related protein 2/3 complex subunit 5
ENSG0000	4.218841	0.062839	0.233961	0.34335	0.749745	CACNB4	785	calcium voltage-gated channel auxiliary subunit beta 4
ENSG0000	8.636281	0.056836	0.232191	0.343458	0.749745	KCNMB3	27094	potassium calcium-activated channel subfamily M regulatory beta subunit 3
ENSG0000	16.50299	0.070739	0.231091	0.343479	0.749745	GIN1	54826	gypsy retrotransposon integrase 1
ENSG0000	10.54039	-0.01981	0.233676	0.343444	0.749745	LINC0247(	1.01E+08	long intergenic non-protein coding RNA 2470
ENSG0000	10.34367	-0.08147	0.229945	0.34352	0.749745	TNFSF9	8744	TNF superfamily member 9
ENSG0000	119.7069	-0.10745	0.206285	0.343679	0.749822	KIFC1	3833	kinesin family member C1
ENSG0000	419.0319	0.092207	0.112743	0.343649	0.749822	PPP1R35	221908	protein phosphatase 1 regulatory subunit 35
ENSG0000	8.504214	-0.08161	0.230812	0.34365	0.749822	TRAJ3	28752	T cell receptor alpha joining 3
ENSG0000	122.1939	0.110453	0.152188	0.3438	0.749997	ZBTB24	9841	zinc finger and BTB domain containing 24
ENSG0000	76.56969	0.091579	0.221755	0.343853	0.750023	ZNF354A	6940	zinc finger protein 354A
ENSG0000	6.615817	0.059459	0.23239	0.344023	0.750304	LOC12490	1.25E+08	uncharacterized LOC124909475
ENSG0000	3.697282	-0.06062	0.234168	0.344243	0.750527	PLGLB2	5342	plasminogen like B2
ENSG0000	2280.308	-0.11449	0.177584	0.344205	0.750527	MYO18A	399687	myosin XVIIIA
ENSG0000	4.119026	-0.03562	0.232998	0.344248	0.750527	NA	NA	NA
ENSG0000	14.69946	-0.07877	0.229332	0.3447	0.750805	LOC10192	1.02E+08	uncharacterized LOC101926907
ENSG0000	3.758648	-0.04876	0.233832	0.344647	0.750805	GRASLND	386597	glycosaminoglycan regulatory associated long non-coding RNA
ENSG0000	258.7638	-0.11225	0.162221	0.344861	0.750805	CHIC2	26511	cysteine rich hydrophobic domain 2
ENSG0000	17.34033	-0.08465	0.228222	0.344837	0.750805	NA	NA	NA
ENSG0000	17.65684	0.081308	0.229556	0.344908	0.750805	NA	NA	NA
ENSG0000	99.67175	0.113906	0.175945	0.34483	0.750805	EPHA1	2041	EPH receptor A1
ENSG0000	64.88487	-0.11227	0.161754	0.344885	0.750805	IPPK	64768	inositol-pentakisphosphate 2-kinase
ENSG0000	9.64159	-0.09103	0.229279	0.344431	0.750805	TRAV25	28658	T cell receptor alpha variable 25
ENSG0000	101.3028	0.099402	0.232645	0.344608	0.750805	IGHV3-64[	1.03E+08	immunoglobulin heavy variable 3-64D
ENSG0000	8.894031	0.071845	0.231386	0.344867	0.750805	CBX2	84733	chromobox 2
ENSG0000	20664.19	-0.11266	0.175123	0.34467	0.750805	R3HDM4	91300	R3H domain containing 4
ENSG0000	142.9226	-0.10978	0.15187	0.344897	0.750805	ZNF324	25799	zinc finger protein 324
ENSG0000	14.92716	0.102006	0.221575	0.344532	0.750805	PIK3IP1-D	1.02E+08	PIK3IP1 divergent transcript
ENSG0000	67.00511	-0.10633	0.208212	0.344961	0.750831	ODF2L	57489	outer dense fiber of sperm tails 2 like
ENSG0000	73.33131	-0.10939	0.152727	0.345133	0.751027	HSD17B7	51478	hydroxysteroid 17-beta dehydrogenase 7
ENSG0000	2015.699	0.094752	0.115401	0.34513	0.751027	CBX7	23492	chromobox 7
ENSG0000	5.330009	-0.06497	0.233249	0.345209	0.751104	OXT	5020	oxytocin/neurophysin I prepropeptide
ENSG0000	11.25209	0.089326	0.230217	0.345391	0.751265	RPL7P18	1E+08	ribosomal protein L7 pseudogene 18
ENSG0000	3957.571	-0.10569	0.14089	0.345332	0.751265	ATP6V1B2	526	ATPase H+ transporting V1 subunit B2
ENSG0000	2.777156	0.046364	0.233364	0.345406	0.751265	NA	NA	NA
ENSG0000	15.78246	0.10802	0.21564	0.345475	0.751324	PTMAP5	150928	prothymosin alpha pseudogene 5
ENSG0000	16.90101	0.079037	0.228755	0.345585	0.751387	HYAL1	3373	hyaluronidase 1
ENSG0000	26.11885	-0.09504	0.222305	0.345577	0.751387	NA	NA	NA
ENSG0000	11.16608	0.098578	0.224359	0.345791	0.751656	PTS	5805	6-pyruvoyltetrahydropterin synthase
ENSG0000	9.891491	0.091032	0.227871	0.345764	0.751656	EFCAB11	90141	EF-hand calcium binding domain 11
ENSG0000	4552.397	-0.1106	0.158589	0.345967	0.75179	ARHGAP2!	9938	Rho GTPase activating protein 25
ENSG0000	2753.876	-0.11134	0.15607	0.345976	0.75179	ILK	3611	integrin linked kinase
ENSG0000	33.86084	0.110054	0.209449	0.345929	0.75179	PCGF2	7703	polycomb group ring finger 2
ENSG0000	123.912	-0.1055	0.207842	0.346152	0.751817	CEPT1	10390	choline/ethanolamine phosphotransferase 1
ENSG0000	6.802817	-0.08298	0.230774	0.346152	0.751817	FGF17	8822	fibroblast growth factor 17
ENSG0000	16.36888	0.104891	0.219765	0.346101	0.751817	GKAP1	80318	G kinase anchoring protein 1
ENSG0000	924.6013	-0.11321	0.141444	0.346076	0.751817	STK35	140901	serine/threonine kinase 35
ENSG0000	626.3341	-0.10722	0.169894	0.346373	0.751922	TNFRSF25	8718	TNF receptor superfamily member 25
ENSG0000	95.18445	-0.11392	0.176977	0.346396	0.751922	ZNF518B	85460	zinc finger protein 518B
ENSG0000	36.93334	-0.10151	0.215229	0.346471	0.751922	NA	NA	NA
ENSG0000	3.177992	0.045251	0.233677	0.346513	0.751922	CRB2	286204	crumbs cell polarity complex component 2
ENSG0000	15.58301	-0.09825	0.222863	0.346596	0.751922	NUTM2A	728118	NUT family member 2A
ENSG0000	6.04947	-0.03994	0.232642	0.346494	0.751922	ANKRD2	26287	ankyrin repeat domain 2
ENSG0000	17.99179	-0.09022	0.225775	0.346293	0.751922	DSTNP2	171220	DSTN pseudogene 2
ENSG0000	1234.762	0.112985	0.181483	0.346531	0.751922	FKBP11	51303	FKBP prolyl isomerase 11
ENSG0000	31.12077	-0.0991	0.218991	0.346425	0.751922	NA	NA	NA
ENSG0000	3.712412	0.046535	0.233961	0.346611	0.751922	NA	NA	NA
ENSG0000	9169.11	-0.10288	0.134087	0.34695	0.752202	TMSB10	9168	thymosin beta 10
ENSG0000	48.03088	0.103014	0.215563	0.346784	0.752202	ANKRD36I	645784	ankyrin repeat domain 36B pseudogene 2
ENSG0000	414.1112	0.112755	0.16313	0.347147	0.752202	NOP14	8602	NOP14 nucleolar protein

ENSG0000	25.31629	0.106167	0.2155	0.347021	0.752202	ACSL6	23305	acyl-CoA synthetase long chain family member 6
ENSG0000	2937.819	0.112741	0.205418	0.34695	0.752202	LCN2	3934	lipocalin 2
ENSG0000	28.69275	-0.08976	0.22556	0.347231	0.752202	MRGPRE	116534	MAS related GPR family member E
ENSG0000	315.5954	0.105802	0.140052	0.347274	0.752202	PDXDC1	23042	pyridoxal dependent decarboxylase domain containing 1
ENSG0000	2.582054	0.023567	0.233679	0.347257	0.752202	SP2-AS1	1.01E+08	SP2 antisense RNA 1
ENSG0000	577.9807	-0.10447	0.205789	0.34688	0.752202	MPO	4353	myeloperoxidase
ENSG0000	6.03169	0.066221	0.232291	0.347225	0.752202	NA	NA	NA
ENSG0000	47.39081	0.111394	0.201153	0.34705	0.752202	NAA20	51126	N-alpha-ac NatB catalytic subunit
ENSG0000	4.254365	-0.0543	0.233098	0.34697	0.752202	RFPL2	10739	ret finger protein like 2
ENSG0000	190.5617	-0.11267	0.172814	0.347271	0.752202	MCTS1	28985	MCTS1 re-initiation and release factor
ENSG0000	327.7518	-0.08778	0.219879	0.347357	0.752203	JAK2	3717	Janus kinase 2
ENSG0000	28.86795	0.105302	0.213674	0.347331	0.752203	NA	NA	NA
ENSG0000	1827.455	0.095281	0.117767	0.347992	0.752531	SFPQ	6421	splicing factor proline and glutamine rich
ENSG0000	238.9783	-0.11288	0.175192	0.347597	0.752531	TMEM40	55287	transmembrane protein 40
ENSG0000	6.093806	-0.05394	0.232911	0.347971	0.752531	PRLR	5618	prolactin receptor
ENSG0000	32.26564	-0.10338	0.213473	0.347575	0.752531	NA	NA	NA
ENSG0000	221.6044	-0.10394	0.204373	0.348023	0.752531	MCTP1	79772	multiple C2 and transmembrane domain containing 1
ENSG0000	19.30362	-0.08303	0.227081	0.347703	0.752531	NA	NA	NA
ENSG0000	1458.469	-0.11002	0.156645	0.347734	0.752531	GFUS	7264	GDP-L-fucose synthase
ENSG0000	167.2867	-0.10601	0.142329	0.348042	0.752531	TTF1	7270	transcription termination factor 1
ENSG0000	121.6628	-0.1011	0.213181	0.347742	0.752531	FRS2	10818	fibroblast growth factor receptor substrate 2
ENSG0000	4.290964	-0.06018	0.23375	0.347774	0.752531	NA	NA	NA
ENSG0000	108.8109	0.112332	0.174128	0.347986	0.752531	CES4A	283848	carboxylesterase 4A
ENSG0000	13.16225	0.099775	0.223276	0.347962	0.752531	NA	NA	NA
ENSG0000	44.4017	0.090774	0.223032	0.347865	0.752531	POLI	11201	DNA polymerase iota
ENSG0000	3.005126	0.053864	0.234841	0.348178	0.752646	HCG23	414764	HLA complex group 23
ENSG0000	240.4513	-0.11006	0.188434	0.348139	0.752646	EPB41L3	23136	erythrocyte membrane protein band 4.1 like 3
ENSG0000	4.678131	0.050726	0.232671	0.348236	0.752646	TRBV6-4	28603	T cell receptor beta variable 6-4
ENSG0000	353.1654	-0.11286	0.190117	0.34826	0.752646	PTGIR	5739	prostaglandin I2 receptor
ENSG0000	164.7317	0.110093	0.152746	0.348428	0.752826	POMZP3	22932	POM121 and ZP3 fusion
ENSG0000	4117.246	-0.10966	0.156547	0.348403	0.752826	UBE2R2	54926	ubiquitin conjugating enzyme E2 R2
ENSG0000	71.52938	0.113439	0.184019	0.348467	0.752826	WDR4	10785	WD repeat domain 4
ENSG0000	37.03857	0.089586	0.223435	0.348596	0.753016	SLC39A10	57181	solute carrier family 39 member 10
ENSG0000	2.533574	0.045213	0.235483	0.348696	0.753144	NA	NA	NA
ENSG0000	19.67465	0.095384	0.223291	0.348779	0.753234	PALS2	51678	protein as: MAGUK p55 family member
ENSG0000	2.753752	0.037626	0.233198	0.349107	0.753587	NA	NA	NA
ENSG0000	143.3926	0.11102	0.192739	0.349079	0.753587	TRPM7	54822	transient receptor potential cation channel subfamily M member 7
ENSG0000	61.51317	0.111713	0.197894	0.349107	0.753587	RPSAP58	388524	ribosomal protein SA pseudogene 58
ENSG0000	3.138734	-0.03745	0.234006	0.349059	0.753587	NA	NA	NA
ENSG0000	197.0069	0.104156	0.132938	0.3493	0.753753	VPS45	11311	vacuolar protein sorting 45 homolog
ENSG0000	1070.372	-0.11104	0.163432	0.349372	0.753753	RBMS1	5937	RNA binding motif single stranded interacting protein 1
ENSG0000	1145.433	0.09934	0.124539	0.349379	0.753753	PPARD	5467	peroxisome proliferator activated receptor delta
ENSG0000	1302.612	-0.11514	0.187147	0.349307	0.753753	BEST1	7439	bestrophin 1
ENSG0000	1849.252	0.035809	0.231548	0.349389	0.753753	NA	NA	NA
ENSG0000	1592.953	0.100526	0.128868	0.349553	0.753939	TRAF7	84231	TNF receptor associated factor 7
ENSG0000	7897.727	-0.10609	0.201316	0.349599	0.753939	STXBP2	6813	syntaxin binding protein 2
ENSG0000	57.86667	0.114505	0.176854	0.34959	0.753939	FRG1CP	1E+08	FSHD regio pseudogene
ENSG0000	4203.165	-0.10791	0.157566	0.349643	0.753946	ARAF	369	A-Raf prot serine/threonine kinase
ENSG0000	507.5305	0.097892	0.129646	0.349921	0.753995	ARID1B	57492	AT-rich interaction domain 1B
ENSG0000	4.328689	0.053794	0.232731	0.349924	0.753995	PSD3	23362	pleckstrin and Sec7 domain containing 3
ENSG0000	824.5155	0.10733	0.149597	0.349886	0.753995	CYB561A3	220002	cytochrome b561 family member A3
ENSG0000	644.827	-0.11232	0.187351	0.349995	0.753995	TESC	54997	tescalcin
ENSG0000	42.25088	0.110544	0.199027	0.349967	0.753995	MORN3	283385	MORN repeat containing 3
ENSG0000	27.47888	0.107274	0.210933	0.349804	0.753995	CMTM4	146223	CKLF like MARVEL transmembrane domain containing 4
ENSG0000	6022.65	-0.11143	0.178866	0.349849	0.753995	ACAP1	9744	ArfGAP wi ankyrin repeat and PH domains 1
ENSG0000	2.919785	0.036083	0.23453	0.3498	0.753995	LOC10050	1.01E+08	uncharacterized LOC100505664
ENSG0000	5.734424	0.06209	0.232441	0.350076	0.75408	TBCE	6905	tubulin folding cofactor E
ENSG0000	380.6949	-0.102	0.133341	0.350161	0.754137	DHDDS	79947	dehydrodolichyl diphosphate synthase subunit
ENSG0000	22.88626	-0.09739	0.220311	0.350278	0.754137	NA	NA	NA
ENSG0000	51.45177	0.114546	0.187473	0.350221	0.754137	MRPL48	51642	mitochondrial ribosomal protein L48
ENSG0000	8.185126	0.072866	0.231649	0.35031	0.754137	NA	NA	NA
ENSG0000	31.96158	0.111868	0.200742	0.35035	0.754137	GINS3	64785	GINS complex subunit 3
ENSG0000	280.1686	-0.09976	0.129073	0.350312	0.754137	DERL2	51009	derlin 2
ENSG0000	25.95154	0.106854	0.212695	0.350423	0.754207	NA	NA	NA
ENSG0000	20.35563	0.103664	0.215342	0.351001	0.75421	DNAAF10	116143	dynein axonemal assembly factor 10
ENSG0000	179.3089	0.099386	0.130038	0.350687	0.75421	GLT8D1	55830	glycosyltransferase 8 domain containing 1
ENSG0000	408.2724	0.06926	0.229695	0.350933	0.75421	SUB1	10923	SUB1 regulator of transcription
ENSG0000	31.89391	0.115363	0.184487	0.350495	0.75421	POP1	10940	POP1 hom ribonuclease P/MRP subunit
ENSG0000	2.836343	0.044948	0.23305	0.350973	0.75421	NA	NA	NA
ENSG0000	826.8212	-0.11036	0.186782	0.350903	0.75421	UBE2D1	7321	ubiquitin conjugating enzyme E2 D1
ENSG0000	1885.015	0.099913	0.126431	0.35076	0.75421	CPSF7	79869	cleavage and polyadenylation specific factor 7
ENSG0000	59.13588	-0.11297	0.189946	0.350659	0.75421	JAM3	83700	junctional adhesion molecule 3
ENSG0000	2.985108	-0.04588	0.233749	0.350812	0.75421	NA	NA	NA
ENSG0000	18.62462	-0.099	0.220457	0.350524	0.75421	CLBA1	122616	clathrin binding box of aftiphilin containing 1
ENSG0000	300.8917	0.106384	0.148621	0.350625	0.75421	MPV17L2	84769	MPV17 mitochondrial inner membrane protein like 2
ENSG0000	638.2976	-0.10038	0.126922	0.350921	0.75421	BLOC1S3	388552	biogenesis of lysosomal organelles complex 1 subunit 3
ENSG0000	445.8916	0.107649	0.149441	0.350848	0.75421	EIF4ENIF1	56478	eukaryotic translation initiation factor 4E nuclear import factor 1
ENSG0000	133.5983	-0.10962	0.151141	0.350837	0.75421	EFNB1	1947	ephrin B1
ENSG0000	48.61876	-0.1016	0.213018	0.351182	0.754216	SS18L2	51188	SS18 like 2
ENSG0000	994.9604	0.091818	0.11876	0.35121	0.754216	EIF4H	7458	eukaryotic translation initiation factor 4H
ENSG0000	4.968132	-0.03818	0.233571	0.351168	0.754216	ACER2	340485	alkaline ceramidase 2
ENSG0000	141.9019	0.115317	0.170438	0.351247	0.754216	ANTKMT	65990	adenine nucleotide translocase lysine methyltransferase
ENSG0000	193.8179	0.107005	0.18188	0.35126	0.754216	GEMIN4	50628	gem nuclear organelle associated protein 4
ENSG0000	270.4228	-0.11166	0.172699	0.351274	0.754216	ABHD3	171586	abhydrola: phospholipase
ENSG0000	423.6412	0.089245	0.108175	0.351292	0.754216	YTHDF1	54915	YTH N6-methyladenosine RNA binding protein 1
ENSG0000	8.609777	-0.06854	0.229107	0.351378	0.754313	NA	NA	NA
ENSG0000	269.961	0.105509	0.14182	0.351527	0.754315	ERI3	79033	ERI1 exoribonuclease family member 3
ENSG0000	3001.965	-0.11201	0.167993	0.351429	0.754315	RAB11FIP1	80223	RAB11 family interacting protein 1
ENSG0000	3756.07	0.100848	0.130469	0.351544	0.754315	CTDSP2	10106	CTD small phosphatase 2
ENSG0000	62.44287	-0.10686	0.205309	0.351506	0.754315	CD70	970	CD70 molecule
ENSG0000	582.3594	0.110571	0.176102	0.35174	0.754647	ISYNA1	51477	inositol-3-phosphate synthase 1
ENSG0000	83.14344	0.106279	0.206964	0.351899	0.754855	RYK	6259	receptor like tyrosine kinase
ENSG0000	69.7756	-0.11044	0.179419	0.352031	0.754855	ZBTB42	1E+08	zinc finger and BTB domain containing 42
ENSG0000	2765.838	-0.11097	0.177733	0.352043	0.754855	ATP6V0A1	535	ATPase H+ transporting V0 subunit a1
ENSG0000	290.6759	-0.11428	0.177205	0.351974	0.754855	CTU1	90353	cytosolic thiouridylase subunit 1
ENSG0000	1073.735	-0.1091	0.164174	0.351931	0.754855	SCAND1	51282	SCAN domain containing 1

ENSG0000	64.12044	0.113764	0.169817	0.352678	0.754977	MECR	51102	mitochondrial trans-2-enoyl-CoA reductase
ENSG0000	79.16859	0.112099	0.186397	0.352575	0.754977	ALMS1	7840	ALMS1 centrosome and basal body associated protein
ENSG0000	9.24426	0.042584	0.234874	0.352207	0.754977	IGKV1D-17	28900	immunoglobulin kappa variable 1D-17
ENSG0000	10.25109	-0.06814	0.2305	0.352506	0.754977	NA	NA	NA
ENSG0000	50.39058	0.108471	0.201188	0.352661	0.754977	LCORL	254251	ligand dependent nuclear receptor corepressor like
ENSG0000	634.3568	0.108994	0.15475	0.352416	0.754977	POLE3	54107	DNA polyr accessory subunit
ENSG0000	2.703906	0.032637	0.233016	0.352341	0.754977	NA	NA	NA
ENSG0000	4.590276	0.059622	0.2324	0.352574	0.754977	NA	NA	NA
ENSG0000	110.4752	0.118027	0.192589	0.352268	0.754977	CAPRIN2	65981	caprin family member 2
ENSG0000	294.7915	0.108779	0.152578	0.352514	0.754977	ACAD10	80724	acyl-CoA dehydrogenase family member 10
ENSG0000	78.36859	-0.11288	0.166596	0.352572	0.754977	NHLRC3	387921	NHL repeat containing 3
ENSG0000	10.74192	0.087147	0.228222	0.35276	0.754977	NA	NA	NA
ENSG0000	44.07343	0.108346	0.203492	0.352747	0.754977	AP4E1	23431	adaptor related protein complex 4 subunit epsilon 1
ENSG0000	27.34782	0.10388	0.214126	0.352437	0.754977	SNRPD1	6632	small nuclear ribonucleoprotein D1 polypeptide
ENSG0000	2.697845	-0.05008	0.233255	0.352743	0.754977	NA	NA	NA
ENSG0000	264.5321	-0.11095	0.178168	0.352254	0.754977	PGRMC1	10857	progesterone receptor membrane component 1
ENSG0000	327.3195	-0.10843	0.153864	0.352823	0.755024	SBF2	81846	SET binding factor 2
ENSG0000	323.0074	0.105053	0.203932	0.35301	0.755072	IGKV1-27	28935	immunoglobulin kappa variable 1-27
ENSG0000	291.8992	0.111598	0.164486	0.352972	0.755072	ABCC5	10057	ATP binding cassette subfamily C member 5
ENSG0000	117.4991	-0.1111	0.181215	0.35296	0.755072	NA	NA	NA
ENSG0000	275.6184	0.102995	0.134177	0.352978	0.755072	NEURL4	84461	neuralized E3 ubiquitin protein ligase 4
ENSG0000	11.05307	0.081356	0.229037	0.353268	0.755258	PLPP3	8613	phospholipid phosphatase 3
ENSG0000	1118.794	-0.09848	0.126787	0.35321	0.755258	PSMD4	5710	proteasom non-ATPase 4
ENSG0000	12.70079	0.085884	0.2277	0.35323	0.755258	NA	NA	NA
ENSG0000	289.9245	-0.11031	0.174448	0.353303	0.755258	NLRP3	114548	NLR family pyrin domain containing 3
ENSG0000	22.18641	-0.08461	0.225216	0.353239	0.755258	ZNF613	79898	zinc finger protein 613
ENSG0000	1643.318	-0.10824	0.154776	0.353375	0.755323	BIN1	274	bridging integrator 1
ENSG0000	393.9693	0.106117	0.145065	0.353757	0.755619	SLBP	7884	stem-loop binding protein
ENSG0000	448.9828	-0.10674	0.146135	0.353842	0.755619	SAP30L	79685	SAP30 like
ENSG0000	6.489273	0.044927	0.232232	0.353876	0.755619	NACAD	23148	NAC alpha domain containing
ENSG0000	52.81919	-0.11245	0.18494	0.353909	0.755619	LOC10099	1.01E+08	uncharacterized LOC100996437
ENSG0000	17257.25	-0.1101	0.181297	0.353636	0.755619	ADAM8	101	ADAM metallopeptidase domain 8
ENSG0000	7233.029	-0.11038	0.172408	0.353725	0.755619	FBRS	64319	fibrosin
ENSG0000	18.81115	0.095214	0.222337	0.353653	0.755619	RPL26	6154	ribosomal protein L26
ENSG0000	7692.92	-0.11222	0.175264	0.353926	0.755619	EPN1	29924	epsin 1
ENSG0000	2573.896	0.109036	0.193563	0.353803	0.755619	PLXNB2	23654	plexin B2
ENSG0000	733.8874	-0.10386	0.136941	0.353617	0.755619	RTL8A	26071	retrotransposon Gag like 8A
ENSG0000	140.9047	0.111918	0.169178	0.354097	0.755707	DCAF16	54876	DDB1 and CUL4 associated factor 16
ENSG0000	156.0476	-0.10907	0.196643	0.354052	0.755707	ZFYVE16	9765	zinc finger FYVE-type containing 16
ENSG0000	130.9738	0.110843	0.182372	0.354132	0.755707	HSF4	3299	heat shock transcription factor 4
ENSG0000	710.7704	-0.1105	0.181858	0.354131	0.755707	ACSS2	55902	acyl-CoA synthetase short chain family member 2
ENSG0000	8.915857	0.079222	0.22973	0.354401	0.756194	NA	NA	NA
ENSG0000	448.8526	-0.09441	0.118593	0.354445	0.756198	PLEKHJ1	55111	pleckstrin homology domain containing J1
ENSG0000	17.08645	0.099478	0.220666	0.354769	0.756803	RN7SKP16	1.06E+08	RN7SK pseudogene 16
ENSG0000	2144.863	-0.10861	0.158513	0.3549	0.756992	LDLRAP1	26119	low density lipoprotein receptor adaptor protein 1
ENSG0000	8.977009	0.046586	0.23175	0.355205	0.757556	ZNF181	339318	zinc finger protein 181
ENSG0000	1689.67	-0.1077	0.182986	0.35526	0.757585	PGS1	9489	phosphatidylglycerophosphate synthase 1
ENSG0000	167.8368	0.100426	0.132739	0.355434	0.757782	ZNF3	7551	zinc finger protein 3
ENSG0000	539.775	0.109172	0.161832	0.355508	0.757782	CPQ	10404	carboxypeptidase Q
ENSG0000	1834.197	0.098223	0.126765	0.355518	0.757782	SH2B3	10019	SH2B adaptor protein 3
ENSG0000	2556.478	-0.11014	0.175077	0.355478	0.757782	MPP1	4354	MAGUK p55 scaffold protein 1
ENSG0000	244.7584	-0.10642	0.151969	0.355783	0.757905	KDM4A	9682	lysine demethylase 4A
ENSG0000	3.942988	0.051851	0.234476	0.355643	0.757905	FAM107A	11170	family with sequence similarity 107 member A
ENSG0000	567.5212	0.104746	0.144532	0.355779	0.757905	SLC39A7	7922	solute carrier family 39 member 7
ENSG0000	713.4117	0.088379	0.108028	0.355701	0.757905	IKBKB	3551	inhibitor of nuclear factor kappa B kinase subunit beta
ENSG0000	133.0664	0.109649	0.158021	0.355746	0.757905	KDM4C	23081	lysine demethylase 4C
ENSG0000	19.94624	-0.07157	0.22828	0.355932	0.758136	LINC02325	1.02E+08	long intergenic non-protein coding RNA 2325
ENSG0000	5.994544	0.054659	0.232396	0.356101	0.758304	NA	NA	NA
ENSG0000	464.5605	-0.10194	0.20826	0.356343	0.758304	GPBP1	65056	GC-rich promoter binding protein 1
ENSG0000	36.79362	0.111854	0.190697	0.35633	0.758304	ZSCAN21	7589	zinc finger and SCAN domain containing 21
ENSG0000	310.0926	-0.10873	0.158448	0.356146	0.758304	SUSD1	64420	sushi domain containing 1
ENSG0000	898.0183	0.107561	0.16439	0.356242	0.758304	ATP11A	23250	ATPase phospholipid transporting 11A
ENSG0000	3.057427	0.048086	0.233153	0.356186	0.758304	NA	NA	NA
ENSG0000	442.6283	0.100513	0.153427	0.35612	0.758304	SNHG16	1.01E+08	small nucleolar RNA host gene 16
ENSG0000	3054.947	-0.1036	0.141468	0.35634	0.758304	DGCR2	9993	DiGeorge syndrome critical region gene 2
ENSG0000	1993.78	-0.09503	0.117963	0.356423	0.758388	BET1L	51272	Bet1 golgi vesicular membrane trafficking protein like
ENSG0000	80.89441	0.111527	0.182503	0.356548	0.758566	GTF3C4	9329	general transcription factor IIIC subunit 4
ENSG0000	40012.61	-0.09495	0.212935	0.356624	0.758569	B2M	567	beta-2-microglobulin
ENSG0000	2765.651	-0.11415	0.182119	0.356674	0.758569	MED16	10025	mediator complex subunit 16
ENSG0000	69.5113	-0.11228	0.181673	0.356658	0.758569	PDGFB	5155	platelet derived growth factor subunit B
ENSG0000	3.188167	-0.0338	0.232945	0.356799	0.758747	NA	NA	NA
ENSG0000	863.7079	0.104937	0.140252	0.35704	0.759072	SUMF2	25870	sulfatase modifying factor 2
ENSG0000	58.4895	0.101068	0.214219	0.357046	0.759072	ZNF93	81931	zinc finger protein 93
ENSG0000	22.28989	-0.10758	0.208635	0.357077	0.759072	CFAP298	56683	cilia and flagella associated protein 298
ENSG0000	1740.382	-0.10938	0.170053	0.357248	0.75935	SLC45A4	57210	solute carrier family 45 member 4
ENSG0000	51.91581	-0.10561	0.204698	0.357492	0.759691	LOC10028	1E+08	uncharacterized LOC100289230
ENSG0000	115.001	0.110453	0.183394	0.357483	0.759691	FUT8	2530	fucosyltransferase 8
ENSG0000	2.702238	0.031856	0.234132	0.357589	0.759711	LINC01635	1.02E+08	long intergenic non-protein coding RNA 1635
ENSG0000	61.82434	0.112491	0.177696	0.357626	0.759711	ZW10	9183	zw10 kinetochore protein
ENSG0000	249.962	-0.10454	0.139529	0.357608	0.759711	PEDS1	387521	plasmanylethanolamine desaturase 1
ENSG0000	254.6043	-0.11007	0.174521	0.358067	0.759802	ACSL3	2181	acyl-CoA synthetase long chain family member 3
ENSG0000	498.2159	-0.10427	0.203434	0.357945	0.759802	MGLL	11343	monoglyceride lipase
ENSG0000	15.69018	-0.07296	0.228229	0.358212	0.759802	NA	NA	NA
ENSG0000	10.3873	0.080858	0.228537	0.358744	0.759802	NA	NA	NA
ENSG0000	8.119913	0.083856	0.229055	0.358732	0.759802	NA	NA	NA
ENSG0000	187.257	0.105699	0.196538	0.358756	0.759802	E2F3	1871	E2F transcription factor 3
ENSG0000	3098.9	-0.10464	0.199055	0.358596	0.759802	HSP90AB1	3326	heat shock protein 90 alpha family class B member 1
ENSG0000	75.43044	0.111366	0.182465	0.358573	0.759802	ELOC	6921	elongin C
ENSG0000	2.45218	-0.04709	0.234955	0.357883	0.759802	NA	NA	NA
ENSG0000	18.20201	0.085969	0.223344	0.358738	0.759802	MSANTD3	91283	Myb/SANT DNA binding domain containing 3
ENSG0000	315.5964	-0.0854	0.117423	0.358162	0.759802	ECPAS	23392	Ecm29 proteasome adaptor and scaffold
ENSG0000	2.478718	-0.02453	0.233062	0.358044	0.759802	COL5A1	1289	collagen type V alpha 1 chain
ENSG0000	37.0542	-0.10402	0.209012	0.358779	0.759802	ZFAND4	93550	zinc finger AN1-type containing 4
ENSG0000	2747.076	-0.07277	0.228155	0.358533	0.759802	NEAT1	283131	nuclear paraspeckle assembly transcript 1
ENSG0000	1854.902	-0.10571	0.188516	0.358554	0.759802	SART1	9092	spliceoson recruiter of U4/U6.U5 tri-snRNP
ENSG0000	3.685256	-0.05124	0.233266	0.3578	0.759802	NA	NA	NA

ENSG0000	2087.546	-0.10836	0.162249	0.357906	0.759802	PICALM	8301	phosphatidylinositol binding clathrin assembly protein
ENSG0000	58.12264	-0.10977	0.192647	0.358511	0.759802	NCAM1	4684	neural cell adhesion molecule 1
ENSG0000	110.2859	0.107991	0.17203	0.358053	0.759802	MRPS35	60488	mitochondrial ribosomal protein S35
ENSG0000	13.97666	-0.08689	0.225236	0.35839	0.759802	NA	NA	NA
ENSG0000	2.414645	0.054684	0.236822	0.357742	0.759802	PRKD1	5587	protein kinase D1
ENSG0000	170.4965	-0.11122	0.169688	0.358789	0.759802	EEF2KMT	196483	eukaryotic elongation factor 2 lysine methyltransferase
ENSG0000	24.42817	0.110686	0.198799	0.358265	0.759802	NA	NA	NA
ENSG0000	1232.576	0.101068	0.135226	0.358252	0.759802	TMEM94	9772	transmembrane protein 94
ENSG0000	2624.122	-0.10676	0.15921	0.358748	0.759802	MARCHF2	51257	membrane associated ring-CH-type finger 2
ENSG0000	55.7069	0.100712	0.212768	0.358335	0.759802	NA	NA	NA
ENSG0000	1790.778	-0.10496	0.147173	0.358706	0.759802	TRPC4AP	26133	transient receptor potential cation channel subfamily C member 4 associated protein
ENSG0000	276.9098	-0.1034	0.140315	0.358861	0.759869	ANAPC7	51434	anaphase promoting complex subunit 7
ENSG0000	46.02937	-0.10286	0.210095	0.359352	0.760178	RPL23AP2	728481	ribosomal protein L23a pseudogene 21
ENSG0000	10.20067	-0.04109	0.23186	0.3591	0.760178	NA	NA	NA
ENSG0000	7.398976	0.059357	0.231527	0.359351	0.760178	NA	NA	NA
ENSG0000	54.19484	0.101753	0.209851	0.359169	0.760178	NET1	10276	neuroepithelial cell transforming 1
ENSG0000	49.17993	0.096374	0.217009	0.359301	0.760178	BMI1	648	BMI1 prot polycomb ring finger
ENSG0000	42.29487	-0.10469	0.206832	0.359066	0.760178	DRD4	1815	dopamine receptor D4
ENSG0000	5.525558	0.069241	0.237594	0.359282	0.760178	TRAV35	28647	T cell receptor alpha variable 35
ENSG0000	9.64462	0.060717	0.231575	0.359381	0.760178	NPTX1	4884	neuronal pentraxin 1
ENSG0000	1973.852	0.092897	0.119658	0.359189	0.760178	EIF3D	8664	eukaryotic translation initiation factor 3 subunit D
ENSG0000	12.34845	0.08238	0.22869	0.359671	0.760704	NA	NA	NA
ENSG0000	40.10589	-0.09108	0.219392	0.359996	0.760787	TRMT10C	54931	tRNA meth mitochondrial RNase P subunit
ENSG0000	11.4651	0.06556	0.230391	0.35985	0.760787	BEND3	57673	BEN domain containing 3
ENSG0000	52.49966	0.095089	0.217166	0.359941	0.760787	SPAG1	6674	sperm associated antigen 1
ENSG0000	16.30239	0.085384	0.22633	0.359766	0.760787	HKDC1	80201	hexokinase domain containing 1
ENSG0000	2753.461	-0.10793	0.162877	0.360001	0.760787	CDK2AP2	10263	cyclin dependent kinase 2 associated protein 2
ENSG0000	571.7837	0.100377	0.136743	0.36	0.760787	SUPT16H	11198	SPT16 hon facilitates chromatin remodeling subunit
ENSG0000	775.1126	-0.09622	0.123273	0.359915	0.760787	SYNRG	11276	synergin gamma
ENSG0000	275.5392	0.098831	0.128975	0.360228	0.760827	NSUN2	54888	NOP2/Sun RNA methyltransferase 2
ENSG0000	1245.135	-0.10034	0.134989	0.360169	0.760827	IK	3550	IK cytokine
ENSG0000	760.367	0.107893	0.159175	0.36019	0.760827	SSRP1	6749	structure specific recognition protein 1
ENSG0000	438.5188	-0.10347	0.14161	0.360134	0.760827	CINP	51550	cyclin dependent kinase 2 interacting protein
ENSG0000	19.56263	-0.09052	0.222231	0.360124	0.760827	NUDT7	283927	nudix hydrolase 7
ENSG0000	12.26931	0.086521	0.22683	0.360312	0.76083	HDGFL3	50810	HDGF like 3
ENSG0000	42.84294	0.104893	0.207421	0.360289	0.76083	SHCBP1	79801	SHC binding and spindle associated 1
ENSG0000	22.62316	-0.07551	0.22861	0.360398	0.760923	CYB5R2	51700	cytochrome b5 reductase 2
ENSG0000	5.164175	-0.06109	0.231706	0.36055	0.760954	NA	NA	NA
ENSG0000	6.951165	0.057389	0.231848	0.360492	0.760954	NA	NA	NA
ENSG0000	5.321862	-0.07766	0.231407	0.360625	0.760954	NA	NA	NA
ENSG0000	245.4323	-0.09793	0.127755	0.360662	0.760954	UVRAG	7405	UV radiation resistance associated
ENSG0000	455.5446	0.108073	0.159299	0.360609	0.760954	AAR2	25980	AAR2 splicing factor
ENSG0000	7.952156	0.056335	0.231216	0.360563	0.760954	LOC12490	1.25E+08	uncharacterized LOC124904917
ENSG0000	817.0788	0.096807	0.125215	0.360783	0.761122	SF3B5	83443	splicing factor 3b subunit 5
ENSG0000	398.7379	-0.1077	0.152088	0.36094	0.761365	FAM120B	84498	family with sequence similarity 120B
ENSG0000	899.2718	0.105342	0.148454	0.361098	0.761612	SH3BGRL	6451	SH3 domain binding glutamate rich protein like
ENSG0000	95.90307	-0.0919	0.215145	0.361162	0.761658	CHST8	64377	carbohydrate sulfotransferase 8
ENSG0000	872.0751	0.101704	0.137915	0.361213	0.761679	SLC25A45	283130	solute carrier family 25 member 45
ENSG0000	117.306	0.110264	0.176701	0.361297	0.761767	GCHFR	2644	GTP cyclohydrolase I feedback regulator
ENSG0000	105.3131	-0.10794	0.193094	0.36165	0.762423	ITGA3	3675	integrin subunit alpha 3
ENSG0000	2.484717	-0.02595	0.232548	0.361793	0.762639	PIK3R2	5296	phosphoinositide-3-kinase regulatory subunit 2
ENSG0000	11.82069	0.080826	0.227904	0.361906	0.762788	NA	NA	NA
ENSG0000	3.519768	0.058455	0.233342	0.362051	0.763007	NA	NA	NA
ENSG0000	83.63696	0.110077	0.185334	0.36226	0.763112	CLIP4	79745	CAP-Gly domain containing linker protein family member 4
ENSG0000	6.437374	-0.05476	0.2317	0.362276	0.763112	DYNLT2	6991	dynein light chain Tctex-type 2
ENSG0000	127.0571	0.111473	0.176977	0.362353	0.763112	DLD	1738	dihydrolipoamide dehydrogenase
ENSG0000	318.5245	0.096507	0.128502	0.36237	0.763112	NA	NA	NA
ENSG0000	263.3639	-0.10538	0.150256	0.362393	0.763112	ANO6	196527	anoctamin 6
ENSG0000	10.794	0.092559	0.224959	0.362225	0.763112	GATM	2628	glycine amidinotransferase
ENSG0000	590.6375	-0.10029	0.205667	0.362324	0.763112	BACH1	571	BTB domain and CNC homolog 1
ENSG0000	2.778349	0.034801	0.233442	0.362451	0.763147	NA	NA	NA
ENSG0000	3.472385	-0.04012	0.232644	0.362528	0.763221	NA	NA	NA
ENSG0000	199373.8	-0.08173	0.227936	0.362694	0.763482	HBA1	3039	hemoglobin subunit alpha 1
ENSG0000	4.570845	0.0591	0.234908	0.362774	0.763564	NA	NA	NA
ENSG0000	50.15584	-0.1095	0.185274	0.363564	0.763909	NA	NA	NA
ENSG0000	1545.06	-0.10256	0.140773	0.363487	0.763909	C1orf43	25912	chromosome 1 open reading frame 43
ENSG0000	304.5358	0.105689	0.155069	0.363093	0.763909	PCNX2	80003	pecanex 2
ENSG0000	57.64776	0.107662	0.194965	0.363234	0.763909	GPR55	9290	G protein-coupled receptor 55
ENSG0000	1486.491	-0.10367	0.144817	0.363495	0.763909	CNBP	7555	CCHC-type zinc finger nucleic acid binding protein
ENSG0000	51.28622	-0.08117	0.222044	0.363177	0.763909	IFRD1	3475	interferon related developmental regulator 1
ENSG0000	1665.32	0.106961	0.158239	0.363554	0.763909	NDUFS8	4728	NADH:ubiquinone oxidoreductase core subunit S8
ENSG0000	111.6545	-0.10888	0.183427	0.363174	0.763909	MINDY2	54629	MINDY lysine 48 deubiquitinase 2
ENSG0000	276.7628	0.096162	0.131676	0.363563	0.763909	ZNF263	10127	zinc finger protein 263
ENSG0000	4.776167	0.037671	0.232486	0.363364	0.763909	NA	NA	NA
ENSG0000	586.9302	0.108916	0.174492	0.363417	0.763909	MYBBP1A	10514	MYB binding protein 1a
ENSG0000	10.82654	0.069504	0.229532	0.363235	0.763909	PIGW	284098	phosphatidylinositol glycan anchor biosynthesis class W
ENSG0000	5.469607	-0.06396	0.232563	0.363144	0.763909	NA	NA	NA
ENSG0000	28.00349	-0.08679	0.222745	0.363518	0.763909	ZNF396	252884	zinc finger protein 396
ENSG0000	18.68009	0.090807	0.222013	0.363132	0.763909	ZFP28	140612	ZFP28 zinc finger protein
ENSG0000	217.5508	0.093457	0.215802	0.363656	0.763928	FSCN1	6624	fascin actin-bundling protein 1
ENSG0000	4979.677	-0.10879	0.183868	0.36364	0.763928	CMIP	80790	c-Maf inducing protein
ENSG0000	127.8065	-0.10707	0.156978	0.36376	0.764058	PILRB	29990	paired immunoglobin like type 2 receptor beta
ENSG0000	138.0268	0.104559	0.145827	0.363918	0.764216	PSRC1	84722	proline and serine rich coiled-coil 1
ENSG0000	4.891675	0.055758	0.232155	0.363878	0.764216	ZNF850	342892	zinc finger protein 850
ENSG0000	26.29867	-0.08545	0.223457	0.364122	0.764512	ANKRD44-	1.02E+08	ANKRD44 intronic transcript 1
ENSG0000	25.01571	-0.07464	0.227002	0.364143	0.764512	NA	NA	NA
ENSG0000	435.239	0.098262	0.208467	0.364187	0.764517	IGKV1-16	28938	immunoglobulin kappa variable 1-16
ENSG0000	24.04051	0.091988	0.22057	0.364345	0.76459	SANBR	84542	SANT and BTB domain regulator of CSR
ENSG0000	56.53006	0.107364	0.191253	0.364368	0.76459	NA	NA	NA
ENSG0000	682.0384	-0.10256	0.14216	0.364389	0.76459	ORMDL3	94103	ORMDL sphingolipid biosynthesis regulator 3
ENSG0000	14.23885	-0.07921	0.225505	0.364274	0.76459	ZNF543	125919	zinc finger protein 543
ENSG0000	133.3339	-0.10681	0.172561	0.364563	0.764714	ZBTB20	26137	zinc finger and BTB domain containing 20
ENSG0000	6.704526	-0.05548	0.231607	0.364637	0.764714	NCS1	23413	neuronal calcium sensor 1
ENSG0000	161.1995	-0.10291	0.142505	0.364581	0.764714	EXOSC1	51013	exosome component 1
ENSG0000	582.6048	-0.09749	0.12948	0.364671	0.764714	HIF1AN	55662	hypoxia inducible factor 1 subunit alpha inhibitor
ENSG0000	41.90954	0.110607	0.185595	0.364654	0.764714	C16orf91	283951	chromosome 16 open reading frame 91

ENSG0000	1609.285	-0.1033	0.145908	0.364698	0.764714	TAOK2	9344	TAO kinase 2
ENSG0000	2.32762	-0.02546	0.23254	0.364876	0.764957	NA	NA	NA
ENSG0000	14.41721	0.073195	0.229571	0.364939	0.764957	IFITM10	402778	interferon induced transmembrane protein 10
ENSG0000	20.20968	-0.08152	0.224993	0.3649	0.764957	ZNF570	148268	zinc finger protein 570
ENSG0000	4.582226	-0.05068	0.232267	0.365071	0.765145	LOC12490	1.25E+08	uncharacterized LOC124906209
ENSG0000	44.35522	-0.10573	0.200957	0.365438	0.76545	DRAXIN	374946	dorsal inhibitory axon guidance protein
ENSG0000	43.96602	-0.11044	0.180855	0.365502	0.76545	LINC00467	84791	long intergenic non-protein coding RNA 467
ENSG0000	54.17383	0.108353	0.191016	0.365592	0.76545	QPRT	23475	quinolinate phosphoribosyltransferase
ENSG0000	66.63064	0.10926	0.188061	0.365462	0.76545	PYCARD-A	1.01E+08	PYCARD antisense RNA 1
ENSG0000	73.84972	0.109383	0.180867	0.365339	0.76545	SPAG5	10615	sperm associated antigen 5
ENSG0000	5.700323	-0.0665	0.231522	0.365402	0.76545	CCNP	79935	cyclin P
ENSG0000	7.263273	-0.06594	0.231143	0.365578	0.76545	NA	NA	NA
ENSG0000	6.772561	-0.0744	0.23036	0.365551	0.76545	NA	NA	NA
ENSG0000	113.644	-0.11137	0.173674	0.365336	0.76545	NA	NA	NA
ENSG0000	2626.817	-0.10604	0.15848	0.365719	0.76554	ZSWIM8	23053	zinc finger SWIM-type containing 8
ENSG0000	455.9627	-0.09714	0.129339	0.365683	0.76554	TTI1	9675	TELO2 interacting protein 1
ENSG0000	3.010937	0.050637	0.232886	0.365989	0.765605	NA	NA	NA
ENSG0000	429.0803	-0.10789	0.174938	0.366034	0.765605	AGFG2	3268	ArfGAP with FG repeats 2
ENSG0000	11.48696	0.07284	0.229114	0.365793	0.765605	TRPC2	7221	transient receptor potential cation channel subfamily C member 2 (pseudogene)
ENSG0000	521.9109	-0.10771	0.170846	0.365982	0.765605	TSPAN18	90139	tetraspanin 18
ENSG0000	3.239816	0.025687	0.233184	0.366014	0.765605	NA	NA	NA
ENSG0000	4.714614	-0.07228	0.231541	0.366043	0.765605	NA	NA	NA
ENSG0000	36.6851	0.109788	0.193024	0.365856	0.765605	ZFP92	139735	ZFP92 zinc finger protein
ENSG0000	3.123474	-0.03098	0.233776	0.366223	0.765772	NA	NA	NA
ENSG0000	401.5946	0.101695	0.140188	0.366173	0.765772	EIF2B4	8890	eukaryotic translation initiation factor 2B subunit delta
ENSG0000	5338.908	-0.10736	0.179332	0.366248	0.765772	PELATON	1.01E+08	plaque enriched lncRNA in atherosclerotic and inflammatory bowel macrophage regulation
ENSG0000	39.24804	-0.10498	0.204106	0.366332	0.76586	NA	NA	NA
ENSG0000	50.60794	-0.10984	0.17465	0.366411	0.765939	LOC12490	1.25E+08	uncharacterized LOC124900513
ENSG0000	616.3037	0.09401	0.122628	0.366492	0.76602	HNRNPAB	3182	heterogeneous nuclear ribonucleoprotein A/B
ENSG0000	48.76666	0.109524	0.186144	0.366931	0.766108	PTCH2	8643	patched 2
ENSG0000	21.31572	0.096794	0.21726	0.366772	0.766108	BCL2L15	440603	BCL2 like 15
ENSG0000	119.6738	0.085457	0.221984	0.366922	0.766108	DNAJB14	79982	DnaJ heat shock protein family (Hsp40) member B14
ENSG0000	42.95726	-0.08802	0.221313	0.367036	0.766108	LLCFC1	135927	LLLL and CFNLAS motif containing 1
ENSG0000	2423.006	-0.11175	0.142418	0.366814	0.766108	DOCK8	81704	dedicator of cytokinesis 8
ENSG0000	26.99479	-0.08655	0.222333	0.367006	0.766108	LOC10537	1.05E+08	uncharacterized LOC105376244
ENSG0000	215.781	-0.0972	0.133565	0.366927	0.766108	PRR5L	79899	proline rich 5 like
ENSG0000	72.43487	0.10135	0.205373	0.366725	0.766108	CUL5	8065	cullin 5
ENSG0000	4.60581	0.04465	0.232085	0.366983	0.766108	NA	NA	NA
ENSG0000	91.83751	0.110818	0.189048	0.366862	0.766108	NETO2	81831	neuropilin and tolloid like 2
ENSG0000	168.5719	0.099191	0.133556	0.366926	0.766108	AFG3L1P	172	AFG3 like pseudogene
ENSG0000	244.9069	0.092964	0.118898	0.366659	0.766108	MYO19	80179	myosin XIX
ENSG0000	41.49035	-0.11058	0.184807	0.367111	0.766177	NA	NA	NA
ENSG0000	65.67646	0.108606	0.187619	0.367258	0.766307	EIF2B3	8891	eukaryotic translation initiation factor 2B subunit gamma
ENSG0000	71.05648	-0.10713	0.197734	0.367299	0.766307	TSTD2	158427	thiosulfate sulfurtransferase like domain containing 2
ENSG0000	33.23357	0.110489	0.186776	0.367296	0.766307	ZNF234	10780	zinc finger protein 234
ENSG0000	4494.193	-0.09964	0.143046	0.367468	0.766503	PGAP6	58986	post-GPI attachment to proteins 6
ENSG0000	1055.872	-0.11115	0.154983	0.367476	0.766503	GTF2F1	2962	general transcription factor IIF subunit 1
ENSG0000	19.48334	0.083478	0.224403	0.367616	0.766534	MREG	55686	melanoregulin
ENSG0000	14.70471	0.081056	0.226943	0.367582	0.766534	SH3RF2	153769	SH3 domain containing ring finger 2
ENSG0000	31.34006	-0.10239	0.208614	0.367539	0.766534	TRAV8-2	28684	T cell receptor alpha variable 8-2
ENSG0000	10.69672	0.090461	0.224077	0.367874	0.766982	TIMM8A	1678	translocase of inner mitochondrial membrane 8A
ENSG0000	121.029	-0.09949	0.206817	0.36793	0.767013	EGR1	1958	early growth response 1
ENSG0000	15.83612	0.062517	0.229204	0.367995	0.76706	NA	NA	NA
ENSG0000	60.29437	0.099814	0.209884	0.368051	0.767091	TMEM263	90488	transmembrane protein 263
ENSG0000	3.569386	-0.04343	0.232659	0.368217	0.767221	NA	NA	NA
ENSG0000	106.8891	0.107648	0.165381	0.368272	0.767221	CACFD1	11094	calcium channel flower domain containing 1
ENSG0000	11.88504	0.036583	0.231652	0.368204	0.767221	JRKL	8690	JRK like
ENSG0000	3.741213	-0.03592	0.233218	0.368323	0.767221	NA	NA	NA
ENSG0000	2.90455	-0.03977	0.23247	0.368302	0.767221	ZNF564	163050	zinc finger protein 564
ENSG0000	260.1635	0.105822	0.150433	0.368656	0.767248	PLCH2	9651	phospholipase C eta 2
ENSG0000	248.4208	0.105887	0.192257	0.36881	0.767248	CMPK1	51727	cytidine/uridine monophosphate kinase 1
ENSG0000	17.78832	-0.0901	0.222626	0.36898	0.767248	RUSC1-AS	284618	RUSC1 antisense RNA 1
ENSG0000	413.773	0.105816	0.159051	0.368994	0.767248	PAICS	10606	phosphoribosylaminoimidazole carboxylase and phosphoribosylaminoimidazolesuccinocarboxamide synthase
ENSG0000	198.004	-0.10626	0.164622	0.3685	0.767248	EXOC2	55770	exocyst complex component 2
ENSG0000	10033.44	-0.11003	0.173112	0.368792	0.767248	RIPOR2	9750	RHO family interacting cell polarization regulator 2
ENSG0000	5.974754	-0.0818	0.228783	0.368858	0.767248	NA	NA	NA
ENSG0000	66.82485	-0.10649	0.193918	0.368958	0.767248	NA	NA	NA
ENSG0000	79.20298	0.108897	0.182064	0.368737	0.767248	DNAJC2	27000	DnaJ heat shock protein family (Hsp40) member C2
ENSG0000	62.9251	0.108956	0.186581	0.368583	0.767248	LRRC56	115399	leucine rich repeat containing 56
ENSG0000	580.1741	0.108025	0.151579	0.369006	0.767248	FOXJ2	55810	forkhead box J2
ENSG0000	60.2571	0.107679	0.191878	0.368687	0.767248	RPL3P4	326307	ribosomal protein L3 pseudogene 4
ENSG0000	6.160022	-0.05363	0.231734	0.368394	0.767248	TEDC2-AS	729652	TEDC2 antisense RNA 1
ENSG0000	903.761	0.096769	0.127136	0.368459	0.767248	ZDHHC7	55625	zinc finger DHHC-type palmitoyltransferase 7
ENSG0000	407.8291	0.10222	0.196768	0.368813	0.767248	ROCK1	6093	Rho associated coiled-coil containing protein kinase 1
ENSG0000	45.48006	0.105903	0.195745	0.368598	0.767248	NUDT19	390916	nudix hydrolase 19
ENSG0000	170.886	0.103999	0.148558	0.369056	0.767264	DNAJB11	51726	DnaJ heat shock protein family (Hsp40) member B11
ENSG0000	3.002852	0.048626	0.234165	0.369129	0.76733	NA	NA	NA
ENSG0000	376.6899	-0.10247	0.141164	0.369178	0.767344	COMMD7	149951	COMM domain containing 7
ENSG0000	83.4256	0.105107	0.198551	0.369247	0.767399	VSIG1	340547	V-set and immunoglobulin domain containing 1
ENSG0000	22.29215	0.101894	0.211935	0.369393	0.767617	PITPNA-AS	1E+08	PITPNA antisense RNA 1
ENSG0000	3.2519	0.054687	0.23352	0.369583	0.767925	NA	NA	NA
ENSG0000	4090.064	-0.10711	0.184762	0.369966	0.768111	NIBAN1	116496	niban apoptosis regulator 1
ENSG0000	4.53807	0.031306	0.233995	0.369846	0.768111	KIF14	9928	kinesin family member 14
ENSG0000	129.6032	-0.10064	0.141875	0.369958	0.768111	POLR1H	30834	RNA polymerase I subunit H
ENSG0000	1128.491	-0.10163	0.139487	0.369959	0.768111	CLSTN3	9746	calsyntenin 3
ENSG0000	3.647914	0.029614	0.232951	0.369966	0.768111	PDE6H	5149	phosphodiesterase 6H
ENSG0000	37.18729	-0.09184	0.217801	0.36993	0.768111	TRAV26-1	28657	T cell receptor alpha variable 26-1
ENSG0000	855.6356	-0.10088	0.140865	0.369799	0.768111	FBXL19	54620	F-box and leucine rich repeat protein 19
ENSG0000	9.070404	0.057772	0.231601	0.370098	0.768123	NA	NA	NA
ENSG0000	20.30226	0.096879	0.21641	0.370017	0.768123	FER	2241	FER tyrosine kinase
ENSG0000	102.0973	0.105094	0.193078	0.370083	0.768123	RNF6	6049	ring finger protein 6
ENSG0000	33.91929	-0.10793	0.196855	0.370212	0.768274	PRSS30P	124221	serine pro pseudogene
ENSG0000	8644.278	-0.1058	0.166144	0.370333	0.76835	ATG16L2	89849	autophagy related 16 like 2
ENSG0000	341.6932	0.10599	0.159455	0.370302	0.76835	ITPKC	80271	inositol-trisphosphate 3-kinase C
ENSG0000	4.201279	0.061523	0.233397	0.370537	0.768687	VARS2	57176	valyl-tRNA mitochondrial
ENSG0000	4.977363	-0.06927	0.231014	0.370593	0.768716	NA	NA	NA

ENSG0000	1559.986	-0.09977	0.131317	0.37065	0.768746	MAN2B2	23324	mannosidase alpha class 2B member 2
ENSG0000	25.8709	0.081495	0.224147	0.370821	0.768843	THUMPD2	80745	THUMP domain containing 2
ENSG0000	3.478642	0.023629	0.232571	0.370782	0.768843	NA	NA	NA
ENSG0000	43.37854	-0.107	0.193871	0.370822	0.768843	CAB39L	81617	calcium binding protein 39 like
ENSG0000	6.601388	0.066055	0.230682	0.370968	0.768884	NA	NA	NA
ENSG0000	25.31547	0.085108	0.222697	0.370915	0.768884	NFXL1	152518	nuclear tra X-box binding like 1
ENSG0000	114.7813	-0.10712	0.162559	0.370957	0.768884	LINC02361	1.01E+08	long intergenic non-protein coding RNA 2361
ENSG0000	46.19074	0.098002	0.211042	0.371073	0.768888	SELENOI	85465	selenoprotein I
ENSG0000	156.572	0.108285	0.188047	0.371021	0.768888	DMAC1	90871	distal membrane arm assembly component 1
ENSG0000	192.4202	0.105434	0.155809	0.371096	0.768888	BMS1	9790	BMS1 ribosome biogenesis factor
ENSG0000	50.21312	0.107695	0.171858	0.371259	0.769138	GTF2H3	2967	general transcription factor IIH subunit 3
ENSG0000	2.400385	0.041703	0.234156	0.371325	0.769188	NA	NA	NA
ENSG0000	1553.533	0.08764	0.113101	0.371615	0.769703	CBX4	8535	chromobox 4
ENSG0000	7.643362	0.050788	0.231251	0.371916	0.770239	NA	NA	NA
ENSG0000	61.93578	-0.09886	0.208765	0.372109	0.77055	NA	NA	NA
ENSG0000	136.0583	0.107374	0.169529	0.37233	0.770921	MRPS10	55173	mitochondrial ribosomal protein S10
ENSG0000	1321.88	-0.08899	0.115297	0.372584	0.771011	UBE2Q1	55585	ubiquitin conjugating enzyme E2 Q1
ENSG0000	3.013977	-0.03607	0.232384	0.372578	0.771011	SLC12A9-A	1.05E+08	SLC12A9 antisense RNA 1
ENSG0000	139.4946	0.104522	0.195101	0.372564	0.771011	CCDC102A	92922	coiled-coil domain containing 102A
ENSG0000	23.38229	0.098568	0.214132	0.372549	0.771011	P2RX5-TAX	1.01E+08	P2RX5-TAX1BP3 readthrough (NMD candidate)
ENSG0000	460.1356	0.098373	0.1353	0.37244	0.771011	SAE1	10055	SUMO1 activating enzyme subunit 1
ENSG0000	4257.091	-0.10577	0.174251	0.372684	0.771043	CDC42EP3	10602	CDC42 effector protein 3
ENSG0000	20.65226	0.099487	0.213982	0.372645	0.771043	C14orf132	56967	chromosome 14 open reading frame 132
ENSG0000	5.980374	-0.04144	0.232017	0.372735	0.771063	CRYGN	155051	crystallin gamma N
ENSG0000	82.67715	-0.10773	0.177244	0.372863	0.771075	COQ10B	80219	coenzyme Q10B
ENSG0000	37.53749	0.098624	0.21096	0.372839	0.771075	CHCHD4	131474	coiled-coil-helix-coiled-coil-helix domain containing 4
ENSG0000	184.6193	0.097847	0.210014	0.372867	0.771075	KCNE1	3753	potassium voltage-gated channel subfamily E regulatory subunit 1
ENSG0000	45.81148	-0.09442	0.213805	0.37299	0.771174	INKA1	389119	inka box actin regulator 1
ENSG0000	4.178441	0.066247	0.232193	0.372999	0.771174	NA	NA	NA
ENSG0000	2619.242	-0.10555	0.177049	0.373062	0.771217	UBN1	29855	ubinuclein 1
ENSG0000	16.56039	0.100939	0.211587	0.373126	0.771263	NA	NA	NA
ENSG0000	27.23804	0.105497	0.199645	0.373237	0.771405	TNFRSF10A	389641	TNFRSF10A divergent transcript
ENSG0000	325.6879	0.0874	0.121054	0.373421	0.771441	HSPA4	3308	heat shock protein family A (Hsp70) member 4
ENSG0000	91.54355	0.105642	0.162229	0.373373	0.771441	PSMG3	84262	proteasome assembly chaperone 3
ENSG0000	24.07143	0.109091	0.196244	0.373421	0.771441	KIAA1328	57536	KIAA1328
ENSG0000	243.0499	0.106115	0.183535	0.373423	0.771441	PAFAH1B3	5050	platelet activating factor acetylhydrolase 1b catalytic subunit 3
ENSG0000	24.32159	-0.09862	0.213	0.373633	0.7717	TRBV2	28620	T cell receptor beta variable 2
ENSG0000	2167.367	-0.0886	0.13455	0.373598	0.7717	MCRIP1	348262	MAPK regulated corepressor interacting protein 1
ENSG0000	654.3548	-0.08787	0.112921	0.373683	0.771717	LMBR1L	55716	limb development membrane protein 1 like
ENSG0000	245.7915	-0.10571	0.186165	0.373895	0.77176	TRIB2	28951	tribbles pseudokinase 2
ENSG0000	7.175785	0.061842	0.230567	0.373832	0.77176	RPSAP36	1E+08	ribosomal protein SA pseudogene 36
ENSG0000	67.3004	-0.09867	0.207135	0.373797	0.77176	BRIX1	55299	biogenesis of ribosomes BRX1
ENSG0000	127.099	-0.10622	0.170886	0.373915	0.77176	AKTIP	64400	AKT interacting protein
ENSG0000	87.69339	0.10522	0.190425	0.373844	0.77176	GINS2	51659	GINS complex subunit 2
ENSG0000	127.709	-0.09869	0.143226	0.373957	0.77176	NA	NA	NA
ENSG0000	3.8694	0.056149	0.232579	0.374051	0.771868	PLA2G4B	1E+08	phospholipase A2 group IVB
ENSG0000	2.650877	-0.01899	0.233422	0.374106	0.771894	NA	NA	NA
ENSG0000	9.210098	0.06717	0.230079	0.374558	0.77274	NA	NA	NA
ENSG0000	1702.387	0.09256	0.122591	0.374679	0.77277	CERS2	29956	ceramide synthase 2
ENSG0000	9.607844	-0.07889	0.227114	0.374699	0.77277	ABLIM2	84448	actin binding LIM protein family member 2
ENSG0000	8.089741	0.073809	0.229111	0.374644	0.77277	ZNF823	55552	zinc finger protein 823
ENSG0000	3.040124	0.034951	0.232176	0.374935	0.772907	NA	NA	NA
ENSG0000	25.2694	0.102213	0.207757	0.374895	0.772907	AMZ2P1	201283	AMZ2 pseudogene 1
ENSG0000	8.521117	0.063418	0.23031	0.374884	0.772907	DSEL	92126	dermatan sulfate epimerase like
ENSG0000	9.950199	0.062997	0.22983	0.374907	0.772907	NA	NA	NA
ENSG0000	467.564	-0.1007	0.137996	0.375089	0.773138	HPS6	79803	HPS6 biogenesis of lysosomal organelles complex 2 subunit 3
ENSG0000	25.53797	0.100261	0.209792	0.375259	0.773151	PIGF	5281	phosphatidylinositol glycan anchor biosynthesis class F
ENSG0000	17.21115	0.074075	0.228022	0.375335	0.773151	SPC25	57405	SPC25 component of NDC80 kinetochore complex
ENSG0000	270.7904	0.106389	0.175622	0.375217	0.773151	GPR35	2859	G protein-coupled receptor 35
ENSG0000	10.48013	0.051396	0.230796	0.37519	0.773151	NA	NA	NA
ENSG0000	338.5681	-0.09753	0.13406	0.375402	0.773151	ZPR1	8882	ZPR1 zinc finger
ENSG0000	162.6518	0.106585	0.178695	0.375433	0.773151	ARID2	196528	AT-rich interaction domain 2
ENSG0000	6.609024	-0.0413	0.231411	0.3754	0.773151	NA	NA	NA
ENSG0000	15.71641	0.091962	0.220565	0.375306	0.773151	GINS1	9837	GINS complex subunit 1
ENSG0000	536.337	-0.10662	0.16617	0.375585	0.773377	PCK2	5106	phosphoe mitochondrial
ENSG0000	800.6151	0.082499	0.110561	0.376407	0.773445	PRDM2	7799	PR/SET domain 2
ENSG0000	32.14974	-0.09349	0.217907	0.375935	0.773445	THNSL2	55258	threonine synthase like 2
ENSG0000	1266.704	0.105182	0.185357	0.376303	0.773445	ARL6IP5	10550	ADP ribosylation factor like GTPase 6 interacting protein 5
ENSG0000	8.856801	0.081032	0.227148	0.376781	0.773445	SRD5A3	79644	steroid 5 alpha-reductase 3
ENSG0000	17.63927	0.083683	0.224222	0.376795	0.773445	PPAT	5471	phosphoribosyl pyrophosphate amidotransferase
ENSG0000	157.5384	0.101261	0.145507	0.376524	0.773445	NNT	23530	nicotinamide nucleotide transhydrogenase
ENSG0000	44.25678	0.063843	0.227859	0.376732	0.773445	RBAK	57786	RB associated KRAB zinc finger
ENSG0000	3.11173	0.034872	0.232389	0.37607	0.773445	LHFPL3-AS	723809	LHFPL3 antisense RNA 2
ENSG0000	185.8899	0.106305	0.18116	0.376678	0.773445	NA	NA	NA
ENSG0000	3.738244	0.057171	0.233317	0.37602	0.773445	CTAGE4	1E+08	CTAGE family member 4
ENSG0000	5.427722	0.071295	0.230877	0.376611	0.773445	NA	NA	NA
ENSG0000	65.95305	0.106242	0.189185	0.376257	0.773445	ZNF252P	286101	zinc finger pseudogene
ENSG0000	341.7895	-0.10653	0.16848	0.376686	0.773445	CEMIP2	23670	cell migration inducing hyaluronidase 2
ENSG0000	67.16705	0.107048	0.171483	0.376285	0.773445	PARG	8505	poly(ADP-ribose) glycohydrolase
ENSG0000	14.24504	0.078389	0.226579	0.376171	0.773445	PAPSS2	9060	3'-phosphoadenosine 5'-phosphosulfate synthase 2
ENSG0000	31.06436	-0.08935	0.219955	0.376383	0.773445	NA	NA	NA
ENSG0000	63.46923	0.106266	0.188355	0.3768	0.773445	SFXN4	119559	sideroflexin 4
ENSG0000	19.70432	-0.09289	0.218153	0.376335	0.773445	KLRK1	22914	killer cell lectin like receptor K1
ENSG0000	3214.942	-0.10474	0.181335	0.376231	0.773445	TECPR2	9895	tectonin beta-propeller repeat containing 2
ENSG0000	1949.645	-0.10286	0.151427	0.375893	0.773445	PSMB3	5691	proteasome 20S subunit beta 3
ENSG0000	736.2603	-0.09529	0.131766	0.375746	0.773445	HSD17B1-.	1.09E+08	HSD17B1 antisense RNA 1
ENSG0000	21.22712	-0.07295	0.227027	0.376396	0.773445	NA	NA	NA
ENSG0000	2538.166	0.100506	0.145236	0.376638	0.773445	LILRA1	11024	leukocyte immunoglobulin like receptor A1
ENSG0000	21.19132	-0.09994	0.208118	0.376057	0.773445	NA	NA	NA
ENSG0000	2.693185	0.039373	0.236182	0.376551	0.773445	NA	NA	NA
ENSG0000	5166.968	-0.09719	0.157054	0.37633	0.773445	TRIOBP	11078	TRIO and F-actin binding protein
ENSG0000	985.4106	0.103794	0.165477	0.376529	0.773445	NAGA	4668	alpha-N-acetylgalactosaminidase
ENSG0000	179.3612	-0.10627	0.176836	0.376397	0.773445	YIPF6	286451	Yip1 domain family member 6
ENSG0000	1269.952	0.098639	0.19056	0.376845	0.77345	SF3B1	23451	splicing factor 3b subunit 1
ENSG0000	2.698475	0.030998	0.234353	0.376912	0.773502	NA	NA	NA
ENSG0000	308.4274	-0.09665	0.132803	0.376957	0.773508	PIP5K1A	8394	phosphatidylinositol-4-phosphate 5-kinase type 1 alpha

ENSG0000	377.5225	-0.09527	0.127588	0.377091	0.773696	CIAO2B	51647	cytosolic iron-sulfur assembly component 2B
ENSG0000	9618.783	-0.104	0.183503	0.377141	0.773711	TNIP1	10318	TNFAIP3 interacting protein 1
ENSG0000	3.920873	0.038303	0.23296	0.377237	0.773814	LINC0135:	1.01E+08	long intergenic non-protein coding RNA 1353
ENSG0000	113.4772	0.095641	0.209228	0.377275	0.773814	GALNT7	51809	polypeptide N-acetylgalactosaminyltransferase 7
ENSG0000	3.30386	0.030476	0.233969	0.377363	0.773906	LINC0176!	1.05E+08	long intergenic non-protein coding RNA 1765
ENSG0000	97.87201	0.096502	0.213764	0.377406	0.773908	GOLGA4	2803	golgin A4
ENSG0000	11.14301	0.065118	0.229309	0.377478	0.773969	FFAR4	338557	free fatty acid receptor 4
ENSG0000	515.4542	-0.09313	0.125504	0.377758	0.774409	COPS7A	50813	COP9 signalosome subunit 7A
ENSG0000	6160.909	-0.1012	0.158288	0.377777	0.774409	MLF2	8079	myeloid leukemia factor 2
ENSG0000	2.862229	-0.03644	0.232657	0.377939	0.774415	ARTN	9048	artemin
ENSG0000	641.8891	0.104109	0.155916	0.378034	0.774415	IPO9	55705	importin 9
ENSG0000	6.596555	-0.05389	0.230733	0.378015	0.774415	CALHM5	254228	calcium homeostasis modulator family member 5
ENSG0000	11.89167	-0.07803	0.226488	0.377878	0.774415	NA	NA	NA
ENSG0000	1347.764	-0.09804	0.138534	0.37794	0.774415	ERP29	10961	endoplasmic reticulum protein 29
ENSG0000	373.6459	-0.1048	0.170289	0.377993	0.774415	INTS6	26512	integrator complex subunit 6
ENSG0000	154.0653	-0.10535	0.188434	0.378192	0.77448	RPS15P4	401019	ribosomal protein S15 pseudogene 4
ENSG0000	2.81867	0.03952	0.23386	0.37816	0.77448	NA	NA	NA
ENSG0000	15.84388	0.062585	0.229213	0.378136	0.77448	NA	NA	NA
ENSG0000	228.8209	0.103298	0.165532	0.378432	0.774626	ZNF593	51042	zinc finger protein 593
ENSG0000	201.3533	-0.11031	0.146143	0.378353	0.774626	AGBL5	60509	AGBL carboxypeptidase 5
ENSG0000	68.71665	0.106384	0.182631	0.378393	0.774626	PPARGC1E	133522	PPARG coactivator 1 beta
ENSG0000	3.512423	-0.03442	0.233148	0.378377	0.774626	NA	NA	NA
ENSG0000	60.29554	0.106272	0.179577	0.378492	0.774661	RSRC1	51319	arginine and serine rich coiled-coil 1
ENSG0000	1932.141	-0.0968	0.135204	0.37869	0.774699	NRDC	4898	nardilysin convertase
ENSG0000	3.058363	0.038629	0.232442	0.37869	0.774699	LINGO4	339398	leucine rich repeat and Ig domain containing 4
ENSG0000	187.616	0.103085	0.154186	0.378878	0.774699	PTCD3	55037	pentatricopeptide repeat domain 3
ENSG0000	4.30321	-0.02022	0.232804	0.37921	0.774699	ANKRD44-	1.02E+08	ANKRD44 antisense RNA 1
ENSG0000	114.8368	0.104699	0.171174	0.378861	0.774699	DTYMK	1841	deoxythymidylate kinase
ENSG0000	6.891607	-0.04246	0.231422	0.378972	0.774699	LOC10272	1.03E+08	uncharacterized LOC102723663
ENSG0000	7.079355	0.064563	0.23182	0.379356	0.774699	ZNF860	344787	zinc finger protein 860
ENSG0000	2.668567	-0.04459	0.232891	0.379316	0.774699	RPS15P5	729789	ribosomal protein S15 pseudogene 5
ENSG0000	3.948431	-0.02778	0.232266	0.379399	0.774699	NA	NA	NA
ENSG0000	137.2431	-0.1022	0.151104	0.379189	0.774699	RSPH3	83861	radial spoke head 3
ENSG0000	140.515	0.103138	0.192322	0.379152	0.774699	MDFIC	29969	MyoD family inhibitor domain containing
ENSG0000	74.16958	0.103956	0.19763	0.379126	0.774699	SMIM27	1E+08	small integral membrane protein 27
ENSG0000	2641.983	0.096033	0.132501	0.378652	0.774699	RAPGEF1	2889	Rap guanine nucleotide exchange factor 1
ENSG0000	13.36129	0.096683	0.216876	0.379238	0.774699	EIF5AL1	143244	eukaryotic translation initiation factor 5A like 1
ENSG0000	106.9215	-0.10588	0.171289	0.378563	0.774699	VWA5A	4013	von Willebrand factor A domain containing 5A
ENSG0000	2.642821	0.037489	0.233087	0.379379	0.774699	TAS2R20	259295	taste 2 receptor member 20
ENSG0000	7.168863	0.06363	0.230795	0.379171	0.774699	WASF3	10810	WASP family member 3
ENSG0000	25.94877	-0.09182	0.218522	0.378812	0.774699	TRAV19	28664	T cell receptor alpha variable 19
ENSG0000	31.43204	0.105175	0.19776	0.378745	0.774699	NA	NA	NA
ENSG0000	39.16933	0.115228	0.18922	0.378945	0.774699	NA	NA	NA
ENSG0000	2.698844	0.034753	0.233629	0.379357	0.774699	NA	NA	NA
ENSG0000	29.80971	0.084519	0.221427	0.379461	0.774741	CARF	79800	calcium responsive transcription factor
ENSG0000	1144.869	-0.09588	0.131183	0.38	0.774774	C2orf68	388969	chromosome 2 open reading frame 68
ENSG0000	87.78363	0.078277	0.22235	0.379795	0.774774	IGKV2D-2!	28882	immunoglobulin kappa variable 2D-29
ENSG0000	15.22197	-0.09161	0.219103	0.379998	0.774774	NA	NA	NA
ENSG0000	8.325089	0.071469	0.228876	0.380026	0.774774	SERPINE2	5270	serpin family E member 2
ENSG0000	169.652	0.105525	0.177087	0.379666	0.774774	FXR1	8087	FMR1 autosomal homolog 1
ENSG0000	8.049285	-0.07086	0.229119	0.379864	0.774774	NA	NA	NA
ENSG0000	85.25384	0.105174	0.161851	0.379592	0.774774	AP4M1	9179	adaptor related protein complex 4 subunit mu 1
ENSG0000	41.14855	0.096125	0.211004	0.379646	0.774774	MTERF3	51001	mitochondrial transcription termination factor 3
ENSG0000	203.9892	0.10614	0.177551	0.379746	0.774774	RIC1	57589	RIC1 homc RAB6A GEF complex partner 1
ENSG0000	3172.078	0.10168	0.194887	0.38007	0.774774	CD27	939	CD27 molecule
ENSG0000	22.48801	0.062466	0.228728	0.38005	0.774774	ACTR6	64431	actin related protein 6
ENSG0000	36.64833	-0.10307	0.198193	0.379524	0.774774	NA	NA	NA
ENSG0000	642.3907	0.09561	0.129822	0.379935	0.774774	PIH1D1	55011	PIH1 domain containing 1
ENSG0000	1126.788	-0.10352	0.180098	0.380016	0.774774	RANGAP1	5905	Ran GTPase activating protein 1
ENSG0000	5.338791	0.042492	0.23169	0.380138	0.774827	FZD6	8323	frizzled class receptor 6
ENSG0000	6.015853	0.057363	0.231214	0.380241	0.774865	NA	NA	NA
ENSG0000	82.13172	-0.10738	0.18419	0.380211	0.774865	PROSER2	254427	proline and serine rich 2
ENSG0000	3.04185	-0.04742	0.232666	0.380804	0.774987	DAB1	1600	DAB adaptor protein 1
ENSG0000	23.88194	0.103224	0.20302	0.380546	0.774987	NA	NA	NA
ENSG0000	1028.425	-0.10504	0.155099	0.380903	0.774987	LBH	81606	LBH regulator of WNT signaling pathway
ENSG0000	1424.045	0.100538	0.135971	0.380965	0.774987	GORASP2	26003	golgi reassembly stacking protein 2
ENSG0000	18.74407	-0.08909	0.21967	0.380437	0.774987	NA	NA	NA
ENSG0000	4.837924	-0.06102	0.231235	0.380492	0.774987	NA	NA	NA
ENSG0000	3.838811	0.046362	0.232551	0.380978	0.774987	PRELID2	153768	PRELI domain containing 2
ENSG0000	192.0322	0.105638	0.173321	0.380884	0.774987	TCEA1	6917	transcription elongation factor A1
ENSG0000	4.692679	0.043579	0.232096	0.380512	0.774987	SLC28A3	64078	solute carrier family 28 member 3
ENSG0000	6.926321	-0.07381	0.228886	0.380953	0.774987	ST6GALNA	30815	ST6 N-ace! 6-sialyltransferase 6
ENSG0000	2.763642	-0.02872	0.233415	0.380692	0.774987	CSTF3-DT	338739	CSTF3 divergent transcript
ENSG0000	42.75729	0.105801	0.18968	0.380771	0.774987	POLA2	23649	DNA polyr accessory subunit
ENSG0000	155.8016	-0.10463	0.165729	0.380437	0.774987	LENG9	94059	leukocyte receptor cluster member 9
ENSG0000	91.85708	-0.10356	0.191762	0.380742	0.774987	NA	NA	NA
ENSG0000	44.02657	0.101642	0.201231	0.380729	0.774987	NA	NA	NA
ENSG0000	426.8413	-0.0995	0.144303	0.380625	0.774987	TCF20	6942	transcription factor 20
ENSG0000	29.57369	-0.07865	0.223289	0.381088	0.775125	MTERF2	80298	mitochondrial transcription termination factor 2
ENSG0000	561.191	0.099324	0.145535	0.381156	0.775142	AGO1	26523	argonaute RISC component 1
ENSG0000	29.03352	0.085112	0.220842	0.381216	0.775142	NA	NA	NA
ENSG0000	48.33093	0.099795	0.205075	0.381224	0.775142	PC	5091	pyruvate carboxylase
ENSG0000	3.558736	-0.02325	0.232694	0.381349	0.775273	GPR83	10888	G protein-coupled receptor 83
ENSG0000	2.788293	0.03959	0.232419	0.381373	0.775273	HSD17B6	8630	hydroxysteroid 17-beta dehydrogenase 6
ENSG0000	726.6768	0.098294	0.146352	0.381548	0.775372	IMMT	10989	inner membrane mitochondrial protein
ENSG0000	3.183598	-0.04869	0.232572	0.381473	0.775372	FAM21EP	1E+08	family witl member A pseudogene
ENSG0000	27.97956	-0.1027	0.200175	0.381515	0.775372	PBLD	64081	phenazine biosynthesis like protein domain containing
ENSG0000	6.072348	-0.04929	0.231462	0.381736	0.775386	NA	NA	NA
ENSG0000	825.3014	0.104251	0.168481	0.381759	0.775386	CALM2	805	calmodulin 2
ENSG0000	31.26849	0.089498	0.216969	0.38174	0.775386	ZNF721	170960	zinc finger protein 721
ENSG0000	204.9057	-0.10105	0.148931	0.381611	0.775386	NA	NA	NA
ENSG0000	590.648	-0.09172	0.125399	0.381852	0.775386	NUDCD3	23386	NudC domain containing 3
ENSG0000	101.6374	0.097498	0.19979	0.381846	0.775386	CCDC186	55088	coiled-coil domain containing 186
ENSG0000	3.365925	0.04258	0.234212	0.381816	0.775386	IFT81	28981	intraflagellar transport 81
ENSG0000	27.37026	-0.09738	0.210135	0.382134	0.775681	H2AC20	8338	H2A clustered histone 20
ENSG0000	2.901859	0.049045	0.233572	0.382166	0.775681	FBXO36	130888	F-box protein 36

ENSG0000	102.7217	-0.10389	0.159758	0.382094	0.775681	NA	NA	NA
ENSG0000	2.856764	0.032373	0.23297	0.382124	0.775681	NA	NA	NA
ENSG0000	29.62865	-0.09775	0.208799	0.382375	0.775914	DNM3	26052	dynamin 3
ENSG0000	743.1826	0.096392	0.144115	0.382328	0.775914	NUBP2	10101	NUBP iron cytosolic
ENSG0000	1887.03	-0.08454	0.110288	0.382408	0.775914	CNOT1	23019	CCR4-NOT transcription complex subunit 1
ENSG0000	154.4204	-0.10331	0.188044	0.382477	0.775967	PKMYT1	9088	protein kir membrane associated tyrosine/threonine 1
ENSG0000	270.7843	0.100397	0.150351	0.382644	0.776061	SERTAD2	9792	SERTA domain containing 2
ENSG0000	1014.729	-0.10129	0.154021	0.38265	0.776061	GTF3C1	2975	general transcription factor IIIC subunit 1
ENSG0000	96.83231	-0.09918	0.202207	0.382598	0.776061	C1GALT1C	29071	C1GALT1 specific chaperone 1
ENSG0000	448.8985	0.096784	0.135837	0.382858	0.776386	HNRNPR	10236	heterogeneous nuclear ribonucleoprotein R
ENSG0000	15.09382	-0.08822	0.220605	0.383288	0.776386	NA	NA	NA
ENSG0000	5.808952	-0.05994	0.231352	0.38297	0.776386	TMEM163	81615	transmembrane protein 163
ENSG0000	481.1015	0.099247	0.166008	0.383277	0.776386	STK11IP	114790	serine/threonine kinase 11 interacting protein
ENSG0000	6.790775	0.063046	0.231894	0.383195	0.776386	NA	NA	NA
ENSG0000	4.937122	-0.05651	0.233392	0.383319	0.776386	RCAN2	10231	regulator of calcineurin 2
ENSG0000	14.26237	0.071084	0.22792	0.383313	0.776386	PRKAG2-A	644090	PRKAG2 antisense RNA 2
ENSG0000	47.29689	0.098773	0.205858	0.383247	0.776386	AGPAT5	55326	1-acylglycerol-3-phosphate O-acyltransferase 5
ENSG0000	452.4595	-0.10293	0.166383	0.383234	0.776386	NA	NA	NA
ENSG0000	104.5026	0.097682	0.205045	0.383224	0.776386	PCNX4	64430	pecanex 4
ENSG0000	1251.466	0.104041	0.167672	0.382898	0.776386	FHOD1	29109	formin homology 2 domain containing 1
ENSG0000	22.38535	-0.09085	0.216801	0.38303	0.776386	ZNF441	126068	zinc finger protein 441
ENSG0000	53.88387	-0.1022	0.197053	0.383585	0.776839	BAIAP2L2	80115	BAR/IMD domain containing adaptor protein 2 like 2
ENSG0000	22723.22	-0.10155	0.157529	0.384048	0.776895	FGR	2268	FGR proto Src family tyrosine kinase
ENSG0000	14.97381	0.08178	0.224113	0.384242	0.776895	FLACC1	130540	flagellum associated containing coiled-coil domains 1
ENSG0000	107.1636	0.103894	0.173846	0.384215	0.776895	ITPR1	3708	inositol 1 4 5-trisphosphate receptor type 1
ENSG0000	44.85848	0.102502	0.197975	0.384094	0.776895	COX17	10063	cytochrome c oxidase copper chaperone COX17
ENSG0000	40.99755	0.102771	0.198611	0.384007	0.776895	PTCD1	26024	pentatricopeptide repeat domain 1
ENSG0000	605.1973	0.097586	0.200994	0.384167	0.776895	GNAQ	2776	G protein subunit alpha q
ENSG0000	37.27044	-0.1063	0.192481	0.383973	0.776895	CUBN	8029	cubilin
ENSG0000	2.930275	0.043057	0.233692	0.383774	0.776895	NA	NA	NA
ENSG0000	218.314	-0.10237	0.189579	0.384184	0.776895	RB1	5925	RB transcriptional corepressor 1
ENSG0000	24.33395	-0.09424	0.214244	0.383665	0.776895	TRAV21	28662	T cell receptor alpha variable 21
ENSG0000	131.8516	-0.10243	0.162684	0.383933	0.776895	FBXO34	55030	F-box protein 34
ENSG0000	118.3946	-0.10086	0.180836	0.384291	0.776895	PCSK6	5046	proprotein convertase subtilisin/kexin type 6
ENSG0000	3.852704	0.046809	0.232155	0.38371	0.776895	NA	NA	NA
ENSG0000	6.483158	0.057836	0.230709	0.384256	0.776895	NA	NA	NA
ENSG0000	20.23457	0.09978	0.209387	0.383867	0.776895	SLC24A3	57419	solute carrier family 24 member 3
ENSG0000	4.691389	-0.03244	0.232901	0.384241	0.776895	H2AB1	474382	H2A.B variant histone 1
ENSG0000	87.74454	0.103821	0.188083	0.384369	0.776951	CCNB2	9133	cyclin B2
ENSG0000	42.78918	0.098657	0.205931	0.384404	0.776951	FLJ40194	124871	uncharacterized FLJ40194
ENSG0000	36.06886	-0.10389	0.192038	0.384484	0.777027	NAPEPLD	222236	N-acyl phosphatidylethanolamine phospholipase D
ENSG0000	657.7163	-0.09682	0.138655	0.384546	0.777067	SERPINB9	5272	serpin family B member 9
ENSG0000	3396.279	0.09935	0.148281	0.384644	0.777176	MAZ	4150	MYC associated zinc finger protein
ENSG0000	1311.686	-0.10046	0.150531	0.384685	0.777176	NARF	26502	nuclear prelamin A recognition factor
ENSG0000	84.44133	-0.10455	0.182227	0.384806	0.777215	CCDC28B	79140	coiled-coil domain containing 28B
ENSG0000	407.7331	0.100809	0.148275	0.384831	0.777215	VPS35L	57020	VPS35 endosomal protein sorting factor like
ENSG0000	4.565131	0.058191	0.231665	0.384781	0.777215	SERHL2	253190	serine hydrolase like 2
ENSG0000	785.5581	0.099925	0.158742	0.384968	0.777217	AGO2	27161	argonaute RISC catalytic component 2
ENSG0000	3.559122	0.05844	0.235236	0.384896	0.777217	LOC64236	642361	uncharacterized LOC642361
ENSG0000	3581.474	-0.0973	0.140802	0.38495	0.777217	OTUB1	55611	OTU deub ubiquitin aldehyde binding 1
ENSG0000	28.05911	0.095829	0.212015	0.385002	0.777217	SLC16A11	162515	solute carrier family 16 member 11
ENSG0000	360.6544	0.094338	0.137271	0.385108	0.777292	P3H1	64175	prolyl 3-hydroxylase 1
ENSG0000	32.38223	-0.09682	0.208297	0.385167	0.777292	NA	NA	NA
ENSG0000	11.8623	0.080804	0.225207	0.385136	0.777292	NA	NA	NA
ENSG0000	2386.496	-0.09868	0.145946	0.385299	0.777388	ANKRD13/	88455	ankyrin repeat domain 13A
ENSG0000	2.887952	0.046948	0.233524	0.385272	0.777388	IQCD	115811	IQ motif containing D
ENSG0000	2.722102	-0.03827	0.232317	0.38549	0.777555	DYNLT2B	255758	dynein light chain Tctex-type 2B
ENSG0000	195.1423	0.103971	0.174055	0.385472	0.777555	CYCS	54205	cytochrom somatic
ENSG0000	15.27522	0.080964	0.224498	0.385764	0.777555	CEP126	57562	centrosomal protein 126
ENSG0000	41.91972	-0.10264	0.19882	0.385725	0.777555	FAM234B	57613	family with sequence similarity 234 member B
ENSG0000	3.242753	0.039755	0.23261	0.385757	0.777555	FGF14-AS2	283481	FGF14 antisense RNA 2
ENSG0000	610.5894	0.103898	0.163065	0.385647	0.777555	IRF8	3394	interferon regulatory factor 8
ENSG0000	25.76364	-0.09777	0.208943	0.385652	0.777555	NA	NA	NA
ENSG0000	301.4391	0.095234	0.134831	0.385572	0.777555	CSTF1	1477	cleavage stimulation factor subunit 1
ENSG0000	5.572672	-0.05371	0.23112	0.38566	0.777555	LINC01644	1.02E+08	long intergenic non-protein coding RNA 1644
ENSG0000	7.640017	0.080054	0.226657	0.385873	0.777689	NA	NA	NA
ENSG0000	252.1732	-0.09882	0.198466	0.386208	0.778193	TREML3P	340206	triggering pseudogene
ENSG0000	80.35934	-0.10388	0.171481	0.386167	0.778193	NMT2	9397	N-myristoyltransferase 2
ENSG0000	4.983853	0.059068	0.230918	0.386311	0.77826	CRHBP	1393	corticotropin releasing hormone binding protein
ENSG0000	114.3616	0.103456	0.171437	0.386334	0.77826	LRRC57	255252	leucine rich repeat containing 57
ENSG0000	565.308	-0.09763	0.143752	0.386411	0.77826	STX4	6810	syntaxin 4
ENSG0000	663.1978	-0.08934	0.12095	0.386396	0.77826	MAPRE1	22919	microtubule associated protein RP/EB family member 1
ENSG0000	5.718707	-0.04762	0.231687	0.386527	0.778322	UNC45B	146862	unc-45 myosin chaperone B
ENSG0000	833.1328	-0.09989	0.146054	0.386499	0.778322	CYTH2	9266	cytohesin 2
ENSG0000	3.79547	-0.04187	0.23237	0.386791	0.778726	CCSER1	401145	coiled-coil serine rich protein 1
ENSG0000	86.03644	0.095414	0.207523	0.386812	0.778726	GABPA	2551	GA binding protein transcription factor subunit alpha
ENSG0000	428.9505	-0.1022	0.158854	0.386893	0.778804	PDZD4	57595	PDZ domain containing 4
ENSG0000	38.64519	0.097913	0.206195	0.387018	0.778884	NA	NA	NA
ENSG0000	4.701414	-0.06141	0.230864	0.387007	0.778884	NA	NA	NA
ENSG0000	2.594626	-0.0493	0.23398	0.387227	0.779218	CPNE4	131034	copine 4
ENSG0000	4.561088	0.052621	0.232032	0.387426	0.779448	PIERCE1	138162	piercer of microtubule wall 1
ENSG0000	93.40409	0.105314	0.176464	0.38739	0.779448	PARD6A	50855	par-6 family cell polarity regulator alpha
ENSG0000	310.3022	-0.09793	0.14364	0.387605	0.779712	NTAN1	123803	N-terminal asparagine amidase
ENSG0000	336.4123	-0.09568	0.136147	0.387685	0.779712	JMJD6	23210	jumonji dc arginine demethylase and lysine hydroxylase
ENSG0000	10.56287	0.070613	0.227706	0.387677	0.779712	ZCCHC18	644353	zinc finger CCHC-type containing 18
ENSG0000	27.97758	-0.10158	0.201137	0.38778	0.779817	RAD51B	5890	RAD51 paralog B
ENSG0000	79.22393	-0.10005	0.195739	0.387841	0.779855	MLLT3	4300	MLLT3 super elongation complex subunit
ENSG0000	5.176618	0.071006	0.230226	0.387915	0.779917	FAM88B	728673	family with sequence similarity 88 member B
ENSG0000	2.773739	0.047314	0.233152	0.388045	0.779922	SLCO2B1	11309	solute carrier organic anion transporter family member 2B1
ENSG0000	219.1561	0.102061	0.165337	0.387974	0.779922	PTGER2	5732	prostaglandin E receptor 2
ENSG0000	5.340949	-0.02783	0.231972	0.388023	0.779922	NA	NA	NA
ENSG0000	2.426429	0.030786	0.232472	0.388127	0.780002	LOC12490	1.25E+08	uncharacterized LOC124909451
ENSG0000	13.27961	-0.08263	0.223972	0.388404	0.780472	RHOBTB3	22836	Rho related BTB domain containing 3
ENSG0000	656.3342	-0.08608	0.120651	0.388573	0.780727	FAM219A	203259	family with sequence similarity 219 member A
ENSG0000	46.32358	0.101912	0.195038	0.388627	0.780749	NUP37	79023	nucleoporin 37
ENSG0000	151.1805	-0.10285	0.17282	0.38876	0.780932	CAVIN2	8436	caveolae associated protein 2

ENSG0000	23.02705	-0.09901	0.206841	0.388811	0.780947	NA	NA	NA	
ENSG0000	463.6826	-0.09651	0.133965	0.388856	0.780952	ZSWIM1	90204	zinc finger SWIM-type containing 1	
ENSG0000	2149.866	0.107869	0.189234	0.389069	0.780981	SRM	6723	spermidine synthase	
ENSG0000	434.4306	-0.09459	0.13463	0.389083	0.780981	TAF12	6883	TATA-box binding protein associated factor 12	
ENSG0000	1486.813	-0.09714	0.142848	0.388973	0.780981	MICU1	10367	mitochondrial calcium uptake 1	
ENSG0000	317.5505	0.089303	0.123292	0.388936	0.780981	POLR2G	5436	RNA polymerase II subunit G	
ENSG0000	9.329234	-0.07452	0.227412	0.389001	0.780981	NA	NA	NA	
ENSG0000	884.7595	-0.09891	0.165065	0.389163	0.781054	HSD17B11	51170	hydroxysteroid 17-beta dehydrogenase 11	
ENSG0000	4.598679	0.031996	0.232644	0.389286	0.781216	HOXA10-A	1.01E+08	HOXA10 antisense RNA	
ENSG0000	2041.963	-0.10069	0.157261	0.390012	0.781329	PLEKHM2	23207	pleckstrin homology and RUN domain containing M2	
ENSG0000	1100.86	-0.09865	0.145839	0.389831	0.781329	NDUFAF3	25915	NADH:ubiquinone oxidoreductase complex assembly factor 3	
ENSG0000	35.54257	0.094505	0.211074	0.389422	0.781329	FBXO45	200933	F-box protein 45	
ENSG0000	3.549236	0.037656	0.234361	0.390057	0.781329	NA	NA	NA	
ENSG0000	156.272	0.102049	0.177216	0.390153	0.781329	UPK3B	1.05E+08	uroplakin 3B	
ENSG0000	2.926615	0.034025	0.233977	0.389927	0.781329	NA	NA	NA	
ENSG0000	13.13966	0.083283	0.223051	0.389804	0.781329	LOC10536	1.05E+08	uncharacterized LOC105369363	
ENSG0000	181.4327	0.099043	0.201044	0.390131	0.781329	ETNK1	55500	ethanolamine kinase 1	
ENSG0000	41.62454	-0.09327	0.21003	0.38988	0.781329	CIT	11113	citron rho-interacting serine/threonine kinase	
ENSG0000	533.8205	0.099553	0.212809	0.3899	0.781329	ZDHHC20	253832	zinc finger DHHC-type palmitoyltransferase 20	
ENSG0000	681.2301	0.100291	0.152832	0.389893	0.781329	ZNF652	22834	zinc finger protein 652	
ENSG0000	10.85272	-0.08381	0.222818	0.390105	0.781329	NPB	256933	neuropeptide B	
ENSG0000	57.58237	0.103695	0.185012	0.389531	0.781329	ATP8B3	148229	ATPase phospholipid transporting 8B3	
ENSG0000	5.769457	0.057296	0.230592	0.389758	0.781329	NA	NA	NA	
ENSG0000	89.59458	0.102939	0.165351	0.389609	0.781329	HAUS8	93323	HAUS augmin like complex subunit 8	
ENSG0000	23.62872	-0.09888	0.206496	0.39014	0.781329	NA	NA	NA	
ENSG0000	25.15232	0.09311	0.214259	0.389578	0.781329	NA	NA	NA	
ENSG0000	8.744112	0.064451	0.228849	0.39001	0.781329	NA	NA	NA	
ENSG0000	172.3796	-0.10202	0.153501	0.389652	0.781329	LAGE3	8270	L antigen family member 3	
ENSG0000	429.3973	0.10214	0.181899	0.390215	0.781368	MKI67	4288	marker of proliferation Ki-67	
ENSG0000	7.198311	0.060423	0.230043	0.390263	0.78138	B4GALNT4	338707	beta-1	4-N-acetyl-galactosaminyltransferase 4
ENSG0000	244.987	0.099431	0.151514	0.390493	0.781508	INPP5E	56623	inositol polyphosphate-5-phosphatase E	
ENSG0000	67.48151	-0.10296	0.182779	0.390498	0.781508	CNTNAP1	8506	contactin associated protein 1	
ENSG0000	10.45967	-0.08201	0.225485	0.390377	0.781508	LINC0018	193629	long intergenic non-protein coding RNA 189	
ENSG0000	153.3775	0.10267	0.182069	0.390456	0.781508	SMTN	6525	smoothelin	
ENSG0000	8.644203	0.079534	0.226238	0.390563	0.781552	CCDC15	80071	coiled-coil domain containing 15	
ENSG0000	567.9051	-0.09614	0.135093	0.39062	0.781581	XYLT2	64132	xylosyltransferase 2	
ENSG0000	135.5269	0.091895	0.210583	0.390702	0.78166	NA	NA	NA	
ENSG0000	3.537401	0.0501	0.232129	0.390748	0.781668	NA	NA	NA	
ENSG0000	71.93119	0.094104	0.204535	0.390889	0.781693	ZNF776	284309	zinc finger protein 776	
ENSG0000	858.0798	0.090678	0.207813	0.390817	0.781693	IGLV8-61	28774	immunoglobulin lambda variable 8-61	
ENSG0000	3.290418	-0.03213	0.232973	0.39085	0.781693	LOC10537	1.05E+08	uncharacterized LOC105373175	
ENSG0000	559.7191	0.090422	0.123719	0.391028	0.781886	UBE2G2	7327	ubiquitin conjugating enzyme E2 G2	
ENSG0000	188.6906	0.101961	0.184448	0.391159	0.781901	HLA-DQA1	3117	major hist class II	DQ alpha 1
ENSG0000	273.4268	-0.10199	0.175201	0.391164	0.781901	TAF3	83860	TATA-box binding protein associated factor 3	
ENSG0000	121.1123	0.101563	0.1788	0.391082	0.781901	DYRK4	8798	dual specificity tyrosine phosphorylation regulated kinase 4	
ENSG0000	1305.047	0.101545	0.164574	0.391271	0.782029	TP53	7157	tumor protein p53	
ENSG0000	19191.16	-0.10122	0.172921	0.391419	0.782239	NBEAL2	23218	neurobeachin like 2	
ENSG0000	28.65212	0.090206	0.214321	0.391528	0.782372	DCTN6	10671	dynactin subunit 6	
ENSG0000	9.126016	0.072821	0.227081	0.391667	0.782565	ANAPC1P2	285074	ANAPC1 pseudogene 2	
ENSG0000	1897.614	-0.08715	0.120449	0.392397	0.782764	SSU72	29101	SSU72 hor RNA polymerase II CTD phosphatase	
ENSG0000	97.50831	0.097221	0.141711	0.391991	0.782764	DNAJC16	23341	DnaJ heat shock protein family (Hsp40) member C16	
ENSG0000	10.11089	-0.06821	0.227452	0.392506	0.782764	PDCD6IP-I	1.05E+08	PDCD6IP divergent transcript	
ENSG0000	43.57826	-0.07634	0.223761	0.392562	0.782764	C4orf33	132321	chromosome 4 open reading frame 33	
ENSG0000	44.54931	-0.09967	0.199359	0.392249	0.782764	SEPTIN8	23176	septin 8	
ENSG0000	339.7645	0.101272	0.149889	0.392246	0.782764	MCM3	4172	minichromosome maintenance complex component 3	
ENSG0000	47.25238	-0.08538	0.216405	0.392234	0.782764	MYBL1	4603	MYB proto-oncogene like 1	
ENSG0000	28.72959	0.090375	0.214891	0.392412	0.782764	WNT1	7471	Wnt family member 1	
ENSG0000	60.8762	-0.10234	0.19034	0.392575	0.782764	SLC9A7P1	121456	solute carrier family 9 member 7 pseudogene 1	
ENSG0000	57.7219	0.102383	0.187415	0.392072	0.782764	MAPKAPK	51275	MAPKAPK5 antisense RNA 1	
ENSG0000	61.09445	0.099269	0.199237	0.392245	0.782764	STX2	2054	syntaxin 2	
ENSG0000	2891.716	-0.09865	0.152017	0.392195	0.782764	SLC12A6	9990	solute carrier family 12 member 6	
ENSG0000	106.166	0.102969	0.175764	0.392	0.782764	PEAK1	79834	pseudopodium enriched atypical kinase 1	
ENSG0000	382.6137	-0.10038	0.153405	0.391973	0.782764	SKAP1	8631	src kinase associated phosphoprotein 1	
ENSG0000	16.86688	0.084264	0.220863	0.392578	0.782764	DLX4	1748	distal-less homeobox 4	
ENSG0000	5.388243	-0.06199	0.23006	0.392342	0.782764	NA	NA	NA	
ENSG0000	7732.627	-0.10074	0.164878	0.392247	0.782764	PPP6R1	22870	protein phosphatase 6 regulatory subunit 1	
ENSG0000	20.31142	-0.07751	0.223546	0.391962	0.782764	TMPRSS3	64699	transmembrane serine protease 3	
ENSG0000	56.74299	0.100834	0.193088	0.392555	0.782764	LINC0020	642852	long intergenic non-protein coding RNA 205	
ENSG0000	8.381982	0.079571	0.225295	0.392667	0.78277	NA	NA	NA	
ENSG0000	10.00099	0.070305	0.227662	0.392642	0.78277	NA	NA	NA	
ENSG0000	97.66257	0.102323	0.166751	0.392733	0.782817	NUP155	9631	nucleoporin 155	
ENSG0000	36.46729	-0.1008	0.197796	0.392812	0.782888	NDUFC2	4718	NADH:ubiquinone oxidoreductase subunit C2	
ENSG0000	1047.162	-0.08466	0.114063	0.392961	0.78293	BSDC1	55108	BSD domain containing 1	
ENSG0000	39.28442	-0.09422	0.208662	0.392958	0.78293	DOK4	55715	docking protein 4	
ENSG0000	6.025382	0.042461	0.231277	0.392942	0.78293	ZNF571-A	1.01E+08	ZNF571 antisense RNA 1	
ENSG0000	18.71415	0.053779	0.229008	0.393135	0.782957	NA	NA	NA	
ENSG0000	8.853655	0.069875	0.227828	0.393083	0.782957	NA	NA	NA	
ENSG0000	246.2883	-0.09865	0.147445	0.393145	0.782957	COA1	55744	cytochrome c oxidase assembly factor 1	
ENSG0000	56.95991	0.100886	0.18556	0.393103	0.782957	MTPAP	55149	mitochondrial poly(A) polymerase	
ENSG0000	112.3886	0.095828	0.141224	0.393201	0.782983	C1orf35	79169	chromosome 1 open reading frame 35	
ENSG0000	8.723195	-0.07602	0.226815	0.39325	0.782996	ZFYVE9	9372	zinc finger FYVE-type containing 9	
ENSG0000	11.15051	0.082887	0.223391	0.393341	0.78309	HHAT	55733	hedgehog acyltransferase	
ENSG0000	10.51323	0.069482	0.227159	0.393483	0.78319	LMLN	89782	leishmanolysin like peptidase	
ENSG0000	11.86635	-0.07946	0.224306	0.393519	0.78319	PSME2P1	257093	proteasome activator subunit 2 pseudogene 1	
ENSG0000	34.5234	0.090054	0.216295	0.393478	0.78319	TBCEL	219899	tubulin folding cofactor E like	
ENSG0000	2302.9	0.099594	0.154738	0.393612	0.783291	XRCC5	7520	X-ray repair cross complementing 5	
ENSG0000	60.52245	0.097137	0.203654	0.393738	0.783455	IGHE	3497	immunoglobulin heavy constant epsilon	
ENSG0000	90.00042	0.100844	0.190064	0.393801	0.783497	CCNB1IP1	57820	cyclin B1 interacting protein 1	
ENSG0000	197.5516	0.09366	0.132559	0.393889	0.783527	MKRN2	23609	makorin ring finger protein 2	
ENSG0000	799.0839	-0.10128	0.176266	0.393902	0.783527	HAPSTR1	29035	HUWE1 associated protein modifying stress responses	
ENSG0000	1638.773	0.102468	0.163336	0.393954	0.783544	XRCC6	2547	X-ray repair cross complementing 6	
ENSG0000	6.541443	0.05803	0.230538	0.394097	0.78359	MAP4K3-D	728730	MAP4K3 divergent transcript	
ENSG0000	39.08113	0.073301	0.224254	0.394158	0.78359	CHRNA2	1135	cholinergic receptor nicotinic alpha 2 subunit	
ENSG0000	1837.599	-0.10107	0.176884	0.394091	0.78359	ST6GALNA	27090	ST6 N-ace 6-sialyltransferase 4	
ENSG0000	22.64367	0.088186	0.216731	0.394191	0.78359	HSD11B1L	374875	hydroxysteroid 11-beta dehydrogenase 1 like	
ENSG0000	932.3011	0.095043	0.165723	0.394155	0.78359	PES1	23481	pescadillo ribosomal biogenesis factor 1	

ENSG0000	102.5096	0.08731	0.215518	0.394374	0.783701	USP1	7398	ubiquitin specific peptidase 1
ENSG0000	62.92583	0.10314	0.181487	0.394313	0.783701	KIAA0753	9851	KIAA0753
ENSG0000	11.58544	0.076173	0.225743	0.394344	0.783701	NA	NA	NA
ENSG0000	3.115306	-0.04663	0.232004	0.394729	0.783884	NA	NA	NA
ENSG0000	142.9592	0.100016	0.188669	0.395094	0.783884	EPC2	26122	enhancer of polycomb homolog 2
ENSG0000	1052.234	-0.07755	0.102301	0.394994	0.783884	CASP10	843	caspase 10
ENSG0000	1177.004	0.093179	0.126424	0.394687	0.783884	QRICH1	54870	glutamine rich 1
ENSG0000	3.326946	0.049761	0.233614	0.394882	0.783884	PCOLCE2	26577	procollagen C-endopeptidase enhancer 2
ENSG0000	7.586278	-0.05822	0.229273	0.394704	0.783884	NA	NA	NA
ENSG0000	2.476433	0.037399	0.232583	0.394881	0.783884	RN7SL403	1.06E+08	RNA 7SL cytoplasm pseudogene
ENSG0000	9.893738	0.070035	0.227648	0.394987	0.783884	LOC12490	1.25E+08	uncharacterized LOC124902027
ENSG0000	2026.555	-0.09806	0.148998	0.395165	0.783884	MARCHF8	220972	membrane associated ring-CH-type finger 8
ENSG0000	15.68393	0.089031	0.217442	0.39521	0.783884	TOLLIP-DT	255512	TOLLIP divergent transcript
ENSG0000	1149.287	-0.09813	0.153019	0.394863	0.783884	RBM14	10432	RNA binding motif protein 14
ENSG0000	253.0295	0.100471	0.169767	0.395139	0.783884	TPP2	7174	tripeptidyl peptidase 2
ENSG0000	10.34986	0.076985	0.225854	0.394989	0.783884	CILP	8483	cartilage intermediate layer protein
ENSG0000	9754.679	-0.09351	0.162412	0.395237	0.783884	CSK	1445	C-terminal Src kinase
ENSG0000	600.6097	-0.08563	0.120292	0.395143	0.783884	TMEM11	8834	transmembrane protein 11
ENSG0000	10332.4	0.097023	0.145084	0.394623	0.783884	SEPTIN9	10801	septin 9
ENSG0000	70.3551	-0.09761	0.199186	0.394675	0.783884	LRRC3	81543	leucine rich repeat containing 3
ENSG0000	7.239998	0.063089	0.229094	0.394747	0.783884	CNKSR2	22866	connector enhancer of kinase suppressor of Ras 2
ENSG0000	689.5266	-0.10021	0.179386	0.395429	0.783888	CCNL1	57018	cyclin L1
ENSG0000	8489.542	-0.09906	0.163417	0.395552	0.783888	RGS14	10636	regulator of G protein signaling 14
ENSG0000	71.25369	0.102026	0.1692	0.395617	0.783888	ZNF853	54753	zinc finger protein 853
ENSG0000	4.555385	0.064314	0.230802	0.395595	0.783888	NA	NA	NA
ENSG0000	261.2753	-0.08864	0.208688	0.395538	0.783888	TGFBR1	7046	transforming growth factor beta receptor 1
ENSG0000	11.26114	-0.07404	0.225694	0.395566	0.783888	DIP2C	22982	disco interacting protein 2 homolog C
ENSG0000	60.82541	0.101277	0.169277	0.395624	0.783888	TESMIN	9633	testis expressed metallothionein like protein
ENSG0000	738.0103	0.091586	0.129548	0.395436	0.783888	PSKH1	5681	protein serine kinase H1
ENSG0000	36.64343	-0.10092	0.194108	0.395357	0.783888	RALY-AS1	1.02E+08	RALY antisense RNA 1
ENSG0000	68.01511	0.098446	0.197441	0.395723	0.784	NA	NA	NA
ENSG0000	328.5275	0.098357	0.150217	0.395785	0.784037	MPND	84954	MPN domain containing
ENSG0000	362.1438	-0.09991	0.183516	0.395857	0.784095	COL6A2	1292	collagen type VI alpha 2 chain
ENSG0000	175.3127	0.099457	0.187118	0.396008	0.784309	UBE3A	7337	ubiquitin protein ligase E3A
ENSG0000	95.55742	0.102184	0.17835	0.396208	0.78462	ZNF431	170959	zinc finger protein 431
ENSG0000	3.634783	0.024801	0.232689	0.396381	0.784644	NA	NA	NA
ENSG0000	237.8913	0.096797	0.208943	0.396554	0.784644	CBX3	11335	chromobox 3
ENSG0000	63.62683	0.096151	0.203026	0.396518	0.784644	LSM1	27257	LSM1 hom mRNA degradation associated
ENSG0000	704.2486	-0.08596	0.118889	0.396486	0.784644	TRAF1	7185	TNF receptor associated factor 1
ENSG0000	1773.036	-0.09453	0.138172	0.396606	0.784644	BANF1	8815	BAF nuclear assembly factor 1
ENSG0000	209.5515	-0.10017	0.160226	0.396549	0.784644	DHRS1	115817	dehydrogenase/reductase 1
ENSG0000	3.993996	0.052114	0.231583	0.396604	0.784644	RPS27AP1	643358	RPS27A pseudogene 16
ENSG0000	47.24533	0.102084	0.188584	0.396494	0.784644	WWOX	51741	WW domain containing oxidoreductase
ENSG0000	32.44819	-0.10336	0.180635	0.396565	0.784644	HOXB-AS1	1.01E+08	HOXB cluster antisense RNA 1
ENSG0000	7297.267	0.098758	0.153269	0.396661	0.784669	HMGA1	3159	high mobility group AT-hook 1
ENSG0000	2011.7	0.100731	0.176914	0.396726	0.784714	TMEM214	54867	transmembrane protein 214
ENSG0000	83.83003	-0.08148	0.217085	0.397017	0.784792	LOC10537	1.05E+08	uncharacterized LOC105374981
ENSG0000	193.7548	-0.09225	0.137751	0.396887	0.784792	TBC1D22B	55633	TBC1 domain family member 22B
ENSG0000	104.0096	0.099466	0.196242	0.397016	0.784792	XPO4	64328	exportin 4
ENSG0000	180.4269	0.095416	0.139953	0.396976	0.784792	ZNF785	146540	zinc finger protein 785
ENSG0000	49.04257	0.095667	0.201902	0.396963	0.784792	NAE1	8883	NEDD8 activating enzyme E1 subunit 1
ENSG0000	8.524441	-0.06612	0.228486	0.397023	0.784792	COL1A1	1277	collagen type I alpha 1 chain
ENSG0000	334.6823	-0.08709	0.120413	0.397126	0.784803	CZIB	54987	CXXC motif containing zinc binding protein
ENSG0000	76.97815	-0.09516	0.202676	0.397142	0.784803	NA	NA	NA
ENSG0000	3.562506	0.045	0.232601	0.397157	0.784803	NA	NA	NA
ENSG0000	3067.271	-0.09545	0.143034	0.397213	0.784804	CNPPD1	27013	cyclin Pas1/PHO80 domain containing 1
ENSG0000	1428.818	0.087304	0.126436	0.397243	0.784804	SIN3B	23309	SIN3 transcription regulator family member B
ENSG0000	4.380755	0.048347	0.232208	0.397336	0.784903	NA	NA	NA
ENSG0000	688.0766	-0.10078	0.17217	0.397413	0.784948	SGF29	112869	SAGA complex associated factor 29
ENSG0000	53.3831	0.096558	0.201686	0.397445	0.784948	ZNF606	80095	zinc finger protein 606
ENSG0000	300.9829	-0.09775	0.172967	0.397499	0.784971	EGLN1	54583	egl-9 family hypoxia inducible factor 1
ENSG0000	19.61285	-0.07867	0.22366	0.397555	0.784996	NA	NA	NA
ENSG0000	127.2256	-0.09955	0.157348	0.397651	0.785101	UBA6-DT	550112	UBA6 divergent transcript
ENSG0000	40.94817	0.100852	0.191543	0.397747	0.785206	PACC1	55248	proton activated chloride channel 1
ENSG0000	67.08539	-0.10045	0.188445	0.398117	0.785767	KATNA1	11104	katanin catalytic subunit A1
ENSG0000	7.976427	-0.0816	0.22432	0.398077	0.785767	NA	NA	NA
ENSG0000	14.52279	0.081998	0.221624	0.398286	0.785803	CENPL	91687	centromere protein L
ENSG0000	1611.617	0.089715	0.125922	0.398306	0.785803	LPCAT1	79888	lysophosphatidylcholine acyltransferase 1
ENSG0000	338.6121	0.093117	0.133426	0.398272	0.785803	PEX26	55670	peroxisomal biogenesis factor 26
ENSG0000	6.125913	-0.07004	0.228321	0.398266	0.785803	FAAH2	158584	fatty acid amide hydrolase 2
ENSG0000	5290.495	-0.09687	0.160528	0.398384	0.785871	APOBEC3C	27350	apolipoprotein B mRNA editing enzyme catalytic subunit 3C
ENSG0000	217.7685	0.102378	0.195865	0.398503	0.786021	F2RL1	2150	F2R like trypsin receptor 1
ENSG0000	4.891459	0.047784	0.231265	0.398547	0.786024	RTN4RL1	146760	reticulon 4 receptor like 1
ENSG0000	4.916102	0.063862	0.230417	0.398624	0.78609	NA	NA	NA
ENSG0000	48.10948	0.102463	0.17847	0.398863	0.786392	POLR1G	10849	RNA polymerase I subunit G
ENSG0000	8.4597	-0.07534	0.226961	0.398837	0.786392	AIRE	326	autoimmune regulator
ENSG0000	4.994844	0.039916	0.231259	0.398989	0.786555	NA	NA	NA
ENSG0000	2.337044	0.036903	0.232729	0.399055	0.786601	MTND4P9	1.01E+08	MT-ND4 pseudogene 9
ENSG0000	2269.014	-0.09072	0.205442	0.399176	0.786755	NA	NA	NA
ENSG0000	120.8816	-0.09637	0.144697	0.399248	0.786812	R3HCC1L	27291	R3H domain and coiled-coil containing 1 like
ENSG0000	3.878374	-0.05931	0.23128	0.399538	0.787299	NA	NA	NA
ENSG0000	2.662033	-0.03565	0.231921	0.399656	0.78739	NA	NA	NA
ENSG0000	4.13417	0.049047	0.231591	0.39967	0.78739	NA	NA	NA
ENSG0000	22.92025	-0.1007	0.192106	0.399761	0.787486	NA	NA	NA
ENSG0000	25.67152	0.090514	0.212388	0.399927	0.787727	HDHD2	84064	haloacid dehalogenase like hydrolase domain containing 2
ENSG0000	474.5905	-0.09976	0.168256	0.399988	0.787763	IQSEC2	23096	IQ motif and Sec7 domain ArfGEF 2
ENSG0000	3166.243	-0.09581	0.159849	0.400595	0.788084	HPCAL1	3241	hippocalcin like 1
ENSG0000	83.39204	0.10106	0.171097	0.400619	0.788084	PCYOX1	51449	prenylcysteine oxidase 1
ENSG0000	2.857122	-0.04145	0.232889	0.400509	0.788084	LOC40112	401127	WD repeat domain 5 pseudogene
ENSG0000	143.3597	0.086858	0.212296	0.400624	0.788084	SLC25A46	91137	solute carrier family 25 member 46
ENSG0000	154.2323	0.100058	0.179908	0.400531	0.788084	AGK	55750	acylglycerol kinase
ENSG0000	73.41217	0.089798	0.209958	0.400494	0.788084	SSPOP	23145	SCO-sponc pseudogene
ENSG0000	1187.482	0.098004	0.154059	0.400394	0.788084	SLC4A2	6522	solute carrier family 4 member 2
ENSG0000	834.599	-0.09195	0.133053	0.400547	0.788084	TUBA1C	84790	tubulin alpha 1c
ENSG0000	2.382035	-0.02743	0.232743	0.400294	0.788084	RPL7AP60	1E+08	ribosomal protein L7a pseudogene 60
ENSG0000	10.62465	-0.06303	0.228114	0.400267	0.788084	MPP2	4355	MAGUK p55 scaffold protein 2

ENSG0000	94.6248	-0.10067	0.176755	0.400581	0.788084	OLFM2	93145	olfactomedin 2
ENSG0000	2113.228	-0.09254	0.136208	0.400709	0.788165	RETREG2	79137	reticulophagy regulator family member 2
ENSG0000	351.298	0.092278	0.134705	0.400851	0.788192	VAMP8	8673	vesicle associated membrane protein 8
ENSG0000	2.561281	-0.02185	0.233716	0.400809	0.788192	A4GALT	53947	alpha 1 4-galactosyltransferase (P blood group)
ENSG0000	6.342764	-0.06138	0.229578	0.400789	0.788192	AMOT	154796	angiomotin
ENSG0000	3.044128	-0.05615	0.233422	0.401019	0.788206	NA	NA	NA
ENSG0000	33.87021	0.094679	0.205922	0.401246	0.788206	RRP15	51018	ribosomal RNA processing 15 homolog
ENSG0000	782.7052	-0.09403	0.132898	0.401156	0.788206	RBM6	10180	RNA binding motif protein 6
ENSG0000	487.937	-0.09931	0.166277	0.401048	0.788206	PDE7A	5150	phosphodiesterase 7A
ENSG0000	61.76429	-0.09353	0.205911	0.401072	0.788206	NA	NA	NA
ENSG0000	3.89196	0.04668	0.231649	0.401168	0.788206	CHST1	8534	carbohydrate sulfotransferase 1
ENSG0000	93.46152	0.104228	0.176343	0.401155	0.788206	CRIP1	1396	cysteine rich protein 1
ENSG0000	15.45628	-0.07321	0.224323	0.400931	0.788206	ATAD5	79915	ATPase family AAA domain containing 5
ENSG0000	746.4535	0.095081	0.142914	0.401245	0.788206	TCEA2	6919	transcription elongation factor A2
ENSG0000	646.3058	-0.09852	0.168817	0.40131	0.788219	IGSF8	93185	immunoglobulin superfamily member 8
ENSG0000	562.1363	-0.05879	0.22718	0.401339	0.788219	ANXA3	306	annexin A3
ENSG0000	15.23751	0.080341	0.221906	0.401513	0.788289	TMEM198	130612	transmembrane protein 198
ENSG0000	81.77209	0.099809	0.187193	0.401546	0.788289	HJURP	55355	Holliday junction recognition protein
ENSG0000	7.607818	0.053967	0.229862	0.40153	0.788289	NA	NA	NA
ENSG0000	25.91175	0.095449	0.205774	0.401514	0.788289	MIR4539	1.01E+08	microRNA 4539
ENSG0000	60.49813	0.095593	0.201097	0.401664	0.788351	PGM3	5238	phosphoglucomutase 3
ENSG0000	700.7941	0.091427	0.145047	0.401659	0.788351	LINC0108(	338799	long intergenic non-protein coding RNA 1089
ENSG0000	176.0918	-0.08977	0.205545	0.40171	0.788356	AZI2	64343	5-azacytidine induced 2
ENSG0000	7.575464	0.075784	0.226301	0.401906	0.788657	ADAMTS4	9507	ADAM metallopeptidase with thrombospondin type 1 motif 4
ENSG0000	33503.11	0.099209	0.16425	0.402175	0.788952	VIM	7431	vimentin
ENSG0000	10.99871	0.064455	0.228823	0.402229	0.788952	IFNG-AS1	1.01E+08	IFNG antisense RNA 1
ENSG0000	467.6864	0.090778	0.133235	0.402117	0.788952	CIAO3	64428	cytosolic iron-sulfur assembly component 3
ENSG0000	1671.433	0.099862	0.154499	0.402206	0.788952	MARK4	57787	microtubule affinity regulating kinase 4
ENSG0000	140.3358	0.095413	0.144208	0.402379	0.789078	TRNAU1AI	54952	tRNA selenocysteine 1 associated protein 1
ENSG0000	170.0579	-0.09968	0.177639	0.402492	0.789078	ELK4	2005	ETS transcription factor ELK4
ENSG0000	1840.73	-0.09469	0.140877	0.402458	0.789078	DPP9	91039	dipeptidyl peptidase 9
ENSG0000	808.0964	-0.08465	0.122055	0.402508	0.789078	ST13	6767	ST13 Hsp70 interacting protein
ENSG0000	57.74838	0.099875	0.18953	0.402438	0.789078	TCEAL8	90843	transcription elongation factor A like 8
ENSG0000	2.432613	-0.04892	0.232437	0.402675	0.789321	NA	NA	NA
ENSG0000	114.0513	-0.09617	0.195459	0.402831	0.789381	SH2D1B	117157	SH2 domain containing 1B
ENSG0000	286.6339	-0.08911	0.126916	0.402835	0.789381	RETSAT	54884	retinol saturase
ENSG0000	1015.873	-0.09057	0.124929	0.402793	0.789381	CDS2	8760	CDP-diacylglycerol synthase 2
ENSG0000	4.751877	0.047647	0.231262	0.403042	0.78966	NA	NA	NA
ENSG0000	1126.581	0.101824	0.154268	0.403064	0.78966	GDE1	51573	glycerophosphodiester phosphodiesterase 1
ENSG0000	467.4689	-0.09378	0.196517	0.403183	0.78981	FOXP3	50943	forkhead box P3
ENSG0000	7.223397	0.049842	0.230803	0.403457	0.789928	DNAJC6	9829	DnaJ heat shock protein family (Hsp40) member C6
ENSG0000	12.26284	-0.06551	0.227119	0.403382	0.789928	NA	NA	NA
ENSG0000	603.3481	0.101689	0.17122	0.403448	0.789928	EEFSEC	60678	eukaryotic selenocysteine-tRNA specific
ENSG0000	74.76188	-0.10061	0.178323	0.403309	0.789928	NSG1	27065	neuronal vesicle trafficking associated 1
ENSG0000	1121.699	0.082458	0.123374	0.403459	0.789928	WIPI2	26100	WD repea phosphoinositide interacting 2
ENSG0000	10.79404	0.079241	0.223737	0.403536	0.789993	NA	NA	NA
ENSG0000	3436.231	-0.09143	0.134676	0.403627	0.790087	MAF1	84232	MAF1 hon negative regulator of RNA polymerase III
ENSG0000	6.391795	0.065576	0.22885	0.403674	0.790096	NA	NA	NA
ENSG0000	13.11094	-0.09093	0.214861	0.403719	0.790099	NA	NA	NA
ENSG0000	31.7454	-0.07743	0.220494	0.403805	0.790115	NA	NA	NA
ENSG0000	5.495144	0.053475	0.230329	0.403857	0.790115	NA	NA	NA
ENSG0000	52.67821	0.070866	0.222275	0.40385	0.790115	ZBTB6	10773	zinc finger and BTB domain containing 6
ENSG0000	25.44684	-0.09515	0.205317	0.404007	0.790325	LOC12253	1.23E+08	RPSAP41-EIF3EP1
ENSG0000	7.990876	0.061789	0.228595	0.40411	0.790383	C2CD4D	1E+08	C2 calcium dependent domain containing 4D
ENSG0000	3.501278	-0.04642	0.232352	0.404136	0.790383	NA	NA	NA
ENSG0000	2429.647	-0.09844	0.167948	0.404253	0.790383	CUX1	1523	cut like homeobox 1
ENSG0000	467.2869	0.100901	0.167727	0.404212	0.790383	CD19	930	CD19 molecule
ENSG0000	5.235277	0.04858	0.23107	0.40423	0.790383	RPL5P34	388907	ribosomal protein L5 pseudogene 34
ENSG0000	20.42906	0.096059	0.205594	0.404306	0.790404	EIF4A1	1973	eukaryotic translation initiation factor 4A1
ENSG0000	2027.585	0.091292	0.133392	0.404365	0.790434	GIGYF1	64599	GRB10 interacting GYF protein 1
ENSG0000	2.402072	0.042293	0.232099	0.404697	0.790542	NA	NA	NA
ENSG0000	1427.577	0.093021	0.138075	0.404721	0.790542	C2CD2L	9854	C2CD2 like
ENSG0000	11716.34	0.099033	0.176854	0.404549	0.790542	PRKACA	5566	protein kinase cAMP-activated catalytic subunit alpha
ENSG0000	10.24575	-0.06163	0.227672	0.404646	0.790542	PRKCG	5582	protein kinase C gamma
ENSG0000	2.890269	0.048096	0.235363	0.404722	0.790542	MIR646	693231	microRNA 646
ENSG0000	587.8068	0.094552	0.1585	0.404494	0.790542	COMT	1312	catechol-O-methyltransferase
ENSG0000	4.288162	0.042071	0.231727	0.4047	0.790542	UXT-AS1	1E+08	UXT antisense RNA 1
ENSG0000	6.548779	-0.06206	0.229134	0.404868	0.790742	SORBS1	10580	sorbin and SH3 domain containing 1
ENSG0000	682.278	-0.09647	0.158286	0.405088	0.790963	ITSN2	50618	intersectin 2
ENSG0000	671.6892	0.095382	0.207353	0.405039	0.790963	ITGA4	3676	integrin subunit alpha 4
ENSG0000	7.517957	0.072858	0.227214	0.405129	0.790963	KRT80	144501	keratin 80
ENSG0000	13.0022	0.067494	0.226085	0.405154	0.790963	NA	NA	NA
ENSG0000	4.2171	-0.05218	0.232006	0.405204	0.790976	TLR8-AS1	349408	TLR8 antisense RNA 1
ENSG0000	11.83512	0.080176	0.222894	0.405292	0.791003	NA	NA	NA
ENSG0000	61.43847	-0.09216	0.205814	0.40538	0.791003	PDE4D	5144	phosphodiesterase 4D
ENSG0000	218.4697	-0.09757	0.156636	0.405402	0.791003	HIVEP1	3096	HIVEP zinc finger 1
ENSG0000	373.2944	0.092018	0.139339	0.405349	0.791003	ATXN7L1	222255	ataxin 7 like 1
ENSG0000	173.5599	-0.09164	0.13309	0.405433	0.791003	TMEM199	147007	transmembrane protein 199
ENSG0000	3.413047	-0.04615	0.232554	0.405502	0.791031	NA	NA	NA
ENSG0000	40.1541	0.100274	0.181348	0.405534	0.791031	MRPL33	9553	mitochondrial ribosomal protein L33
ENSG0000	40.92543	0.074186	0.221973	0.405591	0.791058	NA	NA	NA
ENSG0000	100.0902	0.096214	0.194397	0.405724	0.791136	KYAT3	56267	kynurenine aminotransferase 3
ENSG0000	20.09999	0.084925	0.217109	0.405803	0.791136	ZNF124	7678	zinc finger protein 124
ENSG0000	208.8803	-0.09642	0.155667	0.405683	0.791136	ANAPC13	25847	anaphase promoting complex subunit 13
ENSG0000	65.97028	0.098581	0.178637	0.406106	0.791136	CLOCK	9575	clock circadian regulator
ENSG0000	2.732974	-0.01131	0.23395	0.40597	0.791136	GPR20	2843	G protein-coupled receptor 20
ENSG0000	1129.869	0.095545	0.148512	0.406092	0.791136	CYC1	1537	cytochrome c1
ENSG0000	46.90572	0.09507	0.202935	0.406077	0.791136	SVIP	258010	small VCP interacting protein
ENSG0000	22.72096	0.09186	0.211349	0.405822	0.791136	ARHGAP4:	143872	Rho GTPase activating protein 42
ENSG0000	16.39932	0.061183	0.227428	0.405962	0.791136	NA	NA	NA
ENSG0000	223.2299	-0.08606	0.123512	0.405967	0.791136	SNX12	29934	sorting nexin 12
ENSG0000	334.5287	0.098826	0.164569	0.406018	0.791136	MAGT1	84061	magnesium transporter 1
ENSG0000	3.936475	-0.05127	0.231348	0.406203	0.791157	CYP46A1	10858	cytochrome P450 family 46 subfamily A member 1
ENSG0000	10.50617	0.068689	0.226581	0.406165	0.791157	MGC1291(	84815	uncharacterized protein MGC12916
ENSG0000	55.59856	0.090477	0.209155	0.406695	0.791211	ABCD3	5825	ATP binding cassette subfamily D member 3
ENSG0000	518.8003	-0.09139	0.134399	0.406714	0.791211	POGK	57645	pogo transposable element derived with KRAB domain

ENSG0000	67.08004	0.086951	0.210797	0.406534	0.791211	TSNAX	7257	translin associated factor X
ENSG0000	199.1653	-0.09815	0.16143	0.406433	0.791211	FAM98A	25940	family with sequence similarity 98 member A
ENSG0000	18.16997	0.082691	0.219651	0.406562	0.791211	WNT7A	7476	Wnt family member 7A
ENSG0000	750.1091	-0.08291	0.211033	0.406324	0.791211	CREBRF	153222	CREB3 regulatory factor
ENSG0000	3.156207	-0.04795	0.233077	0.406683	0.791211	TOMM5	401505	translocase of outer mitochondrial membrane 5
ENSG0000	112.0601	0.090318	0.202471	0.406589	0.791211	MPP7	143098	MAGUK p55 scaffold protein 7
ENSG0000	49.93636	0.08085	0.217702	0.406496	0.791211	ARL14EP	120534	ADP ribosylation factor like GTPase 14 effector protein
ENSG0000	21.64752	0.090974	0.211899	0.406714	0.791211	NA	NA	NA
ENSG0000	290.1504	0.090487	0.127907	0.406398	0.791211	CUL4A	8451	cullin 4A
ENSG0000	225792.4	0.053547	0.230288	0.406793	0.791211	IGHA1	3493	immunoglobulin heavy constant alpha 1
ENSG0000	8.93995	0.019654	0.233556	0.406759	0.791211	IGLV3-6	28806	immunoglobulin lambda variable 3-6 (pseudogene)
ENSG0000	314.5937	0.097778	0.159817	0.406907	0.79135	WDR24	84219	WD repeat domain 24
ENSG0000	42.37055	0.09247	0.205477	0.407009	0.79139	CCDC77	84318	coiled-coil domain containing 77
ENSG0000	117.5471	0.09857	0.173437	0.407014	0.79139	ACACA	31	acetyl-CoA carboxylase alpha
ENSG0000	3.556684	0.043551	0.231437	0.407178	0.791497	IPO9-AS1	1.01E+08	IPO9 antisense RNA 1
ENSG0000	2044.171	-0.09488	0.192005	0.407149	0.791497	MGAM	8972	maltase-glucoamylase
ENSG0000	2.781166	-0.0364	0.231838	0.407199	0.791497	MTND1P1	1.01E+08	MT-ND1 pseudogene 11
ENSG0000	189.0039	-0.09643	0.187481	0.407246	0.791505	C5orf22	55322	chromosome 5 open reading frame 22
ENSG0000	237.6242	-0.08611	0.122573	0.40789	0.791588	DCAF6	55827	DDB1 and CUL4 associated factor 6
ENSG0000	263.1495	-0.10067	0.162425	0.407993	0.791588	EIPR1	7260	EARP complex and GARP complex interacting protein 1
ENSG0000	22.72137	0.090567	0.211082	0.407977	0.791588	WDR35	57539	WD repeat domain 35
ENSG0000	14.59144	0.066185	0.225956	0.40742	0.791588	C2orf76	130355	chromosome 2 open reading frame 76
ENSG0000	11.80804	0.041601	0.229956	0.407414	0.791588	LINC01806	1.01E+08	long intergenic non-protein coding RNA 1806
ENSG0000	6960.741	-0.09683	0.183373	0.407486	0.791588	PRKCD	5580	protein kinase C delta
ENSG0000	2026.432	-0.09763	0.171225	0.408237	0.791588	COPG1	22820	COPI coat complex subunit gamma 1
ENSG0000	278.8828	0.078642	0.216494	0.407633	0.791588	C9orf72	203228	C9orf72-SMCR8 complex subunit
ENSG0000	28.78003	0.075883	0.222117	0.407908	0.791588	PRUNE2	158471	prune homolog 2 with BCH domain
ENSG0000	27.38016	0.094735	0.201392	0.408146	0.791588	NDUFB8	4714	NADH:ubiquinone oxidoreductase subunit B8
ENSG0000	584.789	0.085755	0.121449	0.408104	0.791588	ESRRA	2101	estrogen related receptor alpha
ENSG0000	5.386474	-0.06503	0.228667	0.40818	0.791588	NA	NA	NA
ENSG0000	144.2735	0.096044	0.185133	0.40809	0.791588	ZNF664	144348	zinc finger protein 664
ENSG0000	264.6589	0.099986	0.164631	0.408074	0.791588	MMP17	4326	matrix metallopeptidase 17
ENSG0000	44.62572	0.097793	0.192716	0.407881	0.791588	PKD1P6	353511	polycystin transient receptor potential channel interacting pseudogene 6
ENSG0000	42.98516	0.094053	0.202699	0.40824	0.791588	MKS1	54903	MKS transition zone complex subunit 1
ENSG0000	3.706042	0.03208	0.231776	0.407597	0.791588	NA	NA	NA
ENSG0000	1986.491	0.097983	0.156655	0.408133	0.791588	NCLN	56926	nicalin
ENSG0000	96.14329	-0.09882	0.177802	0.407668	0.791588	ZNF585B	92285	zinc finger protein 585B
ENSG0000	179.2526	0.096816	0.190974	0.407714	0.791588	SPIB	6689	Spi-B transcription factor
ENSG0000	20.22268	-0.06444	0.226894	0.407692	0.791588	SIGLEC12	89858	sialic acid binding Ig like lectin 12
ENSG0000	37.92983	0.076796	0.219802	0.408108	0.791588	ZNF154	7710	zinc finger protein 154
ENSG0000	8.97632	0.05765	0.227526	0.408411	0.791754	NA	NA	NA
ENSG0000	51.01396	0.090578	0.207314	0.408502	0.791754	ATL2	64225	atlastin GTPase 2
ENSG0000	7.182603	-0.06346	0.228272	0.408427	0.791754	NA	NA	NA
ENSG0000	82.57926	-0.09624	0.156545	0.408541	0.791754	KDSR	2531	3-ketodihydrosphingosine reductase
ENSG0000	81.38557	0.092758	0.195471	0.408496	0.791754	MTM1	4534	myotubularin 1
ENSG0000	807.9436	0.092698	0.148459	0.408627	0.791837	BORCS8	729991	BLOC-1 related complex subunit 8
ENSG0000	232.1174	0.093418	0.143949	0.408768	0.792026	COX19	90639	cytochrome c oxidase assembly factor COX19
ENSG0000	50.32903	0.083006	0.215407	0.408887	0.792155	CASD1	64921	CAS1 domain containing 1
ENSG0000	1271.076	0.101154	0.169475	0.408921	0.792155	SULT1A1	6817	sulfotransferase family 1A member 1
ENSG0000	566.4187	-0.09595	0.16679	0.409066	0.792268	MAT2B	27430	methionine adenosyltransferase 2B
ENSG0000	365.9729	-0.09757	0.174759	0.409024	0.792268	RNF38	152006	ring finger protein 38
ENSG0000	6.575335	0.054239	0.229723	0.409181	0.792406	NA	NA	NA
ENSG0000	130.9847	-0.10141	0.169477	0.409339	0.792629	METRN	79006	meteorin glial cell differentiation regulator
ENSG0000	57.87252	0.097086	0.195676	0.409503	0.792863	EEIG2	284611	EEIG family member 2
ENSG0000	50.20802	-0.08754	0.201926	0.409565	0.792864	TRAV14DV	28669	T cell receptor alpha variable 14/delta variable 4
ENSG0000	3.40854	-0.02875	0.23226	0.40959	0.792864	NA	NA	NA
ENSG0000	40.77604	-0.07643	0.218774	0.409742	0.792886	CDCA7	83879	cell division cycle associated 7
ENSG0000	123.8887	-0.09105	0.138143	0.409861	0.792886	INTS12	57117	integrator complex subunit 12
ENSG0000	136.3117	0.096729	0.170764	0.409652	0.792886	PWWP2A	114825	PWWP domain containing 2A
ENSG0000	440.6823	0.089714	0.133724	0.409749	0.792886	ESYT2	57488	extended synaptotagmin 2
ENSG0000	45.61978	-0.08356	0.213315	0.409827	0.792886	RMI1	80010	RecQ mediated genome instability 1
ENSG0000	59.42392	0.099108	0.179472	0.409783	0.792886	ZC4H2	55906	zinc finger C4H2-type containing
ENSG0000	175.2389	0.101189	0.1619	0.40995	0.792974	DIS3L2	129563	DIS3 like 3'-5' exoribonuclease 2
ENSG0000	90.36495	0.094891	0.197597	0.409996	0.792979	ASB7	140460	ankyrin repeat and SOCS box containing 7
ENSG0000	1755.371	-0.09362	0.148327	0.41012	0.793135	KLHL21	9903	kelch like family member 21
ENSG0000	2.374341	0.036258	0.2338	0.410234	0.793178	PJVK	494513	pejvakin
ENSG0000	47.66395	0.100577	0.172116	0.410257	0.793178	IQCG	84223	IQ motif containing G
ENSG0000	109.5592	-0.09816	0.1827	0.410272	0.793178	FDXR	2232	ferredoxin reductase
ENSG0000	789.1239	0.083556	0.117824	0.410408	0.793357	CASP2	835	caspase 2
ENSG0000	2.733747	0.03248	0.232638	0.41046	0.793373	MTND5P2	1.01E+08	MT-ND5 pseudogene 2
ENSG0000	77.93942	0.086846	0.209536	0.411225	0.793429	NDC1	55706	NDC1 transmembrane nucleoporin
ENSG0000	134.933	-0.09797	0.169973	0.411179	0.793429	GCLM	2730	glutamate-cysteine ligase modifier subunit
ENSG0000	2303.009	-0.09034	0.136616	0.411011	0.793429	TMBIM1	64114	transmembrane BAX inhibitor motif containing 1
ENSG0000	115.4811	0.095897	0.150923	0.411205	0.793429	SCLY	51540	selenocysteine lyase
ENSG0000	590.6282	-0.09685	0.177061	0.410954	0.793429	UBP1	7342	upstream binding protein 1
ENSG0000	169.5784	0.083934	0.201839	0.411184	0.793429	ABCE1	6059	ATP binding cassette subfamily E member 1
ENSG0000	59.47836	0.09406	0.18359	0.411104	0.793429	EPB41L2	2037	erythrocyte membrane protein band 4.1 like 2
ENSG0000	937.8564	-0.09672	0.173936	0.411224	0.793429	TAB2	23118	TGF-beta activated kinase 1 (MAP3K7) binding protein 2
ENSG0000	2.947881	-0.02162	0.232803	0.410952	0.793429	NA	NA	NA
ENSG0000	84.08884	0.097908	0.161018	0.410644	0.793429	TYW1	55253	tRNA-yW synthesizing protein 1 homolog
ENSG0000	5.875935	0.056189	0.229643	0.411099	0.793429	NA	NA	NA
ENSG0000	4.169672	0.048242	0.2313	0.411036	0.793429	NA	NA	NA
ENSG0000	77.07203	-0.09776	0.163254	0.410926	0.793429	BCDIN3D	144233	BCDIN3 domain containing RNA methyltransferase
ENSG0000	115.051	-0.09648	0.158784	0.410543	0.793429	LINC01599	196913	long intergenic non-protein coding RNA 1599
ENSG0000	2.802679	0.041923	0.232124	0.411146	0.793429	NA	NA	NA
ENSG0000	3127.425	0.093804	0.14547	0.410755	0.793429	GPX4	2879	glutathione peroxidase 4
ENSG0000	9.245497	0.063722	0.227356	0.411103	0.793429	NA	NA	NA
ENSG0000	630.1393	0.088348	0.136189	0.411493	0.793863	SNU13	4809	small nuclear ribonucleoprotein 13
ENSG0000	248.2241	0.090663	0.137122	0.411597	0.793896	MTCH2	23788	mitochondrial carrier 2
ENSG0000	156.9853	-0.09624	0.156686	0.411569	0.793896	SPRYD4	283377	SPRY domain containing 4
ENSG0000	614.461	0.076727	0.103168	0.411641	0.793897	CIAO1	9391	cytosolic iron-sulfur assembly component 1
ENSG0000	3005.765	-0.09627	0.162607	0.411798	0.793949	SLC25A44	9673	solute carrier family 25 member 44
ENSG0000	5.542114	0.036586	0.23135	0.411733	0.793949	GOLGA6L9	440295	golgin A6 family like 9
ENSG0000	15619.53	-0.09602	0.156386	0.411791	0.793949	ARHGAP45	23526	Rho GTPase activating protein 45
ENSG0000	4.161484	0.046593	0.232002	0.411907	0.794076	NA	NA	NA
ENSG0000	156.6773	-0.08731	0.128421	0.411977	0.794128	MRPL20-A	148413	MRPL20 antisense RNA 1

ENSG0000	124.737	-0.09777	0.175339	0.412104	0.794229	TOX	9760	thymocyte selection associated high mobility group box
ENSG0000	705.2791	0.099576	0.18274	0.412116	0.794229	SSH1	54434	slingshot protein phosphatase 1
ENSG0000	2.886939	0.024496	0.232507	0.412326	0.794299	NA	NA	NA
ENSG0000	6.106142	-0.06101	0.229225	0.412238	0.794299	NA	NA	NA
ENSG0000	1929.759	0.108438	0.181808	0.412314	0.794299	PLCG2	5336	phospholipase C gamma 2
ENSG0000	200.5825	-0.08931	0.132868	0.412248	0.794299	DEPDC5	9681	DEP doma GATOR1 subcomplex subunit
ENSG0000	265.9036	-0.09801	0.17576	0.412524	0.794496	PDE4B	5142	phosphodiesterase 4B
ENSG0000	2.51822	0.044521	0.233107	0.412645	0.794496	DCLK2	166614	doublecortin like kinase 2
ENSG0000	457.3743	0.091426	0.19468	0.412639	0.794496	ZNF33A	7581	zinc finger protein 33A
ENSG0000	847.1455	-0.0793	0.11049	0.412545	0.794496	CRTC3	64784	CREB regulated transcription coactivator 3
ENSG0000	3335.616	-0.09727	0.174213	0.412616	0.794496	MINK1	50488	misshapen like kinase 1
ENSG0000	915.836	0.083887	0.137537	0.412865	0.794528	MAT2A	4144	methionine adenosyltransferase 2A
ENSG0000	5.840485	-0.04474	0.230555	0.412868	0.794528	NA	NA	NA
ENSG0000	1623.113	-0.09691	0.162317	0.412922	0.794528	CD63	967	CD63 molecule
ENSG0000	79.2968	0.106549	0.184959	0.412879	0.794528	VEZT	55591	vezatin adherens junctions transmembrane protein
ENSG0000	104.4572	-0.0969	0.184897	0.41279	0.794528	TRIM13	10206	tripartite motif containing 13
ENSG0000	18.36182	-0.06441	0.226069	0.412838	0.794528	NA	NA	NA
ENSG0000	11.10664	0.064838	0.226093	0.413064	0.794634	FIGNL1	63979	fidgetin like 1
ENSG0000	523.2339	-0.08989	0.136403	0.413059	0.794634	XPNPEP1	7511	X-prolyl aminopeptidase 1
ENSG0000	703.068	-0.09844	0.156367	0.413531	0.794782	ZBTB48	3104	zinc finger and BTB domain containing 48
ENSG0000	1460.66	0.095617	0.149104	0.413257	0.794782	NCL	4691	nucleolin
ENSG0000	496.2804	-0.08816	0.123694	0.413409	0.794782	EMC3	55831	ER membrane protein complex subunit 3
ENSG0000	192.0281	-0.08843	0.128189	0.413517	0.794782	ZNF707	286075	zinc finger protein 707
ENSG0000	1917.776	-0.09523	0.184175	0.413497	0.794782	HK1	3098	hexokinase 1
ENSG0000	300.3502	-0.09164	0.142542	0.413444	0.794782	SUDS3	64426	SDS3 hom SIN3A corepressor complex component
ENSG0000	6.775778	0.061655	0.228218	0.413476	0.794782	NA	NA	NA
ENSG0000	4.350631	0.028885	0.232068	0.413207	0.794782	NA	NA	NA
ENSG0000	903.1474	0.097549	0.161816	0.413436	0.794782	GPCPD1	56261	glycerophosphocholine phosphodiesterase 1
ENSG0000	51.0937	0.087724	0.20948	0.4138	0.794798	PPIL3	53938	peptidylprolyl isomerase like 3
ENSG0000	29.48359	0.076436	0.219839	0.413741	0.794798	ZDHHC21	340481	zinc finger DHHC-type palmitoyltransferase 21
ENSG0000	9.728007	0.07721	0.222947	0.413722	0.794798	ZNF420	147923	zinc finger protein 420
ENSG0000	3.174901	0.041435	0.232947	0.413683	0.794798	TMEM145	284339	transmembrane protein 145
ENSG0000	132.0135	-0.09699	0.180596	0.413768	0.794798	ZNF831	128611	zinc finger protein 831
ENSG0000	1786.186	-0.08995	0.164179	0.413798	0.794798	ARSA	410	arylsulfatase A
ENSG0000	14.39385	-0.07682	0.220953	0.414011	0.794955	NKIRAS1	28512	NFKB inhibitor interacting Ras like 1
ENSG0000	18.17648	-0.08454	0.215701	0.414055	0.794955	KLLN	1E+08	killin p53 regulated DNA replication inhibitor
ENSG0000	72.54624	0.095872	0.161299	0.413971	0.794955	LENG8-AS:	1.04E+08	LENG8 antisense RNA 1
ENSG0000	730.9958	-0.0972	0.156146	0.414017	0.794955	ESS2	8220	ess-2 splicing factor homolog
ENSG0000	115.1136	0.093887	0.149961	0.414129	0.795013	EXOSC2	23404	exosome component 2
ENSG0000	11.54941	0.078755	0.221823	0.414185	0.795038	ACTN1-DT	161159	ACTN1 divergent transcript
ENSG0000	5.499871	0.065806	0.228644	0.414428	0.795421	GTSF1L	149699	gametocyte specific factor 1 like
ENSG0000	982.1749	-0.0886	0.135213	0.414764	0.795816	SHC1	6464	SHC adaptor protein 1
ENSG0000	195.1429	0.091086	0.146721	0.414757	0.795816	DPH7	92715	diphthamide biosynthesis 7
ENSG0000	4.883578	-0.06682	0.228539	0.414698	0.795816	KRTAP5-A:	338651	KRTAP5-1/KRTAP5-2 antisense RNA 1
ENSG0000	72.2162	0.096344	0.169839	0.414921	0.796033	MTIF2	4528	mitochondrial translational initiation factor 2
ENSG0000	11.35866	0.063866	0.226493	0.414967	0.796039	RASD1	51655	ras related dexamethasone induced 1
ENSG0000	2236.172	-0.09033	0.146451	0.41508	0.796088	CDIPT	10423	CDP-diacylglycerol--inositol 3-phosphatidyltransferase
ENSG0000	340.2873	0.090321	0.13474	0.41506	0.796088	PROSER3	148137	proline and serine rich 3
ENSG0000	10.36316	0.074307	0.224489	0.415197	0.796229	NSMCE1-D	400512	NSMCE1 divergent transcript
ENSG0000	4236.663	-0.09111	0.179123	0.415247	0.796241	ANXA6	309	annexin A6
ENSG0000	3490.348	-0.0908	0.144443	0.415416	0.796369	UBAP2L	9898	ubiquitin associated protein 2 like
ENSG0000	1855.594	-0.09449	0.186612	0.415488	0.796369	PFKFB4	5210	6-phosphc 6-biphosphatase 4
ENSG0000	4.452562	-0.03403	0.233365	0.415478	0.796369	HCAR1	27198	hydroxycarboxylic acid receptor 1
ENSG0000	965.9795	0.091574	0.15161	0.415403	0.796369	TBC1D22A	25771	TBC1 domain family member 22A
ENSG0000	1259.965	-0.09186	0.149473	0.415565	0.796434	SLC10A3	8273	solute carrier family 10 member 3
ENSG0000	444.5953	0.100157	0.162255	0.415675	0.796479	TET3	200424	tet methylcytosine dioxygenase 3
ENSG0000	50.71594	-0.09688	0.187078	0.415639	0.796479	MTHFD1L	25902	methylenetetrahydrofolate dehydrogenase (NADP+ dependent) 1 like
ENSG0000	43.58459	0.091657	0.202804	0.415892	0.79681	ZNF605	1E+08	zinc finger protein 605
ENSG0000	13.98848	-0.07639	0.221562	0.416107	0.796857	TRGV8	6982	T cell receptor gamma variable 8
ENSG0000	4588.885	0.078129	0.109298	0.416045	0.796857	EEF1D	1936	eukaryotic translation elongation factor 1 delta
ENSG0000	695.8921	0.099167	0.166933	0.416107	0.796857	MROH1	727957	maestro heat like repeat family member 1
ENSG0000	2.638344	0.036059	0.232764	0.41597	0.796857	NA	NA	NA
ENSG0000	9.094116	0.057613	0.229023	0.416134	0.796857	CPNE7	27132	copine 7
ENSG0000	69.06444	0.094003	0.19632	0.416336	0.797161	AURKA	6790	aurora kinase A
ENSG0000	222.5687	-0.08962	0.199134	0.416484	0.797362	PIGB	9488	phosphatidylinositol glycan anchor biosynthesis class B
ENSG0000	855.5685	-0.08781	0.128914	0.41661	0.797436	NA	NA	NA
ENSG0000	32.65003	0.082961	0.21571	0.416606	0.797436	RFC3	5983	replication factor C subunit 3
ENSG0000	15.5543	0.07564	0.222478	0.416737	0.797577	PTX3	5806	pentraxin 3
ENSG0000	8.292119	0.028359	0.23145	0.416908	0.797577	THAP9	79725	THAP domain containing 9
ENSG0000	684.9763	0.090846	0.139163	0.416823	0.797577	SNAPC4	6621	small nuclear RNA activating complex polypeptide 4
ENSG0000	261.6508	0.085575	0.119265	0.416897	0.797577	OSBP	5007	oxysterol binding protein
ENSG0000	1451.665	-0.07641	0.106105	0.416945	0.797577	ARF6	382	ADP ribosylation factor 6
ENSG0000	20774.61	-0.08966	0.137289	0.416894	0.797577	TLE5	166	TLE family transcriptional modulator
ENSG0000	94.4253	0.094327	0.155913	0.417133	0.797854	DIMT1	27292	DIM1 rRNA methyltransferase and ribosome maturation factor
ENSG0000	88.59563	0.092208	0.142156	0.417184	0.797867	PPARA	5465	peroxisome proliferator activated receptor alpha
ENSG0000	400.0178	-0.0847	0.125553	0.417246	0.797904	DCTN5	84516	dynactin subunit 5
ENSG0000	84.18382	0.096218	0.159972	0.417489	0.797949	MRPL36	64979	mitochondrial ribosomal protein L36
ENSG0000	6.12494	-0.04997	0.229775	0.417411	0.797949	NA	NA	NA
ENSG0000	3.695545	-0.04006	0.230002	0.417531	0.797949	NDST2	8509	N-deacetylase and N-sulfotransferase 2
ENSG0000	81.06864	0.095424	0.190943	0.417458	0.797949	LOC10334	1.03E+08	uncharacterized LOC103344931
ENSG0000	34.53248	0.094634	0.186057	0.417446	0.797949	DPP3-DT	1.02E+08	DPP3 divergent transcript
ENSG0000	204.7177	0.094904	0.14609	0.417394	0.797949	ILF3-DT	147727	ILF3 divergent transcript
ENSG0000	2.653388	0.042106	0.23205	0.417723	0.798226	NA	NA	NA
ENSG0000	20.86609	-0.06586	0.223547	0.417807	0.798226	LINC0039{	1.01E+08	long intergenic non-protein coding RNA 398
ENSG0000	18.36711	0.080095	0.218665	0.41779	0.798226	LGALS4	3960	galectin 4
ENSG0000	15.51611	0.084398	0.216049	0.418002	0.798431	TWF2-DT	1.02E+08	TWF2 divergent transcript
ENSG0000	1323.515	-0.08968	0.141249	0.418	0.798431	NDUFA3	4696	NADH:ubiquinone oxidoreductase subunit A3
ENSG0000	2806.883	-0.09633	0.174623	0.418115	0.798564	GLT1D1	144423	glycosyltransferase 1 domain containing 1
ENSG0000	67.83915	0.094663	0.189581	0.418421	0.798632	NBPF8	728841	NBPF member 8
ENSG0000	1741.121	-0.08906	0.134255	0.418359	0.798632	USP4	7375	ubiquitin specific peptidase 4
ENSG0000	77.14684	-0.09428	0.191808	0.418222	0.798632	MSMO1	6307	methylsterol monooxygenase 1
ENSG0000	77.91694	0.0938	0.150077	0.418289	0.798632	MAST4	375449	microtubule associated serine/threonine kinase family member 4
ENSG0000	2121.352	0.094504	0.170457	0.418615	0.798632	UBE2J1	51465	ubiquitin conjugating enzyme E2 J1
ENSG0000	16.65418	-0.07204	0.223115	0.418332	0.798632	TRBV11-2	28581	T cell receptor beta variable 11-2
ENSG0000	1937.249	0.09554	0.160035	0.418476	0.798632	RPS6KA4	8986	ribosomal protein S6 kinase A4
ENSG0000	4.786208	0.0341	0.231179	0.418674	0.798632	NA	NA	NA

ENSG0000	2.527161	0.038904	0.232932	0.418659	0.798632	NA	NA	NA
ENSG0000	161.1255	0.095617	0.159539	0.418501	0.798632	BCL2L12	83596	BCL2 like 12
ENSG0000	301.1455	-0.09174	0.145772	0.418635	0.798632	GART	2618	phosphori phosphor phosphoribosylaminoimidazole synthetase
ENSG0000	317.2889	-0.08826	0.132991	0.418483	0.798632	PI4KAP1	728233	phosphatidylinositol 4-kinase alpha pseudogene 1
ENSG0000	858.5511	0.088606	0.131039	0.418765	0.798724	DOT1L	84444	DOT1 like histone lysine methyltransferase
ENSG0000	24.70323	-0.08706	0.211101	0.418875	0.798849	JAKMIP2	9832	janus kinase and microtubule interacting protein 2
ENSG0000	18.27518	0.073431	0.221355	0.418925	0.798861	PLEKHA8P	51054	pleckstrin homology domain containing A8 pseudogene 1
ENSG0000	1861.373	-0.08879	0.161682	0.419012	0.798945	TAF6	6878	TATA-box binding protein associated factor 6
ENSG0000	4.363645	-0.0469	0.231079	0.41908	0.798992	ZDBF2	57683	zinc finger DBF-type containing 2
ENSG0000	3.172693	0.02611	0.232298	0.419172	0.79901	NA	NA	NA
ENSG0000	49.31097	0.095274	0.190338	0.419177	0.79901	GTPBP10	85865	GTP binding protein 10
ENSG0000	629.9689	-0.08902	0.193601	0.419236	0.799039	SLC40A1	30061	solute carrier family 40 member 1
ENSG0000	9.07846	0.06401	0.227244	0.419281	0.799041	NA	NA	NA
ENSG0000	12.9451	-0.07583	0.221162	0.419385	0.799148	NA	NA	NA
ENSG0000	41.9849	0.091254	0.202997	0.419424	0.799148	HCG11	493812	HLA complex group 11
ENSG0000	135.7534	0.095604	0.162378	0.419551	0.799224	SMIM10L1	1E+08	small integral membrane protein 10 like 1
ENSG0000	134.5362	0.077023	0.217673	0.419545	0.799224	USP16	10600	ubiquitin specific peptidase 16
ENSG0000	12.28552	-0.05239	0.228457	0.419672	0.799371	NA	NA	NA
ENSG0000	100.7002	0.084637	0.208194	0.419746	0.799428	PLEKHF2	79666	pleckstrin homology and FYVE domain containing 2
ENSG0000	155.3401	-0.08629	0.205283	0.419885	0.799492	RNF19A	25897	ring finger RBR E3 ubiquitin protein ligase
ENSG0000	78.64697	0.095313	0.189951	0.41991	0.799492	SLC18A2-A	1.05E+08	SLC18A2 antisense RNA 1
ENSG0000	40.21089	-0.0971	0.176077	0.419894	0.799492	PDPR2P	283922	pyruvate c pseudogene
ENSG0000	108.4713	0.09617	0.165836	0.420049	0.799624	IBA57	200205	iron-sulfur cluster assembly factor IBA57
ENSG0000	5.21786	0.061336	0.228902	0.420067	0.799624	NA	NA	NA
ENSG0000	397.6144	0.096821	0.173029	0.420178	0.799753	RNF11	26994	ring finger protein 11
ENSG0000	34.40872	0.094345	0.196189	0.420264	0.799833	CACNA1F	778	calcium voltage-gated channel subunit alpha1 F
ENSG0000	144.9053	0.095435	0.16086	0.42033	0.799876	CD72	971	CD72 molecule
ENSG0000	30.53043	0.097495	0.18552	0.420454	0.799919	TTN	7273	titin
ENSG0000	5.251464	0.051847	0.230423	0.420483	0.799919	NA	NA	NA
ENSG0000	2.568198	0.036026	0.23227	0.420463	0.799919	NA	NA	NA
ENSG0000	3.682072	0.046555	0.231244	0.420541	0.799945	NA	NA	NA
ENSG0000	2.469275	0.032874	0.232789	0.420732	0.800226	NA	NA	NA
ENSG0000	632.8848	0.092715	0.149149	0.420916	0.800492	RAN	5901	RAN member RAS oncogene family
ENSG0000	3.223336	-0.05121	0.232283	0.421184	0.800503	H4C9	8294	H4 clustered histone 9
ENSG0000	337.2958	0.093554	0.201584	0.421092	0.800503	RNF141	50862	ring finger protein 141
ENSG0000	240.5197	-0.08608	0.12827	0.421075	0.800503	RNF214	257160	ring finger protein 214
ENSG0000	25.29823	-0.09051	0.205115	0.421147	0.800503	TM9SF1	10548	transmembrane 9 superfamily member 1
ENSG0000	8257.509	0.091444	0.142682	0.421073	0.800503	HNRNPUL	11100	heterogeneous nuclear ribonucleoprotein U like 1
ENSG0000	208.6881	-0.09593	0.181369	0.421113	0.800503	LDOC1	23641	LDOC1 regulator of NFKB signaling
ENSG0000	71.53001	0.0968	0.175875	0.421411	0.800769	SLC16A1-A	1.01E+08	SLC16A1 antisense RNA 1
ENSG0000	3.871967	-0.03853	0.231898	0.421449	0.800769	TF	7018	transferrin
ENSG0000	402.7916	0.091737	0.151885	0.421455	0.800769	ABL1	25	ABL proto- non-receptor tyrosine kinase
ENSG0000	10.43352	0.073549	0.223244	0.421532	0.800779	NA	NA	NA
ENSG0000	149.3635	0.09098	0.14096	0.421548	0.800779	UBR7	55148	ubiquitin protein ligase E3 component n-recognin 7
ENSG0000	11.98879	-0.0472	0.229375	0.421824	0.800864	PADI6	353238	peptidyl arginine deiminase 6
ENSG0000	234.651	0.093549	0.156518	0.421716	0.800864	SEC24B	10427	SEC24 hon COPII coat complex component
ENSG0000	25.44879	-0.08714	0.209246	0.42197	0.800864	NA	NA	NA
ENSG0000	111.5981	-0.09572	0.168059	0.421953	0.800864	MTRF1L	54516	mitochondrial translation release factor 1 like
ENSG0000	14.3173	0.063283	0.225661	0.421732	0.800864	GPAM	57678	glycerol-3- mitochondrial
ENSG0000	2361.963	0.094087	0.167842	0.421885	0.800864	EML3	256364	EMAP like 3
ENSG0000	27.28584	0.088105	0.208676	0.421678	0.800864	PRIM1	5557	DNA primase subunit 1
ENSG0000	227.7883	-0.08516	0.124314	0.421981	0.800864	RDH11	51109	retinol dehydrogenase 11
ENSG0000	4.91486	-0.06378	0.228248	0.421986	0.800864	FKBP10	60681	FKBP prolyl isomerase 10
ENSG0000	18.12711	0.074329	0.220712	0.422332	0.801371	NA	NA	NA
ENSG0000	2401.12	-0.09311	0.185336	0.422359	0.801371	NLRP12	91662	NLR family pyrin domain containing 12
ENSG0000	3.688697	0.036637	0.232624	0.422385	0.801371	LCDR	1E+08	lysosome cell death regulator
ENSG0000	465.6334	0.091412	0.146134	0.422524	0.801512	MBD4	8930	methyl-Cp DNA glycosylase
ENSG0000	359.7329	0.08565	0.202599	0.422546	0.801512	S100P	6286	S100 calcium binding protein P
ENSG0000	80.99752	0.087163	0.205783	0.422908	0.801785	ANGEL2	90806	angel homolog 2
ENSG0000	9.632984	-0.07954	0.220286	0.422772	0.801785	PF4	5196	platelet factor 4
ENSG0000	3.661415	0.043821	0.23137	0.422859	0.801785	NA	NA	NA
ENSG0000	136.8752	-0.09425	0.174169	0.422802	0.801785	HOXB4	3214	homeobox B4
ENSG0000	52.05143	0.088741	0.205519	0.422909	0.801785	FRMPD3	84443	FERM and PDZ domain containing 3
ENSG0000	103.1518	0.092188	0.147319	0.422956	0.801791	SH3PXD2A	9644	SH3 and PX domains 2A
ENSG0000	228.0643	0.086611	0.150325	0.42303	0.801849	PEX11B	8799	peroxisomal biogenesis factor 11 beta
ENSG0000	86.00122	0.095363	0.178444	0.423317	0.801897	REV1	51455	REV1 DNA directed polymerase
ENSG0000	26.57415	-0.08637	0.209816	0.423246	0.801897	NA	NA	NA
ENSG0000	10.86871	0.071473	0.223237	0.423269	0.801897	NA	NA	NA
ENSG0000	5.29193	0.045173	0.230443	0.423242	0.801897	NA	NA	NA
ENSG0000	22.25116	0.084304	0.209216	0.4232	0.801897	BMERB1	89927	bMERB domain containing 1
ENSG0000	43.73431	-0.07609	0.217324	0.423318	0.801897	NA	NA	NA
ENSG0000	8.84052	-0.06293	0.226254	0.423711	0.801974	TIE1	7075	tyrosine kinase with immunoglobulin like and EGF like domains 1
ENSG0000	37.67424	-0.09276	0.196432	0.423785	0.801974	HSPB11	51668	heat shock protein family B (small) member 11
ENSG0000	8.242583	0.068264	0.226125	0.423677	0.801974	ASTL	431705	astacin like metalloendopeptidase
ENSG0000	31.0416	0.0879	0.207497	0.423682	0.801974	LOC10029	1E+08	uncharacterized LOC100294145
ENSG0000	227.2848	0.093077	0.148535	0.423694	0.801974	CDC5L	988	cell division cycle 5 like
ENSG0000	31.57151	-0.08808	0.207234	0.423841	0.801974	LPAL2	80350	lipoprotei pseudogene
ENSG0000	74.64576	0.093206	0.193242	0.423826	0.801974	VPS13A	23230	vacuolar protein sorting 13 homolog A
ENSG0000	852.0952	0.092872	0.195345	0.423411	0.801974	MS4A1	931	membrane spanning 4-domains A1
ENSG0000	2.523788	0.03689	0.231691	0.423547	0.801974	NA	NA	NA
ENSG0000	5.171418	0.045315	0.230392	0.423554	0.801974	PRELID3A	10650	PRELI domain containing 3A
ENSG0000	14.41568	0.088472	0.210582	0.423478	0.801974	MAPK12	6300	mitogen-activated protein kinase 12
ENSG0000	2255.455	0.089109	0.173256	0.423913	0.802028	HLA-DRB1	3123	major hist class II DR beta 1
ENSG0000	24.94493	0.095094	0.195266	0.424042	0.802135	LOC10798	1.08E+08	uncharacterized LOC107986412
ENSG0000	82.33306	-0.09522	0.175341	0.424057	0.802135	SLX4IP	128710	SLX4 interacting protein
ENSG0000	241.2592	-0.09027	0.142076	0.424443	0.802207	METTL13	51603	methyltrai eEF1A lysine and N-terminal methyltransferase
ENSG0000	740.2182	0.089941	0.141203	0.424264	0.802207	GALNT2	2590	polypeptide N-acetylgalactosaminyltransferase 2
ENSG0000	89.76596	0.095991	0.182004	0.424391	0.802207	ANAPC1	64682	anaphase promoting complex subunit 1
ENSG0000	382.2904	0.09502	0.158321	0.424445	0.802207	FBXW2	26190	F-box and WD repeat domain containing 2
ENSG0000	24.44012	0.085635	0.211845	0.424168	0.802207	KIF21A	55605	kinesin family member 21A
ENSG0000	65.1008	0.086268	0.207824	0.424355	0.802207	RAP2A	5911	RAP2A member of RAS oncogene family
ENSG0000	517.6634	-0.09117	0.131566	0.42439	0.802207	C19orf47	126526	chromosome 19 open reading frame 47
ENSG0000	74.43502	-0.09536	0.177932	0.424206	0.802207	ZNF71	58491	zinc finger protein 71
ENSG0000	168.2135	-0.09621	0.170343	0.42455	0.802323	PSENEN	55851	presenilin gamma-secretase subunit
ENSG0000	113.2591	0.092707	0.184287	0.424686	0.802323	ACP3	55	acid phosphatase 3
ENSG0000	5.843724	0.062177	0.2281	0.424605	0.802323	NA	NA	NA

ENSG0000	949.6509	-0.09598	0.162759	0.424717	0.802323	PIAS4	51588	protein inhibitor of activated STAT 4
ENSG0000	44.26361	0.096189	0.179493	0.424726	0.802323	ZNF529-AS	1.02E+08	ZNF529 antisense RNA 1
ENSG0000	1595.845	0.093466	0.14385	0.424824	0.802343	INTS11	54973	integrator complex subunit 11
ENSG0000	235.1773	-0.08743	0.131451	0.42481	0.802343	RRP8	23378	ribosomal RNA processing 8
ENSG0000	2950.513	-0.09021	0.192888	0.425221	0.802474	SLC9A1	6548	solute carrier family 9 member A1
ENSG0000	251.9622	-0.09623	0.166143	0.425168	0.802474	SLC5A6	8884	solute carrier family 5 member 6
ENSG0000	326.4946	0.082878	0.122811	0.425065	0.802474	SUCLG1	8802	succinate-CoA ligase GDP/ADP-forming subunit alpha
ENSG0000	4.937463	-0.0535	0.231328	0.425205	0.802474	NA	NA	NA
ENSG0000	5.0302	0.048716	0.230258	0.425273	0.802474	CCDC162P	221262	coiled-coil pseudogene
ENSG0000	30.26364	-0.0833	0.211638	0.425463	0.802474	TRGV7	6981	T cell receptor gamma variable 7 (pseudogene)
ENSG0000	6.697622	0.052639	0.235	0.425267	0.802474	NA	NA	NA
ENSG0000	108.074	0.09511	0.17942	0.425377	0.802474	CHD4	1108	chromodomain helicase DNA binding protein 4
ENSG0000	1252.139	-0.08903	0.126404	0.424973	0.802474	DAD1	1603	defender against cell death 1
ENSG0000	57.9525	-0.09431	0.165695	0.42541	0.802474	CCDC9B	388115	coiled-coil domain containing 9B
ENSG0000	7.007309	0.058578	0.229243	0.425382	0.802474	NA	NA	NA
ENSG0000	4.049775	0.032218	0.232282	0.425185	0.802474	NA	NA	NA
ENSG0000	279.0036	-0.08798	0.131661	0.425439	0.802474	RAE1	8480	ribonucleic acid export 1
ENSG0000	140.911	-0.09387	0.159797	0.425778	0.802512	UTP25	27042	UTP25 small subunit processome component
ENSG0000	48.26383	0.076662	0.215996	0.425981	0.802512	NAT8B	51471	N-acetyltra gene/pseudogene)
ENSG0000	59.95407	0.060292	0.224769	0.425942	0.802512	IGKV2D-40	28878	immunoglobulin kappa variable 2D-40
ENSG0000	20.56494	-0.08126	0.21551	0.425795	0.802512	PAQR3	152559	progestin and adipoQ receptor family member 3
ENSG0000	14.41364	0.06875	0.223421	0.426009	0.802512	CYP21A1P	1590	cytochrom pseudogene
ENSG0000	521.5187	0.090453	0.143826	0.425685	0.802512	COQ4	51117	coenzyme Q4
ENSG0000	92.77468	-0.09575	0.175203	0.425885	0.802512	IL2RA	3559	interleukin 2 receptor subunit alpha
ENSG0000	172.2416	0.093663	0.156985	0.425862	0.802512	TIMM23	1E+08	translocase of inner mitochondrial membrane 23
ENSG0000	103.117	-0.09166	0.147282	0.425886	0.802512	CSTF3	1479	cleavage stimulation factor subunit 3
ENSG0000	866.2071	0.094424	0.17083	0.425813	0.802512	TMED2	10959	transmembrane p24 trafficking protein 2
ENSG0000	125.0224	-0.09363	0.187216	0.425905	0.802512	NA	NA	NA
ENSG0000	1184.998	-0.08837	0.131619	0.425989	0.802512	NA	NA	NA
ENSG0000	1428.75	-0.07497	0.103301	0.426408	0.802687	TMEM250	90120	transmembrane protein 250
ENSG0000	9.271507	0.06517	0.225684	0.426211	0.802687	BMS1P4	729096	BMS1 pseudogene 4
ENSG0000	11.23636	-0.05623	0.227528	0.426254	0.802687	NA	NA	NA
ENSG0000	767.63	0.088883	0.141284	0.426359	0.802687	PIGQ	9091	phosphatidylinositol glycan anchor biosynthesis class Q
ENSG0000	78.03221	-0.09159	0.195076	0.426385	0.802687	ATF7IP2	80063	activating transcription factor 7 interacting protein 2
ENSG0000	210.5459	-0.09301	0.166025	0.426367	0.802687	TP53INP2	58476	tumor protein p53 inducible nuclear protein 2
ENSG0000	99.95825	0.094175	0.164075	0.426365	0.802687	NA	NA	NA
ENSG0000	2.785881	-0.01838	0.232344	0.427035	0.803018	RNU6-536	1.06E+08	RNA U6 small r pseudogene
ENSG0000	15.73214	-0.0787	0.218455	0.426777	0.803018	NEK2	4751	NIMA related kinase 2
ENSG0000	15.2089	-0.07087	0.222455	0.427102	0.803018	NA	NA	NA
ENSG0000	8.475379	0.068452	0.225359	0.427099	0.803018	MIR3936H	553103	MIR3936 host gene
ENSG0000	63.2891	0.093767	0.188211	0.426913	0.803018	RFX3	5991	regulatory factor X3
ENSG0000	169.0337	0.086877	0.197448	0.426893	0.803018	EBLN3P	1.01E+08	endogeno pseudogene
ENSG0000	18.72956	0.078505	0.217805	0.42711	0.803018	ALG11	440138	ALG11 alp 2-mannosyltransferase
ENSG0000	140.5992	0.094666	0.171553	0.426996	0.803018	NA	NA	NA
ENSG0000	146.6493	0.094279	0.164198	0.426874	0.803018	ZFP3	124961	ZFP3 zinc finger protein
ENSG0000	134.4104	0.092276	0.146645	0.426947	0.803018	ZNF562	54811	zinc finger protein 562
ENSG0000	10.9149	0.050541	0.228427	0.426915	0.803018	NA	NA	NA
ENSG0000	3.34224	0.0215	0.233207	0.42672	0.803018	UMODL1-A	150147	UMODL1 antisense RNA 1
ENSG0000	123.482	-0.08733	0.203277	0.427529	0.803476	SF3B6	51639	splicing factor 3b subunit 6
ENSG0000	300.4173	0.093905	0.175011	0.427406	0.803476	SPAG9	9043	sperm associated antigen 9
ENSG0000	8.736885	0.079497	0.220103	0.427485	0.803476	ZNF69	7620	zinc finger protein 69
ENSG0000	38.55592	0.08882	0.205284	0.427489	0.803476	LOC10272	1.03E+08	periodic tryptophan protein 2 homolog
ENSG0000	48.85918	0.09424	0.186431	0.427825	0.803581	WDR53	348793	WD repeat domain 53
ENSG0000	2.3302	0.025324	0.23215	0.427721	0.803581	NA	NA	NA
ENSG0000	6.405337	0.058209	0.228201	0.427789	0.803581	STIM1-AS1	1.05E+08	STIM1 antisense RNA 1
ENSG0000	39.14999	-0.0878	0.210697	0.42765	0.803581	NA	NA	NA
ENSG0000	12906.23	-0.09265	0.161661	0.427682	0.803581	ARHGAP9	64333	Rho GTPase activating protein 9
ENSG0000	186.4812	0.092901	0.155395	0.427848	0.803581	CHST14	113189	carbohydrate sulfotransferase 14
ENSG0000	15.16723	0.083786	0.21397	0.427896	0.803587	SRFBP1	153443	serum response factor binding protein 1
ENSG0000	225.6298	-0.09102	0.150056	0.428128	0.803622	FRYL	285527	FRY like transcription coactivator
ENSG0000	1032.236	0.086544	0.133148	0.428065	0.803622	KDM3B	51780	lysine demethylase 3B
ENSG0000	101.8177	0.092829	0.160198	0.428033	0.803622	ALKBH3	221120	alkB homc alpha-ketoglutarate dependent dioxygenase
ENSG0000	2751.619	-0.08321	0.125138	0.428133	0.803622	NADSYN1	55191	NAD synthetase 1
ENSG0000	5462.242	-0.09279	0.163427	0.428029	0.803622	MAP2K3	5606	mitogen-activated protein kinase kinase 3
ENSG0000	6.28607	0.063782	0.227243	0.429258	0.803759	NA	NA	NA
ENSG0000	94.53352	-0.09363	0.177581	0.42926	0.803759	TM2D1	83941	TM2 domain containing 1
ENSG0000	629.9579	0.087644	0.200099	0.429035	0.803759	STT3B	201595	STT3 oligosaccharyltransferase complex catalytic subunit B
ENSG0000	16.51219	0.088541	0.208155	0.428891	0.803759	NA	NA	NA
ENSG0000	83.35231	0.081332	0.210078	0.42919	0.803759	LYSMD3	116068	LysM domain containing 3
ENSG0000	3.284757	-0.03429	0.232772	0.428523	0.803759	GLP1R	2740	glucagon like peptide 1 receptor
ENSG0000	108.5061	-0.09409	0.16407	0.42914	0.803759	LATS1	9113	large tumor suppressor kinase 1
ENSG0000	19.78625	0.054506	0.226899	0.428662	0.803759	NA	NA	NA
ENSG0000	4.052384	0.031733	0.231707	0.428948	0.803759	NA	NA	NA
ENSG0000	30.37686	0.053297	0.226229	0.428955	0.803759	MRPL50	54534	mitochondrial ribosomal protein L50
ENSG0000	5.762072	-0.04094	0.230658	0.428669	0.803759	PTGR1	22949	prostaglandin reductase 1
ENSG0000	3.822591	0.025765	0.231796	0.429127	0.803759	NA	NA	NA
ENSG0000	400.9644	-0.08307	0.124327	0.428343	0.803759	COX8A	1351	cytochrome c oxidase subunit 8A
ENSG0000	3.855677	0.025993	0.231935	0.428263	0.803759	NA	NA	NA
ENSG0000	660.3331	-0.08651	0.134149	0.428929	0.803759	ASB8	140461	ankyrin repeat and SOCS box containing 8
ENSG0000	64.15636	0.080223	0.212306	0.428863	0.803759	LARP4	113251	La ribonucleoprotein 4
ENSG0000	357.6733	0.083826	0.206567	0.428838	0.803759	RPS29	6235	ribosomal protein S29
ENSG0000	88.71093	-0.09096	0.152823	0.428761	0.803759	MED6	10001	mediator complex subunit 6
ENSG0000	7.79411	0.044262	0.229365	0.428561	0.803759	NA	NA	NA
ENSG0000	1125.964	-0.09084	0.147704	0.429073	0.803759	APBA3	9546	amyloid beta precursor protein binding family A member 3
ENSG0000	2962.766	0.092669	0.141398	0.42847	0.803759	EIF3G	8666	eukaryotic translation initiation factor 3 subunit G
ENSG0000	3193.54	-0.08558	0.159986	0.42917	0.803759	GSK3A	2931	glycogen synthase kinase 3 alpha
ENSG0000	2.772414	-0.0316	0.232906	0.428972	0.803759	BRWD1-AS	1.03E+08	BRWD1 antisense RNA 2
ENSG0000	21.02352	-0.08243	0.213544	0.429215	0.803759	LINC01637	1.02E+08	long intergenic non-protein coding RNA 1637
ENSG0000	16.99969	0.069019	0.222251	0.429467	0.804065	NA	NA	NA
ENSG0000	10.0919	-0.07975	0.218496	0.429556	0.804067	NA	NA	NA
ENSG0000	2.697981	-0.03751	0.233939	0.429513	0.804067	PIWIL2	55124	piwi like RNA-mediated gene silencing 2
ENSG0000	1463.775	0.092309	0.15938	0.429752	0.804278	GPS1	2873	G protein pathway suppressor 1
ENSG0000	578.5246	0.086061	0.129395	0.429756	0.804278	GMEB2	26205	glucocorticoid modulatory element binding protein 2
ENSG0000	94.18772	0.092614	0.157264	0.429843	0.804359	CCDC127	133957	coiled-coil domain containing 127
ENSG0000	272.2857	0.090776	0.149549	0.42989	0.804364	DGCR8	54487	DGCR8 microprocessor complex subunit
ENSG0000	3110.432	0.092793	0.180799	0.429988	0.804466	AP1B1	162	adaptor related protein complex 1 subunit beta 1

ENSG0000	12.40607	0.060388	0.225581	0.430477	0.805063	SND1-IT1	27099	SND1 intronic transcript 1
ENSG0000	5325.619	-0.09324	0.192125	0.43043	0.805063	ITGAM	3684	integrin subunit alpha M
ENSG0000	1193.029	-0.08506	0.123484	0.430406	0.805063	ADCY7	113	adenylate cyclase 7
ENSG0000	172.1292	0.093844	0.177897	0.430483	0.805063	AP5S1	55317	adaptor related protein complex 5 subunit sigma 1
ENSG0000	4574.71	-0.09248	0.166719	0.430534	0.805076	RALY	22913	RALY heterogeneous nuclear ribonucleoprotein
ENSG0000	176.4092	0.092856	0.191044	0.430727	0.805355	SLMAP	7871	sarcolemma associated protein
ENSG0000	1244.074	-0.08993	0.184375	0.430857	0.805433	HYCC2	285172	hyccin PI4KA lipid kinase complex subunit 2
ENSG0000	17.87057	-0.06926	0.222355	0.430835	0.805433	NA	NA	NA
ENSG0000	400.2695	0.091536	0.155065	0.430969	0.805559	IGBP1	3476	immunoglobulin binding protein 1
ENSG0000	12.90153	0.082634	0.215731	0.431071	0.805668	NA	NA	NA
ENSG0000	7.233182	-0.04693	0.229094	0.431268	0.805873	NA	NA	NA
ENSG0000	747.6083	-0.09271	0.173936	0.431241	0.805873	SLC24A4	123041	solute carrier family 24 member 4
ENSG0000	170.5082	0.087372	0.198839	0.431333	0.805891	TMED8	283578	transmembrane p24 trafficking protein family member 8
ENSG0000	521.7727	-0.08927	0.147734	0.431366	0.805891	ZNF526	116115	zinc finger protein 526
ENSG0000	124.2816	-0.09254	0.150738	0.431446	0.805958	LINS1	55180	lines homolog 1
ENSG0000	186.5846	0.085378	0.12923	0.431569	0.805975	NSUN4	387338	NOP2/Sun RNA methyltransferase 4
ENSG0000	99.97582	0.091819	0.188762	0.431631	0.805975	OPN3	23596	opsin 3
ENSG0000	654.8112	-0.09265	0.179277	0.43151	0.805975	UBE2B	7320	ubiquitin conjugating enzyme E2 B
ENSG0000	145.7618	0.091177	0.149562	0.431598	0.805975	MXRA7	439921	matrix remodeling associated 7
ENSG0000	22.42238	0.081903	0.210493	0.431676	0.805976	CNIH3	149111	cornichon family AMPA receptor auxiliary protein 3
ENSG0000	592.1841	0.091571	0.178115	0.431798	0.806122	SLC31A2	1318	solute carrier family 31 member 2
ENSG0000	583.839	-0.08927	0.149082	0.431893	0.806124	ATG9A	79065	autophagy related 9A
ENSG0000	83.28318	0.094043	0.174294	0.431931	0.806124	KANSL2	54934	KAT8 regulatory NSL complex subunit 2
ENSG0000	127.6865	0.092669	0.156555	0.431924	0.806124	ZNF8	7554	zinc finger protein 8
ENSG0000	3.104913	0.019645	0.233345	0.432083	0.806326	NA	NA	NA
ENSG0000	410.3992	-0.09113	0.156171	0.432226	0.806438	KDM5B	10765	lysine demethylase 5B
ENSG0000	1041.013	-0.07583	0.212805	0.432275	0.806438	CES1	1066	carboxylesterase 1
ENSG0000	116.4705	0.078239	0.210696	0.432243	0.806438	MBTD1	54799	mbt domain containing 1
ENSG0000	132.4788	0.092891	0.163925	0.432422	0.806631	MRPL21	219927	mitochondrial ribosomal protein L21
ENSG0000	481.6843	0.088856	0.203266	0.432808	0.806733	SP3	6670	Sp3 transcription factor
ENSG0000	21.10354	0.079724	0.215494	0.432618	0.806733	NDUFAF4	29078	NADH:ubiquinone oxidoreductase complex assembly factor 4
ENSG0000	4.708998	-0.04354	0.231966	0.432829	0.806733	BVES	11149	blood vessel epicardial substance
ENSG0000	6.192178	0.06403	0.227513	0.432683	0.806733	NA	NA	NA
ENSG0000	73.14996	0.089265	0.198307	0.432633	0.806733	NHLRC2	374354	NHL repeat containing 2
ENSG0000	2662.895	0.085854	0.194686	0.432829	0.806733	LRP1	4035	LDL receptor related protein 1
ENSG0000	205.0435	0.092888	0.171909	0.432597	0.806733	HECTD1	25831	HECT domain E3 ubiquitin protein ligase 1
ENSG0000	176.3345	0.089831	0.148858	0.432765	0.806733	TGIF1	7050	TGFB induced factor homeobox 1
ENSG0000	298.6461	-0.09334	0.169912	0.432987	0.806862	SLU7	10569	SLU7 hom splicing factor
ENSG0000	805.4914	-0.0881	0.142427	0.432975	0.806862	PIK3CG	5294	phosphati 5-bisphosphate 3-kinase catalytic subunit gamma
ENSG0000	5.929322	0.059048	0.22769	0.433031	0.806862	PKP2	5318	plakophilin 2
ENSG0000	97.40803	0.090795	0.147921	0.433262	0.807047	RRS1	23212	ribosome biogenesis regulator 1 homolog
ENSG0000	5.428782	0.042836	0.2298	0.433241	0.807047	LOC10042	1E+08	RNA terminal phosphate cyclase like 1 pseudogene
ENSG0000	28.61591	-0.09096	0.197533	0.433201	0.807047	LONRF3	79836	LON peptidase N-terminal domain and ring finger 3
ENSG0000	595.0577	-0.09238	0.156758	0.433392	0.807207	HPSE	10855	heparanase
ENSG0000	40.3678	-0.08065	0.212256	0.433536	0.807241	UGDH	7358	UDP-glucose 6-dehydrogenase
ENSG0000	50.97286	0.087822	0.200154	0.433543	0.807241	MBIP	51562	MAP3K12 binding inhibitory protein 1
ENSG0000	188.9562	-0.08975	0.147934	0.43351	0.807241	TMED1	11018	transmembrane p24 trafficking protein 1
ENSG0000	144.1371	0.090242	0.145154	0.433784	0.807608	ITPRIPL2	162073	ITPRIP like 2
ENSG0000	8.440485	-0.07194	0.222994	0.434083	0.807658	KLKB1	3818	kallikrein B1
ENSG0000	7.74917	-0.05493	0.228204	0.433971	0.807658	NA	NA	NA
ENSG0000	1148.765	-0.08671	0.133998	0.434117	0.807658	C6orf89	221477	chromosome 6 open reading frame 89
ENSG0000	174.0966	-0.08722	0.138554	0.433872	0.807658	ZDHHC4	55146	zinc finger DHHC-type palmitoyltransferase 4
ENSG0000	15.1786	0.073526	0.220766	0.434048	0.807658	NA	NA	NA
ENSG0000	64.27501	-0.07851	0.214838	0.434	0.807658	MYOM2	9172	myomesin 2
ENSG0000	37.00513	0.096904	0.191118	0.434119	0.807658	OCRL	4952	OCRL inositol polyphosphate-5-phosphatase
ENSG0000	587.6703	-0.07789	0.114238	0.434293	0.807898	TRMT112	51504	tRNA methyltransferase activator subunit 11-2
ENSG0000	8.678506	0.068966	0.224448	0.434563	0.80794	RNU6-105	1.06E+08	RNA U6 small r pseudogene
ENSG0000	88.3591	0.0755	0.207871	0.434577	0.80794	ARMT1	79624	acidic residue methyltransferase 1
ENSG0000	18.7417	-0.0814	0.213934	0.43458	0.80794	PARD3	56288	par-3 family cell polarity regulator
ENSG0000	62.81605	-0.08532	0.205253	0.434565	0.80794	B4GALNT3	283358	beta-1 4-N-acetyl-galactosaminyltransferase 3
ENSG0000	5501.465	-0.09436	0.167664	0.434488	0.80794	TIMP2	7077	TIMP metallopeptidase inhibitor 2
ENSG0000	12.28877	-0.04832	0.22824	0.434511	0.80794	INPP5J	27124	inositol polyphosphate-5-phosphatase J
ENSG0000	437.6767	0.086889	0.137551	0.434733	0.808143	PRKAR2A	5576	protein kinase cAMP-dependent type II regulatory subunit alpha
ENSG0000	344.9907	-0.08537	0.134252	0.434937	0.808276	TRA2A	29896	transformer 2 alpha homolog
ENSG0000	5.268941	-0.03454	0.230718	0.434849	0.808276	GNRH2	2797	gonadotropin releasing hormone 2
ENSG0000	13.49291	0.042833	0.228856	0.434909	0.808276	BFSP1	631	beaded filament structural protein 1
ENSG0000	216.567	0.091221	0.187879	0.435145	0.808335	SRSF10	10772	serine and arginine rich splicing factor 10
ENSG0000	128.1636	0.090352	0.190841	0.435127	0.808335	MFN1	55669	mitofusin 1
ENSG0000	106.772	0.090097	0.194596	0.435106	0.808335	TFRC	7037	transferrin receptor
ENSG0000	271.3053	0.089026	0.146715	0.435018	0.808335	GSS	2937	glutathione synthetase
ENSG0000	71.07096	-0.09326	0.17706	0.435225	0.8084	PFKM	5213	phosphofr muscle
ENSG0000	97.23663	0.088393	0.144591	0.435294	0.808447	SNRPA1	6627	small nuclear ribonucleoprotein polypeptide A'
ENSG0000	861.518	0.087583	0.13812	0.435688	0.808462	SLC2A1	6513	solute carrier family 2 member 1
ENSG0000	181.451	-0.08512	0.206228	0.43569	0.808462	IGKV1D-13	28902	immunoglobulin kappa variable 1D-13
ENSG0000	204.3738	-0.0858	0.205435	0.435443	0.808462	PTPN4	5775	protein tyrosine phosphatase non-receptor type 4
ENSG0000	8.699587	0.064054	0.224698	0.435763	0.808462	NA	NA	NA
ENSG0000	165.3864	0.085271	0.128246	0.435554	0.808462	LYAR	55646	Ly1 antibody reactive
ENSG0000	34.24606	0.092759	0.189321	0.435547	0.808462	RPF2	84154	ribosome production factor 2 homolog
ENSG0000	39.44124	0.074608	0.216574	0.435963	0.808462	BET1	10282	Bet1 golgi vesicular membrane trafficking protein
ENSG0000	205.6784	0.086198	0.13206	0.436008	0.808462	RBM28	55131	RNA binding motif protein 28
ENSG0000	14.92056	-0.08218	0.213477	0.435713	0.808462	ADHFE1	137872	alcohol dehydrogenase iron containing 1
ENSG0000	37.10779	0.092452	0.189066	0.435908	0.808462	PHYH	5264	phytanoyl-CoA 2-hydroxylase
ENSG0000	13402.38	-0.08787	0.165909	0.435363	0.808462	MAP3K11	4296	mitogen-activated protein kinase kinase kinase 11
ENSG0000	2816.486	-0.09131	0.159201	0.43584	0.808462	BCL9L	283149	BCL9 like
ENSG0000	15.48002	-0.07596	0.217259	0.435809	0.808462	ZNF19	7567	zinc finger protein 19
ENSG0000	15.22402	-0.07178	0.221226	0.435885	0.808462	NA	NA	NA
ENSG0000	5.152717	0.054742	0.229074	0.436004	0.808462	NA	NA	NA
ENSG0000	1740.816	-0.08841	0.184324	0.435452	0.808462	GAS2L1	10634	growth arrest specific 2 like 1
ENSG0000	198.5494	-0.09206	0.16799	0.436172	0.808517	NBAS	51594	NBAS subunit of NRZ tethering complex
ENSG0000	9.261056	0.067069	0.227575	0.436138	0.808517	CENPK	64105	centromere protein K
ENSG0000	143.1624	-0.08845	0.142213	0.436252	0.808517	NA	NA	NA
ENSG0000	7.892851	-0.03904	0.230542	0.43609	0.808517	HBG2	3048	hemoglobin subunit gamma 2
ENSG0000	4.372337	0.041731	0.230362	0.436302	0.808517	NA	NA	NA
ENSG0000	2722.599	0.089362	0.149388	0.436301	0.808517	DEF8	54849	differentially expressed in FDCP 8 homolog
ENSG0000	4.014576	-0.04544	0.230733	0.436428	0.808587	GSDME	1687	gasdermin E
ENSG0000	71.65537	0.090128	0.192821	0.436406	0.808587	BLM	641	BLM RecQ like helicase

ENSG0000	4.201687	0.046249	0.229887	0.436746	0.808751	PFN1P2	767846	profilin 1 pseudogene 2
ENSG0000	90.40383	0.091722	0.188096	0.436583	0.808751	COX18	285521	cytochrome c oxidase assembly factor COX18
ENSG0000	373.1814	0.083773	0.129383	0.436752	0.808751	SLC35B2	347734	solute carrier family 35 member B2
ENSG0000	29.67186	-0.07441	0.216885	0.436782	0.808751	GPR180	160897	G protein-coupled receptor 180
ENSG0000	166.9704	0.092615	0.175063	0.436757	0.808751	ATG2B	55102	autophagy related 2B
ENSG0000	11.54519	0.062288	0.225983	0.436656	0.808751	NA	NA	NA
ENSG0000	9.485372	-0.05703	0.227405	0.43689	0.808869	NA	NA	NA
ENSG0000	6.271048	-0.0365	0.230578	0.436943	0.808886	KIR2DP1	554300	killer cell i two Ig domains pseudogene 1
ENSG0000	39.6858	-0.08318	0.20719	0.437412	0.809222	FASTKD3	79072	FAST kinase domains 3
ENSG0000	41.9779	0.087538	0.201144	0.437608	0.809222	ZSCAN9	7746	zinc finger and SCAN domain containing 9
ENSG0000	3128.181	-0.08847	0.147575	0.43744	0.809222	ATF6B	1388	activating transcription factor 6 beta
ENSG0000	132.5677	0.0911	0.156498	0.437684	0.809222	BZW2	28969	basic leucine zipper and W2 domains 2
ENSG0000	906.0919	-0.09015	0.146137	0.437544	0.809222	BRMS1	25855	BRMS1 transcriptional repressor and anoikis regulator
ENSG0000	753.3263	-0.09186	0.166769	0.43741	0.809222	CSAD	51380	cysteine sulfinic acid decarboxylase
ENSG0000	5.359925	0.049182	0.230431	0.437639	0.809222	TSFM	10102	Ts translat mitochondrial
ENSG0000	5.884469	0.044274	0.23011	0.437424	0.809222	SDS	10993	serine dehydratase
ENSG0000	928.5406	0.082355	0.137754	0.437699	0.809222	EIF5	1983	eukaryotic translation initiation factor 5
ENSG0000	179.5999	0.087011	0.138228	0.437524	0.809222	SPAG7	9552	sperm associated antigen 7
ENSG0000	211.8004	-0.08924	0.192056	0.437443	0.809222	GIPC3	126326	GIPC PDZ domain containing family member 3
ENSG0000	9.74559	-0.06529	0.226004	0.437447	0.809222	NOTCH3	4854	notch receptor 3
ENSG0000	243.9404	0.091744	0.16807	0.437325	0.809222	FTX	1E+08	FTX transc XIST regulator
ENSG0000	295.3652	-0.09044	0.189107	0.437764	0.809236	ROPN1L	83853	rhophilin associated tail protein 1 like
ENSG0000	125.949	0.093283	0.170106	0.437795	0.809236	EZH2	2146	enhancer of zeste 2 polycomb repressive complex 2 subunit
ENSG0000	116.4937	0.082644	0.206178	0.438062	0.809648	LTK	4058	leukocyte receptor tyrosine kinase
ENSG0000	16.19786	-0.0686	0.221942	0.438273	0.809956	FANCL	55120	FA complementation group L
ENSG0000	73.51433	0.091819	0.164587	0.438393	0.810097	TRMT6	51605	tRNA methyltransferase 6 non-catalytic subunit
ENSG0000	10.39471	0.043458	0.228841	0.438508	0.810219	NA	NA	NA
ENSG0000	487.6708	0.074138	0.133009	0.438548	0.810219	KLHL36	79786	kelch like family member 36
ENSG0000	11.58032	-0.0535	0.227521	0.438834	0.810242	TRNP1	388610	TMF1 regulated nuclear protein 1
ENSG0000	97.92074	0.092231	0.161098	0.438731	0.810242	NAF1	92345	nuclear assembly factor 1 ribonucleoprotein
ENSG0000	15.13515	-0.07913	0.216894	0.43887	0.810242	PTK7	5754	protein tyrosine kinase 7 (inactive)
ENSG0000	26.87246	-0.09168	0.192641	0.438838	0.810242	NA	NA	NA
ENSG0000	179.1957	-0.09138	0.177996	0.438623	0.810242	FAM89B	23625	family with sequence similarity 89 member B
ENSG0000	56.50953	0.079114	0.210701	0.438804	0.810242	SLC16A7	9194	solute carrier family 16 member 7
ENSG0000	219.1296	-0.08726	0.164247	0.438816	0.810242	B9D2	80776	B9 domain containing 2
ENSG0000	87.03362	-0.07632	0.212049	0.438929	0.81027	LAMTOR3	8649	late endos MAPK and MTOR activator 3
ENSG0000	201.7381	-0.07992	0.211812	0.439124	0.810548	DDX11L16	727856	DEAD/H-box helicase 11 like 16 (pseudogene)
ENSG0000	77.73163	-0.09212	0.172438	0.439858	0.81055	SNHG12	85028	small nucleolar RNA host gene 12
ENSG0000	111.2288	-0.08802	0.19328	0.43983	0.81055	PRKCE	5581	protein kinase C epsilon
ENSG0000	2.556091	0.038462	0.231559	0.439183	0.81055	NA	NA	NA
ENSG0000	34.52777	0.072582	0.217785	0.439681	0.81055	IL15	3600	interleukin 15
ENSG0000	66.08064	-0.09236	0.173056	0.439896	0.81055	GPRIN1	114787	G protein regulated inducer of neurite outgrowth 1
ENSG0000	104.1103	0.088864	0.144004	0.439935	0.81055	BMP1	649	bone morphogenetic protein 1
ENSG0000	333.956	0.093677	0.167431	0.439469	0.81055	NANS	54187	N-acetylneuraminate synthase
ENSG0000	90.25728	0.091629	0.181007	0.439546	0.81055	PBX3	5090	PBX homeobox 3
ENSG0000	5.872321	-0.06541	0.226412	0.43941	0.81055	SPOUT1	51490	SPOUT domain containing methyltransferase 1
ENSG0000	2.91279	0.053258	0.233893	0.439614	0.81055	CYP2C8	1558	cytochrome P450 family 2 subfamily C member 8
ENSG0000	61.16548	-0.09228	0.17571	0.439609	0.81055	MTRF1	9617	mitochondrial translation release factor 1
ENSG0000	424.5585	0.075668	0.21172	0.439445	0.81055	ARGLU1	55082	arginine and glutamate rich 1
ENSG0000	7.097297	-0.0524	0.230315	0.439421	0.81055	TRAV13-2	28670	T cell receptor alpha variable 13-2
ENSG0000	22.15579	-0.0807	0.213752	0.439677	0.81055	NA	NA	NA
ENSG0000	13.99628	0.06472	0.223022	0.43975	0.81055	PRC1-AS1	1.01E+08	PRC1 antisense RNA 1
ENSG0000	14.50736	-0.05733	0.226298	0.439675	0.81055	C17orf99	1E+08	chromosome 17 open reading frame 99
ENSG0000	3.864212	0.047691	0.230431	0.439974	0.81055	MIR1250	1E+08	microRNA 1250
ENSG0000	13.04173	0.069293	0.222052	0.439878	0.81055	NA	NA	NA
ENSG0000	1351.261	-0.08559	0.140008	0.439977	0.81055	RALBP1	10928	ralA binding protein 1
ENSG0000	2865.487	-0.0903	0.163011	0.44001	0.81055	MTMR3	8897	myotubularin related protein 3
ENSG0000	558.8639	-0.08654	0.137144	0.440339	0.81097	MAD2L2	10459	mitotic arrest deficient 2 like 2
ENSG0000	7.8138	-0.06157	0.225687	0.440371	0.81097	NA	NA	NA
ENSG0000	68.215	-0.09192	0.176063	0.440321	0.81097	TP53TG1	11257	TP53 target 1
ENSG0000	3.062276	-0.02877	0.233691	0.440458	0.811049	NA	NA	NA
ENSG0000	60.3217	0.091372	0.175879	0.440594	0.811126	PNO1	56902	partner of NOB1 homolog
ENSG0000	62.18397	0.090188	0.188027	0.440766	0.811126	IRS1	3667	insulin receptor substrate 1
ENSG0000	3.039965	0.033942	0.231208	0.440587	0.811126	C5orf34	375444	chromosome 5 open reading frame 34
ENSG0000	34.96457	-0.07844	0.21218	0.440663	0.811126	RPA3	6119	replication protein A3
ENSG0000	17.12669	0.083041	0.211394	0.440707	0.811126	CCNJ	54619	cyclin J
ENSG0000	16.14101	-0.06066	0.22413	0.440729	0.811126	ATL1	51062	atlastin GTPase 1
ENSG0000	7.413103	0.068107	0.224521	0.440817	0.811139	NA	NA	NA
ENSG0000	5826.104	-0.09091	0.146173	0.440881	0.811176	ANKRD13D	338692	ankyrin repeat domain 13D
ENSG0000	279.017	-0.08381	0.138793	0.441156	0.811263	HMGCL	3155	3-hydroxy-3-methylglutaryl-CoA lyase
ENSG0000	103.7589	0.089743	0.151627	0.441094	0.811263	TOE1	114034	target of E exonuclease
ENSG0000	474.0295	-0.08261	0.129998	0.441183	0.811263	FLAD1	80308	flavin adenine dinucleotide synthetase 1
ENSG0000	38.02506	-0.09294	0.178311	0.441146	0.811263	NA	NA	NA
ENSG0000	2.62778	-0.0229	0.232702	0.441195	0.811263	LOC10272	1.03E+08	WAS/WASL-interacting protein family member 2-like
ENSG0000	733.615	-0.07765	0.115839	0.441015	0.811263	TBCB	1155	tubulin folding cofactor B
ENSG0000	5.597982	0.058428	0.227479	0.441343	0.811448	RBM15-AS	440600	RBM15 antisense RNA 1
ENSG0000	7.653264	0.070327	0.226688	0.441392	0.811448	ANKRD35	148741	ankyrin repeat domain 35
ENSG0000	6772.995	-0.09105	0.181278	0.441493	0.811448	BAG6	7917	BAG cochaperone 6
ENSG0000	4.258994	-0.03886	0.232091	0.441479	0.811448	GJB6	10804	gap junction protein beta 6
ENSG0000	78.18338	0.090411	0.157921	0.441517	0.811448	YBEY	54059	ybeY metalloendoribonuclease
ENSG0000	902.9374	-0.0878	0.149094	0.44166	0.811629	SYNGAP1	8831	synaptic Ras GTPase activating protein 1
ENSG0000	6.256201	0.066461	0.225275	0.441812	0.811746	NA	NA	NA
ENSG0000	1723.479	0.079259	0.120472	0.44177	0.811746	CIRBP	1153	cold inducible RNA binding protein
ENSG0000	569.0156	-0.08716	0.146817	0.441879	0.811789	MRPS18A	55168	mitochondrial ribosomal protein S18A
ENSG0000	47.04859	0.074572	0.215338	0.441982	0.811896	ZMAT1	84460	zinc finger matrin-type 1
ENSG0000	36.06193	0.087947	0.199085	0.442333	0.811917	NA	NA	NA
ENSG0000	25.06558	0.090658	0.194813	0.442529	0.811917	EIF3FP3	339799	eukaryotic translation initiation factor 3 subunit F pseudogene 3
ENSG0000	5.581009	-0.05653	0.227736	0.442686	0.811917	NA	NA	NA
ENSG0000	8.557956	-0.0465	0.228898	0.442413	0.811917	MYOSLID	1.05E+08	myocardin inducer of differentiation
ENSG0000	369.9395	0.090986	0.168303	0.442457	0.811917	TOP2B	7155	DNA topoisomerase II beta
ENSG0000	31.05751	0.05528	0.22539	0.442108	0.811917	MRPL47	57129	mitochondrial ribosomal protein L47
ENSG0000	23.47051	-0.06954	0.219468	0.442656	0.811917	NA	NA	NA
ENSG0000	7.752015	-0.05923	0.226379	0.442139	0.811917	POLR3G	10622	RNA polymerase III subunit G
ENSG0000	27.01043	-0.01156	0.233691	0.442164	0.811917	TREML4	285852	triggering receptor expressed on myeloid cells like 4
ENSG0000	64.15141	-0.09024	0.187159	0.442392	0.811917	MAD1L1	8379	mitotic arrest deficient 1 like 1
ENSG0000	9.045742	0.064522	0.225143	0.44274	0.811917	CLDN23	137075	claudin 23

ENSG0000	1023.945	-0.08426	0.126397	0.442293	0.811917	SIGMAR1	10280	sigma non-opioid intracellular receptor 1
ENSG0000	647.6477	-0.09307	0.168031	0.44238	0.811917	TNFSF8	944	TNF superfamily member 8
ENSG0000	74.9751	0.088198	0.193902	0.442633	0.811917	AASDHPPT	60496	aminoadipate-semialdehyde dehydrogenase-phosphopantetheinyl transferase
ENSG0000	317.0913	-0.08551	0.195859	0.442613	0.811917	GPR65	8477	G protein-coupled receptor 65
ENSG0000	20.42875	-0.08285	0.209661	0.442747	0.811917	LOC10798	1.08E+08	uncharacterized LOC107985074
ENSG0000	13.70143	0.06195	0.224097	0.442652	0.811917	NA	NA	NA
ENSG0000	1857.505	0.096078	0.160836	0.442794	0.811923	PIP5K1C	23396	phosphatidylinositol-4-phosphate 5-kinase type 1 gamma
ENSG0000	114.8586	0.090874	0.162171	0.443115	0.812131	TMEM192	201931	transmembrane protein 192
ENSG0000	90.69918	0.068538	0.218647	0.443101	0.812131	UFL1	23376	UFM1 specific ligase 1
ENSG0000	1370.332	-0.08789	0.199532	0.443051	0.812131	HSP90AA1	3320	heat shock protein 90 alpha family class A member 1
ENSG0000	1357.111	-0.08928	0.153776	0.44307	0.812131	NFE2L1	4779	NFE2 like bZIP transcription factor 1
ENSG0000	5.487089	0.043092	0.229944	0.44313	0.812131	NA	NA	NA
ENSG0000	7.809441	-0.04489	0.229031	0.443178	0.812138	NA	NA	NA
ENSG0000	1413.678	0.081803	0.127935	0.443228	0.812149	ARFGAP1	55738	ADP ribosylation factor GTPase activating protein 1
ENSG0000	144.1676	0.08793	0.194284	0.443649	0.812351	DIPK1A	388650	divergent protein kinase domain 1A
ENSG0000	3.982749	0.029874	0.231354	0.443489	0.812351	CFAP276	127003	cilia and flagella associated protein 276
ENSG0000	24.60377	0.074312	0.216878	0.443631	0.812351	EPB41L5	57669	erythrocyte membrane protein band 4.1 like 5
ENSG0000	3682.256	-0.08476	0.146039	0.443543	0.812351	RAF1	5894	Raf-1 prot serine/threonine kinase
ENSG0000	54.62767	0.091298	0.183257	0.443473	0.812351	ZKSCAN4	387032	zinc finger with KRAB and SCAN domains 4
ENSG0000	4.246287	0.042116	0.230864	0.443642	0.812351	NA	NA	NA
ENSG0000	83.53638	0.087969	0.146152	0.44347	0.812351	SETD4	54093	SET domain containing 4
ENSG0000	5.22014	0.05825	0.228176	0.444352	0.812392	MIR4420	1.01E+08	microRNA 4420
ENSG0000	259.1698	-0.08838	0.18804	0.444381	0.812392	DHCR24	1718	24-dehydrocholesterol reductase
ENSG0000	84.21374	0.0783	0.206967	0.443992	0.812392	POGLUT1	56983	protein O-glucosyltransferase 1
ENSG0000	120.1044	0.089557	0.182407	0.444261	0.812392	SCFD2	152579	sec1 family domain containing 2
ENSG0000	19.91946	0.078398	0.213951	0.44409	0.812392	TERT	7015	telomerase reverse transcriptase
ENSG0000	3.905774	0.04091	0.230587	0.444346	0.812392	NA	NA	NA
ENSG0000	698.5839	0.071059	0.103002	0.443803	0.812392	DUSP22	56940	dual specificity phosphatase 22
ENSG0000	709.363	0.086292	0.139773	0.444379	0.812392	CDC123	8872	cell division cycle 123
ENSG0000	4.305753	0.018125	0.232351	0.443951	0.812392	HTR3A	3359	5-hydroxytryptamine receptor 3A
ENSG0000	1326.58	-0.07038	0.11507	0.443755	0.812392	ZNF384	171017	zinc finger protein 384
ENSG0000	11.44201	-0.06002	0.22594	0.444269	0.812392	NA	NA	NA
ENSG0000	6.3329	-0.03824	0.229857	0.444347	0.812392	NA	NA	NA
ENSG0000	1363.615	-0.08889	0.170689	0.44398	0.812392	RAB31	11031	RAB31 member RAS oncogene family
ENSG0000	7.230373	-0.06011	0.226277	0.444211	0.812392	NA	NA	NA
ENSG0000	2.760166	-0.02896	0.23267	0.443961	0.812392	NA	NA	NA
ENSG0000	164.1358	-0.08109	0.12577	0.443867	0.812392	DNAL4	10126	dynein axonemal light chain 4
ENSG0000	324.0951	0.081735	0.12116	0.444557	0.8125	TBC1D5	9779	TBC1 domain family member 5
ENSG0000	193.2729	0.091917	0.177579	0.444573	0.8125	PTTG1	9232	PTTG1 reg securin
ENSG0000	186.3153	0.089461	0.151618	0.444528	0.8125	NUP43	348995	nucleoporin 43
ENSG0000	2.975961	-0.02395	0.232253	0.44468	0.812614	NA	NA	NA
ENSG0000	2.997774	-0.05315	0.230137	0.444794	0.812742	NA	NA	NA
ENSG0000	4.244119	0.039954	0.231142	0.445023	0.812911	NES	10763	nestin
ENSG0000	82.24005	0.045499	0.229107	0.445488	0.812911	SLC51A	200931	solute carrier family 51 subunit alpha
ENSG0000	28.44427	-0.08992	0.197618	0.445375	0.812911	PEX3	8504	peroxisomal biogenesis factor 3
ENSG0000	371.57	0.085444	0.14016	0.445504	0.812911	PDCD2	5134	programmed cell death 2
ENSG0000	5.40711	0.056489	0.228241	0.445215	0.812911	SCIN	85477	scinderin
ENSG0000	23.86173	-0.07734	0.214152	0.445322	0.812911	NA	NA	NA
ENSG0000	1193.183	0.092202	0.153372	0.445065	0.812911	SEC16A	9919	SEC16 hon endoplasmic reticulum export factor
ENSG0000	89.7178	0.090143	0.187641	0.445155	0.812911	ZMYND11	10771	zinc finger MYND-type containing 11
ENSG0000	574.9182	0.084906	0.13751	0.445137	0.812911	NCAPD2	9918	non-SMC condensin I complex subunit D2
ENSG0000	5983.193	-0.08931	0.174126	0.445281	0.812911	OS9	10956	OS9 endoplasmic reticulum lectin
ENSG0000	46.42396	-0.08885	0.19058	0.445373	0.812911	NA	NA	NA
ENSG0000	448.1146	-0.08718	0.17748	0.445294	0.812911	BCAS3	54828	BCAS3 microtubule associated cell migration factor
ENSG0000	3.058416	0.031352	0.232683	0.44534	0.812911	LINC03048	1E+08	long intergenic non-protein coding RNA 3048
ENSG0000	11812.15	-0.08928	0.170054	0.445508	0.812911	SIRPB1	10326	signal regulatory protein beta 1
ENSG0000	15.21724	-0.07456	0.217338	0.445575	0.812952	LINC00654	149837	long intergenic non-protein coding RNA 654
ENSG0000	115.2768	-0.0651	0.219763	0.445701	0.813102	IGKV1D-12	28903	immunoglobulin kappa variable 1D-12
ENSG0000	96.15277	0.090188	0.166287	0.445775	0.813109	ARMC10	83787	armadillo repeat containing 10
ENSG0000	622.4879	-0.07925	0.123599	0.445794	0.813109	UBE2A	7319	ubiquitin conjugating enzyme E2 A
ENSG0000	3704.631	-0.07894	0.122328	0.445999	0.813321	LMAN2	10960	lectin mannose binding 2
ENSG0000	130.5811	0.070948	0.216261	0.445986	0.813321	CREBZF	58487	CREB/ATF bZIP transcription factor
ENSG0000	90.23705	-0.08965	0.160494	0.446076	0.813381	ZFAND2A	90637	zinc finger AN1-type containing 2A
ENSG0000	502.0193	0.08639	0.124603	0.446125	0.813389	SPOP	8405	speckle type BTB/POZ protein
ENSG0000	131.405	0.083848	0.202284	0.446239	0.813517	EFCAB12	90288	EF-hand calcium binding domain 12
ENSG0000	143.6584	-0.08162	0.133284	0.446491	0.813732	ZKSCAN5	23660	zinc finger with KRAB and SCAN domains 5
ENSG0000	1719.707	-0.08532	0.13573	0.446455	0.813732	RELT	84957	RELT TNF receptor
ENSG0000	35.97485	0.091224	0.183094	0.44649	0.813732	ZNF616	90317	zinc finger protein 616
ENSG0000	52.28717	-0.0908	0.185835	0.447089	0.813773	SWT1	54823	SWT1 RNA endoribonuclease homolog
ENSG0000	4.861833	0.040718	0.230162	0.446757	0.813773	NA	NA	NA
ENSG0000	87.73909	0.089479	0.156307	0.447001	0.813773	PCOLCE	5118	procollagen C-endopeptidase enhancer
ENSG0000	2180.391	0.083027	0.144209	0.44681	0.813773	PRRC2B	84726	proline rich coiled-coil 2B
ENSG0000	1536.6	-0.08675	0.147784	0.447135	0.813773	NDOR1	27158	NADPH dependent diflavin oxidoreductase 1
ENSG0000	1040.263	0.083258	0.137018	0.446778	0.813773	SFSWAP	6433	splicing factor SWAP
ENSG0000	172.4843	0.087678	0.154814	0.447042	0.813773	ANGEL1	23357	angel homolog 1
ENSG0000	210.768	-0.0807	0.196088	0.446902	0.813773	CCPG1	9236	cell cycle progression 1
ENSG0000	88.36939	0.090385	0.177294	0.446912	0.813773	METTL2A	339175	methyltrai methylcytidine
ENSG0000	103.7756	0.088782	0.188163	0.447114	0.813773	NAPG	8774	NSF attachment protein gamma
ENSG0000	478.849	0.079153	0.123342	0.447084	0.813773	ELAVL1	1994	ELAV like RNA binding protein 1
ENSG0000	18.949	-0.0695	0.220351	0.447026	0.813773	NA	NA	NA
ENSG0000	59.86434	-0.08106	0.206525	0.446827	0.813773	LILRB5	10990	leukocyte immunoglobulin like receptor B5
ENSG0000	37.18501	0.090384	0.190173	0.446704	0.813773	TMEM191	84222	transmembrane protein 191A (pseudogene)
ENSG0000	6.014729	-0.04212	0.229782	0.447194	0.813798	NA	NA	NA
ENSG0000	609.2299	-0.08693	0.151272	0.447333	0.813971	TNPO3	23534	transportin 3
ENSG0000	1146.87	0.07505	0.113182	0.448175	0.814034	SLC35E2B	728661	solute carrier family 35 member E2B
ENSG0000	10.84648	-0.05742	0.225901	0.447966	0.814034	DDR2	4921	discoidin domain receptor tyrosine kinase 2
ENSG0000	4.087237	0.035669	0.230699	0.448272	0.814034	CLEC20A	400797	C-type lectin domain containing 20A
ENSG0000	3.097689	-0.04067	0.230766	0.448158	0.814034	NA	NA	NA
ENSG0000	6.990075	0.050809	0.228245	0.448143	0.814034	CRIM1	51232	cysteine rich transmembrane BMP regulator 1
ENSG0000	114.8116	0.07655	0.211722	0.447749	0.814034	RIF1	55183	replication timing regulatory factor 1
ENSG0000	4.450486	0.026154	0.231398	0.448064	0.814034	ITPR1-DT	1.01E+08	ITPR1 divergent transcript
ENSG0000	682.1196	0.079838	0.124686	0.448348	0.814034	BRK1	55845	BRICK1 subunit of SCAR/WAVE actin nucleating complex
ENSG0000	218.6935	0.086364	0.138952	0.448293	0.814034	SDAD1	55153	SDA1 domain containing 1
ENSG0000	4.950365	-0.05614	0.227832	0.447923	0.814034	NA	NA	NA
ENSG0000	9.951601	0.055539	0.226548	0.44818	0.814034	C4B	721	complement C4B (Chido blood group)
ENSG0000	1361.276	-0.08629	0.141957	0.447758	0.814034	SRSF3	6428	serine and arginine rich splicing factor 3

ENSG0000	3274.549	0.086948	0.165364	0.447732	0.814034	RNASET2	8635	ribonuclease T2
ENSG0000	119.6262	-0.08114	0.203442	0.447616	0.814034	NA	NA	NA
ENSG0000	73.21527	0.065243	0.218673	0.447894	0.814034	SHLD2	54537	shieldin complex subunit 2
ENSG0000	3962.397	-0.08008	0.118199	0.448261	0.814034	ACIN1	22985	apoptotic chromatin condensation inducer 1
ENSG0000	492.9409	-0.08466	0.195516	0.447448	0.814034	PPM1A	5494	protein ph Mg2+/Mn2+ dependent 1A
ENSG0000	13.00225	-0.04464	0.228224	0.447891	0.814034	KRT18P61	1E+08	keratin 18 pseudogene 61
ENSG0000	3.902946	-0.03397	0.23117	0.447494	0.814034	GAREM1	64762	GRB2 associated regulator of MAPK1 subtype 1
ENSG0000	4.945762	0.051108	0.22859	0.448267	0.814034	NA	NA	NA
ENSG0000	398.6133	-0.0892	0.163083	0.44839	0.814034	UBOX5	22888	U-box domain containing 5
ENSG0000	2.370447	0.027373	0.232859	0.447803	0.814034	ADNP-AS1	1.02E+08	ADNP antisense RNA 1
ENSG0000	5.535199	0.040776	0.22986	0.448308	0.814034	MTMR8	55613	myotubularin related protein 8
ENSG0000	18.75332	0.077804	0.213291	0.448662	0.814239	NA	NA	NA
ENSG0000	120.2877	-0.08529	0.199152	0.448554	0.814239	RALA	5898	RAS like proto-oncogene A
ENSG0000	504.7809	0.086871	0.1514	0.448814	0.814239	PITRM1	10531	pitrilysin metallopeptidase 1
ENSG0000	10.28091	0.063763	0.224	0.448782	0.814239	PDZD7	79955	PDZ domain containing 7
ENSG0000	60.00437	0.083843	0.202199	0.448741	0.814239	FAM76B	143684	family with sequence similarity 76 member B
ENSG0000	39.48931	-0.07507	0.212468	0.448744	0.814239	TAFA2	338811	TAFA chemokine like family member 2
ENSG0000	3.571393	-0.02912	0.232352	0.448649	0.814239	NA	NA	NA
ENSG0000	2.420314	-0.03718	0.232355	0.448896	0.814308	ZNF471	57573	zinc finger protein 471
ENSG0000	41.34736	0.091151	0.176518	0.44908	0.814531	SNHG32	50854	small nucleolar RNA host gene 32
ENSG0000	13.70434	-0.04608	0.227645	0.449152	0.814531	SLC8A3	6547	solute carrier family 8 member A3
ENSG0000	26.52312	0.08582	0.201774	0.449139	0.814531	ZNF551	90233	zinc finger protein 551
ENSG0000	8.934787	0.062249	0.225472	0.449253	0.814633	ATP8A2	51761	ATPase phospholipid transporting 8A2
ENSG0000	38.63842	0.065951	0.219835	0.449456	0.814683	LRRC40	55631	leucine rich repeat containing 40
ENSG0000	4.374042	0.055139	0.22835	0.449489	0.814683	NA	NA	NA
ENSG0000	188.1789	-0.08502	0.141356	0.449503	0.814683	SLC37A4	2542	solute carrier family 37 member 4
ENSG0000	433.9707	0.083162	0.136695	0.449502	0.814683	GOLGA3	2802	golgin A3
ENSG0000	135.0606	-0.08953	0.17676	0.449376	0.814683	RNASEK	440400	ribonuclease K
ENSG0000	132.0906	-0.08509	0.141429	0.449885	0.81471	C1orf174	339448	chromosome 1 open reading frame 174
ENSG0000	3.030238	0.03096	0.231715	0.450047	0.81471	SYNC	81493	syncoilin intermediate filament protein
ENSG0000	563.5129	0.052425	0.226077	0.449879	0.81471	RGS18	64407	regulator of G protein signaling 18
ENSG0000	69.77655	0.083087	0.201972	0.449825	0.81471	UCHL5	51377	ubiquitin C-terminal hydrolase L5
ENSG0000	2.558056	-0.0205	0.231726	0.45018	0.81471	LOC10050	1.01E+08	uncharacterized LOC100506274
ENSG0000	796.3248	-0.08224	0.130601	0.449746	0.81471	FAM193A	8603	family with sequence similarity 193 member A
ENSG0000	5970.906	-0.0857	0.151665	0.450161	0.81471	WDR1	9948	WD repeat domain 1
ENSG0000	32.84827	-0.08283	0.204839	0.449631	0.81471	CCDC126	90693	coiled-coil domain containing 126
ENSG0000	11.97458	0.081179	0.21262	0.450024	0.81471	SEC14L1P1	729799	SEC14 like 1 pseudogene 1
ENSG0000	2.320305	0.032638	0.232015	0.450042	0.81471	DDN-AS1	1.05E+08	DDN and PRKAG1 antisense RNA 1
ENSG0000	34.52012	-0.08502	0.201031	0.450095	0.81471	TRAV8-4	28682	T cell receptor alpha variable 8-4
ENSG0000	3.017203	0.041787	0.231213	0.450185	0.81471	NA	NA	NA
ENSG0000	41.74831	0.091396	0.187354	0.449795	0.81471	ZNF45	7596	zinc finger protein 45
ENSG0000	3.417159	0.034966	0.232035	0.449814	0.81471	PARD6B	84612	par-6 family cell polarity regulator beta
ENSG0000	276.1077	-0.08546	0.140732	0.450095	0.81471	SOD1	6647	superoxide dismutase 1
ENSG0000	304.8521	0.085357	0.146466	0.450233	0.814716	USP48	84196	ubiquitin specific peptidase 48
ENSG0000	5.945374	-0.04884	0.228521	0.450427	0.814744	NA	NA	NA
ENSG0000	9.144115	0.056875	0.226065	0.450419	0.814744	NA	NA	NA
ENSG0000	58.98269	-0.08749	0.177174	0.450423	0.814744	DTHD1	401124	death domain containing 1
ENSG0000	5.355871	0.049201	0.229004	0.450344	0.814744	TMEM67	91147	transmembrane protein 67
ENSG0000	640.5254	0.090503	0.189847	0.450506	0.814807	ITGB1	3688	integrin subunit beta 1
ENSG0000	259.2096	-0.08829	0.189666	0.450555	0.814816	RHOT1	55288	ras homolog family member T1
ENSG0000	851.6326	-0.07633	0.204276	0.450652	0.81491	GNA13	10672	G protein subunit alpha 13
ENSG0000	20.53805	-0.07736	0.213801	0.451019	0.815106	NA	NA	NA
ENSG0000	139.3321	0.093187	0.159326	0.45089	0.815106	S100PBP	64766	S100P binding protein
ENSG0000	8657.065	-0.08652	0.151489	0.451027	0.815106	PTPN18	26469	protein tyrosine phosphatase non-receptor type 18
ENSG0000	44.88825	-0.08843	0.191338	0.45094	0.815106	TBC1D31	93594	TBC1 domain family member 31
ENSG0000	228.9156	0.086371	0.149124	0.451026	0.815106	EI24	9538	EI24 autophagy associated transmembrane protein
ENSG0000	80.68624	0.089394	0.180798	0.450858	0.815106	NA	NA	NA
ENSG0000	440.1438	-0.07905	0.127416	0.451089	0.815138	SECISBP2	79048	SECIS binding protein 2
ENSG0000	1547.08	-0.08708	0.181844	0.451136	0.815141	TLR8	51311	toll like receptor 8
ENSG0000	268.3691	0.084937	0.152752	0.451249	0.815186	FAF1	11124	Fas associated factor 1
ENSG0000	817.2797	0.087111	0.15425	0.451238	0.815186	G6PC3	92579	glucose-6-phosphatase catalytic subunit 3
ENSG0000	10.65595	0.077045	0.216246	0.451403	0.815383	COPB2-DT	1.01E+08	COPB2 divergent transcript
ENSG0000	13.6367	0.072812	0.21854	0.45149	0.815461	NA	NA	NA
ENSG0000	3.681249	-0.04082	0.23004	0.451706	0.815769	NA	NA	NA
ENSG0000	288.0178	0.084902	0.138738	0.451756	0.815779	LETM1	3954	leucine zipper and EF-hand containing transmembrane protein 1
ENSG0000	5.243312	-0.04367	0.228372	0.452053	0.816235	HEATR6-D	1.05E+08	HEATR6 divergent transcript
ENSG0000	20.01588	0.058837	0.223615	0.452334	0.816421	CDK1	983	cyclin dependent kinase 1
ENSG0000	2.521058	0.043701	0.236488	0.452243	0.816421	CABP1	9478	calcium binding protein 1
ENSG0000	165.8249	0.08892	0.156476	0.452276	0.816421	ATP6V0A2	23545	ATPase H+ transporting V0 subunit a2
ENSG0000	13.11574	-0.06623	0.221085	0.4523	0.816421	ZNF287	57336	zinc finger protein 287
ENSG0000	313.7997	-0.08675	0.157046	0.452805	0.817191	AATBC	284837	apoptosis associated transcript in bladder cancer
ENSG0000	1142.966	-0.07674	0.119377	0.453095	0.817307	THRAP3	9967	thyroid hormone receptor associated protein 3
ENSG0000	263.1126	0.088007	0.181045	0.453162	0.817307	EPM2AIP1	9852	EPM2A interacting protein 1
ENSG0000	354.1255	-0.08304	0.139049	0.453049	0.817307	MYCBP2	23077	MYC binding protein 2
ENSG0000	213.5351	-0.08791	0.159971	0.453081	0.817307	ABHD15	116236	abhydrolase domain containing 15
ENSG0000	14.08715	-0.07025	0.219546	0.453053	0.817307	CASKIN2	57513	CASK interacting protein 2
ENSG0000	3459.12	-0.07807	0.144103	0.453198	0.817307	CSNK1D	1453	casein kinase 1 delta
ENSG0000	324.7531	0.081283	0.130849	0.45294	0.817307	RNMT	8731	RNA guanine-7 methyltransferase
ENSG0000	141.9708	0.078816	0.20602	0.453226	0.817307	BRWD1	54014	bromodomain and WD repeat domain containing 1
ENSG0000	146.8083	0.088973	0.159595	0.453296	0.817351	NEPRO	25871	nucleolus and neural progenitor protein
ENSG0000	3.74726	0.042383	0.231917	0.453447	0.817543	PCDHGA6	56109	protocadh 6
ENSG0000	54.53162	-0.08271	0.200324	0.453862	0.81809	NA	NA	NA
ENSG0000	1478.155	-0.0814	0.197133	0.453929	0.81809	LRRK2	120892	leucine rich repeat kinase 2
ENSG0000	59.82779	0.087011	0.193509	0.453829	0.81809	MIR22HG	84981	MIR22 host gene
ENSG0000	5.888087	0.045619	0.229516	0.453899	0.81809	ZNF285	26974	zinc finger protein 285
ENSG0000	339.126	0.087415	0.164198	0.454044	0.818167	ARFGEF1	10565	ADP ribosylation factor guanine nucleotide exchange factor 1
ENSG0000	271.3313	0.087449	0.156212	0.454061	0.818167	ATMIN	23300	ATM interactor
ENSG0000	10.12925	0.065009	0.222604	0.454116	0.818186	RPP40	10799	ribonuclease P/MRP subunit p40
ENSG0000	59.82236	0.08812	0.160587	0.454292	0.818188	C1orf131	128061	chromosome 1 open reading frame 131
ENSG0000	46.28638	0.081115	0.20512	0.454324	0.818188	MRPS18C	51023	mitochondrial ribosomal protein S18C
ENSG0000	660.5205	0.080434	0.130825	0.454321	0.818188	ATXN2	6311	ataxin 2
ENSG0000	75.81243	0.088179	0.180355	0.45434	0.818188	EXOSC8	11340	exosome component 8
ENSG0000	60.77508	0.088525	0.185387	0.454245	0.818188	NA	NA	NA
ENSG0000	208.2513	-0.08329	0.196485	0.454522	0.818434	NA	NA	NA
ENSG0000	5.582391	0.04112	0.230065	0.454684	0.818485	DEPTOR	64798	DEP domain containing MTOR interacting protein
ENSG0000	763.9176	0.076972	0.115825	0.454653	0.818485	MAN1B1	11253	mannosidase alpha class 1B member 1

ENSG0000	74.98299	-0.08981	0.174845	0.454657	0.818485	CHRNA10	57053	cholinergic receptor nicotinic alpha 10 subunit
ENSG0000	350.2075	0.086021	0.155907	0.454815	0.818521	NHP2	55651	NHP2 ribonucleoprotein
ENSG0000	1314.67	-0.08562	0.146548	0.454825	0.818521	CHD8	57680	chromodomain helicase DNA binding protein 8
ENSG0000	30.33075	-0.07627	0.21092	0.454838	0.818521	NA	NA	NA
ENSG0000	2.548808	0.024369	0.232572	0.454918	0.818585	FAM161A	84140	FAM161 centrosomal protein A
ENSG0000	718.338	-0.08394	0.134513	0.455209	0.818786	TNFAIP8L2	79626	TNF alpha induced protein 8 like 2
ENSG0000	187.5	0.088732	0.181308	0.455125	0.818786	STAG1	10274	stromal antigen 1
ENSG0000	348.6854	-0.07366	0.1128	0.455209	0.818786	PPP2R2D	55844	protein phosphatase 2 regulatory subunit Bdelta
ENSG0000	20.82312	-0.07615	0.212915	0.455079	0.818786	PDGFD	80310	platelet derived growth factor D
ENSG0000	283.2977	0.088443	0.169272	0.455449	0.818896	NSUN5P2	260294	NSUN5 pseudogene 2
ENSG0000	101.7982	-0.08445	0.15064	0.455406	0.818896	SAC3D1	29901	SAC3 domain containing 1
ENSG0000	154.4308	-0.08894	0.181011	0.455385	0.818896	SNX21	90203	sorting nexin family member 21
ENSG0000	9.874355	-0.06061	0.224586	0.455423	0.818896	NA	NA	NA
ENSG0000	40.5598	0.08563	0.19566	0.455497	0.818903	APTR	1.01E+08	Alu-mediated CDKN1A/p21 transcriptional regulator
ENSG0000	32.76821	0.087134	0.195447	0.455652	0.819101	PKD1P2	283955	polycystin transient receptor potential channel interacting pseudogene 2
ENSG0000	69.12977	0.090034	0.168897	0.455761	0.819136	DDX31	64794	DEAD-box helicase 31
ENSG0000	46.16015	0.086515	0.190274	0.455734	0.819136	NUFIP1	26747	nuclear FMR1 interacting protein 1
ENSG0000	73.79308	-0.08766	0.180163	0.455829	0.819178	KLHL42	57542	kelch like family member 42
ENSG0000	49.67722	0.056471	0.223246	0.455936	0.819289	RPL9P8	254948	ribosomal protein L9 pseudogene 8
ENSG0000	22666.8	0.063169	0.222799	0.456326	0.819454	ALPL	249	alkaline pł biomineralization associated
ENSG0000	2.89059	-0.03503	0.231573	0.456669	0.819454	TAF12-DT	1.05E+08	TAF12 divergent transcript
ENSG0000	74.01406	0.086388	0.19115	0.456689	0.819454	HENMT1	113802	HEN methyltransferase 1
ENSG0000	101.2857	-0.08224	0.199542	0.456493	0.819454	NA	NA	NA
ENSG0000	71.90436	-0.08822	0.179156	0.456444	0.819454	METAP1D	254042	methionyl mitochondrial
ENSG0000	8.641826	-0.0551	0.226519	0.456164	0.819454	DUBR	344595	DPPA2 upstream binding RNA
ENSG0000	1012.894	-0.08543	0.153921	0.456719	0.819454	SLC12A7	10723	solute carrier family 12 member 7
ENSG0000	16.46852	0.081055	0.208266	0.456093	0.819454	NA	NA	NA
ENSG0000	39.33127	-0.07966	0.207171	0.456682	0.819454	HLA-G	3135	major hist class I G
ENSG0000	2.583644	-0.01466	0.232756	0.456775	0.819454	LOC10798	1.08E+08	uncharacterized LOC107986742
ENSG0000	4.149568	0.04174	0.229854	0.456359	0.819454	NA	NA	NA
ENSG0000	100.3299	0.088269	0.179504	0.456227	0.819454	PAAF1	80227	proteasomal ATPase associated factor 1
ENSG0000	77.66436	0.08867	0.162407	0.456225	0.819454	COQ5	84274	coenzyme methyltransferase
ENSG0000	5.110907	0.040453	0.229468	0.456632	0.819454	NA	NA	NA
ENSG0000	60.00359	0.088227	0.169753	0.456788	0.819454	SLC25A17	10478	solute carrier family 25 member 17
ENSG0000	3542.87	0.081856	0.134639	0.456363	0.819454	PGK1	5230	phosphoglycerate kinase 1
ENSG0000	1195.908	0.0838	0.140972	0.456398	0.819454	ARHGEF6	9459	Rac/Cdc42 guanine nucleotide exchange factor 6
ENSG0000	4.781802	0.056368	0.227186	0.456915	0.819521	NA	NA	NA
ENSG0000	286.9526	-0.08737	0.16644	0.456893	0.819521	SLFN12L	1.01E+08	schlafen family member 12 like
ENSG0000	4.338149	-0.02585	0.231074	0.457028	0.819644	CLEC9A	283420	C-type lectin domain containing 9A
ENSG0000	66.51261	0.087179	0.189463	0.457483	0.81988	NBPF11	200030	NBPF member 11
ENSG0000	72.86155	-0.08264	0.19888	0.457298	0.81988	AFAP1	60312	actin filament associated protein 1
ENSG0000	26.24044	-0.07274	0.211825	0.457518	0.81988	MFAP3L	9848	microfibril associated protein 3 like
ENSG0000	8.10132	-0.06532	0.224731	0.457418	0.81988	NA	NA	NA
ENSG0000	475.7231	-0.08393	0.142814	0.457306	0.81988	BICRAL	23506	BICRA like chromatin remodeling complex associated protein
ENSG0000	106.9679	0.085846	0.189923	0.457489	0.81988	ANKIB1	54467	ankyrin repeat and IBR domain containing 1
ENSG0000	8.932504	-0.06919	0.22208	0.457306	0.81988	TRARG1	286753	trafficking regulator of GLUT4 (SLC2A4) 1
ENSG0000	13784.22	-0.08636	0.159929	0.457482	0.81988	SLC9A3R1	9368	SLC9A3 regulator 1
ENSG0000	138.9846	0.088366	0.166864	0.457621	0.819985	PHLPP1	23239	PH domain and leucine rich repeat protein phosphatase 1
ENSG0000	299.5176	0.087919	0.169822	0.457708	0.820061	MTHFD1	4522	methylene cyclohydrolase and formyltetrahydrofolate synthetase 1
ENSG0000	72.04403	-0.08876	0.162815	0.457809	0.820068	SLC9B2	133308	solute carrier family 9 member B2
ENSG0000	4.211091	0.051951	0.232265	0.457846	0.820068	TMEM139	135932	transmembrane protein 139
ENSG0000	3893.248	0.085531	0.188643	0.457811	0.820068	AATK	9625	apoptosis associated tyrosine kinase
ENSG0000	75.2459	-0.08848	0.167361	0.457959	0.820189	ANKS6	203286	ankyrin repeat and sterile alpha motif domain containing 6
ENSG0000	667.8515	-0.08537	0.126802	0.458155	0.820379	TARDBP	23435	TAR DNA binding protein
ENSG0000	7.486268	0.065635	0.223289	0.458134	0.820379	SPRY2	10253	sprouty RTK signaling antagonist 2
ENSG0000	561.2877	-0.07818	0.125705	0.458255	0.820478	UBAC2	337867	UBA domain containing 2
ENSG0000	11.44444	0.051747	0.226202	0.458301	0.820481	ZNF680	340252	zinc finger protein 680
ENSG0000	13.21264	-0.06731	0.220206	0.458493	0.820744	EDARADD	128178	EDAR associated death domain
ENSG0000	3.857092	-0.03542	0.23074	0.4587	0.820874	GRK7	131890	G protein-coupled receptor kinase 7
ENSG0000	66.61913	-0.07137	0.212757	0.458658	0.820874	ABRAXAS1	84142	abraxas 1 BRCA1 A complex subunit
ENSG0000	1420.677	-0.08641	0.177017	0.458621	0.820874	TMEM120	83862	transmembrane protein 120A
ENSG0000	1747.879	0.086806	0.160439	0.458792	0.820959	EDF1	8721	endothelial differentiation related factor 1
ENSG0000	233.6527	-0.08565	0.184038	0.458934	0.820972	NA	NA	NA
ENSG0000	8.261366	-0.06029	0.227182	0.458931	0.820972	HMGA2-A	1E+08	HMGA2 antisense RNA 1
ENSG0000	3.674798	-0.03677	0.230142	0.45886	0.820972	SNORD4A	26773	small nucl C/D box 4A
ENSG0000	772.3725	0.076815	0.120828	0.459117	0.821036	KHDRBS1	10657	KH RNA bi signal transduction associated 1
ENSG0000	62.5352	0.088392	0.160474	0.459035	0.821036	FAM50B	26240	family with sequence similarity 50 member B
ENSG0000	10.85199	0.061667	0.224083	0.459149	0.821036	ELAPOR2	222223	endosome-lysosome associated apoptosis and autophagy regulator family member 2
ENSG0000	1474.281	0.087151	0.176184	0.459115	0.821036	TBL1X	6907	transducin beta like 1 X-linked
ENSG0000	950.4696	0.067636	0.100495	0.459289	0.82114	VHL	7428	von Hippel-Lindau tumor suppressor
ENSG0000	615.8247	0.082879	0.138945	0.459326	0.82114	NMRAL1	57407	NmrA like redox sensor 1
ENSG0000	658.6152	0.084878	0.148176	0.459342	0.82114	ACP5	54	acid phosp tartrate resistant
ENSG0000	1309.208	-0.08332	0.190747	0.459476	0.821153	IL7R	3575	interleukin 7 receptor
ENSG0000	34.80694	0.086936	0.189894	0.459561	0.821153	CARMIL1	55604	capping protein regulator and myosin 1 linker 1
ENSG0000	3.476088	-0.02184	0.232035	0.459455	0.821153	RHOT1P1	1E+08	ras homolog family member T1 pseudogene 1
ENSG0000	9.316947	-0.05338	0.226457	0.459507	0.821153	ZNF790	388536	zinc finger protein 790
ENSG0000	16.41499	0.072084	0.217183	0.459573	0.821153	ZNF283	284349	zinc finger protein 283
ENSG0000	448.972	0.085276	0.15095	0.459978	0.821155	NUP153	9972	nucleoporin 153
ENSG0000	145.4534	0.082773	0.169258	0.459635	0.821155	HBS1L	10767	HBS1 like translational GTPase
ENSG0000	6.538732	-0.03814	0.229687	0.459928	0.821155	TRBJ1-4	28632	T cell receptor beta joining 1-4
ENSG0000	222.8429	0.080632	0.158659	0.459701	0.821155	ERC1	23085	ELKS/RAB6-interacting/CAST family member 1
ENSG0000	19.0156	-0.06691	0.219764	0.459763	0.821155	NA	NA	NA
ENSG0000	59.70569	0.062539	0.219727	0.459874	0.821155	RCN2	5955	reticulocalbin 2
ENSG0000	35.61425	0.068183	0.215887	0.459967	0.821155	ADPRM	56985	ADP-ribos manganese dependent
ENSG0000	2.774709	-0.02354	0.231944	0.459737	0.821155	NA	NA	NA
ENSG0000	142.6925	0.081597	0.133746	0.459891	0.821155	EOLA2	541578	endothelium and lymphocyte associated ASCH domain 2
ENSG0000	253.5429	-0.08656	0.187297	0.460432	0.821561	BROX	148362	BRO1 domain and CAAX motif containing
ENSG0000	3.569107	0.032627	0.232534	0.460519	0.821561	MAP2	4133	microtubule associated protein 2
ENSG0000	590.5063	0.073373	0.113637	0.460547	0.821561	OGG1	4968	8-oxoguanine DNA glycosylase
ENSG0000	142.078	0.084878	0.147567	0.460339	0.821561	GTPBP4	23560	GTP binding protein 4
ENSG0000	24.74169	-0.08156	0.202817	0.460412	0.821561	NOXRED1	122945	NADP dependent oxidoreductase domain containing 1
ENSG0000	3.406588	0.044663	0.229777	0.460564	0.821561	NA	NA	NA
ENSG0000	3373.623	0.086497	0.179187	0.460463	0.821561	FLII	2314	FLII actin remodeling protein
ENSG0000	3.091543	-0.01933	0.231474	0.460278	0.821561	MIOX	55586	myo-inositol oxygenase
ENSG0000	146.3182	0.083349	0.195081	0.460943	0.821994	STK38L	23012	serine/threonine kinase 38 like
ENSG0000	109.5271	-0.07043	0.212895	0.460957	0.821994	AKAP11	11215	A-kinase anchoring protein 11

ENSG0000	7.216643	0.038276	0.229739	0.460882	0.821994	VASN	114990	vasorin
ENSG0000	15.69047	-0.06971	0.218401	0.461031	0.821994	NA	NA	NA
ENSG0000	674.9033	-0.07895	0.200136	0.461013	0.821994	ACSL4	2182	acyl-CoA synthetase long chain family member 4
ENSG0000	15.70438	0.074075	0.2148	0.461119	0.822036	NA	NA	NA
ENSG0000	620.3972	-0.08431	0.186589	0.461144	0.822036	CLEC2D	29121	C-type lectin domain family 2 member D
ENSG0000	74.46895	-0.08749	0.165178	0.461193	0.822042	C11orf71	54494	chromosome 11 open reading frame 71
ENSG0000	8.428706	-0.06783	0.220843	0.461315	0.822101	NA	NA	NA
ENSG0000	17.7688	-0.06089	0.222965	0.461316	0.822101	CEP295NL	1.01E+08	CEP295 N-terminal like
ENSG0000	11.07719	-0.04346	0.230397	0.461373	0.822124	GPR42	2866	G protein-coupled receptor 42
ENSG0000	29.42142	0.07613	0.209255	0.461502	0.822169	NA	NA	NA
ENSG0000	26.26496	0.078177	0.210752	0.46145	0.822169	NA	NA	NA
ENSG0000	538.5826	-0.08605	0.160997	0.461533	0.822169	IRAK4	51135	interleukin 1 receptor associated kinase 4
ENSG0000	239.3419	0.088723	0.166038	0.46158	0.822172	ZNF512B	57473	zinc finger protein 512B
ENSG0000	14.67552	0.082211	0.205782	0.461681	0.822273	NA	NA	NA
ENSG0000	24.03265	0.085527	0.196753	0.461738	0.822294	SPRYD7	57213	SPRY domain containing 7
ENSG0000	50.13317	0.077865	0.207315	0.462016	0.822312	B3GNT5	84002	UDP-GlcN/ 3-N-acetylglucosaminyltransferase 5
ENSG0000	3939.271	-0.08536	0.178766	0.462018	0.822312	TLR2	7097	toll like receptor 2
ENSG0000	33.47899	0.083384	0.200302	0.461863	0.822312	ZNF25	219749	zinc finger protein 25
ENSG0000	220.4181	-0.08709	0.173706	0.461924	0.822312	BAG3	9531	BAG cochaperone 3
ENSG0000	6.981654	-0.06058	0.224817	0.461938	0.822312	TRAV27	28655	T cell receptor alpha variable 27
ENSG0000	5.986688	0.04751	0.228405	0.461931	0.822312	EVPL	2125	envoplakin
ENSG0000	553.6555	0.086892	0.159167	0.462066	0.822319	CTBP2	1488	C-terminal binding protein 2
ENSG0000	65.29052	0.086314	0.187427	0.462183	0.822448	ZNF43	7594	zinc finger protein 43
ENSG0000	3.084637	0.035144	0.231329	0.462252	0.82249	NA	NA	NA
ENSG0000	7.046462	0.055846	0.226428	0.462407	0.822605	ASS1P2	447	argininosuccinate synthetase 1 pseudogene 2
ENSG0000	169.8028	0.086402	0.16366	0.462393	0.822605	PRPS1	5631	phosphoribosyl pyrophosphate synthetase 1
ENSG0000	3140.748	-0.08263	0.168059	0.462556	0.82271	HGS	9146	hepatocyte growth factor-regulated tyrosine kinase substrate
ENSG0000	2.674558	0.036369	0.230976	0.462536	0.82271	PPEF1	5475	protein phosphatase with EF-hand domain 1
ENSG0000	1739.938	0.077766	0.12597	0.462606	0.82272	URM1	81605	ubiquitin related modifier 1
ENSG0000	4.775165	-0.0391	0.229772	0.462675	0.822763	NA	NA	NA
ENSG0000	12.0177	-0.06152	0.22248	0.462848	0.822911	DOCK3	1795	dedicator of cytokinesis 3
ENSG0000	1307.753	0.085228	0.163721	0.46284	0.822911	ARRB1	408	arrestin beta 1
ENSG0000	392.4378	0.079852	0.12565	0.463046	0.823104	RBM8A	9939	RNA binding motif protein 8A
ENSG0000	5.160006	0.046894	0.228418	0.463003	0.823104	ZFX-AS1	1.01E+08	ZFX antisense RNA 1
ENSG0000	63.16205	-0.06723	0.215745	0.463104	0.823126	NA	NA	NA
ENSG0000	567.899	0.088679	0.161682	0.463213	0.823241	YWHAQ	10971	tyrosine 3-monooxygenase/tryptophan 5-monooxygenase activation protein theta
ENSG0000	308.2971	0.080518	0.136004	0.463397	0.823486	RPA2	6118	replication protein A2
ENSG0000	556.133	0.083571	0.143045	0.463602	0.823504	MZT2B	80097	mitotic spindle organizing protein 2B
ENSG0000	7.980261	0.039435	0.229269	0.463466	0.823504	NA	NA	NA
ENSG0000	236.5595	0.086492	0.177097	0.463548	0.823504	PIK3R1	5295	phosphoinositide-3-kinase regulatory subunit 1
ENSG0000	257.9212	-0.08614	0.175573	0.463631	0.823504	KBTBD2	25948	kelch repeat and BTB domain containing 2
ENSG0000	30.74229	0.077956	0.208574	0.463531	0.823504	ZFP1	162239	ZFP1 zinc finger protein
ENSG0000	1879.904	-0.08309	0.145167	0.463697	0.823541	CHPF2	54480	chondroitin polymerizing factor 2
ENSG0000	47.68472	-0.08294	0.196823	0.463865	0.82376	CLCN5	1184	chloride voltage-gated channel 5
ENSG0000	458.9071	0.078436	0.199724	0.464041	0.823912	TAX1BP1	8887	Tax1 binding protein 1
ENSG0000	921.7249	-0.07574	0.20435	0.464025	0.823912	HIP1	3092	huntingtin interacting protein 1
ENSG0000	92.30206	0.083584	0.144012	0.464128	0.823984	EDRF1	26098	erythroid differentiation regulatory factor 1
ENSG0000	61.8402	0.085867	0.180161	0.464172	0.823984	COIL	8161	coilin
ENSG0000	111.7336	0.086078	0.159978	0.464252	0.824048	CLN8	2055	CLN8 transmembrane ER and ERGIC protein
ENSG0000	8.455085	0.049528	0.226948	0.464351	0.824063	STK32B	55351	serine/threonine kinase 32B
ENSG0000	370.7804	0.078515	0.128265	0.464319	0.824063	GSE1	23199	Gse1 coiled-coil protein
ENSG0000	27.41803	0.087887	0.183655	0.464679	0.824486	NA	NA	NA
ENSG0000	131.7894	-0.08074	0.203013	0.464637	0.824486	GTF2A1	2957	general transcription factor IIA subunit 1
ENSG0000	5356.155	-0.08272	0.13636	0.464727	0.824491	CD44	960	CD44 molecule (Indian blood group)
ENSG0000	311.6478	-0.08534	0.158381	0.464772	0.824491	TFPT	29844	TCF3 fusion partner
ENSG0000	70.52313	0.087379	0.177699	0.464886	0.824564	FCRL2	79368	Fc receptor like 2
ENSG0000	184.1346	-0.08593	0.162062	0.464903	0.824564	VEGFA	7422	vascular endothelial growth factor A
ENSG0000	33.33058	-0.06786	0.215765	0.464984	0.824578	CDC37L1	55664	cell divisio HSP90 cochaperone
ENSG0000	229.8456	-0.08543	0.159612	0.465001	0.824578	PCBD1	5092	pterin-4 alpha-carbinolamine dehydratase 1
ENSG0000	4.550202	0.054211	0.227596	0.465065	0.824582	TMEM269	1E+08	transmembrane protein 269
ENSG0000	64.06919	-0.08688	0.174586	0.465139	0.824582	CMTM8	152189	CKLF like MARVEL transmembrane domain containing 8
ENSG0000	9.897102	0.060216	0.224256	0.465123	0.824582	ZNF85	7639	zinc finger protein 85
ENSG0000	11.63367	-0.05683	0.224517	0.465201	0.824612	NA	NA	NA
ENSG0000	1452.992	-0.08339	0.193501	0.465334	0.824769	STK17B	9262	serine/threonine kinase 17b
ENSG0000	134.3885	0.086064	0.174317	0.465531	0.824958	FBXW11	23291	F-box and WD repeat domain containing 11
ENSG0000	718.8487	-0.07786	0.123719	0.465517	0.824958	PHF3	23469	PHD finger protein 3
ENSG0000	51.87716	0.08713	0.17866	0.465737	0.825164	NA	NA	NA
ENSG0000	2.840218	-0.03351	0.231297	0.465723	0.825164	THAP10	56906	THAP domain containing 10
ENSG0000	373.5013	0.087986	0.158729	0.465862	0.825183	EML4	27436	EMAP like 4
ENSG0000	154.8903	-0.08128	0.198259	0.465882	0.825183	FEM1C	56929	fem-1 homolog C
ENSG0000	7.665015	-0.06609	0.221833	0.465883	0.825183	ACTR3C	653857	actin related protein 3C
ENSG0000	3234.452	-0.08472	0.161423	0.465997	0.825279	MAN2A2	4122	mannosidase alpha class 2A member 2
ENSG0000	3.16001	0.042421	0.230327	0.466027	0.825279	TSPEAR-AS	54082	TSPEAR antisense RNA 1
ENSG0000	6486.083	0.073872	0.182037	0.466116	0.825356	SLC43A2	124935	solute carrier family 43 member 2
ENSG0000	2.542387	0.040377	0.231432	0.46623	0.825373	PSMC1P1	151645	proteasom ATPase 1 pseudogene 1
ENSG0000	8.265389	0.056399	0.225455	0.4662	0.825373	LINC0116	1.02E+08	long intergenic non-protein coding RNA 1163
ENSG0000	19.12248	-0.07769	0.209005	0.466261	0.825373	LAMP5	24141	lysosomal associated membrane protein family member 5
ENSG0000	19.61297	0.055226	0.223874	0.466344	0.825441	NA	NA	NA
ENSG0000	230.6028	0.083321	0.145392	0.466558	0.825624	RCC1	1104	regulator of chromosome condensation 1
ENSG0000	11.84225	0.050102	0.226169	0.466563	0.825624	TBC1D30	23329	TBC1 domain family member 30
ENSG0000	421.2165	0.077941	0.211166	0.466582	0.825624	OSBPL8	114882	oxysterol binding protein like 8
ENSG0000	85.55149	-0.08699	0.172411	0.466806	0.825939	ZBTB8OS	339487	zinc finger and BTB domain containing 8 opposite strand
ENSG0000	4.743409	-0.05467	0.227123	0.46696	0.826132	NA	NA	NA
ENSG0000	277.1719	0.072606	0.114558	0.467183	0.826446	TFCP2	7024	transcription factor CP2
ENSG0000	6857.281	-0.08475	0.164664	0.467287	0.826472	RPS6KA1	6195	ribosomal protein S6 kinase A1
ENSG0000	939.0373	-0.08508	0.147246	0.467246	0.826472	NELFE	7936	negative elongation factor complex member E
ENSG0000	84.47429	0.076167	0.205609	0.467842	0.826546	ACADM	34	acyl-CoA dehydrogenase medium chain
ENSG0000	1277.862	-0.07798	0.13508	0.467847	0.826546	GATAD2B	57459	GATA zinc finger domain containing 2B
ENSG0000	16.39991	0.07303	0.21483	0.467462	0.826546	BGLAP	632	bone gamma-carboxyglutamate protein
ENSG0000	217.9918	0.082426	0.136699	0.467541	0.826546	LSG1	55341	large 60S subunit nuclear export GTPase 1
ENSG0000	5.029839	0.049409	0.227872	0.467786	0.826546	HINT2	84681	histidine triad nucleotide binding protein 2
ENSG0000	2.831059	0.035843	0.231528	0.467918	0.826546	PPP1R26-/	1.01E+08	PPP1R26 antisense RNA 1
ENSG0000	123.6262	0.085805	0.162552	0.467867	0.826546	ACAD8	27034	acyl-CoA dehydrogenase family member 8
ENSG0000	3.552922	0.032776	0.230975	0.46796	0.826546	USP44	84101	ubiquitin specific peptidase 44
ENSG0000	95.15311	0.075808	0.207316	0.467614	0.826546	UBE2Q2	92912	ubiquitin conjugating enzyme E2 Q2
ENSG0000	17.83856	0.064465	0.219851	0.46784	0.826546	SOX8	30812	SRY-box transcription factor 8

ENSG0000	114.5727	0.083592	0.146717	0.467425	0.826546	CDK5RAP3	80279	CDK5 regulatory subunit associated protein 3
ENSG0000	32.3433	0.073211	0.212276	0.467892	0.826546	ZNF136	7695	zinc finger protein 136
ENSG0000	9288.851	0.085368	0.176847	0.467876	0.826546	MYADM	91663	myeloid associated differentiation marker
ENSG0000	423.7946	0.08573	0.164306	0.467961	0.826546	PRPF31	26121	pre-mRNA processing factor 31
ENSG0000	435.2842	-0.08378	0.187244	0.468408	0.826566	KIF1B	23095	kinesin family member 1B
ENSG0000	2.820448	0.032031	0.232751	0.468415	0.826566	NA	NA	NA
ENSG0000	628.4997	0.081413	0.144016	0.468423	0.826566	GSK3B	2932	glycogen synthase kinase 3 beta
ENSG0000	66.62058	0.08248	0.195502	0.468397	0.826566	CCL28	56477	C-C motif chemokine ligand 28
ENSG0000	10.51887	-0.07434	0.215683	0.468303	0.826566	TMEM14A	28978	transmembrane protein 14A
ENSG0000	44.64548	0.083067	0.196186	0.468132	0.826566	FBXO5	26271	F-box protein 5
ENSG0000	12.55948	0.076052	0.216221	0.468278	0.826566	CALD1	800	caldesmon 1
ENSG0000	92.03152	0.084937	0.170544	0.468291	0.826566	FBXO10	26267	F-box protein 10
ENSG0000	46.33135	-0.08574	0.192871	0.468369	0.826566	ZNF845	91664	zinc finger protein 845
ENSG0000	4.280652	0.038136	0.230527	0.46814	0.826566	NA	NA	NA
ENSG0000	158.4188	-0.08335	0.150437	0.468537	0.826686	TMEM184	55751	transmembrane protein 184C
ENSG0000	5411.636	-0.08458	0.167654	0.46859	0.826701	SQSTM1	8878	sequestosome 1
ENSG0000	2122.588	-0.0664	0.136265	0.468742	0.82689	SZT2	23334	SZT2 subunit of KICSTOR complex
ENSG0000	482.7657	-0.07236	0.116014	0.469091	0.827119	USP39	10713	ubiquitin specific peptidase 39
ENSG0000	195.2465	-0.08489	0.160396	0.469149	0.827119	ST3GAL5	8869	ST3 beta-g 3-sialyltransferase 5
ENSG0000	86.59937	0.072606	0.212133	0.469189	0.827119	C5orf24	134553	chromosome 5 open reading frame 24
ENSG0000	136.9795	0.082236	0.145331	0.469037	0.827119	PFDN6	10471	prefoldin subunit 6
ENSG0000	18.97636	-0.07213	0.213731	0.469088	0.827119	NA	NA	NA
ENSG0000	3038.004	-0.08248	0.148202	0.468969	0.827119	SH3GL1	6455	SH3 doma endophilin A2
ENSG0000	1349.554	0.077434	0.127803	0.469106	0.827119	ZNF335	63925	zinc finger protein 335
ENSG0000	302.6137	-0.08329	0.174535	0.46925	0.827148	TMCO4	255104	transmembrane and coiled-coil domains 4
ENSG0000	3.23811	-0.03807	0.23095	0.469675	0.827261	GABRD	2563	gamma-aminobutyric acid type A receptor subunit delta
ENSG0000	29.06211	0.077566	0.206977	0.469694	0.827261	LYPLAL1	127018	lysophospholipase like 1
ENSG0000	4.360474	0.051116	0.231518	0.46946	0.827261	TPO	7173	thyroid peroxidase
ENSG0000	2712.207	0.082609	0.146532	0.469723	0.827261	RNF130	55819	ring finger protein 130
ENSG0000	2460.234	0.084638	0.159159	0.469544	0.827261	SMARCD3	6604	SWI/SNF r matrix ass actin depe subfamily member 3
ENSG0000	4.225641	0.047484	0.228802	0.469766	0.827261	VLDLR	7436	very low density lipoprotein receptor
ENSG0000	149.5816	0.081278	0.141436	0.469555	0.827261	SURF2	6835	surfeit 2
ENSG0000	2.625359	-0.04585	0.231564	0.469581	0.827261	SUGT1P3	283507	SUGT1 pseudogene 3
ENSG0000	82.2476	-0.08535	0.15899	0.46965	0.827261	NA	NA	NA
ENSG0000	965.7587	0.072039	0.110608	0.469581	0.827261	GNPTG	84572	N-acetylglucosamine-1-phosphate transferase subunit gamma
ENSG0000	4.105227	0.037373	0.229948	0.469972	0.827464	NA	NA	NA
ENSG0000	4598.545	-0.07954	0.136929	0.469941	0.827464	GRB2	2885	growth factor receptor bound protein 2
ENSG0000	13.32518	-0.07591	0.211645	0.470052	0.827526	MN1	4330	MN1 proto transcriptional regulator
ENSG0000	5.685821	0.043168	0.228611	0.470199	0.827546	NA	NA	NA
ENSG0000	11.63029	0.058752	0.223495	0.470125	0.827546	SYT17	51760	synaptotagmin 17
ENSG0000	149.8194	0.083025	0.147165	0.470179	0.827546	GLOD4	51031	glyoxalase domain containing 4
ENSG0000	3.754019	0.033527	0.23028	0.470284	0.827617	FKBP2	2286	FKBP prolyl isomerase 2
ENSG0000	131.4836	0.084526	0.165483	0.470544	0.827915	CUL2	8453	cullin 2
ENSG0000	274.1113	-0.08542	0.163338	0.470526	0.827915	PGAP2	27315	post-GPI attachment to proteins 2
ENSG0000	50.93773	0.080472	0.199616	0.470591	0.827919	FKBP3	2287	FKBP prolyl isomerase 3
ENSG0000	1398.44	-0.07992	0.137151	0.470827	0.828174	DCTN2	10540	dynactin subunit 2
ENSG0000	34.91124	0.077215	0.205926	0.470818	0.828174	SNUPN	10073	snurportin 1
ENSG0000	2897.678	0.084705	0.172915	0.471066	0.828435	PRPF8	10594	pre-mRNA processing factor 8
ENSG0000	490.7142	-0.07442	0.204156	0.471035	0.828435	TRPM2	7226	transient receptor potential cation channel subfamily M member 2
ENSG0000	15.14433	-0.06289	0.221327	0.471261	0.82846	AQP1	358	aquaporin 1 (Colton blood group)
ENSG0000	359.1099	-0.07447	0.121374	0.471166	0.82846	ZNF767P	79970	zinc finger pseudogene
ENSG0000	5.797215	0.039821	0.2298	0.471194	0.82846	A2M-AS1	144571	A2M antisense RNA 1
ENSG0000	9.345549	-0.04617	0.227489	0.471221	0.82846	ISM1	140862	isthmin 1
ENSG0000	38.63054	-0.08619	0.179603	0.471368	0.82857	NA	NA	NA
ENSG0000	361.9303	0.07809	0.131045	0.471431	0.8286	KCTD10	83892	potassium channel tetramerization domain containing 10
ENSG0000	490.8178	0.08606	0.172385	0.471566	0.828678	UBXN4	23190	UBX domain protein 4
ENSG0000	27.50151	-0.08219	0.197437	0.471557	0.828678	NR4A2	4929	nuclear receptor subfamily 4 group A member 2
ENSG0000	4.058826	0.043419	0.232249	0.471631	0.828714	NA	NA	NA
ENSG0000	2.413666	0.022177	0.234153	0.471699	0.828754	NECAB1	64168	N-terminal EF-hand calcium binding protein 1
ENSG0000	92.65181	0.083259	0.150682	0.471934	0.828957	PHF14	9678	PHD finger protein 14
ENSG0000	125.8389	-0.08399	0.171595	0.471981	0.828957	SWI5	375757	SWI5 homologous recombination repair protein
ENSG0000	131.5921	0.079249	0.134975	0.471996	0.828957	ARL16	339231	ADP ribosylation factor like GTPase 16
ENSG0000	18691.05	-0.08436	0.174301	0.471989	0.828957	ARHGDIA	396	Rho GDP dissociation inhibitor alpha
ENSG0000	579.0494	-0.06824	0.10733	0.472342	0.829326	PRPF3	9129	pre-mRNA processing factor 3
ENSG0000	2.919706	-0.02552	0.232072	0.472331	0.829326	HPYR1	93668	Helicobacter pylori responsive 1
ENSG0000	112.6145	0.068999	0.213374	0.472314	0.829326	COPS2	9318	COP9 signalosome subunit 2
ENSG0000	5.641247	-0.04596	0.227901	0.472438	0.829342	FBXO16	157574	F-box protein 16
ENSG0000	589.4258	-0.07392	0.204322	0.472441	0.829342	RAB8B	51762	RAB8B member RAS oncogene family
ENSG0000	33.87889	0.051926	0.221117	0.472573	0.829442	NA	NA	NA
ENSG0000	2.76069	-0.03487	0.231133	0.472589	0.829442	ID1	3397	inhibitor of DNA binding 1
ENSG0000	30.47637	-0.0765	0.204777	0.472636	0.829444	TUBD1	51174	tubulin delta 1
ENSG0000	59.22522	0.082353	0.182282	0.472893	0.829499	B3GALNT2	148789	beta-1 3-N-acetylgalactosaminyltransferase 2
ENSG0000	19.96431	-0.07817	0.205383	0.472891	0.829499	LYG1	129530	lysozyme g1
ENSG0000	469.319	0.074696	0.118268	0.472848	0.829499	MRPS11	64963	mitochondrial ribosomal protein S11
ENSG0000	53.22903	0.074954	0.207317	0.472738	0.829499	ZNF302	55900	zinc finger protein 302
ENSG0000	8.472196	0.060456	0.223175	0.472865	0.829499	ELFN2	114794	extracellular leucine rich repeat and fibronectin type III domain containing 2
ENSG0000	536.9857	-0.08386	0.17505	0.473017	0.829636	UNC45A	55898	unc-45 myosin chaperone A
ENSG0000	4.36861	-0.03758	0.230355	0.473126	0.829669	TNPO1P1	1E+08	transportin 1 pseudogene 1
ENSG0000	6.375379	0.052128	0.226267	0.473103	0.829669	NA	NA	NA
ENSG0000	4255.818	-0.07638	0.125023	0.473175	0.829676	SYNGR2	9144	synaptogyrin 2
ENSG0000	9.30891	-0.06494	0.217441	0.473555	0.829897	COLGALT2	23127	collagen beta(1-O)galactosyltransferase 2
ENSG0000	24.51324	-0.06752	0.215828	0.473617	0.829897	ZCCHC4	29063	zinc finger CCHC-type containing 4
ENSG0000	13.74769	0.077033	0.214454	0.473361	0.829897	NA	NA	NA
ENSG0000	97.6438	-0.08311	0.151485	0.473699	0.829897	CNPY4	245812	canopy FGF signaling regulator 4
ENSG0000	956.0185	-0.07511	0.127343	0.473619	0.829897	USP20	10868	ubiquitin specific peptidase 20
ENSG0000	2608.987	-0.07902	0.173434	0.473714	0.829897	C1RL	51279	complement C1r subcomponent like
ENSG0000	26.24015	0.079446	0.203619	0.473631	0.829897	SALL2	6297	spalt like transcription factor 2
ENSG0000	12.35835	0.071674	0.215873	0.473618	0.829897	ZFHX3-AS1	1.02E+08	ZFHX3 antisense RNA 1
ENSG0000	15.09548	0.075408	0.211571	0.473528	0.829897	FLT3LG	2323	fms related receptor tyrosine kinase 3 ligand
ENSG0000	139.5191	-0.08311	0.14936	0.4738	0.829897	HIRA	7290	histone cell cycle regulator
ENSG0000	6.836018	0.056545	0.22551	0.473756	0.829897	SPIN4	139886	spindlin family member 4
ENSG0000	34.38155	0.08181	0.197339	0.473866	0.829934	GATD1-DT	171391	GATD1 divergent transcript
ENSG0000	316.6077	0.074688	0.122698	0.473991	0.829994	GIGYF2	26058	GRB10 interacting GYF protein 2
ENSG0000	17.56107	0.051649	0.224506	0.473987	0.829994	AP1AR	55435	adaptor related protein complex 1 associated regulatory protein
ENSG0000	13.7151	-0.06505	0.219171	0.474106	0.830117	NA	NA	NA
ENSG0000	1973.992	-0.08132	0.143118	0.474228	0.830251	KDM5C	8242	lysine demethylase 5C

ENSG0000	272.7811	-0.07783	0.190986	0.475258	0.830299	MAP3K6	9064	mitogen-activated protein kinase kinase kinase 6
ENSG0000	25.19847	-0.05648	0.221804	0.474948	0.830299	MAB21L3	126868	mab-21 like 3
ENSG0000	802.9886	0.079048	0.136121	0.475101	0.830299	MDM4	4194	MDM4 regulator of p53
ENSG0000	121.8562	0.081719	0.145943	0.47521	0.830299	RPIA	22934	ribose 5-phosphate isomerase A
ENSG0000	118.7264	0.083825	0.155291	0.47517	0.830299	TXK	7294	TXK tyrosine kinase
ENSG0000	6.985957	-0.06386	0.222258	0.475385	0.830299	KHDC1	80759	KH domain containing 1
ENSG0000	357.5146	0.083874	0.162426	0.474917	0.830299	KMT2C	58508	lysine methyltransferase 2C
ENSG0000	2.861567	-0.03719	0.230464	0.475104	0.830299	NA	NA	NA
ENSG0000	10.6394	0.062219	0.222251	0.475237	0.830299	SUV39H2	79723	SUV39H2 histone lysine methyltransferase
ENSG0000	118.311	-0.08506	0.164694	0.474346	0.830299	TMEM254	80195	transmembrane protein 254
ENSG0000	2351.646	-0.08156	0.152499	0.474825	0.830299	MAP4K2	5871	mitogen-activated protein kinase kinase kinase kinase 2
ENSG0000	300.1918	-0.0814	0.175454	0.475379	0.830299	FOXM1	2305	forkhead box M1
ENSG0000	8.418262	0.041322	0.227909	0.475394	0.830299	LOC10537	1.05E+08	uncharacterized LOC105370689
ENSG0000	24.8815	0.063598	0.218492	0.475248	0.830299	NA	NA	NA
ENSG0000	10.02554	-0.06703	0.218844	0.474357	0.830299	NA	NA	NA
ENSG0000	3561.279	-0.08155	0.149387	0.474927	0.830299	PPP4C	5531	protein phosphatase 4 catalytic subunit
ENSG0000	3.223434	0.033227	0.233192	0.474661	0.830299	TEKT3	64518	tektin 3
ENSG0000	646.3548	-0.08195	0.205115	0.475109	0.830299	CPD	1362	carboxypeptidase D
ENSG0000	149.3717	-0.08201	0.186027	0.474847	0.830299	HCN2	610	hyperpolarization activated cyclic nucleotide gated potassium and sodium channel 2
ENSG0000	8261.335	0.081848	0.141623	0.475434	0.830299	RPS15	6209	ribosomal protein S15
ENSG0000	2762.363	-0.08273	0.178201	0.474604	0.830299	SUPT5H	6829	SPT5 homo DSIF elongation factor subunit
ENSG0000	423.1402	-0.07623	0.129238	0.474776	0.830299	RALGAPB	57148	Ral GTPase activating protein non-catalytic subunit beta
ENSG0000	7.453647	0.053933	0.225956	0.474659	0.830299	EEF1A2	1917	eukaryotic translation elongation factor 1 alpha 2
ENSG0000	3.713395	0.045669	0.229671	0.474602	0.830299	NA	NA	NA
ENSG0000	41.62144	-0.08166	0.194139	0.47481	0.830299	NA	NA	NA
ENSG0000	2.456928	0.046237	0.229734	0.475021	0.830299	XRCC6P2	389901	X-ray repair cross complementing 6 pseudogene 2
ENSG0000	314.7158	0.078904	0.136475	0.475571	0.830458	RBBP7	5931	RB binding chromatin remodeling factor
ENSG0000	6.386533	0.049166	0.22697	0.47568	0.83057	SMG1P1	641298	SMG1 pseudogene 1
ENSG0000	232.1587	0.078079	0.131512	0.47592	0.830673	TRIM62	55223	tripartite motif containing 62
ENSG0000	3.026329	-0.03237	0.229804	0.475838	0.830673	THBS4	7060	thrombospondin 4
ENSG0000	96.34778	-0.08249	0.147222	0.475897	0.830673	HPS5	11234	HPS5 biogenesis of lysosomal organelles complex 2 subunit 2
ENSG0000	95.41825	0.079275	0.196137	0.475872	0.830673	ITSN1	6453	intersectin 1
ENSG0000	36.55193	0.064875	0.218164	0.476029	0.830783	FPGT	8790	fucose-1-phosphate guanylyltransferase
ENSG0000	207.0598	0.076096	0.128551	0.476179	0.830808	STX6	10228	syntaxin 6
ENSG0000	3790.063	0.070566	0.110966	0.47615	0.830808	BTG2	7832	BTG anti-proliferation factor 2
ENSG0000	202.0745	-0.0838	0.165035	0.476157	0.830808	TRMT61A	115708	tRNA methyltransferase 61A
ENSG0000	168.8889	0.08147	0.143483	0.476396	0.831107	TMEM223	79064	transmembrane protein 223
ENSG0000	148.966	-0.08374	0.163651	0.476556	0.831138	LOC10099	1.01E+08	uncharacterized LOC100996756
ENSG0000	27.94255	-0.08619	0.184591	0.47692	0.831138	NA	NA	NA
ENSG0000	3.361537	0.038411	0.230238	0.476707	0.831138	LINC0155	202299	long intergenic non-protein coding RNA 1554
ENSG0000	42.61114	0.079344	0.200676	0.476496	0.831138	FRA10AC1	118924	FRA10A associated CGG repeat 1
ENSG0000	6.246801	0.049552	0.227138	0.4769	0.831138	NA	NA	NA
ENSG0000	89.55101	0.078249	0.201252	0.47682	0.831138	TMTC1	83857	transmembrane O-mannosyltransferase targeting cadherins 1
ENSG0000	832.7376	-0.08257	0.176855	0.477185	0.831138	NA	NA	NA
ENSG0000	78.44369	-0.08285	0.184996	0.477156	0.831138	NA	NA	NA
ENSG0000	184.2271	0.072514	0.125917	0.477161	0.831138	WDR73	84942	WD repeat domain 73
ENSG0000	12.05634	-0.04891	0.225555	0.477042	0.831138	NA	NA	NA
ENSG0000	94.95062	0.083645	0.179286	0.47699	0.831138	C17orf80	55028	chromosome 17 open reading frame 80
ENSG0000	3.388102	-0.02176	0.23229	0.477075	0.831138	BEST2	54831	bestrophin 2
ENSG0000	6.554005	0.061783	0.223368	0.477179	0.831138	ZNF573	126231	zinc finger protein 573
ENSG0000	7.002299	0.054975	0.225389	0.476703	0.831138	EID2B	126272	EP300 interacting inhibitor of differentiation 2B
ENSG0000	3.370475	0.037068	0.231747	0.477024	0.831138	NA	NA	NA
ENSG0000	25.31442	0.080194	0.20137	0.476836	0.831138	RPL23AP7	118433	ribosomal protein L23a pseudogene 7
ENSG0000	7.196792	0.053482	0.229442	0.47693	0.831138	XG	7499	Xg glycoprotein (Xg blood group)
ENSG0000	3.238242	-0.04026	0.230016	0.477365	0.83126	NA	NA	NA
ENSG0000	842.1225	0.07302	0.124361	0.47731	0.83126	DNAH1	25981	dynein axonemal heavy chain 1
ENSG0000	298.7493	-0.07551	0.127955	0.477422	0.83126	NA	NA	NA
ENSG0000	567.0447	-0.06792	0.168148	0.477437	0.83126	OSBPL7	114881	oxysterol binding protein like 7
ENSG0000	444.5475	0.081998	0.159487	0.477531	0.831283	CYB5R1	51706	cytochrome b5 reductase 1
ENSG0000	227.0846	-0.08187	0.143141	0.477541	0.831283	POLR2F	5435	RNA polyn I and III subunit F
ENSG0000	3.32825	-0.02126	0.231457	0.477587	0.831284	NA	NA	NA
ENSG0000	35.34645	-0.07781	0.202307	0.477658	0.831295	FCRLB	127943	Fc receptor like B
ENSG0000	60.40201	-0.07332	0.208065	0.477684	0.831295	NA	NA	NA
ENSG0000	14.46984	0.051424	0.225294	0.477878	0.831416	SCOC	60592	short coiled-coil protein
ENSG0000	419.7537	0.071786	0.116893	0.477811	0.831416	NFX1	4799	nuclear tra X-box binding 1
ENSG0000	1202.325	-0.08091	0.141728	0.47789	0.831416	TM9SF2	9375	transmembrane 9 superfamily member 2
ENSG0000	524.2103	0.077678	0.137198	0.47805	0.831539	SF3A3	10946	splicing factor 3a subunit 3
ENSG0000	3.602757	-0.02808	0.230558	0.478051	0.831539	PLS1	5357	plastin 1
ENSG0000	254.7737	-0.08237	0.169659	0.47817	0.831668	LINC0242	1.05E+08	long intergenic non-protein coding RNA 2422
ENSG0000	11.2121	0.067109	0.220007	0.478398	0.831911	NA	NA	NA
ENSG0000	2528.584	-0.08257	0.173719	0.478426	0.831911	CDC25B	994	cell division cycle 25B
ENSG0000	99.10691	0.061455	0.217886	0.478447	0.831911	LTN1	26046	listerin E3 ubiquitin protein ligase 1
ENSG0000	65.75699	0.083768	0.171171	0.478497	0.83192	FDX1	2230	ferredoxin 1
ENSG0000	3.715335	0.029505	0.230796	0.478589	0.831922	NA	NA	NA
ENSG0000	10.07867	0.070273	0.217002	0.478581	0.831922	NA	NA	NA
ENSG0000	31.83668	0.070114	0.212996	0.478702	0.831961	BBOF1	80127	basal body orientation factor 1
ENSG0000	6.208984	0.057834	0.224971	0.478677	0.831961	BCAS1	8537	brain enriched myelin associated protein 1
ENSG0000	72.97184	0.08085	0.192185	0.478922	0.831961	NUP54	53371	nucleoporin 54
ENSG0000	58.99058	0.081458	0.190529	0.478944	0.831961	BYSL	705	bystin like
ENSG0000	554.8206	0.073587	0.12309	0.479109	0.831961	CNOT2	4848	CCR4-NOT transcription complex subunit 2
ENSG0000	16538.12	-0.07756	0.195274	0.478945	0.831961	IGHA2	3494	immunoglobulin heavy constant alpha 2 (A2m marker)
ENSG0000	3.306021	0.033415	0.234534	0.479055	0.831961	IGHD4-17	28494	immunoglobulin heavy diversity 4-17
ENSG0000	1606.977	-0.08173	0.179671	0.479111	0.831961	KRT23	25984	keratin 23
ENSG0000	284.0693	-0.07136	0.118408	0.47881	0.831961	POP4	10775	POP4 hom ribonuclease P/MRP subunit
ENSG0000	2184.757	0.080503	0.16156	0.478907	0.831961	RPN2	6185	ribophorin II
ENSG0000	52.02314	-0.0841	0.184877	0.478994	0.831961	SLCO4A1	28231	solute carrier organic anion transporter family member 4A1
ENSG0000	21.26452	0.068154	0.214908	0.47929	0.832177	NA	NA	NA
ENSG0000	100.9004	-0.08099	0.144331	0.479326	0.832177	ZNF841	284371	zinc finger protein 841
ENSG0000	3.50338	-0.03911	0.230019	0.479513	0.83218	TMEM182	130827	transmembrane protein 182
ENSG0000	897.7212	-0.06908	0.113222	0.479623	0.83218	COPB2	9276	COPI coat complex subunit beta 2
ENSG0000	6.608477	-0.05441	0.225097	0.479564	0.83218	PSME2P2	338099	proteasome activator subunit 2 pseudogene 2
ENSG0000	40.84401	0.080469	0.195699	0.479646	0.83218	PKD1P3	339039	polycystin transient receptor potential channel interacting pseudogene 3
ENSG0000	1232.295	-0.08164	0.161306	0.479641	0.83218	METTL9	51108	methyltransferase like 9
ENSG0000	14.36044	-0.05595	0.222592	0.479521	0.83218	NA	NA	NA
ENSG0000	30553.68	-0.08227	0.16938	0.47957	0.83218	CYBA	1535	cytochrome b-245 alpha chain
ENSG0000	2.641073	0.026333	0.232744	0.479856	0.832358	NA	NA	NA

ENSG0000	91.73419	0.081755	0.187432	0.480184	0.832358	SLC25A12	8604	solute carrier family 25 member 12
ENSG0000	14.05184	-0.05463	0.223554	0.480088	0.832358	ACOX2	8309	acyl-CoA oxidase 2
ENSG0000	43.90059	0.0816	0.193123	0.479959	0.832358	CBR4	84869	carbonyl reductase 4
ENSG0000	24.76626	-0.0775	0.20677	0.480203	0.832358	TRGV3	6976	T cell receptor gamma variable 3
ENSG0000	242.8695	0.074843	0.125558	0.480038	0.832358	HMBS	3145	hydroxymethylbilane synthase
ENSG0000	20.40593	0.075	0.208916	0.479915	0.832358	NA	NA	NA
ENSG0000	93.27337	-0.08303	0.167798	0.480199	0.832358	DICER1-AS	400242	DICER1 antisense RNA 1
ENSG0000	89.96404	0.083936	0.171186	0.480014	0.832358	ACTR5	79913	actin related protein 5
ENSG0000	1720.379	0.081642	0.183052	0.480168	0.832358	ACO2	50	aconitase 2
ENSG0000	413.6637	0.079924	0.142405	0.480281	0.832414	SMAD2	4087	SMAD family member 2
ENSG0000	50.01809	0.084417	0.171675	0.480565	0.832492	EXOG	9941	exo/endonuclease G
ENSG0000	337.0411	0.064101	0.131523	0.480437	0.832492	UBE2K	3093	ubiquitin conjugating enzyme E2 K
ENSG0000	233.4402	0.084095	0.151053	0.480644	0.832492	NA	NA	NA
ENSG0000	284.5415	-0.08171	0.146783	0.480529	0.832492	BCL11B	64919	BAF chromatin remodeling complex subunit BCL11B
ENSG0000	270.2724	0.076586	0.130419	0.480642	0.832492	PHKB	5257	phosphorylase kinase regulatory subunit beta
ENSG0000	595.3245	-0.0663	0.105578	0.480601	0.832492	RETREG3	162427	reticulophagy regulator family member 3
ENSG0000	112.678	-0.08061	0.145481	0.480448	0.832492	PBX4	80714	PBX homeobox 4
ENSG0000	266.2897	0.072194	0.11912	0.480715	0.832536	ZKSCAN1	7586	zinc finger with KRAB and SCAN domains 1
ENSG0000	14377.37	-0.07364	0.197153	0.481081	0.832918	HRH2	3274	histamine receptor H2
ENSG0000	90.27988	0.082215	0.18317	0.481208	0.832918	TNKS1BP1	85456	tankyrase 1 binding protein 1
ENSG0000	12.01202	0.06606	0.218274	0.481129	0.832918	SBNO1-AS	1.12E+08	SBNO1 antisense RNA 1
ENSG0000	291.9738	0.079765	0.185123	0.48118	0.832918	DMXL2	23312	Dmx like 2
ENSG0000	4.075824	-0.04767	0.228028	0.481134	0.832918	NA	NA	NA
ENSG0000	23.5123	-0.08116	0.196662	0.481183	0.832918	MID2	11043	midline 2
ENSG0000	27.68595	0.081628	0.194484	0.481267	0.832941	CEP152	22995	centrosomal protein 152
ENSG0000	188.5003	0.070307	0.123084	0.481328	0.832968	CYB561D2	11068	cytochrome b561 family member D2
ENSG0000	7528.698	0.081594	0.164276	0.481563	0.83306	HCLS1	3059	hematopoietic cell-specific Lyn substrate 1
ENSG0000	36.85129	0.082389	0.190902	0.481548	0.83306	NA	NA	NA
ENSG0000	6.617633	0.056234	0.225057	0.48149	0.83306	ADCY6	112	adenylate cyclase 6
ENSG0000	4041.629	-0.0823	0.165574	0.481464	0.83306	LGALS1	3956	galectin 1
ENSG0000	55.83509	-0.08381	0.17786	0.481687	0.833196	GUCA1B	2979	guanylate cyclase activator 1B
ENSG0000	33.76039	0.082094	0.185815	0.481878	0.833369	BOLA3	388962	bolA family member 3
ENSG0000	585.2458	0.082675	0.175174	0.481858	0.833369	FAM107B	83641	family with sequence similarity 107 member B
ENSG0000	3318.061	0.080052	0.173706	0.481942	0.8334	HM13	81502	histocompatibility minor 13
ENSG0000	15.2226	0.043272	0.227293	0.482051	0.83351	SCML1	6322	Scm polycomb group protein like 1
ENSG0000	573.2436	0.082177	0.163141	0.482126	0.833561	YIF1A	10897	Yip1 intera membrane trafficking protein
ENSG0000	1929.279	0.082244	0.176622	0.482202	0.833614	BCKDK	10295	branched chain keto acid dehydrogenase kinase
ENSG0000	21.54807	-0.08045	0.198246	0.482367	0.833664	NA	NA	NA
ENSG0000	33.19999	0.07757	0.201051	0.482365	0.833664	NA	NA	NA
ENSG0000	17.93214	0.072298	0.21154	0.482316	0.833664	ZBTB46	140685	zinc finger and BTB domain containing 46
ENSG0000	50.71735	0.077902	0.202309	0.482532	0.833797	HIBADH	11112	3-hydroxyisobutyrate dehydrogenase
ENSG0000	27.5205	-0.07451	0.213495	0.48255	0.833797	TRGV5P	6979	T cell receptor gamma variable 5P (pseudogene)
ENSG0000	3.186472	-0.02668	0.231319	0.482672	0.833797	NA	NA	NA
ENSG0000	327.7529	0.075545	0.130777	0.482621	0.833797	RBM17	84991	RNA binding motif protein 17
ENSG0000	3.630433	-0.03864	0.228878	0.482633	0.833797	NA	NA	NA
ENSG0000	261.3451	-0.07731	0.198404	0.482761	0.833858	TGFA	7039	transforming growth factor alpha
ENSG0000	18.83219	0.066409	0.216518	0.482799	0.833858	ICAM5	7087	intercellular adhesion molecule 5
ENSG0000	96.60379	-0.08268	0.167068	0.482872	0.833906	VRK2	7444	VRK serine/threonine kinase 2
ENSG0000	6.751315	-0.05171	0.225914	0.482926	0.833922	LINC00892	1E+08	long intergenic non-protein coding RNA 892
ENSG0000	67.43342	-0.08191	0.155308	0.482977	0.833931	NA	NA	NA
ENSG0000	214.2216	0.071369	0.119277	0.483127	0.834094	ASB1	51665	ankyrin repeat and SOCS box containing 1
ENSG0000	59.29185	0.074871	0.203605	0.483163	0.834094	WDR76	79968	WD repeat domain 76
ENSG0000	7.49267	0.058321	0.223241	0.483372	0.834182	PARL	55486	presenilin associated rhomboid like
ENSG0000	164.0333	0.080786	0.14943	0.483396	0.834182	RFC1	5981	replication factor C subunit 1
ENSG0000	9.134867	0.050332	0.23069	0.483355	0.834182	SFRP5	6425	secreted frizzled related protein 5
ENSG0000	4.754329	-0.03826	0.229377	0.483312	0.834182	NA	NA	NA
ENSG0000	638.1199	0.080931	0.151803	0.483528	0.834331	HNRNPDL	9987	heterogeneous nuclear ribonucleoprotein D like
ENSG0000	261.3263	-0.07977	0.188107	0.48384	0.834792	LRFN3	79414	leucine rich repeat and fibronectin type III domain containing 3
ENSG0000	18.60417	0.063897	0.217972	0.484231	0.834907	NA	NA	NA
ENSG0000	129.5937	-0.08157	0.180671	0.484282	0.834907	LRIF1	55791	ligand dependent nuclear receptor interacting factor 1
ENSG0000	32.56809	0.075241	0.204745	0.484136	0.834907	NA	NA	NA
ENSG0000	19.19812	0.055842	0.222645	0.484222	0.834907	MRPL1	65008	mitochondrial ribosomal protein L1
ENSG0000	76.99806	0.083042	0.164661	0.48419	0.834907	CEP295	85459	centrosomal protein 295
ENSG0000	1765.935	-0.07995	0.147106	0.484155	0.834907	SPRYD3	84926	SPRY domain containing 3
ENSG0000	7.309069	-0.03805	0.228699	0.484014	0.834907	NA	NA	NA
ENSG0000	9.604167	-0.05947	0.222413	0.484317	0.834907	NDUFA7	4701	NADH:ubiquinone oxidoreductase subunit A7
ENSG0000	115.4367	0.082522	0.155905	0.484215	0.834907	RTL6	84247	retrotransposon Gag like 6
ENSG0000	8.399789	-0.06616	0.21944	0.48445	0.835058	NA	NA	NA
ENSG0000	53.85163	0.080244	0.195468	0.484536	0.835127	CEP97	79598	centrosomal protein 97
ENSG0000	9.938412	0.056074	0.223087	0.484648	0.835161	RHEBL1	121268	RHEB like 1
ENSG0000	640.1341	0.082235	0.166908	0.484669	0.835161	TRAP1	10131	TNF receptor associated protein 1
ENSG0000	19.1598	0.076595	0.204896	0.484692	0.835161	LINC01431	1.01E+08	long intergenic non-protein coding RNA 1431
ENSG0000	5.880748	-0.03483	0.229892	0.484742	0.835167	APOD	347	apolipoprotein D
ENSG0000	81.44332	0.082717	0.181896	0.484925	0.835404	RPUSD2	27079	RNA pseudouridine synthase domain containing 2
ENSG0000	84.30667	-0.0816	0.16731	0.484993	0.835443	NA	NA	NA
ENSG0000	1436.981	-0.07703	0.132219	0.485089	0.83553	NUTF2	10204	nuclear transport factor 2
ENSG0000	1401.488	0.081427	0.180188	0.485241	0.83556	ACTR2	10097	actin related protein 2
ENSG0000	3.511604	0.041944	0.230533	0.485178	0.83556	CCDC40	55036	coiled-coil domain containing 40
ENSG0000	1175.58	-0.08112	0.168626	0.485243	0.83556	ADGRE3	84658	adhesion G protein-coupled receptor E3
ENSG0000	72.61282	0.080986	0.188403	0.485418	0.835626	PLEKHA3	65977	pleckstrin homology domain containing A3
ENSG0000	335.9303	-0.07465	0.195831	0.485348	0.835626	C5orf15	56951	chromosome 5 open reading frame 15
ENSG0000	12.38225	0.073721	0.211237	0.485385	0.835626	MIR3677H	1.07E+08	MIR3677 and MIR940 host gene
ENSG0000	34.52181	-0.08019	0.190162	0.485518	0.83572	HES6	55502	hes family bHLH transcription factor 6
ENSG0000	8.930003	-0.06395	0.220083	0.485752	0.836044	LIM2	3982	lens intrinsic membrane protein 2
ENSG0000	5.25112	0.044748	0.227691	0.485815	0.836073	NA	NA	NA
ENSG0000	4957.977	-0.07631	0.135645	0.48632	0.836154	GNB1	2782	G protein subunit beta 1
ENSG0000	92.71137	0.083344	0.152747	0.486013	0.836154	CAMTA1	23261	calmodulin binding transcription activator 1
ENSG0000	9.828167	0.060577	0.221247	0.486296	0.836154	LOC10192	1.02E+08	uncharacterized LOC101929691
ENSG0000	56.44817	-0.06635	0.212129	0.487509	0.836154	NA	NA	NA
ENSG0000	744.1512	0.078835	0.145341	0.486495	0.836154	ANKZF1	55139	ankyrin repeat and zinc finger peptidyl tRNA hydrolase 1
ENSG0000	12.02719	-0.03618	0.228731	0.48778	0.836154	ZNF385D	79750	zinc finger protein 385D
ENSG0000	5.059745	0.033156	0.23002	0.487628	0.836154	TMIE	259236	transmembrane inner ear
ENSG0000	425.856	0.0802	0.162929	0.487349	0.836154	MANF	7873	mesencephalic astrocyte derived neurotrophic factor
ENSG0000	9.263477	0.049972	0.225989	0.485943	0.836154	TMCC1-DT	1.01E+08	TMCC1 divergent transcript
ENSG0000	49.93695	-0.05975	0.217792	0.487197	0.836154	PI4K2B	55300	phosphatidylinositol 4-kinase type 2 beta
ENSG0000	4.134983	-0.02151	0.231346	0.487127	0.836154	LOC10050	1.01E+08	uncharacterized LOC100507053

ENSG0000	97.78183	0.082681	0.156718	0.486131	0.836154	GTF3C6	112495	general transcription factor IIIC subunit 6
ENSG0000	2.452145	-0.01951	0.230836	0.487633	0.836154	TRGJP2	6972	T cell receptor gamma joining P2
ENSG0000	68.28107	-0.08332	0.16996	0.486519	0.836154	HUS1	3364	HUS1 checkpoint clamp component
ENSG0000	274.969	-0.07641	0.14367	0.487662	0.836154	ARPC1B	10095	actin related protein 2/3 complex subunit 1B
ENSG0000	335.7555	-0.07643	0.139002	0.487101	0.836154	NA	NA	NA
ENSG0000	239.8884	-0.08129	0.164412	0.487097	0.836154	BRAF	673	B-Raf prot serine/threonine kinase
ENSG0000	2.933837	-0.03638	0.229867	0.486631	0.836154	NA	NA	NA
ENSG0000	2.746436	-0.02445	0.231958	0.486539	0.836154	NA	NA	NA
ENSG0000	6.884025	0.045668	0.227058	0.487099	0.836154	ZNF239	8187	zinc finger protein 239
ENSG0000	57.59822	0.075982	0.199005	0.486749	0.836154	ZNF32	7580	zinc finger protein 32
ENSG0000	80.57244	0.071751	0.206746	0.487613	0.836154	TFAM	7019	transcripti mitochondrial
ENSG0000	423.3426	-0.07863	0.148551	0.486919	0.836154	SAR1A	56681	secretion associated Ras related GTPase 1A
ENSG0000	41.20364	0.079692	0.188734	0.486644	0.836154	PDE2A	5138	phosphodiesterase 2A
ENSG0000	3.246272	0.043823	0.22895	0.487324	0.836154	NA	NA	NA
ENSG0000	2.431086	-0.01729	0.232224	0.487566	0.836154	NA	NA	NA
ENSG0000	2.702116	0.037276	0.230781	0.487634	0.836154	NA	NA	NA
ENSG0000	53.11118	-0.07957	0.191653	0.487746	0.836154	TMEM255	348013	transmembrane protein 255B
ENSG0000	355.7782	0.080357	0.164	0.486087	0.836154	ARID4A	5926	AT-rich interaction domain 4A
ENSG0000	176.5315	-0.0825	0.170482	0.486747	0.836154	KIFC3	3801	kinesin family member C3
ENSG0000	30.34811	-0.07433	0.205054	0.486921	0.836154	NA	NA	NA
ENSG0000	4.519558	0.034639	0.229602	0.486302	0.836154	NA	NA	NA
ENSG0000	276.0984	0.077639	0.139051	0.487727	0.836154	OXLD1	339229	oxidoreductase like domain containing 1
ENSG0000	171.2249	0.083755	0.191083	0.486475	0.836154	TYMS	7298	thymidylate synthetase
ENSG0000	12643.28	0.081307	0.175663	0.487113	0.836154	CAPNS1	826	calpain small subunit 1
ENSG0000	30.53515	-0.07823	0.198668	0.48756	0.836154	NA	NA	NA
ENSG0000	56.98807	-0.0569	0.220104	0.487732	0.836154	CSF2RBP1	23772	CSF2RB pseudogene 1
ENSG0000	409.246	-0.07193	0.200305	0.487715	0.836154	MOSPD2	158747	motile sperm domain containing 2
ENSG0000	23.99695	-0.06043	0.218502	0.486842	0.836154	KLHL15	80311	kelch like family member 15
ENSG0000	11.15857	0.063532	0.219686	0.48712	0.836154	NA	NA	NA
ENSG0000	114.7841	-0.08133	0.159624	0.487697	0.836154	FHL1	2273	four and a half LIM domains 1
ENSG0000	124.3733	-0.08064	0.188963	0.487652	0.836154	NAA10	8260	N-alpha-a NatA catalytic subunit
ENSG0000	34.28063	-0.06867	0.210906	0.487863	0.83622	SSX2IP	117178	SSX family member 2 interacting protein
ENSG0000	152.3083	0.074726	0.201151	0.488198	0.836715	IGHV3-74	28408	immunoglobulin heavy variable 3-74
ENSG0000	169.5003	0.080408	0.182126	0.48829	0.836795	SGTB	54557	small glutamine rich tetratricopeptide repeat co-chaperone beta
ENSG0000	116.5698	0.081531	0.177277	0.48838	0.836871	MTR	4548	5-methyltetrahydrofolate-homocysteine methyltransferase
ENSG0000	3.111857	-0.02598	0.230988	0.488606	0.83692	NA	NA	NA
ENSG0000	6259.673	-0.07362	0.130353	0.48863	0.83692	IQSEC1	9922	IQ motif and Sec7 domain ArfGEF 1
ENSG0000	2.929999	-0.0429	0.22997	0.488521	0.83692	ROBO4	54538	roundabout guidance receptor 4
ENSG0000	136.2385	0.081502	0.177202	0.488637	0.83692	DNAJC15	29103	DnaJ heat shock protein family (Hsp40) member C15
ENSG0000	10124.56	-0.07764	0.18871	0.488588	0.83692	UBA1	7317	ubiquitin like modifier activating enzyme 1
ENSG0000	9.202979	0.050846	0.225236	0.489048	0.837033	NA	NA	NA
ENSG0000	1001.829	0.070362	0.205853	0.488981	0.837033	ANXA1	301	annexin A1
ENSG0000	21.51242	-0.07087	0.212168	0.488865	0.837033	DPYSL4	10570	dihydropyrimidinase like 4
ENSG0000	3.494515	0.045383	0.228697	0.488874	0.837033	SUPT16HP	400011	SPT16 hon facilitates chromatin remodeling subunit pseudogene 1
ENSG0000	4921.472	0.079096	0.147423	0.488929	0.837033	HDAC7	51564	histone deacetylase 7
ENSG0000	41.80211	0.076437	0.200296	0.488991	0.837033	NA	NA	NA
ENSG0000	28.05945	0.078338	0.198415	0.489069	0.837033	POLR3F	10621	RNA polymerase III subunit F
ENSG0000	6.688178	0.021231	0.231466	0.489047	0.837033	ANOS1	3730	anosmin 1
ENSG0000	8.089889	-0.05017	0.225067	0.489144	0.837083	NA	NA	NA
ENSG0000	285.4169	0.081405	0.168697	0.489227	0.837147	PRDX3	10935	peroxiredoxin 3
ENSG0000	7972.728	-0.06819	0.205755	0.489457	0.837448	SRGN	5552	serglycin
ENSG0000	51.08154	-0.08219	0.172765	0.489586	0.837448	SRP14-DT	1E+08	SRP14 divergent transcript
ENSG0000	536.3637	0.071637	0.131001	0.489576	0.837448	SLC7A6	9057	solute carrier family 7 member 6
ENSG0000	256.2686	-0.0742	0.198511	0.489577	0.837448	MED13	9969	mediator complex subunit 13
ENSG0000	2.990859	-0.03105	0.230318	0.490028	0.837558	ABCB11	8647	ATP binding cassette subfamily B member 11
ENSG0000	50.48937	0.07554	0.202924	0.490007	0.837558	RTP5	285093	receptor transporter protein 5 (putative)
ENSG0000	157.5788	0.07748	0.146848	0.490075	0.837558	SNX30	401548	sorting nexin family member 30
ENSG0000	58.86368	-0.07708	0.171034	0.490089	0.837558	PGM2L1	283209	phosphoglucomutase 2 like 1
ENSG0000	247.5503	0.072003	0.123359	0.490075	0.837558	TM7SF3	51768	transmembrane 7 superfamily member 3
ENSG0000	200.2426	0.081163	0.161292	0.490153	0.837558	XPOT	11260	exportin for tRNA
ENSG0000	78.42885	-0.08168	0.169514	0.489866	0.837558	ZNF830	91603	zinc finger protein 830
ENSG0000	645.144	-0.0715	0.122258	0.490132	0.837558	AMZ2	51321	archaelysin family metallopeptidase 2
ENSG0000	575.3495	0.08377	0.165881	0.490117	0.837558	TBCD	6904	tubulin folding cofactor D
ENSG0000	822.1758	0.080432	0.160197	0.48996	0.837558	DDX39A	10212	DExD-box helicase 39A
ENSG0000	6617.393	-0.07968	0.17536	0.490073	0.837558	DDX17	10521	DEAD-box helicase 17
ENSG0000	88.17501	0.080182	0.168946	0.490406	0.837764	SLC25A26	115286	solute carrier family 25 member 26
ENSG0000	71.19042	0.067976	0.211189	0.490407	0.837764	PUS7L	83448	pseudouridine synthase 7 like
ENSG0000	76.39796	0.080722	0.183406	0.490457	0.837764	MIEF2	125170	mitochondrial elongation factor 2
ENSG0000	7.520845	0.050056	0.22611	0.490419	0.837764	RSPO4	343637	R-spondin 4
ENSG0000	29.85934	0.075432	0.203224	0.490697	0.837856	FAM200A	221786	family with sequence similarity 200 member A
ENSG0000	155.7856	0.079171	0.147921	0.490642	0.837856	PPP2CB	5516	protein phosphatase 2 catalytic subunit beta
ENSG0000	4292.964	0.076195	0.136443	0.490702	0.837856	ATP5F1B	506	ATP synthase F1 subunit beta
ENSG0000	576.7436	0.079288	0.147677	0.490572	0.837856	USP36	57602	ubiquitin specific peptidase 36
ENSG0000	1060.986	0.077781	0.149295	0.490739	0.837856	MBD3	53615	methyl-CpG binding domain protein 3
ENSG0000	13.84218	0.06386	0.217925	0.490809	0.837898	CFAP161	161502	cilia and flagella associated protein 161
ENSG0000	257.2229	0.074752	0.132309	0.49089	0.83794	FKRP	79147	fukutin related protein
ENSG0000	9.739904	-0.06432	0.218435	0.490925	0.83794	MACROD2	140733	mono-ADP ribosylhydrolase 2
ENSG0000	276.8955	-0.0767	0.13925	0.490994	0.83798	AKAP9	10142	A-kinase anchoring protein 9
ENSG0000	1406.804	-0.08014	0.165863	0.491108	0.838096	MAPK13	5603	mitogen-activated protein kinase 13
ENSG0000	6.327596	-0.02636	0.230542	0.491159	0.838105	NA	NA	NA
ENSG0000	16.01428	-0.06263	0.217739	0.491313	0.838289	NSG2	51617	neuronal vesicle trafficking associated 2
ENSG0000	91.41119	0.080066	0.179456	0.491488	0.838353	SREK1IP1	285672	SREK1 interacting protein 1
ENSG0000	378.8187	0.069248	0.119121	0.491399	0.838353	FAM219B	57184	family with sequence similarity 219 member B
ENSG0000	6.901643	0.036209	0.228996	0.491476	0.838353	LOC10537	1.05E+08	uncharacterized LOC105372990
ENSG0000	4.250609	-0.03276	0.230281	0.491594	0.838456	NA	NA	NA
ENSG0000	12.8363	-0.05752	0.221517	0.491777	0.83869	EDA	1896	ectodysplasin A
ENSG0000	985.9884	0.070528	0.126831	0.491833	0.838693	TOR2A	27433	torsin family 2 member A
ENSG0000	147.8961	-0.08013	0.179641	0.49187	0.838693	CD226	10666	CD226 molecule
ENSG0000	2.450438	-0.03385	0.230473	0.492039	0.838903	TUBAP2	399942	tubulin alpha pseudogene 2
ENSG0000	61.54192	0.082198	0.166572	0.492134	0.838909	QTRT2	79691	queuine tRNA-ribosyltransferase accessory subunit 2
ENSG0000	2.643946	-0.01379	0.233117	0.492128	0.838909	NA	NA	NA
ENSG0000	97.44178	-0.08165	0.157793	0.492187	0.83892	SVIL-AS1	1.03E+08	SVIL antisense RNA 1
ENSG0000	1209.95	0.073502	0.136078	0.492275	0.838993	C19orf53	28974	chromosome 19 open reading frame 53
ENSG0000	946.4773	-0.07998	0.171347	0.492563	0.839182	MAP4	4134	microtubule associated protein 4
ENSG0000	2.79643	0.027094	0.231671	0.492483	0.839182	FAM27C	1E+08	family with sequence similarity 27 member C
ENSG0000	447.6042	0.082372	0.186475	0.492472	0.839182	API5	8539	apoptosis inhibitor 5

ENSG0000	88.63503	0.080032	0.185035	0.492569	0.839182	NPIPB13	613037	nuclear pc member B13
ENSG0000	227.9336	0.07659	0.139764	0.492969	0.839609	POLR3C	10623	RNA polymerase III subunit C
ENSG0000	374.8986	0.076248	0.137398	0.492973	0.839609	MANBA	4126	mannosidase beta
ENSG0000	4.567679	0.034168	0.230568	0.493095	0.839609	CENPQ	55166	centromere protein Q
ENSG0000	12.68826	-0.07048	0.212219	0.493079	0.839609	NA	NA	NA
ENSG0000	138.3555	0.080879	0.154601	0.49302	0.839609	TRIP11	9321	thyroid hormone receptor interactor 11
ENSG0000	162.9283	0.079787	0.160598	0.493073	0.839609	BAG5	9529	BAG cochaperone 5
ENSG0000	53.6831	-0.08144	0.165725	0.493168	0.839655	MORN1	79906	MORN repeat containing 1
ENSG0000	193.6358	-0.08089	0.165341	0.493284	0.839774	LBX2-AS1	151534	LBX2 antisense RNA 1
ENSG0000	2.531961	0.024535	0.232618	0.493975	0.839958	NA	NA	NA
ENSG0000	187.0263	-0.07916	0.156383	0.493667	0.839958	MCM6	4175	minichromosome maintenance complex component 6
ENSG0000	93.13161	0.07381	0.201677	0.49354	0.839958	LANCL1	10314	LanC like glutathione S-transferase 1
ENSG0000	345.8964	0.074796	0.133095	0.494107	0.839958	P4HTM	54681	prolyl 4-hy transmembrane
ENSG0000	146.3559	-0.07543	0.136441	0.493803	0.839958	NDUFC1	4717	NADH:ubiquinone oxidoreductase subunit C1
ENSG0000	123.1244	0.067435	0.208028	0.493941	0.839958	TMED7	51014	transmembrane p24 trafficking protein 7
ENSG0000	537.9414	-0.07989	0.159953	0.493687	0.839958	TES	26136	testin LIM domain protein
ENSG0000	226.7653	0.079616	0.186081	0.49355	0.839958	KIF5B	3799	kinesin family member 5B
ENSG0000	2801.35	-0.0764	0.133465	0.49404	0.839958	PTPRE	5791	protein tyrosine phosphatase receptor type E
ENSG0000	13.76602	-0.06688	0.214856	0.493834	0.839958	XNDC1N	1E+08	XRCC1 N-t N-terminal like
ENSG0000	282.5314	-0.07183	0.12521	0.494355	0.839958	RSF1	51773	remodeling and spacing factor 1
ENSG0000	2.655815	-0.03555	0.232548	0.493508	0.839958	NA	NA	NA
ENSG0000	71.48761	0.060197	0.215976	0.494331	0.839958	POLR2M	81488	RNA polymerase II subunit M
ENSG0000	1092.399	0.074266	0.147224	0.494273	0.839958	APRT	353	adenine phosphoribosyltransferase
ENSG0000	1060.301	0.077747	0.145052	0.493678	0.839958	CDK10	8558	cyclin dependent kinase 10
ENSG0000	4.656418	0.042422	0.227907	0.494126	0.839958	EFCAB5	374786	EF-hand calcium binding domain 5
ENSG0000	91.43083	0.079199	0.149678	0.493906	0.839958	INO80C	125476	INO80 complex subunit C
ENSG0000	19.90746	0.068686	0.21362	0.494004	0.839958	ZNF567-D	1.02E+08	ZNF567 divergent transcript
ENSG0000	4.873042	-0.02819	0.230196	0.493985	0.839958	CCM2L	140706	CCM2 like scaffold protein
ENSG0000	37.49739	0.073339	0.204733	0.49424	0.839958	LOC10192	1.02E+08	uncharacterized LOC101927202
ENSG0000	29.74579	0.0681	0.211711	0.494297	0.839958	PIGA	5277	phosphatidylinositol glycan anchor biosynthesis class A
ENSG0000	135.8631	0.078506	0.149931	0.49451	0.840066	EMG1	10436	EMG1 N1-specific pseudouridine methyltransferase
ENSG0000	2009.491	-0.08073	0.150973	0.494468	0.840066	DNAJC5	80331	DnaJ heat shock protein family (Hsp40) member C5
ENSG0000	30.00397	0.075144	0.202534	0.494685	0.840226	KLHL3	26249	kelch like family member 3
ENSG0000	160.2912	0.080836	0.166661	0.494696	0.840226	FLYWCH2	114984	FLYWCH family member 2
ENSG0000	12.46458	-0.06776	0.214495	0.495015	0.840612	RAMP1	10267	receptor activity modifying protein 1
ENSG0000	298.1463	0.074501	0.132058	0.494977	0.840612	SMURF1	57154	SMAD specific E3 ubiquitin protein ligase 1
ENSG0000	3.469363	0.030393	0.232243	0.49521	0.840709	NA	NA	NA
ENSG0000	369.751	0.077941	0.167093	0.49518	0.840709	C19orf48	84798	multidrug resistance-related protein
ENSG0000	189.7138	0.076997	0.173482	0.495125	0.840709	MIRLET7B	400931	MIRLET7B host gene
ENSG0000	7.46585	-0.05042	0.224955	0.495265	0.840724	AURKC	6795	aurora kinase C
ENSG0000	83.11999	-0.07833	0.187649	0.495375	0.840834	GPR153	387509	G protein-coupled receptor 153
ENSG0000	79.18365	-0.07732	0.19115	0.495459	0.840898	SNTA1	6640	syntrophin alpha 1
ENSG0000	2.83832	0.032508	0.230363	0.495596	0.841054	VPS37D	155382	VPS37D subunit of ESCRT-I
ENSG0000	71.68061	0.079599	0.149092	0.495787	0.841222	PMS2CL	441194	PMS2 C-terminal like pseudogene
ENSG0000	242.5129	0.080253	0.1699	0.495752	0.841222	DNAJA2	10294	DnaJ heat shock protein family (Hsp40) member A2
ENSG0000	1772.924	-0.07477	0.141023	0.496243	0.841372	ELOVL1	64834	ELOVL fatty acid elongase 1
ENSG0000	5.290746	-0.05027	0.225991	0.495994	0.841372	NA	NA	NA
ENSG0000	580.1334	0.078702	0.14738	0.496039	0.841372	TRA2B	6434	transformer 2 beta homolog
ENSG0000	3.413848	0.036881	0.229365	0.496138	0.841372	NA	NA	NA
ENSG0000	39.9918	0.081543	0.179392	0.496155	0.841372	TAF5	6877	TATA-box binding protein associated factor 5
ENSG0000	62.81336	0.077971	0.191879	0.495987	0.841372	SLAIN1	122060	SLAIN motif family member 1
ENSG0000	3731.319	-0.07923	0.158437	0.496137	0.841372	STAT5B	6777	signal transducer and activator of transcription 5B
ENSG0000	3432.705	0.07218	0.129546	0.496198	0.841372	PPP1R12C	54776	protein phosphatase 1 regulatory subunit 12C
ENSG0000	7325.629	-0.07943	0.155126	0.496619	0.841732	THEMIS2	9473	thymocyte selection associated family member 2
ENSG0000	202.216	0.069463	0.215633	0.496823	0.841732	NEK7	140609	NIMA related kinase 7
ENSG0000	8753.149	-0.07888	0.164462	0.496755	0.841732	CFLAR	8837	CASP8 and FADD like apoptosis regulator
ENSG0000	365.4493	0.071975	0.126314	0.496746	0.841732	ATP1B3	483	ATPase Na+/K+ transporting subunit beta 3
ENSG0000	174.169	0.064448	0.212435	0.496748	0.841732	NDUFA4	4697	NDUFA4 mitochondrial complex associated
ENSG0000	5.727237	0.046644	0.226473	0.496582	0.841732	NA	NA	NA
ENSG0000	6.963949	0.059581	0.222223	0.496615	0.841732	NA	NA	NA
ENSG0000	51.91774	0.079179	0.174376	0.496808	0.841732	BBS2	583	Bardet-Biedl syndrome 2
ENSG0000	2165.614	-0.07489	0.133111	0.496991	0.841842	ZC3H12A	80149	zinc finger CCCH-type containing 12A
ENSG0000	4330.368	-0.07909	0.156294	0.497072	0.841842	SPOCK2	9806	SPARC (os cwcv and kazal like domains proteoglycan 2
ENSG0000	3.508738	-0.03707	0.229675	0.497054	0.841842	APOE	348	apolipoprotein E
ENSG0000	540.5603	0.078911	0.174656	0.497013	0.841842	TMEM121	27439	transmembrane protein 121B
ENSG0000	5.644718	0.053773	0.224575	0.49731	0.841861	NA	NA	NA
ENSG0000	3841.67	-0.07834	0.180389	0.497162	0.841861	STAB1	23166	stabilin 1
ENSG0000	192.3047	0.080866	0.166879	0.497226	0.841861	SMIM14	201895	small integral membrane protein 14
ENSG0000	5517.548	0.068563	0.11678	0.497396	0.841861	CCDC69	26112	coiled-coil domain containing 69
ENSG0000	90.35422	0.074186	0.199659	0.497387	0.841861	BLOC1S6	26258	biogenesis of lysosomal organelles complex 1 subunit 6
ENSG0000	540.0268	0.076701	0.138315	0.497451	0.841861	E4F1	1877	E4F transcription factor 1
ENSG0000	53.54264	0.064213	0.212343	0.497431	0.841861	ZBTB14	7541	zinc finger and BTB domain containing 14
ENSG0000	2.701814	0.03796	0.22969	0.497385	0.841861	NA	NA	NA
ENSG0000	794.7686	0.079187	0.160035	0.497715	0.842181	HLA-DQA1	3117	major hist class II DQ alpha 1
ENSG0000	23.76274	0.062782	0.215673	0.497732	0.842181	MAGIX	79917	MAGI fam X-linked
ENSG0000	62.65333	-0.07968	0.181388	0.497825	0.842261	TMEM238	388564	transmembrane protein 238
ENSG0000	25.02301	0.075408	0.202773	0.497975	0.842354	DOK7	285489	docking protein 7
ENSG0000	4.160044	-0.02914	0.230519	0.49806	0.842354	HRH4	59340	histamine receptor H4
ENSG0000	10.89226	0.048358	0.225436	0.498064	0.842354	CPXM1	56265	carboxype M14 family member 1
ENSG0000	75.13594	-0.07891	0.183802	0.498041	0.842354	PLTP	5360	phospholipid transfer protein
ENSG0000	689.8496	-0.07523	0.138916	0.498306	0.842686	PSMB8-AS	1.01E+08	PSMB8 antisense RNA 1 (head to head)
ENSG0000	383.797	0.076724	0.145373	0.498868	0.842747	AKR7A2	8574	aldo-keto reductase family 7 member A2
ENSG0000	15.22438	-0.06675	0.213595	0.498624	0.842747	IQGAP3	128239	IQ motif containing GTPase activating protein 3
ENSG0000	4.595145	0.041831	0.229414	0.498481	0.842747	RPL21P28	1E+08	ribosomal protein L21 pseudogene 28
ENSG0000	35.72366	0.072519	0.205734	0.498986	0.842747	ABCC2	1244	ATP binding cassette subfamily C member 2
ENSG0000	363.1737	-0.0774	0.165068	0.498585	0.842747	ZDHHC6	64429	zinc finger DHHC-type palmitoyltransferase 6
ENSG0000	7.305171	-0.04988	0.224889	0.498943	0.842747	ANO5	203859	anoctamin 5
ENSG0000	254.9236	0.070785	0.125148	0.498777	0.842747	RFWD3	55159	ring finger and WD repeat domain 3
ENSG0000	3.520705	-0.05258	0.226175	0.498423	0.842747	MYH10	4628	myosin heavy chain 10
ENSG0000	1665.387	-0.08083	0.158014	0.498755	0.842747	NCOR1	9611	nuclear receptor corepressor 1
ENSG0000	1711.916	-0.06828	0.125496	0.49859	0.842747	GGA3	23163	golgi asso gamma a ARF binding protein 3
ENSG0000	48.92212	0.080971	0.176469	0.49866	0.842747	BSG-AS1	1.05E+08	BSG antisense RNA 1
ENSG0000	50.46719	0.080118	0.165222	0.49892	0.842747	PRR22	163154	proline rich 22
ENSG0000	686.522	0.079325	0.178133	0.498726	0.842747	ETS2	2114	ETS proto- transcription factor
ENSG0000	628.0739	-0.06935	0.121758	0.498933	0.842747	TOB2	10766	transduce 2
ENSG0000	37.94072	-0.07976	0.184533	0.499045	0.842769	SMG1P7	1.01E+08	SMG1 pseudogene 7

ENSG0000	358.8626	0.07293	0.135424	0.499161	0.842887	NOP9	161424	NOP9 nucleolar protein
ENSG0000	610.3429	-0.07106	0.128205	0.49928	0.843009	PLEKHA2	59339	pleckstrin homology domain containing A2
ENSG0000	480.8557	-0.07115	0.12898	0.499739	0.843101	C1orf122	127687	chromosome 1 open reading frame 122
ENSG0000	148.5854	0.0856	0.177143	0.500472	0.843101	DDX59	83479	DEAD-box helicase 59
ENSG0000	90.03571	-0.07836	0.151225	0.50003	0.843101	ACVR1	90	activin A receptor type 1
ENSG0000	12.98864	0.050869	0.223596	0.499632	0.843101	NA	NA	NA
ENSG0000	109.3183	-0.07569	0.145258	0.500066	0.843101	LARS2	23395	leucyl-tRN mitochondrial
ENSG0000	15.40705	0.062119	0.217668	0.500442	0.843101	ZNF718	255403	zinc finger protein 718
ENSG0000	349.9717	-0.07836	0.137584	0.50013	0.843101	PIGG	54872	phosphatidylinositol glycan anchor biosynthesis class G
ENSG0000	73.14614	0.071522	0.203006	0.500368	0.843101	BDP1	55814	B double p subunit of RNA polymerase III transcription initiation factor IIIB
ENSG0000	1152.309	0.080758	0.153867	0.5002	0.843101	MOSPD3	64598	motile sperm domain containing 3
ENSG0000	6.335348	0.05086	0.226674	0.500299	0.843101	CLCN1	1180	chloride voltage-gated channel 1
ENSG0000	393.5047	-0.07368	0.197841	0.500287	0.843101	SHOC2	8036	SHOC2 leucine rich repeat scaffold protein
ENSG0000	6.73976	-0.03876	0.227996	0.499978	0.843101	NA	NA	NA
ENSG0000	11.7993	0.071116	0.210547	0.499806	0.843101	HTR7P1	93164	5-hydroxytryptamine receptor 7 pseudogene 1
ENSG0000	4.268587	-0.03858	0.228933	0.499858	0.843101	NA	NA	NA
ENSG0000	628.3686	-0.08248	0.173157	0.500357	0.843101	NOC4L	79050	nucleolar complex associated 4 homolog
ENSG0000	36.50584	0.069142	0.208903	0.49958	0.843101	SACS	26278	sacsin molecular chaperone
ENSG0000	4.586803	0.043172	0.228297	0.499868	0.843101	LINC0228?	1.02E+08	long intergenic non-protein coding RNA 2287
ENSG0000	64.46602	-0.07854	0.18405	0.500092	0.843101	NIPA1	123606	NIPA magnesium transporter 1
ENSG0000	18.43825	-0.07811	0.195044	0.500218	0.843101	PSMC3IP	29893	PSMC3 interacting protein
ENSG0000	497.8669	-0.07256	0.19592	0.500348	0.843101	KCNJ2	3759	potassium inwardly rectifying channel subfamily J member 2
ENSG0000	2.928511	-0.02822	0.23126	0.500531	0.843101	CYP4F35P	284233	cytochrom pseudogene
ENSG0000	8.636897	0.050657	0.227939	0.500485	0.843101	NA	NA	NA
ENSG0000	3.346851	0.048885	0.228348	0.49968	0.843101	NA	NA	NA
ENSG0000	4578.161	-0.07641	0.147213	0.500308	0.843101	FKBP1A	2280	FKBP prolyl isomerase 1A
ENSG0000	74.97755	0.078667	0.187082	0.500253	0.843101	GTSE1	51512	G2 and S-phase expressed 1
ENSG0000	2284.153	-0.07496	0.137925	0.50053	0.843101	LMF2	91289	lipase maturation factor 2
ENSG0000	7.643696	0.029143	0.230181	0.500641	0.843106	TIMP4	7079	TIMP metallopeptidase inhibitor 4
ENSG0000	74.5724	-0.07909	0.164956	0.500672	0.843106	FAM114A:	10827	family with sequence similarity 114 member A2
ENSG0000	2.535859	-0.02143	0.232342	0.500659	0.843106	NA	NA	NA
ENSG0000	3.63727	0.043697	0.228041	0.500837	0.843306	CATSPER2	440278	cation channel sperm associated 2 pseudogene 1
ENSG0000	543.7207	0.070128	0.124173	0.500918	0.843365	YDJC	150223	YdjC chitooligosaccharide deacetylase homolog
ENSG0000	2.683531	-0.02881	0.231195	0.501211	0.843393	RGS5	8490	regulator of G protein signaling 5
ENSG0000	1142.623	0.074021	0.129442	0.501174	0.843393	CALHM2	51063	calcium homeostasis modulator family member 2
ENSG0000	178.2802	0.076518	0.189562	0.50113	0.843393	TMEM216	51259	transmembrane protein 216
ENSG0000	6.324155	-0.04305	0.227087	0.501041	0.843393	SLC25A10	1468	solute carrier family 25 member 10
ENSG0000	541.2376	-0.07637	0.145788	0.501095	0.843393	YJU2	55702	YJU2 splicing factor homolog
ENSG0000	9.05546	-0.06188	0.222867	0.501131	0.843393	LOC10537	1.05E+08	uncharacterized LOC105372321
ENSG0000	712.5437	-0.06893	0.116708	0.501291	0.843451	SFXN3	81855	sideroflexin 3
ENSG0000	374.8164	-0.07713	0.151493	0.501435	0.843562	COP1	64326	COP1 E3 ubiquitin ligase
ENSG0000	244.4529	-0.07626	0.186152	0.501468	0.843562	ZBTB34	403341	zinc finger and BTB domain containing 34
ENSG0000	130.408	0.076344	0.190648	0.501496	0.843562	CCDC117	150275	coiled-coil domain containing 117
ENSG0000	48.5615	0.064932	0.21038	0.501795	0.843756	WASL	8976	WASP like actin nucleation promoting factor
ENSG0000	35.22126	0.078915	0.187358	0.501772	0.843756	NA	NA	NA
ENSG0000	14.22548	-0.07284	0.206406	0.501787	0.843756	ARL4D	379	ADP ribosylation factor like GTPase 4D
ENSG0000	508.9117	0.078498	0.161428	0.501794	0.843756	UBE2O	63893	ubiquitin conjugating enzyme E2 O
ENSG0000	5.295588	-0.04741	0.22656	0.502183	0.844142	CNN3	1266	calponin 3
ENSG0000	4.218566	-0.00934	0.233827	0.502138	0.844142	LINGO1	84894	leucine rich repeat and Ig domain containing 1
ENSG0000	204.5435	0.077619	0.161146	0.502084	0.844142	DUS2	54920	dihydrouridine synthase 2
ENSG0000	27.36112	-0.07442	0.200184	0.502209	0.844142	NAPSA	9476	napsin A aspartic peptidase
ENSG0000	1489.374	0.076564	0.18915	0.50231	0.844234	KREMEN1	83999	kringle containing transmembrane protein 1
ENSG0000	12.4132	0.065979	0.21456	0.502579	0.844455	NA	NA	NA
ENSG0000	129.512	-0.07875	0.16546	0.502642	0.844455	SLC43A3	29015	solute carrier family 43 member 3
ENSG0000	83.6603	0.079019	0.180643	0.502619	0.844455	DBNDD1	79007	dysbindin domain containing 1
ENSG0000	64.60914	-0.07303	0.201794	0.502652	0.844455	GRIN2D	2906	glutamate ionotropic receptor NMDA type subunit 2D
ENSG0000	2.723866	0.026168	0.230944	0.502672	0.844455	NA	NA	NA
ENSG0000	349.2349	0.069001	0.122542	0.50348	0.844466	LRRC47	57470	leucine rich repeat containing 47
ENSG0000	169.3868	0.079286	0.170626	0.503838	0.844466	CLIC4	25932	chloride intracellular channel 4
ENSG0000	108.069	0.076449	0.155849	0.503713	0.844466	CEP85	64793	centrosomal protein 85
ENSG0000	136.9645	0.079097	0.175668	0.502984	0.844466	COX20	116228	cytochrome c oxidase assembly factor COX20
ENSG0000	111.9504	0.074919	0.191428	0.502847	0.844466	NIF3L1	60491	NGG1 interacting factor 3 like 1
ENSG0000	3.450596	0.048372	0.22706	0.503183	0.844466	RPS3P3	440991	ribosomal protein S3 pseudogene 3
ENSG0000	27.27883	0.078004	0.192985	0.503419	0.844466	DNAJC18	202052	DnaJ heat shock protein family (Hsp40) member C18
ENSG0000	169.05	-0.07571	0.145391	0.50295	0.844466	ZSCAN16-/	1E+08	ZSCAN16 antisense RNA 1
ENSG0000	25.51868	0.067662	0.210033	0.50404	0.844466	RPL27AP6	389435	ribosomal protein L27a pseudogene 6
ENSG0000	946.5236	0.074736	0.14684	0.503775	0.844466	RNF216	54476	ring finger protein 216
ENSG0000	2.751456	-0.0303	0.230526	0.503606	0.844466	INTS4P1	285905	integrator complex subunit 4 pseudogene 1
ENSG0000	6.531188	-0.05225	0.223721	0.503951	0.844466	TRIM73	375593	tripartite motif containing 73
ENSG0000	233.5036	-0.078	0.157882	0.503617	0.844466	PSMD5	5711	proteasom non-ATPase 5
ENSG0000	190.7875	0.069453	0.204094	0.502734	0.844466	RAB18	22931	RAB18 member RAS oncogene family
ENSG0000	6.432678	-0.05245	0.224212	0.503855	0.844466	NA	NA	NA
ENSG0000	437.0671	0.08656	0.170378	0.504061	0.844466	PPP6R3	55291	protein phosphatase 6 regulatory subunit 3
ENSG0000	6579.901	-0.07915	0.165431	0.503684	0.844466	HSPA8	3312	heat shock protein family A (Hsp70) member 8
ENSG0000	1187.496	0.071814	0.13139	0.503377	0.844466	ZNF598	90850	zinc finger E3 ubiquitin ligase
ENSG0000	275.3153	0.070127	0.133933	0.503485	0.844466	EMC8	10328	ER membrane protein complex subunit 8
ENSG0000	3.46192	0.040809	0.228529	0.503145	0.844466	NA	NA	NA
ENSG0000	595.2907	0.077805	0.17387	0.502997	0.844466	TAF15	8148	TATA-box binding protein associated factor 15
ENSG0000	3230.305	-0.07549	0.144978	0.503447	0.844466	ABI3	51225	ABI family member 3
ENSG0000	381.737	-0.0761	0.149738	0.50406	0.844466	TTYH2	94015	tweety family member 2
ENSG0000	459.8764	0.070779	0.127027	0.502893	0.844466	FEM1A	55527	fem-1 homolog A
ENSG0000	63.18127	0.081635	0.169676	0.503943	0.844466	NA	NA	NA
ENSG0000	512.9067	-0.06907	0.121734	0.503612	0.844466	ATG4D	84971	autophagy related 4D cysteine peptidase
ENSG0000	2.858419	-0.03577	0.229853	0.503057	0.844466	LHB	3972	luteinizing hormone subunit beta
ENSG0000	15.56969	-0.05436	0.223086	0.503952	0.844466	LOC10272	1.03E+08	salt inducible kinase 1B (putative)
ENSG0000	71.19862	-0.07828	0.17919	0.503051	0.844466	NPTXR	23467	neuronal pentraxin receptor
ENSG0000	1000.434	-0.06881	0.12107	0.503816	0.844466	PHF8	23133	PHD finger protein 8
ENSG0000	1536.611	-0.07746	0.156114	0.504547	0.844533	SSBP3	23648	single stranded DNA binding protein 3
ENSG0000	671.2879	0.071492	0.131534	0.5047	0.844533	ARNT	405	aryl hydrocarbon receptor nuclear translocator
ENSG0000	215.3071	0.077376	0.149508	0.504516	0.844533	DCAF1	9730	DDB1 and CUL4 associated factor 1
ENSG0000	4.889185	0.040728	0.228018	0.504538	0.844533	SLC26A1	10861	solute carrier family 26 member 1
ENSG0000	7.156297	-0.03752	0.227817	0.504414	0.844533	AK6	1.02E+08	adenylate kinase 6
ENSG0000	448.2063	0.071549	0.128959	0.504503	0.844533	ABT1	29777	activator of basal transcription 1
ENSG0000	31.31433	-0.07719	0.188064	0.504445	0.844533	NA	NA	NA
ENSG0000	2.962995	0.035694	0.230723	0.504369	0.844533	LOC11484	1.15E+08	Uncharacterized protein LOC114841035
ENSG0000	89.13202	0.077956	0.185292	0.504698	0.844533	PRSS36	146547	serine protease 36

ENSG0000	42.40719	-0.07718	0.187813	0.504688	0.844533	NPEPPSP1	440434	NPEPPS pseudogene 1
ENSG0000	5.232871	-0.03852	0.227464	0.504163	0.844533	NA	NA	NA
ENSG0000	40.01073	-0.05839	0.217024	0.504643	0.844533	PLA2G4C	8605	phospholipase A2 group IVC
ENSG0000	3.143918	-0.03095	0.231192	0.504587	0.844533	NA	NA	NA
ENSG0000	1783.214	-0.07298	0.1512	0.504865	0.844613	PRCC	5546	proline rich mitotic checkpoint control factor
ENSG0000	100.5407	-0.07407	0.194537	0.504886	0.844613	PREPL	9581	prolyl endopeptidase like
ENSG0000	9.409384	-0.06477	0.216267	0.504843	0.844613	SLC16A10	117247	solute carrier family 16 member 10
ENSG0000	29.21694	-0.07493	0.196665	0.50494	0.844625	ACKR3	57007	atypical chemokine receptor 3
ENSG0000	102.007	0.077903	0.155078	0.505124	0.844778	LINC0112ε	643837	long intergenic non-protein coding RNA 1128
ENSG0000	212.4279	0.076777	0.184846	0.505088	0.844778	CCT2	10576	chaperonin containing TCP1 subunit 2
ENSG0000	2.975504	0.021743	0.231524	0.505174	0.844785	MAGOH-D	1.01E+08	MAGOH divergent transcript
ENSG0000	281.7513	0.078537	0.171404	0.505233	0.844806	SUV39H1	6839	SUV39H1 histone lysine methyltransferase
ENSG0000	1404.599	0.067047	0.114571	0.505548	0.845179	PSMB4	5692	proteasome 20S subunit beta 4
ENSG0000	171.8117	-0.07649	0.149818	0.50554	0.845179	ZNF337	26152	zinc finger protein 337
ENSG0000	6.037662	-0.05366	0.223864	0.505596	0.845183	NA	NA	NA
ENSG0000	1557.255	0.074459	0.137018	0.505724	0.845319	SNX17	9784	sorting nexin 17
ENSG0000	322.9472	-0.07696	0.154197	0.505977	0.845588	NSMAF	8439	neutral sphingomyelinase activation associated factor
ENSG0000	22.36103	0.074244	0.200803	0.505956	0.845588	NA	NA	NA
ENSG0000	25.55871	0.069977	0.206247	0.506149	0.845798	ANKRD37	353322	ankyrin repeat domain 37
ENSG0000	174.9193	0.07819	0.155139	0.506225	0.845848	HYAL2	8692	hyaluronidase 2
ENSG0000	32.9872	-0.07408	0.19776	0.506286	0.845872	NEO1	4756	neogenin 1
ENSG0000	2919.858	-0.07414	0.144649	0.506352	0.845887	YY1AP1	55249	YY1 associated protein 1
ENSG0000	4.059527	-0.03236	0.229971	0.506444	0.845887	LOC10537	1.05E+08	uncharacterized LOC105375787
ENSG0000	90.86268	0.08116	0.174172	0.506467	0.845887	PDHX	8050	pyruvate dehydrogenase complex component X
ENSG0000	561.9292	0.07385	0.137645	0.506479	0.845887	MZF1	7593	myeloid zinc finger 1
ENSG0000	886.527	-0.0753	0.149917	0.506645	0.845891	S1PR1	1901	sphingosine-1-phosphate receptor 1
ENSG0000	32.38427	0.065286	0.211206	0.506666	0.845891	C4orf46	201725	chromosome 4 open reading frame 46
ENSG0000	53.25278	-0.07839	0.173068	0.506654	0.845891	NA	NA	NA
ENSG0000	90.95503	-0.08267	0.183058	0.506604	0.845891	RGS9	8787	regulator of G protein signaling 9
ENSG0000	34.65819	-0.07033	0.196615	0.506838	0.845999	SUSD4	55061	sushi domain containing 4
ENSG0000	93.66343	0.078384	0.16119	0.506797	0.845999	DOLK	22845	dolichol kinase
ENSG0000	21.74822	-0.0779	0.192731	0.50687	0.845999	RFPL3S	10737	RFPL3 antisense
ENSG0000	42.97206	0.07791	0.188014	0.506923	0.846011	PCSK4	54760	proprotein convertase subtilisin/kexin type 4
ENSG0000	1543.768	-0.06922	0.118805	0.507094	0.846174	SMARCD1	6602	SWI/SNF r matrix ass actin depe subfamily member 1
ENSG0000	10.06648	0.06319	0.217535	0.507113	0.846174	NA	NA	NA
ENSG0000	238.2131	0.074093	0.153517	0.50762	0.846263	KLHL17	339451	kelch like family member 17
ENSG0000	12.6001	0.052151	0.222219	0.507463	0.846263	PRORSD1F	344405	prolyl-tRN pseudogene
ENSG0000	548.3201	0.07622	0.149469	0.507406	0.846263	PPP3R1	5534	protein ph alpha
ENSG0000	754.8871	0.074389	0.181105	0.507767	0.846263	CD38	952	CD38 molecule
ENSG0000	772.5101	-0.07758	0.15452	0.507437	0.846263	NFKB1	4790	nuclear factor kappa B subunit 1
ENSG0000	6.674388	0.052575	0.224073	0.507525	0.846263	BTN1A1	696	butyrophilin subfamily 1 member A1
ENSG0000	328.0246	0.07365	0.194565	0.507399	0.846263	CNOT7	29883	CCR4-NOT transcription complex subunit 7
ENSG0000	10.01179	-0.05947	0.218813	0.507218	0.846263	HPS1-AS1	1.02E+08	HPS1 antisense RNA 1
ENSG0000	61.02752	-0.07855	0.164275	0.507655	0.846263	NA	NA	NA
ENSG0000	41.02397	-0.06791	0.206936	0.507728	0.846263	NA	NA	NA
ENSG0000	2963.706	0.076341	0.151665	0.507671	0.846263	ACADVL	37	acyl-CoA dehydrogenase very long chain
ENSG0000	91.81349	-0.07777	0.159601	0.50738	0.846263	PDE6G	5148	phosphodiesterase 6G
ENSG0000	96.69404	-0.07147	0.197148	0.507734	0.846263	PCP2	126006	Purkinje cell protein 2
ENSG0000	22.21632	0.07326	0.201193	0.508101	0.846743	NBPF1	55672	NBPF member 1
ENSG0000	6549.186	0.077768	0.163951	0.508308	0.8468	SLC6A6	6533	solute carrier family 6 member 6
ENSG0000	105.4854	0.077184	0.183518	0.508239	0.8468	TAF11	6882	TATA-box binding protein associated factor 11
ENSG0000	6356.044	-0.06598	0.17129	0.50832	0.8468	PLPPR2	64748	phospholipid phosphatase related 2
ENSG0000	24.51987	0.073171	0.201232	0.508248	0.8468	LINC01547	84536	long intergenic non-protein coding RNA 1547
ENSG0000	2.541415	-0.03947	0.229211	0.508534	0.846831	TSACC	128229	TSSK6 activating cochaperone
ENSG0000	142.7283	0.075364	0.176262	0.508535	0.846831	PAQR8	85315	progestin and adipoQ receptor family member 8
ENSG0000	646.9895	0.075713	0.191789	0.508617	0.846831	ADD3	120	adducin 3
ENSG0000	121.2469	0.0663	0.207983	0.508432	0.846831	LINC00937	389634	long intergenic non-protein coding RNA 937
ENSG0000	4.11185	0.050908	0.225527	0.508529	0.846831	NA	NA	NA
ENSG0000	661.4085	-0.0677	0.117558	0.508648	0.846831	MRPL10	124995	mitochondrial ribosomal protein L10
ENSG0000	1149.419	0.07194	0.128198	0.508663	0.846831	FAAP100	80233	FA core complex associated protein 100
ENSG0000	348.9968	-0.07234	0.134579	0.50885	0.84693	HEMK1	51409	HemK methyltransferase family member 1
ENSG0000	8.502531	0.051371	0.223581	0.508865	0.84693	NA	NA	NA
ENSG0000	41.91242	0.080076	0.174282	0.508907	0.84693	SLC24A1	9187	solute carrier family 24 member 1
ENSG0000	16.29289	0.046981	0.224166	0.508906	0.84693	NA	NA	NA
ENSG0000	25.17169	-0.07184	0.202272	0.510435	0.847009	NBPF3	84224	NBPF member 3
ENSG0000	26.59893	0.069159	0.206314	0.510437	0.847009	NA	NA	NA
ENSG0000	28476.05	0.077335	0.166544	0.510634	0.847009	TAGLN2	8407	transgelin 2
ENSG0000	45.42091	0.065973	0.208936	0.509869	0.847009	MIXL1	83881	Mix paired-like homeobox
ENSG0000	2.519388	0.022656	0.232383	0.510216	0.847009	LINC0137ε	400945	long intergenic non-protein coding RNA 1376
ENSG0000	37.53426	0.057342	0.217644	0.509775	0.847009	HNMT	3176	histamine N-methyltransferase
ENSG0000	4.546281	0.037919	0.229213	0.50971	0.847009	HNRNPA1I	344697	heterogeneous nuclear ribonucleoprotein A1 pseudogene 21
ENSG0000	25.21467	0.070789	0.205145	0.509155	0.847009	TMEM128	85013	transmembrane protein 128
ENSG0000	765.6626	-0.06801	0.126594	0.510612	0.847009	GALNT10	55568	polypeptide N-acetylgalactosaminyltransferase 10
ENSG0000	143.5236	0.078685	0.163996	0.510625	0.847009	GMDS	2762	GDP-manr 6-dehydratase
ENSG0000	32.7621	0.07806	0.189625	0.509532	0.847009	WAKMAR:	1E+08	wound and keratinocyte migration associated lncRNA 2
ENSG0000	157358.4	-0.07533	0.180149	0.509979	0.847009	ACTB	60	actin beta
ENSG0000	77.28225	-0.0755	0.184169	0.510297	0.847009	ORC5	5001	origin recognition complex subunit 5
ENSG0000	7.185298	0.061514	0.226194	0.510352	0.847009	NA	NA	NA
ENSG0000	2.321311	0.018931	0.233227	0.509928	0.847009	NA	NA	NA
ENSG0000	2260.32	-0.07625	0.167807	0.510506	0.847009	CYRIB	51571	CYFIP related Rac1 interactor B
ENSG0000	2159.702	0.067351	0.116039	0.509538	0.847009	FNBP1	23048	formin binding protein 1
ENSG0000	307.0708	-0.07329	0.189781	0.510045	0.847009	TNKS2	80351	tankyrase 2
ENSG0000	244.9783	0.076989	0.154052	0.509812	0.847009	PPP1R14B	26472	protein phosphatase 1 regulatory inhibitor subunit 14B
ENSG0000	422.9139	0.066584	0.116339	0.509348	0.847009	ADIPOR2	79602	adiponectin receptor 2
ENSG0000	63.77704	-0.06942	0.202631	0.510228	0.847009	NA	NA	NA
ENSG0000	527.6547	0.06531	0.113821	0.510305	0.847009	ZNF740	283337	zinc finger protein 740
ENSG0000	114.4766	-0.07882	0.157343	0.509875	0.847009	GDF11	10220	growth differentiation factor 11
ENSG0000	21.16803	0.069492	0.20786	0.509735	0.847009	CENPJ	55835	centromere protein J
ENSG0000	51.31322	0.078616	0.173715	0.510298	0.847009	SLC7A8	23428	solute carrier family 7 member 8
ENSG0000	42.37871	0.077941	0.174792	0.510019	0.847009	BCL2L2	599	BCL2 like 2
ENSG0000	171.8011	0.078327	0.185413	0.509201	0.847009	FAH	2184	fumarylacetoacetate hydrolase
ENSG0000	3.194834	-0.02623	0.230364	0.509435	0.847009	NA	NA	NA
ENSG0000	45.50654	-0.07818	0.174168	0.509198	0.847009	NA	NA	NA
ENSG0000	5.272716	-0.04122	0.227167	0.509148	0.847009	NA	NA	NA
ENSG0000	36.26683	-0.07577	0.190666	0.510319	0.847009	ZNF566	84924	zinc finger protein 566
ENSG0000	341.3143	0.075524	0.151019	0.510055	0.847009	MRPS12	6183	mitochondrial ribosomal protein S12

ENSG0000	27.16147	-0.07526	0.198888	0.510194	0.847009	NA	NA	NA
ENSG0000	806.531	-0.0759	0.157701	0.509847	0.847009	MAP3K10	4294	mitogen-activated protein kinase kinase kinase 10
ENSG0000	10.20911	-0.04651	0.224844	0.510364	0.847009	CNFN	84518	cornifelin
ENSG0000	5.966776	0.047934	0.225149	0.510665	0.847009	NA	NA	NA
ENSG0000	106.7219	0.075028	0.193113	0.509176	0.847009	TXLNG	55787	taxilin gamma
ENSG0000	2.518298	0.023737	0.231339	0.510753	0.847077	NA	NA	NA
ENSG0000	401.2119	0.07637	0.162678	0.511023	0.847116	SRSF7	6432	serine and arginine rich splicing factor 7
ENSG0000	910.3761	0.065821	0.121705	0.510972	0.847116	STK25	10494	serine/threonine kinase 25
ENSG0000	2.608904	-0.01802	0.232024	0.510984	0.847116	NA	NA	NA
ENSG0000	68.68146	0.065731	0.202203	0.511103	0.847116	POLK	51426	DNA polymerase kappa
ENSG0000	6.969912	-0.05918	0.220173	0.511014	0.847116	MATR3	9782	matrin 3
ENSG0000	65.59616	0.076286	0.186431	0.510923	0.847116	MIOS	54468	meiosis regulator for oocyte development
ENSG0000	9.193532	-0.05965	0.218936	0.511133	0.847116	FOXO3B	2310	forkhead box O3B
ENSG0000	13.30959	0.071077	0.207213	0.511146	0.847116	RPL36A	6173	ribosomal protein L36a
ENSG0000	105.0058	0.076969	0.168267	0.511339	0.847359	NEK4	6787	NIMA related kinase 4
ENSG0000	43.09099	-0.07628	0.186165	0.51142	0.847397	SMAD1	4086	SMAD family member 1
ENSG0000	15.56393	0.060635	0.216919	0.511454	0.847397	KRT73-AS1	1E+08	KRT73 antisense RNA 1
ENSG0000	41.9688	0.059989	0.214772	0.511508	0.847397	COMMD6	170622	COMM domain containing 6
ENSG0000	4.349483	0.033	0.229175	0.511547	0.847397	NA	NA	NA
ENSG0000	98.17192	0.077382	0.166723	0.511649	0.847489	TTL	150465	tubulin tyrosine ligase
ENSG0000	1164.708	-0.06838	0.136461	0.512244	0.847516	TPRG1L	127262	tumor protein p63 regulated 1 like
ENSG0000	273.0787	0.076625	0.156873	0.512138	0.847516	PPIE	10450	peptidylprolyl isomerase E
ENSG0000	16.72257	0.055325	0.219166	0.511946	0.847516	NSRP1P1	1E+08	nuclear speckle splicing regulatory protein 1 pseudogene 1
ENSG0000	21.94868	-0.0757	0.193635	0.512067	0.847516	RTKN	6242	rhotekin
ENSG0000	3.877032	-0.04039	0.229873	0.512069	0.847516	RPL34-DT	285456	RPL34 divergent transcript
ENSG0000	3.118977	0.023096	0.231148	0.512041	0.847516	ANAPC10	10393	anaphase promoting complex subunit 10
ENSG0000	5.530734	-0.04395	0.225859	0.511924	0.847516	NA	NA	NA
ENSG0000	33.24799	-0.06995	0.201961	0.512247	0.847516	RCN1	5954	reticulocalbin 1
ENSG0000	16.40022	0.070202	0.206642	0.511957	0.847516	NA	NA	NA
ENSG0000	360.5872	-0.07437	0.170427	0.512266	0.847516	CD3G	917	CD3 gamma subunit of T-cell receptor complex
ENSG0000	266.1204	0.070979	0.149556	0.512131	0.847516	POU6F1	5463	POU class 6 homeobox 1
ENSG0000	17904.44	-0.07533	0.176718	0.511836	0.847516	UBB	7314	ubiquitin B
ENSG0000	113.0342	-0.07801	0.172015	0.511877	0.847516	ZNF74	7625	zinc finger protein 74
ENSG0000	272.3033	0.070209	0.200193	0.512386	0.847602	TM9SF3	56889	transmembrane 9 superfamily member 3
ENSG0000	10.75048	-0.04958	0.223222	0.512411	0.847602	NA	NA	NA
ENSG0000	7.544107	-0.04723	0.224608	0.512464	0.847613	SOAT2	8435	sterol O-acyltransferase 2
ENSG0000	69.92925	0.075178	0.188533	0.512534	0.847653	FAM209A	200232	family with sequence similarity 209 member A
ENSG0000	12.00002	-0.05985	0.219884	0.512974	0.847788	NA	NA	NA
ENSG0000	41.35513	0.07542	0.19004	0.512855	0.847788	RAB28	9364	RAB28 member RAS oncogene family
ENSG0000	4.207578	-0.01797	0.232225	0.512987	0.847788	NR2E1	7101	nuclear receptor subfamily 2 group E member 1
ENSG0000	5611.044	-0.07538	0.17745	0.512775	0.847788	CAPN1	823	calpain 1
ENSG0000	2.787227	0.030301	0.230754	0.512889	0.847788	KRT86	3892	keratin 86
ENSG0000	5.264592	0.039238	0.227852	0.512764	0.847788	NA	NA	NA
ENSG0000	3463.341	-0.07547	0.157104	0.512942	0.847788	CYBB	1536	cytochrome b-245 beta chain
ENSG0000	8.849784	-0.05754	0.220201	0.512854	0.847788	NA	NA	NA
ENSG0000	2.902059	0.026413	0.230767	0.513095	0.847845	NA	NA	NA
ENSG0000	1257.508	-0.07273	0.141867	0.51316	0.847845	TMEM109	79073	transmembrane protein 109
ENSG0000	140.78	-0.07187	0.135278	0.513129	0.847845	PROCA1	147011	protein interacting with cyclin A1
ENSG0000	224.2589	0.074835	0.139897	0.51327	0.847898	GNPAT	8443	glyceronephosphate O-acyltransferase
ENSG0000	3.70478	0.033457	0.231919	0.513284	0.847898	OOEP	441161	oocyte expressed protein
ENSG0000	4.307649	0.040805	0.227777	0.51337	0.847963	NA	NA	NA
ENSG0000	66.70044	0.073431	0.191968	0.513586	0.848147	CDKN2C	1031	cyclin dependent kinase inhibitor 2C
ENSG0000	89.74953	-0.07636	0.177411	0.51362	0.848147	NA	NA	NA
ENSG0000	224.6359	0.074765	0.174028	0.513536	0.848147	RAPGEFL1	51195	Rap guanine nucleotide exchange factor like 1
ENSG0000	82.29677	0.079022	0.163555	0.513707	0.848214	MCCC1	56922	methylcrotonyl-CoA carboxylase subunit 1
ENSG0000	9.703335	0.049021	0.223835	0.513824	0.848254	LOC15476	154761	family witl member C pseudogene
ENSG0000	43.92334	-0.06822	0.206371	0.513819	0.848254	TXNDC17	84817	thioredoxin domain containing 17
ENSG0000	23.73848	0.076006	0.191484	0.514078	0.848598	RAD51	5888	RAD51 recombinase
ENSG0000	3.848321	0.041878	0.227755	0.514175	0.848681	NA	NA	NA
ENSG0000	37.71395	0.074052	0.19603	0.514236	0.848704	PKHD1L1	93035	PKHD1 like 1
ENSG0000	59.68963	0.067517	0.205285	0.515594	0.848906	SLC16A1	6566	solute carrier family 16 member 1
ENSG0000	30.83179	0.075931	0.186937	0.515674	0.848906	GLRX2	51022	glutaredoxin 2
ENSG0000	843.25	-0.07198	0.189885	0.514895	0.848906	PELI1	57162	pellino E3 ubiquitin protein ligase 1
ENSG0000	13.51662	0.045572	0.224013	0.515464	0.848906	LIPT1	51601	lipoyltransferase 1
ENSG0000	3.357862	-0.02867	0.230548	0.515577	0.848906	OBSL1	23363	obscurin like cytoskeletal adaptor 1
ENSG0000	52.62115	-0.06974	0.20058	0.515887	0.848906	SNORC	389084	secondary ossification center associated regulator of chondrocyte maturation
ENSG0000	147.0543	-0.07512	0.16092	0.515326	0.848906	THOC7	80145	THO complex subunit 7
ENSG0000	93.13776	0.067512	0.204523	0.514702	0.848906	HOPX	84525	HOP homeobox
ENSG0000	133.3953	-0.07459	0.181856	0.515806	0.848906	F12	2161	coagulation factor XII
ENSG0000	2933.413	-0.07216	0.13954	0.515093	0.848906	RGL2	5863	ral guanine nucleotide dissociation stimulator like 2
ENSG0000	3.802405	0.031913	0.230379	0.514794	0.848906	MIR4653	1.01E+08	microRNA 4653
ENSG0000	61.40741	-0.0766	0.163467	0.515759	0.848906	LOXL2	4017	lysyl oxidase like 2
ENSG0000	168.8745	0.076075	0.160051	0.51501	0.848906	RECQL4	9401	RecQ like helicase 4
ENSG0000	866.3173	-0.07579	0.164756	0.51526	0.848906	ABI1	10006	abl interactor 1
ENSG0000	143.1761	0.073772	0.148793	0.515855	0.848906	RBM4B	83759	RNA binding motif protein 4B
ENSG0000	441.512	0.069747	0.124325	0.514587	0.848906	NFRKB	4798	nuclear factor related to kappaB binding protein
ENSG0000	2.774331	-0.0289	0.230598	0.514633	0.848906	NA	NA	NA
ENSG0000	64.95254	0.075204	0.181768	0.514557	0.848906	PLBD1-AS1	1.02E+08	PLBD1 antisense RNA 1
ENSG0000	83.05658	0.072635	0.192351	0.515517	0.848906	MDM1	56890	Mdm1 nuclear protein
ENSG0000	499.0725	0.075062	0.144521	0.515627	0.848906	BRF1	2972	BRF1 RNA polymerase III transcription initiation factor subunit
ENSG0000	2.594094	0.032083	0.23287	0.515645	0.848906	NA	NA	NA
ENSG0000	529.0764	-0.06606	0.121251	0.515058	0.848906	ZNF688	146542	zinc finger protein 688
ENSG0000	14.55601	0.060452	0.216442	0.514878	0.848906	NA	NA	NA
ENSG0000	272.5312	0.072703	0.136461	0.514985	0.848906	OGFOD3	79701	2-oxoglutarate and iron dependent oxygenase domain containing 3
ENSG0000	80.22061	0.075721	0.165625	0.515291	0.848906	TUBB6	84617	tubulin beta 6 class V
ENSG0000	29.95713	-0.07292	0.201143	0.514915	0.848906	PPP1R14A	94274	protein phosphatase 1 regulatory inhibitor subunit 14A
ENSG0000	355.5179	0.07597	0.173747	0.515232	0.848906	LIG1	3978	DNA ligase 1
ENSG0000	2063.125	-0.07655	0.177204	0.51444	0.848906	SIGLEC9	27180	sialic acid binding Ig like lectin 9
ENSG0000	3.524317	0.026851	0.23114	0.515863	0.848906	HM13-AS1	1.01E+08	HM13 antisense RNA 1
ENSG0000	13.72579	0.062718	0.215197	0.515636	0.848906	TTC28	23331	tetratricopeptide repeat domain 28
ENSG0000	1131.591	0.073572	0.144018	0.515616	0.848906	SELENOO	83642	selenoprotein O
ENSG0000	5.930919	-0.05392	0.22299	0.515361	0.848906	EZHIP	340602	EZH inhibitory protein
ENSG0000	7.866738	-0.04434	0.225558	0.514914	0.848906	USP51	158880	ubiquitin specific peptidase 51
ENSG0000	169.8004	0.07629	0.17475	0.515973	0.848962	CCDC50	152137	coiled-coil domain containing 50
ENSG0000	155.4124	-0.07991	0.170209	0.516014	0.848962	ROBO3	64221	roundabout guidance receptor 3
ENSG0000	13.95198	-0.06755	0.208776	0.5161	0.849027	NA	NA	NA

ENSG0000	9.524348	-0.05823	0.218662	0.516336	0.849263	NA	NA	NA
ENSG0000	5.154646	-0.03537	0.228827	0.516295	0.849263	NA	NA	NA
ENSG0000	85.01079	0.077063	0.17605	0.516517	0.849277	FBXL4	26235	F-box and leucine rich repeat protein 4
ENSG0000	3111.787	0.077564	0.163079	0.516495	0.849277	TTYH3	80727	tweety family member 3
ENSG0000	13.53978	0.058547	0.21735	0.516478	0.849277	NA	NA	NA
ENSG0000	1608.211	0.062685	0.114522	0.51653	0.849277	DCAF15	90379	DDB1 and CUL4 associated factor 15
ENSG0000	13.22269	0.055887	0.219454	0.516638	0.849292	CDC42BPA	8476	CDC42 binding protein kinase alpha
ENSG0000	237.0636	0.07342	0.141875	0.516634	0.849292	UBXN7	26043	UBX domain protein 7
ENSG0000	211.5548	0.071438	0.134696	0.516678	0.849292	SLC29A3	55315	solute carrier family 29 member 3
ENSG0000	4.586317	0.04428	0.226537	0.51684	0.849482	LAMA2	3908	laminin subunit alpha 2
ENSG0000	3.267002	0.02898	0.231202	0.517025	0.849553	NA	NA	NA
ENSG0000	1071.001	-0.06674	0.121511	0.517069	0.849553	RXRB	6257	retinoid X receptor beta
ENSG0000	37.26689	0.052369	0.219081	0.517039	0.849553	SLC35A1	10559	solute carrier family 35 member A1
ENSG0000	191.6615	0.06992	0.194626	0.517034	0.849553	PLAGL1	5325	PLAG1 like zinc finger 1
ENSG0000	16.32751	-0.06391	0.212847	0.517239	0.849757	NA	NA	NA
ENSG0000	27.86288	0.065812	0.208459	0.517479	0.84977	CEP57L1	285753	centrosomal protein 57 like 1
ENSG0000	282.4904	0.071235	0.194295	0.517435	0.84977	SESN3	143686	sestrin 3
ENSG0000	91.95909	-0.07621	0.157776	0.517422	0.84977	MTMR2	8898	myotubularin related protein 2
ENSG0000	35.66503	0.07147	0.199918	0.51743	0.84977	CYP4F12	66002	cytochrome P450 family 4 subfamily F member 12
ENSG0000	6.014083	-0.03061	0.229568	0.51743	0.84977	UPK1A	11045	uroplakin 1A
ENSG0000	31.82594	0.075772	0.187491	0.517548	0.849806	NAGS	162417	N-acetylglutamate synthase
ENSG0000	45.91693	-0.07573	0.185303	0.517732	0.849957	INPP5F	22876	inositol polyphosphate-5-phosphatase F
ENSG0000	5.261946	0.041684	0.227483	0.517723	0.849957	NA	NA	NA
ENSG0000	444.2272	0.076594	0.15464	0.517786	0.849969	SLC26A6	65010	solute carrier family 26 member 6
ENSG0000	10.23629	0.047298	0.22394	0.517843	0.849987	GNB3	2784	G protein subunit beta 3
ENSG0000	88.04016	0.077048	0.185251	0.518118	0.850003	CENPO	79172	centromere protein O
ENSG0000	9.559927	-0.04901	0.225811	0.518168	0.850003	SNORD89	692205	small nucl(C/D box 89
ENSG0000	218.8543	-0.07447	0.179746	0.518144	0.850003	RAB5A	5868	RAB5A member RAS oncogene family
ENSG0000	127.9502	0.075819	0.162074	0.517936	0.850003	DLAT	1737	dihydrolipoamide S-acetyltransferase
ENSG0000	12.60356	-0.06469	0.212504	0.517996	0.850003	PRR29-AS1	400612	PRR29 antisense RNA 1
ENSG0000	1234.823	0.075213	0.159899	0.51806	0.850003	PRR12	57479	proline rich 12
ENSG0000	26.53116	0.069833	0.203961	0.518178	0.850003	SEPTIN5	5413	septin 5
ENSG0000	9.200281	0.032559	0.229304	0.518558	0.850146	NEGR1	257194	neuronal growth regulator 1
ENSG0000	170.9341	0.07357	0.186332	0.518898	0.850146	NA	NA	NA
ENSG0000	16.64357	0.064642	0.211956	0.518693	0.850146	LINC01126	1E+08	long intergenic non-protein coding RNA 1126
ENSG0000	28.13957	-0.06704	0.206471	0.518857	0.850146	XCR1	2829	X-C motif chemokine receptor 1
ENSG0000	129.1326	0.073238	0.19034	0.518915	0.850146	MRFAP1L1	114932	Morf4 family associated protein 1 like 1
ENSG0000	13.71309	0.064509	0.212152	0.518708	0.850146	SLC29A4	222962	solute carrier family 29 member 4
ENSG0000	3.898883	-0.03053	0.229273	0.518741	0.850146	AK3P3	1E+08	adenylate kinase 3 pseudogene 3
ENSG0000	37.66858	0.045285	0.223013	0.518873	0.850146	ZNF92	168374	zinc finger protein 92
ENSG0000	3.114341	0.023457	0.230804	0.518622	0.850146	NA	NA	NA
ENSG0000	156.7986	-0.06735	0.136547	0.518671	0.850146	EXT2	2132	exostosin glycosyltransferase 2
ENSG0000	12.28872	-0.05745	0.217826	0.518512	0.850146	ST8SIA1	6489	ST8 alpha- 8-sialyltransferase 1
ENSG0000	4683.7	0.060541	0.105117	0.518768	0.850146	SNRNP70	6625	small nuclear ribonucleoprotein U1 subunit 70
ENSG0000	134.8454	-0.07251	0.141436	0.518531	0.850146	EOLA1	91966	endothelium and lymphocyte associated ASCH domain 1
ENSG0000	96.23163	-0.07492	0.15509	0.518393	0.850146	TMEM185	84548	transmembrane protein 185A
ENSG0000	20.52594	-0.05779	0.216148	0.519037	0.850271	NA	NA	NA
ENSG0000	9.218886	0.046835	0.224107	0.519131	0.850347	NA	NA	NA
ENSG0000	9.291392	0.047812	0.223686	0.519251	0.850468	SMG1P5	595101	SMG1 pseudogene 5
ENSG0000	3.967678	0.041059	0.227428	0.519298	0.85047	NA	NA	NA
ENSG0000	30.07691	-0.0621	0.211107	0.519367	0.850506	PRIMPOL	201973	primase and DNA directed polymerase
ENSG0000	722.0363	0.063925	0.111721	0.519463	0.850588	EIF4E2	9470	eukaryotic translation initiation factor 4E family member 2
ENSG0000	154.443	0.073185	0.134278	0.51965	0.850665	NRF1	4899	nuclear respiratory factor 1
ENSG0000	1076.025	-0.06761	0.1219	0.519575	0.850665	ANKRD52	283373	ankyrin repeat domain 52
ENSG0000	577.2788	0.072027	0.141501	0.519616	0.850665	DDX49	54555	DEAD-box helicase 49
ENSG0000	110.9596	0.071035	0.196336	0.519709	0.850686	PHGDH	26227	phosphoglycerate dehydrogenase
ENSG0000	3.520501	0.038128	0.22887	0.519952	0.850854	NA	NA	NA
ENSG0000	977.9108	-0.07099	0.189931	0.519998	0.850854	CD46	4179	CD46 molecule
ENSG0000	535.4619	0.073965	0.14857	0.519975	0.850854	BRPF1	7862	bromodomain and PHD finger containing 1
ENSG0000	98.17991	-0.06997	0.196825	0.519875	0.850854	PLPP6	403313	phospholipid phosphatase 6
ENSG0000	8.842876	0.056404	0.219738	0.520059	0.850879	LINC03011	1E+08	long intergenic non-protein coding RNA 3011
ENSG0000	175.9545	0.061336	0.210066	0.520327	0.850893	ATP13A3	79572	ATPase 13A3
ENSG0000	19.98322	0.044771	0.223708	0.520246	0.850893	ADRA2A	150	adrenoceptor alpha 2A
ENSG0000	20.5223	-0.06773	0.212073	0.520346	0.850893	LLPH-DT	1.04E+08	LLPH divergent transcript
ENSG0000	147.4066	0.073312	0.183284	0.520319	0.850893	BCL7A	605	BAF chromatin remodeling complex subunit BCL7A
ENSG0000	2.445866	0.025089	0.231161	0.520162	0.850893	NA	NA	NA
ENSG0000	13.90265	0.059222	0.216607	0.520279	0.850893	ZNF382	84911	zinc finger protein 382
ENSG0000	107.8106	0.075256	0.169431	0.520592	0.851061	SNRNP48	154007	small nuclear ribonucleoprotein U11/U12 subunit 48
ENSG0000	125.9943	0.073178	0.144234	0.520544	0.851061	TRIM39	56658	tripartite motif containing 39
ENSG0000	13.72182	-0.06079	0.215303	0.52069	0.851061	KIAA1586	57691	KIAA1586
ENSG0000	7.639111	0.0436	0.225252	0.52095	0.851061	NA	NA	NA
ENSG0000	184.4787	0.076012	0.189597	0.520767	0.851061	TNNT3	7140	troponin T fast skeletal type
ENSG0000	4.613568	-0.03186	0.229176	0.520604	0.851061	NA	NA	NA
ENSG0000	147.3428	-0.07494	0.145737	0.520687	0.851061	GID4	79018	GID complex subunit 4 homolog
ENSG0000	155.6293	-0.07031	0.144039	0.520799	0.851061	DDX52	11056	DExD-box helicase 52
ENSG0000	42.76866	-0.06861	0.202087	0.520945	0.851061	NA	NA	NA
ENSG0000	318.2831	-0.06465	0.117182	0.520856	0.851061	TSR2	90121	TSR2 ribosome maturation factor
ENSG0000	145.1185	-0.06796	0.198531	0.52096	0.851061	INTS6L	203522	integrator complex subunit 6 like
ENSG0000	1544.026	-0.07525	0.160691	0.521107	0.851074	ATP13A2	23400	ATPase cation transporting 13A2
ENSG0000	1668.787	0.069833	0.123937	0.521101	0.851074	CARD19	84270	caspase recruitment domain family member 19
ENSG0000	2952.598	-0.07399	0.157343	0.52102	0.851074	PYCARD	29108	PYD and CARD domain containing
ENSG0000	37.00339	0.072431	0.194134	0.521386	0.851377	CELSR2	1952	cadherin EGF LAG seven-pass G-type receptor 2
ENSG0000	5322.834	0.073803	0.151481	0.521357	0.851377	HLA-DPA1	3113	major hist(class II DP alpha 1
ENSG0000	26.24954	0.067569	0.205354	0.521446	0.851399	TTC30B	150737	tetratricopeptide repeat domain 30B
ENSG0000	510.9738	0.068689	0.133387	0.521808	0.851865	CNOT9	9125	CCR4-NOT transcription complex subunit 9
ENSG0000	348.5084	0.072402	0.189634	0.521824	0.851865	LMBRD1	55788	LMBR1 domain containing 1
ENSG0000	143.2838	-0.07255	0.14664	0.522229	0.852298	INO80D	54891	INO80 complex subunit D
ENSG0000	8.097502	0.031306	0.228242	0.5222	0.852298	ZNF415	55786	zinc finger protein 415
ENSG0000	236.9888	-0.07482	0.171312	0.522202	0.852298	BRWD3	254065	bromodomain and WD repeat domain containing 3
ENSG0000	138.0862	0.071448	0.139669	0.522308	0.852351	MLYCD	23417	malonyl-CoA decarboxylase
ENSG0000	2.640046	0.021882	0.231381	0.52242	0.852457	NA	NA	NA
ENSG0000	7.422326	0.043745	0.225647	0.522576	0.852485	SHLD3	1.12E+08	shieldin complex subunit 3
ENSG0000	5.385282	0.042344	0.22623	0.522559	0.852485	TMPRSS5	80975	transmembrane serine protease 5
ENSG0000	900.9613	-0.06838	0.132852	0.522539	0.852485	M6PR	4074	mannose-(cation dependent
ENSG0000	7.135942	-0.05345	0.221964	0.522754	0.852527	RN7SL834I	1.06E+08	RNA 7SL cytoplasm pseudogene
ENSG0000	6.452388	0.044731	0.229801	0.522788	0.852527	LSMEM2	132228	leucine rich single-pass membrane protein 2

ENSG0000	746.407	-0.07344	0.146508	0.522659	0.852527	PPP2CA	5515	protein phosphatase 2 catalytic subunit alpha
ENSG0000	11.92813	0.062058	0.21507	0.522708	0.852527	CCDC7	79741	coiled-coil domain containing 7
ENSG0000	4.236994	0.039422	0.227998	0.523048	0.852739	PRTFDC1	56952	phosphoribosyl transferase domain containing 1
ENSG0000	207.5223	0.075215	0.163345	0.523058	0.852739	ATL3	25923	atlastin GTPase 3
ENSG0000	4.945859	-0.03139	0.231183	0.523008	0.852739	MIR3685	1.01E+08	microRNA 3685
ENSG0000	38.9143	0.077562	0.169549	0.523206	0.852904	SETMAR	6419	SET domain and mariner transposase fusion gene
ENSG0000	28.09577	-0.06686	0.189678	0.52363	0.852955	MLLT11	10962	MLLT11 transcription factor 7 cofactor
ENSG0000	553.7456	-0.06592	0.121076	0.523559	0.852955	CD84	8832	CD84 molecule
ENSG0000	299.7875	-0.07117	0.145531	0.523843	0.852955	LRRFIP2	9209	LRR binding FLII interacting protein 2
ENSG0000	733.8566	-0.07031	0.137925	0.523858	0.852955	CTNNB1	1499	catenin beta 1
ENSG0000	359.2936	0.068743	0.199806	0.523809	0.852955	CSGALNAC	55454	chondroitin sulfate N-acetylgalactosaminyltransferase 2
ENSG0000	574.1149	0.073548	0.15475	0.523757	0.852955	BLNK	29760	B cell linker
ENSG0000	4.868251	0.032639	0.228838	0.523508	0.852955	CDON	50937	cell adhesi oncogene regulated
ENSG0000	2.40104	-0.02339	0.231259	0.523658	0.852955	NA	NA	NA
ENSG0000	6.939608	0.037021	0.227511	0.523668	0.852955	KRT8P39	1E+08	keratin 8 pseudogene 39
ENSG0000	39.81119	0.061132	0.211506	0.523444	0.852955	LIG4	3981	DNA ligase 4
ENSG0000	163.576	-0.07505	0.151623	0.523835	0.852955	TMEM170	124491	transmembrane protein 170A
ENSG0000	186.168	0.073977	0.15459	0.523889	0.852955	MED11	400569	mediator complex subunit 11
ENSG0000	12.92082	-0.0597	0.214685	0.523844	0.852955	SRRM5	1E+08	serine/arginine repetitive matrix 5
ENSG0000	3.350013	-0.02852	0.229858	0.52345	0.852955	C19orf18	147685	chromosome 19 open reading frame 18
ENSG0000	2.989128	0.023039	0.231189	0.524132	0.853124	NA	NA	NA
ENSG0000	30.65747	-0.04371	0.223096	0.524108	0.853124	SFRP2	6423	secreted frizzled related protein 2
ENSG0000	4626.967	0.069965	0.134781	0.524043	0.853124	TPI1	7167	triosephosphate isomerase 1
ENSG0000	2658.08	-0.07131	0.192424	0.524248	0.853213	EMB	133418	embigin
ENSG0000	161.0396	0.073826	0.150635	0.52428	0.853213	ADCK1	57143	aarF domain containing kinase 1
ENSG0000	8.443931	-0.04531	0.224659	0.524355	0.853259	NA	NA	NA
ENSG0000	1839.253	-0.07281	0.150513	0.524783	0.853329	RHOB	388	ras homolog family member B
ENSG0000	24.66722	0.067117	0.207184	0.524798	0.853329	NEK1	4750	NIMA related kinase 1
ENSG0000	31.64016	-0.0693	0.201482	0.525255	0.853329	HLA-K	3138	major hist class I K (pseudogene)
ENSG0000	220.9062	-0.07528	0.162673	0.524766	0.853329	USP42	84132	ubiquitin specific peptidase 42
ENSG0000	24.60507	0.059129	0.215289	0.525045	0.853329	LSMEM1	286006	leucine rich single-pass membrane protein 1
ENSG0000	12.68051	-0.05672	0.217943	0.525213	0.853329	WEE2-AS1	285962	WEE2 antisense RNA 1
ENSG0000	5.217949	-0.04105	0.226942	0.524454	0.853329	NA	NA	NA
ENSG0000	1190.484	-0.05656	0.117611	0.524844	0.853329	RSU1	6251	Ras suppressor protein 1
ENSG0000	8.309806	0.052961	0.221277	0.52533	0.853329	NA	NA	NA
ENSG0000	54.61855	0.062528	0.209763	0.524608	0.853329	HCFC2	29915	host cell factor C2
ENSG0000	216.4418	0.07102	0.171879	0.525053	0.853329	PUS1	80324	pseudouridine synthase 1
ENSG0000	374.5321	0.075239	0.148976	0.525296	0.853329	BAHD1	22893	bromo adjacent homology domain containing 1
ENSG0000	210.7641	0.072651	0.142482	0.524713	0.853329	DPH1	1801	diphthamide biosynthesis 1
ENSG0000	594.6402	0.064789	0.111921	0.525195	0.853329	DNAJC7	7266	DnaJ heat shock protein family (Hsp40) member C7
ENSG0000	125.3561	-0.07392	0.154927	0.52504	0.853329	HOXB2	3212	homeobox B2
ENSG0000	8.521959	-0.05637	0.219269	0.525246	0.853329	DNAI2	64446	dynein axonemal intermediate chain 2
ENSG0000	121.9179	0.071505	0.196488	0.524564	0.853329	DPY19L3	147991	dpy-19 like C-mannosyltransferase 3
ENSG0000	70.41274	-0.07318	0.176732	0.524885	0.853329	BCAS4	55653	breast carcinoma amplified sequence 4
ENSG0000	758.7476	0.073993	0.174256	0.524561	0.853329	CFP	5199	complement factor properdin
ENSG0000	44.84274	-0.07496	0.17107	0.525097	0.853329	GSPT2	23708	G1 to S phase transition 2
ENSG0000	293.4742	0.069634	0.134143	0.525842	0.853483	CROCCP2	84809	CROCC pseudogene 2
ENSG0000	439.8021	-0.07266	0.167274	0.525736	0.853483	MAST2	23139	microtubule associated serine/threonine kinase 2
ENSG0000	293.3527	-0.07238	0.145401	0.525583	0.853483	TARS2	80222	threonyl-t mitochondrial
ENSG0000	66.07332	0.069188	0.197786	0.525868	0.853483	WNT10A	80326	Wnt family member 10A
ENSG0000	11.37484	0.053492	0.220482	0.525983	0.853483	FAM53A	152877	family with sequence similarity 53 member A
ENSG0000	139.393	-0.07187	0.156201	0.525708	0.853483	ATP6V1H	51606	ATPase H+ transporting V1 subunit H
ENSG0000	6.968642	0.048932	0.223986	0.525837	0.853483	VIRMA-DT	1.02E+08	VIRMA divergent transcript
ENSG0000	3.907097	-0.02808	0.230008	0.525724	0.853483	NA	NA	NA
ENSG0000	52.58386	-0.07358	0.186208	0.525678	0.853483	SLIT1	6585	slit guidance ligand 1
ENSG0000	572.6693	0.073654	0.172918	0.525937	0.853483	STT3A	3703	STT3 oligosaccharyltransferase complex catalytic subunit A
ENSG0000	74.15255	0.071121	0.193513	0.525813	0.853483	LOC12490	1.25E+08	uncharacterized LOC124903372
ENSG0000	427.4681	-0.06908	0.131099	0.525982	0.853483	TMOD3	29766	tropomodulin 3
ENSG0000	46.24378	0.07496	0.172875	0.526931	0.853585	NA	NA	NA
ENSG0000	50059.45	-0.07315	0.170111	0.526422	0.853585	SH3BGRL3	83442	SH3 domain binding glutamate rich protein like 3
ENSG0000	4.042048	0.031716	0.232071	0.527006	0.853585	ADORA1	134	adenosine A1 receptor
ENSG0000	51.66109	-0.07456	0.177847	0.526939	0.853585	FTH1P2	2497	ferritin heavy chain 1 pseudogene 2
ENSG0000	1438.464	0.073132	0.143163	0.527099	0.853585	PREB	10113	prolactin regulatory element binding
ENSG0000	311.9837	-0.06341	0.204933	0.526511	0.853585	GALNT14	79623	polypeptide N-acetylgalactosaminyltransferase 14
ENSG0000	570.0694	-0.07307	0.158816	0.526827	0.853585	HMGN4	10473	high mobility group nucleosomal binding domain 4
ENSG0000	3.669556	-0.02577	0.230004	0.527071	0.853585	NA	NA	NA
ENSG0000	4.867925	-0.01812	0.231589	0.526603	0.853585	NA	NA	NA
ENSG0000	18.83933	0.049978	0.221113	0.526346	0.853585	CKS2	1164	CDC28 protein kinase regulatory subunit 2
ENSG0000	25.55101	-0.06048	0.213259	0.526562	0.853585	HSD17B7P	158160	hydroxysteroid 17-beta dehydrogenase 7 pseudogene 2
ENSG0000	52.99977	0.073889	0.183449	0.52697	0.853585	NA	NA	NA
ENSG0000	9.505596	-0.06394	0.213246	0.526973	0.853585	LRRC51	1.2E+08	leucine rich repeat containing 51
ENSG0000	317.9798	-0.07489	0.161771	0.526802	0.853585	CD3D	915	CD3 delta subunit of T-cell receptor complex
ENSG0000	528.6437	-0.07166	0.166217	0.526929	0.853585	DDX11	1663	DEAD/H-box helicase 11
ENSG0000	1026.334	0.070186	0.145287	0.526465	0.853585	STUB1	10273	STIP1 homology and U-box containing protein 1
ENSG0000	43.19858	0.073795	0.187106	0.526854	0.853585	CENPN	55839	centromere protein N
ENSG0000	286.9049	-0.07135	0.134478	0.527134	0.853585	SAT2	112483	spermidine/spermine N1-acetyltransferase family member 2
ENSG0000	47.80575	-0.07536	0.177266	0.526787	0.853585	ZNF561-AS	284385	ZNF561 antisense RNA 1 (head to head)
ENSG0000	4.8682	-0.0404	0.226959	0.527015	0.853585	NA	NA	NA
ENSG0000	3.56087	0.032666	0.229051	0.526398	0.853585	NA	NA	NA
ENSG0000	203.597	0.07321	0.153384	0.526573	0.853585	SELENOM	140606	selenoprotein M
ENSG0000	212.6459	0.073805	0.138653	0.526344	0.853585	TRMT2B	79979	tRNA methyltransferase 2 homolog B
ENSG0000	406.0488	-0.06807	0.195475	0.527165	0.853585	RAP2C	57826	RAP2C member of RAS oncogene family
ENSG0000	556.3318	0.066642	0.135854	0.527635	0.853658	UBE4B	10277	ubiquitination factor E4B
ENSG0000	129.6948	0.073302	0.166814	0.527612	0.853658	ALDH4A1	8659	aldehyde dehydrogenase 4 family member A1
ENSG0000	114.0588	0.067926	0.195156	0.52778	0.853658	HACD2	201562	3-hydroxyacyl-CoA dehydratase 2
ENSG0000	14.77854	0.067131	0.208001	0.5276	0.853658	NA	NA	NA
ENSG0000	5.762249	0.048923	0.223151	0.527944	0.853658	FAM221A	340277	family with sequence similarity 221 member A
ENSG0000	143.9901	0.074914	0.175043	0.527964	0.853658	INIP	58493	INTS3 and NABP interacting protein
ENSG0000	68.62457	0.068495	0.201678	0.527404	0.853658	BEND7	222389	BEN domain containing 7
ENSG0000	5.682934	0.046545	0.224831	0.52792	0.853658	NA	NA	NA
ENSG0000	2615.37	-0.05925	0.104561	0.527408	0.853658	EIF4EBP2	1979	eukaryotic translation initiation factor 4E binding protein 2
ENSG0000	548.8051	-0.07122	0.183988	0.528049	0.853658	ANO9	338440	anoctamin 9
ENSG0000	327.3644	0.073849	0.16134	0.527576	0.853658	NAT10	55226	N-acetyltransferase 10
ENSG0000	12.81131	0.040853	0.22485	0.527956	0.853658	SLC27A2	11001	solute carrier family 27 member 2
ENSG0000	199.9568	-0.07422	0.16836	0.528034	0.853658	RSPRY1	89970	ring finger and SPRY domain containing 1
ENSG0000	76.48525	0.063806	0.206691	0.527344	0.853658	TOP2A	7153	DNA topoisomerase II alpha

ENSG0000	124.378	-0.07121	0.188425	0.527793	0.853658	SRSF1	6426	serine and arginine rich splicing factor 1
ENSG0000	6.41732	-0.03864	0.226708	0.527964	0.853658	YES1	7525	YES proto- Src family tyrosine kinase
ENSG0000	27.12082	0.052187	0.21854	0.52743	0.853658	IER3IP1	51124	immediate early response 3 interacting protein 1
ENSG0000	12.29306	-0.0656	0.209176	0.527879	0.853658	NKAPP1	158801	NFKB activating protein pseudogene 1
ENSG0000	22.00778	0.071645	0.196535	0.528194	0.853763	NA	NA	NA
ENSG0000	202.968	0.072018	0.140096	0.528207	0.853763	MAP3K12	7786	mitogen-activated protein kinase kinase kinase 12
ENSG0000	26.82768	-0.06674	0.204176	0.528576	0.854283	CPLANE2	79363	ciliogenesis and planar polarity effector complex subunit 2
ENSG0000	28.36885	0.024653	0.232701	0.528715	0.854358	HLA-V	352962	major hist class I V (pseudogene)
ENSG0000	521.8729	0.075835	0.158404	0.528696	0.854358	DDX27	55661	DEAD-box helicase 27
ENSG0000	6.61982	0.055643	0.220659	0.528999	0.854682	NA	NA	NA
ENSG0000	102.9316	0.059434	0.210904	0.529009	0.854682	ICE2	79664	interactor of little elongation complex ELL subunit 2
ENSG0000	5.598343	-0.04159	0.226036	0.529174	0.854798	LRGUK	136332	leucine rich repeats and guanylate kinase domain containing
ENSG0000	40.0542	-0.07031	0.195073	0.529151	0.854798	SCNN1A	6337	sodium channel epithelial 1 subunit alpha
ENSG0000	6.700481	-0.03305	0.228392	0.529409	0.855033	ENPP7	339221	ectonucleotide pyrophosphatase/phosphodiesterase 7
ENSG0000	536.8624	0.076667	0.157763	0.529413	0.855033	ATP5F1E	514	ATP synthase F1 subunit epsilon
ENSG0000	4.010641	0.02572	0.230116	0.52953	0.855146	NA	NA	NA
ENSG0000	409.2877	0.072967	0.152995	0.529586	0.855162	NOL6	65083	nucleolar protein 6
ENSG0000	173.9411	0.073464	0.186641	0.529689	0.855186	BTBD1	53339	BTB domain containing 1
ENSG0000	50.42321	0.060844	0.206181	0.529695	0.855186	ZNF480	147657	zinc finger protein 480
ENSG0000	5.674092	-0.0436	0.225809	0.529813	0.855302	LY6G6F	259215	lymphocyte antigen 6 family member G6F
ENSG0000	208.8479	0.0701	0.138643	0.529977	0.855417	CCDC115	84317	coiled-coil domain containing 115
ENSG0000	19221.56	-0.07142	0.149718	0.529953	0.855417	GNAS	2778	GNAS complex locus
ENSG0000	252.9035	0.06824	0.151457	0.530073	0.85545	PPP1R8	5511	protein phosphatase 1 regulatory subunit 8
ENSG0000	418.5836	-0.07211	0.156165	0.530357	0.85545	FBXO38	81545	F-box protein 38
ENSG0000	2.492262	-0.02666	0.231298	0.530301	0.85545	NA	NA	NA
ENSG0000	839.0751	0.072577	0.156607	0.530371	0.85545	C11orf24	53838	chromosome 11 open reading frame 24
ENSG0000	17.81406	0.04804	0.22147	0.530256	0.85545	ALDH1A2	8854	aldehyde dehydrogenase 1 family member A2
ENSG0000	250.6486	0.068008	0.123754	0.530317	0.85545	LUC7L	55692	LUC7 like
ENSG0000	4.34118	-0.02154	0.231765	0.530135	0.85545	MMP2	4313	matrix metallopeptidase 2
ENSG0000	154.0933	0.074726	0.139204	0.530309	0.85545	UTP6	55813	UTP6 small subunit processome component
ENSG0000	2188.425	-0.06758	0.131504	0.530477	0.855545	EP300	2033	E1A binding protein p300
ENSG0000	6.55053	0.051163	0.222778	0.530589	0.855588	NA	NA	NA
ENSG0000	792.7092	0.064938	0.125549	0.530597	0.855588	PITPNC1	26207	phosphatidylinositol transfer protein cytoplasmic 1
ENSG0000	6.317996	0.045248	0.224415	0.53073	0.855713	MFSD14A	64645	major facilitator superfamily domain containing 14A
ENSG0000	1219.504	-0.08035	0.14525	0.530768	0.855713	TAPBPL	55080	TAP binding protein like
ENSG0000	9.511613	0.040863	0.22586	0.530915	0.855875	SEC14L4	284904	SEC14 like lipid binding 4
ENSG0000	513.9596	0.069558	0.131729	0.531371	0.855974	DMAP1	55929	DNA methyltransferase 1 associated protein 1
ENSG0000	21.4068	0.071747	0.198553	0.53153	0.855974	NA	NA	NA
ENSG0000	5.586031	-0.0271	0.229842	0.531162	0.855974	MST1R	4486	macrophage stimulating 1 receptor
ENSG0000	23.62138	0.046963	0.221351	0.531275	0.855974	HLTF	6596	helicase like transcription factor
ENSG0000	48.70414	0.067318	0.200761	0.531527	0.855974	RASGEF1B	153020	RasGEF domain family member 1B
ENSG0000	403.279	0.068318	0.132148	0.531389	0.855974	KIAA1191	57179	KIAA1191
ENSG0000	34.27112	0.067379	0.201225	0.531434	0.855974	LPAR1	1902	lysophosphatidic acid receptor 1
ENSG0000	14.73316	0.063705	0.210439	0.53129	0.855974	PLPP7	84814	phospholipid phosphatase 7 (inactive)
ENSG0000	3.515179	0.017724	0.231461	0.531538	0.855974	TRAV30	28652	T cell receptor alpha variable 30
ENSG0000	610.5512	0.070661	0.143148	0.531051	0.855974	AREL1	9870	apoptosis resistant E3 ubiquitin protein ligase 1
ENSG0000	284.2375	0.072372	0.174962	0.531192	0.855974	NUP88	4927	nucleoporin 88
ENSG0000	19.68868	-0.06432	0.207357	0.531438	0.855974	FAM210A	125228	family with sequence similarity 210 member A
ENSG0000	5.416407	0.034543	0.228025	0.531932	0.856082	DQX1	165545	DEAQ-box RNA dependent ATPase 1
ENSG0000	3.170859	0.029808	0.230526	0.531927	0.856082	LOC10192	1.02E+08	uncharacterized LOC101927636
ENSG0000	573.5328	-0.06906	0.144874	0.531823	0.856082	RNF145	153830	ring finger protein 145
ENSG0000	313.1825	-0.07629	0.173875	0.531904	0.856082	FXYD6	53826	FXYD domain containing ion transport regulator 6
ENSG0000	27.88845	0.072426	0.190162	0.531757	0.856082	TRMT5	57570	tRNA methyltransferase 5
ENSG0000	21.27597	0.046805	0.221681	0.531784	0.856082	DEGS2	123099	delta 4-de sphingolipid 2
ENSG0000	48.82463	0.057284	0.213727	0.531932	0.856082	ZNF700	90592	zinc finger protein 700
ENSG0000	8.235989	0.041632	0.225621	0.532013	0.856138	NA	NA	NA
ENSG0000	3.837052	-0.04108	0.230203	0.532132	0.856151	ARHGEF28	64283	Rho guanine nucleotide exchange factor 28
ENSG0000	46.13356	0.073332	0.182186	0.532203	0.856151	PDSS1	23590	decaprenyl diphosphate synthase subunit 1
ENSG0000	404.9188	0.067909	0.132741	0.532167	0.856151	ZFP91	80829	ZFP91 zinc atypical E3 ubiquitin ligase
ENSG0000	2.377555	0.033926	0.229569	0.532442	0.856151	APOA1	335	apolipoprotein A1
ENSG0000	9.401618	0.051579	0.221599	0.532292	0.856151	LRP6	4040	LDL receptor related protein 6
ENSG0000	24.85752	0.072728	0.189489	0.532238	0.856151	DNAL1	83544	dynein axonemal light chain 1
ENSG0000	9.410156	0.059226	0.216109	0.532435	0.856151	SPATA7	55812	spermatogenesis associated 7
ENSG0000	879.8259	0.073089	0.141856	0.532328	0.856151	PEPD	5184	peptidase D
ENSG0000	37.8249	-0.07125	0.190952	0.532409	0.856151	CHEK2	11200	checkpoint kinase 2
ENSG0000	79.80336	-0.07035	0.186976	0.532568	0.856171	ALDH5A1	7915	aldehyde dehydrogenase 5 family member A1
ENSG0000	605.5531	-0.06263	0.127849	0.532568	0.856171	BAZ1B	9031	bromodomain adjacent to zinc finger domain 1B
ENSG0000	17.18002	0.057048	0.215798	0.532595	0.856171	ANKK1	255239	ankyrin repeat and kinase domain containing 1
ENSG0000	3.643168	-0.0279	0.231143	0.532643	0.856174	HMGB1P5	10354	high mobility group box 1 pseudogene 5
ENSG0000	15.94449	0.05627	0.217268	0.533142	0.85643	IL10	3586	interleukin 10
ENSG0000	640.4382	0.071298	0.151528	0.533096	0.85643	IDUA	3425	alpha-L-iduronidase
ENSG0000	14.01942	-0.05453	0.217247	0.53294	0.85643	SMN1	6606	survival of telomeric
ENSG0000	6.407919	0.045928	0.224487	0.533173	0.85643	MTCO2P22	1E+08	MT-CO2 pseudogene 22
ENSG0000	425.0842	-0.07178	0.182615	0.532906	0.85643	NCOA2	10499	nuclear receptor coactivator 2
ENSG0000	1760.318	-0.06904	0.127725	0.533059	0.85643	IGSF6	10261	immunoglobulin superfamily member 6
ENSG0000	20.47742	0.069583	0.197754	0.533183	0.85643	ZNF846	162993	zinc finger protein 846
ENSG0000	16190.85	0.072632	0.160392	0.533224	0.85643	FXYD5	53827	FXYD domain containing ion transport regulator 5
ENSG0000	103.5883	0.073914	0.171346	0.532951	0.85643	ZNF211	10520	zinc finger protein 211
ENSG0000	5.189701	-0.04336	0.226	0.533287	0.856457	NA	NA	NA
ENSG0000	64.40493	0.077269	0.174627	0.533347	0.856479	NA	NA	NA
ENSG0000	872.631	0.067239	0.160574	0.533516	0.856675	TTLL12	23170	tubulin tyrosine ligase like 12
ENSG0000	56.92807	0.073572	0.158593	0.533761	0.856994	NA	NA	NA
ENSG0000	19.1554	-0.07077	0.19623	0.533845	0.857053	GPS2	2874	G protein pathway suppressor 2
ENSG0000	772.3902	0.072601	0.174599	0.534116	0.857412	IQCN	80726	IQ motif containing N
ENSG0000	33.66405	-0.07168	0.189433	0.53422	0.85743	ZNF232	7775	zinc finger protein 232
ENSG0000	33.37492	0.060514	0.210596	0.534182	0.85743	PHF6	84295	PHD finger protein 6
ENSG0000	39.35922	0.071913	0.189371	0.534317	0.85751	KANK2	25959	KN motif and ankyrin repeat domains 2
ENSG0000	6.788772	-0.03654	0.226931	0.534575	0.857848	NA	NA	NA
ENSG0000	152.0727	0.075325	0.174311	0.534653	0.857863	SLC35F5	80255	solute carrier family 35 member F5
ENSG0000	3.540987	-0.02874	0.230775	0.534678	0.857863	TRBV21-1	28566	T cell receptor beta variable 21-1 (pseudogene)
ENSG0000	2840.511	-0.05037	0.094337	0.534993	0.857887	HMGN2	3151	high mobility group nucleosomal binding domain 2
ENSG0000	18.47654	0.054754	0.217005	0.534841	0.857887	MSH5	4439	mutS homolog 5
ENSG0000	261.211	0.072068	0.152942	0.534966	0.857887	DDX51	317781	DEAD-box helicase 51
ENSG0000	199.1399	0.071572	0.155926	0.534928	0.857887	SLC7A1	6541	solute carrier family 7 member 1
ENSG0000	873.6571	-0.06433	0.117439	0.534829	0.857887	MIA2	4253	MIA SH3 domain ER export factor 2
ENSG0000	152.3762	-0.06918	0.182157	0.534782	0.857887	HSBP1	3281	heat shock factor binding protein 1

ENSG0000	8.908579	0.038991	0.22598	0.53502	0.857887	CRB3	92359	crumbs cell polarity complex component 3
ENSG0000	18.56723	0.062845	0.209056	0.535126	0.857982	NA	NA	NA
ENSG0000	74.14491	-0.073	0.173564	0.535362	0.857984	ARL15	54622	ADP ribosylation factor like GTPase 15
ENSG0000	27.28309	-0.05567	0.214802	0.535291	0.857984	LYVE1	10894	lymphatic vessel endothelial hyaluronan receptor 1
ENSG0000	2.8113	0.013299	0.233286	0.535292	0.857984	NA	NA	NA
ENSG0000	4.526493	0.032299	0.230106	0.535338	0.857984	NA	NA	NA
ENSG0000	15.83271	-0.05065	0.219384	0.535337	0.857984	CAMSAP3	57662	calmodulin regulated spectrin associated protein family member 3
ENSG0000	392.0103	0.069088	0.129514	0.535433	0.858022	RITA1	84934	RBPJ interacting and tubulin associated 1
ENSG0000	7.65906	0.030626	0.228211	0.535537	0.858115	MACC1	346389	MET transcriptional regulator MACC1
ENSG0000	3.515205	0.036189	0.229271	0.53575	0.858381	NA	NA	NA
ENSG0000	29.57368	0.068684	0.198468	0.535902	0.858473	SIGLEC15	284266	sialic acid binding Ig like lectin 15
ENSG0000	3.246702	-0.0286	0.229577	0.535885	0.858473	LINC01694	1.05E+08	long intergenic non-protein coding RNA 1694
ENSG0000	100.2645	-0.05795	0.209556	0.535987	0.85853	B3GNT2	10678	UDP-GlcN/ 3-N-acetylglucosaminyltransferase 2
ENSG0000	415.2867	0.066762	0.127657	0.536031	0.85853	RPL36AL	6166	ribosomal protein L36a like
ENSG0000	1433.599	0.071187	0.15354	0.536476	0.859168	MRPL4	51073	mitochondrial ribosomal protein L4
ENSG0000	29.42103	0.062848	0.205759	0.53677	0.859223	NA	NA	NA
ENSG0000	27.96408	-0.06127	0.208813	0.536932	0.859223	NA	NA	NA
ENSG0000	1393.366	-0.06823	0.140732	0.536926	0.859223	MAEA	10296	macropha E3 ubiquitin ligase
ENSG0000	2.80855	0.032258	0.229396	0.536698	0.859223	NA	NA	NA
ENSG0000	1366.506	0.074758	0.169486	0.536598	0.859223	VCP	7415	valosin containing protein
ENSG0000	27.13292	-0.05193	0.216865	0.53675	0.859223	NA	NA	NA
ENSG0000	3952.224	-0.07214	0.161016	0.536874	0.859223	DGKA	1606	diacylglycerol kinase alpha
ENSG0000	13.10527	-0.05716	0.209882	0.536812	0.859223	NA	NA	NA
ENSG0000	11.50961	0.054496	0.217476	0.536671	0.859223	CBR3-AS1	1.01E+08	CBR3 antisense RNA 1
ENSG0000	5.261433	0.047099	0.224959	0.537143	0.859485	LINC02615	1.01E+08	long intergenic non-protein coding RNA 2615
ENSG0000	60.26595	-0.07278	0.168933	0.537238	0.859524	NA	NA	NA
ENSG0000	598.3216	0.070706	0.159421	0.537261	0.859524	SRP14	6727	signal recognition particle 14
ENSG0000	21.63078	-0.06882	0.198124	0.537411	0.859538	NPR2	4882	natriuretic peptide receptor 2
ENSG0000	508.5259	0.071779	0.158003	0.537318	0.859538	ALYREF	10189	Aly/REF export factor
ENSG0000	91.58984	0.069693	0.142401	0.537408	0.859538	CETN2	1069	centrin 2
ENSG0000	353.6758	-0.06342	0.121192	0.537557	0.85962	ZNF589	51385	zinc finger protein 589
ENSG0000	101.3735	-0.07085	0.183481	0.537689	0.85962	RBM12B	389677	RNA binding motif protein 12B
ENSG0000	4352.033	0.07206	0.174259	0.537583	0.85962	SORL1	6653	sortilin related receptor 1
ENSG0000	181.4225	-0.06996	0.155705	0.537696	0.85962	CTU2	348180	cytosolic thiouridylase subunit 2
ENSG0000	5.177182	0.037502	0.227905	0.53761	0.85962	LINC01535	1.02E+08	long intergenic non-protein coding RNA 1535
ENSG0000	430.4033	0.064606	0.127611	0.537882	0.859691	AHCYL1	10768	adenosylhomocysteinase like 1
ENSG0000	3560.308	-0.06678	0.155541	0.537798	0.859691	NUMB	8650	NUMB endocytic adaptor protein
ENSG0000	17.68132	0.06435	0.207469	0.537851	0.859691	NA	NA	NA
ENSG0000	382.1491	0.064555	0.121447	0.537943	0.859714	WRAP73	49856	WD repea antisense to TP73
ENSG0000	3.107541	0.037274	0.228216	0.538033	0.859782	NA	NA	NA
ENSG0000	153.5663	-0.06571	0.125875	0.538309	0.859876	PAFAH2	5051	platelet activating factor acetylhydrolase 2
ENSG0000	447.09	-0.06524	0.197075	0.53851	0.859876	SIPA1L2	57568	signal induced proliferation associated 1 like 2
ENSG0000	473.5372	0.063243	0.119982	0.538648	0.859876	CAPN10	11132	calpain 10
ENSG0000	97.67151	0.071863	0.165964	0.538595	0.859876	HMGN3	9324	high mobility group nucleosomal binding domain 3
ENSG0000	16.38489	0.064456	0.206988	0.538262	0.859876	SNHG5	387066	small nucleolar RNA host gene 5
ENSG0000	534.5894	-0.06789	0.139147	0.538416	0.859876	TCP1	6950	t-complex 1
ENSG0000	319.7632	0.063594	0.124867	0.538418	0.859876	EXOC4	60412	exocyst complex component 4
ENSG0000	2.389039	0.016849	0.231501	0.538493	0.859876	ROR2	4920	receptor tyrosine kinase like orphan receptor 2
ENSG0000	5.014012	-0.03788	0.22696	0.53864	0.859876	NA	NA	NA
ENSG0000	32.24164	-0.07148	0.185965	0.538505	0.859876	RIMKLB	57494	ribosomal modification protein rimK like family member B
ENSG0000	51.77199	-0.06955	0.19065	0.5383	0.859876	SPTBN5	51332	spectrin b non-erythrocytic 5
ENSG0000	3.291266	0.028031	0.230093	0.538655	0.859876	NA	NA	NA
ENSG0000	2071.775	-0.07141	0.157179	0.538717	0.8599	ASAP1	50807	ArfGAP wi ankyrin repeat and PH domain 1
ENSG0000	15.84278	0.060826	0.211245	0.538815	0.859982	NA	NA	NA
ENSG0000	183.5708	0.071976	0.169362	0.538987	0.860106	EIF2S1	1965	eukaryotic translation initiation factor 2 subunit alpha
ENSG0000	14.72844	0.063421	0.208876	0.538951	0.860106	ZNF418	147686	zinc finger protein 418
ENSG0000	1786.659	-0.07107	0.16213	0.539075	0.860173	PCNX1	22990	pecanex 1
ENSG0000	4.891027	-0.03953	0.226333	0.53929	0.860362	LOC10537	1.05E+08	uncharacterized LOC105373951
ENSG0000	22.41614	-0.06944	0.196791	0.539382	0.860362	CNTLN	54875	centlein
ENSG0000	209.3418	0.069225	0.139337	0.539346	0.860362	ARFGAP3	26286	ADP ribosylation factor GTPase activating protein 3
ENSG0000	1034.864	-0.05916	0.114008	0.539246	0.860362	EIF2S3	1968	eukaryotic translation initiation factor 2 subunit gamma
ENSG0000	703.8287	0.065738	0.127767	0.539451	0.860397	GGA2	23062	golgi assoc gamma a ARF binding protein 2
ENSG0000	137.4054	0.069779	0.143052	0.539593	0.860474	PDCD5	9141	programmed cell death 5
ENSG0000	23.69265	0.063913	0.206313	0.539562	0.860474	NA	NA	NA
ENSG0000	2082.536	-0.07281	0.167904	0.539665	0.860513	SH3BP1	23616	SH3 domain binding protein 1
ENSG0000	38.21932	0.072946	0.178413	0.540245	0.86054	NA	NA	NA
ENSG0000	654.7123	-0.05875	0.108573	0.54007	0.86054	CYB561D1	284613	cytochrome b561 family member D1
ENSG0000	624.3286	-0.06314	0.122709	0.540199	0.86054	POGZ	23126	pogo transposable element derived with ZNF domain
ENSG0000	1628.073	-0.07263	0.16767	0.539793	0.86054	IP6K1	9807	inositol hexakisphosphate kinase 1
ENSG0000	7.542218	0.044566	0.224519	0.539979	0.86054	NA	NA	NA
ENSG0000	9.511235	0.058287	0.21605	0.540001	0.86054	POLA2	23649	DNA polyr accessory subunit
ENSG0000	206.6044	0.070494	0.170942	0.540119	0.86054	KRAS	3845	KRAS prot GTPase
ENSG0000	1110.942	-0.07082	0.162698	0.539977	0.86054	REM2	161253	RRAD and GEM like GTPase 2
ENSG0000	370.7172	0.046277	0.221005	0.540107	0.86054	IGHV3-7	28452	immunoglobulin heavy variable 3-7
ENSG0000	75.81707	-0.07231	0.170876	0.53993	0.86054	PPIP5K1	9677	diphosphoinositol pentakisphosphate kinase 1
ENSG0000	3.005218	-0.02186	0.231521	0.540157	0.86054	NA	NA	NA
ENSG0000	39.54704	-0.06472	0.202549	0.540081	0.86054	FSD1	79187	fibronectin type III and SPRY domain containing 1
ENSG0000	245.2424	-0.06943	0.140584	0.540326	0.860593	PDLIM5	10611	PDZ and LIM domain 5
ENSG0000	122.2505	0.071339	0.176976	0.540454	0.860723	RSKR	124923	ribosomal protein S6 kinase related
ENSG0000	80.71741	0.061494	0.206252	0.540824	0.860908	PIGK	10026	phosphatidylinositol glycan anchor biosynthesis class K
ENSG0000	652.2479	0.068317	0.1364	0.540956	0.860908	ZXDC	79364	ZXD family zinc finger C
ENSG0000	125.0683	0.059318	0.205698	0.540803	0.860908	PPIP5K2	23262	diphosphoinositol pentakisphosphate kinase 2
ENSG0000	196.1538	-0.07116	0.164306	0.540993	0.860908	SMNDC1	10285	survival motor neuron domain containing 1
ENSG0000	241.7592	-0.06942	0.153544	0.540894	0.860908	TMEM9B	56674	TMEM9 domain family member B
ENSG0000	60.08558	0.070808	0.184324	0.540733	0.860908	GOLT1B	51026	golgi transport 1B
ENSG0000	418.436	0.073274	0.163724	0.540983	0.860908	EIF4A3	9775	eukaryotic translation initiation factor 4A3
ENSG0000	14.82836	0.05193	0.218303	0.540633	0.860908	NA	NA	NA
ENSG0000	450.6405	0.068614	0.139271	0.540731	0.860908	TMEM147	10430	transmembrane protein 147
ENSG0000	335.0179	-0.06676	0.138795	0.541146	0.861077	BTBD6	90135	BTB domain containing 6
ENSG0000	23.97803	0.06722	0.199766	0.541361	0.861228	HCN3	57657	hyperpolarization activated cyclic nucleotide gated potassium channel 3
ENSG0000	681.6471	0.079263	0.147886	0.541383	0.861228	ZNF106	64397	zinc finger protein 106
ENSG0000	5.923227	-0.03675	0.227392	0.541302	0.861228	TUBB8B	260334	tubulin beta 8B
ENSG0000	23.02753	0.069126	0.195955	0.541541	0.861331	NA	NA	NA
ENSG0000	208.0595	0.070617	0.189691	0.541571	0.861331	AVIL	10677	advillin
ENSG0000	143.2292	-0.07289	0.155667	0.541588	0.861331	SUGT1	10910	SGT1 hom MIS12 kinetochore complex assembly cochaperone
ENSG0000	224.3168	-0.07217	0.168844	0.541696	0.861428	UHRF2	115426	ubiquitin like with PHD and ring finger domains 2

ENSG0000	376.8622	-0.06552	0.133141	0.54184	0.861582	PLCL2	23228	phospholipase C like 2
ENSG0000	1304.695	-0.0702	0.139755	0.542063	0.861786	EFCAB14	9813	EF-hand calcium binding domain 14
ENSG0000	37.98395	-0.06942	0.194558	0.54203	0.861786	FBLN5	10516	fibulin 5
ENSG0000	30.46768	-0.06495	0.20313	0.54213	0.861818	NPFF	8620	neuropeptide FF-amide peptide precursor
ENSG0000	891.0424	-0.07014	0.153267	0.542297	0.862009	FHIP2B	64760	FHF complex subunit HOOK interacting protein 2B
ENSG0000	108.3938	0.057135	0.210736	0.54278	0.862054	NIFK	84365	nucleolar protein interacting with the FHA domain of MKI67
ENSG0000	2187.463	-0.07122	0.16784	0.542381	0.862054	PTPN23	25930	protein tyrosine phosphatase non-receptor type 23
ENSG0000	448.4052	-0.06657	0.135264	0.542795	0.862054	PRR7	80758	proline ric synaptic
ENSG0000	3.236474	0.032159	0.229923	0.542633	0.862054	NA	NA	NA
ENSG0000	30.09779	-0.0612	0.207213	0.542607	0.862054	RTKN2	219790	rhotekin 2
ENSG0000	124.9161	-0.06501	0.155824	0.542694	0.862054	SPSB2	84727	splA/ryanodine receptor domain and SOCS box containing 2
ENSG0000	149.0128	0.067604	0.189722	0.54279	0.862054	CRIP2	1397	cysteine rich protein 2
ENSG0000	16.59147	-0.04826	0.220441	0.542447	0.862054	NA	NA	NA
ENSG0000	3.76247	0.03312	0.228711	0.542619	0.862054	C19orf73	55150	chromosome 19 open reading frame 73
ENSG0000	629.3751	0.070244	0.145326	0.542539	0.862054	NUP50	10762	nucleoporin 50
ENSG0000	28.14154	0.065204	0.202889	0.543047	0.862067	NA	NA	NA
ENSG0000	4.54322	0.042468	0.225709	0.543086	0.862067	RPP21	79897	ribonuclease P/MRP subunit p21
ENSG0000	64.53285	-0.07175	0.165321	0.542961	0.862067	PINX1	54984	PIN2 (TERF1) interacting telomerase inhibitor 1
ENSG0000	301.3348	-0.06963	0.178947	0.542861	0.862067	NECAP1	25977	NECAP endocytosis associated 1
ENSG0000	9.002956	0.04231	0.225325	0.543084	0.862067	SMAD6	4091	SMAD family member 6
ENSG0000	24.7167	0.059924	0.208597	0.542917	0.862067	TEDC2	80178	tubulin epsilon and delta complex 2
ENSG0000	103.6948	0.067344	0.136408	0.543635	0.862289	LRIG2	9860	leucine rich repeats and immunoglobulin like domains 2
ENSG0000	109.2329	0.069174	0.142751	0.543547	0.862289	DHX29	54505	DExH-box helicase 29
ENSG0000	2860.029	-0.06648	0.136114	0.543664	0.862289	ADAM19	8728	ADAM metallopeptidase domain 19
ENSG0000	6.371848	-0.03725	0.226784	0.543697	0.862289	BDNF-AS	497258	BDNF antisense RNA
ENSG0000	1635.769	0.066382	0.193209	0.54351	0.862289	USP15	9958	ubiquitin specific peptidase 15
ENSG0000	78.82986	0.072768	0.166761	0.543683	0.862289	COX6A1	1337	cytochrome c oxidase subunit 6A1
ENSG0000	3722.472	0.070365	0.177343	0.543394	0.862289	NCOR2	9612	nuclear receptor corepressor 2
ENSG0000	272.696	-0.06918	0.14491	0.54357	0.862289	WHAMM	123720	WASP hon golgi membranes and microtubules
ENSG0000	16963.9	-0.0696	0.176044	0.543549	0.862289	ITGAX	3687	integrin subunit alpha X
ENSG0000	1087.565	-0.06073	0.155765	0.543678	0.862289	IFNAR1	3454	interferon alpha and beta receptor subunit 1
ENSG0000	41.79264	0.068436	0.195057	0.544093	0.862304	NA	NA	NA
ENSG0000	13.23481	0.061084	0.210396	0.543844	0.862304	NUP35	129401	nucleoporin 35
ENSG0000	1012.519	-0.06421	0.123996	0.54412	0.862304	BRPF3	27154	bromodomain and PHD finger containing 3
ENSG0000	236.1538	-0.06961	0.15105	0.54401	0.862304	POP7	10248	POP7 hom ribonuclease P/MRP subunit
ENSG0000	10217.55	-0.07037	0.161755	0.543926	0.862304	CTSB	1508	cathepsin B
ENSG0000	338.7582	0.062495	0.199539	0.543884	0.862304	FAM91A1	157769	family with sequence similarity 91 member A1
ENSG0000	146.6376	0.070104	0.155654	0.54413	0.862304	POLD3	10714	DNA polyr accessory subunit
ENSG0000	7.767604	0.044609	0.223577	0.544029	0.862304	NA	NA	NA
ENSG0000	269.4637	-0.06887	0.160106	0.543815	0.862304	ZSWIM4	65249	zinc finger SWIM-type containing 4
ENSG0000	5.172505	0.032112	0.228448	0.544338	0.862559	CYP27B1	1594	cytochrome P450 family 27 subfamily B member 1
ENSG0000	5.242238	0.034469	0.228144	0.544676	0.862658	NA	NA	NA
ENSG0000	5735.275	-0.07022	0.156475	0.544532	0.862658	ADD1	118	adducin 1
ENSG0000	381.1497	0.065722	0.128189	0.544608	0.862658	MCCC2	64087	methylcrotonyl-CoA carboxylase subunit 2
ENSG0000	3.258662	0.028434	0.22999	0.544595	0.862658	NA	NA	NA
ENSG0000	4.784461	0.030693	0.228524	0.544512	0.862658	NA	NA	NA
ENSG0000	18.81216	0.051656	0.217825	0.544683	0.862658	PALS1	64398	protein as: MAGUK p55 family member
ENSG0000	390.5903	0.070283	0.177699	0.54484	0.862672	NFKBIZ	64332	NFKB inhibitor zeta
ENSG0000	73.33814	-0.06785	0.169863	0.544859	0.862672	ARL10	285598	ADP ribosylation factor like GTPase 10
ENSG0000	2.955542	0.022387	0.23113	0.544885	0.862672	GFPT2	9945	glutamine-fructose-6-phosphate transaminase 2
ENSG0000	1420.38	-0.07068	0.172253	0.544928	0.862672	C3AR1	719	complement C3a receptor 1
ENSG0000	859.7026	-0.07039	0.158133	0.544828	0.862672	RAVER1	125950	ribonuclec PTB binding 1
ENSG0000	361.6881	-0.07003	0.161083	0.545167	0.862977	FRY	10129	FRY microtubule binding protein
ENSG0000	32.20268	-0.06473	0.200864	0.545239	0.862999	NA	NA	NA
ENSG0000	418.9635	-0.07293	0.150434	0.545322	0.862999	EPC1	80314	enhancer of polycomb homolog 1
ENSG0000	23.82748	0.069189	0.192612	0.545282	0.862999	DHPS	1725	deoxyhypusine synthase
ENSG0000	46.55994	0.073594	0.193144	0.545437	0.863107	NPIPB2	729978	nuclear pore complex interacting protein family member B2
ENSG0000	377.0544	-0.07202	0.150857	0.545485	0.863107	RUNDC1	146923	RUN domain containing 1
ENSG0000	679.5843	-0.06534	0.131212	0.545992	0.863211	ARHGAP1!	55843	Rho GTPase activating protein 15
ENSG0000	26.82293	-0.06536	0.203493	0.545848	0.863211	ZNF717	1E+08	zinc finger protein 717
ENSG0000	102.9463	0.067579	0.172426	0.545909	0.863211	ZBTB9	221504	zinc finger and BTB domain containing 9
ENSG0000	262.4235	-0.06028	0.114264	0.546022	0.863211	PRKRIP1	79706	PRKR interacting protein 1
ENSG0000	1147.204	-0.06787	0.143958	0.546014	0.863211	FIBP	9158	FGF1 intracellular binding protein
ENSG0000	42.44576	0.070193	0.186505	0.545978	0.863211	LRR1	122769	leucine rich repeat protein 1
ENSG0000	3.263922	-0.03102	0.229281	0.545987	0.863211	NA	NA	NA
ENSG0000	138.9625	0.071877	0.162836	0.545702	0.863211	DDX19B	11269	DEAD-box helicase 19B
ENSG0000	21.11765	-0.05414	0.215045	0.545959	0.863211	NA	NA	NA
ENSG0000	2541.063	-0.06902	0.151985	0.545747	0.863211	SH3KBP1	30011	SH3 domain containing kinase binding protein 1
ENSG0000	252.9215	-0.06931	0.158429	0.54695	0.863292	CROCC	9696	ciliary root rootletin
ENSG0000	6452.821	0.070195	0.166744	0.547913	0.863292	MAP7D1	55700	MAP7 domain containing 1
ENSG0000	941.5331	-0.05985	0.11479	0.547264	0.863292	PYGO2	90780	pygopus family PHD finger 2
ENSG0000	2.549062	0.01383	0.232223	0.546728	0.863292	RPS7P3	440732	ribosomal protein S7 pseudogene 3
ENSG0000	37.65717	-0.06766	0.19452	0.547436	0.863292	ZNF669	79862	zinc finger protein 669
ENSG0000	10.27198	0.041416	0.224362	0.546895	0.863292	PLGLB1	5343	plasminogen like B1
ENSG0000	15.44487	0.040978	0.223166	0.547776	0.863292	PRKAR2A-	1.01E+08	PRKAR2A antisense RNA 1
ENSG0000	12.61492	-0.05038	0.219511	0.546606	0.863292	CCDC96	257236	coiled-coil domain containing 96
ENSG0000	19.51176	-0.03541	0.225435	0.547602	0.863292	PLK4	10733	polo like kinase 4
ENSG0000	901.1952	0.070396	0.16887	0.547537	0.863292	NQO2	4835	N-ribosyldihydronicotinamide:quinone reductase 2
ENSG0000	3.927137	0.030118	0.228848	0.547628	0.863292	NA	NA	NA
ENSG0000	4179.037	-0.0729	0.14823	0.5479	0.863292	RAC1	5879	Rac family small GTPase 1
ENSG0000	2418.014	-0.06924	0.156483	0.54736	0.863292	AOAH	313	acyloxyacyl hydrolase
ENSG0000	15.93083	0.053971	0.216051	0.547958	0.863292	SNX16	64089	sorting nexin 16
ENSG0000	453.3844	0.065123	0.133568	0.546187	0.863292	STOML2	30968	stomatin like 2
ENSG0000	28.59874	0.066131	0.199734	0.546175	0.863292	LINC01451	401561	long intergenic non-protein coding RNA 1451
ENSG0000	88.98302	0.067146	0.18552	0.547223	0.863292	NKAPD1	55216	NKAP domain containing 1
ENSG0000	59.18356	0.069653	0.177174	0.547776	0.863292	TTC12	54970	tetratricopeptide repeat domain 12
ENSG0000	2086.693	-0.06778	0.14406	0.546505	0.863292	SIDT2	51092	SID1 transmembrane family member 2
ENSG0000	18.148	0.062638	0.206643	0.547838	0.863292	RAPGEF3	10411	Rap guanine nucleotide exchange factor 3
ENSG0000	23.90078	0.060492	0.209619	0.546319	0.863292	CEP290	80184	centrosomal protein 290
ENSG0000	912.2587	0.064579	0.133064	0.547898	0.863292	SLC8B1	80024	solute carrier family 8 member B1
ENSG0000	4.933268	-0.04498	0.224656	0.546273	0.863292	AKAP6	9472	A-kinase anchoring protein 6
ENSG0000	13.49933	-0.03538	0.225948	0.546696	0.863292	RN7SL587	1.06E+08	RNA 7SL cytoplasm pseudogene
ENSG0000	10.84173	-0.0419	0.223589	0.547098	0.863292	MTHFS	10588	methenyltetrahydrofolate synthetase
ENSG0000	73.05532	0.070359	0.166682	0.54763	0.863292	PDE8A	5151	phosphodiesterase 8A
ENSG0000	9.817468	-0.04965	0.219797	0.547049	0.863292	SYNM	23336	synemin
ENSG0000	3030.652	-0.06066	0.117064	0.546755	0.863292	E2F4	1874	E2F transcription factor 4

ENSG0000	67.59715	0.07103	0.160594	0.54704	0.863292	ARL17A	51326	ADP ribosylation factor like GTPase 17A
ENSG0000	465.5865	-0.07093	0.173233	0.547729	0.863292	ST6GALNA	10610	ST6 N-acet 6-sialyltransferase 2
ENSG0000	2083.775	-0.06463	0.129014	0.547474	0.863292	RANBP3	8498	RAN binding protein 3
ENSG0000	912.5515	0.06661	0.135697	0.547849	0.863292	NDUFB7	4713	NADH:ubiquinone oxidoreductase subunit B7
ENSG0000	31.50568	-0.06531	0.199507	0.54768	0.863292	CATSPERG	57828	cation channel sperm associated auxiliary subunit gamma
ENSG0000	7.463051	-0.05312	0.219244	0.547246	0.863292	IFNL1	282618	interferon lambda 1
ENSG0000	3.011992	0.033624	0.231705	0.547366	0.863292	NA	NA	NA
ENSG0000	71.59275	0.071543	0.174197	0.547313	0.863292	ZNF417	147687	zinc finger protein 417
ENSG0000	393.7538	-0.06902	0.162412	0.546562	0.863292	CTNNBL1	56259	catenin beta like 1
ENSG0000	55.12611	0.065836	0.19226	0.546303	0.863292	DHX35	60625	DEAH-box helicase 35
ENSG0000	135.0817	0.070691	0.164297	0.547879	0.863292	YPEL1	29799	yippee like 1
ENSG0000	1291.922	-0.06762	0.146105	0.547247	0.863292	RTL8C	8933	retrotransposon Gag like 8C
ENSG0000	1230.832	-0.0653	0.134085	0.548055	0.863371	PLEKHM1F	440456	pleckstrin homology and RUN domain containing M1 pseudogene 1
ENSG0000	900.1738	-0.06684	0.140611	0.548162	0.863465	COQ8B	79934	coenzyme Q8B
ENSG0000	5746.376	0.082137	0.156887	0.548215	0.863474	TYK2	7297	tyrosine kinase 2
ENSG0000	5.566988	0.043543	0.225223	0.548419	0.863549	OR7E7P	392752	olfactory receptor family 7 subfamily E member 7 pseudogene
ENSG0000	9.704885	0.031569	0.227393	0.548497	0.863549	TSPYL5	85453	TSPY like 5
ENSG0000	73.56907	-0.05993	0.204957	0.548374	0.863549	ARL5B	221079	ADP ribosylation factor like GTPase 5B
ENSG0000	3690.455	-0.06921	0.166107	0.548545	0.863549	GAB2	9846	GRB2 associated binding protein 2
ENSG0000	105.26	0.074389	0.165198	0.54852	0.863549	TIMELESS	8914	timeless circadian regulator
ENSG0000	3060.811	-0.06841	0.150972	0.548498	0.863549	ABR	29	ABR activator of RhoGEF and GTPase
ENSG0000	491.5735	0.063376	0.127321	0.54865	0.863639	NFATC3	4775	nuclear factor of activated T cells 3
ENSG0000	71.57712	-0.06992	0.158292	0.548878	0.863814	NBPF10	1E+08	NBPF member 10
ENSG0000	92.2301	0.069092	0.180716	0.549022	0.863814	ASB3	51130	ankyrin repeat and SOCS box containing 3
ENSG0000	22.83171	0.067286	0.198223	0.548879	0.863814	NA	NA	NA
ENSG0000	246.1585	0.065825	0.190334	0.549044	0.863814	TNS3	64759	tensin 3
ENSG0000	102.1458	-0.06535	0.144717	0.549091	0.863814	KIAA0586	9786	KIAA0586
ENSG0000	3.572774	0.033434	0.23128	0.548952	0.863814	LINC0126!	1.04E+08	long intergenic non-protein coding RNA 1269
ENSG0000	466.5757	-0.06128	0.1255	0.548952	0.863814	EIF2B2	8892	eukaryotic translation initiation factor 2B subunit beta
ENSG0000	467.7414	-0.06967	0.1377	0.549232	0.863817	ODC1	4953	ornithine decarboxylase 1
ENSG0000	578.2186	0.062594	0.12322	0.549216	0.863817	TRIM44	54765	tripartite motif containing 44
ENSG0000	26.62682	-0.06554	0.196251	0.549234	0.863817	FUNDC1	139341	FUN14 domain containing 1
ENSG0000	4.657454	0.03694	0.227094	0.549361	0.863899	NA	NA	NA
ENSG0000	580.3945	-0.06583	0.148085	0.549381	0.863899	ZNF444	55311	zinc finger protein 444
ENSG0000	156.7804	0.070034	0.163697	0.549655	0.863955	ZMYM4	9202	zinc finger MYM-type containing 4
ENSG0000	2.816588	-0.03579	0.228048	0.549483	0.863955	LOC64445	644456	IK cytokine down-regulator of HLA II pseudogene
ENSG0000	322.0146	0.067778	0.136676	0.549603	0.863955	GRSF1	2926	G-rich RNA sequence binding factor 1
ENSG0000	1020.626	0.064752	0.139165	0.549797	0.863955	CHCHD2	51142	coiled-coil-helix-coiled-coil-helix domain containing 2
ENSG0000	235.5286	-0.06912	0.153846	0.549935	0.863955	COG5	10466	component of oligomeric golgi complex 5
ENSG0000	2.891293	0.029389	0.229512	0.549863	0.863955	ZNF572	137209	zinc finger protein 572
ENSG0000	5147.416	-0.06916	0.173945	0.549907	0.863955	EHBP1L1	254102	EH domain binding protein 1 like 1
ENSG0000	3842.825	-0.06983	0.159922	0.549851	0.863955	CREBBP	1387	CREB binding protein
ENSG0000	495.7995	-0.05661	0.1066	0.549935	0.863955	TNRC6A	27327	trinucleotide repeat containing adaptor 6A
ENSG0000	81.99867	0.069204	0.169932	0.549599	0.863955	NA	NA	NA
ENSG0000	547.3765	-0.06915	0.156231	0.54992	0.863955	HDAC6	10013	histone deacetylase 6
ENSG0000	5042.554	0.066717	0.140558	0.550572	0.86405	WASF2	10163	WASP family member 2
ENSG0000	14.6612	0.043834	0.222871	0.550627	0.86405	INAVA	55765	innate immunity activator
ENSG0000	2.33433	-0.02689	0.229609	0.550739	0.86405	GREB1	9687	growth regulating estrogen receptor binding 1
ENSG0000	36.268	-0.06505	0.200115	0.550711	0.86405	NA	NA	NA
ENSG0000	160.3958	-0.06792	0.148677	0.550668	0.86405	POMGNT2	84892	protein O- 4-)
ENSG0000	38.53178	0.070495	0.182325	0.550529	0.86405	NPHP3	27031	nephrocystin 3
ENSG0000	35.59807	0.058148	0.209201	0.551028	0.86405	ELOVL6	79071	ELOVL fatty acid elongase 6
ENSG0000	722.221	0.059652	0.197438	0.550995	0.86405	HMGB2	3148	high mobility group box 2
ENSG0000	795.1702	0.067842	0.151716	0.551034	0.86405	NRM	11270	nurim
ENSG0000	239.2116	0.070067	0.167048	0.551	0.86405	EXT1	2131	exostosin glycosyltransferase 1
ENSG0000	45.20441	0.071093	0.191451	0.550438	0.86405	AUH	549	AU RNA binding methylglutaconyl-CoA hydratase
ENSG0000	90.65514	0.070496	0.178242	0.550158	0.86405	ZNF33B	7582	zinc finger protein 33B
ENSG0000	4.644312	-0.03743	0.226443	0.550917	0.86405	JAKMIP3	282973	Janus kinase and microtubule interacting protein 3
ENSG0000	4.130088	-0.02726	0.22956	0.550254	0.86405	NA	NA	NA
ENSG0000	2.800058	-0.02794	0.231121	0.550422	0.86405	FBN1	2200	fibrillin 1
ENSG0000	161.2089	0.069222	0.15578	0.550864	0.86405	NEIL1	79661	nei like DNA glycosylase 1
ENSG0000	60.69001	0.057994	0.209044	0.550769	0.86405	ITGAD	3681	integrin subunit alpha D
ENSG0000	142.646	-0.06974	0.170956	0.550501	0.86405	WDR62	284403	WD repeat domain 62
ENSG0000	4.205963	0.033801	0.228127	0.55042	0.86405	PNMA8B	57469	PNMA family member 8B
ENSG0000	15.90027	0.05304	0.216087	0.550767	0.86405	ZNF256	10172	zinc finger protein 256
ENSG0000	129.5763	0.071762	0.176995	0.550708	0.86405	FAM217B	63939	family with sequence similarity 217 member B
ENSG0000	118.041	0.070052	0.162924	0.550312	0.86405	XPNPEP3	63929	X-prolyl aminopeptidase 3
ENSG0000	5.767833	0.048463	0.222443	0.551244	0.864305	NA	NA	NA
ENSG0000	61.95513	0.070273	0.169966	0.551299	0.864318	ACAT2	39	acetyl-CoA acetyltransferase 2
ENSG0000	106.4709	-0.06929	0.171774	0.551361	0.864341	NLK	51701	nemo like kinase
ENSG0000	80.55538	-0.06957	0.149003	0.551779	0.864817	ZNF710-A!	1.1E+08	ZNF710 antisense RNA 1
ENSG0000	44.80494	0.068764	0.181117	0.551861	0.864817	SETD6	79918	SET domai protein lysine methyltransferase
ENSG0000	39.50363	0.047824	0.218443	0.551723	0.864817	NA	NA	NA
ENSG0000	531.6765	0.066922	0.147862	0.5519	0.864817	C19orf54	284325	chromosome 19 open reading frame 54
ENSG0000	5449.512	0.071636	0.149051	0.551847	0.864817	RASSF2	9770	Ras association domain family member 2
ENSG0000	50.00269	0.066167	0.19708	0.551988	0.864881	CEP43	11116	centrosomal protein 43
ENSG0000	113.3255	0.068552	0.152868	0.55209	0.86491	POLR1C	9533	RNA polymerase I and III subunit C
ENSG0000	38.5918	0.059902	0.207342	0.552124	0.86491	DTWD1	56986	DTW domain containing 1
ENSG0000	17.59621	0.055735	0.215715	0.552148	0.86491	BCORP1	286554	BCL6 corepressor pseudogene 1
ENSG0000	2.403802	0.007793	0.233728	0.552293	0.865015	MSLN	10232	mesothelin
ENSG0000	102.8573	0.071326	0.160897	0.552358	0.865015	CRYZL1	9946	crystallin zeta like 1
ENSG0000	120.171	0.069981	0.169706	0.552351	0.865015	CHST7	56548	carbohydrate sulfotransferase 7
ENSG0000	3.578954	0.040548	0.22835	0.552713	0.86505	TMEM52	339456	transmembrane protein 52
ENSG0000	27.55361	0.066997	0.194817	0.552741	0.86505	TMEM169	92691	transmembrane protein 169
ENSG0000	415.5858	-0.06399	0.129477	0.552804	0.86505	TBCC	6903	tubulin folding cofactor C
ENSG0000	110.7282	-0.0712	0.173983	0.552783	0.86505	CCZ1	51622	CCZ1 hom vacuolar protein trafficking and biogenesis associated
ENSG0000	203.3886	0.067272	0.147988	0.552496	0.86505	ALG2	85365	ALG2 alph 3-jan 6-mannosyltransferase
ENSG0000	394.0661	-0.066	0.14377	0.552569	0.86505	ABLIM1	3983	actin binding LIM protein 1
ENSG0000	133.7648	-0.06931	0.169866	0.552629	0.86505	TM7SF2	7108	transmembrane 7 superfamily member 2
ENSG0000	7.03732	0.04498	0.223056	0.552538	0.86505	NA	NA	NA
ENSG0000	1014.57	-0.05703	0.109454	0.552614	0.86505	BTK	695	Bruton tyrosine kinase
ENSG0000	95.23379	0.077708	0.194671	0.553076	0.865401	MAP3K7CI	56911	MAP3K7 C-terminal like
ENSG0000	4.68632	-0.01769	0.231582	0.553162	0.865461	RNU6-117	1.06E+08	RNA U6 small r pseudogene
ENSG0000	110.4102	-0.06835	0.151282	0.553214	0.865469	EXOSC9	5393	exosome component 9
ENSG0000	32.9457	-0.07017	0.174542	0.553315	0.865528	LAPTM4B	55353	lysosomal protein transmembrane 4 beta
ENSG0000	411.5434	0.066175	0.149326	0.553346	0.865528	SERPINB8	5271	serpin family B member 8

ENSG0000	42.57773	0.058779	0.208435	0.55341	0.865553	ZNF615	284370	zinc finger protein 615
ENSG0000	20.69878	-0.05735	0.21106	0.553669	0.865761	CD200R1	131450	CD200 receptor 1
ENSG0000	77.05679	-0.069	0.168763	0.553626	0.865761	YARS2	51067	tyrosyl-tRNA synthetase 2
ENSG0000	259.7424	0.063951	0.157525	0.553684	0.865761	CHAF1A	10036	chromatin assembly factor 1 subunit A
ENSG0000	25.81266	0.057395	0.210554	0.553744	0.86578	HASPIN	83903	histone H3 associated protein kinase
ENSG0000	4.017205	-0.03389	0.227846	0.553922	0.865782	RNF32	140545	ring finger protein 32
ENSG0000	941.1123	-0.0673	0.18067	0.553934	0.865782	SVIL	6840	supervillin
ENSG0000	377.1386	0.069207	0.164629	0.553916	0.865782	NAGPA	51172	N-acetylglucosamine-1-phosphodiester alpha-N-acetylglucosaminidase
ENSG0000	26.46263	0.068246	0.194182	0.553827	0.865782	CLIC2	1193	chloride intracellular channel 2
ENSG0000	590.73	0.071238	0.159679	0.554248	0.866058	SFXN5	94097	sideroflexin 5
ENSG0000	341.4076	0.067373	0.171842	0.554181	0.866058	WDR46	9277	WD repeat domain 46
ENSG0000	39.08215	-0.06493	0.196283	0.554311	0.866058	ARHGAP32	9743	Rho GTPase activating protein 32
ENSG0000	163.1056	-0.0685	0.155441	0.554347	0.866058	TRIP4	9325	thyroid hormone receptor interactor 4
ENSG0000	780.4112	-0.06779	0.154723	0.554274	0.866058	LPIN2	9663	lipin 2
ENSG0000	3204.907	-0.06442	0.137238	0.555388	0.866354	MAPKAPK	9261	MAPK activated protein kinase 2
ENSG0000	2.581828	-0.02801	0.230185	0.554838	0.866354	IL19	29949	interleukin 19
ENSG0000	31.28972	0.054662	0.212421	0.555463	0.866354	IGKV1D-8	28904	immunoglobulin kappa variable 1D-8
ENSG0000	126.0462	-0.06808	0.176363	0.555495	0.866354	DPP4	1803	dipeptidyl peptidase 4
ENSG0000	80.87331	0.0633	0.198491	0.555158	0.866354	NAB1	4664	NGFI-A binding protein 1
ENSG0000	101.2148	-0.06883	0.172649	0.55523	0.866354	FRMD4B	23150	FERM domain containing 4B
ENSG0000	93.66137	0.063836	0.196111	0.554868	0.866354	NA	NA	NA
ENSG0000	186.3614	-0.06868	0.161702	0.555234	0.866354	PELO	53918	pelota mRNA surveillance and ribosome rescue factor
ENSG0000	31.6444	-0.06831	0.187406	0.554767	0.866354	KIF20A	10112	kinesin family member 20A
ENSG0000	1042.371	0.066154	0.141178	0.555341	0.866354	LARP1	23367	La ribonuc translational regulator
ENSG0000	214.7421	-0.06571	0.148436	0.555446	0.866354	VPS41	27072	VPS41 subunit of HOPS complex
ENSG0000	64.21694	-0.06005	0.205783	0.555427	0.866354	ZHX1	11244	zinc fingers and homeoboxes 1
ENSG0000	135.2301	-0.06831	0.16527	0.555449	0.866354	PRKCQ-AS	439949	PRKCQ antisense RNA 1
ENSG0000	4.516561	0.029218	0.228428	0.55559	0.866354	NA	NA	NA
ENSG0000	7.128075	0.048488	0.221408	0.555176	0.866354	NCR3LG1	374383	natural killer cell cytotoxicity receptor 3 ligand 1
ENSG0000	153.7399	0.067175	0.177544	0.554993	0.866354	MS4A4E	643680	membrane spanning 4-domains A4E
ENSG0000	60.53432	-0.06913	0.173532	0.554863	0.866354	ASRGL1	80150	asparaginase and isoaspartyl peptidase 1
ENSG0000	6.419376	0.034152	0.228434	0.554762	0.866354	OVCH1-AS	1.01E+08	OVCH1 antisense RNA 1
ENSG0000	7.188952	0.047974	0.222084	0.555624	0.866354	TMEM30B	161291	transmembrane protein 30B
ENSG0000	5.876961	0.0421	0.225128	0.555616	0.866354	NA	NA	NA
ENSG0000	5.882393	-0.05094	0.22132	0.555315	0.866354	NA	NA	NA
ENSG0000	2.415497	0.023706	0.231894	0.554663	0.866354	IGLC4	3540	immunoglobulin lambda constant 4 (pseudogene)
ENSG0000	9.409784	-0.05404	0.216411	0.554796	0.866354	EOLA1-DT	1E+08	EOLA1 divergent transcript
ENSG0000	3.780075	-0.03205	0.228631	0.555689	0.866381	DYNC2H1	79659	dynein cytoplasmic 2 heavy chain 1
ENSG0000	3.629931	-0.02479	0.229892	0.555881	0.866607	TM4SF19	116211	transmembrane 4 L six family member 19
ENSG0000	9626.515	-0.06658	0.147737	0.555942	0.866628	SH3BP2	6452	SH3 domain binding protein 2
ENSG0000	52.17937	-0.06916	0.173825	0.556062	0.866741	ZNF426	79088	zinc finger protein 426
ENSG0000	20.26047	-0.0627	0.203356	0.55615	0.866805	MIR4453H	54553	MIR4453 host gene
ENSG0000	2.927904	0.035575	0.227966	0.556259	0.866901	LOC44031	440311	NOP53 ribosome biogenesis factor pseudogene
ENSG0000	3.641732	0.028043	0.229286	0.556382	0.867019	RPS3AP18	391706	RPS3A pseudogene 18
ENSG0000	2.999247	-0.02817	0.231465	0.556434	0.867026	ANXA8L1	728113	annexin A8 like 1
ENSG0000	21.43971	0.055512	0.211576	0.556484	0.86703	DTD2	112487	D-aminoacyl-tRNA deacylase 2
ENSG0000	6.516734	-0.02888	0.229183	0.556744	0.867287	ATP1A4	480	ATPase Na+/K+ transporting subunit alpha 4
ENSG0000	20.78225	0.057395	0.21021	0.5567	0.867287	ZNF584	201514	zinc finger protein 584
ENSG0000	36.87759	-0.06961	0.170976	0.556958	0.8674	EXO5	64789	exonuclease 5
ENSG0000	955.2118	-0.06432	0.151795	0.557053	0.8674	TRIP12	9320	thyroid hormone receptor interactor 12
ENSG0000	899.6456	-0.06646	0.168285	0.557039	0.8674	SOS2	6655	SOS Ras/Rho guanine nucleotide exchange factor 2
ENSG0000	78.52159	-0.06834	0.162525	0.557042	0.8674	PDP2	57546	pyruvate dehydrogenase phosphatase catalytic subunit 2
ENSG0000	7417.514	0.067107	0.158176	0.556915	0.8674	ARHGAP4	393	Rho GTPase activating protein 4
ENSG0000	374.5628	-0.0642	0.134666	0.557527	0.867728	POMGNT1	55624	protein O- 2-)
ENSG0000	17.78151	0.057207	0.212047	0.557443	0.867728	TMEM267	64417	transmembrane protein 267
ENSG0000	106.7893	0.035034	0.225943	0.557373	0.867728	PNPLA8	50640	patatin like phospholipase domain containing 8
ENSG0000	214.0631	0.064539	0.134534	0.557445	0.867728	ZNF783	1E+08	zinc finger protein 783
ENSG0000	5.396036	0.044084	0.224269	0.557488	0.867728	CBX3P2	645158	chromobox 3 pseudogene 2
ENSG0000	266.2334	0.068417	0.145207	0.557548	0.867728	PSMG2	56984	proteasome assembly chaperone 2
ENSG0000	19.20969	0.061668	0.203607	0.557787	0.867887	PIK3R3	8503	phosphoinositide-3-kinase regulatory subunit 3
ENSG0000	2.316683	-0.02002	0.233429	0.557816	0.867887	RGPD1	400966	RANBP2 like and GRIP domain containing 1
ENSG0000	6.404752	0.031795	0.22777	0.557839	0.867887	EFNA5	1946	ephrin A5
ENSG0000	96.61057	0.066743	0.18579	0.557834	0.867887	C6orf120	387263	chromosome 6 open reading frame 120
ENSG0000	36.28957	-0.06758	0.181979	0.558081	0.867968	TSTD3	1E+08	thiosulfate sulfurtransferase like domain containing 3
ENSG0000	9.500148	-0.04397	0.223662	0.558029	0.867968	LEP	3952	leptin
ENSG0000	121.9049	0.067977	0.155944	0.558045	0.867968	ZNF212	7988	zinc finger protein 212
ENSG0000	44.52575	-0.06853	0.179819	0.557972	0.867968	ZNF808	388558	zinc finger protein 808
ENSG0000	3.645113	0.026145	0.229636	0.558149	0.867987	PEBP4	157310	phosphatidylethanolamine binding protein 4
ENSG0000	935.3894	-0.05622	0.110128	0.558188	0.867987	UBE2Z	65264	ubiquitin conjugating enzyme E2 Z
ENSG0000	221.7544	0.067853	0.171015	0.558411	0.868124	USO1	8615	USO1 vesicle transport factor
ENSG0000	9.116646	0.039284	0.224968	0.558418	0.868124	CCDC62	84660	coiled-coil domain containing 62
ENSG0000	5.086363	0.039961	0.226607	0.558336	0.868124	DNM1P46	196968	dynamin 1 pseudogene 46
ENSG0000	14.46504	0.05518	0.214505	0.558672	0.868305	PLEKHA6	22874	pleckstrin homology domain containing A6
ENSG0000	99.2614	0.070482	0.16604	0.558986	0.868305	MRPS22	56945	mitochondrial ribosomal protein S22
ENSG0000	862.0452	-0.0645	0.174318	0.558944	0.868305	RBM47	54502	RNA binding motif protein 47
ENSG0000	9.521739	0.052894	0.216819	0.558859	0.868305	SEC24B-AS	1.01E+08	SEC24B antisense RNA 1
ENSG0000	22.6953	-0.06793	0.188139	0.559104	0.868305	NA	NA	NA
ENSG0000	12.04841	0.057072	0.212691	0.558809	0.868305	TCAF1	9747	TRPM8 channel associated factor 1
ENSG0000	197.0639	-0.06488	0.146109	0.559054	0.868305	COX15	1355	cytochrome c oxidase assembly homolog COX15
ENSG0000	2.941329	0.016683	0.231541	0.558695	0.868305	NA	NA	NA
ENSG0000	231.3683	-0.06553	0.13138	0.558829	0.868305	NA	NA	NA
ENSG0000	69.47953	-0.06241	0.198986	0.558899	0.868305	TJP3	27134	tight junction protein 3
ENSG0000	532.3479	0.059386	0.117884	0.55905	0.868305	UQCR10	29796	ubiquinol- complex III subunit X
ENSG0000	77.04255	0.065067	0.193211	0.559077	0.868305	ZNF41	7592	zinc finger protein 41
ENSG0000	672.7321	0.061296	0.176717	0.559235	0.868436	HLA-DRB5	3127	major hist class II DR beta 5
ENSG0000	343.2709	0.060575	0.126722	0.559361	0.868558	GRK5	2869	G protein-coupled receptor kinase 5
ENSG0000	20.48118	-0.04581	0.216409	0.559435	0.868599	CD8B2	927	CD8b2 molecule
ENSG0000	64.28189	-0.05623	0.207488	0.559682	0.868847	DPRXP4	503645	divergent-paired related homeobox pseudogene 4
ENSG0000	29.52476	0.066252	0.19166	0.559689	0.868847	PPAN	56342	peter pan homolog
ENSG0000	8.482782	0.056011	0.214682	0.560457	0.868923	NA	NA	NA
ENSG0000	3.074561	-0.02631	0.230162	0.560574	0.868923	NA	NA	NA
ENSG0000	4.934895	0.033466	0.227365	0.560599	0.868923	DCAF8-DT	1E+08	DCAF8 divergent transcript
ENSG0000	4.830079	-0.0364	0.226605	0.560676	0.868923	NA	NA	NA
ENSG0000	5.072547	-0.03392	0.228133	0.560393	0.868923	KLHL29	114818	kelch like family member 29
ENSG0000	145.1191	-0.06654	0.15662	0.56	0.868923	FEZ2	9637	fasciculation and elongation protein zeta 2
ENSG0000	97.86785	0.068902	0.166061	0.560091	0.868923	BLOC1S4	55330	biogenesis of lysosomal organelles complex 1 subunit 4

ENSG0000	16.30564	0.059894	0.205614	0.560207	0.868923	CEP44	80817	centrosomal protein 44
ENSG0000	1086.242	0.062175	0.127431	0.560045	0.868923	MAML1	9794	mastermind like transcriptional coactivator 1
ENSG0000	123.259	0.065315	0.134985	0.560209	0.868923	CDYL	9425	chromodomain Y like
ENSG0000	33.27084	-0.06655	0.188533	0.560668	0.868923	NA	NA	NA
ENSG0000	116.0438	0.067621	0.157731	0.560693	0.868923	ALKBH4	54784	alkB homc lysine demethylase
ENSG0000	595.1309	0.063181	0.193969	0.560731	0.868923	ENG	2022	endoglin
ENSG0000	2.36021	0.017511	0.232413	0.559892	0.868923	VSIG2	23584	V-set and immunoglobulin domain containing 2
ENSG0000	80.12543	0.060456	0.201823	0.560743	0.868923	NR2C1	7181	nuclear receptor subfamily 2 group C member 1
ENSG0000	5.107805	-0.04036	0.225225	0.560263	0.868923	NA	NA	NA
ENSG0000	43.97829	-0.06748	0.185645	0.560147	0.868923	MYH3	4621	myosin heavy chain 3
ENSG0000	221.5771	0.062898	0.129026	0.560267	0.868923	SCO1	6341	synthesis of cytochrome C oxidase 1
ENSG0000	62.88952	0.065549	0.190581	0.560782	0.868923	SKA2	348235	spindle and kinetochore associated complex subunit 2
ENSG0000	6.191241	0.043642	0.223738	0.560196	0.868923	NA	NA	NA
ENSG0000	34.24029	0.065246	0.19374	0.560496	0.868923	DONSON	29980	DNA replication fork stabilization factor DONSON
ENSG0000	85.86798	0.068162	0.178218	0.559992	0.868923	MCAT	27349	malonyl-CoA-acyl carrier protein transacylase
ENSG0000	571.7002	0.064157	0.132737	0.560986	0.86896	SEC24D	9871	SEC24 hon COPII coat complex component
ENSG0000	519.2729	0.057235	0.111756	0.560904	0.86896	TTC17	55761	tetratricopeptide repeat domain 17
ENSG0000	1008.787	-0.0675	0.144213	0.560912	0.86896	MRPL28	10573	mitochondrial ribosomal protein L28
ENSG0000	934.9791	-0.05653	0.105592	0.560996	0.86896	PTPRA	5786	protein tyrosine phosphatase receptor type A
ENSG0000	7.511039	-0.05226	0.217776	0.56106	0.868985	NA	NA	NA
ENSG0000	255.5725	0.066194	0.185072	0.561222	0.86909	MDH1	4190	malate dehydrogenase 1
ENSG0000	12557.02	-0.0605	0.19757	0.561198	0.86909	ANPEP	290	alanyl ami membrane
ENSG0000	552.2087	-0.06559	0.154172	0.561309	0.869097	TMEM33	55161	transmembrane protein 33
ENSG0000	41.47901	0.050882	0.214733	0.561349	0.869097	NOC3L	64318	NOC3 like DNA replication regulator
ENSG0000	10.9853	0.058021	0.21166	0.561369	0.869097	MYEF2	50804	myelin expression factor 2
ENSG0000	441.1855	0.063844	0.135215	0.561428	0.869114	RFTN1	23180	raftlin lipid raft linker 1
ENSG0000	609.2085	-0.06284	0.133479	0.562095	0.869158	TXLNA	200081	taxilin alpha
ENSG0000	47.24662	-0.06851	0.174395	0.56162	0.869158	CCDC51	79714	coiled-coil domain containing 51
ENSG0000	137.4735	-0.06638	0.17459	0.562072	0.869158	TIPARP	25976	TCDD inducible poly(ADP-ribose) polymerase
ENSG0000	67.94218	0.065968	0.18813	0.5621	0.869158	SKP2	6502	S-phase kinase associated protein 2
ENSG0000	30.13012	-0.06869	0.177191	0.561962	0.869158	ILRUN-AS1	1.02E+08	ILRUN antisense RNA 1
ENSG0000	5.587211	-0.0449	0.222589	0.561909	0.869158	LOC12490	1.25E+08	uncharacterized LOC124901671
ENSG0000	7.973644	-0.04181	0.223284	0.56208	0.869158	NA	NA	NA
ENSG0000	2.567132	-0.00897	0.233759	0.562029	0.869158	LINC0205!	1.08E+08	long intergenic non-protein coding RNA 2055
ENSG0000	225.909	0.06716	0.15435	0.561996	0.869158	TPRN	286262	taperin
ENSG0000	97.36312	-0.06691	0.157299	0.561579	0.869158	PYROXD2	84795	pyridine nucleotide-disulphide oxidoreductase domain 2
ENSG0000	72.60532	0.067406	0.16664	0.562088	0.869158	TMPO-AS1	1E+08	TMPO antisense RNA 1
ENSG0000	12.80732	-0.04425	0.221738	0.56212	0.869158	NA	NA	NA
ENSG0000	5.563925	-0.03707	0.225996	0.56186	0.869158	SULT1A3	6818	sulfotransferase family 1A member 3
ENSG0000	7886.585	-0.06739	0.165309	0.561727	0.869158	MED25	81857	mediator complex subunit 25
ENSG0000	4.139368	-0.02521	0.229611	0.56223	0.869254	NA	NA	NA
ENSG0000	29.32233	0.063922	0.197246	0.562341	0.869353	NA	NA	NA
ENSG0000	6.408279	-0.03758	0.22579	0.562402	0.869373	NA	NA	NA
ENSG0000	316.3792	0.063566	0.136095	0.562457	0.869386	TRIM52-A!	1.01E+08	TRIM52 antisense RNA 1 (head to head)
ENSG0000	4.632807	0.020681	0.231118	0.562521	0.869411	PFDN4	5203	prefoldin subunit 4
ENSG0000	658.5237	0.067172	0.175742	0.562653	0.869449	SPCS3	60559	signal peptidase complex subunit 3
ENSG0000	278.0139	0.064729	0.142843	0.562688	0.869449	NDUFS6	4726	NADH:ubiquinone oxidoreductase subunit S6
ENSG0000	95.96864	-0.06635	0.165676	0.562617	0.869449	BEX4	56271	brain expressed X-linked 4
ENSG0000	12.80236	0.054825	0.213606	0.562885	0.869607	PUS7	54517	pseudouridine synthase 7
ENSG0000	594.2756	-0.06383	0.136057	0.562858	0.869607	ATP6V1E1	529	ATPase H+ transporting V1 subunit E1
ENSG0000	119.6276	-0.06999	0.176822	0.563116	0.869773	SIRT5	23408	sirtuin 5
ENSG0000	3323.101	-0.06329	0.135984	0.563135	0.869773	MMP24OS	1.01E+08	MMP24 opposite strand
ENSG0000	801.9313	-0.06661	0.163104	0.563058	0.869773	THOC5	8563	THO complex subunit 5
ENSG0000	1384.212	-0.06453	0.141685	0.563199	0.869799	DAXX	1616	death domain associated protein
ENSG0000	593.5467	-0.06176	0.143585	0.563317	0.86985	ZNF76	7629	zinc finger protein 76
ENSG0000	17.85356	0.059633	0.206435	0.563327	0.86985	ADPRHL1	113622	ADP-ribosylhydrolase like 1
ENSG0000	901.7545	0.066455	0.153215	0.563534	0.870065	GUSB	2990	glucuronidase beta
ENSG0000	346.43	0.065264	0.152892	0.563704	0.870065	SLC52A2	79581	solute carrier family 52 member 2
ENSG0000	54.67076	0.068145	0.176969	0.56362	0.870065	DHODH	1723	dihydroorotate dehydrogenase (quinone)
ENSG0000	388.8059	0.068289	0.155774	0.563698	0.870065	ADAP2	55803	ArfGAP with dual PH domains 2
ENSG0000	477.5382	0.066598	0.150479	0.563654	0.870065	KDM5D	8284	lysine demethylase 5D
ENSG0000	49.33174	0.067591	0.183691	0.563807	0.870078	NUDT2	318	nudix hydrolase 2
ENSG0000	3.485921	-0.03717	0.226742	0.563761	0.870078	NA	NA	NA
ENSG0000	206.9248	0.063209	0.135544	0.563944	0.870215	C15orf40	123207	chromosome 15 open reading frame 40
ENSG0000	13.5346	-0.04592	0.219871	0.564004	0.870227	NA	NA	NA
ENSG0000	3.145438	-0.03201	0.22817	0.564047	0.870227	DNAJB6P1	387820	DNAJB6 pseudogene 1
ENSG0000	1083.421	-0.06597	0.152621	0.564134	0.870273	TRAPPC1	58485	trafficking protein particle complex subunit 1
ENSG0000	10.39871	-0.04534	0.221416	0.564172	0.870273	ACER1	125981	alkaline ceramidase 1
ENSG0000	11.78336	0.053512	0.215274	0.564313	0.870419	NA	NA	NA
ENSG0000	6.391385	0.048748	0.220437	0.564422	0.870439	LINC0180(	1.02E+08	long intergenic non-protein coding RNA 1800
ENSG0000	345.5657	-0.06646	0.157537	0.564383	0.870439	RUBCNL	80183	rubicon like autophagy enhancer
ENSG0000	2.794511	0.026053	0.230553	0.56469	0.87056	MORN2	729967	MORN repeat containing 2
ENSG0000	564.7521	-0.06216	0.134548	0.564581	0.87056	MEA1	4201	male-enhanced antigen 1
ENSG0000	57.7943	-0.06776	0.16331	0.564681	0.87056	GTSF1	121355	gametocyte specific factor 1
ENSG0000	295.8672	0.067271	0.151875	0.56464	0.87056	PCNT	5116	pericentrin
ENSG0000	120.5192	0.066745	0.172366	0.56474	0.870564	KPNA3	3839	karyopherin subunit alpha 3
ENSG0000	301.584	0.06357	0.135747	0.564801	0.870584	SRP54	6729	signal recognition particle 54
ENSG0000	2930.506	0.060483	0.133615	0.564905	0.870598	MTND2P2	1.01E+08	MT-ND2 pseudogene 28
ENSG0000	4.647592	-0.03616	0.226669	0.56487	0.870598	RPS2P29	646294	ribosomal protein S2 pseudogene 29
ENSG0000	184.8122	-0.0589	0.20131	0.564973	0.870599	TTC14	151613	tetratricopeptide repeat domain 14
ENSG0000	2541.063	0.067009	0.16079	0.565001	0.870599	COL18A1	80781	collagen type XVIII alpha 1 chain
ENSG0000	27.31571	0.066083	0.19015	0.565084	0.870654	ZNF813	126017	zinc finger protein 813
ENSG0000	3.118158	0.009614	0.232395	0.565189	0.870743	NA	NA	NA
ENSG0000	214.0491	0.066038	0.152486	0.565666	0.870909	TESK2	10420	testis associated actin remodelling kinase 2
ENSG0000	26.28947	-0.05033	0.214926	0.565629	0.870909	OLFML2B	25903	olfactomedin like 2B
ENSG0000	16.50296	0.055374	0.212146	0.565361	0.870909	ITGA6-AS1	1.02E+08	ITGA6 antisense RNA 1
ENSG0000	3.244334	-0.02541	0.230355	0.565543	0.870909	MIR4458H	1.01E+08	MIR4458 host gene
ENSG0000	1578.676	-0.06602	0.137112	0.565612	0.870909	DAP	1611	death associated protein
ENSG0000	6.549543	0.046783	0.22166	0.565628	0.870909	NA	NA	NA
ENSG0000	45.55916	-0.06798	0.171733	0.565546	0.870909	MED17	9440	mediator complex subunit 17
ENSG0000	2400.608	-0.06473	0.14755	0.565677	0.870909	CPNE1	8904	copine 1
ENSG0000	2914.388	0.063252	0.138026	0.565777	0.87099	DOK2	9046	docking protein 2
ENSG0000	488.6586	0.06729	0.146267	0.565918	0.871032	AKR1A1	10327	aldo-keto reductase family 1 member A1
ENSG0000	32.67992	0.048668	0.216468	0.566072	0.871032	TRMT13	54482	tRNA methyltransferase 13 homolog
ENSG0000	35.27148	0.061693	0.193201	0.565899	0.871032	NA	NA	NA
ENSG0000	9.134143	-0.0419	0.223656	0.565998	0.871032	LOC12490	1.25E+08	uncharacterized LOC124909396

ENSG0000	4.035096	0.026094	0.229598	0.566114	0.871032	NA	NA	NA
ENSG0000	21.15687	-0.06554	0.192804	0.566088	0.871032	NA	NA	NA
ENSG0000	53.29838	0.067993	0.169672	0.566137	0.871032	LOC10099	1.01E+08	uncharacterized LOC100996842
ENSG0000	162.4655	0.068096	0.167209	0.566208	0.871067	CDK19	23097	cyclin dependent kinase 19
ENSG0000	93.93791	-0.06196	0.194879	0.566329	0.871072	TCEA3	6920	transcription elongation factor A3
ENSG0000	478.3355	-0.06562	0.184383	0.566391	0.871072	UBLCP1	134510	ubiquitin like domain containing CTD phosphatase 1
ENSG0000	118.3207	-0.06805	0.177796	0.566401	0.871072	ARHGAP2	1.09E+08	ARHGAP2 transcribed pseudogene
ENSG0000	1517.199	0.070885	0.1561	0.566284	0.871072	ZNF580	51157	zinc finger protein 580
ENSG0000	718.7105	0.063538	0.138886	0.566525	0.871189	USP34	9736	ubiquitin specific peptidase 34
ENSG0000	15.63114	0.049299	0.216843	0.566814	0.871328	KCNA2	3737	potassium voltage-gated channel subfamily A member 2
ENSG0000	23.91298	0.055761	0.214227	0.566664	0.871328	GNPDA2	132789	glucosamine-6-phosphate deaminase 2
ENSG0000	1758.817	-0.0644	0.144703	0.56675	0.871328	MYL6	4637	myosin light chain 6
ENSG0000	10.72278	0.044752	0.221047	0.566796	0.871328	B3GLCT	145173	beta 3-glucosyltransferase
ENSG0000	1933.043	0.065797	0.151934	0.566853	0.871328	PIEZO1	9780	piezo type mechanosensitive ion channel component 1
ENSG0000	15.66258	0.051118	0.215199	0.567122	0.871377	LAMTOR5-	1.01E+08	LAMTOR5 antisense RNA 1
ENSG0000	1248.155	-0.06576	0.160089	0.567082	0.871377	RNF123	63891	ring finger protein 123
ENSG0000	85.02909	0.062836	0.194366	0.56705	0.871377	SLC39A8	64116	solute carrier family 39 member 8
ENSG0000	31.5296	0.061368	0.201155	0.567119	0.871377	MSANTD4	84437	Myb/SANT DNA binding domain containing 4 with coiled-coils
ENSG0000	4.925069	0.031942	0.227728	0.567123	0.871377	NA	NA	NA
ENSG0000	64224.68	-0.0595	0.197002	0.567233	0.871399	ZYX	7791	zyxin
ENSG0000	5.613022	-0.03933	0.225336	0.567231	0.871399	NA	NA	NA
ENSG0000	31.37771	0.064967	0.191672	0.567284	0.871405	MSS51	118490	MSS51 mitochondrial translational activator
ENSG0000	1375.313	-0.06369	0.137467	0.567335	0.87141	ITPKB	3707	inositol-trisphosphate 3-kinase B
ENSG0000	1200.445	0.067191	0.124951	0.567389	0.87142	ELOB	6923	elongin B
ENSG0000	112.4762	0.066852	0.15928	0.567562	0.871553	ZNF7	7553	zinc finger protein 7
ENSG0000	54.91178	0.068604	0.1708	0.567571	0.871553	ZSCAN22	342945	zinc finger and SCAN domain containing 22
ENSG0000	37.86824	0.068979	0.174322	0.567766	0.871707	TXNDC12	51060	thioredoxin domain containing 12
ENSG0000	95.84784	-0.06325	0.175049	0.567732	0.871707	UNC50	25972	unc-50 inner nuclear membrane RNA binding protein
ENSG0000	2.78915	0.030314	0.2286	0.568014	0.871795	RPS10P11	391833	ribosomal protein S10 pseudogene 11
ENSG0000	14.81528	-0.05429	0.212677	0.56799	0.871795	FIGNL2	401720	fidgetin like 2
ENSG0000	257.9461	0.06882	0.171673	0.567935	0.871795	VPS29	51699	VPS29 retromer complex component
ENSG0000	3.395683	-0.02982	0.229418	0.567888	0.871795	NA	NA	NA
ENSG0000	7.857787	-0.03103	0.226447	0.568137	0.871911	NA	NA	NA
ENSG0000	438.9974	0.065602	0.155884	0.568277	0.871943	BABAM2	9577	BRISC and BRCA1 A complex member 2
ENSG0000	59.84512	-0.06442	0.189443	0.568373	0.871943	FHDC1	85462	FH2 domain containing 1
ENSG0000	339.0521	-0.06482	0.152803	0.568396	0.871943	UBE3C	9690	ubiquitin protein ligase E3C
ENSG0000	3.445415	0.024004	0.229903	0.568377	0.871943	NA	NA	NA
ENSG0000	2.909893	0.016611	0.231134	0.568298	0.871943	NA	NA	NA
ENSG0000	55.11482	0.065321	0.18767	0.568673	0.872295	NA	NA	NA
ENSG0000	6.692247	-0.03483	0.22598	0.568901	0.872573	TRAJ31	28724	T cell receptor alpha joining 31
ENSG0000	2168.389	0.054026	0.203029	0.569139	0.872864	IFNGR1	3459	interferon gamma receptor 1
ENSG0000	5.084352	0.032883	0.227499	0.569314	0.872987	NA	NA	NA
ENSG0000	58.74271	0.068089	0.169043	0.569284	0.872987	PPP1R26	9858	protein phosphatase 1 regulatory subunit 26
ENSG0000	44.36392	0.066398	0.176412	0.569414	0.873067	RAB33A	9363	RAB33A member RAS oncogene family
ENSG0000	17.18349	0.056369	0.211343	0.569777	0.873321	IL17RE	132014	interleukin 17 receptor E
ENSG0000	222.3693	-0.05491	0.205141	0.5698	0.873321	OSTM1	28962	osteoclastogenesis associated transmembrane protein 1
ENSG0000	2.407613	-0.02481	0.230585	0.569769	0.873321	NA	NA	NA
ENSG0000	9.428235	-0.02066	0.230017	0.569866	0.873321	RNU7-181	1.06E+08	RNA U7 small nuclear 181 pseudogene
ENSG0000	72.53302	-0.05548	0.206281	0.569772	0.873321	TBC1D4	9882	TBC1 domain family member 4
ENSG0000	4.781041	0.037375	0.226444	0.569854	0.873321	DNAH17	8632	dynein axonemal heavy chain 17
ENSG0000	294.995	-0.06609	0.163446	0.570034	0.873432	WDR48	57599	WD repeat domain 48
ENSG0000	5.819087	0.033434	0.226886	0.570013	0.873432	NA	NA	NA
ENSG0000	5915.263	0.057623	0.127378	0.570213	0.873513	YBX1	4904	Y-box binding protein 1
ENSG0000	106.1861	-0.06134	0.19397	0.570531	0.873513	PHTF1	10745	putative homeodomain transcription factor 1
ENSG0000	17.35913	0.060422	0.205111	0.570574	0.873513	CENPA	1058	centromere protein A
ENSG0000	36.48164	0.054805	0.208495	0.570594	0.873513	CMSS1	84319	cms1 ribosomal small subunit homolog
ENSG0000	111.6585	0.057418	0.203701	0.570187	0.873513	ZBTB11	27107	zinc finger and BTB domain containing 11
ENSG0000	3.056407	-0.00812	0.233703	0.570312	0.873513	LOC10537	1.05E+08	uncharacterized LOC105377267
ENSG0000	6.262597	0.027475	0.22838	0.570409	0.873513	KRT8P33	1E+08	keratin 8 pseudogene 33
ENSG0000	75.01151	-0.06604	0.179828	0.570253	0.873513	NA	NA	NA
ENSG0000	6.449728	0.035131	0.225674	0.570499	0.873513	KTN1-AS1	1E+08	KTN1 antisense RNA 1
ENSG0000	11.74791	-0.05617	0.211305	0.570513	0.873513	PRSS53	339105	serine protease 53
ENSG0000	26.01452	-0.05339	0.21109	0.570659	0.873513	CMC2	56942	C-X9-C motif containing 2
ENSG0000	3.415788	-0.02532	0.230203	0.570649	0.873513	NA	NA	NA
ENSG0000	304.1453	0.06581	0.159078	0.570862	0.873678	TMCC1	23023	transmembrane and coiled-coil domain family 1
ENSG0000	2.619999	0.024851	0.233923	0.570848	0.873678	NA	NA	NA
ENSG0000	280.8963	0.062913	0.189678	0.570917	0.873688	MTND4P1	1E+08	MT-ND4 pseudogene 12
ENSG0000	9.830305	0.040371	0.223322	0.571129	0.873941	NA	NA	NA
ENSG0000	67.77245	0.059994	0.199783	0.5712	0.873975	E2F5	1875	E2F transcription factor 5
ENSG0000	5497.697	0.062099	0.156194	0.571251	0.873981	CIC	23152	capicua transcriptional repressor
ENSG0000	14.07901	-0.04478	0.219895	0.571307	0.873993	ADORA2B	136	adenosine A2b receptor
ENSG0000	2024.548	0.064546	0.14768	0.571403	0.874052	SNRNP20	23020	small nuclear ribonucleoprotein U5 subunit 200
ENSG0000	1437.123	0.064337	0.147293	0.571441	0.874052	PSMD3	5709	proteasom non-ATPase 3
ENSG0000	574.1948	0.04191	0.220999	0.571645	0.87406	HIPK3	10114	homeodomain interacting protein kinase 3
ENSG0000	5.104298	-0.03392	0.226497	0.57178	0.87406	SCN8A	6334	sodium voltage-gated channel alpha subunit 8
ENSG0000	2.619841	0.02621	0.229476	0.571766	0.87406	NA	NA	NA
ENSG0000	440.2865	0.066513	0.152494	0.571723	0.87406	RABEP2	79874	rabaptin RAB GTPase binding effector protein 2
ENSG0000	680.646	0.051702	0.102088	0.571721	0.87406	CSNK2A1	1457	casein kinase 2 alpha 1
ENSG0000	50.2278	0.06663	0.183288	0.571633	0.87406	ARVCF	421	ARVCF delta catenin family member
ENSG0000	62.15809	-0.06594	0.17688	0.571715	0.87406	FAM104B	90736	family with sequence similarity 104 member B
ENSG0000	230.6224	0.06629	0.17191	0.572718	0.874124	RCAN3	11123	RCAN family member 3
ENSG0000	15.65352	0.056516	0.209185	0.572163	0.874124	DOCK7	85440	dedicator of cytokinesis 7
ENSG0000	13.023	0.048154	0.219628	0.572919	0.874124	TLCD4	148534	TLC domain containing 4
ENSG0000	4.079515	-0.02534	0.229234	0.57269	0.874124	NA	NA	NA
ENSG0000	2163.492	-0.06268	0.138162	0.571988	0.874124	DVL3	1857	dishevelled segment polarity protein 3
ENSG0000	104.2391	-0.04431	0.21553	0.572833	0.874124	RAB33B	83452	RAB33B member RAS oncogene family
ENSG0000	527.2557	-0.06291	0.136894	0.57234	0.874124	MAP3K5	4217	mitogen-activated protein kinase kinase kinase 5
ENSG0000	8.901664	-0.03472	0.225669	0.571931	0.874124	NA	NA	NA
ENSG0000	35.37514	-0.05742	0.204538	0.572812	0.874124	NA	NA	NA
ENSG0000	1160.976	-0.06449	0.173134	0.572788	0.874124	TMEM71	137835	transmembrane protein 71
ENSG0000	80.62235	-0.0663	0.157385	0.572455	0.874124	ZNF79	7633	zinc finger protein 79
ENSG0000	1048.44	-0.0564	0.113937	0.57194	0.874124	WBP1L	54838	WW domain binding protein 1 like
ENSG0000	87.45414	-0.06561	0.174371	0.572224	0.874124	C1RL-AS1	283314	C1RL antisense RNA 1
ENSG0000	5.037772	0.037901	0.225616	0.572738	0.874124	STYK1	55359	serine/threonine/tyrosine kinase 1
ENSG0000	28.84173	0.061729	0.191482	0.572905	0.874124	ATP2B1-A	338758	ATP2B1 antisense RNA 1
ENSG0000	29.03483	-0.06233	0.19656	0.572759	0.874124	VKORC1	79001	vitamin K epoxide reductase complex subunit 1

ENSG0000	180.393	0.067733	0.158046	0.572284	0.874124	FTO	79068	FTO alpha-ketoglutarate dependent dioxygenase
ENSG0000	14.49804	0.035869	0.223991	0.572152	0.874124	NA	NA	NA
ENSG0000	193.5721	0.06469	0.160185	0.572889	0.874124	GARRE1	9710	granule associated Rac and RHOG effector 1
ENSG0000	600.4558	-0.06368	0.133933	0.572137	0.874124	FBXO46	23403	F-box protein 46
ENSG0000	278.3988	0.066926	0.163255	0.572732	0.874124	BCAT2	587	branched chain amino acid transaminase 2
ENSG0000	227.1291	0.067825	0.182732	0.572435	0.874124	MORF4L2	9643	mortality factor 4 like 2
ENSG0000	98827.08	0.062437	0.136104	0.572059	0.874124	NA	NA	NA
ENSG0000	337.3769	0.059304	0.124543	0.573056	0.874128	LIX1L	128077	limb and CNS expressed 1 like
ENSG0000	798.8122	-0.06703	0.155019	0.57303	0.874128	HDAC4	9759	histone deacetylase 4
ENSG0000	205.5158	0.063572	0.187106	0.573165	0.874128	SMC4	10051	structural maintenance of chromosomes 4
ENSG0000	9.550429	0.046378	0.218808	0.573256	0.874128	INHBA	3624	inhibin subunit beta A
ENSG0000	292.2266	0.065617	0.137973	0.573181	0.874128	WDR37	22884	WD repeat domain 37
ENSG0000	1292.299	-0.05648	0.114092	0.573103	0.874128	WASH8P	1E+08	WAS prote pseudogene
ENSG0000	4730.363	-0.06214	0.147994	0.573227	0.874128	SCAMP2	10066	secretory carrier membrane protein 2
ENSG0000	1786.596	-0.06675	0.138443	0.573404	0.874282	CLASRP	11129	CLK4 associating serine/arginine rich protein
ENSG0000	289.4733	0.064983	0.150935	0.573502	0.874358	LAS1L	81887	LAS1 like ribosome biogenesis factor
ENSG0000	8.439252	-0.03915	0.224128	0.573557	0.874369	MAGI3	260425	membran WW and PDZ domain containing 3
ENSG0000	2.401903	0.018916	0.232474	0.573711	0.874517	NIPAL1	152519	NIPA like domain containing 1
ENSG0000	4.055532	0.02615	0.22859	0.573797	0.874517	NA	NA	NA
ENSG0000	40.02952	0.064902	0.186342	0.573764	0.874517	NA	NA	NA
ENSG0000	18.94988	-0.06375	0.198371	0.573894	0.874592	TRIM16	10626	tripartite motif containing 16
ENSG0000	27.42019	0.064864	0.188824	0.574307	0.874719	DISP1	84976	dispatched RND transporter family member 1
ENSG0000	200.5602	0.060882	0.193782	0.574326	0.874719	HAAO	23498	3-hydroxy 4-dioxygenase
ENSG0000	11.14398	-0.05355	0.212982	0.574486	0.874719	NA	NA	NA
ENSG0000	21.71118	-0.05672	0.206903	0.574232	0.874719	TSPAN13	27075	tetraspanin 13
ENSG0000	71.03168	0.066233	0.16605	0.574444	0.874719	NA	NA	NA
ENSG0000	561.5492	0.050599	0.108319	0.574536	0.874719	MAPKAP1	79109	MAPK associated protein 1
ENSG0000	47.63728	-0.05377	0.209274	0.574464	0.874719	LINC0244	1.01E+08	long intergenic non-protein coding RNA 2446
ENSG0000	1391.976	-0.04918	0.097681	0.574295	0.874719	MAPK1IP1	93487	mitogen-activated protein kinase 1 interacting protein 1 like
ENSG0000	1478.134	-0.06412	0.144107	0.574598	0.874719	SUSD6	9766	sushi domain containing 6
ENSG0000	4.075071	-0.02578	0.229294	0.574461	0.874719	C1QL1	10882	complement C1q like 1
ENSG0000	217.1809	-0.0658	0.150462	0.574576	0.874719	TLK2	11011	tousled like kinase 2
ENSG0000	397.0233	0.065258	0.173347	0.574402	0.874719	HOMER3	9454	homer scaffold protein 3
ENSG0000	202.3038	-0.06487	0.144793	0.574028	0.874719	ZNF428	126299	zinc finger protein 428
ENSG0000	22.8385	0.063633	0.194363	0.574937	0.874798	WDPCP	51057	WD repeat containing planar cell polarity effector
ENSG0000	298.558	0.061558	0.192671	0.5749	0.874798	JMJD1C	221037	jumonji domain containing 1C
ENSG0000	1514.765	0.058417	0.129769	0.574776	0.874798	SEC24C	9632	SEC24 hon COPII coat complex component
ENSG0000	179.1701	-0.06381	0.148528	0.574773	0.874798	PDCD7	10081	programmed cell death 7
ENSG0000	1161.729	-0.05722	0.128457	0.574906	0.874798	PIGS	94005	phosphatidylinositol glycan anchor biosynthesis class S
ENSG0000	81.27895	0.066207	0.170787	0.574906	0.874798	NR2C2AP	126382	nuclear receptor 2C2 associated protein
ENSG0000	7.19427	0.032284	0.226793	0.575035	0.874813	IMMP2L	83943	inner mitochondrial membrane peptidase subunit 2
ENSG0000	9.355913	-0.04136	0.222603	0.575042	0.874813	ATP2A1-A	1E+08	ATP2A1 antisense RNA 1
ENSG0000	37.31957	-0.06442	0.186486	0.57528	0.874899	RAB6B	51560	RAB6B member RAS oncogene family
ENSG0000	789.6758	0.065149	0.166054	0.575168	0.874899	NDFIP1	80762	Nedd4 family interacting protein 1
ENSG0000	101.4608	-0.06568	0.166268	0.575228	0.874899	PKN3	29941	protein kinase N3
ENSG0000	939.2657	0.054613	0.111531	0.57529	0.874899	USP22	23326	ubiquitin specific peptidase 22
ENSG0000	6.706025	0.041456	0.223607	0.575491	0.875132	NA	NA	NA
ENSG0000	51.68737	0.055654	0.206522	0.575751	0.87514	KHK	3795	ketohexokinase
ENSG0000	3.175455	0.02414	0.229831	0.575907	0.87514	NA	NA	NA
ENSG0000	2.665958	0.033755	0.228682	0.575663	0.87514	TRIM36	55521	tripartite motif containing 36
ENSG0000	252.378	0.061886	0.195647	0.575846	0.87514	CCNG1	900	cyclin G1
ENSG0000	27.84072	0.062941	0.194715	0.575961	0.87514	BAG2	9532	BAG cochaperone 2
ENSG0000	6.436956	0.032719	0.22681	0.575874	0.87514	RAPGEF5	9771	Rap guanine nucleotide exchange factor 5
ENSG0000	8.670203	0.05536	0.212421	0.575691	0.87514	NA	NA	NA
ENSG0000	227.7283	0.060359	0.184442	0.575896	0.87514	CCDC6	8030	coiled-coil domain containing 6
ENSG0000	6.551145	-0.03485	0.225855	0.575867	0.87514	PARD6G-A	1E+08	PARD6G antisense RNA 1
ENSG0000	13.90548	0.044089	0.220263	0.575974	0.87514	MZF1-AS1	1E+08	MZF1 antisense RNA 1
ENSG0000	73.22646	0.06398	0.184903	0.57621	0.875425	BTLA	151888	B and T lymphocyte associated
ENSG0000	3.281046	0.025594	0.23065	0.576303	0.875425	SMO	6608	smoothen frizzled class receptor
ENSG0000	8.552891	0.0482	0.219219	0.576305	0.875425	WBP2NL	164684	WBP2 N-terminal like
ENSG0000	3.55363	0.023599	0.229435	0.576473	0.875463	MYLK3	91807	myosin light chain kinase 3
ENSG0000	13.15512	-0.05613	0.209133	0.57638	0.875463	NQO1	1728	NAD(P)H quinone dehydrogenase 1
ENSG0000	81.19925	-0.06546	0.175633	0.576448	0.875463	U2AF1	7307	U2 small nuclear RNA auxiliary factor 1
ENSG0000	60.37863	0.065494	0.176154	0.576563	0.875527	PARP2	10038	poly(ADP-ribose) polymerase 2
ENSG0000	3.256624	-0.01962	0.231409	0.576641	0.875572	NA	NA	NA
ENSG0000	3.839943	0.03076	0.228281	0.576698	0.875587	NA	NA	NA
ENSG0000	8.700502	-0.04922	0.217817	0.576779	0.875636	LOC10028	1E+08	uncharacterized LOC100289495
ENSG0000	656.6343	-0.05451	0.11256	0.576916	0.875773	TMED4	222068	transmembrane p24 trafficking protein 4
ENSG0000	130.0723	0.064759	0.150615	0.577215	0.876153	MFSD4B	91749	major facilitator superfamily domain containing 4B
ENSG0000	5041.863	0.061559	0.158481	0.577272	0.876168	MGRN1	23295	mahogunin ring finger 1
ENSG0000	155.9139	-0.05307	0.203403	0.577427	0.876192	OSGIN2	734	oxidative stress induced growth inhibitor family member 2
ENSG0000	55.95628	-0.06523	0.175414	0.577431	0.876192	NA	NA	NA
ENSG0000	89.23717	0.064085	0.148309	0.577414	0.876192	LIME1	54923	Lck interacting transmembrane adaptor 1
ENSG0000	125.4541	0.064627	0.155167	0.577505	0.876231	ATP5ME	521	ATP synthase membrane subunit e
ENSG0000	404.3276	-0.06159	0.141443	0.577757	0.876323	UTRN	7402	utrophin
ENSG0000	442.4788	-0.05971	0.190587	0.577753	0.876323	ATP6V1G1	9550	ATPase H+ transporting V1 subunit G1
ENSG0000	9.170074	0.047871	0.218393	0.577711	0.876323	HERC2P7	1E+08	HERC2 pseudogene 7
ENSG0000	2.390391	-0.02415	0.230351	0.577659	0.876323	NA	NA	NA
ENSG0000	2.548726	0.02174	0.23242	0.577815	0.876339	ACTN3	89	actinin alpha 3
ENSG0000	373.3711	0.064255	0.149118	0.578124	0.876344	TMEM39B	55116	transmembrane protein 39B
ENSG0000	18.61548	-0.04928	0.20922	0.578553	0.876344	NA	NA	NA
ENSG0000	434.9794	-0.0569	0.119756	0.578379	0.876344	STAMBP	10617	STAM binding protein
ENSG0000	52.8328	-0.05408	0.207655	0.578307	0.876344	EIF4E	1977	eukaryotic translation initiation factor 4E
ENSG0000	38.89952	0.062803	0.190519	0.57833	0.876344	HPF1	54969	histone PARylation factor 1
ENSG0000	262.5758	-0.06092	0.13203	0.578435	0.876344	PFDN1	5201	prefoldin subunit 1
ENSG0000	70.40929	0.059812	0.181471	0.578079	0.876344	SPIN1	10927	spindlin 1
ENSG0000	265.7755	-0.06585	0.166632	0.578632	0.876344	TMEM273	170371	transmembrane protein 273
ENSG0000	214.4474	0.061929	0.138304	0.578461	0.876344	NAA40	79829	N-alpha-a NatD catalytic subunit
ENSG0000	311.4289	-0.06192	0.135943	0.578513	0.876344	BAD	572	BCL2 associated agonist of cell death
ENSG0000	2973.675	-0.06611	0.139383	0.578545	0.876344	LAMTOR1	55004	late endos MAPK and MTOR activator 1
ENSG0000	492.8802	0.064603	0.167793	0.578037	0.876344	RN7SL1	6029	RNA component of signal recognition particle 7SL1
ENSG0000	95.10799	0.065234	0.159484	0.577944	0.876344	GNPNAT1	64841	glucosamine-phosphate N-acetyltransferase 1
ENSG0000	17.51434	-0.04201	0.220463	0.578622	0.876344	CDKN3	1033	cyclin dependent kinase inhibitor 3
ENSG0000	1268.185	0.056493	0.11935	0.57828	0.876344	FLYWCH1	84256	FLYWCH-type zinc finger 1
ENSG0000	418.8321	-0.06597	0.153592	0.578513	0.876344	PRMT7	54496	protein arginine methyltransferase 7
ENSG0000	755.28	0.062391	0.137135	0.578501	0.876344	RRP7A	27341	ribosomal RNA processing 7 homolog A

ENSG0000	62.13952	-0.06494	0.16178	0.578866	0.876553	KIFBP	26128	kinesin family binding protein
ENSG0000	150.2122	0.063804	0.184814	0.578822	0.876553	TK1	7083	thymidine kinase 1
ENSG0000	43.61219	0.051521	0.211002	0.579581	0.876838	ATG4C	84938	autophagy related 4C cysteine peptidase
ENSG0000	34.50175	0.053279	0.20912	0.579123	0.876838	CRYZ	1429	crystallin zeta
ENSG0000	4.139087	-0.03586	0.225717	0.579334	0.876838	FAM78B	149297	family with sequence similarity 78 member B
ENSG0000	137.5591	0.06419	0.171531	0.579393	0.876838	DHX36	170506	DEAH-box helicase 36
ENSG0000	1537.027	0.064317	0.158909	0.579334	0.876838	DDX41	51428	DEAD-box helicase 41
ENSG0000	209.617	0.062914	0.149367	0.579229	0.876838	TSNARE1	203062	t-SNARE domain containing 1
ENSG0000	27.55981	-0.05907	0.20086	0.5795	0.876838	KRT18	3875	keratin 18
ENSG0000	78.22477	0.057638	0.200952	0.579471	0.876838	ARHGAP5	394	Rho GTPase activating protein 5
ENSG0000	880.2622	-0.06195	0.185874	0.579269	0.876838	ASGR2	433	asialoglycoprotein receptor 2
ENSG0000	42.59289	0.054264	0.210639	0.579555	0.876838	IGLV5-37	28783	immunoglobulin lambda variable 5-37
ENSG0000	77.87502	-0.06535	0.160546	0.579536	0.876838	PHETA2	150368	PH domain containing endocytic trafficking adaptor 2
ENSG0000	8.012333	-0.0394	0.222708	0.579906	0.876906	FNBP1L	54874	formin binding protein 1 like
ENSG0000	1052.351	-0.06362	0.158675	0.580008	0.876906	NPL	80896	N-acetylneuraminate pyruvate lyase
ENSG0000	637.5217	-0.05584	0.11472	0.579729	0.876906	ANKRD17	26057	ankyrin repeat domain 17
ENSG0000	108.3754	0.062047	0.18958	0.579993	0.876906	RINT1	60561	RAD50 interactor 1
ENSG0000	1824.533	-0.0638	0.187415	0.579874	0.876906	SPTAN1	6709	spectrin al non-erythrocytic 1
ENSG0000	1160.062	-0.0568	0.190693	0.57993	0.876906	FOLR3	2352	folate receptor gamma
ENSG0000	70.25606	-0.06494	0.162198	0.579679	0.876906	HOXC4	3221	homeobox C4
ENSG0000	6.606594	-0.03769	0.224396	0.579855	0.876906	SPIRE2	84501	spire type actin nucleation factor 2
ENSG0000	265.7724	0.059128	0.196368	0.580205	0.876914	PNRC2	55629	proline rich nuclear receptor coactivator 2
ENSG0000	21.24443	0.060261	0.19946	0.580179	0.876914	OSGEPL1	64172	O-sialoglycoprotein endopeptidase like 1
ENSG0000	45.63396	0.061686	0.192967	0.580098	0.876914	TMEM42	131616	transmembrane protein 42
ENSG0000	2.811715	0.033078	0.228943	0.580202	0.876914	NA	NA	NA
ENSG0000	21.4206	0.060783	0.201238	0.580581	0.877149	NA	NA	NA
ENSG0000	589.0377	-0.06111	0.137735	0.580588	0.877149	KEAP1	9817	kelch like ECH associated protein 1
ENSG0000	4.018634	0.031035	0.227677	0.5806	0.877149	ZNF610	162963	zinc finger protein 610
ENSG0000	13.88451	0.05282	0.212724	0.580599	0.877149	ZNF337-AS	1.03E+08	ZNF337 antisense RNA 1
ENSG0000	3.049383	0.030818	0.230233	0.580525	0.877149	OLIG2	10215	oligodendrocyte transcription factor 2
ENSG0000	221.097	-0.06288	0.181431	0.580704	0.877234	TRG-AS1	1.01E+08	T cell receptor gamma locus antisense RNA 1
ENSG0000	9.328475	0.043233	0.220554	0.580807	0.877317	NA	NA	NA
ENSG0000	43.92792	-0.05987	0.196648	0.580948	0.877458	PTPRO	5800	protein tyrosine phosphatase receptor type O
ENSG0000	116.8724	0.064294	0.158742	0.581237	0.877482	SEC23A	10484	SEC23 hon COPII coat complex component
ENSG0000	16.78018	-0.05417	0.209004	0.581173	0.877482	NA	NA	NA
ENSG0000	24.37421	-0.05658	0.205315	0.581156	0.877482	NPIPB6	728741	nuclear pore complex interacting protein family member B6
ENSG0000	2.724877	0.019465	0.232689	0.581251	0.877482	ZNF433	163059	zinc finger protein 433
ENSG0000	5.072289	-0.0265	0.228971	0.581143	0.877482	PHEX	5251	phosphate regulating endopeptidase X-linked
ENSG0000	2610.993	0.062944	0.161924	0.581145	0.877482	ATP6AP1	537	ATPase H+ transporting accessory protein 1
ENSG0000	8.179108	0.02756	0.228659	0.581474	0.877629	IGKJ4	28947	immunoglobulin kappa joining 4
ENSG0000	192.3298	-0.06332	0.168367	0.581463	0.877629	VPS37A	137492	VPS37A subunit of ESCRT-I
ENSG0000	38.35247	0.063747	0.178979	0.581493	0.877629	ERCC4	2072	ERCC excis endonuclease catalytic subunit
ENSG0000	17.44846	-0.06238	0.194626	0.581561	0.87766	NA	NA	NA
ENSG0000	167.7635	0.063395	0.163012	0.581802	0.877716	IKZF5	64376	IKAROS family zinc finger 5
ENSG0000	13.03978	0.052991	0.212048	0.581783	0.877716	OTUB2	78990	OTU deub ubiquitin aldehyde binding 2
ENSG0000	124.6127	0.059084	0.14153	0.581709	0.877716	EFL1	79631	elongation factor like GTPase 1
ENSG0000	6.317842	0.040141	0.223781	0.581673	0.877716	OR4D1	26689	olfactory receptor family 4 subfamily D member 1
ENSG0000	8951.634	-0.0621	0.179577	0.581838	0.877716	CEBPB	1051	CCAAT enhancer binding protein beta
ENSG0000	28.20105	0.059619	0.19866	0.582575	0.877928	AK9	221264	adenylate kinase 9
ENSG0000	18.49962	0.040906	0.220807	0.582543	0.877928	SMIM30	401397	small integral membrane protein 30
ENSG0000	17.02728	-0.04738	0.216699	0.582319	0.877928	TRBV6-1	28606	T cell receptor beta variable 6-1
ENSG0000	64.69399	-0.06489	0.172328	0.582552	0.877928	PTRH1	138428	peptidyl-tRNA hydrolase 1 homolog
ENSG0000	7.759117	-0.02834	0.227805	0.582399	0.877928	NA	NA	NA
ENSG0000	125.6549	0.06064	0.149105	0.582341	0.877928	MRPL51	51258	mitochondrial ribosomal protein L51
ENSG0000	126.3289	0.064138	0.154286	0.582282	0.877928	FBXO21	23014	F-box protein 21
ENSG0000	26.58085	0.058393	0.203157	0.582094	0.877928	ZNF891	1.01E+08	zinc finger protein 891
ENSG0000	5.08057	0.03223	0.22697	0.582243	0.877928	LHFPL6	10186	LHFPL tetraspan subfamily member 6
ENSG0000	1685.391	-0.06235	0.177715	0.582493	0.877928	PAPOLA	10914	poly(A) polymerase alpha
ENSG0000	58.54491	0.064646	0.171823	0.582525	0.877928	ZNF529	57711	zinc finger protein 529
ENSG0000	30.91015	-0.06546	0.182737	0.582601	0.877928	SDCBP2-A	1.01E+08	SDCBP2 antisense RNA 1
ENSG0000	3.664026	-0.02624	0.230322	0.582496	0.877928	TNNC2	7125	troponin C fast skeletal type
ENSG0000	65.09057	-0.06244	0.189209	0.582691	0.877991	NCK1	4690	NCK adaptor protein 1
ENSG0000	61.60009	-0.04589	0.214509	0.582892	0.878102	TWF1	5756	twinfilin actin binding protein 1
ENSG0000	5.051946	-0.03581	0.225465	0.582909	0.878102	NA	NA	NA
ENSG0000	106.5887	0.062827	0.150009	0.582844	0.878102	PRPSAP2	5636	phosphoribosyl pyrophosphate synthetase associated protein 2
ENSG0000	1753.525	-0.05619	0.198698	0.583093	0.878308	GCA	25801	grancalcin
ENSG0000	177.0225	-0.06239	0.146742	0.583231	0.87837	CACYBP	27101	calcyclin binding protein
ENSG0000	15.27044	0.05222	0.211154	0.583185	0.87837	ZDHHC23	254887	zinc finger DHHC-type palmitoyltransferase 23
ENSG0000	495.2702	0.062779	0.176566	0.583307	0.878413	LPGAT1	9926	lysophosphatidylglycerol acyltransferase 1
ENSG0000	2.516773	0.030725	0.23011	0.58341	0.878496	NA	NA	NA
ENSG0000	21.08029	-0.04112	0.220607	0.583459	0.878498	MOSPD1	56180	motile sperm domain containing 1
ENSG0000	10.81501	-0.04749	0.216612	0.583549	0.878517	NA	NA	NA
ENSG0000	2864.593	-0.06105	0.146766	0.583568	0.878517	RAD23A	5886	RAD23 hor nucleotide excision repair protein
ENSG0000	113.9531	-0.06389	0.143308	0.583855	0.878876	EMC9	51016	ER membrane protein complex subunit 9
ENSG0000	643.9697	-0.0607	0.159982	0.584243	0.879047	ZNF692	55657	zinc finger protein 692
ENSG0000	322.0278	0.065378	0.157195	0.584093	0.879047	EHD3	30845	EH domain containing 3
ENSG0000	4.128562	0.032715	0.228427	0.584161	0.879047	IGKJ3	28948	immunoglobulin kappa joining 3
ENSG0000	2.504885	0.026384	0.229658	0.584256	0.879047	RPL26L1-A	1E+08	RPL26L1 antisense RNA 1
ENSG0000	43.56925	0.059023	0.195799	0.584138	0.879047	GVQW3	1.01E+08	GVQW motif containing 3
ENSG0000	61.72044	0.047196	0.214282	0.584212	0.879047	TPTEP1	387590	TPTE pseudogene 1
ENSG0000	535.1248	0.059487	0.132235	0.584305	0.879049	DR1	1810	down-regulator of transcription 1
ENSG0000	1085.643	-0.06287	0.15243	0.584414	0.879141	CTDP1	9150	CTD phosphatase subunit 1
ENSG0000	73.98121	-0.06399	0.167995	0.584493	0.879188	TACO1	51204	translational activator of cytochrome c oxidase I
ENSG0000	101.0573	0.061151	0.184725	0.58457	0.87923	RARS2	57038	arginyl-tRI mitochondrial
ENSG0000	5.535335	0.03196	0.226747	0.584697	0.879256	NA	NA	NA
ENSG0000	146.8535	0.062131	0.156748	0.584731	0.879256	VKORC1L1	154807	vitamin K epoxide reductase complex subunit 1 like 1
ENSG0000	212.7666	-0.06183	0.144897	0.584696	0.879256	GLA	2717	galactosidase alpha
ENSG0000	1554.851	-0.06281	0.181532	0.584805	0.879296	SLC22A18	5002	solute carrier family 22 member 18
ENSG0000	80.97197	-0.06312	0.152556	0.584927	0.879335	TMEM41A	90407	transmembrane protein 41A
ENSG0000	5.707537	0.024519	0.228759	0.584919	0.879335	HNRNPA3	170526	heterogeneous nuclear ribonucleoprotein A3 pseudogene 2
ENSG0000	7.950229	0.046812	0.218437	0.585005	0.879368	ATP1B1	481	ATPase Na+/K+ transporting subunit beta 1
ENSG0000	3.302728	-0.01718	0.230804	0.585198	0.879368	GRHL1	29841	grainyhead like transcription factor 1
ENSG0000	296.483	-0.05884	0.129657	0.585214	0.879368	TARS1	6897	threonyl-tRNA synthetase 1
ENSG0000	17.01993	-0.0536	0.209124	0.585333	0.879368	CROT	54677	carnitine O-octanoyltransferase
ENSG0000	10.54715	-0.04358	0.220269	0.585081	0.879368	NA	NA	NA
ENSG0000	8.949344	-0.03229	0.225265	0.585252	0.879368	NA	NA	NA

ENSG0000	2.727279	0.02695	0.230397	0.585268	0.879368	KREMEN2	79412	kringle containing transmembrane protein 2
ENSG0000	3.507443	-0.01793	0.231795	0.58532	0.879368	DNAH2	146754	dynein axonemal heavy chain 2
ENSG0000	345.0042	-0.05713	0.201097	0.585594	0.879688	CHD1	1105	chromodomain helicase DNA binding protein 1
ENSG0000	246.0746	0.062147	0.180632	0.585911	0.879875	B4GALT2	8704	beta-1 4-galactosyltransferase 2
ENSG0000	6.191367	-0.03883	0.222788	0.585903	0.879875	RPL21P94	1E+08	ribosomal protein L21 pseudogene 94
ENSG0000	8.373103	0.041768	0.221383	0.585858	0.879875	LOH12CR2	503693	loss of het region 2
ENSG0000	9.453245	0.025733	0.227868	0.585903	0.879875	DIAPH3	81624	diaphanous related formin 3
ENSG0000	21.36849	-0.04868	0.212753	0.586042	0.88	EIF4EBP3	8637	eukaryotic translation initiation factor 4E binding protein 3
ENSG0000	5.95758	-0.03976	0.223313	0.586127	0.880055	CYB5D1	124637	cytochrome b5 domain containing 1
ENSG0000	3686.326	-0.06409	0.142732	0.586385	0.88037	ANKRD11	29123	ankyrin repeat domain containing 11
ENSG0000	8.557156	0.026369	0.227589	0.586577	0.880454	IGKV3-7	28915	immunoglobulin kappa variable 3-7 (non-functional)
ENSG0000	100.6291	0.064426	0.165892	0.586521	0.880454	ABRACL	58527	ABRA C-terminal like
ENSG0000	52.15377	0.058358	0.191922	0.586586	0.880454	NA	NA	NA
ENSG0000	2148.863	-0.04895	0.100653	0.586633	0.880454	GUCD1	83606	guanylyl cyclase domain containing 1
ENSG0000	149.6294	0.058339	0.195007	0.587086	0.880485	SLC66A3	130814	solute carrier family 66 member 3
ENSG0000	178.2279	0.061804	0.144547	0.587052	0.880485	MLH1	4292	mutL homolog 1
ENSG0000	9.082718	-0.04046	0.221862	0.586949	0.880485	NA	NA	NA
ENSG0000	107.9804	0.063655	0.159077	0.586725	0.880485	HADH	3033	hydroxyacyl-CoA dehydrogenase
ENSG0000	87.8656	-0.05861	0.19735	0.587055	0.880485	NR4A1	3164	nuclear receptor subfamily 4 group A member 1
ENSG0000	10.11599	0.053363	0.211449	0.586991	0.880485	NA	NA	NA
ENSG0000	20.72604	0.058441	0.199377	0.587063	0.880485	LMO7	4008	LIM domain 7
ENSG0000	99.28191	-0.05726	0.196579	0.586754	0.880485	NA	NA	NA
ENSG0000	771.7278	0.060883	0.1451	0.586862	0.880485	TRMT2A	27037	tRNA methyltransferase 2 homolog A
ENSG0000	9724.396	-0.06136	0.148031	0.587224	0.88052	CAPZB	832	capping actin protein of muscle Z-line subunit beta
ENSG0000	2.512469	0.02901	0.229309	0.587163	0.88052	NA	NA	NA
ENSG0000	207.9143	0.058312	0.130056	0.587446	0.88052	VGLL4	9686	vestigial like family member 4
ENSG0000	59.4589	0.05558	0.204911	0.587374	0.88052	CSNK1G3	1456	casein kinase 1 gamma 3
ENSG0000	30.33334	-0.05147	0.20998	0.587375	0.88052	TRBV12-4	28576	T cell receptor beta variable 12-4
ENSG0000	15.33531	-0.04901	0.214072	0.587296	0.88052	CEBPA-DT	80054	CEBPA divergent transcript
ENSG0000	62.66103	-0.06331	0.168577	0.587422	0.88052	TCEANC	170082	transcription elongation factor A N-terminal and central domain containing
ENSG0000	149.7017	0.061961	0.150911	0.587554	0.880537	GUF1	60558	GTP binding elongation factor GUF1
ENSG0000	7.919755	0.038589	0.223262	0.587509	0.880537	RP9P	441212	RP9 pseudogene
ENSG0000	60.97141	0.063835	0.177683	0.587631	0.880581	AEBP2	121536	AE binding protein 2
ENSG0000	39583.11	-0.05897	0.136068	0.589178	0.880584	LAPTM5	7805	lysosomal protein transmembrane 5
ENSG0000	259.9717	-0.06012	0.15238	0.588041	0.880584	FOXJ3	22887	forkhead box J3
ENSG0000	45.79876	0.062611	0.180449	0.589168	0.880584	CCDC163	126661	CCDC163 homolog
ENSG0000	45.76154	0.062582	0.18797	0.588267	0.880584	TACSTD2	4070	tumor associated calcium signal transducer 2
ENSG0000	114.9621	0.066488	0.165826	0.587702	0.880584	BPNT1	10380	3'(2') 5'-bisphosphate nucleotidase 1
ENSG0000	67.27402	0.063769	0.166107	0.587773	0.880584	ADD2	119	adducin 2
ENSG0000	173.2935	0.060204	0.201279	0.588319	0.880584	SACM1L	22908	SAC1 like phosphatidylinositide phosphatase
ENSG0000	1037.409	0.051226	0.105371	0.588018	0.880584	RASSF1	11186	Ras association domain family member 1
ENSG0000	7.776228	0.042495	0.221391	0.58803	0.880584	NA	NA	NA
ENSG0000	65.67071	-0.06112	0.179958	0.588286	0.880584	NA	NA	NA
ENSG0000	5.161474	-0.02718	0.22782	0.588167	0.880584	MCF2L2	23101	MCF.2 cell line derived transforming sequence-like 2
ENSG0000	43.45716	-0.05883	0.19555	0.588928	0.880584	ZFP62	643836	ZFP62 zinc finger protein
ENSG0000	34.65969	0.063507	0.182216	0.588732	0.880584	DUS4L-BC/	1.15E+08	DUS4L-BCAP29 readthrough
ENSG0000	3.799847	-0.03004	0.227781	0.58808	0.880584	TRBJ2-3	28626	T cell receptor beta joining 2-3
ENSG0000	3.464742	-0.02455	0.230321	0.589036	0.880584	DLC1	10395	DLC1 Rho GTPase activating protein
ENSG0000	40.12167	-0.06212	0.187382	0.588947	0.880584	PSAT1	29968	phosphoserine aminotransferase 1
ENSG0000	239.5613	-0.06374	0.15628	0.588451	0.880584	SPTLC1	10558	serine palmitoyltransferase long chain base subunit 1
ENSG0000	2055.743	0.060392	0.139417	0.58806	0.880584	ARRDC1	92714	arrestin domain containing 1
ENSG0000	60.44928	0.062318	0.184375	0.588501	0.880584	ZWINT	11130	ZW10 interacting kinetochore protein
ENSG0000	185.0482	-0.06365	0.166211	0.588032	0.880584	IPO8	10526	importin 8
ENSG0000	38.26878	-0.06225	0.183712	0.588611	0.880584	NA	NA	NA
ENSG0000	103.7482	0.044676	0.215016	0.588983	0.880584	GMFB	2764	glia maturation factor beta
ENSG0000	5.359126	-0.04105	0.223241	0.588845	0.880584	HSPA2	3306	heat shock protein family A (Hsp70) member 2
ENSG0000	175.4036	-0.05922	0.136256	0.588713	0.880584	NA	NA	NA
ENSG0000	3.356301	0.029563	0.231813	0.589207	0.880584	BCAR1	9564	BCAR1 sca Cas family member
ENSG0000	12.90351	-0.05784	0.203027	0.589138	0.880584	GDPD1	284161	glycerophosphodiester phosphodiesterase domain containing 1
ENSG0000	451.1064	-0.06111	0.134876	0.588702	0.880584	FAM104A	84923	family with sequence similarity 104 member A
ENSG0000	209.0355	0.059038	0.186508	0.588343	0.880584	TRAPPC8	22878	trafficking protein particle complex subunit 8
ENSG0000	5.009326	-0.03721	0.224446	0.58922	0.880584	NA	NA	NA
ENSG0000	172.4562	-0.06176	0.148272	0.589118	0.880584	RPRD1B	58490	regulation of nuclear pre-mRNA domain containing 1B
ENSG0000	2.707301	0.022688	0.230796	0.588347	0.880584	NA	NA	NA
ENSG0000	982.7444	0.057689	0.188617	0.58871	0.880584	CXCR3	2833	C-X-C motif chemokine receptor 3
ENSG0000	253.1818	-0.06117	0.179635	0.588937	0.880584	RLIM	51132	ring finger LIM domain interacting
ENSG0000	7975.386	-0.06187	0.158265	0.589286	0.880596	SOD2	6648	superoxide dismutase 2
ENSG0000	35.67005	0.058495	0.198232	0.589323	0.880596	LIN52	91750	lin-52 DREAM MuvB core complex component
ENSG0000	314.0798	0.059926	0.137293	0.589471	0.880678	FAM43A	131583	family with sequence similarity 43 member A
ENSG0000	384.167	0.061184	0.143778	0.589475	0.880678	ARMC6	93436	armadillo repeat containing 6
ENSG0000	19.62305	0.055077	0.206741	0.589632	0.880842	NA	NA	NA
ENSG0000	2033.876	0.06697	0.119744	0.589688	0.880854	HNRNPU	3192	heterogeneous nuclear ribonucleoprotein U
ENSG0000	4.330803	0.038893	0.224659	0.589756	0.880883	NA	NA	NA
ENSG0000	83.65345	0.059292	0.155132	0.591527	0.880895	CYB5RL	606495	cytochrome b5 reductase like
ENSG0000	71.45764	-0.06306	0.176789	0.591409	0.880895	RORC	6097	RAR related orphan receptor C
ENSG0000	82.15409	0.066206	0.178786	0.590871	0.880895	TSEN15	116461	tRNA splicing endonuclease subunit 15
ENSG0000	4.665765	-0.02765	0.22808	0.589926	0.880895	NA	NA	NA
ENSG0000	106.4759	0.058697	0.193756	0.590689	0.880895	RASA1	5921	RAS p21 protein activator 1
ENSG0000	7.0695	0.028031	0.227736	0.591181	0.880895	HCG9	10255	HLA complex group 9
ENSG0000	10.67042	0.050279	0.213843	0.589919	0.880895	NA	NA	NA
ENSG0000	27.78702	-0.06007	0.198383	0.590188	0.880895	PDGFA-DT	441307	PDGFA divergent transcript
ENSG0000	45.94193	-0.06125	0.18582	0.591364	0.880895	ZCWPW1	55063	zinc finger CW-type and PWWP domain containing 1
ENSG0000	114.4718	-0.06178	0.175108	0.590832	0.880895	KDM7A-D'	1E+08	KDM7A divergent transcript
ENSG0000	7.0446	0.042349	0.222025	0.590922	0.880895	TRBV13	28574	T cell receptor beta variable 13
ENSG0000	3.020327	-0.02685	0.23059	0.59069	0.880895	NA	NA	NA
ENSG0000	85.90722	-0.06216	0.176638	0.59158	0.880895	SQLE	6713	squalene epoxidase
ENSG0000	22.86071	0.05831	0.202232	0.590394	0.880895	ZNF782	158431	zinc finger protein 782
ENSG0000	1490.044	-0.05876	0.131354	0.590736	0.880895	PRCP	5547	prolylcarboxypeptidase
ENSG0000	8.568755	0.04821	0.216407	0.590292	0.880895	NA	NA	NA
ENSG0000	736.6912	-0.05808	0.138673	0.590874	0.880895	KMT2A	4297	lysine methyltransferase 2A
ENSG0000	74.88541	-0.06068	0.190371	0.591337	0.880895	NEMP1	23306	nuclear envelope integral membrane protein 1
ENSG0000	20.50759	0.049923	0.211878	0.591383	0.880895	METTL25	84190	methyltransferase like 25
ENSG0000	10.11262	0.05308	0.210998	0.591469	0.880895	NA	NA	NA
ENSG0000	2.443318	0.022472	0.231139	0.591489	0.880895	CATSPERB	79820	cation channel sperm associated auxiliary subunit beta
ENSG0000	186.0845	-0.05696	0.195707	0.590816	0.880895	RORA	6095	RAR related orphan receptor A
ENSG0000	180.5441	0.060418	0.140368	0.591284	0.880895	ZSCAN32	54925	zinc finger and SCAN domain containing 32

ENSG0000	680.3349	-0.06784	0.140029	0.591014	0.880895	COG4	25839	component of oligomeric golgi complex 4
ENSG0000	2887.24	-0.05548	0.124578	0.590728	0.880895	CTDNEP1	23399	CTD nuclear envelope phosphatase 1
ENSG0000	1732.948	0.053079	0.106721	0.590747	0.880895	ZNF207	7756	zinc finger protein 207
ENSG0000	33.48126	0.058747	0.196399	0.590364	0.880895	SMARCE1	6605	SWI/SNF r matrix ass actin depe subfamily member 1
ENSG0000	885.2525	-0.06296	0.13421	0.59131	0.880895	NPEPPS	9520	aminopeptidase puromycin sensitive
ENSG0000	4022.01	-0.05924	0.138402	0.591143	0.880895	TMC8	147138	transmembrane channel like 8
ENSG0000	7.401646	-0.04739	0.217413	0.591051	0.880895	NA	NA	NA
ENSG0000	1368.335	-0.05859	0.135204	0.591581	0.880895	FZR1	51343	fizzy and cell division cycle 20 related 1
ENSG0000	7.387742	-0.03153	0.22572	0.590751	0.880895	ZNF30	90075	zinc finger protein 30
ENSG0000	16.28115	-0.05221	0.209463	0.591592	0.880895	ZNF829	374899	zinc finger protein 829
ENSG0000	15.76337	0.051832	0.211513	0.589854	0.880895	ANKEF1	63926	ankyrin repeat and EF-hand domain containing 1
ENSG0000	19.6299	0.050873	0.211506	0.591395	0.880895	MIR647	693232	microRNA 647
ENSG0000	3636.714	0.049402	0.104492	0.591186	0.880895	NONO	4841	non-POU domain containing octamer binding
ENSG0000	28.48323	0.044606	0.217494	0.591128	0.880895	NA	NA	NA
ENSG0000	28.25246	0.047495	0.213709	0.590945	0.880895	ZNF449	203523	zinc finger protein 449
ENSG0000	317.766	0.062167	0.157102	0.591909	0.881296	POLR3H	171568	RNA polymerase III subunit H
ENSG0000	3499.83	-0.05465	0.161294	0.592028	0.881401	KMT2B	9757	lysine methyltransferase 2B
ENSG0000	466.8263	0.054948	0.197895	0.592359	0.881636	ATP5F1C	509	ATP synthase F1 subunit gamma
ENSG0000	2.813046	0.024407	0.231326	0.592419	0.881636	TRDJ1	28522	T cell receptor delta joining 1
ENSG0000	119.4808	0.062553	0.167066	0.592409	0.881636	CEP95	90799	centrosomal protein 95
ENSG0000	2759.43	0.066034	0.159671	0.592426	0.881636	UPF1	5976	UPF1 RNA helicase and ATPase
ENSG0000	369.4493	-0.05958	0.138897	0.592404	0.881636	PATZ1	23598	POZ/BTB and AT hook containing zinc finger 1
ENSG0000	1373.433	-0.05872	0.133093	0.592755	0.881693	ACAA1	30	acetyl-CoA acyltransferase 1
ENSG0000	726.8524	-0.06178	0.158075	0.59279	0.881693	ZNF282	8427	zinc finger protein 282
ENSG0000	6.787213	0.033862	0.225283	0.592849	0.881693	NA	NA	NA
ENSG0000	13.62502	0.048646	0.210777	0.592638	0.881693	NA	NA	NA
ENSG0000	9.275536	0.043064	0.219492	0.592819	0.881693	JAG2	3714	jagged canonical Notch ligand 2
ENSG0000	516.2901	0.06124	0.180578	0.592799	0.881693	ADAM10	102	ADAM metallopeptidase domain 10
ENSG0000	2692.05	-0.06278	0.169946	0.592719	0.881693	VAV1	7409	vav guanine nucleotide exchange factor 1
ENSG0000	35.64588	0.061736	0.184564	0.592791	0.881693	EML2-AS1	1E+08	EML2 antisense RNA 1
ENSG0000	49.4649	-0.0604	0.186608	0.593291	0.881725	KIF2C	11004	kinesin family member 2C
ENSG0000	23.92717	-0.04792	0.213959	0.593113	0.881725	SYN2	6854	synapsin II
ENSG0000	18.4212	-0.05396	0.205997	0.593253	0.881725	ST8SIA6	338596	ST8 alpha- 8-sialyltransferase 6
ENSG0000	401.3145	-0.06302	0.167863	0.593448	0.881725	CHID1	66005	chitinase domain containing 1
ENSG0000	66.66827	0.061989	0.1527	0.593278	0.881725	ZBED5-AS1	729013	ZBED5 antisense RNA 1
ENSG0000	5.294852	0.032618	0.225896	0.593408	0.881725	RNU6-118	1.06E+08	RNA U6 small r pseudogene
ENSG0000	1010.584	-0.05832	0.135424	0.593321	0.881725	SCAF11	9169	SR-related CTD associated factor 11
ENSG0000	10.46372	-0.04798	0.215202	0.593137	0.881725	TRAV16	28667	T cell receptor alpha variable 16
ENSG0000	626.8414	0.064469	0.174604	0.593148	0.881725	CORO7	79585	coronin 7
ENSG0000	1960.589	-0.06131	0.137852	0.593309	0.881725	RAP1GAP2	23108	RAP1 GTPase activating protein 2
ENSG0000	431.0846	0.059631	0.176139	0.59296	0.881725	CAMKK1	84254	calcium/calmodulin dependent protein kinase kinase 1
ENSG0000	3.748074	0.030266	0.227486	0.593406	0.881725	SLC17A7	57030	solute carrier family 17 member 7
ENSG0000	326.0477	-0.05924	0.138492	0.593755	0.882005	THEM6	51337	thioesterase superfamily member 6
ENSG0000	390.4791	0.059567	0.139527	0.593746	0.882005	UCKL1	54963	uridine-cytidine kinase 1 like 1
ENSG0000	16.21507	0.049024	0.213482	0.593781	0.882005	LOC10192	1.02E+08	uncharacterized LOC101928415
ENSG0000	4.254623	0.034827	0.225401	0.594144	0.882472	NA	NA	NA
ENSG0000	2501.819	0.059876	0.1439	0.594342	0.882476	MTMR14	64419	myotubularin related protein 14
ENSG0000	18.52077	0.054935	0.205154	0.594258	0.882476	NIPSNAP3	55335	nipsnap homolog 3B
ENSG0000	559.3064	0.058043	0.133546	0.594387	0.882476	ATP2A2	488	ATPase sarcoplasmic/endoplasmic reticulum Ca2+ transporting 2
ENSG0000	6.013074	0.042506	0.220917	0.594346	0.882476	PAPLN	89932	papilin proteoglycan like sulfated glycoprotein
ENSG0000	20.5552	-0.05655	0.201343	0.594196	0.882476	NA	NA	NA
ENSG0000	15.90118	-0.05771	0.20032	0.594474	0.882533	EGR2	1959	early growth response 2
ENSG0000	4.537103	-0.02908	0.227542	0.59454	0.882559	NA	NA	NA
ENSG0000	214.5637	0.057333	0.136777	0.595271	0.882755	ERMAP	114625	erythroblast membrane associated protein (Scianna blood group)
ENSG0000	583.8576	0.058531	0.133644	0.594891	0.882755	FUBP1	8880	far upstream element binding protein 1
ENSG0000	2.617858	-0.02231	0.231608	0.594805	0.882755	ATP2B2	491	ATPase plasma membrane Ca2+ transporting 2
ENSG0000	431.4276	-0.06085	0.153701	0.595309	0.882755	CBLB	868	Cbl proto-oncogene B
ENSG0000	158.0216	-0.0575	0.132498	0.595276	0.882755	PIK3R4	30849	phosphoinositide-3-kinase regulatory subunit 4
ENSG0000	651.9324	-0.0571	0.129784	0.595346	0.882755	PAXX	286257	PAXX non-homologous end joining factor
ENSG0000	1745.604	-0.05845	0.141588	0.595344	0.882755	VCL	7414	vinculin
ENSG0000	3459.905	0.061478	0.146076	0.595076	0.882755	SRPRA	6734	SRP receptor subunit alpha
ENSG0000	54798.98	0.061837	0.166408	0.594892	0.882755	GAPDH	2597	glyceraldehyde-3-phosphate dehydrogenase
ENSG0000	8.029439	0.035533	0.223875	0.595264	0.882755	NA	NA	NA
ENSG0000	14.86925	-0.05246	0.209625	0.595276	0.882755	ZFR2	23217	zinc finger RNA binding protein 2
ENSG0000	1915.856	0.05988	0.132357	0.595271	0.882755	RPL36	25873	ribosomal protein L36
ENSG0000	2294.63	-0.04949	0.104798	0.595116	0.882755	KXD1	79036	KxDL motif containing 1
ENSG0000	68.10573	0.061835	0.155992	0.595116	0.882755	GZF1	64412	GDNF inducible zinc finger protein 1
ENSG0000	399.1637	-0.06127	0.148386	0.595412	0.882781	FBXO9	26268	F-box protein 9
ENSG0000	665.1854	0.057685	0.132524	0.596012	0.882944	TRIM33	51592	tripartite motif containing 33
ENSG0000	3.826785	-0.02143	0.229263	0.596093	0.882944	LYPLAL1-D	643723	LYPLAL1 divergent transcript
ENSG0000	564.429	-0.05598	0.125102	0.5957	0.882944	CNOT11	55571	CCR4-NOT transcription complex subunit 11
ENSG0000	13.38318	0.052102	0.211578	0.595905	0.882944	NA	NA	NA
ENSG0000	797.5144	0.057222	0.126988	0.596142	0.882944	NDUFB4	4710	NADH:ubiquinone oxidoreductase subunit B4
ENSG0000	1015.125	-0.06066	0.124408	0.595693	0.882944	TRIM41	90933	tripartite motif containing 41
ENSG0000	1244.123	-0.06018	0.178003	0.595781	0.882944	NA	NA	NA
ENSG0000	5.576514	-0.02949	0.227004	0.595787	0.882944	LINC02983	1E+08	long intergenic non-protein coding RNA 2983
ENSG0000	915.8584	-0.06001	0.140245	0.595842	0.882944	NSUN5	55695	NOP2/Sun RNA methyltransferase 5
ENSG0000	1078.544	0.061095	0.150462	0.596079	0.882944	AAAS	8086	aladin WD repeat nucleoporin
ENSG0000	1165.913	-0.05881	0.136756	0.595972	0.882944	NME4	4833	NME/NM23 nucleoside diphosphate kinase 4
ENSG0000	10.979	-0.04157	0.219829	0.596138	0.882944	AARSD1	80755	alanyl-tRNA synthetase domain containing 1
ENSG0000	1290.19	-0.05693	0.131458	0.596149	0.882944	PPP6R2	9701	protein phosphatase 6 regulatory subunit 2
ENSG0000	802.5919	-0.04942	0.109838	0.596253	0.883027	UBE2J2	118424	ubiquitin conjugating enzyme E2 J2
ENSG0000	7.941366	-0.03795	0.222367	0.596459	0.883261	NA	NA	NA
ENSG0000	12.83491	-0.04582	0.21661	0.596659	0.883486	NA	NA	NA
ENSG0000	352.3565	0.060417	0.14463	0.596786	0.883531	NCKIPSD	51517	NCK interacting protein with SH3 domain
ENSG0000	1479.987	0.060553	0.142698	0.596764	0.883531	BNIP3L	665	BCL2 interacting protein 3 like
ENSG0000	136.2529	-0.05573	0.195299	0.596937	0.883614	DPH3	285381	diphthamide biosynthesis 3
ENSG0000	8.221592	-0.04041	0.221174	0.596987	0.883614	GARNL3	84253	GTPase activating Rap/RanGAP domain like 3
ENSG0000	71.41238	-0.06055	0.182223	0.59698	0.883614	FAM86C1P	55199	family witl pseudogene
ENSG0000	62.24849	-0.06133	0.165318	0.597071	0.883668	LINC02481	339988	long intergenic non-protein coding RNA 2481
ENSG0000	11.29616	0.0446	0.217733	0.597253	0.883794	NME7	29922	NME/NM23 family member 7
ENSG0000	3338.076	0.060627	0.150648	0.597231	0.883794	CIDEB	27141	cell death inducing DFFA like effector b
ENSG0000	5.045776	-0.03446	0.225162	0.597349	0.883865	MIR3161	1E+08	microRNA 3161
ENSG0000	491.822	0.065276	0.179452	0.597414	0.883879	TMED5	50999	transmembrane p24 trafficking protein 5
ENSG0000	163.753	0.060301	0.143932	0.597456	0.883879	LACTB	114294	lactamase beta
ENSG0000	5790.237	0.061255	0.144064	0.597697	0.883912	KCNAB2	8514	potassium voltage-gated channel subfamily A regulatory beta subunit 2

ENSG0000	66.48163	-0.05883	0.185802	0.597891	0.883912	COL8A2	1296	collagen type VIII alpha 2 chain
ENSG0000	74.53839	0.056093	0.192714	0.598003	0.883912	VPS54	51542	VPS54 subunit of GARP complex
ENSG0000	308.0236	-0.05845	0.141583	0.597977	0.883912	SLC25A38	54977	solute carrier family 25 member 38
ENSG0000	218.9432	-0.05953	0.17409	0.597591	0.883912	EXOC1	55763	exocyst complex component 1
ENSG0000	136.824	-0.05688	0.128988	0.597868	0.883912	LRBA	987	LPS responsive beige-like anchor protein
ENSG0000	4.358815	0.035895	0.226058	0.598104	0.883912	NA	NA	NA
ENSG0000	456.2353	0.059417	0.142449	0.597645	0.883912	FRMD8	83786	FERM domain containing 8
ENSG0000	1939.124	-0.05846	0.139531	0.598105	0.883912	VPS37B	79720	VPS37B subunit of ESCRT-I
ENSG0000	38.50713	-0.05546	0.199261	0.597873	0.883912	AXIN2	8313	axin 2
ENSG0000	66.0602	0.05853	0.192043	0.597778	0.883912	CENPM	79019	centromere protein M
ENSG0000	199.9	-0.0523	0.191101	0.598073	0.883912	ITM2A	9452	integral membrane protein 2A
ENSG0000	19.98796	0.045051	0.217175	0.597803	0.883912	SLITRK4	139065	SLIT and NTRK like family member 4
ENSG0000	3.257934	-0.03215	0.22663	0.598452	0.884353	RGS5	8490	regulator of G protein signaling 5
ENSG0000	2.931849	0.022595	0.230997	0.598643	0.884492	PTPRH	5794	protein tyrosine phosphatase receptor type H
ENSG0000	108.9866	0.061544	0.16053	0.59863	0.884492	ZNF134	7693	zinc finger protein 134
ENSG0000	3.300481	-0.02419	0.229151	0.598753	0.884584	PARK7P1	1E+08	PARK7 pseudogene 1
ENSG0000	1149.927	-0.05581	0.129654	0.598895	0.884722	ZFAND5	7763	zinc finger AN1-type containing 5
ENSG0000	157.491	-0.06081	0.173748	0.599029	0.884737	USPL1	10208	ubiquitin specific peptidase like 1
ENSG0000	5.014427	0.034498	0.225621	0.598996	0.884737	LOC14578	145783	uncharacterized LOC145783
ENSG0000	242.7546	-0.05425	0.12344	0.599049	0.884737	C1QTNF6	114904	C1q and TNF related 6
ENSG0000	43.76606	0.048094	0.212582	0.599112	0.884757	NMD3	51068	NMD3 ribosome export adaptor
ENSG0000	71.91521	-0.06247	0.17302	0.599306	0.884759	C11orf54	28970	chromosome 11 open reading frame 54
ENSG0000	12.75128	0.041585	0.219535	0.599301	0.884759	NA	NA	NA
ENSG0000	342.5079	0.056772	0.193405	0.599242	0.884759	SAMSN1	64092	SAM doma SH3 domain and nuclear localization signals 1
ENSG0000	966.1851	-0.05933	0.147749	0.599178	0.884759	FOXO4	4303	forkhead box O4
ENSG0000	31.33553	0.048996	0.210537	0.599595	0.885112	TRAPPC13	80006	trafficking protein particle complex subunit 13
ENSG0000	36.16037	0.05021	0.208935	0.599642	0.885112	E2F8	79733	E2F transcription factor 8
ENSG0000	99.25122	-0.05981	0.14642	0.599771	0.885232	ZNF407	55628	zinc finger protein 407
ENSG0000	324.5267	0.060687	0.164346	0.599832	0.88525	KPNA2	3838	karyopherin subunit alpha 2
ENSG0000	43.98799	0.045507	0.213872	0.599969	0.885381	ZNF708	7562	zinc finger protein 708
ENSG0000	376.9665	-0.05965	0.164825	0.600149	0.885575	NLGN3	54413	neuroligin 3
ENSG0000	287.8553	0.057426	0.134002	0.600422	0.885906	PGAM5	192111	PGAM fam mitochondrial serine/threonine protein phosphatase
ENSG0000	26.049	-0.05279	0.204458	0.600707	0.886015	NA	NA	NA
ENSG0000	65.50288	0.048649	0.209662	0.600656	0.886015	LMBRD2	92255	LMBR1 domain containing 2
ENSG0000	40.48478	-0.04323	0.215238	0.600737	0.886015	ASAP1-IT2	1.01E+08	ASAP1 intronic transcript 2
ENSG0000	6.941863	-0.04064	0.222171	0.600583	0.886015	NA	NA	NA
ENSG0000	3.492755	-0.02364	0.228863	0.600595	0.886015	NA	NA	NA
ENSG0000	133.6111	-0.05997	0.185786	0.600864	0.886019	EPHX1	2052	epoxide hydrolase 1
ENSG0000	37.34813	0.060177	0.176962	0.601116	0.886019	TMEM242	729515	transmembrane protein 242
ENSG0000	66.96952	-0.05994	0.181116	0.600994	0.886019	ERMP1	79956	endoplasmic reticulum metallopeptidase 1
ENSG0000	3.598844	-0.02181	0.229601	0.600894	0.886019	MIR210HG	1.01E+08	MIR210 host gene
ENSG0000	8.946903	-0.03775	0.222239	0.600938	0.886019	NA	NA	NA
ENSG0000	2557.821	-0.05408	0.124373	0.601102	0.886019	TNFSF12	8742	TNF superfamily member 12
ENSG0000	7.098791	0.02497	0.228376	0.601127	0.886019	NA	NA	NA
ENSG0000	3.095238	0.026531	0.228201	0.600812	0.886019	USP27X-D	158572	USP27X divergent transcript
ENSG0000	149.0701	-0.06195	0.179535	0.601205	0.886062	REST	5978	RE1 silencing transcription factor
ENSG0000	812.3261	-0.05993	0.185879	0.601288	0.886114	CD300C	10871	CD300c molecule
ENSG0000	49.71814	0.058347	0.153955	0.602052	0.88614	C2orf74-D	339803	C2orf74 divergent transcript
ENSG0000	153.2311	0.057165	0.187602	0.601898	0.88614	SH3RF3	344558	SH3 domain containing ring finger 3
ENSG0000	25.41182	-0.04729	0.21259	0.601608	0.88614	PPARG	5468	peroxisome proliferator activated receptor gamma
ENSG0000	335.8613	-0.05474	0.195343	0.602052	0.88614	CLDND1	56650	claudin domain containing 1
ENSG0000	13.3994	-0.02731	0.225712	0.60208	0.88614	NA	NA	NA
ENSG0000	87.30458	0.057824	0.191567	0.601667	0.88614	LINC00243	401247	long intergenic non-protein coding RNA 243
ENSG0000	68.79101	0.058193	0.190718	0.60192	0.88614	ASF1A	25842	anti-silencing function 1A histone chaperone
ENSG0000	18.11854	0.050714	0.209222	0.601985	0.88614	SEM1	7979	SEM1 26S proteasome subunit
ENSG0000	17.22855	0.053504	0.207585	0.601422	0.88614	MEST	4232	mesoderm specific transcript
ENSG0000	5.388303	0.031672	0.226209	0.602028	0.88614	RIDA	10247	reactive intermediate imine deaminase A homolog
ENSG0000	187.6505	0.046327	0.16125	0.601984	0.88614	TCTN3	26123	tectonic family member 3
ENSG0000	691.9556	-0.04839	0.118619	0.601721	0.88614	MUS81	80198	MUS81 structure-specific endonuclease subunit
ENSG0000	4.022318	-0.03302	0.22569	0.601841	0.88614	EIF2S3B	255308	eukaryotic translation initiation factor 2 subunit gamma B
ENSG0000	30.20747	-0.05635	0.196493	0.601422	0.88614	FAM161B	145483	FAM161 centrosomal protein B
ENSG0000	1332.866	0.056249	0.128214	0.601465	0.88614	REXO1	57455	RNA exonuclease 1 homolog
ENSG0000	1749.167	-0.0598	0.170426	0.602006	0.88614	RELB	5971	RELB prot NF-kB subunit
ENSG0000	187.9935	-0.05715	0.13544	0.602132	0.886145	ZNF143	7702	zinc finger protein 143
ENSG0000	19.49589	-0.04931	0.210855	0.602215	0.886186	NA	NA	NA
ENSG0000	11672.92	-0.05673	0.188172	0.602257	0.886186	ACTN4	81	actinin alpha 4
ENSG0000	30.41524	-0.05055	0.207002	0.603512	0.886263	NA	NA	NA
ENSG0000	14.75332	0.051485	0.209029	0.602961	0.886263	TNFSF4	7292	TNF superfamily member 4
ENSG0000	53.1418	0.061269	0.166055	0.60354	0.886263	NA	NA	NA
ENSG0000	63.28398	0.061968	0.163041	0.603028	0.886263	HDAC11	79885	histone deacetylase 11
ENSG0000	53.57857	0.060045	0.180709	0.603556	0.886263	HMGCS1	3157	3-hydroxy-3-methylglutaryl-CoA synthase 1
ENSG0000	572.4762	-0.05136	0.115227	0.603296	0.886263	WDR55	54853	WD repeat domain 55
ENSG0000	4.425407	-0.02981	0.227168	0.603076	0.886263	NA	NA	NA
ENSG0000	13.16726	-0.04612	0.215367	0.603261	0.886263	TRBV10-2	28584	T cell receptor beta variable 10-2
ENSG0000	16.92307	0.053332	0.206053	0.602527	0.886263	NA	NA	NA
ENSG0000	2.692019	-0.01621	0.229602	0.602603	0.886263	NRIP3	56675	nuclear receptor interacting protein 3
ENSG0000	24.69908	0.051004	0.209551	0.602761	0.886263	NA	NA	NA
ENSG0000	183.7007	-0.06037	0.159884	0.602515	0.886263	BLOC1S1	2647	biogenesis of lysosomal organelles complex 1 subunit 1
ENSG0000	140.5076	0.060811	0.167298	0.603119	0.886263	RBMS2	5939	RNA binding motif single stranded interacting protein 2
ENSG0000	214.9065	-0.05672	0.189802	0.602864	0.886263	CEBPE	1053	CCAAT enhancer binding protein epsilon
ENSG0000	214.472	0.059242	0.145422	0.603555	0.886263	NSMCE3	56160	NSE3 hom SMC5-SMC6 complex component
ENSG0000	831.0215	0.051175	0.108717	0.603558	0.886263	ULK3	25989	unc-51 like kinase 3
ENSG0000	193.0429	0.060486	0.172655	0.602553	0.886263	ZNF500	26048	zinc finger protein 500
ENSG0000	15.21814	-0.04878	0.210975	0.603566	0.886263	REXO5	81691	RNA exonuclease 5
ENSG0000	92.57791	0.06025	0.153255	0.602872	0.886263	NA	NA	NA
ENSG0000	13.52739	-0.04631	0.215631	0.602787	0.886263	LINC02132	1.08E+08	long intergenic non-protein coding RNA 2132
ENSG0000	2021.401	-0.05796	0.143575	0.603088	0.886263	SGTA	6449	small glutamine rich tetratricopeptide repeat co-chaperone alpha
ENSG0000	1382.682	-0.05961	0.161888	0.603063	0.886263	YIF1B	90522	Yip1 intera membrane trafficking protein
ENSG0000	16.51232	0.046469	0.215764	0.60344	0.886263	ZNF530	348327	zinc finger protein 530
ENSG0000	79.19965	0.061569	0.173011	0.602479	0.886263	EVA1C	59271	eva-1 homolog C
ENSG0000	310.5931	-0.05924	0.17391	0.602786	0.886263	MORC3	23515	MORC family CW-type zinc finger 3
ENSG0000	2.871209	0.033731	0.23076	0.603185	0.886263	IGLL1	3543	immunoglobulin lambda like polypeptide 1
ENSG0000	8.078865	-0.04696	0.215896	0.603632	0.886288	NA	NA	NA
ENSG0000	7.334936	-0.01626	0.230445	0.603756	0.886399	SPP1	6696	secreted phosphoprotein 1
ENSG0000	580.6614	-0.05701	0.187634	0.603836	0.886445	AARS1	16	alanyl-tRNA synthetase 1
ENSG0000	14.49898	0.03896	0.223672	0.603898	0.886466	MXRA8	54587	matrix remodeling associated 8

ENSG0000	543.1237	-0.05369	0.122743	0.604016	0.886558	YTHDC1	91746	YTH domain containing 1
ENSG0000	366.0946	0.059319	0.150643	0.604058	0.886558	ANKS1A	23294	ankyrin repeat and sterile alpha motif domain containing 1A
ENSG0000	50.67726	0.058907	0.186816	0.604202	0.886627	PCSK5	5125	proprotein convertase subtilisin/kexin type 5
ENSG0000	22.89556	0.050513	0.209041	0.604202	0.886627	ZNF626	199777	zinc finger protein 626
ENSG0000	9.164305	-0.0468	0.215541	0.604376	0.886812	RPS2P55	286444	ribosomal protein S2 pseudogene 55
ENSG0000	2.993099	-0.0216	0.230804	0.605823	0.88713	NA	NA	NA
ENSG0000	2.426619	-0.01592	0.23024	0.604865	0.88713	CD164L2	388611	CD164 molecule like 2
ENSG0000	139.5435	0.060518	0.173474	0.605969	0.88713	DCK	1633	deoxycytidine kinase
ENSG0000	4.202641	0.015794	0.230438	0.605834	0.88713	TRIM2	23321	tripartite motif containing 2
ENSG0000	220.2492	-0.05814	0.140115	0.604657	0.88713	DROSHA	29102	drosha ribonuclease III
ENSG0000	3.360687	0.021838	0.23197	0.605928	0.88713	NA	NA	NA
ENSG0000	34.07554	0.05857	0.188047	0.605298	0.88713	SUPT3H	8464	SPT3 homo SAGA and STAGA complex component
ENSG0000	32.60609	-0.05703	0.191818	0.605708	0.88713	ZNF736	728927	zinc finger protein 736
ENSG0000	4574.145	-0.05681	0.137205	0.605192	0.88713	GSTK1	373156	glutathione S-transferase kappa 1
ENSG0000	28.33551	0.058564	0.191376	0.605724	0.88713	MCM10	55388	minichromosome maintenance 10 replication initiation factor
ENSG0000	9.99409	-0.04374	0.216797	0.605149	0.88713	TSPAN15	23555	tetraspanin 15
ENSG0000	5.242282	-0.01965	0.229621	0.60601	0.88713	PPIAP45	1.01E+08	peptidylprolyl isomerase A pseudogene 45
ENSG0000	883.5863	-0.0599	0.173276	0.606045	0.88713	LINC0017	1E+08	long intergenic non-protein coding RNA 173
ENSG0000	18.32722	-0.05439	0.203064	0.60549	0.88713	NA	NA	NA
ENSG0000	4.152564	0.033882	0.224832	0.605495	0.88713	SAMD15	161394	sterile alpha motif domain containing 15
ENSG0000	6.184681	-0.02878	0.226211	0.60572	0.88713	MTND5P3	1.07E+08	MT-ND5 pseudogene 32
ENSG0000	210.2895	0.06031	0.16065	0.604801	0.88713	NFAT5	10725	nuclear factor of activated T cells 5
ENSG0000	180.2606	0.057597	0.139485	0.604719	0.88713	PPM1D	8493	protein ph Mg2+/Mn2+ dependent 1D
ENSG0000	877.0921	-0.05708	0.187202	0.605748	0.88713	FASN	2194	fatty acid synthase
ENSG0000	251.7061	0.060702	0.162975	0.605801	0.88713	PIK3C3	5289	phosphatidylinositol 3-kinase catalytic subunit type 3
ENSG0000	15983.65	-0.05792	0.179396	0.605579	0.88713	ADGRE5	976	adhesion G protein-coupled receptor E5
ENSG0000	35.76557	0.060588	0.179365	0.605121	0.88713	ALKBH6	84964	alkB homolog 6
ENSG0000	5.635667	0.031621	0.225955	0.605972	0.88713	SLC13A3	64849	solute carrier family 13 member 3
ENSG0000	73.08875	0.060389	0.159182	0.605564	0.88713	MOCS3	27304	molybdenum cofactor synthesis 3
ENSG0000	476.3366	-0.05678	0.131242	0.60511	0.88713	VPS26C	10311	VPS26 endosomal protein sorting factor C
ENSG0000	3.267571	0.02368	0.231423	0.605926	0.88713	NA	NA	NA
ENSG0000	964.5622	0.056451	0.134325	0.605774	0.88713	PPIL2	23759	peptidylprolyl isomerase like 2
ENSG0000	33.56944	-0.05994	0.183826	0.605605	0.88713	NA	NA	NA
ENSG0000	1033.234	0.057265	0.137899	0.604764	0.88713	NDUFB11	54539	NADH:ubiquinone oxidoreductase subunit B11
ENSG0000	49.57657	0.056244	0.201677	0.605305	0.88713	RTL5	340526	retrotransposon Gag like 5
ENSG0000	5.910707	0.033607	0.225032	0.606108	0.88715	RNU6-646	1.06E+08	RNA U6 small r pseudogene
ENSG0000	2.604623	-0.0131	0.231704	0.606347	0.887429	NA	NA	NA
ENSG0000	94.88631	-0.06233	0.162138	0.606504	0.887501	STK36	27148	serine/threonine kinase 36
ENSG0000	114.0799	0.047437	0.209617	0.606541	0.887501	SNX13	23161	sorting nexin 13
ENSG0000	255.8088	0.058745	0.140476	0.606536	0.887501	HAUS5	23354	HAUS augmin like complex subunit 5
ENSG0000	1572.328	-0.05937	0.146258	0.606617	0.88754	SELENON	57190	selenoprotein N
ENSG0000	54.40068	-0.05823	0.183751	0.606926	0.88769	KMO	8564	kynurenine 3-monooxygenase
ENSG0000	56.35979	0.060291	0.17663	0.606961	0.88769	MRPL2	51069	mitochondrial ribosomal protein L2
ENSG0000	44.45001	0.053386	0.201595	0.606928	0.88769	BBS10	79738	Bardet-Biedl syndrome 10
ENSG0000	248.675	0.055921	0.133565	0.606876	0.88769	CDC16	8881	cell division cycle 16
ENSG0000	2351.923	0.06136	0.160586	0.606792	0.88769	IDH3G	3421	isocitrate dehydrogenase (NAD(+)) 3 non-catalytic subunit gamma
ENSG0000	15.26216	-0.04015	0.21884	0.607678	0.888147	MMEL1	79258	membrane metalloendopeptidase like 1
ENSG0000	526.7463	-0.05872	0.159763	0.607706	0.888147	IPO13	9670	importin 13
ENSG0000	201.8617	0.055167	0.19477	0.607425	0.888147	CCNG2	901	cyclin G2
ENSG0000	605.546	0.059745	0.164935	0.607528	0.888147	MARCHF6	10299	membrane associated ring-CH-type finger 6
ENSG0000	31.02981	-0.06029	0.174419	0.607462	0.888147	CDK20	23552	cyclin dependent kinase 20
ENSG0000	2.789216	0.018337	0.230457	0.607672	0.888147	NA	NA	NA
ENSG0000	2.75072	-0.02354	0.22911	0.607703	0.888147	NA	NA	NA
ENSG0000	5.832757	0.037843	0.222872	0.60771	0.888147	NA	NA	NA
ENSG0000	899.4769	-0.05432	0.122536	0.607546	0.888147	NBR1	4077	NBR1 autophagy cargo receptor
ENSG0000	6.759303	-0.04027	0.220526	0.607796	0.8882	MCC	4163	MCC regulator of WNT signaling pathway
ENSG0000	2740.402	-0.0566	0.184763	0.607843	0.8882	CREB5	9586	cAMP responsive element binding protein 5
ENSG0000	2290.052	-0.058	0.144556	0.607934	0.888261	ORAI1	84876	ORAI calcium release-activated calcium modulator 1
ENSG0000	30.58125	-0.0589	0.185614	0.608021	0.888317	RNASEH1-	1.01E+08	RNASEH1 divergent transcript
ENSG0000	78.87291	0.057837	0.187041	0.608113	0.888382	DMXL1	1657	Dmx like 1
ENSG0000	141.4404	0.059044	0.149591	0.608168	0.88839	LMBR1	64327	limb development membrane protein 1
ENSG0000	183.4649	0.05589	0.129896	0.608282	0.888415	AP4B1	10717	adaptor related protein complex 4 subunit beta 1
ENSG0000	74.52689	0.059914	0.170279	0.608272	0.888415	NAA35	60560	N-alpha-ac NatC auxiliary subunit
ENSG0000	4.590183	0.029743	0.227762	0.608332	0.888418	NA	NA	NA
ENSG0000	154.1874	-0.05851	0.157828	0.608442	0.888508	ZFP64	55734	ZFP64 zinc finger protein
ENSG0000	16.06723	0.029967	0.224709	0.60852	0.888551	TRIM23	373	tripartite motif containing 23
ENSG0000	500.1783	-0.05902	0.162184	0.608627	0.888636	GTF2IP12	1.02E+08	general transcription factor IIi pseudogene 12
ENSG0000	7.972915	0.035826	0.222667	0.608702	0.888675	COL19A1	1310	collagen type XIX alpha 1 chain
ENSG0000	788.5715	-0.0557	0.134714	0.60883	0.888763	TADA2B	93624	transcriptional adaptor 2B
ENSG0000	281.1271	0.05894	0.176342	0.608859	0.888763	C11orf58	10944	chromosome 11 open reading frame 58
ENSG0000	9.377612	-0.04518	0.216037	0.609485	0.888813	EVC2	132884	EvC ciliary complex subunit 2
ENSG0000	4859.921	-0.05137	0.116865	0.609567	0.888813	CCNI	10983	cyclin I
ENSG0000	5.962798	0.024493	0.227996	0.609238	0.888813	GUSBP2	387036	GUSB pseudogene 2
ENSG0000	40.94344	0.055184	0.201404	0.609573	0.888813	HSF2	3298	heat shock transcription factor 2
ENSG0000	19.79174	-0.05776	0.19088	0.609375	0.888813	MBLAC1	255374	metallo-beta-lactamase domain containing 1
ENSG0000	50.90958	0.059492	0.179225	0.609042	0.888813	NA	NA	NA
ENSG0000	3466.279	-0.05083	0.142396	0.609563	0.888813	NCKAP1L	3071	NCK associated protein 1 like
ENSG0000	409.3267	-0.05793	0.182686	0.609537	0.888813	FBXL16	146330	F-box and leucine rich repeat protein 16
ENSG0000	5.212171	-0.03701	0.223494	0.609203	0.888813	NPIPB7	440350	nuclear pore complex interacting protein family member B7
ENSG0000	88.70284	0.059355	0.176587	0.609026	0.888813	DGKE	8526	diacylglycerol kinase epsilon
ENSG0000	126.3297	-0.059	0.169498	0.60945	0.888813	NA	NA	NA
ENSG0000	5.314711	0.027752	0.227191	0.609032	0.888813	CD209	30835	CD209 molecule
ENSG0000	3076.824	-0.05253	0.192318	0.609498	0.888813	MAP1S	55201	microtubule associated protein 1S
ENSG0000	1010.062	-0.05799	0.178265	0.609166	0.888813	TCN2	6948	transcobalamin 2
ENSG0000	20.44171	-0.05724	0.193897	0.609719	0.888852	EMC1-AS1	1.02E+08	EMC1 antisense RNA 1
ENSG0000	68.15462	0.060927	0.162836	0.609965	0.888852	ANKRD34	284615	ankyrin repeat domain 34A
ENSG0000	3.279454	0.028748	0.227181	0.610085	0.888852	RPL23AP3	1E+08	ribosomal protein L23a pseudogene 34
ENSG0000	22.41692	-0.04528	0.213422	0.610024	0.888852	ANKRD36	400986	ankyrin repeat domain 36C
ENSG0000	2.451615	-0.01205	0.232132	0.610021	0.888852	IL1A	3552	interleukin 1 alpha
ENSG0000	4.268097	0.037365	0.223161	0.609797	0.888852	NA	NA	NA
ENSG0000	38.3587	0.05525	0.196979	0.610072	0.888852	FAM171A	221061	family with sequence similarity 171 member A1
ENSG0000	6036.72	0.059198	0.169284	0.610034	0.888852	PLCB2	5330	phospholipase C beta 2
ENSG0000	11.73089	0.048421	0.211741	0.609687	0.888852	NA	NA	NA
ENSG0000	3.090055	0.020632	0.230528	0.609795	0.888852	RBM11	54033	RNA binding motif protein 11
ENSG0000	8.259714	0.03218	0.224553	0.610176	0.888913	NA	NA	NA
ENSG0000	71.59585	-0.06205	0.167979	0.610284	0.888981	FAM76A	199870	family with sequence similarity 76 member A

ENSG0000	234.556	0.055388	0.19141	0.610319	0.888981	TMEM30A	55754	transmembrane protein 30A
ENSG0000	165.4499	0.057166	0.149035	0.610395	0.889021	FCF1	51077	FCF1 rRNA-processing protein
ENSG0000	81.38103	0.05868	0.15014	0.6105	0.889035	AGA	175	aspartylglucosaminidase
ENSG0000	271.4133	0.057275	0.137286	0.610534	0.889035	ZBTB2	57621	zinc finger and BTB domain containing 2
ENSG0000	156.2204	-0.05916	0.157727	0.61055	0.889035	SLC25A35	399512	solute carrier family 25 member 35
ENSG0000	19.52351	0.048756	0.21038	0.610702	0.889115	UQCRHL	440567	ubiquinol-cytochrome c reductase hinge protein like
ENSG0000	91.99218	-0.05858	0.151753	0.610678	0.889115	RNF144A	9781	ring finger protein 144A
ENSG0000	5.159942	-0.02527	0.23097	0.610881	0.889304	LINC0095ε	1.01E+08	long intergenic non-protein coding RNA 958
ENSG0000	50.57637	0.057412	0.188666	0.611636	0.889768	CCDC66	285331	coiled-coil domain containing 66
ENSG0000	65.21482	0.055741	0.193815	0.611542	0.889768	SKIL	6498	SKI like proto-oncogene
ENSG0000	997.3508	0.055142	0.12704	0.611496	0.889768	CLTB	1212	clathrin light chain B
ENSG0000	723.5002	0.056409	0.135963	0.611335	0.889768	DHX16	8449	DEAH-box helicase 16
ENSG0000	20.63907	0.056578	0.194528	0.611368	0.889768	CFAP70	118491	cilia and flagella associated protein 70
ENSG0000	2721.758	-0.0584	0.165132	0.611441	0.889768	ARHGAP1	392	Rho GTPase activating protein 1
ENSG0000	33.66133	0.059612	0.17832	0.611402	0.889768	DTD1	92675	D-aminoacyl-tRNA deacylase 1
ENSG0000	222.8022	0.058409	0.146443	0.611448	0.889768	CDK5RAP1	51654	CDK5 regulatory subunit associated protein 1
ENSG0000	15.12691	-0.04376	0.215599	0.611622	0.889768	SEC14L2	23541	SEC14 like lipid binding 2
ENSG0000	236.7893	0.058967	0.158976	0.611802	0.889796	DUT	1854	deoxyuridine triphosphatase
ENSG0000	62.63835	-0.05947	0.170926	0.611783	0.889796	POLR3K	51728	RNA polymerase III subunit K
ENSG0000	139.1666	0.054601	0.127427	0.611756	0.889796	RRP7BP	91695	ribosomal pseudogene
ENSG0000	716.5067	-0.05405	0.129988	0.612039	0.89	PDAP1	11333	PDGFA associated protein 1
ENSG0000	1018.54	-0.05913	0.176459	0.612006	0.89	RRP12	23223	ribosomal RNA processing 12 homolog
ENSG0000	280.0849	0.057285	0.182438	0.612123	0.890007	CERT1	10087	ceramide transporter 1
ENSG0000	955.058	-0.05831	0.133368	0.612141	0.890007	ARFGAP2	84364	ADP ribosylation factor GTPase activating protein 2
ENSG0000	3.169455	-0.03036	0.225796	0.612305	0.890175	CD80	941	CD80 molecule
ENSG0000	66.35363	0.051796	0.201143	0.612385	0.890221	NA	NA	NA
ENSG0000	108.9463	0.058702	0.152131	0.612535	0.890368	BRD3OS	266655	BRD3 opposite strand
ENSG0000	90.47978	0.058269	0.181881	0.612595	0.890385	NA	NA	NA
ENSG0000	4.405453	0.017417	0.230097	0.612889	0.89053	GAS2	2620	growth arrest specific 2
ENSG0000	17.72406	-0.05661	0.193766	0.612871	0.89053	NA	NA	NA
ENSG0000	2642.26	-0.05593	0.159669	0.612848	0.89053	SEPTIN1	1731	septin 1
ENSG0000	48.79466	-0.05329	0.196873	0.612776	0.89053	NA	NA	NA
ENSG0000	4.149558	0.026657	0.22812	0.613096	0.89076	NA	NA	NA
ENSG0000	203.7379	0.054008	0.128095	0.613254	0.890918	C8orf33	65265	chromosome 8 open reading frame 33
ENSG0000	5.841371	-0.03005	0.226211	0.613477	0.890951	TTC4	7268	tetratricopeptide repeat domain 4
ENSG0000	271.7672	0.05929	0.169484	0.613397	0.890951	UBE2E1	7324	ubiquitin conjugating enzyme E2 E1
ENSG0000	4.351666	-0.02875	0.223989	0.61352	0.890951	CCR8	1237	C-C motif chemokine receptor 8
ENSG0000	89.27395	0.058819	0.175737	0.613431	0.890951	TMEM14B	81853	transmembrane protein 14B
ENSG0000	82.86088	0.047622	0.208037	0.613358	0.890951	DPM1	8813	dolichyl-pl catalytic
ENSG0000	27.80187	-0.04765	0.208902	0.613724	0.891106	LOC10798	1.08E+08	uncharacterized LOC107985216
ENSG0000	385.7064	-0.05889	0.167584	0.613694	0.891106	SMYD5	10322	SMYD family member 5
ENSG0000	61.63221	-0.06048	0.17366	0.614108	0.891179	EFNA4	1945	ephrin A4
ENSG0000	8.29223	0.042544	0.218242	0.614319	0.891179	EFHD1	80303	EF-hand domain family member D1
ENSG0000	12.54555	-0.03527	0.221198	0.614146	0.891179	ITIH4	3700	inter-alpha-trypsin inhibitor heavy chain 4
ENSG0000	20.42102	0.048607	0.208447	0.614219	0.891179	CHCHD6	84303	coiled-coil-helix-coiled-coil-helix domain containing 6
ENSG0000	298.51	0.054928	0.134407	0.614091	0.891179	PHC3	80012	polyhomeotic homolog 3
ENSG0000	7.212456	0.029543	0.226184	0.614358	0.891179	CFAP99	402160	cilia and flagella associated protein 99
ENSG0000	13.43621	0.049542	0.20985	0.614229	0.891179	DTWD2	285605	DTW domain containing 2
ENSG0000	254.714	0.058614	0.166268	0.614247	0.891179	ABHD11	83451	abhydrolase domain containing 11
ENSG0000	92.04309	0.059042	0.158861	0.614071	0.891179	FAM118B	79607	family with sequence similarity 118 member B
ENSG0000	943.1742	-0.06533	0.140759	0.614323	0.891179	ADPGK	83440	ADP dependent glucokinase
ENSG0000	166.2938	-0.05294	0.196356	0.613933	0.891179	RETN	56729	resistin
ENSG0000	65.87082	0.058436	0.183885	0.614271	0.891179	KRBOX4	55634	KRAB box domain containing 4
ENSG0000	949.6919	0.057828	0.158398	0.614507	0.891324	POLRMT	5442	RNA polymerase mitochondrial
ENSG0000	4.453865	-0.02564	0.227931	0.614603	0.891393	NA	NA	NA
ENSG0000	3.308212	0.028194	0.227624	0.614873	0.891453	NA	NA	NA
ENSG0000	208.3525	-0.05535	0.161653	0.614887	0.891453	PPP2R3C	55012	protein phosphatase 2 regulatory subunit B''gamma
ENSG0000	40.86304	-0.05615	0.190439	0.614836	0.891453	P3H4	10609	prolyl 3-hydroxylase family member 4 (inactive)
ENSG0000	116.6636	0.052494	0.201703	0.614875	0.891453	RELCH	57614	RAB11 bin coiled-coil and HEAT repeat containing
ENSG0000	1253.098	-0.05582	0.136908	0.614778	0.891453	WIZ	58525	WIZ zinc finger
ENSG0000	355.0134	-0.05775	0.164853	0.615048	0.891616	KAT2B	8850	lysine acetyltransferase 2B
ENSG0000	3.82495	-0.03062	0.22624	0.615296	0.89173	NA	NA	NA
ENSG0000	890.242	-0.05416	0.127772	0.615377	0.89173	MPV17	4358	mitochondrial inner membrane protein MPV17
ENSG0000	6.312587	-0.02842	0.226348	0.615237	0.89173	TMEM183	653659	transmem pseudogene
ENSG0000	4.548169	-0.03321	0.224799	0.615403	0.89173	NA	NA	NA
ENSG0000	67.23917	-0.0583	0.179126	0.615419	0.89173	NIPSNAP3	25934	nipsnap homolog 3A
ENSG0000	166.8649	0.05149	0.130487	0.615287	0.89173	DNAJC14	85406	DnaJ heat shock protein family (Hsp40) member C14
ENSG0000	25.42196	0.051357	0.203656	0.615509	0.89179	TIMM8B	26521	translocase of inner mitochondrial membrane 8 homolog B
ENSG0000	7.727198	0.032376	0.224374	0.615603	0.891855	NA	NA	NA
ENSG0000	686.7474	0.053184	0.186909	0.615679	0.891894	MEF2A	4205	myocyte enhancer factor 2A
ENSG0000	16.62386	-0.05748	0.194896	0.616025	0.892325	NA	NA	NA
ENSG0000	893.0524	-0.05326	0.129841	0.616185	0.892487	PRMT2	3275	protein arginine methyltransferase 2
ENSG0000	161.9177	-0.05848	0.154353	0.616247	0.892506	NA	NA	NA
ENSG0000	660.2171	-0.0555	0.181388	0.616403	0.892661	RC3H1	149041	ring finger and CCCH-type domains 1
ENSG0000	65.39869	0.057499	0.15097	0.616551	0.892763	DUSP10	11221	dual specificity phosphatase 10
ENSG0000	394.1425	-0.05396	0.195468	0.616571	0.892763	TANK	10010	TRAF family member associated NFKB activator
ENSG0000	291.2745	0.05738	0.166789	0.617013	0.892805	SLC66A1	54896	solute carrier family 66 member 1
ENSG0000	1622.849	0.059781	0.162379	0.617098	0.892805	UQCRC1	7384	ubiquinol-cytochrome c reductase core protein 1
ENSG0000	173.508	-0.05703	0.177352	0.617163	0.892805	GPRIN3	285513	GPRIN family member 3
ENSG0000	102.2922	-0.05478	0.181483	0.61703	0.892805	F2R	2149	coagulation factor II thrombin receptor
ENSG0000	105.5556	-0.05778	0.150596	0.617233	0.892805	TBC1D7	51256	TBC1 domain family member 7
ENSG0000	8.155947	-0.04335	0.216842	0.616707	0.892805	PKD1L1	168507	polycystin transient receptor potential channel interacting
ENSG0000	76.90134	0.053187	0.197118	0.616953	0.892805	MAPK8	5599	mitogen-activated protein kinase 8
ENSG0000	147.2264	0.055468	0.156922	0.616735	0.892805	ASCC1	51008	activating signal cointegrator 1 complex subunit 1
ENSG0000	215.6229	0.05746	0.141191	0.617087	0.892805	EIF3J	8669	eukaryotic translation initiation factor 3 subunit J
ENSG0000	32.66375	-0.06394	0.19818	0.617198	0.892805	NDC80	10403	NDC80 kinetochore complex component
ENSG0000	369.4888	0.058176	0.159571	0.616921	0.892805	LMNB2	84823	lamin B2
ENSG0000	561.6191	-0.05701	0.151347	0.616937	0.892805	DAPK3	1613	death associated protein kinase 3
ENSG0000	449.8976	0.053304	0.128509	0.617093	0.892805	MRI1	84245	methylthioribose-1-phosphate isomerase 1
ENSG0000	937.4457	-0.0555	0.137354	0.617301	0.892833	RTF1	23168	RTF1 hom Paf1/RNA polymerase II complex component
ENSG0000	130.0965	0.057287	0.152482	0.6175	0.89301	DDI2	84301	DNA damage inducible 1 homolog 2
ENSG0000	7.370142	-0.01843	0.230951	0.617522	0.89301	ZNF890P	645700	zinc finger pseudogene
ENSG0000	171.2147	0.056765	0.146422	0.617594	0.893028	ARPC5L	81873	actin related protein 2/3 complex subunit 5 like
ENSG0000	26.44036	-0.05722	0.186228	0.617632	0.893028	ARL17A	51326	ADP ribosylation factor like GTPase 17A
ENSG0000	2030.55	-0.05224	0.199455	0.617829	0.893244	CAPZA1	829	capping actin protein of muscle Z-line subunit alpha 1
ENSG0000	41.53182	0.055225	0.191865	0.618361	0.893278	WARS2	10352	tryptophai mitochondrial

ENSG0000	12.13326	0.053175	0.203578	0.618162	0.893278	NA	NA	NA
ENSG0000	286.6859	0.048827	0.203268	0.618147	0.893278	TMEM167	153339	transmembrane protein 167A
ENSG0000	29.52369	-0.05176	0.201711	0.618102	0.893278	ZKSCAN3	80317	zinc finger with KRAB and SCAN domains 3
ENSG0000	4.48483	-0.02257	0.230385	0.618438	0.893278	SPATA6L	55064	spermatogenesis associated 6 like
ENSG0000	195.5902	0.056335	0.141376	0.618136	0.893278	PIGO	84720	phosphatidylinositol glycan anchor biosynthesis class O
ENSG0000	459.9258	0.05312	0.127369	0.61835	0.893278	SUFU	51684	SUFU negative regulator of hedgehog signaling
ENSG0000	1950.541	-0.05778	0.14825	0.618206	0.893278	UBALD1	124402	UBA like domain containing 1
ENSG0000	9.005517	-0.04759	0.212863	0.618419	0.893278	GSDMA	284110	gasdermin A
ENSG0000	23.07625	-0.04993	0.20486	0.618203	0.893278	MEOX1	4222	mesenchyme homeobox 1
ENSG0000	5.846626	0.031416	0.22479	0.618399	0.893278	LOC10042	1E+08	glyoxylate reductase 1 homolog (Arabidopsis) pseudogene
ENSG0000	66.55662	-0.05903	0.174851	0.618342	0.893278	ZXDB	158586	zinc finger X-linked duplicated B
ENSG0000	2237.592	-0.05468	0.137275	0.618655	0.893521	ATG13	9776	autophagy related 13
ENSG0000	4.14821	-0.02909	0.226397	0.618901	0.893562	NA	NA	NA
ENSG0000	84.31451	-0.05028	0.194118	0.618788	0.893562	ZNRF2	223082	zinc and ring finger 2
ENSG0000	129.5679	0.057109	0.15292	0.618976	0.893562	LMNTD2	256329	lamin tail domain containing 2
ENSG0000	8.865368	0.035903	0.221956	0.618814	0.893562	GRTP1	79774	growth hormone regulated TBC protein 1
ENSG0000	19.01629	-0.05278	0.202063	0.618921	0.893562	SMG1P3	1E+08	SMG1 pseudogene 3
ENSG0000	572.894	-0.0561	0.146407	0.618953	0.893562	KATNIP	23247	katanin interacting protein
ENSG0000	121.9765	0.056108	0.184416	0.619098	0.893597	C7orf31	136895	chromosome 7 open reading frame 31
ENSG0000	41.7012	-0.05334	0.195311	0.619064	0.893597	STXBP1	6812	syntaxin binding protein 1
ENSG0000	4.781571	0.019809	0.229703	0.619166	0.893624	NA	NA	NA
ENSG0000	132.3651	0.055792	0.186963	0.619225	0.89364	GAB1	2549	GRB2 associated binding protein 1
ENSG0000	115.5133	-0.05595	0.175088	0.619384	0.893727	ERI1	90459	exoribonuclease 1
ENSG0000	446.0321	-0.05291	0.13026	0.619366	0.893727	DGCR6L	85359	DiGeorge syndrome critical region gene 6 like
ENSG0000	40.22882	-0.04543	0.21104	0.619562	0.893774	CXCR6	10663	C-X-C motif chemokine receptor 6
ENSG0000	888.6841	-0.05687	0.154681	0.619467	0.893774	ACVR1B	91	activin A receptor type 1B
ENSG0000	7.630445	0.036071	0.222121	0.619557	0.893774	SCN4A	6329	sodium voltage-gated channel alpha subunit 4
ENSG0000	620.0347	0.054757	0.185813	0.619719	0.89393	STAG2	10735	stromal antigen 2
ENSG0000	373.4609	0.056376	0.147758	0.6198	0.893977	ATRX	546	ATRX chromatin remodeler
ENSG0000	16.87011	0.041434	0.21586	0.62001	0.894209	NA	NA	NA
ENSG0000	211.3424	-0.05427	0.129253	0.620579	0.894284	SNAPIN	23557	SNAP associated protein
ENSG0000	20.86284	-0.05108	0.203686	0.62012	0.894284	XCL1	6375	X-C motif chemokine ligand 1
ENSG0000	3.156562	0.019688	0.230175	0.620713	0.894284	MFF-DT	654841	MFF divergent transcript
ENSG0000	4.078363	0.022868	0.229175	0.620941	0.894284	LOC12490	1.25E+08	uncharacterized LOC124906237
ENSG0000	239.7285	0.055258	0.178525	0.620703	0.894284	OSBPL11	114885	oxysterol binding protein like 11
ENSG0000	74.42082	0.044638	0.209095	0.620917	0.894284	THAP9-AS	1E+08	THAP9 antisense RNA 1
ENSG0000	161.5812	0.052754	0.196003	0.620897	0.894284	TRIM52	84851	tripartite motif containing 52
ENSG0000	17.05717	0.014785	0.232587	0.62032	0.894284	RNF182	221687	ring finger protein 182
ENSG0000	540.2139	-0.05823	0.147787	0.620277	0.894284	ATXN1	6310	ataxin 1
ENSG0000	15.14671	0.044184	0.214871	0.620481	0.894284	SAMD12	401474	sterile alpha motif domain containing 12
ENSG0000	60.20957	-0.05735	0.176806	0.620238	0.894284	IDNK	414328	IDNK gluconokinase
ENSG0000	71.70334	-0.05641	0.149844	0.620695	0.894284	RALGDS	5900	ral guanine nucleotide dissociation stimulator
ENSG0000	1704.718	-0.04792	0.107394	0.620377	0.894284	HNRNPF	3185	heterogeneous nuclear ribonucleoprotein F
ENSG0000	16.12818	-0.05334	0.199603	0.620323	0.894284	MOK	5891	MOK protein kinase
ENSG0000	1811.083	-0.0512	0.130251	0.620743	0.894284	GSPT1	2935	G1 to S phase transition 1
ENSG0000	28519.29	-0.05509	0.155872	0.620941	0.894284	PFN1	5216	profilin 1
ENSG0000	1021.674	0.057048	0.172118	0.6207	0.894284	SLC25A23	79085	solute carrier family 25 member 23
ENSG0000	67.91498	0.054265	0.188796	0.62087	0.894284	RBBP9	10741	RB binding serine hydrolase
ENSG0000	1618.574	-0.04189	0.110834	0.620993	0.894288	CBX6	23466	chromobox 6
ENSG0000	328.1417	0.0576	0.147161	0.621148	0.894315	HMCES	56941	5-hydroxy ES cell specific
ENSG0000	1966.184	-0.05434	0.142768	0.621207	0.894315	RHBDD2	57414	rhomboid domain containing 2
ENSG0000	190.9357	0.055596	0.183504	0.621106	0.894315	UBA2	10054	ubiquitin like modifier activating enzyme 2
ENSG0000	280.2493	0.053946	0.132934	0.621171	0.894315	HPS4	89781	HPS4 biogenesis of lysosomal organelles complex 3 subunit 2
ENSG0000	930.5042	-0.05649	0.151209	0.621379	0.894492	HHEX	3087	hematopoietically expressed homeobox
ENSG0000	686.5008	0.050295	0.146319	0.621958	0.894497	IMP4	92856	IMP U3 small nucleolar ribonucleoprotein 4
ENSG0000	47.38087	0.050769	0.202283	0.62176	0.894497	MOB1B	92597	MOB kinase activator 1B
ENSG0000	447.2645	0.054347	0.135855	0.622114	0.894497	ETF1	2107	eukaryotic translation termination factor 1
ENSG0000	13.4044	-0.02918	0.224713	0.621587	0.894497	PCDH12	51294	protocadherin 12
ENSG0000	79.44293	-0.04932	0.201573	0.622009	0.894497	CMAHP	8418	cytidine m pseudogene
ENSG0000	3.753798	-0.02171	0.230077	0.621842	0.894497	NA	NA	NA
ENSG0000	18.33304	0.047383	0.210225	0.622102	0.894497	NA	NA	NA
ENSG0000	1470.97	0.057745	0.151158	0.621614	0.894497	GPAA1	8733	glycosylphosphatidylinositol anchor attachment 1
ENSG0000	6.417126	-0.03474	0.222753	0.621818	0.894497	MCAM	4162	melanoma cell adhesion molecule
ENSG0000	75.03205	0.05168	0.198629	0.62201	0.894497	SC5D	6309	sterol-C5-desaturase
ENSG0000	24455.49	-0.05402	0.185822	0.622024	0.894497	PKM	5315	pyruvate kinase M1/2
ENSG0000	314.0634	0.056895	0.149784	0.622032	0.894497	MRPL34	64981	mitochondrial ribosomal protein L34
ENSG0000	1423.318	0.055256	0.140594	0.621446	0.894497	AKT1S1	84335	AKT1 substrate 1
ENSG0000	197.6973	-0.05369	0.132964	0.622084	0.894497	KIF3B	9371	kinesin family member 3B
ENSG0000	181.0007	0.056395	0.151825	0.621605	0.894497	TMEM50B	757	transmembrane protein 50B
ENSG0000	13.57372	-0.03302	0.222732	0.62227	0.894539	RPL10AP6	1E+08	ribosomal protein L10a pseudogene 6
ENSG0000	9.049262	0.039638	0.218804	0.622237	0.894539	NA	NA	NA
ENSG0000	7.235397	0.031136	0.224619	0.62229	0.894539	C17orf113	1.11E+08	chromosome 17 open reading frame 113
ENSG0000	1259.809	-0.05545	0.147503	0.622348	0.894551	GIMAP6	474344	GTPase IMAP family member 6
ENSG0000	6.958708	0.03306	0.223866	0.622396	0.894551	PARP11-A:	1.05E+08	PARP11 antisense RNA 1
ENSG0000	26.61792	-0.04999	0.204612	0.622457	0.894568	ETNK2	55224	ethanolamine kinase 2
ENSG0000	454.9353	-0.05767	0.166064	0.622704	0.894747	PGM1	5236	phosphoglucomutase 1
ENSG0000	33746.52	0.057383	0.167587	0.622934	0.894747	TXNIP	10628	thioredoxin interacting protein
ENSG0000	6.385156	0.034518	0.223389	0.622949	0.894747	LINC0280:	1.08E+08	long intergenic non-protein coding RNA 2803
ENSG0000	167.0137	-0.05615	0.168337	0.62283	0.894747	CISD2	493856	CDGSH iron sulfur domain 2
ENSG0000	387.7753	-0.05078	0.121731	0.622955	0.894747	LSM2	57819	LSM2 hom U6 small nuclear RNA and mRNA degradation associated
ENSG0000	220.6248	0.056867	0.153666	0.622972	0.894747	CCDC28A	25901	coiled-coil domain containing 28A
ENSG0000	5.418664	0.031688	0.224741	0.622637	0.894747	NA	NA	NA
ENSG0000	903.2693	0.063536	0.169844	0.622873	0.894747	LRRC59	55379	leucine rich repeat containing 59
ENSG0000	4.882383	-0.02807	0.226964	0.623194	0.894924	MMP23A	8511	matrix metallopeptidase 23A (pseudogene)
ENSG0000	1422.725	0.056945	0.157131	0.623175	0.894924	TLR6	10333	toll like receptor 6
ENSG0000	3.26543	-0.02222	0.22999	0.623242	0.894924	IGF2	3481	insulin like growth factor 2
ENSG0000	133.2501	0.055805	0.14867	0.623381	0.894997	MDN1	23195	midasin AAA ATPase 1
ENSG0000	116.3847	0.055451	0.144718	0.62344	0.894997	ZNF517	340385	zinc finger protein 517
ENSG0000	591.2089	0.056561	0.150785	0.62342	0.894997	SRP68	6730	signal recognition particle 68
ENSG0000	1419.124	0.045548	0.107664	0.623537	0.895066	SON	6651	SON DNA and RNA binding protein
ENSG0000	342.1804	-0.0545	0.168803	0.62363	0.89513	DPP3	10072	dipeptidyl peptidase 3
ENSG0000	498.7141	0.06095	0.159471	0.623688	0.895143	NA	NA	NA
ENSG0000	400.0103	-0.05105	0.128711	0.624085	0.895161	RFX5	5993	regulatory factor X5
ENSG0000	38.11827	-0.05176	0.197916	0.623929	0.895161	TAPT1-AS:	202020	TAPT1 antisense RNA 1 (head to head)
ENSG0000	310.2866	0.055194	0.144707	0.623864	0.895161	INTS9	55756	integrator complex subunit 9
ENSG0000	159.6429	-0.04265	0.209495	0.623937	0.895161	EXOC6	54536	exocyst complex component 6

ENSG0000	2983.354	0.056771	0.158795	0.624091	0.895161	SCYL1	57410	SCY1 like pseudokinase 1
ENSG0000	107.2583	-0.05618	0.164052	0.624011	0.895161	ABCA3	21	ATP binding cassette subfamily A member 3
ENSG0000	1966.39	-0.05703	0.15876	0.623754	0.895161	ZC3H18	124245	zinc finger CCCH-type containing 18
ENSG0000	9.380398	-0.04033	0.218345	0.623984	0.895161	GTSE1-DT	150384	GTSE1 divergent transcript
ENSG0000	5.87209	0.025665	0.228208	0.624194	0.895169	NMNAT2	23057	nicotinamide nucleotide adenylyltransferase 2
ENSG0000	5.18855	-0.02579	0.227182	0.624181	0.895169	NA	NA	NA
ENSG0000	4818.562	-0.05313	0.187887	0.624327	0.895289	CCDC88B	283234	coiled-coil domain containing 88B
ENSG0000	45.53804	0.057564	0.176401	0.624472	0.895427	CDC26	246184	cell division cycle 26
ENSG0000	22.12186	0.049947	0.203729	0.624558	0.895479	SNRPE	6635	small nuclear ribonucleoprotein polypeptide E
ENSG0000	5.975009	-0.03412	0.2232	0.624646	0.895536	ZFP69B	65243	ZFP69 zinc finger protein B
ENSG0000	3921.634	-0.05482	0.144523	0.624772	0.895548	SYTL1	84958	synaptotagmin like 1
ENSG0000	7.158664	-0.02033	0.22909	0.624915	0.895548	NA	NA	NA
ENSG0000	3.05811	0.019897	0.230101	0.624894	0.895548	KLHL32	114792	kelch like family member 32
ENSG0000	39.81936	-0.05502	0.190324	0.625094	0.895548	NA	NA	NA
ENSG0000	4.315172	0.020896	0.229131	0.624792	0.895548	FLJ46284	441369	uncharacterized LOC441369
ENSG0000	31.15689	-0.04736	0.206778	0.625041	0.895548	LINC0150	1.01E+08	long intergenic non-protein coding RNA 1504
ENSG0000	2389.799	0.05359	0.134178	0.625089	0.895548	CALCOCO	57658	calcium binding and coiled-coil domain 1
ENSG0000	78.51751	-0.05355	0.184934	0.624999	0.895548	KIF18B	146909	kinesin family member 18B
ENSG0000	3.127771	-0.02471	0.228994	0.624867	0.895548	TBC1D8B	54885	TBC1 domain family member 8B
ENSG0000	369.8881	0.053105	0.136945	0.625355	0.895641	MEAF6	64769	MYST/Esa1 associated factor 6
ENSG0000	175.0463	0.046541	0.207448	0.62534	0.895641	IL1RAP	3556	interleukin 1 receptor accessory protein
ENSG0000	7.074289	0.034228	0.223053	0.625222	0.895641	DDO	8528	D-aspartate oxidase
ENSG0000	401.1631	0.049861	0.121662	0.625332	0.895641	FAM118A	55007	family with sequence similarity 118 member A
ENSG0000	5.847221	-0.03809	0.22351	0.625553	0.895854	LOC65164	651644	LIM zinc finger domain containing 2 pseudogene
ENSG0000	124.7571	0.056375	0.148877	0.625636	0.895903	CSTPP1	79096	centriolar satellite-associated tubulin polyglutamylase complex regulator 1
ENSG0000	57.1448	-0.05198	0.195389	0.625698	0.895923	NA	NA	NA
ENSG0000	21.22562	0.048837	0.207134	0.625925	0.896177	ODAD1	93233	outer dynein arm docking complex subunit 1
ENSG0000	113.4509	0.055962	0.150349	0.626119	0.896207	NOL10	79954	nucleolar protein 10
ENSG0000	34034.44	-0.05476	0.177324	0.62607	0.896207	MYO1F	4542	myosin IF
ENSG0000	25.49973	0.043319	0.213245	0.626141	0.896207	ZNF180	7733	zinc finger protein 180
ENSG0000	396.6222	0.059353	0.15643	0.626051	0.896207	ABHD12	26090	abhydrola lysophospholipase
ENSG0000	71.55425	-0.05738	0.178022	0.626313	0.896313	TRIM32	22954	tripartite motif containing 32
ENSG0000	1132.387	-0.05347	0.183051	0.626305	0.896313	ALDOA	226	aldolase fructose-bisphosphate A
ENSG0000	2.458725	-0.02242	0.230282	0.626421	0.896331	RGS7	6000	regulator of G protein signaling 7
ENSG0000	5.697851	0.022664	0.228289	0.626424	0.896331	NA	NA	NA
ENSG0000	6.062586	0.024294	0.228232	0.62661	0.896527	NA	NA	NA
ENSG0000	97.52018	0.056307	0.152508	0.626864	0.89675	LINC0100	1E+08	long intergenic non-protein coding RNA 1003
ENSG0000	71.49101	0.056412	0.158985	0.626858	0.89675	DHX33	56919	DEAH-box helicase 33
ENSG0000	222.4907	-0.05643	0.164748	0.626918	0.896758	DRAM1	55332	DNA damage regulated autophagy modulator 1
ENSG0000	437.8927	-0.05154	0.132594	0.627038	0.89679	FAM168A	23201	family with sequence similarity 168 member A
ENSG0000	2.383494	0.020189	0.230929	0.62703	0.89679	NA	NA	NA
ENSG0000	53.46577	-0.05352	0.189831	0.627237	0.896941	DUSP12	11266	dual specificity phosphatase 12
ENSG0000	63.1653	0.058776	0.168314	0.627401	0.896941	DPY30	84661	dpy-30 histone methyltransferase complex regulatory subunit
ENSG0000	95.03643	-0.05566	0.160421	0.627358	0.896941	ASTE1	28990	asteroid homolog 1
ENSG0000	19.23277	-0.05134	0.202175	0.627437	0.896941	NR3C2	4306	nuclear receptor subfamily 3 group C member 2
ENSG0000	3.172787	0.018147	0.231228	0.627347	0.896941	NANOS3	342977	nanos C2HC-type zinc finger 3
ENSG0000	3.326094	-0.01861	0.230945	0.627355	0.896941	CBS	875	cystathionine beta-synthase
ENSG0000	564.0939	-0.03433	0.221522	0.628434	0.896979	RAP1GAP	5909	RAP1 GTPase activating protein
ENSG0000	11.18869	-0.03247	0.222528	0.628188	0.896979	DNAJB4	11080	DnaJ heat shock protein family (Hsp40) member B4
ENSG0000	9.626187	-0.03869	0.218593	0.627926	0.896979	GPR75	10936	G protein-coupled receptor 75
ENSG0000	50.42924	-0.04294	0.210387	0.62826	0.896979	MOB4	25843	MOB fami phocein
ENSG0000	572.2146	0.055449	0.143454	0.628352	0.896979	CUTA	51596	cutA divalent cation tolerance homolog
ENSG0000	4.9702	0.028191	0.227276	0.627715	0.896979	DDX43	55510	DEAD-box helicase 43
ENSG0000	290.2224	-0.05544	0.163436	0.628514	0.896979	INTS8	55656	integrator complex subunit 8
ENSG0000	631.6359	0.052805	0.116212	0.627802	0.896979	UBQLN1	29979	ubiquilin 1
ENSG0000	261.4611	0.055793	0.146091	0.628221	0.896979	COMTD1	118881	catechol-O-methyltransferase domain containing 1
ENSG0000	51.74392	0.054254	0.19064	0.628131	0.896979	ACVRL1	94	activin A receptor like type 1
ENSG0000	12.39428	0.036201	0.220136	0.628498	0.896979	TNS2	23371	tensin 2
ENSG0000	2402.083	0.051322	0.168302	0.627566	0.896979	CKAP4	10970	cytoskeleton associated protein 4
ENSG0000	2.546673	0.020415	0.230368	0.628542	0.896979	NA	NA	NA
ENSG0000	171.6771	0.059095	0.188177	0.627949	0.896979	RILPL1	353116	Rab interacting lysosomal protein like 1
ENSG0000	170.9841	-0.04732	0.201624	0.628158	0.896979	AP5M1	55745	adaptor related protein complex 5 subunit mu 1
ENSG0000	23.5981	0.049955	0.203575	0.628517	0.896979	L3HYPDH	112849	trans-L-3-hydroxyproline dehydratase
ENSG0000	418.2393	0.053701	0.138686	0.6279	0.896979	IVD	3712	isovaleryl-CoA dehydrogenase
ENSG0000	685.4789	0.05283	0.137528	0.6278	0.896979	CARHSP1	23589	calcium regulated heat stable protein 1
ENSG0000	16.90523	0.050927	0.202581	0.627684	0.896979	RNF227	284023	ring finger protein 227
ENSG0000	19.67104	0.045678	0.210655	0.628324	0.896979	PYY2	23615	peptide YY 2 (pseudogene)
ENSG0000	725.0056	0.054289	0.151919	0.628237	0.896979	SAMD1	90378	sterile alpha motif domain containing 1
ENSG0000	2257.712	-0.05453	0.177598	0.628217	0.896979	SLC19A1	6573	solute carrier family 19 member 1
ENSG0000	765.3519	0.056296	0.167842	0.628685	0.896984	ZBTB18	10472	zinc finger and BTB domain containing 18
ENSG0000	945.1489	-0.04575	0.10762	0.628667	0.896984	YKT6	10652	YKT6 v-SNARE homolog
ENSG0000	8.486058	0.027805	0.225441	0.628692	0.896984	TRAV10	28676	T cell receptor alpha variable 10
ENSG0000	11.83747	-0.03588	0.220631	0.629137	0.897312	REEP2	51308	receptor accessory protein 2
ENSG0000	48.46186	0.054944	0.183518	0.629142	0.897312	PRPF40B	25766	pre-mRNA processing factor 40 homolog B
ENSG0000	5688.805	-0.05462	0.177034	0.629145	0.897312	IL4R	3566	interleukin 4 receptor
ENSG0000	26.63262	0.054927	0.189629	0.629167	0.897312	ZNF56P	7608	zinc finger pseudogene
ENSG0000	12.09916	0.048362	0.208614	0.629037	0.897312	ZNF8-DT	1.05E+08	ZNF8 divergent transcript
ENSG0000	969.2696	-0.05177	0.1328	0.629263	0.89736	RAD23B	5887	RAD23 ho nucleotide excision repair protein
ENSG0000	102.0698	0.057291	0.163223	0.629298	0.89736	RPP30	10556	ribonuclease P/MRP subunit p30
ENSG0000	1034.654	0.050672	0.126093	0.629399	0.897411	HDAC1	3065	histone deacetylase 1
ENSG0000	2.636904	0.019964	0.231578	0.629432	0.897411	DLGAP1-A	201477	DLGAP1 antisense RNA 3
ENSG0000	74.73712	0.038908	0.215369	0.629907	0.897441	ZNF644	84146	zinc finger protein 644
ENSG0000	4078.57	-0.05146	0.15068	0.630076	0.897441	PLEKHO1	51177	pleckstrin homology domain containing O1
ENSG0000	372.5608	0.054781	0.153937	0.629958	0.897441	CD86	942	CD86 molecule
ENSG0000	3626.731	0.051747	0.143939	0.630006	0.897441	PNRC1	10957	proline rich nuclear receptor coactivator 1
ENSG0000	55.71342	0.057587	0.176374	0.630062	0.897441	CEP41	95681	centrosomal protein 41
ENSG0000	6.52926	-0.02846	0.224472	0.629696	0.897441	NA	NA	NA
ENSG0000	11.56093	-0.03407	0.221459	0.630013	0.897441	LCNL1	401562	lipocalin like 1
ENSG0000	9.31714	0.028372	0.225127	0.629824	0.897441	RPL21P16	729402	ribosomal protein L21 pseudogene 16
ENSG0000	2.987546	-0.02023	0.22926	0.629574	0.897441	NA	NA	NA
ENSG0000	182.1876	-0.0531	0.129308	0.629536	0.897441	LSM12	124801	LSM12 homolog
ENSG0000	6.649879	0.028678	0.231028	0.629977	0.897441	BCRP2	400892	BCR pseudogene 2
ENSG0000	634.1233	-0.05164	0.135283	0.63009	0.897441	MPST	4357	mercaptopyruvate sulfurtransferase
ENSG0000	222.2302	0.055306	0.150187	0.629837	0.897441	DIAPH2	1730	diaphanous related formin 2
ENSG0000	163.0808	0.053475	0.177342	0.63074	0.897617	GFI1	2672	growth factor independent 1 transcriptional repressor
ENSG0000	7486.522	0.05427	0.148413	0.630844	0.897617	CTSS	1520	cathepsin S

ENSG0000	50.3135	-0.05572	0.177643	0.630562	0.897617	NHEJ1	79840	non-homologous end joining factor 1
ENSG0000	13.97697	0.04401	0.213548	0.630547	0.897617	NA	NA	NA
ENSG0000	972.3332	0.053436	0.138996	0.630777	0.897617	INTS15	79034	integrator complex subunit 15
ENSG0000	68.88012	0.054225	0.182061	0.6307	0.897617	RALGAPA1	253959	Ral GTPase activating protein catalytic subunit alpha 1
ENSG0000	373.1232	0.060906	0.182288	0.630341	0.897617	GOLM2	113201	golgi membrane protein 2
ENSG0000	1417.64	0.055561	0.157969	0.630868	0.897617	FUS	2521	FUS RNA binding protein
ENSG0000	1451.884	-0.05713	0.15957	0.630899	0.897617	VAC14	55697	VAC14 component of PIKFYVE complex
ENSG0000	388.3074	-0.05319	0.143565	0.630425	0.897617	ATP5PD	10476	ATP synthase peripheral stalk subunit d
ENSG0000	258.8154	0.057165	0.143493	0.63052	0.897617	U2AF1L4	199746	U2 small nuclear RNA auxiliary factor 1 like 4
ENSG0000	4.988147	-0.02137	0.228626	0.63052	0.897617	NA	NA	NA
ENSG0000	17.88076	0.044781	0.212608	0.630609	0.897617	NA	NA	NA
ENSG0000	2.94497	-0.02796	0.227459	0.630837	0.897617	SCML2	10389	Scm polycomb group protein like 2
ENSG0000	84.24218	-0.05611	0.15786	0.630988	0.897673	ZNF557	79230	zinc finger protein 557
ENSG0000	5554.134	-0.05391	0.145568	0.631103	0.897768	GDI1	2664	GDP dissociation inhibitor 1
ENSG0000	25.16221	-0.04949	0.2034	0.631186	0.897815	NA	NA	NA
ENSG0000	3.113074	-0.02655	0.227078	0.631279	0.897878	NA	NA	NA
ENSG0000	101.4416	-0.04887	0.199453	0.631428	0.897987	THEMIS	387357	thymocyte selection associated
ENSG0000	160.3201	0.055699	0.151756	0.631453	0.897987	ZC3H7A	29066	zinc finger CCCH-type containing 7A
ENSG0000	43.83636	-0.05147	0.194157	0.631593	0.898046	NA	NA	NA
ENSG0000	2501.256	-0.05296	0.142274	0.631587	0.898046	PCNX3	399909	pecanex 3
ENSG0000	1619.926	0.049573	0.120993	0.63166	0.898071	SPSB3	90864	splA/ryanodine receptor domain and SOCS box containing 3
ENSG0000	586.4768	0.054182	0.143688	0.63171	0.898073	TRERF1	55809	transcriptional regulating factor 1
ENSG0000	354.8961	-0.05728	0.124478	0.632248	0.898208	PUM1	9698	pumilio RNA binding family member 1
ENSG0000	117.0552	-0.05236	0.189965	0.632289	0.898208	CHRM3-AS	1.01E+08	CHRM3 antisense RNA 2
ENSG0000	6.531455	0.027571	0.225966	0.632111	0.898208	NA	NA	NA
ENSG0000	808.5344	-0.05118	0.169038	0.632018	0.898208	CTNNA1	1495	catenin alpha 1
ENSG0000	70.43738	-0.05559	0.164674	0.631889	0.898208	CDKAL1	54901	CDK5 regulatory subunit associated protein 1 like 1
ENSG0000	57.88777	0.057698	0.18529	0.632192	0.898208	PPIL1	51645	peptidylprolyl isomerase like 1
ENSG0000	601.8396	-0.04874	0.128966	0.63219	0.898208	RSBN1L	222194	round spermatid basic protein 1 like
ENSG0000	1701.443	-0.05444	0.152183	0.632296	0.898208	ARF3	377	ADP ribosylation factor 3
ENSG0000	4.243304	0.022368	0.228156	0.631933	0.898208	NA	NA	NA
ENSG0000	6.803654	0.035135	0.222415	0.632025	0.898208	NDUFA13	51079	NADH:ubiquinone oxidoreductase subunit A13
ENSG0000	2.557982	0.019288	0.230623	0.632416	0.898253	GALNT8	26290	polypeptide N-acetylgalactosaminyltransferase 8
ENSG0000	191.2303	0.053055	0.1324	0.632426	0.898253	VAPB	9217	VAMP associated protein B and C
ENSG0000	2006.815	0.049586	0.121741	0.632635	0.898342	JAK1	3716	Janus kinase 1
ENSG0000	212.7295	-0.05382	0.143953	0.632589	0.898342	FNTB	2342	farnesyltra CAAX box beta
ENSG0000	16.86717	-0.0402	0.21633	0.632594	0.898342	NA	NA	NA
ENSG0000	129.0049	0.057577	0.163233	0.632747	0.898432	CEP78	84131	centrosomal protein 78
ENSG0000	3.588457	-0.02154	0.229155	0.633023	0.898439	NA	NA	NA
ENSG0000	28.19825	0.050112	0.201291	0.633047	0.898439	EBF1	1879	EBF transcription factor 1
ENSG0000	5.846926	0.029728	0.225039	0.632869	0.898439	NA	NA	NA
ENSG0000	24.27773	0.03213	0.222041	0.632902	0.898439	CD69	969	CD69 molecule
ENSG0000	509.0136	-0.05466	0.15654	0.633005	0.898439	MVD	4597	mevalonate diphosphate decarboxylase
ENSG0000	5.558678	-0.03498	0.222534	0.633001	0.898439	NA	NA	NA
ENSG0000	45.31463	0.052753	0.191985	0.633191	0.898559	NDUFAF7	55471	NADH:ubiquinone oxidoreductase complex assembly factor 7
ENSG0000	610.1975	0.051487	0.192436	0.633229	0.898559	TUT7	79670	terminal uridylyl transferase 7
ENSG0000	163.1261	0.055296	0.146778	0.633396	0.898656	ADRB2	154	adrenoceptor beta 2
ENSG0000	327.3044	0.053118	0.167645	0.63337	0.898656	SLC27A4	10999	solute carrier family 27 member 4
ENSG0000	840.605	-0.05387	0.149856	0.634099	0.898715	TMEM59	9528	transmembrane protein 59
ENSG0000	143.8036	0.049119	0.190241	0.634063	0.898715	TSPAN2	10100	tetraspanin 2
ENSG0000	54.80748	0.05181	0.193839	0.633959	0.898715	LCLAT1	253558	lysocardiolipin acyltransferase 1
ENSG0000	53.98439	0.049885	0.200615	0.633959	0.898715	HIGD1A	25994	HIG1 hypoxia inducible domain family member 1A
ENSG0000	10.66709	0.027802	0.224827	0.633578	0.898715	BDH2	56898	3-hydroxybutyrate dehydrogenase 2
ENSG0000	17.98355	-0.05262	0.19618	0.634124	0.898715	ATG10	83734	autophagy related 10
ENSG0000	51.24879	0.054055	0.186833	0.633904	0.898715	HLA-DQB2	3120	major hist class II DQ beta 2
ENSG0000	288.6037	-0.05419	0.174421	0.634023	0.898715	NA	NA	NA
ENSG0000	148.4239	-0.05365	0.147148	0.633765	0.898715	ZC3HC1	51530	zinc finger C3HC-type containing 1
ENSG0000	225.6192	0.053365	0.174326	0.634099	0.898715	AZIN1	51582	antizyme inhibitor 1
ENSG0000	362.6154	-0.05253	0.137231	0.633555	0.898715	INTS4	92105	integrator complex subunit 4
ENSG0000	89.41228	0.048763	0.199994	0.633844	0.898715	CTDSPL2	51496	CTD small phosphatase like 2
ENSG0000	630.286	-0.05301	0.181213	0.633723	0.898715	NUFIP2	57532	nuclear FMR1 interacting protein 2
ENSG0000	160.1157	-0.05121	0.192576	0.633907	0.898715	CARD8-AS	1.01E+08	CARD8 antisense RNA 1
ENSG0000	75.88686	0.047596	0.201721	0.634206	0.898719	COPS4	51138	COP9 signalosome subunit 4
ENSG0000	333.2326	-0.05472	0.158305	0.634225	0.898719	PCTP	58488	phosphatidylcholine transfer protein
ENSG0000	154.7869	-0.05037	0.194189	0.634418	0.898742	GTF2B	2959	general transcription factor IIB
ENSG0000	5.554852	0.026449	0.226562	0.634345	0.898742	NA	NA	NA
ENSG0000	256.5647	0.053536	0.168316	0.634487	0.898742	RAPGEF2	9693	Rap guanine nucleotide exchange factor 2
ENSG0000	9.315621	0.038333	0.218661	0.634369	0.898742	LINC02937	1.01E+08	long intergenic non-protein coding RNA 2937
ENSG0000	3063.376	0.053539	0.18107	0.634484	0.898742	POU2AF1	5450	POU class 2 homeobox associating factor 1
ENSG0000	54.23742	0.055182	0.166551	0.634588	0.898815	LIN7C	55327	lin-7 homc crumbs cell polarity complex component
ENSG0000	9.974134	-0.03273	0.221606	0.634651	0.898835	LOC10272	1.03E+08	neuroblastoma breakpoint family member 1-like
ENSG0000	262.4348	0.055157	0.165382	0.634751	0.898863	RHOBTB2	23221	Rho related BTB domain containing 2
ENSG0000	3232.746	-0.05208	0.129911	0.634768	0.898863	UNC119	9094	unc-119 lipid binding chaperone
ENSG0000	25.36576	0.052273	0.195308	0.634879	0.898949	FAM86C2I	645332	family witl member A pseudogene
ENSG0000	17.71646	0.040349	0.215711	0.635014	0.899071	NA	NA	NA
ENSG0000	483.5478	-0.05645	0.172103	0.635137	0.899134	ARL8B	55207	ADP ribosylation factor like GTPase 8B
ENSG0000	3.351503	0.028048	0.228391	0.635205	0.899134	NA	NA	NA
ENSG0000	1234.036	-0.04896	0.128671	0.635157	0.899134	TBC1D1	23216	TBC1 domain family member 1
ENSG0000	3.508089	-0.00653	0.232167	0.635333	0.899186	NA	NA	NA
ENSG0000	2099.191	0.04447	0.132098	0.63534	0.899186	ANP32A	8125	acidic nuclear phosphoprotein 32 family member A
ENSG0000	127.5622	0.054591	0.147658	0.635559	0.899357	AGPAT4	56895	1-acylglycerol-3-phosphate O-acyltransferase 4
ENSG0000	104.5545	-0.05337	0.151434	0.635536	0.899357	TTBK2	146057	tau tubulin kinase 2
ENSG0000	38.83721	0.049897	0.19945	0.63583	0.899373	DNAJC19	131118	DnaJ heat shock protein family (Hsp40) member C19
ENSG0000	203.3902	0.054378	0.140554	0.635867	0.899373	SENP5	205564	SUMO specific peptidase 5
ENSG0000	23.52965	0.048516	0.202595	0.635861	0.899373	ARMC2	84071	armadillo repeat containing 2
ENSG0000	21.99157	-0.0489	0.202289	0.635894	0.899373	MTFR2	113115	mitochondrial fission regulator 2
ENSG0000	5.004959	0.021839	0.228163	0.635946	0.899373	NA	NA	NA
ENSG0000	17.02336	0.04228	0.213496	0.636258	0.899373	ZBTB10	65986	zinc finger and BTB domain containing 10
ENSG0000	2913.853	-0.05284	0.192725	0.636258	0.899373	GSN	2934	gelsolin
ENSG0000	225.4536	-0.05281	0.18202	0.636143	0.899373	LGALS12	85329	galectin 12
ENSG0000	162.1099	0.057739	0.184787	0.636048	0.899373	DHCR7	1717	7-dehydrocholesterol reductase
ENSG0000	46.06946	-0.04676	0.189202	0.636049	0.899373	RAD52	5893	RAD52 hoi DNA repair protein
ENSG0000	103.9034	-0.05437	0.153151	0.635815	0.899373	TMEM19	55266	transmembrane protein 19
ENSG0000	21.94832	-0.04935	0.201852	0.635859	0.899373	NA	NA	NA
ENSG0000	5.682461	-0.02427	0.226862	0.636195	0.899373	NA	NA	NA
ENSG0000	603.2462	0.053837	0.147269	0.636107	0.899373	THAP7	80764	THAP domain containing 7

ENSG0000	304.685	0.048346	0.198808	0.636352	0.899437	MMP8	4317	matrix metallopeptidase 8
ENSG0000	2170.601	-0.05036	0.142952	0.636473	0.899469	ST3GAL1	6482	ST3 beta-g 3-sialyltransferase 1
ENSG0000	57.7037	0.043881	0.209393	0.636432	0.899469	COG6	57511	component of oligomeric golgi complex 6
ENSG0000	548.1469	-0.05277	0.144719	0.63672	0.899748	SLC35C1	55343	solute carrier family 35 member C1
ENSG0000	7.724382	-0.02898	0.224225	0.636875	0.899827	CXCL2	2920	C-X-C motif chemokine ligand 2
ENSG0000	194.6805	0.043882	0.207839	0.636923	0.899827	RB1CC1	9821	RB1 inducible coiled-coil 1
ENSG0000	90.83144	0.054199	0.148194	0.636861	0.899827	HSD17B12	51144	hydroxysteroid 17-beta dehydrogenase 12
ENSG0000	87.16223	0.051831	0.190868	0.637123	0.899936	RAPGEF6	51735	Rap guanine nucleotide exchange factor 6
ENSG0000	158.4959	-0.05377	0.176339	0.637197	0.899936	TAF2	6873	TATA-box binding protein associated factor 2
ENSG0000	48.29039	0.067607	0.177315	0.63706	0.899936	SAAL1	113174	serum amyloid A like 1
ENSG0000	13.89315	0.043456	0.211996	0.637187	0.899936	ZNF599	148103	zinc finger protein 599
ENSG0000	1476.049	-0.04454	0.110963	0.637533	0.900235	RNF4	6047	ring finger protein 4
ENSG0000	117.2353	0.053287	0.1742	0.637502	0.900235	CPEB2	132864	cytoplasmic polyadenylation element binding protein 2
ENSG0000	83.84374	0.055378	0.159217	0.637605	0.900235	ZNF37A	7587	zinc finger protein 37A
ENSG0000	61.54402	-0.04087	0.212577	0.637598	0.900235	VBP1	7411	VHL binding protein 1
ENSG0000	9.302311	0.032499	0.223007	0.637658	0.90024	NA	NA	NA
ENSG0000	887.9273	0.053301	0.139635	0.637772	0.900263	TWF2	11344	twinfilin actin binding protein 2
ENSG0000	65.13889	0.056284	0.157782	0.637732	0.900263	NR2F6	2063	nuclear receptor subfamily 2 group F member 6
ENSG0000	3505.51	0.052971	0.181222	0.639404	0.900268	CD52	1043	CD52 molecule
ENSG0000	638.1514	0.054082	0.140713	0.638979	0.900268	DNAJC8	22826	DnaJ heat shock protein family (Hsp40) member C8
ENSG0000	209.9495	0.054238	0.151926	0.638352	0.900268	SCMH1	22955	Scm polycomb group protein homolog 1
ENSG0000	29.01642	-0.04708	0.20339	0.639132	0.900268	NA	NA	NA
ENSG0000	8.13796	0.020441	0.228287	0.639612	0.900268	NA	NA	NA
ENSG0000	9.306976	0.019808	0.228713	0.639386	0.900268	LRRN2	10446	leucine rich repeat neuronal 2
ENSG0000	27.91557	-0.04514	0.206755	0.638689	0.900268	FAM89A	375061	family with sequence similarity 89 member A
ENSG0000	19.4141	-0.0478	0.203946	0.638386	0.900268	RBKS	64080	ribokinase
ENSG0000	5.687017	0.032581	0.223201	0.638385	0.900268	NA	NA	NA
ENSG0000	239.4554	-0.05389	0.16005	0.639643	0.900268	TRAK2	66008	trafficking kinesin protein 2
ENSG0000	26.20015	0.04057	0.216048	0.639124	0.900268	GP5	2814	glycoprotein V platelet
ENSG0000	553.7598	0.055852	0.148561	0.638995	0.900268	ANTXR2	118429	ANTXR cell adhesion molecule 2
ENSG0000	2324.483	-0.05096	0.125997	0.638724	0.900268	CAST	831	calpastatin
ENSG0000	6.964874	0.034023	0.222136	0.638551	0.900268	PRSS16	10279	serine protease 16
ENSG0000	40.23479	0.053188	0.183756	0.638917	0.900268	NA	NA	NA
ENSG0000	2.469004	-0.02915	0.228408	0.638151	0.900268	TAS2R5	54429	taste 2 receptor member 5
ENSG0000	16.24483	-0.03725	0.217892	0.639249	0.900268	NA	NA	NA
ENSG0000	16.60528	-0.03603	0.218352	0.639557	0.900268	C8G	733	complement C8 gamma chain
ENSG0000	176.2864	0.053816	0.168795	0.637982	0.900268	TCF7L2	6934	transcription factor 7 like 2
ENSG0000	111.1397	0.054384	0.168515	0.637836	0.900268	RRM1	6240	ribonucleotide reductase catalytic subunit M1
ENSG0000	15.06611	0.043326	0.211002	0.639045	0.900268	NUDT8	254552	nudix hydrolase 8
ENSG0000	37.73691	-0.04423	0.209144	0.639005	0.900268	KRT73	319101	keratin 73
ENSG0000	12.12025	0.044294	0.211448	0.638772	0.900268	RDH16	8608	retinol dehydrogenase 16
ENSG0000	780.8052	0.05423	0.165669	0.639475	0.900268	PLBD2	196463	phospholipase B domain containing 2
ENSG0000	2.361324	0.007178	0.233617	0.639069	0.900268	GOLGA6L4	643707	golgin A6 family like 4
ENSG0000	40.71506	-0.05059	0.195766	0.638994	0.900268	GDPGP1	390637	GDP-D-glucose phosphorylase 1
ENSG0000	175.721	-0.05369	0.148512	0.639457	0.900268	JPT2	90861	Jupiter microtubule associated homolog 2
ENSG0000	1682.007	0.055082	0.148023	0.638291	0.900268	RUSF1	64755	RUS family member 1
ENSG0000	2.425263	0.026534	0.230613	0.638602	0.900268	LINC00304	283860	long intergenic non-protein coding RNA 304
ENSG0000	4.105346	-0.03262	0.224313	0.638108	0.900268	NA	NA	NA
ENSG0000	178.1322	0.04952	0.124776	0.639122	0.900268	RANGRF	29098	RAN guanine nucleotide release factor
ENSG0000	126.9102	-0.05376	0.147591	0.638861	0.900268	LINC00910	1E+08	long intergenic non-protein coding RNA 910
ENSG0000	217.7222	0.052856	0.141859	0.639233	0.900268	PWWP3A	84939	PWWP do DNA repair factor
ENSG0000	8616.255	-0.0536	0.160689	0.638869	0.900268	S1PR4	8698	sphingosine-1-phosphate receptor 4
ENSG0000	40.21826	0.057434	0.171808	0.639257	0.900268	ODAD3	115948	outer dynein arm docking complex subunit 3
ENSG0000	23.37668	0.049171	0.201577	0.639568	0.900268	NA	NA	NA
ENSG0000	14.79242	0.045408	0.209755	0.63905	0.900268	SMIM26	388789	small integral membrane protein 26
ENSG0000	6.222261	0.02641	0.226989	0.63948	0.900268	NA	NA	NA
ENSG0000	138.6605	-0.05423	0.143583	0.639719	0.900305	RABGAP1	23637	RAB GTPase activating protein 1
ENSG0000	14.20236	-0.03339	0.216286	0.639863	0.900439	PAIP2B	400961	poly(A) binding protein interacting protein 2B
ENSG0000	57.3351	0.04134	0.212893	0.640192	0.900449	NA	NA	NA
ENSG0000	14.80262	0.0406	0.214211	0.640028	0.900449	NA	NA	NA
ENSG0000	458.9607	-0.05088	0.14029	0.640286	0.900449	NIPBL	25836	NIPBL cohesin loading factor
ENSG0000	2612.223	0.047559	0.117962	0.640271	0.900449	FAM193B	54540	family with sequence similarity 193 member B
ENSG0000	1000.113	0.050642	0.131502	0.640345	0.900449	GTF3C5	9328	general transcription factor IIIC subunit 5
ENSG0000	69.1714	0.054961	0.159727	0.640133	0.900449	TMEM25	84866	transmembrane protein 25
ENSG0000	6.62603	0.026054	0.226548	0.640362	0.900449	NA	NA	NA
ENSG0000	257.4776	-0.04773	0.19796	0.640189	0.900449	PSMA4	5685	proteasome 20S subunit alpha 4
ENSG0000	4790.03	-0.04966	0.155278	0.640245	0.900449	BRD4	23476	bromodomain containing 4
ENSG0000	926.7666	0.054705	0.160058	0.640178	0.900449	CCDC124	115098	coiled-coil domain containing 124
ENSG0000	32.68936	-0.04757	0.201699	0.640471	0.900464	NA	NA	NA
ENSG0000	35.0321	0.050746	0.19254	0.64043	0.900464	ZNF528	84436	zinc finger protein 528
ENSG0000	283.5954	0.049262	0.119692	0.640651	0.900579	ARHGEF7	8874	Rho guanine nucleotide exchange factor 7
ENSG0000	4.787062	0.034292	0.2231	0.640622	0.900579	CDRT4	284040	CMT1A duplicated region transcript 4
ENSG0000	359.6562	-0.05341	0.173246	0.64155	0.900651	LRRC8C	84230	leucine rich repeat containing 8 VRAC subunit C
ENSG0000	150.0561	-0.05392	0.159118	0.640845	0.900651	SOX13	9580	SRY-box transcription factor 13
ENSG0000	147.4956	-0.05376	0.171717	0.640973	0.900651	CNST	163882	consortin connexin sorting protein
ENSG0000	18.35684	0.048038	0.203802	0.641667	0.900651	ASAP2	8853	ArfGAP wi ankyrin repeat and PH domain 2
ENSG0000	100.5261	0.054883	0.158002	0.640878	0.900651	KCTD18	130535	potassium channel tetramerization domain containing 18
ENSG0000	16.11338	0.039941	0.214994	0.64113	0.900651	ARSK	153642	arylsulfatase family member K
ENSG0000	507.9082	-0.06933	0.170229	0.641061	0.900651	RAB32	10981	RAB32 member RAS oncogene family
ENSG0000	879.437	-0.04762	0.120103	0.641579	0.900651	GIMAP1	170575	GTPase IMAP family member 1
ENSG0000	55.29156	0.056494	0.18127	0.641174	0.900651	IL11RA	3590	interleukin 11 receptor subunit alpha
ENSG0000	150.2083	-0.05278	0.177714	0.641119	0.900651	ISCA1	81689	iron-sulfur cluster assembly 1
ENSG0000	166.4341	0.056182	0.156994	0.641767	0.900651	RPL18AP3	390354	ribosomal protein L18a pseudogene 3
ENSG0000	210.876	-0.05413	0.167591	0.64178	0.900651	P2RX7	5027	purinergic receptor P2X 7
ENSG0000	139.5483	0.051858	0.137999	0.641358	0.900651	ZNF764	92595	zinc finger protein 764
ENSG0000	19.74184	-0.05113	0.195143	0.64117	0.900651	MPHOSPH	10200	M-phase phosphoprotein 6
ENSG0000	28.90199	-0.05308	0.188116	0.641587	0.900651	EPOP	1E+08	elongin BC and polycomb repressive complex 2 associated protein
ENSG0000	417.4893	-0.05255	0.144115	0.641478	0.900651	TMEM106	113277	transmembrane protein 106A
ENSG0000	70.07307	-0.0522	0.180245	0.641463	0.900651	ZNF121	7675	zinc finger protein 121
ENSG0000	356.1744	0.051399	0.133334	0.641745	0.900651	GADD45G	90480	GADD45G interacting protein 1
ENSG0000	76.03384	-0.04891	0.198875	0.641581	0.900651	BCAM	4059	basal cell adhesion molecule (Lutheran blood group)
ENSG0000	1617.011	-0.04929	0.127358	0.641296	0.900651	CHMP4B	128866	charged multivesicular body protein 4B
ENSG0000	902.463	-0.05133	0.165748	0.641808	0.900651	SLC25A1	6576	solute carrier family 25 member 1
ENSG0000	117.1611	-0.05183	0.138672	0.641833	0.900651	NOL12	79159	nucleolar protein 12
ENSG0000	41.87025	0.054467	0.177601	0.640885	0.900651	ADSL	158	adenylosuccinate lyase
ENSG0000	983.0503	0.052391	0.145791	0.641907	0.900686	PCYT1A	5130	phosphate choline

ENSG0000	7.11447	0.038032	0.219122	0.642175	0.900879	NA	NA	NA
ENSG0000	124.2964	0.054749	0.151408	0.642192	0.900879	ABRAXAS2	23172	abraxas 2 BRISC complex subunit
ENSG0000	375.341	-0.05005	0.168542	0.642128	0.900879	SPINDOC	144097	spindlin interactor and repressor of chromatin binding
ENSG0000	134.597	0.052085	0.186037	0.64244	0.900894	PRKD3	23683	protein kinase D3
ENSG0000	40.99471	0.054053	0.180141	0.642616	0.900894	POPDC2	64091	popeye domain containing 2
ENSG0000	18.59222	-0.05025	0.198631	0.642503	0.900894	RPS2P32	256355	ribosomal protein S2 pseudogene 32
ENSG0000	489.7807	-0.04839	0.121607	0.642588	0.900894	WASHC2C	253725	WASH complex subunit 2C
ENSG0000	10.00913	0.040954	0.218628	0.642459	0.900894	AGAP6	414189	ArfGAP wi ankyrin repeat and PH domain 6
ENSG0000	11.74525	-0.02902	0.222931	0.642258	0.900894	NA	NA	NA
ENSG0000	15.98452	0.047844	0.204395	0.642478	0.900894	ZNF345	25850	zinc finger protein 345
ENSG0000	43.74565	0.050272	0.194501	0.642646	0.900894	PSMG1	8624	proteasome assembly chaperone 1
ENSG0000	41.30948	0.052097	0.188825	0.642585	0.900894	CTPS2	56474	CTP synthase 2
ENSG0000	1952.95	0.059517	0.155315	0.642762	0.900988	LRPAP1	4043	LDL receptor related protein associated protein 1
ENSG0000	84.05086	0.053502	0.17824	0.643022	0.901266	DNAJC9	23234	DnaJ heat shock protein family (Hsp40) member C9
ENSG0000	147.8953	0.050911	0.193377	0.643058	0.901266	C2CD5	9847	C2 calcium dependent domain containing 5
ENSG0000	927.7053	0.053182	0.134081	0.643202	0.901332	CELF1	10658	CUGBP Elav-like family member 1
ENSG0000	12.06925	-0.03956	0.215988	0.643204	0.901332	NGFR	4804	nerve growth factor receptor
ENSG0000	665.6346	-0.05277	0.156336	0.643407	0.901547	LINC0017	285908	long intergenic non-protein coding RNA 174
ENSG0000	338.5789	-0.05402	0.172882	0.643499	0.901608	SIL1	64374	SIL1 nucleotide exchange factor
ENSG0000	43.81322	0.054611	0.168668	0.643574	0.901643	ZNF765	91661	zinc finger protein 765
ENSG0000	18.21119	-0.04851	0.200837	0.643681	0.901725	KCNQ1OT	10984	KCNQ1 opposite strand/antisense transcript 1
ENSG0000	5.96907	-0.02623	0.225963	0.644258	0.901776	AUNIP	79000	aurora kinase A and ninein interacting protein
ENSG0000	312.5816	0.049429	0.133952	0.644255	0.901776	SNRNP40	9410	small nuclear ribonucleoprotein U5 subunit 40
ENSG0000	5184.103	-0.05321	0.156463	0.644047	0.901776	SF3B4	10262	splicing factor 3b subunit 4
ENSG0000	19.97786	-0.03638	0.21738	0.643846	0.901776	RGS16	6004	regulator of G protein signaling 16
ENSG0000	580.4733	-0.05284	0.169839	0.644327	0.901776	MTARC1	64757	mitochondrial amidoxime reducing component 1
ENSG0000	233.6956	-0.05221	0.148084	0.644353	0.901776	RUVBL1	8607	RuvB like AAA ATPase 1
ENSG0000	8.044324	-0.0371	0.218789	0.643858	0.901776	NA	NA	NA
ENSG0000	5.011167	0.028928	0.225333	0.644358	0.901776	NA	NA	NA
ENSG0000	237.973	-0.05307	0.155434	0.643933	0.901776	DIS3	22894	DIS3 homc exosome endoribonuclease and 3'-5' exoribonuclease
ENSG0000	14.88099	0.042991	0.212337	0.644077	0.901776	SNRPN	6638	small nuclear ribonucleoprotein polypeptide N
ENSG0000	2213.66	-0.05285	0.158627	0.644302	0.901776	CLCN7	1186	chloride voltage-gated channel 7
ENSG0000	240.3829	0.053444	0.155265	0.64402	0.901776	TMEM204	79652	transmembrane protein 204
ENSG0000	12.73089	-0.04476	0.209177	0.644009	0.901776	ATP2C2-A	1.05E+08	ATP2C2 antisense RNA 1
ENSG0000	20.51021	0.052411	0.193987	0.64441	0.901781	XKR6	286046	XK related 6
ENSG0000	11.48917	-0.03596	0.218955	0.644606	0.901916	ZNF367	195828	zinc finger protein 367
ENSG0000	93.179	0.048851	0.192382	0.644593	0.901916	ZMYND15	84225	zinc finger MYND-type containing 15
ENSG0000	4.624999	-0.03133	0.223812	0.644685	0.901958	SLC23A3	151295	solute carrier family 23 member 3
ENSG0000	4.505195	0.02427	0.227874	0.644854	0.902126	NA	NA	NA
ENSG0000	5.996441	-0.03542	0.220509	0.645081	0.902236	LIX1L-AS1	1.05E+08	LIX1L antisense RNA 1
ENSG0000	193.6732	-0.05114	0.154368	0.645065	0.902236	HIVEP2	3097	HIVEP zinc finger 2
ENSG0000	212.2664	0.050459	0.134323	0.644994	0.902236	RSAD1	55316	radical S-adenosyl methionine domain containing 1
ENSG0000	18.47452	0.044638	0.212117	0.645499	0.902697	TWIST2	117581	twist family bHLH transcription factor 2
ENSG0000	42.67023	0.054318	0.167329	0.645755	0.902697	PSMA1	5682	proteasome 20S subunit alpha 1
ENSG0000	8.481316	0.036134	0.219107	0.6457	0.902697	L2HGDH	79944	L-2-hydroxyglutarate dehydrogenase
ENSG0000	5.303415	0.023593	0.227347	0.645551	0.902697	RNU1-103	1.06E+08	RNA U1 small r pseudogene
ENSG0000	685.7521	0.050503	0.14332	0.64575	0.902697	SIPA1L3	23094	signal induced proliferation associated 1 like 3
ENSG0000	5.469234	0.039742	0.217815	0.645739	0.902697	CFAP298-T	1.1E+08	CFAP298-TCP10L readthrough
ENSG0000	5.79022	0.027152	0.22514	0.645646	0.902697	NA	NA	NA
ENSG0000	156.1544	-0.05236	0.14833	0.646218	0.902821	PPIEL	728448	peptidylprolyl isomerase E like pseudogene
ENSG0000	89.73268	0.053231	0.154474	0.646241	0.902821	POLE4	56655	DNA polyr accessory subunit
ENSG0000	2281.761	-0.05213	0.151247	0.646058	0.902821	PPM1M	132160	protein ph Mg2+/Mn2+ dependent 1M
ENSG0000	65.46095	0.04868	0.196999	0.646313	0.902821	KIAA0319	9856	KIAA0319
ENSG0000	60.01402	0.052357	0.181852	0.646373	0.902821	RMND1	55005	required for meiotic nuclear division 1 homolog
ENSG0000	16.44864	0.039439	0.215211	0.646032	0.902821	NA	NA	NA
ENSG0000	5.289062	-0.02341	0.227082	0.646087	0.902821	CCZ1P1	1.1E+08	CCZ1 pseudogene 1
ENSG0000	854.4455	-0.04815	0.124754	0.646019	0.902821	ZNHIT1	10467	zinc finger HIT-type containing 1
ENSG0000	391.1998	0.052713	0.157539	0.646104	0.902821	NA	NA	NA
ENSG0000	70.54891	0.047248	0.201591	0.646386	0.902821	RBBP8	5932	RB binding endonuclease
ENSG0000	548.5666	-0.04653	0.162109	0.646162	0.902821	TSPYL2	64061	TSPY like 2
ENSG0000	3.018136	0.017135	0.230258	0.646572	0.902831	NA	NA	NA
ENSG0000	34.42585	0.050738	0.192458	0.646626	0.902831	C1orf109	54955	chromosome 1 open reading frame 109
ENSG0000	16.99409	-0.04275	0.21048	0.646864	0.902831	NA	NA	NA
ENSG0000	43.75698	0.052615	0.183227	0.646726	0.902831	TMEM177	80775	transmembrane protein 177
ENSG0000	8.166269	0.033786	0.220488	0.64691	0.902831	NA	NA	NA
ENSG0000	843.6948	-0.05175	0.145157	0.646454	0.902831	ATP6V0E2	155066	ATPase H+ transporting V0 subunit e2
ENSG0000	40.36556	-0.04859	0.195044	0.646745	0.902831	CKLF	51192	chemokine like factor
ENSG0000	1808.392	-0.05102	0.144122	0.646723	0.902831	UBTF	7343	upstream binding transcription factor
ENSG0000	60.73336	-0.0509	0.187667	0.646862	0.902831	NA	NA	NA
ENSG0000	46.31401	0.051282	0.190857	0.646935	0.902831	ZSCAN30	1E+08	zinc finger and SCAN domain containing 30
ENSG0000	52.86798	0.053566	0.170248	0.64677	0.902831	NA	NA	NA
ENSG0000	150.3707	-0.05029	0.142126	0.647072	0.902953	THAP8	199745	THAP domain containing 8
ENSG0000	323.4288	0.049708	0.157259	0.647123	0.902955	DPH2	1802	diphthamide biosynthesis 2
ENSG0000	913.4835	-0.04981	0.189876	0.647253	0.902975	CHD7	55636	chromodomain helicase DNA binding protein 7
ENSG0000	303.0749	-0.0512	0.152652	0.647285	0.902975	FBXO31	79791	F-box protein 31
ENSG0000	4.235023	-0.01761	0.22933	0.647256	0.902975	TRAPPC2B	10597	trafficking protein particle complex subunit 2B
ENSG0000	326.8101	0.052682	0.175648	0.64743	0.903086	ZFR	51663	zinc finger RNA binding protein
ENSG0000	335.2666	0.045043	0.110913	0.647463	0.903086	TXNL4A	10907	thioredoxin like 4A
ENSG0000	575.8834	-0.04389	0.109311	0.64752	0.903097	CLTA	1211	clathrin light chain A
ENSG0000	6.190108	0.034394	0.222075	0.647857	0.903335	SLFNL1	200172	schlafen like 1
ENSG0000	609.114	0.049825	0.13194	0.648184	0.903335	TMEM183	92703	transmembrane protein 183A
ENSG0000	868.8056	0.052771	0.168549	0.647996	0.903335	PARP1	142	poly(ADP-ribose) polymerase 1
ENSG0000	4.942743	-0.02283	0.227156	0.648234	0.903335	NA	NA	NA
ENSG0000	96.40684	0.052678	0.150006	0.647844	0.903335	SDHAP1	255812	SDHA pseudogene 1
ENSG0000	446.7108	0.050899	0.131471	0.648172	0.903335	GNL1	2794	G protein nucleolar 1 (putative)
ENSG0000	5928.261	-0.05298	0.163212	0.648219	0.903335	MICAL1	64780	microtubu calponin and LIM domain containing 1
ENSG0000	4521.12	-0.05169	0.151129	0.648064	0.903335	LAT2	7462	linker for activation of T cells family member 2
ENSG0000	189.2778	0.051928	0.145732	0.647841	0.903335	MCPH1	79648	microcephalin 1
ENSG0000	374.938	-0.05159	0.130102	0.648131	0.903335	ATXN7L3B	552889	ataxin 7 like 3B
ENSG0000	155.7763	0.050902	0.139812	0.647907	0.903335	TSHZ1	10194	teashirt zinc finger homeobox 1
ENSG0000	2.620837	0.021073	0.228909	0.648361	0.903372	NA	NA	NA
ENSG0000	4.059444	-0.02959	0.225857	0.648357	0.903372	NDST2	8509	N-deacetylase and N-sulfotransferase 2
ENSG0000	16.28528	0.04609	0.20727	0.648408	0.903372	NA	NA	NA
ENSG0000	409.6746	-0.0581	0.13711	0.648473	0.903395	TSEN54	283989	tRNA splicing endonuclease subunit 54
ENSG0000	310.9971	-0.05061	0.140981	0.648638	0.903417	IWS1	55677	interacts v CTD assembly factor 1
ENSG0000	27.46298	-0.05046	0.194609	0.648625	0.903417	CRYGS	1427	crystallin gamma S

ENSG0000	2.871604	-0.0183	0.230396	0.648579	0.903417	NA	NA	NA
ENSG0000	66.80592	0.052354	0.181833	0.648933	0.903546	NA	NA	NA
ENSG0000	22.95656	0.043777	0.208636	0.648935	0.903546	MIRLET7A	1.13E+08	miRlet-7a-1/let-7f-1/let-7d cluster host gene
ENSG0000	438.0126	-0.04788	0.129036	0.648977	0.903546	FMNL3	91010	formin like 3
ENSG0000	403.0418	-0.04935	0.187126	0.648864	0.903546	BNIP2	663	BCL2 interacting protein 2
ENSG0000	13212.73	-0.0509	0.179264	0.648916	0.903546	HCK	3055	HCK proto Src family tyrosine kinase
ENSG0000	23.75397	0.048324	0.199994	0.649063	0.903571	NA	NA	NA
ENSG0000	73.13589	-0.05139	0.191497	0.649093	0.903571	TPPP3	51673	tubulin polymerization promoting protein family member 3
ENSG0000	70.51334	-0.05297	0.168079	0.64932	0.903769	HYAL3	8372	hyaluronidase 3
ENSG0000	2390.761	0.052293	0.16918	0.649334	0.903769	MAP4K1	11184	mitogen-activated protein kinase kinase kinase kinase 1
ENSG0000	11.29108	-0.03497	0.218608	0.649965	0.903823	MIR3605	1.01E+08	microRNA 3605
ENSG0000	28.41177	0.036434	0.216498	0.649807	0.903823	ITGB3BP	23421	integrin subunit beta 3 binding protein
ENSG0000	8.492352	0.0359	0.219884	0.649603	0.903823	MAP3K21	84451	mitogen-activated protein kinase kinase kinase 21
ENSG0000	17.08845	0.044666	0.207373	0.649938	0.903823	NA	NA	NA
ENSG0000	38.81797	0.050101	0.194116	0.649448	0.903823	EEF1DP2	442429	eukaryotic translation elongation factor 1 delta pseudogene 2
ENSG0000	3.162185	0.028712	0.226473	0.649946	0.903823	NA	NA	NA
ENSG0000	18.27831	-0.03839	0.214148	0.649719	0.903823	NA	NA	NA
ENSG0000	69656.86	0.049964	0.18615	0.649656	0.903823	IGHM	3507	immunoglobulin heavy constant mu
ENSG0000	557.8053	0.04544	0.112779	0.649886	0.903823	HERC1	8925	HECT and RLD domain containing E3 ubiquitin protein ligase family member 1
ENSG0000	652.187	0.052203	0.154675	0.64971	0.903823	KCNK6	9424	potassium two pore domain channel subfamily K member 6
ENSG0000	1842.177	0.045977	0.114571	0.649858	0.903823	HNRNPL	3191	heterogeneous nuclear ribonucleoprotein L
ENSG0000	7.168498	-0.03812	0.217979	0.649873	0.903823	BEX5	340542	brain expressed X-linked 5
ENSG0000	8.091764	-0.02488	0.225443	0.650341	0.90386	RHCE	6006	Rh blood group CcEe antigens
ENSG0000	7.264235	0.028384	0.224185	0.650156	0.90386	NA	NA	NA
ENSG0000	73.77423	0.044627	0.20405	0.650096	0.90386	SCAMP1	9522	secretory carrier membrane protein 1
ENSG0000	37.65588	-0.04211	0.20701	0.650387	0.90386	NA	NA	NA
ENSG0000	18.48085	0.042108	0.209687	0.650143	0.90386	EPM2A	7957	EPM2A glu laforin
ENSG0000	27.46742	-0.04957	0.195402	0.65025	0.90386	NA	NA	NA
ENSG0000	286.0665	0.052406	0.172608	0.650333	0.90386	PDCD11	22984	programmed cell death 11
ENSG0000	10.58581	0.026338	0.224888	0.650264	0.90386	KIF18A	81930	kinesin family member 18A
ENSG0000	21.33606	0.033706	0.218074	0.650459	0.903892	NA	NA	NA
ENSG0000	112.9279	0.05575	0.162657	0.650669	0.904047	THUMPD3	25917	THUMP domain containing 3
ENSG0000	87.67688	-0.05167	0.144087	0.650659	0.904047	MAP3K13	9175	mitogen-activated protein kinase kinase kinase 13
ENSG0000	155.3503	-0.05243	0.164406	0.65077	0.904118	ARHGAP21	57584	Rho GTPase activating protein 21
ENSG0000	12.81316	0.041977	0.211367	0.650876	0.90416	CABLES1	91768	Cdk5 and Abl enzyme substrate 1
ENSG0000	188.1612	0.049877	0.136363	0.650898	0.90416	FECH	2235	ferrochelatase
ENSG0000	23.24507	0.040796	0.211251	0.651171	0.904196	ANKHD1	54882	ankyrin repeat and KH domain containing 1
ENSG0000	27.9315	0.040413	0.211111	0.651027	0.904196	NDUFA5	4698	NADH:ubiquinone oxidoreductase subunit A5
ENSG0000	461.304	-0.04423	0.112934	0.651027	0.904196	TBRG1	84897	transforming growth factor beta regulator 1
ENSG0000	10.46235	-0.03507	0.219763	0.651119	0.904196	CNN1	1264	calponin 1
ENSG0000	1198.525	0.044661	0.113203	0.65117	0.904196	TXN2	25828	thioredoxin 2
ENSG0000	5.43402	-0.01964	0.228916	0.651333	0.904352	FSD1L	83856	fibronectin type III and SPRY domain containing 1 like
ENSG0000	3194.583	0.050968	0.144134	0.651525	0.904481	KHSRP	8570	KH-type splicing regulatory protein
ENSG0000	5.138912	0.025225	0.229371	0.651505	0.904481	IGLV1-50	28821	immunoglobulin lambda variable 1-50 (non-functional)
ENSG0000	221.4205	0.05267	0.143742	0.651679	0.904592	LIG3	3980	DNA ligase 3
ENSG0000	31.76657	-0.04733	0.198388	0.651703	0.904592	ZNF614	80110	zinc finger protein 614
ENSG0000	400.8969	-0.05146	0.170029	0.65177	0.904616	APOBEC3B	9582	apolipoprotein B mRNA editing enzyme catalytic subunit 3B
ENSG0000	11074.54	-0.05115	0.172434	0.651905	0.904736	PTPN6	5777	protein tyrosine phosphatase non-receptor type 6
ENSG0000	1153.746	0.053936	0.158995	0.652133	0.904866	MOGS	7841	mannosyl-oligosaccharide glucosidase
ENSG0000	126.3824	0.052472	0.153925	0.652213	0.904866	PCGF3-AS1	1E+08	PCGF3 antisense RNA 1
ENSG0000	73.51706	0.051199	0.186999	0.652246	0.904866	RAD1	5810	RAD1 checkpoint DNA exonuclease
ENSG0000	69.16392	0.051067	0.162356	0.652191	0.904866	CTSF	8722	cathepsin F
ENSG0000	11.82629	0.039407	0.214338	0.652195	0.904866	SSC5D	284297	scavenger receptor cysteine rich family member with 5 domains
ENSG0000	126.4275	0.052769	0.154358	0.65241	0.904928	SSBP1	6742	single stranded DNA binding protein 1
ENSG0000	16.04373	0.039	0.214262	0.652353	0.904928	WDR86-AS	1E+08	WDR86 antisense RNA 1
ENSG0000	2.754209	-0.02067	0.228669	0.652439	0.904928	LINC00562	1.01E+08	long intergenic non-protein coding RNA 562
ENSG0000	308.7847	0.049074	0.13424	0.652502	0.904947	LLGL1	3996	LLGL scribble cell polarity complex component 1
ENSG0000	10.8322	0.043576	0.211547	0.652581	0.904988	NDFIP2	54602	Nedd4 family interacting protein 2
ENSG0000	125.1891	0.04697	0.181913	0.65265	0.905015	CWC22	57703	CWC22 spliceosome associated protein homolog
ENSG0000	218.6315	-0.05024	0.177064	0.652847	0.90515	TBK1	29110	TANK binding kinase 1
ENSG0000	3.507845	-0.02643	0.227615	0.65284	0.90515	LINC02324	1E+08	long intergenic non-protein coding RNA 2324
ENSG0000	75.38654	0.052395	0.164848	0.653157	0.905175	PHF13	148479	PHD finger protein 13
ENSG0000	132.0792	0.051186	0.150517	0.653082	0.905175	DSTYK	25778	dual serine/threonine and tyrosine protein kinase
ENSG0000	173.5504	-0.04609	0.185487	0.653161	0.905175	SAMD8	142891	sterile alpha motif domain containing 8
ENSG0000	172.765	0.049281	0.19039	0.65298	0.905175	SENP1	29843	SUMO specific peptidase 1
ENSG0000	8.215168	0.036681	0.218078	0.652999	0.905175	CCDC102B	79839	coiled-coil domain containing 102B
ENSG0000	13926.98	-0.05129	0.160346	0.653079	0.905175	IL17RA	23765	interleukin 17 receptor A
ENSG0000	12.55964	0.033314	0.220371	0.653287	0.905252	SLC22A14	9389	solute carrier family 22 member 14
ENSG0000	2476.507	-0.04597	0.194248	0.653315	0.905252	RESF1	55196	retroelement silencing factor 1
ENSG0000	37.03582	0.052061	0.181719	0.653394	0.905292	RXYLT1	10329	ribitol xylosyltransferase 1
ENSG0000	6.491341	-0.03383	0.222248	0.653445	0.905295	EFHB	151651	EF-hand domain family member B
ENSG0000	403.4256	0.0501	0.139619	0.653548	0.905301	ARL2BP	23568	ADP ribosylation factor like GTPase 2 binding protein
ENSG0000	2.799824	-0.01994	0.228371	0.653508	0.905301	NA	NA	NA
ENSG0000	11.76612	0.038161	0.216217	0.653614	0.905323	SNORD62B	692093	small nucl C/D box 62B
ENSG0000	123.3696	0.051779	0.175394	0.653669	0.905332	CDK6	1021	cyclin dependent kinase 6
ENSG0000	104.0319	0.05275	0.157161	0.653828	0.905346	ETFDH	2110	electron transfer flavoprotein dehydrogenase
ENSG0000	4.009005	0.026271	0.227676	0.653735	0.905346	FAM184A	79632	family with sequence similarity 184 member A
ENSG0000	68.46804	0.041838	0.206385	0.653827	0.905346	TRIQK	286144	triple QxxK/R motif containing
ENSG0000	632.1675	0.051426	0.162914	0.654043	0.905454	PIP4P2	55529	phosphati 5-bisphosphate 4-phosphatase 2
ENSG0000	248.9265	-0.04293	0.117603	0.654054	0.905454	CTR9	9646	CTR9 hom Paf1/RNA polymerase II complex component
ENSG0000	330.2348	-0.04752	0.131235	0.654053	0.905454	NDUFA12	55967	NADH:ubiquinone oxidoreductase subunit A12
ENSG0000	1733.552	-0.04807	0.139958	0.654128	0.905488	MYL12B	103910	myosin light chain 12B
ENSG0000	43.81991	-0.04604	0.19723	0.654242	0.905509	RECK	8434	reversion inducing cysteine rich protein with kazal motifs
ENSG0000	1244.277	-0.04939	0.142209	0.65422	0.905509	SIK3	23387	SIK family kinase 3
ENSG0000	73.25877	-0.04823	0.188042	0.654331	0.905561	CDKN2AIP	55602	CDKN2A interacting protein
ENSG0000	26.1545	-0.04425	0.203927	0.654428	0.905561	TUBE1	51175	tubulin epsilon 1
ENSG0000	3.586297	0.027199	0.22968	0.654382	0.905561	MFSD6L	162387	major facilitator superfamily domain containing 6 like
ENSG0000	120.9013	0.028557	0.222034	0.654492	0.905581	PDCD10	11235	programmed cell death 10
ENSG0000	726.3011	0.042751	0.108057	0.65557	0.905799	SDHB	6390	succinate dehydrogenase complex iron sulfur subunit B
ENSG0000	13.21056	0.032092	0.22097	0.655639	0.905799	NA	NA	NA
ENSG0000	1120.97	-0.06093	0.182436	0.65543	0.905799	NABP1	64859	nucleic acid binding protein 1
ENSG0000	129.3148	0.050798	0.181193	0.655097	0.905799	TMA7	51372	translation machinery associated 7 homolog
ENSG0000	110.9187	-0.05387	0.167404	0.655222	0.905799	THAP6	152815	THAP domain containing 6
ENSG0000	16.82358	0.032255	0.219899	0.655213	0.905799	CENPU	79682	centromere protein U
ENSG0000	206.4012	-0.05116	0.156683	0.65532	0.905799	NFYA	4800	nuclear transcription factor Y subunit alpha
ENSG0000	4.27267	-0.02035	0.228168	0.655605	0.905799	DLK2	65989	delta like non-canonical Notch ligand 2

ENSG0000	224.3556	-0.04855	0.132867	0.654812	0.905799	TTI2	80185	TELO2 interacting protein 2
ENSG0000	3515.066	0.038865	0.11554	0.654934	0.905799	IL10RA	3587	interleukin 10 receptor subunit alpha
ENSG0000	52.10901	0.045888	0.198007	0.655219	0.905799	MAP3K9	4293	mitogen-activated protein kinase kinase kinase 9
ENSG0000	240.726	-0.04638	0.118137	0.655278	0.905799	ABCD4	5826	ATP binding cassette subfamily D member 4
ENSG0000	11436.92	-0.04862	0.135599	0.654976	0.905799	TNFAIP2	7127	TNF alpha induced protein 2
ENSG0000	308.5347	0.041256	0.137059	0.654776	0.905799	GFOD2	81577	glucose-fructose oxidoreductase domain containing 2
ENSG0000	3.367696	-0.02632	0.228064	0.655543	0.905799	SCGB1C2	653486	secretoglobin family 1C member 2
ENSG0000	9.70588	0.031258	0.221855	0.655532	0.905799	NA	NA	NA
ENSG0000	2.382991	0.031326	0.230376	0.655423	0.905799	NA	NA	NA
ENSG0000	346.7935	0.050849	0.176195	0.654902	0.905799	CSE1L	1434	chromosome segregation 1 like
ENSG0000	13.20096	0.035812	0.213914	0.655272	0.905799	TMEM191	645426	transmembrane protein 191C
ENSG0000	186.453	0.051706	0.163154	0.654845	0.905799	AIFM1	9131	apoptosis inducing factor mitochondria associated 1
ENSG0000	201.4613	0.050942	0.145814	0.655771	0.905913	PSMD12	5718	proteasom non-ATPase 12
ENSG0000	339.2503	0.050309	0.17478	0.656106	0.905976	CREB1	1385	cAMP responsive element binding protein 1
ENSG0000	86.77004	-0.02684	0.223878	0.656113	0.905976	ZNF654	55279	zinc finger protein 654
ENSG0000	10.29565	-0.03915	0.214314	0.65588	0.905976	C8orf76	84933	chromosome 8 open reading frame 76
ENSG0000	167.8074	0.047893	0.127025	0.655979	0.905976	PRPF4	9128	pre-mRNA processing factor 4
ENSG0000	5.380448	0.028708	0.224314	0.656008	0.905976	NA	NA	NA
ENSG0000	68.76557	-0.0514	0.172907	0.656033	0.905976	NA	NA	NA
ENSG0000	209.5521	0.04891	0.18663	0.656358	0.906041	LGALSL	29094	galectin like
ENSG0000	1446.961	-0.0499	0.152749	0.656215	0.906041	MFSD10	10227	major facilitator superfamily domain containing 10
ENSG0000	105.6137	-0.05175	0.149169	0.656293	0.906041	TMEM107	84314	transmembrane protein 107
ENSG0000	2.756244	0.020918	0.230226	0.656347	0.906041	NA	NA	NA
ENSG0000	4.525777	0.038038	0.226238	0.656507	0.906095	DISP3	57540	dispatched RND transporter family member 3
ENSG0000	66.18998	0.048391	0.193302	0.656545	0.906095	CCNA2	890	cyclin A2
ENSG0000	3.422889	0.019329	0.229081	0.656536	0.906095	NA	NA	NA
ENSG0000	24.65254	-0.0434	0.204499	0.656603	0.906106	FAM111A-	1.02E+08	FAM111A divergent transcript
ENSG0000	290.4529	-0.04901	0.128448	0.656755	0.906248	TIRAP	114609	TIR domain containing adaptor protein
ENSG0000	4.562129	-0.02413	0.22678	0.656892	0.906368	MAP3K4-A	1.18E+08	MAP3K4 antisense RNA 1
ENSG0000	11.1525	0.039643	0.214115	0.656949	0.906378	NA	NA	NA
ENSG0000	26.8718	-0.05056	0.190132	0.657325	0.906829	C13orf46	1.01E+08	chromosome 13 open reading frame 46
ENSG0000	12.17794	0.029663	0.222193	0.657382	0.90684	NA	NA	NA
ENSG0000	9.186639	0.031913	0.221589	0.657484	0.906912	ZNF502	91392	zinc finger protein 502
ENSG0000	550.5666	0.046572	0.203451	0.657795	0.907159	SULT1B1	27284	sulfotransferase family 1B member 1
ENSG0000	4.584969	0.025097	0.227075	0.657818	0.907159	ARHGAP39	80728	Rho GTPase activating protein 39
ENSG0000	283.6139	-0.04729	0.129313	0.657772	0.907159	PYM1	84305	PYM homo exon junction complex associated factor
ENSG0000	305.5733	-0.04886	0.139553	0.657861	0.907159	INAFM2	1.01E+08	InaF motif containing 2
ENSG0000	46.34066	-0.04884	0.188381	0.658166	0.907238	DPH5	51611	diphthamide biosynthesis 5
ENSG0000	32.13056	-0.04677	0.196271	0.658155	0.907238	NA	NA	NA
ENSG0000	255.375	-0.04924	0.132164	0.658139	0.907238	NA	NA	NA
ENSG0000	106.5111	-0.0511	0.166959	0.658144	0.907238	ZNF827	152485	zinc finger protein 827
ENSG0000	24.42238	-0.04857	0.19364	0.658128	0.907238	MIR762HG	1.02E+08	MIR762 host gene
ENSG0000	9.521063	-0.03869	0.214247	0.659341	0.907349	NA	NA	NA
ENSG0000	8.640192	0.025553	0.224574	0.659163	0.907349	LOC10042	1E+08	FKBP prolyl isomerase 8 pseudogene
ENSG0000	7.325953	-0.03856	0.215245	0.659252	0.907349	RPL10P6	285176	ribosomal protein L10 pseudogene 6
ENSG0000	2.772277	0.02225	0.229647	0.658945	0.907349	TRIM61	391712	tripartite motif containing 61
ENSG0000	915.3039	-0.04981	0.181671	0.659256	0.907349	SRPK1	6732	SRSF protein kinase 1
ENSG0000	18.66746	-0.03091	0.22028	0.65865	0.907349	ENPP5	59084	ectonucleotide pyrophosphatase/phosphodiesterase family member 5
ENSG0000	1030.414	-0.04923	0.178866	0.65838	0.907349	MTPN	136319	myotrophin
ENSG0000	1629.819	0.043783	0.113686	0.659386	0.907349	SURF4	6836	surfeit 4
ENSG0000	86.02849	-0.05025	0.152909	0.658627	0.907349	BTRC	8945	beta-transducin repeat containing E3 ubiquitin protein ligase
ENSG0000	5.931278	0.034964	0.219858	0.659176	0.907349	LINC01619	256021	long intergenic non-protein coding RNA 1619
ENSG0000	14.56937	-0.0386	0.213549	0.659028	0.907349	POLE2	5427	DNA polyr accessory subunit
ENSG0000	195.7929	0.047086	0.129551	0.659139	0.907349	NEMF	9147	nuclear export mediator factor
ENSG0000	334.3536	0.054836	0.144976	0.658947	0.907349	MAPKBP1	23005	mitogen-activated protein kinase binding protein 1
ENSG0000	8.594948	-0.03383	0.219929	0.658485	0.907349	RRN3P2	653390	RRN3 pseudogene 2
ENSG0000	54.50226	-0.04337	0.203209	0.658479	0.907349	ARHGAP23	57636	Rho GTPase activating protein 23
ENSG0000	943.5878	-0.05054	0.156528	0.658528	0.907349	MED24	9862	mediator complex subunit 24
ENSG0000	291.1108	-0.05096	0.144549	0.658809	0.907349	PCYT2	5833	phosphate ethanolamine
ENSG0000	411.4043	0.051664	0.167007	0.658986	0.907349	HDGFL2	84717	HDGF like 2
ENSG0000	936.1891	-0.0508	0.151262	0.659295	0.907349	DNAJB1	3337	DnaJ heat shock protein family (Hsp40) member B1
ENSG0000	63.52605	0.050661	0.182175	0.659341	0.907349	LINC00662	148189	long intergenic non-protein coding RNA 662
ENSG0000	231.1642	-0.04575	0.122026	0.65896	0.907349	ZNF875	284459	zinc finger protein 875
ENSG0000	35.51284	-0.04489	0.201274	0.659006	0.907349	TSPY26P	128854	testis spec pseudogene
ENSG0000	147.2019	-0.04341	0.201787	0.658681	0.907349	UPB1	51733	beta-ureidopropionase 1
ENSG0000	150.8645	0.051395	0.170668	0.659562	0.907387	SNRNP27	11017	small nuclear ribonucleoprotein U4/U6.U5 subunit 27
ENSG0000	2.366588	0.024358	0.229857	0.659544	0.907387	PDE6B-AS1	1.02E+08	PDE6B antisense RNA 1
ENSG0000	9.547327	-0.02877	0.222938	0.659523	0.907387	NA	NA	NA
ENSG0000	297.5691	-0.04695	0.116393	0.659612	0.907388	ZNF506	440515	zinc finger protein 506
ENSG0000	321.7494	-0.05005	0.170256	0.659718	0.907459	RABGAP1L	9910	RAB GTPase activating protein 1 like
ENSG0000	11.75809	0.032531	0.220224	0.659763	0.907459	MICALL2-D	1.08E+08	MICALL2 divergent transcript
ENSG0000	23.46977	0.052081	0.188019	0.659817	0.907465	WDR5B-DT	1.03E+08	WDR5B divergent transcript
ENSG0000	12.70667	0.039453	0.212869	0.660028	0.907648	TIMD4	91937	T cell immunoglobulin and mucin domain containing 4
ENSG0000	3.135023	-0.02927	0.224967	0.660049	0.907648	TAX1BP3	30851	Tax1 binding protein 3
ENSG0000	116.9484	0.043708	0.202373	0.660248	0.907717	DCUN1D1	54165	defective in cullin neddylation 1 domain containing 1
ENSG0000	8.780336	0.033448	0.220433	0.66022	0.907717	PGBD1	84547	piggyBac transposable element derived 1
ENSG0000	23.05272	0.042841	0.208079	0.660178	0.907717	ZNF699	374879	zinc finger protein 699
ENSG0000	269.5512	-0.04428	0.116066	0.660311	0.907735	ZNF346	23567	zinc finger protein 346
ENSG0000	6.288007	-0.02062	0.226779	0.660869	0.907754	NA	NA	NA
ENSG0000	170.0307	-0.04713	0.190902	0.660682	0.907754	C3orf38	285237	chromosome 3 open reading frame 38
ENSG0000	834.7639	0.051579	0.160664	0.660557	0.907754	TBC1D14	57533	TBC1 domain family member 14
ENSG0000	186.1425	-0.05288	0.142206	0.660805	0.907754	FNTA	2339	farnesyltra CAAX box alpha
ENSG0000	174.657	0.046725	0.192278	0.660589	0.907754	PIK3C2A	5286	phosphatidylinositol-4-phosphate 3-kinase catalytic subunit type 2 alpha
ENSG0000	6845.791	-0.05003	0.16382	0.660768	0.907754	FURIN	5045	furin paired basic amino acid cleaving enzyme
ENSG0000	4.210354	-0.03184	0.222046	0.660631	0.907754	NA	NA	NA
ENSG0000	498.5812	0.041361	0.107616	0.660821	0.907754	AMFR	267	autocrine motility factor receptor
ENSG0000	17.78276	-0.04698	0.19844	0.660694	0.907754	SP2-DT	1.03E+08	SP2 divergent transcript
ENSG0000	63.53924	0.05138	0.15945	0.660459	0.907754	SLC2A11	66035	solute carrier family 2 member 11
ENSG0000	1215.419	0.043457	0.117532	0.660415	0.907754	CDK16	5127	cyclin dependent kinase 16
ENSG0000	57.24903	-0.0508	0.174105	0.660982	0.90784	SRP19	6728	signal recognition particle 19
ENSG0000	84.69463	0.053726	0.186569	0.661081	0.907858	RBPMS2	348093	RNA bindi mRNA processing factor 2
ENSG0000	396.1115	-0.04432	0.19773	0.661094	0.907858	ABCC3	8714	ATP binding cassette subfamily C member 3
ENSG0000	48.73779	0.051434	0.169876	0.661199	0.907934	SPNS2	124976	sphingolipid transporter 2
ENSG0000	165.7318	0.050212	0.172965	0.661252	0.907939	AK3	50808	adenylate kinase 3
ENSG0000	867.9772	0.050654	0.157677	0.661497	0.908207	GCN1	10985	GCN1 activator of EIF2AK4
ENSG0000	669.2907	0.050584	0.153564	0.661615	0.908302	RNF122	79845	ring finger protein 122

ENSG0000	24.15183	-0.03373	0.217163	0.662033	0.908739	CDC7	8317	cell division cycle 7
ENSG0000	20.63104	-0.04258	0.206495	0.661986	0.908739	MORC4	79710	MORC family CW-type zinc finger 4
ENSG0000	18.9543	-0.04409	0.205317	0.662087	0.908745	NA	NA	NA
ENSG0000	5591.213	-0.04966	0.166181	0.662499	0.90897	DENND3	22898	DENN domain containing 3
ENSG0000	287.0083	-0.04781	0.143515	0.662357	0.90897	VTI1B	10490	vesicle transport through interaction with t-SNAREs 1B
ENSG0000	11.8807	-0.03505	0.216444	0.662399	0.90897	NA	NA	NA
ENSG0000	93.83783	0.047315	0.191398	0.66247	0.90897	CCR10	2826	C-C motif chemokine receptor 10
ENSG0000	2.750085	0.018183	0.229558	0.662418	0.90897	NA	NA	NA
ENSG0000	253.138	-0.04653	0.134102	0.662584	0.909018	ZNF18	7566	zinc finger protein 18
ENSG0000	743.4425	0.049295	0.144465	0.662693	0.9091	MRPS2	51116	mitochondrial ribosomal protein S2
ENSG0000	93.77041	-0.04684	0.195869	0.663054	0.909221	EXOC8	149371	exocyst complex component 8
ENSG0000	89.827	-0.04997	0.150351	0.662906	0.909221	ABI2	10152	abl interactor 2
ENSG0000	9.485793	0.036741	0.216717	0.663129	0.909221	BBS12	166379	Bardet-Biedl syndrome 12
ENSG0000	155.1615	0.05001	0.17321	0.662859	0.909221	OSBPL3	26031	oxysterol binding protein like 3
ENSG0000	93.00495	-0.04633	0.190775	0.663075	0.909221	SLC22A17	51310	solute carrier family 22 member 17
ENSG0000	237.6255	-0.04908	0.179223	0.663116	0.909221	GALC	2581	galactosylceramidase
ENSG0000	5.080959	0.029203	0.224125	0.662946	0.909221	NA	NA	NA
ENSG0000	90.17406	0.048809	0.171205	0.663242	0.909309	IBTK	25998	inhibitor of Bruton tyrosine kinase
ENSG0000	240.1407	0.052855	0.183036	0.663398	0.909454	SLK	9748	STE20 like kinase
ENSG0000	2666.456	-0.05116	0.167489	0.663515	0.909546	DIAPH1	1729	diaphanous related formin 1
ENSG0000	53.34167	0.04767	0.190635	0.66366	0.909677	TCAIM	285343	T cell activ mitochondrial
ENSG0000	5.719469	0.026421	0.224954	0.663811	0.909816	LOC10537	1.05E+08	uncharacterized LOC105375743
ENSG0000	218.2465	-0.04749	0.186656	0.663996	0.910001	ALG13	79868	ALG13 UDP-N-acetylglucosaminyltransferase subunit
ENSG0000	145.0083	0.04899	0.142105	0.664082	0.910051	DHRS4	10901	dehydrogenase/reductase 4
ENSG0000	289.1929	-0.04838	0.176221	0.664317	0.910129	HIVEP3	59269	HIVEP zinc finger 3
ENSG0000	1069.317	-0.0518	0.161281	0.664268	0.910129	PXK	54899	PX domain containing serine/threonine kinase like
ENSG0000	1792.043	-0.05126	0.137354	0.664211	0.910129	IRF5	3663	interferon regulatory factor 5
ENSG0000	12930.54	-0.0472	0.13565	0.664337	0.910129	BIN2	51411	bridging integrator 2
ENSG0000	115.2628	-0.04937	0.165474	0.664461	0.91023	SRD5A1	6715	steroid 5 alpha-reductase 1
ENSG0000	116.57	-0.03779	0.20891	0.664518	0.91024	TMX1	81542	thioredoxin related transmembrane protein 1
ENSG0000	368.814	0.049041	0.140331	0.664605	0.910292	LARS1	51520	leucyl-tRNA synthetase 1
ENSG0000	106.4387	-0.04989	0.150666	0.664722	0.910383	C1orf56	54964	chromosome 1 open reading frame 56
ENSG0000	906.978	-0.0388	0.100301	0.664906	0.910567	CCT5	22948	chaperonin containing TCP1 subunit 5
ENSG0000	21.0765	-0.04747	0.194426	0.665285	0.910821	NA	NA	NA
ENSG0000	8.98677	-0.03494	0.218065	0.665258	0.910821	CAND2	23066	cullin associated and neddylation dissociated 2 (putative)
ENSG0000	5376.925	-0.04765	0.144579	0.665375	0.910821	DBNL	28988	drebrin like
ENSG0000	13.52459	0.043931	0.206413	0.66539	0.910821	SMIM35	1.01E+08	small integral membrane protein 35
ENSG0000	1729.937	-0.04805	0.146761	0.665209	0.910821	PDXK	8566	pyridoxal kinase
ENSG0000	679.3816	-0.05103	0.123865	0.665348	0.910821	ELK1	2002	ETS transcription factor ELK1
ENSG0000	105.7285	0.049221	0.181795	0.665464	0.910851	POC5	134359	POC5 centriolar protein
ENSG0000	44.76219	-0.04986	0.178486	0.665511	0.910851	CDC45	8318	cell division cycle 45
ENSG0000	3.108147	0.015143	0.230516	0.66568	0.910899	RNU4-82P	1.06E+08	RNA U4 small r pseudogene
ENSG0000	575.3641	0.046643	0.124874	0.665745	0.910899	THAP11	57215	THAP domain containing 11
ENSG0000	649.6816	0.047685	0.143919	0.665697	0.910899	DRG2	1819	developmentally regulated GTP binding protein 2
ENSG0000	3853.885	-0.04064	0.162935	0.665702	0.910899	SF3A2	8175	splicing factor 3a subunit 2
ENSG0000	5.339377	-0.02015	0.227521	0.665913	0.911061	MYLK4	340156	myosin light chain kinase family member 4
ENSG0000	639.1601	0.047788	0.139099	0.666033	0.911089	SAP130	79595	Sin3A associated protein 130
ENSG0000	6.888977	-0.03183	0.22086	0.666026	0.911089	CCND1	595	cyclin D1
ENSG0000	8.459897	-0.03386	0.218915	0.666212	0.911266	EPDR1	54749	ependymin related 1
ENSG0000	11.00914	0.037364	0.214662	0.66637	0.911277	NA	NA	NA
ENSG0000	7.865514	-0.03921	0.213972	0.666297	0.911277	TBC1D32	221322	TBC1 domain family member 32
ENSG0000	3328.783	-0.04477	0.123599	0.666369	0.911277	HNRNPK	3190	heterogeneous nuclear ribonucleoprotein K
ENSG0000	2.716093	0.013204	0.230675	0.666797	0.911288	NA	NA	NA
ENSG0000	982.7304	0.049306	0.171462	0.666715	0.911288	GMPPA	29926	GDP-mannose pyrophosphorylase A
ENSG0000	103.682	0.050225	0.162953	0.666939	0.911288	THUMPD3	440944	THUMPD3 antisense RNA 1
ENSG0000	7.919763	-0.02895	0.221116	0.666729	0.911288	MOCS2-D	257396	MOCS2 divergent transcript
ENSG0000	420.029	-0.04579	0.131724	0.666911	0.911288	SKP1	6500	S-phase kinase associated protein 1
ENSG0000	55.17252	0.042233	0.203969	0.666597	0.911288	KCTD9	54793	potassium channel tetramerization domain containing 9
ENSG0000	40.92381	-0.04966	0.179719	0.666531	0.911288	ERCC6L2-/	1E+08	ERCC6L2 antisense RNA 1
ENSG0000	1649.851	-0.0494	0.140463	0.666975	0.911288	RHOF	54509	ras homolo filopodia associated
ENSG0000	21.86858	-0.04843	0.191431	0.666585	0.911288	NA	NA	NA
ENSG0000	155.2863	0.047944	0.131462	0.666669	0.911288	MED26	9441	mediator complex subunit 26
ENSG0000	56.41323	-0.04989	0.170273	0.666494	0.911288	NDUFA1	4694	NADH:ubiquinone oxidoreductase subunit A1
ENSG0000	7.763466	0.030047	0.222151	0.666882	0.911288	NA	NA	NA
ENSG0000	2090.103	0.049933	0.165178	0.667145	0.911453	EIF4G1	1981	eukaryotic translation initiation factor 4 gamma 1
ENSG0000	2.875267	-0.01104	0.232022	0.667207	0.911469	APLP1	333	amyloid beta precursor like protein 1
ENSG0000	39.80973	0.044405	0.200168	0.667503	0.911712	ZMYM6	9204	zinc finger MYM-type containing 6
ENSG0000	79.48812	-0.04977	0.171079	0.667584	0.911712	C3orf18	51161	chromosome 3 open reading frame 18
ENSG0000	177.5847	-0.05011	0.147804	0.667509	0.911712	TBP	6908	TATA-box binding protein
ENSG0000	329.73	0.045376	0.125066	0.667537	0.911712	SURF1	6834	SURF1 cytochrome c oxidase assembly factor
ENSG0000	394.6022	-0.04438	0.119642	0.667996	0.9118	SMIM12	113444	small integral membrane protein 12
ENSG0000	248.1344	0.047407	0.131373	0.667766	0.9118	PPCS	79717	phosphopantothenoylcysteine synthetase
ENSG0000	13.71717	-0.03696	0.213931	0.66795	0.9118	LAMB3	3914	laminin subunit beta 3
ENSG0000	6.504256	-0.03589	0.217332	0.66793	0.9118	NA	NA	NA
ENSG0000	427.1629	-0.04904	0.157843	0.667728	0.9118	NA	NA	NA
ENSG0000	3728.995	0.048534	0.144169	0.6678	0.9118	LRCH4	4034	leucine rich repeats and calponin homology domain containing 4
ENSG0000	3.541211	-0.02684	0.225112	0.667891	0.9118	PTENP1	11191	phosphatase and tensin homolog pseudogene 1
ENSG0000	3.671333	0.020444	0.227714	0.66835	0.911801	NA	NA	NA
ENSG0000	490.6073	0.044328	0.156165	0.668402	0.911801	ERICH1	157697	glutamate rich 1
ENSG0000	89.16333	-0.04826	0.181343	0.668395	0.911801	BLOC1S2	282991	biogenesis of lysosomal organelles complex 1 subunit 2
ENSG0000	838.959	-0.04909	0.168647	0.668379	0.911801	RAB6A	5870	RAB6A member RAS oncogene family
ENSG0000	27.86983	-0.03957	0.208561	0.668436	0.911801	NA	NA	NA
ENSG0000	82.15403	0.0316	0.216233	0.66841	0.911801	IGHV3-43	28426	immunoglobulin heavy variable 3-43
ENSG0000	57.18791	0.048973	0.178334	0.668142	0.911801	EIF3J-DT	645212	EIF3J divergent transcript
ENSG0000	438.7404	0.048337	0.151388	0.668445	0.911801	COG1	9382	component of oligomeric golgi complex 1
ENSG0000	291.0552	0.049524	0.166707	0.668423	0.911801	PTPN2	5771	protein tyrosine phosphatase non-receptor type 2
ENSG0000	3.961885	0.024991	0.225713	0.66853	0.911848	NA	NA	NA
ENSG0000	2.69504	-0.01965	0.228171	0.668628	0.911915	SOX30	11063	SRY-box transcription factor 30
ENSG0000	3.397759	0.02036	0.229406	0.668713	0.91192	LRRC39	127495	leucine rich repeat containing 39
ENSG0000	383.0739	-0.04898	0.156592	0.668736	0.91192	HSPD1	3329	heat shock protein family D (Hsp60) member 1
ENSG0000	206.1102	-0.04425	0.193406	0.668799	0.91192	RASA2	5922	RAS p21 protein activator 2
ENSG0000	92.168	0.047002	0.192714	0.668831	0.91192	RFX7	64864	regulatory factor X7
ENSG0000	42.23499	-0.04692	0.183096	0.669039	0.912111	BICD1	636	BICD cargo adaptor 1
ENSG0000	681.5464	-0.03432	0.111684	0.669071	0.912111	SNAP29	9342	synaptosome associated protein 29
ENSG0000	99.04982	-0.04866	0.179987	0.669209	0.912164	TUBB2A	7280	tubulin beta 2A class IIa
ENSG0000	137.6429	-0.04918	0.177745	0.669201	0.912164	USP47	55031	ubiquitin specific peptidase 47

ENSG0000	360.0501	-0.04603	0.190507	0.669459	0.912395	RICTOR	253260	RPTOR independent companion of MTOR complex 2
ENSG0000	125.5101	-0.04658	0.168189	0.669478	0.912395	CDK14	5218	cyclin dependent kinase 14
ENSG0000	42.19167	0.04673	0.191848	0.669611	0.91244	CYB561D2	11068	cytochrome b561 family member D2
ENSG0000	218.5056	-0.04957	0.17306	0.669574	0.91244	TRBV29-1	28558	T cell receptor beta variable 29-1
ENSG0000	16.43787	0.042341	0.20538	0.669773	0.912594	NA	NA	NA
ENSG0000	7.422455	-0.03656	0.215956	0.669868	0.912655	NA	NA	NA
ENSG0000	27.45908	0.046847	0.192924	0.670118	0.912845	DHFR2	200895	dihydrofolate reductase 2
ENSG0000	3.704693	0.022734	0.227116	0.67011	0.912845	NA	NA	NA
ENSG0000	1276.745	0.051746	0.134069	0.670157	0.912845	ARPC3	10094	actin related protein 2/3 complex subunit 3
ENSG0000	9.998874	0.039598	0.209653	0.670314	0.912855	CAMSAP2	23271	calmodulin regulated spectrin associated protein family member 2
ENSG0000	4.355366	-0.02599	0.225009	0.670225	0.912855	LINC00639	283547	long intergenic non-protein coding RNA 639
ENSG0000	11.35289	0.036082	0.217792	0.670282	0.912855	NA	NA	NA
ENSG0000	1943.95	0.048424	0.166531	0.671844	0.912933	PLOD1	5351	procollage 2-oxoglutarate 5-dioxygenase 1
ENSG0000	225.8708	-0.04797	0.158367	0.672258	0.912933	FUCA1	2517	alpha-L-fucosidase 1
ENSG0000	2.516992	0.016838	0.230002	0.67249	0.912933	PTGFR	5737	prostaglandin F receptor
ENSG0000	737.4296	0.047634	0.163767	0.670932	0.912933	SORT1	6272	sortilin 1
ENSG0000	2.600604	0.021617	0.232246	0.672797	0.912933	NA	NA	NA
ENSG0000	647.0631	0.045907	0.130244	0.670668	0.912933	HADHB	3032	hydroxyacyl-CoA dehydrogenase trifunctional multienzyme complex subunit beta
ENSG0000	6.40278	0.031241	0.22204	0.670738	0.912933	C2orf81	388963	chromosome 2 open reading frame 81
ENSG0000	96.45435	0.048779	0.161808	0.672898	0.912933	AMMECR1	83607	AMMECR1 like
ENSG0000	3661.436	-0.05034	0.143117	0.672502	0.912933	CXCR4	7852	C-X-C motif chemokine receptor 4
ENSG0000	43.61017	-0.04765	0.184679	0.670876	0.912933	RBM45	129831	RNA binding motif protein 45
ENSG0000	127.6857	-0.06025	0.168052	0.673103	0.912933	PLXNA1	5361	plexin A1
ENSG0000	3.619384	0.022806	0.226853	0.672248	0.912933	NA	NA	NA
ENSG0000	188.1432	-0.04789	0.143891	0.672106	0.912933	ZFYVE28	57732	zinc finger FYVE-type containing 28
ENSG0000	8.662438	-0.03606	0.215542	0.672559	0.912933	NA	NA	NA
ENSG0000	162.6098	0.046233	0.184117	0.671665	0.912933	SKIC3	9652	SKI3 subunit of superkiller complex
ENSG0000	9.9331	-0.03004	0.220078	0.672302	0.912933	WWC1	23286	WW and C2 domain containing 1
ENSG0000	5144.798	-0.04811	0.14073	0.673103	0.912933	TUBB	203068	tubulin beta class I
ENSG0000	15.12212	-0.03614	0.213544	0.673026	0.912933	USP45	85015	ubiquitin specific peptidase 45
ENSG0000	12.57554	-0.02857	0.220307	0.672222	0.912933	NA	NA	NA
ENSG0000	250.1066	-0.04267	0.196447	0.673212	0.912933	SNX10	29887	sorting nexin 10
ENSG0000	752.6988	0.045603	0.164752	0.670841	0.912933	POLD2	5425	DNA polyr accessory subunit
ENSG0000	797.2618	0.046362	0.173193	0.672388	0.912933	PLOD3	8985	procollage 2-oxoglutarate 5-dioxygenase 3
ENSG0000	65.04276	0.047045	0.191029	0.671353	0.912933	THAP5	168451	THAP domain containing 5
ENSG0000	4.002758	0.019174	0.229002	0.672591	0.912933	NA	NA	NA
ENSG0000	2.979928	0.017239	0.229558	0.671415	0.912933	NXNL2	158046	nucleoredoxin like 2
ENSG0000	365.13	0.042217	0.113734	0.671099	0.912933	TSC1	7248	TSC complex subunit 1
ENSG0000	3.598201	-0.02123	0.227431	0.672202	0.912933	NA	NA	NA
ENSG0000	20.05578	-0.03774	0.211185	0.671272	0.912933	INTS6-AS1	1.01E+08	INTS6 antisense RNA 1
ENSG0000	35.46703	-0.04959	0.187002	0.671039	0.912933	NA	NA	NA
ENSG0000	801.8974	-0.04807	0.151934	0.672771	0.912933	RABGGTA	5875	Rab geranylgeranyltransferase subunit alpha
ENSG0000	429.2703	-0.0469	0.139434	0.671032	0.912933	SYNE2	23224	spectrin repeat containing nuclear envelope protein 2
ENSG0000	243.0085	-0.04335	0.19565	0.672978	0.912933	CHURC1	91612	churchill domain containing 1
ENSG0000	27.71797	-0.04393	0.19869	0.67291	0.912933	DRAIC	145837	downregu inhibitor of cell invasion and migration
ENSG0000	2.661796	0.023616	0.227267	0.671465	0.912933	UBE2Q2P1	388165	UBE2Q2 pseudogene 1
ENSG0000	41.9528	0.048765	0.179158	0.673201	0.912933	FAM174B	400451	family with sequence similarity 174 member B
ENSG0000	5.878083	-0.02206	0.229732	0.672994	0.912933	TUBB8P7	197331	tubulin beta 8 class VIII pseudogene 7
ENSG0000	12570.77	-0.04815	0.158331	0.6723	0.912933	ATP2A3	489	ATPase sarcoplasmic/endoplasmic reticulum Ca2+ transporting 3
ENSG0000	28.06518	0.046981	0.189729	0.672876	0.912933	DUSP14	11072	dual specificity phosphatase 14
ENSG0000	8.567655	0.036936	0.215079	0.672374	0.912933	NA	NA	NA
ENSG0000	181.2689	-0.04735	0.146301	0.673167	0.912933	STRADA	92335	STE20 related adaptor alpha
ENSG0000	128.8866	0.045957	0.187564	0.671081	0.912933	CEP131	22994	centrosomal protein 131
ENSG0000	3.109897	-0.02354	0.225863	0.671068	0.912933	MALT1-AS	1.02E+08	MALT1 antisense RNA 1
ENSG0000	27.26604	-0.04426	0.199975	0.672887	0.912933	ZNF554	115196	zinc finger protein 554
ENSG0000	1872.725	0.047271	0.1395	0.671784	0.912933	COLGALT1	79709	collagen beta(1-O)galactosyltransferase 1
ENSG0000	14.70991	0.032351	0.218297	0.672681	0.912933	ZNF569	148266	zinc finger protein 569
ENSG0000	4.709004	-0.02067	0.22737	0.673036	0.912933	SPTBN4	57731	spectrin b non-erythrocytic 4
ENSG0000	704.6228	0.04751	0.139235	0.670641	0.912933	SPHK2	56848	sphingosine kinase 2
ENSG0000	2.566785	-0.00715	0.232243	0.672093	0.912933	TULP2	7288	TUB like protein 2
ENSG0000	117.7378	0.046645	0.136953	0.672267	0.912933	FASTKD5	60493	FAST kinase domains 5
ENSG0000	9.841953	0.034286	0.218889	0.672343	0.912933	SRMS	6725	src-related kinase lacking C-terminal regulatory tyrosine and N-terminal myristylation sites
ENSG0000	134.5593	0.048043	0.143978	0.671259	0.912933	TIAM1	7074	TIAM Rac1 associated GEF 1
ENSG0000	101.9788	0.041948	0.198745	0.672528	0.912933	PAXBP1	94104	PAX3 and PAX7 binding protein 1
ENSG0000	470.2163	-0.05107	0.175107	0.672808	0.912933	DYRK1A	1859	dual specificity tyrosine phosphorylation regulated kinase 1A
ENSG0000	3.119718	0.028762	0.230847	0.672049	0.912933	NA	NA	NA
ENSG0000	3.364821	0.015409	0.230795	0.671704	0.912933	GAL3ST1	9514	galactose-3-O-sulfotransferase 1
ENSG0000	217.6954	-0.04865	0.173556	0.67239	0.912933	TAB3	257397	TGF-beta activated kinase 1 (MAP3K7) binding protein 3
ENSG0000	114.0381	-0.04789	0.184643	0.671794	0.912933	ATP7A	538	ATPase copper transporting alpha
ENSG0000	4.799185	0.030106	0.223285	0.673312	0.913001	NA	NA	NA
ENSG0000	4.978927	0.025596	0.225031	0.673442	0.91311	NA	NA	NA
ENSG0000	4.747163	0.031954	0.221717	0.673803	0.913114	CTH	1491	cystathionine gamma-lyase
ENSG0000	5.021665	0.023853	0.225912	0.673894	0.913114	NA	NA	NA
ENSG0000	23.16073	-0.04631	0.197765	0.673598	0.913114	EPHA4	2043	EPH receptor A4
ENSG0000	34.2551	0.047996	0.185195	0.673694	0.913114	SMIM20	389203	small integral membrane protein 20
ENSG0000	66.00552	0.04923	0.162417	0.673681	0.913114	SERAC1	84947	serine active site containing 1
ENSG0000	916.3354	-0.0494	0.153371	0.673877	0.913114	CPT1A	1374	carnitine palmitoyltransferase 1A
ENSG0000	942.3444	0.035761	0.144824	0.673751	0.913114	NA	NA	NA
ENSG0000	67.16318	0.048276	0.166407	0.673566	0.913114	TTLL1	25809	TTL family tubulin polyglutamylase complex subunit L1
ENSG0000	94.70718	0.050371	0.149417	0.673658	0.913114	EBP	10682	EBP cholestenol delta-isomerase
ENSG0000	603.5338	-0.04856	0.161702	0.674757	0.913146	LEPROT	54741	leptin receptor overlapping transcript
ENSG0000	5.935065	-0.02769	0.223914	0.674028	0.913146	NA	NA	NA
ENSG0000	89.3492	-0.04765	0.148852	0.674297	0.913146	MTLN	205251	mitoregulin
ENSG0000	11.62807	0.038737	0.211472	0.675606	0.913146	RN7SL32P	1.06E+08	RNA 7SL cytoplasm pseudogene
ENSG0000	655.093	-0.04778	0.147927	0.674416	0.913146	TMEM43	79188	transmembrane protein 43
ENSG0000	131.9391	0.045856	0.132718	0.674534	0.913146	FIP1L1	81608	factor interacting with PAPOLA and CPSF1
ENSG0000	55.60431	0.047985	0.180369	0.675728	0.913146	LARP1B	55132	La ribonucleoprotein 1B
ENSG0000	14.24477	-0.03954	0.209515	0.675117	0.913146	CCNI2	645121	cyclin I family member 2
ENSG0000	9.882849	-0.03247	0.216784	0.675857	0.913146	NA	NA	NA
ENSG0000	27.33792	0.046093	0.196753	0.675596	0.913146	NA	NA	NA
ENSG0000	11.53586	0.028371	0.223557	0.6757	0.913146	NA	NA	NA
ENSG0000	129.8014	-0.04778	0.149429	0.675351	0.913146	SLC17A5	26503	solute carrier family 17 member 5
ENSG0000	154.6154	0.043365	0.196815	0.676027	0.913146	RWDD1	51389	RWD domain containing 1
ENSG0000	6.645277	0.032871	0.221198	0.675493	0.913146	NA	NA	NA
ENSG0000	9.140442	0.023511	0.225346	0.675456	0.913146	CHN2	1124	chimerin 2
ENSG0000	73.07093	-0.04252	0.196962	0.676061	0.913146	DPY19L1	23333	dpy-19 like C-mannosyltransferase 1

ENSG0000	26.16856	-0.04441	0.197	0.674151	0.913146	ABCB1	5243	ATP binding cassette subfamily B member 1
ENSG0000	12.96576	0.039778	0.209874	0.674562	0.913146	ATP6V1FN	1E+08	ATP6V1F neighbor
ENSG0000	287.3124	0.047609	0.168473	0.674354	0.913146	PPP3CC	5533	protein phosphatase 3 catalytic subunit gamma
ENSG0000	2075.803	-0.04316	0.121293	0.674675	0.913146	PCSK7	9159	proprotein convertase subtilisin/kexin type 7
ENSG0000	527.7333	-0.0541	0.136685	0.675514	0.913146	KDM5A	5927	lysine demethylase 5A
ENSG0000	10.86573	0.025307	0.225009	0.675668	0.913146	KLRC1	3821	killer cell lectin like receptor C1
ENSG0000	2.39188	0.023978	0.230608	0.675908	0.913146	NA	NA	NA
ENSG0000	11.55702	0.034929	0.216235	0.674913	0.913146	ERI2	112479	ERI1 exoribonuclease family member 2
ENSG0000	2.709909	-0.01786	0.229139	0.675592	0.913146	NA	NA	NA
ENSG0000	135.3109	-0.04891	0.171112	0.674914	0.913146	CNEP1R1	255919	CTD nuclear envelope phosphatase 1 regulatory subunit 1
ENSG0000	41.40044	0.047454	0.187482	0.675627	0.913146	CLEC18A	348174	C-type lectin domain family 18 member A
ENSG0000	39.5418	-0.04609	0.190991	0.675091	0.913146	SRR	63826	serine racemase
ENSG0000	798.5496	-0.04047	0.106274	0.675012	0.913146	PAFAH1B1	5048	platelet activating factor acetylhydrolase 1b regulatory subunit 1
ENSG0000	388.5263	0.04544	0.128988	0.676043	0.913146	HEXIM1	10614	HEXIM P-TEFb complex subunit 1
ENSG0000	185.753	0.048362	0.158926	0.675457	0.913146	NAT9	26151	N-acetyltransferase 9 (putative)
ENSG0000	9.378315	-0.02669	0.222939	0.674539	0.913146	MTCO2P2	1.01E+08	MT-CO2 pseudogene 2
ENSG0000	152.8042	0.045811	0.132386	0.676001	0.913146	VMAC	400673	vimentin type intermediate filament associated coiled-coil protein
ENSG0000	312.3712	-0.04682	0.141663	0.675672	0.913146	UBL5	59286	ubiquitin like 5
ENSG0000	3.352772	-0.02642	0.225646	0.675353	0.913146	LOC72848	728485	uncharacterized LOC728485
ENSG0000	3.641934	-0.01463	0.228295	0.674511	0.913146	MIR499B	1.01E+08	microRNA 499b
ENSG0000	227.5435	-0.04816	0.16111	0.674901	0.913146	CD40	958	CD40 molecule
ENSG0000	2.59046	0.020493	0.228125	0.675017	0.913146	NA	NA	NA
ENSG0000	4.530553	-0.02413	0.226054	0.676013	0.913146	RN7SL385	1.06E+08	RNA 7SL cytoplasm pseudogene
ENSG0000	84.0578	0.048931	0.160587	0.675584	0.913146	RAB9A	9367	RAB9A member RAS oncogene family
ENSG0000	5.814595	-0.02939	0.22234	0.674448	0.913146	NAP1L3	4675	nucleosome assembly protein 1 like 3
ENSG0000	76.95051	-0.04942	0.158221	0.675654	0.913146	SLC25A53	401612	solute carrier family 25 member 53
ENSG0000	1776.03	-0.03735	0.149623	0.675361	0.913146	BCAP31	10134	B cell receptor associated protein 31
ENSG0000	453.2961	0.041914	0.11816	0.676966	0.913169	MRPL20	55052	mitochondrial ribosomal protein L20
ENSG0000	2.96088	-0.02233	0.229096	0.676968	0.913169	RPL7AP16	1E+08	ribosomal protein L7a pseudogene 16
ENSG0000	427.5313	0.044394	0.127679	0.676973	0.913169	HARS1	3035	histidyl-tRNA synthetase 1
ENSG0000	6027.614	-0.03695	0.098017	0.67642	0.913169	BRD2	6046	bromodomain containing 2
ENSG0000	7.066645	0.026306	0.223153	0.676818	0.913169	NA	NA	NA
ENSG0000	4.168544	-0.02304	0.228106	0.676228	0.913169	KCNA6	3742	potassium voltage-gated channel subfamily A member 6
ENSG0000	2.868805	-0.01543	0.230959	0.676516	0.913169	ANO2	57101	anoctamin 2
ENSG0000	2.651587	0.023862	0.227034	0.676976	0.913169	C12orf60	144608	chromosome 12 open reading frame 60
ENSG0000	29.55691	0.047822	0.185055	0.67634	0.913169	ATP23	91419	ATP23 metallopeptidase and ATP synthase assembly factor homolog
ENSG0000	6.282518	-0.02947	0.222144	0.67631	0.913169	NA	NA	NA
ENSG0000	331.3972	-0.04572	0.135672	0.676555	0.913169	PSMC1	5700	proteasom ATPase 1
ENSG0000	75.19594	-0.04517	0.161195	0.676237	0.913169	LCMT2	9836	leucine carboxyl methyltransferase 2
ENSG0000	8.311236	-0.03476	0.218786	0.676464	0.913169	RNF112	7732	ring finger protein 112
ENSG0000	231.4225	0.04887	0.177076	0.676803	0.913169	SLFN11	91607	schlafen family member 11
ENSG0000	9.38644	-0.02862	0.221392	0.676951	0.913169	MIR548AA	1.01E+08	microRNA 548aa-2
ENSG0000	12.34698	-0.03323	0.216617	0.676784	0.913169	NA	NA	NA
ENSG0000	5.76392	0.032288	0.220133	0.67674	0.913169	NA	NA	NA
ENSG0000	33.32293	-0.04034	0.205455	0.676936	0.913169	DYNLT3	6990	dynein light chain Tctex-type 3
ENSG0000	4.60921	-0.0213	0.22713	0.677231	0.913389	SLC35F3	148641	solute carrier family 35 member F3
ENSG0000	2.794628	0.019573	0.228256	0.677288	0.913389	MYG1	60314	MYG1 exonuclease
ENSG0000	133.2075	-0.04429	0.190493	0.677277	0.913389	P2RY10	27334	P2Y receptor family member 10
ENSG0000	870.9051	0.044322	0.122273	0.67769	0.913433	TPR	7175	translocat nuclear basket protein
ENSG0000	1221.601	0.048065	0.164704	0.677666	0.913433	CYP27A1	1593	cytochrome P450 family 27 subfamily A member 1
ENSG0000	50.85367	-0.04501	0.191605	0.677418	0.913433	SHQ1	55164	SHQ1 H/ACA ribonucleoprotein assembly factor
ENSG0000	312.3817	-0.04147	0.115745	0.677711	0.913433	COMMD5	28991	COMM domain containing 5
ENSG0000	71.76313	0.041861	0.200299	0.677463	0.913433	TMEM41B	440026	transmembrane protein 41B
ENSG0000	8.865295	0.033861	0.217766	0.677561	0.913433	NA	NA	NA
ENSG0000	10.57574	-0.03718	0.212912	0.677719	0.913433	ZNF222	7673	zinc finger protein 222
ENSG0000	4.396911	0.021242	0.23077	0.677644	0.913433	NA	NA	NA
ENSG0000	77.80611	0.0458	0.172178	0.678333	0.913489	BCAS2	10286	BCAS2 pre-mRNA processing factor
ENSG0000	3.711131	0.019621	0.22761	0.678162	0.913489	MRNIP-DT	1.01E+08	MRNIP divergent transcript
ENSG0000	168.9016	-0.0256	0.222341	0.678034	0.913489	RNA5SP20	1.01E+08	RNA 5S ribosomal pseudogene 207
ENSG0000	136.3968	0.0368	0.210217	0.678235	0.913489	PCMTD1	115294	protein-L-isoaspartate (D-aspartate) O-methyltransferase domain containing 1
ENSG0000	2.801022	-0.01097	0.229555	0.678362	0.913489	STAM-DT	1.03E+08	STAM divergent transcript
ENSG0000	18.44667	0.034615	0.215168	0.678018	0.913489	RNLS	55328	renalase FAD dependent amine oxidase
ENSG0000	159.7007	-0.04263	0.193621	0.678262	0.913489	JKAMP	51528	JNK1/MAPK8 associated membrane protein
ENSG0000	4.994997	0.023173	0.226653	0.678218	0.913489	MTA1-DT	1.01E+08	MTA1 divergent transcript
ENSG0000	972.887	-0.04292	0.122735	0.678409	0.913489	CHD2	1106	chromodomain helicase DNA binding protein 2
ENSG0000	222.2013	0.048564	0.159835	0.678247	0.913489	ASGR1	432	asialoglycoprotein receptor 1
ENSG0000	59.62537	0.047854	0.172883	0.677946	0.913489	KRBA2	124751	KRAB-A domain containing 2
ENSG0000	65.3818	0.047633	0.17946	0.678259	0.913489	NA	NA	NA
ENSG0000	4079.589	0.044057	0.126212	0.678181	0.913489	JUND	3727	JunD prot AP-1 transcription factor subunit
ENSG0000	68.49654	-0.04727	0.151362	0.679006	0.913545	C1orf216	127703	chromosome 1 open reading frame 216
ENSG0000	2.325541	0.013419	0.230441	0.678963	0.913545	NA	NA	NA
ENSG0000	26.31113	0.03572	0.213004	0.679042	0.913545	GRAMD2B	65983	GRAM domain containing 2B
ENSG0000	145.7723	0.039401	0.206043	0.67905	0.913545	RRM2B	50484	ribonucleotide reductase regulatory TP53 inducible subunit M2B
ENSG0000	5.393505	-0.02296	0.229237	0.679015	0.913545	NEBL	10529	nebulette
ENSG0000	70.83901	0.050079	0.18128	0.678905	0.913545	PRDM10	56980	PR/SET domain 10
ENSG0000	2.586649	0.016894	0.232745	0.678939	0.913545	FAIM2	23017	Fas apoptotic inhibitory molecule 2
ENSG0000	2.733824	-0.01027	0.231235	0.678833	0.913545	CELA1	1990	chymotrypsin like elastase 1
ENSG0000	8.91934	0.020092	0.22661	0.678611	0.913545	KCNH4	23415	potassium voltage-gated channel subfamily H member 4
ENSG0000	147.6103	0.048001	0.171949	0.678696	0.913545	NOL11	25926	nucleolar protein 11
ENSG0000	794.6376	-0.04811	0.143482	0.678772	0.913545	SCAF4	57466	SR-related CTD associated factor 4
ENSG0000	526.8455	-0.04692	0.132793	0.678893	0.913545	NBDY	550643	negative regulator of P-body association
ENSG0000	18.92616	-0.03991	0.207087	0.679288	0.913664	GRK4	2868	G protein-coupled receptor kinase 4
ENSG0000	296.5425	0.040362	0.185029	0.679224	0.913664	DEK	7913	DEK proto-oncogene
ENSG0000	206.606	-0.04757	0.160629	0.679288	0.913664	SNRPB2	6629	small nuclear ribonucleoprotein polypeptide B2
ENSG0000	170.137	-0.04777	0.174682	0.679473	0.913693	HNRNPLL	92906	heterogeneous nuclear ribonucleoprotein L like
ENSG0000	143.6468	-0.04281	0.140852	0.67957	0.913693	ZC3H6	376940	zinc finger CCCH-type containing 6
ENSG0000	425.4213	0.048662	0.147015	0.679708	0.913693	CUL3	8452	cullin 3
ENSG0000	253.8189	0.046178	0.176285	0.679605	0.913693	U2SURP	23350	U2 snRNP associated SURP domain containing
ENSG0000	11.79101	-0.02243	0.225231	0.679656	0.913693	NA	NA	NA
ENSG0000	2.820073	0.012011	0.231688	0.679585	0.913693	NA	NA	NA
ENSG0000	226.4647	0.043419	0.12122	0.679399	0.913693	TRPT1	83707	tRNA phosphotransferase 1
ENSG0000	274.8422	-0.04353	0.138797	0.679677	0.913693	MTMR1	8776	myotubularin related protein 1
ENSG0000	4.89692	0.014329	0.229744	0.679774	0.913715	NA	NA	NA
ENSG0000	4.700801	0.02471	0.225799	0.679934	0.913717	FOXD4L1	200350	forkhead box D4 like 1
ENSG0000	26.53031	0.045587	0.188423	0.679927	0.913717	FAM86HP	729375	family witl member A pseudogene
ENSG0000	12.88805	-0.03394	0.21596	0.679891	0.913717	ADAMTS7	390660	ADAMTS7 pseudogene 1

ENSG0000	2.576839	0.013134	0.230532	0.680006	0.913717	MIR6865	1.02E+08	microRNA 6865
ENSG0000	500.8933	0.045402	0.133997	0.680025	0.913717	OPA3	80207	outer mitochondrial membrane lipid metabolism regulator OPA3
ENSG0000	17.98559	0.039149	0.208037	0.680099	0.913723	NA	NA	NA
ENSG0000	7.334606	0.032695	0.218538	0.68013	0.913723	NA	NA	NA
ENSG0000	132.5867	0.047846	0.161299	0.68026	0.913832	ZBTB25	7597	zinc finger and BTB domain containing 25
ENSG0000	10.32781	-0.02651	0.226029	0.68038	0.913926	NUP210L	91181	nucleoporin 210 like
ENSG0000	159.3702	0.048099	0.170722	0.680531	0.914061	MTND1P2	1.01E+08	MT-ND1 pseudogene 23
ENSG0000	612.868	0.042759	0.123149	0.68059	0.914073	TIAL1	7073	TIA1 cytotoxic granule associated RNA binding protein like 1
ENSG0000	106.3689	0.042676	0.199158	0.680787	0.914271	STYX	6815	serine/threonine/tyrosine interacting protein
ENSG0000	9.578219	0.032495	0.218605	0.681026	0.914526	FKBP1C	642489	FKBP prolyl isomerase family member 1C
ENSG0000	752.478	0.043662	0.124487	0.681206	0.9147	LUC7L3	51747	LUC7 like 3 pre-mRNA splicing factor
ENSG0000	384.9348	0.037857	0.205079	0.681454	0.914952	PPP1CB	5500	protein phosphatase 1 catalytic subunit beta
ENSG0000	10923.1	-0.04669	0.164703	0.681494	0.914952	CEBPD	1052	CCAAT enhancer binding protein delta
ENSG0000	4.766415	0.02391	0.224916	0.681613	0.915045	NA	NA	NA
ENSG0000	11.61885	0.031878	0.218406	0.681665	0.915048	SPX	80763	spexin hormone
ENSG0000	845.865	0.045772	0.156027	0.68192	0.915164	MIB2	142678	MIB E3 ubiquitin protein ligase 2
ENSG0000	4423.927	-0.04455	0.176729	0.681956	0.915164	NFKB2	4791	nuclear factor kappa B subunit 2
ENSG0000	11.0894	-0.03316	0.216992	0.682043	0.915164	NA	NA	NA
ENSG0000	67.1398	-0.04686	0.172463	0.682051	0.915164	TBX6	6911	T-box transcription factor 6
ENSG0000	5.786049	0.029632	0.220923	0.681884	0.915164	SLC47A1	55244	solute carrier family 47 member 1
ENSG0000	8.917013	0.03632	0.214224	0.681839	0.915164	WTIP	126374	WT1 interacting protein
ENSG0000	5.908095	0.024394	0.225071	0.682118	0.915187	DLGAP4-A	1.02E+08	DLGAP4 antisense RNA 1
ENSG0000	1044.744	0.046061	0.171518	0.682178	0.915199	EFTUD2	9343	elongation factor Tu GTP binding domain containing 2
ENSG0000	218.7605	-0.04717	0.169958	0.68223	0.915202	CCNT1	904	cyclin T1
ENSG0000	3.104486	0.030473	0.227957	0.68228	0.915203	MMP1	4312	matrix metallopeptidase 1
ENSG0000	474.4579	-0.04198	0.126149	0.682371	0.915258	UBL4A	8266	ubiquitin like 4A
ENSG0000	5.657851	-0.0331	0.218273	0.682472	0.915262	TUBA4B	80086	tubulin alpha 4b
ENSG0000	97.85672	-0.0454	0.182787	0.682575	0.915262	DOP1A	23033	DOP1 leucine zipper like protein A
ENSG0000	5.069479	-0.02395	0.225553	0.682519	0.915262	NA	NA	NA
ENSG0000	912.171	-0.04674	0.158634	0.682561	0.915262	SIRT6	51548	sirtuin 6
ENSG0000	320.8701	0.046269	0.14469	0.682736	0.915412	MRNIP	51149	MRN complex interacting protein
ENSG0000	2326.506	-0.0461	0.156322	0.682813	0.915448	UBL7	84993	ubiquitin like 7
ENSG0000	199.6257	-0.04607	0.145503	0.682946	0.915493	CD244	51744	CD244 molecule
ENSG0000	24.63377	-0.04607	0.197351	0.682922	0.915493	BATF3	55509	basic leucine zipper ATF-like transcription factor 3
ENSG0000	6.138713	-0.02686	0.223241	0.683202	0.915706	NA	NA	NA
ENSG0000	12.70048	0.031624	0.218223	0.68327	0.915706	CKAP2L	150468	cytoskeleton associated protein 2 like
ENSG0000	322.7336	0.047329	0.164725	0.683305	0.915706	NR2C2	7182	nuclear receptor subfamily 2 group C member 2
ENSG0000	69.33009	0.042961	0.20049	0.683231	0.915706	POT1	25913	protection of telomeres 1
ENSG0000	2516.716	0.048254	0.169849	0.683462	0.91574	SPN	6693	sialophorin
ENSG0000	78.83688	-0.04744	0.17536	0.68348	0.91574	C17orf97	400566	chromosome 17 open reading frame 97
ENSG0000	346.1943	0.047355	0.170815	0.683455	0.91574	ALDOC	230	aldolase fructose-bisphosphate C
ENSG0000	326.9874	0.045805	0.155966	0.683602	0.915759	ATIC	471	5-aminoimidazole-4-carboxamide ribonucleotide formyltransferase/IMP cyclohydrolase
ENSG0000	86.68882	0.05324	0.164206	0.683628	0.915759	ZNF747	65988	zinc finger protein 747
ENSG0000	1347.387	-0.0391	0.140672	0.683645	0.915759	COASY	80347	Coenzyme A synthase
ENSG0000	75.59502	-0.0481	0.163848	0.683756	0.915827	NA	NA	NA
ENSG0000	11.62596	0.026716	0.225422	0.683796	0.915827	SIK1	150094	salt inducible kinase 1
ENSG0000	331.7171	0.046902	0.1614	0.683982	0.915943	RBM26	64062	RNA binding motif protein 26
ENSG0000	280.1524	0.040996	0.121612	0.683953	0.915943	ADAT1	23536	adenosine deaminase tRNA specific 1
ENSG0000	140.9827	0.04299	0.197436	0.684138	0.916085	DBI	1622	diazepam acyl-CoA binding protein
ENSG0000	10.12554	0.031417	0.219552	0.684428	0.916221	NA	NA	NA
ENSG0000	116.7161	0.047105	0.16448	0.68454	0.916221	MFSD6	54842	major facilitator superfamily domain containing 6
ENSG0000	3437.864	-0.04573	0.147628	0.684434	0.916221	GAK	2580	cyclin G associated kinase
ENSG0000	506.5752	0.030753	0.126713	0.684444	0.916221	MRFAP1	93621	Morf4 family associated protein 1
ENSG0000	220.6879	0.045857	0.179082	0.684307	0.916221	VPS13C	54832	vacuolar protein sorting 13 homolog C
ENSG0000	11.36294	-0.03698	0.211634	0.684524	0.916221	NA	NA	NA
ENSG0000	8.484609	0.032245	0.218494	0.684791	0.916282	DENND2C	163259	DENN domain containing 2C
ENSG0000	11.53796	0.034402	0.215312	0.684791	0.916282	NA	NA	NA
ENSG0000	227.1267	-0.04762	0.15914	0.684836	0.916282	ATG101	60673	autophagy related 101
ENSG0000	66.2506	-0.04668	0.175762	0.684753	0.916282	KLHL25	64410	kelch like family member 25
ENSG0000	184.0831	0.043533	0.140761	0.684715	0.916282	UQCC1	55245	ubiquinol-cytochrome c reductase complex assembly factor 1
ENSG0000	5.746508	-0.0181	0.228215	0.684919	0.916326	NA	NA	NA
ENSG0000	233.7615	0.046667	0.175595	0.685024	0.9164	ZBTB32	27033	zinc finger and BTB domain containing 32
ENSG0000	5.137938	0.020084	0.227702	0.685108	0.916412	IGHV1-17	28469	immunoglobulin heavy variable 1-17 (pseudogene)
ENSG0000	138.5747	0.048412	0.163756	0.685133	0.916412	ARRDC5	645432	arrestin domain containing 5
ENSG0000	3.044883	0.0202	0.230534	0.685408	0.916449	NA	NA	NA
ENSG0000	267.3109	-0.04647	0.170486	0.68542	0.916449	ELF2	1998	E74 like ETS transcription factor 2
ENSG0000	2.463496	-0.0096	0.231829	0.685276	0.916449	NA	NA	NA
ENSG0000	21.15215	-0.04218	0.20015	0.685362	0.916449	NA	NA	NA
ENSG0000	102.0963	0.049087	0.155263	0.685511	0.916449	FOCAD	54914	focadhesin
ENSG0000	141.6767	-0.0469	0.165527	0.685403	0.916449	SPTY2D1	144108	SPT2 chromatin protein domain containing 1
ENSG0000	689.3308	0.044723	0.177639	0.685504	0.916449	CLEC12A	160364	C-type lectin domain family 12 member A
ENSG0000	72.99048	0.046088	0.144163	0.685709	0.916648	SVBP	374969	small vasohibin binding protein
ENSG0000	7.666985	-0.03207	0.217443	0.685885	0.916748	LINC0088(	339894	long intergenic non-protein coding RNA 880
ENSG0000	2.695215	0.020365	0.227904	0.68586	0.916748	ZNF8-ERVI	1.09E+08	ZNF8-ERVK3-1 readthrough
ENSG0000	103.0485	-0.0467	0.161854	0.686205	0.91705	NBPF15	284565	NBPF member 15
ENSG0000	146.1068	0.04598	0.142042	0.68621	0.91705	POLR3A	11128	RNA polymerase III subunit A
ENSG0000	4.696573	-0.01591	0.229077	0.686461	0.917318	NA	NA	NA
ENSG0000	9.161399	0.027693	0.221709	0.686856	0.917323	SLFNL1-AS	1.01E+08	SLFNL1 antisense RNA 1
ENSG0000	67.91729	0.046625	0.17613	0.68674	0.917323	RBM15	64783	RNA binding motif protein 15
ENSG0000	83.39536	-0.04519	0.182831	0.686789	0.917323	NA	NA	NA
ENSG0000	9.220429	-0.03105	0.218937	0.686692	0.917323	NA	NA	NA
ENSG0000	45.00001	0.044143	0.191197	0.686589	0.917323	ATP5MK	84833	ATP synthase membrane subunit k
ENSG0000	20.81267	-0.03779	0.209753	0.686865	0.917323	JHY	79864	junctional cadherin complex regulator
ENSG0000	131.0051	0.046124	0.145252	0.686677	0.917323	ZNHIT3	9326	zinc finger HIT-type containing 3
ENSG0000	473.1979	-0.04413	0.13576	0.686851	0.917323	DDRGK1	65992	DDRGK domain containing 1
ENSG0000	322.1535	0.044885	0.148888	0.687003	0.917373	THBS3	7059	thrombospondin 3
ENSG0000	96.95049	-0.0446	0.185393	0.68699	0.917373	TSHZ2	128553	teashirt zinc finger homeobox 2
ENSG0000	30.83182	-0.04443	0.190938	0.687185	0.917482	MTERF1	7978	mitochondrial transcription termination factor 1
ENSG0000	136.5137	-0.04698	0.160241	0.687181	0.917482	MRPL52	122704	mitochondrial ribosomal protein L52
ENSG0000	24.47951	0.046893	0.184236	0.687247	0.917498	BRSK1	84446	BR serine/threonine kinase 1
ENSG0000	6.556783	0.016939	0.229842	0.687398	0.917503	FAM83A	84985	family with sequence similarity 83 member A
ENSG0000	167.0759	0.047898	0.17104	0.687401	0.917503	ACSF2	80221	acyl-CoA synthetase family member 2
ENSG0000	291.7046	-0.04127	0.116711	0.68734	0.917503	ZNF274	10782	zinc finger protein 274
ENSG0000	8.749986	-0.01941	0.226836	0.687658	0.917713	GJA4	2701	gap junction protein alpha 4
ENSG0000	9.098576	0.030565	0.218885	0.68761	0.917713	NA	NA	NA
ENSG0000	15.73983	0.042733	0.199694	0.687768	0.917793	NA	NA	NA

ENSG0000	31.39313	-0.04549	0.185102	0.687944	0.91796	IFT43	112752	intraflagellar transport 43
ENSG0000	102.6712	0.045591	0.17991	0.68827	0.917983	KCNH2	3757	potassium voltage-gated channel subfamily H member 2
ENSG0000	281.4107	0.043467	0.131403	0.688066	0.917983	SBNO1	55206	strawberry notch homolog 1
ENSG0000	460.8381	-0.04826	0.151879	0.688256	0.917983	RNF111	54778	ring finger protein 111
ENSG0000	9812.872	-0.044	0.138683	0.688362	0.917983	MKNK2	2872	MAPK interacting serine/threonine kinase 2
ENSG0000	2486.615	-0.04201	0.142138	0.688353	0.917983	STX10	8677	syntaxin 10
ENSG0000	289.7685	-0.04564	0.14716	0.688292	0.917983	POLR2I	5438	RNA polymerase II subunit I
ENSG0000	162.191	0.044669	0.183544	0.688054	0.917983	CLEC11A	6320	C-type lectin domain containing 11A
ENSG0000	472.3154	-0.0464	0.167337	0.68813	0.917983	HSPBP1	23640	HSPA (Hsp70) binding protein 1
ENSG0000	285.2165	0.044705	0.132529	0.688522	0.917996	ERCC3	2071	ERCC excis TFIIH core complex helicase subunit
ENSG0000	642.617	-0.04096	0.123071	0.688473	0.917996	TMEM80	283232	transmembrane protein 80
ENSG0000	761.869	-0.04625	0.164994	0.688458	0.917996	ZFPM1	161882	zinc finger FOG family member 1
ENSG0000	1588.273	-0.04415	0.17586	0.688627	0.918004	CD8A	925	CD8a molecule
ENSG0000	485.5508	0.042944	0.141207	0.688628	0.918004	BUD23	114049	BUD23 rRNA methyltransferase and ribosome maturation factor
ENSG0000	39.42792	-0.03718	0.204113	0.68869	0.918019	RAB27B	5874	RAB27B member RAS oncogene family
ENSG0000	79.17254	-0.04628	0.17703	0.688792	0.918022	MCM9	254394	minichromosome maintenance 9 homologous recombination repair factor
ENSG0000	7671.309	0.046361	0.154836	0.688783	0.918022	MPEG1	219972	macrophage expressed 1
ENSG0000	56.82446	0.037936	0.206105	0.688994	0.918163	SUCO	51430	SUN domain containing ossification factor
ENSG0000	18.8697	0.04058	0.203723	0.688998	0.918163	PAWR	5074	pro-apoptotic WT1 regulator
ENSG0000	8839.468	-0.03277	0.123647	0.689902	0.918498	ARF1	375	ADP ribosylation factor 1
ENSG0000	275.8347	-0.04675	0.164522	0.689425	0.918498	SMARCAL1	50485	SWI/SNF r matrix ass actin depe subfamily a like 1
ENSG0000	114.3944	-0.04616	0.160745	0.689776	0.918498	AP3M2	10947	adaptor related protein complex 3 subunit mu 2
ENSG0000	3.495064	-0.0253	0.224741	0.689777	0.918498	RHPN1-AS	78998	RHPN1 antisense RNA 1 (head to head)
ENSG0000	7.767172	0.024158	0.224434	0.689406	0.918498	NA	NA	NA
ENSG0000	3406.037	-0.04522	0.155641	0.689845	0.918498	NOTCH1	4851	notch receptor 1
ENSG0000	6.604451	0.025596	0.223677	0.689651	0.918498	NA	NA	NA
ENSG0000	629.6988	0.04438	0.169334	0.689619	0.918498	TMPO	7112	thymopoietin
ENSG0000	18.53193	0.036676	0.211445	0.689891	0.918498	SRXN1	140809	sulfiredoxin 1
ENSG0000	894.4453	0.042539	0.133454	0.68947	0.918498	PCED1A	64773	PC-esterase domain containing 1A
ENSG0000	5.633454	0.018899	0.227055	0.689425	0.918498	NA	NA	NA
ENSG0000	86.63634	0.045518	0.148198	0.689755	0.918498	MIR3667H	348645	MIR3667 host gene
ENSG0000	14.86885	-0.03201	0.21594	0.689664	0.918498	BEX2	84707	brain expressed X-linked 2
ENSG0000	71.65903	-0.04269	0.194654	0.690015	0.918516	CCDC24	149473	coiled-coil domain containing 24
ENSG0000	3.265425	-0.01194	0.230842	0.689979	0.918516	COL1A2	1278	collagen type I alpha 2 chain
ENSG0000	143.504	0.041673	0.162504	0.690162	0.918645	TOMM70	9868	translocase of outer mitochondrial membrane 70
ENSG0000	42.1682	-0.03793	0.205481	0.690414	0.918779	CRTAM	56253	cytotoxic and regulatory T cell molecule
ENSG0000	5.516461	-0.03019	0.221782	0.690412	0.918779	RRAD	6236	RRAD Ras related glycolysis inhibitor and calcium channel regulator
ENSG0000	3.010796	0.016076	0.229771	0.690348	0.918779	NA	NA	NA
ENSG0000	513.4114	0.039141	0.112432	0.691584	0.918838	GPN2	54707	GPN-loop GTPase 2
ENSG0000	226.6573	-0.04253	0.129703	0.691557	0.918838	MGST3	4259	microsomal glutathione S-transferase 3
ENSG0000	8.141454	-0.03732	0.211671	0.690982	0.918838	RHEX	440712	regulator of hemoglobinization and erythroid cell expansion
ENSG0000	697.7686	-0.03839	0.181429	0.691017	0.918838	MAL	4118	mal T cell differentiation protein
ENSG0000	283.1038	0.04533	0.183562	0.691436	0.918838	BZW1	9689	basic leucine zipper and W2 domains 1
ENSG0000	56.07014	0.043751	0.189251	0.691596	0.918838	MOCS2	4338	molybdenum cofactor synthesis 2
ENSG0000	2.487464	0.019567	0.23137	0.690991	0.918838	NA	NA	NA
ENSG0000	6.999781	-0.03112	0.218623	0.691513	0.918838	THBS4-AS1	1.02E+08	THBS4 antisense RNA 1
ENSG0000	187.9719	0.044125	0.185946	0.690842	0.918838	COX7A2	1347	cytochrome c oxidase subunit 7A2
ENSG0000	4.533369	0.019796	0.226898	0.690924	0.918838	NA	NA	NA
ENSG0000	236.8437	-0.04675	0.147816	0.691471	0.918838	USP28	57646	ubiquitin specific peptidase 28
ENSG0000	255.5414	0.040986	0.158491	0.69132	0.918838	GALNT6	11226	polypeptide N-acetylgalactosaminyltransferase 6
ENSG0000	15.10158	0.033909	0.214366	0.691424	0.918838	TRAV2	28691	T cell receptor alpha variable 2
ENSG0000	1018.346	-0.0453	0.157064	0.690832	0.918838	ARPP19	10776	cAMP regulated phosphoprotein 19
ENSG0000	7.625254	-0.03091	0.222361	0.69065	0.918838	NA	NA	NA
ENSG0000	257.4055	0.042101	0.122919	0.69144	0.918838	WDR59	79726	WD repeat domain 59
ENSG0000	876.5226	-0.04373	0.144856	0.690619	0.918838	RFFL	117584	ring finger and FYVE like domain containing E3 ubiquitin protein ligase
ENSG0000	3.594005	0.020997	0.228021	0.691128	0.918838	NA	NA	NA
ENSG0000	1374.757	0.044754	0.153299	0.691539	0.918838	AP1M1	8907	adaptor related protein complex 1 subunit mu 1
ENSG0000	135.0234	-0.03995	0.187134	0.691611	0.918838	MIR646HG	284757	MIR646 host gene
ENSG0000	539.283	0.039744	0.11334	0.690641	0.918838	GID8	54994	GID complex subunit 8 homolog
ENSG0000	18.40209	-0.03853	0.201663	0.691109	0.918838	GCNA	93953	germ cell nuclear acidic peptidase
ENSG0000	3.30621	0.016245	0.22999	0.691538	0.918838	DGCR6	8214	DiGeorge syndrome critical region gene 6
ENSG0000	1125.689	-0.04784	0.137362	0.691798	0.918888	BOD1L1	259282	biorientation of chromosomes in cell division 1 like 1
ENSG0000	288.4592	0.042935	0.129291	0.691942	0.918888	SCAF8	22828	SR-related CTD associated factor 8
ENSG0000	3.493285	0.014153	0.229793	0.691822	0.918888	SGCE	8910	sarcoglycan epsilon
ENSG0000	16.26411	-0.02324	0.222728	0.691819	0.918888	NA	NA	NA
ENSG0000	31.56903	-0.0436	0.188151	0.691891	0.918888	NA	NA	NA
ENSG0000	38.0378	-0.04309	0.193265	0.69195	0.918888	GPRASP1	9737	G protein-coupled receptor associated sorting protein 1
ENSG0000	3.328066	0.023971	0.229826	0.692158	0.918964	H1-4	3008	H1.4 linker cluster member
ENSG0000	2.622967	0.022615	0.231195	0.692079	0.918964	SH2D4B	387694	SH2 domain containing 4B
ENSG0000	4.185215	0.021338	0.227632	0.692125	0.918964	RN7SL138	1.06E+08	RNA 7SL cytoplasm pseudogene
ENSG0000	422.4342	0.043634	0.169717	0.692213	0.918971	MTMR10	54893	myotubularin related protein 10
ENSG0000	38.917	-0.03689	0.207741	0.692305	0.918974	CCDC154	645811	coiled-coil domain containing 154
ENSG0000	4035.835	-0.04044	0.121296	0.692331	0.918974	CYTH1	9267	cytohesin 1
ENSG0000	25.01214	-0.04396	0.190322	0.692366	0.918974	NA	NA	NA
ENSG0000	3.797264	0.020642	0.226861	0.692493	0.91901	NA	NA	NA
ENSG0000	49.67158	0.04445	0.180358	0.692455	0.91901	ZNF430	80264	zinc finger protein 430
ENSG0000	16.7529	0.033251	0.215248	0.692606	0.919093	TMEM44	93109	transmembrane protein 44
ENSG0000	173.3738	0.046356	0.169421	0.692835	0.919196	AEBP1	165	AE binding protein 1
ENSG0000	6.49061	-0.0331	0.217006	0.692983	0.919196	NA	NA	NA
ENSG0000	11.82431	0.032616	0.216239	0.693077	0.919196	NA	NA	NA
ENSG0000	24.48654	0.042584	0.196472	0.693152	0.919196	PRICKLE1	144165	prickle planar cell polarity protein 1
ENSG0000	231.9945	-0.04247	0.187946	0.693186	0.919196	MTMR6	9107	myotubularin related protein 6
ENSG0000	146.9477	-0.0451	0.160896	0.693078	0.919196	WDR20	91833	WD repeat domain 20
ENSG0000	7.531577	0.022443	0.225379	0.692974	0.919196	NA	NA	NA
ENSG0000	14.23801	-0.03821	0.20674	0.6931	0.919196	NA	NA	NA
ENSG0000	2.807934	0.017627	0.228639	0.692965	0.919196	NA	NA	NA
ENSG0000	221.1391	0.044437	0.142195	0.693167	0.919196	CENPBD2P	65996	CENPB DN pseudogene
ENSG0000	2.539854	0.011431	0.230832	0.69326	0.919224	IRGM	345611	immunity related GTPase M
ENSG0000	331.6003	0.045614	0.165542	0.693307	0.919224	FTSJ3	117246	FtsJ RNA 2'-O-methyltransferase 3
ENSG0000	2.385111	0.016377	0.230093	0.693493	0.919244	NECTIN3	25945	nectin cell adhesion molecule 3
ENSG0000	43.83646	0.045102	0.181414	0.69344	0.919244	ZNF189	7743	zinc finger protein 189
ENSG0000	347.2622	0.044494	0.142215	0.693523	0.919244	RBM19	9904	RNA binding motif protein 19
ENSG0000	6.593174	-0.02993	0.21957	0.693376	0.919244	NDN	4692	necdin MAGE family member
ENSG0000	242.3044	-0.03856	0.117436	0.693626	0.919314	NIPSNAP1	8508	nipsnap homolog 1
ENSG0000	80.20663	0.040371	0.194546	0.693755	0.919419	CAPN7	23473	calpain 7
ENSG0000	1197.088	-0.03714	0.110672	0.694751	0.919439	RER1	11079	retention in endoplasmic reticulum sorting receptor 1

ENSG0000	362.5209	-0.04375	0.134522	0.693878	0.919439	MTFR1L	56181	mitochondrial fission regulator 1 like
ENSG0000	8804.78	0.044412	0.139445	0.694453	0.919439	C1orf162	128346	chromosome 1 open reading frame 162
ENSG0000	87.85141	0.034842	0.209213	0.694301	0.919439	NDUFB3	4709	NADH:ubiquinone oxidoreductase subunit B3
ENSG0000	66.4495	0.045863	0.169007	0.694728	0.919439	MUC20	200958	mucin 20 cell surface associated
ENSG0000	240.9381	0.044146	0.140404	0.694605	0.919439	SERINC5	256987	serine incorporator 5
ENSG0000	5.318631	0.029256	0.220894	0.694484	0.919439	EPM2A-DT	1.01E+08	EPM2A divergent transcript
ENSG0000	122.8825	-0.04379	0.172356	0.693873	0.919439	TRPM6	140803	transient receptor potential cation channel subfamily M member 6
ENSG0000	9.946723	0.031848	0.217335	0.694825	0.919439	WNK2	65268	WNK lysine deficient protein kinase 2
ENSG0000	4.481218	0.024011	0.224951	0.69422	0.919439	KCNT1	57582	potassium sodium-activated channel subfamily T member 1
ENSG0000	2.571846	-0.00517	0.233563	0.694694	0.919439	NA	NA	NA
ENSG0000	2.741035	-0.01472	0.22954	0.694671	0.919439	NA	NA	NA
ENSG0000	78.69717	-0.04418	0.173533	0.69467	0.919439	TIGAR	57103	TP53 induced glycolysis regulatory phosphatase
ENSG0000	24.90417	0.038584	0.204591	0.694297	0.919439	C12orf73	728568	chromosome 12 open reading frame 73
ENSG0000	27.28952	0.03294	0.213542	0.694563	0.919439	DACH1	1602	dachshund family transcription factor 1
ENSG0000	79.25991	0.045737	0.167475	0.69459	0.919439	RRN3	54700	RRN3 hom RNA polymerase I transcription factor
ENSG0000	243.3203	-0.04119	0.186431	0.694778	0.919439	CMTM2	146225	CKLF like MARVEL transmembrane domain containing 2
ENSG0000	19.69186	-0.0376	0.207254	0.694154	0.919439	KRT10-AS1	147184	KRT10 antisense RNA 1
ENSG0000	822.6026	-0.04405	0.135303	0.694615	0.919439	SFI1	9814	SFI1 centrin binding protein
ENSG0000	2.819467	-0.01421	0.231096	0.694483	0.919439	NEXMIF	340533	neurite extension and migration factor
ENSG0000	10.15379	-0.03411	0.217787	0.694045	0.919439	TTTY14	83869	testis-spec Y-linked 14
ENSG0000	23.76132	-0.04152	0.198991	0.694933	0.91945	TFAP2E	339488	transcription factor AP-2 epsilon
ENSG0000	2.826597	0.016667	0.231291	0.694899	0.91945	GOLGA7B-	1.01E+08	GOLGA7B divergent transcript
ENSG0000	999.7957	0.044795	0.175411	0.695374	0.919589	TRAM1	23471	translocation associated membrane protein 1
ENSG0000	2.37553	0.010862	0.231185	0.695359	0.919589	SNORD14A	26822	small nucl C/D box 14A
ENSG0000	3116.559	-0.04427	0.140572	0.695242	0.919589	GANAB	23193	glucosidase II alpha subunit
ENSG0000	2.952894	-0.01162	0.230914	0.69517	0.919589	NA	NA	NA
ENSG0000	30.22963	-0.03889	0.202877	0.695313	0.919589	NA	NA	NA
ENSG0000	6.243578	-0.03152	0.220185	0.695194	0.919589	NA	NA	NA
ENSG0000	22.81094	0.038648	0.206042	0.695389	0.919589	C16orf87	388272	chromosome 16 open reading frame 87
ENSG0000	2.439782	0.025988	0.227827	0.695475	0.919636	NA	NA	NA
ENSG0000	4.342001	0.029005	0.222218	0.695641	0.919789	NA	NA	NA
ENSG0000	3.902622	-0.02712	0.223156	0.695834	0.919963	NA	NA	NA
ENSG0000	53.12762	0.044828	0.182341	0.695873	0.919963	S100B	6285	S100 calcium binding protein B
ENSG0000	6.950347	0.031674	0.218621	0.696041	0.920002	NA	NA	NA
ENSG0000	126.2437	-0.04029	0.196882	0.696955	0.920002	FBXO28	23219	F-box protein 28
ENSG0000	43.85089	0.045498	0.178236	0.696645	0.920002	H2AC25	92815	H2A clustered histone 25
ENSG0000	54.16204	-0.0314	0.212477	0.695963	0.920002	HECW2	57520	HECT C2 and WW domain containing E3 ubiquitin protein ligase 2
ENSG0000	5.02454	0.015569	0.228788	0.696705	0.920002	HECW2-AS	1.02E+08	HECW2 antisense RNA 1
ENSG0000	3.196208	-0.0207	0.227074	0.696762	0.920002	NA	NA	NA
ENSG0000	14.86621	-0.03453	0.211605	0.697159	0.920002	STIMATE	375346	STIM activating enhancer
ENSG0000	5.458915	0.023997	0.224175	0.696273	0.920002	NA	NA	NA
ENSG0000	90.4574	0.042991	0.18651	0.696625	0.920002	OXCT1	5019	3-oxoacid CoA-transferase 1
ENSG0000	187.0221	-0.04581	0.16046	0.697139	0.920002	CCNH	902	cyclin H
ENSG0000	25.88861	-0.02538	0.215045	0.696818	0.920002	SCGB3A1	92304	secretoglobin family 3A member 1
ENSG0000	16.23448	0.037924	0.20718	0.69645	0.920002	CCT6P1	643253	chaperonin containing TCP1 subunit 6 pseudogene 1
ENSG0000	2.481432	-0.02125	0.228735	0.696294	0.920002	LOC10537	1.05E+08	uncharacterized LOC105375713
ENSG0000	63.05641	0.041432	0.189496	0.697101	0.920002	FKTN	2218	fukutin
ENSG0000	7.688127	0.03146	0.215771	0.696575	0.920002	NA	NA	NA
ENSG0000	42.88736	0.045031	0.182688	0.696605	0.920002	DGKZP1	400126	diacylglycerol kinase zeta pseudogene 1
ENSG0000	1017.408	0.046583	0.134271	0.697056	0.920002	MORF4L1	10933	mortality factor 4 like 1
ENSG0000	3.683933	-0.0217	0.228264	0.69682	0.920002	KIF7	374654	kinesin family member 7
ENSG0000	16.92976	0.038856	0.20425	0.697089	0.920002	ZNF747-DT	1.08E+08	ZNF747 divergent transcript
ENSG0000	2.448238	0.016118	0.229213	0.696844	0.920002	NA	NA	NA
ENSG0000	88.93094	-0.04516	0.179857	0.696237	0.920002	NA	NA	NA
ENSG0000	42.06733	0.044962	0.179098	0.697026	0.920002	RBFA	79863	ribosome binding factor A
ENSG0000	53.351	-0.04567	0.166099	0.696666	0.920002	PPM1N	147699	protein ph Mg2+/Mn2+ dependent 1N (putative)
ENSG0000	547.7852	0.04152	0.126499	0.697023	0.920002	NCOA5	57727	nuclear receptor coactivator 5
ENSG0000	3314.895	-0.04311	0.165418	0.696912	0.920002	ASCC2	84164	activating signal cointegrator 1 complex subunit 2
ENSG0000	99.40902	0.041444	0.192114	0.697219	0.920016	CLEC10A	10462	C-type lectin domain containing 10A
ENSG0000	85.18174	0.044577	0.173866	0.69789	0.920049	SCNN1D	6339	sodium channel epithelial 1 subunit delta
ENSG0000	132.3385	0.041724	0.190489	0.697825	0.920049	RLF	6018	RLF zinc finger
ENSG0000	930.1464	0.038691	0.113445	0.698098	0.920049	TEX264	51368	testis expr ER-phagy receptor
ENSG0000	3.860622	0.010835	0.231666	0.697777	0.920049	DNAH12	201625	dynein axonemal heavy chain 12
ENSG0000	2027.921	0.041178	0.132022	0.697761	0.920049	FGD2	221472	FYVE RhoGEF and PH domain containing 2
ENSG0000	5.44543	-0.02732	0.222147	0.697905	0.920049	GRID2IP	392862	Grid2 interacting protein
ENSG0000	2.568954	0.016741	0.229413	0.697622	0.920049	NA	NA	NA
ENSG0000	594.3146	-0.04693	0.173472	0.698102	0.920049	TSPAN33	340348	tetraspanin 33
ENSG0000	5.243522	0.022175	0.225369	0.697793	0.920049	EEF1AKMT	399818	EEF1A lysine methyltransferase 2
ENSG0000	549.2316	0.043162	0.12575	0.698026	0.920049	CBX5	23468	chromobox 5
ENSG0000	72.67511	0.044727	0.178167	0.698101	0.920049	DOCK9	23348	dedicator of cytokinesis 9
ENSG0000	3.223162	-0.02085	0.227867	0.697986	0.920049	NA	NA	NA
ENSG0000	22.5591	-0.04472	0.185631	0.697585	0.920049	NA	NA	NA
ENSG0000	790.685	0.05174	0.150363	0.697843	0.920049	LRRK1	79705	leucine rich repeat kinase 1
ENSG0000	34.99704	0.035547	0.208833	0.697332	0.920049	ZNF260	339324	zinc finger protein 260
ENSG0000	448.4236	-0.0442	0.148115	0.697585	0.920049	HMGN1	3150	high mobility group nucleosome binding domain 1
ENSG0000	276.0505	-0.04243	0.123114	0.698148	0.920049	SYAP1	94056	synapse associated protein 1
ENSG0000	727.9707	-0.04389	0.173173	0.697939	0.920049	USP11	8237	ubiquitin specific peptidase 11
ENSG0000	6.684092	-0.0183	0.227828	0.698272	0.920085	NA	NA	NA
ENSG0000	29813.31	0.044683	0.154426	0.698276	0.920085	COTL1	23406	coactosin like F-actin binding protein 1
ENSG0000	5.399847	-0.02727	0.222731	0.698341	0.920104	CCDC183	84960	coiled-coil domain containing 183
ENSG0000	187.6022	-0.0394	0.125764	0.698605	0.92013	NBPF14	25832	NBPF member 14
ENSG0000	249.3208	0.044497	0.163528	0.698709	0.92013	BCL11A	53335	BAF chromatin remodeling complex subunit BCL11A
ENSG0000	480.5803	0.044416	0.138116	0.698863	0.92013	RMND5B	64777	required for meiotic nuclear division 5 homolog B
ENSG0000	31.24801	0.02655	0.221008	0.698714	0.92013	ENPP4	22875	ectonucleotide pyrophosphatase/phosphodiesterase 4
ENSG0000	3.252963	0.017825	0.228239	0.698852	0.92013	NA	NA	NA
ENSG0000	26.27784	-0.04308	0.189158	0.698731	0.92013	CCDC180	1E+08	coiled-coil domain containing 180
ENSG0000	501.5386	0.040933	0.118838	0.698813	0.92013	LRRC8A	56262	leucine rich repeat containing 8 VRAC subunit A
ENSG0000	20.49127	0.043272	0.190937	0.698629	0.92013	CDIN1	84529	CDAN1 interacting nuclease 1
ENSG0000	180.0391	-0.04728	0.167753	0.698771	0.92013	LMTK3	114783	lemur tyrosine kinase 3
ENSG0000	539.5872	-0.04913	0.146637	0.698668	0.92013	L3MBTL2	83746	L3MBTL histone methyl-lysine binding protein 2
ENSG0000	1305.663	0.043894	0.150696	0.698967	0.920135	WDR6	11180	WD repeat domain 6
ENSG0000	75.82689	0.044288	0.181588	0.698939	0.920135	LOC10192	1.02E+08	uncharacterized LOC101928143
ENSG0000	2.455175	-0.00471	0.2335	0.699019	0.920137	TMEM51-A	200197	TMEM51 antisense RNA 1
ENSG0000	1403.28	0.036059	0.10577	0.699113	0.920195	UBE2D2	7322	ubiquitin conjugating enzyme E2 D2
ENSG0000	10772.45	-0.0437	0.168873	0.699264	0.920328	ANXA11	311	annexin A11
ENSG0000	11.13552	0.03126	0.216562	0.699556	0.920646	MIR3150B	1.05E+08	MIR3150B host gene

ENSG0000	1410.623	0.043238	0.141552	0.699739	0.920821	GYS1	2997	glycogen synthase 1
ENSG0000	14.33194	-0.0329	0.213661	0.699834	0.920866	SGO1	151648	shugoshin 1
ENSG0000	338.069	0.044391	0.172507	0.699874	0.920866	MYO1C	4641	myosin IC
ENSG0000	123.3908	0.044224	0.172891	0.700278	0.920868	KYNU	8942	kynureninase
ENSG0000	12.58611	-0.03667	0.209958	0.700077	0.920868	MED12L	116931	mediator complex subunit 12L
ENSG0000	680.679	-0.0439	0.184887	0.700065	0.920868	TRAPPC9	83696	trafficking protein particle complex subunit 9
ENSG0000	9.739639	-0.02948	0.216354	0.700223	0.920868	SLC2A13	114134	solute carrier family 2 member 13
ENSG0000	72.21848	0.040294	0.197912	0.70023	0.920868	KRR1	11103	KRR1 small subunit processome component homolog
ENSG0000	5.414968	-0.01965	0.226072	0.700045	0.920868	NA	NA	NA
ENSG0000	24.22266	0.040288	0.198022	0.700099	0.920868	ALKBH2	121642	alkB homc alpha-ketoglutarate dependent dioxygenase
ENSG0000	58.08284	0.043742	0.182859	0.700257	0.920868	LIN7B	64130	lin-7 homc crumbs cell polarity complex component
ENSG0000	2132.135	-0.04331	0.131114	0.700451	0.921029	MAU2	23383	MAU2 sister chromatid cohesion factor
ENSG0000	1037.811	0.04468	0.165335	0.700504	0.921033	EXTL3	2137	exostosin like glycosyltransferase 3
ENSG0000	160.7774	-0.04349	0.145449	0.700571	0.921056	NA	NA	NA
ENSG0000	175.664	-0.03986	0.180702	0.700773	0.921189	FYTTD1	84248	forty-two-three domain containing 1
ENSG0000	3386.886	-0.041	0.177727	0.700763	0.921189	SCAF1	58506	SR-related CTD associated factor 1
ENSG0000	280.922	0.043401	0.142247	0.700928	0.921326	FBXL14	144699	F-box and leucine rich repeat protein 14
ENSG0000	59.56072	0.044521	0.174426	0.701143	0.921541	MEMO1	51072	mediator of cell motility 1
ENSG0000	41.1173	-0.04521	0.181485	0.701192	0.921541	NA	NA	NA
ENSG0000	39700.4	-0.04312	0.170777	0.701338	0.921661	VSIR	64115	V-set immunoregulatory receptor
ENSG0000	773.1797	0.042041	0.1696	0.701485	0.921661	SLC48A1	55652	solute carrier family 48 member 1
ENSG0000	517.5063	0.042954	0.183634	0.701407	0.921661	DICER1	23405	dicer 1 ribonuclease III
ENSG0000	66.50894	0.044268	0.176226	0.701466	0.921661	MAGEH1	28986	MAGE family member H1
ENSG0000	83.45894	-0.04163	0.188268	0.701596	0.921675	TMEM70	54968	transmembrane protein 70
ENSG0000	94.87961	-0.04383	0.182455	0.701581	0.921675	LGMN	5641	legumain
ENSG0000	434.1653	0.04805	0.137823	0.701899	0.92184	NPRL2	10641	NPR2 like GATOR1 complex subunit
ENSG0000	12.39889	0.038543	0.204842	0.701844	0.92184	NA	NA	NA
ENSG0000	608.4645	0.040797	0.128846	0.701923	0.92184	SCPEP1	59342	serine carboxypeptidase 1
ENSG0000	10.43741	0.03822	0.206748	0.701898	0.92184	ZNF677	342926	zinc finger protein 677
ENSG0000	309.5061	0.040006	0.121947	0.702259	0.922178	EMC7	56851	ER membrane protein complex subunit 7
ENSG0000	7.669592	0.02267	0.224256	0.702281	0.922178	PEX11A	8800	peroxisomal biogenesis factor 11 alpha
ENSG0000	14.70539	-0.0214	0.224275	0.70235	0.922202	TMEM38B	55151	transmembrane protein 38B
ENSG0000	3.790305	0.02299	0.224567	0.70243	0.922241	NA	NA	NA
ENSG0000	16.09595	-0.0378	0.213959	0.703371	0.922415	NA	NA	NA
ENSG0000	288.0579	-0.04313	0.175627	0.703071	0.922415	STRN	6801	striatin
ENSG0000	229.0897	-0.04017	0.153547	0.703245	0.922415	SUMO1	7341	small ubiquitin like modifier 1
ENSG0000	451.3229	0.041357	0.189022	0.702866	0.922415	UBA3	9039	ubiquitin like modifier activating enzyme 3
ENSG0000	2393.761	-0.04324	0.160551	0.702855	0.922415	TMEM154	201799	transmembrane protein 154
ENSG0000	1492.717	-0.04325	0.159663	0.703056	0.922415	NEDD9	4739	neural pre developmentally down-regulated 9
ENSG0000	34.26744	0.04443	0.173999	0.703232	0.922415	ZFAND2A-	1.02E+08	ZFAND2A divergent transcript
ENSG0000	43.79342	-0.03561	0.204881	0.703441	0.922415	SDHAF3	57001	succinate dehydrogenase complex assembly factor 3
ENSG0000	4.63767	-0.0161	0.228305	0.703476	0.922415	LOC12490	1.25E+08	uncharacterized LOC124901909
ENSG0000	123.4285	0.043032	0.181948	0.703238	0.922415	ADAM9	8754	ADAM metallopeptidase domain 9
ENSG0000	11.10057	0.036731	0.209593	0.703563	0.922415	PRPF18	8559	pre-mRNA processing factor 18
ENSG0000	118.4973	-0.04249	0.137533	0.703453	0.922415	SUPV3L1	6832	Suv3 like RNA helicase
ENSG0000	3.008462	0.020096	0.227399	0.703569	0.922415	LOC10192	1.02E+08	uncharacterized LOC101928053
ENSG0000	52.47145	0.043383	0.182139	0.703565	0.922415	ZNF140	7699	zinc finger protein 140
ENSG0000	995.8339	-0.04291	0.134816	0.703361	0.922415	DNASE2	1777	deoxyribo lysosomal
ENSG0000	224.805	0.043234	0.143334	0.70304	0.922415	KIZ	55857	kizuna centrosomal protein
ENSG0000	329.0627	0.039054	0.122164	0.703303	0.922415	TRMU	55687	tRNA mitochondrial 2-thiouridylase
ENSG0000	451.9246	0.046345	0.156571	0.702696	0.922415	PQBP1	10084	polyglutamine binding protein 1
ENSG0000	23.04705	-0.04006	0.198341	0.703476	0.922415	USP27X	389856	ubiquitin specific peptidase 27 X-linked
ENSG0000	5.069315	-0.02082	0.22604	0.702906	0.922415	ERCC6L	54821	ERCC excis spindle assembly checkpoint helicase
ENSG0000	241.7956	-0.04483	0.156651	0.703635	0.922435	CDC27	996	cell division cycle 27
ENSG0000	20.39365	0.041597	0.194597	0.703722	0.922484	GLIDR	389741	glioblastoma down-regulated RNA
ENSG0000	2.444788	0.017556	0.229013	0.70388	0.922492	NA	NA	NA
ENSG0000	30.83812	-0.04328	0.176218	0.703788	0.922492	NA	NA	NA
ENSG0000	5.127724	0.027419	0.221781	0.703871	0.922492	SHANK3	85358	SH3 and multiple ankyrin repeat domains 3
ENSG0000	11.40248	-0.0321	0.21376	0.710596	0.922597	NA	NA	NA
ENSG0000	228.8371	0.043814	0.171604	0.708527	0.922597	ATAD3A	55210	ATPase family AAA domain containing 3A
ENSG0000	11.57717	0.03647	0.208483	0.705852	0.922597	LOC10099	1.01E+08	uncharacterized LOC100996583
ENSG0000	495.8486	-0.04129	0.132839	0.710275	0.922597	FBXO42	54455	F-box protein 42
ENSG0000	6.822073	-0.01208	0.229773	0.705162	0.922597	KIF17	57576	kinesin family member 17
ENSG0000	201.8264	-0.04414	0.148823	0.705648	0.922597	MAN1C1	57134	mannosidase alpha class 1C member 1
ENSG0000	30.82085	-0.03727	0.20193	0.708223	0.922597	STIL	6491	STIL centriolar assembly protein
ENSG0000	27.96121	-0.04157	0.190415	0.706559	0.922597	AK5	26289	adenylate kinase 5
ENSG0000	7.574506	0.019024	0.22662	0.710011	0.922597	RPL7P9	653702	ribosomal protein L7 pseudogene 9
ENSG0000	3.970994	0.023878	0.224525	0.705604	0.922597	NA	NA	NA
ENSG0000	2.598312	-0.01834	0.229413	0.709182	0.922597	CD101-AS	1.02E+08	CD101 antisense RNA 1
ENSG0000	1807.612	-0.03391	0.107891	0.711267	0.922597	APH1A	51107	aph-1 hom gamma-secretase subunit
ENSG0000	607.4103	-0.03698	0.11356	0.706737	0.922597	CHTOP	26097	chromatin target of PRMT1
ENSG0000	75.83553	0.045263	0.155037	0.704173	0.922597	KRTCAP2	200185	keratinocyte associated protein 2
ENSG0000	17.19966	0.035588	0.208407	0.709175	0.922597	NA	NA	NA
ENSG0000	183.2072	-0.04669	0.158225	0.707716	0.922597	KLHL12	59349	kelch like family member 12
ENSG0000	2.536768	0.011466	0.230928	0.704139	0.922597	NA	NA	NA
ENSG0000	5.903744	-0.02932	0.218867	0.709461	0.922597	NA	NA	NA
ENSG0000	737.5294	-0.03691	0.121925	0.708979	0.922597	PYCR2	29920	pyrroline-5-carboxylate reductase 2
ENSG0000	10.12204	-0.02931	0.217355	0.709172	0.922597	GJC2	57165	gap junction protein gamma 2
ENSG0000	466.8259	0.043034	0.170869	0.709334	0.922597	ADAM17	6868	ADAM metallopeptidase domain 17
ENSG0000	45.79754	0.03874	0.197388	0.709808	0.922597	MSH2	4436	mutS homolog 2
ENSG0000	590.7161	0.040803	0.128981	0.707834	0.922597	CCT4	10575	chaperonin containing TCP1 subunit 4
ENSG0000	197.5621	0.040496	0.130457	0.706602	0.922597	CCDC142	84865	coiled-coil domain containing 142
ENSG0000	4.359156	-0.0263	0.222354	0.70776	0.922597	LINC0196	1E+08	long intergenic non-protein coding RNA 1960
ENSG0000	21.22701	-0.03998	0.196278	0.708282	0.922597	LINC0261	151174	long intergenic non-protein coding RNA 2610
ENSG0000	59.6529	0.032648	0.211089	0.706132	0.922597	SLC4A7	9497	solute carrier family 4 member 7
ENSG0000	41.00664	-0.04194	0.183874	0.708601	0.922597	ZNF197	10168	zinc finger protein 197
ENSG0000	17.8462	-0.02946	0.215992	0.708947	0.922597	CCR9	10803	C-C motif chemokine receptor 9
ENSG0000	5.686915	-0.02934	0.223404	0.708651	0.922597	DPPA4	55211	developmental pluripotency associated 4
ENSG0000	109.109	0.037698	0.198189	0.710868	0.922597	ABHD10	55347	abhydrola depalmitoylase
ENSG0000	35.37897	0.043893	0.179458	0.704765	0.922597	CFAP44	55779	cilia and flagella associated protein 44
ENSG0000	152.7497	0.043582	0.150498	0.706882	0.922597	USF3	205717	upstream transcription factor family member 3
ENSG0000	13.11753	-0.02984	0.215322	0.710449	0.922597	KALRN	8997	kalirin RhoGEF kinase
ENSG0000	6.159512	-0.01509	0.225112	0.708835	0.922597	NA	NA	NA
ENSG0000	84.21257	0.043734	0.156457	0.70592	0.922597	GFM1	85476	G elongation factor mitochondrial 1
ENSG0000	419.1148	0.042179	0.13961	0.705341	0.922597	NELFA	7469	negative elongation factor complex member A
ENSG0000	386.0438	0.041499	0.142546	0.708921	0.922597	STIM2	57620	stromal interaction molecule 2

ENSG0000	261.8974	0.047402	0.17523	0.705724	0.922597	PGM2	55276	phosphoglucomutase 2
ENSG0000	27.53636	-0.03539	0.209113	0.706541	0.922597	UCHL1	7345	ubiquitin C-terminal hydrolase L1
ENSG0000	401.5439	-0.04016	0.126487	0.707672	0.922597	SLAIN2	57606	SLAIN motif family member 2
ENSG0000	74.66361	0.042707	0.179966	0.707966	0.922597	SLC10A7	84068	solute carrier family 10 member 7
ENSG0000	14.61365	0.025121	0.221378	0.70978	0.922597	PDGFC	56034	platelet derived growth factor C
ENSG0000	3.484852	0.015038	0.230201	0.709769	0.922597	NA	NA	NA
ENSG0000	4.920029	-0.02201	0.224593	0.711027	0.922597	CCDC152	1E+08	coiled-coil domain containing 152
ENSG0000	192.0815	-0.03891	0.192495	0.708581	0.922597	GZMK	3003	granzyme K
ENSG0000	70.62266	0.041389	0.165127	0.709456	0.922597	CDK7	1022	cyclin dependent kinase 7
ENSG0000	25.3949	0.027986	0.217984	0.704323	0.922597	GCNT4	51301	glucosaminyl (N-acetyl) transferase 4
ENSG0000	4.814088	-0.02346	0.224333	0.70719	0.922597	NA	NA	NA
ENSG0000	99.14364	0.036717	0.20307	0.711196	0.922597	CEP120	153241	centrosomal protein 120
ENSG0000	15.57753	-0.03805	0.202155	0.709772	0.922597	MTND4LP	1.07E+08	MT-ND4L pseudogene 30
ENSG0000	412.5346	-0.03956	0.125426	0.706349	0.922597	HMGXB3	22993	HMG-box containing 3
ENSG0000	68.24106	-0.04301	0.179422	0.704469	0.922597	SLC35B3	51000	solute carrier family 35 member B3
ENSG0000	826.1641	0.041828	0.14205	0.710614	0.922597	MDC1	9656	mediator of DNA damage checkpoint 1
ENSG0000	66.7209	0.043214	0.155984	0.709375	0.922597	MMUT	4594	methylmalonyl-CoA mutase
ENSG0000	329.8444	-0.03769	0.115983	0.70677	0.922597	SMAP1	60682	small ArfGAP 1
ENSG0000	49.26057	0.044288	0.161021	0.705611	0.922597	PDSS2	57107	decaprenyl diphosphate synthase subunit 2
ENSG0000	71.7217	0.043317	0.157601	0.708086	0.922597	ZDHHC14	79683	zinc finger DHHC-type palmitoyltransferase 14
ENSG0000	149.6099	-0.04246	0.149828	0.709286	0.922597	PHF10	55274	PHD finger protein 10
ENSG0000	54.54291	0.042359	0.178307	0.710625	0.922597	NA	NA	NA
ENSG0000	695.5349	0.040299	0.143185	0.708578	0.922597	DAGLB	221955	diacylglycerol lipase beta
ENSG0000	689.4218	-0.03731	0.113293	0.705465	0.922597	JAZF1	221895	JAZF zinc finger 1
ENSG0000	44.84979	0.040029	0.176689	0.709975	0.922597	NA	NA	NA
ENSG0000	15.02726	-0.03313	0.2115	0.704157	0.922597	METTL27	155368	methyltransferase like 27
ENSG0000	570.3471	0.042774	0.135616	0.705082	0.922597	COPS6	10980	COP9 signalosome subunit 6
ENSG0000	4222.238	-0.04244	0.164718	0.70865	0.922597	SLC12A9	56996	solute carrier family 12 member 9
ENSG0000	2.432672	0.023176	0.226304	0.711414	0.922597	NA	NA	NA
ENSG0000	41.81091	0.043456	0.174472	0.709884	0.922597	MINCR	1.01E+08	MYC-induced long non-coding RNA
ENSG0000	55.9832	0.042081	0.163311	0.705821	0.922597	NA	NA	NA
ENSG0000	70.4541	0.045051	0.153707	0.709793	0.922597	SLC25A51	92014	solute carrier family 25 member 51
ENSG0000	647.9963	0.041846	0.13914	0.705592	0.922597	PPP6C	5537	protein phosphatase 6 catalytic subunit
ENSG0000	23.96779	0.04288	0.183966	0.71049	0.922597	NA	NA	NA
ENSG0000	98.62491	-0.0428	0.171156	0.711303	0.922597	ACBD5	91452	acyl-CoA binding domain containing 5
ENSG0000	27.73262	0.042269	0.187661	0.70553	0.922597	ELOVL3	83401	ELOVL fatty acid elongase 3
ENSG0000	1197.603	0.039968	0.136344	0.706181	0.922597	LHPP	64077	phospholysine phosphohistidine inorganic pyrophosphate phosphatase
ENSG0000	13.73789	-0.03377	0.211861	0.706786	0.922597	PAOX	196743	polyamine oxidase
ENSG0000	141.516	0.043278	0.166435	0.707095	0.922597	HRAS	3265	HRas prot GTPase
ENSG0000	2721.197	0.037704	0.129312	0.705669	0.922597	LDHA	3939	lactate dehydrogenase A
ENSG0000	136.356	-0.03969	0.190044	0.708996	0.922597	FBXO3	26273	F-box protein 3
ENSG0000	12.07476	0.02741	0.220038	0.707995	0.922597	NA	NA	NA
ENSG0000	402.7728	0.043905	0.163661	0.709367	0.922597	WDR74	54663	WD repeat domain 74
ENSG0000	87.4914	-0.04164	0.19315	0.706822	0.922597	KCNK7	10089	potassium two pore domain channel subfamily K member 7
ENSG0000	74.54195	0.038041	0.18457	0.70674	0.922597	MSANTD2	79684	Myb/SANT DNA binding domain containing 2
ENSG0000	181.1392	-0.04175	0.135631	0.705475	0.922597	DUSP16	80824	dual specificity phosphatase 16
ENSG0000	2.469432	-0.01652	0.230029	0.704775	0.922597	DDX11-AS	1.01E+08	DDX11 antisense RNA 1
ENSG0000	11969.42	0.043145	0.148131	0.705612	0.922597	STAT6	6778	signal transducer and activator of transcription 6
ENSG0000	51.25104	-0.03996	0.192731	0.707668	0.922597	MSRB3	253827	methionine sulfoxide reductase B3
ENSG0000	3.417384	0.009243	0.231497	0.710698	0.922597	IFNG	3458	interferon gamma
ENSG0000	503.8319	0.044301	0.163335	0.707386	0.922597	PPP1R12A	4659	protein phosphatase 1 regulatory subunit 12A
ENSG0000	89.54385	0.043207	0.148078	0.706757	0.922597	CCDC59	29080	coiled-coil domain containing 59
ENSG0000	288.1943	0.042091	0.140408	0.705842	0.922597	RSRC2	65117	arginine and serine rich coiled-coil 2
ENSG0000	125.9642	-0.04281	0.151086	0.710835	0.922597	DENR	8562	density regulated re-initiation and release factor
ENSG0000	512.3029	-0.03467	0.107212	0.7114	0.922597	SAP18	10284	Sin3A associated protein 18
ENSG0000	9.90058	0.026431	0.219406	0.709118	0.922597	LINC0053	1.01E+08	long intergenic non-protein coding RNA 539
ENSG0000	12.81929	0.026167	0.221598	0.707637	0.922597	FLT1	2321	fms related receptor tyrosine kinase 1
ENSG0000	30.40543	0.038116	0.20162	0.70643	0.922597	RPL13AP2	1E+08	ribosomal protein L13a pseudogene 25
ENSG0000	34.45747	-0.03918	0.196249	0.708827	0.922597	BORA	79866	BORA aurora kinase A activator
ENSG0000	55.88908	0.042897	0.178889	0.705758	0.922597	TTC5	91875	tetratricopeptide repeat domain 5
ENSG0000	188.4739	-0.04026	0.185637	0.711263	0.922597	DDHD1	80821	DDHD domain containing 1
ENSG0000	218.0846	0.044062	0.155563	0.707882	0.922597	HERC2	8924	HECT and RLD domain containing E3 ubiquitin protein ligase 2
ENSG0000	1480.557	0.043591	0.162101	0.705214	0.922597	ZNF710	374655	zinc finger protein 710
ENSG0000	5.588841	-0.01651	0.228189	0.707965	0.922597	MIR5587	1.01E+08	microRNA 5587
ENSG0000	144.9417	0.042634	0.183806	0.70661	0.922597	CCNF	899	cyclin F
ENSG0000	11.87162	-0.02372	0.22411	0.708476	0.922597	NA	NA	NA
ENSG0000	6914.685	-0.04206	0.170506	0.708877	0.922597	MAPK3	5595	mitogen-activated protein kinase 3
ENSG0000	3.080549	0.018927	0.228892	0.71114	0.922597	NA	NA	NA
ENSG0000	169.1773	-0.04106	0.170037	0.70873	0.922597	CBFB	865	core-binding factor subunit beta
ENSG0000	137.3851	0.04296	0.153494	0.704761	0.922597	ELMO3	79767	engulfment and cell motility 3
ENSG0000	60.34554	0.043261	0.158683	0.710836	0.922597	PLEKHG4	25894	pleckstrin homology and RhoGEF domain containing G4
ENSG0000	84.41056	0.043574	0.16846	0.709305	0.922597	RPH3AL	9501	rabphilin 3A like (without C2 domains)
ENSG0000	7.521111	0.031621	0.216398	0.704581	0.922597	NA	NA	NA
ENSG0000	103.4652	0.036883	0.18037	0.70575	0.922597	PFAS	5198	phosphoribosylformylglycinamidine synthase
ENSG0000	8.856202	0.030147	0.218241	0.705848	0.922597	NA	NA	NA
ENSG0000	249.5717	-0.04222	0.153915	0.711119	0.922597	AKAP10	11216	A-kinase anchoring protein 10
ENSG0000	2.565968	-0.01461	0.231864	0.705702	0.922597	TMEM98	26022	transmembrane protein 98
ENSG0000	195.5627	-0.04223	0.176798	0.707965	0.922597	AOC2	314	amine oxidase copper containing 2
ENSG0000	3.988112	-0.0264	0.222844	0.707952	0.922597	HOXB7	3217	homeobox B7
ENSG0000	395.9162	0.041382	0.163238	0.711155	0.922597	ATP5MC1	516	ATP synthase membrane subunit c locus 1
ENSG0000	5138.781	0.042479	0.151397	0.711394	0.922597	MAP3K3	4215	mitogen-activated protein kinase kinase kinase 3
ENSG0000	43.81288	-0.04266	0.18274	0.705889	0.922597	NA	NA	NA
ENSG0000	8.641837	-0.02604	0.220517	0.705044	0.922597	FOXJ1	2302	forkhead box J1
ENSG0000	30134.54	-0.04212	0.153599	0.709667	0.922597	ACTG1	71	actin gamma 1
ENSG0000	1900.202	-0.03854	0.122844	0.70679	0.922597	NPLOC4	55666	NPL4 hom ubiquitin recognition factor
ENSG0000	44.44017	0.03876	0.196182	0.710786	0.922597	ZNF407-A	400657	ZNF407 antisense RNA 1
ENSG0000	47.32361	0.043217	0.171095	0.709115	0.922597	ZNF236-D	1E+08	ZNF236 divergent transcript
ENSG0000	31.93176	0.030839	0.214584	0.710236	0.922597	KCNG2	26251	potassium voltage-gated channel modifier subfamily G member 2
ENSG0000	8.203245	-0.01514	0.228402	0.7073	0.922597	NA	NA	NA
ENSG0000	526.2444	0.040121	0.140168	0.709737	0.922597	CACTIN	58509	cactin spliceosome C complex subunit
ENSG0000	270.4804	0.043856	0.164948	0.710439	0.922597	TMEM161	54929	transmembrane protein 161A
ENSG0000	10.84604	0.034058	0.212331	0.705698	0.922597	ZNF566-A	728752	ZNF566 antisense RNA 1
ENSG0000	17.62639	-0.03206	0.211777	0.710104	0.922597	ZNF571	51276	zinc finger protein 571
ENSG0000	1576.26	-0.04236	0.149482	0.707656	0.922597	SNRPA	6626	small nuclear ribonucleoprotein polypeptide A
ENSG0000	15.34529	-0.03776	0.204351	0.706062	0.922597	PRRG2	5639	proline rich and Gla domain 2
ENSG0000	3.452362	0.021565	0.22639	0.707223	0.922597	NA	NA	NA

ENSG0000	9989.558	-0.04236	0.162419	0.709844	0.922597	LILRB2	10288	leukocyte immunoglobulin like receptor B2
ENSG0000	5.646663	-0.02483	0.222945	0.70942	0.922597	NLRP7	199713	NLR family pyrin domain containing 7
ENSG0000	565.9222	0.042286	0.172718	0.710806	0.922597	ISOC2	79763	isochorismatase domain containing 2
ENSG0000	285.0108	-0.04013	0.134391	0.710497	0.922597	ZSCAN18	65982	zinc finger and SCAN domain containing 18
ENSG0000	17.70225	-0.03214	0.212396	0.710891	0.922597	NA	NA	NA
ENSG0000	825.7604	-0.04152	0.147551	0.704594	0.922597	CEP250	11190	centrosomal protein 250
ENSG0000	107.3616	-0.04195	0.153755	0.70882	0.922597	NFS1	9054	NFS1 cysteine desulfurase
ENSG0000	16.43349	-0.03645	0.205138	0.70635	0.922597	ERG	2078	ETS transcription factor ERG
ENSG0000	7.541937	-0.03096	0.217525	0.704123	0.922597	NA	NA	NA
ENSG0000	74.26975	-0.04115	0.189235	0.704275	0.922597	FOXRED2	80020	FAD dependent oxidoreductase domain containing 2
ENSG0000	29.92108	0.031817	0.21344	0.71036	0.922597	SYN1	6853	synapsin I
ENSG0000	54.51446	-0.04388	0.17243	0.710286	0.922597	FAM120C	54954	family with sequence similarity 120C
ENSG0000	7.437919	-0.02952	0.21772	0.711048	0.922597	EIF4A1P1C	1E+08	eukaryotic translation initiation factor 4A1 pseudogene 10
ENSG0000	26.87717	-0.03804	0.201337	0.70628	0.922597	NA	NA	NA
ENSG0000	20.76318	-0.0375	0.202295	0.711489	0.922629	NA	NA	NA
ENSG0000	131.8393	-0.04257	0.172881	0.711681	0.922813	TEKT4P2	1E+08	tektin 4 pseudogene 2
ENSG0000	5625.047	-0.03371	0.151186	0.711799	0.922901	CTSZ	1522	cathepsin Z
ENSG0000	3757.682	0.038013	0.117294	0.711913	0.922983	B4GALT1	2683	beta-1 4-galactosyltransferase 1
ENSG0000	43.32084	0.038639	0.197736	0.711973	0.922995	RFK	55312	riboflavin kinase
ENSG0000	50.16986	0.040585	0.186179	0.712165	0.923114	NBPF9	400818	NBPF member 9
ENSG0000	6.069128	0.021878	0.225152	0.712142	0.923114	DCAF4L1	285429	DDB1 and CUL4 associated factor 4 like 1
ENSG0000	304.0335	0.041204	0.124705	0.712373	0.923187	FBXL6	26233	F-box and leucine rich repeat protein 6
ENSG0000	1990.076	0.038979	0.123812	0.712373	0.923187	EZH1	2145	enhancer of zeste 1 polycomb repressive complex 2 subunit
ENSG0000	109.2473	0.042537	0.16968	0.712363	0.923187	PHLDB3	653583	pleckstrin homology like domain family B member 3
ENSG0000	2284.812	-0.03718	0.14769	0.712474	0.923252	SMARCD2	6603	SWI/SNF r matrix ass actin depe subfamily member 2
ENSG0000	4.116066	-0.018	0.227316	0.712609	0.923363	NA	NA	NA
ENSG0000	402.1557	0.041603	0.169561	0.712666	0.92337	VEZF1	7716	vascular endothelial zinc finger 1
ENSG0000	123.5396	-0.04242	0.163655	0.712721	0.923377	TGS1	96764	trimethylguanosine synthase 1
ENSG0000	7.385975	0.03069	0.216743	0.712807	0.923423	PCCA-DT	1.05E+08	PCCA divergent transcript
ENSG0000	15.68722	-0.03113	0.213582	0.712993	0.923478	SPEF2	79925	sperm flagellar 2
ENSG0000	66.98149	0.042263	0.182831	0.713051	0.923478	PPIL4	85313	peptidylprolyl isomerase like 4
ENSG0000	2.785219	0.018986	0.227764	0.712944	0.923478	C1S	716	complement C1s
ENSG0000	85.38855	-0.04244	0.170283	0.713028	0.923478	UNG	7374	uracil DNA glycosylase
ENSG0000	1304.592	0.04232	0.155094	0.713664	0.923517	SARS1	6301	seryl-tRNA synthetase 1
ENSG0000	3.876761	-0.01346	0.229478	0.71336	0.923517	RASAL2	9462	RAS protein activator like 2
ENSG0000	164.7522	0.042101	0.156925	0.713686	0.923517	PGRMC2	10424	progesterone receptor membrane component 2
ENSG0000	11.66125	0.033705	0.211939	0.713626	0.923517	NA	NA	NA
ENSG0000	20.70413	-0.03064	0.215968	0.71337	0.923517	TG	7038	thyroglobulin
ENSG0000	7.915307	0.025181	0.222139	0.713448	0.923517	BNC2	54796	basonuclin 2
ENSG0000	49.57347	-0.03999	0.179944	0.713402	0.923517	EPS8L2	64787	EPS8 like 2
ENSG0000	7.040661	-0.02542	0.221645	0.713413	0.923517	NA	NA	NA
ENSG0000	3.873356	-0.01645	0.228456	0.713676	0.923517	NA	NA	NA
ENSG0000	401.4193	-0.03965	0.133799	0.713281	0.923517	NUDT16L1	84309	nudix hydrolase 16 like 1
ENSG0000	13.32084	-0.02276	0.221842	0.713149	0.923517	ZNF799	90576	zinc finger protein 799
ENSG0000	573.5032	0.041979	0.153637	0.713636	0.923517	TIMM50	92609	translocase of inner mitochondrial membrane 50
ENSG0000	34.32308	-0.0321	0.193168	0.714611	0.923572	CLSPN	63967	claspin
ENSG0000	10.37634	-0.0354	0.208087	0.714676	0.923572	FOXD2	2306	forkhead box D2
ENSG0000	512.4566	-0.0357	0.110553	0.714278	0.923572	LMO4	8543	LIM domain only 4
ENSG0000	27.28139	-0.03954	0.194194	0.714727	0.923572	TTC24	164118	tetratricopeptide repeat domain 24
ENSG0000	14.7092	0.033877	0.21069	0.714814	0.923572	NA	NA	NA
ENSG0000	8.996122	0.021368	0.224232	0.714785	0.923572	NA	NA	NA
ENSG0000	29.89086	0.031895	0.21366	0.714735	0.923572	DCUN1D4	23142	defective in cullin neddylation 1 domain containing 4
ENSG0000	20.38725	-0.03709	0.206486	0.714504	0.923572	ING2	3622	inhibitor of growth family member 2
ENSG0000	6.767864	0.028603	0.220328	0.714454	0.923572	LINC0214	401172	long intergenic non-protein coding RNA 2145
ENSG0000	3.068074	0.016424	0.231973	0.714888	0.923572	NA	NA	NA
ENSG0000	3.098895	0.021841	0.227255	0.713878	0.923572	H3C3	8352	H3 clustered histone 3
ENSG0000	80.1395	0.042287	0.171481	0.714244	0.923572	FAM220A	84792	family with sequence similarity 220 member A
ENSG0000	1911.527	-0.03814	0.135767	0.714561	0.923572	FASTK	10922	Fas activated serine/threonine kinase
ENSG0000	461.351	0.043221	0.15113	0.714668	0.923572	CREB3	10488	cAMP responsive element binding protein 3
ENSG0000	16.82079	0.034139	0.208747	0.713809	0.923572	CDHR1	92211	cadherin related family member 1
ENSG0000	27.03269	0.041438	0.177736	0.714429	0.923572	PTPMT1	114971	protein tyrosine phosphatase mitochondrial 1
ENSG0000	106.5492	0.042397	0.154789	0.714471	0.923572	NUP160	23279	nucleoporin 160
ENSG0000	5.09238	-0.01352	0.229239	0.713891	0.923572	AMHR2	269	anti-Mullerian hormone receptor type 2
ENSG0000	2.772389	0.011915	0.231153	0.7143	0.923572	HIF1A-AS3	1.05E+08	HIF1A antisense RNA 3
ENSG0000	3.183067	0.014208	0.229297	0.713976	0.923572	NA	NA	NA
ENSG0000	7.957866	-0.02096	0.224446	0.714868	0.923572	NFE2L1-DT	1.16E+08	NFE2L1 divergent transcript
ENSG0000	1626.841	0.043112	0.138846	0.714195	0.923572	DUS1L	64118	dihydrouridine synthase 1 like
ENSG0000	61.04799	-0.03981	0.188798	0.71477	0.923572	LINC0167	1.02E+08	long intergenic non-protein coding RNA 1679
ENSG0000	326.24	0.041961	0.168757	0.714973	0.923579	AHSA2P	130872	activator c pseudogene
ENSG0000	17.81724	0.037196	0.205258	0.715045	0.923579	SLC12A2	6558	solute carrier family 12 member 2
ENSG0000	20.41133	-0.03909	0.196601	0.71502	0.923579	ZNF528-A	1.03E+08	ZNF528 antisense RNA 1
ENSG0000	581.1627	0.039257	0.133569	0.715607	0.923783	ZNF655	79027	zinc finger protein 655
ENSG0000	321.6769	-0.03964	0.119038	0.715481	0.923783	PABIR1	116224	PP2A Aalpha (PPP2R1A) and B55A (PPP2R2A) interacting phosphatase regulator 1
ENSG0000	438.2711	-0.04282	0.158928	0.715605	0.923783	CACUL1	143384	CDK2 associated cullin domain 1
ENSG0000	2778.852	0.040401	0.172708	0.715323	0.923783	CD4	920	CD4 molecule
ENSG0000	3.142303	0.013889	0.229674	0.715559	0.923783	NID2	22795	nidogen 2
ENSG0000	360.6406	0.041971	0.142304	0.715464	0.923783	UTP4	84916	UTP4 small subunit processome component
ENSG0000	2.389065	0.01917	0.228039	0.71543	0.923783	NA	NA	NA
ENSG0000	72.59376	-0.04232	0.165278	0.715274	0.923783	CLCN4	1183	chloride voltage-gated channel 4
ENSG0000	1592.56	-0.04183	0.152828	0.715658	0.923785	STX5	6811	syntaxin 5
ENSG0000	2.76932	0.016917	0.230151	0.715711	0.923788	NA	NA	NA
ENSG0000	6.107208	-0.02174	0.22304	0.71637	0.924069	NA	NA	NA
ENSG0000	644.0877	-0.03907	0.135234	0.716298	0.924069	ENTREP3	10712	endosomal transmembrane epsin interactor 3
ENSG0000	12.19999	-0.03314	0.208657	0.716186	0.924069	NA	NA	NA
ENSG0000	412.2109	0.040291	0.181006	0.716484	0.924069	RYBP	23429	RING1 and YY1 binding protein
ENSG0000	595.4406	0.040083	0.18466	0.716476	0.924069	RNASE1	6035	ribonuclea pancreatic
ENSG0000	64.08808	0.042201	0.181958	0.716334	0.924069	CCP110	9738	centriolar coiled-coil protein 110
ENSG0000	256.1725	0.039601	0.133489	0.716293	0.924069	TK2	7084	thymidine kinase 2
ENSG0000	313.6465	0.037037	0.123262	0.716468	0.924069	ZNF317	57693	zinc finger protein 317
ENSG0000	1154.287	-0.03882	0.129986	0.716302	0.924069	NFKBID	84807	NFKB inhibitor delta
ENSG0000	195.2029	-0.04171	0.158538	0.716067	0.924069	GGT7	2686	gamma-glutamyltransferase 7
ENSG0000	163.665	-0.04166	0.147973	0.716264	0.924069	TP53RK	112858	TP53 regulating kinase
ENSG0000	1326.988	-0.04036	0.142405	0.716562	0.924104	CCNDBP1	23582	cyclin D1 binding protein 1
ENSG0000	6.992526	0.019779	0.225716	0.716625	0.92412	NA	NA	NA
ENSG0000	16.73667	0.035049	0.207481	0.716694	0.92413	NA	NA	NA
ENSG0000	19.48094	-0.0354	0.207395	0.716733	0.92413	ZXDA	7789	zinc finger X-linked duplicated A

ENSG0000	4061.466	-0.04107	0.157552	0.716883	0.924194	IL6R	3570	interleukin 6 receptor
ENSG0000	363.2336	-0.03651	0.117185	0.716859	0.924194	ARMH3	79591	armadillo like helical domain containing 3
ENSG0000	559.7941	-0.04145	0.163958	0.717061	0.924332	PCF11	51585	PCF11 cleavage and polyadenylation factor subunit
ENSG0000	15117.59	-0.04082	0.167913	0.717091	0.924332	PHOSPHO	162466	phosphoethanolamine/phosphocholine phosphatase 1
ENSG0000	1085.533	-0.0319	0.120945	0.717191	0.924396	BAX	581	BCL2 assoc apoptosis regulator
ENSG0000	125.1328	-0.0396	0.185537	0.717243	0.924397	ZNF703	80139	zinc finger protein 703
ENSG0000	51.97248	0.041651	0.180353	0.717326	0.924398	DLG5	9231	discs large MAGUK scaffold protein 5
ENSG0000	671.8823	-0.04585	0.153562	0.717348	0.924398	LPCAT3	10162	lysophosphatidylcholine acyltransferase 3
ENSG0000	206.7652	-0.04311	0.149515	0.717395	0.924398	LZTR1	8216	leucine zipper like transcription regulator 1
ENSG0000	42.64123	0.043531	0.168032	0.717669	0.924678	WARS2-AS	1.02E+08	WARS2 antisense RNA 1
ENSG0000	8.328514	0.02891	0.217528	0.717895	0.924678	NA	NA	NA
ENSG0000	9.369708	0.03247	0.213012	0.717915	0.924678	POLQ	10721	DNA polymerase theta
ENSG0000	3.72093	0.014185	0.228975	0.717915	0.924678	RPS3AP26	644972	RPS3A pseudogene 26
ENSG0000	128.7556	0.040394	0.159294	0.717867	0.924678	NUDT21	11051	nudix hydrolase 21
ENSG0000	51.36328	0.040264	0.190446	0.717854	0.924678	F8	2157	coagulation factor VIII
ENSG0000	7.494658	0.026663	0.220683	0.718074	0.924745	NA	NA	NA
ENSG0000	5.032634	0.015546	0.228189	0.718119	0.924745	NA	NA	NA
ENSG0000	15.81128	-0.01765	0.21555	0.718054	0.924745	FAM222A	84915	family with sequence similarity 222 member A
ENSG0000	675.7588	0.039811	0.138526	0.718245	0.924772	SERPINB6	5269	serpin family B member 6
ENSG0000	570.3026	0.041141	0.173947	0.718293	0.924772	WTAP	9589	WT1 associated protein
ENSG0000	105.0265	0.040495	0.142457	0.718342	0.924772	AHCYL2	23382	adenosylhomocysteinase like 2
ENSG0000	516.6645	-0.04176	0.154037	0.718216	0.924772	CA5BP1	340591	carbonic anhydrase 5B pseudogene 1
ENSG0000	110.7337	0.039234	0.151179	0.718459	0.924843	TAF5L	27097	TATA-box binding protein associated factor 5 like
ENSG0000	139.9533	0.044291	0.162522	0.718497	0.924843	HIRIP3	8479	HIRA interacting protein 3
ENSG0000	4.57012	-0.01963	0.226042	0.718687	0.925021	NA	NA	NA
ENSG0000	4.550253	-0.01656	0.227938	0.718855	0.925108	TRBJ1-3	28633	T cell receptor beta joining 1-3
ENSG0000	156.4604	0.038925	0.126114	0.71883	0.925108	RTL10	79680	retrotransposon Gag like 10
ENSG0000	8335.165	0.044164	0.170347	0.718957	0.925174	IMPDH1	3614	inosine monophosphate dehydrogenase 1
ENSG0000	710.9663	-0.03946	0.136884	0.719226	0.925181	PEX14	5195	peroxisomal biogenesis factor 14
ENSG0000	99.96569	-0.04134	0.172643	0.719317	0.925181	LRP8	7804	LDL receptor related protein 8
ENSG0000	663.0064	0.041842	0.167582	0.719271	0.925181	RAP1A	5906	RAP1A member of RAS oncogene family
ENSG0000	1190.252	-0.03964	0.148904	0.719373	0.925181	CCT7	10574	chaperonin containing TCP1 subunit 7
ENSG0000	15.97547	-0.02016	0.224162	0.719161	0.925181	ZFP57	346171	ZFP57 zinc finger protein
ENSG0000	171.8691	0.04152	0.144829	0.719022	0.925181	TTLL5	23093	tubulin tyrosine ligase like 5
ENSG0000	87.3868	-0.04162	0.149147	0.719417	0.925181	ZSCAN29	146050	zinc finger and SCAN domain containing 29
ENSG0000	11.5939	0.037483	0.203055	0.719176	0.925181	LOC10798	1.08E+08	uncharacterized LOC107985075
ENSG0000	11.06433	-0.03093	0.214668	0.719385	0.925181	TNS4	84951	tensin 4
ENSG0000	66.24134	0.042088	0.169692	0.719607	0.925329	AIFM2	84883	apoptosis inducing factor mitochondria associated 2
ENSG0000	2777.632	-0.04172	0.160326	0.719684	0.925329	TBC1D10B	26000	TBC1 domain family member 10B
ENSG0000	187.2372	-0.03963	0.135685	0.719664	0.925329	NSRP1	84081	nuclear speckle splicing regulatory protein 1
ENSG0000	15.93244	-0.03627	0.202746	0.719815	0.925382	NA	NA	NA
ENSG0000	15.15694	0.029936	0.216275	0.719876	0.925382	BAZ2B	29994	bromodomain adjacent to zinc finger domain 2B
ENSG0000	21.80161	0.037459	0.201346	0.719839	0.925382	HELLS	3070	helicase lymphoid specific
ENSG0000	174.2393	-0.04054	0.147991	0.71997	0.925437	NINJ2-AS1	1E+08	NINJ2 antisense RNA 1
ENSG0000	39.19457	0.040484	0.16454	0.720552	0.925471	PMS2P2	5380	PMS1 hom mismatch repair system component pseudogene 2
ENSG0000	1989.47	-0.03615	0.147039	0.720505	0.925471	DGAT1	8694	diacylglycerol O-acyltransferase 1
ENSG0000	441.1669	0.044171	0.143524	0.720234	0.925471	UBAP2	55833	ubiquitin associated protein 2
ENSG0000	111.878	-0.03592	0.19785	0.720426	0.925471	LIPN	643418	lipase family member N
ENSG0000	114.4938	-0.04149	0.151506	0.720384	0.925471	CWC15	51503	CWC15 spliceosome associated protein homolog
ENSG0000	3313.257	-0.03935	0.140029	0.720543	0.925471	FLI1	2313	Fli-1 proto ETS transcription factor
ENSG0000	3.274971	0.014652	0.230612	0.72045	0.925471	NA	NA	NA
ENSG0000	111.4632	0.039504	0.191049	0.720535	0.925471	POC1B	282809	POC1 centriolar protein B
ENSG0000	214.4623	0.039415	0.13446	0.720177	0.925471	PABPN1	8106	poly(A) binding protein nuclear 1
ENSG0000	468.2059	0.039775	0.172456	0.720254	0.925471	SEL1L	6400	SEL1L adaptor subunit of ERAD E3 ubiquitin ligase
ENSG0000	5.275864	-0.01975	0.22567	0.720287	0.925471	NA	NA	NA
ENSG0000	1195.499	-0.04166	0.164605	0.720654	0.925537	VAPA	9218	VAMP associated protein A
ENSG0000	12.7926	-0.03367	0.20981	0.720704	0.925537	NA	NA	NA
ENSG0000	396.8946	0.035287	0.134172	0.720808	0.925605	DHX15	1665	DEAH-box helicase 15
ENSG0000	425.413	-0.04517	0.167212	0.720998	0.925654	ADGRG5	221188	adhesion G protein-coupled receptor G5
ENSG0000	19.75111	-0.0315	0.210369	0.72096	0.925654	ZNF665	79788	zinc finger protein 665
ENSG0000	47.07073	-0.02534	0.21827	0.720995	0.925654	IGLV4-3	28786	immunoglobulin lambda variable 4-3
ENSG0000	461.9523	0.040673	0.148187	0.721209	0.925691	STK16	8576	serine/threonine kinase 16
ENSG0000	1314.05	-0.04092	0.153285	0.721301	0.925691	SSR1	6745	signal sequence receptor subunit 1
ENSG0000	20.33947	-0.02842	0.216438	0.721138	0.925691	NA	NA	NA
ENSG0000	37.90843	0.041708	0.173685	0.721329	0.925691	NA	NA	NA
ENSG0000	5.900758	-0.02268	0.223578	0.721268	0.925691	NA	NA	NA
ENSG0000	10020.46	-0.04047	0.160175	0.721235	0.925691	GMIP	51291	GEM interacting protein
ENSG0000	5.565388	0.017062	0.226735	0.721938	0.925714	OXCT2	64064	3-oxoacid CoA-transferase 2
ENSG0000	3.520868	-0.01442	0.229567	0.72253	0.925714	CIART	148523	circadian associated repressor of transcription
ENSG0000	609.565	-0.03682	0.127154	0.722417	0.925714	USP21	27005	ubiquitin specific peptidase 21
ENSG0000	9.72324	0.023006	0.22242	0.72195	0.925714	NA	NA	NA
ENSG0000	22.3692	-0.03625	0.201051	0.721868	0.925714	NA	NA	NA
ENSG0000	364.3206	0.042149	0.159605	0.722573	0.925714	FBXO11	80204	F-box protein 11
ENSG0000	2.344295	0.013771	0.230204	0.722734	0.925714	COL5A2	1290	collagen type V alpha 2 chain
ENSG0000	1249.501	0.042333	0.138151	0.722447	0.925714	ATG4B	23192	autophagy related 4B cysteine peptidase
ENSG0000	253.4735	0.041928	0.184518	0.721654	0.925714	PCNP	57092	PEST proteolytic signal containing nuclear protein
ENSG0000	3.902264	-0.02111	0.225157	0.722763	0.925714	ZBED8	63920	zinc finger BED-type containing 8
ENSG0000	87.15976	-0.04219	0.167462	0.721761	0.925714	PSMG4	389362	proteasome assembly chaperone 4
ENSG0000	2.515652	-0.0175	0.228597	0.721542	0.925714	HMGN3-A	1E+08	HMGN3 antisense RNA 1
ENSG0000	51.57586	0.039006	0.187379	0.721405	0.925714	NCAPG2	54892	non-SMC condensin II complex subunit G2
ENSG0000	12.08856	0.025922	0.218648	0.722503	0.925714	NA	NA	NA
ENSG0000	5.534514	-0.02264	0.224096	0.722593	0.925714	NA	NA	NA
ENSG0000	3.691366	-0.0238	0.223032	0.722685	0.925714	NA	NA	NA
ENSG0000	203.3271	0.043372	0.146437	0.722206	0.925714	CDK2AP1	8099	cyclin dependent kinase 2 associated protein 1
ENSG0000	7.088153	-0.02348	0.220736	0.722501	0.925714	CTCF-DT	1.08E+08	CTCF divergent transcript
ENSG0000	2.870553	0.015367	0.228743	0.722659	0.925714	NA	NA	NA
ENSG0000	3.173612	-0.01219	0.229905	0.722378	0.925714	NA	NA	NA
ENSG0000	57.14659	-0.04074	0.158917	0.722372	0.925714	SLFN12	55106	schlafen family member 12
ENSG0000	189.7332	-0.04105	0.156196	0.721968	0.925714	ZNF561	93134	zinc finger protein 561
ENSG0000	278.8909	0.04846	0.165227	0.721914	0.925714	CCDC61	729440	coiled-coil domain containing 61
ENSG0000	2.342049	0.010043	0.231493	0.722207	0.925714	NKILA	1.05E+08	NF-kappaB interacting lncRNA
ENSG0000	94.90694	-0.0371	0.19387	0.722378	0.925714	C21orf91	54149	chromosome 21 open reading frame 91
ENSG0000	3.238536	0.026044	0.228941	0.721632	0.925714	NA	NA	NA
ENSG0000	1101.006	-0.03602	0.118112	0.721668	0.925714	TIMM17B	10245	translocase of inner mitochondrial membrane 17B
ENSG0000	50.56292	-0.04157	0.163473	0.722684	0.925714	AMER1	139285	APC membrane recruitment protein 1
ENSG0000	2677.992	-0.03615	0.127794	0.722859	0.925735	NISCH	11188	nischarin

ENSG0000	381.5965	-0.04051	0.166089	0.722886	0.925735	INTS5	80789	integrator complex subunit 5
ENSG0000	311.214	-0.04109	0.155331	0.722981	0.925735	PPHLN1	51535	periphilin 1
ENSG0000	3.904604	0.015584	0.228385	0.722931	0.925735	NA	NA	NA
ENSG0000	46.61692	0.041376	0.172653	0.723248	0.925936	EHBP1	23301	EH domain binding protein 1
ENSG0000	7.754078	-0.02824	0.217223	0.723436	0.925936	MB21D2	151963	Mab-21 domain containing 2
ENSG0000	8.06084	0.032463	0.214618	0.723593	0.925936	NA	NA	NA
ENSG0000	3852.394	-0.04028	0.159099	0.723247	0.925936	CIZ1	25792	CDKN1A interacting zinc finger protein 1
ENSG0000	2.646316	-0.01	0.231345	0.723561	0.925936	SCN2B	6327	sodium voltage-gated channel beta subunit 2
ENSG0000	250.2353	-0.03955	0.180597	0.723489	0.925936	PELI2	57161	pellino E3 ubiquitin protein ligase family member 2
ENSG0000	7.276777	0.023098	0.222606	0.723526	0.925936	NA	NA	NA
ENSG0000	111.4654	0.040812	0.167608	0.723368	0.925936	LOC10537	1.05E+08	uncharacterized LOC105370792
ENSG0000	7.426351	0.024405	0.221623	0.723446	0.925936	PLLP	51090	plasmolipin
ENSG0000	65.31669	0.040787	0.166374	0.723928	0.926117	SDHA	6389	succinate dehydrogenase complex flavoprotein subunit A
ENSG0000	1208.659	-0.04084	0.150584	0.724038	0.926117	CBL	867	Cbl proto-oncogene
ENSG0000	366.2926	0.039498	0.141464	0.723997	0.926117	GOT2	2806	glutamic-oxaloacetic transaminase 2
ENSG0000	7.354838	-0.0259	0.220132	0.723927	0.926117	LOC10537	1.05E+08	uncharacterized LOC105371414
ENSG0000	251.1776	0.04094	0.156631	0.723869	0.926117	ZNF266	10781	zinc finger protein 266
ENSG0000	321.8213	-0.0403	0.157754	0.724	0.926117	PAK4	10298	p21 (RAC1) activated kinase 4
ENSG0000	322.5493	0.040557	0.163738	0.724136	0.926178	NUP58	9818	nucleoporin 58
ENSG0000	358.2127	-0.03927	0.159412	0.724272	0.926287	RGS10	6001	regulator of G protein signaling 10
ENSG0000	44.62336	-0.04073	0.173573	0.724355	0.926329	EXOC6B	23233	exocyst complex component 6B
ENSG0000	151.263	-0.04024	0.148334	0.724413	0.926338	GNB5	10681	G protein subunit beta 5
ENSG0000	852.1454	0.040382	0.134535	0.724578	0.926485	FAAP20	199990	FA core complex associated protein 20
ENSG0000	275.5435	-0.04022	0.154332	0.724692	0.926501	SFT2D2	375035	SFT2 domain containing 2
ENSG0000	28.09587	0.035781	0.200862	0.724681	0.926501	RTN4RL2	349667	reticulon 4 receptor like 2
ENSG0000	750.6755	0.040286	0.175498	0.724837	0.926511	CAB39	51719	calcium binding protein 39
ENSG0000	102.1724	-0.0365	0.191051	0.724862	0.926511	GCSAM	257144	germinal center associated signaling and motility
ENSG0000	210.5573	-0.03412	0.203986	0.724891	0.926511	FAR1	84188	fatty acyl-CoA reductase 1
ENSG0000	121.9186	0.041236	0.160166	0.724902	0.926511	TAF1	6872	TATA-box binding protein associated factor 1
ENSG0000	5.574389	0.015363	0.228697	0.725054	0.926641	RPL36AP3	729362	ribosomal protein L36a pseudogene 37
ENSG0000	35.57315	0.039918	0.184241	0.725122	0.926663	NA	NA	NA
ENSG0000	148.6237	0.04372	0.136855	0.725452	0.926743	SNHG3	8420	small nucleolar RNA host gene 3
ENSG0000	972.6336	0.04598	0.15221	0.725402	0.926743	ATF6	22926	activating transcription factor 6
ENSG0000	9.65957	0.024397	0.219524	0.72538	0.926743	SCD5	79966	stearoyl-CoA desaturase 5
ENSG0000	14.08582	-0.0331	0.208636	0.725779	0.926743	SLC23A1	9963	solute carrier family 23 member 1
ENSG0000	4.782706	0.025906	0.223901	0.725659	0.926743	TPI1P2	286016	triosephosphate isomerase 1 pseudogene 2
ENSG0000	102.6448	-0.04131	0.175682	0.725381	0.926743	CD248	57124	CD248 molecule
ENSG0000	5.710179	0.020949	0.224085	0.72584	0.926743	NA	NA	NA
ENSG0000	941.785	0.039228	0.171634	0.72591	0.926743	HAL	3034	histidine ammonia-lyase
ENSG0000	1307.95	-0.03453	0.115915	0.725505	0.926743	KHNYN	23351	KH and NYN domain containing
ENSG0000	118.5649	0.037921	0.189576	0.725923	0.926743	CNIH1	10175	cornichon family AMPA receptor auxiliary protein 1
ENSG0000	4.868859	-0.02131	0.227735	0.725697	0.926743	NA	NA	NA
ENSG0000	882.2263	0.038346	0.134269	0.725571	0.926743	ZC3H4	23211	zinc finger CCCH-type containing 4
ENSG0000	20.43042	0.032744	0.208275	0.725944	0.926743	LOC10272	1.03E+08	trafficking protein particle complex subunit 10-like
ENSG0000	17.43246	0.031267	0.211297	0.725261	0.926743	ZNF674-AS	401588	ZNF674 antisense RNA 1 (head to head)
ENSG0000	46.84061	0.036232	0.198588	0.725797	0.926743	NXT2	55916	nuclear transport factor 2 like export factor 2
ENSG0000	148.1638	0.042544	0.184984	0.726102	0.92688	PPTC7	160760	protein phosphatase targeting COQ7
ENSG0000	917.5013	-0.03998	0.141221	0.726156	0.926885	ZC3H7B	23264	zinc finger CCCH-type containing 7B
ENSG0000	1607.639	0.040609	0.151428	0.726358	0.927079	DUSP6	1848	dual specificity phosphatase 6
ENSG0000	44.31332	-0.04025	0.172672	0.726575	0.927162	ZNF436	80818	zinc finger protein 436
ENSG0000	58.1652	0.035434	0.200192	0.726571	0.927162	MFSD8	256471	major facilitator superfamily domain containing 8
ENSG0000	1429.839	0.038614	0.136001	0.726518	0.927162	WNK1	65125	WNK lysine deficient protein kinase 1
ENSG0000	4256.957	-0.03745	0.135426	0.726643	0.927183	TMC6	11322	transmembrane channel like 6
ENSG0000	789.317	-0.03905	0.159486	0.726946	0.927312	NUDC	10726	nuclear di: dynein complex regulator
ENSG0000	89.87271	0.040639	0.172347	0.726908	0.927312	CDCA8	55143	cell division cycle associated 8
ENSG0000	47.93125	0.037175	0.171633	0.726927	0.927312	TTC28-AS1	284900	TTC28 antisense RNA 1
ENSG0000	890.1017	0.038934	0.138916	0.726904	0.927312	WDR13	64743	WD repeat domain 13
ENSG0000	6.958494	0.028532	0.217115	0.727742	0.927608	LOC10192	1.02E+08	uncharacterized LOC101928626
ENSG0000	3014.491	-0.03093	0.129363	0.727601	0.927608	TGOLN2	10618	trans-golgi network protein 2
ENSG0000	321.2905	0.038894	0.14033	0.727668	0.927608	ZBTB47	92999	zinc finger and BTB domain containing 47
ENSG0000	764.5862	-0.03564	0.119317	0.727609	0.927608	SETD2	29072	SET domai histone lysine methyltransferase
ENSG0000	153.635	-0.03848	0.178937	0.727735	0.927608	STARD3NL	83930	STARD3 N-terminal like
ENSG0000	2.758072	0.016504	0.228972	0.727709	0.927608	NA	NA	NA
ENSG0000	24.30391	-0.03714	0.19453	0.727534	0.927608	AGBL2	79841	AGBL carboxypeptidase 2
ENSG0000	30.69969	-0.03504	0.201036	0.727716	0.927608	SKA3	221150	spindle and kinetochore associated complex subunit 3
ENSG0000	11.91774	0.032285	0.208059	0.727256	0.927608	NA	NA	NA
ENSG0000	61.45161	-0.04037	0.16723	0.727391	0.927608	LINC0269:	339263	long intergenic non-protein coding RNA 2693
ENSG0000	8.182201	0.029735	0.214685	0.727321	0.927608	NA	NA	NA
ENSG0000	361.9261	0.040231	0.158306	0.727786	0.927608	PRR14L	253143	proline rich 14 like
ENSG0000	18.23041	-0.02967	0.212358	0.727896	0.927619	NA	NA	NA
ENSG0000	700.2312	0.040214	0.169846	0.727892	0.927619	CHMP1B	57132	charged multivesicular body protein 1B
ENSG0000	8.501188	-0.01577	0.22717	0.728143	0.927623	UTS2	10911	urotensin 2
ENSG0000	1578.687	-0.04252	0.157491	0.728184	0.927623	ATP1A1	476	ATPase Na+/K+ transporting subunit alpha 1
ENSG0000	234.8054	0.030713	0.206058	0.728254	0.927623	EDEM3	80267	ER degradation enhancing alpha-mannosidase like protein 3
ENSG0000	7.724357	0.031186	0.212932	0.727956	0.927623	MYORG	57462	myogenesis regulating glycosidase (putative)
ENSG0000	40.95217	-0.03828	0.187953	0.728008	0.927623	METTL1	4234	methyltra tRNA methylguanosine
ENSG0000	3.56116	0.014619	0.231257	0.728213	0.927623	NA	NA	NA
ENSG0000	470.7701	-0.03618	0.129509	0.728226	0.927623	CLEC16A	23274	C-type lectin domain containing 16A
ENSG0000	120.0455	-0.03608	0.193786	0.728354	0.92767	HEMGN	55363	hemogen
ENSG0000	10.77582	-0.03133	0.211526	0.728391	0.92767	MRPL46	26589	mitochondrial ribosomal protein L46
ENSG0000	425.4737	-0.03772	0.147431	0.728514	0.927761	PPP4R3A	55671	protein phosphatase 4 regulatory subunit 3A
ENSG0000	120.8903	-0.03879	0.179455	0.728804	0.927973	WDR75	84128	WD repeat domain 75
ENSG0000	48.63463	0.035603	0.19976	0.728891	0.927973	BMT2	154743	base methyltransferase of 25S rRNA 2 homolog
ENSG0000	17.47908	-0.03419	0.205279	0.728914	0.927973	DISP2	85455	dispatched RND transporter family member 2
ENSG0000	196.1345	-0.03843	0.184118	0.728933	0.927973	SCAMP5	192683	secretory carrier membrane protein 5
ENSG0000	14.33677	-0.02723	0.217207	0.728775	0.927973	SEC14L5	9717	SEC14 like lipid binding 5
ENSG0000	220.072	0.038345	0.136702	0.729023	0.928022	TARBP2	6895	TARBP2 subunit of RISC loading complex
ENSG0000	226.6608	0.040172	0.138469	0.729096	0.928036	FYCO1	79443	FYVE and coiled-coil domain autophagy adaptor 1
ENSG0000	899.8257	0.038394	0.166516	0.729243	0.928036	NLRX1	79671	NLR family member X1
ENSG0000	4.231391	-0.01585	0.229652	0.7292	0.928036	NA	NA	NA
ENSG0000	25.98601	0.033826	0.208838	0.729219	0.928036	LINC0040	1.01E+08	long intergenic non-protein coding RNA 402
ENSG0000	1018.923	-0.03891	0.177457	0.729287	0.928036	AATF	26574	apoptosis antagonizing transcription factor
ENSG0000	262.358	0.039012	0.188697	0.729342	0.928042	PRKAA1	5562	protein kinase AMP-activated catalytic subunit alpha 1
ENSG0000	30.90827	0.039999	0.191671	0.729661	0.928358	NA	NA	NA
ENSG0000	317.0675	0.035628	0.120504	0.729692	0.928358	CHCHD5	84269	coiled-coil-helix-coiled-coil-helix domain containing 5
ENSG0000	194.7089	-0.03864	0.145022	0.729797	0.928362	TAF10	6881	TATA-box binding protein associated factor 10

ENSG0000	121.6998	0.023186	0.22357	0.729795	0.928362	LINC00671	388387	long intergenic non-protein coding RNA 671
ENSG0000	20.71926	0.029463	0.212641	0.730012	0.928443	WDFY3-AS	404201	WDFY3 antisense RNA 2
ENSG0000	23.62656	-0.03192	0.207498	0.729981	0.928443	NA	NA	NA
ENSG0000	4.230762	0.030037	0.225065	0.729943	0.928443	NHIP	1.05E+08	neuronal h placenta associated
ENSG0000	81.55767	0.038422	0.154465	0.730064	0.928445	UPF3B	65109	UPF3B regulator of nonsense mediated mRNA decay
ENSG0000	176.8025	-0.03994	0.162552	0.730354	0.928748	PRNP	5621	prion protein
ENSG0000	255.2508	0.037861	0.134192	0.730631	0.928779	TP53BP2	7159	tumor protein p53 binding protein 2
ENSG0000	236.7915	0.04016	0.153949	0.730537	0.928779	SNAP47	116841	synaptosome associated protein 47
ENSG0000	21.86685	0.035744	0.190542	0.730445	0.928779	LOC84214	84214	Putative uncharacterized protein FLJ92257
ENSG0000	52.17942	0.039828	0.161291	0.730608	0.928779	NA	NA	NA
ENSG0000	8.761988	-0.03008	0.213475	0.730527	0.928779	TMPRSS6	164656	transmembrane serine protease 6
ENSG0000	178.3073	-0.03919	0.162969	0.730805	0.928835	GCC2	9648	GRIP and coiled-coil domain containing 2
ENSG0000	93.90067	0.039081	0.164193	0.730878	0.928835	NA	NA	NA
ENSG0000	3.670499	-0.00972	0.231517	0.730834	0.928835	NA	NA	NA
ENSG0000	660.2896	-0.03637	0.133615	0.730741	0.928835	PABPC1L	80336	poly(A) binding protein cytoplasmic 1 like
ENSG0000	74.04258	-0.03888	0.151668	0.730988	0.928845	LRRC27	80313	leucine rich repeat containing 27
ENSG0000	882.1357	0.036269	0.123711	0.730981	0.928845	NCOA3	8202	nuclear receptor coactivator 3
ENSG0000	9.992065	-0.02289	0.221073	0.731607	0.928857	MIR3671	1.01E+08	microRNA 3671
ENSG0000	60.63019	0.031108	0.207934	0.731682	0.928857	MERTK	10461	MER protc tyrosine kinase
ENSG0000	41.07777	0.037947	0.189679	0.731707	0.928857	TSEN2	80746	tRNA splicing endonuclease subunit 2
ENSG0000	3.059682	-0.00775	0.23233	0.731424	0.928857	C1QTNF7-	1.02E+08	C1QTNF7 antisense RNA 1
ENSG0000	284.9552	-0.03866	0.147533	0.731628	0.928857	RANBP9	10048	RAN binding protein 9
ENSG0000	3.382836	-0.01797	0.225986	0.731537	0.928857	NA	NA	NA
ENSG0000	2.386484	-0.00948	0.231117	0.731215	0.928857	RAD54B	25788	RAD54 homolog B
ENSG0000	490.6987	0.040218	0.153476	0.731404	0.928857	GLUD1	2746	glutamate dehydrogenase 1
ENSG0000	8.097011	-0.02439	0.22157	0.731143	0.928857	NA	NA	NA
ENSG0000	8.384798	-0.0304	0.213496	0.731612	0.928857	NA	NA	NA
ENSG0000	381.927	0.039377	0.154473	0.731203	0.928857	YLPM1	56252	YLP motif containing 1
ENSG0000	749.3344	-0.0326	0.108284	0.73113	0.928857	NFATC2IP	84901	nuclear factor of activated T cells 2 interacting protein
ENSG0000	175.0903	0.03861	0.140794	0.731329	0.928857	NPC1	4864	NPC intracellular cholesterol transporter 1
ENSG0000	904.3212	0.03991	0.162165	0.731277	0.928857	NA	NA	NA
ENSG0000	7.480742	-0.02468	0.219987	0.731811	0.928925	PHLDB1	23187	pleckstrin homology like domain family B member 1
ENSG0000	79.59117	-0.04061	0.175558	0.731883	0.928952	FAM174A	345757	family with sequence similarity 174 member A
ENSG0000	25.49611	-0.03261	0.205272	0.732193	0.929281	MTND5P1	1.01E+08	MT-ND5 pseudogene 11
ENSG0000	588.8407	-0.03609	0.134512	0.73233	0.929326	PPP1R7	5510	protein phosphatase 1 regulatory subunit 7
ENSG0000	142.4785	-0.04243	0.157114	0.73233	0.929326	GTF2H1	2965	general transcription factor IIH subunit 1
ENSG0000	8.063677	0.021994	0.222302	0.732468	0.929398	FTH1P22	1E+08	ferritin heavy chain 1 pseudogene 22
ENSG0000	241.8605	0.040286	0.156549	0.732488	0.929398	DYNC2I2	89891	dynein 2 intermediate chain 2
ENSG0000	18.05447	0.031973	0.208948	0.732545	0.929406	NA	NA	NA
ENSG0000	16.25377	0.03009	0.212245	0.732899	0.929429	METTL18	92342	methyltransferase like 18
ENSG0000	28.58263	0.038028	0.189625	0.732889	0.929429	CHST10	9486	carbohydrate sulfotransferase 10
ENSG0000	64.01397	0.039093	0.18011	0.732696	0.929429	PSMD14	10213	proteasom non-ATPase 14
ENSG0000	43.14797	0.038046	0.18136	0.732831	0.929429	ERCC8	1161	ERCC excis CSA ubiquitin ligase complex subunit
ENSG0000	61.64192	-0.03849	0.185779	0.732735	0.929429	TCAF2	285966	TRPM8 channel associated factor 2
ENSG0000	38.5263	-0.034	0.20108	0.732933	0.929429	AVPI1	60370	arginine vasopressin induced 1
ENSG0000	146.6393	-0.03776	0.181524	0.732969	0.929429	NPIPB3	23117	nuclear pore complex interacting protein family member B3
ENSG0000	4.103084	0.012799	0.229077	0.732709	0.929429	FANCB	2187	FA complementation group B
ENSG0000	34.58041	-0.03931	0.175851	0.733043	0.929458	CCDC153	283152	coiled-coil domain containing 153
ENSG0000	75.51803	0.038986	0.17226	0.733153	0.929534	RAD18	56852	RAD18 E3 ubiquitin protein ligase
ENSG0000	20.44094	-0.03573	0.198323	0.733411	0.929552	NA	NA	NA
ENSG0000	6.875948	-0.0154	0.226234	0.733355	0.929552	NA	NA	NA
ENSG0000	1374.842	0.038902	0.172978	0.733472	0.929552	AQP3	360	aquaporin 3 (Gill blood group)
ENSG0000	12.4126	-0.03177	0.208821	0.733448	0.929552	NA	NA	NA
ENSG0000	2412.686	-0.03853	0.166518	0.733364	0.929552	RAB3D	9545	RAB3D member RAS oncogene family
ENSG0000	232.4253	-0.03883	0.153838	0.733377	0.929552	ZNF333	84449	zinc finger protein 333
ENSG0000	89.99905	0.037572	0.186626	0.733663	0.929601	COA5	493753	cytochrome c oxidase assembly factor 5
ENSG0000	1388.217	0.037347	0.136262	0.733619	0.929601	TBC1D9B	23061	TBC1 domain family member 9B
ENSG0000	536.0526	-0.03469	0.120919	0.733612	0.929601	USP8	9101	ubiquitin specific peptidase 8
ENSG0000	72.36391	0.035978	0.183967	0.733772	0.929674	CDC14A	8556	cell division cycle 14A
ENSG0000	61.90978	-0.03969	0.157682	0.733882	0.92975	WDCP	80304	WD repeat and coiled coil containing
ENSG0000	11.52572	0.029011	0.214188	0.733935	0.929753	NA	NA	NA
ENSG0000	8.115877	0.028943	0.214406	0.733994	0.929764	NA	NA	NA
ENSG0000	89.20726	-0.04067	0.166077	0.734223	0.929791	NTPCR	84284	nucleoside cancer-related
ENSG0000	6940.784	-0.03841	0.156133	0.734221	0.929791	INPP5D	3635	inositol polyphosphate-5-phosphatase D
ENSG0000	386.0327	0.036318	0.191951	0.734241	0.929791	TET2	54790	tet methylcytosine dioxygenase 2
ENSG0000	73.4532	0.039549	0.171948	0.734625	0.929791	REV3L	5980	REV3 like DNA directed polymerase zeta catalytic subunit
ENSG0000	3.372476	-0.00667	0.233499	0.734352	0.929791	NA	NA	NA
ENSG0000	25.01783	-0.03768	0.190023	0.734535	0.929791	NA	NA	NA
ENSG0000	39.29238	0.033172	0.202357	0.73458	0.929791	PKD1P4	353512	polycystin transient receptor potential channel interacting pseudogene 4
ENSG0000	397.0988	0.039095	0.157211	0.734493	0.929791	SLC38A7	55238	solute carrier family 38 member 7
ENSG0000	600.5079	0.039153	0.155353	0.734315	0.929791	CUEDC1	404093	CUE domain containing 1
ENSG0000	97.52004	0.038589	0.144453	0.73438	0.929791	ROMO1	140823	reactive oxygen species modulator 1
ENSG0000	152.5674	0.034797	0.198687	0.734104	0.929791	RP2	6102	RP2 activator of ARL3 GTPase
ENSG0000	250.8768	-0.03664	0.131634	0.734511	0.929791	RNF113A	7737	ring finger protein 113A
ENSG0000	442.168	-0.04079	0.16695	0.735468	0.929948	CTBS	1486	chitobiase
ENSG0000	134.0943	0.038398	0.182	0.73534	0.929948	SLC22A15	55356	solute carrier family 22 member 15
ENSG0000	13.38745	-0.0353	0.199502	0.735282	0.929948	NBPF20	1E+08	NBPF member 20
ENSG0000	16.73837	0.030677	0.210833	0.73544	0.929948	LINC01814	1.02E+08	long intergenic non-protein coding RNA 1814
ENSG0000	154.8727	-0.03768	0.174629	0.735194	0.929948	MFSD2B	388931	major facilitator superfamily domain containing 2B
ENSG0000	320.1183	0.039118	0.164999	0.735474	0.929948	ZNF445	353274	zinc finger protein 445
ENSG0000	11.7142	-0.02972	0.212999	0.735457	0.929948	FAM86JP	1E+08	family witl member A pseudogene
ENSG0000	13.09612	0.030033	0.211919	0.735191	0.929948	LOC10537	1.05E+08	uncharacterized LOC105377730
ENSG0000	1167.494	0.039888	0.156936	0.734886	0.929948	CCAR2	57805	cell cycle and apoptosis regulator 2
ENSG0000	2.556606	0.017029	0.227479	0.735511	0.929948	NA	NA	NA
ENSG0000	82.22988	0.038698	0.146134	0.735131	0.929948	POP5	51367	POP5 hom ribonuclease P/MRP subunit
ENSG0000	2.361476	0.011825	0.230255	0.735384	0.929948	NA	NA	NA
ENSG0000	34.61005	0.021998	0.220182	0.735587	0.929948	IGHV3-66	28412	immunoglobulin heavy variable 3-66
ENSG0000	2985.345	-0.03826	0.162338	0.734951	0.929948	LOC40049	400499	putative uncharacterized protein LOC400499
ENSG0000	2.921321	0.019	0.2265	0.734898	0.929948	NA	NA	NA
ENSG0000	29.7965	-0.03865	0.179953	0.735663	0.929948	ZNF559	84527	zinc finger protein 559
ENSG0000	83.52392	0.037977	0.159954	0.735616	0.929948	LOC10012	1E+08	uncharacterized LOC100128398
ENSG0000	338.4878	0.038567	0.153482	0.735387	0.929948	KCTD17	79734	potassium channel tetramerization domain containing 17
ENSG0000	63.02316	0.036538	0.191512	0.735881	0.930159	SOCS4	122809	suppressor of cytokine signaling 4
ENSG0000	36.96417	-0.03852	0.181119	0.736208	0.930204	TAF13	6884	TATA-box binding protein associated factor 13
ENSG0000	15.59613	0.031369	0.209371	0.736221	0.930204	NA	NA	NA
ENSG0000	212.3465	0.039801	0.173514	0.736207	0.930204	ARMC8	25852	armadillo repeat containing 8

ENSG0000	2.907608	0.009778	0.230481	0.73609	0.930204	AIF1L	83543	allograft inflammatory factor 1 like
ENSG0000	3.429568	0.015825	0.228671	0.736113	0.930204	MYO16	23026	myosin XVI
ENSG0000	5.23641	-0.01719	0.226514	0.736042	0.930204	MIR4324	1E+08	microRNA 4324
ENSG0000	49.88744	0.037234	0.188867	0.736284	0.93022	WEE1	7465	WEE1 G2 checkpoint kinase
ENSG0000	14.3978	-0.03442	0.202771	0.736388	0.930287	LEPR	3953	leptin receptor
ENSG0000	5.135131	-0.01584	0.22736	0.736603	0.930429	NA	NA	NA
ENSG0000	65.87637	0.038829	0.172069	0.736553	0.930429	ZNF786	136051	zinc finger protein 786
ENSG0000	44.95732	-0.04058	0.172792	0.736678	0.930461	SLC39A14	23516	solute carrier family 39 member 14
ENSG0000	9.455323	0.021743	0.221823	0.73682	0.930576	ZNF90	7643	zinc finger protein 90
ENSG0000	50.6458	-0.03875	0.173437	0.736922	0.93064	GAR1	54433	GAR1 ribonucleoprotein
ENSG0000	4.628432	0.018504	0.226074	0.73705	0.930738	NA	NA	NA
ENSG0000	11.96673	-0.02605	0.215761	0.737232	0.930818	MIR3142H	1.07E+08	MIR3142 host gene
ENSG0000	12.88722	0.025594	0.217971	0.737317	0.930818	NA	NA	NA
ENSG0000	110.1887	-0.03941	0.15261	0.737279	0.930818	DDX55	57696	DEAD-box helicase 55
ENSG0000	466.2583	-0.03284	0.113099	0.737239	0.930818	KCTD5	54442	potassium channel tetramerization domain containing 5
ENSG0000	37.69637	0.022855	0.220657	0.737486	0.930967	IGHV3-72	28410	immunoglobulin heavy variable 3-72
ENSG0000	30.21787	0.03908	0.180448	0.737549	0.930983	TIGD1	200765	tigger transposable element derived 1
ENSG0000	46.6957	0.039302	0.17417	0.737688	0.931094	LOC10272	1.03E+08	U2 small nuclear RNA auxiliary factor 1 like 5
ENSG0000	153.2409	0.038753	0.158218	0.73781	0.93112	LOXL3	84695	lysyl oxidase like 3
ENSG0000	1473.01	-0.03801	0.166477	0.737786	0.93112	SF3B3	23450	splicing factor 3b subunit 3
ENSG0000	206.7263	-0.03319	0.121674	0.737895	0.931163	ILKAP	80895	ILK associated serine/threonine phosphatase
ENSG0000	21.30335	0.030842	0.209332	0.738029	0.931166	LBX2	85474	ladybird homeobox 2
ENSG0000	3.006772	0.016128	0.227203	0.737963	0.931166	LOC11856	1.19E+08	uncharacterized LOC118567325
ENSG0000	18.19997	0.036163	0.195507	0.73805	0.931166	NA	NA	NA
ENSG0000	134.3343	0.038382	0.146916	0.738125	0.931175	MALSU1	115416	mitochondrial assembly of ribosomal large subunit 1
ENSG0000	11.12032	-0.03229	0.207896	0.738159	0.931175	NA	NA	NA
ENSG0000	97.70821	0.039143	0.165761	0.73836	0.931226	AMIGO1	57463	adhesion molecule with Ig like domain 1
ENSG0000	913.9314	0.038165	0.118766	0.73843	0.931226	TMED9	54732	transmembrane p24 trafficking protein 9
ENSG0000	63.75199	0.037745	0.182008	0.738438	0.931226	TTC19	54902	tetratricopeptide repeat domain 19
ENSG0000	17.46035	0.024005	0.218986	0.738453	0.931226	NA	NA	NA
ENSG0000	308.217	-0.04004	0.162263	0.738277	0.931226	TST	7263	thiosulfate sulfurtransferase
ENSG0000	11.97044	-0.03425	0.202564	0.738951	0.931463	NA	NA	NA
ENSG0000	13.21017	-0.02777	0.213818	0.73888	0.931463	LINC02611	1.02E+08	long intergenic non-protein coding RNA 2611
ENSG0000	47.02234	0.037698	0.183252	0.739354	0.931463	EEF1AKMT	1.11E+08	EEF1A lysine methyltransferase 4
ENSG0000	122.8316	0.03693	0.18173	0.739342	0.931463	RUFY3	22902	RUN and FYVE domain containing 3
ENSG0000	33.68251	-0.03599	0.191778	0.738873	0.931463	FTH1P10	2502	ferritin heavy chain 1 pseudogene 10
ENSG0000	3.96	-0.02169	0.224137	0.739103	0.931463	LOC10192	1.02E+08	uncharacterized LOC101927609
ENSG0000	3.659258	0.014509	0.228034	0.739185	0.931463	VGF	7425	VGF nerve growth factor inducible
ENSG0000	2.550743	-0.01148	0.230389	0.739355	0.931463	HTRA4	203100	HtrA serine peptidase 4
ENSG0000	21.63602	-0.02883	0.210357	0.738812	0.931463	NA	NA	NA
ENSG0000	88.67978	-0.02564	0.176981	0.739113	0.931463	LTBP2	4053	latent transforming growth factor beta binding protein 2
ENSG0000	2506.985	-0.03668	0.139137	0.739061	0.931463	GIT1	28964	GIT ArfGAP 1
ENSG0000	2031.523	0.040438	0.116278	0.739404	0.931463	DAZAP1	26528	DAZ associated protein 1
ENSG0000	13.92034	0.033904	0.204111	0.738999	0.931463	DIRAS1	148252	DIRAS family GTPase 1
ENSG0000	730.3651	-0.03766	0.160624	0.739208	0.931463	NOP56	10528	NOP56 ribonucleoprotein
ENSG0000	3.165357	0.014327	0.232259	0.738792	0.931463	PAGE2B	389860	PAGE family member 2B
ENSG0000	7.810007	0.019913	0.224049	0.739456	0.931464	NA	NA	NA
ENSG0000	362.7815	0.031089	0.205147	0.739587	0.931566	SLC38A2	54407	solute carrier family 38 member 2
ENSG0000	121.3995	0.038989	0.16453	0.739666	0.9316	TSC22D2	9819	TSC22 domain family member 2
ENSG0000	72.90423	-0.03807	0.171066	0.739947	0.931837	FAM228B	375190	family with sequence similarity 228 member B
ENSG0000	7.022179	-0.02162	0.222954	0.739956	0.931837	RAB9B	51209	RAB9B member RAS oncogene family
ENSG0000	137.7517	-0.03603	0.131417	0.740076	0.931842	SEMA4C	54910	semaphorin 4C
ENSG0000	104.2901	0.037644	0.150766	0.740056	0.931842	ZNF623	9831	zinc finger protein 623
ENSG0000	547.8936	-0.03642	0.136413	0.740112	0.931842	GPR132	29933	G protein-coupled receptor 132
ENSG0000	526.2232	-0.03761	0.155732	0.740524	0.932041	PRKCZ	5590	protein kinase C zeta
ENSG0000	3.099418	0.023027	0.223456	0.740369	0.932041	NA	NA	NA
ENSG0000	4.03698	0.018808	0.227298	0.740507	0.932041	NA	NA	NA
ENSG0000	21.39795	0.03227	0.206258	0.740373	0.932041	SOX6	55553	SRY-box transcription factor 6
ENSG0000	158.5715	0.038701	0.170054	0.740478	0.932041	NPAT	4863	nuclear pr coactivator of histone transcription
ENSG0000	721.6231	0.037518	0.146763	0.741034	0.932121	FDPS	2224	farnesyl diphosphate synthase
ENSG0000	461.8588	-0.03717	0.146282	0.740991	0.932121	DNPEP	23549	aspartyl aminopeptidase
ENSG0000	8095.127	0.036125	0.132652	0.740971	0.932121	RHOA	387	ras homolog family member A
ENSG0000	13.95138	-0.03307	0.203613	0.740822	0.932121	NA	NA	NA
ENSG0000	131.0291	-0.03678	0.149169	0.740728	0.932121	ZSCAN25	221785	zinc finger and SCAN domain containing 25
ENSG0000	2661.865	-0.03888	0.18147	0.740684	0.932121	PLXNC1	10154	plexin C1
ENSG0000	303.1128	-0.02863	0.206371	0.741046	0.932121	WASHC4	23325	WASH complex subunit 4
ENSG0000	2.785121	-0.01296	0.230737	0.740776	0.932121	RAMP2	10266	receptor activity modifying protein 2
ENSG0000	4.578761	0.019623	0.225027	0.740965	0.932121	NA	NA	NA
ENSG0000	335.7306	-0.0375	0.154232	0.741177	0.932158	SLC23A2	9962	solute carrier family 23 member 2
ENSG0000	7.564461	-0.02958	0.212628	0.741169	0.932158	NA	NA	NA
ENSG0000	3.158279	0.010946	0.230203	0.741282	0.932162	NA	NA	NA
ENSG0000	18.69545	0.036241	0.197757	0.741238	0.932162	LAIR2	3904	leukocyte associated immunoglobulin like receptor 2
ENSG0000	6.034616	-0.02154	0.222768	0.741351	0.932182	MTCO1P1	1.07E+08	MT-CO1 pseudogene 11
ENSG0000	28.82143	-0.0331	0.200816	0.7414	0.932182	CKB	1152	creatine kinase B
ENSG0000	435.2723	0.037542	0.175626	0.741474	0.932212	PLK1	5347	polo like kinase 1
ENSG0000	220.1744	0.037713	0.147886	0.743364	0.932407	JMJD4	65094	jumonji domain containing 4
ENSG0000	914.1666	-0.03264	0.115168	0.742496	0.932407	INPP4A	3631	inositol polyphosphate-4-phosphatase type I A
ENSG0000	7.238559	0.024044	0.218482	0.743045	0.932407	RGPD8	727851	RANBP2 like and GRIP domain containing 8
ENSG0000	887.6859	0.031298	0.135496	0.742793	0.932407	APEH	327	acylaminoacyl-peptide hydrolase
ENSG0000	247.2298	0.033033	0.19393	0.743005	0.932407	GPR160	26996	G protein-coupled receptor 160
ENSG0000	238.6469	0.0351	0.188726	0.743212	0.932407	TBL1XR1	79718	TBL1X/Y related 1
ENSG0000	22.29914	-0.03386	0.199282	0.743359	0.932407	FAM86EP	348926	family witl member A pseudogene
ENSG0000	2.522573	0.010973	0.232168	0.742343	0.932407	NA	NA	NA
ENSG0000	139.7664	0.035316	0.190985	0.743318	0.932407	PGGT1B	5229	protein geranylgeranyltransferase type I subunit beta
ENSG0000	733.7003	-0.03465	0.134059	0.742978	0.932407	PRDM1	639	PR/SET domain 1
ENSG0000	271.6075	-0.03612	0.158499	0.743322	0.932407	NA	NA	NA
ENSG0000	4.863197	-0.0211	0.223572	0.742514	0.932407	ASAH1-AS	1.02E+08	ASAH1 antisense RNA 1
ENSG0000	133.3458	0.03755	0.158648	0.74201	0.932407	RMDN1	51115	regulator of microtubule dynamics 1
ENSG0000	22.45115	0.03162	0.205368	0.742507	0.932407	TMEM64	169200	transmembrane protein 64
ENSG0000	3.088245	0.009835	0.231686	0.743126	0.932407	NA	NA	NA
ENSG0000	46.78768	0.034141	0.197331	0.742885	0.932407	KCNIP2	30819	potassium voltage-gated channel interacting protein 2
ENSG0000	23.30647	-0.0227	0.219031	0.743223	0.932407	TRUB1	142940	TruB pseudouridine synthase family member 1
ENSG0000	852.0086	0.03945	0.162757	0.741729	0.932407	PHRF1	57661	PHD and ring finger domains 1
ENSG0000	354.5814	0.037215	0.16327	0.742551	0.932407	BMAL1	406	basic helix-loop-helix ARNT like 1
ENSG0000	9.684952	0.028474	0.213692	0.742587	0.932407	CTNND1	1500	catenin delta 1
ENSG0000	225.676	0.034696	0.13832	0.743395	0.932407	HNRNPUL	221092	heterogeneous nuclear ribonucleoprotein U like 2

ENSG0000	4238.46	-0.03673	0.175633	0.741874	0.932407	SF3B2	10992	splicing factor 3b subunit 2
ENSG0000	5.455568	0.017123	0.225807	0.741937	0.932407	NA	NA	NA
ENSG0000	35.08509	-0.03266	0.200912	0.742636	0.932407	DNAJC3-D	1E+08	DNAJC3 divergent transcript
ENSG0000	8.811769	-0.02198	0.221615	0.743564	0.932407	TRAV36DV	28646	T cell receptor alpha variable 36/delta variable 7
ENSG0000	32.92307	0.035175	0.190407	0.743134	0.932407	PRORP	9692	protein only RNase P catalytic subunit
ENSG0000	1236.502	-0.03666	0.176796	0.741889	0.932407	INF2	64423	inverted formin 2
ENSG0000	22.22842	0.031204	0.208058	0.743187	0.932407	NA	NA	NA
ENSG0000	5.671594	0.019715	0.223858	0.742748	0.932407	NA	NA	NA
ENSG0000	839.5923	0.032698	0.115816	0.743463	0.932407	ACSF3	197322	acyl-CoA synthetase family member 3
ENSG0000	37.68218	-0.02236	0.217912	0.741758	0.932407	KCNJ2-AS1	400617	KCNJ2 antisense RNA 1
ENSG0000	7247.356	0.03747	0.175904	0.742242	0.932407	LRRC25	126364	leucine rich repeat containing 25
ENSG0000	2294.026	-0.03784	0.135266	0.742703	0.932407	SSBP4	170463	single stranded DNA binding protein 4
ENSG0000	282.9246	-0.03403	0.124236	0.742367	0.932407	ANKRD27	84079	ankyrin repeat domain 27
ENSG0000	45453.1	-0.03441	0.141537	0.743547	0.932407	RPL28	6158	ribosomal protein L28
ENSG0000	141.7549	-0.03573	0.155819	0.742918	0.932407	MRPS26	64949	mitochondrial ribosomal protein S26
ENSG0000	5.162951	0.0211	0.223432	0.74225	0.932407	NA	NA	NA
ENSG0000	29.37126	0.03146	0.205554	0.74347	0.932407	CHIC1	53344	cysteine rich hydrophobic domain 1
ENSG0000	6.191407	0.020741	0.223117	0.743647	0.932447	NA	NA	NA
ENSG0000	176.1861	0.035232	0.182863	0.743859	0.932548	DYRK2	8445	dual specificity tyrosine phosphorylation regulated kinase 2
ENSG0000	1637.635	-0.03869	0.163348	0.74388	0.932548	DDX24	57062	DEAD-box helicase 24
ENSG0000	3.49172	0.017985	0.226002	0.743828	0.932548	TSPAN10	83882	tetraspanin 10
ENSG0000	83.19147	-0.03913	0.163347	0.745437	0.932581	CROCCP3	114819	CROCC pseudogene 3
ENSG0000	312.4896	0.037745	0.143708	0.745053	0.932581	GMEB1	10691	glucocorticoid modulatory element binding protein 1
ENSG0000	27.25531	-0.03666	0.185342	0.744375	0.932581	ZSCAN20	7579	zinc finger and SCAN domain containing 20
ENSG0000	6.961596	-0.02248	0.221134	0.744807	0.932581	ANXA9	8416	annexin A9
ENSG0000	2.936051	-0.00792	0.230985	0.745348	0.932581	RPS14P1	1E+08	ribosomal protein S14 pseudogene 1
ENSG0000	2.351933	-0.00893	0.229508	0.74445	0.932581	CREG2	200407	cellular repressor of E1A stimulated genes 2
ENSG0000	1630.467	-0.03691	0.159568	0.745536	0.932581	GPBAR1	151306	G protein-coupled bile acid receptor 1
ENSG0000	581.27	0.032022	0.112004	0.745062	0.932581	TUSC2	11334	tumor sup mitochondrial calcium regulator
ENSG0000	34.60138	-0.03696	0.183767	0.744759	0.932581	ZMYND10	51364	zinc finger MYND-type containing 10
ENSG0000	33.54132	-0.03079	0.206	0.74406	0.932581	GPR15	2838	G protein-coupled receptor 15
ENSG0000	41.11936	0.033583	0.197726	0.74487	0.932581	WDR5B	54554	WD repeat domain 5B
ENSG0000	11.19872	-0.02458	0.217998	0.744665	0.932581	TBC1D19	55296	TBC1 domain family member 19
ENSG0000	154.7349	0.036208	0.181345	0.744204	0.932581	PLRG1	5356	pleiotropic regulator 1
ENSG0000	8.449183	-0.02474	0.21802	0.744816	0.932581	NA	NA	NA
ENSG0000	10.78457	-0.03161	0.208916	0.745206	0.932581	NA	NA	NA
ENSG0000	7.27989	0.02695	0.216108	0.744915	0.932581	DUS4L	11062	dihydrouridine synthase 4 like
ENSG0000	375.7117	-0.03741	0.166834	0.745153	0.932581	KDM7A	80853	lysine demethylase 7A
ENSG0000	15.40568	-0.0347	0.197533	0.745074	0.932581	ATP6V0E2	401431	ATP6V0E2 antisense RNA 1
ENSG0000	2268.838	0.038177	0.159467	0.745464	0.932581	SARAF	51669	store-operated calcium entry associated regulatory factor
ENSG0000	2.596667	0.006494	0.233461	0.74514	0.932581	RN7SL329	1.06E+08	RNA 7SL cytoplasm pseudogene
ENSG0000	457.1485	-0.03671	0.157216	0.745026	0.932581	GOLGA2	2801	golgin A2
ENSG0000	3.756993	-0.01471	0.228177	0.744043	0.932581	NRP1	8829	neuropilin 1
ENSG0000	4.064689	0.009272	0.230617	0.744133	0.932581	NA	NA	NA
ENSG0000	5.136901	0.026629	0.217759	0.745041	0.932581	NA	NA	NA
ENSG0000	29.35672	-0.0335	0.198991	0.74428	0.932581	TMPRSS13	84000	transmembrane serine protease 13
ENSG0000	3.265738	0.012693	0.230195	0.745509	0.932581	NA	NA	NA
ENSG0000	6.528442	0.024624	0.219377	0.744484	0.932581	NA	NA	NA
ENSG0000	4841.984	0.03696	0.136027	0.744111	0.932581	IL16	3603	interleukin 16
ENSG0000	7.945854	0.021669	0.221542	0.745434	0.932581	NA	NA	NA
ENSG0000	450.1962	-0.03701	0.150346	0.744279	0.932581	FIZ1	84922	FLT3 interacting zinc finger 1
ENSG0000	12.36386	0.0301	0.210435	0.745378	0.932581	ZNF583	147949	zinc finger protein 583
ENSG0000	2.514894	-0.00869	0.231137	0.74436	0.932581	MIR645	693230	microRNA 645
ENSG0000	467.2145	-0.03383	0.123	0.745645	0.932655	LSM7	51690	LSM7 hom U6 small nuclear RNA and mRNA degradation associated
ENSG0000	7.729364	0.02701	0.219377	0.745736	0.932704	MAMSTR	284358	MEF2 activating motif and SAP domain containing transcriptional regulator
ENSG0000	53.50192	0.035632	0.184081	0.745802	0.932724	ARV1	64801	ARV1 hom fatty acid homeostasis modulator
ENSG0000	79.03028	-0.0372	0.180874	0.745861	0.932733	CLCF1	23529	cardiotrophin like cytokine factor 1
ENSG0000	81.73431	0.037722	0.162488	0.74598	0.932755	SBDS	51119	SBDS ribosome maturation factor
ENSG0000	6.966419	-0.01524	0.226671	0.745957	0.932755	NA	NA	NA
ENSG0000	3.394875	-0.01712	0.226401	0.746057	0.932787	AKAP5	9495	A-kinase anchoring protein 5
ENSG0000	31.4637	-0.03323	0.19697	0.746251	0.93284	ADAT2	134637	adenosine deaminase tRNA specific 2
ENSG0000	4829.646	0.037588	0.152657	0.746208	0.93284	ZMIZ2	83637	zinc finger MIZ-type containing 2
ENSG0000	2.85045	-0.01421	0.229153	0.746212	0.93284	NA	NA	NA
ENSG0000	3.638157	0.01831	0.225972	0.746515	0.933106	NA	NA	NA
ENSG0000	123.5628	0.036793	0.167112	0.746596	0.933143	GFPT1	2673	glutamine--fructose-6-phosphate transaminase 1
ENSG0000	6.729686	0.024154	0.21923	0.746716	0.933229	NA	NA	NA
ENSG0000	39.08272	0.037837	0.164769	0.746879	0.933242	NA	NA	NA
ENSG0000	545.9445	0.035671	0.144737	0.746868	0.933242	CENPT	80152	centromere protein T
ENSG0000	11.18338	-0.02909	0.212171	0.746801	0.933242	ZNF470	388566	zinc finger protein 470
ENSG0000	118.1299	0.035412	0.178686	0.747056	0.933399	C20orf96	140680	chromosome 20 open reading frame 96
ENSG0000	22.42841	-0.02913	0.207342	0.74715	0.9334	NA	NA	NA
ENSG0000	233.9127	0.033004	0.175395	0.747158	0.9334	ZNF579	163033	zinc finger protein 579
ENSG0000	1729.528	-0.04048	0.156308	0.747349	0.933447	AGPAT2	10555	1-acylglycerol-3-phosphate O-acyltransferase 2
ENSG0000	1497.188	-0.03637	0.163278	0.747346	0.933447	CXXC1	30827	CXXC finger protein 1
ENSG0000	3.133065	-0.01676	0.225819	0.747334	0.933447	NA	NA	NA
ENSG0000	4.203929	0.009886	0.229879	0.747492	0.933562	LOC28509	285097	uncharacterized FLJ38379
ENSG0000	8.029211	-0.02608	0.218326	0.747552	0.933574	NA	NA	NA
ENSG0000	2.671849	0.024288	0.228309	0.747632	0.93361	PFKFB1	5207	6-phosphc 6-biphosphatase 1
ENSG0000	42.889	-0.02827	0.209056	0.748035	0.933846	NCAPG	64151	non-SMC condensin I complex subunit G
ENSG0000	3.298123	0.014713	0.22848	0.748022	0.933846	NA	NA	NA
ENSG0000	58.17675	-0.03683	0.17572	0.748127	0.933846	PPP2R2B	5521	protein phosphatase 2 regulatory subunit Bbeta
ENSG0000	10.83498	-0.02671	0.214451	0.74807	0.933846	NA	NA	NA
ENSG0000	79.99495	0.038437	0.176144	0.748112	0.933846	NPIPB12	440353	nuclear pore complex interacting protein family member B12
ENSG0000	648.1814	-0.03375	0.130561	0.748081	0.933846	RNF135	84282	ring finger protein 135
ENSG0000	54.94919	0.036586	0.181637	0.74823	0.933868	MED31	51003	mediator complex subunit 31
ENSG0000	136.1956	0.036661	0.144293	0.748247	0.933868	GPATCH1	55094	G-patch domain containing 1
ENSG0000	2169.318	-0.03635	0.153946	0.748415	0.933888	FAM120A	23196	family with sequence similarity 120A
ENSG0000	109.8944	0.032851	0.197681	0.748373	0.933888	ZBTB1	22890	zinc finger and BTB domain containing 1
ENSG0000	51.71803	0.0357	0.184914	0.748382	0.933888	ZNF440	126070	zinc finger protein 440
ENSG0000	31.2118	0.033689	0.184526	0.748675	0.934148	NEMP2	1E+08	nuclear envelope integral membrane protein 2
ENSG0000	56.98404	0.033641	0.194665	0.748994	0.934269	GTF2E1	2960	general transcription factor IIE subunit 1
ENSG0000	49.59931	-0.03626	0.183019	0.748974	0.934269	FKBPL	63943	FKBP prolyl isomerase like
ENSG0000	22.04927	-0.02854	0.208143	0.749078	0.934269	TVP23B	51030	trans-golgi network vesicle protein 23 homolog B
ENSG0000	179.4725	0.033523	0.130243	0.749064	0.934269	NA	NA	NA
ENSG0000	907.0855	0.037129	0.153222	0.748854	0.934269	RBM39	9584	RNA binding motif protein 39
ENSG0000	36.93289	-0.03655	0.177943	0.748942	0.934269	APOBEC3D	140564	apolipoprotein B mRNA editing enzyme catalytic subunit 3D

ENSG0000	37.07189	0.027796	0.210501	0.749346	0.934539	TMEM161	153396	transmembrane protein 161B
ENSG0000	28.72019	-0.02947	0.205051	0.749503	0.934672	P2RY12	64805	purinergic receptor P2Y12
ENSG0000	13388.09	0.035468	0.13794	0.749558	0.934677	GPX1	2876	glutathione peroxidase 1
ENSG0000	6.206206	-0.01656	0.225811	0.749714	0.934779	NA	NA	NA
ENSG0000	158.9228	0.035574	0.145661	0.749777	0.934779	ICE1	23379	interactor of little elongation complex ELL subunit 1
ENSG0000	40.41856	0.034562	0.190355	0.75001	0.934779	NA	NA	NA
ENSG0000	2790.649	0.033117	0.122147	0.74994	0.934779	ATP6V1F	9296	ATPase H+ transporting V1 subunit F
ENSG0000	361.7971	-0.03648	0.152209	0.750014	0.934779	PNN	5411	pinin desmosome associated protein
ENSG0000	163.9194	-0.03697	0.154656	0.750048	0.934779	WDR25	79446	WD repeat domain 25
ENSG0000	26.27706	0.033206	0.196925	0.749927	0.934779	ZNF597	146434	zinc finger protein 597
ENSG0000	3.614027	0.015975	0.22751	0.749938	0.934779	TSPAN7	7102	tetraspanin 7
ENSG0000	3.851483	-0.01767	0.225778	0.750117	0.934801	LINC0261	1.02E+08	long intergenic non-protein coding RNA 2614
ENSG0000	244.6354	-0.03379	0.127641	0.750242	0.934893	SLC7A6OS	84138	solute carrier family 7 member 6 opposite strand
ENSG0000	13.8336	-0.02589	0.213924	0.750309	0.934913	MTUS1	57509	microtubule associated scaffold protein 1
ENSG0000	771.7702	0.036401	0.151529	0.75056	0.93502	SETDB1	9869	SET domain bifurcated histone lysine methyltransferase 1
ENSG0000	4.236251	-0.02354	0.219866	0.750599	0.93502	HNRNPKP	644063	heterogeneous nuclear ribonucleoprotein K pseudogene 4
ENSG0000	6.080393	0.020607	0.221685	0.750472	0.93502	LOC10798	1.08E+08	uncharacterized LOC107986639
ENSG0000	10.12	0.019017	0.224101	0.750504	0.93502	RPL32P32	1E+08	ribosomal protein L32 pseudogene 32
ENSG0000	6.366844	-0.02661	0.215039	0.750664	0.935038	SCOC-AS1	1E+08	SCOC antisense RNA 1
ENSG0000	2.434031	-0.00974	0.231174	0.751809	0.935182	RN7SL444	1.06E+08	RNA 7SL cytoplasm pseudogene
ENSG0000	1216.575	-0.03652	0.156326	0.751701	0.935182	SCAMP3	10067	secretory carrier membrane protein 3
ENSG0000	4.202633	-0.01972	0.224671	0.751148	0.935182	ZNF662	389114	zinc finger protein 662
ENSG0000	5.813157	-0.02486	0.218351	0.751174	0.935182	ZNF501	115560	zinc finger protein 501
ENSG0000	20.06809	0.033796	0.197295	0.751602	0.935182	NA	NA	NA
ENSG0000	26.47813	-0.03409	0.193214	0.751996	0.935182	MMAA	166785	metabolism of cobalamin associated A
ENSG0000	8.529846	0.019647	0.22275	0.751589	0.935182	NUDT19P5	1E+08	NUDT19 pseudogene 5
ENSG0000	108.9385	0.040412	0.153708	0.752109	0.935182	WDR70	55100	WD repeat domain 70
ENSG0000	126.6871	0.034105	0.189311	0.751863	0.935182	KEL	3792	Kell metallo-endopeptidase (Kell blood group)
ENSG0000	185.5625	-0.03285	0.189341	0.751862	0.935182	UBE2W	55284	ubiquitin conjugating enzyme E2 W
ENSG0000	534.4034	0.038526	0.140452	0.750942	0.935182	NDUFB9	4715	NADH:ubiquinone oxidoreductase subunit B9
ENSG0000	5.545508	-0.01318	0.22734	0.751479	0.935182	MIR5194	1.01E+08	microRNA 5194
ENSG0000	762.5618	-0.04144	0.141318	0.752048	0.935182	YME1L1	10730	YME1 like 1 ATPase
ENSG0000	139.0255	-0.03776	0.162282	0.751183	0.935182	SGMS1	259230	sphingomyelin synthase 1
ENSG0000	2.587943	-0.00761	0.230363	0.751768	0.935182	GPRC5D-A	1.01E+08	GPRC5D and HEBP1 antisense RNA 1
ENSG0000	264.3394	-0.03606	0.156645	0.752158	0.935182	NUDT4	11163	nudix hydrolase 4
ENSG0000	68.41967	0.037372	0.167574	0.751199	0.935182	WBP4	11193	WW domain binding protein 4
ENSG0000	22.52116	-0.03095	0.203855	0.751878	0.935182	TRAV9-2	28677	T cell receptor alpha variable 9-2
ENSG0000	340.8245	-0.03186	0.13358	0.751828	0.935182	ZFYVE19	84936	zinc finger FYVE-type containing 19
ENSG0000	279.4926	-0.03475	0.136874	0.751472	0.935182	MAF	4094	MAF bZIP transcription factor
ENSG0000	207.9998	0.031033	0.19444	0.750839	0.935182	LGALS9C	654346	galectin 9C
ENSG0000	13.3574	0.026689	0.215619	0.751643	0.935182	NA	NA	NA
ENSG0000	4.520971	-0.01985	0.226331	0.751722	0.935182	EFCAB13	124989	EF-hand calcium binding domain 13
ENSG0000	1030.701	-0.03792	0.16466	0.752143	0.935182	RALGAPA2	57186	Ral GTPase activating protein catalytic subunit alpha 2
ENSG0000	1261.829	-0.03308	0.12498	0.751259	0.935182	SLC35C2	51006	solute carrier family 35 member C2
ENSG0000	382.9901	0.037828	0.15034	0.752026	0.935182	DIP2A	23181	disco interacting protein 2 homolog A
ENSG0000	2.605167	0.018004	0.22872	0.751443	0.935182	RHOXF1	158800	Rhox homeobox family member 1
ENSG0000	35.80831	-0.02608	0.207111	0.752469	0.935187	ATAD3C	219293	ATPase family AAA domain containing 3C
ENSG0000	2.497874	0.010196	0.230798	0.752389	0.935187	NA	NA	NA
ENSG0000	84.56712	-0.02685	0.210535	0.752235	0.935187	TRAT1	50852	T cell receptor associated transmembrane adaptor 1
ENSG0000	2.938981	0.01599	0.227209	0.752467	0.935187	NA	NA	NA
ENSG0000	54.7592	0.035049	0.183072	0.752349	0.935187	ETFRF1	144363	electron transfer flavoprotein regulatory factor 1
ENSG0000	31.91821	-0.03313	0.195332	0.752457	0.935187	NA	NA	NA
ENSG0000	400.999	-0.0356	0.158124	0.752571	0.935251	APBA2	321	amyloid beta precursor protein binding family A member 2
ENSG0000	20.68777	-0.03482	0.189687	0.753149	0.935455	NA	NA	NA
ENSG0000	275.042	-0.03308	0.143854	0.753078	0.935455	RHOH	399	ras homolog family member H
ENSG0000	6.24841	-0.01454	0.227003	0.753195	0.935455	NA	NA	NA
ENSG0000	349.0708	0.035812	0.147518	0.753097	0.935455	PURB	5814	purine rich element binding protein B
ENSG0000	37.54974	0.024956	0.214411	0.752919	0.935455	RANBP6	26953	RAN binding protein 6
ENSG0000	104.3607	0.034766	0.136187	0.753109	0.935455	POMT2	29954	protein O-mannosyltransferase 2
ENSG0000	465.5941	0.035149	0.147446	0.752826	0.935455	BANP	54971	BTG3 associated nuclear protein
ENSG0000	64.10075	-0.03286	0.1591	0.752957	0.935455	NA	NA	NA
ENSG0000	145.3109	-0.03461	0.136523	0.753089	0.935455	RBMX2	51634	RNA binding motif protein X-linked 2
ENSG0000	538.696	-0.03335	0.189013	0.753332	0.935561	MARCHF7	64844	membrane associated ring-CH-type finger 7
ENSG0000	34.55063	-0.03604	0.176601	0.753503	0.935583	NA	NA	NA
ENSG0000	465.3163	0.03504	0.172272	0.753411	0.935583	PPP4R3B	57223	protein phosphatase 4 regulatory subunit 3B
ENSG0000	2.891158	0.014292	0.229685	0.753465	0.935583	NA	NA	NA
ENSG0000	1177.507	-0.03333	0.129995	0.753614	0.935658	KARS1	3735	lysyl-tRNA synthetase 1
ENSG0000	5694.965	-0.035	0.17432	0.7537	0.935701	ZNF467	168544	zinc finger protein 467
ENSG0000	338.1989	-0.03651	0.132596	0.753975	0.93574	METTL25B	51093	methyltransferase like 25B
ENSG0000	144.3272	0.034311	0.186577	0.753907	0.93574	SLC30A6	55676	solute carrier family 30 member 6
ENSG0000	88.39608	-0.03635	0.150556	0.753989	0.93574	ZNF330	27309	zinc finger protein 330
ENSG0000	614.7962	0.03393	0.135517	0.754021	0.93574	NUDT22	84304	nudix hydrolase 22
ENSG0000	6.767067	-0.01851	0.224348	0.754037	0.93574	ETFBKMT	254013	electron transfer flavoprotein subunit beta lysine methyltransferase
ENSG0000	6.598199	0.024783	0.217851	0.753993	0.93574	LPIN3	64900	lipin 3
ENSG0000	77.09669	-0.02454	0.210694	0.754423	0.935907	TFEC	22797	transcription factor EC
ENSG0000	883.1021	0.03353	0.129824	0.754462	0.935907	PSMD13	5719	proteasom non-ATPase 13
ENSG0000	217.2388	-0.03262	0.123093	0.754479	0.935907	CRNKL1	51340	crooked neck pre-mRNA splicing factor 1
ENSG0000	4.089917	-0.01862	0.226895	0.754471	0.935907	NA	NA	NA
ENSG0000	576.7906	-0.03588	0.149306	0.754364	0.935907	PRICKLE3	4007	prickle planar cell polarity protein 3
ENSG0000	12.01501	0.015993	0.226037	0.754341	0.935907	SHROOM4	57477	shroom family member 4
ENSG0000	394.9196	-0.03164	0.120006	0.75463	0.936031	TAF8	129685	TATA-box binding protein associated factor 8
ENSG0000	657.9086	0.035813	0.154422	0.754776	0.936149	STMN3	50861	stathmin 3
ENSG0000	360.0294	0.034255	0.138287	0.75493	0.936214	OCIAD1	54940	OCIA domain containing 1
ENSG0000	65.72187	0.035254	0.181505	0.754881	0.936214	PRANCR	1.02E+08	progenitor renewal associated non-coding RNA
ENSG0000	2.479264	-0.01068	0.23148	0.75504	0.936272	SLCO2A1	6578	solute carrier organic anion transporter family member 2A1
ENSG0000	346.0139	0.033532	0.153413	0.755158	0.936272	CKAP5	9793	cytoskeleton associated protein 5
ENSG0000	18.28938	0.02962	0.209679	0.755223	0.936272	OTUD7A	161725	OTU deubiquitinase 7A
ENSG0000	3664.369	0.03541	0.1546	0.755188	0.936272	IQGAP1	8826	IQ motif containing GTPase activating protein 1
ENSG0000	21.2914	-0.02711	0.212482	0.755233	0.936272	PWP2	5822	PWP2 small subunit processome component
ENSG0000	14.42719	-0.0262	0.209465	0.755292	0.936282	JAG1	182	jagged canonical Notch ligand 1
ENSG0000	188.6759	-0.03422	0.15476	0.755499	0.936415	HEATR5B	54497	HEAT repeat containing 5B
ENSG0000	139.7153	0.035534	0.186893	0.755502	0.936415	IGLV2-18	28814	immunoglobulin lambda variable 2-18
ENSG0000	137.6388	0.034953	0.184799	0.75573	0.936456	GPX7	2882	glutathione peroxidase 7
ENSG0000	54.85001	-0.0353	0.176017	0.75579	0.936456	SLC22A5	6584	solute carrier family 22 member 5
ENSG0000	42.6603	0.035316	0.185316	0.755641	0.936456	GTF2F2	2963	general transcription factor IIF subunit 2
ENSG0000	4.280465	0.025141	0.219045	0.755775	0.936456	NA	NA	NA

ENSG0000	6.206419	0.023002	0.219972	0.755622	0.936456	SMIM10L2	399668	small integral membrane protein 10 like 2A
ENSG0000	9.870577	0.027838	0.212528	0.755983	0.936619	EIF5A2	56648	eukaryotic translation initiation factor 5A2
ENSG0000	9.533789	0.030238	0.206363	0.756178	0.936619	NA	NA	NA
ENSG0000	113.9626	0.039325	0.153841	0.756108	0.936619	MGA	23269	MAX dimerization protein MGA
ENSG0000	4.247803	0.019793	0.222421	0.756124	0.936619	CHTF8	54921	chromosome transmission fidelity factor 8
ENSG0000	400.8768	-0.03535	0.15075	0.756157	0.936619	CTNS	1497	cystinosin lysosomal cystine transporter
ENSG0000	874.96	-0.02779	0.110506	0.75645	0.936656	CLK2	1196	CDC like kinase 2
ENSG0000	41.46083	0.0346	0.189309	0.756463	0.936656	CRYBG3	131544	crystallin beta-gamma domain containing 3
ENSG0000	35.3841	0.03329	0.193133	0.756387	0.936656	NUDCD2	134492	NudC domain containing 2
ENSG0000	147.2873	-0.03631	0.147954	0.756353	0.936656	ELP1	8518	elongator acetyltransferase complex subunit 1
ENSG0000	3.918287	0.018772	0.224575	0.756314	0.936656	ZNF491	126069	zinc finger protein 491
ENSG0000	8.361273	0.020731	0.220112	0.756601	0.936672	NA	NA	NA
ENSG0000	251.7096	0.038234	0.153818	0.756629	0.936672	COA4	51287	cytochrome c oxidase assembly factor 4 homolog
ENSG0000	170.3269	0.036066	0.163748	0.756617	0.936672	QTRT1	81890	queuine tRNA-ribosyltransferase catalytic subunit 1
ENSG0000	6.281477	-0.0216	0.220657	0.756904	0.936846	NA	NA	NA
ENSG0000	84.1593	-0.03729	0.157443	0.757462	0.936846	KRT8P12	90133	keratin 8 pseudogene 12
ENSG0000	963.1704	0.03626	0.145292	0.757536	0.936846	HEBP2	23593	heme binding protein 2
ENSG0000	54.40813	-0.03572	0.165554	0.7575	0.936846	PTCH1	5727	patched 1
ENSG0000	5.057542	-0.01687	0.222952	0.756854	0.936846	NA	NA	NA
ENSG0000	38.95791	0.03566	0.180262	0.757495	0.936846	LINC02723	1.08E+08	long intergenic non-protein coding RNA 2723
ENSG0000	3.980976	0.019811	0.22955	0.757206	0.936846	NA	NA	NA
ENSG0000	120.6784	-0.03807	0.18189	0.757394	0.936846	FBXL9P	26231	F-box and pseudogene
ENSG0000	3.533825	-0.01435	0.227944	0.757477	0.936846	NA	NA	NA
ENSG0000	286.1517	-0.03223	0.124121	0.75726	0.936846	FBXL12	54850	F-box and leucine rich repeat protein 12
ENSG0000	21.05786	0.030855	0.203209	0.757518	0.936846	NA	NA	NA
ENSG0000	21.75476	-0.0284	0.206846	0.757537	0.936846	ZNF738	148203	zinc finger protein 738
ENSG0000	86.58238	-0.03468	0.176326	0.75704	0.936846	NA	NA	NA
ENSG0000	293.633	0.035387	0.156187	0.757537	0.936846	SAMD10	140700	sterile alpha motif domain containing 10
ENSG0000	572.3114	-0.03531	0.174155	0.757234	0.936846	CCDC22	28952	coiled-coil domain containing 22
ENSG0000	31.82248	-0.03378	0.181662	0.757598	0.936858	LACTB2	51110	lactamase beta 2
ENSG0000	1298.802	0.032771	0.12309	0.757723	0.936887	TMEM222	84065	transmembrane protein 222
ENSG0000	142.5378	-0.03376	0.183484	0.75769	0.936887	XPR1	9213	xenotropic and polytropic retrovirus receptor 1
ENSG0000	3.802459	-0.01938	0.223381	0.757859	0.936924	LOC10537	1.05E+08	uncharacterized LOC105374101
ENSG0000	273.5431	-0.03698	0.153225	0.757907	0.936924	IGFBP7	3490	insulin like growth factor binding protein 7
ENSG0000	148.1986	-0.03132	0.152326	0.757809	0.936924	PIGU	128869	phosphatidylinositol glycan anchor biosynthesis class U
ENSG0000	1396.066	-0.03278	0.135733	0.761095	0.936959	UBR4	23352	ubiquitin protein ligase E3 component n-recognin 4
ENSG0000	4.572531	0.021011	0.222094	0.760651	0.936959	OSCP1	127700	organic solute carrier partner 1
ENSG0000	38.94812	0.032331	0.195961	0.759721	0.936959	GPAT2	150763	glycerol-3- mitochondrial
ENSG0000	80.10141	0.037094	0.166727	0.758201	0.936959	C2orf49	79074	chromosome 2 open reading frame 49
ENSG0000	122.1908	0.035323	0.154365	0.760954	0.936959	ORC2	4999	origin recognition complex subunit 2
ENSG0000	8.824134	-0.01545	0.225028	0.760571	0.936959	NA	NA	NA
ENSG0000	43.34628	0.035407	0.176118	0.758177	0.936959	NA	NA	NA
ENSG0000	76.14538	0.035638	0.172683	0.760935	0.936959	FAM162A	26355	family with sequence similarity 162 member A
ENSG0000	49.7124	0.032591	0.194195	0.758118	0.936959	LXN	56925	latexin
ENSG0000	3.600622	0.01967	0.229348	0.759879	0.936959	SLC4A4	8671	solute carrier family 4 member 4
ENSG0000	93.63854	0.035123	0.175143	0.759653	0.936959	PPA2	27068	inorganic pyrophosphatase 2
ENSG0000	129.0126	0.034674	0.181856	0.759854	0.936959	TMEM161	1.01E+08	TMEM161B divergent transcript
ENSG0000	22.79064	0.031566	0.199059	0.760858	0.936959	OCM	654231	oncomodulin
ENSG0000	277.0502	-0.0361	0.133165	0.758999	0.936959	RFC2	5982	replication factor C subunit 2
ENSG0000	3.361608	0.016868	0.230409	0.759333	0.936959	NA	NA	NA
ENSG0000	4.546017	-0.01742	0.224666	0.75907	0.936959	NYAP1	222950	neuronal tyrosine phosphorylated phosphoinositide-3-kinase adaptor 1
ENSG0000	15.2996	-0.02797	0.208348	0.759696	0.936959	NA	NA	NA
ENSG0000	338.1451	0.031936	0.127048	0.758648	0.936959	VDAC3	7419	voltage dependent anion channel 3
ENSG0000	10.4859	0.023298	0.217819	0.760894	0.936959	LOC12490	1.25E+08	uncharacterized LOC124902026
ENSG0000	50.98665	-0.03407	0.184732	0.760174	0.936959	ZNF250	58500	zinc finger protein 250
ENSG0000	82.42018	0.031036	0.198496	0.758386	0.936959	ZNF510	22869	zinc finger protein 510
ENSG0000	1485.14	-0.03462	0.167792	0.760323	0.936959	TBC1D2	55357	TBC1 domain family member 2
ENSG0000	285.7175	-0.03474	0.171076	0.760652	0.936959	MCU	90550	mitochondrial calcium uniporter
ENSG0000	15.75327	0.033186	0.194309	0.761158	0.936959	BMPR1A	657	bone morphogenetic protein receptor type 1A
ENSG0000	120.9646	-0.03478	0.168711	0.759933	0.936959	MARCHF5	54708	membrane associated ring-CH-type finger 5
ENSG0000	10104.42	0.035096	0.158199	0.760909	0.936959	DGKZ	8525	diacylglycerol kinase zeta
ENSG0000	135.5058	-0.03951	0.160727	0.758823	0.936959	TUT1	64852	terminal u U6 snRNA-specific
ENSG0000	235.6579	0.034794	0.152294	0.758752	0.936959	LRFN4	78999	leucine rich repeat and fibronectin type III domain containing 4
ENSG0000	10.86595	-0.02128	0.219292	0.760408	0.936959	LRRC32	2615	leucine rich repeat containing 32
ENSG0000	103.0807	-0.0357	0.172517	0.760865	0.936959	TULP3	7289	TUB like protein 3
ENSG0000	80.68651	-0.02937	0.197595	0.759509	0.936959	PYROXD1	79912	pyridine nucleotide-disulphide oxidoreductase domain 1
ENSG0000	157.0216	-0.02892	0.194524	0.760257	0.936959	ERGIC2	51290	ERGIC and golgi 2
ENSG0000	6.181677	-0.02244	0.220175	0.760301	0.936959	NA	NA	NA
ENSG0000	132.4206	0.03732	0.164334	0.759365	0.936959	CDK2	1017	cyclin dependent kinase 2
ENSG0000	7.198791	0.023491	0.221693	0.760633	0.936959	MYO1A	4640	myosin IA
ENSG0000	51.04786	-0.03326	0.18324	0.760414	0.936959	NEDD1	121441	NEDD1 gamma-tubulin ring complex targeting factor
ENSG0000	121.1811	0.034374	0.174322	0.760498	0.936959	C12orf75	387882	chromosome 12 open reading frame 75
ENSG0000	229.1322	0.033736	0.136322	0.759003	0.936959	EMC4	51234	ER membrane protein complex subunit 4
ENSG0000	32.82516	-0.03517	0.180739	0.760063	0.936959	CATSPER2	117155	cation channel sperm associated 2
ENSG0000	426.694	-0.03296	0.138318	0.75969	0.936959	EDC3	80153	enhancer of mRNA decapping 3
ENSG0000	411.0985	0.032599	0.128592	0.75985	0.936959	GFER	2671	growth fac augmenter of liver regeneration
ENSG0000	314.7155	0.035301	0.147421	0.759232	0.936959	PDPK1	5170	3-phosphoinositide dependent protein kinase 1
ENSG0000	2.744587	0.014772	0.227681	0.758183	0.936959	NA	NA	NA
ENSG0000	2.695582	-0.01027	0.229169	0.758268	0.936959	MT1DP	326343	metallothi pseudogene
ENSG0000	5.781355	0.021847	0.22133	0.759591	0.936959	NA	NA	NA
ENSG0000	574.2131	0.033808	0.162411	0.759701	0.936959	ELAC2	60528	elaC ribonuclease Z 2
ENSG0000	73.95698	-0.03514	0.155498	0.7609	0.936959	METTL23	124512	methyltransferase like 23
ENSG0000	476.3304	-0.0321	0.15605	0.759573	0.936959	B3GNTL1	146712	UDP-GlcNA 3-N-acetylglucosaminyltransferase like 1
ENSG0000	6.84091	0.02506	0.217094	0.760803	0.936959	L3MBTL4	91133	L3MBTL histone methyl-lysine binding protein 4
ENSG0000	217.413	-0.03279	0.187968	0.758877	0.936959	SLC14A1	6563	solute carrier family 14 member 1 (Kidd blood group)
ENSG0000	75.11874	-0.02433	0.211819	0.7592	0.936959	TMX3	54495	thioredoxin related transmembrane protein 3
ENSG0000	18.31457	0.029458	0.204043	0.760117	0.936959	ZNF57	126295	zinc finger protein 57
ENSG0000	12.90698	0.020078	0.221509	0.758414	0.936959	GNA15-DT	1.01E+08	GNA15 divergent transcript
ENSG0000	10.47934	0.029208	0.208244	0.759644	0.936959	PODNL1	79883	podocan like 1
ENSG0000	3469.829	-0.03469	0.161852	0.75966	0.936959	CLPTM1	1209	CLPTM1 regulator of GABA type A receptor forward trafficking
ENSG0000	125.4001	-0.02956	0.185688	0.759014	0.936959	GP6	51206	glycoprotein VI platelet
ENSG0000	10.74913	0.02384	0.215198	0.759017	0.936959	ZNF772	400720	zinc finger protein 772
ENSG0000	961.389	0.03159	0.141115	0.76115	0.936959	SNRPB	6628	small nuclear ribonucleoprotein polypeptides B and B1
ENSG0000	4.608953	-0.01113	0.228861	0.759797	0.936959	NA	NA	NA
ENSG0000	2958.76	-0.03186	0.130055	0.761059	0.936959	EWSR1	2130	EWS RNA binding protein 1
ENSG0000	1451.545	-0.02939	0.156891	0.759892	0.936959	PACSIN2	11252	protein kinase C and casein kinase substrate in neurons 2

ENSG0000	172.6891	0.033767	0.149863	0.758816	0.936959	HCCS	3052	holocytochrome c synthase
ENSG0000	30.43235	-0.03257	0.186351	0.760906	0.936959	APOO	79135	apolipoprotein O
ENSG0000	1303.357	0.034636	0.144309	0.761928	0.937001	B4GALT3	8703	beta-1 4-galactosyltransferase 3
ENSG0000	76.00339	0.034674	0.168294	0.761968	0.937001	TATDN3	128387	TatD DNase domain containing 3
ENSG0000	87.11095	0.03298	0.19427	0.762247	0.937001	NID1	4811	nidogen 1
ENSG0000	10.76798	-0.02716	0.212037	0.762317	0.937001	NA	NA	NA
ENSG0000	65.52034	-0.03519	0.164786	0.762173	0.937001	ITPRIPL1	150771	ITPRIP like 1
ENSG0000	286.2263	-0.0347	0.166542	0.762229	0.937001	TLK1	9874	tousled like kinase 1
ENSG0000	269.2072	-0.03296	0.172852	0.762299	0.937001	PARP15	165631	poly(ADP-ribose) polymerase family member 15
ENSG0000	5.642978	-0.01559	0.225882	0.761818	0.937001	LINC01259	1.06E+08	long intergenic non-protein coding RNA 1259
ENSG0000	65.55935	0.03459	0.184834	0.762025	0.937001	OCIAD2	132299	OCIA domain containing 2
ENSG0000	345.0052	-0.03305	0.179028	0.76151	0.937001	BTNL8	79908	butyrophilin like 8
ENSG0000	2677.585	-0.03415	0.168792	0.761843	0.937001	SERPINB1	1992	serpin family B member 1
ENSG0000	10.45692	0.027133	0.211044	0.761937	0.937001	PEX7	5191	peroxisomal biogenesis factor 7
ENSG0000	1090.438	0.032494	0.128047	0.761699	0.937001	GDI2	2665	GDP dissociation inhibitor 2
ENSG0000	383.1482	0.033724	0.141428	0.761796	0.937001	TRIM66	9866	tripartite motif containing 66
ENSG0000	118.2908	-0.03412	0.137634	0.761642	0.937001	MED19	219541	mediator complex subunit 19
ENSG0000	239.1786	-0.03483	0.156371	0.761941	0.937001	ZBTB44	29068	zinc finger and BTB domain containing 44
ENSG0000	11.81781	0.03063	0.204096	0.761627	0.937001	EEF1AKMT	221143	EEF1A lysine methyltransferase 1
ENSG0000	29.65186	0.03443	0.182667	0.761683	0.937001	TGM1	7051	transglutaminase 1
ENSG0000	70.94953	-0.03498	0.163087	0.76224	0.937001	THOC1	9984	THO complex subunit 1
ENSG0000	2744.663	0.031575	0.134222	0.761558	0.937001	PTBP1	5725	polypyrimidine tract binding protein 1
ENSG0000	4.098671	-0.014	0.227804	0.762173	0.937001	B3GNT3	10331	UDP-GlcN/ 3-N-acetylglucosaminyltransferase 3
ENSG0000	11968.39	-0.03388	0.170773	0.762129	0.937001	RASGRP4	115727	RAS guanyl releasing protein 4
ENSG0000	13.04521	-0.02579	0.212274	0.762749	0.937339	ENTPD3-A	285266	ENTPD3 antisense RNA 1
ENSG0000	69.6019	-0.03304	0.188434	0.762746	0.937339	LONRF1	91694	LON peptidase N-terminal domain and ring finger 1
ENSG0000	402.6628	0.033515	0.141065	0.762848	0.937339	POLE	5426	DNA polyr catalytic subunit
ENSG0000	30.31258	0.033899	0.186733	0.762803	0.937339	PIGP	51227	phosphatidylinositol glycan anchor biosynthesis class P
ENSG0000	152.4769	-0.03265	0.13229	0.762729	0.937339	HIC2	23119	HIC ZBTB transcriptional repressor 2
ENSG0000	7160.837	-0.03373	0.17326	0.763031	0.937501	ADA2	51816	adenosine deaminase 2
ENSG0000	148.6197	0.034974	0.182378	0.76316	0.937509	KCNA3	3738	potassium voltage-gated channel subfamily A member 3
ENSG0000	619.9417	0.033379	0.137253	0.763191	0.937509	NAP1L4	4676	nucleosome assembly protein 1 like 4
ENSG0000	2.481273	0.011043	0.229609	0.763177	0.937509	NA	NA	NA
ENSG0000	2.894016	-0.01048	0.229963	0.763283	0.937559	NA	NA	NA
ENSG0000	53.19803	-0.03223	0.175075	0.763502	0.937709	PATJ	10207	PATJ crumbs cell polarity complex component
ENSG0000	356.8225	-0.03192	0.132521	0.763508	0.937709	POFUT2	23275	protein O-fucosyltransferase 2
ENSG0000	19.00444	-0.01996	0.220274	0.763807	0.937761	PTGFRN	5738	prostaglandin F2 receptor inhibitor
ENSG0000	956.828	-0.03326	0.140738	0.763741	0.937761	IKBKE	9641	inhibitor of nuclear factor kappa B kinase subunit epsilon
ENSG0000	6.838922	-0.02161	0.220264	0.76372	0.937761	BBS1	582	Bardet-Biedl syndrome 1
ENSG0000	78.19537	0.030684	0.193201	0.763857	0.937761	TRAPPC6B	122553	trafficking protein particle complex subunit 6B
ENSG0000	1149.531	0.034852	0.143383	0.763714	0.937761	ERO1A	30001	endoplasmic reticulum oxidoreductase 1 alpha
ENSG0000	5.770393	0.021187	0.221243	0.763793	0.937761	MINAR1	23251	membrane integral NOTCH2 associated receptor 1
ENSG0000	6.164128	0.020014	0.221816	0.764358	0.938313	NA	NA	NA
ENSG0000	76.13761	0.034706	0.160318	0.764458	0.938373	CAMKMT	79823	calmodulin-lysine N-methyltransferase
ENSG0000	60.97617	-0.03344	0.173168	0.764539	0.93841	ATP8B1-A!	1.01E+08	ATP8B1 antisense RNA 1
ENSG0000	74.23199	0.034228	0.1462	0.764824	0.938697	LOC11974	1.2E+08	uncharacterized LOC119746555
ENSG0000	3.987625	-0.01885	0.223374	0.764918	0.938743	NA	NA	NA
ENSG0000	129.0481	0.032653	0.166077	0.764965	0.938743	HSPA1L	3305	heat shock protein family A (Hsp70) member 1 like
ENSG0000	4.163137	-0.01733	0.22575	0.765351	0.939004	NA	NA	NA
ENSG0000	14.37204	0.029638	0.204417	0.765383	0.939004	SNX15	29907	sorting nexin 15
ENSG0000	14.41504	0.028472	0.205732	0.765283	0.939004	ALG9	79796	ALG9 alph 2-mannosyltransferase
ENSG0000	34.51015	0.031714	0.19512	0.765433	0.939004	CORO6	84940	coronin 6
ENSG0000	7.757288	0.025655	0.215238	0.765318	0.939004	NA	NA	NA
ENSG0000	518.3105	-0.03361	0.146897	0.765779	0.939014	SPTBN1	6711	spectrin b non-erythrocytic 1
ENSG0000	9.644215	-0.02371	0.216513	0.765582	0.939014	NA	NA	NA
ENSG0000	69.21924	0.027915	0.175184	0.765604	0.939014	CENPC	1060	centromere protein C
ENSG0000	153.5341	0.033705	0.166134	0.765835	0.939014	MGST2	4258	microsomal glutathione S-transferase 2
ENSG0000	9.944734	0.022175	0.217834	0.765899	0.939014	ZNF483	158399	zinc finger protein 483
ENSG0000	4.739937	-0.02003	0.222983	0.765903	0.939014	FGF8	2253	fibroblast growth factor 8
ENSG0000	12.04842	0.025959	0.213049	0.765673	0.939014	DPH6	89978	diphthamine biosynthesis 6
ENSG0000	1402.913	-0.03302	0.149017	0.765521	0.939014	UBE2M	9040	ubiquitin conjugating enzyme E2 M
ENSG0000	445.7152	0.034059	0.160913	0.765855	0.939014	MIF	4282	macrophage migration inhibitory factor
ENSG0000	89.02664	-0.03399	0.150078	0.765981	0.939047	ZBTB49	166793	zinc finger and BTB domain containing 49
ENSG0000	46.39669	0.035099	0.184637	0.766391	0.939487	AMY2B	280	amylase alpha 2B
ENSG0000	216.7581	-0.03281	0.170172	0.766705	0.939572	PCMT1	5110	protein-L-isoaspartate (D-aspartate) O-methyltransferase
ENSG0000	17.51142	0.03229	0.194691	0.766697	0.939572	TIMM23B	1.01E+08	translocase of inner mitochondrial membrane 23 homolog B
ENSG0000	6.19625	-0.02229	0.222761	0.766637	0.939572	OIP5	11339	Opa interacting protein 5
ENSG0000	771.2783	-0.03231	0.156395	0.766668	0.939572	PHKG2	5261	phosphorylase kinase catalytic subunit gamma 2
ENSG0000	17.84669	0.028849	0.1981	0.766717	0.939572	EME1	146956	essential meiotic structure-specific endonuclease 1
ENSG0000	330.0371	0.034248	0.161931	0.766883	0.939576	SLAMF1	6504	signaling lymphocytic activation molecule family member 1
ENSG0000	332.7985	0.03479	0.171136	0.766922	0.939576	PLCB3	5331	phospholipase C beta 3
ENSG0000	119.9263	0.035217	0.162895	0.766985	0.939576	COG3	83548	component of oligomeric golgi complex 3
ENSG0000	4.683162	0.012797	0.228806	0.766966	0.939576	NA	NA	NA
ENSG0000	4.66355	0.017603	0.227223	0.766901	0.939576	NA	NA	NA
ENSG0000	22.08794	-0.02167	0.217079	0.767028	0.939576	SDR42E1	93517	short chai member 1
ENSG0000	10.50196	0.020962	0.219697	0.767121	0.939628	ZNF540	163255	zinc finger protein 540
ENSG0000	10.60002	0.024894	0.213775	0.767359	0.939855	NA	NA	NA
ENSG0000	42.75498	-0.03363	0.179346	0.767422	0.939871	UNC5CL	222643	unc-5 family C-terminal like
ENSG0000	2.545135	0.011307	0.229429	0.767546	0.939959	NA	NA	NA
ENSG0000	335.2516	-0.03345	0.15584	0.767664	0.940041	CCS	9973	copper chaperone for superoxide dismutase
ENSG0000	32.1369	0.023577	0.214584	0.767717	0.940043	LRRC46	90506	leucine rich repeat containing 46
ENSG0000	66.71039	0.033828	0.168904	0.767777	0.940053	LDAH	60526	lipid droplet associated hydrolase
ENSG0000	149.4188	-0.03387	0.172237	0.767977	0.940177	TTC13	79573	tetratricopeptide repeat domain 13
ENSG0000	211.3251	-0.03348	0.163428	0.768092	0.940177	PAPOLG	64895	poly(A) polymerase gamma
ENSG0000	1268.583	-0.03133	0.128682	0.768044	0.940177	GABBR1	2550	gamma-aminobutyric acid type B receptor subunit 1
ENSG0000	194.1163	0.031474	0.140782	0.768134	0.940177	ELP3	55140	elongator acetyltransferase complex subunit 3
ENSG0000	184.1288	0.032907	0.137847	0.768067	0.940177	BABAM1	29086	BRISC and BRCA1 A complex member 1
ENSG0000	294.4388	-0.03318	0.180483	0.768405	0.940445	TMEM167	56900	transmembrane protein 167B
ENSG0000	14.66679	-0.02871	0.205382	0.768529	0.940534	SHF	90525	Src homology 2 domain containing F
ENSG0000	5.426547	-0.01798	0.224151	0.768722	0.940644	ADAMTS7	642935	ADAMTS7 pseudogene 4
ENSG0000	7.31941	-0.01247	0.222052	0.768714	0.940644	MYH11	4629	myosin heavy chain 11
ENSG0000	7.8104	-0.00951	0.229093	0.769113	0.940746	SNORA77	677843	small nucl H/ACA box 77
ENSG0000	38.68133	-0.02648	0.206632	0.769093	0.940746	SLF1	84250	SMC5-SMC6 complex localization factor 1
ENSG0000	125.6688	-0.03042	0.192846	0.769048	0.940746	BBIP1	92482	BBSome interacting protein 1
ENSG0000	18.78686	0.024341	0.209468	0.769021	0.940746	CFL2	1073	cofilin 2
ENSG0000	6.805928	-0.02421	0.21687	0.769091	0.940746	RPL17	6139	ribosomal protein L17

ENSG0000	30.40531	0.032978	0.185644	0.769011	0.940746	ZNF343	79175	zinc finger protein 343
ENSG0000	44.58231	0.032031	0.190489	0.769211	0.940803	PEX12	5193	peroxisomal biogenesis factor 12
ENSG0000	7.780626	-0.01841	0.222444	0.769278	0.940823	NA	NA	NA
ENSG0000	19.39598	-0.02255	0.219801	0.769334	0.940828	OVOS2	144203	alpha-2-macroglobulin like 1 pseudogene
ENSG0000	147.5355	0.0331	0.140348	0.769396	0.940841	TBCA	6902	tubulin folding cofactor A
ENSG0000	113.9649	-0.0325	0.140119	0.769487	0.940888	ZNF778	197320	zinc finger protein 778
ENSG0000	971.5833	-0.03271	0.130193	0.769589	0.940888	CTC1	80169	CST telomere replication complex component 1
ENSG0000	2258.267	-0.03194	0.13625	0.769541	0.940888	KDELR1	10945	KDEL endoplasmic reticulum protein retention receptor 1
ENSG0000	6.999863	0.020429	0.220789	0.769663	0.940917	NA	NA	NA
ENSG0000	4.18429	0.015602	0.22613	0.770209	0.941161	GSTM5	2949	glutathione S-transferase mu 5
ENSG0000	120.5517	0.028472	0.202529	0.770301	0.941161	TBC1D23	55773	TBC1 domain family member 23
ENSG0000	867.2116	-0.03614	0.141163	0.770232	0.941161	PPP2R5D	5528	protein phosphatase 2 regulatory subunit B'delta
ENSG0000	3.900536	-0.01503	0.227211	0.769965	0.941161	NA	NA	NA
ENSG0000	437.4096	0.031888	0.133591	0.769975	0.941161	ARFIP2	23647	ADP ribosylation factor interacting protein 2
ENSG0000	4.8879	-0.01558	0.225194	0.770087	0.941161	NA	NA	NA
ENSG0000	36.73987	0.028102	0.203377	0.77016	0.941161	TGDS	23483	TDP-gluco 6-dehydratase
ENSG0000	7.889234	0.020769	0.219991	0.770326	0.941161	HYKK	123688	hydroxylysine kinase
ENSG0000	2.870212	0.016566	0.225973	0.77032	0.941161	NA	NA	NA
ENSG0000	255.5842	-0.03313	0.163436	0.77048	0.941223	PTGER4	5734	prostaglandin E receptor 4
ENSG0000	2339.897	-0.03378	0.146313	0.770432	0.941223	SMARCC2	6601	SWI/SNF r matrix as: actin dependent regulator of chromatin subfamily c member 2
ENSG0000	482.0693	0.033773	0.160533	0.770676	0.941337	DPYSL2	1808	dihydropyrimidinase like 2
ENSG0000	4.145092	-0.01819	0.223611	0.77064	0.941337	NA	NA	NA
ENSG0000	1018.495	-0.02721	0.10512	0.770813	0.941441	DIDO1	11083	death inducer-obliterator 1
ENSG0000	241.5668	-0.03163	0.132889	0.770864	0.941441	NUP50-DT	1.01E+08	NUP50 divergent transcript
ENSG0000	2.435552	-0.01556	0.227211	0.77103	0.941581	NA	NA	NA
ENSG0000	35.14992	-0.02858	0.201318	0.77115	0.941665	ZKSCAN7	55888	zinc finger with KRAB and SCAN domains 7
ENSG0000	43.59146	0.032803	0.185438	0.771213	0.94168	TRIP13	9319	thyroid hormone receptor interactor 13
ENSG0000	17.49233	-0.02375	0.202113	0.771366	0.941741	STAG3L5P	1.02E+08	stromal antigen 3-like 5 pseudogene
ENSG0000	27.47444	0.031123	0.194702	0.771337	0.941741	NA	NA	NA
ENSG0000	33.09041	-0.03118	0.192262	0.771759	0.941851	ECE1-AS1	1.01E+08	ECE1 antisense RNA 1
ENSG0000	1144.419	-0.03126	0.172392	0.771779	0.941851	DCTN1	1639	dynactin subunit 1
ENSG0000	30.69172	-0.03153	0.191324	0.771674	0.941851	NA	NA	NA
ENSG0000	2.321886	0.01177	0.231046	0.771674	0.941851	GPR141BP	1E+08	G protein- pseudogene
ENSG0000	230.3099	-0.03099	0.12953	0.771816	0.941851	SRI	6717	sorcin
ENSG0000	8.150718	0.025488	0.212832	0.771534	0.941851	ZNF252P-/	286103	ZNF252P antisense RNA 1
ENSG0000	654.6294	-0.03283	0.151629	0.77166	0.941851	CDIP1	29965	cell death inducing p53 target 1
ENSG0000	5.851255	-0.01933	0.222557	0.772035	0.941993	LOC10192	1.02E+08	uncharacterized LOC101929200
ENSG0000	2.485258	0.007379	0.233479	0.772005	0.941993	NA	NA	NA
ENSG0000	389.9037	-0.03216	0.195248	0.772208	0.942141	ERBIN	55914	erbb2 interacting protein
ENSG0000	822.5687	-0.03087	0.182618	0.772673	0.942269	TMCC2	9911	transmembrane and coiled-coil domain family 2
ENSG0000	2943.5	0.029497	0.145593	0.772594	0.942269	SEC61A1	29927	SEC61 translocon subunit alpha 1
ENSG0000	569.7765	-0.03225	0.166119	0.772533	0.942269	SMG1	23049	SMG1 nonsense mediated mRNA decay associated PI3K related kinase
ENSG0000	681.8181	-0.03121	0.148586	0.772461	0.942269	MATK	4145	megakaryocyte-associated tyrosine kinase
ENSG0000	180.3956	0.0319	0.144764	0.772656	0.942269	POFUT1	23509	protein O-fucosyltransferase 1
ENSG0000	539.3227	0.030459	0.123454	0.772473	0.942269	NDRG3	57446	NDRG family member 3
ENSG0000	141.9185	-0.03414	0.159385	0.772408	0.942269	UBE2C	11065	ubiquitin conjugating enzyme E2 C
ENSG0000	494.2608	0.032382	0.179829	0.772745	0.942295	LSR	51599	lipolysis stimulated lipoprotein receptor
ENSG0000	50.82492	0.028338	0.201822	0.772926	0.942327	IMPA1	3612	inositol monophosphatase 1
ENSG0000	398.8321	-0.03187	0.164261	0.77292	0.942327	DPAGT1	1798	dolichyl-phosphate N-acetylglucosaminephosphotransferase 1
ENSG0000	76.70507	-0.03038	0.190938	0.7729	0.942327	ATP8B4	79895	ATPase phospholipid transporting 8B4 (putative)
ENSG0000	89.72128	0.031097	0.190679	0.773245	0.942401	RNF2	6045	ring finger protein 2
ENSG0000	582.4401	-0.03297	0.157903	0.773212	0.942401	CNOT8	9337	CCR4-NOT transcription complex subunit 8
ENSG0000	3.922297	-0.01366	0.229362	0.773154	0.942401	EEF1E1	9521	eukaryotic translation elongation factor 1 epsilon 1
ENSG0000	990.1604	0.033414	0.143515	0.773295	0.942401	NELFB	25920	negative elongation factor complex member B
ENSG0000	14.4008	-0.03015	0.198671	0.773274	0.942401	NA	NA	NA
ENSG0000	774.3295	0.031777	0.144843	0.773142	0.942401	TOP3A	7156	DNA topoisomerase III alpha
ENSG0000	103.1517	0.025707	0.207922	0.773415	0.942433	YOD1	55432	YOD1 deubiquitinase
ENSG0000	16.77431	0.024983	0.211819	0.773515	0.942433	NA	NA	NA
ENSG0000	277.399	0.031748	0.143502	0.773444	0.942433	PLCD1	5333	phospholipase C delta 1
ENSG0000	139.5707	-0.03043	0.133445	0.773528	0.942433	SMUG1	23583	single-strand-selective monofunctional uracil-DNA glycosylase 1
ENSG0000	274.6142	-0.03248	0.142474	0.773647	0.942516	RHBDD1	84236	rhomboid domain containing 1
ENSG0000	228.6589	0.032008	0.182648	0.773704	0.942523	APC	324	APC regulator of WNT signaling pathway
ENSG0000	3072.636	-0.02947	0.121239	0.773795	0.942557	AUP1	550	AUP1 lipid droplet regulating VLDL assembly factor
ENSG0000	369.6984	-0.02943	0.120392	0.773938	0.942557	DALRD3	55152	DALR anticodon binding domain containing 3
ENSG0000	16.98416	-0.01985	0.213995	0.773895	0.942557	NA	NA	NA
ENSG0000	4.740298	-0.01126	0.230052	0.773848	0.942557	NA	NA	NA
ENSG0000	127.2023	0.033442	0.173165	0.77405	0.942631	DHX40	79665	DEAH-box helicase 40
ENSG0000	91.17687	0.033507	0.157317	0.774443	0.942671	MRPS9	64965	mitochondrial ribosomal protein S9
ENSG0000	241.8899	-0.03033	0.13783	0.774287	0.942671	POLH	5429	DNA polymerase eta
ENSG0000	447.9787	-0.03031	0.188204	0.774432	0.942671	CYB5R4	51167	cytochrome b5 reductase 4
ENSG0000	287.3061	0.030079	0.124556	0.774148	0.942671	TYSND1	219743	trypsin like peroxisomal matrix peptidase 1
ENSG0000	8.594551	-0.01624	0.224489	0.774314	0.942671	NA	NA	NA
ENSG0000	19.87948	0.026997	0.206758	0.774439	0.942671	AMH	268	anti-Mullerian hormone
ENSG0000	29.16928	0.032709	0.183381	0.774288	0.942671	NA	NA	NA
ENSG0000	76.20149	0.032912	0.151497	0.774501	0.942678	RALGPS1	9649	Ral GEF with PH domain and SH3 binding motif 1
ENSG0000	912.5952	-0.03085	0.153213	0.774598	0.942734	ATP8B2	57198	ATPase phospholipid transporting 8B2
ENSG0000	701.403	0.029458	0.122971	0.774739	0.942843	OIP5-AS1	729082	OIP5 antisense RNA 1
ENSG0000	111.1643	0.03242	0.179043	0.774907	0.942985	GNA11	2767	G protein subunit alpha 11
ENSG0000	9.83514	0.022387	0.217497	0.775697	0.942995	NA	NA	NA
ENSG0000	137.9278	0.032778	0.175374	0.775566	0.942995	COPS8	10920	COP9 signalosome subunit 8
ENSG0000	8.269654	0.015949	0.224353	0.77543	0.942995	NA	NA	NA
ENSG0000	192.1442	0.029663	0.185419	0.77539	0.942995	NA	NA	NA
ENSG0000	2439.013	0.03249	0.167412	0.775358	0.942995	INTS1	26173	integrator complex subunit 1
ENSG0000	8.56698	-0.02427	0.214757	0.775231	0.942995	NA	NA	NA
ENSG0000	352.506	-0.03137	0.133646	0.775594	0.942995	CRY2	1408	cryptochrome circadian regulator 2
ENSG0000	9836.063	-0.0327	0.14262	0.775564	0.942995	UCP2	7351	uncoupling protein 2
ENSG0000	99.89929	-0.02969	0.192416	0.775719	0.942995	PSMA3	5684	proteasome 20S subunit alpha 3
ENSG0000	116.0733	-0.03263	0.158286	0.775304	0.942995	SEMA7A	8482	semaphorin 7A (John Milton Hagen blood group)
ENSG0000	11.56415	-0.01685	0.222925	0.775584	0.942995	NA	NA	NA
ENSG0000	7.610879	-0.0208	0.219371	0.775335	0.942995	ZNF257	113835	zinc finger protein 257
ENSG0000	1139.803	0.031849	0.15049	0.775062	0.942995	LTBP4	8425	latent transforming growth factor beta binding protein 4
ENSG0000	37.98613	0.02887	0.199752	0.775323	0.942995	SLC5A3	6526	solute carrier family 5 member 3
ENSG0000	311.3653	0.02961	0.141299	0.775388	0.942995	TBC1D10A	83874	TBC1 domain family member 10A
ENSG0000	6.342579	-0.0194	0.22128	0.775739	0.942995	ARMCX1	51309	armadillo repeat containing X-linked 1
ENSG0000	8.21609	0.022901	0.216456	0.775831	0.943043	NA	NA	NA
ENSG0000	115.7731	-0.02813	0.195676	0.775927	0.943098	BMX	660	BMX non-receptor tyrosine kinase

ENSG0000	27.05951	-0.03478	0.198774	0.776052	0.943124	C11orf1	64776	chromosome 11 open reading frame 1
ENSG0000	13.01541	-0.0294	0.200403	0.77603	0.943124	CYSLTR2	57105	cysteinyl leukotriene receptor 2
ENSG0000	2.893453	-0.00369	0.231757	0.776147	0.943178	DSCAML1	57453	DS cell adhesion molecule like 1
ENSG0000	840.2992	-0.0281	0.114049	0.776243	0.943209	HNRNPA3	220988	heterogeneous nuclear ribonucleoprotein A3
ENSG0000	307.1566	-0.02856	0.123843	0.776276	0.943209	PRR3	80742	proline rich 3
ENSG0000	5.452202	0.017566	0.225521	0.776504	0.943361	NA	NA	NA
ENSG0000	4.491089	-0.01023	0.229814	0.776481	0.943361	NALF2	27112	NALCN channel auxiliary factor 2
ENSG0000	1005.886	0.03136	0.171224	0.776564	0.943372	SLC15A4	121260	solute carrier family 15 member 4
ENSG0000	135.0199	0.031696	0.168459	0.776655	0.94342	NOL8	55035	nucleolar protein 8
ENSG0000	89.37526	0.031487	0.14963	0.776835	0.943537	APTX	54840	aprataxin
ENSG0000	43.47168	-0.03188	0.181425	0.776854	0.943537	CDYL2	124359	chromodomain Y like 2
ENSG0000	135.7649	0.033369	0.16506	0.777018	0.94361	ETV3	2117	ETS variant transcription factor 3
ENSG0000	5.088393	0.017899	0.223093	0.776973	0.94361	MCF2	4168	MCF.2 cell line derived transforming sequence
ENSG0000	4619.288	-0.03102	0.138976	0.777225	0.943759	CRTC2	200186	CREB regulated transcription coactivator 2
ENSG0000	335.8079	0.031761	0.176759	0.777295	0.943759	SH3BP5-A!	1.01E+08	SH3BP5 antisense RNA 1
ENSG0000	6.718998	-0.01987	0.220465	0.777269	0.943759	NA	NA	NA
ENSG0000	749.877	0.026875	0.10776	0.777412	0.943839	TMEM129	92305	transmem E3 ubiquitin ligase
ENSG0000	66.72588	0.030788	0.193132	0.77771	0.944082	LRRC8B	23507	leucine rich repeat containing 8 VRAC subunit B
ENSG0000	138.9897	-0.03092	0.182141	0.777816	0.944082	ITGA6	3655	integrin subunit alpha 6
ENSG0000	175.8424	0.025191	0.207439	0.777847	0.944082	IDI1	3422	isopentenyl-diphosphate delta isomerase 1
ENSG0000	22.63675	-0.01351	0.225378	0.777922	0.944082	NA	NA	NA
ENSG0000	42.07046	-0.03109	0.184521	0.777877	0.944082	KICS2	144577	KICSTOR subunit 2
ENSG0000	154.2943	0.032816	0.149122	0.777887	0.944082	SOWAHD	347454	sosondowah ankyrin repeat domain family member D
ENSG0000	59.28772	-0.03397	0.176307	0.777975	0.944084	ZNF514	84874	zinc finger protein 514
ENSG0000	32.43026	0.029604	0.196239	0.779016	0.94413	RAD54L	8438	RAD54 like
ENSG0000	13.40995	0.027798	0.204736	0.778966	0.94413	EFNA3	1944	ephrin A3
ENSG0000	4.677432	-0.01238	0.227803	0.778426	0.94413	NA	NA	NA
ENSG0000	29.10573	0.031125	0.188935	0.779071	0.94413	TAF1B	9014	TATA-box RNA polymerase I subunit B
ENSG0000	117.2004	-0.02893	0.192649	0.778252	0.94413	ORC4	5000	origin recognition complex subunit 4
ENSG0000	44.19456	0.031954	0.177137	0.77879	0.94413	ZNF35	7584	zinc finger protein 35
ENSG0000	2.510015	-0.01247	0.22945	0.779007	0.94413	NA	NA	NA
ENSG0000	10.52512	0.02833	0.219661	0.779147	0.94413	TEAD3	7005	TEA domain transcription factor 3
ENSG0000	256.3596	-0.02914	0.123378	0.778544	0.94413	VIRMA	25962	vir like m6A methyltransferase associated
ENSG0000	5163.833	-0.0277	0.163188	0.778943	0.94413	RXRA	6256	retinoid X receptor alpha
ENSG0000	2.900227	-0.01043	0.230212	0.779096	0.94413	NA	NA	NA
ENSG0000	2582.044	0.032272	0.168959	0.778483	0.94413	RNH1	6050	ribonuclease/angiogenin inhibitor 1
ENSG0000	10.87052	-0.02159	0.216756	0.778642	0.94413	NA	NA	NA
ENSG0000	43.63155	-0.02821	0.199216	0.778186	0.94413	NA	NA	NA
ENSG0000	40.13143	-0.03197	0.177744	0.779067	0.94413	RTTN	25914	rotatin
ENSG0000	4.971701	-0.00383	0.233298	0.77847	0.94413	NA	NA	NA
ENSG0000	4.493952	0.015367	0.225527	0.778782	0.94413	ZNF442	79973	zinc finger protein 442
ENSG0000	265.215	-0.02832	0.127582	0.778644	0.94413	ZNF264	9422	zinc finger protein 264
ENSG0000	68.19941	0.033093	0.181809	0.778322	0.94413	RBL1	5933	RB transcriptional corepressor like 1
ENSG0000	74.27769	0.032681	0.159048	0.779004	0.94413	RWDD2B	10069	RWD domain containing 2B
ENSG0000	106.6098	-0.02989	0.158795	0.779045	0.94413	MRPS6	64968	mitochondrial ribosomal protein S6
ENSG0000	3.713235	-0.01264	0.227737	0.778182	0.94413	NA	NA	NA
ENSG0000	15.28755	-0.02836	0.201527	0.779316	0.944147	PXDC1	221749	PX domain containing 1
ENSG0000	2.394132	-0.00982	0.230369	0.779249	0.944147	NA	NA	NA
ENSG0000	474.4917	0.029511	0.12636	0.77928	0.944147	CHMP6	79643	charged multivesicular body protein 6
ENSG0000	65.71782	0.030047	0.18945	0.779622	0.944394	CASP8AP2	9994	caspase 8 associated protein 2
ENSG0000	2007.483	0.033885	0.152588	0.779586	0.944394	SRRT	51593	serrate RNA effector molecule
ENSG0000	249.2735	0.033874	0.151072	0.779744	0.944479	TATDN2	9797	TatD DNase domain containing 2
ENSG0000	62.70098	-0.03185	0.155652	0.779964	0.944638	YRDC	79693	yrdC N6-threonylcarbamoyltransferase domain containing
ENSG0000	154.9023	-0.03044	0.183831	0.779979	0.944638	PPBP	5473	pro-platelet basic protein
ENSG0000	357.1676	-0.02735	0.107816	0.7801	0.94466	CCDC12	151903	coiled-coil domain containing 12
ENSG0000	1237.757	-0.03336	0.143081	0.780069	0.94466	POLL	27343	DNA polymerase lambda
ENSG0000	989.2069	0.029099	0.193607	0.780436	0.944678	PLB1	151056	phospholipase B1
ENSG0000	409.9193	0.03174	0.129234	0.780476	0.944678	KANSL3	55683	KAT8 regulatory NSL complex subunit 3
ENSG0000	230.0977	-0.02534	0.203191	0.780281	0.944678	GZMA	3001	granzyme A
ENSG0000	25.39007	0.030193	0.193333	0.780305	0.944678	SMIM8	57150	small integral membrane protein 8
ENSG0000	32.06056	-0.02677	0.202508	0.780383	0.944678	PM20D2	135293	peptidase M20 domain containing 2
ENSG0000	622.2157	0.031376	0.158195	0.78033	0.944678	RUVBL2	10856	RuvB like AAA ATPase 2
ENSG0000	72.89782	-0.03116	0.169339	0.780346	0.944678	ZNF805	390980	zinc finger protein 805
ENSG0000	11362.57	-0.03096	0.173241	0.780601	0.944767	PACS1	55690	phosphofurin acidic cluster sorting protein 1
ENSG0000	38.35249	0.031365	0.186468	0.780653	0.944768	HAUS3	79441	HAUS augmin like complex subunit 3
ENSG0000	63.25874	0.029705	0.191708	0.780864	0.944804	POLR1F	221830	RNA polymerase I subunit F
ENSG0000	20.69554	0.02841	0.200842	0.780824	0.944804	PHKG1	5260	phosphorylase kinase catalytic subunit gamma 1
ENSG0000	1486.225	0.032387	0.146642	0.780889	0.944804	LIMK1	3984	LIM domain kinase 1
ENSG0000	7.407141	-0.01702	0.214033	0.780755	0.944804	RAB38	23682	RAB38 member RAS oncogene family
ENSG0000	3479.004	0.031728	0.14762	0.78101	0.944815	ORAI2	80228	ORAI calcium release-activated calcium modulator 2
ENSG0000	4.082421	0.014729	0.225061	0.78105	0.944815	FAM227B	196951	family with sequence similarity 227 member B
ENSG0000	10.93348	-0.02528	0.210793	0.781053	0.944815	NA	NA	NA
ENSG0000	8149.271	0.034681	0.178321	0.781247	0.944961	ENO1	2023	enolase 1
ENSG0000	344.8187	0.025581	0.198074	0.781463	0.944961	ZRANB2	9406	zinc finger RANBP2-type containing 2
ENSG0000	245.9086	-0.03005	0.132614	0.781284	0.944961	MPC2	25874	mitochondrial pyruvate carrier 2
ENSG0000	13.73391	-0.02764	0.203158	0.781586	0.944961	ZNF804A	91752	zinc finger protein 804A
ENSG0000	226.2223	0.02785	0.153177	0.781582	0.944961	TRIM4	89122	tripartite motif containing 4
ENSG0000	168.2327	0.031842	0.138625	0.781494	0.944961	FGFR1	2260	fibroblast growth factor receptor 1
ENSG0000	18.79845	-0.02749	0.202574	0.781573	0.944961	NA	NA	NA
ENSG0000	6.984433	-0.0156	0.224447	0.781445	0.944961	NA	NA	NA
ENSG0000	21.51317	0.030284	0.197669	0.78175	0.945096	ZMYM1	79830	zinc finger MYM-type containing 1
ENSG0000	892.598	0.030827	0.144939	0.781985	0.945209	MPPE1	65258	metallophosphoesterase 1
ENSG0000	5.51171	0.014467	0.224871	0.781952	0.945209	NA	NA	NA
ENSG0000	8.463659	-0.01989	0.219311	0.781998	0.945209	ADORA2A	135	adenosine A2a receptor
ENSG0000	65.17643	0.03171	0.150675	0.782194	0.945364	DDX10	1662	DEAD-box helicase 10
ENSG0000	43.49273	-0.02951	0.186488	0.782229	0.945364	NHLRC4	283948	NHL repeat containing 4
ENSG0000	8.79374	0.018757	0.221372	0.782407	0.945516	SLC22A16	85413	solute carrier family 22 member 16
ENSG0000	22.9889	0.019119	0.220557	0.782901	0.94552	ASPM	259266	assembly factor for spindle microtubules
ENSG0000	3.68804	0.011714	0.227475	0.782685	0.94552	MFSD4A	148808	major facilitator superfamily domain containing 4A
ENSG0000	51.6232	-0.03211	0.172156	0.782633	0.94552	LOC12490	1.25E+08	uncharacterized LOC124907726
ENSG0000	186.2223	-0.03066	0.124029	0.782811	0.94552	GRPEL1	80273	GrpE like 1 mitochondrial
ENSG0000	624.4315	-0.03295	0.143311	0.782641	0.94552	NA	NA	NA
ENSG0000	207.8462	0.031839	0.15872	0.782743	0.94552	CUTALP	253039	cutA divalı pseudogene
ENSG0000	17.94841	-0.02289	0.213299	0.782798	0.94552	NEDD4	4734	NEDD4 E3 ubiquitin protein ligase
ENSG0000	11.77505	0.027461	0.206075	0.782871	0.94552	NA	NA	NA
ENSG0000	1029.717	0.032359	0.169035	0.782813	0.94552	PRPF6	24148	pre-mRNA processing factor 6

ENSG0000	132.8035	-0.02931	0.189064	0.782926	0.94552	ZNF81	347344	zinc finger protein 81
ENSG0000	9.77406	0.021696	0.217835	0.782995	0.94554	DNAH10	196385	dynein axonemal heavy chain 10
ENSG0000	13.74672	-0.02774	0.203997	0.783113	0.94562	LINC02166	1.05E+08	long intergenic non-protein coding RNA 2166
ENSG0000	2620.263	0.028873	0.120706	0.783266	0.94568	YWHAE	7531	tyrosine 3-monooxygenase/tryptophan 5-monooxygenase activation protein epsilon
ENSG0000	74.78715	0.031841	0.170826	0.783265	0.94568	ZNF780A	284323	zinc finger protein 780A
ENSG0000	437.9217	-0.03066	0.160482	0.78338	0.945715	TRABD2A	129293	TraB domain containing 2A
ENSG0000	49.2986	0.031782	0.174531	0.783428	0.945715	TRNT1	51095	tRNA nucleotidyl transferase 1
ENSG0000	18.2978	0.021817	0.21505	0.783449	0.945715	TCEAL1	9338	transcription elongation factor A like 1
ENSG0000	16440.08	-0.02561	0.130213	0.783602	0.945837	KLF2	10365	KLF transcription factor 2
ENSG0000	97.41667	0.031105	0.169035	0.783689	0.945879	FAM149B1	317662	family with sequence similarity 149 member B1
ENSG0000	87.55862	0.030372	0.185016	0.784358	0.945957	VAMP4	8674	vesicle associated membrane protein 4
ENSG0000	1735.753	-0.0281	0.199617	0.784128	0.945957	IL18RAP	8807	interleukin 18 receptor accessory protein
ENSG0000	53.40877	0.031656	0.159067	0.784429	0.945957	DBR1	51163	debranching RNA lariats 1
ENSG0000	266.871	-0.02835	0.148654	0.784101	0.945957	AFF4	27125	ALF transcription elongation factor 4
ENSG0000	56.82635	0.027846	0.198696	0.784277	0.945957	GOPC	57120	golgi associated PDZ and coiled-coil motif containing
ENSG0000	31.06882	0.028315	0.198922	0.784325	0.945957	SBDSP1	155370	SBDS pseudogene 1
ENSG0000	2.518413	0.011449	0.231734	0.78397	0.945957	RN7SL521	1.06E+08	RNA 7SL cytoplasm pseudogene
ENSG0000	1441.222	0.023213	0.104997	0.784406	0.945957	POLR1D	51082	RNA polymerase I and III subunit D
ENSG0000	301.5636	0.030968	0.147319	0.784077	0.945957	EAPP	55837	E2F associated phosphoprotein
ENSG0000	13.04549	0.023481	0.213492	0.783969	0.945957	SPIRE1	56907	spire type actin nucleation factor 1
ENSG0000	69.86328	-0.02789	0.195353	0.784044	0.945957	AIFM3	150209	apoptosis inducing factor mitochondria associated 3
ENSG0000	6486.075	0.030689	0.140368	0.784476	0.945957	PARVG	64098	parvin gamma
ENSG0000	5044.498	0.031283	0.150082	0.784341	0.945957	KIAA0930	23313	KIAA0930
ENSG0000	43.52535	-0.02867	0.195517	0.784264	0.945957	KLF8	11279	KLF transcription factor 8
ENSG0000	4.656788	-0.01503	0.224919	0.78453	0.945959	NA	NA	NA
ENSG0000	694.9775	0.028143	0.117293	0.784728	0.946136	ATP5IF1	93974	ATP synthase inhibitory factor subunit 1
ENSG0000	2197.88	-0.03	0.17648	0.785067	0.946483	CRISPLD2	83716	cysteine rich secretory protein LCCL domain containing 2
ENSG0000	827.0266	0.028785	0.154441	0.786308	0.94692	EXOSC10	5394	exosome component 10
ENSG0000	10.51409	0.01835	0.220328	0.786159	0.94692	CLHC1	130162	clathrin heavy chain linker domain containing 1
ENSG0000	20.57799	0.020888	0.213461	0.785627	0.94692	SGO2	151246	shugoshin 2
ENSG0000	256.004	0.030304	0.183022	0.78625	0.94692	TASOR	23272	transcription activation suppressor
ENSG0000	4.653113	-0.02025	0.220816	0.786001	0.94692	NA	NA	NA
ENSG0000	37.03982	0.031022	0.180874	0.785796	0.94692	MRPS36	92259	mitochondrial ribosomal protein S36
ENSG0000	35.3902	0.025065	0.211552	0.786278	0.94692	ZNF184	7738	zinc finger protein 184
ENSG0000	11.41776	-0.02474	0.208788	0.78594	0.94692	NA	NA	NA
ENSG0000	32.3508	0.030966	0.183408	0.785519	0.94692	SCAI	286205	suppressor of cancer cell invasion
ENSG0000	323.1312	0.028836	0.125309	0.78617	0.94692	SGPL1	8879	sphingosine-1-phosphate lyase 1
ENSG0000	4.021082	0.016125	0.223282	0.785689	0.94692	KCNJ5-AS1	219833	KCNJ5 antisense RNA 1
ENSG0000	326.3992	-0.03027	0.130985	0.785781	0.94692	MESD	23184	mesoderm development LRP chaperone
ENSG0000	131.0275	-0.02508	0.204411	0.786191	0.94692	ZNF267	10308	zinc finger protein 267
ENSG0000	87.26385	-0.03262	0.176377	0.785704	0.94692	NIP7	51388	nucleolar pre-rRNA processing protein NIP7
ENSG0000	80.64199	0.027681	0.20176	0.785785	0.94692	HS3ST3B1	9953	heparan sulfate-glucosamine 3-sulfotransferase 3B1
ENSG0000	578.2405	0.028776	0.139336	0.785845	0.94692	LSM14A	26065	LSM14A mRNA processing body assembly factor
ENSG0000	559.2011	-0.03052	0.16654	0.7861	0.94692	ZNF574	64763	zinc finger protein 574
ENSG0000	15.29606	0.024385	0.212712	0.786366	0.946927	NA	NA	NA
ENSG0000	23.28023	0.030334	0.187316	0.786564	0.947041	TPRKB	51002	TP53RK binding protein
ENSG0000	169.6095	0.029029	0.142923	0.786545	0.947041	SLC39A11	201266	solute carrier family 39 member 11
ENSG0000	42.58505	-0.02078	0.212909	0.786928	0.947043	LOC12490	1.25E+08	uncharacterized LOC124909446
ENSG0000	2.769641	0.011973	0.228795	0.786813	0.947043	TRAPPC3L	1E+08	trafficking protein particle complex subunit 3L
ENSG0000	26.40377	-0.02752	0.198459	0.786718	0.947043	NA	NA	NA
ENSG0000	347.8672	-0.02981	0.139012	0.786881	0.947043	GBGT1	26301	globoside 3-N-acetylgalactosaminyltransferase 1 (FORS blood group)
ENSG0000	35.34846	0.02883	0.195563	0.786743	0.947043	CADM1	23705	cell adhesion molecule 1
ENSG0000	2.329448	-0.00819	0.230435	0.78688	0.947043	NA	NA	NA
ENSG0000	2931.296	0.027181	0.194989	0.786637	0.947043	IGLV1-44	28823	immunoglobulin lambda variable 1-44
ENSG0000	158.0601	0.032142	0.163824	0.787038	0.947068	CREBL2	1389	cAMP responsive element binding protein like 2
ENSG0000	2.445976	0.005161	0.231763	0.787052	0.947068	LINC02321	1.05E+08	long intergenic non-protein coding RNA 2321
ENSG0000	13.64209	-0.02372	0.21147	0.787543	0.947527	RGPD2	729857	RANBP2 like and GRIP domain containing 2
ENSG0000	3.345408	-0.01275	0.229021	0.78768	0.947527	NA	NA	NA
ENSG0000	23.24619	-0.02612	0.202725	0.787692	0.947527	RPL14P1	144581	ribosomal protein L14 pseudogene 1
ENSG0000	1368.555	-0.0307	0.151405	0.787675	0.947527	GLG1	2734	golgi glycoprotein 1
ENSG0000	27.99011	0.023327	0.210993	0.787586	0.947527	SLC22A31	146429	solute carrier family 22 member 31
ENSG0000	172.1532	0.031329	0.161161	0.787866	0.947675	ZNF131	7690	zinc finger protein 131
ENSG0000	12.03709	0.026031	0.206089	0.787968	0.947686	ASH1L-AS1	645676	ASH1L antisense RNA 1
ENSG0000	914.5008	-0.03053	0.155984	0.787993	0.947686	CCT3	7203	chaperonin containing TCP1 subunit 3
ENSG0000	487.7727	-0.0306	0.160134	0.788031	0.947686	CAD	790	carbamoyl aspartate and dihydroorotase
ENSG0000	7.960369	0.01958	0.219845	0.788169	0.94779	RTEL1	51750	regulator of telomere elongation helicase 1
ENSG0000	103.4162	0.029797	0.139937	0.788294	0.947877	ZFYVE21	79038	zinc finger FYVE-type containing 21
ENSG0000	1033.055	0.031553	0.151056	0.789003	0.94805	EXOC3	11336	exocyst complex component 3
ENSG0000	11.64434	0.025265	0.208112	0.788866	0.94805	SEMA6A	57556	semaphorin 6A
ENSG0000	177.3691	-0.03139	0.154745	0.788864	0.94805	URGCP	55665	upregulator of cell proliferation
ENSG0000	6.857774	0.020478	0.217502	0.78885	0.94805	POU5F1B	5462	POU class 5 homeobox 1B
ENSG0000	90.27022	0.032554	0.15834	0.788809	0.94805	MAP4K5	11183	mitogen-activated protein kinase kinase kinase kinase 5
ENSG0000	2.95422	0.01252	0.228049	0.788613	0.94805	LINC02285	1.02E+08	long intergenic non-protein coding RNA 2285
ENSG0000	1268.478	-0.02962	0.147931	0.788857	0.94805	SNAI3	333929	snail family transcriptional repressor 3
ENSG0000	93.89932	-0.03049	0.172053	0.789112	0.94805	GAMT	2593	guanidinoacetate N-methyltransferase
ENSG0000	54.02505	0.0295	0.16743	0.789027	0.94805	ZNF792	126375	zinc finger protein 792
ENSG0000	3.658093	-0.015	0.226036	0.789008	0.94805	DACT3	147906	dishevelled binding antagonist of beta catenin 3
ENSG0000	29.97238	-0.03109	0.19035	0.788508	0.94805	IFNAR2	3455	interferon alpha and beta receptor subunit 2
ENSG0000	15.95566	0.021898	0.214296	0.789108	0.94805	RIMBP3	85376	RIMS binding protein 3
ENSG0000	173.4752	-0.03084	0.161283	0.789003	0.94805	CCDC120	90060	coiled-coil domain containing 120
ENSG0000	125.7663	0.031365	0.151108	0.789162	0.94805	PJA1	64219	praja ring finger ubiquitin ligase 1
ENSG0000	171.78	0.030955	0.153365	0.789231	0.94807	PIK3C2B	5287	phosphatidylinositol-4-phosphate 3-kinase catalytic subunit type 2 beta
ENSG0000	3085.572	0.031011	0.170225	0.789348	0.948146	SLC38A10	124565	solute carrier family 38 member 10
ENSG0000	149.1172	0.029254	0.133837	0.789397	0.948146	CEP89	84902	centrosomal protein 89
ENSG0000	134.3321	-0.02787	0.193402	0.789672	0.948352	NA	NA	NA
ENSG0000	3.785616	0.011881	0.228595	0.789642	0.948352	NA	NA	NA
ENSG0000	11.56283	0.02223	0.213751	0.790732	0.948382	HSPE1	3336	heat shock protein family E (Hsp10) member 1
ENSG0000	37.77914	0.028624	0.192449	0.790682	0.948382	NA	NA	NA
ENSG0000	111.5159	0.032743	0.164864	0.79007	0.948382	MRPL14	64928	mitochondrial ribosomal protein L14
ENSG0000	8.14874	-0.02031	0.223923	0.790362	0.948382	RHAG	6005	Rh associated glycoprotein
ENSG0000	3770.862	0.026785	0.115213	0.7898	0.948382	ARF5	381	ADP ribosylation factor 5
ENSG0000	16.48205	-0.02402	0.208749	0.790476	0.948382	TRBJ1-5	28631	T cell receptor beta joining 1-5
ENSG0000	377.27	0.030855	0.180893	0.790671	0.948382	ATP6V1C1	528	ATPase H+ transporting V1 subunit C1
ENSG0000	568.6468	-0.02973	0.170722	0.79037	0.948382	RAD21	5885	RAD21 cohesin complex component
ENSG0000	724.8111	-0.03066	0.169086	0.790533	0.948382	PYGM	5837	glycogen p muscle associated
ENSG0000	2553.446	0.030448	0.170369	0.790351	0.948382	ALDH3B1	221	aldehyde dehydrogenase 3 family member B1

ENSG0000	218.0605	-0.02719	0.140217	0.790401	0.948382	NA	NA	NA	
ENSG0000	12.9817	0.022933	0.209986	0.790167	0.948382	SLC30A4	7782	solute carrier family 30 member 4	
ENSG0000	175.6771	-0.02923	0.136802	0.79029	0.948382	LCMT1	51451	leucine carboxyl methyltransferase 1	
ENSG0000	13.225	-0.01793	0.222353	0.790493	0.948382	CES1P1	51716	carboxylesterase 1 pseudogene 1	
ENSG0000	75.87559	-0.03059	0.164118	0.790328	0.948382	IFT20	90410	intraflagellar transport 20	
ENSG0000	35.02286	0.029881	0.185353	0.790073	0.948382	INTS2	57508	integrator complex subunit 2	
ENSG0000	6.512226	0.020381	0.223486	0.789763	0.948382	NA	NA	NA	
ENSG0000	232.7322	0.031194	0.160998	0.790362	0.948382	SMAD4	4089	SMAD family member 4	
ENSG0000	153.8099	-0.02963	0.1541	0.790154	0.948382	A1BG-AS1	503538	A1BG antisense RNA 1	
ENSG0000	17.03734	-0.02262	0.211776	0.790653	0.948382	XKR7	343702	XK related 7	
ENSG0000	28.53223	0.02701	0.199381	0.790961	0.948594	RAB40B	10966	RAB40B	member RAS oncogene family
ENSG0000	303.4122	-0.02983	0.169881	0.791181	0.948741	ITPRID2	6744	ITPR interacting domain containing 2	
ENSG0000	77.0541	0.020461	0.215466	0.791223	0.948741	IPMK	253430	inositol polyphosphate multikinase	
ENSG0000	25.37882	0.016011	0.221354	0.791239	0.948741	DOC2B	8447	double C2 domain beta	
ENSG0000	78.06262	-0.0252	0.200658	0.791392	0.948771	PRKAB2	5565	protein kinase AMP-activated non-catalytic subunit beta 2	
ENSG0000	69.13131	-0.02847	0.183684	0.791549	0.948771	ARHGAP18	93663	Rho GTPase activating protein 18	
ENSG0000	11.83797	0.019426	0.217954	0.791425	0.948771	NA	NA	NA	
ENSG0000	528.7362	0.029328	0.119513	0.791577	0.948771	BORCS6	54785	BLOC-1 related complex subunit 6	
ENSG0000	623.2216	0.028759	0.153257	0.791324	0.948771	TRMT1	55621	tRNA methyltransferase 1	
ENSG0000	76.75751	0.030686	0.164363	0.791627	0.948771	ZNF91	7644	zinc finger protein 91	
ENSG0000	676.6076	-0.03019	0.149865	0.791477	0.948771	MORC2	22880	MORC family CW-type zinc finger 2	
ENSG0000	80.79217	-0.02833	0.178605	0.791768	0.948878	CCDC90B	60492	coiled-coil domain containing 90B	
ENSG0000	817.3575	-0.02991	0.161793	0.791823	0.948882	ABCF1	23	ATP binding cassette subfamily F member 1	
ENSG0000	833.6482	-0.02833	0.131907	0.792203	0.948992	KAT5	10524	lysine acetyltransferase 5	
ENSG0000	922.4667	0.028928	0.120062	0.792031	0.948992	VPS26B	112936	VPS26 retromer complex component B	
ENSG0000	7.537128	-0.0208	0.216716	0.792094	0.948992	NA	NA	NA	
ENSG0000	317.8197	-0.02986	0.141798	0.792001	0.948992	RRP1B	23076	ribosomal RNA processing 1B	
ENSG0000	211.1093	0.029712	0.144354	0.792225	0.948992	PI4KAP2	375133	phosphatidylinositol 4-kinase alpha pseudogene 2	
ENSG0000	25.31385	-0.02645	0.209216	0.792223	0.948992	LGALS2	3957	galectin 2	
ENSG0000	2.848887	-0.00671	0.228826	0.7924	0.949054	NA	NA	NA	
ENSG0000	1911.97	0.029515	0.16393	0.792433	0.949054	ASAH1	427	N-acylsphingosine amidohydrolase 1	
ENSG0000	184.8253	0.027515	0.172899	0.792423	0.949054	WAPL	23063	WAPL cohesin release factor	
ENSG0000	716.4518	-0.02665	0.119825	0.793036	0.949231	PLEKHB2	55041	pleckstrin homology domain containing B2	
ENSG0000	69.82277	-0.0284	0.184958	0.792993	0.949231	LY75	4065	lymphocyte antigen 75	
ENSG0000	135.5353	0.028807	0.182167	0.79303	0.949231	CDC40	51362	cell division cycle 40	
ENSG0000	5.470645	-0.0189	0.220867	0.792887	0.949231	NA	NA	NA	
ENSG0000	131.2267	-0.03074	0.160179	0.793047	0.949231	KCNAB3	9196	potassium voltage-gated channel subfamily A regulatory beta subunit 3	
ENSG0000	1144.568	0.029327	0.167851	0.792951	0.949231	CC2D1A	54862	coiled-coil and C2 domain containing 1A	
ENSG0000	16.61838	0.021993	0.21266	0.792706	0.949231	ZNF568	374900	zinc finger protein 568	
ENSG0000	1656.677	0.030276	0.161693	0.792955	0.949231	HUWE1	10075	HECT	UBA and WWE domain containing E3 ubiquitin protein ligase 1
ENSG0000	8448.003	-0.02776	0.186073	0.792647	0.949231	G6PD	2539	glucose-6-phosphate dehydrogenase	
ENSG0000	33.34723	0.027791	0.195071	0.793108	0.949242	ZNF594-DT	1E+08	ZNF594 divergent transcript	
ENSG0000	166.5048	0.027326	0.18672	0.793192	0.94928	PRELID3B	51012	PRELI domain containing 3B	
ENSG0000	405.3731	0.027881	0.147585	0.793263	0.949304	MARCHF9	92979	membrane associated ring-CH-type finger 9	
ENSG0000	180775.3	0.030323	0.161703	0.793323	0.949314	ND4	4538	NADH dehydrogenase subunit 4	
ENSG0000	169.3118	-0.02668	0.133076	0.793646	0.949472	NA	NA	NA	
ENSG0000	3.21866	0.009891	0.229218	0.793517	0.949472	LOC10012	1E+08	four and a half LIM domains 1 pseudogene	
ENSG0000	3.209918	0.014441	0.227803	0.79377	0.949472	LOC10050	1.01E+08	uncharacterized LOC100507547	
ENSG0000	110.8576	0.030057	0.159831	0.793819	0.949472	OAT	4942	ornithine aminotransferase	
ENSG0000	225.4401	0.028147	0.190725	0.79375	0.949472	IPO7	10527	importin 7	
ENSG0000	43.79167	-0.02975	0.160803	0.793748	0.949472	TSSK6	83983	testis specific serine kinase 6	
ENSG0000	14.04975	0.023909	0.210405	0.793626	0.949472	ZNF790-AS	284408	ZNF790 antisense RNA 1	
ENSG0000	27.33254	0.029126	0.186682	0.794118	0.949477	NDUFB2-A	1E+08	NDUFB2 antisense RNA 1	
ENSG0000	2.472152	0.016987	0.227763	0.794033	0.949477	GAS1	2619	growth arrest specific 1	
ENSG0000	479.2356	0.026347	0.118982	0.793977	0.949477	DDB2	1643	damage specific DNA binding protein 2	
ENSG0000	179.1891	0.026575	0.189451	0.793889	0.949477	FGD4	121512	FYVE	RhoGEF and PH domain containing 4
ENSG0000	8.249258	0.024082	0.210583	0.794134	0.949477	ZNF426-DT	1.02E+08	ZNF426 divergent transcript	
ENSG0000	2026.473	0.026002	0.116489	0.794063	0.949477	MAVS	57506	mitochondrial antiviral signaling protein	
ENSG0000	10.17861	-0.02259	0.212125	0.794238	0.949539	NA	NA	NA	
ENSG0000	3.692879	0.015194	0.22496	0.794307	0.94956	PROM2	150696	prominin 2	
ENSG0000	1328.836	-0.02916	0.171847	0.794725	0.949595	EHMT2	10919	euchromatic histone lysine methyltransferase 2	
ENSG0000	15.88837	-0.01785	0.220022	0.794891	0.949595	ANLN	54443	anillin	actin binding protein
ENSG0000	42.76069	0.029274	0.181089	0.794803	0.949595	GPHN	10243	gephyrin	
ENSG0000	16.40696	0.022129	0.211627	0.794659	0.949595	NA	NA	NA	
ENSG0000	101.5626	-0.02984	0.157807	0.794688	0.949595	CHRNE	1145	cholinergic receptor nicotinic epsilon subunit	
ENSG0000	108.81	-0.02914	0.140547	0.794814	0.949595	CCDC47	57003	coiled-coil domain containing 47	
ENSG0000	99.52695	0.029233	0.140514	0.794906	0.949595	MFSD11	79157	major facilitator superfamily domain containing 11	
ENSG0000	74.19521	-0.03001	0.185559	0.794628	0.949595	COL5A3	50509	collagen type V alpha 3 chain	
ENSG0000	1188.285	0.029477	0.14564	0.794671	0.949595	AP2S1	1175	adaptor related protein complex 2 subunit sigma 1	
ENSG0000	3381.029	-0.03016	0.151528	0.794566	0.949595	CNOT3	4849	CCR4-NOT transcription complex subunit 3	
ENSG0000	39.46655	-0.02963	0.178945	0.794618	0.949595	RTN4R	65078	reticulon 4 receptor	
ENSG0000	258.3535	0.026426	0.136188	0.794964	0.949602	MRPL9	65005	mitochondrial ribosomal protein L9	
ENSG0000	30.39288	-0.02414	0.205458	0.795269	0.949697	CTLA4	1493	cytotoxic T-lymphocyte associated protein 4	
ENSG0000	309.1217	-0.02822	0.134822	0.795267	0.949697	YEATS2	55689	YEATS domain containing 2	
ENSG0000	1067.537	0.028537	0.131746	0.795273	0.949697	NDUFB10	4716	NADH:ubiquinone oxidoreductase subunit B10	
ENSG0000	149.5021	-0.02893	0.143376	0.795302	0.949697	HEATR6	63897	HEAT repeat containing 6	
ENSG0000	18.34573	0.026727	0.199876	0.79518	0.949697	SLC25A14	9016	solute carrier family 25 member 14	
ENSG0000	2670.508	0.029368	0.176198	0.795567	0.949703	SLC15A3	51296	solute carrier family 15 member 3	
ENSG0000	937.6307	-0.02756	0.130136	0.795516	0.949703	LPCAT4	254531	lysophosphatidylcholine acyltransferase 4	
ENSG0000	6.482277	0.019305	0.21937	0.795392	0.949703	NA	NA	NA	
ENSG0000	22.98942	-0.02682	0.196839	0.795466	0.949703	KIF23	9493	kinesin family member 23	
ENSG0000	643.5083	0.028855	0.13848	0.795559	0.949703	PIP4K2B	8396	phosphatidylinositol-5-phosphate 4-kinase type 2 beta	
ENSG0000	50.74536	-0.02892	0.179486	0.795688	0.949786	PFDN2	5202	prefoldin subunit 2	
ENSG0000	35.12243	0.027349	0.19044	0.795793	0.94985	NA	NA	NA	
ENSG0000	3.205755	-0.01513	0.224942	0.795864	0.949867	NA	NA	NA	
ENSG0000	10.77604	0.019612	0.217971	0.795911	0.949867	NA	NA	NA	
ENSG0000	59.08706	0.029183	0.179598	0.795976	0.949882	SLC25A43	203427	solute carrier family 25 member 43	
ENSG0000	2.562477	0.015443	0.225285	0.796235	0.950129	PLAC8L1	153770	PLAC8 like 1	
ENSG0000	528.4101	0.028097	0.132649	0.796374	0.950179	OSBPL9	114883	oxysterol binding protein like 9	
ENSG0000	30.97796	0.027515	0.19072	0.796505	0.950179	FAM153A	285596	family with sequence similarity 153 member A	
ENSG0000	2.889927	0.010876	0.22897	0.796588	0.950179	OR7E38P	389537	olfactory receptor family 7 subfamily E member 38 pseudogene	
ENSG0000	44.82846	0.026955	0.196178	0.796575	0.950179	MMP14	4323	matrix metallopeptidase 14	
ENSG0000	1779.759	0.027317	0.124078	0.79649	0.950179	CCDC88C	440193	coiled-coil domain containing 88C	
ENSG0000	135.3768	0.029506	0.153747	0.796448	0.950179	ZFP90	146198	ZFP90 zinc finger protein	
ENSG0000	2032.983	-0.02889	0.113601	0.796813	0.950261	PCBP2	5094	poly(rC) binding protein 2	
ENSG0000	78.14186	-0.02989	0.15962	0.79681	0.950261	AACS	65985	acetoacetyl-CoA synthetase	

ENSG0000	3.198865	0.008137	0.230446	0.796764	0.950261	NA	NA	NA
ENSG0000	904.7365	-0.02895	0.147105	0.796924	0.950277	PDIA6	10130	protein disulfide isomerase family A member 6
ENSG0000	238.2542	-0.03148	0.177033	0.79693	0.950277	RASGRP1	10125	RAS guanyl releasing protein 1
ENSG0000	2.567243	-0.01123	0.229874	0.797524	0.950319	DYNC2LI1	51626	dynein cytoplasmic 2 light intermediate chain 1
ENSG0000	91.74188	0.024209	0.203717	0.797484	0.950319	IGFBP2	3485	insulin like growth factor binding protein 2
ENSG0000	4.239446	-0.01529	0.219339	0.797536	0.950319	ADGRA3	166647	adhesion G protein-coupled receptor A3
ENSG0000	5.28219	-0.02092	0.216623	0.79729	0.950319	NA	NA	NA
ENSG0000	11.62755	-0.02174	0.212772	0.797115	0.950319	SAP30-DT	1.05E+08	SAP30 divergent transcript
ENSG0000	3.532205	-0.01697	0.223017	0.797276	0.950319	HOXA3	3200	homeobox A3
ENSG0000	36.89674	0.023927	0.205726	0.797383	0.950319	CPNE8	144402	copine 8
ENSG0000	329.0321	-0.0279	0.121041	0.797198	0.950319	SART3	9733	spliceoson U4/U6 recycling protein
ENSG0000	1093.082	-0.03099	0.15461	0.797266	0.950319	SAFB2	9667	scaffold attachment factor B2
ENSG0000	1312.72	0.015367	0.136747	0.797456	0.950319	CLPP	8192	caseinolytic mitochondrial matrix peptidase proteolytic subunit
ENSG0000	3.336505	0.010246	0.228287	0.797062	0.950319	NA	NA	NA
ENSG0000	156.9412	0.026733	0.191293	0.797786	0.950482	CTSO	1519	cathepsin O
ENSG0000	4.687486	0.017389	0.224662	0.797761	0.950482	SUGT1P4-!	1E+08	SUGT1P4-STRA6LP readthrough
ENSG0000	42.66984	-0.02524	0.199154	0.797835	0.950482	VPS9D1-A!	1E+08	VPS9D1 antisense RNA 1
ENSG0000	31.88903	-0.02542	0.191463	0.79788	0.950482	NA	NA	NA
ENSG0000	673.3676	0.027659	0.13434	0.798019	0.950586	NAXE	128240	NAD(P)HX epimerase
ENSG0000	625.4977	0.029629	0.133344	0.79819	0.950607	NDUFS2	4720	NADH:ubiquinone oxidoreductase core subunit S2
ENSG0000	913.5667	0.034717	0.135645	0.798191	0.950607	MCMBP	79892	minichromosome maintenance complex binding protein
ENSG0000	64.78835	-0.02956	0.168422	0.798192	0.950607	BNIP3	664	BCL2 interacting protein 3
ENSG0000	405.3386	-0.02876	0.165402	0.798476	0.950883	PHIP	55023	pleckstrin homology domain interacting protein
ENSG0000	2.819004	-0.00886	0.229833	0.798711	0.950888	NA	NA	NA
ENSG0000	558.4604	-0.02465	0.117136	0.798646	0.950888	TSPYL1	7259	TSPY like 1
ENSG0000	49.45445	-0.02533	0.198342	0.79874	0.950888	SP4	6671	Sp4 transcription factor
ENSG0000	87.41335	-0.02467	0.1995	0.798589	0.950888	SINHCAF	58516	SIN3-HDAC complex associated factor
ENSG0000	19.06308	-0.02338	0.207537	0.798692	0.950888	NA	NA	NA
ENSG0000	5.23985	0.017022	0.221899	0.798856	0.950964	NA	NA	NA
ENSG0000	21.74366	0.025721	0.202014	0.798942	0.951006	ZNG1F	644019	Zn regulated GTPase metalloprotein activator 1F
ENSG0000	42.99656	-0.03032	0.186587	0.799333	0.95109	SFN	2810	stratifin
ENSG0000	10.48051	-0.02041	0.215379	0.799176	0.95109	NA	NA	NA
ENSG0000	189.8225	0.027959	0.181649	0.799426	0.95109	VAV3	10451	vav guanine nucleotide exchange factor 3
ENSG0000	411.5404	0.029308	0.15172	0.799496	0.95109	MTX1	4580	metaxin 1
ENSG0000	78.33731	0.02709	0.172981	0.79966	0.95109	BMP6	654	bone morphogenetic protein 6
ENSG0000	18.95754	-0.01985	0.215126	0.799677	0.95109	TRGV5	6978	T cell receptor gamma variable 5
ENSG0000	18.24645	0.024116	0.206915	0.799373	0.95109	LOC12490	1.25E+08	uncharacterized LOC124902114
ENSG0000	25.44189	-0.02796	0.188267	0.799688	0.95109	NA	NA	NA
ENSG0000	22.63334	0.026642	0.198413	0.799492	0.95109	NA	NA	NA
ENSG0000	56.80469	-0.03043	0.164965	0.799167	0.95109	ALDH6A1	4329	aldehyde dehydrogenase 6 family member A1
ENSG0000	2632.886	0.026807	0.117396	0.799573	0.95109	RNPS1	10921	RNA binding protein with serine rich domain 1
ENSG0000	3.987087	-0.00815	0.22975	0.799338	0.95109	CDIPTOSP	440356	CDIP trans pseudogene
ENSG0000	242.6374	-0.02871	0.158145	0.799357	0.95109	NF1	4763	neurofibromin 1
ENSG0000	8.838467	0.024267	0.208671	0.799757	0.951111	LOC10050	1.01E+08	uncharacterized LOC100506235
ENSG0000	6.975768	-0.01638	0.224893	0.799935	0.95126	NA	NA	NA
ENSG0000	4650.465	0.028797	0.179451	0.800078	0.951368	VMP1	81671	vacuole membrane protein 1
ENSG0000	56.90731	-0.02857	0.176698	0.800219	0.951475	PBDC1	51260	polysaccharide biosynthesis domain containing 1
ENSG0000	232.1664	-0.02991	0.152239	0.801364	0.951674	IFFO2	126917	intermediate filament family orphan 2
ENSG0000	47.83628	0.028236	0.179934	0.800684	0.951674	FASTKD1	79675	FAST kinase domains 1
ENSG0000	763.747	0.032318	0.164712	0.801625	0.951674	CD180	4064	CD180 molecule
ENSG0000	561.0887	0.026651	0.139829	0.800606	0.951674	ITPR3	3710	inositol 1 4 5-trisphosphate receptor type 3
ENSG0000	65.07847	-0.02874	0.181365	0.801577	0.951674	DBF4	10926	DBF4 zinc finger
ENSG0000	4.267239	-0.01315	0.226261	0.800844	0.951674	MTATP6P1	1.07E+08	MT-ATP6 pseudogene 11
ENSG0000	9.769386	0.022184	0.212559	0.801528	0.951674	NA	NA	NA
ENSG0000	3.266018	-0.01547	0.226859	0.801309	0.951674	NA	NA	NA
ENSG0000	1566.409	-0.02796	0.158765	0.801383	0.951674	GBF1	8729	golgi brefeldin A resistant guanine nucleotide exchange factor 1
ENSG0000	57.33937	-0.02877	0.174231	0.801542	0.951674	CNNM2	54805	cyclin and CBS domain divalent metal cation transport mediator 2
ENSG0000	1319.46	0.023403	0.131278	0.801506	0.951674	UBXN1	51035	UBX domain protein 1
ENSG0000	318.741	0.027313	0.127911	0.800648	0.951674	NIPA2	81614	NIPA magnesium transporter 2
ENSG0000	102.4367	-0.02587	0.189705	0.801631	0.951674	SECISBP2L	9728	SECIS binding protein 2 like
ENSG0000	1513.343	0.025379	0.115777	0.801067	0.951674	SH2B1	25970	SH2B adaptor protein 1
ENSG0000	3.773516	0.018663	0.223772	0.800736	0.951674	MPP3	4356	MAGUK p55 scaffold protein 3
ENSG0000	801.9742	-0.02683	0.129812	0.801289	0.951674	DCAF7	10238	DDB1 and CUL4 associated factor 7
ENSG0000	34.99591	0.023178	0.206184	0.801241	0.951674	NA	NA	NA
ENSG0000	1070.624	0.025866	0.178383	0.80119	0.951674	SMARCA4	6597	SWI/SNF r matrix ass actin depe subfamily member 4
ENSG0000	2638.947	0.028638	0.179387	0.800637	0.951674	CEACAM4	1089	CEA cell adhesion molecule 4
ENSG0000	3209.781	0.028821	0.160432	0.800966	0.951674	AP2A1	160	adaptor related protein complex 2 subunit alpha 1
ENSG0000	3.158463	0.012126	0.227104	0.801471	0.951674	MIR6813	1.02E+08	microRNA 6813
ENSG0000	6.037298	-0.02173	0.214251	0.801506	0.951674	CHADL	150356	chondroadherin like
ENSG0000	9537.273	-0.02703	0.181963	0.801634	0.951674	TIMP1	7076	TIMP metallopeptidase inhibitor 1
ENSG0000	39.67119	-0.02758	0.186374	0.80044	0.951674	SYP	6855	synaptophysin
ENSG0000	408.5773	-0.02721	0.18319	0.80212	0.951881	ZBTB37	84614	zinc finger and BTB domain containing 37
ENSG0000	153.9491	-0.0245	0.19864	0.802055	0.951881	VWA7	80737	von Willebrand factor A domain containing 7
ENSG0000	1231.814	-0.02698	0.184116	0.802088	0.951881	NIBAN2	64855	niban apoptosis regulator 2
ENSG0000	13.57668	-0.02258	0.205963	0.801867	0.951881	ALG10	84920	ALG10 alp 2-glucosyltransferase
ENSG0000	217.5522	0.029139	0.173373	0.802015	0.951881	LOC10192	1.02E+08	uncharacterized LOC101929107
ENSG0000	2.807361	0.013231	0.22899	0.802106	0.951881	NA	NA	NA
ENSG0000	77.17272	0.030072	0.188196	0.802652	0.95189	SH3YL1	26751	SH3 and SYLF domain containing 1
ENSG0000	7.344895	-0.02126	0.213789	0.80222	0.95189	EFCC1	79825	EF-hand and coiled-coil domain containing 1
ENSG0000	14.46706	0.023125	0.209401	0.802691	0.95189	NA	NA	NA
ENSG0000	11.29948	-0.02391	0.206735	0.80269	0.95189	NA	NA	NA
ENSG0000	34.2347	-0.02645	0.193656	0.802307	0.95189	GMNN	51053	geminin DNA replication inhibitor
ENSG0000	12.31884	-0.02141	0.212871	0.802667	0.95189	NHSL1	57224	NHS like 1
ENSG0000	4707.381	-0.02099	0.112075	0.802633	0.95189	LDB1	8861	LIM domain binding 1
ENSG0000	6.480978	-0.0179	0.219537	0.802699	0.95189	NA	NA	NA
ENSG0000	8.292009	-0.01883	0.217017	0.802374	0.95189	NME2	4831	NME/NM23 nucleoside diphosphate kinase 2
ENSG0000	594.9966	-0.02831	0.140024	0.802635	0.95189	YIPF2	78992	Yip1 domain family member 2
ENSG0000	226.6382	0.029808	0.155955	0.802547	0.95189	ZNF784	147808	zinc finger protein 784
ENSG0000	25.73325	-0.02522	0.19361	0.802784	0.951929	NUP153-A	1.05E+08	NUP153 antisense RNA 1
ENSG0000	1628.455	0.027259	0.133696	0.803783	0.952056	MFN2	9927	mitofusin 2
ENSG0000	48.67748	-0.02871	0.171273	0.803894	0.952056	ELOA-AS1	1.01E+08	ELOA antisense RNA 1
ENSG0000	72.82356	-0.02474	0.197794	0.803936	0.952056	GPA33	10223	glycoprotein A33
ENSG0000	3.319102	0.016708	0.225777	0.804014	0.952056	NA	NA	NA
ENSG0000	101.7424	0.028491	0.172874	0.803578	0.952056	ZNF141	7700	zinc finger protein 141
ENSG0000	61.91123	0.023633	0.203854	0.803092	0.952056	INPP4B	8821	inositol polyphosphate-4-phosphatase type II B
ENSG0000	16.39195	0.024519	0.204346	0.803475	0.952056	ARHGEF37	389337	Rho guanine nucleotide exchange factor 37

ENSG0000	369.8329	-0.0227	0.125596	0.803979	0.952056	ATP5MF	9551	ATP synthase membrane subunit f
ENSG0000	3.532224	-0.01598	0.223482	0.803448	0.952056	LOC10192	1.02E+08	uncharacterized LOC101927588
ENSG0000	1057.552	-0.02861	0.182943	0.803715	0.952056	MROH6	642475	maestro heat like repeat family member 6
ENSG0000	38.43038	0.026952	0.18786	0.804011	0.952056	NSUN6	221078	NOP2/Sun RNA methyltransferase 6
ENSG0000	1920.335	-0.02666	0.132511	0.803671	0.952056	PGAM1	5223	phosphoglycerate mutase 1
ENSG0000	17.66898	0.026097	0.201888	0.802987	0.952056	CSKMT	751071	citrate synthase lysine methyltransferase
ENSG0000	14.0216	0.018642	0.218425	0.803202	0.952056	ABCD2	225	ATP binding cassette subfamily D member 2
ENSG0000	33.0423	0.029101	0.172044	0.803432	0.952056	CLYBL	171425	citramalyl-CoA lyase
ENSG0000	168.712	-0.02882	0.145525	0.804083	0.952056	TPM1	7168	tropomyosin 1
ENSG0000	11.64306	-0.01291	0.225443	0.804025	0.952056	NA	NA	NA
ENSG0000	231.1377	0.026214	0.12958	0.803827	0.952056	CIAPIN1	57019	cytokine induced apoptosis inhibitor 1
ENSG0000	8.172734	0.013964	0.224171	0.804087	0.952056	NA	NA	NA
ENSG0000	4.842201	0.012921	0.224942	0.803966	0.952056	PNMT	5409	phenylethanolamine N-methyltransferase
ENSG0000	391.7426	-0.0276	0.141828	0.803744	0.952056	ACBD4	79777	acyl-CoA binding domain containing 4
ENSG0000	3039.821	-0.02822	0.158082	0.80308	0.952056	PCIF1	63935	phosphorylated CTD interacting factor 1
ENSG0000	112.1181	-0.0281	0.146843	0.803163	0.952056	ARMCX3	51566	armadillo repeat containing X-linked 3
ENSG0000	127.332	-0.0266	0.150728	0.804404	0.95237	ZNHIT2	741	zinc finger HIT-type containing 2
ENSG0000	6.749168	0.016572	0.22218	0.80451	0.952434	RAD51AP1	10635	RAD51 associated protein 1
ENSG0000	4966.541	-0.02744	0.14625	0.804945	0.95247	KIAA2013	90231	KIAA2013
ENSG0000	237.1659	-0.0286	0.153787	0.804657	0.95247	ATXN7L2	127002	ataxin 7 like 2
ENSG0000	3.015894	0.010197	0.228799	0.805032	0.95247	NA	NA	NA
ENSG0000	60.05958	0.025569	0.192087	0.804954	0.95247	RCHY1	25898	ring finger and CHY zinc finger domain containing 1
ENSG0000	70.50884	-0.02716	0.172142	0.805023	0.95247	OARD1	221443	O-acyl-ADP-ribose deacylase 1
ENSG0000	467.8845	0.027565	0.182281	0.804717	0.95247	STX7	8417	syntaxin 7
ENSG0000	276.6839	0.026115	0.140323	0.804816	0.95247	SURF6	6838	surfeit 6
ENSG0000	168.7322	0.02752	0.170577	0.80506	0.95247	SLC43A1	8501	solute carrier family 43 member 1
ENSG0000	180.4159	0.028308	0.16855	0.80496	0.95247	USP14	9097	ubiquitin specific peptidase 14
ENSG0000	224.6106	-0.02683	0.183062	0.804706	0.95247	TSHZ3	57616	teashirt zinc finger homeobox 3
ENSG0000	120.1243	-0.02867	0.157719	0.805121	0.952481	WDR77	79084	WD repeat domain 77
ENSG0000	42.87505	0.027566	0.187485	0.805184	0.952493	GIPR	2696	gastric inhibitory polypeptide receptor
ENSG0000	5.572879	-0.01755	0.2249	0.805473	0.952774	NA	NA	NA
ENSG0000	273.4878	0.028895	0.148896	0.805718	0.952818	MRPL24	79590	mitochondrial ribosomal protein L24
ENSG0000	16.05588	0.023056	0.206995	0.806653	0.952818	MTX2	10651	metaxin 2
ENSG0000	52.21085	0.028617	0.163933	0.80667	0.952818	PPM1L	151742	protein ph Mg2+/Mn2+ dependent 1L
ENSG0000	35.62865	0.026656	0.187894	0.806756	0.952818	STX18-AS1	1.01E+08	STX18 antisense RNA 1 (head to head)
ENSG0000	51.64115	-0.02833	0.161315	0.805696	0.952818	NA	NA	NA
ENSG0000	172.5099	-0.02944	0.159573	0.806622	0.952818	HLA-DOB	3112	major hist class II DO beta
ENSG0000	36.8316	0.024485	0.203124	0.805859	0.952818	ANKMY2	57037	ankyrin repeat and MYND domain containing 2
ENSG0000	110.4841	-0.02811	0.154238	0.806007	0.952818	SCRN1	9805	secernin 1
ENSG0000	7.132462	0.015403	0.223711	0.806775	0.952818	NA	NA	NA
ENSG0000	51.0465	-0.02825	0.160431	0.806862	0.952818	NA	NA	NA
ENSG0000	53.54546	0.029217	0.162522	0.806328	0.952818	C9orf64	84267	chromosome 9 open reading frame 64
ENSG0000	402.2579	-0.02343	0.152578	0.806769	0.952818	CUEDC2	79004	CUE domain containing 2
ENSG0000	4.167078	-0.01089	0.227059	0.806661	0.952818	DKK3	27122	dickkopf WNT signaling pathway inhibitor 3
ENSG0000	182.5374	-0.03065	0.152652	0.805769	0.952818	NAALADL1	10004	N-acetylated alpha-linked acidic dipeptidase like 1
ENSG0000	22.18124	0.025707	0.194914	0.80605	0.952818	BUB1B	701	BUB1 mitotic checkpoint serine/threonine kinase B
ENSG0000	5563.401	0.027992	0.156162	0.806815	0.952818	ANXA2	302	annexin A2
ENSG0000	129.438	0.027672	0.135603	0.806195	0.952818	B3GNT9	84752	UDP-GlcN/ 3-N-acetylglucosaminyltransferase 9
ENSG0000	542.1655	0.027134	0.142346	0.806789	0.952818	FBXL20	84961	F-box and leucine rich repeat protein 20
ENSG0000	2.642523	-0.00967	0.230403	0.806714	0.952818	MIR1203	1E+08	microRNA 1203
ENSG0000	25.05401	0.0197	0.201434	0.80597	0.952818	SERPINB1(	5273	serpin family B member 10
ENSG0000	3.823404	-0.01352	0.226488	0.805648	0.952818	NA	NA	NA
ENSG0000	497.3756	-0.02786	0.159713	0.805985	0.952818	EVI5L	115704	ecotropic viral integration site 5 like
ENSG0000	9909.139	-0.02339	0.138408	0.80602	0.952818	DNM2	1785	dynamin 2
ENSG0000	232.7716	0.029814	0.161401	0.806104	0.952818	NA	NA	NA
ENSG0000	94.17915	-0.02612	0.169646	0.806212	0.952818	ZSWIM9	374920	zinc finger SWIM-type containing 9
ENSG0000	62.09219	-0.02787	0.170012	0.806647	0.952818	AMMECR1	9949	AMMECR nuclear protein 1
ENSG0000	72.06833	-0.02706	0.185077	0.807169	0.952968	SDHAP3	728609	SDHA pseudogene 3
ENSG0000	68.53777	0.028727	0.172272	0.807314	0.952968	FBXL17	64839	F-box and leucine rich repeat protein 17
ENSG0000	256.959	-0.02676	0.143329	0.807406	0.952968	MED27	9442	mediator complex subunit 27
ENSG0000	11.53814	0.014823	0.222882	0.807291	0.952968	PARPBP	55010	PARP1 binding protein
ENSG0000	398.5252	-0.02646	0.12487	0.807334	0.952968	SNF8	11267	SNF8 subunit of ESCRT-II
ENSG0000	157.5723	0.027935	0.167329	0.807311	0.952968	TANC2	26115	tetratricop ankyrin repeat and coiled-coil containing 2
ENSG0000	4.08378	0.014206	0.225015	0.807275	0.952968	CILP2	148113	cartilage intermediate layer protein 2
ENSG0000	9.865615	0.019709	0.215818	0.807381	0.952968	ZNF582	147948	zinc finger protein 582
ENSG0000	5.43838	-0.01785	0.221715	0.80752	0.953014	TTLL7	79739	tubulin tyrosine ligase like 7
ENSG0000	44.39899	-0.0255	0.191201	0.807549	0.953014	NOS3	4846	nitric oxide synthase 3
ENSG0000	47.38433	0.020265	0.179353	0.807671	0.953058	HERC2P9	440248	HERC2 pseudogene 9
ENSG0000	219.5859	-0.02767	0.147748	0.80769	0.953058	GEMIN7	79760	gem nuclear organelle associated protein 7
ENSG0000	12.53124	0.022434	0.20734	0.807758	0.953076	NA	NA	NA
ENSG0000	696.4943	0.025074	0.129463	0.814292	0.953231	MTHFR	4524	methylenetetrahydrofolate reductase
ENSG0000	2538.634	0.027514	0.159925	0.811096	0.953231	ARID1A	8289	AT-rich interaction domain 1A
ENSG0000	16.85779	-0.02106	0.208731	0.814299	0.953231	ADGRB2	576	adhesion G protein-coupled receptor B2
ENSG0000	1501.729	-0.02544	0.148683	0.814106	0.953231	PTP4A2	8073	protein tyrosine phosphatase 4A2
ENSG0000	235.9489	-0.02602	0.136711	0.813725	0.953231	TCEANC2	127428	transcription elongation factor A N-terminal and central domain containing 2
ENSG0000	22.30734	0.018821	0.214712	0.812038	0.953231	NA	NA	NA
ENSG0000	5812.413	-0.02562	0.135145	0.812959	0.953231	HDGF	3068	heparin binding growth factor
ENSG0000	6.744546	-0.01721	0.219028	0.808203	0.953231	LINC01686	284648	long intergenic non-protein coding RNA 1686
ENSG0000	158.1057	-0.02392	0.200545	0.811286	0.953231	RO60	6738	Ro60 Y RNA binding protein
ENSG0000	197.0719	-0.02594	0.134838	0.813064	0.953231	RABIF	5877	RAB interacting factor
ENSG0000	9.304321	0.017876	0.215913	0.811248	0.953231	NA	NA	NA
ENSG0000	27.71386	-0.0272	0.180055	0.811145	0.953231	STON1	11037	stonin 1
ENSG0000	828.6109	-0.02672	0.150589	0.811721	0.953231	AAK1	22848	AP2 associated kinase 1
ENSG0000	285.7356	-0.01975	0.189533	0.8108	0.953231	TIA1	7072	TIA1 cytotoxic granule associated RNA binding protein
ENSG0000	14.22226	-0.00126	0.23308	0.810794	0.953231	CYP26B1	56603	cytochrome P450 family 26 subfamily B member 1
ENSG0000	2.735979	0.011569	0.231166	0.814105	0.953231	NA	NA	NA
ENSG0000	149.8558	0.027292	0.143066	0.808967	0.953231	CNNM4	26504	cyclin and CBS domain divalent metal cation transport mediator 4
ENSG0000	47.39452	0.026942	0.168311	0.809765	0.953231	NA	NA	NA
ENSG0000	426.6113	0.023762	0.216505	0.812579	0.953231	CLK1	1195	CDC like kinase 1
ENSG0000	16.39947	-0.01752	0.215402	0.81315	0.953231	AP1S3	130340	adaptor related protein complex 1 subunit sigma 3
ENSG0000	11653.69	-0.02626	0.150671	0.814429	0.953231	RNPEPL1	57140	arginyl aminopeptidase like 1
ENSG0000	2086.854	-0.02819	0.149656	0.810029	0.953231	NUP210	23225	nucleoporin 210
ENSG0000	2004.261	-0.02185	0.101609	0.809857	0.953231	PDCD6IP	10015	programmed cell death 6 interacting protein
ENSG0000	1395.466	-0.02904	0.124035	0.811196	0.953231	ZDHHC3	51304	zinc finger DHHC-type palmitoyltransferase 3
ENSG0000	258.4493	0.025217	0.123454	0.811045	0.953231	KLHL18	23276	kelch like family member 18
ENSG0000	21.88134	0.026453	0.193249	0.808418	0.953231	FSTL1	11167	follistatin like 1

ENSG0000	6.836471	0.021399	0.214635	0.812041	0.953231	NA	NA	NA
ENSG0000	194.7639	-0.02342	0.197714	0.814454	0.953231	PIK3CA	5290	phosphati 5-bisphosphate 3-kinase catalytic subunit alpha
ENSG0000	568.835	-0.0262	0.138877	0.811402	0.953231	VPS8	23355	VPS8 subunit of CORVET complex
ENSG0000	1740.554	0.026924	0.146323	0.812617	0.953231	ST6GAL1	6480	ST6 beta-g 6-sialyltransferase 1
ENSG0000	5.127477	0.010014	0.227962	0.812953	0.953231	NA	NA	NA
ENSG0000	50.0321	-0.02239	0.20475	0.809117	0.953231	PKD2	5311	polycystin transient receptor potential cation channel
ENSG0000	16.47619	-0.02356	0.20311	0.811797	0.953231	TIGD2	166815	tigger transposable element derived 2
ENSG0000	2072.467	-0.02755	0.143304	0.810336	0.953231	ANXA5	308	annexin A5
ENSG0000	5.493592	-0.01794	0.219185	0.809091	0.953231	NA	NA	NA
ENSG0000	290.9168	0.027271	0.1749	0.808551	0.953231	TENT2	167153	terminal nucleotidyltransferase 2
ENSG0000	8.818679	-0.0179	0.216616	0.814074	0.953231	NA	NA	NA
ENSG0000	7.753074	-0.01511	0.222717	0.809781	0.953231	SEMA6A-A	1.02E+08	SEMA6A antisense RNA 1
ENSG0000	2027.299	0.024869	0.15632	0.812357	0.953231	FAM53C	51307	family with sequence similarity 53 member C
ENSG0000	260.1301	-0.02521	0.137667	0.814412	0.953231	NDUFA2	4695	NADH:ubiquinone oxidoreductase subunit A2
ENSG0000	785.5551	0.027195	0.167832	0.814268	0.953231	SLC36A1	206358	solute carrier family 36 member 1
ENSG0000	49.21016	-0.02634	0.183398	0.812669	0.953231	BOD1	91272	biorientation of chromosomes in cell division 1
ENSG0000	1186.784	-0.02257	0.138287	0.808239	0.953231	NSD1	64324	nuclear receptor binding SET domain protein 1
ENSG0000	94.21482	0.027306	0.164336	0.811867	0.953231	TPMT	7172	thiopurine S-methyltransferase
ENSG0000	86.50811	-0.02757	0.169315	0.809535	0.953231	CSNK2B	1460	casein kinase 2 beta
ENSG0000	6.129725	-0.01336	0.225035	0.811162	0.953231	NA	NA	NA
ENSG0000	1091.508	-0.02503	0.153492	0.808399	0.953231	UBR2	23304	ubiquitin protein ligase E3 component n-recognin 2
ENSG0000	88.75795	-0.02049	0.205434	0.813332	0.953231	ATG5	9474	autophagy related 5
ENSG0000	21.49713	-0.02189	0.209295	0.808047	0.953231	ABCA13	154664	ATP binding cassette subfamily A member 13
ENSG0000	452.9456	-0.02573	0.130195	0.813755	0.953231	RABGEF1P	493754	RABGEF1 pseudogene 1
ENSG0000	96.49276	-0.02658	0.182672	0.81192	0.953231	AUTS2	26053	activator of transcription and developmental regulator AUTS2
ENSG0000	4.245198	-0.00835	0.228736	0.81352	0.953231	GTF2IRD1	9569	GTF2I repeat domain containing 1
ENSG0000	8.682326	-0.01479	0.220506	0.810005	0.953231	MAGI2-AS	1.01E+08	MAGI2 antisense RNA 3
ENSG0000	64.27176	0.025633	0.192183	0.808908	0.953231	VPS50	55610	VPS50 subunit of EARP/GARPII complex
ENSG0000	66.16731	-0.02725	0.164718	0.809939	0.953231	NA	NA	NA
ENSG0000	35.75665	-0.02784	0.167373	0.81093	0.953231	TRMT12	55039	tRNA methyltransferase 12 homolog
ENSG0000	47.36108	0.024307	0.188762	0.812949	0.953231	KANK1	23189	KN motif and ankyrin repeat domains 1
ENSG0000	21.68388	-0.02401	0.196907	0.814054	0.953231	GALNT12	79695	polypeptide N-acetylgalactosaminyltransferase 12
ENSG0000	156.5549	0.026565	0.177762	0.808754	0.953231	ABITRAM	54942	actin binding transcription modulator
ENSG0000	89.90953	-0.02679	0.140504	0.810466	0.953231	NA	NA	NA
ENSG0000	32.03343	0.022547	0.205668	0.808073	0.953231	ZNF618	114991	zinc finger protein 618
ENSG0000	208.0381	-0.02991	0.14973	0.813734	0.953231	RC3H2	54542	ring finger and CCCH-type domains 2
ENSG0000	62.05972	-0.02731	0.161697	0.812972	0.953231	ZBTB43	23099	zinc finger and BTB domain containing 43
ENSG0000	312.0697	0.027223	0.178125	0.809266	0.953231	LINC0150З	1.01E+08	long intergenic non-protein coding RNA 1503
ENSG0000	39.60683	-0.02461	0.197523	0.813227	0.953231	SARDH	1757	sarcosine dehydrogenase
ENSG0000	85.44737	-0.02726	0.150113	0.808907	0.953231	SFMBT2	57713	Scm like with four mbt domains 2
ENSG0000	11.49588	0.019738	0.214905	0.812843	0.953231	HACD1	9200	3-hydroxyacyl-CoA dehydratase 1
ENSG0000	6.515866	-0.01251	0.226319	0.809425	0.953231	RET	5979	ret proto-oncogene
ENSG0000	25.65957	-0.02422	0.198117	0.813309	0.953231	CEP55	55165	centrosomal protein 55
ENSG0000	19.09929	0.018435	0.215992	0.810185	0.953231	DCLRE1A	9937	DNA cross-link repair 1A
ENSG0000	150.6796	0.027729	0.166017	0.810402	0.953231	GLRX3	10539	glutaredoxin 3
ENSG0000	1173.782	0.024814	0.119424	0.809545	0.953231	C11orf21	29125	chromosome 11 open reading frame 21
ENSG0000	1196.559	-0.02728	0.142571	0.809811	0.953231	NUP98	4928	nucleoporin 98 and 96 precursor
ENSG0000	91.08353	-0.03646	0.163913	0.814066	0.953231	TRIM68	55128	tripartite motif containing 68
ENSG0000	10263.1	-0.02517	0.130381	0.813015	0.953231	SF1	7536	splicing factor 1
ENSG0000	3000.35	0.027754	0.13754	0.808544	0.953231	PITPNM1	9600	phosphatidylinositol transfer protein membrane associated 1
ENSG0000	4.109687	0.015439	0.223746	0.814056	0.953231	NA	NA	NA
ENSG0000	443.6254	0.028955	0.153113	0.814498	0.953231	NINJ2	4815	ninjurin 2
ENSG0000	3.241812	-0.01131	0.229183	0.810163	0.953231	NA	NA	NA
ENSG0000	118.3297	-0.02639	0.178626	0.812451	0.953231	TROAP	10024	trophinin associated protein
ENSG0000	89.47812	0.026459	0.186281	0.811061	0.953231	KRT72	140807	keratin 72
ENSG0000	81.88067	0.027756	0.16984	0.81228	0.953231	ESPL1	9700	extra spin separase
ENSG0000	143.8184	0.027438	0.181247	0.810203	0.953231	PTPN11	5781	protein tyrosine phosphatase non-receptor type 11
ENSG0000	28.66009	0.025391	0.193978	0.814181	0.953231	PIBF1	10464	progesterone immunomodulatory binding factor 1
ENSG0000	134.7282	-0.02675	0.185443	0.812772	0.953231	KLF12	11278	KLF transcription factor 12
ENSG0000	5.454472	0.014463	0.223039	0.813617	0.953231	LOC10537	1.05E+08	uncharacterized LOC105370259
ENSG0000	37.51879	0.022157	0.207441	0.812991	0.953231	OBI1	79596	ORC ubiquitin ligase 1
ENSG0000	3.351733	0.009813	0.230575	0.810285	0.953231	MIR8075	1.02E+08	microRNA 8075
ENSG0000	18.15959	0.024152	0.20346	0.808789	0.953231	PSMA6	5687	proteasome 20S subunit alpha 6
ENSG0000	149.3677	-0.02523	0.187138	0.813968	0.953231	FBXO33	254170	F-box protein 33
ENSG0000	90.46245	-0.02537	0.18671	0.810039	0.953231	GSKIP	51527	GSK3B interacting protein
ENSG0000	272.8353	0.027866	0.1588	0.80822	0.953231	CDC42BPE	9578	CDC42 binding protein kinase beta
ENSG0000	41.22265	0.027499	0.173239	0.812916	0.953231	TEX22	647310	testis expressed 22
ENSG0000	66.69609	0.023368	0.202082	0.811344	0.953231	ZNF770	54989	zinc finger protein 770
ENSG0000	142.192	0.025997	0.162995	0.810673	0.953231	DIS3L	115752	DIS3 like exosome 3'-5' exoribonuclease
ENSG0000	169.4426	-0.02622	0.145315	0.813769	0.953231	LRRC28	123355	leucine rich repeat containing 28
ENSG0000	114.9731	-0.02392	0.140396	0.812275	0.953231	FAHD1	81889	fumarylacetoacetate hydrolase domain containing 1
ENSG0000	3.912561	0.015768	0.222402	0.81342	0.953231	NA	NA	NA
ENSG0000	5.03474	0.011537	0.2261	0.812398	0.953231	C17orf114	1.19E+08	chromosome 17 open reading frame 114
ENSG0000	95.78164	-0.02723	0.178069	0.809244	0.953231	ENO3	2027	enolase 3
ENSG0000	1554.256	-0.02476	0.127627	0.813404	0.953231	PSME3	10197	proteasome activator subunit 3
ENSG0000	8.421094	0.018345	0.217129	0.810063	0.953231	ABCA10	10349	ATP binding cassette subfamily A member 10
ENSG0000	2.58798	0.012746	0.22798	0.811508	0.953231	RPL23AP8	388574	ribosomal protein L23a pseudogene 87
ENSG0000	68.21791	-0.0201	0.205507	0.812935	0.953231	RAB12	201475	RAB12 member RAS oncogene family
ENSG0000	4.318326	0.010151	0.227645	0.813646	0.953231	NA	NA	NA
ENSG0000	2.450998	0.009971	0.229585	0.809538	0.953231	SCARNA17	677769	small Cajal body-specific RNA 17
ENSG0000	30.51095	-0.02614	0.19135	0.809834	0.953231	SKA1	220134	spindle and kinetochore associated complex subunit 1
ENSG0000	8.113088	0.017132	0.218944	0.809861	0.953231	CIRBP-AS1	148046	CIRBP antisense RNA 1
ENSG0000	2928.575	-0.02263	0.112291	0.80953	0.953231	TRIR	79002	telomerase RNA component interacting RNase
ENSG0000	1444.649	0.02924	0.159846	0.808678	0.953231	EPS15L1	58513	epidermal growth factor receptor pathway substrate 15 like 1
ENSG0000	133.6999	0.027997	0.172728	0.810917	0.953231	TMEM38A	79041	transmembrane protein 38A
ENSG0000	8.551789	0.015288	0.2218	0.810287	0.953231	ZNF681	148213	zinc finger protein 681
ENSG0000	2.34068	-0.01029	0.229447	0.814101	0.953231	KIRREL2	84063	kirre like nephrin family adhesion molecule 2
ENSG0000	859.661	-0.02485	0.123594	0.810328	0.953231	DMAC2	55101	distal membrane arm assembly component 2
ENSG0000	6.975765	-0.01714	0.219362	0.811712	0.953231	ZNF497	162968	zinc finger protein 497
ENSG0000	152.1082	0.02657	0.135461	0.809192	0.953231	NA	NA	NA
ENSG0000	27.99831	-0.02498	0.195416	0.811699	0.953231	SDCBP2	27111	syndecan binding protein 2
ENSG0000	444.2704	0.026262	0.138424	0.810028	0.953231	EIF2S2	8894	eukaryotic translation initiation factor 2 subunit beta
ENSG0000	309.0606	0.025379	0.127885	0.813388	0.953231	TGIF2	60436	TGFB induced factor homeobox 2
ENSG0000	9.20177	-0.02215	0.207709	0.813112	0.953231	NA	NA	NA
ENSG0000	24.81004	0.017789	0.216997	0.810963	0.953231	C20orf204	284739	chromosome 20 open reading frame 204
ENSG0000	238.3269	-0.02923	0.151288	0.81079	0.953231	SLX9	85395	SLX9 ribosome biogenesis factor

ENSG0000	2421.121	-0.0221	0.11199	0.81184	0.953231	ATF4	468	activating transcription factor 4
ENSG0000	3.747123	0.013975	0.224611	0.808904	0.953231	NA	NA	NA
ENSG0000	4.761959	-0.00873	0.22909	0.812913	0.953231	NA	NA	NA
ENSG0000	65.51376	-0.02746	0.178685	0.810856	0.953231	SLC9A6	10479	solute carrier family 9 member A6
ENSG0000	5.145517	0.015495	0.224428	0.810578	0.953231	TTTY10	246119	testis-spec Y-linked 10
ENSG0000	4.187959	-0.0151	0.222664	0.814588	0.953276	NA	NA	NA
ENSG0000	1047.753	0.026817	0.142788	0.814754	0.953409	POLDIP2	26073	DNA polymerase delta interacting protein 2
ENSG0000	632.1891	0.0256	0.180955	0.815118	0.953469	PUM2	23369	pumilio RNA binding family member 2
ENSG0000	178.7262	0.024653	0.175062	0.815019	0.953469	SNAPC3	6619	small nuclear RNA activating complex polypeptide 3
ENSG0000	419.7879	-0.02638	0.17308	0.815057	0.953469	NR1D1	9572	nuclear receptor subfamily 1 group D member 1
ENSG0000	2518.464	0.026791	0.150976	0.815077	0.953469	MYO15B	80022	myosin XVB
ENSG0000	391.2968	0.026959	0.153247	0.814926	0.953469	RECQL5	9400	RecQ like helicase 5
ENSG0000	28.88069	-0.02092	0.20878	0.814948	0.953469	KIF16B	55614	kinesin family member 16B
ENSG0000	147.9562	0.027018	0.164999	0.81534	0.953485	NUDT14	256281	nudix hydrolase 14
ENSG0000	8.170898	-0.01767	0.217179	0.815328	0.953485	NA	NA	NA
ENSG0000	144.0428	0.026568	0.139843	0.815334	0.953485	BLMH	642	bleomycin hydrolase
ENSG0000	12.25789	-0.02013	0.21143	0.815281	0.953485	NA	NA	NA
ENSG0000	1854.427	-0.02565	0.137957	0.815515	0.953517	RTN4	57142	reticulon 4
ENSG0000	98.29922	-0.03236	0.154091	0.815615	0.953517	CWC27	10283	CWC27 spliceosome associated cyclophilin
ENSG0000	9.491372	-0.01975	0.213284	0.815528	0.953517	NA	NA	NA
ENSG0000	61.3961	-0.02635	0.175277	0.815627	0.953517	GPT2	84706	glutamic--pyruvic transaminase 2
ENSG0000	69.1149	-0.02204	0.190067	0.815517	0.953517	PSMD10	5716	proteason non-ATPase 10
ENSG0000	69.43426	0.029619	0.155278	0.815725	0.953547	C15orf61	145853	chromosome 15 open reading frame 61
ENSG0000	190.0777	0.028446	0.15071	0.815757	0.953547	TUBG2	27175	tubulin gamma 2
ENSG0000	548.0622	-0.02543	0.177388	0.815879	0.953622	ST8SIA4	7903	ST8 alpha- 8-sialyltransferase 4
ENSG0000	255.1229	0.025315	0.132702	0.815925	0.953622	HCG18	414777	HLA complex group 18
ENSG0000	5579.911	-0.02709	0.135347	0.8161	0.953765	RAB11B	9230	RAB11B member RAS oncogene family
ENSG0000	954.6522	-0.02658	0.146746	0.816946	0.953826	YARS1	8565	tyrosyl-tRNA synthetase 1
ENSG0000	661.9115	-0.02275	0.112267	0.816929	0.953826	DHX9	1660	DExH-box helicase 9
ENSG0000	4.5337	-0.01303	0.224689	0.816901	0.953826	DUSP19	142679	dual specificity phosphatase 19
ENSG0000	907.0578	-0.02449	0.128765	0.816649	0.953826	ZNF142	7701	zinc finger protein 142
ENSG0000	17.11499	0.023387	0.199041	0.816308	0.953826	TEC	7006	tec protein tyrosine kinase
ENSG0000	88.92214	-0.02424	0.191279	0.816802	0.953826	METTL14	57721	methyltra N6-adenosine-methyltransferase subunit
ENSG0000	2.690737	0.009932	0.230479	0.817066	0.953826	LOC10192	1.02E+08	uncharacterized LOC101929004
ENSG0000	2644.797	0.025393	0.128839	0.816777	0.953826	SH3GLB2	56904	SH3 doma endophilin B2
ENSG0000	51.91228	-0.02605	0.170094	0.816803	0.953826	MSANTD7	1E+08	Myb/SANT DNA binding domain containing 7
ENSG0000	1581.937	0.029935	0.149024	0.816727	0.953826	TOLLIP	54472	toll interacting protein
ENSG0000	2.527111	0.012814	0.228877	0.817077	0.953826	PIH1D2	120379	PIH1 domain containing 2
ENSG0000	1084.459	0.026276	0.174134	0.816966	0.953826	NCKAP5L	57701	NCK associated protein 5 like
ENSG0000	167.3151	0.026479	0.176319	0.816468	0.953826	VASH1	22846	vasohibin 1
ENSG0000	410.1396	-0.02613	0.152349	0.816882	0.953826	EHD4	30844	EH domain containing 4
ENSG0000	3.472078	-0.00376	0.22858	0.816666	0.953826	RASL10B	91608	RAS like family 10 member B
ENSG0000	17.78607	-0.01614	0.224195	0.816237	0.953826	EEF1DP7	1E+08	eukaryotic translation elongation factor 1 delta pseudogene 7
ENSG0000	5.762214	0.018395	0.217371	0.816984	0.953826	RPS6P25	729389	ribosomal protein S6 pseudogene 25
ENSG0000	9407.941	0.017101	0.141367	0.817089	0.953826	ARHGEF1	9138	Rho guanine nucleotide exchange factor 1
ENSG0000	7.679559	0.020639	0.214956	0.817235	0.953837	TLN2	83660	talin 2
ENSG0000	1025.993	0.026824	0.144193	0.817215	0.953837	HNRNPM	4670	heterogeneous nuclear ribonucleoprotein M
ENSG0000	65.07613	-0.02446	0.187204	0.817255	0.953837	CLIP3	25999	CAP-Gly domain containing linker protein 3
ENSG0000	4.938443	-0.01371	0.224794	0.817397	0.953905	NA	NA	NA
ENSG0000	4.340667	-0.01608	0.221239	0.817417	0.953905	RPS9P4	388556	ribosomal protein S9 pseudogene 4
ENSG0000	8.472526	-0.02054	0.211361	0.817762	0.953933	NA	NA	NA
ENSG0000	196.7721	0.025955	0.142921	0.81771	0.953933	NME6	10201	NME/NM23 nucleoside diphosphate kinase 6
ENSG0000	3.706191	-0.0097	0.228287	0.817673	0.953933	RN7SKP29	1.06E+08	RN7SK pseudogene 292
ENSG0000	1037.526	0.029836	0.150716	0.817806	0.953933	AP2A2	161	adaptor related protein complex 2 subunit alpha 2
ENSG0000	61.55585	-0.02422	0.183743	0.817801	0.953933	ZDHHC13	54503	zinc finger DHHC-type palmitoyltransferase 13
ENSG0000	6.532058	-0.01981	0.213605	0.817801	0.953933	NA	NA	NA
ENSG0000	9.640663	0.02012	0.213028	0.817506	0.953933	NA	NA	NA
ENSG0000	175.0248	-0.02014	0.199726	0.81811	0.954227	CEROX1	1.16E+08	cytoplasmic endogenous regulator of oxidative phosphorylation 1
ENSG0000	34.64974	-0.02492	0.188641	0.818255	0.954335	ZNF14	7561	zinc finger protein 14
ENSG0000	228.6439	0.027438	0.160437	0.818334	0.954367	NRBP2	340371	nuclear receptor binding protein 2
ENSG0000	9.922424	0.013864	0.222634	0.818469	0.954396	NA	NA	NA
ENSG0000	7.254673	-0.02245	0.213379	0.818776	0.954396	ASNS	440	asparagine synthetase (glutamine-hydrolyzing)
ENSG0000	26.58929	-0.02548	0.176946	0.818572	0.954396	PEG13	359809	paternally expressed 13
ENSG0000	10.29486	-0.00279	0.233526	0.818756	0.954396	NA	NA	NA
ENSG0000	71.55134	0.025771	0.165124	0.818759	0.954396	ZNF202	7753	zinc finger protein 202
ENSG0000	50232.85	-0.02473	0.135428	0.818648	0.954396	ARHGDIB	397	Rho GDP dissociation inhibitor beta
ENSG0000	679.2031	-0.02536	0.150151	0.818438	0.954396	DHX8	1659	DEAH-box helicase 8
ENSG0000	14.76843	0.022502	0.204874	0.818739	0.954396	NA	NA	NA
ENSG0000	74.30451	-0.02148	0.199968	0.818912	0.954494	ARMC1	55156	armadillo repeat containing 1
ENSG0000	560.0376	0.022233	0.119255	0.819168	0.954732	BAG1	573	BAG cochaperone 1
ENSG0000	14.66689	-0.01891	0.213633	0.819345	0.954816	WDHD1	11169	WD repeat and HMG-box DNA binding protein 1
ENSG0000	575.7444	-0.02879	0.142548	0.819303	0.954816	PSMC5	5705	proteason ATPase 5
ENSG0000	83.18762	-0.02568	0.175229	0.819422	0.954846	CWF19L2	143884	CWF19 like cell cycle control factor 2
ENSG0000	466.4677	0.027749	0.13505	0.819567	0.954953	CCT8	10694	chaperonin containing TCP1 subunit 8
ENSG0000	601.5819	0.024051	0.135556	0.819707	0.955057	TRRAP	8295	transformation/transcription domain associated protein
ENSG0000	13462.27	-0.02464	0.179078	0.819892	0.95512	PHC2	1912	polyhomeotic homolog 2
ENSG0000	71.58942	-0.01888	0.171424	0.819874	0.95512	MICALL2	79778	MICAL like 2
ENSG0000	544.3117	0.025693	0.155085	0.81997	0.95512	HGH1	51236	HGH1 homolog
ENSG0000	164.7978	-0.02582	0.143107	0.819954	0.95512	TMEM143	55260	transmembrane protein 143
ENSG0000	56.47601	-0.02422	0.189701	0.820026	0.955124	ASB2	51676	ankyrin repeat and SOCS box containing 2
ENSG0000	82.02024	0.025842	0.17254	0.820392	0.955489	PRDM11	56981	PR/SET domain 11
ENSG0000	21.59732	-0.01722	0.216729	0.820785	0.955887	NA	NA	NA
ENSG0000	346.2659	0.026263	0.152824	0.821043	0.956036	DHX37	57647	DEAH-box helicase 37
ENSG0000	2.38995	-0.00444	0.233408	0.82107	0.956036	RIMBP2	23504	RIMS binding protein 2
ENSG0000	90.29661	0.026287	0.156769	0.821038	0.956036	ZGPAT	84619	zinc finger CCCH-type and G-patch domain containing
ENSG0000	335.3692	0.026836	0.169225	0.82122	0.956056	PAQR6	79957	progestin and adipoQ receptor family member 6
ENSG0000	15.78307	0.02411	0.197392	0.821185	0.956056	NEPRO-AS	1.08E+08	NEPRO antisense RNA 1
ENSG0000	292.8361	0.025115	0.140481	0.821243	0.956056	STRAP	11171	serine/threonine kinase receptor associated protein
ENSG0000	25.59168	-0.02493	0.179566	0.821349	0.956118	GPRC5C	55890	G protein-coupled receptor class C group 5 member C
ENSG0000	6.226599	-0.01741	0.217863	0.821552	0.956174	SMG7-AS1	284649	SMG7 antisense RNA 1
ENSG0000	10.59425	0.018195	0.212908	0.821509	0.956174	CCDC13	152206	coiled-coil domain containing 13
ENSG0000	2.687937	-0.00948	0.228807	0.821554	0.956174	NA	NA	NA
ENSG0000	750.454	0.022006	0.190573	0.821696	0.956279	SELENOT	51714	selenoprotein T
ENSG0000	618.875	-0.01992	0.120222	0.821764	0.956297	PRMT5	10419	protein arginine methyltransferase 5
ENSG0000	46.92109	0.025542	0.167332	0.821835	0.956303	TMEM186	25880	transmembrane protein 186
ENSG0000	16.20336	0.02015	0.209808	0.821978	0.956303	NA	NA	NA

ENSG0000	553.2568	0.02622	0.16417	0.82194	0.956303	WDR18	57418	WD repeat domain 18
ENSG0000	3.035172	0.008518	0.232746	0.821913	0.956303	OR7D2	162998	olfactory receptor family 7 subfamily D member 2
ENSG0000	350.9878	0.024569	0.130388	0.822164	0.956398	KIAA2026	158358	KIAA2026
ENSG0000	152.0544	0.02386	0.161162	0.822112	0.956398	SLC25A19	60386	solute carrier family 25 member 19
ENSG0000	17.84281	-0.019	0.210265	0.822239	0.956417	ZNF596	169270	zinc finger protein 596
ENSG0000	4.266353	-0.0125	0.224522	0.822285	0.956417	DIXDC1	85458	DIX domain containing 1
ENSG0000	22.01816	-0.02044	0.205441	0.822632	0.95676	NDUFAF5	79133	NADH:ubiquinone oxidoreductase complex assembly factor 5
ENSG0000	2.827007	-0.00868	0.229042	0.822748	0.956834	EEF1A1P1(	1E+08	eukaryotic translation elongation factor 1 alpha 1 pseudogene 10
ENSG0000	7.145718	0.019193	0.213719	0.823739	0.956902	NA	NA	NA
ENSG0000	39.21798	-0.02539	0.175381	0.823253	0.956902	CPOX	1371	coproporphyrinogen oxidase
ENSG0000	2.794786	-0.01257	0.225638	0.823699	0.956902	OTULIN-D'	728178	OTULIN divergent transcript
ENSG0000	106.994	-0.02103	0.198918	0.823472	0.956902	TMEM106	54664	transmembrane protein 106B
ENSG0000	121.1328	-0.02598	0.172811	0.823505	0.956902	ZFP41	286128	ZFP41 zinc finger protein
ENSG0000	126.0195	0.022757	0.169562	0.82346	0.956902	TMEM8B	51754	transmembrane protein 8B
ENSG0000	3.727381	0.007438	0.229234	0.823569	0.956902	LOC64416	644169	voltage dependent anion channel 1 pseudogene
ENSG0000	47.96302	0.026024	0.159489	0.82372	0.956902	PANX1	24145	pannexin 1
ENSG0000	837.9144	-0.02518	0.158522	0.822957	0.956902	DDX6	1656	DEAD-box helicase 6
ENSG0000	39.98129	-0.0243	0.163379	0.823517	0.956902	NDUFA9	4704	NADH:ubiquinone oxidoreductase subunit A9
ENSG0000	63.53962	-0.02548	0.170317	0.823122	0.956902	FBXO22	26263	F-box protein 22
ENSG0000	6.552043	-0.01769	0.217203	0.823421	0.956902	RPGRIP1L	23322	RPGRIP1 like
ENSG0000	35.95961	-0.02542	0.181464	0.823076	0.956902	TMEM241	85019	transmembrane protein 241
ENSG0000	28.76345	-0.02232	0.197307	0.823747	0.956902	NA	NA	NA
ENSG0000	7569.654	0.023809	0.189782	0.823363	0.956902	PRAM1	84106	PML-RARA regulated adaptor molecule 1
ENSG0000	1152.212	0.02553	0.170671	0.823356	0.956902	CCDC159	126075	coiled-coil domain containing 159
ENSG0000	6719.812	-0.02503	0.167004	0.822945	0.956902	MYO9B	4650	myosin IXB
ENSG0000	10.89364	-0.02121	0.206798	0.823469	0.956902	NANP	140838	N-acetylneuraminic acid phosphatase
ENSG0000	490.1536	-0.02545	0.161425	0.82381	0.956907	PPP1R15B	84919	protein phosphatase 1 regulatory subunit 15B
ENSG0000	128.0249	-0.02478	0.178656	0.823855	0.956907	CRIPT	9419	CXXC repeat containing interactor of PDZ3 domain
ENSG0000	390.1282	0.02086	0.150862	0.824018	0.956999	FCRL5	83416	Fc receptor like 5
ENSG0000	257.9341	-0.02405	0.188307	0.824091	0.956999	VCPIP1	80124	valosin containing protein interacting protein 1
ENSG0000	245.8188	-0.02431	0.136313	0.824071	0.956999	MRPL16	54948	mitochondrial ribosomal protein L16
ENSG0000	584.6153	0.024411	0.134459	0.82426	0.957013	G3BP1	10146	G3BP stress granule assembly factor 1
ENSG0000	974.0495	-0.0239	0.140576	0.824251	0.957013	NIN	51199	ninein
ENSG0000	123.5713	0.025349	0.153186	0.824233	0.957013	UTP18	51096	UTP18 small subunit processome component
ENSG0000	4.859357	0.011783	0.225494	0.824483	0.957156	RNF175	285533	ring finger protein 175
ENSG0000	214.2159	0.026403	0.176507	0.824487	0.957156	SOGA1	140710	suppresso autophagy associated 1
ENSG0000	1081.972	0.020572	0.133818	0.824577	0.957175	BICRA	29998	BRD4 interacting chromatin remodeling complex associated protein
ENSG0000	3.186048	-0.01147	0.225872	0.824608	0.957175	NA	NA	NA
ENSG0000	234.0704	-0.02312	0.140739	0.824825	0.957255	MRPS30	10884	mitochondrial ribosomal protein S30
ENSG0000	478.7251	0.023936	0.130071	0.824808	0.957255	B4GALT7	11285	beta-1 4-galactosyltransferase 7
ENSG0000	7.069801	0.014618	0.222341	0.824834	0.957255	NA	NA	NA
ENSG0000	3128.805	-0.02456	0.146289	0.824979	0.957274	ATP6V0B	533	ATPase H+ transporting V0 subunit b
ENSG0000	24.11612	-0.01493	0.21922	0.825027	0.957274	NA	NA	NA
ENSG0000	113.7896	-0.0253	0.175265	0.825404	0.957274	SPAST	6683	spastin
ENSG0000	173.117	0.024183	0.141361	0.825407	0.957274	MCUR1	63933	mitochondrial calcium uniporter regulator 1
ENSG0000	260.9334	-0.02189	0.161304	0.82493	0.957274	INSIG1	3638	insulin induced gene 1
ENSG0000	12.52286	-0.01746	0.214988	0.825251	0.957274	NA	NA	NA
ENSG0000	529.4501	-0.02443	0.141398	0.825289	0.957274	TSSC4	10078	tumor suppressing subtransferable candidate 4
ENSG0000	2.567478	-0.01005	0.22875	0.825132	0.957274	F2	2147	coagulatio thrombin
ENSG0000	20.35602	0.019446	0.210552	0.825322	0.957274	TMEM126	84233	transmembrane protein 126A
ENSG0000	68.39065	-0.02558	0.171218	0.825223	0.957274	EBPL	84650	EBP like
ENSG0000	5.951972	0.016297	0.220097	0.825425	0.957274	NA	NA	NA
ENSG0000	227.4493	-0.0226	0.124836	0.82553	0.957286	ATG16L1	55054	autophagy related 16 like 1
ENSG0000	597.5526	0.023138	0.144032	0.82554	0.957286	COX5A	9377	cytochrome c oxidase subunit 5A
ENSG0000	2.429586	-0.00754	0.232065	0.82566	0.957364	NA	NA	NA
ENSG0000	1031.797	0.022639	0.126428	0.826307	0.957375	RCC2	55920	regulator of chromosome condensation 2
ENSG0000	486.0512	0.015068	0.112889	0.826677	0.957375	YTHDF2	51441	YTH N6-methyladenosine RNA binding protein 2
ENSG0000	416.6588	-0.02316	0.129764	0.826355	0.957375	DNMT3A	1788	DNA methyltransferase 3 alpha
ENSG0000	128.2867	-0.01978	0.20354	0.826491	0.957375	CCNT2	905	cyclin T2
ENSG0000	253.1659	-0.02482	0.143651	0.826974	0.957375	CYP20A1	57404	cytochrome P450 family 20 subfamily A member 1
ENSG0000	45.35635	0.023886	0.189632	0.825887	0.957375	RPE	6120	ribulose-5-phosphate-3-epimerase
ENSG0000	128.0747	0.022251	0.17257	0.826976	0.957375	NAA15	80155	N-alpha-ac NatA auxiliary subunit
ENSG0000	122.5674	-0.02457	0.154939	0.826592	0.957375	CLCN3	1182	chloride voltage-gated channel 3
ENSG0000	3.87609	0.006703	0.229473	0.826143	0.957375	NA	NA	NA
ENSG0000	4.827499	0.017194	0.218726	0.826527	0.957375	NA	NA	NA
ENSG0000	12.29041	0.019373	0.212532	0.826498	0.957375	FAM135A	57579	family with sequence similarity 135 member A
ENSG0000	2.764181	-0.0093	0.227977	0.826841	0.957375	SRRM1P1	401475	serine/arginine repetitive matrix 1 pseudogene 1
ENSG0000	4.49203	-0.01204	0.225048	0.826615	0.957375	NA	NA	NA
ENSG0000	377.977	-0.02121	0.114329	0.826932	0.957375	SEC31B	25956	SEC31 hon COPII coat complex component
ENSG0000	489.4753	0.023698	0.130224	0.826399	0.957375	PEX16	9409	peroxisomal biogenesis factor 16
ENSG0000	65.33252	0.024957	0.179754	0.826018	0.957375	CDCA3	83461	cell division cycle associated 3
ENSG0000	2970.705	0.025119	0.146545	0.825815	0.957375	CHST11	50515	carbohydrate sulfotransferase 11
ENSG0000	2.450879	0.006517	0.231746	0.826525	0.957375	NA	NA	NA
ENSG0000	48.93895	-0.02532	0.171678	0.826907	0.957375	CFDP1	10428	craniofacial development protein 1
ENSG0000	722.0136	-0.02258	0.130039	0.826446	0.957375	MBTPS1	8720	membrane site 1
ENSG0000	50.86208	-0.02481	0.177218	0.825989	0.957375	MRPS23	51649	mitochondrial ribosomal protein S23
ENSG0000	461.3706	0.023466	0.135308	0.825804	0.957375	PPP1R37	284352	protein phosphatase 1 regulatory subunit 37
ENSG0000	13.62775	0.017841	0.214486	0.826125	0.957375	ZNF525	170958	zinc finger protein 525
ENSG0000	384.1164	0.024963	0.149871	0.826153	0.957375	NCAPH2	29781	non-SMC condensin II complex subunit H2
ENSG0000	7.685679	-0.0166	0.218315	0.826812	0.957375	RIBC1	158787	RIB43A domain with coiled-coils 1
ENSG0000	5.146644	0.013263	0.223562	0.827291	0.957679	KIAA1614	57710	KIAA1614
ENSG0000	9.131811	0.018441	0.213722	0.827454	0.957686	CHRM4	1132	cholinergic receptor muscarinic 4
ENSG0000	14.9441	0.021271	0.206008	0.827427	0.957686	KIF26A	26153	kinesin family member 26A
ENSG0000	433.8252	-0.01887	0.142283	0.827444	0.957686	GIPC1	10755	GIPC PDZ domain containing family member 1
ENSG0000	185.8987	0.025412	0.166253	0.82771	0.957891	EMC1	23065	ER membrane protein complex subunit 1
ENSG0000	14.49708	-0.02072	0.205426	0.827735	0.957891	NA	NA	NA
ENSG0000	125.2578	0.026325	0.158541	0.827865	0.957981	TEDC1	283643	tubulin epsilon and delta complex 1
ENSG0000	506.1189	-0.02411	0.162184	0.828056	0.958141	ZNF628	89887	zinc finger protein 628
ENSG0000	627.9975	0.026397	0.144656	0.828272	0.958185	HP1BP3	50809	heterochromatin protein 1 binding protein 3
ENSG0000	9.346309	0.013579	0.221705	0.828542	0.958185	TAF1A	9015	TATA-box RNA polymerase I subunit A
ENSG0000	9.725806	0.014849	0.220864	0.828476	0.958185	NA	NA	NA
ENSG0000	3.080036	0.01105	0.226696	0.828774	0.958185	NA	NA	NA
ENSG0000	4.165768	-0.01038	0.226369	0.828742	0.958185	NA	NA	NA
ENSG0000	1042.271	-0.02124	0.113326	0.828226	0.958185	TACC1	6867	transforming acidic coiled-coil containing protein 1
ENSG0000	384.6551	0.020828	0.10646	0.828701	0.958185	DCTN3	11258	dynactin subunit 3
ENSG0000	67.70856	-0.02474	0.156926	0.828522	0.958185	AGAP9	642517	ArfGAP wi ankyrin repeat and PH domain 9

ENSG0000	253.8408	-0.02142	0.17475	0.828738	0.958185	ERLIN1	10613	ER lipid raft associated 1
ENSG0000	11.05185	0.01945	0.210417	0.828506	0.958185	NA	NA	NA
ENSG0000	56.28564	0.023072	0.190122	0.828548	0.958185	ZMYM5	9205	zinc finger MYM-type containing 5
ENSG0000	163.7665	0.023967	0.1526	0.82845	0.958185	NA	NA	NA
ENSG0000	4.710581	0.0111	0.225786	0.82863	0.958185	NA	NA	NA
ENSG0000	209.6102	0.024256	0.167566	0.828867	0.958232	PAPSS1	9061	3'-phosphoadenosine 5'-phosphosulfate synthase 1
ENSG0000	3.176194	-0.0093	0.229078	0.828966	0.958285	NA	NA	NA
ENSG0000	109.0815	-0.022	0.189438	0.829099	0.958319	SIKE1	80143	suppressor of IKBKE 1
ENSG0000	54.33734	-0.02327	0.169988	0.829076	0.958319	L3MBTL1	26013	L3MBTL histone methyl-lysine binding protein 1
ENSG0000	64.89964	-0.02457	0.166345	0.829355	0.958433	ALS2	57679	alsin Rho guanine nucleotide exchange factor ALS2
ENSG0000	21.17792	-0.02207	0.196872	0.829256	0.958433	PHYHD1	254295	phytanoyl-CoA dioxygenase domain containing 1
ENSG0000	8.073581	0.017475	0.217534	0.829304	0.958433	LOC12490	1.25E+08	EF-hand calcium-binding domain-containing protein 3-like
ENSG0000	565.4839	-0.025	0.171003	0.829721	0.958727	HECA	51696	hdc homol cell cycle regulator
ENSG0000	73.18293	0.020024	0.205224	0.829762	0.958727	SIRT1	23411	sirtuin 1
ENSG0000	197.8504	-0.02467	0.161398	0.829767	0.958727	DNM1L	10059	dynamin 1 like
ENSG0000	36.49652	0.023762	0.187153	0.829822	0.958731	NA	NA	NA
ENSG0000	47.53888	0.0252	0.157088	0.8301	0.958992	HDAC8	55869	histone deacetylase 8
ENSG0000	79.67253	0.026544	0.169405	0.830191	0.959036	ATG4A	115201	autophagy related 4A cysteine peptidase
ENSG0000	73.72119	-0.02482	0.162989	0.830276	0.959073	LEO1	123169	LEO1 hom Paf1/RNA polymerase II complex component
ENSG0000	44.89198	-0.02467	0.168191	0.830611	0.959135	CASP6	839	caspase 6
ENSG0000	791.4775	-0.02414	0.152216	0.830511	0.959135	KIFC2	90990	kinesin family member C2
ENSG0000	1388.173	-0.02775	0.155757	0.830643	0.959135	H2AX	3014	H2A.X variant histone
ENSG0000	23.42297	-0.02086	0.198886	0.830569	0.959135	MAGOHB	55110	mago hom exon junction complex subunit
ENSG0000	47.62254	-0.02405	0.176538	0.830504	0.959135	KIF19	124602	kinesin family member 19
ENSG0000	521.3677	0.023851	0.138152	0.830398	0.959135	WDR45	11152	WD repeat domain 45
ENSG0000	213.8218	0.024185	0.157817	0.83099	0.959173	TIMM10B	26515	translocase of inner mitochondrial membrane 10B
ENSG0000	158.2774	-0.02366	0.192107	0.830837	0.959173	FGFR1OP2	26127	FGFR1 oncogene partner 2
ENSG0000	208.7938	0.025036	0.162722	0.830773	0.959173	HMG20A	10363	high mobility group 20A
ENSG0000	250.8797	-0.01221	0.221638	0.830945	0.959173	EVI2A	2123	ecotropic viral integration site 2A
ENSG0000	291.2739	-0.02091	0.115705	0.830982	0.959173	MED1	5469	mediator complex subunit 1
ENSG0000	2511.685	0.023366	0.159396	0.830937	0.959173	TECR	9524	trans-2 3-enoyl-CoA reductase
ENSG0000	1923.418	0.024809	0.147996	0.831049	0.95918	ELL	8178	elongation factor for RNA polymerase II
ENSG0000	5.447639	-0.01242	0.224424	0.831175	0.959266	NA	NA	NA
ENSG0000	123.8135	0.023887	0.159928	0.831385	0.959392	XRRA1	143570	X-ray radiation resistance associated 1
ENSG0000	486.8727	-0.02398	0.155332	0.831389	0.959392	PAFAH1B2	5049	platelet activating factor acetylhydrolase 1b catalytic subunit 2
ENSG0000	249.946	0.025137	0.160031	0.831484	0.95944	CAND1	55832	cullin associated and neddylation dissociated 1
ENSG0000	327.6026	-0.02366	0.153909	0.831676	0.959603	CLUHP3	1E+08	clustered mitochondria homolog pseudogene 3
ENSG0000	6.091439	0.013177	0.22381	0.831764	0.959643	LINC02981	441204	long intergenic non-protein coding RNA 2981
ENSG0000	20.49209	-0.02181	0.196563	0.831922	0.959765	CEP72	55722	centrosomal protein 72
ENSG0000	2.526363	-0.00437	0.23074	0.832036	0.959775	NA	NA	NA
ENSG0000	80.37407	0.024755	0.153864	0.832017	0.959775	TULP4	56995	TUB like protein 4
ENSG0000	15.5858	0.020685	0.205414	0.832323	0.959823	KCTD3	51133	potassium channel tetramerization domain containing 3
ENSG0000	693.8975	0.019954	0.115423	0.832391	0.959823	SMARCC1	6599	SWI/SNF r matrix ass actin dependent regulator of chromatin subfamily c member 1
ENSG0000	1086.332	-0.02354	0.168064	0.832334	0.959823	PARP8	79668	poly(ADP-ribose) polymerase family member 8
ENSG0000	249.2275	-0.02264	0.135757	0.83234	0.959823	TRIM35	23087	tripartite motif containing 35
ENSG0000	178.1597	-0.02809	0.142321	0.832294	0.959823	COA3	28958	cytochrome c oxidase assembly factor 3
ENSG0000	55.39834	0.024246	0.179829	0.832218	0.959823	NAT14	57106	N-acetyltransferase 14 (putative)
ENSG0000	11.22019	0.015612	0.217717	0.832638	0.959874	SCN9A	6335	sodium voltage-gated channel alpha subunit 9
ENSG0000	190.7499	0.024571	0.1404	0.832648	0.959874	YIPF5	81555	Yip1 domain family member 5
ENSG0000	11.64169	-0.02198	0.200631	0.83257	0.959874	LINC0101	1.01E+08	long intergenic non-protein coding RNA 1012
ENSG0000	5139.365	0.024477	0.146014	0.832569	0.959874	PPP1CA	5499	protein phosphatase 1 catalytic subunit alpha
ENSG0000	3.650724	-0.00879	0.227345	0.832698	0.959874	NA	NA	NA
ENSG0000	92.85307	0.024544	0.159854	0.832849	0.959988	PPP1R13L	10848	protein phosphatase 1 regulatory subunit 13 like
ENSG0000	121.8642	-0.02862	0.179112	0.832973	0.960071	KLHL9	55958	kelch like family member 9
ENSG0000	97.83022	0.018173	0.197853	0.833317	0.960303	DCAF17	80067	DDB1 and CUL4 associated factor 17
ENSG0000	90.80863	0.023976	0.149757	0.833227	0.960303	NPM3	10360	nucleophosmin/nucleoplasmin 3
ENSG0000	141.0051	0.023435	0.17609	0.833332	0.960303	HERC2P2	400322	HERC2 pseudogene 2
ENSG0000	2233.762	-0.02374	0.16881	0.833514	0.960305	HSPA5	3309	heat shock protein family A (Hsp70) member 5
ENSG0000	1873.276	-0.02277	0.132497	0.833516	0.960305	TOR4A	54863	torsin family 4 member A
ENSG0000	891.4674	-0.02437	0.133661	0.833544	0.960305	RANBP10	57610	RAN binding protein 10
ENSG0000	230.4708	-0.02291	0.14323	0.833529	0.960305	PKNOX1	5316	PBX/knotted 1 homeobox 1
ENSG0000	422.3238	-0.02317	0.138045	0.833724	0.960453	CWF19L1	55280	CWF19 like cell cycle control factor 1
ENSG0000	49.11218	-0.02422	0.173978	0.833989	0.960609	OLMALINC	90271	oligodendrocyte maturation-associated long intergenic non-coding RNA
ENSG0000	111.5753	0.01891	0.205935	0.834019	0.960609	RECQL	5965	RecQ like helicase
ENSG0000	4.055122	0.008775	0.22895	0.834069	0.960609	NA	NA	NA
ENSG0000	73.89641	0.024329	0.159676	0.833943	0.960609	ZBTB21	49854	zinc finger and BTB domain containing 21
ENSG0000	314.9388	0.02183	0.137918	0.834223	0.96072	ZKSCAN8	7745	zinc finger with KRAB and SCAN domains 8
ENSG0000	456.2482	0.022832	0.132651	0.834271	0.96072	PHF2	5253	PHD finger protein 2
ENSG0000	6.850944	0.018999	0.210636	0.8347	0.96087	NA	NA	NA
ENSG0000	3.829606	0.009524	0.227351	0.83491	0.96087	NA	NA	NA
ENSG0000	2.93347	-1.67E-04	0.231255	0.834916	0.96087	RP1L1	94137	RP1 like 1
ENSG0000	146.4461	-0.02196	0.125934	0.834869	0.96087	RPP38	10557	ribonuclease P/MRP subunit p38
ENSG0000	1051.271	0.023353	0.139738	0.834904	0.96087	RILPL2	196383	Rab interacting lysosomal protein like 2
ENSG0000	203.9682	-0.02426	0.175109	0.834885	0.96087	ACTR10	55860	actin related protein 10
ENSG0000	4.830262	-0.00807	0.228067	0.834844	0.96087	NOXO1	124056	NADPH oxidase organizer 1
ENSG0000	11.84457	-0.01926	0.208202	0.834911	0.96087	NA	NA	NA
ENSG0000	734.7734	-0.02417	0.162981	0.834544	0.96087	IRF2BP1	26145	interferon regulatory factor 2 binding protein 1
ENSG0000	1130.965	0.023715	0.146447	0.834975	0.96087	BCR	613	BCR activator of RhoGEF and GTPase
ENSG0000	1726.644	0.020786	0.171713	0.834978	0.96087	HMOX1	3162	heme oxygenase 1
ENSG0000	71.08502	0.024367	0.162697	0.835053	0.960882	FBXO48	554251	F-box protein 48
ENSG0000	18.37362	0.017343	0.212527	0.835162	0.960882	KIF15	56992	kinesin family member 15
ENSG0000	9.185912	0.020562	0.205981	0.835153	0.960882	C3orf14	57415	chromosome 3 open reading frame 14
ENSG0000	19.13658	-0.01379	0.219224	0.835198	0.960882	NA	NA	NA
ENSG0000	3.271607	-0.00607	0.229584	0.835369	0.961018	NA	NA	NA
ENSG0000	3.068269	0.006369	0.232432	0.835497	0.961105	NA	NA	NA
ENSG0000	1089.63	0.028219	0.152673	0.836087	0.961228	MRPL37	51253	mitochondrial ribosomal protein L37
ENSG0000	2.640682	-0.00992	0.227034	0.836041	0.961228	NA	NA	NA
ENSG0000	119.4822	0.026153	0.155472	0.836062	0.961228	TERF1	7013	telomeric repeat binding factor 1
ENSG0000	5.937963	0.016859	0.216582	0.836129	0.961228	MIR6753	1.02E+08	microRNA 6753
ENSG0000	3.502269	0.009798	0.227677	0.835787	0.961228	CCND2-AS	1.04E+08	CCND2 antisense RNA 1
ENSG0000	1573.691	-0.0223	0.13271	0.836004	0.961228	MARS1	4141	methionyl-tRNA synthetase 1
ENSG0000	8.821819	-0.01027	0.225853	0.835664	0.961228	LOC64666	646665	golgin A6 family member A pseudogene
ENSG0000	3.276573	0.006969	0.230197	0.836056	0.961228	CRYM	1428	crystallin mu
ENSG0000	195.6329	0.023138	0.174143	0.83595	0.961228	RABEP1	9135	rabaptin RAB GTPase binding effector protein 1
ENSG0000	1342.02	-0.02357	0.145531	0.835897	0.961228	TUG1	55000	taurine up-regulated 1
ENSG0000	251.6155	0.0254	0.127145	0.837304	0.961252	NIPAL3	57185	NIPA like domain containing 3

ENSG0000	207.0837	0.022277	0.191334	0.836912	0.961252	ZMPSTE24	10269	zinc metallopeptidase STE24
ENSG0000	1444.24	-0.02175	0.128092	0.837142	0.961252	BAP1	8314	BRCA1 associated protein 1
ENSG0000	306.1113	0.018676	0.19412	0.836409	0.961252	FNIP1	96459	folliculin interacting protein 1
ENSG0000	2.453553	0.010949	0.230732	0.837304	0.961252	NA	NA	NA
ENSG0000	307.7713	0.023447	0.161277	0.836927	0.961252	PCM1	5108	pericentriolar material 1
ENSG0000	193.5981	-0.02157	0.125117	0.836308	0.961252	HMBOX1	79618	homeobox containing 1
ENSG0000	75.87067	0.022196	0.190194	0.836934	0.961252	UBE2V2	7336	ubiquitin conjugating enzyme E2 V2
ENSG0000	9.134482	0.017077	0.21509	0.836653	0.961252	DENND2B	6764	DENN domain containing 2B
ENSG0000	4.208696	-0.00557	0.225052	0.837296	0.961252	NA	NA	NA
ENSG0000	6323.85	0.022276	0.129191	0.83651	0.961252	RASGRP2	10235	RAS guanyl releasing protein 2
ENSG0000	3.212584	0.012722	0.226658	0.837281	0.961252	NA	NA	NA
ENSG0000	31.51721	0.02358	0.181083	0.836685	0.961252	GPN3	51184	GPN-loop GTPase 3
ENSG0000	2.705273	-8.63E-04	0.231566	0.837272	0.961252	GCATP1	319140	glycine C-acetyltransferase pseudogene 1
ENSG0000	11.68621	0.019146	0.208009	0.837083	0.961252	TMEM202	1.05E+08	TMEM202 antisense RNA 1
ENSG0000	67.9087	-0.02198	0.185988	0.836496	0.961252	NA	NA	NA
ENSG0000	5.339579	0.011614	0.226431	0.837264	0.961252	PABPN1L	390748	PABPN1 lil cytoplasmic
ENSG0000	575.5236	0.026662	0.15008	0.836238	0.961252	KRI1	65095	KRI1 homolog
ENSG0000	1054.206	-0.0209	0.150249	0.837172	0.961252	IL27RA	9466	interleukin 27 receptor subunit alpha
ENSG0000	3032.18	0.02325	0.138583	0.836716	0.961252	PGLS	25796	6-phosphogluconolactonase
ENSG0000	113.4108	0.024392	0.142583	0.836716	0.961252	IRGQ	126298	immunity related GTPase Q
ENSG0000	391.9886	-0.02167	0.146325	0.836682	0.961252	ZMYM3	9203	zinc finger MYM-type containing 3
ENSG0000	3.208127	0.009651	0.229501	0.837916	0.961386	CCDC150	284992	coiled-coil domain containing 150
ENSG0000	526.5338	0.022884	0.142072	0.837704	0.961386	SATB1	6304	SATB homeobox 1
ENSG0000	5.161624	-0.01225	0.223976	0.837998	0.961386	RNU6-761	1.06E+08	RNA U6 small r pseudogene
ENSG0000	846.7659	0.022407	0.136037	0.83769	0.961386	TNFAIP3	7128	TNF alpha induced protein 3
ENSG0000	2.593051	0.014255	0.229421	0.837734	0.961386	SMKR1	1E+08	small lysine rich protein 1
ENSG0000	140.6938	-0.0235	0.160183	0.837989	0.961386	UBN2	254048	ubinuclein 2
ENSG0000	31.21831	0.023577	0.176733	0.837803	0.961386	NEIL2	252969	nei like DNA glycosylase 2
ENSG0000	710.8751	-0.01945	0.13261	0.837893	0.961386	DERL1	79139	derlin 1
ENSG0000	45.91925	-0.02053	0.186963	0.837515	0.961386	LRP5	4041	LDL receptor related protein 5
ENSG0000	91.14217	-0.02175	0.155949	0.837805	0.961386	FUT4	2526	fucosyltransferase 4
ENSG0000	4.207853	0.014394	0.221175	0.837894	0.961386	SGK2	10110	serum/glucocorticoid regulated kinase 2
ENSG0000	28.71613	-0.02199	0.194564	0.838172	0.961491	SLC35G5	83650	solute carrier family 35 member G5
ENSG0000	4.677306	-0.01247	0.223468	0.838195	0.961491	NA	NA	NA
ENSG0000	5.175753	-0.00773	0.228498	0.838266	0.961513	NA	NA	NA
ENSG0000	211.9737	0.018371	0.137981	0.83833	0.961526	DDX18	8886	DEAD-box helicase 18
ENSG0000	82.64522	0.0237	0.157245	0.838406	0.961553	HSD17B8	7923	hydroxysteroid 17-beta dehydrogenase 8
ENSG0000	188.0039	-0.02325	0.143173	0.838673	0.961731	GOLPH3L	55204	golgi phosphoprotein 3 like
ENSG0000	255.954	-0.0227	0.149728	0.838998	0.961731	BTBD9	114781	BTB domain containing 9
ENSG0000	13.92265	0.021427	0.208353	0.838992	0.961731	PLAG1	5324	PLAG1 zinc finger
ENSG0000	10.13818	0.019102	0.207724	0.838781	0.961731	ZDHHC12-	1.01E+08	ZDHHC12 divergent transcript
ENSG0000	11.78941	0.015711	0.216289	0.839296	0.961731	AKR1C1	1645	aldo-keto reductase family 1 member C1
ENSG0000	7.712778	-0.01684	0.213386	0.838886	0.961731	NA	NA	NA
ENSG0000	462.6366	0.023818	0.151117	0.839035	0.961731	SNAP23	8773	synaptosome associated protein 23
ENSG0000	142.6286	-0.02262	0.177422	0.839207	0.961731	HACD3	51495	3-hydroxyacyl-CoA dehydratase 3
ENSG0000	259.8079	0.022632	0.14914	0.83884	0.961731	COMMD4	54939	COMM domain containing 4
ENSG0000	1154.457	-0.02018	0.134629	0.839203	0.961731	MARF1	9665	meiosis regulator and mRNA stability factor 1
ENSG0000	578.7352	0.021864	0.121499	0.838926	0.961731	BCL7C	9274	BAF chromatin remodeling complex subunit BCL7C
ENSG0000	525.3022	-0.02934	0.14595	0.839227	0.961731	DHX38	9785	DEAH-box helicase 38
ENSG0000	200.7257	0.020952	0.13733	0.839039	0.961731	DSTN	11034	destrin actin depolymerizing factor
ENSG0000	7.920347	0.017715	0.212362	0.839269	0.961731	HDX	139324	highly divergent homeobox
ENSG0000	12.22348	0.016134	0.214262	0.839484	0.961766	NA	NA	NA
ENSG0000	2.734118	-0.00745	0.229011	0.839387	0.961766	NA	NA	NA
ENSG0000	117.1896	0.018841	0.168182	0.839445	0.961766	CEP192	55125	centrosomal protein 192
ENSG0000	6.537372	0.016142	0.216346	0.839598	0.961836	ZNF460	10794	zinc finger protein 460
ENSG0000	185.1526	-0.02418	0.140446	0.83968	0.96187	CSRNP2	81566	cysteine and serine rich nuclear protein 2
ENSG0000	314.6393	0.02305	0.161486	0.839824	0.961954	DENND10	404636	DENN domain containing 10
ENSG0000	4.24379	0.006966	0.228943	0.839858	0.961954	C16orf95	1.01E+08	chromosome 16 open reading frame 95
ENSG0000	22.31198	0.018006	0.208581	0.839962	0.962012	ARHGEF39	84904	Rho guanine nucleotide exchange factor 39
ENSG0000	1560.675	-0.01968	0.179349	0.840033	0.962034	RCN3	57333	reticulocalbin 3
ENSG0000	108.3101	0.023225	0.170661	0.840447	0.962065	HYI	81888	hydroxypyruvate isomerase (putative)
ENSG0000	225.0355	0.022412	0.134504	0.840481	0.962065	TMEM9	252839	transmembrane protein 9
ENSG0000	38.22654	-0.01861	0.205143	0.840329	0.962065	POU5F1	5460	POU class 5 homeobox 1
ENSG0000	4.015881	0.012696	0.222391	0.840364	0.962065	KIF5A	3798	kinesin family member 5A
ENSG0000	29.7983	0.022514	0.186291	0.840416	0.962065	SLC25A30	253512	solute carrier family 25 member 30
ENSG0000	136.4393	0.025828	0.160635	0.84047	0.962065	NA	NA	NA
ENSG0000	4.493733	0.010264	0.228255	0.84019	0.962065	DNAAF8	146562	dynein axonemal assembly factor 8
ENSG0000	1091.11	0.024664	0.143387	0.840209	0.962065	NDUFS7	374291	NADH:ubiquinone oxidoreductase core subunit S7
ENSG0000	35.7033	0.020282	0.197756	0.840753	0.96207	NA	NA	NA
ENSG0000	71.95889	0.022359	0.194002	0.840853	0.96207	GK5	256356	glycerol kinase 5
ENSG0000	3069.201	0.022601	0.150756	0.840777	0.96207	DOCK2	1794	dedicator of cytokinesis 2
ENSG0000	6.008367	0.014075	0.220442	0.840839	0.96207	RNU7-41P	1E+08	RNA U7 small nuclear 41 pseudogene
ENSG0000	326.7338	-0.02252	0.145405	0.840684	0.96207	NSMCE1	197370	NSE1 hom SMC5-SMC6 complex component
ENSG0000	6.983978	0.013846	0.219352	0.840698	0.96207	NA	NA	NA
ENSG0000	313.0743	0.023327	0.159423	0.840851	0.96207	POLD1	5424	DNA polyr catalytic subunit
ENSG0000	9.012819	0.009778	0.225793	0.841172	0.962375	NA	NA	NA
ENSG0000	67.22665	0.023308	0.164115	0.841298	0.96246	TPM3P9	147804	tropomyosin 3 pseudogene 9
ENSG0000	239.5368	0.021697	0.133488	0.841609	0.962529	NSL1	25936	NSL1 component of MIS12 kinetochore complex
ENSG0000	2.960707	0.009725	0.227141	0.841621	0.962529	BCAP29	55973	B cell receptor associated protein 29
ENSG0000	67.41159	-0.0234	0.175606	0.841483	0.962529	SERPINH1	871	serpin family H member 1
ENSG0000	5.149955	-0.00724	0.224366	0.841518	0.962529	BRMS1L	84312	BRMS1 like transcriptional repressor
ENSG0000	11.81032	-0.01343	0.219646	0.841596	0.962529	NXN	64359	nucleoredoxin
ENSG0000	4.105099	0.011282	0.228094	0.841717	0.962578	MCOLN3	55283	mucolipin TRP cation channel 3
ENSG0000	5.342611	0.013258	0.223618	0.842152	0.96276	RPL19P6	1E+08	ribosomal protein L19 pseudogene 6
ENSG0000	1413.687	0.022813	0.151697	0.842181	0.96276	MGAT4B	11282	alpha-1 3-mannosyl-glycoprotein 4-beta-N-acetylglucosaminyltransferase B
ENSG0000	1134.675	0.021493	0.123953	0.842005	0.96276	NA	NA	NA
ENSG0000	131.8725	-0.02257	0.147739	0.842029	0.96276	TRAPPC4	51399	trafficking protein particle complex subunit 4
ENSG0000	11.78903	-0.01872	0.207448	0.84211	0.96276	TEPP	374739	testis prostate and placenta expressed
ENSG0000	2228.711	0.023043	0.158257	0.842245	0.96276	KIAA0513	9764	KIAA0513
ENSG0000	13.02091	0.01979	0.204626	0.842212	0.96276	ZNF527	84503	zinc finger protein 527
ENSG0000	269.0934	0.022531	0.163697	0.842484	0.962784	POLR1A	25885	RNA polymerase I subunit A
ENSG0000	124.5186	-0.02281	0.171047	0.842729	0.962784	MFAP3	4238	microfibril associated protein 3
ENSG0000	152.9949	-0.02322	0.167381	0.842588	0.962784	ADO	84890	2-aminoethanethiol dioxygenase
ENSG0000	89.11347	-0.02282	0.158456	0.842739	0.962784	ZBTB3	79842	zinc finger and BTB domain containing 3
ENSG0000	13.91624	0.020382	0.200466	0.842666	0.962784	ATP6V0C	527	ATPase H+ transporting V0 subunit c
ENSG0000	17.2375	-0.02085	0.195937	0.842351	0.962784	MYO15A	51168	myosin XVA

ENSG0000	3.23827	-0.00892	0.228338	0.842394	0.962784	NA	NA	NA
ENSG0000	13.77169	0.018336	0.208715	0.842434	0.962784	RN7SKP80	1.06E+08	RN7SK pseudogene 80
ENSG0000	2.418922	-0.00336	0.230471	0.842689	0.962784	NA	NA	NA
ENSG0000	113.7591	-0.02302	0.169861	0.842827	0.962825	HEXIM2	124790	HEXIM P-TEFb complex subunit 2
ENSG0000	511.238	0.018198	0.147734	0.843515	0.96286	ARMH1	339541	armadillo like helical domain containing 1
ENSG0000	2798.616	0.021681	0.13248	0.843264	0.96286	NOTCH2	4853	notch receptor 2
ENSG0000	1024.972	0.02177	0.157037	0.843137	0.96286	SLAMF7	57823	SLAM family member 7
ENSG0000	10.49969	-0.01768	0.20865	0.843683	0.96286	GPR17	2840	G protein-coupled receptor 17
ENSG0000	25.02731	-0.01466	0.207685	0.84303	0.96286	LOC10272	1.03E+08	uncharacterized LOC102724104
ENSG0000	166.7172	0.021851	0.134269	0.843762	0.96286	DAG1	1605	dystroglycan 1
ENSG0000	89.60718	0.023263	0.169446	0.84333	0.96286	LRIG1	26018	leucine rich repeats and immunoglobulin like domains 1
ENSG0000	75.21897	-0.01629	0.173418	0.843929	0.96286	NDUFB5	4711	NADH:ubiquinone oxidoreductase subunit B5
ENSG0000	771.0036	-0.02251	0.182133	0.843149	0.96286	ACAP2	23527	ArfGAP wi ankyrin repeat and PH domains 2
ENSG0000	192.5758	-0.0142	0.2181	0.843878	0.96286	NA	NA	NA
ENSG0000	4.973281	0.011615	0.224598	0.84324	0.96286	AK8	158067	adenylate kinase 8
ENSG0000	4083.106	0.022782	0.151855	0.843263	0.96286	TRIM8	81603	tripartite motif containing 8
ENSG0000	2.723549	-0.00654	0.230535	0.843796	0.96286	C10orf88B	399815	C10orf88B (pseudogene)
ENSG0000	346.1644	-0.02065	0.174675	0.843515	0.96286	CRACR2B	283229	calcium release activated channel regulator 2B
ENSG0000	1450.116	0.017275	0.156021	0.842967	0.96286	DYNC1H1	1778	dynein cytoplasmic 1 heavy chain 1
ENSG0000	499.5668	0.020772	0.128343	0.843961	0.96286	SNAPC2	6618	small nuclear RNA activating complex polypeptide 2
ENSG0000	95.06656	-0.02057	0.194241	0.84337	0.96286	FCGBP	8857	Fc gamma binding protein
ENSG0000	49.57215	-0.01939	0.177563	0.843806	0.96286	NA	NA	NA
ENSG0000	799.3734	-0.02695	0.134721	0.843917	0.96286	NOL4L	140688	nucleolar protein 4 like
ENSG0000	5.526574	-0.01334	0.221	0.843696	0.96286	NA	NA	NA
ENSG0000	87.4932	0.025944	0.167643	0.843514	0.96286	GNB1L	54584	G protein subunit beta 1 like
ENSG0000	4.773589	0.015063	0.218876	0.84412	0.962922	NA	NA	NA
ENSG0000	2.959784	0.00594	0.23024	0.844089	0.962922	TRIM72	493829	tripartite motif containing 72
ENSG0000	108.1296	0.022631	0.172423	0.844526	0.963324	CNOT6	57472	CCR4-NOT transcription complex subunit 6
ENSG0000	6069.376	-0.02032	0.184699	0.844774	0.963362	PTPRC	5788	protein tyrosine phosphatase receptor type C
ENSG0000	611.6936	-0.02286	0.162402	0.844892	0.963362	ATG7	10533	autophagy related 7
ENSG0000	8.564216	0.009079	0.221641	0.845032	0.963362	NA	NA	NA
ENSG0000	2210.084	0.022661	0.171149	0.844843	0.963362	ATG2A	23130	autophagy related 2A
ENSG0000	1422.028	-0.02188	0.161458	0.844817	0.963362	RASSF3	283349	Ras association domain family member 3
ENSG0000	676.0925	-0.02095	0.139558	0.844846	0.963362	KATNB1	10300	katanin regulatory subunit B1
ENSG0000	22.75579	-0.01911	0.203433	0.844757	0.963362	PIPOX	51268	pipecolic acid and sarcosine oxidase
ENSG0000	124.4821	0.022411	0.167401	0.845007	0.963362	LIPE	3991	lipase E hormone sensitive type
ENSG0000	126.3402	0.022071	0.136912	0.844943	0.963362	CRYBB2P1	1416	crystallin beta B2 pseudogene 1
ENSG0000	4.271586	0.013416	0.222995	0.845364	0.963381	FOXD4	2298	forkhead box D4
ENSG0000	28.82543	-0.02271	0.172342	0.845146	0.963381	NA	NA	NA
ENSG0000	4.66518	-0.00994	0.227968	0.84521	0.963381	LIPC-AS1	1.02E+08	LIPC antisense RNA 1
ENSG0000	36.44499	0.017815	0.20708	0.845291	0.963381	LOXL1	4016	lysyl oxidase like 1
ENSG0000	2.376079	0.004882	0.230661	0.845364	0.963381	NA	NA	NA
ENSG0000	6.530211	-0.01265	0.221667	0.845202	0.963381	NA	NA	NA
ENSG0000	955.3563	0.021943	0.141915	0.84551	0.963455	GARS1	2617	glycyl-tRNA synthetase 1
ENSG0000	3.531781	0.007548	0.229158	0.845535	0.963455	TPBGL	1.01E+08	trophoblast glycoprotein like
ENSG0000	765.0601	0.022264	0.145895	0.845731	0.963619	DHRS7	51635	dehydrogenase/reductase 7
ENSG0000	807.1567	-0.01846	0.196471	0.845922	0.963777	SERINC1	57515	serine incorporator 1
ENSG0000	1155.818	-0.02236	0.141654	0.846247	0.963832	DEDD	9191	death effector domain containing
ENSG0000	114.5403	0.021609	0.153492	0.84655	0.963832	ACOX3	8310	acyl-CoA c pristanoyl
ENSG0000	850.6993	0.024311	0.16381	0.846322	0.963832	SKIC2	6499	SKI2 subunit of superkiller complex
ENSG0000	172.5909	0.023423	0.151642	0.846498	0.963832	UBE2D4	51619	ubiquitin conjugating enzyme E2 D4 (putative)
ENSG0000	3.309521	0.010442	0.226541	0.846447	0.963832	NA	NA	NA
ENSG0000	508.5018	-0.02161	0.130433	0.846188	0.963832	GLTP	51228	glycolipid transfer protein
ENSG0000	50.54167	-0.01834	0.201671	0.846529	0.963832	NA	NA	NA
ENSG0000	2125.727	-0.02218	0.158318	0.846524	0.963832	SYMPK	8189	symplekin scaffold protein
ENSG0000	24.16346	-0.0197	0.20008	0.846369	0.963832	N6AMT1	29104	N-6 adenine-specific DNA methyltransferase 1
ENSG0000	25.2985	-0.02065	0.190152	0.846229	0.963832	NA	NA	NA
ENSG0000	82280.18	0.022173	0.171901	0.846193	0.963832	ND5	4540	NADH dehydrogenase subunit 5
ENSG0000	13.97438	-0.01896	0.202212	0.846686	0.963867	NA	NA	NA
ENSG0000	5.898711	-0.00976	0.225385	0.846676	0.963867	NA	NA	NA
ENSG0000	4.343737	0.009125	0.226799	0.846849	0.963993	NA	NA	NA
ENSG0000	24.29201	0.027658	0.178491	0.847772	0.964002	CDK11A	728642	cyclin dependent kinase 11A
ENSG0000	195.6517	0.019678	0.132246	0.847373	0.964002	LZIC	84328	leucine zipper and CTNNBIP1 domain containing
ENSG0000	9.340274	-0.0126	0.220511	0.847821	0.964002	POTEE	445582	POTE ankyrin domain family member E
ENSG0000	3.100058	0.010882	0.229683	0.847321	0.964002	KRT8P15	645834	keratin 8 pseudogene 15
ENSG0000	156.7355	-0.02041	0.138753	0.847314	0.964002	MPLKIP	136647	M-phase specific PLK1 interacting protein
ENSG0000	8.108201	0.014631	0.218187	0.847166	0.964002	NA	NA	NA
ENSG0000	71.70736	0.021781	0.157772	0.84795	0.964002	CHCHD7	79145	coiled-coil-helix-coiled-coil-helix domain containing 7
ENSG0000	44.1907	0.020826	0.18861	0.847493	0.964002	NA	NA	NA
ENSG0000	664.4859	0.021296	0.137307	0.847999	0.964002	RNF26	79102	ring finger protein 26
ENSG0000	2.446903	0.007331	0.230048	0.847627	0.964002	KLRK1-AS1	1.02E+08	KLRK1 antisense RNA 1
ENSG0000	81.13809	0.019589	0.197134	0.848016	0.964002	ZDHHC17	23390	zinc finger DHHC-type palmitoyltransferase 17
ENSG0000	3321.644	0.018258	0.137953	0.847662	0.964002	FBRSL1	57666	fibrosin like 1
ENSG0000	255.7729	0.020661	0.193325	0.847132	0.964002	GPR183	1880	G protein-coupled receptor 183
ENSG0000	84.74473	-0.00648	0.181156	0.847606	0.964002	LINC00641	283624	long intergenic non-protein coding RNA 641
ENSG0000	5.597815	-0.01054	0.224472	0.847768	0.964002	NA	NA	NA
ENSG0000	725.7852	-0.02164	0.11492	0.848119	0.964002	RBBP6	5930	RB binding ubiquitin ligase
ENSG0000	321.4056	-0.02141	0.143352	0.847231	0.964002	EMC6	83460	ER membrane protein complex subunit 6
ENSG0000	52.31473	0.0177	0.200931	0.847496	0.964002	TRIM37	4591	tripartite motif containing 37
ENSG0000	2.876742	-0.01069	0.228277	0.847908	0.964002	NA	NA	NA
ENSG0000	9.86891	0.015963	0.211701	0.847418	0.964002	NKPD1	284353	NTPase KAP family P-loop domain containing 1
ENSG0000	13.97555	-0.01883	0.202481	0.848094	0.964002	NA	NA	NA
ENSG0000	134.873	-0.02204	0.155516	0.847206	0.964002	URB1	9875	URB1 ribosome biogenesis homolog
ENSG0000	198.5397	0.022791	0.138921	0.848058	0.964002	CCDC134	79879	coiled-coil domain containing 134
ENSG0000	164.7424	-0.01876	0.120463	0.847755	0.964002	NKAP	79576	NFKB activating protein
ENSG0000	163.9698	0.022144	0.168596	0.84836	0.964186	TSPYL4	23270	TSPY like 4
ENSG0000	18.0504	-0.0207	0.18944	0.848387	0.964186	ZNF132	7691	zinc finger protein 132
ENSG0000	15.46587	-0.01821	0.205321	0.848567	0.964331	RNF212	285498	ring finger protein 212
ENSG0000	33.09264	-0.01946	0.197568	0.848839	0.964429	MMP23B	8510	matrix metallopeptidase 23B
ENSG0000	3.107139	-0.00776	0.230049	0.848864	0.964429	ACVR1C	130399	activin A receptor type 1C
ENSG0000	175.021	0.02056	0.173594	0.848816	0.964429	AP3S1	1176	adaptor related protein complex 3 subunit sigma 1
ENSG0000	7.028113	0.013876	0.220609	0.848743	0.964429	PBK	55872	PDZ binding kinase
ENSG0000	20.24317	-0.01849	0.205734	0.848959	0.964477	LINC0113	1E+08	long intergenic non-protein coding RNA 1134
ENSG0000	976.0024	0.021458	0.164169	0.849521	0.964883	NOC2L	26155	NOC2 like nucleolar associated transcriptional repressor
ENSG0000	1174.065	-0.01771	0.159094	0.849663	0.964883	AGFG1	3267	ArfGAP with FG repeats 1
ENSG0000	78.70643	0.021021	0.167454	0.84958	0.964883	SELENOK	58515	selenoprotein K

ENSG0000	8.57863	0.017372	0.210317	0.84967	0.964883	NA	NA	NA
ENSG0000	225.1343	0.023041	0.182288	0.849654	0.964883	PAN3	255967	poly(A) specific ribonuclease subunit PAN3
ENSG0000	62.83833	0.021861	0.163729	0.849685	0.964883	ACOT2	10965	acyl-CoA thioesterase 2
ENSG0000	4.561863	0.01147	0.22342	0.849448	0.964883	DBNDD2	55861	dysbindin domain containing 2
ENSG0000	205.7032	0.020799	0.128054	0.849757	0.964906	NDUFA11	126328	NADH:ubiquinone oxidoreductase subunit A11
ENSG0000	14.3361	0.018329	0.206139	0.849892	0.964939	NA	NA	NA
ENSG0000	9.040203	-0.01116	0.222503	0.849858	0.964939	ZNF624	57547	zinc finger protein 624
ENSG0000	347.7609	-0.02128	0.179062	0.849969	0.964966	PTPN22	26191	protein tyrosine phosphatase non-receptor type 22
ENSG0000	7.869798	-0.01088	0.223516	0.85011	0.965066	MT1G	4495	metallothionein 1G
ENSG0000	2804.588	-0.02074	0.142736	0.850477	0.965081	PBXIP1	57326	PBX homeobox interacting protein 1
ENSG0000	60.53785	0.021581	0.169237	0.850544	0.965081	LYRM2	57226	LYR motif containing 2
ENSG0000	77.04122	0.021812	0.162768	0.850227	0.965081	RNF170	81790	ring finger protein 170
ENSG0000	78.34083	-0.02052	0.180721	0.85051	0.965081	REXO2	25996	RNA exonuclease 2
ENSG0000	175.1849	0.019951	0.19291	0.850288	0.965081	UBL3	5412	ubiquitin like 3
ENSG0000	5.569481	0.009648	0.226779	0.850274	0.965081	NA	NA	NA
ENSG0000	721.8052	-0.02022	0.132602	0.850472	0.965081	POLG	5428	DNA polyr catalytic subunit
ENSG0000	7.709814	-0.00578	0.22199	0.850512	0.965081	NA	NA	NA
ENSG0000	121.7794	0.018894	0.193136	0.850669	0.965088	PIKFYVE	200576	phosphoin FYVE-type zinc finger containing
ENSG0000	3.782603	0.010021	0.226572	0.850709	0.965088	NA	NA	NA
ENSG0000	14.76758	-0.02019	0.19611	0.850684	0.965088	NA	NA	NA
ENSG0000	886.8009	0.02296	0.152741	0.85086	0.9652	FAM234A	83986	family with sequence similarity 234 member A
ENSG0000	7.388482	0.014776	0.216679	0.85099	0.965228	NA	NA	NA
ENSG0000	509.7791	-0.02212	0.154389	0.850984	0.965228	FAM50A	9130	family with sequence similarity 50 member A
ENSG0000	17.16506	-0.0196	0.196577	0.851125	0.965262	UFSP1	402682	UFM1 specific peptidase 1 (inactive)
ENSG0000	86.97925	-0.02088	0.179153	0.85109	0.965262	USP12	219333	ubiquitin specific peptidase 12
ENSG0000	3.53017	-0.00242	0.232662	0.85131	0.965342	NA	NA	NA
ENSG0000	19.64794	-0.01941	0.198485	0.851453	0.965342	NA	NA	NA
ENSG0000	440.448	-0.02222	0.172038	0.851356	0.965342	PHF20L1	51105	PHD finger protein 20 like 1
ENSG0000	425.0695	-0.01626	0.151124	0.851277	0.965342	APBB1	322	amyloid beta precursor protein binding family B member 1
ENSG0000	2.846907	-0.00942	0.227865	0.851459	0.965342	NA	NA	NA
ENSG0000	16.53041	-0.01692	0.208487	0.851854	0.965392	CSRP1-AS1	1.08E+08	CSRP1 antisense RNA 1
ENSG0000	12.44927	-0.01716	0.208903	0.851873	0.965392	SGCB	6443	sarcoglycan beta
ENSG0000	1328.481	-0.02148	0.145256	0.851767	0.965392	PTPRJ	5795	protein tyrosine phosphatase receptor type J
ENSG0000	90.67684	0.020194	0.192036	0.851813	0.965392	ACSS3	79611	acyl-CoA synthetase short chain family member 3
ENSG0000	19.14296	-0.01794	0.200544	0.851798	0.965392	ALDH1L2	160428	aldehyde dehydrogenase 1 family member L2
ENSG0000	1251.754	0.021007	0.152051	0.851758	0.965392	HCFC1R1	54985	host cell factor C1 regulator 1
ENSG0000	14.8059	-0.01831	0.203108	0.851794	0.965392	ADM5	199800	adrenomedullin 5 (putative)
ENSG0000	5.446124	-0.00874	0.227519	0.852079	0.965567	PLEKHD1	400224	pleckstrin homology and coiled-coil domain containing D1
ENSG0000	43.67798	-0.02027	0.167097	0.852293	0.965689	ZNF34	80778	zinc finger protein 34
ENSG0000	220.305	-0.01816	0.195787	0.852272	0.965689	ZBED5	58486	zinc finger BED-type containing 5
ENSG0000	2637.879	-0.02081	0.162061	0.852499	0.965864	PINK1	65018	PTEN induced kinase 1
ENSG0000	76.14922	-0.01973	0.191325	0.852558	0.965871	RAB4A	5867	RAB4A member RAS oncogene family
ENSG0000	2.620563	0.00644	0.231007	0.852756	0.965915	NA	NA	NA
ENSG0000	80.42965	-0.02065	0.185054	0.852708	0.965915	UEVLD	55293	UEV and lactate/malate dehyrogenase domains
ENSG0000	6.114488	-0.00967	0.224154	0.85266	0.965915	FAM66C	440078	family with sequence similarity 66 member C
ENSG0000	581.3029	-0.01397	0.129867	0.853373	0.966495	TSR3	115939	TSR3 ribosome maturation factor
ENSG0000	19022.62	-0.01992	0.178464	0.853365	0.966495	SHKBP1	92799	SH3KBP1 binding protein 1
ENSG0000	46.306	-0.02146	0.173634	0.853759	0.966573	ZNF619	285267	zinc finger protein 619
ENSG0000	3.036632	-0.00526	0.228955	0.853727	0.966573	SUGCT	79783	succinyl-CoA:glutarate-CoA transferase
ENSG0000	4.553287	-0.01312	0.219736	0.8538	0.966573	NA	NA	NA
ENSG0000	3.158265	-0.00521	0.22402	0.853812	0.966573	MIR3677	1.01E+08	microRNA 3677
ENSG0000	6.741139	-0.0137	0.218057	0.853636	0.966573	TMEM231	79583	transmembrane protein 231
ENSG0000	598.9389	0.021651	0.162319	0.853752	0.966573	ASPSCR1	79058	ASPSCR1 t UBX domain containing
ENSG0000	764.8978	0.021546	0.150056	0.853561	0.966573	SMG9	56006	SMG9 nonsense mediated mRNA decay factor
ENSG0000	1051.828	0.021081	0.154868	0.853935	0.966598	VPS11	55823	VPS11 core subunit of CORVET and HOPS complexes
ENSG0000	741.3462	0.021214	0.156697	0.853939	0.966598	CDKN1B	1027	cyclin dependent kinase inhibitor 1B
ENSG0000	5.787592	0.008648	0.226239	0.854053	0.966631	LINC0054{	400123	long intergenic non-protein coding RNA 548
ENSG0000	304.365	0.019741	0.129132	0.854074	0.966631	ZCCHC3	85364	zinc finger CCHC-type containing 3
ENSG0000	3.047461	-0.00677	0.229508	0.854188	0.966701	PARVA	55742	parvin alpha
ENSG0000	84.47116	-0.02062	0.17491	0.854332	0.966733	TMEM156	80008	transmembrane protein 156
ENSG0000	878.366	-0.01788	0.111229	0.85427	0.966733	CHFR	55743	checkpoint with forkhead and ring finger domains
ENSG0000	6.477658	0.015509	0.220047	0.854405	0.966733	MAPT	4137	microtubule associated protein tau
ENSG0000	15.64903	0.019641	0.195314	0.854427	0.966733	NA	NA	NA
ENSG0000	41.22442	0.021267	0.172608	0.854775	0.966747	OTUD3	23252	OTU deubiquitinase 3
ENSG0000	1212.342	-0.02033	0.160204	0.855119	0.966747	WDR26	80232	WD repeat domain 26
ENSG0000	94.55339	-0.02194	0.167054	0.855166	0.966747	AKT3	10000	AKT serine/threonine kinase 3
ENSG0000	135.393	-0.02087	0.17385	0.85525	0.966747	BUB1	699	BUB1 mitotic checkpoint serine/threonine kinase
ENSG0000	242.0934	-0.02191	0.149441	0.855267	0.966747	FARSB	10056	phenylalanyl-tRNA synthetase subunit beta
ENSG0000	246.7298	0.019957	0.140133	0.854887	0.966747	IFRD2	7866	interferon related developmental regulator 2
ENSG0000	3.341413	0.010192	0.228373	0.854863	0.966747	NA	NA	NA
ENSG0000	13.21559	-0.01834	0.199691	0.854941	0.966747	NA	NA	NA
ENSG0000	80.54365	0.020297	0.152015	0.854879	0.966747	MOAP1	64112	modulator of apoptosis 1
ENSG0000	305.4056	-0.01991	0.137559	0.855088	0.966747	RAD51-AS	1.01E+08	RAD51 antisense RNA 1
ENSG0000	77.1655	0.021379	0.1663	0.855263	0.966747	RRN3P3	1E+08	RRN3 pseudogene 3
ENSG0000	348.5981	0.020453	0.176867	0.855285	0.966747	MAP2K4	6416	mitogen-activated protein kinase kinase 4
ENSG0000	8.30635	-0.01628	0.210921	0.85459	0.966747	ZNF565	147929	zinc finger protein 565
ENSG0000	66.21909	-0.02062	0.177966	0.855	0.966747	NA	NA	NA
ENSG0000	4.728444	-0.01071	0.224361	0.855147	0.966747	NA	NA	NA
ENSG0000	14.62923	-0.01763	0.207593	0.854875	0.966747	NA	NA	NA
ENSG0000	32.23199	-0.02039	0.182657	0.855426	0.966848	ZNF549	256051	zinc finger protein 549
ENSG0000	1232.241	-0.01923	0.125189	0.855503	0.966875	WBP11	51729	WW domain binding protein 11
ENSG0000	16.57193	0.017592	0.204816	0.855757	0.967043	TMPPE	643853	transmembrane protein with metallophosphoesterase domain
ENSG0000	118.7265	-0.02109	0.172356	0.85574	0.967043	POLG2	11232	DNA polyr accessory subunit
ENSG0000	114.3753	0.02097	0.154481	0.855957	0.967208	NA	NA	NA
ENSG0000	133.4339	0.020549	0.169214	0.856079	0.967227	MYCBP	26292	MYC binding protein
ENSG0000	1714.231	-0.01638	0.147748	0.856059	0.967227	NDE1	54820	nudE neurodevelopment protein 1
ENSG0000	24.24821	-0.0189	0.186793	0.856136	0.967232	NA	NA	NA
ENSG0000	317.9907	0.019222	0.19131	0.856313	0.967248	DCP2	167227	decapping mRNA 2
ENSG0000	199.0615	0.019899	0.161177	0.856226	0.967248	NA	NA	NA
ENSG0000	8.926755	0.012906	0.218894	0.856361	0.967248	NA	NA	NA
ENSG0000	146.1727	-0.02232	0.174122	0.856326	0.967248	PRPS2	5634	phosphoribosyl pyrophosphate synthetase 2
ENSG0000	146.1767	-0.02307	0.158571	0.856658	0.967523	MVK	4598	mevalonate kinase
ENSG0000	7.721328	-0.01127	0.222792	0.856917	0.967578	YAE1	57002	YAE1 maturation factor of ABCE1
ENSG0000	3.576735	-0.00742	0.228806	0.856905	0.967578	NA	NA	NA
ENSG0000	82.1916	0.019003	0.192114	0.856917	0.967578	NA	NA	NA
ENSG0000	46.89204	0.019513	0.192886	0.856848	0.967578	FAAP24	91442	FA core complex associated protein 24

ENSG0000	91.20657	-0.02065	0.152603	0.857456	0.967582	NA	NA	NA
ENSG0000	844.3519	-0.02434	0.166595	0.857311	0.967582	CLSTN1	22883	calsyntenin 1
ENSG0000	308.5293	0.020746	0.17026	0.857399	0.967582	CEP350	9857	centrosomal protein 350
ENSG0000	74.90363	-0.02368	0.176337	0.8575	0.967582	ALS2CL	259173	ALS2 C-terminal like
ENSG0000	15.35584	-0.01792	0.200458	0.857308	0.967582	NA	NA	NA
ENSG0000	76.75693	-0.01501	0.204521	0.857386	0.967582	DIPK2A	205428	divergent protein kinase domain 2A
ENSG0000	68.31859	0.019731	0.188274	0.857336	0.967582	HAUS6	54801	HAUS augmin like complex subunit 6
ENSG0000	97.51197	0.017578	0.194177	0.857528	0.967582	ZNF22	7570	zinc finger protein 22
ENSG0000	18.18893	0.01326	0.212386	0.857103	0.967582	RAPSN	5913	receptor associated protein of the synapse
ENSG0000	33.76175	0.018074	0.199461	0.857051	0.967582	LAT	27040	linker for activation of T cells
ENSG0000	2704.815	-0.02195	0.14904	0.857555	0.967582	DDX5	1655	DEAD-box helicase 5
ENSG0000	584.8655	-0.01914	0.16525	0.857476	0.967582	PRKAR1A	5573	protein kinase cAMP-dependent type I regulatory subunit alpha
ENSG0000	330.128	-0.01842	0.132516	0.85763	0.967607	ZDHHC16	84287	zinc finger DHHC-type palmitoyltransferase 16
ENSG0000	132.152	-0.01911	0.191632	0.857706	0.967633	DNTTIP2	30836	deoxynucleotidyltransferase terminal interacting protein 2
ENSG0000	30.50283	0.018956	0.179293	0.857816	0.967698	NA	NA	NA
ENSG0000	2114.915	0.020194	0.149259	0.858112	0.967723	COPA	1314	COPI coat complex subunit alpha
ENSG0000	11.85851	0.011969	0.220239	0.858133	0.967723	NA	NA	NA
ENSG0000	968.4704	-0.02008	0.173218	0.858035	0.967723	TCOF1	6949	treacle ribosome biogenesis factor 1
ENSG0000	3165.992	0.021527	0.145986	0.858083	0.967723	DDX39B	7919	DExD-box helicase 39B
ENSG0000	28.12262	-0.0153	0.207097	0.858108	0.967723	PIANP	196500	PILR alpha associated neural protein
ENSG0000	83.31998	0.020546	0.171536	0.858156	0.967723	WDR44	54521	WD repeat domain 44
ENSG0000	54.21422	-0.01667	0.202582	0.858211	0.967726	ZNG1B	150472	Zn regulated GTPase metalloprotein activator 1B
ENSG0000	18.06167	0.017609	0.201873	0.85913	0.967749	NA	NA	NA
ENSG0000	348.8769	0.015836	0.121326	0.858967	0.967749	AP3B1	8546	adaptor related protein complex 3 subunit beta 1
ENSG0000	11.02909	-0.01318	0.217437	0.85866	0.967749	TRAF3IP2-	643749	TRAF3IP2 antisense RNA 1
ENSG0000	453.9571	-0.0176	0.195779	0.858919	0.967749	OPLAH	26873	5-oxoproli ATP-hydrolysing
ENSG0000	98.28723	0.019635	0.179484	0.858459	0.967749	DDX50	79009	DExD-box helicase 50
ENSG0000	32.11022	0.019781	0.184388	0.858707	0.967749	C10orf143	387723	chromosome 10 open reading frame 143
ENSG0000	1100.824	0.019758	0.13534	0.858969	0.967749	TSPAN32	10077	tetraspanin 32
ENSG0000	4.801137	0.010409	0.223994	0.85912	0.967749	HPX	3263	hemopexin
ENSG0000	63.05776	0.01601	0.184023	0.858778	0.967749	NAA16	79612	N-alpha-ac NatA auxiliary subunit
ENSG0000	3483.154	-0.01901	0.133796	0.859083	0.967749	C16orf54	283897	chromosome 16 open reading frame 54
ENSG0000	11.62972	0.014641	0.213368	0.858645	0.967749	PRRT2	112476	proline rich transmembrane protein 2
ENSG0000	190.6142	-0.01982	0.144977	0.858622	0.967749	ANKRD40	91369	ankyrin repeat domain 40
ENSG0000	8.266688	0.01331	0.217736	0.858608	0.967749	ZFP30	22835	ZFP30 zinc finger protein
ENSG0000	1705.687	0.019513	0.162642	0.858466	0.967749	NSFL1C	55968	NSFL1 cofactor
ENSG0000	44.68498	-0.01873	0.191662	0.858855	0.967749	LZTS3	9762	leucine zipper tumor suppressor family member 3
ENSG0000	3742.919	-0.02041	0.149945	0.858993	0.967749	CYB5R3	1727	cytochrome b5 reductase 3
ENSG0000	651.1285	0.017705	0.147713	0.858845	0.967749	EMD	2010	emerin
ENSG0000	59.8708	-0.02028	0.163987	0.859318	0.967901	RIC8B	55188	RIC8 guanine nucleotide exchange factor B
ENSG0000	24.96401	0.016955	0.204393	0.859389	0.967922	ZUP1	221302	zinc finger containing ubiquitin peptidase 1
ENSG0000	3.004083	0.007326	0.227668	0.859877	0.967939	RPSAP18	388707	ribosomal protein SA pseudogene 18
ENSG0000	57.65856	-0.02016	0.177509	0.860076	0.967939	PLD1	5337	phospholipase D1
ENSG0000	67.98777	0.023779	0.153551	0.859975	0.967939	GALNT11	63917	polypeptide N-acetylgalactosaminyltransferase 11
ENSG0000	13.42856	-0.01433	0.213683	0.859695	0.967939	STPG3-AS1	1E+08	STPG3 antisense RNA 1
ENSG0000	121.4421	-0.01788	0.192068	0.859978	0.967939	ZEB1	6935	zinc finger E-box binding homeobox 1
ENSG0000	2166.045	0.019063	0.129291	0.859639	0.967939	PA2G4	5036	proliferation-associated 2G4
ENSG0000	3.644878	0.005463	0.229227	0.859984	0.967939	NA	NA	NA
ENSG0000	5.555616	-0.00962	0.227735	0.860059	0.967939	GOLGA2P7	388152	GOLGA2 pseudogene 7
ENSG0000	209.5118	-0.01782	0.128331	0.859749	0.967939	COX10	1352	cytochrome c oxidase assembly factor heme A:farnesyltransferase COX10
ENSG0000	360.284	0.02018	0.162704	0.860091	0.967939	CRLF3	51379	cytokine receptor like factor 3
ENSG0000	1487.068	0.019287	0.161933	0.859988	0.967939	RBM42	79171	RNA binding motif protein 42
ENSG0000	57.06112	-0.01897	0.188439	0.859843	0.967939	ZNF780B	163131	zinc finger protein 780B
ENSG0000	3944.864	-0.01857	0.184301	0.859628	0.967939	MYL9	10398	myosin light chain 9
ENSG0000	189.06	-0.01938	0.132564	0.860939	0.967947	PITHD1	57095	PITH domain containing 1
ENSG0000	1187.402	-0.01902	0.135937	0.860801	0.967947	PSMB2	5690	proteasome 20S subunit beta 2
ENSG0000	7.017889	0.010345	0.224143	0.860654	0.967947	NA	NA	NA
ENSG0000	219.3851	-0.01491	0.205306	0.860915	0.967947	FOXN2	3344	forkhead box N2
ENSG0000	21.23685	-0.0151	0.204312	0.860758	0.967947	DCBLD1	285761	discoidin CUB and LCCL domain containing 1
ENSG0000	72.90083	-0.02041	0.162614	0.86055	0.967947	SNHG15	285958	small nucleolar RNA host gene 15
ENSG0000	215.3428	0.021725	0.169551	0.86047	0.967947	ERV3-1	2086	endogeno envelope
ENSG0000	4.545741	-0.01236	0.220561	0.860681	0.967947	TRBV7-4	28594	T cell receptor beta variable 7-4
ENSG0000	627.5546	0.020246	0.171466	0.86082	0.967947	BOP1	23246	BOP1 ribosomal biogenesis factor
ENSG0000	1476.735	-0.01946	0.149687	0.860854	0.967947	PLXDC2	84898	plexin domain containing 2
ENSG0000	1497.201	-0.01703	0.150461	0.860869	0.967947	CD81	975	CD81 molecule
ENSG0000	19802.94	-0.01949	0.148137	0.860557	0.967947	GRK2	156	G protein-coupled receptor kinase 2
ENSG0000	4.982879	-0.00957	0.22825	0.86026	0.967947	NA	NA	NA
ENSG0000	1130.929	0.019591	0.167529	0.860752	0.967947	CARMIL2	146206	capping protein regulator and myosin 1 linker 2
ENSG0000	1926.334	-0.01846	0.15404	0.860943	0.967947	SREBF2	6721	sterol regulatory element binding transcription factor 2
ENSG0000	294.3101	0.019902	0.144659	0.860811	0.967947	SLC35A2	7355	solute carrier family 35 member A2
ENSG0000	367.1309	-0.01869	0.135184	0.861547	0.96799	ASH1L	55870	ASH1 like histone lysine methyltransferase
ENSG0000	493.8425	0.016899	0.143798	0.861322	0.96799	THAP4	51078	THAP domain containing 4
ENSG0000	319.8738	0.019287	0.158613	0.86107	0.96799	CUL7	9820	cullin 7
ENSG0000	55.58782	-0.02	0.155269	0.861321	0.96799	ZNF789	285989	zinc finger protein 789
ENSG0000	13.84389	0.014002	0.214168	0.861496	0.96799	IFT74	80173	intraflagellar transport 74
ENSG0000	3.055931	-0.01026	0.228269	0.861563	0.96799	LINC02561	1.11E+08	long intergenic non-protein coding RNA 2561
ENSG0000	2.546206	-0.00893	0.226976	0.861261	0.96799	NA	NA	NA
ENSG0000	2153.078	0.01856	0.165906	0.861369	0.96799	TSC2	7249	TSC complex subunit 2
ENSG0000	650.1264	0.019984	0.160292	0.861474	0.96799	RPTOR	57521	regulatory associated protein of MTOR complex 1
ENSG0000	95.58625	-0.01934	0.170864	0.861314	0.96799	ADNP2	22850	ADNP homeobox 2
ENSG0000	11.86005	0.017729	0.201651	0.861389	0.96799	CASTOR1	652968	cytosolic arginine sensor for mTORC1 subunit 1
ENSG0000	7.211537	0.007553	0.226916	0.86167	0.96805	EPHX3	79852	epoxide hydrolase 3
ENSG0000	3.3084	-0.00968	0.228121	0.861874	0.968131	LINC02884	1.05E+08	long intergenic non-protein coding RNA 2884
ENSG0000	84.44713	0.015705	0.207928	0.862255	0.968131	SENP7	57337	SUMO specific peptidase 7
ENSG0000	135.3435	-0.01879	0.185173	0.86232	0.968131	OTUD4	54726	OTU deubiquitinase 4
ENSG0000	83.30641	0.020871	0.17251	0.862154	0.968131	SH3BGRL2	83699	SH3 domain binding glutamate rich protein like 2
ENSG0000	11.85903	0.011607	0.220153	0.862323	0.968131	CDHR3	222256	cadherin related family member 3
ENSG0000	7.969521	-0.01199	0.219358	0.862	0.968131	AASS	10157	aminoadipate-semialdehyde synthase
ENSG0000	114.067	0.020002	0.151139	0.862065	0.968131	ZNF775	285971	zinc finger protein 775
ENSG0000	35.47789	-0.01849	0.189271	0.862081	0.968131	NA	NA	NA
ENSG0000	152.6522	-0.01933	0.173633	0.862133	0.968131	FAM83G	644815	family with sequence similarity 83 member G
ENSG0000	19.68284	-0.01434	0.210533	0.862195	0.968131	NA	NA	NA
ENSG0000	11.8653	-0.01151	0.219808	0.862172	0.968131	NA	NA	NA
ENSG0000	124.3356	-0.02662	0.175092	0.86262	0.968146	DUSP2	1844	dual specificity phosphatase 2
ENSG0000	91.58893	0.02051	0.151328	0.862503	0.968146	INPP1	3628	inositol polyphosphate-1-phosphatase
ENSG0000	6.703398	-0.01447	0.213949	0.862653	0.968146	BHLHE40-/	1.01E+08	BHLHE40 antisense RNA 1

ENSG0000	2975.072	0.019463	0.131082	0.862549	0.968146	CYFIP2	26999	cytoplasmic FMR1 interacting protein 2
ENSG0000	7.143813	0.012477	0.218474	0.862437	0.968146	LRRC69	1E+08	leucine rich repeat containing 69
ENSG0000	4.667125	-0.00477	0.232523	0.862515	0.968146	NA	NA	NA
ENSG0000	4.045126	-0.01042	0.225348	0.862731	0.968174	PPP1R3G	648791	protein phosphatase 1 regulatory subunit 3G
ENSG0000	666.7676	-0.02136	0.131363	0.862923	0.968271	SNRPC	6631	small nuclear ribonucleoprotein polypeptide C
ENSG0000	1080.855	0.017748	0.192447	0.862921	0.968271	RRBP1	6238	ribosome binding protein 1
ENSG0000	111.2281	0.021071	0.176571	0.863005	0.968304	AIMP1	9255	aminoacyl tRNA synthetase complex interacting multifunctional protein 1
ENSG0000	310.3479	0.017686	0.118526	0.86337	0.968357	SDHAP2	727956	SDHA pseudogene 2
ENSG0000	444.3302	-0.01857	0.137205	0.863412	0.968357	CLIP1	6249	CAP-Gly domain containing linker protein 1
ENSG0000	465.6045	0.019647	0.156279	0.863154	0.968357	TUBG1	7283	tubulin gamma 1
ENSG0000	52.45422	0.01881	0.189256	0.863422	0.968357	RNFT1	51136	ring finger transmembrane 1
ENSG0000	3590.717	0.019789	0.157402	0.863416	0.968357	PPP2R1A	5518	protein phosphatase 2 scaffold subunit Aalpha
ENSG0000	21.09806	0.015587	0.207032	0.863195	0.968357	ZNF816	125893	zinc finger protein 816
ENSG0000	96.69833	-0.01644	0.201891	0.863266	0.968357	NA	NA	NA
ENSG0000	2182.491	-0.01943	0.144	0.86359	0.968367	TGFBR2	7048	transforming growth factor beta receptor 2
ENSG0000	30.3172	0.018631	0.195166	0.863587	0.968367	UTP15	84135	UTP15 small subunit processome component
ENSG0000	116.7171	0.01994	0.164866	0.863529	0.968367	CAMLG	819	calcium modulating ligand
ENSG0000	56.28822	-0.01899	0.169612	0.863865	0.968408	ACYP2	98	acylphosphatase 2
ENSG0000	33.6945	-0.02017	0.184857	0.863694	0.968408	NA	NA	NA
ENSG0000	15.75176	-0.01329	0.215459	0.863891	0.968408	NA	NA	NA
ENSG0000	96.50953	-0.01933	0.162948	0.863854	0.968408	CAPN3	825	calpain 3
ENSG0000	2.520468	-0.00523	0.229616	0.863839	0.968408	KDELR3	11015	KDEL endoplasmic reticulum protein retention receptor 3
ENSG0000	8.16486	-0.01292	0.217553	0.86396	0.968426	AFG1L	246269	AFG1 like ATPase
ENSG0000	3.032331	-0.0052	0.230247	0.864095	0.96844	NA	NA	NA
ENSG0000	23.25817	0.019591	0.182971	0.864131	0.96844	PET100	1E+08	PET100 cytochrome c oxidase chaperone
ENSG0000	255.7089	0.018599	0.147836	0.864046	0.96844	NA	NA	NA
ENSG0000	382.8776	-0.01855	0.165812	0.864384	0.968486	MSRA	4482	methionine sulfoxide reductase A
ENSG0000	503.246	0.019371	0.163815	0.864306	0.968486	AP3M1	26985	adaptor related protein complex 3 subunit mu 1
ENSG0000	43.73565	0.015876	0.169791	0.86424	0.968486	LLPH	84298	LLP homol long-term synaptic facilitation factor
ENSG0000	410.3042	-0.01771	0.124053	0.864356	0.968486	KLHDC4	54758	kelch domain containing 4
ENSG0000	284.635	-0.01691	0.192612	0.864509	0.968523	MBOAT2	129642	membrane bound O-acyltransferase domain containing 2
ENSG0000	1128.835	0.017983	0.129383	0.864523	0.968523	ADAP1	11033	ArfGAP with dual PH domains 1
ENSG0000	433.3786	-0.02197	0.138199	0.86497	0.96875	UBIAD1	29914	UbiA prenyltransferase domain containing 1
ENSG0000	144.8224	-0.01829	0.136648	0.865347	0.96875	PIGV	55650	phosphatidylinositol glycan anchor biosynthesis class V
ENSG0000	380.9509	-0.01899	0.13436	0.86537	0.96875	TUT4	23318	terminal uridylyl transferase 4
ENSG0000	485.9559	0.017451	0.149486	0.865945	0.96875	USP24	23358	ubiquitin specific peptidase 24
ENSG0000	2.569349	0.006758	0.231579	0.865132	0.96875	ILDR2	387597	immunoglobulin like domain containing receptor 2
ENSG0000	380.1878	0.017924	0.123282	0.865427	0.96875	BIRC6	57448	baculoviral IAP repeat containing 6
ENSG0000	231.27	-0.02055	0.168599	0.866384	0.96875	MGAT4A	11320	alpha-1 3-mannosyl-glycoprotein 4-beta-N-acetylglucosaminyltransferase A
ENSG0000	2.870333	0.007137	0.229316	0.865819	0.96875	NA	NA	NA
ENSG0000	20.5315	0.01606	0.206629	0.866224	0.96875	CIP2A	57650	cellular inhibitor of PP2A
ENSG0000	2.348779	0.007575	0.229526	0.866603	0.96875	NA	NA	NA
ENSG0000	474.8508	-0.01755	0.127174	0.866227	0.96875	TPRA1	131601	transmembrane protein adipocyte associated 1
ENSG0000	283.1462	0.020123	0.169459	0.865531	0.96875	KLHL24	54800	kelch like family member 24
ENSG0000	88.55997	0.020514	0.176372	0.866619	0.96875	RGS12	6002	regulator of G protein signaling 12
ENSG0000	245.7032	-0.01771	0.168668	0.86497	0.96875	ATG12	9140	autophagy related 12
ENSG0000	12.059	0.011446	0.220548	0.866225	0.96875	MIR1244-2	1E+08	microRNA 1244-2
ENSG0000	2672.866	-0.01917	0.143151	0.865143	0.96875	STING1	340061	stimulator of interferon response cGAMP interactor 1
ENSG0000	325.7181	-0.02207	0.159621	0.865639	0.96875	UIMC1	51720	ubiquitin interaction motif containing 1
ENSG0000	67.18462	-0.01939	0.165273	0.865156	0.96875	UMAD1	729852	UBAP1-MVB12-associated (UMA) domain containing 1
ENSG0000	19.81399	-0.0181	0.193876	0.864898	0.96875	GNRH1	2796	gonadotropin releasing hormone 1
ENSG0000	3.846421	-0.00708	0.227546	0.866267	0.96875	PXDNL	137902	peroxidasin like
ENSG0000	185.8573	-0.01723	0.182412	0.865854	0.96875	NA	NA	NA
ENSG0000	779.5661	-0.01915	0.127948	0.86632	0.96875	PHF19	26147	PHD finger protein 19
ENSG0000	198.3234	0.020503	0.158663	0.865427	0.96875	TRAF2	7186	TNF receptor associated factor 2
ENSG0000	92.78709	-0.01718	0.193144	0.865701	0.96875	P4HA1	5033	prolyl 4-hydroxylase subunit alpha 1
ENSG0000	12331.75	-0.01463	0.160091	0.864926	0.96875	PNPLA2	57104	patatin like phospholipase domain containing 2
ENSG0000	3.797868	0.006661	0.228881	0.866304	0.96875	NA	NA	NA
ENSG0000	47.1942	-0.01544	0.204711	0.86581	0.96875	STRN3	29966	striatin 3
ENSG0000	21.67393	-0.01561	0.207996	0.865631	0.96875	PAK6	56924	p21 (RAC1) activated kinase 6
ENSG0000	7.691264	0.013938	0.215003	0.865981	0.96875	PCLAF	9768	PCNA clamp associated factor
ENSG0000	18.48969	0.017169	0.199034	0.866443	0.96875	NA	NA	NA
ENSG0000	345.0132	0.018534	0.134313	0.866179	0.96875	MIF4GD	57409	MIF4G domain containing
ENSG0000	2528.444	0.01935	0.151841	0.866682	0.96875	GPR108	56927	G protein-coupled receptor 108
ENSG0000	118.7841	0.019324	0.158393	0.866118	0.96875	RABAC1	10567	Rab acceptor 1
ENSG0000	3.138606	0.009017	0.225642	0.86613	0.96875	NA	NA	NA
ENSG0000	139.5887	0.018891	0.156097	0.866612	0.96875	TTPAL	79183	alpha tocopherol transfer protein like
ENSG0000	61.37955	-0.01542	0.204324	0.866639	0.96875	GPR174	84636	G protein-coupled receptor 174
ENSG0000	129.0969	-0.01917	0.156887	0.86597	0.96875	VMA21	203547	vacuolar ATPase assembly factor VMA21
ENSG0000	3.03809	-0.00542	0.22898	0.866877	0.968764	NEU4	129807	neuraminidase 4
ENSG0000	593.3186	0.01309	0.142657	0.86682	0.968764	SEC62	7095	SEC62 hon preprotein translocation factor
ENSG0000	64.69434	-0.01884	0.175556	0.866826	0.968764	TTLL11	158135	tubulin tyrosine ligase like 11
ENSG0000	36.16003	-0.00703	0.227955	0.866906	0.968764	TRPV4	59341	transient receptor potential cation channel subfamily V member 4
ENSG0000	4.964481	-0.00953	0.224166	0.86705	0.968778	IL31RA	133396	interleukin 31 receptor A
ENSG0000	34.52616	-0.01872	0.178158	0.867253	0.968778	NA	NA	NA
ENSG0000	634.1059	0.021195	0.157131	0.867122	0.968778	KIF22	3835	kinesin family member 22
ENSG0000	32.34505	-0.01565	0.205299	0.867288	0.968778	NOG	9241	noggin
ENSG0000	551.2657	0.01945	0.14939	0.867263	0.968778	TMEM230	29058	transmembrane protein 230
ENSG0000	1346.453	-0.01732	0.140926	0.867247	0.968778	DNASE1L1	1774	deoxyribonuclease 1 like 1
ENSG0000	3.893713	0.009152	0.226965	0.867015	0.968778	NA	NA	NA
ENSG0000	3152.856	-0.01835	0.170036	0.868131	0.968821	MKNK1	8569	MAPK interacting serine/threonine kinase 1
ENSG0000	3.334446	-0.00582	0.230946	0.867655	0.968821	NA	NA	NA
ENSG0000	164.4078	0.017744	0.190731	0.867677	0.968821	C1D	10438	C1D nuclear receptor corepressor
ENSG0000	2.449887	-0.00814	0.228731	0.867472	0.968821	NA	NA	NA
ENSG0000	13.98488	-0.01426	0.21154	0.868371	0.968821	MAD2L1	4085	mitotic arrest deficient 2 like 1
ENSG0000	13.36854	-0.01508	0.207727	0.86779	0.968821	TXLNB	167838	taxilin beta
ENSG0000	508.5703	0.017788	0.166973	0.868269	0.968821	IQCE	23288	IQ motif containing E
ENSG0000	6.620942	0.011541	0.221449	0.867605	0.968821	TRBV14	28573	T cell receptor beta variable 14
ENSG0000	24.17659	0.014887	0.209722	0.868076	0.968821	CTNNAL1	8727	catenin alpha like 1
ENSG0000	4.834978	-0.00822	0.225968	0.867936	0.968821	NA	NA	NA
ENSG0000	88.59694	0.019077	0.158119	0.867865	0.968821	FICD	11153	FIC domain protein adenylyltransferase
ENSG0000	3.236281	-0.0089	0.225745	0.868145	0.968821	NA	NA	NA
ENSG0000	15.2501	0.012586	0.218279	0.867813	0.968821	NA	NA	NA
ENSG0000	4.75425	-0.00763	0.229002	0.868385	0.968821	SLC2A4	6517	solute carrier family 2 member 4
ENSG0000	2352.434	0.017273	0.175853	0.867694	0.968821	NFIC	4782	nuclear factor I C
ENSG0000	20.20761	0.016803	0.19579	0.867758	0.968821	ZNF607	84775	zinc finger protein 607

ENSG0000	12.15027	0.014903	0.210492	0.86819	0.968821	DNMT3B	1789	DNA methyltransferase 3 beta
ENSG0000	6.592979	-0.00362	0.225682	0.868092	0.968821	XKR3	150165	XK related 3
ENSG0000	15.77755	0.014405	0.211426	0.86767	0.968821	SHISA8	440829	shisa family member 8
ENSG0000	25.50358	-0.01738	0.192452	0.86834	0.968821	MBTPS2	51360	membrane site 2
ENSG0000	2.472528	0.004961	0.230395	0.868481	0.968869	NA	NA	NA
ENSG0000	44.58643	0.018865	0.188094	0.868546	0.968883	ANKDD1A	348094	ankyrin repeat and death domain containing 1A
ENSG0000	328.682	-0.01745	0.12746	0.868806	0.969023	AP1S1	1174	adaptor related protein complex 1 subunit sigma 1
ENSG0000	135.0899	-0.01846	0.167255	0.868835	0.969023	NCBP1	4686	nuclear cap binding protein subunit 1
ENSG0000	383.5258	-0.01948	0.129578	0.868929	0.969023	R3HDM2	22864	R3H domain containing 2
ENSG0000	157.2527	-0.01868	0.152656	0.868767	0.969023	WASHC3	51019	WASH complex subunit 3
ENSG0000	160.9258	-0.01882	0.165041	0.868936	0.969023	BCL2	596	BCL2 apoptosis regulator
ENSG0000	2.75975	0.008116	0.228448	0.869082	0.969127	NA	NA	NA
ENSG0000	8505.412	-0.01812	0.149212	0.869204	0.969204	CTDSP1	58190	CTD small phosphatase 1
ENSG0000	748.0797	0.017159	0.123966	0.869453	0.969258	SYNE1	23345	spectrin repeat containing nuclear envelope protein 1
ENSG0000	48.78867	0.016707	0.194689	0.869496	0.969258	ATAD2	29028	ATPase family AAA domain containing 2
ENSG0000	12.01828	0.01342	0.213609	0.869429	0.969258	GIHCG	1.01E+08	GIHCG inhibitor of miR-200b/200a/429 expression
ENSG0000	4.988772	-0.00976	0.222169	0.869518	0.969258	NA	NA	NA
ENSG0000	50.98917	-0.01509	0.204072	0.869455	0.969258	ZNF304	57343	zinc finger protein 304
ENSG0000	654.1819	-0.01896	0.13899	0.869742	0.969337	NA	NA	NA
ENSG0000	5886.934	0.018783	0.148664	0.869747	0.969337	COPE	11316	COPI coat complex subunit epsilon
ENSG0000	11924.69	-0.01807	0.165391	0.869726	0.969337	PREX1	57580	phosphati 4 5-trisphosphate dependent Rac exchange factor 1
ENSG0000	13.275	0.016329	0.207482	0.869844	0.969386	CACNA1C-	1.01E+08	CACNA1C antisense RNA 2
ENSG0000	37.2817	-0.01655	0.191283	0.869982	0.969425	RGMB	285704	repulsive guidance molecule BMP co-receptor b
ENSG0000	2.763193	0.005877	0.229187	0.870091	0.969425	BRSK2	9024	BR serine/threonine kinase 2
ENSG0000	4345.378	-0.01684	0.10644	0.870021	0.969425	TPM4	7171	tropomyosin 4
ENSG0000	7.32794	-0.0112	0.219766	0.870048	0.969425	ZC3H12B	340554	zinc finger CCCH-type containing 12B
ENSG0000	36.64148	-0.01668	0.193182	0.870227	0.969483	RUFY2	55680	RUN and FYVE domain containing 2
ENSG0000	31.92578	-0.01807	0.180263	0.870248	0.969483	ZNF461	92283	zinc finger protein 461
ENSG0000	33.57889	0.017868	0.184084	0.870461	0.969579	LOC10012	1E+08	uncharacterized LOC100128494
ENSG0000	81.23227	0.018753	0.151067	0.870477	0.969579	TANGO6	79613	transport and golgi organization 6 homolog
ENSG0000	2.471852	-0.00583	0.230526	0.870493	0.969579	LOC10537	1.05E+08	uncharacterized LOC105372832
ENSG0000	2160.33	0.019591	0.161426	0.870691	0.969741	AP3D1	8943	adaptor related protein complex 3 subunit delta 1
ENSG0000	15.46233	0.016289	0.201273	0.870866	0.969773	NA	NA	NA
ENSG0000	112.7863	0.017784	0.164018	0.870786	0.969773	CCZ1B	221960	CCZ1 hom vacuolar protein trafficking and biogenesis associated
ENSG0000	7.613237	-0.00979	0.222545	0.870902	0.969773	PODXL	5420	podocalyxin like
ENSG0000	521.2676	0.018503	0.131835	0.871038	0.969773	P2RX4	5025	purinergic receptor P2X 4
ENSG0000	6.217304	-0.00822	0.225967	0.870961	0.969773	ADPGK-AS	1E+08	ADPGK antisense RNA 1
ENSG0000	954.8535	-0.01417	0.101067	0.87103	0.969773	MED29	55588	mediator complex subunit 29
ENSG0000	10.7756	-0.01048	0.218066	0.871262	0.969898	SLC19A2	10560	solute carrier family 19 member 2
ENSG0000	4.670279	0.008863	0.225525	0.871309	0.969898	CATIP	375307	ciliogenesis associated TTC17 interacting protein
ENSG0000	156.4832	-0.01596	0.180188	0.871231	0.969898	RNF146	81847	ring finger protein 146
ENSG0000	985.9692	0.018886	0.147479	0.871422	0.969928	PPM1G	5496	protein ph Mg2+/Mn2+ dependent 1G
ENSG0000	5.409824	0.0105	0.222295	0.871547	0.969928	PTGES2-A!	389791	PTGES2 antisense RNA 1 (head to head)
ENSG0000	441.7149	0.018238	0.132573	0.871538	0.969928	PWWP2B	170394	PWWP domain containing 2B
ENSG0000	344.1188	0.015339	0.142347	0.871518	0.969928	ARHGAP3:	115703	Rho GTPase activating protein 33
ENSG0000	18.26429	0.015554	0.204034	0.871603	0.969931	ACSM3	6296	acyl-CoA synthetase medium chain family member 3
ENSG0000	31.463	-0.01507	0.206847	0.871685	0.969963	ZNF28	7576	zinc finger protein 28
ENSG0000	8.439529	-0.00311	0.226843	0.871819	0.970039	NA	NA	NA
ENSG0000	7.382301	0.011991	0.217839	0.87186	0.970039	NA	NA	NA
ENSG0000	3.006243	-0.00819	0.226007	0.872012	0.970091	CAP1P2	399748	CAP1 pseudogene 2
ENSG0000	279.5992	0.018228	0.163799	0.872001	0.970091	MALT1	10892	MALT1 paracaspase
ENSG0000	4.664484	0.003591	0.233504	0.872096	0.970126	SPACA3	124912	sperm acrosome associated 3
ENSG0000	79.13102	0.015823	0.201497	0.872222	0.970207	SLC25A24	29957	solute carrier family 25 member 24
ENSG0000	5.579561	-0.00226	0.229774	0.872464	0.970251	NA	NA	NA
ENSG0000	134.8396	0.018303	0.165833	0.872453	0.970251	GPN1	11321	GPN-loop GTPase 1
ENSG0000	54.27728	0.018001	0.183845	0.872474	0.970251	FNIP2	57600	folliculin interacting protein 2
ENSG0000	91.75368	0.013557	0.176111	0.872392	0.970251	WDR7	23335	WD repeat domain 7
ENSG0000	6.649381	0.018208	0.222107	0.872529	0.970253	NA	NA	NA
ENSG0000	6.820763	0.01061	0.221287	0.872595	0.970267	NA	NA	NA
ENSG0000	6.912946	0.013391	0.214136	0.873074	0.970549	NA	NA	NA
ENSG0000	8.606714	-0.01566	0.205438	0.873018	0.970549	LOC72986	729867	uncharacterized LOC729867
ENSG0000	84.54521	-0.01971	0.163423	0.873183	0.970549	DDR1	780	discoidin domain receptor tyrosine kinase 1
ENSG0000	3.508266	0.005792	0.228884	0.872989	0.970549	TTC36-AS1	1.02E+08	TTC36 and KMT2A antisense RNA 1
ENSG0000	46.19839	-0.01916	0.173949	0.873218	0.970549	KANSL1-A!	644246	KANSL1 antisense RNA 1
ENSG0000	1039.299	-0.01727	0.181836	0.872959	0.970549	EMILIN2	84034	elastin microfibril interfacer 2
ENSG0000	23616.54	-0.01774	0.152677	0.873144	0.970549	MSN	4478	moesin
ENSG0000	17.02983	-0.01574	0.203604	0.873332	0.970557	NA	NA	NA
ENSG0000	133.8225	0.018007	0.157547	0.873325	0.970557	CDR2	1039	cerebellar degeneration related protein 2
ENSG0000	211.1534	0.018754	0.173378	0.873443	0.970622	NECTIN1	5818	nectin cell adhesion molecule 1
ENSG0000	122.8621	-0.01805	0.175254	0.873506	0.970633	TASOR2	54906	transcription activation suppressor family member 2
ENSG0000	1355.279	0.01641	0.119604	0.873593	0.97067	GET3	439	guided ent ATPase
ENSG0000	3.249908	-0.01025	0.222924	0.873782	0.970801	NA	NA	NA
ENSG0000	13.73049	0.015276	0.205906	0.874626	0.970801	BTBD8	284697	BTB domain containing 8
ENSG0000	33.93395	-0.01728	0.186946	0.874332	0.970801	ANKRD36	375248	ankyrin repeat domain 36
ENSG0000	451.2698	-0.01666	0.120971	0.874048	0.970801	RPL32P3	132241	ribosomal protein L32 pseudogene 3
ENSG0000	4.589028	-0.01082	0.221622	0.873941	0.970801	NIM1K	167359	NIM1 serine/threonine protein kinase
ENSG0000	9.084197	0.015303	0.207311	0.874584	0.970801	POLR2J2	246721	RNA polymerase II subunit J2
ENSG0000	10.91247	-0.0123	0.216721	0.874213	0.970801	TRBV23-1	28564	T cell receptor beta variable 23-1 (non-functional)
ENSG0000	20.14071	-0.01236	0.212859	0.874445	0.970801	TMEM68	137695	transmembrane protein 68
ENSG0000	3104.56	-0.01579	0.158112	0.874665	0.970801	VPS28	51160	VPS28 subunit of ESCRT-I
ENSG0000	828.948	-0.01639	0.162754	0.874451	0.970801	NUP188	23511	nucleoporin 188
ENSG0000	28.6511	-0.01691	0.18974	0.874177	0.970801	APOLD1	81575	apolipoprotein L domain containing 1
ENSG0000	29.31886	-0.0157	0.197867	0.874227	0.970801	NA	NA	NA
ENSG0000	11.82582	-0.01593	0.201977	0.87389	0.970801	TRAV41	28640	T cell receptor alpha variable 41
ENSG0000	1536.463	-0.01687	0.13944	0.87462	0.970801	PITPNA	5306	phosphatidylinositol transfer protein alpha
ENSG0000	92.64117	-0.01791	0.173334	0.874429	0.970801	MRM1	79922	mitochondrial rRNA methyltransferase 1
ENSG0000	8.696823	0.007605	0.221723	0.874227	0.970801	NA	NA	NA
ENSG0000	43.91792	0.017563	0.182431	0.874342	0.970801	NA	NA	NA
ENSG0000	2546.601	0.016214	0.132739	0.874718	0.970801	PSMD8	5714	proteasom non-ATPase 8
ENSG0000	292.146	0.017971	0.145525	0.874642	0.970801	KLHL22	84861	kelch like family member 22
ENSG0000	252.8122	0.017265	0.141072	0.875535	0.970908	ATPAF1	64756	ATP synthase mitochondrial F1 complex assembly factor 1
ENSG0000	324.9032	0.018361	0.128128	0.874895	0.970908	SLC50A1	55974	solute carrier family 50 member 1
ENSG0000	151.9652	0.016778	0.174949	0.875477	0.970908	ACBD3	64746	acyl-CoA binding domain containing 3
ENSG0000	3.79896	-0.00584	0.228235	0.875464	0.970908	NEMP2-DT	1.05E+08	NEMP2 divergent transcript
ENSG0000	9.285143	0.013604	0.213396	0.875193	0.970908	NA	NA	NA
ENSG0000	30.15728	-0.01613	0.194873	0.875556	0.970908	CHEK1	1111	checkpoint kinase 1

ENSG0000	87.82925	0.017424	0.168102	0.875164	0.970908	COQ10A	93058	coenzyme Q10A
ENSG0000	226.5943	-0.01818	0.163581	0.875404	0.970908	IKBIP	121457	IKBKB interacting protein
ENSG0000	7.430782	0.010273	0.220423	0.875298	0.970908	NA	NA	NA
ENSG0000	18079.3	-0.01731	0.144397	0.875164	0.970908	SRRM2	23524	serine/arginine repetitive matrix 2
ENSG0000	2.942892	0.010244	0.22686	0.875065	0.970908	NA	NA	NA
ENSG0000	159.3773	-0.01722	0.143443	0.875051	0.970908	SPECC1L	23384	sperm antigen with calponin homology and coiled-coil domains 1 like
ENSG0000	5.869954	-0.00999	0.221909	0.875029	0.970908	NA	NA	NA
ENSG0000	1765.839	0.017294	0.184814	0.875454	0.970908	OGT	8473	O-linked N-acetylglucosamine (GlcNAc) transferase
ENSG0000	118.6597	-0.01999	0.161613	0.875711	0.970963	NDUFB1	4707	NADH:ubiquinone oxidoreductase subunit B1
ENSG0000	431.9049	-0.01612	0.118788	0.875692	0.970963	TXNL1	9352	thioredoxin like 1
ENSG0000	3235.497	-0.01712	0.140857	0.875829	0.971035	NUP214	8021	nucleoporin 214
ENSG0000	333.9283	-0.01388	0.139488	0.875889	0.971043	CACNB1	782	calcium voltage-gated channel auxiliary subunit beta 1
ENSG0000	25.18619	0.009914	0.220387	0.876013	0.971121	RPL11P3	1E+08	ribosomal protein L11 pseudogene 3
ENSG0000	51.04325	0.016029	0.197202	0.876141	0.971151	SLC33A1	9197	solute carrier family 33 member 1
ENSG0000	253.4279	-0.01607	0.187044	0.876146	0.971151	KIF2A	3796	kinesin family member 2A
ENSG0000	102.8497	-0.01743	0.175091	0.876602	0.971422	MMACHC	25974	metabolism of cobalamin associated C
ENSG0000	452.7321	0.018574	0.154265	0.87649	0.971422	AMD1	262	adenosylmethionine decarboxylase 1
ENSG0000	37.39642	-0.01782	0.176439	0.876569	0.971422	AKIP1	56672	A-kinase interacting protein 1
ENSG0000	3.932107	-0.01057	0.221145	0.876592	0.971422	KRT18P4	391256	keratin 18 pseudogene 4
ENSG0000	7.844607	-0.0046	0.226124	0.876819	0.971427	IL6	3569	interleukin 6
ENSG0000	20.78438	-0.01439	0.205828	0.876785	0.971427	ANKRD42-	1.01E+08	ANKRD42 divergent transcript
ENSG0000	712.8052	0.017605	0.176768	0.876689	0.971427	SPINT1	6692	serine pep Kunitz type 1
ENSG0000	82.84107	0.016748	0.165353	0.876726	0.971427	SLC9A3R2	9351	SLC9A3 regulator 2
ENSG0000	4.994984	-0.00357	0.225738	0.876886	0.971443	MTCO3P1	1.07E+08	MT-CO3 pseudogene 11
ENSG0000	6.389364	0.015839	0.224289	0.87695	0.971454	LINC0168	1.05E+08	long intergenic non-protein coding RNA 1684
ENSG0000	16.42143	0.015736	0.200702	0.877116	0.971579	SPIN2B	474343	spindlin family member 2B
ENSG0000	114.8694	-0.01698	0.141486	0.87758	0.97183	LINC0086	57234	long intergenic non-protein coding RNA 869
ENSG0000	3.201405	-0.00291	0.224638	0.877543	0.97183	NA	NA	NA
ENSG0000	4044.142	-0.01415	0.164563	0.877607	0.97183	OGDH	4967	oxoglutarate dehydrogenase
ENSG0000	104.114	-0.01756	0.143506	0.87753	0.97183	CFAP119	90835	cilia and flagella associated protein 119
ENSG0000	4.723644	-0.00968	0.223871	0.877513	0.97183	NA	NA	NA
ENSG0000	283.4238	0.018944	0.163852	0.877776	0.971945	PRPF4B	8899	pre-mRNA processing factor 4B
ENSG0000	229.7576	0.017827	0.161221	0.877818	0.971945	MMGT1	93380	membrane magnesium transporter 1
ENSG0000	6694.002	-0.01724	0.148152	0.87791	0.971988	FCMR	9214	Fc mu receptor
ENSG0000	3.448432	0.006167	0.228923	0.878021	0.972016	NA	NA	NA
ENSG0000	14.59364	-0.01414	0.204903	0.878041	0.972016	ME3	10873	malic enzyme 3
ENSG0000	15.19757	-0.01467	0.204076	0.878101	0.972024	HORMAD1	84072	HORMA domain containing 1
ENSG0000	1617.915	-0.01693	0.122464	0.878611	0.972097	PI4KB	5298	phosphatidylinositol 4-kinase beta
ENSG0000	187.3717	-0.01691	0.141516	0.878658	0.972097	TIMMDC1	51300	translocase of inner mitochondrial membrane domain containing 1
ENSG0000	9.598527	-0.01135	0.217207	0.878665	0.972097	HERC3	8916	HECT and RLD domain containing E3 ubiquitin protein ligase 3
ENSG0000	1529.404	0.013108	0.118693	0.878697	0.972097	GBA2	57704	glucosylceramidase beta 2
ENSG0000	8.153967	0.010277	0.221094	0.878629	0.972097	BCO2	83875	beta-carotene oxygenase 2
ENSG0000	6.05859	0.010651	0.220417	0.878397	0.972097	NA	NA	NA
ENSG0000	77.04942	-0.01499	0.193076	0.878466	0.972097	TDRD9	122402	tudor domain containing 9
ENSG0000	251.8827	-0.01589	0.126637	0.878687	0.972097	DEXI	28955	Dexi homolog
ENSG0000	56.37326	0.017291	0.168058	0.878577	0.972097	NA	NA	NA
ENSG0000	603.6531	0.017122	0.147402	0.878681	0.972097	FTSJ1	24140	FtsJ RNA 2'-O-methyltransferase 1
ENSG0000	74.22557	0.018141	0.153864	0.878757	0.972104	GET4	51608	guided entry of tail-anchored proteins factor 4
ENSG0000	1082.747	0.016735	0.165763	0.878829	0.972125	PRPF19	27339	pre-mRNA processing factor 19
ENSG0000	7305.271	0.017663	0.163665	0.879126	0.972336	PRRC2A	7916	proline rich coiled-coil 2A
ENSG0000	316.8229	-0.01585	0.128831	0.879091	0.972336	ING4	51147	inhibitor of growth family member 4
ENSG0000	874.1008	-0.0169	0.172516	0.879413	0.972432	POU2F1	5451	POU class 2 homeobox 1
ENSG0000	122.0206	-0.01788	0.148926	0.879313	0.972432	CEP68	23177	centrosomal protein 68
ENSG0000	169.2718	-0.0169	0.17527	0.880511	0.972432	GTDC1	79712	glycosyltransferase like domain containing 1
ENSG0000	707.9194	-0.01471	0.125361	0.880072	0.972432	COPS7B	64708	COP9 signalosome subunit 7B
ENSG0000	206.3618	-0.01734	0.168291	0.879519	0.972432	RAD54L2	23132	RAD54 like 2
ENSG0000	550.8426	-0.01692	0.152357	0.87951	0.972432	ABCF3	55324	ATP binding cassette subfamily F member 3
ENSG0000	3.25662	0.003937	0.233288	0.880407	0.972432	FOXQ1	94234	forkhead box Q1
ENSG0000	83.78589	0.017123	0.157107	0.880558	0.972432	FANCE	2178	FA complementation group E
ENSG0000	18.92386	0.015783	0.199086	0.880403	0.972432	DTX2P1-U	441263	DTX2P1-U transcribed pseudogene
ENSG0000	598.7695	0.015072	0.115105	0.880241	0.972432	XPO7	23039	exportin 7
ENSG0000	5.302288	-0.00644	0.227144	0.880498	0.972432	NA	NA	NA
ENSG0000	467.2688	-0.01615	0.140984	0.880199	0.972432	C8orf82	414919	chromosome 8 open reading frame 82
ENSG0000	771.2119	0.016318	0.135332	0.880299	0.972432	CDK9	1025	cyclin dependent kinase 9
ENSG0000	1104.979	-0.01908	0.165921	0.880401	0.972432	PDLIM1	9124	PDZ and LIM domain 1
ENSG0000	70.88484	-0.0209	0.201	0.880381	0.972432	PLEKHA1	59338	pleckstrin homology domain containing A1
ENSG0000	1161.462	-0.01752	0.169871	0.880114	0.972432	LTBP3	4054	latent transforming growth factor beta binding protein 3
ENSG0000	10.58532	0.014706	0.206126	0.880075	0.972432	NA	NA	NA
ENSG0000	1099.679	-0.01618	0.150045	0.879635	0.972432	KCNE3	10008	potassium voltage-gated channel subfamily E regulatory subunit 3
ENSG0000	4.223311	0.007379	0.226432	0.880544	0.972432	LINC02731	1E+08	long intergenic non-protein coding RNA 2731
ENSG0000	40.63462	0.017007	0.18577	0.880376	0.972432	NA	NA	NA
ENSG0000	4.622318	0.001616	0.228659	0.879681	0.972432	WSCD2	9671	WSC domain containing 2
ENSG0000	11.54684	-0.00918	0.220418	0.879726	0.972432	NA	NA	NA
ENSG0000	174.5785	0.016567	0.135017	0.880593	0.972432	METTL16	79066	methyltra N6-methyladenosine
ENSG0000	2.794748	-0.00846	0.225639	0.880282	0.972432	NA	NA	NA
ENSG0000	727.5935	0.014549	0.145353	0.879596	0.972432	ZNF414	84330	zinc finger protein 414
ENSG0000	449.116	0.016194	0.122772	0.879365	0.972432	IFT27	11020	intraflagellar transport 27
ENSG0000	37.96871	-0.01617	0.186643	0.880759	0.972556	NA	NA	NA
ENSG0000	119.316	-0.01721	0.170003	0.880842	0.972568	ASCC3	10973	activating signal cointegrator 1 complex subunit 3
ENSG0000	58.89341	-0.01395	0.202729	0.880875	0.972568	RPS27L	51065	ribosomal protein S27 like
ENSG0000	602.1381	-0.01561	0.122351	0.881	0.972631	MOB2	81532	MOB kinase activator 2
ENSG0000	415.4407	-0.02011	0.127698	0.881039	0.972631	ETHE1	23474	ETHE1 persulfide dioxygenase
ENSG0000	493.6547	-0.01609	0.131721	0.881211	0.972763	VDAC2	7417	voltage dependent anion channel 2
ENSG0000	307.9778	-0.01768	0.170547	0.881377	0.972887	TAOK1	57551	TAO kinase 1
ENSG0000	30.95138	0.01593	0.195174	0.881698	0.973124	TPPP	11076	tubulin polymerization promoting protein
ENSG0000	291.6033	-0.01715	0.161617	0.881675	0.973124	RGCC	28984	regulator of cell cycle
ENSG0000	21.87515	0.015996	0.193355	0.881754	0.973127	SGSM1	129049	small G protein signaling modulator 1
ENSG0000	28.18587	0.016732	0.186057	0.882566	0.97313	NA	NA	NA
ENSG0000	106.9357	0.017683	0.146396	0.882554	0.97313	SDCCAG8	10806	SHH signaling and ciliogenesis regulator SDCCAG8
ENSG0000	108.3991	0.01693	0.16326	0.882491	0.97313	PRKRA	8575	protein activator of interferon induced protein kinase EIF2AK2
ENSG0000	6.524834	0.007544	0.224793	0.882042	0.97313	NA	NA	NA
ENSG0000	10.83379	0.009623	0.221304	0.882589	0.97313	NEIL3	55247	nei like DNA glycosylase 3
ENSG0000	3.003337	-0.00581	0.228704	0.881912	0.97313	LINC0136	257358	long intergenic non-protein coding RNA 1366
ENSG0000	204.8392	0.017259	0.175168	0.881982	0.97313	MAP3K7	6885	mitogen-activated protein kinase kinase kinase 7
ENSG0000	39.71773	-0.01664	0.177096	0.882302	0.97313	NA	NA	NA
ENSG0000	15.63849	-0.01448	0.203101	0.882224	0.97313	NA	NA	NA

ENSG0000	1551.585	0.016618	0.184753	0.882236	0.97313	CLEC4E	26253	C-type lectin domain family 4 member E
ENSG0000	5.74505	0.012911	0.219642	0.882606	0.97313	TRDV2	28517	T cell receptor delta variable 2
ENSG0000	41.8786	-0.01742	0.180456	0.882505	0.97313	IFI27L1	122509	interferon alpha inducible protein 27 like 1
ENSG0000	179.4205	-0.01674	0.144553	0.881904	0.97313	NA	NA	NA
ENSG0000	307.111	0.010575	0.135217	0.88229	0.97313	SUMO2	6613	small ubiquitin like modifier 2
ENSG0000	8.808404	0.012392	0.214101	0.88203	0.97313	NA	NA	NA
ENSG0000	44.21727	-0.0168	0.173626	0.882409	0.97313	TREX2	11219	three prime repair exonuclease 2
ENSG0000	31.12096	-0.0132	0.208431	0.883013	0.973335	IGKV1D-3:	28896	immunoglobulin kappa variable 1D-33
ENSG0000	70.24729	0.016776	0.182323	0.883412	0.973335	UMPS	7372	uridine monophosphate synthetase
ENSG0000	102.6247	-0.0169	0.174118	0.883248	0.973335	RNGTT	8732	RNA guanylyltransferase and 5'-phosphatase
ENSG0000	24.2263	0.013097	0.204785	0.883299	0.973335	FANCC	2176	FA complementation group C
ENSG0000	71.88734	0.013965	0.203666	0.882849	0.973335	C12orf4	57102	chromosome 12 open reading frame 4
ENSG0000	4.967697	0.01082	0.220102	0.883397	0.973335	GOLGA8R	1.01E+08	golgin A8 family member R
ENSG0000	338.694	0.017792	0.144538	0.883098	0.973335	PLA2G15	23659	phospholipase A2 group XV
ENSG0000	1996.271	0.016817	0.147173	0.883262	0.973335	MNT	4335	MAX network transcriptional repressor
ENSG0000	791.5411	-0.01617	0.150811	0.88343	0.973335	TMEM104	54868	transmembrane protein 104
ENSG0000	33.05788	0.014118	0.19996	0.882962	0.973335	TWSG1	57045	twisted gastrulation BMP signaling modulator 1
ENSG0000	45.18059	0.017758	0.175423	0.883206	0.973335	C19orf44	84167	chromosome 19 open reading frame 44
ENSG0000	645.6272	0.01572	0.181491	0.883415	0.973335	DOCK11	139818	dedicator of cytokinesis 11
ENSG0000	4605.841	0.015945	0.130599	0.8835	0.973354	USF2	7392	upstream c-fos interacting
ENSG0000	95.81053	-0.01638	0.168626	0.88362	0.973369	NA	NA	NA
ENSG0000	1399.059	0.017521	0.14547	0.883568	0.973369	C9orf78	51759	chromosome 9 open reading frame 78
ENSG0000	175.4975	0.017773	0.172092	0.883797	0.973451	USP38	84640	ubiquitin specific peptidase 38
ENSG0000	3.839635	-0.00624	0.228015	0.883801	0.973451	NA	NA	NA
ENSG0000	2.379534	-0.00805	0.228555	0.88397	0.9735	C2orf50	130813	chromosome 2 open reading frame 50
ENSG0000	34.43987	-0.01582	0.184186	0.884004	0.9735	CLCN2	1181	chloride voltage-gated channel 2
ENSG0000	620.9655	-0.01347	0.116288	0.883932	0.9735	DYNLL1	8655	dynein light chain LC8-type 1
ENSG0000	18.39345	0.013785	0.197399	0.884111	0.973553	PER3	8863	period circadian regulator 3
ENSG0000	7.767622	0.01001	0.219789	0.884159	0.973553	CYP2B7P	1556	cytochrom pseudogene
ENSG0000	57.1772	0.018493	0.161111	0.884405	0.973765	ZCCHC9	84240	zinc finger CCHC-type containing 9
ENSG0000	516.1671	0.013938	0.109243	0.885858	0.973816	NA	NA	NA
ENSG0000	200.6617	0.015175	0.188907	0.88636	0.973816	CD58	965	CD58 molecule
ENSG0000	407.8312	0.016969	0.146912	0.886208	0.973816	TOR3A	64222	torsin family 3 member A
ENSG0000	304.262	0.015583	0.152215	0.885207	0.973816	RCOR3	55758	REST corepressor 3
ENSG0000	19.48327	0.008755	0.215249	0.885049	0.973816	IGKJ1	28950	immunoglobulin kappa joining 1
ENSG0000	563.0789	-0.01557	0.147515	0.88619	0.973816	EIF5B	9669	eukaryotic translation initiation factor 5B
ENSG0000	159.2609	-0.00934	0.196031	0.886373	0.973816	CRBN	51185	cereblon
ENSG0000	2.454632	-0.00219	0.231426	0.886155	0.973816	NA	NA	NA
ENSG0000	4.542265	0.009042	0.224394	0.884727	0.973816	SMIM14-D	1.05E+08	SMIM14 divergent transcript
ENSG0000	515.0503	-0.01674	0.15496	0.885453	0.973816	SNX2	6643	sorting nexin 2
ENSG0000	26.28265	0.012744	0.209928	0.886195	0.973816	IGIP	492311	IgA inducing protein
ENSG0000	5.99201	-0.00477	0.228859	0.885374	0.973816	INSYN2B	1E+08	inhibitory synaptic factor family member 2B
ENSG0000	476.0354	-0.01542	0.125539	0.88452	0.973816	C6orf136	221545	chromosome 6 open reading frame 136
ENSG0000	4.727971	-0.01053	0.217853	0.88631	0.973816	NA	NA	NA
ENSG0000	9.792557	0.01175	0.214055	0.886229	0.973816	NA	NA	NA
ENSG0000	183.42	-0.01597	0.150249	0.885481	0.973816	TNKS	8658	tankyrase
ENSG0000	111.0845	-0.0157	0.154797	0.885212	0.973816	STAU2	27067	staufen double-stranded RNA binding protein 2
ENSG0000	5.791437	-0.00668	0.225316	0.886418	0.973816	EPPK1	83481	epiplakin 1
ENSG0000	72.20597	0.018295	0.168118	0.885842	0.973816	TRDMT1	1787	tRNA aspartic acid methyltransferase 1
ENSG0000	8983.983	-0.0157	0.170141	0.886217	0.973816	TALDO1	6888	transaldolase 1
ENSG0000	764.0432	0.016976	0.147	0.88544	0.973816	MADD	8567	MAP kinase activating death domain
ENSG0000	287.1183	0.016529	0.148885	0.885284	0.973816	ZNRD2	10534	zinc ribbon domain containing 2
ENSG0000	3.177875	0.012295	0.22949	0.885051	0.973816	LAYN	143903	layilin
ENSG0000	1392.374	0.015709	0.183491	0.886093	0.973816	HYOU1	10525	hypoxia up-regulated 1
ENSG0000	620.5376	-0.0161	0.154415	0.885919	0.973816	VDR	7421	vitamin D receptor
ENSG0000	1262.407	0.013288	0.168842	0.885317	0.973816	ESYT1	23344	extended synaptotagmin 1
ENSG0000	40.92324	-0.01579	0.189493	0.884754	0.973816	SAV1	60485	salvador family WW domain containing protein 1
ENSG0000	42.30173	-0.0162	0.181532	0.885271	0.973816	MIR4537	1.01E+08	microRNA 4537
ENSG0000	485.9954	0.014768	0.130997	0.884691	0.973816	IMP3	55272	IMP U3 small nucleolar ribonucleoprotein 3
ENSG0000	2.51533	-3.11E-04	0.228673	0.88596	0.973816	NA	NA	NA
ENSG0000	32.51739	-0.0155	0.191362	0.886142	0.973816	PRSS27	83886	serine protease 27
ENSG0000	3.626009	0.008778	0.223831	0.886269	0.973816	NA	NA	NA
ENSG0000	4.806384	-0.00983	0.220997	0.886039	0.973816	NA	NA	NA
ENSG0000	66.02031	-0.0161	0.184219	0.884992	0.973816	MT1E	4493	metallothionein 1E
ENSG0000	34.34702	0.013676	0.203582	0.885859	0.973816	RAD51C	5889	RAD51 paralog C
ENSG0000	36.76188	0.014871	0.202393	0.885462	0.973816	ZNF507	22847	zinc finger protein 507
ENSG0000	20.8796	0.018702	0.191454	0.88586	0.973816	ZNF347	84671	zinc finger protein 347
ENSG0000	24.34706	0.015214	0.193053	0.887017	0.974416	FRRS1	391059	ferric chelate reductase 1
ENSG0000	547.3828	-0.016	0.16187	0.887275	0.974431	ZNF777	27153	zinc finger protein 777
ENSG0000	1596.564	0.016085	0.150374	0.887093	0.974431	ZNF746	155061	zinc finger protein 746
ENSG0000	31.82925	0.015669	0.189263	0.887243	0.974431	LNX2	222484	ligand of numb-protein X 2
ENSG0000	17.08646	0.014694	0.199453	0.88741	0.974431	LOC10537	1.05E+08	uncharacterized LOC105370203
ENSG0000	13.85769	0.01186	0.213795	0.887217	0.974431	NA	NA	NA
ENSG0000	3.808402	-0.00461	0.229817	0.88738	0.974431	NA	NA	NA
ENSG0000	18.20597	0.010755	0.215533	0.88742	0.974431	PTPRM	5797	protein tyrosine phosphatase receptor type M
ENSG0000	169.733	-0.015	0.139885	0.887457	0.974431	RAB22A	57403	RAB22A member RAS oncogene family
ENSG0000	627.885	-0.01432	0.143137	0.887554	0.97448	MPI	4351	mannose phosphate isomerase
ENSG0000	129.0278	-0.0168	0.166582	0.887674	0.974504	SLC29A1	2030	solute carrier family 29 member 1 (Augustine blood group)
ENSG0000	981.4814	0.018316	0.150701	0.887683	0.974504	NCOA6	23054	nuclear receptor coactivator 6
ENSG0000	85.79564	-0.01605	0.164825	0.887855	0.974529	C1orf52	148423	chromosome 1 open reading frame 52
ENSG0000	166.5925	0.016952	0.130725	0.888075	0.974529	ATAT1	79969	alpha tubulin acetyltransferase 1
ENSG0000	22.35156	-0.01571	0.184171	0.887892	0.974529	LINC0300€	441242	long intergenic non-protein coding RNA 3006
ENSG0000	62.00021	-0.01426	0.192462	0.887928	0.974529	HGF	3082	hepatocyte growth factor
ENSG0000	45.72794	0.014862	0.192673	0.8883	0.974529	LOC10272	1.03E+08	uncharacterized LOC102723566
ENSG0000	157.9301	0.016711	0.144018	0.888401	0.974529	DOLPP1	57171	dolichyldiphosphatase 1
ENSG0000	34.02234	0.015529	0.188592	0.88828	0.974529	ANKRD26	22852	ankyrin repeat domain containing 26
ENSG0000	3.840527	0.010071	0.224323	0.888273	0.974529	NA	NA	NA
ENSG0000	3.971793	-0.00561	0.227699	0.888357	0.974529	GRIK4	2900	glutamate ionotropic receptor kainate type subunit 4
ENSG0000	47.82258	0.014516	0.194642	0.888257	0.974529	FBF1	85302	Fas binding factor 1
ENSG0000	6.097994	0.010307	0.218917	0.888169	0.974529	CDK3	1018	cyclin dependent kinase 3
ENSG0000	1013.043	-0.01708	0.153766	0.88825	0.974529	SLC1A5	6510	solute carrier family 1 member 5
ENSG0000	27.73849	-0.0139	0.197086	0.888451	0.974529	FAM209B	388799	family with sequence similarity 209 member B
ENSG0000	3.304901	-0.0074	0.225599	0.888393	0.974529	LOC10192	1.02E+08	uncharacterized LOC101927745
ENSG0000	614.3948	0.01592	0.142193	0.888659	0.974641	DNAJB6	10049	DnaJ heat shock protein family (Hsp40) member B6
ENSG0000	44.14509	0.015942	0.179049	0.888632	0.974641	ICAM4	3386	intercellular adhesion molecule 4 (Landsteiner-Wiener blood group)
ENSG0000	27.1204	-0.00946	0.216747	0.888978	0.974683	DYRK3	8444	dual specificity tyrosine phosphorylation regulated kinase 3

ENSG0000	384.6306	-0.02289	0.140101	0.889017	0.974683	KIDINS220	57498	kinase D interacting substrate 220
ENSG0000	85.17285	-0.01744	0.165175	0.88887	0.974683	MRPL19	9801	mitochondrial ribosomal protein L19
ENSG0000	10.20502	0.010861	0.218069	0.888928	0.974683	BBS5	129880	Bardet-Biedl syndrome 5
ENSG0000	375.949	0.014272	0.142254	0.888987	0.974683	BUB3	9184	BUB3 mitotic checkpoint protein
ENSG0000	20.319	0.014495	0.192404	0.888912	0.974683	UBE2V1	7335	ubiquitin conjugating enzyme E2 V1
ENSG0000	3.085114	0.007364	0.226302	0.889095	0.974711	NA	NA	NA
ENSG0000	67.64188	-0.01403	0.1937	0.889235	0.974742	KANSL1L	151050	KAT8 regulatory NSL complex subunit 1 like
ENSG0000	375.2172	0.018216	0.137722	0.889229	0.974742	NOLC1	9221	nucleolar and coiled-body phosphoprotein 1
ENSG0000	11.96012	-0.0141	0.201315	0.889283	0.974742	TINCR	257000	TINCR ubiquitin domain containing
ENSG0000	242.1097	-0.01271	0.125609	0.889432	0.974842	ICMT	23463	isoprenylcysteine carboxyl methyltransferase
ENSG0000	4.584787	0.006035	0.226837	0.889605	0.974842	LNP1	348801	leukemia NUP98 fusion partner 1
ENSG0000	259.4516	0.015426	0.178298	0.889694	0.974842	SHROOM1	134549	shroom family member 1
ENSG0000	14.04881	-0.01284	0.205827	0.889607	0.974842	PLSCR3	57048	phospholipid scramblase 3
ENSG0000	5.805488	0.007828	0.224387	0.889693	0.974842	NA	NA	NA
ENSG0000	65.75525	-0.01597	0.164449	0.889496	0.974842	TRAPPC2	6399	trafficking protein particle complex subunit 2
ENSG0000	2653.391	-0.01408	0.175736	0.889785	0.974844	QSOX1	5768	quiescin sulfhydryl oxidase 1
ENSG0000	565.3675	0.015564	0.137901	0.889802	0.974844	FBXL15	79176	F-box and leucine rich repeat protein 15
ENSG0000	5.82716	0.001474	0.230148	0.890068	0.974902	IGKJ5	28946	immunoglobulin kappa joining 5
ENSG0000	115.9136	0.025864	0.184304	0.889975	0.974902	TMEM87B	84910	transmembrane protein 87B
ENSG0000	144.8361	0.015878	0.193409	0.889956	0.974902	ATF2	1386	activating transcription factor 2
ENSG0000	3.075019	-0.00781	0.224824	0.890066	0.974902	NA	NA	NA
ENSG0000	3.409413	0.00456	0.228202	0.890152	0.974936	ADAMDEC	27299	ADAM like decysin 1
ENSG0000	2.803185	0.004093	0.231499	0.890374	0.97512	CGREF1	10669	cell growth regulator with EF-hand domain 1
ENSG0000	34.5053	-0.01636	0.178302	0.890496	0.975196	SOCS2-AS:	144481	SOCS2 antisense RNA 1
ENSG0000	9.781888	0.01276	0.209048	0.890811	0.975482	FAM72A	729533	family with sequence similarity 72 member A
ENSG0000	9.460229	0.014935	0.209197	0.890903	0.975525	TRAV22	28661	T cell receptor alpha variable 22
ENSG0000	11.23277	0.012391	0.209121	0.891432	0.975583	NA	NA	NA
ENSG0000	3.95491	0.004888	0.2282	0.891662	0.975583	ZMYND12	84217	zinc finger MYND-type containing 12
ENSG0000	124.2779	-0.01374	0.153416	0.891643	0.975583	RTCA	8634	RNA 3'-terminal phosphate cyclase
ENSG0000	106.7255	0.015769	0.156644	0.891232	0.975583	FAM86DP	692099	family witl pseudogene
ENSG0000	185.3403	-0.01933	0.159111	0.891229	0.975583	PIK3CB	5291	phosphati 5-bisphosphate 3-kinase catalytic subunit beta
ENSG0000	5.002962	-0.00929	0.220544	0.891442	0.975583	NA	NA	NA
ENSG0000	75.66345	0.016458	0.149804	0.891594	0.975583	ERCC6	2074	ERCC excis chromatin remodeling factor
ENSG0000	342.9338	0.01467	0.127523	0.891558	0.975583	TMEM138	51524	transmembrane protein 138
ENSG0000	11.38833	0.011463	0.212962	0.891087	0.975583	LOC10024	1E+08	uncharacterized LOC100240735
ENSG0000	1056.439	0.014747	0.157501	0.891384	0.975583	ACOX1	51	acyl-CoA oxidase 1
ENSG0000	2.58545	0.009306	0.230773	0.891701	0.975583	LINC0197:	400624	long intergenic non-protein coding RNA 1973
ENSG0000	227.2334	0.016236	0.152237	0.891577	0.975583	GCDH	2639	glutaryl-CoA dehydrogenase
ENSG0000	5.633367	0.009395	0.224433	0.891234	0.975583	NA	NA	NA
ENSG0000	742.4557	0.013912	0.11793	0.891187	0.975583	FAM3A	60343	FAM3 metabolism regulating signaling molecule A
ENSG0000	385.7563	-0.01483	0.137547	0.891828	0.975663	RASSF7	8045	Ras association domain family member 7
ENSG0000	20.44859	-0.01287	0.203805	0.89195	0.975688	E2F6	1876	E2F transcription factor 6
ENSG0000	373.1299	0.015235	0.181901	0.892007	0.975688	CGGBP1	8545	CGG triplet repeat binding protein 1
ENSG0000	272.886	-0.01715	0.141801	0.892011	0.975688	CABLES2	81928	Cdk5 and Abl enzyme substrate 2
ENSG0000	4.737953	0.005458	0.228401	0.892149	0.975708	NRG2	9542	neuregulin 2
ENSG0000	3.917754	-0.00621	0.230128	0.892126	0.975708	CLEC18C	283971	C-type lectin domain family 18 member C
ENSG0000	2568.789	0.014421	0.128244	0.892188	0.975708	EMC10	284361	ER membrane protein complex subunit 10
ENSG0000	16.79423	-0.0125	0.205225	0.892277	0.975746	RAC3	5881	Rac family small GTPase 3
ENSG0000	747.7305	0.013543	0.124748	0.892786	0.975779	DCAF8	50717	DDB1 and CUL4 associated factor 8
ENSG0000	7.193887	0.008693	0.221431	0.892693	0.975779	ZNF670	93474	zinc finger protein 670
ENSG0000	2.351341	-0.00663	0.229168	0.892684	0.975779	NA	NA	NA
ENSG0000	2.584782	-0.00756	0.228543	0.892475	0.975779	NA	NA	NA
ENSG0000	116.4666	-0.01535	0.168487	0.892465	0.975779	NA	NA	NA
ENSG0000	15.7927	0.013211	0.204109	0.892551	0.975779	NA	NA	NA
ENSG0000	169.4801	0.016187	0.161277	0.892635	0.975779	NA	NA	NA
ENSG0000	563.4019	-0.01381	0.128773	0.892748	0.975779	SEC23B	10483	SEC23 hon COPII coat complex component
ENSG0000	465.3214	-0.01406	0.130059	0.892423	0.975779	CRKL	1399	CRK like pı adaptor protein
ENSG0000	12.01722	0.011725	0.211944	0.893654	0.975935	CD34	947	CD34 molecule
ENSG0000	11.76799	-0.01276	0.210395	0.893119	0.975935	RPL18AP6	285053	ribosomal protein L18a pseudogene 6
ENSG0000	61.72165	-0.01562	0.176256	0.893322	0.975935	ZNF2	7549	zinc finger protein 2
ENSG0000	7.885607	-0.0081	0.222492	0.893299	0.975935	NA	NA	NA
ENSG0000	103.1102	-0.01483	0.175094	0.893614	0.975935	ANKRD28	23243	ankyrin repeat domain 28
ENSG0000	94.48225	0.015721	0.162731	0.894182	0.975935	ST3GAL6	10402	ST3 beta-g 3-sialyltransferase 6
ENSG0000	17.78298	-0.01202	0.203693	0.89357	0.975935	NA	NA	NA
ENSG0000	29.3161	0.015018	0.186287	0.893249	0.975935	NOTCH4	4855	notch receptor 4
ENSG0000	3.728186	-0.00387	0.230877	0.893571	0.975935	RNF216-IT	1.01E+08	RNF216 intronic transcript 1
ENSG0000	299.3447	0.015645	0.153302	0.893884	0.975935	AVL9	23080	AVL9 cell migration associated
ENSG0000	53.40166	0.015088	0.169188	0.893904	0.975935	NA	NA	NA
ENSG0000	157.3812	-0.01426	0.167728	0.893471	0.975935	TOPORS	10210	TOP1 bind E3 ubiquitin ligase
ENSG0000	11.46597	-0.01057	0.212104	0.894009	0.975935	NUTM2B	729262	NUT family member 2B
ENSG0000	1068.055	0.011405	0.1058	0.894208	0.975935	CAPRIN1	4076	cell cycle associated protein 1
ENSG0000	371.7219	0.015089	0.147828	0.894138	0.975935	TXNRD1	7296	thioredoxin reductase 1
ENSG0000	83.55033	-0.01217	0.198651	0.893398	0.975935	NA	NA	NA
ENSG0000	3.666962	-0.0061	0.227595	0.894035	0.975935	ARHGAP1:	89839	Rho GTPase activating protein 11B
ENSG0000	42.85613	0.015723	0.172378	0.893035	0.975935	AVEN	57099	apoptosis and caspase activation inhibitor
ENSG0000	51.6193	0.014313	0.191193	0.894173	0.975935	NLGN2	57555	neuroligin 2
ENSG0000	179.5262	-0.0147	0.159522	0.894118	0.975935	NEK8	284086	NIMA related kinase 8
ENSG0000	310.7336	0.017157	0.122918	0.893436	0.975935	NT5C	30833	5' 3'-nucleot cytosolic
ENSG0000	16.8816	-0.00926	0.216846	0.893144	0.975935	ZNF17	7565	zinc finger protein 17
ENSG0000	50.36206	-0.01486	0.15965	0.893988	0.975935	ZNF329	79673	zinc finger protein 329
ENSG0000	17.84455	0.010431	0.215146	0.894166	0.975935	IGLVI-70	28763	immunoglobulin lambda variable (I)-70 (pseudogene)
ENSG0000	1423.131	-0.01429	0.134232	0.894317	0.975997	TMEM175	84286	transmembrane protein 175
ENSG0000	453.8875	0.015136	0.169934	0.895219	0.976204	SERINC2	347735	serine incorporator 2
ENSG0000	543.7076	0.020535	0.145278	0.89469	0.976204	EPS15	2060	epidermal growth factor receptor pathway substrate 15
ENSG0000	79.25443	-0.01404	0.177593	0.895748	0.976204	PRDX6-AS	1.02E+08	PRDX6 antisense RNA 1
ENSG0000	2.705177	0.006409	0.226964	0.895348	0.976204	GREM2	64388	gremlin 2 DAN family BMP antagonist
ENSG0000	141.5105	-0.01473	0.183298	0.896619	0.976204	ARL6IP6	151188	ADP ribosylation factor like GTPase 6 interacting protein 6
ENSG0000	13.34807	-0.01078	0.213202	0.895251	0.976204	RN7SL172	1.06E+08	RNA 7SL cytoplasm pseudogene
ENSG0000	41.89613	-0.01315	0.197111	0.896153	0.976204	SEPSECS	51091	Sep (O-phosphoserine) tRNA:Sec (selenocysteine) tRNA synthase
ENSG0000	220.4431	-0.01412	0.160826	0.895753	0.976204	LARP7	51574	La ribonuc transcriptional regulator
ENSG0000	538.5574	0.013076	0.137435	0.895862	0.976204	CUL9	23113	cullin 9
ENSG0000	6.592395	0.007236	0.224511	0.895265	0.976204	NA	NA	NA
ENSG0000	166.3496	-0.01794	0.167808	0.895219	0.976204	MED23	9439	mediator complex subunit 23
ENSG0000	4041.058	0.015264	0.167253	0.895657	0.976204	TNRC18	84629	trinucleotide repeat containing 18
ENSG0000	4.721774	0.009836	0.219104	0.896411	0.976204	NA	NA	NA
ENSG0000	4.485579	-0.00507	0.228104	0.896639	0.976204	LINC03022	1.02E+08	long intergenic non-protein coding RNA 3022

ENSG0000	10.21151	0.010776	0.215641	0.89653	0.976204	NA	NA	NA
ENSG0000	2.886207	-0.00121	0.232254	0.895409	0.976204	NA	NA	NA
ENSG0000	84.95728	0.015139	0.171815	0.894707	0.976204	CHCHD1	118487	coiled-coil-helix-coiled-coil-helix domain containing 1
ENSG0000	77.24023	0.013929	0.192849	0.894832	0.976204	CUTC	51076	cutC copper transporter
ENSG0000	27.56793	0.014962	0.194181	0.896259	0.976204	PLEKHA7	144100	pleckstrin homology domain containing A7
ENSG0000	1191.187	-0.01491	0.153641	0.895188	0.976204	ETS1	2113	ETS proto- transcription factor
ENSG0000	219.9857	0.014384	0.188543	0.895978	0.976204	FBXL3	26224	F-box and leucine rich repeat protein 3
ENSG0000	33.28086	-0.01429	0.181271	0.895818	0.976204	TRAV29DV	28653	T cell receptor alpha variable 29/delta variable 5
ENSG0000	24.33789	-0.01336	0.191282	0.895635	0.976204	LYSET	26175	lysosomal enzyme trafficking factor
ENSG0000	612.1662	0.009721	0.121085	0.895363	0.976204	SMAD3	4088	SMAD family member 3
ENSG0000	133.5399	-0.01536	0.149746	0.896014	0.976204	RPP25	54913	ribonuclease P and MRP subunit p25
ENSG0000	278.4369	0.017494	0.154694	0.895456	0.976204	ABAT	18	4-aminobutyrate aminotransferase
ENSG0000	310.8979	0.012739	0.138503	0.896313	0.976204	BFAR	51283	bifunctional apoptosis regulator
ENSG0000	11.53112	-0.00741	0.222614	0.895329	0.976204	GPRC5B	51704	G protein-coupled receptor class C group 5 member B
ENSG0000	162.6756	-0.01716	0.128198	0.895584	0.976204	CFAP20	29105	cilia and flagella associated protein 20
ENSG0000	3.387923	-0.00644	0.226956	0.895411	0.976204	NA	NA	NA
ENSG0000	14.66539	-0.00947	0.217984	0.895808	0.976204	ADGRE4P	326342	adhesion (pseudogene
ENSG0000	23.61102	-0.01325	0.201376	0.895723	0.976204	NA	NA	NA
ENSG0000	5.606604	0.009989	0.217881	0.896633	0.976204	BRME1	79173	break repair meiotic recombinase recruitment factor 1
ENSG0000	1116.993	-0.01444	0.130776	0.896634	0.976204	SUGP1	57794	SURP and G-patch domain containing 1
ENSG0000	2.853055	0.008331	0.224351	0.895974	0.976204	NA	NA	NA
ENSG0000	737.2114	0.014181	0.173106	0.894756	0.976204	TMEM91	641649	transmembrane protein 91
ENSG0000	16.01453	0.013596	0.197712	0.896507	0.976204	PINLYP	390940	phospholipase A2 inhibitor and LY6/PLAUR domain containing
ENSG0000	3.377635	-2.83E-04	0.226873	0.896285	0.976204	NA	NA	NA
ENSG0000	9.234161	0.011688	0.210157	0.896158	0.976204	DDTL	1E+08	D-dopachrome tautomerase like
ENSG0000	192118	-0.01459	0.155166	0.895405	0.976204	ND1	4535	NADH dehydrogenase subunit 1
ENSG0000	358.6211	-0.01682	0.154177	0.896759	0.976209	PDS5A	23244	PDS5 cohesin associated factor A
ENSG0000	980.6352	-0.01454	0.165313	0.896803	0.976209	QKI	9444	QKI KH domain containing RNA binding
ENSG0000	1289.82	0.015456	0.154605	0.896776	0.976209	CHERP	10523	calcium homeostasis endoplasmic reticulum protein
ENSG0000	87.08445	0.013636	0.193172	0.897058	0.976254	PANK3	79646	pantothenate kinase 3
ENSG0000	13.30411	0.014101	0.200591	0.897053	0.976254	LOC12490	1.25E+08	uncharacterized LOC124901151
ENSG0000	81.9268	0.008273	0.222424	0.896943	0.976254	ZNF518A	9849	zinc finger protein 518A
ENSG0000	446.8502	0.013134	0.114953	0.896996	0.976254	ZDHHC24	254359	zinc finger DHHC-type containing 24
ENSG0000	45.5873	-0.01487	0.170332	0.897155	0.976301	RCCD1	91433	RCC1 domain containing 1
ENSG0000	30.92896	-0.01098	0.209141	0.897378	0.976394	DPH5-DT	1.03E+08	DPH5 divergent transcript
ENSG0000	2.778825	-0.00362	0.229138	0.897453	0.976394	NA	NA	NA
ENSG0000	7.574696	-0.00936	0.219554	0.897413	0.976394	NEURL1B	54492	neuralized E3 ubiquitin protein ligase 1B
ENSG0000	11.19661	-0.0099	0.215429	0.897358	0.976394	MTCO2P3	1.07E+08	MT-CO2 pseudogene 33
ENSG0000	279.2483	0.012644	0.191779	0.897629	0.976498	CNOT6L	246175	CCR4-NOT transcription complex subunit 6 like
ENSG0000	32.11477	-0.01043	0.207856	0.897708	0.976498	MSR1	4481	macrophage scavenger receptor 1
ENSG0000	172.0029	-0.01382	0.145864	0.8977	0.976498	DRG1	4733	developmentally regulated GTP binding protein 1
ENSG0000	160.7086	0.016707	0.150586	0.897884	0.97663	STAM	8027	signal transducing adaptor molecule
ENSG0000	11.25186	0.013172	0.215008	0.898467	0.976766	PM20D1	148811	peptidase M20 domain containing 1
ENSG0000	2.615809	0.004197	0.228923	0.898298	0.976766	PLOD2	5352	procollage 2-oxoglutarate 5-dioxygenase 2
ENSG0000	9.03901	-0.00943	0.217484	0.898574	0.976766	NA	NA	NA
ENSG0000	20.47829	0.012146	0.206034	0.898361	0.976766	LINC0236	728175	long intergenic non-protein coding RNA 2363
ENSG0000	1950.894	-0.01382	0.13901	0.898595	0.976766	ELMO1	9844	engulfment and cell motility 1
ENSG0000	308.6151	-0.01353	0.129033	0.898276	0.976766	MFSD14CI	84278	major facil pseudogene
ENSG0000	23.94942	-0.01286	0.198227	0.898398	0.976766	C12orf29	91298	chromosome 12 open reading frame 29
ENSG0000	2.86204	0.005533	0.228617	0.898492	0.976766	ZNF23	7571	zinc finger protein 23
ENSG0000	264.1573	-0.00977	0.132308	0.898546	0.976766	GOSR2	9570	golgi SNAP receptor complex member 2
ENSG0000	166.9865	-0.01395	0.136123	0.898169	0.976766	PNPO	55163	pyridoxamine 5'-phosphate oxidase
ENSG0000	39.97593	0.01456	0.176093	0.898433	0.976766	ZNF416	55659	zinc finger protein 416
ENSG0000	9.958504	-0.01053	0.211568	0.898866	0.976915	FDXACB1	91893	ferredoxin-fold anticodon binding domain containing 1
ENSG0000	8.773392	0.00957	0.213749	0.898892	0.976915	NA	NA	NA
ENSG0000	932.9051	0.015182	0.150875	0.898803	0.976915	GRAP	10750	GRB2 related adaptor protein
ENSG0000	4.994563	0.007663	0.226331	0.89919	0.977011	NA	NA	NA
ENSG0000	19.49253	0.013147	0.198619	0.899069	0.977011	RNF139-D	1.02E+08	RNF139 divergent transcript
ENSG0000	349.5944	0.015598	0.16212	0.899193	0.977011	GABARAPI	23710	GABA type A receptor associated protein like 1
ENSG0000	1761.741	-0.01598	0.117191	0.899187	0.977011	SNX1	6642	sorting nexin 1
ENSG0000	7.260897	0.007592	0.223514	0.899339	0.97708	ZNF300	91975	zinc finger protein 300
ENSG0000	644.9395	0.020476	0.144467	0.899364	0.97708	UNKL	64718	unk like zinc finger
ENSG0000	3257.558	0.022251	0.136666	0.901368	0.977161	SDF4	51150	stromal cell derived factor 4
ENSG0000	310.5321	-0.01375	0.168691	0.901137	0.977161	AHDC1	27245	AT-hook DNA binding motif containing 1
ENSG0000	259.8522	0.014618	0.131322	0.899972	0.977161	INPP5B	3633	inositol polyphosphate-5-phosphatase B
ENSG0000	35.90144	-0.01312	0.197388	0.901263	0.977161	MCOLN2	255231	mucolipin TRP cation channel 2
ENSG0000	2457.869	0.009477	0.120787	0.899729	0.977161	MEF2D	4209	myocyte enhancer factor 2D
ENSG0000	10.05742	-0.0105	0.211609	0.900022	0.977161	NA	NA	NA
ENSG0000	15.22478	0.011727	0.205023	0.901332	0.977161	NA	NA	NA
ENSG0000	6.301132	-0.00924	0.219598	0.900433	0.977161	NA	NA	NA
ENSG0000	7.131795	-0.0058	0.224873	0.900514	0.977161	NA	NA	NA
ENSG0000	3.123455	0.007182	0.22481	0.900103	0.977161	WWTR1	25937	WW domain containing transcription regulator 1
ENSG0000	538.4635	-0.01363	0.151453	0.900735	0.977161	PPP1R2	5504	protein phosphatase 1 regulatory inhibitor subunit 2
ENSG0000	125.051	0.014195	0.147811	0.901137	0.977161	PHAX	51808	phosphorylated adaptor for RNA export
ENSG0000	727.3212	-0.0125	0.189185	0.901149	0.977161	CDC42SE2	56990	CDC42 small effector 2
ENSG0000	3.390907	-0.0056	0.227569	0.900354	0.977161	FAM183BI	340286	acyloxyacyl hydrolase (neutrophil)
ENSG0000	268.7777	-0.01395	0.180842	0.899668	0.977161	DMTF1	9988	cyclin D binding myb like transcription factor 1
ENSG0000	8.490999	0.007493	0.223002	0.900588	0.977161	TRBJ2-7	28622	T cell receptor beta joining 2-7
ENSG0000	4.50938	-0.01011	0.218864	0.899529	0.977161	LOC10041	1E+08	zinc finger protein 41 pseudogene
ENSG0000	1781.313	0.014035	0.157244	0.900424	0.977161	CAMK2G	818	calcium/calmodulin dependent protein kinase II gamma
ENSG0000	1455.753	-0.015	0.167132	0.901002	0.977161	ENTPD1	953	ectonucleoside triphosphate diphosphohydrolase 1
ENSG0000	13.18952	-0.01048	0.212019	0.899749	0.977161	PSTK	118672	phosphoseryl-tRNA kinase
ENSG0000	12.49668	0.010693	0.211932	0.901357	0.977161	B4GAT1-D	1.03E+08	B4GAT1 divergent transcript
ENSG0000	3420.764	-0.014	0.164075	0.900234	0.977161	NUMA1	4926	nuclear mitotic apparatus protein 1
ENSG0000	2.860053	0.006618	0.228372	0.900763	0.977161	CLEC6A	93978	C-type lectin domain containing 6A
ENSG0000	4.66861	-0.00583	0.227082	0.900959	0.977161	SORD2P	653381	sorbitol de pseudogene
ENSG0000	141.7053	0.008769	0.15418	0.901063	0.977161	IREB2	3658	iron responsive element binding protein 2
ENSG0000	2.596933	0.007263	0.225345	0.900961	0.977161	PLIN1	5346	perilipin 1
ENSG0000	5288.448	-0.01274	0.119314	0.900995	0.977161	RNF166	115992	ring finger protein 166
ENSG0000	74.43441	-0.01413	0.164325	0.900313	0.977161	SPATA33	124045	spermatogenesis associated 33
ENSG0000	2.917352	0.005771	0.227032	0.900332	0.977161	NA	NA	NA
ENSG0000	2.666544	-0.00627	0.227972	0.90042	0.977161	NA	NA	NA
ENSG0000	141.5533	0.014192	0.176252	0.900464	0.977161	RGL3	57139	ral guanine nucleotide dissociation stimulator like 3
ENSG0000	24.83628	-0.01129	0.200458	0.901352	0.977161	PPFIA3	8541	PTPRF interacting protein alpha 3
ENSG0000	12.47251	0.013205	0.198812	0.900535	0.977161	SLC27A5	10998	solute carrier family 27 member 5
ENSG0000	79.76881	-0.01335	0.15588	0.900145	0.977161	PDRG1	81572	p53 and DNA damage regulated 1

ENSG0000	396.6092	0.013644	0.171425	0.900965	0.977161	SDF2L1	23753	stromal cell derived factor 2 like 1
ENSG0000	357.2784	0.012847	0.116873	0.901412	0.977161	RPS19BP1	91582	ribosomal protein S19 binding protein 1
ENSG0000	262.452	-0.01314	0.15036	0.899578	0.977161	HNRNPH2	3188	heterogeneous nuclear ribonucleoprotein H2
ENSG0000	54.71467	0.013327	0.171781	0.901618	0.977326	HIBCH	26275	3-hydroxyisobutyryl-CoA hydrolase
ENSG0000	6.390827	0.010647	0.213628	0.901778	0.977384	ZNF436-A!	148898	ZNF436 antisense RNA 1
ENSG0000	471.7727	-0.01426	0.164372	0.901727	0.977384	ANO10	55129	anoctamin 10
ENSG0000	32.80887	0.013836	0.190204	0.901913	0.977414	SRGAP2B	647135	SLIT-ROBO Rho GTPase activating protein 2B
ENSG0000	8.082385	-0.00906	0.218488	0.901867	0.977414	NA	NA	NA
ENSG0000	5.499902	0.010942	0.219339	0.902007	0.977459	ZNF582-D'	386758	ZNF582 divergent transcript
ENSG0000	96.65648	0.014414	0.162828	0.90222	0.977516	ATR	545	ATR serine/threonine kinase
ENSG0000	138.722	0.012879	0.164844	0.902175	0.977516	AARS2	57505	alanyl-tRN mitochondrial
ENSG0000	1230.322	0.012752	0.127627	0.902197	0.977516	TAOK3	51347	TAO kinase 3
ENSG0000	33.0862	-0.01374	0.180194	0.902299	0.977544	LRRC23	10233	leucine rich repeat containing 23
ENSG0000	967.6676	-0.01504	0.178054	0.902427	0.977625	PTBP3	9991	polypyrimidine tract binding protein 3
ENSG0000	45.48927	-0.01392	0.175488	0.902559	0.977656	NA	NA	NA
ENSG0000	9649.554	-0.01347	0.155929	0.902562	0.977656	SLC44A2	57153	solute carrier family 44 member 2
ENSG0000	62.42773	0.0148	0.17834	0.902807	0.977756	ING3	54556	inhibitor of growth family member 3
ENSG0000	2547.653	-0.01449	0.147431	0.902818	0.977756	MBP	4155	myelin basic protein
ENSG0000	134.2411	-0.01171	0.149105	0.902846	0.977756	TIMM29	90580	translocase of inner mitochondrial membrane 29
ENSG0000	11.71309	-0.00853	0.218456	0.902868	0.977756	DRICH1	51233	aspartate rich 1
ENSG0000	1380.818	-0.01275	0.181068	0.903168	0.978023	TLR4	7099	toll like receptor 4
ENSG0000	10.36421	0.008285	0.218846	0.903287	0.978045	NA	NA	NA
ENSG0000	556.5379	-0.01144	0.120029	0.903295	0.978045	RAP2B	5912	RAP2B member of RAS oncogene family
ENSG0000	262.0603	0.013456	0.164915	0.903365	0.978063	HK2	3099	hexokinase 2
ENSG0000	414.1581	0.010231	0.141943	0.903595	0.978097	DFFA	1676	DNA fragmentation factor subunit alpha
ENSG0000	602.5101	0.012741	0.122513	0.903643	0.978097	ENTPD4	9583	ectonucleoside triphosphate diphosphohydrolase 4
ENSG0000	11767.9	-0.01341	0.167683	0.903534	0.978097	PLEC	5339	plectin
ENSG0000	944.9978	-0.01277	0.128631	0.903506	0.978097	LPXN	9404	leupaxin
ENSG0000	15.54662	0.011184	0.207007	0.903663	0.978097	NA	NA	NA
ENSG0000	10.87385	0.010659	0.213545	0.903735	0.978117	NA	NA	NA
ENSG0000	33.62352	-0.01359	0.178646	0.903841	0.978173	PLEKHA4	57664	pleckstrin homology domain containing A4
ENSG0000	78.15787	0.0145	0.150089	0.904017	0.978306	TRMT10B	158234	tRNA methyltransferase 10B
ENSG0000	463.8927	-0.00939	0.182258	0.904126	0.978366	SNRK	54861	SNF related kinase
ENSG0000	69.86229	0.014459	0.174956	0.904408	0.978439	ERO1B	56605	endoplasmic reticulum oxidoreductase 1 beta
ENSG0000	299.1181	0.013764	0.142889	0.904499	0.978439	DYNC1LI1	51143	dynein cytoplasmic 1 light intermediate chain 1
ENSG0000	6.425169	0.005661	0.225835	0.904567	0.978439	PFN2	5217	profilin 2
ENSG0000	15.06164	0.011462	0.205833	0.904539	0.978439	MIR4451	1.01E+08	microRNA 4451
ENSG0000	9.691293	0.008778	0.218592	0.904447	0.978439	KRT79	338785	keratin 79
ENSG0000	164.0883	0.013401	0.143232	0.904533	0.978439	DHRS7B	25979	dehydrogenase/reductase 7B
ENSG0000	1802.206	-0.01309	0.140655	0.904268	0.978439	MID1IP1	58526	MID1 interacting protein 1
ENSG0000	2.359267	0.004044	0.23156	0.904695	0.978519	LINC02541	1.02E+08	long intergenic non-protein coding RNA 2541
ENSG0000	8542.222	-0.01924	0.165639	0.904788	0.978562	ARAP1	116985	ArfGAP wi ankyrin repeat and PH domain 1
ENSG0000	52.23578	0.013882	0.175101	0.904943	0.978634	TTC27	55622	tetratricopeptide repeat domain 27
ENSG0000	54.59732	-0.01369	0.167373	0.905108	0.978634	TFB1M	51106	transcripti mitochondrial
ENSG0000	3.881022	-0.00411	0.225983	0.905122	0.978634	HOXA4	3201	homeobox A4
ENSG0000	3.655447	-0.0071	0.224037	0.904987	0.978634	FLJ13224	79857	uncharacterized LOC79857
ENSG0000	32.32375	0.013376	0.177562	0.905039	0.978634	ZNF714	148206	zinc finger protein 714
ENSG0000	41.31863	0.013622	0.179572	0.905219	0.978681	NOTCH2N	1.01E+08	notch 2 N-terminal like C
ENSG0000	1858.951	-0.01297	0.158091	0.905355	0.978771	ECH1	1891	enoyl-CoA hydratase 1
ENSG0000	37.42901	0.013109	0.182897	0.90554	0.978787	POLR3GL	84265	RNA polymerase III subunit GL
ENSG0000	3.897484	0.011865	0.226101	0.905583	0.978787	NA	NA	NA
ENSG0000	4.252057	-0.00558	0.229329	0.905576	0.978787	KLRC4	8302	killer cell lectin like receptor C4
ENSG0000	510.4198	0.007204	0.154119	0.90547	0.978787	NLRC3	197358	NLR family CARD domain containing 3
ENSG0000	23.144	0.012822	0.19277	0.905787	0.978949	ZGLP1	1E+08	zinc finger GATA like protein 1
ENSG0000	267.7075	-0.01325	0.145199	0.905876	0.978987	PTGR3	284273	prostaglandin reductase 3
ENSG0000	820.4556	-0.01191	0.186276	0.906054	0.979064	RNF13	11342	ring finger protein 13
ENSG0000	435.5775	0.013004	0.150994	0.906054	0.979064	LONP1	9361	lon peptid mitochondrial
ENSG0000	157.9133	0.013624	0.14609	0.906163	0.979124	ALG8	79053	ALG8 alph 3-glucosyltransferase
ENSG0000	7.626254	-0.00742	0.221399	0.906263	0.979175	CBR3	874	carbonyl reductase 3
ENSG0000	21.73567	0.011088	0.205865	0.906378	0.979241	GON7	84520	GON7 subunit of KEOPS complex
ENSG0000	72.55369	0.013994	0.155821	0.906452	0.979263	DFFB	1677	DNA fragmentation factor subunit beta
ENSG0000	737.1141	0.002311	0.11034	0.906565	0.979272	WDR82	80335	WD repeat domain 82
ENSG0000	1877.643	-0.01059	0.101937	0.906674	0.979272	HNRNPA2I	3181	heterogeneous nuclear ribonucleoprotein A2/B1
ENSG0000	8.092576	-0.00647	0.223393	0.90665	0.979272	PDE3A	5139	phosphodiesterase 3A
ENSG0000	40.01953	0.011686	0.198406	0.906602	0.979272	ZNF558	148156	zinc finger protein 558
ENSG0000	3.050534	-5.06E-04	0.227448	0.906818	0.979369	SLC25A32	81034	solute carrier family 25 member 32
ENSG0000	10.73717	0.010473	0.209677	0.906945	0.979391	NA	NA	NA
ENSG0000	78.56848	0.011933	0.198927	0.906936	0.979391	PPWD1	23398	peptidylprolyl isomerase domain and WD repeat containing 1
ENSG0000	3.852985	0.008333	0.22057	0.907046	0.979443	NA	NA	NA
ENSG0000	12.91113	-0.00928	0.222974	0.907866	0.979555	GNB1-DT	1.05E+08	GNB1 divergent transcript
ENSG0000	2.530717	0.004491	0.23054	0.90814	0.979555	NA	NA	NA
ENSG0000	120.8335	0.012846	0.141571	0.907863	0.979555	QDPR	5860	quinoid dihydropteridine reductase
ENSG0000	127.0176	0.012998	0.161906	0.908509	0.979555	ADH5	128	alcohol de chi polypeptide
ENSG0000	14.13764	0.010948	0.207879	0.908275	0.979555	LEAP2	116842	liver enriched antimicrobial peptide 2
ENSG0000	7.158097	-0.00698	0.223418	0.907866	0.979555	HEY2	23493	hes related family bHLH transcription factor with YRPW motif 2
ENSG0000	14.69488	0.011971	0.201049	0.908036	0.979555	NA	NA	NA
ENSG0000	117.6927	0.014459	0.160164	0.908255	0.979555	ST7	7982	suppression of tumorigenicity 7
ENSG0000	34.57645	-0.01276	0.174902	0.90854	0.979555	ZNNT1	1.16E+08	ZNF706 neighboring transcript 1
ENSG0000	402.9665	0.011114	0.108706	0.907922	0.979555	WASHC5	9897	WASH complex subunit 5
ENSG0000	11.27978	0.01104	0.20723	0.907889	0.979555	RFX3-DT	1.02E+08	RFX3 divergent transcript
ENSG0000	3.557509	-0.00362	0.229887	0.908013	0.979555	NA	NA	NA
ENSG0000	359.6578	0.013081	0.164061	0.907251	0.979555	HSDL2	84263	hydroxysteroid dehydrogenase like 2
ENSG0000	789.642	-0.01202	0.131513	0.908415	0.979555	MMS19	64210	MMS19 hc cytosolic iron-sulfur assembly component
ENSG0000	143.1846	0.011355	0.158298	0.908506	0.979555	PCID2	55795	PCI domain containing 2
ENSG0000	5.2143	-0.0074	0.219959	0.907924	0.979555	GPR135	64582	G protein-coupled receptor 135
ENSG0000	8.20709	0.00747	0.220257	0.908509	0.979555	NA	NA	NA
ENSG0000	2448.313	-0.01228	0.181368	0.907381	0.979555	CBFA2T3	863	CBFA2/RUNX1 partner transcriptional co-repressor 3
ENSG0000	415.7662	0.014084	0.157322	0.908221	0.979555	DLG4	1742	discs large MAGUK scaffold protein 4
ENSG0000	166.4854	-0.01093	0.125495	0.908001	0.979555	TMEM101	84336	transmembrane protein 101
ENSG0000	749.7485	0.013529	0.149039	0.907646	0.979555	NFATC1	4772	nuclear factor of activated T cells 1
ENSG0000	1401.179	0.012722	0.143963	0.908295	0.979555	KLF16	83855	KLF transcription factor 16
ENSG0000	1165.913	-0.01284	0.168971	0.90808	0.979555	XAB2	56949	XPA binding protein 2
ENSG0000	11.3153	-0.00993	0.21113	0.907828	0.979555	SARS2	54938	seryl-tRNA mitochondrial
ENSG0000	20.4057	-0.01022	0.200674	0.908455	0.979555	ARMCX4	1E+08	armadillo repeat containing X-linked 4
ENSG0000	134.494	-0.01293	0.167479	0.90827	0.979555	NSDHL	50814	NAD(P) dependent steroid dehydrogenase-like
ENSG0000	170.3074	0.013405	0.168187	0.908609	0.979572	ALDH18A1	5832	aldehyde dehydrogenase 18 family member A1

ENSG0000	1218.062	0.015144	0.128491	0.90868	0.97959	ZZEF1	23140	zinc finger ZZ-type and EF-hand domain containing 1
ENSG0000	284.9496	0.00997	0.20172	0.908939	0.979666	UHMK1	127933	U2AF homology motif kinase 1
ENSG0000	10.02459	0.010864	0.208117	0.908871	0.979666	HLA-J	3137	major hist class I J (pseudogene)
ENSG0000	12.69377	0.008525	0.216083	0.908964	0.979666	IGHV4-28	28400	immunoglobulin heavy variable 4-28
ENSG0000	325.3582	0.012552	0.171972	0.908817	0.979666	RPAP1	26015	RNA polymerase II associated protein 1
ENSG0000	8.498265	0.008013	0.221274	0.909126	0.979737	TCF7L1	83439	transcription factor 7 like 1
ENSG0000	681.0618	-0.0102	0.103437	0.909137	0.979737	DLST	1743	dihydrolipoamide S-succinyltransferase
ENSG0000	90.8787	-0.01108	0.194843	0.909231	0.97978	LSM8	51691	LSM8 hom U6 small nuclear RNA associated
ENSG0000	3.538464	7.93E-04	0.228483	0.909289	0.979786	SPRR4	163778	small proline rich protein 4
ENSG0000	42.60693	-0.01062	0.200223	0.909351	0.979795	AKR1C3	8644	aldo-keto reductase family 1 member C3
ENSG0000	9.407241	-0.00593	0.22465	0.909693	0.9799	DOCK1	1793	dedicator of cytokinesis 1
ENSG0000	7.752859	0.010794	0.210866	0.909562	0.9799	NA	NA	NA
ENSG0000	48.5231	0.019902	0.167149	0.909716	0.9799	GAS8	2622	growth arrest specific 8
ENSG0000	1012.123	0.013205	0.154785	0.909601	0.9799	XRCC1	7515	X-ray repair cross complementing 1
ENSG0000	447.5267	-0.01275	0.167337	0.909696	0.9799	ITCH	83737	itchy E3 ubiquitin protein ligase
ENSG0000	7.105853	0.008846	0.215142	0.909784	0.979916	EXTL3-AS1	1.02E+08	EXTL3 antisense RNA 1
ENSG0000	6.285472	0.001184	0.225975	0.910212	0.980048	SLC16A4	9122	solute carrier family 16 member 4
ENSG0000	284.1535	-0.01275	0.136429	0.910133	0.980048	PCYOX1L	78991	prenylcysteine oxidase 1 like
ENSG0000	38.75426	0.011802	0.198522	0.910228	0.980048	PLEKHA8	84725	pleckstrin homology domain containing A8
ENSG0000	2625.903	-0.01171	0.111816	0.910164	0.980048	FAM78A	286336	family with sequence similarity 78 member A
ENSG0000	224.4968	0.012568	0.132532	0.910026	0.980048	SMIM7	79086	small integral membrane protein 7
ENSG0000	4.56527	-3.65E-04	0.223457	0.91011	0.980048	LOC10012	1E+08	uncharacterized LOC100128310
ENSG0000	187.591	-0.01236	0.143737	0.910358	0.980068	RBSN	64145	rabenosyn RAB effector
ENSG0000	1322.268	-0.01	0.142814	0.910407	0.980068	EZR	7430	ezrin
ENSG0000	3.414395	0.004994	0.227087	0.910351	0.980068	NA	NA	NA
ENSG0000	1208.798	0.012721	0.152991	0.910463	0.98007	ALG3	10195	ALG3 alph 3- mannosyltransferase
ENSG0000	490.8089	0.008505	0.150826	0.910898	0.980482	GIMAP7	168537	GTPase IMAP family member 7
ENSG0000	17.12012	0.011654	0.196127	0.910959	0.980489	NA	NA	NA
ENSG0000	28.76781	-0.0125	0.179237	0.911046	0.980526	NA	NA	NA
ENSG0000	75.18043	0.013287	0.167368	0.911253	0.980684	NA	NA	NA
ENSG0000	187.5356	0.01797	0.15597	0.911354	0.980684	MTIF3	219402	mitochondrial translational initiation factor 3
ENSG0000	4.641866	0.00837	0.225333	0.91135	0.980684	NA	NA	NA
ENSG0000	70.88486	-0.01267	0.152844	0.911427	0.980705	DBT	1629	dihydrolipoamide branched chain transacylase E2
ENSG0000	2660.881	-0.01288	0.158329	0.911554	0.980758	CPNE5	57699	copine 5
ENSG0000	11.86481	0.00727	0.217427	0.911637	0.980758	TET1	80312	tet methylcytosine dioxygenase 1
ENSG0000	242.0808	0.011715	0.125938	0.911633	0.980758	RNASEH2E	79621	ribonuclease H2 subunit B
ENSG0000	29.81353	0.009647	0.203659	0.91178	0.980806	NAT1	9	N-acetyltransferase 1
ENSG0000	224.424	-0.0115	0.137799	0.911788	0.980806	PPP1R13B	23368	protein phosphatase 1 regulatory subunit 13B
ENSG0000	6.522447	0.007418	0.220634	0.912101	0.981085	IGSF22	283284	immunoglobulin superfamily member 22
ENSG0000	3.355862	-0.00348	0.227386	0.912362	0.981307	LAMA5-AS	1.02E+08	LAMA5 antisense RNA 1
ENSG0000	4.333588	-0.00603	0.224275	0.912521	0.98142	NA	NA	NA
ENSG0000	2.557429	0.002016	0.231041	0.912881	0.981439	SERPINC1	462	serpin family C member 1
ENSG0000	285.5112	-0.0114	0.121534	0.912917	0.981439	UXS1	80146	UDP-glucuronate decarboxylase 1
ENSG0000	268.7267	0.011103	0.139489	0.912967	0.981439	DYNC1I2	1781	dynein cytoplasmic 1 intermediate chain 2
ENSG0000	254.5757	-0.01515	0.120238	0.912907	0.981439	JAGN1	84522	jagunal homolog 1
ENSG0000	4.003572	0.00759	0.22874	0.912809	0.981439	TTC41P	253724	tetratricop pseudogene
ENSG0000	5.59627	0.008858	0.216871	0.912909	0.981439	TMEM132	124842	transmembrane protein 132E
ENSG0000	13.04716	-0.00991	0.210697	0.912787	0.981439	NA	NA	NA
ENSG0000	15.99571	0.009579	0.213621	0.912944	0.981439	IGLV3-12	28802	immunoglobulin lambda variable 3-12
ENSG0000	4628.177	-0.01048	0.139617	0.913114	0.98152	ARHGEF2	9181	Rho/Rac guanine nucleotide exchange factor 2
ENSG0000	30.6663	-0.01147	0.187193	0.913649	0.98152	COA6	388753	cytochrome c oxidase assembly factor 6
ENSG0000	4.006908	0.005672	0.226127	0.913549	0.98152	SERF1A	8293	small EDRK-rich factor 1A
ENSG0000	282.7725	0.010163	0.130406	0.913899	0.98152	FAF2	23197	Fas associated factor family member 2
ENSG0000	843.4501	0.012921	0.150356	0.913876	0.98152	PNISR	25957	PNN interacting serine and arginine rich protein
ENSG0000	21.53588	0.011402	0.192905	0.913884	0.98152	STXBP5-AS	729178	STXBP5 antisense RNA 1
ENSG0000	1539.361	-0.01178	0.144254	0.913573	0.98152	MTA2	9219	metastasis associated 1 family member 2
ENSG0000	145.4533	-0.014	0.168477	0.913869	0.98152	TMEM106	79022	transmembrane protein 106C
ENSG0000	6.337416	0.006636	0.222782	0.913754	0.98152	MIR17HG	407975	miR-17-92a-1 cluster host gene
ENSG0000	34.86568	-0.01193	0.182315	0.913458	0.98152	TMTC4	84899	transmembrane O-mannosyltransferase targeting cadherins 4
ENSG0000	144.2331	-0.01103	0.143443	0.9137	0.98152	SORD	6652	sorbitol dehydrogenase
ENSG0000	65.1142	0.011507	0.188664	0.913263	0.98152	MAPK6	5597	mitogen-activated protein kinase 6
ENSG0000	1439.85	0.014574	0.156977	0.913183	0.98152	ICAM1	3383	intercellular adhesion molecule 1
ENSG0000	730.2194	0.012576	0.135374	0.913678	0.98152	ZNF524	147807	zinc finger protein 524
ENSG0000	122.5017	-0.01272	0.161186	0.913577	0.98152	NA	NA	NA
ENSG0000	2.662204	0.007505	0.231616	0.913729	0.98152	NA	NA	NA
ENSG0000	18.53652	0.012264	0.184993	0.914226	0.981593	KRTCAP3	200634	keratinocyte associated protein 3
ENSG0000	3.494947	0.005396	0.226624	0.915189	0.981593	NA	NA	NA
ENSG0000	3.971268	-0.00672	0.222309	0.914373	0.981593	ACKR2	1238	atypical chemokine receptor 2
ENSG0000	238.1204	0.011726	0.169543	0.915404	0.981593	FLNB	2317	filamin B
ENSG0000	844.0585	-0.01165	0.122423	0.914342	0.981593	HEXB	3074	hexosaminidase subunit beta
ENSG0000	3.749419	0.006897	0.222922	0.915088	0.981593	ARRDC3-A	1E+08	ARRDC3 antisense RNA 1
ENSG0000	435.087	-0.01144	0.190875	0.914356	0.981593	PJA2	9867	praja ring finger ubiquitin ligase 2
ENSG0000	10.96009	0.008846	0.218461	0.914788	0.981593	NA	NA	NA
ENSG0000	4481.202	0.011247	0.123396	0.915414	0.981593	PRELID1	27166	PRELI domain containing 1
ENSG0000	6.391458	-0.00707	0.220632	0.914996	0.981593	VIPR2	7434	vasoactive intestinal peptide receptor 2
ENSG0000	44.79565	-0.0108	0.189964	0.914332	0.981593	ABHD17B	51104	abhydrola depalmitoylase
ENSG0000	24.73539	-0.01169	0.188728	0.915291	0.981593	HSPA14	51182	heat shock protein family A (Hsp70) member 14
ENSG0000	52.70196	-0.01014	0.199817	0.915097	0.981593	STAMBPL1	57559	STAM binding protein like 1
ENSG0000	567.1248	-0.01204	0.155582	0.914371	0.981593	VEGFB	7423	vascular endothelial growth factor B
ENSG0000	56.90035	0.012623	0.19315	0.915404	0.981593	KLRB1	3820	killer cell lectin like receptor B1
ENSG0000	62.33853	-0.01266	0.169886	0.915191	0.981593	CMAS	55907	cytidine monophosphate N-acetylneuraminic acid synthetase
ENSG0000	144.0374	-0.01158	0.176301	0.914081	0.981593	FNDC3A	22862	fibronectin type III domain containing 3A
ENSG0000	6.757363	-0.00789	0.216904	0.915412	0.981593	RPS4XP16	220433	ribosomal protein S4X pseudogene 16
ENSG0000	23.82714	0.009007	0.21567	0.91513	0.981593	KNL1	57082	kinetochore scaffold 1
ENSG0000	430.1741	-0.01263	0.176004	0.91538	0.981593	ARL6IP1	23204	ADP ribosylation factor like GTPase 6 interacting protein 1
ENSG0000	9.592926	-0.00703	0.219568	0.914601	0.981593	NA	NA	NA
ENSG0000	1388.048	-0.01128	0.147374	0.915357	0.981593	RBL2	5934	RB transcriptional corepressor like 2
ENSG0000	4859.781	0.011534	0.128152	0.915027	0.981593	EIF5A	1984	eukaryotic translation initiation factor 5A
ENSG0000	26.68227	-0.00976	0.204692	0.915328	0.981593	COX10-DT	1.01E+08	COX10 divergent transcript
ENSG0000	46.40387	-0.01217	0.177848	0.915057	0.981593	CCNE1	898	cyclin E1
ENSG0000	17.02806	0.01037	0.204104	0.915102	0.981593	NA	NA	NA
ENSG0000	22151.6	0.01182	0.185014	0.915368	0.981593	FLNA	2316	filamin A
ENSG0000	20.8492	-0.00522	0.216825	0.915484	0.981611	DNA2	1763	DNA replication helicase/nuclease 2
ENSG0000	87.24456	0.009998	0.170508	0.915678	0.981761	NA	NA	NA
ENSG0000	661.7268	0.012018	0.143568	0.915733	0.981763	PKD1	5310	polycystin transient receptor potential channel interacting
ENSG0000	6.45998	-0.00865	0.214773	0.916302	0.982028	NA	NA	NA

ENSG0000	30.02149	0.012525	0.172302	0.916267	0.982028	SLC4A8	9498	solute carrier family 4 member 8
ENSG0000	2984.814	-0.0125	0.176838	0.91616	0.982028	FES	2242	FES proto- tyrosine kinase
ENSG0000	11.98049	-0.00972	0.205889	0.916294	0.982028	COPZ2	51226	COPI coat complex subunit zeta 2
ENSG0000	805.5196	0.00959	0.12307	0.916187	0.982028	CLTC	1213	clathrin heavy chain
ENSG0000	434.6618	0.011529	0.158092	0.916101	0.982028	SLC39A3	29985	solute carrier family 39 member 3
ENSG0000	11.62048	0.009945	0.207633	0.916394	0.982041	MYLK-AS1	1.01E+08	MYLK antisense RNA 1
ENSG0000	756.1943	-0.01113	0.163902	0.916475	0.982041	ELOVL5	60481	ELOVL fatty acid elongase 5
ENSG0000	20.49348	-0.01048	0.200176	0.916429	0.982041	KIF24	347240	kinesin family member 24
ENSG0000	90.00778	-0.01158	0.158996	0.916634	0.982057	SRGAP2C	653464	SLIT-ROBO Rho GTPase activating protein 2C
ENSG0000	97.58337	-0.00971	0.169369	0.91665	0.982057	SEPTIN11	55752	septin 11
ENSG0000	44.77486	-0.01204	0.180385	0.916651	0.982057	NA	NA	NA
ENSG0000	18.35013	0.009421	0.212717	0.916719	0.982072	NA	NA	NA
ENSG0000	293.2637	-0.01007	0.172212	0.916799	0.9821	ZNF148	7707	zinc finger protein 148
ENSG0000	366.571	-0.01141	0.186439	0.916991	0.982101	OBSCN	84033	obscurin cytoskeletal calmodulin and titin-interacting RhoGEF
ENSG0000	82.35142	-0.01169	0.185209	0.917151	0.982101	B4GALT4	8702	beta-1 4-galactosyltransferase 4
ENSG0000	506.6966	-8.77E-04	0.187499	0.917119	0.982101	CLIP2	7461	CAP-Gly domain containing linker protein 2
ENSG0000	3.432074	0.002979	0.229098	0.917185	0.982101	MUC12-AS	1.03E+08	MUC12 antisense RNA 1
ENSG0000	780.0064	0.012243	0.12568	0.917355	0.982101	WASHC1	1E+08	WASH complex subunit 1
ENSG0000	358.5038	-0.01235	0.156623	0.917389	0.982101	IARS1	3376	isoleucyl-tRNA synthetase 1
ENSG0000	275.8207	0.01261	0.128167	0.91693	0.982101	ESD	2098	esterase D
ENSG0000	11.90114	0.006416	0.222146	0.917108	0.982101	NA	NA	NA
ENSG0000	3.437146	-0.00457	0.227586	0.91731	0.982101	NA	NA	NA
ENSG0000	5746.614	0.012005	0.137936	0.917341	0.982101	SUN2	25777	Sad1 and UNC84 domain containing 2
ENSG0000	29.09723	-0.01084	0.192899	0.917271	0.982101	LOC11226	1.12E+08	translation machinery-associated protein 7 -like
ENSG0000	7396.444	-0.0111	0.132564	0.917465	0.982125	ARPC2	10109	actin related protein 2/3 complex subunit 2
ENSG0000	169.7963	-0.00358	0.176132	0.917672	0.982258	TRAF5	7188	TNF receptor associated factor 5
ENSG0000	8.915568	-0.00769	0.217337	0.91775	0.982258	ZNF22-AS1	220979	ZNF22 antisense RNA 1
ENSG0000	117.5228	0.009586	0.187677	0.917747	0.982258	RNF138	51444	ring finger protein 138
ENSG0000	71.35641	0.012552	0.157531	0.917974	0.98244	AP4S1	11154	adaptor related protein complex 4 subunit sigma 1
ENSG0000	374.4436	-0.01196	0.168576	0.91844	0.982767	RRM2	6241	ribonucleotide reductase regulatory subunit M2
ENSG0000	50.90861	-0.00793	0.21082	0.918393	0.982767	NA	NA	NA
ENSG0000	6.398546	-0.00779	0.218057	0.918387	0.982767	TLCD1	116238	TLC domain containing 1
ENSG0000	309.4542	0.007689	0.215916	0.918519	0.982774	IGHV4-61	28391	immunoglobulin heavy variable 4-61
ENSG0000	124.5214	-0.01	0.145816	0.918646	0.982774	ADCY9	115	adenylate cyclase 9
ENSG0000	3.253071	0.010665	0.227804	0.9186	0.982774	NA	NA	NA
ENSG0000	297.5505	0.011804	0.129538	0.918661	0.982774	RABL2B	11158	RAB member of RAS oncogene family like 2B
ENSG0000	69.54571	0.011955	0.159101	0.918803	0.982811	ZNF236	7776	zinc finger protein 236
ENSG0000	297.3598	0.0101	0.132505	0.918783	0.982811	PDHA1	5160	pyruvate dehydrogenase E1 subunit alpha 1
ENSG0000	253.6393	-0.01101	0.142242	0.919002	0.982852	SOAT1	6646	sterol O-acyltransferase 1
ENSG0000	62.22446	0.00989	0.198587	0.9189	0.982852	ICOS	29851	inducible T cell costimulator
ENSG0000	116.0354	0.012027	0.144081	0.918956	0.982852	BORCS5	118426	BLOC-1 related complex subunit 5
ENSG0000	15.64855	-0.00473	0.219949	0.919226	0.982942	C1orf220	400798	chromosome 1 putative open reading frame 220
ENSG0000	88.54662	0.017918	0.202953	0.919545	0.982942	PTGS2	5743	prostaglandin-endoperoxide synthase 2
ENSG0000	182.8598	0.012717	0.138213	0.919472	0.982942	HEATR1	55127	HEAT repeat containing 1
ENSG0000	5.945645	-0.00511	0.225092	0.919623	0.982942	NA	NA	NA
ENSG0000	339.0771	-0.01228	0.157021	0.919421	0.982942	KLC4	89953	kinesin light chain 4
ENSG0000	15946.15	-0.0107	0.177285	0.919329	0.982942	RIN3	79890	Ras and Rab interactor 3
ENSG0000	68.52948	-0.0115	0.170833	0.919598	0.982942	ZNF320	162967	zinc finger protein 320
ENSG0000	116.0743	0.011751	0.148004	0.919234	0.982942	ZNF671	79891	zinc finger protein 671
ENSG0000	89.98602	0.011587	0.149957	0.919447	0.982942	ZNF133	7692	zinc finger protein 133
ENSG0000	670.9238	0.012172	0.150018	0.919402	0.982942	GAB3	139716	GRB2 associated binding protein 3
ENSG0000	30.54377	-0.0113	0.182277	0.91975	0.982963	PIWIL4	143689	piwi like RNA-mediated gene silencing 4
ENSG0000	36.22513	-0.00832	0.209221	0.919736	0.982963	SGPP1	81537	sphingosine-1-phosphate phosphatase 1
ENSG0000	49.33323	0.011817	0.166229	0.91983	0.982991	PIGN	23556	phosphatidylinositol glycan anchor biosynthesis class N
ENSG0000	3.797169	0.005533	0.225245	0.920169	0.983107	NA	NA	NA
ENSG0000	11.99668	0.009851	0.205774	0.920315	0.983107	NA	NA	NA
ENSG0000	6498.951	-0.01102	0.163905	0.9201	0.983107	STK10	6793	serine/threonine kinase 10
ENSG0000	4.910471	0.00675	0.221432	0.920059	0.983107	LRRC1	55227	leucine rich repeat containing 1
ENSG0000	5.204041	0.006528	0.222718	0.920188	0.983107	TMEM252	1.05E+08	TMEM252 divergent transcript
ENSG0000	3745.938	0.011506	0.178773	0.920291	0.983107	PYGL	5836	glycogen phosphorylase L
ENSG0000	9388.97	0.009627	0.106688	0.920254	0.983107	LENG8	114823	leukocyte receptor cluster member 8
ENSG0000	108.526	-0.00885	0.202584	0.920452	0.983197	ATP8A1	10396	ATPase phospholipid transporting 8A1
ENSG0000	252.4541	0.011424	0.148413	0.920556	0.98325	NRDE2	55051	NRDE-2 necessary domain containing
ENSG0000	462.622	-0.01054	0.130714	0.920641	0.983284	DEAF1	10522	DEAF1 transcription factor
ENSG0000	286.5661	0.010588	0.140163	0.920734	0.983326	PPP1R16A	84988	protein phosphatase 1 regulatory subunit 16A
ENSG0000	431.6311	-0.01123	0.166841	0.920866	0.98341	LOC12245	1.22E+08	testis-specific serine/proline-rich protein
ENSG0000	3.514096	0.003131	0.228154	0.921056	0.983498	NA	NA	NA
ENSG0000	69.14305	0.010407	0.200322	0.921012	0.983498	CCN3	4856	cellular communication network factor 3
ENSG0000	199.5575	-0.01145	0.168081	0.921343	0.98355	RPF1	80135	ribosome production factor 1 homolog
ENSG0000	3.793439	0.00514	0.225749	0.921413	0.98355	NA	NA	NA
ENSG0000	584.9323	0.008639	0.137329	0.921376	0.98355	RING1	6015	ring finger protein 1
ENSG0000	14.19732	0.008551	0.20974	0.921534	0.98355	TRMT11	60487	tRNA methyltransferase 11 homolog
ENSG0000	622.0869	0.011148	0.114759	0.921372	0.98355	RNF41	10193	ring finger protein 41
ENSG0000	230.0672	-0.01045	0.157591	0.921531	0.98355	APPL2	55198	adaptor pr phosphotyrosine interacting with PH domain and leucine zipper 2
ENSG0000	48.90868	0.01293	0.161079	0.921356	0.98355	SNAPC5	10302	small nuclear RNA activating complex polypeptide 5
ENSG0000	1590.94	-0.01067	0.17633	0.921531	0.98355	MSL3	10943	MSL complex subunit 3
ENSG0000	284.2046	0.01296	0.146535	0.927097	0.983706	ATAD3B	83858	ATPase family AAA domain containing 3B
ENSG0000	11.17718	-0.00823	0.212573	0.922279	0.983706	NA	NA	NA
ENSG0000	252.7457	0.009496	0.12698	0.925502	0.983706	CEP104	9731	centrosomal protein 104
ENSG0000	39.71325	0.011017	0.180335	0.925157	0.983706	LINC0033	29092	long intergenic non-protein coding RNA 339
ENSG0000	43.02623	-0.01049	0.189339	0.927615	0.983706	HPCAL4	51440	hippocalcin like 4
ENSG0000	843.0299	-0.01092	0.15041	0.922181	0.983706	LRRC41	10489	leucine rich repeat containing 41
ENSG0000	298.4707	-0.01032	0.143536	0.925958	0.983706	SHISAL2A	348378	shisa like 2A
ENSG0000	24.41008	0.009392	0.199028	0.927151	0.983706	CCDC18	343099	coiled-coil domain containing 18
ENSG0000	108.7611	-0.00546	0.17851	0.927207	0.983706	SMYD2	56950	SET and MYND domain containing 2
ENSG0000	9.436186	0.007992	0.213004	0.925309	0.983706	OBSCN-AS	574407	OBSCN antisense RNA 1
ENSG0000	50.76231	0.01064	0.186161	0.924252	0.983706	ABCB10	23456	ATP binding cassette subfamily B member 10
ENSG0000	3.053615	0.004945	0.225277	0.922495	0.983706	RBM34	23029	RNA binding motif protein 34
ENSG0000	404.2309	-0.00974	0.139335	0.925122	0.983706	HTRA2	27429	HtrA serine peptidase 2
ENSG0000	171.4892	-0.01045	0.140655	0.927261	0.983706	RABL2A	11159	RAB member of RAS oncogene family like 2A
ENSG0000	3.388114	0.00145	0.227811	0.925793	0.983706	NA	NA	NA
ENSG0000	25.69166	0.011665	0.191097	0.925043	0.983706	AGAP1	116987	ArfGAP wi ankyrin repeat and PH domain 1
ENSG0000	28.45101	0.006894	0.215392	0.927445	0.983706	NA	NA	NA
ENSG0000	2699.947	0.010522	0.133649	0.922301	0.983706	MAPKAPK	7867	MAPK activated protein kinase 3
ENSG0000	167.0694	0.015634	0.160943	0.922004	0.983706	ATP2C1	27032	ATPase secretory pathway Ca2+ transporting 1
ENSG0000	974.0556	-0.00955	0.153779	0.922972	0.983706	PSMD2	5708	proteason non-ATPase 2

ENSG0000	104.857	0.010742	0.165976	0.922432	0.983706	SDHAP4	220729	SDHA pseudogene 4
ENSG0000	8.635823	-0.00661	0.219018	0.92585	0.983706	BEND4	389206	BEN domain containing 4
ENSG0000	1433.94	0.011952	0.160376	0.922751	0.983706	SEC31A	22872	SEC31 hon COPII coat complex component
ENSG0000	5.911236	-0.00651	0.219889	0.92338	0.983706	NA	NA	NA
ENSG0000	35.48765	0.006592	0.218215	0.924933	0.983706	MIER3	166968	MIER family member 3
ENSG0000	2.330942	0.003418	0.232231	0.921788	0.983706	MAP1B	4131	microtubule associated protein 1B
ENSG0000	51.8137	-0.01087	0.169173	0.923787	0.983706	MARCHF3	115123	membrane associated ring-CH-type finger 3
ENSG0000	13.15329	0.009909	0.195344	0.92673	0.983706	NA	NA	NA
ENSG0000	391.8733	-0.00995	0.182007	0.92733	0.983706	NR3C1	2908	nuclear receptor subfamily 3 group C member 1
ENSG0000	77.02777	0.009057	0.180705	0.926292	0.983706	RBM27	54439	RNA binding motif protein 27
ENSG0000	116.7089	0.011599	0.133981	0.923875	0.983706	FARS2	10667	phenylalar mitochondrial
ENSG0000	12.311	0.007555	0.215816	0.925827	0.983706	H2AC11	8969	H2A clustered histone 11
ENSG0000	172.2267	-0.01009	0.163528	0.922369	0.983706	CCHCR1	54535	coiled-coil alpha-helical rod protein 1
ENSG0000	311.2414	-0.01098	0.119302	0.927538	0.983706	NFKBIL1	4795	NFKB inhibitor like 1
ENSG0000	32.01519	-0.01033	0.184006	0.925931	0.983706	HCG25	414765	HLA complex group 25
ENSG0000	67.91062	0.010531	0.160318	0.927242	0.983706	BLTP3A	54887	bridge-like lipid transfer protein family member 3A
ENSG0000	892.3884	-0.01138	0.130175	0.92298	0.983706	TJAP1	93643	tight junction associated protein 1
ENSG0000	92.21335	0.011183	0.174104	0.927601	0.983706	ZNF451	26036	zinc finger protein 451
ENSG0000	3.271433	0.005688	0.225448	0.924779	0.983706	KHDRBS2	202559	KH RNA bi signal transduction associated 2
ENSG0000	889.3516	-0.00755	0.138285	0.924087	0.983706	DDX56	54606	DEAD-box helicase 56
ENSG0000	386.3713	0.008655	0.155764	0.924891	0.983706	NSUN5P1	155400	NSUN5 pseudogene 1
ENSG0000	25.24776	0.010536	0.195999	0.921993	0.983706	GPC2	221914	glypican 2
ENSG0000	180.0343	0.011562	0.154697	0.92618	0.983706	RHEB	6009	Ras homol mTORC1 binding
ENSG0000	27.31618	-0.0109	0.188543	0.924714	0.983706	NA	NA	NA
ENSG0000	43.63375	-0.00945	0.194856	0.925009	0.983706	EMC2	9694	ER membrane protein complex subunit 2
ENSG0000	105.3818	0.008099	0.190158	0.923871	0.983706	TMEM65	157378	transmembrane protein 65
ENSG0000	3.373233	0.004474	0.231027	0.923641	0.983706	NA	NA	NA
ENSG0000	38.78268	0.010548	0.182556	0.925822	0.983706	NA	NA	NA
ENSG0000	47.00806	-0.01079	0.162647	0.926006	0.983706	ZNF169	169841	zinc finger protein 169
ENSG0000	46.27059	0.005095	0.15963	0.926838	0.983706	SLC35D2	11046	solute carrier family 35 member D2
ENSG0000	366.435	-0.0102	0.143443	0.926437	0.983706	ODF2	4957	outer dense fiber of sperm tails 2
ENSG0000	7.521965	-0.0072	0.213817	0.926372	0.983706	NA	NA	NA
ENSG0000	7.805383	-0.00633	0.218422	0.926926	0.983706	NA	NA	NA
ENSG0000	48.07222	0.010517	0.176956	0.92746	0.983706	LINC00863	439994	long intergenic non-protein coding RNA 863
ENSG0000	16.26901	-0.00837	0.206199	0.926896	0.983706	SFR1	119392	SWI5 dependent homologous recombination repair protein 1
ENSG0000	40.23891	0.010131	0.180072	0.926228	0.983706	SERGEF	26297	secretion regulating guanine nucleotide exchange factor
ENSG0000	2.932192	0.003237	0.231203	0.9247	0.983706	NA	NA	NA
ENSG0000	3.955293	0.00336	0.230971	0.92754	0.983706	ARHGEF17	1E+08	ARHGEF17 antisense RNA 1
ENSG0000	26.29368	0.010101	0.196575	0.922625	0.983706	LOC10012	1E+08	uncharacterized LOC100129203
ENSG0000	462.7402	-0.00959	0.157961	0.927465	0.983706	UBE4A	9354	ubiquitination factor E4A
ENSG0000	10.58971	0.008507	0.207848	0.925322	0.983706	MSANTD2	1.01E+08	MSANTD2 antisense RNA 1
ENSG0000	152.987	-0.01063	0.151561	0.92389	0.983706	NCAPD3	23310	non-SMC condensin II complex subunit D3
ENSG0000	9.636228	0.007359	0.216101	0.92564	0.983706	LINC00987	1E+08	long intergenic non-protein coding RNA 987
ENSG0000	4.593032	0.004009	0.226748	0.922631	0.983706	PRB3	5544	proline rich protein BstNI subfamily 3
ENSG0000	35.46035	0.008153	0.200071	0.927179	0.983706	MED21	9412	mediator complex subunit 21
ENSG0000	5.043055	0.005988	0.230891	0.925344	0.983706	NA	NA	NA
ENSG0000	5.550526	0.006268	0.227491	0.923314	0.983706	NA	NA	NA
ENSG0000	372.4783	0.010716	0.141947	0.92336	0.983706	CDK4	1019	cyclin dependent kinase 4
ENSG0000	136.8982	0.010548	0.162565	0.92494	0.983706	TMBIM4	51643	transmembrane BAX inhibitor motif containing 4
ENSG0000	305.9564	0.009764	0.173271	0.922449	0.983706	UBE2N	7334	ubiquitin conjugating enzyme E2 N
ENSG0000	5155.273	0.009541	0.197039	0.927075	0.983706	RPH3A	22895	rabphilin 3A
ENSG0000	89.21278	0.011287	0.19223	0.926723	0.983706	UFM1	51569	ubiquitin fold modifier 1
ENSG0000	2.333892	0.004427	0.230187	0.92522	0.983706	SERP2	387923	stress associated endoplasmic reticulum protein family member 2
ENSG0000	77.70221	0.009778	0.196259	0.925059	0.983706	ABHD13	84945	abhydrolase domain containing 13
ENSG0000	2.881805	0.0036	0.228777	0.923165	0.983706	RPL4P1	650808	ribosomal protein L4 pseudogene 1
ENSG0000	40.92832	0.01067	0.184041	0.924597	0.983706	DAAM1	23002	dishevelled associated activator of morphogenesis 1
ENSG0000	193.7951	0.009399	0.195642	0.924812	0.983706	TC2N	123036	tandem C2 nuclear
ENSG0000	202.6107	-0.00892	0.127155	0.92501	0.983706	TUBGCP4	27229	tubulin gamma complex associated protein 4
ENSG0000	9.660245	-0.00503	0.221459	0.927411	0.983706	MYO5C	55930	myosin VC
ENSG0000	49.73711	0.010854	0.183314	0.925387	0.983706	NA	NA	NA
ENSG0000	3.098543	-0.00562	0.224196	0.926494	0.983706	ZG16B	124220	zymogen granule protein 16B
ENSG0000	3.029759	0.006229	0.224151	0.923933	0.983706	NA	NA	NA
ENSG0000	100.4458	-0.01088	0.168423	0.922453	0.983706	TENT4B	64282	terminal nucleotidyltransferase 4B
ENSG0000	747.1833	-0.01305	0.162236	0.922899	0.983706	EDC4	23644	enhancer of mRNA decapping 4
ENSG0000	407.2598	0.009546	0.114628	0.924483	0.983706	NCBP3	55421	nuclear cap binding subunit 3
ENSG0000	8.184085	-0.00665	0.212369	0.925372	0.983706	TMEM11-I	1.08E+08	TMEM11 divergent transcript
ENSG0000	85.63424	0.008055	0.145125	0.926753	0.983706	RAD51D	5892	RAD51 paralog D
ENSG0000	14.23193	-0.00784	0.211633	0.924256	0.983706	NA	NA	NA
ENSG0000	85.47636	-0.01199	0.181869	0.927347	0.983706	ESCO1	114799	establishment of sister chromatid cohesion N-acetyltransferase 1
ENSG0000	5.638832	0.004897	0.224584	0.925996	0.983706	CABYR	26256	calcium binding tyrosine phosphorylation regulated
ENSG0000	4.441982	-0.00444	0.227231	0.922501	0.983706	NA	NA	NA
ENSG0000	283.557	0.00995	0.160592	0.924207	0.983706	THOP1	7064	thimet oligopeptidase 1
ENSG0000	26.18927	-0.00992	0.187889	0.926847	0.983706	STAP2	55620	signal transducing adaptor family member 2
ENSG0000	107.7113	-0.00691	0.209737	0.926493	0.983706	SEMA6B	10501	semaphorin 6B
ENSG0000	8.776257	-0.00659	0.219877	0.92211	0.983706	LOC10537	1.05E+08	uncharacterized LOC105372273
ENSG0000	1222.151	0.010094	0.164583	0.924294	0.983706	RFX1	5989	regulatory factor X1
ENSG0000	626.2045	0.01002	0.126429	0.925575	0.983706	REX1BD	55049	required for excision 1-B domain containing
ENSG0000	64.86636	-0.01027	0.178184	0.925794	0.983706	ZNF737	1E+08	zinc finger protein 737
ENSG0000	19.56837	-0.0094	0.202904	0.922084	0.983706	ZFP14	57677	ZFP14 zinc finger protein
ENSG0000	18.56208	0.009341	0.197897	0.927642	0.983706	ZNF225	7768	zinc finger protein 225
ENSG0000	46.36695	0.009693	0.192663	0.925009	0.983706	ZNF468	90333	zinc finger protein 468
ENSG0000	20.70461	-0.01078	0.191672	0.922618	0.983706	NA	NA	NA
ENSG0000	55.45336	0.012736	0.164594	0.927482	0.983706	SHLD1	149840	shieldin complex subunit 1
ENSG0000	19.66916	0.009482	0.210566	0.923362	0.983706	ABCC13	150000	ATP binding cassette subfamily C member 13 (pseudogene)
ENSG0000	286.246	0.009896	0.14595	0.925402	0.983706	TRAPPC10	7109	trafficking protein particle complex subunit 10
ENSG0000	17.58554	-0.00916	0.203881	0.923186	0.983706	NA	NA	NA
ENSG0000	13.23986	-0.00979	0.197532	0.92457	0.983706	NA	NA	NA
ENSG0000	73.84671	0.009409	0.201508	0.925563	0.983706	COX7B	1349	cytochrome c oxidase subunit 7B
ENSG0000	65.73721	-0.00865	0.198159	0.926992	0.983706	CHM	1121	CHM Rab escort protein
ENSG0000	2.757651	0.005434	0.225034	0.924174	0.983706	NA	NA	NA
ENSG0000	119032.7	-0.01067	0.152736	0.9227	0.983706	ND2	4536	NADH dehydrogenase subunit 2
ENSG0000	368.5295	-0.00933	0.121794	0.92775	0.983707	PARK7	11315	Parkinsonism associated deglycase
ENSG0000	14.74337	0.00804	0.213007	0.927742	0.983707	MTATP8P2	1.06E+08	MT-ATP8 pseudogene 2
ENSG0000	731.8097	-0.00949	0.135821	0.92785	0.983757	TBRG4	9238	transforming growth factor beta regulator 4
ENSG0000	576.0511	-0.00995	0.15076	0.927982	0.983783	ABCB8	11194	ATP binding cassette subfamily B member 8
ENSG0000	14.00658	-0.00841	0.20375	0.927981	0.983783	NA	NA	NA

ENSG0000	26.57796	-0.00771	0.206101	0.928411	0.983806	ZFP69	339559	ZFP69 zinc finger protein
ENSG0000	2.690833	0.005472	0.231658	0.928367	0.983806	SSBP3-AS1	619518	SSBP3 antisense RNA 1
ENSG0000	3.705641	0.011606	0.225376	0.928278	0.983806	IBA57-DT	574432	IBA57 divergent transcript
ENSG0000	87.79086	0.010316	0.187178	0.928237	0.983806	LINC00211	1.02E+08	long intergenic non-protein coding RNA 211
ENSG0000	363.8354	0.008676	0.125789	0.92826	0.983806	PSMD6	9861	proteasom non-ATPase 6
ENSG0000	161.3077	-0.01036	0.134371	0.928168	0.983806	ISY1	57461	ISY1 splicing factor homolog
ENSG0000	192.7936	-0.01166	0.146707	0.928242	0.983806	POLR3D	661	RNA polymerase III subunit D
ENSG0000	3.864229	0.004998	0.22231	0.928581	0.983806	NA	NA	NA
ENSG0000	11.22906	0.008312	0.209818	0.928512	0.983806	NA	NA	NA
ENSG0000	672.4219	-0.0108	0.141315	0.928511	0.983806	ENTPD6	955	ectonucleoside triphosphate diphosphohydrolase 6
ENSG0000	775.2564	-0.00753	0.101395	0.928595	0.983806	PSMA7	5688	proteasome 20S subunit alpha 7
ENSG0000	182.9986	-0.01105	0.154485	0.928717	0.983878	TNFSF13	8741	TNF superfamily member 13
ENSG0000	34.54183	-0.00884	0.194895	0.928838	0.983887	CFAP36	112942	cilia and flagella associated protein 36
ENSG0000	59.77628	-0.00815	0.184251	0.928822	0.983887	ZNF277	11179	zinc finger protein 277
ENSG0000	91.46523	-0.00735	0.206574	0.928994	0.983887	ANKRD49	54851	ankyrin repeat domain 49
ENSG0000	116.391	0.009874	0.18835	0.928976	0.983887	RPAP3	79657	RNA polymerase II associated protein 3
ENSG0000	6.847146	-0.00651	0.21836	0.928959	0.983887	SGSM3	27352	small G protein signaling modulator 3
ENSG0000	231.3211	-0.00966	0.185309	0.929086	0.983927	FZD2	2535	frizzled class receptor 2
ENSG0000	198.6534	0.009798	0.127528	0.929348	0.984091	NA	NA	NA
ENSG0000	250.4074	-0.01414	0.165932	0.929356	0.984091	CDT1	81620	chromatin licensing and DNA replication factor 1
ENSG0000	323.526	-0.00809	0.159858	0.929401	0.984091	NF2	4771	NF2 moesin-ezrin-radixin like (MERLIN) tumor suppressor
ENSG0000	90.01609	0.010168	0.171377	0.930761	0.984144	NMNAT1	64802	nicotinamide nucleotide adenylyltransferase 1
ENSG0000	47.76868	0.01007	0.172327	0.931235	0.984144	MAGOH	4116	mago hom exon junction complex subunit
ENSG0000	679.0074	-0.01243	0.174591	0.930431	0.984144	SELENOF	9403	selenoprotein F
ENSG0000	19.42579	0.008198	0.197706	0.930265	0.984144	NA	NA	NA
ENSG0000	216.7702	-0.00791	0.189987	0.931463	0.984144	STXBP3	6814	syntaxin binding protein 3
ENSG0000	322.1523	0.009061	0.146987	0.931012	0.984144	LAMTOR2	28956	late endos MAPK and MTOR activator 2
ENSG0000	73.76726	0.007568	0.20057	0.931094	0.984144	SMC6	79677	structural maintenance of chromosomes 6
ENSG0000	726.6281	-0.00943	0.129651	0.929788	0.984144	WDR33	55339	WD repeat domain 33
ENSG0000	311.5956	-0.00826	0.127396	0.930619	0.984144	VILL	50853	villin like
ENSG0000	46.4333	-0.00854	0.195917	0.931407	0.984144	NA	NA	NA
ENSG0000	3013.508	0.008958	0.160158	0.930669	0.984144	TACC3	10460	transforming acidic coiled-coil containing protein 3
ENSG0000	117.9584	-0.00915	0.189652	0.930865	0.984144	UBA6	55236	ubiquitin like modifier activating enzyme 6
ENSG0000	17.86998	-0.00744	0.209069	0.930624	0.984144	ZGRF1	55345	zinc finger GRF-type containing 1
ENSG0000	5.726068	0.005481	0.220802	0.929554	0.984144	FAM169A	26049	family with sequence similarity 169 member A
ENSG0000	12.28043	-0.00818	0.205164	0.930893	0.984144	CDC25C	995	cell division cycle 25C
ENSG0000	2.520174	-0.00315	0.229423	0.930814	0.984144	RN7SL336	1.06E+08	RNA 7SL cytoplasm pseudogene
ENSG0000	331.4709	0.009957	0.15064	0.93143	0.984144	NA	NA	NA
ENSG0000	635.1777	-0.00553	0.162725	0.930223	0.984144	HBP1	26959	HMG-box transcription factor 1
ENSG0000	138.1784	-0.00831	0.143622	0.931076	0.984144	METTL2B	55798	methyltra methylcytidine
ENSG0000	3.341452	0.004259	0.227448	0.930342	0.984144	NA	NA	NA
ENSG0000	283.8533	-0.00954	0.111857	0.92984	0.984144	PRKAG2	51422	protein kinase AMP-activated non-catalytic subunit gamma 2
ENSG0000	32.87822	-0.00654	0.194244	0.930404	0.984144	WRN	7486	WRN RecQ like helicase
ENSG0000	4.528415	0.00571	0.220955	0.930539	0.984144	NA	NA	NA
ENSG0000	97.67643	0.009435	0.146279	0.931319	0.984144	FANCG	2189	FA complementation group G
ENSG0000	6.16628	-0.00582	0.219329	0.929854	0.984144	NA	NA	NA
ENSG0000	131.3393	0.010194	0.167966	0.931009	0.984144	FADS1	3992	fatty acid desaturase 1
ENSG0000	164.9459	0.009946	0.158784	0.929842	0.984144	XRCC3	7517	X-ray repair cross complementing 3
ENSG0000	2.653904	-0.00384	0.228679	0.931479	0.984144	NA	NA	NA
ENSG0000	503.9599	-0.00949	0.172184	0.931342	0.984144	TBL3	10607	transducin beta like 3
ENSG0000	30.06583	-0.01041	0.179493	0.929955	0.984144	KRBOX5	124411	KRAB box domain containing 5
ENSG0000	134.9895	0.01097	0.176642	0.930821	0.984144	AOC3	8639	amine oxidase copper containing 3
ENSG0000	2135.812	0.009522	0.165869	0.930899	0.984144	ARID3A	1820	AT-rich interaction domain 3A
ENSG0000	107.402	0.010036	0.160557	0.931493	0.984144	LINC00665	1.01E+08	long intergenic non-protein coding RNA 665
ENSG0000	27.35504	0.009962	0.183922	0.929984	0.984144	ZNF321P	399669	zinc finger pseudogene
ENSG0000	2485.179	0.010481	0.135995	0.93006	0.984144	U2AF2	11338	U2 small nuclear RNA auxiliary factor 2
ENSG0000	12.60066	-0.00747	0.20852	0.931225	0.984144	NRSN2-AS	1.01E+08	NRSN2 antisense RNA 1
ENSG0000	132.947	-0.00924	0.177499	0.931204	0.984144	ZFX	7543	zinc finger protein X-linked
ENSG0000	5.050254	0.005487	0.224003	0.929961	0.984144	NA	NA	NA
ENSG0000	4341.395	0.010018	0.159329	0.931952	0.984173	S100A4	6275	S100 calcium binding protein A4
ENSG0000	7741.734	-0.0088	0.180383	0.932779	0.984173	SEMA4A	64218	semaphorin 4A
ENSG0000	75.6309	0.008864	0.162437	0.933015	0.984173	RPS6KC1	26750	ribosomal protein S6 kinase C1
ENSG0000	2960.561	-0.00925	0.144716	0.932137	0.984173	NA	NA	NA
ENSG0000	232.5129	0.015562	0.164376	0.932802	0.984173	PASK	23178	PAS domain containing serine/threonine kinase
ENSG0000	613.507	-0.0086	0.157489	0.931917	0.984173	DHX30	22907	DExH-box helicase 30
ENSG0000	5.338745	-0.00477	0.227519	0.933089	0.984173	NEK11	79858	NIMA related kinase 11
ENSG0000	24.2558	-0.00877	0.193197	0.933034	0.984173	TRIM59	286827	tripartite motif containing 59
ENSG0000	47.78742	-0.00634	0.212515	0.93162	0.984173	BASP1-AS1	285696	BASP1 antisense RNA 1
ENSG0000	4868.396	0.007531	0.169595	0.933176	0.984173	LST1	7940	leukocyte specific transcript 1
ENSG0000	60.33345	-0.00808	0.188	0.932039	0.984173	TNXB	7148	tenascin XB
ENSG0000	4.358312	2.19E-04	0.233426	0.932522	0.984173	NA	NA	NA
ENSG0000	35.24886	-0.0099	0.171386	0.931785	0.984173	POLR2J3-U	1.23E+08	POLR2J3-UPK3BL2 readthrough
ENSG0000	7.699051	-0.00449	0.224687	0.932407	0.984173	NA	NA	NA
ENSG0000	25.6442	0.007992	0.204769	0.932392	0.984173	ZNF248	57209	zinc finger protein 248
ENSG0000	71.10511	0.009538	0.157269	0.932534	0.984173	CACNB3	784	calcium voltage-gated channel auxiliary subunit beta 3
ENSG0000	252.2312	0.009349	0.144019	0.932133	0.984173	ZCCHC8	55596	zinc finger CCHC-type containing 8
ENSG0000	5.237191	0.004773	0.224312	0.932438	0.984173	CFAP97D2	1.02E+08	CFAP97 domain containing 2
ENSG0000	1208.082	0.010188	0.146901	0.932276	0.984173	RBM25	58517	RNA binding motif protein 25
ENSG0000	3.287985	-0.00494	0.228527	0.933108	0.984173	ESRRB	2103	estrogen related receptor beta
ENSG0000	7.817446	0.008139	0.213362	0.933186	0.984173	MESP1	55897	mesoderm posterior bHLH transcription factor 1
ENSG0000	25.4589	-0.00904	0.189978	0.931776	0.984173	ZNF771	51333	zinc finger protein 771
ENSG0000	387.3012	0.009652	0.132342	0.931818	0.984173	SARM1	23098	sterile alpha and TIR motif containing 1
ENSG0000	90.44016	-0.00897	0.177125	0.932838	0.984173	PLXDC1	57125	plexin domain containing 1
ENSG0000	696.9813	-0.007	0.122043	0.931902	0.984173	KAT7	11143	lysine acetyltransferase 7
ENSG0000	157.3254	-0.00966	0.172695	0.932983	0.984173	APPBP2	10513	amyloid beta precursor protein binding protein 2
ENSG0000	80.22531	-0.00984	0.162183	0.932596	0.984173	NA	NA	NA
ENSG0000	29.61575	0.007642	0.200936	0.933106	0.984173	NTN5	126147	netrin 5
ENSG0000	2916.01	0.009902	0.156473	0.932898	0.984173	ZNF865	1.01E+08	zinc finger protein 865
ENSG0000	57.85529	0.009109	0.193672	0.932165	0.984173	ATP9A	10079	ATPase phospholipid transporting 9A (putative)
ENSG0000	21.84147	-0.0056	0.216838	0.932796	0.984173	SEPT5-GP1	1.01E+08	SEPT5-GP1BB readthrough
ENSG0000	33.17495	-0.00757	0.199933	0.933721	0.984221	C1QTNF12	388581	C1q and TNF related 12
ENSG0000	1884.865	0.009692	0.171566	0.933992	0.984221	FHL3	2275	four and a half LIM domains 3
ENSG0000	116.5941	0.01022	0.177381	0.93436	0.984221	IAH1	285148	isoamyl acetate hydrolyzing esterase 1 (putative)
ENSG0000	5.032877	-0.0075	0.224327	0.933734	0.984221	NA	NA	NA
ENSG0000	854.3068	-0.01045	0.150158	0.933562	0.984221	HTT	3064	huntingtin
ENSG0000	110.9438	-0.00995	0.160045	0.933851	0.984221	GEMIN5	25929	gem nuclear organelle associated protein 5

ENSG0000	12.49862	0.006534	0.216399	0.934206	0.984221	NKAPL	222698	NFKB activating protein like
ENSG0000	103.5874	-0.00932	0.158153	0.93426	0.984221	NUDT1	4521	nudix hydrolase 1
ENSG0000	482.4549	-0.00921	0.163571	0.933951	0.984221	EPHB4	2050	EPH receptor B4
ENSG0000	869.5039	0.010639	0.162506	0.933988	0.984221	SCRIB	23513	scribble planar cell polarity protein
ENSG0000	176.8813	0.007769	0.14394	0.934228	0.984221	FUT11	170384	fucosyltransferase 11
ENSG0000	559.7592	0.007986	0.141603	0.933574	0.984221	NPTN	27020	neuroplastin
ENSG0000	273.0759	-0.00885	0.132797	0.934125	0.984221	DNAJA3	9093	DnaJ heat shock protein family (Hsp40) member A3
ENSG0000	173.2878	0.010372	0.169077	0.93436	0.984221	LCAT	3931	lecithin-cholesterol acyltransferase
ENSG0000	11.6816	0.002569	0.233401	0.933506	0.984221	TBC1D3D	1.01E+08	TBC1 domain family member 3D
ENSG0000	376.2245	0.009289	0.130708	0.933786	0.984221	TSPOAP1	9256	TSPO associated protein 1
ENSG0000	157.3726	-0.00971	0.152172	0.933832	0.984221	NA	NA	NA
ENSG0000	7.974942	0.004997	0.222975	0.933802	0.984221	LINC0186	284365	long intergenic non-protein coding RNA 1869
ENSG0000	52.85018	0.010041	0.185783	0.934298	0.984221	NRSN2	80023	neurensin 2
ENSG0000	5.917008	0.003367	0.228558	0.933533	0.984221	C22orf15	150248	chromosome 22 open reading frame 15
ENSG0000	15135.64	-0.00845	0.184265	0.933824	0.984221	MYH9	4627	myosin heavy chain 9
ENSG0000	25.74805	0.008995	0.18365	0.934568	0.984384	FBLN7	129804	fibulin 7
ENSG0000	72.7251	-0.00936	0.17716	0.934745	0.984497	ALG6	29929	ALG6 alph 3-glucosyltransferase
ENSG0000	188.0707	0.010144	0.165515	0.934883	0.984497	RNPC3	55599	RNA bindi RRM) containing 3
ENSG0000	433.427	-0.00913	0.157022	0.934841	0.984497	PDE1B	5153	phosphodiesterase 1B
ENSG0000	72.52411	-0.01307	0.178172	0.934891	0.984497	NA	NA	NA
ENSG0000	672.6703	0.009671	0.172036	0.934982	0.984537	PTPN12	5782	protein tyrosine phosphatase non-receptor type 12
ENSG0000	306.7243	0.00929	0.148344	0.935687	0.984757	LRRC8D	55144	leucine rich repeat containing 8 VRAC subunit D
ENSG0000	2.487357	-0.00351	0.224688	0.935604	0.984757	RPS2P17	400963	ribosomal protein S2 pseudogene 17
ENSG0000	13043.23	0.009765	0.165899	0.935705	0.984757	ABTB1	80325	ankyrin repeat and BTB domain containing 1
ENSG0000	2076.65	-0.00894	0.156491	0.935575	0.984757	EEIG1	399665	estrogen-induced osteoclastogenesis regulator 1
ENSG0000	116.2938	0.008511	0.169163	0.935368	0.984757	SEC61A2	55176	SEC61 translocon subunit alpha 2
ENSG0000	328.097	-0.01507	0.190161	0.935444	0.984757	LIN7A	8825	lin-7 homc crumbs cell polarity complex component
ENSG0000	115.1776	-0.00536	0.159705	0.935729	0.984757	ENOSF1	55556	enolase superfamily member 1
ENSG0000	16.93597	-0.00822	0.197153	0.935331	0.984757	ZNF433-A	1.02E+08	ZNF433 and ZNF878 antisense RNA 1
ENSG0000	10.30475	-0.00432	0.225953	0.935478	0.984757	CKM	1158	creatine ki M-type
ENSG0000	4.4437	-0.00335	0.226917	0.935538	0.984757	LINC0065	200261	long intergenic non-protein coding RNA 656
ENSG0000	221.6057	-0.01095	0.162901	0.935805	0.984781	ZNF48	197407	zinc finger protein 48
ENSG0000	115.6977	-0.00856	0.138536	0.936196	0.984807	NVL	4931	nuclear VCP like
ENSG0000	4.367538	-0.00474	0.223136	0.936245	0.984807	NA	NA	NA
ENSG0000	14.40665	0.006587	0.209876	0.936134	0.984807	LINC0096	1.01E+08	long intergenic non-protein coding RNA 968
ENSG0000	156.9319	0.009297	0.139735	0.936249	0.984807	RABEPK	10244	Rab9 effector protein with kelch motifs
ENSG0000	3.265257	0.004089	0.226512	0.936306	0.984807	NA	NA	NA
ENSG0000	4.741347	0.004583	0.224737	0.935981	0.984807	CLEC4A	50856	C-type lectin domain family 4 member A
ENSG0000	9758.134	0.008339	0.148853	0.936285	0.984807	MBD6	114785	methyl-CpG binding domain protein 6
ENSG0000	1008.679	-0.00813	0.151716	0.936314	0.984807	METTL26	84326	methyltransferase like 26
ENSG0000	1074.565	0.009735	0.13223	0.935936	0.984807	DYRK1B	9149	dual specificity tyrosine phosphorylation regulated kinase 1B
ENSG0000	3.25889	-0.00242	0.22931	0.936654	0.985108	TPBG	7162	trophoblast glycoprotein
ENSG0000	13.75102	0.006956	0.210619	0.936796	0.985201	NA	NA	NA
ENSG0000	32.18939	0.007092	0.209095	0.936945	0.985245	MYO10	4651	myosin X
ENSG0000	7.518314	-0.00518	0.220924	0.936901	0.985245	SLC6A13	6540	solute carrier family 6 member 13
ENSG0000	1570.873	0.009054	0.14353	0.937234	0.98547	INPP5K	51763	inositol polyphosphate-5-phosphatase K
ENSG0000	27.62623	0.009408	0.201612	0.93736	0.98547	ZNF667-A	1E+08	ZNF667 antisense RNA 1 (head to head)
ENSG0000	100.7127	0.009504	0.162135	0.937375	0.98547	STARD8	9754	StAR related lipid transfer domain containing 8
ENSG0000	362.9445	-0.00819	0.171771	0.937281	0.98547	CUL4B	8450	cullin 4B
ENSG0000	129.1398	-0.00737	0.188807	0.937551	0.985542	GTF3C3	9330	general transcription factor IIIC subunit 3
ENSG0000	61.76574	0.008744	0.157786	0.937504	0.985542	TMEM116	89894	transmembrane protein 116
ENSG0000	383.4286	-0.01607	0.134265	0.937837	0.985643	MTERF4	130916	mitochondrial transcription termination factor 4
ENSG0000	652.6791	-0.00904	0.153247	0.937751	0.985643	ATM	472	ATM serine/threonine kinase
ENSG0000	10.10574	0.00695	0.211812	0.937862	0.985643	NA	NA	NA
ENSG0000	423.8254	0.008036	0.19137	0.937804	0.985643	IGLV10-54	28772	immunoglobulin lambda variable 10-54
ENSG0000	9584.373	0.008802	0.135462	0.938	0.985731	OAZ1	4946	ornithine decarboxylase antizyme 1
ENSG0000	2.80729	0.004023	0.229189	0.938132	0.985814	NA	NA	NA
ENSG0000	33.18748	-0.00794	0.194948	0.938546	0.985953	EBLN2	55096	endogenous Bornavirus like nucleoprotein 2
ENSG0000	48.81209	-0.008	0.186555	0.938514	0.985953	PDE6B	5158	phosphodiesterase 6B
ENSG0000	144.1978	-0.00845	0.149743	0.938395	0.985953	FRG1	2483	FSHD region gene 1
ENSG0000	6.393054	-0.00446	0.225002	0.938588	0.985953	RN7SL200	1.06E+08	RNA 7SL cytoplasm pseudogene
ENSG0000	206.7074	-0.0013	0.152264	0.938377	0.985953	SIRT3	23410	sirtuin 3
ENSG0000	317.4537	0.00845	0.147484	0.938579	0.985953	RCOR1	23186	REST corepressor 1
ENSG0000	347.1732	0.007581	0.193895	0.938841	0.985993	IL6ST	3572	interleukin 6 cytokine family signal transducer
ENSG0000	6.292739	0.005132	0.221264	0.938816	0.985993	FAM86B1	85002	family with sequence similarity 86 member B1
ENSG0000	212.3186	0.009769	0.154853	0.938824	0.985993	NA	NA	NA
ENSG0000	18.13457	-0.00697	0.205531	0.938687	0.985993	ZNF793	390927	zinc finger protein 793
ENSG0000	51.8246	-0.00817	0.185789	0.939019	0.986124	HPRT1	3251	hypoxanthine phosphoribosyltransferase 1
ENSG0000	26.20494	0.006129	0.215102	0.93914	0.986137	PLA2G7	7941	phospholipase A2 group VII
ENSG0000	686.6493	0.008453	0.134734	0.939106	0.986137	PGPEP1	54858	pyroglutamyl-peptidase I
ENSG0000	1348.432	0.008767	0.171983	0.9398	0.98624	RNPEP	6051	arginyl aminopeptidase
ENSG0000	618.6017	-0.01044	0.166664	0.939531	0.98624	ABHD5	51099	abhydrola lysophosphatidic acid acyltransferase
ENSG0000	3.40331	-0.0012	0.227188	0.939757	0.98624	AREG	374	amphiregulin
ENSG0000	6.770672	-0.00484	0.222646	0.939904	0.98624	PTPN13	5783	protein tyrosine phosphatase non-receptor type 13
ENSG0000	484.8381	-0.00799	0.123275	0.939816	0.98624	BRD8	10902	bromodomain containing 8
ENSG0000	26.75533	0.008178	0.192676	0.939799	0.98624	SDHAF4	135154	succinate dehydrogenase complex assembly factor 4
ENSG0000	144.8801	0.008532	0.185599	0.939875	0.98624	SLC46A2	57864	solute carrier family 46 member 2
ENSG0000	1216.003	-0.00891	0.144624	0.939938	0.98624	POLR2L	5441	RNA polyn I and III subunit L
ENSG0000	110.7473	0.008531	0.158078	0.939711	0.98624	MTRFR	91574	mitochondrial translation release factor in rescue
ENSG0000	2.466286	0.001399	0.230123	0.939738	0.98624	PROX2	283571	prospero homeobox 2
ENSG0000	11.17953	0.0062	0.214372	0.939329	0.98624	NA	NA	NA
ENSG0000	688.6097	0.008401	0.151863	0.93968	0.98624	METRNL	284207	meteorin l glial cell differentiation regulator
ENSG0000	6123.627	-0.0083	0.15001	0.939483	0.98624	ZBTB7A	51341	zinc finger and BTB domain containing 7A
ENSG0000	548.7683	0.008501	0.146244	0.940162	0.98638	UBQLN4	56893	ubiquilin 4
ENSG0000	3141.641	-0.0079	0.176404	0.940179	0.98638	CD82	3732	CD82 molecule
ENSG0000	7.664337	-0.00472	0.220653	0.940294	0.986388	NA	NA	NA
ENSG0000	89.9766	-0.00551	0.156139	0.940273	0.986388	PKIG	11142	cAMP-dependent protein kinase inhibitor gamma
ENSG0000	2107.111	-0.00825	0.124784	0.940484	0.986531	GLYR1	84656	glyoxylate reductase 1 homolog
ENSG0000	194.7456	0.007144	0.204673	0.940675	0.986655	TMCO1	54499	transmembrane and coiled-coil domains 1
ENSG0000	4.938568	0.006736	0.223747	0.94071	0.986655	SENP8	123228	SUMO pe NEDD8 specific
ENSG0000	422.5327	-0.00825	0.167461	0.940838	0.986732	KLC2	64837	kinesin light chain 2
ENSG0000	7.76747	-0.00428	0.223045	0.941007	0.986853	NA	NA	NA
ENSG0000	59.57957	-0.00765	0.189169	0.94111	0.986859	MINDY3	80013	MINDY lysine 48 deubiquitinase 3
ENSG0000	3.533326	-0.00383	0.225959	0.94112	0.986859	LOC10042	1E+08	nucleosome assembly protein 1 like 1 pseudogene
ENSG0000	10.74496	0.005307	0.215034	0.942129	0.986916	NA	NA	NA
ENSG0000	3.810555	-0.00452	0.223362	0.941937	0.986916	NFASC	23114	neurofascin

ENSG0000	239.917	-0.00719	0.191591	0.941949	0.986916	CYP1B1	1545	cytochrome P450 family 1 subfamily B member 1
ENSG0000	7.28676	-0.00482	0.219877	0.941851	0.986916	DYTN	391475	dystrotelin
ENSG0000	293.8366	-0.00831	0.162185	0.941347	0.986916	MCUB	55013	mitochondrial calcium uniporter dominant negative subunit beta
ENSG0000	28.87139	-0.00623	0.205612	0.942145	0.986916	AKAP7	9465	A-kinase anchoring protein 7
ENSG0000	70.65067	0.008841	0.16501	0.941541	0.986916	MED30	90390	mediator complex subunit 30
ENSG0000	93.35348	-0.00778	0.154572	0.941713	0.986916	ATE1	11101	arginyltransferase 1
ENSG0000	26950.18	-0.00771	0.135902	0.941846	0.986916	LYZ	4069	lysozyme
ENSG0000	2.769182	-0.00437	0.230017	0.941795	0.986916	NA	NA	NA
ENSG0000	2167.253	-0.00854	0.15578	0.942106	0.986916	ZNF592	9640	zinc finger protein 592
ENSG0000	4950.083	-0.00304	0.130289	0.941361	0.986916	PIK3R5	23533	phosphoinositide-3-kinase regulatory subunit 5
ENSG0000	2.524533	-0.00133	0.230321	0.942041	0.986916	NA	NA	NA
ENSG0000	4.594891	0.005007	0.22271	0.94146	0.986916	NA	NA	NA
ENSG0000	1545.025	0.008489	0.174162	0.941467	0.986916	GPI	2821	glucose-6-phosphate isomerase
ENSG0000	102.9096	-0.00707	0.175753	0.941961	0.986916	ZNF600	162966	zinc finger protein 600
ENSG0000	687.3078	0.012116	0.144653	0.941635	0.986916	VPS16	64601	VPS16 core subunit of CORVET and HOPS complexes
ENSG0000	6.234624	-0.00283	0.223342	0.941615	0.986916	NA	NA	NA
ENSG0000	30.21777	-0.00764	0.193355	0.942281	0.987002	ACTL10	170487	actin like 10
ENSG0000	313.0474	-0.00835	0.13909	0.942408	0.987023	ITFG1	81533	integrin alpha FG-GAP repeat containing 1
ENSG0000	3289.643	0.007724	0.147899	0.942382	0.987023	HDAC5	10014	histone deacetylase 5
ENSG0000	76.26859	0.007188	0.191276	0.942509	0.987072	TMEM209	84928	transmembrane protein 209
ENSG0000	214.0667	-0.00796	0.150627	0.94262	0.987132	DUSP11	8446	dual specificity phosphatase 11
ENSG0000	52.77023	0.008423	0.174316	0.942679	0.987138	LOC10537	1.05E+08	uncharacterized LOC105376805
ENSG0000	3.613943	0.002675	0.225931	0.942777	0.987184	NA	NA	NA
ENSG0000	10.04304	-0.00362	0.224181	0.94352	0.987241	TMEM54	113452	transmembrane protein 54
ENSG0000	4.017973	-0.00404	0.226227	0.943883	0.987241	HEYL	26508	hes related family bHLH transcription factor with YRPW motif like
ENSG0000	86.91736	-0.00677	0.195843	0.9433	0.987241	GADD45A	1647	growth arrest and DNA damage inducible alpha
ENSG0000	67.99714	-0.00791	0.176518	0.943459	0.987241	WDR47	22911	WD repeat domain 47
ENSG0000	1256.945	-0.00738	0.166856	0.944032	0.987241	LYST	1130	lysosomal trafficking regulator
ENSG0000	203.1087	-0.01026	0.149196	0.944079	0.987241	ASXL2	55252	ASXL transcriptional regulator 2
ENSG0000	3.029143	0.002424	0.229542	0.944202	0.987241	NA	NA	NA
ENSG0000	132.9694	0.008208	0.143861	0.943054	0.987241	OTULIN	90268	OTU deubiquitinase with linear linkage specificity
ENSG0000	6.419197	0.002311	0.225054	0.944044	0.987241	NA	NA	NA
ENSG0000	6.241529	-0.0046	0.221324	0.944054	0.987241	NA	NA	NA
ENSG0000	16.88127	-0.00667	0.202401	0.943358	0.987241	MYO6	4646	myosin VI
ENSG0000	427.5112	0.007289	0.129225	0.943804	0.987241	REPS1	85021	RALBP1 associated Eps domain containing 1
ENSG0000	61.66471	0.007744	0.19312	0.942917	0.987241	FBXO30	84085	F-box protein 30
ENSG0000	899.4372	-0.00618	0.17381	0.943988	0.987241	SORBS3	10174	sorbin and SH3 domain containing 3
ENSG0000	38.07273	0.007307	0.19212	0.944203	0.987241	CPEB3	22849	cytoplasmic polyadenylation element binding protein 3
ENSG0000	3.293677	0.004408	0.223773	0.943621	0.987241	ZBTB44-D'	646383	ZBTB44 divergent transcript
ENSG0000	639.9427	-0.00785	0.169906	0.942956	0.987241	USP5	8078	ubiquitin specific peptidase 5
ENSG0000	17.69796	0.006096	0.212697	0.943915	0.987241	NA	NA	NA
ENSG0000	2.57804	-0.00334	0.230782	0.94327	0.987241	NA	NA	NA
ENSG0000	150.9638	0.007617	0.194045	0.943341	0.987241	EXOC5	10640	exocyst complex component 5
ENSG0000	11.4465	0.006151	0.204055	0.943752	0.987241	MIR4538	1.01E+08	microRNA 4538
ENSG0000	26.82515	-0.00745	0.195042	0.944197	0.987241	PDF	64146	peptide de mitochondrial
ENSG0000	327.2061	0.007812	0.122817	0.943255	0.987241	FAM98C	147965	family with sequence similarity 98 member C
ENSG0000	35.06451	0.00784	0.176938	0.943967	0.987241	ZNF419	79744	zinc finger protein 419
ENSG0000	20.27675	0.006176	0.209976	0.944233	0.987241	NA	NA	NA
ENSG0000	387.0672	3.28E-04	0.233067	0.943569	0.987241	XIST	7503	X inactive specific transcript
ENSG0000	182.0879	0.008159	0.150979	0.944526	0.987311	STX12	23673	syntaxin 12
ENSG0000	4.942087	0.004266	0.222185	0.944407	0.987311	NA	NA	NA
ENSG0000	406.7243	0.007475	0.127073	0.944839	0.987311	ZNF513	130557	zinc finger protein 513
ENSG0000	265.1442	-0.00751	0.175316	0.94472	0.987311	ZNF638	27332	zinc finger protein 638
ENSG0000	29.0288	-0.00751	0.188178	0.944822	0.987311	BTN2A3P	54718	butyrophil pseudogene
ENSG0000	138.7892	0.008745	0.165933	0.944806	0.987311	PPP1R32	220004	protein phosphatase 1 regulatory subunit 32
ENSG0000	2.901499	-0.00318	0.229832	0.9448	0.987311	LOC10537	1.05E+08	uncharacterized LOC105370174
ENSG0000	11.07568	-0.00449	0.218547	0.944761	0.987311	NA	NA	NA
ENSG0000	8.846815	0.005701	0.212738	0.944431	0.987311	PIMREG	54478	PICALM interacting mitotic regulator
ENSG0000	2.689533	0.002974	0.229068	0.944484	0.987311	NA	NA	NA
ENSG0000	4.721318	0.001497	0.231382	0.94505	0.987449	DNAI4	79819	dynein axonemal intermediate chain 4
ENSG0000	29.64479	-0.00599	0.19129	0.945237	0.987449	ZNG1C	445571	Zn regulated GTPase metalloprotein activator 1C
ENSG0000	239.7306	-0.00772	0.140911	0.945087	0.987449	SEC11A	23478	SEC11 hon signal peptidase complex subunit
ENSG0000	111.8575	-0.00807	0.15892	0.945247	0.987449	LRRC75A	388341	leucine rich repeat containing 75A
ENSG0000	243.2625	0.010362	0.178195	0.94519	0.987449	MISP3	113230	MISP family member 3
ENSG0000	3.095218	-0.00463	0.223963	0.945294	0.987449	GPR173	54328	G protein-coupled receptor 173
ENSG0000	149.5255	0.011299	0.150895	0.945394	0.987455	SFXN1	94081	sideroflexin 1
ENSG0000	2.533149	0.002441	0.233476	0.945408	0.987455	CLDN3	1365	claudin 3
ENSG0000	1088.398	-0.00797	0.179733	0.945477	0.987471	CMTM6	54918	CKLF like MARVEL transmembrane domain containing 6
ENSG0000	30.38356	-0.00559	0.206688	0.945636	0.98758	SNAI1	6615	snail family transcriptional repressor 1
ENSG0000	578.4555	0.008561	0.157943	0.945841	0.987655	GOLGA7	51125	golgin A7
ENSG0000	28.3586	0.006847	0.211825	0.945924	0.987655	NA	NA	NA
ENSG0000	297.1773	-0.00757	0.154304	0.945913	0.987655	GRWD1	83743	glutamate rich WD repeat containing 1
ENSG0000	10.37088	-0.00637	0.212716	0.945781	0.987655	NA	NA	NA
ENSG0000	9.727284	0.005473	0.215111	0.946259	0.987671	NA	NA	NA
ENSG0000	6.14167	-0.00445	0.221412	0.946459	0.987671	NA	NA	NA
ENSG0000	49.95103	0.007844	0.190953	0.946063	0.987671	USP37	57695	ubiquitin specific peptidase 37
ENSG0000	256.8146	-0.00719	0.146724	0.946414	0.987671	FAM131A	131408	family with sequence similarity 131 member A
ENSG0000	1123.96	0.001325	0.138699	0.946478	0.987671	PAK2	5062	p21 (RAC1) activated kinase 2
ENSG0000	2.463071	0.002803	0.228682	0.946364	0.987671	CYTL1	54360	cytokine like 1
ENSG0000	72.84482	-0.00754	0.177768	0.946039	0.987671	NME8	51314	NME/NM23 family member 8
ENSG0000	294.5203	0.007167	0.170258	0.946428	0.987671	PSMA3-AS	379025	PSMA3 antisense RNA 1
ENSG0000	5276.025	-0.00724	0.141564	0.946471	0.987671	CFD	1675	complement factor D
ENSG0000	424.0681	0.009454	0.154626	0.946204	0.987671	CD320	51293	CD320 molecule
ENSG0000	52.90538	-0.00642	0.19301	0.946576	0.987681	ISOC1	51015	isochorismatase domain containing 1
ENSG0000	17.78139	-0.00484	0.216059	0.946595	0.987681	LOC10536	1.05E+08	uncharacterized LOC105369344
ENSG0000	5.37447	0.002649	0.229432	0.946705	0.98774	NA	NA	NA
ENSG0000	41.73889	0.007329	0.18459	0.94677	0.987751	NBL1	4681	NBL1 DAN family BMP antagonist
ENSG0000	76.47939	0.007253	0.18409	0.946866	0.987765	ATAD1	84896	ATPase family AAA domain containing 1
ENSG0000	112.0575	0.007777	0.163296	0.946909	0.987765	DUSP8	1850	dual specificity phosphatase 8
ENSG0000	281.8601	-0.0073	0.14672	0.946945	0.987765	TNRC6C	57690	trinucleotide repeat containing adaptor 6C
ENSG0000	3.789306	-0.00326	0.227623	0.947223	0.987773	HSD11B1-,	1.02E+08	HSD11B1 antisense RNA 1
ENSG0000	177.7344	0.005041	0.146646	0.947161	0.987773	MBOAT1	154141	membrane bound O-acyltransferase domain containing 1
ENSG0000	19.71545	-0.00656	0.196987	0.947218	0.987773	METTL15	196074	methyltransferase like 15
ENSG0000	40.47116	0.007809	0.179023	0.947132	0.987773	NA	NA	NA
ENSG0000	785.0644	0.016865	0.177571	0.947014	0.987773	ABCG1	9619	ATP binding cassette subfamily G member 1
ENSG0000	252.1952	0.008043	0.173697	0.947371	0.987816	DTX1	1840	deltex E3 ubiquitin ligase 1

ENSG0000	471.3987	0.007513	0.150211	0.947349	0.987816	ERAL1	26284	Era like 12S mitochondrial rRNA chaperone 1
ENSG0000	237.7579	-0.00729	0.180615	0.947698	0.9881	FAM13B	51306	family with sequence similarity 13 member B
ENSG0000	3.511716	0.001194	0.233443	0.947758	0.988107	NA	NA	NA
ENSG0000	99.09251	-0.00593	0.199685	0.948034	0.988176	KRCC1	51315	lysine rich coiled-coil 1
ENSG0000	46.45638	0.007575	0.175249	0.948311	0.988176	SLC15A2	6565	solute carrier family 15 member 2
ENSG0000	893.3725	0.008729	0.164007	0.948304	0.988176	RBPJ	3516	recombination signal binding protein for immunoglobulin kappa J region
ENSG0000	13.72448	-0.00587	0.205496	0.948268	0.988176	ZNF273	10793	zinc finger protein 273
ENSG0000	76.26417	-0.0071	0.180298	0.948051	0.988176	NA	NA	NA
ENSG0000	618.6916	0.007508	0.174205	0.948158	0.988176	CXCR5	643	C-X-C motif chemokine receptor 5
ENSG0000	280.1645	0.0087	0.139147	0.948247	0.988176	HINFP	25988	histone H4 transcription factor
ENSG0000	22.50951	-0.00691	0.192336	0.948056	0.988176	NA	NA	NA
ENSG0000	16.79944	-0.00548	0.205499	0.948034	0.988176	KLK14	43847	kallikrein related peptidase 14
ENSG0000	34.89664	-0.00721	0.187847	0.948715	0.988205	NA	NA	NA
ENSG0000	2.561494	0.00128	0.228417	0.948716	0.988205	NA	NA	NA
ENSG0000	16.61151	-0.00559	0.208087	0.948449	0.988205	NA	NA	NA
ENSG0000	18.81019	0.006644	0.207029	0.948573	0.988205	NA	NA	NA
ENSG0000	5.70612	0.003833	0.225115	0.948516	0.988205	NA	NA	NA
ENSG0000	5.127089	-0.00197	0.228141	0.948683	0.988205	PRR36	80164	proline rich 36
ENSG0000	3.374071	0.001612	0.230257	0.948653	0.988205	NA	NA	NA
ENSG0000	3.200959	0.003032	0.225887	0.948898	0.988226	NA	NA	NA
ENSG0000	2645.267	-0.00823	0.156059	0.948848	0.988226	C19orf38	255809	chromosome 19 open reading frame 38
ENSG0000	3738.978	0.006425	0.135077	0.948876	0.988226	SBF1	6305	SET binding factor 1
ENSG0000	528.6735	0.007402	0.13488	0.948972	0.988247	GIMAP8	155038	GTPase IMAP family member 8
ENSG0000	15.12556	0.005411	0.205623	0.94906	0.988282	IQCC	55721	IQ motif containing C
ENSG0000	88.98113	-0.00612	0.188346	0.949205	0.988378	NA	NA	NA
ENSG0000	146.4281	0.006597	0.19572	0.949269	0.988388	OSGIN1	29948	oxidative stress induced growth inhibitor 1
ENSG0000	161.356	0.006129	0.126816	0.949385	0.988396	PARP16	54956	poly(ADP-ribose) polymerase family member 16
ENSG0000	173.5518	-0.0075	0.166945	0.949366	0.988396	SPHK1	8877	sphingosine kinase 1
ENSG0000	142.1955	0.008154	0.152781	0.949519	0.988479	QSOX2	169714	quiescin sulfhydryl oxidase 2
ENSG0000	5.800336	-0.00387	0.222116	0.949619	0.98851	NA	NA	NA
ENSG0000	9.438639	-0.0058	0.206444	0.94971	0.98851	NA	NA	NA
ENSG0000	66.06681	0.007258	0.164352	0.949696	0.98851	TMLHE	55217	trimethyll epsilon
ENSG0000	532.6596	0.007322	0.156205	0.950399	0.988675	AKIRIN1	79647	akirin 1
ENSG0000	345.2686	-0.00584	0.136861	0.95003	0.988675	SDHC	6391	succinate dehydrogenase complex subunit C
ENSG0000	1584.386	0.003043	0.146763	0.950378	0.988675	RBM33	155435	RNA binding motif protein 33
ENSG0000	21.59395	-0.00586	0.199814	0.950358	0.988675	PKIA	5569	cAMP-dependent protein kinase inhibitor alpha
ENSG0000	2.418544	-0.00185	0.230368	0.950413	0.988675	NA	NA	NA
ENSG0000	11.68878	0.006022	0.208833	0.950624	0.988675	RPL7AP50	87688	ribosomal protein L7a pseudogene 50
ENSG0000	2332.016	-0.00704	0.147749	0.950615	0.988675	KMT2D	8085	lysine methyltransferase 2D
ENSG0000	3.88389	-0.00396	0.221488	0.950282	0.988675	NA	NA	NA
ENSG0000	182.8202	0.007189	0.146188	0.950601	0.988675	INTS14	81556	integrator complex subunit 14
ENSG0000	11.54217	-0.00453	0.215302	0.950087	0.988675	LOC11269	1.13E+08	uncharaterized LOC112694756
ENSG0000	384.6517	-0.007	0.18709	0.950593	0.988675	ZNF24	7572	zinc finger protein 24
ENSG0000	7.425991	0.005148	0.21797	0.95006	0.988675	NA	NA	NA
ENSG0000	132.2408	-0.00644	0.154812	0.950198	0.988675	ETFB	2109	electron transfer flavoprotein subunit beta
ENSG0000	23.63773	-0.00679	0.185998	0.950336	0.988675	CHKB	1120	choline kinase beta
ENSG0000	416.6157	-0.00699	0.143963	0.950719	0.988718	ARFRP1	10139	ADP ribosylation factor related protein 1
ENSG0000	4002.874	0.007357	0.171604	0.950854	0.988801	MLLT1	4298	MLLT1 super elongation complex subunit
ENSG0000	30.0857	0.005647	0.186375	0.950967	0.988823	NA	NA	NA
ENSG0000	205.4238	-0.0076	0.164369	0.950982	0.988823	SRPRB	58477	SRP receptor subunit beta
ENSG0000	491.2056	-0.00697	0.151913	0.951248	0.988987	PSMD1	5707	proteasom non-ATPase 1
ENSG0000	109.8685	-0.00703	0.148012	0.951221	0.988987	ALKBH1	8846	alkB homo histone H2A dioxygenase
ENSG0000	104.7261	0.010508	0.175073	0.952415	0.989241	DDX20	11218	DEAD-box helicase 20
ENSG0000	104.0647	-0.00623	0.167855	0.952238	0.989241	FASTKD2	22868	FAST kinase domains 2
ENSG0000	14.23798	0.006289	0.199696	0.952083	0.989241	NA	NA	NA
ENSG0000	341.1121	-0.00681	0.168424	0.95248	0.989241	G3BP2	9908	G3BP stress granule assembly factor 2
ENSG0000	15.00605	0.00583	0.197737	0.952455	0.989241	ZBTB12	221527	zinc finger and BTB domain containing 12
ENSG0000	4489.065	0.007236	0.167472	0.951969	0.989241	TFEB	7942	transcription factor EB
ENSG0000	374.3744	-0.00828	0.162323	0.952518	0.989241	CPNE3	8895	copine 3
ENSG0000	41.32821	-0.0059	0.195422	0.952334	0.989241	NDUFAF6	137682	NADH:ubiquinone oxidoreductase complex assembly factor 6
ENSG0000	1548.973	-0.00664	0.156973	0.951729	0.989241	PUF60	22827	poly(U) binding splicing factor 60
ENSG0000	73.44639	0.007009	0.183973	0.952293	0.989241	LRMDA	83938	leucine rich melanocyte differentiation associated
ENSG0000	4378.439	-0.00643	0.159224	0.951731	0.989241	RTN3	10313	reticulon 3
ENSG0000	91.60506	0.006243	0.192301	0.951939	0.989241	SLC29A2	3177	solute carrier family 29 member 2
ENSG0000	77.30102	0.006461	0.197426	0.952368	0.989241	LEMD3	23592	LEM domain containing 3
ENSG0000	685.4435	0.005667	0.114174	0.951921	0.989241	SRSF9	8683	serine and arginine rich splicing factor 9
ENSG0000	3541.721	-0.00715	0.157934	0.951976	0.989241	MSL1	339287	MSL complex subunit 1
ENSG0000	76.73451	0.008576	0.15264	0.951775	0.989241	MRPL27	51264	mitochondrial ribosomal protein L27
ENSG0000	1072.531	0.007089	0.155255	0.952146	0.989241	PSMC4	5704	proteasom ATPase 4
ENSG0000	23.10322	0.006514	0.194751	0.952477	0.989241	ZNF749	388567	zinc finger protein 749
ENSG0000	448.7138	-0.00649	0.126886	0.952177	0.989241	CXorf38	159013	chromosome X open reading frame 38
ENSG0000	292.8029	-0.00587	0.141627	0.952622	0.989277	CSNK1A1	1452	casein kinase 1 alpha 1
ENSG0000	389.0927	0.006194	0.161079	0.952661	0.989277	ZNF358	140467	zinc finger protein 358
ENSG0000	711.2811	-0.00523	0.194835	0.952809	0.989318	ATP11B	23200	ATPase phospholipid transporting 11B (putative)
ENSG0000	80.40598	-0.00568	0.170406	0.952778	0.989318	ATP10D	57205	ATPase phospholipid transporting 10D (putative)
ENSG0000	3.955707	-0.00309	0.22833	0.95305	0.989428	NRARP	441478	NOTCH regulated ankyrin repeat protein
ENSG0000	12.84801	0.0066	0.211529	0.953077	0.989428	OR52N4	390072	olfactory receptor family 52 subfamily N member 4
ENSG0000	84.81752	-0.00608	0.168464	0.952992	0.989428	HDAC10	83933	histone deacetylase 10
ENSG0000	32.30725	-0.00577	0.199882	0.953268	0.98957	RPL6P27	645387	ribosomal protein L6 pseudogene 27
ENSG0000	182.0761	-0.00649	0.153912	0.95359	0.989625	PMVK	10654	phosphomevalonate kinase
[truncated: 147,103 more chars]
