# Supplementary material for: Dexamethasone attenuates interferon-related cytokine hyperresponsiveness in COVID-19 patients
Source: Front Immunol. 2023 Aug 8;14:1233318. doi: 10.3389/fimmu.2023.1233318 (PMC10442808; doi:10.3389/fimmu.2023.1233318)
Supplement: Supplementary file 1 [file DataSheet_1.zip › Supplementary Table 1.pdf]

Supplementary Table 1. Demographic characteristics of healthy volunteer samples.

	Healthy volunteer samples		
**Demographic characteristics**	**Plasma proteomics**	**PBMCs cytokines ELISA**	**PBMCs RNAseq**
Female – No. (%)	1 (12.5%)	0 (0%)	0 (0%)
Male – No. (%)	7 (87.5%)	6 (100%)	5 (100%)
Age (years) – Mean ± SD	58.9 ± 6.6	48.6 ± 15.7	49.2 ± 9.2
Height (cm) – Mean ± SD	179 ± 9.0[1]	187 ± 6.7	184 ± 3.5
Weight (kg) – Mean ± SD	75.2 ± 9.4[1]	82.8 ± 13.7	83.4 ± 11.8
BMI (kg/m2) – Mean ± SD	23.5 ± 2.6[1]	23.9 ± 4.3	24.6 ± 3.3
Reported corticosteroid use	0 (0%)[1]	0 (0%)	0 (0%)
**COVID-19 status**			
COVID-19 symptoms at the time of sampling (self-reported)	N.A.[2]	N.A.[3]	0 (0%)
Molecular test positive for SARS-CoV-2 (self-reported)	N.A.[2]	N.A.[3]	0 (0%)
Had suspected COVID-19 in the past	N.A.[2]	0 (0%)	1 (20%)
Had confirmed COVID-19 in the past	N.A.[2]	0 (0%)	0 (0%)
Hospital admission for COVID-19 in the past	N.A.[2]	0 (0%)	0 (0%)
Vaccinated against SARS-CoV-2	0 (0%)[2,4]	0 (0%)[3,4]	4 (80%)

[1] Data on height, weight, and reported corticosteroid use were missing for three out of eight volunteers in this group.

[2] Samples were collected before COVID-19 outbreak in the Netherlands.

[3] This was not part of the questionnaire at the time samples were collected from these volunteers.

[4] Vaccines against SARS-CoV-2 were not available at the time these samples were collected.
