# Supplementary material for: Dexamethasone attenuates interferon-related cytokine hyperresponsiveness in COVID-19 patients
Source: Front Immunol. 2023 Aug 8;14:1233318. doi: 10.3389/fimmu.2023.1233318 (PMC10442808; doi:10.3389/fimmu.2023.1233318)
Supplement: Supplementary file 1 [file DataSheet_1.zip › Supplementary Table 2.pdf]

Supplementary Table 2. Characteristics of COVID-19 patients included in the study before dexamethasone treatment

Demographic characteristics	Expressed as		
Sex			
Female	Frequency	2 (15.4%)	
Male	Frequency	11 (84.6%)	
Age (years)	Mean ± SD	56.77 ± 9.1	
Length (cm)	Mean ± SD	176.4 ± 7.8	
Weight (kg)	Mean ± SD	86.2 ± 13.4	
BMI (kg/m2)	Mean ± SD	27.6 ± 4.1	
		**Timepoints**	
**Clinical characteristics**	**Expressed as**	Screening	Timepoint 1
Systolic blood pressure (mmHg)	Mean ± SD	137.0 ± 14.5	N.A.
Diastolic blood pressure (mmHg)	Mean ± SD	80.4 ± 8.2	N.A.
qSOFA score	Median (range)	0 (0-1)	N.A.
Respiratory rate	Mean ± SD	20.7 ± 2.9	20.8 ± 2.9
Duration of illness	Mean ± SD	8.8 ± 2.9	8.9 ± 2.9
Supplemental oxygen volume (L/min)	Mean ± SD	3.6 ± 2.9	4.2 ± 3.1
Type of supplemental oxygen			
Nasal cannula	Frequency	11 (84.6%)	9 (69.2%)
Venturi mask (40%)	Frequency	0 (0%)	1 (7.7%)
Venturi mask (60%)	Frequency	2 (15.4%)	3 (23.1%)
Non-rebreathing mask	Frequency	0 (0%)	0 (0.0%)
High-flow nasal cannula / Non-invasive ventilation mask	Frequency	0 (0%)	0 (0%)
Mechanical ventilation	Frequency	0 (0%)	0 (0%)
**Clinical outcome**	**Expressed as**		
Length of hospital stay (days)	Mean ± SD	7.9 ± 3.5	
Required ICU admission	Frequency	1 (7.7%)	
In-hospital mortality	Frequency	0 (0.0%)	
