# Supplementary material for: Dexamethasone attenuates interferon-related cytokine hyperresponsiveness in COVID-19 patients
Source: Front Immunol. 2023 Aug 8;14:1233318. doi: 10.3389/fimmu.2023.1233318 (PMC10442808; doi:10.3389/fimmu.2023.1233318)
Supplement: Supplementary file 1 [file DataSheet_1.zip › Supplementary Table 3.pdf]

Supplementary Table 3. Characteristics of the patients of whom PBMCs were isolated for RNA sequencing

Demographic characteristics	Expressed as	
Sex		
Female	Frequency	0 (0%)
Male	Frequency	5 (100%)
Age (years)	Mean ± SD	52.40 ± 11.5
Length (cm)	Mean ± SD	176.0 ± 3.4
Weight (kg)	Mean ± SD	85.4 ± 8.4
BMI (kg/m^2)	Mean ± SD	27.5 ± 1.8
**Clinical characteristics**	**Expressed as**	
Systolic blood pressure (mmHg)	Mean ± SD	135.0 ± 15.0
Diastolic blood pressure (mmHg)	Mean ± SD	79.4 ± 7.2
qSOFA score	Median (range)	0 (0-1)
Respiratory rate	Mean ± SD	19.8 ± 1.8
Duration of illness	Mean ± SD	8.8 ± 3.0
Supplemental oxygen volume (L/min)	Mean ± SD	3.8 ± 3.5
Type of supplemental oxygen		
Nasal cannula	Frequency (*percentage*)	4 (80%)
Venturi mask (40%)	Frequency (*percentage*)	0 (0%)
Venturi mask (60%)	Frequency (*percentage*)	1 (20%)
Non-rebreathing mask	Frequency (*percentage*)	0 (0%)
High-flow nasal cannula / Non-invasive ventilation mask	Frequency (*percentage*)	0 (0%)
Mechanical ventilation	Frequency (*percentage*)	0 (0%)
**Clinical outcome**	**Expressed as**	
Length of hospital stay (days)	Mean ± SD	6.8 ± 2.2
In-hospital mortality	Frequency (*percentage*)	0 (0.0%)
